# Quantitative Proteomic Analysis of Human Lung Tumor Xenografts Treated with the Ectopic ATP Synthase Inhibitor Citreoviridin

**DOI:** 10.1371/journal.pone.0070642

**Published:** 2013-08-21

**Authors:** Yi-Hsuan Wu, Chia-Wei Hu, Chih-Wei Chien, Yu-Ju Chen, Hsuan-Cheng Huang, Hsueh-Fen Juan

**Affiliations:** 1 Institute of Molecular and Cellular Biology, Department of Life Science, National Taiwan University, Taipei, Taiwan; 2 Institute of Chemistry, Academia Sinica, Taipei, Taiwan; 3 Institute of Biomedical Informatics, Center for Systems and Synthetic Biology, National Yang-Ming University, Taipei, Taiwan; Virginia Commonwealth University, United States of America

## Abstract

ATP synthase is present on the plasma membrane of several types of cancer cells. Citreoviridin, an ATP synthase inhibitor, selectively suppresses the proliferation and growth of lung cancer without affecting normal cells. However, the global effects of targeting ectopic ATP synthase *in vivo* have not been well defined. In this study, we performed quantitative proteomic analysis using isobaric tags for relative and absolute quantitation (iTRAQ) and provided a comprehensive insight into the complicated regulation by citreoviridin in a lung cancer xenograft model. With high reproducibility of the quantitation, we obtained quantitative proteomic profiling with 2,659 proteins identified. Bioinformatics analysis of the 141 differentially expressed proteins selected by their relative abundance revealed that citreoviridin induces alterations in the expression of glucose metabolism-related enzymes in lung cancer. The up-regulation of enzymes involved in gluconeogenesis and storage of glucose indicated that citreoviridin may reduce the glycolytic intermediates for macromolecule synthesis and inhibit cell proliferation. Using comprehensive proteomics, the results identify metabolic aspects that help explain the antitumorigenic effect of citreoviridin in lung cancer, which may lead to a better understanding of the links between metabolism and tumorigenesis in cancer therapy.

## Introduction

Lung cancer is the leading cause of cancer-related death worldwide, with nearly 1.4 million people dying from lung cancer each year [1]. One of the treatment strategies for lung cancer is based on the discovery that subsets of lung cancer harbor specific mutations in genes coding crucial proteins involved in signaling pathways of cell survival and proliferation. For example, gefitinib (Iressa) and erlotinib (Tarceva), two drugs inhibiting epidermal growth factor receptor (EGFR) tyrosine kinase, are effective therapies for non-small cell lung cancer (NSCLC) [2]–[4]. However, unavoidable drug resistance eventually develops in patients with objective responses to gefitinib or erlotinib initially [5], [6]. Furthermore, 30% of patients receiving gefitinib showed not much change from pre-treatment conditions [7], [8].

The oncogenic gene expression in cancer causes alterations to metabolism besides affecting signaling pathways. Nutrients are converted to biosynthetic building blocks, which are further converted to macromolecules for constituting new cells [9]. Glucose is the major source of cellular energy and building blocks for new cells. Hence, glucose metabolism and dependence are altered in cancer cells. In 1924, Otto Warburg observed that rapidly proliferating cancer cells exhibited higher glucose consumption than normal cells through higher levels of glycolysis, which turns glucose into lactate even in the presence of oxygen [10], [11]. This phenomenon is known as the Warburg effect, and is also called aerobic glycolysis to distinguish from traditional anaerobic glycolysis, where glucose is converted to lactate when limited oxygen is available. The major advantage of aerobic glycolysis is maintaining the level of glycolytic intermediates to sustain continuous building blocks for macromolecular synthesis, including generating nucleotides, lipids, and amino acids [12], [13]. The understanding of cancer metabolism showed that aerobic glycolysis is a promising target for cancer therapies.

ATP synthase is nature's smallest motor that is important in producing energy to drive many processes in cells. Although ATP synthase has been thought to be exclusively located on the inner membrane of mitochondria, several reports have showed that components of ATP synthase also exist on the plasma membrane of several types of cells. ATP synthase located on the plasma membrane is called ectopic ATP synthase or ecto-ATP synthase. In tumor cells, ectopic ATP synthase was recognized as a ligand of a cytolytic pathway used by naive natural killer (NK) and lymphokine-activated killer (LAK) cells [14], [15]. Furthermore, ATP synthase was also found on the surface of breast cancer cells and was involved in cell proliferation, which showed that it could be a target for cancer therapy [16]. Diverse categories of ATP synthase inhibitors have been discovered and investigated, including peptides, polyphenolic phytochemicals, polyketides, polyenic α-pyrone derivatives, and so on [17]. One of the compounds called citreoviridin is a polyene mycotoxin produced by several molds of genera, such as *Penicillium* and *Aspergillus*. It consists of an α-pyrone ring conjugated to a furan ring. Citreoviridin inhibits the activity of ATP synthase by interacting with the β subunit of F1 ATP synthase [18], [19]. It was shown to affect several metabolic enzymes, including glycogen synthase, glutamic-oxaloacetic transaminase and transketolase [20]–[22]. Citreoviridin has been proved to inhibit the proliferation of the lung adenocarcinoma cell lines A549 and CL1-0 by activating the unfolded protein response [23].

Proteomics, which measures mature proteins, could be used to closely observe biological functions in cells. There are two major methods available for mass spectrometry (MS) quantitation, the stable isotope-based and the label-free approaches [24]. A well-established and widely used stable isotope-based method is isobaric tags for relative and absolute quantitation (iTRAQ) [25]. iTRAQ reagents are amide reactive and covalently link to the N terminus and side chain of lysine residues of peptides. It provides multiplex protein quantitation by labeling peptides from different samples with different iTRAQ reagents. One of the most significant advantages of iTRAQ quantitation is that the intensities of peptide precursor ions in MS and fragment ions in MS/MS are enhanced by combination of all iTRAQ-labeled samples prior to MS analysis, which increases the accuracy of quantitation. However, global biases can arise from the sample preparation, reducing the accuracy of protein quantitation [26]. Therefore, a good normalization method is of significant importance and should be performed to access accurate quantitation. Another key concern about iTRAQ is the integration of peptide-level information into the measurement of protein abundance [27]. A variety of algorithms were proposed and many software packages are also available for estimation of protein expression.

In this study, our major objective was to elucidate the effect induced by citreoviridin in a lung cancer xenograft model. Applying proteomic analysis, we investigated the proteomic changes and pathways leading to cell proliferation inhibition caused by citreoviridin in lung cancer. First, the reproducibility of the iTRAQ-based proteomic strategies was assessed, followed by the acquisition of the proteomic profiling of citreoviridin-treated tumors with iTRAQ proteomic experiments. For data analysis, we optimized the normalization of iTRAQ signals and quantified the expression of proteins identified. After selecting differentially expressed human proteins between control and citreoviridin-treated tumors, we investigated the pathways induced by citreoviridin in lung cancer xenograft tumors. Finally, the most significant pathway elucidated by bioinformatics methods was subsequently validated.

## Results

### The effect of an ATP synthase inhibitor on tumor xenografts

**Figure 1:**
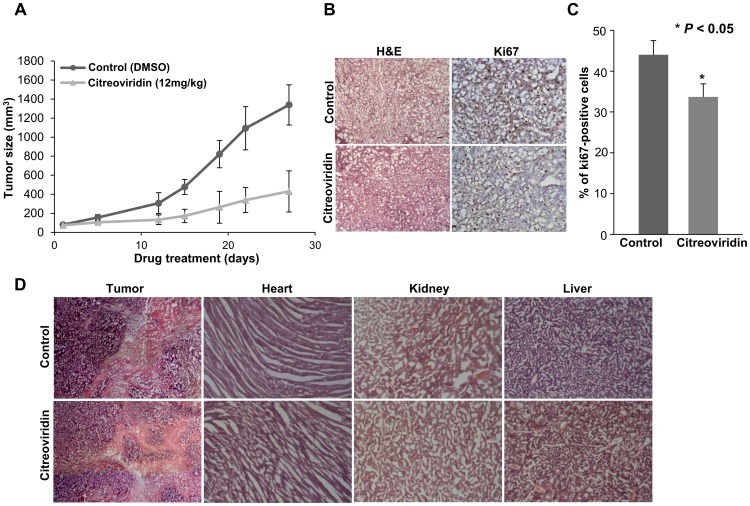
Tumor growth and cell proliferation analysis in the CL1-0 xenograft model. (A) Tumor regression in a xenograft model. The tumor volume was decreased after treatment with citreoviridin. 5×10^6^ CL1-0 cells were implanted subcutaneously in SCID mice and the abdominal injection of citreoviridin was performed after tumor size reached 100 mm^3^. (B) The histology (left, H&E, 100×) and Ki67 staining (right, Ki67, 100×) within the same area of tumor tissues. (C) The percentage of proliferating cells in tumor sections using Ki67-immunohistochemistry. Ki67 staining showed a lower percentage of proliferating cells in citreoviridin-treated tumors. (D) Histological analysis of tumor tissues and mice organs. The histology of tumor and organ tissue sections was analyzed by H&E staining (tumor sections, 40×; organ sections, 100×). No obvious histological damages were observed in citreoviridin-treated organ sections, including the heart, kidney and liver. All the staining was performed in 10 µm cryostat sections. H&E, hematoxylin and eosin.

We investigated the effect of an ATP synthase inhibitor on lung cancer growth *in vivo* by monitoring the growth rate of tumors in a xenograft model. By measuring the tumor volume, our study showed a reduced tumor growth rate in citreoviridin-treated mice (Figure 1A). We further studied cell proliferation by immunohistochemical analysis of Ki67, an important marker of proliferating cells [28]. Our results showed that the percentage of Ki67 positive cells was significantly lower in citreoviridin-treated tumor tissues (Figure 1B and Figure 1C). All these results suggest a role for ATP synthase inhibitors that suppresses the malignant development of tumors. Moreover, the histological analysis of citreoviridin-treated tumor tissues and other organs (heart, kidney, and liver) also revealed a less serious condition of tumor development with low toxicity to major organs of mice (Figure 1D).

### Reproducibility assessment by analysis of the iTRAQ duplicate experiment

**Figure 2:**
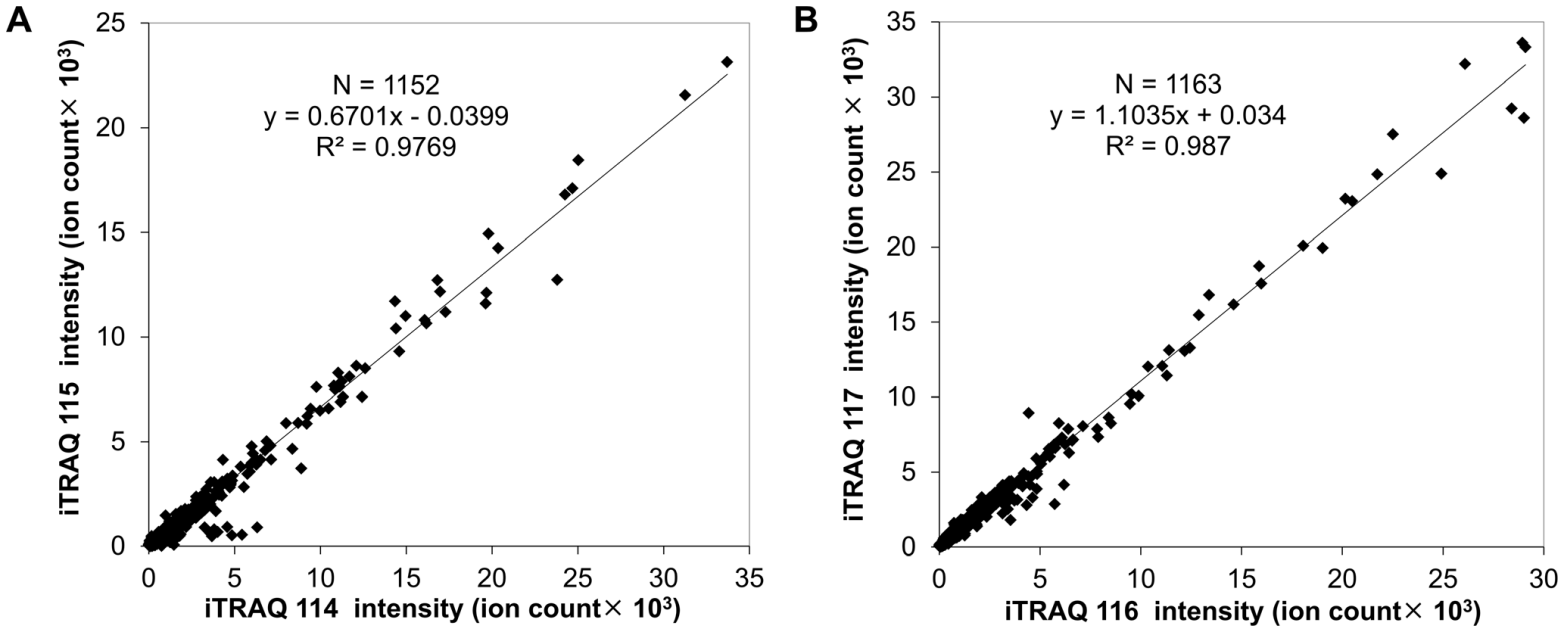
iTRAQ quantitative proteomic experiments showed high reproducibility and accuracy. (A) Scattering plot of two replicate control tumor samples, iTRAQ 114-labeled C_1α_ and iTRAQ 115-labeled C_1β_. (B) Scattering plot of two replicate citreoviridin-treated tumor samples, iTRAQ 116-labeled T_1α_ and iTRAQ 117-labeled T_1β_.

We applied proteomic analysis to investigate the effects of targeting ectopic ATP synthase in a human lung cancer xenograft model. Reproducibility is an important concern for quantitative proteomic studies. Any procedure, including protein extraction, reduction, alkylation, trypsin digestion and iTRAQ labeling, may affect the reproducibility and accuracy of quantitation. In order to check the reproducibility, two replicate preparations of proteins from both controls (C_1α_ and C_1β_) and citreoviridin-treated (T_1α_ and T_1β_) tumor samples were analyzed (Figure S1). Two replicate proteins were independently extracted from tumors and separately subjected to reduction, alkylation and trypsin digestion. For iTRAQ labeling, equal amounts of peptides from each sample were labeled with iTRAQ. Sample C_1α_ was labeled with iTRAQ 114 tag while sample C_1β_ was labeled with iTRAQ 115 tag. Sample T_1α_ was labeled with iTRAQ 116 tag while sample T_1β_ was labeled with iTRAQ 117 tag. All iTRAQ-labeled peptides were combined and analyzed by LC-MS/MS. The proteomic data were provided in Table S1 and the information of single-peptide-based protein identifications was in Spectra S1. After protein identification and peptide selection, the original intensity of each of the iTRAQ signature ions was plotted in Figure 2. There was a high correlation (the correlation coefficient R^2^ = 0.9769) between two replicate control tumor samples, iTRAQ 114-labeled C_1α_ and iTRAQ 115-labeled C_1β_ (Figure 2A). Two replicate citreoviridin-treated tumor samples, iTRAQ 116-labeled T_1α_ and iTRAQ 117-labeled T_1β_, also showed high correlation (the correlation coefficient R^2^ = 0.987, Figure 2B). The high correlation of peptide iTRAQ signature ion intensity between duplicate samples indicated that the iTRAQ quantitative proteomic experiment has high reproducibility and accuracy.

### Proteomic profiling of citreoviridin-treated tumors

To investigate the proteomic change induced by citreoviridin, proteins of two control tumors (C_1_ and C_2_) and two citreoviridin-treated tumors (T_1_ and T_2_) from a total of four different mice were analyzed (Figure S2). We first performed a small-scale experiment, which analyzed 5 µg peptides of each sample. For iTRAQ labeling, peptides from samples C_1_, C_2_, T_1_ and T_2_ were labeled with iTRAQ 114, 115, 116 and 117 tags, respectively. The proteomic data of the small-scale experiment were provided in Table S2 and the information of single-peptide-based protein identifications was in Spectra S2. We identified 277 proteins with a false discovery rate (FDR) of 3.51%. It was confirmed that 99.72% of identified peptides were labeled with iTRAQ and a total of 1,185 peptides were qualified for protein quantitation (Table 1).

**Table 1:** Statistics of three iTRAQ quantitative proteomic experiments.

iTRAQ quantitative proteomic experiment	Number of proteins identified	FDR (%)^a^	Percentage of iTRAQ-labeled peptides (%)	Number of qualified peptides^b^	Percentage of quantified proteins (%)
Duplicate experiment	296	2.14	99.67	1,159	93.92
Small-scale experiment	277	3.51	99.72	1,185	98.56
Large-scale experiment	2,659	2.22	99.53	28,894	94.55

a FDR (false discovery rate) was calculated by the formula: *D*/*R*×100%, where *D* and *R* are the number of matches above identity threshold determined by searching decoy and real databases, respectively.

b Qualified peptides are peptides satisfying all the four criteria (see Methods for details) and were used for protein quantitation.

**Figure 3:**
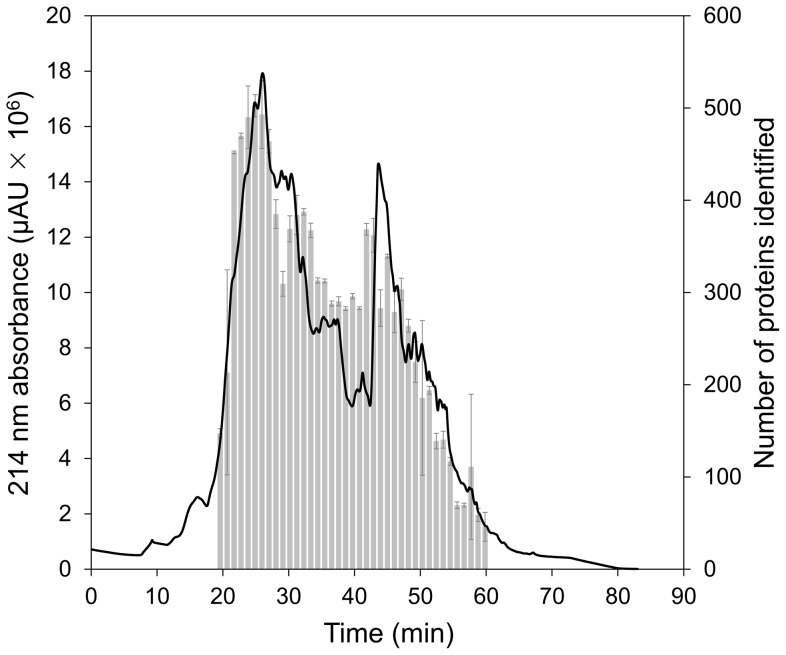
Strong cation exchange (SCX) chromatogram. The absorbance of peptide bonds occurs at 214 nm. Therefore, the left axis represents the contents of combined iTRAQ-labeled peptides. Fractions were collected every minute. The right axis is the number of identified proteins in each fraction. Error bars represent standard deviation of the replicate analysis of LC-MS/MS. Fraction 19: n = 5; fraction 47: n = 3; fraction 54: n = 4; other fractions: n = 2.

To identify more proteins, a large-scale experiment, which analyzed 150 µg peptides of each sample, was performed to acquire the proteomic profiling of two control tumors (C_1_ and C_2_) and two citreoviridin-treated tumors (T_1_ and T_2_) from a total of four different mice (Figure S2). Peptides from samples C_1_, C_2_, T_1_ and T_2_ were also labeled with iTRAQ 114, 115, 116 and 117 tags, respectively. To reduce the sample complexity and increase the possibility of detecting low abundance proteins, the combined iTRAQ-labeled peptides were fractioned by strong cation exchange (SCX) chromatography. A total of 39 fractions were individually analyzed by LC-MS/MS. The SCX chromatogram and the number of proteins identified in each fraction were shown in Figure 3. The proteomic data of the large-scale experiment were provided in Table S3 and the information of single-peptide-based protein identifications was in Spectra S3, Spectra S4 and Spectra S5. In this large-scale experiment, we identified a total of 2,659 proteins with FDR of 2.22% (Table 1). Compared to the results of the small-scale experiment, SCX chromatography reduced the sample complexity and enhanced protein identification. It was also confirmed that 99.53% of identified peptides were labeled with iTRAQ and a total of 28,894 peptides were qualified for protein quantitation (Table 1).

### Optimization of peptide iTRAQ signal normalization

After protein identification, the peptides with qualified intensities of iTRAQ signature ions were selected for further quantitative analysis. Deviation of the iTRAQ signature ions intensities of peptides (hereafter referred to as peptide iTRAQ signals) may exist due to measurement errors in the experiments and individual variations from biological replicates of samples. Therefore, normalization is necessary for accuracy in protein quantitation. We have tried seven different normalization methods. The details of the normalization methods and evaluation were described in Method S1. First, we use the dataset from the duplicate experiment (Figure S1), which contained 296 identified proteins and 1,159 qualified peptides for protein quantitation (Table 1). Briefly, the normalized peptide iTRAQ signals were used for calculation of the *S* values, which represent the errors of the protein abundance ratios, followed by the calculation of mean of all *S* values (Table S4). Considering normalization performed directly on the level of peptide iTRAQ signals, the methods 1 and 2, which had relative small mean of *S* values (Table 2) were selected.

**Table 2:** The *S* values calculated by seven different normalization methods in the duplicate experiment.

			*S* value^b^	
Normalization level	Method	Description^a^	Mean	S.D.^c^
Peptide iTRAQ signal	1	Equal summation of peptide iTRAQ signals	−0.02026	0.34727
	2	Median of log_2_ (peptide iTRAQ ratio) to zero	0.01946	0.34727
	3	Trend line of peptide iTRAQ signals	0.16265	0.29973
	4	Trend line of log_2_ (peptide iTRAQ signal)	0.04154	0.33903
	5	Multi-Q normalization factor performing on peptide iTRAQ signals	0.02810	0.34727
Protein abundance ratio	6	Multi-Q normalization factor performing on protein abundance ratio	0.02810	0.34727
	7	Median of log_2_ (protein abundance ratio) to zero	0.00816	0.34727

a Detailed calculation methods are described in Method S1.

b *S* value is in log_2_ scale: 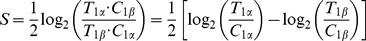
where *T*_1α_/*C*_1α_ is the protein abundance ratio of citreoviridin-treated tumor sample T_1α_ to control sample C_1α_, while *T*_1β_/*C*_1β_ is the protein abundance ratio of citreoviridin-treated tumor sample T_1β_ to control sample C_1β_.

c S.D.: standard deviation.

**Figure 4:**
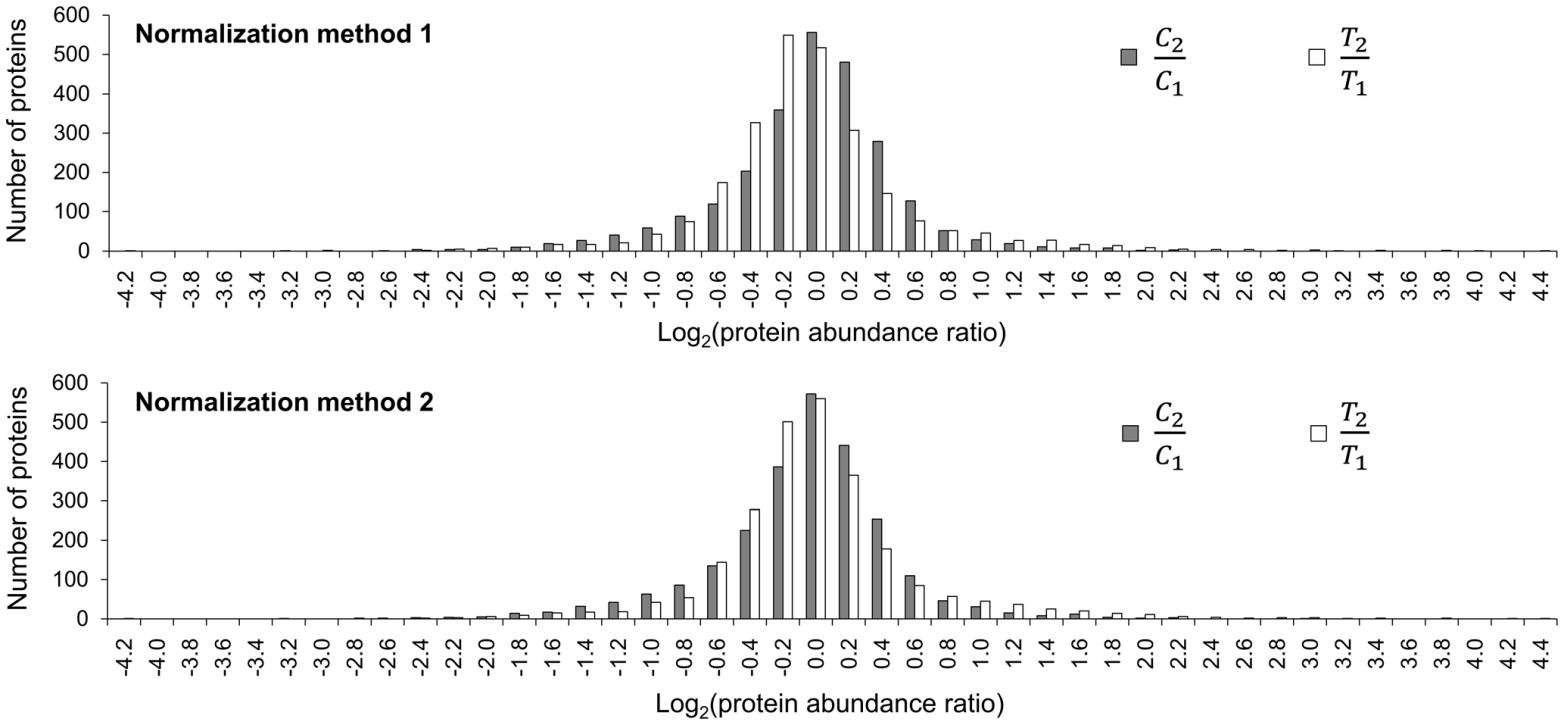
The distribution of log_2_ (*C*_2_/*C*_1_) and log_2_ (*T*_2_/*T*_1_) in the large-scale experiment by using normalization method 1 and method 2. *C*_2_/*C*_1_ and *T*_2_/*T*_1_ were the protein abundance ratios of sample C_2_ to C_1_ and sample T_2_ to T_1_, respectively.

To test whether method 1 and method 2 had the capability of normalizing larger datasets, we applied these two normalization methods to the dataset of the large-scale experiment (Figure S2), which contained 2,659 identified proteins and 28,894 qualified peptides for protein quantitation. Similarly, the mean of *S* values was also calculated from the normalized peptide iTRAQ signals (Table S5). It was shown that method 1 had a smaller mean of *S* values than method 2 (Table S6). Next, normalized peptide iTRAQ signals were used for the calculation of the protein abundance ratios, *C*_2_/*C*_1_ and *T*_2_/*T*_1_, which should be close to 1 with a good normalization method. The results showed that the mode of log_2_ (*T*_2_/*T*_1_) was −0.2 with normalization method 1, while the mode of log_2_ (*C*_2_/*C*_1_) and log_2_ (*T*_2_/*T*_1_) were both 0 with normalization method 2 (Figure 4). Compared with method 1, method 2 was able to correct both the protein abundance ratios, *C*_2_/*C*_1_ and *T*_2_/*T*_1_. Therefore, we chose method 2 as the optimal normalization approach for our data.

### Quantitation of protein expression by iTRAQ signals

**Figure 5:**
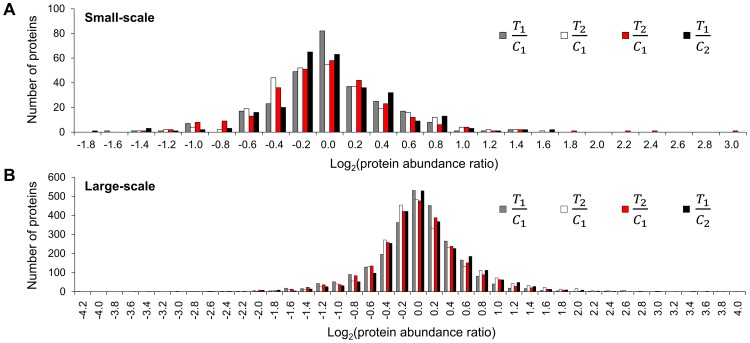
The distribution of four sets of treatment to control log_2_ protein abundance ratios. (A) Small-scale experiment. (B) Large-scale experiment. *T*_1_/*C*_1_, *T*_2_/*C*_2_, *T*_2_/*C*_1_ and *T*_1_/*C*_2_ were the protein abundance ratios of sample T_1_ to C_1_, sample T_2_ to C_2_, sample T_2_ to C_1_ and sample T_1_ to C_2_, respectively.

**Figure 6:**
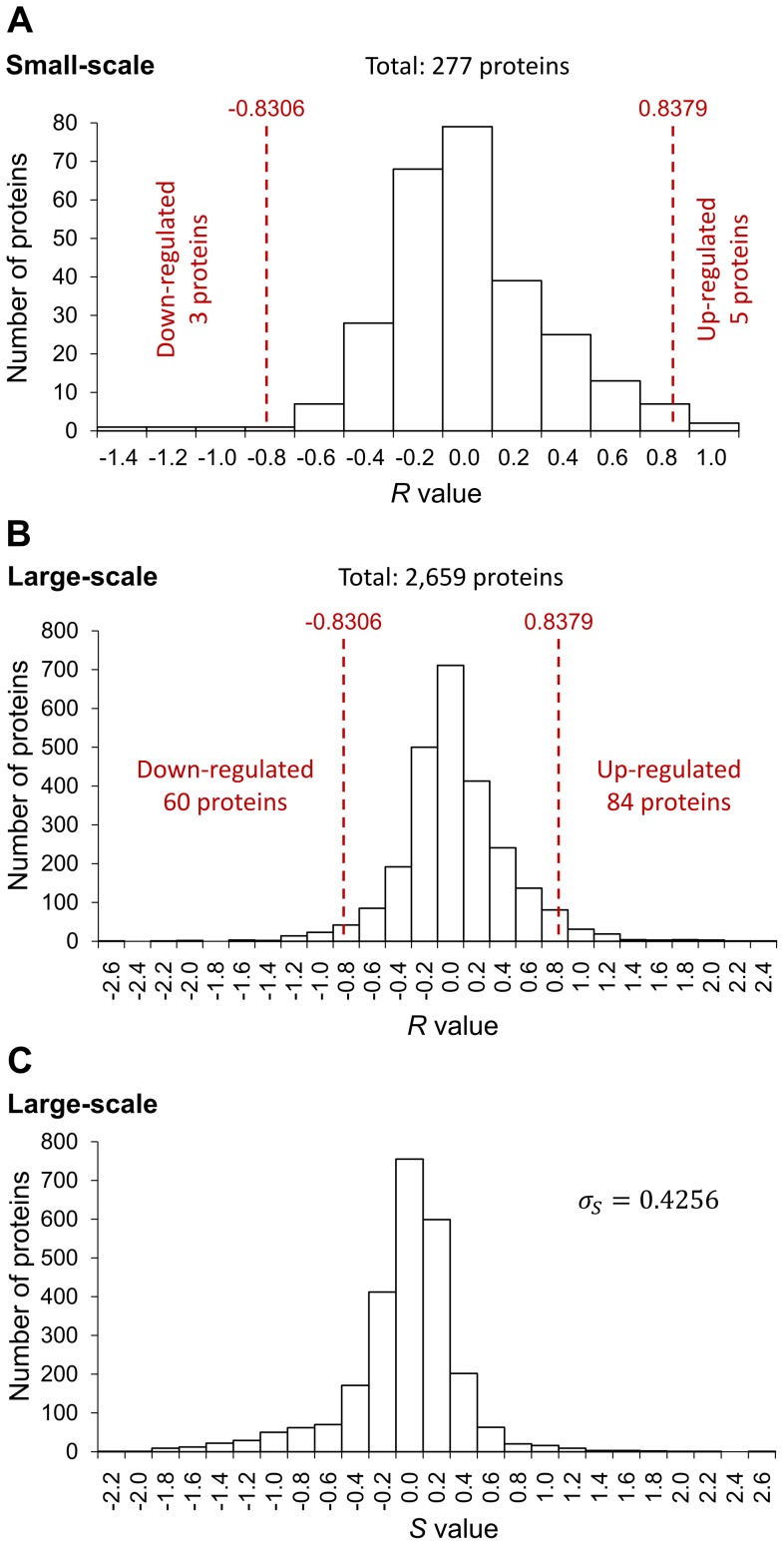
Quantitation of differentially expressed proteins. (A) The distribution of the *R* values in the small-scale experiment. The *R* value of each protein was calculated by the protein abundance ratios *T*_1_/*C*_1_, *T*_2_/*C*_2_, *T*_2_/*C*_1_ and *T*_1_/*C*_2_. There were 277 proteins identified and 8 proteins were differentially expressed as determined by comparing with the cut-off values. (B) The distribution of the *R* values in the large-scale experiment. There were 2,659 proteins identified and 144 proteins were differentially expressed as determined by comparing with the cut-off values. (C) The distribution of the *S* values in the large-scale experiment. The *S* value of each protein was calculated by the protein abundance ratios *T*_1_/*C*_1_ and *T*_2_/*C*_2_. The standard deviation of the *S* values was 0.4256. *σ_S_*: standard deviation of the *S* values.

The normalized peptide iTRAQ signals were used for the quantitation of proteins identified in the small-scale and large-scale experiments. We applied the sum of intensities in protein quantitation. The iTRAQ signature ion intensities of peptides matching the protein were summed and the protein abundance ratio was calculated as dividing a sample's summation of intensities to another sample's summation of intensities. This is a weighted calculation because the larger intensity has more contribution to the protein abundance ratio. For each protein, we calculated four protein abundance ratios, *T*_1_/*C*_1_, *T*_2_/*C*_2_, *T*_2_/*C*_1_ and *T*_1_/*C*_2_. There were over 90% of proteins quantified in both experiments (Table 1). The distribution of these four sets of protein abundance ratios in both experiments was shown in Figure 5, indicating that the expression level of most proteins remained unchanged with the treatment of citreoviridin. The *R* value of each protein, which represents the relative abundance of the protein, was calculated with the four protein abundance ratios, *T*_1_/*C*_1_, *T*_2_/*C*_2_, *T*_2_/*C*_1_ and *T*_1_/*C*_2_. The distribution of *R* values in the small-scale and large-scale experiments was shown in Figure 6A and Figure 6B, respectively. The median of *R* values (*M_R_*) in the large-scale experiment was 0.0037.

### Cut-off value calculation for selecting differentially expressed proteins

In order to elucidate the proteomic change induced by citreoviridin in lung cancer xenograft tumors, differentially expressed proteins were selected by their relative protein abundance between control and citreoviridin-treated tumors. However, differences observed between control and treatment groups may arise from measurement errors in experiments and individual variations among tumors from different mice. Therefore, to positively select the differentially expressed proteins, we first calculated the cut-off values that indicate a significant degree of up-regulation or down-regulation. The large-scale experiment, which contains two biological replicates for both control and citreoviridin-treated tumor samples, is suitable for measuring the errors. The *S* value of each protein, which represents the error of protein abundance ratios, was calculated by its protein abundance ratios, *T*_1_/*C*_1_ and *T*_2_/*C*_2_. Each protein had one *S* value and the distribution of *S* values can be deemed as the distribution of errors (Figure 6C). Assuming that the errors follow a normal distribution, a 1.96-fold of the standard deviation (1.96 S.D.) of *S* values is statistically significant (*P*<0.05) and can be taken as the cut-off value. The standard deviation of *S* values (*σ_S_*) calculated from the large-scale experiment was 0.4256. The median of *R* values (*M_R_*) in the large-scale experiment was 0.0037. The cut-off values were defined as *M_R_*±1.96 *σ_S_* . Hence, we took 0.8379 and −0.8306 as cut-off values for selecting differentially expressed proteins. The data of cut-off value calculation were provided in Table S7.

To select the differentially expressed proteins from the datasets of small-scale and large-scale experiments, the *R* value of each protein was compared with the cut-off values. In the small-scale experiment that identified 277 proteins, there were five proteins with *R* values larger than the cut-off value 0.8379 and can be taken as up-regulated proteins. On the other hand, three proteins, which had *R* values smaller than the cut-off value −0.8306, were down-regulated (Figure 6A). Among the 2,659 identified proteins in the large-scale experiment, 84 proteins with *R* values larger than 0.8379 were up-regulated, while 60 proteins with *R* values smaller than −0.8306 were down-regulated (Figure 6B).

The standard deviation of *S* values (*σ_S_*) calculated from the large-scale experiment was the estimation of the errors from both of the experimental measurements and the individual variations among biological replicates of samples. We were also able to determine the errors only from the experimental measurements. The *S* value of each protein identified in the duplicate experiment was calculated by its protein abundance ratios, *T*_1α_/*C*_1α_ and *T*_1β_/*C*_1β_. Samples C_1α_ and C_1β_ were from the same control tumor and T_1α_ and T_1β_ were from the same citreoviridin-treated tumor. In theory, *T*_1α_/*C*_1α_ and *T*_1β_/*C*_1β_ were free from the error caused by the individual variations of biological replicates of samples. Therefore, the standard deviation of *S* values from the duplicate experiment (*σ_S_*_(*t*)_) can be deemed as the errors arising only from the experimental measurements. The distribution of *S* values and the *σ_S_*_(*t*)_ calculated from the duplicate experiment were shown in Figure S3. With the *σ_S_*, which represented the total errors of experimental measurements and individual variations among tumors, and the *σ_S_*_(*t*)_, which represented the errors only from the experimental measurements, the errors arising from the individual variations of biological replicate of samples (*σ_S_*_(*b*)_) can be estimated:
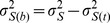
The *σ_S_*_(*b*)_ calculated by the above equation was 0.2461 (Table S7).

### Bioinformatics analysis of human differential proteomic profiling induced by citreoviridin

To elucidate the pathways induced by the ATP synthase inhibitor citreoviridin in tumors of lung cancer xenografts, we applied bioinformatics analysis to the differentially expressed proteins between control and citreoviridin-treated tumors. In the xenograft mouse model, mouse cells may be present in the subcutaneous tumors of human lung cancer. To exclude the contaminants of mouse proteins in our analysis, we selected only human proteins from the differential proteomes in the small-scale and large-scale experiments acquired previously. A total of 141 differentially expressed human proteins were selected, including 78 proteins with identified peptides only matched to human proteins and 63 proteins with identified peptides matched to both human and mouse proteins (Table S8).

To characterize the biological functions of differentially expressed proteins, first we performed functional annotation with Gene Ontology biological process by using DAVID Bioinformatics Resources [29], [30]. The functional annotation clustering enriched in our dataset was shown in Table 3 and Table S9. The top two GO biological process clusters were related to glucose metabolism, indicating that several citreoviridin-regulated proteins were involved in glucose metabolism pathways. The protein ubiquitination process was also enriched in the differentially expressed protein dataset. In the proteomic profiling, we also identified ubiquitin to be up-regulated 3.31-fold in tumors treated with citreoviridin (Table S8).

**Table 3:** Gene Ontology biological process clustering enrichment analysis of the differential proteome induced by citreoviridin in humans.

GO biological process cluster	Enrichment score^a^
Carbohydrate catabolic process	6.20
Glucose metabolic process	5.96
Protein ubiquitination	5.25
Macromolecule catabolic process	3.15
Regulation of developmental growth	2.83
Intermediate filament organization	2.40
Cellular amide metabolic process	2.34
Protein modification by small protein conjugation	2.19
Regulation of programmed cell death	1.67
Cellular polysaccharide metabolic process	1.57
Macromolecular complex assembly	1.50
Negative regulation of apoptosis	1.47

a The enrichment score is the geometric mean (in −log scale) of the p-values of the members in the annotation cluster. It represents the significance of relevance between the group of annotations and the experimental dataset.

**Figure 7:**
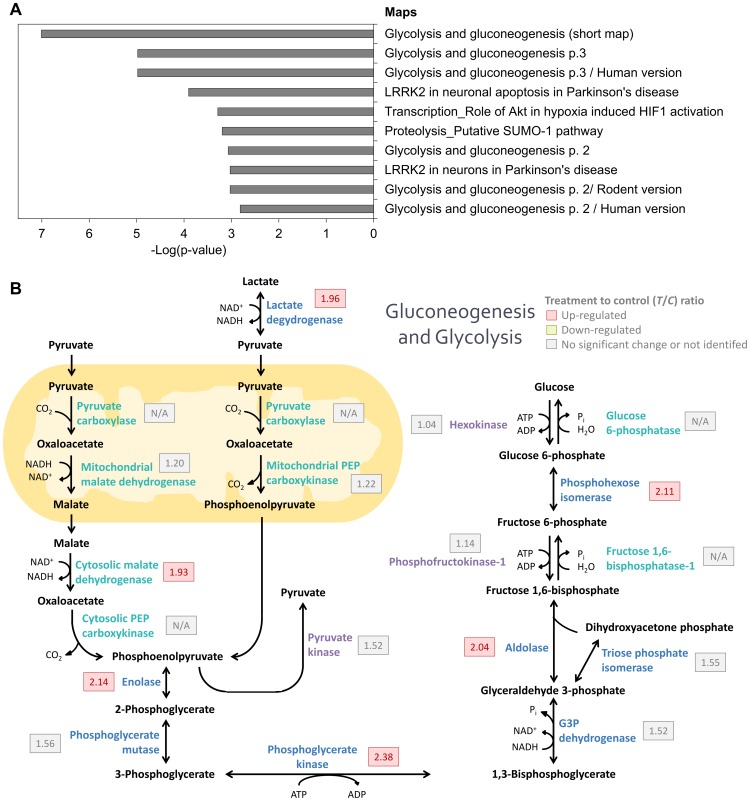
Bioinformatics analysis of human differential proteomic profiling induced by citreoviridin. (A) Pathways associated with differentially expressed human proteins by MetaCore pathway map analysis. The top associated pathway was the glycolysis and gluconeogenesis pathway and there were eight differentially expressed human proteins involved in the glycolysis and gluconeogenesis pathway. (B) The expression level of enzymes involved in gluconeogenesis and glycolysis in the large-scale experiment. Several enzymes were up-regulated with citreoviridin treatment. Enzymes specific for gluconeogenesis and glycolysis are shown in light blue and purple, respectively.

Next, we used MetaCore to analyze the pathway maps that differentially expressed proteins were involved in. The level of intersection between pre-existing pathway maps in the MetaCore database and our differentially expressed protein dataset was calculated and ordered based on the significance of relevance (Figure 7A and Table S10). The top pathway map enriched in our data was the glycolysis and gluconeogenesis pathway map (p-value = 9.76×10^−8^). Besides, six of the ten most enriched pathway maps were related to glycolysis and gluconeogenesis. The eight differentially expressed proteins involved in the glycolysis and gluconeogenesis pathway map were all up-regulated in tumors treated with citreoviridin. The change in expression level of enzymes involved in gluconeogenesis and glycolysis in the large-scale experiment were summarized in Figure 7B. The list of proteins involved in gluconeogenesis and glycolysis processes were in Table 4 and most enzymes were up-regulated with the treatment of citreoviridin.

**Table 4:** List of proteins in gluconeogenesis and glycolysis identified in the large-scale experiment.

No.	Accession	Gene symbol	Description	Score	No. of unique peptides^a^	Coverage (%)^b^	log_2_ (*T*_1_/*C*_1_)	log_2_ (*T*_2_/*C*_2_)	log_2_ (*T*_2_/*C*_1_)	log_2_ (*T*_1_/*C*_2_)	*R* value^c^	*σ_R_* d	*T*/*C* ratio^e^	M^f^
**Both in gluconeogenesis and glycolysis**														
Lactate dehydrogenase														
1	P00338	LDHA	L-lactate dehydrogenase A chain	1740	15	48.2	1.06	0.95	0.81	1.20	1.01	0.14	2.01	
2	P07195	LDHB	L-lactate dehydrogenase B chain	2132	15	46.7	1.28	0.58	0.75	1.11	0.93	0.28	1.91	
Enolase														
3	P06733	ENO1	Alpha-enolase	9055	20	61.1	1.32	0.88	0.75	1.45	1.10	0.29	2.14	
Phosphoglycerate mutase														
4	P18669	PGAM1	Phosphoglycerate mutase 1	1599	9	57.9	0.51	0.77	0.56	0.72	0.64	0.11	1.56	**+**
Phosphoglycerate kinase														
5	P00558	PGK1	Phosphoglycerate kinase 1	2588	18	52	1.08	1.43	1.35	1.16	1.25	0.14	2.38	
Glyceraldehyde-3-phosphate dehydrogenase														
6	P04406	GAPDH	Glyceraldehyde-3-phosphate dehydrogenase	11276	15	63.3	0.58	0.61	0.79	0.41	0.60	0.14	1.52	
Triose phosphate isomerase														
7	P60174	TPI1	Triose phosphate isomerase	2671	13	53.8	0.61	0.66	0.66	0.61	0.64	0.03	1.55	
Aldolase														
8	P04075	ALDOA	Fructose-bisphosphate aldolase A	3775	21	68.4	1.01	1.37	1.01	1.37	1.19	0.18	2.28	
9	P09972	ALDOC	Fructose-bisphosphate aldolase C	2223	7	28.3	0.74	0.94	0.49	1.19	0.84	0.26	1.79	
Phosphohexose isomerase														
10	P06744	GPI	Glucose-6-phosphate isomerase	3426	17	42.3	1.09	1.06	0.94	1.20	1.07	0.09	2.11	
**Gluconeogenesis**														
Phosphoenolpyruvate carboxykinase														
11	Q16822	PCK2	Phosphoenolpyruvate carboxykinase [GTP], mitochondrial (PEPCK-M)	73	1	3.1	−0.19	0.77	1.14	−0.56	0.29	0.69	1.22	
Mitochondrial malate dehydrogenase														
12	P40926	MDH2	Malate dehydrogenase, mitochondrial	2910	11	42	0.24	0.28	0.08	0.44	0.26	0.13	1.20	
Cytosolic malate dehydrogenase														
13	P40925	MDH1	Malate dehydrogenase, cytoplasmic	1272	8	30.5	0.83	1.07	0.78	1.12	0.95	0.15	1.93	
**Glycolysis**														
Pyruvate kinase														
14	P14618	PKM2	Pyruvate kinase isozymes M1/M2	6203	29	62	0.49	0.72	1.05	0.15	0.60	0.33	1.52	
Phosphofructokinase-1														
15	P08237	PFKM	6-phosphofructokinase, muscle type	1069	13	22.4	0.20	0.03	0.05	0.18	0.12	0.07	1.08	
16	P17858	PFKL	6-phosphofructokinase, liver type	834	7	12.2	−0.06	0.39	0.41	−0.08	0.16	0.24	1.12	
17	Q01813	PFKP	6-phosphofructokinase type C	1661	12	20.3	0.04	0.53	0.48	0.08	0.28	0.22	1.22	
Hexokinase														
18	P19367	HK1	Hexokinase-1	539	6	7.7	0.08	0.30	0.10	0.28	0.19	0.10	1.14	
19	P52789	HK2	Hexokinase-2	226	7	10.1	0.01	−0.20	0.11	−0.30	−0.09	0.17	0.94	

a Number of unique peptides: peptides that uniquely matched to the protein.

b Coverage (%): percentage of protein sequence covered by assigned peptide matches.

c *R* value was calculated by the protein abundance ratios *T*_1_/*C*_1_, *T*_2_/*C*_2_, *T*_2_/*C*_1_ and *T*_1_/*C*_2_. 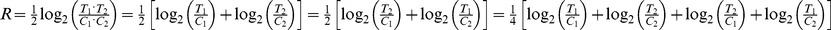.

d *σ_R_*: the standard deviation of *R* values. 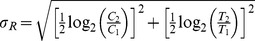.

e *T*/*C* ratio, the linear treatment to control ratio calculated by: *T*/*C* ratio = 2*^R^.*

f M: labeled with “**+**” if the peptides matching this human protein also matched to mouse proteins.

We found that differentially expressed proteins involved in the glycolysis and gluconeogenesis pathway were all up-regulated with the treatment of citreoviridin. To investigate the functions of the 62 down-regulated proteins, they were annotated with Gene Ontology biological process by using DAVID. It was shown that several down-regulated proteins were involved in macromolecular complex assembly and mitosis (Table S9 and Table S11). To further investigate the interactions between the differentially expressed human proteins in citreoviridin treatment tumors, we performed network analysis with these proteins as seed nodes by using MetaCore. The top five networks related to the differentially expressed proteins were shown in Table S10 and Table S12. The top network was related to the macromolecule catabolic process and ubiquitin-regulated cell cycle (Figure S4).

In summary, three major pathways, i.e. glucose metabolism, protein ubiquitination and cell cycle regulation, were involved in the citreoviridin-induced effects on lung cancer xenograft tumors. Of the three major pathways induced by citreoviridin, glucose metabolism had the most prominent role. We identified and quantified most of the enzymes catalyzing glycolysis and gluconeogenesis (Figure 7B). Besides, enzymes involved in glucose metabolism were identified with high confidence and their expression levels were significantly changed by citreoviridin (Table 4). Furthermore, gluconeogenic enzymes and the enzyme catalyzing the reaction of converting glucose to *myo*-inositol was also up-regulated (Table 5). Therefore, we focused on the citreoviridin-induced gluconeogenesis process.

**Table 5:** List of glucose metabolism-related proteins identified in the large-scale experiment.

No.	Accession	Gene symbol	Description	Score	No. of unique peptides^a^	Coverage (%)^b^	log_2_ (*T*_1_/*C*_1_)	log_2_ (*T*_2_/*C*_2_)	log_2_ (*T*_2_/*C*_1_)	log_2_ (*T*_1_/*C*_2_)	*R* value^c^	*σ_R_* d	*T*/*C* ratio^e^	M^f^
**Glucose transporter GLUT-3**														
1	P11169	SLC2A3	Solute carrier family 2	1216	6	12.5	−0.25	−0.77	−0.95	−0.07	−0.51	0.36	0.70	
**Conversion of glucose to myo-inositol**														
2	Q9NPH2	ISYNA1	Inositol-3-phosphate synthase 1	354	2	3.9	1.23	1.03	1.32	0.94	1.13	0.15	2.18	
**Conversion of glucose to sorbitol**														
3	P15121	AKR1B1	Aldose reductase	609	8	38.6	0.79	0.79	0.90	0.69	0.79	0.07	1.73	
**Glycogen synthesis**														
4	Q16851	UGP2	UTP-glucose-1-phosphate uridylyltransferase	343	3	10.8	0.35	1.07	0.63	0.79	0.71	0.26	1.64	**+**
5	P13807	GYS1	Glycogen [starch] synthase, muscle	147	2	4.3	0.51	−0.29	−0.04	0.25	0.11	0.30	1.08	**+**
6	P36871	PGM1	Phosphoglucomutase-1	179	3	8.2	0.93	0.61	0.70	0.85	0.77	0.12	1.71	
7	Q96G03	PGM2	Phosphoglucomutase-2	324	4	10.5	0.90	0.46	0.22	1.13	0.68	0.36	1.60	
**Glycogen breakdown**														
8	P06737	PYGL	Glycogen phosphorylase, liver form	565	6	9.3	0.43	1.25	0.63	1.05	0.84	0.32	1.79	
9	P11216	PYGB	Glycogen phosphorylase, brain form	518	5	9.7	−0.02	0.14	0.07	0.06	0.06	0.06	1.04	
10	P35573	AGL	Glycogen debranching enzyme	109	1	1.1	0.16	−0.47	−0.47	0.16	−0.16	0.32	0.90	

a Number of unique peptides: peptides that uniquely matched to the protein.

b Coverage (%): percentage of protein sequence covered by assigned peptide matches.

c *R* value was calculated by the protein abundance ratios *T*_1_/*C*_1_, *T*_2_/*C*_2_, *T*_2_/*C*_1_ and *T*_1_/*C*_2_. 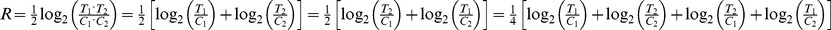.

d *σ_R_*: the standard deviation of *R* values. 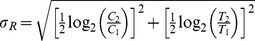.

e *T*/*C* ratio, the linear treatment to control ratio calculated by: *T*/*C* ratio = 2*^R^.*

f M: labeled with “**+**” if the peptides matching this human protein also matched to mouse proteins.

### Validation of citreoviridin-induced gluconeogenesis in lung cancer xenograft tumors

**Figure 8:**
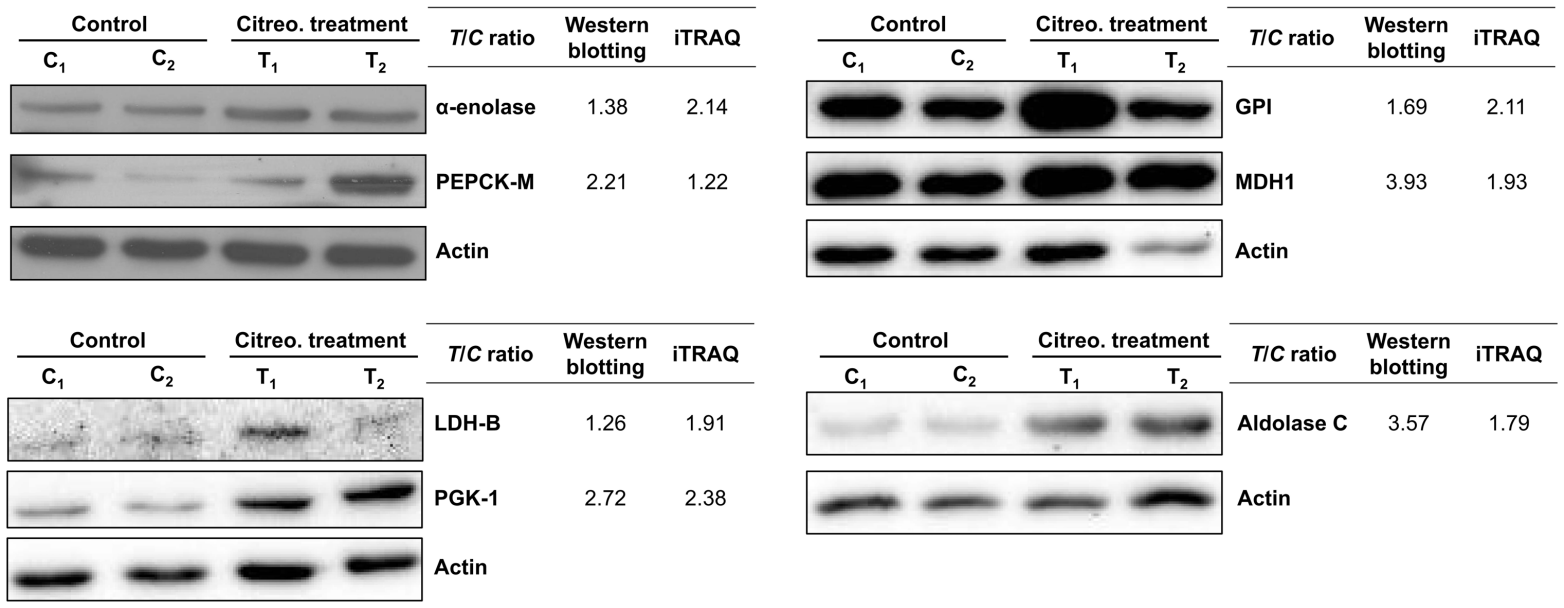
Citreoviridin induced the up-regulation of proteins catalyzing glucose metabolism. Protein expressions of seven proteins involved in glucose metabolism, α-enolase, PEPCK-M, GPI, MDH1, LDH-B, PGK-1 and aldolase C, were measured by western blotting. Actin was used as an internal loading control. The expression levels of two control tumors (C_1_ and C_2_) and two citreoviridin-treated tumors (T_1_ and T_2_) from a total of four different mice were quantified. The band intensities were normalized to actin, and the averages of intensities from two control samples and two treatment samples were calculated, respectively. Fold change of protein expression shown was obtained by dividing the intensity of control group by the intensity of treatment group. The *T*/*C* (treatment to control) ratios from the iTRAQ large-scale experiment were listed for comparison.

To confirm the regulation of gluconeogenesis by citreoviridin in lung cancer xenograft tumors, we measured the protein expression level of seven proteins involved in glucose metabolism. L-lactate dehydrogenase B chain (LDH-B), α-enolase, phosphoglycerate kinase 1 (PGK-1), fructose-bisphosphate aldolase C (aldolase C) glucose-6-phosphate isomerase (GPI) are enzymes that shared by glycolysis and gluconeogenesis. On the other hand, mitochondrial phosphoenolpyruvate carboxykinase (PEPCK-M) and cytoplasmic malate dehydrogenase (MDH1) are two the key eznymes catalyzing gluconeogenesis. The expression levels of these seven enzymes were all higher in citreoviridin-treated tumors than in control tumors (Table 4). We analyzed the proteins previously extracted from two control (C_1_ and C_2_) and two citreoviridin-treated (T_1_ and T_2_) biological repeated tumor samples for proteomic analysis by western blotting, and the protein expression levels of the enzymes were measured. Quantitation of the western blots showed that expressions of the seven glucose-metabolism-related proteins were all up-regulated in citreoviridin-treated tumor samples, which confirmed the results of the proteomic analysis (Figure 8). The up-regulation of both PEPCK-M and MDH1 also indicated the activation of gluconeogenesis in citreoviridin-treated tumors.

## Discussion

Shotgun proteomics is a powerful strategy for large-scale studies of the proteome. However, the peptide-centric nature of it raises the protein inference problem and complicates the interpretation of the data [31]. A set of peptides might be assigned to multiple different proteins or protein isoforms, making the determination of protein identity ambiguous. In studies with xenograft models, tumor samples often contain both human and mouse cells and this complicates the protein inference problem. Many human and mouse proteins share a high degree of sequence homology, so it is hard to distinguish conserved human proteins from mouse proteins. The problem was also addressed and the assignment of human proteins was performed by the criteria of at least one peptide uniquely mapping to human entry [32]. Another similar method is searching the putative human peptides against the mouse sequence using BLAST and removing the peptides matching the mouse sequences [33], [34]. A method combined searching the mouse database with BLAST and was also used to distinguished human proteins from mouse proteins [35]. Except the methods described above, most of the proteomic studies in xenograft models neglected to consider the protein inference problem of human and mouse proteins. In this study, we noticed the problem and adopted a more conservative strategy. For the protein identification step, the combined sequence database of the Swiss-Prot human database and Swiss-Prot mouse database was searched, and proteins matched only to human proteins or to both human and mouse ones were selected. Because we could not exclude the possibility that double-matched proteins were of mouse-origin, the proteins were labeled in the protein identification table. By this way, proteins that may be of human-origin were not completely excluded and noting of this protein inference was retainable during the following analysis.

For protein quantitation, the intensities of iTRAQ signature ions should be normalized to diminish the global bias. We have tried seven methods of normalization (Method S1) and making the median of log_2_ (peptide iTRAQ ratio) equal to zero is the best way to minimize the errors. The optimal normalization method may depend on the structure of the dataset. For the calculation of protein abundance ratios, several algorithms and software tools are available [27] and there are three major algorithms used by the current software tools. ProteinPilot (AB Sciex, Foster, CA, USA), ProQuant (AB Sciex), Multi-Q [36], PEAKS (Bioinformatics Solutions Inc., Waterloo, ON, Canada) and MassTRAQ [37] apply the weighted average of peptide ratios; Phenyx (GeneBio, Geneva, Switzerland), VEMS [38] and Proteome Discoverer (Thermo Fisher Scientific, Waltham, MA, USA) apply the median of peptide ratios; Spectrum Mill (Agilent Technologies, Santa Clara, CA, USA) and Libra (Institute for Systems Biology, Seattle, WA, USA) apply the mean of peptide ratios as protein ratios. Mascot (Matrix Science) offers all the three major methods described above, while i-Tracker [39] only provide data in peptide level. We applied the sum of intensities in protein quantitation, which has similar idea as the weighted average. A previous study showed that compared to others, the sum of intensities (or the weighted average) provides lower errors, especially with the existence of outliers [40]. Besides, the sum of intensities has the advantage of being computationally simple. In this study, we provided the criteria for selecting peptides and a simple method for calculating protein abundance ratios. Furthermore, we proposed a robust workflow for selecting differentially expressed proteins by also considering measurement errors in experiments and individual variations among samples.

With the quantitative proteome, we found that citreoviridin-regulated proteins in lung cancer were associated with glucose metabolism, especially gluconeogenesis and glycolysis. We identified and quantified most of the enzymes involved in gluconeogenesis and glycolysis processes (Figure 7B). The confidence for identification of these proteins was high so as to ensure the existence of these proteins in the samples (Table 4). Furthermore, the expression level of several proteins in the processes was significantly up-regulated with treatment of citreoviridin (Figure 7B), suggesting two possible results: the activation of gluconeogenesis or the activation of glycolysis. These two processes share almost the same set of enzymes except some catalyzing the irreversible reactions. We identified all eight enzymes shared by gluconeogenesis and glycolysis. All three enzymes catalyzing the irreversible steps in glycolysis were also identified, and we noticed that all of these enzymes were not up-regulated by citreoviridin. Regarding the major seven enzymes catalyzing the irreversible steps in gluconeogenesis, we identified and quantified three enzymes in our proteomic analysis, including PEPCK-M, MDH1 and mitochondrial malate dehydrogenase (MDH2). MDH1 was significantly up-regulated 1.93-fold with treatment of citreoviridin. Although the expression levels of MDH2 and PEPCK-M showed no significant up-regulation, these two enzymes had higher expression levels in citreoviridin-treated tumors than control tumors.

Is it possible that gluconeogenesis occurs in cancer cells when treated with citreoviridin? The whole proteomic profiling of control and citreoviridin-treated tumors may provide some hints. The expression level of several other proteins related to glucose metabolism was changed with citreoviridin treatment (Table 5). These proteins are involved in synthesis of glycogen from glucose, conversion of glucose to inositol or sorbitol (a sugar alcohol that the human body metabolizes slowly) and glucose transport. The expression levels of three enzymes, which convert glucose to other compounds, were higher in the citreoviridin-treated tumors. The first one is UTP-glucose-1-phosphate uridylyltransferase (UDP-glucose pyrophosphorylase, UDPGP), which catalyzes the reaction of converting glucose 1-phosphate to UDP-glucose, the immediate donor of glucose for glycogen synthesis. The second one is inositol-3-phosphate synthase 1 (IPS 1), which catalyzes the conversion of glucose 6-phosphate to 1-*myo*-inositol 3-phosphate. Third, aldose reductase reduces glucose to sorbitol, which accumulated in the cells in response to hyperosmotic stress that causes shrinkage of the cells [41], [42]. Surplus glucose enters the polyol pathway by converting to sorbitol catalyzed by aldose reductase. From the above observations, glucose might be overproduced in cancer cells with treatment of citreoviridin. We also noticed that the expression level of glucose transporter GLUT-3 was lower (0.70-fold) with the treatment of citreoviridin, which indicated that excess glucose mainly came from gluconeogenesis.

Citreoviridin was shown to suppress lung adenocarcinoma growth by targeting ectopic ATP-synthase [23]. The observation of activated gluconeogenesis by citreoviridin in the proteomic profiling raised the question of whether there is a relationship between gluconeogenesis and inhibition of lung cancer cell proliferation. There are only limited literatures describing the effects of gluconeogenesis on cancer and most of them were reported in the 1970s. The role of gluconeogenesis in cancer cells can vary depending on the gluconeogenic precursors, including lactate, pyruvate, amino acids and other metabolites. It was suggested that gluconeogenesis from alanine is increased in cancer patients with cachexia, a syndrome with significant loss of appetite resulting in weakness and loss of weight [43], [44]. A recent report showed that gluconeogenesis was down-regulated in hepatocellular carcinoma and the reduced gluconeogenesis may facilitate tumorigenesis by accumulation of glucose 6-phosphate, the precursor for nucleotide synthesis [45].

The expression profile of proteomes in control and citreoviridin-treated tumors provides novel implications for understanding the antitumorigenic effect by activation of gluconeogenesis in cancer cells. First, the glucose synthesized could be converted into *myo*-inositol, which has anti-cancer activity. We observed the up-regulation of the enzyme IPS 1 with treatment of citreoviridin (Table 5). This enzyme catalyzes the key rate-limiting step in the *myo*-inositol biosynthesis pathway. The level of *myo*-inositol was found to be higher in normal tissue compared to breast cancer tissue [46] but lower in lung tumors [47]. Besides, *myo*-inositol was shown to have anti-cancer activity by inhibiting tumor formation of colon, mammary, soft tissue and lung cancers. The phosphorylated *myo*-inositol, inositol hexaphosphate (IP_6_) was also recognized for its effectiveness in cancer prevention [48]. IP_6_ is able to induce G_1_ cell cycle arrest by modulating cyclins, CDKs, p27^Kip1^, p21^CIP1/WAF1^, and pRb in prostate cancer and breast cancer [49]–[52].

With the treatment of citreoviridin, the glucose synthesized from gluconeogenesis may also be converted to other compounds and escape from utilization by glycolysis. The reduction in glycolysis flux results in the decrease of glycolytic intermediates to sustain the continuous building blocks for macromolecular synthesis [12], [13] and thereby inhibits the proliferation of cancer cells. We found that the expression level of aldose reductase that converts glucose to sorbitol was higher in citreoviridin-treated tumors (Table 5). The increased intracellular glucose results in its conversion to sorbitol. Although sorbitol entering the polyol pathway can be converted to fructose by sorbitol dehydrogenase, high glucose levels still favors the production of sorbitol.

Glucose synthesized from gluconeogenesis may also be polymerized into glycogen for storage. Thus, the decrease of glucose influx into glycolysis inhibits proliferation of cancer cells. A previous report showed that the expression level of UDPGP, activities of phosphoglucomutase (PGM) and glycogen synthase were all decreased in tumor tissues, so the defective glycogen synthesis process is unable to compete with glycolysis [53]. In our proteomic profiling data, we observed that the expression levels of PGM and UDPGP were higher with citreoviridin treatment in lung cancer (Table 5). Regarding glycogen breakdown, previous studies suggested that glycogen phosphorylase was expressed in tumor tissues and served as a target for anticancer therapy [54], [55]. In our proteomic profiling data, we found that the glycogen phosphorylase liver form was up-regulated by citreoviridin. Why does there seem to be a contradiction between the expression levels of enzymes involved in glycogen synthesis and glycogen breakdown? In fact, glycogen synthase and glycogen phosphorylase are both allosterically regulated by kinases and phosphatases, the activities of which are also post-translationally regulated. Therefore, the activities of glycogen synthase and glycogen phosphorylase in citreoviridin-treated tumors remain unknown. However, it is still possible that glucose from gluconeogenesis is converted into UDP-glucose in citreoviridin-treated lung cancer xenograft tumors.

**Figure 9:**
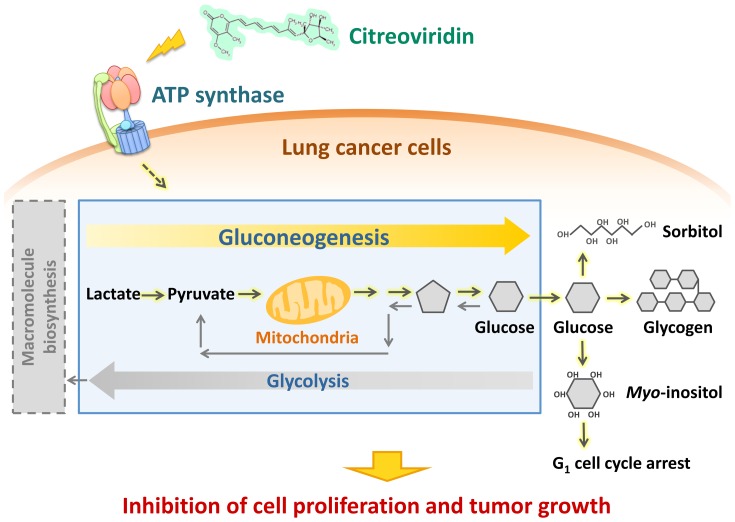
Citreoviridin affects the glucose metabolism in lung cancer xenograft tumors. Proteomic analysis of lung cancer xenograft tumors treated with citreoviridin indicated that differentially expressed proteins were involved in gluconeogenesis and the conversion of glucose to other compounds. The activation of these pathways may decrease glycolysis and thus cause the inhibition of cell proliferation and tumor growth.

In conclusion, proteomic analysis provided novel implications about targeting ectopic ATP synthase by the inhibitor citreoviridin in a lung cancer xenograft model (Figure 9). With high reproducibility of quantitation, we identified 2,659 proteins in the proteomic profiling of the tumors. We applied an optimized normalization method and an appropriate calculation of protein abundance ratio, and over 90% of identified proteins were quantified. Besides, we were able to estimate the errors arising from experimental measurements and individual variations individually. Based on the errors estimated, we calculated the cut-off values and selected 141 human proteins as differentially expressed. Bioinformatics analysis of the differentially expressed human proteins illustrated the enrichment of glucose metabolism-related processes. We found that citreoviridin may activate gluconeogenesis by up-regulation of the expression levels of gluconeogenic enzymes. Furthermore, the change in expression levels of other glucose metabolism-related enzymes may link gluconeogenesis to cell proliferation inhibition. This study helps to achieve a better understanding of the complexity of metabolic regulations and the plasticity of cancer cells, which may shed light on improvements to cancer therapy.

## Materials and Methods

### Reagents

Citreoviridin was obtained from Enzo Life Sciences (Farmingdale, NY, USA). Dimethyl sulfoxide (DMSO, cell culture grade) was purchased from AppliChem (Darmstadt, Germany). Optimal cutting temperature (O.C.T.) compound was obtained from Exibo Research (Taipei, Taiwan). 3,3′-Diaminobenzidine was purchased from Kirkegaard & Perry Laboratories (Gaithersburg, MD, USA). Protease inhibitor was obtained from Bioman Scientific (Taipei, Taiwan). Matrigel™ was from BD Biosciences (Bedford, MA, USA). Glycerol was purchased from Scharlau (Barcelona, Spain). BCA™ Protein Assay Reagent kit was obtained from Pierce (Rockford, IL, USA). Triethylammonium bicarbonate buffer (TEABC), tris(2-carboxyethyl)phosphine hydrochloride (TCEP), *S*-Methyl methanethiosulfonate (MMTS), *N,N,N′,N′*-Tetramethylethylenediamine (TEMED), trifluoroacetic acid (TFA), Tween 20, anti-rabbit IgG-HRP and anti-mouse IgG-HRP were purchased from Sigma-Aldrich (St Louis, MO, USA). Acrylamide/bisacrylamide (40%, v/v, 37.5∶1) was obtained from Bioshop (Burlington, ON, Canada). Ammonium persulfate (APS, ACS grade) was from Amresco (Solon, OH, USA). Acetonitrile (ACN) was obtained from Lab-Scan (Dublin, Ireland). Sequencing grade modified trypsin was purchased from Promega (Madison, WI, USA). iTRAQ® Reagent kit (including iTRAQ Reagent 114, iTRAQ Reagent 115, iTRAQ Reagent 116, iTRAQ Reagent 117 and iTRAQ Dissolution buffer) was obtained from Applied Biosystems (Forster City, CA, USA). Ki67 antibody was purchased from Abcam (Cambridge, MA, USA). ENO1, PCK2, GPI, MDH1, PGK1, LDHB and ALDOC antibodies were purchased from GeneTex (Irvine, CA, USA). Anti-actin antibody clone 4 and Immobilon Chemiluminescent HRP substrate were from Millipore (Bedford, MA, USA).

### Cell culture

The human lung adenocarcinoma cell line CL1-0 was kindly provided by Dr. Pan-Chyr Yang (Department of Internal Medicine, National Taiwan University Hospital, Taiwan) [56]. Cells were grown as previously described [56]. Briefly, cells were cultured in Dulbecco's modified Eagle's medium (Gibco, NY, USA) containing 10% fetal bovine serum (Gibco) at 37°C and 5% CO_2_.

### Tumorigenicity assays in athymic mice

This study had been approved by the animal care and use committee of National Taiwan University (Permit Numbers: 97-47). All animal work was performed in accordance with NIH animal use guidelines. *NOD.CB17-Prkdcscid* female mice (4–5 weeks old) were purchased from National Taiwan University hospital and housed in an isolator and fed *ad libitum* with autoclaved food. For tumor growth in animals, 5×10^6^ CL1-0 cells resuspended in 0.1 ml Hanks' balanced salt solution (HBSS) were mixed with matrigel and injected subcutaneously into mice. The tumor masses were measured every two days and the tumor volume was calculated as 1/2 width^2^×length in mm^3^. After tumor volumes reached 100 mm^3^, animals were randomly assigned into two groups: those receiving intraperitoneal injection with the vehicle control (DMSO, N = 5) or the ATP synthase inhibitor (citreoviridin, N = 4). The citreoviridin treatment (1 mg/kg) was administered three times a week to a final dosage of 12 mg/kg for 27 days. Tumor size was measured after treatment and the mice weights were monitored as a health indicator. Animals were sacrificed when the tumor volume of control animals reached 1000 mm^3^. At sacrifice, mice were anesthetized and cervical dislocation was performed. Tumors were excised and stored at −80°C for further analysis. For immunohistochemistry, dissected organs and tumors were fixed in 10% paraformaldehyde.

### Immunohistochemistry

Immunohistochemistry was performed with 3,3′-Diaminobenzidine (DAB) as a detection agent. Cryostat sections prepared from O.C.T. compound embedded tissues were treated with blocking buffer (10% normal goat serum) to inhibit endogenous enzyme activity. Sections were then incubated with Ki67 antibody at 4°C overnight. A horseradish peroxidase containing polymer conjugated with IgG antibody was applied at room temperature for 1 h. The enzymatic reaction was developed in freshly DAB solution as a chromogen for horseradish peroxidase. The sections were then counterstained with hematoxylin and mounted in xylene. The negative control, where the primary antibody was substituted with Tris-HCl buffer, was also included in each staining case.

### Protein preparation

Tumor tissues were ground into a fine powder using a mortar and pestle in liquid nitrogen. Liquid nitrogen was added to the mortar frequently to ensure that the tissues did not thaw during grinding. Tissue power was then suspended in lysis buffer containing 1% (v/v) SDS, 50 mM Tris-HCl, 10% (v/v) glycerol and protease inhibitor. The amount of lysis buffer added was based on the amount of tissue powder. The solution containing tissue powder was resuspended by pipetting until there was almost no visible pellet. The sample solution was homogenized on ice using a homogenizer (LABSONIC® M ultrasonic homogenizer; Sartorius AG, Göttingen, Germany) with 60% amplitude, cycle = 0.6 (operated 0.6 s every 1 s) for 4–5 min. The lysate was centrifuged at 17,000 *g*, 4°C for 30 min. The supernatant, which contained the crude extract proteins, was collected. The concentration of proteins was measured with the BCA™ Protein Assay Reagent kit.

### Reduction, alkylation and digestion of proteins

We followed the methods of a previous study by Han et al. for protein reduction, alkylation and digestion [57]. Equivalent amounts of proteins in control and treatment samples were used for further processing: 100 µg/sample was used for the duplicate experiment, while 400 µg/sample was used for the small-scale experiment and the large-scale experiment. Each sample was adjusted to have the same protein concentration by adding lysis buffer. We added 1 M TEABC to make the final concentration of 50 mM TEABC (pH was about 8.5) for every sample. Proteins were reduced by 5 mM TCEP in a dry bath at 37°C for 30 min and then alkylated by 2 mM MMTS at room temperature avoiding light for 30 min.

Next, we applied gel-assisted digestion for proteins. Acrylamide/bisacrylamide (40%, v/v, 37.5∶1), 10% APS (w/v) and TEMED were mixed with the protein solution (protein solution: acrylamide/bisacrylamide: APS: TEMED = 14∶5∶0.3∶0.3, v/v) to allow gel polymerization. The gel was cut into small pieces and washed with 25 mM TEABC and 25 mM TEABC/50% (v/v) ACN several times. Briefly, buffer was added to the gel and then the solution was vortexed. The gel was further dehydrated with 100% ACN and dried completely with a centrifugal evaporator (CVE-2000; Eyela, Tokyo, Japan). 25 mM TEABC was added to rehydrate the gel and trypsin (protein: trypsin = 10∶1, w/w) was subsequently added to the rehydrated gel. After ensuring the gel was fully covered by 25 mM TEABC, the sample was incubated at 37°C overnight (at least 16 h). Peptides were extracted from the gel with 0.1% (v/v) TFA, 50% (v/v) ACN/0.1% (v/v) TFA, and 100% ACN sequentially. Like the gel washing step, buffer was added to the gel and the gel solution was vortexed. The liquid part of the sample was collected and combined. The extracted peptide solution was dried with a sample concentrator (miVac Duo Concentrator; Genevac, Ipswich, UK).

### iTRAQ labeling of peptides

The peptides were resuspended in iTRAQ Dissolution buffer. We confirmed that the solution was basic (about pH 8.5). The concentration of peptide was measured with the BCA™ Protein Assay Reagent kit. For the duplicate experiment and small-scale experiment, 5 µg of peptides from each sample were required for iTRAQ labeling. For the large-scale experiment, 150 µg of peptides from each sample were required. Each vial (1 unit) of iTRAQ Reagent was brought to room temperature and dissolved in 70 µL absolute ethanol by vortexing for 1 min. Equal amounts of peptides from different samples were labeled by adding iTRAQ Reagent 114, iTRAQ Reagent 115, iTRAQ Reagent 116, or iTRAQ Reagent 117, and vortexing at room temperature for 1 h. Labeled peptides were combined and dried with a centrifugal evaporator (CVE-2000; Eyela, Tokyo, Japan).

### Strong cation exchange (SCX) chromatography

For the large-scale experiment, SCX chromatography was performed after iTRAQ labeling. The labeled peptides were resuspended in 2 mL buffer A (5 mM KH_2_PO_4_ and 25% (v/v) ACN, pH 3) and fractioned by SCX chromatography. The peptide solution was subjected to a 2.1×200 mm PolySULFOETHYL A™ column containing 5 µm particles with 200 Å pore size (PolyLC, Columbia, MD, USA). A flow rate of 200 µl/min with a gradient of 0–25% buffer B (5 mM KH_2_PO_4_, 350 mM KCl and 25% ACN (v/v), pH 3) for 30 min followed by a gradient of 25–100% buffer B for 20 min was applied for peptide elution. The eluate was monitored by the absorbance of the peptide bond at 214 nm, and fractions were collected every 1 min. Each fraction was dried with a sample concentrator (Savant SpeedVac® Plus SC210A Concentrator; Thermo Fisher Scientific).

### ZipTip desalting

For the duplicate experiment and small-scale experiment, iTRAQ-labeled peptides were directly subjected to the desalting step. For the large-scale experiment, each fraction of peptides was desalted individually. We performed desalting using ZipTip® Pipette Tips (Millipore, Bedford, MA, USA). Dried peptides were resuspended in 20–30 µL of 0.1% (v/v) TFA, and 10% TFA was added to adjust the pH of the solution to about pH 2–3. The ZipTip was first wetted with 50% (v/v) ACN/0.1% (v/v) TFA and then equilibrated in 0.1% (v/v) TFA. The peptides were bound to the ZipTip by aspirating and dispensing the peptide solution for 20 cycles. Subsequently, the ZipTip was washed with 0.1% (v/v) TFA. At last, peptides were eluted with 20 µL of 50% (v/v) ACN/0.1% (v/v) by aspirating and dispensing the eluate for 10 cycles. The eluate was dried with a centrifugal evaporator (CVE-2000; Eyela, Tokyo, Japan).

### LC-MS/MS analysis

After ZipTip desalting, samples containing about 3 µg peptides were reconstituted in 24 µL buffer A (0.1% (v/v) formic acid (FA) in H_2_O) and analyzed by LC-ESI-Q-TOF mass spectrometry (Waters SYNAPT® G2 HDMS; Waters Corp., Milford, MA, USA). Samples were injected into a 180 µm×2 cm capillary trap column and separated by a 75 µm×25 cm nanoACQUITY UPLC™ 1.7 µm Ethylene Bridged Hybrid (BEH) C18 column using a nanoACQUITY Ultra Performance LC™ System (Waters Corp.). The column was maintained at 35°C. Buffer A was 0.1% FA in H_2_O and buffer B was 0.1% FA in ACN. Bound peptides were eluted with a linear gradient of 6 to 50% buffer B at a flow rate of 300 µl/min for 100 min. MS was operated in electrospray ionization sensitivity mode. NanoLockSpray™ source (Waters Corp.) was used for accurate mass measurements, and the lock mass channel was sampled every 30 s. The mass spectrometer was calibrated with a synthetic human [Glu^1^]-Fibrinopeptide B solution (1 pmol/µl; Sigma-Aldrich) delivered through the NanoLockSpray source. Data acquisition was performed using data directed analysis (DDA). The DDA method included one full MS scan (*m*/*z* 350–1700, 1 s) and three MS/MS scans (*m*/*z* 100–1990, 1.5 s for each scan) sequentially on the three most intense ions present in the full scan mass spectrum. Each sample was analyzed in duplicate.

### Protein identification

The peak list resulting from MS/MS spectra was exported to mgf format by Mascot Distiller v2.3.2 (Matrix Science, London, United Kingdom) with charge state set to 2+, 3+, 4+, 5+, and other default parameters. Data files were merged and searched against the combined sequence database (containing 36,774 sequence entries) of the Swiss-Prot human database (April 2, 2012) and the Swiss-Prot mouse database (April 2, 2012) using Mascot search engine v2.3.02 (Matrix Science, London, United Kingdom). Search parameters for peptide and MS/MS mass tolerance were both ±0.3 Da with allowance for two missed cleavages made from the trypsin digestion. Variable modifications of deamidated (NQ), oxidation (M), iTRAQ4plex (K), iTRAQ4plex (N-term), and methylthio (C) were selected and none of the fixed modifications was selected. Peptide charge was set to Mr, instrument was set to ESI-QUAD-TOF and decoy database was searched. Mascot search results were filtered using “Significance threshold” set at *P*<0.05 and “Ions score or expect cut-off” set at 0.05. Only peptides with ion scores higher than the Mascot identity score (*P*<0.05) were confidently assigned. A protein hit required at least one “bold red” peptide match to have the most likely assignment. The peptide shown in “red” indicates the highest scoring match for the spectrum. When the peptide matched to the spectrum does not appear in any higher scoring protein, it is shown in “bold”. Thus, the “bold red” match is the highest scoring match to a particular spectrum listed under the highest scoring protein containing that match. To evaluate the false discovery rate (FDR), we performed a decoy database search against a randomized decoy database created by Mascot using identical search parameters and validation criteria. FDR was calculated as *D*/*R*×100%, where *D* and *R* are the number of matches above identity threshold using the decoy and real databases, respectively.

### Selecting peptides for quantitation

Mascot search results were exported in XML format and raw data files from Waters SYNAPT® G2 HDMS mass spectrometer were converted to mzXML format using massWolf (Institute for Systems Biology, Seattle, WA, USA). Data files in XML and mzXML formats were analyzed using Multi-Q software (v1.6.5.4) [36] to detect signature ions (*m/z* = 114, 115, 116 and 117) and select peptides satisfying all the following four criteria: 1) the peptide is labeled with iTRAQ tags; 2) the peptide is considered as confidently identified (the peptide has ion score higher than the Mascot identity score (*P*<0.05)); 3) the peptide is unique (non-degenerate); 4) the iTRAQ signature ion peak intensity (ion count) of the peptide is within the dynamic range (the peak intensity of each iTRAQ signature ion must be >0, the average of the peak intensities of all iTRAQ signature ions must be ≥30). Only peptides satisfying the above four criteria were considered as qualified peptides and applied for further analysis. We ensured that the percentage of peptides indeed labeled with iTRAQ tags in every dataset was over 99%.

### iTRAQ signal normalization

Before quantitation of the expression of each protein, we first normalized the peak intensity (ion count) of the iTRAQ signature ion (hereafter referred to as peptide iTRAQ signal), which was used for calculating protein abundance ratios. We have tried seven different normalization methods (Method 1 and 3–7 in Method S1) and chose the optimal normalization method (Method 2), as described below.

Each of the proteomic experiments performed in this study contained four different cell states, A, B, C, and D labeled with iTRAQ tags *m/z* = 114, 115, 116, and 117, respectively. For peptide *i* in cell state X, the original peptide iTRAQ ratio 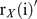 is calculated as follows:
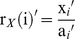
where 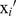 and 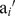 denote the original peptide iTRAQ signal representing the abundance of peptide *i* in cell state X and A , respectively. The peptide iTRAQ ratio represents the relative peak intensity of the iTRAQ signature ion *m/z* 114, 115, 116 or 117 to the iTRAQ signature ion *m/z* 114.

Next, the original log_2_ peptide iTRAQ ratio of peptide *i*, 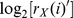 is calculated.

The normalization is performed to make
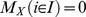
where 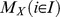 denotes the median of the normalized log_2_ peptide iTRAQ ratio 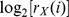 of all peptides *i* belonging to *I* in cell state X. *I* is the set containing all qualified peptides in the dataset, which satisfy the four criteria described above; 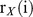 is the normalized peptide iTRAQ ratio calculated from the normalized iTRAQ peak intensity *x_i_*.

To achieve the above normalization, the normalized peptide iTRAQ signal *x_i_* is calculated as follows:
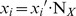
where *N_X_* is the normalization factor, which is expressed as
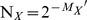
where 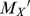 denotes the median of 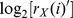 of all peptides *i* belonging to *I*. Normalized peptide iTRAQ signals were used for calculating protein abundance ratios.

### Protein quantitation

For determination of the relative expression of proteins in two different cell states, C and T, the relative protein abundance ratio *T*/*C* of protein *p* in two different cell states, C and T, is expressed as
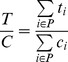
where *c_i_* and *t_i_* denote the normalized peptide iTRAQ signal representing the abundance of peptide *i* in cell state C and T, respectively; *P* is the set containing qualified peptides assigned to protein *p*.

We have checked that over 90% of proteins in each dataset were quantified and had the protein abundance ratios.

### Selection of differentially expressed proteins

For both the small-scale and the large-scale experiments, there were two biological sample replicates in every condition. In other words, two control tumors (C_1_ and C_2_) and two citreoviridin-treated tumors (T_1_ and T_2_) from a total of four different mice were analyzed. Therefore, each protein identified had four protein abundance ratios, *T*_1_/*C*_1_, *T*_2_/*C*_2_, *T*_2_/*C*_1_ and *T*_1_/*C*_2_, in an experiment. We calculated the *R* value of each protein, which is expressed as
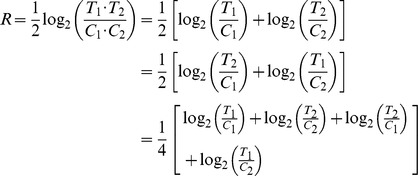
The *R* value is also the average of the log_2_ of these protein abundance ratios. Besides, we calculated the median of the *R* value (*M_R_*) in the large-scale experiments. On the other hand, the standard deviation of the *R* value (*σ_R_*) of each protein can be calculated as follows:
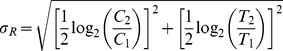
where *C*_2_/*C*_1_ and *T*_2_/*T*_1_ are the protein abundance ratios of two control tumors (C_1_ and C_2_) and two citreoviridin-treated tumors (T_1_ and T_2_), respectively.

To select proteins which are differentially expressed between control and citreoviridin-treated tumors, errors in the *R* value should be first considered. The measurement errors in experiments and individual variations among tumors from different mice cause errors in the *R* value. These errors cannot be accounted for by the difference between the proteomes of control and citreoviridin-treated tumors. To estimate the errors, *C*_2_ and *T*_2_ of the *R* value equation are exchanged to calculate the *S* value of each protein, which is given by:
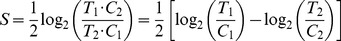
If there are no measurement errors and individual variations, the *S* value should be equal to zero. The *S* value of each protein quantified in the large-scale experiment was calculated to estimate the measurement errors in experiments and individual variations. In reality, the *S* values are not zero due to the errors. Therefore, the distribution of all the *S* values can be deemed as the distribution of errors. Assuming that the errors follow a normal distribution, 1.96-fold of the standard deviation (1.96 S.D.) of the *S* values (1.96 *σ_S_*) calculated from the large-scale experiment is statistically significant (*P*<0.05) and can be taken as the cut-off value for selecting differentially expressed proteins. In the small-scale and large-scale experiments, proteins with *R* value larger than (*M_R_* + 1.96 *σ_S_*) (up-regulated) or smaller than (*M_R_* - 1.96 *σ_S_*) (down-regulated) were selected as differentially expressed. *M_R_* is the median of the *R* value and *σ_S_* is the standard deviation of the *S* values calculated by the data in the large-scale experiment.

The duplicate experiment, containing two replicate preparations of both controls (C_1α_ and C_1β_) and citreoviridin-treated (T_1α_ and T_1β_) tumor samples, is suitable for estimation of the measurement errors in experiments. Subsequently, the individual variations between biological replicates of samples can be measured. The *S* value of each protein in the duplicate experiment was also calculated as follows: 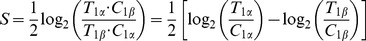 The standard deviation (S.D.) of *S* values from the duplicate experiment (*σ_S_*_(*t*)_) was calculated. The individual variation resulting from biological replicates of samples (*σ_S_*_(*b*)_)was expressed as:
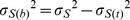
where *σ_S_* is the S.D. of *S* values calculated from the large-scale experiment, which represents the total measurement errors and individual variations among biological replicates of samples.

### Integration of differentially expressed human proteins

Proteomic data was searched against the combined sequence database of the Swiss-Prot human database and the Swiss-Prot mouse database, followed by selecting differentially expressed proteins as described previously. To select only human proteins from all the differentially expressed proteins, protein inferencing from three situations was performed as described: 1) if a set of peptides matched only human protein, then this protein was selected; 2) if a set of peptides matched only mouse protein, then this protein was excluded; 3) if a set of peptides matched both human and mouse proteins (these proteins are conserved between human and mouse), then this protein was selected. With the above strategy, differentially expressed human proteins were selected. Subsequently, we took the union of differentially expressed human proteins acquired from both small-scale and large-scale experiments to form the human differential proteomic profiling induced by citreoviridin, which was later analyzed by bioinformatics methods.

### Bioinformatics analysis

We analyzed the human differential proteomic profiling induced by citreoviridin using bioinformatics methods to investigate the effects of citreoviridin on lung cancer xenografts. For Gene Ontology (GO) analysis, DAVID Bioinformatics Resources version 6.7 [29], [30] was used for functional annotation of GO terms biological processes (GOTERM_BP_FAT). The differentially expressed proteins were uploaded and analyzed using functional annotation clustering with human background, high stringent clustering option and enrichment threshold set as 0.05. The functional annotation clustering is designed to reduce the redundancy of GO annotations. For the analysis of only down-regulated proteins, the medium stringent clustering option was set and enrichment threshold was set as 0.05.

MetaCore™ databases and software version 6.10 build 31731 (GeneGo, St. Joseph, MI, USA) were used to map the differentially expressed human proteins. For pathway map analysis, the list and expression level data of differentially expressed proteins was uploaded and the intersection between the proteins in our data and the proteins of all pre-existing pathway maps was calculated. The pathway maps were ordered based on the significance of associating with our protein dataset. We also used the “Analyze network” option to build sub-networks enriched with the differentially expressed proteins as seed nodes. Sub-networks were ranked by p-value and interpreted in terms of Gene Ontology processes.

### Western blotting

Proteins for western blotting were extracted from two biological replicates of control and citreoviridin-treated tumors. For electrophoresis, 20 µg protein for each sample was mixed with 5× sample buffer (250 mM Tris-HCl, pH 6.8, 20% (w/v) SDS, 50% (v/v) glycerol, 40% (v/v) β-mercaptoethanol, 0.25% (w/v) bromophenol blue) and incubated at 95°C for 5 min. Proteins were subjected to an 8% polyacrylamide gel in SDS-PAGE and the electrophoresis was run at 100 V for 90 min. After electrophoresis, proteins were transferred to a 0.45 µm polyvinylidene fluoride (PVDF) membrane (Millipore, Bedford, MA, USA) at 20 V for 45 min. Blocking was performed in 5% (w/v) nonfat milk in PBS containing 0.1% (v/v) Tween 20 (PBST) at room temperature for 1 h. Subsequently, the membrane was incubated with ENO1 antibody (1∶2500) for α-enolase, PCK2 antibody (1∶1000) for PEPCK-M, GPI antibody (1∶1250), MDH1 antibody (1∶25), PGK1 antibody (1∶125), ALDOC antibody (1∶250) and LDHB antibody (1∶50) at 4°C overnight. The membrane was then incubated with anti-rabbit IgG-HRP (1∶100000) at room temperature for 1 h. For actin, the membrane was incubated with anti-actin antibody (1∶5000) at room temperature for 2 h and incubated with anti-mouse IgG-HRP (1∶100000) at room temperature for 1 h. The immunoblots were all visualized using Immobilon Chemiluminescent HRP substrate. The membrane of ENO1 and PCK2 was exposed to X-ray film (Fuji, Tokyo, Japan), while the images of others were captured on FluorChem M (Proteinsimple, Santa Clara, CA, USA). Scanned X-ray films or captured images of western blots were quantified by Kodak 1D image analysis software (Eastman Kodak Co, Rochester, NY, USA). The band intensities were first normalized to the intensity of actin, followed by averaging the intensities of two biological replicates of control samples and treatment samples, respectively. Fold change of protein expression (*T*/*C*) was calculated by dividing the intensity of control group by the intensity of treatment group.

## Supporting Information

Method S1 **Seven different normalization methods and evaluation process of the seven different normalization methods.**
(PDF)

Figure S1 **The experimental design and data analysis process of the iTRAQ duplicate experiment.** Tumor samples from control and citreoviridin treatment mice were both separated into two samples and labeled with different iTRAQ tags. For reproducibility assessment, the intensities of iTRAQ signature ions of selected peptides were plotted.
(TIF)

Figure S2 **The experimental design and data analysis process of the iTRAQ small-scale experiment and large-scale experiment.** Two biological replicates of both control and citreoviridin-treated tumor samples were labeled with different iTRAQ tags. For the small-scale experiment, combined iTRAQ-labeled peptides were directly analyzed by LC-MS/MS. For the large-scale experiment, combined iTRAQ-labeled peptides were first fractioned by SCX chromatography and each fraction was individually analyzed by LC-MS/MS. The cut-off values for selecting differentially expressed proteins were calculated from the *S* values acquired from the large-scale experiment. Subsequently, the protein abundance ratios were used for calculating the *R* values and selecting differentially expressed proteins by comparing with the cut-off values.
(TIF)

Figure S3 **The distribution of the** ***S*** **values in the duplicate experiment.** The *S* value of each protein was calculated by the protein abundance ratios *T*_1α_/*C*_1α_ and *T*_1β_/*C*_1β_ in the duplicate experiment. The standard deviation of the *S* value was 0.3473. *σ_S_*_(*t*)_: standard deviation of the *S* values.
(TIF)

Figure S4 **Top network: macromolecule catabolic process and ubiquitin-regulated cell cycle network.** p-value = 3.38×10^−11^. Blue circles indicate differentially expressed proteins, which were identified in experiments.
(TIF)

Table S1 **The proteomic data of the duplicate experiment.** S1-1: Information of proteins identified in the duplicate experiment. S1-2: The peptide iTRAQ signals of proteins identified in the duplicate experiment.
(XLSX)

Table S2 **The proteomic data of the small-scale experiment.** S2-1: Information of proteins identified in the small-scale experiment. S2-2: The protein abundance ratios of proteins identified in the small-scale experiment. S2-3: The *R* values of proteins identified in the small-scale experiment.
(XLSX)

Table S3 **The proteomic data of the large-scale experiment.** S3-1: Information of proteins identified in the large-scale experiment. S3-2: The protein abundance ratios of proteins identified in the large-scale experiment. S3-3: The *R* values of proteins identified in the large-scale experiment.
(XLSX)

Table S4 **Normalized data of the duplicate experiment obtained from seven different normalization methods.** The *S* values of proteins identified in the duplicate experiment obtained by using the normalization methods.
(XLSX)

Table S5 **Normalized data of the large-scale experiment obtained using normalization method 1 and method 2.** S5-1: The *S* values of proteins identified in the large-scale experiment by using normalization method 1. S5-2: The protein abundance ratios *C*_2_/*C*_1_ and *T*_2_/*T*_1_ of proteins identified in the large-scale experiment by using normalization method 1. S5-3: The *S* values of proteins identified in the large-scale experiment by using normalization method 2. S5-4: The protein abundance ratios *C*_2_/*C*_1_ and *T*_2_/*T*_1_ of proteins identified in the large-scale experiment by using normalization method 2.
(XLSX)

Table S6 **The** ***S*** **value calculated by normalization method 1 and 2 in the large-scale experiment.**
(PDF)

Table S7 **Calculation of cut-off values and estimation of individual variations among biological replicates of samples.** S7-1: Calculation of cut-off values for selecting differentially expressed proteins. S7-2: Calculation of the variations resulting from the biological replicates of samples.
(XLSX)

Table S8 **List of differentially expressed human proteins between control and citreoviridin-treated tumors.**
(XLSX)

Table S9 **The results of Gene Ontology biological process clustering enrichment analysis.** S9-1: Gene Ontology biological process clustering enrichment analysis of the differential proteome induced by citreoviridin in humans. S9-2: Gene Ontology biological process clustering enrichment analysis of down-regulated human proteins with citreoviridin treatment.
(XLSX)

Table S10 **The results of pathway map analysis and network analysis.** S10-1: Pathways associated with differentially expressed human proteins determined by MetaCore pathway map analysis. S10-2: Networks associated with differentially expressed human proteins determined by MetaCore network analysis.
(XLSX)

Table S11 **Gene Ontology biological process clustering enrichment analysis of down-regulated proteins with citreoviridin treatment.**
(PDF)

Table S12 **Top 5 networks of the differential proteome induced by citreoviridin in humans.**
(PDF)

Spectra S1 **The information of single-peptide-based protein identifications in the duplicate experiment.**
(PDF)

Spectra S2 **The information of single-peptide-based protein identifications in the small-scale experiment.**
(PDF)

Spectra S3 **The information of single-peptide-based protein identifications in the large-scale experiment (part 1).**
(PDF)

Spectra S4 **The information of single-peptide-based protein identifications in the large-scale experiment (part 2).**
(PDF)

Spectra S5 **The information of single-peptide-based protein identifications in the large-scale experiment (part 3).**
(PDF)
