# Supplementary material for: Quantitative Proteomic Analysis of Human Lung Tumor Xenografts Treated with the Ectopic ATP Synthase Inhibitor Citreoviridin
Source: PLoS One. 2013 Aug 21;8(8):e70642. doi: 10.1371/journal.pone.0070642 (PMC3749231; doi:10.1371/journal.pone.0070642)
Supplement: Method S1 — Seven different normalization methods and evaluation process of the seven different normalization methods. (PDF) [file pone.0070642.s001.pdf]

Method S1

Seven different normalization methods

For normalization of the peak intensity (ion count) of iTRAQ signature ion (hereafter referred to as peptide iTRAQ signal), we have tried seven different normalization methods. Method 2 was described in "Materials and Methods: iTRAQ signal normalization" while method 1 and 3-7 were described below.

Method 1: Equal summation of peptide iTRAQ signals

For determination of the relative expression of proteins in two different cell states, C and T, the normalization was performed to make

$$\sum_{i\in I} c_i = \sum_{i\in I} t_i$$

where c_i and t_i denote the normalized peak intensity (ion count) of iTRAQ signature ion representing the abundance of peptide i in cell state C and T, respectively; I is the set containing all qualified peptides in the dataset which satisfy the four criteria described above.

To achieve the above normalization, the peak intensity of each iTRAQ signature ion in cell state T was multiplied by a normalization factor N which is expressed as

$$N = \frac{\sum_{i\in I} c_i{}'}{\sum_{i\in I} t_i{}'}$$

where $c_i{}'$ and $t_i{}'$ denote the original peak intensity.

Method 3: Trend line of peptide iTRAQ signals

In this method, scatter plot of original peptide iTRAQ signals from two different cell states, C and T, was produced and the equation of the trend line was expressed as

$$t' = \mathrm{A}c' + B$$

where c' and t' represent the original peptide iTRAQ signal of cell state C and T,

respectively.

For normalization, the original peptide iTRAQ signals of cell state C were subjected to the equation of the trend line.

$$c = \mathrm{A}c' + B$$

where c and c' represent the normalized and original peptide iTRAQ signal of cell state C, respectively.

The normalized peptide iTRAQ signals of cell state T were equal to the original ones. At last, protein abundance ratio was calculated by the normalized peptide iTRAQ signals.

Method 4: Trend line of $\log_2$(peptide iTRAQ signals)

As method 5, we first calculated the $\log_2$ (peptide iTRAQ signals) and constructed the scatter plot of original $\log_2$ (peptide iTRAQ signals) from two different cell states, C and T. The equation of trend line was expressed as

$$t' = \mathrm{A}c' + B$$

where c and c' represent the original and normalized $\log_2$ (peptide iTRAQ signals) of cell state C, respectively.

Next, the original $\log_2$ (peptide iTRAQ signals) of cell state C were subjected to the equation of the trend line.

$$c = \mathrm{A}c' + B$$

where c and c' represent the normalized and original $\log_2$ (peptide iTRAQ signals) of cell state C, respectively.

The normalized $\log_2$ (peptide iTRAQ signals) of cell state T were equal to the original ones. At last, protein abundance ratio was calculated by the normalized peptide iTRAQ signals.

Method 5: Multi-Q normalization factor performing on peptide iTRAQ signals

Multi-Q performed normalization on peptide abundance ratio level. In Multi-Q, the normalized peptide ratio is calculated by

$$\frac{Original\,peptide\,ratio}{Normalization\,factor} = Normalized\,peptide\,ratio$$

That is

$$\left(\frac{t}{c}\right)' \times \frac{1}{Normalization\,factor} = \frac{t}{c}$$

where $\left(\frac{t}{c}\right)'$ is the original while $\frac{t}{c}$ is the normalized peptide abundance ratio in two different cell states, C and T.

We used the normalization factor outputted from Multi-Q to normalize the peptide iTRAQ signals. The peptide iTRAQ signals of cell state C were multiplied by the normalization factor from Multi-Q while the peptide iTRAQ signals of cell state T were multiplied by 1.

Method 6: Multi-Q normalization factor performing on protein abundance ratio

In this method, original protein abundance ratio was first calculated from original peptide iTRAQ signals. The normalized protein abundance ratio was calculated with normalization factor outputted from Multi-Q as follows:

$$\left(\frac{T}{C}\right)' \times \frac{1}{Normalization\,factor} = \frac{T}{C}$$

where $\left(\frac{T}{C}\right)'$ is the original while $\frac{T}{C}$ is the normalized protein abundance ratio in two different cell states, C and T.

Method 7: Log_2 (protein abundance ratio) median to zero

First, original protein abundance ratio was calculated from original peptide iTRAQ signals. The median of $\log_2$ (original protein abundance ratio) M was calculated.

Normalization factor was calculated as follows:

$$Normalization\ factor = 2^{-\mathrm{M}}$$

The normalized protein abundance ratio was expressed as

$$\left(\frac{T}{C}\right)' \times Normalization\ factor = \frac{T}{C}$$

where $\left(\frac{T}{C}\right)'$ is the original while $\frac{T}{C}$ is the normalized protein abundance ratio in two different cell states, C and T.

Evaluation process of the seven different normalization methods

To select the optimized normalization method, first we used the dataset from the duplicate experiment (Figure S1), which contained 296 identified proteins and 1,159 qualified peptides for protein quantitation (Table 1). For the normalization performed on the level of peptide iTRAQ signals (methods 1 to 5), normalized peptide iTRAQ signals were used for the calculation of the protein abundance ratios, $T_{1\alpha}/C_{1\alpha}$ and $T_{1\beta}/C_{1\beta}$. Next, we calculated the S values, which represent the errors of the protein abundance ratios, followed by the mean of all S values. For the normalization performed on the level of protein abundance ratios (methods 6 and 7), the protein abundance ratios, $T_{1\alpha}/C_{1\alpha}$ and $T_{1\beta}/C_{1\beta}$ were calculated from the original peptide iTRAQ signals followed by normalization of these protein abundance ratios. Subsequently, the S values and the mean of all S values were also calculated. The smaller mean of S values indicates less deviation from the actual protein abundance ratios. We selected methods that not only have smaller mean of S values but also directly correct the peptide iTRAQ signals, which were the origin of the deviations.

Next, we applied the selected normalization methods to the dataset of the large-scale experiment (Figure S2), which contained 2,659 identified proteins and 28,894 qualified

peptides for protein quantitation. We calculated T_1/C_1 and T_2/C_2 from the normalized peptide iTRAQ signals, followed by the mean of S values. On the other hand, normalized peptide iTRAQ signals were also used for the calculation of the protein abundance ratios, C_2/C_1 and T_2/T_1, which represent the relative protein expression in two biological replicates of control tumor samples (C_1 and C_2) and two biological replicates of citreoviridin-treated tumor samples (T_2 and T_1), respectively. In theory, the C_2/C_1 and T_2/T_1 protein abundance ratios of each protein should be equal to 1. However, measurement errors in experiments and individual variations in biological replicates of samples may cause deviation from 1. Therefore, a good normalization method is necessary for minimizing the deviation to obtain the accurate protein abundance ratios. Finally, we chose the method that has the capacity to correct both the protein abundance ratios, C_2/C_1 and T_2/T_1.
