# Supplementary material for: Quantitative Proteomic Analysis of Human Lung Tumor Xenografts Treated with the Ectopic ATP Synthase Inhibitor Citreoviridin
Source: PLoS One. 2013 Aug 21;8(8):e70642. doi: 10.1371/journal.pone.0070642 (PMC3749231; doi:10.1371/journal.pone.0070642)
Supplement: Table S6 — The S value calculated by normalization method 1 and 2 in the large-scale experiment. (PDF) [file pone.0070642.s011.pdf]

Table S6. The *S* value calculated by normalization method 1 and 2 in the large-scale experiment.

Method	Description[a]	*S* value [b]	
		Mean	S.D. [c]
1	Equal summation of peptide iTRAQ signals	0.00574	0.34727
2	Median of $\log_2$(peptide iTRAQ ratio) to zero	-0.03737	0.42563

[a] Detail calculation are described in Method S1.

[b] *S* value is in $\log_2$ scale:

$$S = \frac{1}{2}\ \log_2\left(\frac{T_1 \cdot C_2}{T_2 \cdot C_1}\right) = \frac{1}{2}\ \left[\log_2\left(\frac{T_1}{C_1}\right) - \log_2\left(\frac{T_2}{C_2}\right)\right]$$

where T_1/C_1 is the protein abundance ratio of citreoviridin-treated tumor sample T_1 to control sample C_1, while T_2/C_2 is the protein abundance ratio of citreoviridin-treated tumor sample T_2 to control sample C_2.

[c] S.D.: standard deviation.
