# Supplementary material for: Quantitative Proteomic Analysis of Human Lung Tumor Xenografts Treated with the Ectopic ATP Synthase Inhibitor Citreoviridin
Source: PLoS One. 2013 Aug 21;8(8):e70642. doi: 10.1371/journal.pone.0070642 (PMC3749231; doi:10.1371/journal.pone.0070642)
Supplement: Table S11 — Gene Ontology biological process clustering enrichment analysis of down-regulated proteins with citreoviridin treatment. (PDF) [file pone.0070642.s016.pdf]

Table S11. Gene Ontology biological process clustering enrichment analysis of down-regulated proteins with citreoviridin treatment.

GO biological process cluster	Enrichment score[a]	GO biological process term	Count[b]	p-Value
**Macromolecular complex assembly**	**1.66**			
		macromolecular complex assembly	8	0.01
		macromolecular complex subunit organization	8	0.01
		cellular macromolecular complex assembly	5	0.03
		protein complex assembly	6	0.03
		protein complex biogenesis	6	0.03
		cellular macromolecular complex subunit organization	5	0.04
**Mitosis**	**1.35**			
		nuclear division	4	0.04
		mitosis	4	0.04
		M phase of mitotic cell cycle	4	0.04
		organelle fission	4	0.05

[a] The enrichment score is the geometric mean (in –log scale) of the p-values of the members in the annotation cluster. It represents the significance of relevance between the group of annotations and the experimental dataset.

[b] Count: the number of proteins annotated with the GO term.
