# Supplementary material for: Quantitative Proteomic Analysis of Human Lung Tumor Xenografts Treated with the Ectopic ATP Synthase Inhibitor Citreoviridin
Source: PLoS One. 2013 Aug 21;8(8):e70642. doi: 10.1371/journal.pone.0070642 (PMC3749231; doi:10.1371/journal.pone.0070642)
Supplement: Table S12 — Top 5 networks of the differential proteome induced by citreoviridin in humans. (PDF) [file pone.0070642.s017.pdf]

Table S12. Top 5 networks of the differential proteome induced by citreoviridin in humans.

No.	Network	GO processes	Seed nodes[a]	p-Value[b]	zScore[c]	gScore[d]
1	ANAPC1, TMEM48, PSMB3, TMF1, RBM3	cellular macromolecule catabolic process (65.3%; 1.217e-35), macromolecule catabolic process (65.3%; 1.209e-33), negative regulation of ubiquitin-protein ligase activity involved in mitotic cell cycle (36.7%; 8.645e-33), negative regulation of ligase activity (36.7%; 7.969e-32), negative regulation of ubiquitin-protein ligase activity (36.7%; 7.969e-32)	7	3.38E-11	19.99	19.99
2	14-3-3 zeta/delta, NF-kB2 (p52), RBBP5, KIF1C, Alpha-actinin 4	positive regulation of biological process (75.0%; 7.781e-17), positive regulation of cellular process (70.8%; 5.017e-16), positive regulation of gene expression (47.9%; 1.318e-15), nervous system development (56.2%; 7.887e-15), system development (70.8%; 9.013e-15)	6	2.08E-09	17.27	17.27
3	LDHA, LEDGF, DJ-1, SP1, LRP5	S phase of mitotic cell cycle (36.0%; 4.053e-27), S phase (36.0%; 8.632e-27), regulation of cellular amino acid metabolic process (28.0%; 6.385e-25), mitotic cell cycle G1/S transition DNA damage checkpoint (30.0%; 8.107e-25), M/G1 transition of mitotic cell cycle (30.0%; 9.714e-25)	6	2.08E-09	17.27	17.27
4	OGT (GlcNAc transferase), ACOX1, MKLP2, FKBP12, Alpha-actinin 1	protein polymerization (27.1%; 5.633e-23), 'de novo' protein folding (25.0%; 3.937e-20), microtubule-based movement (29.2%; 6.046e-19), 'de novo' posttranslational protein folding (22.9%; 2.327e-18), platelet degranulation (25.0%; 8.506e-18)	6	2.36E-09	17.09	17.09
5	NEFM, ATP6M, SUPT5H, 14-3-3 zeta/delta, OGT (GlcNAc transferase)	response to organic substance (65.3%; 6.354e-18), gene expression (73.5%; 8.784e-18), regulation of cellular macromolecule biosynthetic process (73.5%; 1.315e-17), regulation of transcription, DNA-dependent (71.4%; 1.426e-17), regulation of RNA biosynthetic process (71.4%; 1.840e-17)	6	2.36E-09	17.09	17.09

[a] Seed nodes: the number of differentially expressed proteins identified in the experiments in the network.

[b] The p-value represents a measure of relevance of the intersection between the experimental dataset and the network.

[c] A high Z-score means the network is highly saturated with proteins from the experiment.

[d] A high G-score means the network is saturated with proteins from the experiment (from Z-score) and it contains many Canonical Pathways.
