# Supplementary material for: Quantitative Proteomic Analysis of Human Lung Tumor Xenografts Treated with the Ectopic ATP Synthase Inhibitor Citreoviridin
Source: PLoS One. 2013 Aug 21;8(8):e70642. doi: 10.1371/journal.pone.0070642 (PMC3749231; doi:10.1371/journal.pone.0070642)
Supplement: Spectra S1 — The information of single-peptide-based protein identifications in the duplicate experiment. (PDF) [file pone.0070642.s018.pdf]

Spectra S1: The information of single-peptide-based protein identifications in the duplicate experiment.

Query 2113 Hit 1

MS/MS Fragmentation of **LEQFVSILMASIPLPDK**
Found in **sp|P00491|PNPH_HUMAN**, Purine nucleoside phosphorylase OS=Homo sapiens GN=PNP PE=1 SV=2
Match to Query 2113: 2188.256from(730.4258,3+)
Title: 1440: Sum of 2 scans in range 3510 (rt=87.2714, f=4, i=953) to 3511 (rt=87.2969, f=4, i=954)
[D:\lab212\membrane\Grace\20120214_iTRAQ_2.raw]
Data File:
Monoisotopic mass of neutral peptide Mr(calc): 2188.256
Variable modifications:
K17 :iTRAQ4plex (K)
Ions Score: 40.59 **Expect:** 0.037

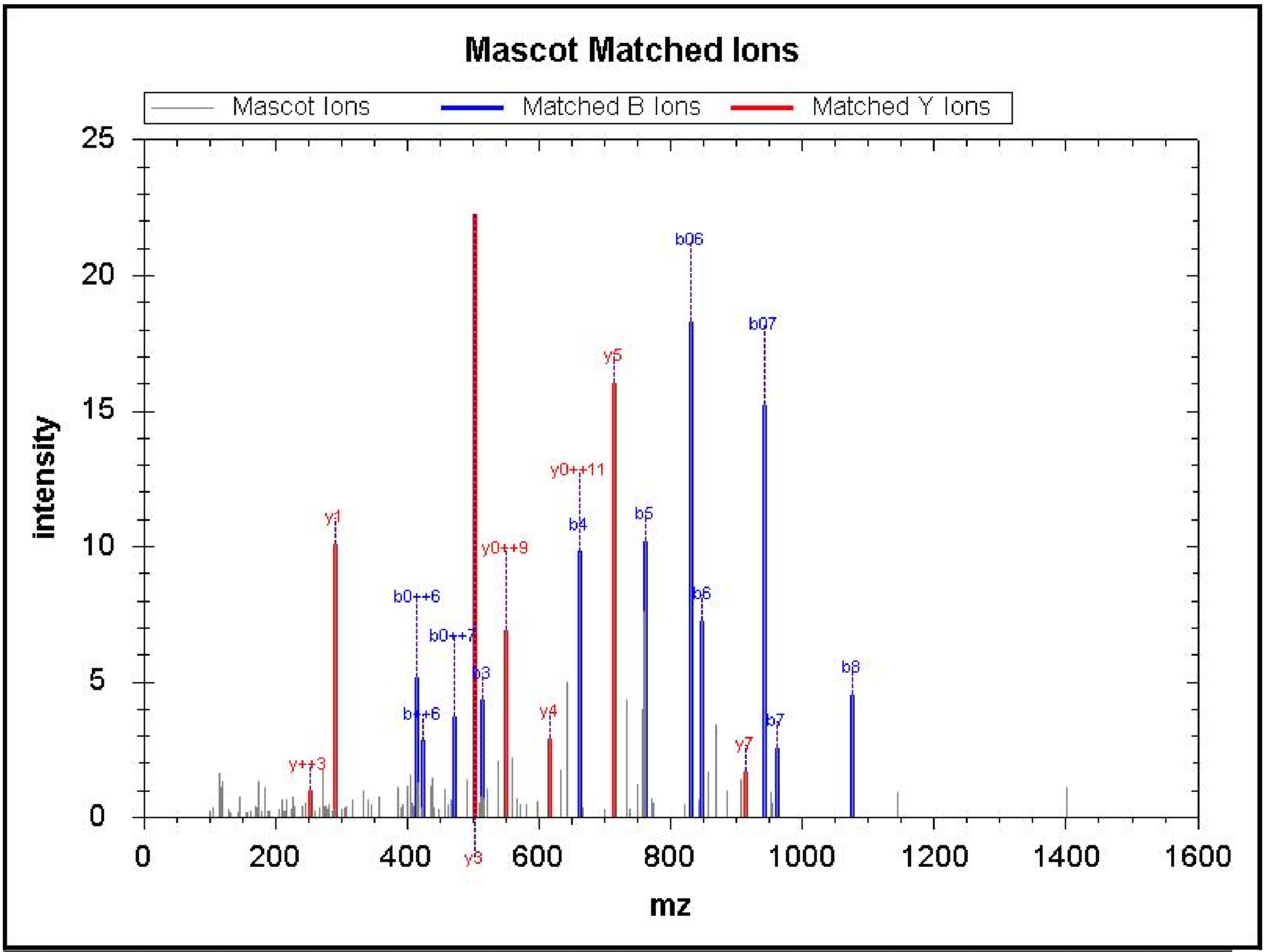

No	b	b++	b*	b*++	b0	b0++	Seq	y	y++	y*	y*++	y0	y0++	RevNo
1	258.19	129.60					L							17
2	387.24	194.12			369.23	185.12	E	1,932.06	966.54	1,915.04	958.02	1,914.05	957.53	16
3	**515.29**	258.15	498.27	249.64	497.28	249.15	Q	1,803.02	902.01	1,785.99	893.50	1,785.01	893.01	15
4	**662.36**	331.69	645.34	323.17	644.35	322.68	F	1,674.96	837.98	1,657.94	829.47	1,656.95	828.98	14
5	**761.43**	381.22	744.40	372.71	743.42	372.21	V	1,527.89	764.45	1,510.87	755.94	1,509.88	755.45	13
6	**848.46**	**424.74**	831.44	416.22	**830.45**	**415.73**	S	1,428.83	714.92	1,411.80	706.40	1,410.81	705.91	12
7	**961.55**	481.28	944.52	472.76	**943.54**	**472.27**	I	1,341.79	671.40	1,324.77	662.89	1,323.78	**662.40**	11
8	**1,074.63**	537.82	1,057.61	529.31	1,056.62	528.81	L	1,228.71	614.86	1,211.68	606.35	1,210.70	605.85	10
9	1,205.67	603.34	1,188.65	594.83	1,187.66	594.33	M	1,115.63	558.32	1,098.60	549.80	1,097.61	**549.31**	9
10	1,276.71	638.86	1,259.68	630.35	1,258.70	629.85	A	984.58	492.80	967.56	484.28	966.57	483.79	8
11	1,363.74	682.37	1,346.71	673.86	1,345.73	673.37	S	**913.55**	457.28	896.52	448.76	895.54	448.27	7
12	1,476.83	738.92	1,459.80	730.40	1,458.81	729.91	I	826.52	413.76	809.49	405.25	808.50	404.76	6
13	1,573.88	787.44	1,556.85	778.93	1,555.87	778.44	P	**713.43**	357.22	696.40	348.71	695.42	348.21	5

No	b	b++	b*	b*++	b0	b0++	Seq	y	y++	y*	y*++	RevNo
14	1,686.96	843.98	1,669.94	835.47	1,668.95	834.98	L	**616.38**	308.69	599.35	300.18	598.37	299.69	4
15	1,784.01	892.51	1,766.99	884.00	1,766.00	883.51	P	**503.29**	**252.15**	486.27	243.64	485.28	243.15	3
16	1,899.04	950.02	1,882.02	941.51	1,881.03	941.02	D	406.24	203.62	389.22	195.11	388.23	194.62	2
17							K	**291.21**	146.11	274.19	137.60			1

Query 2241 Hit 1

MS/MS Fragmentation of **EGKPHLVLGLLWQIIK**
Found in **sp|Q14651|PLSI_HUMAN**, Plastin-1 OS=Homo sapiens GN=PLS1 PE=1 SV=2
Match to Query 2241: 2276.322from(759.7813,3+)
Title: 801: Scan 2111 (rt=55.8202, f=2, i=330) [D:\lab212\membrane\Grace\20120214_iTRAQ_2.raw]
Data File:
Monoisotopic mass of neutral peptide Mr(calc): 2276.322
Variable modifications:
K3 :iTRAQ4plex (K)
Q13 :Deamidated (NQ)
K16 :iTRAQ4plex (K)
Ions Score: 41.17 **Expect:** 0.025

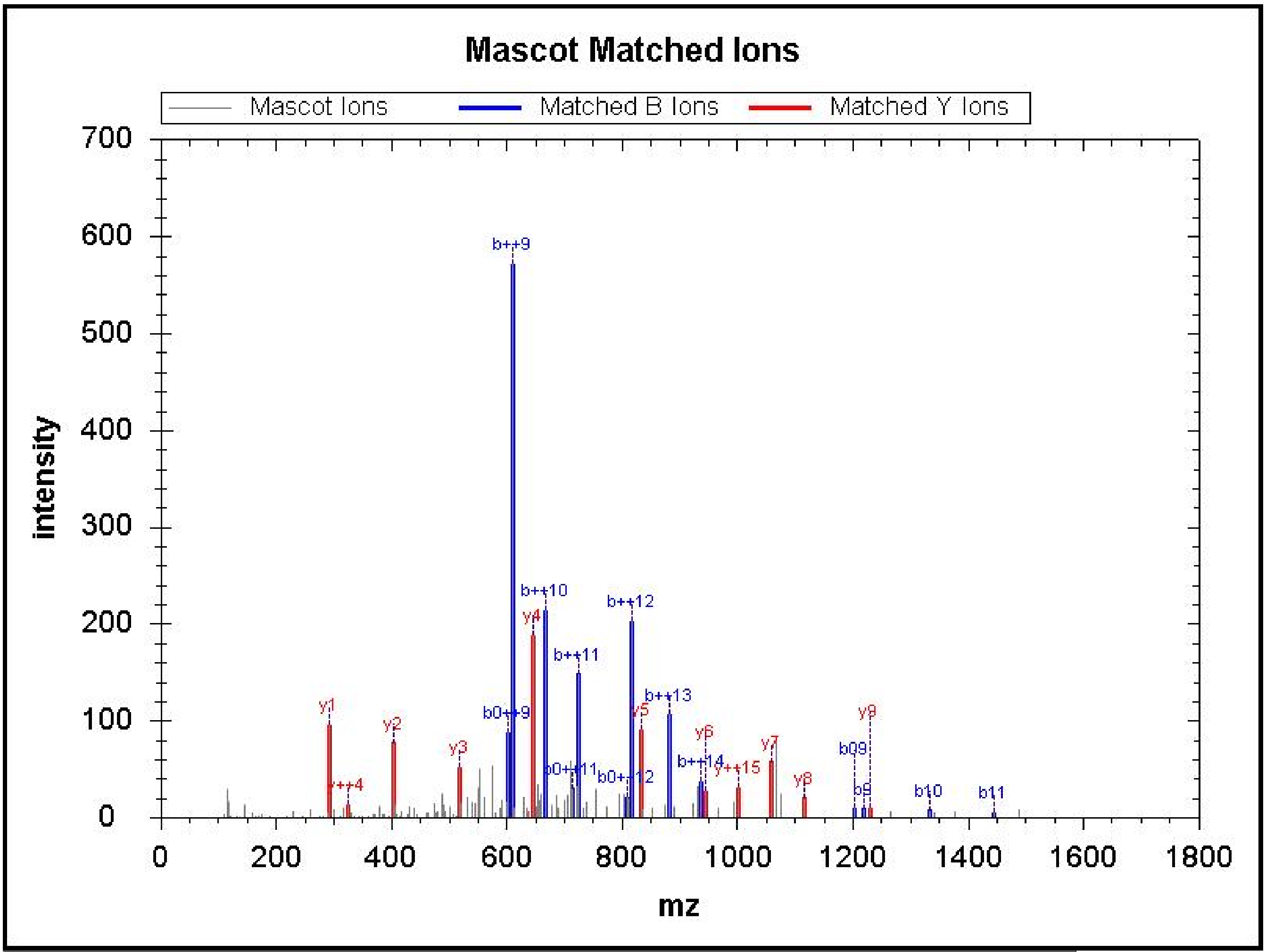

No	b	b++	b*	b*++	b0	b0++	Seq	y	y++	y*	y*++	RevNo
1	274.15	137.58			256.14	128.57	E					16
2	331.17	166.09			313.16	157.09	G	2,004.26	**1,002.63**	1,987.23	994.12	15
3	603.37	302.19	586.34	293.68	585.36	293.18	K	1,947.24	974.12	1,930.21	965.61	14
4	700.42	350.72	683.40	342.20	682.41	341.71	P	1,675.04	838.03	1,658.02	829.51	13
5	837.48	419.24	820.46	410.73	819.47	410.24	H	1,577.99	789.50	1,560.96	780.99	12
6	950.57	475.79	933.54	467.27	932.56	466.78	L	1,440.93	720.97	1,423.90	712.46	11
7	1,049.63	525.32	1,032.61	516.81	1,031.62	516.32	V	1,327.85	664.43	1,310.82	655.91	10
8	1,162.72	581.86	1,145.69	573.35	1,144.71	572.86	L	**1,228.78**	614.89	1,211.75	606.38	9

9	1,219.74	610.37	1,202.71	601.86	1,201.73	601.37	G	1,115.69	558.35	1,098.67	549.84		8
10	1,332.82	666.92	1,315.80	658.40	1,314.81	657.91	L	1,058.67	529.84	1,041.65	521.33		7
11	1,445.91	723.46	1,428.88	714.94	1,427.90	714.45	L	945.59	473.30	928.56	464.78		6
12	1,631.99	816.50	1,614.96	807.98	1,613.98	807.49	W	832.50	416.76	815.48	408.24		5
13	1,761.03	881.02	1,744.00	872.51	1,743.02	872.01	Q	646.43	323.72	629.40	315.20		4
14	1,874.11	937.56	1,857.09	929.05	1,856.10	928.56	I	517.38	259.20	500.36	250.68		3
15	1,987.20	994.10	1,970.17	985.59	1,969.19	985.10	I	404.30	202.65	387.27	194.14		2
16							K	291.21	146.11	274.19	137.60		1

Query 278 Hit 1

MS/MS Fragmentation of **RIQDLER**
Found in **sp|Q6ZQ06|QN1_MOUSE**, Protein QN1 homolog OS=Mus musculus GN=Kiaa1009 PE=1 SV=2
Match to Query 278: 1073.595from(358.8724,3+)
Title: 202: Scan 858 (rt=27.5877, f=2, i=128) [D:\lab212\membrane\Grace\20120214_iTRAQ_2.raw]
Data File:
Monoisotopic mass of neutral peptide Mr(calc): 1073.595
Variable modifications:
Q3 :Deamidated (NQ)
Ions Score: 41.27 **Expect:** 0.058

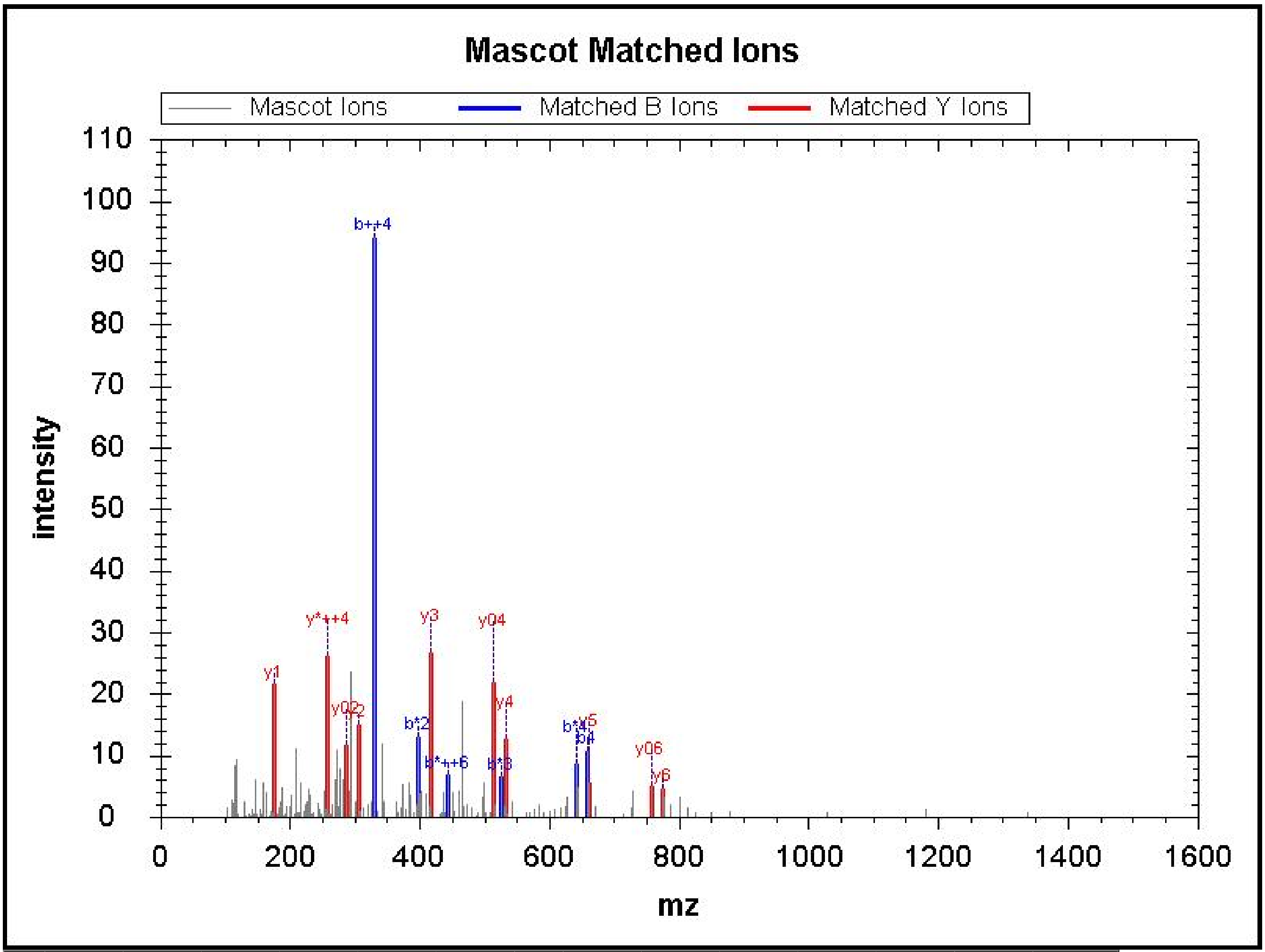

No	b	b++	b*	b*++	b0	b0++	Seq	y	y++	y*	y*++	y0	y0++	RevNo
1	301.21	151.11	284.18	142.60			R							7
2	414.29	207.65	397.27	199.14			I	774.40	387.70	757.37	379.19	756.39	378.70	6
3	543.34	272.17	526.31	263.66			Q	661.32	331.16	644.29	322.65	643.30	322.16	5
4	658.36	329.69	641.34	321.17	640.35	320.68	D	532.27	266.64	515.25	258.13	514.26	257.63	4
5	771.45	386.23	754.42	377.71	753.44	377.22	L	417.25	209.13	400.22	200.61	399.24	200.12	3

6	900.49	450.75	883.46	442.24	882.48	441.74	E	304.16	152.58	287.13	144.07	286.15	143.58	2
7							R	175.12	88.06	158.09	79.55			1

Query 849 Hit 1

MS/MS Fragmentation of **LWTLVSEQTR**
Found in **sp|P46776|RL27A_HUMAN**, 60S ribosomal protein L27a OS=Homo sapiens GN=RPL27A PE=1 SV=2
Match to Query 849: 1375.76from(688.8874,2+)
Title: 731: Scan 1963 (rt=52.4888, f=3, i=271) [D:\lab212\membrane\Grace\20120214_iTRAQ_2.raw]
Data File:
Monoisotopic mass of neutral peptide Mr(calc): 1375.76
Variable modifications:
Ions Score: 42.32 **Expect:** 0.050

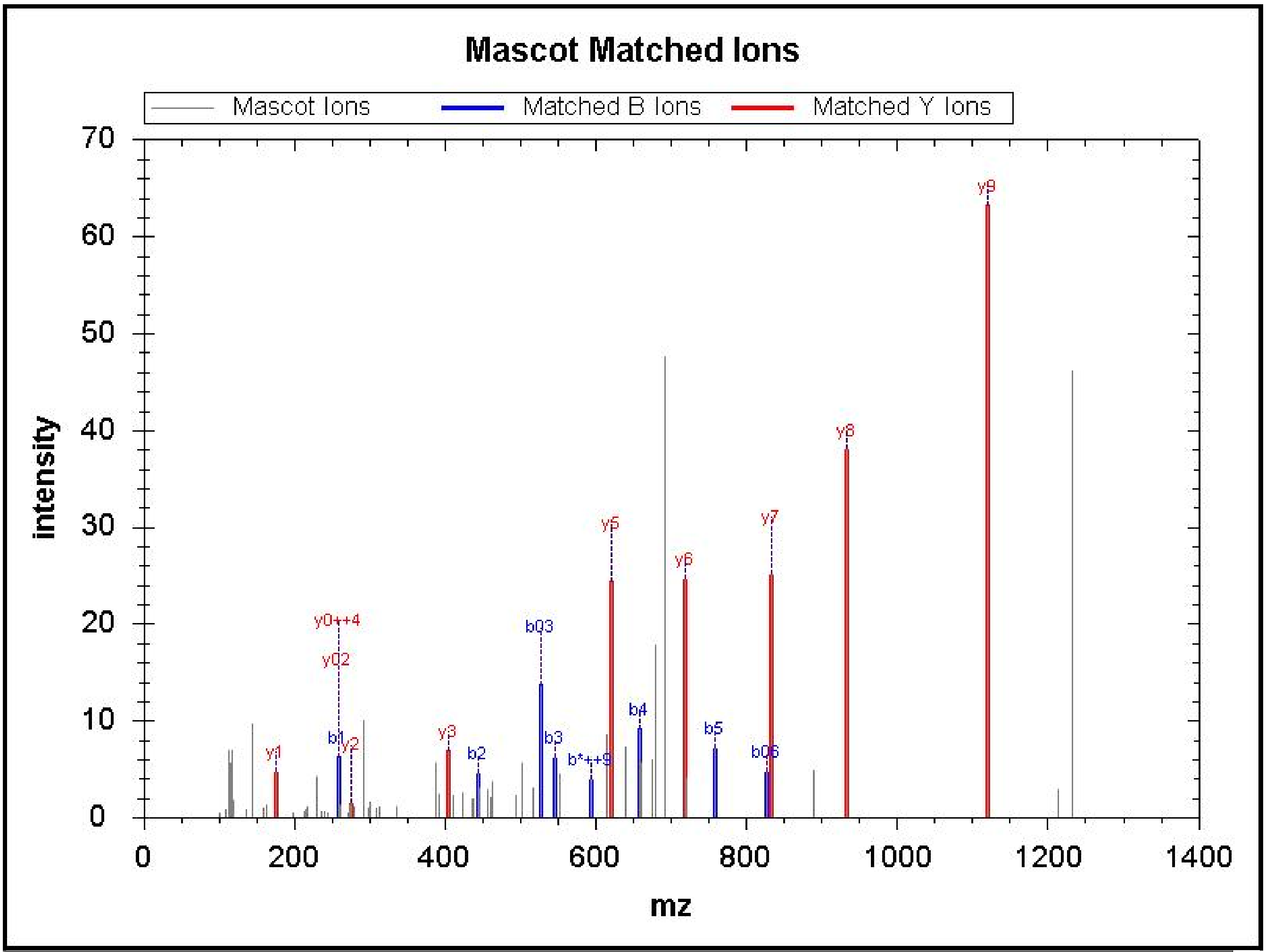

No	b	b++	b*	b*++	b0	b0++	Seq	y	y++	y*	y*++	y0	y0++	RevNo
1	258.19	129.60					L							10
2	444.27	222.64					W	1,119.58	560.29	1,102.55	551.78	1,101.57	551.29	9
3	545.32	273.16			527.31	264.16	T	933.50	467.25	916.47	458.74	915.49	458.25	8
4	658.40	329.71			640.39	320.70	L	832.45	416.73	815.43	408.22	814.44	407.72	7
5	757.47	379.24			739.46	370.23	V	719.37	360.19	702.34	351.67	701.36	351.18	6
6	844.50	422.76			826.49	413.75	S	620.30	310.65	603.27	302.14	602.29	301.65	5
7	973.55	487.28			955.54	478.27	E	533.27	267.14	516.24	258.62	515.26	258.13	4
8	1,101.61	551.31	1,084.58	542.79	1,083.60	542.30	Q	404.23	202.62	387.20	194.10	386.21	193.61	3
9	1,202.65	601.83	1,185.63	593.32	1,184.64	592.83	T	276.17	138.59	259.14	130.07	258.16	129.58	2
10							R	175.12	88.06	158.09	79.55			1

Query 2257 Hit 1

MS/MS Fragmentation of **NVGNAILYETVLTIMDIK**
Found in **sp|O43747|AP1G1_HUMAN**, AP-1 complex subunit gamma-1 OS=Homo sapiens GN=AP1G1 PE=1 SV=5
Match to Query 2257: 2294.279from(765.7671,3+)
Title: 1384: Sum of 2 scans in range 3354 (rt=83.8282, f=4, i=927) to 3355 (rt=83.8536, f=4, i=928) [D:\lab212\membrane\Grace\20120214_iTRAQ_2.raw]
Data File:
Monoisotopic mass of neutral peptide Mr(calc): 2294.279
Variable modifications:
K18 :iTRAQ4plex (K)
Ions Score: 42.43 **Expect:** 0.032

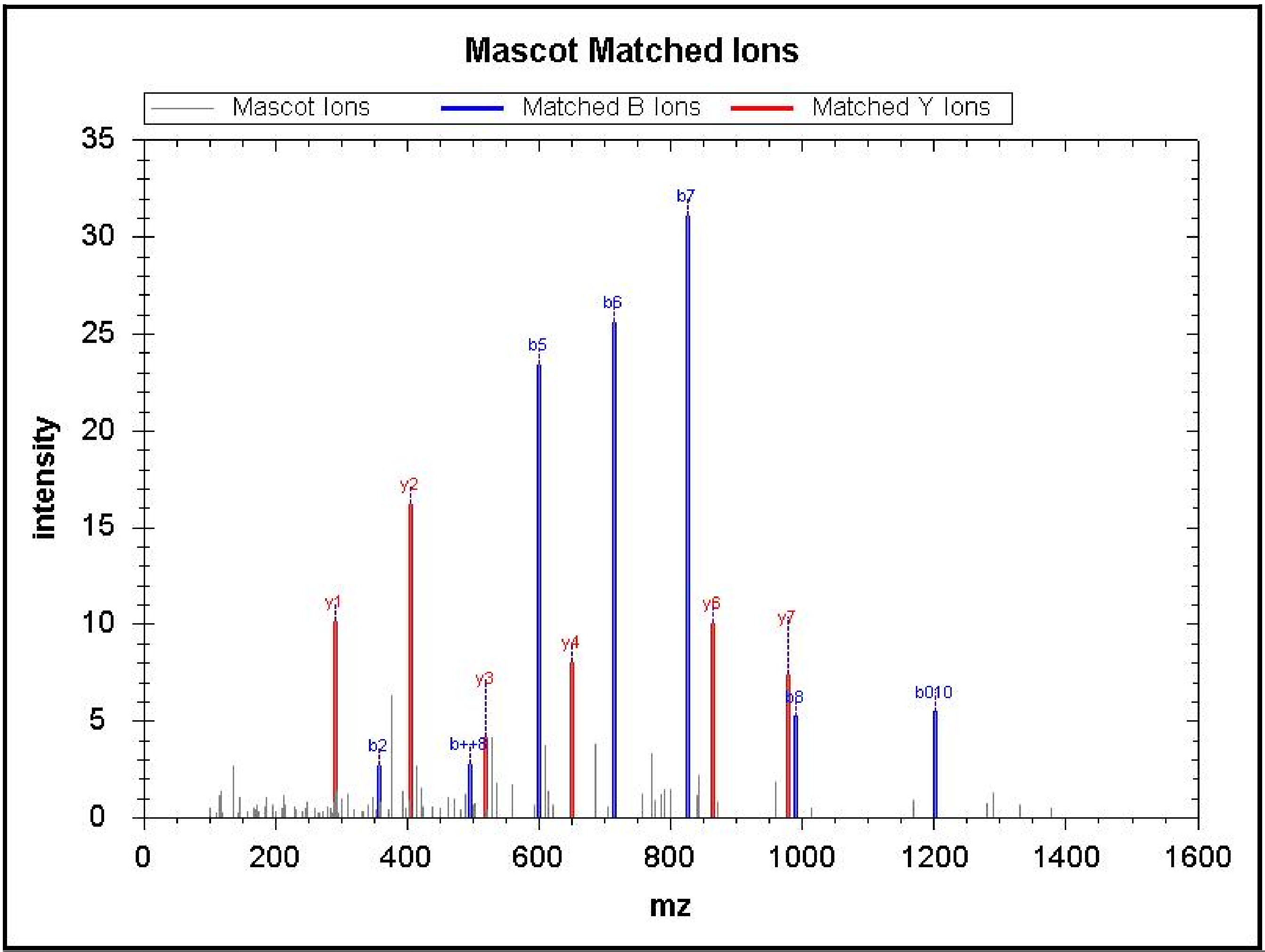

No	b	b++	b*	b*++	b0	b0++	Seq	y	y++	y*	y*++	y0	y0++	RevNo
1	259.15	130.08	242.13	121.57			N							18
2	**358.22**	179.61	341.19	171.10			V	2,037.14	1,019.07	2,020.12	1,010.56	2,019.13	1,010.07	17
3	415.24	208.12	398.22	199.61			G	1,938.07	969.54	1,921.05	961.03	1,920.06	960.54	16
4	529.29	265.15	512.26	256.63			N	1,881.05	941.03	1,864.03	932.52	1,863.04	932.02	15
5	**600.32**	300.66	583.30	292.15			A	1,767.01	884.01	1,749.98	875.50	1,749.00	875.00	14
6	**713.41**	357.21	696.38	348.69			I	1,695.97	848.49	1,678.95	839.98	1,677.96	839.48	13
7	**826.49**	413.75	809.46	405.24			L	1,582.89	791.95	1,565.86	783.43	1,564.88	782.94	12
8	**989.55**	**495.28**	972.53	486.77			Y	1,469.80	735.41	1,452.78	726.89	1,451.79	726.40	11
9	1,118.60	559.80	1,101.57	551.29	1,100.59	550.80	E	1,306.74	653.87	1,289.71	645.36	1,288.73	644.87	10
10	1,219.64	610.33	1,202.62	601.81	**1,201.63**	601.32	T	1,177.70	589.35	1,160.67	580.84	1,159.69	580.35	9
11	1,318.71	659.86	1,301.69	651.35	1,300.70	650.85	V	1,076.65	538.83	1,059.62	530.32	1,058.64	529.82	8
12	1,431.80	716.40	1,414.77	707.89	1,413.79	707.40	L	**977.58**	489.29	960.56	480.78	959.57	480.29	7
13	1,532.84	766.93	1,515.82	758.41	1,514.83	757.92	T	**864.50**	432.75	847.47	424.24	846.49	423.75	6
14	1,645.93	823.47	1,628.90	814.95	1,627.92	814.46	I	763.45	382.23	746.42	373.72	745.44	373.22	5
15	1,776.97	888.99	1,759.94	880.47	1,758.96	879.98	M	**650.37**	325.69	633.34	317.17	632.36	316.68	4

16	1,892.00	946.50	1,874.97	937.99	1,873.99	937.50	D	519.33	260.17	502.30	251.65	501.32	251.16	3
17	2,005.08	1,003.04	1,988.05	994.53	1,987.07	994.04	I	404.30	202.65	387.27	194.14			2
18							K	291.21	146.11	274.19	137.60			1

Query 1845 Hit 1

MS/MS Fragmentation of **KLDLEAWFPGSGAFR**
Found in **sp|P49591|SYSC_HUMAN**, Serine--tRNA ligase
Match to Query 1845: 1981.071from(661.3644,3+)
Title: 902: Sum of 2 scans in range 2361 (rt=61.3745, f=4, i=614) to 2362 (rt=61.3999, f=4, i=615) [D:\lab212\membrane\Grace\20120214_iTRAQ_1.raw]
Data File:
Monoisotopic mass of neutral peptide Mr(calc): 1981.071
Variable modifications:
K1 :iTRAQ4plex (K)
Ions Score: 42.63 **Expect:** 0.044

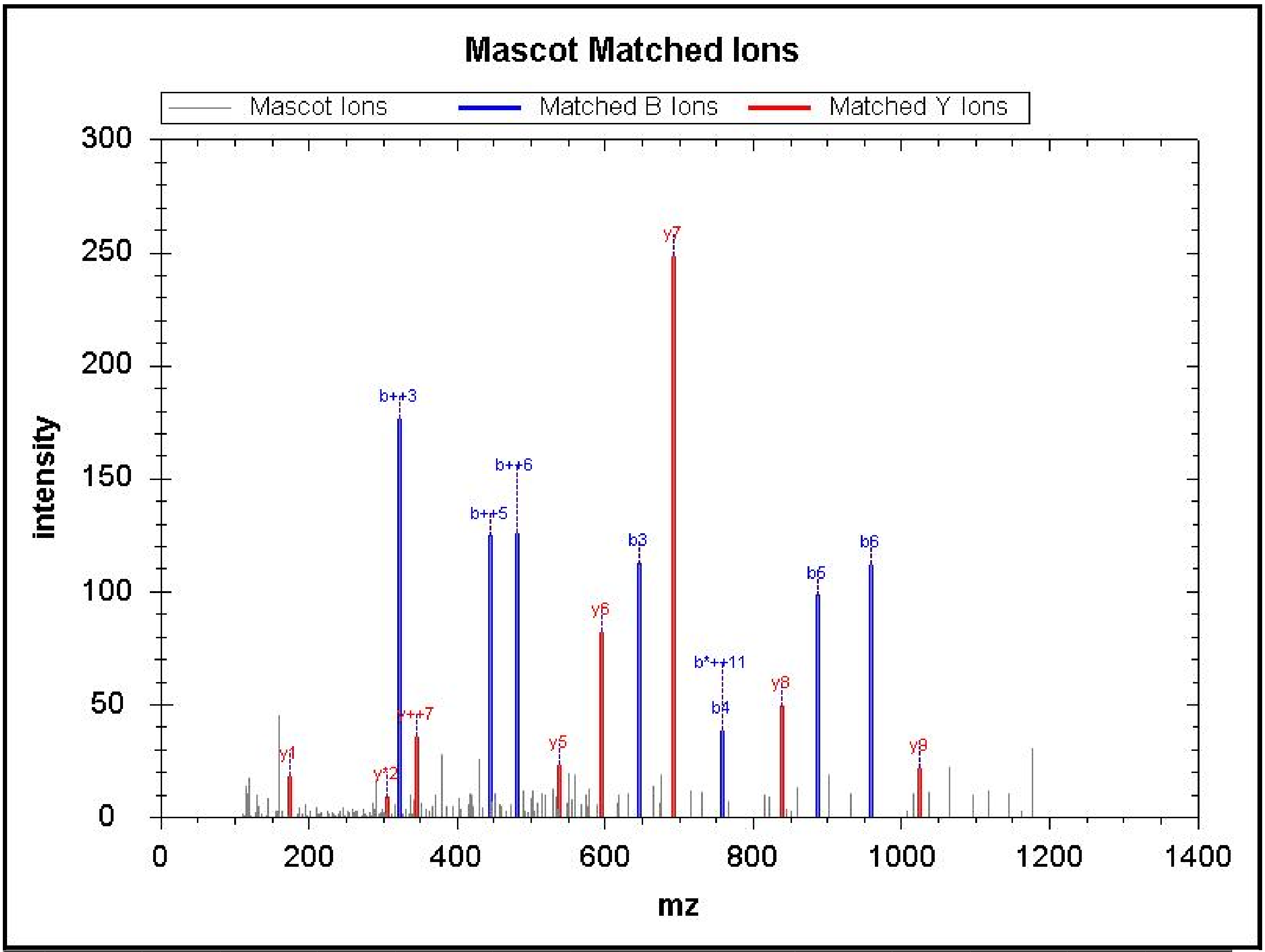

No	b	b++	b*	b*++	b0	b0++	Seq	y	y++	y*	y*++	y0	y0++	RevNo
1	417.31	209.16	400.28	200.64			K							15
2	530.39	265.70	513.36	257.19			L	1,565.77	783.39	1,548.75	774.88	1,547.76	774.39	14
3	645.42	323.21	628.39	314.70	627.41	314.21	D	1,452.69	726.85	1,435.66	718.34	1,434.68	717.84	13
4	758.50	379.75	741.47	371.24	740.49	370.75	L	1,337.66	669.34	1,320.64	660.82	1,319.65	660.33	12
5	887.54	444.28	870.52	435.76	869.53	435.27	E	1,224.58	612.79	1,207.55	604.28	1,206.57	603.79	11
6	958.58	479.79	941.55	471.28	940.57	470.79	A	1,095.54	548.27	1,078.51	539.76	1,077.53	539.27	10
7	1,144.66	572.83	1,127.63	564.32	1,126.65	563.83	W	1,024.50	512.75	1,007.47	504.24	1,006.49	503.75	9
8	1,291.73	646.37	1,274.70	637.85	1,273.72	637.36	F	838.42	419.71	821.39	411.20	820.41	410.71	8
9	1,388.78	694.89	1,371.76	686.38	1,370.77	685.89	P	691.35	346.18	674.33	337.67	673.34	337.17	7
10	1,445.80	723.41	1,428.78	714.89	1,427.79	714.40	G	594.30	297.65	577.27	289.14	576.29	288.65	6

11	1,532.83	766.92	1,515.81	758.41	1,514.82	757.92	S	537.28	269.14	520.25	260.63	519.27	260.14	5
12	1,589.86	795.43	1,572.83	786.92	1,571.85	786.43	G	450.25	225.63	433.22	217.11			4
13	1,660.89	830.95	1,643.87	822.44	1,642.88	821.95	A	393.22	197.12	376.20	188.60			3
14	1,807.96	904.48	1,790.94	895.97	1,789.95	895.48	F	322.19	161.60	305.16	153.08			2
15							R	175.12	88.06	158.09	79.55			1

Query 1008 Hit 1

MS/MS Fragmentation of VMVQPINLIFR
Found in sp|P62304|RUXE_HUMAN, Small nuclear ribonucleoprotein E OS=Homo sapiens GN=SNRPE PE=1 SV=1
Match to Query 1008: 1472.866from(737.4403,2+)
Title: 955: Scan 2471 (rt=63.852, f=3, i=340) [D:\lab212\membrane\Grace\20120214_iTRAQ_1.raw]
Data File:
Monoisotopic mass of neutral peptide Mr(calc): 1472.866
Variable modifications:
Ions Score: 42.72 **Expect:** 0.032

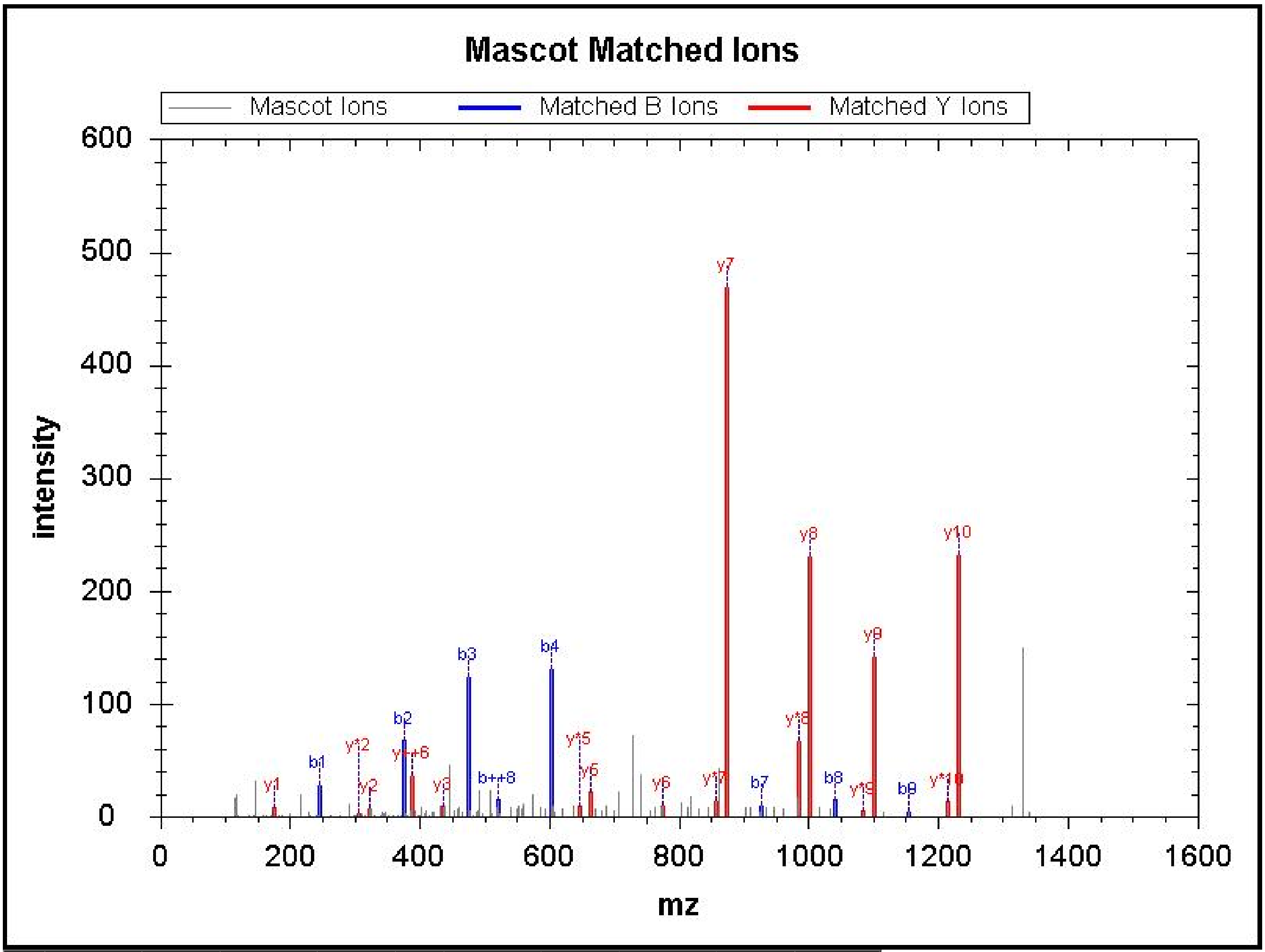

No	b	b++	b*	b*++	Seq	y	y++	y*	y*++	RevNo
1	244.18	122.59			V					11
2	375.22	188.11			M	1,230.70	615.85	1,213.68	607.34	10
3	474.29	237.65			V	1,099.66	550.33	1,082.64	541.82	9
4	602.35	301.68	585.32	293.16	Q	1,000.59	500.80	983.57	492.29	8
5	699.40	350.20	682.37	341.69	P	872.54	436.77	855.51	428.26	7
6	812.48	406.74	795.46	398.23	I	775.48	388.24	758.46	379.73	6
7	926.52	463.77	909.50	455.25	N	662.40	331.70	645.37	323.19	5
8	1,039.61	520.31	1,022.58	511.79	L	548.36	274.68	531.33	266.17	4
9	1,152.69	576.85	1,135.67	568.34	I	435.27	218.14	418.24	209.63	3

10	1,299.76	650.38	1,282.74	641.87	F	322.19	161.60	305.16	153.08	2
11					R	175.12	88.06	158.09	79.55	1

Query 2359 Hit 1

MS/MS Fragmentation of **DMEALLPLMNMVIYSIDK**
Found in **sp|Q96A33|CCD47_HUMAN**, Coiled-coil domain-containing protein 47 OS=Homo sapiens GN=CCDC47 PE=1 SV=1
Match to Query 2359: 2383.238from(795.42,3+)
Title: 1415: Scan 3499 (rt=86.9833, f=3, i=509) [D:\lab212\membrane\Grace\20120214_iTRAQ_1.raw]
Data File:
Monoisotopic mass of neutral peptide Mr(calc): 2383.238
Variable modifications:
K18 :iTRAQ4plex (K)
Ions Score: 42.94 **Expect:** 0.046

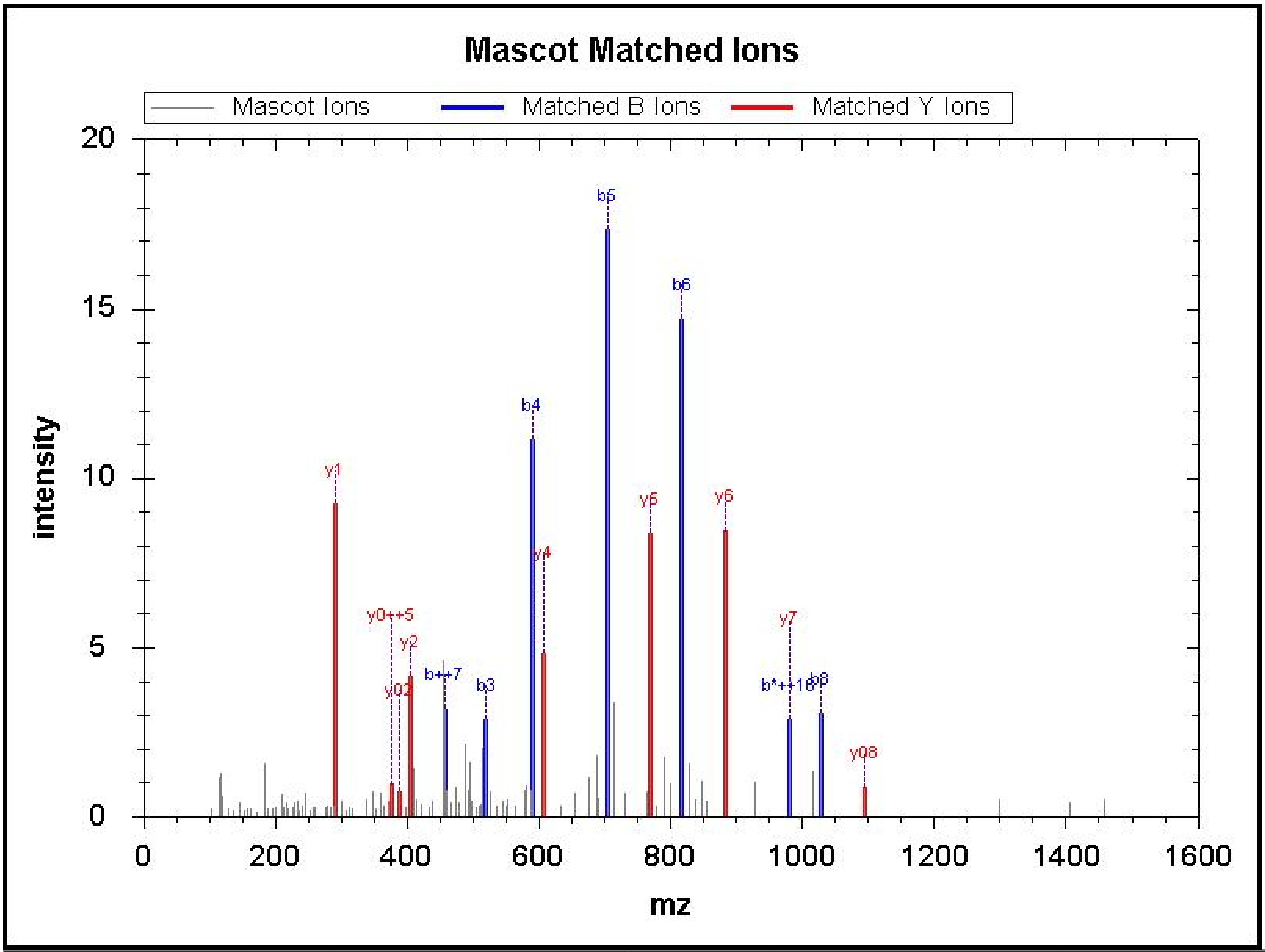

No	b	b++	b*	b*++	b0	b0++	Seq	y	y++	y*	y*++	y0	y0++	RevNo
1	260.14	130.57			242.13	121.57	D							18
2	391.18	196.09			373.17	187.09	M	2,125.12	1,063.07	2,108.10	1,054.55	2,107.11	1,054.06	17
3	520.22	260.61			502.21	251.61	E	1,994.08	997.54	1,977.06	989.03	1,976.07	988.54	16
4	591.26	296.13			573.25	287.13	A	1,865.04	933.02	1,848.01	924.51	1,847.03	924.02	15
5	704.34	352.67			686.33	343.67	L	1,794.00	897.51	1,776.98	888.99	1,775.99	888.50	14
6	817.42	409.22			799.41	400.21	L	1,680.92	840.96	1,663.89	832.45	1,662.91	831.96	13
7	914.48	457.74			896.47	448.74	P	1,567.83	784.42	1,550.81	775.91	1,549.82	775.42	12
8	1,027.56	514.28			1,009.55	505.28	L	1,470.78	735.89	1,453.76	727.38	1,452.77	726.89	11
9	1,158.60	579.80			1,140.59	570.80	M	1,357.70	679.35	1,340.67	670.84	1,339.69	670.35	10
10	1,272.65	636.83	1,255.62	628.31	1,254.63	627.82	N	1,226.66	613.83	1,209.63	605.32	1,208.65	604.83	9
11	1,403.69	702.35	1,386.66	693.83	1,385.67	693.34	M	1,112.61	556.81	1,095.59	548.30	1,094.60	547.81	8
12	1,502.75	751.88	1,485.73	743.37	1,484.74	742.88	V	981.57	491.29	964.55	482.78	963.56	482.29	7

13	1,615.84	808.42	1,598.81	799.91	1,597.83	799.42	I	882.51	441.76	865.48	433.24	864.49	432.75	6
14	1,778.90	889.95	1,761.87	881.44	1,760.89	880.95	Y	769.42	385.21	752.39	376.70	751.41	376.21	5
15	1,865.93	933.47	1,848.91	924.96	1,847.92	924.47	S	606.36	303.68	589.33	295.17	588.35	294.68	4
16	1,979.02	990.01	1,961.99	981.50	1,961.01	981.01	I	519.33	260.17	502.30	251.65	501.32	251.16	3
17	2,094.04	1,047.53	2,077.02	1,039.01	2,076.03	1,038.52	D	406.24	203.62	389.22	195.11	388.23	194.62	2
18							K	291.21	146.11	274.19	137.60			1

Query 61 Hit 1

MS/MS Fragmentation of SGLDGVK
Found in sp|Q9H0U9|TSYL1_HUMAN, Testis-specific Y-encoded-like protein 1 OS=Homo sapiens GN=TSPYL1 PE=1 SV=3
Match to Query 61: 818.5082 from(410.2614,2+)
Title: 169: Scan 834 (rt=26.9606, f=2, i=124) [D:\lab212\membrane\Grace\20120214_iTRAQ_1.raw]
Data File:
Monoisotopic mass of neutral peptide Mr(calc): 818.5082
Variable modifications:
K7 :iTRAQ4plex (K)
Ions Score: 43.07 **Expect:** 0.039

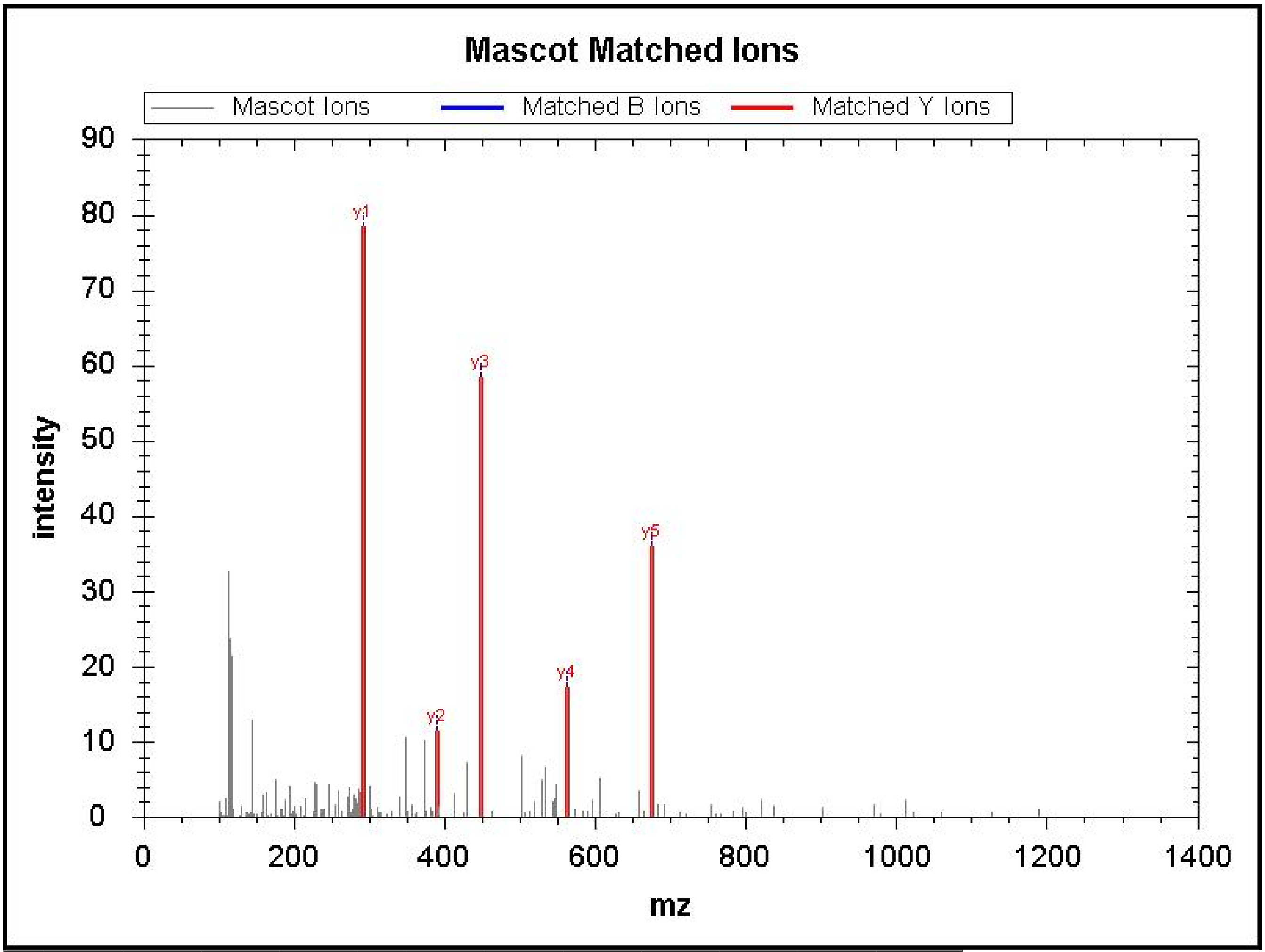

No	b	b++	b0	b0++	Seq	y	y++	y*	y*++	y0	y0++	RevNo
1	88.04	44.52	70.03	35.52	S							7
2	145.06	73.03	127.05	64.03	G	732.44	366.72	715.41	358.21	714.43	357.72	6
3	258.14	129.58	240.13	120.57	L	675.42	338.21	658.39	329.70	657.41	329.21	5
4	373.17	187.09	355.16	178.08	D	562.33	281.67	545.31	273.16	544.32	272.66	4
5	430.19	215.60	412.18	206.59	G	447.30	224.16	430.28	215.64			3
6	529.26	265.13	511.25	256.13	V	390.28	195.65	373.26	187.13			2
7					K	291.21	146.11	274.19	137.60			1

Query 866 Hit 1

MS/MS Fragmentation of **AGNLGGGVVTIER**
Found in **sp|P35268|RL22_HUMAN**, 60S ribosomal protein L22 OS=Homo sapiens GN=RPL22 PE=1 SV=2
Match to Query 866: 1385.774from(693.8942,2+)
Title: 414: Sum of 2 scans in range 1344 (rt=38.4592, f=4, i=286) to 1345 (rt=38.4846, f=4, i=287) [D:\lab212\membrane\Grace\20120214_iTRAQ_1.raw]
Data File:
Monoisotopic mass of neutral peptide Mr(calc): 1385.774
Variable modifications:
Ions Score: 43.07 **Expect:** 0.034

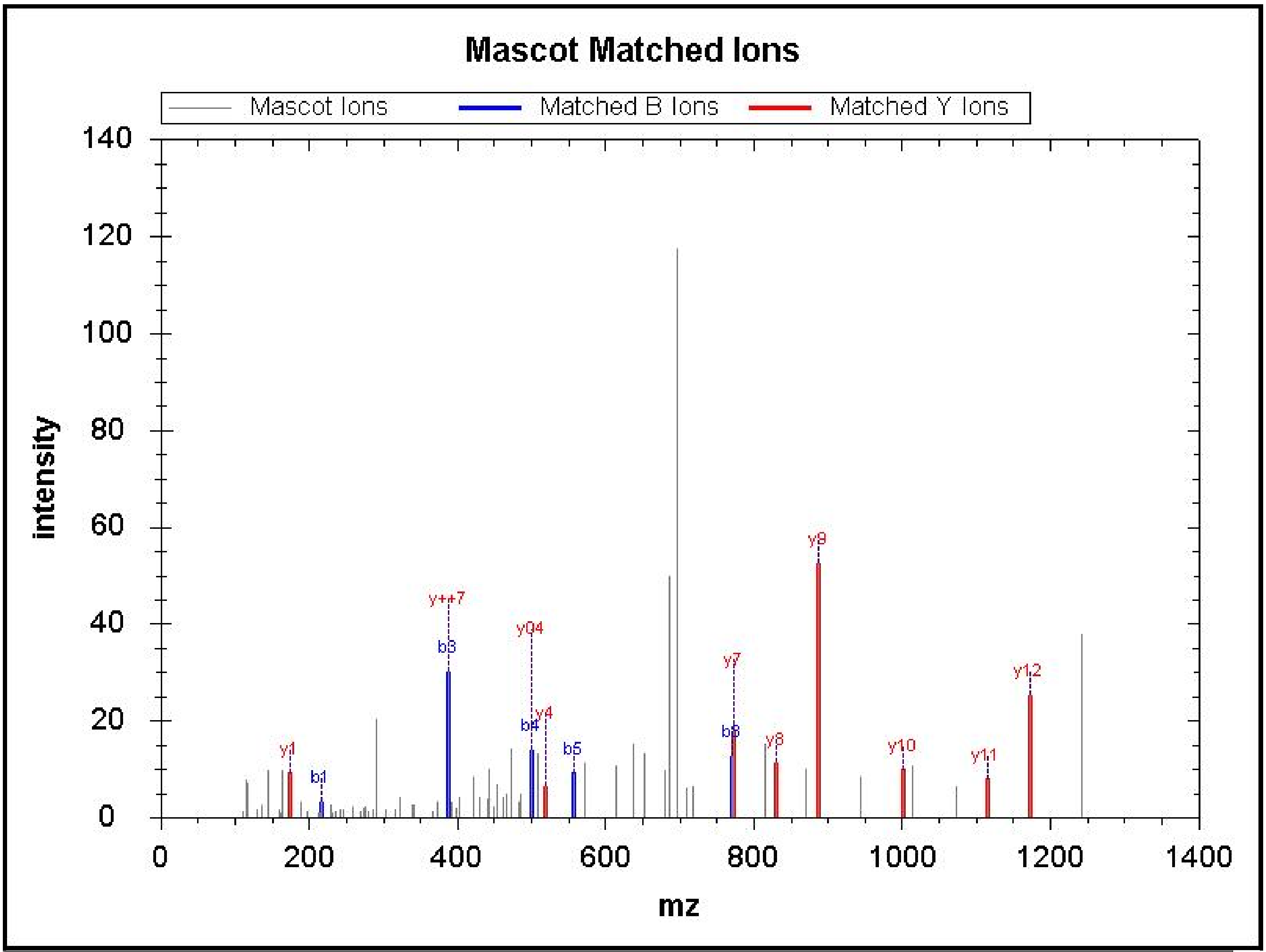

No	b	b++	b*	b*++	b0	b0++	Seq	y	y++	y*	y*++	y0	y0++	RevNo
1	216.15	108.58					A							13
2	273.17	137.09					G	1,171.64	586.33	1,154.62	577.81	1,153.63	577.32	12
3	387.21	194.11	370.18	185.60			N	1,114.62	557.81	1,097.59	549.30	1,096.61	548.81	11
4	500.29	250.65	483.27	242.14			L	1,000.58	500.79	983.55	492.28	982.57	491.79	10
5	557.32	279.16	540.29	270.65			G	887.49	444.25	870.47	435.74	869.48	435.25	9
6	614.34	307.67	597.31	299.16			G	830.47	415.74	813.45	407.23	812.46	406.73	8
7	671.36	336.18	654.33	327.67			G	773.45	387.23	756.42	378.72	755.44	378.22	7
8	770.43	385.72	753.40	377.20			V	716.43	358.72	699.40	350.21	698.42	349.71	6
9	869.50	435.25	852.47	426.74			V	617.36	309.18	600.34	300.67	599.35	300.18	5
10	970.54	485.78	953.52	477.26	952.53	476.77	T	518.29	259.65	501.27	251.14	500.28	250.64	4
11	1,083.63	542.32	1,066.60	533.80	1,065.62	533.31	I	417.25	209.13	400.22	200.61	399.24	200.12	3
12	1,212.67	606.84	1,195.64	598.33	1,194.66	597.83	E	304.16	152.58	287.13	144.07	286.15	143.58	2
13							R	175.12	88.06	158.09	79.55			1

Query 2415 Hit 1

MS/MS Fragmentation of **YMLLPNQVWDSIIQQATK**
Found in **sp|O14980|XPO1_HUMAN**, Exportin-1 OS=Homo sapiens GN=XPO1 PE=1 SV=1
Match to Query 2415: 2435.307from(812.7763,3+)
Title: 1200: Scan 2992 (rt=75.5913, f=3, i=424) [D:\lab212\membrane\Grace\20120214_iTRAQ_1.raw]
Data File:
Monoisotopic mass of neutral peptide Mr(calc): 2435.307
Variable modifications:
K18 :iTRAQ4plex (K)
Ions Score: 43.29 **Expect:** 0.036

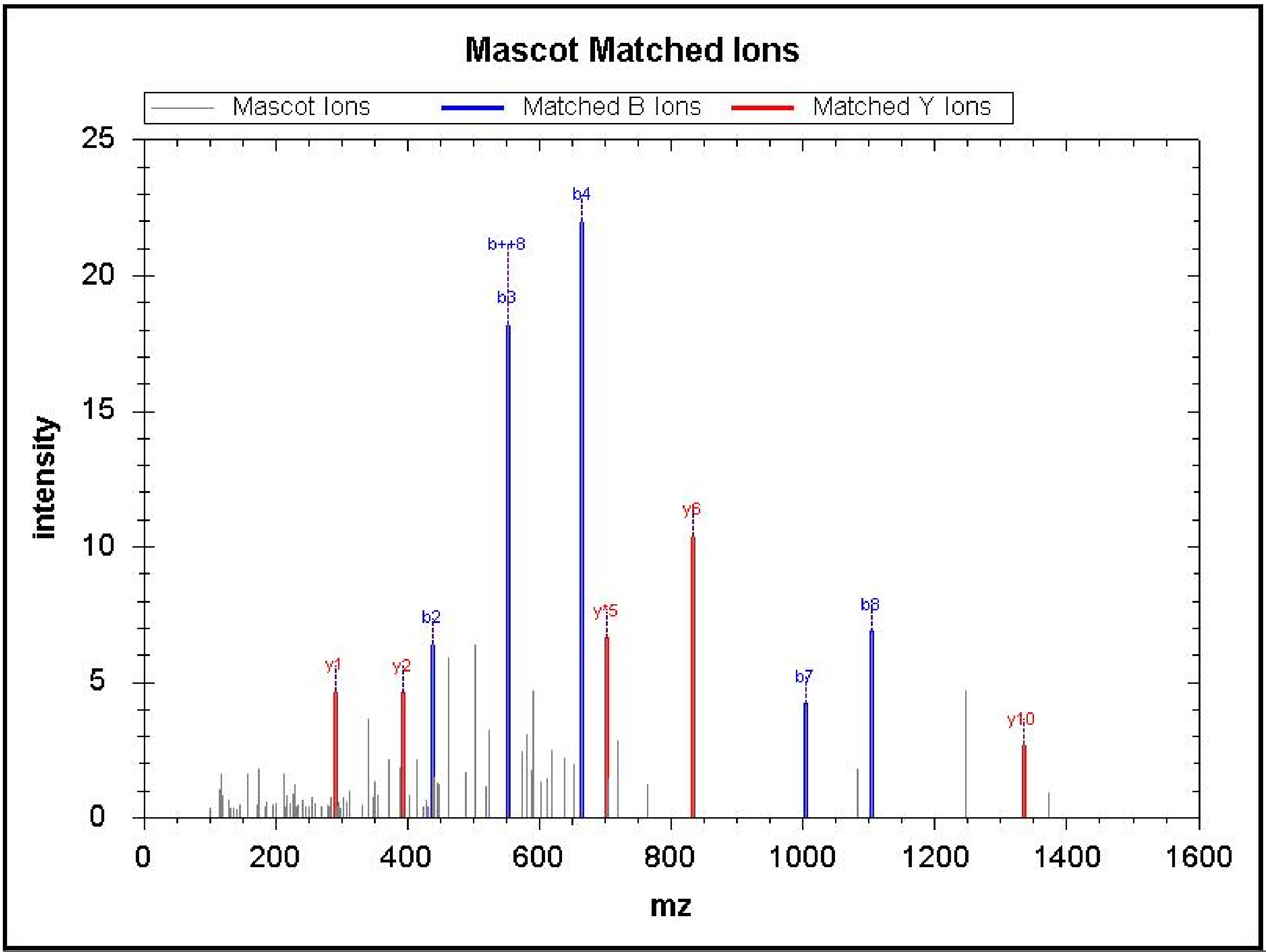

No	b	b++	b*	b*++	b0	b0++	Seq	y	y++	y*	y*++	y0	y0++	RevNo
1	308.17	154.59					Y							18
2	439.21	220.11					M	2,129.15	1,065.08	2,112.13	1,056.57	2,111.14	1,056.08	17
3	552.30	276.65					L	1,998.11	999.56	1,981.09	991.05	1,980.10	990.56	16
4	665.38	333.19					L	1,885.03	943.02	1,868.00	934.51	1,867.02	934.01	15
5	762.43	381.72					P	1,771.95	886.48	1,754.92	877.96	1,753.94	877.47	14
6	876.48	438.74	859.45	430.23			N	1,674.89	837.95	1,657.87	829.44	1,656.88	828.94	13
7	1,004.54	502.77	987.51	494.26			Q	1,560.85	780.93	1,543.82	772.42	1,542.84	771.92	12
8	1,103.60	552.31	1,086.58	543.79			V	1,432.79	716.90	1,415.77	708.39	1,414.78	707.89	11
9	1,289.68	645.35	1,272.66	636.83			W	1,333.72	667.37	1,316.70	658.85	1,315.71	658.36	10
10	1,404.71	702.86	1,387.68	694.35	1,386.70	693.85	D	1,147.64	574.33	1,130.62	565.81	1,129.63	565.32	9
11	1,491.74	746.37	1,474.72	737.86	1,473.73	737.37	S	1,032.62	516.81	1,015.59	508.30	1,014.61	507.81	8
12	1,604.83	802.92	1,587.80	794.40	1,586.82	793.91	I	945.58	473.30	928.56	464.78	927.57	464.29	7
13	1,717.91	859.46	1,700.88	850.95	1,699.90	850.45	I	832.50	416.75	815.47	408.24	814.49	407.75	6
14	1,845.97	923.49	1,828.94	914.97	1,827.96	914.48	Q	719.42	360.21	702.39	351.70	701.41	351.21	5

15	1,974.03	987.52	1,957.00	979.00	1,956.02	978.51	Q	591.36	296.18	574.33	287.67	573.35	287.18	4
16	2,045.06	1,023.04	2,028.04	1,014.52	2,027.05	1,014.03	A	463.30	232.15	446.27	223.64	445.29	223.15	3
17	2,146.11	1,073.56	2,129.09	1,065.05	2,128.10	1,064.55	T	**392.26**	196.63	375.24	188.12	374.25	187.63	2
18							K	**291.21**	146.11	274.19	137.60			1

Query 2119 Hit 1

MS/MS Fragmentation of **YVATLGVEVHPLVFHTNR**
Found in **sp|P62826|RAN_HUMAN**, GTP-binding nuclear protein Ran OS=Homo sapiens GN=RAN PE=1 SV=3
Match to Query 2119: 2195.195from(549.806,4+)
Title: 652: Scan 1845 (rt=49.746, f=3, i=239) [D:\lab212\membrane\Grace\20120214_iTRAQ_1.raw]
Data File:
Monoisotopic mass of neutral peptide Mr(calc): 2195.195
Variable modifications:
Ions Score: 43.39 **Expect:** 0.034

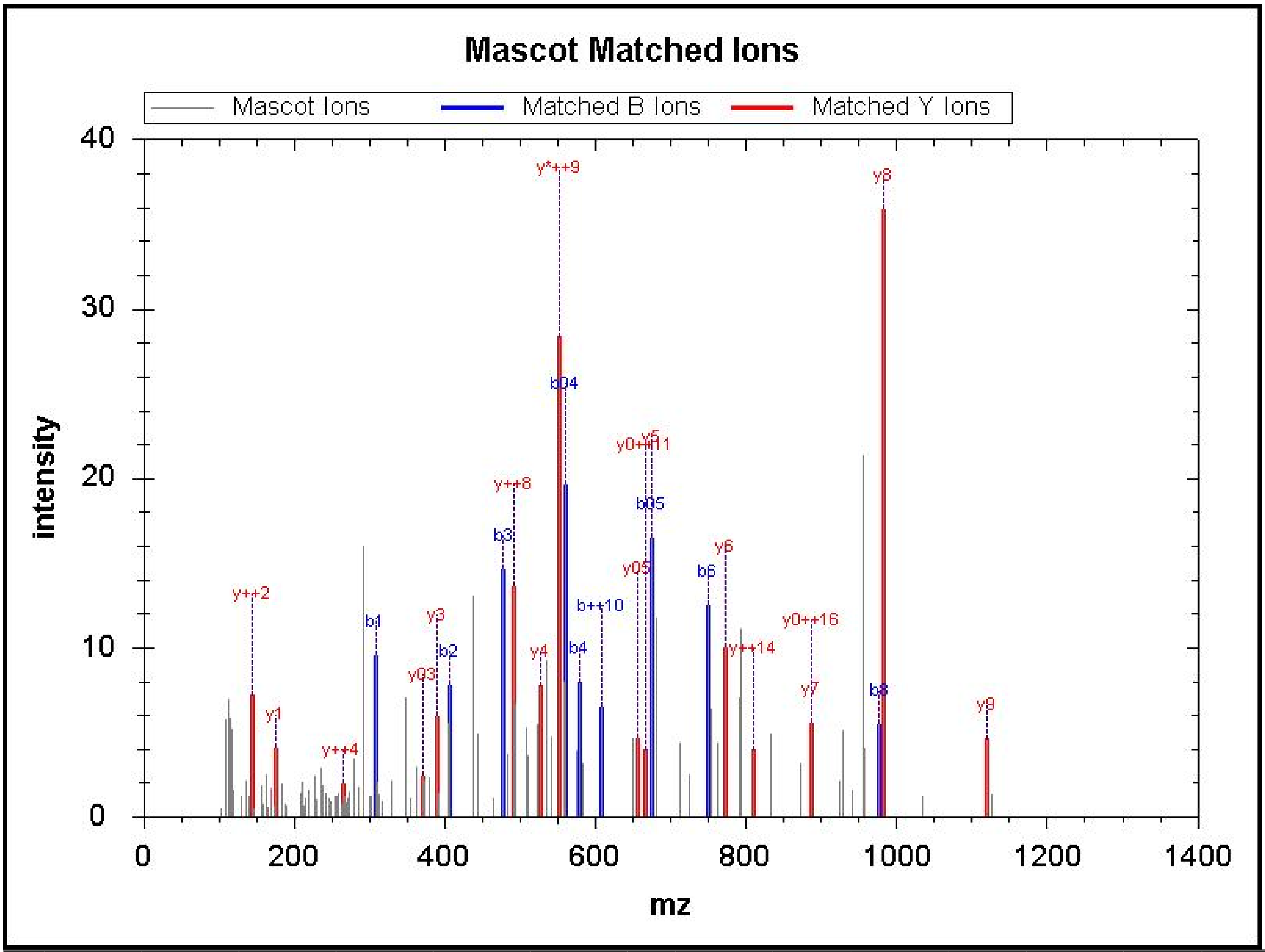

No	b	b++	b*	b*++	b0	b0++	Seq	y	y++	y*	y*++	y0	y0++	RevNo
1	**308.17**	154.59					Y							18
2	**407.24**	204.12					V	1,889.04	945.02	1,872.01	936.51	1,871.03	936.02	17
3	**478.28**	239.64					A	1,789.97	895.49	1,772.94	886.98	1,771.96	**886.48**	16
4	**579.33**	290.17			**561.32**	281.16	T	1,718.93	859.97	1,701.91	851.46	1,700.92	850.97	15
5	692.41	346.71			**674.40**	337.70	L	1,617.89	**809.45**	1,600.86	800.93	1,599.88	800.44	14
6	**749.43**	375.22			731.42	366.21	G	1,504.80	752.90	1,487.78	744.39	1,486.79	743.90	13
7	848.50	424.75			830.49	415.75	V	1,447.78	724.39	1,430.75	715.88	1,429.77	715.39	12
8	**977.54**	489.27			959.53	480.27	E	1,348.71	674.86	1,331.69	666.35	1,330.70	**665.85**	11
9	1,076.61	538.81			1,058.60	529.80	V	1,219.67	610.34	1,202.64	601.83	1,201.66	601.33	10
10	1,213.67	**607.34**			1,195.66	598.33	H	**1,120.60**	560.80	1,103.57	**552.29**	1,102.59	551.80	9

11	1,310.72	655.86			1,292.71	646.86	P	983.54	492.27	966.52	483.76	965.53	483.27	8
12	1,423.81	712.41			1,405.80	703.40	L	886.49	443.75	869.46	435.24	868.48	434.74	7
13	1,522.88	761.94			1,504.86	752.94	V	773.41	387.21	756.38	378.69	755.39	378.20	6
14	1,669.94	835.48			1,651.93	826.47	F	674.34	337.67	657.31	329.16	656.33	328.67	5
15	1,807.00	904.00			1,788.99	895.00	H	527.27	264.14	510.24	255.62	509.26	255.13	4
16	1,908.05	954.53			1,890.04	945.52	T	390.21	195.61	373.18	187.10	372.20	186.60	3
17	2,022.09	1,011.55	2,005.07	1,003.04	2,004.08	1,002.54	N	289.16	145.08	272.14	136.57			2
18							R	175.12	88.06	158.09	79.55			1

Query 2258 Hit 1

MS/MS Fragmentation of **LKQEYFVVAATLQDIIR**
Found in **sp|P11216|PYGB_HUMAN**, Glycogen phosphorylase
Match to Query 2258: 2294.32from(765.7806,3+)
Title: 1126: Sum of 2 scans in range 2826 (rt=71.8524, f=4, i=764) to 2827 (rt=71.8778, f=4, i=765) [D:\lab212\membrane\Grace\20120214_iTRAQ_1.raw]
Data File:
Monoisotopic mass of neutral peptide Mr(calc): 2294.32
Variable modifications:
K2 :iTRAQ4plex (K)
Ions Score: 43.62 **Expect:** 0.016

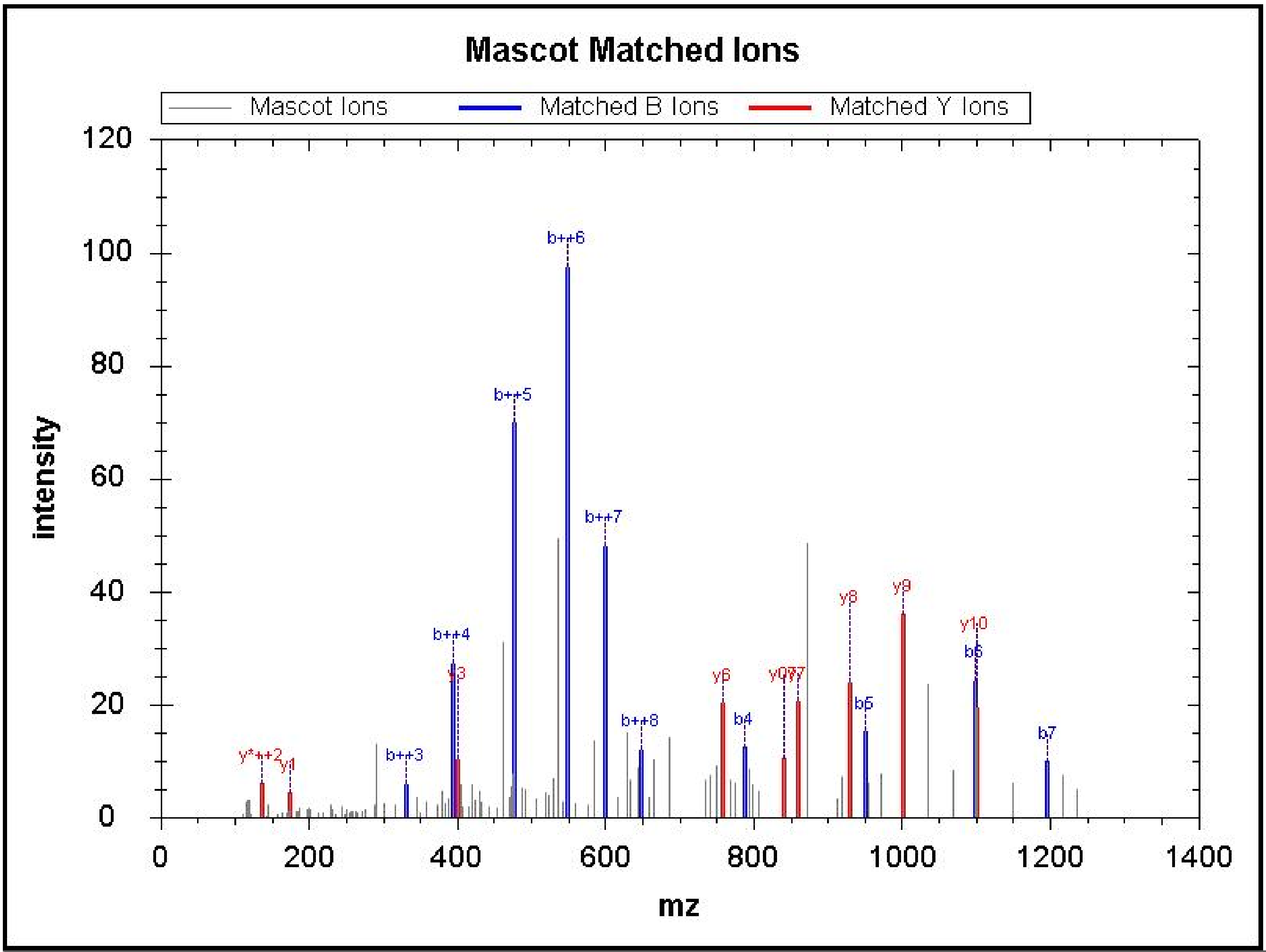

No	b	b++	b*	b*++	b0	b0++	Seq	y	y++	y*	y*++	y0	y0++	RevNo
1	258.19	129.60					L							17
2	530.39	265.70	513.36	257.19			K	2,038.15	1,019.58	2,021.12	1,011.06	2,020.13	1,010.57	16
3	658.45	329.73	641.42	321.21			Q	1,765.95	883.48	1,748.92	874.96	1,747.94	874.47	15
4	787.49	394.25	770.47	385.74	769.48	385.24	E	1,637.89	819.45	1,620.86	810.94	1,619.88	810.44	14
5	950.55	475.78	933.53	467.27	932.54	466.78	Y	1,508.85	754.93	1,491.82	746.41	1,490.84	745.92	13

6	1,097.62	549.32	1,080.60	540.80	1,079.61	540.31	F	1,345.78	673.40	1,328.76	664.88	1,327.77	664.39	12
7	1,196.69	598.85	1,179.67	590.34	1,178.68	589.84	V	1,198.72	599.86	1,181.69	591.35	1,180.70	590.86	11
8	1,295.76	648.38	1,278.73	639.87	1,277.75	639.38	V	1,099.65	550.33	1,082.62	541.81	1,081.64	541.32	10
9	1,366.80	683.90	1,349.77	675.39	1,348.79	674.90	A	1,000.58	500.79	983.55	492.28	982.57	491.79	9
10	1,437.83	719.42	1,420.81	710.91	1,419.82	710.42	A	929.54	465.27	912.51	456.76	911.53	456.27	8
11	1,538.88	769.94	1,521.86	761.43	1,520.87	760.94	T	858.50	429.76	841.48	421.24	840.49	420.75	7
12	1,651.97	826.49	1,634.94	817.97	1,633.96	817.48	L	757.46	379.23	740.43	370.72	739.45	370.23	6
13	1,780.02	890.52	1,763.00	882.00	1,762.01	881.51	Q	644.37	322.69	627.35	314.18	626.36	313.68	5
14	1,895.05	948.03	1,878.03	939.52	1,877.04	939.02	D	516.31	258.66	499.29	250.15	498.30	249.66	4
15	2,008.14	1,004.57	1,991.11	996.06	1,990.13	995.57	I	401.29	201.15	384.26	192.63			3
16	2,121.22	1,061.11	2,104.19	1,052.60	2,103.21	1,052.11	I	288.20	144.61	271.18	136.09			2
17							R	175.12	88.06	158.09	79.55			1

Query 2342 Hit 1

MS/MS Fragmentation of **QFGFIVLTTSAGIMDHEEAR**
Found in **sp|P62244|RS15A_HUMAN**, 40S ribosomal protein S15a OS=Homo sapiens GN=RPS15A PE=1 SV=2
Match to Query 2342: 2365.185from(789.4022,3+)
Title: 940: Sum of 2 scans in range 2398 (rt=62.2933, f=4, i=623) to 2399 (rt=62.3187, f=4, i=624) [D:\lab212\membrane\Grace\20120214_iTRAQ_2.raw]
Data File:
Monoisotopic mass of neutral peptide Mr(calc): 2365.185
Variable modifications:
Ions Score: 44.6 **Expect:** 0.033

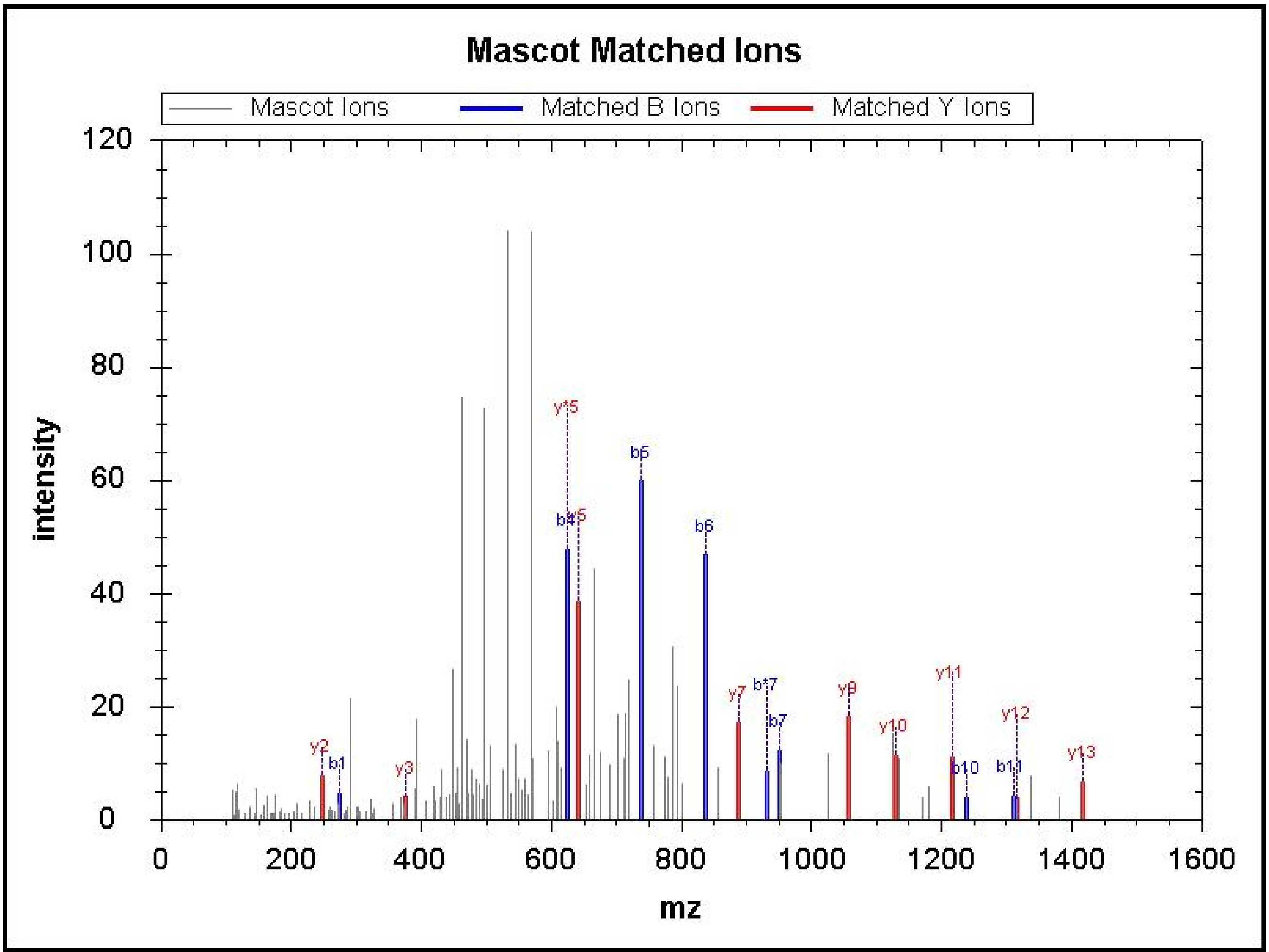

No	b	b++	b*	b*++	b0	b0++	Seq	y	y++	y*	y*++	y0	y0++	RevNo
1	273.17	137.09	256.14	128.57			Q							20
2	420.24	210.62	403.21	202.11			F	2,094.03	1,047.52	2,077.01	1,039.01	2,076.02	1,038.51	19

3	477.26	239.13	460.23	230.62			G	1,946.96	973.99	1,929.94	965.47	1,928.95	964.98	18
4	**624.33**	312.67	607.30	304.15			F	1,889.94	945.47	1,872.92	936.96	1,871.93	936.47	17
5	**737.41**	369.21	720.38	360.70			I	1,742.87	871.94	1,725.85	863.43	1,724.86	862.94	16
6	**836.48**	418.74	819.45	410.23			V	1,629.79	815.40	1,612.76	806.89	1,611.78	806.39	15
7	**949.56**	475.29	**932.54**	466.77			L	1,530.72	765.86	1,513.70	757.35	1,512.71	756.86	14
8	1,050.61	525.81	1,033.58	517.30	1,032.60	516.80	T	**1,417.64**	709.32	1,400.61	700.81	1,399.63	700.32	13
9	1,151.66	576.33	1,134.63	567.82	1,133.65	567.33	T	**1,316.59**	658.80	1,299.56	650.29	1,298.58	649.79	12
10	**1,238.69**	619.85	1,221.66	611.34	1,220.68	610.84	S	**1,215.54**	608.27	1,198.52	599.76	1,197.53	599.27	11
11	**1,309.73**	655.37	1,292.70	646.85	1,291.72	646.36	A	**1,128.51**	564.76	1,111.48	556.25	1,110.50	555.75	10
12	1,366.75	683.88	1,349.72	675.36	1,348.74	674.87	G	**1,057.47**	529.24	1,040.45	520.73	1,039.46	520.23	9
13	1,479.83	740.42	1,462.81	731.91	1,461.82	731.41	I	1,000.45	500.73	983.42	492.22	982.44	491.72	8
14	1,610.87	805.94	1,593.85	797.43	1,592.86	796.94	M	**887.37**	444.19	870.34	435.67	869.36	435.18	7
15	1,725.90	863.45	1,708.87	854.94	1,707.89	854.45	D	756.33	378.67	739.30	370.15	738.32	369.66	6
16	1,862.96	931.98	1,845.93	923.47	1,844.95	922.98	H	**641.30**	321.15	**624.27**	312.64	623.29	312.15	5
17	1,992.00	996.50	1,974.98	987.99	1,973.99	987.50	E	504.24	252.62	487.21	244.11	486.23	243.62	4
18	2,121.04	1,061.03	2,104.02	1,052.51	2,103.03	1,052.02	E	**375.20**	188.10	358.17	179.59	357.19	179.10	3
19	2,192.08	1,096.54	2,175.05	1,088.03	2,174.07	1,087.54	A	**246.16**	123.58	229.13	115.07			2
20							R	175.12	88.06	158.09	79.55			1

Query 2175 Hit 1

MS/MS Fragmentation of **FVSSPQTIVELFFQEVAR**
Found in **sp|Q12769|NU160_HUMAN**, Nuclear pore complex protein Nup160 OS=Homo sapiens GN=NUP160 PE=1 SV=3
Match to Query 2175: 2240.198from(747.7398,3+)
Title: 1427: Scan 3471 (rt=86.403, f=3, i=521) [D:\lab212\membrane\Grace\20120214_iTRAQ_2.raw]
Data File:
Monoisotopic mass of neutral peptide Mr(calc): 2240.198
Variable modifications:
Ions Score: 44.62 **Expect:** 0.029

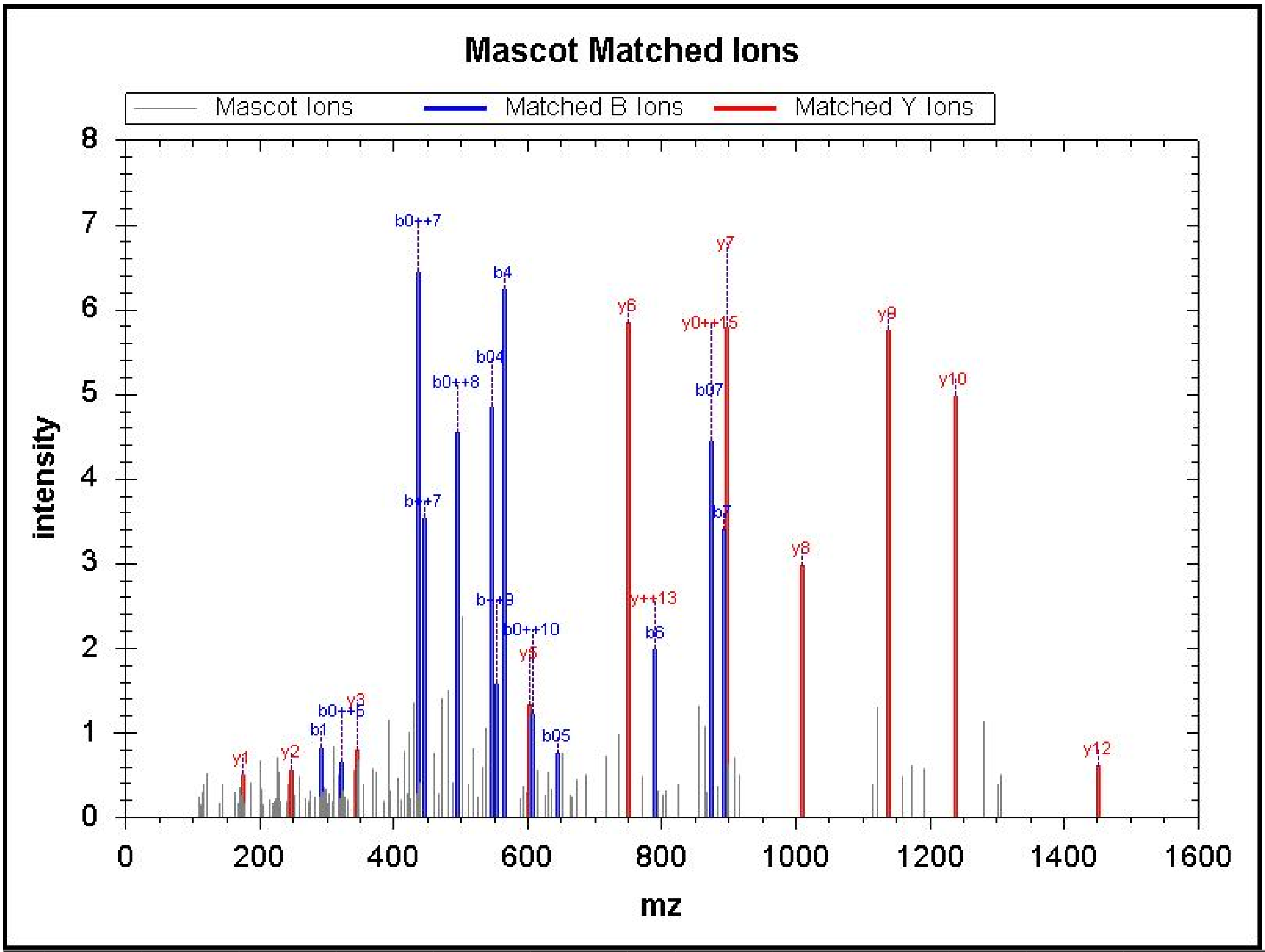

No	b	b++	b*	b*++	b0	b0++	Seq	y	y++	y*	y*++	y0	y0++	RevNo
1	**292.18**	146.59					F							18
2	391.25	196.13					V	1,950.03	975.52	1,933.01	967.01	1,932.02	966.51	17
3	478.28	239.64			460.27	230.64	S	1,850.96	925.99	1,833.94	917.47	1,832.95	916.98	16
4	**565.31**	283.16			**547.30**	274.15	S	1,763.93	882.47	1,746.91	873.96	1,745.92	**873.46**	15
5	662.36	331.69			**644.35**	**322.68**	P	1,676.90	838.95	1,659.87	830.44	1,658.89	829.95	14
6	**790.42**	395.71	773.40	387.20	772.41	386.71	Q	1,579.85	**790.43**	1,562.82	781.91	1,561.84	781.42	13
7	**891.47**	**446.24**	874.44	437.72	**873.46**	**437.23**	T	**1,451.79**	726.40	1,434.76	717.89	1,433.78	717.39	12
8	1,004.55	502.78	987.53	494.27	986.54	**493.77**	I	1,350.74	675.87	1,333.72	667.36	1,332.73	666.87	11
9	1,103.62	**552.31**	1,086.60	543.80	1,085.61	543.31	V	**1,237.66**	619.33	1,220.63	610.82	1,219.65	610.33	10
10	1,232.66	616.84	1,215.64	608.32	1,214.65	**607.83**	E	**1,138.59**	569.80	1,121.56	561.28	1,120.58	560.79	9
11	1,345.75	673.38	1,328.72	664.86	1,327.74	664.37	L	**1,009.55**	505.28	992.52	496.76	991.54	496.27	8
12	1,492.82	746.91	1,475.79	738.40	1,474.81	737.91	F	**896.46**	448.73	879.44	440.22	878.45	439.73	7
13	1,639.89	820.45	1,622.86	811.93	1,621.87	811.44	F	**749.39**	375.20	732.37	366.69	731.38	366.20	6
14	1,767.94	884.48	1,750.92	875.96	1,749.93	875.47	Q	**602.33**	301.67	585.30	293.15	584.32	292.66	5
15	1,896.99	949.00	1,879.96	940.48	1,878.98	939.99	E	474.27	237.64	457.24	229.12	456.26	228.63	4
16	1,996.05	998.53	1,979.03	990.02	1,978.04	989.53	V	**345.22**	173.12	328.20	164.60			3
17	2,067.09	1,034.05	2,050.07	1,025.54	2,049.08	1,025.04	A	**246.16**	123.58	229.13	115.07			2
18							R	**175.12**	88.06	158.09	79.55			1

Query 1942 Hit 1

MS/MS Fragmentation of **VAIIEELVVGYETSLK**
Found in **sp|Q9BXJ9|NAA15_HUMAN**, N-alpha-acetyltransferase 15
Match to Query 1942: 2050.174from(684.3984,3+)
Title: 1209: Scan 3011 (rt=76.0182, f=3, i=427) [D:\lab212\membrane\Grace\20120214_iTRAQ_1.raw]

Data File:
Monoisotopic mass of neutral peptide Mr(calc): 2050.174
Variable modifications:
K16 :iTRAQ4plex (K)
Ions Score: 44.97 **Expect:** 0.016

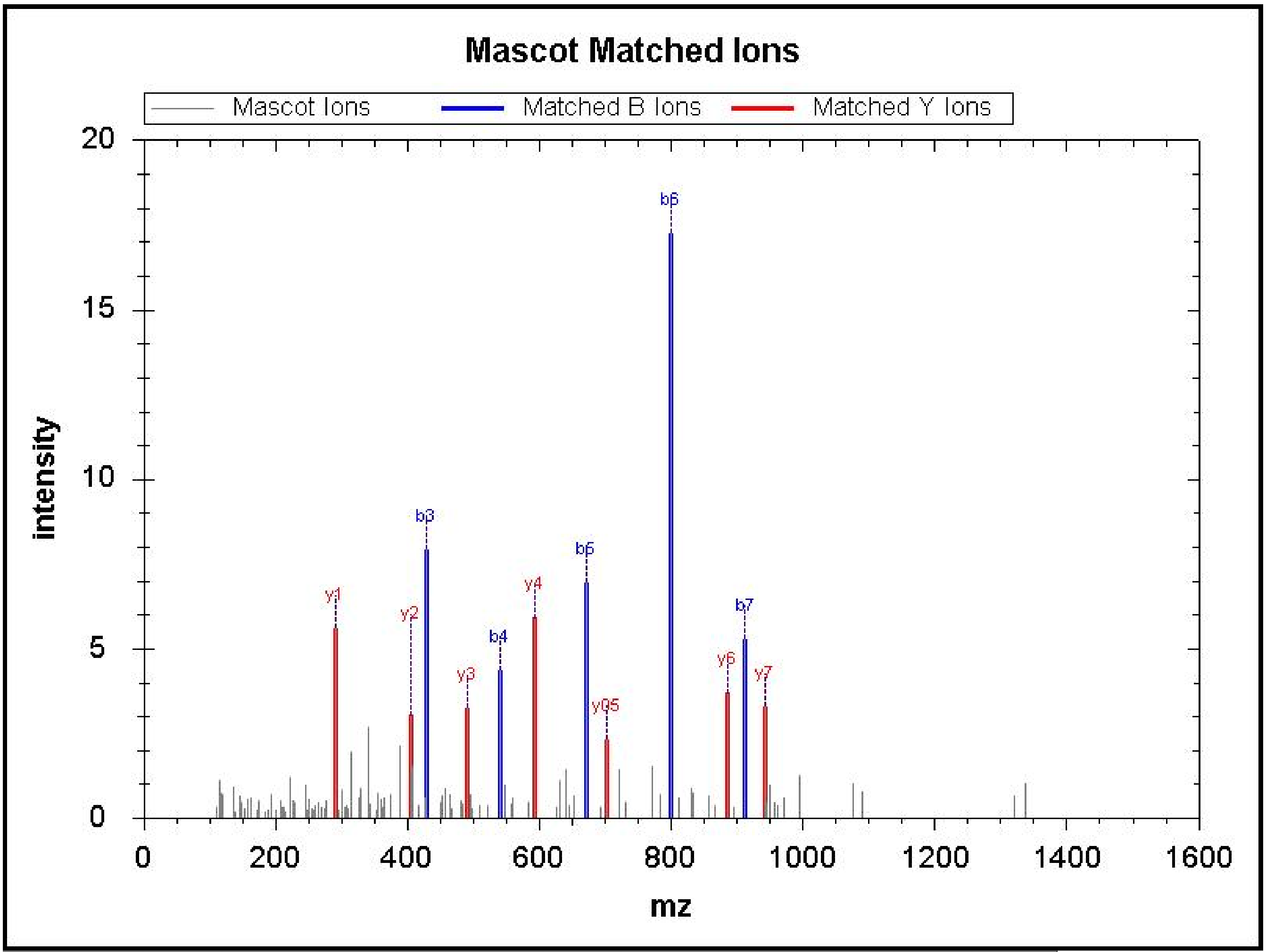

No	b	b++	b0	b0++	Seq	y	y++	y*	y*++	y0	y0++	RevNo
1	244.18	122.59			V							16
2	315.21	158.11			A	1,808.02	904.51	1,790.99	896.00	1,790.01	895.51	15
3	428.30	214.65			I	1,736.98	868.99	1,719.95	860.48	1,718.97	859.99	14
4	541.38	271.20			I	1,623.90	812.45	1,606.87	803.94	1,605.89	803.45	13
5	670.43	335.72	652.41	326.71	E	1,510.81	755.91	1,493.79	747.40	1,492.80	746.90	12
6	799.47	400.24	781.46	391.23	E	1,381.77	691.39	1,364.74	682.88	1,363.76	682.38	11
7	912.55	456.78	894.54	447.77	L	1,252.73	626.87	1,235.70	618.35	1,234.72	617.86	10
8	1,011.62	506.31	993.61	497.31	V	1,139.64	570.33	1,122.62	561.81	1,121.63	561.32	9
9	1,110.69	555.85	1,092.68	546.84	V	1,040.57	520.79	1,023.55	512.28	1,022.56	511.79	8
10	1,167.71	584.36	1,149.70	575.35	G	941.51	471.26	924.48	462.74	923.50	462.25	7
11	1,330.77	665.89	1,312.76	656.89	Y	884.48	442.75	867.46	434.23	866.47	433.74	6
12	1,459.82	730.41	1,441.81	721.41	E	721.42	361.21	704.39	352.70	703.41	352.21	5
13	1,560.86	780.94	1,542.85	771.93	T	592.38	296.69	575.35	288.18	574.37	287.69	4
14	1,647.90	824.45	1,629.89	815.45	S	491.33	246.17	474.30	237.66	473.32	237.16	3
15	1,760.98	880.99	1,742.97	871.99	L	404.30	202.65	387.27	194.14			2
16					K	291.21	146.11	274.19	137.60			1

Query 1778 Hit 1

MS/MS Fragmentation of **DISEASVFDAYVLPK**

Found in sp|P62854|RS26_HUMAN, 40S ribosomal protein S26 OS=Homo sapiens GN=RPS26 PE=1 SV=3
Match to Query 1778: 1941.03from(648.0173,3+)
Title: 810: Scan 2173 (rt=57.1326, f=2, i=340) [D:\lab212\membrane\Grace\20120214_iTRAQ_1.raw]
Data File:
Monoisotopic mass of neutral peptide Mr(calc): 1941.03
Variable modifications:
K15 :iTRAQ4plex (K)
Ions Score: 45.07 **Expect:** 0.027

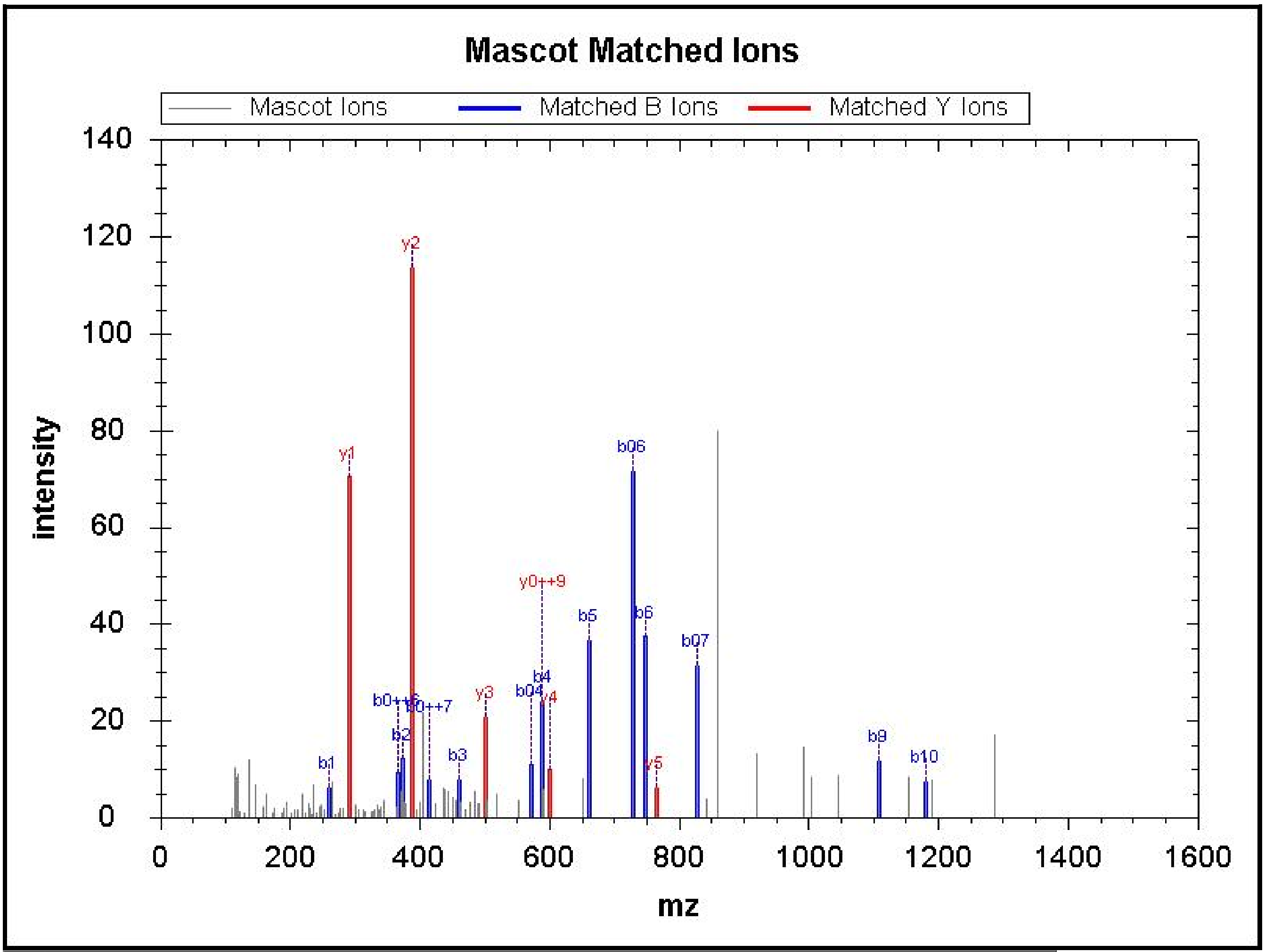

No	b	b++	b0	b0++	Seq	y	y++	y*	y*++	y0	y0++	RevNo
1	260.14	130.57	242.13	121.57	D							15
2	373.22	187.11	355.21	178.11	I	1,682.91	841.96	1,665.89	833.45	1,664.90	832.95	14
3	460.25	230.63	442.24	221.62	S	1,569.83	785.42	1,552.80	776.90	1,551.82	776.41	13
4	589.29	295.15	571.28	286.15	E	1,482.80	741.90	1,465.77	733.39	1,464.79	732.90	12
5	660.33	330.67	642.32	321.66	A	1,353.75	677.38	1,336.73	668.87	1,335.74	668.38	11
6	747.36	374.19	729.35	365.18	S	1,282.72	641.86	1,265.69	633.35	1,264.71	632.86	10
7	846.43	423.72	828.42	414.71	V	1,195.68	598.35	1,178.66	589.83	1,177.67	589.34	9
8	993.50	497.25	975.49	488.25	F	1,096.62	548.81	1,079.59	540.30	1,078.61	539.81	8
9	1,108.53	554.77	1,090.52	545.76	D	949.55	475.28	932.52	466.76	931.54	466.27	7
10	1,179.57	590.29	1,161.55	581.28	A	834.52	417.76	817.49	409.25			6
11	1,342.63	671.82	1,324.62	662.81	Y	763.48	382.25	746.46	373.73			5
12	1,441.70	721.35	1,423.69	712.35	V	600.42	300.71	583.39	292.20			4
13	1,554.78	777.89	1,536.77	768.89	L	501.35	251.18	484.33	242.67			3
14	1,651.83	826.42	1,633.82	817.42	P	388.27	194.64	371.24	186.12			2
15					K	291.21	146.11	274.19	137.60			1

Query 130 Hit 1

MS/MS Fragmentation of **SGEIINK**
Found in **sp|Q8R4U0|STAB2_MOUSE**, Stabilin-2 OS=Mus musculus GN=Stab2 PE=1 SV=1
Match to Query 130: 904.5448 from(453.2797,2+)
Title: 243: Scan 989 (rt=30.4532, f=2, i=149) [D:\lab212\membrane\Grace\20120214_iTRAQ_1.raw]
Data File:
Monoisotopic mass of neutral peptide Mr(calc): 904.5448
Variable modifications:
N6 :Deamidated (NQ)
K7 :iTRAQ4plex (K)
Ions Score: 45.9 **Expect:** 0.022

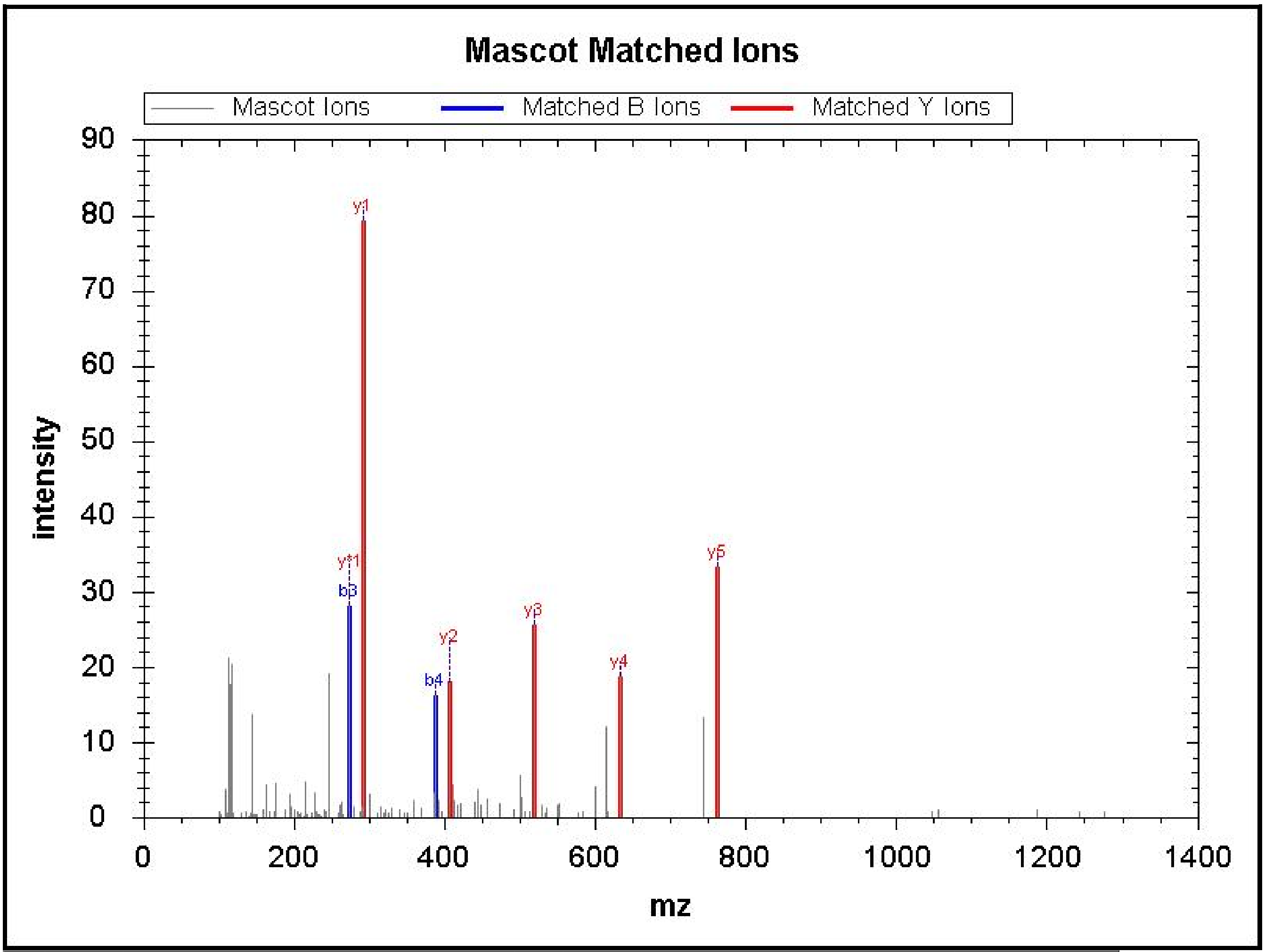

No	b	b++	b*	b*++	b0	b0++	Seq	y	y++	y*	y*++	y0	y0++	RevNo
1	88.04	44.52			70.03	35.52	S							7
2	145.06	73.03			127.05	64.03	G	818.47	409.74	801.45	401.23	800.46	400.74	6
3	274.10	137.56			256.09	128.55	E	761.45	381.23	744.43	372.72	743.44	372.22	5
4	387.19	194.10			369.18	185.09	I	632.41	316.71	615.38	308.20			4
5	500.27	250.64			482.26	241.63	I	519.33	260.17	502.30	251.65			3
6	615.30	308.15	598.27	299.64	597.29	299.15	N	406.24	203.62	389.22	195.11			2
7							K	291.21	146.11	274.19	137.60			1

Query 40 Hit 1

MS/MS Fragmentation of **GSINLKR**
Found in **sp|Q7Z5Q5|DPOLN_HUMAN**, DNA polymerase nu OS=Homo sapiens GN=POLN PE=1 SV=2
Match to Query 40: 787.4691 from(394.7418,2+)
Title: 314: Scan 1094 (rt=32.9043, f=2, i=166) [D:\lab212\membrane\Grace\20120214_iTRAQ_2.raw]
Data File:
Monoisotopic mass of neutral peptide Mr(calc): 787.4691
Variable modifications:
N4 :Deamidated (NQ)

Ions Score: 46.18 **Expect:** 0.017

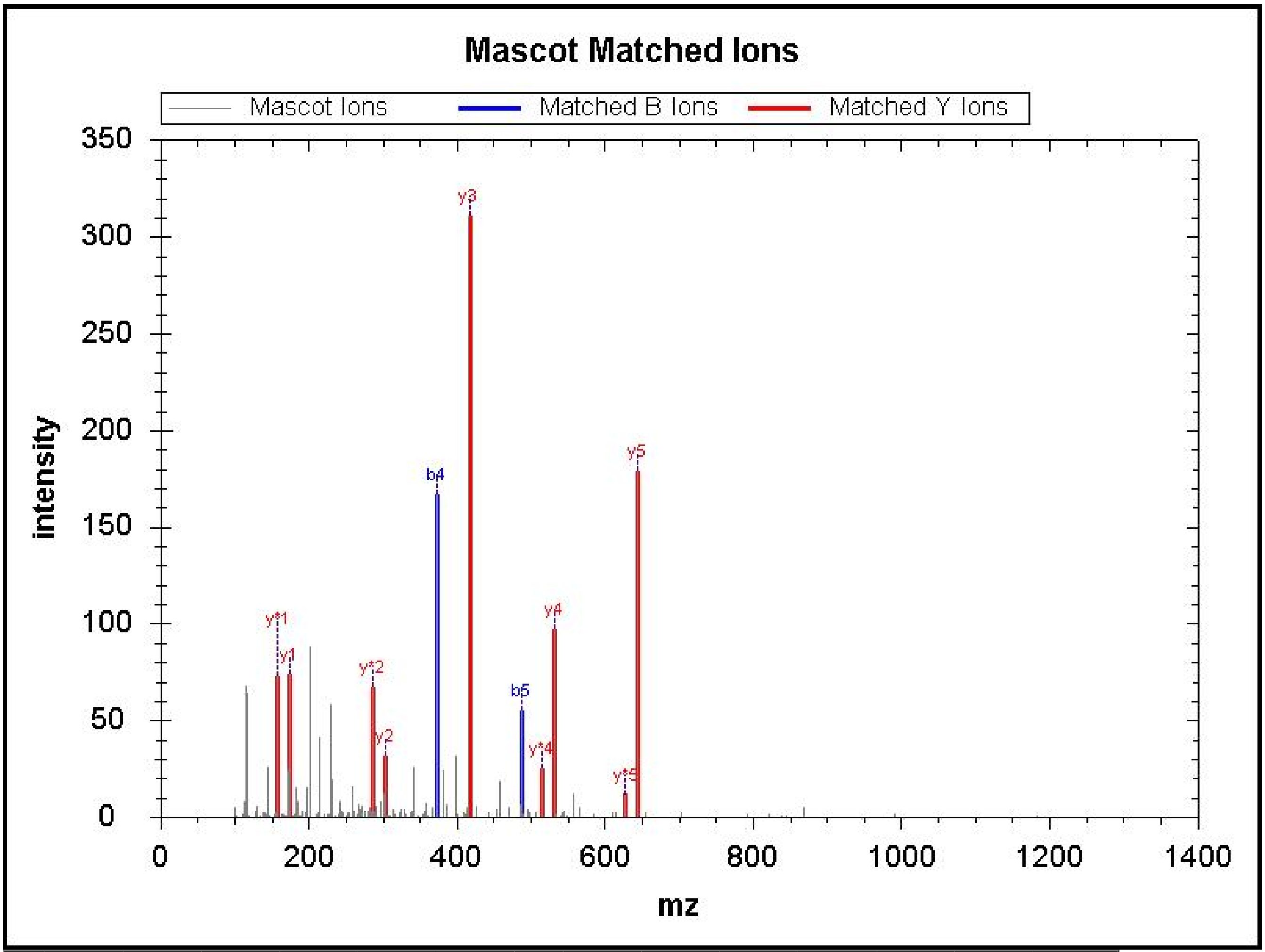

No	b	b++	b*	b*++	b0	b0++	Seq	y	y++	y*	y*++	y0	y0++	RevNo
1	58.03	29.52					G							7
2	145.06	73.03			127.05	64.03	S	731.44	366.22	714.41	357.71	713.43	357.22	6
3	258.14	129.58			240.13	120.57	I	**644.41**	322.71	**627.38**	314.19			5
4	**373.17**	187.09	356.15	178.58	355.16	178.08	N	**531.32**	266.17	**514.30**	257.65			4
5	**486.26**	243.63	469.23	235.12	468.25	234.63	L	**416.30**	208.65	399.27	200.14			3
6	614.35	307.68	597.32	299.17	596.34	298.67	K	**303.21**	152.11	**286.19**	143.60			2
7							R	**175.12**	88.06	**158.09**	79.55			1

Query 2457 Hit 1

MS/MS Fragmentation of **KEELMFFLWAPELAPLK**
Found in **sp|P60981|DEST_HUMAN**, Destrin OS=Homo sapiens GN=DSTN PE=1 SV=3
Match to Query 2457: 2493.401 from(832.1409,3+)
Title: 1152: Scan 2887 (rt=73.224, f=3, i=407) [D:\lab212\membrane\Grace\20120214_iTRAQ_1.raw]
Data File:
Monoisotopic mass of neutral peptide Mr(calc): 2493.401
Variable modifications:
K1 :iTRAQ4plex (K)
K17 :iTRAQ4plex (K)
Ions Score: 46.45 **Expect:** 0.010

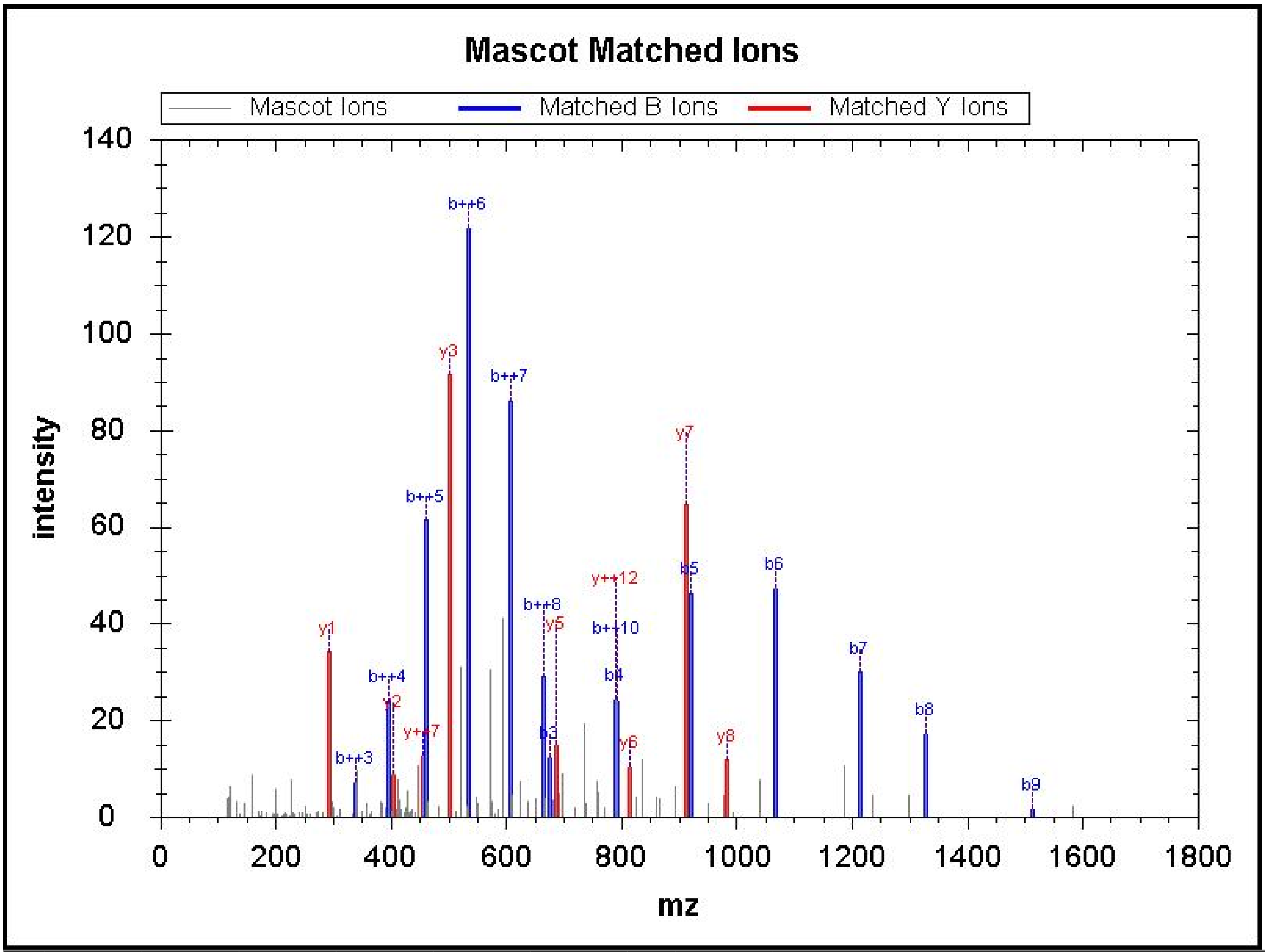

No	b	b++	b*	b*++	b0	b0++	Seq	y	y++	y*	y*++	y0	y0++	RevNo
1	417.31	209.16	400.28	200.64			K							17
2	546.35	273.68	529.32	265.16	528.34	264.67	E	2,078.12	1,039.56	2,061.09	1,031.05	2,060.10	1,030.56	16
3	675.39	338.20	658.36	329.69	657.38	329.19	E	1,949.07	975.04	1,932.05	966.53	1,931.06	966.03	15
4	788.48	394.74	771.45	386.23	770.47	385.74	L	1,820.03	910.52	1,803.00	902.01	1,802.02	901.51	14
5	919.52	460.26	902.49	451.75	901.51	451.26	M	1,706.95	853.98	1,689.92	845.46	1,688.94	844.97	13
6	1,066.58	533.80	1,049.56	525.28	1,048.57	524.79	F	1,575.91	788.46	1,558.88	779.94	1,557.89	779.45	12
7	1,213.65	607.33	1,196.63	598.82	1,195.64	598.32	F	1,428.84	714.92	1,411.81	706.41	1,410.83	705.92	11
8	1,326.74	663.87	1,309.71	655.36	1,308.73	654.87	L	1,281.77	641.39	1,264.74	632.87	1,263.76	632.38	10
9	1,512.82	756.91	1,495.79	748.40	1,494.81	747.91	W	1,168.68	584.85	1,151.66	576.33	1,150.67	575.84	9
10	1,583.85	792.43	1,566.83	783.92	1,565.84	783.43	A	982.61	491.81	965.58	483.29	964.59	482.80	8
11	1,680.91	840.96	1,663.88	832.44	1,662.90	831.95	P	911.57	456.29	894.54	447.77	893.56	447.28	7
12	1,809.95	905.48	1,792.92	896.96	1,791.94	896.47	E	814.52	407.76	797.49	399.25	796.50	398.76	6
13	1,923.03	962.02	1,906.01	953.51	1,905.02	953.01	L	685.47	343.24	668.45	334.73			5
14	1,994.07	997.54	1,977.04	989.03	1,976.06	988.53	A	572.39	286.70	555.36	278.18			4
15	2,091.12	1,046.07	2,074.10	1,037.55	2,073.11	1,037.06	P	501.35	251.18	484.33	242.67			3
16	2,204.21	1,102.61	2,187.18	1,094.09	2,186.20	1,093.60	L	404.30	202.65	387.27	194.14			2
17							K	291.21	146.11	274.19	137.60			1

Query 2495 Hit 1

MS/MS Fragmentation of **EAPFVPVGIAGFAAIVAYGLYK**
Found in **sp|Q9Y241|HIG1A_HUMAN**, HIG1 domain family member 1A OS=Homo sapiens GN=HIGD1A PE=1 SV=1
Match to Query 2495: 2540.418from(847.8133,3+)
Title: 1410: Sum of 2 scans in range 3420 (rt=85.2935, f=4, i=939) to 3421 (rt=85.3189, f=4, i=940) [D:\lab212\membrane\Grace\20120214_iTRAQ_2.raw]
Data File:

Monoisotopic mass of neutral peptide Mr(calc): 2540.418
Variable modifications:
K22 :iTRAQ4plex (K)
Ions Score: 46.54 **Expect:** 0.011

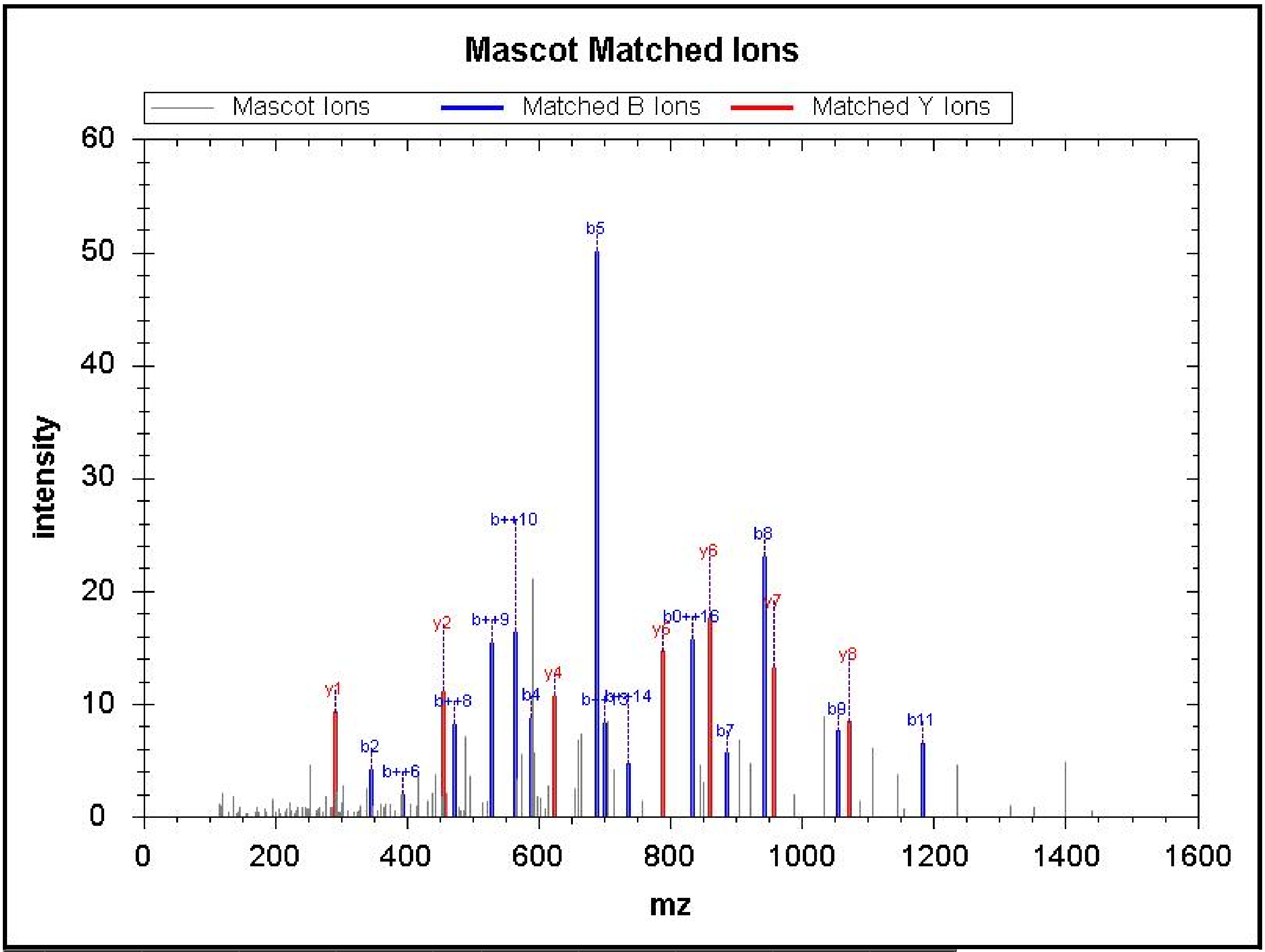

No	b	b++	b0	b0++	Seq	y	y++	y*	y*++	RevNo
1	274.15	137.58	256.14	128.57	E					22
2	345.19	173.10	327.18	164.09	A	2,268.29	1,134.65	2,251.26	1,126.14	21
3	442.24	221.62	424.23	212.62	P	2,197.25	1,099.13	2,180.23	1,090.62	20
4	589.31	295.16	571.30	286.15	F	2,100.20	1,050.60	2,083.17	1,042.09	19
5	688.38	344.69	670.37	335.69	V	1,953.13	977.07	1,936.11	968.56	18
6	785.43	393.22	767.42	384.21	P	1,854.06	927.54	1,837.04	919.02	17
7	884.50	442.75	866.49	433.75	V	1,757.01	879.01	1,739.99	870.50	16
8	941.52	471.26	923.51	462.26	G	1,657.94	829.48	1,640.92	820.96	15
9	1,054.61	527.81	1,036.59	518.80	I	1,600.92	800.96	1,583.90	792.45	14
10	1,125.64	563.32	1,107.63	554.32	A	1,487.84	744.42	1,470.81	735.91	13
11	1,182.66	591.84	1,164.65	582.83	G	1,416.80	708.90	1,399.77	700.39	12
12	1,329.73	665.37	1,311.72	656.36	F	1,359.78	680.39	1,342.75	671.88	11
13	1,400.77	700.89	1,382.76	691.88	A	1,212.71	606.86	1,195.68	598.35	10
14	1,471.81	736.41	1,453.80	727.40	A	1,141.67	571.34	1,124.65	562.83	9
15	1,584.89	792.95	1,566.88	783.94	I	1,070.64	535.82	1,053.61	527.31	8
16	1,683.96	842.48	1,665.95	833.48	V	957.55	479.28	940.53	470.77	7
17	1,755.00	878.00	1,736.99	869.00	A	858.48	429.75	841.46	421.23	6
18	1,918.06	959.53	1,900.05	950.53	Y	787.45	394.23	770.42	385.71	5
19	1,975.08	988.04	1,957.07	979.04	G	624.38	312.70	607.36	304.18	4
20	2,088.17	1,044.59	2,070.15	1,035.58	L	567.36	284.18	550.34	275.67	3

21	2,251.23	1,126.12	2,233.22	1,117.11	Y	454.28	227.64	437.25	219.13	2
22					K	291.21	146.11	274.19	137.60	1

Query 2219 Hit 1

MS/MS Fragmentation of **DKSFLAMVVDIVQELK**
Found in **sp|O00764|PDXK_HUMAN**, Pyridoxal kinase OS=Homo sapiens GN=PDXK PE=1 SV=1
Match to Query 2219: 2266.288from(756.4368,3+)
Title: 1408: Sum of 2 scans in range 3475 (rt=86.446, f=3, i=504) to 3476 (rt=86.4714, f=3, i=505) [D:\lab212\membrane\Grace\20120214_iTRAQ_1.raw]
Data File:
Monoisotopic mass of neutral peptide Mr(calc): 2266.288
Variable modifications:
K2 :iTRAQ4plex (K)
K16 :iTRAQ4plex (K)
Ions Score: 47.16 **Expect:** 0.008

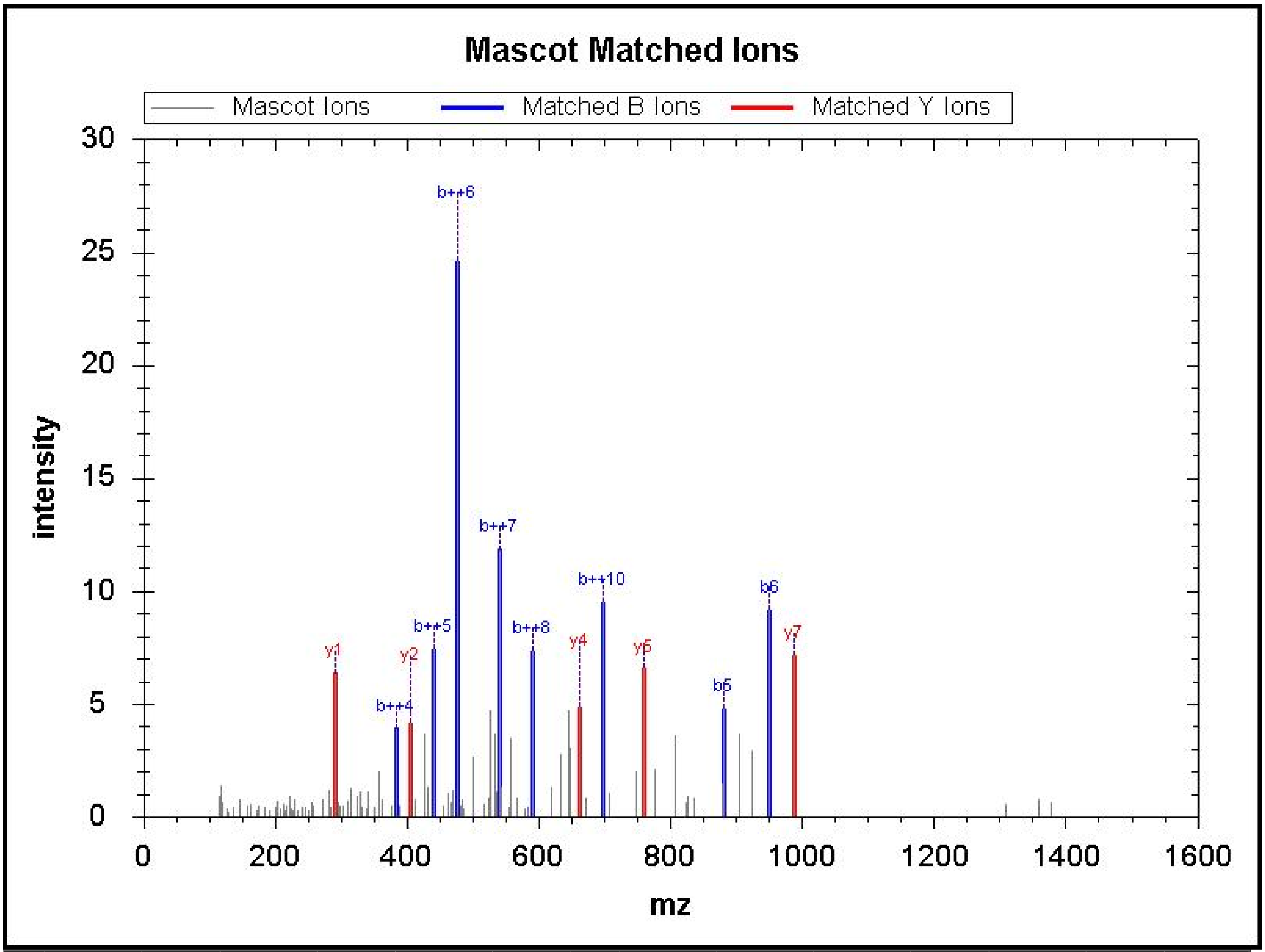

No	b	b++	b*	b*++	b0	b0++	Seq	y	y++	y*	y*++	y0	y0++	RevNo
1	260.14	130.57			242.13	121.57	D							16
2	532.33	266.67	515.31	258.16	514.32	257.67	K	2,008.18	1,004.59	1,991.15	996.08	1,990.16	995.59	15
3	619.37	310.19	602.34	301.67	601.35	301.18	S	1,735.98	868.49	1,718.95	859.98	1,717.97	859.49	14
4	766.43	383.72	749.41	375.21	748.42	374.72	F	1,648.95	824.98	1,631.92	816.46	1,630.94	815.97	13
5	879.52	440.26	862.49	431.75	861.51	431.26	L	1,501.88	751.44	1,484.85	742.93	1,483.87	742.44	12
6	950.55	475.78	933.53	467.27	932.54	466.78	A	1,388.79	694.90	1,371.77	686.39	1,370.78	685.90	11
7	1,081.60	541.30	1,064.57	532.79	1,063.58	532.30	M	1,317.76	659.38	1,300.73	650.87	1,299.75	650.38	10
8	1,180.66	590.84	1,163.64	582.32	1,162.65	581.83	V	1,186.72	593.86	1,169.69	585.35	1,168.71	584.86	9
9	1,279.73	640.37	1,262.71	631.86	1,261.72	631.36	V	1,087.65	544.33	1,070.62	535.81	1,069.64	535.32	8
10	1,394.76	697.88	1,377.73	689.37	1,376.75	688.88	D	988.58	494.79	971.55	486.28	970.57	485.79	7
11	1,507.84	754.43	1,490.82	745.91	1,489.83	745.42	I	873.55	437.28	856.53	428.77	855.54	428.27	6

**12**	1,606.91	803.96	1,589.89	795.45	1,588.90	794.95	**V**	**760.47**	380.74	743.44	372.22	742.46	371.73	**5**
**13**	1,734.97	867.99	1,717.94	859.48	1,716.96	858.98	**Q**	**661.40**	331.20	644.37	322.69	643.39	322.20	**4**
**14**	1,864.01	932.51	1,846.99	924.00	1,846.00	923.50	**E**	533.34	267.17	516.31	258.66	515.33	258.17	**3**
**15**	1,977.10	989.05	1,960.07	980.54	1,959.09	980.05	**L**	**404.30**	202.65	387.27	194.14			**2**
**16**							**K**	**291.21**	146.11	274.19	137.60			**1**

Query 1862 Hit 1

MS/MS Fragmentation of **VQALTTDISLIFAALK**
Found in **sp|Q6PKG0|LARP1_HUMAN**, La-related protein 1 OS=Homo sapiens GN=LARP1 PE=1 SV=2
Match to Query 1862: 1991.196from(664.7392,3+)
Title: 1382: Scan 3352 (rt=83.7774, f=2, i=532) [D:\lab212\membrane\Grace\20120214_iTRAQ_2.raw]
Data File:
Monoisotopic mass of neutral peptide Mr(calc): 1991.196
Variable modifications:
K16 :iTRAQ4plex (K)
Ions Score: 47.54 **Expect:** 0.005

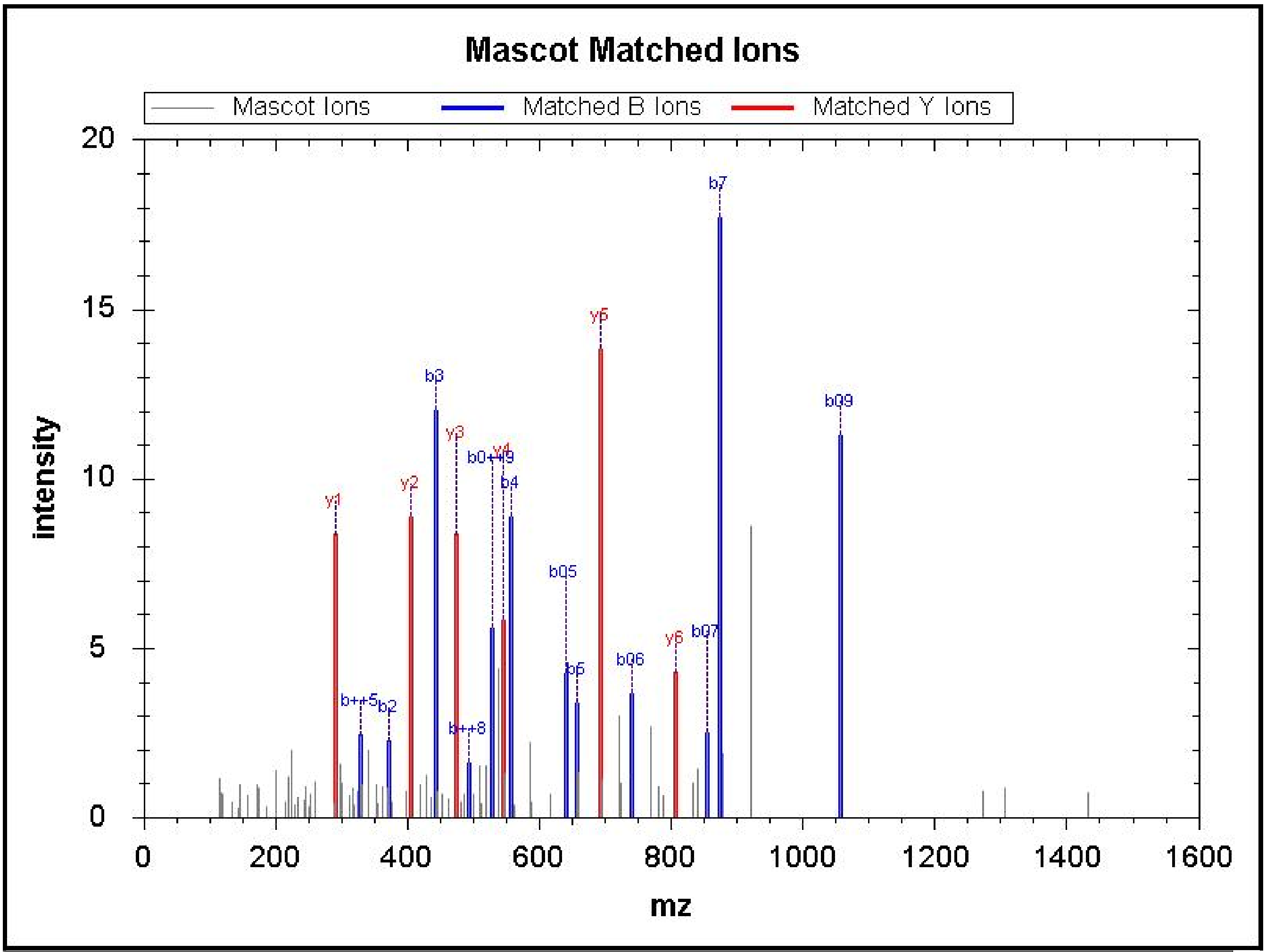

No	b	b++	b*	b*++	b0	b0++	Seq	y	y++	y*	y*++	y0	y0++	RevNo
**1**	244.18	122.59					**V**							**16**
**2**	**372.24**	186.62	355.21	178.11			**Q**	1,749.03	875.02	1,732.00	866.50	1,731.02	866.01	**15**
**3**	**443.27**	222.14	426.25	213.63			**A**	1,620.97	810.99	1,603.94	802.48	1,602.96	801.98	**14**
**4**	**556.36**	278.68	539.33	270.17			**L**	1,549.93	775.47	1,532.91	766.96	1,531.92	766.46	**13**
**5**	**657.41**	**329.21**	640.38	320.69	**639.39**	320.20	**T**	1,436.85	718.93	1,419.82	710.41	1,418.84	709.92	**12**
**6**	758.45	379.73	741.43	371.22	**740.44**	370.72	**T**	1,335.80	668.40	1,318.77	659.89	1,317.79	659.40	**11**
**7**	**873.48**	437.24	856.45	428.73	**855.47**	428.24	**D**	1,234.75	617.88	1,217.73	609.37	1,216.74	608.87	**10**
**8**	986.56	**493.79**	969.54	485.27	968.55	484.78	**I**	1,119.73	560.37	1,102.70	551.85	1,101.72	551.36	**9**
**9**	1,073.60	537.30	1,056.57	528.79	**1,055.59**	**528.30**	**S**	1,006.64	503.82	989.62	495.31	988.63	494.82	**8**

10	1,186.68	593.84	1,169.65	585.33	1,168.67	584.84	L	919.61	460.31	902.58	451.80			7
11	1,299.76	650.39	1,282.74	641.87	1,281.75	641.38	I	806.53	403.77	789.50	395.25			6
12	1,446.83	723.92	1,429.81	715.41	1,428.82	714.91	F	693.44	347.22	676.41	338.71			5
13	1,517.87	759.44	1,500.84	750.93	1,499.86	750.43	A	546.37	273.69	529.35	265.18			4
14	1,588.91	794.96	1,571.88	786.44	1,570.90	785.95	A	475.34	238.17	458.31	229.66			3
15	1,701.99	851.50	1,684.96	842.99	1,683.98	842.49	L	404.30	202.65	387.27	194.14			2
16							K	291.21	146.11	274.19	137.60			1

Query 2827 Hit 1

MS/MS Fragmentation of **KLEAADLVIFQFPLQWFGVPAILK**
Found in **sp|P15559|NQO1_HUMAN**, NAD(P)H dehydrogenase [quinone] 1 OS=Homo sapiens GN=NQO1 PE=1 SV=1
Match to Query 2827: 3174.85from(794.7199,4+)
Title: 1393: Sum of 2 scans in range 3429 (rt=85.4304, f=4, i=956) to 3430 (rt=85.4558, f=4, i=957) [D:\lab212\membrane\Grace\20120214_iTRAQ_1.raw]
Data File:
Monoisotopic mass of neutral peptide Mr(calc): 3174.85
Variable modifications:
K1 :iTRAQ4plex (K)
K24 :iTRAQ4plex (K)
Ions Score: 47.58 **Expect:** 0.002

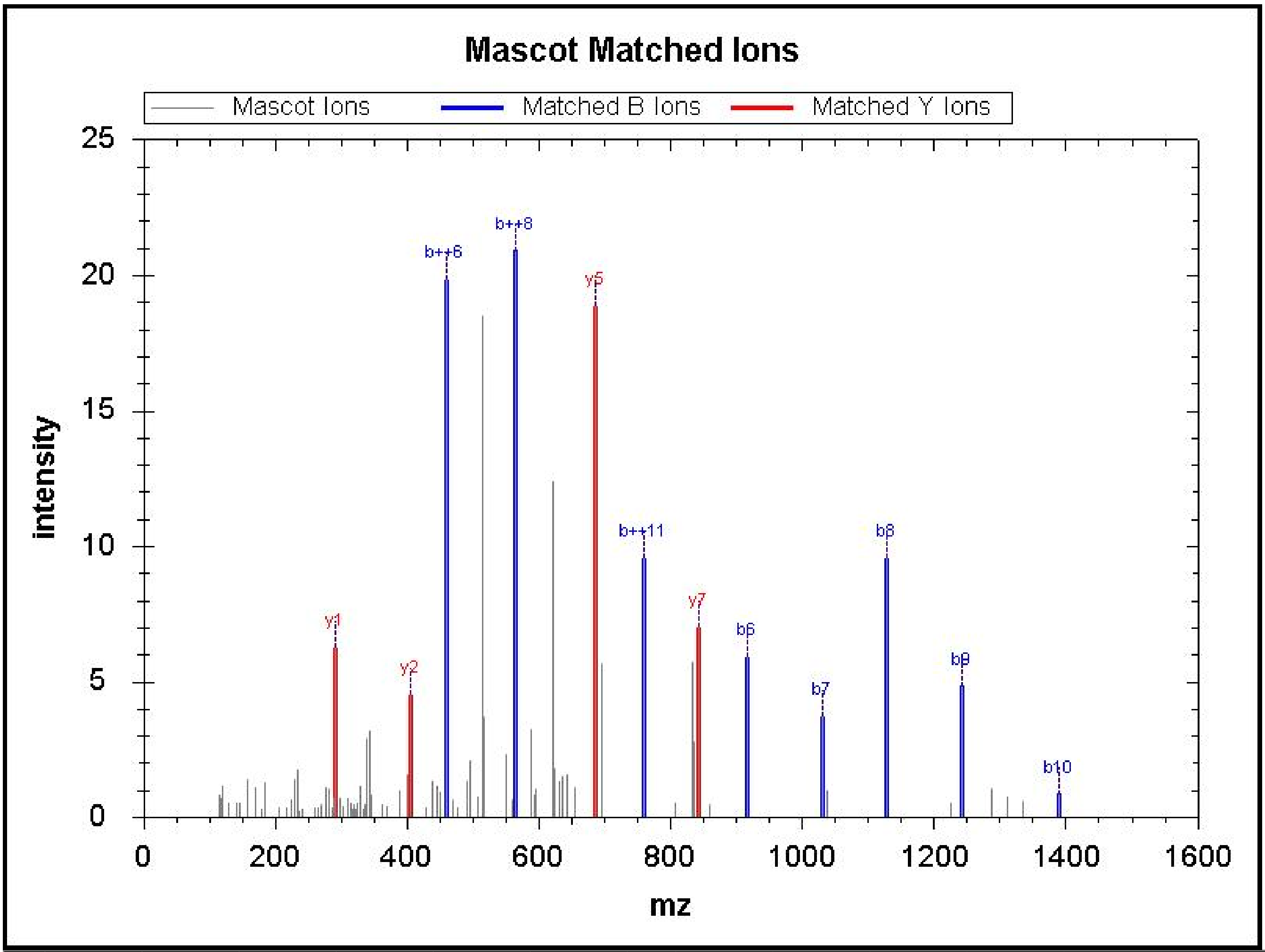

No	b	b++	b*	b*++	b0	b0++	Seq	y	y++	y*	y*++	y0	y0++	RevNo
1	417.31	209.16	400.28	200.64			K							24
2	530.39	265.70	513.36	257.19			L	2,759.57	1,380.29	2,742.54	1,371.77	2,741.55	1,371.28	23
3	659.43	330.22	642.41	321.71	641.42	321.21	E	2,646.48	1,323.74	2,629.45	1,315.23	2,628.47	1,314.74	22
4	730.47	365.74	713.44	357.23	712.46	356.73	A	2,517.44	1,259.22	2,500.41	1,250.71	2,499.43	1,250.22	21
5	801.51	401.26	784.48	392.74	783.50	392.25	A	2,446.40	1,223.70	2,429.38	1,215.19	2,428.39	1,214.70	20
6	916.53	458.77	899.51	450.26	898.52	449.77	D	2,375.36	1,188.19	2,358.34	1,179.67	2,357.35	1,179.18	19

7	1,029.62	515.31	1,012.59	506.80	1,011.61	506.31	L	2,260.34	1,130.67	2,243.31	1,122.16			18
8	1,128.69	564.85	1,111.66	556.33	1,110.68	555.84	V	2,147.25	1,074.13	2,130.23	1,065.62			17
9	1,241.77	621.39	1,224.74	612.88	1,223.76	612.38	I	2,048.19	1,024.60	2,031.16	1,016.08			16
10	1,388.84	694.92	1,371.81	686.41	1,370.83	685.92	F	1,935.10	968.05	1,918.07	959.54			15
11	1,516.90	758.95	1,499.87	750.44	1,498.89	749.95	Q	1,788.03	894.52	1,771.01	886.01			14
12	1,663.97	832.49	1,646.94	823.97	1,645.96	823.48	F	1,659.97	830.49	1,642.95	821.98			13
13	1,761.02	881.01	1,743.99	872.50	1,743.01	872.01	P	1,512.91	756.96	1,495.88	748.44			12
14	1,874.10	937.56	1,857.08	929.04	1,856.09	928.55	L	1,415.85	708.43	1,398.83	699.92			11
15	2,002.16	1,001.58	1,985.14	993.07	1,984.15	992.58	Q	1,302.77	651.89	1,285.74	643.37			10
16	2,188.24	1,094.62	2,171.21	1,086.11	2,170.23	1,085.62	W	1,174.71	587.86	1,157.68	579.35			9
17	2,335.31	1,168.16	2,318.28	1,159.64	2,317.30	1,159.15	F	988.63	494.82	971.60	486.31			8
18	2,392.33	1,196.67	2,375.30	1,188.16	2,374.32	1,187.66	G	841.56	421.28	824.54	412.77			7
19	2,491.40	1,246.20	2,474.37	1,237.69	2,473.39	1,237.20	V	784.54	392.77	767.51	384.26			6
20	2,588.45	1,294.73	2,571.43	1,286.22	2,570.44	1,285.72	P	685.47	343.24	668.45	334.73			5
21	2,659.49	1,330.25	2,642.46	1,321.73	2,641.48	1,321.24	A	588.42	294.71	571.39	286.20			4
22	2,772.57	1,386.79	2,755.55	1,378.28	2,754.56	1,377.78	I	517.38	259.20	500.36	250.68			3
23	2,885.66	1,443.33	2,868.63	1,434.82	2,867.65	1,434.33	L	404.30	202.65	387.27	194.14			2
24							K	291.21	146.11	274.19	137.60			1

Query 721 Hit 1

MS/MS Fragmentation of **LYLILDFLR**
Found in **sp|Q15418|KS6A1_HUMAN**, Ribosomal protein S6 kinase alpha-1 OS=Homo sapiens GN=RPS6KA1 PE=1 SV=2
Match to Query 721: 1308.791 from(655.4027,2+)
Title: 1277: Scan 3160 (rt=79.3751, f=3, i=451) [D:\lab212\membrane\Grace\20120214_iTRAQ_1.raw]
Data File:
Monoisotopic mass of neutral peptide Mr(calc): 1308.791
Variable modifications:
Ions Score: 48.55 **Expect:** 0.007

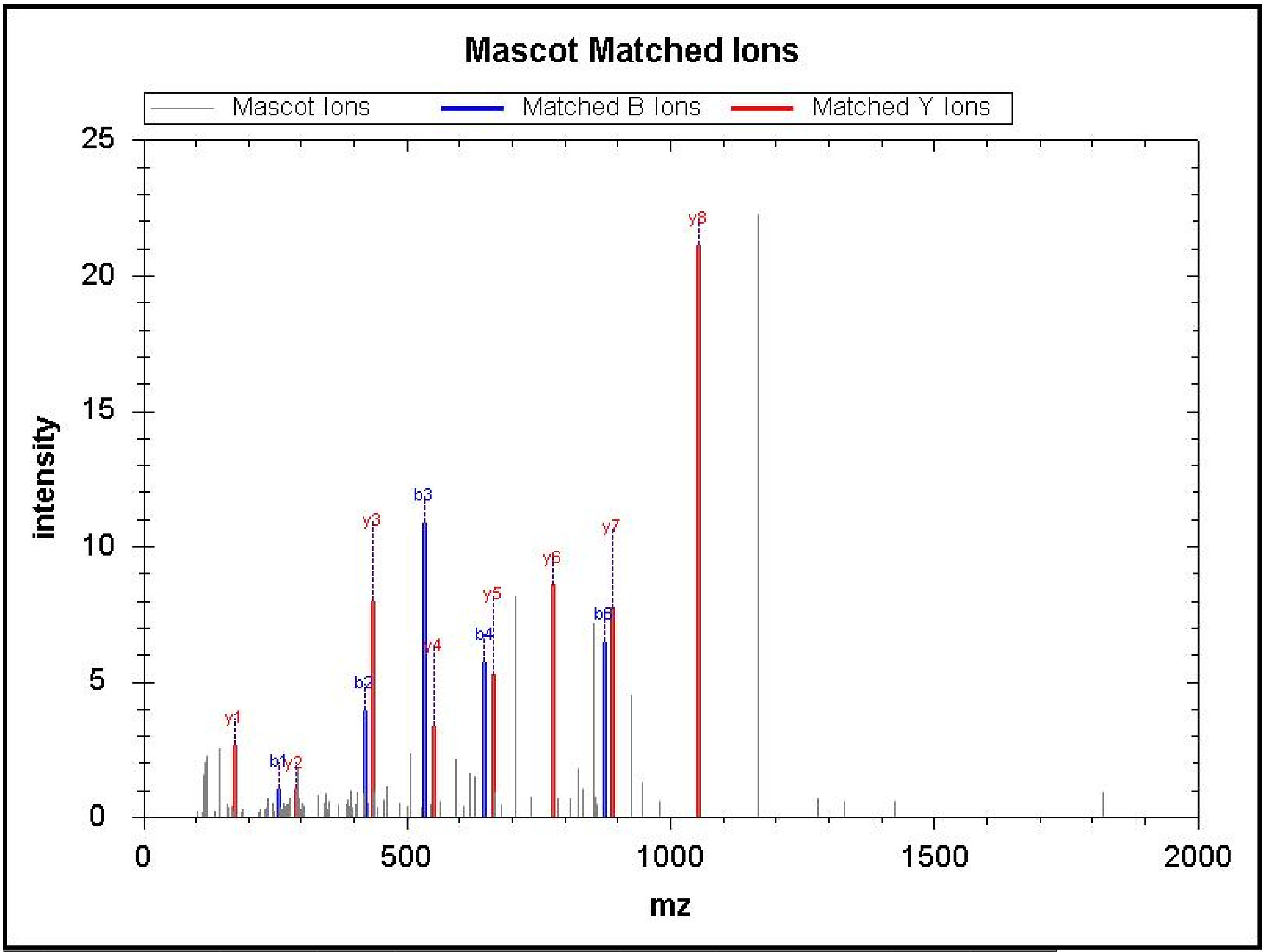

No	b	b++	b0	b0++	Seq	y	y++	y*	y*++	y0	y0++	RevNo
1	258.19	129.60			L							9
2	421.26	211.13			Y	1,052.61	526.81	1,035.59	518.30	1,034.60	517.81	8
3	534.34	267.67			L	889.55	445.28	872.52	436.77	871.54	436.27	7
4	647.42	324.22			I	776.47	388.74	759.44	380.22	758.46	379.73	6
5	760.51	380.76			L	663.38	332.19	646.36	323.68	645.37	323.19	5
6	875.54	438.27	857.53	429.27	D	550.30	275.65	533.27	267.14	532.29	266.65	4
7	1,022.60	511.81	1,004.59	502.80	F	435.27	218.14	418.24	209.63			3
8	1,135.69	568.35	1,117.68	559.34	L	288.20	144.61	271.18	136.09			2
9					R	175.12	88.06	158.09	79.55			1

Query 2156 Hit 1

MS/MS Fragmentation of **SIVSELAGLLSAMEYVQK**
Found in **sp|P40763|STAT3_HUMAN**, Signal transducer and activator of transcription 3 OS=Homo sapiens GN=STAT3 PE=1 SV=2
Match to Query 2156: 2225.225from(742.749,3+)
Title: 1430: Scan 3482 (rt=86.6492, f=3, i=524) [D:\lab212\membrane\Grace\20120214_iTRAQ_2.raw]
Data File:
Monoisotopic mass of neutral peptide Mr(calc): 2225.225
Variable modifications:
K18 :iTRAQ4plex (K)
Ions Score: 48.6 **Expect:** 0.009

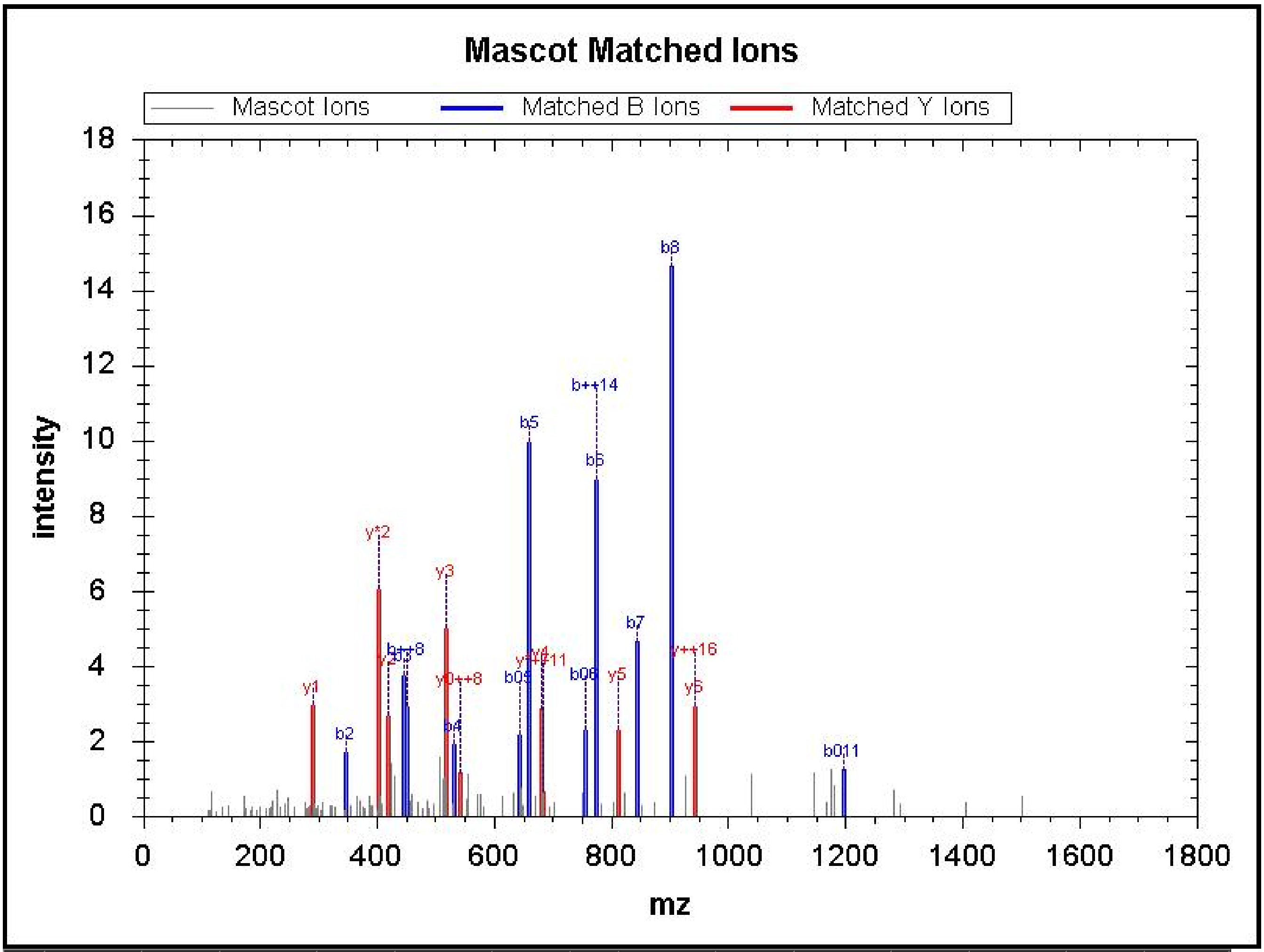

No	b	b++	b*	b*++	b0	b0++	Seq	y	y++	y*	y*++	y0	y0++	RevNo
1	232.14	116.57			214.13	107.57	S							18
2	**345.23**	173.12			327.21	164.11	I	1,995.10	998.05	1,978.07	989.54	1,977.08	989.05	17
3	**444.29**	222.65			426.28	213.65	V	1,882.01	**941.51**	1,864.98	933.00	1,864.00	932.50	16
4	**531.33**	266.17			513.32	257.16	S	1,782.94	891.98	1,765.92	883.46	1,764.93	882.97	15
5	**660.37**	330.69			**642.36**	321.68	E	1,695.91	848.46	1,678.88	839.95	1,677.90	839.45	14
6	**773.45**	387.23			**755.44**	378.22	L	1,566.87	783.94	1,549.84	775.42	1,548.86	774.93	13
7	**844.49**	422.75			826.48	413.74	A	1,453.78	727.40	1,436.76	718.88	1,435.77	718.39	12
8	**901.51**	**451.26**			883.50	442.25	G	1,382.75	691.88	1,365.72	**683.36**	1,364.74	682.87	11
9	1,014.60	507.80			996.58	498.80	L	1,325.73	663.37	1,308.70	654.85	1,307.71	654.36	10
10	1,127.68	564.34			1,109.67	555.34	L	1,212.64	606.82	1,195.61	598.31	1,194.63	597.82	9
11	1,214.71	607.86			**1,196.70**	598.85	S	1,099.56	550.28	1,082.53	541.77	1,081.55	**541.28**	8
12	1,285.75	643.38			1,267.74	634.37	A	1,012.53	506.77	995.50	498.25	994.51	497.76	7
13	1,416.79	708.90			1,398.78	699.89	M	**941.49**	471.25	924.46	462.73	923.48	462.24	6
14	1,545.83	**773.42**			1,527.82	764.41	E	**810.45**	405.73	793.42	397.21	792.44	396.72	5
15	1,708.89	854.95			1,690.88	845.95	Y	**681.41**	341.21	664.38	332.69			4
16	1,807.96	904.49			1,789.95	895.48	V	**518.34**	259.67	501.32	251.16			3
17	1,936.02	968.51	1,919.00	960.00	1,918.01	959.51	Q	**419.27**	210.14	**402.25**	201.63			2
18							K	**291.21**	146.11	274.19	137.60			1

Query 854 Hit 1

MS/MS Fragmentation of **IETIEVMEDR**
Found in **sp|P51991|ROA3_HUMAN**, Heterogeneous nuclear ribonucleoprotein A3 OS=Homo sapiens GN=HNRNPA3 PE=1 SV=2
Match to Query 854: 1377.7from(689.8572,2+)
Title: 543: Sum of 2 scans in range 1617 (rt=44.6101, f=4, i=374) to 1618 (rt=44.6355, f=4, i=375) [D:\lab212\membrane\Grace\20120214_iTRAQ_1.raw]

Data File:
Monoisotopic mass of neutral peptide Mr(calc): 1377.7
Variable modifications:
Ions Score: 49.11 **Expect:** 0.011

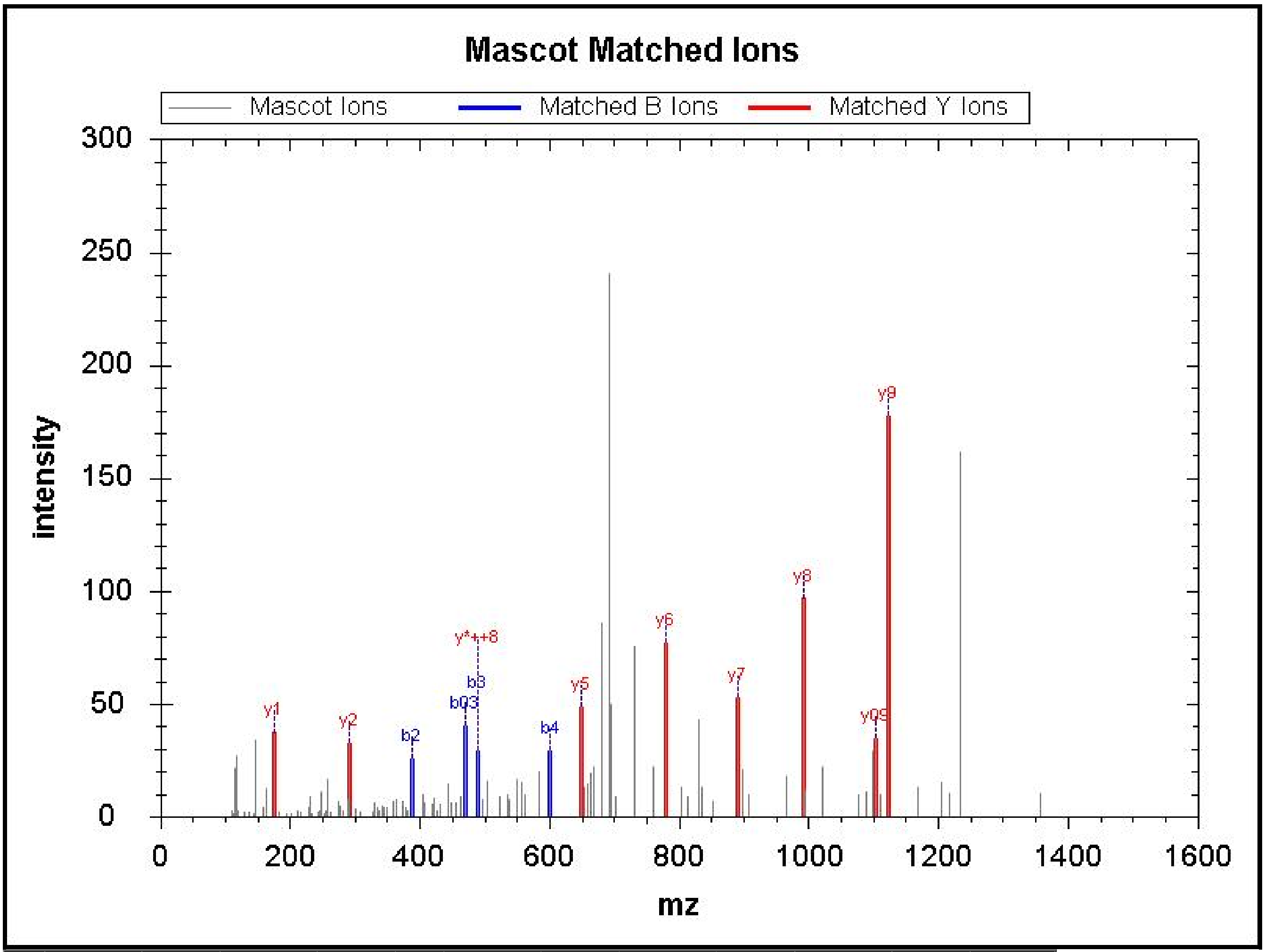

No	b	b++	b0	b0++	Seq	y	y++	y*	y*++	y0	y0++	RevNo
1	258.19	129.60			I							10
2	387.24	194.12	369.23	185.12	E	1,121.51	561.26	1,104.49	552.75	1,103.50	552.26	9
3	488.28	244.65	470.27	235.64	T	992.47	496.74	975.45	488.23	974.46	487.73	8
4	601.37	301.19	583.36	292.18	I	891.42	446.22	874.40	437.70	873.41	437.21	7
5	730.41	365.71	712.40	356.70	E	778.34	389.67	761.31	381.16	760.33	380.67	6
6	829.48	415.24	811.47	406.24	V	649.30	325.15	632.27	316.64	631.29	316.15	5
7	960.52	480.76	942.51	471.76	M	550.23	275.62	533.20	267.10	532.22	266.61	4
8	1,089.56	545.28	1,071.55	536.28	E	419.19	210.10	402.16	201.58	401.18	201.09	3
9	1,204.59	602.80	1,186.58	593.79	D	290.15	145.58	273.12	137.06	272.14	136.57	2
10					R	175.12	88.06	158.09	79.55			1

Query 1816 Hit 1

MS/MS Fragmentation of **YLMEEDEDAYKK**
Found in **sp|P46777|RL5_HUMAN**, 60S ribosomal protein L5 OS=Homo sapiens GN=RPL5 PE=1 SV=3
Match to Query 1816: 1964.98from(656.0007,3+)
Title: 378: Sum of 2 scans in range 1270 (rt=36.7906, f=4, i=262) to 1271 (rt=36.8161, f=4, i=263) [D:\lab212\membrane\Grace\20120214_iTRAQ_1.raw]
Data File:
Monoisotopic mass of neutral peptide Mr(calc): 1964.98
Variable modifications:
K11 :iTRAQ4plex (K)
K12 :iTRAQ4plex (K)

Ions Score: 49.12 **Expect:** 0.011

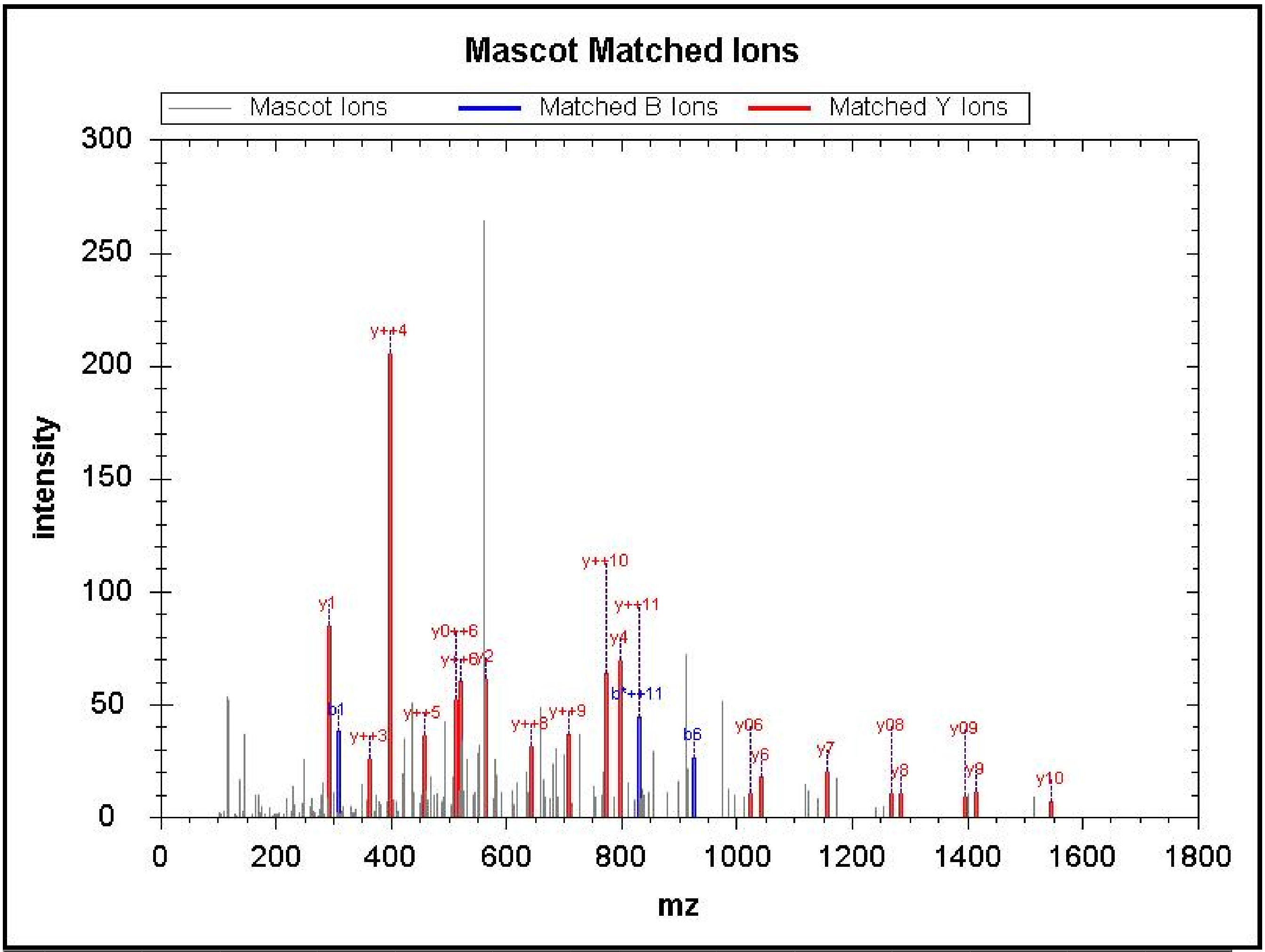

No	b	b++	b*	b*++	b0	b0++	Seq	y	y++	y*	y*++	y0	y0++	RevNo
1	**308.17**	154.59					Y							12
2	421.26	211.13					L	1,658.82	**829.91**	1,641.79	821.40	1,640.81	820.91	11
3	552.30	276.65					M	**1,545.73**	**773.37**	1,528.71	764.86	1,527.72	764.37	10
4	681.34	341.17			663.33	332.17	E	**1,414.69**	**707.85**	1,397.67	699.34	**1,396.68**	698.85	9
5	810.38	405.69			792.37	396.69	E	**1,285.65**	**643.33**	1,268.62	634.82	**1,267.64**	634.32	8
6	**925.41**	463.21			907.40	454.20	D	**1,156.61**	578.81	1,139.58	570.29	1,138.60	569.80	7
7	1,054.45	527.73			1,036.44	518.72	E	**1,041.58**	**521.29**	1,024.56	512.78	**1,023.57**	**512.29**	6
8	1,169.48	585.24			1,151.47	576.24	D	912.54	**456.77**	895.51	448.26	894.53	447.77	5
9	1,240.52	620.76			1,222.51	611.76	A	**797.51**	**399.26**	780.49	390.75			4
10	1,403.58	702.29			1,385.57	693.29	Y	726.48	**363.74**	709.45	355.23			3
11	1,675.78	838.39	1,658.75	**829.88**	1,657.77	829.39	K	**563.41**	282.21	546.39	273.70			2
12							K	**291.21**	146.11	274.19	137.60			1

Query 2067 Hit 1

MS/MS Fragmentation of **GGPNIITLADIVKDPVSR**
Found in **sp|P68400|CSK21_HUMAN**, Casein kinase II subunit alpha OS=Homo sapiens GN=CSNK2A1 PE=1 SV=1
Match to Query 2067: 2152.236from(718.4191,3+)
Title: 844: Scan 2242 (rt=58.6908, f=3, i=303) [D:\lab212\membrane\Grace\20120214_iTRAQ_1.raw]
Data File:
Monoisotopic mass of neutral peptide Mr(calc): 2152.236
Variable modifications:
K13 :iTRAQ4plex (K)
Ions Score: 49.49 **Expect:** 0.005

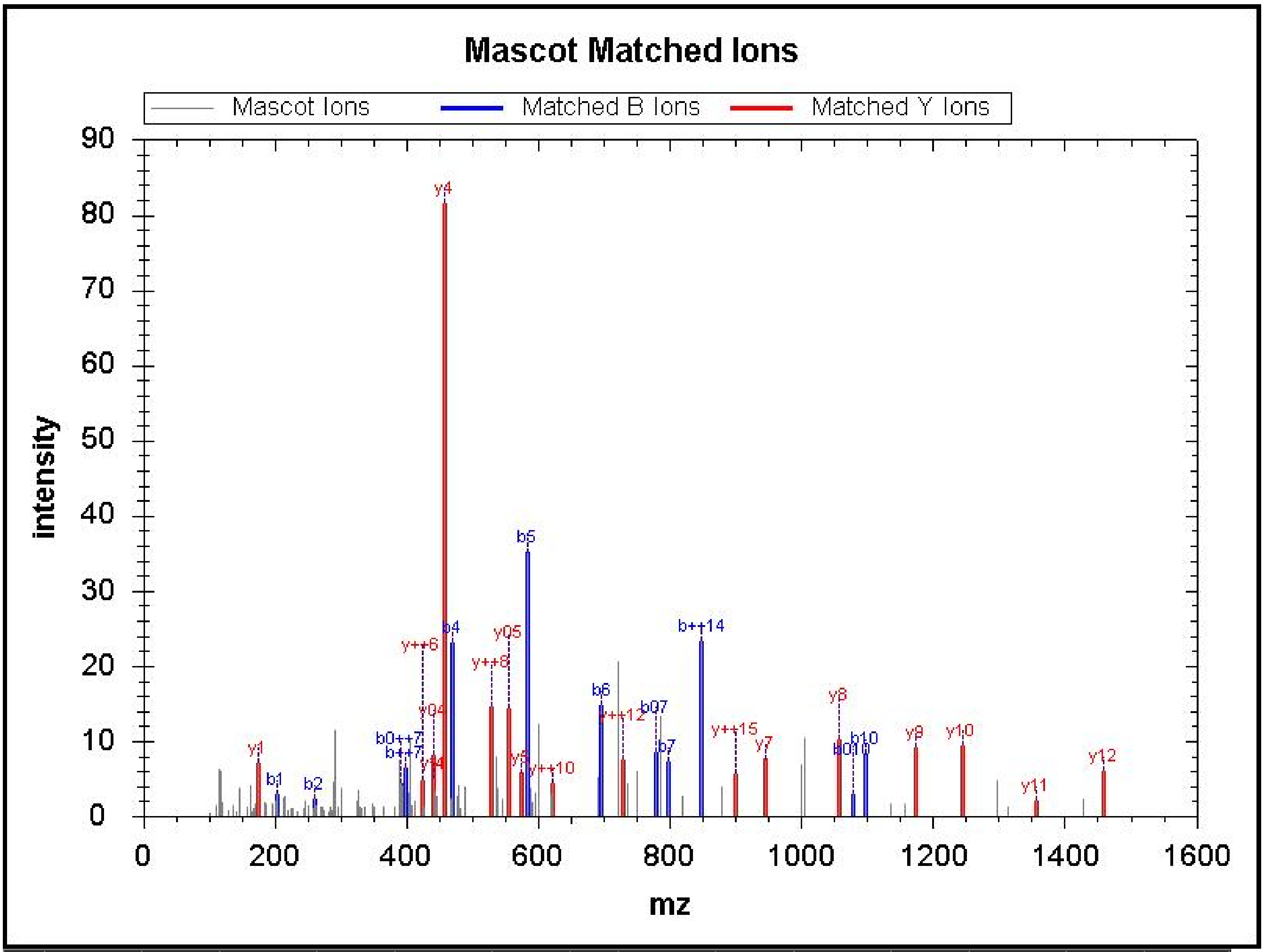

No	b	b++	b*	b*++	b0	b0++	Seq	y	y++	y*	y*++	y0	y0++	RevNo
1	**202.13**	101.57					G							18
2	**259.15**	130.08					G	1,952.13	976.57	1,935.10	968.06	1,934.12	967.56	17
3	356.21	178.61					P	1,895.11	948.06	1,878.08	939.54	1,877.10	939.05	16
4	**470.25**	235.63	453.22	227.11			N	1,798.06	**899.53**	1,781.03	891.02	1,780.05	890.53	15
5	**583.33**	292.17	566.31	283.66			I	1,684.01	842.51	1,666.99	834.00	1,666.00	833.50	14
6	**696.42**	348.71	679.39	340.20			I	1,570.93	785.97	1,553.90	777.45	1,552.92	776.96	13
7	**797.46**	**399.24**	780.44	390.72	**779.45**	**390.23**	T	**1,457.84**	**729.43**	1,440.82	720.91	1,439.83	720.42	12
8	910.55	455.78	893.52	447.26	892.54	446.77	L	**1,356.80**	678.90	1,339.77	670.39	1,338.79	669.90	11
9	981.58	491.30	964.56	482.78	963.57	482.29	A	**1,243.71**	**622.36**	1,226.69	613.85	1,225.70	613.35	10
10	**1,096.61**	548.81	1,079.59	540.30	**1,078.60**	539.80	D	**1,172.68**	586.84	1,155.65	578.33	1,154.66	577.84	9
11	1,209.70	605.35	1,192.67	596.84	1,191.69	596.35	I	**1,057.65**	**529.33**	1,040.62	520.81	1,039.64	520.32	8
12	1,308.76	654.89	1,291.74	646.37	1,290.75	645.88	V	**944.56**	472.79	927.54	464.27	926.55	463.78	7
13	1,580.96	790.98	1,563.93	782.47	1,562.95	781.98	K	845.50	**423.25**	828.47	414.74	827.49	414.25	6
14	1,695.99	**848.50**	1,678.96	839.98	1,677.98	839.49	D	**573.30**	287.15	556.27	278.64	**555.29**	278.15	5
15	1,793.04	897.02	1,776.01	888.51	1,775.03	888.02	P	**458.27**	229.64	**441.25**	221.13	**440.26**	220.63	4
16	1,892.11	946.56	1,875.08	938.05	1,874.10	937.55	V	361.22	181.11	344.19	172.60	343.21	172.11	3
17	1,979.14	990.07	1,962.11	981.56	1,961.13	981.07	S	262.15	131.58	245.12	123.07	244.14	122.57	2
18							R	**175.12**	88.06	158.09	79.55			1

Query 2418 Hit 1

MS/MS Fragmentation of **VEENFVILFSDLTMHELK**
Found in **sp|O00267|SPT5H_HUMAN**, Transcription elongation factor SPT5 OS=Homo sapiens GN=SUPT5H PE=1 SV=1
Match to Query 2418: 2451.291 from(818.1041,3+)
Title: 1238: Sum of 2 scans in range 3074 (rt=77.4407, f=4, i=844) to 3075 (rt=77.4661, f=4, i=845)

[D:\lab212\membrane\Grace\20120214_iTRAQ_1.raw]
Data File:
Monoisotopic mass of neutral peptide Mr(calc): 2451.291
Variable modifications:
K18 :iTRAQ4plex (K)
Ions Score: 50.46 **Expect:** 0.008

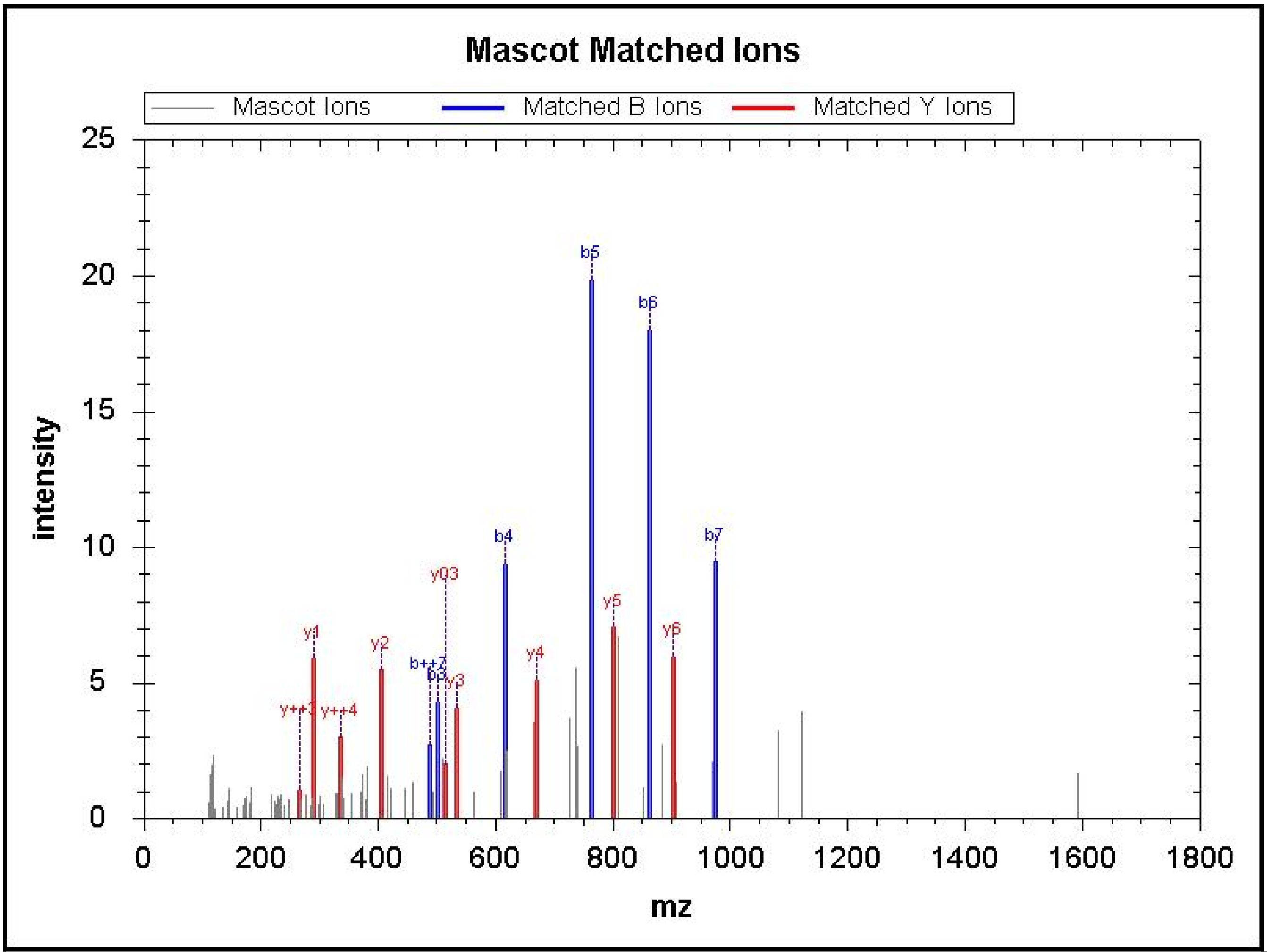

No	b	b++	b*	b*++	b0	b0++	Seq	y	y++	y*	y*++	y0	y0++	RevNo
1	244.18	122.59					V							18
2	373.22	187.11			355.21	178.11	E	2,209.13	1,105.07	2,192.11	1,096.56	2,191.12	1,096.06	17
3	**502.26**	251.64			484.25	242.63	E	2,080.09	1,040.55	2,063.06	1,032.04	2,062.08	1,031.54	16
4	**616.31**	308.66	599.28	300.14	598.30	299.65	N	1,951.05	976.03	1,934.02	967.51	1,933.04	967.02	15
5	**763.37**	382.19	746.35	373.68	745.36	373.19	F	1,837.00	919.01	1,819.98	910.49	1,818.99	910.00	14
6	**862.44**	431.72	845.42	423.21	844.43	422.72	V	1,689.94	845.47	1,672.91	836.96	1,671.93	836.47	13
7	**975.53**	**488.27**	958.50	479.75	957.52	479.26	I	1,590.87	795.94	1,573.84	787.42	1,572.86	786.93	12
8	1,088.61	544.81	1,071.58	536.30	1,070.60	535.80	L	1,477.78	739.40	1,460.76	730.88	1,459.77	730.39	11
9	1,235.68	618.34	1,218.65	609.83	1,217.67	609.34	F	1,364.70	682.85	1,347.67	674.34	1,346.69	673.85	10
10	1,322.71	661.86	1,305.68	653.35	1,304.70	652.85	S	1,217.63	609.32	1,200.60	600.81	1,199.62	600.31	9
11	1,437.74	719.37	1,420.71	710.86	1,419.73	710.37	D	1,130.60	565.80	1,113.57	557.29	1,112.59	556.80	8
12	1,550.82	775.91	1,533.80	767.40	1,532.81	766.91	L	1,015.57	508.29	998.55	499.78	997.56	499.28	7
13	1,651.87	826.44	1,634.84	817.93	1,633.86	817.43	T	**902.49**	451.75	885.46	443.23	884.48	442.74	6
14	1,782.91	891.96	1,765.88	883.45	1,764.90	882.95	M	**801.44**	401.22	784.41	392.71	783.43	392.22	5
15	1,919.97	960.49	1,902.94	951.98	1,901.96	951.48	H	**670.40**	**335.70**	653.37	327.19	652.39	326.70	4
16	2,049.01	1,025.01	2,031.99	1,016.50	2,031.00	1,016.00	E	**533.34**	**267.17**	516.31	258.66	**515.33**	258.17	3
17	2,162.10	1,081.55	2,145.07	1,073.04	2,144.09	1,072.55	L	**404.30**	202.65	387.27	194.14			2
18							K	**291.21**	146.11	274.19	137.60			1

Query 555 Hit 1

MS/MS Fragmentation of **YLNFFTK**
Found in **sp|P12004|PCNA_HUMAN**, Proliferating cell nuclear antigen OS=Homo sapiens GN=PCNA PE=1 SV=1
Match to Query 555: 1219.684from(610.8495,2+)
Title: 746: Sum of 2 scans in range 2038 (rt=54.0983, f=4, i=510) to 2039 (rt=54.1237, f=4, i=511)
[D:\lab212\membrane\Grace\20120214_iTRAQ_1.raw]
Data File:
Monoisotopic mass of neutral peptide Mr(calc): 1219.684
Variable modifications:
K7 :iTRAQ4plex (K)
Ions Score: 51.16 **Expect:** 0.006

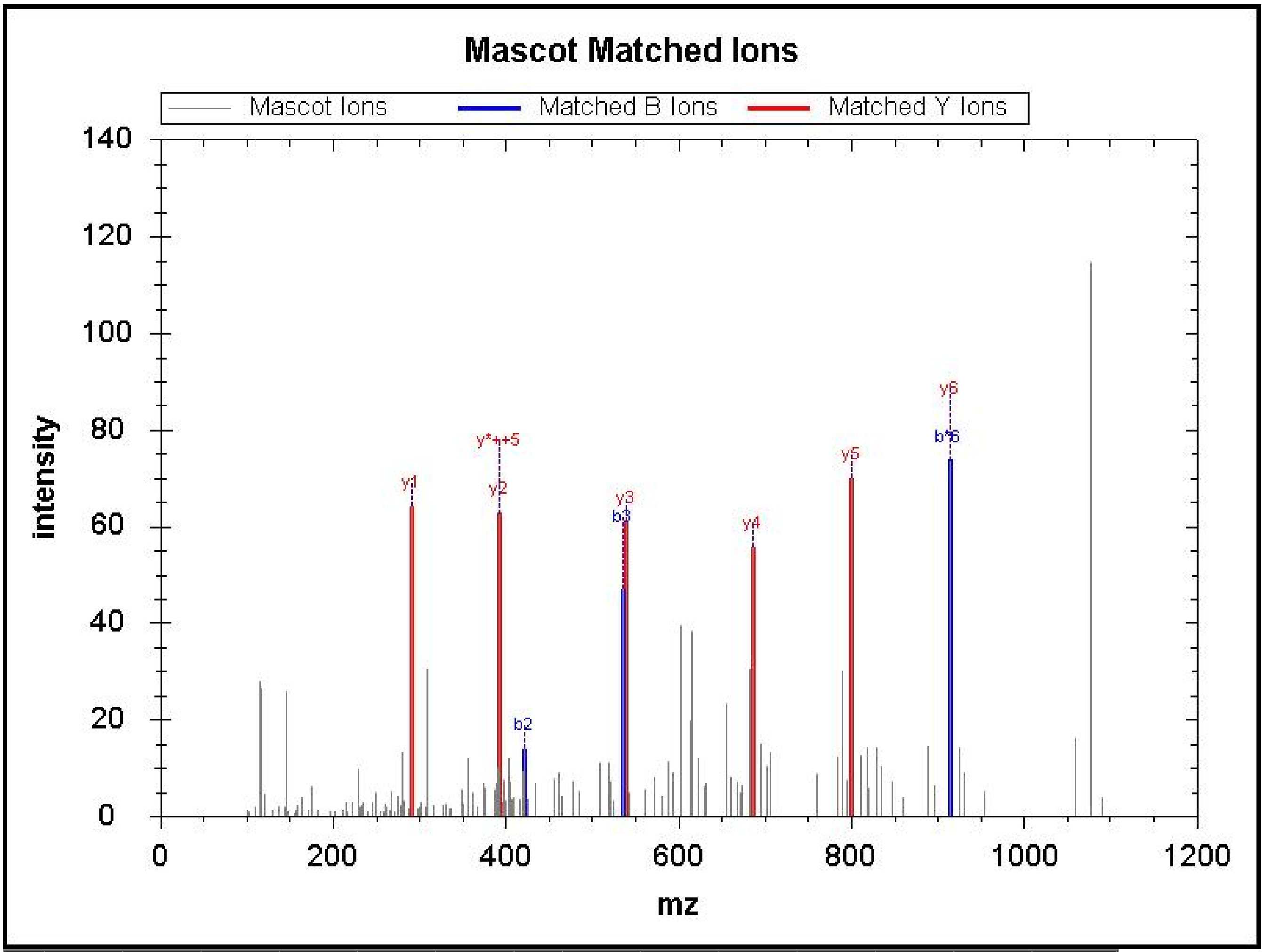

No	b	b++	b*	b*++	b0	b0++	Seq	y	y++	y*	y*++	y0	y0++	RevNo
1	308.17	154.59					Y							7
2	421.26	211.13					L	913.53	457.27	896.50	448.75	895.52	448.26	6
3	535.30	268.15	518.27	259.64			N	800.44	400.72	783.42	392.21	782.43	391.72	5
4	682.37	341.69	665.34	333.17			F	686.40	343.70	669.37	335.19	668.39	334.70	4
5	829.44	415.22	812.41	406.71			F	539.33	270.17	522.30	261.66	521.32	261.16	3
6	930.48	465.75	913.46	457.23	912.47	456.74	T	392.26	196.63	375.24	188.12	374.25	187.63	2
7							K	291.21	146.11	274.19	137.60			1

Query 2606 Hit 1

MS/MS Fragmentation of **LLTAPELILDQWFQLSSSGPNSR**
Found in **sp|Q9BSJ8|ESYT1_HUMAN**, Extended synaptotagmin-1 OS=Homo sapiens GN=ESYT1 PE=1 SV=1
Match to Query 2606: 2715.431from(906.1509,3+)
Title: 1404: Sum of 2 scans in range 3404 (rt=84.9428, f=4, i=937) to 3405 (rt=84.9682, f=4, i=938)
[D:\lab212\membrane\Grace\20120214_iTRAQ_2.raw]
Data File:

Monoisotopic mass of neutral peptide Mr(calc): 2715.431
Variable modifications:
Ions Score: 52.53 **Expect:** 0.004

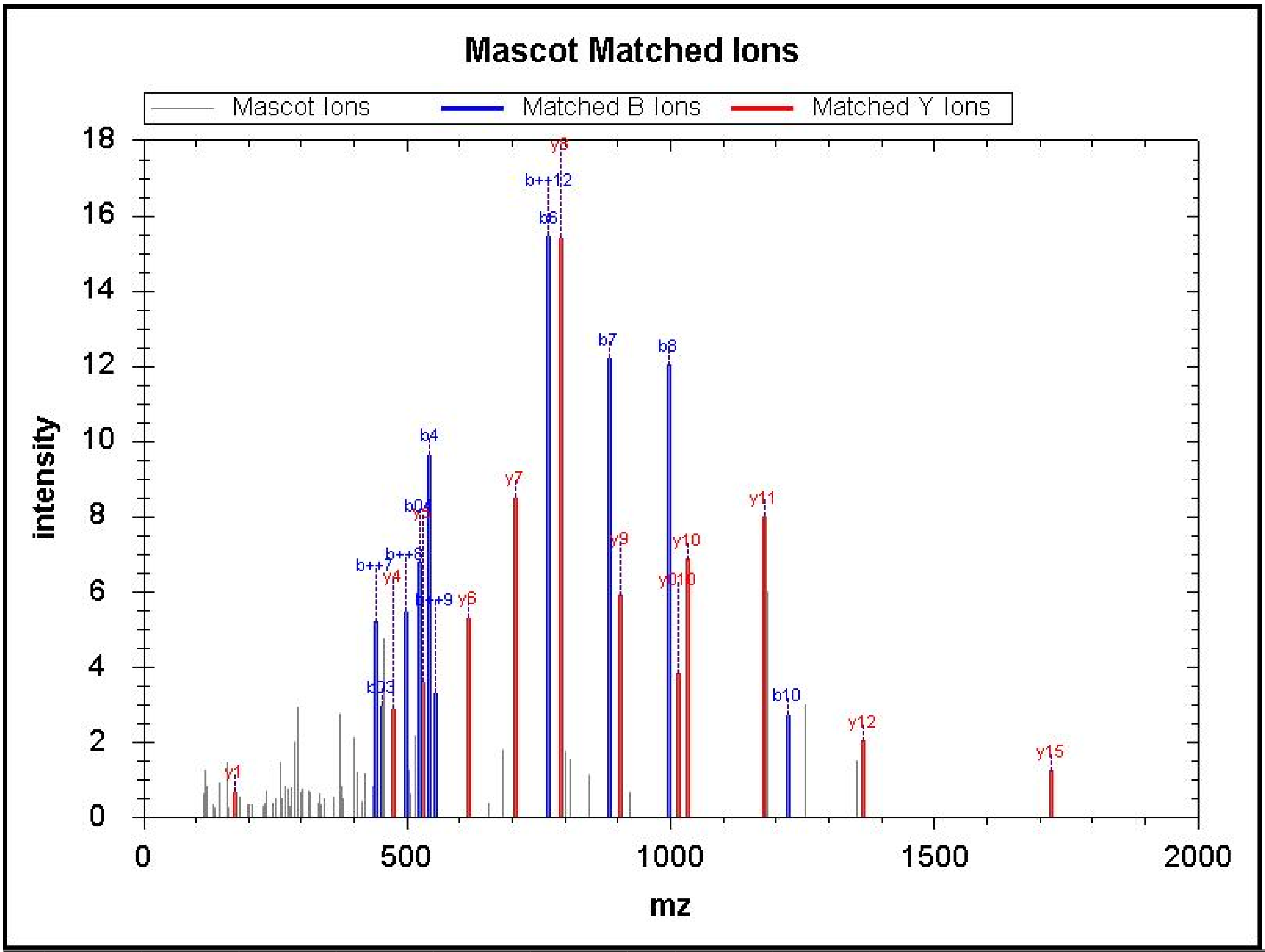

No	b	b++	b*	b*++	b0	b0++	Seq	y	y++	y*	y*++	y0	y0++	RevNo
1	258.19	129.60					L							23
2	371.28	186.14					L	2,459.26	1,230.13	2,442.23	1,221.62	2,441.25	1,221.13	22
3	472.33	236.67			**454.31**	227.66	T	2,346.17	1,173.59	2,329.15	1,165.08	2,328.16	1,164.58	21
4	**543.36**	272.18			**525.35**	263.18	A	2,245.12	1,123.07	2,228.10	1,114.55	2,227.11	1,114.06	20
5	640.41	320.71			622.40	311.71	P	2,174.09	1,087.55	2,157.06	1,079.03	2,156.08	1,078.54	19
6	**769.46**	385.23			751.45	376.23	E	2,077.03	1,039.02	2,060.01	1,030.51	2,059.02	1,030.02	18
7	**882.54**	**441.77**			864.53	432.77	L	1,947.99	974.50	1,930.97	965.99	1,929.98	965.49	17
8	**995.63**	**498.32**			977.62	489.31	I	1,834.91	917.96	1,817.88	909.44	1,816.90	908.95	16
9	1,108.71	**554.86**			1,090.70	545.85	L	**1,721.82**	861.42	1,704.80	852.90	1,703.81	852.41	15
10	**1,223.74**	612.37			1,205.73	603.37	D	1,608.74	804.87	1,591.71	796.36	1,590.73	795.87	14
11	1,351.80	676.40	1,334.77	667.89	1,333.78	667.40	Q	1,493.71	747.36	1,476.69	738.85	1,475.70	738.35	13
12	1,537.87	**769.44**	1,520.85	760.93	1,519.86	760.44	W	**1,365.65**	683.33	1,348.63	674.82	1,347.64	674.33	12
13	1,684.94	842.98	1,667.92	834.46	1,666.93	833.97	F	**1,179.58**	590.29	1,162.55	581.78	1,161.56	581.29	11
14	1,813.00	907.00	1,795.98	898.49	1,794.99	898.00	Q	**1,032.51**	516.76	1,015.48	508.24	**1,014.50**	507.75	10
15	1,926.09	963.55	1,909.06	955.03	1,908.08	954.54	L	**904.45**	452.73	887.42	444.21	886.44	443.72	9
16	2,013.12	1,007.06	1,996.09	998.55	1,995.11	998.06	S	**791.36**	396.19	774.34	387.67	773.35	387.18	8
17	2,100.15	1,050.58	2,083.12	1,042.07	2,082.14	1,041.57	S	**704.33**	352.67	687.31	344.16	686.32	343.66	7
18	2,187.18	1,094.09	2,170.16	1,085.58	2,169.17	1,085.09	S	**617.30**	309.15	600.27	300.64	599.29	300.15	6
19	2,244.20	1,122.61	2,227.18	1,114.09	2,226.19	1,113.60	G	**530.27**	265.64	513.24	257.12	512.26	256.63	5
20	2,341.26	1,171.13	2,324.23	1,162.62	2,323.25	1,162.13	P	**473.25**	237.13	456.22	228.61	455.24	228.12	4

21	2,455.30	1,228.15	2,438.27	1,219.64	2,437.29	1,219.15	N	376.19	188.60	359.17	180.09	358.18	179.60	3
22	2,542.33	1,271.67	2,525.30	1,263.16	2,524.32	1,262.66	S	262.15	131.58	245.12	123.07	244.14	122.57	2
23							R	175.12	88.06	158.09	79.55			1

Query 1732 Hit 1

MS/MS Fragmentation of **GLGTDEDTIIDIITHR**
Found in **sp|P08133|ANXA6_HUMAN**, Annexin A6 OS=Homo sapiens GN=ANXA6 PE=1 SV=3
Match to Query 1732: 1911.998from(638.34,3+)
Title: 933: Scan 2384 (rt=61.9767, f=3, i=339) [D:\lab212\membrane\Grace\20120214_iTRAQ_2.raw]
Data File:
Monoisotopic mass of neutral peptide Mr(calc): 1911.998
Variable modifications:
Ions Score: 54.24 **Expect:** 0.003

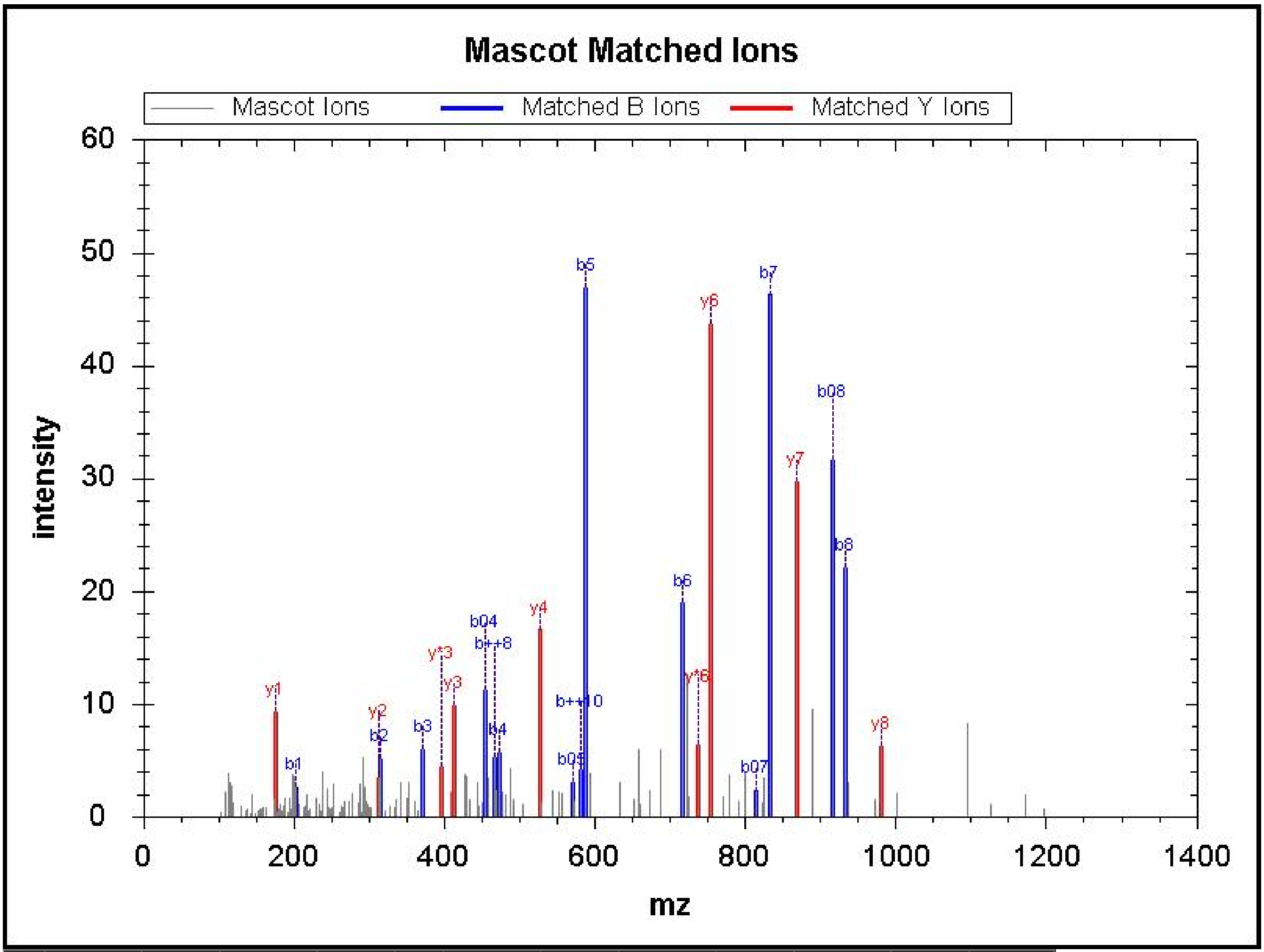

No	b	b++	b0	b0++	Seq	y	y++	y*	y*++	y0	y0++	RevNo
1	202.13	101.57			G							16
2	315.21	158.11			L	1,711.89	856.45	1,694.86	847.93	1,693.88	847.44	15
3	372.24	186.62			G	1,598.80	799.90	1,581.78	791.39	1,580.79	790.90	14
4	473.28	237.15	455.27	228.14	T	1,541.78	771.39	1,524.75	762.88	1,523.77	762.39	13
5	588.31	294.66	570.30	285.65	D	1,440.73	720.87	1,423.71	712.36	1,422.72	711.86	12
6	717.35	359.18	699.34	350.18	E	1,325.71	663.36	1,308.68	654.84	1,307.70	654.35	11
7	832.38	416.69	814.37	407.69	D	1,196.66	598.84	1,179.64	590.32	1,178.65	589.83	10
8	933.43	467.22	915.42	458.21	T	1,081.64	541.32	1,064.61	532.81	1,063.63	532.32	9
9	1,046.51	523.76	1,028.50	514.75	I	980.59	490.80	963.56	482.28	962.58	481.79	8
10	1,159.60	580.30	1,141.59	571.30	I	867.50	434.26	850.48	425.74	849.49	425.25	7
11	1,274.62	637.82	1,256.61	628.81	D	754.42	377.71	737.39	369.20	736.41	368.71	6
12	1,387.71	694.36	1,369.70	685.35	I	639.39	320.20	622.37	311.69	621.38	311.20	5

13	1,500.79	750.90	1,482.78	741.89	I	526.31	263.66	509.28	255.15	508.30	254.65	4
14	1,601.84	801.42	1,583.83	792.42	T	413.23	207.12	396.20	198.60	395.21	198.11	3
15	1,738.90	869.95	1,720.89	860.95	H	312.18	156.59	295.15	148.08			2
16					R	175.12	88.06	158.09	79.55			1

Query 2571 Hit 1

MS/MS Fragmentation of **VAMQDATAQMAMLQFISSGLSK**
Found in **sp|Q99798|ACON_HUMAN**, Aconitate hydratase
Match to Query 2571: 2615.333from(872.7852,3+)
Title: 1130: Sum of 2 scans in range 2795 (rt=71.2381, f=4, i=751) to 2796 (rt=71.2635, f=4, i=752) [D:\lab212\membrane\Grace\20120214_iTRAQ_2.raw]
Data File:
Monoisotopic mass of neutral peptide Mr(calc): 2615.333
Variable modifications:
K22 :iTRAQ4plex (K)
Ions Score: 54.33 **Expect:** 0.003

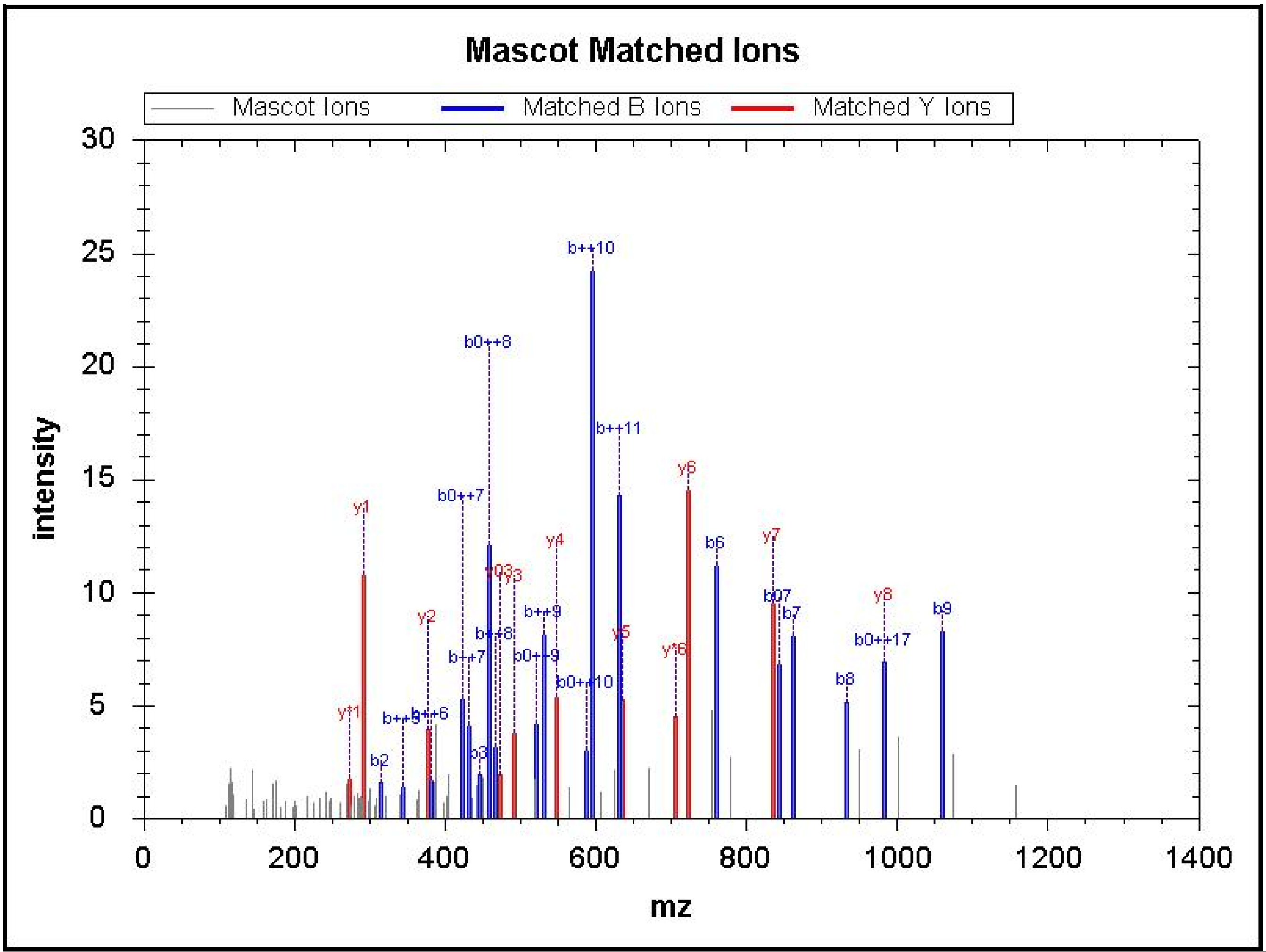

No	b	b++	b*	b*++	b0	b0++	Seq	y	y++	y*	y*++	y0	y0++	RevNo
1	244.18	122.59					V							22
2	315.21	158.11					A	2,373.17	1,187.09	2,356.15	1,178.58	2,355.16	1,178.09	21
3	446.26	223.63					M	2,302.14	1,151.57	2,285.11	1,143.06	2,284.13	1,142.57	20
4	574.31	287.66	557.29	279.15			Q	2,171.10	1,086.05	2,154.07	1,077.54	2,153.09	1,077.05	19
5	689.34	345.17	672.31	336.66	671.33	336.17	D	2,043.04	1,022.02	2,026.01	1,013.51	2,025.03	1,013.02	18
6	760.38	380.69	743.35	372.18	742.37	371.69	A	1,928.01	964.51	1,910.98	956.00	1,910.00	955.50	17
7	861.43	431.22	844.40	422.70	843.42	422.21	T	1,856.97	928.99	1,839.95	920.48	1,838.96	919.98	16
8	932.46	466.73	915.44	458.22	914.45	457.73	A	1,755.93	878.47	1,738.90	869.95	1,737.91	869.46	15
9	1,060.52	530.76	1,043.49	522.25	1,042.51	521.76	Q	1,684.89	842.95	1,667.86	834.43	1,666.88	833.94	14

10	1,191.56	596.28	1,174.54	587.77	1,173.55	587.28	M	1,556.83	778.92	1,539.80	770.41	1,538.82	769.91	13
11	1,262.60	631.80	1,245.57	623.29	1,244.59	622.80	A	1,425.79	713.40	1,408.76	704.89	1,407.78	704.39	12
12	1,393.64	697.32	1,376.61	688.81	1,375.63	688.32	M	1,354.75	677.88	1,337.73	669.37	1,336.74	668.87	11
13	1,506.72	753.87	1,489.70	745.35	1,488.71	744.86	L	1,223.71	612.36	1,206.69	603.85	1,205.70	603.35	10
14	1,634.78	817.89	1,617.76	809.38	1,616.77	808.89	Q	1,110.63	555.82	1,093.60	547.30	1,092.62	546.81	9
15	1,781.85	891.43	1,764.82	882.92	1,763.84	882.42	F	982.57	491.79	965.54	483.27	964.56	482.78	8
16	1,894.93	947.97	1,877.91	939.46	1,876.92	938.97	I	835.50	418.25	818.47	409.74	817.49	409.25	7
17	1,981.97	991.49	1,964.94	982.97	1,963.96	982.48	S	722.42	361.71	705.39	353.20	704.41	352.71	6
18	2,069.00	1,035.00	2,051.97	1,026.49	2,050.99	1,026.00	S	635.38	318.20	618.36	309.68	617.37	309.19	5
19	2,126.02	1,063.51	2,108.99	1,055.00	2,108.01	1,054.51	G	548.35	274.68	531.33	266.17	530.34	265.67	4
20	2,239.10	1,120.06	2,222.08	1,111.54	2,221.09	1,111.05	L	491.33	246.17	474.30	237.66	473.32	237.16	3
21	2,326.14	1,163.57	2,309.11	1,155.06	2,308.13	1,154.57	S	378.25	189.63	361.22	181.11	360.24	180.62	2
22							K	291.21	146.11	274.19	137.60			1

Query 545 Hit 1

MS/MS Fragmentation of **GALKKVLAK**
Found in **sp|Q8NGZ5|OR2G2_HUMAN**, Olfactory receptor 2G2 OS=Homo sapiens GN=OR2G2 PE=2 SV=1
Match to Query 545: 1214.76from(608.3871,2+)
Title: 333: Scan 1176 (rt=34.6697, f=3, i=131) [D:\lab212\membrane\Grace\20120214_iTRAQ_1.raw]
Data File:
Monoisotopic mass of neutral peptide Mr(calc): 1214.76
Variable modifications:
K9 :iTRAQ4plex (K)
Ions Score: 56.14 **Expect:** 0.001

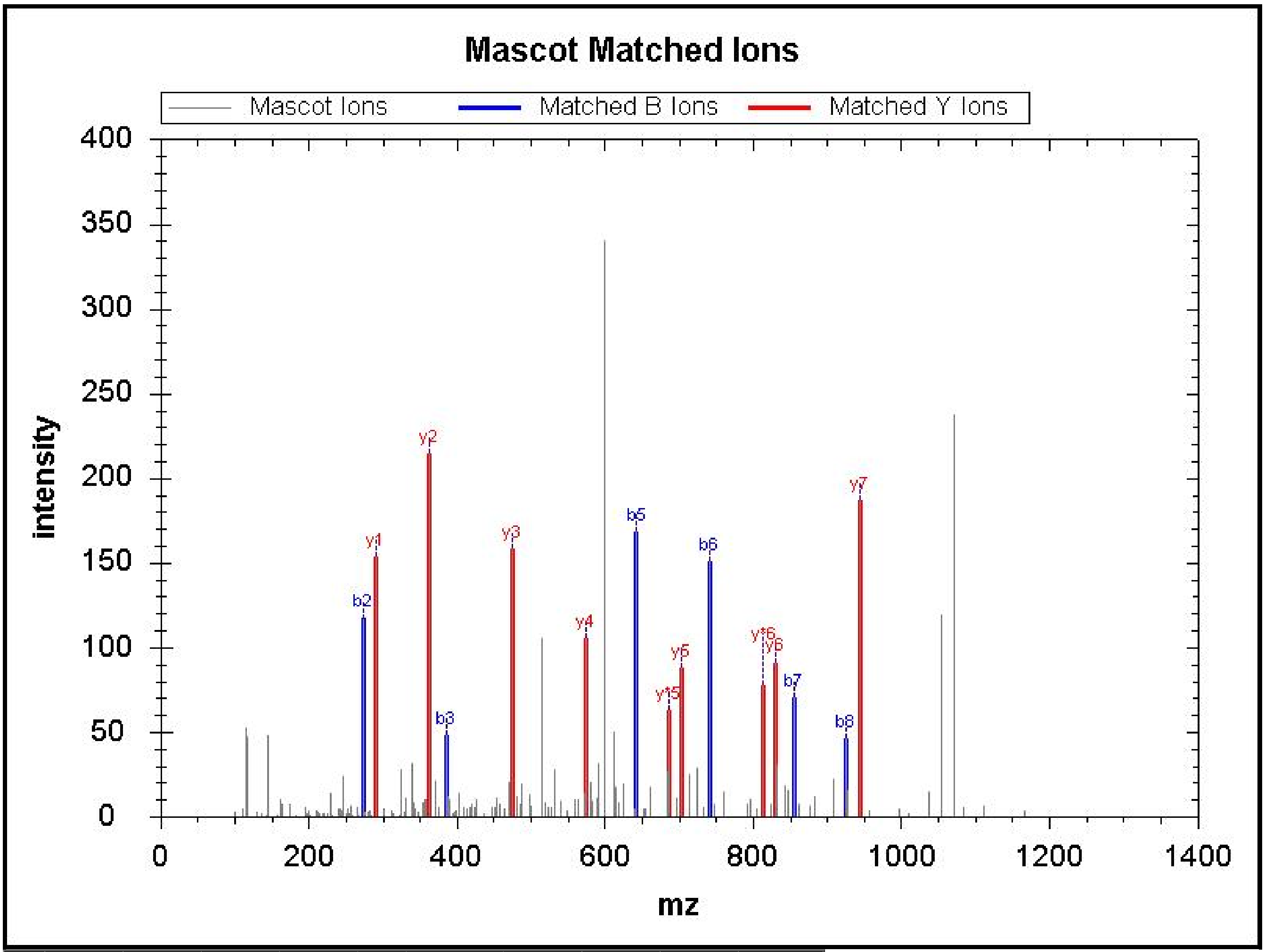

No	b	b++	b*	b*++	Seq	y	y++	y*	y*++	RevNo
1	202.13	101.57			G					9

2	273.17	137.09			A	1,014.72	507.86	997.69	499.35	8
3	386.25	193.63			L	943.68	472.34	926.65	463.83	7
4	514.35	257.68	497.32	249.16	K	830.59	415.80	813.57	407.29	6
5	642.44	321.72	625.42	313.21	K	702.50	351.75	685.47	343.24	5
6	741.51	371.26	724.48	362.75	V	574.40	287.71	557.38	279.19	4
7	854.59	427.80	837.57	419.29	L	475.34	238.17	458.31	229.66	3
8	925.63	463.32	908.60	454.81	A	362.25	181.63	345.23	173.12	2
9					K	291.21	146.11	274.19	137.60	1

Query 2215 Hit 1

MS/MS Fragmentation of **QQDAQEFFLHLINMVER**
Found in **sp|P45974|UBP5_HUMAN**, Ubiquitin carboxyl-terminal hydrolase 5 OS=Homo sapiens GN=USP5 PE=1 SV=2
Match to Query 2215: 2261.142from(754.7213,3+)
Title: 1211: Scan 2973 (rt=75.2483, f=3, i=434) [D:\lab212\membrane\Grace\20120214_iTRAQ_2.raw]
Data File:
Monoisotopic mass of neutral peptide Mr(calc): 2261.142
Variable modifications:
Ions Score: 56.33 **Expect:** 0.002

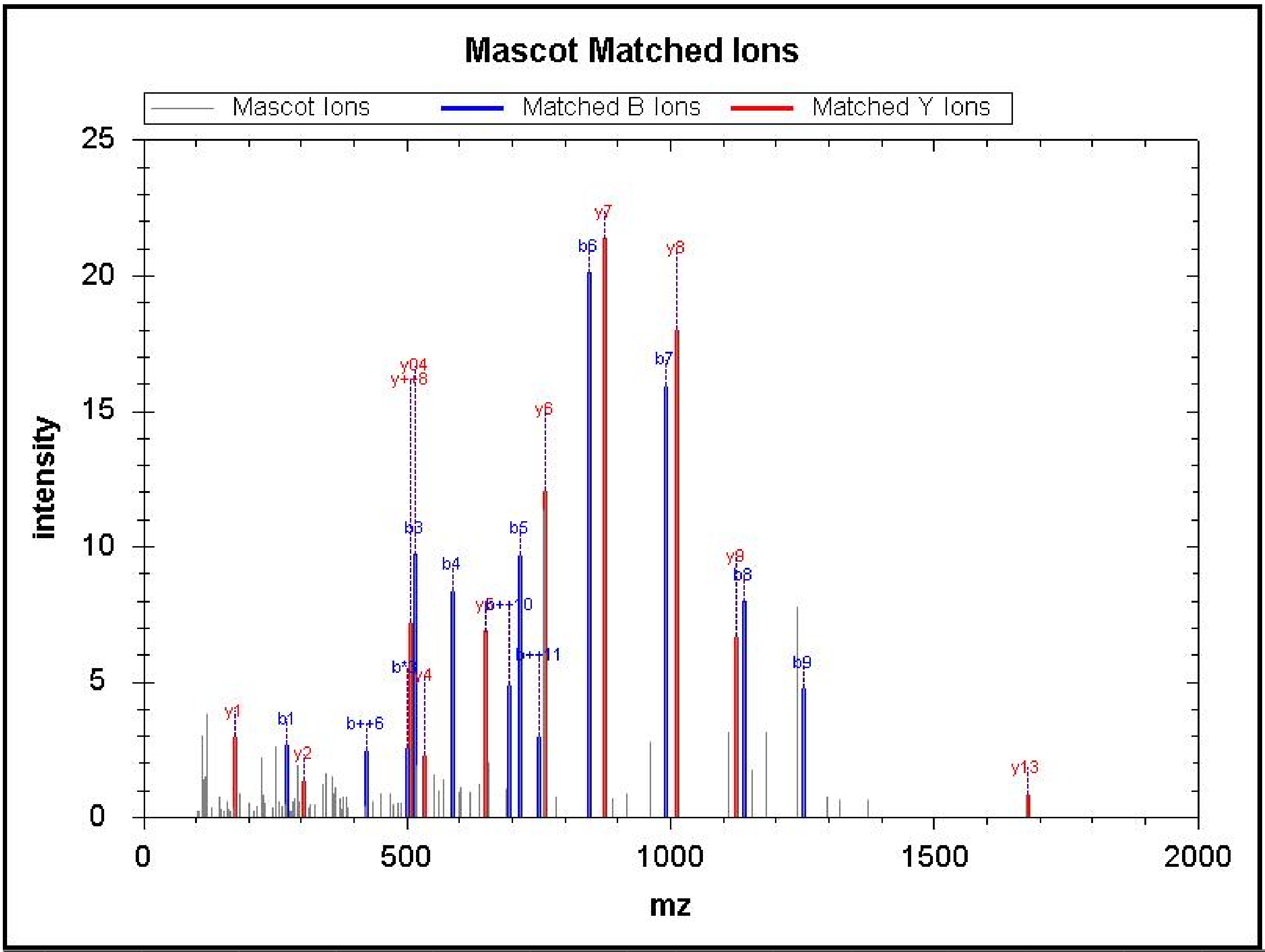

No	b	b++	b*	b*++	b0	b0++	Seq	y	y++	y*	y*++	y0	y0++	RevNo
1	273.17	137.09	256.14	128.57			Q							17
2	401.23	201.12	384.20	192.60			Q	1,989.99	995.50	1,972.96	986.98	1,971.97	986.49	16
3	516.25	258.63	499.23	250.12	498.24	249.63	D	1,861.93	931.47	1,844.90	922.95	1,843.92	922.46	15
4	587.29	294.15	570.26	285.64	569.28	285.14	A	1,746.90	873.95	1,729.87	865.44	1,728.89	864.95	14
5	715.35	358.18	698.32	349.66	697.34	349.17	Q	1,675.86	838.43	1,658.84	829.92	1,657.85	829.43	13
6	844.39	422.70	827.37	414.19	826.38	413.69	E	1,547.80	774.41	1,530.78	765.89	1,529.79	765.40	12
7	991.46	496.23	974.43	487.72	973.45	487.23	F	1,418.76	709.88	1,401.73	701.37	1,400.75	700.88	11

8	1,138.53	569.77	1,121.50	561.25	1,120.52	560.76	F	1,271.69	636.35	1,254.67	627.84	1,253.68	627.34	10
9	1,251.61	626.31	1,234.59	617.80	1,233.60	617.30	L	1,124.62	562.82	1,107.60	554.30	1,106.61	553.81	9
10	1,388.67	694.84	1,371.65	686.33	1,370.66	685.83	H	1,011.54	506.27	994.51	497.76	993.53	497.27	8
11	1,501.76	751.38	1,484.73	742.87	1,483.75	742.38	L	874.48	437.74	857.45	429.23	856.47	428.74	7
12	1,614.84	807.92	1,597.81	799.41	1,596.83	798.92	I	761.40	381.20	744.37	372.69	743.39	372.20	6
13	1,728.88	864.95	1,711.86	856.43	1,710.87	855.94	N	648.31	324.66	631.29	316.15	630.30	315.65	5
14	1,859.92	930.47	1,842.90	921.95	1,841.91	921.46	M	534.27	267.64	517.24	259.13	516.26	258.63	4
15	1,958.99	980.00	1,941.97	971.49	1,940.98	970.99	V	403.23	202.12	386.20	193.61	385.22	193.11	3
16	2,088.03	1,044.52	2,071.01	1,036.01	2,070.02	1,035.52	E	304.16	152.58	287.13	144.07	286.15	143.58	2
17							R	175.12	88.06	158.09	79.55			1

Query 2450 Hit 1

MS/MS Fragmentation of **LAIQEALSMMVGAYSTLEGAQR**
Found in **sp|Q5VYK3|ECM29_HUMAN**, Proteasome-associated protein ECM29 homolog OS=Homo sapiens GN=ECM29 PE=1 SV=2
Match to Query 2450: 2482.265from(828.4288,3+)
Title: 1381: Sum of 2 scans in range 3400 (rt=84.7827, f=4, i=948) to 3401 (rt=84.8081, f=4, i=949) [D:\lab212\membrane\Grace\20120214_iTRAQ_1.raw]
Data File:
Monoisotopic mass of neutral peptide Mr(calc): 2482.265
Variable modifications:
Ions Score: 57.2 **Expect:** 0.002

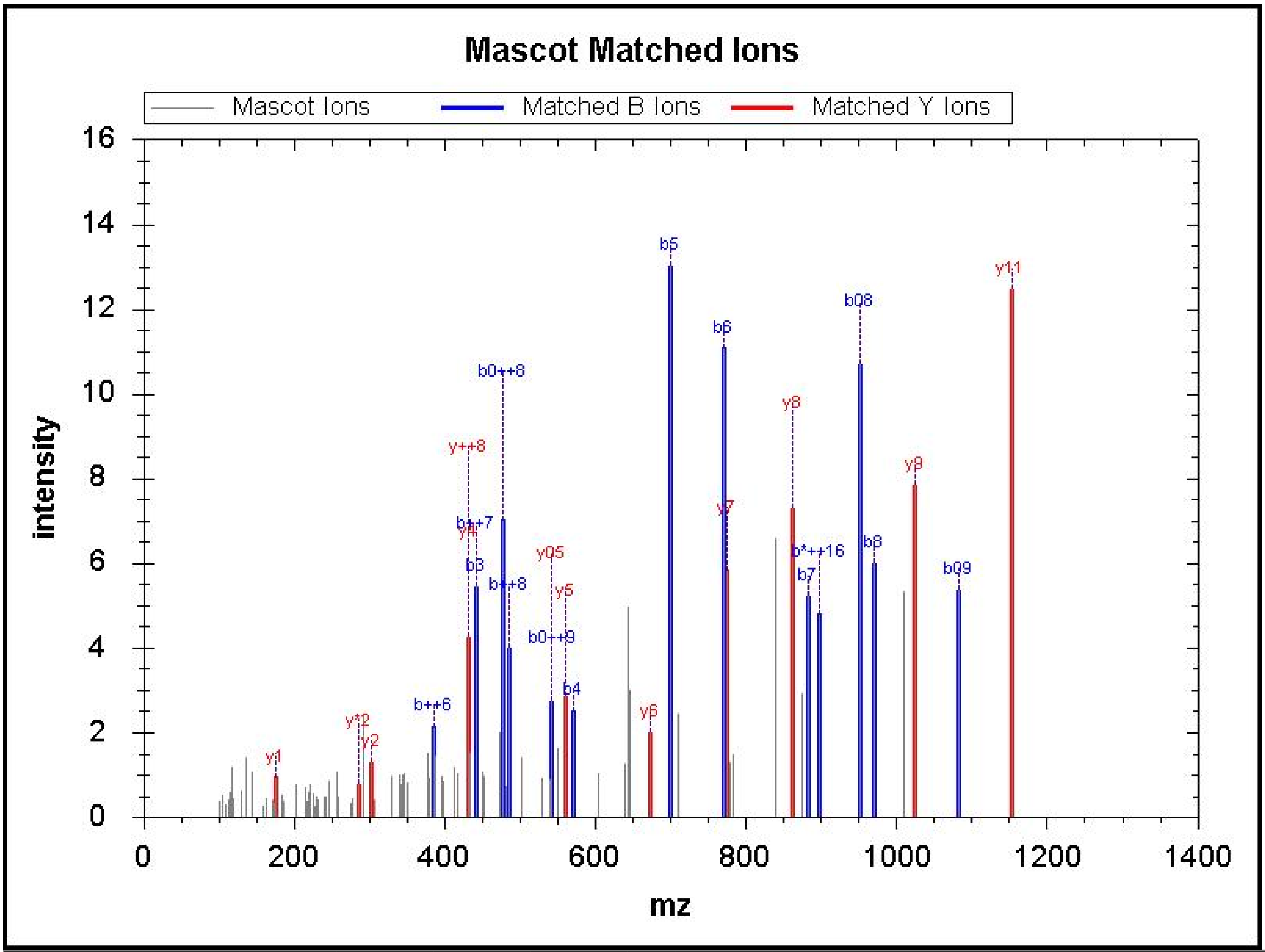

No	b	b++	b*	b*++	b0	b0++	Seq	y	y++	y*	y*++	y0	y0++	RevNo
1	258.19	129.60					L							22
2	329.23	165.12					A	2,226.09	1,113.55	2,209.06	1,105.04	2,208.08	1,104.54	21
3	442.31	221.66					I	2,155.05	1,078.03	2,138.03	1,069.52	2,137.04	1,069.02	20

**4**	**570.37**	285.69	553.35	277.18			**Q**	2,041.97	1,021.49	2,024.94	1,012.97	2,023.96	1,012.48	**19**
**5**	**699.42**	350.21	682.39	341.70	681.41	341.21	**E**	1,913.91	957.46	1,896.88	948.95	1,895.90	948.45	**18**
**6**	**770.45**	**385.73**	753.43	377.22	752.44	376.72	**A**	1,784.87	892.94	1,767.84	884.42	1,766.86	883.93	**17**
**7**	**883.54**	**442.27**	866.51	433.76	865.53	433.27	**L**	1,713.83	857.42	1,696.80	848.91	1,695.82	848.41	**16**
**8**	**970.57**	**485.79**	953.54	477.27	**952.56**	**476.78**	**S**	1,600.75	800.88	1,583.72	792.36	1,582.74	791.87	**15**
**9**	1,101.61	551.31	1,084.58	542.80	**1,083.60**	**542.30**	**M**	1,513.71	757.36	1,496.69	748.85	1,495.70	748.36	**14**
**10**	1,232.65	616.83	1,215.62	608.32	1,214.64	607.82	**M**	1,382.67	691.84	1,365.65	683.33	1,364.66	682.83	**13**
**11**	1,331.72	666.36	1,314.69	657.85	1,313.71	657.36	**V**	1,251.63	626.32	1,234.61	617.81	1,233.62	617.31	**12**
**12**	1,388.74	694.87	1,371.71	686.36	1,370.73	685.87	**G**	**1,152.56**	576.79	1,135.54	568.27	1,134.55	567.78	**11**
**13**	1,459.78	730.39	1,442.75	721.88	1,441.77	721.39	**A**	1,095.54	548.28	1,078.52	539.76	1,077.53	539.27	**10**
**14**	1,622.84	811.92	1,605.81	803.41	1,604.83	802.92	**Y**	**1,024.51**	512.76	1,007.48	504.24	1,006.50	503.75	**9**
**15**	1,709.87	855.44	1,692.85	846.93	1,691.86	846.43	**S**	**861.44**	**431.22**	844.42	422.71	843.43	422.22	**8**
**16**	1,810.92	905.96	1,793.89	**897.45**	1,792.91	896.96	**T**	**774.41**	387.71	757.38	379.20	756.40	378.70	**7**
**17**	1,924.00	962.51	1,906.98	953.99	1,905.99	953.50	**L**	**673.36**	337.18	656.34	328.67	655.35	328.18	**6**
**18**	2,053.05	1,027.03	2,036.02	1,018.51	2,035.04	1,018.02	**E**	**560.28**	280.64	543.25	272.13	**542.27**	271.64	**5**
**19**	2,110.07	1,055.54	2,093.04	1,047.02	2,092.06	1,046.53	**G**	**431.24**	216.12	414.21	207.61			**4**
**20**	2,181.11	1,091.06	2,164.08	1,082.54	2,163.09	1,082.05	**A**	374.21	187.61	357.19	179.10			**3**
**21**	2,309.16	1,155.09	2,292.14	1,146.57	2,291.15	1,146.08	**Q**	**303.18**	152.09	**286.15**	143.58			**2**
**22**							**R**	**175.12**	88.06	158.09	79.55			**1**

Query 1037 Hit 1

MS/MS Fragmentation of **LVAIVDVIDQNR**
Found in **sp|P50914|RL14_HUMAN**, 60S ribosomal protein L14 OS=Homo sapiens GN=RPL14 PE=1 SV=4
Match to Query 1037: 1497.863 from(749.9385,2+)
Title: 877: Sum of 2 scans in range 2268 (rt=59.3635, f=4, i=581) to 2269 (rt=59.3889, f=4, i=582) [D:\lab212\membrane\Grace\20120214_iTRAQ_2.raw]
Data File:
Monoisotopic mass of neutral peptide Mr(calc): 1497.863
Variable modifications:
Ions Score: 57.88 **Expect:** 0.001

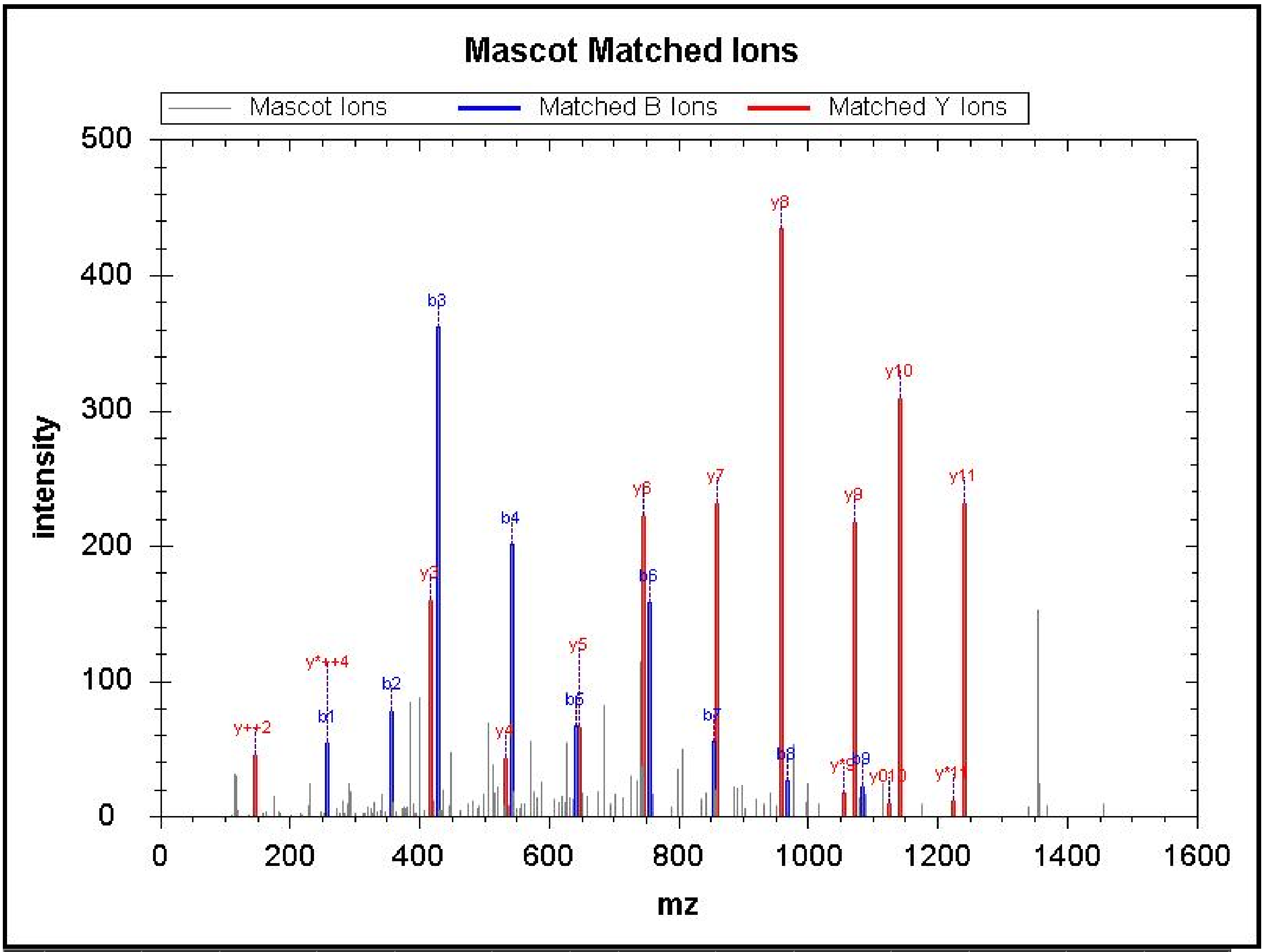

No	b	b++	b*	b*++	b0	b0++	Seq	y	y++	y*	y*++	y0	y0++	RevNo
1	258.19	129.60					L							12
2	357.26	179.13					V	1,241.68	621.35	1,224.66	612.83	1,223.67	612.34	11
3	428.30	214.65					A	1,142.62	571.81	1,125.59	563.30	1,124.61	562.81	10
4	541.38	271.20					I	1,071.58	536.29	1,054.55	527.78	1,053.57	527.29	9
5	640.45	320.73					V	958.50	479.75	941.47	471.24	940.48	470.75	8
6	755.48	378.24			737.47	369.24	D	859.43	430.22	842.40	421.70	841.42	421.21	7
7	854.55	427.78			836.54	418.77	V	744.40	372.70	727.37	364.19	726.39	363.70	6
8	967.63	484.32			949.62	475.31	I	645.33	323.17	628.30	314.66	627.32	314.16	5
9	1,082.66	541.83			1,064.65	532.83	D	532.25	266.63	515.22	258.11	514.24	257.62	4
10	1,210.72	605.86	1,193.69	597.35	1,192.71	596.86	Q	417.22	209.11	400.19	200.60			3
11	1,324.76	662.88	1,307.73	654.37	1,306.75	653.88	N	289.16	145.08	272.14	136.57			2
12							R	175.12	88.06	158.09	79.55			1

Query 2554 Hit 1

MS/MS Fragmentation of EPGQDLVVLSLPITPEFIPSFR
Found in sp|P01027|CO3_MOUSE, Complement C3 OS=Mus musculus GN=C3 PE=1 SV=3
Match to Query 2554: 2597.417from(866.8129,3+)
Title: 1195: Scan 2980 (rt=75.3197, f=3, i=422) [D:\lab212\membrane\Grace\20120214_iTRAQ_1.raw]
Data File:
Monoisotopic mass of neutral peptide Mr(calc): 2597.417
Variable modifications:
Ions Score: 57.96 **Expect:** 0.001

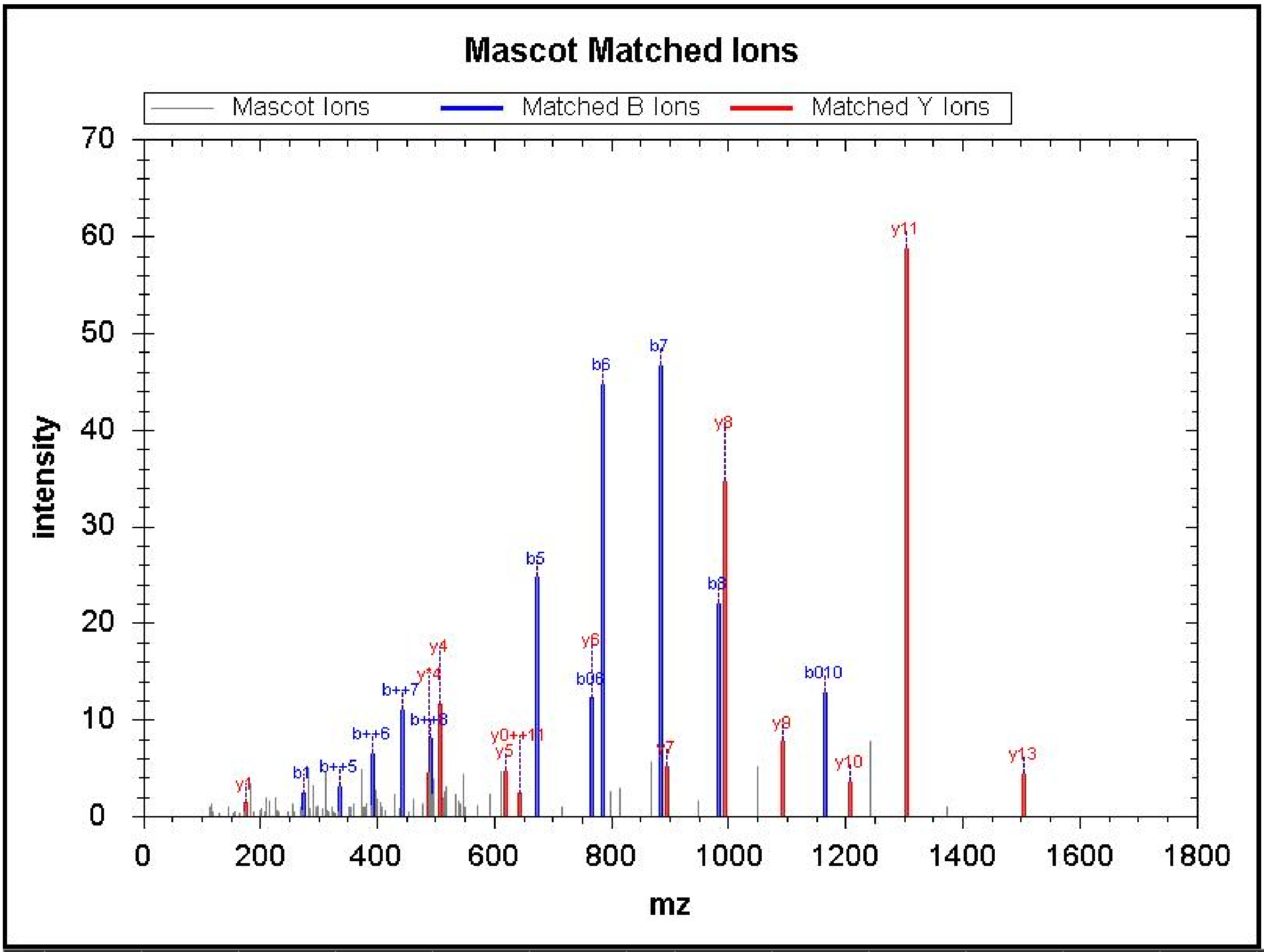

No	b	b++	b*	b*++	b0	b0++	Seq	y	y++	y*	y*++	y0	y0++	RevNo
1	**274.15**	137.58			256.14	128.57	E							22
2	371.20	186.11			353.19	177.10	P	2,325.29	1,163.15	2,308.26	1,154.63	2,307.27	1,154.14	21
3	428.23	214.62			410.22	205.61	G	2,228.23	1,114.62	2,211.21	1,106.11	2,210.22	1,105.61	20
4	556.28	278.65	539.26	270.13	538.27	269.64	Q	2,171.21	1,086.11	2,154.18	1,077.60	2,153.20	1,077.10	19
5	**671.31**	**336.16**	654.29	327.65	653.30	327.15	D	2,043.15	1,022.08	2,026.13	1,013.57	2,025.14	1,013.07	18
6	**784.40**	**392.70**	767.37	384.19	**766.39**	383.70	L	1,928.13	964.57	1,911.10	956.05	1,910.11	955.56	17
7	**883.46**	**442.24**	866.44	433.72	865.45	433.23	V	1,815.04	908.02	1,798.02	899.51	1,797.03	899.02	16
8	**982.53**	**491.77**	965.51	483.26	964.52	482.76	V	1,715.97	858.49	1,698.95	849.98	1,697.96	849.48	15
9	1,095.62	548.31	1,078.59	539.80	1,077.61	539.31	L	1,616.90	808.96	1,599.88	800.44	1,598.89	799.95	14
10	1,182.65	591.83	1,165.62	583.31	**1,164.64**	582.82	S	**1,503.82**	752.41	1,486.79	743.90	1,485.81	743.41	13
11	1,295.73	648.37	1,278.71	639.86	1,277.72	639.36	L	1,416.79	708.90	1,399.76	700.38	1,398.78	699.89	12
12	1,392.79	696.90	1,375.76	688.38	1,374.77	687.89	P	**1,303.70**	652.36	1,286.68	643.84	1,285.69	**643.35**	11
13	1,505.87	753.44	1,488.84	744.93	1,487.86	744.43	I	**1,206.65**	603.83	1,189.63	595.32	1,188.64	594.82	10
14	1,606.92	803.96	1,589.89	795.45	1,588.91	794.96	T	**1,093.57**	547.29	1,076.54	538.77	1,075.56	538.28	9
15	1,703.97	852.49	1,686.94	843.98	1,685.96	843.48	P	**992.52**	496.76	975.49	488.25	974.51	487.76	8
16	1,833.01	917.01	1,815.99	908.50	1,815.00	908.00	E	**895.47**	448.24	878.44	439.72	877.46	439.23	7
17	1,980.08	990.54	1,963.05	982.03	1,962.07	981.54	F	**766.42**	383.72	749.40	375.20	748.41	374.71	6
18	2,093.17	1,047.09	2,076.14	1,038.57	2,075.15	1,038.08	I	**619.36**	310.18	602.33	301.67	601.35	301.18	5
19	2,190.22	1,095.61	2,173.19	1,087.10	2,172.21	1,086.61	P	**506.27**	253.64	**489.25**	245.13	488.26	244.63	4
20	2,277.25	1,139.13	2,260.22	1,130.62	2,259.24	1,130.12	S	409.22	205.11	392.19	196.60	391.21	196.11	3
21	2,424.32	1,212.66	2,407.29	1,204.15	2,406.31	1,203.66	F	322.19	161.60	305.16	153.08			2
22							R	**175.12**	88.06	158.09	79.55			1

Query 2974 Hit 1

MS/MS Fragmentation of **GVDNTFADELVELSTALEHQEYITFLEDLK**
Found in **sp|Q07021|C1QBP_HUMAN**, Complement component 1 Q subcomponent-binding protein
Match to Query 2974: 3726.873from(932.7254,4+)
Title: 1431: Sum of 2 scans in range 3483 (rt=86.6746, f=4, i=949) to 3484 (rt=86.7, f=4, i=950)
[D:\lab212\membrane\Grace\20120214_iTRAQ_2.raw]
Data File:
Monoisotopic mass of neutral peptide Mr(calc): 3726.873
Variable modifications:
K30 :iTRAQ4plex (K)
Ions Score: 58.39 **Expect:** 0.001

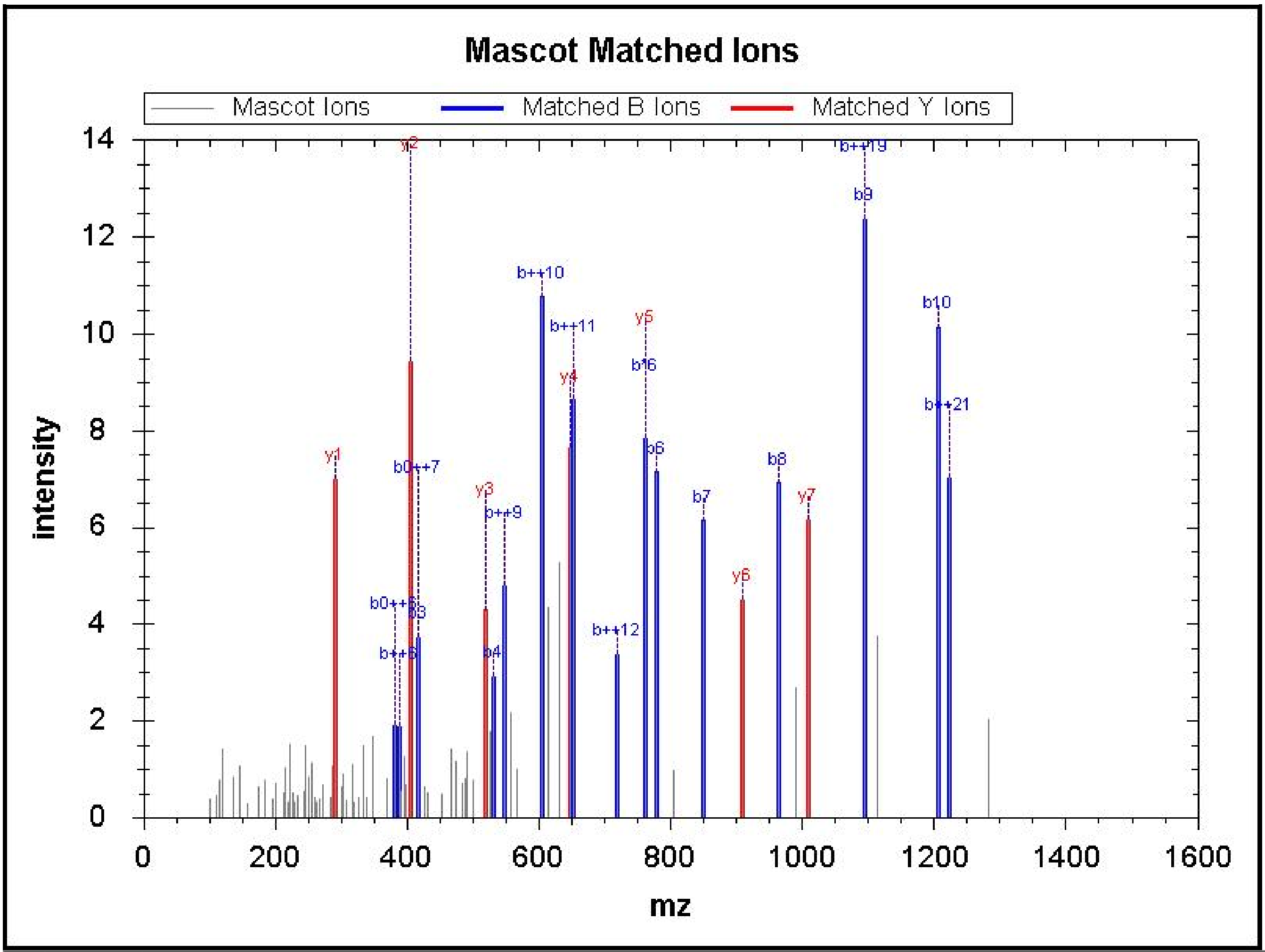

No	b	b++	b*	b*++	b0	b0++	Seq	y	y++	y*	y*++	y0	y0++	RevNo
1	202.13	101.57					G							30
2	301.20	151.10					V	3,526.76	1,763.88	3,509.73	1,755.37	3,508.75	1,754.88	29
3	416.23	208.62			398.22	199.61	D	3,427.69	1,714.35	3,410.66	1,705.84	3,409.68	1,705.34	28
4	530.27	265.64	513.24	257.12	512.26	256.63	N	3,312.66	1,656.84	3,295.64	1,648.32	3,294.65	1,647.83	27
5	631.32	316.16	614.29	307.65	613.31	307.16	T	3,198.62	1,599.81	3,181.59	1,591.30	3,180.61	1,590.81	26
6	778.39	389.70	761.36	381.18	760.37	380.69	F	3,097.57	1,549.29	3,080.55	1,540.78	3,079.56	1,540.29	25
7	849.42	425.21	832.40	416.70	831.41	416.21	A	2,950.50	1,475.76	2,933.48	1,467.24	2,932.49	1,466.75	24
8	964.45	482.73	947.42	474.21	946.44	473.72	D	2,879.47	1,440.24	2,862.44	1,431.72	2,861.46	1,431.23	23
9	1,093.49	547.25	1,076.47	538.74	1,075.48	538.24	E	2,764.44	1,382.72	2,747.41	1,374.21	2,746.43	1,373.72	22
10	1,206.58	603.79	1,189.55	595.28	1,188.57	594.79	L	2,635.40	1,318.20	2,618.37	1,309.69	2,617.39	1,309.20	21
11	1,305.64	653.33	1,288.62	644.81	1,287.63	644.32	V	2,522.31	1,261.66	2,505.29	1,253.15	2,504.30	1,252.66	20
12	1,434.69	717.85	1,417.66	709.33	1,416.68	708.84	E	2,423.25	1,212.13	2,406.22	1,203.61	2,405.24	1,203.12	19
13	1,547.77	774.39	1,530.74	765.88	1,529.76	765.38	L	2,294.20	1,147.61	2,277.18	1,139.09	2,276.19	1,138.60	18
14	1,634.80	817.91	1,617.78	809.39	1,616.79	808.90	S	2,181.12	1,091.06	2,164.09	1,082.55	2,163.11	1,082.06	17
15	1,735.85	868.43	1,718.82	859.92	1,717.84	859.42	T	2,094.09	1,047.55	2,077.06	1,039.03	2,076.08	1,038.54	16

16	1,806.89	903.95	1,789.86	895.43	1,788.88	894.94	A	1,993.04	997.02	1,976.01	988.51	1,975.03	988.02	15
17	1,919.97	960.49	1,902.95	951.98	1,901.96	951.48	L	1,922.00	961.51	1,904.98	952.99	1,903.99	952.50	14
18	2,049.01	1,025.01	2,031.99	1,016.50	2,031.00	1,016.01	E	1,808.92	904.96	1,791.89	896.45	1,790.91	895.96	13
19	2,186.07	**1,093.54**	2,169.05	1,085.03	2,168.06	1,084.54	H	1,679.88	840.44	1,662.85	831.93	1,661.87	831.44	12
20	2,314.13	1,157.57	2,297.11	1,149.06	2,296.12	1,148.56	Q	1,542.82	771.91	1,525.79	763.40	1,524.81	762.91	11
21	2,443.17	**1,222.09**	2,426.15	1,213.58	2,425.16	1,213.09	E	1,414.76	707.88	1,397.73	699.37	1,396.75	698.88	10
22	2,606.24	1,303.62	2,589.21	1,295.11	2,588.23	1,294.62	Y	1,285.72	643.36	1,268.69	634.85	1,267.71	634.36	9
23	2,719.32	1,360.16	2,702.29	1,351.65	2,701.31	1,351.16	I	1,122.65	561.83	1,105.63	553.32	1,104.64	552.82	8
24	2,820.37	1,410.69	2,803.34	1,402.17	2,802.36	1,401.68	T	**1,009.57**	505.29	992.54	496.77	991.56	496.28	7
25	2,967.44	1,484.22	2,950.41	1,475.71	2,949.43	1,475.22	F	**908.52**	454.76	891.49	446.25	890.51	445.76	6
26	3,080.52	1,540.76	3,063.49	1,532.25	3,062.51	1,531.76	L	**761.45**	381.23	744.43	372.72	743.44	372.22	5
27	3,209.56	1,605.29	3,192.54	1,596.77	3,191.55	1,596.28	E	**648.37**	324.69	631.34	316.17	630.36	315.68	4
28	3,324.59	1,662.80	3,307.56	1,654.29	3,306.58	1,653.79	D	**519.33**	260.17	502.30	251.65	501.32	251.16	3
29	3,437.67	1,719.34	3,420.65	1,710.83	3,419.66	1,710.34	L	**404.30**	202.65	387.27	194.14			2
30							K	**291.21**	146.11	274.19	137.60			1

Query 2295 Hit 1

MS/MS Fragmentation of **TWWNQFSVTALQLLQANR**
Found in **sp|Q99536|VAT1_HUMAN**, Synaptic vesicle membrane protein VAT-1 homolog OS=Homo sapiens GN=VAT1 PE=1 SV=2
Match to Query 2295: 2319.229from(774.0835,3+)
Title: 1229: Scan 3054 (rt=76.9883, f=3, i=434) [D:\lab212\membrane\Grace\20120214_iTRAQ_1.raw]
Data File:
Monoisotopic mass of neutral peptide Mr(calc): 2319.229
Variable modifications:
Ions Score: 59.23 **Expect:** 0.001

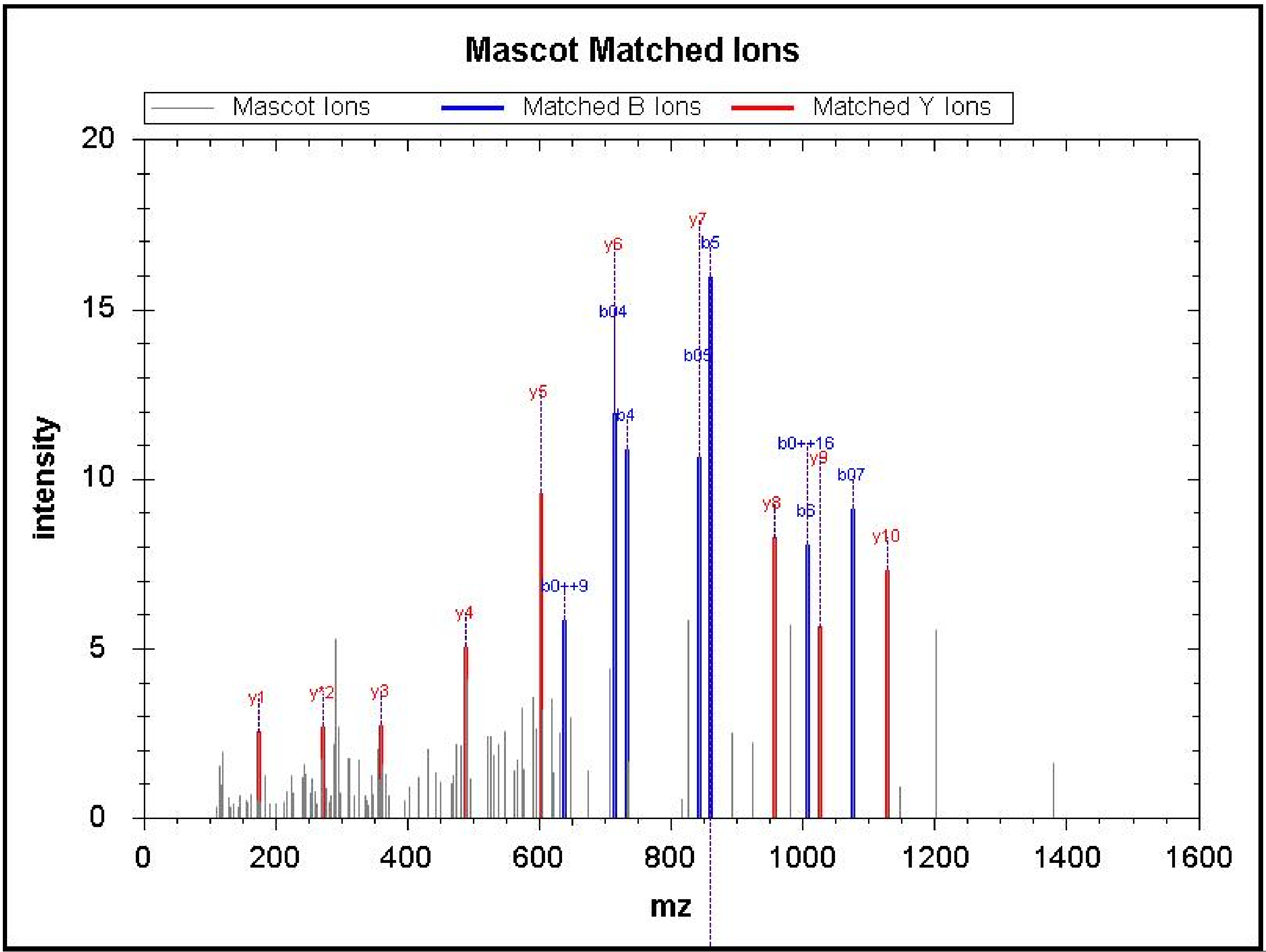

No	b	b++	b*	b*++	b0	b0++	Seq	y	y++	y*	y*++	y0	y0++	RevNo

1	246.16	123.58			228.15	114.58	T							18
2	432.24	216.62			414.23	207.62	W	2,075.08	1,038.04	2,058.06	1,029.53	2,057.07	1,029.04	17
3	618.32	309.66			600.31	300.66	W	1,889.00	945.01	1,871.98	936.49	1,870.99	936.00	16
4	**732.36**	366.68	715.33	358.17	**714.35**	357.68	N	1,702.92	851.97	1,685.90	843.45	1,684.91	842.96	15
5	**860.42**	430.71	843.39	422.20	**842.41**	421.71	Q	1,588.88	794.94	1,571.85	786.43	1,570.87	785.94	14
6	**1,007.49**	504.25	990.46	495.73	989.47	495.24	F	1,460.82	730.91	1,443.80	722.40	1,442.81	721.91	13
7	1,094.52	547.76	1,077.49	539.25	**1,076.51**	538.76	S	1,313.75	657.38	1,296.73	648.87	1,295.74	648.38	12
8	1,193.59	597.30	1,176.56	588.78	1,175.58	588.29	V	1,226.72	613.86	1,209.69	605.35	1,208.71	604.86	11
9	1,294.63	647.82	1,277.61	639.31	1,276.62	**638.82**	T	**1,127.65**	564.33	1,110.63	555.82	1,109.64	555.32	10
10	1,365.67	683.34	1,348.64	674.83	1,347.66	674.33	A	**1,026.61**	513.81	1,009.58	505.29			9
11	1,478.75	739.88	1,461.73	731.37	1,460.74	730.88	L	**955.57**	478.29	938.54	469.77			8
12	1,606.81	803.91	1,589.79	795.40	1,588.80	794.91	Q	**842.48**	421.75	825.46	413.23			7
13	1,719.90	**860.45**	1,702.87	851.94	1,701.89	851.45	L	**714.43**	357.72	697.40	349.20			6
14	1,832.98	916.99	1,815.96	908.48	1,814.97	907.99	L	**601.34**	301.17	584.32	292.66			5
15	1,961.04	981.02	1,944.01	972.51	1,943.03	972.02	Q	**488.26**	244.63	471.23	236.12			4
16	2,032.08	1,016.54	2,015.05	1,008.03	2,014.07	**1,007.54**	A	**360.20**	180.60	343.17	172.09			3
17	2,146.12	1,073.56	2,129.09	1,065.05	2,128.11	1,064.56	N	289.16	145.08	**272.14**	136.57			2
18							R	**175.12**	88.06	158.09	79.55			1

Query 547 Hit 1

MS/MS Fragmentation of **GFGFILFK**
Found in **sp|Q99729|ROAA_HUMAN**, Heterogeneous nuclear ribonucleoprotein A/B OS=Homo sapiens GN=HNRNPAB PE=1 SV=2
Match to Query 547: 1215.729from(608.8715,2+)
Title: 952: Scan 2465 (rt=63.7162, f=3, i=339) [D:\lab212\membrane\Grace\20120214_iTRAQ_1.raw]
Data File:
Monoisotopic mass of neutral peptide Mr(calc): 1215.729
Variable modifications:
K8 :iTRAQ4plex (K)
Ions Score: 59.85 **Expect:** 0.001

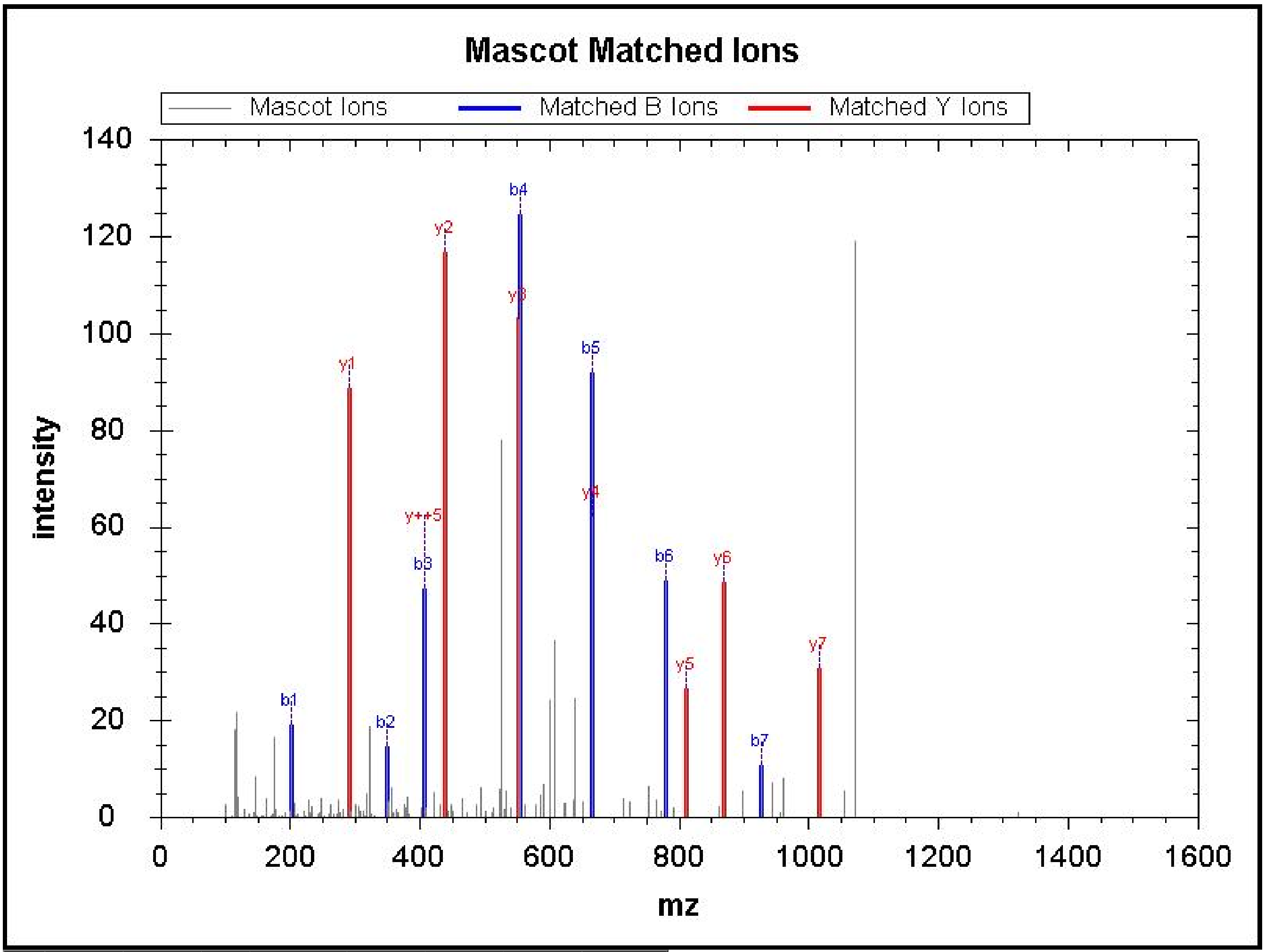

No	b	b++	Seq	y	y++	y*	y*++	RevNo
1	202.13	101.57	G					8
2	349.20	175.10	F	1,015.61	508.31	998.58	499.80	7
3	406.22	203.61	G	868.54	434.77	851.51	426.26	6
4	553.29	277.15	F	811.52	406.26	794.49	397.75	5
5	666.37	333.69	I	664.45	332.73	647.42	324.22	4
6	779.46	390.23	L	551.37	276.19	534.34	267.67	3
7	926.53	463.77	F	438.28	219.65	421.26	211.13	2
8			K	291.21	146.11	274.19	137.60	1

Query 2094 Hit 1

MS/MS Fragmentation of **TLMELLNQMDGFDTLHR**
Found in **sp|P62333|PRS10_HUMAN**, 26S protease regulatory subunit 10B OS=Homo sapiens GN=PSMC6 PE=1 SV=1
Match to Query 2094: 2177.073from(726.6984,3+)
Title: 1135: Scan 2806 (rt=71.4842, f=3, i=407) [D:\lab212\membrane\Grace\20120214_iTRAQ_2.raw]
Data File:
Monoisotopic mass of neutral peptide Mr(calc): 2177.073
Variable modifications:
Ions Score: 59.86 **Expect:** 0.001

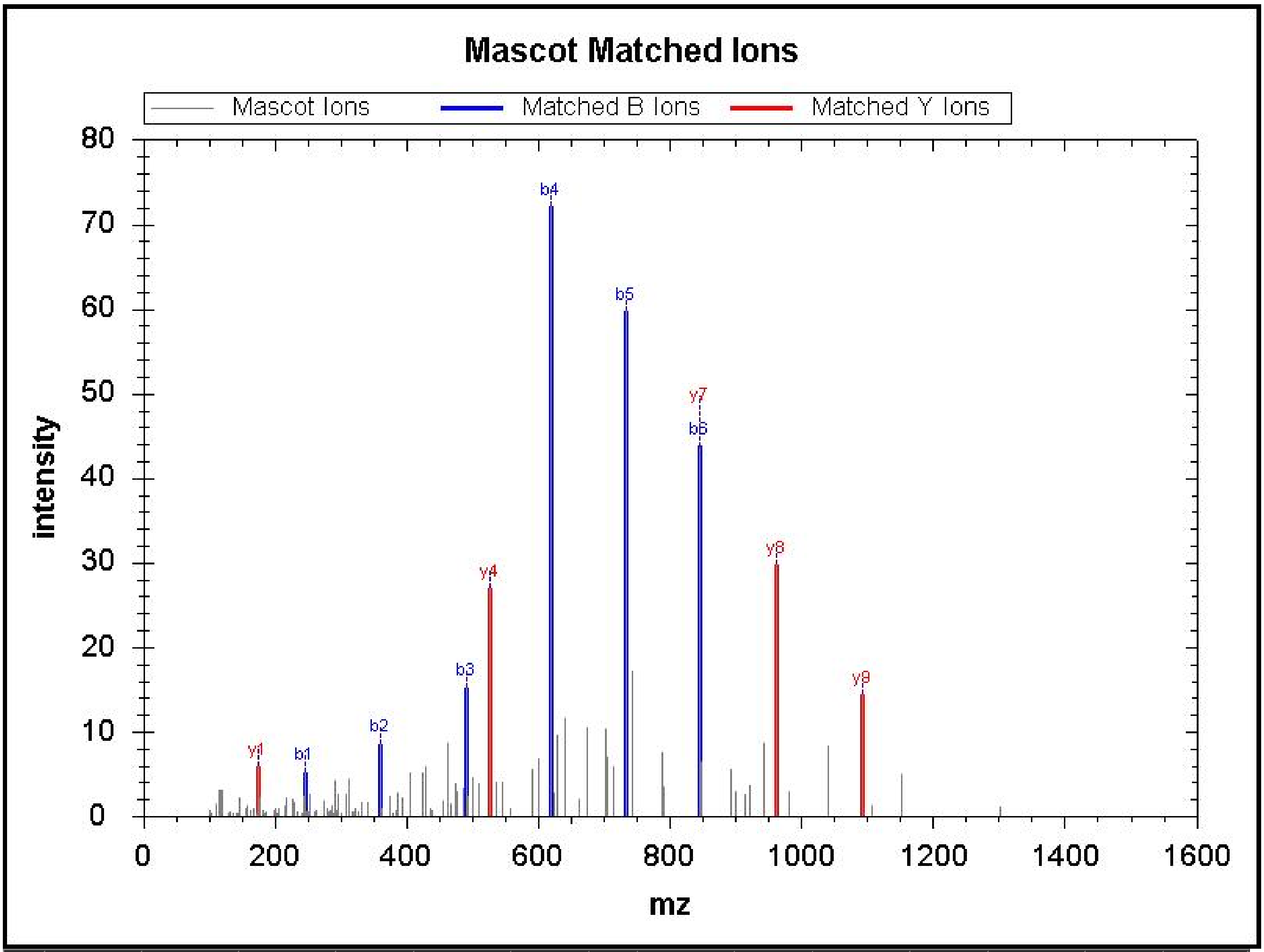

No	b	b++	b*	b*++	b0	b0++	Seq	y	y++	y*	y*++	y0	y0++	RevNo
1	**246.16**	123.58			228.15	114.58	T							17
2	**359.24**	180.12			341.23	171.12	L	1,932.93	966.97	1,915.90	958.46	1,914.92	957.96	16
3	**490.28**	245.64			472.27	236.64	M	1,819.85	910.43	1,802.82	901.91	1,801.84	901.42	15
4	**619.32**	310.17			601.31	301.16	E	1,688.81	844.91	1,671.78	836.39	1,670.80	835.90	14
5	**732.41**	366.71			714.40	357.70	L	1,559.76	780.39	1,542.74	771.87	1,541.75	771.38	13
6	**845.49**	423.25			827.48	414.24	L	1,446.68	723.84	1,429.65	715.33	1,428.67	714.84	12
7	959.54	480.27	942.51	471.76	941.52	471.27	N	1,333.60	667.30	1,316.57	658.79	1,315.58	658.30	11
8	1,087.59	544.30	1,070.57	535.79	1,069.58	535.30	Q	1,219.55	610.28	1,202.53	601.77	1,201.54	601.27	10
9	1,218.63	609.82	1,201.61	601.31	1,200.62	600.82	M	**1,091.49**	546.25	1,074.47	537.74	1,073.48	537.25	9
10	1,333.66	667.33	1,316.63	658.82	1,315.65	658.33	D	**960.45**	480.73	943.43	472.22	942.44	471.73	8
11	1,390.68	695.85	1,373.66	687.33	1,372.67	686.84	G	**845.43**	423.22	828.40	414.70	827.42	414.21	7
12	1,537.75	769.38	1,520.72	760.87	1,519.74	760.37	F	788.40	394.71	771.38	386.19	770.39	385.70	6
13	1,652.78	826.89	1,635.75	818.38	1,634.77	817.89	D	641.34	321.17	624.31	312.66	623.33	312.17	5
14	1,753.83	877.42	1,736.80	868.90	1,735.82	868.41	T	**526.31**	263.66	509.28	255.15	508.30	254.65	4
15	1,866.91	933.96	1,849.88	925.45	1,848.90	924.95	L	425.26	213.13	408.24	204.62			3
16	2,003.97	1,002.49	1,986.94	993.97	1,985.96	993.48	H	312.18	156.59	295.15	148.08			2
17							R	**175.12**	88.06	158.09	79.55			1

Query 2901 Hit 1

MS/MS Fragmentation of **FISDKDASIVGFFDDSFSEAHSEFLK**
Found in **sp|P30101|PDIA3_HUMAN**, Protein disulfide-isomerase A3 OS=Homo sapiens GN=PDIA3 PE=1 SV=4
Match to Query 2901: 3369.674from(843.4258,4+)
Title: 1025: Sum of 2 scans in range 2615 (rt=67.0984, f=4, i=696) to 2616 (rt=67.1238, f=4, i=697) [D:\lab212\membrane\Grace\20120214_iTRAQ_1.raw]

Data File:
Monoisotopic mass of neutral peptide Mr(calc): 3369.674
Variable modifications:
K5 :iTRAQ4plex (K)
K26 :iTRAQ4plex (K)
Ions Score: 60.72 **Expect:** 0.001

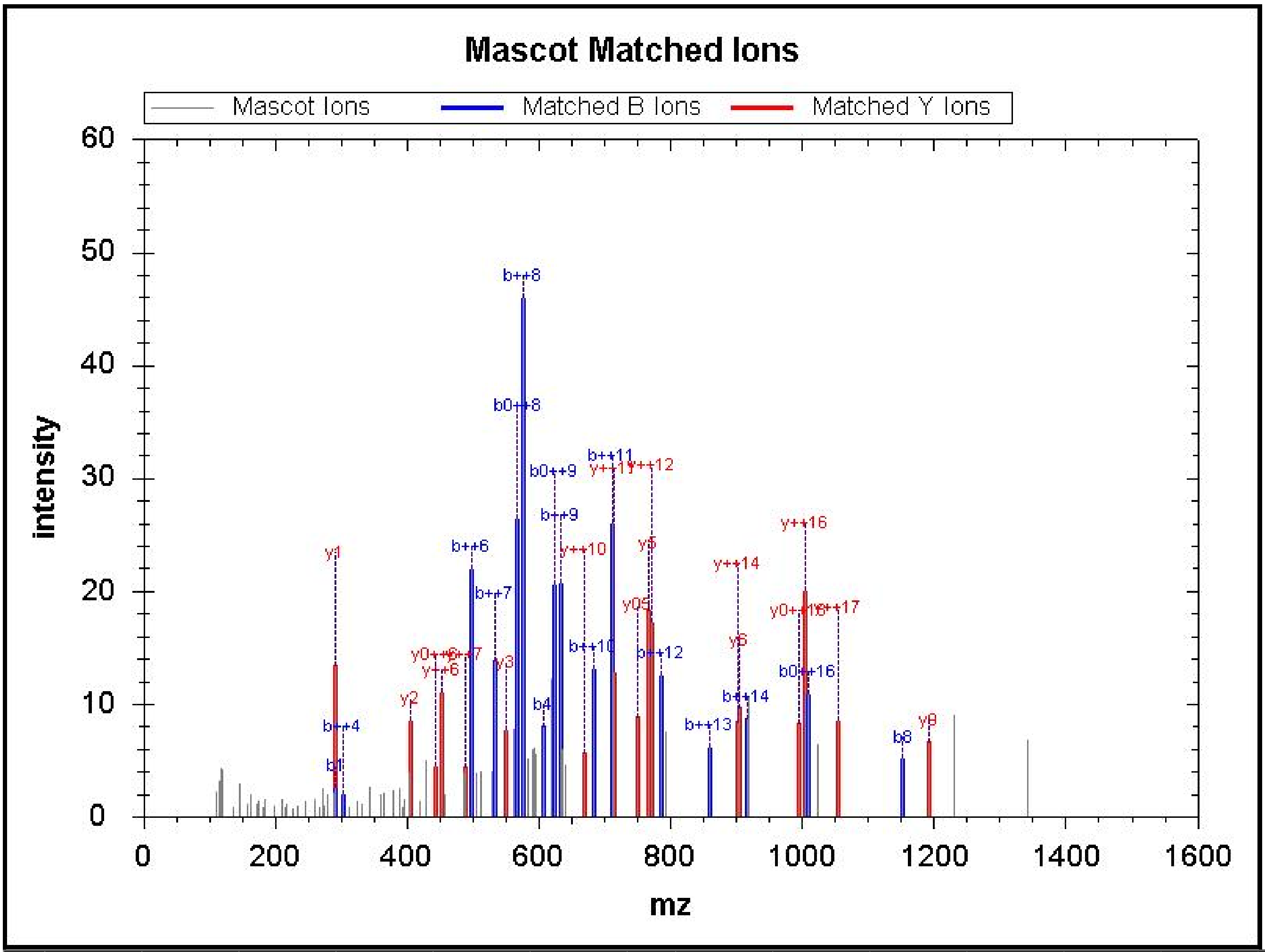

No	b	b++	b*	b*++	b0	b0++	Seq	y	y++	y*	y*++	y0	y0++	RevNo
1	**292.18**	146.59					F							26
2	405.26	203.13					I	3,079.51	1,540.26	3,062.49	1,531.75	3,061.50	1,531.26	25
3	492.29	246.65			474.28	237.65	S	2,966.43	1,483.72	2,949.40	1,475.20	2,948.42	1,474.71	24
4	**607.32**	**304.16**			589.31	295.16	D	2,879.40	1,440.20	2,862.37	1,431.69	2,861.39	1,431.20	23
5	879.52	440.26	862.49	431.75	861.51	431.26	K	2,764.37	1,382.69	2,747.34	1,374.18	2,746.36	1,373.68	22
6	994.54	**497.78**	977.52	489.26	976.53	488.77	D	2,492.17	1,246.59	2,475.15	1,238.08	2,474.16	1,237.59	21
7	1,065.58	**533.29**	1,048.56	524.78	1,047.57	524.29	A	2,377.15	1,189.08	2,360.12	1,180.56	2,359.14	1,180.07	20
8	**1,152.61**	**576.81**	1,135.59	568.30	1,134.60	**567.81**	S	2,306.11	1,153.56	2,289.08	1,145.05	2,288.10	1,144.55	19
9	1,265.70	**633.35**	1,248.67	624.84	1,247.69	**624.35**	I	2,219.08	1,110.04	2,202.05	1,101.53	2,201.07	1,101.04	18
10	1,364.77	**682.89**	1,347.74	674.37	1,346.76	673.88	V	2,105.99	**1,053.50**	2,088.97	1,044.99	2,087.98	1,044.50	17
11	1,421.79	**711.40**	1,404.76	702.88	1,403.78	702.39	G	2,006.93	**1,003.97**	1,989.90	995.45	1,988.91	**994.96**	16
12	1,568.86	**784.93**	1,551.83	776.42	1,550.85	775.93	F	1,949.90	975.46	1,932.88	966.94	1,931.89	966.45	15
13	1,715.92	**858.47**	1,698.90	849.95	1,697.91	849.46	F	1,802.84	**901.92**	1,785.81	893.41	1,784.82	892.92	14
14	1,830.95	**915.98**	1,813.93	907.47	1,812.94	906.97	D	1,655.77	828.39	1,638.74	819.87	1,637.76	819.38	13
15	1,945.98	973.49	1,928.95	964.98	1,927.97	964.49	D	1,540.74	**770.87**	1,523.71	762.36	1,522.73	761.87	12
16	2,033.01	1,017.01	2,015.98	1,008.50	2,015.00	**1,008.00**	S	1,425.71	**713.36**	1,408.69	704.85	1,407.70	704.35	11
17	2,180.08	1,090.54	2,163.05	1,082.03	2,162.07	1,081.54	F	1,338.68	**669.84**	1,321.65	661.33	1,320.67	660.84	10
18	2,267.11	1,134.06	2,250.08	1,125.55	2,249.10	1,125.05	S	**1,191.61**	596.31	1,174.59	587.80	1,173.60	587.30	9

19	2,396.15	1,198.58	2,379.13	1,190.07	2,378.14	1,189.58	E	1,104.58	552.79	1,087.55	544.28	1,086.57	543.79	8
20	2,467.19	1,234.10	2,450.16	1,225.59	2,449.18	1,225.09	A	975.54	488.27	958.51	479.76	957.53	479.27	7
21	2,604.25	1,302.63	2,587.22	1,294.11	2,586.24	1,293.62	H	904.50	452.75	887.47	444.24	886.49	443.75	6
22	2,691.28	1,346.14	2,674.25	1,337.63	2,673.27	1,337.14	S	767.44	384.22	750.42	375.71	749.43	375.22	5
23	2,820.32	1,410.67	2,803.30	1,402.15	2,802.31	1,401.66	E	680.41	340.71	663.38	332.20	662.40	331.70	4
24	2,967.39	1,484.20	2,950.37	1,475.69	2,949.38	1,475.19	F	551.37	276.19	534.34	267.67			3
25	3,080.48	1,540.74	3,063.45	1,532.23	3,062.47	1,531.74	L	404.30	202.65	387.27	194.14			2
26							K	291.21	146.11	274.19	137.60			1

Query 2549 Hit 1

MS/MS Fragmentation of **GLAFIQDPDGYWIEILNPNK**
Found in **sp|Q04760|LGUL_HUMAN**, Lactoylglutathione lyase OS=Homo sapiens GN=GLO1 PE=1 SV=4
Match to Query 2549: 2590.372from(864.4646,3+)
Title: 1105: Scan 2744 (rt=70.0872, f=3, i=397) [D:\lab212\membrane\Grace\20120214_iTRAQ_2.raw]
Data File:
Monoisotopic mass of neutral peptide Mr(calc): 2590.372
Variable modifications:
K20 :iTRAQ4plex (K)
Ions Score: 61.62 **Expect:** 0.001

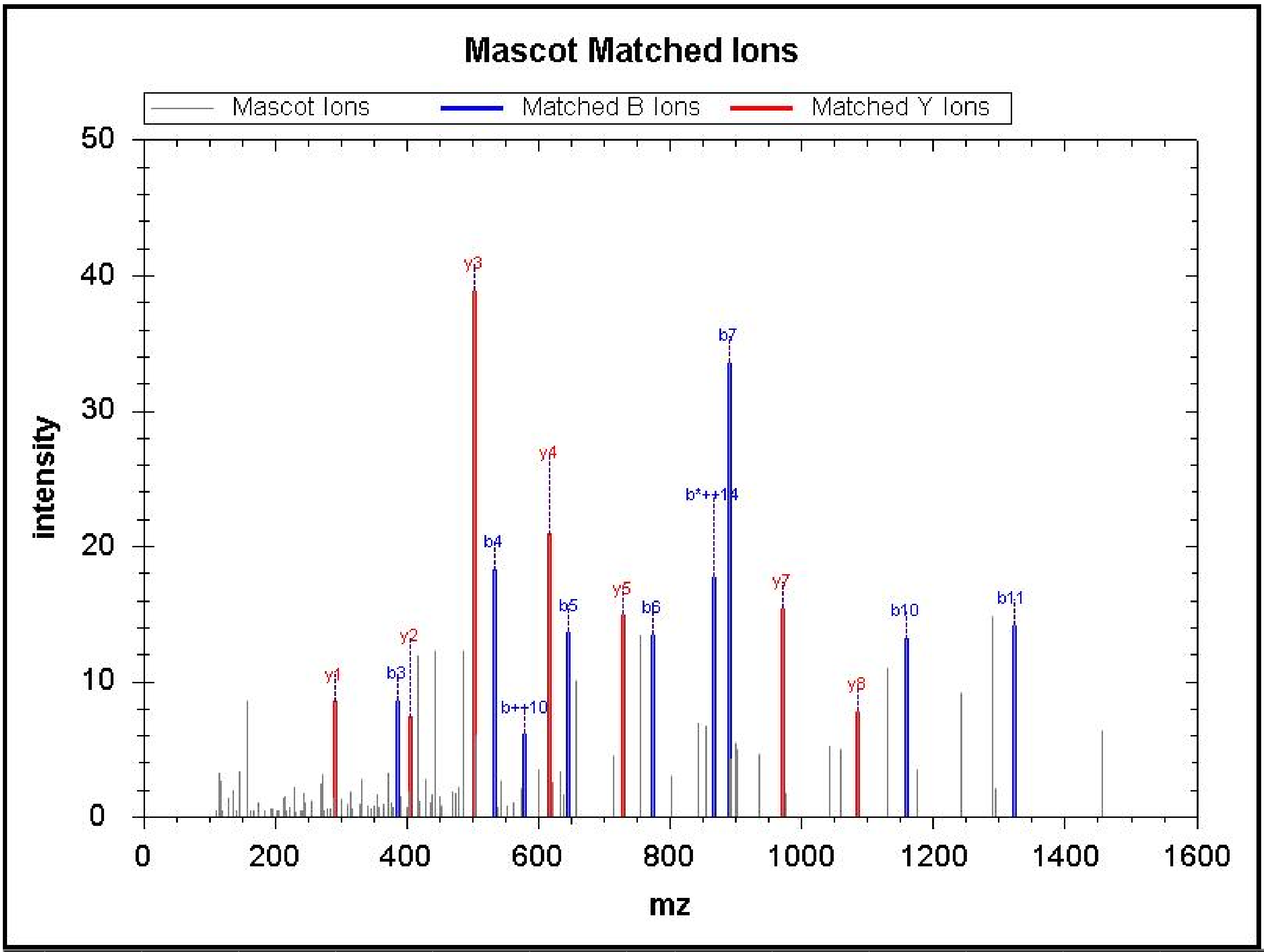

No	b	b++	b*	b*++	b0	b0++	Seq	y	y++	y*	y*++	y0	y0++	RevNo
1	202.13	101.57					G							20
2	315.21	158.11					L	2,390.25	1,195.63	2,373.22	1,187.12	2,372.24	1,186.62	19
3	386.25	193.63					A	2,277.17	1,139.09	2,260.14	1,130.57	2,259.16	1,130.08	18
4	533.32	267.16					F	2,206.13	1,103.57	2,189.10	1,095.06	2,188.12	1,094.56	17
5	646.40	323.71					I	2,059.06	1,030.03	2,042.04	1,021.52	2,041.05	1,021.03	16
6	774.46	387.74	757.44	379.22			Q	1,945.98	973.49	1,928.95	964.98	1,927.97	964.49	15

7	889.49	445.25	872.46	436.74	871.48	436.24	D	1,817.92	909.46	1,800.89	900.95	1,799.91	900.46	14
8	986.54	493.77	969.52	485.26	968.53	484.77	P	1,702.89	851.95	1,685.87	843.44	1,684.88	842.94	13
9	1,101.57	551.29	1,084.54	542.78	1,083.56	542.28	D	1,605.84	803.42	1,588.81	794.91	1,587.83	794.42	12
10	1,158.59	579.80	1,141.56	571.29	1,140.58	570.79	G	1,490.81	745.91	1,473.79	737.40	1,472.80	736.90	11
11	1,321.65	661.33	1,304.63	652.82	1,303.64	652.33	Y	1,433.79	717.40	1,416.76	708.89	1,415.78	708.39	10
12	1,507.73	754.37	1,490.71	745.86	1,489.72	745.37	W	1,270.73	635.87	1,253.70	627.35	1,252.72	626.86	9
13	1,620.82	810.91	1,603.79	802.40	1,602.81	801.91	I	1,084.65	542.83	1,067.62	534.31	1,066.64	533.82	8
14	1,749.86	875.43	1,732.83	866.92	1,731.85	866.43	E	971.56	486.29	954.54	477.77	953.55	477.28	7
15	1,862.94	931.98	1,845.92	923.46	1,844.93	922.97	I	842.52	421.76	825.49	413.25			6
16	1,976.03	988.52	1,959.00	980.00	1,958.02	979.51	L	729.44	365.22	712.41	356.71			5
17	2,090.07	1,045.54	2,073.05	1,037.03	2,072.06	1,036.53	N	616.35	308.68	599.33	300.17			4
18	2,187.12	1,094.07	2,170.10	1,085.55	2,169.11	1,085.06	P	502.31	251.66	485.28	243.15			3
19	2,301.17	1,151.09	2,284.14	1,142.57	2,283.16	1,142.08	N	405.26	203.13	388.23	194.62			2
20							K	291.21	146.11	274.19	137.60			1

Query 608 Hit 1

MS/MS Fragmentation of **VFQFLNAK**
Found in **sp|P83731|RL24_HUMAN**, 60S ribosomal protein L24 OS=Homo sapiens GN=RPL24 PE=1 SV=1
Match to Query 608: 1253.735from(627.875,2+)
Title: 716: Scan 1932 (rt=51.7903, f=3, i=266) [D:\lab212\membrane\Grace\20120214_iTRAQ_2.raw]
Data File:
Monoisotopic mass of neutral peptide Mr(calc): 1253.735
Variable modifications:
K8 :iTRAQ4plex (K)
Ions Score: 62.57 **Expect:** 0.000

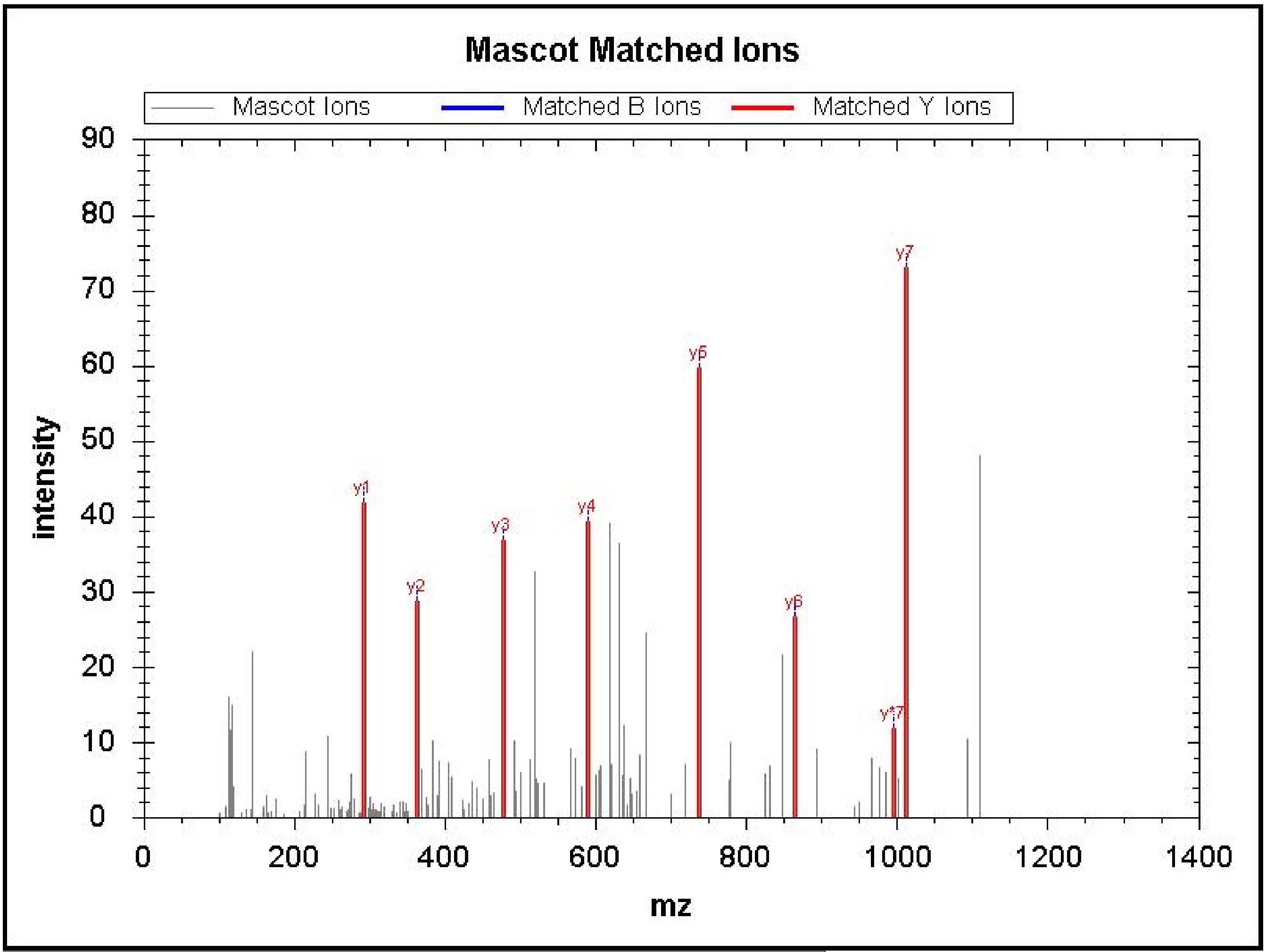

No	b	b++	b*	b*++	Seq	y	y++	y*	y*++	RevNo

1	244.18	122.59			V					8
2	391.25	196.13			F	1,011.57	506.29	994.55	497.78	7
3	519.30	260.16	502.28	251.64	Q	864.51	432.76	847.48	424.24	6
4	666.37	333.69	649.35	325.18	F	736.45	368.73	719.42	360.21	5
5	779.46	390.23	762.43	381.72	L	589.38	295.19	572.35	286.68	4
6	893.50	447.25	876.47	438.74	N	476.29	238.65	459.27	230.14	3
7	964.54	482.77	947.51	474.26	A	362.25	181.63	345.23	173.12	2
8					K	291.21	146.11	274.19	137.60	1

Query 2838 Hit 1

MS/MS Fragmentation of **VDNMIIQSISLLDQLDKDINTFSMR**
Found in **sp|O00567|NOP56_HUMAN**, Nucleolar protein 56 OS=Homo sapiens GN=NOP56 PE=1 SV=4
Match to Query 2838: 3196.672from(1066.564,3+)
Title: 1458: Scan 3671 (rt=90.6937, f=3, i=539) [D:\lab212\membrane\Grace\20120214_iTRAQ_1.raw]
Data File:
Monoisotopic mass of neutral peptide Mr(calc): 3196.672
Variable modifications:
K17 :iTRAQ4plex (K)
Ions Score: 62.98 **Expect:** 0.000

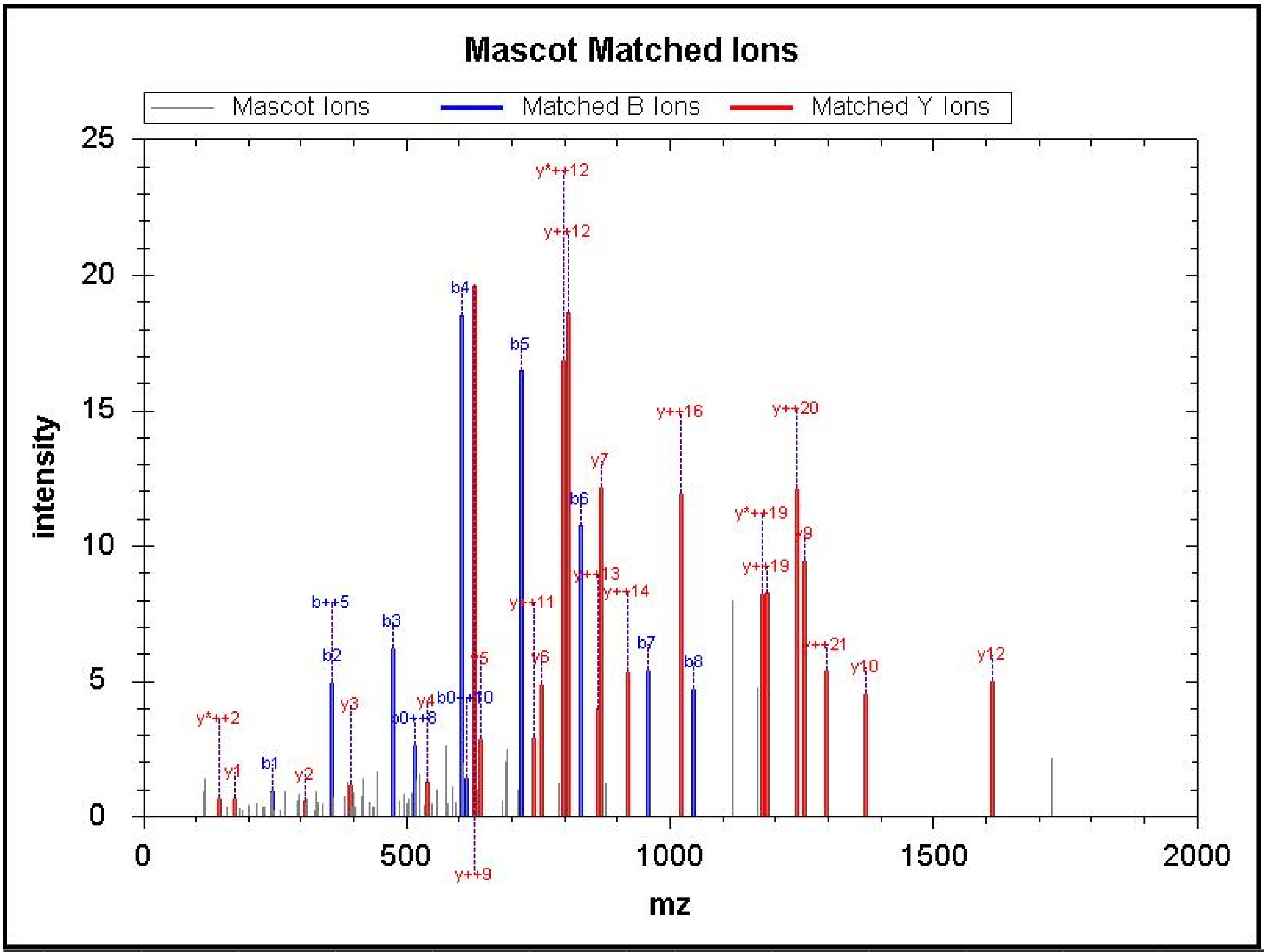

No	b	b++	b*	b*++	b0	b0++	Seq	y	y++	y*	y*++	y0	y0++	RevNo
1	244.18	122.59					V							25
2	359.20	180.11			341.19	171.10	D	2,954.51	1,477.76	2,937.48	1,469.24	2,936.50	1,468.75	24
3	473.25	237.13	456.22	228.61	455.24	228.12	N	2,839.48	1,420.24	2,822.45	1,411.73	2,821.47	1,411.24	23
4	604.29	302.65	587.26	294.13	586.28	293.64	M	2,725.44	1,363.22	2,708.41	1,354.71	2,707.43	1,354.22	22
5	717.37	359.19	700.35	350.68	699.36	350.18	I	2,594.40	1,297.70	2,577.37	1,289.19	2,576.39	1,288.70	21
6	830.46	415.73	813.43	407.22	812.45	406.73	I	2,481.31	1,241.16	2,464.29	1,232.65	2,463.30	1,232.16	20

7	958.51	479.76	941.49	471.25	940.50	470.76	Q	2,368.23	1,184.62	2,351.20	1,176.11	2,350.22	1,175.61	19
8	1,045.55	523.28	1,028.52	514.76	1,027.54	514.27	S	2,240.17	1,120.59	2,223.14	1,112.08	2,222.16	1,111.58	18
9	1,158.63	579.82	1,141.60	571.31	1,140.62	570.81	I	2,153.14	1,077.07	2,136.11	1,068.56	2,135.13	1,068.07	17
10	1,245.66	623.34	1,228.64	614.82	1,227.65	614.33	S	2,040.06	1,020.53	2,023.03	1,012.02	2,022.04	1,011.53	16
11	1,358.75	679.88	1,341.72	671.36	1,340.74	670.87	L	1,953.02	977.02	1,936.00	968.50	1,935.01	968.01	15
12	1,471.83	736.42	1,454.80	727.91	1,453.82	727.41	L	1,839.94	920.47	1,822.91	911.96	1,821.93	911.47	14
13	1,586.86	793.93	1,569.83	785.42	1,568.85	784.93	D	1,726.86	863.93	1,709.83	855.42	1,708.84	854.93	13
14	1,714.92	857.96	1,697.89	849.45	1,696.91	848.96	Q	1,611.83	806.42	1,594.80	797.90	1,593.82	797.41	12
15	1,828.00	914.50	1,810.97	905.99	1,809.99	905.50	L	1,483.77	742.39	1,466.74	733.88	1,465.76	733.38	11
16	1,943.03	972.02	1,926.00	963.50	1,925.02	963.01	D	1,370.69	685.85	1,353.66	677.33	1,352.67	676.84	10
17	2,215.22	1,108.12	2,198.20	1,099.60	2,197.21	1,099.11	K	1,255.66	628.33	1,238.63	619.82	1,237.65	619.33	9
18	2,330.25	1,165.63	2,313.23	1,157.12	2,312.24	1,156.62	D	983.46	492.23	966.43	483.72	965.45	483.23	8
19	2,443.34	1,222.17	2,426.31	1,213.66	2,425.33	1,213.17	I	868.43	434.72	851.41	426.21	850.42	425.72	7
20	2,557.38	1,279.19	2,540.35	1,270.68	2,539.37	1,270.19	N	755.35	378.18	738.32	369.67	737.34	369.17	6
21	2,658.43	1,329.72	2,641.40	1,321.20	2,640.42	1,320.71	T	641.31	321.16	624.28	312.64	623.30	312.15	5
22	2,805.49	1,403.25	2,788.47	1,394.74	2,787.48	1,394.25	F	540.26	270.63	523.23	262.12	522.25	261.63	4
23	2,892.53	1,446.77	2,875.50	1,438.25	2,874.52	1,437.76	S	393.19	197.10	376.16	188.59	375.18	188.09	3
24	3,023.57	1,512.29	3,006.54	1,503.77	3,005.56	1,503.28	M	306.16	153.58	289.13	145.07			2
25							R	175.12	88.06	158.09	79.55			1

Query 2133 Hit 1

MS/MS Fragmentation of **RPFGISALIVGFDFDGTPR**
Found in **sp|O14818|PSA7_HUMAN**, Proteasome subunit alpha type-7 OS=Homo sapiens GN=PSMA7 PE=1 SV=1
Match to Query 2133: 2208.18from(737.0673,3+)
Title: 1041: Scan 2613 (rt=67.132, f=2, i=411) [D:\lab212\membrane\Grace\20120214_iTRAQ_2.raw]
Data File:
Monoisotopic mass of neutral peptide Mr(calc): 2208.18
Variable modifications:
Ions Score: 63.81 **Expect:** 0.000

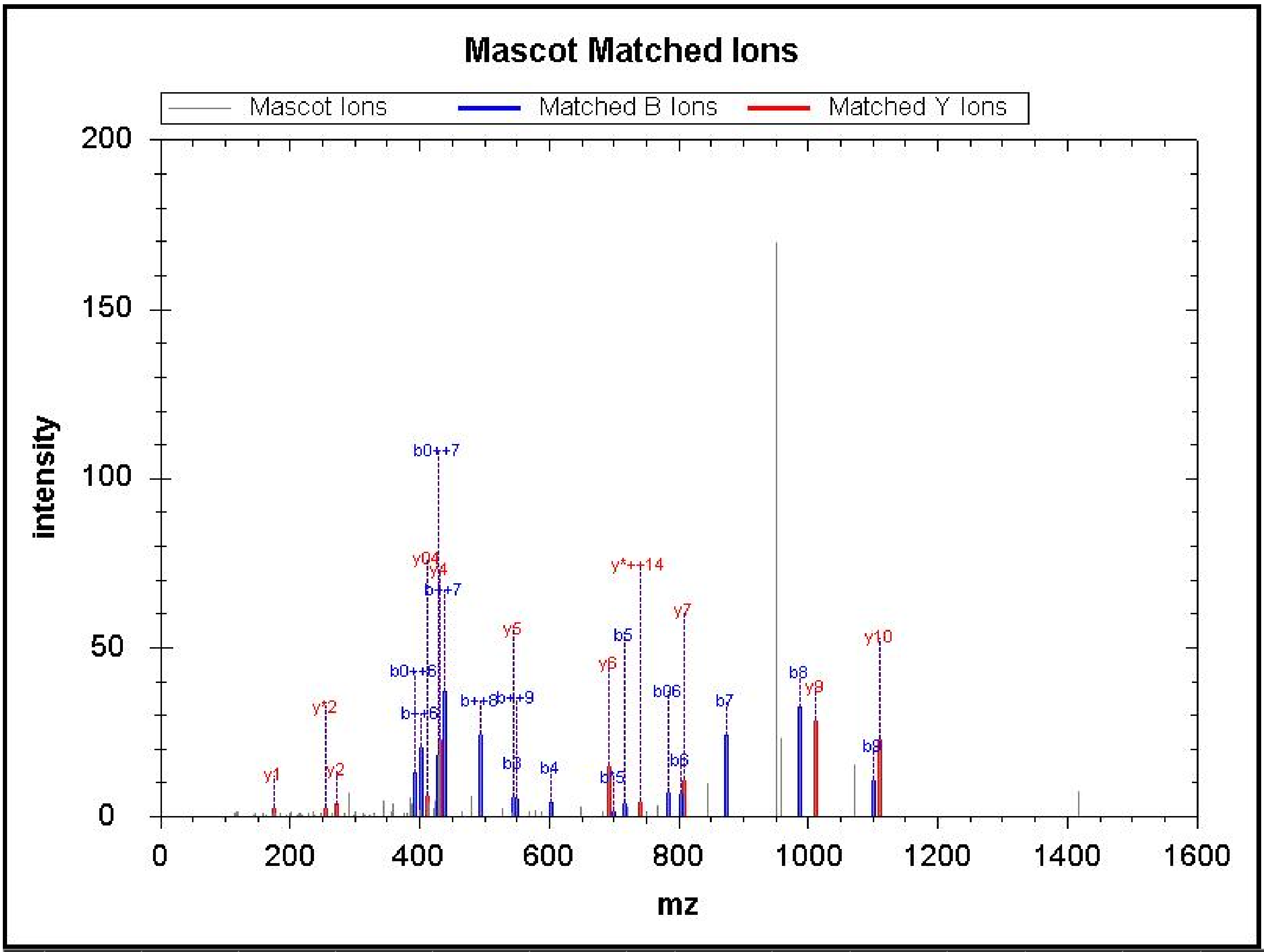

No	b	b++	b*	b*++	b0	b0++	Seq	y	y++	y*	y*++	y0	y0++	RevNo
1	301.21	151.11	284.18	142.60			R							19
2	398.26	199.64	381.24	191.12			P	1,908.99	955.00	1,891.96	946.48	1,890.97	945.99	18
3	**545.33**	273.17	528.31	264.66			F	1,811.93	906.47	1,794.91	897.96	1,793.92	897.46	17
4	**602.35**	301.68	585.33	293.17			G	1,664.86	832.94	1,647.84	824.42	1,646.85	823.93	16
5	**715.44**	358.22	**698.41**	349.71			I	1,607.84	804.43	1,590.82	795.91	1,589.83	795.42	15
6	**802.47**	**401.74**	785.44	393.22	**784.46**	**392.73**	S	1,494.76	747.88	1,477.73	**739.37**	1,476.75	738.88	14
7	**873.51**	**437.26**	856.48	428.74	855.50	**428.25**	A	1,407.73	704.37	1,390.70	695.85	1,389.72	695.36	13
8	**986.59**	**493.80**	969.56	485.29	968.58	484.79	L	1,336.69	668.85	1,319.66	660.34	1,318.68	659.84	12
9	**1,099.67**	**550.34**	1,082.65	541.83	1,081.66	541.34	I	1,223.61	612.31	1,206.58	603.79	1,205.59	603.30	11
10	1,198.74	599.88	1,181.72	591.36	1,180.73	590.87	V	**1,110.52**	555.76	1,093.49	547.25	1,092.51	546.76	10
11	1,255.76	628.39	1,238.74	619.87	1,237.75	619.38	G	**1,011.45**	506.23	994.43	497.72	993.44	497.22	9
12	1,402.83	701.92	1,385.81	693.41	1,384.82	692.91	F	954.43	477.72	937.40	469.21	936.42	468.71	8
13	1,517.86	759.43	1,500.83	750.92	1,499.85	750.43	D	**807.36**	404.19	790.34	395.67	789.35	395.18	7
14	1,664.93	832.97	1,647.90	824.45	1,646.92	823.96	F	**692.34**	346.67	675.31	338.16	674.33	337.67	6
15	1,779.96	890.48	1,762.93	881.97	1,761.94	881.48	D	**545.27**	273.14	528.24	264.62	527.26	264.13	5
16	1,836.98	918.99	1,819.95	910.48	1,818.97	909.99	G	**430.24**	215.62	413.21	207.11	**412.23**	206.62	4
17	1,938.02	969.52	1,921.00	961.00	1,920.01	960.51	T	373.22	187.11	356.19	178.60	355.21	178.11	3
18	2,035.08	1,018.04	2,018.05	1,009.53	2,017.07	1,009.04	P	**272.17**	136.59	**255.15**	128.08			2
19							R	**175.12**	88.06	158.09	79.55			1

Query 1137 Hit 1

MS/MS Fragmentation of **NIPGITLLNVSK**
Found in **sp|P36578|RL4_HUMAN**, 60S ribosomal protein L4 OS=Homo sapiens GN=RPL4 PE=1 SV=5
Match to Query 1137: 1555.953from(778.9838,2+)

Title: 740: Sum of 2 scans in range 2026 (rt=53.8267, f=4, i=506) to 2027 (rt=53.8521, f=4, i=507) [D:\lab212\membrane\Grace\20120214_iTRAQ_1.raw]
Data File:
Monoisotopic mass of neutral peptide Mr(calc): 1555.953
Variable modifications:
K12 :iTRAQ4plex (K)
Ions Score: 64.09 **Expect:** 0.000

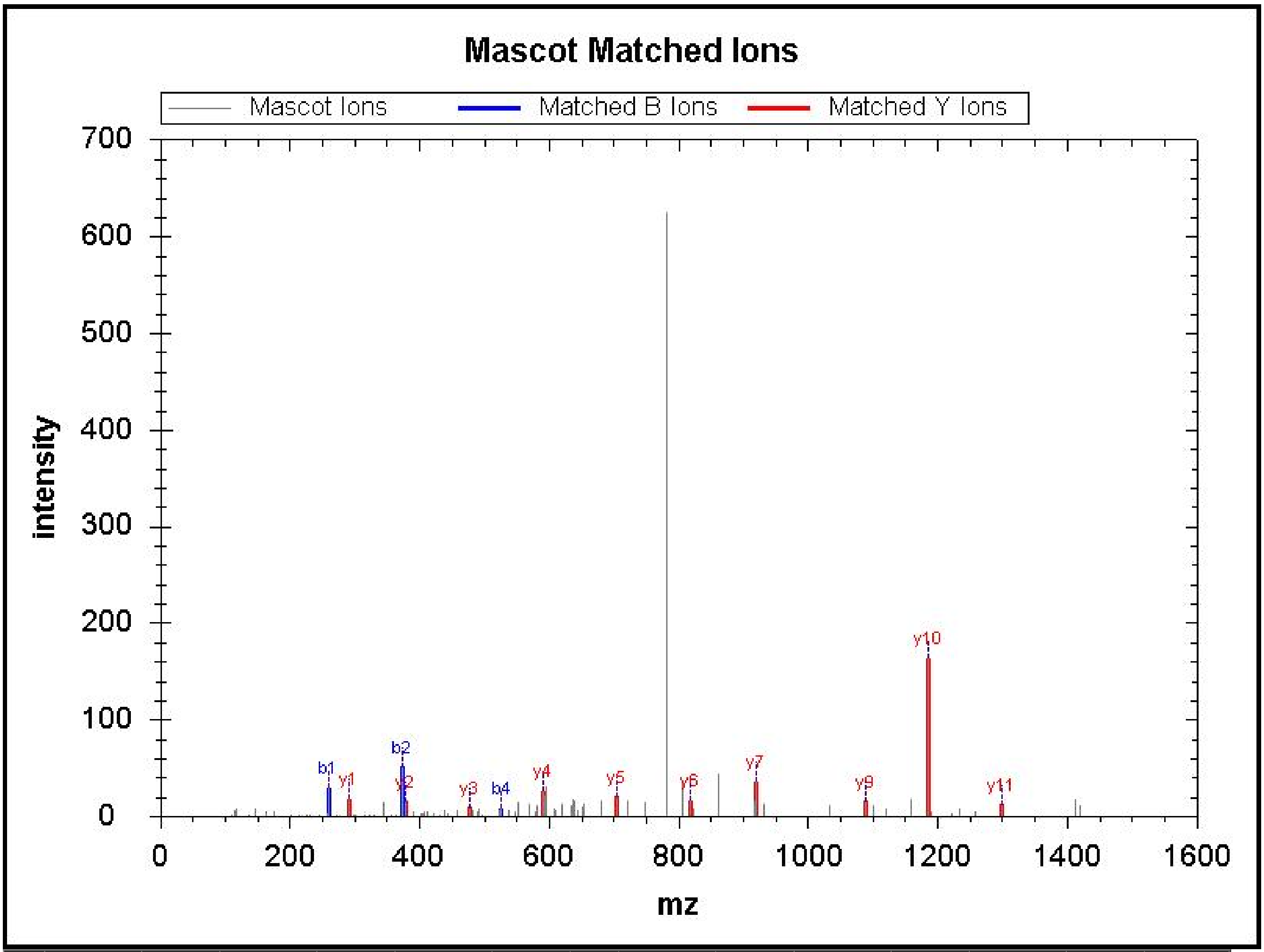

No	b	b++	b*	b*++	b0	b0++	Seq	y	y++	y*	y*++	y0	y0++	RevNo
1	259.15	130.08	242.13	121.57			N							12
2	372.24	186.62	355.21	178.11			I	1,298.82	649.91	1,281.79	641.40	1,280.81	640.91	11
3	469.29	235.15	452.26	226.63			P	1,185.73	593.37	1,168.71	584.86	1,167.72	584.36	10
4	526.31	263.66	509.28	255.15			G	1,088.68	544.84	1,071.65	536.33	1,070.67	535.84	9
5	639.39	320.20	622.37	311.69			I	1,031.66	516.33	1,014.63	507.82	1,013.65	507.33	8
6	740.44	370.72	723.42	362.21	722.43	361.72	T	918.57	459.79	901.55	451.28	900.56	450.79	7
7	853.53	427.27	836.50	418.75	835.52	418.26	L	817.53	409.27	800.50	400.75	799.52	400.26	6
8	966.61	483.81	949.58	475.30	948.60	474.80	L	704.44	352.72	687.42	344.21	686.43	343.72	5
9	1,080.65	540.83	1,063.63	532.32	1,062.64	531.83	N	591.36	296.18	574.33	287.67	573.35	287.18	4
10	1,179.72	590.36	1,162.70	581.85	1,161.71	581.36	V	477.32	239.16	460.29	230.65	459.30	230.16	3
11	1,266.75	633.88	1,249.73	625.37	1,248.74	624.88	S	378.25	189.63	361.22	181.11	360.24	180.62	2
12							K	291.21	146.11	274.19	137.60			1

Query 1878 Hit 1

MS/MS Fragmentation of **KIEEIKDFLLTAR**
Found in **sp|P63173|RL38_HUMAN**, 60S ribosomal protein L38 OS=Homo sapiens GN=RPL38 PE=1 SV=2
Match to Query 1878: 2007.206from(670.076,3+)
Title: 810: Scan 2129 (rt=56.2275, f=2, i=333) [D:\lab212\membrane\Grace\20120214_iTRAQ_2.raw]
Data File:

Monoisotopic mass of neutral peptide Mr(calc): 2007.206
Variable modifications:
K1 :iTRAQ4plex (K)
K6 :iTRAQ4plex (K)
Ions Score: 64.72 **Expect:** 0.000

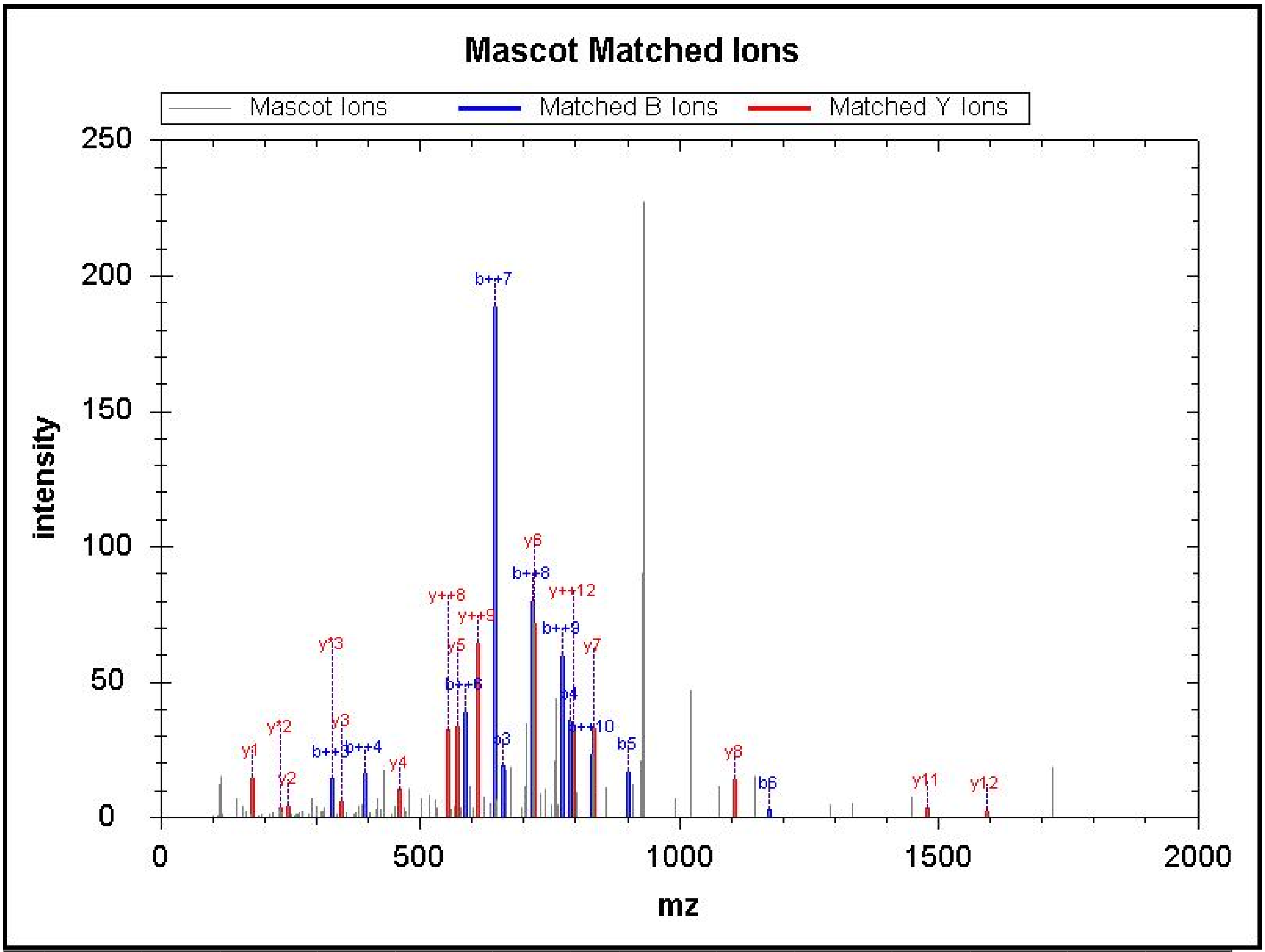

No	b	b++	b*	b*++	b0	b0++	Seq	y	y++	y*	y*++	y0	y0++	RevNo
1	417.31	209.16	400.28	200.64			K							13
2	530.39	265.70	513.36	257.19			I	**1,591.92**	**796.46**	1,574.89	787.95	1,573.91	787.46	12
3	**659.43**	**330.22**	642.41	321.71	641.42	321.21	E	**1,478.83**	739.92	1,461.81	731.41	1,460.82	730.92	11
4	**788.48**	**394.74**	771.45	386.23	770.47	385.74	E	1,349.79	675.40	1,332.76	666.89	1,331.78	666.39	10
5	**901.56**	451.28	884.53	442.77	883.55	442.28	I	1,220.75	**610.88**	1,203.72	602.36	1,202.74	601.87	9
6	**1,173.76**	**587.38**	1,156.73	578.87	1,155.75	578.38	K	**1,107.66**	**554.34**	1,090.64	545.82	1,089.65	545.33	8
7	1,288.78	**644.90**	1,271.76	636.38	1,270.77	635.89	D	**835.47**	418.24	818.44	409.72	817.46	409.23	7
8	1,435.85	**718.43**	1,418.83	709.92	1,417.84	709.42	F	**720.44**	360.72	703.41	352.21	702.43	351.72	6
9	1,548.94	**774.97**	1,531.91	766.46	1,530.93	765.97	L	**573.37**	287.19	556.35	278.68	555.36	278.18	5
10	1,662.02	**831.51**	1,644.99	823.00	1,644.01	822.51	L	**460.29**	230.65	443.26	222.13	442.28	221.64	4
11	1,763.07	882.04	1,746.04	873.52	1,745.06	873.03	T	**347.20**	174.11	**330.18**	165.59	329.19	165.10	3
12	1,834.11	917.56	1,817.08	909.04	1,816.09	908.55	A	**246.16**	123.58	**229.13**	115.07			2
13							R	**175.12**	88.06	158.09	79.55			1

Query 989 Hit 1

MS/MS Fragmentation of **LGELPSWILMR**
Found in **sp|P56134|ATPK_HUMAN**, ATP synthase subunit f
Match to Query 989: 1457.821from(729.918,2+)
Title: 1021: Scan 2570 (rt=66.1677, f=3, i=369) [D:\lab212\membrane\Grace\20120214_iTRAQ_2.raw]
Data File:

Monoisotopic mass of neutral peptide Mr(calc): 1457.821
Variable modifications:
Ions Score: 65.89 **Expect:** 0.000

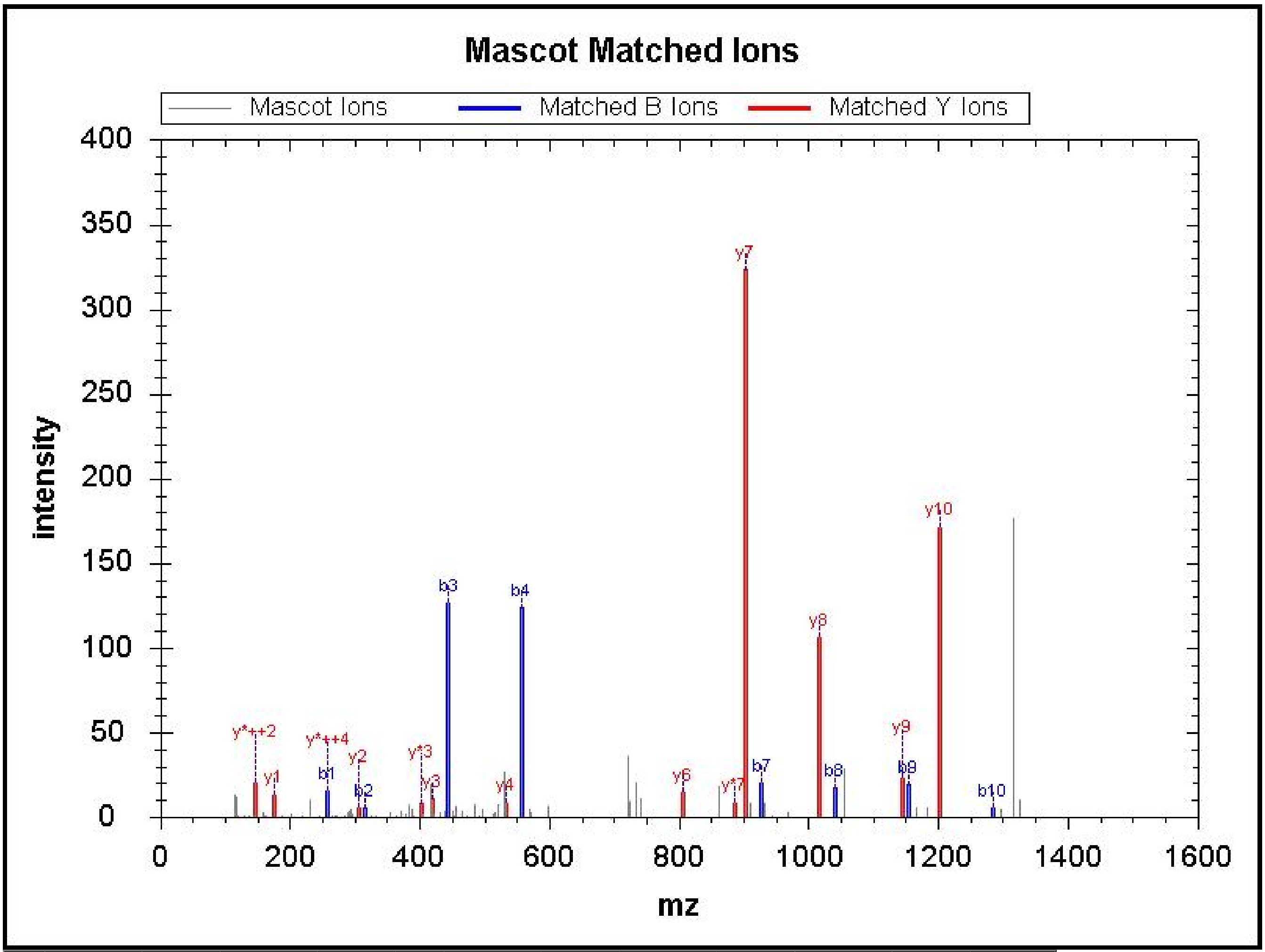

No	b	b++	b0	b0++	Seq	y	y++	y*	y*++	y0	y0++	RevNo
1	258.19	129.60			L							11
2	315.21	158.11			G	1,201.64	601.32	1,184.61	592.81	1,183.63	592.32	10
3	444.26	222.63	426.25	213.63	E	1,144.62	572.81	1,127.59	564.30	1,126.61	563.81	9
4	557.34	279.17	539.33	270.17	L	1,015.58	508.29	998.55	499.78	997.57	499.29	8
5	654.39	327.70	636.38	318.70	P	902.49	451.75	885.47	443.24	884.48	442.74	7
6	741.43	371.22	723.42	362.21	S	805.44	403.22	788.41	394.71	787.43	394.22	6
7	927.51	464.26	909.49	455.25	W	718.41	359.71	701.38	351.19			5
8	1,040.59	520.80	1,022.58	511.79	I	532.33	266.67	515.30	258.15			4
9	1,153.67	577.34	1,135.66	568.34	L	419.24	210.13	402.22	201.61			3
10	1,284.71	642.86	1,266.70	633.86	M	306.16	153.58	289.13	145.07			2
11					R	175.12	88.06	158.09	79.55			1

Query 2882 Hit 1

MS/MS Fragmentation of IMPLEDMNEFTTHILEVINAHMVLSK
Found in sp|P15927|RFA2_HUMAN, Replication protein A 32 kDa subunit OS=Homo sapiens GN=RPA2 PE=1 SV=1
Match to Query 2882: 3312.71 from(829.1848,4+)
Title: 1372: Scan 3377 (rt=84.265, f=3, i=486) [D:\lab212\membrane\Grace\20120214_iTRAQ_1.raw]
Data File:
Monoisotopic mass of neutral peptide Mr(calc): 3312.71
Variable modifications:
K26 :iTRAQ4plex (K)
Ions Score: 66.08 **Expect:** 0.000

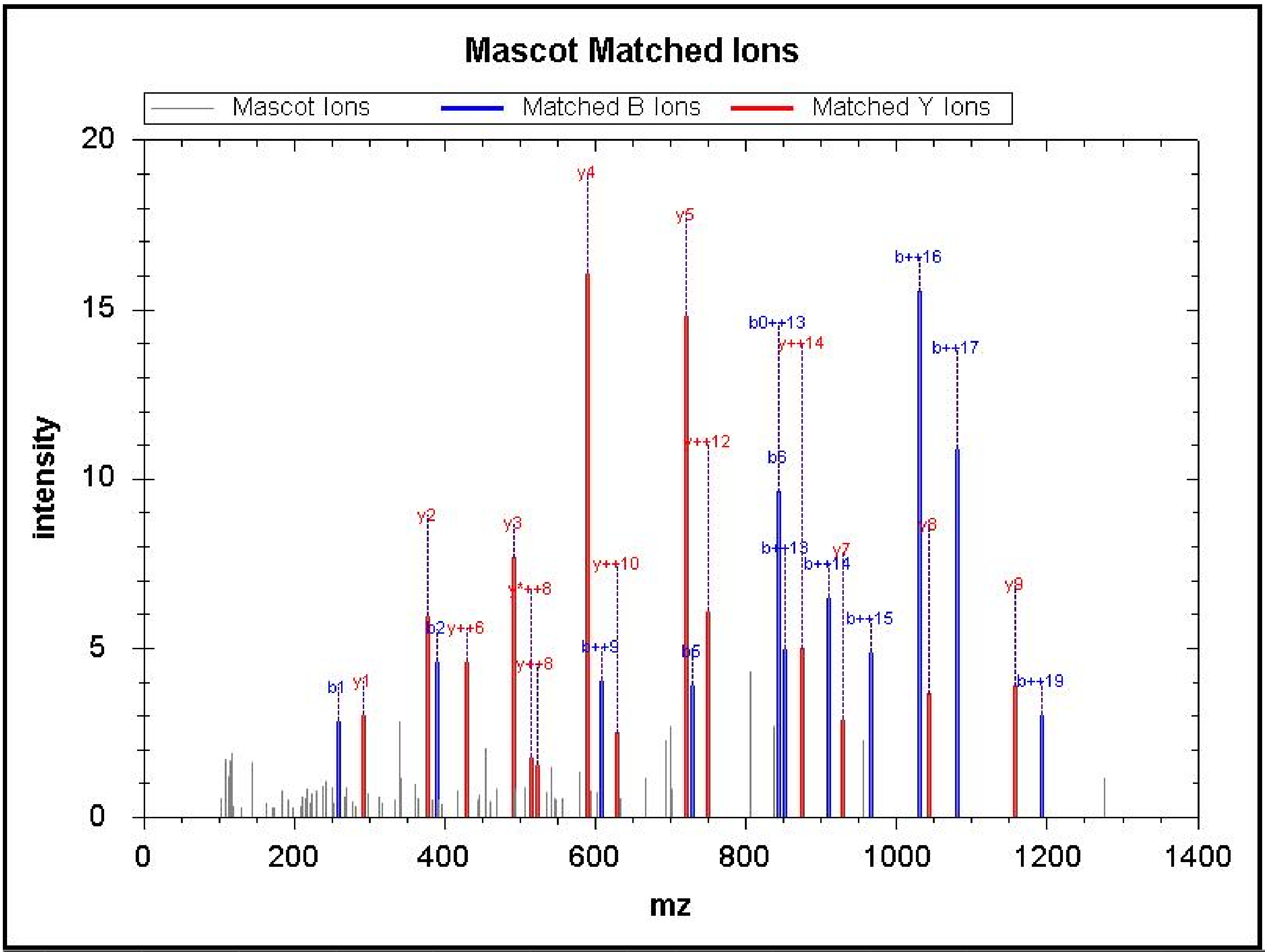

No	b	b++	b*	b*++	b0	b0++	Seq	y	y++	y*	y*++	y0	y0++	RevNo
1	258.19	129.60					I							26
2	389.23	195.12					M	3,056.54	1,528.77	3,039.51	1,520.26	3,038.53	1,519.77	25
3	486.29	243.65					P	2,925.50	1,463.25	2,908.47	1,454.74	2,907.49	1,454.25	24
4	599.37	300.19					L	2,828.44	1,414.73	2,811.42	1,406.21	2,810.43	1,405.72	23
5	728.41	364.71			710.40	355.71	E	2,715.36	1,358.18	2,698.33	1,349.67	2,697.35	1,349.18	22
6	843.44	422.22			825.43	413.22	D	2,586.32	1,293.66	2,569.29	1,285.15	2,568.31	1,284.66	21
7	974.48	487.74			956.47	478.74	M	2,471.29	1,236.15	2,454.26	1,227.64	2,453.28	1,227.14	20
8	1,088.52	544.77	1,071.50	536.25	1,070.51	535.76	N	2,340.25	1,170.63	2,323.22	1,162.12	2,322.24	1,161.62	19
9	1,217.57	609.29	1,200.54	600.77	1,199.56	600.28	E	2,226.21	1,113.61	2,209.18	1,105.09	2,208.20	1,104.60	18
10	1,364.63	682.82	1,347.61	674.31	1,346.62	673.82	F	2,097.16	1,049.09	2,080.14	1,040.57	2,079.15	1,040.08	17
11	1,465.68	733.34	1,448.66	724.83	1,447.67	724.34	T	1,950.10	975.55	1,933.07	967.04	1,932.09	966.55	16
12	1,566.73	783.87	1,549.70	775.36	1,548.72	774.86	T	1,849.05	925.03	1,832.02	916.51	1,831.04	916.02	15
13	1,703.79	852.40	1,686.76	843.89	1,685.78	843.39	H	1,748.00	874.50	1,730.97	865.99	1,729.99	865.50	14
14	1,816.87	908.94	1,799.85	900.43	1,798.86	899.93	I	1,610.94	805.97	1,593.92	797.46	1,592.93	796.97	13
15	1,929.96	965.48	1,912.93	956.97	1,911.95	956.48	L	1,497.86	749.43	1,480.83	740.92	1,479.85	740.43	12
16	2,059.00	1,030.00	2,041.97	1,021.49	2,040.99	1,021.00	E	1,384.77	692.89	1,367.75	684.38	1,366.76	683.89	11
17	2,158.07	1,079.54	2,141.04	1,071.02	2,140.06	1,070.53	V	1,255.73	628.37	1,238.70	619.86	1,237.72	619.36	10
18	2,271.15	1,136.08	2,254.13	1,127.57	2,253.14	1,127.07	I	1,156.66	578.84	1,139.64	570.32	1,138.65	569.83	9
19	2,385.20	1,193.10	2,368.17	1,184.59	2,367.18	1,184.10	N	1,043.58	522.29	1,026.55	513.78	1,025.57	513.29	8
20	2,456.23	1,228.62	2,439.21	1,220.11	2,438.22	1,219.61	A	929.54	465.27	912.51	456.76	911.53	456.27	7
21	2,593.29	1,297.15	2,576.26	1,288.64	2,575.28	1,288.14	H	858.50	429.75	841.47	421.24	840.49	420.75	6
22	2,724.33	1,362.67	2,707.31	1,354.16	2,706.32	1,353.66	M	721.44	361.22	704.41	352.71	703.43	352.22	5
23	2,823.40	1,412.20	2,806.37	1,403.69	2,805.39	1,403.20	V	590.40	295.70	573.37	287.19	572.39	286.70	4
24	2,936.48	1,468.75	2,919.46	1,460.23	2,918.47	1,459.74	L	491.33	246.17	474.30	237.66	473.32	237.16	3

25	3,023.52	1,512.26	3,006.49	1,503.75	3,005.51	1,503.26	S	378.25	189.63	361.22	181.11	360.24	180.62	2
26							K	291.21	146.11	274.19	137.60			1

Query 999 Hit 1

MS/MS Fragmentation of **AAVESLGFILFR**
Found in **sp|Q99460|PSMD1_HUMAN**, 26S proteasome non-ATPase regulatory subunit 1 OS=Homo sapiens GN=PSMD1 PE=1 SV=2
Match to Query 999: 1465.844from(733.9293,2+)
Title: 1052: Scan 2675 (rt=68.4448, f=2, i=421) [D:\lab212\membrane\Grace\20120214_iTRAQ_1.raw]
Data File:
Monoisotopic mass of neutral peptide Mr(calc): 1465.844
Variable modifications:
Ions Score: 66.24 **Expect:** 0.000

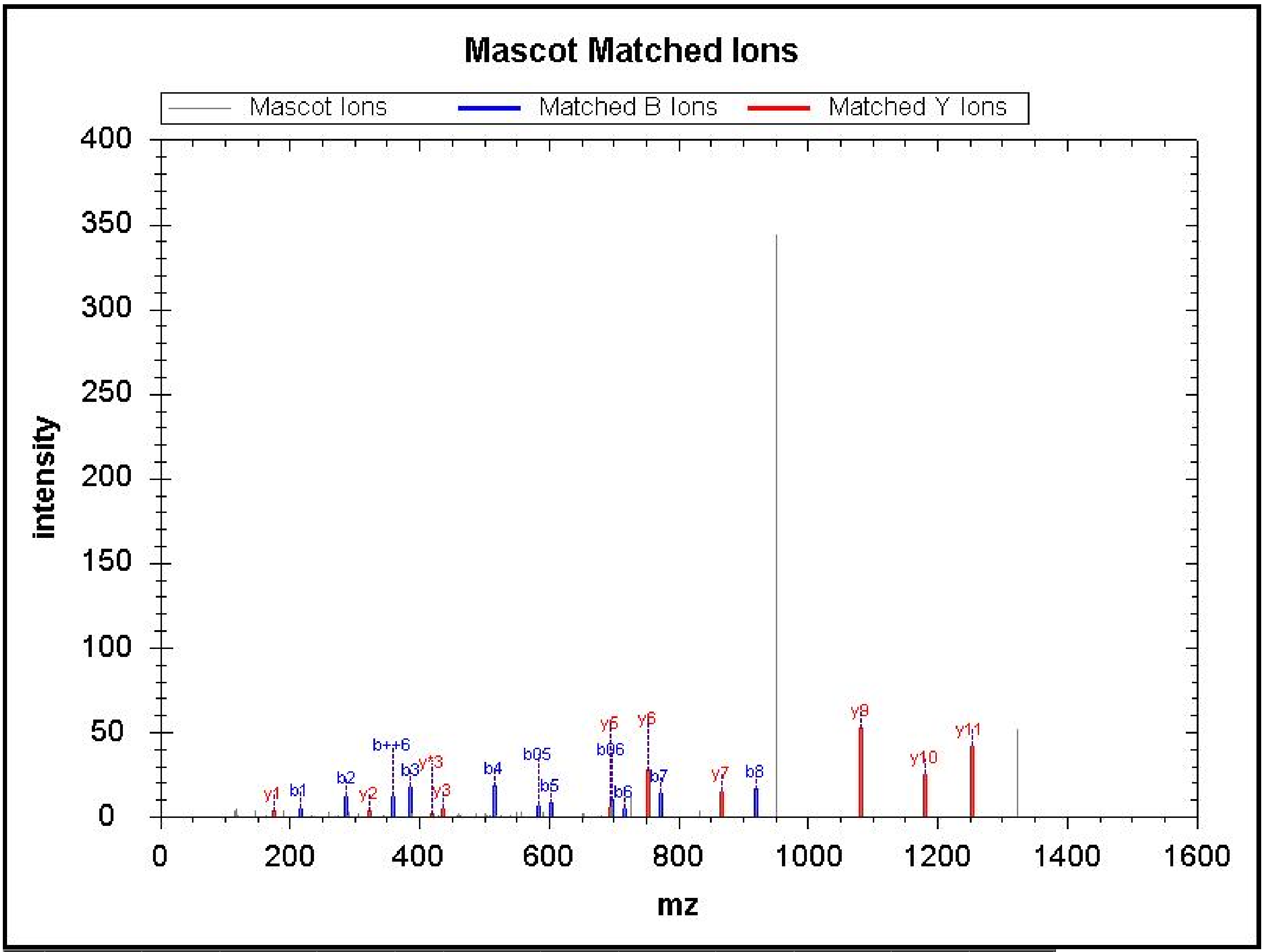

No	b	b++	b0	b0++	Seq	y	y++	y*	y*++	y0	y0++	RevNo
1	216.15	108.58			A							12
2	287.18	144.10			A	1,251.71	626.36	1,234.68	617.85	1,233.70	617.35	11
3	386.25	193.63			V	1,180.67	590.84	1,163.65	582.33	1,162.66	581.83	10
4	515.29	258.15	497.28	249.15	E	1,081.60	541.31	1,064.58	532.79	1,063.59	532.30	9
5	602.33	301.67	584.32	292.66	S	952.56	476.78	935.53	468.27	934.55	467.78	8
6	715.41	358.21	697.40	349.20	L	865.53	433.27	848.50	424.76			7
7	772.43	386.72	754.42	377.71	G	752.45	376.73	735.42	368.21			6
8	919.50	460.25	901.49	451.25	F	695.42	348.22	678.40	339.70			5
9	1,032.58	516.80	1,014.57	507.79	I	548.36	274.68	531.33	266.17			4
10	1,145.67	573.34	1,127.66	564.33	L	435.27	218.14	418.24	209.63			3
11	1,292.74	646.87	1,274.73	637.87	F	322.19	161.60	305.16	153.08			2
12					R	175.12	88.06	158.09	79.55			1

Query 2326 Hit 1

MS/MS Fragmentation of **AQMALWTVLAAPLLMSTDLR**
Found in **sp|P17050|NAGAB_HUMAN**, Alpha-N-acetylgalactosaminidase OS=Homo sapiens GN=NAGA PE=1 SV=2
Match to Query 2326: 2344.27from(782.4304,3+)
Title: 1422: Scan 3519 (rt=87.4273, f=2, i=565) [D:\lab212\membrane\Grace\20120214_iTRAQ_1.raw]
Data File:
Monoisotopic mass of neutral peptide Mr(calc): 2344.27
Variable modifications:
Ions Score: 66.34 **Expect:** 0.000

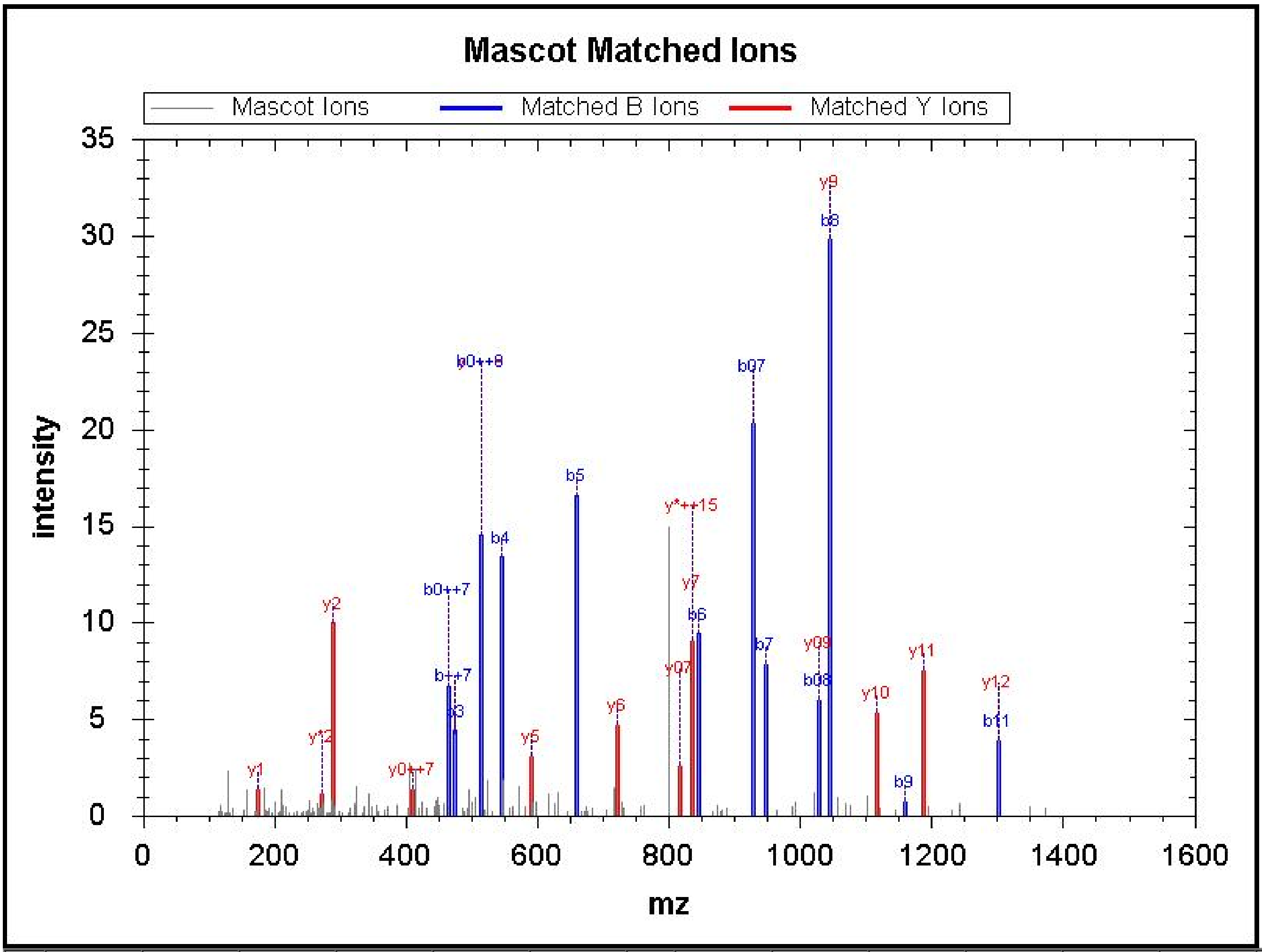

No	b	b++	b*	b*++	b0	b0++	Seq	y	y++	y*	y*++	y0	y0++	RevNo
1	216.15	108.58					A							20
2	344.21	172.61	327.18	164.09			Q	2,130.15	1,065.58	2,113.12	1,057.06	2,112.13	1,056.57	19
3	**475.25**	238.13	458.22	229.61			M	2,002.09	1,001.55	1,985.06	993.03	1,984.08	992.54	18
4	**546.28**	273.64	529.26	265.13			A	1,871.05	936.03	1,854.02	927.51	1,853.04	927.02	17
5	**659.37**	330.19	642.34	321.67			L	1,800.01	900.51	1,782.98	891.99	1,782.00	891.50	16
6	**845.45**	423.23	828.42	414.71			W	1,686.92	843.97	1,669.90	**835.45**	1,668.91	834.96	15
7	**946.49**	**473.75**	929.47	465.24	**928.48**	**464.75**	T	1,500.85	750.93	1,483.82	742.41	1,482.83	741.92	14
8	**1,045.56**	523.28	1,028.54	514.77	**1,027.55**	**514.28**	V	1,399.80	700.40	1,382.77	691.89	1,381.79	691.40	13
9	**1,158.65**	579.83	1,141.62	571.31	1,140.64	570.82	L	**1,300.73**	650.87	1,283.70	642.36	1,282.72	641.86	12
10	1,229.68	615.35	1,212.66	606.83	1,211.67	606.34	A	**1,187.65**	594.33	1,170.62	585.81	1,169.63	585.32	11
11	**1,300.72**	650.86	1,283.69	642.35	1,282.71	641.86	A	**1,116.61**	558.81	1,099.58	550.29	1,098.60	549.80	10
12	1,397.77	699.39	1,380.75	690.88	1,379.76	690.38	P	**1,045.57**	523.29	1,028.54	514.78	**1,027.56**	**514.28**	9
13	1,510.86	755.93	1,493.83	747.42	1,492.85	746.93	L	948.52	474.76	931.49	466.25	930.51	465.76	8
14	1,623.94	812.47	1,606.91	803.96	1,605.93	803.47	L	**835.43**	418.22	818.41	409.71	**817.42**	**409.22**	7
15	1,754.98	877.99	1,737.96	869.48	1,736.97	868.99	M	**722.35**	361.68	705.32	353.17	704.34	352.67	6

16	1,842.01	921.51	1,824.99	913.00	1,824.00	912.51	S	591.31	296.16	574.28	287.65	573.30	287.15	5
17	1,943.06	972.03	1,926.04	963.52	1,925.05	963.03	T	504.28	252.64	487.25	244.13	486.27	243.64	4
18	2,058.09	1,029.55	2,041.06	1,021.03	2,040.08	1,020.54	D	403.23	202.12	386.20	193.61	385.22	193.11	3
19	2,171.17	1,086.09	2,154.15	1,077.58	2,153.16	1,077.08	L	288.20	144.61	271.18	136.09			2
20							R	175.12	88.06	158.09	79.55			1

Query 1050 Hit 1

MS/MS Fragmentation of **DLLSWMESIIR**
Found in **sp|P11277|SPTB1_HUMAN**, Spectrin beta chain
Match to Query 1050: 1505.799from(753.907,2+)
Title: 1396: Sum of 2 scans in range 3384 (rt=84.5013, f=4, i=935) to 3385 (rt=84.5267, f=4, i=936) [D:\lab212\membrane\Grace\20120214_iTRAQ_2.raw]
Data File:
Monoisotopic mass of neutral peptide Mr(calc): 1505.799
Variable modifications:
Ions Score: 66.49 **Expect:** 0.000

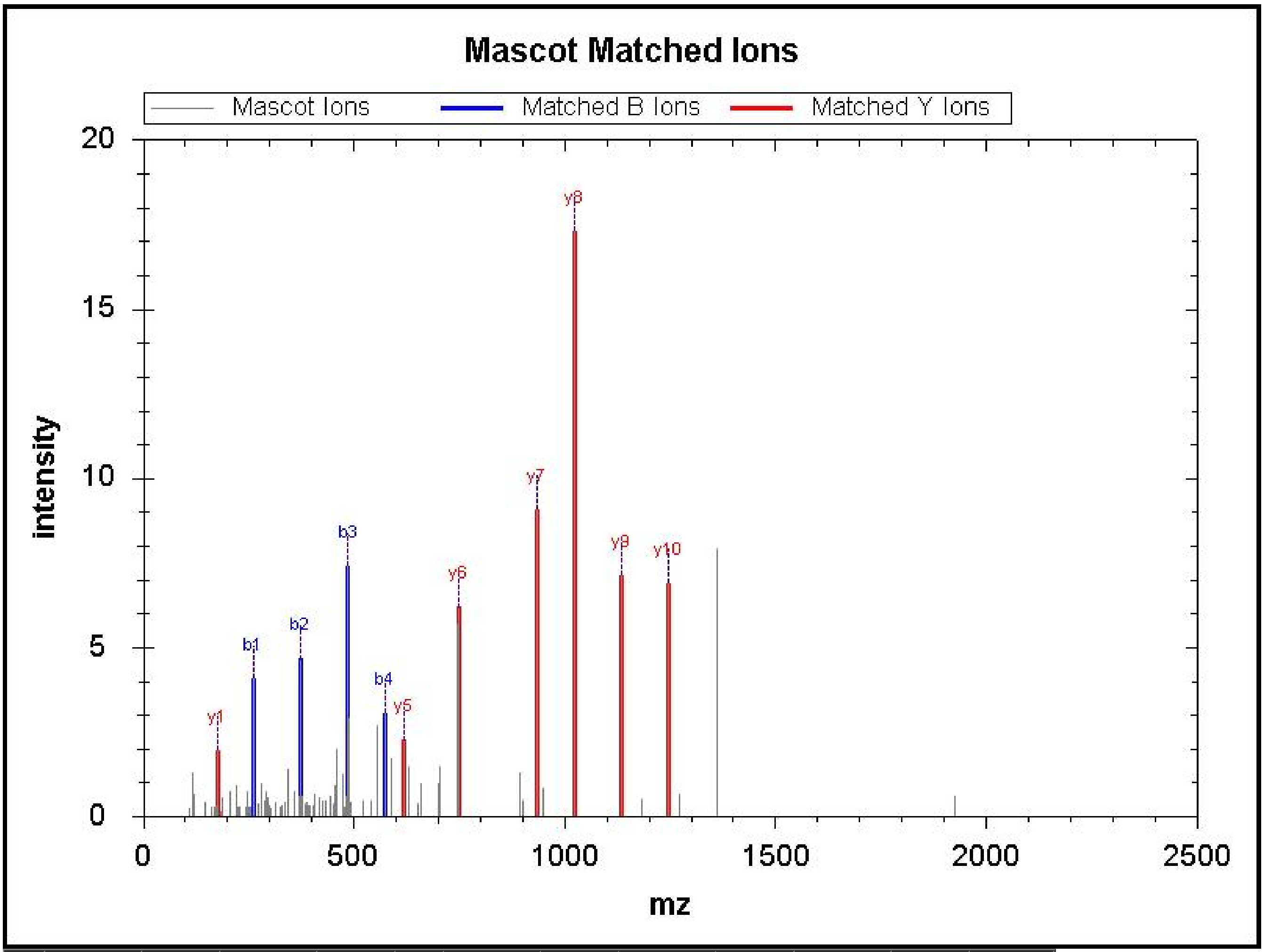

No	b	b++	b0	b0++	Seq	y	y++	y*	y*++	y0	y0++	RevNo
1	260.14	130.57	242.13	121.57	D							11
2	373.22	187.11	355.21	178.11	L	1,247.68	624.34	1,230.66	615.83	1,229.67	615.34	10
3	486.30	243.66	468.29	234.65	L	1,134.60	567.80	1,117.57	559.29	1,116.59	558.80	9
4	573.34	287.17	555.33	278.17	S	1,021.51	511.26	1,004.49	502.75	1,003.50	502.26	8
5	759.42	380.21	741.41	371.21	W	934.48	467.74	917.45	459.23	916.47	458.74	7
6	890.46	445.73	872.45	436.73	M	748.40	374.70	731.38	366.19	730.39	365.70	6
7	1,019.50	510.25	1,001.49	501.25	E	617.36	309.18	600.34	300.67	599.35	300.18	5
8	1,106.53	553.77	1,088.52	544.76	S	488.32	244.66	471.29	236.15	470.31	235.66	4

9	1,219.61	610.31	1,201.60	601.31	I	401.29	201.15	384.26	192.63			3
10	1,332.70	666.85	1,314.69	657.85	I	288.20	144.61	271.18	136.09			2
11					R	**175.12**	88.06	158.09	79.55			1

Query 2222 Hit 1

MS/MS Fragmentation of **VPSTEAEALASSLMGLFEK**
Found in **sp|P50395|GDIB_HUMAN**, Rab GDP dissociation inhibitor beta OS=Homo sapiens GN=GDI2 PE=1 SV=2
Match to Query 2222: 2267.195from(756.739,3+)
Title: 1282: Scan 3171 (rt=79.6213, f=2, i=501) [D:\lab212\membrane\Grace\20120214_iTRAQ_1.raw]
Data File:
Monoisotopic mass of neutral peptide Mr(calc): 2267.195
Variable modifications:
K19 :iTRAQ4plex (K)
Ions Score: 66.55 **Expect:** 0.000

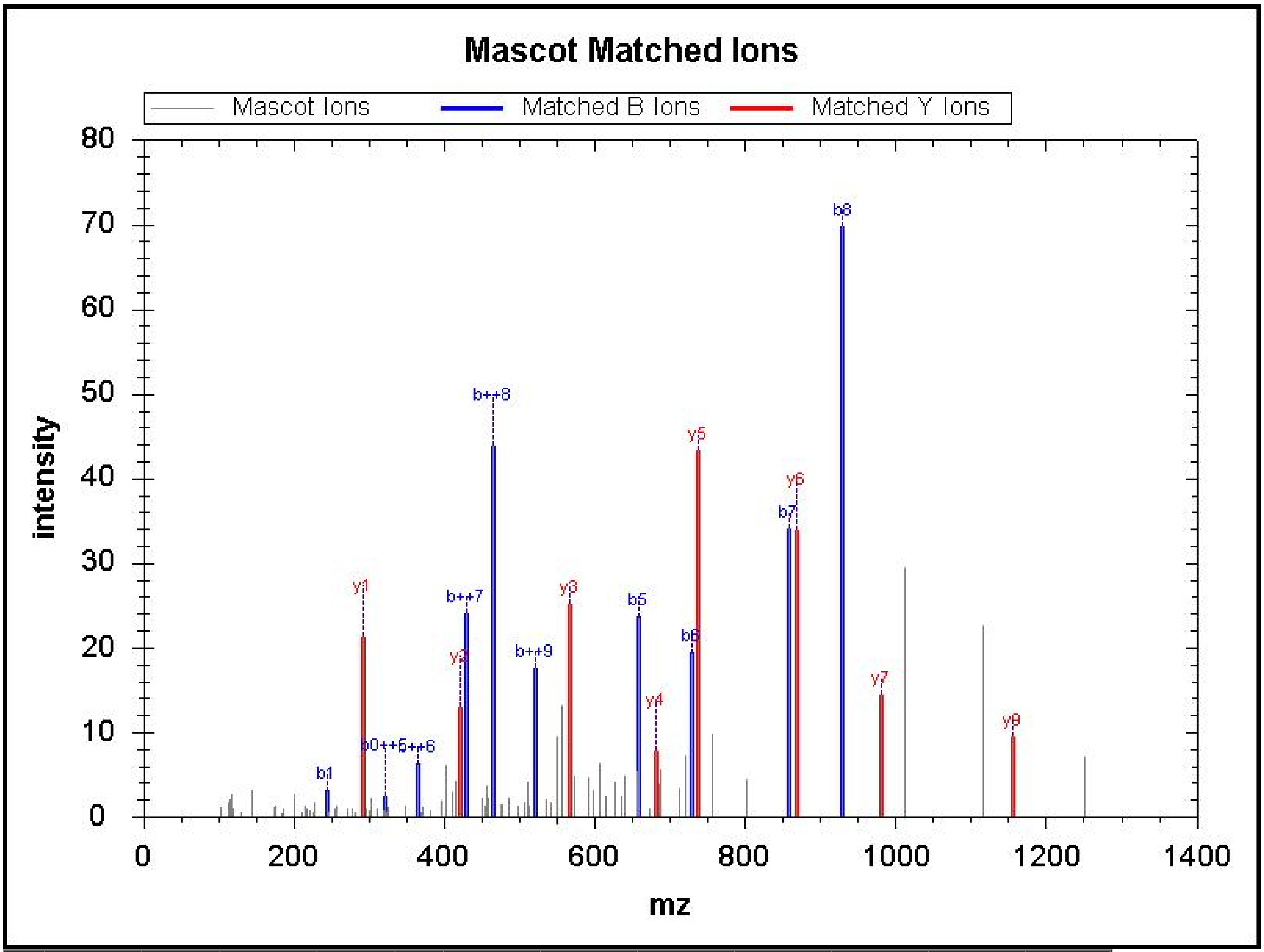

No	b	b++	b0	b0++	Seq	y	y++	y*	y*++	y0	y0++	RevNo
1	**244.18**	122.59			V							19
2	341.23	171.12			P	2,025.03	1,013.02	2,008.01	1,004.51	2,007.02	1,004.01	18
3	428.26	214.63	410.25	205.63	S	1,927.98	964.49	1,910.95	955.98	1,909.97	955.49	17
4	529.31	265.16	511.30	256.15	T	1,840.95	920.98	1,823.92	912.46	1,822.94	911.97	16
5	**658.35**	329.68	640.34	**320.67**	E	1,739.90	870.45	1,722.87	861.94	1,721.89	861.45	15
6	**729.39**	**365.20**	711.38	356.19	A	1,610.86	805.93	1,593.83	797.42	1,592.85	796.93	14
7	**858.43**	**429.72**	840.42	420.71	E	1,539.82	770.41	1,522.79	761.90	1,521.81	761.41	13
8	**929.47**	**465.24**	911.46	456.23	A	1,410.78	705.89	1,393.75	697.38	1,392.77	696.89	12
9	1,042.55	**521.78**	1,024.54	512.78	L	1,339.74	670.37	1,322.71	661.86	1,321.73	661.37	11
10	1,113.59	557.30	1,095.58	548.29	A	1,226.66	613.83	1,209.63	605.32	1,208.65	604.83	10

11	1,200.62	600.82	1,182.61	591.81	S	1,155.62	578.31	1,138.59	569.80	1,137.61	569.31	9
12	1,287.65	644.33	1,269.64	635.33	S	1,068.59	534.80	1,051.56	526.28	1,050.58	525.79	8
13	1,400.74	700.87	1,382.73	691.87	L	981.56	491.28	964.53	482.77	963.55	482.28	7
14	1,531.78	766.39	1,513.77	757.39	M	868.47	434.74	851.45	426.23	850.46	425.73	6
15	1,588.80	794.90	1,570.79	785.90	G	737.43	369.22	720.40	360.71	719.42	360.21	5
16	1,701.89	851.45	1,683.87	842.44	L	680.41	340.71	663.38	332.20	662.40	331.70	4
17	1,848.95	924.98	1,830.94	915.98	F	567.33	284.17	550.30	275.65	549.32	275.16	3
18	1,978.00	989.50	1,959.99	980.50	E	420.26	210.63	403.23	202.12	402.25	201.63	2
19					K	291.21	146.11	274.19	137.60			1

Query 2358 Hit 1

MS/MS Fragmentation of **ADLEMQIESLTEELAYLK**
Found in **sp|P13645|K1C10_HUMAN**, Keratin
Match to Query 2358: 2383.237from(795.4197,3+)
Title: 1251: Sum of 2 scans in range 3105 (rt=78.1393, f=4, i=854) to 3106 (rt=78.1647, f=4, i=855) [D:\lab212\membrane\Grace\20120214_iTRAQ_1.raw]
Data File:
Monoisotopic mass of neutral peptide Mr(calc): 2383.237
Variable modifications:
K18 :iTRAQ4plex (K)
Ions Score: 66.61 **Expect:** 0.000

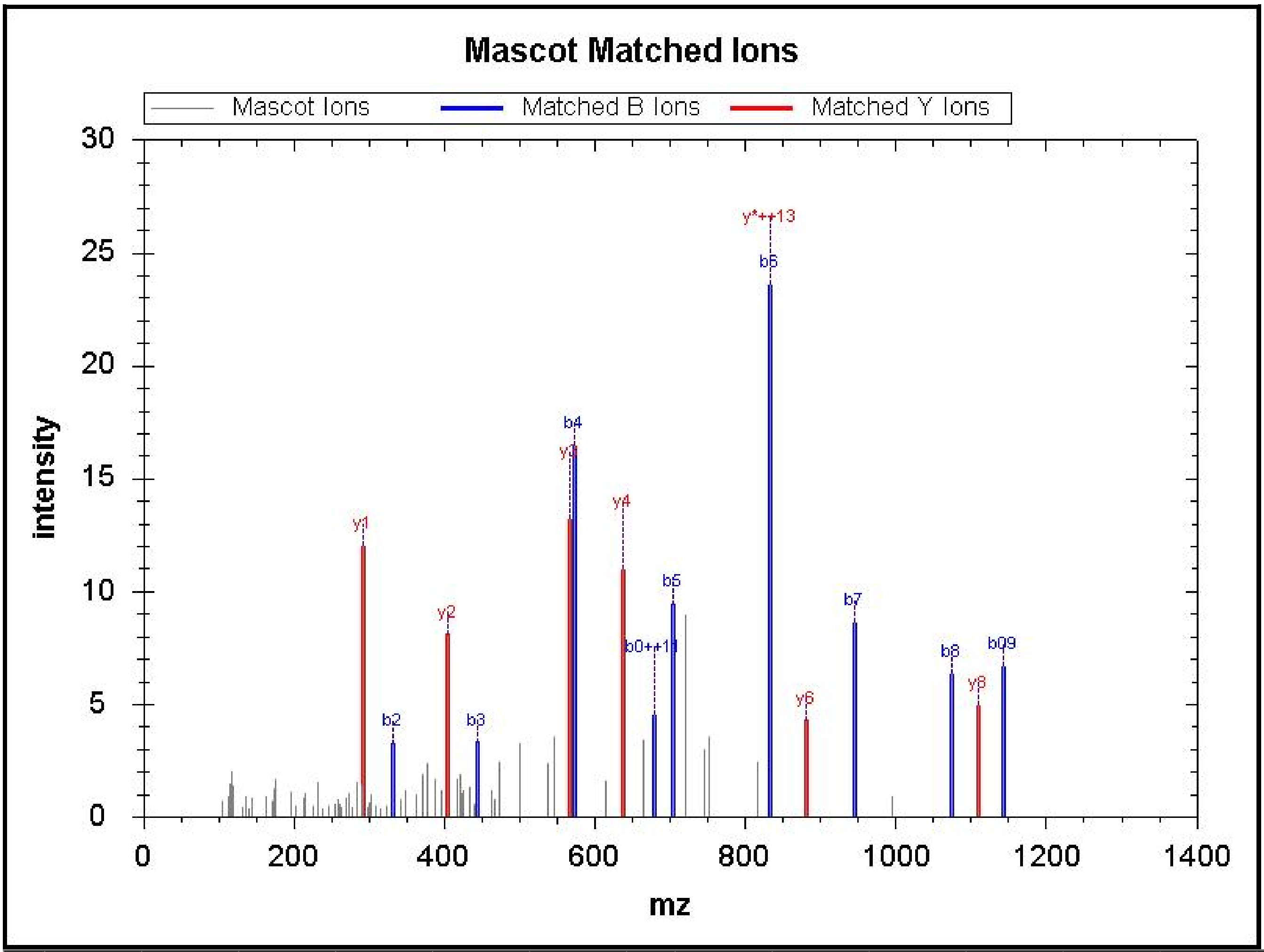

No	b	b++	b*	b*++	b0	b0++	Seq	y	y++	y*	y*++	y0	y0++	RevNo
1	216.15	108.58					A							18
2	331.17	166.09			313.16	157.09	D	2,169.11	1,085.06	2,152.09	1,076.55	2,151.10	1,076.05	17
3	444.26	222.63			426.25	213.63	L	2,054.08	1,027.55	2,037.06	1,019.03	2,036.07	1,018.54	16
4	573.30	287.15			555.29	278.15	E	1,941.00	971.00	1,923.97	962.49	1,922.99	962.00	15

5	704.34	352.67			686.33	343.67	M	1,811.96	906.48	1,794.93	897.97	1,793.95	897.48	14
6	832.40	416.70	815.37	408.19	814.39	407.70	Q	1,680.92	840.96	1,663.89	832.45	1,662.91	831.96	13
7	945.48	473.25	928.46	464.73	927.47	464.24	I	1,552.86	776.93	1,535.83	768.42	1,534.85	767.93	12
8	1,074.53	537.77	1,057.50	529.25	1,056.52	528.76	E	1,439.77	720.39	1,422.75	711.88	1,421.76	711.39	11
9	1,161.56	581.28	1,144.53	572.77	1,143.55	572.28	S	1,310.73	655.87	1,293.71	647.36	1,292.72	646.86	10
10	1,274.64	637.82	1,257.62	629.31	1,256.63	628.82	L	1,223.70	612.35	1,206.67	603.84	1,205.69	603.35	9
11	1,375.69	688.35	1,358.66	679.84	1,357.68	679.34	T	1,110.62	555.81	1,093.59	547.30	1,092.61	546.81	8
12	1,504.73	752.87	1,487.71	744.36	1,486.72	743.86	E	1,009.57	505.29	992.54	496.77	991.56	496.28	7
13	1,633.77	817.39	1,616.75	808.88	1,615.76	808.39	E	880.53	440.77	863.50	432.25	862.52	431.76	6
14	1,746.86	873.93	1,729.83	865.42	1,728.85	864.93	L	751.48	376.25	734.46	367.73			5
15	1,817.90	909.45	1,800.87	900.94	1,799.89	900.45	A	638.40	319.70	621.37	311.19			4
16	1,980.96	990.98	1,963.93	982.47	1,962.95	981.98	Y	567.36	284.18	550.34	275.67			3
17	2,094.04	1,047.53	2,077.02	1,039.01	2,076.03	1,038.52	L	404.30	202.65	387.27	194.14			2
18							K	291.21	146.11	274.19	137.60			1

Query 2368 Hit 1

MS/MS Fragmentation of **ISDGVVLFIDAAEGVMLNTER**
Found in **sp|Q15029|U5S1_HUMAN**, 116 kDa U5 small nuclear ribonucleoprotein component OS=Homo sapiens GN=EFTUD2 PE=1 SV=1
Match to Query 2368: 2392.235from(798.4189,3+)
Title: 1414: Sum of 2 scans in range 3494 (rt=86.8729, f=4, i=966) to 3495 (rt=86.8984, f=4, i=967) [D:\lab212\membrane\Grace\20120214_iTRAQ_1.raw]
Data File:
Monoisotopic mass of neutral peptide Mr(calc): 2392.235
Variable modifications:
Ions Score: 67.68 **Expect:** 0.000

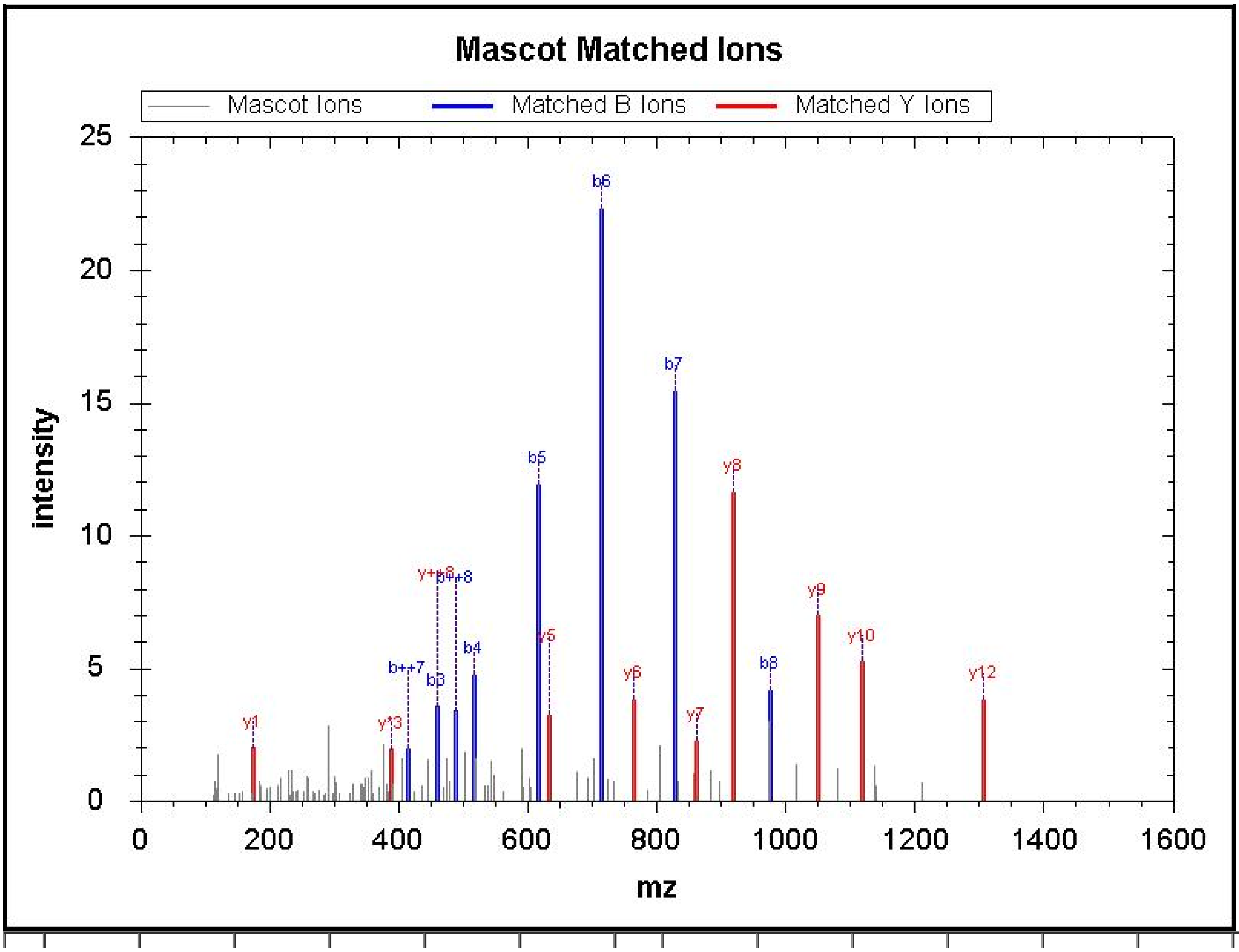

No	b	b++	b*	b*++	b0	b0++	Seq	y	y++	y*	y*++	y0	y0++	RevNo
1	258.19	129.60					I							21
2	345.23	173.12			327.21	164.11	S	2,136.06	1,068.54	2,119.04	1,060.02	2,118.05	1,059.53	20
3	460.25	230.63			442.24	221.62	D	2,049.03	1,025.02	2,032.01	1,016.51	2,031.02	1,016.01	19
4	517.27	259.14			499.26	250.14	G	1,934.01	967.51	1,916.98	958.99	1,915.99	958.50	18
5	616.34	308.67			598.33	299.67	V	1,876.98	939.00	1,859.96	930.48	1,858.97	929.99	17
6	715.41	358.21			697.40	349.20	V	1,777.92	889.46	1,760.89	880.95	1,759.90	880.46	16
7	828.49	414.75			810.48	405.75	L	1,678.85	839.93	1,661.82	831.41	1,660.84	830.92	15
8	975.56	488.29			957.55	479.28	F	1,565.76	783.39	1,548.74	774.87	1,547.75	774.38	14
9	1,088.65	544.83			1,070.64	535.82	I	1,418.69	709.85	1,401.67	701.34	1,400.68	700.85	13
10	1,203.67	602.34			1,185.66	593.34	D	1,305.61	653.31	1,288.58	644.80	1,287.60	644.30	12
11	1,274.71	637.86			1,256.70	628.85	A	1,190.58	595.80	1,173.56	587.28	1,172.57	586.79	11
12	1,345.75	673.38			1,327.74	664.37	A	1,119.55	560.28	1,102.52	551.76	1,101.54	551.27	10
13	1,474.79	737.90			1,456.78	728.89	E	1,048.51	524.76	1,031.48	516.24	1,030.50	515.75	9
14	1,531.81	766.41			1,513.80	757.40	G	919.47	460.24	902.44	451.72	901.46	451.23	8
15	1,630.88	815.94			1,612.87	806.94	V	862.45	431.73	845.42	423.21	844.43	422.72	7
16	1,761.92	881.46			1,743.91	872.46	M	763.38	382.19	746.35	373.68	745.37	373.19	6
17	1,875.01	938.01			1,856.99	929.00	L	632.34	316.67	615.31	308.16	614.33	307.67	5
18	1,989.05	995.03	1,972.02	986.51	1,971.04	986.02	N	519.25	260.13	502.23	251.62	501.24	251.12	4
19	2,090.10	1,045.55	2,073.07	1,037.04	2,072.09	1,036.55	T	405.21	203.11	388.18	194.59	387.20	194.10	3
20	2,219.14	1,110.07	2,202.11	1,101.56	2,201.13	1,101.07	E	304.16	152.58	287.13	144.07	286.15	143.58	2
21							R	175.12	88.06	158.09	79.55			1

Query 2962 Hit 1

MS/MS Fragmentation of **AFAMIIDKLEEDISSSMTNSTAASRPPVTLR**
Found in **sp|Q15366|PCBP2_HUMAN**, Poly(rC)-binding protein 2 OS=Homo sapiens GN=PCBP2 PE=1 SV=1
Match to Query 2962: 3638.889from(910.7295,4+)
Title: 1295: Scan 3153 (rt=79.3036, f=3, i=463) [D:\lab212\membrane\Grace\20120214_iTRAQ_2.raw]
Data File:
Monoisotopic mass of neutral peptide Mr(calc): 3638.889
Variable modifications:
K8 :iTRAQ4plex (K)
Ions Score: 70.84 **Expect:** 0.000

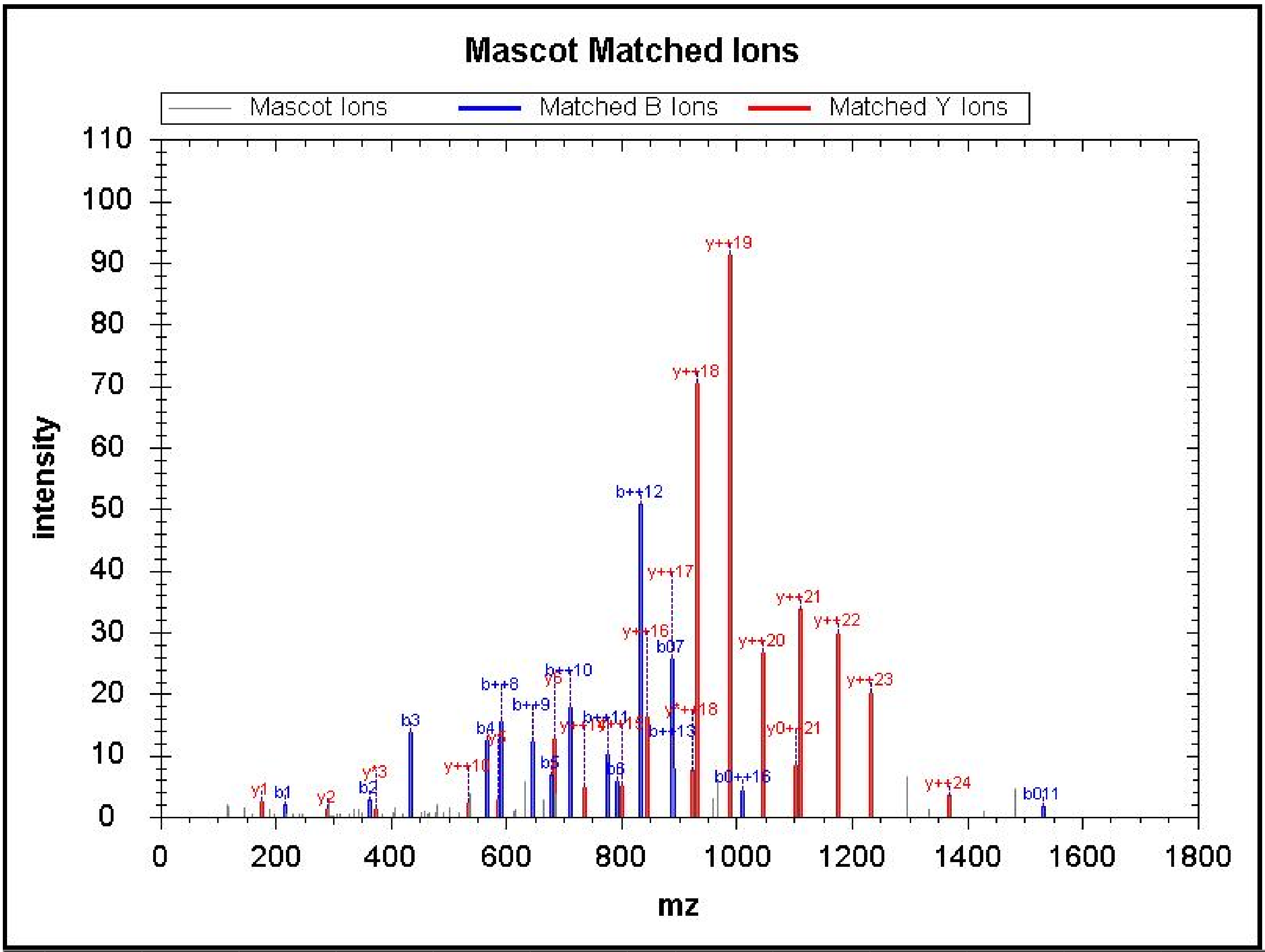

No	b	b++	b*	b*++	b0	b0++	Seq	y	y++	y*	y*++	y0	y0++	RevNo
1	216.15	108.58					A							31
2	363.21	182.11					F	3,424.76	1,712.88	3,407.73	1,704.37	3,406.75	1,703.88	30
3	434.25	217.63					A	3,277.69	1,639.35	3,260.66	1,630.83	3,259.68	1,630.34	29
4	565.29	283.15					M	3,206.65	1,603.83	3,189.62	1,595.32	3,188.64	1,594.82	28
5	678.38	339.69					I	3,075.61	1,538.31	3,058.58	1,529.80	3,057.60	1,529.30	27
6	791.46	396.23					I	2,962.53	1,481.77	2,945.50	1,473.25	2,944.52	1,472.76	26
7	906.49	453.75			888.48	444.74	D	2,849.44	1,425.22	2,832.42	1,416.71	2,831.43	1,416.22	25
8	1,178.68	589.85	1,161.66	581.33	1,160.67	580.84	K	2,734.42	1,367.71	2,717.39	1,359.20	2,716.41	1,358.71	24
9	1,291.77	646.39	1,274.74	637.87	1,273.76	637.38	L	2,462.22	1,231.61	2,445.19	1,223.10	2,444.21	1,222.61	23
10	1,420.81	710.91	1,403.78	702.40	1,402.80	701.90	E	2,349.13	1,175.07	2,332.11	1,166.56	2,331.12	1,166.07	22
11	1,549.85	775.43	1,532.83	766.92	1,531.84	766.43	E	2,220.09	1,110.55	2,203.07	1,102.04	2,202.08	1,101.54	21
12	1,664.88	832.94	1,647.85	824.43	1,646.87	823.94	D	2,091.05	1,046.03	2,074.02	1,037.52	2,073.04	1,037.02	20
13	1,777.96	889.49	1,760.94	880.97	1,759.95	880.48	I	1,976.02	988.52	1,959.00	980.00	1,958.01	979.51	19
14	1,865.00	933.00	1,847.97	924.49	1,846.99	924.00	S	1,862.94	931.97	1,845.91	923.46	1,844.93	922.97	18
15	1,952.03	976.52	1,935.00	968.00	1,934.02	967.51	S	1,775.91	888.46	1,758.88	879.94	1,757.90	879.45	17
16	2,039.06	1,020.03	2,022.03	1,011.52	2,021.05	1,011.03	S	1,688.87	844.94	1,671.85	836.43	1,670.86	835.94	16
17	2,170.10	1,085.55	2,153.07	1,077.04	2,152.09	1,076.55	M	1,601.84	801.43	1,584.82	792.91	1,583.83	792.42	15
18	2,271.15	1,136.08	2,254.12	1,127.56	2,253.14	1,127.07	T	1,470.80	735.90	1,453.78	727.39	1,452.79	726.90	14
19	2,385.19	1,193.10	2,368.17	1,184.59	2,367.18	1,184.09	N	1,369.75	685.38	1,352.73	676.87	1,351.74	676.38	13
20	2,472.22	1,236.62	2,455.20	1,228.10	2,454.21	1,227.61	S	1,255.71	628.36	1,238.69	619.85	1,237.70	619.35	12
21	2,573.27	1,287.14	2,556.24	1,278.63	2,555.26	1,278.13	T	1,168.68	584.84	1,151.65	576.33	1,150.67	575.84	11
22	2,644.31	1,322.66	2,627.28	1,314.14	2,626.30	1,313.65	A	1,067.63	534.32	1,050.61	525.81	1,049.62	525.31	10
23	2,715.35	1,358.18	2,698.32	1,349.66	2,697.34	1,349.17	A	996.59	498.80	979.57	490.29	978.58	489.80	9

24	2,802.38	1,401.69	2,785.35	1,393.18	2,784.37	1,392.69	S	925.56	463.28	908.53	454.77	907.55	454.28	8
25	2,958.48	1,479.74	2,941.45	1,471.23	2,940.47	1,470.74	R	838.53	419.77	821.50	411.25	820.52	410.76	7
26	3,055.53	1,528.27	3,038.50	1,519.76	3,037.52	1,519.26	P	682.42	341.72	665.40	333.20	664.41	332.71	6
27	3,152.58	1,576.80	3,135.56	1,568.28	3,134.57	1,567.79	P	585.37	293.19	568.35	284.68	567.36	284.18	5
28	3,251.65	1,626.33	3,234.63	1,617.82	3,233.64	1,617.32	V	488.32	244.66	471.29	236.15	470.31	235.66	4
29	3,352.70	1,676.85	3,335.67	1,668.34	3,334.69	1,667.85	T	389.25	195.13	372.22	186.62	371.24	186.12	3
30	3,465.78	1,733.40	3,448.76	1,724.88	3,447.77	1,724.39	L	288.20	144.61	271.18	136.09			2
31							R	175.12	88.06	158.09	79.55			1

Query 2505 Hit 1

MS/MS Fragmentation of **LIPEMDQIFTEVEMTTLEK**
Found in **sp|Q9Y4L1|HYOU1_HUMAN**, Hypoxia up-regulated protein 1 OS=Homo sapiens GN=HYOU1 PE=1 SV=1
Match to Query 2505: 2554.313from(852.4451,3+)
Title: 1298: Sum of 2 scans in range 3204 (rt=80.3707, f=4, i=886) to 3205 (rt=80.3961, f=4, i=887) [D:\lab212\membrane\Grace\20120214_iTRAQ_1.raw]
Data File:
Monoisotopic mass of neutral peptide Mr(calc): 2554.313
Variable modifications:
K19 :iTRAQ4plex (K)
Ions Score: 74.34 **Expect:** 0.000

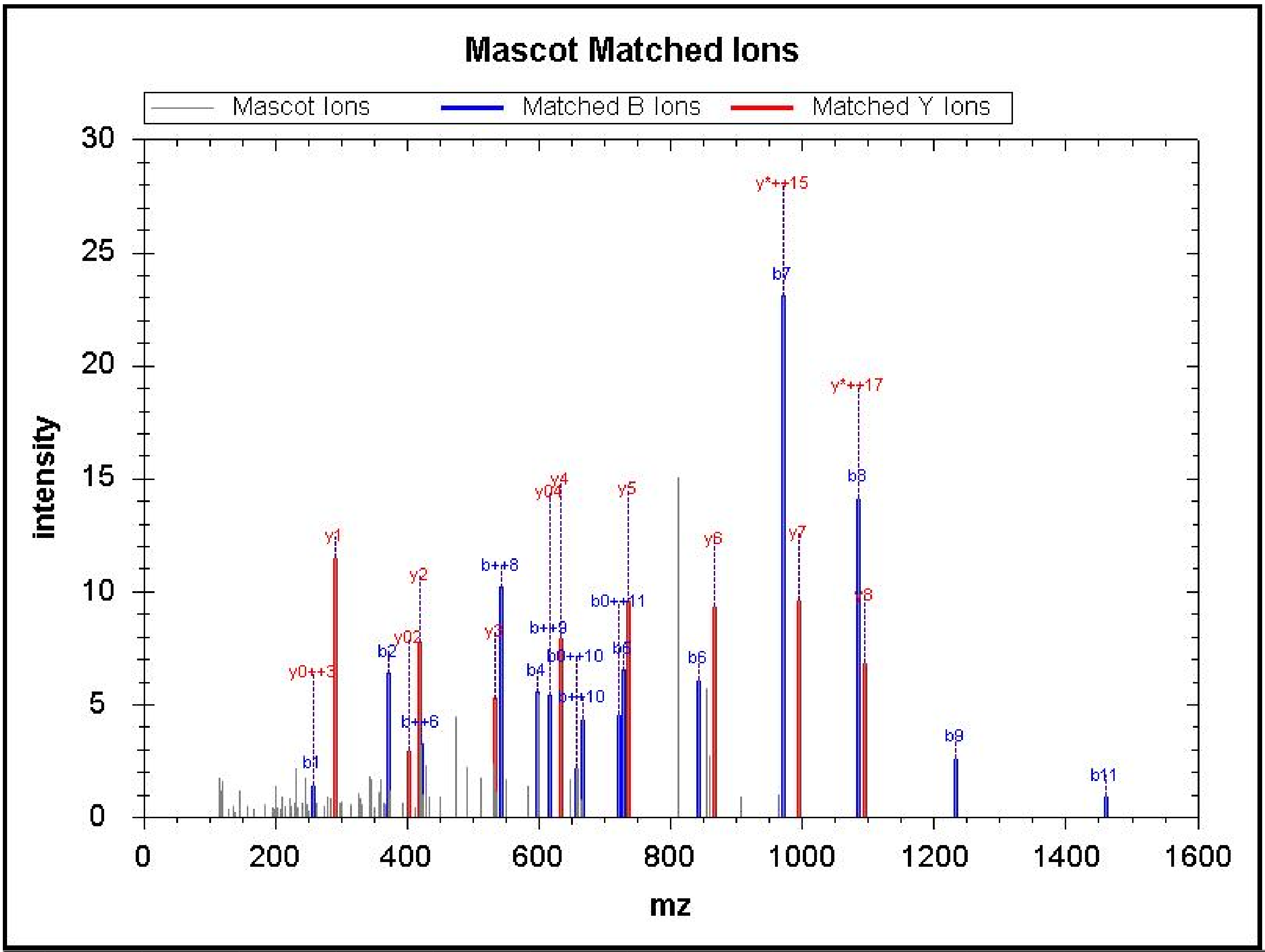

No	b	b++	b*	b*++	b0	b0++	Seq	y	y++	y*	y*++	y0	y0++	RevNo
1	258.19	129.60					L							19
2	371.28	186.14					I	2,298.14	1,149.57	2,281.11	1,141.06	2,280.13	1,140.57	18
3	468.33	234.67					P	2,185.05	1,093.03	2,168.03	1,084.52	2,167.04	1,084.02	17
4	597.37	299.19			579.36	290.18	E	2,088.00	1,044.50	2,070.97	1,035.99	2,069.99	1,035.50	16
5	728.41	364.71			710.40	355.70	M	1,958.96	979.98	1,941.93	971.47	1,940.95	970.98	15

6	843.44	422.22			825.43	413.22	D	1,827.92	914.46	1,810.89	905.95	1,809.91	905.46	14
7	971.50	486.25	954.47	477.74	953.49	477.25	Q	1,712.89	856.95	1,695.86	848.44	1,694.88	847.94	13
8	1,084.58	542.80	1,067.56	534.28	1,066.57	533.79	I	1,584.83	792.92	1,567.80	784.41	1,566.82	783.91	12
9	1,231.65	616.33	1,214.62	607.82	1,213.64	607.32	F	1,471.75	736.38	1,454.72	727.86	1,453.74	727.37	11
10	1,332.70	666.85	1,315.67	658.34	1,314.69	657.85	T	1,324.68	662.84	1,307.65	654.33	1,306.67	653.84	10
11	1,461.74	731.37	1,444.72	722.86	1,443.73	722.37	E	1,223.63	612.32	1,206.60	603.81	1,205.62	603.31	9
12	1,560.81	780.91	1,543.78	772.40	1,542.80	771.90	V	1,094.59	547.80	1,077.56	539.28	1,076.58	538.79	8
13	1,689.85	845.43	1,672.83	836.92	1,671.84	836.42	E	995.52	498.26	978.49	489.75	977.51	489.26	7
14	1,820.89	910.95	1,803.87	902.44	1,802.88	901.94	M	866.48	433.74	849.45	425.23	848.47	424.74	6
15	1,921.94	961.47	1,904.91	952.96	1,903.93	952.47	T	735.44	368.22	718.41	359.71	717.43	359.22	5
16	2,022.99	1,012.00	2,005.96	1,003.48	2,004.98	1,002.99	T	634.39	317.70	617.36	309.18	616.38	308.69	4
17	2,136.07	1,068.54	2,119.05	1,060.03	2,118.06	1,059.53	L	533.34	267.17	516.31	258.66	515.33	258.17	3
18	2,265.12	1,133.06	2,248.09	1,124.55	2,247.10	1,124.06	E	420.26	210.63	403.23	202.12	402.25	201.63	2
19							K	291.21	146.11	274.19	137.60			1

Query 2665 Hit 1

MS/MS Fragmentation of **AQNVPLPVSTLVEFVIAATDCTAK**
Found in **sp|P29699|FETUA_MOUSE**, Alpha-2-HS-glycoprotein OS=Mus musculus GN=Ahsg PE=1 SV=1
Match to Query 2665: 2820.506from(941.176,3+)
Title: 1487: Sum of 2 scans in range 3933 (rt=96.0486, f=2, i=671) to 3934 (rt=96.074, f=2, i=672) [D:\lab212\membrane\Grace\20120214_iTRAQ_1.raw]
Data File:
Monoisotopic mass of neutral peptide Mr(calc): 2820.506
Variable modifications:
C21 :Methylthio (C)
K24 :iTRAQ4plex (K)
Ions Score: 75.07 **Expect:** 0.000

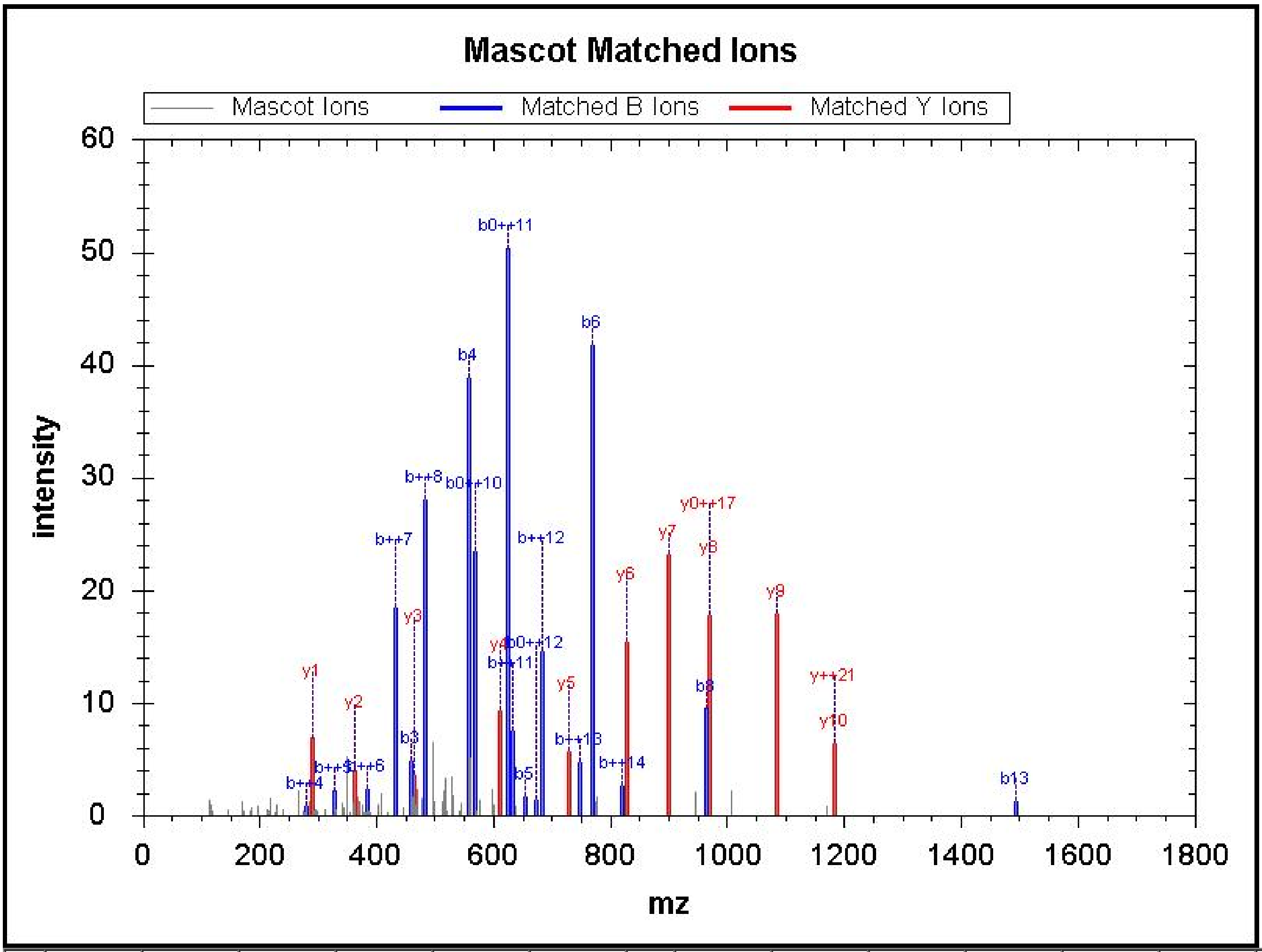

No	b	b++	b*	b*++	b0	b0++	Seq	y	y++	y*	y*++	y0	y0++	RevNo
1	216.15	108.58					A							24
2	344.21	172.61	327.18	164.09			Q	2,606.37	1,303.69	2,589.34	1,295.17	2,588.36	1,294.68	23
3	458.25	229.63	441.22	221.11			N	2,478.31	1,239.66	2,461.28	1,231.15	2,460.30	1,230.65	22
4	557.32	279.16	540.29	270.65			V	2,364.27	1,182.64	2,347.24	1,174.12	2,346.26	1,173.63	21
5	654.37	327.69	637.34	319.17			P	2,265.20	1,133.10	2,248.17	1,124.59	2,247.19	1,124.10	20
6	767.45	384.23	750.43	375.72			L	2,168.15	1,084.58	2,151.12	1,076.06	2,150.14	1,075.57	19
7	864.51	432.76	847.48	424.24			P	2,055.06	1,028.03	2,038.04	1,019.52	2,037.05	1,019.03	18
8	963.57	482.29	946.55	473.78			V	1,958.01	979.51	1,940.98	971.00	1,940.00	970.50	17
9	1,050.61	525.81	1,033.58	517.29	1,032.60	516.80	S	1,858.94	929.97	1,841.91	921.46	1,840.93	920.97	16
10	1,151.65	576.33	1,134.63	567.82	1,133.64	567.33	T	1,771.91	886.46	1,754.88	877.94	1,753.90	877.45	15
11	1,264.74	632.87	1,247.71	624.36	1,246.73	623.87	L	1,670.86	835.93	1,653.83	827.42	1,652.85	826.93	14
12	1,363.81	682.41	1,346.78	673.89	1,345.80	673.40	V	1,557.78	779.39	1,540.75	770.88	1,539.77	770.39	13
13	1,492.85	746.93	1,475.82	738.42	1,474.84	737.92	E	1,458.71	729.86	1,441.68	721.34	1,440.70	720.85	12
14	1,639.92	820.46	1,622.89	811.95	1,621.91	811.46	F	1,329.67	665.34	1,312.64	656.82	1,311.66	656.33	11
15	1,738.99	870.00	1,721.96	861.48	1,720.98	860.99	V	1,182.60	591.80	1,165.57	583.29	1,164.59	582.80	10
16	1,852.07	926.54	1,835.04	918.03	1,834.06	917.53	I	1,083.53	542.27	1,066.50	533.76	1,065.52	533.26	9
17	1,923.11	962.06	1,906.08	953.54	1,905.10	953.05	A	970.45	485.73	953.42	477.21	952.43	476.72	8
18	1,994.14	997.58	1,977.12	989.06	1,976.13	988.57	A	899.41	450.21	882.38	441.69	881.40	441.20	7
19	2,095.19	1,048.10	2,078.17	1,039.59	2,077.18	1,039.09	T	828.37	414.69	811.34	406.18	810.36	405.68	6
20	2,210.22	1,105.61	2,193.19	1,097.10	2,192.21	1,096.61	D	727.32	364.17	710.30	355.65	709.31	355.16	5
21	2,359.22	1,180.11	2,342.19	1,171.60	2,341.21	1,171.11	C	612.30	306.65	595.27	298.14	594.29	297.65	4
22	2,460.26	1,230.64	2,443.24	1,222.12	2,442.25	1,221.63	T	463.30	232.15	446.27	223.64	445.29	223.15	3
23	2,531.30	1,266.15	2,514.27	1,257.64	2,513.29	1,257.15	A	362.25	181.63	345.23	173.12			2

No	b	b++	b*	b*++	b0	b0++	Seq	y	y++	y*	y*++	y0	y0++	RevNo
24							K	291.21	146.11	274.19	137.60			1

Query 2009 Hit 1

MS/MS Fragmentation of **TMLELLNQLDGFEATK**
Found in **sp|P62195|PRS8_HUMAN**, 26S protease regulatory subunit 8 OS=Homo sapiens GN=PSMC5 PE=1 SV=1
Match to Query 2009: 2110.124from(704.382,3+)
Title: 1136: Sum of 2 scans in range 2807 (rt=71.5096, f=4, i=755) to 2808 (rt=71.535, f=4, i=756) [D:\lab212\membrane\Grace\20120214_iTRAQ_2.raw]
Data File:
Monoisotopic mass of neutral peptide Mr(calc): 2110.124
Variable modifications:
K16 :iTRAQ4plex (K)
Ions Score: 75.67 **Expect:** 0.000

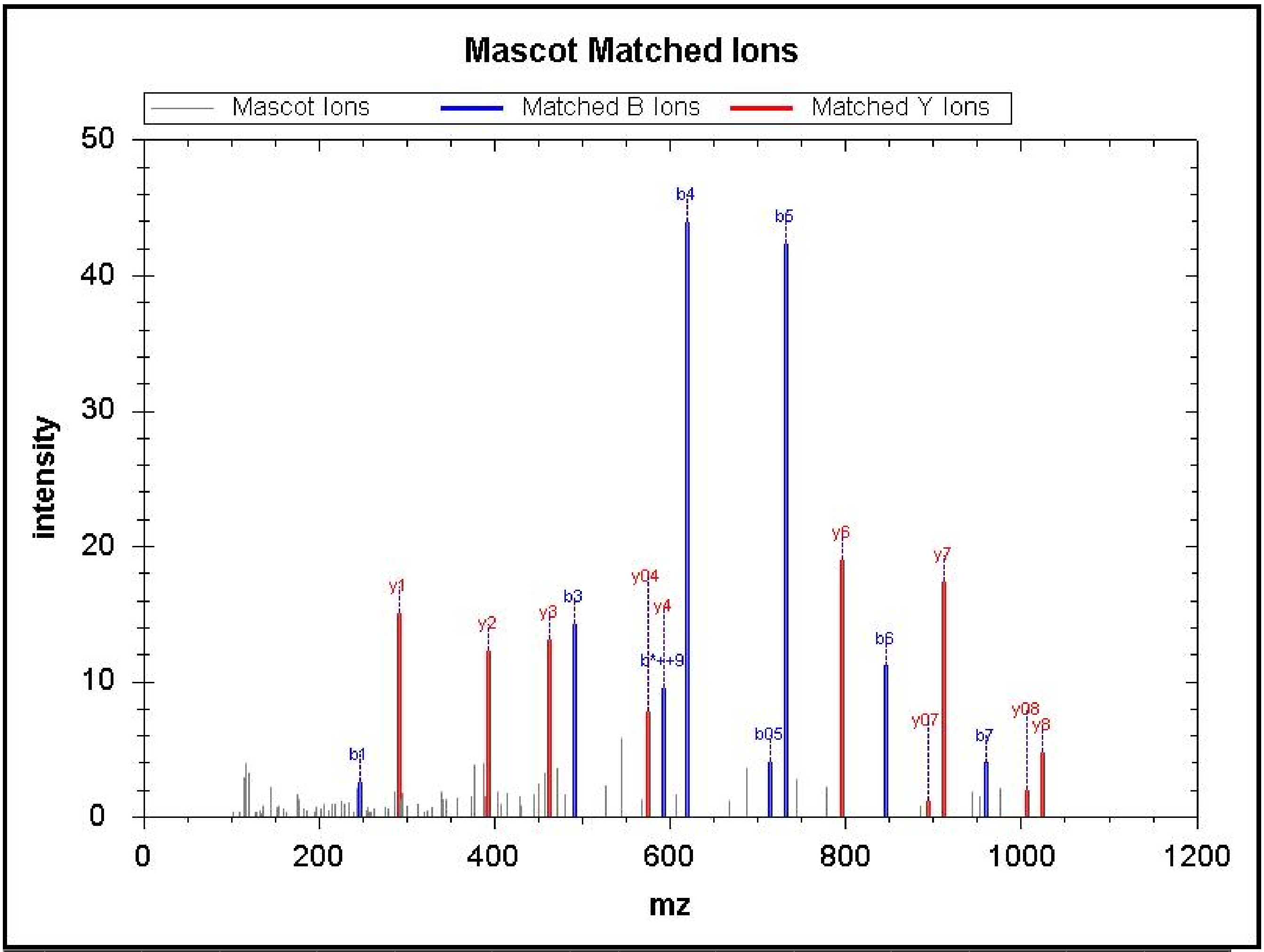

No	b	b++	b*	b*++	b0	b0++	Seq	y	y++	y*	y*++	y0	y0++	RevNo
1	246.16	123.58			228.15	114.58	T							16
2	377.20	189.10			359.19	180.10	M	1,865.98	933.49	1,848.95	924.98	1,847.97	924.49	15
3	490.28	245.64			472.27	236.64	L	1,734.94	867.97	1,717.91	859.46	1,716.93	858.97	14
4	619.32	310.17			601.31	301.16	E	1,621.86	811.43	1,604.83	802.92	1,603.84	802.43	13
5	732.41	366.71			714.40	357.70	L	1,492.81	746.91	1,475.79	738.40	1,474.80	737.90	12
6	845.49	423.25			827.48	414.24	L	1,379.73	690.37	1,362.70	681.85	1,361.72	681.36	11
7	959.54	480.27	942.51	471.76	941.52	471.27	N	1,266.64	633.83	1,249.62	625.31	1,248.63	624.82	10
8	1,087.59	544.30	1,070.57	535.79	1,069.58	535.30	Q	1,152.60	576.80	1,135.58	568.29	1,134.59	567.80	9
9	1,200.68	600.84	1,183.65	592.33	1,182.67	591.84	L	1,024.54	512.78	1,007.52	504.26	1,006.53	503.77	8
10	1,315.70	658.36	1,298.68	649.84	1,297.69	649.35	D	911.46	456.23	894.43	447.72	893.45	447.23	7
11	1,372.73	686.87	1,355.70	678.35	1,354.72	677.86	G	796.43	398.72	779.41	390.21	778.42	389.71	6
12	1,519.79	760.40	1,502.77	751.89	1,501.78	751.40	F	739.41	370.21	722.38	361.70	721.40	361.20	5

13	1,648.84	824.92	1,631.81	816.41	1,630.83	815.92	E	592.34	296.67	575.32	288.16	574.33	287.67	4
14	1,719.87	860.44	1,702.85	851.93	1,701.86	851.44	A	463.30	232.15	446.27	223.64	445.29	223.15	3
15	1,820.92	910.96	1,803.90	902.45	1,802.91	901.96	T	392.26	196.63	375.24	188.12	374.25	187.63	2
16							K	291.21	146.11	274.19	137.60			1

Query 1132 Hit 1

MS/MS Fragmentation of ALDTMNFDVIK
Found in sp|P11940|PABP1_HUMAN, Polyadenylate-binding protein 1 OS=Homo sapiens GN=PABPC1 PE=1 SV=2
Match to Query 1132: 1553.835from(777.9249,2+)
Title: 710: Sum of 2 scans in range 1964 (rt=52.4297, f=4, i=486) to 1965 (rt=52.4551, f=4, i=487) [D:\lab212\membrane\Grace\20120214_iTRAQ_1.raw]
Data File:
Monoisotopic mass of neutral peptide Mr(calc): 1553.835
Variable modifications:
K11 :iTRAQ4plex (K)
Ions Score: 76.68 **Expect:** 0.000

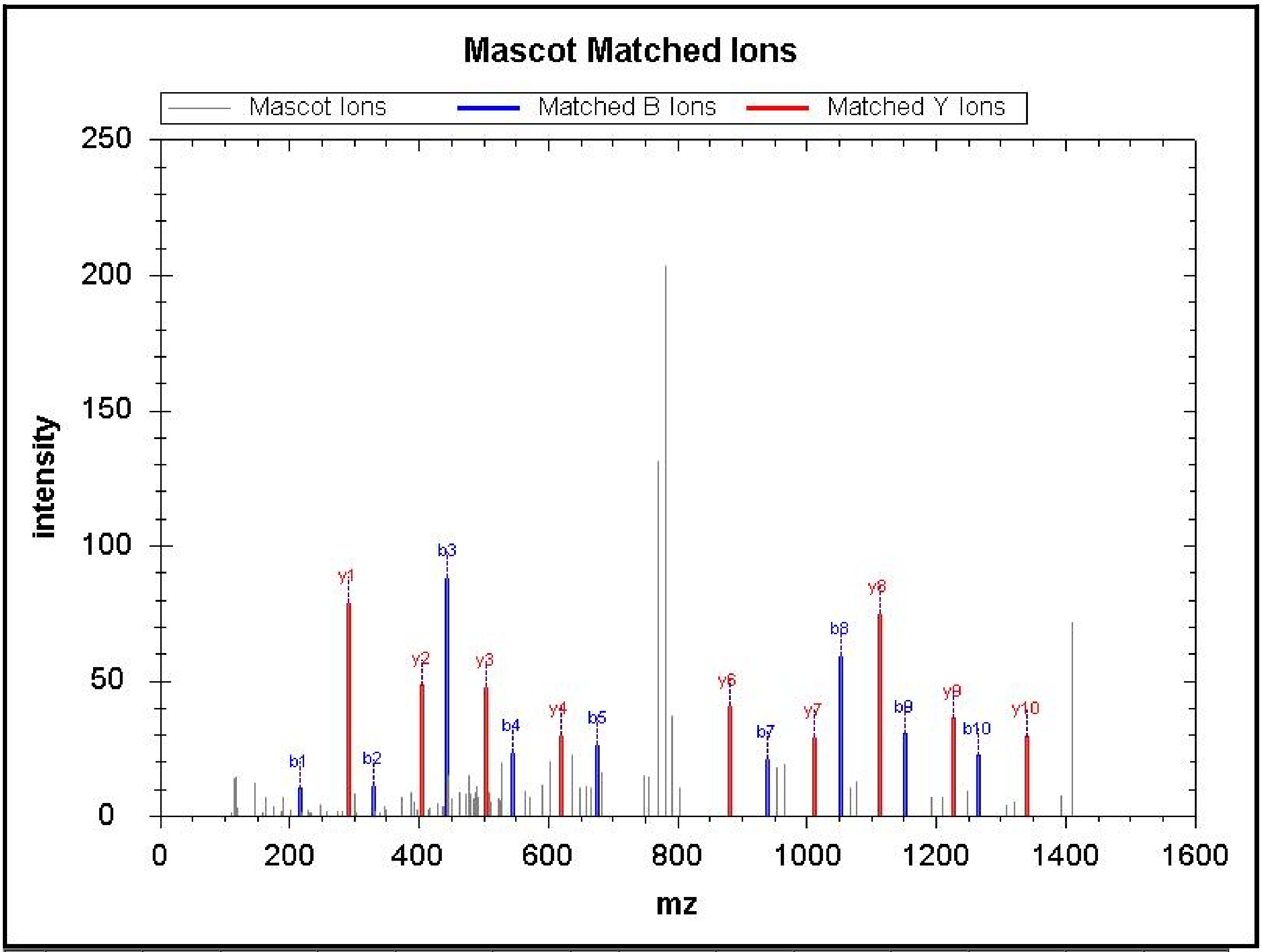

No	b	b++	b*	b*++	b0	b0++	Seq	y	y++	y*	y*++	y0	y0++	RevNo
1	216.15	108.58					A							11
2	329.23	165.12					L	1,339.70	670.36	1,322.68	661.84	1,321.69	661.35	10
3	444.26	222.63			426.25	213.63	D	1,226.62	613.81	1,209.59	605.30	1,208.61	604.81	9
4	545.31	273.16			527.29	264.15	T	1,111.59	556.30	1,094.57	547.79	1,093.58	547.30	8
5	676.35	338.68			658.34	329.67	M	1,010.55	505.78	993.52	497.26	992.54	496.77	7
6	790.39	395.70	773.36	387.18	772.38	386.69	N	879.51	440.26	862.48	431.74	861.49	431.25	6
7	937.46	469.23	920.43	460.72	919.45	460.23	F	765.46	383.23	748.44	374.72	747.45	374.23	5
8	1,052.48	526.75	1,035.46	518.23	1,034.47	517.74	D	618.39	309.70	601.37	301.19	600.38	300.70	4
9	1,151.55	576.28	1,134.53	567.77	1,133.54	567.27	V	503.37	252.19	486.34	243.67			3

10	1,264.64	632.82	1,247.61	624.31	1,246.63	623.82	I	404.30	202.65	387.27	194.14			2
11							K	291.21	146.11	274.19	137.60			1

Query 2302 Hit 1

MS/MS Fragmentation of **VTDPVGDIVSFMHSFEEK**
Found in **sp|Q96CS3|FAF2_HUMAN**, FAS-associated factor 2 OS=Homo sapiens GN=FAF2 PE=1 SV=2
Match to Query 2302: 2324.16from(775.7274,3+)
Title: 1182: Sum of 2 scans in range 2950 (rt=74.6464, f=4, i=804) to 2951 (rt=74.6718, f=4, i=805) [D:\lab212\membrane\Grace\20120214_iTRAQ_1.raw]
Data File:
Monoisotopic mass of neutral peptide Mr(calc): 2324.16
Variable modifications:
K18 :iTRAQ4plex (K)
Ions Score: 82.57 **Expect:** 0.000

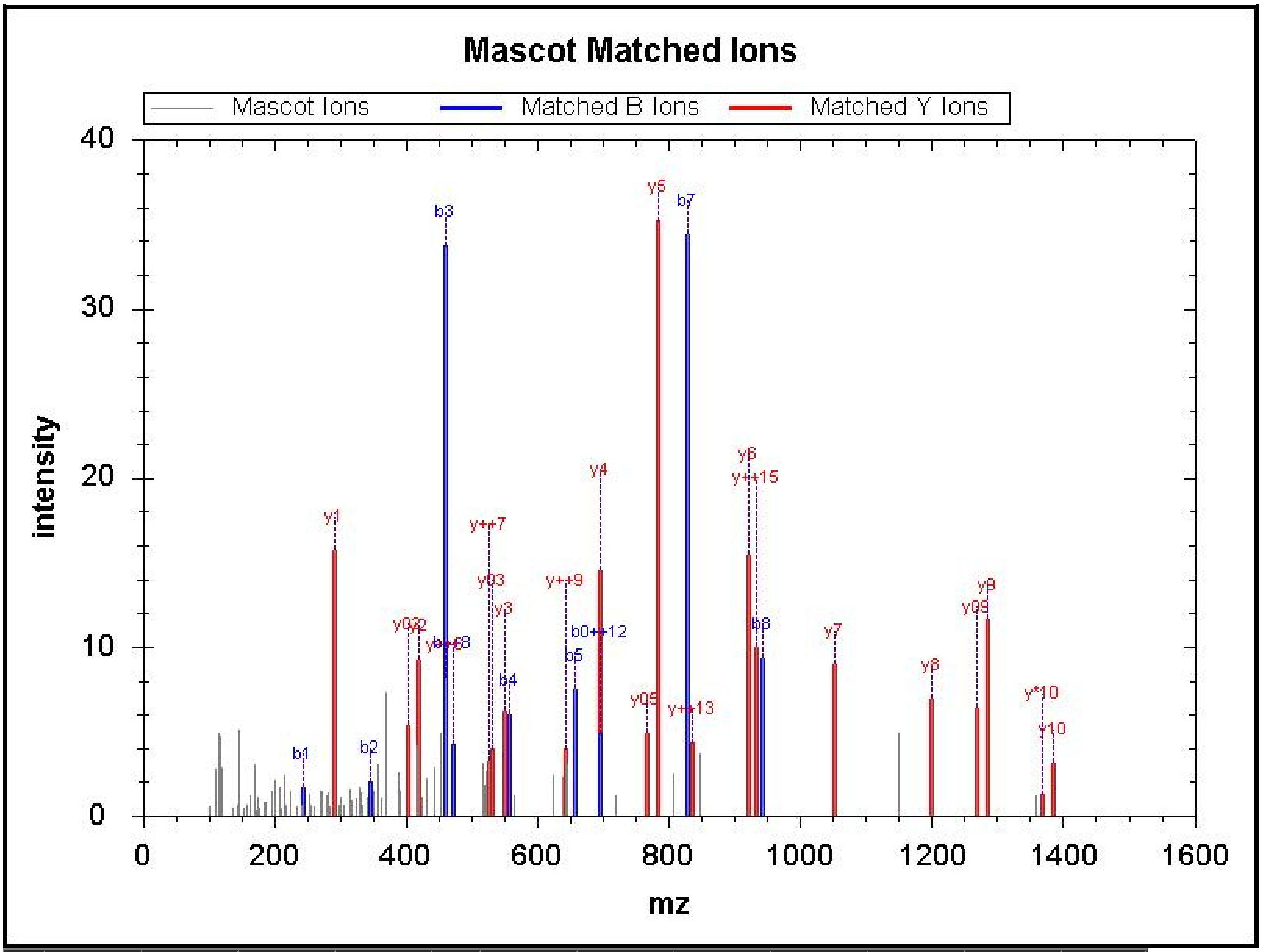

No	b	b++	b0	b0++	Seq	y	y++	y*	y*++	y0	y0++	RevNo
1	244.18	122.59			V							18
2	345.23	173.12	327.21	164.11	T	2,082.00	1,041.50	2,064.97	1,032.99	2,063.99	1,032.50	17
3	460.25	230.63	442.24	221.62	D	1,980.95	990.98	1,963.92	982.47	1,962.94	981.97	16
4	557.31	279.16	539.29	270.15	P	1,865.92	933.46	1,848.90	924.95	1,847.91	924.46	15
5	656.37	328.69	638.36	319.69	V	1,768.87	884.94	1,751.84	876.43	1,750.86	875.93	14
6	713.40	357.20	695.38	348.20	G	1,669.80	835.40	1,652.77	826.89	1,651.79	826.40	13
7	828.42	414.71	810.41	405.71	D	1,612.78	806.89	1,595.75	798.38	1,594.77	797.89	12
8	941.51	471.26	923.50	462.25	I	1,497.75	749.38	1,480.73	740.87	1,479.74	740.37	11
9	1,040.57	520.79	1,022.56	511.79	V	1,384.67	692.84	1,367.64	684.32	1,366.66	683.83	10
10	1,127.61	564.31	1,109.60	555.30	S	1,285.60	643.30	1,268.57	634.79	1,267.59	634.30	9
11	1,274.67	637.84	1,256.66	628.84	F	1,198.57	599.79	1,181.54	591.27	1,180.56	590.78	8

12	1,405.72	703.36	1,387.70	**694.36**	M	**1,051.50**	**526.25**	1,034.47	517.74	1,033.49	517.25	7
13	1,542.77	771.89	1,524.76	762.89	H	**920.46**	**460.73**	903.43	452.22	902.45	451.73	6
14	1,629.81	815.41	1,611.80	806.40	S	**783.40**	392.20	766.37	383.69	**765.39**	383.20	5
15	1,776.87	888.94	1,758.86	879.94	F	**696.37**	348.69	679.34	340.17	678.36	339.68	4
16	1,905.92	953.46	1,887.91	944.46	E	**549.30**	275.15	532.27	266.64	**531.29**	266.15	3
17	2,034.96	1,017.98	2,016.95	1,008.98	E	**420.26**	210.63	403.23	202.12	**402.25**	201.63	2
18					K	**291.21**	146.11	274.19	137.60			1

Query 2454 Hit 1

MS/MS Fragmentation of **ILLELLNQMDGFDQNVNVK**
Found in **sp|P43686|PRS6B_HUMAN**, 26S protease regulatory subunit 6B OS=Homo sapiens GN=PSMC4 PE=1 SV=2
Match to Query 2454: 2490.334from(831.1185,3+)
Title: 1257: Sum of 2 scans in range 3117 (rt=78.4109, f=4, i=858) to 3118 (rt=78.4363, f=4, i=859)
[D:\lab212\membrane\Grace\20120214_iTRAQ_1.raw]
Data File:
Monoisotopic mass of neutral peptide Mr(calc): 2490.334
Variable modifications:
K19 :iTRAQ4plex (K)
Ions Score: 82.92 **Expect:** 0.000

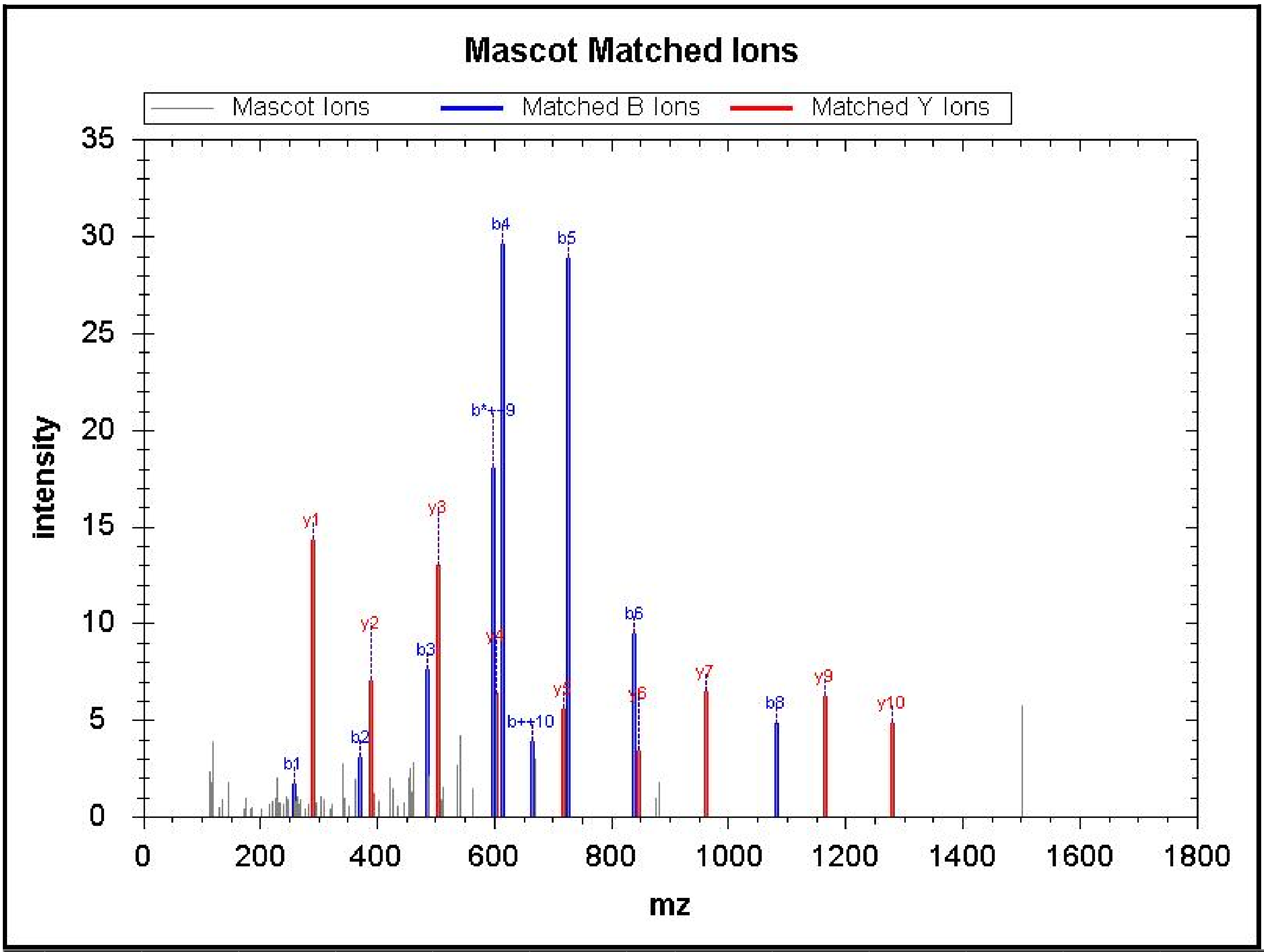

No	b	b++	b*	b*++	b0	b0++	Seq	y	y++	y*	y*++	y0	y0++	RevNo
1	**258.19**	129.60					I							19
2	**371.28**	186.14					L	2,234.16	1,117.58	2,217.13	1,109.07	2,216.15	1,108.58	18
3	**484.36**	242.68					L	2,121.08	1,061.04	2,104.05	1,052.53	2,103.07	1,052.04	17
4	**613.40**	307.21			595.39	298.20	E	2,007.99	1,004.50	1,990.97	995.99	1,989.98	995.49	16
5	**726.49**	363.75			708.48	354.74	L	1,878.95	939.98	1,861.92	931.47	1,860.94	930.97	15
6	**839.57**	420.29			821.56	411.28	L	1,765.87	883.44	1,748.84	874.92	1,747.86	874.43	14

7	953.62	477.31	936.59	468.80	935.60	468.31	N	1,652.78	826.89	1,635.76	818.38	1,634.77	817.89	13
8	1,081.67	541.34	1,064.65	532.83	1,063.66	532.34	Q	1,538.74	769.87	1,521.71	761.36	1,520.73	760.87	12
9	1,212.71	606.86	1,195.69	598.35	1,194.70	597.86	M	1,410.68	705.84	1,393.65	697.33	1,392.67	696.84	11
10	1,327.74	664.37	1,310.71	655.86	1,309.73	655.37	D	1,279.64	640.32	1,262.61	631.81	1,261.63	631.32	10
11	1,384.76	692.89	1,367.74	684.37	1,366.75	683.88	G	1,164.61	582.81	1,147.59	574.30	1,146.60	573.80	9
12	1,531.83	766.42	1,514.80	757.91	1,513.82	757.41	F	1,107.59	554.30	1,090.56	545.79	1,089.58	545.29	8
13	1,646.86	823.93	1,629.83	815.42	1,628.85	814.93	D	960.52	480.77	943.50	472.25	942.51	471.76	7
14	1,774.92	887.96	1,757.89	879.45	1,756.91	878.96	Q	845.50	423.25	828.47	414.74			6
15	1,888.96	944.98	1,871.93	936.47	1,870.95	935.98	N	717.44	359.22	700.41	350.71			5
16	1,988.03	994.52	1,971.00	986.00	1,970.02	985.51	V	603.39	302.20	586.37	293.69			4
17	2,102.07	1,051.54	2,085.04	1,043.03	2,084.06	1,042.53	N	504.33	252.67	487.30	244.15			3
18	2,201.14	1,101.07	2,184.11	1,092.56	2,183.13	1,092.07	V	390.28	195.65	373.26	187.13			2
19							K	291.21	146.11	274.19	137.60			1

Query 847 Hit 1

MS/MS Fragmentation of **STESLQANVQR**
Found in **sp|P26373|RL13_HUMAN**, 60S ribosomal protein L13 OS=Homo sapiens GN=RPL13 PE=1 SV=4
Match to Query 847: 1375.719from(688.8668,2+)
Title: 183: Sum of 2 scans in range 860 (rt=27.5545, f=4, i=130) to 861 (rt=27.5799, f=4, i=131)
[D:\lab212\membrane\Grace\20120214_iTRAQ_1.raw]
Data File:
Monoisotopic mass of neutral peptide Mr(calc): 1375.719
Variable modifications:
Ions Score: 83.23 **Expect:** 0.000

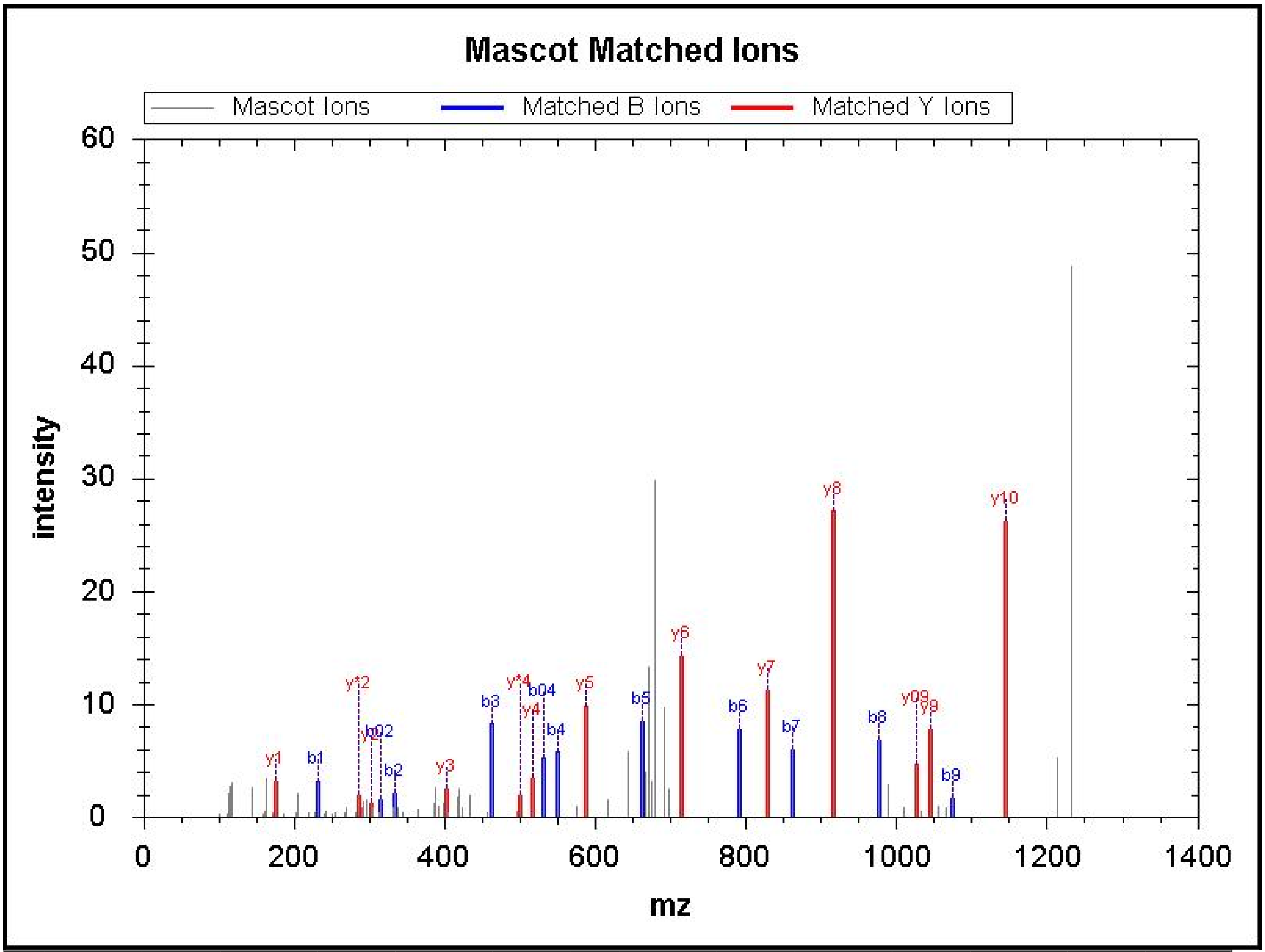

No	b	b++	b*	b*++	b0	b0++	Seq	y	y++	y*	y*++	y0	y0++	RevNo
1	232.14	116.57			214.13	107.57	S							11

2	333.19	167.10			315.18	158.09	T	1,145.59	573.30	1,128.56	564.79	1,127.58	564.29	10
3	462.23	231.62			444.22	222.61	E	1,044.54	522.78	1,027.52	514.26	1,026.53	513.77	9
4	549.26	275.14			531.25	266.13	S	915.50	458.25	898.47	449.74	897.49	449.25	8
5	662.35	331.68			644.34	322.67	L	828.47	414.74	811.44	406.22			7
6	790.41	395.71	773.38	387.19	772.40	386.70	Q	715.38	358.20	698.36	349.68			6
7	861.44	431.23	844.42	422.71	843.43	422.22	A	587.33	294.17	570.30	285.65			5
8	975.49	488.25	958.46	479.73	957.48	479.24	N	516.29	258.65	499.26	250.13			4
9	1,074.55	537.78	1,057.53	529.27	1,056.54	528.78	V	402.25	201.63	385.22	193.11			3
10	1,202.61	601.81	1,185.59	593.30	1,184.60	592.81	Q	303.18	152.09	286.15	143.58			2
11							R	175.12	88.06	158.09	79.55			1

Query 1334 Hit 1

MS/MS Fragmentation of **LLSSFDFFLTDAR**
Found in **sp|O76021|RL1D1_HUMAN**, Ribosomal L1 domain-containing protein 1 OS=Homo sapiens GN=RSL1D1 PE=1 SV=3
Match to Query 1334: 1674.875from(838.4446,2+)
Title: 1166: Scan 2874 (rt=73.017, f=3, i=418) [D:\lab212\membrane\Grace\20120214_iTRAQ_2.raw]
Data File:
Monoisotopic mass of neutral peptide Mr(calc): 1674.875
Variable modifications:
Ions Score: 83.44 **Expect:** 0.000

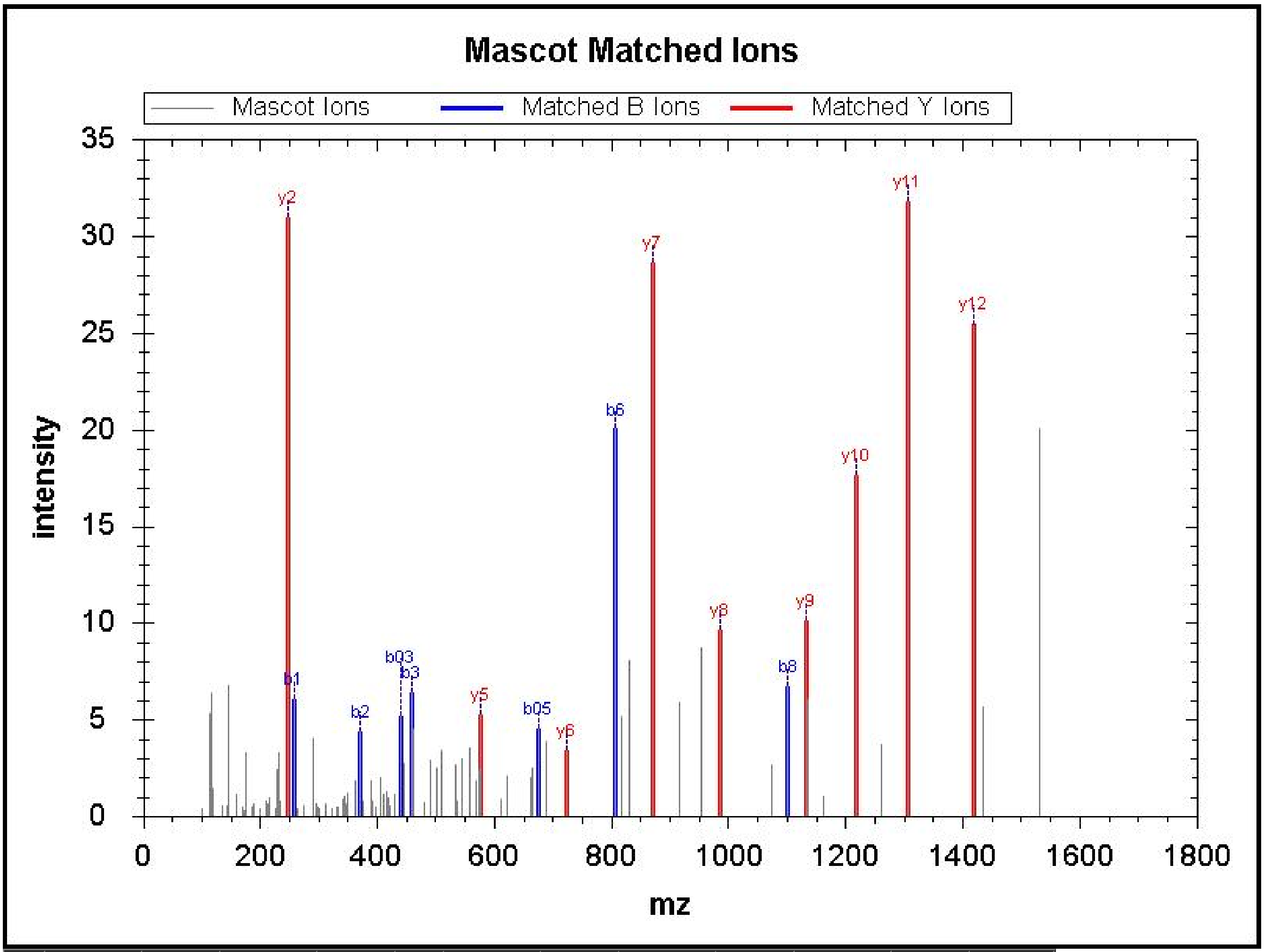

No	b	b++	b0	b0++	Seq	y	y++	y*	y*++	y0	y0++	RevNo
1	258.19	129.60			L							13
2	371.28	186.14			L	1,418.69	709.85	1,401.67	701.34	1,400.68	700.85	12
3	458.31	229.66	440.30	220.65	S	1,305.61	653.31	1,288.58	644.80	1,287.60	644.30	11
4	545.34	273.17	527.33	264.17	S	1,218.58	609.79	1,201.55	601.28	1,200.57	600.79	10
5	692.41	346.71	674.40	337.70	F	1,131.55	566.28	1,114.52	557.76	1,113.54	557.27	9

6	807.44	404.22	789.43	395.22	D	984.48	492.74	967.45	484.23	966.47	483.74	8
7	954.51	477.76	936.49	468.75	F	869.45	435.23	852.42	426.72	851.44	426.22	7
8	1,101.57	551.29	1,083.56	542.29	F	722.38	361.70	705.36	353.18	704.37	352.69	6
9	1,214.66	607.83	1,196.65	598.83	L	575.31	288.16	558.29	279.65	557.30	279.16	5
10	1,315.71	658.36	1,297.69	649.35	T	462.23	231.62	445.20	223.11	444.22	222.61	4
11	1,430.73	715.87	1,412.72	706.86	D	361.18	181.10	344.16	172.58	343.17	172.09	3
12	1,501.77	751.39	1,483.76	742.38	A	246.16	123.58	229.13	115.07			2
13					R	175.12	88.06	158.09	79.55			1

Query 2799 Hit 1

MS/MS Fragmentation of **LLVSNLDFGVSDADIQELFAEFGTLK**
Found in **sp|Q86V81|THOC4_HUMAN**, THO complex subunit 4 OS=Homo sapiens GN=ALYREF PE=1 SV=3
Match to Query 2799: 3128.645from(1043.889,3+)
Title: 1374: Sum of 2 scans in range 3384 (rt=84.4262, f=4, i=944) to 3385 (rt=84.4516, f=4, i=945)
[D:\lab212\membrane\Grace\20120214_iTRAQ_1.raw]
Data File:
Monoisotopic mass of neutral peptide Mr(calc): 3128.645
Variable modifications:
K26 :iTRAQ4plex (K)
Ions Score: 90.52 **Expect:** 0.000

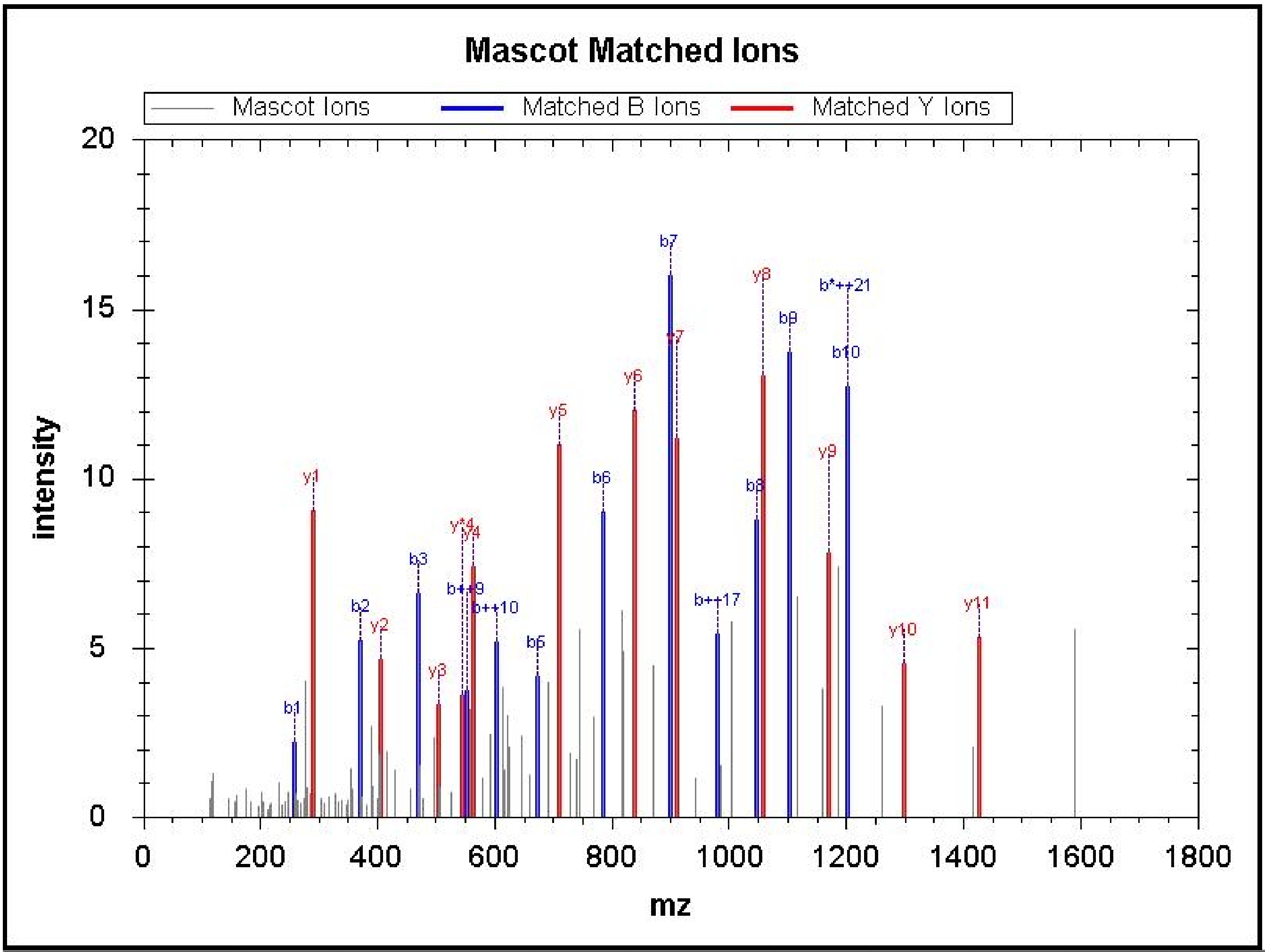

No	b	b++	b*	b*++	b0	b0++	Seq	y	y++	y*	y*++	y0	y0++	RevNo
1	258.19	129.60					L							26
2	371.28	186.14					L	2,872.47	1,436.74	2,855.45	1,428.23	2,854.46	1,427.74	25
3	470.35	235.68					V	2,759.39	1,380.20	2,742.36	1,371.69	2,741.38	1,371.19	24
4	557.38	279.19			539.37	270.19	S	2,660.32	1,330.66	2,643.29	1,322.15	2,642.31	1,321.66	23
5	671.42	336.21	654.39	327.70	653.41	327.21	N	2,573.29	1,287.15	2,556.26	1,278.63	2,555.28	1,278.14	22

**6**	**784.50**	392.76	767.48	384.24	766.49	383.75	**L**	2,459.25	1,230.13	2,442.22	1,221.61	2,441.24	1,221.12	**21**
**7**	**899.53**	450.27	882.51	441.76	881.52	441.26	**D**	2,346.16	1,173.58	2,329.14	1,165.07	2,328.15	1,164.58	**20**
**8**	**1,046.60**	523.80	1,029.57	515.29	1,028.59	514.80	**F**	2,231.14	1,116.07	2,214.11	1,107.56	2,213.12	1,107.07	**19**
**9**	**1,103.62**	**552.31**	1,086.60	543.80	1,085.61	543.31	**G**	2,084.07	1,042.54	2,067.04	1,034.02	2,066.06	1,033.53	**18**
**10**	**1,202.69**	**601.85**	1,185.66	593.34	1,184.68	592.84	**V**	2,027.05	1,014.03	2,010.02	1,005.51	2,009.03	1,005.02	**17**
**11**	1,289.72	645.36	1,272.70	636.85	1,271.71	636.36	**S**	1,927.98	964.49	1,910.95	955.98	1,909.97	955.49	**16**
**12**	1,404.75	702.88	1,387.72	694.36	1,386.74	693.87	**D**	1,840.95	920.98	1,823.92	912.46	1,822.93	911.97	**15**
**13**	1,475.79	738.40	1,458.76	729.88	1,457.78	729.39	**A**	1,725.92	863.46	1,708.89	854.95	1,707.91	854.46	**14**
**14**	1,590.81	795.91	1,573.79	787.40	1,572.80	786.90	**D**	1,654.88	827.94	1,637.85	819.43	1,636.87	818.94	**13**
**15**	1,703.90	852.45	1,686.87	843.94	1,685.89	843.45	**I**	1,539.85	770.43	1,522.83	761.92	1,521.84	761.43	**12**
**16**	1,831.96	916.48	1,814.93	907.97	1,813.95	907.48	**Q**	**1,426.77**	713.89	1,409.74	705.38	1,408.76	704.88	**11**
**17**	1,961.00	**981.00**	1,943.97	972.49	1,942.99	972.00	**E**	**1,298.71**	649.86	1,281.68	641.35	1,280.70	640.85	**10**
**18**	2,074.08	1,037.54	2,057.06	1,029.03	2,056.07	1,028.54	**L**	**1,169.67**	585.34	1,152.64	576.82	1,151.66	576.33	**9**
**19**	2,221.15	1,111.08	2,204.12	1,102.57	2,203.14	1,102.07	**F**	**1,056.58**	528.80	1,039.56	520.28	1,038.57	519.79	**8**
**20**	2,292.19	1,146.60	2,275.16	1,138.08	2,274.18	1,137.59	**A**	**909.52**	455.26	892.49	446.75	891.51	446.26	**7**
**21**	2,421.23	1,211.12	2,404.20	**1,202.61**	2,403.22	1,202.11	**E**	**838.48**	419.74	821.45	411.23	820.47	410.74	**6**
**22**	2,568.30	1,284.65	2,551.27	1,276.14	2,550.29	1,275.65	**F**	**709.44**	355.22	692.41	346.71	691.43	346.22	**5**
**23**	2,625.32	1,313.16	2,608.29	1,304.65	2,607.31	1,304.16	**G**	**562.37**	281.69	**545.34**	273.17	544.36	272.68	**4**
**24**	2,726.37	1,363.69	2,709.34	1,355.17	2,708.36	1,354.68	**T**	**505.35**	253.18	488.32	244.66	487.34	244.17	**3**
**25**	2,839.45	1,420.23	2,822.43	1,411.72	2,821.44	1,411.22	**L**	**404.30**	202.65	387.27	194.14			**2**
**26**							**K**	**291.21**	146.11	274.19	137.60			**1**

Query 2733 Hit 1

MS/MS Fragmentation of **SMAGNIIPAIATTNAVIAGLIVLEGLK**
Found in **sp|Q9UBT2|SAE2_HUMAN**, SUMO-activating enzyme subunit 2 OS=Homo sapiens GN=UBA2 PE=1 SV=2
Match to Query 2733: 2937.707from(980.243,3+)
Title: 1474: Sum of 2 scans in range 3818 (rt=93.6581, f=2, i=637) to 3819 (rt=93.6835, f=2, i=638) [D:\lab212\membrane\Grace\20120214_iTRAQ_1.raw]
Data File:
Monoisotopic mass of neutral peptide Mr(calc): 2937.707
Variable modifications:
K27 :iTRAQ4plex (K)
Ions Score: 90.95 **Expect:** 0.000

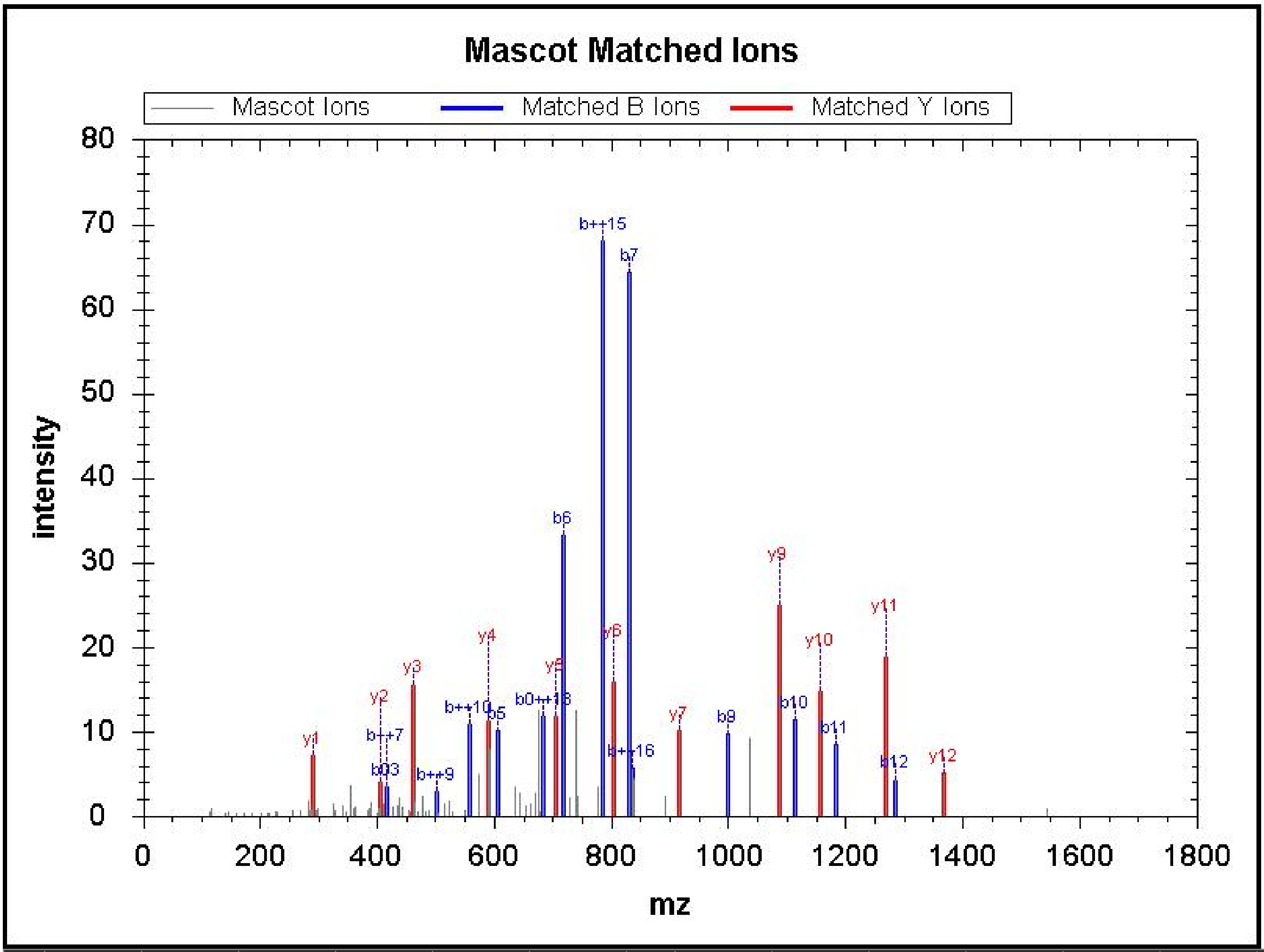

No	b	b++	b*	b*++	b0	b0++	Seq	y	y++	y*	y*++	y0	y0++	RevNo
1	232.14	116.57			214.13	107.57	S							27
2	363.18	182.09			345.17	173.09	M	2,707.59	1,354.30	2,690.56	1,345.79	2,689.58	1,345.29	26
3	434.22	217.61			416.21	208.61	A	2,576.55	1,288.78	2,559.52	1,280.27	2,558.54	1,279.77	25
4	491.24	246.12			473.23	237.12	G	2,505.51	1,253.26	2,488.49	1,244.75	2,487.50	1,244.26	24
5	605.28	303.15	588.26	294.63	587.27	294.14	N	2,448.49	1,224.75	2,431.47	1,216.24	2,430.48	1,215.74	23
6	718.37	359.69	701.34	351.17	700.36	350.68	I	2,334.45	1,167.73	2,317.42	1,159.21	2,316.44	1,158.72	22
7	831.45	416.23	814.42	407.72	813.44	407.22	I	2,221.37	1,111.19	2,204.34	1,102.67	2,203.35	1,102.18	21
8	928.50	464.76	911.48	456.24	910.49	455.75	P	2,108.28	1,054.64	2,091.25	1,046.13	2,090.27	1,045.64	20
9	999.54	500.27	982.51	491.76	981.53	491.27	A	2,011.23	1,006.12	1,994.20	997.60	1,993.22	997.11	19
10	1,112.63	556.82	1,095.60	548.30	1,094.61	547.81	I	1,940.19	970.60	1,923.16	962.09	1,922.18	961.59	18
11	1,183.66	592.33	1,166.64	583.82	1,165.65	583.33	A	1,827.11	914.06	1,810.08	905.54	1,809.10	905.05	17
12	1,284.71	642.86	1,267.68	634.35	1,266.70	633.85	T	1,756.07	878.54	1,739.04	870.03	1,738.06	869.53	16
13	1,385.76	693.38	1,368.73	684.87	1,367.75	684.38	T	1,655.02	828.01	1,638.00	819.50	1,637.01	819.01	15
14	1,499.80	750.40	1,482.77	741.89	1,481.79	741.40	N	1,553.97	777.49	1,536.95	768.98	1,535.96	768.49	14
15	1,570.84	785.92	1,553.81	777.41	1,552.83	776.92	A	1,439.93	720.47	1,422.91	711.96	1,421.92	711.46	13
16	1,669.91	835.46	1,652.88	826.94	1,651.90	826.45	V	1,368.89	684.95	1,351.87	676.44	1,350.88	675.95	12
17	1,782.99	892.00	1,765.96	883.49	1,764.98	882.99	I	1,269.83	635.42	1,252.80	626.90	1,251.82	626.41	11
18	1,854.03	927.52	1,837.00	919.00	1,836.02	918.51	A	1,156.74	578.87	1,139.72	570.36	1,138.73	569.87	10
19	1,911.05	956.03	1,894.02	947.52	1,893.04	947.02	G	1,085.70	543.36	1,068.68	534.84	1,067.69	534.35	9
20	2,024.13	1,012.57	2,007.11	1,004.06	2,006.12	1,003.57	L	1,028.68	514.85	1,011.66	506.33	1,010.67	505.84	8
21	2,137.22	1,069.11	2,120.19	1,060.60	2,119.21	1,060.11	I	915.60	458.30	898.57	449.79	897.59	449.30	7
22	2,236.29	1,118.65	2,219.26	1,110.13	2,218.28	1,109.64	V	802.52	401.76	785.49	393.25	784.50	392.76	6
23	2,349.37	1,175.19	2,332.34	1,166.68	2,331.36	1,166.18	L	703.45	352.23	686.42	343.71	685.44	343.22	5

24	2,478.41	1,239.71	2,461.39	1,231.20	2,460.40	1,230.70	E	**590.36**	295.69	573.34	287.17	572.35	286.68	4
25	2,535.43	1,268.22	2,518.41	1,259.71	2,517.42	1,259.22	G	**461.32**	231.16	444.29	222.65			3
26	2,648.52	1,324.76	2,631.49	1,316.25	2,630.51	1,315.76	L	**404.30**	202.65	387.27	194.14			2
27							K	**291.21**	146.11	274.19	137.60			1

Query 2513 Hit 1

MS/MS Fragmentation of **VVGFHVLGPNAGEVTQGFAAALK**
Found in **sp|Q16881|TRXR1_HUMAN**, Thioredoxin reductase 1
Match to Query 2513: 2569.419from(857.4802,3+)
Title: 849: Scan 2210 (rt=58.0515, f=2, i=346) [D:\lab212\membrane\Grace\20120214_iTRAQ_2.raw]
Data File:
Monoisotopic mass of neutral peptide Mr(calc): 2569.419
Variable modifications:
K23 :iTRAQ4plex (K)
Ions Score: 113.66 **Expect:** 0.000

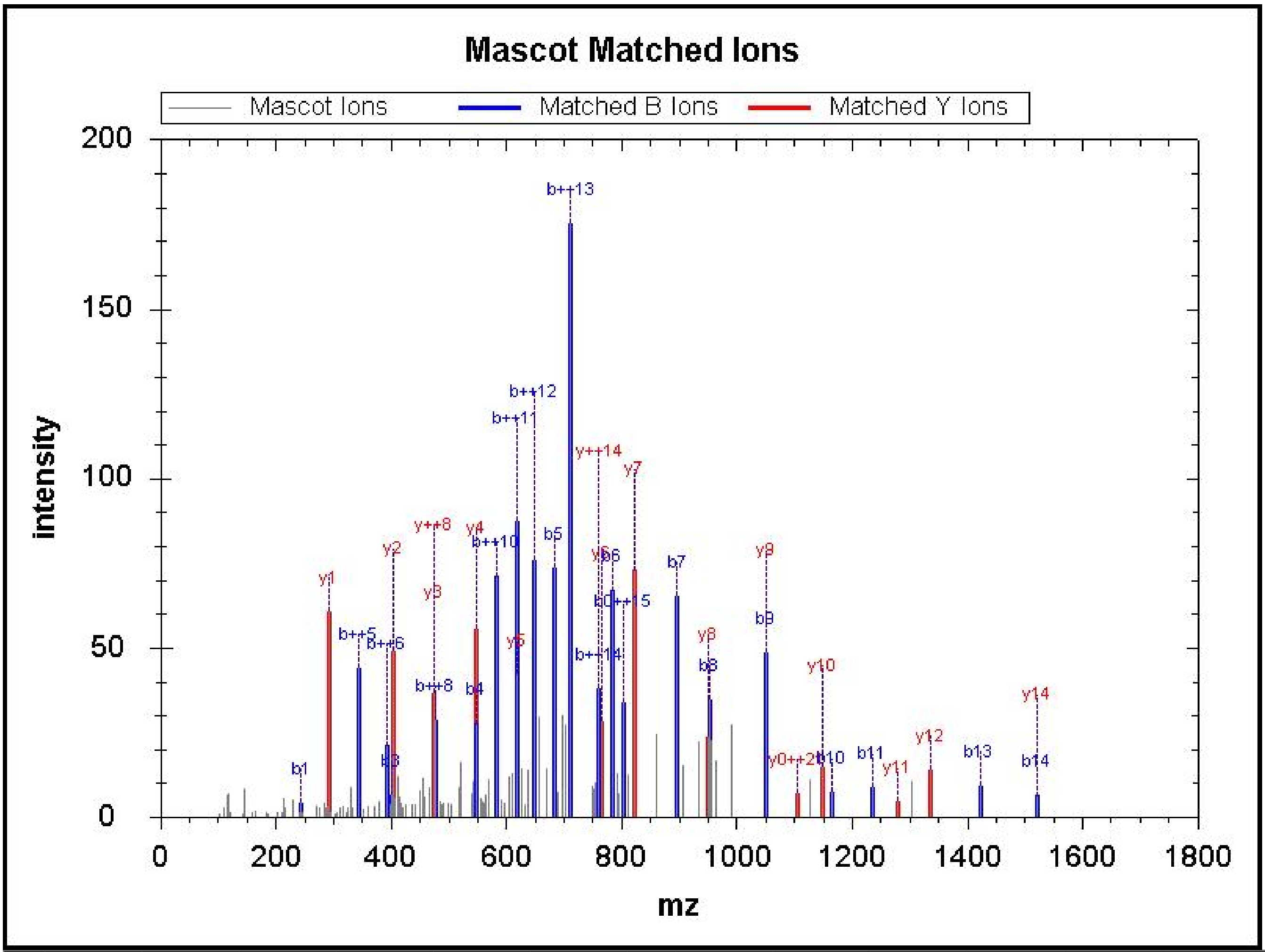

No	b	b++	b*	b*++	b0	b0++	Seq	y	y++	y*	y*++	y0	y0++	RevNo
1	**244.18**	122.59					V							23
2	343.25	172.13					V	2,327.26	1,164.14	2,310.24	1,155.62	2,309.25	1,155.13	22
3	**400.27**	200.64					G	2,228.19	1,114.60	2,211.17	1,106.09	2,210.18	**1,105.60**	21
4	**547.34**	274.17					F	2,171.17	1,086.09	2,154.15	1,077.58	2,153.16	1,077.08	20
5	**684.39**	**342.70**					H	2,024.10	1,012.56	2,007.08	1,004.04	2,006.09	1,003.55	19
6	**783.46**	**392.24**					V	1,887.05	944.03	1,870.02	935.51	1,869.04	935.02	18
7	**896.55**	448.78					L	1,787.98	894.49	1,770.95	885.98	1,769.97	885.49	17
8	**953.57**	**477.29**					G	1,674.89	837.95	1,657.87	829.44	1,656.88	828.94	16
9	**1,050.62**	525.81					P	1,617.87	809.44	1,600.85	800.93	1,599.86	800.43	15

10	1,164.66	582.84	1,147.64	574.32			N	1,520.82	760.91	1,503.79	752.40	1,502.81	751.91	14
11	1,235.70	618.35	1,218.68	609.84			A	1,406.78	703.89	1,389.75	695.38	1,388.77	694.89	13
12	1,292.72	646.87	1,275.70	638.35			G	1,335.74	668.37	1,318.71	659.86	1,317.73	659.37	12
13	1,421.77	711.39	1,404.74	702.87	1,403.76	702.38	E	1,278.72	639.86	1,261.69	631.35	1,260.71	630.86	11
14	1,520.83	760.92	1,503.81	752.41	1,502.82	751.92	V	1,149.67	575.34	1,132.65	566.83	1,131.66	566.34	10
15	1,621.88	811.44	1,604.86	802.93	1,603.87	802.44	T	1,050.61	525.81	1,033.58	517.29	1,032.60	516.80	9
16	1,749.94	875.47	1,732.91	866.96	1,731.93	866.47	Q	949.56	475.28	932.53	466.77			8
17	1,806.96	903.98	1,789.94	895.47	1,788.95	894.98	G	821.50	411.25	804.47	402.74			7
18	1,954.03	977.52	1,937.00	969.01	1,936.02	968.51	F	764.48	382.74	747.45	374.23			6
19	2,025.07	1,013.04	2,008.04	1,004.52	2,007.06	1,004.03	A	617.41	309.21	600.38	300.70			5
20	2,096.10	1,048.56	2,079.08	1,040.04	2,078.09	1,039.55	A	546.37	273.69	529.35	265.18			4
21	2,167.14	1,084.07	2,150.12	1,075.56	2,149.13	1,075.07	A	475.34	238.17	458.31	229.66			3
22	2,280.23	1,140.62	2,263.20	1,132.10	2,262.22	1,131.61	L	404.30	202.65	387.27	194.14			2
23							K	291.21	146.11	274.19	137.60			1

Query 2906 Hit 1

MS/MS Fragmentation of **IDLGVHVDGFIANVAHTFVVDVAQGTQVTGR**
Found in **sp|Q9UQ80|PA2G4_HUMAN**, Proliferation-associated protein 2G4 OS=Homo sapiens GN=PA2G4 PE=1 SV=3
Match to Query 2906: 3378.782from(845.7027,4+)
Title: 1268: Scan 3097 (rt=78.0423, f=3, i=454) [D:\lab212\membrane\Grace\20120214_iTRAQ_2.raw]
Data File:
Monoisotopic mass of neutral peptide Mr(calc): 3378.782
Variable modifications:
Ions Score: 132.71 **Expect:** 0.000

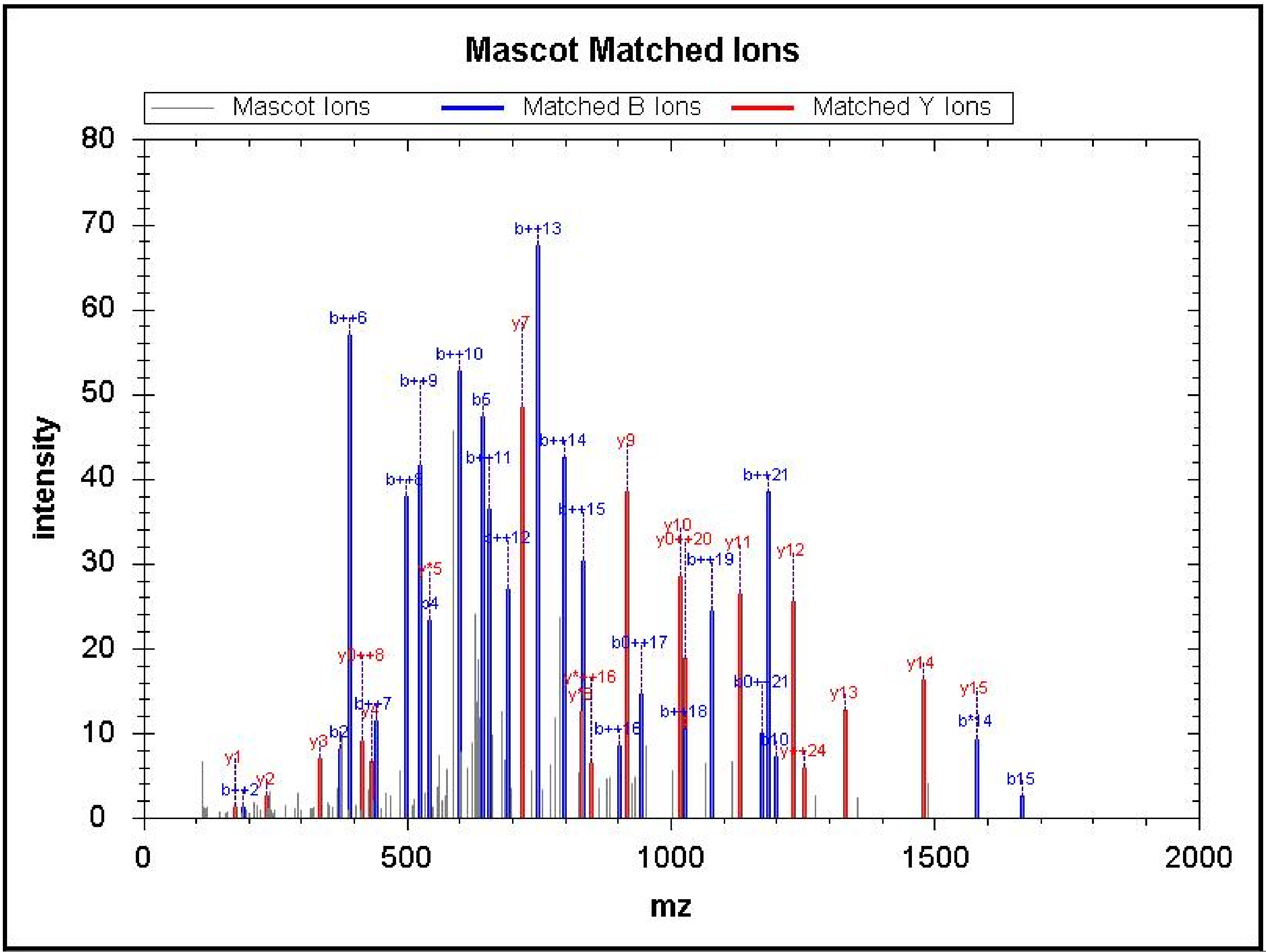

No	b	b++	b*	b*++	b0	b0++	Seq	y	y++	y*	y*++	y0	y0++	RevNo

1	258.19	129.60					I						31
2	**373.22**	**187.11**			355.21	178.11	D	3,122.60	1,561.80	3,105.57	1,553.29	3,104.59	1,552.80	30
3	486.30	243.66			468.29	234.65	L	3,007.57	1,504.29	2,990.55	1,495.78	2,989.56	1,495.29	29
4	**543.33**	272.17			525.32	263.16	G	2,894.49	1,447.75	2,877.46	1,439.24	2,876.48	1,438.74	28
5	**642.39**	321.70			624.38	312.70	V	2,837.47	1,419.24	2,820.44	1,410.72	2,819.46	1,410.23	27
6	779.45	**390.23**			761.44	381.22	H	2,738.40	1,369.70	2,721.37	1,361.19	2,720.39	1,360.70	26
7	878.52	**439.76**			860.51	430.76	V	2,601.34	1,301.17	2,584.32	1,292.66	2,583.33	1,292.17	25
8	993.55	**497.28**			975.54	488.27	D	2,502.27	**1,251.64**	2,485.25	1,243.13	2,484.26	1,242.64	24
9	1,050.57	**525.79**			1,032.56	516.78	G	2,387.25	1,194.13	2,370.22	1,185.61	2,369.24	1,185.12	23
10	**1,197.64**	**599.32**			1,179.63	590.32	F	2,330.23	1,165.62	2,313.20	1,157.10	2,312.21	1,156.61	22
11	1,310.72	**655.86**			1,292.71	646.86	I	2,183.16	1,092.08	2,166.13	1,083.57	2,165.15	1,083.08	21
12	1,381.76	**691.38**			1,363.75	682.38	A	2,070.07	1,035.54	2,053.05	1,027.03	2,052.06	**1,026.53**	20
13	1,495.80	**748.40**	1,478.78	739.89	1,477.79	739.40	N	1,999.04	1,000.02	1,982.01	991.51	1,981.03	991.02	19
14	1,594.87	**797.94**	**1,577.84**	789.43	1,576.86	788.93	V	1,884.99	943.00	1,867.97	934.49	1,866.98	933.99	18
15	**1,665.91**	**833.46**	1,648.88	824.94	1,647.90	824.45	A	1,785.92	893.47	1,768.90	884.95	1,767.91	884.46	17
16	1,802.97	**901.99**	1,785.94	893.47	1,784.96	892.98	H	1,714.89	857.95	1,697.86	**849.43**	1,696.88	848.94	16
17	1,904.01	952.51	1,886.99	944.00	1,886.00	**943.51**	T	**1,577.83**	789.42	1,560.80	780.90	1,559.82	780.41	15
18	2,051.08	**1,026.05**	2,034.06	1,017.53	2,033.07	1,017.04	F	**1,476.78**	738.89	1,459.75	730.38	1,458.77	729.89	14
19	2,150.15	**1,075.58**	2,133.13	1,067.07	2,132.14	1,066.57	V	**1,329.71**	665.36	1,312.69	656.85	1,311.70	656.35	13
20	2,249.22	1,125.11	2,232.19	1,116.60	2,231.21	1,116.11	V	**1,230.64**	615.83	1,213.62	607.31	1,212.63	606.82	12
21	2,364.25	**1,182.63**	2,347.22	1,174.11	2,346.24	**1,173.62**	D	**1,131.58**	566.29	1,114.55	557.78	1,113.56	557.29	11
22	2,463.32	1,232.16	2,446.29	1,223.65	2,445.30	1,223.16	V	**1,016.55**	508.78	999.52	500.26	998.54	499.77	10
23	2,534.35	1,267.68	2,517.33	1,259.17	2,516.34	1,258.67	A	**917.48**	459.24	900.45	450.73	899.47	450.24	9
24	2,662.41	1,331.71	2,645.38	1,323.20	2,644.40	1,322.70	Q	846.44	423.73	**829.42**	415.21	828.43	**414.72**	8
25	2,719.43	1,360.22	2,702.41	1,351.71	2,701.42	1,351.21	G	**718.38**	359.70	701.36	351.18	700.37	350.69	7
26	2,820.48	1,410.74	2,803.45	1,402.23	2,802.47	1,401.74	T	661.36	331.18	644.34	322.67	643.35	322.18	6
27	2,948.54	1,474.77	2,931.51	1,466.26	2,930.53	1,465.77	Q	560.32	280.66	**543.29**	272.15	542.30	271.66	5
28	3,047.61	1,524.31	3,030.58	1,515.79	3,029.60	1,515.30	V	**432.26**	216.63	415.23	208.12	414.25	207.63	4
29	3,148.65	1,574.83	3,131.63	1,566.32	3,130.64	1,565.83	T	**333.19**	167.10	316.16	158.58	315.18	158.09	3
30	3,205.68	1,603.34	3,188.65	1,594.83	3,187.67	1,594.34	G	**232.14**	116.57	215.11	108.06			2
31							R	**175.12**	88.06	158.09	79.55			1
