# Supplementary material for: Quantitative Proteomic Analysis of Human Lung Tumor Xenografts Treated with the Ectopic ATP Synthase Inhibitor Citreoviridin
Source: PLoS One. 2013 Aug 21;8(8):e70642. doi: 10.1371/journal.pone.0070642 (PMC3749231; doi:10.1371/journal.pone.0070642)
Supplement: Spectra S2 — The information of single-peptide-based protein identifications in the small-scale experiment. (PDF) [file pone.0070642.s019.pdf]

Spectra S2: The information of single-peptide-based protein identifications in the small-scale experiment.

Query 2912 Hit 1

MS/MS Fragmentation of **GLIDGVVEADLVEALQEFGPISYVVVMPK**
Found in **sp|P14866|HNRPL_HUMAN**, Heterogeneous nuclear ribonucleoprotein L OS=Homo sapiens GN=HNRNPL PE=1 SV=2
Match to Query 2912: 3374.849from(1125.957,3+)
Title: 1430: Sum of 2 scans in range 3580 (rt=89.1048, f=4, i=1046) to 3581 (rt=89.1302, f=4, i=1047) [D:\lab212\membrane\Grace\20120305-iTRAQ-2.raw]
Data File:
Monoisotopic mass of neutral peptide Mr(calc): 3374.849
Variable modifications:
K29 :iTRAQ4plex (K)
Ions Score: 138.16 **Expect:** 0.000

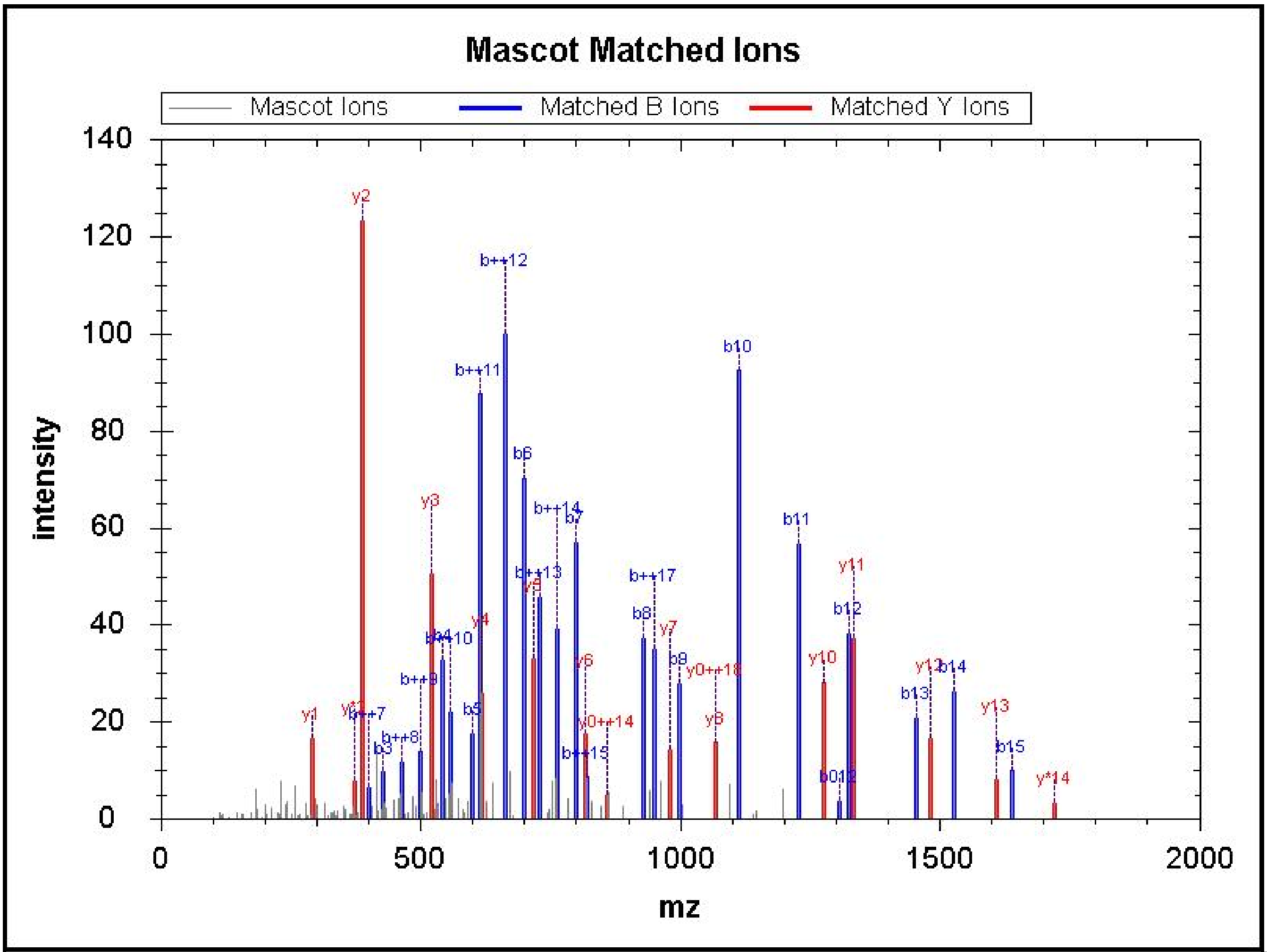

No	b	b++	b*	b*++	b0	b0++	Seq	y	y++	y*	y*++	y0	y0++	RevNo
1	202.13	101.57					G							29
2	315.21	158.11					L	3,174.71	1,587.86	3,157.69	1,579.35	3,156.70	1,578.85	28
3	428.30	214.65					I	3,061.63	1,531.32	3,044.60	1,522.80	3,043.62	1,522.31	27
4	543.33	272.17			525.32	263.16	D	2,948.54	1,474.78	2,931.52	1,466.26	2,930.53	1,465.77	26
5	600.35	300.68			582.34	291.67	G	2,833.52	1,417.26	2,816.49	1,408.75	2,815.51	1,408.26	25
6	699.42	350.21			681.41	341.21	V	2,776.50	1,388.75	2,759.47	1,380.24	2,758.49	1,379.75	24
7	798.48	399.75			780.47	390.74	V	2,677.43	1,339.22	2,660.40	1,330.70	2,659.42	1,330.21	23
8	927.53	464.27			909.52	455.26	E	2,578.36	1,289.68	2,561.33	1,281.17	2,560.35	1,280.68	22
9	998.56	499.79			980.55	490.78	A	2,449.32	1,225.16	2,432.29	1,216.65	2,431.31	1,216.16	21
10	1,113.59	557.30			1,095.58	548.29	D	2,378.28	1,189.64	2,361.25	1,181.13	2,360.27	1,180.64	20
11	1,226.67	613.84			1,208.66	604.84	L	2,263.25	1,132.13	2,246.23	1,123.62	2,245.24	1,123.12	19
12	1,325.74	663.38			1,307.73	654.37	V	2,150.17	1,075.59	2,133.14	1,067.07	2,132.16	1,066.58	18
13	1,454.79	727.90			1,436.78	718.89	E	2,051.10	1,026.05	2,034.07	1,017.54	2,033.09	1,017.05	17

14	**1,525.82**	**763.42**			1,507.81	754.41	A	1,922.06	961.53	1,905.03	953.02	1,904.05	952.53	16
15	**1,638.91**	**819.96**			1,620.90	810.95	L	1,851.02	926.01	1,833.99	917.50	1,833.01	917.01	15
16	1,766.97	883.99	1,749.94	875.47	1,748.96	874.98	Q	1,737.94	869.47	**1,720.91**	860.96	1,719.93	**860.47**	14
17	1,896.01	**948.51**	1,878.98	939.99	1,878.00	939.50	E	**1,609.88**	805.44	1,592.85	796.93	1,591.87	796.44	13
18	2,043.08	1,022.04	2,026.05	1,013.53	2,025.07	1,013.04	F	**1,480.84**	740.92	1,463.81	732.41	1,462.82	731.92	12
19	2,100.10	1,050.55	2,083.07	1,042.04	2,082.09	1,041.55	G	**1,333.77**	667.39	1,316.74	658.87	1,315.76	658.38	11
20	2,197.15	1,099.08	2,180.12	1,090.57	2,179.14	1,090.07	P	**1,276.75**	638.88	1,259.72	630.36	1,258.73	629.87	10
21	2,310.23	1,155.62	2,293.21	1,147.11	2,292.22	1,146.62	I	1,179.69	590.35	1,162.67	581.84	1,161.68	581.34	9
22	2,397.27	1,199.14	2,380.24	1,190.62	2,379.26	1,190.13	S	**1,066.61**	533.81	1,049.58	525.29	1,048.60	524.80	8
23	2,560.33	1,280.67	2,543.30	1,272.16	2,542.32	1,271.66	Y	**979.58**	490.29	962.55	481.78			7
24	2,659.40	1,330.20	2,642.37	1,321.69	2,641.39	1,321.20	V	**816.51**	408.76	799.49	400.25			6
25	2,758.47	1,379.74	2,741.44	1,371.22	2,740.46	1,370.73	V	**717.44**	359.23	700.42	350.71			5
26	2,857.54	1,429.27	2,840.51	1,420.76	2,839.52	1,420.27	V	**618.38**	309.69	601.35	301.18			4
27	2,988.58	1,494.79	2,971.55	1,486.28	2,970.57	1,485.79	M	**519.31**	260.16	502.28	251.64			3
28	3,085.63	1,543.32	3,068.60	1,534.80	3,067.62	1,534.31	P	**388.27**	194.64	**371.24**	186.12			2
29							K	**291.21**	146.11	274.19	137.60			1

Query 2629 Hit 1

MS/MS Fragmentation of **DLSAAGIGLLAAATQSLSMPASLGR**
Found in **sp|P43243|MATR3_HUMAN**, Matrin-3 OS=Homo sapiens GN=MATR3 PE=1 SV=2
Match to Query 2629: 2514.369from(839.1303,3+)
Title: 1158: Sum of 2 scans in range 2964 (rt=74.7768, f=4, i=814) to 2965 (rt=74.8022, f=4, i=815) [D:\lab212\membrane\Grace\20120305-iTRAQ-1.raw]
Data File:
Monoisotopic mass of neutral peptide Mr(calc): 2514.369
Variable modifications:
Ions Score: 125.9 **Expect:** 0.000

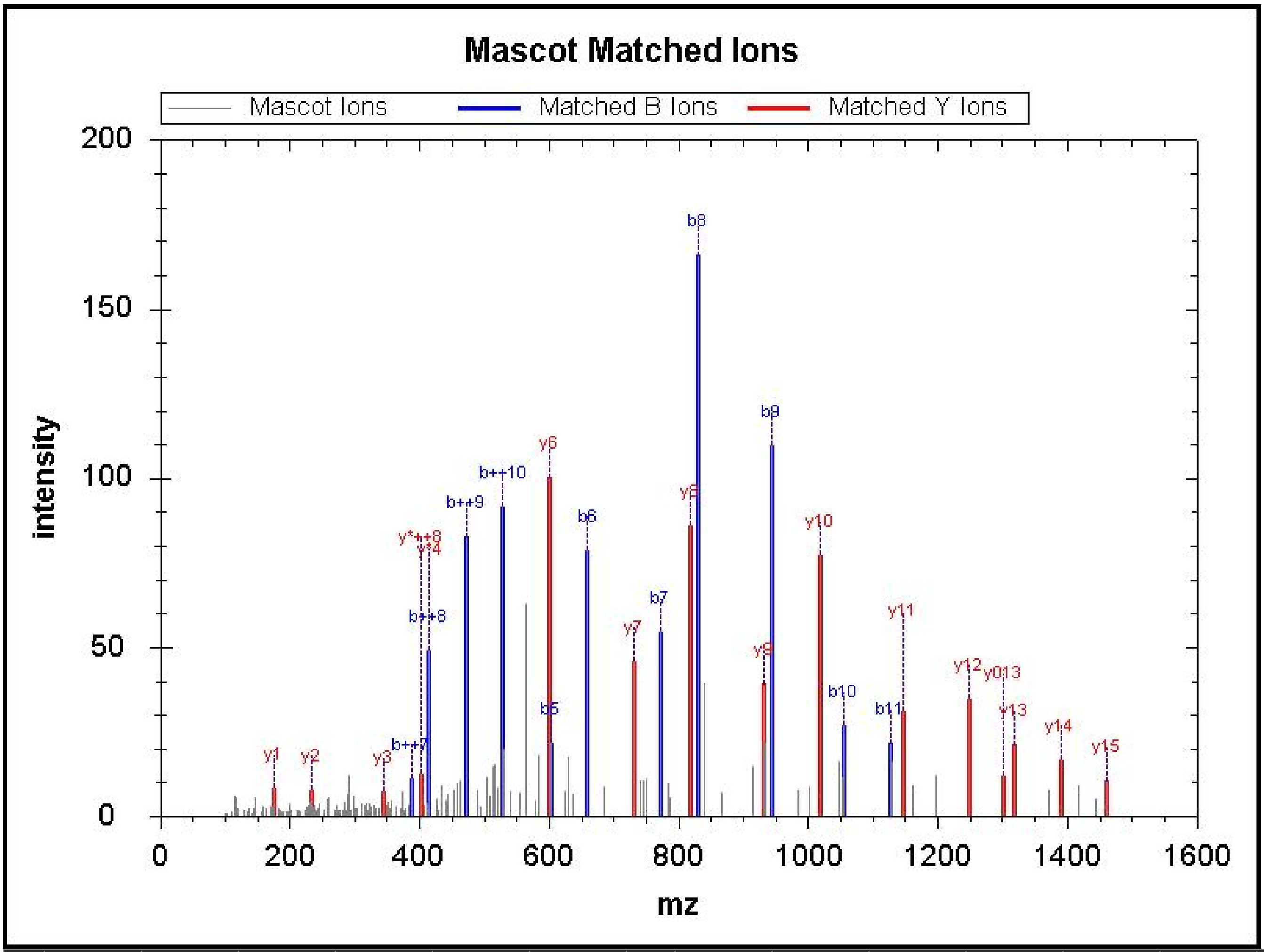

No	b	b++	b*	b*++	b0	b0++	Seq	y	y++	y*	y*++	y0	y0++	RevNo
1	260.14	130.57			242.13	121.57	D							25
2	373.22	187.11			355.21	178.11	L	2,256.24	1,128.62	2,239.21	1,120.11	2,238.23	1,119.62	24
3	460.25	230.63			442.24	221.62	S	2,143.15	1,072.08	2,126.13	1,063.57	2,125.14	1,063.08	23
4	531.29	266.15			513.28	257.14	A	2,056.12	1,028.56	2,039.10	1,020.05	2,038.11	1,019.56	22
5	602.33	301.67			584.32	292.66	A	1,985.08	993.05	1,968.06	984.53	1,967.07	984.04	21
6	659.35	330.18			641.34	321.17	G	1,914.05	957.53	1,897.02	949.01	1,896.04	948.52	20
7	772.43	386.72			754.42	377.71	I	1,857.03	929.02	1,840.00	920.50	1,839.02	920.01	19
8	829.45	415.23			811.44	406.23	G	1,743.94	872.47	1,726.92	863.96	1,725.93	863.47	18
9	942.54	471.77			924.53	462.77	L	1,686.92	843.96	1,669.89	835.45	1,668.91	834.96	17
10	1,055.62	528.31			1,037.61	519.31	L	1,573.84	787.42	1,556.81	778.91	1,555.83	778.42	16
11	1,126.66	563.83			1,108.65	554.83	A	1,460.75	730.88	1,443.73	722.37	1,442.74	721.87	15
12	1,197.70	599.35			1,179.69	590.35	A	1,389.72	695.36	1,372.69	686.85	1,371.70	686.36	14
13	1,268.73	634.87			1,250.72	625.86	A	1,318.68	659.84	1,301.65	651.33	1,300.67	650.84	13
14	1,369.78	685.39			1,351.77	676.39	T	1,247.64	624.32	1,230.61	615.81	1,229.63	615.32	12
15	1,497.84	749.42	1,480.81	740.91	1,479.83	740.42	Q	1,146.59	573.80	1,129.57	565.29	1,128.58	564.80	11
16	1,584.87	792.94	1,567.84	784.43	1,566.86	783.93	S	1,018.53	509.77	1,001.51	501.26	1,000.52	500.77	10
17	1,697.96	849.48	1,680.93	840.97	1,679.94	840.48	L	931.50	466.26	914.48	457.74	913.49	457.25	9
18	1,784.99	893.00	1,767.96	884.48	1,766.98	883.99	S	818.42	409.71	801.39	401.20	800.41	400.71	8
19	1,916.03	958.52	1,899.00	950.00	1,898.02	949.51	M	731.39	366.20	714.36	357.68	713.38	357.19	7
20	2,013.08	1,007.04	1,996.05	998.53	1,995.07	998.04	P	600.35	300.68	583.32	292.16	582.34	291.67	6
21	2,084.12	1,042.56	2,067.09	1,034.05	2,066.11	1,033.56	A	503.29	252.15	486.27	243.64	485.28	243.15	5
22	2,171.15	1,086.08	2,154.12	1,077.57	2,153.14	1,077.07	S	432.26	216.63	415.23	208.12	414.25	207.63	4
23	2,284.23	1,142.62	2,267.21	1,134.11	2,266.22	1,133.62	L	345.22	173.12	328.20	164.60			3

24	2,341.26	1,171.13	2,324.23	1,162.62	2,323.24	1,162.13	G	**232.14**	116.57	215.11	108.06			2
25							R	**175.12**	88.06	158.09	79.55			1

Query 2964 Hit 1

MS/MS Fragmentation of **GVDNTFADELVELSTALEHQEYITFLEDLK**
Found in **sp|Q07021|C1QBP_HUMAN**, Complement component 1 Q subcomponent-binding protein
Match to Query 2964: 3726.892from(932.7303,4+)
Title: 1331: Scan 3428 (rt=85.2295, f=3, i=491) [D:\lab212\membrane\Grace\20120305-iTRAQ-1.raw]
Data File:
Monoisotopic mass of neutral peptide Mr(calc): 3726.892
Variable modifications:
K30 :iTRAQ4plex (K)
Ions Score: 118.68 **Expect:** 0.000

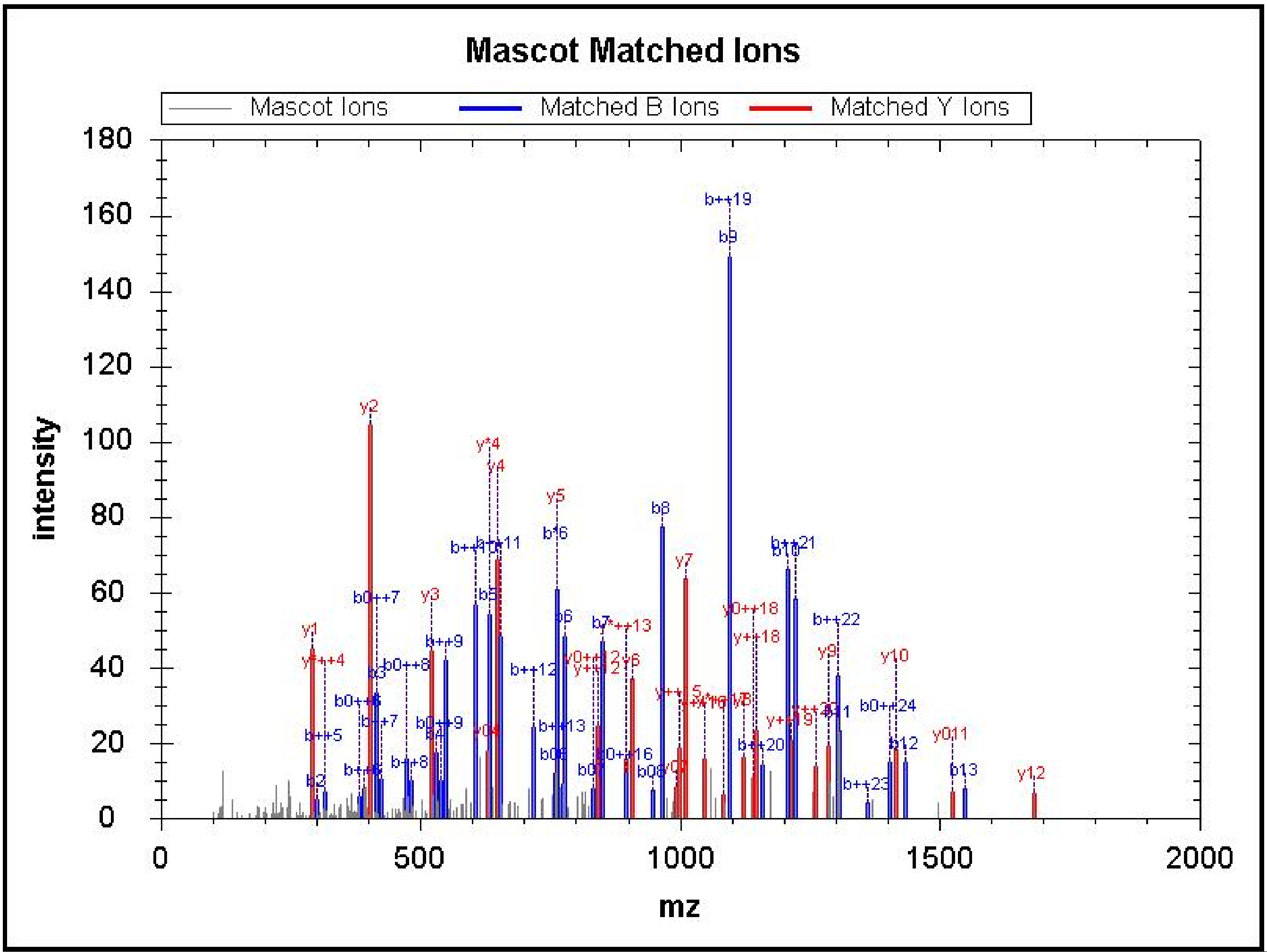

No	b	b++	b*	b*++	b0	b0++	Seq	y	y++	y*	y*++	y0	y0++	RevNo
1	202.13	101.57					G							30
2	**301.20**	151.10					V	3,526.76	1,763.88	3,509.73	1,755.37	3,508.75	1,754.88	29
3	**416.23**	208.62			398.22	199.61	D	3,427.69	1,714.35	3,410.66	1,705.84	3,409.68	1,705.34	28
4	**530.27**	265.64	513.24	257.12	512.26	256.63	N	3,312.66	1,656.84	3,295.64	1,648.32	3,294.65	1,647.83	27
5	**631.32**	**316.16**	614.29	307.65	613.31	307.16	T	3,198.62	1,599.81	3,181.59	1,591.30	3,180.61	1,590.81	26
6	**778.39**	**389.70**	**761.36**	381.18	**760.37**	**380.69**	F	3,097.57	1,549.29	3,080.55	1,540.78	3,079.56	1,540.29	25
7	**849.42**	**425.21**	832.40	416.70	**831.41**	**416.21**	A	2,950.50	1,475.76	2,933.48	1,467.24	2,932.49	1,466.75	24
8	**964.45**	**482.73**	947.42	474.21	**946.44**	**473.72**	D	2,879.47	1,440.24	2,862.44	1,431.72	2,861.46	1,431.23	23
9	**1,093.49**	**547.25**	1,076.47	538.74	1,075.48	**538.24**	E	2,764.44	1,382.72	2,747.41	1,374.21	2,746.43	1,373.72	22
10	**1,206.58**	**603.79**	1,189.55	595.28	1,188.57	594.79	L	2,635.40	1,318.20	2,618.37	1,309.69	2,617.39	1,309.20	21
11	**1,305.64**	**653.33**	1,288.62	644.81	1,287.63	644.32	V	2,522.31	**1,261.66**	2,505.29	1,253.15	2,504.30	1,252.66	20

**12**	**1,434.69**	**717.85**	1,417.66	709.33	1,416.68	708.84	**E**	2,423.25	**1,212.13**	2,406.22	1,203.61	2,405.24	1,203.12	**19**
**13**	**1,547.77**	**774.39**	1,530.74	765.88	1,529.76	765.38	**L**	2,294.20	**1,147.61**	2,277.18	1,139.09	2,276.19	**1,138.60**	**18**
**14**	1,634.80	817.91	1,617.78	809.39	1,616.79	808.90	**S**	2,181.12	1,091.06	2,164.09	**1,082.55**	2,163.11	1,082.06	**17**
**15**	1,735.85	868.43	1,718.82	859.92	1,717.84	859.42	**T**	2,094.09	**1,047.55**	2,077.06	1,039.03	2,076.08	1,038.54	**16**
**16**	1,806.89	903.95	1,789.86	895.43	1,788.88	**894.94**	**A**	1,993.04	**997.02**	1,976.01	988.51	1,975.03	988.02	**15**
**17**	1,919.97	960.49	1,902.95	951.98	1,901.96	951.48	**L**	1,922.00	961.51	1,904.98	952.99	1,903.99	952.50	**14**
**18**	2,049.01	1,025.01	2,031.99	1,016.50	2,031.00	1,016.01	**E**	1,808.92	904.96	1,791.89	**896.45**	1,790.91	895.96	**13**
**19**	2,186.07	**1,093.54**	2,169.05	1,085.03	2,168.06	1,084.54	**H**	**1,679.88**	**840.44**	1,662.85	831.93	1,661.87	**831.44**	**12**
**20**	2,314.13	**1,157.57**	2,297.11	1,149.06	2,296.12	1,148.56	**Q**	1,542.82	771.91	1,525.79	763.40	**1,524.81**	762.91	**11**
**21**	2,443.17	**1,222.09**	2,426.15	1,213.58	2,425.16	1,213.09	**E**	**1,414.76**	707.88	1,397.73	699.37	1,396.75	698.88	**10**
**22**	2,606.24	**1,303.62**	2,589.21	1,295.11	2,588.23	1,294.62	**Y**	**1,285.72**	643.36	1,268.69	634.85	1,267.71	634.36	**9**
**23**	2,719.32	**1,360.16**	2,702.29	1,351.65	2,701.31	1,351.16	**I**	**1,122.65**	561.83	1,105.63	553.32	1,104.64	552.82	**8**
**24**	2,820.37	1,410.69	2,803.34	1,402.17	2,802.36	**1,401.68**	**T**	**1,009.57**	505.29	992.54	496.77	**991.56**	496.28	**7**
**25**	2,967.44	1,484.22	2,950.41	1,475.71	2,949.43	1,475.22	**F**	**908.52**	454.76	891.49	446.25	890.51	445.76	**6**
**26**	3,080.52	1,540.76	3,063.49	1,532.25	3,062.51	1,531.76	**L**	**761.45**	381.23	744.43	372.72	743.44	372.22	**5**
**27**	3,209.56	1,605.29	3,192.54	1,596.77	3,191.55	1,596.28	**E**	**648.37**	324.69	**631.34**	**316.17**	**630.36**	315.68	**4**
**28**	3,324.59	1,662.80	3,307.56	1,654.29	3,306.58	1,653.79	**D**	**519.33**	260.17	502.30	251.65	501.32	251.16	**3**
**29**	3,437.67	1,719.34	3,420.65	1,710.83	3,419.66	1,710.34	**L**	**404.30**	202.65	387.27	194.14			**2**
**30**							**K**	**291.21**	146.11	274.19	137.60			**1**

Query 2977 Hit 1

MS/MS Fragmentation of **IPNFWVTTFVNHPQVSALLGEEDEEALHYLTR**
Found in **sp|Q01105|SET_HUMAN**, Protein SET OS=Homo sapiens GN=SET PE=1 SV=3
Match to Query 2977: 3868.96from(968.2472,4+)
Title: 1098: Scan 2819 (rt=71.505, f=2, i=420) [D:\lab212\membrane\Grace\20120305-iTRAQ-1.raw]
Data File:
Monoisotopic mass of neutral peptide Mr(calc): 3868.96
Variable modifications:
Ions Score: 107.9 **Expect:** 0.000

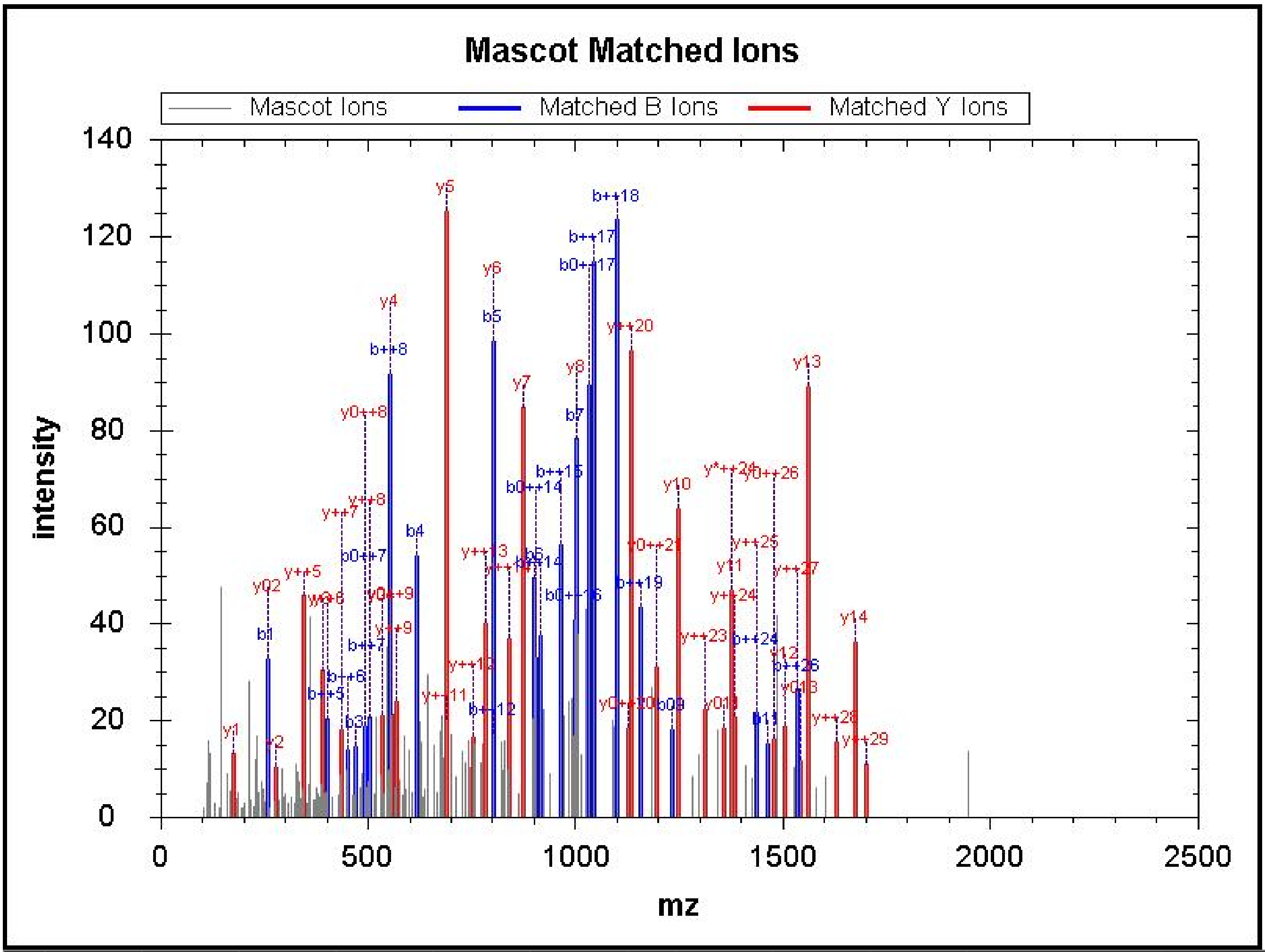

No	b	b++	b*	b*++	b0	b0++	Seq	y	y++	y*	y*++	y0	y0++	RevNo
1	258.19	129.60					I							32
2	355.25	178.13					P	3,612.78	1,806.89	3,595.75	1,798.38	3,594.76	1,797.89	31
3	469.29	235.15	452.26	226.63			N	3,515.72	1,758.36	3,498.70	1,749.85	3,497.71	1,749.36	30
4	616.36	308.68	599.33	300.17			F	3,401.68	1,701.34	3,384.65	1,692.83	3,383.67	1,692.34	29
5	802.44	401.72	785.41	393.21			W	3,254.61	1,627.81	3,237.58	1,619.30	3,236.60	1,618.80	28
6	901.51	451.26	884.48	442.74			V	3,068.53	1,534.77	3,051.51	1,526.26	3,050.52	1,525.76	27
7	1,002.55	501.78	985.53	493.27	984.54	492.77	T	2,969.46	1,485.24	2,952.44	1,476.72	2,951.45	1,476.23	26
8	1,103.60	552.30	1,086.57	543.79	1,085.59	543.30	T	2,868.42	1,434.71	2,851.39	1,426.20	2,850.41	1,425.71	25
9	1,250.67	625.84	1,233.64	617.33	1,232.66	616.83	F	2,767.37	1,384.19	2,750.34	1,375.67	2,749.36	1,375.18	24
10	1,349.74	675.37	1,332.71	666.86	1,331.73	666.37	V	2,620.30	1,310.65	2,603.27	1,302.14	2,602.29	1,301.65	23
11	1,463.78	732.39	1,446.75	723.88	1,445.77	723.39	N	2,521.23	1,261.12	2,504.21	1,252.61	2,503.22	1,252.11	22
12	1,600.84	800.92	1,583.81	792.41	1,582.83	791.92	H	2,407.19	1,204.10	2,390.16	1,195.58	2,389.18	1,195.09	21
13	1,697.89	849.45	1,680.87	840.94	1,679.88	840.44	P	2,270.13	1,135.57	2,253.10	1,127.06	2,252.12	1,126.56	20
14	1,825.95	913.48	1,808.92	904.97	1,807.94	904.47	Q	2,173.08	1,087.04	2,156.05	1,078.53	2,155.07	1,078.04	19
15	1,925.02	963.01	1,907.99	954.50	1,907.01	954.01	V	2,045.02	1,023.01	2,027.99	1,014.50	2,027.01	1,014.01	18
16	2,012.05	1,006.53	1,995.02	998.02	1,994.04	997.52	S	1,945.95	973.48	1,928.92	964.97	1,927.94	964.47	17
17	2,083.09	1,042.05	2,066.06	1,033.53	2,065.08	1,033.04	A	1,858.92	929.96	1,841.89	921.45	1,840.91	920.96	16
18	2,196.17	1,098.59	2,179.15	1,090.08	2,178.16	1,089.58	L	1,787.88	894.44	1,770.85	885.93	1,769.87	885.44	15
19	2,309.26	1,155.13	2,292.23	1,146.62	2,291.25	1,146.13	L	1,674.80	837.90	1,657.77	829.39	1,656.79	828.90	14
20	2,366.28	1,183.64	2,349.25	1,175.13	2,348.27	1,174.64	G	1,561.71	781.36	1,544.69	772.85	1,543.70	772.35	13
21	2,495.32	1,248.16	2,478.29	1,239.65	2,477.31	1,239.16	E	1,504.69	752.85	1,487.66	744.34	1,486.68	743.84	12
22	2,624.36	1,312.69	2,607.34	1,304.17	2,606.35	1,303.68	E	1,375.65	688.33	1,358.62	679.81	1,357.64	679.32	11
23	2,739.39	1,370.20	2,722.36	1,361.69	2,721.38	1,361.19	D	1,246.61	623.81	1,229.58	615.29	1,228.60	614.80	10

24	2,868.43	1,434.72	2,851.41	1,426.21	2,850.42	1,425.71	E	1,131.58	566.29	1,114.55	557.78	1,113.57	557.29	9
25	2,997.47	1,499.24	2,980.45	1,490.73	2,979.46	1,490.24	E	1,002.54	501.77	985.51	493.26	984.53	492.77	8
26	3,068.51	1,534.76	3,051.49	1,526.25	3,050.50	1,525.75	A	873.49	437.25	856.47	428.74	855.48	428.25	7
27	3,181.60	1,591.30	3,164.57	1,582.79	3,163.59	1,582.30	L	802.46	401.73	785.43	393.22	784.45	392.73	6
28	3,318.65	1,659.83	3,301.63	1,651.32	3,300.64	1,650.83	H	689.37	345.19	672.35	336.68	671.36	336.18	5
29	3,481.72	1,741.36	3,464.69	1,732.85	3,463.71	1,732.36	Y	552.31	276.66	535.29	268.15	534.30	267.66	4
30	3,594.80	1,797.90	3,577.78	1,789.39	3,576.79	1,788.90	L	389.25	195.13	372.22	186.62	371.24	186.12	3
31	3,695.85	1,848.43	3,678.82	1,839.92	3,677.84	1,839.42	T	276.17	138.59	259.14	130.07	258.16	129.58	2
32							R	175.12	88.06	158.09	79.55			1

Query 2511 Hit 1

MS/MS Fragmentation of **MLIAMVDVIFADVAQPDQTR**
Found in **sp|P22087|FBRL_HUMAN**, rRNA 2'-O-methyltransferase fibrillarin OS=Homo sapiens GN=FBL PE=1 SV=2
Match to Query 2511: 2376.245from(793.0888,3+)
Title: 1399: Scan 3478 (rt=86.8031, f=2, i=536) [D:\lab212\membrane\Grace\20120305-iTRAQ-2.raw]
Data File:
Monoisotopic mass of neutral peptide Mr(calc): 2376.245
Variable modifications:
Ions Score: 103.99 **Expect:** 0.000

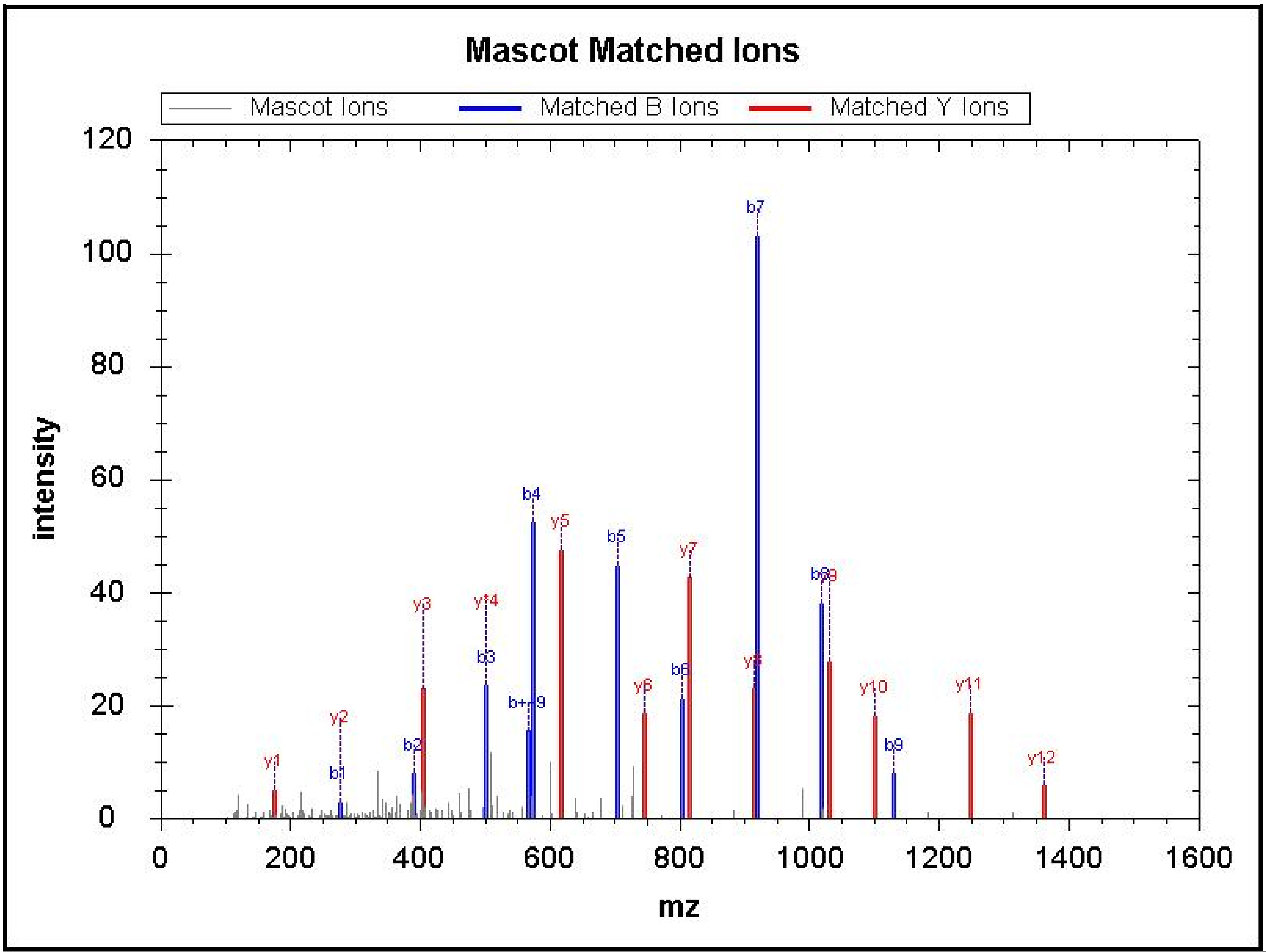

No	b	b++	b*	b*++	b0	b0++	Seq	y	y++	y*	y*++	y0	y0++	RevNo
1	276.15	138.58					M							20
2	389.23	195.12					L	2,102.10	1,051.55	2,085.07	1,043.04	2,084.08	1,042.55	19
3	502.32	251.66					I	1,989.01	995.01	1,971.98	986.50	1,971.00	986.00	18
4	573.36	287.18					A	1,875.93	938.47	1,858.90	929.95	1,857.92	929.46	17
5	704.40	352.70					M	1,804.89	902.95	1,787.86	894.44	1,786.88	893.94	16

**6**	**803.46**	402.24					**V**	1,673.85	837.43	1,656.82	828.92	1,655.84	828.42	**15**
**7**	**918.49**	459.75			900.48	450.74	**D**	1,574.78	787.89	1,557.75	779.38	1,556.77	778.89	**14**
**8**	**1,017.56**	509.28			999.55	500.28	**V**	1,459.75	730.38	1,442.73	721.87	1,441.74	721.38	**13**
**9**	**1,130.64**	**565.83**			1,112.63	556.82	**I**	**1,360.69**	680.85	1,343.66	672.33	1,342.68	671.84	**12**
**10**	1,277.71	639.36			1,259.70	630.35	**F**	**1,247.60**	624.30	1,230.58	615.79	1,229.59	615.30	**11**
**11**	1,348.75	674.88			1,330.74	665.87	**A**	**1,100.53**	550.77	1,083.51	542.26	1,082.52	541.76	**10**
**12**	1,463.78	732.39			1,445.77	723.39	**D**	**1,029.50**	515.25	1,012.47	506.74	1,011.49	506.25	**9**
**13**	1,562.84	781.93			1,544.83	772.92	**V**	**914.47**	457.74	897.44	449.22	896.46	448.73	**8**
**14**	1,633.88	817.44			1,615.87	808.44	**A**	**815.40**	408.20	798.37	399.69	797.39	399.20	**7**
**15**	1,761.94	881.47	1,744.91	872.96	1,743.93	872.47	**Q**	**744.36**	372.69	727.34	364.17	726.35	363.68	**6**
**16**	1,858.99	930.00	1,841.97	921.49	1,840.98	920.99	**P**	**616.30**	308.66	599.28	300.14	598.29	299.65	**5**
**17**	1,974.02	987.51	1,956.99	979.00	1,956.01	978.51	**D**	519.25	260.13	**502.23**	251.62	501.24	251.12	**4**
**18**	2,102.08	1,051.54	2,085.05	1,043.03	2,084.07	1,042.54	**Q**	**404.23**	202.62	387.20	194.10	386.21	193.61	**3**
**19**	2,203.13	1,102.07	2,186.10	1,093.55	2,185.12	1,093.06	**T**	**276.17**	138.59	259.14	130.07	258.16	129.58	**2**
**20**							**R**	**175.12**	88.06	158.09	79.55			**1**

Query 2602 Hit 1

MS/MS Fragmentation of **YNEDLELEDAIHTAILTLK**
Found in **sp|P25787|PSA2_HUMAN**, Proteasome subunit alpha type-2 OS=Homo sapiens GN=PSMA2 PE=1 SV=2
Match to Query 2602: 2488.347from(830.4564,3+)
Title: 1116: Scan 2711 (rt=69.5208, f=3, i=386) [D:\lab212\membrane\Grace\20120305-iTRAQ-2.raw]
Data File:
Monoisotopic mass of neutral peptide Mr(calc): 2488.347
Variable modifications:
K19 :iTRAQ4plex (K)
Ions Score: 97.39 **Expect:** 0.000

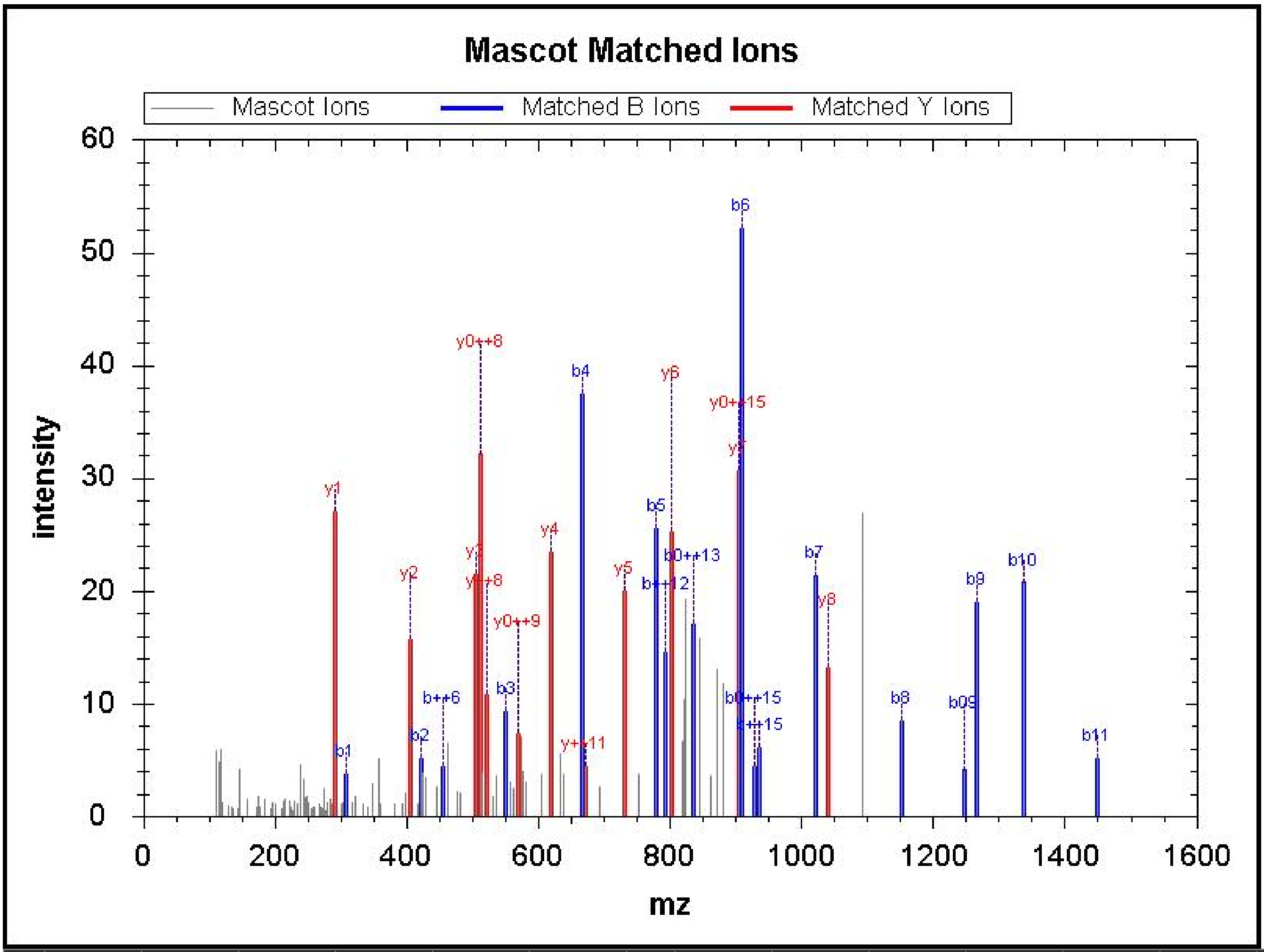

No	b	b++	b*	b*++	b0	b0++	Seq	y	y++	y*	y*++	y0	y0++	RevNo
1	308.17	154.59					Y							19
2	422.22	211.61	405.19	203.10			N	2,182.17	1,091.59	2,165.15	1,083.08	2,164.16	1,082.58	18
3	551.26	276.13	534.23	267.62	533.25	267.13	E	2,068.13	1,034.57	2,051.10	1,026.06	2,050.12	1,025.56	17
4	666.29	333.65	649.26	325.13	648.27	324.64	D	1,939.09	970.05	1,922.06	961.53	1,921.08	961.04	16
5	779.37	390.19	762.34	381.67	761.36	381.18	L	1,824.06	912.53	1,807.03	904.02	1,806.05	903.53	15
6	908.41	454.71	891.39	446.20	890.40	445.70	E	1,710.98	855.99	1,693.95	847.48	1,692.97	846.99	14
7	1,021.50	511.25	1,004.47	502.74	1,003.49	502.25	L	1,581.93	791.47	1,564.91	782.96	1,563.92	782.46	13
8	1,150.54	575.77	1,133.51	567.26	1,132.53	566.77	E	1,468.85	734.93	1,451.82	726.41	1,450.84	725.92	12
9	1,265.57	633.29	1,248.54	624.77	1,247.55	624.28	D	1,339.81	670.41	1,322.78	661.89	1,321.80	661.40	11
10	1,336.60	668.80	1,319.58	660.29	1,318.59	659.80	A	1,224.78	612.89	1,207.75	604.38	1,206.77	603.89	10
11	1,449.69	725.35	1,432.66	716.83	1,431.68	716.34	I	1,153.74	577.37	1,136.72	568.86	1,135.73	568.37	9
12	1,586.75	793.88	1,569.72	785.36	1,568.73	784.87	H	1,040.66	520.83	1,023.63	512.32	1,022.65	511.83	8
13	1,687.79	844.40	1,670.77	835.89	1,669.78	835.40	T	903.60	452.30	886.57	443.79	885.59	443.30	7
14	1,758.83	879.92	1,741.80	871.41	1,740.82	870.91	A	802.55	401.78	785.53	393.27	784.54	392.77	6
15	1,871.91	936.46	1,854.89	927.95	1,853.90	927.46	I	731.51	366.26	714.49	357.75	713.50	357.26	5
16	1,985.00	993.00	1,967.97	984.49	1,966.99	984.00	L	618.43	309.72	601.40	301.21	600.42	300.71	4
17	2,086.05	1,043.53	2,069.02	1,035.01	2,068.04	1,034.52	T	505.35	253.18	488.32	244.66	487.34	244.17	3
18	2,199.13	1,100.07	2,182.10	1,091.56	2,181.12	1,091.06	L	404.30	202.65	387.27	194.14			2
19							K	291.21	146.11	274.19	137.60			1

Query 1896 Hit 1

MS/MS Fragmentation of **GFLEFVEDFIQVPR**
Found in **sp|P51114|FXR1_HUMAN**, Fragile X mental retardation syndrome-related protein 1 OS=Homo sapiens GN=FXR1 PE=1 SV=3

Match to Query 1896: 1838.974from(920.4941,2+)
Title: 1297: Sum of 2 scans in range 3293 (rt=82.1891, f=4, i=920) to 3294 (rt=82.2145, f=4, i=921) [D:\lab212\membrane\Grace\20120305-iTRAQ-1.raw]
Data File:
Monoisotopic mass of neutral peptide Mr(calc): 1838.974
Variable modifications:
Ions Score: 97.35 **Expect:** 0.000

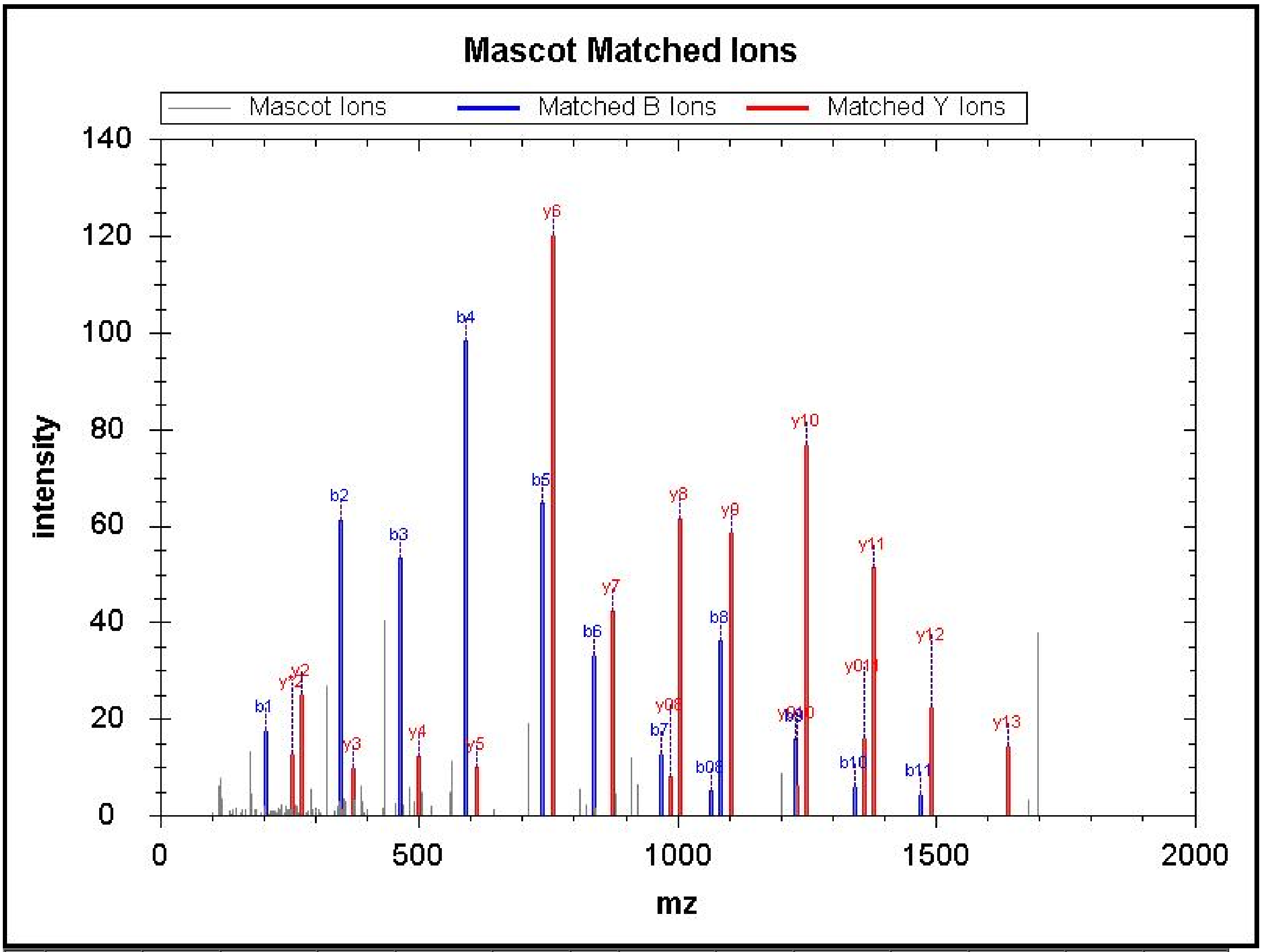

No	b	b++	b*	b*++	b0	b0++	Seq	y	y++	y*	y*++	y0	y0++	RevNo
1	**202.13**	101.57					G							14
2	**349.20**	175.10					F	**1,638.85**	819.93	1,621.83	811.42	1,620.84	810.92	13
3	**462.28**	231.65					L	**1,491.78**	746.40	1,474.76	737.88	1,473.77	737.39	12
4	**591.33**	296.17			573.32	287.16	E	**1,378.70**	689.85	1,361.67	681.34	**1,360.69**	680.85	11
5	**738.39**	369.70			720.38	360.70	F	**1,249.66**	625.33	1,232.63	616.82	**1,231.65**	616.33	10
6	**837.46**	419.23			819.45	410.23	V	**1,102.59**	551.80	1,085.56	543.28	1,084.58	542.79	9
7	**966.51**	483.76			948.49	474.75	E	**1,003.52**	502.26	986.49	493.75	**985.51**	493.26	8
8	**1,081.53**	541.27			**1,063.52**	532.26	D	**874.48**	437.74	857.45	429.23	856.47	428.74	7
9	**1,228.60**	614.80			1,210.59	605.80	F	**759.45**	380.23	742.42	371.72			6
10	**1,341.68**	671.35			1,323.67	662.34	I	**612.38**	306.70	595.36	298.18			5
11	**1,469.74**	735.38	1,452.72	726.86	1,451.73	726.37	Q	**499.30**	250.15	482.27	241.64			4
12	1,568.81	784.91	1,551.79	776.40	1,550.80	775.90	V	**371.24**	186.12	354.21	177.61			3
13	1,665.86	833.44	1,648.84	824.92	1,647.85	824.43	P	**272.17**	136.59	**255.15**	128.08			2
14							R	175.12	88.06	158.09	79.55			1

Query 1984 Hit 1

MS/MS Fragmentation of **LQAILEDIQVTLFTR**
Found in **sp|P07814|SYEP_HUMAN**, Bifunctional glutamate/proline--tRNA ligase OS=Homo sapiens GN=EPRS PE=1 SV=5

Match to Query 1984: 1903.095from(952.5549,2+)
Title: 1312: Scan 3335 (rt=83.1339, f=3, i=476) [D:\lab212\membrane\Grace\20120305-iTRAQ-1.raw]
Data File:
Monoisotopic mass of neutral peptide Mr(calc): 1903.095
Variable modifications:
Ions Score: 92.2 **Expect:** 0.000

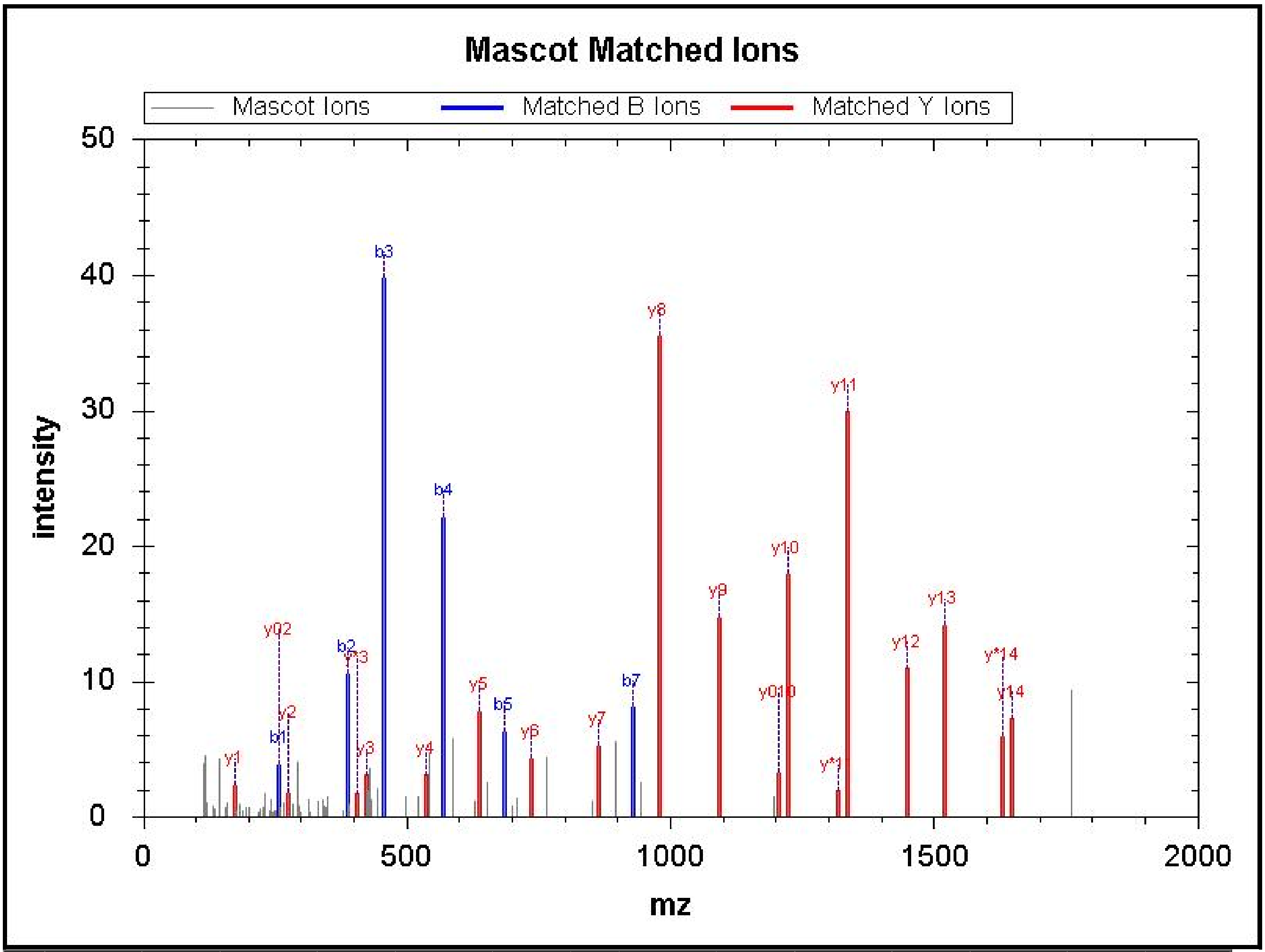

No	b	b++	b*	b*++	b0	b0++	Seq	y	y++	y*	y*++	y0	y0++	RevNo
1	**258.19**	129.60					L							15
2	**386.25**	193.63	369.23	185.12			Q	**1,646.91**	823.96	**1,629.88**	815.45	1,628.90	814.95	14
3	**457.29**	229.15	440.26	220.63			A	**1,518.85**	759.93	1,501.83	751.42	1,500.84	750.92	13
4	**570.37**	285.69	553.35	277.18			I	**1,447.82**	724.41	1,430.79	715.90	1,429.80	715.41	12
5	**683.46**	342.23	666.43	333.72			L	**1,334.73**	667.87	**1,317.70**	659.36	1,316.72	658.86	11
6	812.50	406.75	795.47	398.24	794.49	397.75	E	**1,221.65**	611.33	1,204.62	602.81	**1,203.64**	602.32	10
7	**927.53**	464.27	910.50	455.75	909.52	455.26	D	**1,092.60**	546.81	1,075.58	538.29	1,074.59	537.80	9
8	1,040.61	520.81	1,023.58	512.30	1,022.60	511.80	I	**977.58**	489.29	960.55	480.78	959.57	480.29	8
9	1,168.67	584.84	1,151.64	576.33	1,150.66	575.83	Q	**864.49**	432.75	847.47	424.24	846.48	423.75	7
10	1,267.74	634.37	1,250.71	625.86	1,249.73	625.37	V	**736.44**	368.72	719.41	360.21	718.42	359.72	6
11	1,368.79	684.90	1,351.76	676.38	1,350.77	675.89	T	**637.37**	319.19	620.34	310.67	619.36	310.18	5
12	1,481.87	741.44	1,464.84	732.93	1,463.86	732.43	L	**536.32**	268.66	519.29	260.15	518.31	259.66	4
13	1,628.94	814.97	1,611.91	806.46	1,610.93	805.97	F	**423.24**	212.12	**406.21**	203.61	405.22	203.12	3
14	1,729.99	865.50	1,712.96	856.98	1,711.98	856.49	T	**276.17**	138.59	259.14	130.07	**258.16**	129.58	2
15							R	**175.12**	88.06	158.09	79.55			1

Query 1877 Hit 1

MS/MS Fragmentation of **ILEPGLNILIPVLDR**

Found in **sp|Q9UJZ1|STML2_HUMAN**, Stomatin-like protein 2 OS=Homo sapiens GN=STOML2 PE=1 SV=1
Match to Query 1877: 1818.116from(910.0654,2+)
Title: 1149: Sum of 2 scans in range 2792 (rt=71.3506, f=4, i=792) to 2793 (rt=71.376, f=4, i=793) [D:\lab212\membrane\Grace\20120305-iTRAQ-2.raw]
Data File:
Monoisotopic mass of neutral peptide Mr(calc): 1818.116
Variable modifications:
Ions Score: 88.19 **Expect:** 0.000

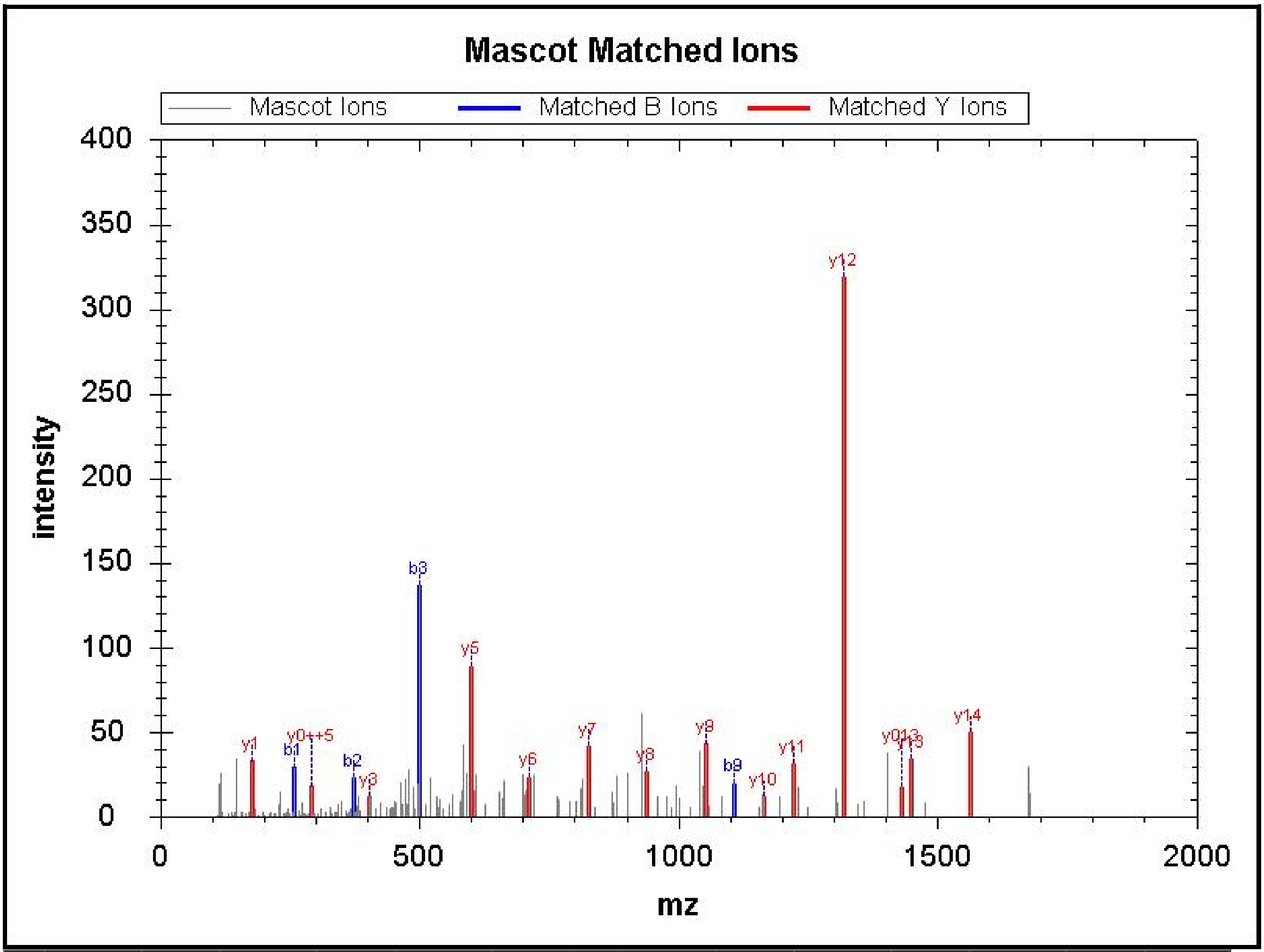

No	b	b++	b*	b*++	b0	b0++	Seq	y	y++	y*	y*++	y0	y0++	RevNo
1	258.19	129.60					I							15
2	371.28	186.14					L	1,561.93	781.47	1,544.90	772.96	1,543.92	772.46	14
3	500.32	250.66			482.31	241.66	E	1,448.85	724.93	1,431.82	716.41	1,430.84	715.92	13
4	597.37	299.19			579.36	290.18	P	1,319.80	660.41	1,302.78	651.89	1,301.79	651.40	12
5	654.39	327.70			636.38	318.70	G	1,222.75	611.88	1,205.73	603.37	1,204.74	602.87	11
6	767.48	384.24			749.47	375.24	L	1,165.73	583.37	1,148.70	574.86	1,147.72	574.36	10
7	881.52	441.26	864.49	432.75	863.51	432.26	N	1,052.65	526.83	1,035.62	518.31	1,034.64	517.82	9
8	994.61	497.81	977.58	489.29	976.59	488.80	I	938.60	469.81	921.58	461.29	920.59	460.80	8
9	1,107.69	554.35	1,090.66	545.84	1,089.68	545.34	L	825.52	413.26	808.49	404.75	807.51	404.26	7
10	1,220.77	610.89	1,203.75	602.38	1,202.76	601.89	I	712.44	356.72	695.41	348.21	694.42	347.72	6
11	1,317.83	659.42	1,300.80	650.90	1,299.82	650.41	P	599.35	300.18	582.32	291.67	581.34	291.17	5
12	1,416.89	708.95	1,399.87	700.44	1,398.88	699.95	V	502.30	251.65	485.27	243.14	484.29	242.65	4
13	1,529.98	765.49	1,512.95	756.98	1,511.97	756.49	L	403.23	202.12	386.20	193.61	385.22	193.11	3
14	1,645.01	823.01	1,627.98	814.49	1,627.00	814.00	D	290.15	145.58	273.12	137.06	272.14	136.57	2
15							R	175.12	88.06	158.09	79.55			1

Query 2488 Hit 1

MS/MS Fragmentation of **EIADLGEALATAVIPQWQK**
Found in **sp|P49588|SYAC_HUMAN**, Alanine--tRNA ligase
Match to Query 2488: 2340.3from(781.1071,3+)
Title: 1071: Sum of 2 scans in range 2753 (rt=70.023, f=4, i=746) to 2754 (rt=70.0484, f=4, i=747) [D:\lab212\membrane\Grace\20120305-iTRAQ-1.raw]
Data File:
Monoisotopic mass of neutral peptide Mr(calc): 2340.3
Variable modifications:
K19 :iTRAQ4plex (K)
Ions Score: 87.59 **Expect:** 0.000

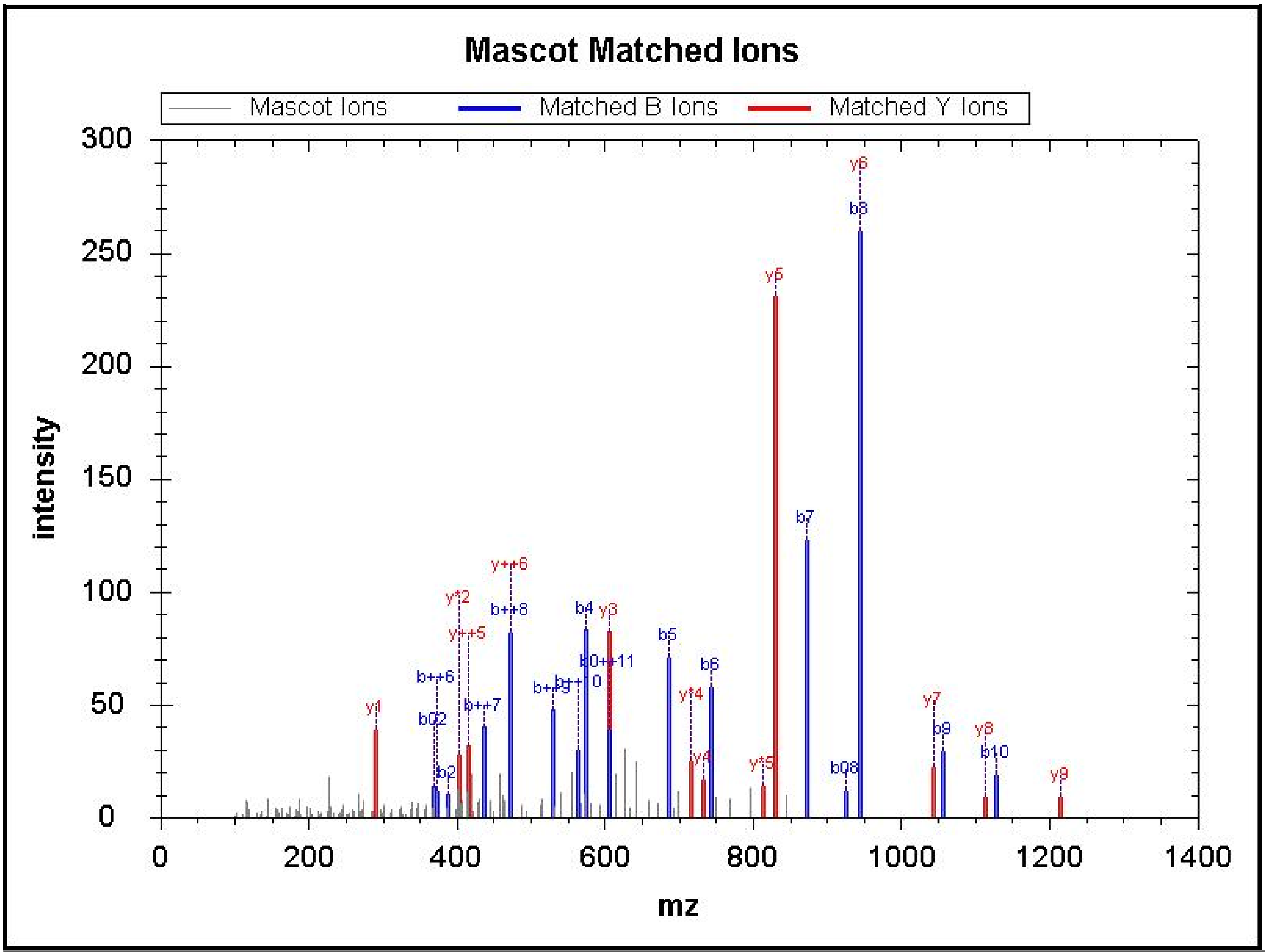

No	b	b++	b*	b*++	b0	b0++	Seq	y	y++	y*	y*++	y0	y0++	RevNo
1	274.15	137.58			256.14	128.57	E							19
2	387.24	194.12			369.23	185.12	I	2,068.16	1,034.58	2,051.13	1,026.07	2,050.15	1,025.58	18
3	458.27	229.64			440.26	220.63	A	1,955.07	978.04	1,938.05	969.53	1,937.06	969.03	17
4	573.30	287.15			555.29	278.15	D	1,884.03	942.52	1,867.01	934.01	1,866.02	933.52	16
5	686.38	343.70			668.37	334.69	L	1,769.01	885.01	1,751.98	876.49	1,751.00	876.00	15
6	743.41	372.21			725.39	363.20	G	1,655.92	828.47	1,638.90	819.95	1,637.91	819.46	14
7	872.45	436.73			854.44	427.72	E	1,598.90	799.95	1,581.88	791.44	1,580.89	790.95	13
8	943.49	472.25			925.47	463.24	A	1,469.86	735.43	1,452.83	726.92	1,451.85	726.43	12
9	1,056.57	528.79			1,038.56	519.78	L	1,398.82	699.91	1,381.80	691.40	1,380.81	690.91	11
10	1,127.61	564.31			1,109.60	555.30	A	1,285.74	643.37	1,268.71	634.86	1,267.73	634.37	10
11	1,228.65	614.83			1,210.64	605.83	T	1,214.70	607.85	1,197.67	599.34	1,196.69	598.85	9
12	1,299.69	650.35			1,281.68	641.34	A	1,113.65	557.33	1,096.63	548.82			8
13	1,398.76	699.88			1,380.75	690.88	V	1,042.62	521.81	1,025.59	513.30			7
14	1,511.84	756.43			1,493.83	747.42	I	943.55	472.28	926.52	463.76			6
15	1,608.90	804.95			1,590.89	795.95	P	830.46	415.74	813.44	407.22			5

16	1,736.96	868.98	1,719.93	860.47	1,718.94	859.98	Q	**733.41**	367.21	**716.38**	358.70			4
17	1,923.03	962.02	1,906.01	953.51	1,905.02	953.02	W	**605.35**	303.18	588.33	294.67			3
18	2,051.09	1,026.05	2,034.07	1,017.54	2,033.08	1,017.04	Q	419.27	210.14	**402.25**	201.63			2
19							K	**291.21**	146.11	274.19	137.60			1

Query 2722 Hit 1

MS/MS Fragmentation of **EQHDALEFFNSLVDSLDEALK**
Found in **sp|Q93008|USP9X_HUMAN**, Probable ubiquitin carboxyl-terminal hydrolase FAF-X OS=Homo sapiens GN=USP9X PE=1 SV=3
Match to Query 2722: 2707.373from(903.4651,3+)
Title: 1306: Scan 3323 (rt=82.8623, f=3, i=474) [D:\lab212\membrane\Grace\20120305-iTRAQ-1.raw]
Data File:
Monoisotopic mass of neutral peptide Mr(calc): 2707.373
Variable modifications:
K21 :iTRAQ4plex (K)
Ions Score: 85.86 **Expect:** 0.000

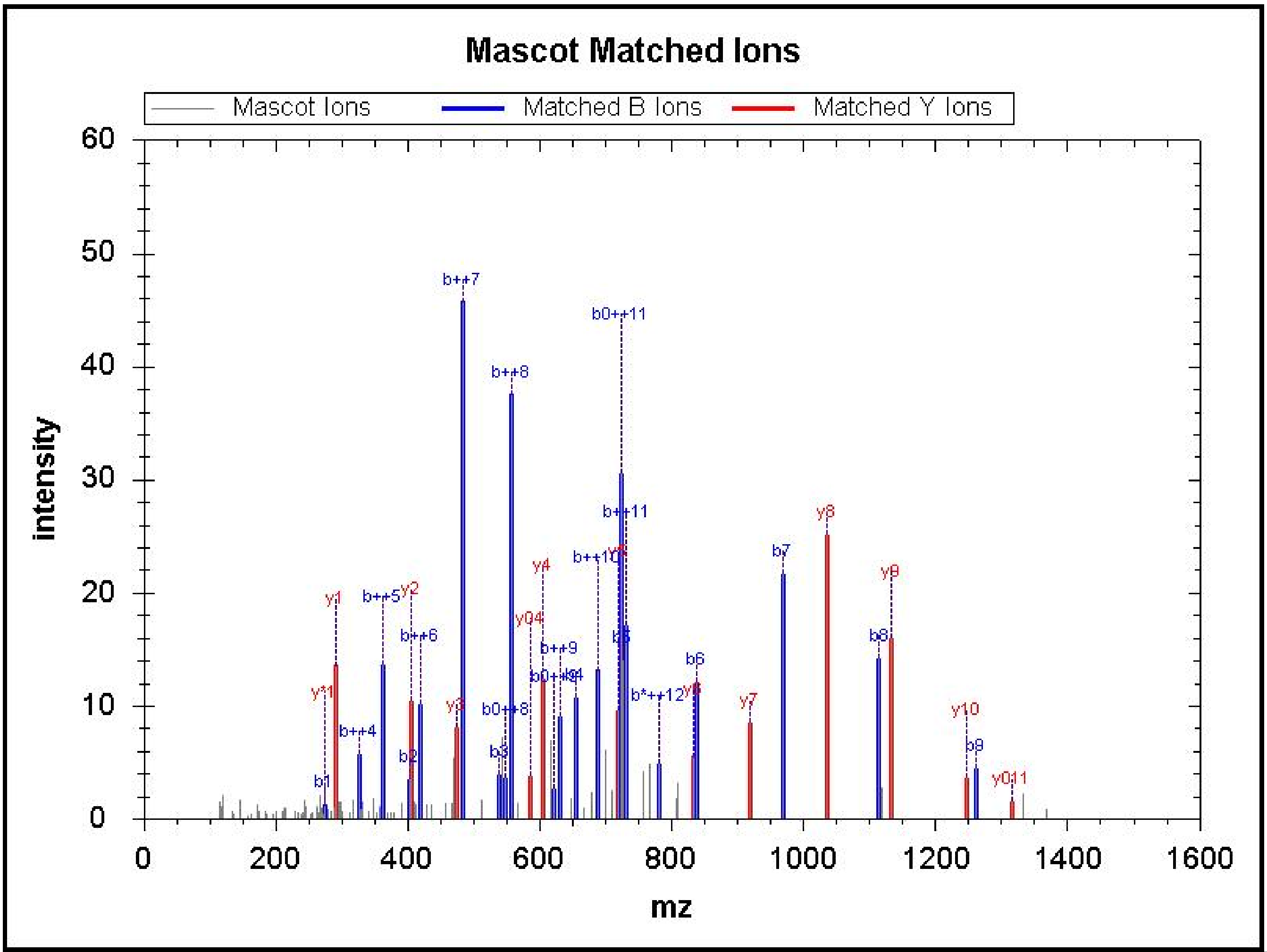

No	b	b++	b*	b*++	b0	b0++	Seq	y	y++	y*	y*++	y0	y0++	RevNo
1	**274.15**	137.58			256.14	128.57	E							21
2	**402.21**	201.61	385.18	193.10	384.20	192.60	Q	2,435.22	1,218.11	2,418.19	1,209.60	2,417.21	1,209.11	20
3	**539.27**	270.14	522.24	261.63	521.26	261.13	H	2,307.16	1,154.08	2,290.14	1,145.57	2,289.15	1,145.08	19
4	**654.30**	**327.65**	637.27	319.14	636.29	318.65	D	2,170.10	1,085.56	2,153.08	1,077.04	2,152.09	1,076.55	18
5	**725.33**	**363.17**	708.31	354.66	707.32	354.17	A	2,055.08	1,028.04	2,038.05	1,019.53	2,037.07	1,019.04	17
6	**838.42**	**419.71**	821.39	411.20	820.41	410.71	L	1,984.04	992.52	1,967.01	984.01	1,966.03	983.52	16
7	**967.46**	**484.23**	950.43	475.72	949.45	475.23	E	1,870.96	935.98	1,853.93	927.47	1,852.94	926.98	15
8	**1,114.53**	**557.77**	1,097.50	549.25	1,096.52	**548.76**	F	1,741.91	871.46	1,724.89	862.95	1,723.90	862.45	14
9	**1,261.60**	**631.30**	1,244.57	622.79	1,243.59	**622.30**	F	1,594.84	797.93	1,577.82	789.41	1,576.83	788.92	13

**10**	1,375.64	**688.32**	1,358.61	679.81	1,357.63	679.32	**N**	1,447.78	724.39	1,430.75	715.88	1,429.77	715.39	**12**
**11**	1,462.67	**731.84**	1,445.65	723.33	1,444.66	**722.83**	**S**	1,333.73	667.37	1,316.71	658.86	**1,315.72**	658.36	**11**
**12**	1,575.76	788.38	1,558.73	**779.87**	1,557.75	779.38	**L**	**1,246.70**	623.85	1,229.67	615.34	1,228.69	614.85	**10**
**13**	1,674.82	837.92	1,657.80	829.40	1,656.81	828.91	**V**	**1,133.62**	567.31	1,116.59	558.80	1,115.61	558.31	**9**
**14**	1,789.85	895.43	1,772.82	886.92	1,771.84	886.42	**D**	**1,034.55**	517.78	1,017.52	509.26	1,016.54	508.77	**8**
**15**	1,876.88	938.95	1,859.86	930.43	1,858.87	929.94	**S**	**919.52**	460.26	902.50	451.75	901.51	451.26	**7**
**16**	1,989.97	995.49	1,972.94	986.97	1,971.96	986.48	**L**	**832.49**	416.75	815.46	408.24	814.48	407.74	**6**
**17**	2,104.99	1,053.00	2,087.97	1,044.49	2,086.98	1,044.00	**D**	**719.41**	360.21	702.38	351.69	701.39	351.20	**5**
**18**	2,234.04	1,117.52	2,217.01	1,109.01	2,216.03	1,108.52	**E**	**604.38**	302.69	587.35	294.18	**586.37**	293.69	**4**
**19**	2,305.07	1,153.04	2,288.05	1,144.53	2,287.06	1,144.04	**A**	**475.34**	238.17	458.31	229.66			**3**
**20**	2,418.16	1,209.58	2,401.13	1,201.07	2,400.15	1,200.58	**L**	**404.30**	202.65	387.27	194.14			**2**
**21**							**K**	**291.21**	146.11	**274.19**	137.60			**1**

Query 2148 Hit 1

MS/MS Fragmentation of **VPADLGAEAGLQQLLGALR**
Found in **sp|P35270|SPRE_HUMAN**, Sepiapterin reductase OS=Homo sapiens GN=SPR PE=1 SV=1
Match to Query 2148: 2035.164from(679.3952,3+)
Title: 1073: Scan 2758 (rt=70.1334, f=3, i=383) [D:\lab212\membrane\Grace\20120305-iTRAQ-1.raw]
Data File:
Monoisotopic mass of neutral peptide Mr(calc): 2035.164
Variable modifications:
Ions Score: 83.99 **Expect:** 0.000

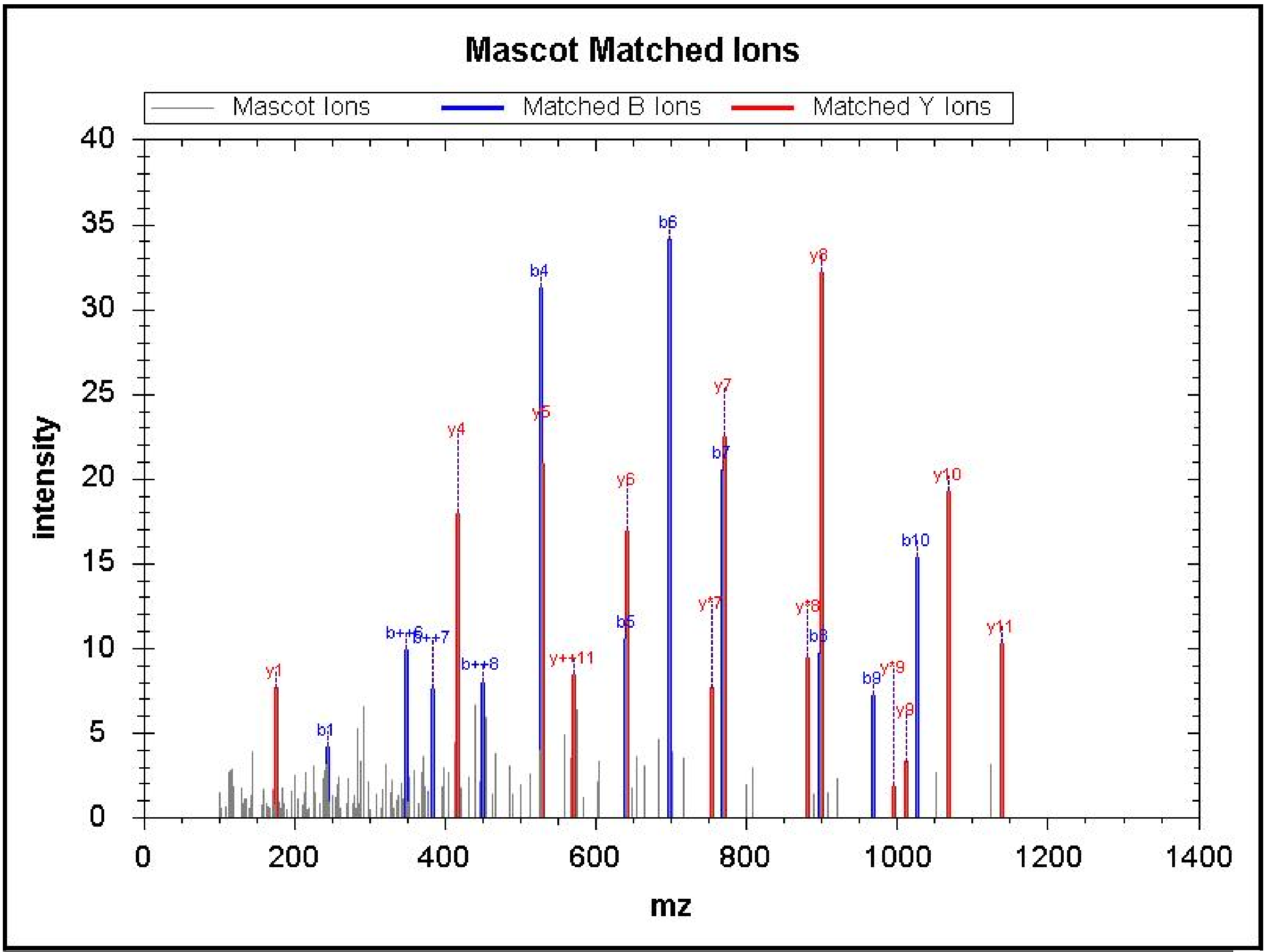

No	b	b++	b*	b*++	b0	b0++	Seq	y	y++	y*	y*++	y0	y0++	RevNo
**1**	**244.18**	122.59					**V**							**19**
**2**	341.23	171.12					**P**	1,792.99	897.00	1,775.97	888.49	1,774.98	887.99	**18**
**3**	412.27	206.64					**A**	1,695.94	848.47	1,678.91	839.96	1,677.93	839.47	**17**

**4**	**527.29**	264.15			509.28	255.15	**D**	1,624.90	812.95	1,607.88	804.44	1,606.89	803.95	**16**
**5**	**640.38**	320.69			622.37	311.69	**L**	1,509.87	755.44	1,492.85	746.93	1,491.86	746.44	**15**
**6**	**697.40**	**349.20**			679.39	340.20	**G**	1,396.79	698.90	1,379.76	690.39	1,378.78	689.89	**14**
**7**	**768.44**	**384.72**			750.43	375.72	**A**	1,339.77	670.39	1,322.74	661.88	1,321.76	661.38	**13**
**8**	**897.48**	**449.24**			879.47	440.24	**E**	1,268.73	634.87	1,251.71	626.36	1,250.72	625.86	**12**
**9**	**968.52**	484.76			950.51	475.76	**A**	**1,139.69**	**570.35**	1,122.66	561.84			**11**
**10**	**1,025.54**	513.27			1,007.53	504.27	**G**	**1,068.65**	534.83	1,051.63	526.32			**10**
**11**	1,138.62	569.81			1,120.61	560.81	**L**	**1,011.63**	506.32	**994.60**	497.81			**9**
**12**	1,266.68	633.84	1,249.65	625.33	1,248.67	624.84	**Q**	**898.55**	449.78	**881.52**	441.26			**8**
**13**	1,394.74	697.87	1,377.71	689.36	1,376.73	688.87	**Q**	**770.49**	385.75	**753.46**	377.23			**7**
**14**	1,507.82	754.42	1,490.80	745.90	1,489.81	745.41	**L**	**642.43**	321.72	625.40	313.21			**6**
**15**	1,620.91	810.96	1,603.88	802.44	1,602.90	801.95	**L**	**529.35**	265.18	512.32	256.66			**5**
**16**	1,677.93	839.47	1,660.90	830.96	1,659.92	830.46	**G**	**416.26**	208.63	399.24	200.12			**4**
**17**	1,748.97	874.99	1,731.94	866.47	1,730.96	865.98	**A**	359.24	180.12	342.21	171.61			**3**
**18**	1,862.05	931.53	1,845.02	923.02	1,844.04	922.52	**L**	288.20	144.61	271.18	136.09			**2**
**19**							**R**	**175.12**	88.06	158.09	79.55			**1**

Query 2630 Hit 1

MS/MS Fragmentation of **FLVLDEADGLLSQGYSDFINR**
Found in **sp|Q92499|DDX1_HUMAN**, ATP-dependent RNA helicase DDX1 OS=Homo sapiens GN=DDX1 PE=1 SV=2
Match to Query 2630: 2515.292from(839.4378,3+)
Title: 1236: Sum of 2 scans in range 2997 (rt=75.9688, f=4, i=858) to 2998 (rt=75.9942, f=4, i=859) [D:\lab212\membrane\Grace\20120305-iTRAQ-2.raw]
Data File:
Monoisotopic mass of neutral peptide Mr(calc): 2515.292
Variable modifications:
Ions Score: 83.31 **Expect:** 0.000

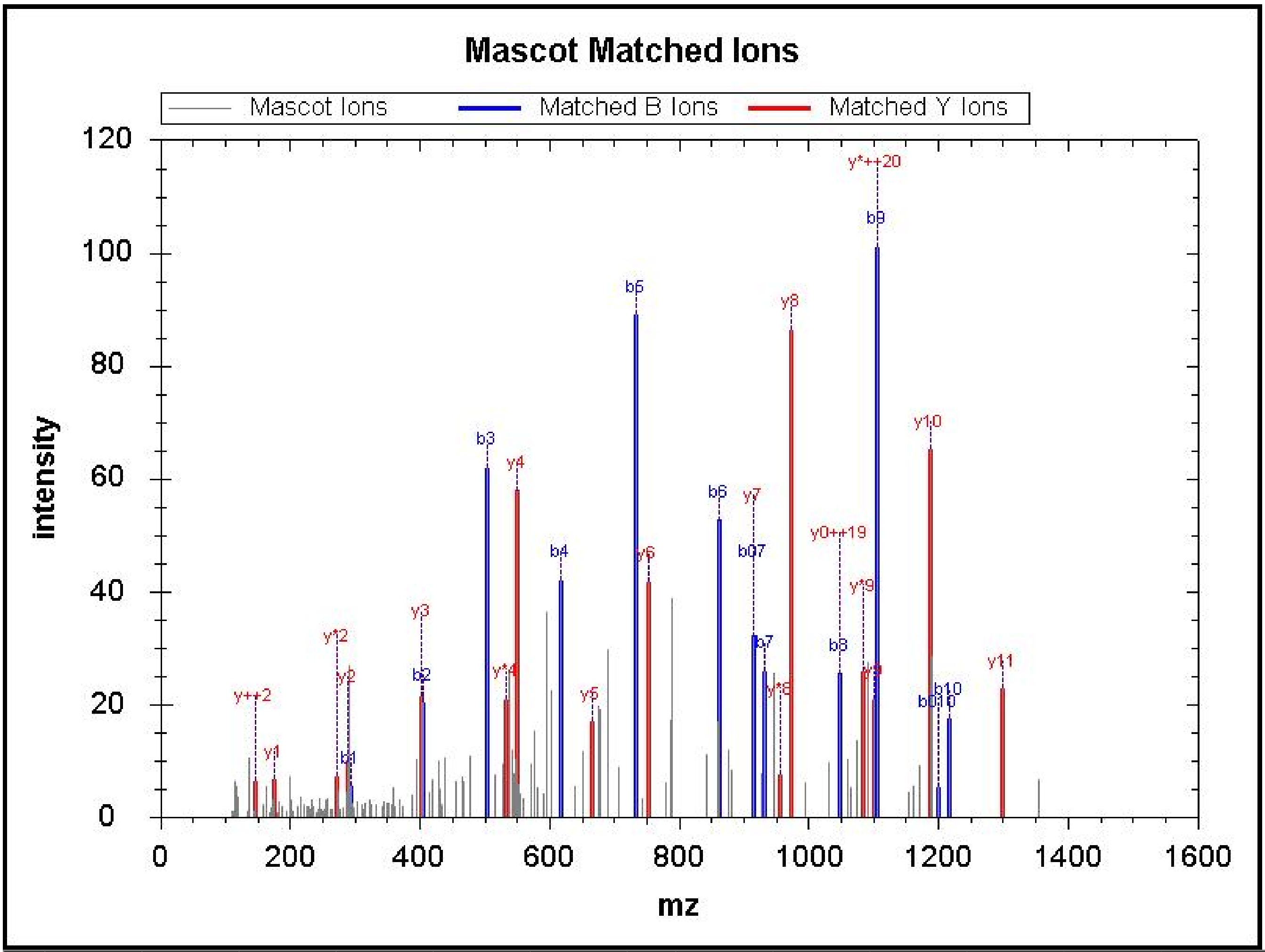

No	b	b++	b*	b*++	b0	b0++	Seq	y	y++	y*	y*++	y0	y0++	RevNo
1	292.18	146.59					F							21
2	405.26	203.13					L	2,225.11	1,113.06	2,208.08	1,104.54	2,207.10	1,104.05	20
3	504.33	252.67					V	2,112.02	1,056.52	2,095.00	1,048.00	2,094.01	1,047.51	19
4	617.41	309.21					L	2,012.96	1,006.98	1,995.93	998.47	1,994.95	997.98	18
5	732.44	366.72			714.43	357.72	D	1,899.87	950.44	1,882.85	941.93	1,881.86	941.43	17
6	861.48	431.25			843.47	422.24	E	1,784.85	892.93	1,767.82	884.41	1,766.83	883.92	16
7	932.52	466.76			914.51	457.76	A	1,655.80	828.40	1,638.78	819.89	1,637.79	819.40	15
8	1,047.55	524.28			1,029.54	515.27	D	1,584.77	792.89	1,567.74	784.37	1,566.75	783.88	14
9	1,104.57	552.79			1,086.56	543.78	G	1,469.74	735.37	1,452.71	726.86	1,451.73	726.37	13
10	1,217.65	609.33			1,199.64	600.33	L	1,412.72	706.86	1,395.69	698.35	1,394.71	697.86	12
11	1,330.74	665.87			1,312.73	656.87	L	1,299.63	650.32	1,282.61	641.81	1,281.62	641.31	11
12	1,417.77	709.39			1,399.76	700.38	S	1,186.55	593.78	1,169.52	585.26	1,168.54	584.77	10
13	1,545.83	773.42	1,528.80	764.90	1,527.82	764.41	Q	1,099.52	550.26	1,082.49	541.75	1,081.51	541.26	9
14	1,602.85	801.93	1,585.82	793.42	1,584.84	792.92	G	971.46	486.23	954.43	477.72	953.45	477.23	8
15	1,765.91	883.46	1,748.89	874.95	1,747.90	874.45	Y	914.44	457.72	897.41	449.21	896.43	448.72	7
16	1,852.94	926.98	1,835.92	918.46	1,834.93	917.97	S	751.37	376.19	734.35	367.68	733.36	367.18	6
17	1,967.97	984.49	1,950.95	975.98	1,949.96	975.48	D	664.34	332.67	647.31	324.16	646.33	323.67	5
18	2,115.04	1,058.02	2,098.01	1,049.51	2,097.03	1,049.02	F	549.31	275.16	532.29	266.65			4
19	2,228.12	1,114.57	2,211.10	1,106.05	2,210.11	1,105.56	I	402.25	201.63	385.22	193.11			3
20	2,342.17	1,171.59	2,325.14	1,163.07	2,324.16	1,162.58	N	289.16	145.08	272.14	136.57			2
21							R	175.12	88.06	158.09	79.55			1

Query 1421 Hit 1

MS/MS Fragmentation of LIALLEVLSQK

Found in **sp|P21333|FLNA_HUMAN**, Filamin-A OS=Homo sapiens GN=FLNA PE=1 SV=4
Match to Query 1421: 1513.98from(757.9973,2+)
Title: 1173: Scan 2852 (rt=72.6968, f=2, i=435) [D:\lab212\membrane\Grace\20120305-iTRAQ-2.raw]
Data File:
Monoisotopic mass of neutral peptide Mr(calc): 1513.98
Variable modifications:
K11 :iTRAQ4plex (K)
Ions Score: 77.64 **Expect:** 0.000

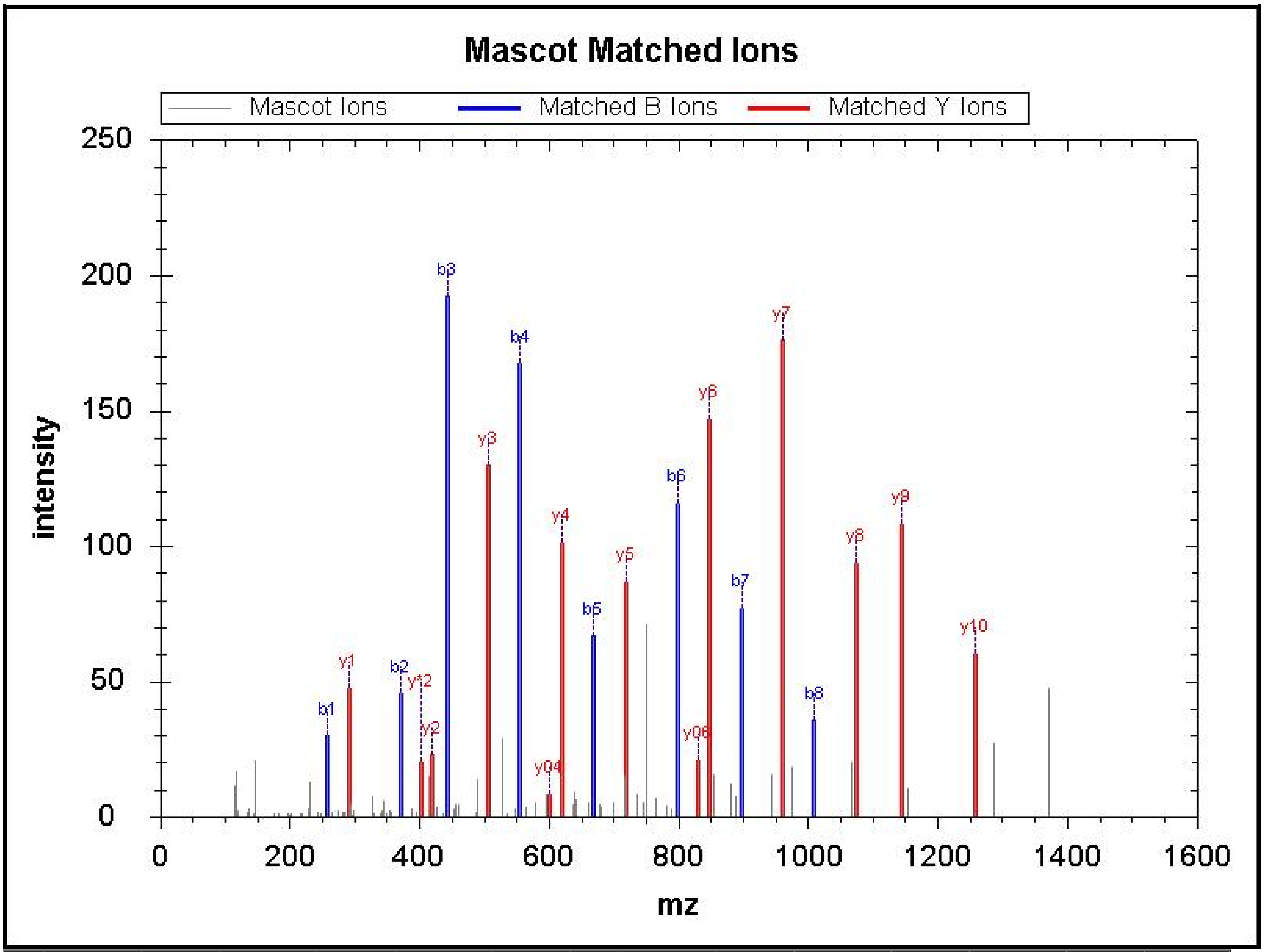

No	b	b++	b*	b*++	b0	b0++	Seq	y	y++	y*	y*++	y0	y0++	RevNo
1	**258.19**	129.60					L							11
2	**371.28**	186.14					I	**1,257.79**	629.40	1,240.76	620.89	1,239.78	620.39	10
3	**442.31**	221.66					A	**1,144.71**	572.86	1,127.68	564.34	1,126.70	563.85	9
4	**555.40**	278.20					L	**1,073.67**	537.34	1,056.64	528.82	1,055.66	528.33	8
5	**668.48**	334.74					L	**960.58**	480.80	943.56	472.28	942.57	471.79	7
6	**797.53**	399.27			779.51	390.26	E	**847.50**	424.25	830.47	415.74	**829.49**	415.25	6
7	**896.59**	448.80			878.58	439.80	V	**718.46**	359.73	701.43	351.22	700.45	350.73	5
8	**1,009.68**	505.34			991.67	496.34	L	**619.39**	310.20	602.36	301.69	**601.38**	301.19	4
9	1,096.71	548.86			1,078.70	539.85	S	**506.31**	253.66	489.28	245.14	488.29	244.65	3
10	1,224.77	612.89	1,207.74	604.37	1,206.76	603.88	Q	**419.27**	210.14	**402.25**	201.63			2
11							K	**291.21**	146.11	274.19	137.60			1

Query 1638 Hit 1

MS/MS Fragmentation of **IIDFLSALEGFK**
Found in **sp|P52701|MSH6_HUMAN**, DNA mismatch repair protein Msh6 OS=Homo sapiens GN=MSH6 PE=1 SV=2
Match to Query 1638: 1639.943from(820.9789,2+)
Title: 1196: Sum of 2 scans in range 3057 (rt=76.8723, f=4, i=844) to 3058 (rt=76.8977, f=4, i=845) [D:\lab212\membrane\Grace\20120305-iTRAQ-1.raw]

Data File:
Monoisotopic mass of neutral peptide Mr(calc): 1639.943
Variable modifications:
K12 :iTRAQ4plex (K)
Ions Score: 76.84 **Expect:** 0.000

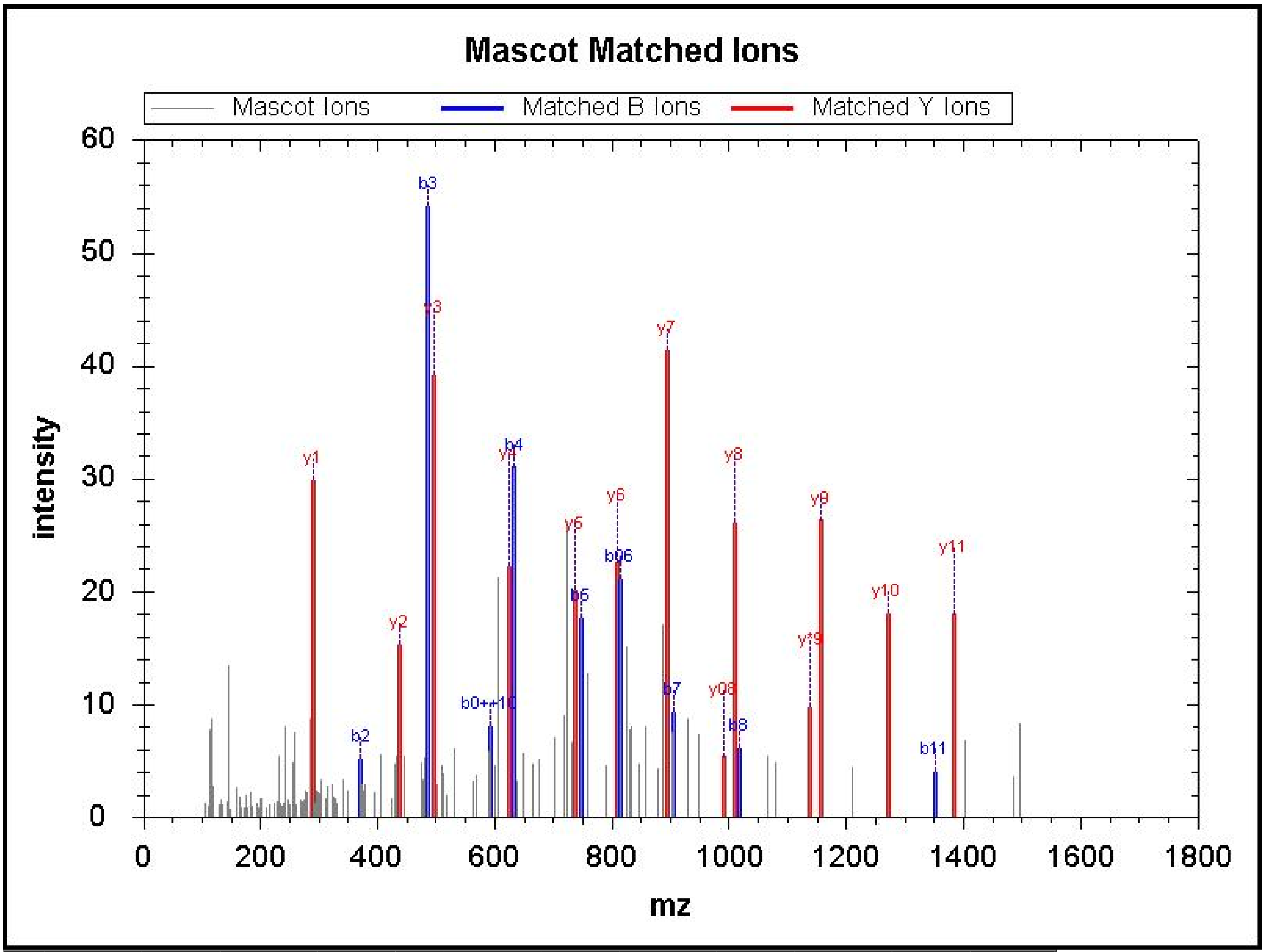

No	b	b++	b0	b0++	Seq	y	y++	y*	y*++	y0	y0++	RevNo
1	258.19	129.60			I							12
2	371.28	186.14			I	1,383.76	692.39	1,366.74	683.87	1,365.75	683.38	11
3	486.30	243.66	468.29	234.65	D	1,270.68	635.84	1,253.65	627.33	1,252.67	626.84	10
4	633.37	317.19	615.36	308.18	F	1,155.65	578.33	1,138.63	569.82	1,137.64	569.32	9
5	746.46	373.73	728.45	364.73	L	1,008.58	504.80	991.56	496.28	990.57	495.79	8
6	833.49	417.25	815.48	408.24	S	895.50	448.25	878.47	439.74	877.49	439.25	7
7	904.53	452.77	886.52	443.76	A	808.47	404.74	791.44	396.22	790.46	395.73	6
8	1,017.61	509.31	999.60	500.30	L	737.43	369.22	720.40	360.71	719.42	360.21	5
9	1,146.65	573.83	1,128.64	564.82	E	624.35	312.68	607.32	304.16	606.34	303.67	4
10	1,203.67	602.34	1,185.66	593.34	G	495.30	248.16	478.28	239.64			3
11	1,350.74	675.88	1,332.73	666.87	F	438.28	219.65	421.26	211.13			2
12					K	291.21	146.11	274.19	137.60			1

Query 2266 Hit 1

MS/MS Fragmentation of **SLVEIIEHGLVDEQQK**
Found in **sp|O75533|SF3B1_HUMAN**, Splicing factor 3B subunit 1 OS=Homo sapiens GN=SF3B1 PE=1 SV=3
Match to Query 2266: 2124.173 from(709.0649,3+)
Title: 872: Scan 2331 (rt=60.5096, f=3, i=314) [D:\lab212\membrane\Grace\20120305-iTRAQ-1.raw]
Data File:
Monoisotopic mass of neutral peptide Mr(calc): 2124.173
Variable modifications:

K16 :iTRAQ4plex (K)
Ions Score: 76.48 **Expect:** 0.000

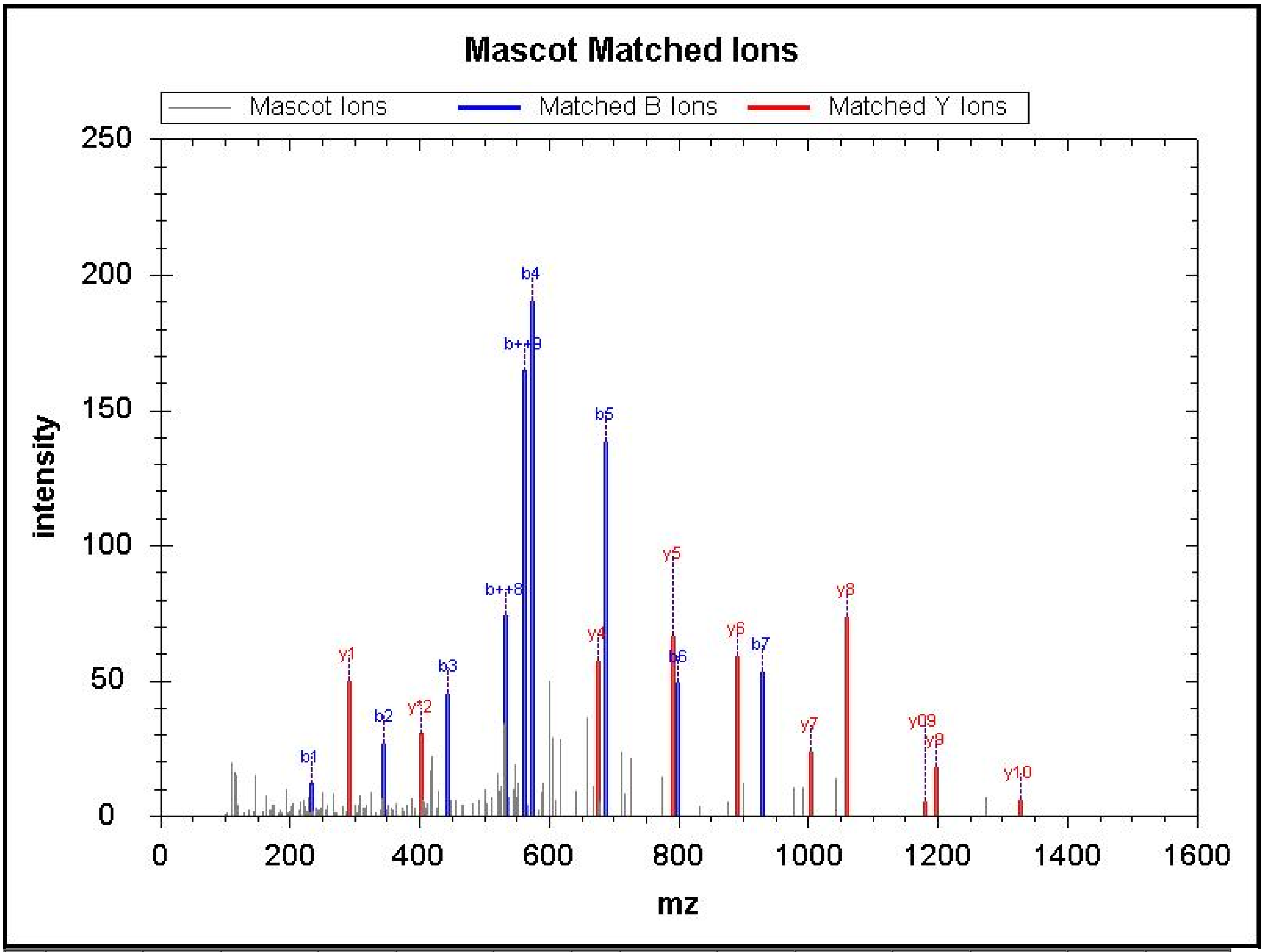

No	b	b++	b*	b*++	b0	b0++	Seq	y	y++	y*	y*++	y0	y0++	RevNo
1	**232.14**	116.57			214.13	107.57	S							16
2	**345.23**	173.12			327.21	164.11	L	1,894.04	947.52	1,877.01	939.01	1,876.03	938.52	15
3	**444.29**	222.65			426.28	213.65	V	1,780.96	890.98	1,763.93	882.47	1,762.95	881.98	14
4	**573.34**	287.17			555.33	278.17	E	1,681.89	841.45	1,664.86	832.93	1,663.88	832.44	13
5	**686.42**	343.71			668.41	334.71	I	1,552.85	776.93	1,535.82	768.41	1,534.83	767.92	12
6	**799.50**	400.26			781.49	391.25	I	1,439.76	720.38	1,422.73	711.87	1,421.75	711.38	11
7	**928.55**	464.78			910.54	455.77	E	**1,326.68**	663.84	1,309.65	655.33	1,308.67	654.84	10
8	1,065.61	**533.31**			1,047.60	524.30	H	**1,197.63**	599.32	1,180.61	590.81	**1,179.62**	590.32	9
9	1,122.63	**561.82**			1,104.62	552.81	G	**1,060.58**	530.79	1,043.55	522.28	1,042.56	521.79	8
10	1,235.71	618.36			1,217.70	609.35	L	**1,003.55**	502.28	986.53	493.77	985.54	493.28	7
11	1,334.78	667.89			1,316.77	658.89	V	**890.47**	445.74	873.44	437.23	872.46	436.73	6
12	1,449.81	725.41			1,431.80	716.40	D	**791.40**	396.20	774.38	387.69	773.39	387.20	5
13	1,578.85	789.93			1,560.84	780.92	E	**676.37**	338.69	659.35	330.18	658.36	329.69	4
14	1,706.91	853.96	1,689.88	845.44	1,688.90	844.95	Q	547.33	274.17	530.31	265.66			3
15	1,834.97	917.99	1,817.94	909.47	1,816.96	908.98	Q	419.27	210.14	**402.25**	201.63			2
16							K	**291.21**	146.11	274.19	137.60			1

Query 2187 Hit 1

MS/MS Fragmentation of **IMEIVDAITTTAQSHQR**
Found in **sp|P08237|K6PF_HUMAN**, 6-phosphofructokinase
Match to Query 2187: 2057.081 from(686.7009,3+)
Title: 941: Sum of 2 scans in range 2352 (rt=61.4357, f=4, i=650) to 2353 (rt=61.4611, f=4, i=651)

[D:\lab212\membrane\Grace\20120305-iTRAQ-2.raw]
Data File:
Monoisotopic mass of neutral peptide Mr(calc): 2057.081
Variable modifications:
Ions Score: 75.88 **Expect:** 0.000

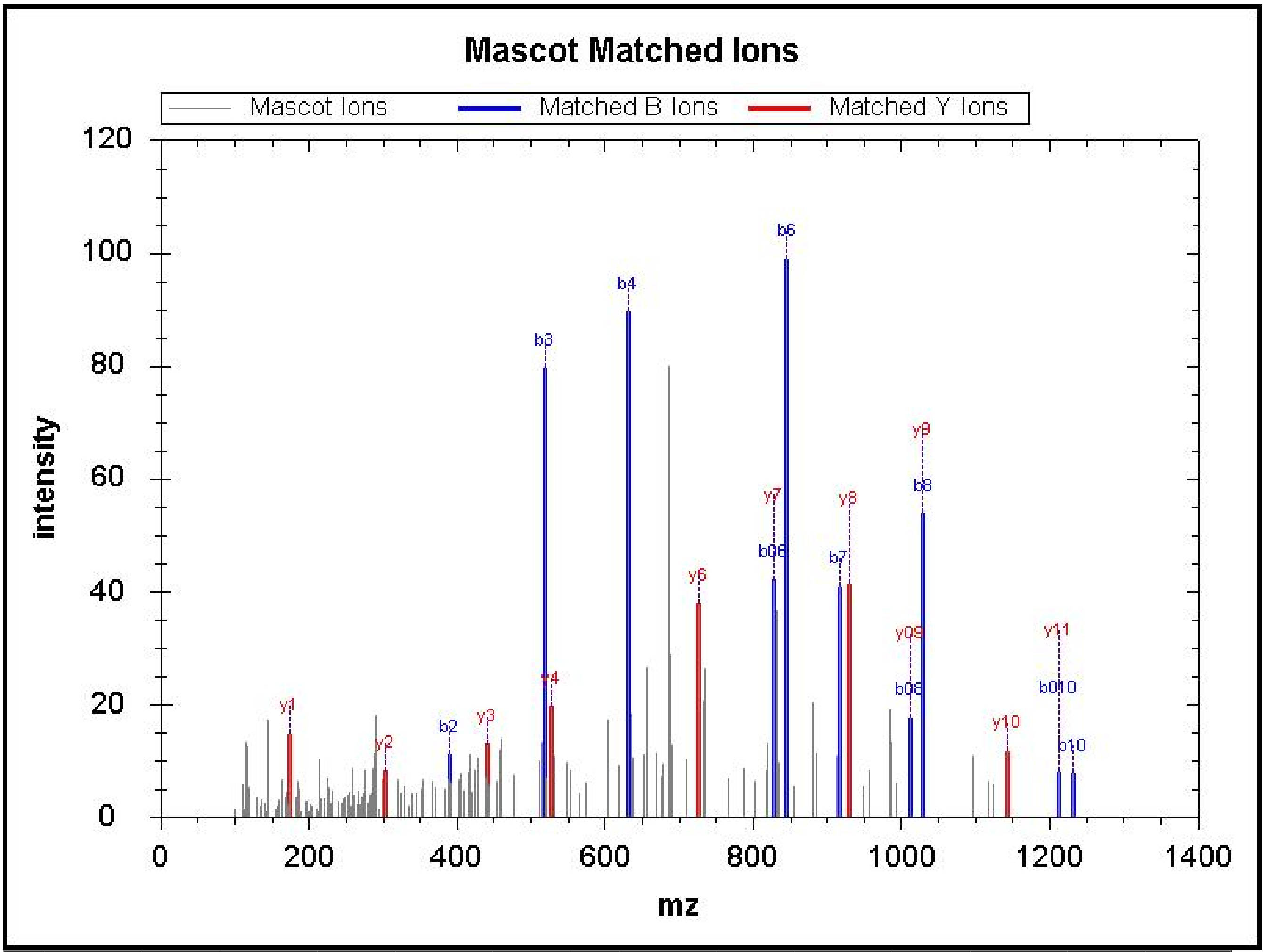

No	b	b++	b*	b*++	b0	b0++	Seq	y	y++	y*	y*++	y0	y0++	RevNo
1	258.19	129.60					I							17
2	389.23	195.12					M	1,800.89	900.95	1,783.86	892.44	1,782.88	891.94	16
3	518.28	259.64			500.27	250.64	E	1,669.85	835.43	1,652.82	826.92	1,651.84	826.42	15
4	631.36	316.18			613.35	307.18	I	1,540.81	770.91	1,523.78	762.39	1,522.80	761.90	14
5	730.43	365.72			712.42	356.71	V	1,427.72	714.37	1,410.70	705.85	1,409.71	705.36	13
6	845.46	423.23			827.45	414.23	D	1,328.66	664.83	1,311.63	656.32	1,310.64	655.83	12
7	916.49	458.75			898.48	449.74	A	1,213.63	607.32	1,196.60	598.80	1,195.62	598.31	11
8	1,029.58	515.29			1,011.57	506.29	I	1,142.59	571.80	1,125.56	563.29	1,124.58	562.79	10
9	1,130.62	565.82			1,112.61	556.81	T	1,029.51	515.26	1,012.48	506.74	1,011.50	506.25	9
10	1,231.67	616.34			1,213.66	607.33	T	928.46	464.73	911.43	456.22	910.45	455.73	8
11	1,332.72	666.86			1,314.71	657.86	T	827.41	414.21	810.39	405.70	809.40	405.20	7
12	1,403.76	702.38			1,385.75	693.38	A	726.36	363.69	709.34	355.17	708.35	354.68	6
13	1,531.82	766.41	1,514.79	757.90	1,513.81	757.41	Q	655.33	328.17	638.30	319.65	637.32	319.16	5
14	1,618.85	809.93	1,601.82	801.41	1,600.84	800.92	S	527.27	264.14	510.24	255.62	509.26	255.13	4
15	1,755.91	878.46	1,738.88	869.94	1,737.90	869.45	H	440.24	220.62	423.21	212.11			3
16	1,883.97	942.49	1,866.94	933.97	1,865.95	933.48	Q	303.18	152.09	286.15	143.58			2
17							R	175.12	88.06	158.09	79.55			1

Query 2375 Hit 1

MS/MS Fragmentation of **SIVSELAGLLSAMEYVQK**

Found in **sp|P40763|STAT3_HUMAN**, Signal transducer and activator of transcription 3 OS=Homo sapiens GN=STAT3 PE=1 SV=2

Match to Query 2375: 2225.236from(742.7526,3+)

Title: 1390: Sum of 2 scans in range 3400 (rt=85.0496, f=4, i=988) to 3401 (rt=85.075, f=4, i=989) [D:\lab212\membrane\Grace\20120305-iTRAQ-2.raw]

Data File:

Monoisotopic mass of neutral peptide Mr(calc): 2225.236

Variable modifications:

K18 :iTRAQ4plex (K)

Ions Score: 75.38 **Expect:** 0.000

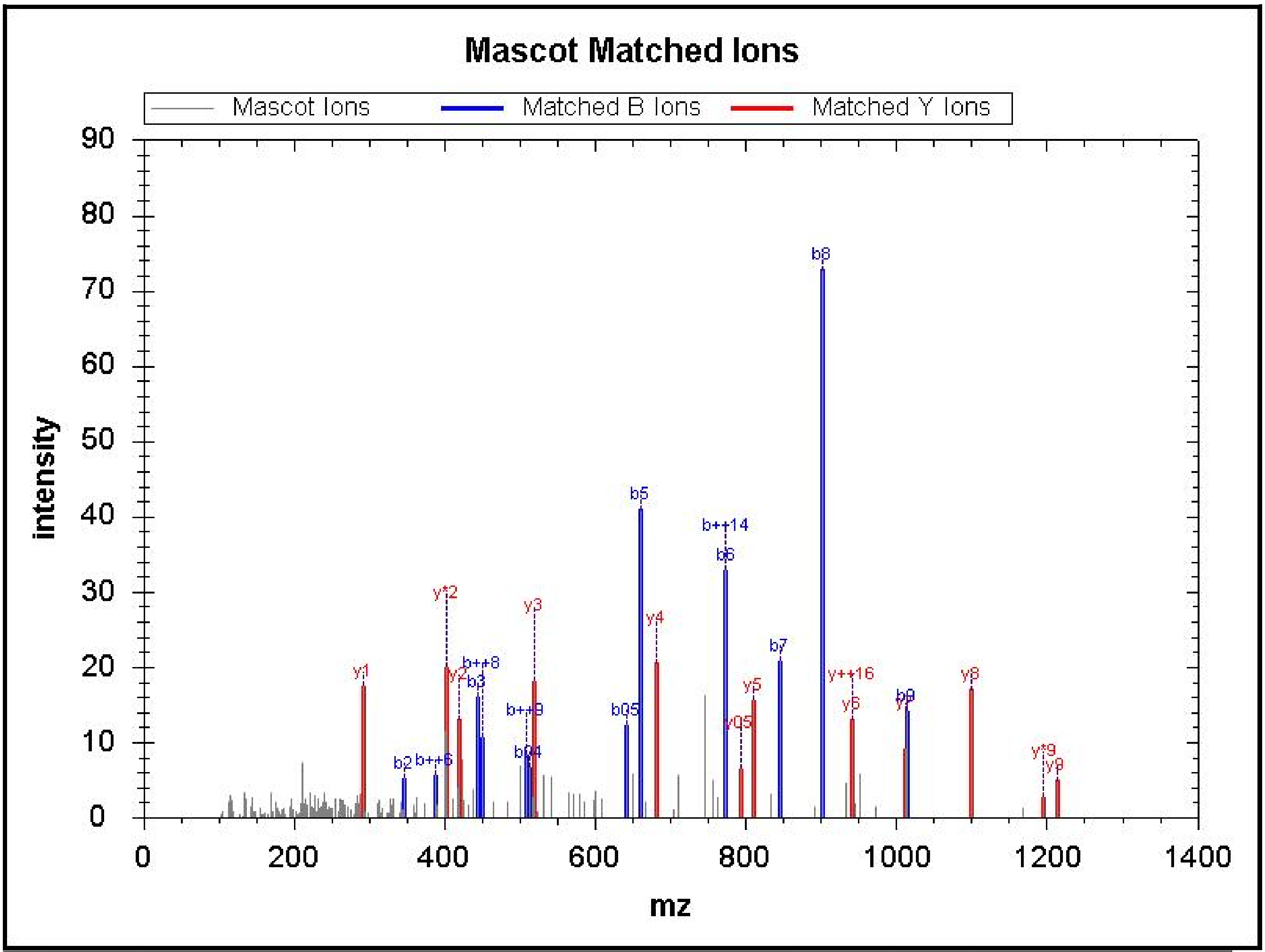

No	b	b++	b*	b*++	b0	b0++	Seq	y	y++	y*	y*++	y0	y0++	RevNo
1	232.14	116.57			214.13	107.57	S							18
2	**345.23**	173.12			327.21	164.11	I	1,995.10	998.05	1,978.07	989.54	1,977.08	989.05	17
3	**444.29**	222.65			426.28	213.65	V	1,882.01	**941.51**	1,864.98	933.00	1,864.00	932.50	16
4	531.33	266.17			**513.32**	257.16	S	1,782.94	891.98	1,765.92	883.46	1,764.93	882.97	15
5	**660.37**	330.69			**642.36**	321.68	E	1,695.91	848.46	1,678.88	839.95	1,677.90	839.45	14
6	**773.45**	**387.23**			755.44	378.22	L	1,566.87	783.94	1,549.84	775.42	1,548.86	774.93	13
7	**844.49**	422.75			826.48	413.74	A	1,453.78	727.40	1,436.76	718.88	1,435.77	718.39	12
8	**901.51**	**451.26**			883.50	442.25	G	1,382.75	691.88	1,365.72	683.36	1,364.74	682.87	11
9	**1,014.60**	**507.80**			996.58	498.80	L	1,325.73	663.37	1,308.70	654.85	1,307.71	654.36	10
10	1,127.68	564.34			1,109.67	555.34	L	**1,212.64**	606.82	**1,195.61**	598.31	1,194.63	597.82	9
11	1,214.71	607.86			1,196.70	598.85	S	**1,099.56**	550.28	1,082.53	541.77	1,081.55	541.28	8
12	1,285.75	643.38			1,267.74	634.37	A	**1,012.53**	506.77	995.50	498.25	994.51	497.76	7
13	1,416.79	708.90			1,398.78	699.89	M	**941.49**	471.25	924.46	462.73	923.48	462.24	6
14	1,545.83	**773.42**			1,527.82	764.41	E	**810.45**	405.73	793.42	397.21	**792.44**	396.72	5
15	1,708.89	854.95			1,690.88	845.95	Y	**681.41**	341.21	664.38	332.69			4

No	b	b++	b*	b*++	b0	b0++	Seq	y	y++	y*	y*++	y0	y0++	RevNo
16	1,807.96	904.49			1,789.95	895.48	V	**518.34**	259.67	501.32	251.16			3
17	1,936.02	968.51	1,919.00	960.00	1,918.01	959.51	Q	**419.27**	210.14	**402.25**	201.63			2
18							K	**291.21**	146.11	274.19	137.60			1

Query 2585 Hit 1

MS/MS Fragmentation of **KLELSDNIISGGLEVLAEK**
Found in **sp|Q9BTT0|AN32E_HUMAN**, Acidic leucine-rich nuclear phosphoprotein 32 family member E OS=Homo sapiens GN=ANP32E PE=1 SV=1
Match to Query 2585: 2459.429from(820.8171,3+)
Title: 879: Scan 2349 (rt=60.9169, f=3, i=317) [D:\lab212\membrane\Grace\20120305-iTRAQ-1.raw]
Data File:
Monoisotopic mass of neutral peptide Mr(calc): 2459.429
Variable modifications:
K1 :iTRAQ4plex (K)
K19 :iTRAQ4plex (K)
Ions Score: 74.41 **Expect:** 0.000

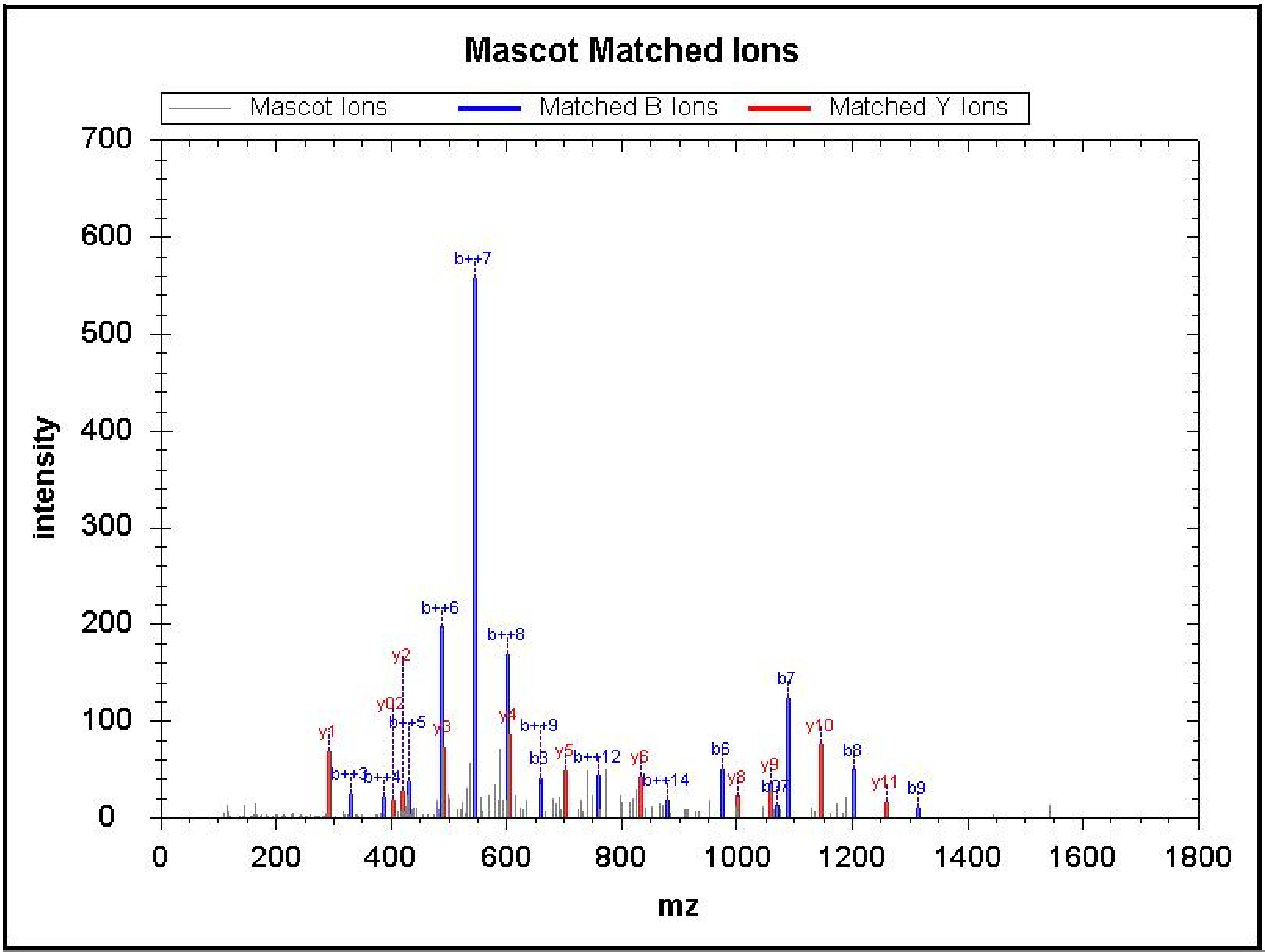

No	b	b++	b*	b*++	b0	b0++	Seq	y	y++	y*	y*++	y0	y0++	RevNo
1	417.31	209.16	400.28	200.64			K							19
2	530.39	265.70	513.36	257.19			L	2,044.13	1,022.57	2,027.10	1,014.06	2,026.12	1,013.56	18
3	**659.43**	**330.22**	642.41	321.71	641.42	321.21	E	1,931.05	966.03	1,914.02	957.51	1,913.03	957.02	17
4	772.52	**386.76**	755.49	378.25	754.51	377.76	L	1,802.00	901.51	1,784.98	892.99	1,783.99	892.50	16
5	859.55	**430.28**	842.52	421.76	841.54	421.27	S	1,688.92	844.96	1,671.89	836.45	1,670.91	835.96	15
6	**974.58**	**487.79**	957.55	479.28	956.57	478.79	D	1,601.89	801.45	1,584.86	792.93	1,583.88	792.44	14
7	**1,088.62**	**544.81**	1,071.59	536.30	**1,070.61**	535.81	N	1,486.86	743.93	1,469.83	735.42	1,468.85	734.93	13
8	**1,201.70**	**601.36**	1,184.68	592.84	1,183.69	592.35	I	1,372.82	686.91	1,355.79	678.40	1,354.81	677.91	12
9	**1,314.79**	**657.90**	1,297.76	649.38	1,296.78	648.89	I	**1,259.73**	630.37	1,242.71	621.86	1,241.72	621.36	11

10	1,401.82	701.41	1,384.79	692.90	1,383.81	692.41	S	1,146.65	573.83	1,129.62	565.31	1,128.64	564.82	10
11	1,458.84	729.92	1,441.81	721.41	1,440.83	720.92	G	1,059.62	530.31	1,042.59	521.80	1,041.61	521.31	9
12	1,515.86	758.43	1,498.84	749.92	1,497.85	749.43	G	1,002.60	501.80	985.57	493.29	984.58	492.80	8
13	1,628.95	814.98	1,611.92	806.46	1,610.94	805.97	L	945.57	473.29	928.55	464.78	927.56	464.29	7
14	1,757.99	879.50	1,740.96	870.98	1,739.98	870.49	E	832.49	416.75	815.46	408.24	814.48	407.74	6
15	1,857.06	929.03	1,840.03	920.52	1,839.05	920.03	V	703.45	352.23	686.42	343.71	685.44	343.22	5
16	1,970.14	985.57	1,953.11	977.06	1,952.13	976.57	L	604.38	302.69	587.35	294.18	586.37	293.69	4
17	2,041.18	1,021.09	2,024.15	1,012.58	2,023.17	1,012.09	A	491.29	246.15	474.27	237.64	473.28	237.15	3
18	2,170.22	1,085.61	2,153.19	1,077.10	2,152.21	1,076.61	E	420.26	210.63	403.23	202.12	402.25	201.63	2
19							K	291.21	146.11	274.19	137.60			1

Query 2419 Hit 1

MS/MS Fragmentation of **QQDAQEFFLHLINMVER**
Found in **sp|P45974|UBP5_HUMAN**, Ubiquitin carboxyl-terminal hydrolase 5 OS=Homo sapiens GN=USP5 PE=1 SV=2
Match to Query 2419: 2261.157from(754.7263,3+)
Title: 1193: Sum of 2 scans in range 2898 (rt=73.7373, f=4, i=826) to 2899 (rt=73.7627, f=4, i=827) [D:\lab212\membrane\Grace\20120305-iTRAQ-2.raw]
Data File:
Monoisotopic mass of neutral peptide Mr(calc): 2261.157
Variable modifications:
Ions Score: 74.32 **Expect:** 0.000

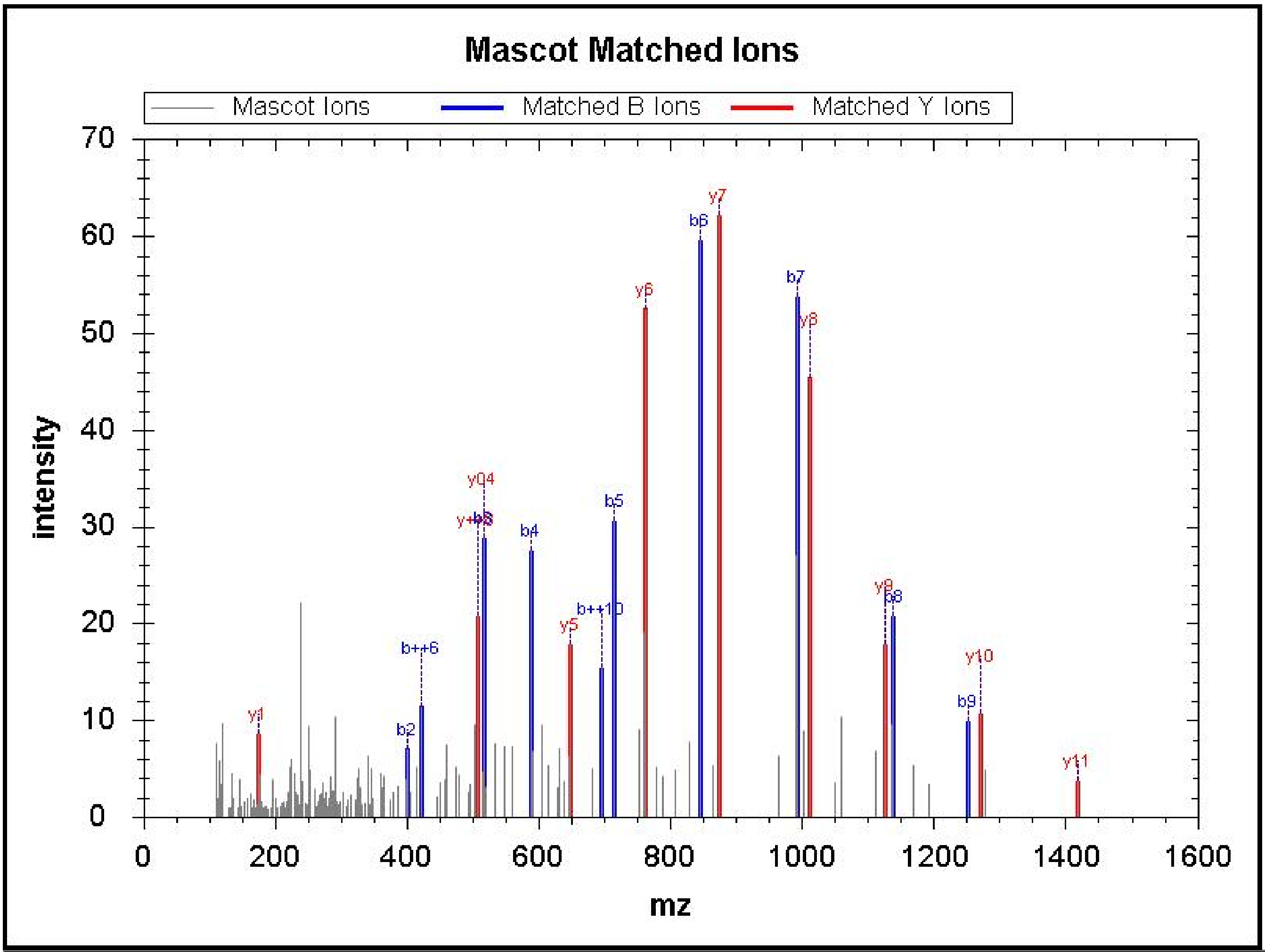

No	b	b++	b*	b*++	b0	b0++	Seq	y	y++	y*	y*++	y0	y0++	RevNo
1	273.17	137.09	256.14	128.57			Q							17
2	401.23	201.12	384.20	192.60			Q	1,989.99	995.50	1,972.96	986.98	1,971.97	986.49	16
3	516.25	258.63	499.23	250.12	498.24	249.63	D	1,861.93	931.47	1,844.90	922.95	1,843.92	922.46	15

4	587.29	294.15	570.26	285.64	569.28	285.14	A	1,746.90	873.95	1,729.87	865.44	1,728.89	864.95	14
5	715.35	358.18	698.32	349.66	697.34	349.17	Q	1,675.86	838.43	1,658.84	829.92	1,657.85	829.43	13
6	844.39	422.70	827.37	414.19	826.38	413.69	E	1,547.80	774.41	1,530.78	765.89	1,529.79	765.40	12
7	991.46	496.23	974.43	487.72	973.45	487.23	F	1,418.76	709.88	1,401.73	701.37	1,400.75	700.88	11
8	1,138.53	569.77	1,121.50	561.25	1,120.52	560.76	F	1,271.69	636.35	1,254.67	627.84	1,253.68	627.34	10
9	1,251.61	626.31	1,234.59	617.80	1,233.60	617.30	L	1,124.62	562.82	1,107.60	554.30	1,106.61	553.81	9
10	1,388.67	694.84	1,371.65	686.33	1,370.66	685.83	H	1,011.54	506.27	994.51	497.76	993.53	497.27	8
11	1,501.76	751.38	1,484.73	742.87	1,483.75	742.38	L	874.48	437.74	857.45	429.23	856.47	428.74	7
12	1,614.84	807.92	1,597.81	799.41	1,596.83	798.92	I	761.40	381.20	744.37	372.69	743.39	372.20	6
13	1,728.88	864.95	1,711.86	856.43	1,710.87	855.94	N	648.31	324.66	631.29	316.15	630.30	315.65	5
14	1,859.92	930.47	1,842.90	921.95	1,841.91	921.46	M	534.27	267.64	517.24	259.13	516.26	258.63	4
15	1,958.99	980.00	1,941.97	971.49	1,940.98	970.99	V	403.23	202.12	386.20	193.61	385.22	193.11	3
16	2,088.03	1,044.52	2,071.01	1,036.01	2,070.02	1,035.52	E	304.16	152.58	287.13	144.07	286.15	143.58	2
17							R	175.12	88.06	158.09	79.55			1

Query 2000 Hit 1

MS/MS Fragmentation of **GLGTDEDTIIDIITHR**
Found in **sp|P08133|ANXA6_HUMAN**, Annexin A6 OS=Homo sapiens GN=ANXA6 PE=1 SV=3
Match to Query 2000: 1912.012from(638.3445,3+)
Title: 922: Sum of 2 scans in range 2309 (rt=60.4656, f=4, i=636) to 2310 (rt=60.491, f=4, i=637) [D:\lab212\membrane\Grace\20120305-iTRAQ-2.raw]
Data File:
Monoisotopic mass of neutral peptide Mr(calc): 1912.012
Variable modifications:
Ions Score: 71.53 **Expect:** 0.000

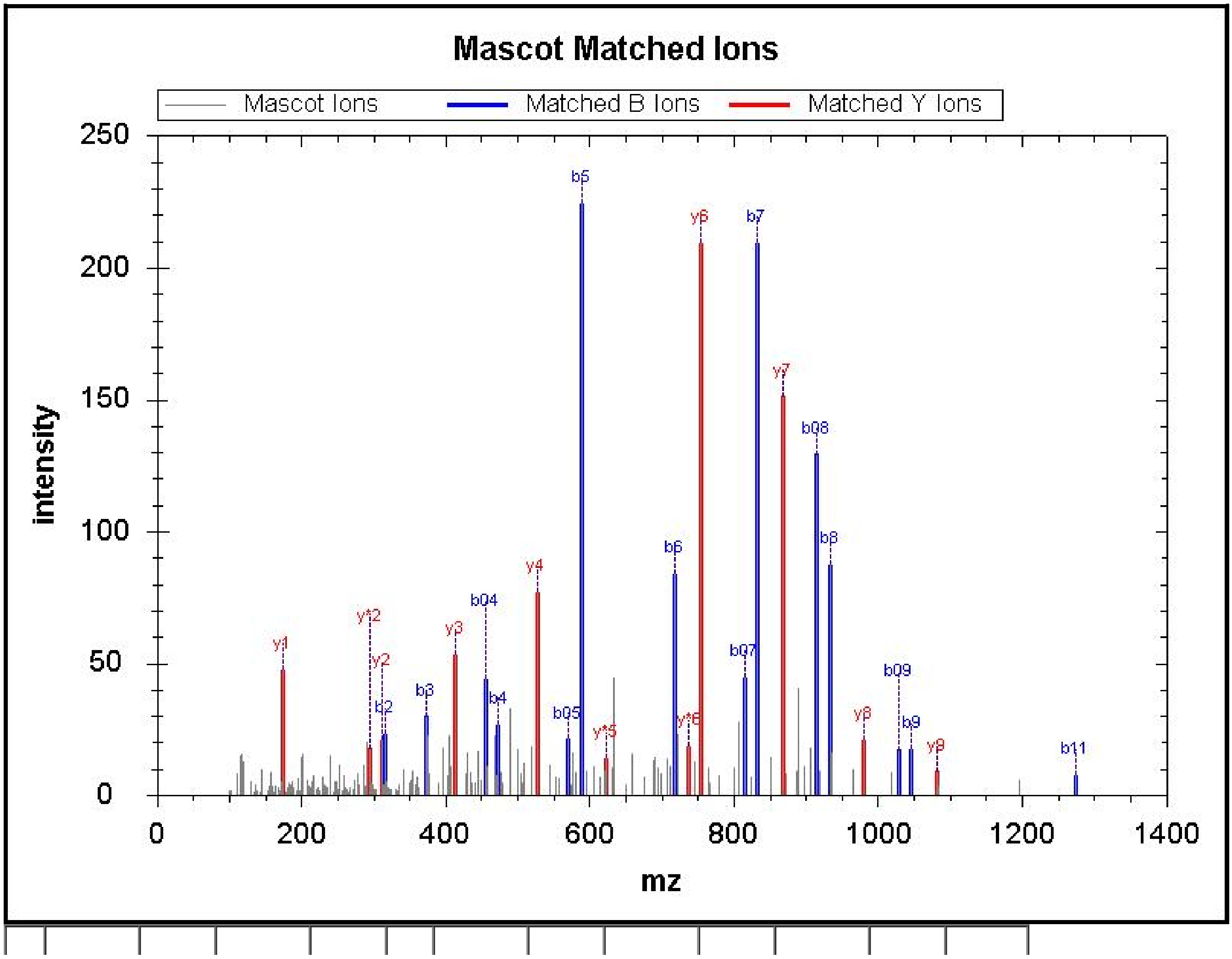

No	b	b++	b0	b0++	Seq	y	y++	y*	y*++	y0	y0++	RevNo
1	202.13	101.57			G							16
2	**315.21**	158.11			L	1,711.89	856.45	1,694.86	847.93	1,693.88	847.44	15
3	**372.24**	186.62			G	1,598.80	799.90	1,581.78	791.39	1,580.79	790.90	14
4	**473.28**	237.15	**455.27**	228.14	T	1,541.78	771.39	1,524.75	762.88	1,523.77	762.39	13
5	**588.31**	294.66	**570.30**	285.65	D	1,440.73	720.87	1,423.71	712.36	1,422.72	711.86	12
6	**717.35**	359.18	699.34	350.18	E	1,325.71	663.36	1,308.68	654.84	1,307.70	654.35	11
7	**832.38**	416.69	**814.37**	407.69	D	1,196.66	598.84	1,179.64	590.32	1,178.65	589.83	10
8	**933.43**	467.22	**915.42**	458.21	T	**1,081.64**	541.32	1,064.61	532.81	1,063.63	532.32	9
9	**1,046.51**	523.76	**1,028.50**	514.75	I	**980.59**	490.80	963.56	482.28	962.58	481.79	8
10	1,159.60	580.30	1,141.59	571.30	I	**867.50**	434.26	850.48	425.74	849.49	425.25	7
11	**1,274.62**	637.82	1,256.61	628.81	D	**754.42**	377.71	**737.39**	369.20	736.41	368.71	6
12	1,387.71	694.36	1,369.70	685.35	I	639.39	320.20	**622.37**	311.69	621.38	311.20	5
13	1,500.79	750.90	1,482.78	741.89	I	**526.31**	263.66	509.28	255.15	508.30	254.65	4
14	1,601.84	801.42	1,583.83	792.42	T	**413.23**	207.12	396.20	198.60	395.21	198.11	3
15	1,738.90	869.95	1,720.89	860.95	H	**312.18**	156.59	**295.15**	148.08			2
16					R	**175.12**	88.06	158.09	79.55			1

Query 1785 Hit 1

MS/MS Fragmentation of **ILTFDQLALDSPK**
Found in **sp|Q07020|RL18_HUMAN**, 60S ribosomal protein L18 OS=Homo sapiens GN=RPL18 PE=1 SV=2
Match to Query 1785: 1747.995from(875.0048,2+)
Title: 775: Sum of 2 scans in range 2121 (rt=55.7811, f=4, i=542) to 2122 (rt=55.8065, f=4, i=543) [D:\lab212\membrane\Grace\20120305-iTRAQ-1.raw]
Data File:
Monoisotopic mass of neutral peptide Mr(calc): 1747.995
Variable modifications:
K13 :iTRAQ4plex (K)
Ions Score: 70.96 **Expect:** 0.000

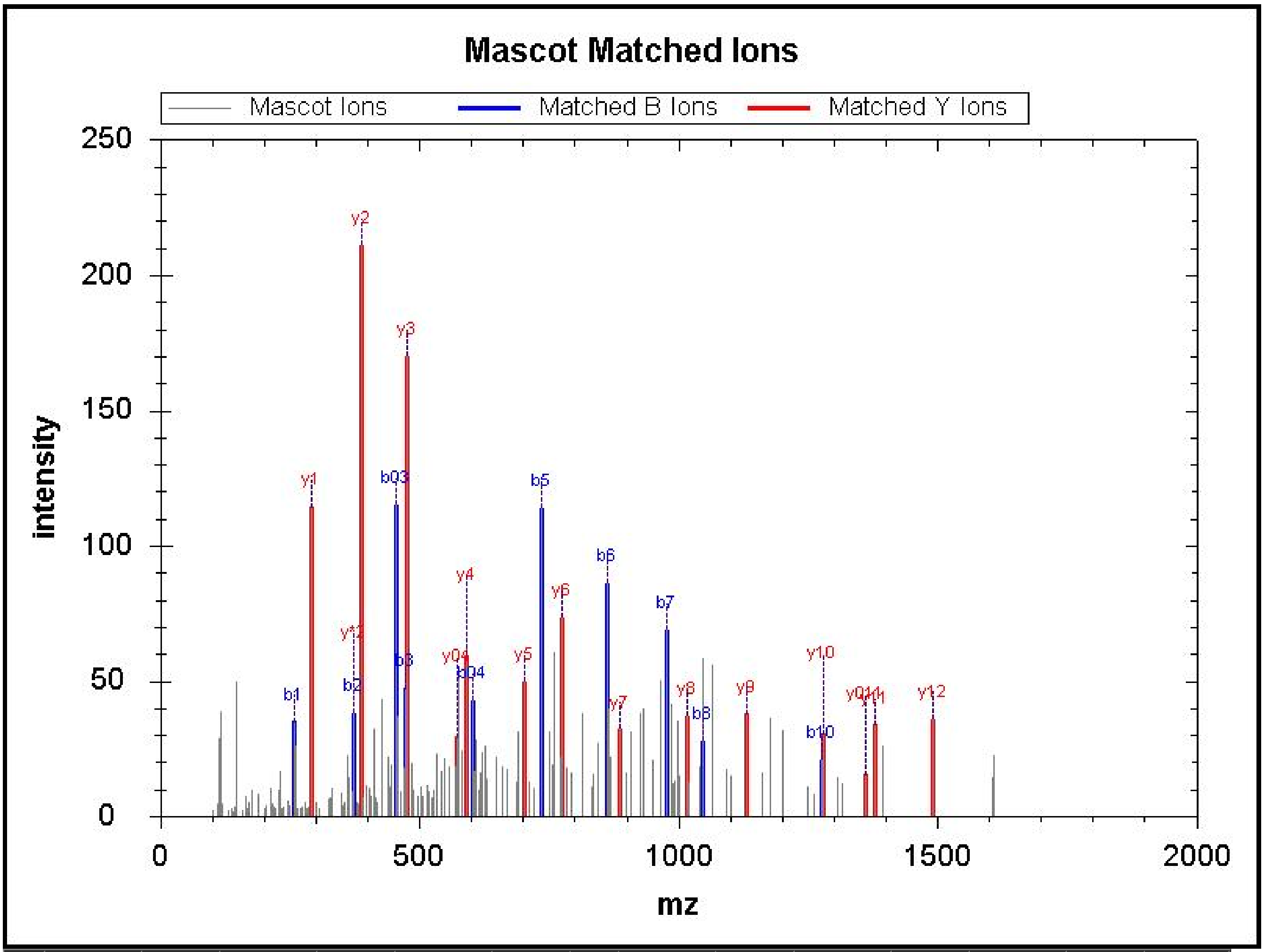

No	b	b++	b*	b*++	b0	b0++	Seq	y	y++	y*	y*++	y0	y0++	RevNo
1	**258.19**	129.60					I							13
2	**371.28**	186.14					L	**1,491.82**	746.41	1,474.79	737.90	1,473.81	737.41	12
3	**472.33**	236.67			**454.31**	227.66	T	**1,378.73**	689.87	1,361.71	681.36	**1,360.72**	680.87	11
4	619.39	310.20			**601.38**	301.20	F	**1,277.69**	639.35	1,260.66	630.83	1,259.68	630.34	10
5	**734.42**	367.71			716.41	358.71	D	**1,130.62**	565.81	1,113.59	557.30	1,112.61	556.81	9
6	**862.48**	431.74	845.45	423.23	844.47	422.74	Q	**1,015.59**	508.30	998.56	499.79	997.58	499.29	8
7	**975.56**	488.29	958.54	479.77	957.55	479.28	L	**887.53**	444.27	870.51	435.76	869.52	435.26	7
8	**1,046.60**	523.80	1,029.57	515.29	1,028.59	514.80	A	**774.45**	387.73	757.42	379.21	756.44	378.72	6
9	1,159.68	580.35	1,142.66	571.83	1,141.67	571.34	L	**703.41**	352.21	686.38	343.70	685.40	343.20	5
10	**1,274.71**	637.86	1,257.68	629.35	1,256.70	628.85	D	**590.33**	295.67	573.30	287.15	**572.32**	286.66	4
11	1,361.74	681.38	1,344.72	672.86	1,343.73	672.37	S	**475.30**	238.15	458.27	229.64	457.29	229.15	3
12	1,458.80	729.90	1,441.77	721.39	1,440.79	720.90	P	**388.27**	194.64	**371.24**	186.12			2
13							K	**291.21**	146.11	274.19	137.60			1

Query 1866 Hit 1

MS/MS Fragmentation of **LLNNAFEELVAFQR**
Found in **sp|P25205|MCM3_HUMAN**, DNA replication licensing factor MCM3 OS=Homo sapiens GN=MCM3 PE=1 SV=3
Match to Query 1866: 1806.977from(904.4958,2+)
Title: 1008: Sum of 2 scans in range 2629 (rt=67.229, f=4, i=706) to 2630 (rt=67.2544, f=4, i=707) [D:\lab212\membrane\Grace\20120305-iTRAQ-1.raw]
Data File:
Monoisotopic mass of neutral peptide Mr(calc): 1806.977
Variable modifications:
Ions Score: 69.62 **Expect:** 0.000

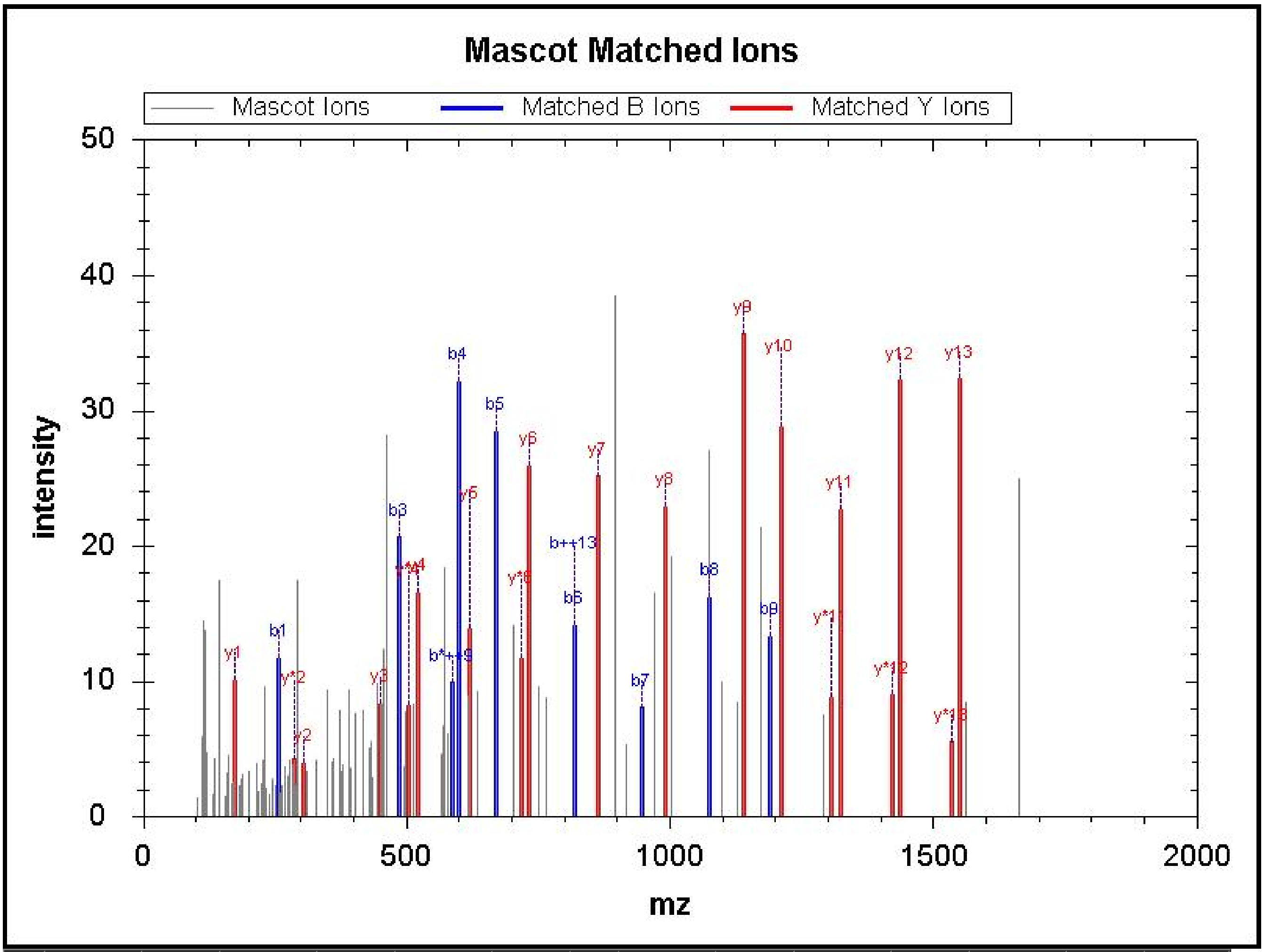

No	b	b++	b*	b*++	b0	b0++	Seq	y	y++	y*	y*++	y0	y0++	RevNo
1	**258.19**	129.60					L							14
2	371.28	186.14					L	**1,550.80**	775.90	**1,533.77**	767.39	1,532.79	766.90	13
3	**485.32**	243.16	468.29	234.65			N	**1,437.71**	719.36	**1,420.69**	710.85	1,419.70	710.35	12
4	**599.36**	300.19	582.34	291.67			N	**1,323.67**	662.34	**1,306.64**	653.83	1,305.66	653.33	11
5	**670.40**	335.70	653.37	327.19			A	**1,209.63**	605.32	1,192.60	596.80	1,191.62	596.31	10
6	**817.47**	409.24	800.44	400.72			F	**1,138.59**	569.80	1,121.56	561.28	1,120.58	560.79	9
7	**946.51**	473.76	929.48	465.25	928.50	464.75	E	**991.52**	496.26	974.49	487.75	973.51	487.26	8
8	**1,075.55**	538.28	1,058.53	529.77	1,057.54	529.28	E	**862.48**	431.74	845.45	423.23	844.47	422.74	7
9	**1,188.64**	594.82	1,171.61	**586.31**	1,170.63	585.82	L	**733.44**	367.22	**716.41**	358.71			6
10	1,287.71	644.36	1,270.68	635.84	1,269.70	635.35	V	**620.35**	310.68	603.32	302.17			5
11	1,358.74	679.88	1,341.72	671.36	1,340.73	670.87	A	**521.28**	261.15	**504.26**	252.63			4
12	1,505.81	753.41	1,488.79	744.90	1,487.80	744.40	F	**450.25**	225.63	433.22	217.11			3
13	1,633.87	**817.44**	1,616.84	808.93	1,615.86	808.43	Q	**303.18**	152.09	**286.15**	143.58			2
14							R	**175.12**	88.06	158.09	79.55			1

Query 2474 Hit 1

MS/MS Fragmentation of **TWWNQFSVTALQLLQANR**
Found in **sp|Q99536|VAT1_HUMAN**, Synaptic vesicle membrane protein VAT-1 homolog OS=Homo sapiens GN=VAT1 PE=1 SV=2
Match to Query 2474: 2320.234from(774.4186,3+)
Title: 1223: Scan 2971 (rt=75.3806, f=3, i=428) [D:\lab212\membrane\Grace\20120305-iTRAQ-2.raw]
Data File:
Monoisotopic mass of neutral peptide Mr(calc): 2320.234
Variable modifications:
Q5 :Deamidated (NQ)
Ions Score: 66.23 **Expect:** 0.000

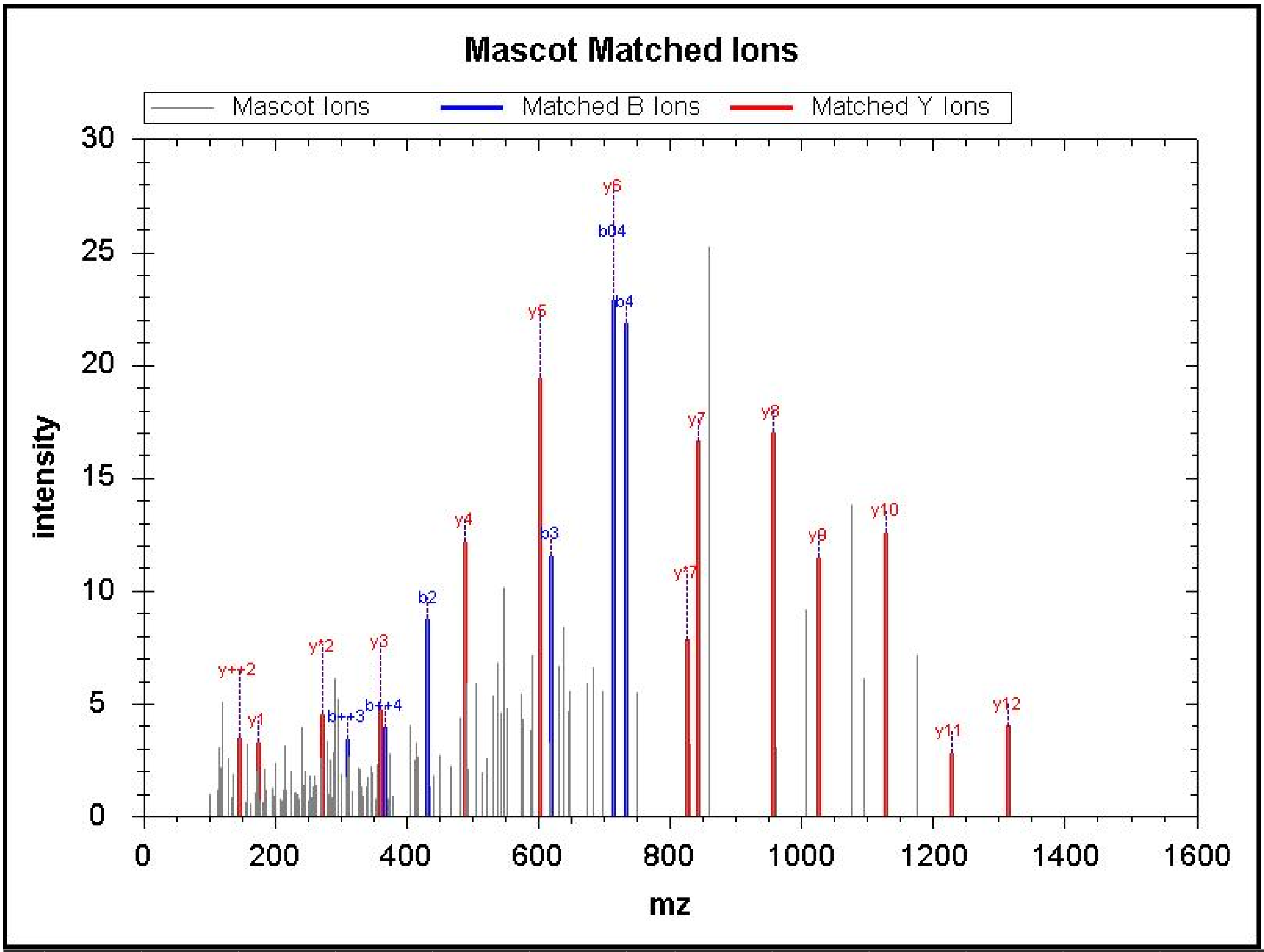

No	b	b++	b*	b*++	b0	b0++	Seq	y	y++	y*	y*++	y0	y0++	RevNo
1	246.16	123.58			228.15	114.58	T							18
2	432.24	216.62			414.23	207.62	W	2,076.07	1,038.54	2,059.04	1,030.02	2,058.06	1,029.53	17
3	618.32	309.66			600.31	300.66	W	1,889.99	945.50	1,872.96	936.98	1,871.98	936.49	16
4	732.36	366.68	715.33	358.17	714.35	357.68	N	1,703.91	852.46	1,686.88	843.94	1,685.90	843.45	15
5	861.40	431.20	844.37	422.69	843.39	422.20	Q	1,589.86	795.44	1,572.84	786.92	1,571.85	786.43	14
6	1,008.47	504.74	991.44	496.23	990.46	495.73	F	1,460.82	730.91	1,443.80	722.40	1,442.81	721.91	13
7	1,095.50	548.25	1,078.48	539.74	1,077.49	539.25	S	1,313.75	657.38	1,296.73	648.87	1,295.74	648.38	12
8	1,194.57	597.79	1,177.54	589.28	1,176.56	588.78	V	1,226.72	613.86	1,209.69	605.35	1,208.71	604.86	11
9	1,295.62	648.31	1,278.59	639.80	1,277.61	639.31	T	1,127.65	564.33	1,110.63	555.82	1,109.64	555.32	10
10	1,366.65	683.83	1,349.63	675.32	1,348.64	674.83	A	1,026.61	513.81	1,009.58	505.29			9
11	1,479.74	740.37	1,462.71	731.86	1,461.73	731.37	L	955.57	478.29	938.54	469.77			8
12	1,607.80	804.40	1,590.77	795.89	1,589.79	795.40	Q	842.48	421.75	825.46	413.23			7
13	1,720.88	860.94	1,703.86	852.43	1,702.87	851.94	L	714.43	357.72	697.40	349.20			6
14	1,833.97	917.49	1,816.94	908.97	1,815.96	908.48	L	601.34	301.17	584.32	292.66			5
15	1,962.02	981.52	1,945.00	973.00	1,944.01	972.51	Q	488.26	244.63	471.23	236.12			4
16	2,033.06	1,017.03	2,016.03	1,008.52	2,015.05	1,008.03	A	360.20	180.60	343.17	172.09			3
17	2,147.10	1,074.06	2,130.08	1,065.54	2,129.09	1,065.05	N	289.16	145.08	272.14	136.57			2
18							R	175.12	88.06	158.09	79.55			1

Query 995 Hit 1

MS/MS Fragmentation of **LITEDVQGK**
Found in **sp|P61247|RS3A_HUMAN**, 40S ribosomal protein S3a OS=Homo sapiens GN=RPS3A PE=1 SV=2
Match to Query 995: 1289.75from(645.8821,2+)

Title: 323: Sum of 2 scans in range 1147 (rt=33.8356, f=4, i=228) to 1148 (rt=33.861, f=4, i=229) [D:\lab212\membrane\Grace\20120305-iTRAQ-1.raw]
Data File:
Monoisotopic mass of neutral peptide Mr(calc): 1289.75
Variable modifications:
K9 :iTRAQ4plex (K)
Ions Score: 66.2 **Expect:** 0.000

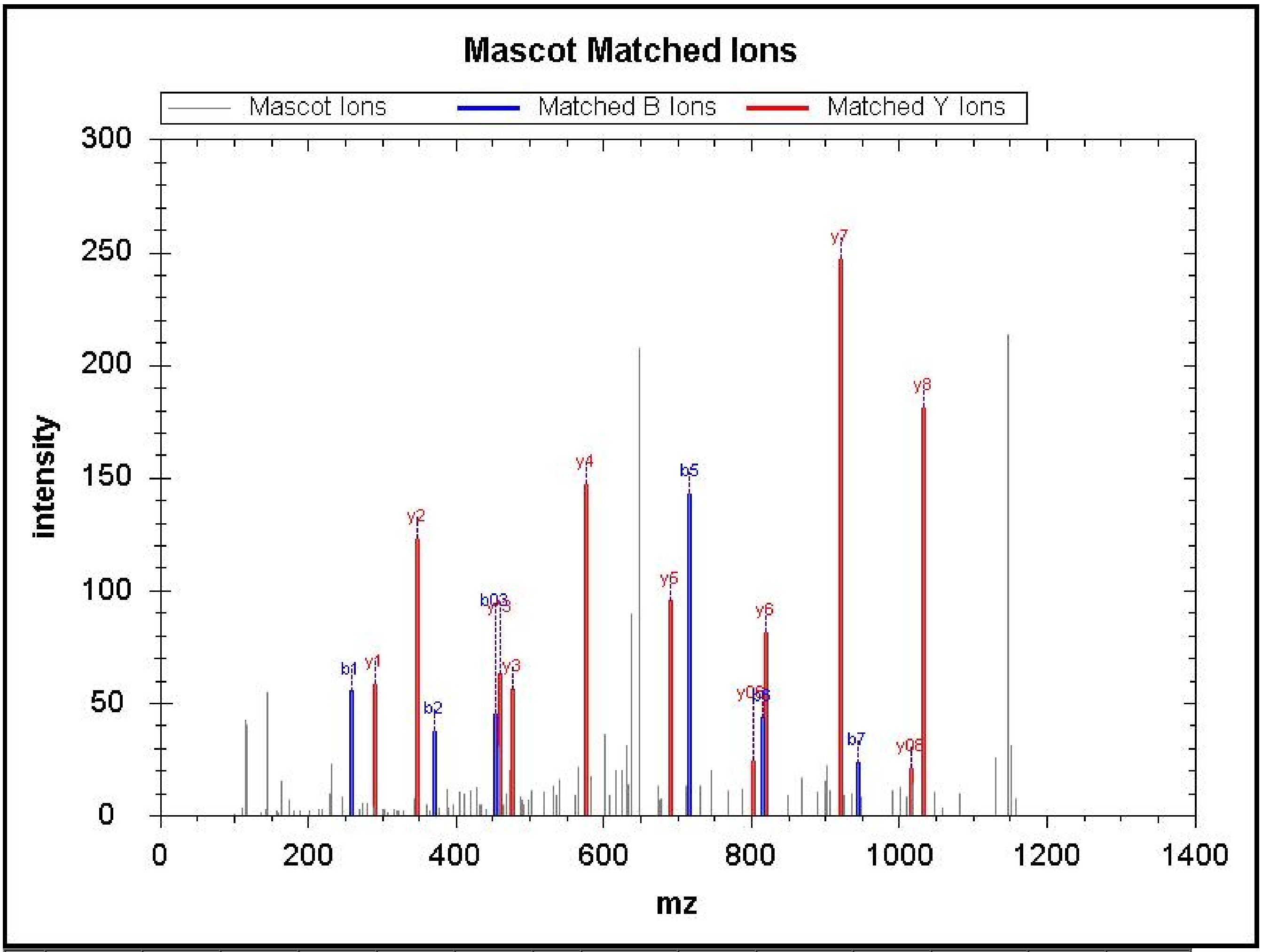

No	b	b++	b*	b*++	b0	b0++	Seq	y	y++	y*	y*++	y0	y0++	RevNo
1	258.19	129.60					L							9
2	371.28	186.14					I	1,033.56	517.29	1,016.54	508.77	1,015.55	508.28	8
3	472.33	236.67			454.31	227.66	T	920.48	460.74	903.45	452.23	902.47	451.74	7
4	601.37	301.19			583.36	292.18	E	819.43	410.22	802.41	401.71	801.42	401.21	6
5	716.39	358.70			698.38	349.70	D	690.39	345.70	673.36	337.19	672.38	336.69	5
6	815.46	408.24			797.45	399.23	V	575.36	288.19	558.34	279.67			4
7	943.52	472.26	926.50	463.75	925.51	463.26	Q	476.29	238.65	459.27	230.14			3
8	1,000.54	500.78	983.52	492.26	982.53	491.77	G	348.24	174.62	331.21	166.11			2
9							K	291.21	146.11	274.19	137.60			1

Query 2490 Hit 1

MS/MS Fragmentation of **LASEILMQNWDAAMEDLTR**
Found in **sp|P60228|EIF3E_HUMAN**, Eukaryotic translation initiation factor 3 subunit E OS=Homo sapiens GN=EIF3E PE=1 SV=1
Match to Query 2490: 2350.149from(784.3903,3+)
Title: 1190: Sum of 2 scans in range 3045 (rt=76.6007, f=4, i=840) to 3046 (rt=76.6262, f=4, i=841) [D:\lab212\membrane\Grace\20120305-iTRAQ-1.raw]
Data File:
Monoisotopic mass of neutral peptide Mr(calc): 2350.149
Variable modifications:
Ions Score: 66.06 **Expect:** 0.000

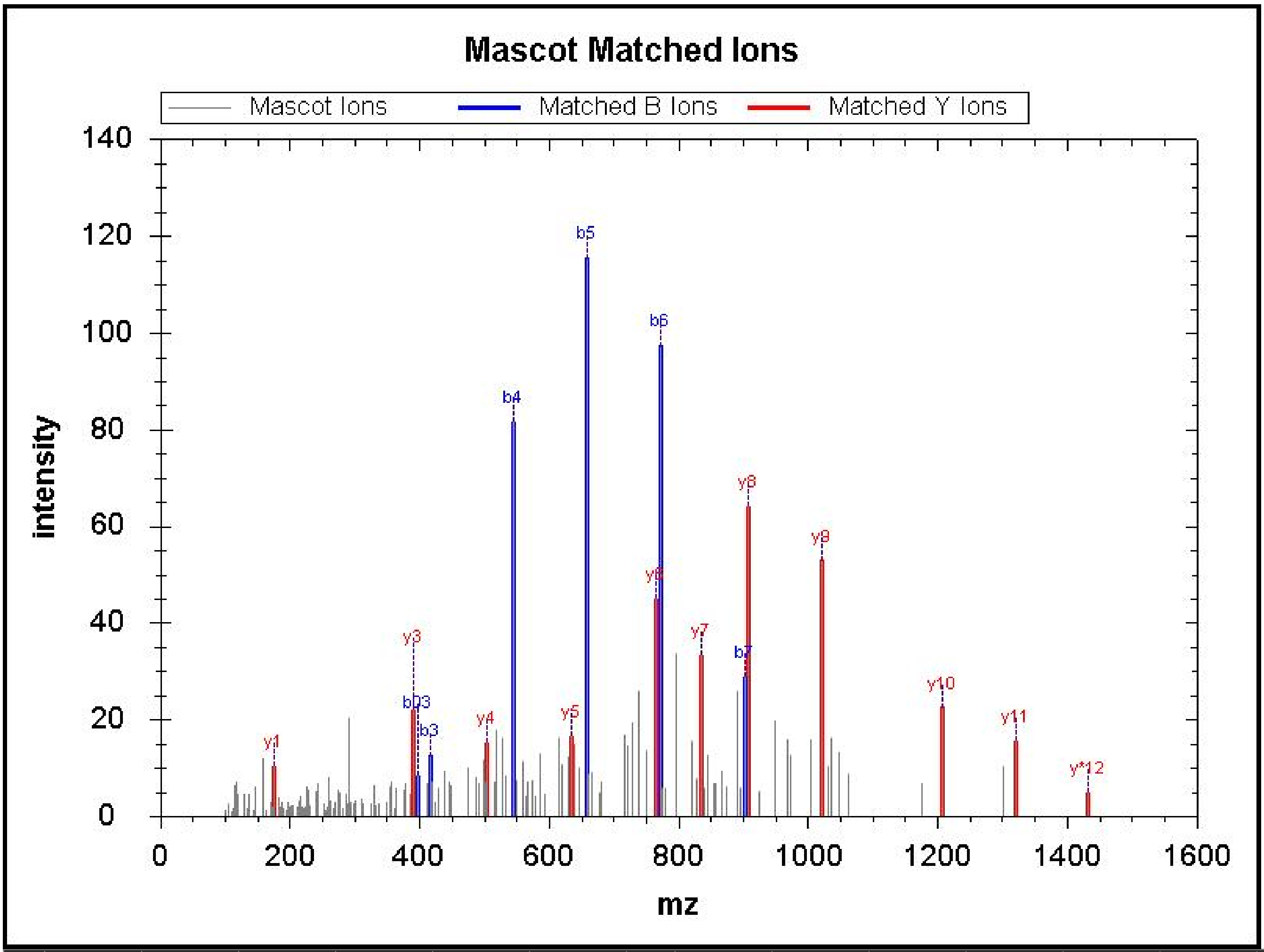

No	b	b++	b*	b*++	b0	b0++	Seq	y	y++	y*	y*++	y0	y0++	RevNo
1	258.19	129.60					L							19
2	329.23	165.12					A	2,093.96	1,047.49	2,076.94	1,038.97	2,075.95	1,038.48	18
3	**416.26**	208.63			**398.25**	199.63	S	2,022.93	1,011.97	2,005.90	1,003.45	2,004.92	1,002.96	17
4	**545.31**	273.16			527.29	264.15	E	1,935.89	968.45	1,918.87	959.94	1,917.88	959.45	16
5	**658.39**	329.70			640.38	320.69	I	1,806.85	903.93	1,789.82	895.42	1,788.84	894.92	15
6	**771.47**	386.24			753.46	377.23	L	1,693.77	847.39	1,676.74	838.87	1,675.76	838.38	14
7	**902.51**	451.76			884.50	442.76	M	1,580.68	790.85	1,563.66	782.33	1,562.67	781.84	13
8	1,030.57	515.79	1,013.55	507.28	1,012.56	506.78	Q	1,449.64	725.33	**1,432.62**	716.81	1,431.63	716.32	12
9	1,144.62	572.81	1,127.59	564.30	1,126.60	563.81	N	**1,321.58**	661.30	1,304.56	652.78	1,303.57	652.29	11
10	1,330.69	665.85	1,313.67	657.34	1,312.68	656.85	W	**1,207.54**	604.27	1,190.51	595.76	1,189.53	595.27	10
11	1,445.72	723.36	1,428.70	714.85	1,427.71	714.36	D	**1,021.46**	511.23	1,004.44	502.72	1,003.45	502.23	9
12	1,516.76	758.88	1,499.73	750.37	1,498.75	749.88	A	**906.43**	453.72	889.41	445.21	888.42	444.72	8
13	1,587.80	794.40	1,570.77	785.89	1,569.79	785.40	A	**835.40**	418.20	818.37	409.69	817.39	409.20	7
14	1,718.84	859.92	1,701.81	851.41	1,700.83	850.92	M	**764.36**	382.68	747.33	374.17	746.35	373.68	6
15	1,847.88	924.44	1,830.85	915.93	1,829.87	915.44	E	**633.32**	317.16	616.29	308.65	615.31	308.16	5
16	1,962.91	981.96	1,945.88	973.44	1,944.90	972.95	D	**504.28**	252.64	487.25	244.13	486.27	243.64	4
17	2,075.99	1,038.50	2,058.96	1,029.99	2,057.98	1,029.49	L	**389.25**	195.13	372.22	186.62	371.24	186.12	3
18	2,177.04	1,089.02	2,160.01	1,080.51	2,159.03	1,080.02	T	276.17	138.59	259.14	130.07	258.16	129.58	2
19							R	**175.12**	88.06	158.09	79.55			1

Query 1341 Hit 1

MS/MS Fragmentation of **TLNNDIMLIK**
Found in **sp|P07477|TRY1_HUMAN**, Trypsin-1 OS=Homo sapiens GN=PRSS1 PE=1 SV=1

Match to Query 1341: 1462.833from(732.4239,2+)
Title: 692: Sum of 2 scans in range 1819 (rt=49.4253, f=4, i=478) to 1820 (rt=49.4507, f=4, i=479) [D:\lab212\membrane\Grace\20120305-iTRAQ-2.raw]
Data File:
Monoisotopic mass of neutral peptide Mr(calc): 1462.833
Variable modifications:
N3 :Deamidated (NQ)
K10 :iTRAQ4plex (K)
Ions Score: 64.81 **Expect:** 0.000

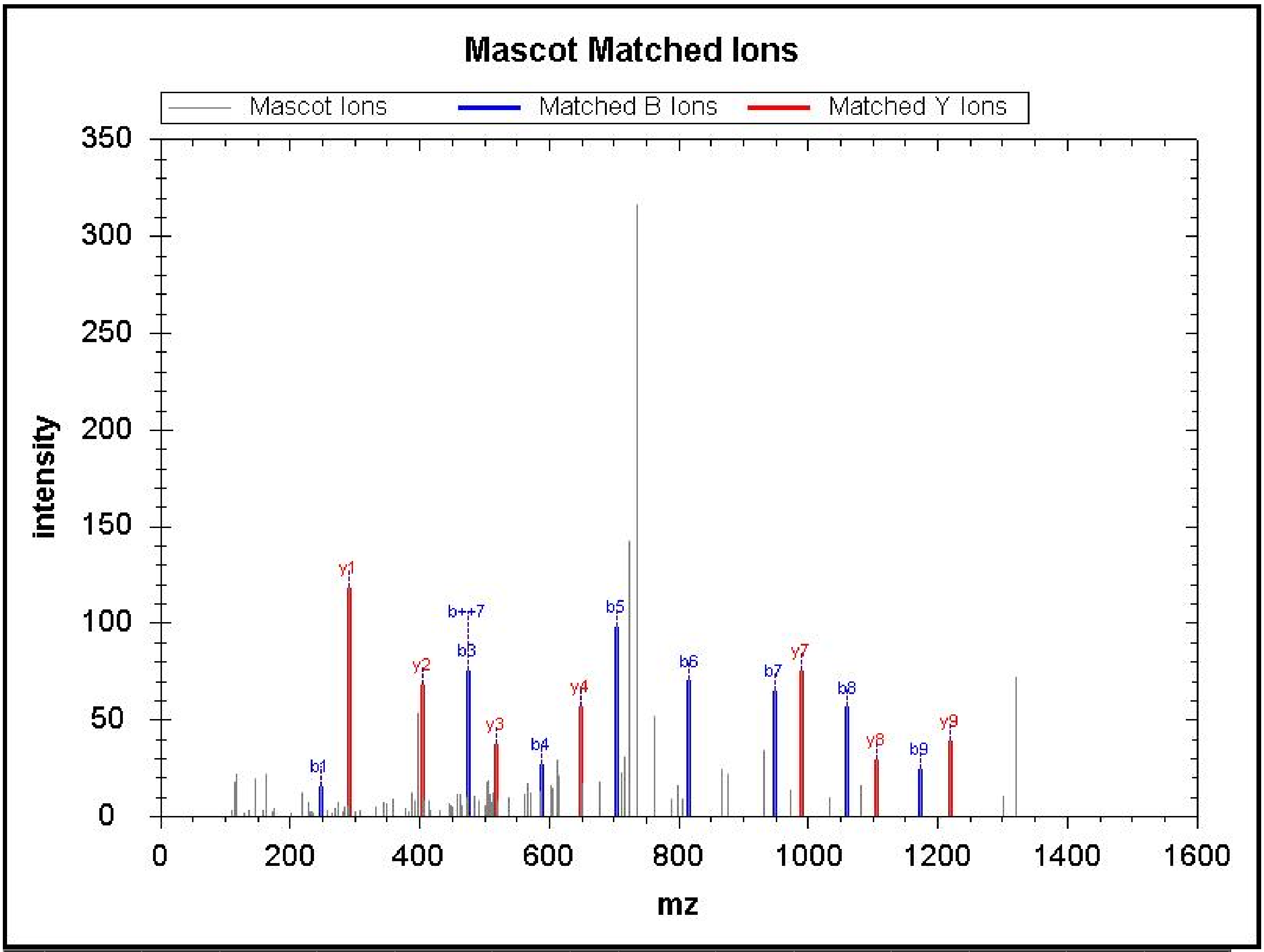

No	b	b++	b*	b*++	b0	b0++	Seq	y	y++	y*	y*++	y0	y0++	RevNo
1	246.16	123.58			228.15	114.58	T							10
2	359.24	180.12			341.23	171.12	L	1,218.69	609.85	1,201.66	601.33	1,200.68	600.84	9
3	474.27	237.64	457.24	229.12	456.26	228.63	N	1,105.60	553.31	1,088.58	544.79	1,087.59	544.30	8
4	588.31	294.66	571.28	286.15	570.30	285.65	N	990.58	495.79	973.55	487.28	972.57	486.79	7
5	703.34	352.17	686.31	343.66	685.33	343.17	D	876.53	438.77	859.51	430.26	858.52	429.77	6
6	816.42	408.71	799.40	400.20	798.41	399.71	I	761.51	381.26	744.48	372.74			5
7	947.46	474.23	930.44	465.72	929.45	465.23	M	648.42	324.72	631.40	316.20			4
8	1,060.55	530.78	1,043.52	522.26	1,042.54	521.77	L	517.38	259.20	500.36	250.68			3
9	1,173.63	587.32	1,156.60	578.81	1,155.62	578.31	I	404.30	202.65	387.27	194.14			2
10							K	291.21	146.11	274.19	137.60			1

Query 2212 Hit 1

MS/MS Fragmentation of **VLGILAMIDEGETDWK**
Found in **sp|Q15181|IPYR_HUMAN**, Inorganic pyrophosphatase OS=Homo sapiens GN=PPA1 PE=1 SV=2
Match to Query 2212: 2077.115from(693.3789,3+)
Title: 1121: Sum of 2 scans in range 2871 (rt=72.6813, f=4, i=784) to 2872 (rt=72.7067, f=4, i=785) [D:\lab212\membrane\Grace\20120305-iTRAQ-1.raw]

Data File:
Monoisotopic mass of neutral peptide Mr(calc): 2077.115
Variable modifications:
K16 :iTRAQ4plex (K)
Ions Score: 62.8 **Expect:** 0.000

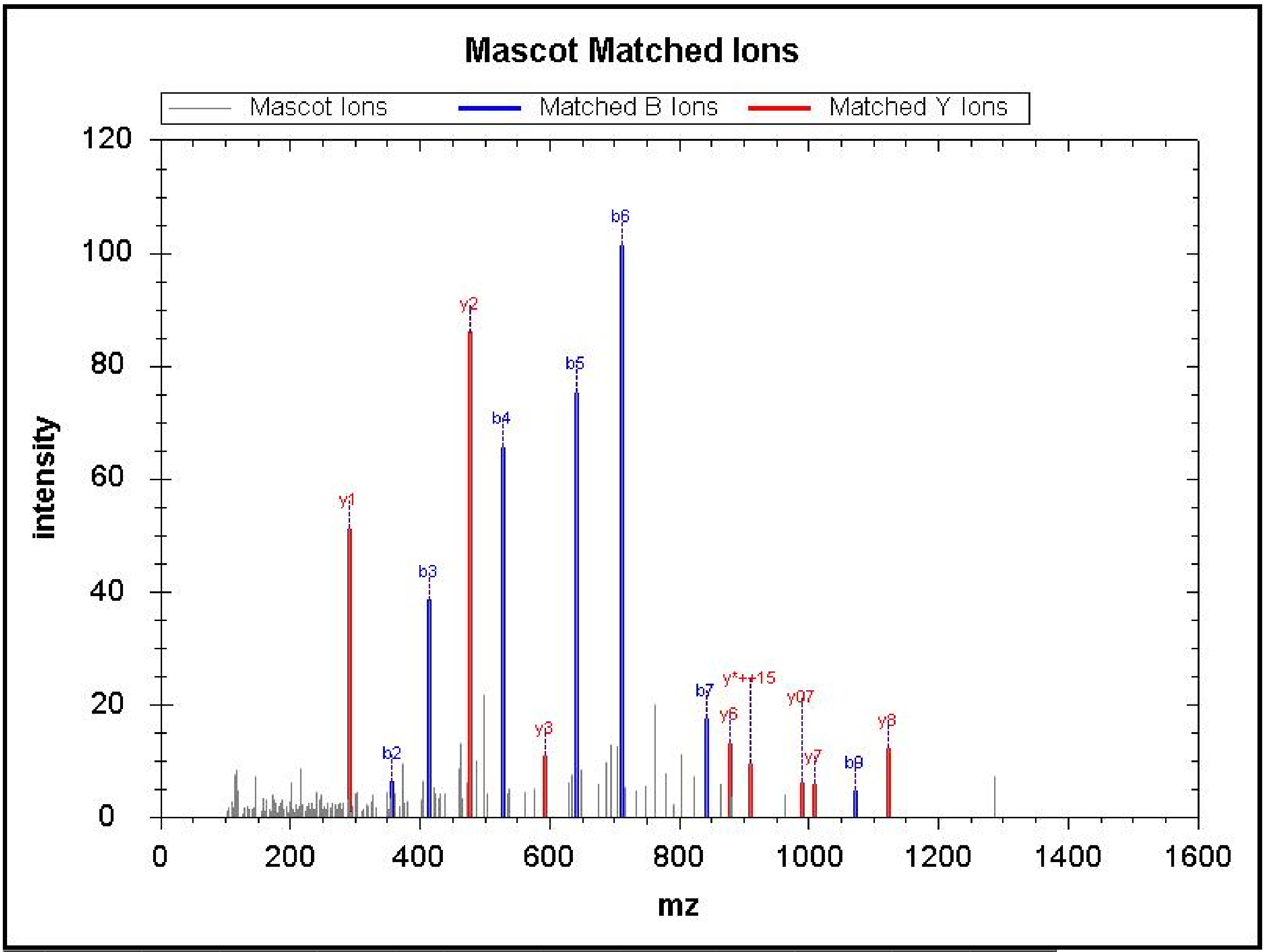

No	b	b++	b0	b0++	Seq	y	y++	y*	y*++	y0	y0++	RevNo
1	244.18	122.59			V							16
2	357.26	179.13			L	1,834.94	917.97	1,817.91	909.46	1,816.93	908.97	15
3	414.28	207.65			G	1,721.85	861.43	1,704.83	852.92	1,703.84	852.43	14
4	527.37	264.19			I	1,664.83	832.92	1,647.81	824.41	1,646.82	823.91	13
5	640.45	320.73			L	1,551.75	776.38	1,534.72	767.86	1,533.74	767.37	12
6	711.49	356.25			A	1,438.66	719.84	1,421.64	711.32	1,420.65	710.83	11
7	842.53	421.77			M	1,367.63	684.32	1,350.60	675.80	1,349.62	675.31	10
8	955.61	478.31			I	1,236.59	618.80	1,219.56	610.28	1,218.58	609.79	9
9	1,070.64	535.82	1,052.63	526.82	D	1,123.50	562.25	1,106.48	553.74	1,105.49	553.25	8
10	1,199.68	600.34	1,181.67	591.34	E	1,008.48	504.74	991.45	496.23	990.46	495.74	7
11	1,256.70	628.86	1,238.69	619.85	G	879.43	440.22	862.41	431.71	861.42	431.21	6
12	1,385.75	693.38	1,367.74	684.37	E	822.41	411.71	805.38	403.20	804.40	402.70	5
13	1,486.79	743.90	1,468.78	734.90	T	693.37	347.19	676.34	338.67	675.36	338.18	4
14	1,601.82	801.41	1,583.81	792.41	D	592.32	296.66	575.29	288.15	574.31	287.66	3
15	1,787.90	894.45	1,769.89	885.45	W	477.29	239.15	460.27	230.64			2
16					K	291.21	146.11	274.19	137.60			1

Query 2416 Hit 1

MS/MS Fragmentation of **LYSTWIGGSILASLDTFK**

Found in sp|P42025|ACTY_HUMAN, Beta-centractin OS=Homo sapiens GN=ACTR1B PE=1 SV=1
Match to Query 2416: 2259.252from(754.0912,3+)
Title: 1241: Sum of 2 scans in range 3009 (rt=76.2404, f=4, i=862) to 3010 (rt=76.2658, f=4, i=863) [D:\lab212\membrane\Grace\20120305-iTRAQ-2.raw]
Data File:
Monoisotopic mass of neutral peptide Mr(calc): 2259.252
Variable modifications:
K18 :iTRAQ4plex (K)
Ions Score: 62.63 **Expect:** 0.000

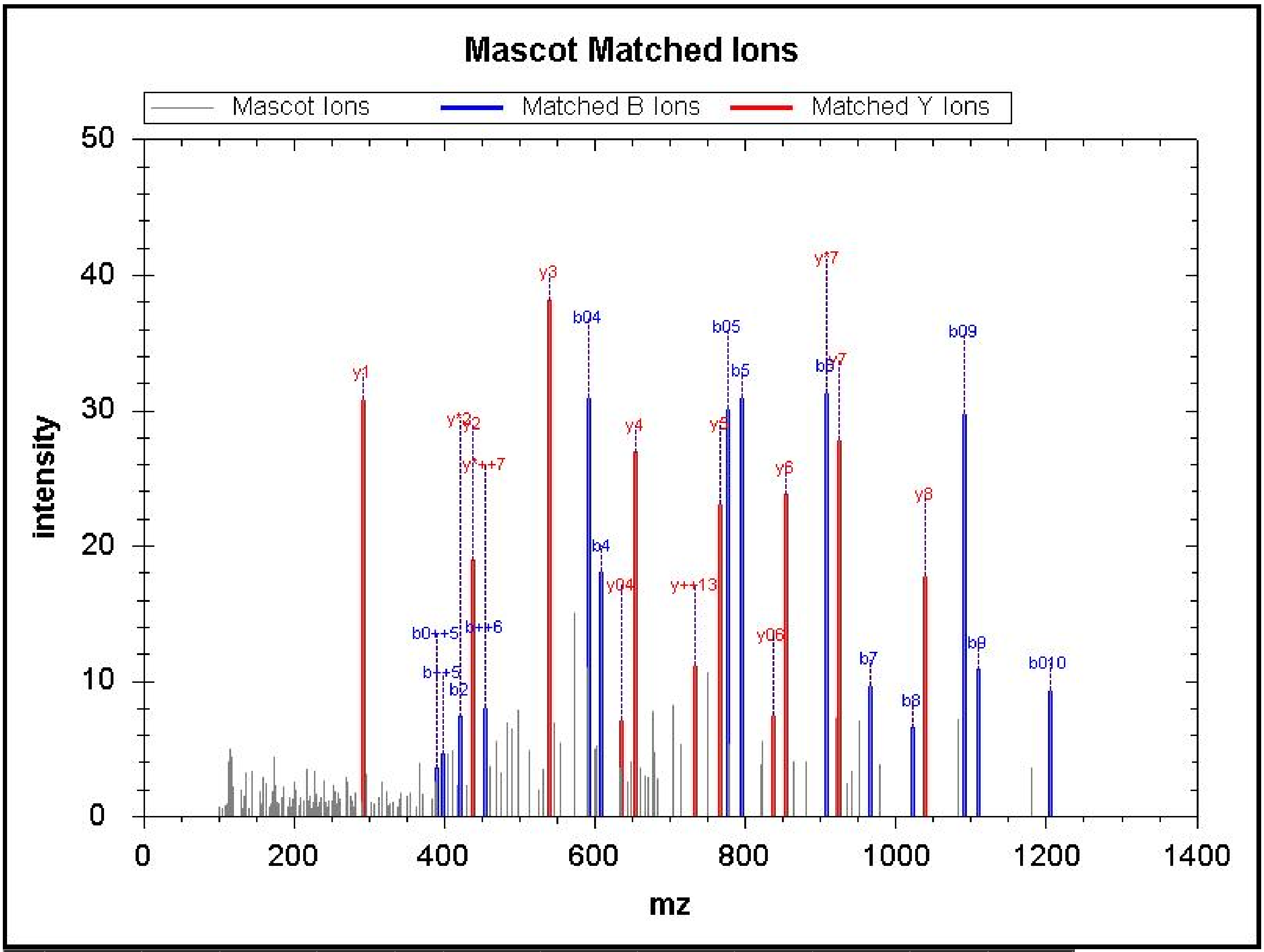

No	b	b++	b0	b0++	Seq	y	y++	y*	y*++	y0	y0++	RevNo
1	258.19	129.60			L							18
2	421.26	211.13			Y	2,003.06	1,002.03	1,986.03	993.52	1,985.05	993.03	17
3	508.29	254.65	490.28	245.64	S	1,840.00	920.50	1,822.97	911.99	1,821.99	911.50	16
4	609.34	305.17	591.33	296.17	T	1,752.97	876.99	1,735.94	868.47	1,734.95	867.98	15
5	795.42	398.21	777.41	389.21	W	1,651.92	826.46	1,634.89	817.95	1,633.91	817.46	14
6	908.50	454.75	890.49	445.75	I	1,465.84	733.42	1,448.81	724.91	1,447.83	724.42	13
7	965.52	483.26	947.51	474.26	G	1,352.75	676.88	1,335.73	668.37	1,334.74	667.88	12
8	1,022.54	511.77	1,004.53	502.77	G	1,295.73	648.37	1,278.71	639.86	1,277.72	639.36	11
9	1,109.57	555.29	1,091.56	546.29	S	1,238.71	619.86	1,221.68	611.35	1,220.70	610.85	10
10	1,222.66	611.83	1,204.65	602.83	I	1,151.68	576.34	1,134.65	567.83	1,133.67	567.34	9
11	1,335.74	668.38	1,317.73	659.37	L	1,038.60	519.80	1,021.57	511.29	1,020.58	510.80	8
12	1,406.78	703.89	1,388.77	694.89	A	925.51	463.26	908.48	454.75	907.50	454.25	7
13	1,493.81	747.41	1,475.80	738.40	S	854.47	427.74	837.45	419.23	836.46	418.74	6
14	1,606.90	803.95	1,588.89	794.95	L	767.44	384.22	750.42	375.71	749.43	375.22	5
15	1,721.92	861.47	1,703.91	852.46	D	654.36	327.68	637.33	319.17	636.35	318.68	4
16	1,822.97	911.99	1,804.96	902.98	T	539.33	270.17	522.30	261.66	521.32	261.16	3

**17**	1,970.04	985.52	1,952.03	976.52	**F**	**438.28**	219.65	**421.26**	211.13			**2**
**18**					**K**	**291.21**	146.11	274.19	137.60			**1**

Query 2116 Hit 1

MS/MS Fragmentation of **ALFEEVPELLTEAEK**
Found in **sp|P31939|PUR9_HUMAN**, Bifunctional purine biosynthesis protein PURH OS=Homo sapiens GN=ATIC PE=1 SV=3
Match to Query 2116: 2005.098from(669.3732,3+)
Title: 1028: Sum of 2 scans in range 2544 (rt=65.7625, f=4, i=712) to 2545 (rt=65.7879, f=4, i=713) [D:\lab212\membrane\Grace\20120305-iTRAQ-2.raw]
Data File:
Monoisotopic mass of neutral peptide Mr(calc): 2005.098
Variable modifications:
K15 :iTRAQ4plex (K)
Ions Score: 62.54 **Expect:** 0.000

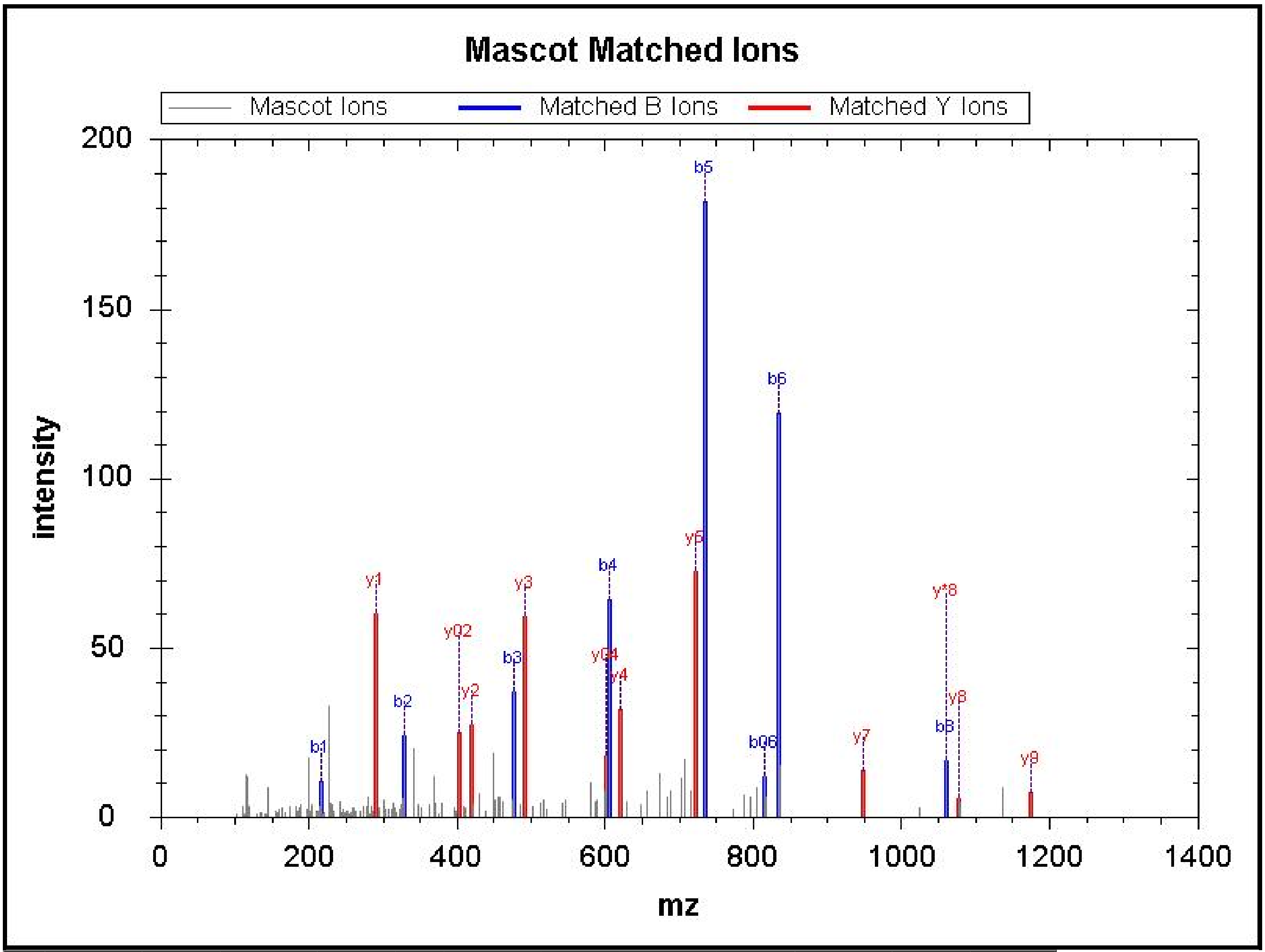

No	b	b++	b0	b0++	Seq	y	y++	y*	y*++	y0	y0++	RevNo
**1**	**216.15**	108.58			**A**							**15**
**2**	**329.23**	165.12			**L**	1,790.95	895.98	1,773.93	887.47	1,772.94	886.98	**14**
**3**	**476.30**	238.65			**F**	1,677.87	839.44	1,660.84	830.93	1,659.86	830.43	**13**
**4**	**605.34**	303.17	587.33	294.17	**E**	1,530.80	765.90	1,513.78	757.39	1,512.79	756.90	**12**
**5**	**734.38**	367.70	716.37	358.69	**E**	1,401.76	701.38	1,384.73	692.87	1,383.75	692.38	**11**
**6**	**833.45**	417.23	**815.44**	408.22	**V**	1,272.72	636.86	1,255.69	628.35	1,254.71	627.86	**10**
**7**	930.51	465.76	912.49	456.75	**P**	**1,173.65**	587.33	1,156.62	578.81	1,155.64	578.32	**9**
**8**	**1,059.55**	530.28	1,041.54	521.27	**E**	**1,076.60**	538.80	**1,059.57**	530.29	1,058.58	529.80	**8**
**9**	1,172.63	586.82	1,154.62	577.81	**L**	**947.55**	474.28	930.53	465.77	929.54	465.27	**7**
**10**	1,285.72	643.36	1,267.71	634.36	**L**	834.47	417.74	817.44	409.22	816.46	408.73	**6**
**11**	1,386.76	693.89	1,368.75	684.88	**T**	**721.38**	361.20	704.36	352.68	703.37	352.19	**5**

12	1,515.81	758.41	1,497.80	749.40	E	**620.34**	310.67	603.31	302.16	**602.33**	301.67	4
13	1,586.84	793.93	1,568.83	784.92	A	**491.29**	246.15	474.27	237.64	473.28	237.15	3
14	1,715.89	858.45	1,697.88	849.44	E	**420.26**	210.63	403.23	202.12	**402.25**	201.63	2
15					K	**291.21**	146.11	274.19	137.60			1

Query 1840 Hit 1

MS/MS Fragmentation of **HDADGQATLLNLLLR**
Found in **sp|O43242|PSMD3_HUMAN**, 26S proteasome non-ATPase regulatory subunit 3 OS=Homo sapiens GN=PSMD3 PE=1 SV=2
Match to Query 1840: 1792.999from(598.6736,3+)
Title: 946: Scan 2363 (rt=61.6818, f=3, i=330) [D:\lab212\membrane\Grace\20120305-iTRAQ-2.raw]
Data File:
Monoisotopic mass of neutral peptide Mr(calc): 1792.999
Variable modifications:
Ions Score: 60.84 **Expect:** 0.001

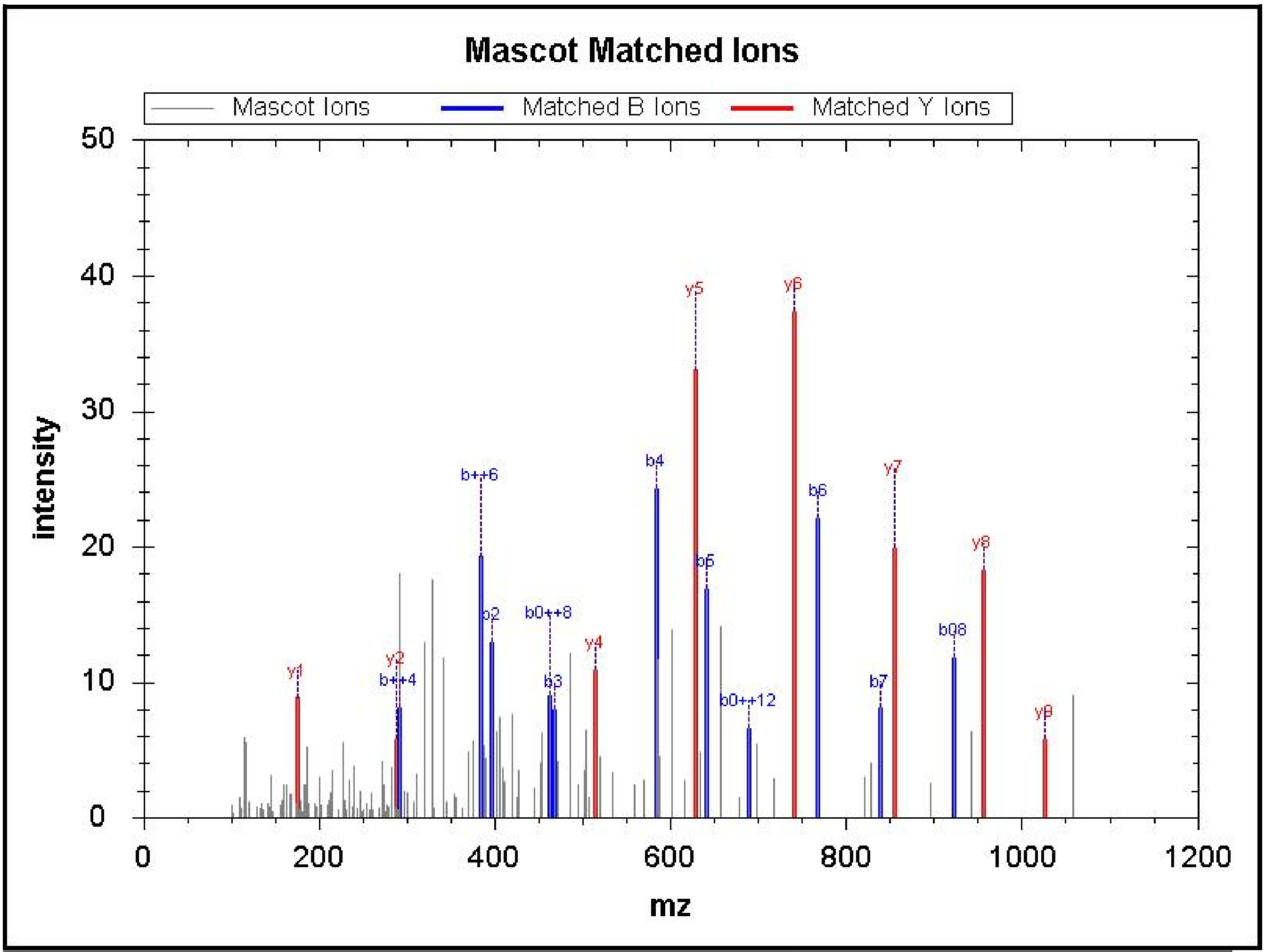

No	b	b++	b*	b*++	b0	b0++	Seq	y	y++	y*	y*++	y0	y0++	RevNo
1	282.17	141.59					H							15
2	**397.20**	199.10			379.18	190.10	D	1,512.84	756.92	1,495.81	748.41	1,494.83	747.92	14
3	**468.23**	234.62			450.22	225.61	A	1,397.81	699.41	1,380.78	690.90	1,379.80	690.40	13
4	**583.26**	**292.13**			565.25	283.13	D	1,326.77	663.89	1,309.75	655.38	1,308.76	654.89	12
5	**640.28**	320.64			622.27	311.64	G	1,211.75	606.38	1,194.72	597.86	1,193.74	597.37	11
6	**768.34**	**384.67**	751.31	376.16	750.33	375.67	Q	1,154.73	577.87	1,137.70	569.35	1,136.71	568.86	10
7	**839.38**	420.19	822.35	411.68	821.37	411.19	A	**1,026.67**	513.84	1,009.64	505.32	1,008.66	504.83	9
8	940.42	470.72	923.40	462.20	**922.41**	**461.71**	T	**955.63**	478.32	938.60	469.81	937.62	469.31	8
9	1,053.51	527.26	1,036.48	518.74	1,035.50	518.25	L	**854.58**	427.79	837.56	419.28			7

10	1,166.59	583.80	1,149.57	575.29	1,148.58	574.79	L	741.50	371.25	724.47	362.74			6
11	1,280.64	640.82	1,263.61	632.31	1,262.62	631.82	N	628.41	314.71	611.39	306.20			5
12	1,393.72	697.36	1,376.69	688.85	1,375.71	688.36	L	514.37	257.69	497.34	249.18			4
13	1,506.80	753.91	1,489.78	745.39	1,488.79	744.90	L	401.29	201.15	384.26	192.63			3
14	1,619.89	810.45	1,602.86	801.93	1,601.88	801.44	L	288.20	144.61	271.18	136.09			2
15							R	175.12	88.06	158.09	79.55			1

Query 2576 Hit 1

MS/MS Fragmentation of **ELNELVSAIEEHFFQPQK**
Found in **sp|Q96G03|PGM2_HUMAN**, Phosphoglucomutase-2 OS=Homo sapiens GN=PGM2 PE=1 SV=4
Match to Query 2576: 2445.285from(816.1022,3+)
Title: 1152: Sum of 2 scans in range 2952 (rt=74.5052, f=4, i=810) to 2953 (rt=74.5306, f=4, i=811)
[D:\lab212\membrane\Grace\20120305-iTRAQ-1.raw]
Data File:
Monoisotopic mass of neutral peptide Mr(calc): 2445.285
Variable modifications:
K18 :iTRAQ4plex (K)
Ions Score: 60.54 **Expect:** 0.001

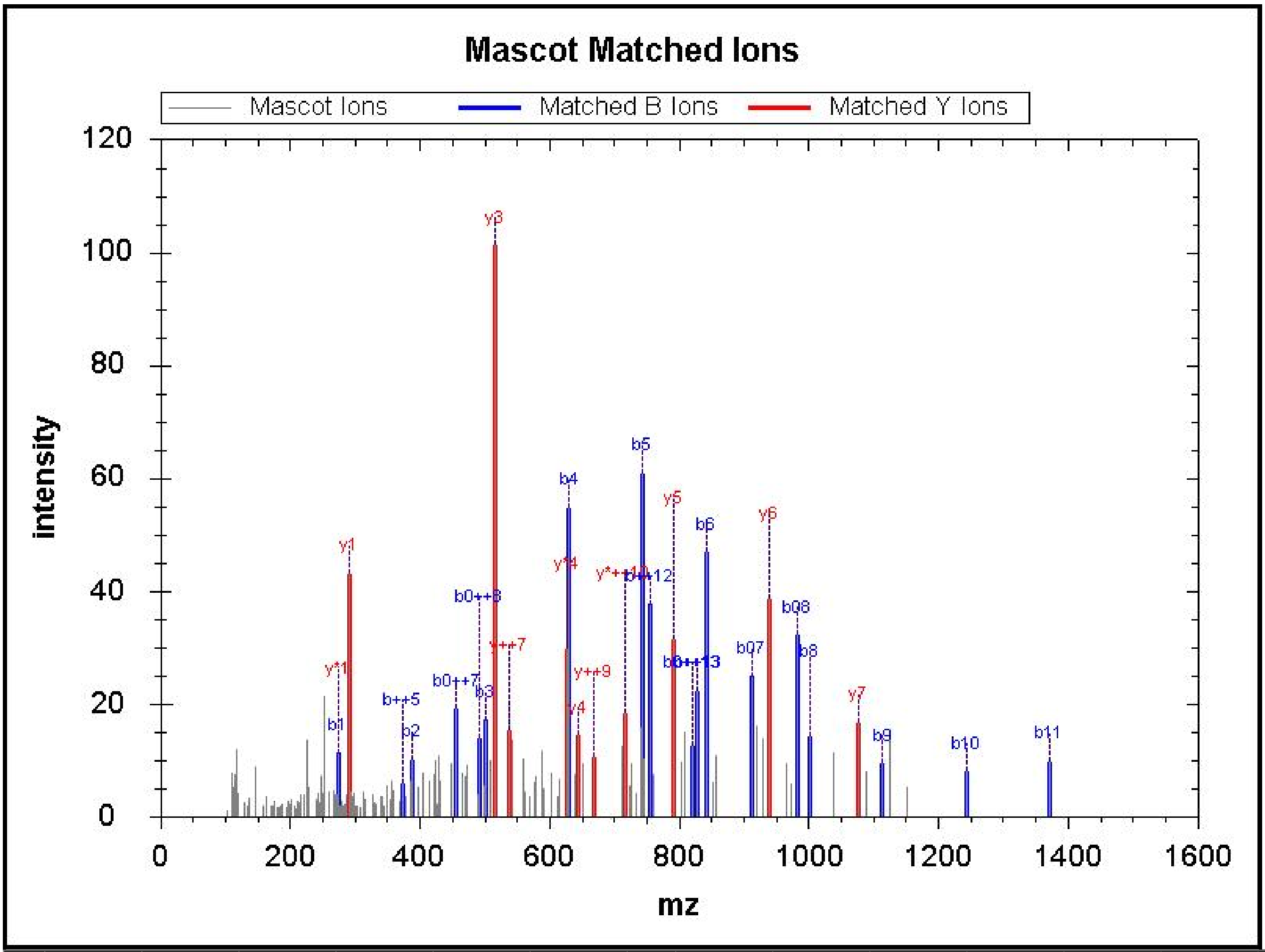

No	b	b++	b*	b*++	b0	b0++	Seq	y	y++	y*	y*++	y0	y0++	RevNo
1	274.15	137.58			256.14	128.57	E							18
2	387.24	194.12			369.23	185.12	L	2,173.14	1,087.07	2,156.11	1,078.56	2,155.13	1,078.07	17
3	501.28	251.14	484.25	242.63	483.27	242.14	N	2,060.06	1,030.53	2,043.03	1,022.02	2,042.05	1,021.53	16
4	630.32	315.66	613.29	307.15	612.31	306.66	E	1,946.01	973.51	1,928.99	965.00	1,928.00	964.51	15
5	743.41	372.21	726.38	363.69	725.39	363.20	L	1,816.97	908.99	1,799.94	900.48	1,798.96	899.98	14
6	842.47	421.74	825.45	413.23	824.46	412.74	V	1,703.89	852.45	1,686.86	843.93	1,685.88	843.44	13
7	929.51	465.26	912.48	456.74	911.50	456.25	S	1,604.82	802.91	1,587.79	794.40	1,586.81	793.91	12

8	1,000.54	500.78	983.52	492.26	982.53	491.77	A	1,517.79	759.40	1,500.76	750.88	1,499.78	750.39	11
9	1,113.63	557.32	1,096.60	548.80	1,095.62	548.31	I	1,446.75	723.88	1,429.72	715.37	1,428.74	714.87	10
10	1,242.67	621.84	1,225.64	613.33	1,224.66	612.83	E	1,333.67	667.34	1,316.64	658.82	1,315.66	658.33	9
11	1,371.71	686.36	1,354.69	677.85	1,353.70	677.35	E	1,204.62	602.82	1,187.60	594.30	1,186.61	593.81	8
12	1,508.77	754.89	1,491.74	746.38	1,490.76	745.88	H	1,075.58	538.29	1,058.55	529.78			7
13	1,655.84	828.42	1,638.81	819.91	1,637.83	819.42	F	938.52	469.76	921.50	461.25			6
14	1,802.91	901.96	1,785.88	893.44	1,784.90	892.95	F	791.45	396.23	774.43	387.72			5
15	1,930.97	965.99	1,913.94	957.47	1,912.96	956.98	Q	644.38	322.70	627.36	314.18			4
16	2,028.02	1,014.51	2,010.99	1,006.00	2,010.01	1,005.51	P	516.33	258.67	499.30	250.15			3
17	2,156.08	1,078.54	2,139.05	1,070.03	2,138.07	1,069.54	Q	419.27	210.14	402.25	201.63			2
18							K	291.21	146.11	274.19	137.60			1

Query 551 Hit 1

MS/MS Fragmentation of **VEEIAASK**
Found in **sp|Q02543|RL18A_HUMAN**, 60S ribosomal protein L18a OS=Homo sapiens GN=RPL18A PE=1 SV=2
Match to Query 551: 1133.658from(567.8362,2+)
Title: 268: Sum of 2 scans in range 901 (rt=28.7413, f=4, i=182) to 902 (rt=28.7667, f=4, i=183) [D:\lab212\membrane\Grace\20120305-iTRAQ-2.raw]
Data File:
Monoisotopic mass of neutral peptide Mr(calc): 1133.658
Variable modifications:
K8 :iTRAQ4plex (K)
Ions Score: 60.48 **Expect:** 0.001

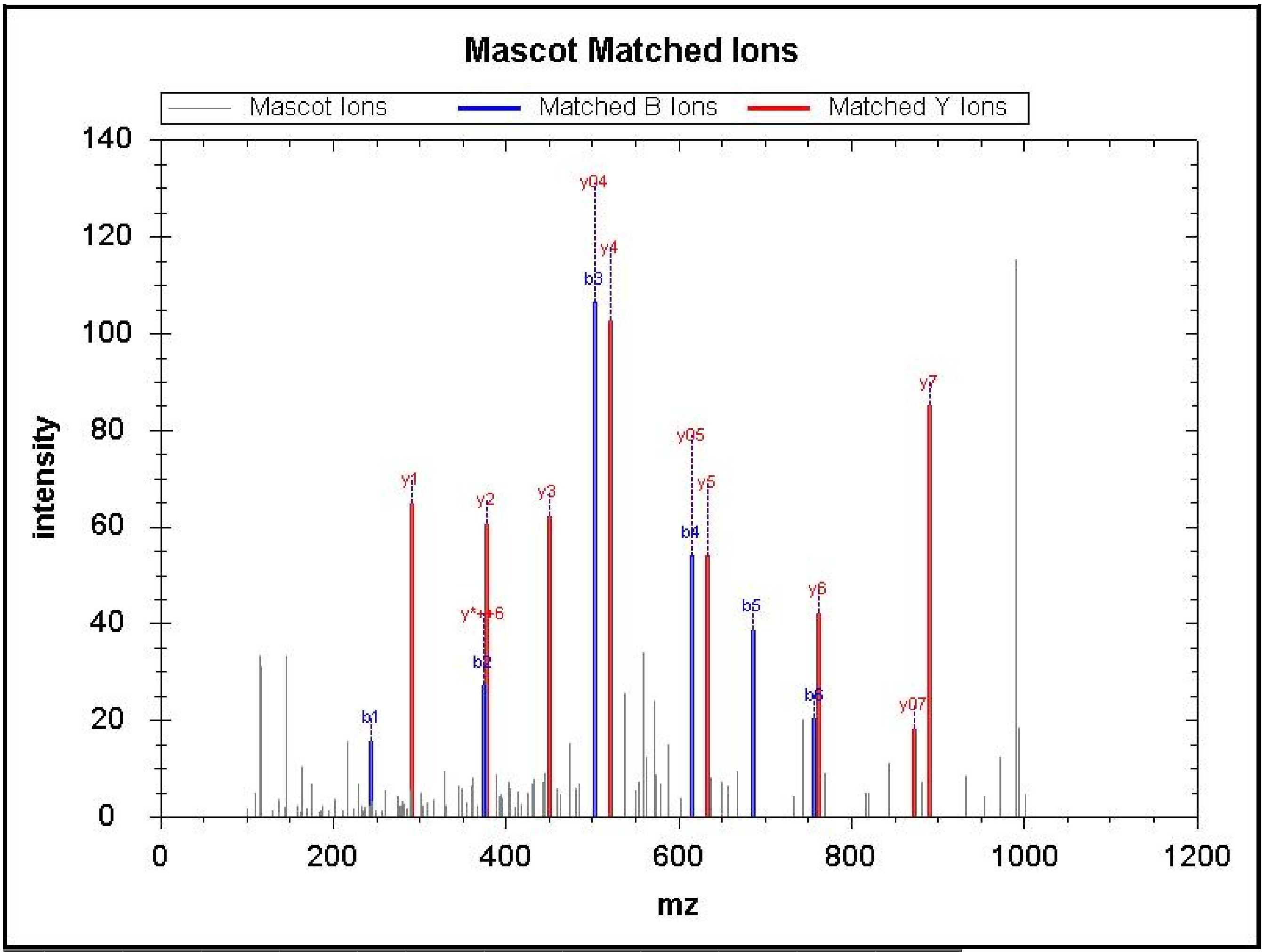

No	b	b++	b0	b0++	Seq	y	y++	y*	y*++	y0	y0++	RevNo
1	244.18	122.59			V							8
2	373.22	187.11	355.21	178.11	E	891.49	446.25	874.46	437.74	873.48	437.24	7

3	**502.26**	251.64	484.25	242.63	E	**762.45**	381.73	745.42	**373.21**	744.44	372.72	6
4	**615.35**	308.18	597.34	299.17	I	**633.41**	317.21	616.38	308.69	**615.39**	308.20	5
5	**686.38**	343.70	668.37	334.69	A	**520.32**	260.66	503.29	252.15	**502.31**	251.66	4
6	**757.42**	379.21	739.41	370.21	A	**449.28**	225.15	432.26	216.63	431.27	216.14	3
7	844.45	422.73	826.44	413.72	S	**378.25**	189.63	361.22	181.11	360.24	180.62	2
8					K	**291.21**	146.11	274.19	137.60			1

Query 2301 Hit 1

MS/MS Fragmentation of **YELPLVIQALTNIEDK**
Found in **sp|Q96QD8|S38A2_HUMAN**, Sodium-coupled neutral amino acid transporter 2 OS=Homo sapiens GN=SLC38A2 PE=1 SV=2
Match to Query 2301: 2146.227from(716.4163,3+)
Title: 1214: Scan 2946 (rt=74.8177, f=3, i=424) [D:\lab212\membrane\Grace\20120305-iTRAQ-2.raw]
Data File:
Monoisotopic mass of neutral peptide Mr(calc): 2146.227
Variable modifications:
K16 :iTRAQ4plex (K)
Ions Score: 59.98 **Expect:** 0.000

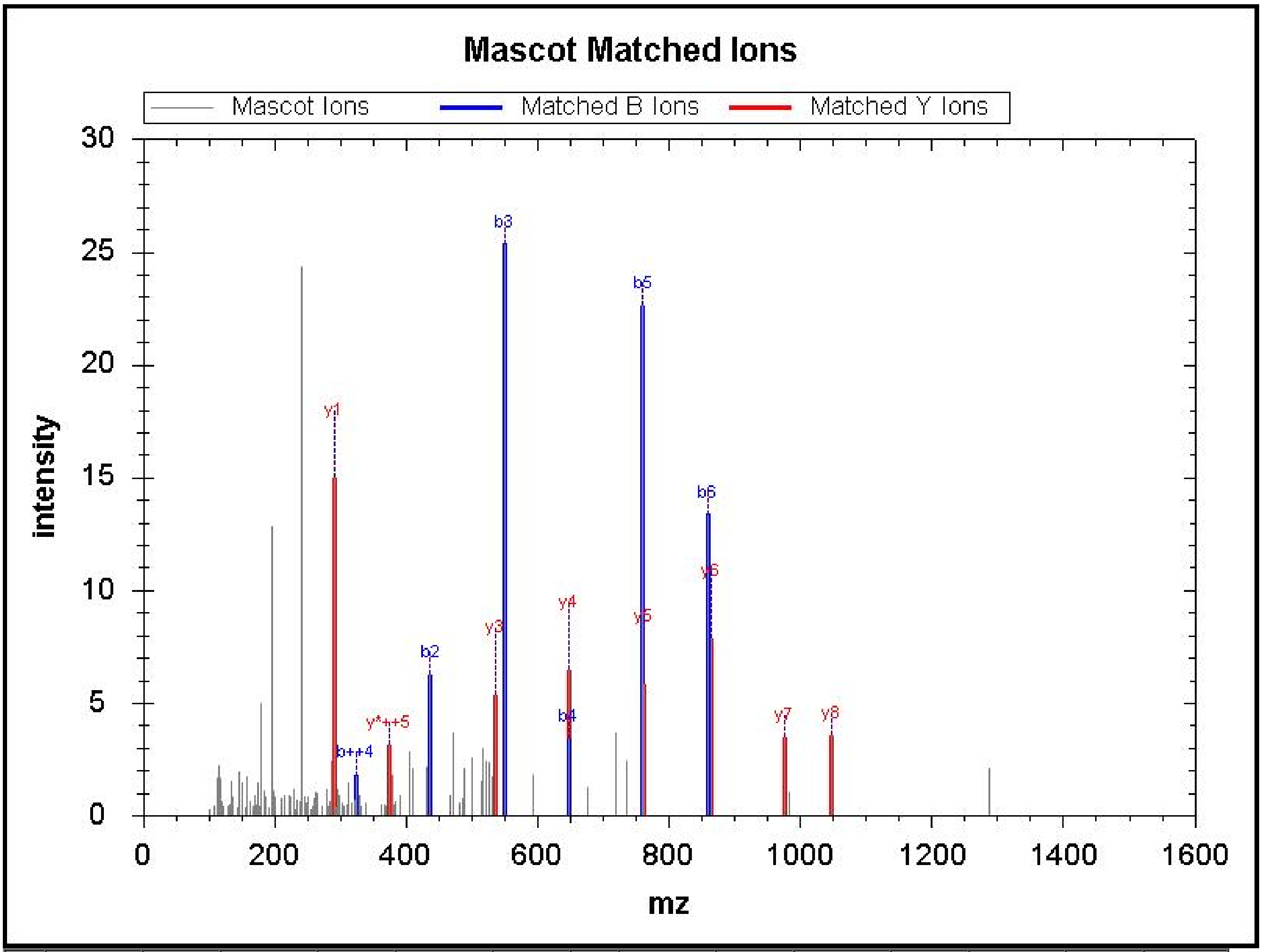

No	b	b++	b*	b*++	b0	b0++	Seq	y	y++	y*	y*++	y0	y0++	RevNo
1	308.17	154.59					Y							16
2	**437.22**	219.11			419.20	210.11	E	1,840.05	920.53	1,823.03	912.02	1,822.04	911.53	15
3	**550.30**	275.65			532.29	266.65	L	1,711.01	856.01	1,693.99	847.50	1,693.00	847.00	14
4	**647.35**	**324.18**			629.34	315.17	P	1,597.93	799.47	1,580.90	790.95	1,579.92	790.46	13
5	**760.44**	380.72			742.43	371.72	L	1,500.88	750.94	1,483.85	742.43	1,482.86	741.94	12
6	**859.50**	430.26			841.49	421.25	V	1,387.79	694.40	1,370.76	685.89	1,369.78	685.39	11
7	972.59	486.80			954.58	477.79	I	1,288.72	644.87	1,271.70	636.35	1,270.71	635.86	10

8	1,100.65	550.83	1,083.62	542.31	1,082.64	541.82	Q	1,175.64	588.32	1,158.61	579.81	1,157.63	579.32	9
9	1,171.68	586.35	1,154.66	577.83	1,153.67	577.34	A	1,047.58	524.29	1,030.55	515.78	1,029.57	515.29	8
10	1,284.77	642.89	1,267.74	634.37	1,266.76	633.88	L	976.54	488.78	959.52	480.26	958.53	479.77	7
11	1,385.82	693.41	1,368.79	684.90	1,367.81	684.41	T	863.46	432.23	846.43	423.72	845.45	423.23	6
12	1,499.86	750.43	1,482.83	741.92	1,481.85	741.43	N	762.41	381.71	745.38	373.20	744.40	372.70	5
13	1,612.94	806.98	1,595.92	798.46	1,594.93	797.97	I	648.37	324.69	631.34	316.17	630.36	315.68	4
14	1,741.99	871.50	1,724.96	862.98	1,723.98	862.49	E	535.28	268.15	518.26	259.63	517.27	259.14	3
15	1,857.01	929.01	1,839.99	920.50	1,839.00	920.00	D	406.24	203.62	389.22	195.11	388.23	194.62	2
16							K	291.21	146.11	274.19	137.60			1

Query 2318 Hit 1

MS/MS Fragmentation of GQNLLLTNLQTIQGILER
Found in sp|P12270|TPR_HUMAN, Nucleoprotein TPR OS=Homo sapiens GN=TPR PE=1 SV=3
Match to Query 2318: 2167.252from(723.4245,3+)
Title: 1209: Sum of 2 scans in range 2935 (rt=74.5716, f=4, i=838) to 2936 (rt=74.597, f=4, i=839) [D:\lab212\membrane\Grace\20120305-iTRAQ-2.raw]
Data File:
Monoisotopic mass of neutral peptide Mr(calc): 2167.252
Variable modifications:
Ions Score: 59.53 **Expect:** 0.000

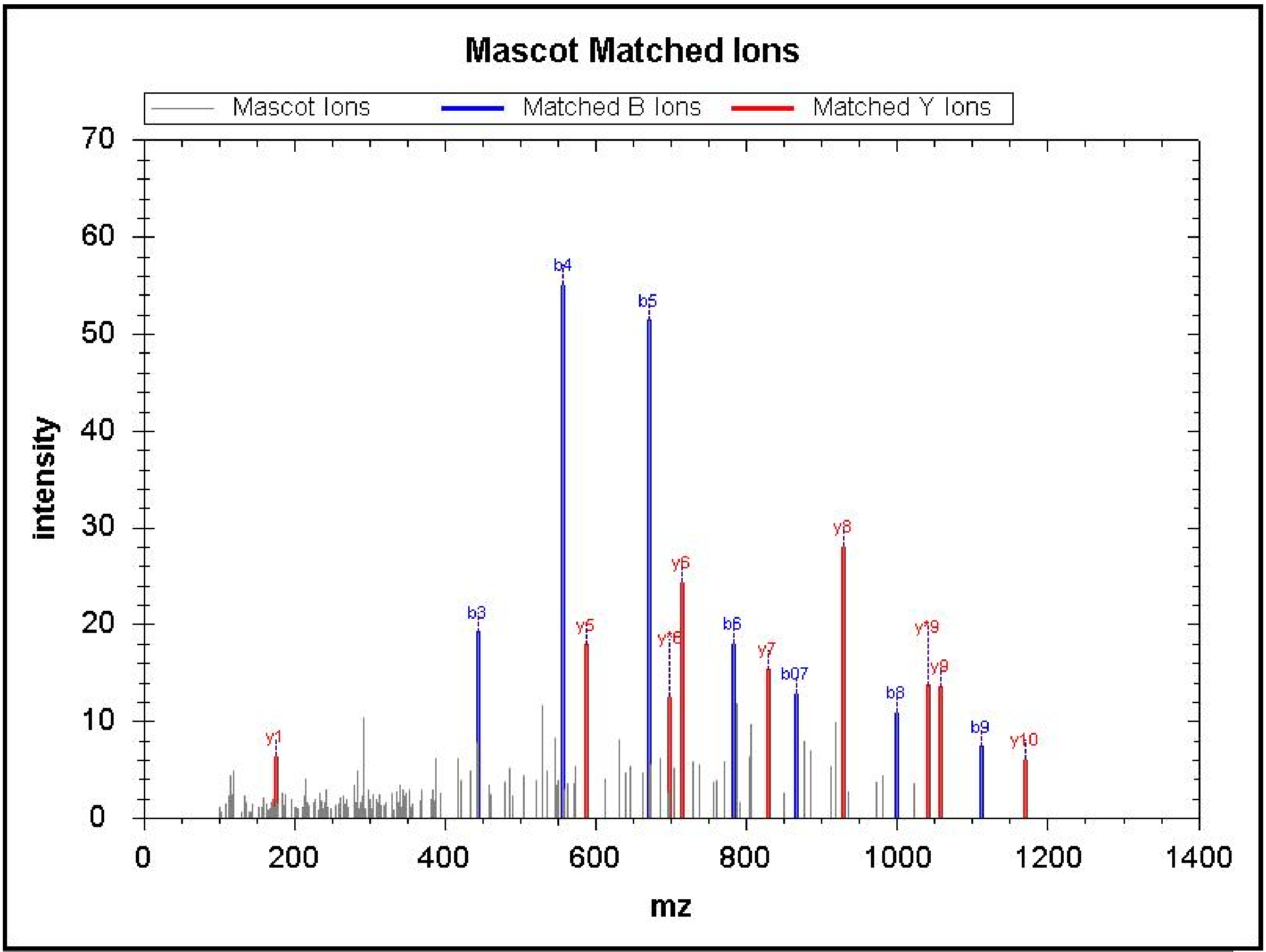

No	b	b++	b*	b*++	b0	b0++	Seq	y	y++	y*	y*++	y0	y0++	RevNo
1	202.13	101.57					G							18
2	330.19	165.60	313.16	157.09			Q	1,967.13	984.07	1,950.10	975.55	1,949.12	975.06	17
3	444.23	222.62	427.21	214.11			N	1,839.07	920.04	1,822.04	911.53	1,821.06	911.03	16
4	557.32	279.16	540.29	270.65			L	1,725.03	863.02	1,708.00	854.50	1,707.02	854.01	15
5	670.40	335.70	653.37	327.19			L	1,611.94	806.48	1,594.92	797.96	1,593.93	797.47	14

6	783.48	392.25	766.46	383.73			L	1,498.86	749.93	1,481.83	741.42	1,480.85	740.93	13
7	884.53	442.77	867.51	434.26	866.52	433.76	T	1,385.77	693.39	1,368.75	684.88	1,367.76	684.39	12
8	998.58	499.79	981.55	491.28	980.56	490.79	N	1,284.73	642.87	1,267.70	634.35	1,266.72	633.86	11
9	1,111.66	556.33	1,094.63	547.82	1,093.65	547.33	L	1,170.68	585.85	1,153.66	577.33	1,152.67	576.84	10
10	1,239.72	620.36	1,222.69	611.85	1,221.71	611.36	Q	1,057.60	529.30	1,040.57	520.79	1,039.59	520.30	9
11	1,340.77	670.89	1,323.74	662.37	1,322.75	661.88	T	929.54	465.27	912.51	456.76	911.53	456.27	8
12	1,453.85	727.43	1,436.82	718.92	1,435.84	718.42	I	828.49	414.75	811.47	406.24	810.48	405.75	7
13	1,581.91	791.46	1,564.88	782.94	1,563.90	782.45	Q	715.41	358.21	698.38	349.70	697.40	349.20	6
14	1,638.93	819.97	1,621.90	811.46	1,620.92	810.96	G	587.35	294.18	570.32	285.67	569.34	285.17	5
15	1,752.01	876.51	1,734.99	868.00	1,734.00	867.51	I	530.33	265.67	513.30	257.16	512.32	256.66	4
16	1,865.10	933.05	1,848.07	924.54	1,847.09	924.05	L	417.25	209.13	400.22	200.61	399.24	200.12	3
17	1,994.14	997.57	1,977.11	989.06	1,976.13	988.57	E	304.16	152.58	287.13	144.07	286.15	143.58	2
18							R	175.12	88.06	158.09	79.55			1

Query 2140 Hit 1

MS/MS Fragmentation of **LQNIFLGLVNIIEEK**
Found in **sp|O15042|SR140_HUMAN**, U2 snRNP-associated SURP motif-containing protein OS=Homo sapiens GN=U2SURP PE=1 SV=2
Match to Query 2140: 2030.205from(677.7423,3+)
Title: 1314: Scan 3347 (rt=83.4054, f=3, i=478) [D:\lab212\membrane\Grace\20120305-iTRAQ-1.raw]
Data File:
Monoisotopic mass of neutral peptide Mr(calc): 2030.205
Variable modifications:
K15 :iTRAQ4plex (K)
Ions Score: 59.09 **Expect:** 0.000

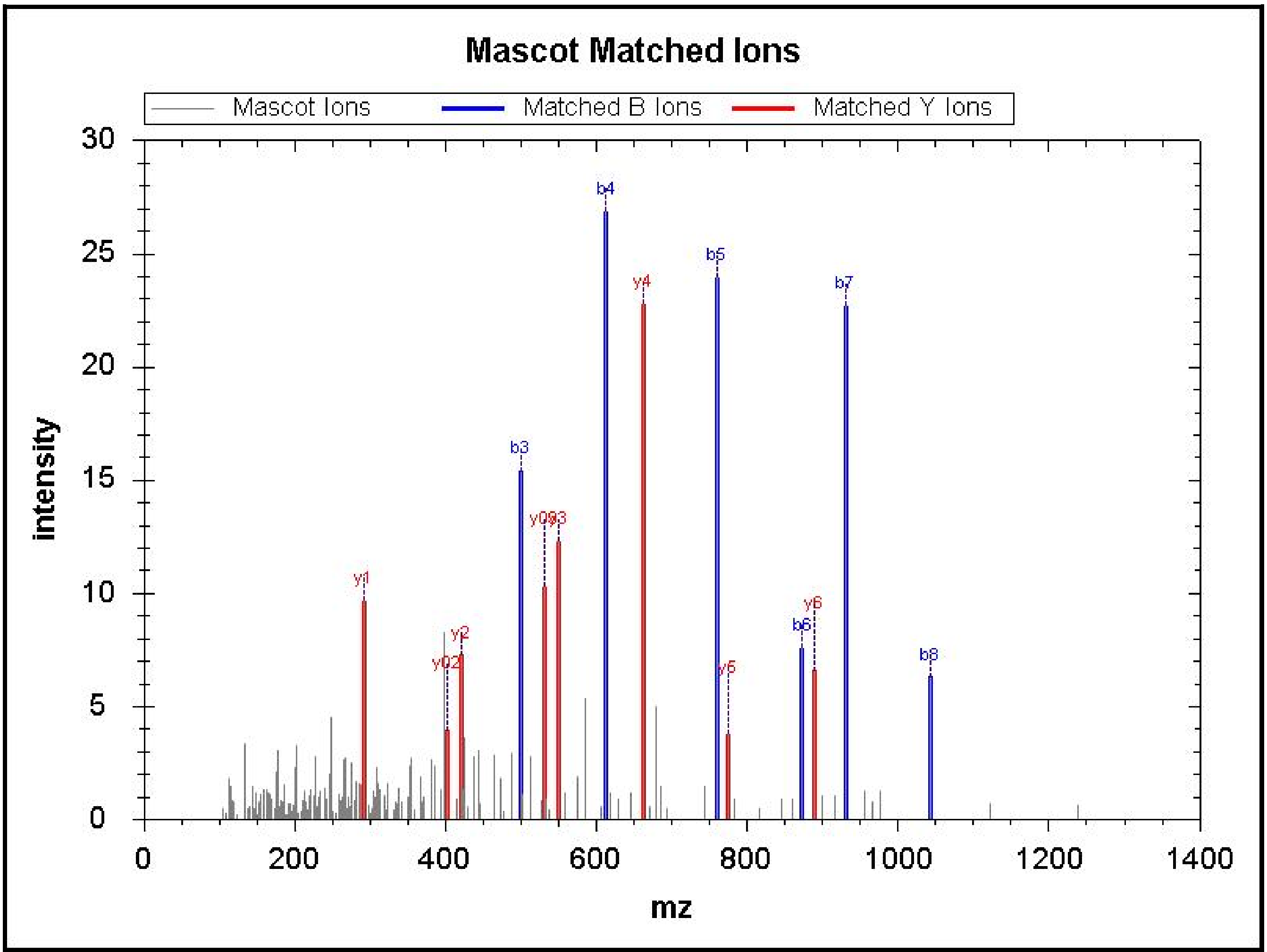

No	b	b++	b*	b*++	b0	b0++	Seq	y	y++	y*	y*++	y0	y0++	RevNo

**1**	258.19	129.60					**L**							**15**
**2**	386.25	193.63	369.23	185.12			**Q**	1,774.02	887.52	1,757.00	879.00	1,756.01	878.51	**14**
**3**	**500.29**	250.65	483.27	242.14			**N**	1,645.96	823.49	1,628.94	814.97	1,627.95	814.48	**13**
**4**	**613.38**	307.19	596.35	298.68			**I**	1,531.92	766.46	1,514.90	757.95	1,513.91	757.46	**12**
**5**	**760.45**	380.73	743.42	372.21			**F**	1,418.84	709.92	1,401.81	701.41	1,400.83	700.92	**11**
**6**	**873.53**	437.27	856.50	428.76			**L**	1,271.77	636.39	1,254.74	627.88	1,253.76	627.38	**10**
**7**	**930.55**	465.78	913.53	457.27			**G**	1,158.69	579.85	1,141.66	571.33	1,140.67	570.84	**9**
**8**	**1,043.64**	522.32	1,026.61	513.81			**L**	1,101.66	551.34	1,084.64	542.82	1,083.65	542.33	**8**
**9**	1,142.71	571.86	1,125.68	563.34			**V**	988.58	494.79	971.55	486.28	970.57	485.79	**7**
**10**	1,256.75	628.88	1,239.72	620.36			**N**	**889.51**	445.26	872.48	436.75	871.50	436.25	**6**
**11**	1,369.83	685.42	1,352.81	676.91			**I**	**775.47**	388.24	758.44	379.72	757.46	379.23	**5**
**12**	1,482.92	741.96	1,465.89	733.45			**I**	**662.38**	331.70	645.36	323.18	644.37	322.69	**4**
**13**	1,611.96	806.48	1,594.93	797.97	1,593.95	797.48	**E**	**549.30**	275.15	532.27	266.64	**531.29**	266.15	**3**
**14**	1,741.00	871.00	1,723.98	862.49	1,722.99	862.00	**E**	**420.26**	210.63	403.23	202.12	**402.25**	201.63	**2**
**15**							**K**	**291.21**	146.11	274.19	137.60			**1**

Query 2079 Hit 1

MS/MS Fragmentation of **LLFEYLTLFGIDDK**
Found in **sp|P12081|SYHC_HUMAN**, Histidine--tRNA ligase
Match to Query 2079: 1974.111from(659.0444,3+)
Title: 1260: Sum of 2 scans in range 3206 (rt=80.2292, f=4, i=892) to 3207 (rt=80.2546, f=4, i=893) [D:\lab212\membrane\Grace\20120305-iTRAQ-1.raw]
Data File:
Monoisotopic mass of neutral peptide Mr(calc): 1974.111
Variable modifications:
K14 :iTRAQ4plex (K)
Ions Score: 58.97 **Expect:** 0.001

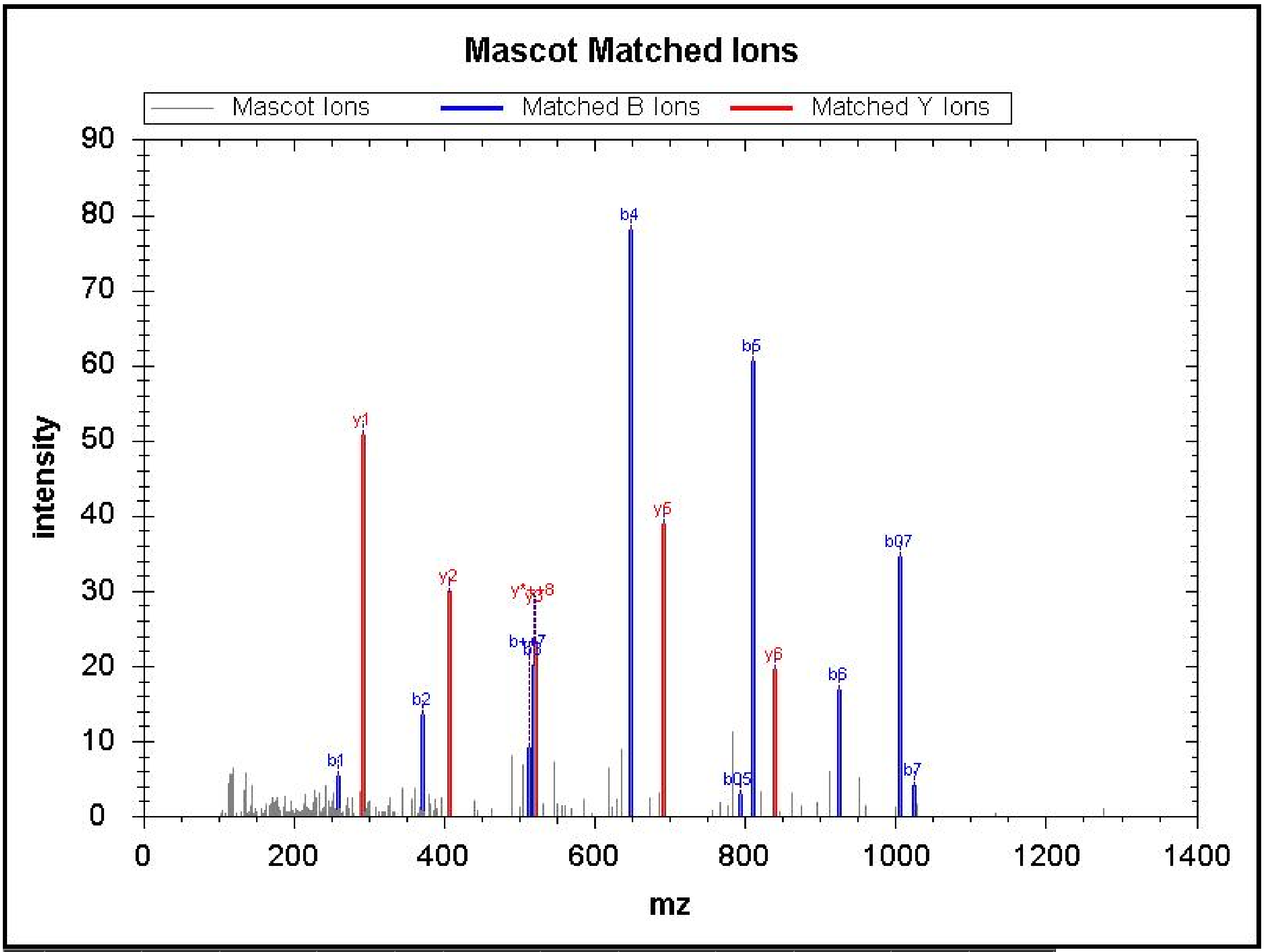

No	b	b++	b0	b0++	Seq	y	y++	y*	y*++	y0	y0++	RevNo
1	258.19	129.60			L							14
2	371.28	186.14			L	1,717.92	859.46	1,700.89	850.95	1,699.91	850.46	13
3	518.35	259.68			F	1,604.83	802.92	1,587.81	794.41	1,586.82	793.91	12
4	647.39	324.20	629.38	315.19	E	1,457.76	729.39	1,440.74	720.87	1,439.75	720.38	11
5	810.45	405.73	792.44	396.72	Y	1,328.72	664.86	1,311.70	656.35	1,310.71	655.86	10
6	923.54	462.27	905.53	453.27	L	1,165.66	583.33	1,148.63	574.82	1,147.65	574.33	9
7	1,024.58	512.80	1,006.57	503.79	T	1,052.57	526.79	1,035.55	518.28	1,034.56	517.79	8
8	1,137.67	569.34	1,119.66	560.33	L	951.53	476.27	934.50	467.75	933.52	467.26	7
9	1,284.74	642.87	1,266.73	633.87	F	838.44	419.72	821.42	411.21	820.43	410.72	6
10	1,341.76	671.38	1,323.75	662.38	G	691.37	346.19	674.35	337.68	673.36	337.19	5
11	1,454.84	727.92	1,436.83	718.92	I	634.35	317.68	617.33	309.17	616.34	308.67	4
12	1,569.87	785.44	1,551.86	776.43	D	521.27	261.14	504.24	252.62	503.26	252.13	3
13	1,684.90	842.95	1,666.89	833.95	D	406.24	203.62	389.22	195.11	388.23	194.62	2
14					K	291.21	146.11	274.19	137.60			1

Query 2823 Hit 1

MS/MS Fragmentation of **LGDPAEYAHLVQAIIENPFLNGEVIR**
Found in **sp|Q99714|HCD2_HUMAN**, 3-hydroxyacyl-CoA dehydrogenase type-2 OS=Homo sapiens GN=HSD17B10 PE=1 SV=3
Match to Query 2823: 3021.615from(756.4109,4+)
Title: 1243: Sum of 2 scans in range 3169 (rt=79.3949, f=4, i=880) to 3170 (rt=79.4203, f=4, i=881) [D:\lab212\membrane\Grace\20120305-iTRAQ-1.raw]
Data File:
Monoisotopic mass of neutral peptide Mr(calc): 3021.615
Variable modifications:
Ions Score: 55.42 **Expect:** 0.002

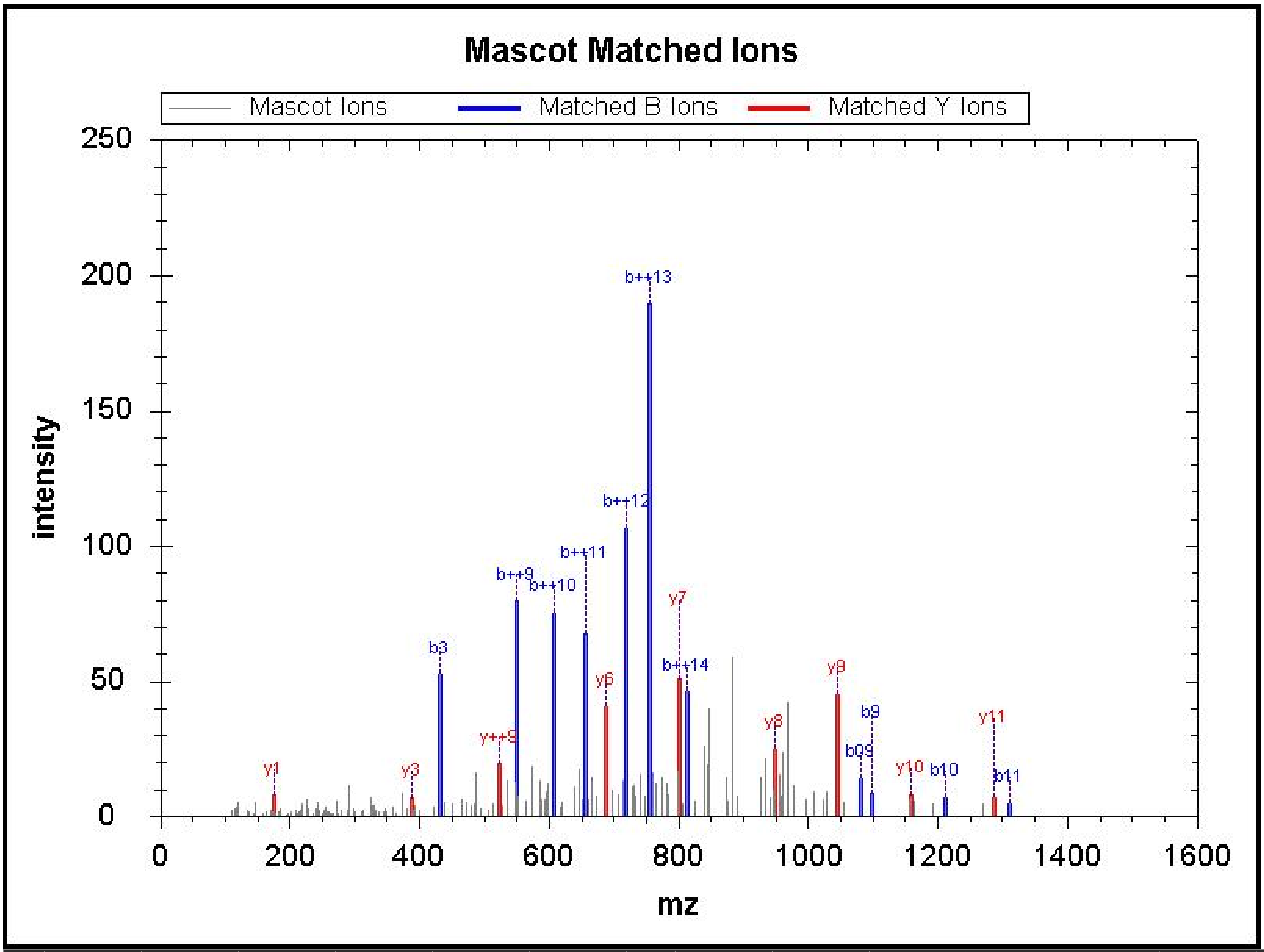

No	b	b++	b*	b*++	b0	b0++	Seq	y	y++	y*	y*++	y0	y0++	RevNo
1	258.19	129.60					L							26
2	315.21	158.11					G	2,765.43	1,383.22	2,748.40	1,374.70	2,747.42	1,374.21	25
3	430.24	215.62			412.23	206.62	D	2,708.40	1,354.71	2,691.38	1,346.19	2,690.39	1,345.70	24
4	527.29	264.15			509.28	255.15	P	2,593.38	1,297.19	2,576.35	1,288.68	2,575.37	1,288.19	23
5	598.33	299.67			580.32	290.66	A	2,496.32	1,248.67	2,479.30	1,240.15	2,478.31	1,239.66	22
6	727.37	364.19			709.36	355.19	E	2,425.29	1,213.15	2,408.26	1,204.63	2,407.28	1,204.14	21
7	890.44	445.72			872.43	436.72	Y	2,296.24	1,148.63	2,279.22	1,140.11	2,278.23	1,139.62	20
8	961.47	481.24			943.46	472.24	A	2,133.18	1,067.09	2,116.16	1,058.58	2,115.17	1,058.09	19
9	1,098.53	549.77			1,080.52	540.77	H	2,062.14	1,031.58	2,045.12	1,023.06	2,044.13	1,022.57	18
10	1,211.62	606.31			1,193.61	597.31	L	1,925.09	963.05	1,908.06	954.53	1,907.07	954.04	17
11	1,310.69	655.85			1,292.68	646.84	V	1,812.00	906.50	1,794.97	897.99	1,793.99	897.50	16
12	1,438.74	719.88	1,421.72	711.36	1,420.73	710.87	Q	1,712.93	856.97	1,695.91	848.46	1,694.92	847.96	15
13	1,509.78	755.39	1,492.76	746.88	1,491.77	746.39	A	1,584.87	792.94	1,567.85	784.43	1,566.86	783.94	14
14	1,622.87	811.94	1,605.84	803.42	1,604.86	802.93	I	1,513.84	757.42	1,496.81	748.91	1,495.83	748.42	13
15	1,735.95	868.48	1,718.92	859.97	1,717.94	859.47	I	1,400.75	700.88	1,383.73	692.37	1,382.74	691.88	12
16	1,864.99	933.00	1,847.97	924.49	1,846.98	923.99	E	1,287.67	644.34	1,270.64	635.83	1,269.66	635.33	11
17	1,979.04	990.02	1,962.01	981.51	1,961.03	981.02	N	1,158.63	579.82	1,141.60	571.30	1,140.62	570.81	10
18	2,076.09	1,038.55	2,059.06	1,030.03	2,058.08	1,029.54	P	1,044.58	522.80	1,027.56	514.28	1,026.57	513.79	9
19	2,223.16	1,112.08	2,206.13	1,103.57	2,205.15	1,103.08	F	947.53	474.27	930.50	465.76	929.52	465.26	8
20	2,336.24	1,168.62	2,319.21	1,160.11	2,318.23	1,159.62	L	800.46	400.73	783.44	392.22	782.45	391.73	7
21	2,450.28	1,225.65	2,433.26	1,217.13	2,432.27	1,216.64	N	687.38	344.19	670.35	335.68	669.37	335.19	6
22	2,507.31	1,254.16	2,490.28	1,245.64	2,489.29	1,245.15	G	573.34	287.17	556.31	278.66	555.32	278.17	5
23	2,636.35	1,318.68	2,619.32	1,310.16	2,618.34	1,309.67	E	516.31	258.66	499.29	250.15	498.30	249.66	4

**24**	2,735.42	1,368.21	2,718.39	1,359.70	2,717.41	1,359.21	**V**	**387.27**	194.14	370.24	185.63			**3**
**25**	2,848.50	1,424.75	2,831.47	1,416.24	2,830.49	1,415.75	**I**	288.20	144.61	271.18	136.09			**2**
**26**							**R**	**175.12**	88.06	158.09	79.55			**1**

Query 1374 Hit 1

MS/MS Fragmentation of **SAEFLLHMLK**
Found in **sp|P18621|RL17_HUMAN**, 60S ribosomal protein L17 OS=Homo sapiens GN=RPL17 PE=1 SV=3
Match to Query 1374: 1475.849from(492.9569,3+)
Title: 751: Sum of 2 scans in range 2071 (rt=54.6555, f=4, i=526) to 2072 (rt=54.6809, f=4, i=527) [D:\lab212\membrane\Grace\20120305-iTRAQ-1.raw]
Data File:
Monoisotopic mass of neutral peptide Mr(calc): 1475.849
Variable modifications:
K10 :iTRAQ4plex (K)
Ions Score: 53.93 **Expect:** 0.003

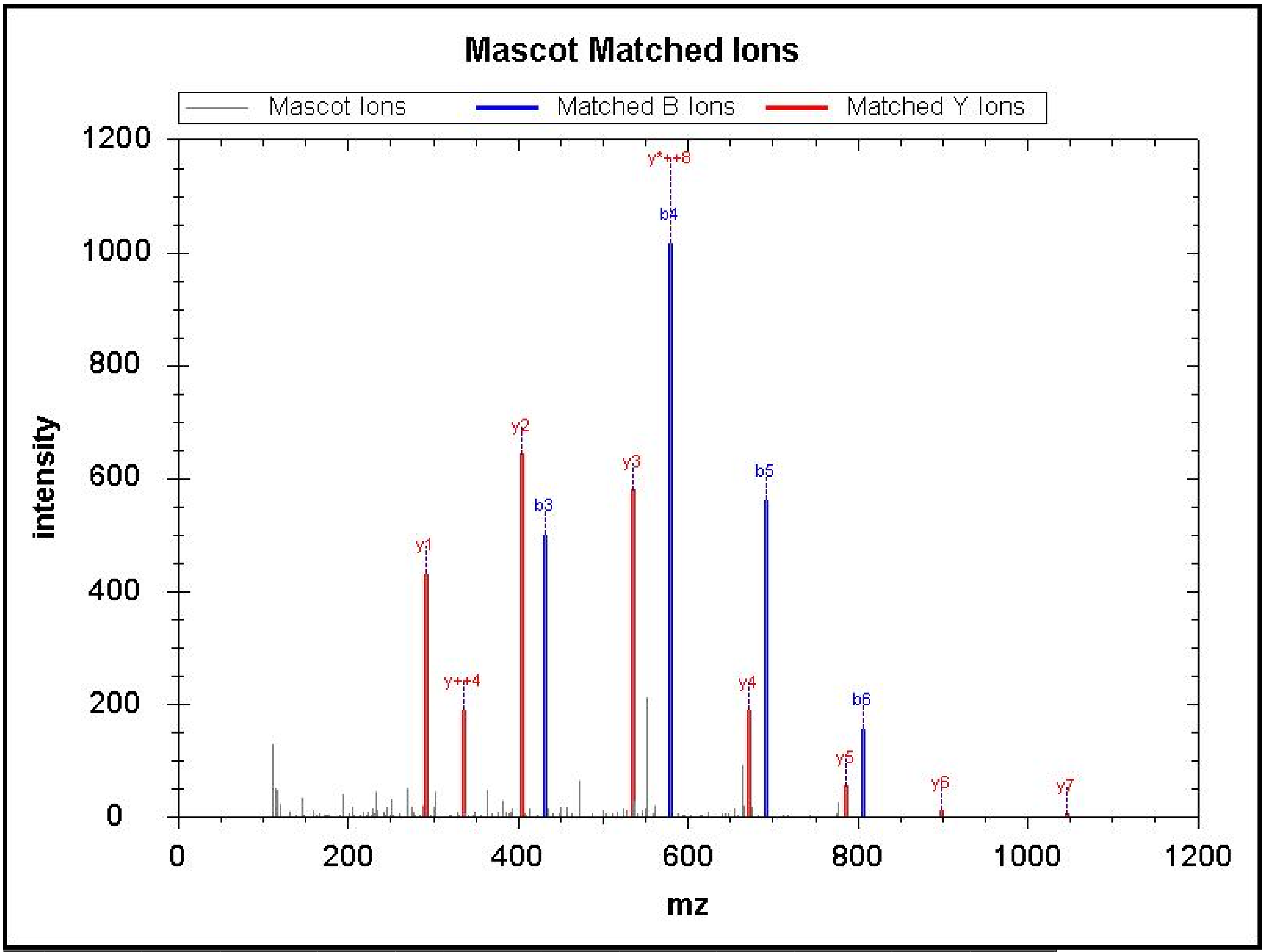

No	b	b++	b0	b0++	Seq	y	y++	y*	y*++	y0	y0++	RevNo
**1**	232.14	116.57	214.13	107.57	**S**							**10**
**2**	303.18	152.09	285.17	143.09	**A**	1,245.71	623.36	1,228.69	614.85	1,227.70	614.36	**9**
**3**	**432.22**	216.61	414.21	207.61	**E**	1,174.68	587.84	1,157.65	**579.33**	1,156.67	578.84	**8**
**4**	**579.29**	290.15	561.28	281.14	**F**	**1,045.63**	523.32	1,028.61	514.81			**7**
**5**	**692.37**	346.69	674.36	337.69	**L**	**898.57**	449.79	881.54	441.27			**6**
**6**	**805.46**	403.23	787.45	394.23	**L**	**785.48**	393.24	768.46	384.73			**5**
**7**	942.52	471.76	924.51	462.76	**H**	**672.40**	**336.70**	655.37	328.19			**4**
**8**	1,073.56	537.28	1,055.55	528.28	**M**	**535.34**	268.17	518.31	259.66			**3**
**9**	1,186.64	593.82	1,168.63	584.82	**L**	**404.30**	202.65	387.27	194.14			**2**
**10**					**K**	**291.21**	146.11	274.19	137.60			**1**

Query 1684 Hit 1

MS/MS Fragmentation of **GAYIYNALIEFIR**
Found in **sp|P26639|SYTC_HUMAN**, Threonine--tRNA ligase
Match to Query 1684: 1685.935from(843.9747,2+)
Title: 1178: Sum of 2 scans in range 2860 (rt=72.8834, f=4, i=814) to 2861 (rt=72.9088, f=4, i=815) [D:\lab212\membrane\Grace\20120305-iTRAQ-2.raw]
Data File:
Monoisotopic mass of neutral peptide Mr(calc): 1685.935
Variable modifications:
Ions Score: 53.57 **Expect:** 0.003

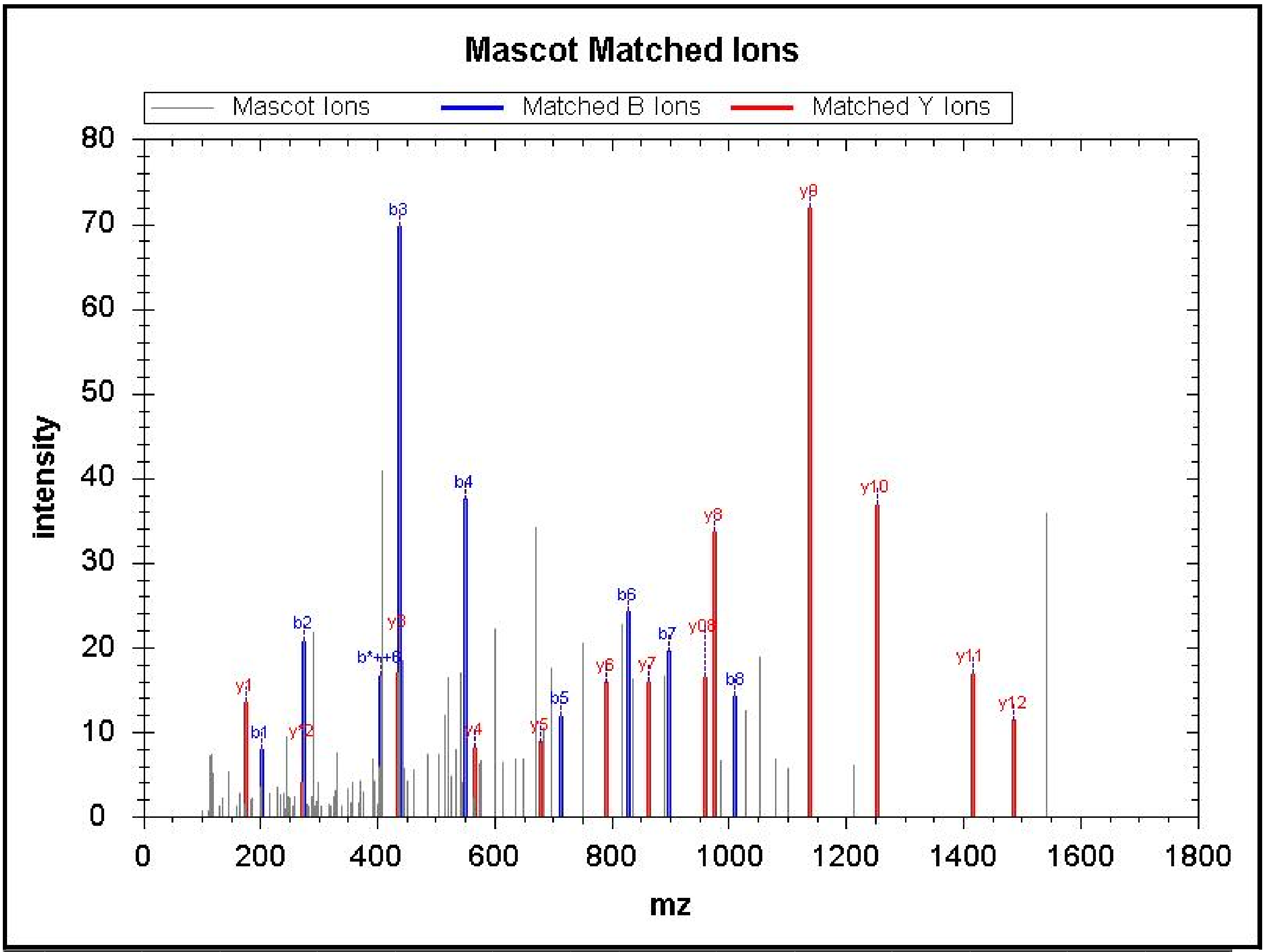

No	b	b++	b*	b*++	b0	b0++	Seq	y	y++	y*	y*++	y0	y0++	RevNo
1	202.13	101.57					G							13
2	273.17	137.09					A	1,485.81	743.41	1,468.78	734.90	1,467.80	734.40	12
3	436.23	218.62					Y	1,414.77	707.89	1,397.75	699.38	1,396.76	698.88	11
4	549.32	275.16					I	1,251.71	626.36	1,234.68	617.85	1,233.70	617.35	10
5	712.38	356.69					Y	1,138.63	569.82	1,121.60	561.30	1,120.61	560.81	9
6	826.42	413.71	809.39	405.20			N	975.56	488.28	958.54	479.77	957.55	479.28	8
7	897.46	449.23	880.43	440.72			A	861.52	431.26	844.49	422.75	843.51	422.26	7
8	1,010.54	505.77	993.52	497.26			L	790.48	395.74	773.46	387.23	772.47	386.74	6
9	1,123.63	562.32	1,106.60	553.80			I	677.40	339.20	660.37	330.69	659.39	330.20	5
10	1,252.67	626.84	1,235.64	618.33	1,234.66	617.83	E	564.31	282.66	547.29	274.15	546.30	273.66	4
11	1,399.74	700.37	1,382.71	691.86	1,381.73	691.37	F	435.27	218.14	418.24	209.63			3
12	1,512.82	756.91	1,495.80	748.40	1,494.81	747.91	I	288.20	144.61	271.18	136.09			2
13							R	175.12	88.06	158.09	79.55			1

Query 1308 Hit 1

MS/MS Fragmentation of **ILVTLLHTLER**
Found in **sp|Q9BWD1|THIC_HUMAN**, Acetyl-CoA acetyltransferase
Match to Query 1308: 1450.909from(484.6436,3+)
Title: 1018: Scan 2525 (rt=65.3297, f=3, i=356) [D:\lab212\membrane\Grace\20120305-iTRAQ-2.raw]
Data File:
Monoisotopic mass of neutral peptide Mr(calc): 1450.909
Variable modifications:
Ions Score: 52.76 **Expect:** 0.001

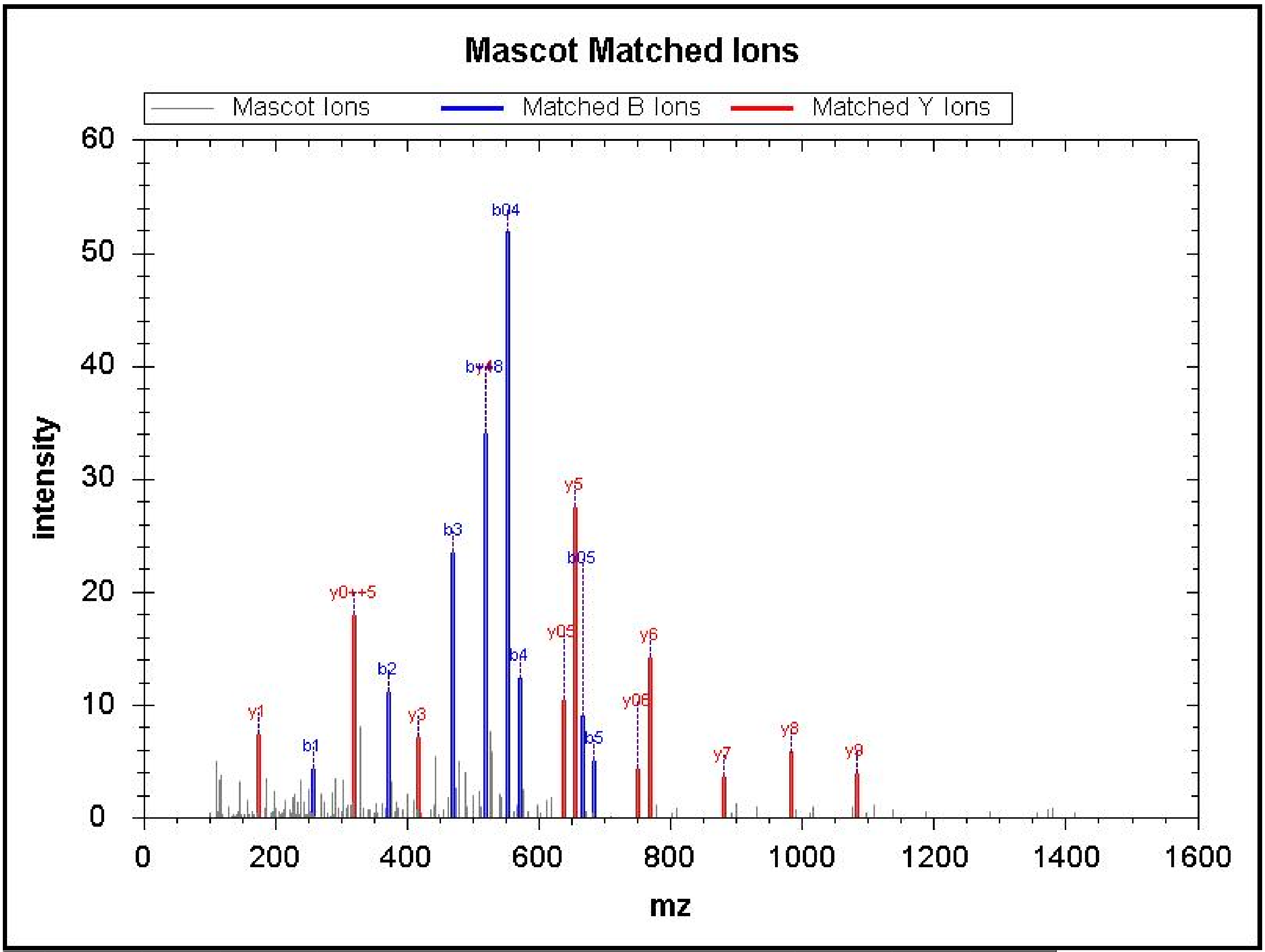

No	b	b++	b0	b0++	Seq	y	y++	y*	y*++	y0	y0++	RevNo
1	258.19	129.60			I							11
2	371.28	186.14			L	1,194.72	597.86	1,177.69	589.35	1,176.71	588.86	10
3	470.35	235.68			V	1,081.64	541.32	1,064.61	532.81	1,063.63	532.32	9
4	571.39	286.20	553.38	277.20	T	982.57	491.79	965.54	483.27	964.56	482.78	8
5	684.48	342.74	666.47	333.74	L	881.52	441.26	864.49	432.75	863.51	432.26	7
6	797.56	399.28	779.55	390.28	L	768.44	384.72	751.41	376.21	750.43	375.72	6
7	934.62	467.81	916.61	458.81	H	655.35	328.18	638.33	319.67	637.34	319.17	5
8	1,035.67	518.34	1,017.66	509.33	T	518.29	259.65	501.27	251.14	500.28	250.64	4
9	1,148.75	574.88	1,130.74	565.87	L	417.25	209.13	400.22	200.61	399.24	200.12	3
10	1,277.80	639.40	1,259.78	630.40	E	304.16	152.58	287.13	144.07	286.15	143.58	2
11					R	175.12	88.06	158.09	79.55			1

Query 1376 Hit 1

MS/MS Fragmentation of **VANVSLLALYK**
Found in **sp|P62266|RS23_HUMAN**, 40S ribosomal protein S23 OS=Homo sapiens GN=RPS23 PE=1 SV=3
Match to Query 1376: 1477.917from(739.9656,2+)

Title: 769: Sum of 2 scans in range 1980 (rt=53.0536, f=4, i=530) to 1981 (rt=53.079, f=4, i=531) [D:\lab212\membrane\Grace\20120305-iTRAQ-2.raw]
Data File:
Monoisotopic mass of neutral peptide Mr(calc): 1477.917
Variable modifications:
K11 :iTRAQ4plex (K)
Ions Score: 51.68 **Expect:** 0.002

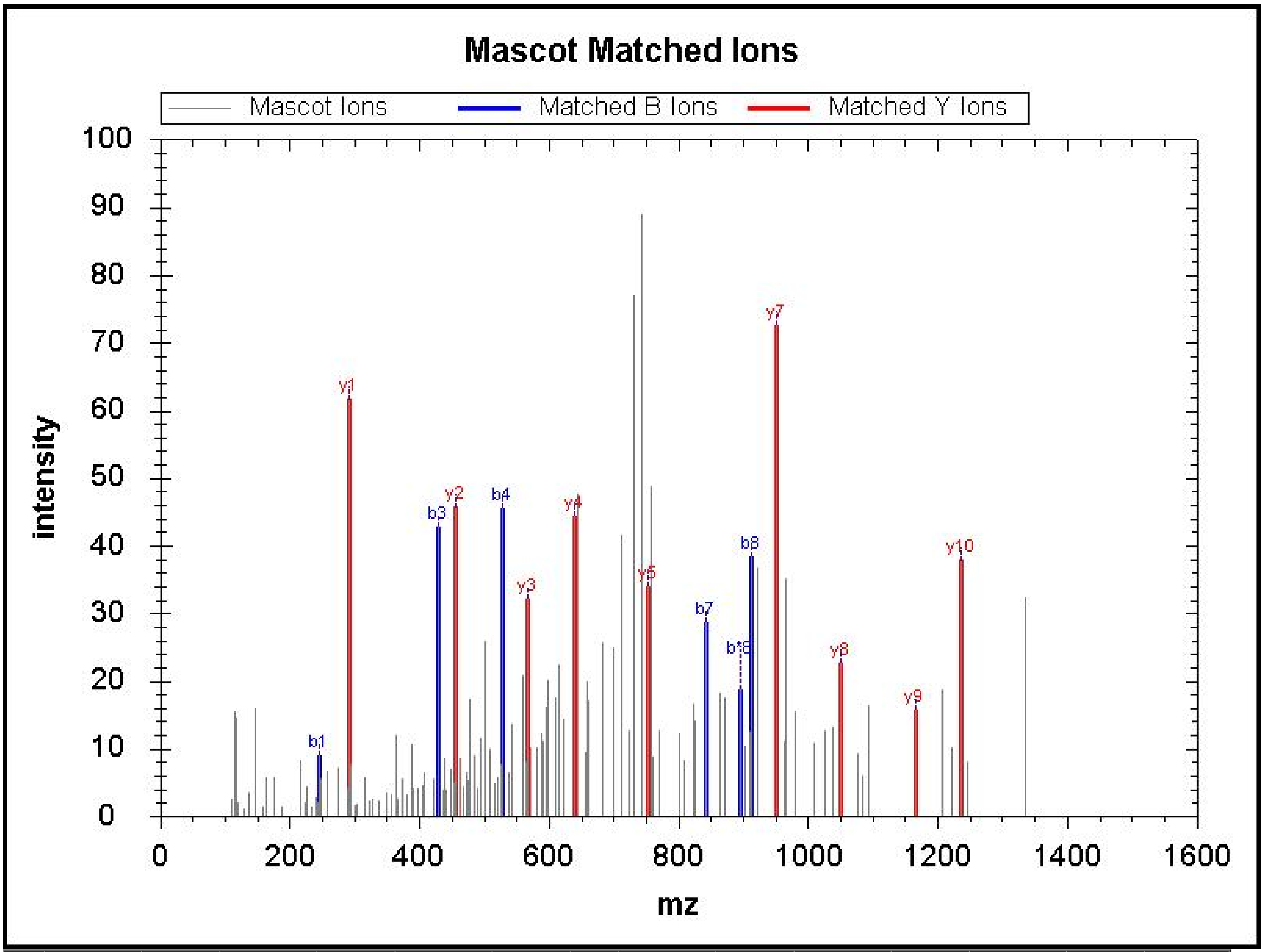

No	b	b++	b*	b*++	b0	b0++	Seq	y	y++	y*	y*++	y0	y0++	RevNo
1	244.18	122.59					V							11
2	315.21	158.11					A	1,235.75	618.38	1,218.72	609.86	1,217.74	609.37	10
3	429.26	215.13	412.23	206.62			N	1,164.71	582.86	1,147.68	574.35	1,146.70	573.85	9
4	528.33	264.67	511.30	256.15			V	1,050.67	525.84	1,033.64	517.32	1,032.66	516.83	8
5	615.36	308.18	598.33	299.67	597.35	299.18	S	951.60	476.30	934.57	467.79	933.59	467.30	7
6	728.44	364.72	711.42	356.21	710.43	355.72	L	864.57	432.79	847.54	424.27			6
7	841.53	421.27	824.50	412.75	823.52	412.26	L	751.48	376.25	734.46	367.73			5
8	912.56	456.79	895.54	448.27	894.55	447.78	A	638.40	319.70	621.37	311.19			4
9	1,025.65	513.33	1,008.62	504.81	1,007.64	504.32	L	567.36	284.18	550.34	275.67			3
10	1,188.71	594.86	1,171.68	586.35	1,170.70	585.85	Y	454.28	227.64	437.25	219.13			2
11							K	291.21	146.11	274.19	137.60			1

Query 37 Hit 1

MS/MS Fragmentation of **GSINLKR**
Found in **sp|Q7Z5Q5|DPOLN_HUMAN**, DNA polymerase nu OS=Homo sapiens GN=POLN PE=1 SV=2
Match to Query 37: 787.4729from(394.7437,2+)
Title: 323: Scan 1017 (rt=31.3488, f=2, i=139) [D:\lab212\membrane\Grace\20120305-iTRAQ-2.raw]
Data File:
Monoisotopic mass of neutral peptide Mr(calc): 787.4729

Variable modifications:
N4 :Deamidated (NQ)
Ions Score: 49.69 **Expect:** 0.007

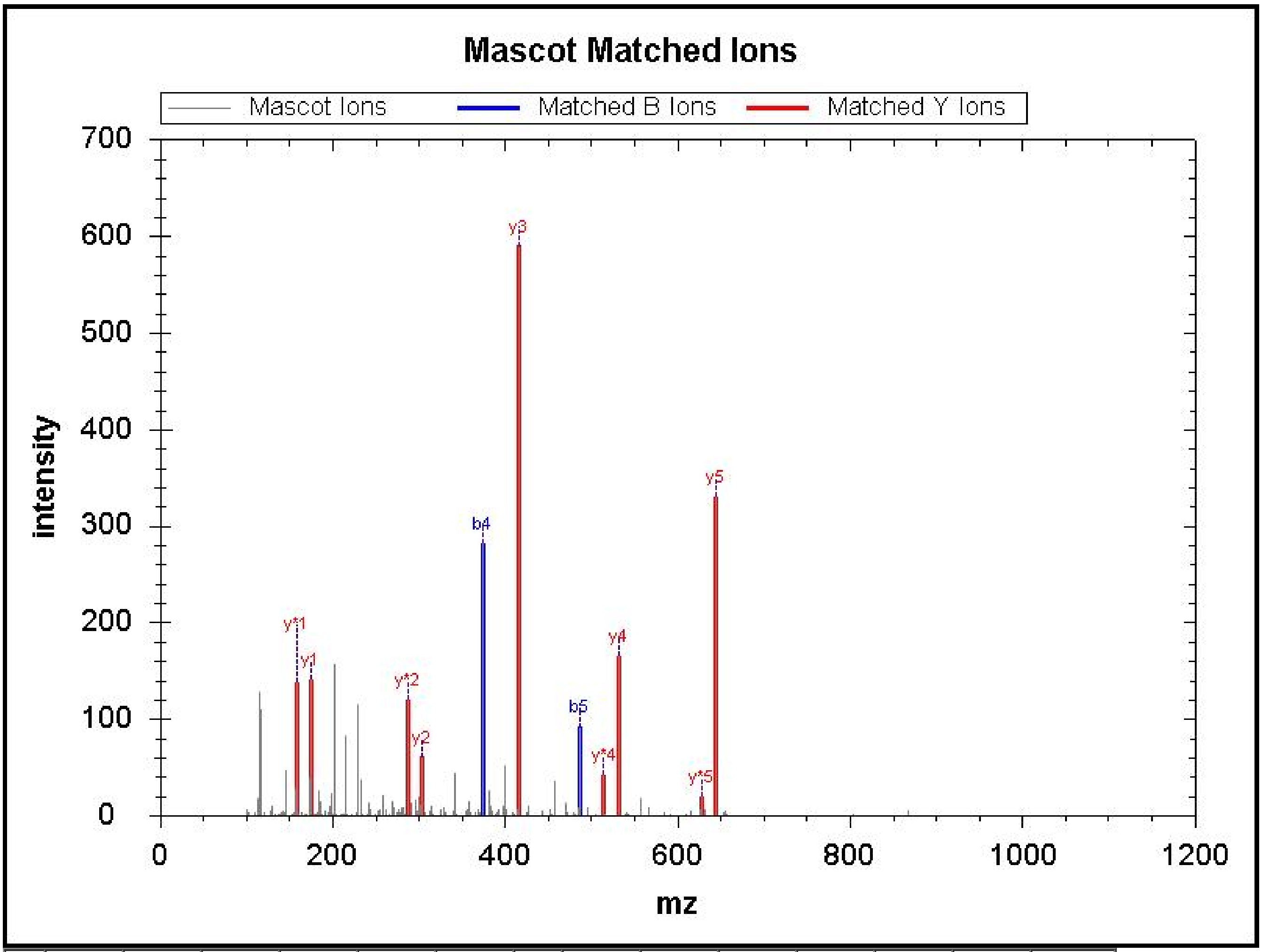

No	b	b++	b*	b*++	b0	b0++	Seq	y	y++	y*	y*++	y0	y0++	RevNo
1	58.03	29.52					G							7
2	145.06	73.03			127.05	64.03	S	731.44	366.22	714.41	357.71	713.43	357.22	6
3	258.14	129.58			240.13	120.57	I	**644.41**	322.71	**627.38**	314.19			5
4	**373.17**	187.09	356.15	178.58	355.16	178.08	N	**531.32**	266.17	**514.30**	257.65			4
5	**486.26**	243.63	469.23	235.12	468.25	234.63	L	**416.30**	208.65	399.27	200.14			3
6	614.35	307.68	597.32	299.17	596.34	298.67	K	**303.21**	152.11	**286.19**	143.60			2
7							R	**175.12**	88.06	**158.09**	79.55			1

Query 2432 Hit 1

MS/MS Fragmentation of **FVDHVFDEQVIDSLTVK**
Found in **sp|P04843|RPN1_HUMAN**, Dolichyl-diphosphooligosaccharide--protein glycosyltransferase subunit 1 OS=Homo sapiens GN=RPN1 PE=1 SV=1
Match to Query 2432: 2278.222from(760.4145,3+)
Title: 845: Sum of 2 scans in range 2141 (rt=56.6819, f=4, i=582) to 2142 (rt=56.7074, f=4, i=583) [D:\lab212\membrane\Grace\20120305-iTRAQ-2.raw]
Data File:
Monoisotopic mass of neutral peptide Mr(calc): 2278.222
Variable modifications:
K17 :iTRAQ4plex (K)
Ions Score: 49.17 **Expect:** 0.010

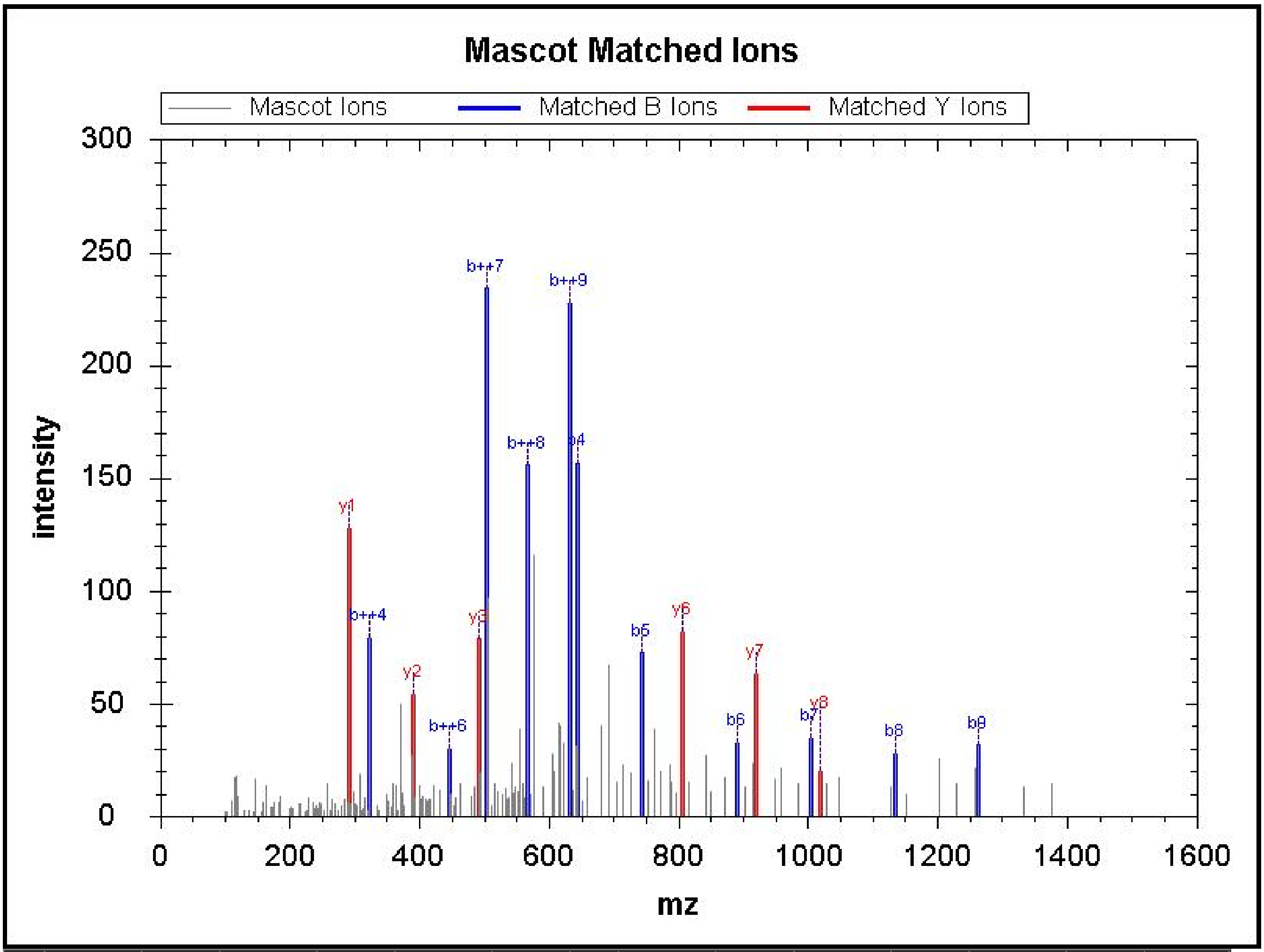

No	b	b++	b*	b*++	b0	b0++	Seq	y	y++	y*	y*++	y0	y0++	RevNo
1	292.18	146.59					F							17
2	391.25	196.13					V	1,988.05	994.53	1,971.02	986.01	1,970.04	985.52	16
3	506.27	253.64			488.26	244.63	D	1,888.98	944.99	1,871.95	936.48	1,870.97	935.99	15
4	643.33	322.17			625.32	313.16	H	1,773.95	887.48	1,756.92	878.97	1,755.94	878.47	14
5	742.40	371.70			724.39	362.70	V	1,636.89	818.95	1,619.86	810.44	1,618.88	809.94	13
6	889.47	445.24			871.46	436.23	F	1,537.82	769.42	1,520.80	760.90	1,519.81	760.41	12
7	1,004.50	502.75			986.49	493.75	D	1,390.75	695.88	1,373.73	687.37	1,372.74	686.88	11
8	1,133.54	567.27			1,115.53	558.27	E	1,275.73	638.37	1,258.70	629.85	1,257.72	629.36	10
9	1,261.60	631.30	1,244.57	622.79	1,243.59	622.30	Q	1,146.68	573.85	1,129.66	565.33	1,128.67	564.84	9
10	1,360.67	680.84	1,343.64	672.32	1,342.65	671.83	V	1,018.63	509.82	1,001.60	501.30	1,000.62	500.81	8
11	1,473.75	737.38	1,456.72	728.87	1,455.74	728.37	I	919.56	460.28	902.53	451.77	901.55	451.28	7
12	1,588.78	794.89	1,571.75	786.38	1,570.77	785.89	D	806.47	403.74	789.45	395.23	788.46	394.74	6
13	1,675.81	838.41	1,658.78	829.89	1,657.80	829.40	S	691.45	346.23	674.42	337.71	673.44	337.22	5
14	1,788.89	894.95	1,771.87	886.44	1,770.88	885.94	L	604.41	302.71	587.39	294.20	586.40	293.71	4
15	1,889.94	945.47	1,872.91	936.96	1,871.93	936.47	T	491.33	246.17	474.30	237.66	473.32	237.16	3
16	1,989.01	995.01	1,971.98	986.49	1,971.00	986.00	V	390.28	195.65	373.26	187.13			2
17							K	291.21	146.11	274.19	137.60			1

Query 2459 Hit 1

MS/MS Fragmentation of **DAEEVISQTIDTIVDMIK**
Found in **sp|Q04857|CO6A1_MOUSE**, Collagen alpha-1(VI) chain OS=Mus musculus GN=Col6a1 PE=2 SV=1
Match to Query 2459: 2308.229from(770.4171,3+)
Title: 1398: Scan 3904 (rt=94.9306, f=2, i=603) [D:\lab212\membrane\Grace\20120305-iTRAQ-1.raw]
Data File:
Monoisotopic mass of neutral peptide Mr(calc): 2308.229

Variable modifications:
Q8 :Deamidated (NQ)
K18 :iTRAQ4plex (K)
Ions Score: 48.66 **Expect:** 0.011

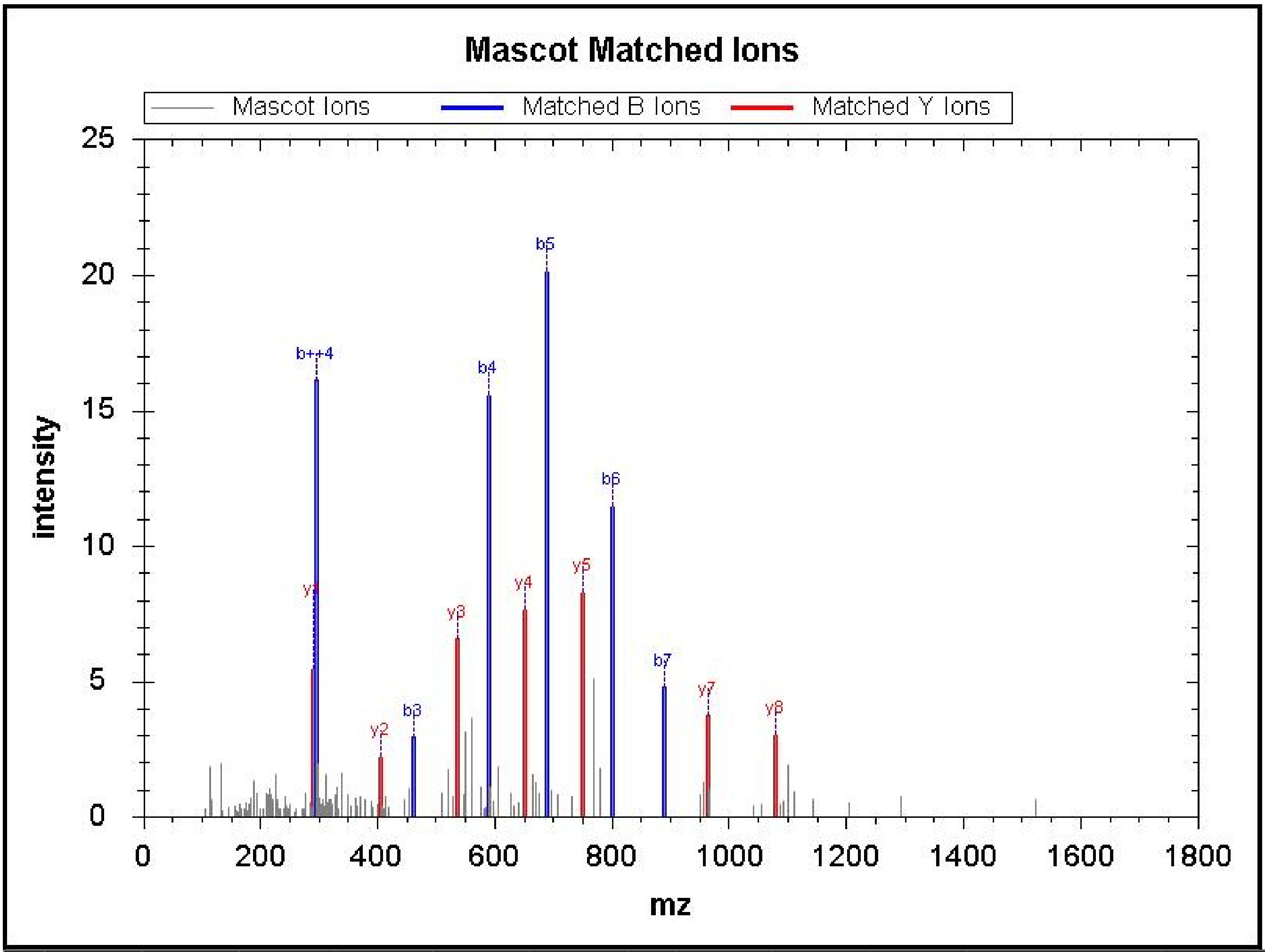

No	b	b++	b*	b*++	b0	b0++	Seq	y	y++	y*	y*++	y0	y0++	RevNo
1	260.14	130.57			242.13	121.57	D							18
2	331.17	166.09			313.16	157.09	A	2,050.07	1,025.54	2,033.05	1,017.03	2,032.06	1,016.54	17
3	460.22	230.61			442.21	221.61	E	1,979.04	990.02	1,962.01	981.51	1,961.03	981.02	16
4	589.26	295.13			571.25	286.13	E	1,849.99	925.50	1,832.97	916.99	1,831.98	916.50	15
5	688.33	344.67			670.32	335.66	V	1,720.95	860.98	1,703.93	852.47	1,702.94	851.97	14
6	801.41	401.21			783.40	392.20	I	1,621.88	811.45	1,604.86	802.93	1,603.87	802.44	13
7	888.44	444.73			870.43	435.72	S	1,508.80	754.90	1,491.77	746.39	1,490.79	745.90	12
8	1,017.49	509.25	1,000.46	500.73	999.48	500.24	Q	1,421.77	711.39	1,404.74	702.87	1,403.76	702.38	11
9	1,118.53	559.77	1,101.51	551.26	1,100.52	550.77	T	1,292.73	646.87	1,275.70	638.35	1,274.71	637.86	10
10	1,231.62	616.31	1,214.59	607.80	1,213.61	607.31	I	1,191.68	596.34	1,174.65	587.83	1,173.67	587.34	9
11	1,346.64	673.83	1,329.62	665.31	1,328.63	664.82	D	1,078.59	539.80	1,061.57	531.29	1,060.58	530.80	8
12	1,447.69	724.35	1,430.67	715.84	1,429.68	715.34	T	963.57	482.29	946.54	473.77	945.56	473.28	7
13	1,560.78	780.89	1,543.75	772.38	1,542.77	771.89	I	862.52	431.76	845.49	423.25	844.51	422.76	6
14	1,659.84	830.43	1,642.82	821.91	1,641.83	821.42	V	749.43	375.22	732.41	366.71	731.42	366.22	5
15	1,774.87	887.94	1,757.85	879.43	1,756.86	878.93	D	650.37	325.69	633.34	317.17	632.36	316.68	4
16	1,905.91	953.46	1,888.89	944.95	1,887.90	944.45	M	535.34	268.17	518.31	259.66			3
17	2,019.00	1,010.00	2,001.97	1,001.49	2,000.99	1,001.00	I	404.30	202.65	387.27	194.14			2
18							K	291.21	146.11	274.19	137.60			1

Query 1123 Hit 1

MS/MS Fragmentation of **ALLANALTSALR**
Found in **sp|P57088|TMM33_HUMAN**, Transmembrane protein 33 OS=Homo sapiens GN=TMEM33 PE=1 SV=2
Match to Query 1123: 1356.825from(679.4199,2+)
Title: 840: Scan 2133 (rt=56.4953, f=2, i=319) [D:\lab212\membrane\Grace\20120305-iTRAQ-2.raw]
Data File:
Monoisotopic mass of neutral peptide Mr(calc): 1356.825
Variable modifications:
Ions Score: 47.7 **Expect:** 0.007

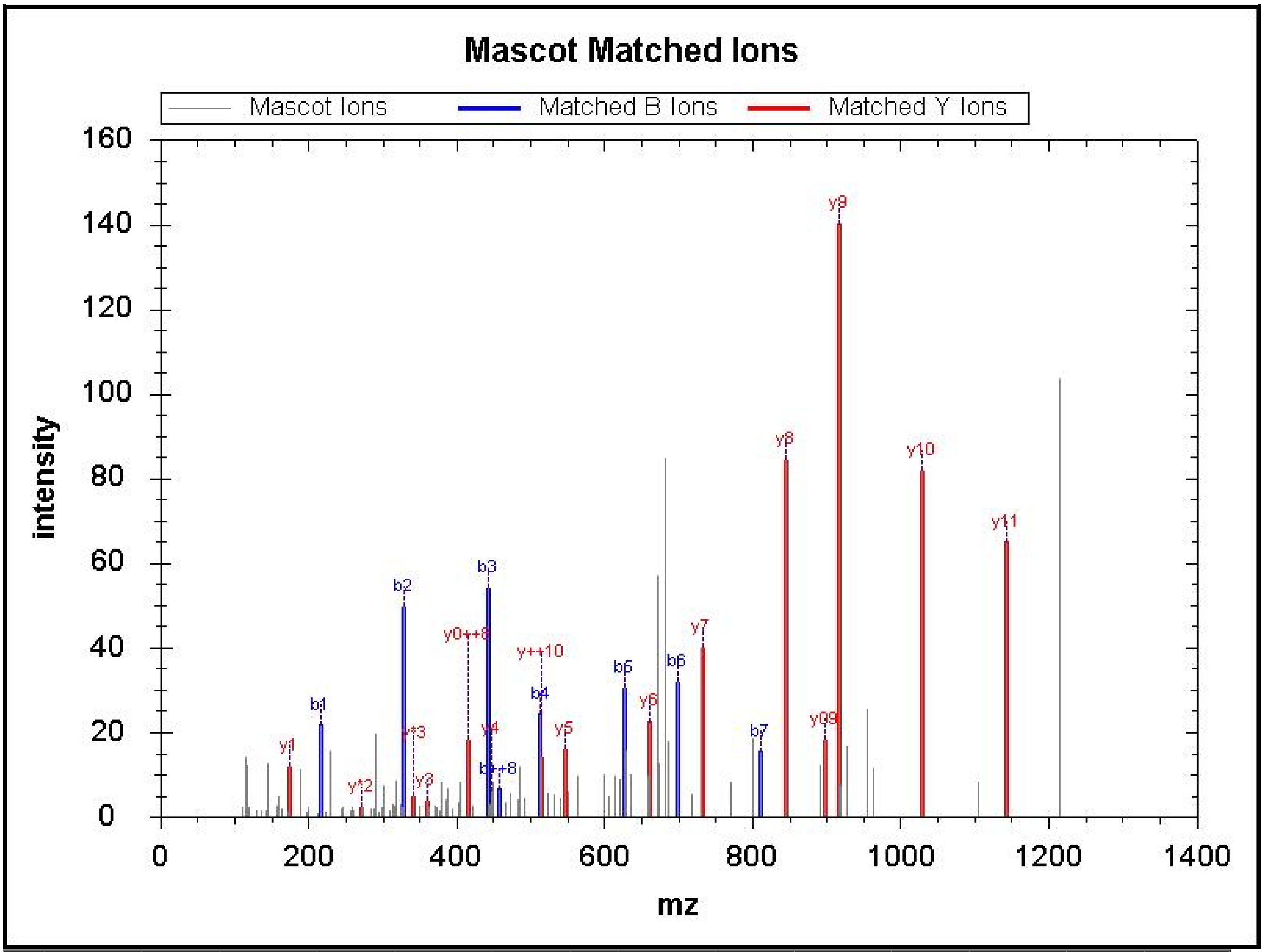

No	b	b++	b*	b*++	b0	b0++	Seq	y	y++	y*	y*++	y0	y0++	RevNo
1	216.15	108.58					A							12
2	329.23	165.12					L	1,142.69	571.85	1,125.66	563.33	1,124.68	562.84	11
3	442.31	221.66					L	1,029.61	515.31	1,012.58	506.79	1,011.59	506.30	10
4	513.35	257.18					A	916.52	458.76	899.49	450.25	898.51	449.76	9
5	627.39	314.20	610.37	305.69			N	845.48	423.25	828.46	414.73	827.47	414.24	8
6	698.43	349.72	681.41	341.21			A	731.44	366.22	714.41	357.71	713.43	357.22	7
7	811.52	406.26	794.49	397.75			L	660.40	330.71	643.38	322.19	642.39	321.70	6
8	912.56	456.79	895.54	448.27	894.55	447.78	T	547.32	274.16	530.29	265.65	529.31	265.16	5
9	999.60	500.30	982.57	491.79	981.58	491.30	S	446.27	223.64	429.25	215.13	428.26	214.63	4
10	1,070.63	535.82	1,053.61	527.31	1,052.62	526.81	A	359.24	180.12	342.21	171.61			3
11	1,183.72	592.36	1,166.69	583.85	1,165.71	583.36	L	288.20	144.61	271.18	136.09			2
12							R	175.12	88.06	158.09	79.55			1

Query 88 Hit 1

MS/MS Fragmentation of **SGNLPSKR**
Found in **sp|P46013|KI67_HUMAN**, Antigen KI-67 OS=Homo sapiens GN=MKI67 PE=1 SV=2
Match to Query 88: 858.5085from(430.2615,2+)
Title: 201: Scan 892 (rt=28.0862, f=3, i=82) [D:\lab212\membrane\Grace\20120305-iTRAQ-1.raw]

Data File:
Monoisotopic mass of neutral peptide Mr(calc): 858.5085
Variable modifications:
N3 :Deamidated (NQ)
Ions Score: 47.12 **Expect:** 0.012

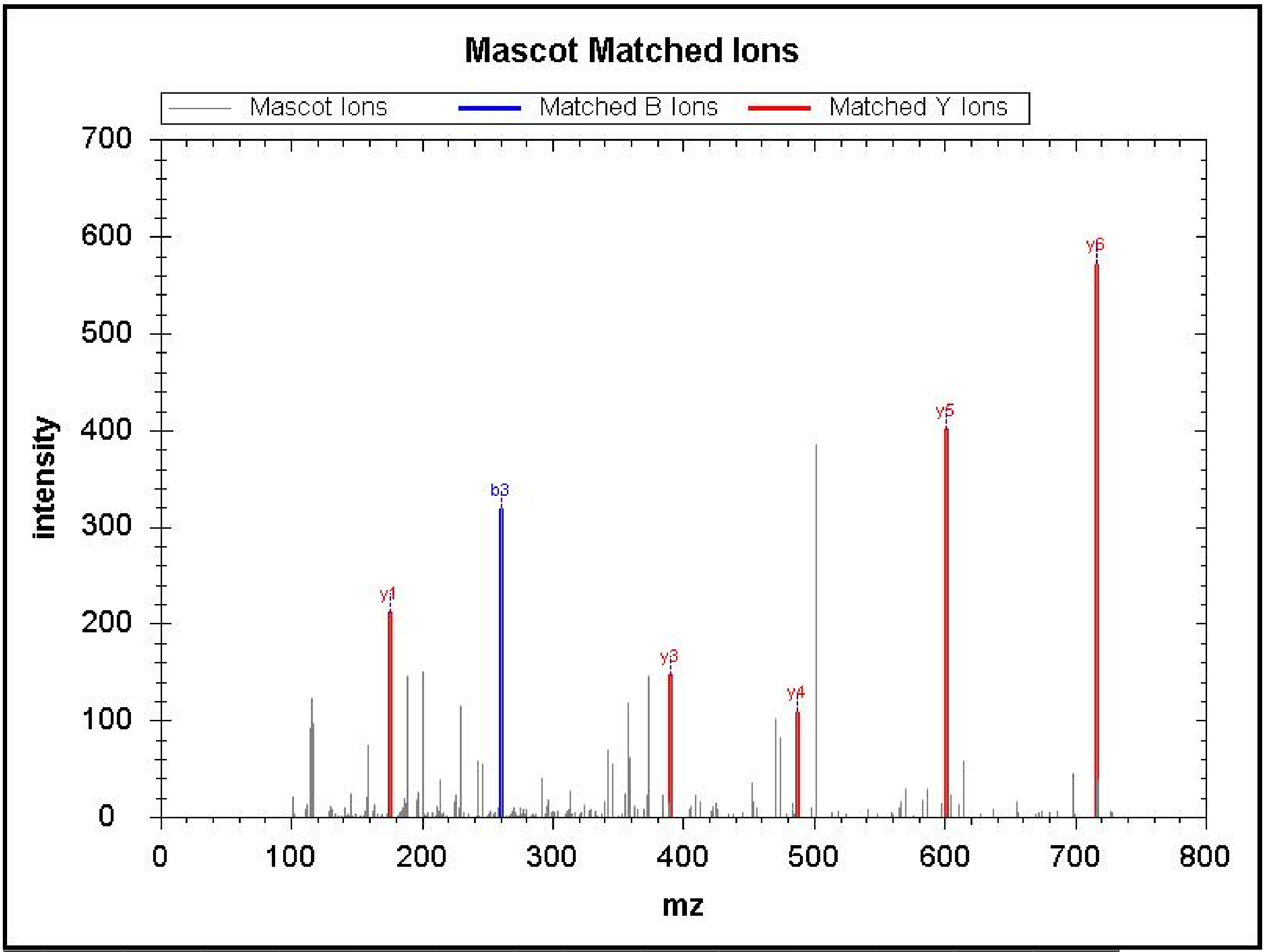

No	b	b++	b*	b*++	b0	b0++	Seq	y	y++	y*	y*++	y0	y0++	RevNo
1	88.04	44.52			70.03	35.52	S							8
2	145.06	73.03			127.05	64.03	G	772.43	386.72	755.40	378.21	754.42	377.71	7
3	**260.09**	130.55	243.06	122.03	242.08	121.54	N	**715.41**	358.21	698.38	349.70	697.40	349.20	6
4	373.17	187.09	356.15	178.58	355.16	178.08	L	**600.38**	300.70	583.36	292.18	582.37	291.69	5
5	470.22	235.62	453.20	227.10	452.21	226.61	P	**487.30**	244.15	470.27	235.64	469.29	235.15	4
6	557.26	279.13	540.23	270.62	539.25	270.13	S	**390.25**	195.63	373.22	187.11	372.24	186.62	3
7	685.35	343.18	668.32	334.67	667.34	334.17	K	303.21	152.11	286.19	143.60			2
8							R	**175.12**	88.06	158.09	79.55			1

Query 2302 Hit 1

MS/MS Fragmentation of **NLLSSWDEVIHIADQLR**
Found in **sp|Q15813|TBCE_HUMAN**, Tubulin-specific chaperone E OS=Homo sapiens GN=TBCE PE=1 SV=1
Match to Query 2302: 2152.149from(718.3903,3+)
Title: 1133: Sum of 2 scans in range 2902 (rt=73.3798, f=4, i=794) to 2903 (rt=73.4052, f=4, i=795) [D:\lab212\membrane\Grace\20120305-iTRAQ-1.raw]
Data File:
Monoisotopic mass of neutral peptide Mr(calc): 2152.149
Variable modifications:
Ions Score: 46.84 **Expect:** 0.018

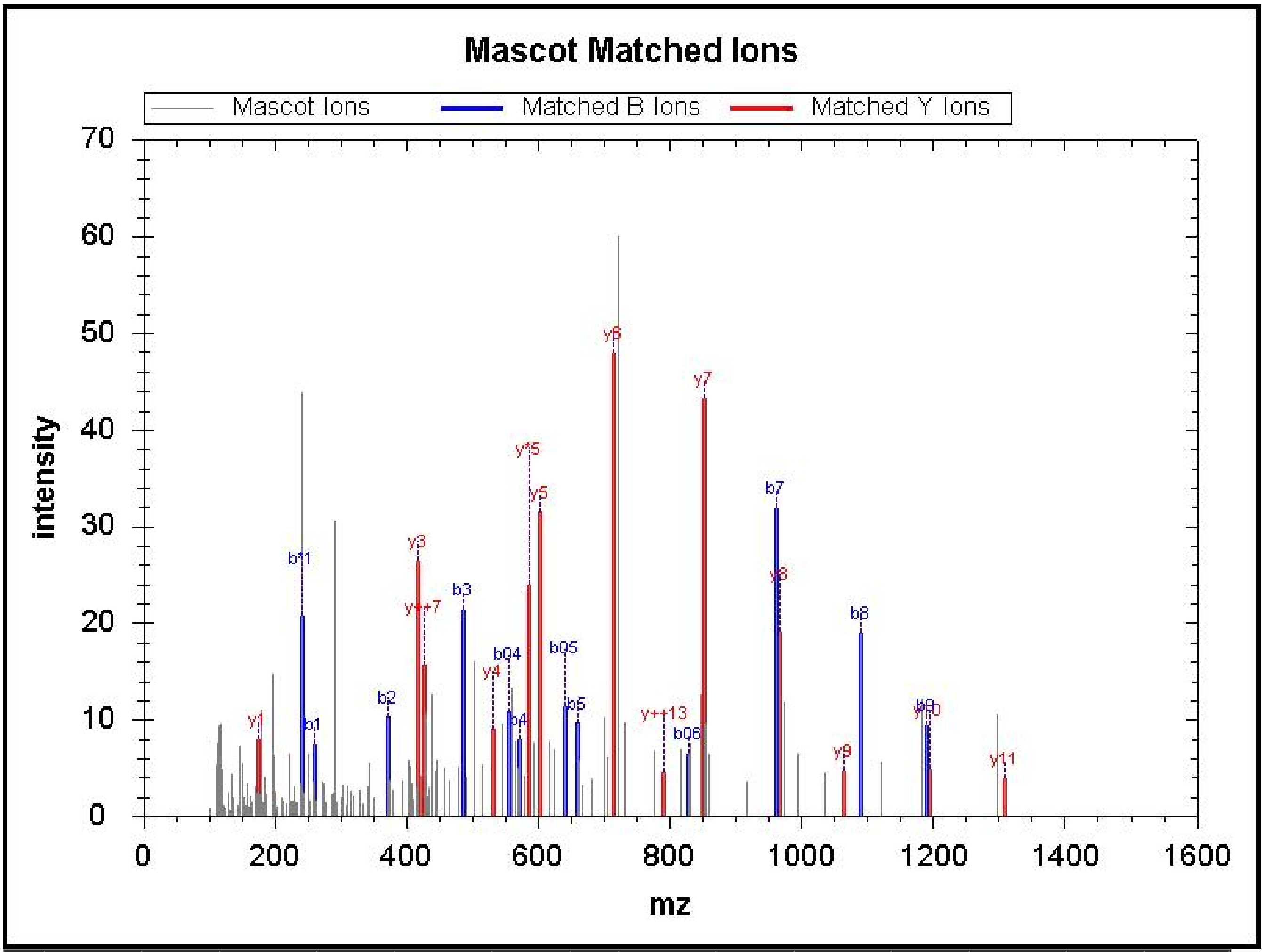

No	b	b++	b*	b*++	b0	b0++	Seq	y	y++	y*	y*++	y0	y0++	RevNo
1	259.15	130.08	242.13	121.57			N							17
2	372.24	186.62	355.21	178.11			L	1,895.00	948.00	1,877.98	939.49	1,876.99	939.00	16
3	485.32	243.16	468.29	234.65			L	1,781.92	891.46	1,764.89	882.95	1,763.91	882.46	15
4	572.35	286.68	555.33	278.17	554.34	277.67	S	1,668.83	834.92	1,651.81	826.41	1,650.82	825.92	14
5	659.38	330.20	642.36	321.68	641.37	321.19	S	1,581.80	791.40	1,564.78	782.89	1,563.79	782.40	13
6	845.46	423.24	828.44	414.72	827.45	414.23	W	1,494.77	747.89	1,477.74	739.38	1,476.76	738.88	12
7	960.49	480.75	943.46	472.24	942.48	471.74	D	1,308.69	654.85	1,291.66	646.34	1,290.68	645.84	11
8	1,089.53	545.27	1,072.51	536.76	1,071.52	536.27	E	1,193.66	597.34	1,176.64	588.82	1,175.65	588.33	10
9	1,188.60	594.80	1,171.58	586.29	1,170.59	585.80	V	1,064.62	532.81	1,047.59	524.30	1,046.61	523.81	9
10	1,301.69	651.35	1,284.66	642.83	1,283.68	642.34	I	965.55	483.28	948.53	474.77	947.54	474.27	8
11	1,438.74	719.88	1,421.72	711.36	1,420.73	710.87	H	852.47	426.74	835.44	418.22	834.46	417.73	7
12	1,551.83	776.42	1,534.80	767.90	1,533.82	767.41	I	715.41	358.21	698.38	349.70	697.40	349.20	6
13	1,622.87	811.94	1,605.84	803.42	1,604.86	802.93	A	602.33	301.67	585.30	293.15	584.32	292.66	5
14	1,737.89	869.45	1,720.87	860.94	1,719.88	860.44	D	531.29	266.15	514.26	257.63	513.28	257.14	4
15	1,865.95	933.48	1,848.93	924.97	1,847.94	924.47	Q	416.26	208.63	399.24	200.12			3
16	1,979.04	990.02	1,962.01	981.51	1,961.03	981.02	L	288.20	144.61	271.18	136.09			2
17							R	175.12	88.06	158.09	79.55			1

Query 1948 Hit 1

MS/MS Fragmentation of **KSEIEYYAMLAK**
Found in **sp|P62888|RL30_HUMAN**, 60S ribosomal protein L30 OS=Homo sapiens GN=RPL30 PE=1 SV=2
Match to Query 1948: 1877.041 from(626.6876,3+)
Title: 535: Scan 1617 (rt=44.418, f=2, i=226) [D:\lab212\membrane\Grace\20120305-iTRAQ-1.raw]
Data File:
Monoisotopic mass of neutral peptide Mr(calc): 1877.041

Variable modifications:
K1 :iTRAQ4plex (K)
K12 :iTRAQ4plex (K)
Ions Score: 46.59 **Expect:** 0.017

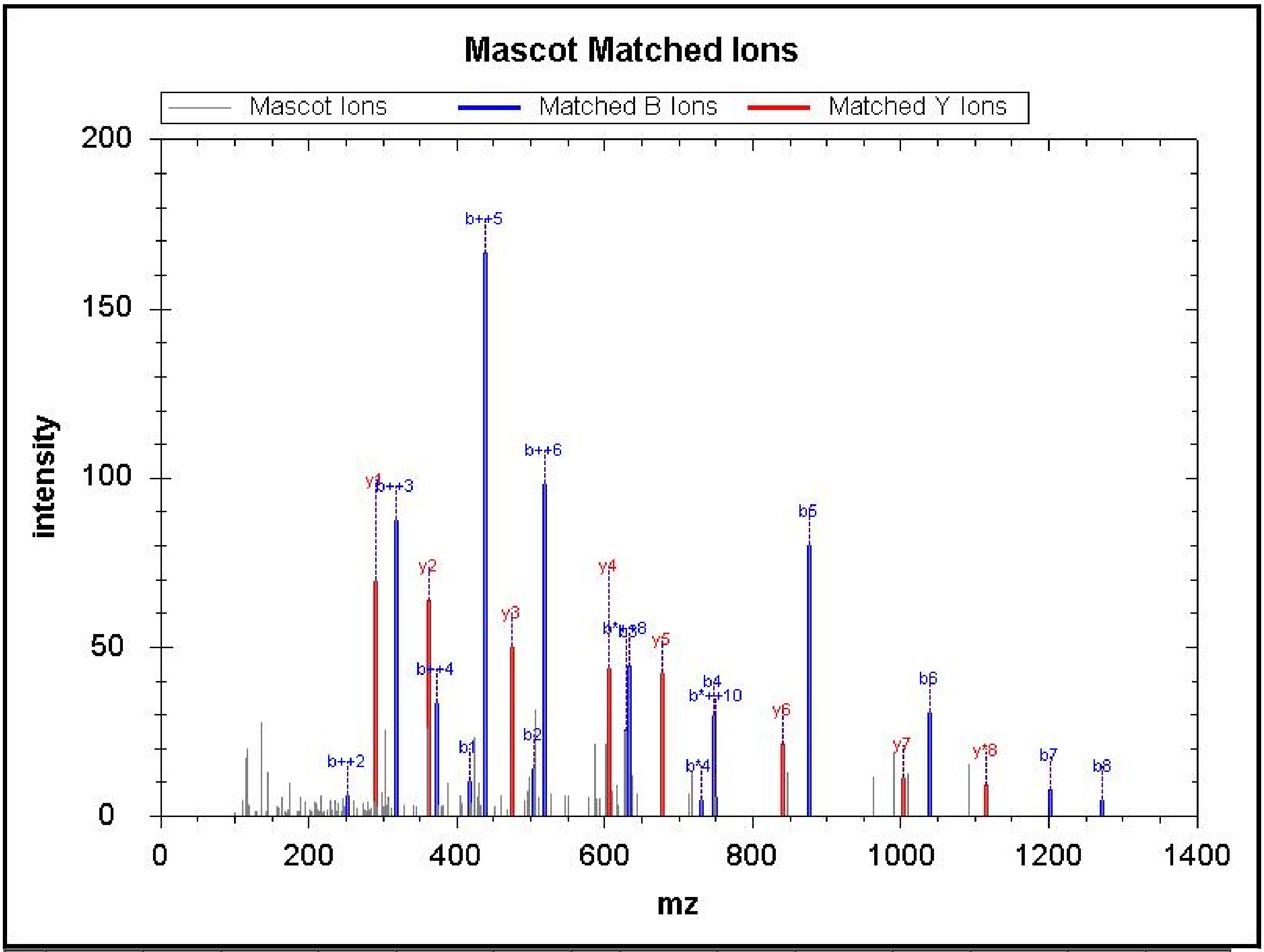

No	b	b++	b*	b*++	b0	b0++	Seq	y	y++	y*	y*++	y0	y0++	RevNo
1	417.31	209.16	400.28	200.64			K							12
2	504.34	252.67	487.31	244.16	486.33	243.67	S	1,461.74	731.37	1,444.72	722.86	1,443.73	722.37	11
3	633.38	317.19	616.35	308.68	615.37	308.19	E	1,374.71	687.86	1,357.68	679.35	1,356.70	678.85	10
4	746.47	373.74	729.44	365.22	728.45	364.73	I	1,245.67	623.34	1,228.64	614.82	1,227.66	614.33	9
5	875.51	438.26	858.48	429.74	857.50	429.25	E	1,132.58	566.80	1,115.56	558.28	1,114.57	557.79	8
6	1,038.57	519.79	1,021.54	511.28	1,020.56	510.78	Y	1,003.54	502.27	986.51	493.76			7
7	1,201.63	601.32	1,184.61	592.81	1,183.62	592.32	Y	840.48	420.74	823.45	412.23			6
8	1,272.67	636.84	1,255.65	628.33	1,254.66	627.83	A	677.41	339.21	660.39	330.70			5
9	1,403.71	702.36	1,386.69	693.85	1,385.70	693.35	M	606.38	303.69	589.35	295.18			4
10	1,516.80	758.90	1,499.77	750.39	1,498.79	749.90	L	475.34	238.17	458.31	229.66			3
11	1,587.83	794.42	1,570.81	785.91	1,569.82	785.41	A	362.25	181.63	345.23	173.12			2
12							K	291.21	146.11	274.19	137.60			1

Query 2362 Hit 1

MS/MS Fragmentation of **IDLRPVLGEGVPILASFLR**
Found in **sp|Q86VP6|CAND1_HUMAN**, Cullin-associated NEDD8-dissociated protein 1 OS=Homo sapiens GN=CAND1 PE=1 SV=2
Match to Query 2362: 2208.309from(737.1104,3+)
Title: 1159: Scan 2969 (rt=74.8872, f=3, i=417) [D:\lab212\membrane\Grace\20120305-iTRAQ-1.raw]
Data File:
Monoisotopic mass of neutral peptide Mr(calc): 2208.309
Variable modifications:
Ions Score: 46.27 **Expect:** 0.006

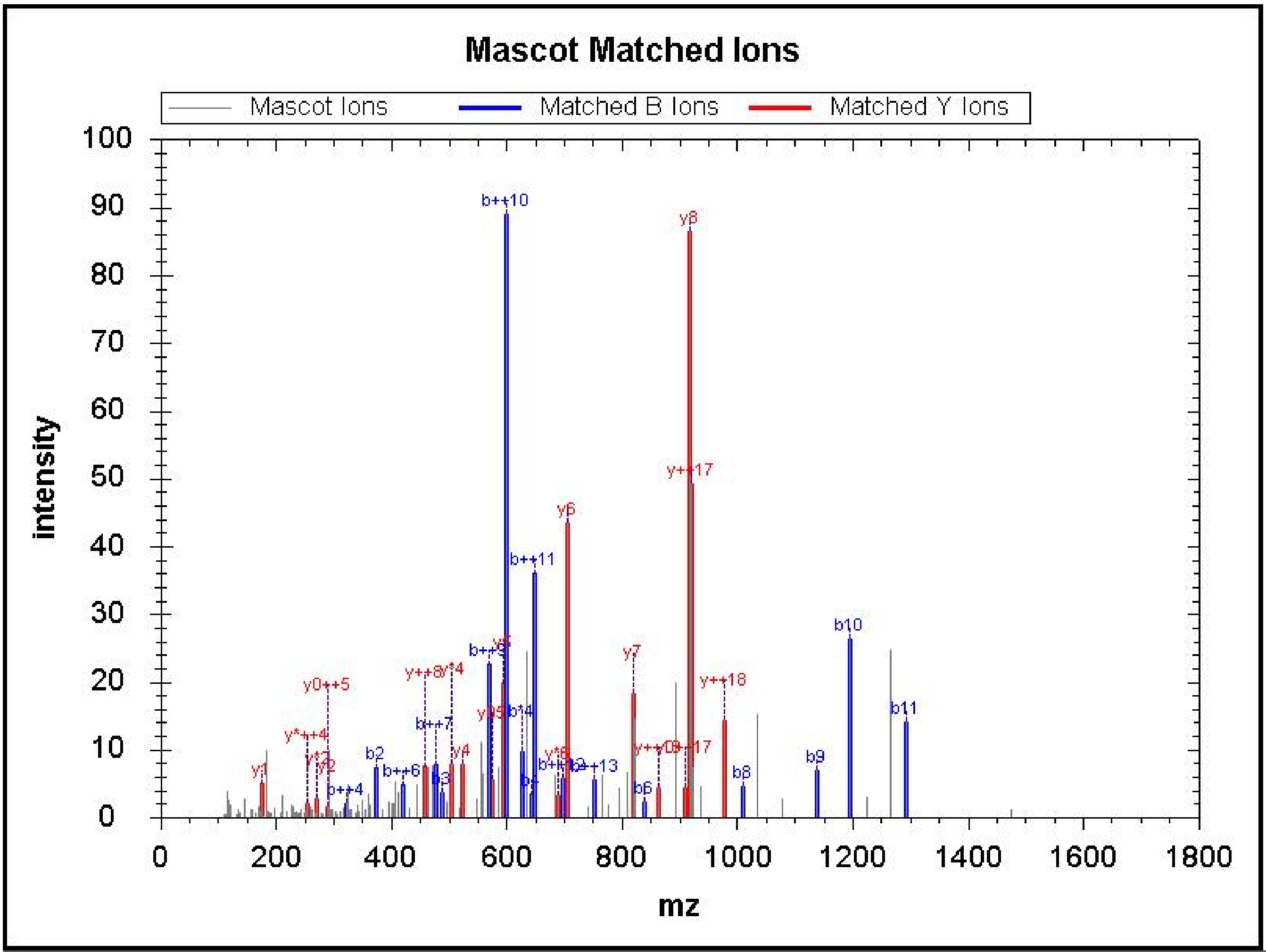

No	b	b++	b*	b*++	b0	b0++	Seq	y	y++	y*	y*++	y0	y0++	RevNo
1	258.19	129.60					I							19
2	373.22	187.11			355.21	178.11	D	1,952.13	976.57	1,935.11	968.06	1,934.12	967.56	18
3	486.30	243.66			468.29	234.65	L	1,837.11	919.06	1,820.08	910.54	1,819.10	910.05	17
4	642.41	321.71	625.38	313.19	624.39	312.70	R	1,724.02	862.51	1,707.00	854.00	1,706.01	853.51	16
5	739.46	370.23	722.43	361.72	721.45	361.23	P	1,567.92	784.46	1,550.89	775.95	1,549.91	775.46	15
6	838.53	419.77	821.50	411.25	820.52	410.76	V	1,470.87	735.94	1,453.84	727.42	1,452.86	726.93	14
7	951.61	476.31	934.58	467.80	933.60	467.30	L	1,371.80	686.40	1,354.77	677.89	1,353.79	677.40	13
8	1,008.63	504.82	991.61	496.31	990.62	495.81	G	1,258.72	629.86	1,241.69	621.35	1,240.70	620.86	12
9	1,137.67	569.34	1,120.65	560.83	1,119.66	560.34	E	1,201.69	601.35	1,184.67	592.84	1,183.68	592.35	11
10	1,194.70	597.85	1,177.67	589.34	1,176.69	588.85	G	1,072.65	536.83	1,055.62	528.32	1,054.64	527.82	10
11	1,293.76	647.39	1,276.74	638.87	1,275.75	638.38	V	1,015.63	508.32	998.60	499.81	997.62	499.31	9
12	1,390.82	695.91	1,373.79	687.40	1,372.81	686.91	P	916.56	458.78	899.53	450.27	898.55	449.78	8
13	1,503.90	752.45	1,486.88	743.94	1,485.89	743.45	I	819.51	410.26	802.48	401.74	801.50	401.25	7
14	1,616.99	809.00	1,599.96	800.48	1,598.97	799.99	L	706.42	353.72	689.40	345.20	688.41	344.71	6
15	1,688.02	844.52	1,671.00	836.00	1,670.01	835.51	A	593.34	297.17	576.31	288.66	575.33	288.17	5
16	1,775.05	888.03	1,758.03	879.52	1,757.04	879.03	S	522.30	261.66	505.28	253.14	504.29	252.65	4
17	1,922.12	961.57	1,905.10	953.05	1,904.11	952.56	F	435.27	218.14	418.24	209.63			3
18	2,035.21	1,018.11	2,018.18	1,009.59	2,017.20	1,009.10	L	288.20	144.61	271.18	136.09			2
19							R	175.12	88.06	158.09	79.55			1

Query 1940 Hit 1

MS/MS Fragmentation of **IEVFVQTLLHLAAK**
Found in **sp|Q09161|NCBP1_HUMAN**, Nuclear cap-binding protein subunit 1 OS=Homo sapiens GN=NCBP1 PE=1 SV=1
Match to Query 1940: 1869.144from(624.0551,3+)

Title: 1255: Sum of 2 scans in range 3040 (rt=76.939, f=4, i=872) to 3041 (rt=76.9644, f=4, i=873) [D:\lab212\membrane\Grace\20120305-iTRAQ-2.raw]
Data File:
Monoisotopic mass of neutral peptide Mr(calc): 1869.144
Variable modifications:
K14 :iTRAQ4plex (K)
Ions Score: 45.88 **Expect:** 0.005

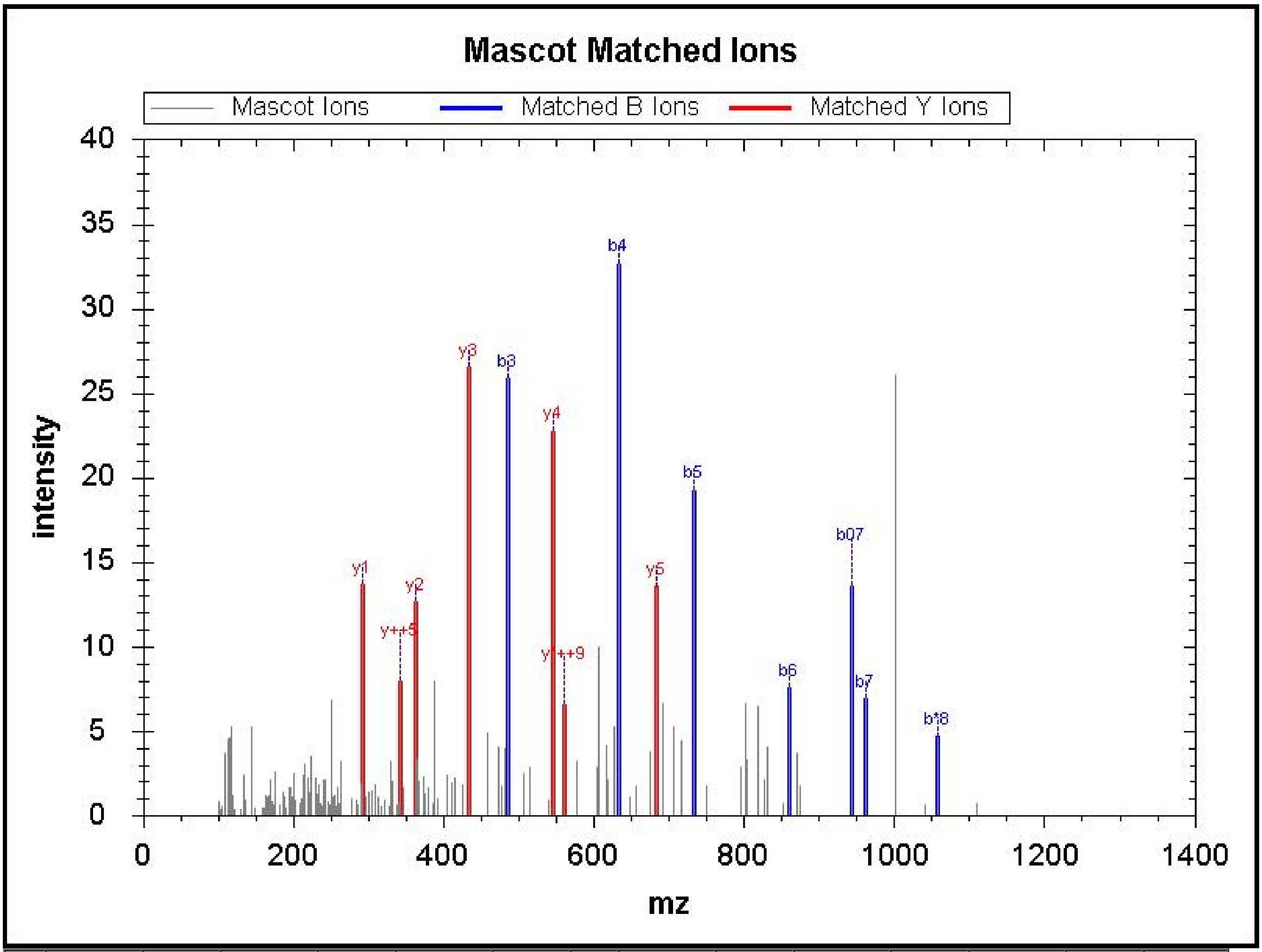

No	b	b++	b*	b*++	b0	b0++	Seq	y	y++	y*	y*++	y0	y0++	RevNo
1	258.19	129.60					I							14
2	387.24	194.12			369.23	185.12	E	1,612.95	806.98	1,595.93	798.47	1,594.94	797.98	13
3	**486.30**	243.66			468.29	234.65	V	1,483.91	742.46	1,466.88	733.95	1,465.90	733.45	12
4	**633.37**	317.19			615.36	308.18	F	1,384.84	692.93	1,367.82	684.41	1,366.83	683.92	11
5	**732.44**	366.72			714.43	357.72	V	1,237.77	619.39	1,220.75	610.88	1,219.76	610.39	10
6	**860.50**	430.75	843.47	422.24	842.49	421.75	Q	1,138.71	569.86	1,121.68	**561.34**	1,120.70	560.85	9
7	**961.55**	481.28	944.52	472.76	**943.54**	472.27	T	1,010.65	505.83	993.62	497.31	992.64	496.82	8
8	1,074.63	537.82	**1,057.61**	529.31	1,056.62	528.81	L	909.60	455.30	892.57	446.79			7
9	1,187.72	594.36	1,170.69	585.85	1,169.71	585.36	L	796.52	398.76	779.49	390.25			6
10	1,324.77	662.89	1,307.75	654.38	1,306.76	653.89	H	**683.43**	**342.22**	666.41	333.71			5
11	1,437.86	719.43	1,420.83	710.92	1,419.85	710.43	L	**546.37**	273.69	529.35	265.18			4
12	1,508.90	754.95	1,491.87	746.44	1,490.89	745.95	A	**433.29**	217.15	416.26	208.63			3
13	1,579.93	790.47	1,562.91	781.96	1,561.92	781.46	A	**362.25**	181.63	345.23	173.12			2
14							K	**291.21**	146.11	274.19	137.60			1

Query 2567 Hit 1

MS/MS Fragmentation of **SLQELFLAHILSPWGAEVK**
Found in **sp|P09874|PARP1_HUMAN**, Poly [ADP-ribose] polymerase 1 OS=Homo sapiens GN=PARP1 PE=1 SV=4

Match to Query 2567: 2425.354from(809.4587,3+)
Title: 1099: Scan 2820 (rt=71.5304, f=3, i=393) [D:\lab212\membrane\Grace\20120305-iTRAQ-1.raw]
Data File:
Monoisotopic mass of neutral peptide Mr(calc): 2425.354
Variable modifications:
K19 :iTRAQ4plex (K)
Ions Score: 45.25 **Expect:** 0.015

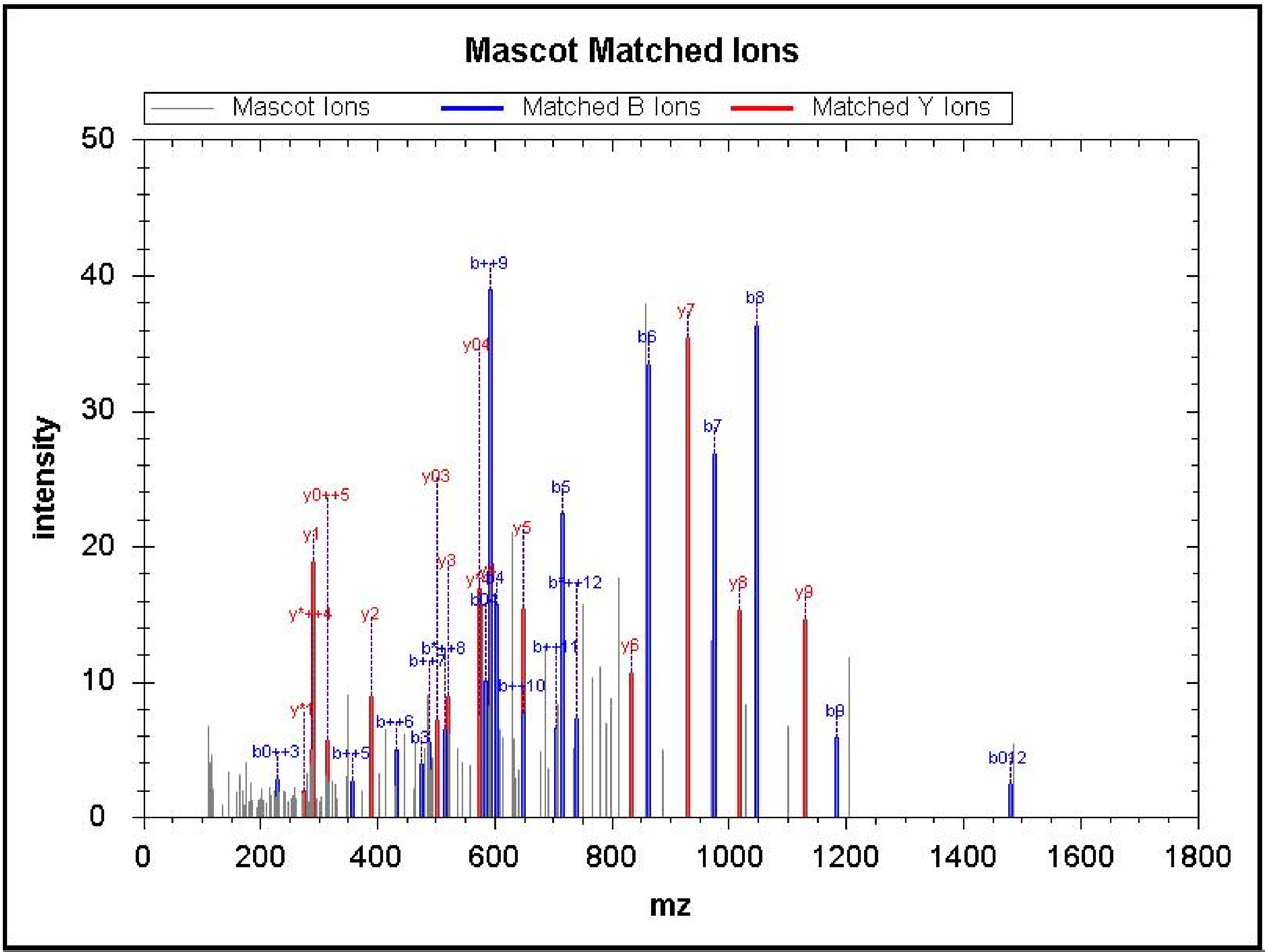

No	b	b++	b*	b*++	b0	b0++	Seq	y	y++	y*	y*++	y0	y0++	RevNo
1	232.14	116.57			214.13	107.57	S							19
2	345.23	173.12			327.21	164.11	L	2,195.23	1,098.12	2,178.21	1,089.61	2,177.22	1,089.12	18
3	**473.28**	237.15	456.26	228.63	455.27	**228.14**	Q	2,082.15	1,041.58	2,065.12	1,033.07	2,064.14	1,032.57	17
4	**602.33**	301.67	585.30	293.15	**584.32**	292.66	E	1,954.09	977.55	1,937.07	969.04	1,936.08	968.54	16
5	**715.41**	**358.21**	698.38	349.70	697.40	349.20	L	1,825.05	913.03	1,808.02	904.52	1,807.04	904.02	15
6	**862.48**	**431.74**	845.45	423.23	844.47	422.74	F	1,711.97	856.49	1,694.94	847.97	1,693.95	847.48	14
7	**975.56**	**488.29**	958.54	479.77	957.55	479.28	L	1,564.90	782.95	1,547.87	774.44	1,546.89	773.95	13
8	**1,046.60**	523.80	1,029.57	**515.29**	1,028.59	514.80	A	1,451.81	726.41	1,434.79	717.90	1,433.80	717.40	12
9	**1,183.66**	**592.33**	1,166.63	583.82	1,165.65	583.33	H	1,380.78	690.89	1,363.75	682.38	1,362.77	681.89	11
10	1,296.74	**648.88**	1,279.72	640.36	1,278.73	639.87	I	1,243.72	622.36	1,226.69	613.85	1,225.71	613.36	10
11	1,409.83	**705.42**	1,392.80	696.90	1,391.82	696.41	L	**1,130.63**	565.82	1,113.61	557.31	1,112.62	556.81	9
12	1,496.86	748.93	1,479.83	**740.42**	**1,478.85**	739.93	S	**1,017.55**	509.28	1,000.52	500.76	999.54	500.27	8
13	1,593.91	797.46	1,576.89	788.95	1,575.90	788.45	P	**930.52**	465.76	913.49	457.25	912.51	456.76	7
14	1,779.99	890.50	1,762.97	881.99	1,761.98	881.49	W	**833.46**	417.24	816.44	408.72	815.45	408.23	6
15	1,837.01	919.01	1,819.99	910.50	1,819.00	910.00	G	**647.38**	324.20	630.36	315.68	629.37	**315.19**	5
16	1,908.05	954.53	1,891.02	946.02	1,890.04	945.52	A	**590.36**	295.69	**573.34**	**287.17**	**572.35**	286.68	4
17	2,037.09	1,019.05	2,020.07	1,010.54	2,019.08	1,010.04	E	**519.33**	260.17	502.30	251.65	**501.32**	251.16	3
18	2,136.16	1,068.58	2,119.13	1,060.07	2,118.15	1,059.58	V	**390.28**	195.65	373.26	187.13			2

19							K	291.21	146.11	274.19	137.60			1

Query 1322 Hit 1

MS/MS Fragmentation of IQAIELEDLLR
Found in sp|P21810|PGS1_HUMAN, Biglycan OS=Homo sapiens GN=BGN PE=1 SV=2
Match to Query 1322: 1455.845from(728.9296,2+)
Title: 883: Sum of 2 scans in range 2356 (rt=61.0782, f=4, i=618) to 2357 (rt=61.1036, f=4, i=619) [D:\lab212\membrane\Grace\20120305-iTRAQ-1.raw]
Data File:
Monoisotopic mass of neutral peptide Mr(calc): 1455.845
Variable modifications:
Ions Score: 45.11 **Expect:** 0.018

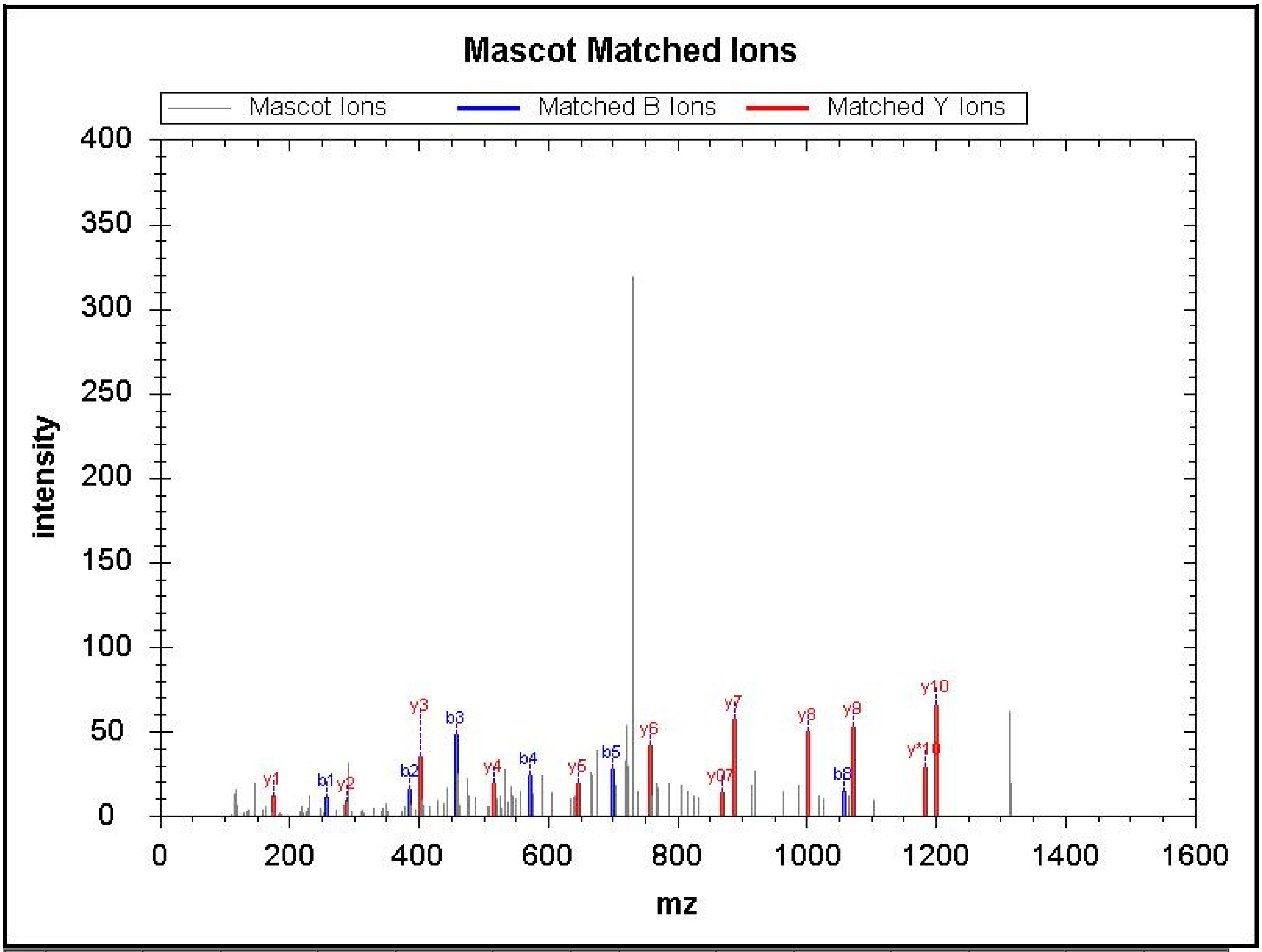

No	b	b++	b*	b*++	b0	b0++	Seq	y	y++	y*	y*++	y0	y0++	RevNo
1	258.19	129.60					I							11
2	386.25	193.63	369.23	185.12			Q	1,199.66	600.34	1,182.64	591.82	1,181.65	591.33	10
3	457.29	229.15	440.26	220.63			A	1,071.60	536.31	1,054.58	527.79	1,053.59	527.30	9
4	570.37	285.69	553.35	277.18			I	1,000.57	500.79	983.54	492.27	982.56	491.78	8
5	699.42	350.21	682.39	341.70	681.41	341.21	E	887.48	444.25	870.46	435.73	869.47	435.24	7
6	812.50	406.75	795.47	398.24	794.49	397.75	L	758.44	379.72	741.41	371.21	740.43	370.72	6
7	941.54	471.27	924.52	462.76	923.53	462.27	E	645.36	323.18	628.33	314.67	627.35	314.18	5
8	1,056.57	528.79	1,039.54	520.28	1,038.56	519.78	D	516.31	258.66	499.29	250.15	498.30	249.66	4
9	1,169.65	585.33	1,152.63	576.82	1,151.64	576.33	L	401.29	201.15	384.26	192.63			3
10	1,282.74	641.87	1,265.71	633.36	1,264.73	632.87	L	288.20	144.61	271.18	136.09			2
11							R	175.12	88.06	158.09	79.55			1

Query 856 Hit 1

MS/MS Fragmentation of **LMDVGLIAIR**
Found in **sp|P31948|STIP1_HUMAN**, Stress-induced-phosphoprotein 1 OS=Homo sapiens GN=STIP1 PE=1 SV=1
Match to Query 856: 1243.751from(622.8829,2+)
Title: 853: Scan 2159 (rt=57.0835, f=3, i=297) [D:\lab212\membrane\Grace\20120305-iTRAQ-2.raw]
Data File:
Monoisotopic mass of neutral peptide Mr(calc): 1243.751
Variable modifications:
Ions Score: 44.76 **Expect:** 0.019

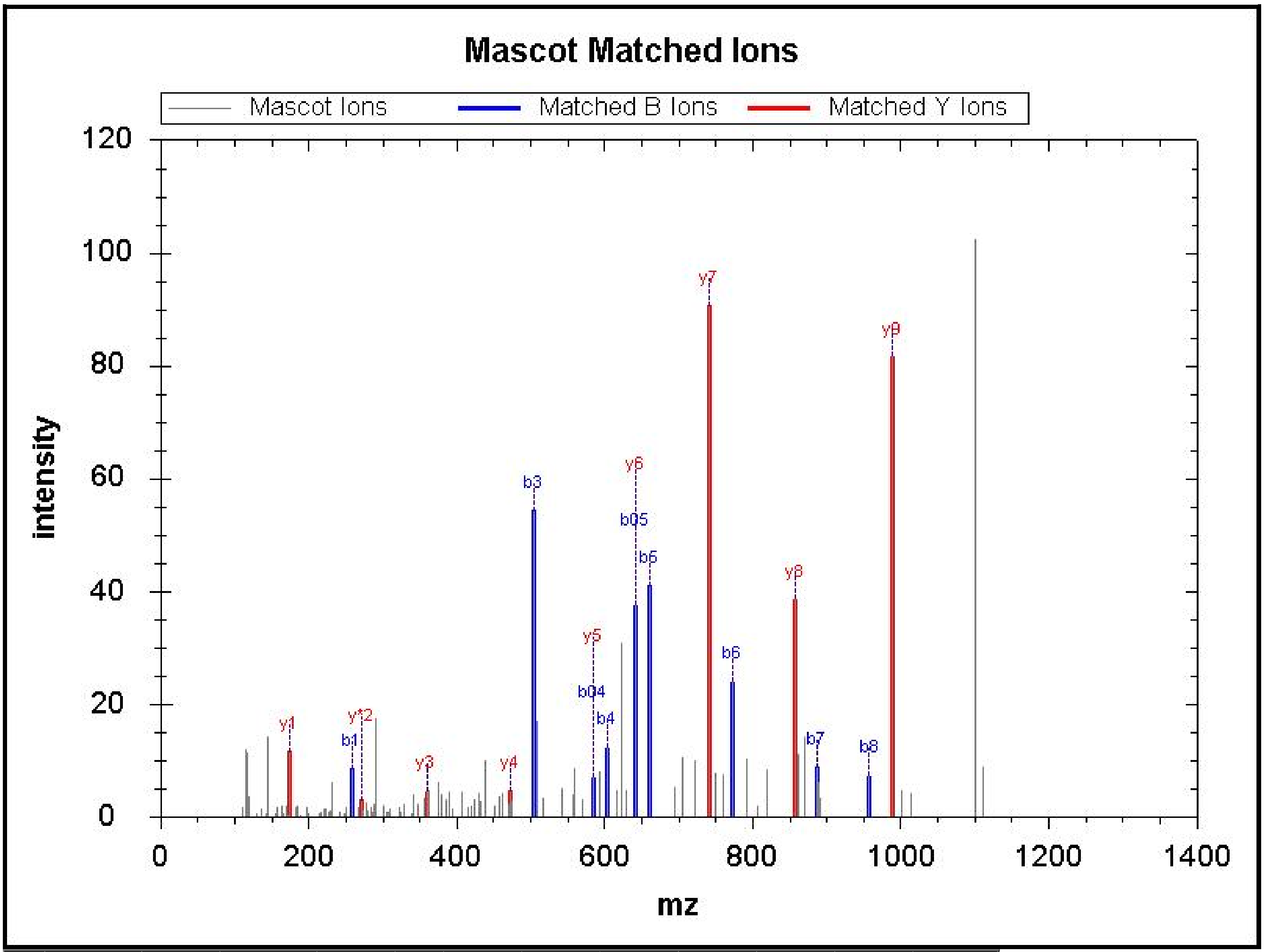

No	b	b++	b0	b0++	Seq	y	y++	y*	y*++	y0	y0++	RevNo
1	258.19	129.60			L							10
2	389.23	195.12			M	987.57	494.29	970.54	485.77	969.55	485.28	9
3	504.26	252.63	486.25	243.63	D	856.53	428.77	839.50	420.25	838.51	419.76	8
4	603.33	302.17	585.32	293.16	V	741.50	371.25	724.47	362.74			7
5	660.35	330.68	642.34	321.67	G	642.43	321.72	625.40	313.21			6
6	773.43	387.22	755.42	378.22	L	585.41	293.21	568.38	284.69			5
7	886.52	443.76	868.51	434.76	I	472.32	236.67	455.30	228.15			4
8	957.56	479.28	939.55	470.28	A	359.24	180.12	342.21	171.61			3
9	1,070.64	535.82	1,052.63	526.82	I	288.20	144.61	271.18	136.09			2
10					R	175.12	88.06	158.09	79.55			1

Query 1906 Hit 1

MS/MS Fragmentation of **ITFVDFIAYDVLER**
Found in **sp|P28161|GSTM2_HUMAN**, Glutathione S-transferase Mu 2 OS=Homo sapiens GN=GSTM2 PE=1 SV=2
Match to Query 1906: 1843.994from(923.0045,2+)
Title: 1184: Sum of 2 scans in range 3026 (rt=76.1738, f=4, i=834) to 3027 (rt=76.1992, f=4, i=835) [D:\lab212\membrane\Grace\20120305-iTRAQ-1.raw]

Data File:
Monoisotopic mass of neutral peptide Mr(calc): 1843.994
Variable modifications:
Ions Score: 44.08 **Expect:** 0.032

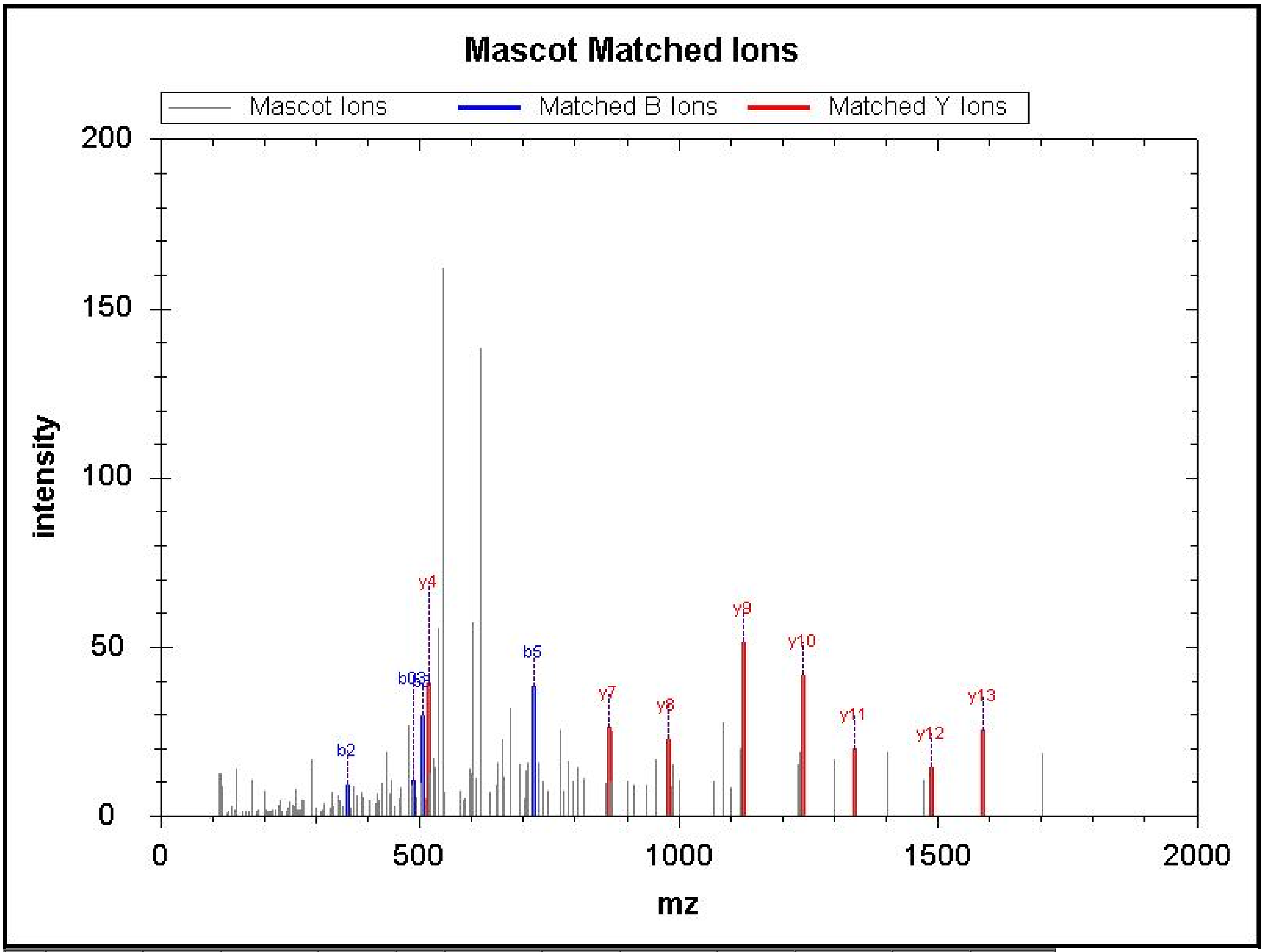

No	b	b++	b0	b0++	Seq	y	y++	y*	y*++	y0	y0++	RevNo
1	258.19	129.60			I							14
2	**359.24**	180.12	341.23	171.12	T	**1,587.81**	794.41	1,570.78	785.89	1,569.79	785.40	13
3	**506.31**	253.66	**488.30**	244.65	F	**1,486.76**	743.88	1,469.73	735.37	1,468.75	734.88	12
4	605.38	303.19	587.37	294.19	V	**1,339.69**	670.35	1,322.66	661.83	1,321.68	661.34	11
5	**720.40**	360.71	702.39	351.70	D	**1,240.62**	620.81	1,223.59	612.30	1,222.61	611.81	10
6	867.47	434.24	849.46	425.23	F	**1,125.59**	563.30	1,108.57	554.79	1,107.58	554.30	9
7	980.56	490.78	962.55	481.78	I	**978.53**	489.77	961.50	481.25	960.51	480.76	8
8	1,051.59	526.30	1,033.58	517.30	A	**865.44**	433.22	848.41	424.71	847.43	424.22	7
9	1,214.66	607.83	1,196.65	598.83	Y	794.40	397.71	777.38	389.19	776.39	388.70	6
10	1,329.68	665.35	1,311.67	656.34	D	631.34	316.17	614.31	307.66	613.33	307.17	5
11	1,428.75	714.88	1,410.74	705.87	V	**516.31**	258.66	499.29	250.15	498.30	249.66	4
12	1,541.84	771.42	1,523.83	762.42	L	417.25	209.13	400.22	200.61	399.24	200.12	3
13	1,670.88	835.94	1,652.87	826.94	E	304.16	152.58	287.13	144.07	286.15	143.58	2
14					R	175.12	88.06	158.09	79.55			1

Query 906 Hit 1

MS/MS Fragmentation of **LWWLDLK**
Found in **sp|P02790|HEMO_HUMAN**, Hemopexin OS=Homo sapiens GN=HPX PE=1 SV=2
Match to Query 906: 1260.756from(631.3855,2+)
Title: 943: Scan 2492 (rt=64.138, f=3, i=340) [D:\lab212\membrane\Grace\20120305-iTRAQ-1.raw]
Data File:

Monoisotopic mass of neutral peptide Mr(calc): 1260.756
Variable modifications:
K7 :iTRAQ4plex (K)
Ions Score: 43.71 **Expect:** 0.027

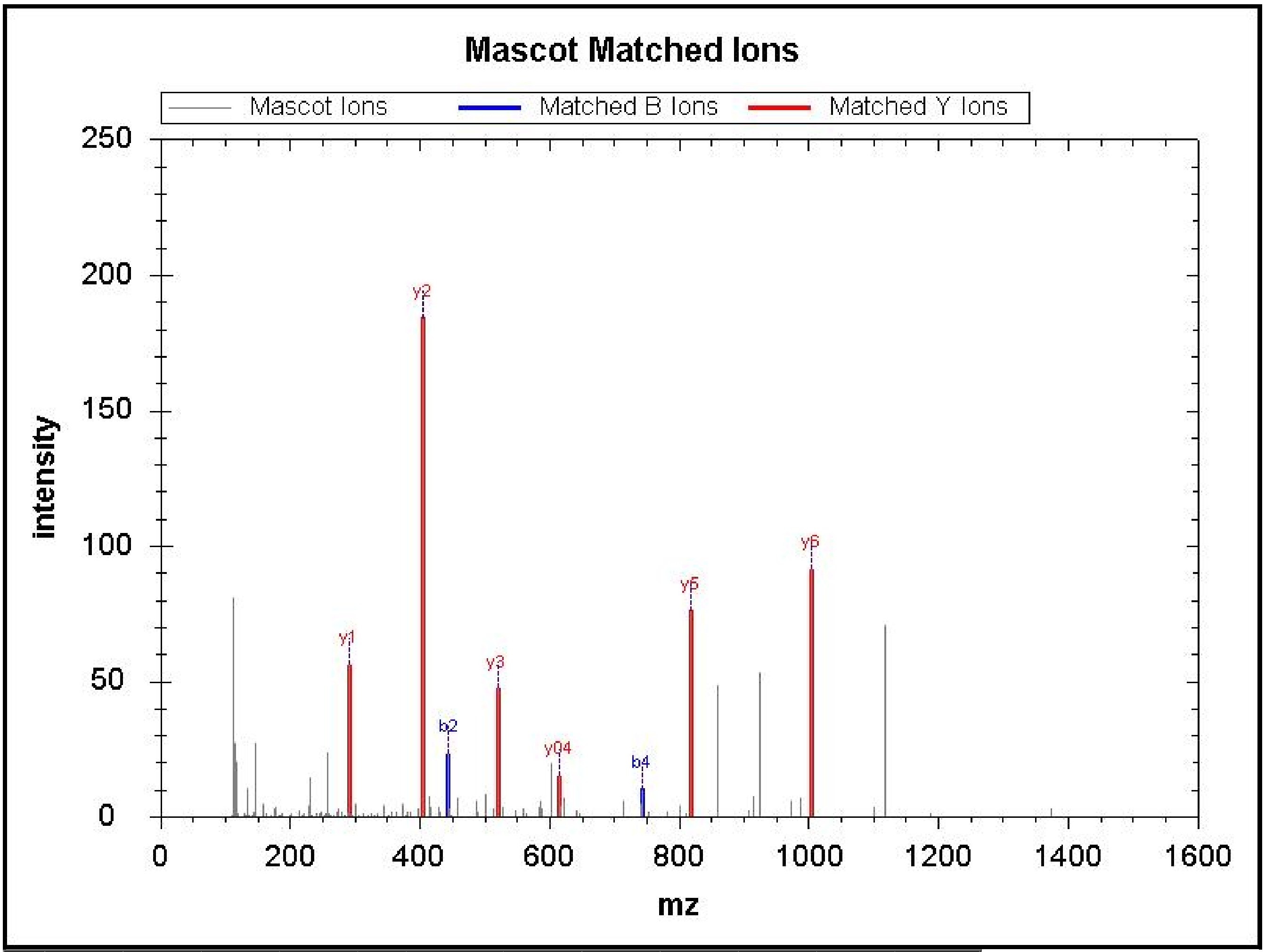

No	b	b++	b0	b0++	Seq	y	y++	y*	y*++	y0	y0++	RevNo
1	258.19	129.60			L							7
2	444.27	222.64			W	1,004.57	502.79	987.54	494.27	986.56	493.78	6
3	630.35	315.68			W	818.49	409.75	801.46	401.23	800.48	400.74	5
4	743.44	372.22			L	632.41	316.71	615.38	308.20	614.40	307.70	4
5	858.46	429.74	840.45	420.73	D	519.33	260.17	502.30	251.65	501.32	251.16	3
6	971.55	486.28	953.54	477.27	L	404.30	202.65	387.27	194.14			2
7					K	291.21	146.11	274.19	137.60			1

Query 1489 Hit 1

MS/MS Fragmentation of NIPGITLLNVSK
Found in sp|P36578|RL4_HUMAN, 60S ribosomal protein L4 OS=Homo sapiens GN=RPL4 PE=1 SV=5
Match to Query 1489: 1555.964from(778.9894,2+)
Title: 751: Sum of 2 scans in range 1943 (rt=52.2193, f=4, i=518) to 1944 (rt=52.2447, f=4, i=519) [D:\lab212\membrane\Grace\20120305-iTRAQ-2.raw]
Data File:
Monoisotopic mass of neutral peptide Mr(calc): 1555.964
Variable modifications:
K12 :iTRAQ4plex (K)
Ions Score: 42.92 **Expect:** 0.014

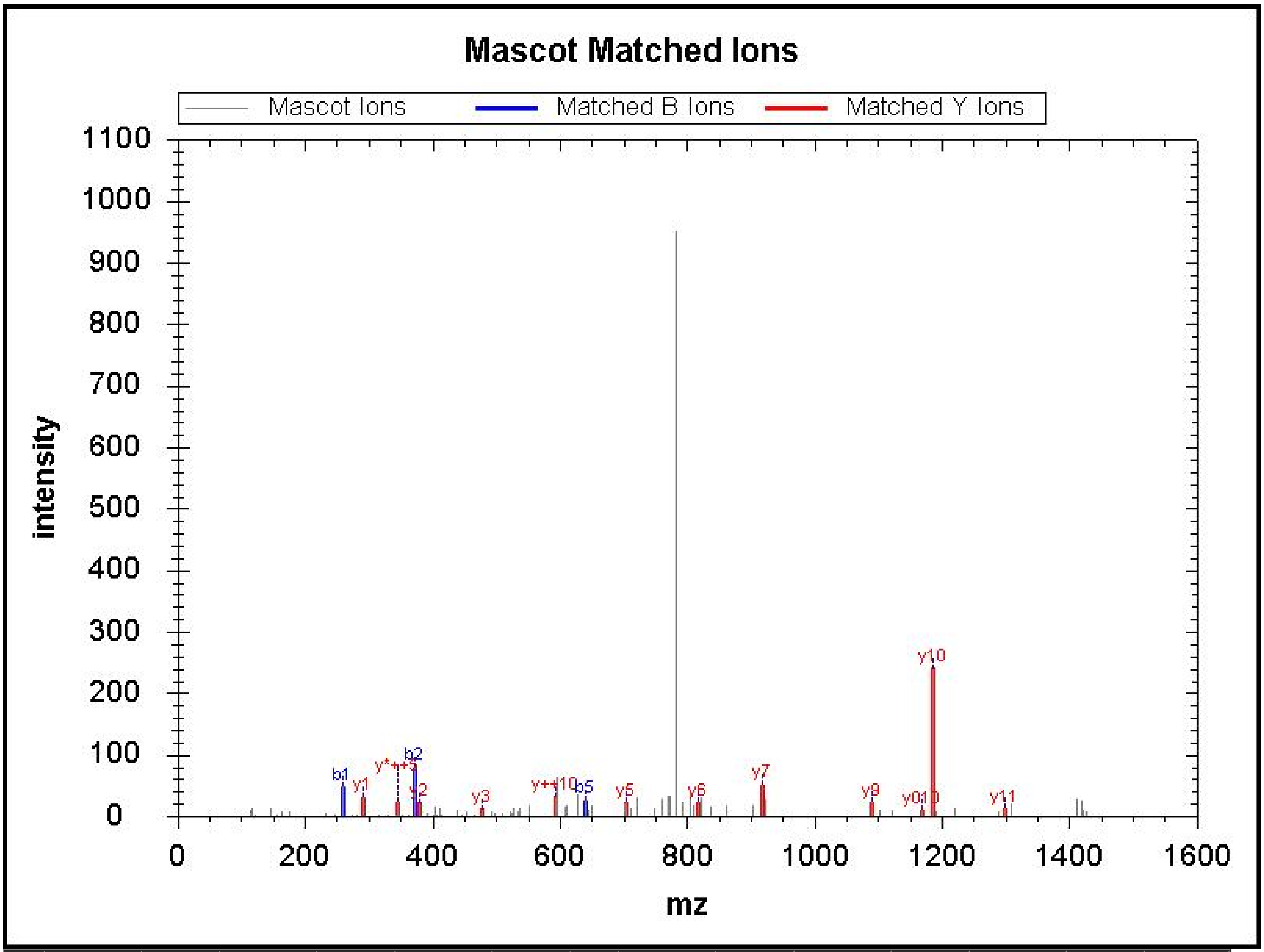

No	b	b++	b*	b*++	b0	b0++	Seq	y	y++	y*	y*++	y0	y0++	RevNo
1	259.15	130.08	242.13	121.57			N							12
2	372.24	186.62	355.21	178.11			I	1,298.82	649.91	1,281.79	641.40	1,280.81	640.91	11
3	469.29	235.15	452.26	226.63			P	1,185.73	593.37	1,168.71	584.86	1,167.72	584.36	10
4	526.31	263.66	509.28	255.15			G	1,088.68	544.84	1,071.65	536.33	1,070.67	535.84	9
5	639.39	320.20	622.37	311.69			I	1,031.66	516.33	1,014.63	507.82	1,013.65	507.33	8
6	740.44	370.72	723.42	362.21	722.43	361.72	T	918.57	459.79	901.55	451.28	900.56	450.79	7
7	853.53	427.27	836.50	418.75	835.52	418.26	L	817.53	409.27	800.50	400.75	799.52	400.26	6
8	966.61	483.81	949.58	475.30	948.60	474.80	L	704.44	352.72	687.42	344.21	686.43	343.72	5
9	1,080.65	540.83	1,063.63	532.32	1,062.64	531.83	N	591.36	296.18	574.33	287.67	573.35	287.18	4
10	1,179.72	590.36	1,162.70	581.85	1,161.71	581.36	V	477.32	239.16	460.29	230.65	459.30	230.16	3
11	1,266.75	633.88	1,249.73	625.37	1,248.74	624.88	S	378.25	189.63	361.22	181.11	360.24	180.62	2
12							K	291.21	146.11	274.19	137.60			1

Query 1838 Hit 1

MS/MS Fragmentation of FFEVILIDPFHK
Found in sp|P61313|RL15_HUMAN, 60S ribosomal protein L15 OS=Homo sapiens GN=RPL15 PE=1 SV=2
Match to Query 1838: 1792.025from(598.3489,3+)
Title: 1000: Scan 2615 (rt=66.9066, f=2, i=387) [D:\lab212\membrane\Grace\20120305-iTRAQ-1.raw]
Data File:
Monoisotopic mass of neutral peptide Mr(calc): 1792.025
Variable modifications:
K12 :iTRAQ4plex (K)
Ions Score: 42.89 **Expect:** 0.032

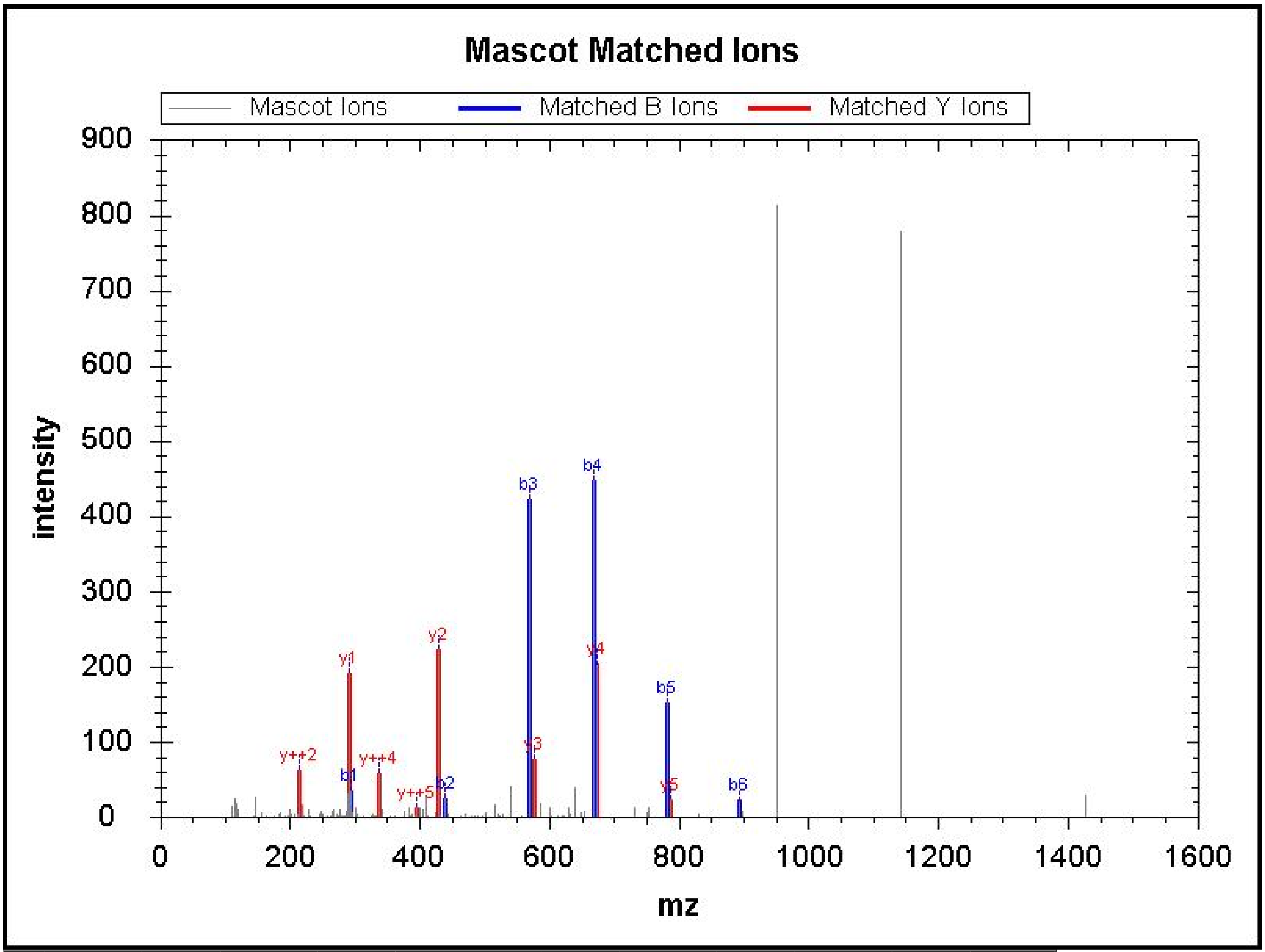

No	b	b++	b0	b0++	Seq	y	y++	y*	y*++	y0	y0++	RevNo
1	292.18	146.59			F							12
2	439.25	220.13			F	1,501.85	751.43	1,484.83	742.92	1,483.84	742.42	11
3	568.29	284.65	550.28	275.64	E	1,354.78	677.90	1,337.76	669.38	1,336.77	668.89	10
4	667.36	334.18	649.35	325.18	V	1,225.74	613.37	1,208.72	604.86	1,207.73	604.37	9
5	780.44	390.72	762.43	381.72	I	1,126.67	563.84	1,109.65	555.33	1,108.66	554.84	8
6	893.53	447.27	875.51	438.26	L	1,013.59	507.30	996.56	498.79	995.58	498.29	7
7	1,006.61	503.81	988.60	494.80	I	900.51	450.76	883.48	442.24	882.50	441.75	6
8	1,121.64	561.32	1,103.63	552.32	D	787.42	394.21	770.40	385.70	769.41	385.21	5
9	1,218.69	609.85	1,200.68	600.84	P	672.39	336.70	655.37	328.19			4
10	1,365.76	683.38	1,347.75	674.38	F	575.34	288.17	558.32	279.66			3
11	1,502.82	751.91	1,484.81	742.91	H	428.27	214.64	411.25	206.13			2
12					K	291.21	146.11	274.19	137.60			1

Query 687 Hit 1

MS/MS Fragmentation of ALVMLLEVR
Found in sp|Q92973|TNPO1_HUMAN, Transportin-1 OS=Homo sapiens GN=TNPO1 PE=1 SV=2
Match to Query 687: 1186.727from(594.3709,2+)
Title: 916: Sum of 2 scans in range 2296 (rt=60.1744, f=4, i=632) to 2297 (rt=60.1999, f=4, i=633) [D:\lab212\membrane\Grace\20120305-iTRAQ-2.raw]
Data File:
Monoisotopic mass of neutral peptide Mr(calc): 1186.727
Variable modifications:
Ions Score: 42.82 **Expect:** 0.027

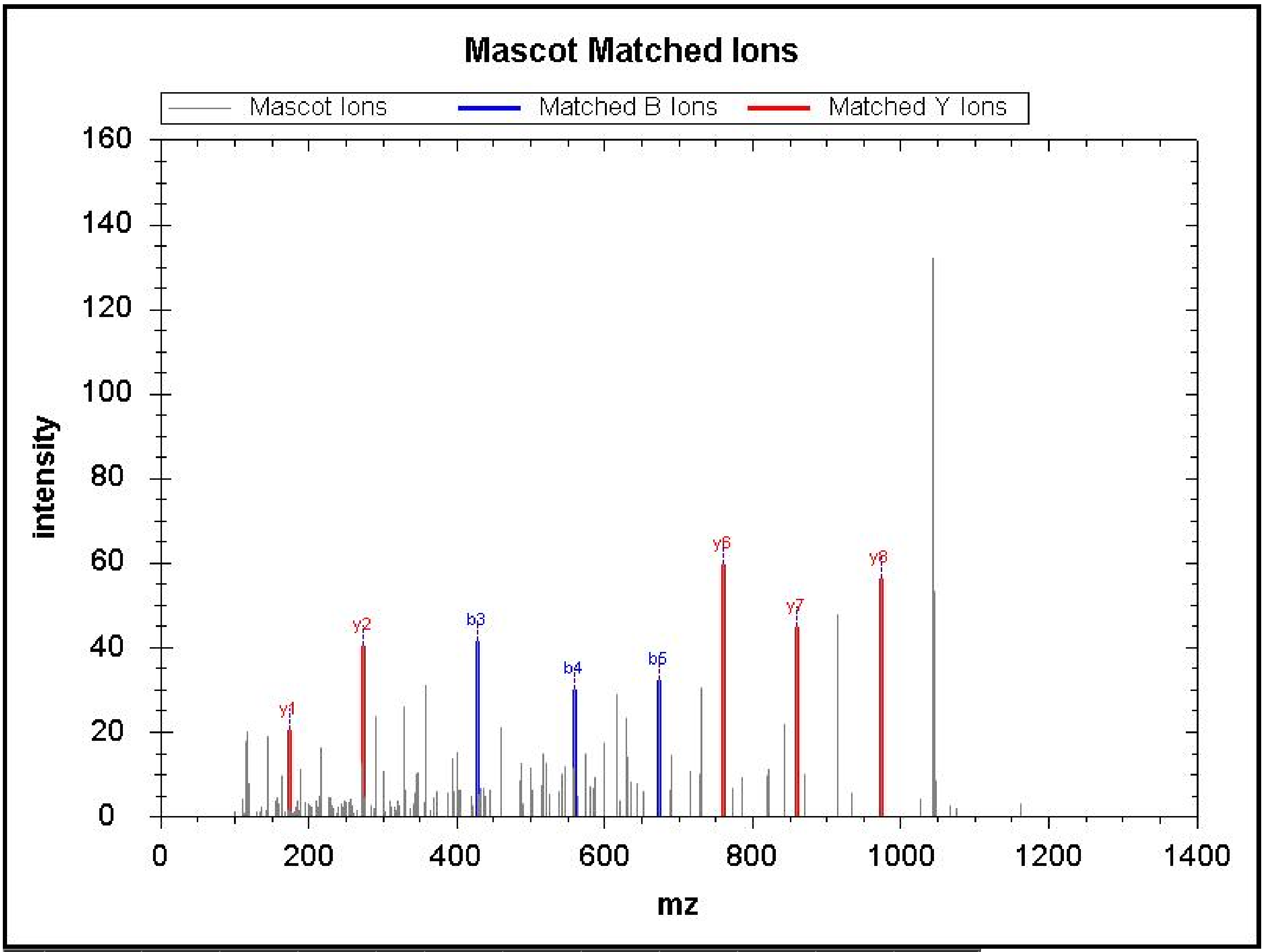

No	b	b++	b0	b0++	Seq	y	y++	y*	y*++	y0	y0++	RevNo
1	216.15	108.58			A							9
2	329.23	165.12			L	972.59	486.80	955.56	478.29	954.58	477.79	8
3	428.30	214.65			V	859.51	430.26	842.48	421.74	841.50	421.25	7
4	559.34	280.17			M	760.44	380.72	743.41	372.21	742.43	371.72	6
5	672.42	336.72			L	629.40	315.20	612.37	306.69	611.39	306.20	5
6	785.51	393.26			L	516.31	258.66	499.29	250.15	498.30	249.66	4
7	914.55	457.78	896.54	448.77	E	403.23	202.12	386.20	193.61	385.22	193.11	3
8	1,013.62	507.31	995.61	498.31	V	274.19	137.60	257.16	129.08			2
9					R	175.12	88.06	158.09	79.55			1

Query 1365 Hit 1

MS/MS Fragmentation of VMVQPINLIFR
Found in sp|P62304|RUXE_HUMAN, Small nuclear ribonucleoprotein E OS=Homo sapiens GN=SNRPE PE=1 SV=1
Match to Query 1365: 1472.871 from(737.4426,2+)
Title: 903: Scan 2399 (rt=62.0425, f=3, i=325) [D:\lab212\membrane\Grace\20120305-iTRAQ-1.raw]
Data File:
Monoisotopic mass of neutral peptide Mr(calc): 1472.871
Variable modifications:
Ions Score: 42.71 **Expect:** 0.031

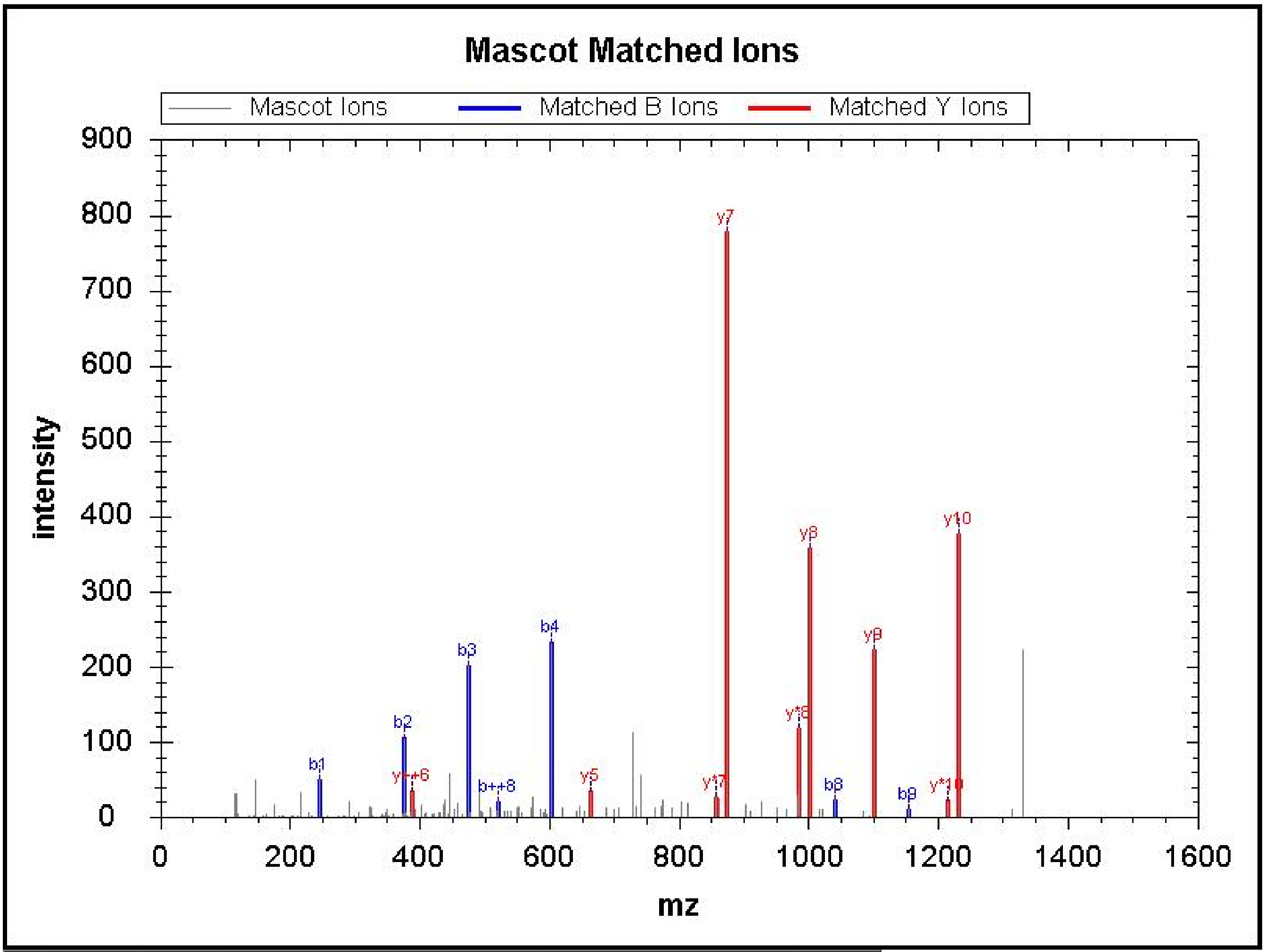

No	b	b++	b*	b*++	Seq	y	y++	y*	y*++	RevNo
1	244.18	122.59			V					11
2	375.22	188.11			M	1,230.70	615.85	1,213.68	607.34	10
3	474.29	237.65			V	1,099.66	550.33	1,082.64	541.82	9
4	602.35	301.68	585.32	293.16	Q	1,000.59	500.80	983.57	492.29	8
5	699.40	350.20	682.37	341.69	P	872.54	436.77	855.51	428.26	7
6	812.48	406.74	795.46	398.23	I	775.48	388.24	758.46	379.73	6
7	926.52	463.77	909.50	455.25	N	662.40	331.70	645.37	323.19	5
8	1,039.61	520.31	1,022.58	511.79	L	548.36	274.68	531.33	266.17	4
9	1,152.69	576.85	1,135.67	568.34	I	435.27	218.14	418.24	209.63	3
10	1,299.76	650.38	1,282.74	641.87	F	322.19	161.60	305.16	153.08	2
11					R	175.12	88.06	158.09	79.55	1

Query 867 Hit 1

MS/MS Fragmentation of **ILVATNLFGR**
Found in **sp|O00148|DX39A_HUMAN**, ATP-dependent RNA helicase DDX39A OS=Homo sapiens GN=DDX39A PE=1 SV=2
Match to Query 867: 1246.755from(624.3847,2+)
Title: 798: Sum of 2 scans in range 2042 (rt=54.4506, f=4, i=550) to 2043 (rt=54.476, f=4, i=551) [D:\lab212\membrane\Grace\20120305-iTRAQ-2.raw]
Data File:
Monoisotopic mass of neutral peptide Mr(calc): 1246.755
Variable modifications:
Ions Score: 42.29 **Expect:** 0.037

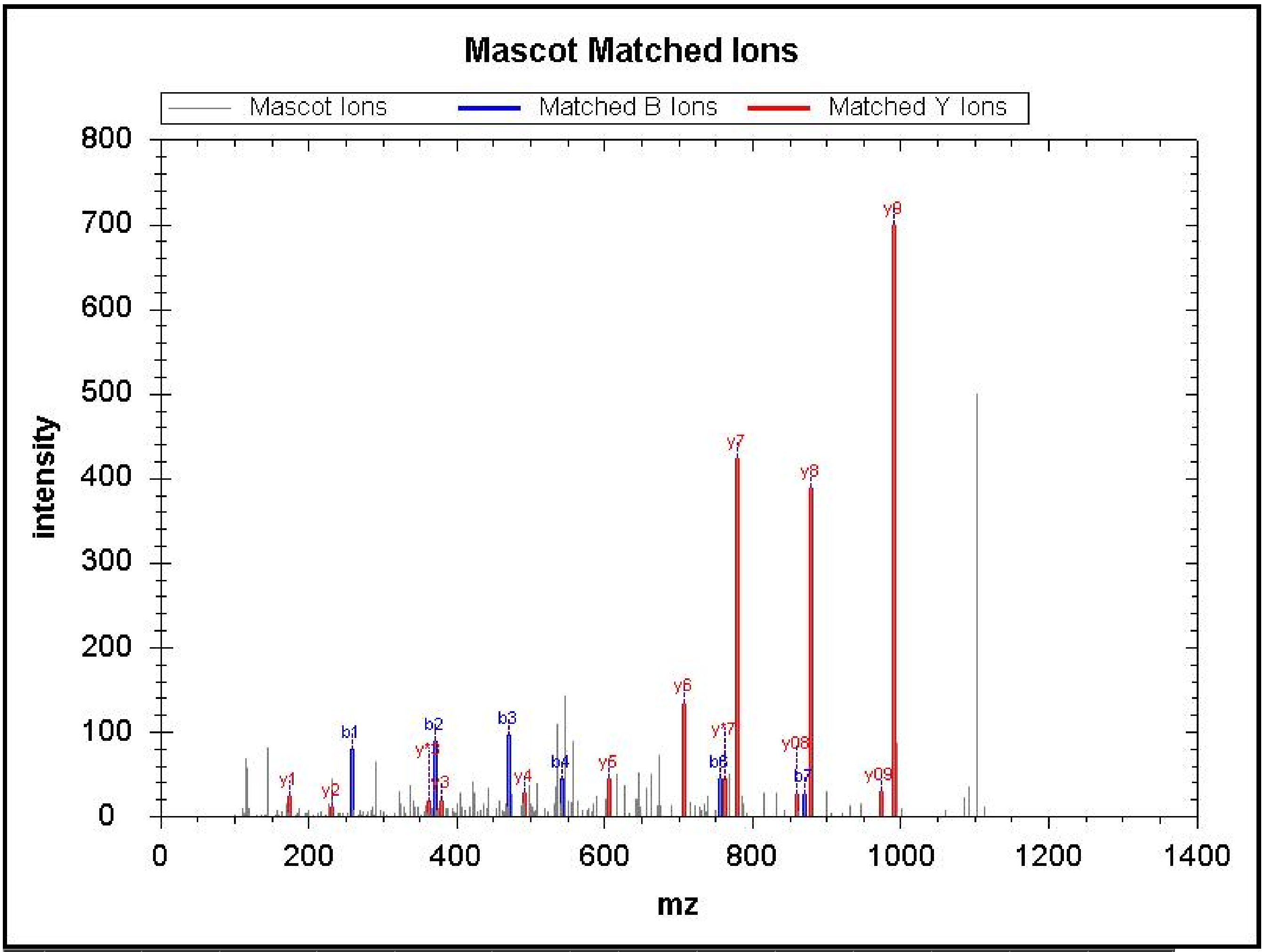

No	b	b++	b*	b*++	b0	b0++	Seq	y	y++	y*	y*++	y0	y0++	RevNo
1	**258.19**	129.60					I							10
2	**371.28**	186.14					L	**990.57**	495.79	973.55	487.28	**972.56**	486.78	9
3	**470.35**	235.68					V	**877.49**	439.25	860.46	430.73	**859.48**	430.24	8
4	**541.38**	271.20					A	**778.42**	389.71	**761.39**	381.20	760.41	380.71	7
5	642.43	321.72			624.42	312.71	T	**707.38**	354.20	690.36	345.68	689.37	345.19	6
6	**756.47**	378.74	739.45	370.23	738.46	369.74	N	**606.34**	303.67	589.31	295.16			5
7	**869.56**	435.28	852.53	426.77	851.55	426.28	L	**492.29**	246.65	475.27	238.14			4
8	1,016.63	508.82	999.60	500.30	998.62	499.81	F	**379.21**	190.11	**362.18**	181.59			3
9	1,073.65	537.33	1,056.62	528.81	1,055.64	528.32	G	**232.14**	116.57	215.11	108.06			2
10							R	**175.12**	88.06	158.09	79.55			1

Query 884 Hit 1

MS/MS Fragmentation of **ILEFFGLK**
Found in **sp|P07237|PDIA1_HUMAN**, Protein disulfide-isomerase OS=Homo sapiens GN=P4HB PE=1 SV=3
Match to Query 884: 1253.772from(627.8932,2+)
Title: 1010: Scan 2506 (rt=64.9028, f=3, i=353) [D:\lab212\membrane\Grace\20120305-iTRAQ-2.raw]
Data File:
Monoisotopic mass of neutral peptide Mr(calc): 1253.772
Variable modifications:
K8 :iTRAQ4plex (K)
Ions Score: 42.11 **Expect:** 0.022

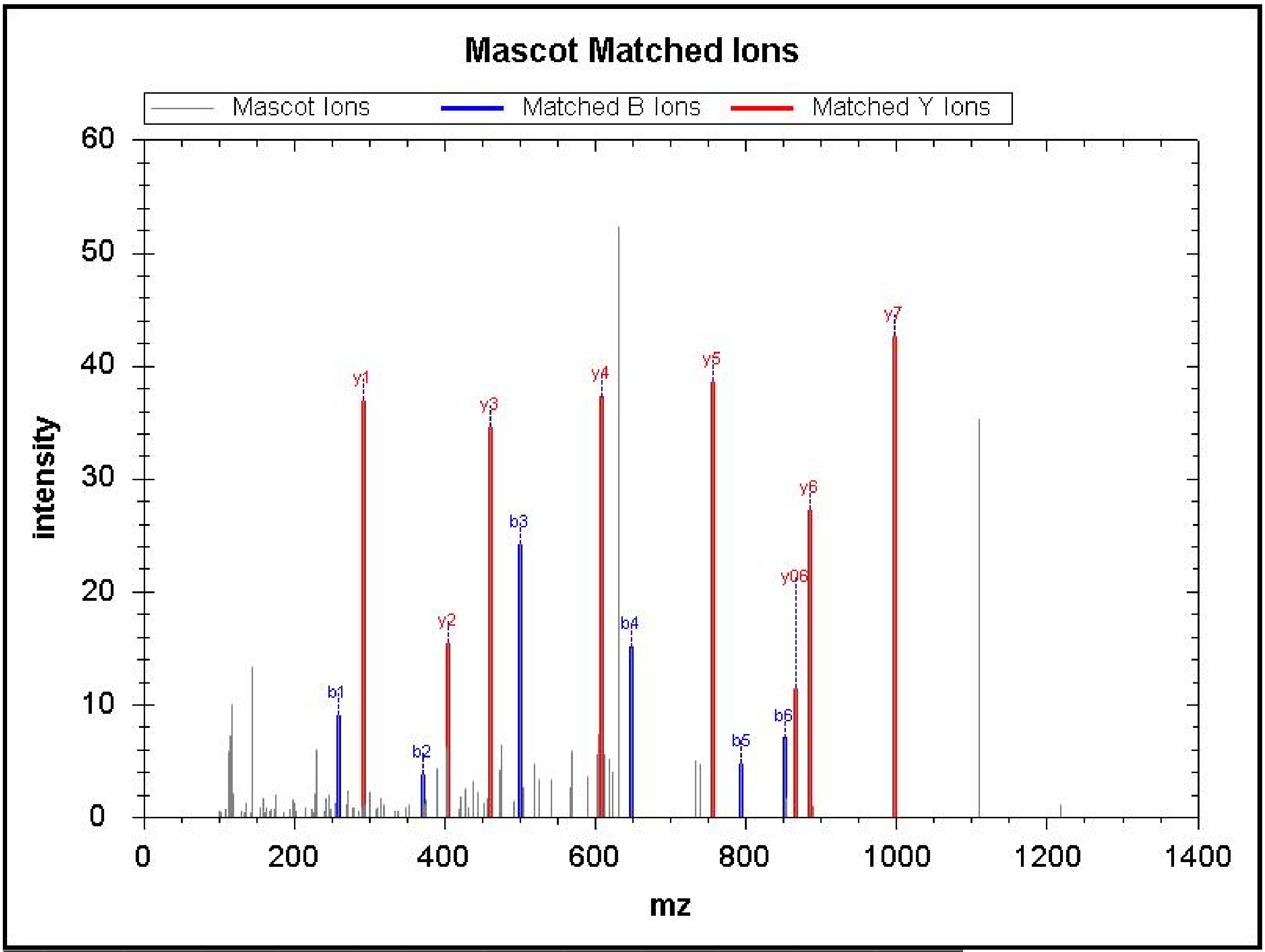

No	b	b++	b0	b0++	Seq	y	y++	y*	y*++	y0	y0++	RevNo
1	258.19	129.60			I							8
2	371.28	186.14			L	997.58	499.30	980.56	490.78	979.57	490.29	7
3	500.32	250.66	482.31	241.66	E	884.50	442.75	867.47	434.24	866.49	433.75	6
4	647.39	324.20	629.38	315.19	F	755.46	378.23	738.43	369.72			5
5	794.46	397.73	776.45	388.73	F	608.39	304.70	591.36	296.18			4
6	851.48	426.24	833.47	417.24	G	461.32	231.16	444.29	222.65			3
7	964.56	482.78	946.55	473.78	L	404.30	202.65	387.27	194.14			2
8					K	291.21	146.11	274.19	137.60			1

Query 2844 Hit 1

MS/MS Fragmentation of **LLVSNLDFGVSDADIQELFAEFGTLK**
Found in **sp|Q86V81|THOC4_HUMAN**, THO complex subunit 4 OS=Homo sapiens GN=ALYREF PE=1 SV=3
Match to Query 2844: 3129.667from(1044.23,3+)
Title: 1307: Sum of 2 scans in range 3324 (rt=82.8877, f=4, i=930) to 3325 (rt=82.9131, f=4, i=931) [D:\lab212\membrane\Grace\20120305-iTRAQ-1.raw]
Data File:
Monoisotopic mass of neutral peptide Mr(calc): 3129.667
Variable modifications:
Q16 :Deamidated (NQ)
K26 :iTRAQ4plex (K)
Ions Score: 41.48 **Expect:** 0.042

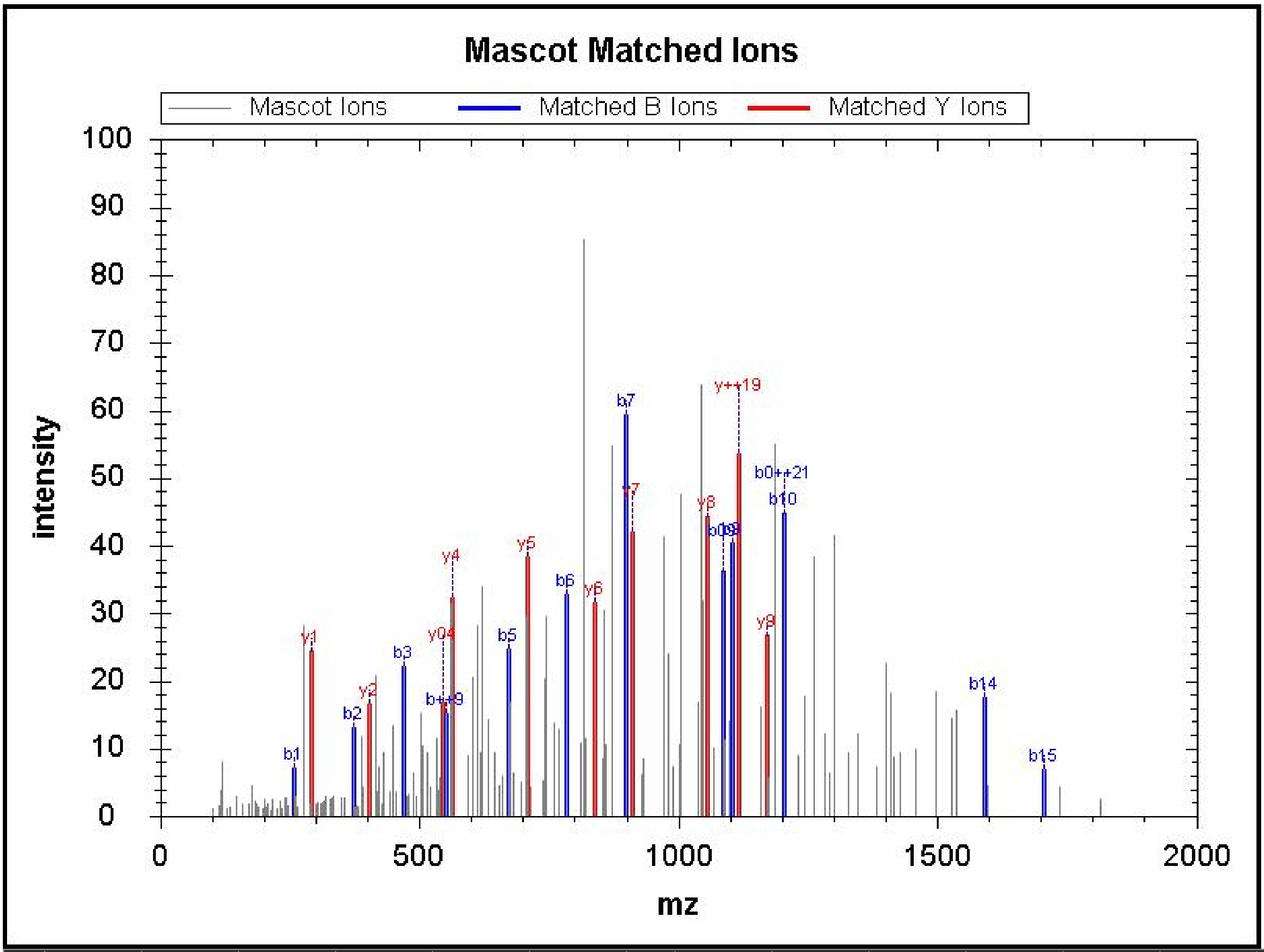

No	b	b++	b*	b*++	b0	b0++	Seq	y	y++	y*	y*++	y0	y0++	RevNo
1	258.19	129.60					L							26
2	371.28	186.14					L	2,873.46	1,437.23	2,856.43	1,428.72	2,855.45	1,428.23	25
3	470.35	235.68					V	2,760.37	1,380.69	2,743.35	1,372.18	2,742.36	1,371.69	24
4	557.38	279.19			539.37	270.19	S	2,661.30	1,331.16	2,644.28	1,322.64	2,643.29	1,322.15	23
5	671.42	336.21	654.39	327.70	653.41	327.21	N	2,574.27	1,287.64	2,557.25	1,279.13	2,556.26	1,278.63	22
6	784.50	392.76	767.48	384.24	766.49	383.75	L	2,460.23	1,230.62	2,443.20	1,222.11	2,442.22	1,221.61	21
7	899.53	450.27	882.51	441.76	881.52	441.26	D	2,347.15	1,174.08	2,330.12	1,165.56	2,329.14	1,165.07	20
8	1,046.60	523.80	1,029.57	515.29	1,028.59	514.80	F	2,232.12	1,116.56	2,215.09	1,108.05	2,214.11	1,107.56	19
9	1,103.62	552.31	1,086.60	543.80	1,085.61	543.31	G	2,085.05	1,043.03	2,068.02	1,034.52	2,067.04	1,034.02	18
10	1,202.69	601.85	1,185.66	593.34	1,184.68	592.84	V	2,028.03	1,014.52	2,011.00	1,006.01	2,010.02	1,005.51	17
11	1,289.72	645.36	1,272.70	636.85	1,271.71	636.36	S	1,928.96	964.98	1,911.93	956.47	1,910.95	955.98	16
12	1,404.75	702.88	1,387.72	694.36	1,386.74	693.87	D	1,841.93	921.47	1,824.90	912.95	1,823.92	912.46	15
13	1,475.79	738.40	1,458.76	729.88	1,457.78	729.39	A	1,726.90	863.95	1,709.88	855.44	1,708.89	854.95	14
14	1,590.81	795.91	1,573.79	787.40	1,572.80	786.90	D	1,655.86	828.44	1,638.84	819.92	1,637.85	819.43	13
15	1,703.90	852.45	1,686.87	843.94	1,685.89	843.45	I	1,540.84	770.92	1,523.81	762.41	1,522.83	761.92	12
16	1,832.94	916.97	1,815.91	908.46	1,814.93	907.97	Q	1,427.75	714.38	1,410.73	705.87	1,409.74	705.38	11
17	1,961.98	981.49	1,944.96	972.98	1,943.97	972.49	E	1,298.71	649.86	1,281.68	641.35	1,280.70	640.85	10
18	2,075.07	1,038.04	2,058.04	1,029.52	2,057.06	1,029.03	L	1,169.67	585.34	1,152.64	576.82	1,151.66	576.33	9
19	2,222.14	1,111.57	2,205.11	1,103.06	2,204.12	1,102.57	F	1,056.58	528.80	1,039.56	520.28	1,038.57	519.79	8
20	2,293.17	1,147.09	2,276.15	1,138.58	2,275.16	1,138.08	A	909.52	455.26	892.49	446.75	891.51	446.26	7
21	2,422.21	1,211.61	2,405.19	1,203.10	2,404.20	1,202.61	E	838.48	419.74	821.45	411.23	820.47	410.74	6
22	2,569.28	1,285.15	2,552.26	1,276.63	2,551.27	1,276.14	F	709.44	355.22	692.41	346.71	691.43	346.22	5
23	2,626.30	1,313.66	2,609.28	1,305.14	2,608.29	1,304.65	G	562.37	281.69	545.34	273.17	544.36	272.68	4

No	b	b++	b*	b*++	b0	b0++	Seq	y	y++	y*	y*++	y0	y0++	RevNo
24	2,727.35	1,364.18	2,710.33	1,355.67	2,709.34	1,355.17	T	505.35	253.18	488.32	244.66	487.34	244.17	3
25	2,840.44	1,420.72	2,823.41	1,412.21	2,822.43	1,411.72	L	**404.30**	202.65	387.27	194.14			2
26							K	**291.21**	146.11	274.19	137.60			1

Query 2100 Hit 1

MS/MS Fragmentation of **VQALTTDISLIFAALK**
Found in **sp|Q6PKG0|LARP1_HUMAN**, La-related protein 1 OS=Homo sapiens GN=LARP1 PE=1 SV=2
Match to Query 2100: 1991.206from(664.7427,3+)
Title: 1354: Scan 3275 (rt=82.2301, f=3, i=477) [D:\lab212\membrane\Grace\20120305-iTRAQ-2.raw]
Data File:
Monoisotopic mass of neutral peptide Mr(calc): 1991.206
Variable modifications:
K16 :iTRAQ4plex (K)
Ions Score: 41.14 **Expect:** 0.017

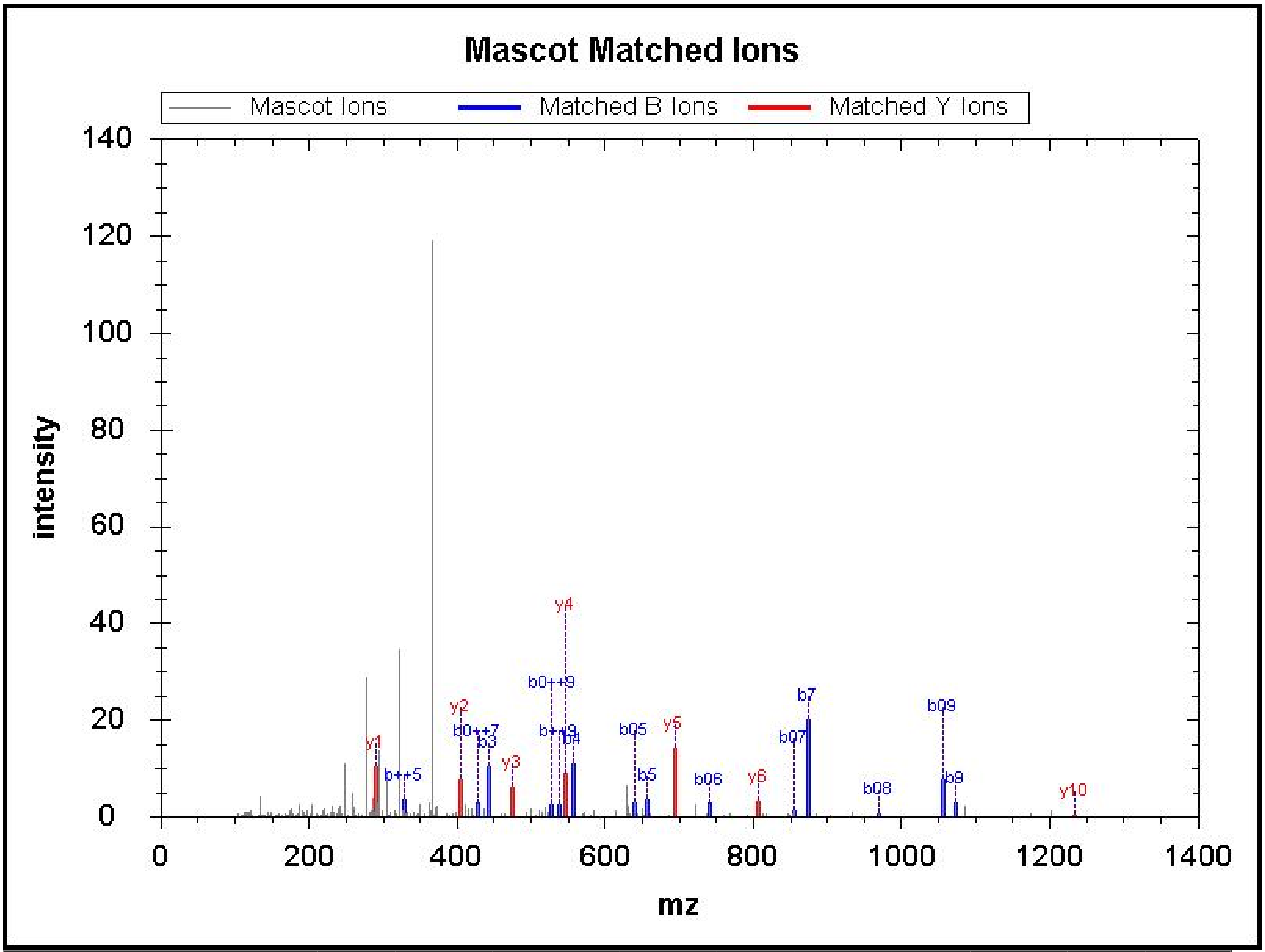

No	b	b++	b*	b*++	b0	b0++	Seq	y	y++	y*	y*++	y0	y0++	RevNo
1	244.18	122.59					V							16
2	372.24	186.62	355.21	178.11			Q	1,749.03	875.02	1,732.00	866.50	1,731.02	866.01	15
3	**443.27**	222.14	426.25	213.63			A	1,620.97	810.99	1,603.94	802.48	1,602.96	801.98	14
4	**556.36**	278.68	539.33	270.17			L	1,549.93	775.47	1,532.91	766.96	1,531.92	766.46	13
5	**657.41**	**329.21**	640.38	320.69	**639.39**	320.20	T	1,436.85	718.93	1,419.82	710.41	1,418.84	709.92	12
6	758.45	379.73	741.43	371.22	**740.44**	370.72	T	1,335.80	668.40	1,318.77	659.89	1,317.79	659.40	11
7	**873.48**	437.24	856.45	428.73	**855.47**	**428.24**	D	**1,234.75**	617.88	1,217.73	609.37	1,216.74	608.87	10
8	986.56	493.79	969.54	485.27	**968.55**	484.78	I	1,119.73	560.37	1,102.70	551.85	1,101.72	551.36	9
9	**1,073.60**	**537.30**	1,056.57	528.79	**1,055.59**	**528.30**	S	1,006.64	503.82	989.62	495.31	988.63	494.82	8
10	1,186.68	593.84	1,169.65	585.33	1,168.67	584.84	L	919.61	460.31	902.58	451.80			7
11	1,299.76	650.39	1,282.74	641.87	1,281.75	641.38	I	**806.53**	403.77	789.50	395.25			6

**12**	1,446.83	723.92	1,429.81	715.41	1,428.82	714.91	**F**	**693.44**	347.22	676.41	338.71			**5**
**13**	1,517.87	759.44	1,500.84	750.93	1,499.86	750.43	**A**	**546.37**	273.69	529.35	265.18			**4**
**14**	1,588.91	794.96	1,571.88	786.44	1,570.90	785.95	**A**	**475.34**	238.17	458.31	229.66			**3**
**15**	1,701.99	851.50	1,684.96	842.99	1,683.98	842.49	**L**	**404.30**	202.65	387.27	194.14			**2**
**16**							**K**	**291.21**	146.11	274.19	137.60			**1**

Query 2214 Hit 1

MS/MS Fragmentation of **LTGDLSHLAAIVILLLK**
Found in **sp|P33947|ERD22_HUMAN**, ER lumen protein retaining receptor 2 OS=Homo sapiens GN=KDELR2 PE=1 SV=1
Match to Query 2214: 2077.323from(693.4484,3+)
Title: 1421: Scan 3553 (rt=88.4912, f=2, i=548) [D:\lab212\membrane\Grace\20120305-iTRAQ-2.raw]
Data File:
Monoisotopic mass of neutral peptide Mr(calc): 2077.323
Variable modifications:
K17 :iTRAQ4plex (K)
Ions Score: 40.95 **Expect:** 0.004

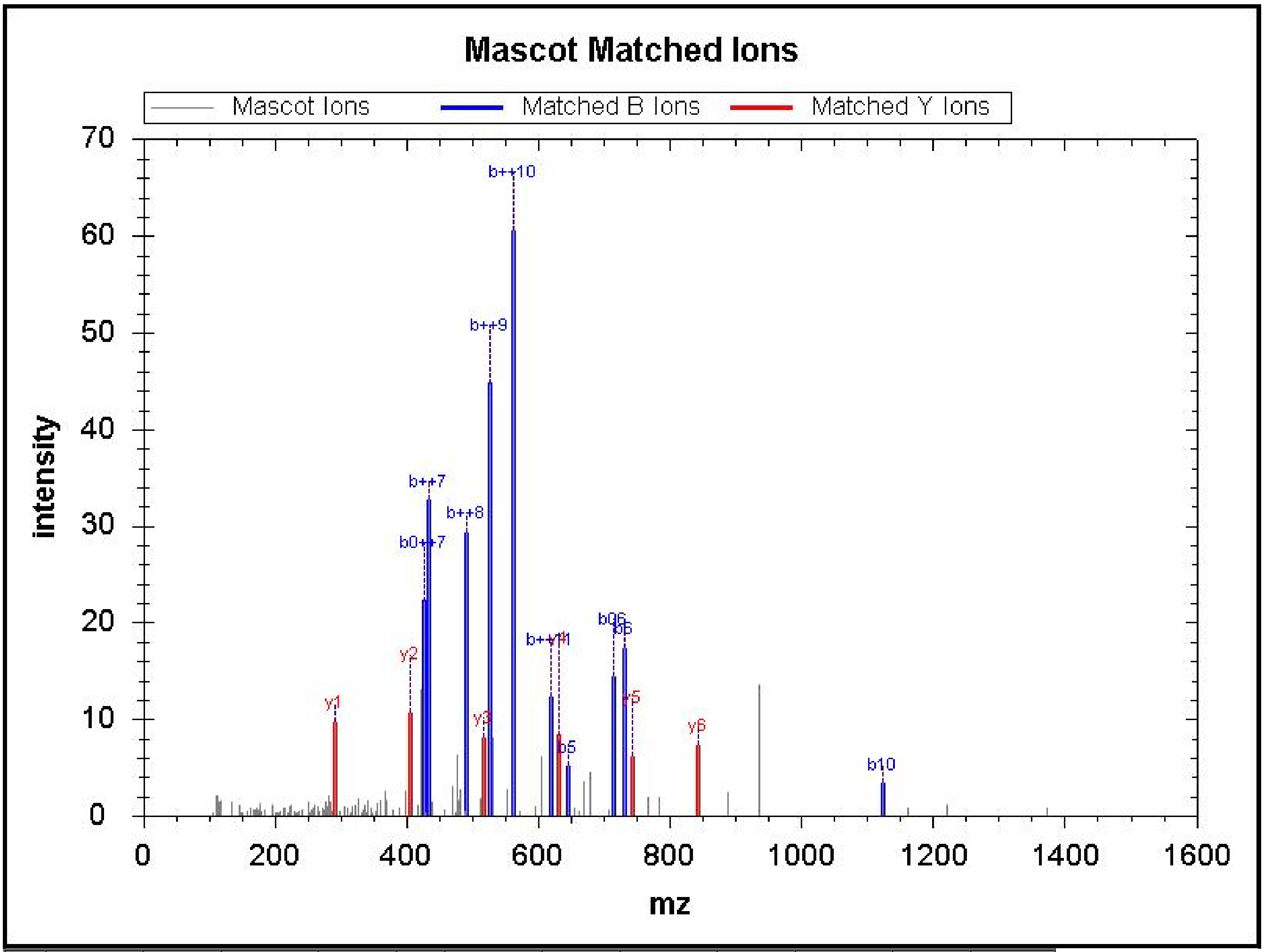

No	b	b++	b0	b0++	Seq	y	y++	y*	y*++	y0	y0++	RevNo
**1**	258.19	129.60			**L**							**17**
**2**	359.24	180.12	341.23	171.12	**T**	1,821.13	911.07	1,804.11	902.56	1,803.12	902.06	**16**
**3**	416.26	208.63	398.25	199.63	**G**	1,720.09	860.55	1,703.06	852.03	1,702.07	851.54	**15**
**4**	531.29	266.15	513.28	257.14	**D**	1,663.06	832.04	1,646.04	823.52	1,645.05	823.03	**14**
**5**	**644.37**	322.69	626.36	313.69	**L**	1,548.04	774.52	1,531.01	766.01	1,530.03	765.52	**13**
**6**	**731.41**	366.21	**713.39**	357.20	**S**	1,434.95	717.98	1,417.93	709.47	1,416.94	708.97	**12**
**7**	868.46	**434.74**	850.45	**425.73**	**H**	1,347.92	674.46	1,330.89	665.95			**11**
**8**	981.55	**491.28**	963.54	482.27	**L**	1,210.86	605.93	1,193.84	597.42			**10**
**9**	1,052.59	**526.80**	1,034.58	517.79	**A**	1,097.78	549.39	1,080.75	540.88			**9**

**10**	**1,123.62**	**562.32**	1,105.61	553.31	**A**	1,026.74	513.87	1,009.71	505.36			**8**
**11**	1,236.71	**618.86**	1,218.70	609.85	**I**	955.70	478.36	938.68	469.84			**7**
**12**	1,335.78	668.39	1,317.76	659.39	**V**	**842.62**	421.81	825.59	413.30			**6**
**13**	1,448.86	724.93	1,430.85	715.93	**I**	**743.55**	372.28	726.52	363.77			**5**
**14**	1,561.94	781.48	1,543.93	772.47	**L**	**630.47**	315.74	613.44	307.22			**4**
**15**	1,675.03	838.02	1,657.02	829.01	**L**	**517.38**	259.20	500.36	250.68			**3**
**16**	1,788.11	894.56	1,770.10	885.55	**L**	**404.30**	202.65	387.27	194.14			**2**
**17**					**K**	**291.21**	146.11	274.19	137.60			**1**

Query 841 Hit 1

MS/MS Fragmentation of **NLYIISVK**
Found in **sp|P62829|RL23_HUMAN**, 60S ribosomal protein L23 OS=Homo sapiens GN=RPL23 PE=1 SV=1
Match to Query 841: 1236.768from(619.3913,2+)
Title: 612: Sum of 2 scans in range 1651 (rt=45.6413, f=4, i=424) to 1652 (rt=45.6667, f=4, i=425)
[D:\lab212\membrane\Grace\20120305-iTRAQ-2.raw]
Data File:
Monoisotopic mass of neutral peptide Mr(calc): 1236.768
Variable modifications:
K8 :iTRAQ4plex (K)
Ions Score: 40.33 **Expect:** 0.038

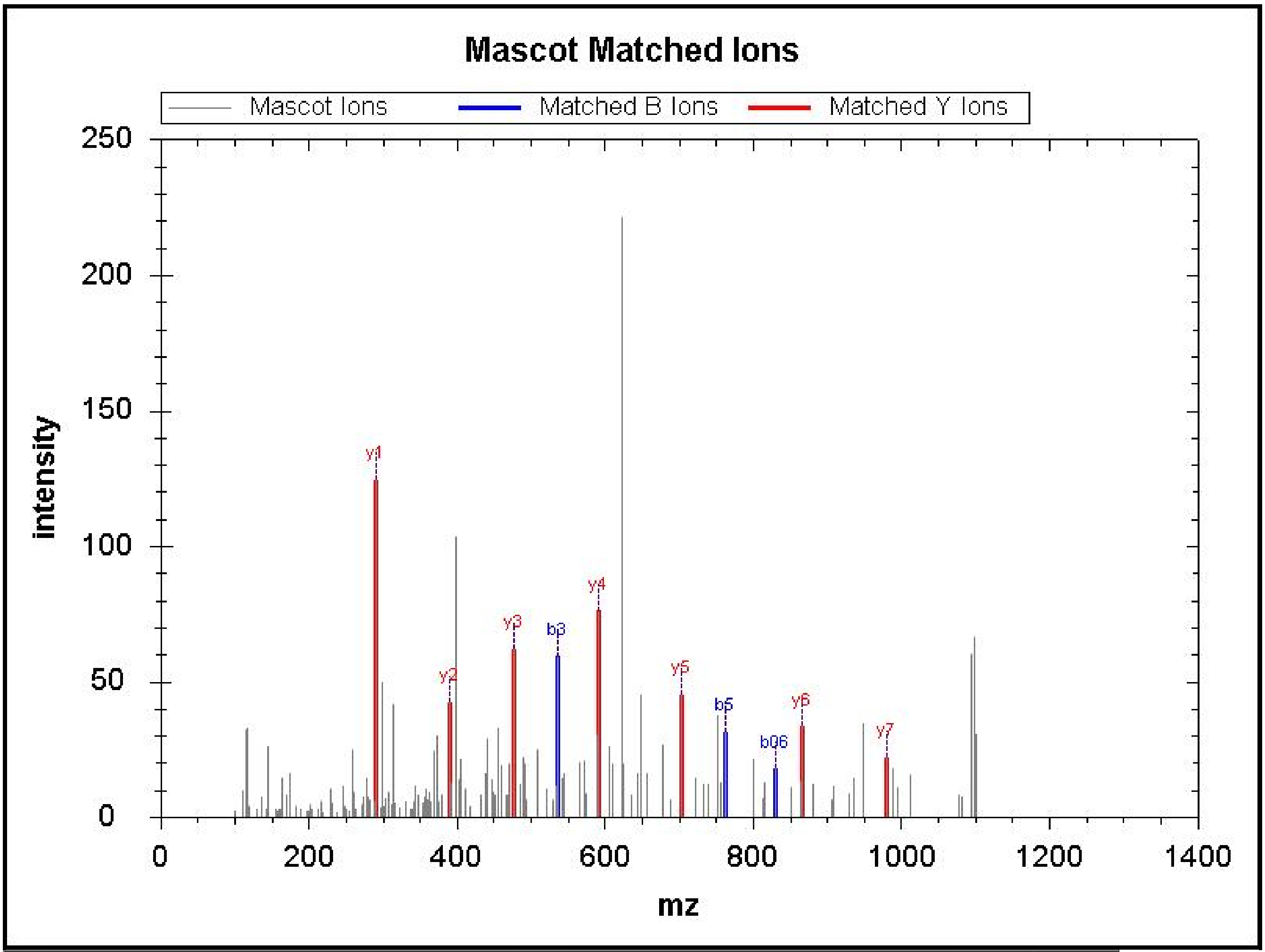

No	b	b++	b*	b*++	b0	b0++	Seq	y	y++	y*	y*++	y0	y0++	RevNo
**1**	259.15	130.08	242.13	121.57			**N**							**8**
**2**	372.24	186.62	355.21	178.11			**L**	**979.63**	490.32	962.60	481.81	961.62	481.31	**7**
**3**	**535.30**	268.15	518.27	259.64			**Y**	**866.55**	433.78	849.52	425.26	848.54	424.77	**6**
**4**	648.38	324.70	631.36	316.18			**I**	**703.48**	352.25	686.46	343.73	685.47	343.24	**5**
**5**	**761.47**	381.24	744.44	372.72			**I**	**590.40**	295.70	573.37	287.19	572.39	286.70	**4**

6	848.50	424.75	831.47	416.24	830.49	415.75	S	477.32	239.16	460.29	230.65	459.30	230.16	3
7	947.57	474.29	930.54	465.77	929.56	465.28	V	390.28	195.65	373.26	187.13			2
8							K	291.21	146.11	274.19	137.60			1

Query 1167 Hit 1

MS/MS Fragmentation of **FLILPDMLK**
Found in **sp|P62318|SMD3_HUMAN**, Small nuclear ribonucleoprotein Sm D3 OS=Homo sapiens GN=SNRPD3 PE=1 SV=1
Match to Query 1167: 1376.843from(689.4288,2+)
Title: 980: Sum of 2 scans in range 2439 (rt=63.3954, f=4, i=678) to 2440 (rt=63.4208, f=4, i=679) [D:\lab212\membrane\Grace\20120305-iTRAQ-2.raw]
Data File:
Monoisotopic mass of neutral peptide Mr(calc): 1376.843
Variable modifications:
K9 :iTRAQ4plex (K)
Ions Score: 39.56 **Expect:** 0.054

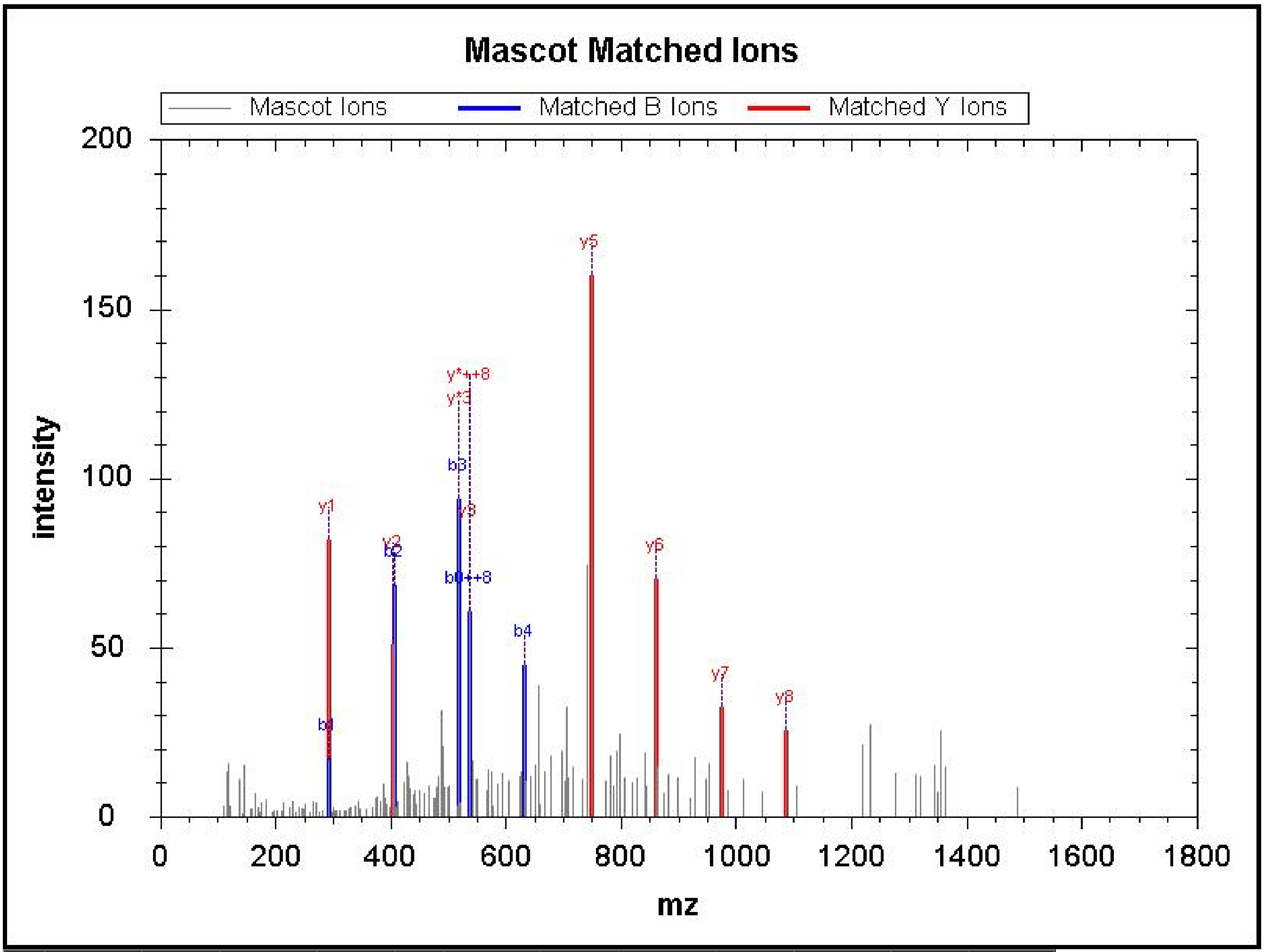

No	b	b++	b0	b0++	Seq	y	y++	y*	y*++	y0	y0++	RevNo
1	292.18	146.59			F							9
2	405.26	203.13			L	1,086.67	543.84	1,069.64	535.33	1,068.66	534.83	8
3	518.35	259.68			I	973.59	487.30	956.56	478.78	955.58	478.29	7
4	631.43	316.22			L	860.50	430.76	843.48	422.24	842.49	421.75	6
5	728.48	364.74			P	747.42	374.21	730.39	365.70	729.41	365.21	5
6	843.51	422.26	825.50	413.25	D	650.37	325.69	633.34	317.17	632.36	316.68	4
7	974.55	487.78	956.54	478.77	M	535.34	268.17	518.31	259.66			3
8	1,087.63	544.32	1,069.62	535.32	L	404.30	202.65	387.27	194.14			2
9					K	291.21	146.11	274.19	137.60			1

Query 1029 Hit 1

MS/MS Fragmentation of **LYLILDFLR**
Found in **sp|Q15418|KS6A1_HUMAN**, Ribosomal protein S6 kinase alpha-1 OS=Homo sapiens GN=RPS6KA1 PE=1 SV=2
Match to Query 1029: 1308.797from(655.4058,2+)
Title: 1208: Scan 3087 (rt=77.5454, f=3, i=436) [D:\lab212\membrane\Grace\20120305-iTRAQ-1.raw]
Data File:
Monoisotopic mass of neutral peptide Mr(calc): 1308.797
Variable modifications:
Ions Score: 39.55 **Expect:** 0.049

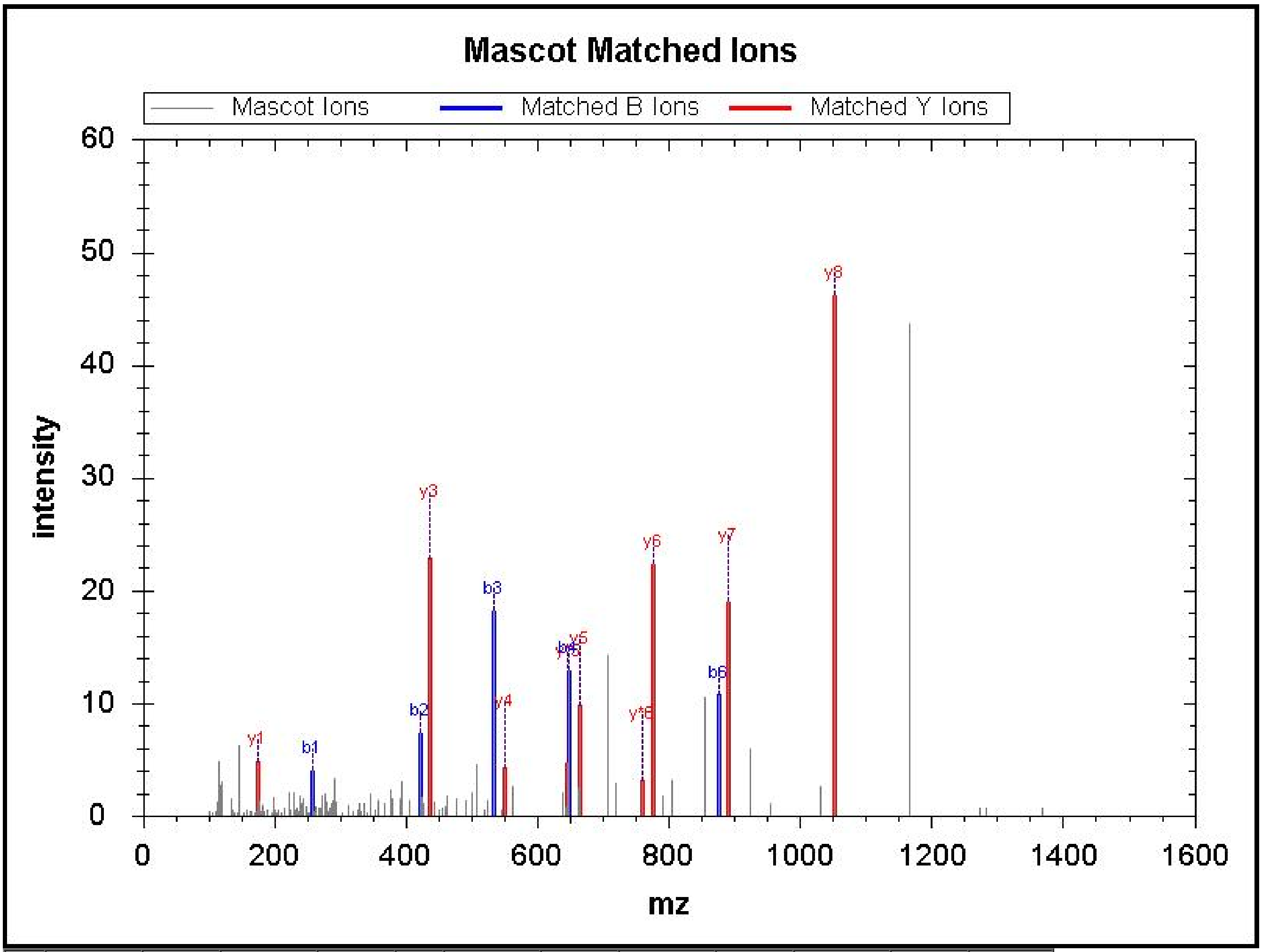

No	b	b++	b0	b0++	Seq	y	y++	y*	y*++	y0	y0++	RevNo
1	**258.19**	129.60			L							9
2	**421.26**	211.13			Y	**1,052.61**	526.81	1,035.59	518.30	1,034.60	517.81	8
3	**534.34**	267.67			L	**889.55**	445.28	872.52	436.77	871.54	436.27	7
4	**647.42**	324.22			I	**776.47**	388.74	**759.44**	380.22	758.46	379.73	6
5	760.51	380.76			L	**663.38**	332.19	**646.36**	323.68	645.37	323.19	5
6	**875.54**	438.27	857.53	429.27	D	**550.30**	275.65	533.27	267.14	532.29	266.65	4
7	1,022.60	511.81	1,004.59	502.80	F	**435.27**	218.14	418.24	209.63			3
8	1,135.69	568.35	1,117.68	559.34	L	288.20	144.61	271.18	136.09			2
9					R	**175.12**	88.06	158.09	79.55			1

Query 329 Hit 1

MS/MS Fragmentation of **LLLFLK**
Found in **sp|Q7L1Q6|BZW1_HUMAN**, Basic leucine zipper and W2 domain-containing protein 1 OS=Homo sapiens GN=BZW1 PE=1 SV=1
Match to Query 329: 1033.72from(517.8672,2+)
Title: 904: Sum of 2 scans in range 2271 (rt=59.6117, f=4, i=624) to 2272 (rt=59.6371, f=4, i=625)

[D:\lab212\membrane\Grace\20120305-iTRAQ-2.raw]
Data File:
Monoisotopic mass of neutral peptide Mr(calc): 1033.72
Variable modifications:
K6 :iTRAQ4plex (K)
Ions Score: 37.61 **Expect:** 0.034

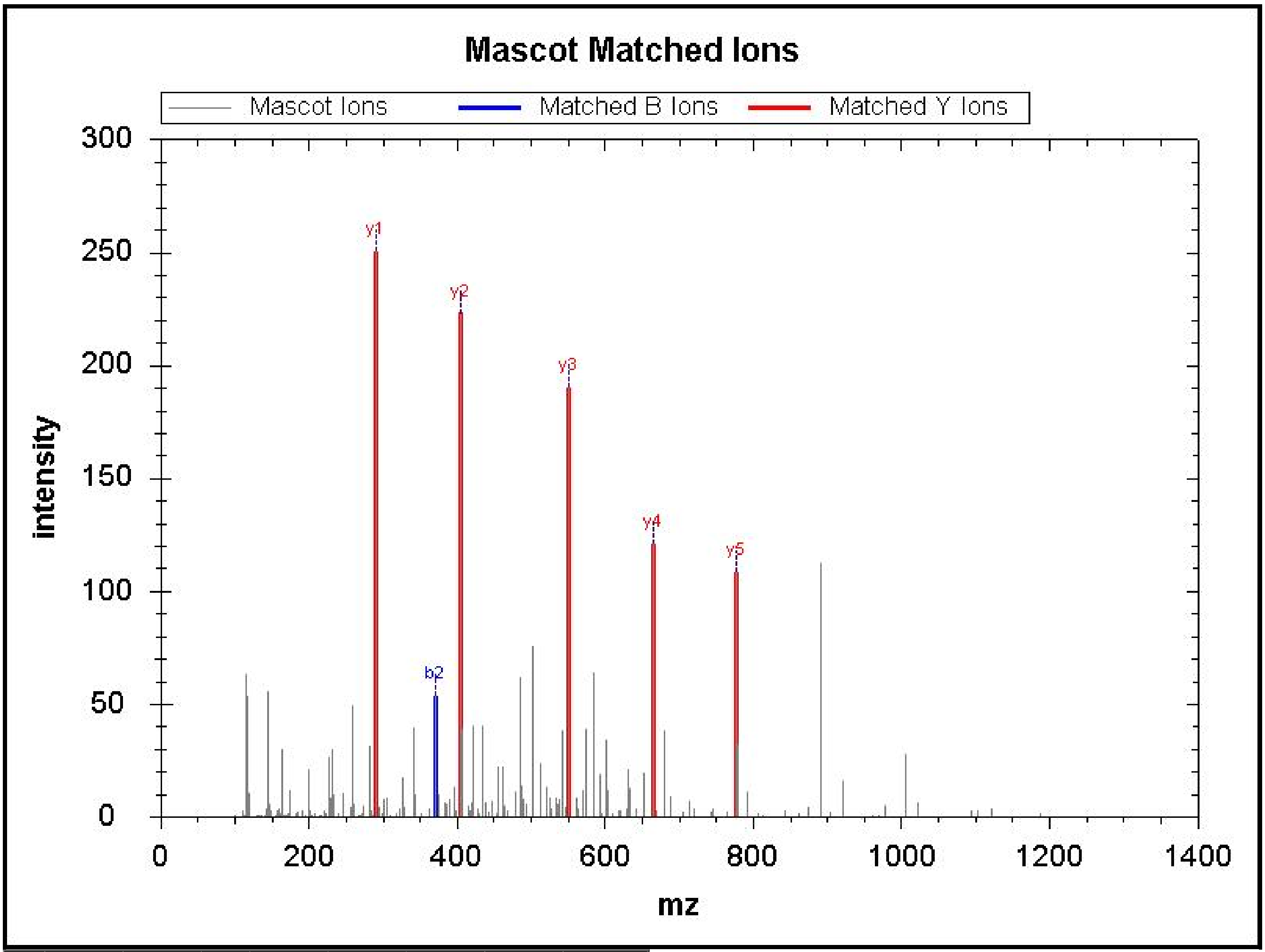

No	b	b++	Seq	y	y++	y*	y*++	RevNo
1	258.19	129.60	L					6
2	**371.28**	186.14	L	**777.54**	389.27	760.51	380.76	5
3	484.36	242.68	L	**664.45**	332.73	647.42	324.22	4
4	631.43	316.22	F	**551.37**	276.19	534.34	267.67	3
5	744.51	372.76	L	**404.30**	202.65	387.27	194.14	2
6			K	**291.21**	146.11	274.19	137.60	1

Query 1143 Hit 1

MS/MS Fragmentation of **LLGLLFPLLAR**
Found in **sp|Q8TEX9|IPO4_HUMAN**, Importin-4 OS=Homo sapiens GN=IPO4 PE=1 SV=2
Match to Query 1143: 1368.897from(685.4559,2+)
Title: 1371: Sum of 2 scans in range 3332 (rt=83.5168, f=4, i=966) to 3333 (rt=83.5422, f=4, i=967)
[D:\lab212\membrane\Grace\20120305-iTRAQ-2.raw]
Data File:
Monoisotopic mass of neutral peptide Mr(calc): 1368.897
Variable modifications:
Ions Score: 36.01 **Expect:** 0.035

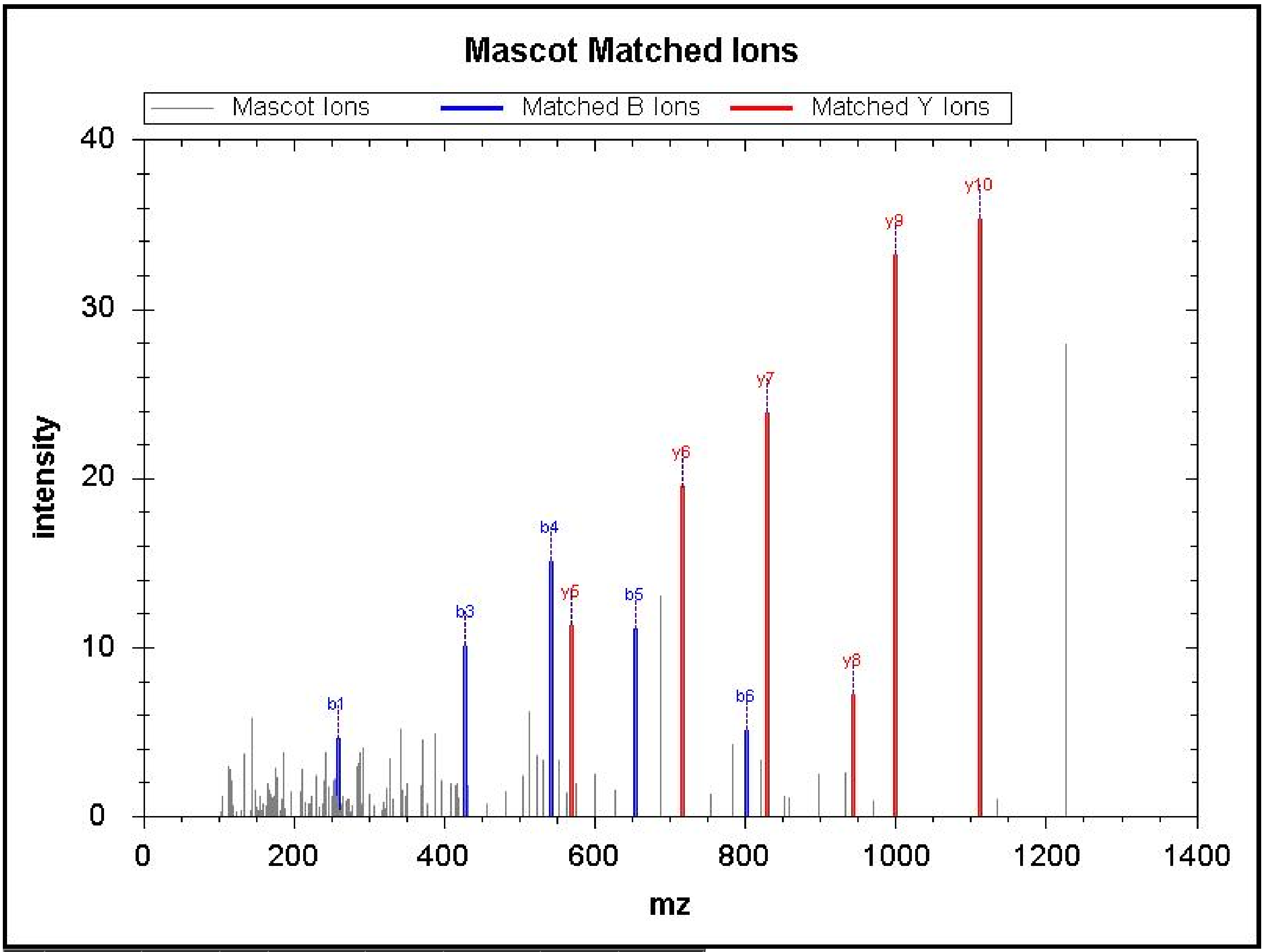

No	b	b++	Seq	y	y++	y*	y*++	RevNo
1	258.19	129.60	L					11
2	371.28	186.14	L	1,112.72	556.86	1,095.69	548.35	10
3	428.30	214.65	G	999.63	500.32	982.61	491.81	9
4	541.38	271.20	L	942.61	471.81	925.59	463.30	8
5	654.47	327.74	L	829.53	415.27	812.50	406.76	7
6	801.54	401.27	F	716.45	358.73	699.42	350.21	6
7	898.59	449.80	P	569.38	285.19	552.35	276.68	5
8	1,011.67	506.34	L	472.32	236.67	455.30	228.15	4
9	1,124.76	562.88	L	359.24	180.12	342.21	171.61	3
10	1,195.79	598.40	A	246.16	123.58	229.13	115.07	2
11			R	175.12	88.06	158.09	79.55	1
