# Supplementary material for: Quantitative Proteomic Analysis of Human Lung Tumor Xenografts Treated with the Ectopic ATP Synthase Inhibitor Citreoviridin
Source: PLoS One. 2013 Aug 21;8(8):e70642. doi: 10.1371/journal.pone.0070642 (PMC3749231; doi:10.1371/journal.pone.0070642)
Supplement: Spectra S3 — The information of single-peptide-based protein identifications in the large-scale experiment (part 1). (PDF) [file pone.0070642.s020.pdf]

Spectra S3: The information of single-peptide-based protein identifications in the large-scale experiment (part 1).

Query 63537 Hit 1

MS/MS Fragmentation of KLELQVESMLSEIK
Found in sp|Q96CV9|OPTN_HUMAN, Optineurin OS=Homo sapiens GN=OPTN PE=1 SV=2
Match to Query 63537: 2078.208from(693.7432,3+)
Title: 975: Sum of 2 scans in range 2104 (rt=57.0943, f=4, i=655) to 2105 (rt=57.1198, f=4, i=656)
[D:\lab212\membrane\Grace\Joyce\iTRAQ_45_1.raw]
Data File:Submitted from 20120508-1(merge) by Mascot Daemon on JOYCE-VAIO
Monoisotopic mass of neutral peptide Mr(calc): 2078.208
Variable modifications:
K1 :iTRAQ4plex (K)
K14 :iTRAQ4plex (K)
Ions Score: 45.39 Expect: 0.011

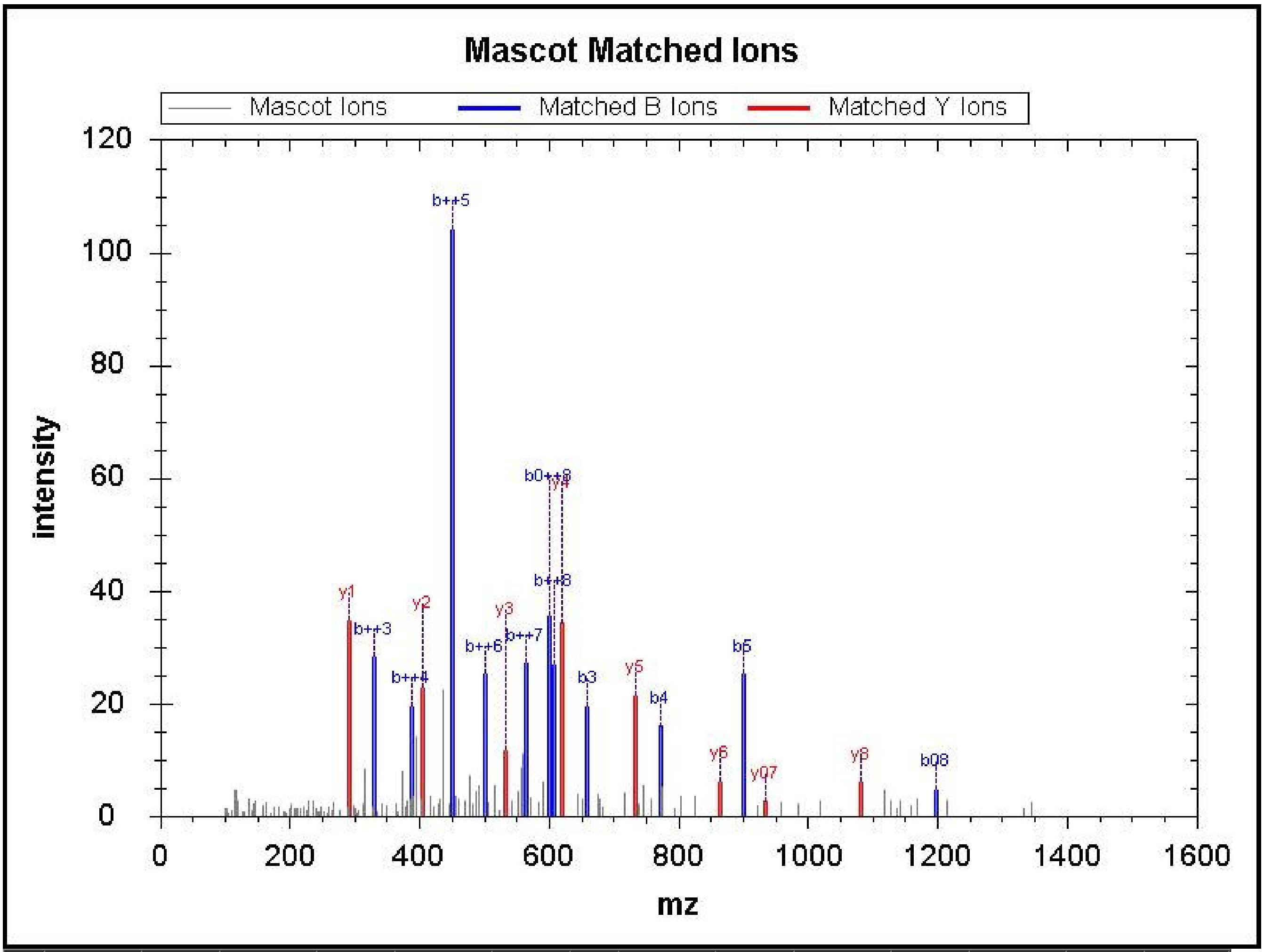

No	b	b++	b*	b*++	b0	b0++	Seq	y	y++	y*	y*++	y0	y0++	RevNo
1	417.31	209.16	400.28	200.64			K							14
2	530.39	265.70	513.36	257.19			L	1,662.91	831.96	1,645.88	823.45	1,644.90	822.95	13
3	659.43	330.22	642.41	321.71	641.42	321.21	E	1,549.83	775.42	1,532.80	766.90	1,531.82	766.41	12
4	772.52	386.76	755.49	378.25	754.51	377.76	L	1,420.78	710.90	1,403.76	702.38	1,402.77	701.89	11
5	900.58	450.79	883.55	442.28	882.57	441.79	Q	1,307.70	654.35	1,290.67	645.84	1,289.69	645.35	10
6	999.64	500.33	982.62	491.81	981.63	491.32	V	1,179.64	590.32	1,162.61	581.81	1,161.63	581.32	9
7	1,128.69	564.85	1,111.66	556.33	1,110.68	555.84	E	1,080.57	540.79	1,063.55	532.28	1,062.56	531.78	8
8	1,215.72	608.36	1,198.69	599.85	1,197.71	599.36	S	951.53	476.27	934.50	467.76	933.52	467.26	7
9	1,346.76	673.88	1,329.73	665.37	1,328.75	664.88	M	864.50	432.75	847.47	424.24	846.49	423.75	6
10	1,459.84	730.43	1,442.82	721.91	1,441.83	721.42	L	733.46	367.23	716.43	358.72	715.45	358.23	5
11	1,546.88	773.94	1,529.85	765.43	1,528.86	764.94	S	620.37	310.69	603.35	302.18	602.36	301.69	4
12	1,675.92	838.46	1,658.89	829.95	1,657.91	829.46	E	533.34	267.17	516.31	258.66	515.33	258.17	3
13	1,789.00	895.00	1,771.98	886.49	1,770.99	886.00	I	404.30	202.65	387.27	194.14			2

14							K	291.21	146.11	274.19	137.60			1

Query 88653 Hit 1

MS/MS Fragmentation of EERPLFPQILSSIELLQHSLPK
Found in sp|P04049|RAF1_HUMAN, RAF proto-oncogene serine/threonine-protein kinase OS=Homo sapiens GN=RAF1 PE=1 SV=1
Match to Query 88653: 2861.626from(716.4138,4+)
Title: 1214: Sum of 2 scans in range 2640 (rt=69.0675, f=4, i=801) to 2641 (rt=69.0929, f=4, i=802)
[D:\lab212\membrane\Grace\Joyce\iTRAQ_45_2.raw]
Data File:Submitted from 20120508-1(merge) by Mascot Daemon on JOYCE-VAIO
Monoisotopic mass of neutral peptide Mr(calc): 2861.626
Variable modifications:
K22 :iTRAQ4plex (K)
Ions Score: 45.36 Expect: 0.007

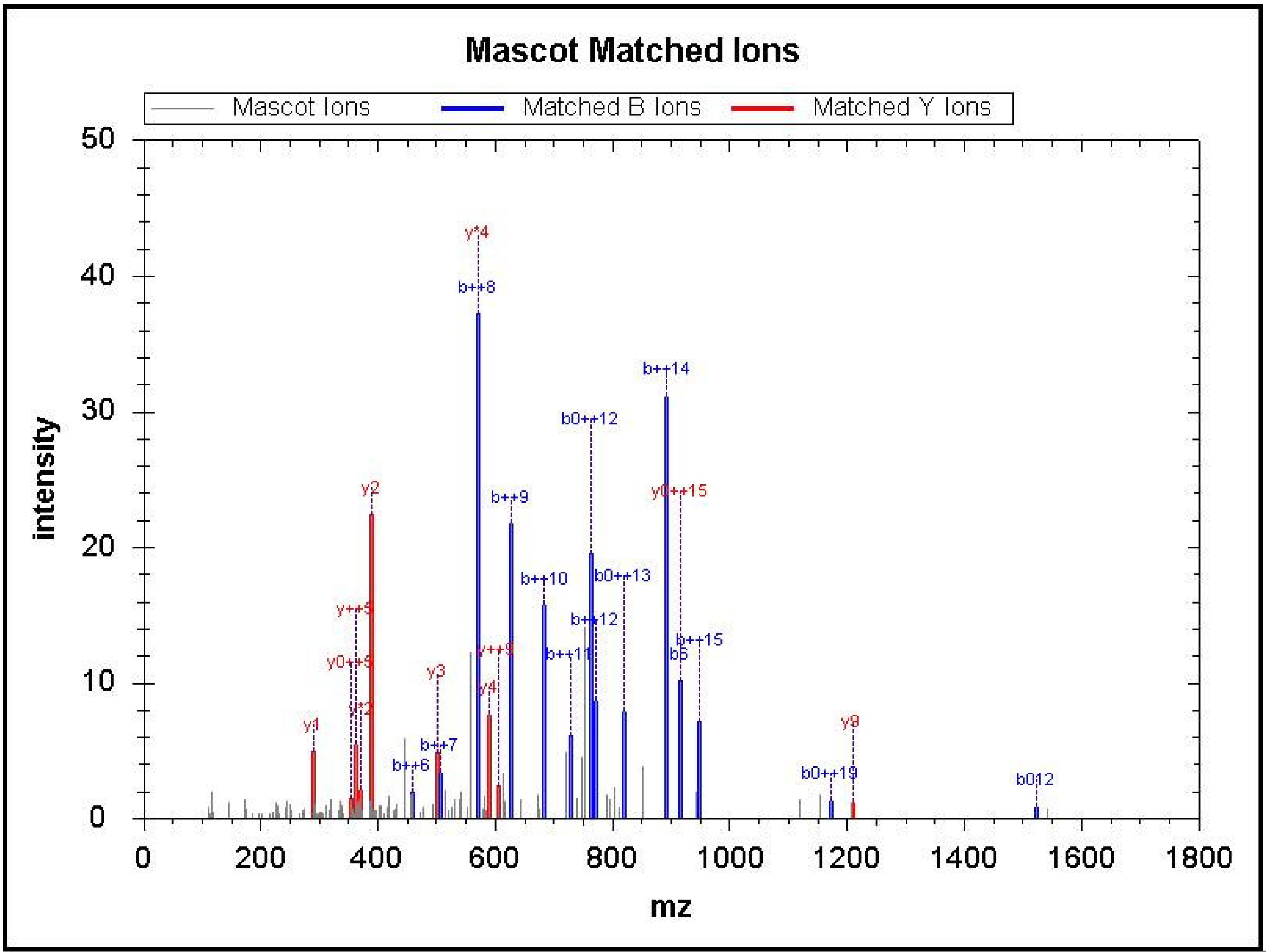

No	b	b++	b*	b*++	b0	b0++	Seq	y	y++	y*	y*++	y0	y0++	RevNo
1	274.15	137.58			256.14	128.57	E							22
2	403.19	202.10			385.18	193.10	E	2,589.49	1,295.25	2,572.46	1,286.73	2,571.48	1,286.24	21
3	559.30	280.15	542.27	271.64	541.29	271.15	R	2,460.45	1,230.73	2,443.42	1,222.21	2,442.44	1,221.72	20
4	656.35	328.68	639.32	320.16	638.34	319.67	P	2,304.34	1,152.68	2,287.32	1,144.16	2,286.33	1,143.67	19
5	769.43	385.22	752.41	376.71	751.42	376.21	L	2,207.29	1,104.15	2,190.27	1,095.64	2,189.28	1,095.14	18
6	916.50	458.75	899.47	450.24	898.49	449.75	F	2,094.21	1,047.61	2,077.18	1,039.09	2,076.20	1,038.60	17
7	1,013.55	507.28	996.53	498.77	995.54	498.28	P	1,947.14	974.07	1,930.11	965.56	1,929.13	965.07	16
8	1,141.61	571.31	1,124.59	562.80	1,123.60	562.30	Q	1,850.09	925.55	1,833.06	917.03	1,832.08	916.54	15
9	1,254.70	627.85	1,237.67	619.34	1,236.69	618.85	I	1,722.03	861.52	1,705.00	853.00	1,704.02	852.51	14
10	1,367.78	684.39	1,350.75	675.88	1,349.77	675.39	L	1,608.94	804.98	1,591.92	796.46	1,590.93	795.97	13
11	1,454.81	727.91	1,437.79	719.40	1,436.80	718.90	S	1,495.86	748.43	1,478.83	739.92	1,477.85	739.43	12
12	1,541.84	771.43	1,524.82	762.91	1,523.83	762.42	S	1,408.83	704.92	1,391.80	696.40	1,390.82	695.91	11

13	1,654.93	827.97	1,637.90	819.45	1,636.92	818.96	I	1,321.80	661.40	1,304.77	652.89	1,303.79	652.40	10
14	1,783.97	892.49	1,766.94	883.98	1,765.96	883.48	E	1,208.71	604.86	1,191.69	596.35	1,190.70	595.85	9
15	1,897.06	949.03	1,880.03	940.52	1,879.04	940.03	L	1,079.67	540.34	1,062.64	531.83	1,061.66	531.33	8
16	2,010.14	1,005.57	1,993.11	997.06	1,992.13	996.57	L	966.59	483.80	949.56	475.28	948.57	474.79	7
17	2,138.20	1,069.60	2,121.17	1,061.09	2,120.19	1,060.60	Q	853.50	427.25	836.47	418.74	835.49	418.25	6
18	2,275.26	1,138.13	2,258.23	1,129.62	2,257.25	1,129.13	H	725.44	363.22	708.42	354.71	707.43	354.22	5
19	2,362.29	1,181.65	2,345.26	1,173.13	2,344.28	1,172.64	S	588.38	294.70	571.36	286.18	570.37	285.69	4
20	2,475.37	1,238.19	2,458.35	1,229.68	2,457.36	1,229.18	L	501.35	251.18	484.33	242.67			3
21	2,572.43	1,286.72	2,555.40	1,278.20	2,554.41	1,277.71	P	388.27	194.64	371.24	186.12			2
22							K	291.21	146.11	274.19	137.60			1

Query 66076 Hit 1

MS/MS Fragmentation of VLLDHGAGINTHSNEFK
Found in sp|Q8IWZ3|ANKH1_HUMAN, Ankyrin repeat and KH domain-containing protein 1 OS=Homo sapiens GN=ANKHD1 PE=1 SV=1
Match to Query 66076: 2139.132from(714.0513,3+)
Title: 422: Sum of 2 scans in range 965 (rt=31.4388, f=4, i=286) to 966 (rt=31.4642, f=4, i=287) [D:\lab212\membrane\Grace\Joyce\iTRAQ_49_1.raw]
Data File:Submitted from 20120508-1(merge) by Mascot Daemon on JOYCE-VAIO
Monoisotopic mass of neutral peptide Mr(calc): 2139.132
Variable modifications:
K17 :iTRAQ4plex (K)
Ions Score: 45.31 Expect: 0.026

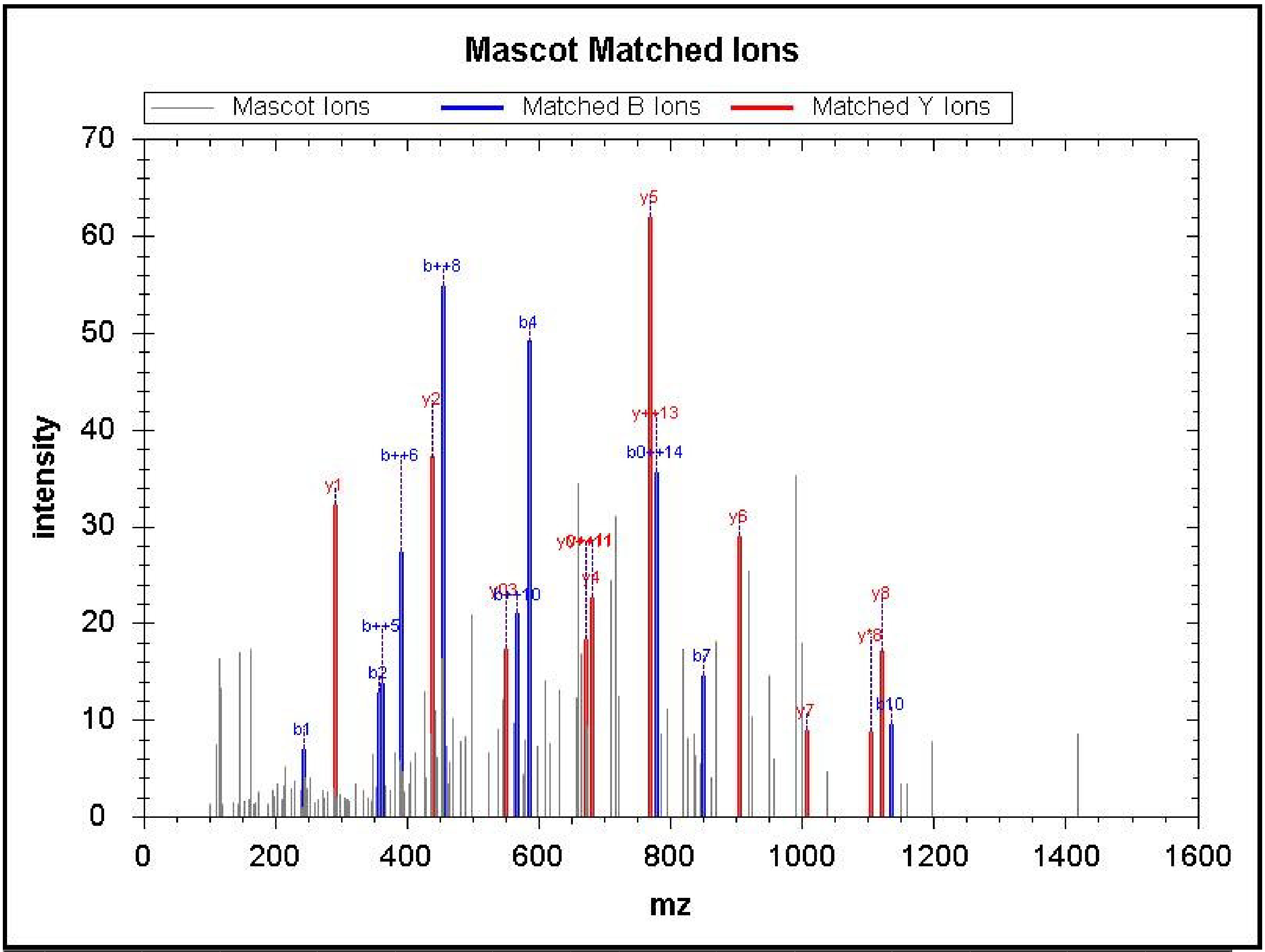

No	b	b++	b*	b*++	b0	b0++	Seq	y	y++	y*	y*++	y0	y0++	RevNo
1	244.18	122.59					V							17
2	357.26	179.13					L	1,896.97	948.99	1,879.94	940.47	1,878.96	939.98	16

3	470.35	235.68					L	1,783.88	892.45	1,766.86	883.93	1,765.87	883.44	15
4	585.37	293.19			567.36	284.18	D	1,670.80	835.90	1,653.77	827.39	1,652.79	826.90	14
5	722.43	361.72			704.42	352.71	H	1,555.77	778.39	1,538.75	769.88	1,537.76	769.39	13
6	779.45	390.23			761.44	381.22	G	1,418.71	709.86	1,401.69	701.35	1,400.70	700.86	12
7	850.49	425.75			832.48	416.74	A	1,361.69	681.35	1,344.67	672.84	1,343.68	672.34	11
8	907.51	454.26			889.50	445.25	G	1,290.66	645.83	1,273.63	637.32	1,272.65	636.83	10
9	1,020.60	510.80			1,002.59	501.80	I	1,233.63	617.32	1,216.61	608.81	1,215.62	608.32	9
10	1,134.64	567.82	1,117.61	559.31	1,116.63	558.82	N	1,120.55	560.78	1,103.52	552.27	1,102.54	551.77	8
11	1,235.69	618.35	1,218.66	609.83	1,217.68	609.34	T	1,006.51	503.76	989.48	495.24	988.50	494.75	7
12	1,372.75	686.88	1,355.72	678.36	1,354.73	677.87	H	905.46	453.23	888.43	444.72	887.45	444.23	6
13	1,459.78	730.39	1,442.75	721.88	1,441.77	721.39	S	768.40	384.70	751.37	376.19	750.39	375.70	5
14	1,573.82	787.41	1,556.79	778.90	1,555.81	778.41	N	681.37	341.19	664.34	332.67	663.36	332.18	4
15	1,702.86	851.94	1,685.84	843.42	1,684.85	842.93	E	567.33	284.17	550.30	275.65	549.32	275.16	3
16	1,849.93	925.47	1,832.90	916.96	1,831.92	916.46	F	438.28	219.65	421.26	211.13			2
17							K	291.21	146.11	274.19	137.60			1

Query 15396 Hit 1

MS/MS Fragmentation of TASVPLDAVR
Found in sp|Q96DV4|RM38_HUMAN, 39S ribosomal protein L38
Match to Query 15396: 1171.67from(586.8423,2+)
Title: 337: Scan 899 (rt=29.2986, f=3, i=121) [D:\lab212\membrane\Grace\Joyce\iTRAQ_23_1.raw]
Data File:Submitted from 20120508-1(merge) by Mascot Daemon on JOYCE-VAIO
Monoisotopic mass of neutral peptide Mr(calc): 1171.67
Variable modifications:
Ions Score: 45.27 Expect: 0.018

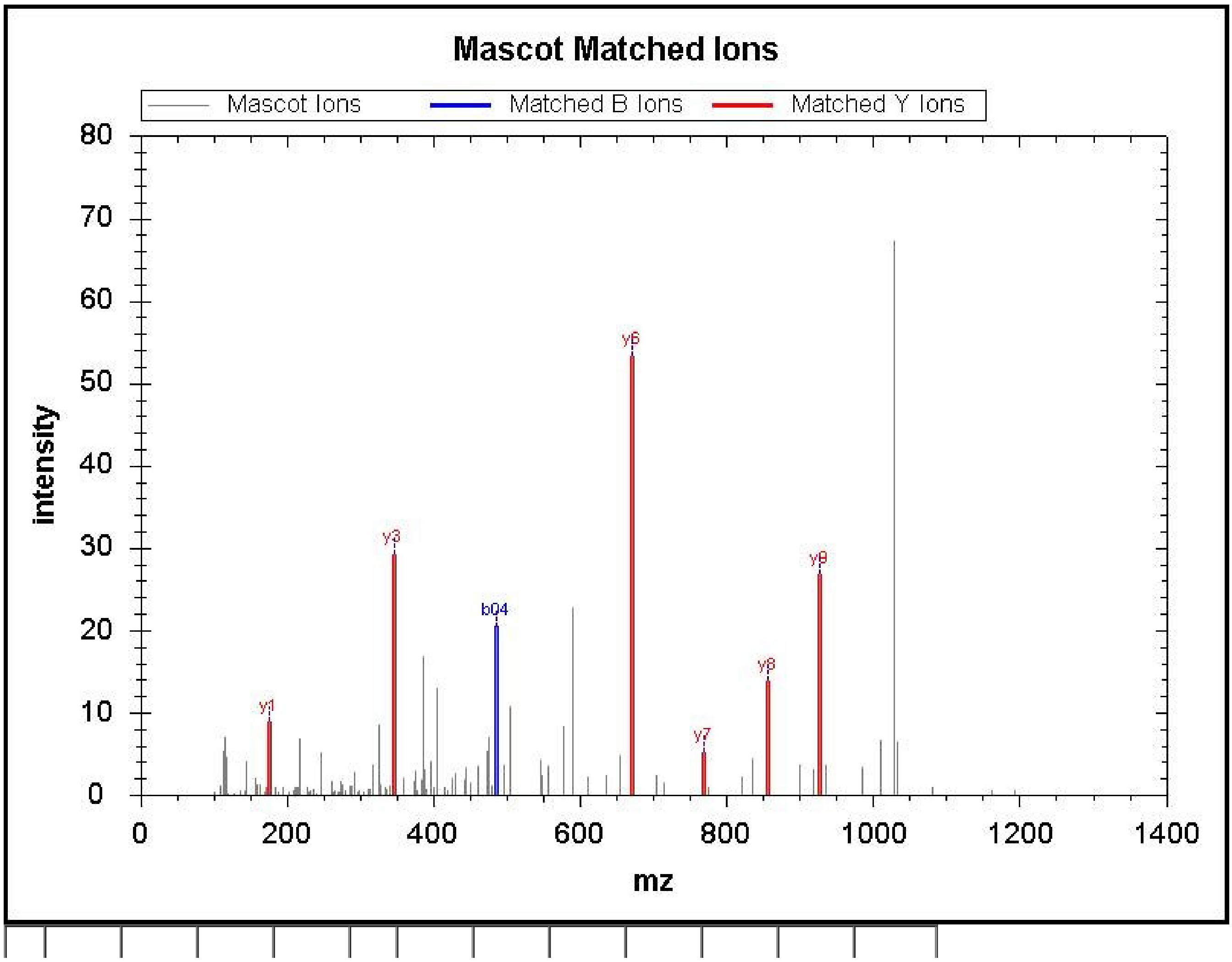

No	b	b++	b0	b0++	Seq	y	y++	y*	y*++	y0	y0++	RevNo
1	246.16	123.58	228.15	114.58	T							10
2	317.19	159.10	299.18	150.10	A	927.53	464.27	910.50	455.75	909.52	455.26	9
3	404.23	202.62	386.22	193.61	S	856.49	428.75	839.46	420.23	838.48	419.74	8
4	503.29	252.15	485.28	243.15	V	769.46	385.23	752.43	376.72	751.45	376.23	7
5	600.35	300.68	582.34	291.67	P	670.39	335.70	653.36	327.18	652.38	326.69	6
6	713.43	357.22	695.42	348.21	L	573.34	287.17	556.31	278.66	555.32	278.17	5
7	828.46	414.73	810.45	405.73	D	460.25	230.63	443.22	222.12	442.24	221.62	4
8	899.50	450.25	881.48	441.25	A	345.22	173.12	328.20	164.60			3
9	998.56	499.79	980.55	490.78	V	274.19	137.60	257.16	129.08			2
10					R	175.12	88.06	158.09	79.55			1

Query 64697 Hit 1

MS/MS Fragmentation of LSLNNGSITHLVIRPNGR
Found in sp|Q96HS1|PGAM5_HUMAN, Serine/threonine-protein phosphatase PGAM5
Match to Query 64697: 2106.172from(527.5504,4+)
Title: 667: Sum of 2 scans in range 1466 (rt=42.7169, f=4, i=449) to 1467 (rt=42.7423, f=4, i=450)
[D:\lab212\membrane\Grace\Joyce\iTRAQ_45_1.raw]
Data File:Submitted from 20120508-1(merge) by Mascot Daemon on JOYCE-VAIO
Monoisotopic mass of neutral peptide Mr(calc): 2106.172
Variable modifications:
N5 :Deamidated (NQ)
N16 :Deamidated (NQ)
Ions Score: 45.25 Expect: 0.020

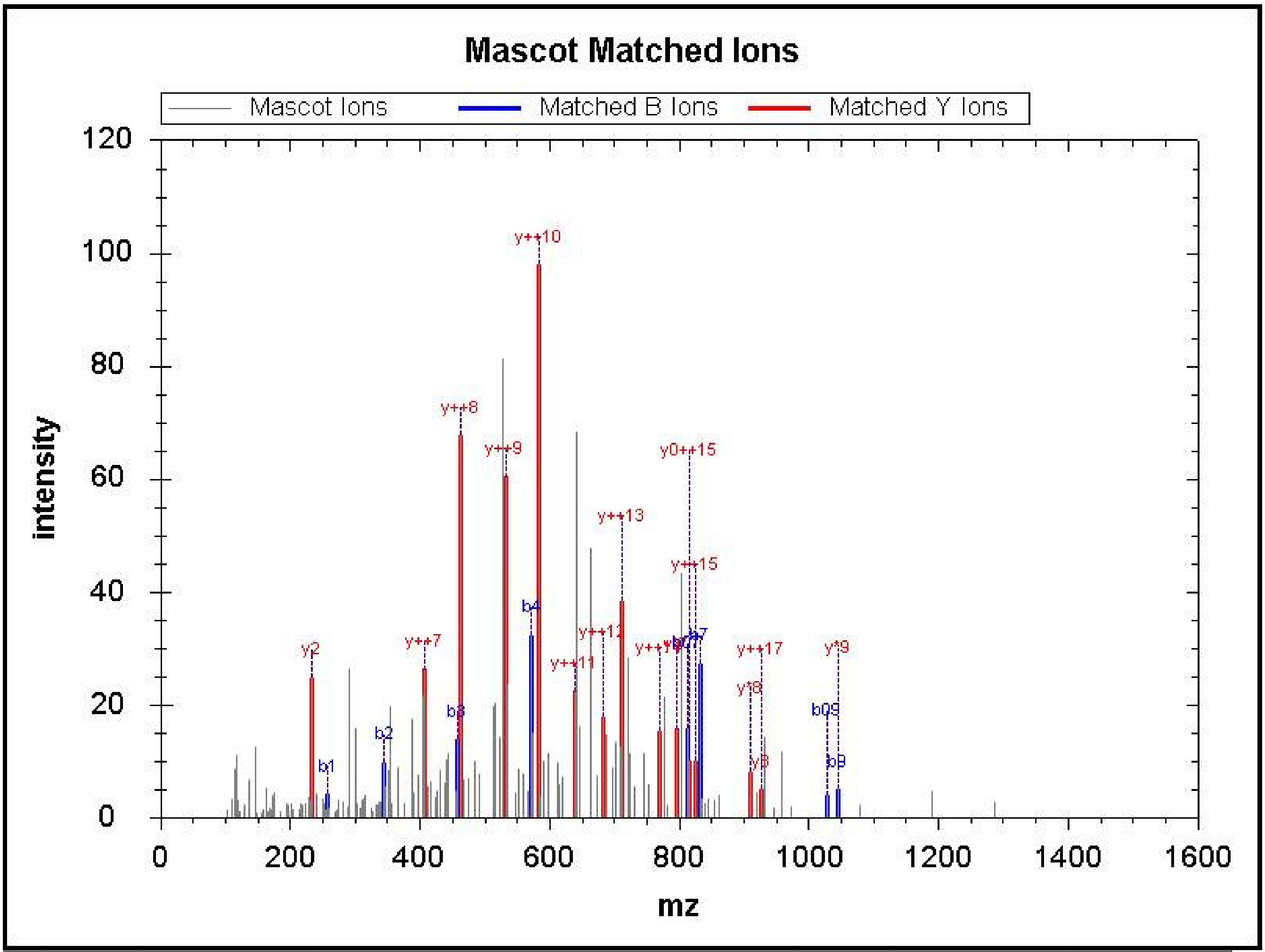

No	b	b++	b*	b*++	b0	b0++	Seq	y	y++	y*	y*++	y0	y0++	RevNo

1	258.19	129.60					L							18
2	345.23	173.12			327.21	164.11	S	1,849.99	925.50	1,832.96	916.98	1,831.98	916.49	17
3	458.31	229.66			440.30	220.65	L	1,762.96	881.98	1,745.93	873.47	1,744.95	872.98	16
4	572.35	286.68	555.33	278.17	554.34	277.67	N	1,649.87	825.44	1,632.85	816.93	1,631.86	816.43	15
5	687.38	344.19	670.35	335.68	669.37	335.19	N	1,535.83	768.42	1,518.80	759.90	1,517.82	759.41	14
6	744.40	372.70	727.37	364.19	726.39	363.70	G	1,420.80	710.90	1,403.78	702.39	1,402.79	701.90	13
7	831.43	416.22	814.41	407.71	813.42	407.21	S	1,363.78	682.39	1,346.75	673.88	1,345.77	673.39	12
8	944.52	472.76	927.49	464.25	926.51	463.76	I	1,276.75	638.88	1,259.72	630.36	1,258.74	629.87	11
9	1,045.56	523.29	1,028.54	514.77	1,027.55	514.28	T	1,163.66	582.34	1,146.64	573.82	1,145.65	573.33	10
10	1,182.62	591.82	1,165.60	583.30	1,164.61	582.81	H	1,062.62	531.81	1,045.59	523.30			9
11	1,295.71	648.36	1,278.68	639.84	1,277.70	639.35	L	925.56	463.28	908.53	454.77			8
12	1,394.78	697.89	1,377.75	689.38	1,376.77	688.89	V	812.47	406.74	795.45	398.23			7
13	1,507.86	754.43	1,490.83	745.92	1,489.85	745.43	I	713.41	357.21	696.38	348.69			6
14	1,663.96	832.48	1,646.93	823.97	1,645.95	823.48	R	600.32	300.66	583.29	292.15			5
15	1,761.01	881.01	1,743.99	872.50	1,743.00	872.01	P	444.22	222.61	427.19	214.10			4
16	1,876.04	938.52	1,859.01	930.01	1,858.03	929.52	N	347.17	174.09	330.14	165.57			3
17	1,933.06	967.03	1,916.04	958.52	1,915.05	958.03	G	232.14	116.57	215.11	108.06			2
18							R	175.12	88.06	158.09	79.55			1

Query 73146 Hit 1

MS/MS Fragmentation of SVFDIPIFTEEFLNHSK
Found in sp|Q9NP66|HM20A_HUMAN, High mobility group protein 20A OS=Homo sapiens GN=HMG20A PE=1 SV=1
Match to Query 73146: 2310.217from(771.0797,3+)
Title: 1136: Sum of 2 scans in range 2501 (rt=65.7265, f=4, i=749) to 2502 (rt=65.7519, f=4, i=750)
[D:\lab212\membrane\Grace\Joyce\iTRAQ_36_1.raw]
Data File:Submitted from 20120508-1(merge) by Mascot Daemon on JOYCE-VAIO
Monoisotopic mass of neutral peptide Mr(calc): 2310.217
Variable modifications:
K17 :iTRAQ4plex (K)
Ions Score: 45.02 Expect: 0.027

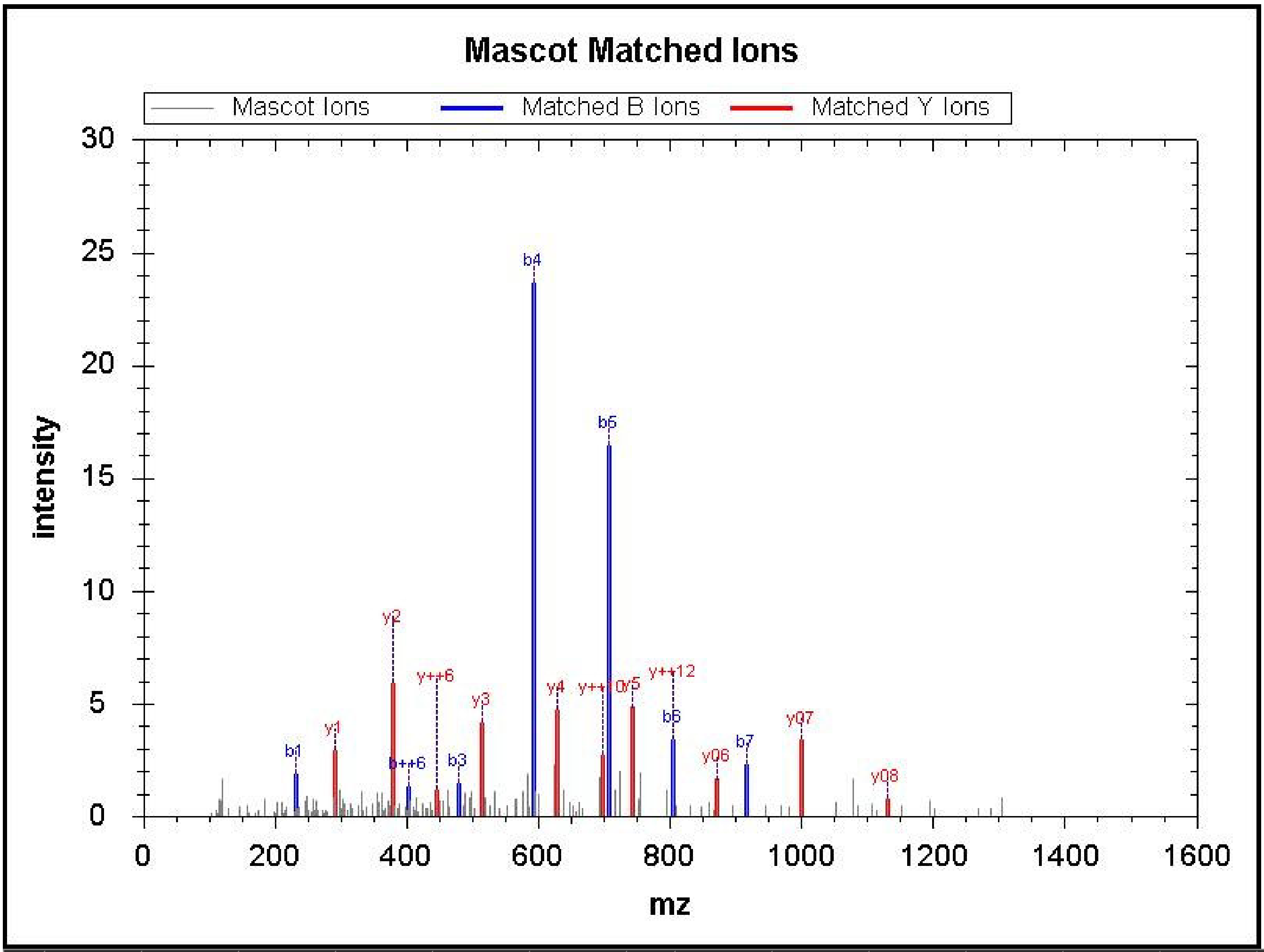

No	b	b++	b*	b*++	b0	b0++	Seq	y	y++	y*	y*++	y0	y0++	RevNo
1	232.14	116.57			214.13	107.57	S							17
2	331.21	166.11			313.20	157.10	V	2,080.09	1,040.55	2,063.06	1,032.03	2,062.08	1,031.54	16
3	478.28	239.64			460.27	230.64	F	1,981.02	991.01	1,963.99	982.50	1,963.01	982.01	15
4	593.31	297.16			575.29	288.15	D	1,833.95	917.48	1,816.92	908.97	1,815.94	908.47	14
5	706.39	353.70			688.38	344.69	I	1,718.92	859.97	1,701.90	851.45	1,700.91	850.96	13
6	803.44	402.22			785.43	393.22	P	1,605.84	803.42	1,588.81	794.91	1,587.83	794.42	12
7	916.53	458.77			898.52	449.76	I	1,508.79	754.90	1,491.76	746.38	1,490.78	745.89	11
8	1,063.59	532.30			1,045.58	523.30	F	1,395.70	698.35	1,378.68	689.84	1,377.69	689.35	10
9	1,164.64	582.82			1,146.63	573.82	T	1,248.63	624.82	1,231.61	616.31	1,230.62	615.82	9
10	1,293.68	647.35			1,275.67	638.34	E	1,147.59	574.30	1,130.56	565.78	1,129.58	565.29	8
11	1,422.73	711.87			1,404.72	702.86	E	1,018.54	509.78	1,001.52	501.26	1,000.53	500.77	7
12	1,569.80	785.40			1,551.79	776.40	F	889.50	445.25	872.47	436.74	871.49	436.25	6
13	1,682.88	841.94			1,664.87	832.94	L	742.43	371.72	725.41	363.21	724.42	362.71	5
14	1,796.92	898.97	1,779.90	890.45	1,778.91	889.96	N	629.35	315.18	612.32	306.66	611.34	306.17	4
15	1,933.98	967.49	1,916.96	958.98	1,915.97	958.49	H	515.31	258.16	498.28	249.64	497.30	249.15	3
16	2,021.01	1,011.01	2,003.99	1,002.50	2,003.00	1,002.01	S	378.25	189.63	361.22	181.11	360.24	180.62	2
17							K	291.21	146.11	274.19	137.60			1

Query 60932 Hit 1

MS/MS Fragmentation of FKPGYLEATLNWFR
Found in sp|Q9H2U2|IPYR2_HUMAN, Inorganic pyrophosphatase 2
Match to Query 60932: 2029.105from(677.3757,3+)
Title: 1000: Sum of 2 scans in range 2209 (rt=59.2822, f=4, i=663) to 2210 (rt=59.3076, f=4, i=664)
[D:\lab212\membrane\Grace\Joyce\iTRAQ_41_2.raw]
Data File:Submitted from 20120508-1(merge) by Mascot Daemon on JOYCE-VAIO

Monoisotopic mass of neutral peptide Mr(calc): 2029.105
Variable modifications:
K2 :iTRAQ4plex (K)
Ions Score: 45.02 Expect: 0.025

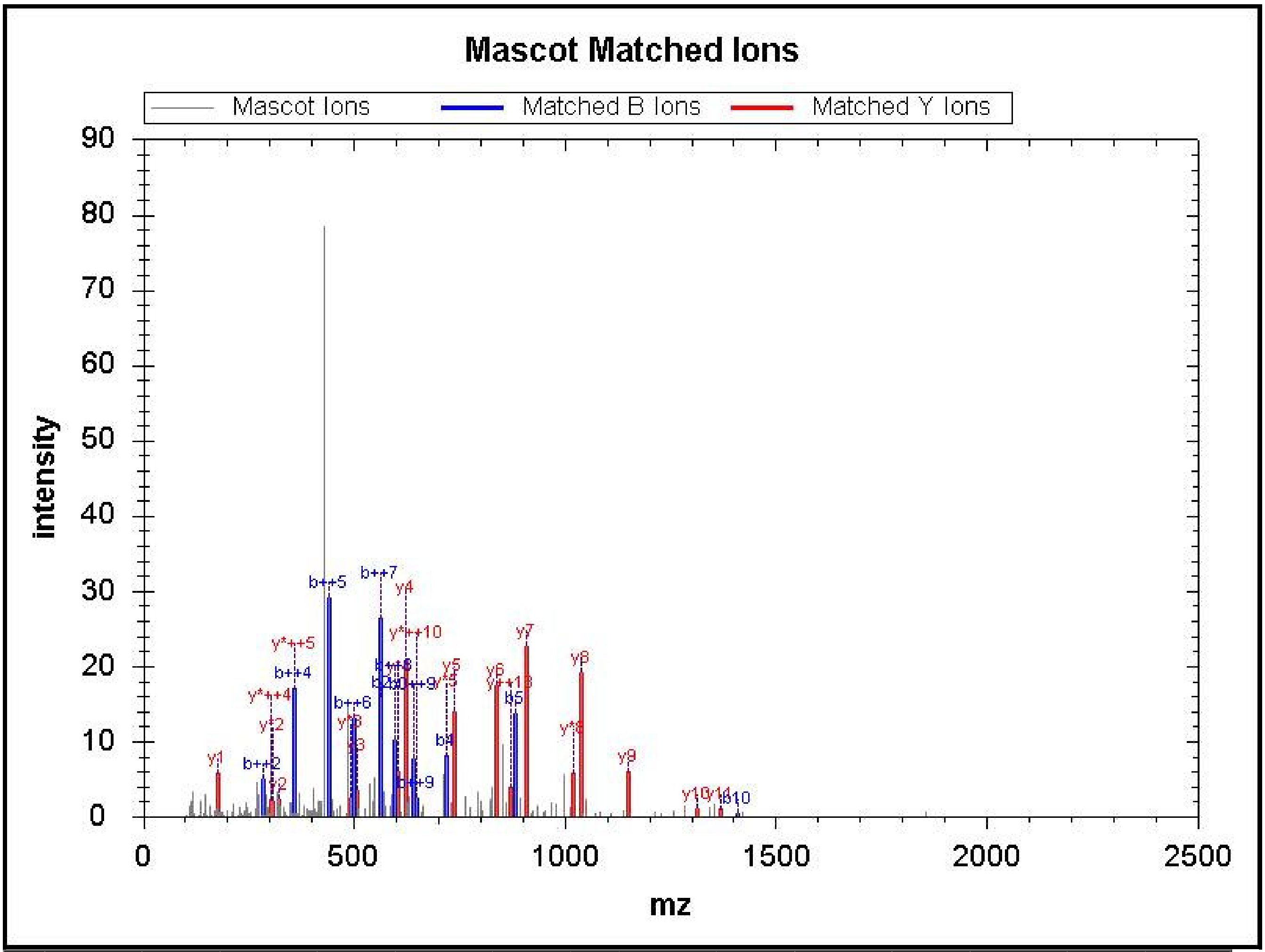

No	b	b++	b*	b*++	b0	b0++	Seq	y	y++	y*	y*++	y0	y0++	RevNo
1	292.18	146.59					F							14
2	564.37	282.69	547.35	274.18			K	1,738.94	869.97	1,721.91	861.46	1,720.93	860.97	13
3	661.43	331.22	644.40	322.70			P	1,466.74	733.88	1,449.72	725.36	1,448.73	724.87	12
4	718.45	359.73	701.42	351.21			G	1,369.69	685.35	1,352.66	676.84	1,351.68	676.34	11
5	881.51	441.26	864.49	432.75			Y	1,312.67	656.84	1,295.64	648.32	1,294.66	647.83	10
6	994.60	497.80	977.57	489.29			L	1,149.61	575.31	1,132.58	566.79	1,131.59	566.30	9
7	1,123.64	562.32	1,106.61	553.81	1,105.63	553.32	E	1,036.52	518.76	1,019.49	510.25	1,018.51	509.76	8
8	1,194.68	597.84	1,177.65	589.33	1,176.67	588.84	A	907.48	454.24	890.45	445.73	889.47	445.24	7
9	1,295.72	648.37	1,278.70	639.85	1,277.71	639.36	T	836.44	418.72	819.41	410.21	818.43	409.72	6
10	1,408.81	704.91	1,391.78	696.39	1,390.80	695.90	L	735.39	368.20	718.37	359.69			5
11	1,522.85	761.93	1,505.82	753.42	1,504.84	752.92	N	622.31	311.66	605.28	303.15			4
12	1,708.93	854.97	1,691.90	846.46	1,690.92	845.96	W	508.27	254.64	491.24	246.12			3
13	1,856.00	928.50	1,838.97	919.99	1,837.99	919.50	F	322.19	161.60	305.16	153.08			2
14							R	175.12	88.06	158.09	79.55			1

Query 35799 Hit 1

MS/MS Fragmentation of VGPGNHGTEGSGGER
Found in sp|Q99442|SEC62_HUMAN, Translocation protein SEC62 OS=Homo sapiens GN=SEC62 PE=1 SV=1
Match to Query 35799: 1553.729from(518.9169,3+)
Title: 13: Scan 138 (rt=12.6659, f=3, i=8) [D:\lab212\membrane\Grace\Joyce\iTRAQ_42_1.raw]
Data File:Submitted from 20120508-1(merge) by Mascot Daemon on JOYCE-VAIO

Monoisotopic mass of neutral peptide Mr(calc): 1553.729
Variable modifications:
Ions Score: 45.02 Expect: 0.019

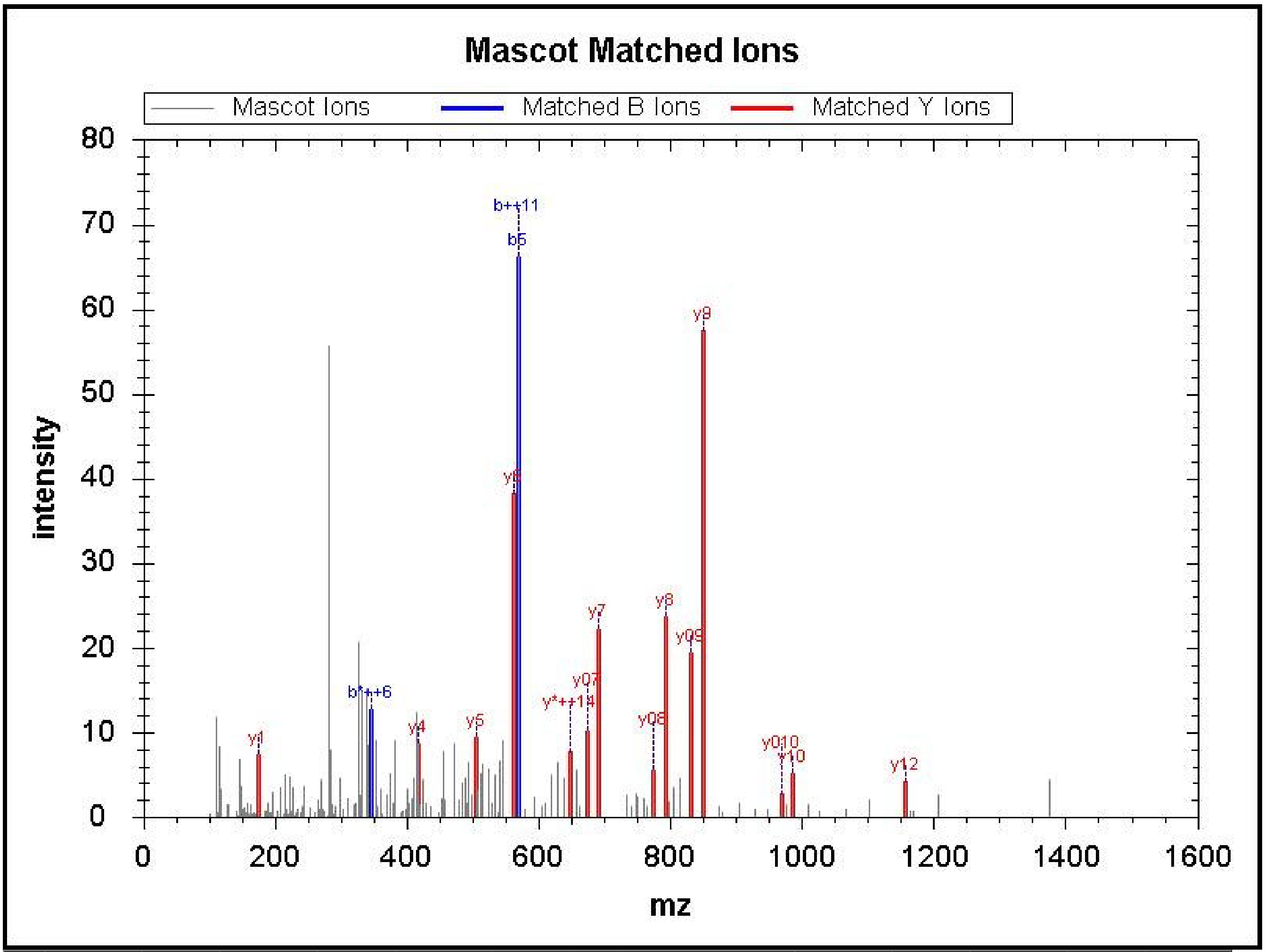

No	b	b++	b*	b*++	b0	b0++	Seq	y	y++	y*	y*++	y0	y0++	RevNo
1	244.18	122.59					V							15
2	301.20	151.10					G	1,311.57	656.29	1,294.54	647.77	1,293.56	647.28	14
3	398.25	199.63					P	1,254.55	627.78	1,237.52	619.26	1,236.54	618.77	13
4	455.27	228.14					G	1,157.49	579.25	1,140.47	570.74	1,139.48	570.24	12
5	569.32	285.16	552.29	276.65			N	1,100.47	550.74	1,083.45	542.23	1,082.46	541.73	11
6	706.38	353.69	689.35	345.18			H	986.43	493.72	969.40	485.20	968.42	484.71	10
7	763.40	382.20	746.37	373.69			G	849.37	425.19	832.34	416.68	831.36	416.18	9
8	864.44	432.73	847.42	424.21	846.43	423.72	T	792.35	396.68	775.32	388.16	774.34	387.67	8
9	993.49	497.25	976.46	488.73	975.48	488.24	E	691.30	346.15	674.27	337.64	673.29	337.15	7
10	1,050.51	525.76	1,033.48	517.24	1,032.50	516.75	G	562.26	281.63	545.23	273.12	544.25	272.63	6
11	1,137.54	569.27	1,120.51	560.76	1,119.53	560.27	S	505.24	253.12	488.21	244.61	487.23	244.12	5
12	1,194.56	597.78	1,177.54	589.27	1,176.55	588.78	G	418.20	209.61	401.18	201.09	400.19	200.60	4
13	1,251.58	626.30	1,234.56	617.78	1,233.57	617.29	G	361.18	181.10	344.16	172.58	343.17	172.09	3
14	1,380.63	690.82	1,363.60	682.30	1,362.62	681.81	E	304.16	152.58	287.13	144.07	286.15	143.58	2
15							R	175.12	88.06	158.09	79.55			1

Query 86005 Hit 1

MS/MS Fragmentation of LVQLIEDYSLVSFIPLNIQDK
Found in sp|Q9H9Y4|GPN2_HUMAN, GPN-loop GTPase 2 OS=Homo sapiens GN=GPN2 PE=2 SV=2
Match to Query 86005: 2734.536from(912.5194,3+)
Title: 1182: Scan 3219 (rt=78.712, f=3, i=399) [D:\lab212\membrane\Grace\Joyce\iTRAQ_24_2.raw]

Data File:Submitted from 20120508-1(merge) by Mascot Daemon on JOYCE-VAIO
Monoisotopic mass of neutral peptide Mr(calc): 2734.536
Variable modifications:
K21 :iTRAQ4plex (K)
Ions Score: 45 Expect: 0.011

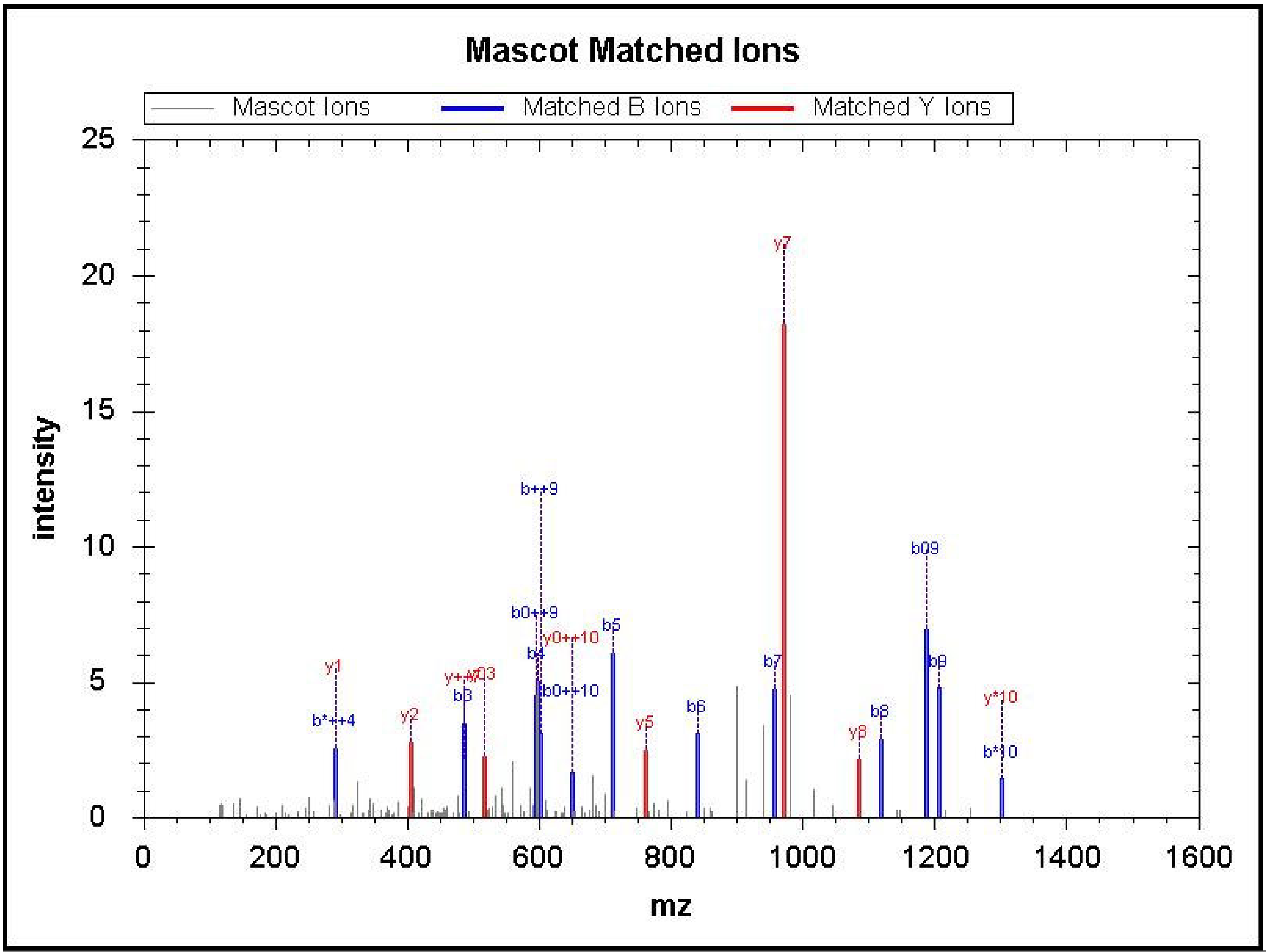

No	b	b++	b*	b*++	b0	b0++	Seq	y	y++	y*	y*++	y0	y0++	RevNo
1	258.19	129.60					L							21
2	357.26	179.13					V	2,478.36	1,239.68	2,461.33	1,231.17	2,460.35	1,230.68	20
3	485.32	243.16	468.29	234.65			Q	2,379.29	1,190.15	2,362.27	1,181.64	2,361.28	1,181.14	19
4	598.40	299.71	581.38	291.19			L	2,251.23	1,126.12	2,234.21	1,117.61	2,233.22	1,117.12	18
5	711.49	356.25	694.46	347.73			I	2,138.15	1,069.58	2,121.12	1,061.07	2,120.14	1,060.57	17
6	840.53	420.77	823.50	412.26	822.52	411.76	E	2,025.07	1,013.04	2,008.04	1,004.52	2,007.06	1,004.03	16
7	955.56	478.28	938.53	469.77	937.55	469.28	D	1,896.02	948.52	1,879.00	940.00	1,878.01	939.51	15
8	1,118.62	559.81	1,101.59	551.30	1,100.61	550.81	Y	1,781.00	891.00	1,763.97	882.49	1,762.99	882.00	14
9	1,205.65	603.33	1,188.63	594.82	1,187.64	594.33	S	1,617.93	809.47	1,600.91	800.96	1,599.92	800.46	13
10	1,318.74	659.87	1,301.71	651.36	1,300.73	650.87	L	1,530.90	765.95	1,513.87	757.44	1,512.89	756.95	12
11	1,417.81	709.41	1,400.78	700.89	1,399.80	700.40	V	1,417.82	709.41	1,400.79	700.90	1,399.81	700.41	11
12	1,504.84	752.92	1,487.81	744.41	1,486.83	743.92	S	1,318.75	659.88	1,301.72	651.36	1,300.74	650.87	10
13	1,651.91	826.46	1,634.88	817.94	1,633.90	817.45	F	1,231.72	616.36	1,214.69	607.85	1,213.71	607.36	9
14	1,764.99	883.00	1,747.96	874.49	1,746.98	873.99	I	1,084.65	542.83	1,067.62	534.31	1,066.64	533.82	8
15	1,862.04	931.53	1,845.02	923.01	1,844.03	922.52	P	971.56	486.29	954.54	477.77	953.55	477.28	7
16	1,975.13	988.07	1,958.10	979.55	1,957.12	979.06	L	874.51	437.76	857.48	429.25	856.50	428.75	6
17	2,089.17	1,045.09	2,072.14	1,036.58	2,071.16	1,036.08	N	761.43	381.22	744.40	372.70	743.42	372.21	5
18	2,202.25	1,101.63	2,185.23	1,093.12	2,184.24	1,092.63	I	647.38	324.20	630.36	315.68	629.37	315.19	4
19	2,330.31	1,165.66	2,313.29	1,157.15	2,312.30	1,156.65	Q	534.30	267.65	517.27	259.14	516.29	258.65	3

20	2,445.34	1,223.17	2,428.31	1,214.66	2,427.33	1,214.17	D	406.24	203.62	389.22	195.11	388.23	194.62	2
21							K	291.21	146.11	274.19	137.60			1

Query 4036 Hit 1

MS/MS Fragmentation of SLSPQGR
Found in sp|Q148V8|FA83H_MOUSE, Protein FAM83H OS=Mus musculus GN=Fam83h PE=1 SV=1
Match to Query 4036: 888.5192from(445.2669,2+)
Title: 225: Scan 601 (rt=22.8593, f=3, i=81) [D:\lab212\membrane\Grace\Joyce\iTRAQ_27_1.raw]
Data File:Submitted from 20120508-1(merge) by Mascot Daemon on JOYCE-VAIO
Monoisotopic mass of neutral peptide Mr(calc): 888.5192
Variable modifications:
Q5 :Deamidated (NQ)
Ions Score: 44.97 Expect: 0.028

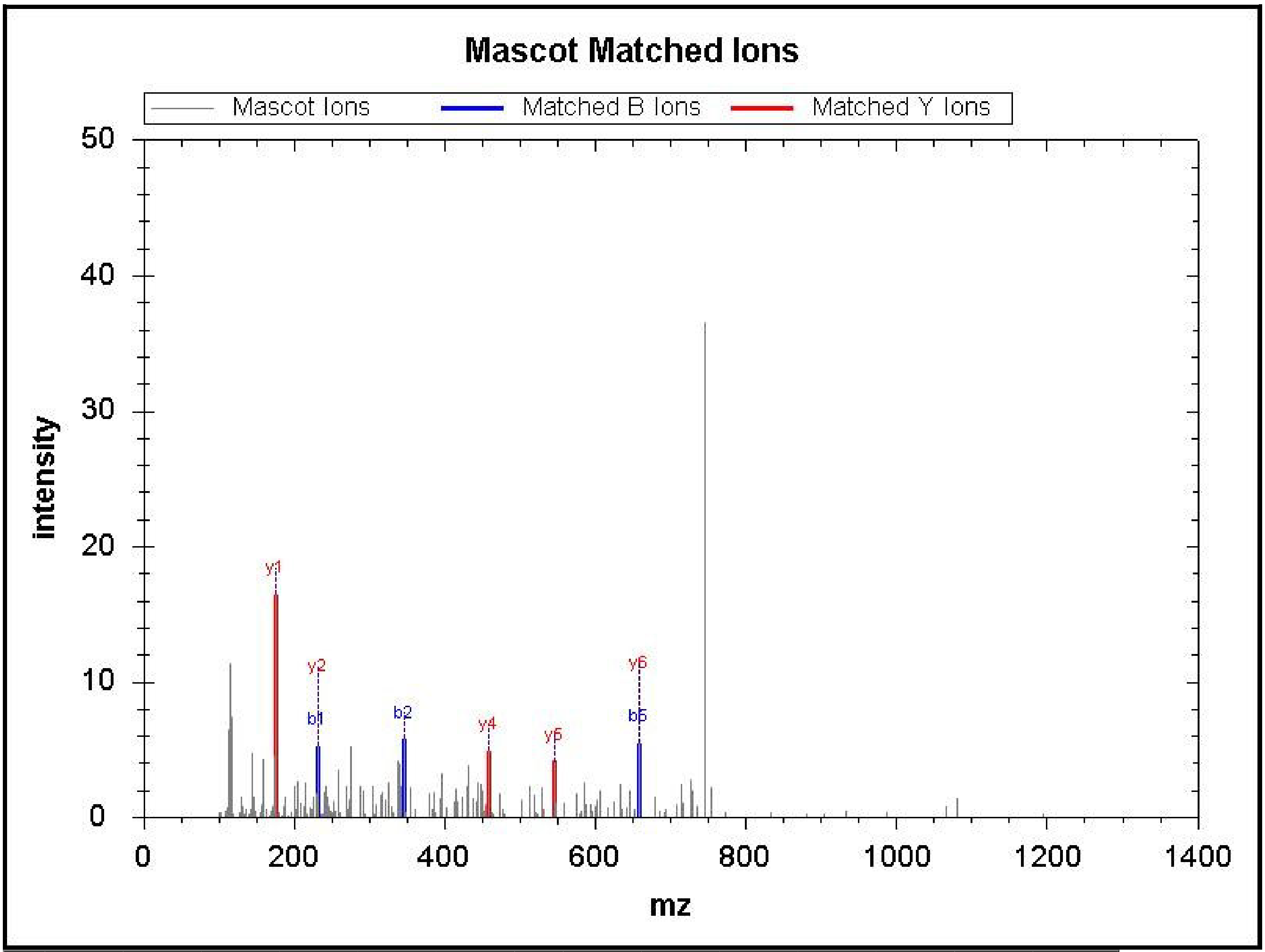

No	b	b++	b*	b*++	b0	b0++	Seq	y	y++	y*	y*++	y0	y0++	RevNo
1	232.14	116.57			214.13	107.57	S							7
2	345.23	173.12			327.21	164.11	L	658.35	329.68	641.33	321.17	640.34	320.67	6
3	432.26	216.63			414.25	207.63	S	545.27	273.14	528.24	264.62	527.26	264.13	5
4	529.31	265.16			511.30	256.15	P	458.24	229.62	441.21	221.11			4
5	658.35	329.68	641.33	321.17	640.34	320.67	Q	361.18	181.10	344.16	172.58			3
6	715.37	358.19	698.35	349.68	697.36	349.19	G	232.14	116.57	215.11	108.06			2
7							R	175.12	88.06	158.09	79.55			1

Query 53530 Hit 1

MS/MS Fragmentation of ILDIGLAYINHLVER
Found in sp|P49754|VPS41_HUMAN, Vacuolar protein sorting-associated protein 41 homolog OS=Homo sapiens GN=VPS41 PE=1 SV=3
Match to Query 53530: 1882.077from(628.3663,3+)

Title: 1198: Sum of 2 scans in range 2655 (rt=69.1936, f=4, i=773) to 2656 (rt=69.219, f=4, i=774) [D:\lab212\membrane\Grace\Joyce\iTRAQ_34_2.raw]
Data File:Submitted from 20120508-1(merge) by Mascot Daemon on JOYCE-VAIO
Monoisotopic mass of neutral peptide Mr(calc): 1882.077
Variable modifications:
Ions Score: 44.97 Expect: 0.017

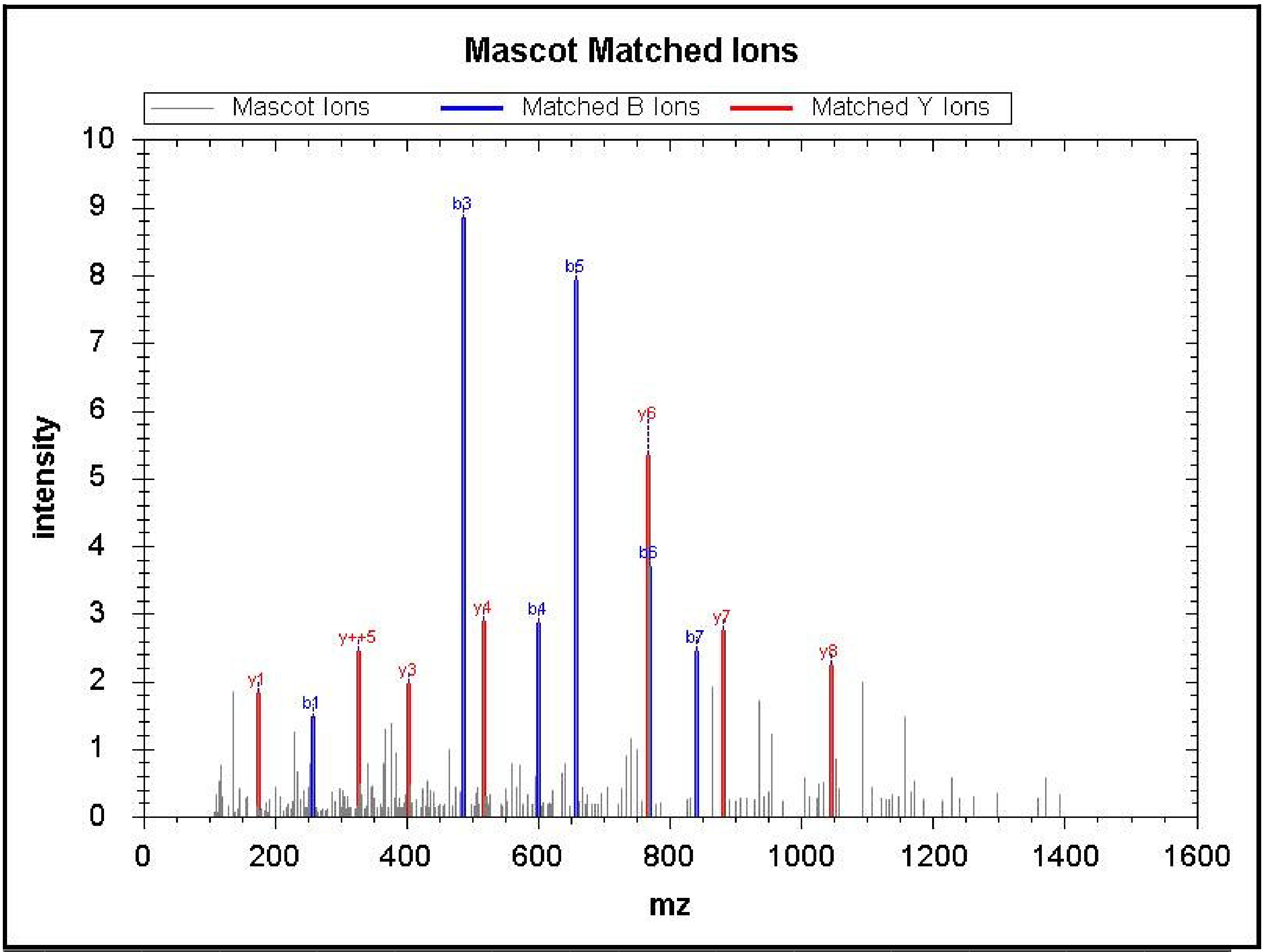

No	b	b++	b*	b*++	b0	b0++	Seq	y	y++	y*	y*++	y0	y0++	RevNo
1	258.19	129.60					I							15
2	371.28	186.14					L	1,625.90	813.45	1,608.87	804.94	1,607.89	804.45	14
3	486.30	243.66			468.29	234.65	D	1,512.82	756.91	1,495.79	748.40	1,494.81	747.91	13
4	599.39	300.20			581.38	291.19	I	1,397.79	699.40	1,380.76	690.89	1,379.78	690.39	12
5	656.41	328.71			638.40	319.70	G	1,284.71	642.86	1,267.68	634.34	1,266.70	633.85	11
6	769.49	385.25			751.48	376.25	L	1,227.68	614.35	1,210.66	605.83	1,209.67	605.34	10
7	840.53	420.77			822.52	411.76	A	1,114.60	557.80	1,097.57	549.29	1,096.59	548.80	9
8	1,003.59	502.30			985.58	493.30	Y	1,043.56	522.29	1,026.54	513.77	1,025.55	513.28	8
9	1,116.68	558.84			1,098.67	549.84	I	880.50	440.75	863.47	432.24	862.49	431.75	7
10	1,230.72	615.86	1,213.70	607.35	1,212.71	606.86	N	767.42	384.21	750.39	375.70	749.41	375.21	6
11	1,367.78	684.39	1,350.75	675.88	1,349.77	675.39	H	653.37	327.19	636.35	318.68	635.36	318.18	5
12	1,480.86	740.94	1,463.84	732.42	1,462.85	731.93	L	516.31	258.66	499.29	250.15	498.30	249.66	4
13	1,579.93	790.47	1,562.91	781.96	1,561.92	781.46	V	403.23	202.12	386.20	193.61	385.22	193.11	3
14	1,708.98	854.99	1,691.95	846.48	1,690.96	845.99	E	304.16	152.58	287.13	144.07	286.15	143.58	2
15							R	175.12	88.06	158.09	79.55			1

Query 36506 Hit 1

MS/MS Fragmentation of KVPGEGLPLPK

Found in sp|P25685|DNJB1_HUMAN, DnaJ homolog subfamily B member 1 OS=Homo sapiens GN=DNAJB1 PE=1 SV=4
Match to Query 36506: 1565.988from(523.0034,3+)
Title: 322: Scan 982 (rt=30.8396, f=3, i=112) [D:\lab212\membrane\Grace\Joyce\iTRAQ_46_2.raw]
Data File:Submitted from 20120508-1(merge) by Mascot Daemon on JOYCE-VAIO
Monoisotopic mass of neutral peptide Mr(calc): 1565.988
Variable modifications:
K1 :iTRAQ4plex (K)
K11 :iTRAQ4plex (K)
Ions Score: 44.96 Expect: 0.006

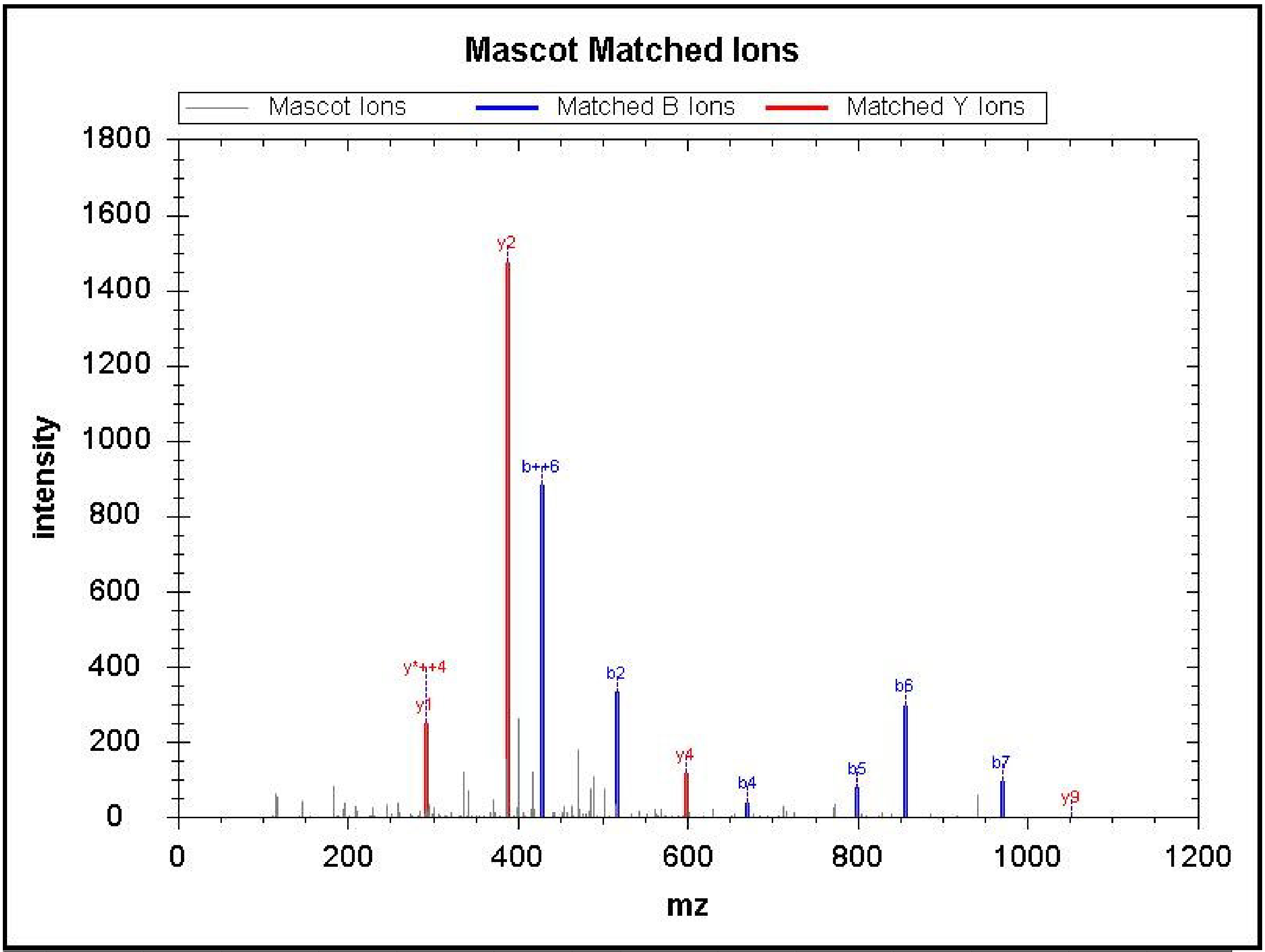

No	b	b++	b*	b*++	b0	b0++	Seq	y	y++	y*	y*++	y0	y0++	RevNo
1	417.31	209.16	400.28	200.64			K							11
2	516.37	258.69	499.35	250.18			V	1,150.70	575.85	1,133.67	567.34	1,132.68	566.85	10
3	613.43	307.22	596.40	298.70			P	1,051.63	526.32	1,034.60	517.80	1,033.62	517.31	9
4	670.45	335.73	653.42	327.21			G	954.57	477.79	937.55	469.28	936.56	468.79	8
5	799.49	400.25	782.47	391.74	781.48	391.24	E	897.55	449.28	880.53	440.77	879.54	440.27	7
6	856.51	428.76	839.49	420.25	838.50	419.75	G	768.51	384.76	751.48	376.25			6
7	969.60	485.30	952.57	476.79	951.59	476.30	L	711.49	356.25	694.46	347.73			5
8	1,066.65	533.83	1,049.62	525.32	1,048.64	524.82	P	598.40	299.71	581.38	291.19			4
9	1,179.73	590.37	1,162.71	581.86	1,161.72	581.37	L	501.35	251.18	484.33	242.67			3
10	1,276.79	638.90	1,259.76	630.38	1,258.78	629.89	P	388.27	194.64	371.24	186.12			2
11							K	291.21	146.11	274.19	137.60			1

Query 43588 Hit 1

MS/MS Fragmentation of THQLSLDAFLVALK
Found in sp|Q5JVF3|PCID2_HUMAN, PCI domain-containing protein 2 OS=Homo sapiens GN=PCID2 PE=1 SV=2
Match to Query 43588: 1698.979from(567.3336,3+)
Title: 1019: Sum of 2 scans in range 2243 (rt=60.0398, f=4, i=681) to 2244 (rt=60.0653, f=4, i=682)

[D:\lab212\membrane\Grace\Joyce\iTRAQ_39_1.raw]
Data File:Submitted from 20120508-1(merge) by Mascot Daemon on JOYCE-VAIO
Monoisotopic mass of neutral peptide Mr(calc): 1698.979
Variable modifications:
K14 :iTRAQ4plex (K)
Ions Score: 44.9 Expect: 0.018

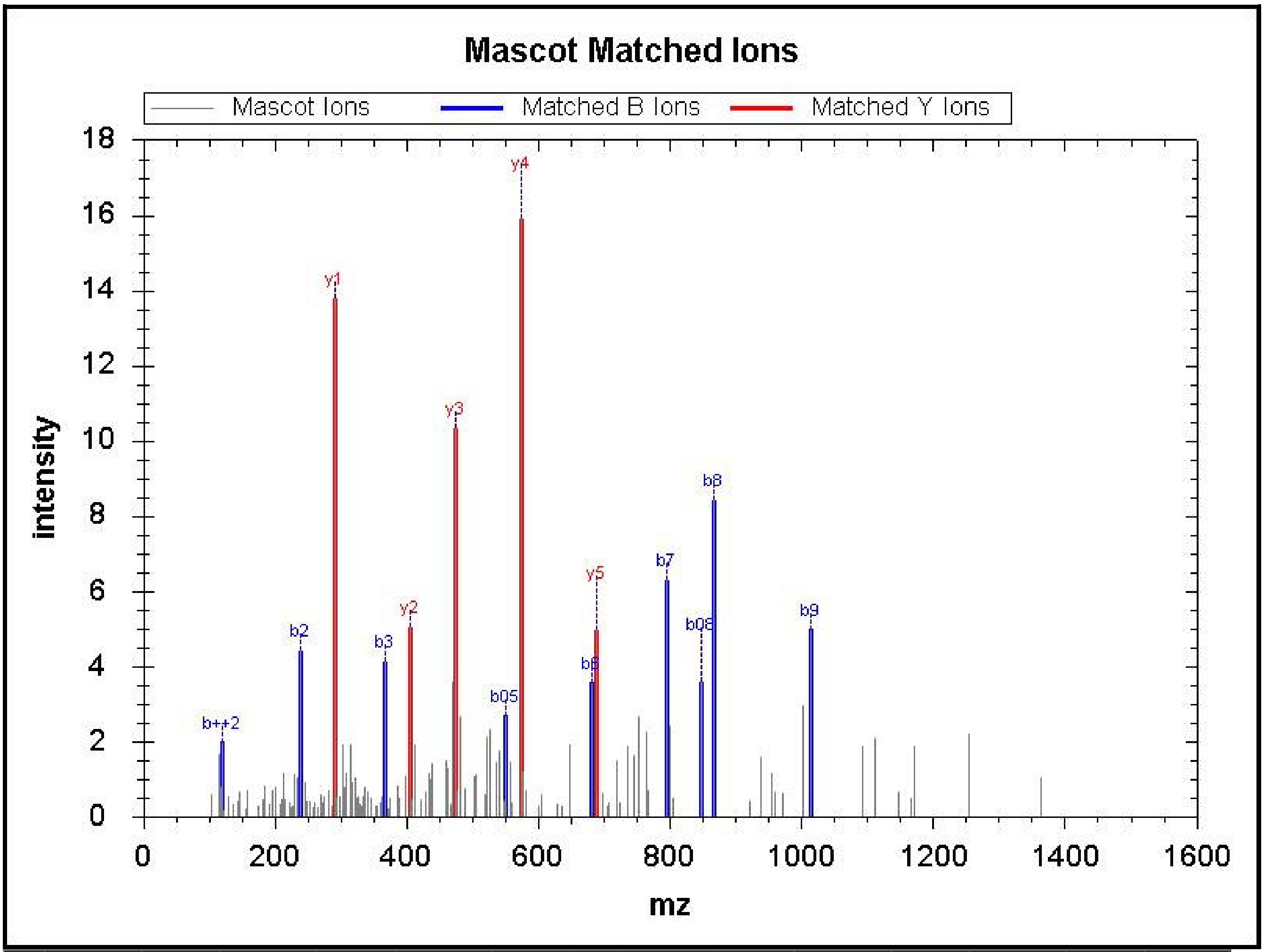

No	b	b++	b*	b*++	b0	b0++	Seq	y	y++	y*	y*++	y0	y0++	RevNo
1	102.05	51.53			84.04	42.53	T							14
2	239.11	120.06			221.10	111.06	H	1,598.94	799.97	1,581.91	791.46	1,580.93	790.97	13
3	367.17	184.09	350.15	175.58	349.16	175.08	Q	1,461.88	731.44	1,444.85	722.93	1,443.87	722.44	12
4	480.26	240.63	463.23	232.12	462.25	231.63	L	1,333.82	667.41	1,316.79	658.90	1,315.81	658.41	11
5	567.29	284.15	550.26	275.63	549.28	275.14	S	1,220.74	610.87	1,203.71	602.36	1,202.73	601.87	10
6	680.37	340.69	663.35	332.18	662.36	331.68	L	1,133.70	567.36	1,116.68	558.84	1,115.69	558.35	9
7	795.40	398.20	778.37	389.69	777.39	389.20	D	1,020.62	510.81	1,003.59	502.30	1,002.61	501.81	8
8	866.44	433.72	849.41	425.21	848.43	424.72	A	905.59	453.30	888.57	444.79			7
9	1,013.51	507.26	996.48	498.74	995.49	498.25	F	834.56	417.78	817.53	409.27			6
10	1,126.59	563.80	1,109.56	555.28	1,108.58	554.79	L	687.49	344.25	670.46	335.73			5
11	1,225.66	613.33	1,208.63	604.82	1,207.65	604.33	V	574.40	287.71	557.38	279.19			4
12	1,296.69	648.85	1,279.67	640.34	1,278.68	639.85	A	475.34	238.17	458.31	229.66			3
13	1,409.78	705.39	1,392.75	696.88	1,391.77	696.39	L	404.30	202.65	387.27	194.14			2
14							K	291.21	146.11	274.19	137.60			1

Query 38631 Hit 1

MS/MS Fragmentation of VDSEPLAILTLHR
Found in sp|Q62230|SN_MOUSE, Sialoadhesin OS=Mus musculus GN=Siglec1 PE=1 SV=2
Match to Query 38631: 1606.91 from(536.6439,3+)

Title: 730: Scan 1653 (rt=46.6169, f=2, i=255) [D:\lab212\membrane\Grace\Joyce\iTRAQ_35_1.raw]
Data File:Submitted from 20120508-1(merge) by Mascot Daemon on JOYCE-VAIO
Monoisotopic mass of neutral peptide Mr(calc): 1606.91
Variable modifications:
Ions Score: 44.86 Expect: 0.024

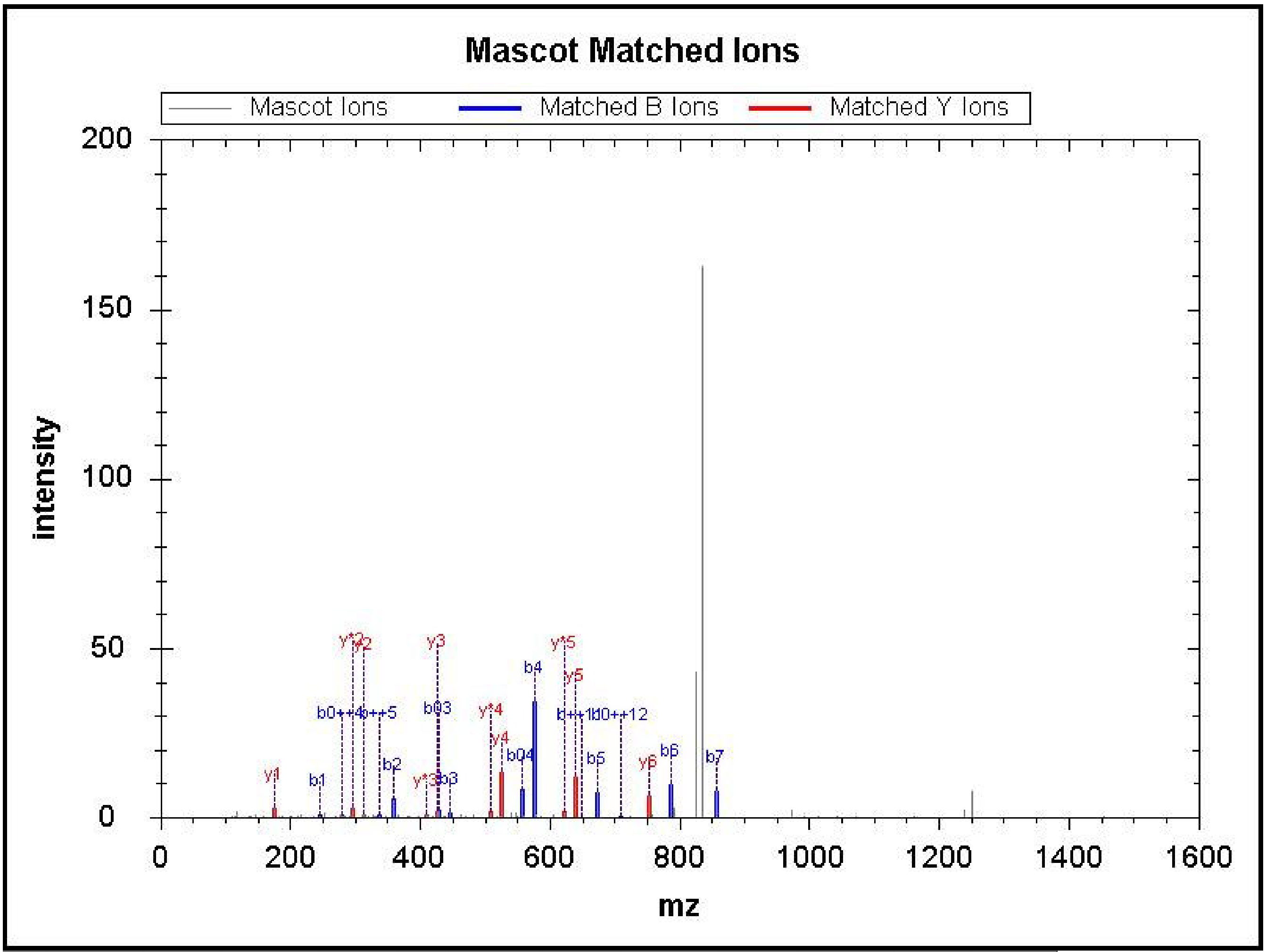

No	b	b++	b0	b0++	Seq	y	y++	y*	y*++	y0	y0++	RevNo
1	244.18	122.59			V							13
2	359.20	180.11	341.19	171.10	D	1,364.75	682.88	1,347.73	674.37	1,346.74	673.87	12
3	446.24	223.62	428.23	214.62	S	1,249.73	625.37	1,232.70	616.85	1,231.72	616.36	11
4	575.28	288.14	557.27	279.14	E	1,162.69	581.85	1,145.67	573.34	1,144.68	572.85	10
5	672.33	336.67	654.32	327.66	P	1,033.65	517.33	1,016.63	508.82	1,015.64	508.32	9
6	785.42	393.21	767.41	384.21	L	936.60	468.80	919.57	460.29	918.59	459.80	8
7	856.45	428.73	838.44	419.72	A	823.51	412.26	806.49	403.75	805.50	403.26	7
8	969.54	485.27	951.53	476.27	I	752.48	376.74	735.45	368.23	734.47	367.74	6
9	1,082.62	541.81	1,064.61	532.81	L	639.39	320.20	622.37	311.69	621.38	311.20	5
10	1,183.67	592.34	1,165.66	583.33	T	526.31	263.66	509.28	255.15	508.30	254.65	4
11	1,296.75	648.88	1,278.74	639.88	L	425.26	213.13	408.24	204.62			3
12	1,433.81	717.41	1,415.80	708.40	H	312.18	156.59	295.15	148.08			2
13					R	175.12	88.06	158.09	79.55			1

Query 16694 Hit 1

MS/MS Fragmentation of AALEGTATYR
Found in sp|Q9NRV9|HEBP1_HUMAN, Heme-binding protein 1 OS=Homo sapiens GN=HEBP1 PE=1 SV=1
Match to Query 16694: 1195.637from(598.8259,2+)
Title: 12: Sum of 2 scans in range 730 (rt=22.6933, f=2, i=20) to 731 (rt=22.7187, f=2, i=21)
[D:\lab212\membrane\Grace\Joyce\iTRAQ_26_1.raw]

Data File:Submitted from 20120508-1(merge) by Mascot Daemon on JOYCE-VAIO
Monoisotopic mass of neutral peptide Mr(calc): 1195.637
Variable modifications:
Ions Score: 44.83 Expect: 0.023

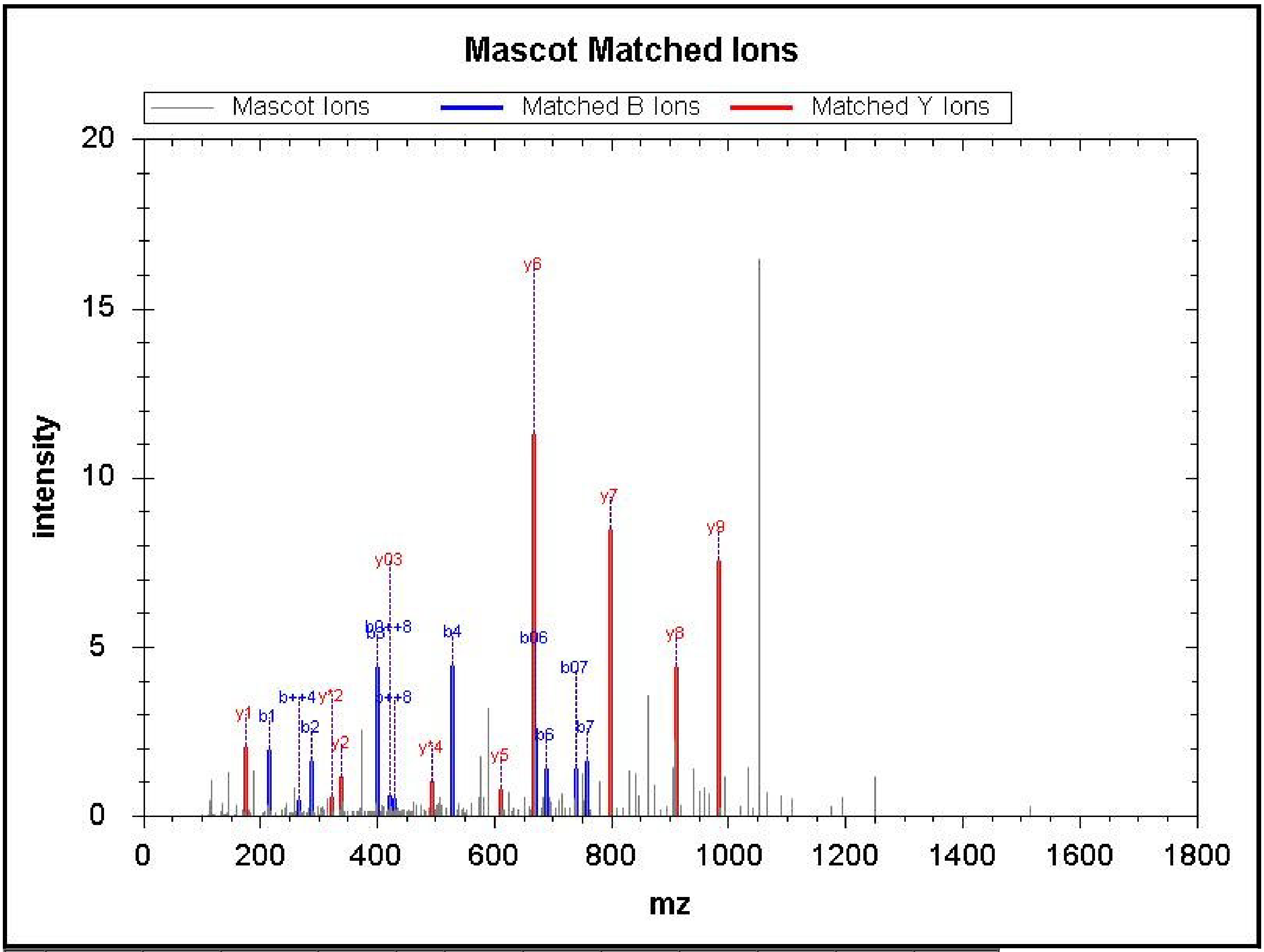

No	b	b++	b0	b0++	Seq	y	y++	y*	y*++	y0	y0++	RevNo
1	216.15	108.58			A							10
2	287.18	144.10			A	981.50	491.25	964.47	482.74	963.49	482.25	9
3	400.27	200.64			L	910.46	455.74	893.44	447.22	892.45	446.73	8
4	529.31	265.16	511.30	256.15	E	797.38	399.19	780.35	390.68	779.37	390.19	7
5	586.33	293.67	568.32	284.66	G	668.34	334.67	651.31	326.16	650.33	325.67	6
6	687.38	344.19	669.37	335.19	T	611.31	306.16	594.29	297.65	593.30	297.16	5
7	758.42	379.71	740.41	370.71	A	510.27	255.64	493.24	247.12	492.26	246.63	4
8	859.46	430.24	841.45	421.23	T	439.23	220.12	422.20	211.61	421.22	211.11	3
9	1,022.53	511.77	1,004.52	502.76	Y	338.18	169.59	321.16	161.08			2
10					R	175.12	88.06	158.09	79.55			1

Query 32200 Hit 1

MS/MS Fragmentation of EVLEGLDYLHR
Found in sp|Q9UEW8|STK39_HUMAN, STE20/SPS1-related proline-alanine-rich protein kinase OS=Homo sapiens GN=STK39 PE=1 SV=3
Match to Query 32200: 1486.791 from(496.6041,3+)
Title: 610: Scan 1400 (rt=40.9519, f=3, i=209) [D:\lab212\membrane\Grace\Joyce\iTRAQ_36_2.raw]
Data File:Submitted from 20120508-1(merge) by Mascot Daemon on JOYCE-VAIO
Monoisotopic mass of neutral peptide Mr(calc): 1486.791
Variable modifications:
Ions Score: 44.82 Expect: 0.027

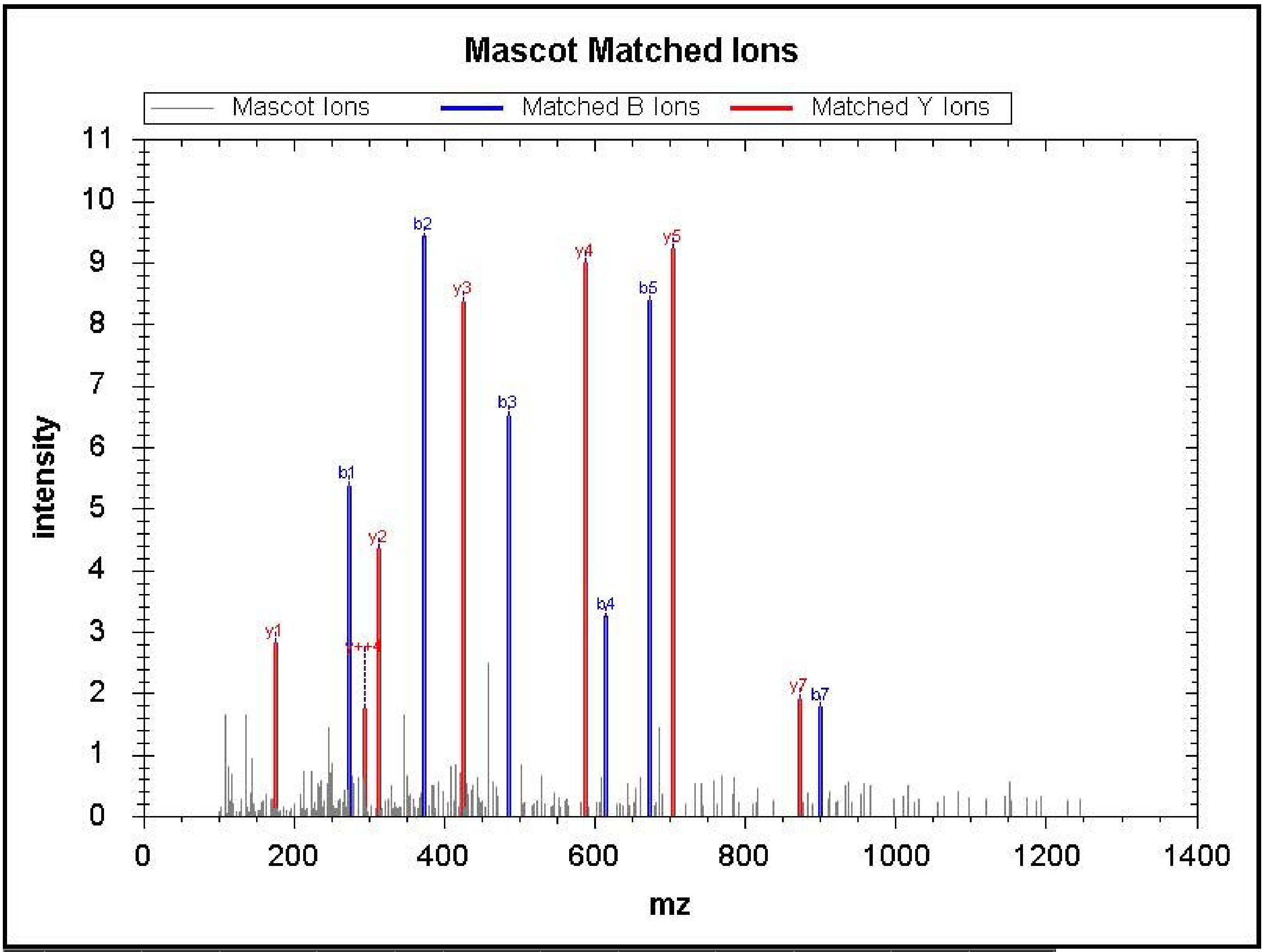

No	b	b++	b0	b0++	Seq	y	y++	y*	y*++	y0	y0++	RevNo
1	274.15	137.58	256.14	128.57	E							11
2	373.22	187.11	355.21	178.11	V	1,214.65	607.83	1,197.63	599.32	1,196.64	598.82	10
3	486.30	243.66	468.29	234.65	L	1,115.58	558.30	1,098.56	549.78	1,097.57	549.29	9
4	615.35	308.18	597.34	299.17	E	1,002.50	501.75	985.47	493.24	984.49	492.75	8
5	672.37	336.69	654.36	327.68	G	873.46	437.23	856.43	428.72	855.45	428.23	7
6	785.45	393.23	767.44	384.22	L	816.44	408.72	799.41	400.21	798.43	399.72	6
7	900.48	450.74	882.47	441.74	D	703.35	352.18	686.33	343.67	685.34	343.17	5
8	1,063.54	532.28	1,045.53	523.27	Y	588.33	294.67	571.30	286.15			4
9	1,176.63	588.82	1,158.62	579.81	L	425.26	213.13	408.24	204.62			3
10	1,313.69	657.35	1,295.68	648.34	H	312.18	156.59	295.15	148.08			2
11					R	175.12	88.06	158.09	79.55			1

Query 70308 Hit 1

MS/MS Fragmentation of LMAKCRMLIQENQELGR
Found in sp|Q15007|FL2D_HUMAN, Pre-mRNA-splicing regulator WTAP OS=Homo sapiens GN=WTAP PE=1 SV=2
Match to Query 70308: 2240.088from(747.7034,3+)
Title: 716: Scan 1592 (rt=45.3874, f=3, i=245) [D:\lab212\membrane\Grace\Joyce\iTRAQ_34_2.raw]
Data File:Submitted from 20120508-1(merge) by Mascot Daemon on JOYCE-VAIO
Monoisotopic mass of neutral peptide Mr(calc): 2240.088
Variable modifications:
M2 :Oxidation (M), 63.99828
K4 :iTRAQ4plex (K)
C5 :Methylthio (C)
N12 :Deamidated (NQ)
Q13 :Deamidated (NQ)
Ions Score: 44.71 Expect: 0.030

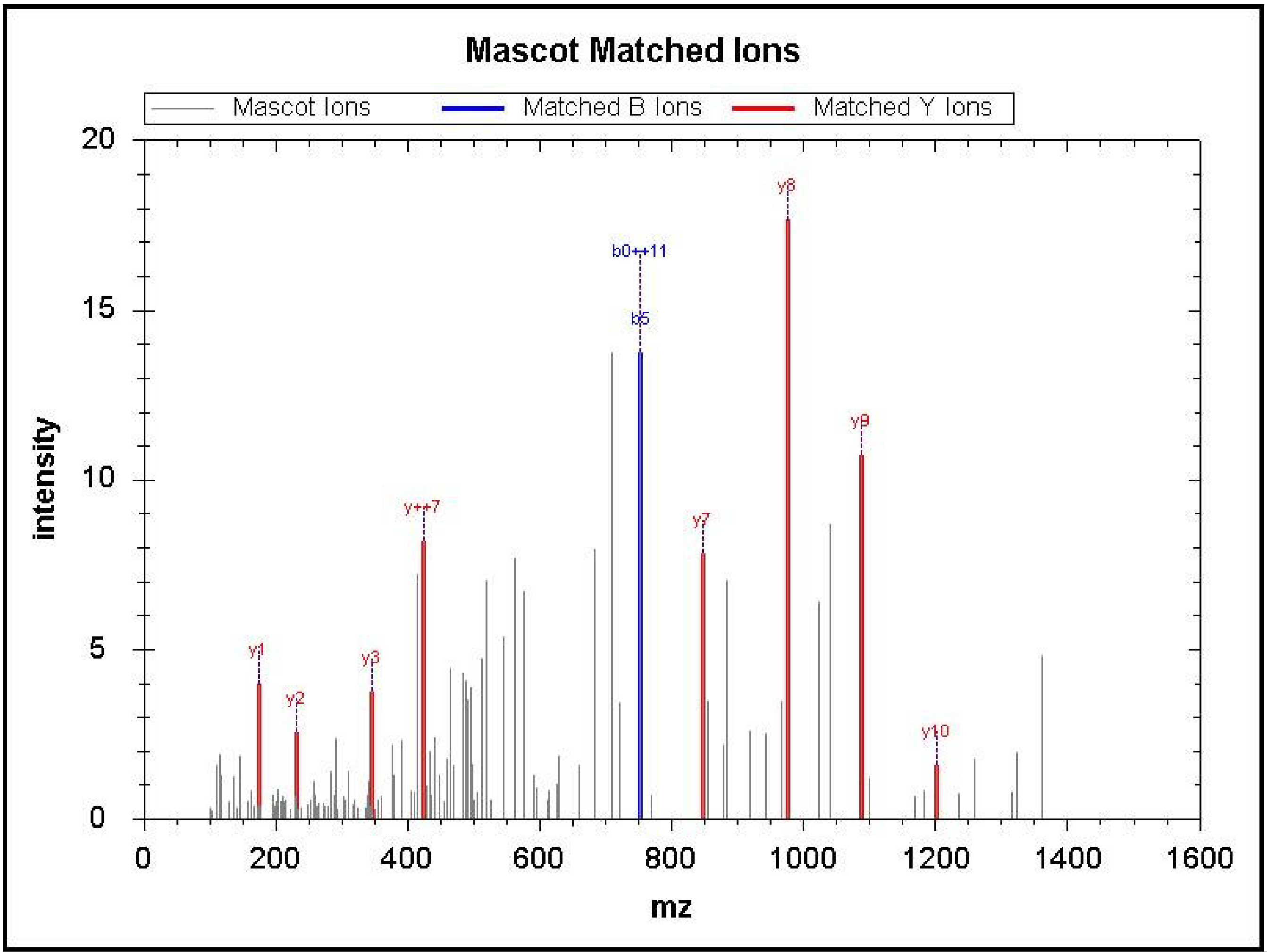

No	b	b++	b*	b*++	b0	b0++	Seq	y	y++	y*	y*++	y0	y0++	RevNo
1	114.09	57.55					L							17
2	261.13	131.07					M	2,128.01	1,064.51	2,110.99	1,056.00	2,110.00	1,055.51	16
3	332.16	166.59					A	1,980.98	990.99	1,963.95	982.48	1,962.97	981.99	15
4	604.36	302.68	587.33	294.17			K	1,909.94	955.47	1,892.92	946.96	1,891.93	946.47	14
5	753.36	377.18	736.33	368.67			C	1,637.74	819.38	1,620.72	810.86	1,619.73	810.37	13
6	909.46	455.23	892.43	446.72			R	1,488.75	744.88	1,471.72	736.36	1,470.74	735.87	12
7	1,040.50	520.75	1,023.47	512.24			M	1,332.65	666.83	1,315.62	658.31	1,314.64	657.82	11
8	1,153.58	577.30	1,136.56	568.78			L	1,201.61	601.31	1,184.58	592.79	1,183.60	592.30	10
9	1,266.67	633.84	1,249.64	625.32			I	1,088.52	544.76	1,071.50	536.25	1,070.51	535.76	9
10	1,394.73	697.87	1,377.70	689.35			Q	975.44	488.22	958.41	479.71	957.43	479.22	8
11	1,523.77	762.39	1,506.74	753.87	1,505.76	753.38	E	847.38	424.19	830.35	415.68	829.37	415.19	7
12	1,638.80	819.90	1,621.77	811.39	1,620.79	810.90	N	718.34	359.67	701.31	351.16	700.33	350.67	6
13	1,767.84	884.42	1,750.81	875.91	1,749.83	875.42	Q	603.31	302.16	586.28	293.65	585.30	293.15	5
14	1,896.88	948.94	1,879.85	940.43	1,878.87	939.94	E	474.27	237.64	457.24	229.12	456.26	228.63	4
15	2,009.97	1,005.49	1,992.94	996.97	1,991.95	996.48	L	345.22	173.12	328.20	164.60			3
16	2,066.99	1,034.00	2,049.96	1,025.48	2,048.98	1,024.99	G	232.14	116.57	215.11	108.06			2
17							R	175.12	88.06	158.09	79.55			1

Query 75437 Hit 1

MS/MS Fragmentation of FIIDEELFGQTHQHELK
Found in sp|P46934|NEDD4_HUMAN, E3 ubiquitin-protein ligase NEDD4 OS=Homo sapiens GN=NEDD4 PE=1 SV=4
Match to Query 75437: 2371.246from(593.8188,4+)
Title: 746: Scan 1729 (rt=48.4058, f=2, i=267) [D:\lab212\membrane\Grace\Joyce\47_1.raw]
Data File:Submitted from 20120508-1(merge) by Mascot Daemon on JOYCE-VAIO
Monoisotopic mass of neutral peptide Mr(calc): 2371.246

Variable modifications:
K17 :iTRAQ4plex (K)
Ions Score: 44.68 Expect: 0.030

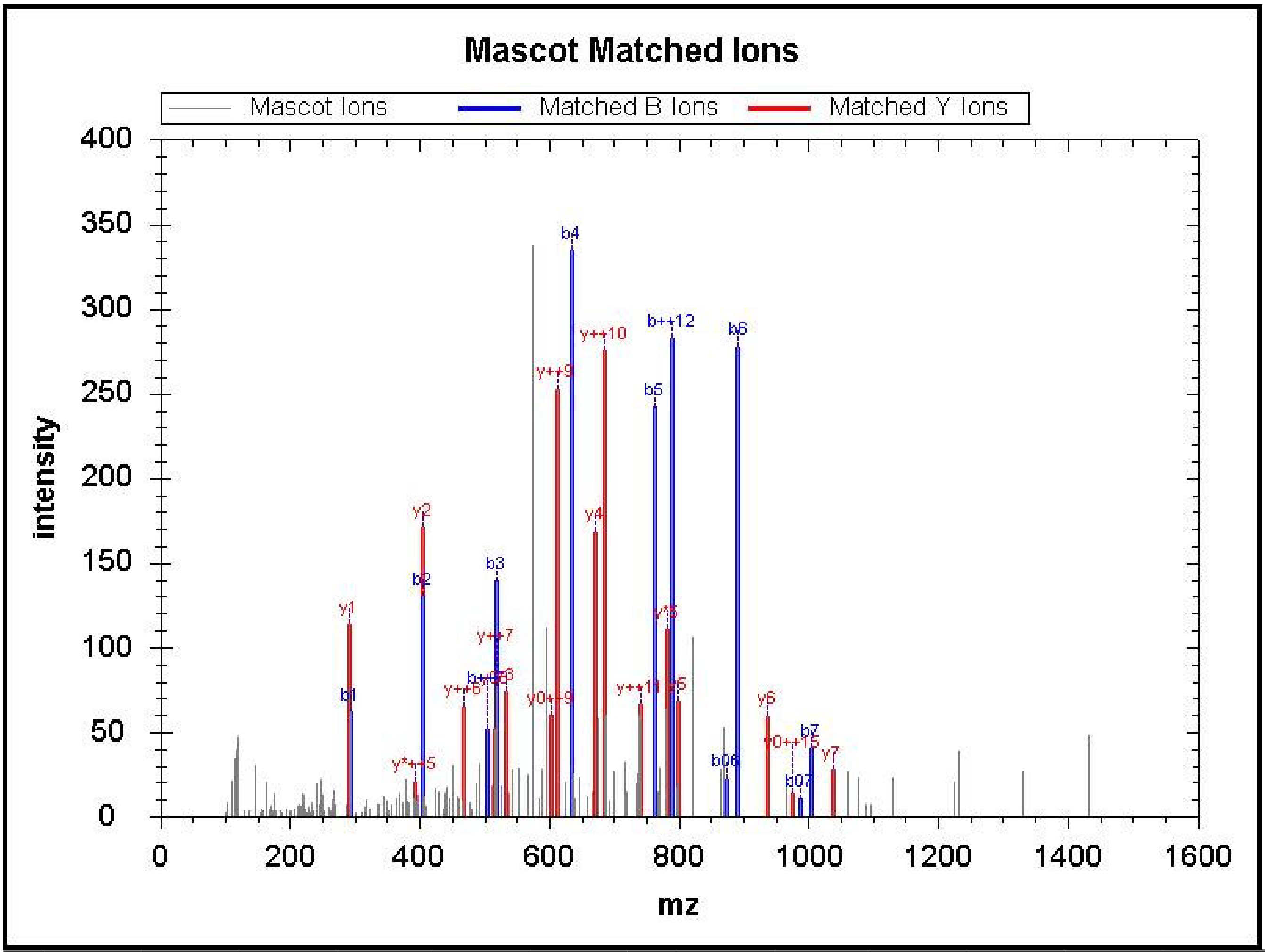

No	b	b++	b*	b*++	b0	b0++	Seq	y	y++	y*	y*++	y0	y0++	RevNo
1	292.18	146.59					F							17
2	405.26	203.13					I	2,081.08	1,041.04	2,064.05	1,032.53	2,063.07	1,032.04	16
3	518.35	259.68					I	1,967.99	984.50	1,950.97	975.99	1,949.98	975.50	15
4	633.37	317.19			615.36	308.18	D	1,854.91	927.96	1,837.88	919.45	1,836.90	918.95	14
5	762.42	381.71			744.40	372.71	E	1,739.88	870.45	1,722.86	861.93	1,721.87	861.44	13
6	891.46	446.23			873.45	437.23	E	1,610.84	805.92	1,593.81	797.41	1,592.83	796.92	12
7	1,004.54	502.77			986.53	493.77	L	1,481.80	741.40	1,464.77	732.89	1,463.79	732.40	11
8	1,151.61	576.31			1,133.60	567.30	F	1,368.71	684.86	1,351.69	676.35	1,350.70	675.86	10
9	1,208.63	604.82			1,190.62	595.81	G	1,221.65	611.33	1,204.62	602.81	1,203.64	602.32	9
10	1,336.69	668.85	1,319.66	660.34	1,318.68	659.84	Q	1,164.62	582.82	1,147.60	574.30	1,146.61	573.81	8
11	1,437.74	719.37	1,420.71	710.86	1,419.73	710.37	T	1,036.57	518.79	1,019.54	510.27	1,018.55	509.78	7
12	1,574.80	787.90	1,557.77	779.39	1,556.79	778.90	H	935.52	468.26	918.49	459.75	917.51	459.26	6
13	1,702.86	851.93	1,685.83	843.42	1,684.85	842.93	Q	798.46	399.73	781.43	391.22	780.45	390.73	5
14	1,839.91	920.46	1,822.89	911.95	1,821.90	911.46	H	670.40	335.70	653.37	327.19	652.39	326.70	4
15	1,968.96	984.98	1,951.93	976.47	1,950.95	975.98	E	533.34	267.17	516.31	258.66	515.33	258.17	3
16	2,082.04	1,041.52	2,065.01	1,033.01	2,064.03	1,032.52	L	404.30	202.65	387.27	194.14			2
17							K	291.21	146.11	274.19	137.60			1

Query 67801 Hit 1

MS/MS Fragmentation of EMSPLWEMVQDGIDLK
Found in sp|Q9Y697|NFS1_HUMAN, Cysteine desulfurase

Match to Query 67801: 2178.093from(727.0382,3+)
Title: 1157: Sum of 2 scans in range 2574 (rt=67.3198, f=4, i=781) to 2575 (rt=67.3452, f=4, i=782) [D:\lab212\membrane\Grace\Joyce\iTRAQ_27_1.raw]
Data File:Submitted from 20120508-1(merge) by Mascot Daemon on JOYCE-VAIO
Monoisotopic mass of neutral peptide Mr(calc): 2178.093
Variable modifications:
K16 :iTRAQ4plex (K)
Ions Score: 44.62 Expect: 0.032

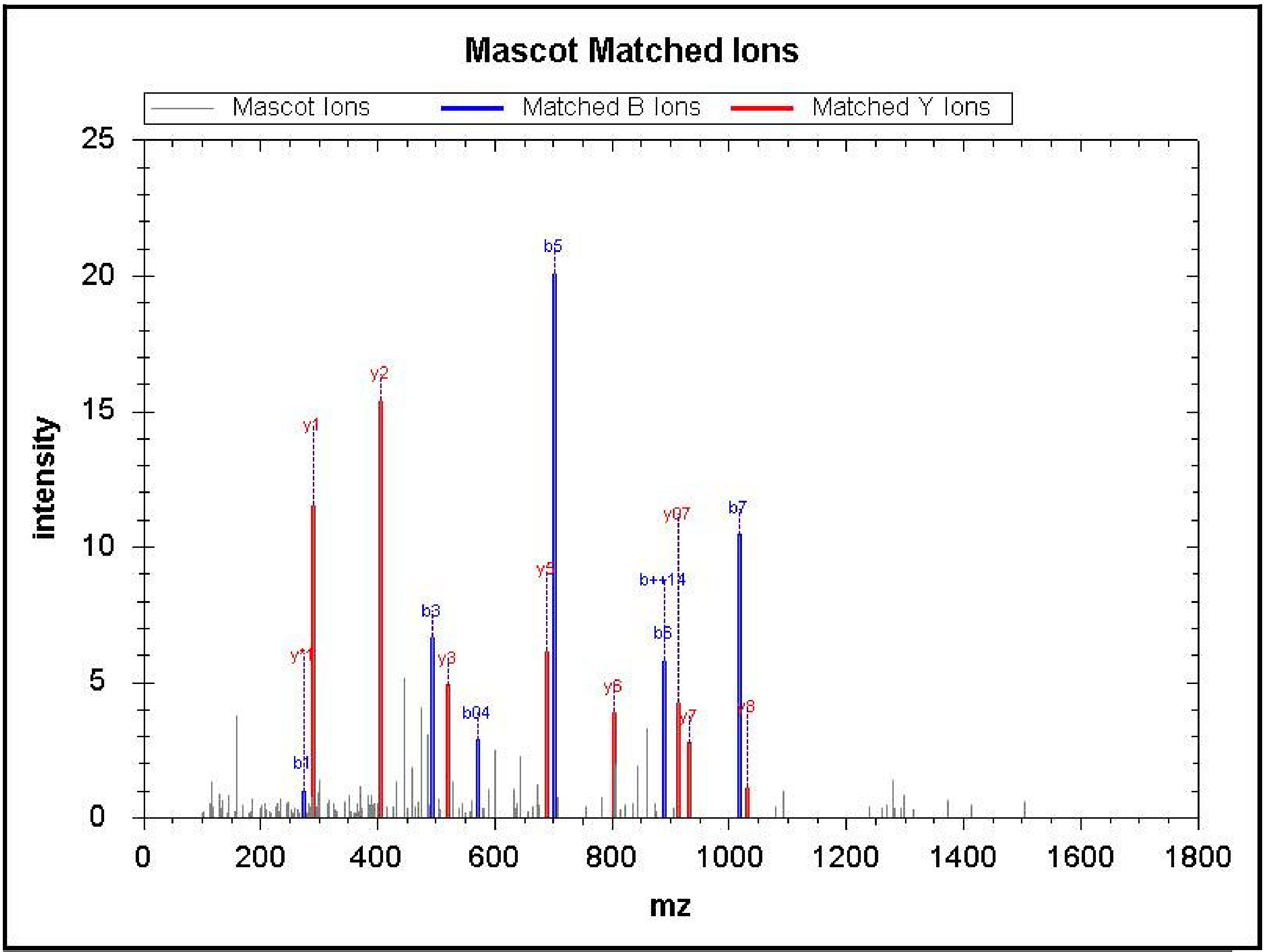

No	b	b++	b*	b*++	b0	b0++	Seq	y	y++	y*	y*++	y0	y0++	RevNo
1	274.15	137.58			256.14	128.57	E							16
2	405.19	203.10			387.18	194.09	M	1,905.96	953.48	1,888.93	944.97	1,887.95	944.48	15
3	492.22	246.62			474.21	237.61	S	1,774.92	887.96	1,757.89	879.45	1,756.91	878.96	14
4	589.28	295.14			571.27	286.14	P	1,687.88	844.45	1,670.86	835.93	1,669.87	835.44	13
5	702.36	351.68			684.35	342.68	L	1,590.83	795.92	1,573.81	787.41	1,572.82	786.91	12
6	888.44	444.72			870.43	435.72	W	1,477.75	739.38	1,460.72	730.86	1,459.74	730.37	11
7	1,017.48	509.25			999.47	500.24	E	1,291.67	646.34	1,274.64	637.82	1,273.66	637.33	10
8	1,148.52	574.77			1,130.51	565.76	M	1,162.63	581.82	1,145.60	573.30	1,144.62	572.81	9
9	1,247.59	624.30			1,229.58	615.29	V	1,031.59	516.30	1,014.56	507.78	1,013.57	507.29	8
10	1,375.65	688.33	1,358.62	679.82	1,357.64	679.32	Q	932.52	466.76	915.49	458.25	914.51	457.76	7
11	1,490.68	745.84	1,473.65	737.33	1,472.67	736.84	D	804.46	402.73	787.43	394.22	786.45	393.73	6
12	1,547.70	774.35	1,530.67	765.84	1,529.69	765.35	G	689.43	345.22	672.40	336.71	671.42	336.21	5
13	1,660.78	830.90	1,643.76	822.38	1,642.77	821.89	I	632.41	316.71	615.38	308.20	614.40	307.70	4
14	1,775.81	888.41	1,758.78	879.90	1,757.80	879.40	D	519.33	260.17	502.30	251.65	501.32	251.16	3
15	1,888.89	944.95	1,871.87	936.44	1,870.88	935.95	L	404.30	202.65	387.27	194.14			2
16							K	291.21	146.11	274.19	137.60			1

Query 19521 Hit 1

MS/MS Fragmentation of LLDFLNVK
Found in sp|Q8NDZ4|CC058_HUMAN, UPF0672 protein C3orf58 OS=Homo sapiens GN=C3orf58 PE=1 SV=1
Match to Query 19521: 1248.77from(625.3923,2+)
Title: 915: Scan 2090 (rt=56.2476, f=3, i=310) [D:\lab212\membrane\Grace\Joyce\iTRAQ_30_1.raw]
Data File:Submitted from 20120508-1(merge) by Mascot Daemon on JOYCE-VAIO
Monoisotopic mass of neutral peptide Mr(calc): 1248.77
Variable modifications:
K8 :iTRAQ4plex (K)
Ions Score: 44.61 Expect: 0.018

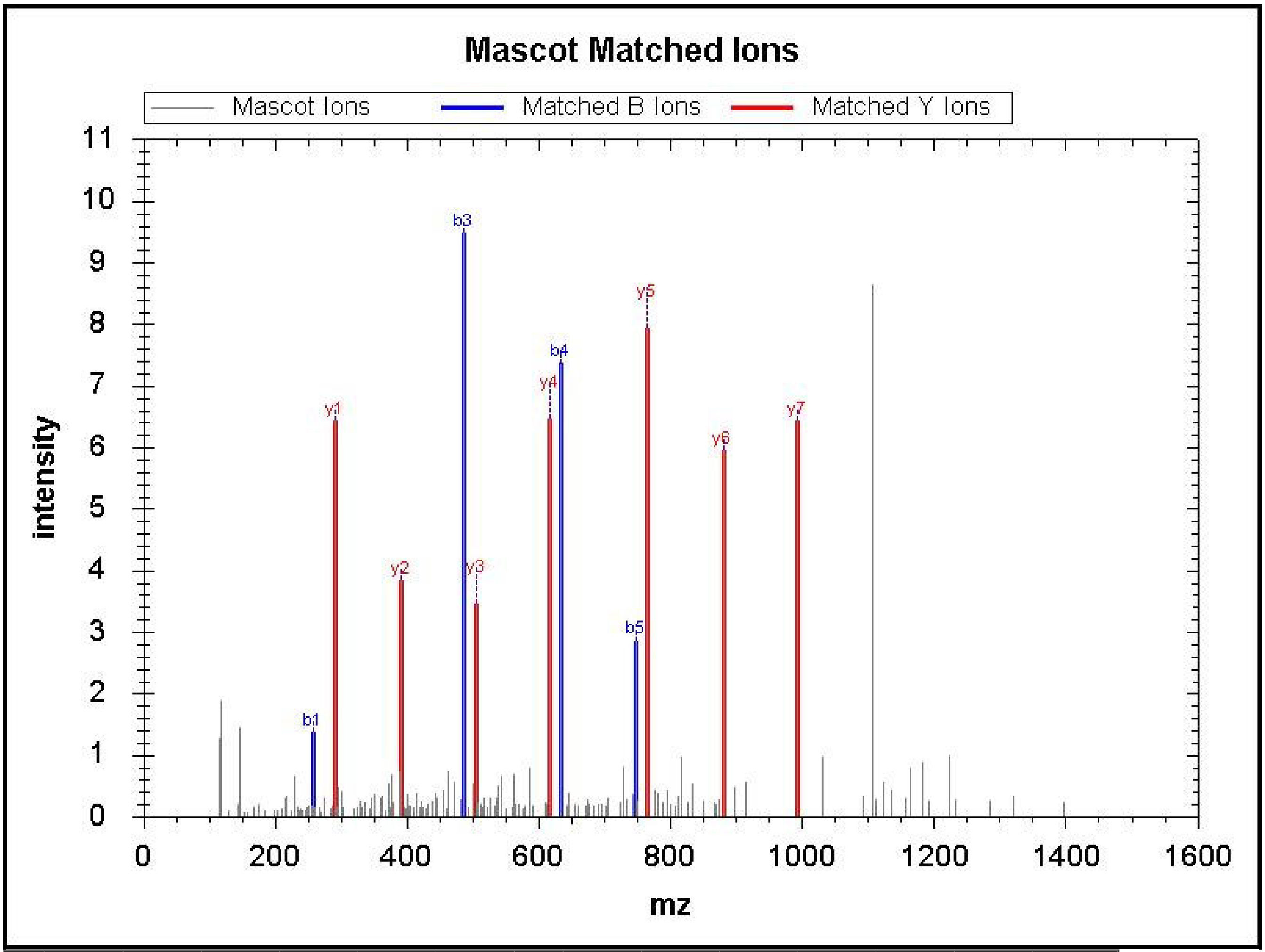

No	b	b++	b*	b*++	b0	b0++	Seq	y	y++	y*	y*++	y0	y0++	RevNo
1	258.19	129.60					L							8
2	371.28	186.14					L	992.59	496.80	975.56	488.29	974.58	487.79	7
3	486.30	243.66			468.29	234.65	D	879.51	440.26	862.48	431.74	861.50	431.25	6
4	633.37	317.19			615.36	308.18	F	764.48	382.74	747.45	374.23			5
5	746.46	373.73			728.45	364.73	L	617.41	309.21	600.38	300.70			4
6	860.50	430.75	843.47	422.24	842.49	421.75	N	504.33	252.67	487.30	244.15			3
7	959.57	480.29	942.54	471.77	941.56	471.28	V	390.28	195.65	373.26	187.13			2
8							K	291.21	146.11	274.19	137.60			1

Query 65934 Hit 1

MS/MS Fragmentation of EELMAPTLLPELHLLK
Found in sp|Q9H1A4|APC1_HUMAN, Anaphase-promoting complex subunit 1 OS=Homo sapiens GN=ANAPC1 PE=1 SV=1
Match to Query 65934: 2134.233from(712.4184,3+)
Title: 915: Sum of 2 scans in range 2206 (rt=58.1632, f=4, i=609) to 2207 (rt=58.1886, f=4, i=610)
[D:\lab212\membrane\Grace\Joyce\iTRAQ_34_1.raw]
Data File:Submitted from 20120508-1(merge) by Mascot Daemon on JOYCE-VAIO

Monoisotopic mass of neutral peptide Mr(calc): 2134.233
Variable modifications:
K16 :iTRAQ4plex (K)
Ions Score: 44.57 Expect: 0.014

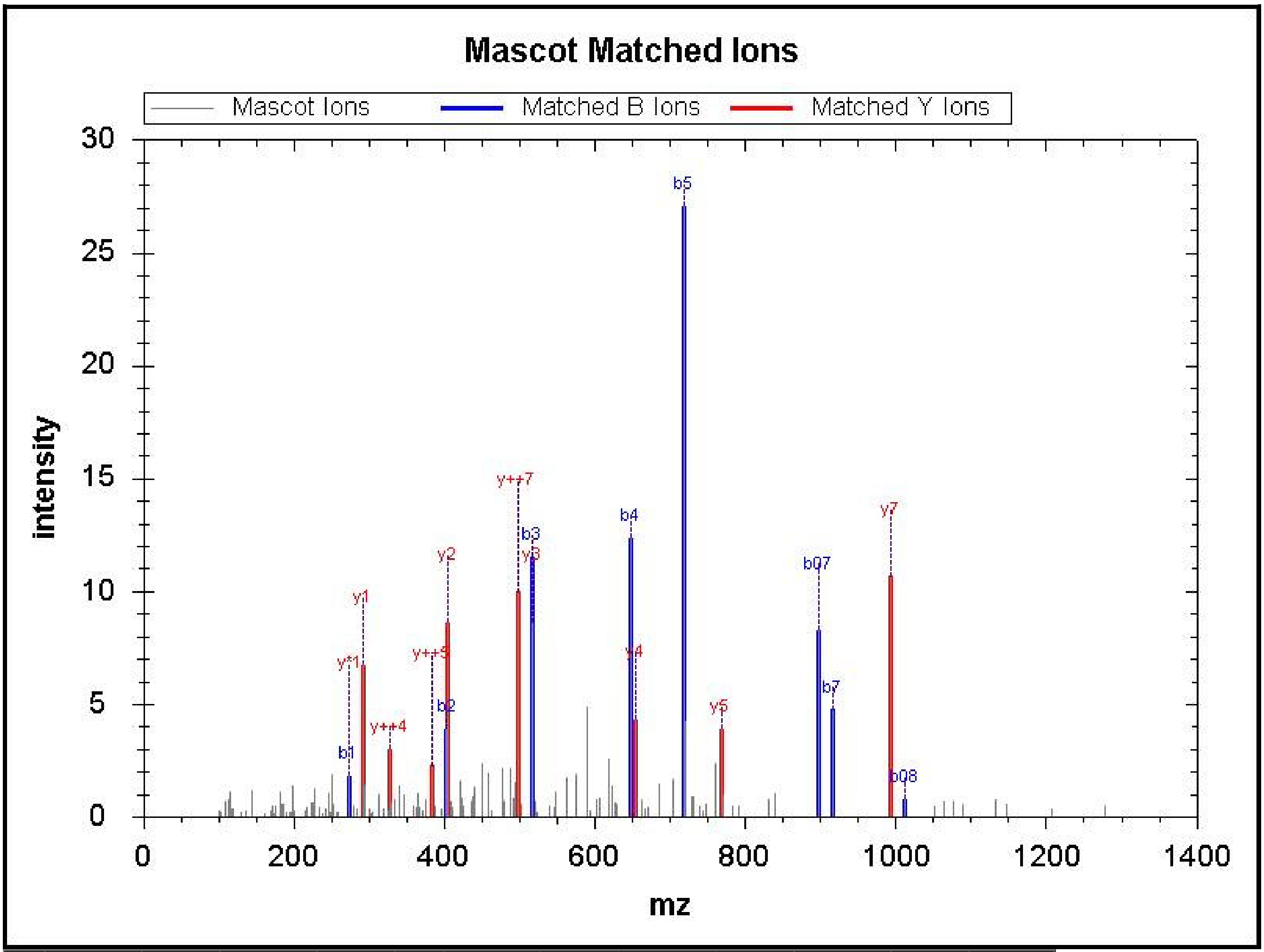

No	b	b++	b0	b0++	Seq	y	y++	y*	y*++	y0	y0++	RevNo
1	274.15	137.58	256.14	128.57	E							16
2	403.19	202.10	385.18	193.10	E	1,862.09	931.55	1,845.07	923.04	1,844.08	922.55	15
3	516.28	258.64	498.27	249.64	L	1,733.05	867.03	1,716.03	858.52	1,715.04	858.02	14
4	647.32	324.16	629.31	315.16	M	1,619.97	810.49	1,602.94	801.97	1,601.96	801.48	13
5	718.36	359.68	700.35	350.68	A	1,488.93	744.97	1,471.90	736.45	1,470.92	735.96	12
6	815.41	408.21	797.40	399.20	P	1,417.89	709.45	1,400.86	700.94	1,399.88	700.44	11
7	916.46	458.73	898.45	449.73	T	1,320.84	660.92	1,303.81	652.41	1,302.83	651.92	10
8	1,029.54	515.27	1,011.53	506.27	L	1,219.79	610.40	1,202.76	601.89	1,201.78	601.39	9
9	1,142.62	571.82	1,124.61	562.81	L	1,106.71	553.86	1,089.68	545.34	1,088.69	544.85	8
10	1,239.68	620.34	1,221.67	611.34	P	993.62	497.31	976.59	488.80	975.61	488.31	7
11	1,368.72	684.86	1,350.71	675.86	E	896.57	448.79	879.54	440.27	878.56	439.78	6
12	1,481.80	741.41	1,463.79	732.40	L	767.53	384.27	750.50	375.75			5
13	1,618.86	809.94	1,600.85	800.93	H	654.44	327.72	637.42	319.21			4
14	1,731.95	866.48	1,713.94	857.47	L	517.38	259.20	500.36	250.68			3
15	1,845.03	923.02	1,827.02	914.01	L	404.30	202.65	387.27	194.14			2
16					K	291.21	146.11	274.19	137.60			1

Query 16624 Hit 1

MS/MS Fragmentation of GSFLKEKNK
Found in sp|Q96QE3|ATAD5_HUMAN, ATPase family AAA domain-containing protein 5 OS=Homo sapiens GN=ATAD5 PE=1 SV=4

Match to Query 16624: 1193.662from(597.8385,2+)
Title: 197: Scan 543 (rt=21.5808, f=2, i=77) [D:\lab212\membrane\Grace\Joyce\iTRAQ_31_2.raw]
Data File:Submitted from 20120508-1(merge) by Mascot Daemon on JOYCE-VAIO
Monoisotopic mass of neutral peptide Mr(calc): 1193.662
Variable modifications:
K9 :iTRAQ4plex (K)
Ions Score: 44.47 Expect: 0.028

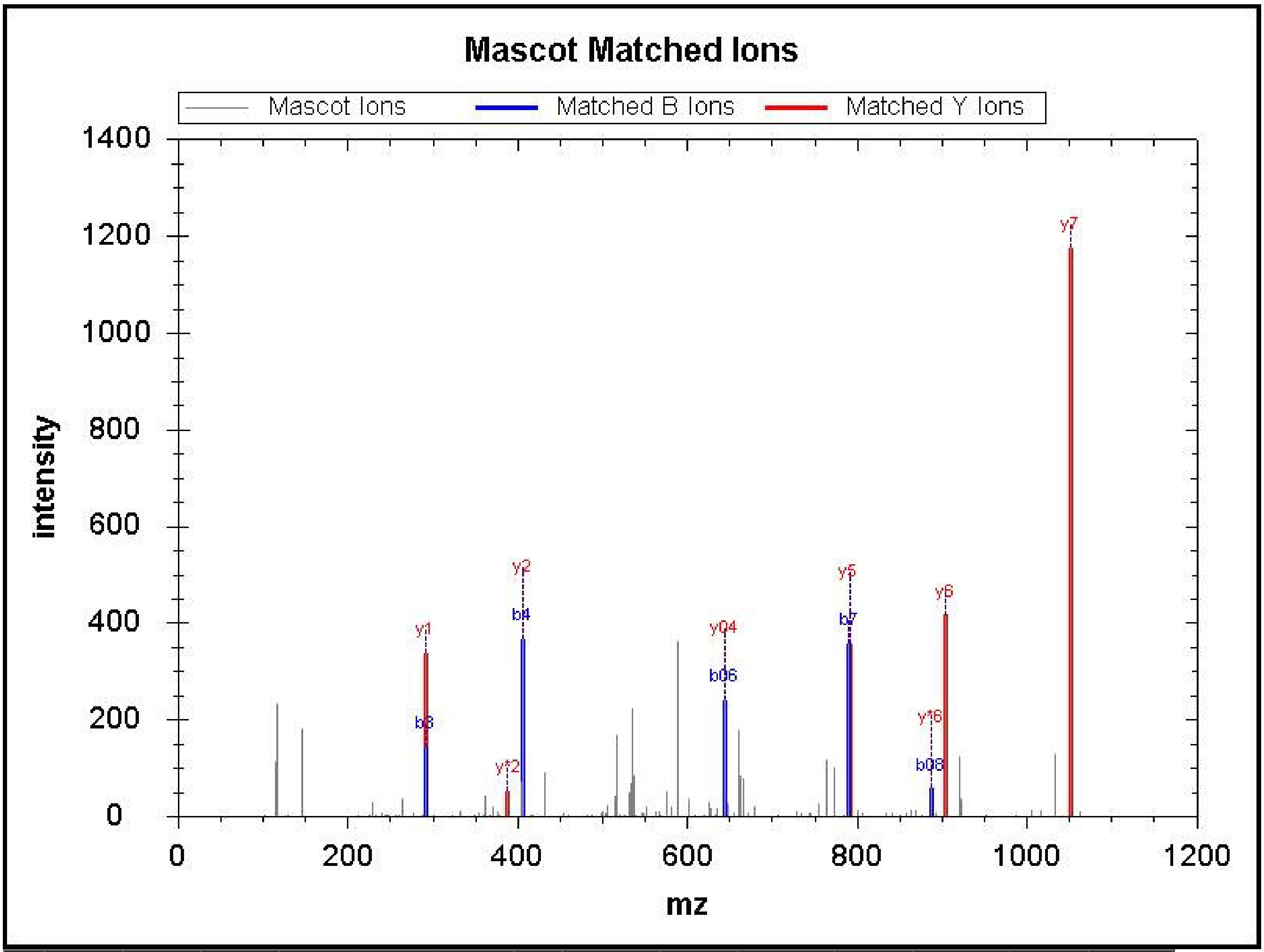

No	b	b++	b*	b*++	b0	b0++	Seq	y	y++	y*	y*++	y0	y0++	RevNo
1	58.03	29.52					G							9
2	145.06	73.03			127.05	64.03	S	1,137.67	569.34	1,120.65	560.83	1,119.66	560.34	8
3	292.13	146.57			274.12	137.56	F	1,050.64	525.83	1,033.62	517.31	1,032.63	516.82	7
4	405.21	203.11			387.20	194.10	L	903.57	452.29	886.55	443.78	885.56	443.29	6
5	533.31	267.16	516.28	258.64	515.30	258.15	K	790.49	395.75	773.46	387.24	772.48	386.74	5
6	662.35	331.68	645.32	323.17	644.34	322.67	E	662.40	331.70	645.37	323.19	644.38	322.70	4
7	790.45	395.73	773.42	387.21	772.44	386.72	K	533.35	267.18	516.33	258.67			3
8	904.49	452.75	887.46	444.23	886.48	443.74	N	405.26	203.13	388.23	194.62			2
9							K	291.21	146.11	274.19	137.60			1

Query 32304 Hit 1

MS/MS Fragmentation of LGFDVVFNYK
Found in sp|Q14914|PTGR1_HUMAN, Prostaglandin reductase 1 OS=Homo sapiens GN=PTGR1 PE=1 SV=2
Match to Query 32304: 1488.82from(745.4172,2+)
Title: 839: Sum of 2 scans in range 1930 (rt=52.6445, f=4, i=563) to 1931 (rt=52.6699, f=4, i=564)
[D:\lab212\membrane\Grace\Joyce\iTRAQ_30_1.raw]
Data File:Submitted from 20120508-1(merge) by Mascot Daemon on JOYCE-VAIO
Monoisotopic mass of neutral peptide Mr(calc): 1488.82
Variable modifications:
K10 :iTRAQ4plex (K)

Ions Score: 44.33 Expect: 0.031

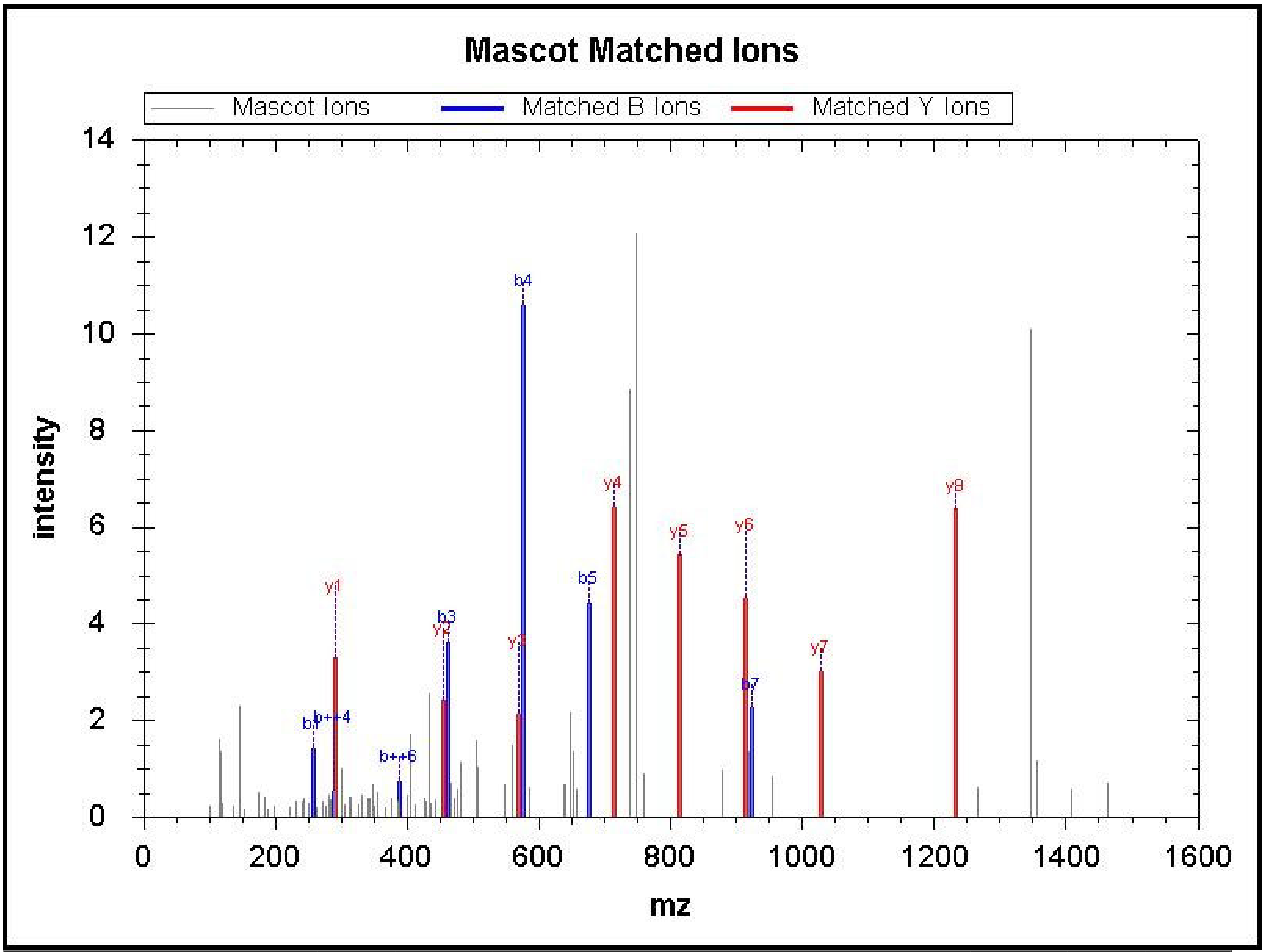

No	b	b++	b*	b*++	b0	b0++	Seq	y	y++	y*	y*++	y0	y0++	RevNo
1	258.19	129.60					L							10
2	315.21	158.11					G	1,232.64	616.83	1,215.62	608.31	1,214.63	607.82	9
3	462.28	231.65					F	1,175.62	588.31	1,158.60	579.80	1,157.61	579.31	8
4	577.31	289.16			559.30	280.15	D	1,028.55	514.78	1,011.53	506.27	1,010.54	505.78	7
5	676.38	338.69			658.37	329.69	V	913.53	457.27	896.50	448.75			6
6	775.45	388.23			757.44	379.22	V	814.46	407.73	797.43	399.22			5
7	922.52	461.76			904.50	452.76	F	715.39	358.20	698.36	349.69			4
8	1,036.56	518.78	1,019.53	510.27	1,018.55	509.78	N	568.32	284.66	551.29	276.15			3
9	1,199.62	600.31	1,182.60	591.80	1,181.61	591.31	Y	454.28	227.64	437.25	219.13			2
10							K	291.21	146.11	274.19	137.60			1

Query 74270 Hit 1

MS/MS Fragmentation of ILYSHLVDYFPEFDGPQR
Found in sp|P29992|GNA11_HUMAN, Guanine nucleotide-binding protein subunit alpha-11 OS=Homo sapiens GN=GNA11 PE=1 SV=2
Match to Query 74270: 2339.17from(780.7308,3+)
Title: 937: Scan 2087 (rt=56.3967, f=3, i=317) [D:\lab212\membrane\Grace\Joyce\iTRAQ_33_1.raw]
Data File:Submitted from 20120508-1(merge) by Mascot Daemon on JOYCE-VAIO
Monoisotopic mass of neutral peptide Mr(calc): 2339.17
Variable modifications:
Ions Score: 44.32 Expect: 0.035

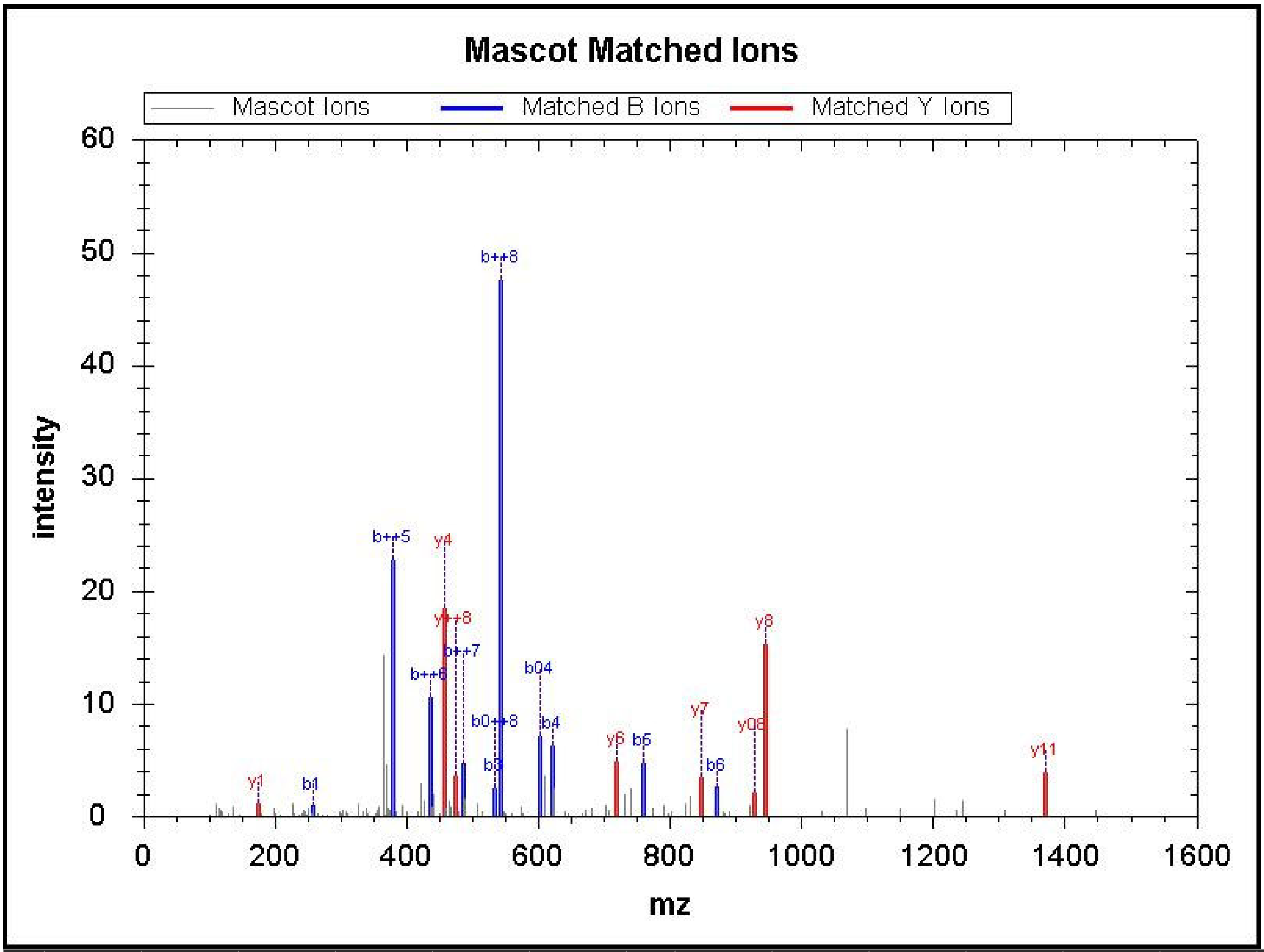

No	b	b++	b*	b*++	b0	b0++	Seq	y	y++	y*	y*++	y0	y0++	RevNo
1	258.19	129.60					I							18
2	371.28	186.14					L	2,082.99	1,042.00	2,065.97	1,033.49	2,064.98	1,032.99	17
3	534.34	267.67					Y	1,969.91	985.46	1,952.88	976.94	1,951.90	976.45	16
4	621.37	311.19			603.36	302.18	S	1,806.84	903.93	1,789.82	895.41	1,788.83	894.92	15
5	758.43	379.72			740.42	370.71	H	1,719.81	860.41	1,702.79	851.90	1,701.80	851.40	14
6	871.52	436.26			853.51	427.26	L	1,582.75	791.88	1,565.73	783.37	1,564.74	782.88	13
7	970.58	485.80			952.57	476.79	V	1,469.67	735.34	1,452.64	726.83	1,451.66	726.33	12
8	1,085.61	543.31			1,067.60	534.30	D	1,370.60	685.80	1,353.57	677.29	1,352.59	676.80	11
9	1,248.67	624.84			1,230.66	615.84	Y	1,255.57	628.29	1,238.55	619.78	1,237.56	619.29	10
10	1,395.74	698.38			1,377.73	689.37	F	1,092.51	546.76	1,075.48	538.25	1,074.50	537.75	9
11	1,492.80	746.90			1,474.79	737.90	P	945.44	473.22	928.42	464.71	927.43	464.22	8
12	1,621.84	811.42			1,603.83	802.42	E	848.39	424.70	831.36	416.19	830.38	415.69	7
13	1,768.91	884.96			1,750.90	875.95	F	719.35	360.18	702.32	351.66	701.34	351.17	6
14	1,883.93	942.47			1,865.92	933.47	D	572.28	286.64	555.25	278.13	554.27	277.64	5
15	1,940.96	970.98			1,922.94	961.98	G	457.25	229.13	440.23	220.62			4
16	2,038.01	1,019.51			2,020.00	1,010.50	P	400.23	200.62	383.20	192.11			3
17	2,166.07	1,083.54	2,149.04	1,075.02	2,148.06	1,074.53	Q	303.18	152.09	286.15	143.58			2
18							R	175.12	88.06	158.09	79.55			1

Query 63891 Hit 1

MS/MS Fragmentation of LPLQMALTELETLAEK
Found in sp|O15013|ARHGA_HUMAN, Rho guanine nucleotide exchange factor 10 OS=Homo sapiens GN=ARHGEF10 PE=1 SV=4
Match to Query 63891: 2087.181from(696.7342,3+)
Title: 1418: Sum of 2 scans in range 3154 (rt=80.1871, f=2, i=527) to 3155 (rt=80.2125, f=2, i=528)

[D:\lab212\membrane\Grace\Joyce\iTRAQ_26_2.raw]
Data File:Submitted from 20120508-1(merge) by Mascot Daemon on JOYCE-VAIO
Monoisotopic mass of neutral peptide Mr(calc): 2087.181
Variable modifications:
K16 :iTRAQ4plex (K)
Ions Score: 44.31 Expect: 0.020

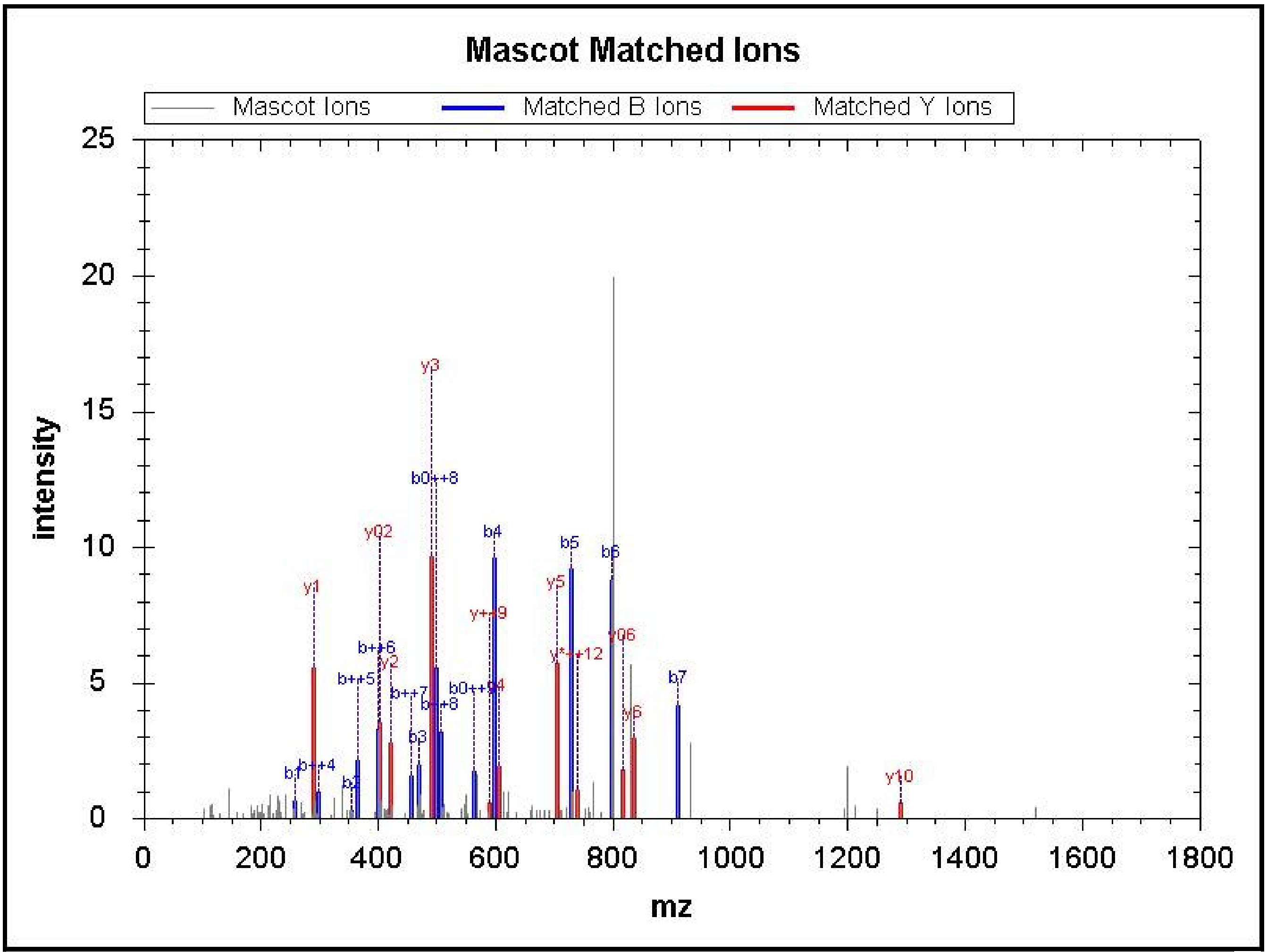

No	b	b++	b*	b*++	b0	b0++	Seq	y	y++	y*	y*++	y0	y0++	RevNo
1	258.19	129.60					L							16
2	355.25	178.13					P	1,831.00	916.00	1,813.97	907.49	1,812.99	907.00	15
3	468.33	234.67					L	1,733.95	867.48	1,716.92	858.96	1,715.94	858.47	14
4	596.39	298.70	579.36	290.18			Q	1,620.86	810.94	1,603.84	802.42	1,602.85	801.93	13
5	727.43	364.22	710.40	355.70			M	1,492.80	746.91	1,475.78	738.39	1,474.79	737.90	12
6	798.47	399.74	781.44	391.22			A	1,361.76	681.39	1,344.74	672.87	1,343.75	672.38	11
7	911.55	456.28	894.52	447.77			L	1,290.73	645.87	1,273.70	637.35	1,272.72	636.86	10
8	1,012.60	506.80	995.57	498.29	994.59	497.80	T	1,177.64	589.33	1,160.62	580.81	1,159.63	580.32	9
9	1,141.64	571.32	1,124.61	562.81	1,123.63	562.32	E	1,076.60	538.80	1,059.57	530.29	1,058.58	529.80	8
10	1,254.72	627.87	1,237.70	619.35	1,236.71	618.86	L	947.55	474.28	930.53	465.77	929.54	465.27	7
11	1,383.77	692.39	1,366.74	683.87	1,365.76	683.38	E	834.47	417.74	817.44	409.22	816.46	408.73	6
12	1,484.82	742.91	1,467.79	734.40	1,466.80	733.91	T	705.43	353.22	688.40	344.70	687.42	344.21	5
13	1,597.90	799.45	1,580.87	790.94	1,579.89	790.45	L	604.38	302.69	587.35	294.18	586.37	293.69	4
14	1,668.94	834.97	1,651.91	826.46	1,650.93	825.97	A	491.29	246.15	474.27	237.64	473.28	237.15	3
15	1,797.98	899.49	1,780.95	890.98	1,779.97	890.49	E	420.26	210.63	403.23	202.12	402.25	201.63	2
16							K	291.21	146.11	274.19	137.60			1

Query 22141 Hit 1

MS/MS Fragmentation of FLVGPDGVPVR
Found in sp|P11352|GPX1_MOUSE, Glutathione peroxidase 1 OS=Mus musculus GN=Gpx1 PE=1 SV=2
Match to Query 22141: 1298.747from(650.3807,2+)
Title: 537: Scan 1367 (rt=39.9765, f=3, i=200) [D:\lab212\membrane\Grace\Joyce\iTRAQ_21_2.raw]
Data File:Submitted from 20120508-1(merge) by Mascot Daemon on JOYCE-VAIO
Monoisotopic mass of neutral peptide Mr(calc): 1298.747
Variable modifications:
Ions Score: 44.3 Expect: 0.021

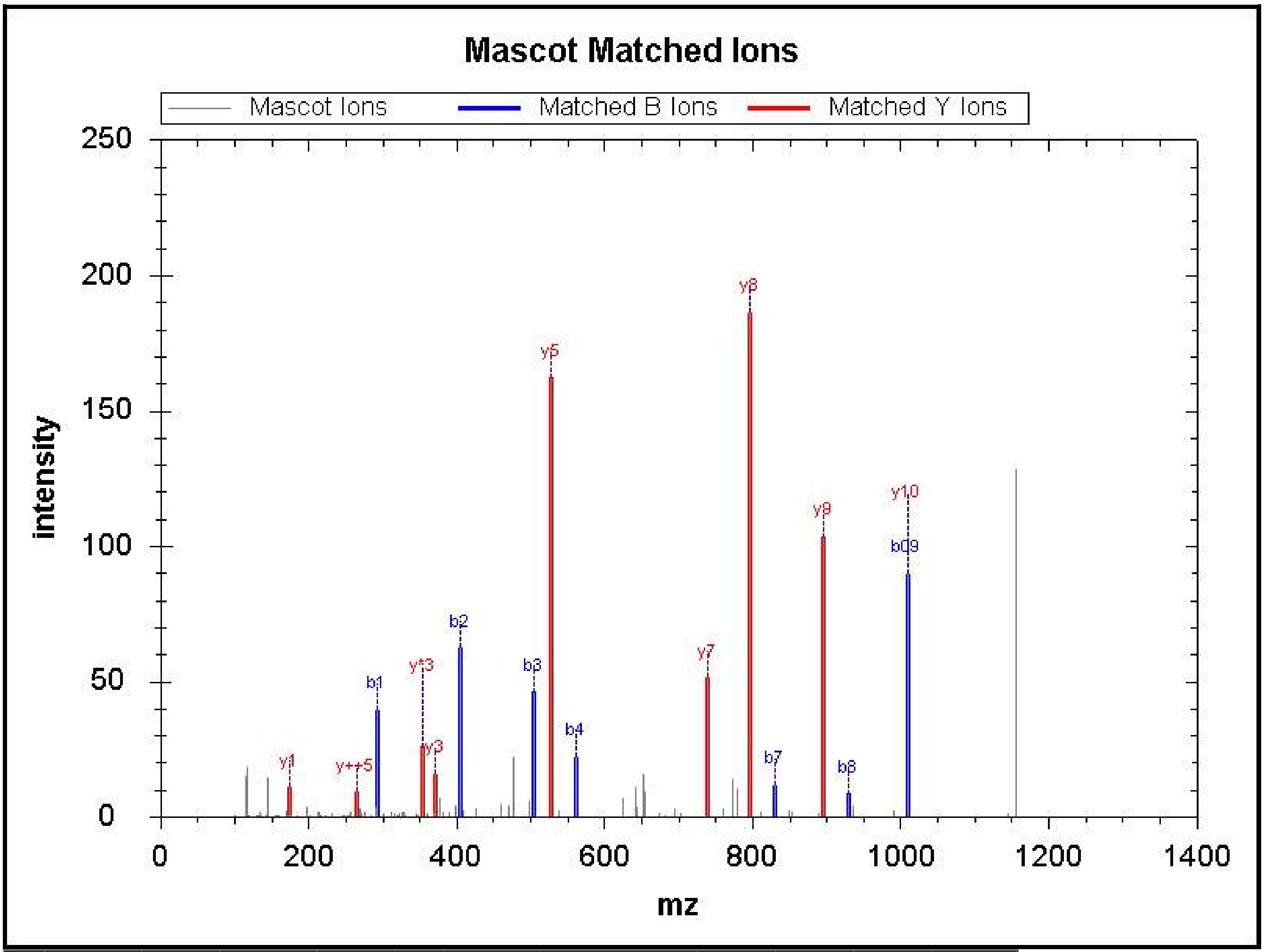

No	b	b++	b0	b0++	Seq	y	y++	y*	y*++	y0	y0++	RevNo
1	292.18	146.59			F							11
2	405.26	203.13			L	1,008.58	504.80	991.56	496.28	990.57	495.79	10
3	504.33	252.67			V	895.50	448.25	878.47	439.74	877.49	439.25	9
4	561.35	281.18			G	796.43	398.72	779.40	390.21	778.42	389.71	8
5	658.40	329.71			P	739.41	370.21	722.38	361.70	721.40	361.20	7
6	773.43	387.22	755.42	378.21	D	642.36	321.68	625.33	313.17	624.35	312.68	6
7	830.45	415.73	812.44	406.72	G	527.33	264.17	510.30	255.66			5
8	929.52	465.26	911.51	456.26	V	470.31	235.66	453.28	227.14			4
9	1,026.57	513.79	1,008.56	504.79	P	371.24	186.12	354.21	177.61			3
10	1,125.64	563.32	1,107.63	554.32	V	274.19	137.60	257.16	129.08			2
11					R	175.12	88.06	158.09	79.55			1

Query 12875 Hit 1

MS/MS Fragmentation of LPVGPSHK
Found in sp|O95182|NDUA7_HUMAN, NADH dehydrogenase [ubiquinone] 1 alpha subcomplex subunit 7 OS=Homo sapiens GN=NDUFA7 PE=1 SV=3
Match to Query 12875: 1121.68from(374.9007,3+)

Title: 194: Scan 499 (rt=20.8528, f=3, i=69) [D:\lab212\membrane\Grace\Joyce\iTRAQ_45_2.raw]
Data File:Submitted from 20120508-1(merge) by Mascot Daemon on JOYCE-VAIO
Monoisotopic mass of neutral peptide Mr(calc): 1121.68
Variable modifications:
K8 :iTRAQ4plex (K)
Ions Score: 44.25 Expect: 0.022

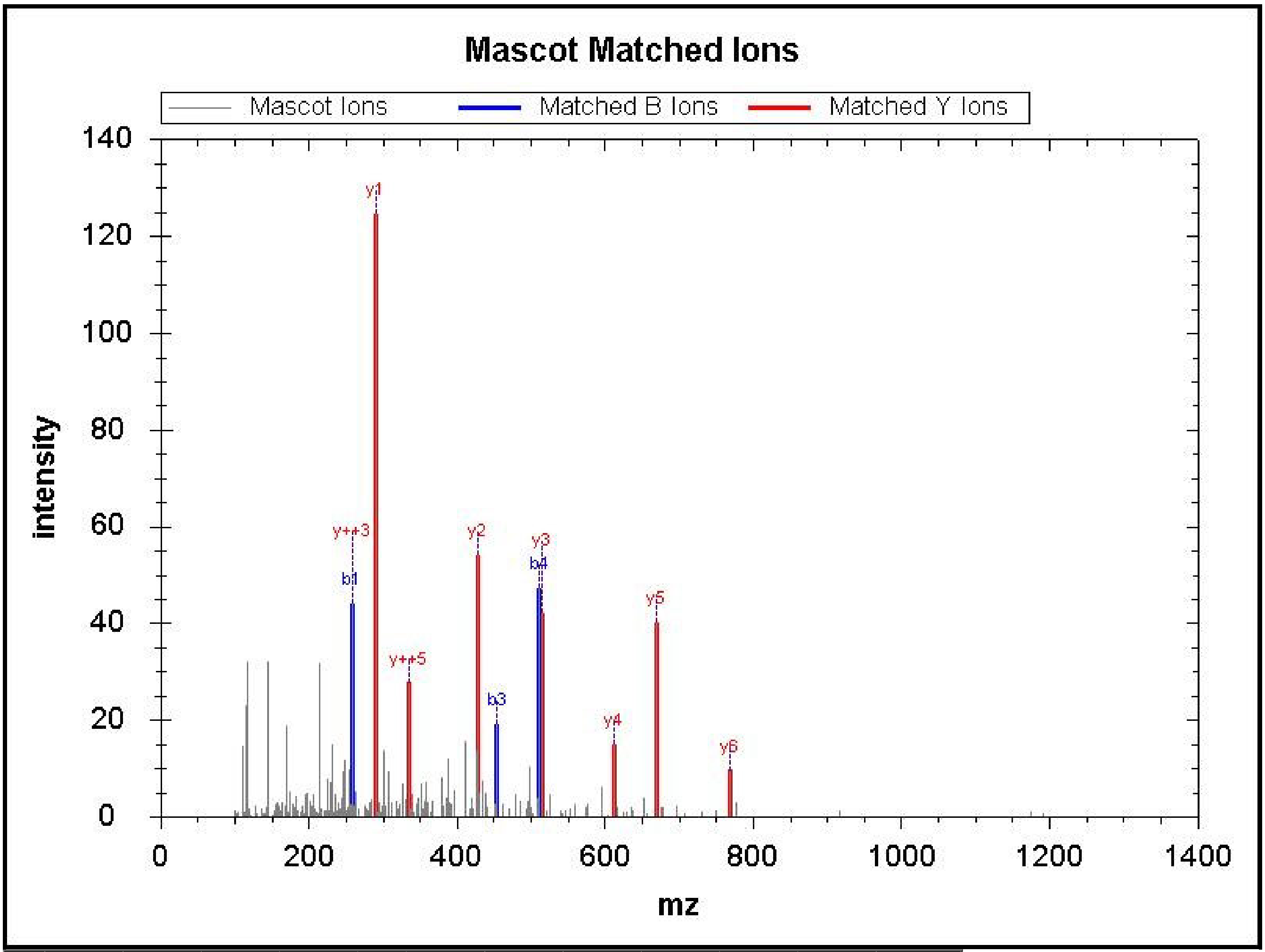

No	b	b++	b0	b0++	Seq	y	y++	y*	y*++	y0	y0++	RevNo
1	258.19	129.60			L							8
2	355.25	178.13			P	865.50	433.25	848.47	424.74	847.49	424.25	7
3	454.31	227.66			V	768.45	384.73	751.42	376.21	750.44	375.72	6
4	511.34	256.17			G	669.38	335.19	652.35	326.68	651.37	326.19	5
5	608.39	304.70			P	612.36	306.68	595.33	298.17	594.35	297.68	4
6	695.42	348.21	677.41	339.21	S	515.31	258.16	498.28	249.64	497.30	249.15	3
7	832.48	416.74	814.47	407.74	H	428.27	214.64	411.25	206.13			2
8					K	291.21	146.11	274.19	137.60			1

Query 53101 Hit 1

MS/MS Fragmentation of SFLINFIHTLENQR
Found in sp|O15031|PLXB2_HUMAN, Plexin-B2 OS=Homo sapiens GN=PLXNB2 PE=1 SV=3
Match to Query 53101: 1875.014from(626.012,3+)
Title: 1131: Sum of 2 scans in range 2495 (rt=65.5814, f=4, i=743) to 2496 (rt=65.6068, f=4, i=744) [D:\lab212\membrane\Grace\Joyce\iTRAQ_36_2.raw]
Data File:Submitted from 20120508-1(merge) by Mascot Daemon on JOYCE-VAIO
Monoisotopic mass of neutral peptide Mr(calc): 1875.014
Variable modifications:
Ions Score: 44.22 Expect: 0.032

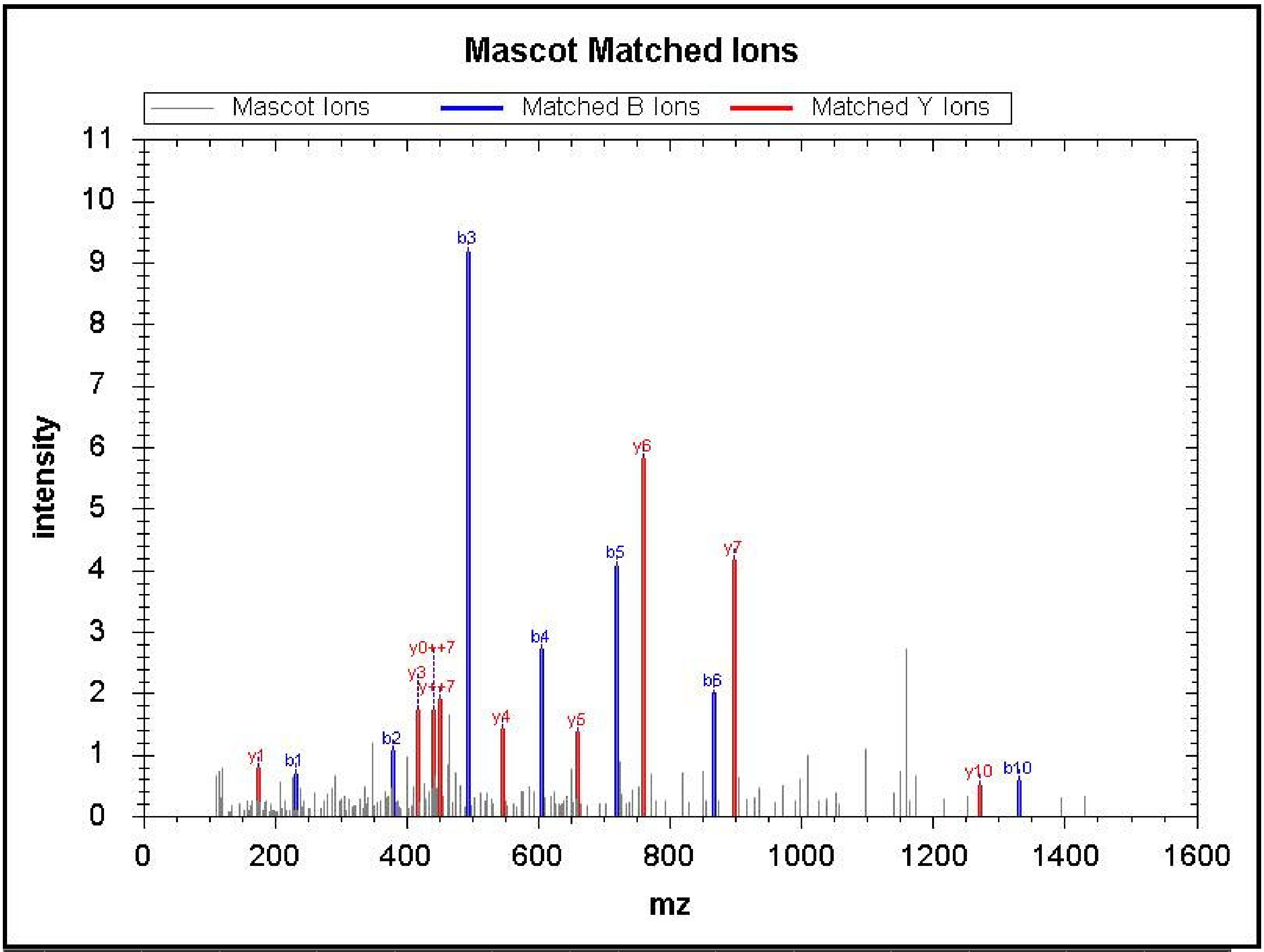

No	b	b++	b*	b*++	b0	b0++	Seq	y	y++	y*	y*++	y0	y0++	RevNo
1	232.14	116.57			214.13	107.57	S							14
2	379.21	190.11			361.20	181.10	F	1,644.89	822.95	1,627.86	814.43	1,626.88	813.94	13
3	492.29	246.65			474.28	237.65	L	1,497.82	749.41	1,480.79	740.90	1,479.81	740.41	12
4	605.38	303.19			587.37	294.19	I	1,384.73	692.87	1,367.71	684.36	1,366.72	683.86	11
5	719.42	360.21	702.39	351.70	701.41	351.21	N	1,271.65	636.33	1,254.62	627.81	1,253.64	627.32	10
6	866.49	433.75	849.46	425.23	848.48	424.74	F	1,157.61	579.31	1,140.58	570.79	1,139.60	570.30	9
7	979.57	490.29	962.55	481.78	961.56	481.28	I	1,010.54	505.77	993.51	497.26	992.53	496.77	8
8	1,116.63	558.82	1,099.61	550.31	1,098.62	549.81	H	897.45	449.23	880.43	440.72	879.44	440.23	7
9	1,217.68	609.34	1,200.65	600.83	1,199.67	600.34	T	760.39	380.70	743.37	372.19	742.38	371.70	6
10	1,330.76	665.89	1,313.74	657.37	1,312.75	656.88	L	659.35	330.18	642.32	321.66	641.34	321.17	5
11	1,459.81	730.41	1,442.78	721.89	1,441.80	721.40	E	546.26	273.64	529.24	265.12	528.25	264.63	4
12	1,573.85	787.43	1,556.82	778.92	1,555.84	778.42	N	417.22	209.11	400.19	200.60			3
13	1,701.91	851.46	1,684.88	842.94	1,683.90	842.45	Q	303.18	152.09	286.15	143.58			2
14							R	175.12	88.06	158.09	79.55			1

Query 14126 Hit 1

MS/MS Fragmentation of AVGAQASVGSR
Found in sp|Q9NWV8|BABA1_HUMAN, BRISC and BRCA1-A complex member 1 OS=Homo sapiens GN=BABAM1 PE=1 SV=1
Match to Query 14126: 1145.625from(573.8197,2+)
Title: 53: Scan 235 (rt=14.6797, f=2, i=38) [D:\lab212\membrane\Grace\Joyce\iTRAQ_26_2.raw]
Data File:Submitted from 20120508-1(merge) by Mascot Daemon on JOYCE-VAIO
Monoisotopic mass of neutral peptide Mr(calc): 1145.625
Variable modifications:
Ions Score: 44.16 Expect: 0.032

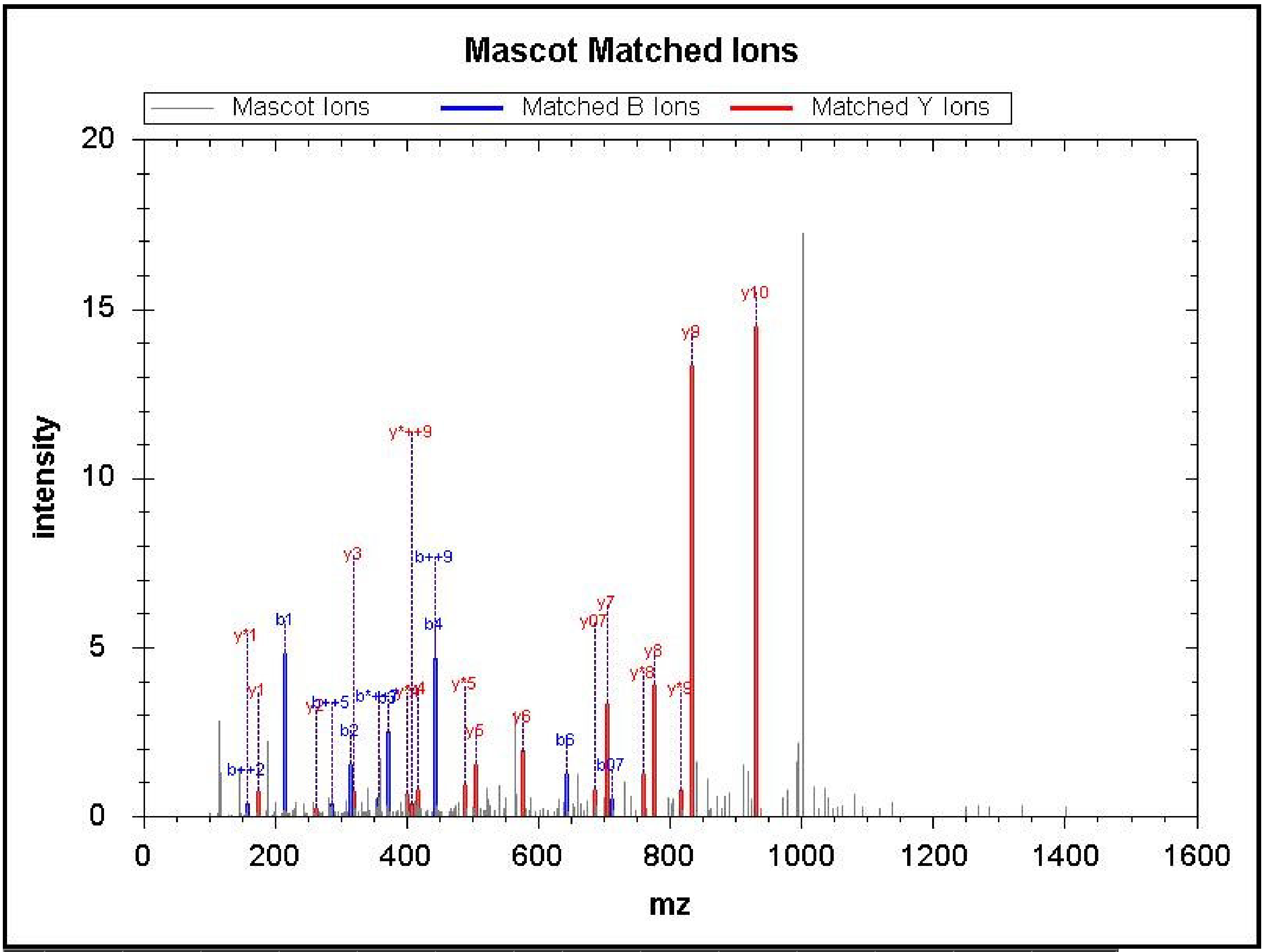

No	b	b++	b*	b*++	b0	b0++	Seq	y	y++	y*	y*++	y0	y0++	RevNo
1	216.15	108.58					A							11
2	315.21	158.11					V	931.50	466.25	914.47	457.74	913.49	457.25	10
3	372.24	186.62					G	832.43	416.72	815.40	408.20	814.42	407.71	9
4	443.27	222.14					A	775.41	388.21	758.38	379.69	757.40	379.20	8
5	571.33	286.17	554.31	277.66			Q	704.37	352.69	687.34	344.17	686.36	343.68	7
6	642.37	321.69	625.34	313.17			A	576.31	288.66	559.28	280.15	558.30	279.65	6
7	729.40	365.20	712.37	356.69	711.39	356.20	S	505.27	253.14	488.25	244.63	487.26	244.13	5
8	828.47	414.74	811.44	406.23	810.46	405.73	V	418.24	209.62	401.21	201.11	400.23	200.62	4
9	885.49	443.25	868.46	434.74	867.48	434.24	G	319.17	160.09	302.15	151.58	301.16	151.08	3
10	972.52	486.77	955.50	478.25	954.51	477.76	S	262.15	131.58	245.12	123.07	244.14	122.57	2
11							R	175.12	88.06	158.09	79.55			1

Query 39098 Hit 1

MS/MS Fragmentation of MLSFQGLAELAHR
Found in sp|O15294|OGT1_HUMAN, UDP-N-acetylglucosamine--peptide N-acetylglucosaminyltransferase 110 kDa subunit OS=Homo sapiens GN=OGT PE=1 SV=3
Match to Query 39098: 1615.863from(539.6282,3+)
Title: 827: Scan 1843 (rt=50.9264, f=3, i=281) [D:\lab212\membrane\Grace\Joyce\iTRAQ_36_1.raw]
Data File:Submitted from 20120508-1(merge) by Mascot Daemon on JOYCE-VAIO
Monoisotopic mass of neutral peptide Mr(calc): 1615.863
Variable modifications:
Ions Score: 44.15 Expect: 0.032

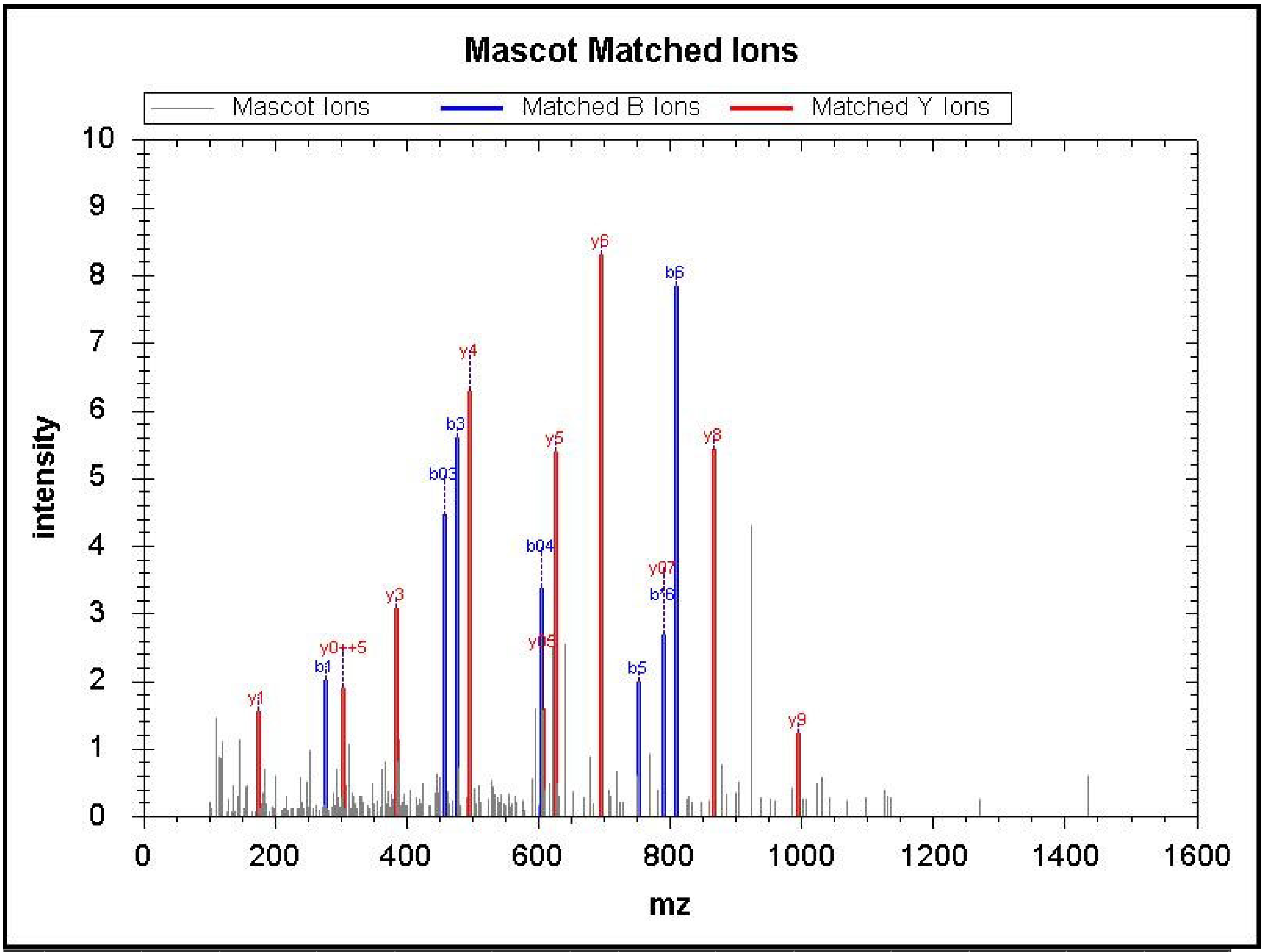

No	b	b++	b*	b*++	b0	b0++	Seq	y	y++	y*	y*++	y0	y0++	RevNo
1	276.15	138.58					M							13
2	389.23	195.12					L	1,341.73	671.37	1,324.70	662.85	1,323.72	662.36	12
3	476.27	238.64			458.26	229.63	S	1,228.64	614.83	1,211.62	606.31	1,210.63	605.82	11
4	623.33	312.17			605.32	303.17	F	1,141.61	571.31	1,124.58	562.80	1,123.60	562.30	10
5	751.39	376.20	734.37	367.69	733.38	367.19	Q	994.54	497.78	977.52	489.26	976.53	488.77	9
6	808.41	404.71	791.39	396.20	790.40	395.71	G	866.48	433.75	849.46	425.23	848.47	424.74	8
7	921.50	461.25	904.47	452.74	903.49	452.25	L	809.46	405.24	792.44	396.72	791.45	396.23	7
8	992.54	496.77	975.51	488.26	974.53	487.77	A	696.38	348.69	679.35	340.18	678.37	339.69	6
9	1,121.58	561.29	1,104.55	552.78	1,103.57	552.29	E	625.34	313.17	608.32	304.66	607.33	304.17	5
10	1,234.66	617.83	1,217.64	609.32	1,216.65	608.83	L	496.30	248.65	479.27	240.14			4
11	1,305.70	653.35	1,288.67	644.84	1,287.69	644.35	A	383.21	192.11	366.19	183.60			3
12	1,442.76	721.88	1,425.73	713.37	1,424.75	712.88	H	312.18	156.59	295.15	148.08			2
13							R	175.12	88.06	158.09	79.55			1

Query 3139 Hit 1

MS/MS Fragmentation of SGSPLEK
Found in sp|Q9NRA8|4ET_HUMAN, Eukaryotic translation initiation factor 4E transporter OS=Homo sapiens GN=EIF4ENIF1 PE=1 SV=2
Match to Query 3139: 860.5255from(431.27,2+)
Title: 107: Sum of 2 scans in range 327 (rt=16.8334, f=4, i=67) to 328 (rt=16.8588, f=4, i=68) [D:\lab212\membrane\Grace\Joyce\iTRAQ_32_1.raw]
Data File:Submitted from 20120508-1(merge) by Mascot Daemon on JOYCE-VAIO
Monoisotopic mass of neutral peptide Mr(calc): 860.5255
Variable modifications:
K7 :iTRAQ4plex (K)
Ions Score: 44.15 Expect: 0.031

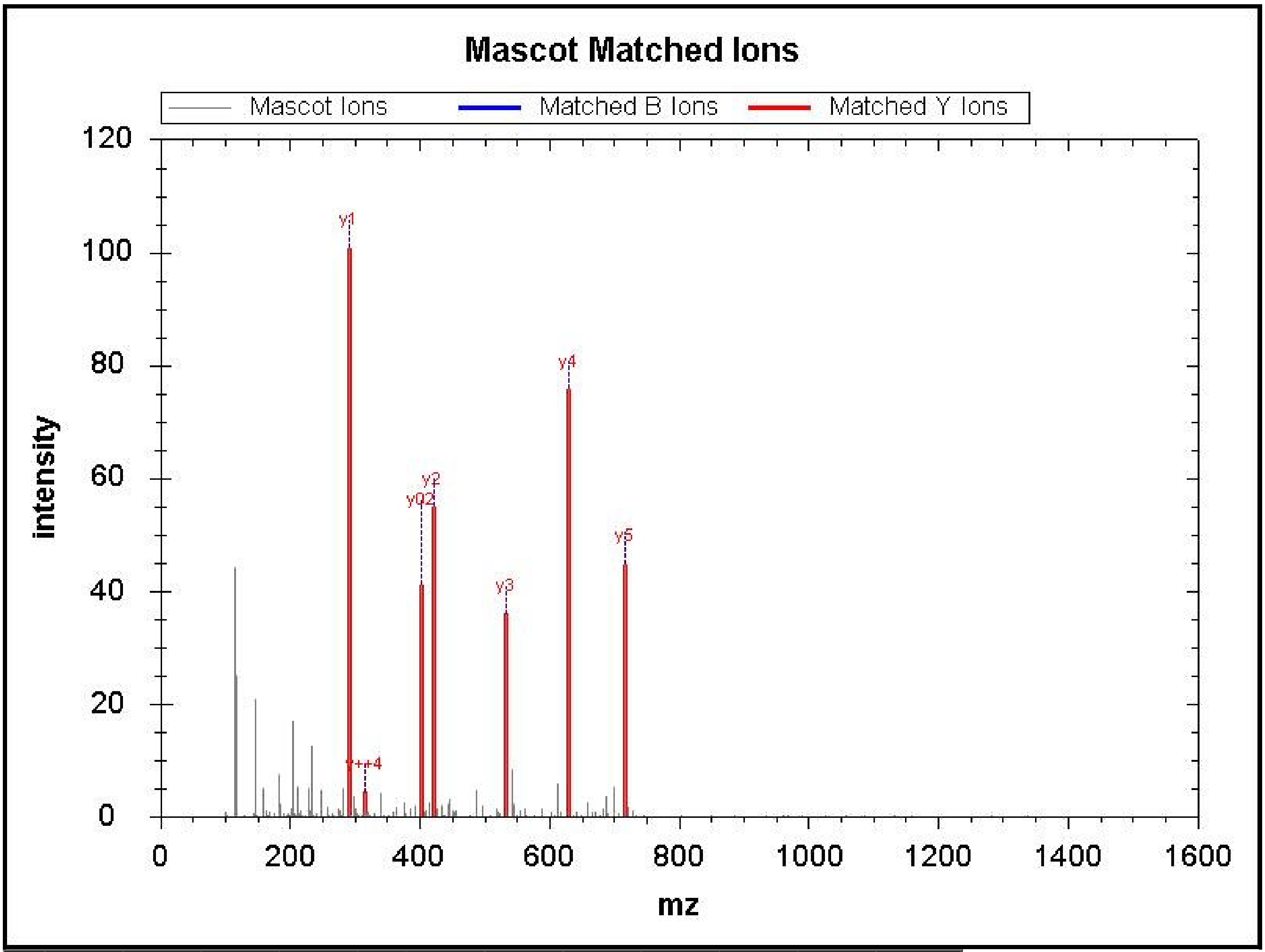

No	b	b++	b0	b0++	Seq	y	y++	y*	y*++	y0	y0++	RevNo
1	88.04	44.52	70.03	35.52	S							7
2	145.06	73.03	127.05	64.03	G	774.45	387.73	757.42	379.21	756.44	378.72	6
3	232.09	116.55	214.08	107.54	S	717.43	359.22	700.40	350.70	699.42	350.21	5
4	329.15	165.08	311.14	156.07	P	630.39	315.70	613.37	307.19	612.38	306.70	4
5	442.23	221.62	424.22	212.61	L	533.34	267.17	516.31	258.66	515.33	258.17	3
6	571.27	286.14	553.26	277.13	E	420.26	210.63	403.23	202.12	402.25	201.63	2
7					K	291.21	146.11	274.19	137.60			1

Query 60971 Hit 1

MS/MS Fragmentation of GFDQTINLILDESHER
Found in sp|O95777|NAA38_HUMAN, N-alpha-acetyltransferase 38
Match to Query 60971: 2030.021from(677.681,3+)
Title: 866: Sum of 2 scans in range 1936 (rt=53.0221, f=4, i=581) to 1937 (rt=53.0475, f=4, i=582)
[D:\lab212\membrane\Grace\Joyce\iTRAQ_35_2.raw]
Data File:Submitted from 20120508-1(merge) by Mascot Daemon on JOYCE-VAIO
Monoisotopic mass of neutral peptide Mr(calc): 2030.021
Variable modifications:
Ions Score: 44.12 Expect: 0.035

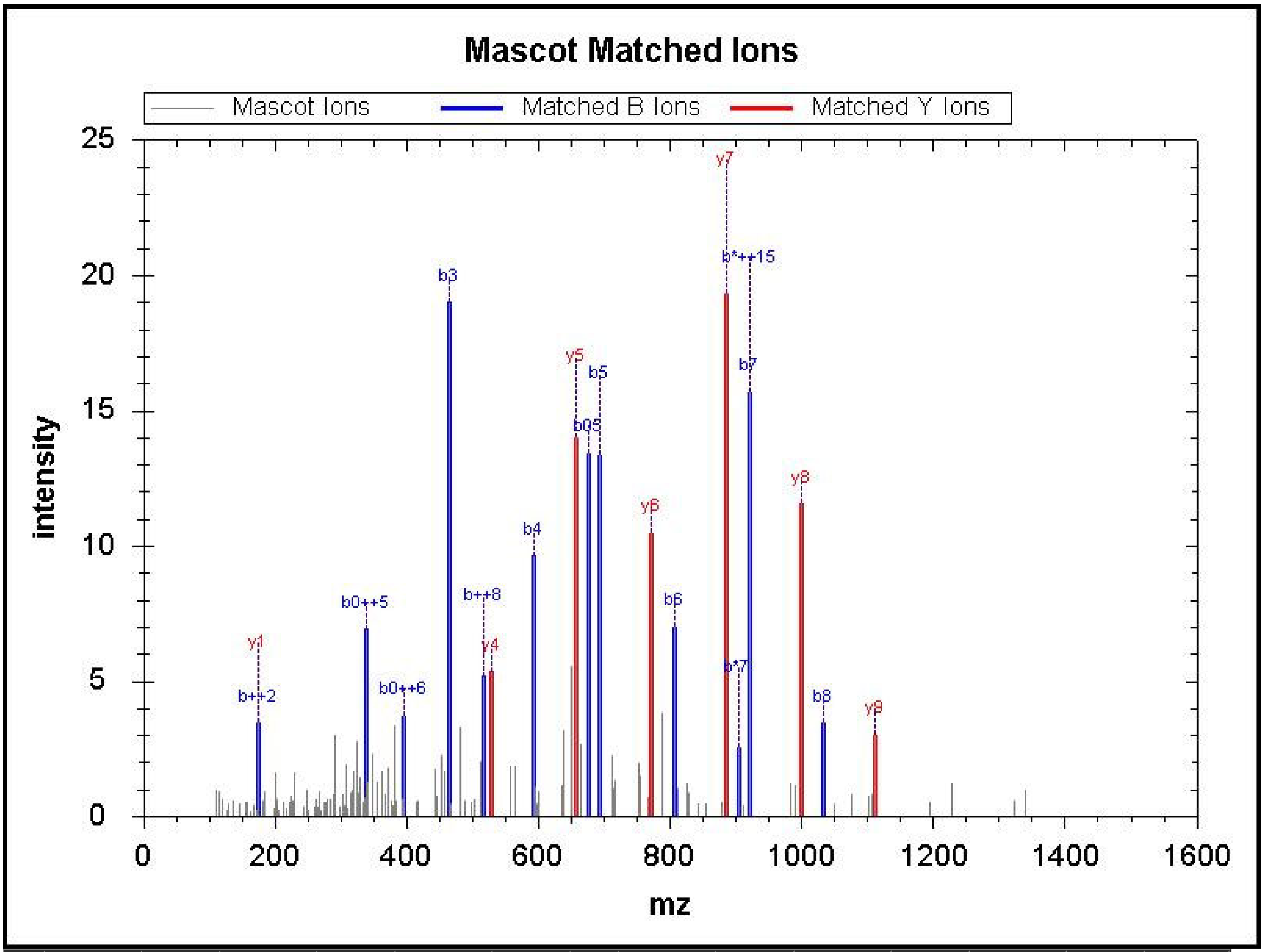

No	b	b++	b*	b*++	b0	b0++	Seq	y	y++	y*	y*++	y0	y0++	RevNo
1	202.13	101.57					G							16
2	349.20	175.10					F	1,829.90	915.46	1,812.88	906.94	1,811.89	906.45	15
3	464.23	232.62			446.22	223.61	D	1,682.83	841.92	1,665.81	833.41	1,664.82	832.92	14
4	592.28	296.65	575.26	288.13	574.27	287.64	Q	1,567.81	784.41	1,550.78	775.89	1,549.80	775.40	13
5	693.33	347.17	676.31	338.66	675.32	338.16	T	1,439.75	720.38	1,422.72	711.86	1,421.74	711.37	12
6	806.42	403.71	789.39	395.20	788.41	394.71	I	1,338.70	669.85	1,321.67	661.34	1,320.69	660.85	11
7	920.46	460.73	903.43	452.22	902.45	451.73	N	1,225.62	613.31	1,208.59	604.80	1,207.61	604.31	10
8	1,033.54	517.28	1,016.52	508.76	1,015.53	508.27	L	1,111.57	556.29	1,094.55	547.78	1,093.56	547.29	9
9	1,146.63	573.82	1,129.60	565.30	1,128.62	564.81	I	998.49	499.75	981.46	491.24	980.48	490.74	8
10	1,259.71	630.36	1,242.69	621.85	1,241.70	621.35	L	885.41	443.21	868.38	434.69	867.40	434.20	7
11	1,374.74	687.87	1,357.71	679.36	1,356.73	678.87	D	772.32	386.66	755.30	378.15	754.31	377.66	6
12	1,503.78	752.39	1,486.75	743.88	1,485.77	743.39	E	657.30	329.15	640.27	320.64	639.28	320.15	5
13	1,590.81	795.91	1,573.79	787.40	1,572.80	786.90	S	528.25	264.63	511.23	256.12	510.24	255.62	4
14	1,727.87	864.44	1,710.85	855.93	1,709.86	855.43	H	441.22	221.11	424.19	212.60	423.21	212.11	3
15	1,856.91	928.96	1,839.89	920.45	1,838.90	919.96	E	304.16	152.58	287.13	144.07	286.15	143.58	2
16							R	175.12	88.06	158.09	79.55			1

Query 60986 Hit 1

MS/MS Fragmentation of VSIAELAQASNSLIAWGR
Found in sp|Q96HY6|DDRGK_HUMAN, DDRGK domain-containing protein 1 OS=Homo sapiens GN=DDRGK1 PE=1 SV=2
Match to Query 60986: 2030.097from(677.7061,3+)
Title: 1030: Sum of 2 scans in range 2466 (rt=63.9498, f=4, i=677) to 2467 (rt=63.9752, f=4, i=678)
[D:\lab212\membrane\Grace\Joyce\iTRAQ_23_2.raw]
Data File:Submitted from 20120508-1(merge) by Mascot Daemon on JOYCE-VAIO
Monoisotopic mass of neutral peptide Mr(calc): 2030.097

Variable modifications:
Q8 :Deamidated (NQ)
Ions Score: 44.12 Expect: 0.031

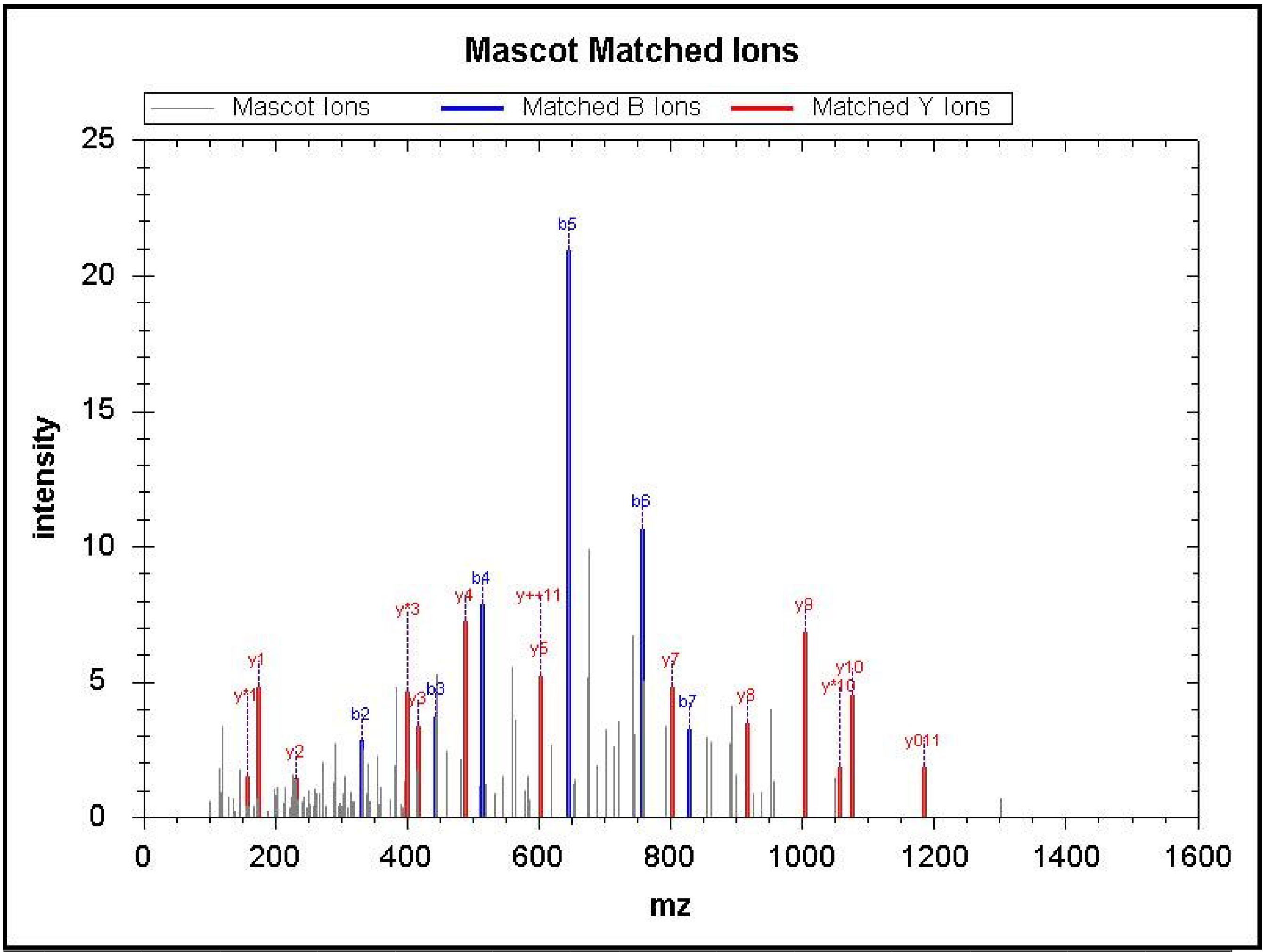

No	b	b++	b*	b*++	b0	b0++	Seq	y	y++	y*	y*++	y0	y0++	RevNo
1	244.18	122.59					V							18
2	331.21	166.11			313.20	157.10	S	1,787.93	894.47	1,770.90	885.95	1,769.92	885.46	17
3	444.29	222.65			426.28	213.65	I	1,700.90	850.95	1,683.87	842.44	1,682.89	841.95	16
4	515.33	258.17			497.32	249.16	A	1,587.81	794.41	1,570.79	785.90	1,569.80	785.40	15
5	644.37	322.69			626.36	313.69	E	1,516.78	758.89	1,499.75	750.38	1,498.76	749.89	14
6	757.46	379.23			739.45	370.23	L	1,387.73	694.37	1,370.71	685.86	1,369.72	685.36	13
7	828.49	414.75			810.48	405.75	A	1,274.65	637.83	1,257.62	629.31	1,256.64	628.82	12
8	957.54	479.27	940.51	470.76	939.53	470.27	Q	1,203.61	602.31	1,186.59	593.80	1,185.60	593.30	11
9	1,028.57	514.79	1,011.55	506.28	1,010.56	505.79	A	1,074.57	537.79	1,057.54	529.27	1,056.56	528.78	10
10	1,115.61	558.31	1,098.58	549.79	1,097.60	549.30	S	1,003.53	502.27	986.51	493.76	985.52	493.26	9
11	1,229.65	615.33	1,212.62	606.82	1,211.64	606.32	N	916.50	458.75	899.47	450.24	898.49	449.75	8
12	1,316.68	658.84	1,299.65	650.33	1,298.67	649.84	S	802.46	401.73	785.43	393.22	784.45	392.73	7
13	1,429.77	715.39	1,412.74	706.87	1,411.75	706.38	L	715.42	358.22	698.40	349.70			6
14	1,542.85	771.93	1,525.82	763.42	1,524.84	762.92	I	602.34	301.67	585.31	293.16			5
15	1,613.89	807.45	1,596.86	798.93	1,595.88	798.44	A	489.26	245.13	472.23	236.62			4
16	1,799.97	900.49	1,782.94	891.97	1,781.96	891.48	W	418.22	209.61	401.19	201.10			3
17	1,856.99	929.00	1,839.96	920.48	1,838.98	919.99	G	232.14	116.57	215.11	108.06			2
18							R	175.12	88.06	158.09	79.55			1

Query 27675 Hit 1

MS/MS Fragmentation of SSSQPSPSDPK
Found in sp|Q9H320|VCX1_HUMAN, Variable charge X-linked protein 1 OS=Homo sapiens GN=VCX PE=2 SV=1
Match to Query 27675: 1403.717from(702.8657,2+)
Title: 4: Sum of 2 scans in range 159 (rt=12.7685, f=2, i=7) to 160 (rt=12.7939, f=2, i=8)
[D:\lab212\membrane\Grace\Joyce\iTRAQ_30_2.raw]
Data File:Submitted from 20120508-1(merge) by Mascot Daemon on JOYCE-VAIO
Monoisotopic mass of neutral peptide Mr(calc): 1403.717
Variable modifications:
K11 :iTRAQ4plex (K)
Ions Score: 44.1 Expect: 0.032

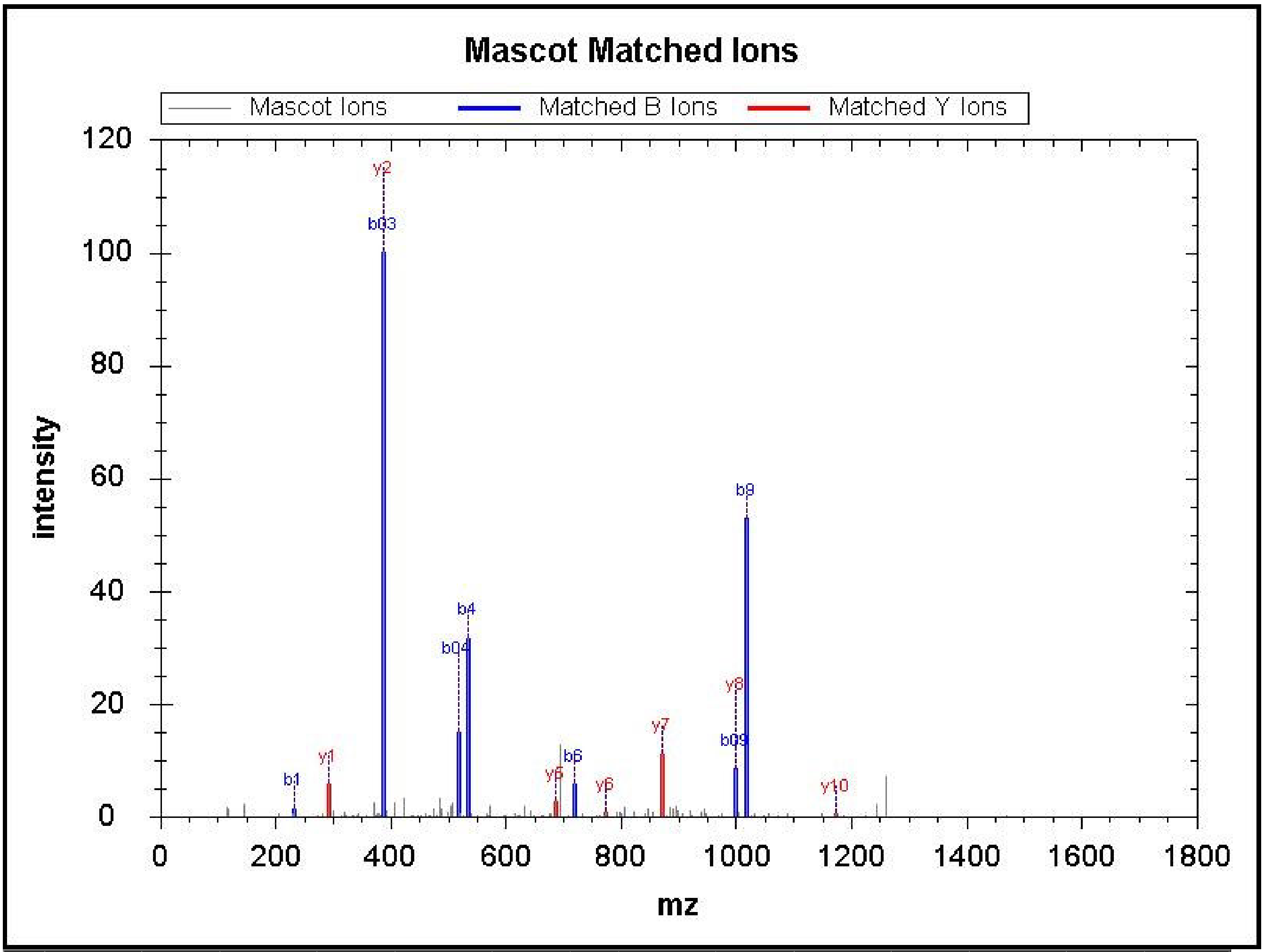

No	b	b++	b*	b*++	b0	b0++	Seq	y	y++	y*	y*++	y0	y0++	RevNo
1	232.14	116.57			214.13	107.57	S							11
2	319.17	160.09			301.16	151.09	S	1,173.59	587.30	1,156.56	578.78	1,155.58	578.29	10
3	406.21	203.61			388.19	194.60	S	1,086.55	543.78	1,069.53	535.27	1,068.54	534.78	9
4	534.26	267.64	517.24	259.12	516.25	258.63	Q	999.52	500.27	982.50	491.75	981.51	491.26	8
5	631.32	316.16	614.29	307.65	613.31	307.16	P	871.46	436.24	854.44	427.72	853.45	427.23	7
6	718.35	359.68	701.32	351.16	700.34	350.67	S	774.41	387.71	757.38	379.20	756.40	378.70	6
7	815.40	408.20	798.38	399.69	797.39	399.20	P	687.38	344.19	670.35	335.68	669.37	335.19	5
8	902.43	451.72	885.41	443.21	884.42	442.72	S	590.33	295.67	573.30	287.15	572.32	286.66	4
9	1,017.46	509.23	1,000.43	500.72	999.45	500.23	D	503.29	252.15	486.27	243.64	485.28	243.15	3
10	1,114.51	557.76	1,097.49	549.25	1,096.50	548.76	P	388.27	194.64	371.24	186.12			2
11							K	291.21	146.11	274.19	137.60			1

Query 67078 Hit 1

MS/MS Fragmentation of SLLEPLMQGYWEWLVR
Found in sp|Q9Y6K0|CEPT1_HUMAN, Choline/ethanolaminephosphotransferase 1 OS=Homo sapiens GN=CEPT1 PE=1 SV=1
Match to Query 67078: 2163.126from(722.0492,3+)

Title: 1341: Sum of 2 scans in range 3057 (rt=77.9569, f=2, i=499) to 3058 (rt=77.9823, f=2, i=500) [D:\lab212\membrane\Grace\Joyce\iTRAQ_22_1.raw]
Data File:Submitted from 20120508-1(merge) by Mascot Daemon on JOYCE-VAIO
Monoisotopic mass of neutral peptide Mr(calc): 2163.126
Variable modifications:
Ions Score: 44.08 Expect: 0.036

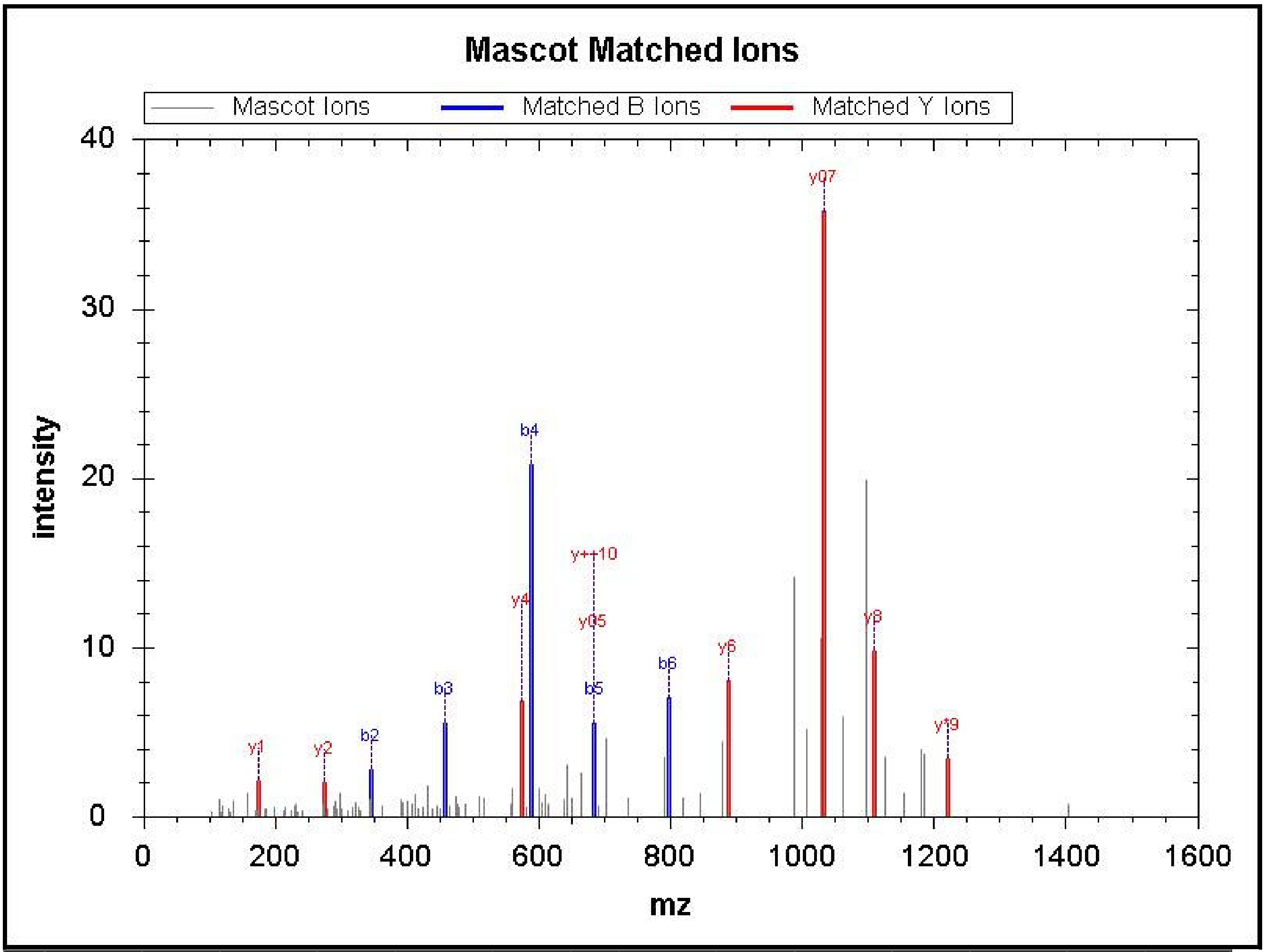

No	b	b++	b*	b*++	b0	b0++	Seq	y	y++	y*	y*++	y0	y0++	RevNo
1	232.14	116.57			214.13	107.57	S							16
2	345.23	173.12			327.21	164.11	L	1,933.00	967.01	1,915.98	958.49	1,914.99	958.00	15
3	458.31	229.66			440.30	220.65	L	1,819.92	910.46	1,802.89	901.95	1,801.91	901.46	14
4	587.35	294.18			569.34	285.17	E	1,706.84	853.92	1,689.81	845.41	1,688.83	844.92	13
5	684.40	342.71			666.39	333.70	P	1,577.79	789.40	1,560.77	780.89	1,559.78	780.40	12
6	797.49	399.25			779.48	390.24	L	1,480.74	740.87	1,463.71	732.36	1,462.73	731.87	11
7	928.53	464.77			910.52	455.76	M	1,367.66	684.33	1,350.63	675.82	1,349.65	675.33	10
8	1,056.59	528.80	1,039.56	520.28	1,038.58	519.79	Q	1,236.62	618.81	1,219.59	610.30	1,218.61	609.81	9
9	1,113.61	557.31	1,096.58	548.80	1,095.60	548.30	G	1,108.56	554.78	1,091.53	546.27	1,090.55	545.78	8
10	1,276.67	638.84	1,259.65	630.33	1,258.66	629.83	Y	1,051.54	526.27	1,034.51	517.76	1,033.53	517.27	7
11	1,462.75	731.88	1,445.73	723.37	1,444.74	722.87	W	888.47	444.74	871.45	436.23	870.46	435.73	6
12	1,591.79	796.40	1,574.77	787.89	1,573.78	787.40	E	702.39	351.70	685.37	343.19	684.38	342.70	5
13	1,777.87	889.44	1,760.85	880.93	1,759.86	880.44	W	573.35	287.18	556.32	278.67			4
14	1,890.96	945.98	1,873.93	937.47	1,872.95	936.98	L	387.27	194.14	370.24	185.63			3
15	1,990.03	995.52	1,973.00	987.00	1,972.02	986.51	V	274.19	137.60	257.16	129.08			2
16							R	175.12	88.06	158.09	79.55			1

Query 42068 Hit 1

MS/MS Fragmentation of KIDDLIGDLVQR
Found in sp|Q9Y6X5|ENPP4_HUMAN, Ectonucleotide pyrophosphatase/phosphodiesterase family member 4 OS=Homo sapiens GN=ENPP4 PE=1 SV=3
Match to Query 42068: 1671.977from(558.3328,3+)
Title: 841: Sum of 2 scans in range 1860 (rt=51.5027, f=4, i=564) to 1861 (rt=51.5281, f=4, i=565) [D:\lab212\membrane\Grace\Joyce\iTRAQ_44_2.raw]
Data File:Submitted from 20120508-1(merge) by Mascot Daemon on JOYCE-VAIO
Monoisotopic mass of neutral peptide Mr(calc): 1671.977
Variable modifications:
K1 :iTRAQ4plex (K)
Ions Score: 44.06 Expect: 0.021

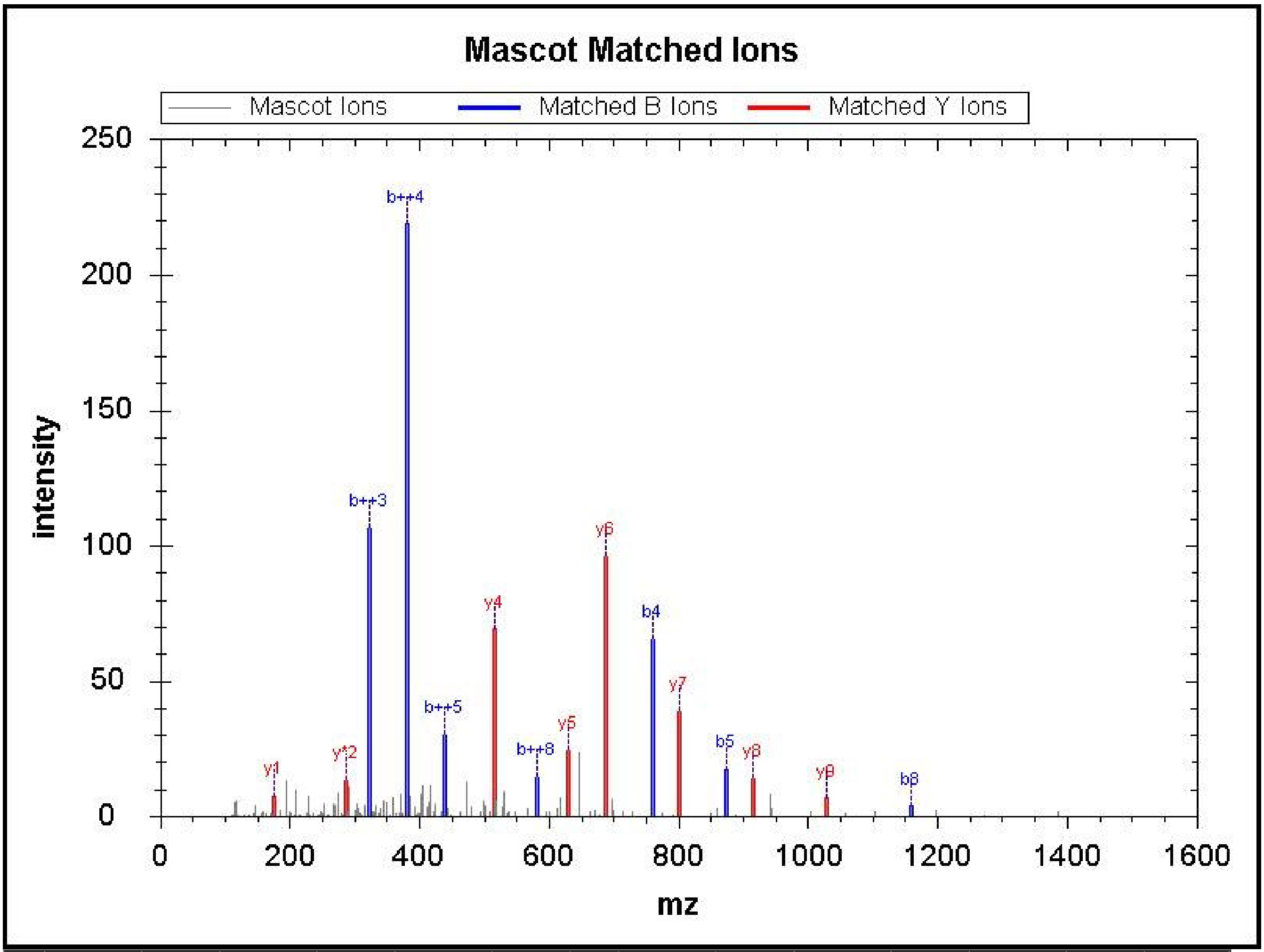

No	b	b++	b*	b*++	b0	b0++	Seq	y	y++	y*	y*++	y0	y0++	RevNo
1	417.31	209.16	400.28	200.64			K							12
2	530.39	265.70	513.36	257.19			I	1,256.68	628.85	1,239.66	620.33	1,238.67	619.84	11
3	645.42	323.21	628.39	314.70	627.41	314.21	D	1,143.60	572.30	1,126.57	563.79	1,125.59	563.30	10
4	760.44	380.73	743.42	372.21	742.43	371.72	D	1,028.57	514.79	1,011.55	506.28	1,010.56	505.79	9
5	873.53	437.27	856.50	428.75	855.52	428.26	L	913.55	457.28	896.52	448.76	895.54	448.27	8
6	986.61	493.81	969.59	485.30	968.60	484.80	I	800.46	400.73	783.44	392.22	782.45	391.73	7
7	1,043.63	522.32	1,026.61	513.81	1,025.62	513.32	G	687.38	344.19	670.35	335.68	669.37	335.19	6
8	1,158.66	579.83	1,141.63	571.32	1,140.65	570.83	D	630.36	315.68	613.33	307.17	612.35	306.68	5
9	1,271.74	636.38	1,254.72	627.86	1,253.73	627.37	L	515.33	258.17	498.30	249.66			4
10	1,370.81	685.91	1,353.79	677.40	1,352.80	676.90	V	402.25	201.63	385.22	193.11			3
11	1,498.87	749.94	1,481.85	741.43	1,480.86	740.93	Q	303.18	152.09	286.15	143.58			2
12							R	175.12	88.06	158.09	79.55			1

Query 58645 Hit 1

MS/MS Fragmentation of NEVSFVIHNLPIIAK

Found in sp|Q99611|SPS2_HUMAN, Selenide
Match to Query 58645: 1981.157from(661.3929,3+)
Title: 872: Sum of 2 scans in range 1934 (rt=52.9877, f=4, i=581) to 1935 (rt=53.0131, f=4, i=582)
[D:\lab212\membrane\Grace\Joyce\iTRAQ_36_2.raw]
Data File:Submitted from 20120508-1(merge) by Mascot Daemon on JOYCE-VAIO
Monoisotopic mass of neutral peptide Mr(calc): 1981.157
Variable modifications:
K15 :iTRAQ4plex (K)
Ions Score: 43.99 Expect: 0.016

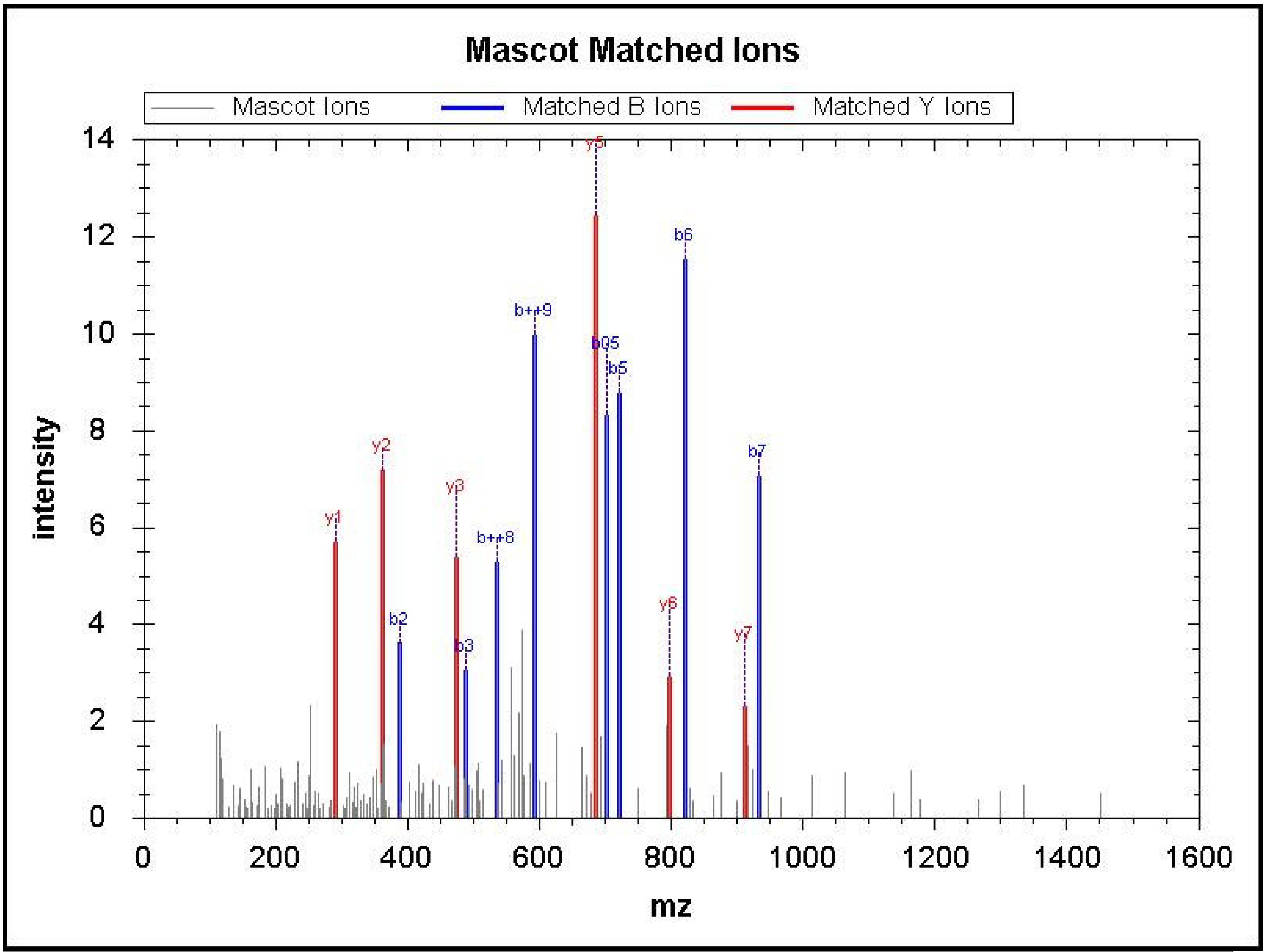

No	b	b++	b*	b*++	b0	b0++	Seq	y	y++	y*	y*++	y0	y0++	RevNo
1	259.15	130.08	242.13	121.57			N							15
2	388.19	194.60	371.17	186.09	370.18	185.60	E	1,724.02	862.51	1,707.00	854.00	1,706.01	853.51	14
3	487.26	244.14	470.24	235.62	469.25	235.13	V	1,594.98	797.99	1,577.95	789.48	1,576.97	788.99	13
4	574.30	287.65	557.27	279.14	556.28	278.65	S	1,495.91	748.46	1,478.89	739.95	1,477.90	739.45	12
5	721.36	361.19	704.34	352.67	703.35	352.18	F	1,408.88	704.94	1,391.85	696.43			11
6	820.43	410.72	803.41	402.21	802.42	401.71	V	1,261.81	631.41	1,244.78	622.90			10
7	933.52	467.26	916.49	458.75	915.51	458.26	I	1,162.74	581.88	1,145.72	573.36			9
8	1,070.58	535.79	1,053.55	527.28	1,052.56	526.79	H	1,049.66	525.33	1,032.63	516.82			8
9	1,184.62	592.81	1,167.59	584.30	1,166.61	583.81	N	912.60	456.80	895.57	448.29			7
10	1,297.70	649.35	1,280.68	640.84	1,279.69	640.35	L	798.56	399.78	781.53	391.27			6
11	1,394.76	697.88	1,377.73	689.37	1,376.74	688.88	P	685.47	343.24	668.45	334.73			5
12	1,507.84	754.42	1,490.81	745.91	1,489.83	745.42	I	588.42	294.71	571.39	286.20			4
13	1,620.92	810.97	1,603.90	802.45	1,602.91	801.96	I	475.34	238.17	458.31	229.66			3
14	1,691.96	846.48	1,674.93	837.97	1,673.95	837.48	A	362.25	181.63	345.23	173.12			2
15							K	291.21	146.11	274.19	137.60			1

Query 22667 Hit 1

MS/MS Fragmentation of WFLDALEK
Found in sp|Q13042|CDC16_HUMAN, Cell division cycle protein 16 homolog OS=Homo sapiens GN=CDC16 PE=1 SV=2
Match to Query 22667: 1308.736from(655.3754,2+)
Title: 844: Scan 1960 (rt=53.1007, f=3, i=283) [D:\lab212\membrane\Grace\Joyce\iTRAQ_29_1.raw]
Data File:Submitted from 20120508-1(merge) by Mascot Daemon on JOYCE-VAIO
Monoisotopic mass of neutral peptide Mr(calc): 1308.736
Variable modifications:
K8 :iTRAQ4plex (K)
Ions Score: 43.98 Expect: 0.029

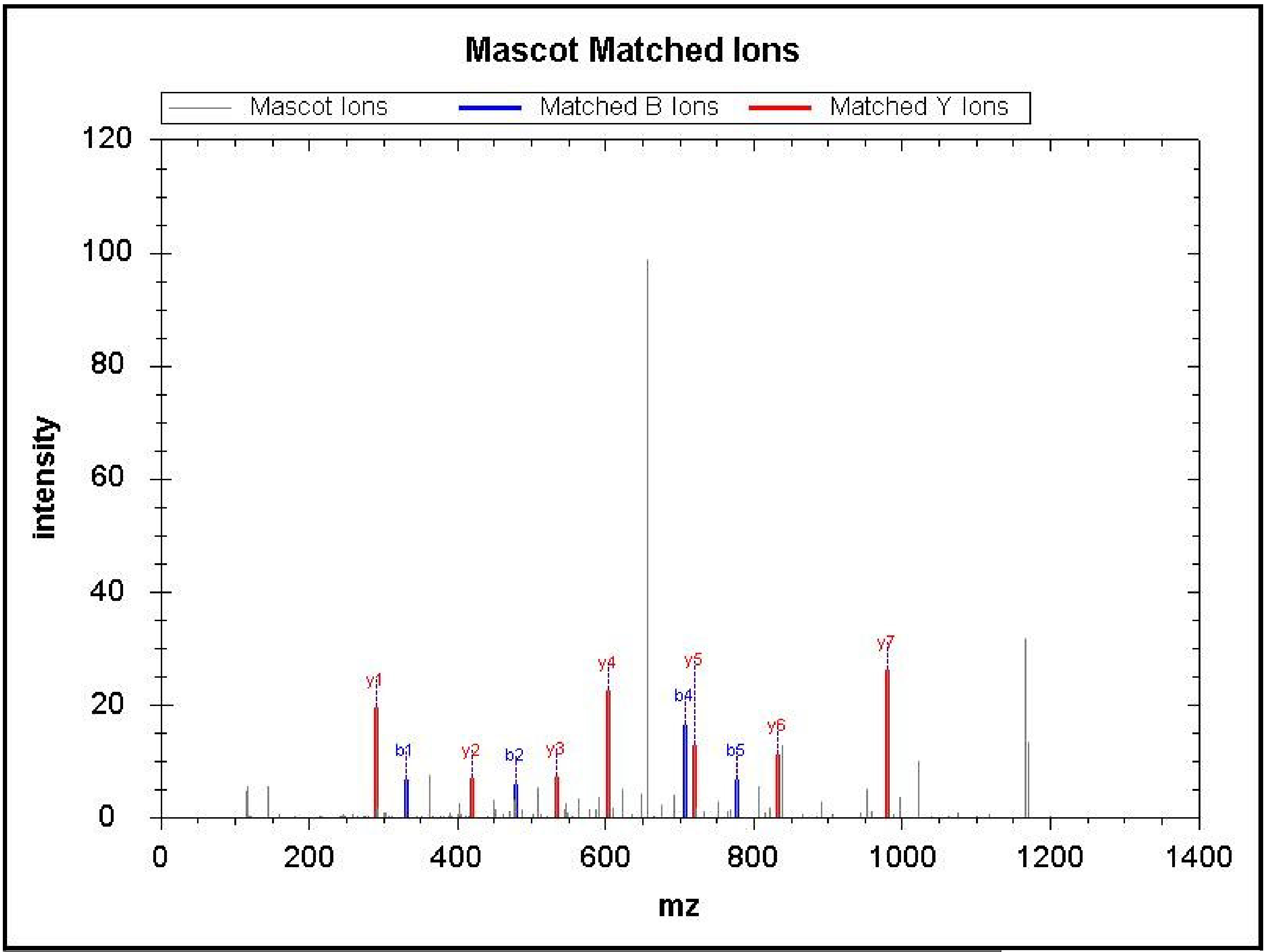

No	b	b++	b0	b0++	Seq	y	y++	y*	y*++	y0	y0++	RevNo
1	331.19	166.10			W							8
2	478.26	239.63			F	979.56	490.28	962.53	481.77	961.55	481.28	7
3	591.34	296.17			L	832.49	416.75	815.46	408.24	814.48	407.74	6
4	706.37	353.69	688.36	344.68	D	719.41	360.21	702.38	351.69	701.39	351.20	5
5	777.41	389.21	759.39	380.20	A	604.38	302.69	587.35	294.18	586.37	293.69	4
6	890.49	445.75	872.48	436.74	L	533.34	267.17	516.31	258.66	515.33	258.17	3
7	1,019.53	510.27	1,001.52	501.26	E	420.26	210.63	403.23	202.12	402.25	201.63	2
8					K	291.21	146.11	274.19	137.60			1

Query 46716 Hit 1

MS/MS Fragmentation of DFPDEVLQLIHER
Found in sp|Q96SZ6|CK5P1_HUMAN, CDK5 regulatory subunit-associated protein 1 OS=Homo sapiens GN=CDK5RAP1 PE=1 SV=2
Match to Query 46716: 1753.911from(585.6442,3+)
Title: 901: Scan 2179 (rt=57.5496, f=2, i=316) [D:\lab212\membrane\Grace\Joyce\iTRAQ_34_1.raw]
Data File:Submitted from 20120508-1(merge) by Mascot Daemon on JOYCE-VAIO

Monoisotopic mass of neutral peptide Mr(calc): 1753.911
Variable modifications:
Ions Score: 43.97 Expect: 0.035

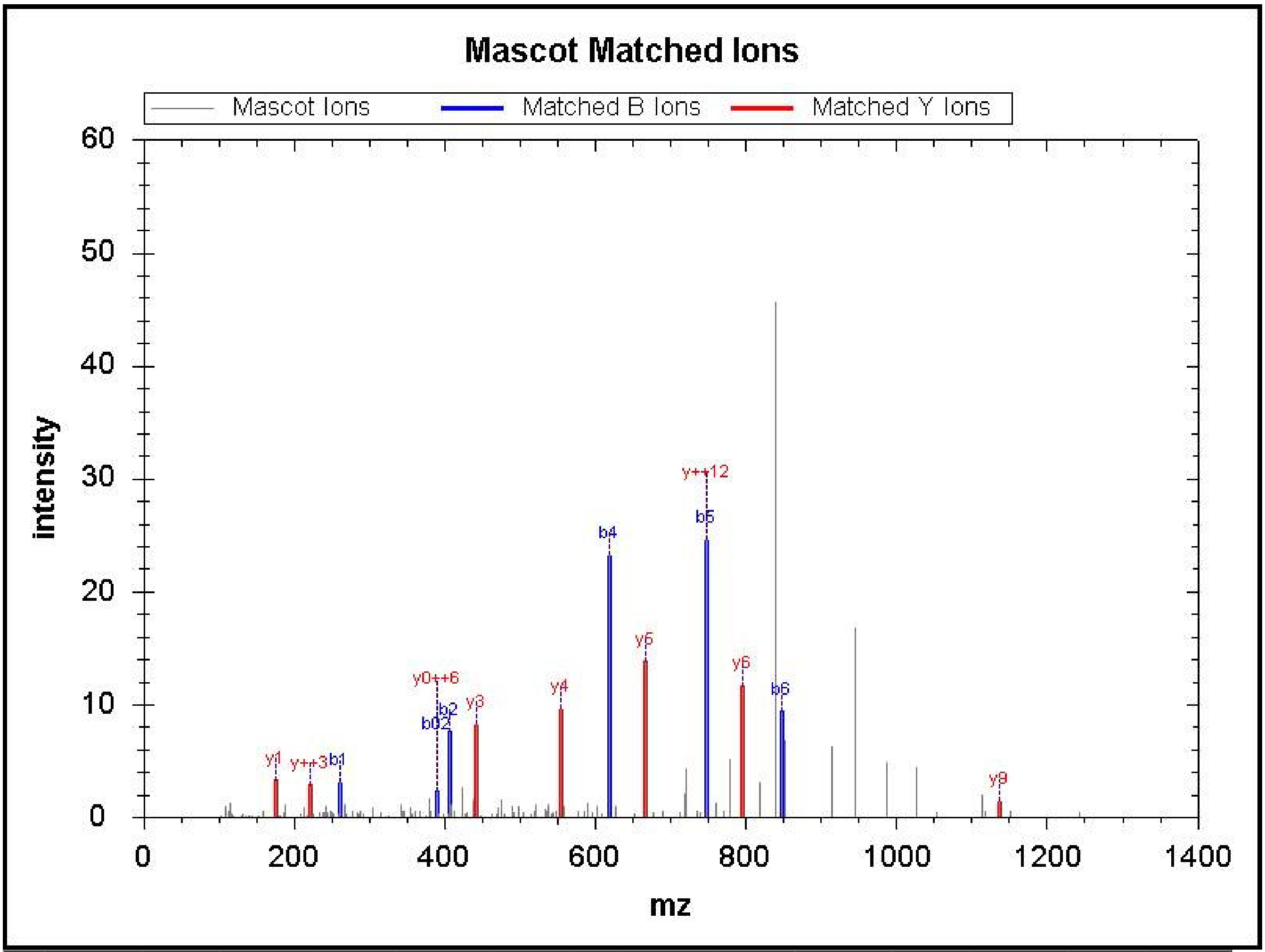

No	b	b++	b*	b*++	b0	b0++	Seq	y	y++	y*	y*++	y0	y0++	RevNo
1	260.14	130.57			242.13	121.57	D							13
2	407.20	204.11			389.19	195.10	F	1,495.79	748.40	1,478.76	739.89	1,477.78	739.39	12
3	504.26	252.63			486.25	243.63	P	1,348.72	674.86	1,331.70	666.35	1,330.71	665.86	11
4	619.28	310.15			601.27	301.14	D	1,251.67	626.34	1,234.64	617.82	1,233.66	617.33	10
5	748.33	374.67			730.32	365.66	E	1,136.64	568.82	1,119.62	560.31	1,118.63	559.82	9
6	847.40	424.20			829.38	415.20	V	1,007.60	504.30	990.57	495.79	989.59	495.30	8
7	960.48	480.74			942.47	471.74	L	908.53	454.77	891.50	446.26	890.52	445.76	7
8	1,088.54	544.77	1,071.51	536.26	1,070.53	535.77	Q	795.45	398.23	778.42	389.71	777.44	389.22	6
9	1,201.62	601.31	1,184.60	592.80	1,183.61	592.31	L	667.39	334.20	650.36	325.68	649.38	325.19	5
10	1,314.71	657.86	1,297.68	649.34	1,296.70	648.85	I	554.30	277.66	537.28	269.14	536.29	268.65	4
11	1,451.77	726.39	1,434.74	717.87	1,433.75	717.38	H	441.22	221.11	424.19	212.60	423.21	212.11	3
12	1,580.81	790.91	1,563.78	782.39	1,562.80	781.90	E	304.16	152.58	287.13	144.07	286.15	143.58	2
13							R	175.12	88.06	158.09	79.55			1

Query 3621 Hit 1

MS/MS Fragmentation of LPSSGSGK
Found in sp|P57078|RIPK4_HUMAN, Receptor-interacting serine/threonine-protein kinase 4 OS=Homo sapiens GN=RIPK4 PE=1 SV=1
Match to Query 3621: 875.527from(438.7708,2+)
Title: 82: Scan 265 (rt=15.4862, f=3, i=31) [D:\lab212\membrane\Grace\Joyce\iTRAQ_34_2.raw]
Data File:Submitted from 20120508-1(merge) by Mascot Daemon on JOYCE-VAIO

Monoisotopic mass of neutral peptide Mr(calc): 875.527
Variable modifications:
Ions Score: 43.87 Expect: 0.037

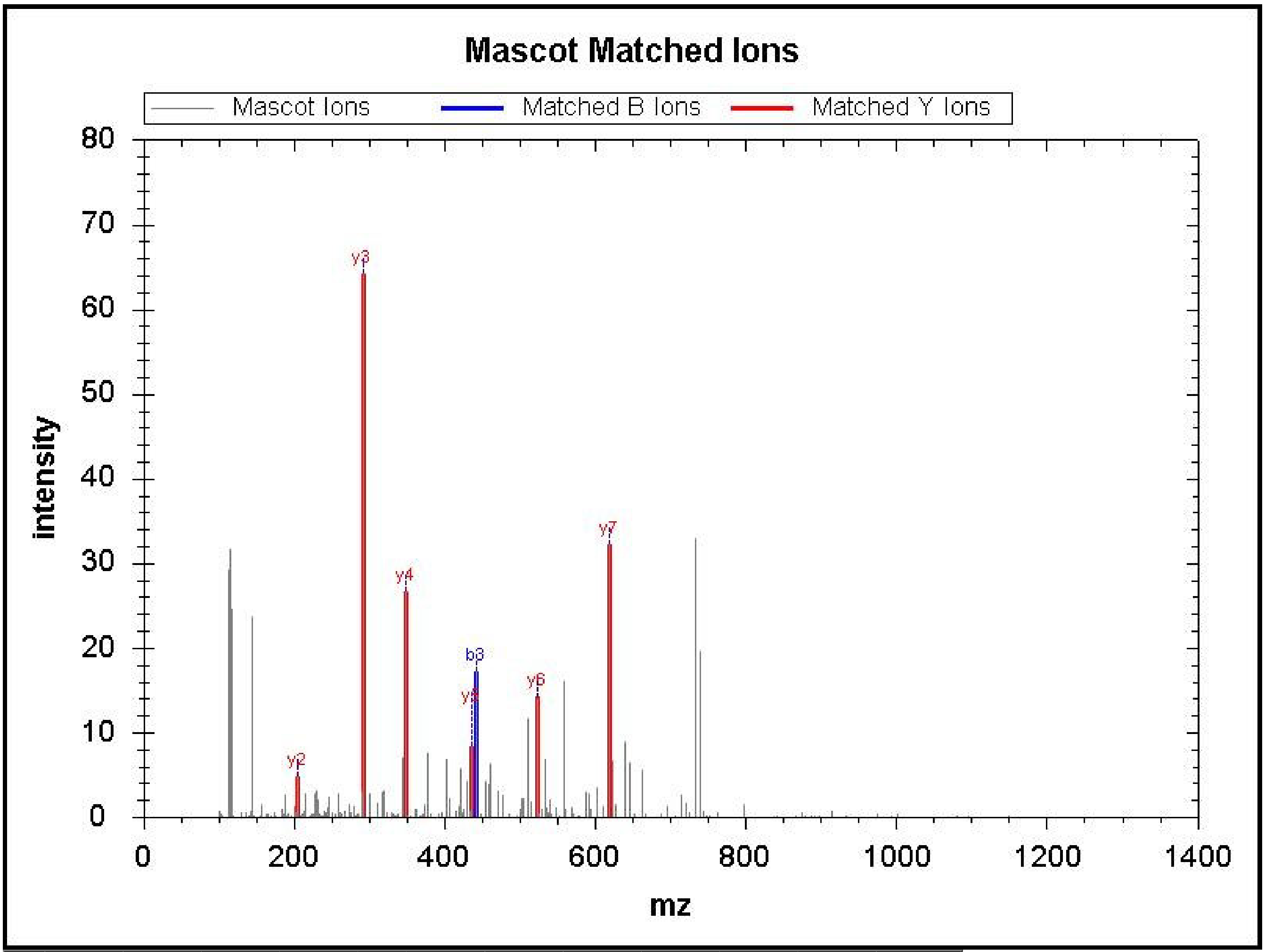

No	b	b++	b0	b0++	Seq	y	y++	y*	y*++	y0	y0++	RevNo
1	258.19	129.60			L							8
2	355.25	178.13			P	619.30	310.16	602.28	301.64	601.29	301.15	7
3	442.28	221.64	424.27	212.64	S	522.25	261.63	505.23	253.12	504.24	252.62	6
4	529.31	265.16	511.30	256.15	S	435.22	218.11	418.19	209.60	417.21	209.11	5
5	586.33	293.67	568.32	284.66	G	348.19	174.60	331.16	166.08	330.18	165.59	4
6	673.36	337.19	655.35	328.18	S	291.17	146.09	274.14	137.57	273.16	137.08	3
7	730.39	365.70	712.37	356.69	G	204.13	102.57	187.11	94.06			2
8					K	147.11	74.06	130.09	65.55			1

Query 6566 Hit 1

MS/MS Fragmentation of GTSSFGK
Found in sp|P61927|RL37_HUMAN, 60S ribosomal protein L37 OS=Homo sapiens GN=RPL37 PE=1 SV=2
Match to Query 6566: 970.5325 from(486.2735,2+)
Title: 77: Scan 253 (rt=15.2147, f=3, i=29) [D:\lab212\membrane\Grace\Joyce\iTRAQ_34_2.raw]
Data File:Submitted from 20120508-1(merge) by Mascot Daemon on JOYCE-VAIO
Monoisotopic mass of neutral peptide Mr(calc): 970.5325
Variable modifications:
K7 :iTRAQ4plex (K)
Ions Score: 43.63 Expect: 0.022

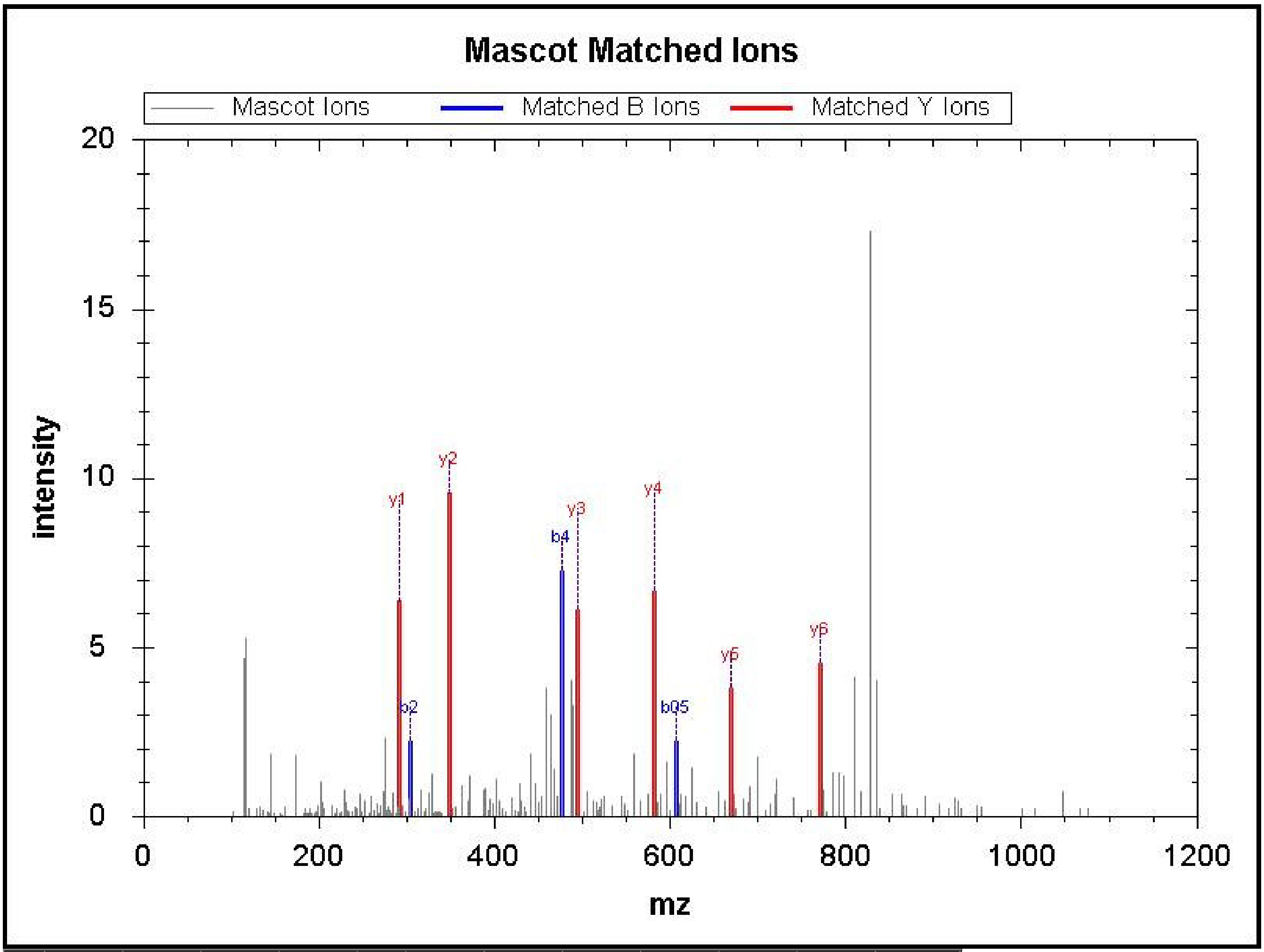

No	b	b++	b0	b0++	Seq	y	y++	y*	y*++	y0	y0++	RevNo
1	202.13	101.57			G							7
2	303.18	152.09	285.17	143.09	T	770.42	385.71	753.39	377.20	752.41	376.71	6
3	390.21	195.61	372.20	186.60	S	669.37	335.19	652.34	326.67	651.36	326.18	5
4	477.24	239.12	459.23	230.12	S	582.34	291.67	565.31	283.16	564.33	282.67	4
5	624.31	312.66	606.30	303.65	F	495.30	248.16	478.28	239.64			3
6	681.33	341.17	663.32	332.16	G	348.24	174.62	331.21	166.11			2
7					K	291.21	146.11	274.19	137.60			1

Query 13672 Hit 1

MS/MS Fragmentation of AGIFQSVK
Found in sp|P09669|COX6C_HUMAN, Cytochrome c oxidase subunit 6C OS=Homo sapiens GN=COX6C PE=1 SV=2
Match to Query 13672: 1136.68from(569.3474,2+)
Title: 382: Sum of 2 scans in range 912 (rt=29.9613, f=4, i=251) to 913 (rt=29.9867, f=4, i=252) [D:\lab212\membrane\Grace\Joyce\iTRAQ_32_2.raw]
Data File:Submitted from 20120508-1(merge) by Mascot Daemon on JOYCE-VAIO
Monoisotopic mass of neutral peptide Mr(calc): 1136.68
Variable modifications:
K8 :iTRAQ4plex (K)
Ions Score: 43.63 Expect: 0.025

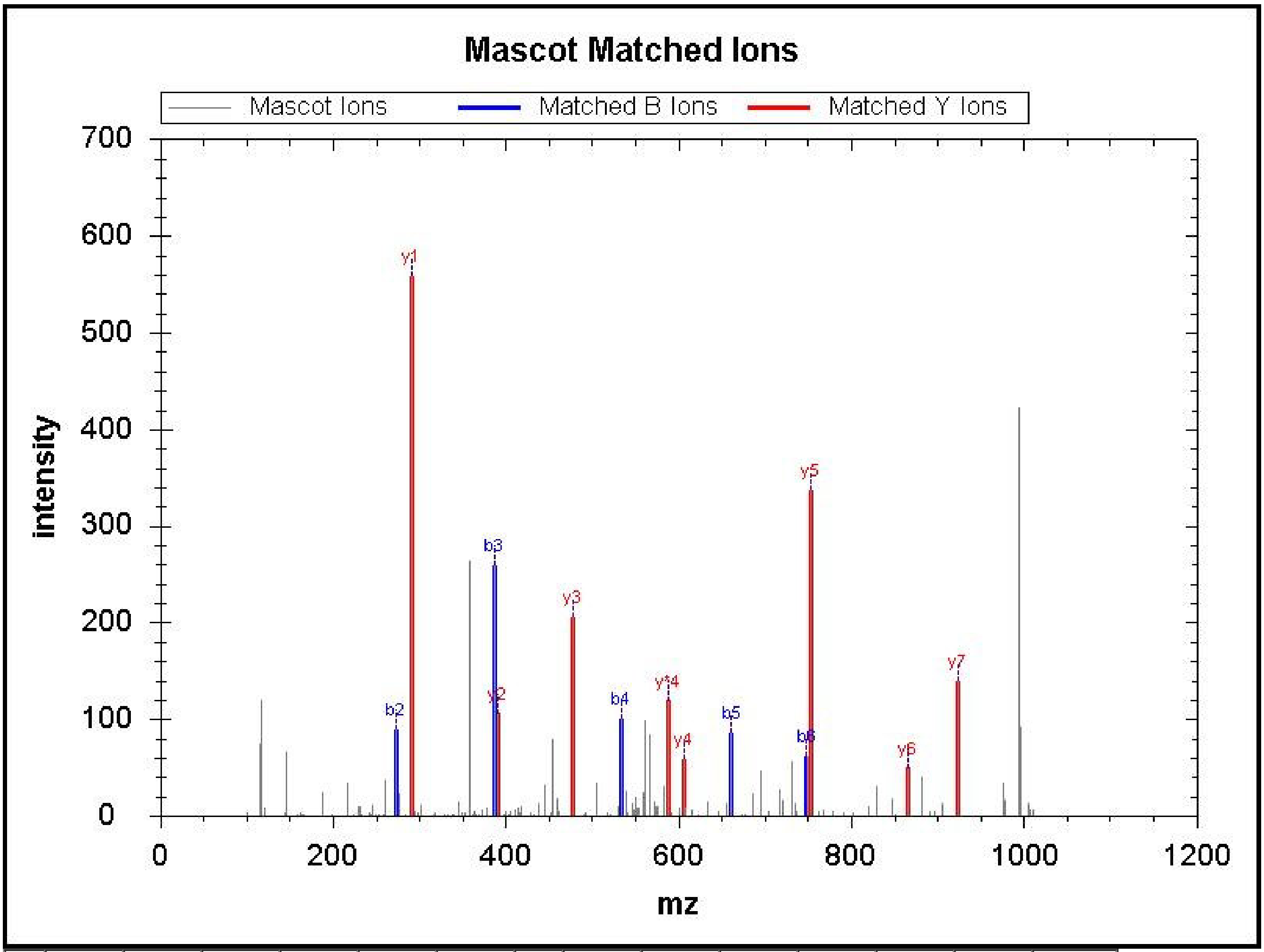

No	b	b++	b*	b*++	b0	b0++	Seq	y	y++	y*	y*++	y0	y0++	RevNo
1	216.15	108.58					A							8
2	273.17	137.09					G	922.55	461.78	905.52	453.26	904.54	452.77	7
3	386.25	193.63					I	865.53	433.27	848.50	424.75	847.52	424.26	6
4	533.32	267.16					F	752.44	376.72	735.42	368.21	734.43	367.72	5
5	661.38	331.19	644.35	322.68			Q	605.37	303.19	588.35	294.68	587.36	294.19	4
6	748.41	374.71	731.38	366.20	730.40	365.70	S	477.32	239.16	460.29	230.65	459.30	230.16	3
7	847.48	424.24	830.45	415.73	829.47	415.24	V	390.28	195.65	373.26	187.13			2
8							K	291.21	146.11	274.19	137.60			1

Query 9047 Hit 1

MS/MS Fragmentation of MLGGVFK
Found in sp|Q96MU7|YTDC1_HUMAN, YTH domain-containing protein 1 OS=Homo sapiens GN=YTHDC1 PE=1 SV=3
Match to Query 9047: 1038.614from(520.3143,2+)
Title: 533: Scan 1240 (rt=37.3482, f=3, i=185) [D:\lab212\membrane\Grace\Joyce\iTRAQ_32_2.raw]
Data File:Submitted from 20120508-1(merge) by Mascot Daemon on JOYCE-VAIO
Monoisotopic mass of neutral peptide Mr(calc): 1038.614
Variable modifications:
K7 :iTRAQ4plex (K)
Ions Score: 43.53 Expect: 0.022

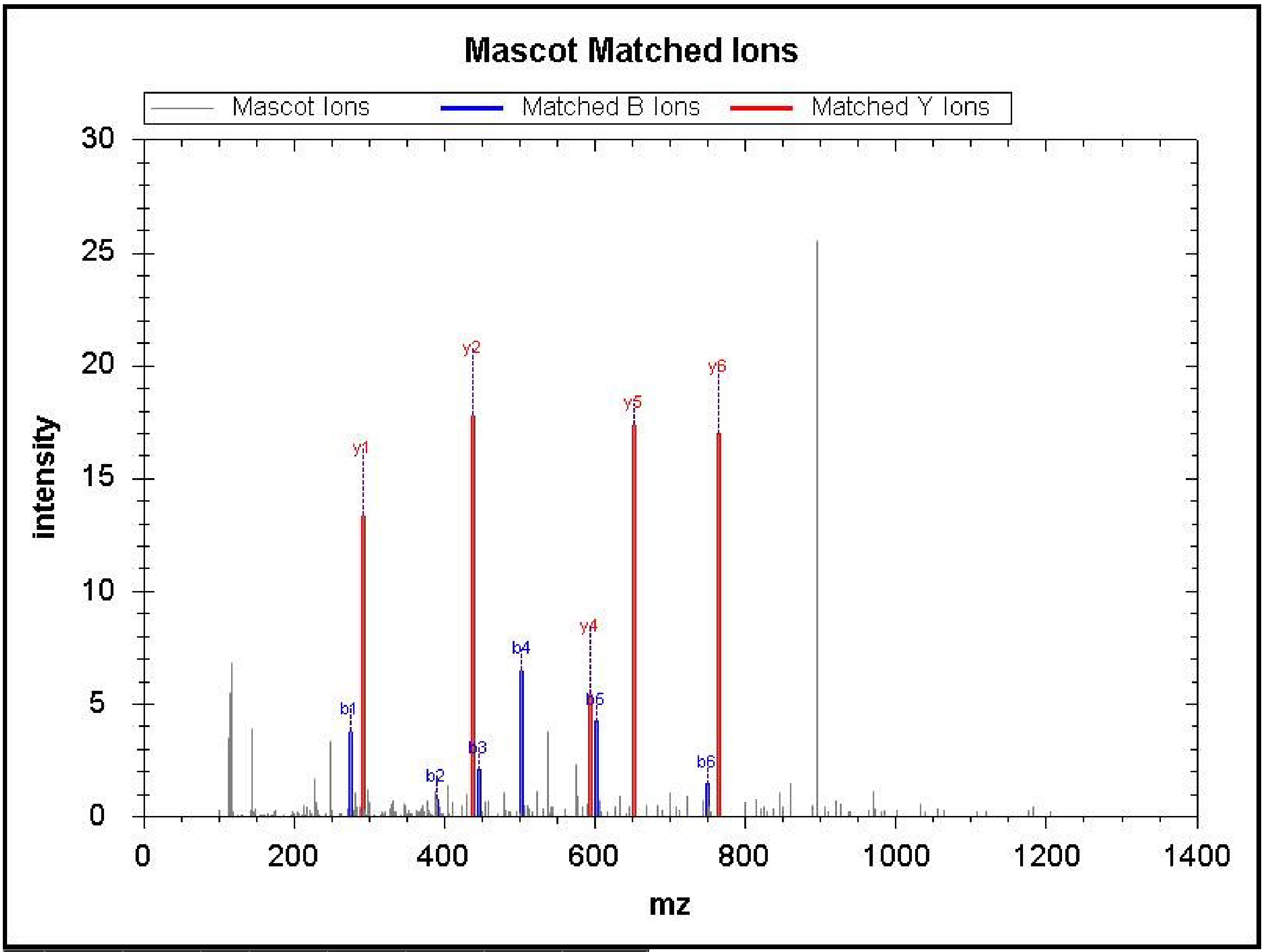

No	b	b++	Seq	y	y++	y*	y*++	RevNo
1	276.15	138.58	M					7
2	389.23	195.12	L	764.48	382.74	747.45	374.23	6
3	446.26	223.63	G	651.39	326.20	634.37	317.69	5
4	503.28	252.14	G	594.37	297.69	577.35	289.18	4
5	602.35	301.68	V	537.35	269.18	520.33	260.67	3
6	749.41	375.21	F	438.28	219.65	421.26	211.13	2
7			K	291.21	146.11	274.19	137.60	1

Query 74786 Hit 1

MS/MS Fragmentation of HVVLPVDDDSDLNVVASFDR
Found in sp|Q15291|RBBP5_HUMAN, Retinoblastoma-binding protein 5 OS=Homo sapiens GN=RBBP5 PE=1 SV=2
Match to Query 74786: 2355.182from(786.068,3+)
Title: 771: Sum of 2 scans in range 1705 (rt=47.9352, f=4, i=513) to 1706 (rt=47.9607, f=4, i=514) [D:\lab212\membrane\Grace\Joyce\iTRAQ_34_2.raw]
Data File:Submitted from 20120508-1(merge) by Mascot Daemon on JOYCE-VAIO
Monoisotopic mass of neutral peptide Mr(calc): 2355.182
Variable modifications:
Ions Score: 43.52 Expect: 0.043

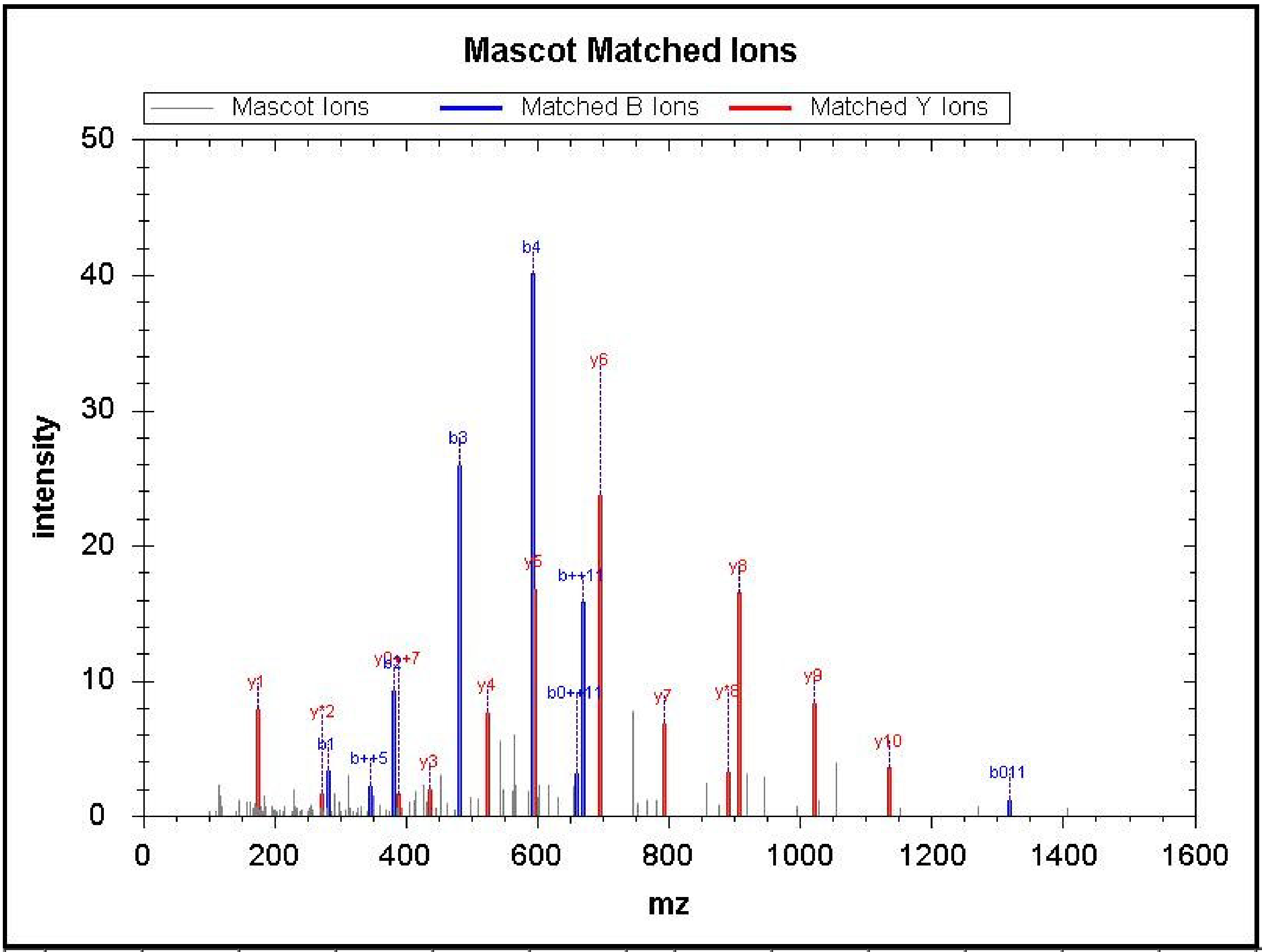

No	b	b++	b*	b*++	b0	b0++	Seq	y	y++	y*	y*++	y0	y0++	RevNo
1	282.17	141.59					H							20
2	381.24	191.12					V	2,075.03	1,038.02	2,058.00	1,029.50	2,057.02	1,029.01	19
3	480.31	240.66					V	1,975.96	988.48	1,958.93	979.97	1,957.95	979.48	18
4	593.39	297.20					L	1,876.89	938.95	1,859.87	930.44	1,858.88	929.94	17
5	690.44	345.72					P	1,763.81	882.41	1,746.78	873.89	1,745.80	873.40	16
6	789.51	395.26					V	1,666.76	833.88	1,649.73	825.37	1,648.74	824.88	15
7	904.54	452.77			886.53	443.77	D	1,567.69	784.35	1,550.66	775.83	1,549.68	775.34	14
8	1,019.56	510.29			1,001.55	501.28	D	1,452.66	726.83	1,435.63	718.32	1,434.65	717.83	13
9	1,134.59	567.80			1,116.58	558.79	D	1,337.63	669.32	1,320.61	660.81	1,319.62	660.31	12
10	1,221.62	611.32			1,203.61	602.31	S	1,222.61	611.81	1,205.58	603.29	1,204.60	602.80	11
11	1,336.65	668.83			1,318.64	659.82	D	1,135.57	568.29	1,118.55	559.78	1,117.56	559.29	10
12	1,449.73	725.37			1,431.72	716.37	L	1,020.55	510.78	1,003.52	502.26	1,002.54	501.77	9
13	1,563.78	782.39	1,546.75	773.88	1,545.77	773.39	N	907.46	454.24	890.44	445.72	889.45	445.23	8
14	1,662.85	831.93	1,645.82	823.41	1,644.83	822.92	V	793.42	397.21	776.39	388.70	775.41	388.21	7
15	1,761.91	881.46	1,744.89	872.95	1,743.90	872.46	V	694.35	347.68	677.33	339.17	676.34	338.67	6
16	1,832.95	916.98	1,815.92	908.47	1,814.94	907.97	A	595.28	298.15	578.26	289.63	577.27	289.14	5
17	1,919.98	960.50	1,902.96	951.98	1,901.97	951.49	S	524.25	262.63	507.22	254.11	506.24	253.62	4
18	2,067.05	1,034.03	2,050.02	1,025.52	2,049.04	1,025.02	F	437.21	219.11	420.19	210.60	419.20	210.11	3
19	2,182.08	1,091.54	2,165.05	1,083.03	2,164.07	1,082.54	D	290.15	145.58	273.12	137.06	272.14	136.57	2
20							R	175.12	88.06	158.09	79.55			1

Query 17872 Hit 1

MS/MS Fragmentation of SIKTRLGR

Found in sp|A8MXV6|CD15L_HUMAN, CMT1A duplicated region transcript 15 protein-like protein OS=Homo sapiens GN=CDRT15L2 PE=2 SV=1
Match to Query 17872: 1217.727from(609.8707,2+)
Title: 797: Sum of 2 scans in range 1811 (rt=50.1527, f=4, i=525) to 1812 (rt=50.1781, f=4, i=526) [D:\lab212\membrane\Grace\Joyce\iTRAQ_25_2.raw]
Data File:Submitted from 20120508-1(merge) by Mascot Daemon on JOYCE-VAIO
Monoisotopic mass of neutral peptide Mr(calc): 1217.727
Variable modifications:
K3 :iTRAQ4plex (K)
Ions Score: 43.46 Expect: 0.032

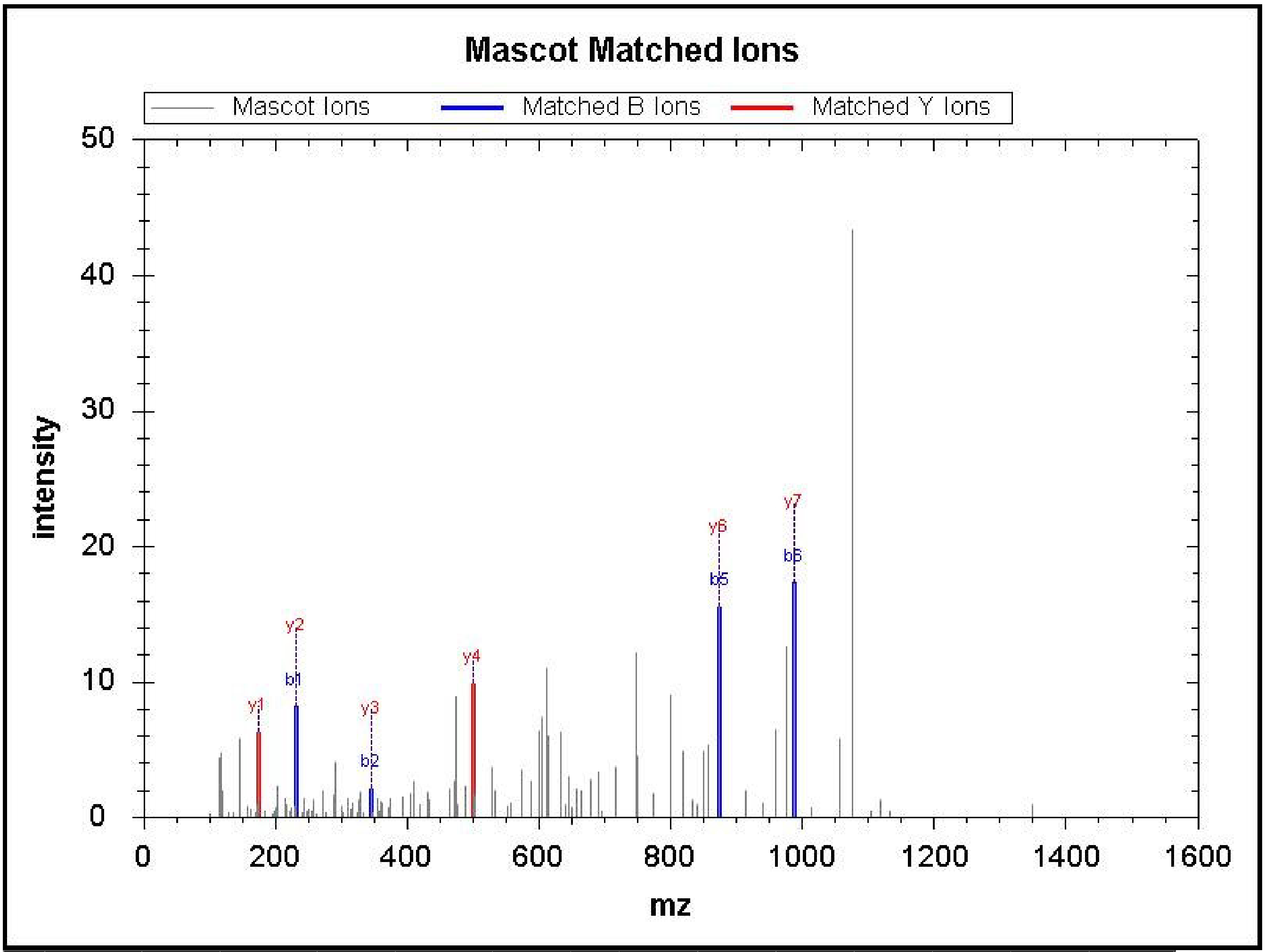

No	b	b++	b*	b*++	b0	b0++	Seq	y	y++	y*	y*++	y0	y0++	RevNo
1	232.14	116.57			214.13	107.57	S							8
2	345.23	173.12			327.21	164.11	I	987.65	494.33	970.63	485.82	969.64	485.33	7
3	617.42	309.21	600.40	300.70	599.41	300.21	K	874.57	437.79	857.54	429.28	856.56	428.78	6
4	718.47	359.74	701.44	351.23	700.46	350.73	T	602.37	301.69	585.35	293.18	584.36	292.68	5
5	874.57	437.79	857.54	429.28	856.56	428.78	R	501.33	251.17	484.30	242.65			4
6	987.66	494.33	970.63	485.82	969.64	485.33	L	345.22	173.12	328.20	164.60			3
7	1,044.68	522.84	1,027.65	514.33	1,026.67	513.84	G	232.14	116.57	215.11	108.06			2
8							R	175.12	88.06	158.09	79.55			1

Query 90838 Hit 1

MS/MS Fragmentation of VLSVDESIIKPEQEFFTAPFEK
Found in sp|Q4KMQ2|ANO6_HUMAN, Anoctamin-6 OS=Homo sapiens GN=ANO6 PE=1 SV=2
Match to Query 90838: 2984.604from(747.1582,4+)
Title: 967: Scan 2141 (rt=57.6398, f=2, i=338) [D:\lab212\membrane\Grace\Joyce\iTRAQ_36_2.raw]
Data File:Submitted from 20120508-1(merge) by Mascot Daemon on JOYCE-VAIO
Monoisotopic mass of neutral peptide Mr(calc): 2984.604
Variable modifications:

K10 :iTRAQ4plex (K)
K22 :iTRAQ4plex (K)
Ions Score: 43.4 Expect: 0.026

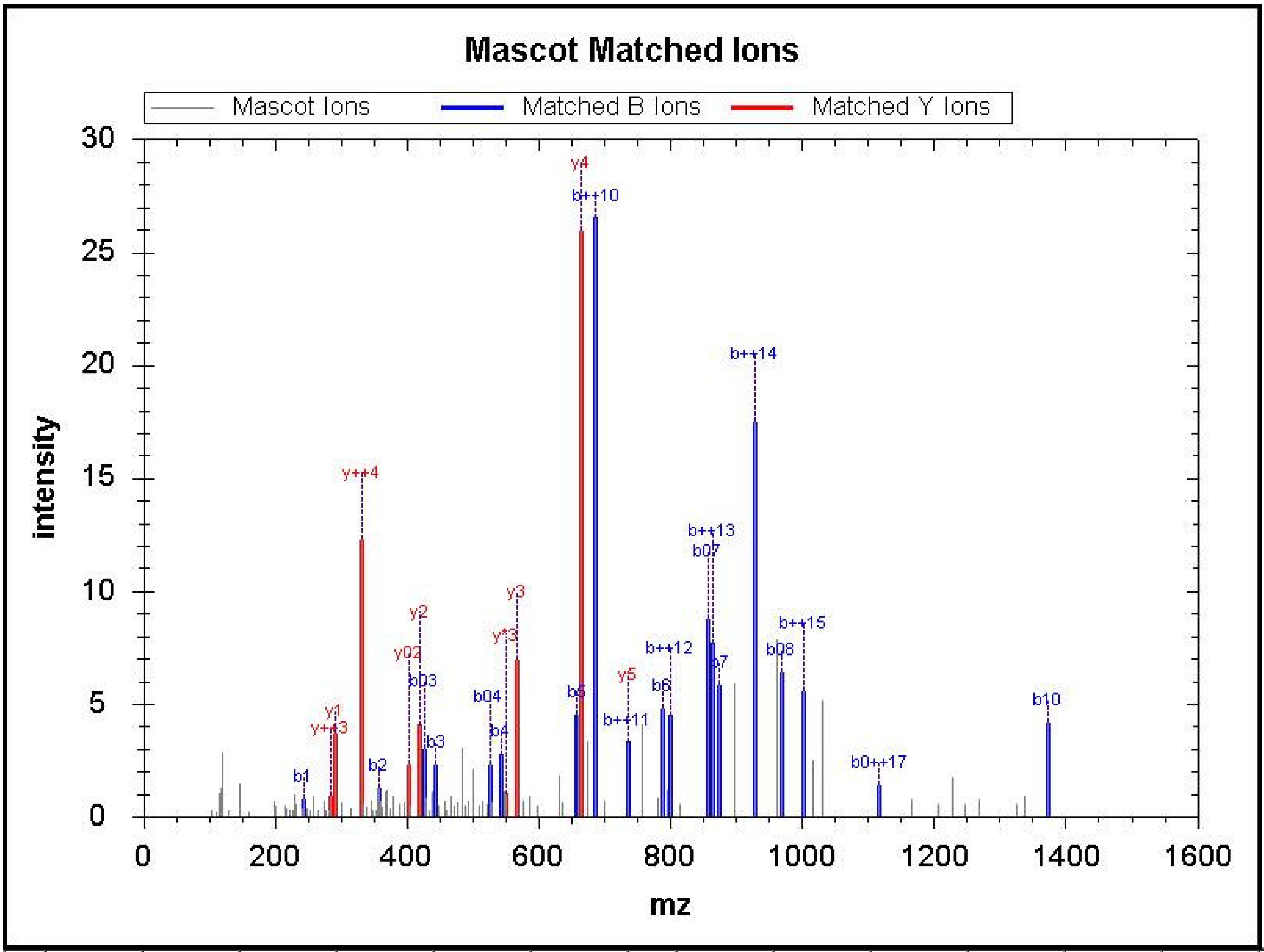

No	b	b++	b*	b*++	b0	b0++	Seq	y	y++	y*	y*++	y0	y0++	RevNo
1	244.18	122.59					V							22
2	357.26	179.13					L	2,742.45	1,371.73	2,725.42	1,363.21	2,724.44	1,362.72	21
3	444.29	222.65			426.28	213.65	S	2,629.36	1,315.19	2,612.34	1,306.67	2,611.35	1,306.18	20
4	543.36	272.18			525.35	263.18	V	2,542.33	1,271.67	2,525.31	1,263.16	2,524.32	1,262.66	19
5	658.39	329.70			640.38	320.69	D	2,443.26	1,222.14	2,426.24	1,213.62	2,425.25	1,213.13	18
6	787.43	394.22			769.42	385.21	E	2,328.24	1,164.62	2,311.21	1,156.11	2,310.23	1,155.62	17
7	874.46	437.74			856.45	428.73	S	2,199.19	1,100.10	2,182.17	1,091.59	2,181.18	1,091.10	16
8	987.55	494.28			969.54	485.27	I	2,112.16	1,056.58	2,095.14	1,048.07	2,094.15	1,047.58	15
9	1,100.63	550.82			1,082.62	541.81	I	1,999.08	1,000.04	1,982.05	991.53	1,981.07	991.04	14
10	1,372.83	686.92	1,355.80	678.40	1,354.82	677.91	K	1,885.99	943.50	1,868.97	934.99	1,867.98	934.50	13
11	1,469.88	735.44	1,452.86	726.93	1,451.87	726.44	P	1,613.80	807.40	1,596.77	798.89	1,595.79	798.40	12
12	1,598.92	799.97	1,581.90	791.45	1,580.91	790.96	E	1,516.74	758.88	1,499.72	750.36	1,498.73	749.87	11
13	1,726.98	864.00	1,709.96	855.48	1,708.97	854.99	Q	1,387.70	694.35	1,370.68	685.84	1,369.69	685.35	10
14	1,856.03	928.52	1,839.00	920.00	1,838.01	919.51	E	1,259.64	630.33	1,242.62	621.81	1,241.63	621.32	9
15	2,003.09	1,002.05	1,986.07	993.54	1,985.08	993.05	F	1,130.60	565.80	1,113.57	557.29	1,112.59	556.80	8
16	2,150.16	1,075.58	2,133.14	1,067.07	2,132.15	1,066.58	F	983.53	492.27	966.51	483.76	965.52	483.26	7
17	2,251.21	1,126.11	2,234.18	1,117.60	2,233.20	1,117.10	T	836.46	418.74	819.44	410.22	818.45	409.73	6
18	2,322.25	1,161.63	2,305.22	1,153.11	2,304.24	1,152.62	A	735.42	368.21	718.39	359.70	717.41	359.21	5
19	2,419.30	1,210.15	2,402.27	1,201.64	2,401.29	1,201.15	P	664.38	332.69	647.35	324.18	646.37	323.69	4
20	2,566.37	1,283.69	2,549.34	1,275.17	2,548.36	1,274.68	F	567.33	284.17	550.30	275.65	549.32	275.16	3
21	2,695.41	1,348.21	2,678.38	1,339.70	2,677.40	1,339.20	E	420.26	210.63	403.23	202.12	402.25	201.63	2

22							K	291.21	146.11	274.19	137.60			1

Query 78638 Hit 1

MS/MS Fragmentation of DLHEEMNYITAIIEEQPK
Found in sp|P49354|FNTA_HUMAN, Protein farnesyltransferase/geranylgeranyltransferase type-1 subunit alpha OS=Homo sapiens GN=FNTA PE=1 SV=1
Match to Query 78638: 2460.249from(821.0902,3+)
Title: 978: Sum of 2 scans in range 2177 (rt=58.2996, f=4, i=633) to 2178 (rt=58.325, f=4, i=634) [D:\lab212\membrane\Grace\Joyce\iTRAQ_38_2.raw]
Data File:Submitted from 20120508-1(merge) by Mascot Daemon on JOYCE-VAIO
Monoisotopic mass of neutral peptide Mr(calc): 2460.249
Variable modifications:
K18 :iTRAQ4plex (K)
Ions Score: 43.35 Expect: 0.045

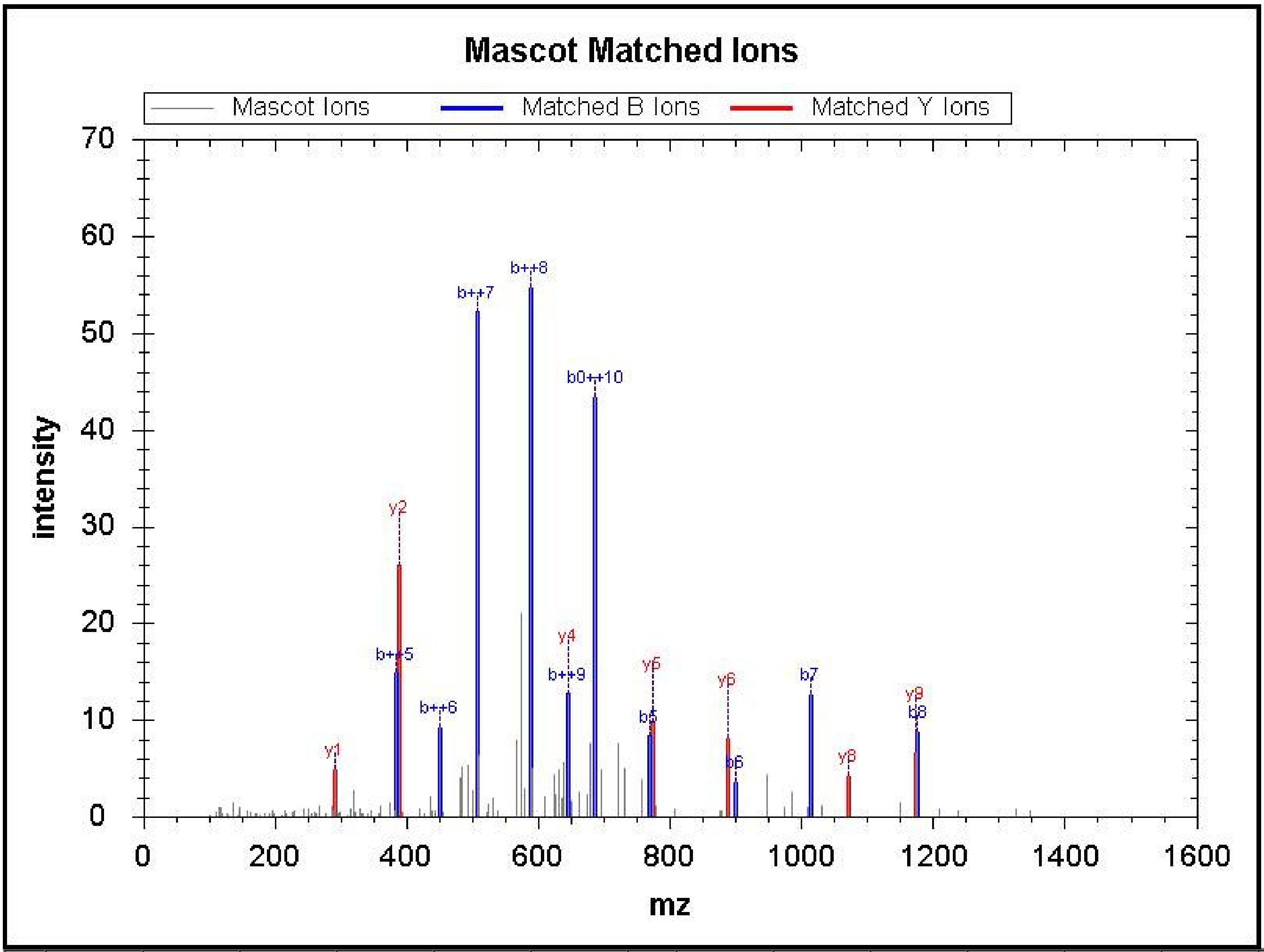

No	b	b++	b*	b*++	b0	b0++	Seq	y	y++	y*	y*++	y0	y0++	RevNo
1	260.14	130.57			242.13	121.57	D							18
2	373.22	187.11			355.21	178.11	L	2,202.12	1,101.57	2,185.10	1,093.05	2,184.11	1,092.56	17
3	510.28	255.64			492.27	246.64	H	2,089.04	1,045.02	2,072.01	1,036.51	2,071.03	1,036.02	16
4	639.32	320.16			621.31	311.16	E	1,951.98	976.49	1,934.95	967.98	1,933.97	967.49	15
5	768.36	384.69			750.35	375.68	E	1,822.94	911.97	1,805.91	903.46	1,804.93	902.97	14
6	899.40	450.21			881.39	441.20	M	1,693.90	847.45	1,676.87	838.94	1,675.88	838.45	13
7	1,013.45	507.23	996.42	498.71	995.44	498.22	N	1,562.85	781.93	1,545.83	773.42	1,544.84	772.93	12
8	1,176.51	588.76	1,159.48	580.25	1,158.50	579.75	Y	1,448.81	724.91	1,431.79	716.40	1,430.80	715.90	11
9	1,289.60	645.30	1,272.57	636.79	1,271.58	636.30	I	1,285.75	643.38	1,268.72	634.86	1,267.74	634.37	10
10	1,390.64	695.83	1,373.62	687.31	1,372.63	686.82	T	1,172.66	586.84	1,155.64	578.32	1,154.65	577.83	9
11	1,461.68	731.34	1,444.65	722.83	1,443.67	722.34	A	1,071.62	536.31	1,054.59	527.80	1,053.61	527.31	8
12	1,574.76	787.89	1,557.74	779.37	1,556.75	778.88	I	1,000.58	500.79	983.55	492.28	982.57	491.79	7

13	1,687.85	844.43	1,670.82	835.91	1,669.84	835.42	I	887.50	444.25	870.47	435.74	869.48	435.25	6
14	1,816.89	908.95	1,799.86	900.44	1,798.88	899.94	E	774.41	387.71	757.38	379.20	756.40	378.70	5
15	1,945.93	973.47	1,928.91	964.96	1,927.92	964.47	E	645.37	323.19	628.34	314.67	627.36	314.18	4
16	2,073.99	1,037.50	2,056.97	1,028.99	2,055.98	1,028.49	Q	516.33	258.67	499.30	250.15			3
17	2,171.04	1,086.03	2,154.02	1,077.51	2,153.03	1,077.02	P	388.27	194.64	371.24	186.12			2
18							K	291.21	146.11	274.19	137.60			1

Query 71359 Hit 1

MS/MS Fragmentation of QRPADAEDLPAAPGQSIDK
Found in sp|Q8N6R0|MTL13_HUMAN, Methyltransferase-like protein 13 OS=Homo sapiens GN=METTL13 PE=1 SV=1
Match to Query 71359: 2266.171 from(756.3976,3+)
Title: 339: Sum of 2 scans in range 772 (rt=27.1417, f=4, i=229) to 773 (rt=27.1671, f=4, i=230)
[D:\lab212\membrane\Grace\Joyce\iTRAQ40.raw]
Data File:Submitted from 20120508-1(merge) by Mascot Daemon on JOYCE-VAIO
Monoisotopic mass of neutral peptide Mr(calc): 2266.171
Variable modifications:
K19 :iTRAQ4plex (K)
Ions Score: 43.32 Expect: 0.044

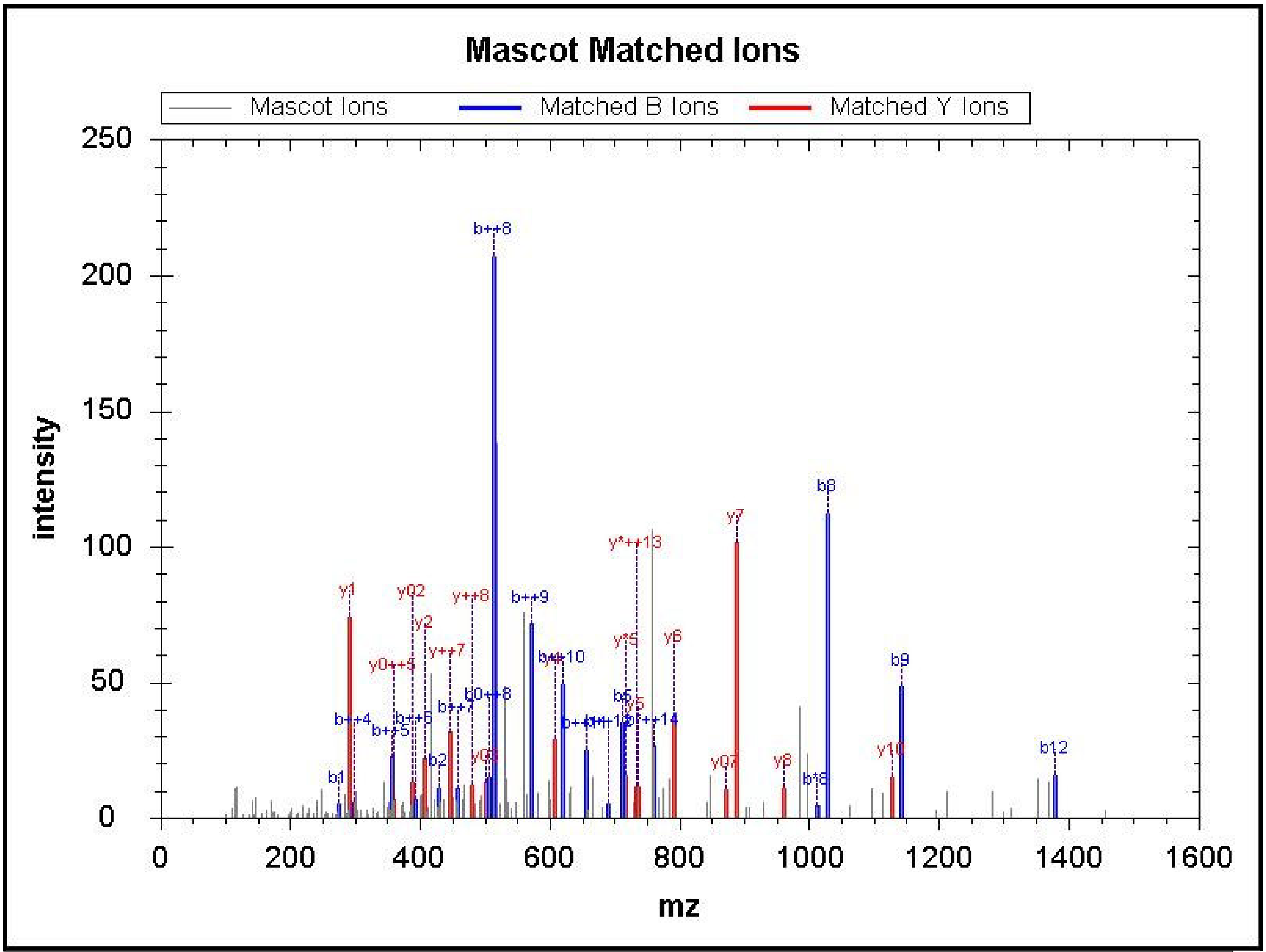

No	b	b++	b*	b*++	b0	b0++	Seq	y	y++	y*	y*++	y0	y0++	RevNo
1	273.17	137.09	256.14	128.57			Q							19
2	429.27	215.14	412.24	206.62			R	1,995.03	998.02	1,978.00	989.50	1,977.02	989.01	18
3	526.32	263.66	509.30	255.15			P	1,838.93	919.97	1,821.90	911.45	1,820.91	910.96	17
4	597.36	299.18	580.33	290.67			A	1,741.87	871.44	1,724.85	862.93	1,723.86	862.43	16
5	712.39	356.70	695.36	348.18	694.38	347.69	D	1,670.84	835.92	1,653.81	827.41	1,652.82	826.92	15
6	783.42	392.22	766.40	383.70	765.41	383.21	A	1,555.81	778.41	1,538.78	769.89	1,537.80	769.40	14
7	912.47	456.74	895.44	448.22	894.45	447.73	E	1,484.77	742.89	1,467.74	734.38	1,466.76	733.88	13

8	1,027.49	514.25	1,010.47	505.74	1,009.48	505.24	D	1,355.73	678.37	1,338.70	669.85	1,337.72	669.36	12
9	1,140.58	570.79	1,123.55	562.28	1,122.57	561.79	L	1,240.70	620.85	1,223.68	612.34	1,222.69	611.85	11
10	1,237.63	619.32	1,220.60	610.81	1,219.62	610.31	P	1,127.62	564.31	1,110.59	555.80	1,109.61	555.31	10
11	1,308.67	654.84	1,291.64	646.32	1,290.66	645.83	A	1,030.56	515.79	1,013.54	507.27	1,012.55	506.78	9
12	1,379.70	690.36	1,362.68	681.84	1,361.69	681.35	A	959.53	480.27	942.50	471.75	941.52	471.26	8
13	1,476.76	738.88	1,459.73	730.37	1,458.75	729.88	P	888.49	444.75	871.46	436.24	870.48	435.74	7
14	1,533.78	767.39	1,516.75	758.88	1,515.77	758.39	G	791.44	396.22	774.41	387.71	773.43	387.22	6
15	1,661.84	831.42	1,644.81	822.91	1,643.83	822.42	Q	734.42	367.71	717.39	359.20	716.41	358.71	5
16	1,748.87	874.94	1,731.84	866.42	1,730.86	865.93	S	606.36	303.68	589.33	295.17	588.35	294.68	4
17	1,861.95	931.48	1,844.93	922.97	1,843.94	922.47	I	519.33	260.17	502.30	251.65	501.32	251.16	3
18	1,976.98	988.99	1,959.95	980.48	1,958.97	979.99	D	406.24	203.62	389.22	195.11	388.23	194.62	2
19							K	291.21	146.11	274.19	137.60			1

Query 64947 Hit 1

MS/MS Fragmentation of VQVVDEEGDQQHQEGK
Found in sp|Q02447|SP3_HUMAN, Transcription factor Sp3 OS=Homo sapiens GN=SP3 PE=1 SV=3
Match to Query 64947: 2112.036from(705.0193,3+)
Title: 194: Sum of 2 scans in range 477 (rt=20.4235, f=4, i=125) to 478 (rt=20.449, f=4, i=126) [D:\lab212\membrane\Grace\Joyce\iTRAQ_40_1.raw]
Data File:Submitted from 20120508-1(merge) by Mascot Daemon on JOYCE-VAIO
Monoisotopic mass of neutral peptide Mr(calc): 2112.036
Variable modifications:
K16 :iTRAQ4plex (K)
Ions Score: 43.32 Expect: 0.041

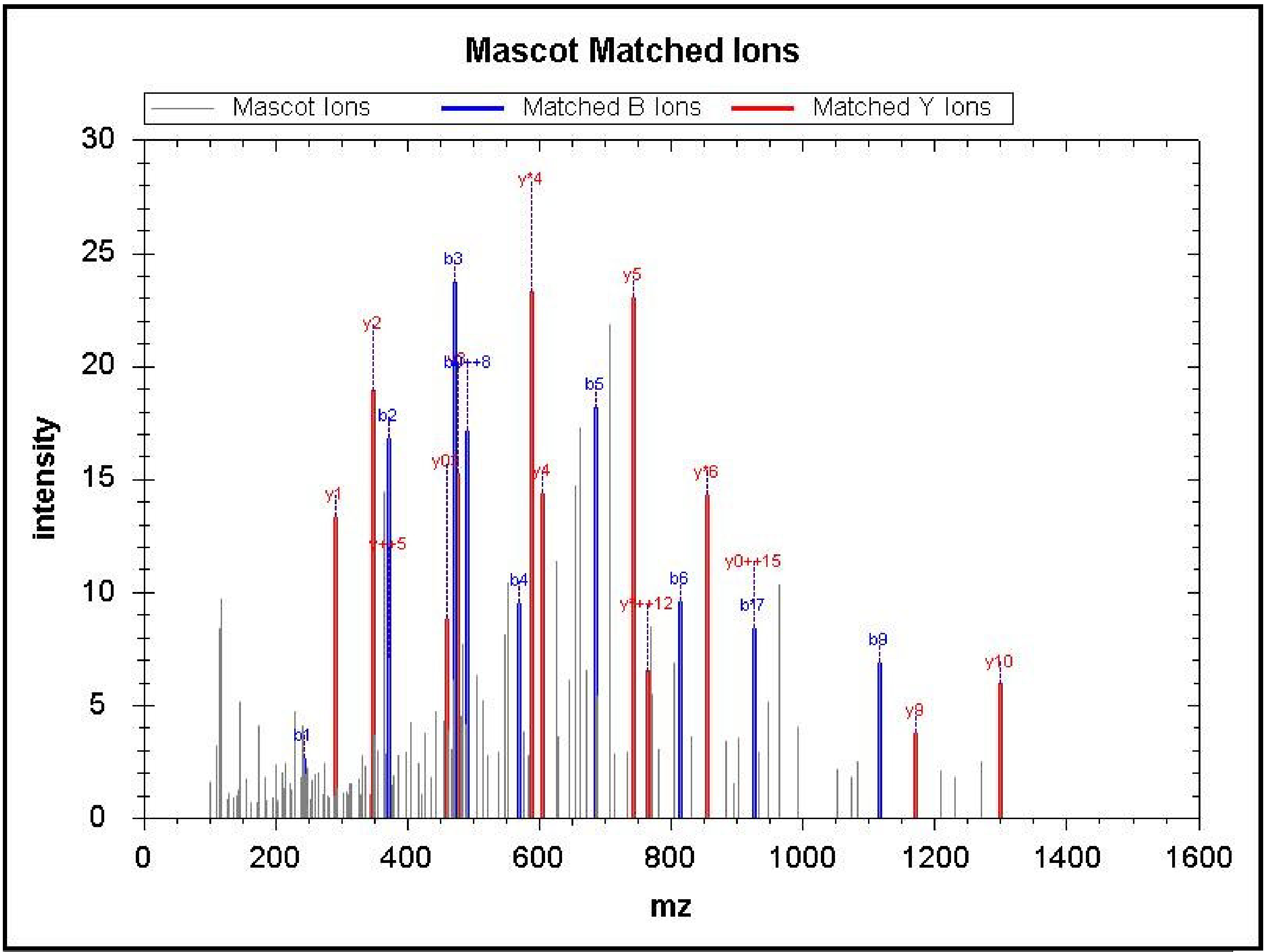

No	b	b++	b*	b*++	b0	b0++	Seq	y	y++	y*	y*++	y0	y0++	RevNo
1	244.18	122.59					V							16

2	372.24	186.62	355.21	178.11			Q	1,869.87	935.44	1,852.84	926.93	1,851.86	926.43	15
3	471.30	236.16	454.28	227.64			V	1,741.81	871.41	1,724.78	862.90	1,723.80	862.40	14
4	570.37	285.69	553.35	277.18			V	1,642.74	821.87	1,625.72	813.36	1,624.73	812.87	13
5	685.40	343.20	668.37	334.69	667.39	334.20	D	1,543.67	772.34	1,526.65	763.83	1,525.66	763.34	12
6	814.44	407.72	797.42	399.21	796.43	398.72	E	1,428.65	714.83	1,411.62	706.31	1,410.64	705.82	11
7	943.49	472.25	926.46	463.73	925.47	463.24	E	1,299.60	650.31	1,282.58	641.79	1,281.59	641.30	10
8	1,000.51	500.76	983.48	492.24	982.50	491.75	G	1,170.56	585.78	1,153.54	577.27	1,152.55	576.78	9
9	1,115.53	558.27	1,098.51	549.76	1,097.52	549.27	D	1,113.54	557.27	1,096.51	548.76	1,095.53	548.27	8
10	1,243.59	622.30	1,226.57	613.79	1,225.58	613.29	Q	998.51	499.76	981.49	491.25	980.50	490.76	7
11	1,371.65	686.33	1,354.62	677.82	1,353.64	677.32	Q	870.45	435.73	853.43	427.22	852.44	426.73	6
12	1,508.71	754.86	1,491.68	746.35	1,490.70	745.85	H	742.40	371.70	725.37	363.19	724.39	362.70	5
13	1,636.77	818.89	1,619.74	810.37	1,618.76	809.88	Q	605.34	303.17	588.31	294.66	587.33	294.17	4
14	1,765.81	883.41	1,748.78	874.90	1,747.80	874.40	E	477.28	239.14	460.25	230.63	459.27	230.14	3
15	1,822.83	911.92	1,805.81	903.41	1,804.82	902.91	G	348.24	174.62	331.21	166.11			2
16							K	291.21	146.11	274.19	137.60			1

Query 77235 Hit 1

MS/MS Fragmentation of WEVLIIGPPDTLYEGGVFK
Found in sp|P62253|UB2G1_HUMAN, Ubiquitin-conjugating enzyme E2 G1 OS=Homo sapiens GN=UBE2G1 PE=1 SV=3
Match to Query 77235: 2420.318from(807.7798,3+)
Title: 1071: Sum of 2 scans in range 2476 (rt=64.5887, f=4, i=701) to 2477 (rt=64.6141, f=4, i=702)
[D:\lab212\membrane\Grace\Joyce\iTRAQ_24_1.raw]
Data File:Submitted from 20120508-1(merge) by Mascot Daemon on JOYCE-VAIO
Monoisotopic mass of neutral peptide Mr(calc): 2420.318
Variable modifications:
K19 :iTRAQ4plex (K)
Ions Score: 43.24 Expect: 0.033

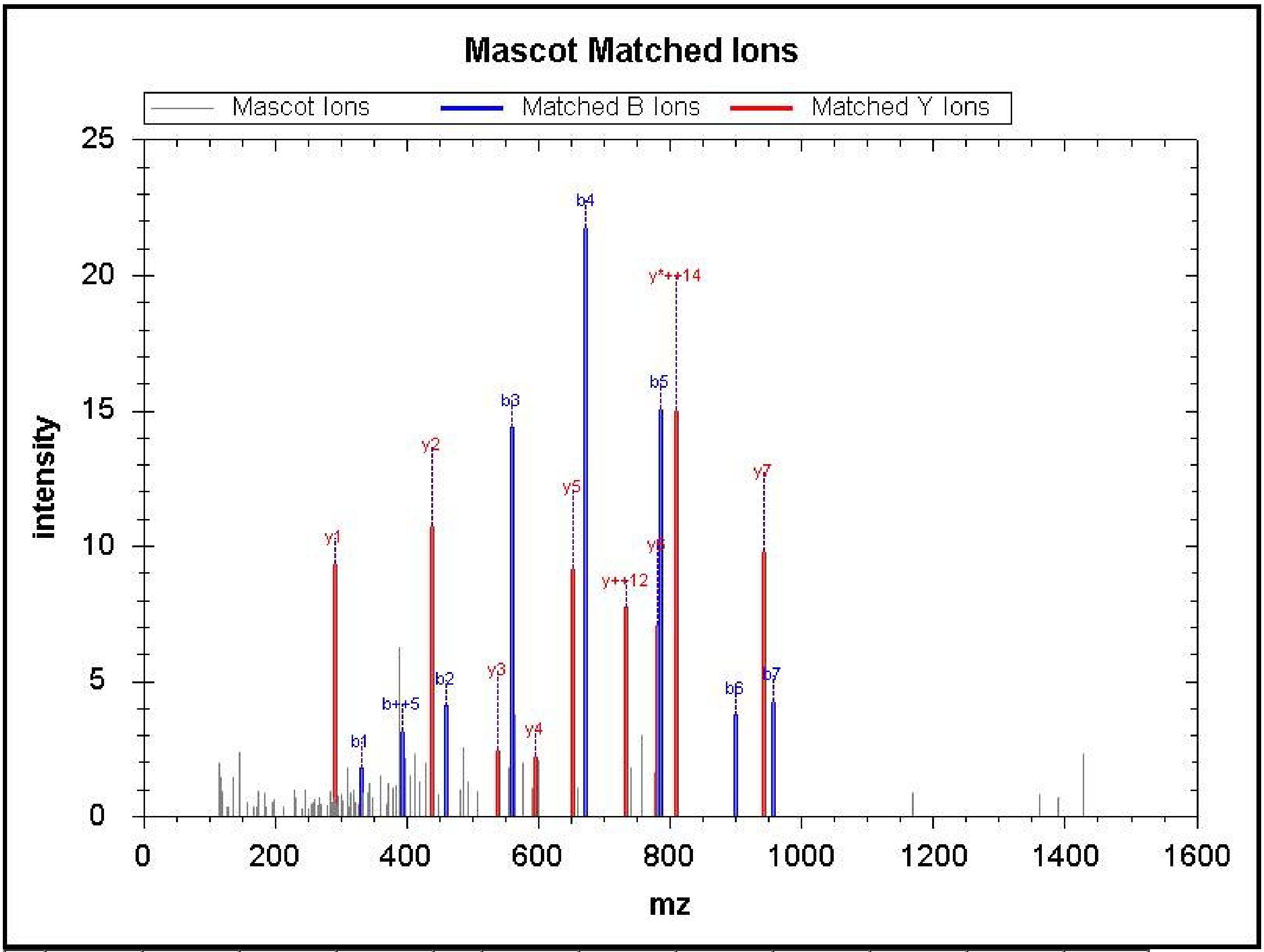

No	b	b++	b0	b0++	Seq	y	y++	y*	y*++	y0	y0++	RevNo
1	331.19	166.10			W							19
2	460.23	230.62	442.22	221.61	E	2,091.15	1,046.08	2,074.12	1,037.57	2,073.14	1,037.07	18
3	559.30	280.15	541.29	271.15	V	1,962.11	981.56	1,945.08	973.04	1,944.10	972.55	17
4	672.38	336.70	654.37	327.69	L	1,863.04	932.02	1,846.01	923.51	1,845.03	923.02	16
5	785.47	393.24	767.46	384.23	I	1,749.95	875.48	1,732.93	866.97	1,731.94	866.48	15
6	898.55	449.78	880.54	440.77	I	1,636.87	818.94	1,619.84	810.43	1,618.86	809.93	14
7	955.57	478.29	937.56	469.29	G	1,523.79	762.40	1,506.76	753.88	1,505.78	753.39	13
8	1,052.63	526.82	1,034.62	517.81	P	1,466.76	733.89	1,449.74	725.37	1,448.75	724.88	12
9	1,149.68	575.34	1,131.67	566.34	P	1,369.71	685.36	1,352.69	676.85	1,351.70	676.35	11
10	1,264.71	632.86	1,246.70	623.85	D	1,272.66	636.83	1,255.63	628.32	1,254.65	627.83	10
11	1,365.75	683.38	1,347.74	674.38	T	1,157.63	579.32	1,140.61	570.81	1,139.62	570.31	9
12	1,478.84	739.92	1,460.83	730.92	L	1,056.58	528.80	1,039.56	520.28	1,038.57	519.79	8
13	1,641.90	821.45	1,623.89	812.45	Y	943.50	472.25	926.47	463.74	925.49	463.25	7
14	1,770.94	885.98	1,752.93	876.97	E	780.44	390.72	763.41	382.21	762.43	381.72	6
15	1,827.97	914.49	1,809.95	905.48	G	651.39	326.20	634.37	317.69			5
16	1,884.99	943.00	1,866.98	933.99	G	594.37	297.69	577.35	289.18			4
17	1,984.05	992.53	1,966.04	983.53	V	537.35	269.18	520.33	260.67			3
18	2,131.12	1,066.07	2,113.11	1,057.06	F	438.28	219.65	421.26	211.13			2
19					K	291.21	146.11	274.19	137.60			1

Query 78769 Hit 1

MS/MS Fragmentation of GLAIPYLEHIIHVWEETGSR
Found in sp|Q96JC1|VPS39_HUMAN, Vam6/Vps39-like protein OS=Homo sapiens GN=VPS39 PE=1 SV=2
Match to Query 78769: 2463.3from(822.1072,3+)

Title: 1105: Sum of 2 scans in range 2644 (rt=68.1961, f=2, i=438) to 2645 (rt=68.2215, f=2, i=439) [D:\lab212\membrane\Grace\Joyce\iTRAQ_46_2.raw]
Data File:Submitted from 20120508-1(merge) by Mascot Daemon on JOYCE-VAIO
Monoisotopic mass of neutral peptide Mr(calc): 2463.3
Variable modifications:
Ions Score: 43.21 Expect: 0.041

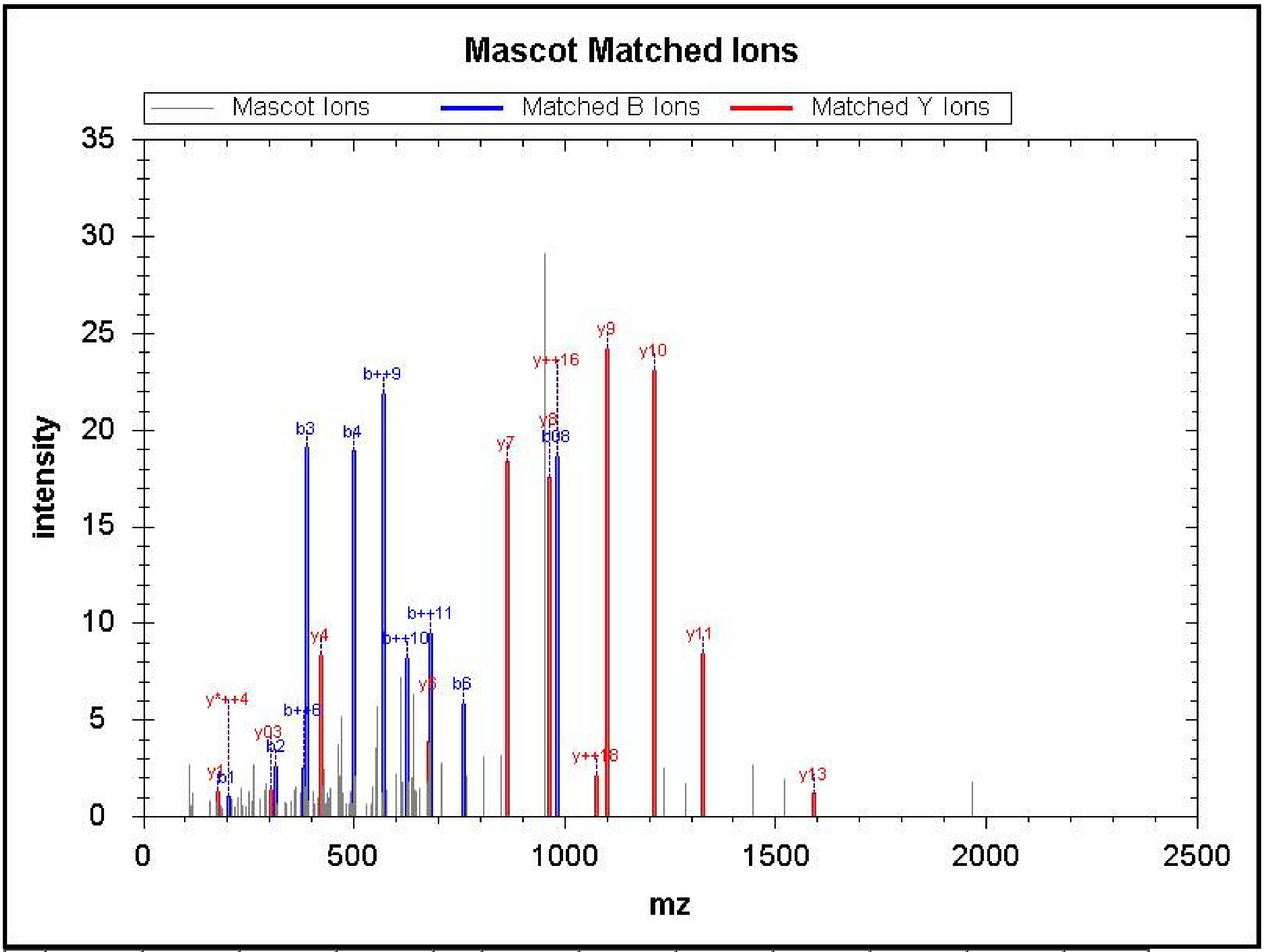

No	b	b++	b0	b0++	Seq	y	y++	y*	y*++	y0	y0++	RevNo
1	202.13	101.57			G							20
2	315.21	158.11			L	2,263.19	1,132.10	2,246.16	1,123.58	2,245.18	1,123.09	19
3	386.25	193.63			A	2,150.10	1,075.56	2,133.08	1,067.04	2,132.09	1,066.55	18
4	499.34	250.17			I	2,079.07	1,040.04	2,062.04	1,031.52	2,061.06	1,031.03	17
5	596.39	298.70			P	1,965.98	983.49	1,948.96	974.98	1,947.97	974.49	16
6	759.45	380.23			Y	1,868.93	934.97	1,851.90	926.46	1,850.92	925.96	15
7	872.54	436.77			L	1,705.87	853.44	1,688.84	844.92	1,687.86	844.43	14
8	1,001.58	501.29	983.57	492.29	E	1,592.78	796.89	1,575.76	788.38	1,574.77	787.89	13
9	1,138.64	569.82	1,120.63	560.82	H	1,463.74	732.37	1,446.71	723.86	1,445.73	723.37	12
10	1,251.72	626.36	1,233.71	617.36	I	1,326.68	663.84	1,309.65	655.33	1,308.67	654.84	11
11	1,364.81	682.91	1,346.80	673.90	I	1,213.60	607.30	1,196.57	598.79	1,195.59	598.30	10
12	1,501.86	751.44	1,483.85	742.43	H	1,100.51	550.76	1,083.49	542.25	1,082.50	541.75	9
13	1,600.93	800.97	1,582.92	791.96	V	963.45	482.23	946.43	473.72	945.44	473.22	8
14	1,787.01	894.01	1,769.00	885.00	W	864.38	432.70	847.36	424.18	846.37	423.69	7
15	1,916.06	958.53	1,898.04	949.53	E	678.31	339.66	661.28	331.14	660.29	330.65	6
16	2,045.10	1,023.05	2,027.09	1,014.05	E	549.26	275.13	532.24	266.62	531.25	266.13	5
17	2,146.15	1,073.58	2,128.14	1,064.57	T	420.22	210.61	403.19	202.10	402.21	201.61	4
18	2,203.17	1,102.09	2,185.16	1,093.08	G	319.17	160.09	302.15	151.58	301.16	151.08	3

19	2,290.20	1,145.60	2,272.19	1,136.60	S	262.15	131.58	245.12	123.07	244.14	122.57	2
20					R	175.12	88.06	158.09	79.55			1

Query 37217 Hit 1

MS/MS Fragmentation of DLGQPAAAAGHFQR
Found in sp|P23610|F8I2_HUMAN, Factor VIII intron 22 protein OS=Homo sapiens GN=F8A1 PE=1 SV=2
Match to Query 37217: 1581.809from(528.277,3+)
Title: 267: Scan 666 (rt=24.4496, f=3, i=97) [D:\lab212\membrane\Grace\Joyce\iTRAQ_37_2.raw]
Data File:Submitted from 20120508-1(merge) by Mascot Daemon on JOYCE-VAIO
Monoisotopic mass of neutral peptide Mr(calc): 1581.809
Variable modifications:
Ions Score: 43.18 Expect: 0.040

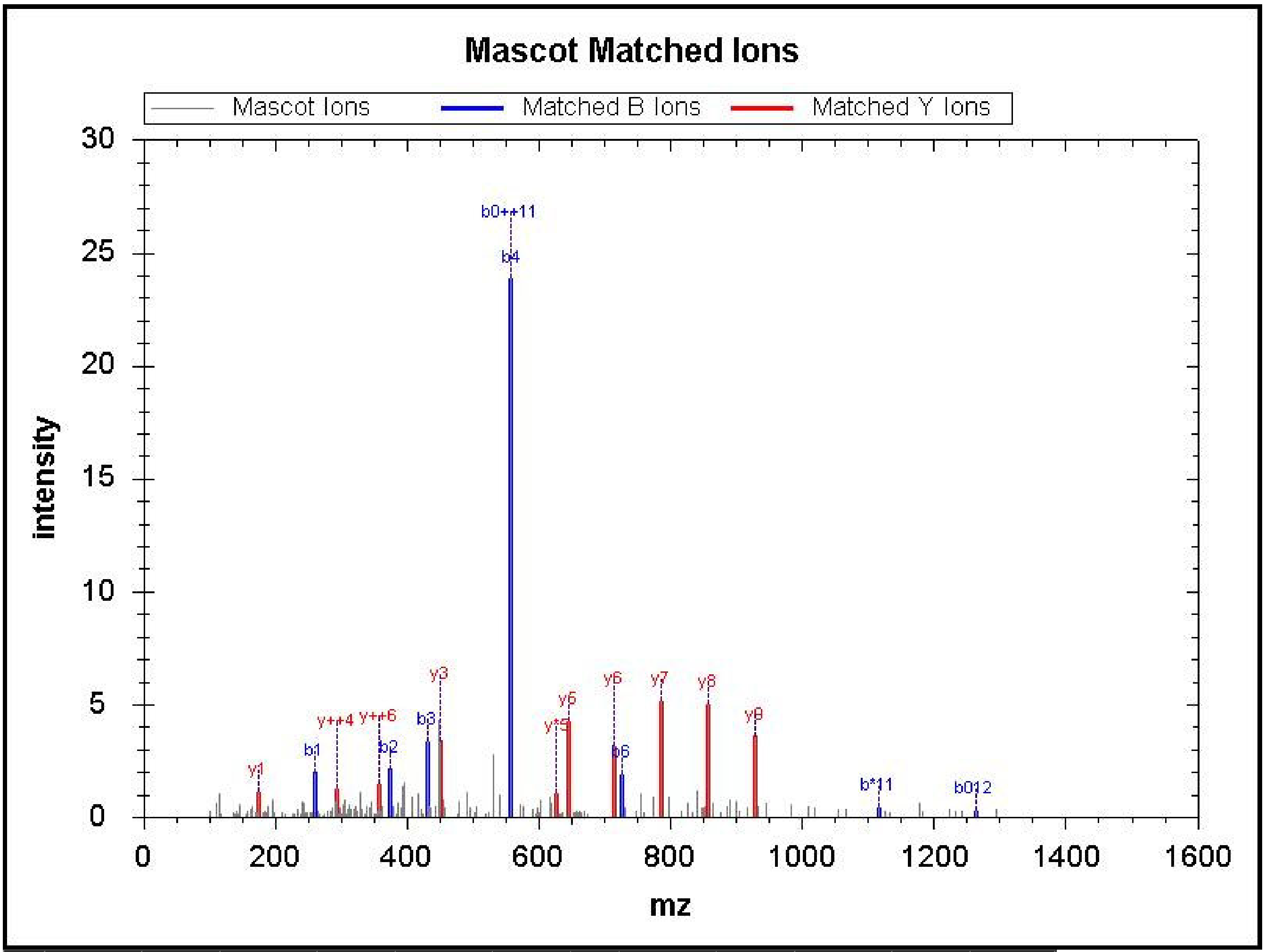

No	b	b++	b*	b*++	b0	b0++	Seq	y	y++	y*	y*++	RevNo
1	260.14	130.57			242.13	121.57	D					14
2	373.22	187.11			355.21	178.11	L	1,323.69	662.35	1,306.67	653.84	13
3	430.24	215.62			412.23	206.62	G	1,210.61	605.81	1,193.58	597.29	12
4	558.30	279.65	541.27	271.14	540.29	270.65	Q	1,153.59	577.30	1,136.56	568.78	11
5	655.35	328.18	638.33	319.67	637.34	319.17	P	1,025.53	513.27	1,008.50	504.75	10
6	726.39	363.70	709.36	355.19	708.38	354.69	A	928.47	464.74	911.45	456.23	9
7	797.43	399.22	780.40	390.70	779.42	390.21	A	857.44	429.22	840.41	420.71	8
8	868.46	434.74	851.44	426.22	850.45	425.73	A	786.40	393.70	769.37	385.19	7
9	939.50	470.25	922.47	461.74	921.49	461.25	A	715.36	358.19	698.34	349.67	6
10	996.52	498.77	979.50	490.25	978.51	489.76	G	644.33	322.67	627.30	314.15	5
11	1,133.58	567.29	1,116.56	558.78	1,115.57	558.29	H	587.30	294.16	570.28	285.64	4
12	1,280.65	640.83	1,263.62	632.32	1,262.64	631.82	F	450.25	225.63	433.22	217.11	3
13	1,408.71	704.86	1,391.68	696.34	1,390.70	695.85	Q	303.18	152.09	286.15	143.58	2

14							R	175.12	88.06	158.09	79.55	1

Query 54392 Hit 1

MS/MS Fragmentation of ILALLDALSTVHSQK
Found in sp|Q14692|BMS1_HUMAN, Ribosome biogenesis protein BMS1 homolog OS=Homo sapiens GN=BMS1 PE=1 SV=1
Match to Query 54392: 1896.125from(633.0489,3+)
Title: 1066: Scan 2331 (rt=61.958, f=2, i=365) [D:\lab212\membrane\Grace\Joyce\iTRAQ_37_2.raw]
Data File:Submitted from 20120508-1(merge) by Mascot Daemon on JOYCE-VAIO
Monoisotopic mass of neutral peptide Mr(calc): 1896.125
Variable modifications:
K15 :iTRAQ4plex (K)
Ions Score: 43.16 Expect: 0.016

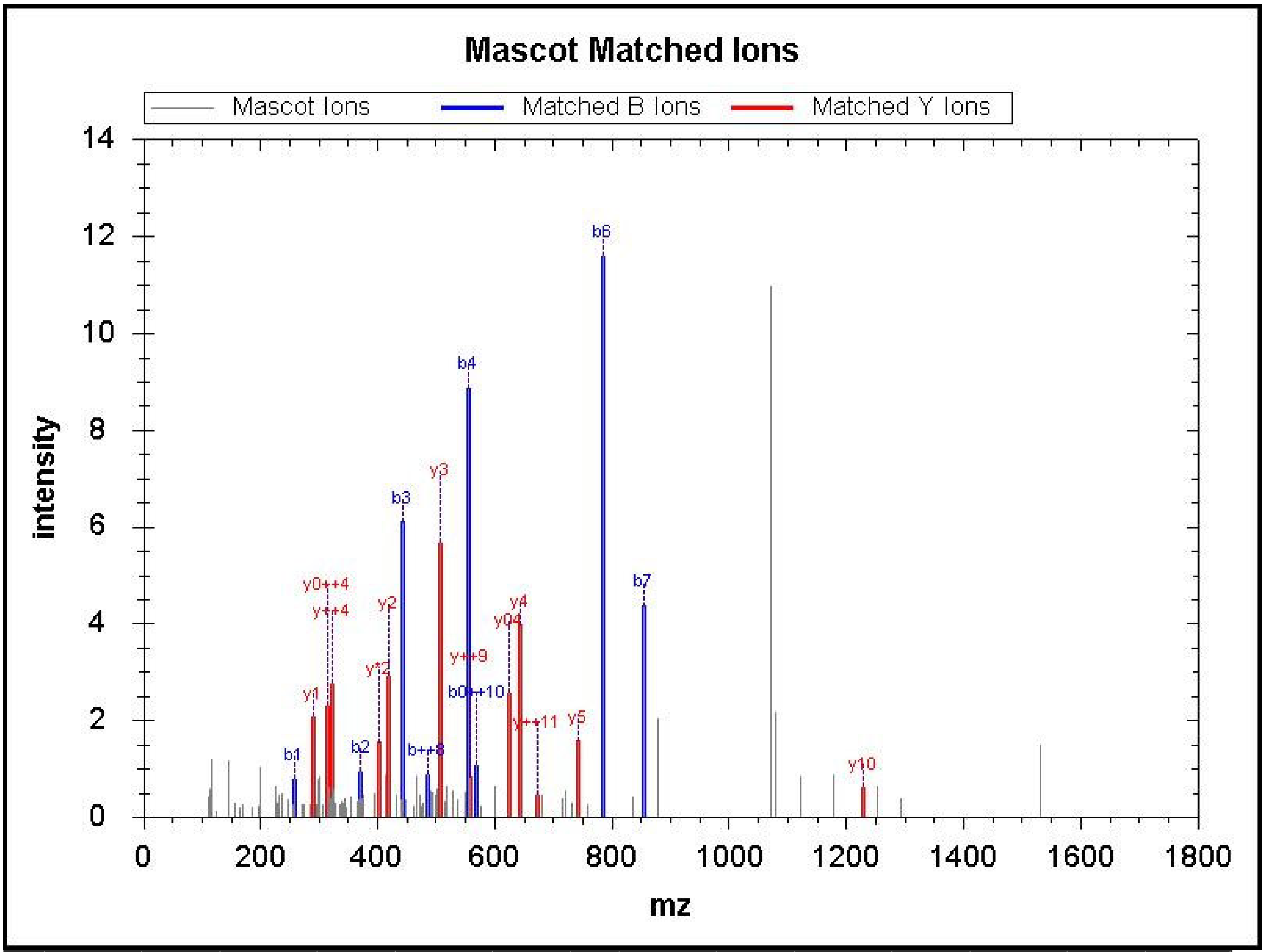

No	b	b++	b*	b*++	b0	b0++	Seq	y	y++	y*	y*++	y0	y0++	RevNo
1	258.19	129.60					I							15
2	371.28	186.14					L	1,639.95	820.48	1,622.92	811.97	1,621.94	811.47	14
3	442.31	221.66					A	1,526.87	763.94	1,509.84	755.42	1,508.86	754.93	13
4	555.40	278.20					L	1,455.83	728.42	1,438.80	719.90	1,437.82	719.41	12
5	668.48	334.74					L	1,342.74	671.88	1,325.72	663.36	1,324.73	662.87	11
6	783.51	392.26			765.50	383.25	D	1,229.66	615.33	1,212.63	606.82	1,211.65	606.33	10
7	854.55	427.78			836.54	418.77	A	1,114.63	557.82	1,097.61	549.31	1,096.62	548.82	9
8	967.63	484.32			949.62	475.31	L	1,043.60	522.30	1,026.57	513.79	1,025.59	513.30	8
9	1,054.66	527.84			1,036.65	518.83	S	930.51	465.76	913.49	457.25	912.50	456.75	7
10	1,155.71	578.36			1,137.70	569.35	T	843.48	422.24	826.45	413.73	825.47	413.24	6
11	1,254.78	627.89			1,236.77	618.89	V	742.43	371.72	725.41	363.21	724.42	362.71	5
12	1,391.84	696.42			1,373.83	687.42	H	643.36	322.19	626.34	313.67	625.35	313.18	4
13	1,478.87	739.94			1,460.86	730.93	S	506.31	253.66	489.28	245.14	488.29	244.65	3

14	1,606.93	803.97	1,589.90	795.45	1,588.92	794.96	Q	419.27	210.14	402.25	201.63			2
15							K	291.21	146.11	274.19	137.60			1

Query 22783 Hit 1

MS/MS Fragmentation of EVYEEAGVK
Found in sp|Q8NFP7|NUD10_HUMAN, Diphosphoinositol polyphosphate phosphohydrolase 3-alpha OS=Homo sapiens GN=NUDT10 PE=1 SV=1
Match to Query 22783: 1310.698from(656.3563,2+)
Title: 23: Sum of 2 scans in range 752 (rt=23.2104, f=3, i=8) to 753 (rt=23.2358, f=3, i=9) [D:\lab212\membrane\Grace\Joyce\iTRAQ_29_2.raw]
Data File:Submitted from 20120508-1(merge) by Mascot Daemon on JOYCE-VAIO
Monoisotopic mass of neutral peptide Mr(calc): 1310.698
Variable modifications:
K9 :iTRAQ4plex (K)
Ions Score: 43.11 Expect: 0.036

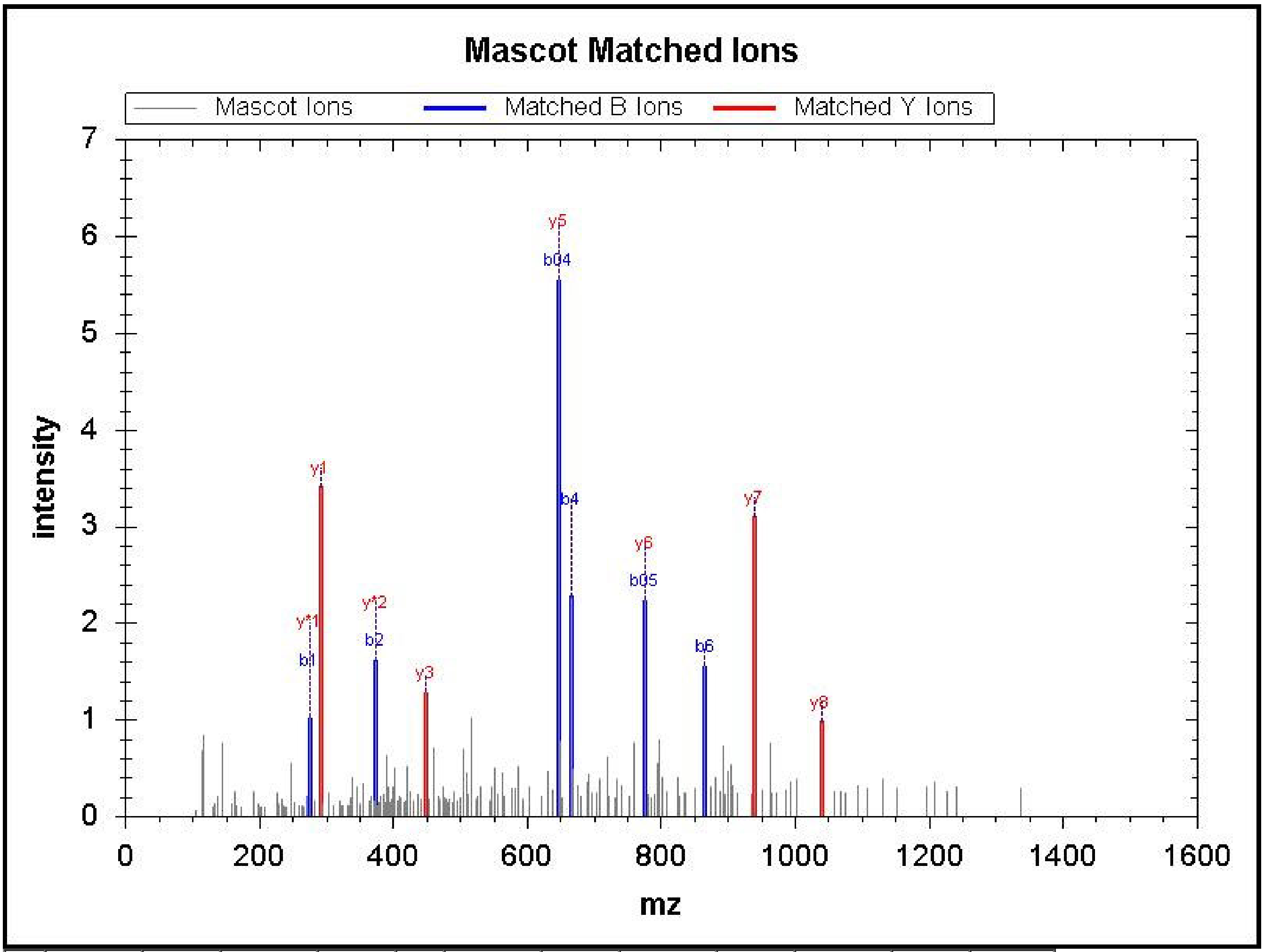

No	b	b++	b0	b0++	Seq	y	y++	y*	y*++	y0	y0++	RevNo
1	274.15	137.58	256.14	128.57	E							9
2	373.22	187.11	355.21	178.11	V	1,038.56	519.78	1,021.53	511.27	1,020.55	510.78	8
3	536.28	268.65	518.27	259.64	Y	939.49	470.25	922.46	461.74	921.48	461.24	7
4	665.33	333.17	647.32	324.16	E	776.43	388.72	759.40	380.20	758.42	379.71	6
5	794.37	397.69	776.36	388.68	E	647.38	324.20	630.36	315.68	629.37	315.19	5
6	865.41	433.21	847.40	424.20	A	518.34	259.67	501.32	251.16			4
7	922.43	461.72	904.42	452.71	G	447.30	224.16	430.28	215.64			3
8	1,021.50	511.25	1,003.49	502.25	V	390.28	195.65	373.26	187.13			2
9					K	291.21	146.11	274.19	137.60			1

Query 6670 Hit 1

MS/MS Fragmentation of LIELAK
Found in sp|P41218|MNDA_HUMAN, Myeloid cell nuclear differentiation antigen OS=Homo sapiens GN=MNDA PE=1 SV=1
Match to Query 6670: 973.6475from(487.831,2+)
Title: 445: Scan 1050 (rt=33.0145, f=3, i=153) [D:\lab212\membrane\Grace\Joyce\iTRAQ_31_1.raw]
Data File:Submitted from 20120508-1(merge) by Mascot Daemon on JOYCE-VAIO
Monoisotopic mass of neutral peptide Mr(calc): 973.6475
Variable modifications:
K6 :iTRAQ4plex (K)
Ions Score: 43.1 Expect: 0.022

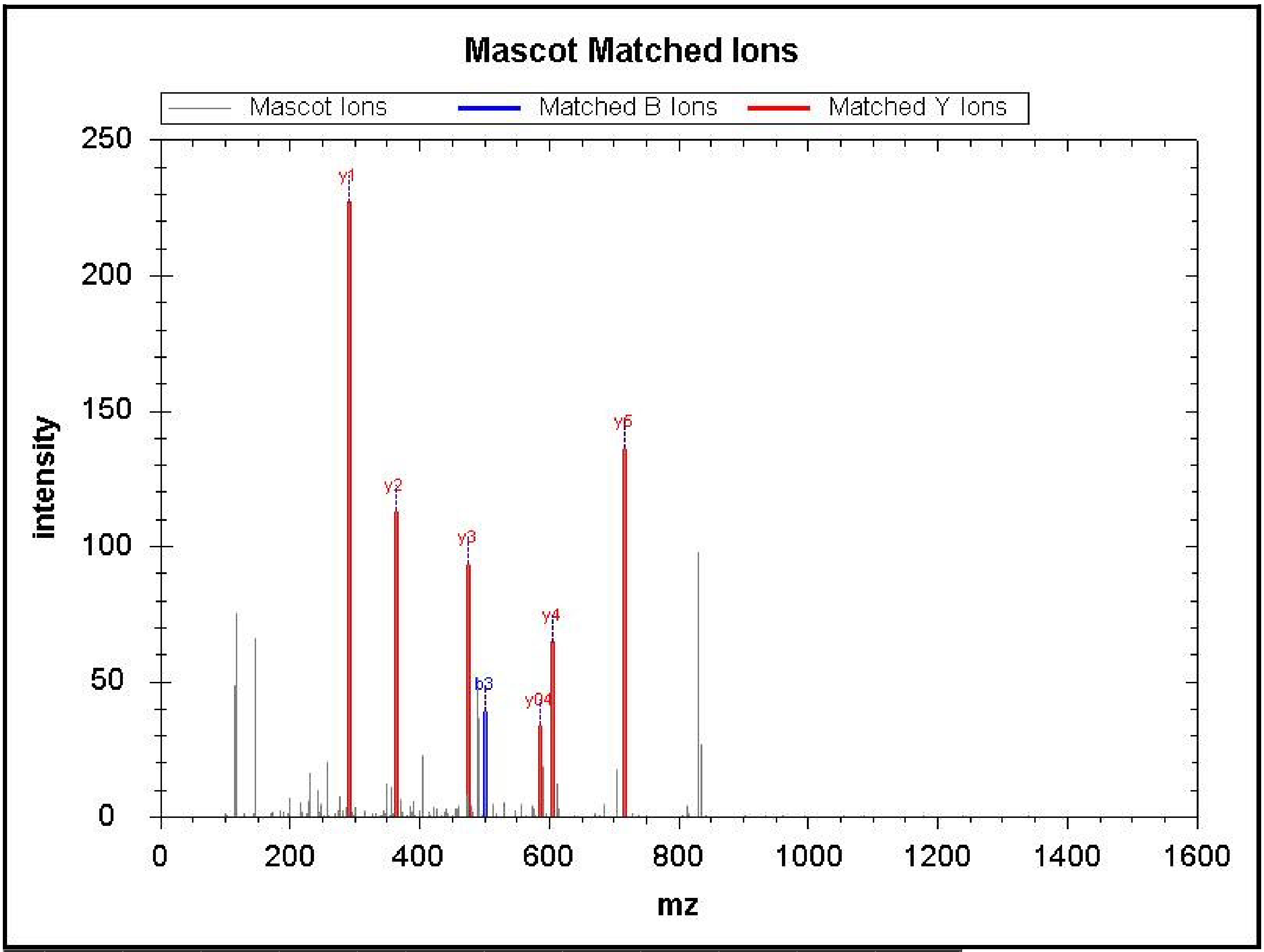

No	b	b++	b0	b0++	Seq	y	y++	y*	y*++	y0	y0++	RevNo
1	258.19	129.60			L							6
2	371.28	186.14			I	717.46	359.23	700.44	350.72	699.45	350.23	5
3	500.32	250.66	482.31	241.66	E	604.38	302.69	587.35	294.18	586.37	293.69	4
4	613.40	307.21	595.39	298.20	L	475.34	238.17	458.31	229.66			3
5	684.44	342.72	666.43	333.72	A	362.25	181.63	345.23	173.12			2
6					K	291.21	146.11	274.19	137.60			1

Query 25466 Hit 1

MS/MS Fragmentation of DSVLTWLLK
Found in sp|Q9H1H9|KI13A_HUMAN, Kinesin-like protein KIF13A OS=Homo sapiens GN=KIF13A PE=1 SV=2
Match to Query 25466: 1361.819from(681.9171,2+)
Title: 823: Sum of 2 scans in range 2419 (rt=60.7785, f=4, i=539) to 2420 (rt=60.8039, f=4, i=540) [D:\lab212\membrane\Grace\Joyce\iTRAQ_29_2.raw]
Data File:Submitted from 20120508-1(merge) by Mascot Daemon on JOYCE-VAIO
Monoisotopic mass of neutral peptide Mr(calc): 1361.819
Variable modifications:
K9 :iTRAQ4plex (K)
Ions Score: 43.05 Expect: 0.030

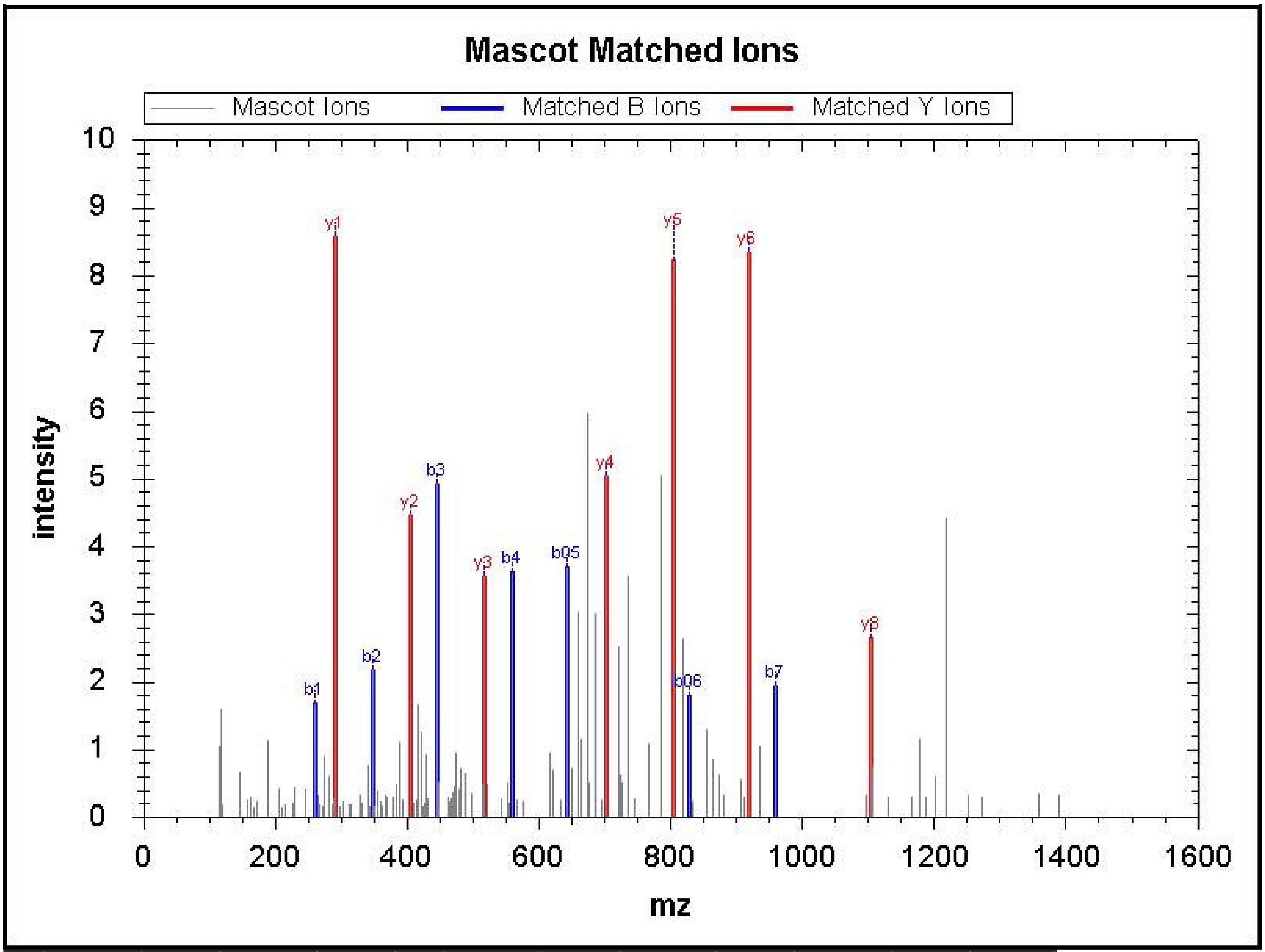

No	b	b++	b0	b0++	Seq	y	y++	y*	y*++	y0	y0++	RevNo
1	260.14	130.57	242.13	121.57	D							9
2	347.17	174.09	329.16	165.08	S	1,103.69	552.35	1,086.67	543.84	1,085.68	543.35	8
3	446.24	223.62	428.23	214.62	V	1,016.66	508.83	999.64	500.32	998.65	499.83	7
4	559.32	280.16	541.31	271.16	L	917.59	459.30	900.57	450.79	899.58	450.30	6
5	660.37	330.69	642.36	321.68	T	804.51	402.76	787.48	394.25	786.50	393.75	5
6	846.45	423.73	828.44	414.72	W	703.46	352.23	686.44	343.72			4
7	959.53	480.27	941.52	471.26	L	517.38	259.20	500.36	250.68			3
8	1,072.62	536.81	1,054.61	527.81	L	404.30	202.65	387.27	194.14			2
9					K	291.21	146.11	274.19	137.60			1

Query 87694 Hit 1

MS/MS Fragmentation of EKEELIEEWQPEPLVPPVPK
Found in sp|O15269|SPTC1_HUMAN, Serine palmitoyltransferase 1 OS=Homo sapiens GN=SPTLC1 PE=1 SV=1
Match to Query 87694: 2817.554from(940.1921,3+)
Title: 810: Sum of 2 scans in range 1809 (rt=50.2603, f=4, i=541) to 1810 (rt=50.2858, f=4, i=542) [D:\lab212\membrane\Grace\Joyce\iTRAQ_39_1.raw]
Data File:Submitted from 20120508-1(merge) by Mascot Daemon on JOYCE-VAIO
Monoisotopic mass of neutral peptide Mr(calc): 2817.554
Variable modifications:
K2 :iTRAQ4plex (K)
K20 :iTRAQ4plex (K)
Ions Score: 43.05 Expect: 0.023

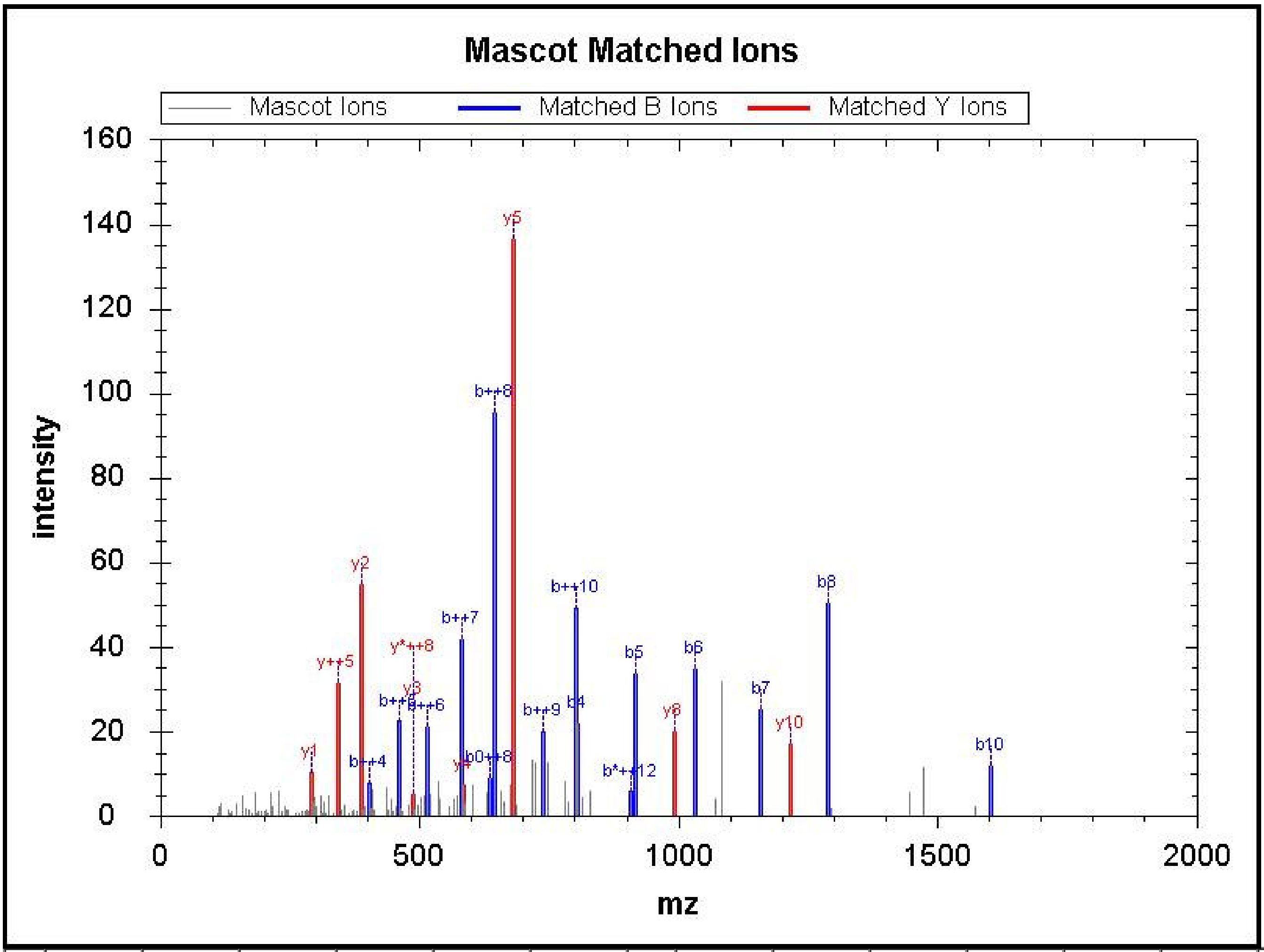

No	b	b++	b*	b*++	b0	b0++	Seq	y	y++	y*	y*++	y0	y0++	RevNo
1	274.15	137.58			256.14	128.57	E							20
2	546.35	273.68	529.32	265.16	528.34	264.67	K	2,545.42	1,273.21	2,528.39	1,264.70	2,527.41	1,264.21	19
3	675.39	338.20	658.36	329.69	657.38	329.19	E	2,273.22	1,137.11	2,256.19	1,128.60	2,255.21	1,128.11	18
4	804.43	402.72	787.41	394.21	786.42	393.72	E	2,144.18	1,072.59	2,127.15	1,064.08	2,126.17	1,063.59	17
5	917.52	459.26	900.49	450.75	899.51	450.26	L	2,015.13	1,008.07	1,998.11	999.56	1,997.12	999.07	16
6	1,030.60	515.80	1,013.58	507.29	1,012.59	506.80	I	1,902.05	951.53	1,885.02	943.02	1,884.04	942.52	15
7	1,159.64	580.33	1,142.62	571.81	1,141.63	571.32	E	1,788.97	894.99	1,771.94	886.47	1,770.95	885.98	14
8	1,288.69	644.85	1,271.66	636.33	1,270.68	635.84	E	1,659.92	830.46	1,642.90	821.95	1,641.91	821.46	13
9	1,474.77	737.89	1,457.74	729.37	1,456.76	728.88	W	1,530.88	765.94	1,513.85	757.43	1,512.87	756.94	12
10	1,602.83	801.92	1,585.80	793.40	1,584.81	792.91	Q	1,344.80	672.90	1,327.77	664.39	1,326.79	663.90	11
11	1,699.88	850.44	1,682.85	841.93	1,681.87	841.44	P	1,216.74	608.87	1,199.72	600.36	1,198.73	599.87	10
12	1,828.92	914.96	1,811.89	906.45	1,810.91	905.96	E	1,119.69	560.35	1,102.66	551.84	1,101.68	551.34	9
13	1,925.97	963.49	1,908.95	954.98	1,907.96	954.49	P	990.65	495.83	973.62	487.31			8
14	2,039.06	1,020.03	2,022.03	1,011.52	2,021.05	1,011.03	L	893.59	447.30	876.57	438.79			7
15	2,138.13	1,069.57	2,121.10	1,061.05	2,120.12	1,060.56	V	780.51	390.76	763.48	382.25			6
16	2,235.18	1,118.09	2,218.15	1,109.58	2,217.17	1,109.09	P	681.44	341.22	664.41	332.71			5
17	2,332.23	1,166.62	2,315.20	1,158.11	2,314.22	1,157.61	P	584.39	292.70	567.36	284.18			4
18	2,431.30	1,216.15	2,414.27	1,207.64	2,413.29	1,207.15	V	487.34	244.17	470.31	235.66			3
19	2,528.35	1,264.68	2,511.33	1,256.17	2,510.34	1,255.67	P	388.27	194.64	371.24	186.12			2
20							K	291.21	146.11	274.19	137.60			1

Query 61360 Hit 1

MS/MS Fragmentation of ETDLQELFRPFGSISR
Found in sp|O75821|EIF3G_HUMAN, Eukaryotic translation initiation factor 3 subunit G OS=Homo sapiens GN=EIF3G PE=1 SV=2

Match to Query 61360: 2038.055from(680.3589,3+)
Title: 900: Scan 2009 (rt=54.6595, f=2, i=316) [D:\lab212\membrane\Grace\Joyce\iTRAQ_35_2.raw]
Data File:Submitted from 20120508-1(merge) by Mascot Daemon on JOYCE-VAIO
Monoisotopic mass of neutral peptide Mr(calc): 2038.055
Variable modifications:
Ions Score: 43 Expect: 0.047

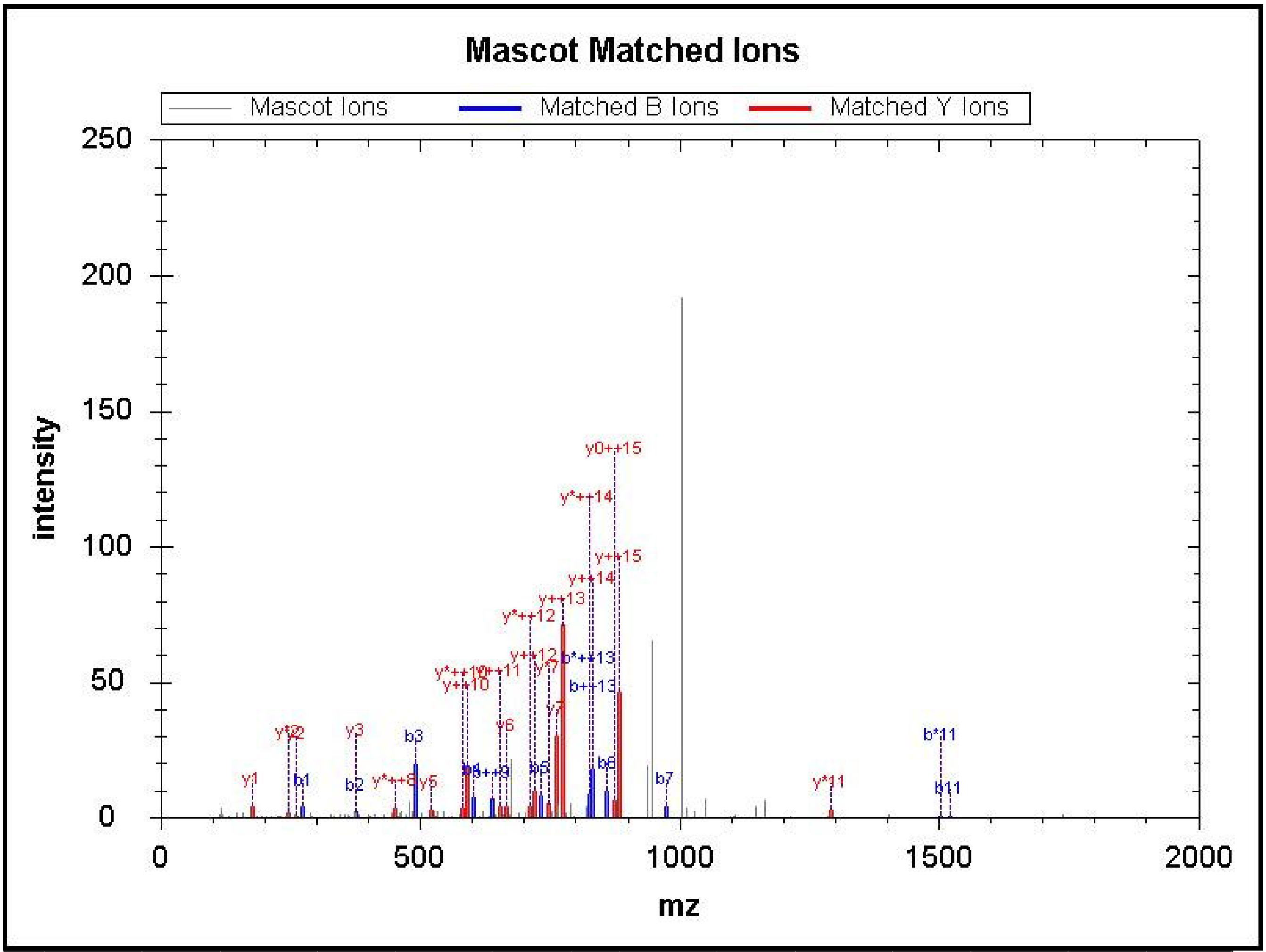

No	b	b++	b*	b*++	b0	b0++	Seq	y	y++	y*	y*++	y0	y0++	RevNo
1	274.15	137.58			256.14	128.57	E							16
2	375.20	188.10			357.19	179.10	T	1,765.92	883.47	1,748.90	874.95	1,747.91	874.46	15
3	490.23	245.62			472.22	236.61	D	1,664.88	832.94	1,647.85	824.43	1,646.86	823.94	14
4	603.31	302.16			585.30	293.15	L	1,549.85	775.43	1,532.82	766.91	1,531.84	766.42	13
5	731.37	366.19	714.34	357.67	713.36	357.18	Q	1,436.76	718.89	1,419.74	710.37	1,418.75	709.88	12
6	860.41	430.71	843.39	422.20	842.40	421.70	E	1,308.71	654.86	1,291.68	646.34	1,290.70	645.85	11
7	973.50	487.25	956.47	478.74	955.49	478.25	L	1,179.66	590.34	1,162.64	581.82	1,161.65	581.33	10
8	1,120.56	560.79	1,103.54	552.27	1,102.55	551.78	F	1,066.58	533.79	1,049.55	525.28	1,048.57	524.79	9
9	1,276.67	638.84	1,259.64	630.32	1,258.65	629.83	R	919.51	460.26	902.48	451.75	901.50	451.25	8
10	1,373.72	687.36	1,356.69	678.85	1,355.71	678.36	P	763.41	382.21	746.38	373.70	745.40	373.20	7
11	1,520.79	760.90	1,503.76	752.38	1,502.78	751.89	F	666.36	333.68	649.33	325.17	648.35	324.68	6
12	1,577.81	789.41	1,560.78	780.89	1,559.80	780.40	G	519.29	260.15	502.26	251.63	501.28	251.14	5
13	1,664.84	832.92	1,647.81	824.41	1,646.83	823.92	S	462.27	231.64	445.24	223.12	444.26	222.63	4
14	1,777.92	889.47	1,760.90	880.95	1,759.91	880.46	I	375.24	188.12	358.21	179.61	357.22	179.12	3
15	1,864.96	932.98	1,847.93	924.47	1,846.95	923.98	S	262.15	131.58	245.12	123.07	244.14	122.57	2
16							R	175.12	88.06	158.09	79.55			1

Query 19893 Hit 1

MS/MS Fragmentation of NVELTEVGPR
Found in sp|Q96G21|IMP4_HUMAN, U3 small nucleolar ribonucleoprotein protein IMP4 OS=Homo sapiens GN=IMP4 PE=1 SV=1
Match to Query 19893: 1256.686from(629.3502,2+)
Title: 187: Scan 980 (rt=29.1326, f=3, i=65) [D:\lab212\membrane\Grace\Joyce\iTRAQ_22_2.raw]
Data File:Submitted from 20120508-1(merge) by Mascot Daemon on JOYCE-VAIO
Monoisotopic mass of neutral peptide Mr(calc): 1256.686
Variable modifications:
Ions Score: 43 Expect: 0.033

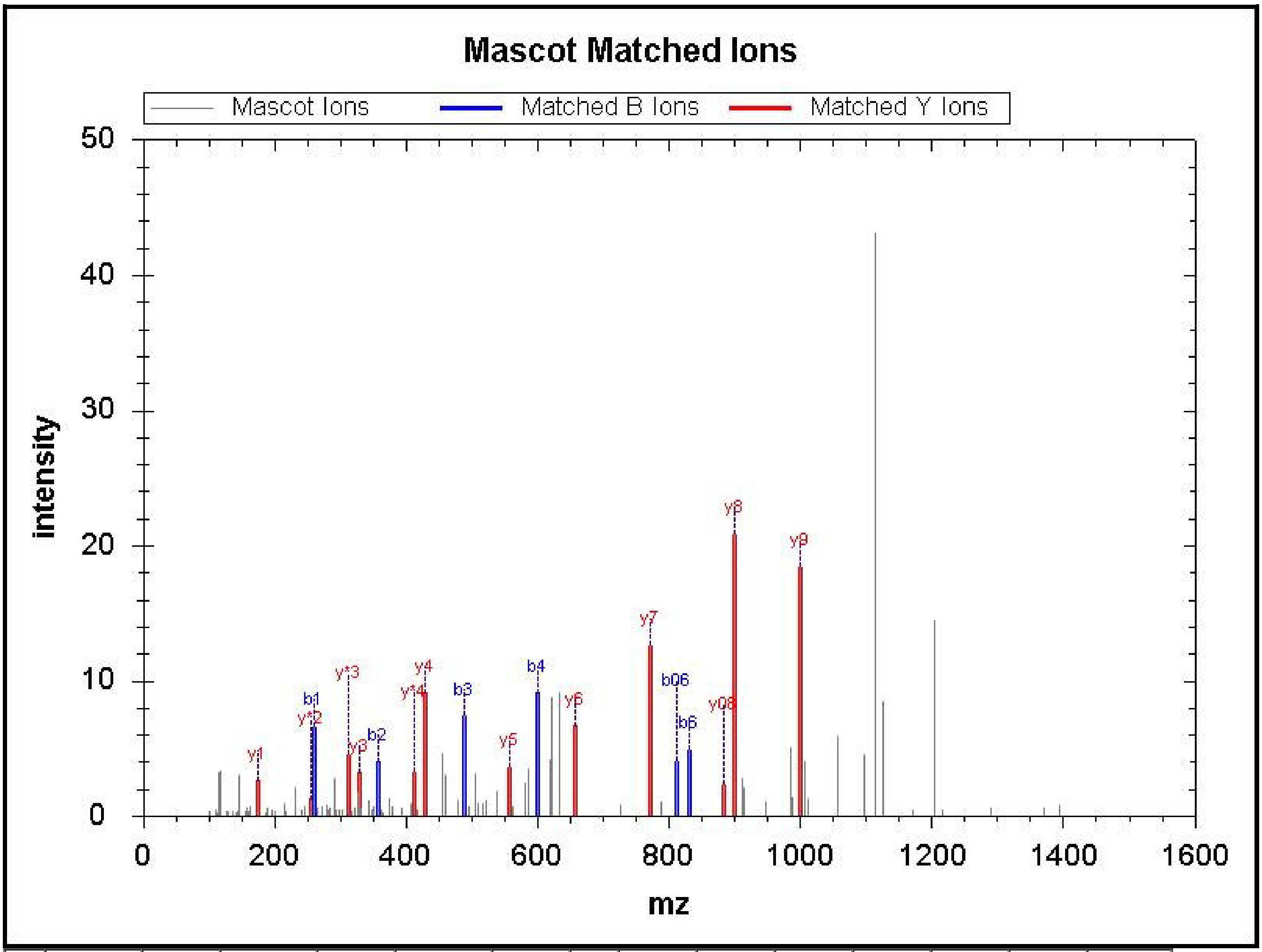

No	b	b++	b*	b*++	b0	b0++	Seq	y	y++	y*	y*++	y0	y0++	RevNo
1	259.15	130.08	242.13	121.57			N							10
2	358.22	179.61	341.19	171.10			V	999.55	500.28	982.52	491.76	981.54	491.27	9
3	487.26	244.14	470.24	235.62	469.25	235.13	E	900.48	450.74	883.45	442.23	882.47	441.74	8
4	600.35	300.68	583.32	292.16	582.34	291.67	L	771.44	386.22	754.41	377.71	753.43	377.22	7
5	701.39	351.20	684.37	342.69	683.38	342.20	T	658.35	329.68	641.33	321.17	640.34	320.67	6
6	830.44	415.72	813.41	407.21	812.43	406.72	E	557.30	279.16	540.28	270.64	539.29	270.15	5
7	929.51	465.26	912.48	456.74	911.50	456.25	V	428.26	214.63	411.24	206.12			4
8	986.53	493.77	969.50	485.25	968.52	484.76	G	329.19	165.10	312.17	156.59			3
9	1,083.58	542.29	1,066.55	533.78	1,065.57	533.29	P	272.17	136.59	255.15	128.08			2
10							R	175.12	88.06	158.09	79.55			1

Query 21250 Hit 1

MS/MS Fragmentation of SALGITVHQGR
Found in sp|Q14145|KEAP1_HUMAN, Kelch-like ECH-associated protein 1 OS=Homo sapiens GN=KEAP1 PE=1 SV=2
Match to Query 21250: 1281.723from(428.2484,3+)
Title: 277: Scan 656 (rt=24.4536, f=3, i=99) [D:\lab212\membrane\Grace\Joyce\iTRAQ_40_1.raw]
Data File:Submitted from 20120508-1(merge) by Mascot Daemon on JOYCE-VAIO

Monoisotopic mass of neutral peptide Mr(calc): 1281.723
Variable modifications:
Ions Score: 42.88 Expect: 0.033

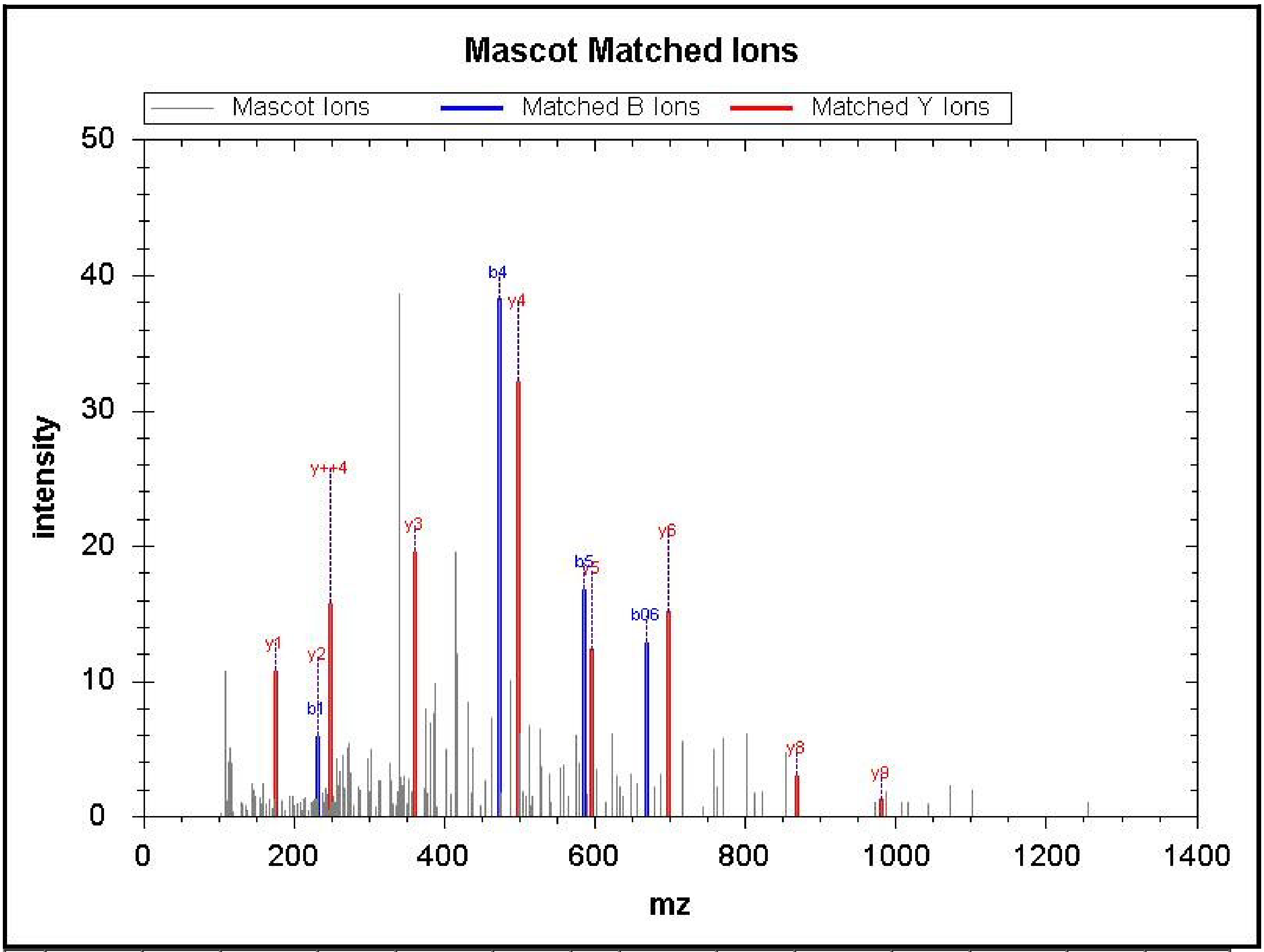

No	b	b++	b*	b*++	b0	b0++	Seq	y	y++	y*	y*++	y0	y0++	RevNo
1	232.14	116.57			214.13	107.57	S							11
2	303.18	152.09			285.17	143.09	A	1,051.60	526.30	1,034.57	517.79	1,033.59	517.30	10
3	416.26	208.63			398.25	199.63	L	980.56	490.79	963.54	482.27	962.55	481.78	9
4	473.28	237.15			455.27	228.14	G	867.48	434.24	850.45	425.73	849.47	425.24	8
5	586.37	293.69			568.36	284.68	I	810.46	405.73	793.43	397.22	792.45	396.73	7
6	687.42	344.21			669.41	335.21	T	697.37	349.19	680.35	340.68	679.36	340.19	6
7	786.48	393.75			768.47	384.74	V	596.33	298.67	579.30	290.15			5
8	923.54	462.28			905.53	453.27	H	497.26	249.13	480.23	240.62			4
9	1,051.60	526.30	1,034.58	517.79	1,033.59	517.30	Q	360.20	180.60	343.17	172.09			3
10	1,108.62	554.82	1,091.60	546.30	1,090.61	545.81	G	232.14	116.57	215.11	108.06			2
11							R	175.12	88.06	158.09	79.55			1

Query 87751 Hit 1

MS/MS Fragmentation of LVAVGGFSPNVNHGELLNAAIEALK
Found in sp|Q9NXV6|CARF_HUMAN, CDKN2A-interacting protein OS=Homo sapiens GN=CDKN2AIP PE=1 SV=3
Match to Query 87751: 2820.568from(706.1492,4+)
Title: 1008: Sum of 2 scans in range 2181 (rt=58.8092, f=4, i=677) to 2182 (rt=58.8346, f=4, i=678)
[D:\lab212\membrane\Grace\Joyce\iTRAQ_33_2.raw]
Data File:Submitted from 20120508-1(merge) by Mascot Daemon on JOYCE-VAIO
Monoisotopic mass of neutral peptide Mr(calc): 2820.568
Variable modifications:
K25 :iTRAQ4plex (K)
Ions Score: 42.86 Expect: 0.021

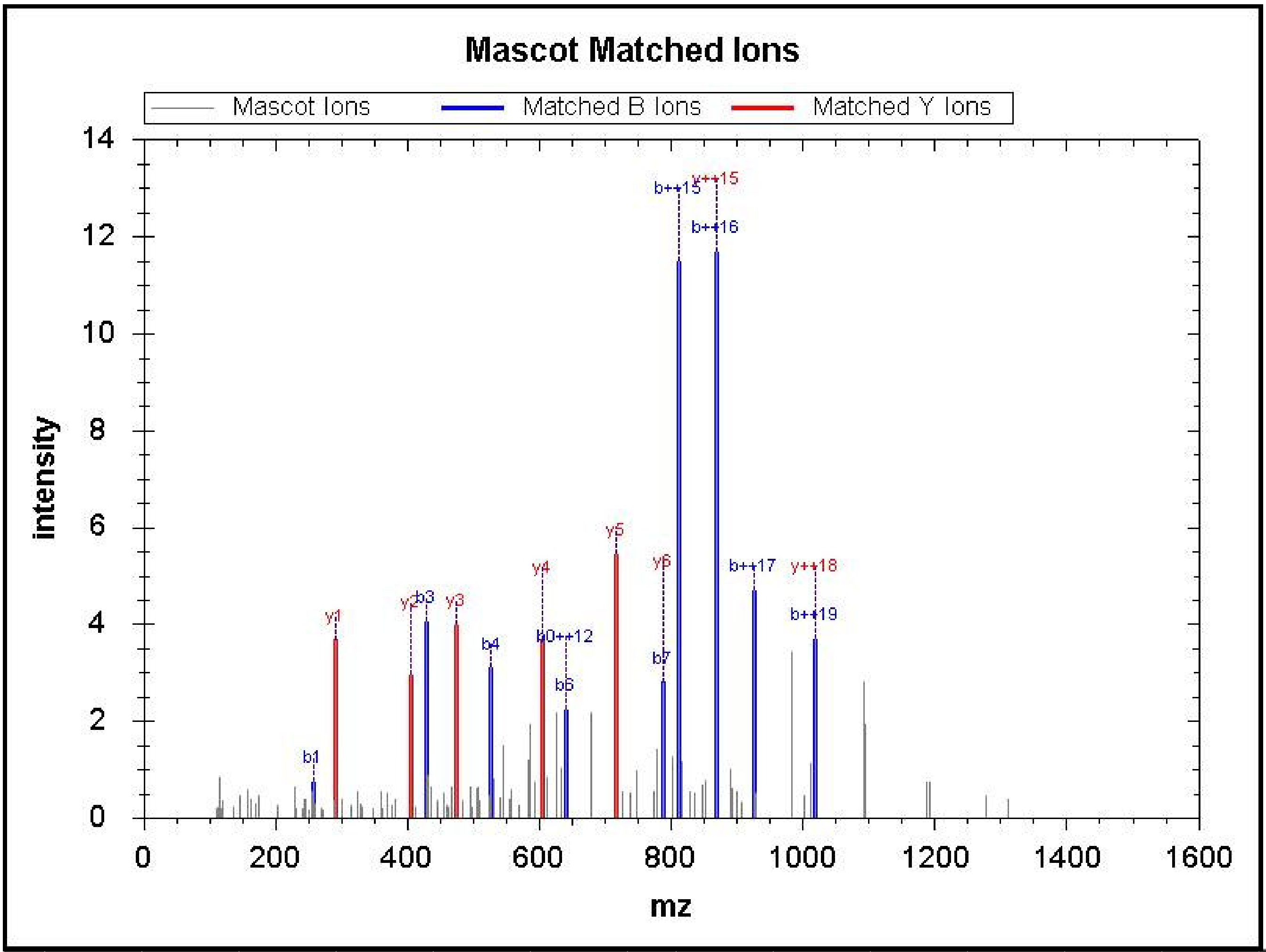

No	b	b++	b*	b*++	b0	b0++	Seq	y	y++	y*	y*++	y0	y0++	RevNo
1	258.19	129.60					L							25
2	357.26	179.13					V	2,564.40	1,282.70	2,547.37	1,274.19	2,546.38	1,273.70	24
3	428.30	214.65					A	2,465.33	1,233.17	2,448.30	1,224.65	2,447.32	1,224.16	23
4	527.37	264.19					V	2,394.29	1,197.65	2,377.26	1,189.14	2,376.28	1,188.64	22
5	584.39	292.70					G	2,295.22	1,148.11	2,278.19	1,139.60	2,277.21	1,139.11	21
6	641.41	321.21					G	2,238.20	1,119.60	2,221.17	1,111.09	2,220.19	1,110.60	20
7	788.48	394.74					F	2,181.18	1,091.09	2,164.15	1,082.58	2,163.17	1,082.09	19
8	875.51	438.26			857.50	429.25	S	2,034.11	1,017.56	2,017.08	1,009.05	2,016.10	1,008.55	18
9	972.56	486.79			954.55	477.78	P	1,947.08	974.04	1,930.05	965.53	1,929.07	965.04	17
10	1,086.61	543.81	1,069.58	535.29	1,068.60	534.80	N	1,850.03	925.52	1,833.00	917.00	1,832.01	916.51	16
11	1,185.67	593.34	1,168.65	584.83	1,167.66	584.34	V	1,735.98	868.49	1,718.96	859.98	1,717.97	859.49	15
12	1,299.72	650.36	1,282.69	641.85	1,281.71	641.36	N	1,636.91	818.96	1,619.89	810.45	1,618.90	809.96	14
13	1,436.78	718.89	1,419.75	710.38	1,418.77	709.89	H	1,522.87	761.94	1,505.84	753.43	1,504.86	752.93	13
14	1,493.80	747.40	1,476.77	738.89	1,475.79	738.40	G	1,385.81	693.41	1,368.79	684.90	1,367.80	684.40	12
15	1,622.84	811.92	1,605.81	803.41	1,604.83	802.92	E	1,328.79	664.90	1,311.76	656.39	1,310.78	655.89	11
16	1,735.93	868.47	1,718.90	859.95	1,717.91	859.46	L	1,199.75	600.38	1,182.72	591.86	1,181.74	591.37	10
17	1,849.01	925.01	1,831.98	916.49	1,831.00	916.00	L	1,086.66	543.84	1,069.64	535.32	1,068.65	534.83	9
18	1,963.05	982.03	1,946.03	973.52	1,945.04	973.02	N	973.58	487.29	956.55	478.78	955.57	478.29	8
19	2,034.09	1,017.55	2,017.06	1,009.04	2,016.08	1,008.54	A	859.54	430.27	842.51	421.76	841.53	421.27	7
20	2,105.13	1,053.07	2,088.10	1,044.55	2,087.12	1,044.06	A	788.50	394.75	771.47	386.24	770.49	385.75	6
21	2,218.21	1,109.61	2,201.18	1,101.10	2,200.20	1,100.60	I	717.46	359.23	700.44	350.72	699.45	350.23	5
22	2,347.25	1,174.13	2,330.23	1,165.62	2,329.24	1,165.12	E	604.38	302.69	587.35	294.18	586.37	293.69	4
23	2,418.29	1,209.65	2,401.26	1,201.14	2,400.28	1,200.64	A	475.34	238.17	458.31	229.66			3

24	2,531.37	1,266.19	2,514.35	1,257.68	2,513.36	1,257.19	L	404.30	202.65	387.27	194.14			2
25							K	291.21	146.11	274.19	137.60			1

Query 27499 Hit 1

MS/MS Fragmentation of TILFIDEIHR
Found in sp|Q96S55|WRIP1_HUMAN, ATPase WRNIP1 OS=Homo sapiens GN=WRNIP1 PE=1 SV=2
Match to Query 27499: 1399.791 from(467.6041,3+)
Title: 676: Scan 1536 (rt=44.0175, f=3, i=231) [D:\lab212\membrane\Grace\Joyce\iTRAQ_36_2.raw]
Data File:Submitted from 20120508-1(merge) by Mascot Daemon on JOYCE-VAIO
Monoisotopic mass of neutral peptide Mr(calc): 1399.791
Variable modifications:
Ions Score: 42.83 Expect: 0.035

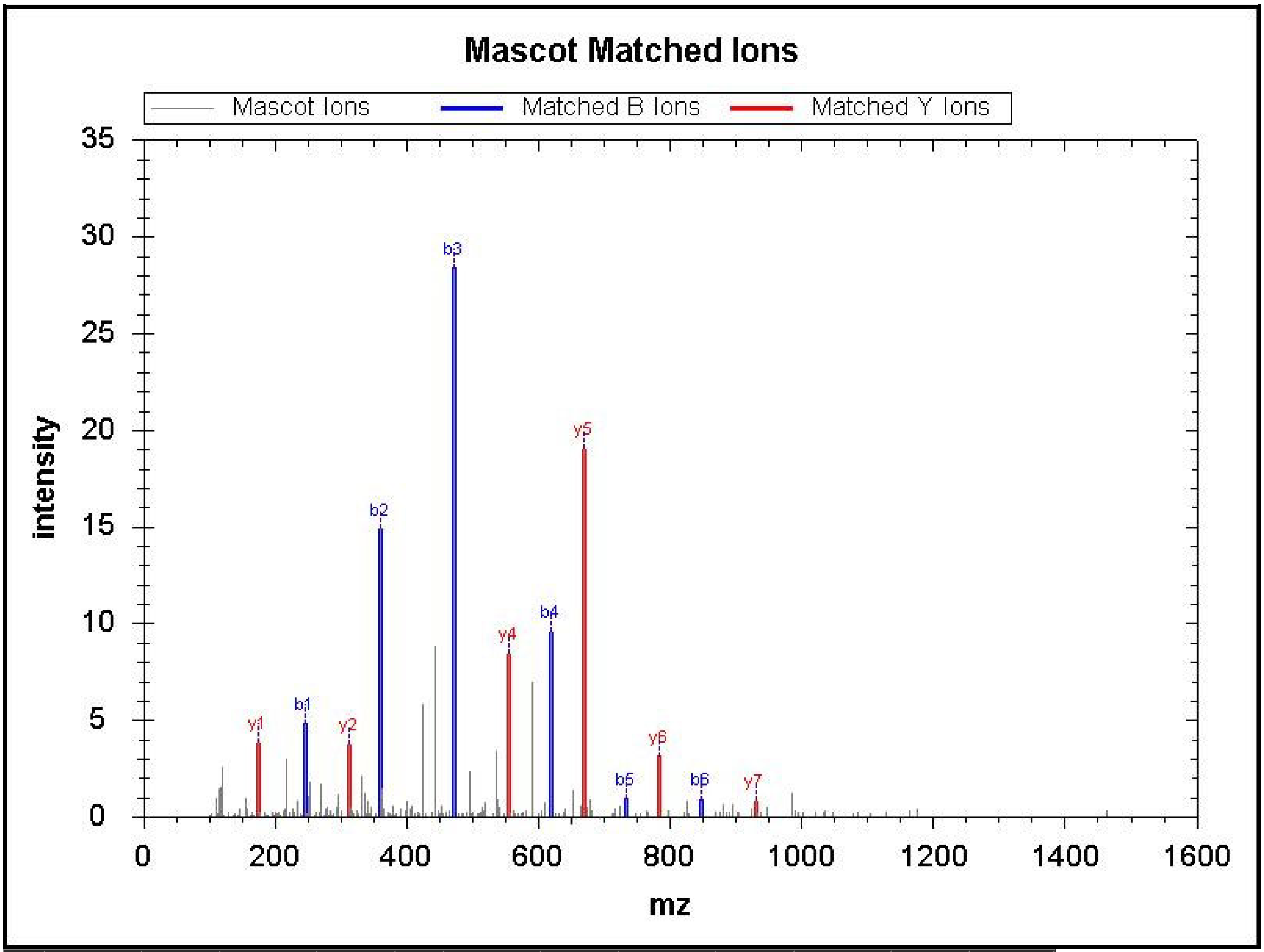

No	b	b++	b0	b0++	Seq	y	y++	y*	y*++	y0	y0++	RevNo
1	246.16	123.58	228.15	114.58	T							10
2	359.24	180.12	341.23	171.12	I	1,155.65	578.33	1,138.63	569.82	1,137.64	569.32	9
3	472.33	236.67	454.31	227.66	L	1,042.57	521.79	1,025.54	513.27	1,024.56	512.78	8
4	619.39	310.20	601.38	301.20	F	929.48	465.25	912.46	456.73	911.47	456.24	7
5	732.48	366.74	714.47	357.74	I	782.42	391.71	765.39	383.20	764.40	382.71	6
6	847.50	424.26	829.49	415.25	D	669.33	335.17	652.30	326.66	651.32	326.16	5
7	976.55	488.78	958.54	479.77	E	554.30	277.66	537.28	269.14	536.29	268.65	4
8	1,089.63	545.32	1,071.62	536.31	I	425.26	213.13	408.24	204.62			3
9	1,226.69	613.85	1,208.68	604.84	H	312.18	156.59	295.15	148.08			2
10					R	175.12	88.06	158.09	79.55			1

Query 3720 Hit 1

MS/MS Fragmentation of SGQQEGK

Found in sp|P09838|TDT_MOUSE, DNA nucleotidylexotransferase OS=Mus musculus GN=Dntt PE=1 SV=3
Match to Query 3720: 878.4721 from(440.2433,2+)
Title: 128: Scan 370 (rt=17.7808, f=2, i=48) [D:\lab212\membrane\Grace\Joyce\iTRAQ_37_2.raw]
Data File:Submitted from 20120508-1(merge) by Mascot Daemon on JOYCE-VAIO
Monoisotopic mass of neutral peptide Mr(calc): 878.4721
Variable modifications:
Q3 :Deamidated (NQ)
Q4 :Deamidated (NQ)
K7 :iTRAQ4plex (K)
Ions Score: 42.81 Expect: 0.037

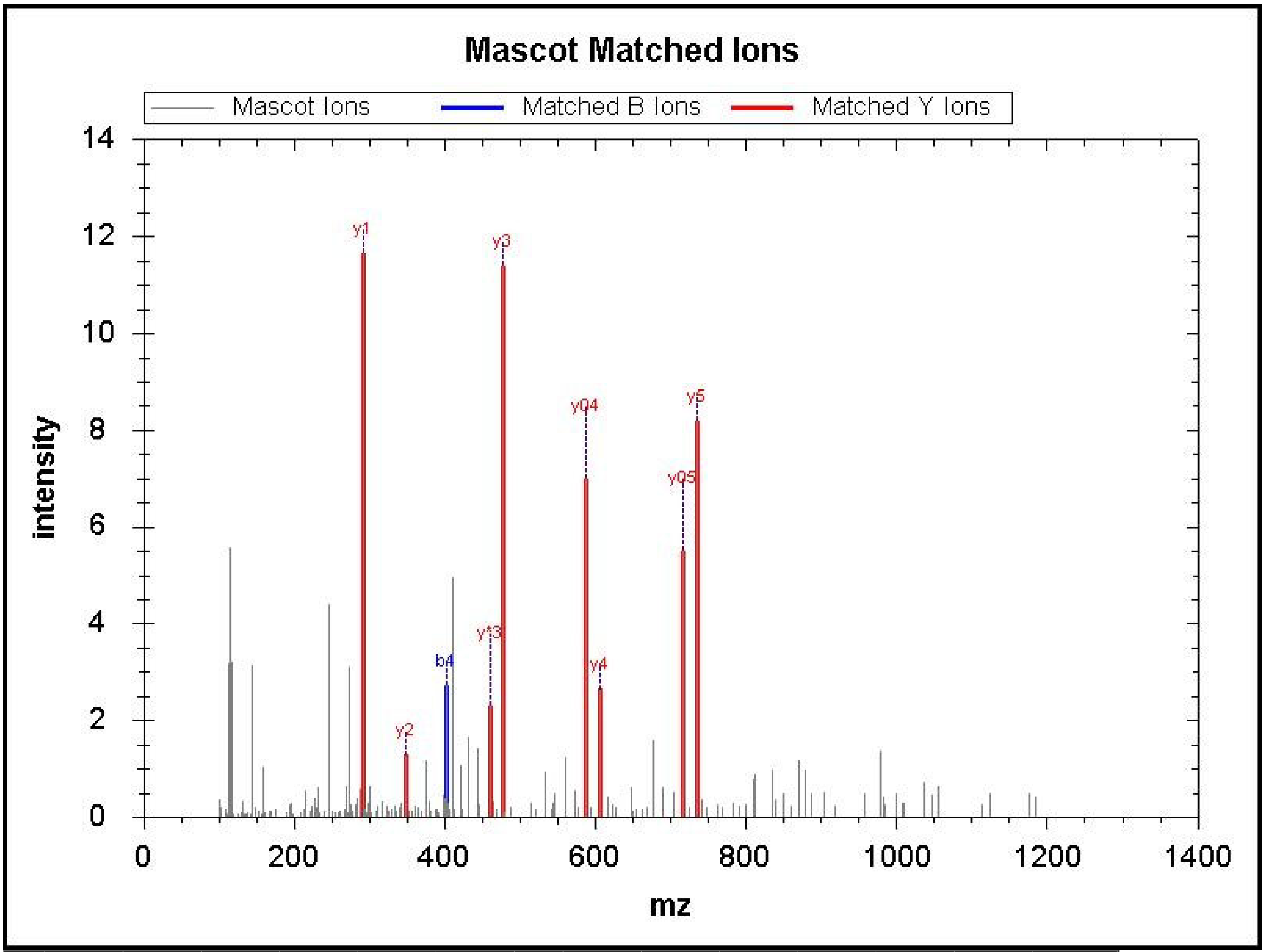

No	b	b++	b*	b*++	b0	b0++	Seq	y	y++	y*	y*++	y0	y0++	RevNo
1	88.04	44.52			70.03	35.52	S							7
2	145.06	73.03			127.05	64.03	G	792.39	396.70	775.36	388.18	774.38	387.69	6
3	274.10	137.56	257.08	129.04	256.09	128.55	Q	735.36	368.19	718.34	359.67	717.35	359.18	5
4	403.15	202.08	386.12	193.56	385.14	193.07	Q	606.32	303.66	589.29	295.15	588.31	294.66	4
5	532.19	266.60	515.16	258.08	514.18	257.59	E	477.28	239.14	460.25	230.63	459.27	230.14	3
6	589.21	295.11	572.18	286.60	571.20	286.10	G	348.24	174.62	331.21	166.11			2
7							K	291.21	146.11	274.19	137.60			1

Query 3179 Hit 1

MS/MS Fragmentation of SGAKVEK
Found in sp|Q8VDC0|SYLM_MOUSE, Probable leucine--tRNA ligase
Match to Query 3179: 861.5121 from(431.7633,2+)
Title: 14: Scan 122 (rt=12.2595, f=2, i=13) [D:\lab212\membrane\Grace\Joyce\iTRAQ_34_2.raw]
Data File:Submitted from 20120508-1(merge) by Mascot Daemon on JOYCE-VAIO
Monoisotopic mass of neutral peptide Mr(calc): 861.5121
Variable modifications:
K7 :iTRAQ4plex (K)

Ions Score: 42.81 Expect: 0.045

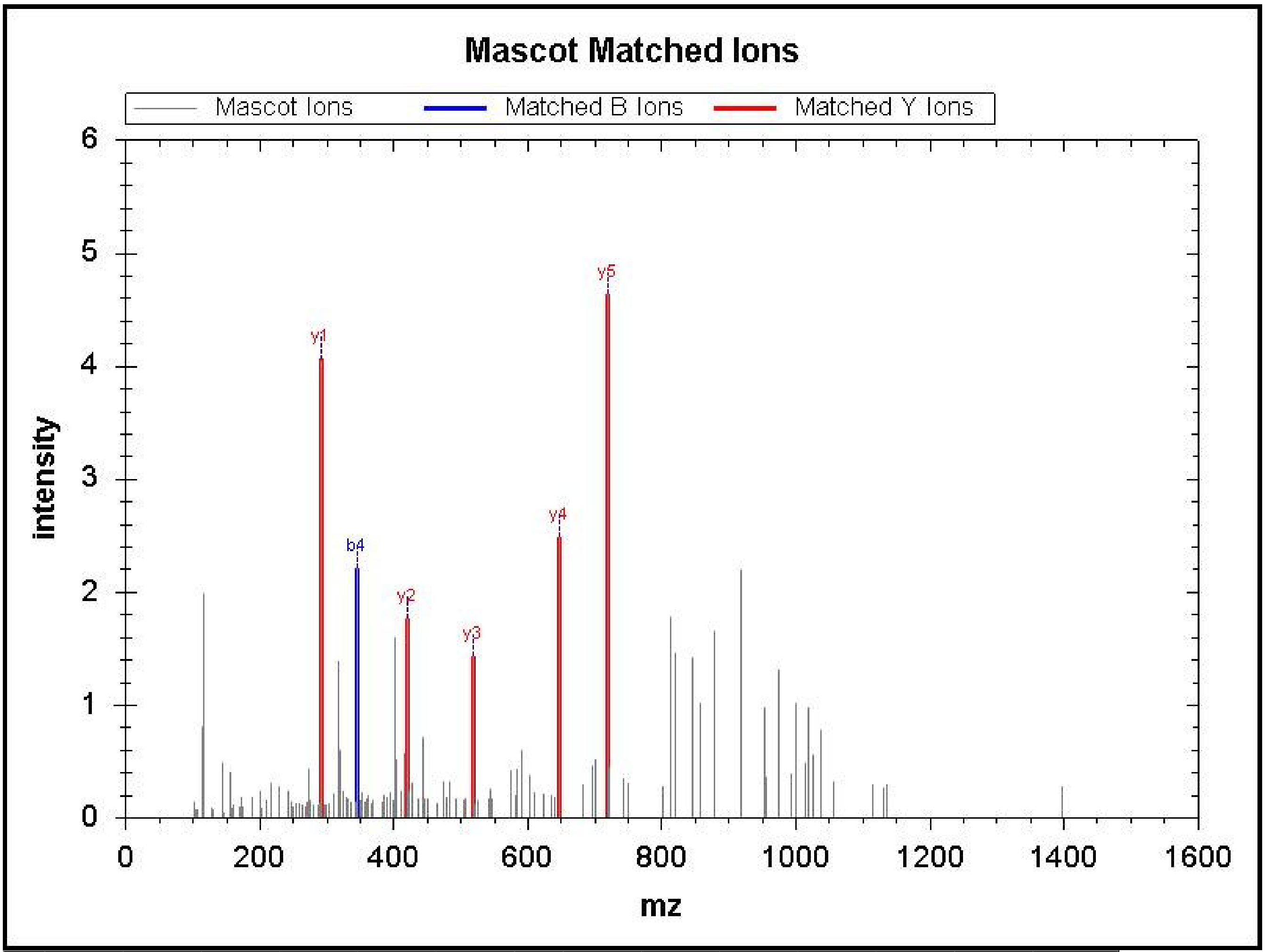

No	b	b++	b*	b*++	b0	b0++	Seq	y	y++	y*	y*++	y0	y0++	RevNo
1	88.04	44.52			70.03	35.52	S							7
2	145.06	73.03			127.05	64.03	G	775.48	388.24	758.45	379.73	757.47	379.24	6
3	216.10	108.55			198.09	99.55	A	718.46	359.73	701.43	351.22	700.45	350.73	5
4	344.19	172.60	327.17	164.09	326.18	163.59	K	647.42	324.21	630.39	315.70	629.41	315.21	4
5	443.26	222.13	426.23	213.62	425.25	213.13	V	519.33	260.17	502.30	251.65	501.32	251.16	3
6	572.30	286.66	555.28	278.14	554.29	277.65	E	420.26	210.63	403.23	202.12	402.25	201.63	2
7							K	291.21	146.11	274.19	137.60			1

Query 48649 Hit 1

MS/MS Fragmentation of AGEGLWVILHLYK
Found in sp|Q9H2J4|PDCL3_HUMAN, Phosducin-like protein 3 OS=Homo sapiens GN=PDCL3 PE=1 SV=1
Match to Query 48649: 1786.039from(596.3535,3+)
Title: 1034: Scan 2277 (rt=60.8643, f=3, i=353) [D:\lab212\membrane\Grace\Joyce\iTRAQ_42_1.raw]
Data File:Submitted from 20120508-1(merge) by Mascot Daemon on JOYCE-VAIO
Monoisotopic mass of neutral peptide Mr(calc): 1786.039
Variable modifications:
K13 :iTRAQ4plex (K)
Ions Score: 42.79 Expect: 0.026

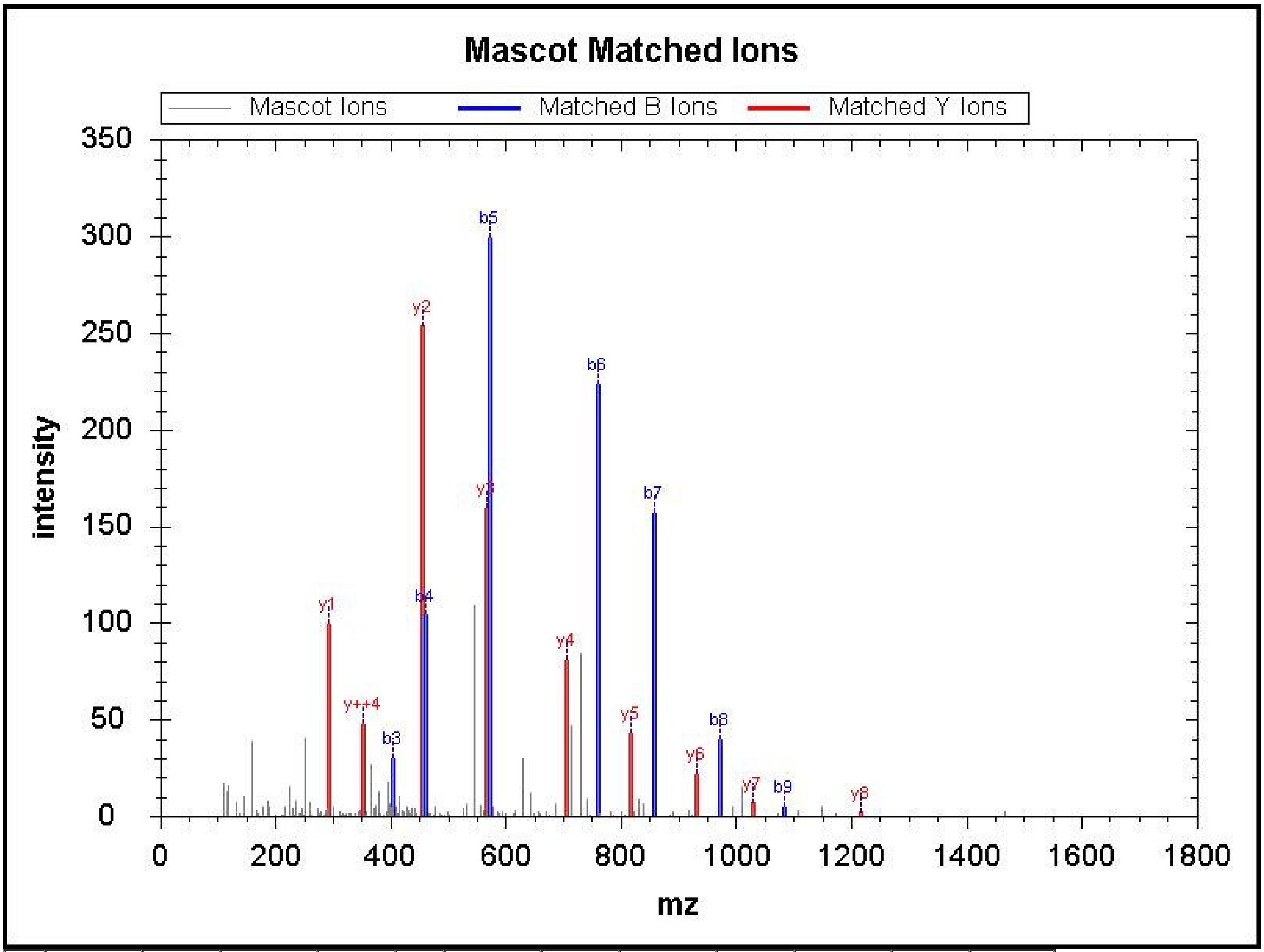

No	b	b++	b0	b0++	Seq	y	y++	y*	y*++	y0	y0++	RevNo
1	216.15	108.58			A							13
2	273.17	137.09			G	1,571.91	786.46	1,554.88	777.94	1,553.90	777.45	12
3	402.21	201.61	384.20	192.60	E	1,514.89	757.95	1,497.86	749.43	1,496.87	748.94	11
4	459.23	230.12	441.22	221.11	G	1,385.84	693.42	1,368.82	684.91			10
5	572.32	286.66	554.31	277.66	L	1,328.82	664.91	1,311.79	656.40			9
6	758.40	379.70	740.38	370.70	W	1,215.74	608.37	1,198.71	599.86			8
7	857.46	429.24	839.45	420.23	V	1,029.66	515.33	1,012.63	506.82			7
8	970.55	485.78	952.54	476.77	I	930.59	465.80	913.56	457.28			6
9	1,083.63	542.32	1,065.62	533.31	L	817.51	409.26	800.48	400.74			5
10	1,220.69	610.85	1,202.68	601.84	H	704.42	352.71	687.39	344.20			4
11	1,333.78	667.39	1,315.76	658.39	L	567.36	284.18	550.34	275.67			3
12	1,496.84	748.92	1,478.83	739.92	Y	454.28	227.64	437.25	219.13			2
13					K	291.21	146.11	274.19	137.60			1

Query 1250 Hit 1

MS/MS Fragmentation of SAIVVR
Found in sp|Q8R367|RERG_MOUSE, Ras-related and estrogen-regulated growth inhibitor OS=Mus musculus GN=Rerg PE=2 SV=1
Match to Query 1250: 787.4655 from(394.74,2+)
Title: 100: Scan 282 (rt=15.9874, f=2, i=44) [D:\lab212\membrane\Grace\Joyce\iTRAQ_27_2_327.raw]
Data File:Submitted from 20120508-1(merge) by Mascot Daemon on JOYCE-VAIO
Monoisotopic mass of neutral peptide Mr(calc): 787.4655
Variable modifications:
Ions Score: 42.75 Expect: 0.037

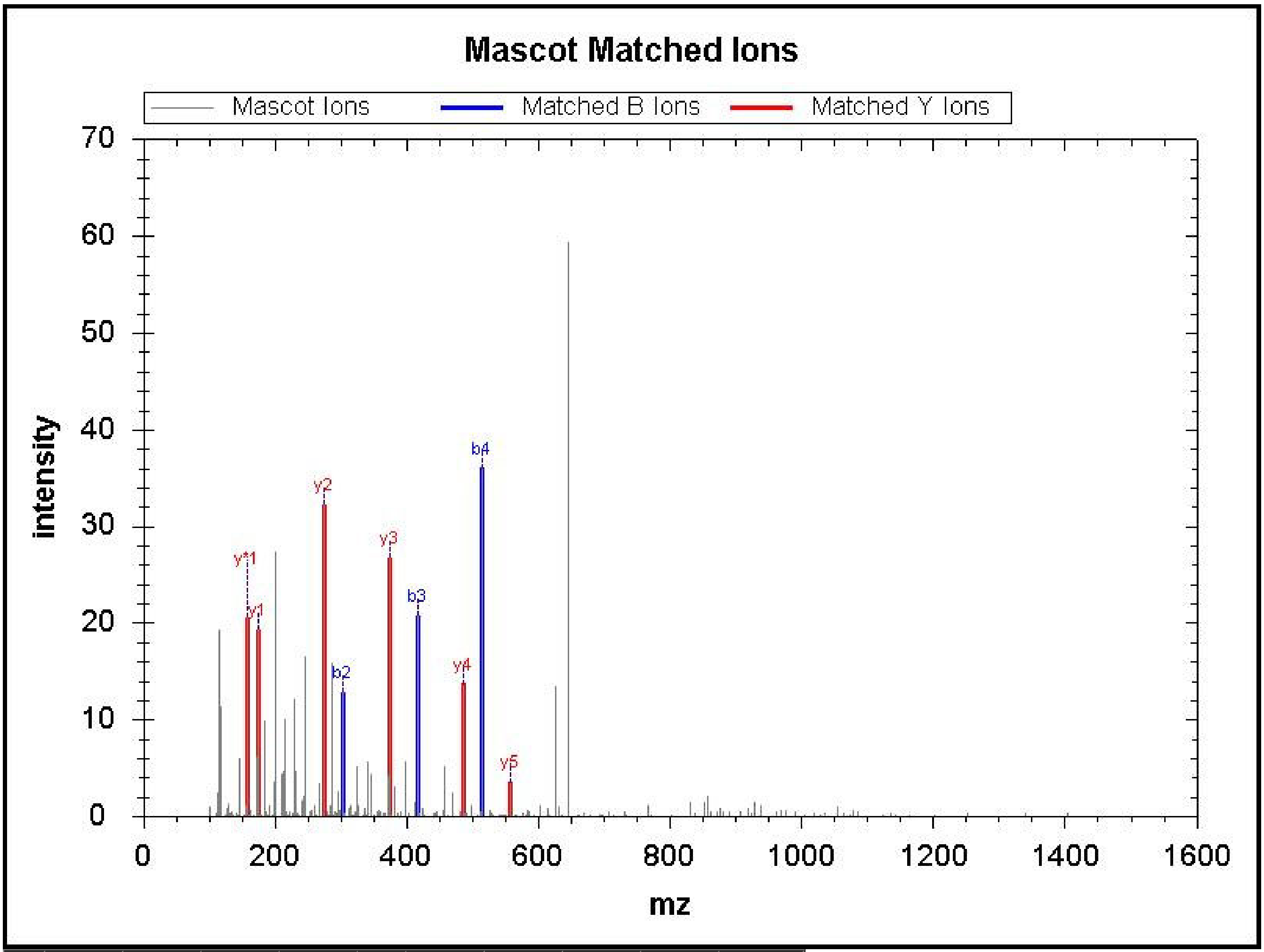

No	b	b++	b0	b0++	Seq	y	y++	y*	y*++	RevNo
1	232.14	116.57	214.13	107.57	S					6
2	303.18	152.09	285.17	143.09	A	557.38	279.19	540.35	270.68	5
3	416.26	208.63	398.25	199.63	I	486.34	243.67	469.31	235.16	4
4	515.33	258.17	497.32	249.16	V	373.26	187.13	356.23	178.62	3
5	614.40	307.70	596.39	298.70	V	274.19	137.60	257.16	129.08	2
6					R	175.12	88.06	158.09	79.55	1

Query 90602 Hit 1

MS/MS Fragmentation of SLVHAGVPASYLLIPAASYVLPEVSK
Found in sp|Q9UI10|EI2BD_HUMAN, Translation initiation factor eIF-2B subunit delta OS=Homo sapiens GN=EIF2B4 PE=1 SV=2
Match to Query 90602: 2968.682from(743.1777,4+)
Title: 1042: Sum of 2 scans in range 2280 (rt=60.8409, f=4, i=697) to 2281 (rt=60.8663, f=4, i=698)
[D:\lab212\membrane\Grace\Joyce\iTRAQ_32_1.raw]
Data File:Submitted from 20120508-1(merge) by Mascot Daemon on JOYCE-VAIO
Monoisotopic mass of neutral peptide Mr(calc): 2968.682
Variable modifications:
K26 :iTRAQ4plex (K)
Ions Score: 42.74 Expect: 0.013

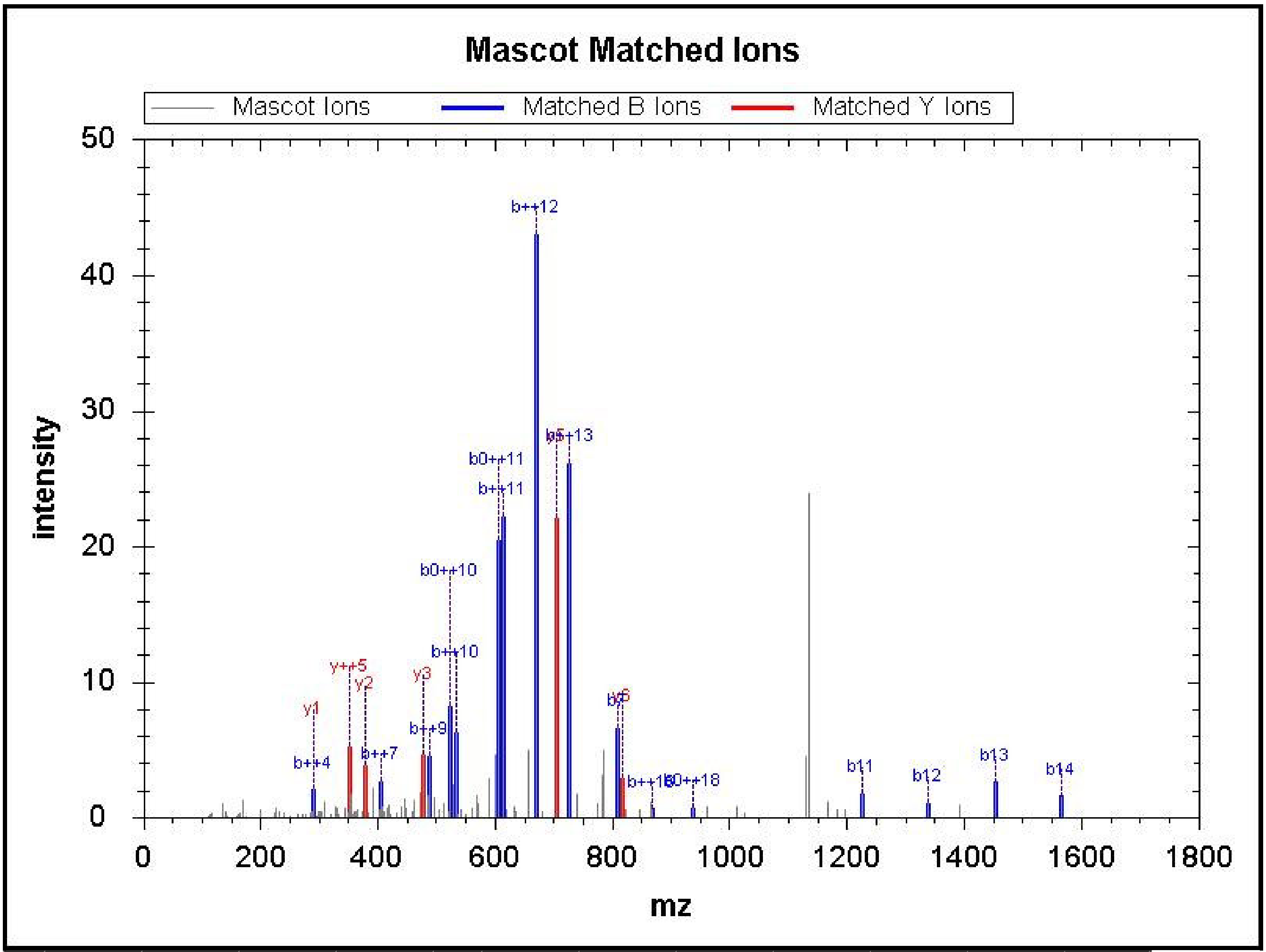

No	b	b++	b0	b0++	Seq	y	y++	y*	y*++	y0	y0++	RevNo
1	232.14	116.57	214.13	107.57	S							26
2	345.23	173.12	327.21	164.11	L	2,738.56	1,369.78	2,721.53	1,361.27	2,720.55	1,360.78	25
3	444.29	222.65	426.28	213.65	V	2,625.48	1,313.24	2,608.45	1,304.73	2,607.47	1,304.24	24
4	581.35	291.18	563.34	282.17	H	2,526.41	1,263.71	2,509.38	1,255.19	2,508.40	1,254.70	23
5	652.39	326.70	634.38	317.69	A	2,389.35	1,195.18	2,372.32	1,186.67	2,371.34	1,186.17	22
6	709.41	355.21	691.40	346.20	G	2,318.31	1,159.66	2,301.29	1,151.15	2,300.30	1,150.65	21
7	808.48	404.74	790.47	395.74	V	2,261.29	1,131.15	2,244.26	1,122.64	2,243.28	1,122.14	20
8	905.53	453.27	887.52	444.26	P	2,162.22	1,081.62	2,145.20	1,073.10	2,144.21	1,072.61	19
9	976.57	488.79	958.56	479.78	A	2,065.17	1,033.09	2,048.14	1,024.58	2,047.16	1,024.08	18
10	1,063.60	532.30	1,045.59	523.30	S	1,994.13	997.57	1,977.11	989.06	1,976.12	988.56	17
11	1,226.67	613.84	1,208.65	604.83	Y	1,907.10	954.05	1,890.07	945.54	1,889.09	945.05	16
12	1,339.75	670.38	1,321.74	661.37	L	1,744.04	872.52	1,727.01	864.01	1,726.03	863.52	15
13	1,452.83	726.92	1,434.82	717.91	L	1,630.95	815.98	1,613.93	807.47	1,612.94	806.98	14
14	1,565.92	783.46	1,547.91	774.46	I	1,517.87	759.44	1,500.84	750.93	1,499.86	750.43	13
15	1,662.97	831.99	1,644.96	822.98	P	1,404.79	702.90	1,387.76	694.38	1,386.77	693.89	12
16	1,734.01	867.51	1,716.00	858.50	A	1,307.73	654.37	1,290.71	645.86	1,289.72	645.36	11
17	1,805.04	903.03	1,787.03	894.02	A	1,236.70	618.85	1,219.67	610.34	1,218.68	609.85	10
18	1,892.08	946.54	1,874.07	937.54	S	1,165.66	583.33	1,148.63	574.82	1,147.65	574.33	9
19	2,055.14	1,028.07	2,037.13	1,019.07	Y	1,078.63	539.82	1,061.60	531.30	1,060.62	530.81	8
20	2,154.21	1,077.61	2,136.20	1,068.60	V	915.56	458.29	898.54	449.77	897.55	449.28	7
21	2,267.29	1,134.15	2,249.28	1,125.14	L	816.49	408.75	799.47	400.24	798.48	399.75	6
22	2,364.34	1,182.68	2,346.33	1,173.67	P	703.41	352.21	686.38	343.70	685.40	343.20	5
23	2,493.39	1,247.20	2,475.38	1,238.19	E	606.36	303.68	589.33	295.17	588.35	294.68	4
24	2,592.46	1,296.73	2,574.45	1,287.73	V	477.32	239.16	460.29	230.65	459.30	230.16	3

25	2,679.49	1,340.25	2,661.48	1,331.24	S	378.25	189.63	361.22	181.11	360.24	180.62	2
26					K	291.21	146.11	274.19	137.60			1

Query 30374 Hit 1

MS/MS Fragmentation of KHEIEAAIVR
Found in sp|Q13618|CUL3_HUMAN, Cullin-3 OS=Homo sapiens GN=CUL3 PE=1 SV=2
Match to Query 30374: 1452.872from(485.298,3+)
Title: 239: Scan 578 (rt=22.6143, f=2, i=88) [D:\lab212\membrane\Grace\Joyce\iTRAQ_53_1.raw]
Data File:Submitted from 20120508-1(merge) by Mascot Daemon on JOYCE-VAIO
Monoisotopic mass of neutral peptide Mr(calc): 1452.872
Variable modifications:
K1 :iTRAQ4plex (K)
Ions Score: 42.67 Expect: 0.024

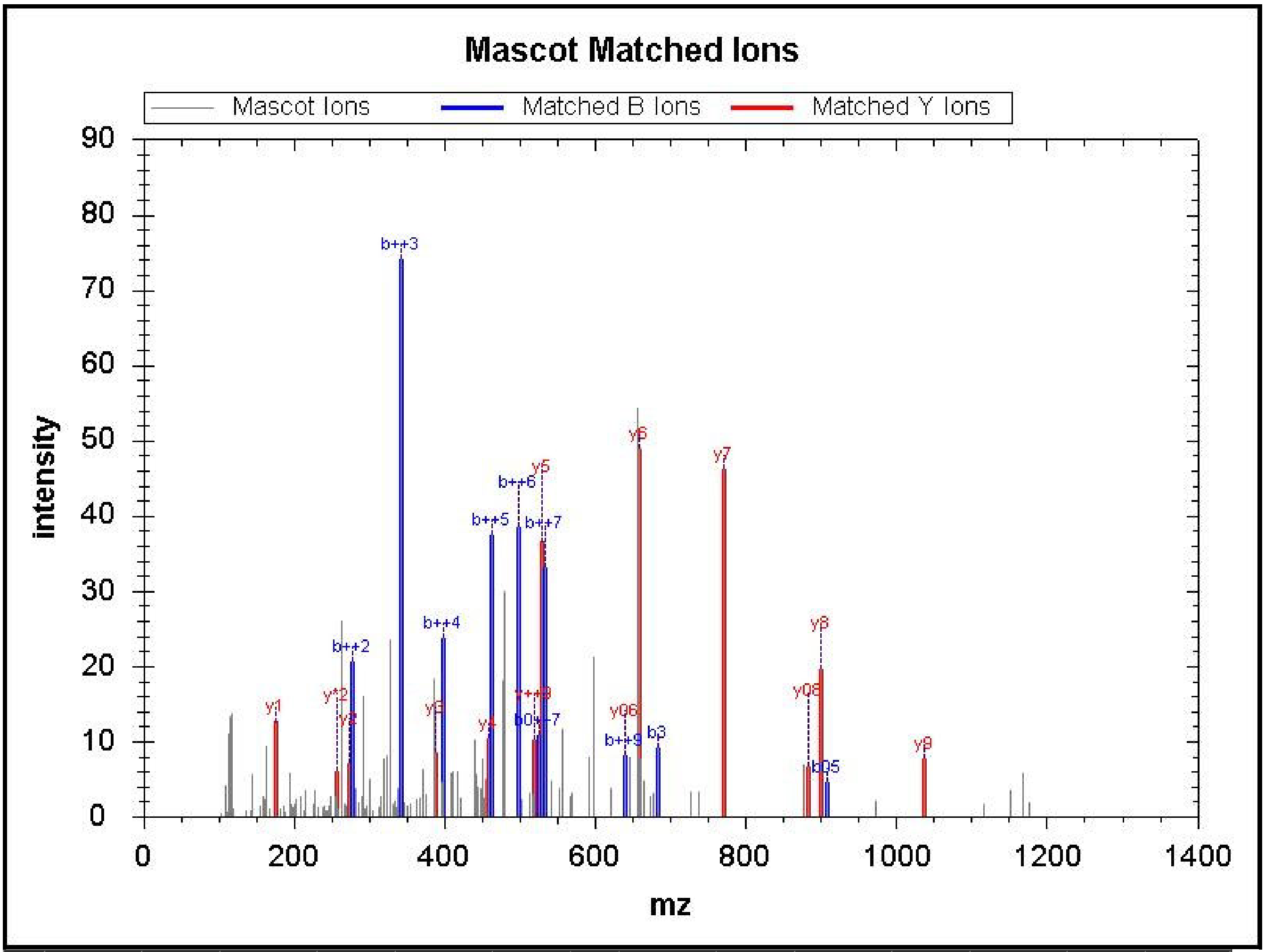

No	b	b++	b*	b*++	b0	b0++	Seq	y	y++	y*	y*++	y0	y0++	RevNo
1	417.31	209.16	400.28	200.64			K							10
2	554.37	277.69	537.34	269.17			H	1,037.57	519.29	1,020.55	510.78	1,019.56	510.29	9
3	683.41	342.21	666.38	333.69	665.40	333.20	E	900.51	450.76	883.49	442.25	882.50	441.76	8
4	796.49	398.75	779.47	390.24	778.48	389.74	I	771.47	386.24	754.45	377.73	753.46	377.23	7
5	925.53	463.27	908.51	454.76	907.52	454.27	E	658.39	329.70	641.36	321.18	640.38	320.69	6
6	996.57	498.79	979.55	490.28	978.56	489.78	A	529.35	265.18	512.32	256.66			5
7	1,067.61	534.31	1,050.58	525.79	1,049.60	525.30	A	458.31	229.66	441.28	221.14			4
8	1,180.69	590.85	1,163.67	582.34	1,162.68	581.84	I	387.27	194.14	370.24	185.63			3
9	1,279.76	640.38	1,262.73	631.87	1,261.75	631.38	V	274.19	137.60	257.16	129.08			2
10							R	175.12	88.06	158.09	79.55			1

Query 51833 Hit 1

MS/MS Fragmentation of AGAEDDEEKGEGTPR
Found in sp|Q86VM9|ZCH18_HUMAN, Zinc finger CCCH domain-containing protein 18 OS=Homo sapiens GN=ZC3H18 PE=1 SV=2
Match to Query 51833: 1847.877from(616.9664,3+)
Title: 46: Scan 197 (rt=14.0282, f=3, i=19) [D:\lab212\membrane\Grace\Joyce\iTRAQ_44_2.raw]
Data File:Submitted from 20120508-1(merge) by Mascot Daemon on JOYCE-VAIO
Monoisotopic mass of neutral peptide Mr(calc): 1847.877
Variable modifications:
K9 :iTRAQ4plex (K)
Ions Score: 42.67 Expect: 0.040

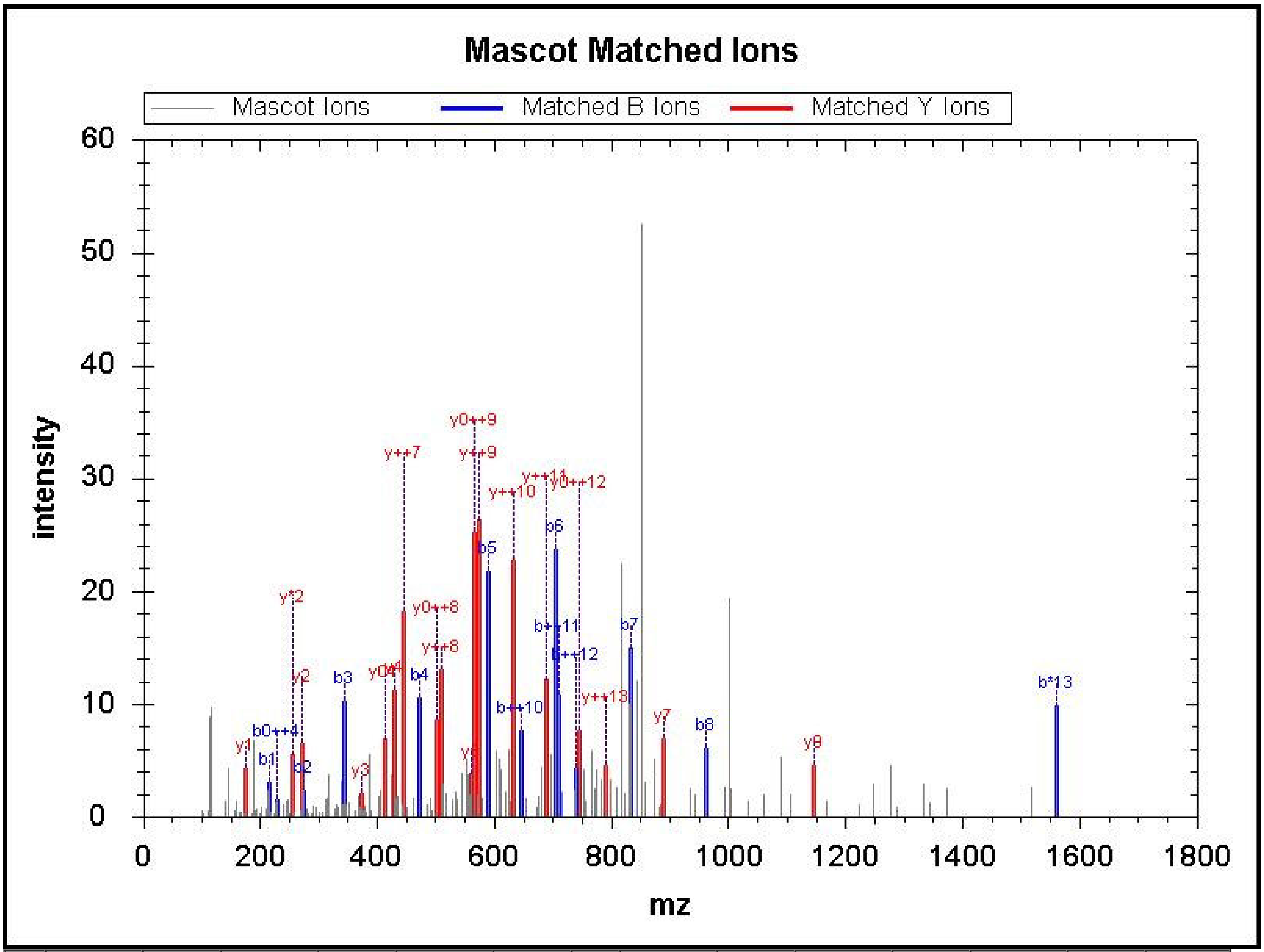

No	b	b++	b*	b*++	b0	b0++	Seq	y	y++	y*	y*++	y0	y0++	RevNo
1	216.15	108.58					A							15
2	273.17	137.09					G	1,633.74	817.37	1,616.72	808.86	1,615.73	808.37	14
3	344.21	172.61					A	1,576.72	788.86	1,559.69	780.35	1,558.71	779.86	13
4	473.25	237.13			455.24	228.12	E	1,505.68	753.35	1,488.66	744.83	1,487.67	744.34	12
5	588.27	294.64			570.26	285.64	D	1,376.64	688.82	1,359.61	680.31	1,358.63	679.82	11
6	703.30	352.15			685.29	343.15	D	1,261.61	631.31	1,244.59	622.80	1,243.60	622.31	10
7	832.34	416.68			814.33	407.67	E	1,146.59	573.80	1,129.56	565.28	1,128.58	564.79	9
8	961.39	481.20			943.38	472.19	E	1,017.54	509.28	1,000.52	500.76	999.53	500.27	8
9	1,233.58	617.30	1,216.56	608.78	1,215.57	608.29	K	888.50	444.75	871.48	436.24	870.49	435.75	7
10	1,290.61	645.81	1,273.58	637.29	1,272.59	636.80	G	616.30	308.66	599.28	300.14	598.29	299.65	6
11	1,419.65	710.33	1,402.62	701.81	1,401.64	701.32	E	559.28	280.15	542.26	271.63	541.27	271.14	5
12	1,476.67	738.84	1,459.64	730.33	1,458.66	729.83	G	430.24	215.62	413.21	207.11	412.23	206.62	4
13	1,577.72	789.36	1,560.69	780.85	1,559.71	780.36	T	373.22	187.11	356.19	178.60	355.21	178.11	3
14	1,674.77	837.89	1,657.74	829.38	1,656.76	828.88	P	272.17	136.59	255.15	128.08			2
15							R	175.12	88.06	158.09	79.55			1

Query 87580 Hit 1

MS/MS Fragmentation of GDLSGHFEHVMVALVTAPALFDAK
Found in sp|O35639|ANXA3_MOUSE, Annexin A3 OS=Mus musculus GN=Anxa3 PE=1 SV=4
Match to Query 87580: 2812.483from(704.1281,4+)
Title: 1135: Scan 2451 (rt=64.8336, f=2, i=399) [D:\lab212\membrane\Grace\Joyce\iTRAQ_45_2.raw]
Data File:Submitted from 20120508-1(merge) by Mascot Daemon on JOYCE-VAIO
Monoisotopic mass of neutral peptide Mr(calc): 2812.483
Variable modifications:
K24 :iTRAQ4plex (K)
Ions Score: 42.67 Expect: 0.041

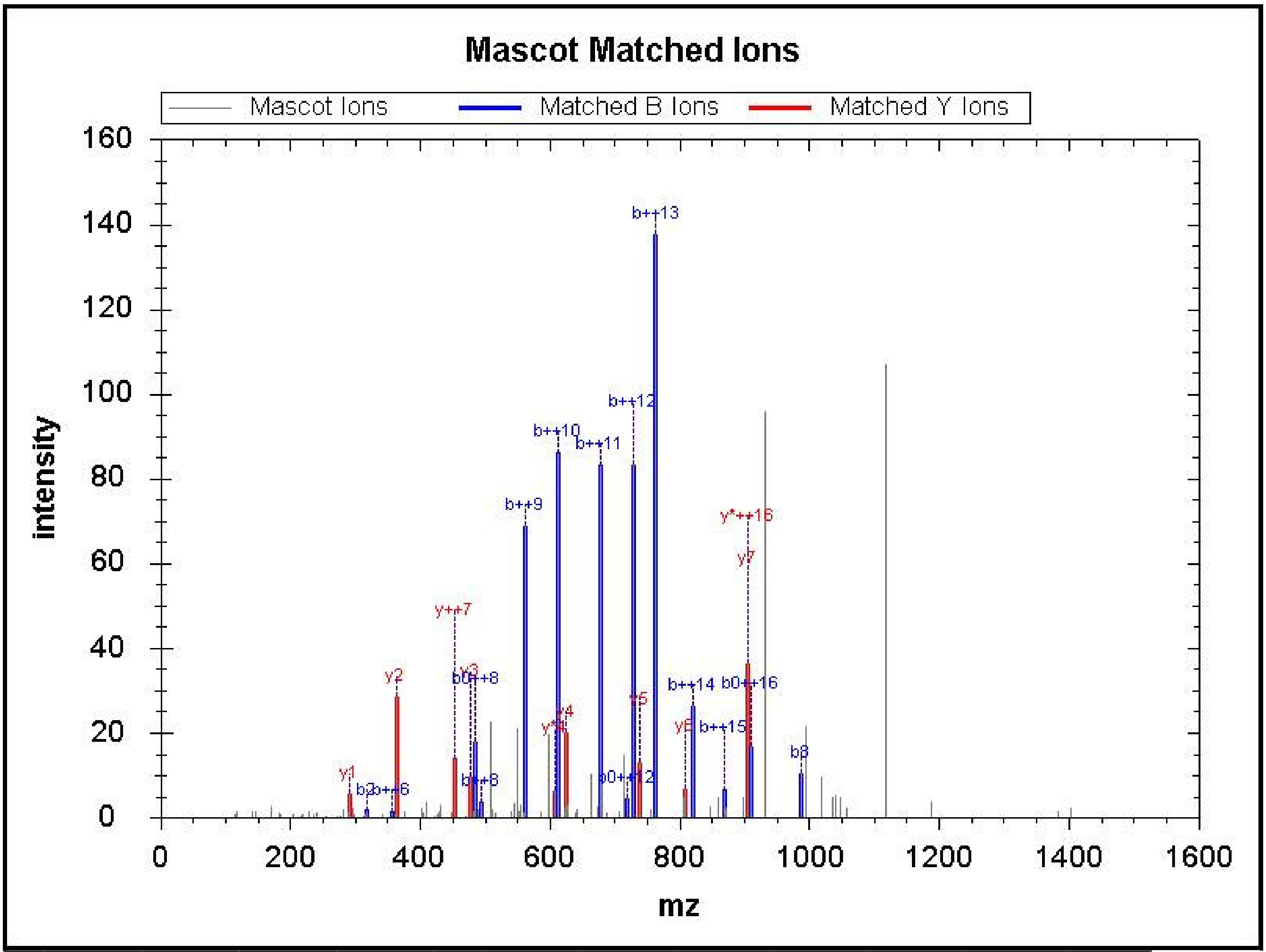

No	b	b++	b0	b0++	Seq	y	y++	y*	y*++	y0	y0++	RevNo
1	202.13	101.57			G							24
2	317.16	159.08	299.15	150.08	D	2,612.37	1,306.69	2,595.34	1,298.17	2,594.36	1,297.68	23
3	430.24	215.62	412.23	206.62	L	2,497.34	1,249.17	2,480.31	1,240.66	2,479.33	1,240.17	22
4	517.27	259.14	499.26	250.14	S	2,384.26	1,192.63	2,367.23	1,184.12	2,366.24	1,183.63	21
5	574.30	287.65	556.28	278.65	G	2,297.22	1,149.12	2,280.20	1,140.60	2,279.21	1,140.11	20
6	711.35	356.18	693.34	347.18	H	2,240.20	1,120.60	2,223.18	1,112.09	2,222.19	1,111.60	19
7	858.42	429.71	840.41	420.71	F	2,103.14	1,052.08	2,086.12	1,043.56	2,085.13	1,043.07	18
8	987.47	494.24	969.45	485.23	E	1,956.07	978.54	1,939.05	970.03	1,938.06	969.54	17
9	1,124.52	562.77	1,106.51	553.76	H	1,827.03	914.02	1,810.01	905.51	1,809.02	905.01	16
10	1,223.59	612.30	1,205.58	603.29	V	1,689.97	845.49	1,672.95	836.98	1,671.96	836.48	15
11	1,354.63	677.82	1,336.62	668.81	M	1,590.90	795.96	1,573.88	787.44	1,572.89	786.95	14
12	1,453.70	727.35	1,435.69	718.35	V	1,459.86	730.44	1,442.84	721.92	1,441.85	721.43	13
13	1,524.74	762.87	1,506.73	753.87	A	1,360.80	680.90	1,343.77	672.39	1,342.79	671.90	12
14	1,637.82	819.42	1,619.81	810.41	L	1,289.76	645.38	1,272.73	636.87	1,271.75	636.38	11

15	1,736.89	868.95	1,718.88	859.94	V	1,176.67	588.84	1,159.65	580.33	1,158.66	579.84	10
16	1,837.94	919.47	1,819.93	910.47	T	1,077.61	539.31	1,060.58	530.79	1,059.60	530.30	9
17	1,908.98	954.99	1,890.97	945.99	A	976.56	488.78	959.53	480.27	958.55	479.78	8
18	2,006.03	1,003.52	1,988.02	994.51	P	905.52	453.26	888.49	444.75	887.51	444.26	7
19	2,077.07	1,039.04	2,059.06	1,030.03	A	808.47	404.74	791.44	396.22	790.46	395.73	6
20	2,190.15	1,095.58	2,172.14	1,086.57	L	737.43	369.22	720.40	360.71	719.42	360.21	5
21	2,337.22	1,169.11	2,319.21	1,160.11	F	624.35	312.68	607.32	304.16	606.34	303.67	4
22	2,452.25	1,226.63	2,434.23	1,217.62	D	477.28	239.14	460.25	230.63	459.27	230.14	3
23	2,523.28	1,262.14	2,505.27	1,253.14	A	362.25	181.63	345.23	173.12			2
24					K	291.21	146.11	274.19	137.60			1

Query 55727 Hit 1

MS/MS Fragmentation of VLLVIDEPHTDWAK
Found in sp|P17600|SYN1_HUMAN, Synapsin-1 OS=Homo sapiens GN=SYN1 PE=1 SV=3
Match to Query 55727: 1923.07from(642.0306,3+)
Title: 677: Sum of 2 scans in range 1538 (rt=43.9855, f=4, i=443) to 1539 (rt=44.0109, f=4, i=444)
[D:\lab212\membrane\Grace\Joyce\iTRAQ_38_1.raw]
Data File:Submitted from 20120508-1(merge) by Mascot Daemon on JOYCE-VAIO
Monoisotopic mass of neutral peptide Mr(calc): 1923.07
Variable modifications:
K14 :iTRAQ4plex (K)
Ions Score: 42.64 Expect: 0.039

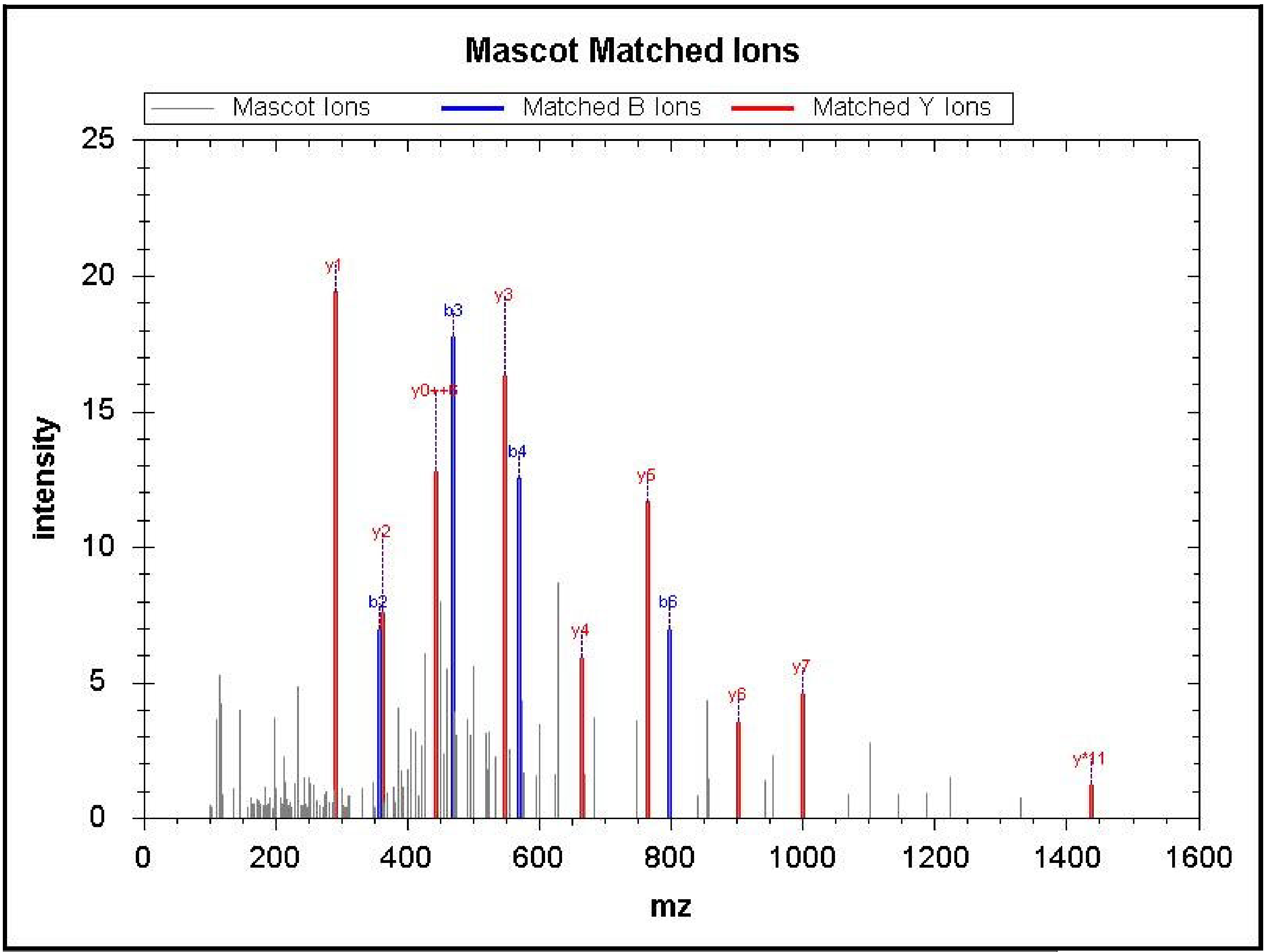

No	b	b++	b0	b0++	Seq	y	y++	y*	y*++	y0	y0++	RevNo
1	244.18	122.59			V							14
2	357.26	179.13			L	1,680.91	840.96	1,663.88	832.44	1,662.90	831.95	13
3	470.35	235.68			L	1,567.82	784.42	1,550.80	775.90	1,549.81	775.41	12

4	569.41	285.21			V	1,454.74	727.87	1,437.71	719.36	1,436.73	718.87	11
5	682.50	341.75			I	1,355.67	678.34	1,338.64	669.83	1,337.66	669.33	10
6	797.53	399.27	779.51	390.26	D	1,242.59	621.80	1,225.56	613.28	1,224.58	612.79	9
7	926.57	463.79	908.56	454.78	E	1,127.56	564.28	1,110.53	555.77	1,109.55	555.28	8
8	1,023.62	512.31	1,005.61	503.31	P	998.52	499.76	981.49	491.25	980.51	490.76	7
9	1,160.68	580.84	1,142.67	571.84	H	901.46	451.24	884.44	442.72	883.45	442.23	6
10	1,261.73	631.37	1,243.72	622.36	T	764.41	382.71	747.38	374.19	746.40	373.70	5
11	1,376.75	688.88	1,358.74	679.88	D	663.36	332.18	646.33	323.67	645.35	323.18	4
12	1,562.83	781.92	1,544.82	772.92	W	548.33	274.67	531.30	266.16			3
13	1,633.87	817.44	1,615.86	808.43	A	362.25	181.63	345.23	173.12			2
14					K	291.21	146.11	274.19	137.60			1

Query 13431 Hit 1

MS/MS Fragmentation of SPIAEAVFR
Found in sp|P24666|PPAC_HUMAN, Low molecular weight phosphotyrosine protein phosphatase OS=Homo sapiens GN=ACP1 PE=1 SV=3
Match to Query 13431: 1132.638from(567.3264,2+)
Title: 326: Sum of 2 scans in range 1408 (rt=37.9587, f=4, i=197) to 1409 (rt=37.9841, f=4, i=198) [D:\lab212\membrane\Grace\Joyce\iTRAQ_24_2.raw]
Data File:Submitted from 20120508-1(merge) by Mascot Daemon on JOYCE-VAIO
Monoisotopic mass of neutral peptide Mr(calc): 1132.638
Variable modifications:
Ions Score: 42.58 Expect: 0.046

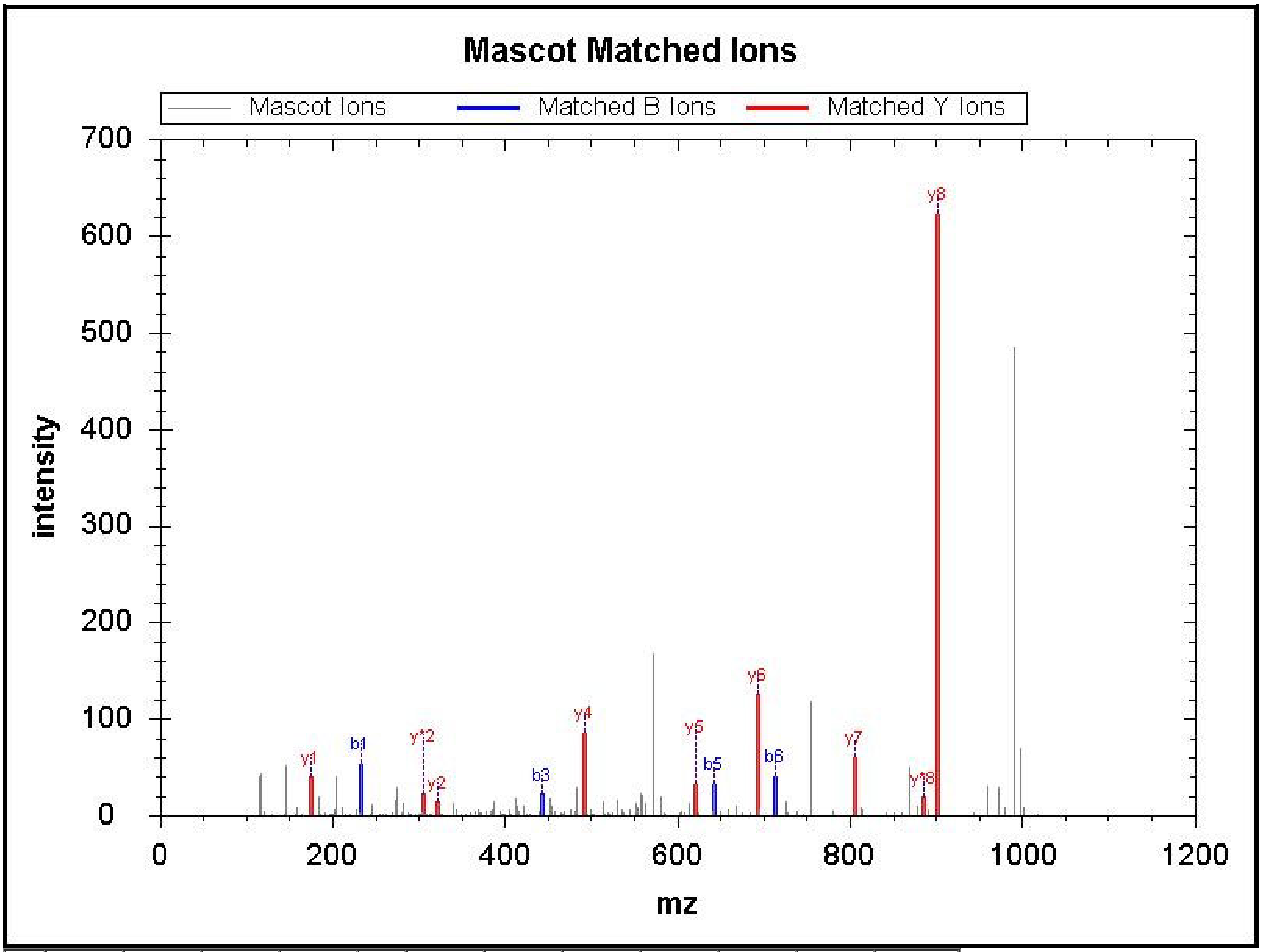

No	b	b++	b0	b0++	Seq	y	y++	y*	y*++	y0	y0++	RevNo
1	232.14	116.57	214.13	107.57	S							9
2	329.19	165.10	311.18	156.10	P	902.51	451.76	885.48	443.25	884.50	442.75	8

3	442.28	221.64	424.27	212.64	I	805.46	403.23	788.43	394.72	787.45	394.23	7
4	513.32	257.16	495.30	248.16	A	692.37	346.69	675.35	338.18	674.36	337.68	6
5	642.36	321.68	624.35	312.68	E	621.34	311.17	604.31	302.66	603.32	302.17	5
6	713.40	357.20	695.38	348.20	A	492.29	246.65	475.27	238.14			4
7	812.46	406.74	794.45	397.73	V	421.26	211.13	404.23	202.62			3
8	959.53	480.27	941.52	471.26	F	322.19	161.60	305.16	153.08			2
9					R	175.12	88.06	158.09	79.55			1

Query 63358 Hit 1

MS/MS Fragmentation of IIEDLGVHFLQYVLK
Found in sp|Q5XG71|UTP20_MOUSE, Small subunit processome component 20 homolog OS=Mus musculus GN=Utp20 PE=2 SV=2
Match to Query 63358: 2074.209from(692.4103,3+)
Title: 1210: Sum of 2 scans in range 2877 (rt=73.2533, f=2, i=491) to 2878 (rt=73.2787, f=2, i=492)
[D:\lab212\membrane\Grace\Joyce\iTRAQ_36_2.raw]
Data File:Submitted from 20120508-1(merge) by Mascot Daemon on JOYCE-VAIO
Monoisotopic mass of neutral peptide Mr(calc): 2074.209
Variable modifications:
K15 :iTRAQ4plex (K)
Ions Score: 42.55 Expect: 0.021

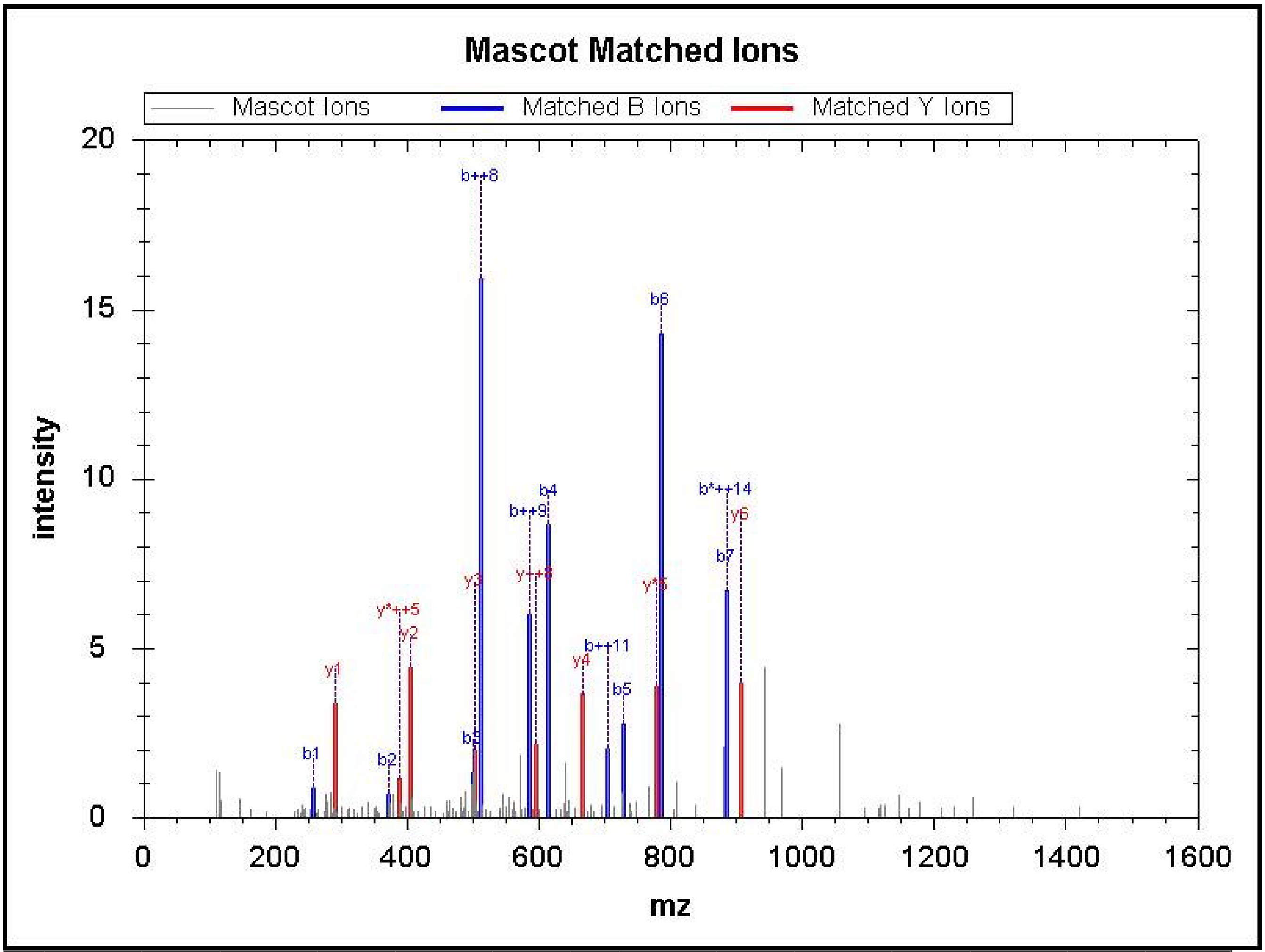

No	b	b++	b*	b*++	b0	b0++	Seq	y	y++	y*	y*++	y0	y0++	RevNo
1	258.19	129.60					I							15
2	371.28	186.14					I	1,818.03	909.52	1,801.00	901.00	1,800.02	900.51	14
3	500.32	250.66			482.31	241.66	E	1,704.94	852.98	1,687.92	844.46	1,686.93	843.97	13
4	615.35	308.18			597.34	299.17	D	1,575.90	788.45	1,558.88	779.94	1,557.89	779.45	12
5	728.43	364.72			710.42	355.71	L	1,460.87	730.94	1,443.85	722.43			11
6	785.45	393.23			767.44	384.22	G	1,347.79	674.40	1,330.76	665.89			10

7	884.52	442.76			866.51	433.76	V	1,290.77	645.89	1,273.74	637.37			9
8	1,021.58	511.29			1,003.57	502.29	H	1,191.70	596.35	1,174.67	587.84			8
9	1,168.65	584.83			1,150.64	575.82	F	1,054.64	527.82	1,037.62	519.31			7
10	1,281.73	641.37			1,263.72	632.36	L	907.57	454.29	890.55	445.78			6
11	1,409.79	705.40	1,392.76	696.89	1,391.78	696.39	Q	794.49	397.75	777.46	389.23			5
12	1,572.85	786.93	1,555.83	778.42	1,554.84	777.93	Y	666.43	333.72	649.40	325.21			4
13	1,671.92	836.47	1,654.90	827.95	1,653.91	827.46	V	503.37	252.19	486.34	243.67			3
14	1,785.01	893.01	1,767.98	884.49	1,767.00	884.00	L	404.30	202.65	387.27	194.14			2
15							K	291.21	146.11	274.19	137.60			1

Query 33544 Hit 1

MS/MS Fragmentation of HVGDLGNVTADK
Found in sp|P00441|SODC_HUMAN, Superoxide dismutase [Cu-Zn] OS=Homo sapiens GN=SOD1 PE=1 SV=2
Match to Query 33544: 1512.816from(505.2793,3+)
Title: 260: Scan 635 (rt=23.9183, f=3, i=91) [D:\lab212\membrane\Grace\Joyce\iTRAQ_45_2.raw]
Data File:Submitted from 20120508-1(merge) by Mascot Daemon on JOYCE-VAIO
Monoisotopic mass of neutral peptide Mr(calc): 1512.816
Variable modifications:
K12 :iTRAQ4plex (K)
Ions Score: 42.51 Expect: 0.043

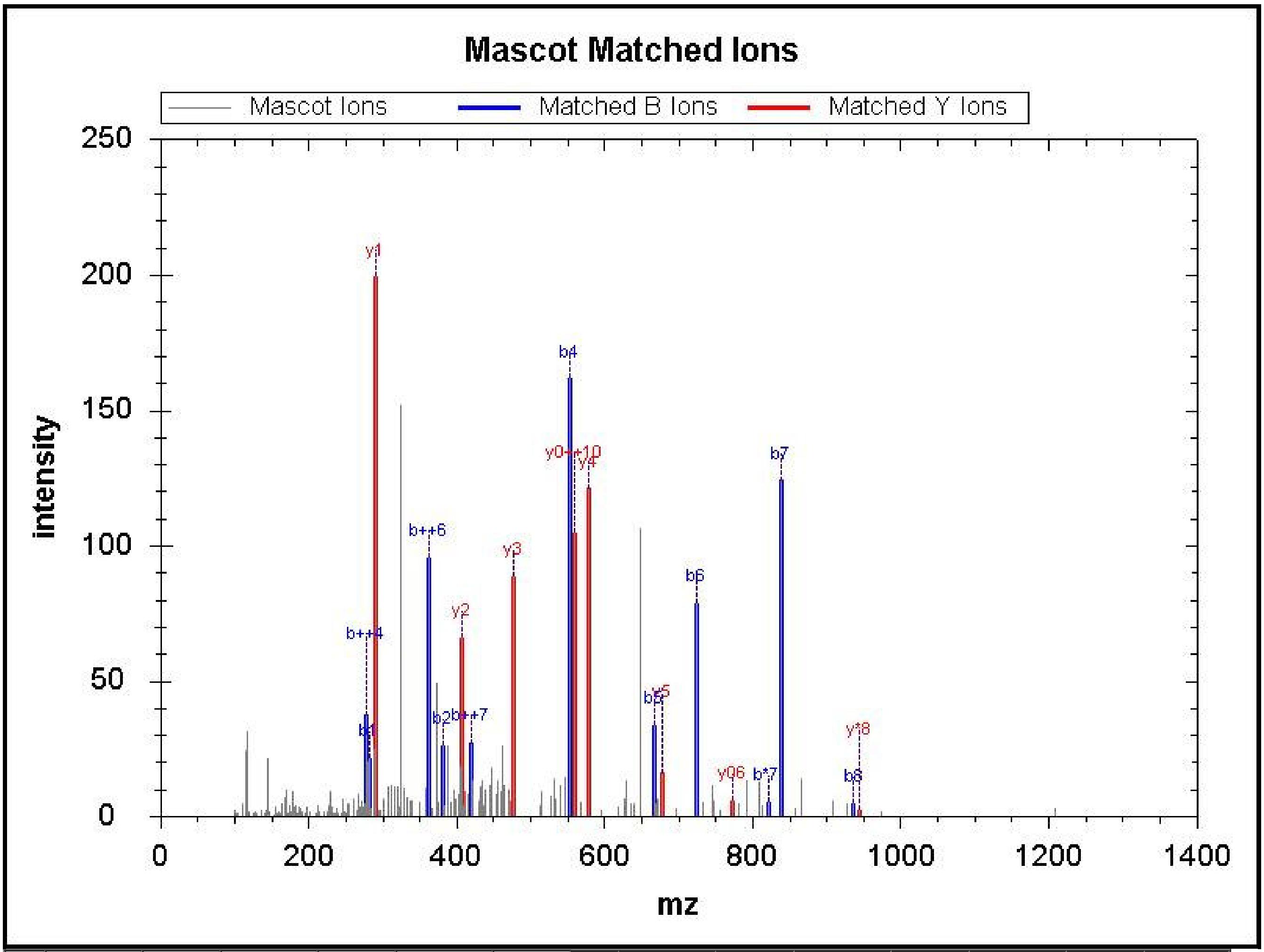

No	b	b++	b*	b*++	b0	b0++	Seq	y	y++	y*	y*++	y0	y0++	RevNo
1	282.17	141.59					H							12
2	381.24	191.12					V	1,232.66	616.83	1,215.63	608.32	1,214.65	607.83	11
3	438.26	219.63					G	1,133.59	567.30	1,116.57	558.79	1,115.58	558.29	10
4	553.29	277.15			535.27	268.14	D	1,076.57	538.79	1,059.54	530.28	1,058.56	529.78	9
5	666.37	333.69			648.36	324.68	L	961.54	481.28	944.52	472.76	943.53	472.27	8

6	723.39	362.20			705.38	353.19	G	848.46	424.73	831.43	416.22	830.45	415.73	7
7	837.43	419.22	820.41	410.71	819.42	410.22	N	791.44	396.22	774.41	387.71	773.43	387.22	6
8	936.50	468.75	919.48	460.24	918.49	459.75	V	677.40	339.20	660.37	330.69	659.38	330.20	5
9	1,037.55	519.28	1,020.52	510.77	1,019.54	510.27	T	578.33	289.67	561.30	281.15	560.32	280.66	4
10	1,108.59	554.80	1,091.56	546.28	1,090.58	545.79	A	477.28	239.14	460.25	230.63	459.27	230.14	3
11	1,223.61	612.31	1,206.59	603.80	1,205.60	603.31	D	406.24	203.62	389.22	195.11	388.23	194.62	2
12							K	291.21	146.11	274.19	137.60			1

Query 80106 Hit 1

MS/MS Fragmentation of EDLIIPHHHSFYDFIVTK
Found in sp|Q14320|FA50A_HUMAN, Protein FAM50A OS=Homo sapiens GN=FAM50A PE=1 SV=2
Match to Query 80106: 2498.319from(625.5871,4+)
Title: 765: Scan 1623 (rt=46.1862, f=3, i=250) [D:\lab212\membrane\Grace\Joyce\iTRAQ_53_2.raw]
Data File:Submitted from 20120508-1(merge) by Mascot Daemon on JOYCE-VAIO
Monoisotopic mass of neutral peptide Mr(calc): 2498.319
Variable modifications:
K18 :iTRAQ4plex (K)
Ions Score: 42.51 Expect: 0.046

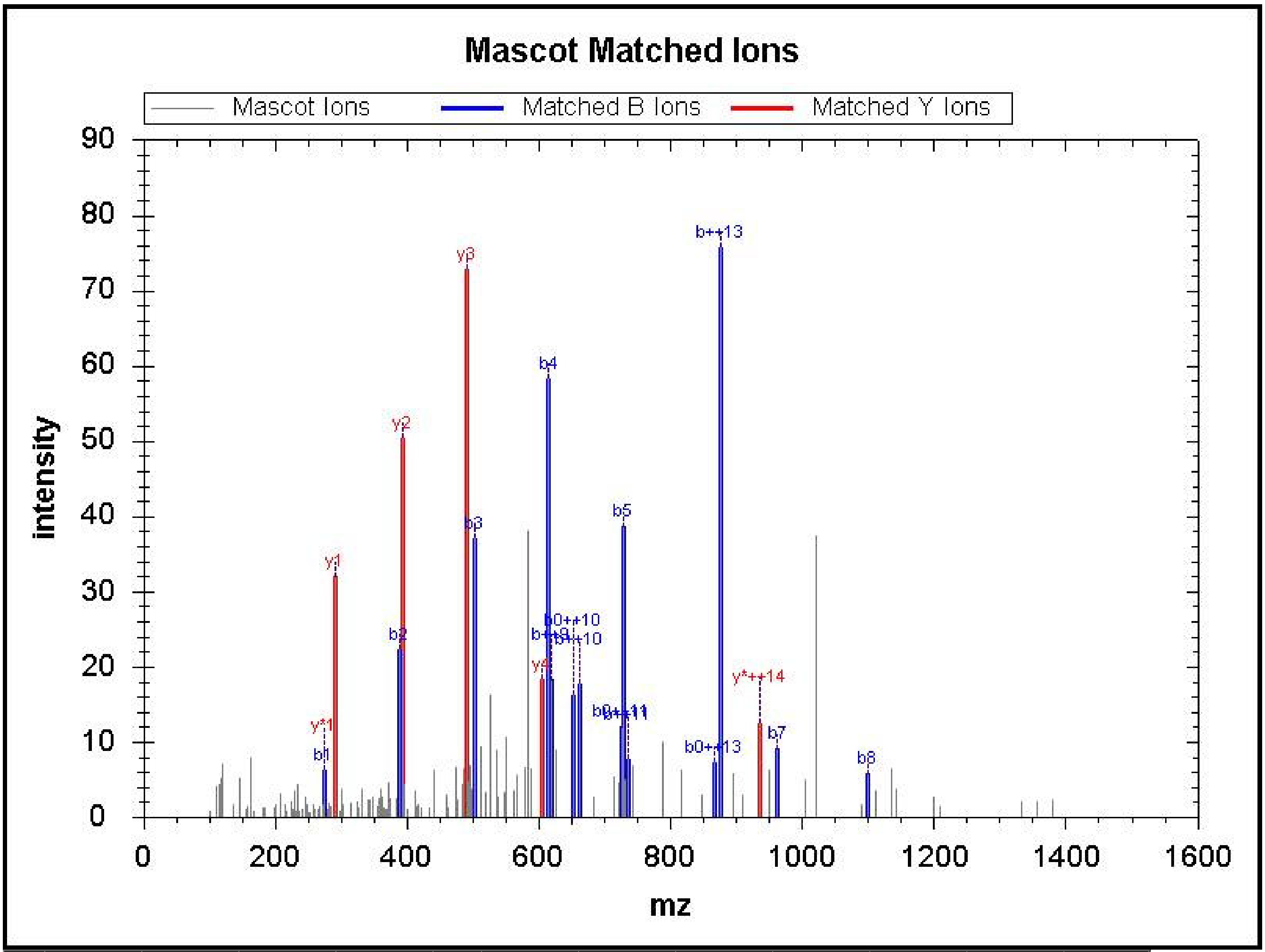

No	b	b++	b0	b0++	Seq	y	y++	y*	y*++	y0	y0++	RevNo
1	274.15	137.58	256.14	128.57	E							18
2	389.18	195.09	371.17	186.09	D	2,226.18	1,113.60	2,209.16	1,105.08	2,208.17	1,104.59	17
3	502.26	251.64	484.25	242.63	L	2,111.16	1,056.08	2,094.13	1,047.57	2,093.15	1,047.08	16
4	615.35	308.18	597.34	299.17	I	1,998.07	999.54	1,981.05	991.03	1,980.06	990.53	15
5	728.43	364.72	710.42	355.71	I	1,884.99	943.00	1,867.96	934.48	1,866.98	933.99	14
6	825.48	413.25	807.47	404.24	P	1,771.90	886.46	1,754.88	877.94	1,753.89	877.45	13
7	962.54	481.77	944.53	472.77	H	1,674.85	837.93	1,657.82	829.42	1,656.84	828.92	12

8	1,099.60	550.30	1,081.59	541.30	H	1,537.79	769.40	1,520.77	760.89	1,519.78	760.39	11
9	1,236.66	618.83	1,218.65	609.83	H	1,400.73	700.87	1,383.71	692.36	1,382.72	691.86	10
10	1,323.69	662.35	1,305.68	653.34	S	1,263.67	632.34	1,246.65	623.83	1,245.66	623.34	9
11	1,470.76	735.88	1,452.75	726.88	F	1,176.64	588.82	1,159.62	580.31	1,158.63	579.82	8
12	1,633.82	817.42	1,615.81	808.41	Y	1,029.57	515.29	1,012.55	506.78	1,011.56	506.29	7
13	1,748.85	874.93	1,730.84	865.92	D	866.51	433.76	849.48	425.25	848.50	424.75	6
14	1,895.92	948.46	1,877.91	939.46	F	751.48	376.25	734.46	367.73	733.47	367.24	5
15	2,009.00	1,005.01	1,990.99	996.00	I	604.41	302.71	587.39	294.20	586.40	293.71	4
16	2,108.07	1,054.54	2,090.06	1,045.53	V	491.33	246.17	474.30	237.66	473.32	237.16	3
17	2,209.12	1,105.06	2,191.11	1,096.06	T	392.26	196.63	375.24	188.12	374.25	187.63	2
18					K	291.21	146.11	274.19	137.60			1

Query 34688 Hit 1

MS/MS Fragmentation of ELVIPLIQNGHR
Found in sp|Q92917|GPKOW_HUMAN, G patch domain and KOW motifs-containing protein OS=Homo sapiens GN=GPKOW PE=1 SV=2
Match to Query 34688: 1532.875from(511.9655,3+)
Title: 671: Scan 1499 (rt=43.2919, f=3, i=230) [D:\lab212\membrane\Grace\Joyce\iTRAQ_34_2.raw]
Data File:Submitted from 20120508-1(merge) by Mascot Daemon on JOYCE-VAIO
Monoisotopic mass of neutral peptide Mr(calc): 1532.875
Variable modifications:
N9 :Deamidated (NQ)
Ions Score: 42.5 Expect: 0.040

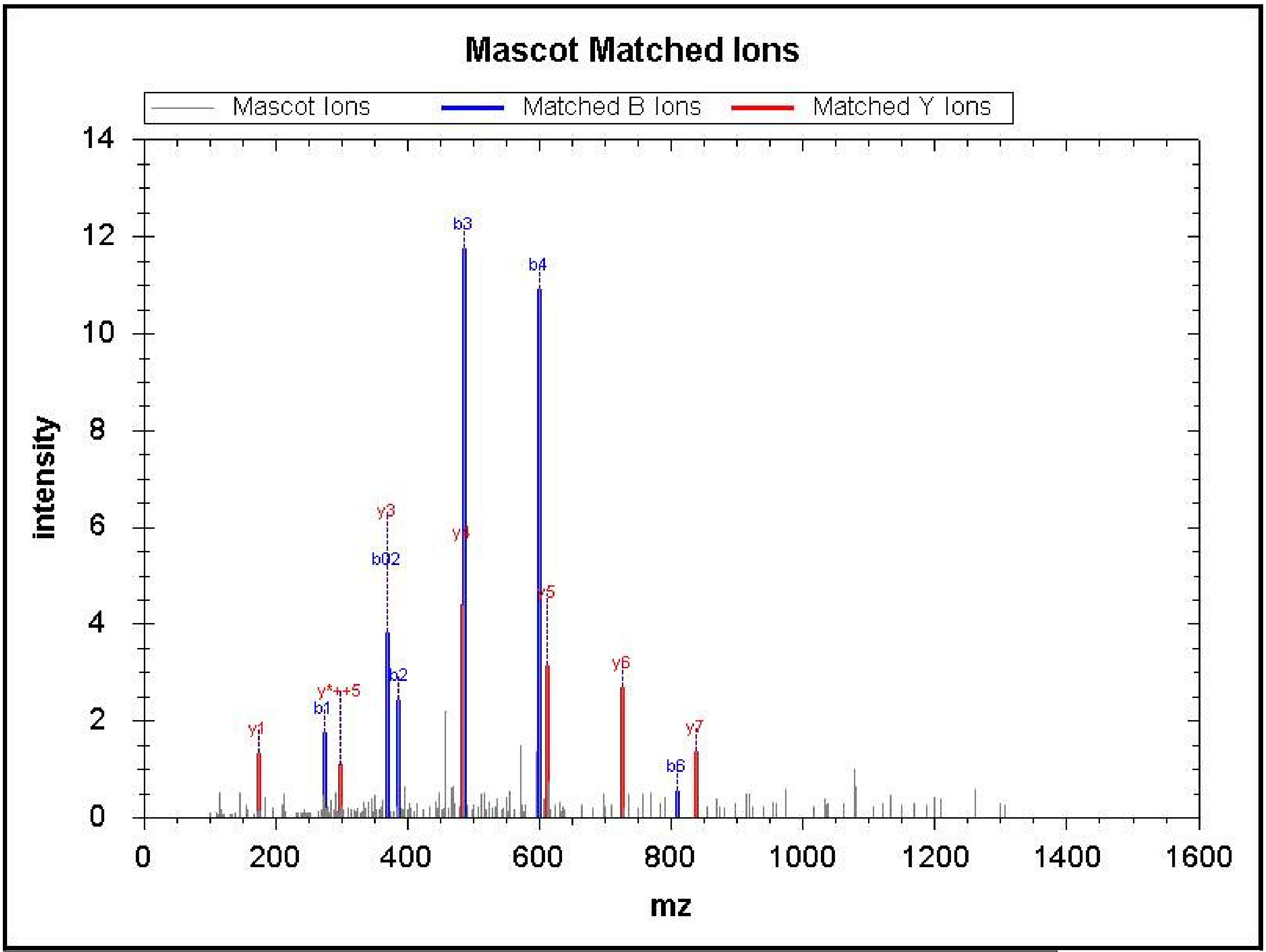

No	b	b++	b*	b*++	b0	b0++	Seq	y	y++	y*	y*++	RevNo
1	274.15	137.58			256.14	128.57	E					12
2	387.24	194.12			369.23	185.12	L	1,260.74	630.87	1,243.72	622.36	11

3	486.30	243.66			468.29	234.65	V	1,147.66	574.33	1,130.63	565.82	10
4	599.39	300.20			581.38	291.19	I	1,048.59	524.80	1,031.56	516.29	9
5	696.44	348.72			678.43	339.72	P	935.51	468.26	918.48	459.74	8
6	809.53	405.27			791.51	396.26	L	838.45	419.73	821.43	411.22	7
7	922.61	461.81			904.60	452.80	I	725.37	363.19	708.34	354.67	6
8	1,050.67	525.84	1,033.64	517.32	1,032.66	516.83	Q	612.28	306.65	595.26	298.13	5
9	1,165.69	583.35	1,148.67	574.84	1,147.68	574.35	N	484.23	242.62	467.20	234.10	4
10	1,222.72	611.86	1,205.69	603.35	1,204.71	602.86	G	369.20	185.10	352.17	176.59	3
11	1,359.78	680.39	1,342.75	671.88	1,341.76	671.39	H	312.18	156.59	295.15	148.08	2
12							R	175.12	88.06	158.09	79.55	1

Query 26847 Hit 1

MS/MS Fragmentation of ILMLGLDAAGK
Found in sp|P62330|ARF6_HUMAN, ADP-ribosylation factor 6 OS=Homo sapiens GN=ARF6 PE=1 SV=2
Match to Query 26847: 1388.832from(695.4232,2+)
Title: 744: Scan 1689 (rt=47.3927, f=2, i=263) [D:\lab212\membrane\Grace\Joyce\iTRAQ_28_1_327.raw]
Data File:Submitted from 20120508-1(merge) by Mascot Daemon on JOYCE-VAIO
Monoisotopic mass of neutral peptide Mr(calc): 1388.832
Variable modifications:
K11 :iTRAQ4plex (K)
Ions Score: 42.47 Expect: 0.032

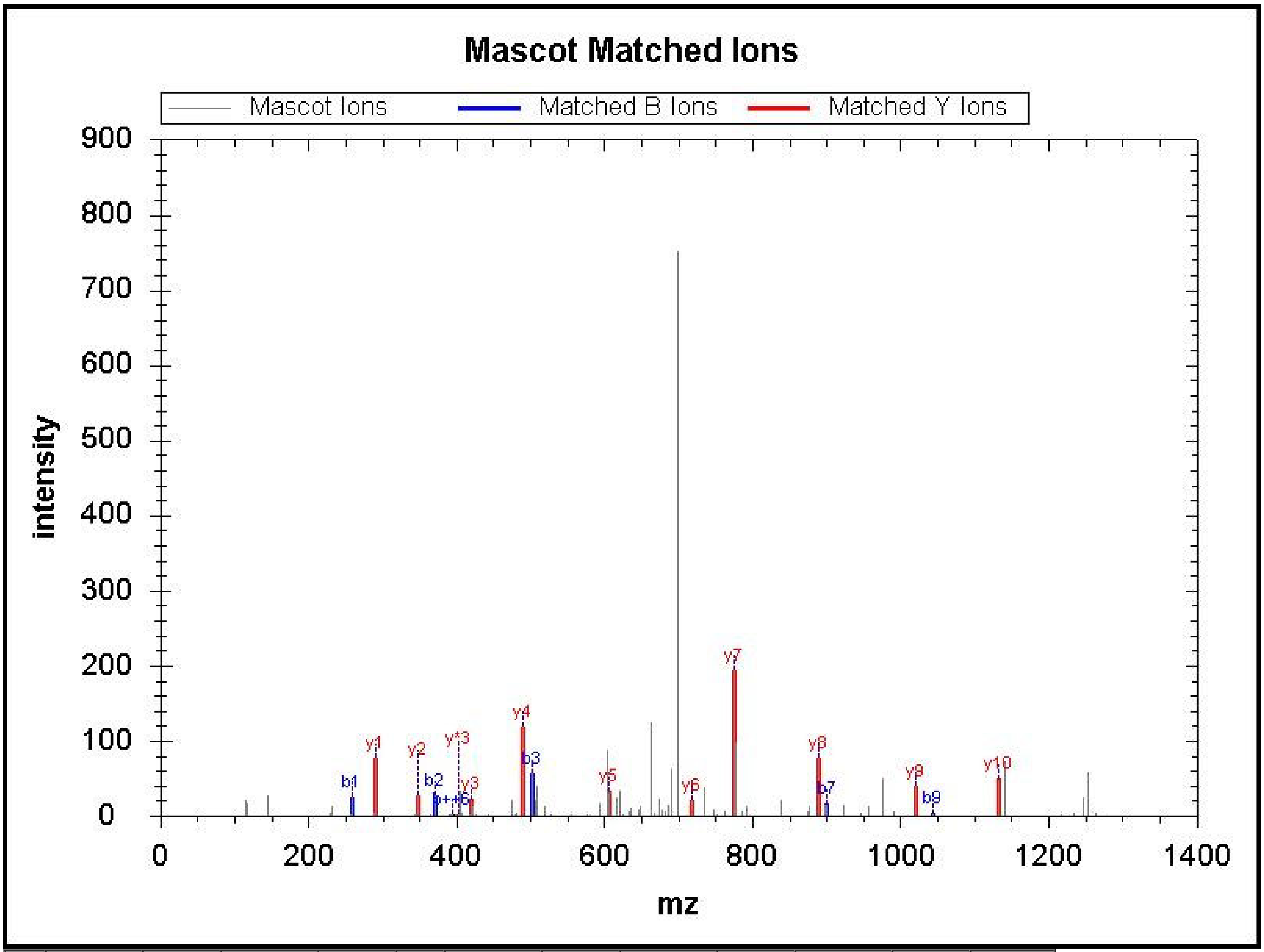

No	b	b++	b0	b0++	Seq	y	y++	y*	y*++	y0	y0++	RevNo
1	258.19	129.60			I							11
2	371.28	186.14			L	1,132.65	566.83	1,115.63	558.32	1,114.64	557.82	10
3	502.32	251.66			M	1,019.57	510.29	1,002.54	501.77	1,001.56	501.28	9
4	615.40	308.20			L	888.53	444.77	871.50	436.25	870.52	435.76	8

5	672.42	336.72			G	775.44	388.23	758.42	379.71	757.43	379.22	7
6	785.51	393.26			L	718.42	359.71	701.39	351.20	700.41	350.71	6
7	900.53	450.77	882.52	441.77	D	605.34	303.17	588.31	294.66	587.33	294.17	5
8	971.57	486.29	953.56	477.28	A	490.31	245.66	473.28	237.15			4
9	1,042.61	521.81	1,024.60	512.80	A	419.27	210.14	402.25	201.63			3
10	1,099.63	550.32	1,081.62	541.31	G	348.24	174.62	331.21	166.11			2
11					K	291.21	146.11	274.19	137.60			1

Query 74034 Hit 1

MS/MS Fragmentation of LEMDAQHIKDEFFHER
Found in sp|Q8IWJ2|GCC2_HUMAN, GRIP and coiled-coil domain-containing protein 2 OS=Homo sapiens GN=GCC2 PE=1 SV=4
Match to Query 74034: 2332.157from(584.0466,4+)
Title: 832: Scan 1776 (rt=49.662, f=3, i=276) [D:\lab212\membrane\Grace\Joyce\54-2.raw]
Data File:Submitted from 20120508-1(merge) by Mascot Daemon on JOYCE-VAIO
Monoisotopic mass of neutral peptide Mr(calc): 2332.157
Variable modifications:
K9 :iTRAQ4plex (K)
Ions Score: 42.45 Expect: 0.054

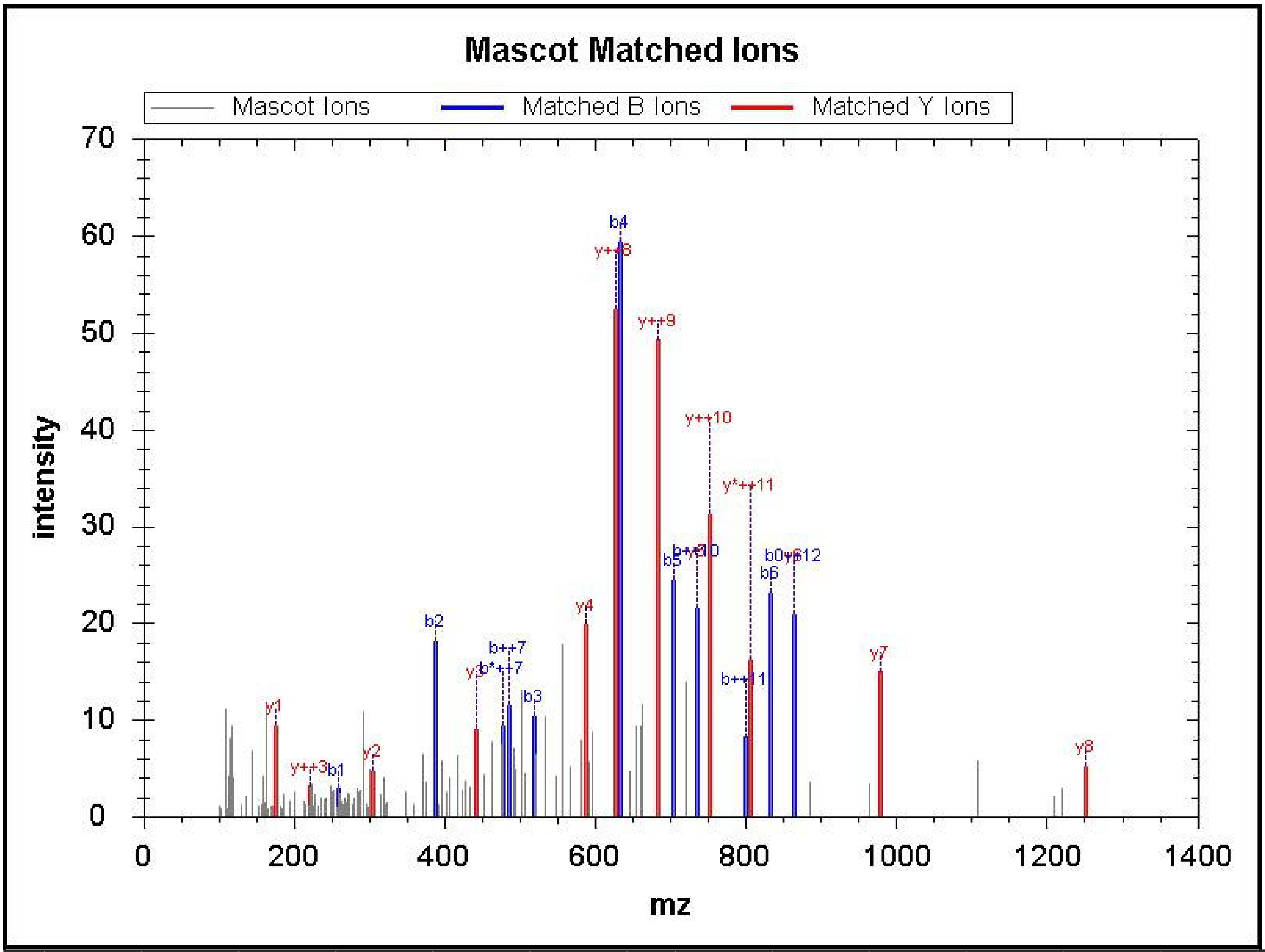

No	b	b++	b*	b*++	b0	b0++	Seq	y	y++	y*	y*++	y0	y0++	RevNo
1	258.19	129.60					L							16
2	387.24	194.12			369.23	185.12	E	2,075.97	1,038.49	2,058.95	1,029.98	2,057.96	1,029.48	15
3	518.28	259.64			500.27	250.64	M	1,946.93	973.97	1,929.90	965.46	1,928.92	964.96	14
4	633.30	317.16			615.29	308.15	D	1,815.89	908.45	1,798.86	899.94	1,797.88	899.44	13
5	704.34	352.67			686.33	343.67	A	1,700.86	850.93	1,683.84	842.42	1,682.85	841.93	12
6	832.40	416.70	815.37	408.19	814.39	407.70	Q	1,629.83	815.42	1,612.80	806.90	1,611.81	806.41	11
7	969.46	485.23	952.43	476.72	951.45	476.23	H	1,501.77	751.39	1,484.74	742.87	1,483.76	742.38	10

8	1,082.54	541.77	1,065.52	533.26	1,064.53	532.77	I	1,364.71	682.86	1,347.68	674.34	1,346.70	673.85	9
9	1,354.74	677.87	1,337.71	669.36	1,336.73	668.87	K	1,251.62	626.32	1,234.60	617.80	1,233.61	617.31	8
10	1,469.77	735.39	1,452.74	726.87	1,451.76	726.38	D	979.43	490.22	962.40	481.70	961.42	481.21	7
11	1,598.81	799.91	1,581.78	791.39	1,580.80	790.90	E	864.40	432.70	847.37	424.19	846.39	423.70	6
12	1,745.88	873.44	1,728.85	864.93	1,727.87	864.44	F	735.36	368.18	718.33	359.67	717.35	359.18	5
13	1,892.95	946.98	1,875.92	938.46	1,874.93	937.97	F	588.29	294.65	571.26	286.13	570.28	285.64	4
14	2,030.00	1,015.51	2,012.98	1,006.99	2,011.99	1,006.50	H	441.22	221.11	424.19	212.60	423.21	212.11	3
15	2,159.05	1,080.03	2,142.02	1,071.51	2,141.04	1,071.02	E	304.16	152.58	287.13	144.07	286.15	143.58	2
16							R	175.12	88.06	158.09	79.55			1

Query 7850 Hit 1

MS/MS Fragmentation of SGLTFLPK
Found in sp|Q75T13|PGAP1_HUMAN, GPI inositol-deacylase OS=Homo sapiens GN=PGAP1 PE=2 SV=1
Match to Query 7850: 1005.616from(503.8153,2+)
Title: 357: Sum of 2 scans in range 920 (rt=29.8845, f=4, i=237) to 921 (rt=29.9099, f=4, i=238)
[D:\lab212\membrane\Grace\Joyce\iTRAQ_30_1.raw]
Data File:Submitted from 20120508-1(merge) by Mascot Daemon on JOYCE-VAIO
Monoisotopic mass of neutral peptide Mr(calc): 1005.616
Variable modifications:
K8 :iTRAQ4plex (K)
Ions Score: 42.38 Expect: 0.041

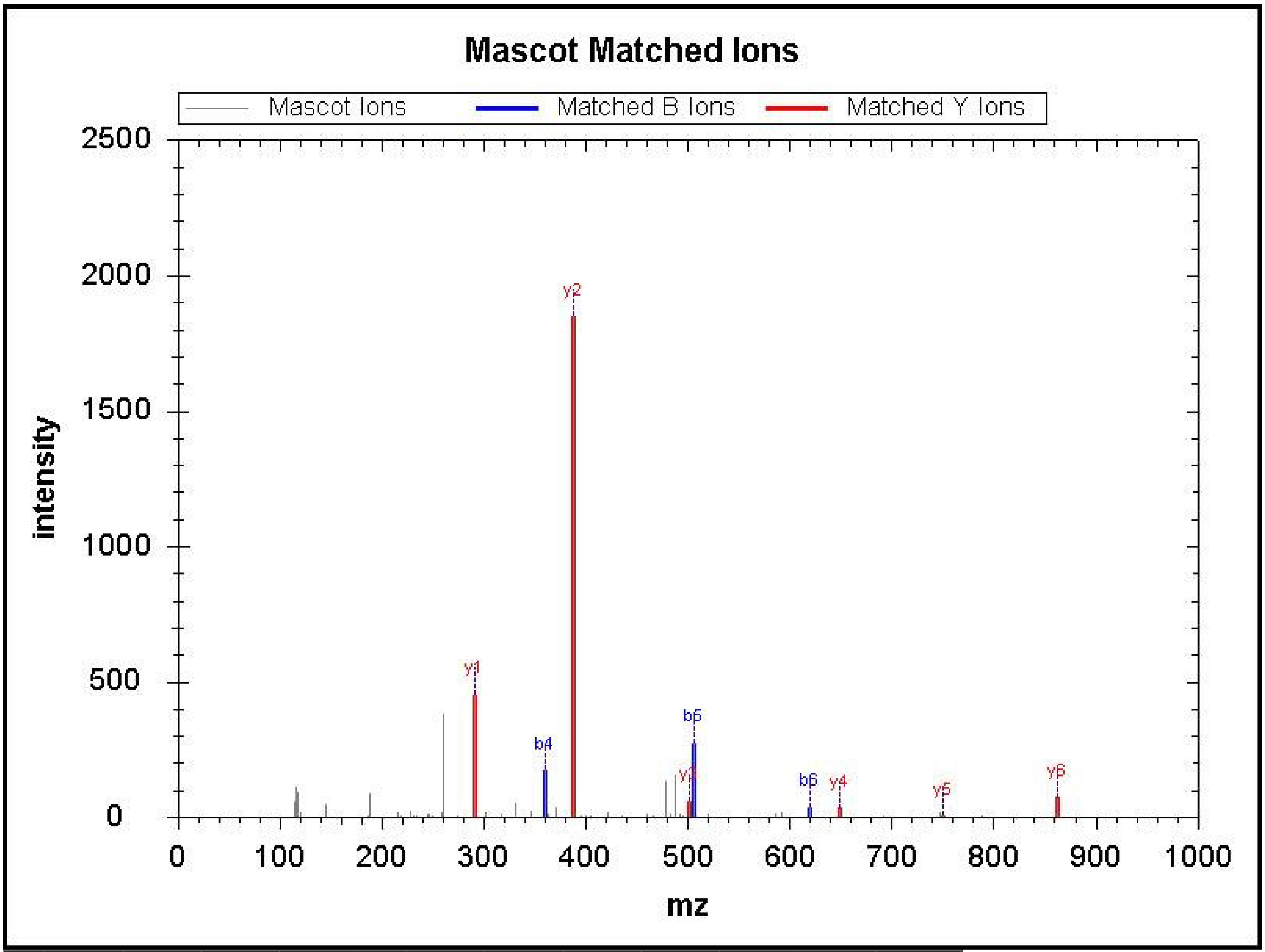

No	b	b++	b0	b0++	Seq	y	y++	y*	y*++	y0	y0++	RevNo
1	88.04	44.52	70.03	35.52	S							8
2	145.06	73.03	127.05	64.03	G	919.57	460.29	902.55	451.78	901.56	451.29	7
3	258.14	129.58	240.13	120.57	L	862.55	431.78	845.53	423.27	844.54	422.77	6
4	359.19	180.10	341.18	171.09	T	749.47	375.24	732.44	366.72	731.46	366.23	5

5	506.26	253.63	488.25	244.63	F	648.42	324.71	631.39	316.20			4
6	619.34	310.18	601.33	301.17	L	501.35	251.18	484.33	242.67			3
7	716.40	358.70	698.39	349.70	P	388.27	194.64	371.24	186.12			2
8					K	291.21	146.11	274.19	137.60			1

Query 5983 Hit 1

MS/MS Fragmentation of GSFGTVLK
Found in sp|Q96LW2|SG494_HUMAN, Uncharacterized serine/threonine-protein kinase SgK494 OS=Homo sapiens GN=SGK494 PE=2 SV=1
Match to Query 5983: 951.6027 from(476.8087,2+)
Title: 334: Scan 819 (rt=27.8083, f=2, i=124) [D:\lab212\membrane\Grace\Joyce\iTRAQ_31_1.raw]
Data File:Submitted from 20120508-1(merge) by Mascot Daemon on JOYCE-VAIO
Monoisotopic mass of neutral peptide Mr(calc): 951.6027
Variable modifications:
K8 :iTRAQ4plex (K)
Ions Score: 42.36 Expect: 0.023

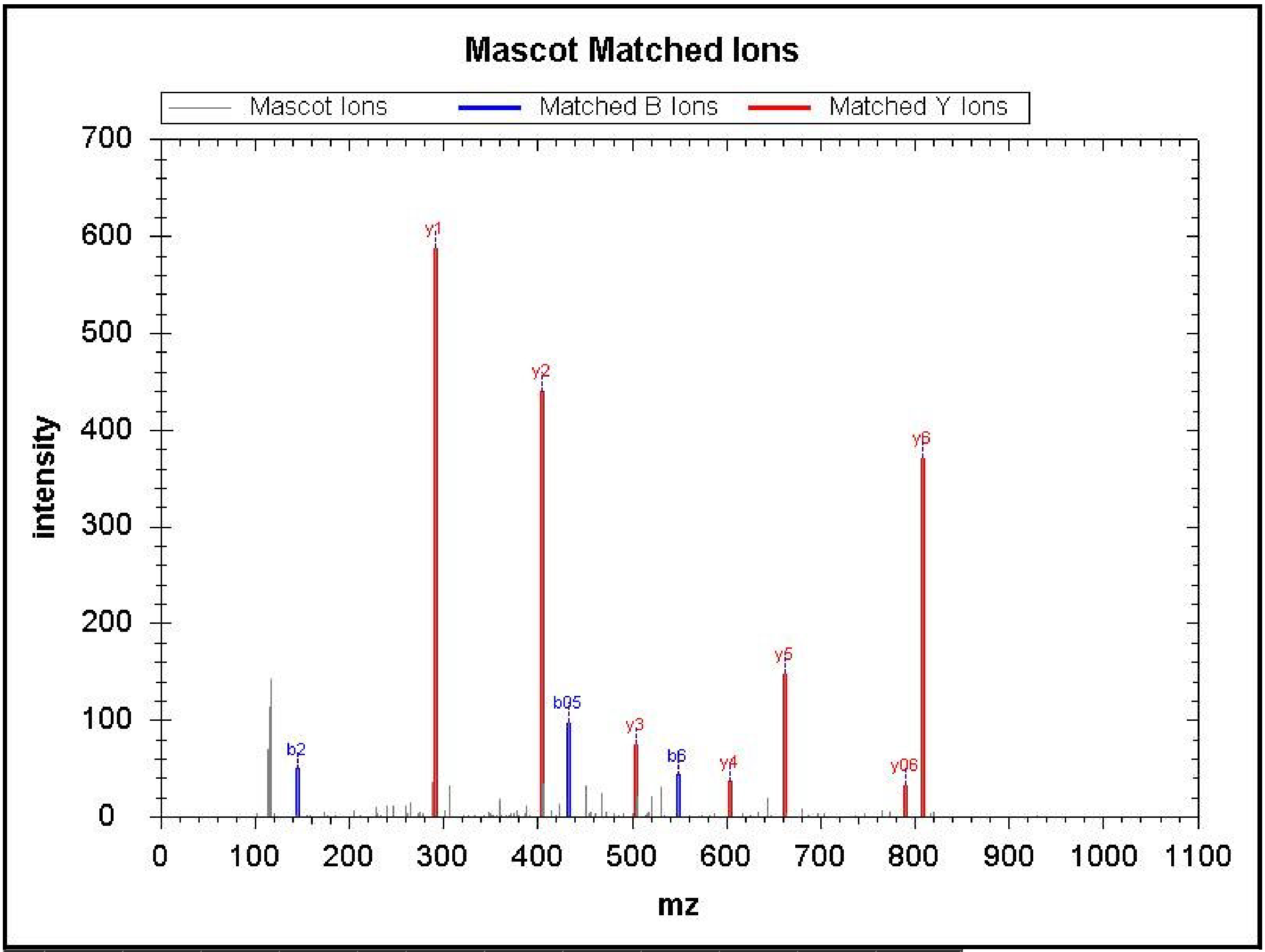

No	b	b++	b0	b0++	Seq	y	y++	y*	y*++	y0	y0++	RevNo
1	58.03	29.52			G							8
2	145.06	73.03	127.05	64.03	S	895.54	448.27	878.51	439.76	877.53	439.27	7
3	292.13	146.57	274.12	137.56	F	808.50	404.76	791.48	396.24	790.49	395.75	6
4	349.15	175.08	331.14	166.07	G	661.44	331.22	644.41	322.71	643.43	322.22	5
5	450.20	225.60	432.19	216.60	T	604.41	302.71	587.39	294.20	586.40	293.71	4
6	549.27	275.14	531.26	266.13	V	503.37	252.19	486.34	243.67			3
7	662.35	331.68	644.34	322.67	L	404.30	202.65	387.27	194.14			2
8					K	291.21	146.11	274.19	137.60			1

Query 43911 Hit 1

MS/MS Fragmentation of DGNLGLAVGEVGMDSK
Found in sp|Q8IVF2|AHNK2_HUMAN, Protein AHNAK2 OS=Homo sapiens GN=AHNAK2 PE=1 SV=2
Match to Query 43911: 1705.877from(853.9457,2+)
Title: 127: Scan 1211 (rt=33.5136, f=3, i=64) [D:\lab212\membrane\Grace\Joyce\iTRAQ_19_2_330.raw]
Data File:Submitted from 20120508-1(merge) by Mascot Daemon on JOYCE-VAIO
Monoisotopic mass of neutral peptide Mr(calc): 1705.877
Variable modifications:
N3 :Deamidated (NQ)
Ions Score: 42.36 Expect: 0.053

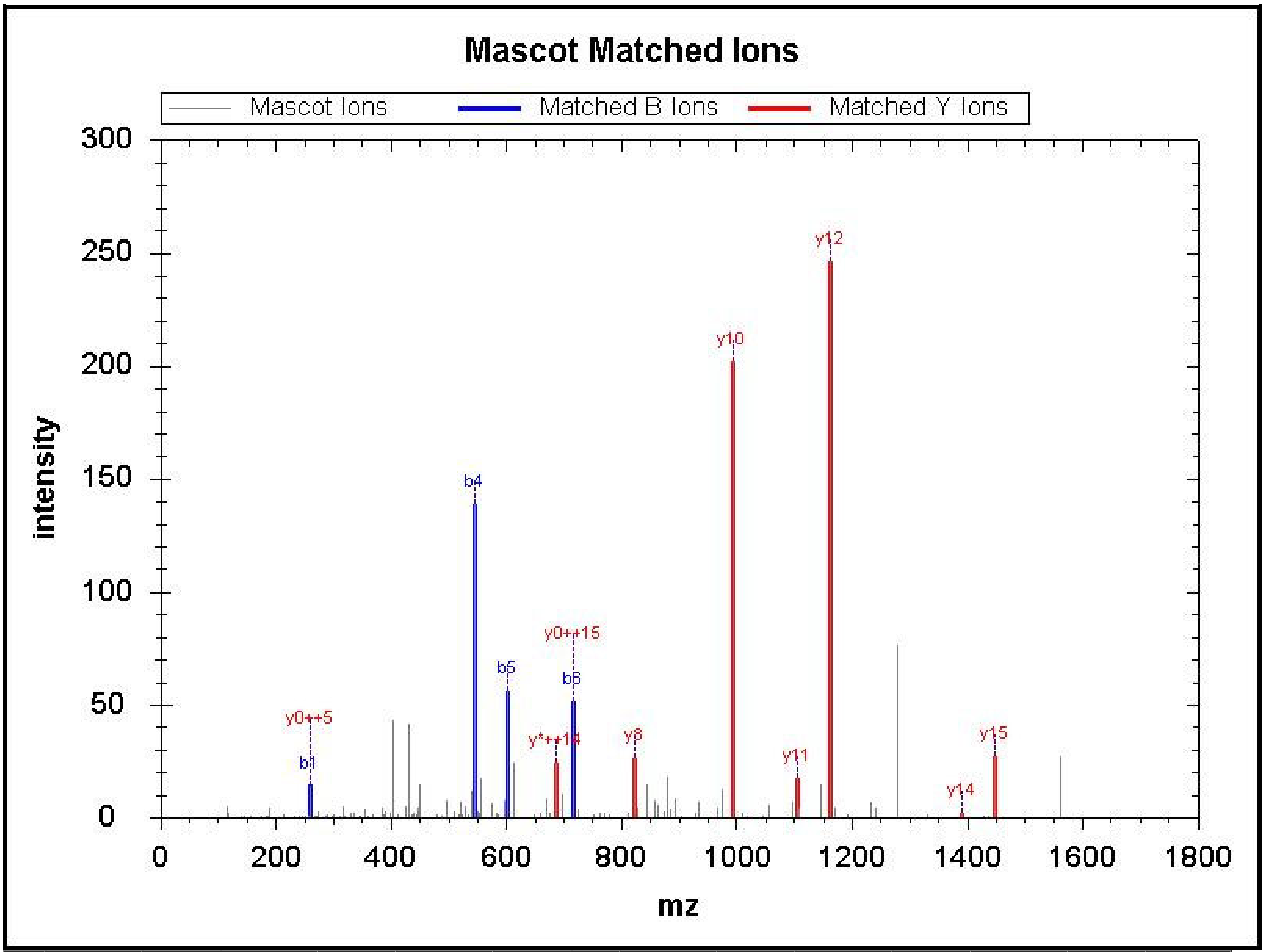

No	b	b++	b*	b*++	b0	b0++	Seq	y	y++	y*	y*++	y0	y0++	RevNo
1	260.14	130.57			242.13	121.57	D							16
2	317.16	159.08			299.15	150.08	G	1,447.71	724.36	1,430.68	715.85	1,429.70	715.35	15
3	432.18	216.60	415.16	208.08	414.17	207.59	N	1,390.69	695.85	1,373.66	687.33	1,372.68	686.84	14
4	545.27	273.14	528.24	264.62	527.26	264.13	L	1,275.66	638.33	1,258.63	629.82	1,257.65	629.33	13
5	602.29	301.65	585.26	293.14	584.28	292.64	G	1,162.58	581.79	1,145.55	573.28	1,144.57	572.79	12
6	715.37	358.19	698.35	349.68	697.36	349.19	L	1,105.56	553.28	1,088.53	544.77	1,087.55	544.28	11
7	786.41	393.71	769.38	385.20	768.40	384.70	A	992.47	496.74	975.45	488.23	974.46	487.73	10
8	885.48	443.24	868.45	434.73	867.47	434.24	V	921.43	461.22	904.41	452.71	903.42	452.22	9
9	942.50	471.75	925.47	463.24	924.49	462.75	G	822.37	411.69	805.34	403.17	804.36	402.68	8
10	1,071.54	536.28	1,054.52	527.76	1,053.53	527.27	E	765.34	383.18	748.32	374.66	747.33	374.17	7
11	1,170.61	585.81	1,153.59	577.30	1,152.60	576.80	V	636.30	318.65	619.28	310.14	618.29	309.65	6
12	1,227.63	614.32	1,210.61	605.81	1,209.62	605.32	G	537.23	269.12	520.21	260.61	519.22	260.12	5
13	1,358.67	679.84	1,341.65	671.33	1,340.66	670.84	M	480.21	240.61	463.19	232.10	462.20	231.60	4
14	1,473.70	737.35	1,456.67	728.84	1,455.69	728.35	D	349.17	175.09	332.15	166.58	331.16	166.08	3
15	1,560.73	780.87	1,543.71	772.36	1,542.72	771.86	S	234.14	117.58	217.12	109.06	216.13	108.57	2
16							K	147.11	74.06	130.09	65.55			1

Query 6571 Hit 1

MS/MS Fragmentation of LLEAIIR
Found in sp|Q96H55|MYO19_HUMAN, Unconventional myosin-XIX OS=Homo sapiens GN=MYO19 PE=2 SV=2
Match to Query 6571: 970.5917from(486.3031,2+)
Title: 153: Sum of 2 scans in range 1099 (rt=30.6661, f=4, i=79) to 1100 (rt=30.6915, f=4, i=80)
[D:\lab212\membrane\Grace\Joyce\iTRAQ_26_1.raw]
Data File:Submitted from 20120508-1(merge) by Mascot Daemon on JOYCE-VAIO
Monoisotopic mass of neutral peptide Mr(calc): 970.5917
Variable modifications:
Ions Score: 42.35 Expect: 0.023

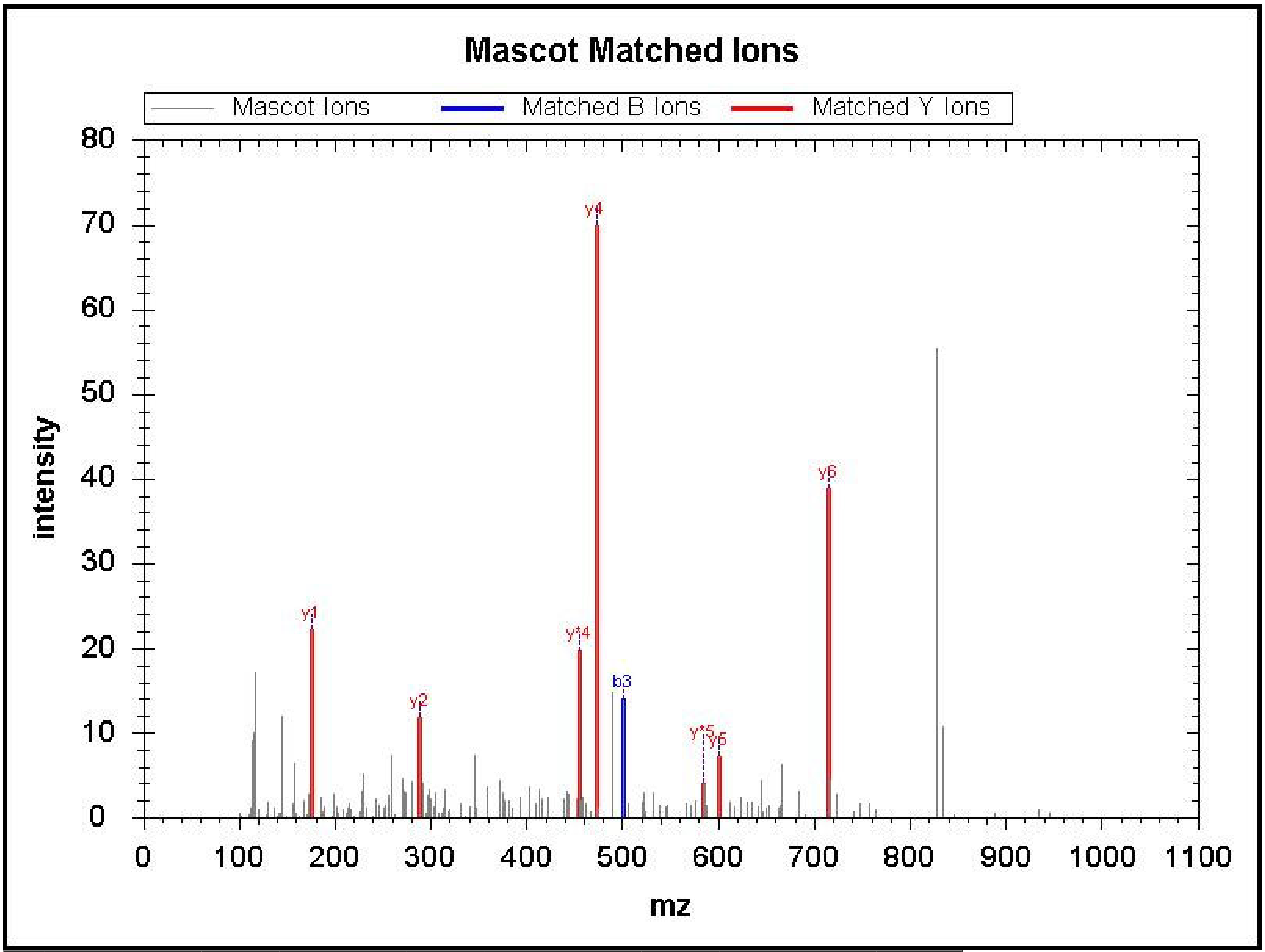

No	b	b++	b0	b0++	Seq	y	y++	y*	y*++	y0	y0++	RevNo
1	258.19	129.60			L							7
2	371.28	186.14			L	714.45	357.73	697.42	349.22	696.44	348.72	6
3	500.32	250.66	482.31	241.66	E	601.37	301.19	584.34	292.67	583.36	292.18	5
4	571.36	286.18	553.35	277.18	A	472.32	236.67	455.30	228.15			4
5	684.44	342.72	666.43	333.72	I	401.29	201.15	384.26	192.63			3
6	797.53	399.27	779.51	390.26	I	288.20	144.61	271.18	136.09			2
7					R	175.12	88.06	158.09	79.55			1

Query 84445 Hit 1

MS/MS Fragmentation of EQLEQELEELTASLFEEAHK
Found in sp|Q8TBN0|R3GEF_HUMAN, Guanine nucleotide exchange factor for Rab-3A OS=Homo sapiens GN=RAB3IL1 PE=1 SV=1
Match to Query 84445: 2660.336from(887.7858,3+)
Title: 1152: Sum of 2 scans in range 2806 (rt=71.309, f=3, i=405) to 2807 (rt=71.3344, f=3, i=406)
[D:\lab212\membrane\Grace\Joyce\iTRAQ_34_1.raw]
Data File:Submitted from 20120508-1(merge) by Mascot Daemon on JOYCE-VAIO

Monoisotopic mass of neutral peptide Mr(calc): 2660.336
Variable modifications:
K20 :iTRAQ4plex (K)
Ions Score: 42.32 Expect: 0.055

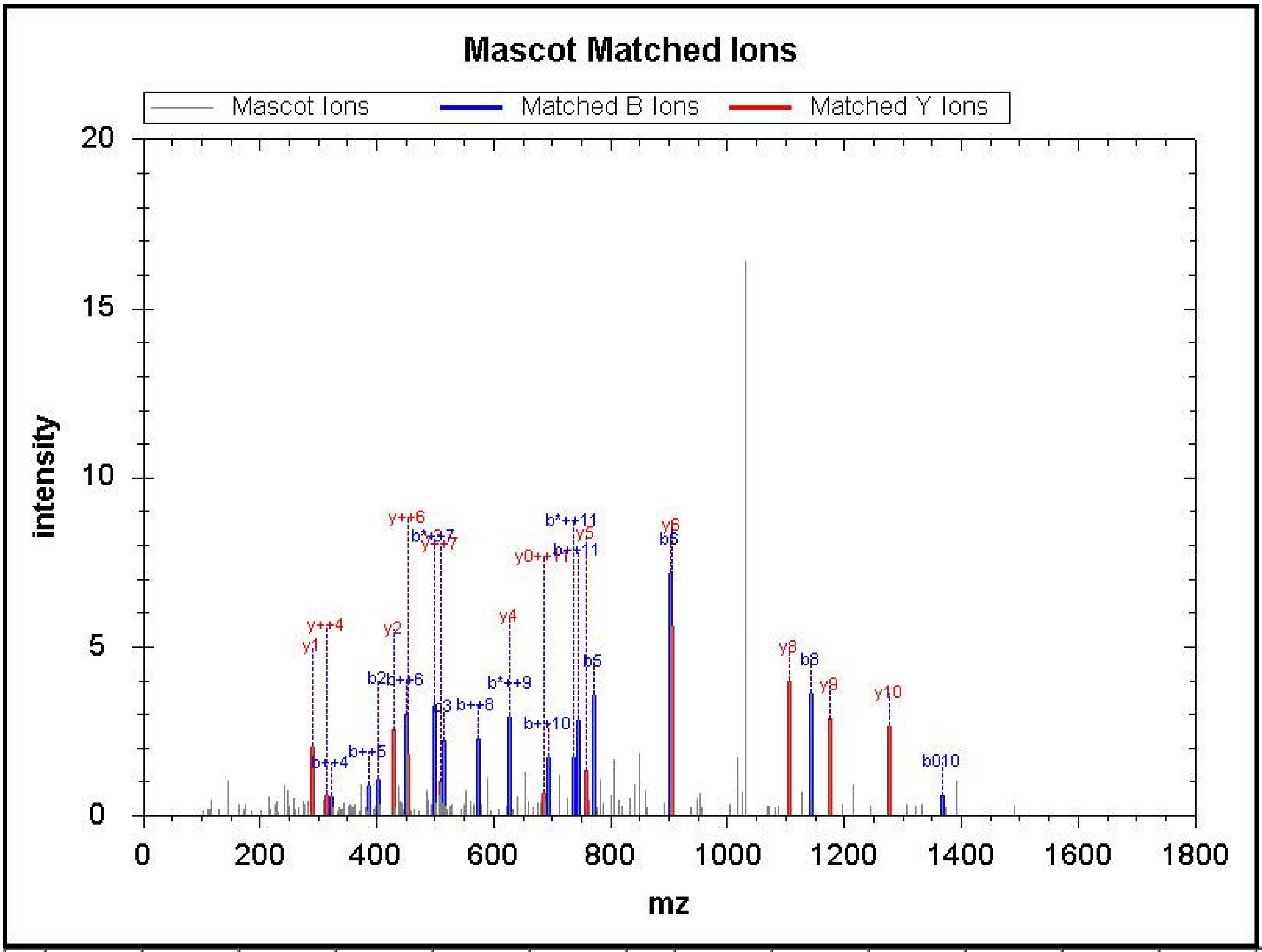

No	b	b++	b*	b*++	b0	b0++	Seq	y	y++	y*	y*++	y0	y0++	RevNo
1	274.15	137.58			256.14	128.57	E							20
2	402.21	201.61	385.18	193.10	384.20	192.60	Q	2,388.21	1,194.61	2,371.18	1,186.09	2,370.19	1,185.60	19
3	515.29	258.15	498.27	249.64	497.28	249.15	L	2,260.15	1,130.58	2,243.12	1,122.06	2,242.14	1,121.57	18
4	644.34	322.67	627.31	314.16	626.33	313.67	E	2,147.06	1,074.03	2,130.04	1,065.52	2,129.05	1,065.03	17
5	772.40	386.70	755.37	378.19	754.39	377.70	Q	2,018.02	1,009.51	2,000.99	1,001.00	2,000.01	1,000.51	16
6	901.44	451.22	884.41	442.71	883.43	442.22	E	1,889.96	945.48	1,872.93	936.97	1,871.95	936.48	15
7	1,014.52	507.76	997.50	499.25	996.51	498.76	L	1,760.92	880.96	1,743.89	872.45	1,742.91	871.96	14
8	1,143.57	572.29	1,126.54	563.77	1,125.55	563.28	E	1,647.83	824.42	1,630.81	815.91	1,629.82	815.42	13
9	1,272.61	636.81	1,255.58	628.29	1,254.60	627.80	E	1,518.79	759.90	1,501.77	751.39	1,500.78	750.89	12
10	1,385.69	693.35	1,368.67	684.84	1,367.68	684.34	L	1,389.75	695.38	1,372.72	686.87	1,371.74	686.37	11
11	1,486.74	743.87	1,469.71	735.36	1,468.73	734.87	T	1,276.67	638.84	1,259.64	630.32	1,258.65	629.83	10
12	1,557.78	779.39	1,540.75	770.88	1,539.77	770.39	A	1,175.62	588.31	1,158.59	579.80	1,157.61	579.31	9
13	1,644.81	822.91	1,627.78	814.39	1,626.80	813.90	S	1,104.58	552.79	1,087.55	544.28	1,086.57	543.79	8
14	1,757.89	879.45	1,740.87	870.94	1,739.88	870.44	L	1,017.55	509.28	1,000.52	500.76	999.54	500.27	7
15	1,904.96	952.98	1,887.93	944.47	1,886.95	943.98	F	904.46	452.74	887.44	444.22	886.45	443.73	6
16	2,034.00	1,017.51	2,016.98	1,008.99	2,015.99	1,008.50	E	757.40	379.20	740.37	370.69	739.39	370.20	5
17	2,163.05	1,082.03	2,146.02	1,073.51	2,145.04	1,073.02	E	628.35	314.68	611.33	306.17	610.34	305.68	4
18	2,234.08	1,117.55	2,217.06	1,109.03	2,216.07	1,108.54	A	499.31	250.16	482.28	241.65			3
19	2,371.14	1,186.07	2,354.12	1,177.56	2,353.13	1,177.07	H	428.27	214.64	411.25	206.13			2
20							K	291.21	146.11	274.19	137.60			1

Query 48000 Hit 1

MS/MS Fragmentation of ITESSLVEITEHK
Found in sp|Q99590|SCAFB_HUMAN, Protein SCAF11 OS=Homo sapiens GN=SCAF11 PE=1 SV=2
Match to Query 48000: 1772.978from(592.0001,3+)
Title: 583: Scan 1317 (rt=39.2685, f=3, i=203) [D:\lab212\membrane\Grace\Joyce\iTRAQ_39_2.raw]
Data File:Submitted from 20120508-1(merge) by Mascot Daemon on JOYCE-VAIO
Monoisotopic mass of neutral peptide Mr(calc): 1772.978
Variable modifications:
K13 :iTRAQ4plex (K)
Ions Score: 42.29 Expect: 0.046

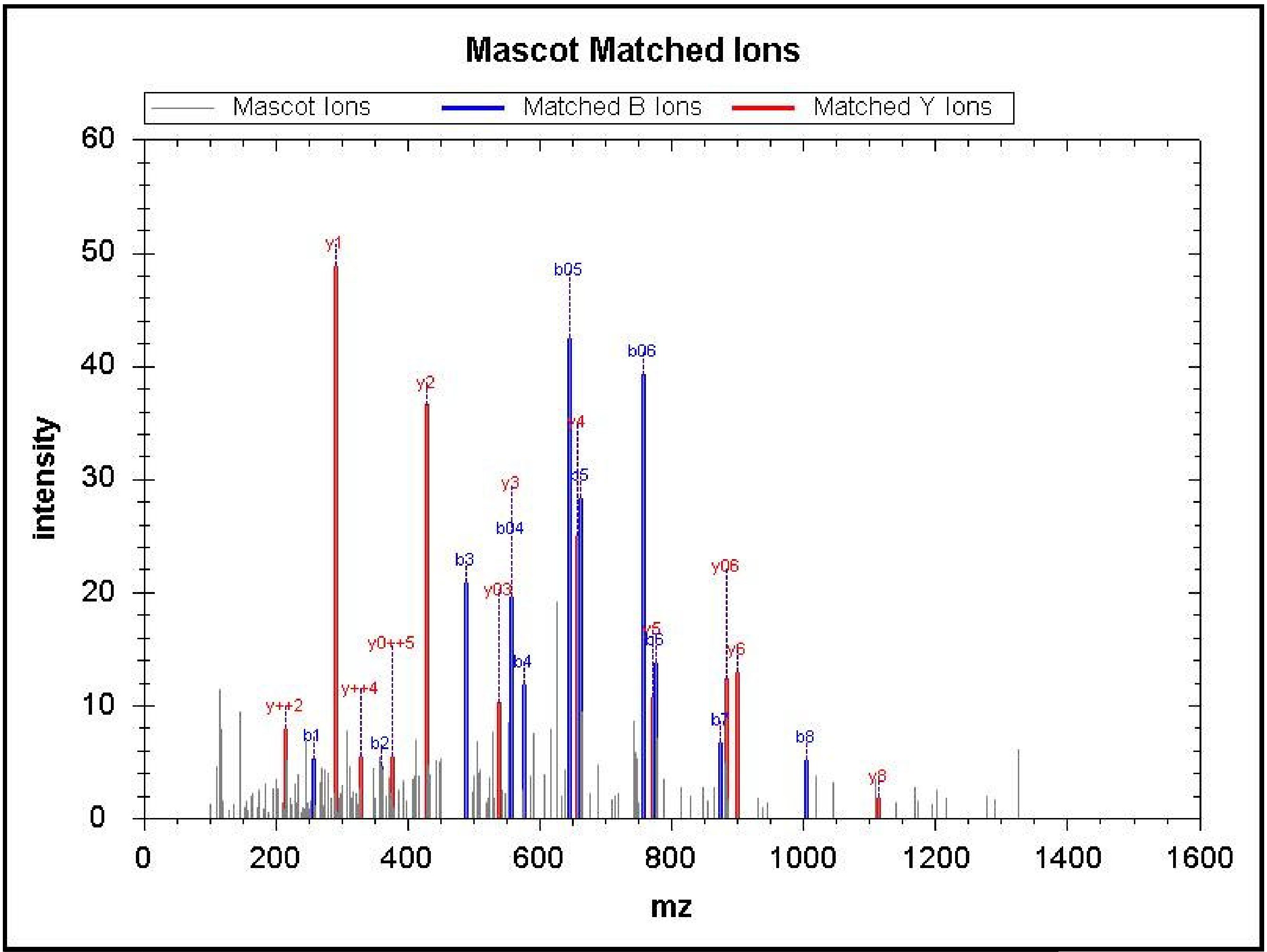

No	b	b++	b0	b0++	Seq	y	y++	y*	y*++	y0	y0++	RevNo
1	258.19	129.60			I							13
2	359.24	180.12	341.23	171.12	T	1,516.80	758.90	1,499.77	750.39	1,498.79	749.90	12
3	488.28	244.65	470.27	235.64	E	1,415.75	708.38	1,398.72	699.87	1,397.74	699.37	11
4	575.32	288.16	557.31	279.16	S	1,286.71	643.86	1,269.68	635.34	1,268.70	634.85	10
5	662.35	331.68	644.34	322.67	S	1,199.68	600.34	1,182.65	591.83	1,181.66	591.34	9
6	775.43	388.22	757.42	379.21	L	1,112.64	556.83	1,095.62	548.31	1,094.63	547.82	8
7	874.50	437.75	856.49	428.75	V	999.56	500.28	982.53	491.77	981.55	491.28	7
8	1,003.54	502.28	985.53	493.27	E	900.49	450.75	883.46	442.24	882.48	441.74	6
9	1,116.63	558.82	1,098.62	549.81	I	771.45	386.23	754.42	377.71	753.44	377.22	5
10	1,217.67	609.34	1,199.66	600.34	T	658.36	329.69	641.34	321.17	640.35	320.68	4
11	1,346.72	673.86	1,328.71	664.86	E	557.32	279.16	540.29	270.65	539.31	270.16	3
12	1,483.78	742.39	1,465.77	733.39	H	428.27	214.64	411.25	206.13			2
13					K	291.21	146.11	274.19	137.60			1

Query 4507 Hit 1

MS/MS Fragmentation of SGLDQIK
Found in sp|Q9Y4D8|K0614_HUMAN, Probable E3 ubiquitin-protein ligase C12orf51 OS=Homo sapiens GN=C12orf51 PE=1 SV=5
Match to Query 4507: 904.5459from(453.2802,2+)
Title: 273: Sum of 2 scans in range 643 (rt=24.1546, f=4, i=181) to 644 (rt=24.18, f=4, i=182)
[D:\lab212\membrane\Grace\Joyce\iTRAQ_33_2.raw]
Data File:Submitted from 20120508-1(merge) by Mascot Daemon on JOYCE-VAIO
Monoisotopic mass of neutral peptide Mr(calc): 904.5459
Variable modifications:
Q5 :Deamidated (NQ)
K7 :iTRAQ4plex (K)
Ions Score: 42.29 Expect: 0.051

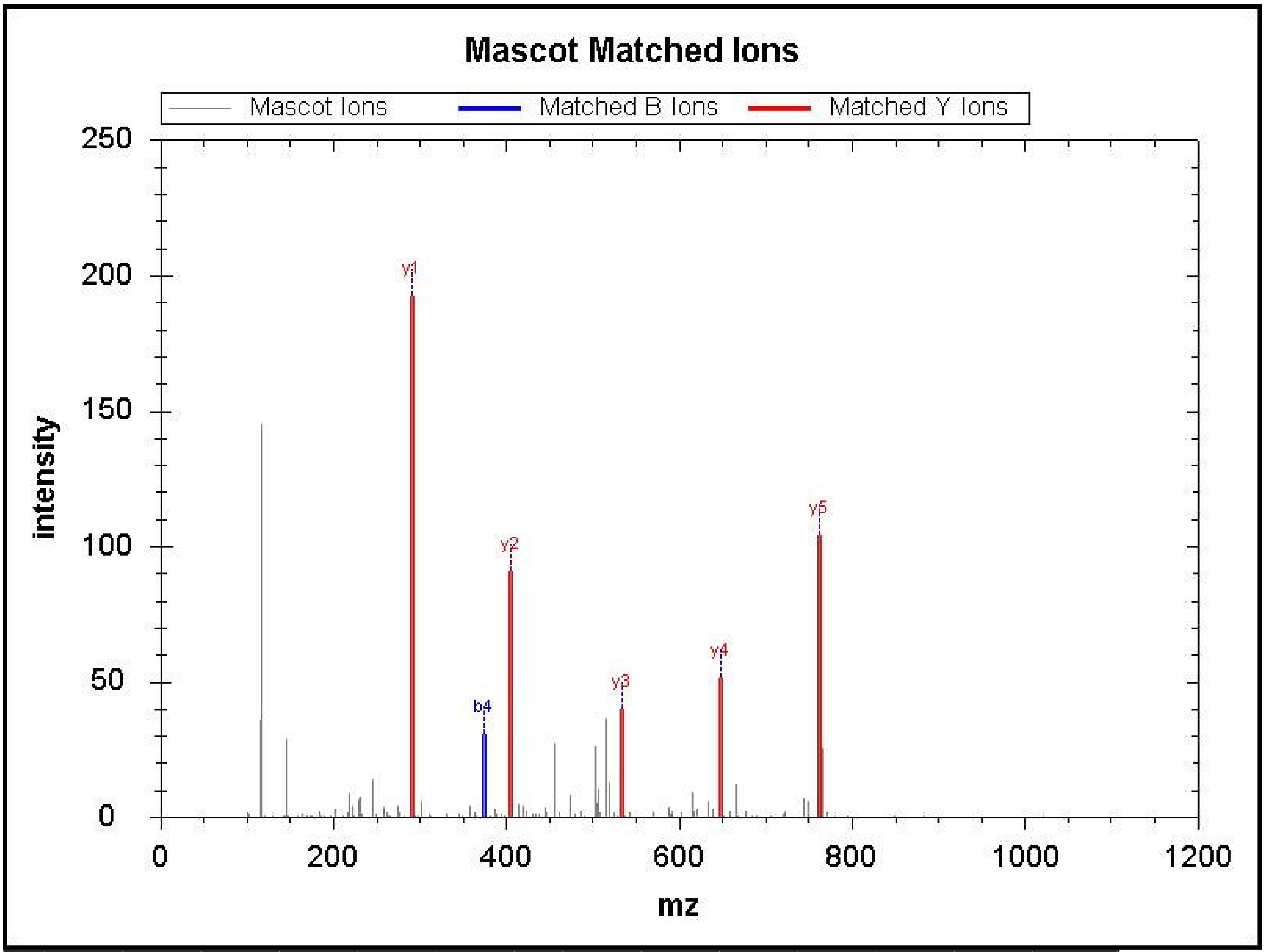

No	b	b++	b*	b*++	b0	b0++	Seq	y	y++	y*	y*++	y0	y0++	RevNo
1	88.04	44.52			70.03	35.52	S							7
2	145.06	73.03			127.05	64.03	G	818.47	409.74	801.45	401.23	800.46	400.74	6
3	258.14	129.58			240.13	120.57	L	761.45	381.23	744.43	372.72	743.44	372.22	5
4	373.17	187.09			355.16	178.08	D	648.37	324.69	631.34	316.17	630.36	315.68	4
5	502.21	251.61	485.19	243.10	484.20	242.61	Q	533.34	267.17	516.31	258.66			3
6	615.30	308.15	598.27	299.64	597.29	299.15	I	404.30	202.65	387.27	194.14			2
7							K	291.21	146.11	274.19	137.60			1

Query 41486 Hit 1

MS/MS Fragmentation of ISPEAFKPLVK
Found in sp|P28654|PGS2_MOUSE, Decorin OS=Mus musculus GN=Dcn PE=2 SV=1
Match to Query 41486: 1660.017from(554.3464,3+)
Title: 601: Scan 1362 (rt=40.2757, f=2, i=222) [D:\lab212\membrane\Grace\Joyce\iTRAQ_44_2.raw]
Data File:Submitted from 20120508-1(merge) by Mascot Daemon on JOYCE-VAIO
Monoisotopic mass of neutral peptide Mr(calc): 1660.017
Variable modifications:

K7 :iTRAQ4plex (K)
K11 :iTRAQ4plex (K)
Ions Score: 42.26 Expect: 0.020

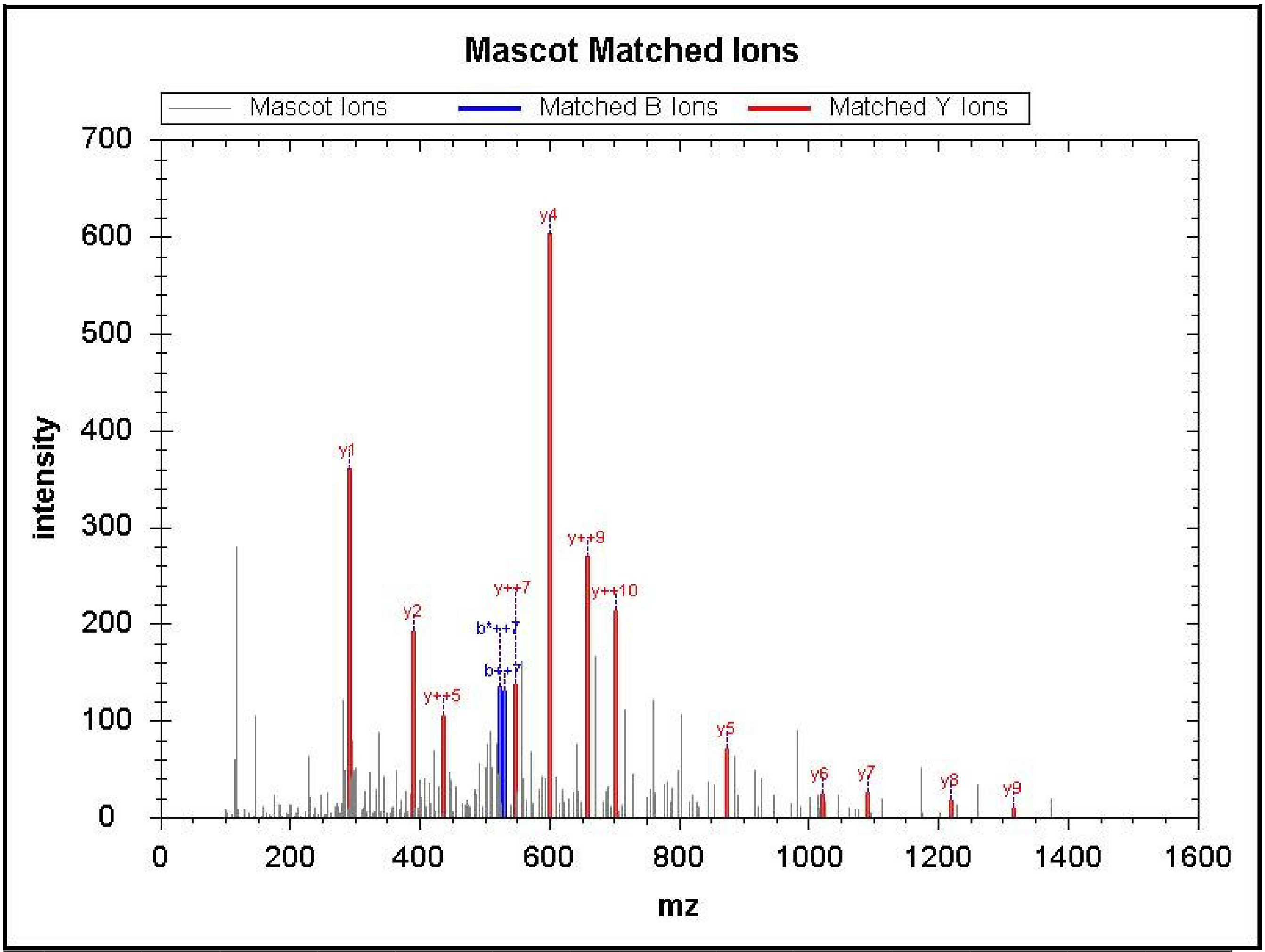

No	b	b++	b*	b*++	b0	b0++	Seq	y	y++	y*	y*++	y0	y0++	RevNo
1	258.19	129.60					I							11
2	345.23	173.12			327.21	164.11	S	1,403.85	702.43	1,386.82	693.92	1,385.84	693.42	10
3	442.28	221.64			424.27	212.64	P	1,316.82	658.91	1,299.79	650.40	1,298.81	649.91	9
4	571.32	286.16			553.31	277.16	E	1,219.77	610.39	1,202.74	601.87	1,201.75	601.38	8
5	642.36	321.68			624.35	312.68	A	1,090.72	545.86	1,073.70	537.35			7
6	789.43	395.22			771.42	386.21	F	1,019.69	510.35	1,002.66	501.83			6
7	1,061.62	531.32	1,044.60	522.80	1,043.61	522.31	K	872.62	436.81	855.59	428.30			5
8	1,158.68	579.84	1,141.65	571.33	1,140.67	570.84	P	600.42	300.71	583.39	292.20			4
9	1,271.76	636.38	1,254.73	627.87	1,253.75	627.38	L	503.37	252.19	486.34	243.67			3
10	1,370.83	685.92	1,353.80	677.40	1,352.82	676.91	V	390.28	195.65	373.26	187.13			2
11							K	291.21	146.11	274.19	137.60			1

Query 68815 Hit 1

MS/MS Fragmentation of DILMVVGNEIIEAPMAWR
Found in sp|Q9D964|GATM_MOUSE, Glycine amidinotransferase
Match to Query 68815: 2200.151 from(734.3909,3+)
Title: 1127: Scan 2939 (rt=73.5423, f=3, i=384) [D:\lab212\membrane\Grace\Joyce\20120320_iTRAQ_20-2.raw]
Data File:Submitted from 20120508-1(merge) by Mascot Daemon on JOYCE-VAIO
Monoisotopic mass of neutral peptide Mr(calc): 2200.151
Variable modifications:
Ions Score: 42.22 Expect: 0.054

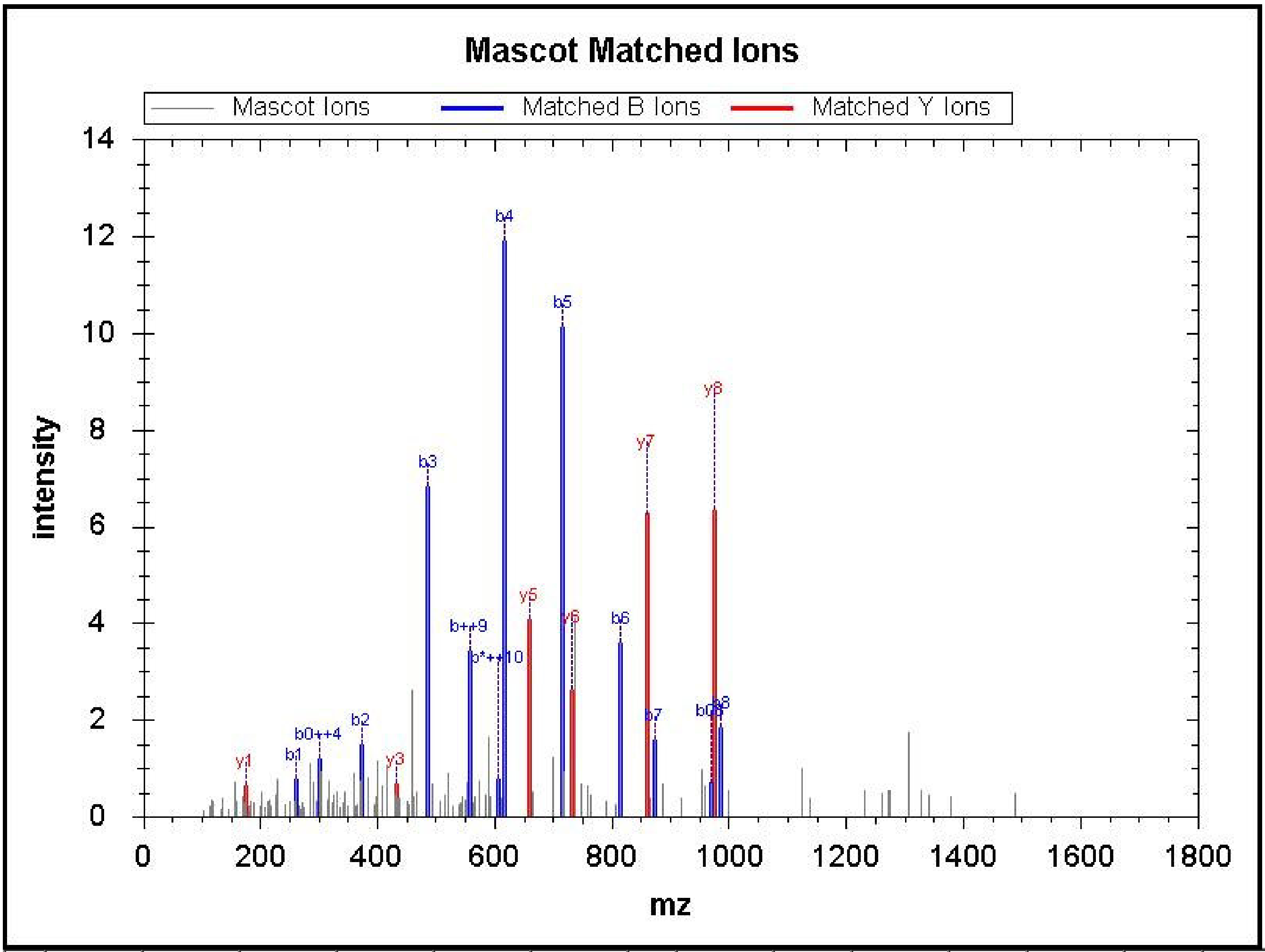

No	b	b++	b*	b*++	b0	b0++	Seq	y	y++	y*	y*++	y0	y0++	RevNo
1	260.14	130.57			242.13	121.57	D							18
2	373.22	187.11			355.21	178.11	I	1,942.03	971.52	1,925.00	963.00	1,924.02	962.51	17
3	486.30	243.66			468.29	234.65	L	1,828.94	914.98	1,811.92	906.46	1,810.93	905.97	16
4	617.34	309.18			599.33	300.17	M	1,715.86	858.43	1,698.83	849.92	1,697.85	849.43	15
5	716.41	358.71			698.40	349.70	V	1,584.82	792.91	1,567.79	784.40	1,566.81	783.91	14
6	815.48	408.24			797.47	399.24	V	1,485.75	743.38	1,468.73	734.87	1,467.74	734.37	13
7	872.50	436.76			854.49	427.75	G	1,386.68	693.85	1,369.66	685.33	1,368.67	684.84	12
8	986.55	493.78	969.52	485.26	968.54	484.77	N	1,329.66	665.33	1,312.64	656.82	1,311.65	656.33	11
9	1,115.59	558.30	1,098.56	549.78	1,097.58	549.29	E	1,215.62	608.31	1,198.59	599.80	1,197.61	599.31	10
10	1,228.67	614.84	1,211.65	606.33	1,210.66	605.83	I	1,086.58	543.79	1,069.55	535.28	1,068.57	534.79	9
11	1,341.76	671.38	1,324.73	662.87	1,323.75	662.38	I	973.49	487.25	956.47	478.74	955.48	478.24	8
12	1,470.80	735.90	1,453.77	727.39	1,452.79	726.90	E	860.41	430.71	843.38	422.19	842.40	421.70	7
13	1,541.84	771.42	1,524.81	762.91	1,523.83	762.42	A	731.37	366.19	714.34	357.67			6
14	1,638.89	819.95	1,621.86	811.44	1,620.88	810.94	P	660.33	330.67	643.30	322.15			5
15	1,769.93	885.47	1,752.90	876.96	1,751.92	876.46	M	563.28	282.14	546.25	273.63			4
16	1,840.97	920.99	1,823.94	912.47	1,822.96	911.98	A	432.24	216.62	415.21	208.11			3
17	2,027.05	1,014.03	2,010.02	1,005.51	2,009.04	1,005.02	W	361.20	181.10	344.17	172.59			2
18							R	175.12	88.06	158.09	79.55			1

Query 52871 Hit 1

MS/MS Fragmentation of TPLEQEIFNLLHK
Found in sp|Q9BVJ6|UT14A_HUMAN, U3 small nucleolar RNA-associated protein 14 homolog A OS=Homo sapiens GN=UTP14A PE=1 SV=1
Match to Query 52871: 1869.061 from(624.0277,3+)

Title: 1098: Sum of 2 scans in range 2391 (rt=63.4708, f=4, i=731) to 2392 (rt=63.4962, f=4, i=732) [D:\lab212\membrane\Grace\Joyce\iTRAQ_39_2.raw]
Data File:Submitted from 20120508-1(merge) by Mascot Daemon on JOYCE-VAIO
Monoisotopic mass of neutral peptide Mr(calc): 1869.061
Variable modifications:
K13 :iTRAQ4plex (K)
Ions Score: 42.21 Expect: 0.035

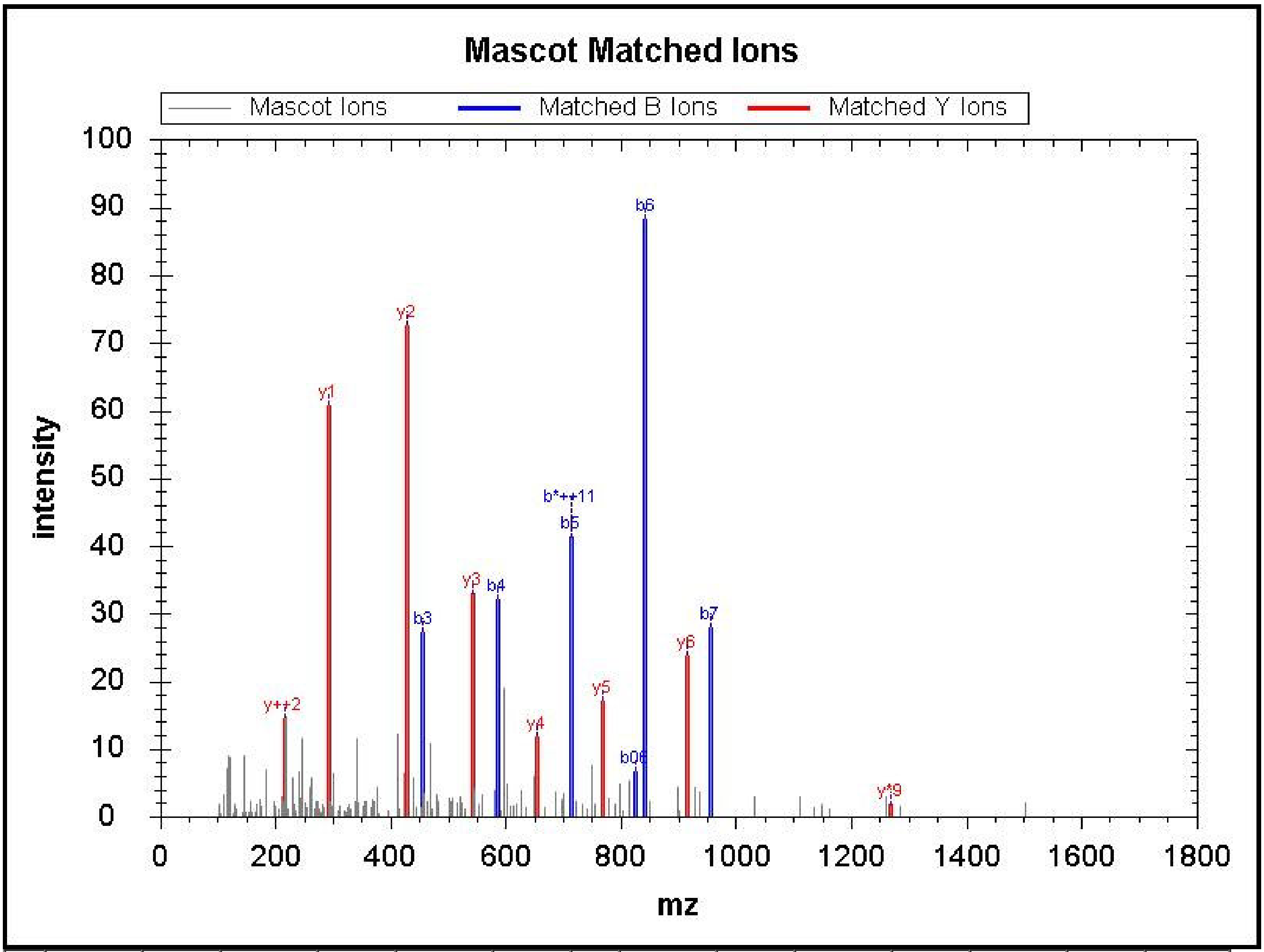

No	b	b++	b*	b*++	b0	b0++	Seq	y	y++	y*	y*++	y0	y0++	RevNo
1	246.16	123.58			228.15	114.58	T							13
2	343.21	172.11			325.20	163.10	P	1,624.92	812.96	1,607.89	804.45	1,606.91	803.96	12
3	456.29	228.65			438.28	219.65	L	1,527.87	764.44	1,510.84	755.92	1,509.85	755.43	11
4	585.34	293.17			567.33	284.17	E	1,414.78	707.89	1,397.75	699.38	1,396.77	698.89	10
5	713.40	357.20	696.37	348.69	695.38	348.20	Q	1,285.74	643.37	1,268.71	634.86	1,267.73	634.37	9
6	842.44	421.72	825.41	413.21	824.43	412.72	E	1,157.68	579.34	1,140.65	570.83	1,139.67	570.34	8
7	955.52	478.26	938.50	469.75	937.51	469.26	I	1,028.64	514.82	1,011.61	506.31			7
8	1,102.59	551.80	1,085.56	543.29	1,084.58	542.79	F	915.55	458.28	898.53	449.77			6
9	1,216.63	608.82	1,199.61	600.31	1,198.62	599.81	N	768.48	384.75	751.46	376.23			5
10	1,329.72	665.36	1,312.69	656.85	1,311.71	656.36	L	654.44	327.72	637.42	319.21			4
11	1,442.80	721.90	1,425.77	713.39	1,424.79	712.90	L	541.36	271.18	524.33	262.67			3
12	1,579.86	790.43	1,562.83	781.92	1,561.85	781.43	H	428.27	214.64	411.25	206.13			2
13							K	291.21	146.11	274.19	137.60			1

Query 66383 Hit 1

MS/MS Fragmentation of LTFLSLLDLPLTIAAEK
Found in sp|P36371|TAP2_MOUSE, Antigen peptide transporter 2 OS=Mus musculus GN=Tap2 PE=2 SV=1
Match to Query 66383: 2145.292from(716.1045,3+)

Title: 1252: Sum of 2 scans in range 3564 (rt=85.6585, f=2, i=512) to 3565 (rt=85.6839, f=2, i=513)
[D:\lab212\membrane\Grace\Joyce\iTRAQ_25_1.raw]
Data File:Submitted from 20120508-1(merge) by Mascot Daemon on JOYCE-VAIO
Monoisotopic mass of neutral peptide Mr(calc): 2145.292
Variable modifications:
K17 :iTRAQ4plex (K)
Ions Score: 42.16 Expect: 0.011

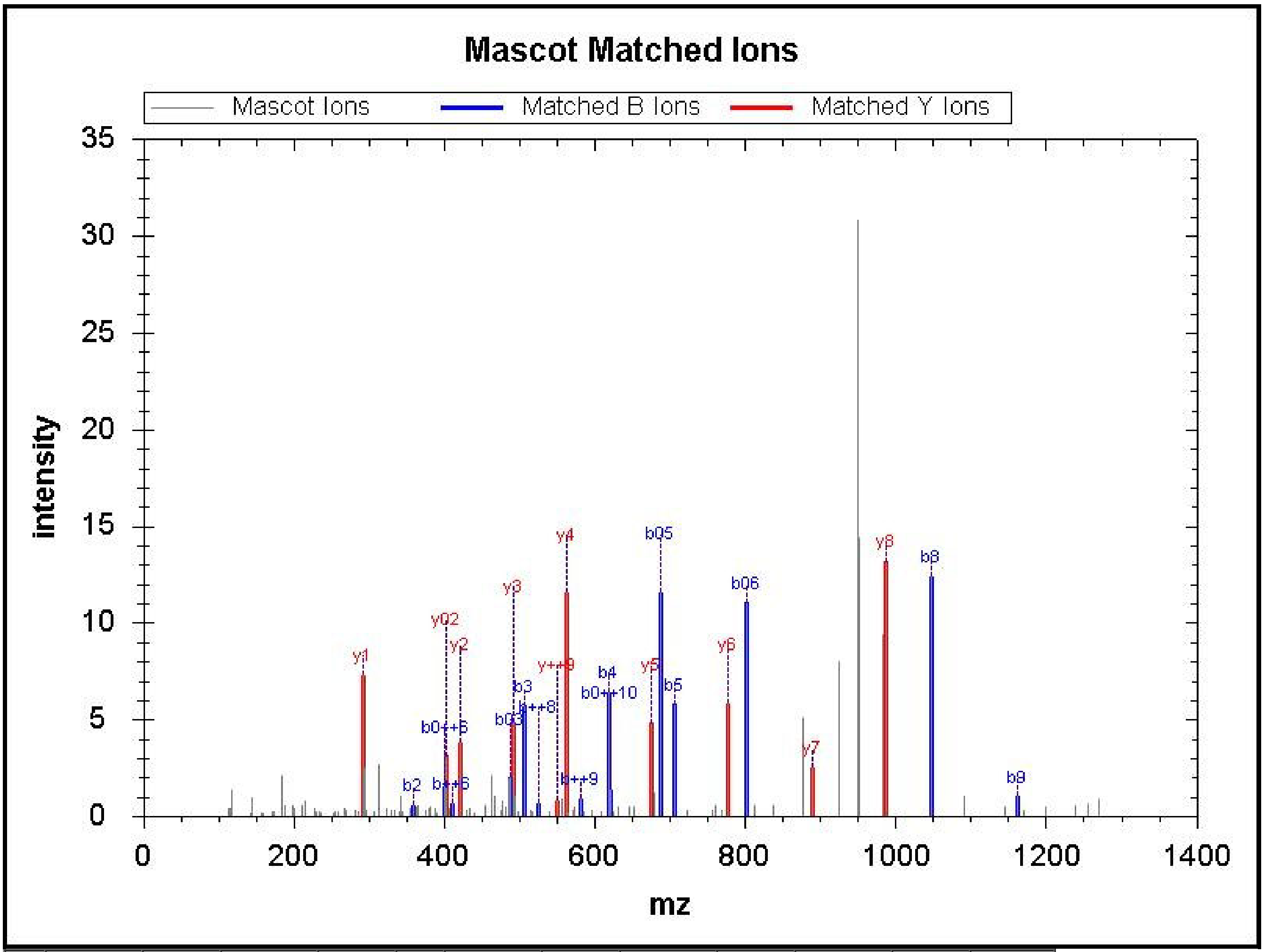

No	b	b++	b0	b0++	Seq	y	y++	y*	y*++	y0	y0++	RevNo
1	258.19	129.60			L							17
2	359.24	180.12	341.23	171.12	T	1,889.11	945.06	1,872.09	936.55	1,871.10	936.05	16
3	506.31	253.66	488.30	244.65	F	1,788.06	894.54	1,771.04	886.02	1,770.05	885.53	15
4	619.39	310.20	601.38	301.20	L	1,641.00	821.00	1,623.97	812.49	1,622.98	812.00	14
5	706.43	353.72	688.42	344.71	S	1,527.91	764.46	1,510.89	755.95	1,509.90	755.45	13
6	819.51	410.26	801.50	401.25	L	1,440.88	720.94	1,423.85	712.43	1,422.87	711.94	12
7	932.59	466.80	914.58	457.80	L	1,327.80	664.40	1,310.77	655.89	1,309.78	655.40	11
8	1,047.62	524.31	1,029.61	515.31	D	1,214.71	607.86	1,197.68	599.35	1,196.70	598.85	10
9	1,160.70	580.86	1,142.69	571.85	L	1,099.68	550.35	1,082.66	541.83	1,081.67	541.34	9
10	1,257.76	629.38	1,239.75	620.38	P	986.60	493.80	969.57	485.29	968.59	484.80	8
11	1,370.84	685.92	1,352.83	676.92	L	889.55	445.28	872.52	436.76	871.54	436.27	7
12	1,471.89	736.45	1,453.88	727.44	T	776.46	388.74	759.44	380.22	758.45	379.73	6
13	1,584.97	792.99	1,566.96	783.99	I	675.42	338.21	658.39	329.70	657.41	329.21	5
14	1,656.01	828.51	1,638.00	819.50	A	562.33	281.67	545.31	273.16	544.32	272.66	4
15	1,727.05	864.03	1,709.04	855.02	A	491.29	246.15	474.27	237.64	473.28	237.15	3
16	1,856.09	928.55	1,838.08	919.54	E	420.26	210.63	403.23	202.12	402.25	201.63	2
17					K	291.21	146.11	274.19	137.60			1

Query 97416 Hit 1

MS/MS Fragmentation of VVVRPVEDGEIQGVWLLTEVDHWNNEK
Found in sp|Q5T0D9|TPRGL_HUMAN, Tumor protein p63-regulated gene 1-like protein OS=Homo sapiens GN=TPRG1L PE=1 SV=1
Match to Query 97416: 3447.796from(862.9563,4+)
Title: 1015: Scan 2218 (rt=59.5686, f=3, i=345) [D:\lab212\membrane\Grace\Joyce\iTRAQ_44_2.raw]
Data File:Submitted from 20120508-1(merge) by Mascot Daemon on JOYCE-VAIO
Monoisotopic mass of neutral peptide Mr(calc): 3447.796
Variable modifications:
K27 :iTRAQ4plex (K)
Ions Score: 42.14 Expect: 0.041

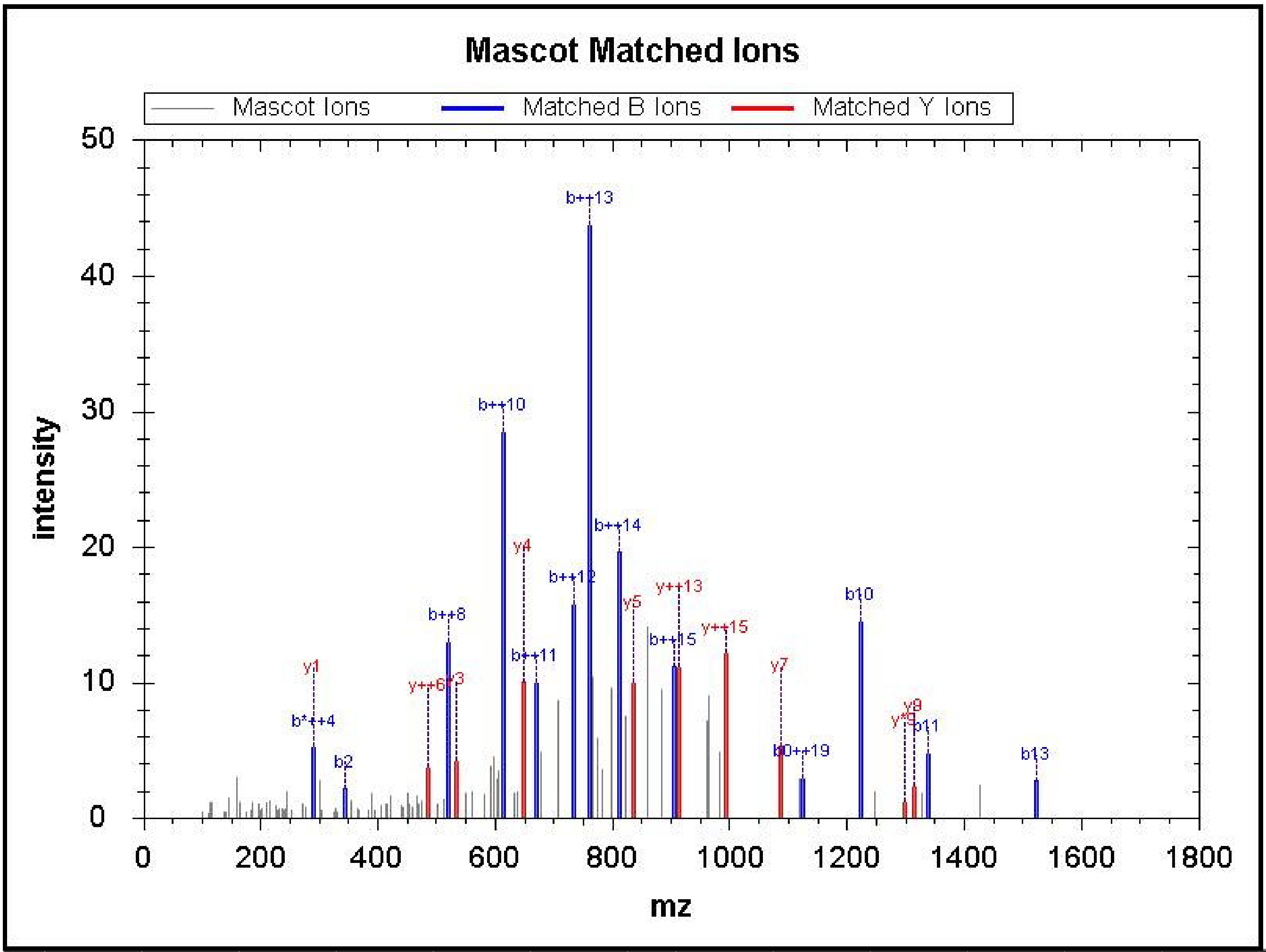

No	b	b++	b*	b*++	b0	b0++	Seq	y	y++	y*	y*++	y0	y0++	RevNo
1	244.18	122.59					V							27
2	343.25	172.13					V	3,205.64	1,603.32	3,188.61	1,594.81	3,187.63	1,594.32	26
3	442.31	221.66					V	3,106.57	1,553.79	3,089.54	1,545.28	3,088.56	1,544.78	25
4	598.42	299.71	581.39	291.20			R	3,007.50	1,504.25	2,990.48	1,495.74	2,989.49	1,495.25	24
5	695.47	348.24	678.44	339.72			P	2,851.40	1,426.20	2,834.37	1,417.69	2,833.39	1,417.20	23
6	794.54	397.77	777.51	389.26			V	2,754.35	1,377.68	2,737.32	1,369.16	2,736.34	1,368.67	22
7	923.58	462.29	906.55	453.78	905.57	453.29	E	2,655.28	1,328.14	2,638.25	1,319.63	2,637.27	1,319.14	21
8	1,038.61	519.81	1,021.58	511.29	1,020.60	510.80	D	2,526.24	1,263.62	2,509.21	1,255.11	2,508.23	1,254.62	20
9	1,095.63	548.32	1,078.60	539.80	1,077.62	539.31	G	2,411.21	1,206.11	2,394.18	1,197.60	2,393.20	1,197.10	19
10	1,224.67	612.84	1,207.64	604.33	1,206.66	603.83	E	2,354.19	1,177.60	2,337.16	1,169.09	2,336.18	1,168.59	18
11	1,337.75	669.38	1,320.73	660.87	1,319.74	660.38	I	2,225.15	1,113.08	2,208.12	1,104.56	2,207.14	1,104.07	17
12	1,465.81	733.41	1,448.79	724.90	1,447.80	724.40	Q	2,112.06	1,056.54	2,095.04	1,048.02	2,094.05	1,047.53	16
13	1,522.83	761.92	1,505.81	753.41	1,504.82	752.92	G	1,984.00	992.51	1,966.98	983.99	1,965.99	983.50	15
14	1,621.90	811.46	1,604.88	802.94	1,603.89	802.45	V	1,926.98	964.00	1,909.96	955.48	1,908.97	954.99	14

15	1,807.98	904.49	1,790.96	895.98	1,789.97	895.49	W	1,827.91	914.46	1,810.89	905.95	1,809.90	905.46	13
16	1,921.07	961.04	1,904.04	952.52	1,903.06	952.03	L	1,641.84	821.42	1,624.81	812.91	1,623.82	812.42	12
17	2,034.15	1,017.58	2,017.12	1,009.07	2,016.14	1,008.57	L	1,528.75	764.88	1,511.72	756.37	1,510.74	755.87	11
18	2,135.20	1,068.10	2,118.17	1,059.59	2,117.19	1,059.10	T	1,415.67	708.34	1,398.64	699.82	1,397.66	699.33	10
19	2,264.24	1,132.62	2,247.21	1,124.11	2,246.23	1,123.62	E	1,314.62	657.81	1,297.59	649.30	1,296.61	648.81	9
20	2,363.31	1,182.16	2,346.28	1,173.64	2,345.30	1,173.15	V	1,185.58	593.29	1,168.55	584.78	1,167.57	584.29	8
21	2,478.34	1,239.67	2,461.31	1,231.16	2,460.33	1,230.67	D	1,086.51	543.76	1,069.48	535.24	1,068.50	534.75	7
22	2,615.39	1,308.20	2,598.37	1,299.69	2,597.38	1,299.20	H	971.48	486.24	954.45	477.73	953.47	477.24	6
23	2,801.47	1,401.24	2,784.45	1,392.73	2,783.46	1,392.24	W	834.42	417.71	817.40	409.20	816.41	408.71	5
24	2,915.52	1,458.26	2,898.49	1,449.75	2,897.51	1,449.26	N	648.34	324.68	631.32	316.16	630.33	315.67	4
25	3,029.56	1,515.28	3,012.53	1,506.77	3,011.55	1,506.28	N	534.30	267.65	517.27	259.14	516.29	258.65	3
26	3,158.60	1,579.80	3,141.58	1,571.29	3,140.59	1,570.80	E	420.26	210.63	403.23	202.12	402.25	201.63	2
27							K	291.21	146.11	274.19	137.60			1

Query 69726 Hit 1

MS/MS Fragmentation of DNTQLLINQLWQLPTER
Found in sp|Q15050|RRS1_HUMAN, Ribosome biogenesis regulatory protein homolog OS=Homo sapiens GN=RRS1 PE=1 SV=2
Match to Query 69726: 2225.193from(742.7382,3+)
Title: 1006: Sum of 2 scans in range 2354 (rt=62.2203, f=4, i=675) to 2355 (rt=62.2458, f=4, i=676) [D:\lab212\membrane\Grace\Joyce\iTRAQ_21_2.raw]
Data File:Submitted from 20120508-1(merge) by Mascot Daemon on JOYCE-VAIO
Monoisotopic mass of neutral peptide Mr(calc): 2225.193
Variable modifications:
Ions Score: 42.13 Expect: 0.050

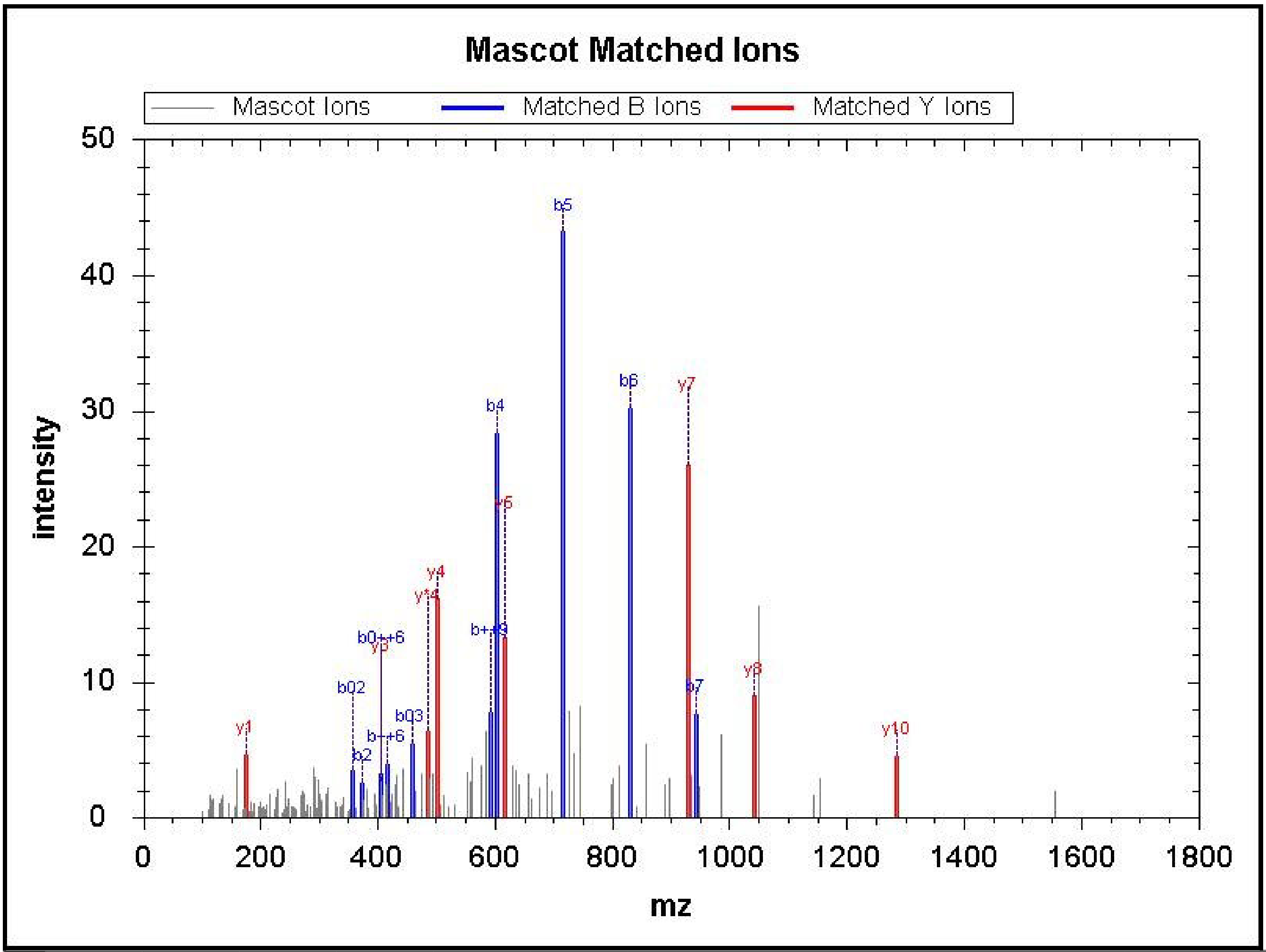

No	b	b++	b*	b*++	b0	b0++	Seq	y	y++	y*	y*++	y0	y0++	RevNo
1	260.14	130.57			242.13	121.57	D							17

2	374.18	187.59	357.15	179.08	356.17	178.59	N	1,967.07	984.04	1,950.04	975.53	1,949.06	975.03	16
3	475.23	238.12	458.20	229.60	457.22	229.11	T	1,853.03	927.02	1,836.00	918.50	1,835.02	918.01	15
4	603.29	302.15	586.26	293.63	585.27	293.14	Q	1,751.98	876.49	1,734.95	867.98	1,733.97	867.49	14
5	716.37	358.69	699.34	350.18	698.36	349.68	L	1,623.92	812.46	1,606.90	803.95	1,605.91	803.46	13
6	829.45	415.23	812.43	406.72	811.44	406.23	L	1,510.84	755.92	1,493.81	747.41	1,492.83	746.92	12
7	942.54	471.77	925.51	463.26	924.53	462.77	I	1,397.75	699.38	1,380.73	690.87	1,379.74	690.38	11
8	1,056.58	528.79	1,039.55	520.28	1,038.57	519.79	N	1,284.67	642.84	1,267.64	634.33	1,266.66	633.83	10
9	1,184.64	592.82	1,167.61	584.31	1,166.63	583.82	Q	1,170.63	585.82	1,153.60	577.30	1,152.62	576.81	9
10	1,297.72	649.37	1,280.70	640.85	1,279.71	640.36	L	1,042.57	521.79	1,025.54	513.27	1,024.56	512.78	8
11	1,483.80	742.40	1,466.78	733.89	1,465.79	733.40	W	929.48	465.25	912.46	456.73	911.47	456.24	7
12	1,611.86	806.43	1,594.83	797.92	1,593.85	797.43	Q	743.40	372.21	726.38	363.69	725.39	363.20	6
13	1,724.95	862.98	1,707.92	854.46	1,706.93	853.97	L	615.35	308.18	598.32	299.66	597.34	299.17	5
14	1,822.00	911.50	1,804.97	902.99	1,803.99	902.50	P	502.26	251.63	485.24	243.12	484.25	242.63	4
15	1,923.05	962.03	1,906.02	953.51	1,905.04	953.02	T	405.21	203.11	388.18	194.59	387.20	194.10	3
16	2,052.09	1,026.55	2,035.06	1,018.03	2,034.08	1,017.54	E	304.16	152.58	287.13	144.07	286.15	143.58	2
17							R	175.12	88.06	158.09	79.55			1

Query 34169 Hit 1

MS/MS Fragmentation of DTGQLYAALHHR
Found in sp|Q9H6Z4|RANB3_HUMAN, Ran-binding protein 3 OS=Homo sapiens GN=RANBP3 PE=1 SV=1
Match to Query 34169: 1524.795from(509.2722,3+)
Title: 345: Scan 789 (rt=27.4434, f=3, i=117) [D:\lab212\membrane\Grace\Joyce\iTRAQ_50_1.raw]
Data File:Submitted from 20120508-1(merge) by Mascot Daemon on JOYCE-VAIO
Monoisotopic mass of neutral peptide Mr(calc): 1524.795
Variable modifications:
Ions Score: 42.12 Expect: 0.051

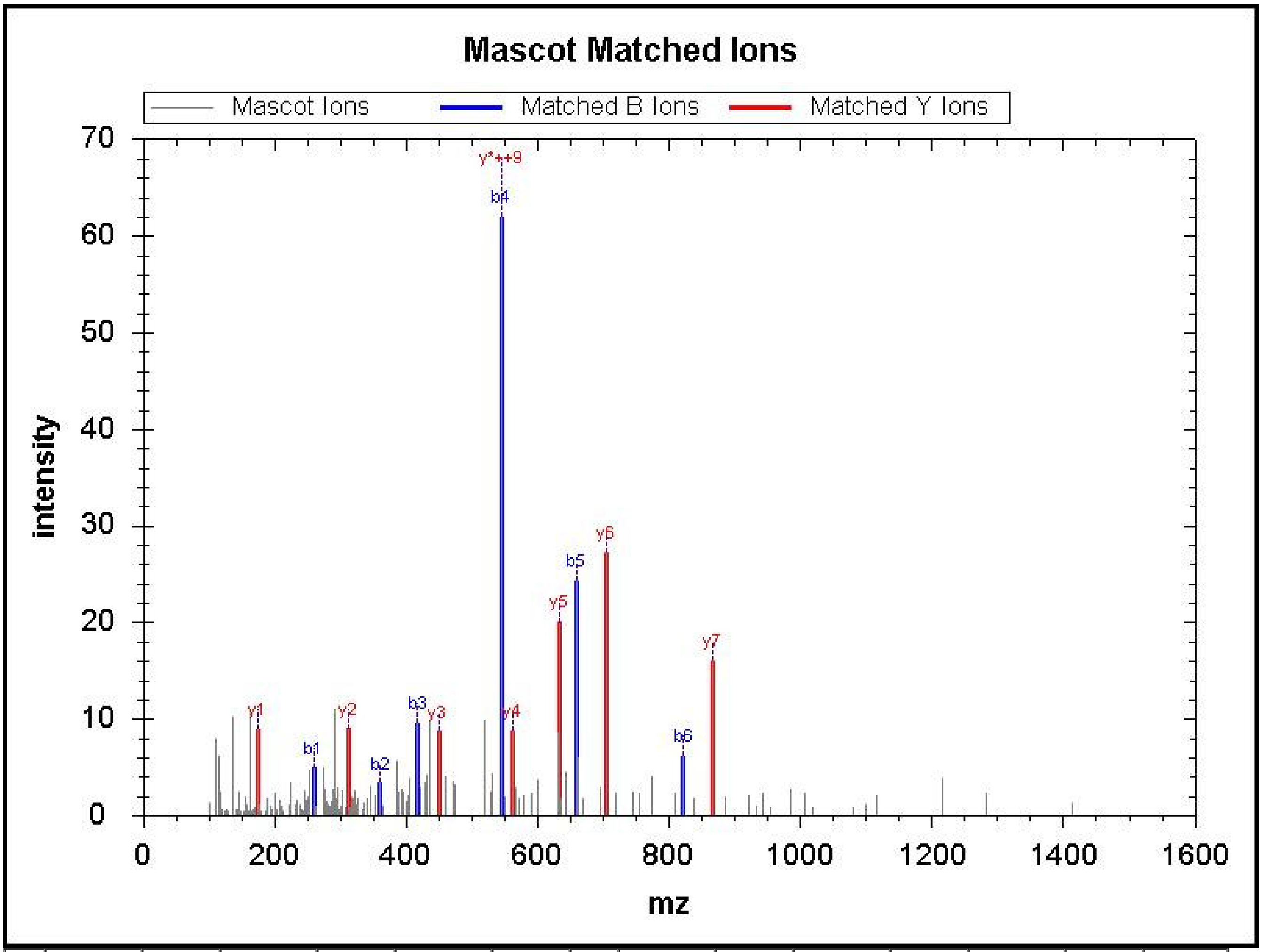

No	b	b++	b*	b*++	b0	b0++	Seq	y	y++	y*	y*++	y0	y0++	RevNo
1	260.14	130.57			242.13	121.57	D							12
2	361.18	181.10			343.17	172.09	T	1,266.67	633.84	1,249.64	625.33	1,248.66	624.83	11
3	418.21	209.61			400.19	200.60	G	1,165.62	583.31	1,148.60	574.80			10
4	546.26	273.64	529.24	265.12	528.25	264.63	Q	1,108.60	554.80	1,091.57	546.29			9
5	659.35	330.18	642.32	321.66	641.34	321.17	L	980.54	490.77	963.52	482.26			8
6	822.41	411.71	805.38	403.20	804.40	402.70	Y	867.46	434.23	850.43	425.72			7
7	893.45	447.23	876.42	438.71	875.44	438.22	A	704.40	352.70	687.37	344.19			6
8	964.49	482.75	947.46	474.23	946.47	473.74	A	633.36	317.18	616.33	308.67			5
9	1,077.57	539.29	1,060.54	530.78	1,059.56	530.28	L	562.32	281.66	545.29	273.15			4
10	1,214.63	607.82	1,197.60	599.30	1,196.62	598.81	H	449.24	225.12	432.21	216.61			3
11	1,351.69	676.35	1,334.66	667.83	1,333.68	667.34	H	312.18	156.59	295.15	148.08			2
12							R	175.12	88.06	158.09	79.55			1

Query 76935 Hit 1

MS/MS Fragmentation of SLTPAVPVESKPDKPSGK
Found in sp|P20810|ICAL_HUMAN, Calpastatin OS=Homo sapiens GN=CAST PE=1 SV=4
Match to Query 76935: 2412.407from(604.109,4+)
Title: 311: Scan 720 (rt=25.8852, f=2, i=115) [D:\lab212\membrane\Grace\Joyce\iTRAQ_50_1.raw]
Data File:Submitted from 20120508-1(merge) by Mascot Daemon on JOYCE-VAIO
Monoisotopic mass of neutral peptide Mr(calc): 2412.407
Variable modifications:
K11 :iTRAQ4plex (K)
K14 :iTRAQ4plex (K)
K18 :iTRAQ4plex (K)
Ions Score: 42.12 Expect: 0.016

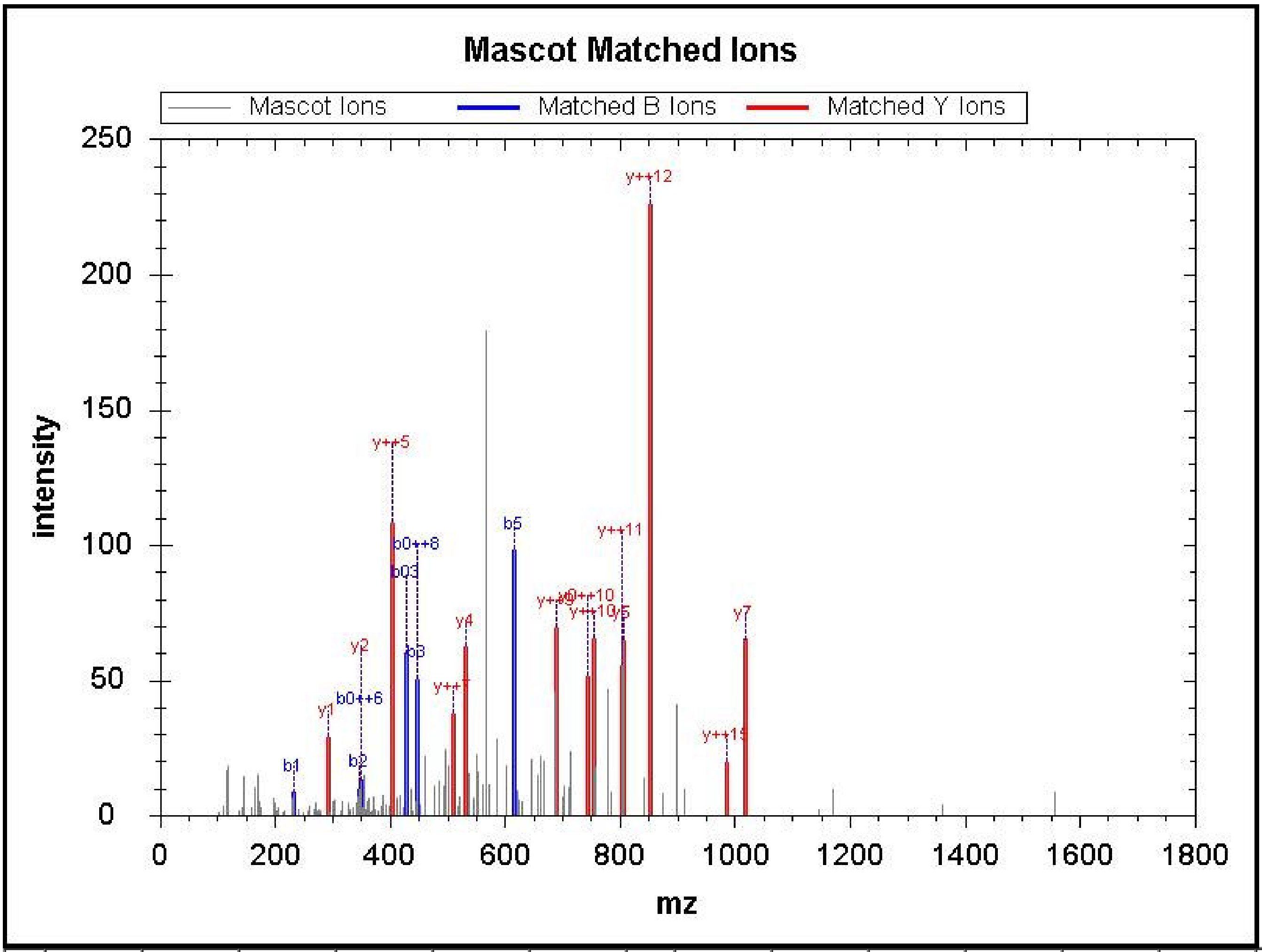

No	b	b++	b*	b*++	b0	b0++	Seq	y	y++	y*	y*++	y0	y0++	RevNo
1	232.14	116.57			214.13	107.57	S							18
2	345.23	173.12			327.21	164.11	L	2,182.28	1,091.64	2,165.25	1,083.13	2,164.27	1,082.64	17
3	446.27	223.64			428.26	214.63	T	2,069.20	1,035.10	2,052.17	1,026.59	2,051.19	1,026.10	16
4	543.33	272.17			525.32	263.16	P	1,968.15	984.58	1,951.12	976.06	1,950.14	975.57	15
5	614.36	307.69			596.35	298.68	A	1,871.10	936.05	1,854.07	927.54	1,853.09	927.05	14
6	713.43	357.22			695.42	348.21	V	1,800.06	900.53	1,783.03	892.02	1,782.05	891.53	13
7	810.48	405.75			792.47	396.74	P	1,700.99	851.00	1,683.96	842.49	1,682.98	841.99	12
8	909.55	455.28			891.54	446.27	V	1,603.94	802.47	1,586.91	793.96	1,585.93	793.47	11
9	1,038.60	519.80			1,020.58	510.80	E	1,504.87	752.94	1,487.84	744.43	1,486.86	743.93	10
10	1,125.63	563.32			1,107.62	554.31	S	1,375.83	688.42	1,358.80	679.90	1,357.82	679.41	9
11	1,397.82	699.42	1,380.80	690.90	1,379.81	690.41	K	1,288.79	644.90	1,271.77	636.39	1,270.78	635.90	8
12	1,494.88	747.94	1,477.85	739.43	1,476.87	738.94	P	1,016.60	508.80	999.57	500.29	998.59	499.80	7
13	1,609.90	805.46	1,592.88	796.94	1,591.89	796.45	D	919.54	460.28	902.52	451.76	901.53	451.27	6
14	1,882.10	941.55	1,865.07	933.04	1,864.09	932.55	K	804.52	402.76	787.49	394.25	786.51	393.76	5
15	1,979.15	990.08	1,962.13	981.57	1,961.14	981.08	P	532.32	266.66	515.29	258.15	514.31	257.66	4
16	2,066.19	1,033.60	2,049.16	1,025.08	2,048.18	1,024.59	S	435.27	218.14	418.24	209.62	417.26	209.13	3
17	2,123.21	1,062.11	2,106.18	1,053.59	2,105.20	1,053.10	G	348.24	174.62	331.21	166.11			2
18							K	291.21	146.11	274.19	137.60			1

Query 19218 Hit 1

MS/MS Fragmentation of EGFLESFK
Found in sp|Q15070|OXA1L_HUMAN, Mitochondrial inner membrane protein OXA1L OS=Homo sapiens GN=OXA1L PE=1 SV=3
Match to Query 19218: 1243.674from(622.8442,2+)
Title: 637: Scan 1446 (rt=41.9401, f=3, i=217) [D:\lab212\membrane\Grace\Joyce\iTRAQ_31_1.raw]

Data File:Submitted from 20120508-1(merge) by Mascot Daemon on JOYCE-VAIO
Monoisotopic mass of neutral peptide Mr(calc): 1243.674
Variable modifications:
K8 :iTRAQ4plex (K)
Ions Score: 42.11 Expect: 0.046

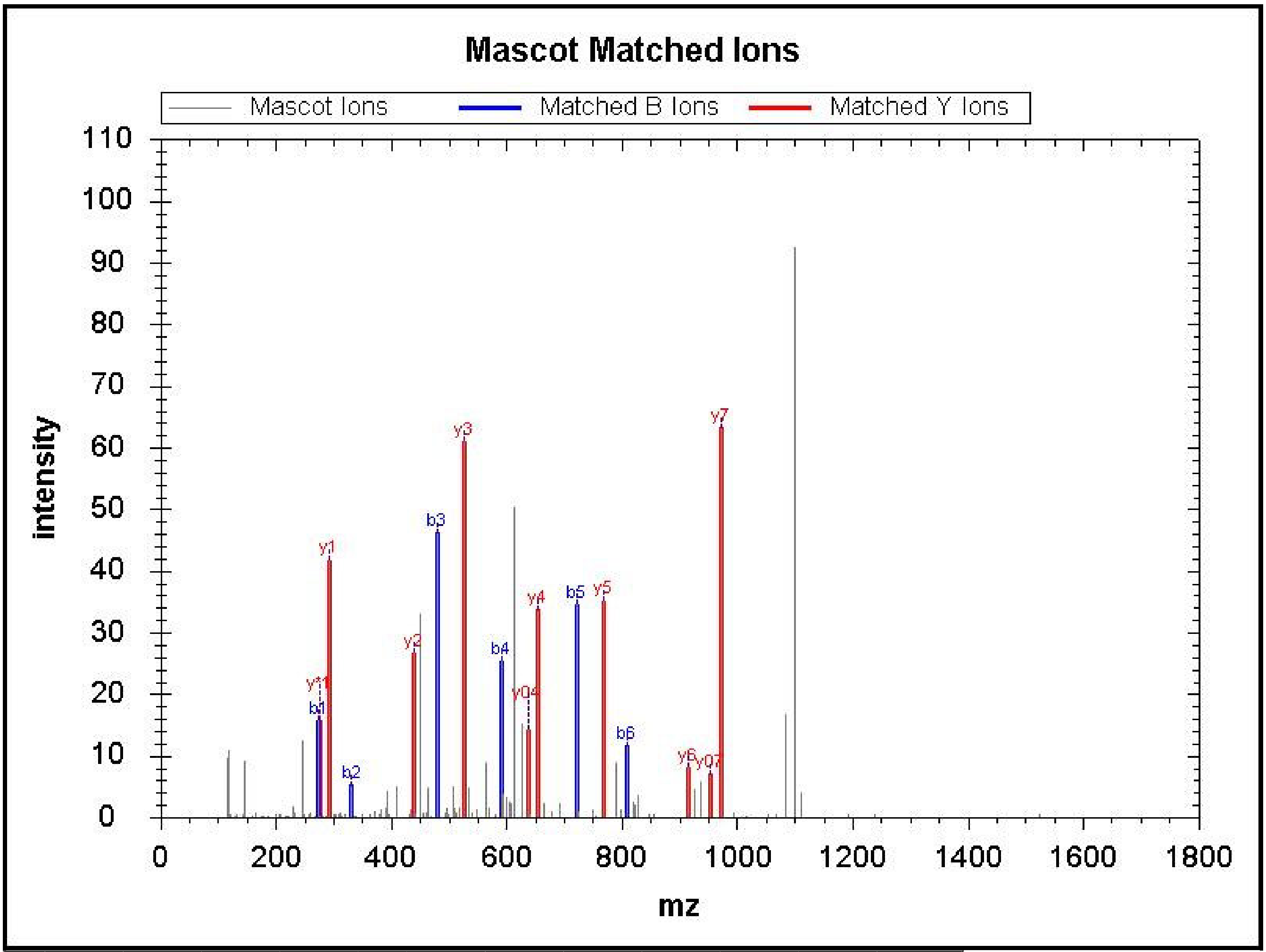

No	b	b++	b0	b0++	Seq	y	y++	y*	y*++	y0	y0++	RevNo
1	274.15	137.58	256.14	128.57	E							8
2	331.17	166.09	313.16	157.09	G	971.53	486.27	954.51	477.76	953.52	477.26	7
3	478.24	239.62	460.23	230.62	F	914.51	457.76	897.48	449.25	896.50	448.75	6
4	591.33	296.17	573.32	287.16	L	767.44	384.22	750.42	375.71	749.43	375.22	5
5	720.37	360.69	702.36	351.68	E	654.36	327.68	637.33	319.17	636.35	318.68	4
6	807.40	404.20	789.39	395.20	S	525.32	263.16	508.29	254.65	507.30	254.16	3
7	954.47	477.74	936.46	468.73	F	438.28	219.65	421.26	211.13			2
8					K	291.21	146.11	274.19	137.60			1

Query 95548 Hit 1

MS/MS Fragmentation of LQLVGSGLHEAEAAGEAQQAVLEGLQGLLSR
Found in sp|Q99K41|EMIL1_MOUSE, EMILIN-1 OS=Mus musculus GN=Emilin1 PE=1 SV=1
Match to Query 95548: 3287.753from(822.9454,4+)
Title: 1183: Sum of 2 scans in range 2705 (rt=69.7515, f=2, i=425) to 2706 (rt=69.7769, f=2, i=426) [D:\lab212\membrane\Grace\Joyce\iTRAQ_29_1.raw]
Data File:Submitted from 20120508-1(merge) by Mascot Daemon on JOYCE-VAIO
Monoisotopic mass of neutral peptide Mr(calc): 3287.753
Variable modifications:
Ions Score: 42.1 Expect: 0.034

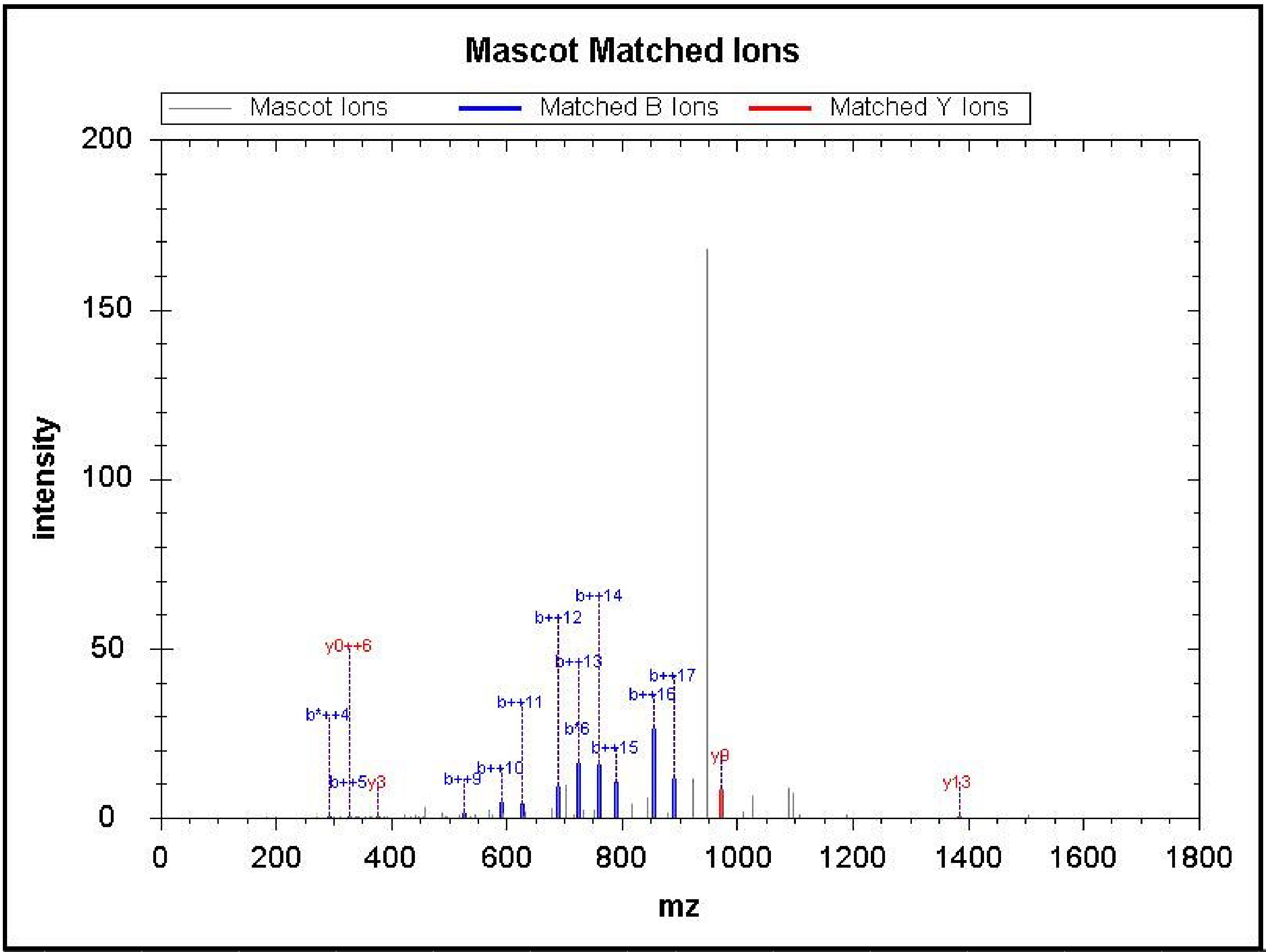

No	b	b++	b*	b*++	b0	b0++	Seq	y	y++	y*	y*++	y0	y0++	RevNo
1	258.19	129.60					L							31
2	386.25	193.63	369.23	185.12			Q	3,031.58	1,516.29	3,014.55	1,507.78	3,013.57	1,507.29	30
3	499.34	250.17	482.31	241.66			L	2,903.52	1,452.26	2,886.50	1,443.75	2,885.51	1,443.26	29
4	598.40	299.71	581.38	291.19			V	2,790.44	1,395.72	2,773.41	1,387.21	2,772.43	1,386.72	28
5	655.43	328.22	638.40	319.70			G	2,691.37	1,346.19	2,674.34	1,337.68	2,673.36	1,337.18	27
6	742.46	371.73	725.43	363.22	724.45	362.73	S	2,634.35	1,317.68	2,617.32	1,309.16	2,616.34	1,308.67	26
7	799.48	400.24	782.45	391.73	781.47	391.24	G	2,547.32	1,274.16	2,530.29	1,265.65	2,529.31	1,265.16	25
8	912.56	456.79	895.54	448.27	894.55	447.78	L	2,490.29	1,245.65	2,473.27	1,237.14	2,472.28	1,236.65	24
9	1,049.62	525.31	1,032.60	516.80	1,031.61	516.31	H	2,377.21	1,189.11	2,360.18	1,180.60	2,359.20	1,180.10	23
10	1,178.67	589.84	1,161.64	581.32	1,160.65	580.83	E	2,240.15	1,120.58	2,223.13	1,112.07	2,222.14	1,111.57	22
11	1,249.70	625.35	1,232.68	616.84	1,231.69	616.35	A	2,111.11	1,056.06	2,094.08	1,047.54	2,093.10	1,047.05	21
12	1,378.74	689.88	1,361.72	681.36	1,360.73	680.87	E	2,040.07	1,020.54	2,023.05	1,012.03	2,022.06	1,011.53	20
13	1,449.78	725.39	1,432.76	716.88	1,431.77	716.39	A	1,911.03	956.02	1,894.00	947.51	1,893.02	947.01	19
14	1,520.82	760.91	1,503.79	752.40	1,502.81	751.91	A	1,839.99	920.50	1,822.97	911.99	1,821.98	911.49	18
15	1,577.84	789.42	1,560.81	780.91	1,559.83	780.42	G	1,768.96	884.98	1,751.93	876.47	1,750.94	875.98	17
16	1,706.88	853.95	1,689.86	845.43	1,688.87	844.94	E	1,711.93	856.47	1,694.91	847.96	1,693.92	847.47	16
17	1,777.92	889.46	1,760.89	880.95	1,759.91	880.46	A	1,582.89	791.95	1,565.86	783.44	1,564.88	782.94	15
18	1,905.98	953.49	1,888.95	944.98	1,887.97	944.49	Q	1,511.85	756.43	1,494.83	747.92	1,493.84	747.43	14
19	2,034.04	1,017.52	2,017.01	1,009.01	2,016.03	1,008.52	Q	1,383.80	692.40	1,366.77	683.89	1,365.78	683.40	13
20	2,105.07	1,053.04	2,088.05	1,044.53	2,087.06	1,044.04	A	1,255.74	628.37	1,238.71	619.86	1,237.73	619.37	12
21	2,204.14	1,102.58	2,187.12	1,094.06	2,186.13	1,093.57	V	1,184.70	592.85	1,167.67	584.34	1,166.69	583.85	11
22	2,317.23	1,159.12	2,300.20	1,150.60	2,299.22	1,150.11	L	1,085.63	543.32	1,068.60	534.81	1,067.62	534.31	10
23	2,446.27	1,223.64	2,429.24	1,215.13	2,428.26	1,214.63	E	972.55	486.78	955.52	478.26	954.54	477.77	9

24	2,503.29	1,252.15	2,486.26	1,243.64	2,485.28	1,243.14	G	843.50	422.26	826.48	413.74	825.49	413.25	8
25	2,616.37	1,308.69	2,599.35	1,300.18	2,598.36	1,299.69	L	786.48	393.75	769.46	385.23	768.47	384.74	7
26	2,744.43	1,372.72	2,727.41	1,364.21	2,726.42	1,363.72	Q	673.40	337.20	656.37	328.69	655.39	328.20	6
27	2,801.45	1,401.23	2,784.43	1,392.72	2,783.44	1,392.23	G	545.34	273.17	528.31	264.66	527.33	264.17	5
28	2,914.54	1,457.77	2,897.51	1,449.26	2,896.53	1,448.77	L	488.32	244.66	471.29	236.15	470.31	235.66	4
29	3,027.62	1,514.32	3,010.60	1,505.80	3,009.61	1,505.31	L	375.24	188.12	358.21	179.61	357.22	179.12	3
30	3,114.65	1,557.83	3,097.63	1,549.32	3,096.64	1,548.83	S	262.15	131.58	245.12	123.07	244.14	122.57	2
31							R	175.12	88.06	158.09	79.55			1

Query 46899 Hit 1

MS/MS Fragmentation of AFDLIVDRPVTLVR
Found in sp|O96000|NDUBA_HUMAN, NADH dehydrogenase [ubiquinone] 1 beta subcomplex subunit 10 OS=Homo sapiens GN=NDUFB10 PE=1 SV=3
Match to Query 46899: 1757.029from(586.6835,3+)
Title: 700: Scan 1752 (rt=47.9255, f=2, i=247) [D:\lab212\membrane\Grace\Joyce\iTRAQ_34_1.raw]
Data File:Submitted from 20120508-1(merge) by Mascot Daemon on JOYCE-VAIO
Monoisotopic mass of neutral peptide Mr(calc): 1757.029
Variable modifications:
Ions Score: 42.08 Expect: 0.030

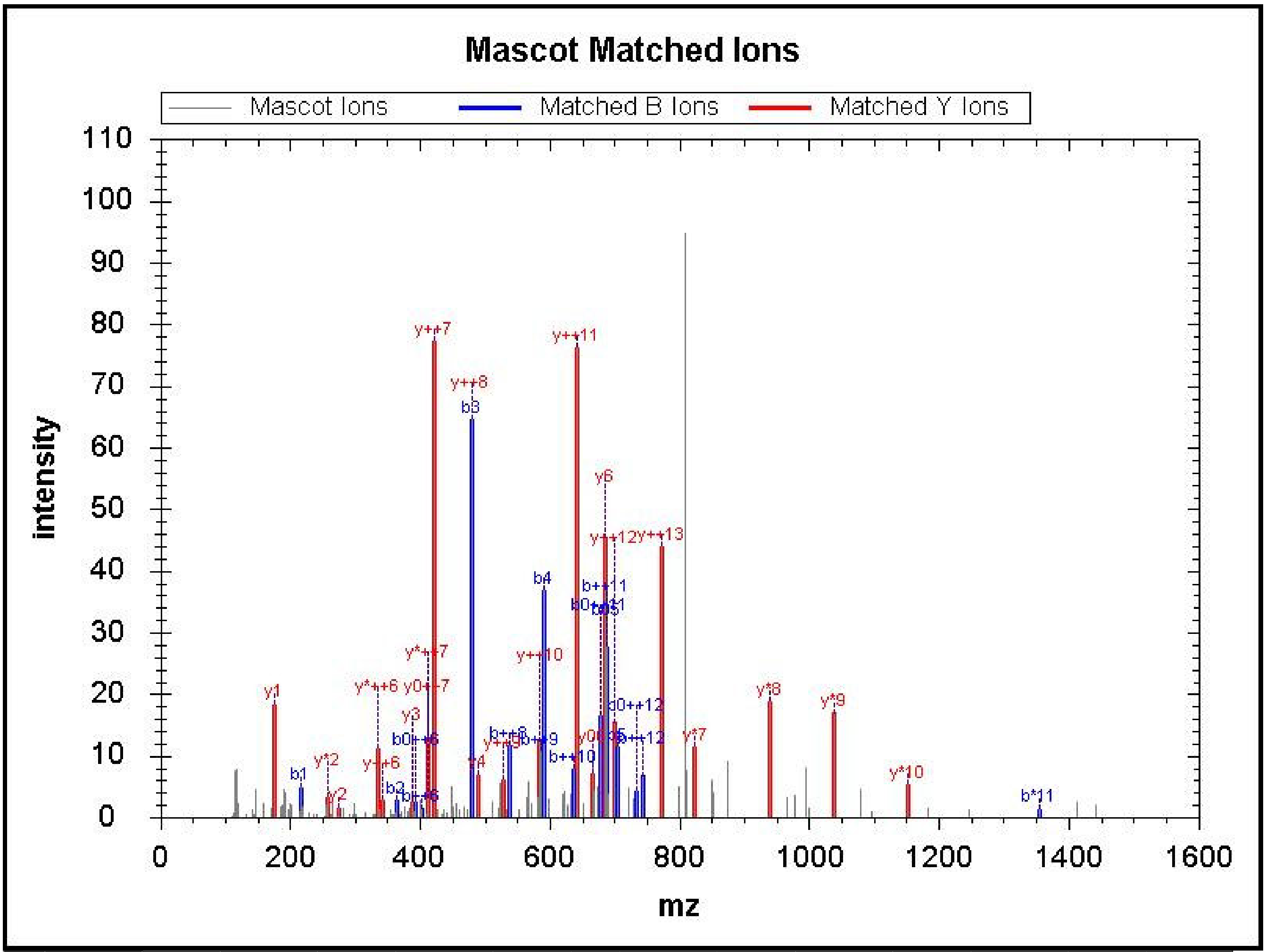

No	b	b++	b*	b*++	b0	b0++	Seq	y	y++	y*	y*++	y0	y0++	RevNo
1	216.15	108.58					A							14
2	363.21	182.11					F	1,542.90	771.95	1,525.87	763.44	1,524.89	762.95	13
3	478.24	239.62			460.23	230.62	D	1,395.83	698.42	1,378.81	689.91	1,377.82	689.41	12
4	591.33	296.17			573.32	287.16	L	1,280.80	640.91	1,263.78	632.39	1,262.79	631.90	11
5	704.41	352.71			686.40	343.70	I	1,167.72	584.36	1,150.69	575.85	1,149.71	575.36	10
6	803.48	402.24			785.47	393.24	V	1,054.64	527.82	1,037.61	519.31	1,036.63	518.82	9

7	918.51	459.76			900.49	450.75	D	955.57	478.29	938.54	469.77	937.56	469.28	8
8	1,074.61	537.81	1,057.58	529.29	1,056.60	528.80	R	840.54	420.77	823.51	412.26	822.53	411.77	7
9	1,171.66	586.33	1,154.63	577.82	1,153.65	577.33	P	684.44	342.72	667.41	334.21	666.43	333.72	6
10	1,270.73	635.87	1,253.70	627.35	1,252.72	626.86	V	587.39	294.20	570.36	285.68	569.38	285.19	5
11	1,371.78	686.39	1,354.75	677.88	1,353.76	677.39	T	488.32	244.66	471.29	236.15	470.31	235.66	4
12	1,484.86	742.93	1,467.83	734.42	1,466.85	733.93	L	387.27	194.14	370.24	185.63			3
13	1,583.93	792.47	1,566.90	783.95	1,565.92	783.46	V	274.19	137.60	257.16	129.08			2
14							R	175.12	88.06	158.09	79.55			1

Query 35248 Hit 1

MS/MS Fragmentation of SLLSMLVEVHK
Found in sp|O75976|CBPD_HUMAN, Carboxypeptidase D OS=Homo sapiens GN=CPD PE=1 SV=2
Match to Query 35248: 1542.904from(515.3088,3+)
Title: 911: Sum of 2 scans in range 1977 (rt=54.2242, f=4, i=609) to 1978 (rt=54.2496, f=4, i=610)
[D:\lab212\membrane\Grace\Joyce\iTRAQ_40_1.raw]
Data File:Submitted from 20120508-1(merge) by Mascot Daemon on JOYCE-VAIO
Monoisotopic mass of neutral peptide Mr(calc): 1542.904
Variable modifications:
K11 :iTRAQ4plex (K)
Ions Score: 42.06 Expect: 0.034

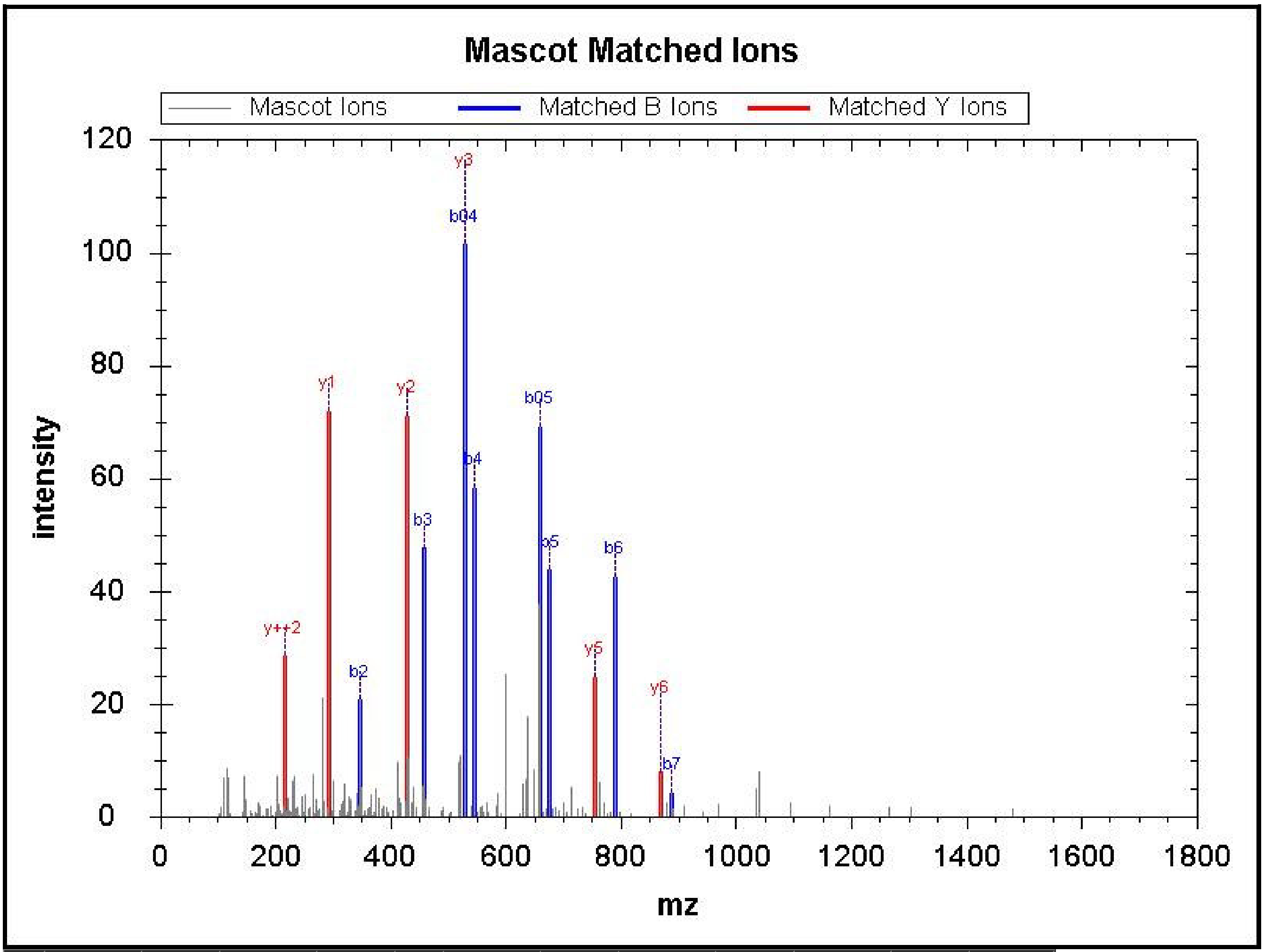

No	b	b++	b0	b0++	Seq	y	y++	y*	y*++	y0	y0++	RevNo
1	232.14	116.57	214.13	107.57	S							11
2	345.23	173.12	327.21	164.11	L	1,312.78	656.89	1,295.75	648.38	1,294.77	647.89	10
3	458.31	229.66	440.30	220.65	L	1,199.69	600.35	1,182.67	591.84	1,181.68	591.35	9
4	545.34	273.17	527.33	264.17	S	1,086.61	543.81	1,069.58	535.30	1,068.60	534.80	8
5	676.38	338.69	658.37	329.69	M	999.58	500.29	982.55	491.78	981.57	491.29	7

6	789.47	395.24	771.46	386.23	L	868.54	434.77	851.51	426.26	850.53	425.77	6
7	888.53	444.77	870.52	435.77	V	755.45	378.23	738.43	369.72	737.44	369.22	5
8	1,017.58	509.29	999.57	500.29	E	656.38	328.70	639.36	320.18	638.37	319.69	4
9	1,116.65	558.83	1,098.63	549.82	V	527.34	264.17	510.32	255.66			3
10	1,253.70	627.36	1,235.69	618.35	H	428.27	214.64	411.25	206.13			2
11					K	291.21	146.11	274.19	137.60			1

Query 97954 Hit 1

MS/MS Fragmentation of KSDLFQDDLYPDTAGPEAALEAEEWVSGR
Found in sp|Q9BR76|COR1B_HUMAN, Coronin-1B OS=Homo sapiens GN=CORO1B PE=1 SV=1
Match to Query 97954: 3496.686from(875.1787,4+)
Title: 984: Sum of 2 scans in range 2158 (rt=58.0562, f=4, i=655) to 2159 (rt=58.0816, f=4, i=656)
[D:\lab212\membrane\Grace\Joyce\iTRAQ_37_1.raw]
Data File:Submitted from 20120508-1(merge) by Mascot Daemon on JOYCE-VAIO
Monoisotopic mass of neutral peptide Mr(calc): 3496.686
Variable modifications:
K1 :iTRAQ4plex (K)
Ions Score: 42.02 Expect: 0.053

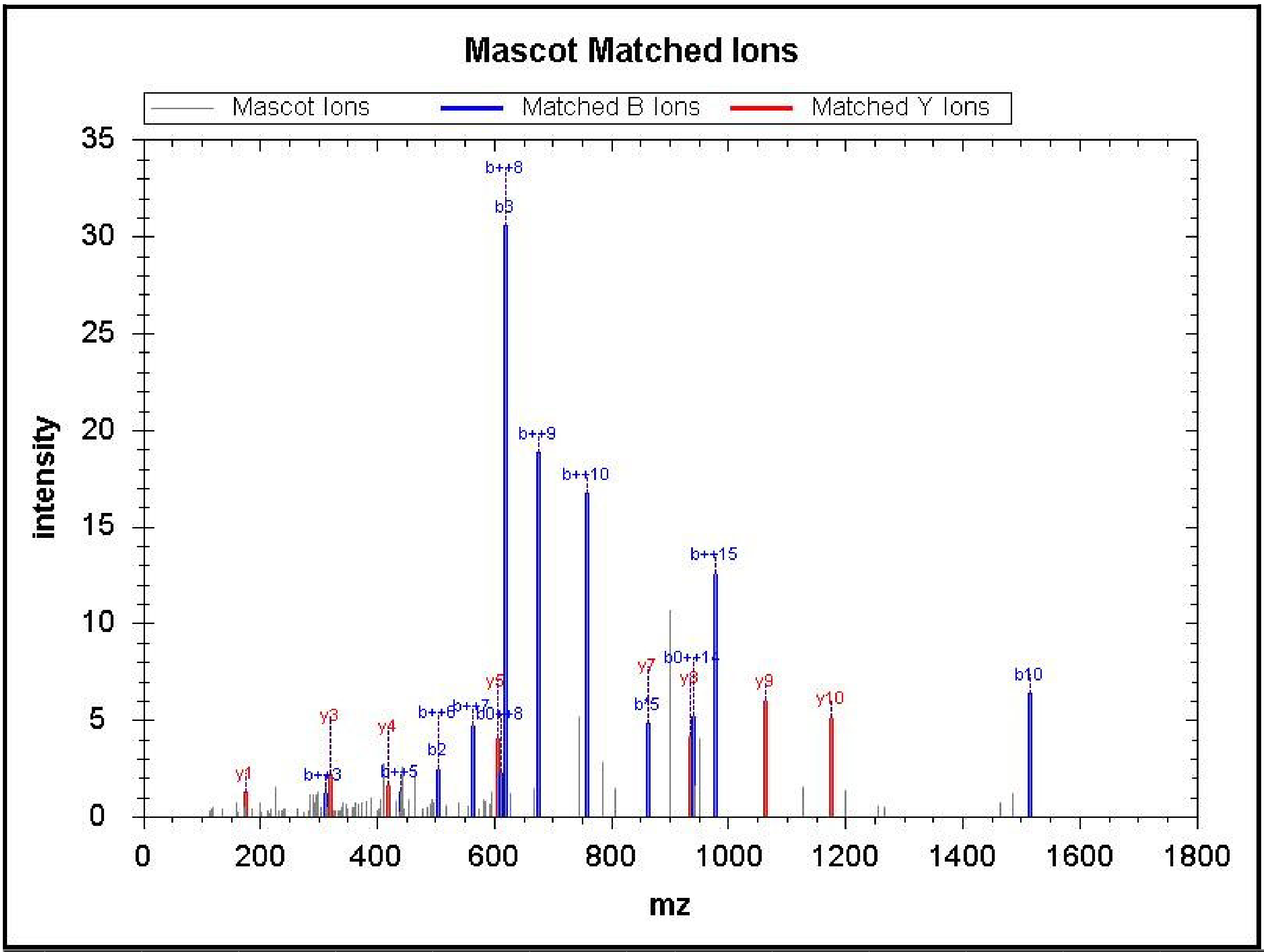

No	b	b++	b*	b*++	b0	b0++	Seq	y	y++	y*	y*++	y0	y0++	RevNo
1	417.31	209.16	400.28	200.64			K							29
2	504.34	252.67	487.31	244.16	486.33	243.67	S	3,081.40	1,541.20	3,064.37	1,532.69	3,063.39	1,532.20	28
3	619.37	310.19	602.34	301.67	601.35	301.18	D	2,994.36	1,497.69	2,977.34	1,489.17	2,976.35	1,488.68	27
4	732.45	366.73	715.42	358.22	714.44	357.72	L	2,879.34	1,440.17	2,862.31	1,431.66	2,861.33	1,431.17	26
5	879.52	440.26	862.49	431.75	861.51	431.26	F	2,766.25	1,383.63	2,749.23	1,375.12	2,748.24	1,374.62	25
6	1,007.58	504.29	990.55	495.78	989.57	495.29	Q	2,619.18	1,310.10	2,602.16	1,301.58	2,601.17	1,301.09	24
7	1,122.60	561.81	1,105.58	553.29	1,104.59	552.80	D	2,491.13	1,246.07	2,474.10	1,237.55	2,473.12	1,237.06	23

8	1,237.63	619.32	1,220.60	610.81	1,219.62	610.31	D	2,376.10	1,188.55	2,359.07	1,180.04	2,358.09	1,179.55	22
9	1,350.71	675.86	1,333.69	667.35	1,332.70	666.86	L	2,261.07	1,131.04	2,244.05	1,122.53	2,243.06	1,122.03	21
10	1,513.78	757.39	1,496.75	748.88	1,495.77	748.39	Y	2,147.99	1,074.50	2,130.96	1,065.98	2,129.98	1,065.49	20
11	1,610.83	805.92	1,593.80	797.41	1,592.82	796.91	P	1,984.92	992.97	1,967.90	984.45	1,966.91	983.96	19
12	1,725.86	863.43	1,708.83	854.92	1,707.85	854.43	D	1,887.87	944.44	1,870.85	935.93	1,869.86	935.43	18
13	1,826.91	913.96	1,809.88	905.44	1,808.89	904.95	T	1,772.85	886.93	1,755.82	878.41	1,754.83	877.92	17
14	1,897.94	949.47	1,880.92	940.96	1,879.93	940.47	A	1,671.80	836.40	1,654.77	827.89	1,653.79	827.40	16
15	1,954.96	977.99	1,937.94	969.47	1,936.95	968.98	G	1,600.76	800.88	1,583.73	792.37	1,582.75	791.88	15
16	2,052.02	1,026.51	2,034.99	1,018.00	2,034.01	1,017.51	P	1,543.74	772.37	1,526.71	763.86	1,525.73	763.37	14
17	2,181.06	1,091.03	2,164.03	1,082.52	2,163.05	1,082.03	E	1,446.69	723.85	1,429.66	715.33	1,428.68	714.84	13
18	2,252.10	1,126.55	2,235.07	1,118.04	2,234.09	1,117.55	A	1,317.64	659.33	1,300.62	650.81	1,299.63	650.32	12
19	2,323.13	1,162.07	2,306.11	1,153.56	2,305.12	1,153.07	A	1,246.61	623.81	1,229.58	615.29	1,228.60	614.80	11
20	2,436.22	1,218.61	2,419.19	1,210.10	2,418.21	1,209.61	L	1,175.57	588.29	1,158.54	579.78	1,157.56	579.28	10
21	2,565.26	1,283.13	2,548.23	1,274.62	2,547.25	1,274.13	E	1,062.49	531.75	1,045.46	523.23	1,044.47	522.74	9
22	2,636.30	1,318.65	2,619.27	1,310.14	2,618.29	1,309.65	A	933.44	467.22	916.42	458.71	915.43	458.22	8
23	2,765.34	1,383.17	2,748.31	1,374.66	2,747.33	1,374.17	E	862.41	431.71	845.38	423.19	844.39	422.70	7
24	2,894.38	1,447.69	2,877.36	1,439.18	2,876.37	1,438.69	E	733.36	367.19	716.34	358.67	715.35	358.18	6
25	3,080.46	1,540.73	3,063.43	1,532.22	3,062.45	1,531.73	W	604.32	302.66	587.29	294.15	586.31	293.66	5
26	3,179.53	1,590.27	3,162.50	1,581.76	3,161.52	1,581.26	V	418.24	209.62	401.21	201.11	400.23	200.62	4
27	3,266.56	1,633.78	3,249.53	1,625.27	3,248.55	1,624.78	S	319.17	160.09	302.15	151.58	301.16	151.08	3
28	3,323.58	1,662.30	3,306.56	1,653.78	3,305.57	1,653.29	G	232.14	116.57	215.11	108.06			2
29							R	175.12	88.06	158.09	79.55			1

Query 47797 Hit 1

MS/MS Fragmentation of ALTLDQWLEQHK
Found in sp|Q9HBL8|NMRL1_HUMAN, NmrA-like family domain-containing protein 1 OS=Homo sapiens GN=NMRAL1 PE=1 SV=1
Match to Query 47797: 1768.974from(590.6654,3+)
Title: 752: Scan 1653 (rt=47.009, f=2, i=265) [D:\lab212\membrane\Grace\Joyce\iTRAQ_42_2.raw]
Data File:Submitted from 20120508-1(merge) by Mascot Daemon on JOYCE-VAIO
Monoisotopic mass of neutral peptide Mr(calc): 1768.974
Variable modifications:
K12 :iTRAQ4plex (K)
Ions Score: 42 Expect: 0.047

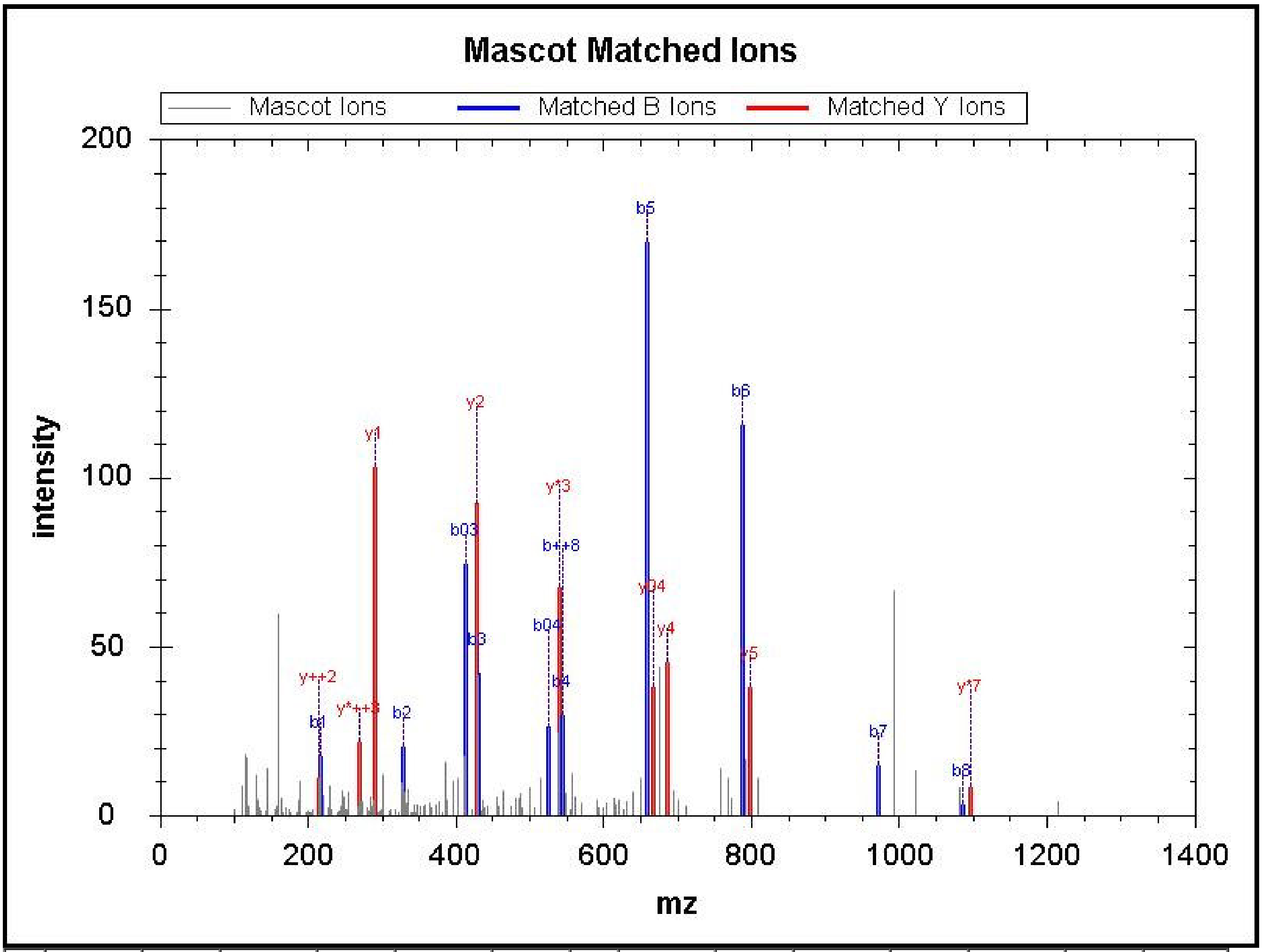

No	b	b++	b*	b*++	b0	b0++	Seq	y	y++	y*	y*++	y0	y0++	RevNo
1	216.15	108.58					A							12
2	329.23	165.12					L	1,554.84	777.92	1,537.81	769.41	1,536.83	768.92	11
3	430.28	215.64			412.27	206.64	T	1,441.76	721.38	1,424.73	712.87	1,423.75	712.38	10
4	543.36	272.18			525.35	263.18	L	1,340.71	670.86	1,323.68	662.34	1,322.70	661.85	9
5	658.39	329.70			640.38	320.69	D	1,227.62	614.32	1,210.60	605.80	1,209.61	605.31	8
6	786.45	393.73	769.42	385.21	768.44	384.72	Q	1,112.60	556.80	1,095.57	548.29	1,094.59	547.80	7
7	972.53	486.77	955.50	478.25	954.52	477.76	W	984.54	492.77	967.51	484.26	966.53	483.77	6
8	1,085.61	543.31	1,068.58	534.80	1,067.60	534.30	L	798.46	399.73	781.43	391.22	780.45	390.73	5
9	1,214.65	607.83	1,197.63	599.32	1,196.64	598.83	E	685.37	343.19	668.35	334.68	667.36	334.19	4
10	1,342.71	671.86	1,325.69	663.35	1,324.70	662.85	Q	556.33	278.67	539.31	270.16			3
11	1,479.77	740.39	1,462.74	731.88	1,461.76	731.38	H	428.27	214.64	411.25	206.13			2
12							K	291.21	146.11	274.19	137.60			1

Query 50244 Hit 1

MS/MS Fragmentation of EDVLWFKPVELR
Found in sp|Q2NL82|TSR1_HUMAN, Pre-rRNA-processing protein TSR1 homolog OS=Homo sapiens GN=TSR1 PE=1 SV=1
Match to Query 50244: 1818.026from(607.0159,3+)
Title: 854: Sum of 2 scans in range 1860 (rt=51.5854, f=4, i=571) to 1861 (rt=51.6108, f=4, i=572)
[D:\lab212\membrane\Grace\Joyce\iTRAQ_40_1.raw]
Data File:Submitted from 20120508-1(merge) by Mascot Daemon on JOYCE-VAIO
Monoisotopic mass of neutral peptide Mr(calc): 1818.026
Variable modifications:
K7 :iTRAQ4plex (K)
Ions Score: 41.92 Expect: 0.045

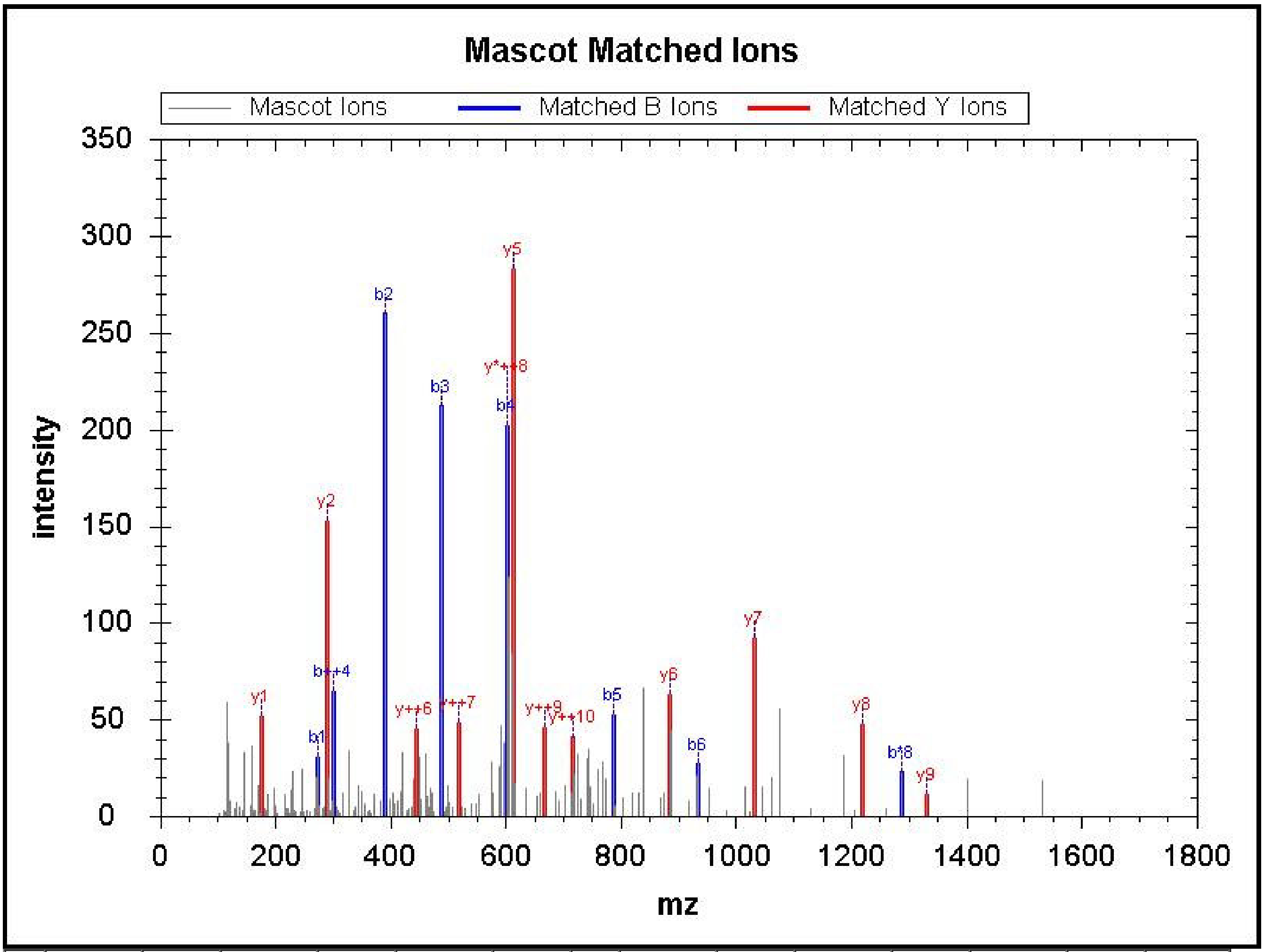

No	b	b++	b*	b*++	b0	b0++	Seq	y	y++	y*	y*++	y0	y0++	RevNo
1	274.15	137.58			256.14	128.57	E							12
2	389.18	195.09			371.17	186.09	D	1,545.89	773.45	1,528.86	764.94	1,527.88	764.44	11
3	488.25	244.63			470.24	235.62	V	1,430.86	715.94	1,413.84	707.42	1,412.85	706.93	10
4	601.33	301.17			583.32	292.16	L	1,331.80	666.40	1,314.77	657.89	1,313.79	657.40	9
5	787.41	394.21			769.40	385.20	W	1,218.71	609.86	1,201.69	601.35	1,200.70	600.85	8
6	934.48	467.74			916.47	458.74	F	1,032.63	516.82	1,015.61	508.31	1,014.62	507.81	7
7	1,206.68	603.84	1,189.65	595.33	1,188.67	594.84	K	885.56	443.29	868.54	434.77	867.55	434.28	6
8	1,303.73	652.37	1,286.70	643.85	1,285.72	643.36	P	613.37	307.19	596.34	298.67	595.36	298.18	5
9	1,402.80	701.90	1,385.77	693.39	1,384.79	692.90	V	516.31	258.66	499.29	250.15	498.30	249.66	4
10	1,531.84	766.42	1,514.81	757.91	1,513.83	757.42	E	417.25	209.13	400.22	200.61	399.24	200.12	3
11	1,644.92	822.97	1,627.90	814.45	1,626.91	813.96	L	288.20	144.61	271.18	136.09			2
12							R	175.12	88.06	158.09	79.55			1

Query 71998 Hit 1

MS/MS Fragmentation of GTLSAELTAAHFGGGGGLLHK
Found in sp|P78316|NOP14_HUMAN, Nucleolar protein 14 OS=Homo sapiens GN=NOP14 PE=1 SV=3
Match to Query 71998: 2281.247from(571.3191,4+)
Title: 651: Scan 1455 (rt=42.4214, f=3, i=224) [D:\lab212\membrane\Grace\Joyce\iTRAQ_54_2.raw]
Data File:Submitted from 20120508-1(merge) by Mascot Daemon on JOYCE-VAIO
Monoisotopic mass of neutral peptide Mr(calc): 2281.247
Variable modifications:
K21 :iTRAQ4plex (K)
Ions Score: 41.86 Expect: 0.045

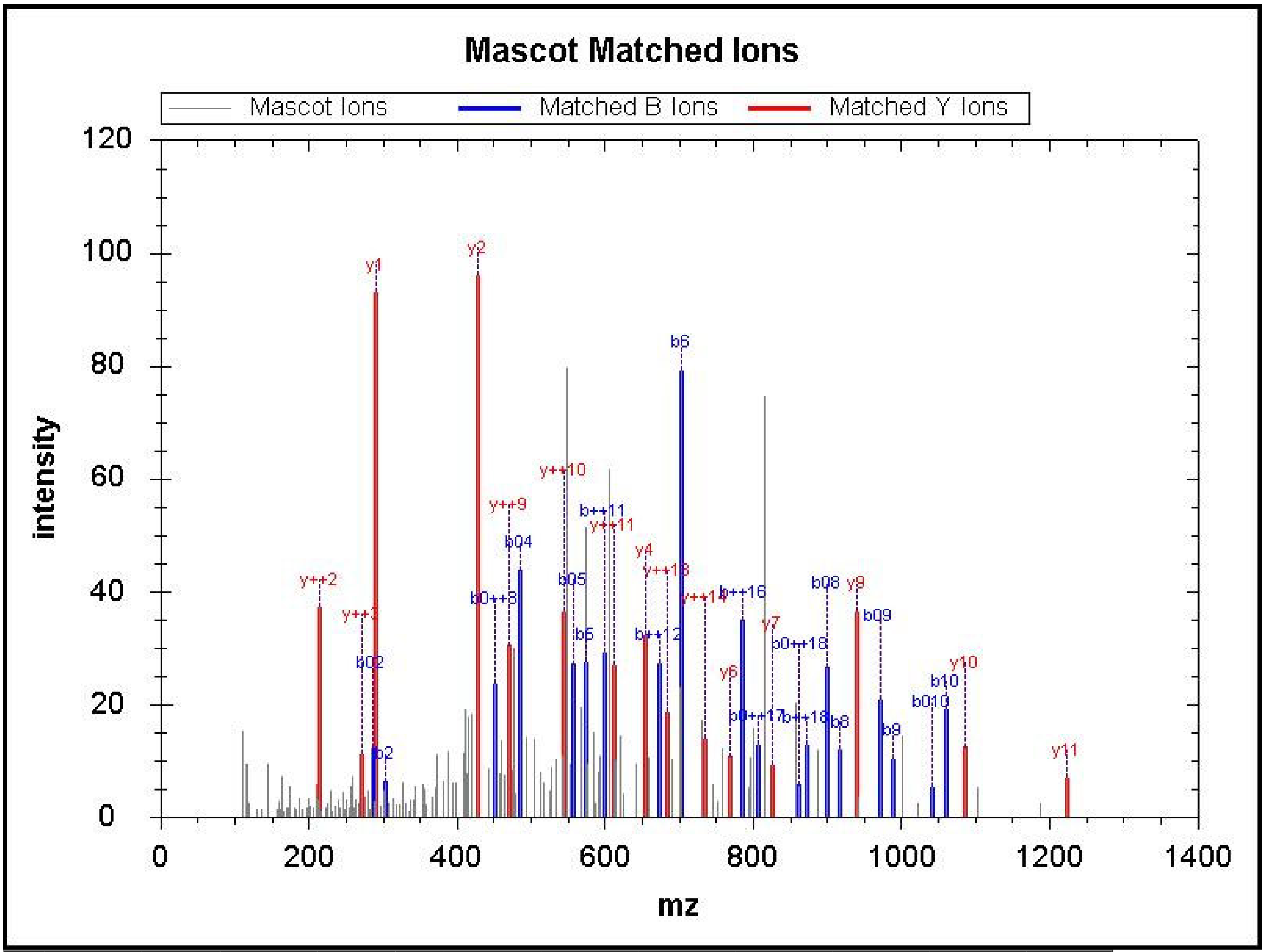

No	b	b++	b0	b0++	Seq	y	y++	y*	y*++	y0	y0++	RevNo
1	202.13	101.57			G							21
2	303.18	152.09	285.17	143.09	T	2,081.13	1,041.07	2,064.10	1,032.55	2,063.12	1,032.06	20
3	416.26	208.63	398.25	199.63	L	1,980.08	990.54	1,963.05	982.03	1,962.07	981.54	19
4	503.29	252.15	485.28	243.15	S	1,866.99	934.00	1,849.97	925.49	1,848.98	925.00	18
5	574.33	287.67	556.32	278.66	A	1,779.96	890.48	1,762.94	881.97	1,761.95	881.48	17
6	703.37	352.19	685.36	343.19	E	1,708.93	854.97	1,691.90	846.45	1,690.91	845.96	16
7	816.46	408.73	798.45	399.73	L	1,579.88	790.45	1,562.86	781.93	1,561.87	781.44	15
8	917.51	459.26	899.50	450.25	T	1,466.80	733.90	1,449.77	725.39	1,448.79	724.90	14
9	988.54	494.78	970.53	485.77	A	1,365.75	683.38	1,348.72	674.87			13
10	1,059.58	530.29	1,041.57	521.29	A	1,294.71	647.86	1,277.69	639.35			12
11	1,196.64	598.82	1,178.63	589.82	H	1,223.68	612.34	1,206.65	603.83			11
12	1,343.71	672.36	1,325.70	663.35	F	1,086.62	543.81	1,069.59	535.30			10
13	1,400.73	700.87	1,382.72	691.86	G	939.55	470.28	922.52	461.76			9
14	1,457.75	729.38	1,439.74	720.37	G	882.53	441.77	865.50	433.25			8

15	1,514.77	757.89	1,496.76	748.88	G	825.51	413.26	808.48	404.74			7
16	1,571.79	786.40	1,553.78	777.40	G	768.48	384.75	751.46	376.23			6
17	1,628.82	814.91	1,610.80	805.91	G	711.46	356.24	694.44	347.72			5
18	1,741.90	871.45	1,723.89	862.45	L	654.44	327.72	637.42	319.21			4
19	1,854.98	928.00	1,836.97	918.99	L	541.36	271.18	524.33	262.67			3
20	1,992.04	996.52	1,974.03	987.52	H	428.27	214.64	411.25	206.13			2
21					K	291.21	146.11	274.19	137.60			1

Query 31317 Hit 1

MS/MS Fragmentation of GGFDWNLVFK
Found in sp|Q10471|GALT2_HUMAN, Polypeptide N-acetylgalactosaminyltransferase 2 OS=Homo sapiens GN=GALNT2 PE=1 SV=1
Match to Query 31317: 1469.791from(735.9025,2+)
Title: 926: Sum of 2 scans in range 2061 (rt=55.8003, f=4, i=615) to 2062 (rt=55.8257, f=4, i=616)
[D:\lab212\membrane\Grace\Joyce\iTRAQ_31_1.raw]
Data File:Submitted from 20120508-1(merge) by Mascot Daemon on JOYCE-VAIO
Monoisotopic mass of neutral peptide Mr(calc): 1469.791
Variable modifications:
K10 :iTRAQ4plex (K)
Ions Score: 41.8 Expect: 0.049

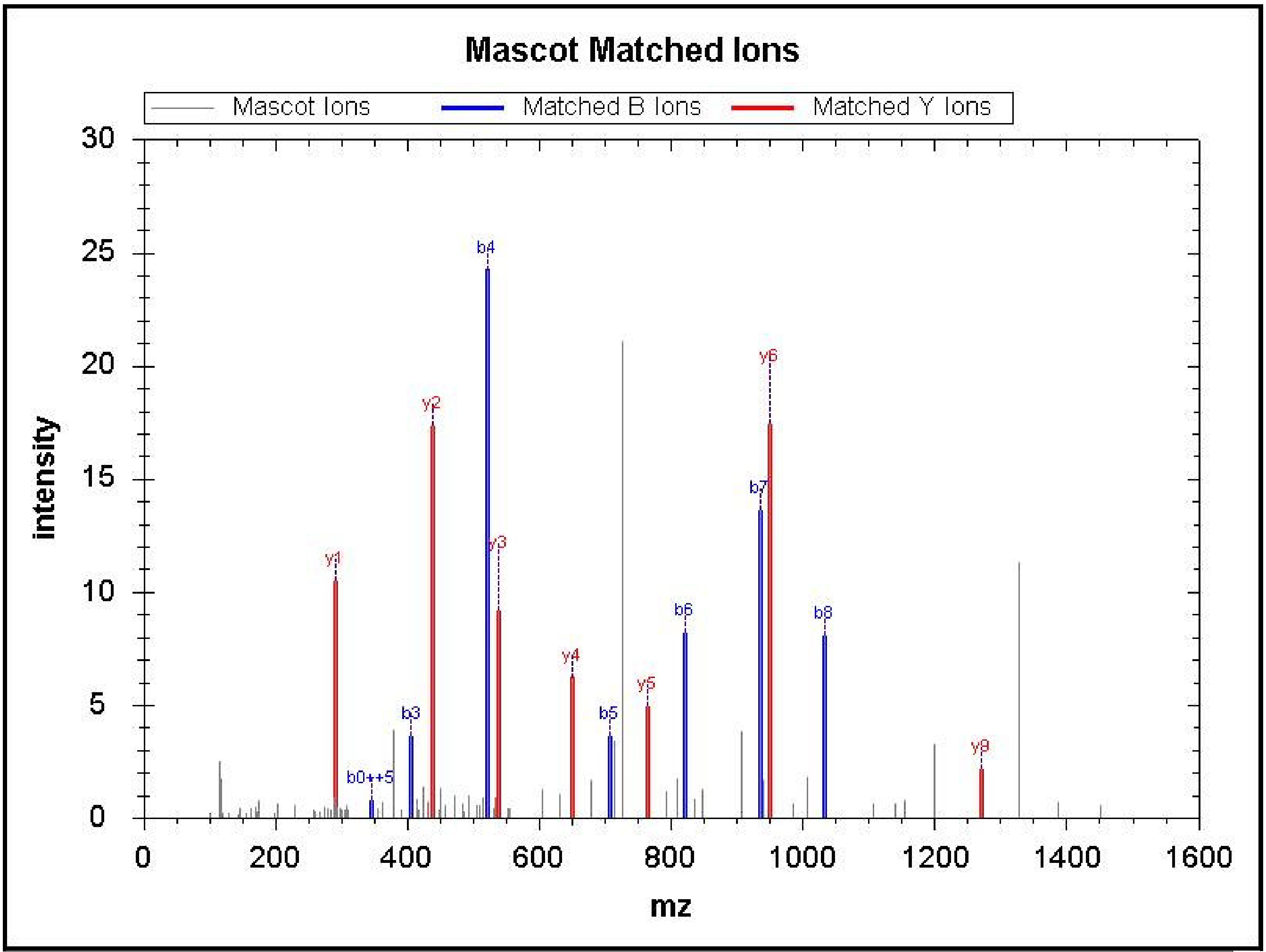

No	b	b++	b*	b*++	b0	b0++	Seq	y	y++	y*	y*++	y0	y0++	RevNo
1	202.13	101.57					G							10
2	259.15	130.08					G	1,269.67	635.34	1,252.65	626.83	1,251.66	626.34	9
3	406.22	203.61					F	1,212.65	606.83	1,195.63	598.32	1,194.64	597.83	8
4	521.25	261.13			503.24	252.12	D	1,065.58	533.30	1,048.56	524.78	1,047.57	524.29	7
5	707.33	354.17			689.32	345.16	W	950.56	475.78	933.53	467.27			6
6	821.37	411.19	804.34	402.68	803.36	402.18	N	764.48	382.74	747.45	374.23			5

7	934.45	467.73	917.43	459.22	916.44	458.73	L	650.44	325.72	633.41	317.21			4
8	1,033.52	517.26	1,016.50	508.75	1,015.51	508.26	V	537.35	269.18	520.33	260.67			3
9	1,180.59	590.80	1,163.56	582.29	1,162.58	581.79	F	438.28	219.65	421.26	211.13			2
10							K	291.21	146.11	274.19	137.60			1

Query 52132 Hit 1

MS/MS Fragmentation of LYKDDQLLDDGK
Found in sp|Q15370|ELOB_HUMAN, Transcription elongation factor B polypeptide 2 OS=Homo sapiens GN=TCEB2 PE=1 SV=1
Match to Query 52132: 1854.017from(619.013,3+)
Title: 182: Sum of 2 scans in range 1046 (rt=31.0314, f=4, i=83) to 1047 (rt=31.0569, f=4, i=84) [D:\lab212\membrane\Grace\Joyce\iTRAQ_46_1.raw]
Data File:Submitted from 20120508-1(merge) by Mascot Daemon on JOYCE-VAIO
Monoisotopic mass of neutral peptide Mr(calc): 1854.017
Variable modifications:
K3 :iTRAQ4plex (K)
K12 :iTRAQ4plex (K)
Ions Score: 41.77 Expect: 0.049

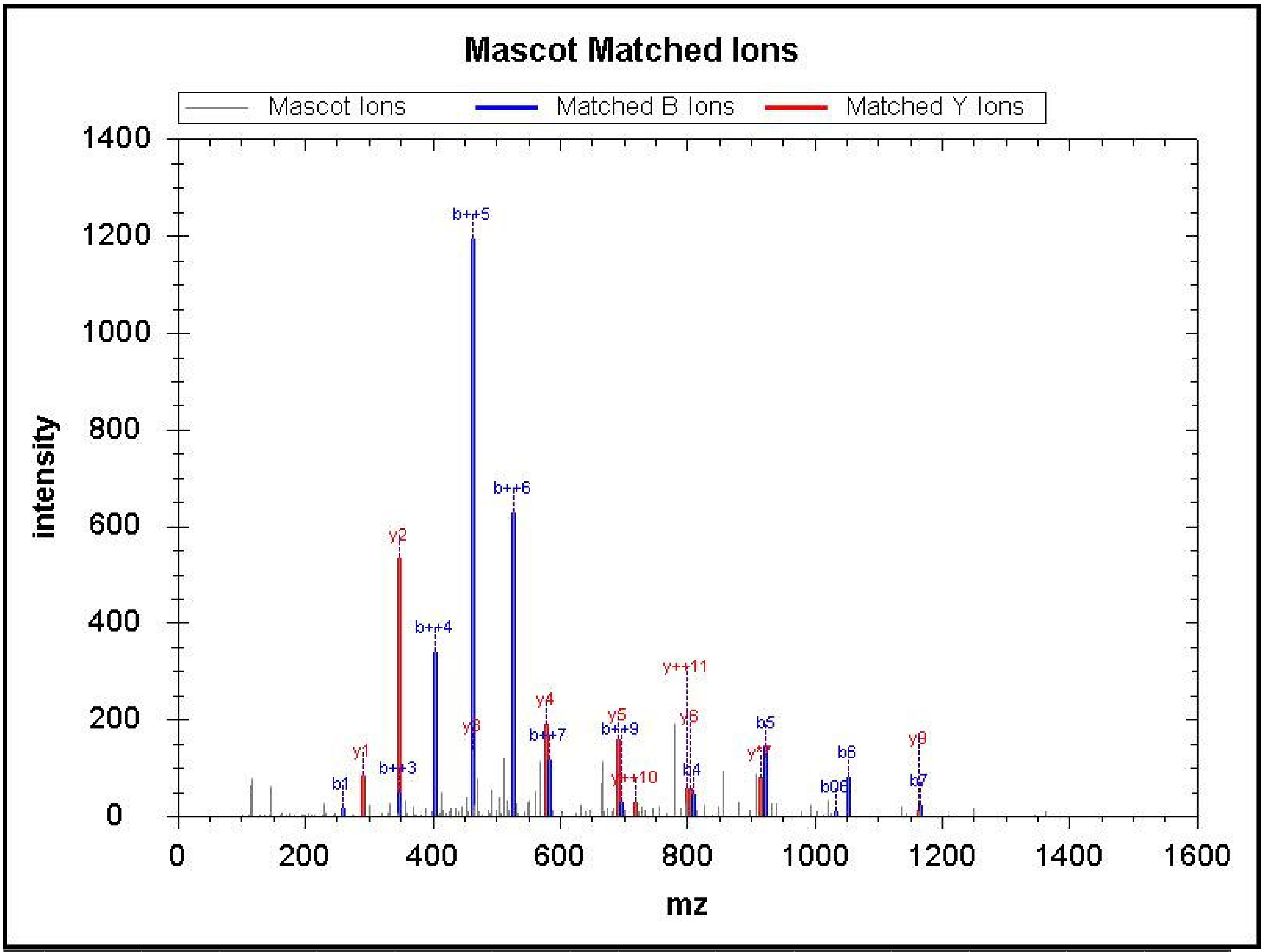

No	b	b++	b*	b*++	b0	b0++	Seq	y	y++	y*	y*++	y0	y0++	RevNo
1	258.19	129.60					L							12
2	421.26	211.13					Y	1,597.83	799.42	1,580.80	790.91	1,579.82	790.41	11
3	693.45	347.23	676.43	338.72			K	1,434.77	717.89	1,417.74	709.37	1,416.76	708.88	10
4	808.48	404.74	791.45	396.23	790.47	395.74	D	1,162.57	581.79	1,145.54	573.28	1,144.56	572.78	9
5	923.51	462.26	906.48	453.74	905.50	453.25	D	1,047.54	524.28	1,030.52	515.76	1,029.53	515.27	8
6	1,051.57	526.29	1,034.54	517.77	1,033.56	517.28	Q	932.52	466.76	915.49	458.25	914.51	457.76	7
7	1,164.65	582.83	1,147.62	574.32	1,146.64	573.82	L	804.46	402.73	787.43	394.22	786.45	393.73	6
8	1,277.73	639.37	1,260.71	630.86	1,259.72	630.37	L	691.37	346.19	674.35	337.68	673.36	337.19	5

9	1,392.76	696.88	1,375.73	688.37	1,374.75	687.88	D	578.29	289.65	561.26	281.14	560.28	280.64	4
10	1,507.79	754.40	1,490.76	745.88	1,489.78	745.39	D	463.26	232.14	446.24	223.62	445.25	223.13	3
11	1,564.81	782.91	1,547.78	774.40	1,546.80	773.90	G	348.24	174.62	331.21	166.11			2
12							K	291.21	146.11	274.19	137.60			1

Query 21104 Hit 1

MS/MS Fragmentation of EKKEQLSK
Found in sp|Q9CPR1|RWDD4_MOUSE, RWD domain-containing protein 4 OS=Mus musculus GN=Rwdd4 PE=2 SV=1
Match to Query 21104: 1277.797from(426.9396,3+)
Title: 234: Scan 572 (rt=22.5785, f=2, i=92) [D:\lab212\membrane\Grace\Joyce\iTRAQ_49_1.raw]
Data File:Submitted from 20120508-1(merge) by Mascot Daemon on JOYCE-VAIO
Monoisotopic mass of neutral peptide Mr(calc): 1277.797
Variable modifications:
Q5 :Deamidated (NQ)
K8 :iTRAQ4plex (K)
Ions Score: 41.77 Expect: 0.030

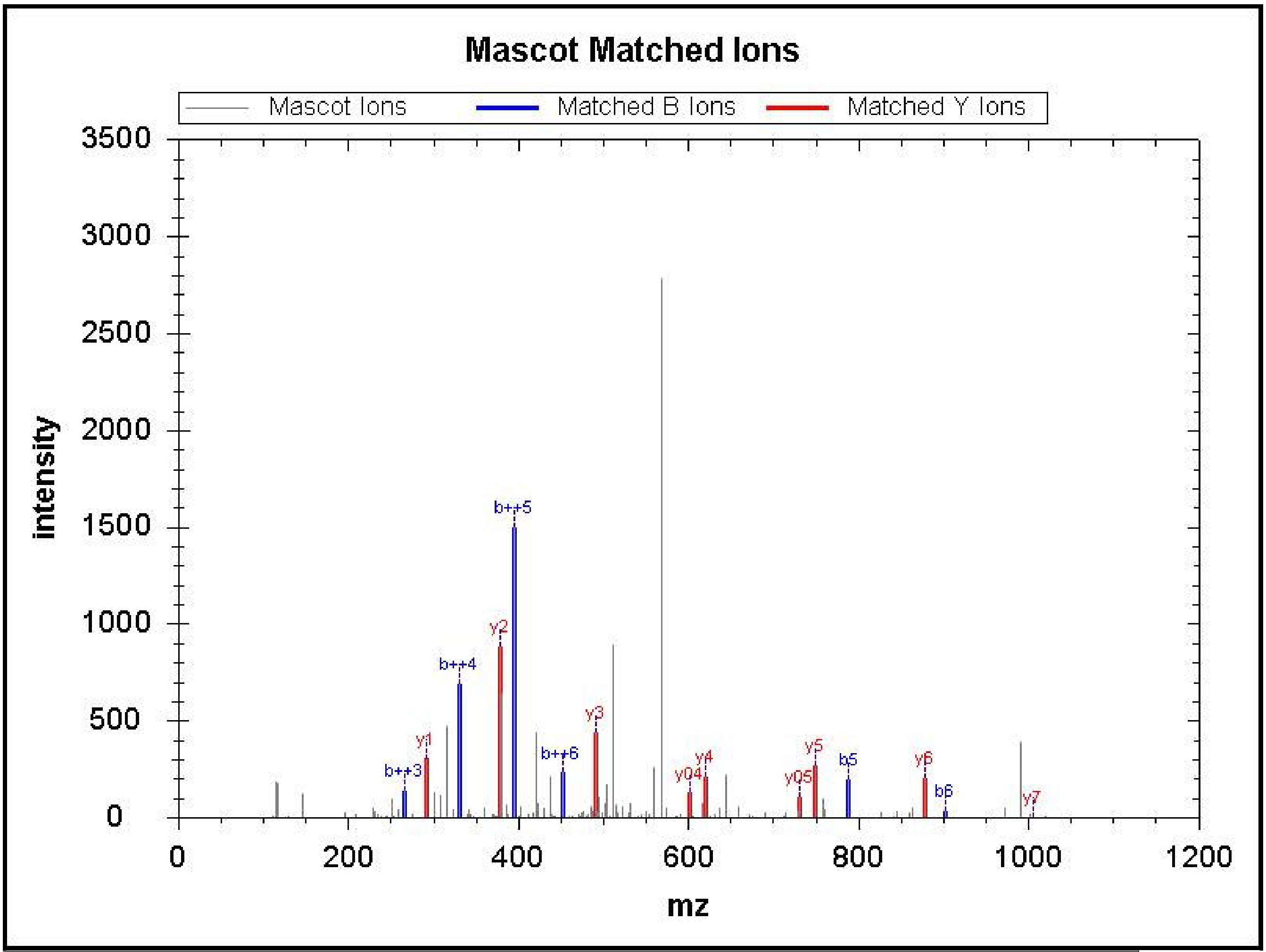

No	b	b++	b*	b*++	b0	b0++	Seq	y	y++	y*	y*++	y0	y0++	RevNo
1	274.15	137.58			256.14	128.57	E							8
2	402.25	201.63	385.22	193.11	384.24	192.62	K	1,005.61	503.31	988.58	494.79	987.60	494.30	7
3	530.34	265.67	513.32	257.16	512.33	256.67	K	877.51	439.26	860.48	430.75	859.50	430.25	6
4	659.38	330.20	642.36	321.68	641.37	321.19	E	749.42	375.21	732.39	366.70	731.41	366.21	5
5	788.43	394.72	771.40	386.20	770.42	385.71	Q	620.37	310.69	603.35	302.18	602.36	301.69	4
6	901.51	451.26	884.48	442.75	883.50	442.25	L	491.33	246.17	474.30	237.66	473.32	237.16	3
7	988.54	494.78	971.52	486.26	970.53	485.77	S	378.25	189.63	361.22	181.11	360.24	180.62	2
8							K	291.21	146.11	274.19	137.60			1

Query 65777 Hit 1

MS/MS Fragmentation of MKQELEAEYLAVFK
Found in sp|Q9Y5X3|SNX5_HUMAN, Sorting nexin-5 OS=Homo sapiens GN=SNX5 PE=1 SV=1
Match to Query 65777: 2130.177from(711.0665,3+)
Title: 898: Scan 1947 (rt=53.551, f=2, i=309) [D:\lab212\membrane\Grace\Joyce\iTRAQ_45_1.raw]
Data File:Submitted from 20120508-1(merge) by Mascot Daemon on JOYCE-VAIO
Monoisotopic mass of neutral peptide Mr(calc): 2130.177
Variable modifications:
K2 :iTRAQ4plex (K)
K14 :iTRAQ4plex (K)
Ions Score: 41.77 Expect: 0.044

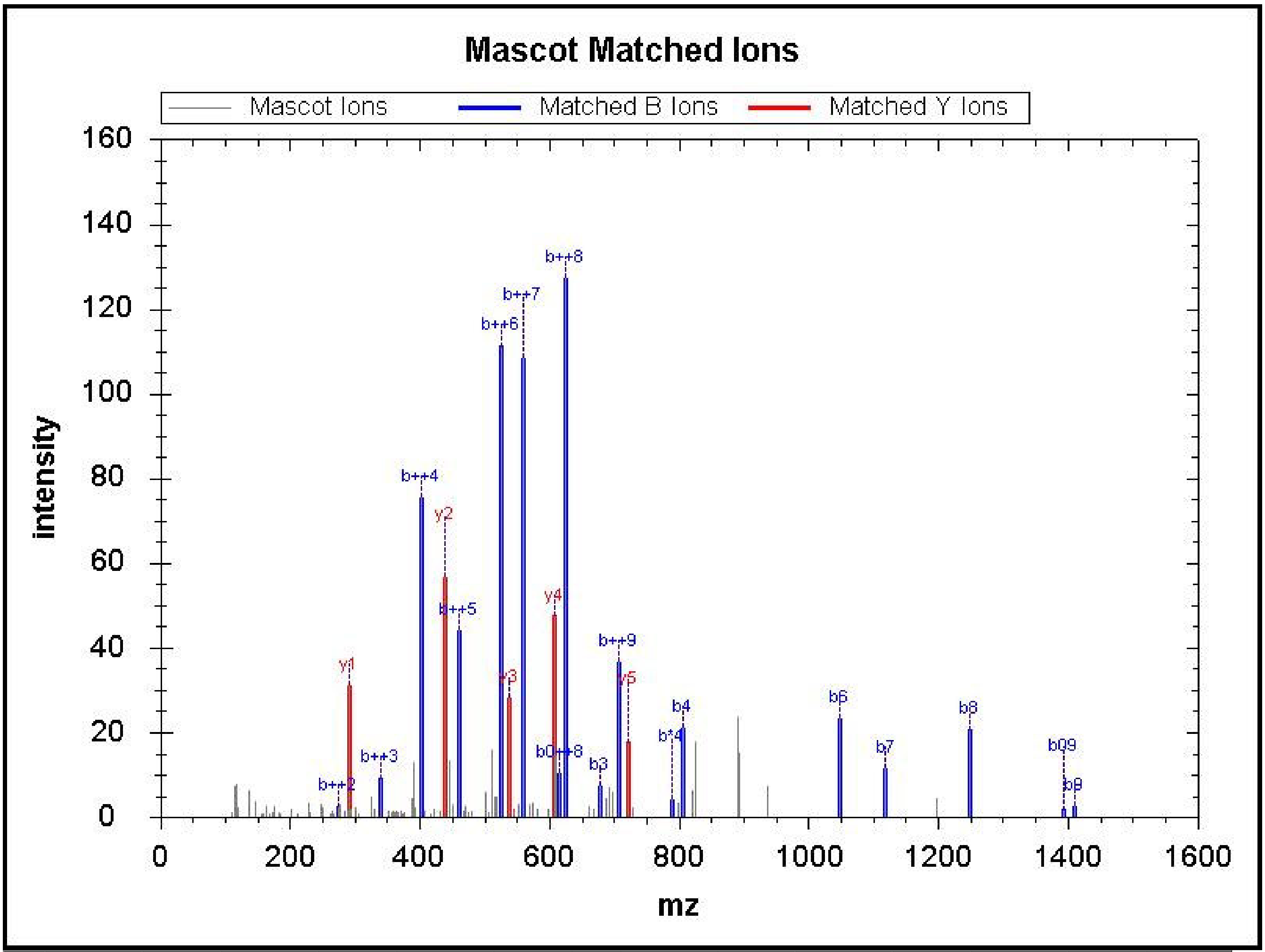

No	b	b++	b*	b*++	b0	b0++	Seq	y	y++	y*	y*++	y0	y0++	RevNo
1	276.15	138.58					M							14
2	548.35	274.68	531.32	266.16			K	1,856.04	928.52	1,839.01	920.01	1,838.03	919.52	13
3	676.41	338.71	659.38	330.19			Q	1,583.84	792.43	1,566.82	783.91	1,565.83	783.42	12
4	805.45	403.23	788.42	394.71	787.44	394.22	E	1,455.79	728.40	1,438.76	719.88	1,437.77	719.39	11
5	918.53	459.77	901.51	451.26	900.52	450.76	L	1,326.74	663.87	1,309.72	655.36	1,308.73	654.87	10
6	1,047.57	524.29	1,030.55	515.78	1,029.56	515.29	E	1,213.66	607.33	1,196.63	598.82	1,195.65	598.33	9
7	1,118.61	559.81	1,101.59	551.30	1,100.60	550.80	A	1,084.62	542.81	1,067.59	534.30	1,066.61	533.81	8
8	1,247.65	624.33	1,230.63	615.82	1,229.64	615.33	E	1,013.58	507.29	996.55	498.78	995.57	498.29	7
9	1,410.72	705.86	1,393.69	697.35	1,392.71	696.86	Y	884.54	442.77	867.51	434.26			6
10	1,523.80	762.40	1,506.78	753.89	1,505.79	753.40	L	721.47	361.24	704.45	352.73			5
11	1,594.84	797.92	1,577.81	789.41	1,576.83	788.92	A	608.39	304.70	591.36	296.18			4
12	1,693.91	847.46	1,676.88	838.94	1,675.90	838.45	V	537.35	269.18	520.33	260.67			3
13	1,840.98	920.99	1,823.95	912.48	1,822.97	911.99	F	438.28	219.65	421.26	211.13			2
14							K	291.21	146.11	274.19	137.60			1

Query 74470 Hit 1

MS/MS Fragmentation of YYELLIVNPIWLVPPTK
Found in sp|Q99LI2|CLCC1_MOUSE, Chloride channel CLIC-like protein 1 OS=Mus musculus GN=Clcc1 PE=1 SV=1
Match to Query 74470: 2345.355from(782.7922,3+)
Title: 1257: Sum of 2 scans in range 2828 (rt=72.7696, f=4, i=827) to 2829 (rt=72.795, f=4, i=828)
[D:\lab212\membrane\Grace\Joyce\iTRAQ_23_1.raw]
Data File:Submitted from 20120508-1(merge) by Mascot Daemon on JOYCE-VAIO
Monoisotopic mass of neutral peptide Mr(calc): 2345.355
Variable modifications:
K17 :iTRAQ4plex (K)
Ions Score: 41.76 Expect: 0.022

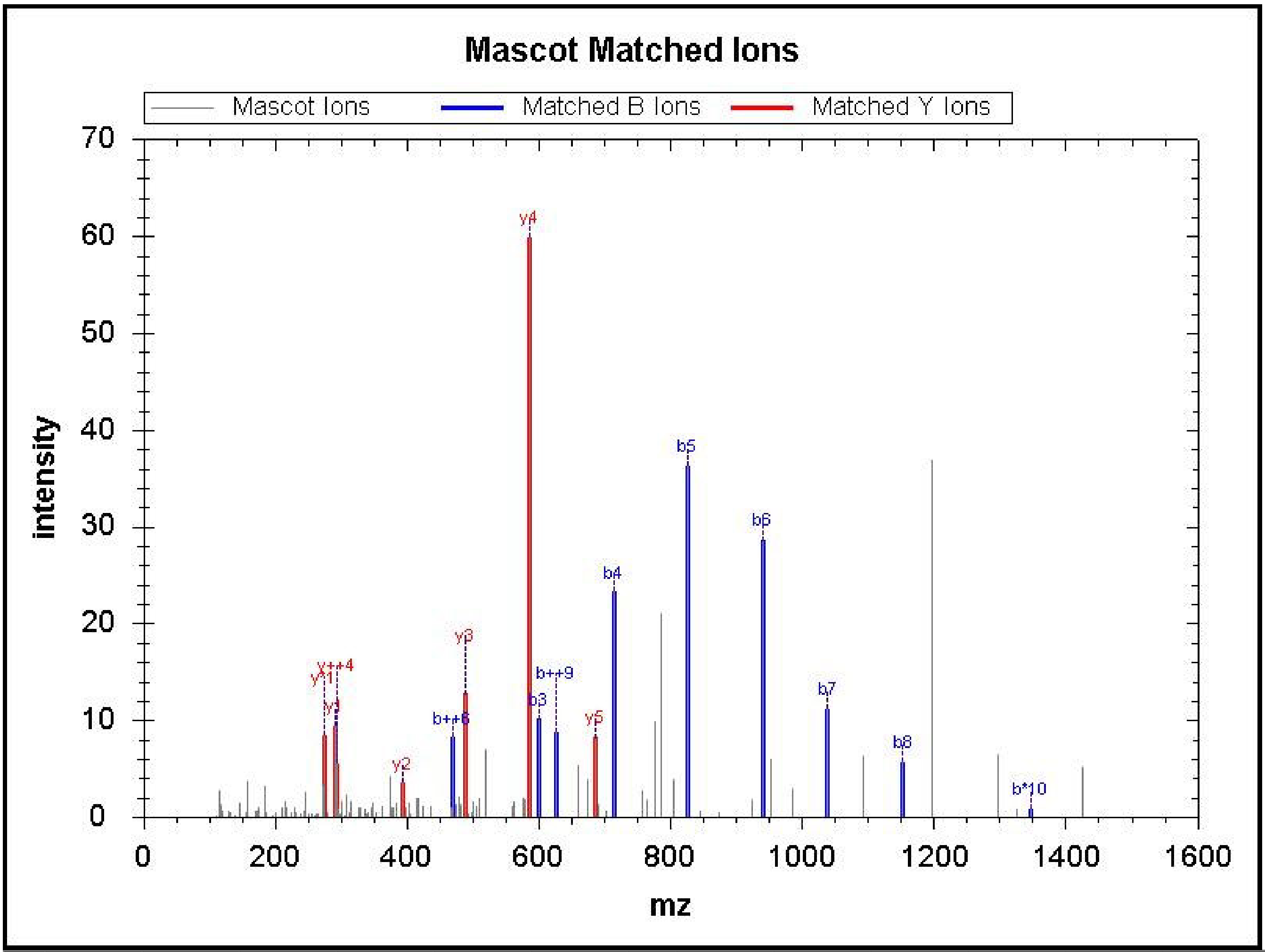

No	b	b++	b*	b*++	b0	b0++	Seq	y	y++	y*	y*++	y0	y0++	RevNo
1	308.17	154.59					Y							17
2	471.24	236.12					Y	2,039.21	1,020.11	2,022.18	1,011.59	2,021.20	1,011.10	16
3	600.28	300.64			582.27	291.64	E	1,876.14	938.58	1,859.12	930.06	1,858.13	929.57	15
4	713.36	357.18			695.35	348.18	L	1,747.10	874.05	1,730.07	865.54	1,729.09	865.05	14
5	826.45	413.73			808.44	404.72	L	1,634.02	817.51	1,616.99	809.00	1,616.01	808.51	13
6	939.53	470.27			921.52	461.26	I	1,520.93	760.97	1,503.91	752.46	1,502.92	751.96	12
7	1,038.60	519.80			1,020.59	510.80	V	1,407.85	704.43	1,390.82	695.91	1,389.84	695.42	11
8	1,152.64	576.82	1,135.62	568.31	1,134.63	567.82	N	1,308.78	654.89	1,291.75	646.38	1,290.77	645.89	10
9	1,249.69	625.35	1,232.67	616.84	1,231.68	616.35	P	1,194.74	597.87	1,177.71	589.36	1,176.73	588.87	9
10	1,362.78	681.89	1,345.75	673.38	1,344.77	672.89	I	1,097.68	549.35	1,080.66	540.83	1,079.67	540.34	8
11	1,548.86	774.93	1,531.83	766.42	1,530.85	765.93	W	984.60	492.80	967.57	484.29	966.59	483.80	7
12	1,661.94	831.47	1,644.92	822.96	1,643.93	822.47	L	798.52	399.76	781.49	391.25	780.51	390.76	6
13	1,761.01	881.01	1,743.98	872.50	1,743.00	872.00	V	685.44	343.22	668.41	334.71	667.43	334.22	5
14	1,858.06	929.54	1,841.04	921.02	1,840.05	920.53	P	586.37	293.69	569.34	285.17	568.36	284.68	4

15	1,955.12	978.06	1,938.09	969.55	1,937.11	969.06	P	489.32	245.16	472.29	236.65	471.30	236.16	3
16	2,056.16	1,028.59	2,039.14	1,020.07	2,038.15	1,019.58	T	392.26	196.63	375.24	188.12	374.25	187.63	2
17							K	291.21	146.11	274.19	137.60			1

Query 4367 Hit 1

MS/MS Fragmentation of EIAGLVR
Found in sp|Q7L5L3|GDPD3_HUMAN, Glycerophosphodiester phosphodiesterase domain-containing protein 3 OS=Homo sapiens GN=GDPD3 PE=2 SV=3
Match to Query 4367: 900.5509from(451.2827,2+)
Title: 277: Sum of 2 scans in range 701 (rt=25.1827, f=4, i=173) to 702 (rt=25.2081, f=4, i=174) [D:\lab212\membrane\Grace\Joyce\iTRAQ_26_2.raw]
Data File:Submitted from 20120508-1(merge) by Mascot Daemon on JOYCE-VAIO
Monoisotopic mass of neutral peptide Mr(calc): 900.5509
Variable modifications:
Ions Score: 41.71 Expect: 0.035

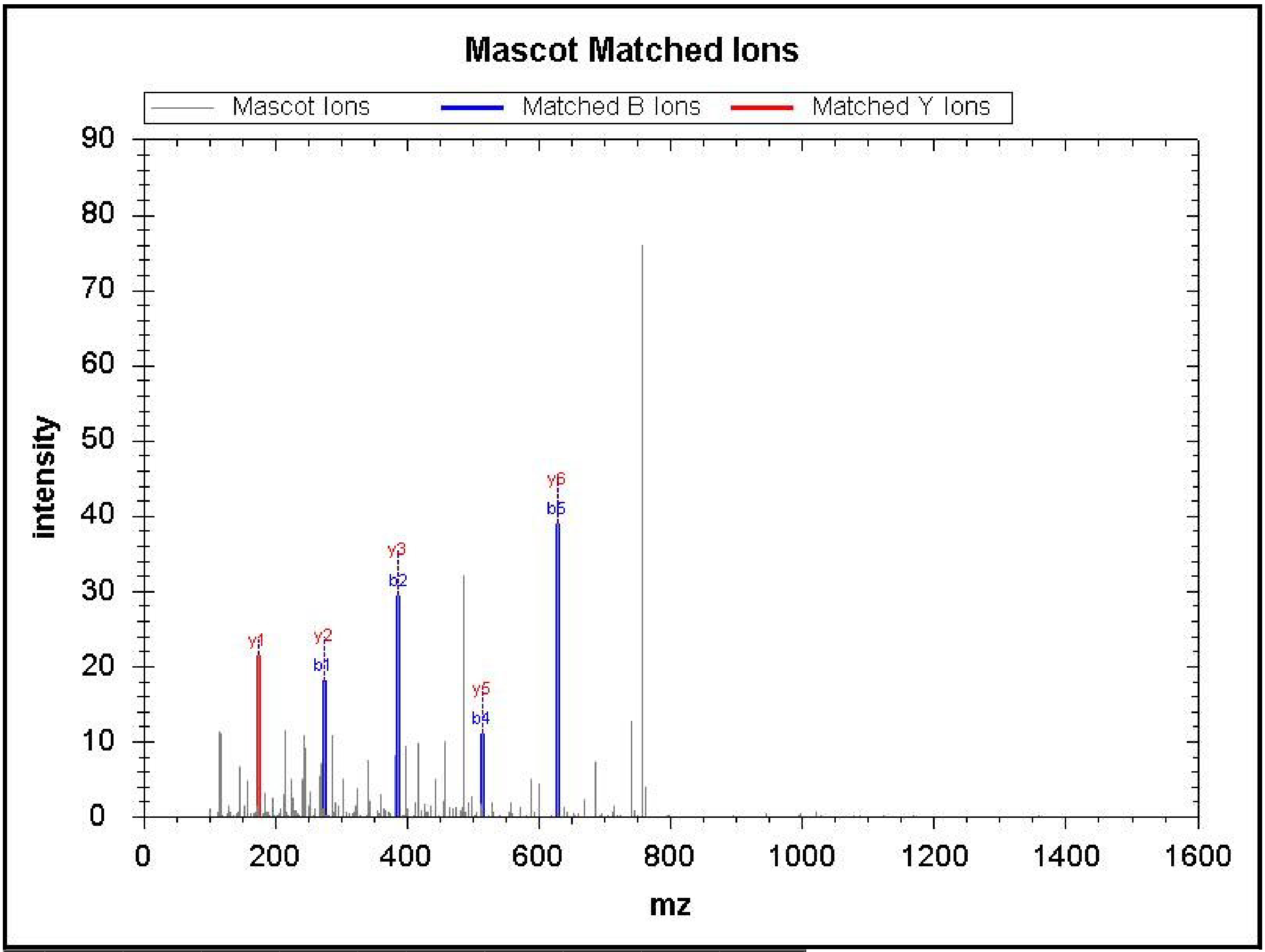

No	b	b++	b0	b0++	Seq	y	y++	y*	y*++	RevNo
1	274.15	137.58	256.14	128.57	E					7
2	387.24	194.12	369.23	185.12	I	628.41	314.71	611.39	306.20	6
3	458.27	229.64	440.26	220.63	A	515.33	258.17	498.30	249.66	5
4	515.29	258.15	497.28	249.15	G	444.29	222.65	427.27	214.14	4
5	628.38	314.69	610.37	305.69	L	387.27	194.14	370.24	185.63	3
6	727.45	364.23	709.44	355.22	V	274.19	137.60	257.16	129.08	2
7					R	175.12	88.06	158.09	79.55	1

Query 25221 Hit 1

MS/MS Fragmentation of KVIEYFK
Found in sp|Q9BTE3|MCMBP_HUMAN, Mini-chromosome maintenance complex-binding protein OS=Homo sapiens GN=MCMBP

PE=1 SV=2
Match to Query 25221: 1357.838from(453.6199,3+)
Title: 286: Scan 1035 (rt=31.5827, f=3, i=97) [D:\lab212\membrane\Grace\Joyce\iTRAQ_48_1.raw]
Data File:Submitted from 20120508-1(merge) by Mascot Daemon on JOYCE-VAIO
Monoisotopic mass of neutral peptide Mr(calc): 1357.838
Variable modifications:
K1 :iTRAQ4plex (K)
K7 :iTRAQ4plex (K)
Ions Score: 41.62 Expect: 0.029

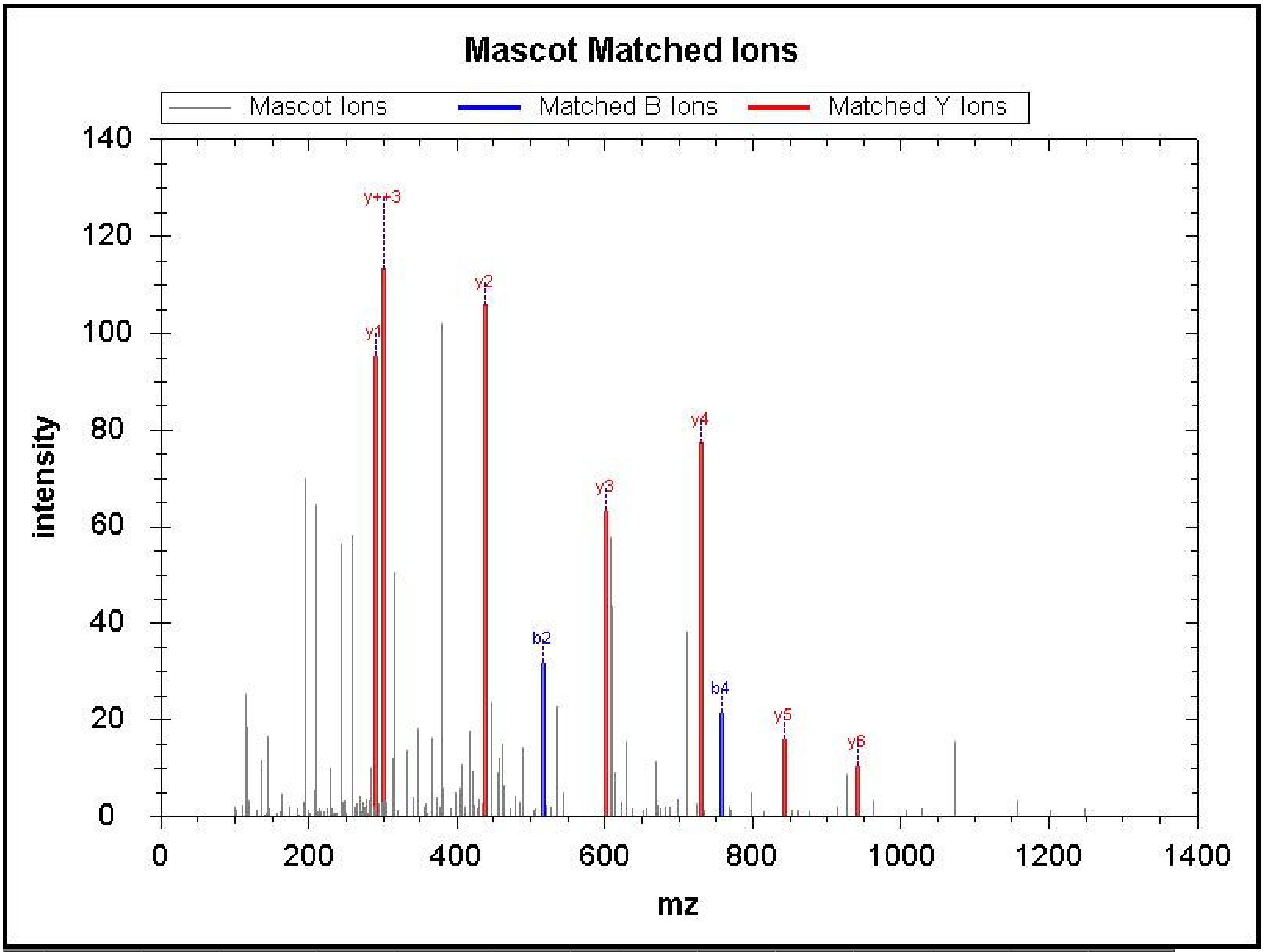

No	b	b++	b*	b*++	b0	b0++	Seq	y	y++	y*	y*++	y0	y0++	RevNo
1	417.31	209.16	400.28	200.64			K							7
2	516.37	258.69	499.35	250.18			V	942.54	471.77	925.52	463.26	924.53	462.77	6
3	629.46	315.23	612.43	306.72			I	843.47	422.24	826.45	413.73	825.46	413.23	5
4	758.50	379.75	741.47	371.24	740.49	370.75	E	730.39	365.70	713.36	357.18	712.38	356.69	4
5	921.56	461.29	904.54	452.77	903.55	452.28	Y	601.35	301.18	584.32	292.66			3
6	1,068.63	534.82	1,051.61	526.31	1,050.62	525.82	F	438.28	219.65	421.26	211.13			2
7							K	291.21	146.11	274.19	137.60			1

Query 55696 Hit 1

MS/MS Fragmentation of ILKEDILNYLEK
Found in sp|P11182|ODB2_HUMAN, Lipoamide acyltransferase component of branched-chain alpha-keto acid dehydrogenase complex
Match to Query 55696: 1922.149from(641.7236,3+)
Title: 896: Scan 1942 (rt=53.4407, f=3, i=307) [D:\lab212\membrane\Grace\Joyce\iTRAQ_45_1.raw]
Data File:Submitted from 20120508-1(merge) by Mascot Daemon on JOYCE-VAIO
Monoisotopic mass of neutral peptide Mr(calc): 1922.149
Variable modifications:
K3 :iTRAQ4plex (K)
K12 :iTRAQ4plex (K)

Ions Score: 41.62 Expect: 0.020

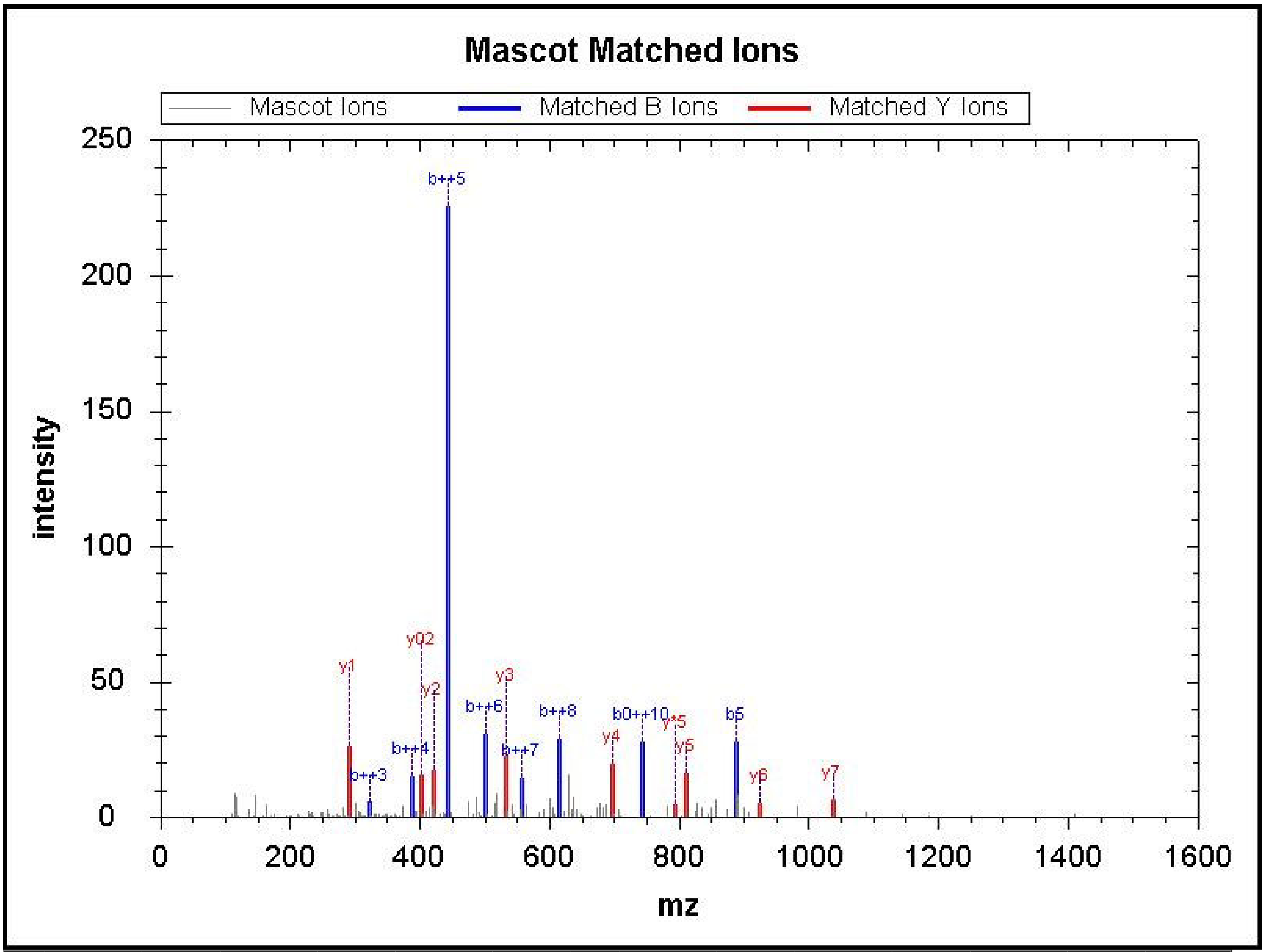

No	b	b++	b*	b*++	b0	b0++	Seq	y	y++	y*	y*++	y0	y0++	RevNo
1	258.19	129.60					I							12
2	371.28	186.14					L	1,665.97	833.49	1,648.94	824.97	1,647.96	824.48	11
3	643.47	322.24	626.45	313.73			K	1,552.88	776.94	1,535.86	768.43	1,534.87	767.94	10
4	772.52	386.76	755.49	378.25	754.51	377.76	E	1,280.69	640.85	1,263.66	632.33	1,262.67	631.84	9
5	887.54	444.28	870.52	435.76	869.53	435.27	D	1,151.64	576.33	1,134.62	567.81	1,133.63	567.32	8
6	1,000.63	500.82	983.60	492.30	982.62	491.81	I	1,036.62	518.81	1,019.59	510.30	1,018.61	509.81	7
7	1,113.71	557.36	1,096.69	548.85	1,095.70	548.35	L	923.53	462.27	906.51	453.76	905.52	453.26	6
8	1,227.76	614.38	1,210.73	605.87	1,209.74	605.38	N	810.45	405.73	793.42	397.21	792.44	396.72	5
9	1,390.82	695.91	1,373.79	687.40	1,372.81	686.91	Y	696.40	348.71	679.38	340.19	678.39	339.70	4
10	1,503.90	752.45	1,486.88	743.94	1,485.89	743.45	L	533.34	267.17	516.31	258.66	515.33	258.17	3
11	1,632.95	816.98	1,615.92	808.46	1,614.93	807.97	E	420.26	210.63	403.23	202.12	402.25	201.63	2
12							K	291.21	146.11	274.19	137.60			1

Query 8489 Hit 1

MS/MS Fragmentation of MEAQGGK
Found in sp|Q8NBP7|PCSK9_HUMAN, Proprotein convertase subtilisin/kexin type 9 OS=Homo sapiens GN=PCSK9 PE=1 SV=3
Match to Query 8489: 1023.591 from(512.8029,2+)
Title: 311: Scan 780 (rt=26.9227, f=3, i=106) [D:\lab212\membrane\Grace\Joyce\iTRAQ_31_2.raw]
Data File:Submitted from 20120508-1(merge) by Mascot Daemon on JOYCE-VAIO
Monoisotopic mass of neutral peptide Mr(calc): 1023.591
Variable modifications:
M1 :Oxidation (M), 63.99828
K7 :iTRAQ4plex (K)
Ions Score: 41.56 Expect: 0.040

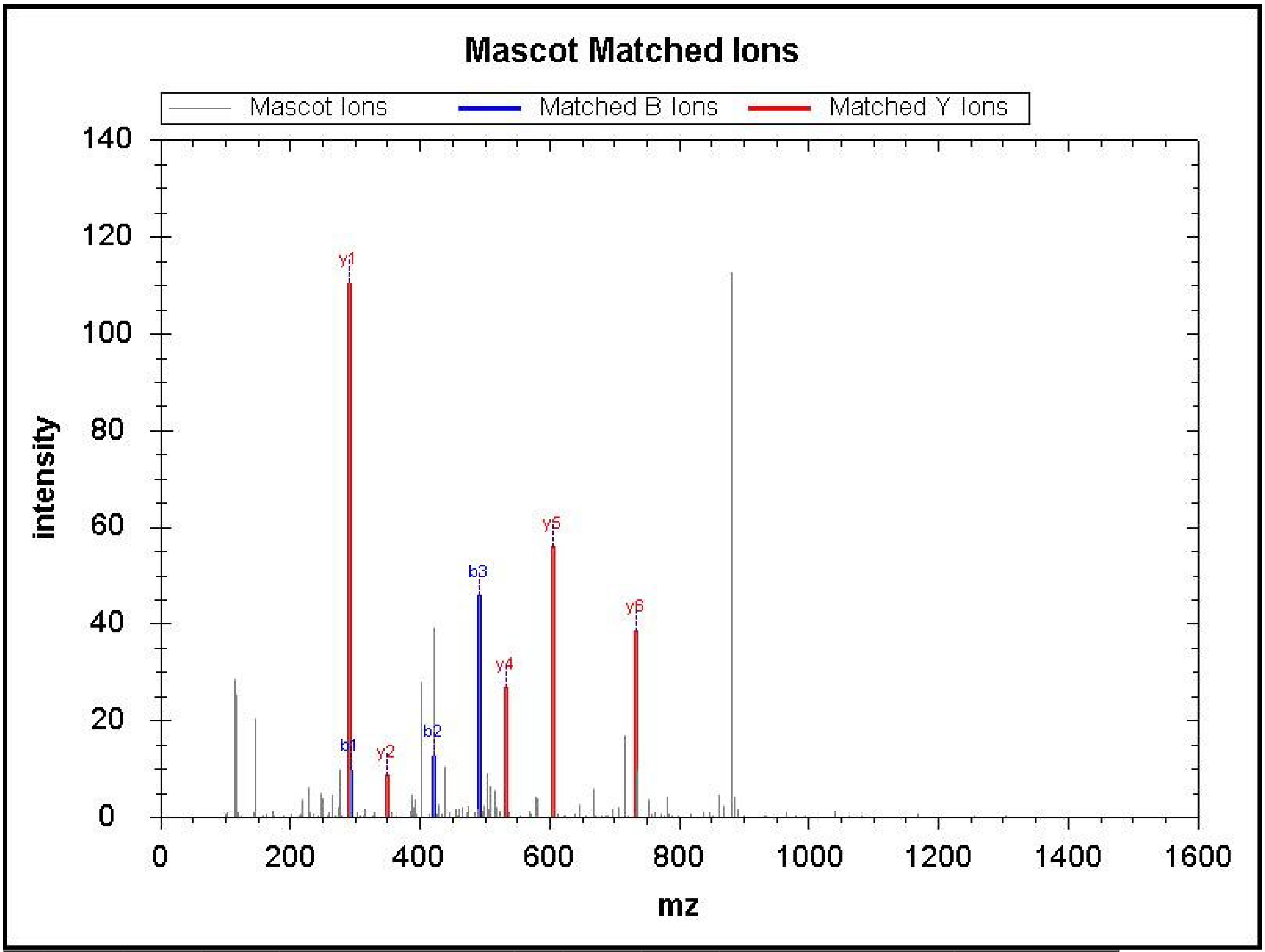

No	b	b++	b*	b*++	b0	b0++	Seq	y	y++	y*	y*++	y0	y0++	RevNo
1	292.14	146.58					M							7
2	421.19	211.10			403.18	202.09	E	733.40	367.20	716.37	358.69	715.39	358.20	6
3	492.22	246.62			474.21	237.61	A	604.35	302.68	587.33	294.17			5
4	620.28	310.65	603.26	302.13	602.27	301.64	Q	533.32	267.16	516.29	258.65			4
5	677.30	339.16	660.28	330.64	659.29	330.15	G	405.26	203.13	388.23	194.62			3
6	734.33	367.67	717.30	359.15	716.32	358.66	G	348.24	174.62	331.21	166.11			2
7							K	291.21	146.11	274.19	137.60			1

Query 10450 Hit 1

MS/MS Fragmentation of RIQDLER
Found in sp|Q6ZQ06|QN1_MOUSE, Protein QN1 homolog OS=Mus musculus GN=Kiaa1009 PE=1 SV=2
Match to Query 10450: 1073.6from(358.8739,3+)
Title: 86: Scan 480 (rt=19.5275, f=3, i=31) [D:\lab212\membrane\Grace\Joyce\iTRAQ_46_2.raw]
Data File:Submitted from 20120508-1(merge) by Mascot Daemon on JOYCE-VAIO
Monoisotopic mass of neutral peptide Mr(calc): 1073.6
Variable modifications:
Q3 :Deamidated (NQ)
Ions Score: 41.53 Expect: 0.053

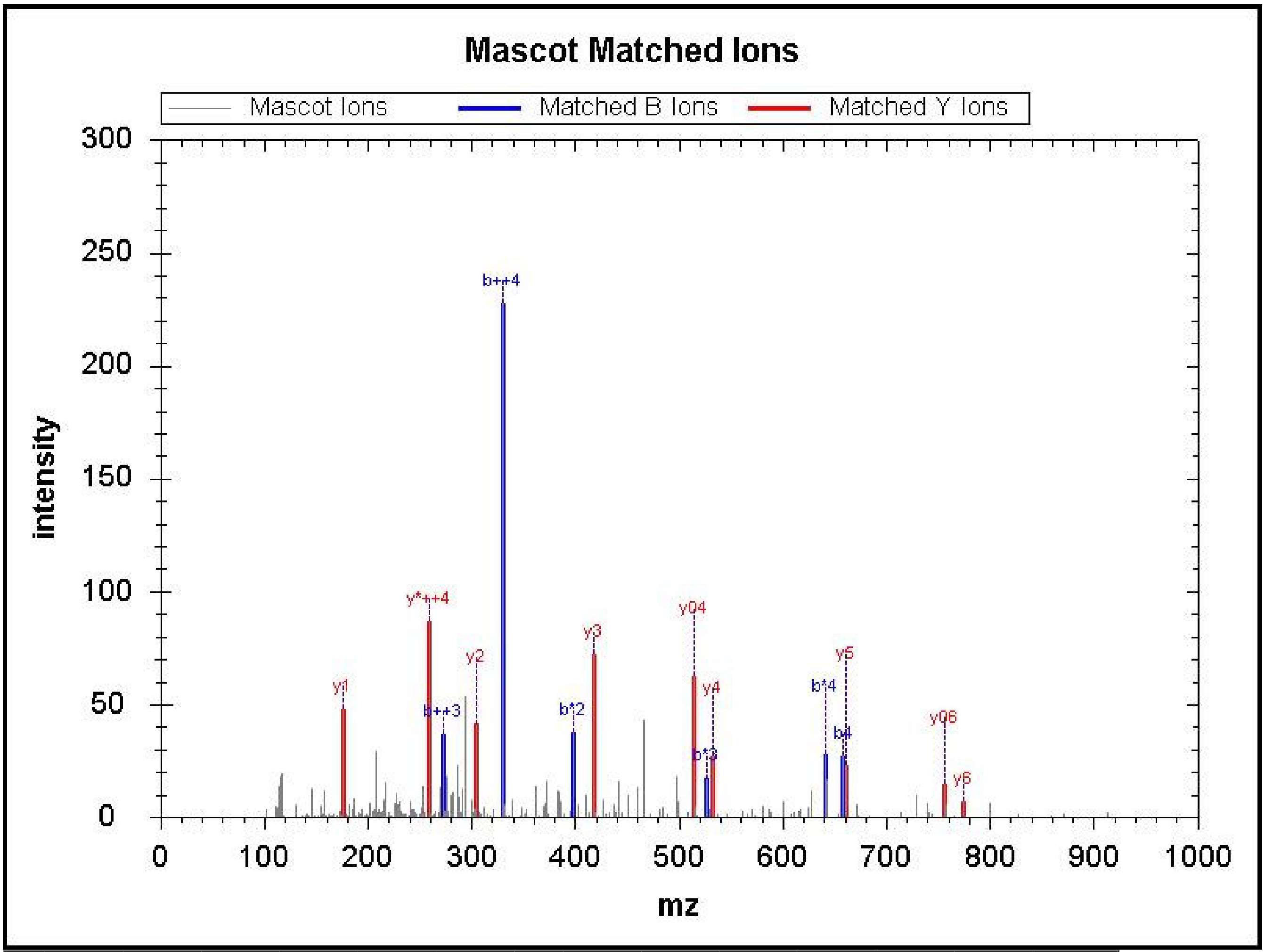

No	b	b++	b*	b*++	b0	b0++	Seq	y	y++	y*	y*++	y0	y0++	RevNo
1	301.21	151.11	284.18	142.60			R							7
2	414.29	207.65	397.27	199.14			I	774.40	387.70	757.37	379.19	756.39	378.70	6
3	543.34	272.17	526.31	263.66			Q	661.32	331.16	644.29	322.65	643.30	322.16	5
4	658.36	329.69	641.34	321.17	640.35	320.68	D	532.27	266.64	515.25	258.13	514.26	257.63	4
5	771.45	386.23	754.42	377.71	753.44	377.22	L	417.25	209.13	400.22	200.61	399.24	200.12	3
6	900.49	450.75	883.46	442.24	882.48	441.74	E	304.16	152.58	287.13	144.07	286.15	143.58	2
7							R	175.12	88.06	158.09	79.55			1

Query 60772 Hit 1

MS/MS Fragmentation of GHPDVATMLNILALVYR
Found in sp|Q9NSK0|KLC4_HUMAN, Kinesin light chain 4 OS=Homo sapiens GN=KLC4 PE=1 SV=3
Match to Query 60772: 2026.119from(676.3803,3+)
Title: 1176: Sum of 2 scans in range 2649 (rt=68.7908, f=2, i=433) to 2650 (rt=68.8162, f=2, i=434) [D:\lab212\membrane\Grace\Joyce\iTRAQ_35_1.raw]
Data File:Submitted from 20120508-1(merge) by Mascot Daemon on JOYCE-VAIO
Monoisotopic mass of neutral peptide Mr(calc): 2026.119
Variable modifications:
Ions Score: 41.51 Expect: 0.050

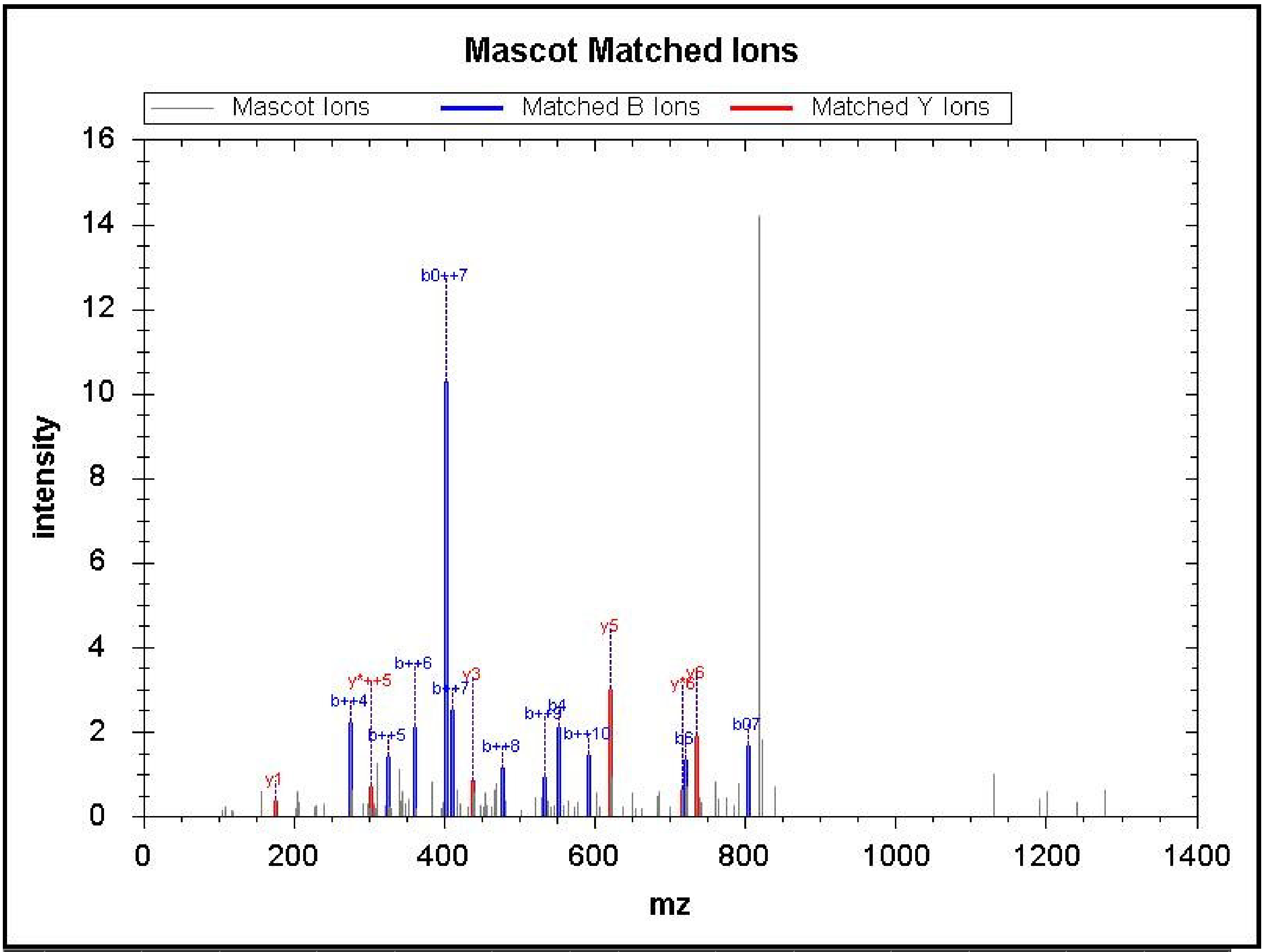

No	b	b++	b*	b*++	b0	b0++	Seq	y	y++	y*	y*++	y0	y0++	RevNo
1	202.13	101.57					G							17
2	339.19	170.10					H	1,826.00	913.50	1,808.97	904.99	1,807.99	904.50	16
3	436.24	218.62					P	1,688.94	844.97	1,671.91	836.46	1,670.93	835.97	15
4	551.27	276.14			533.26	267.13	D	1,591.89	796.45	1,574.86	787.93	1,573.88	787.44	14
5	650.34	325.67			632.33	316.67	V	1,476.86	738.93	1,459.83	730.42	1,458.85	729.93	13
6	721.37	361.19			703.36	352.19	A	1,377.79	689.40	1,360.77	680.89	1,359.78	680.39	12
7	822.42	411.71			804.41	402.71	T	1,306.76	653.88	1,289.73	645.37	1,288.74	644.88	11
8	953.46	477.24			935.45	468.23	M	1,205.71	603.36	1,188.68	594.84			10
9	1,066.55	533.78			1,048.54	524.77	L	1,074.67	537.84	1,057.64	529.32			9
10	1,180.59	590.80	1,163.56	582.29	1,162.58	581.79	N	961.58	481.30	944.56	472.78			8
11	1,293.67	647.34	1,276.65	638.83	1,275.66	638.34	I	847.54	424.27	830.51	415.76			7
12	1,406.76	703.88	1,389.73	695.37	1,388.75	694.88	L	734.46	367.73	717.43	359.22			6
13	1,477.80	739.40	1,460.77	730.89	1,459.78	730.40	A	621.37	311.19	604.35	302.68			5
14	1,590.88	795.94	1,573.85	787.43	1,572.87	786.94	L	550.33	275.67	533.31	267.16			4
15	1,689.95	845.48	1,672.92	836.96	1,671.94	836.47	V	437.25	219.13	420.22	210.62			3
16	1,853.01	927.01	1,835.98	918.50	1,835.00	918.00	Y	338.18	169.59	321.16	161.08			2
17							R	175.12	88.06	158.09	79.55			1

Query 35185 Hit 1

MS/MS Fragmentation of KEQEQLQARLR
Found in sp|Q8TDN1|KCNG4_HUMAN, Potassium voltage-gated channel subfamily G member 4 OS=Homo sapiens GN=KCNG4 PE=2 SV=1
Match to Query 35185: 1541.906from(514.9758,3+)
Title: 367: Scan 873 (rt=29.2602, f=3, i=128) [D:\lab212\membrane\Grace\Joyce\iTRAQ_44_2.raw]

Data File:Submitted from 20120508-1(merge) by Mascot Daemon on JOYCE-VAIO
Monoisotopic mass of neutral peptide Mr(calc): 1541.906
Variable modifications:
K1 :iTRAQ4plex (K)
Ions Score: 41.48 Expect: 0.037

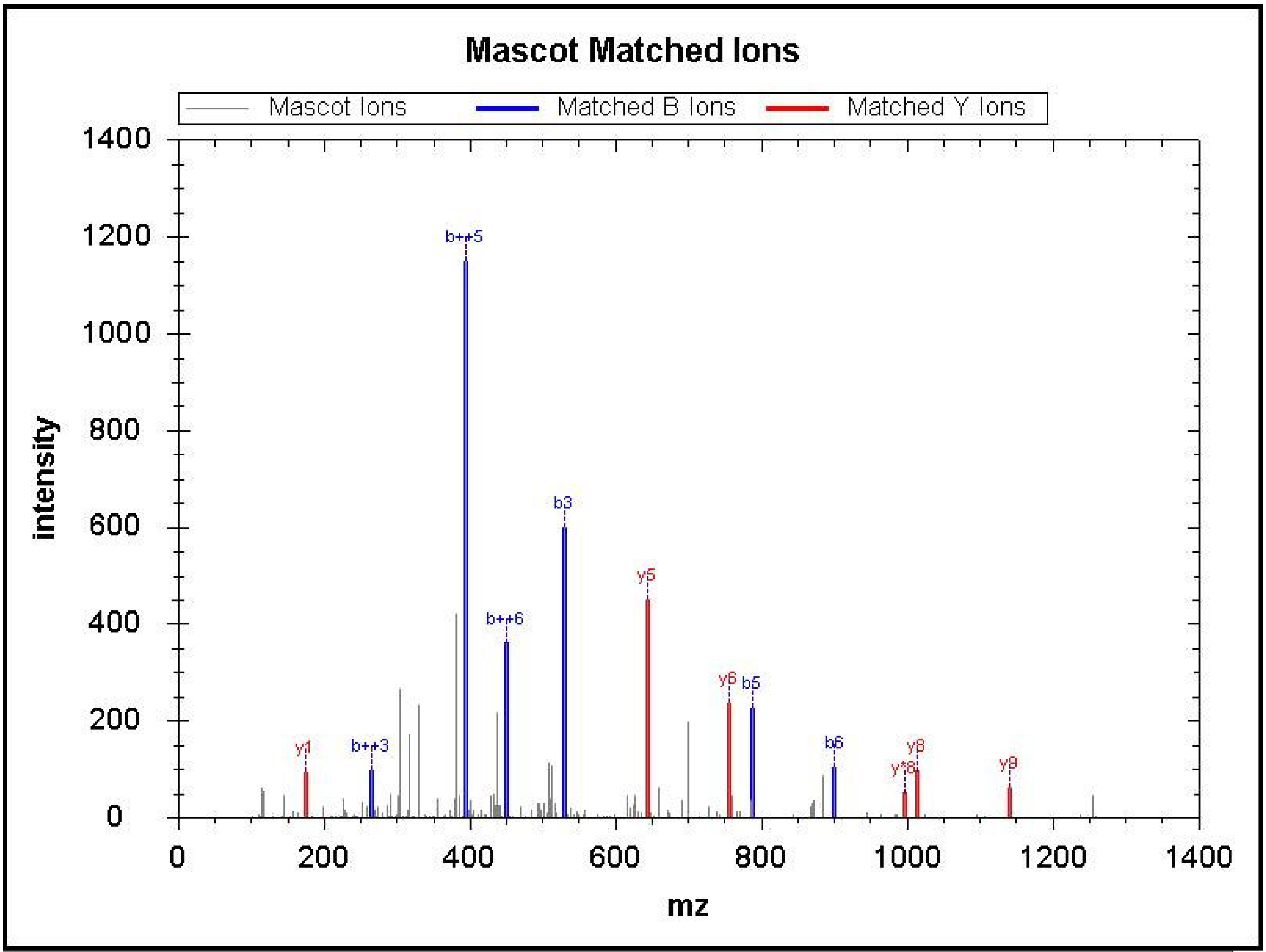

No	b	b++	b*	b*++	b0	b0++	Seq	y	y++	y*	y*++	y0	y0++	RevNo
1	273.20	137.11	256.18	128.59			K							11
2	402.25	201.63	385.22	193.11	384.24	192.62	E	1,270.69	635.85	1,253.66	627.33	1,252.68	626.84	10
3	530.31	265.66	513.28	257.14	512.29	256.65	Q	1,141.64	571.33	1,124.62	562.81	1,123.63	562.32	9
4	659.35	330.18	642.32	321.66	641.34	321.17	E	1,013.59	507.30	996.56	498.78	995.57	498.29	8
5	787.41	394.21	770.38	385.69	769.40	385.20	Q	884.54	442.77	867.52	434.26			7
6	900.49	450.75	883.46	442.24	882.48	441.74	L	756.48	378.75	739.46	370.23			6
7	1,028.55	514.78	1,011.52	506.27	1,010.54	505.77	Q	643.40	322.20	626.37	313.69			5
8	1,099.59	550.30	1,082.56	541.78	1,081.58	541.29	A	515.34	258.17	498.31	249.66			4
9	1,255.69	628.35	1,238.66	619.83	1,237.68	619.34	R	444.30	222.66	427.28	214.14			3
10	1,368.77	684.89	1,351.75	676.38	1,350.76	675.88	L	288.20	144.61	271.18	136.09			2
11							R	175.12	88.06	158.09	79.55			1

Query 12025 Hit 1

MS/MS Fragmentation of QCAQQILR
Found in sp|Q8VEG0|CCD71_MOUSE, Coiled-coil domain-containing protein 71 OS=Mus musculus GN=Ccdc71 PE=2 SV=1
Match to Query 12025: 1105.558from(553.7864,2+)
Title: 233: Scan 620 (rt=23.3111, f=3, i=81) [D:\lab212\membrane\Grace\Joyce\iTRAQ_25_2.raw]
Data File:Submitted from 20120508-1(merge) by Mascot Daemon on JOYCE-VAIO
Monoisotopic mass of neutral peptide Mr(calc): 1105.558
Variable modifications:
Q1 :Deamidated (NQ)

Q4 :Deamidated (NQ)
Q5 :Deamidated (NQ)
Ions Score: 41.41 Expect: 0.057

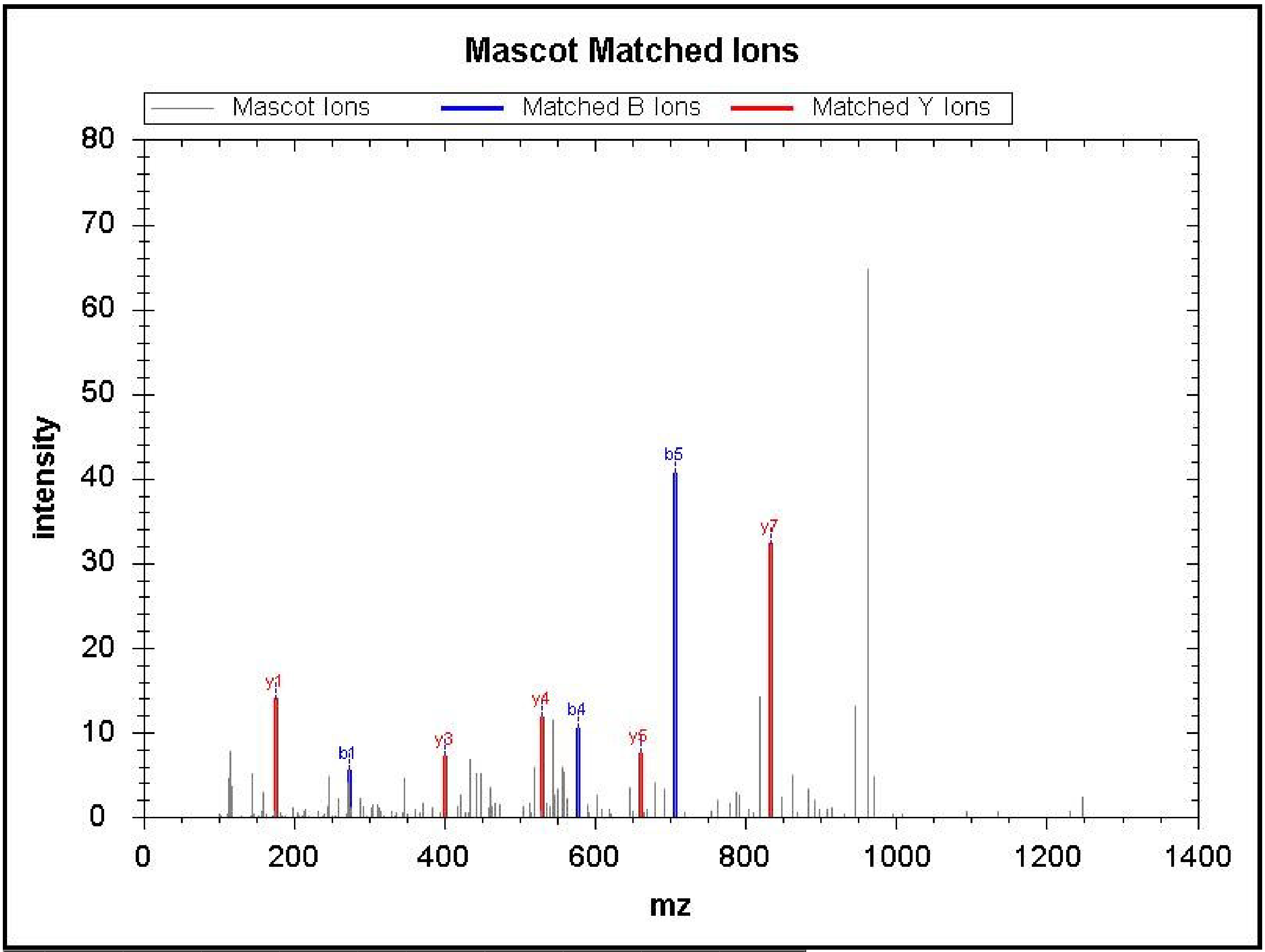

No	b	b++	b*	b*++	Seq	y	y++	y*	y*++	RevNo
1	274.15	137.58	257.13	129.07	Q					8
2	377.16	189.08	360.13	180.57	C	833.42	417.21	816.39	408.70	7
3	448.20	224.60	431.17	216.09	A	730.41	365.71	713.38	357.20	6
4	577.24	289.12	560.21	280.61	Q	659.37	330.19	642.35	321.68	5
5	706.28	353.65	689.26	345.13	Q	530.33	265.67	513.30	257.16	4
6	819.37	410.19	802.34	401.67	I	401.29	201.15	384.26	192.63	3
7	932.45	466.73	915.42	458.22	L	288.20	144.61	271.18	136.09	2
8					R	175.12	88.06	158.09	79.55	1

Query 21964 Hit 1

MS/MS Fragmentation of AEAEAAAVHGAR
Found in sp|Q5T8P6|RBM26_HUMAN, RNA-binding protein 26 OS=Homo sapiens GN=RBM26 PE=1 SV=3
Match to Query 21964: 1295.591from(432.871,3+)
Title: 75: Scan 213 (rt=14.5431, f=3, i=27) [D:\lab212\membrane\Grace\Joyce\iTRAQ40.raw]
Data File:Submitted from 20120508-1(merge) by Mascot Daemon on JOYCE-VAIO
Monoisotopic mass of neutral peptide Mr(calc): 1295.591
Variable modifications:
Ions Score: 41.38 Expect: 0.036

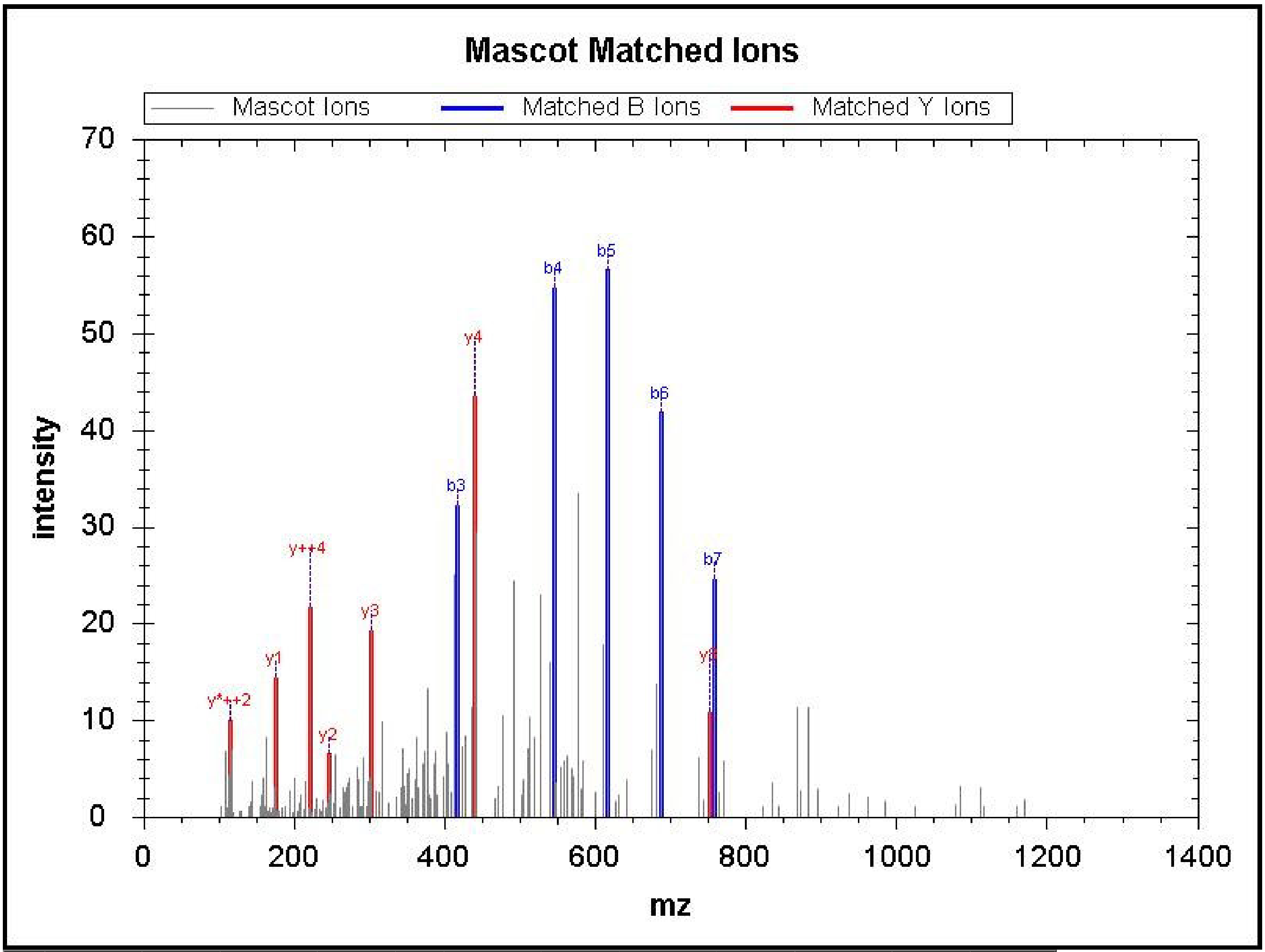

No	b	b++	b0	b0++	Seq	y	y++	y*	y*++	y0	y0++	RevNo
1	216.15	108.58			A							12
2	345.19	173.10	327.18	164.09	E	1,081.54	541.27	1,064.51	532.76	1,063.53	532.27	11
3	416.23	208.62	398.22	199.61	A	952.50	476.75	935.47	468.24	934.49	467.75	10
4	545.27	273.14	527.26	264.13	E	881.46	441.23	864.43	432.72	863.45	432.23	9
5	616.31	308.66	598.30	299.65	A	752.42	376.71	735.39	368.20			8
6	687.34	344.18	669.33	335.17	A	681.38	341.19	664.35	332.68			7
7	758.38	379.69	740.37	370.69	A	610.34	305.67	593.32	297.16			6
8	857.45	429.23	839.44	420.22	V	539.30	270.16	522.28	261.64			5
9	994.51	497.76	976.50	488.75	H	440.24	220.62	423.21	212.11			4
10	1,051.53	526.27	1,033.52	517.26	G	303.18	152.09	286.15	143.58			3
11	1,122.57	561.79	1,104.56	552.78	A	246.16	123.58	229.13	115.07			2
12					R	175.12	88.06	158.09	79.55			1

Query 11927 Hit 1

MS/MS Fragmentation of IAWALNK
Found in sp|Q9UPN6|SCAF8_HUMAN, Protein SCAF8 OS=Homo sapiens GN=SCAF8 PE=1 SV=1
Match to Query 11927: 1102.676from(552.3451,2+)
Title: 550: Scan 1280 (rt=38.209, f=2, i=196) [D:\lab212\membrane\Grace\Joyce\iTRAQ_33_1.raw]
Data File:Submitted from 20120508-1(merge) by Mascot Daemon on JOYCE-VAIO
Monoisotopic mass of neutral peptide Mr(calc): 1102.676
Variable modifications:
K7 :iTRAQ4plex (K)
Ions Score: 41.23 Expect: 0.048

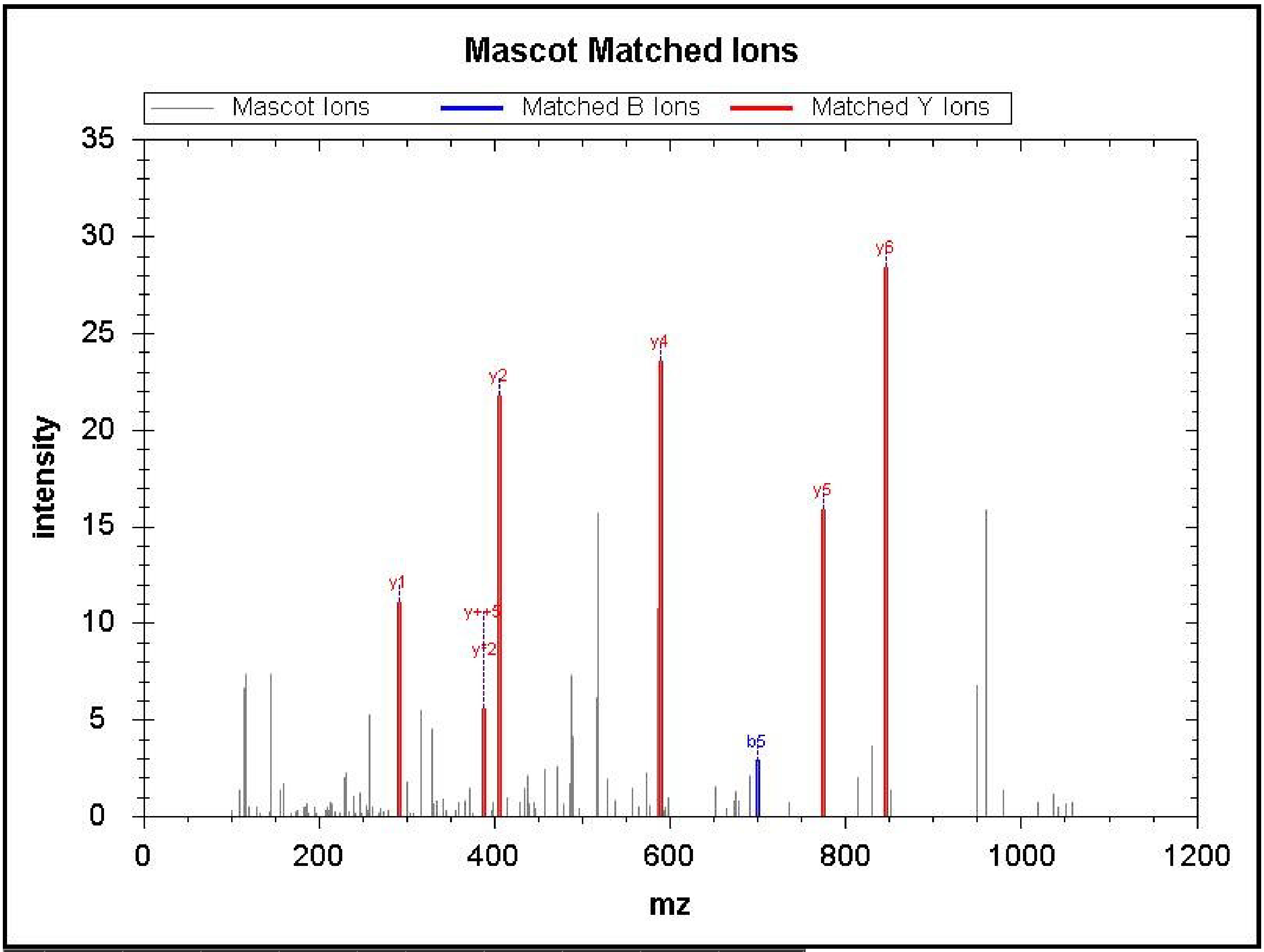

No	b	b++	b*	b*++	Seq	y	y++	y*	y*++	RevNo
1	258.19	129.60			I					7
2	329.23	165.12			A	846.50	423.75	829.47	415.24	6
3	515.31	258.16			W	775.46	388.23	758.43	379.72	5
4	586.35	293.68			A	589.38	295.19	572.35	286.68	4
5	699.43	350.22			L	518.34	259.67	501.32	251.16	3
6	813.47	407.24	796.45	398.73	N	405.26	203.13	388.23	194.62	2
7					K	291.21	146.11	274.19	137.60	1

Query 65983 Hit 1

MS/MS Fragmentation of EISNLNQLIEEFKK
Found in sp|Q14789|GOGB1_HUMAN, Golgin subfamily B member 1 OS=Homo sapiens GN=GOLGB1 PE=1 SV=2
Match to Query 65983: 2136.221 from(713.0811,3+)
Title: 929: Scan 2039 (rt=55.5326, f=3, i=316) [D:\lab212\membrane\Grace\Joyce\iTRAQ_44_2.raw]
Data File:Submitted from 20120508-1(merge) by Mascot Daemon on JOYCE-VAIO
Monoisotopic mass of neutral peptide Mr(calc): 2136.221
Variable modifications:
K13 :iTRAQ4plex (K)
K14 :iTRAQ4plex (K)
Ions Score: 41.13 Expect: 0.037

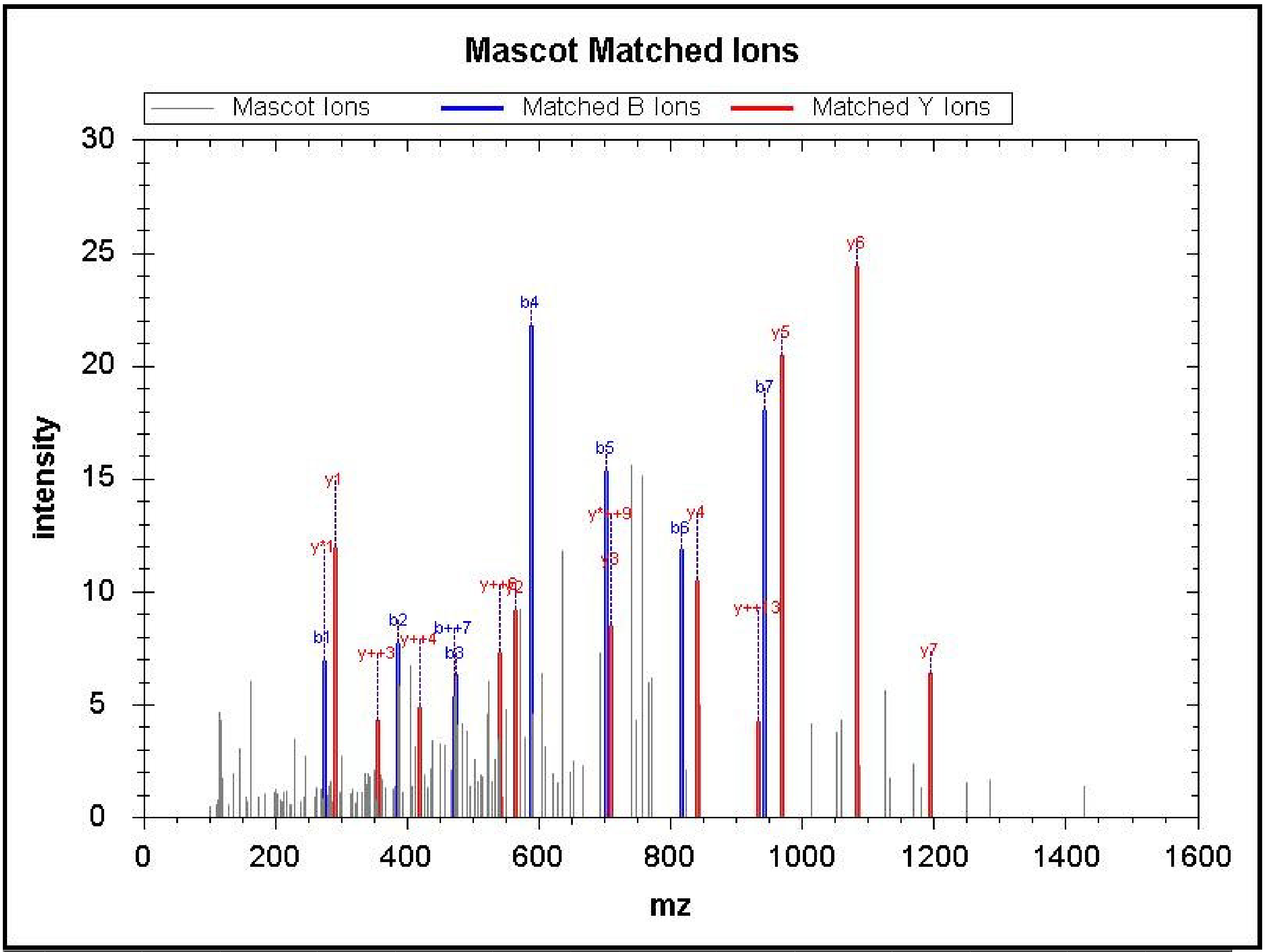

No	b	b++	b*	b*++	b0	b0++	Seq	y	y++	y*	y*++	y0	y0++	RevNo
1	274.15	137.58			256.14	128.57	E							14
2	387.24	194.12			369.23	185.12	I	1,864.08	932.54	1,847.05	924.03	1,846.07	923.54	13
3	474.27	237.64			456.26	228.63	S	1,750.99	876.00	1,733.97	867.49	1,732.98	867.00	12
4	588.31	294.66	571.28	286.15	570.30	285.65	N	1,663.96	832.48	1,646.94	823.97	1,645.95	823.48	11
5	701.39	351.20	684.37	342.69	683.38	342.20	L	1,549.92	775.46	1,532.89	766.95	1,531.91	766.46	10
6	815.44	408.22	798.41	399.71	797.43	399.22	N	1,436.84	718.92	1,419.81	710.41	1,418.82	709.92	9
7	943.50	472.25	926.47	463.74	925.49	463.25	Q	1,322.79	661.90	1,305.77	653.39	1,304.78	652.89	8
8	1,056.58	528.79	1,039.55	520.28	1,038.57	519.79	L	1,194.73	597.87	1,177.71	589.36	1,176.72	588.87	7
9	1,169.66	585.34	1,152.64	576.82	1,151.65	576.33	I	1,081.65	541.33	1,064.62	532.82	1,063.64	532.32	6
10	1,298.71	649.86	1,281.68	641.34	1,280.70	640.85	E	968.57	484.79	951.54	476.27	950.55	475.78	5
11	1,427.75	714.38	1,410.72	705.87	1,409.74	705.37	E	839.52	420.27	822.50	411.75	821.51	411.26	4
12	1,574.82	787.91	1,557.79	779.40	1,556.81	778.91	F	710.48	355.74	693.45	347.23			3
13	1,847.02	924.01	1,829.99	915.50	1,829.00	915.01	K	563.41	282.21	546.39	273.70			2
14							K	291.21	146.11	274.19	137.60			1

Query 7529 Hit 1

MS/MS Fragmentation of HLEESIK
Found in sp|Q8K389|CK5P2_MOUSE, CDK5 regulatory subunit-associated protein 2 OS=Mus musculus GN=Cdk5rap2 PE=1 SV=3
Match to Query 7529: 998.5626from(500.2886,2+)
Title: 94: Scan 264 (rt=15.6106, f=3, i=30) [D:\lab212\membrane\Grace\Joyce\iTRAQ_33_2.raw]
Data File:Submitted from 20120508-1(merge) by Mascot Daemon on JOYCE-VAIO
Monoisotopic mass of neutral peptide Mr(calc): 998.5626
Variable modifications:
K7 :iTRAQ4plex (K)
Ions Score: 41.1 Expect: 0.037

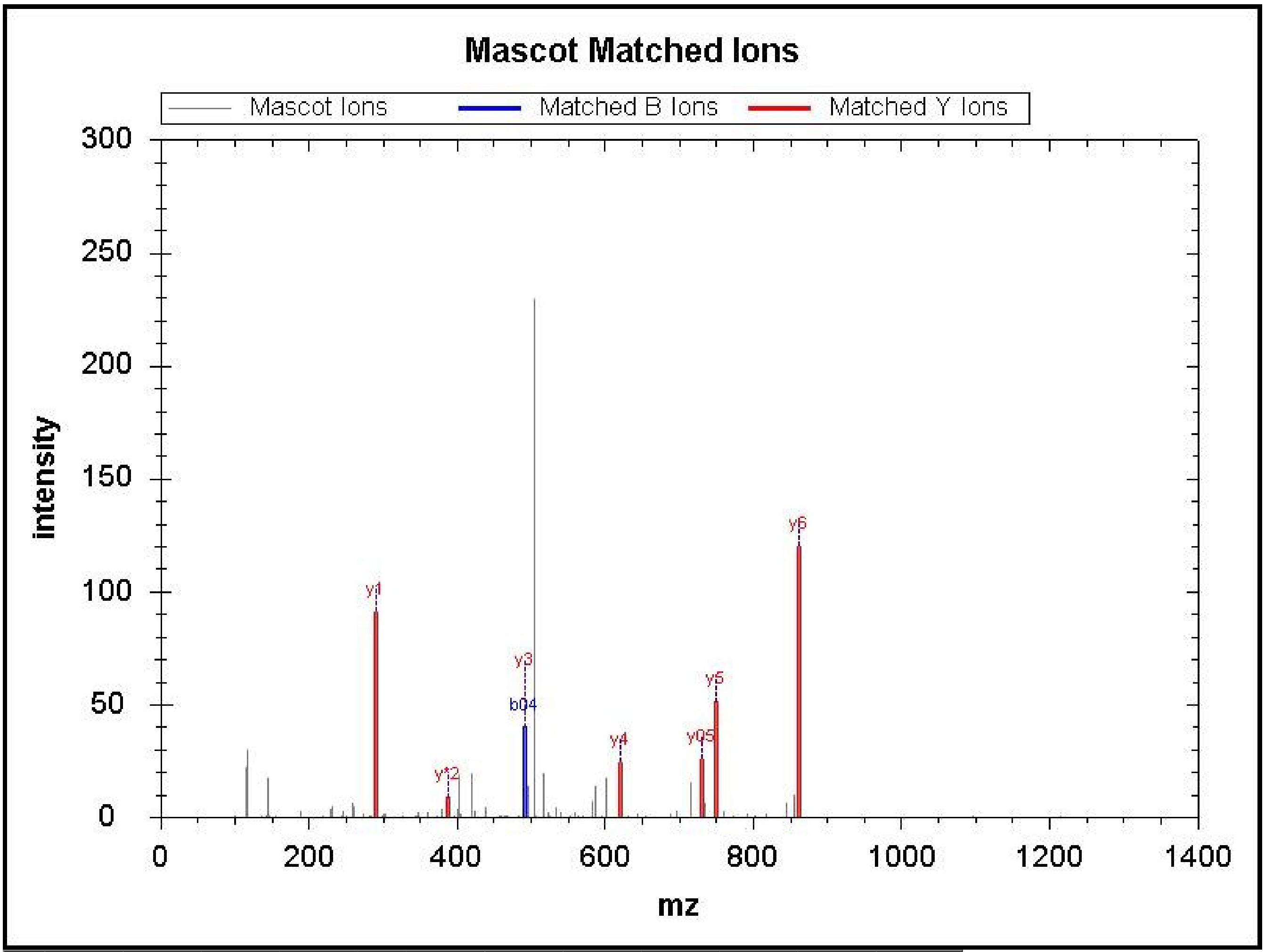

No	b	b++	b0	b0++	Seq	y	y++	y*	y*++	y0	y0++	RevNo
1	138.07	69.54			H							7
2	251.15	126.08			L	862.50	431.75	845.47	423.24	844.49	422.75	6
3	380.19	190.60	362.18	181.59	E	749.42	375.21	732.39	366.70	731.41	366.21	5
4	509.24	255.12	491.22	246.12	E	620.37	310.69	603.35	302.18	602.36	301.69	4
5	596.27	298.64	578.26	289.63	S	491.33	246.17	474.30	237.66	473.32	237.16	3
6	709.35	355.18	691.34	346.17	I	404.30	202.65	387.27	194.14			2
7					K	291.21	146.11	274.19	137.60			1

Query 97485 Hit 1

MS/MS Fragmentation of DALVQPLTSQGVDGVQDVVALMDTYYLMK
Found in sp|P35251|RFC1_HUMAN, Replication factor C subunit 1 OS=Homo sapiens GN=RFC1 PE=1 SV=4
Match to Query 97485: 3456.776from(865.2013,4+)
Title: 1312: Sum of 2 scans in range 2993 (rt=76.5257, f=4, i=869) to 2994 (rt=76.5511, f=4, i=870) [D:\lab212\membrane\Grace\Joyce\iTRAQ_22_1.raw]
Data File:Submitted from 20120508-1(merge) by Mascot Daemon on JOYCE-VAIO
Monoisotopic mass of neutral peptide Mr(calc): 3456.776
Variable modifications:
K29 :iTRAQ4plex (K)
Ions Score: 41.09 Expect: 0.059

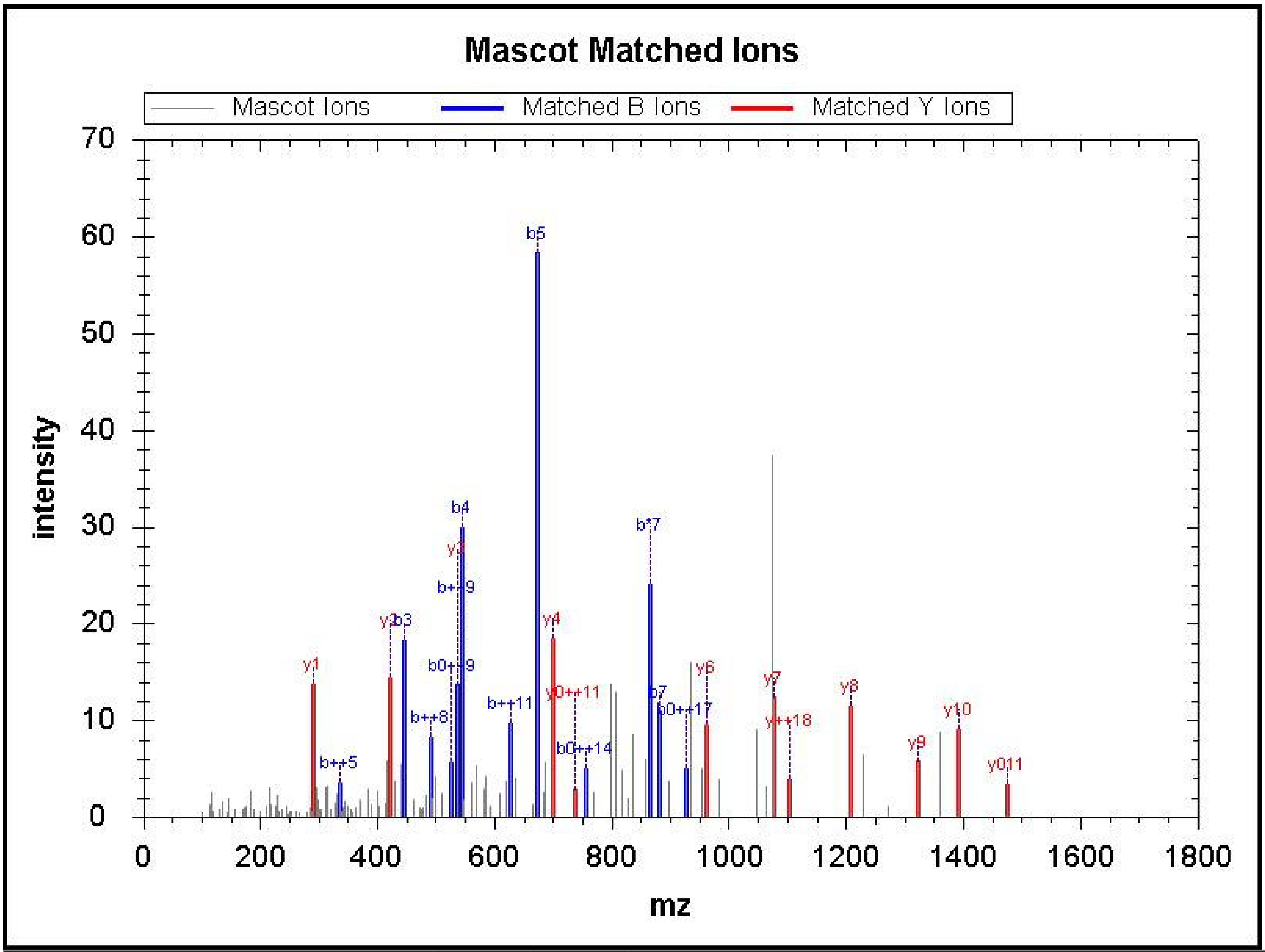

No	b	b++	b*	b*++	b0	b0++	Seq	y	y++	y*	y*++	y0	y0++	RevNo
1	260.14	130.57			242.13	121.57	D							29
2	331.17	166.09			313.16	157.09	A	3,198.65	1,599.83	3,181.63	1,591.32	3,180.64	1,590.83	28
3	444.26	222.63			426.25	213.63	L	3,127.62	1,564.31	3,110.59	1,555.80	3,109.61	1,555.31	27
4	543.33	272.17			525.32	263.16	V	3,014.53	1,507.77	2,997.51	1,499.26	2,996.52	1,498.77	26
5	671.38	336.20	654.36	327.68	653.37	327.19	Q	2,915.46	1,458.24	2,898.44	1,449.72	2,897.45	1,449.23	25
6	768.44	384.72	751.41	376.21	750.43	375.72	P	2,787.41	1,394.21	2,770.38	1,385.69	2,769.40	1,385.20	24
7	881.52	441.26	864.49	432.75	863.51	432.26	L	2,690.35	1,345.68	2,673.33	1,337.17	2,672.34	1,336.68	23
8	982.57	491.79	965.54	483.27	964.56	482.78	T	2,577.27	1,289.14	2,560.24	1,280.63	2,559.26	1,280.13	22
9	1,069.60	535.30	1,052.57	526.79	1,051.59	526.30	S	2,476.22	1,238.61	2,459.20	1,230.10	2,458.21	1,229.61	21
10	1,197.66	599.33	1,180.63	590.82	1,179.65	590.33	Q	2,389.19	1,195.10	2,372.16	1,186.59	2,371.18	1,186.09	20
11	1,254.68	627.84	1,237.65	619.33	1,236.67	618.84	G	2,261.13	1,131.07	2,244.10	1,122.56	2,243.12	1,122.06	19
12	1,353.75	677.38	1,336.72	668.87	1,335.74	668.37	V	2,204.11	1,102.56	2,187.08	1,094.05	2,186.10	1,093.55	18
13	1,468.78	734.89	1,451.75	726.38	1,450.77	725.89	D	2,105.04	1,053.02	2,088.01	1,044.51	2,087.03	1,044.02	17
14	1,525.80	763.40	1,508.77	754.89	1,507.79	754.40	G	1,990.01	995.51	1,972.99	987.00	1,972.00	986.51	16
15	1,624.87	812.94	1,607.84	804.42	1,606.86	803.93	V	1,932.99	967.00	1,915.97	958.49	1,914.98	957.99	15
16	1,752.92	876.97	1,735.90	868.45	1,734.91	867.96	Q	1,833.92	917.47	1,816.90	908.95	1,815.91	908.46	14
17	1,867.95	934.48	1,850.93	925.97	1,849.94	925.47	D	1,705.87	853.44	1,688.84	844.92	1,687.86	844.43	13
18	1,967.02	984.01	1,949.99	975.50	1,949.01	975.01	V	1,590.84	795.92	1,573.81	787.41	1,572.83	786.92	12
19	2,066.09	1,033.55	2,049.06	1,025.03	2,048.08	1,024.54	V	1,491.77	746.39	1,474.74	737.88	1,473.76	737.38	11
20	2,137.13	1,069.07	2,120.10	1,060.55	2,119.12	1,060.06	A	1,392.70	696.85	1,375.68	688.34	1,374.69	687.85	10
21	2,250.21	1,125.61	2,233.18	1,117.10	2,232.20	1,116.60	L	1,321.67	661.34	1,304.64	652.82	1,303.65	652.33	9
22	2,381.25	1,191.13	2,364.22	1,182.62	2,363.24	1,182.12	M	1,208.58	604.79	1,191.55	596.28	1,190.57	595.79	8
23	2,496.28	1,248.64	2,479.25	1,240.13	2,478.27	1,239.64	D	1,077.54	539.27	1,060.51	530.76	1,059.53	530.27	7
24	2,597.32	1,299.17	2,580.30	1,290.65	2,579.31	1,290.16	T	962.51	481.76	945.49	473.25	944.50	472.76	6

25	2,760.39	1,380.70	2,743.36	1,372.18	2,742.38	1,371.69	Y	861.47	431.24	844.44	422.72			5
26	2,923.45	1,462.23	2,906.42	1,453.72	2,905.44	1,453.22	Y	698.40	349.70	681.38	341.19			4
27	3,036.54	1,518.77	3,019.51	1,510.26	3,018.52	1,509.77	L	535.34	268.17	518.31	259.66			3
28	3,167.58	1,584.29	3,150.55	1,575.78	3,149.57	1,575.29	M	422.26	211.63	405.23	203.12			2
29							K	291.21	146.11	274.19	137.60			1

Query 5704 Hit 1

MS/MS Fragmentation of NQKLSVVR
Found in sp|Q14CX7|NAA25_HUMAN, N-alpha-acetyltransferase 25
Match to Query 5704: 944.5411 from(473.2778,2+)
Title: 144: Scan 540 (rt=20.9633, f=3, i=51) [D:\lab212\membrane\Grace\Joyce\iTRAQ_24_1.raw]
Data File:Submitted from 20120508-1(merge) by Mascot Daemon on JOYCE-VAIO
Monoisotopic mass of neutral peptide Mr(calc): 944.5411
Variable modifications:
N1 :Deamidated (NQ)
Q2 :Deamidated (NQ)
Ions Score: 41.08 Expect: 0.055

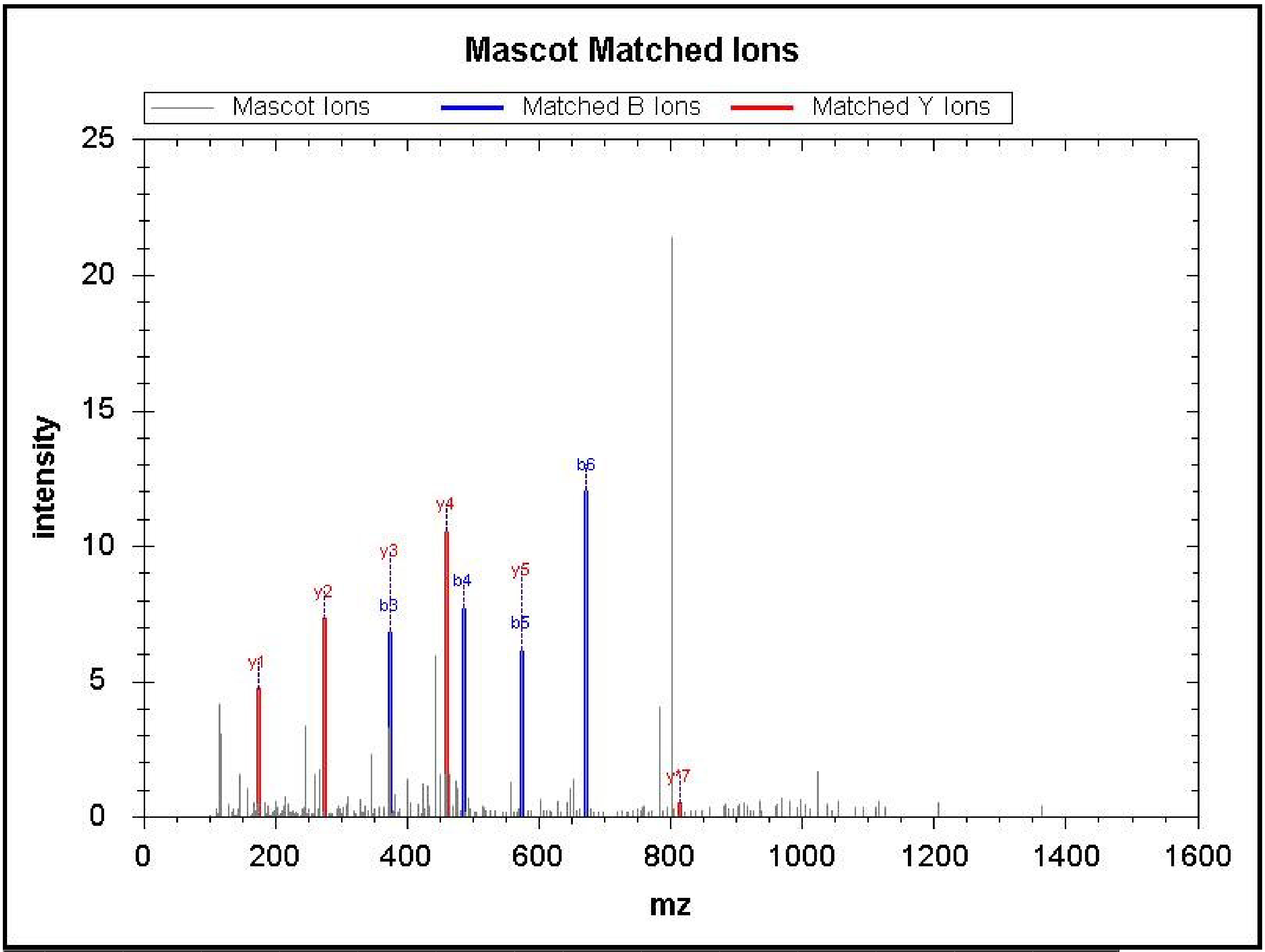

No	b	b++	b*	b*++	b0	b0++	Seq	y	y++	y*	y*++	y0	y0++	RevNo
1	116.03	58.52	99.01	50.01			N							8
2	245.08	123.04	228.05	114.53			Q	830.51	415.76	813.48	407.25	812.50	406.75	7
3	373.17	187.09	356.15	178.58			K	701.47	351.24	684.44	342.72	683.46	342.23	6
4	486.26	243.63	469.23	235.12			L	573.37	287.19	556.35	278.68	555.36	278.18	5
5	573.29	287.15	556.26	278.63	555.28	278.14	S	460.29	230.65	443.26	222.13	442.28	221.64	4
6	672.36	336.68	655.33	328.17	654.35	327.68	V	373.26	187.13	356.23	178.62			3
7	771.42	386.22	754.40	377.70	753.41	377.21	V	274.19	137.60	257.16	129.08			2
8							R	175.12	88.06	158.09	79.55			1

Query 7197 Hit 1

MS/MS Fragmentation of AQDEVQR
Found in sp|Q8NCX0|CC150_HUMAN, Coiled-coil domain-containing protein 150 OS=Homo sapiens GN=CCDC150 PE=2 SV=2
Match to Query 7197: 989.4898from(495.7522,2+)
Title: 6: Scan 111 (rt=11.9608, f=2, i=5) [D:\lab212\membrane\Grace\Joyce\iTRAQ_28_2_327.raw]
Data File:Submitted from 20120508-1(merge) by Mascot Daemon on JOYCE-VAIO
Monoisotopic mass of neutral peptide Mr(calc): 989.4898
Variable modifications:
Q2 :Deamidated (NQ)
Ions Score: 41.08 Expect: 0.056

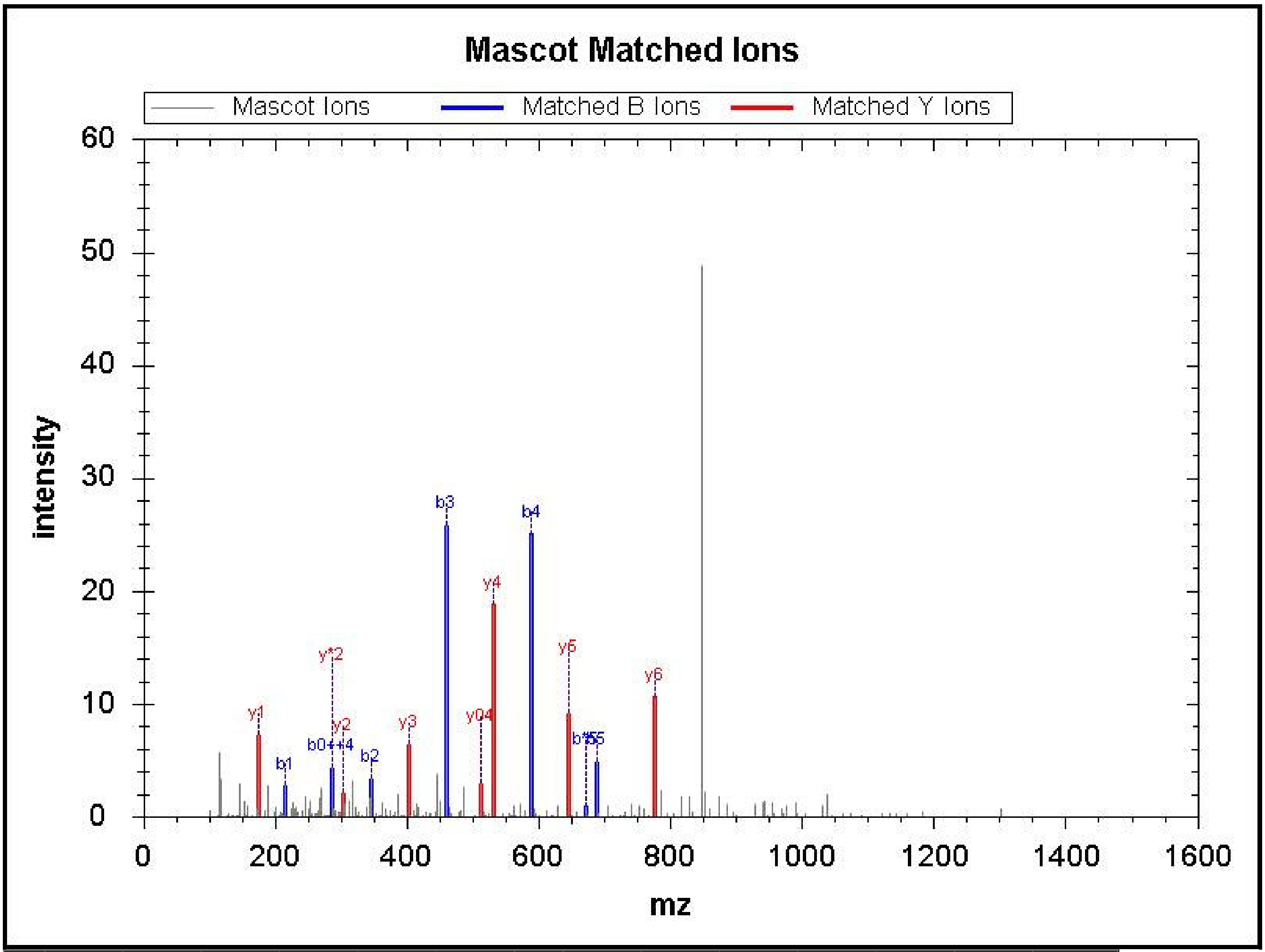

No	b	b++	b*	b*++	b0	b0++	Seq	y	y++	y*	y*++	y0	y0++	RevNo
1	216.15	108.58					A							7
2	345.19	173.10	328.16	164.58			Q	775.36	388.18	758.33	379.67	757.35	379.18	6
3	460.22	230.61	443.19	222.10	442.21	221.61	D	646.32	323.66	629.29	315.15	628.30	314.66	5
4	589.26	295.13	572.23	286.62	571.25	286.13	E	531.29	266.15	514.26	257.63	513.28	257.14	4
5	688.33	344.67	671.30	336.15	670.32	335.66	V	402.25	201.63	385.22	193.11			3
6	816.39	408.70	799.36	400.18	798.38	399.69	Q	303.18	152.09	286.15	143.58			2
7							R	175.12	88.06	158.09	79.55			1

Query 28994 Hit 1

MS/MS Fragmentation of KYFQGLLNR
Found in sp|P31532|SAA4_MOUSE, Serum amyloid A-4 protein OS=Mus musculus GN=Saa4 PE=1 SV=2
Match to Query 28994: 1425.837from(476.2862,3+)
Title: 534: Sum of 2 scans in range 1196 (rt=36.6175, f=4, i=360) to 1197 (rt=36.6429, f=4, i=361)
[D:\lab212\membrane\Grace\Joyce\iTRAQ_47_1.raw]
Data File:Submitted from 20120508-1(merge) by Mascot Daemon on JOYCE-VAIO
Monoisotopic mass of neutral peptide Mr(calc): 1425.837

Variable modifications:
K1 :iTRAQ4plex (K)
Ions Score: 40.96 Expect: 0.041

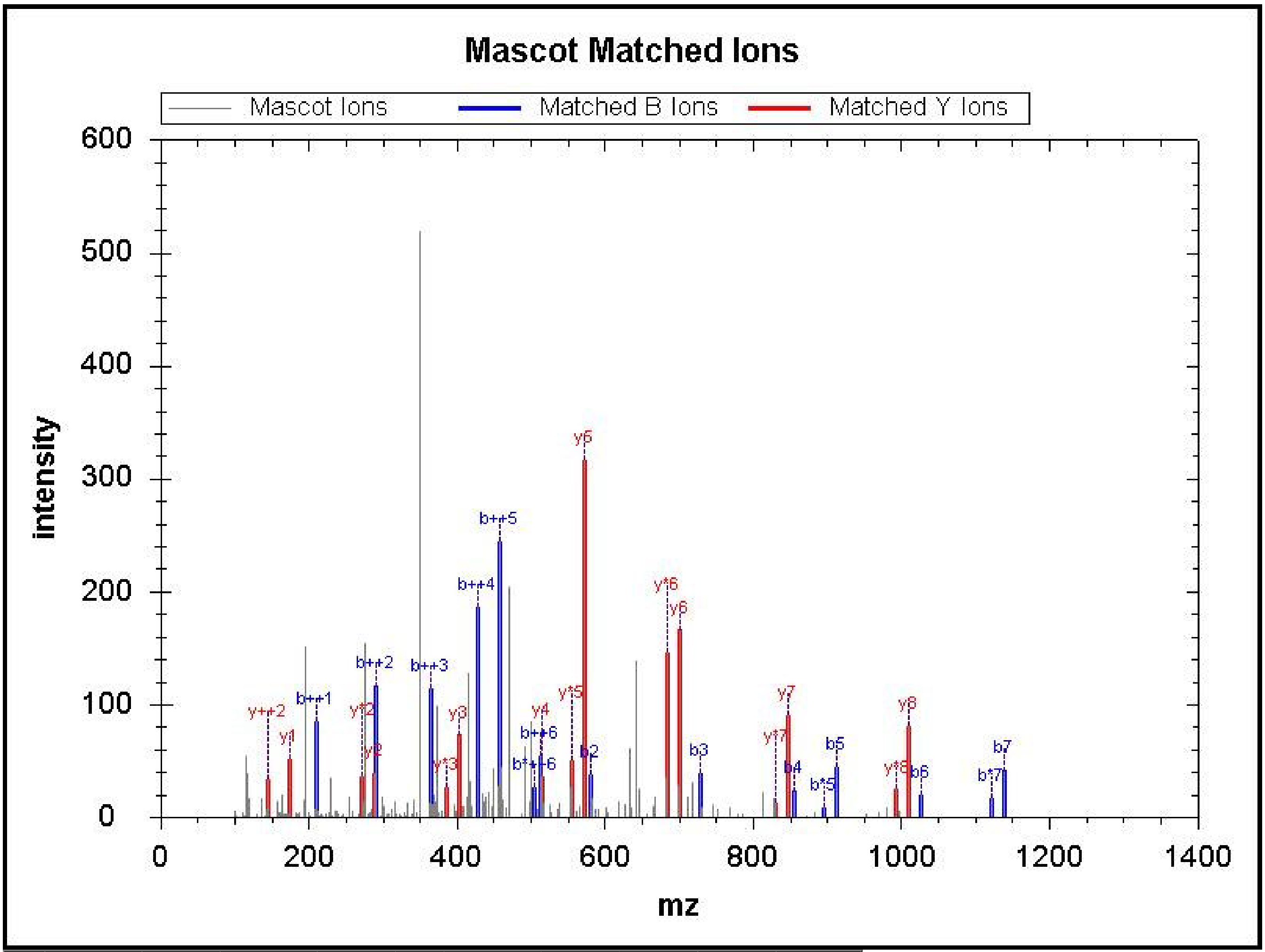

No	b	b++	b*	b*++	Seq	y	y++	y*	y*++	RevNo
1	417.31	209.16	400.28	200.64	K					9
2	580.37	290.69	563.34	282.18	Y	1,010.54	505.77	993.52	497.26	8
3	727.44	364.22	710.41	355.71	F	847.48	424.24	830.45	415.73	7
4	855.50	428.25	838.47	419.74	Q	700.41	350.71	683.38	342.20	6
5	912.52	456.76	895.49	448.25	G	572.35	286.68	555.32	278.17	5
6	1,025.60	513.30	1,008.58	504.79	L	515.33	258.17	498.30	249.66	4
7	1,138.69	569.85	1,121.66	561.33	L	402.25	201.63	385.22	193.11	3
8	1,252.73	626.87	1,235.70	618.36	N	289.16	145.08	272.14	136.57	2
9					R	175.12	88.06	158.09	79.55	1

Query 10271 Hit 1

MS/MS Fragmentation of LWSFTK
Found in sp|Q8IUI8|CRLF3_HUMAN, Cytokine receptor-like factor 3 OS=Homo sapiens GN=CRLF3 PE=1 SV=2
Match to Query 10271: 1068.622from(535.3185,2+)
Title: 645: Scan 1429 (rt=41.8578, f=2, i=232) [D:\lab212\membrane\Grace\Joyce\iTRAQ_33_2.raw]
Data File:Submitted from 20120508-1(merge) by Mascot Daemon on JOYCE-VAIO
Monoisotopic mass of neutral peptide Mr(calc): 1068.622
Variable modifications:
K6 :iTRAQ4plex (K)
Ions Score: 40.92 Expect: 0.039

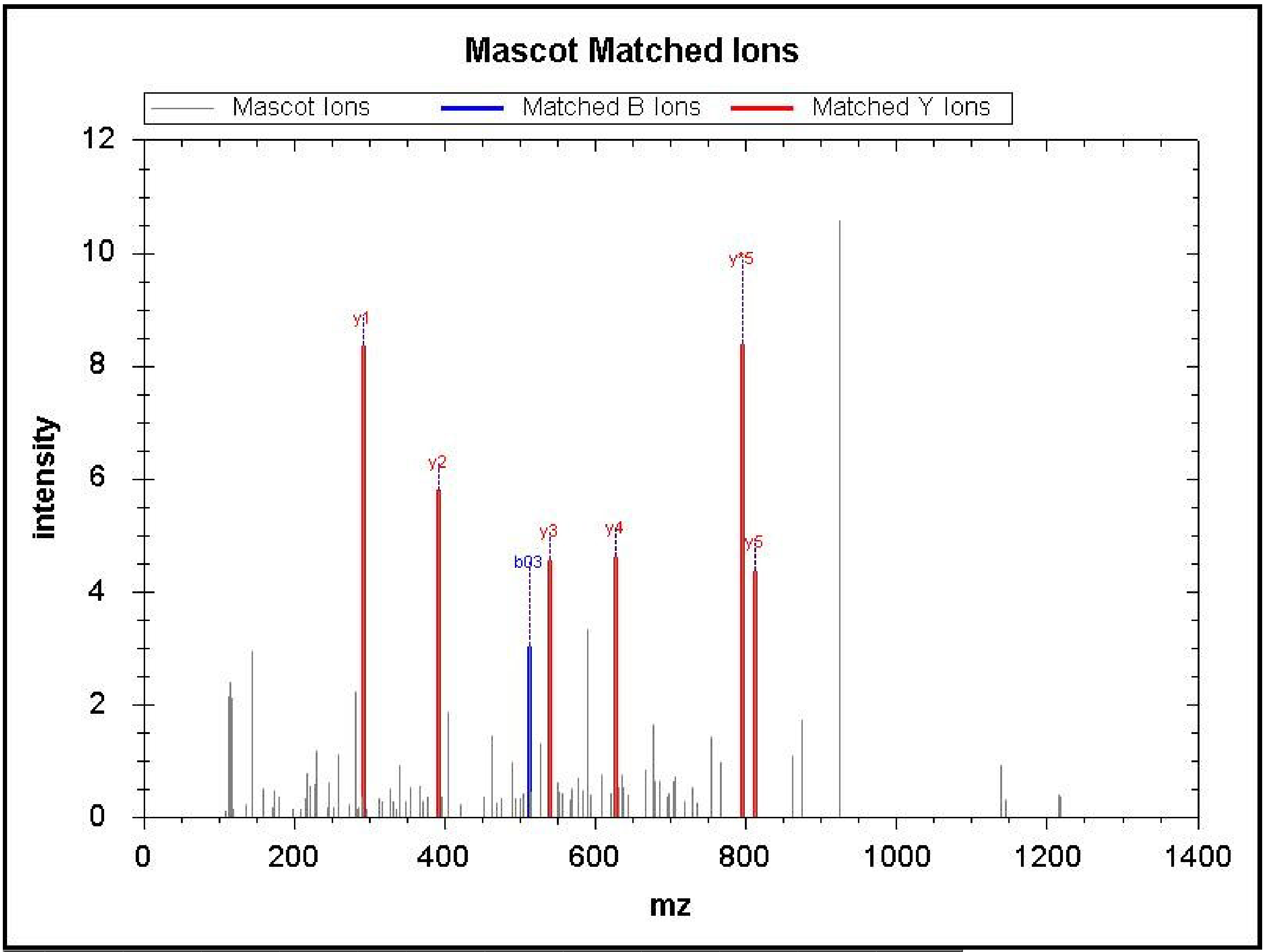

No	b	b++	b0	b0++	Seq	y	y++	y*	y*++	y0	y0++	RevNo
1	258.19	129.60			L							6
2	444.27	222.64			W	812.44	406.72	795.42	398.21	794.43	397.72	5
3	531.30	266.16	513.29	257.15	S	626.36	313.69	609.34	305.17	608.35	304.68	4
4	678.37	339.69	660.36	330.68	F	539.33	270.17	522.30	261.66	521.32	261.16	3
5	779.42	390.21	761.41	381.21	T	392.26	196.63	375.24	188.12	374.25	187.63	2
6					K	291.21	146.11	274.19	137.60			1

Query 56867 Hit 1

MS/MS Fragmentation of TASPLEHILQTLFGK
Found in sp|Q9BTC0|DIDO1_HUMAN, Death-inducer obliterator 1 OS=Homo sapiens GN=DIDO1 PE=1 SV=5
Match to Query 56867: 1942.115from(648.3792,3+)
Title: 1291: Scan 2918 (rt=74.9128, f=2, i=493) [D:\lab212\membrane\Grace\Joyce\iTRAQ_39_1.raw]
Data File:Submitted from 20120508-1(merge) by Mascot Daemon on JOYCE-VAIO
Monoisotopic mass of neutral peptide Mr(calc): 1942.115
Variable modifications:
K15 :iTRAQ4plex (K)
Ions Score: 40.91 Expect: 0.041

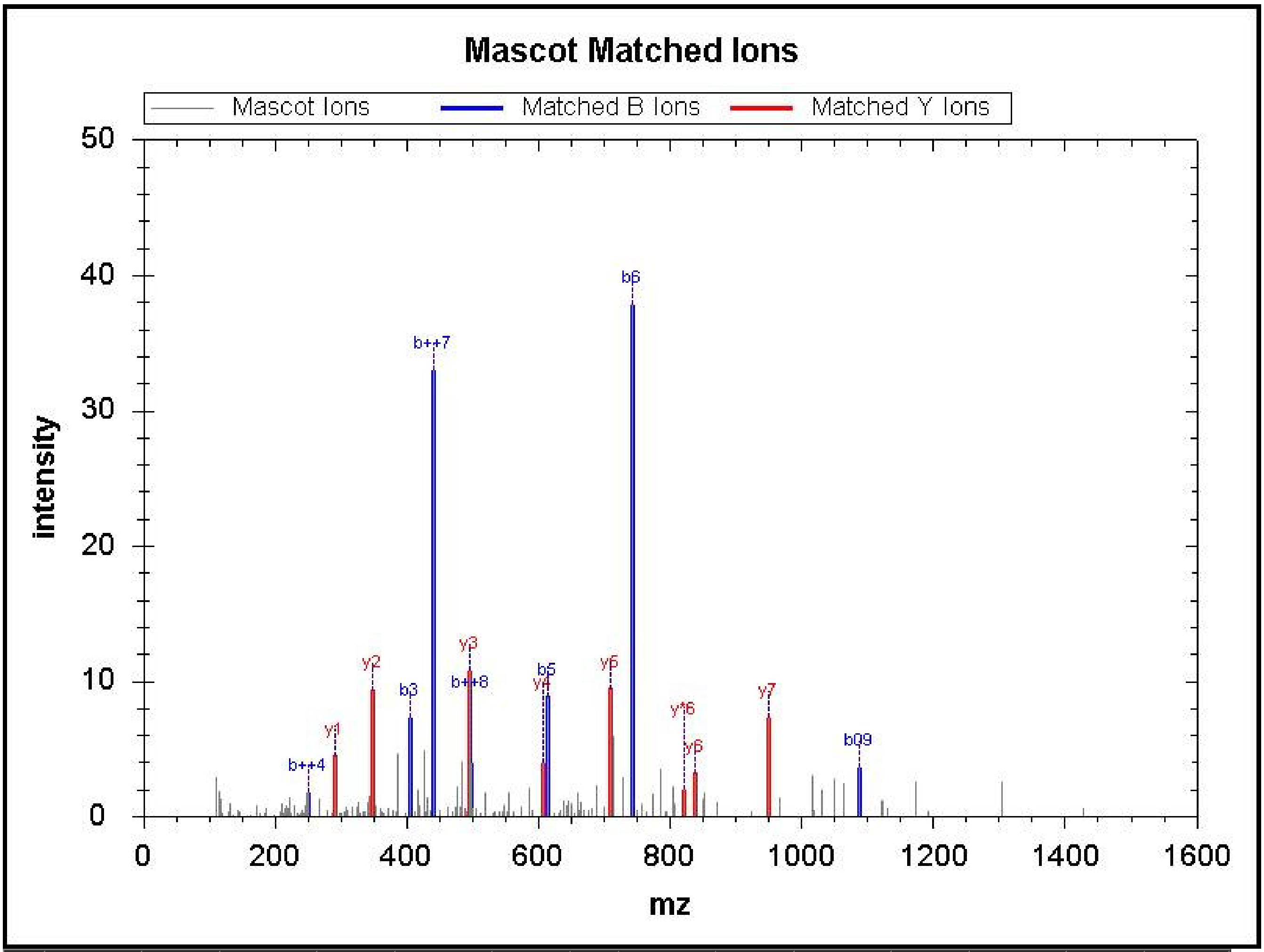

No	b	b++	b*	b*++	b0	b0++	Seq	y	y++	y*	y*++	y0	y0++	RevNo
1	246.16	123.58			228.15	114.58	T							15
2	317.19	159.10			299.18	150.10	A	1,697.97	849.49	1,680.94	840.98	1,679.96	840.48	14
3	404.23	202.62			386.22	193.61	S	1,626.93	813.97	1,609.91	805.46	1,608.92	804.97	13
4	501.28	251.14			483.27	242.14	P	1,539.90	770.45	1,522.88	761.94	1,521.89	761.45	12
5	614.36	307.69			596.35	298.68	L	1,442.85	721.93	1,425.82	713.41	1,424.84	712.92	11
6	743.41	372.21			725.40	363.20	E	1,329.76	665.39	1,312.74	656.87	1,311.75	656.38	10
7	880.46	440.74			862.45	431.73	H	1,200.72	600.86	1,183.70	592.35	1,182.71	591.86	9
8	993.55	497.28			975.54	488.27	I	1,063.66	532.34	1,046.64	523.82	1,045.65	523.33	8
9	1,106.63	553.82			1,088.62	544.81	L	950.58	475.79	933.55	467.28	932.57	466.79	7
10	1,234.69	617.85	1,217.66	609.34	1,216.68	608.84	Q	837.50	419.25	820.47	410.74	819.48	410.25	6
11	1,335.74	668.37	1,318.71	659.86	1,317.73	659.37	T	709.44	355.22	692.41	346.71	691.43	346.22	5
12	1,448.82	724.92	1,431.80	716.40	1,430.81	715.91	L	608.39	304.70	591.36	296.18			4
13	1,595.89	798.45	1,578.87	789.94	1,577.88	789.44	F	495.30	248.16	478.28	239.64			3
14	1,652.91	826.96	1,635.89	818.45	1,634.90	817.95	G	348.24	174.62	331.21	166.11			2
15							K	291.21	146.11	274.19	137.60			1

Query 187 Hit 1

MS/MS Fragmentation of NPNVK
Found in sp|Q9JM99|PRG4_MOUSE, Proteoglycan 4 OS=Mus musculus GN=Prg4 PE=1 SV=2
Match to Query 187: 714.414from(358.2143,2+)
Title: 771: Sum of 2 scans in range 1747 (rt=48.7047, f=4, i=517) to 1748 (rt=48.7301, f=4, i=518) [D:\lab212\membrane\Grace\Joyce\iTRAQ_28_1_327.raw]
Data File:Submitted from 20120508-1(merge) by Mascot Daemon on JOYCE-VAIO
Monoisotopic mass of neutral peptide Mr(calc): 714.414
Variable modifications:

K5 :iTRAQ4plex (K)
Ions Score: 40.9 Expect: 0.028

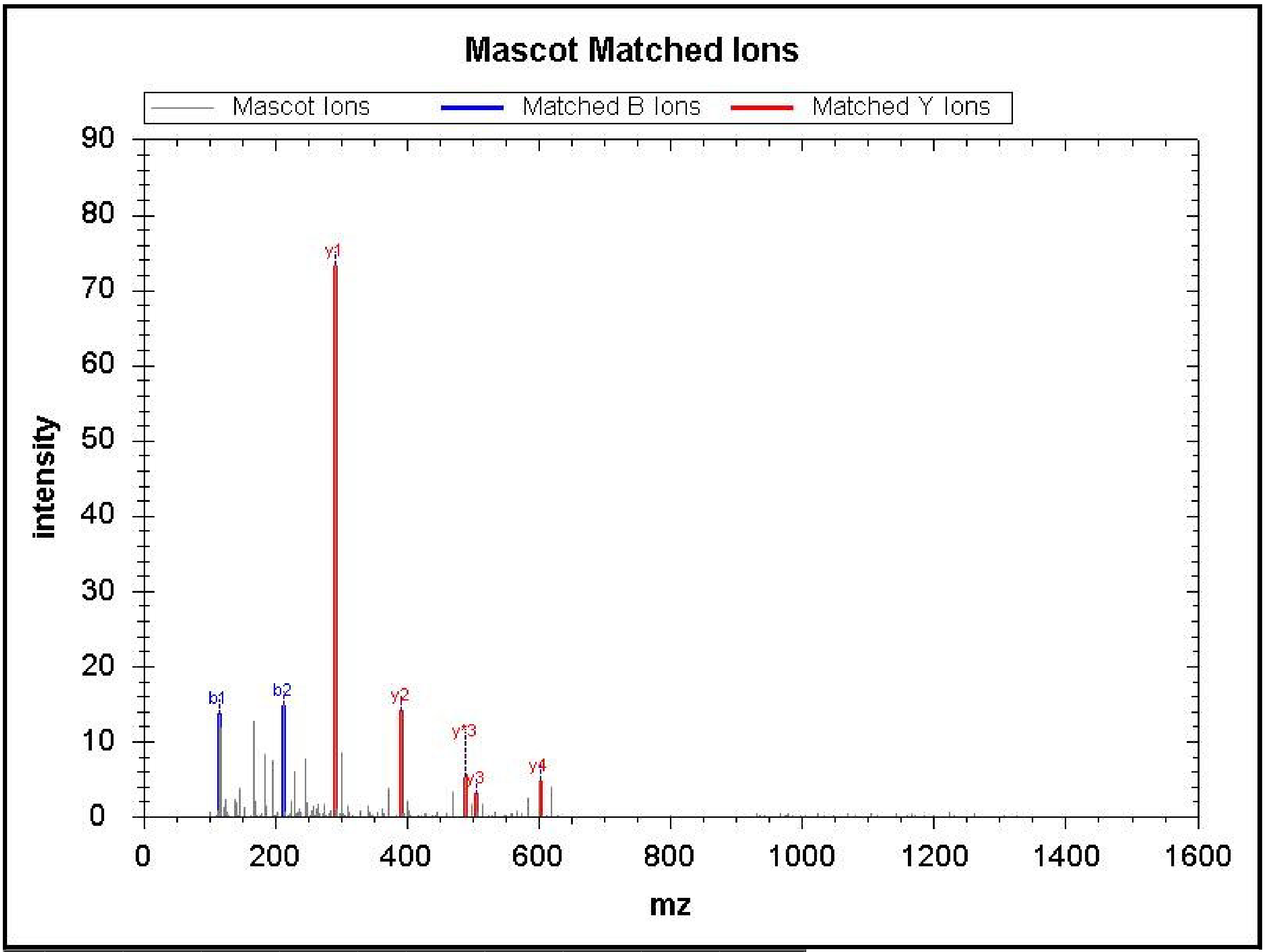

No	b	b++	b*	b*++	Seq	y	y++	y*	y*++	RevNo
1	115.05	58.03	98.02	49.52	N					5
2	212.10	106.56	195.08	98.04	P	601.38	301.19	584.35	292.68	4
3	326.15	163.58	309.12	155.06	N	504.33	252.67	487.30	244.15	3
4	425.21	213.11	408.19	204.60	V	390.28	195.65	373.26	187.13	2
5					K	291.21	146.11	274.19	137.60	1

Query 13484 Hit 1

MS/MS Fragmentation of MILDLLK
Found in sp|Q53EL6|PDCD4_HUMAN, Programmed cell death protein 4 OS=Homo sapiens GN=PDCD4 PE=1 SV=2
Match to Query 13484: 1132.714from(567.3644,2+)
Title: 853: Scan 1979 (rt=53.5277, f=3, i=286) [D:\lab212\membrane\Grace\Joyce\iTRAQ_29_1.raw]
Data File:Submitted from 20120508-1(merge) by Mascot Daemon on JOYCE-VAIO
Monoisotopic mass of neutral peptide Mr(calc): 1132.714
Variable modifications:
K7 :iTRAQ4plex (K)
Ions Score: 40.74 Expect: 0.046

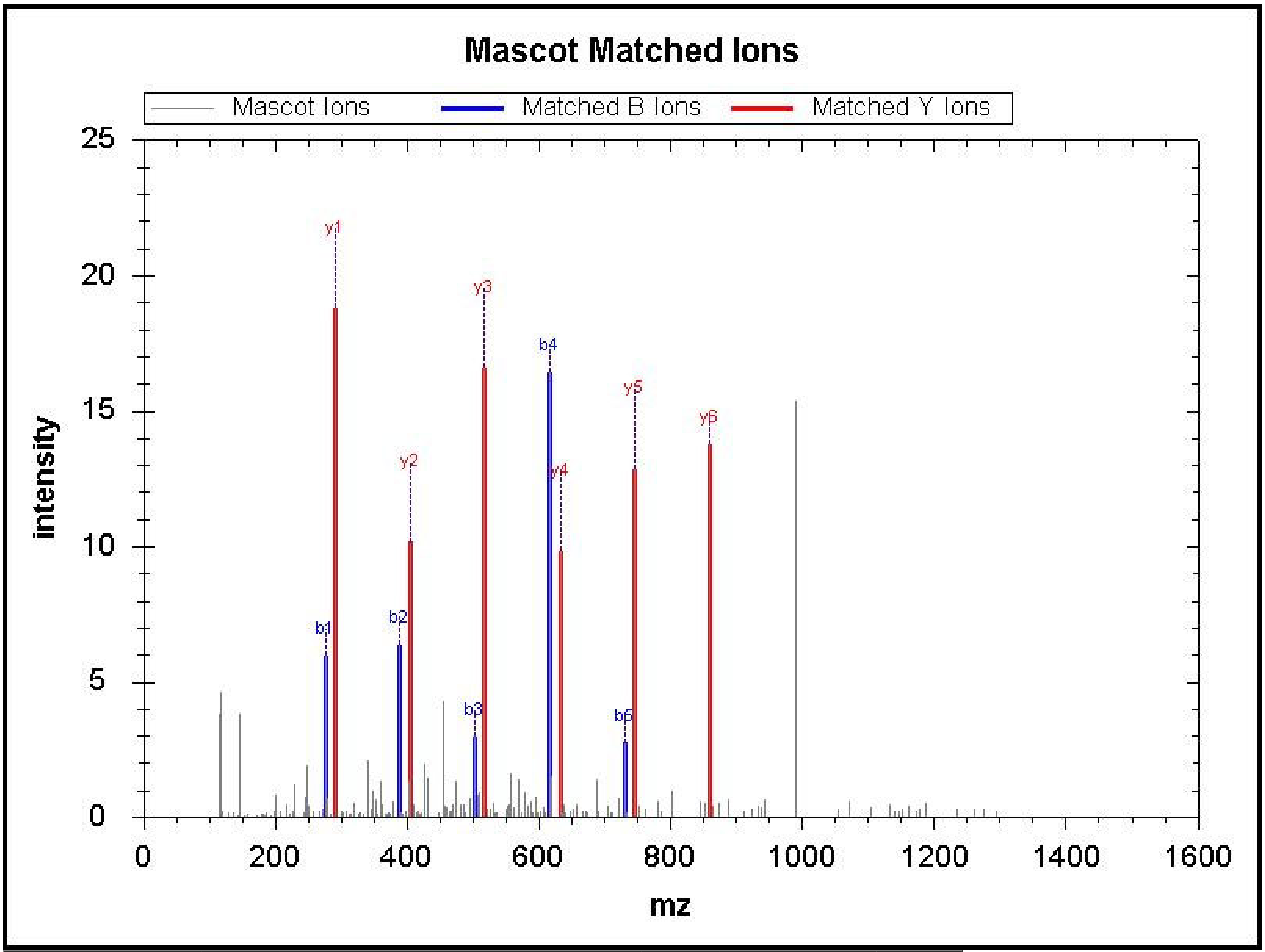

No	b	b++	b0	b0++	Seq	y	y++	y*	y*++	y0	y0++	RevNo
1	276.15	138.58			M							7
2	389.23	195.12			I	858.58	429.79	841.55	421.28	840.57	420.79	6
3	502.32	251.66			L	745.49	373.25	728.47	364.74	727.48	364.25	5
4	617.34	309.18	599.33	300.17	D	632.41	316.71	615.38	308.20	614.40	307.70	4
5	730.43	365.72	712.42	356.71	L	517.38	259.20	500.36	250.68			3
6	843.51	422.26	825.50	413.25	L	404.30	202.65	387.27	194.14			2
7					K	291.21	146.11	274.19	137.60			1

Query 16707 Hit 1

MS/MS Fragmentation of IWGELYK
Found in sp|Q13823|NOG2_HUMAN, Nucleolar GTP-binding protein 2 OS=Homo sapiens GN=GNL2 PE=1 SV=1
Match to Query 16707: 1195.684from(598.8491,2+)
Title: 633: Scan 1435 (rt=41.7938, f=2, i=223) [D:\lab212\membrane\Grace\Joyce\iTRAQ_32_1.raw]
Data File:Submitted from 20120508-1(merge) by Mascot Daemon on JOYCE-VAIO
Monoisotopic mass of neutral peptide Mr(calc): 1195.684
Variable modifications:
K7 :iTRAQ4plex (K)
Ions Score: 40.45 Expect: 0.056

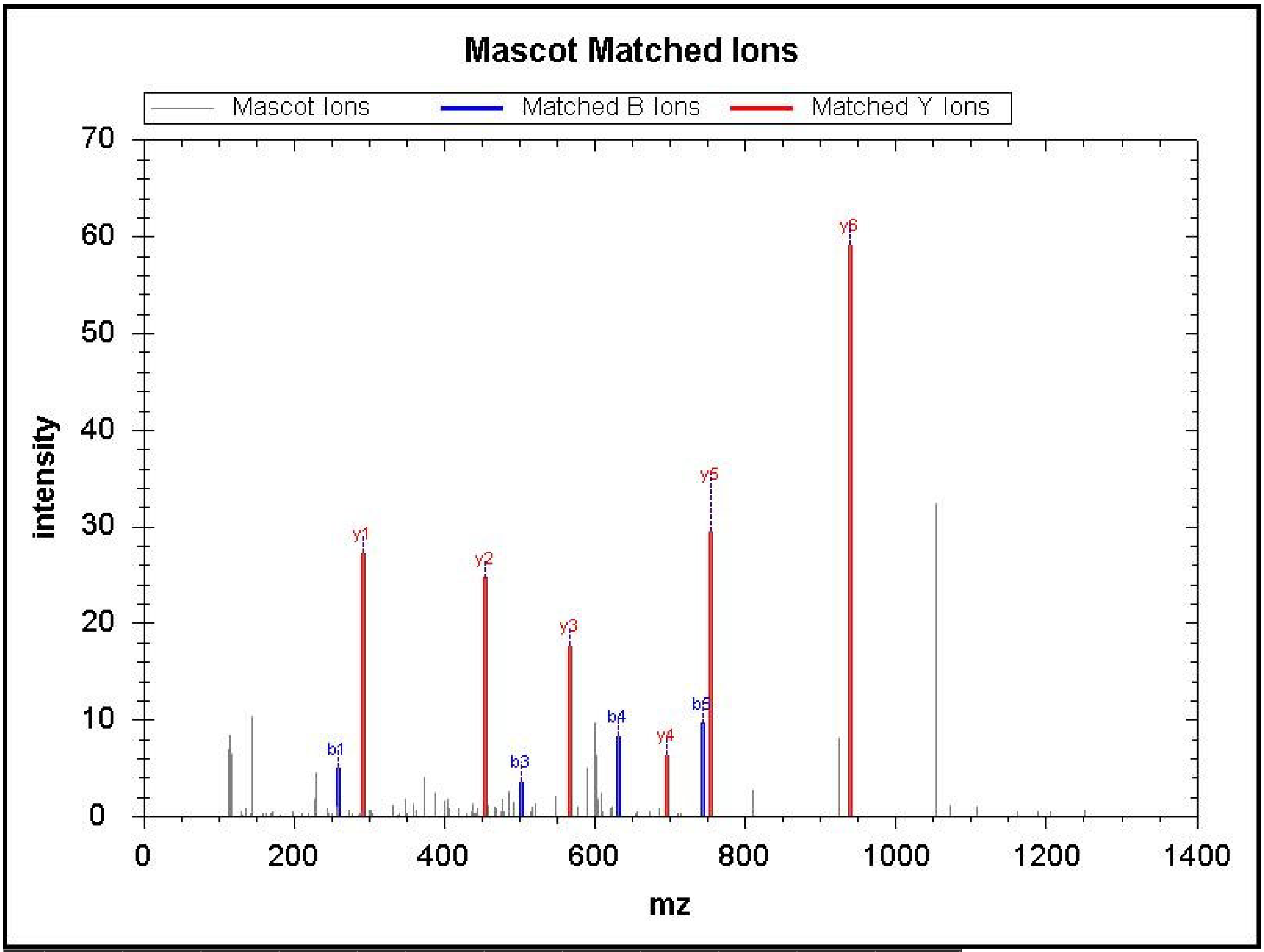

No	b	b++	b0	b0++	Seq	y	y++	y*	y*++	y0	y0++	RevNo
1	258.19	129.60			I							7
2	444.27	222.64			W	939.51	470.26	922.48	461.74	921.50	461.25	6
3	501.29	251.15			G	753.43	377.22	736.40	368.70	735.42	368.21	5
4	630.34	315.67	612.33	306.67	E	696.40	348.71	679.38	340.19	678.39	339.70	4
5	743.42	372.21	725.41	363.21	L	567.36	284.18	550.34	275.67			3
6	906.48	453.75	888.47	444.74	Y	454.28	227.64	437.25	219.13			2
7					K	291.21	146.11	274.19	137.60			1

Query 21717 Hit 1

MS/MS Fragmentation of EKQNEIIK
Found in sp|Q4G0S7|CC152_HUMAN, Coiled-coil domain-containing protein 152 OS=Homo sapiens GN=CCDC152 PE=2 SV=3
Match to Query 21717: 1289.833from(430.9518,3+)
Title: 351: Scan 916 (rt=29.8297, f=2, i=142) [D:\lab212\membrane\Grace\Joyce\iTRAQ_48_2.raw]
Data File:Submitted from 20120508-1(merge) by Mascot Daemon on JOYCE-VAIO
Monoisotopic mass of neutral peptide Mr(calc): 1289.833
Variable modifications:
N4 :Deamidated (NQ)
K8 :iTRAQ4plex (K)
Ions Score: 40.38 Expect: 0.031

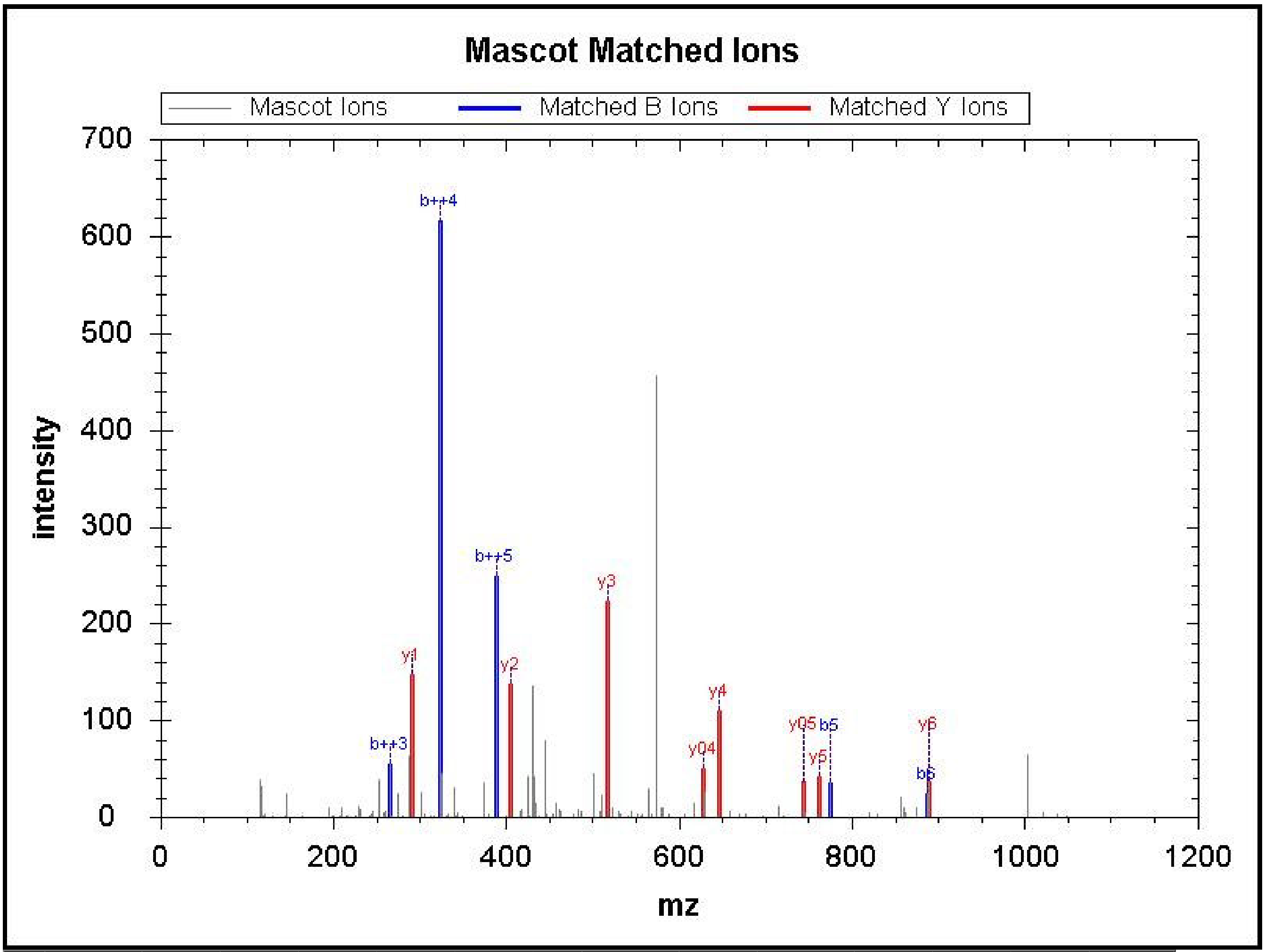

No	b	b++	b*	b*++	b0	b0++	Seq	y	y++	y*	y*++	y0	y0++	RevNo
1	274.15	137.58			256.14	128.57	E							8
2	402.25	201.63	385.22	193.11	384.24	192.62	K	1,017.61	509.31	1,000.58	500.79	999.60	500.30	7
3	530.31	265.66	513.28	257.14	512.29	256.65	Q	889.51	445.26	872.48	436.75	871.50	436.25	6
4	645.33	323.17	628.31	314.66	627.32	314.16	N	761.45	381.23	744.43	372.72	743.44	372.22	5
5	774.38	387.69	757.35	379.18	756.36	378.69	E	646.43	323.72	629.40	315.20	628.41	314.71	4
6	887.46	444.23	870.43	435.72	869.45	435.23	I	517.38	259.20	500.36	250.68			3
7	1,000.54	500.78	983.52	492.26	982.53	491.77	I	404.30	202.65	387.27	194.14			2
8							K	291.21	146.11	274.19	137.60			1

Query 9783 Hit 1

MS/MS Fragmentation of AALGQLEEAR
Found in sp|Q6P566|LIX1_MOUSE, Protein limb expression 1 homolog OS=Mus musculus GN=Lix1 PE=2 SV=1
Match to Query 9783: 1057.63from(529.822,2+)
Title: 325: Scan 1407 (rt=37.9333, f=3, i=101) [D:\lab212\membrane\Grace\Joyce\iTRAQ_24_2.raw]
Data File:Submitted from 20120508-1(merge) by Mascot Daemon on JOYCE-VAIO
Monoisotopic mass of neutral peptide Mr(calc): 1057.63
Variable modifications:
Q5 :Deamidated (NQ)
Ions Score: 40.34 Expect: 0.051

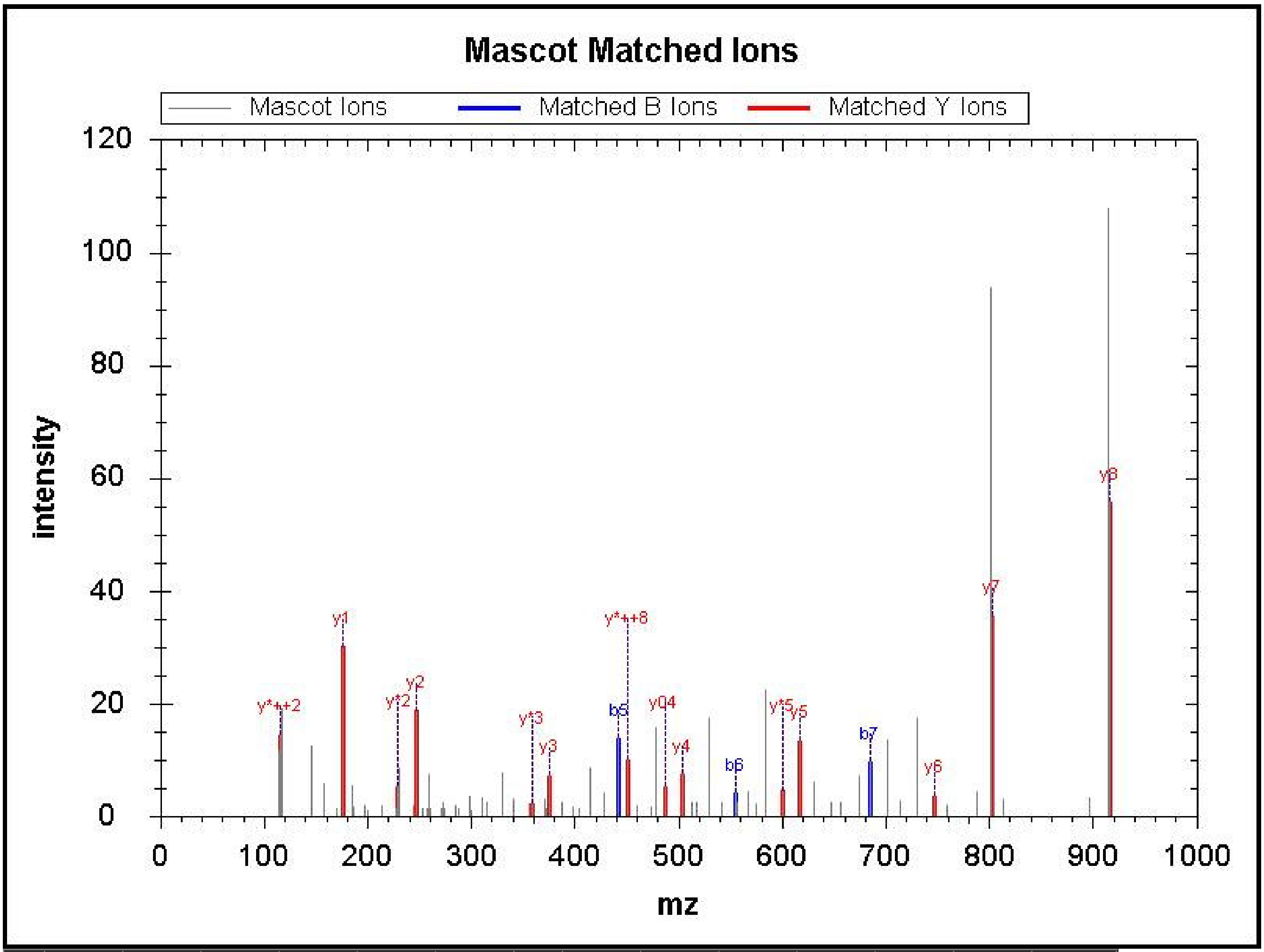

No	b	b++	b*	b*++	b0	b0++	Seq	y	y++	y*	y*++	y0	y0++	RevNo
1	72.04	36.53					A							10
2	143.08	72.04					A	987.51	494.26	970.48	485.75	969.50	485.25	9
3	256.17	128.59					L	916.47	458.74	899.45	450.23	898.46	449.74	8
4	313.19	157.10					G	803.39	402.20	786.36	393.69	785.38	393.19	7
5	442.23	221.62	425.20	213.11			Q	746.37	373.69	729.34	365.17	728.36	364.68	6
6	555.31	278.16	538.29	269.65			L	617.33	309.17	600.30	300.65	599.31	300.16	5
7	684.36	342.68	667.33	334.17	666.35	333.68	E	504.24	252.62	487.21	244.11	486.23	243.62	4
8	813.40	407.20	796.37	398.69	795.39	398.20	E	375.20	188.10	358.17	179.59	357.19	179.10	3
9	884.44	442.72	867.41	434.21	866.43	433.72	A	246.16	123.58	229.13	115.07			2
10							R	175.12	88.06	158.09	79.55			1

Query 65183 Hit 1

MS/MS Fragmentation of YHWLVLPWTSISSLK
Found in sp|Q7Z2E3|APTX_HUMAN, Aprataxin OS=Homo sapiens GN=APTX PE=1 SV=2
Match to Query 65183: 2117.196from(706.7392,3+)
Title: 1099: Scan 2395 (rt=63.5558, f=2, i=390) [D:\lab212\membrane\Grace\Joyce\iTRAQ_39_2.raw]
Data File:Submitted from 20120508-1(merge) by Mascot Daemon on JOYCE-VAIO
Monoisotopic mass of neutral peptide Mr(calc): 2117.196
Variable modifications:
K15 :iTRAQ4plex (K)
Ions Score: 40.33 Expect: 0.051

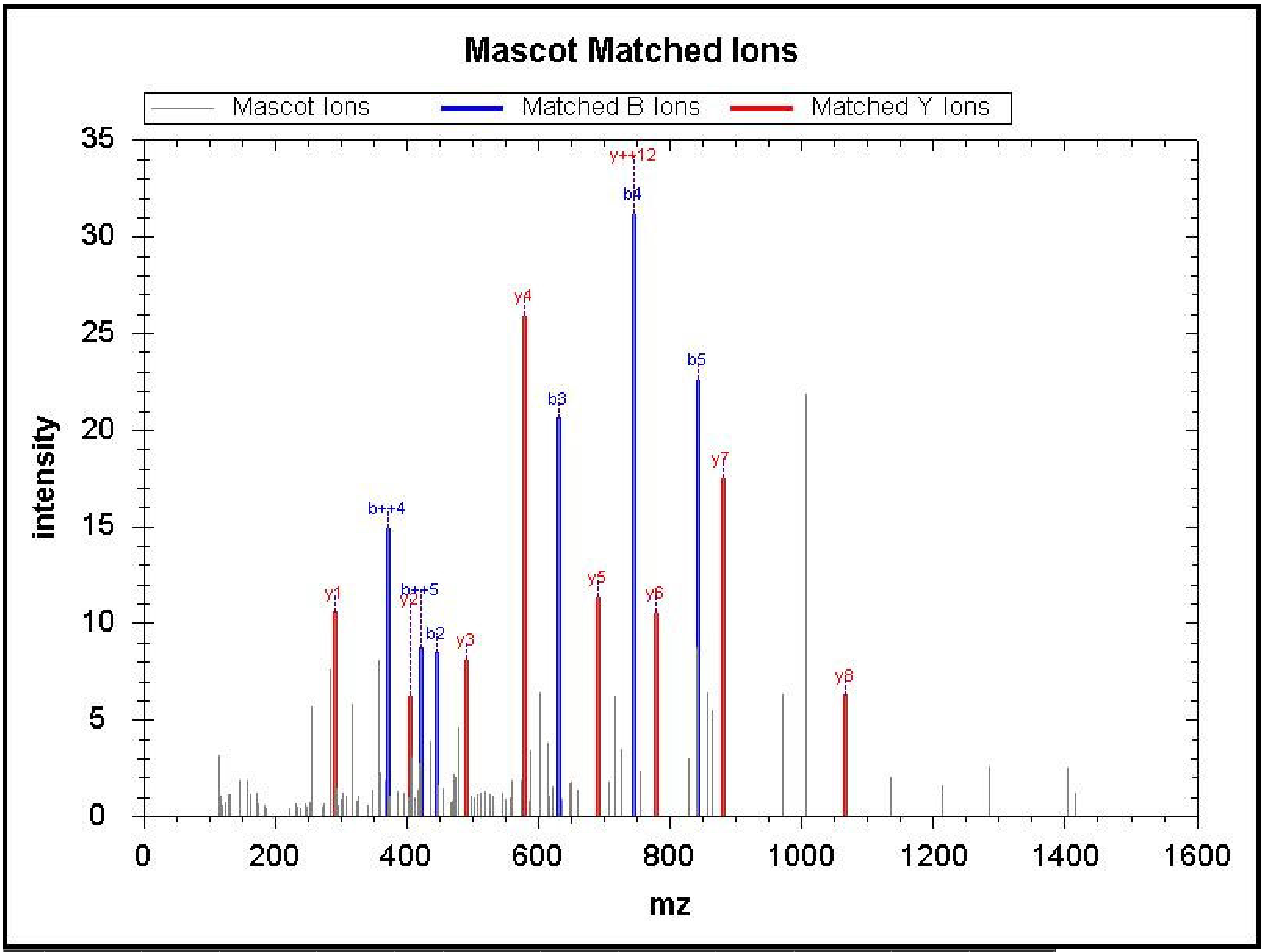

No	b	b++	b0	b0++	Seq	y	y++	y*	y*++	y0	y0++	RevNo
1	308.17	154.59			Y							15
2	445.23	223.12			H	1,811.03	906.02	1,794.01	897.51	1,793.02	897.02	14
3	631.31	316.16			W	1,673.97	837.49	1,656.95	828.98	1,655.96	828.49	13
4	744.39	372.70			L	1,487.90	744.45	1,470.87	735.94	1,469.88	735.45	12
5	843.46	422.24			V	1,374.81	687.91	1,357.78	679.40	1,356.80	678.90	11
6	956.55	478.78			L	1,275.74	638.38	1,258.72	629.86	1,257.73	629.37	10
7	1,053.60	527.30			P	1,162.66	581.83	1,145.63	573.32	1,144.65	572.83	9
8	1,239.68	620.34			W	1,065.61	533.31	1,048.58	524.79	1,047.60	524.30	8
9	1,340.73	670.87	1,322.72	661.86	T	879.53	440.27	862.50	431.75	861.52	431.26	7
10	1,427.76	714.38	1,409.75	705.38	S	778.48	389.74	761.45	381.23	760.47	380.74	6
11	1,540.84	770.93	1,522.83	761.92	I	691.45	346.23	674.42	337.71	673.44	337.22	5
12	1,627.88	814.44	1,609.86	805.44	S	578.36	289.69	561.34	281.17	560.35	280.68	4
13	1,714.91	857.96	1,696.90	848.95	S	491.33	246.17	474.30	237.66	473.32	237.16	3
14	1,827.99	914.50	1,809.98	905.49	L	404.30	202.65	387.27	194.14			2
15					K	291.21	146.11	274.19	137.60			1

Query 33310 Hit 1

MS/MS Fragmentation of YLEKPITMLL
Found in sp|P10515|ODP2_HUMAN, Dihydrolipoyllysine-residue acetyltransferase component of pyruvate dehydrogenase complex
Match to Query 33310: 1507.887from(754.9506,2+)
Title: 945: Sum of 2 scans in range 2082 (rt=56.5528, f=4, i=641) to 2083 (rt=56.5782, f=4, i=642) [D:\lab212\membrane\Grace\Joyce\iTRAQ_27_2_327.raw]
Data File:Submitted from 20120508-1(merge) by Mascot Daemon on JOYCE-VAIO
Monoisotopic mass of neutral peptide Mr(calc): 1507.887
Variable modifications:

K4 :iTRAQ4plex (K)
Ions Score: 40.25 Expect: 0.053

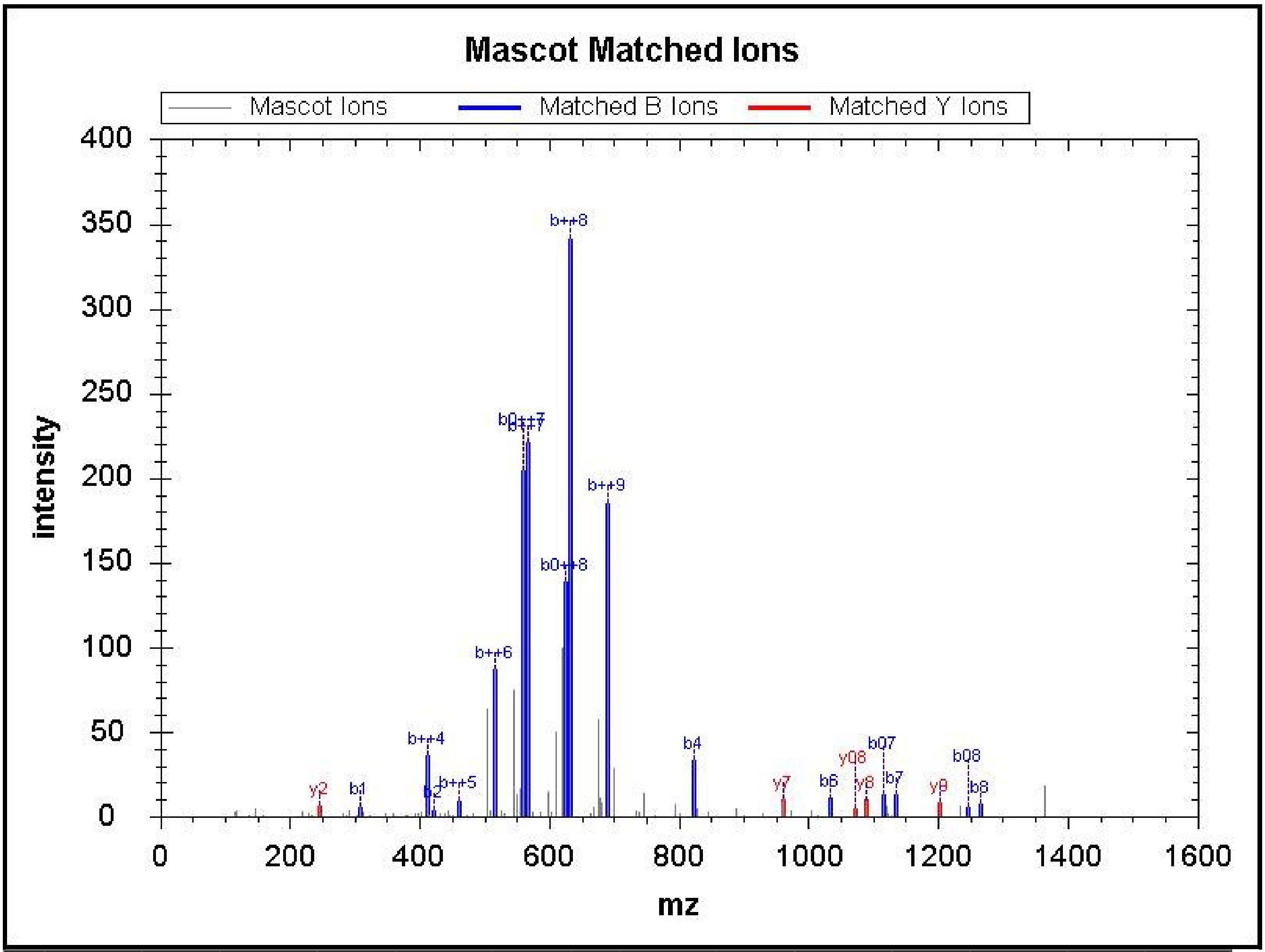

No	b	b++	b*	b*++	b0	b0++	Seq	y	y++	y*	y*++	y0	y0++	RevNo
1	308.17	154.59					Y							10
2	421.26	211.13					L	1,201.73	601.37	1,184.71	592.86	1,183.72	592.37	9
3	550.30	275.65			532.29	266.65	E	1,088.65	544.83	1,071.62	536.32	1,070.64	535.82	8
4	822.50	411.75	805.47	403.24	804.49	402.75	K	959.61	480.31	942.58	471.79	941.60	471.30	7
5	919.55	460.28	902.52	451.76	901.54	451.27	P	687.41	344.21			669.40	335.20	6
6	1,032.63	516.82	1,015.61	508.31	1,014.62	507.82	I	590.36	295.68			572.35	286.68	5
7	1,133.68	567.34	1,116.65	558.83	1,115.67	558.34	T	477.27	239.14			459.26	230.14	4
8	1,264.72	632.86	1,247.70	624.35	1,246.71	623.86	M	376.23	188.62					3
9	1,377.81	689.41	1,360.78	680.89	1,359.80	680.40	L	245.19	123.10					2
10							L	132.10	66.55					1

Query 20774 Hit 1

MS/MS Fragmentation of IAKVETVPK
Found in sp|Q07864|DPOE1_HUMAN, DNA polymerase epsilon catalytic subunit A OS=Homo sapiens GN=POLE PE=1 SV=5
Match to Query 20774: 1271.805from(636.91,2+)
Title: 788: Scan 1824 (rt=50.252, f=3, i=267) [D:\lab212\membrane\Grace\Joyce\iTRAQ_30_1.raw]
Data File:Submitted from 20120508-1(merge) by Mascot Daemon on JOYCE-VAIO
Monoisotopic mass of neutral peptide Mr(calc): 1271.805
Variable modifications:
K9 :iTRAQ4plex (K)
Ions Score: 40.19 Expect: 0.035

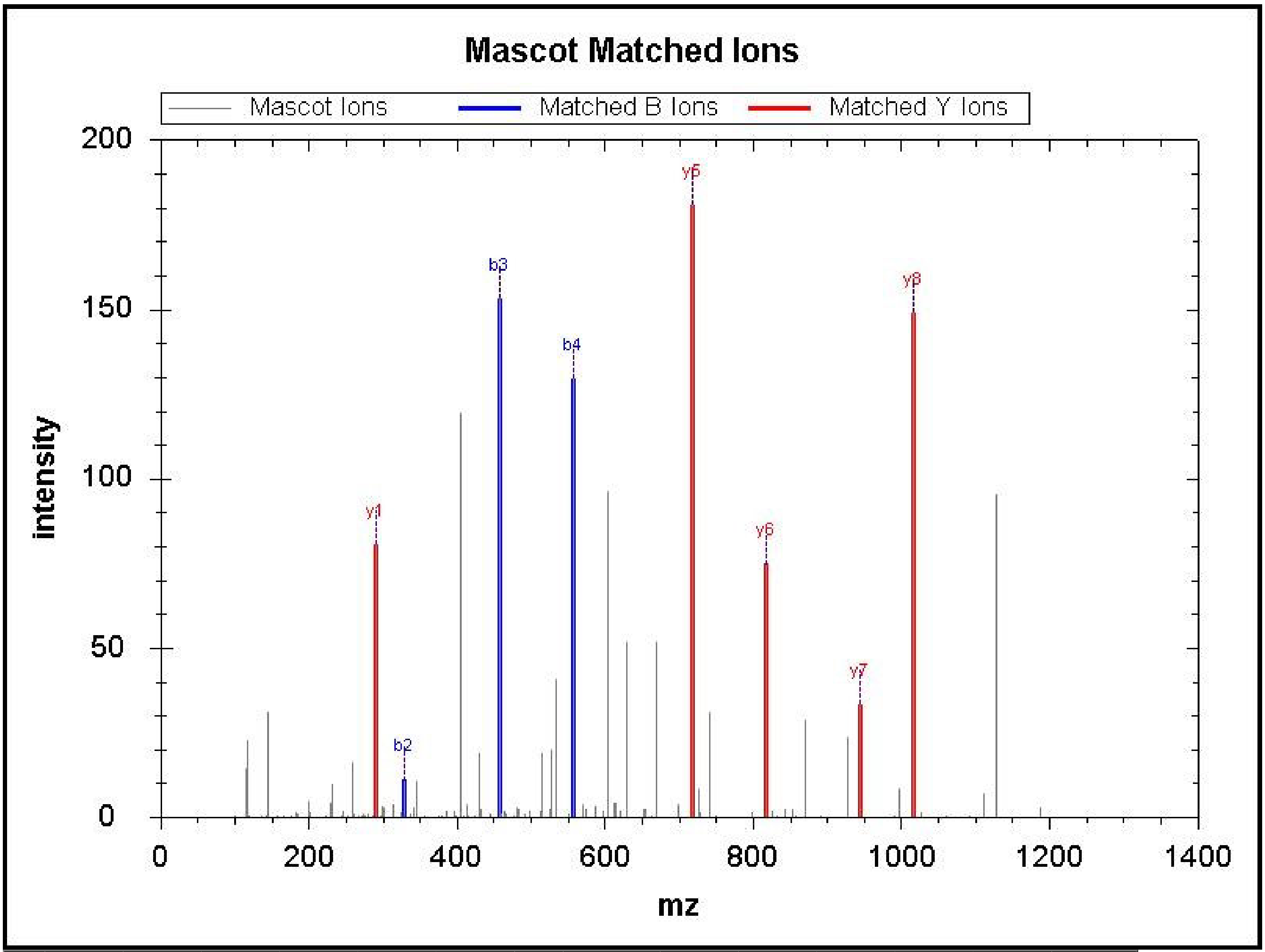

No	b	b++	b*	b*++	b0	b0++	Seq	y	y++	y*	y*++	y0	y0++	RevNo
1	258.19	129.60					I							9
2	329.23	165.12					A	1,015.63	508.32	998.60	499.80	997.62	499.31	8
3	457.33	229.17	440.30	220.65			K	944.59	472.80	927.56	464.29	926.58	463.79	7
4	556.39	278.70	539.37	270.19			V	816.49	408.75	799.47	400.24	798.48	399.75	6
5	685.44	343.22	668.41	334.71	667.43	334.22	E	717.43	359.22	700.40	350.70	699.42	350.21	5
6	786.48	393.75	769.46	385.23	768.47	384.74	T	588.38	294.70	571.36	286.18	570.37	285.69	4
7	885.55	443.28	868.53	434.77	867.54	434.27	V	487.34	244.17	470.31	235.66			3
8	982.61	491.81	965.58	483.29	964.59	482.80	P	388.27	194.64	371.24	186.12			2
9							K	291.21	146.11	274.19	137.60			1

Query 46575 Hit 1

MS/MS Fragmentation of IALLPLLQAETDRR
Found in sp|Q9P0J0|NDUAD_HUMAN, NADH dehydrogenase [ubiquinone] 1 alpha subcomplex subunit 13 OS=Homo sapiens GN=NDUFA13 PE=1 SV=3
Match to Query 46575: 1752.038from(585.0198,3+)
Title: 864: Scan 1934 (rt=52.9713, f=2, i=304) [D:\lab212\membrane\Grace\Joyce\iTRAQ_35_2.raw]
Data File:Submitted from 20120508-1(merge) by Mascot Daemon on JOYCE-VAIO
Monoisotopic mass of neutral peptide Mr(calc): 1752.038
Variable modifications:
Ions Score: 40.15 Expect: 0.039

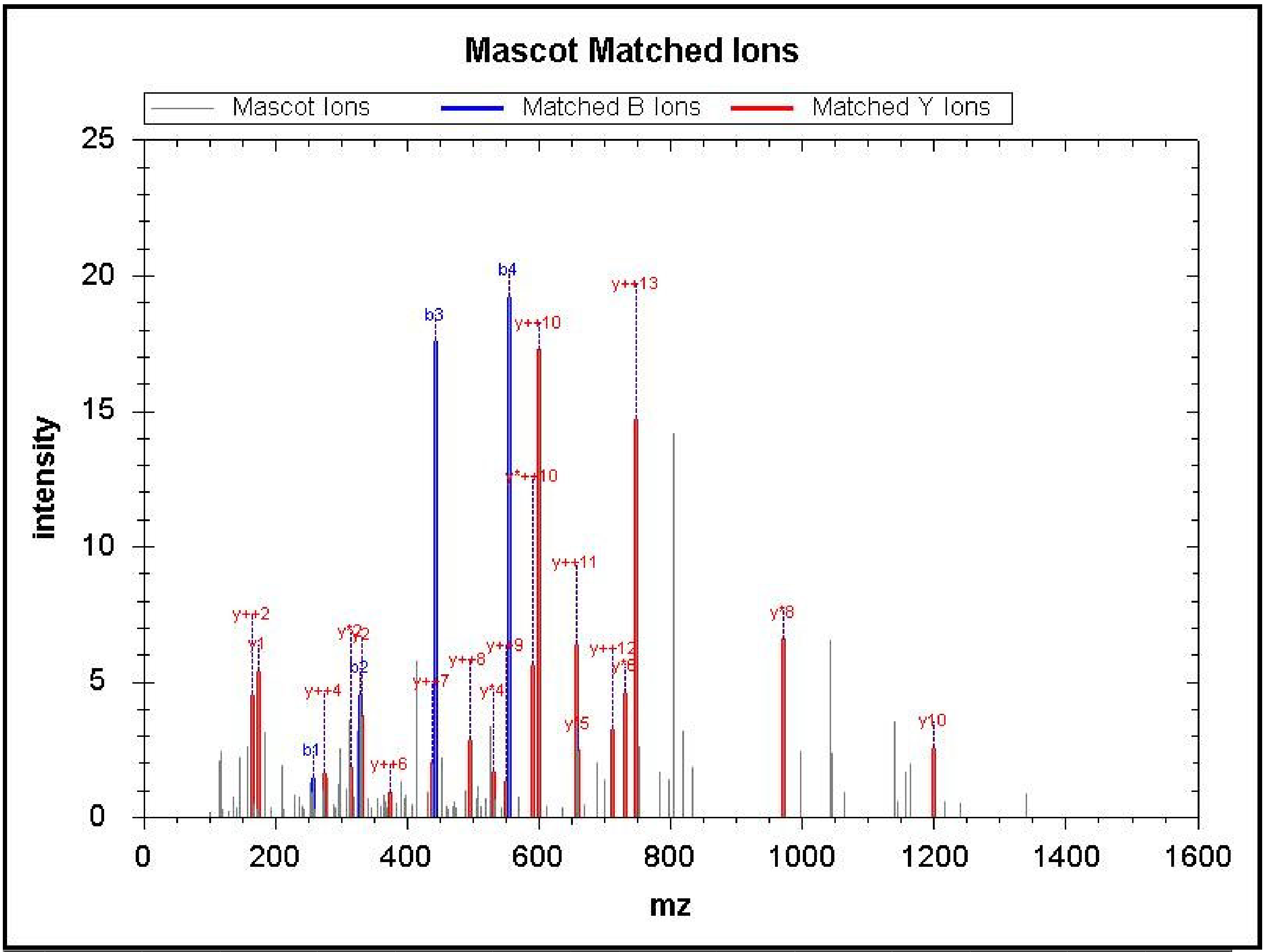

No	b	b++	b*	b*++	b0	b0++	Seq	y	y++	y*	y*++	y0	y0++	RevNo
1	258.19	129.60					I							14
2	329.23	165.12					A	1,495.86	748.43	1,478.83	739.92	1,477.85	739.43	13
3	442.31	221.66					L	1,424.82	712.91	1,407.80	704.40	1,406.81	703.91	12
4	555.40	278.20					L	1,311.74	656.37	1,294.71	647.86	1,293.73	647.37	11
5	652.45	326.73					P	1,198.65	599.83	1,181.63	591.32	1,180.64	590.83	10
6	765.54	383.27					L	1,101.60	551.30	1,084.57	542.79	1,083.59	542.30	9
7	878.62	439.81					L	988.52	494.76	971.49	486.25	970.51	485.76	8
8	1,006.68	503.84	989.65	495.33			Q	875.43	438.22	858.41	429.71	857.42	429.21	7
9	1,077.72	539.36	1,060.69	530.85			A	747.37	374.19	730.35	365.68	729.36	365.19	6
10	1,206.76	603.88	1,189.73	595.37	1,188.75	594.88	E	676.34	338.67	659.31	330.16	658.33	329.67	5
11	1,307.81	654.41	1,290.78	645.89	1,289.79	645.40	T	547.29	274.15	530.27	265.64	529.28	265.15	4
12	1,422.83	711.92	1,405.81	703.41	1,404.82	702.91	D	446.25	223.63	429.22	215.11	428.24	214.62	3
13	1,578.93	789.97	1,561.91	781.46	1,560.92	780.97	R	331.22	166.11	314.19	157.60			2
14							R	175.12	88.06	158.09	79.55			1

Query 43662 Hit 1

MS/MS Fragmentation of IKPSESNVTILGR
Found in sp|O75530|EED_HUMAN, Polycomb protein EED OS=Homo sapiens GN=EED PE=1 SV=2
Match to Query 43662: 1701 from(568.0074,3+)
Title: 163: Sum of 2 scans in range 1079 (rt=31.364, f=4, i=91) to 1080 (rt=31.3894, f=4, i=92)
[D:\lab212\membrane\Grace\Joyce\iTRAQ_43_2.raw]
Data File:Submitted from 20120508-1(merge) by Mascot Daemon on JOYCE-VAIO
Monoisotopic mass of neutral peptide Mr(calc): 1701
Variable modifications:
K2 :iTRAQ4plex (K)

Ions Score: 39.82 Expect: 0.051

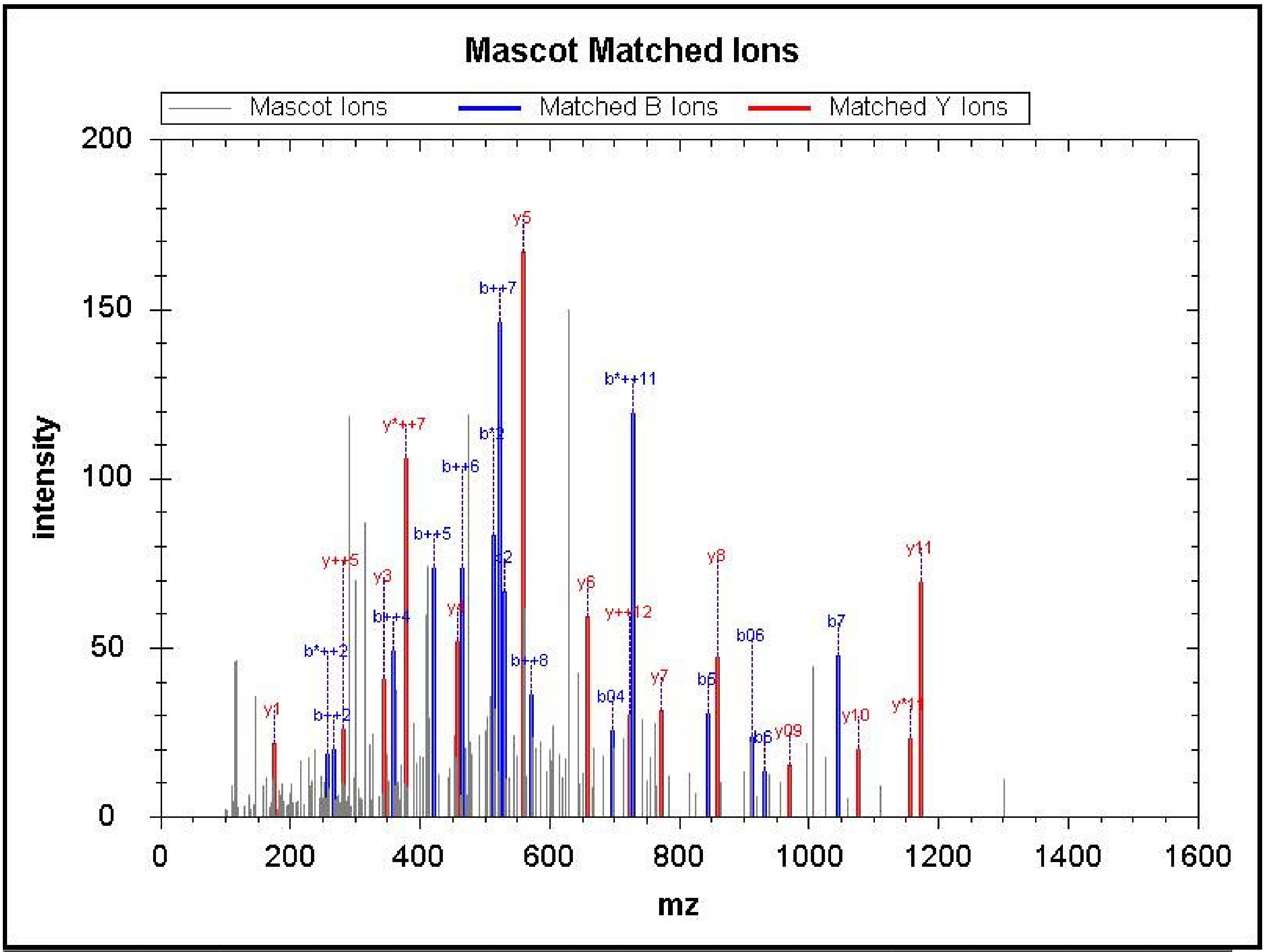

No	b	b++	b*	b*++	b0	b0++	Seq	y	y++	y*	y*++	y0	y0++	RevNo
1	258.19	129.60					I							13
2	530.39	265.70	513.36	257.19			K	1,444.82	722.92	1,427.80	714.40	1,426.81	713.91	12
3	627.44	314.23	610.42	305.71			P	1,172.63	586.82	1,155.60	578.30	1,154.62	577.81	11
4	714.48	357.74	697.45	349.23	696.46	348.74	S	1,075.57	538.29	1,058.55	529.78	1,057.56	529.29	10
5	843.52	422.26	826.49	413.75	825.51	413.26	E	988.54	494.77	971.52	486.26	970.53	485.77	9
6	930.55	465.78	913.52	457.27	912.54	456.77	S	859.50	430.25	842.47	421.74	841.49	421.25	8
7	1,044.59	522.80	1,027.57	514.29	1,026.58	513.79	N	772.47	386.74	755.44	378.22	754.46	377.73	7
8	1,143.66	572.33	1,126.63	563.82	1,125.65	563.33	V	658.42	329.72	641.40	321.20	640.41	320.71	6
9	1,244.71	622.86	1,227.68	614.34	1,226.70	613.85	T	559.36	280.18	542.33	271.67	541.35	271.18	5
10	1,357.79	679.40	1,340.77	670.89	1,339.78	670.39	I	458.31	229.66	441.28	221.14			4
11	1,470.88	735.94	1,453.85	727.43	1,452.87	726.94	L	345.22	173.12	328.20	164.60			3
12	1,527.90	764.45	1,510.87	755.94	1,509.89	755.45	G	232.14	116.57	215.11	108.06			2
13							R	175.12	88.06	158.09	79.55			1

Query 68450 Hit 1

MS/MS Fragmentation of VFPNQVLKPFLEDSK
Found in sp|Q7Z7H8|RM10_HUMAN, 39S ribosomal protein L10
Match to Query 68450: 2192.262from(731.7613,3+)
Title: 847: Sum of 2 scans in range 1847 (rt=51.3867, f=4, i=578) to 1848 (rt=51.4121, f=4, i=579)
[D:\lab212\membrane\Grace\Joyce\iTRAQ_42_2.raw]
Data File:Submitted from 20120508-1(merge) by Mascot Daemon on JOYCE-VAIO
Monoisotopic mass of neutral peptide Mr(calc): 2192.262
Variable modifications:
K8 :iTRAQ4plex (K)

K15 :iTRAQ4plex (K)
Ions Score: 39.32 Expect: 0.047

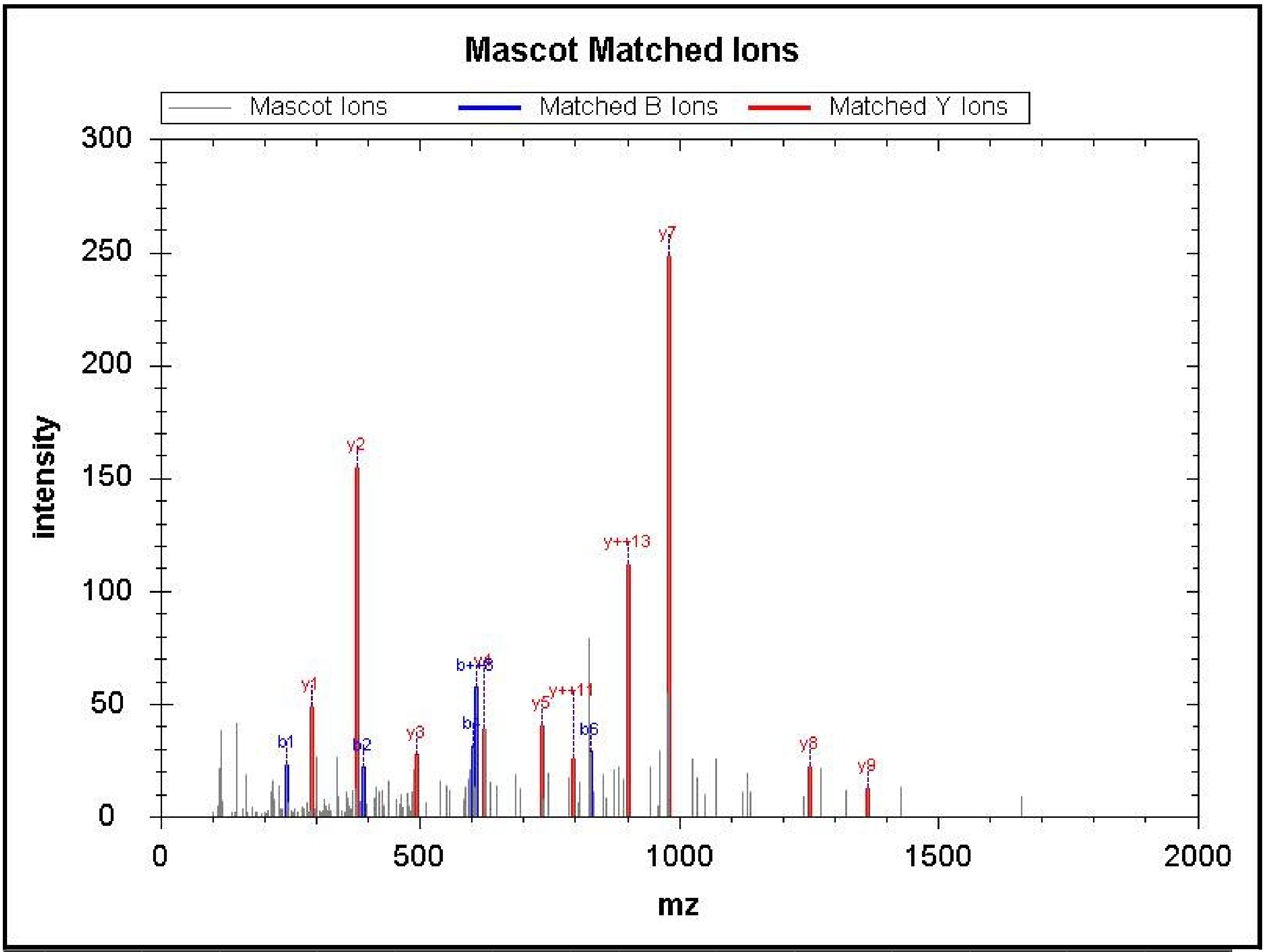

No	b	b++	b*	b*++	b0	b0++	Seq	y	y++	y*	y*++	y0	y0++	RevNo
1	244.18	122.59					V							15
2	391.25	196.13					F	1,950.09	975.55	1,933.07	967.04	1,932.08	966.55	14
3	488.30	244.65					P	1,803.03	902.02	1,786.00	893.50	1,785.01	893.01	13
4	602.34	301.67	585.32	293.16			N	1,705.97	853.49	1,688.95	844.98	1,687.96	844.48	12
5	730.40	365.70	713.37	357.19			Q	1,591.93	796.47	1,574.90	787.96	1,573.92	787.46	11
6	829.47	415.24	812.44	406.72			V	1,463.87	732.44	1,446.84	723.93	1,445.86	723.43	10
7	942.55	471.78	925.53	463.27			L	1,364.80	682.91	1,347.78	674.39	1,346.79	673.90	9
8	1,214.75	607.88	1,197.72	599.37			K	1,251.72	626.36	1,234.69	617.85	1,233.71	617.36	8
9	1,311.80	656.41	1,294.78	647.89			P	979.52	490.26	962.50	481.75	961.51	481.26	7
10	1,458.87	729.94	1,441.84	721.43			F	882.47	441.74	865.44	433.22	864.46	432.73	6
11	1,571.96	786.48	1,554.93	777.97			L	735.40	368.20	718.37	359.69	717.39	359.20	5
12	1,701.00	851.00	1,683.97	842.49	1,682.99	842.00	E	622.32	311.66	605.29	303.15	604.31	302.66	4
13	1,816.02	908.52	1,799.00	900.00	1,798.01	899.51	D	493.27	247.14	476.25	238.63	475.26	238.14	3
14	1,903.06	952.03	1,886.03	943.52	1,885.05	943.03	S	378.25	189.63	361.22	181.11	360.24	180.62	2
15							K	291.21	146.11	274.19	137.60			1

Query 3508 Hit 1

MS/MS Fragmentation of NLIVIR
Found in sp|Q9UDX3|S14L4_HUMAN, SEC14-like protein 4 OS=Homo sapiens GN=SEC14L4 PE=2 SV=1
Match to Query 3508: 871.5637from(436.7891,2+)
Title: 265: Sum of 2 scans in range 1328 (rt=35.8605, f=4, i=163) to 1329 (rt=35.8859, f=4, i=164)
[D:\lab212\membrane\Grace\Joyce\iTRAQ_25_1.raw]

Data File:Submitted from 20120508-1(merge) by Mascot Daemon on JOYCE-VAIO
Monoisotopic mass of neutral peptide Mr(calc): 871.5637
Variable modifications:
N1 :Deamidated (NQ)
Ions Score: 39.18 Expect: 0.046

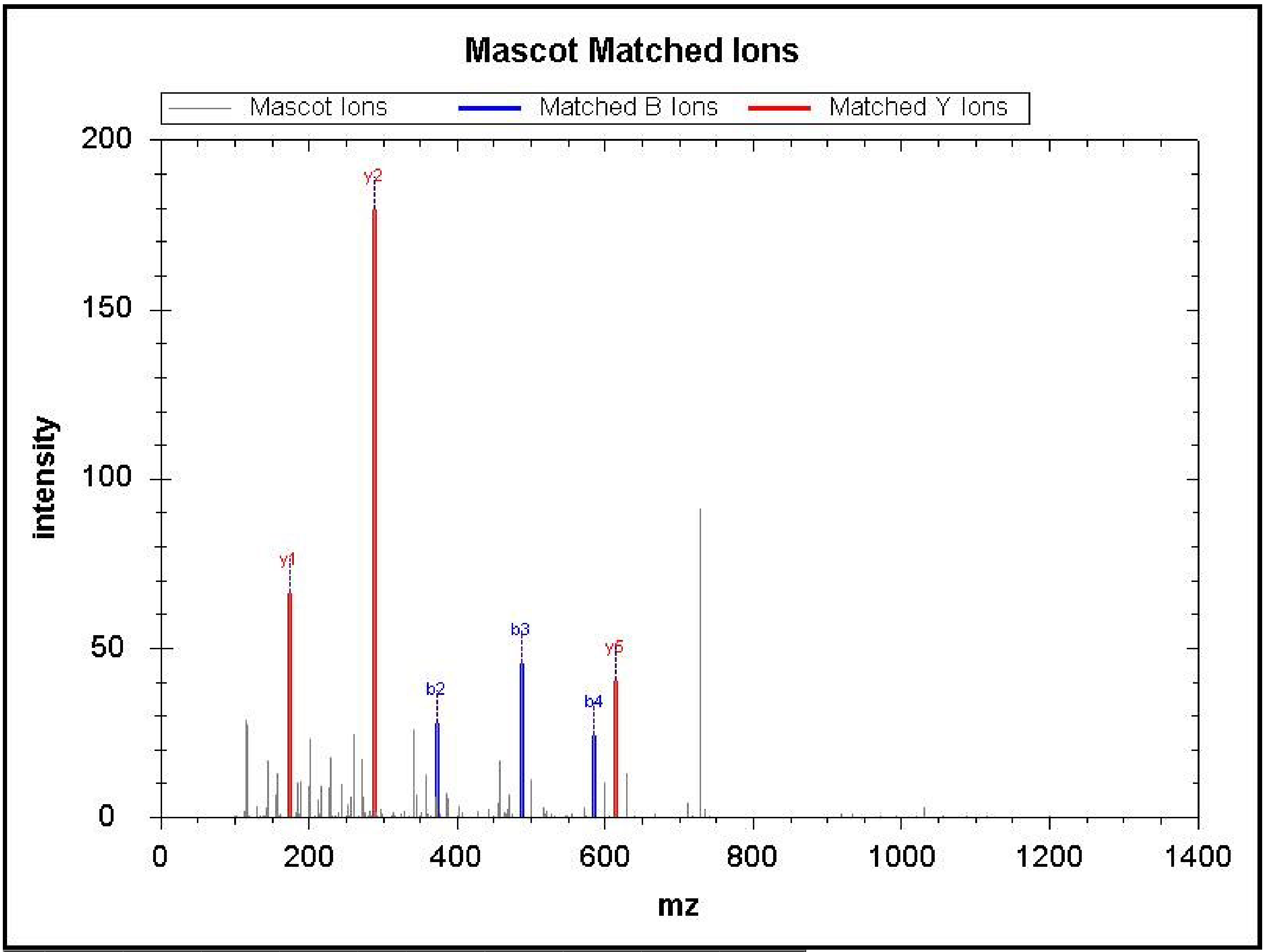

No	b	b++	b*	b*++	Seq	y	y++	y*	y*++	RevNo
1	260.14	130.57	243.11	122.06	N					6
2	373.22	187.11	356.19	178.60	L	613.44	307.22	596.41	298.71	5
3	486.30	243.66	469.28	235.14	I	500.36	250.68	483.33	242.17	4
4	585.37	293.19	568.35	284.68	V	387.27	194.14	370.24	185.63	3
5	698.46	349.73	681.43	341.22	I	288.20	144.61	271.18	136.09	2
6					R	175.12	88.06	158.09	79.55	1

Query 5225 Hit 1

MS/MS Fragmentation of DAALLEAAR
Found in sp|Q9H078|CLPB_HUMAN, Caseinolytic peptidase B protein homolog OS=Homo sapiens GN=CLPB PE=1 SV=1
Match to Query 5225: 928.5833 from(465.2989,2+)
Title: 409: Scan 997 (rt=31.8038, f=2, i=163) [D:\lab212\membrane\Grace\Joyce\iTRAQ_25_2.raw]
Data File:Submitted from 20120508-1(merge) by Mascot Daemon on JOYCE-VAIO
Monoisotopic mass of neutral peptide Mr(calc): 928.5833
Variable modifications:
Ions Score: 39.12 Expect: 0.051

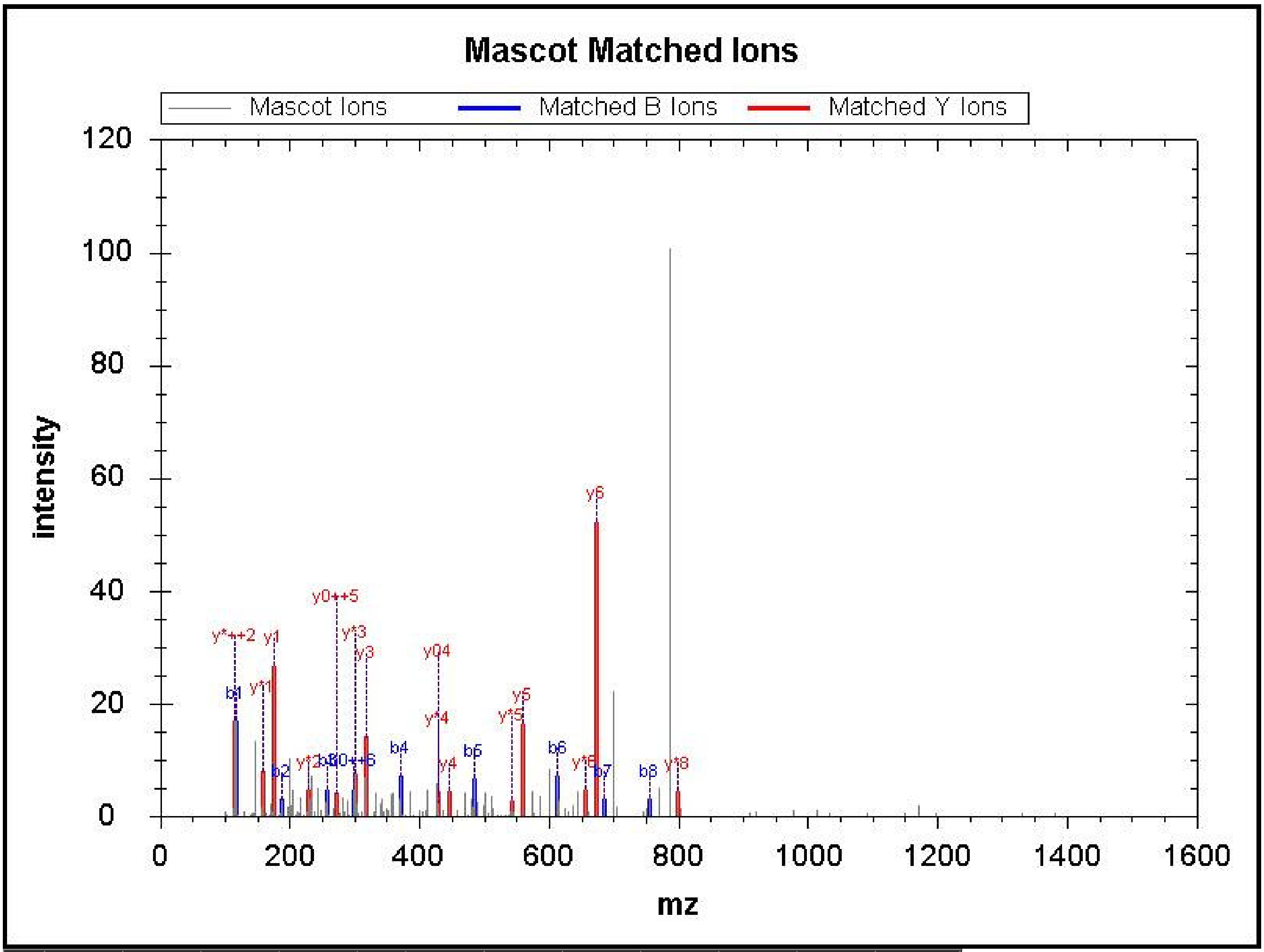

No	b	b++	b0	b0++	Seq	y	y++	y*	y*++	y0	y0++	RevNo
1	116.03	58.52	98.02	49.52	D							9
2	187.07	94.04	169.06	85.03	A	814.48	407.74	797.45	399.23	796.47	398.74	8
3	258.11	129.56	240.10	120.55	A	743.44	372.22	726.41	363.71	725.43	363.22	7
4	371.19	186.10	353.18	177.09	L	672.40	336.71	655.38	328.19	654.39	327.70	6
5	484.28	242.64	466.27	233.64	L	559.32	280.16	542.29	271.65	541.31	271.16	5
6	613.32	307.16	595.31	298.16	E	446.24	223.62	429.21	215.11	428.23	214.62	4
7	684.36	342.68	666.35	333.68	A	317.19	159.10	300.17	150.59			3
8	755.39	378.20	737.38	369.20	A	246.16	123.58	229.13	115.07			2
9					R	175.12	88.06	158.09	79.55			1

Query 45777 Hit 1

MS/MS Fragmentation of ILQLLAETFHHK
Found in sp|Q9NNW5|WDR6_HUMAN, WD repeat-containing protein 6 OS=Homo sapiens GN=WDR6 PE=1 SV=1
Match to Query 45777: 1737.022from(580.0147,3+)
Title: 1016: Scan 2177 (rt=58.7163, f=2, i=350) [D:\lab212\membrane\Grace\Joyce\iTRAQ_50_1.raw]
Data File:Submitted from 20120508-1(merge) by Mascot Daemon on JOYCE-VAIO
Monoisotopic mass of neutral peptide Mr(calc): 1737.022
Variable modifications:
K12 :iTRAQ4plex (K)
Ions Score: 39.11 Expect: 0.056

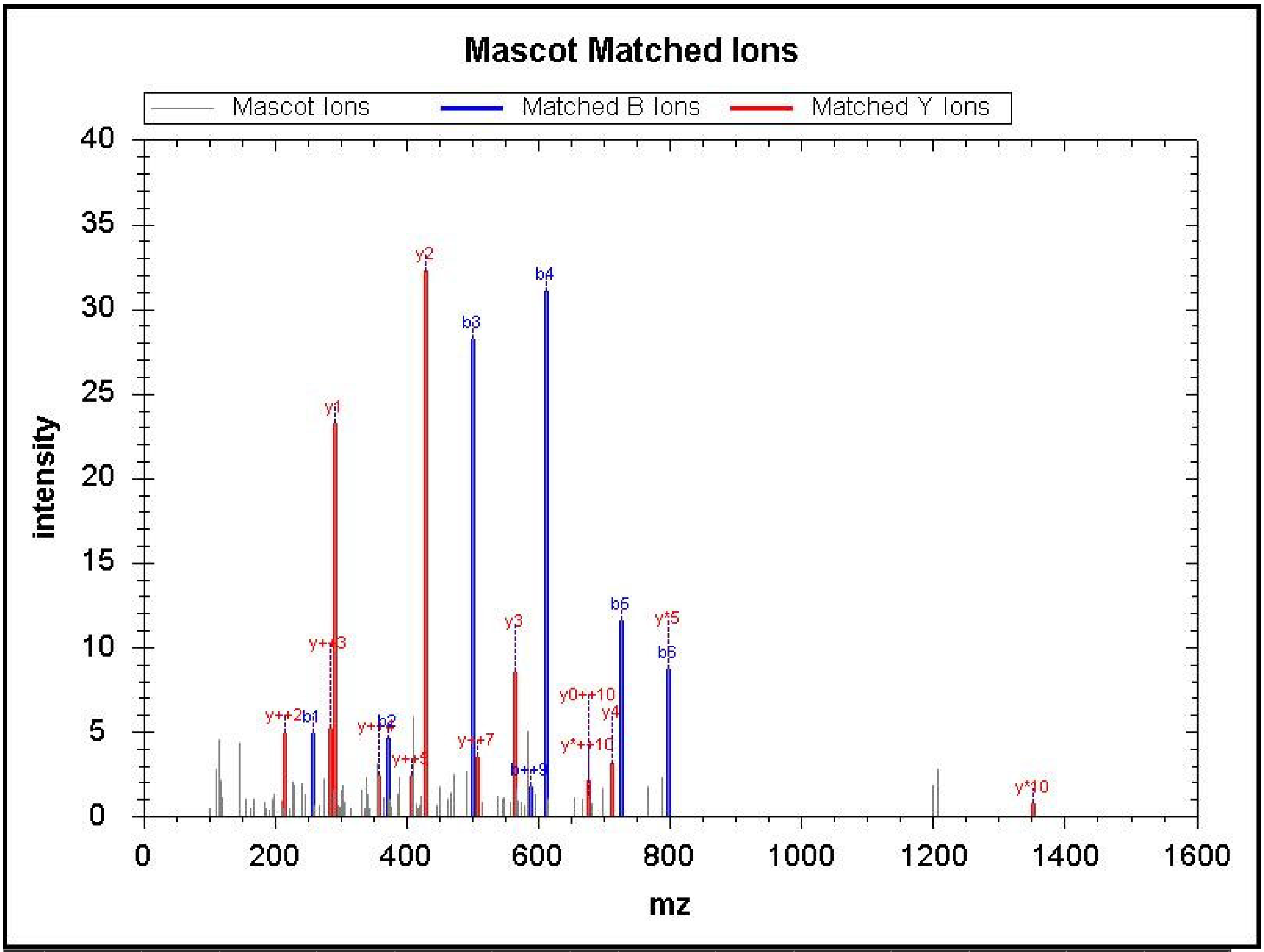

No	b	b++	b*	b*++	b0	b0++	Seq	y	y++	y*	y*++	y0	y0++	RevNo
1	258.19	129.60					I							12
2	371.28	186.14					L	1,480.84	740.92	1,463.81	732.41	1,462.83	731.92	11
3	499.34	250.17	482.31	241.66			Q	1,367.76	684.38	1,350.73	675.87	1,349.74	675.38	10
4	612.42	306.71	595.39	298.20			L	1,239.70	620.35	1,222.67	611.84	1,221.69	611.35	9
5	725.50	363.26	708.48	354.74			L	1,126.61	563.81	1,109.59	555.30	1,108.60	554.80	8
6	796.54	398.77	779.51	390.26			A	1,013.53	507.27	996.50	498.75	995.52	498.26	7
7	925.58	463.30	908.56	454.78	907.57	454.29	E	942.49	471.75	925.46	463.24	924.48	462.74	6
8	1,026.63	513.82	1,009.61	505.31	1,008.62	504.81	T	813.45	407.23	796.42	398.71	795.44	398.22	5
9	1,173.70	587.35	1,156.67	578.84	1,155.69	578.35	F	712.40	356.70	695.37	348.19			4
10	1,310.76	655.88	1,293.73	647.37	1,292.75	646.88	H	565.33	283.17	548.31	274.66			3
11	1,447.82	724.41	1,430.79	715.90	1,429.81	715.41	H	428.27	214.64	411.25	206.13			2
12							K	291.21	146.11	274.19	137.60			1

Query 3400 Hit 1

MS/MS Fragmentation of GSLSSFK
Found in sp|Q8BRE0|RD3_MOUSE, Protein RD3 OS=Mus musculus GN=Rd3 PE=2 SV=1
Match to Query 3400: 868.5229from(435.2687,2+)
Title: 279: Sum of 2 scans in range 707 (rt=25.3047, f=4, i=185) to 708 (rt=25.3301, f=4, i=186)
[D:\lab212\membrane\Grace\Joyce\iTRAQ_35_1.raw]
Data File:Submitted from 20120508-1(merge) by Mascot Daemon on JOYCE-VAIO
Monoisotopic mass of neutral peptide Mr(calc): 868.5229
Variable modifications:
K7 :iTRAQ4plex (K)
Ions Score: 38.97 Expect: 0.043

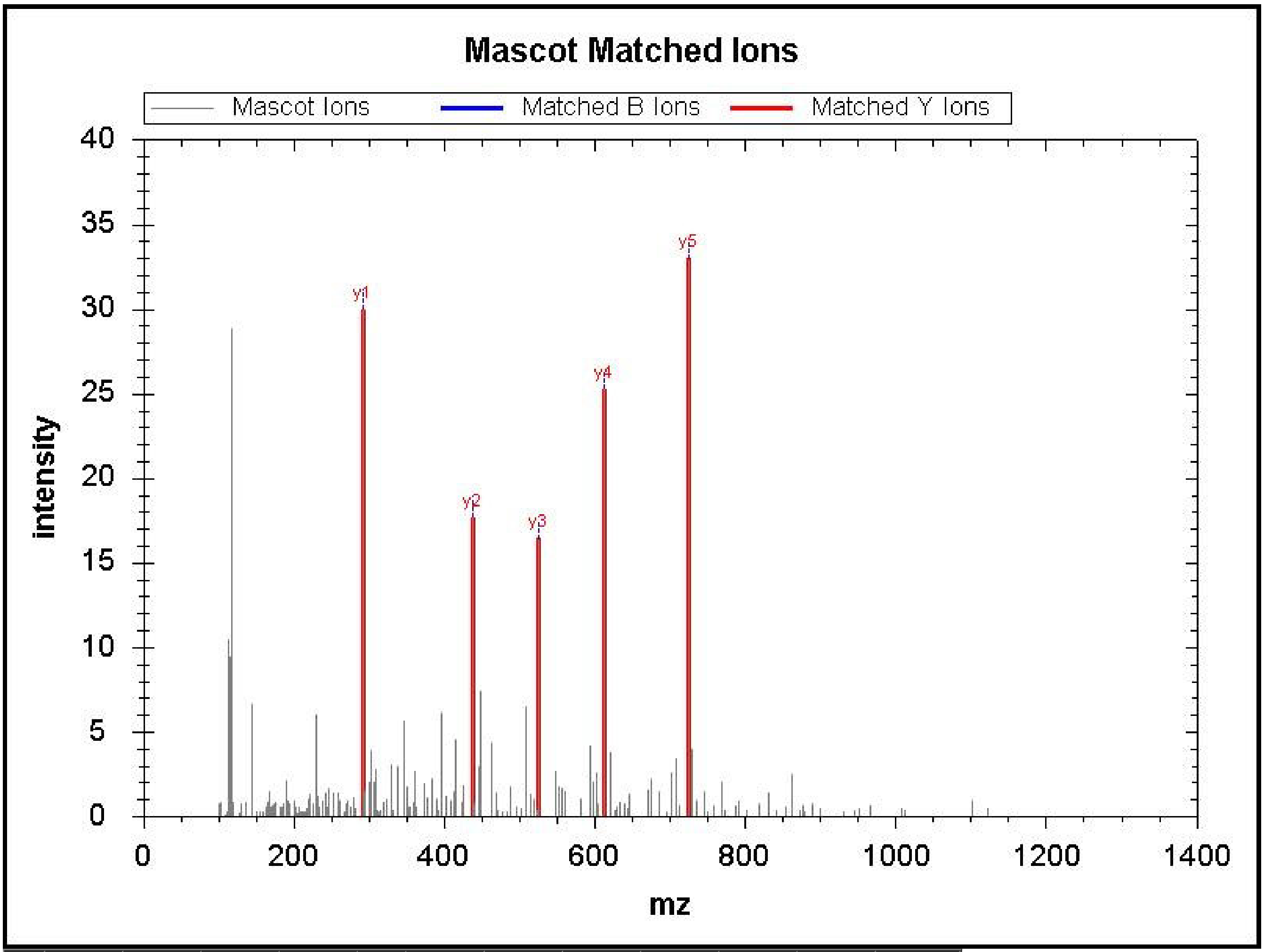

No	b	b++	b0	b0++	Seq	y	y++	y*	y*++	y0	y0++	RevNo
1	58.03	29.52			G							7
2	145.06	73.03	127.05	64.03	S	812.46	406.74	795.44	398.22	794.45	397.73	6
3	258.14	129.58	240.13	120.57	L	725.43	363.22	708.40	354.71	707.42	354.21	5
4	345.18	173.09	327.17	164.09	S	612.35	306.68	595.32	298.16	594.34	297.67	4
5	432.21	216.61	414.20	207.60	S	525.32	263.16	508.29	254.65	507.30	254.16	3
6	579.28	290.14	561.27	281.14	F	438.28	219.65	421.26	211.13			2
7					K	291.21	146.11	274.19	137.60			1

Query 42507 Hit 1

MS/MS Fragmentation of DTILAELLQIHK
Found in sp|P46100|ATRX_HUMAN, Transcriptional regulator ATRX OS=Homo sapiens GN=ATRX PE=1 SV=5
Match to Query 42507: 1681.004from(561.342,3+)
Title: 959: Sum of 2 scans in range 2106 (rt=56.8984, f=4, i=637) to 2107 (rt=56.9239, f=4, i=638) [D:\lab212\membrane\Grace\Joyce\iTRAQ_37_2.raw]
Data File:Submitted from 20120508-1(merge) by Mascot Daemon on JOYCE-VAIO
Monoisotopic mass of neutral peptide Mr(calc): 1681.004
Variable modifications:
K12 :iTRAQ4plex (K)
Ions Score: 38.95 Expect: 0.048

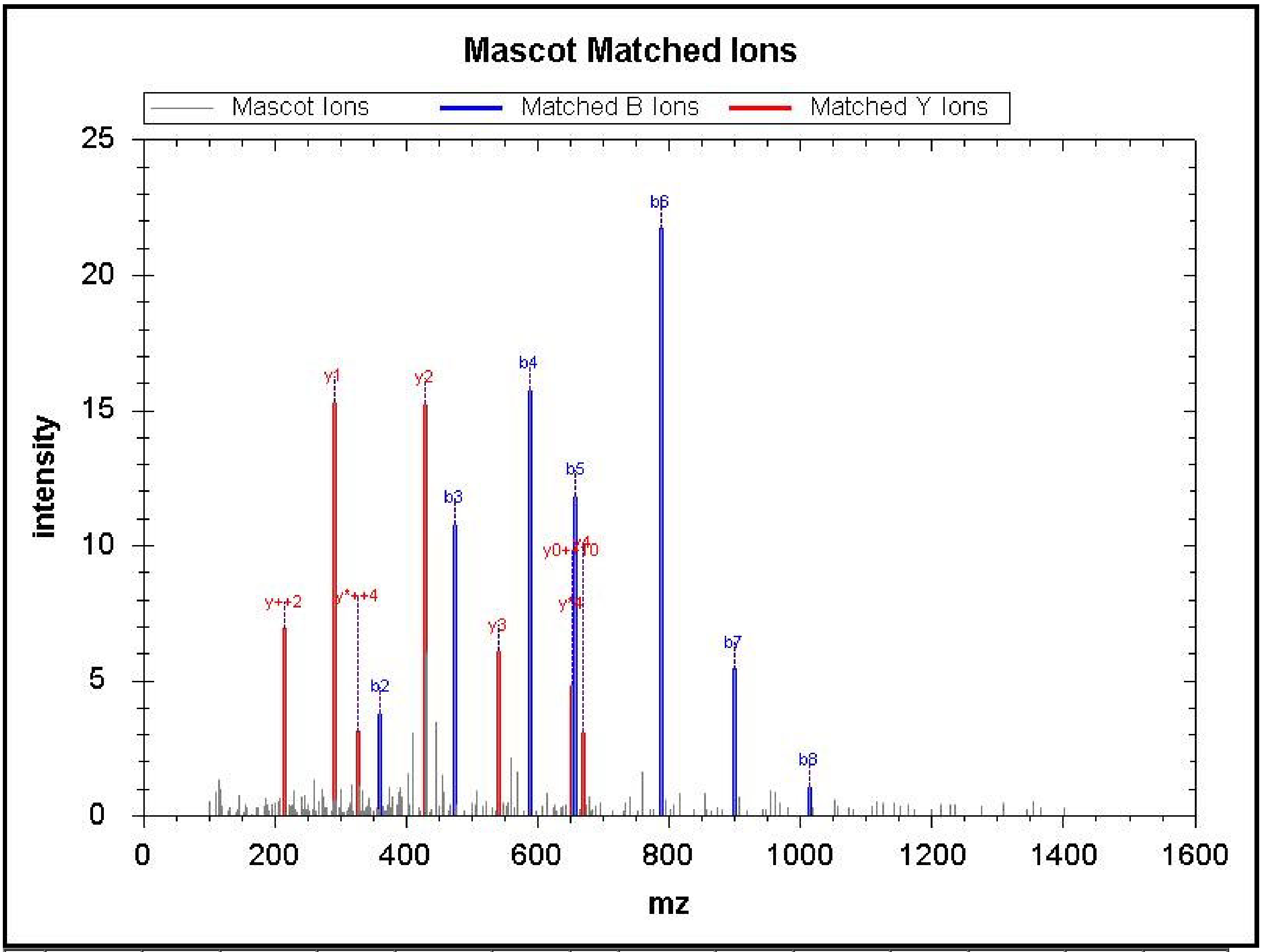

No	b	b++	b*	b*++	b0	b0++	Seq	y	y++	y*	y*++	y0	y0++	RevNo
1	260.14	130.57			242.13	121.57	D							12
2	361.18	181.10			343.17	172.09	T	1,422.88	711.94	1,405.85	703.43	1,404.87	702.94	11
3	474.27	237.64			456.26	228.63	I	1,321.83	661.42	1,304.81	652.91	1,303.82	652.41	10
4	587.35	294.18			569.34	285.17	L	1,208.75	604.88	1,191.72	596.36	1,190.74	595.87	9
5	658.39	329.70			640.38	320.69	A	1,095.66	548.34	1,078.64	539.82	1,077.65	539.33	8
6	787.43	394.22			769.42	385.21	E	1,024.63	512.82	1,007.60	504.30	1,006.62	503.81	7
7	900.52	450.76			882.51	441.76	L	895.58	448.30	878.56	439.78			6
8	1,013.60	507.30			995.59	498.30	L	782.50	391.75	765.47	383.24			5
9	1,141.66	571.33	1,124.63	562.82	1,123.65	562.33	Q	669.42	335.21	652.39	326.70			4
10	1,254.74	627.87	1,237.72	619.36	1,236.73	618.87	I	541.36	271.18	524.33	262.67			3
11	1,391.80	696.40	1,374.78	687.89	1,373.79	687.40	H	428.27	214.64	411.25	206.13			2
12							K	291.21	146.11	274.19	137.60			1

Query 44665 Hit 1

MS/MS Fragmentation of EALLLLEDIKK
Found in sp|O15091|MRRP3_HUMAN, Mitochondrial ribonuclease P protein 3 OS=Homo sapiens GN=KIAA0391 PE=1 SV=2
Match to Query 44665: 1716.078from(573.0331,3+)
Title: 827: Scan 1843 (rt=51.11, f=3, i=286) [D:\lab212\membrane\Grace\Joyce\iTRAQ_44_1.raw]
Data File:Submitted from 20120508-1(merge) by Mascot Daemon on JOYCE-VAIO
Monoisotopic mass of neutral peptide Mr(calc): 1716.078
Variable modifications:
K10 :iTRAQ4plex (K)
K11 :iTRAQ4plex (K)
Ions Score: 38.85 Expect: 0.025

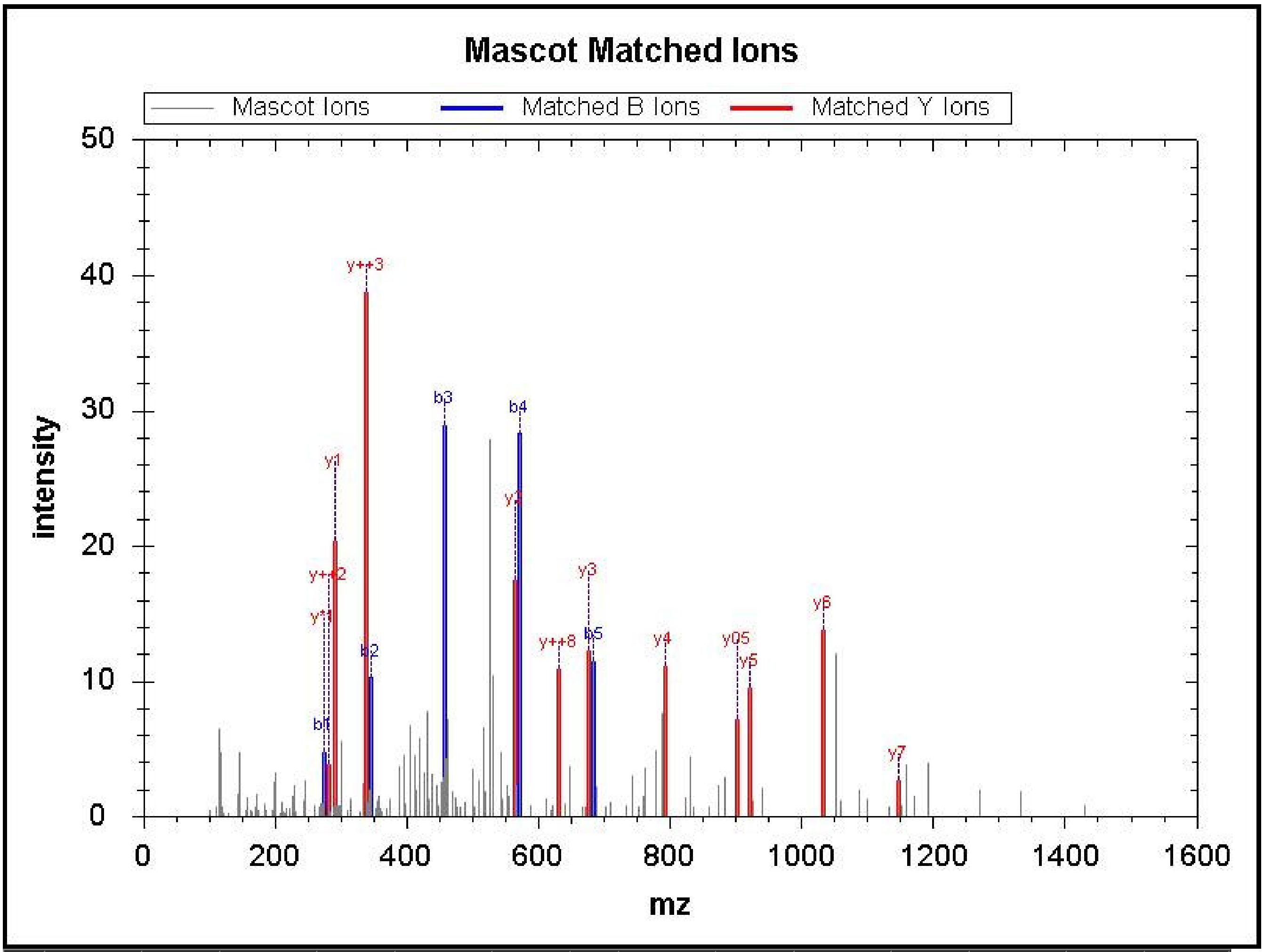

No	b	b++	b*	b*++	b0	b0++	Seq	y	y++	y*	y*++	y0	y0++	RevNo
1	274.15	137.58			256.14	128.57	E							11
2	345.19	173.10			327.18	164.09	A	1,443.94	722.47	1,426.91	713.96	1,425.93	713.47	10
3	458.27	229.64			440.26	220.63	L	1,372.90	686.95	1,355.88	678.44	1,354.89	677.95	9
4	571.36	286.18			553.35	277.18	L	1,259.82	630.41	1,242.79	621.90	1,241.81	621.41	8
5	684.44	342.72			666.43	333.72	L	1,146.73	573.87	1,129.71	565.36	1,128.72	564.87	7
6	797.53	399.27			779.51	390.26	L	1,033.65	517.33	1,016.62	508.82	1,015.64	508.32	6
7	926.57	463.79			908.56	454.78	E	920.57	460.79	903.54	452.27	902.55	451.78	5
8	1,041.59	521.30			1,023.58	512.30	D	791.52	396.27	774.50	387.75	773.51	387.26	4
9	1,154.68	577.84			1,136.67	568.84	I	676.50	338.75	659.47	330.24			3
10	1,426.88	713.94	1,409.85	705.43	1,408.87	704.94	K	563.41	282.21	546.39	273.70			2
11							K	291.21	146.11	274.19	137.60			1

Query 11522 Hit 1

MS/MS Fragmentation of LSSILFK
Found in sp|O60879|DIAP2_HUMAN, Protein diaphanous homolog 2 OS=Homo sapiens GN=DIAPH2 PE=1 SV=1
Match to Query 11522: 1094.69from(548.3524,2+)
Title: 695: Sum of 2 scans in range 1581 (rt=44.974, f=4, i=465) to 1582 (rt=44.9995, f=4, i=466)
[D:\lab212\membrane\Grace\Joyce\iTRAQ_31_2.raw]
Data File:Submitted from 20120508-1(merge) by Mascot Daemon on JOYCE-VAIO
Monoisotopic mass of neutral peptide Mr(calc): 1094.69
Variable modifications:
K7 :iTRAQ4plex (K)
Ions Score: 38.27 Expect: 0.053

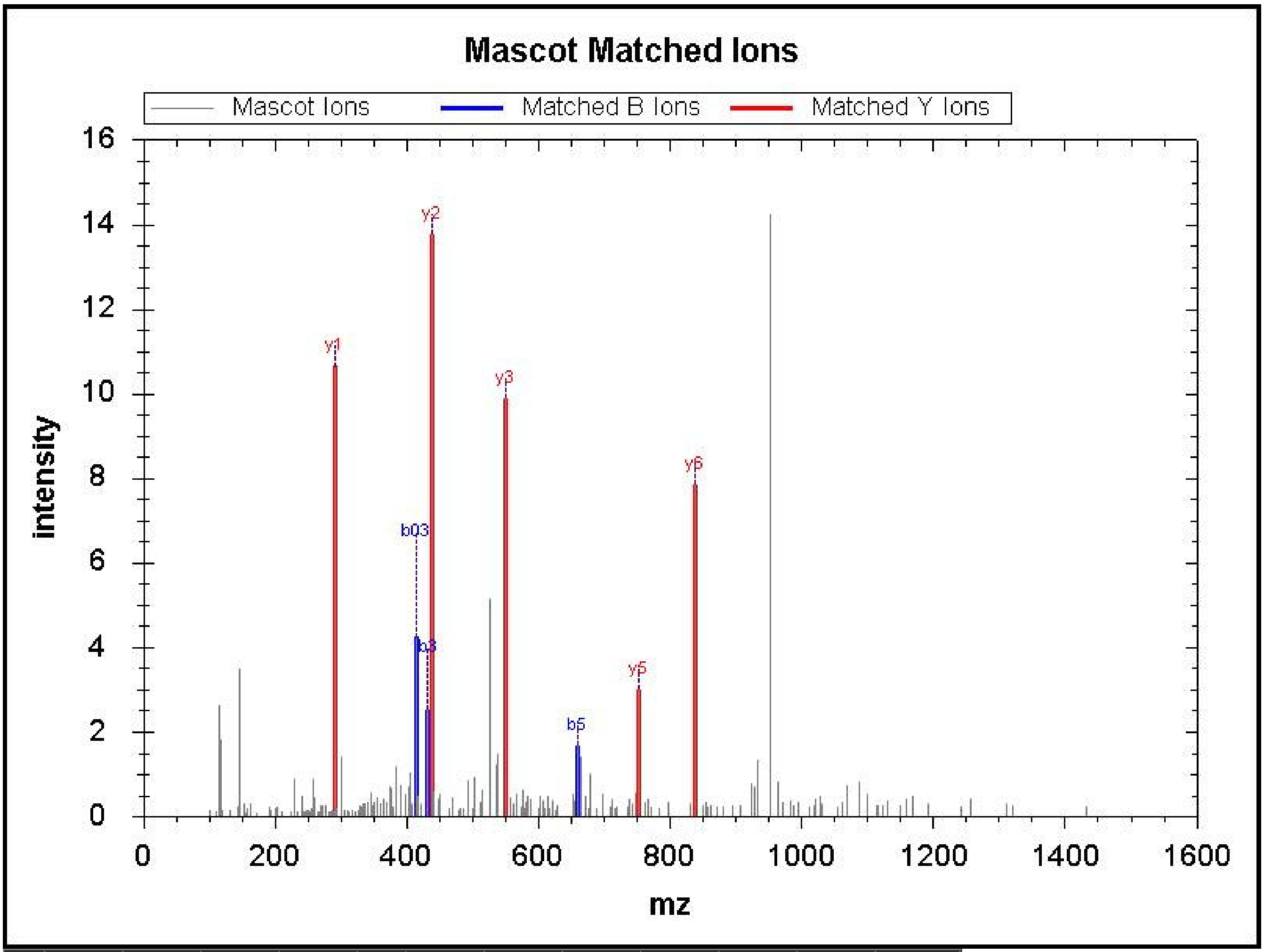

No	b	b++	b0	b0++	Seq	y	y++	y*	y*++	y0	y0++	RevNo
1	258.19	129.60			L							7
2	345.23	173.12	327.21	164.11	S	838.52	419.76	821.49	411.25	820.50	410.76	6
3	432.26	216.63	414.25	207.63	S	751.48	376.25	734.46	367.73	733.47	367.24	5
4	545.34	273.17	527.33	264.17	I	664.45	332.73	647.42	324.22			4
5	658.43	329.72	640.41	320.71	L	551.37	276.19	534.34	267.67			3
6	805.49	403.25	787.48	394.25	F	438.28	219.65	421.26	211.13			2
7					K	291.21	146.11	274.19	137.60			1

Query 48021 Hit 1

MS/MS Fragmentation of ENAEQGEVDMESHR
Found in sp|P14209|CD99_HUMAN, CD99 antigen OS=Homo sapiens GN=CD99 PE=1 SV=1
Match to Query 48021: 1773.777from(592.2662,3+)
Title: 110: Scan 359 (rt=17.4153, f=3, i=41) [D:\lab212\membrane\Grace\Joyce\iTRAQ_38_1.raw]
Data File:Submitted from 20120508-1(merge) by Mascot Daemon on JOYCE-VAIO
Monoisotopic mass of neutral peptide Mr(calc): 1773.777
Variable modifications:
Ions Score: 38.11 Expect: 0.059

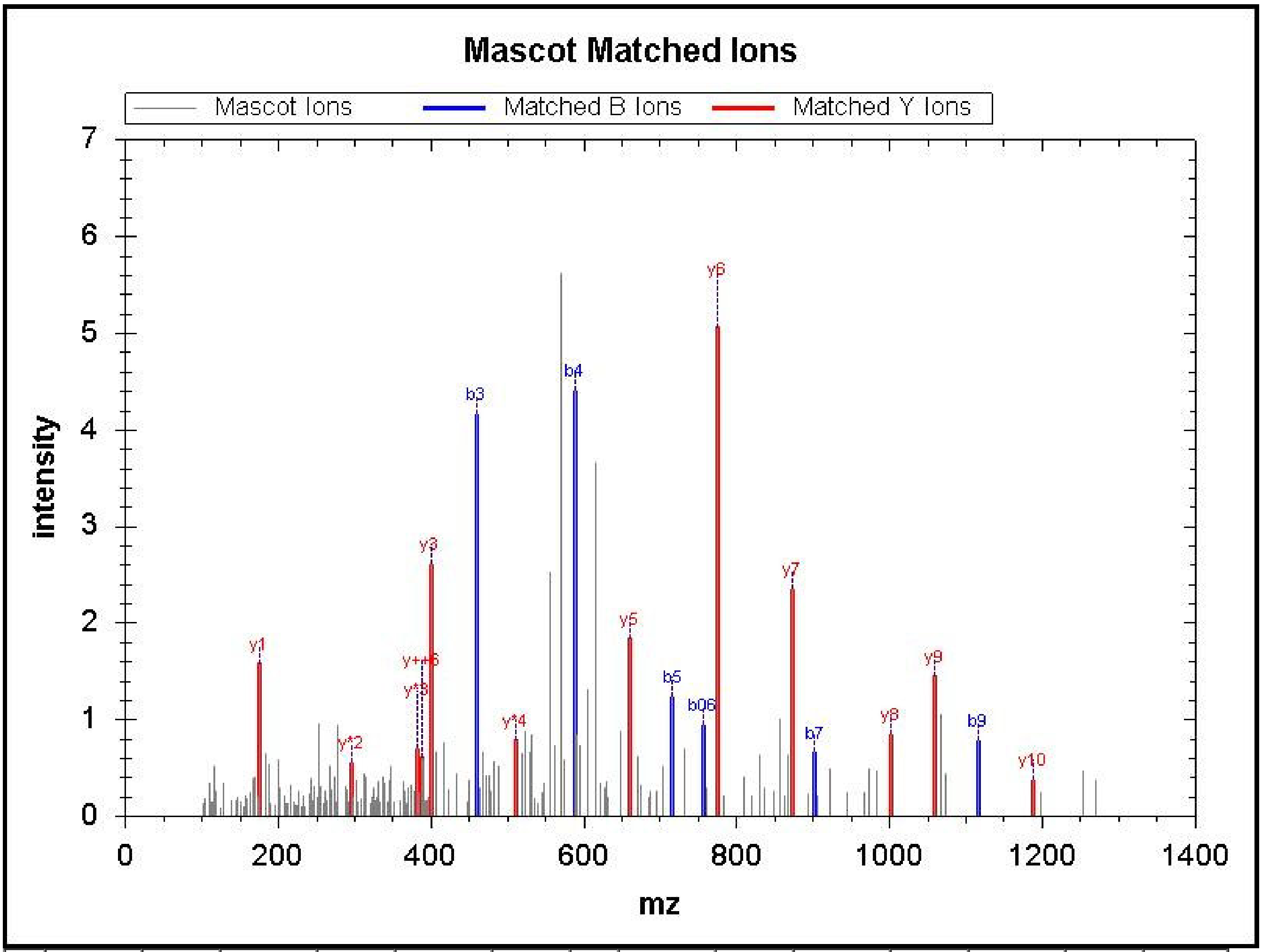

No	b	b++	b*	b*++	b0	b0++	Seq	y	y++	y*	y*++	y0	y0++	RevNo
1	274.15	137.58			256.14	128.57	E							14
2	388.19	194.60	371.17	186.09	370.18	185.60	N	1,501.63	751.32	1,484.61	742.81	1,483.62	742.32	13
3	459.23	230.12	442.21	221.61	441.22	221.11	A	1,387.59	694.30	1,370.56	685.79	1,369.58	685.29	12
4	588.27	294.64	571.25	286.13	570.26	285.64	E	1,316.55	658.78	1,299.53	650.27	1,298.54	649.78	11
5	716.33	358.67	699.31	350.16	698.32	349.66	Q	1,187.51	594.26	1,170.48	585.75	1,169.50	585.25	10
6	773.35	387.18	756.33	378.67	755.34	378.18	G	1,059.45	530.23	1,042.43	521.72	1,041.44	521.22	9
7	902.40	451.70	885.37	443.19	884.39	442.70	E	1,002.43	501.72	985.40	493.21	984.42	492.71	8
8	1,001.47	501.24	984.44	492.72	983.46	492.23	V	873.39	437.20	856.36	428.68	855.38	428.19	7
9	1,116.49	558.75	1,099.47	550.24	1,098.48	549.74	D	774.32	387.66	757.29	379.15	756.31	378.66	6
10	1,247.53	624.27	1,230.51	615.76	1,229.52	615.26	M	659.29	330.15	642.27	321.64	641.28	321.14	5
11	1,376.58	688.79	1,359.55	680.28	1,358.57	679.79	E	528.25	264.63	511.23	256.12	510.24	255.62	4
12	1,463.61	732.31	1,446.58	723.79	1,445.60	723.30	S	399.21	200.11	382.18	191.60	381.20	191.10	3
13	1,600.67	800.84	1,583.64	792.32	1,582.66	791.83	H	312.18	156.59	295.15	148.08			2
14							R	175.12	88.06	158.09	79.55			1

Query 76285 Hit 1

MS/MS Fragmentation of LVHLQTLVLNGNPLLHAQLR
Found in sp|Q13045|FLII_HUMAN, Protein flightless-1 homolog OS=Homo sapiens GN=FLII PE=1 SV=2
Match to Query 76285: 2393.408from(599.3594,4+)
Title: 883: Sum of 2 scans in range 1924 (rt=53.084, f=4, i=596) to 1925 (rt=53.1094, f=4, i=597)
[D:\lab212\membrane\Grace\Joyce\iTRAQ_43_1.raw]
Data File:Submitted from 20120508-1(merge) by Mascot Daemon on JOYCE-VAIO
Monoisotopic mass of neutral peptide Mr(calc): 2393.408
Variable modifications:
N10 :Deamidated (NQ)

Ions Score: 37.21 Expect: 0.039

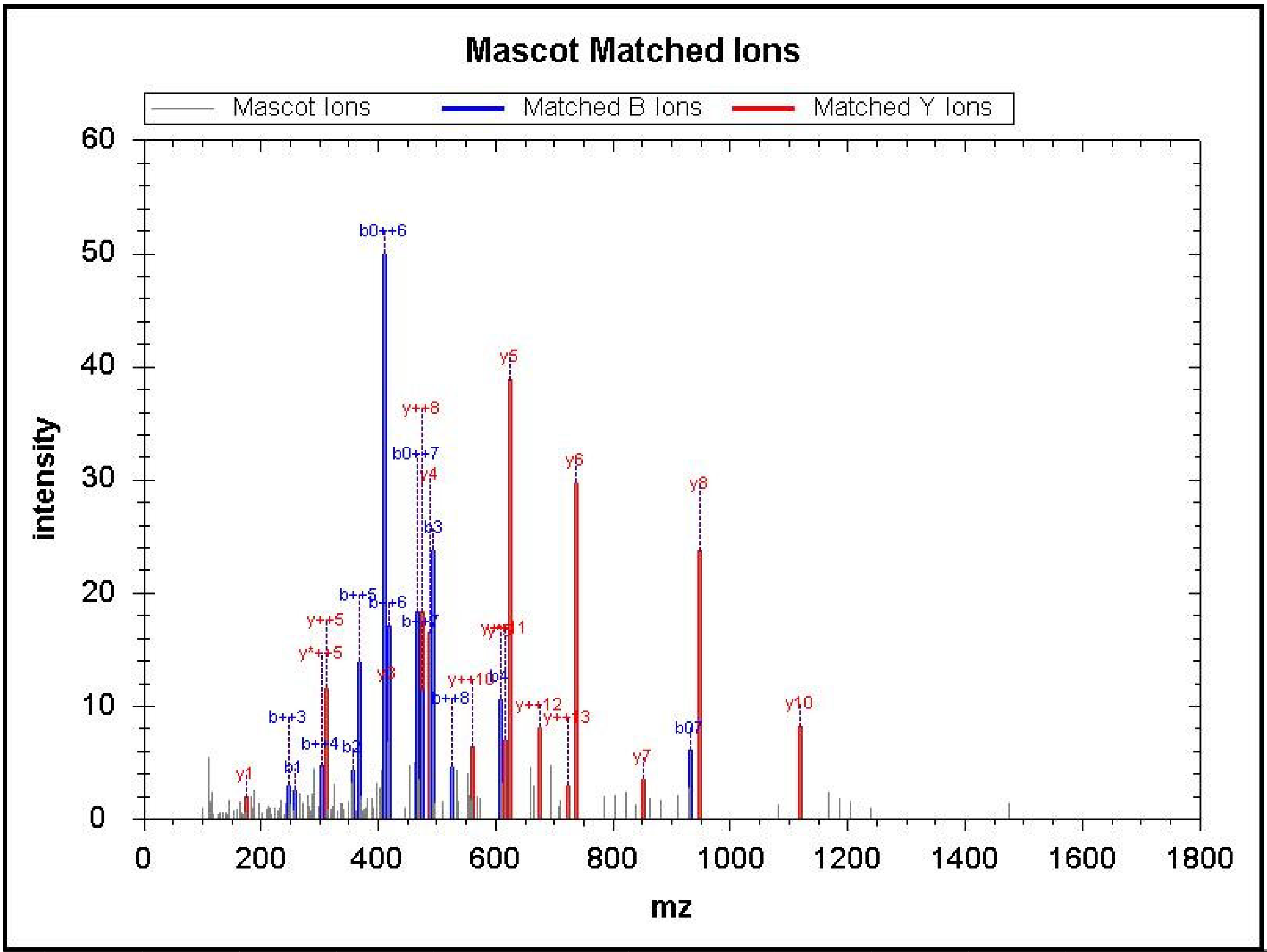

No	b	b++	b*	b*++	b0	b0++	Seq	y	y++	y*	y*++	y0	y0++	RevNo
1	258.19	129.60					L							20
2	357.26	179.13					V	2,137.22	1,069.12	2,120.20	1,060.60	2,119.21	1,060.11	19
3	494.32	247.66					H	2,038.16	1,019.58	2,021.13	1,011.07	2,020.15	1,010.58	18
4	607.40	304.21					L	1,901.10	951.05	1,884.07	942.54	1,883.09	942.05	17
5	735.46	368.24	718.44	359.72			Q	1,788.01	894.51	1,770.99	886.00	1,770.00	885.50	16
6	836.51	418.76	819.48	410.25	818.50	409.75	T	1,659.95	830.48	1,642.93	821.97	1,641.94	821.48	15
7	949.60	475.30	932.57	466.79	931.58	466.30	L	1,558.91	779.96	1,541.88	771.44			14
8	1,048.66	524.84	1,031.64	516.32	1,030.65	515.83	V	1,445.82	723.41	1,428.80	714.90			13
9	1,161.75	581.38	1,144.72	572.86	1,143.74	572.37	L	1,346.75	673.88	1,329.73	665.37			12
10	1,276.77	638.89	1,259.75	630.38	1,258.76	629.89	N	1,233.67	617.34	1,216.64	608.83			11
11	1,333.80	667.40	1,316.77	658.89	1,315.79	658.40	G	1,118.64	559.83	1,101.62	551.31			10
12	1,447.84	724.42	1,430.81	715.91	1,429.83	715.42	N	1,061.62	531.31	1,044.59	522.80			9
13	1,544.89	772.95	1,527.87	764.44	1,526.88	763.94	P	947.58	474.29	930.55	465.78			8
14	1,657.98	829.49	1,640.95	820.98	1,639.97	820.49	L	850.53	425.77	833.50	417.25			7
15	1,771.06	886.03	1,754.03	877.52	1,753.05	877.03	L	737.44	369.22	720.42	360.71			6
16	1,908.12	954.56	1,891.09	946.05	1,890.11	945.56	H	624.36	312.68	607.33	304.17			5
17	1,979.16	990.08	1,962.13	981.57	1,961.15	981.08	A	487.30	244.15	470.27	235.64			4
18	2,107.21	1,054.11	2,090.19	1,045.60	2,089.20	1,045.11	Q	416.26	208.63	399.24	200.12			3
19	2,220.30	1,110.65	2,203.27	1,102.14	2,202.29	1,101.65	L	288.20	144.61	271.18	136.09			2
20							R	175.12	88.06	158.09	79.55			1

Query 66216 Hit 1

MS/MS Fragmentation of KLDTSGFSSILVTLTK
Found in sp|Q969F9|HPS3_HUMAN, Hermansky-Pudlak syndrome 3 protein OS=Homo sapiens GN=HPS3 PE=1 SV=1
Match to Query 66216: 2141.271 from(714.7643,3+)
Title: 964: Scan 2122 (rt=57.3968, f=3, i=331) [D:\lab212\membrane\Grace\Joyce\iTRAQ_44_1.raw]
Data File:Submitted from 20120508-1(merge) by Mascot Daemon on JOYCE-VAIO
Monoisotopic mass of neutral peptide Mr(calc): 2141.271
Variable modifications:
K1 :iTRAQ4plex (K)
K16 :iTRAQ4plex (K)
Ions Score: 36.59 Expect: 0.052

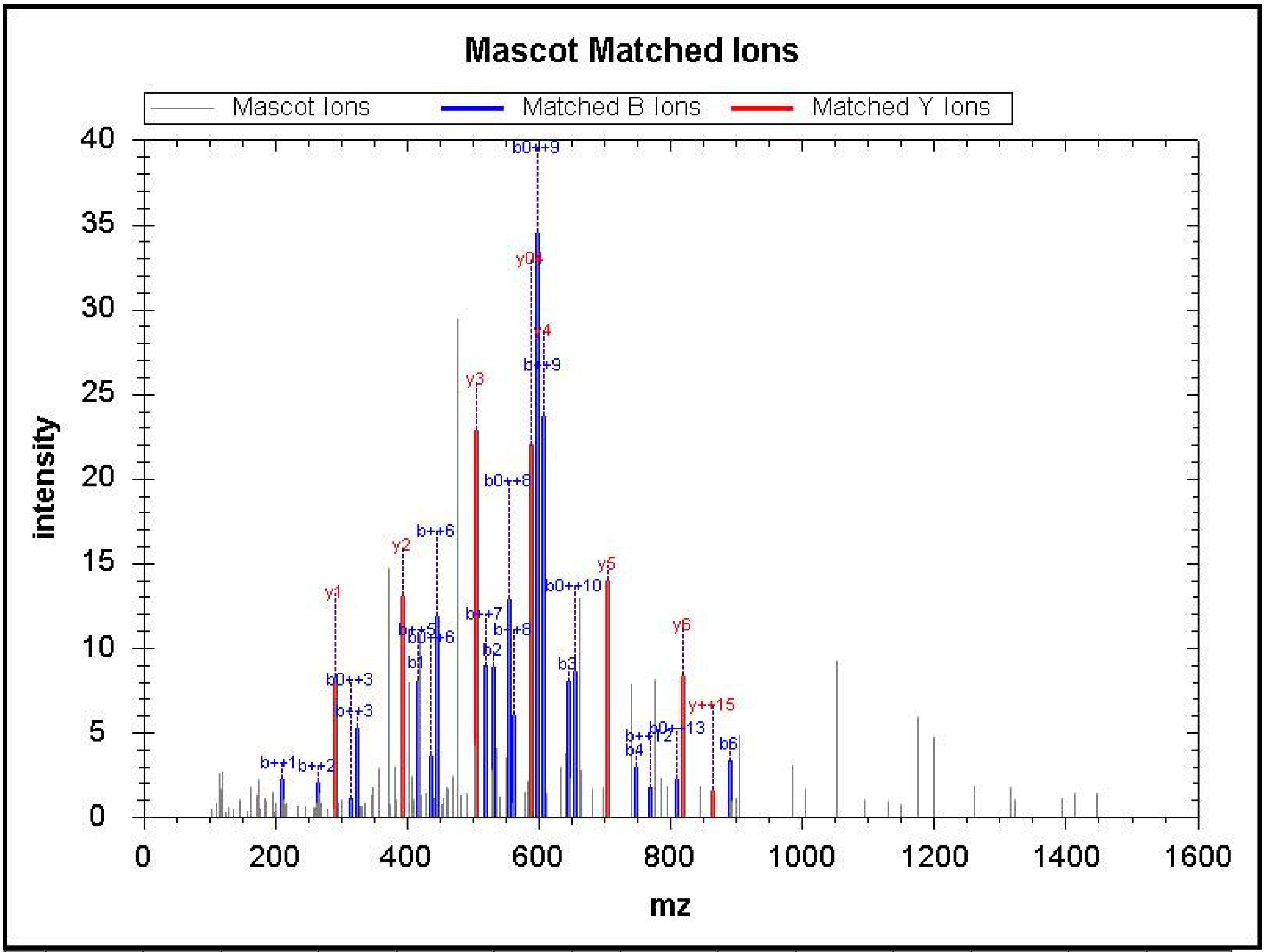

No	b	b++	b*	b*++	b0	b0++	Seq	y	y++	y*	y*++	y0	y0++	RevNo
1	417.31	209.16	400.28	200.64			K							16
2	530.39	265.70	513.36	257.19			L	1,725.98	863.49	1,708.95	854.98	1,707.96	854.49	15
3	645.42	323.21	628.39	314.70	627.41	314.21	D	1,612.89	806.95	1,595.86	798.44	1,594.88	797.94	14
4	746.46	373.74	729.44	365.22	728.45	364.73	T	1,497.86	749.44	1,480.84	740.92	1,479.85	740.43	13
5	833.50	417.25	816.47	408.74	815.49	408.25	S	1,396.82	698.91	1,379.79	690.40	1,378.81	689.91	12
6	890.52	445.76	873.49	437.25	872.51	436.76	G	1,309.78	655.40	1,292.76	646.88	1,291.77	646.39	11
7	1,037.59	519.30	1,020.56	510.78	1,019.58	510.29	F	1,252.76	626.89	1,235.74	618.37	1,234.75	617.88	10
8	1,124.62	562.81	1,107.59	554.30	1,106.61	553.81	S	1,105.69	553.35	1,088.67	544.84	1,087.68	544.35	9
9	1,211.65	606.33	1,194.62	597.82	1,193.64	597.32	S	1,018.66	509.84	1,001.64	501.32	1,000.65	500.83	8
10	1,324.73	662.87	1,307.71	654.36	1,306.72	653.87	I	931.63	466.32	914.60	457.81	913.62	457.31	7
11	1,437.82	719.41	1,420.79	710.90	1,419.81	710.41	L	818.55	409.78	801.52	401.26	800.54	400.77	6
12	1,536.89	768.95	1,519.86	760.43	1,518.88	759.94	V	705.46	353.23	688.44	344.72	687.45	344.23	5
13	1,637.94	819.47	1,620.91	810.96	1,619.92	810.47	T	606.39	303.70	589.37	295.19	588.38	294.70	4
14	1,751.02	876.01	1,733.99	867.50	1,733.01	867.01	L	505.35	253.18	488.32	244.66	487.34	244.17	3
15	1,852.07	926.54	1,835.04	918.02	1,834.06	917.53	T	392.26	196.63	375.24	188.12	374.25	187.63	2

16							K	291.21	146.11	274.19	137.60		1

Query 28812 Hit 1

MS/MS Fragmentation of LLVPILLPEK
Found in sp|O75352|MPU1_HUMAN, Mannose-P-dolichol utilization defect 1 protein OS=Homo sapiens GN=MPDU1 PE=1 SV=2
Match to Query 28812: 1421.95from(711.9821,2+)
Title: 940: Sum of 2 scans in range 2115 (rt=56.9781, f=4, i=633) to 2116 (rt=57.0035, f=4, i=634)
[D:\lab212\membrane\Grace\Joyce\iTRAQ_27_1.raw]
Data File:Submitted from 20120508-1(merge) by Mascot Daemon on JOYCE-VAIO
Monoisotopic mass of neutral peptide Mr(calc): 1421.95
Variable modifications:
K10 :iTRAQ4plex (K)
Ions Score: 36.26 Expect: 0.021

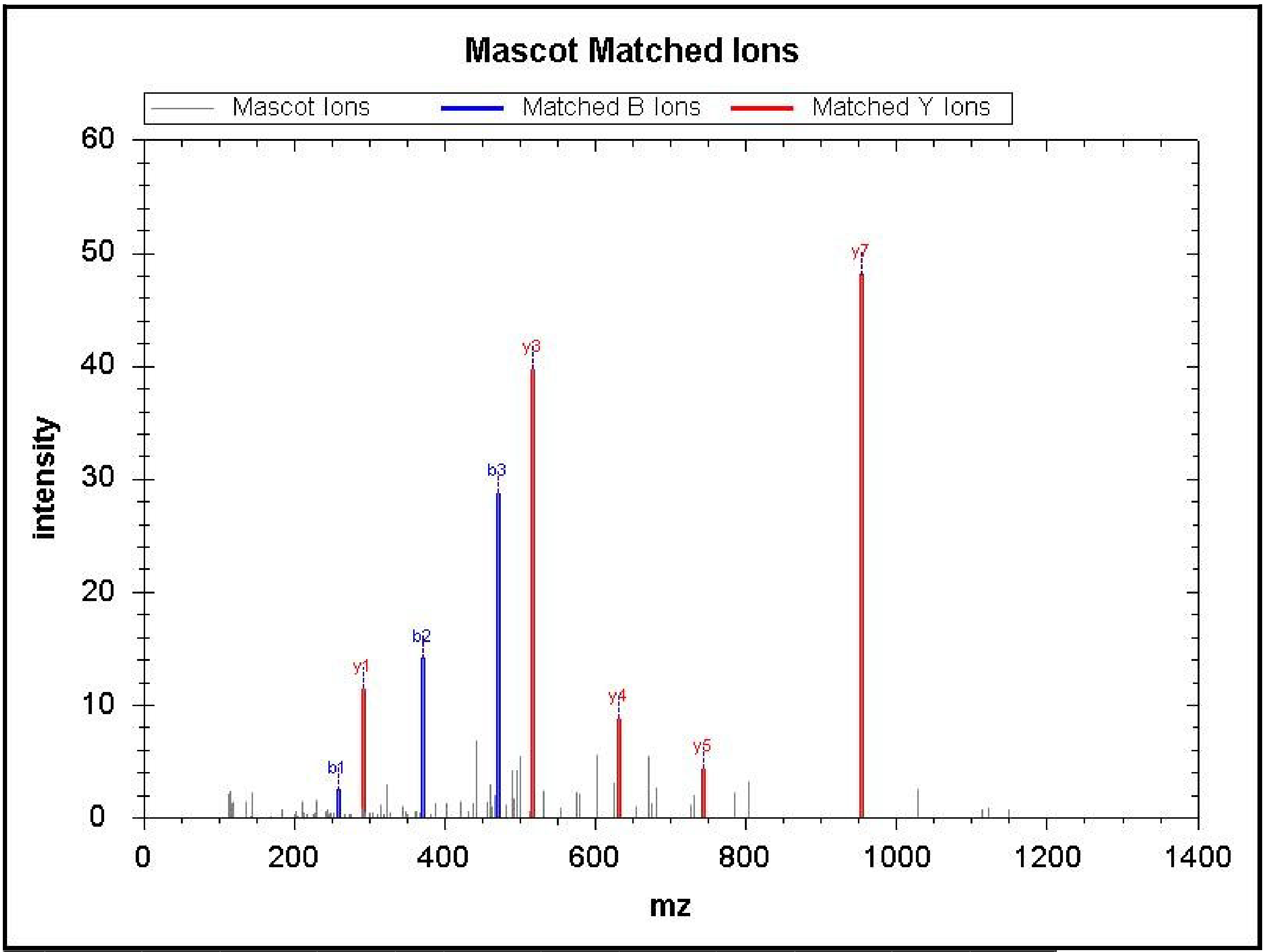

No	b	b++	b0	b0++	Seq	y	y++	y*	y*++	y0	y0++	RevNo
1	258.19	129.60			L							10
2	371.28	186.14			L	1,165.77	583.39	1,148.74	574.87	1,147.76	574.38	9
3	470.35	235.68			V	1,052.68	526.85	1,035.66	518.33	1,034.67	517.84	8
4	567.40	284.20			P	953.62	477.31	936.59	468.80	935.60	468.31	7
5	680.48	340.74			I	856.56	428.78	839.54	420.27	838.55	419.78	6
6	793.57	397.29			L	743.48	372.24	726.45	363.73	725.47	363.24	5
7	906.65	453.83			L	630.39	315.70	613.37	307.19	612.38	306.70	4
8	1,003.70	502.36			P	517.31	259.16	500.28	250.65	499.30	250.15	3
9	1,132.75	566.88	1,114.74	557.87	E	420.26	210.63	403.23	202.12	402.25	201.63	2
10					K	291.21	146.11	274.19	137.60			1

Query 3927 Hit 1

MS/MS Fragmentation of NEIKLLR
Found in sp|P38935|SMBP2_HUMAN, DNA-binding protein SMUBP-2 OS=Homo sapiens GN=IGHMBP2 PE=1 SV=3
Match to Query 3927: 884.6009from(443.3077,2+)
Title: 589: Scan 1363 (rt=40.0265, f=2, i=206) [D:\lab212\membrane\Grace\Joyce\iTRAQ_27_1.raw]
Data File:Submitted from 20120508-1(merge) by Mascot Daemon on JOYCE-VAIO
Monoisotopic mass of neutral peptide Mr(calc): 884.6009
Variable modifications:
Ions Score: 36.17 Expect: 0.050

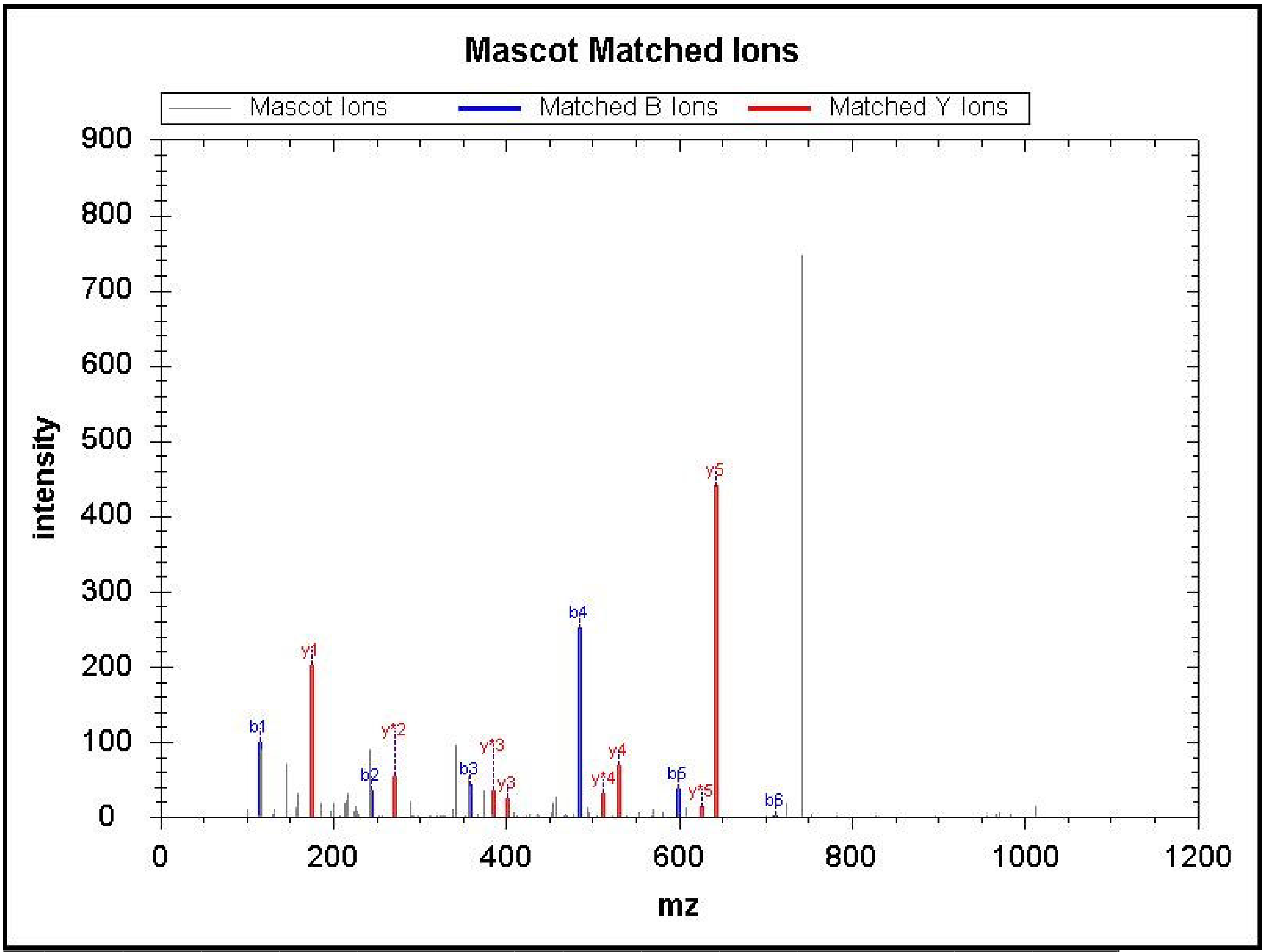

No	b	b++	b*	b*++	b0	b0++	Seq	y	y++	y*	y*++	y0	y0++	RevNo
1	115.05	58.03	98.02	49.52			N							7
2	244.09	122.55	227.07	114.04	226.08	113.54	E	771.51	386.26	754.48	377.74	753.50	377.25	6
3	357.18	179.09	340.15	170.58	339.17	170.09	I	642.47	321.74	625.44	313.22			5
4	485.27	243.14	468.25	234.63	467.26	234.13	K	529.38	265.19	512.36	256.68			4
5	598.36	299.68	581.33	291.17	580.35	290.68	L	401.29	201.15	384.26	192.63			3
6	711.44	356.22	694.41	347.71	693.43	347.22	L	288.20	144.61	271.18	136.09			2
7							R	175.12	88.06	158.09	79.55			1

Query 7002 Hit 1

MS/MS Fragmentation of QQIPILK
Found in sp|Q9JHW2|NIT2_MOUSE, Omega-amidase NIT2 OS=Mus musculus GN=Nit2 PE=1 SV=1
Match to Query 7002: 983.699from(492.8568,2+)
Title: 669: Sum of 2 scans in range 1575 (rt=44.6298, f=4, i=443) to 1576 (rt=44.6552, f=4, i=444) [D:\lab212\membrane\Grace\Joyce\iTRAQ_30_2.raw]
Data File:Submitted from 20120508-1(merge) by Mascot Daemon on JOYCE-VAIO
Monoisotopic mass of neutral peptide Mr(calc): 983.699
Variable modifications:
Q2 :Deamidated (NQ)
K7 :iTRAQ4plex (K)

Ions Score: 36.16 Expect: 0.019

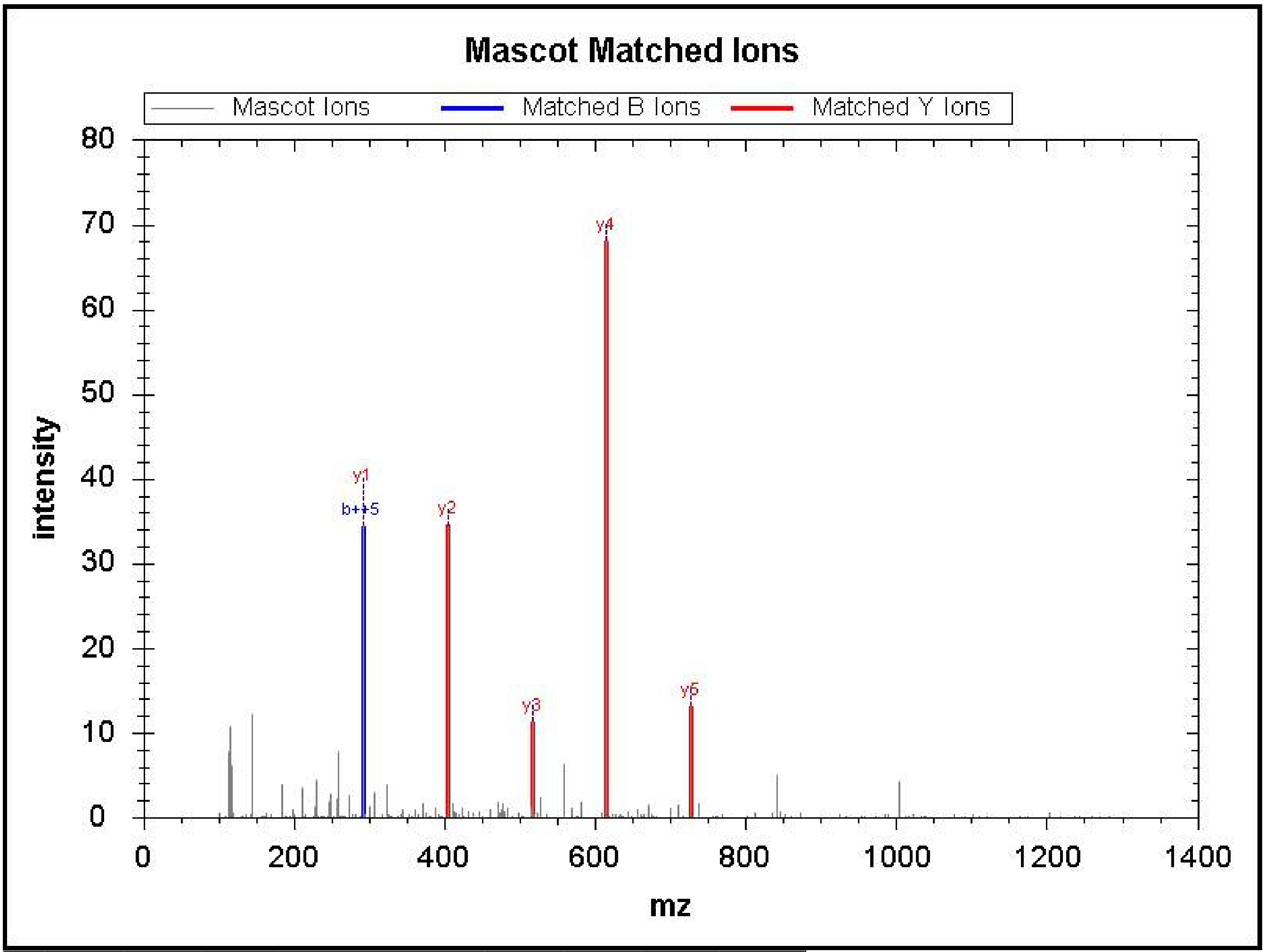

No	b	b++	b*	b*++	Seq	y	y++	y*	y*++	RevNo
1	129.07	65.04	112.04	56.52	Q					7
2	258.11	129.56	241.08	121.04	Q	856.56	428.78	839.54	420.27	6
3	371.19	186.10	354.17	177.59	I	727.52	364.26	710.49	355.75	5
4	468.25	234.63	451.22	226.11	P	614.44	307.72	597.41	299.21	4
5	581.33	291.17	564.30	282.66	I	517.38	259.20	500.36	250.68	3
6	694.41	347.71	677.39	339.20	L	404.30	202.65	387.27	194.14	2
7					K	291.21	146.11	274.19	137.60	1

Query 78407 Hit 1

MS/MS Fragmentation of VALHALTRPPALLLLAAASSGLR
Found in sp|Q8WUY8|NAT14_HUMAN, N-acetyltransferase 14 OS=Homo sapiens GN=NAT14 PE=1 SV=1
Match to Query 78407: 2454.496from(614.6313,4+)
Title: 823: Sum of 2 scans in range 2462 (rt=62.526, f=4, i=537) to 2463 (rt=62.5514, f=4, i=538) [D:\lab212\membrane\Grace\Joyce\iTRAQ_43_2.raw]
Data File:Submitted from 20120508-1(merge) by Mascot Daemon on JOYCE-VAIO
Monoisotopic mass of neutral peptide Mr(calc): 2454.496
Variable modifications:
Ions Score: 35.48 Expect: 0.020

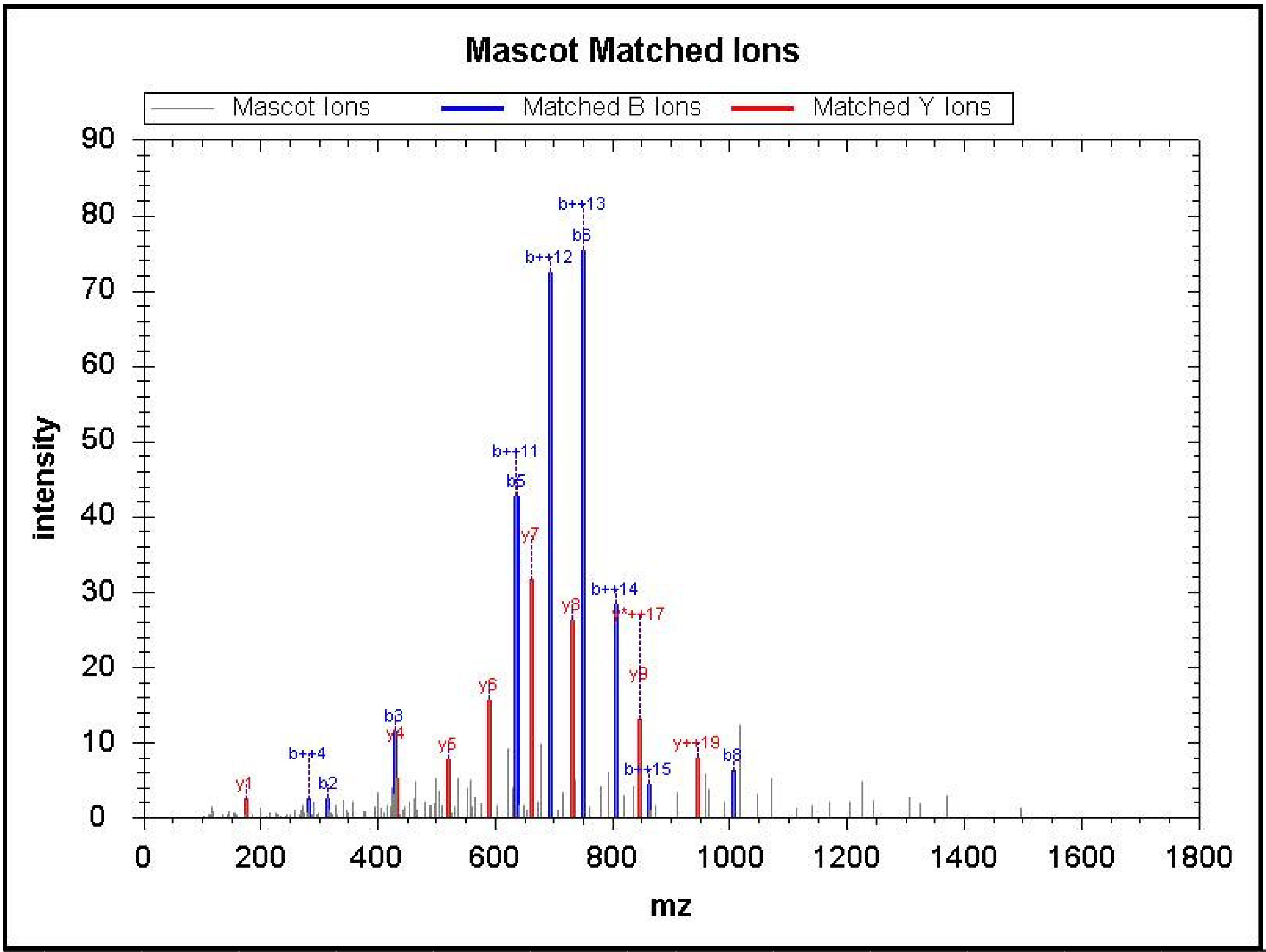

No	b	b++	b*	b*++	b0	b0++	Seq	y	y++	y*	y*++	y0	y0++	RevNo
1	244.18	122.59					V							23
2	315.21	158.11					A	2,212.33	1,106.67	2,195.30	1,098.15	2,194.32	1,097.66	22
3	428.30	214.65					L	2,141.29	1,071.15	2,124.27	1,062.64	2,123.28	1,062.14	21
4	565.36	283.18					H	2,028.21	1,014.61	2,011.18	1,006.09	2,010.20	1,005.60	20
5	636.39	318.70					A	1,891.15	946.08	1,874.12	937.56	1,873.14	937.07	19
6	749.48	375.24					L	1,820.11	910.56	1,803.09	902.05	1,802.10	901.55	18
7	850.53	425.77			832.52	416.76	T	1,707.03	854.02	1,690.00	845.50	1,689.02	845.01	17
8	1,006.63	503.82	989.60	495.30	988.62	494.81	R	1,605.98	803.49	1,588.95	794.98	1,587.97	794.49	16
9	1,103.68	552.34	1,086.65	543.83	1,085.67	543.34	P	1,449.88	725.44	1,432.85	716.93	1,431.87	716.44	15
10	1,200.73	600.87	1,183.71	592.36	1,182.72	591.86	P	1,352.83	676.92	1,335.80	668.40	1,334.82	667.91	14
11	1,271.77	636.39	1,254.74	627.88	1,253.76	627.38	A	1,255.77	628.39	1,238.75	619.88	1,237.76	619.38	13
12	1,384.85	692.93	1,367.83	684.42	1,366.84	683.93	L	1,184.74	592.87	1,167.71	584.36	1,166.73	583.87	12
13	1,497.94	749.47	1,480.91	740.96	1,479.93	740.47	L	1,071.65	536.33	1,054.63	527.82	1,053.64	527.32	11
14	1,611.02	806.02	1,594.00	797.50	1,593.01	797.01	L	958.57	479.79	941.54	471.27	940.56	470.78	10
15	1,724.11	862.56	1,707.08	854.04	1,706.10	853.55	L	845.48	423.25	828.46	414.73	827.47	414.24	9
16	1,795.14	898.08	1,778.12	889.56	1,777.13	889.07	A	732.40	366.70	715.37	358.19	714.39	357.70	8
17	1,866.18	933.59	1,849.15	925.08	1,848.17	924.59	A	661.36	331.18	644.34	322.67	643.35	322.18	7
18	1,937.22	969.11	1,920.19	960.60	1,919.21	960.11	A	590.33	295.67	573.30	287.15	572.32	286.66	6
19	2,024.25	1,012.63	2,007.22	1,004.12	2,006.24	1,003.62	S	519.29	260.15	502.26	251.63	501.28	251.14	5
20	2,111.28	1,056.14	2,094.26	1,047.63	2,093.27	1,047.14	S	432.26	216.63	415.23	208.12	414.25	207.63	4
21	2,168.30	1,084.66	2,151.28	1,076.14	2,150.29	1,075.65	G	345.22	173.12	328.20	164.60			3
22	2,281.39	1,141.20	2,264.36	1,132.68	2,263.38	1,132.19	L	288.20	144.61	271.18	136.09			2
23							R	175.12	88.06	158.09	79.55			1

Query 78756 Hit 1

MS/MS Fragmentation of LSKPTLENLTPVVLRPEIR
Found in sp|P55211|CASP9_HUMAN, Caspase-9 OS=Homo sapiens GN=CASP9 PE=1 SV=3
Match to Query 78756: 2462.485from(616.6287,4+)
Title: 530: Scan 1783 (rt=47.6347, f=3, i=206) [D:\lab212\membrane\Grace\Joyce\iTRAQ_46_1.raw]
Data File:Submitted from 20120508-1(merge) by Mascot Daemon on JOYCE-VAIO
Monoisotopic mass of neutral peptide Mr(calc): 2462.485
Variable modifications:
K3 :iTRAQ4plex (K)
Ions Score: 35.28 Expect: 0.028

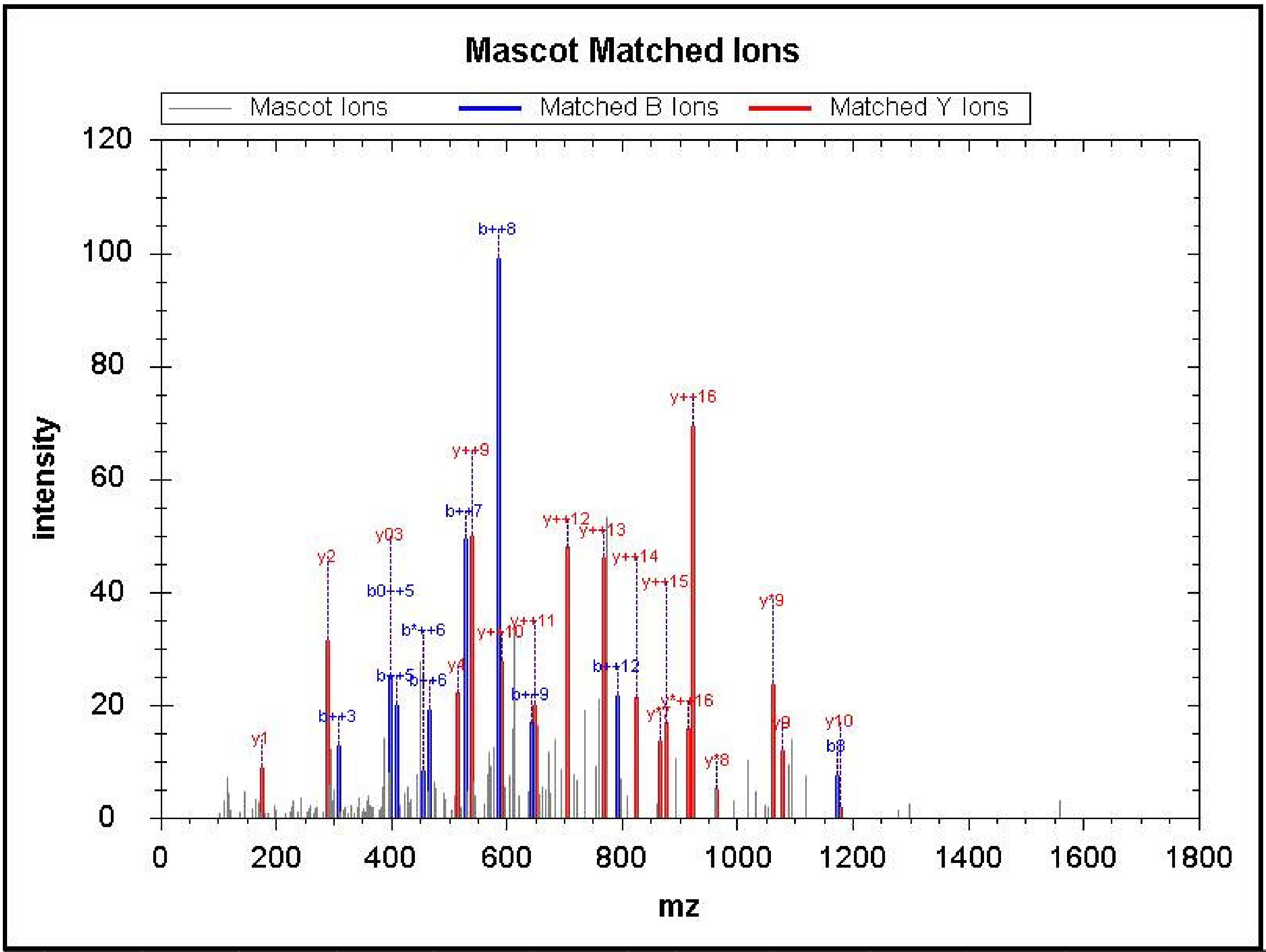

No	b	b++	b*	b*++	b0	b0++	Seq	y	y++	y*	y*++	y0	y0++	RevNo
1	258.19	129.60					L							19
2	345.23	173.12			327.21	164.11	S	2,206.30	1,103.66	2,189.28	1,095.14	2,188.29	1,094.65	18
3	617.42	309.21	600.40	300.70	599.41	300.21	K	2,119.27	1,060.14	2,102.25	1,051.63	2,101.26	1,051.13	17
4	714.48	357.74	697.45	349.23	696.46	348.74	P	1,847.08	924.04	1,830.05	915.53	1,829.06	915.04	16
5	815.52	408.27	798.50	399.75	797.51	399.26	T	1,750.02	875.51	1,733.00	867.00	1,732.01	866.51	15
6	928.61	464.81	911.58	456.29	910.60	455.80	L	1,648.97	824.99	1,631.95	816.48	1,630.96	815.99	14
7	1,057.65	529.33	1,040.62	520.82	1,039.64	520.32	E	1,535.89	768.45	1,518.86	759.94	1,517.88	759.44	13
8	1,171.69	586.35	1,154.67	577.84	1,153.68	577.34	N	1,406.85	703.93	1,389.82	695.41	1,388.84	694.92	12
9	1,284.78	642.89	1,267.75	634.38	1,266.77	633.89	L	1,292.80	646.91	1,275.78	638.39	1,274.79	637.90	11
10	1,385.82	693.42	1,368.80	684.90	1,367.81	684.41	T	1,179.72	590.36	1,162.69	581.85	1,161.71	581.36	10
11	1,482.88	741.94	1,465.85	733.43	1,464.87	732.94	P	1,078.67	539.84	1,061.65	531.33	1,060.66	530.83	9
12	1,581.95	791.48	1,564.92	782.96	1,563.93	782.47	V	981.62	491.31	964.59	482.80	963.61	482.31	8
13	1,681.01	841.01	1,663.99	832.50	1,663.00	832.01	V	882.55	441.78	865.53	433.27	864.54	432.77	7
14	1,794.10	897.55	1,777.07	889.04	1,776.09	888.55	L	783.48	392.25	766.46	383.73	765.47	383.24	6

15	1,950.20	975.60	1,933.17	967.09	1,932.19	966.60	R	670.40	335.70	653.37	327.19	652.39	326.70	5
16	2,047.25	1,024.13	2,030.23	1,015.62	2,029.24	1,015.12	P	514.30	257.65	497.27	249.14	496.29	248.65	4
17	2,176.29	1,088.65	2,159.27	1,080.14	2,158.28	1,079.65	E	417.25	209.13	400.22	200.61	399.24	200.12	3
18	2,289.38	1,145.19	2,272.35	1,136.68	2,271.37	1,136.19	I	288.20	144.61	271.18	136.09			2
19							R	175.12	88.06	158.09	79.55			1

Query 69648 Hit 1

MS/MS Fragmentation of LQEQLPELLEIIKPK
Found in sp|Q14746|COG2_HUMAN, Conserved oligomeric Golgi complex subunit 2 OS=Homo sapiens GN=COG2 PE=1 SV=1
Match to Query 69648: 2222.364from(741.7953,3+)
Title: 1011: Sum of 2 scans in range 2182 (rt=58.8426, f=4, i=675) to 2183 (rt=58.868, f=4, i=676)
[D:\lab212\membrane\Grace\Joyce\iTRAQ_40_1.raw]
Data File:Submitted from 20120508-1(merge) by Mascot Daemon on JOYCE-VAIO
Monoisotopic mass of neutral peptide Mr(calc): 2222.364
Variable modifications:
K13 :iTRAQ4plex (K)
K15 :iTRAQ4plex (K)
Ions Score: 33.89 Expect: 0.038

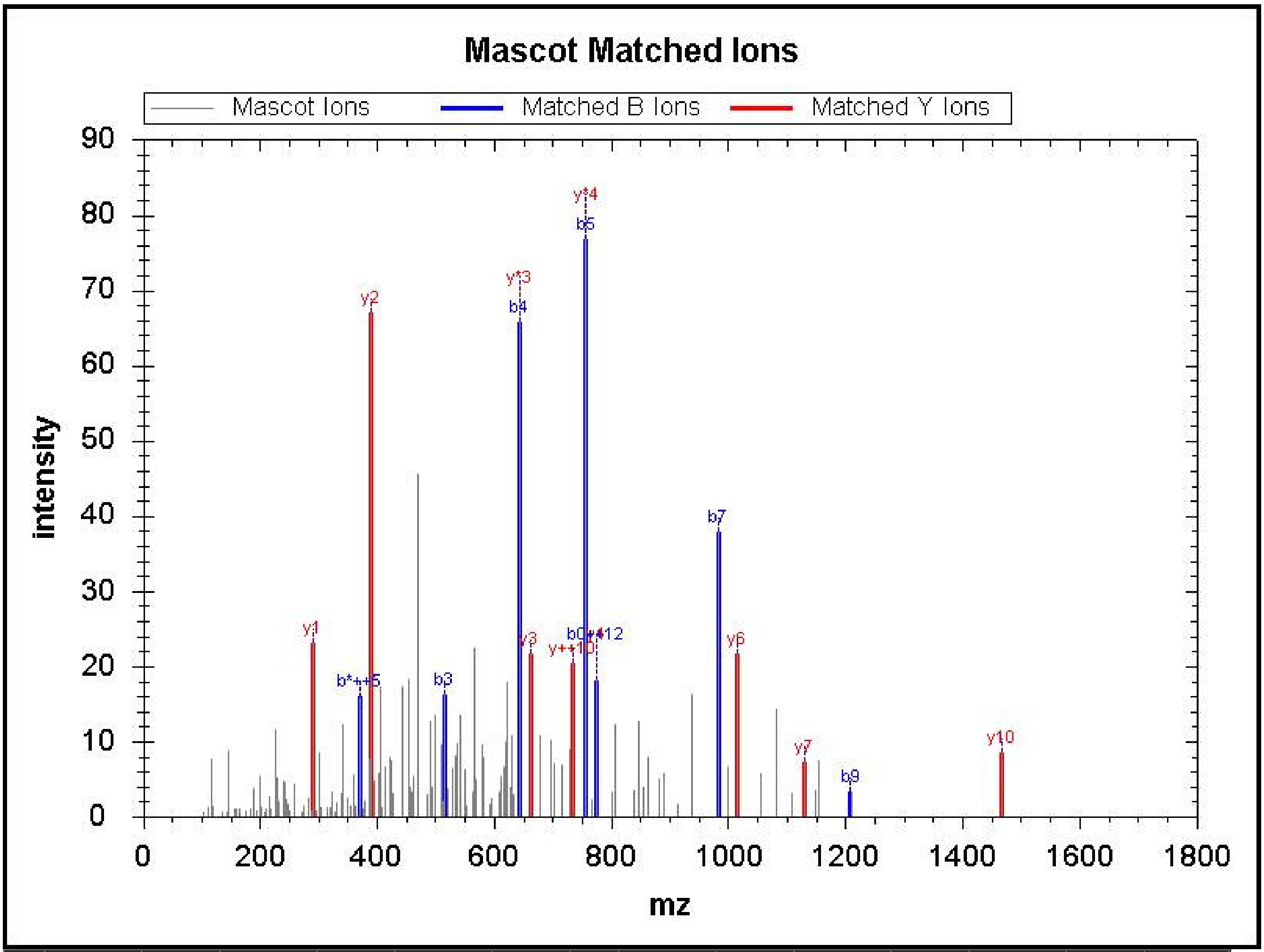

No	b	b++	b*	b*++	b0	b0++	Seq	y	y++	y*	y*++	y0	y0++	RevNo
1	258.19	129.60					L							15
2	386.25	193.63	369.23	185.12			Q	1,966.18	983.60	1,949.16	975.08	1,948.17	974.59	14
3	515.29	258.15	498.27	249.64	497.28	249.15	E	1,838.12	919.57	1,821.10	911.05	1,820.11	910.56	13
4	643.35	322.18	626.33	313.67	625.34	313.17	Q	1,709.08	855.04	1,692.06	846.53	1,691.07	846.04	12
5	756.44	378.72	739.41	370.21	738.43	369.72	L	1,581.02	791.02	1,564.00	782.50	1,563.01	782.01	11
6	853.49	427.25	836.46	418.74	835.48	418.24	P	1,467.94	734.47	1,450.91	725.96	1,449.93	725.47	10
7	982.53	491.77	965.51	483.26	964.52	482.76	E	1,370.89	685.95	1,353.86	677.43	1,352.88	676.94	9

8	1,095.62	548.31	1,078.59	539.80	1,077.61	539.31	L	1,241.84	621.43	1,224.82	612.91	1,223.83	612.42	8
9	1,208.70	604.85	1,191.67	596.34	1,190.69	595.85	L	1,128.76	564.88	1,111.73	556.37	1,110.75	555.88	7
10	1,337.74	669.38	1,320.72	660.86	1,319.73	660.37	E	1,015.68	508.34	998.65	499.83	997.66	499.34	6
11	1,450.83	725.92	1,433.80	717.40	1,432.82	716.91	I	886.63	443.82	869.61	435.31			5
12	1,563.91	782.46	1,546.89	773.95	1,545.90	773.45	I	773.55	387.28	756.52	378.76			4
13	1,836.11	918.56	1,819.08	910.04	1,818.10	909.55	K	660.46	330.74	643.44	322.22			3
14	1,933.16	967.08	1,916.13	958.57	1,915.15	958.08	P	388.27	194.64	371.24	186.12			2
15							K	291.21	146.11	274.19	137.60			1

Query 55697 Hit 1

MS/MS Fragmentation of AKPLLLQLPIGEAK
Found in sp|Q969S9|RRF2M_HUMAN, Ribosome-releasing factor 2
Match to Query 55697: 1922.233from(641.7517,3+)
Title: 537: Sum of 2 scans in range 1860 (rt=48.9628, f=4, i=343) to 1861 (rt=48.9882, f=4, i=344)
[D:\lab212\membrane\Grace\Joyce\iTRAQ_43_2.raw]
Data File:Submitted from 20120508-1(merge) by Mascot Daemon on JOYCE-VAIO
Monoisotopic mass of neutral peptide Mr(calc): 1922.233
Variable modifications:
K2 :iTRAQ4plex (K)
K14 :iTRAQ4plex (K)
Ions Score: 31.06 Expect: 0.040

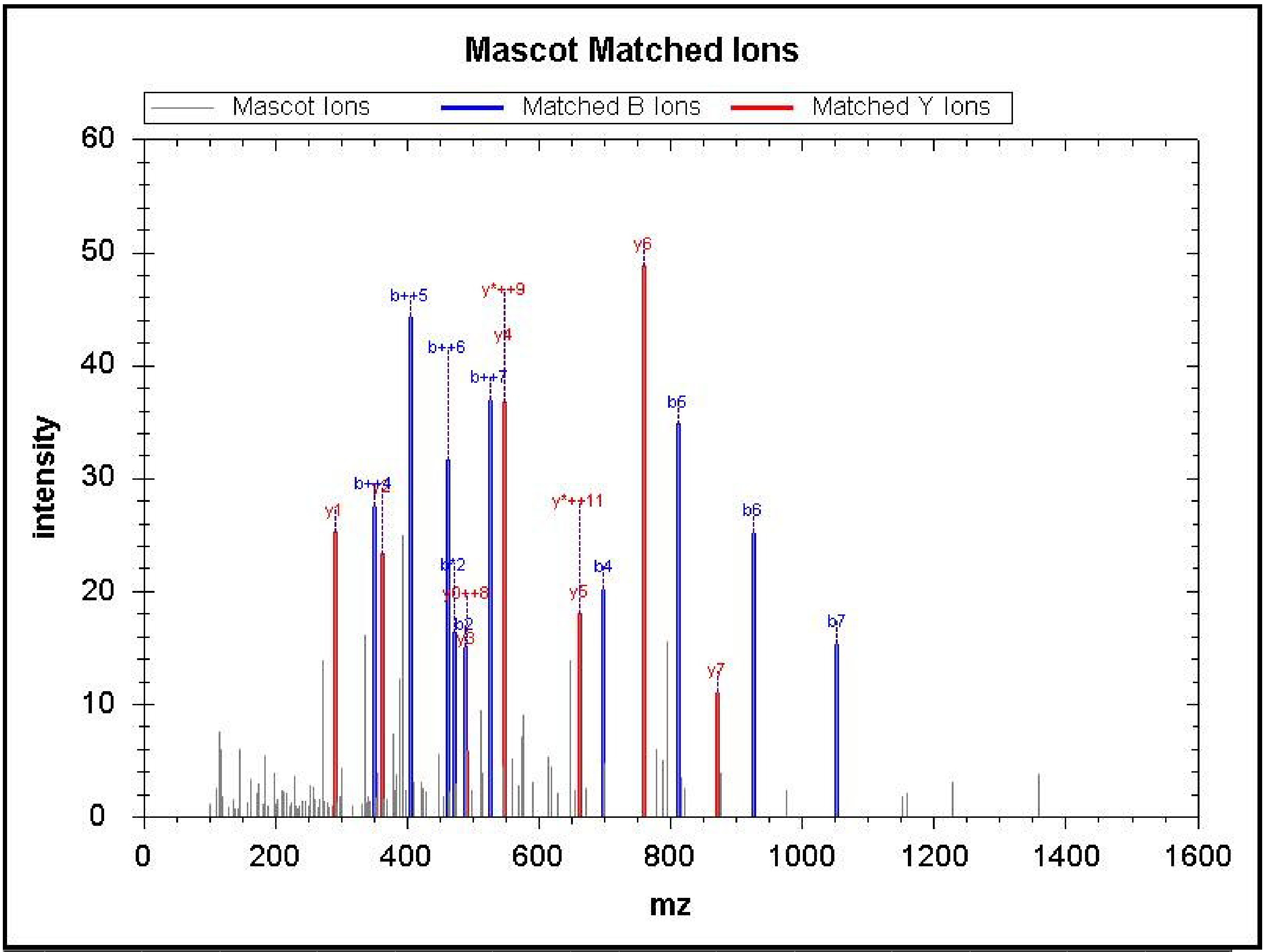

No	b	b++	b*	b*++	b0	b0++	Seq	y	y++	y*	y*++	y0	y0++	RevNo
1	216.15	108.58					A							14
2	488.34	244.68	471.32	236.16			K	1,708.10	854.55	1,691.07	846.04	1,690.09	845.55	13
3	585.40	293.20	568.37	284.69			P	1,435.90	718.45	1,418.87	709.94	1,417.89	709.45	12
4	698.48	349.74	681.45	341.23			L	1,338.85	669.93	1,321.82	661.41	1,320.84	660.92	11

5	811.56	406.29	794.54	397.77			L	1,225.76	613.39	1,208.74	604.87	1,207.75	604.38	10
6	924.65	462.83	907.62	454.31			L	1,112.68	556.84	1,095.65	548.33	1,094.67	547.84	9
7	1,052.71	526.86	1,035.68	518.34			Q	999.60	500.30	982.57	491.79	981.58	491.30	8
8	1,165.79	583.40	1,148.76	574.89			L	871.54	436.27	854.51	427.76	853.53	427.27	7
9	1,262.84	631.93	1,245.82	623.41			P	758.45	379.73	741.43	371.22	740.44	370.72	6
10	1,375.93	688.47	1,358.90	679.95			I	661.40	331.20	644.37	322.69	643.39	322.20	5
11	1,432.95	716.98	1,415.92	708.47			G	548.32	274.66	531.29	266.15	530.31	265.66	4
12	1,561.99	781.50	1,544.97	772.99	1,543.98	772.49	E	491.29	246.15	474.27	237.64	473.28	237.15	3
13	1,633.03	817.02	1,616.00	808.51	1,615.02	808.01	A	362.25	181.63	345.23	173.12			2
14							K	291.21	146.11	274.19	137.60			1
