# Supplementary material for: Quantitative Proteomic Analysis of Human Lung Tumor Xenografts Treated with the Ectopic ATP Synthase Inhibitor Citreoviridin
Source: PLoS One. 2013 Aug 21;8(8):e70642. doi: 10.1371/journal.pone.0070642 (PMC3749231; doi:10.1371/journal.pone.0070642)
Supplement: Spectra S4 — The information of single-peptide-based protein identifications in the large-scale experiment (part 2). (PDF) [file pone.0070642.s021.pdf]

Spectra S4: The information of single-peptide-based protein identifications in the large-scale experiment (part 2).

Query 77577 Hit 1

MS/MS Fragmentation of LGAGYGLLSEAELEVVPIIAK
Found in sp|Q15642|CIP4_HUMAN, Cdc42-interacting protein 4 OS=Homo sapiens GN=TRIP10 PE=1 SV=3
Match to Query 77577: 2429.4from(810.8073,3+)
Title: 1113: Scan 2567 (rt=66.6333, f=2, i=406) [D:\lab212\membrane\Grace\Joyce\iTRAQ_24_1.raw]
Data File:Submitted from 20120508-1(merge) by Mascot Daemon on JOYCE-VAIO
Monoisotopic mass of neutral peptide Mr(calc): 2429.4
Variable modifications:
K21 :iTRAQ4plex (K)
Ions Score: 51.41 Expect: 0.002

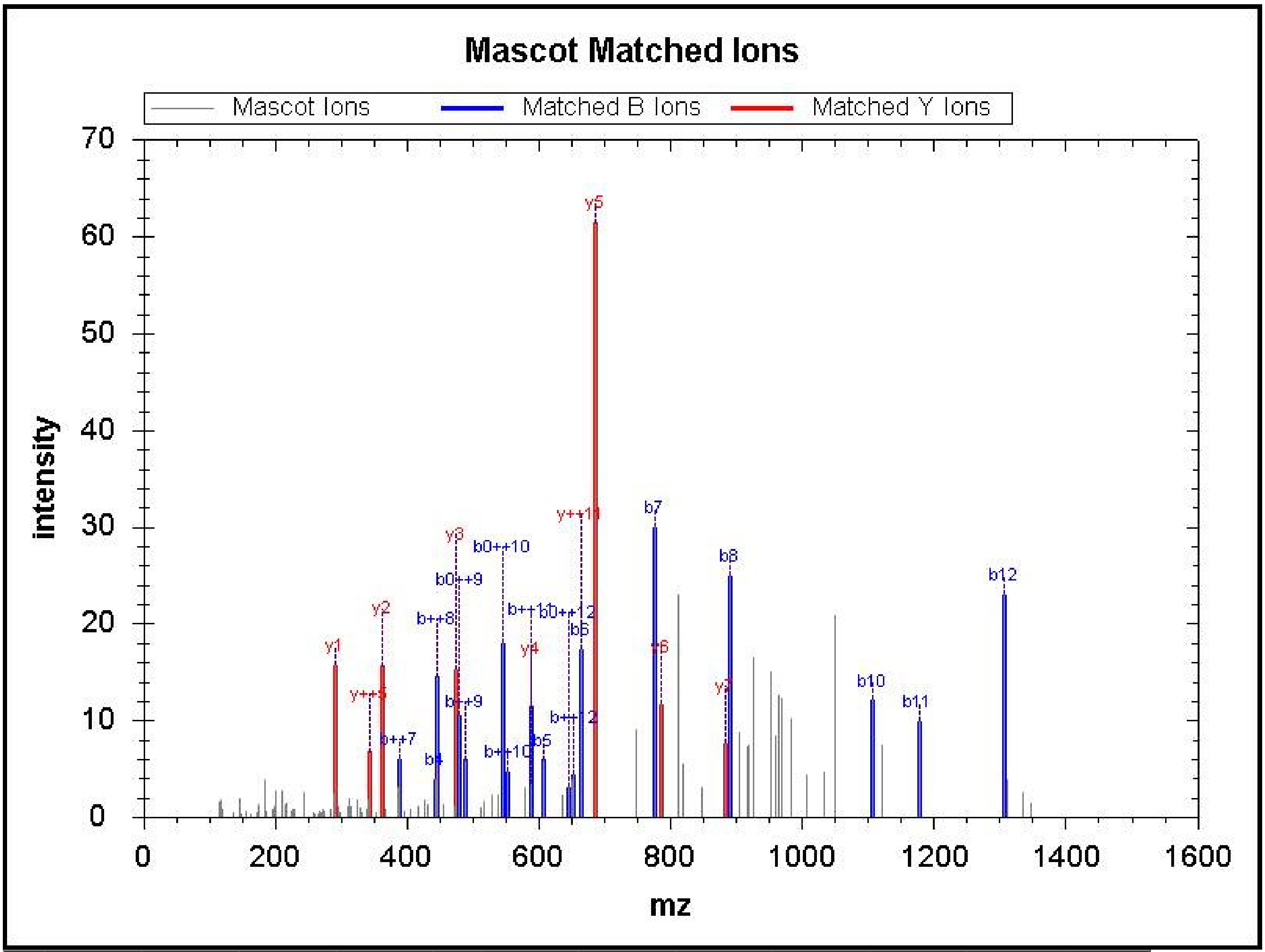

No	b	b++	b0	b0++	Seq	y	y++	y*	y*++	y0	y0++	RevNo
1	258.19	129.60			L							21
2	315.21	158.11			G	2,173.22	1,087.12	2,156.20	1,078.60	2,155.21	1,078.11	20
3	386.25	193.63			A	2,116.20	1,058.60	2,099.18	1,050.09	2,098.19	1,049.60	19
4	443.27	222.14			G	2,045.17	1,023.09	2,028.14	1,014.57	2,027.15	1,014.08	18
5	606.34	303.67			Y	1,988.14	994.58	1,971.12	986.06	1,970.13	985.57	17
6	663.36	332.18			G	1,825.08	913.04	1,808.05	904.53	1,807.07	904.04	16
7	776.44	388.72			L	1,768.06	884.53	1,751.03	876.02	1,750.05	875.53	15
8	889.53	445.27			L	1,654.97	827.99	1,637.95	819.48	1,636.96	818.99	14
9	976.56	488.78	958.55	479.78	S	1,541.89	771.45	1,524.86	762.94	1,523.88	762.44	13
10	1,105.60	553.30	1,087.59	544.30	E	1,454.86	727.93	1,437.83	719.42	1,436.85	718.93	12
11	1,176.64	588.82	1,158.63	579.82	A	1,325.82	663.41	1,308.79	654.90	1,307.81	654.41	11
12	1,305.68	653.34	1,287.67	644.34	E	1,254.78	627.89	1,237.75	619.38	1,236.77	618.89	10
13	1,418.76	709.89	1,400.75	700.88	L	1,125.74	563.37	1,108.71	554.86	1,107.73	554.37	9
14	1,547.81	774.41	1,529.80	765.40	E	1,012.65	506.83	995.63	498.32	994.64	497.82	8

15	1,646.88	823.94	1,628.87	814.94	V	883.61	442.31	866.58	433.80			7
16	1,745.94	873.48	1,727.93	864.47	V	784.54	392.77	767.51	384.26			6
17	1,843.00	922.00	1,824.99	913.00	P	685.47	343.24	668.45	334.73			5
18	1,956.08	978.54	1,938.07	969.54	I	588.42	294.71	571.39	286.20			4
19	2,069.17	1,035.09	2,051.15	1,026.08	I	475.34	238.17	458.31	229.66			3
20	2,140.20	1,070.60	2,122.19	1,061.60	A	362.25	181.63	345.23	173.12			2
21					K	291.21	146.11	274.19	137.60			1

Query 69518 Hit 1

MS/MS Fragmentation of KVSQEILELLNTTTAK
Found in sp|Q86U44|MTA70_HUMAN, N6-adenosine-methyltransferase 70 kDa subunit OS=Homo sapiens GN=METTL3 PE=1 SV=2
Match to Query 69518: 2219.313from(740.7783,3+)
Title: 915: Scan 1987 (rt=54.3811, f=3, i=309) [D:\lab212\membrane\Grace\Joyce\iTRAQ_45_2.raw]
Data File:Submitted from 20120508-1(merge) by Mascot Daemon on JOYCE-VAIO
Monoisotopic mass of neutral peptide Mr(calc): 2219.313
Variable modifications:
K1 :iTRAQ4plex (K)
K16 :iTRAQ4plex (K)
Ions Score: 51.35 Expect: 0.002

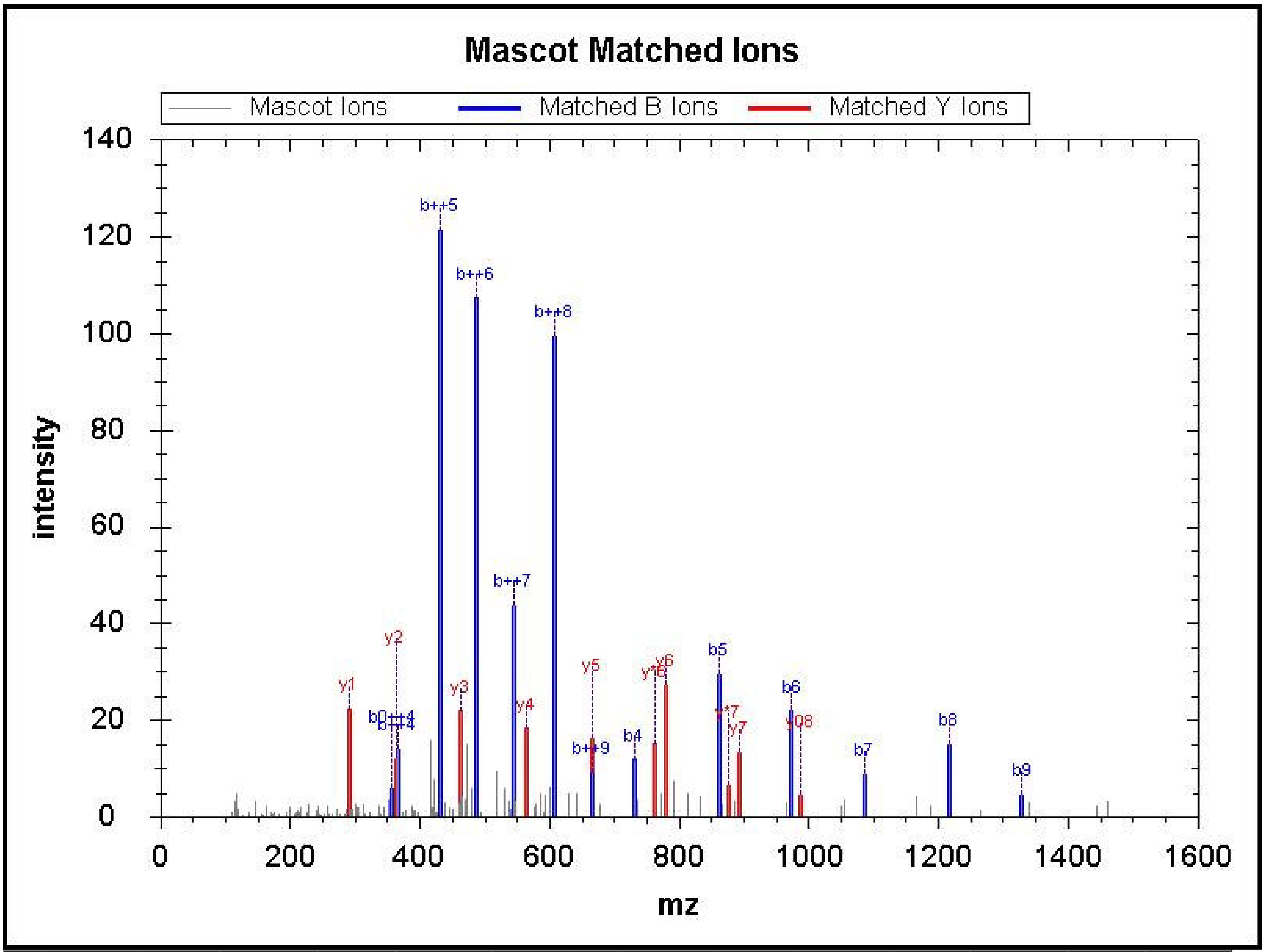

No	b	b++	b*	b*++	b0	b0++	Seq	y	y++	y*	y*++	y0	y0++	RevNo
1	417.31	209.16	400.28	200.64			K							16
2	516.37	258.69	499.35	250.18			V	1,804.02	902.51	1,786.99	894.00	1,786.01	893.51	15
3	603.41	302.21	586.38	293.69	585.40	293.20	S	1,704.95	852.98	1,687.92	844.47	1,686.94	843.97	14
4	731.47	366.24	714.44	357.72	713.45	357.23	Q	1,617.92	809.46	1,600.89	800.95	1,599.91	800.46	13
5	860.51	430.76	843.48	422.24	842.50	421.75	E	1,489.86	745.43	1,472.83	736.92	1,471.85	736.43	12

6	973.59	487.30	956.57	478.79	955.58	478.29	I	1,360.82	680.91	1,343.79	672.40	1,342.81	671.91	11
7	1,086.68	543.84	1,069.65	535.33	1,068.67	534.84	L	1,247.73	624.37	1,230.71	615.86	1,229.72	615.36	10
8	1,215.72	608.36	1,198.69	599.85	1,197.71	599.36	E	1,134.65	567.83	1,117.62	559.31	1,116.64	558.82	9
9	1,328.80	664.91	1,311.78	656.39	1,310.79	655.90	L	1,005.61	503.31	988.58	494.79	987.60	494.30	8
10	1,441.89	721.45	1,424.86	712.93	1,423.88	712.44	L	892.52	446.76	875.50	438.25	874.51	437.76	7
11	1,555.93	778.47	1,538.90	769.96	1,537.92	769.46	N	779.44	390.22	762.41	381.71	761.43	381.22	6
12	1,656.98	828.99	1,639.95	820.48	1,638.97	819.99	T	665.39	333.20	648.37	324.69	647.38	324.20	5
13	1,758.03	879.52	1,741.00	871.00	1,740.01	870.51	T	564.35	282.68	547.32	274.16	546.34	273.67	4
14	1,859.07	930.04	1,842.05	921.53	1,841.06	921.03	T	463.30	232.15	446.27	223.64	445.29	223.15	3
15	1,930.11	965.56	1,913.08	957.05	1,912.10	956.55	A	362.25	181.63	345.23	173.12			2
16							K	291.21	146.11	274.19	137.60			1

Query 65714 Hit 1

MS/MS Fragmentation of AFVEFLTDEIKEEK
Found in sp|O35658|C1QBP_MOUSE, Complement component 1 Q subcomponent-binding protein
Match to Query 65714: 2129.167from(710.7294,3+)
Title: 945: Scan 2053 (rt=55.9887, f=3, i=324) [D:\lab212\membrane\Grace\Joyce\iTRAQ_43_1.raw]
Data File:Submitted from 20120508-1(merge) by Mascot Daemon on JOYCE-VAIO
Monoisotopic mass of neutral peptide Mr(calc): 2129.167
Variable modifications:
K11 :iTRAQ4plex (K)
K14 :iTRAQ4plex (K)
Ions Score: 51.12 Expect: 0.006

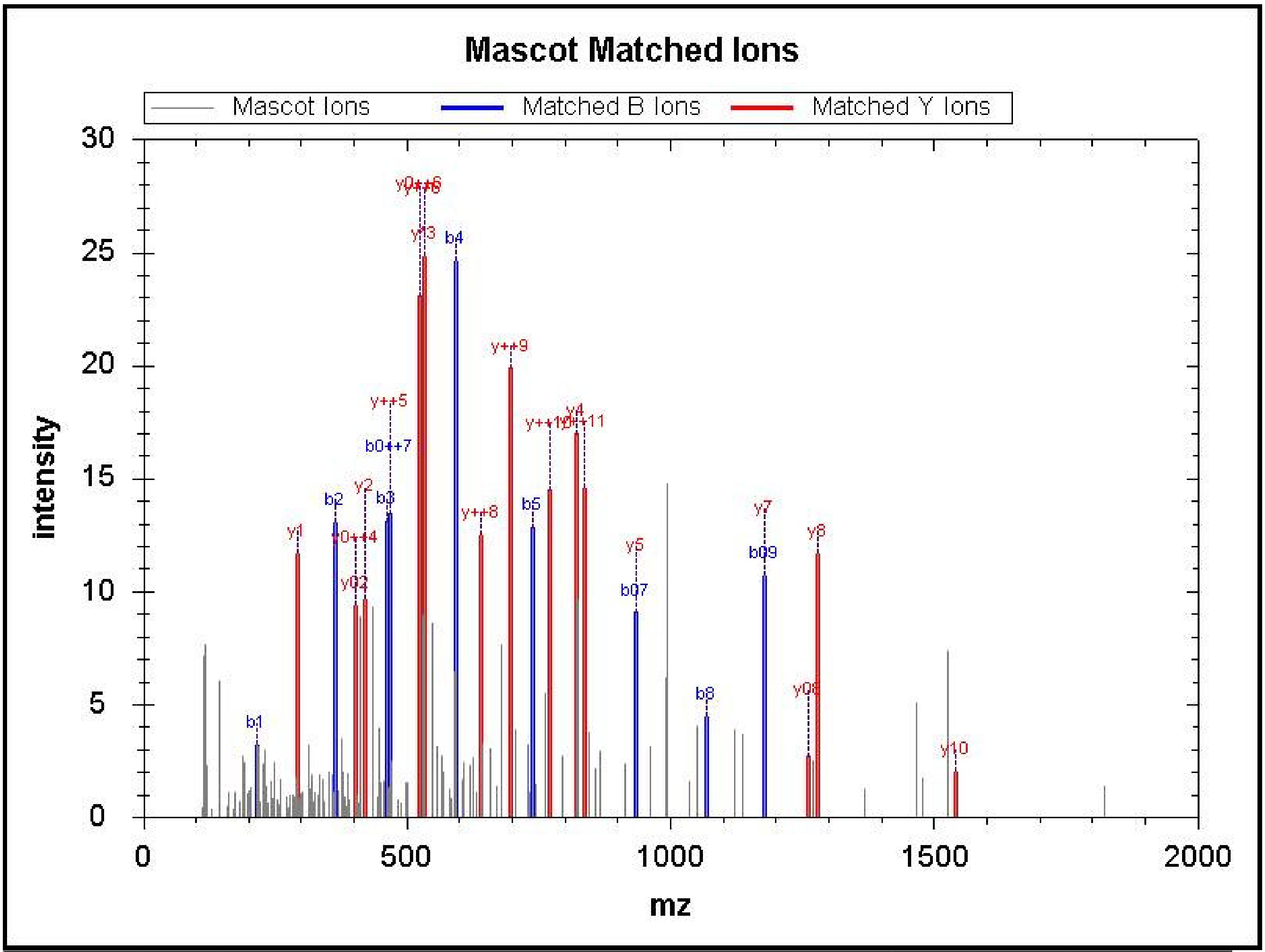

No	b	b++	b*	b*++	b0	b0++	Seq	y	y++	y*	y*++	y0	y0++	RevNo
1	216.15	108.58					A							14
2	363.21	182.11					F	1,915.03	958.02	1,898.00	949.51	1,897.02	949.01	13

3	462.28	231.65					V	1,767.96	884.48	1,750.94	875.97	1,749.95	875.48	12
4	591.33	296.17			573.32	287.16	E	1,668.89	834.95	1,651.87	826.44	1,650.88	825.95	11
5	738.39	369.70			720.38	360.70	F	1,539.85	770.43	1,522.82	761.92	1,521.84	761.42	10
6	851.48	426.24			833.47	417.24	L	1,392.78	696.89	1,375.76	688.38	1,374.77	687.89	9
7	952.53	476.77			934.52	467.76	T	1,279.70	640.35	1,262.67	631.84	1,261.69	631.35	8
8	1,067.55	534.28			1,049.54	525.27	D	1,178.65	589.83	1,161.62	581.32	1,160.64	580.82	7
9	1,196.60	598.80			1,178.58	589.80	E	1,063.62	532.32	1,046.60	523.80	1,045.61	523.31	6
10	1,309.68	655.34			1,291.67	646.34	I	934.58	467.79	917.55	459.28	916.57	458.79	5
11	1,581.88	791.44	1,564.85	782.93	1,563.87	782.44	K	821.50	411.25	804.47	402.74	803.49	402.25	4
12	1,710.92	855.96	1,693.89	847.45	1,692.91	846.96	E	549.30	275.15	532.27	266.64	531.29	266.15	3
13	1,839.96	920.48	1,822.94	911.97	1,821.95	911.48	E	420.26	210.63	403.23	202.12	402.25	201.63	2
14							K	291.21	146.11	274.19	137.60			1

Query 50714 Hit 1

MS/MS Fragmentation of EEGHAGPDDNEEVMR
Found in sp|P29083|T2EA_HUMAN, General transcription factor IIE subunit 1 OS=Homo sapiens GN=GTF2E1 PE=1 SV=2
Match to Query 50714: 1827.782from(610.2681,3+)
Title: 100: Sum of 2 scans in range 337 (rt=16.923, f=4, i=55) to 338 (rt=16.9484, f=4, i=56)
[D:\lab212\membrane\Grace\Joyce\iTRAQ_38_2.raw]
Data File:Submitted from 20120508-1(merge) by Mascot Daemon on JOYCE-VAIO
Monoisotopic mass of neutral peptide Mr(calc): 1827.782
Variable modifications:
Ions Score: 51.11 Expect: 0.003

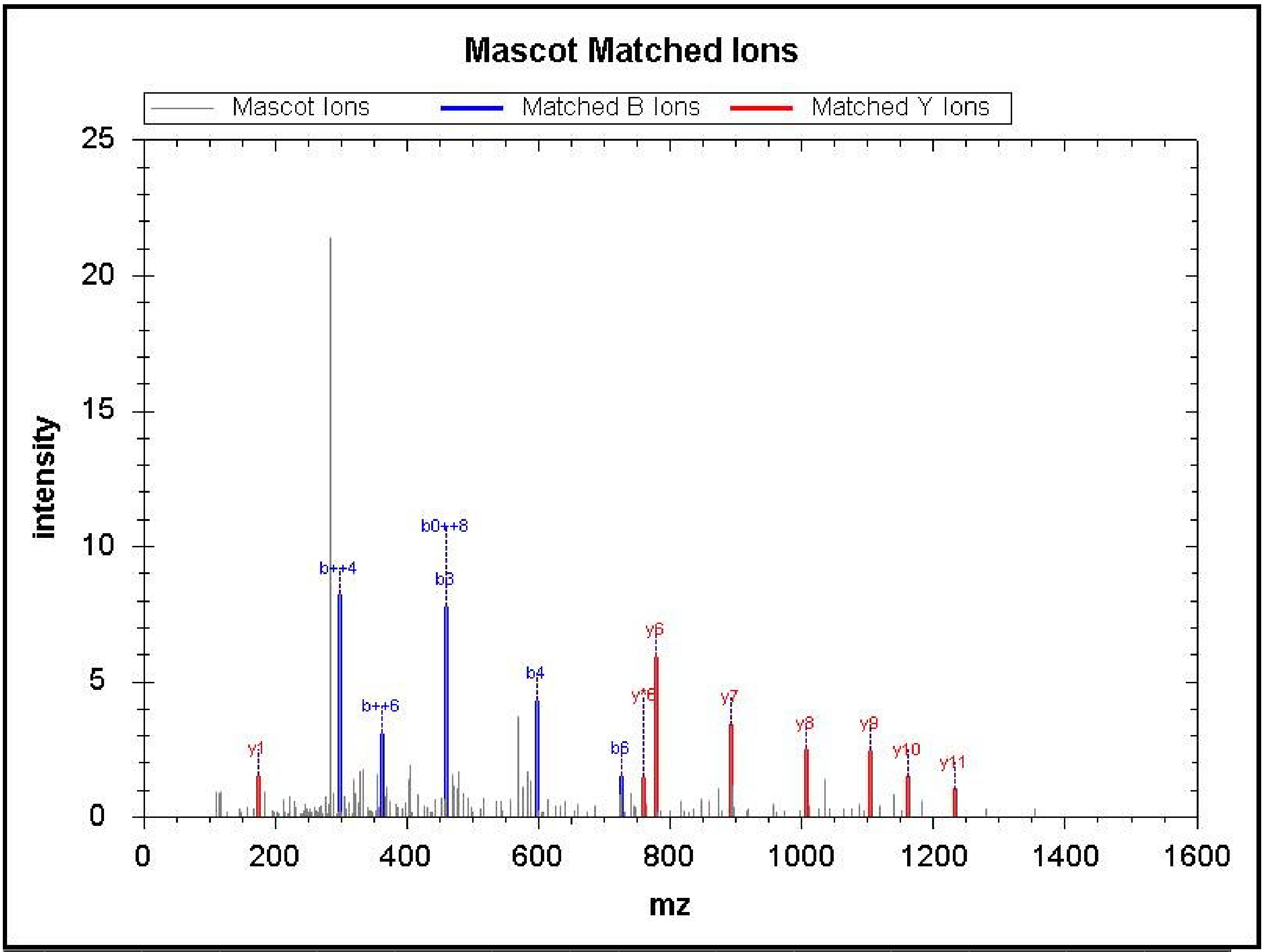

No	b	b++	b*	b*++	b0	b0++	Seq	y	y++	y*	y*++	y0	y0++	RevNo
1	274.15	137.58			256.14	128.57	E							15
2	403.19	202.10			385.18	193.10	E	1,555.64	778.33	1,538.62	769.81	1,537.63	769.32	14

3	460.22	230.61			442.21	221.61	G	1,426.60	713.80	1,409.58	705.29	1,408.59	704.80	13
4	597.27	299.14			579.26	290.14	H	1,369.58	685.29	1,352.55	676.78	1,351.57	676.29	12
5	668.31	334.66			650.30	325.65	A	1,232.52	616.76	1,215.49	608.25	1,214.51	607.76	11
6	725.33	363.17			707.32	354.17	G	1,161.48	581.25	1,144.46	572.73	1,143.47	572.24	10
7	822.39	411.70			804.38	402.69	P	1,104.46	552.73	1,087.44	544.22	1,086.45	543.73	9
8	937.41	469.21			919.40	460.20	D	1,007.41	504.21	990.38	495.70	989.40	495.20	8
9	1,052.44	526.72			1,034.43	517.72	D	892.38	446.70	875.36	438.18	874.37	437.69	7
10	1,166.48	583.75	1,149.46	575.23	1,148.47	574.74	N	777.36	389.18	760.33	380.67	759.35	380.18	6
11	1,295.53	648.27	1,278.50	639.75	1,277.52	639.26	E	663.31	332.16	646.29	323.65	645.30	323.15	5
12	1,424.57	712.79	1,407.54	704.27	1,406.56	703.78	E	534.27	267.64	517.24	259.13	516.26	258.63	4
13	1,523.64	762.32	1,506.61	753.81	1,505.63	753.32	V	405.23	203.12	388.20	194.60			3
14	1,654.68	827.84	1,637.65	819.33	1,636.67	818.84	M	306.16	153.58	289.13	145.07			2
15							R	175.12	88.06	158.09	79.55			1

Query 33852 Hit 1

MS/MS Fragmentation of ALSAETESHIYR
Found in sp|O14569|C56D2_HUMAN, Cytochrome b561 domain-containing protein 2 OS=Homo sapiens GN=CYB561D2 PE=1 SV=1
Match to Query 33852: 1519.774from(507.5987,3+)
Title: 304: Sum of 2 scans in range 729 (rt=26.0218, f=4, i=195) to 730 (rt=26.0472, f=4, i=196)
[D:\lab212\membrane\Grace\Joyce\iTRAQ_39_2.raw]
Data File:Submitted from 20120508-1(merge) by Mascot Daemon on JOYCE-VAIO
Monoisotopic mass of neutral peptide Mr(calc): 1519.774
Variable modifications:
Ions Score: 51.07 Expect: 0.007

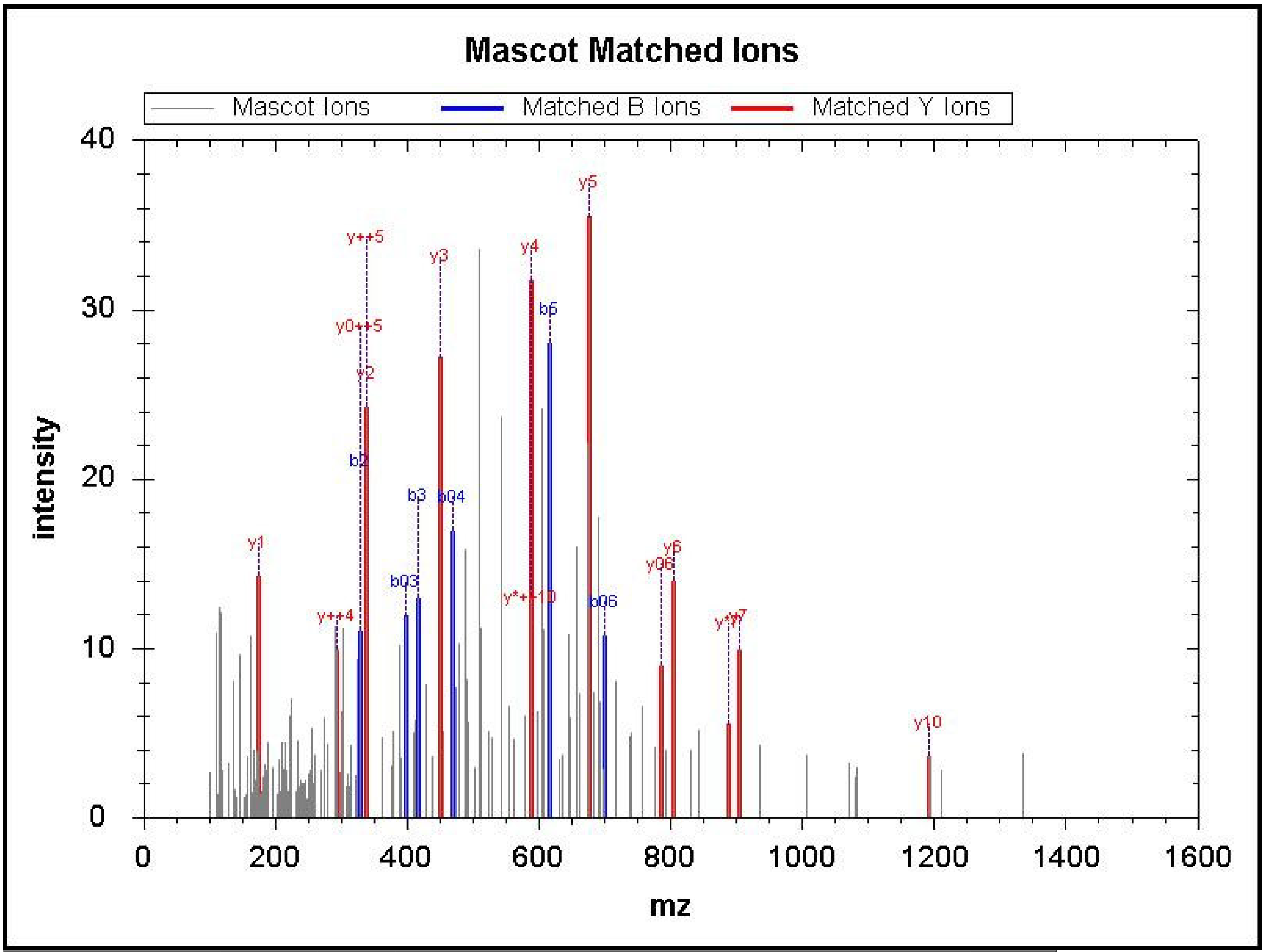

No	b	b++	b0	b0++	Seq	y	y++	y*	y*++	y0	y0++	RevNo

1	216.15	108.58			A							12
2	329.23	165.12			L	1,305.64	653.33	1,288.62	644.81	1,287.63	644.32	11
3	416.26	208.63	398.25	199.63	S	1,192.56	596.78	1,175.53	588.27	1,174.55	587.78	10
4	487.30	244.15	469.29	235.15	A	1,105.53	553.27	1,088.50	544.75	1,087.52	544.26	9
5	616.34	308.67	598.33	299.67	E	1,034.49	517.75	1,017.46	509.24	1,016.48	508.74	8
6	717.39	359.20	699.38	350.19	T	905.45	453.23	888.42	444.71	887.44	444.22	7
7	846.43	423.72	828.42	414.71	E	804.40	402.70	787.37	394.19	786.39	393.70	6
8	933.46	467.24	915.45	458.23	S	675.36	338.18	658.33	329.67	657.35	329.18	5
9	1,070.52	535.77	1,052.51	526.76	H	588.33	294.67	571.30	286.15			4
10	1,183.61	592.31	1,165.60	583.30	I	451.27	226.14	434.24	217.62			3
11	1,346.67	673.84	1,328.66	664.83	Y	338.18	169.59	321.16	161.08			2
12					R	175.12	88.06	158.09	79.55			1

Query 48712 Hit 1

MS/MS Fragmentation of HLVLLDTAQAAAAGHR
Found in sp|O00754|MA2B1_HUMAN, Lysosomal alpha-mannosidase OS=Homo sapiens GN=MAN2B1 PE=1 SV=3
Match to Query 48712: 1786.996from(596.6726,3+)
Title: 466: Scan 1153 (rt=35.1716, f=3, i=163) [D:\lab212\membrane\Grace\Joyce\iTRAQ_48_2.raw]
Data File:Submitted from 20120508-1(merge) by Mascot Daemon on JOYCE-VAIO
Monoisotopic mass of neutral peptide Mr(calc): 1786.996
Variable modifications:
Ions Score: 50.86 Expect: 0.006

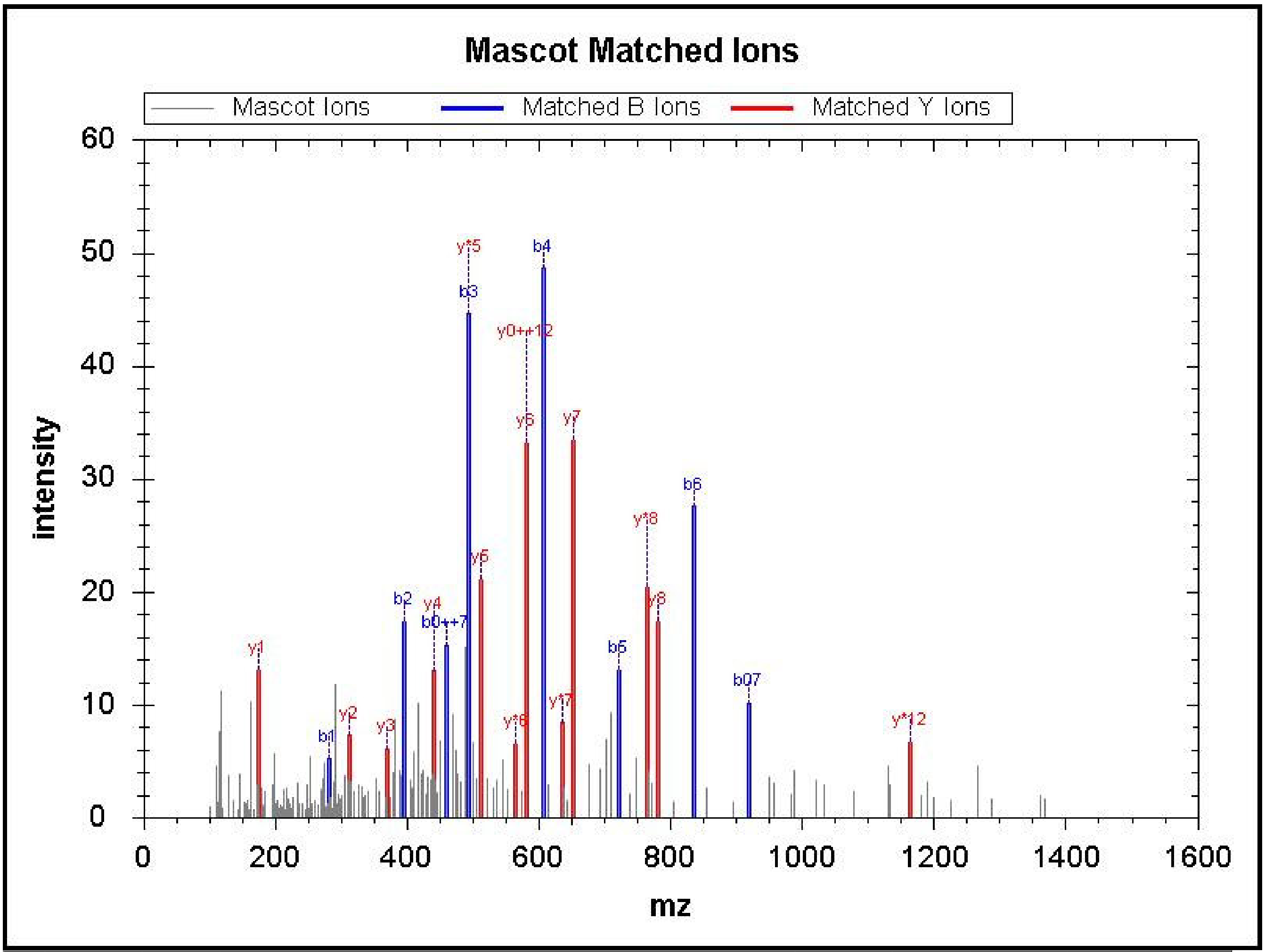

No	b	b++	b*	b*++	b0	b0++	Seq	y	y++	y*	y*++	y0	y0++	RevNo
1	282.17	141.59					H							16
2	395.25	198.13					L	1,506.84	753.92	1,489.81	745.41	1,488.83	744.92	15

3	494.32	247.66					V	1,393.75	697.38	1,376.73	688.87	1,375.74	688.38	14
4	607.40	304.21					L	1,294.69	647.85	1,277.66	639.33	1,276.68	638.84	13
5	720.49	360.75					L	1,181.60	591.30	1,164.58	582.79	1,163.59	582.30	12
6	835.52	418.26			817.51	409.26	D	1,068.52	534.76	1,051.49	526.25	1,050.51	525.76	11
7	936.56	468.79			918.55	459.78	T	953.49	477.25	936.46	468.74	935.48	468.24	10
8	1,007.60	504.30			989.59	495.30	A	852.44	426.73	835.42	418.21			9
9	1,135.66	568.33	1,118.63	559.82	1,117.65	559.33	Q	781.41	391.21	764.38	382.69			8
10	1,206.70	603.85	1,189.67	595.34	1,188.69	594.85	A	653.35	327.18	636.32	318.66			7
11	1,277.73	639.37	1,260.71	630.86	1,259.72	630.37	A	582.31	291.66	565.28	283.15			6
12	1,348.77	674.89	1,331.74	666.38	1,330.76	665.88	A	511.27	256.14	494.25	247.63			5
13	1,419.81	710.41	1,402.78	701.89	1,401.80	701.40	A	440.24	220.62	423.21	212.11			4
14	1,476.83	738.92	1,459.80	730.40	1,458.82	729.91	G	369.20	185.10	352.17	176.59			3
15	1,613.89	807.45	1,596.86	798.93	1,595.88	798.44	H	312.18	156.59	295.15	148.08			2
16							R	175.12	88.06	158.09	79.55			1

Query 94284 Hit 1

MS/MS Fragmentation of LQLFIQEVNHAVEETSHQALQNMPK
Found in sp|P83436|COG7_HUMAN, Conserved oligomeric Golgi complex subunit 7 OS=Homo sapiens GN=COG7 PE=1 SV=1
Match to Query 94284: 3191.669from(1064.897,3+)
Title: 927: Scan 2724 (rt=68.3447, f=2, i=437) [D:\lab212\membrane\Grace\Joyce\iTRAQ_43_2.raw]
Data File:Submitted from 20120508-1(merge) by Mascot Daemon on JOYCE-VAIO
Monoisotopic mass of neutral peptide Mr(calc): 3191.669
Variable modifications:
K25 :iTRAQ4plex (K)
Ions Score: 50.78 Expect: 0.006

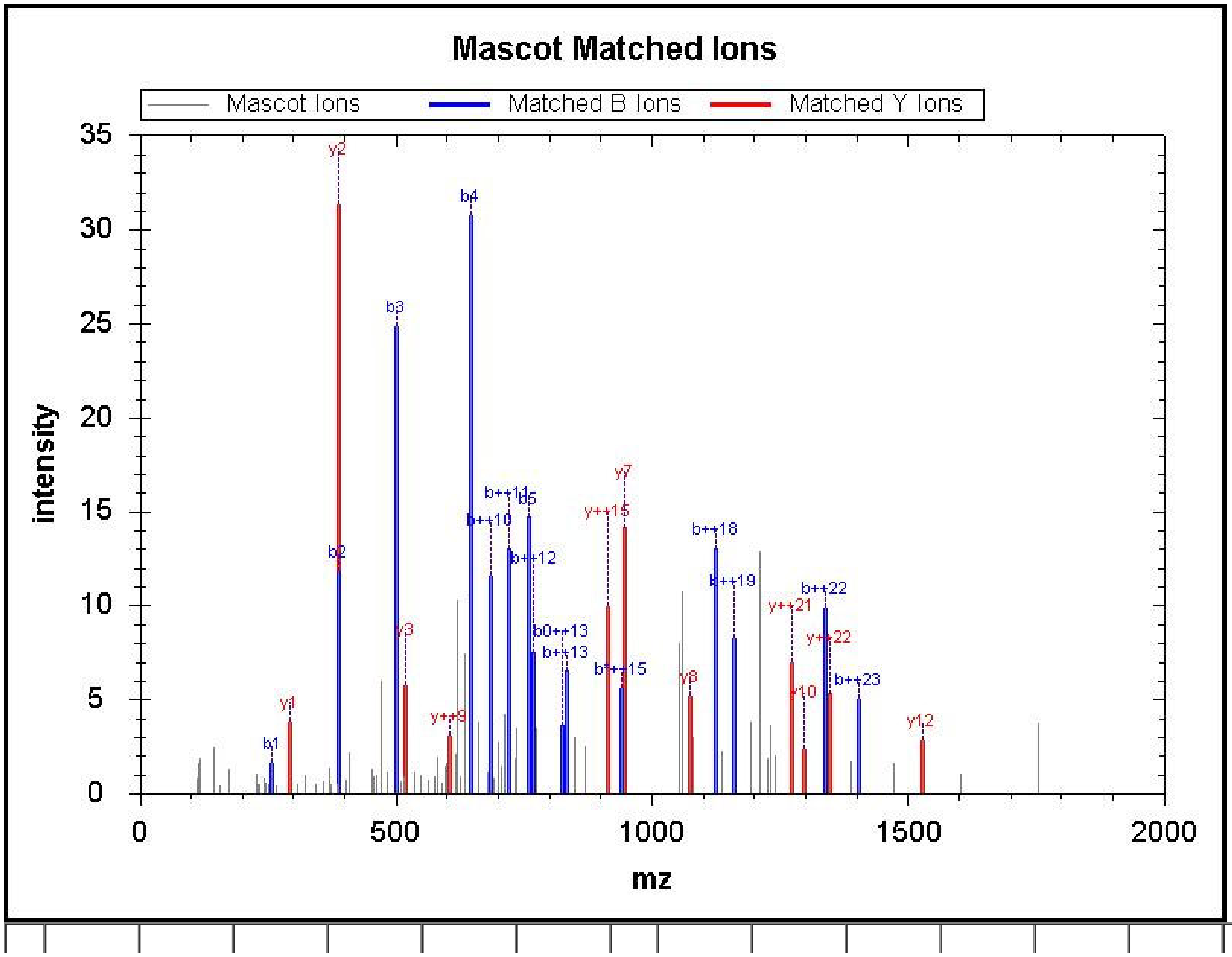

No	b	b++	b*	b*++	b0	b0++	Seq	y	y++	y*	y*++	y0	y0++	RevNo
1	258.19	129.60					L							25
2	386.25	193.63	369.23	185.12			Q	2,935.48	1,468.25	2,918.46	1,459.73	2,917.47	1,459.24	24
3	499.34	250.17	482.31	241.66			L	2,807.43	1,404.22	2,790.40	1,395.70	2,789.42	1,395.21	23
4	646.40	323.71	629.38	315.19			F	2,694.34	1,347.67	2,677.32	1,339.16	2,676.33	1,338.67	22
5	759.49	380.25	742.46	371.73			I	2,547.27	1,274.14	2,530.25	1,265.63	2,529.26	1,265.14	21
6	887.55	444.28	870.52	435.76			Q	2,434.19	1,217.60	2,417.16	1,209.09	2,416.18	1,208.59	20
7	1,016.59	508.80	999.56	500.29	998.58	499.79	E	2,306.13	1,153.57	2,289.10	1,145.06	2,288.12	1,144.56	19
8	1,115.66	558.33	1,098.63	549.82	1,097.65	549.33	V	2,177.09	1,089.05	2,160.06	1,080.53	2,159.08	1,080.04	18
9	1,229.70	615.35	1,212.67	606.84	1,211.69	606.35	N	2,078.02	1,039.51	2,060.99	1,031.00	2,060.01	1,030.51	17
10	1,366.76	683.88	1,349.73	675.37	1,348.75	674.88	H	1,963.98	982.49	1,946.95	973.98	1,945.97	973.49	16
11	1,437.80	719.40	1,420.77	710.89	1,419.79	710.40	A	1,826.92	913.96	1,809.89	905.45	1,808.91	904.96	15
12	1,536.87	768.94	1,519.84	760.42	1,518.85	759.93	V	1,755.88	878.44	1,738.86	869.93	1,737.87	869.44	14
13	1,665.91	833.46	1,648.88	824.94	1,647.90	824.45	E	1,656.81	828.91	1,639.79	820.40	1,638.80	819.90	13
14	1,794.95	897.98	1,777.92	889.47	1,776.94	888.97	E	1,527.77	764.39	1,510.74	755.88	1,509.76	755.38	12
15	1,896.00	948.50	1,878.97	939.99	1,877.99	939.50	T	1,398.73	699.87	1,381.70	691.35	1,380.72	690.86	11
16	1,983.03	992.02	1,966.00	983.51	1,965.02	983.01	S	1,297.68	649.34	1,280.65	640.83	1,279.67	640.34	10
17	2,120.09	1,060.55	2,103.06	1,052.04	2,102.08	1,051.54	H	1,210.65	605.83	1,193.62	597.31			9
18	2,248.15	1,124.58	2,231.12	1,116.06	2,230.14	1,115.57	Q	1,073.59	537.30	1,056.56	528.79			8
19	2,319.19	1,160.10	2,302.16	1,151.58	2,301.17	1,151.09	A	945.53	473.27	928.50	464.76			7
20	2,432.27	1,216.64	2,415.24	1,208.12	2,414.26	1,207.63	L	874.49	437.75	857.47	429.24			6
21	2,560.33	1,280.67	2,543.30	1,272.15	2,542.32	1,271.66	Q	761.41	381.21	744.38	372.70			5
22	2,674.37	1,337.69	2,657.34	1,329.18	2,656.36	1,328.68	N	633.35	317.18	616.32	308.67			4
23	2,805.41	1,403.21	2,788.38	1,394.70	2,787.40	1,394.20	M	519.31	260.16	502.28	251.64			3
24	2,902.46	1,451.74	2,885.44	1,443.22	2,884.45	1,442.73	P	388.27	194.64	371.24	186.12			2
25							K	291.21	146.11	274.19	137.60			1

Query 93980 Hit 1

MS/MS Fragmentation of ALYFYEQAITTPAMAVSHIMLESYK
Found in sp|Q9UNS2|CSN3_HUMAN, COP9 signalosome complex subunit 3 OS=Homo sapiens GN=COPS3 PE=1 SV=3
Match to Query 93980: 3164.616from(792.1612,4+)
Title: 1211: Sum of 2 scans in range 2805 (rt=72.0248, f=2, i=472) to 2806 (rt=72.0503, f=2, i=473) [D:\lab212\membrane\Grace\Joyce\iTRAQ_32_2.raw]
Data File:Submitted from 20120508-1(merge) by Mascot Daemon on JOYCE-VAIO
Monoisotopic mass of neutral peptide Mr(calc): 3164.616
Variable modifications:
K25 :iTRAQ4plex (K)
Ions Score: 50.67 Expect: 0.007

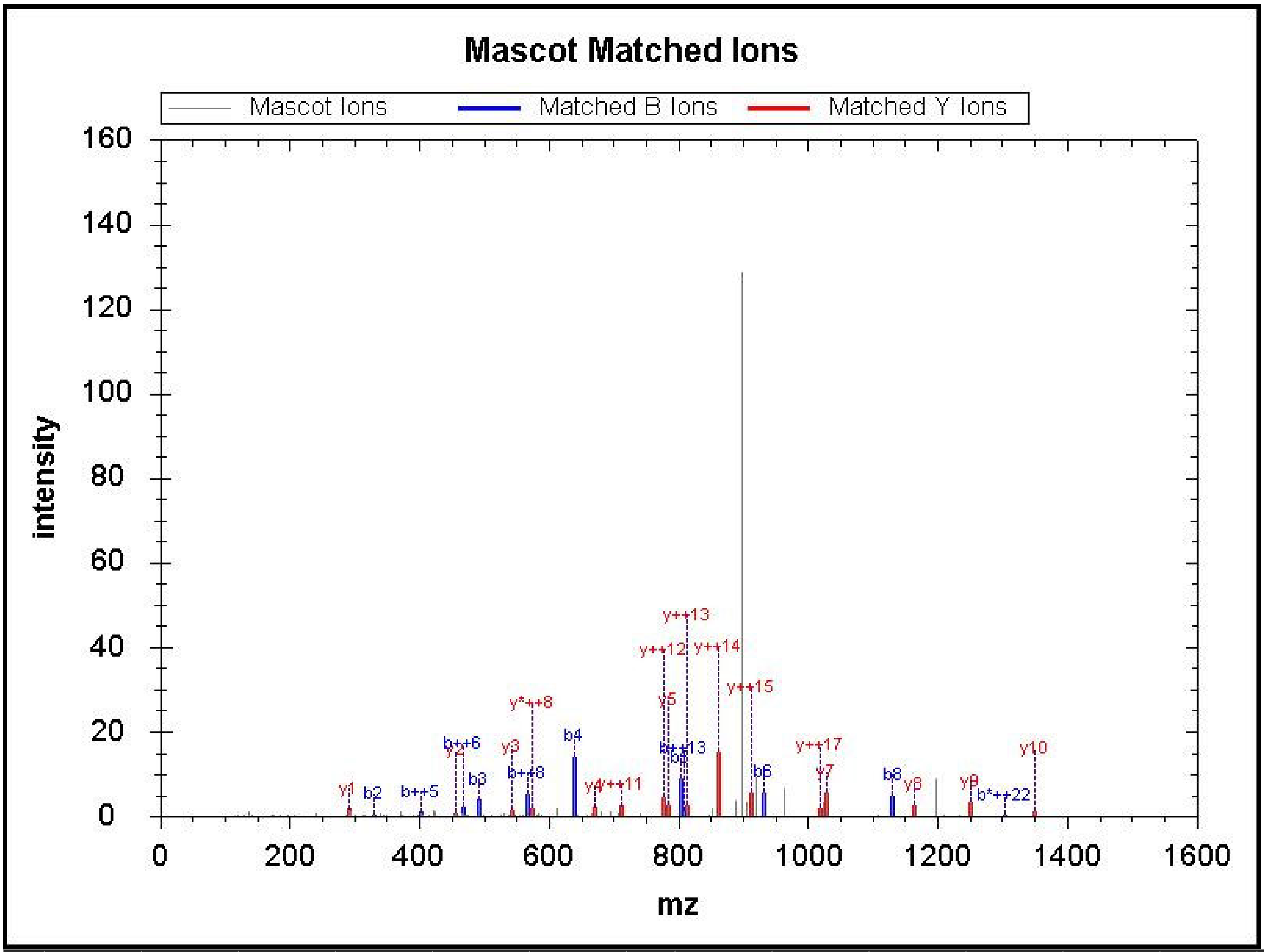

No	b	b++	b*	b*++	b0	b0++	Seq	y	y++	y*	y*++	y0	y0++	RevNo
1	216.15	108.58					A							25
2	329.23	165.12					L	2,950.48	1,475.75	2,933.46	1,467.23	2,932.47	1,466.74	24
3	492.29	246.65					Y	2,837.40	1,419.20	2,820.37	1,410.69	2,819.39	1,410.20	23
4	639.36	320.18					F	2,674.34	1,337.67	2,657.31	1,329.16	2,656.33	1,328.67	22
5	802.43	401.72					Y	2,527.27	1,264.14	2,510.24	1,255.62	2,509.26	1,255.13	21
6	931.47	466.24			913.46	457.23	E	2,364.21	1,182.61	2,347.18	1,174.09	2,346.20	1,173.60	20
7	1,059.53	530.27	1,042.50	521.75	1,041.52	521.26	Q	2,235.16	1,118.09	2,218.14	1,109.57	2,217.15	1,109.08	19
8	1,130.56	565.79	1,113.54	557.27	1,112.55	556.78	A	2,107.10	1,054.06	2,090.08	1,045.54	2,089.09	1,045.05	18
9	1,243.65	622.33	1,226.62	613.81	1,225.64	613.32	I	2,036.07	1,018.54	2,019.04	1,010.02	2,018.06	1,009.53	17
10	1,344.70	672.85	1,327.67	664.34	1,326.69	663.85	T	1,922.98	962.00	1,905.96	953.48	1,904.97	952.99	16
11	1,445.74	723.38	1,428.72	714.86	1,427.73	714.37	T	1,821.94	911.47	1,804.91	902.96	1,803.93	902.47	15
12	1,542.80	771.90	1,525.77	763.39	1,524.79	762.90	P	1,720.89	860.95	1,703.86	852.43	1,702.88	851.94	14
13	1,613.83	807.42	1,596.81	798.91	1,595.82	798.42	A	1,623.84	812.42	1,606.81	803.91	1,605.82	803.42	13
14	1,744.87	872.94	1,727.85	864.43	1,726.86	863.94	M	1,552.80	776.90	1,535.77	768.39	1,534.79	767.90	12
15	1,815.91	908.46	1,798.88	899.95	1,797.90	899.45	A	1,421.76	711.38	1,404.73	702.87	1,403.75	702.38	11
16	1,914.98	957.99	1,897.95	949.48	1,896.97	948.99	V	1,350.72	675.86	1,333.69	667.35	1,332.71	666.86	10
17	2,002.01	1,001.51	1,984.98	993.00	1,984.00	992.50	S	1,251.65	626.33	1,234.63	617.82	1,233.64	617.32	9
18	2,139.07	1,070.04	2,122.04	1,061.53	2,121.06	1,061.03	H	1,164.62	582.81	1,147.59	574.30	1,146.61	573.81	8
19	2,252.15	1,126.58	2,235.13	1,118.07	2,234.14	1,117.58	I	1,027.56	514.28	1,010.53	505.77	1,009.55	505.28	7
20	2,383.19	1,192.10	2,366.17	1,183.59	2,365.18	1,183.10	M	914.48	457.74	897.45	449.23	896.47	448.74	6
21	2,496.28	1,248.64	2,479.25	1,240.13	2,478.27	1,239.64	L	783.44	392.22	766.41	383.71	765.43	383.22	5
22	2,625.32	1,313.16	2,608.29	1,304.65	2,607.31	1,304.16	E	670.35	335.68	653.33	327.17	652.34	326.67	4
23	2,712.35	1,356.68	2,695.33	1,348.17	2,694.34	1,347.68	S	541.31	271.16	524.28	262.65	523.30	262.15	3

24	2,875.42	1,438.21	2,858.39	1,429.70	2,857.41	1,429.21	Y	454.28	227.64	437.25	219.13			2
25							K	291.21	146.11	274.19	137.60			1

Query 15054 Hit 1

MS/MS Fragmentation of LVLLFSGK
Found in sp|Q15126|PMVK_HUMAN, Phosphomevalonate kinase OS=Homo sapiens GN=PMVK PE=1 SV=3
Match to Query 15054: 1163.75from(582.8824,2+)
Title: 769: Sum of 2 scans in range 1800 (rt=49.4978, f=4, i=509) to 1801 (rt=49.5232, f=4, i=510)
[D:\lab212\membrane\Grace\Joyce\iTRAQ_29_1.raw]
Data File:Submitted from 20120508-1(merge) by Mascot Daemon on JOYCE-VAIO
Monoisotopic mass of neutral peptide Mr(calc): 1163.75
Variable modifications:
K8 :iTRAQ4plex (K)
Ions Score: 50.51 Expect: 0.003

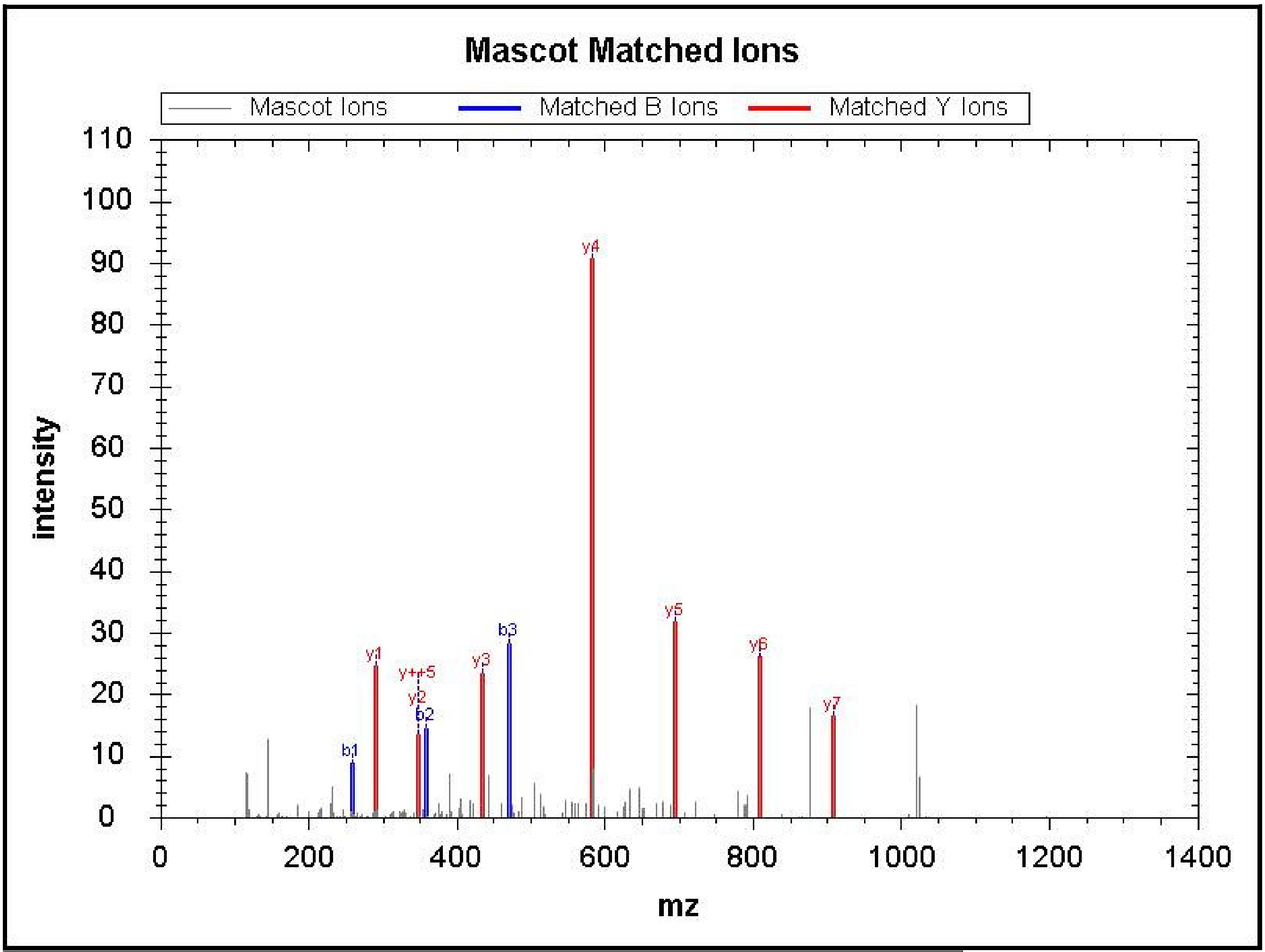

No	b	b++	b0	b0++	Seq	y	y++	y*	y*++	y0	y0++	RevNo
1	258.19	129.60			L							8
2	357.26	179.13			V	907.57	454.29	890.55	445.78	889.56	445.28	7
3	470.35	235.68			L	808.50	404.76	791.48	396.24	790.49	395.75	6
4	583.43	292.22			L	695.42	348.21	678.39	339.70	677.41	339.21	5
5	730.50	365.75			F	582.34	291.67	565.31	283.16	564.33	282.67	4
6	817.53	409.27	799.52	400.26	S	435.27	218.14	418.24	209.62	417.26	209.13	3
7	874.55	437.78	856.54	428.77	G	348.24	174.62	331.21	166.11			2
8					K	291.21	146.11	274.19	137.60			1

Query 70660 Hit 1

MS/MS Fragmentation of MDILVTETEELAENILK

Found in sp|Q9NVX0|HAUS2_HUMAN, HAUS augmin-like complex subunit 2 OS=Homo sapiens GN=HAUS2 PE=1 SV=1
Match to Query 70660: 2248.21 from(750.4106,3+)
Title: 1408: Sum of 2 scans in range 3125 (rt=79.5561, f=2, i=517) to 3126 (rt=79.5815, f=2, i=518) [D:\lab212\membrane\Grace\Joyce\iTRAQ_26_2.raw]
Data File:Submitted from 20120508-1(merge) by Mascot Daemon on JOYCE-VAIO
Monoisotopic mass of neutral peptide Mr(calc): 2248.21
Variable modifications:
K17 :iTRAQ4plex (K)
Ions Score: 50.48 Expect: 0.007

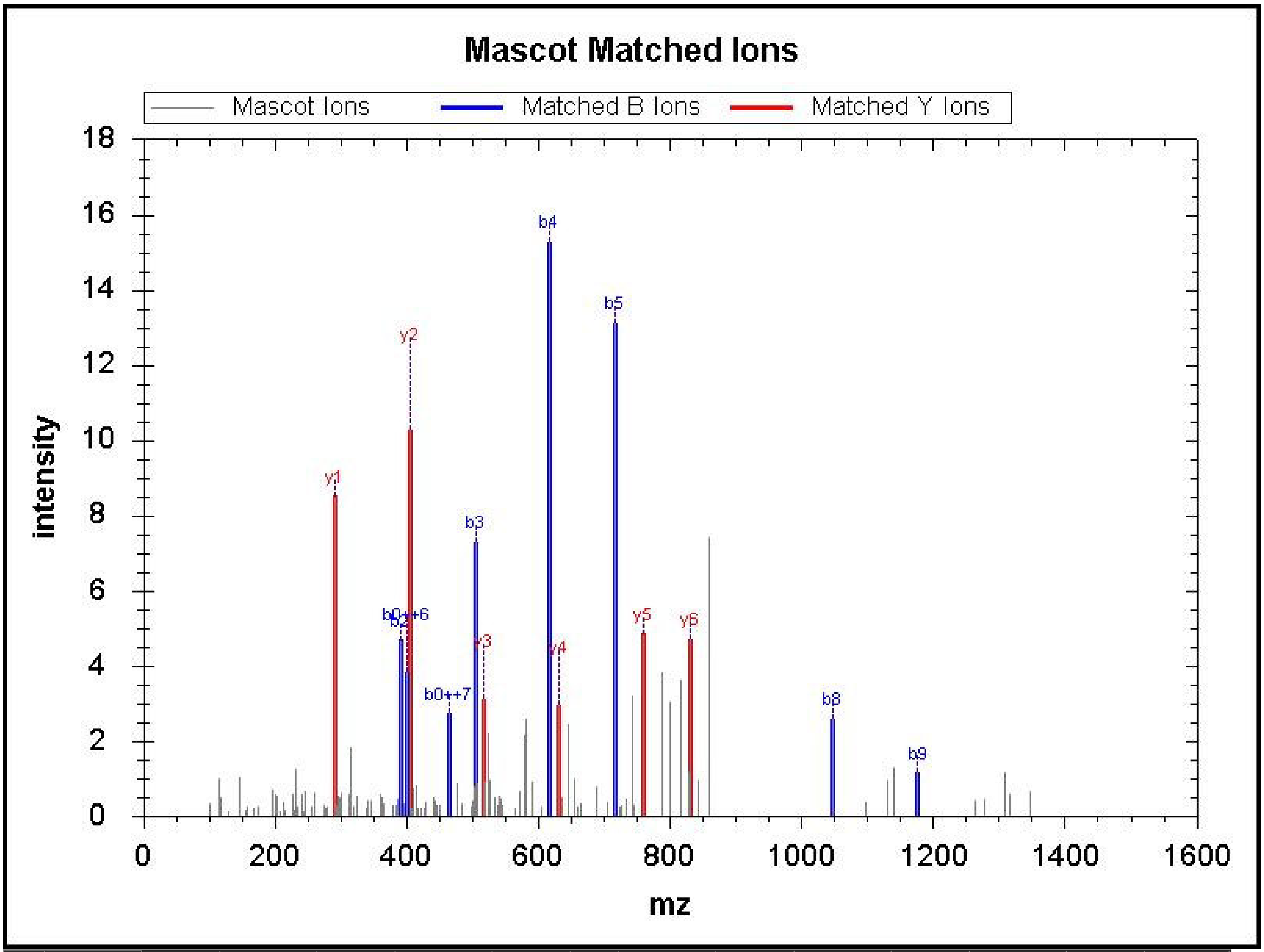

No	b	b++	b*	b*++	b0	b0++	Seq	y	y++	y*	y*++	y0	y0++	RevNo
1	276.15	138.58					M							17
2	391.18	196.09			373.17	187.09	D	1,974.08	987.54	1,957.05	979.03	1,956.07	978.54	16
3	504.26	252.63			486.25	243.63	I	1,859.05	930.03	1,842.02	921.52	1,841.04	921.02	15
4	617.34	309.18			599.33	300.17	L	1,745.97	873.49	1,728.94	864.97	1,727.95	864.48	14
5	716.41	358.71			698.40	349.70	V	1,632.88	816.94	1,615.85	808.43	1,614.87	807.94	13
6	817.46	409.23			799.45	400.23	T	1,533.81	767.41	1,516.79	758.90	1,515.80	758.40	12
7	946.50	473.76			928.49	464.75	E	1,432.77	716.89	1,415.74	708.37	1,414.75	707.88	11
8	1,047.55	524.28			1,029.54	515.27	T	1,303.72	652.36	1,286.70	643.85	1,285.71	643.36	10
9	1,176.59	588.80			1,158.58	579.80	E	1,202.67	601.84	1,185.65	593.33	1,184.66	592.84	9
10	1,305.64	653.32			1,287.63	644.32	E	1,073.63	537.32	1,056.61	528.81	1,055.62	528.31	8
11	1,418.72	709.86			1,400.71	700.86	L	944.59	472.80	927.56	464.29	926.58	463.79	7
12	1,489.76	745.38			1,471.75	736.38	A	831.51	416.26	814.48	407.74	813.49	407.25	6
13	1,618.80	809.90			1,600.79	800.90	E	760.47	380.74	743.44	372.22	742.46	371.73	5
14	1,732.84	866.93	1,715.82	858.41	1,714.83	857.92	N	631.43	316.22	614.40	307.70			4
15	1,845.93	923.47	1,828.90	914.95	1,827.92	914.46	I	517.38	259.20	500.36	250.68			3
16	1,959.01	980.01	1,941.98	971.50	1,941.00	971.00	L	404.30	202.65	387.27	194.14			2

17							K	291.21	146.11	274.19	137.60			1

Query 72926 Hit 1

MS/MS Fragmentation of FIAHVPVPSQQEIEEALVR
Found in sp|Q9ULR0|ISY1_HUMAN, Pre-mRNA-splicing factor ISY1 homolog OS=Homo sapiens GN=ISY1 PE=1 SV=3
Match to Query 72926: 2305.247from(769.423,3+)
Title: 848: Sum of 2 scans in range 1903 (rt=52.2309, f=4, i=569) to 1904 (rt=52.2563, f=4, i=570) [D:\lab212\membrane\Grace\Joyce\iTRAQ_31_2.raw]
Data File:Submitted from 20120508-1(merge) by Mascot Daemon on JOYCE-VAIO
Monoisotopic mass of neutral peptide Mr(calc): 2305.247
Variable modifications:
Ions Score: 50.43 Expect: 0.007

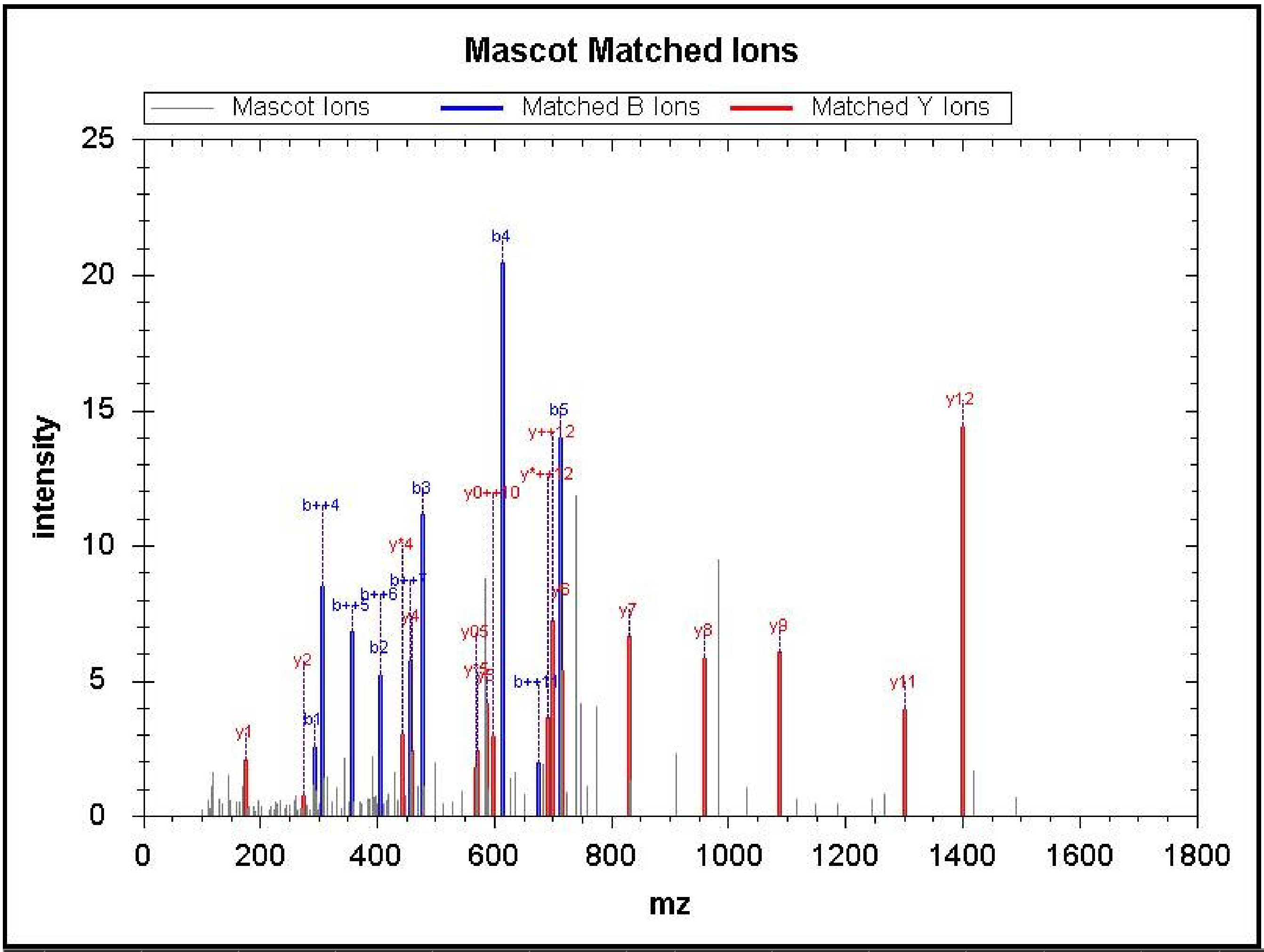

No	b	b++	b*	b*++	b0	b0++	Seq	y	y++	y*	y*++	y0	y0++	RevNo
1	292.18	146.59					F							19
2	405.26	203.13					I	2,015.09	1,008.05	1,998.07	999.54	1,997.08	999.04	18
3	476.30	238.65					A	1,902.01	951.51	1,884.98	942.99	1,884.00	942.50	17
4	613.36	307.18					H	1,830.97	915.99	1,813.94	907.48	1,812.96	906.98	16
5	712.43	356.72					V	1,693.91	847.46	1,676.89	838.95	1,675.90	838.45	15
6	809.48	405.24					P	1,594.84	797.93	1,577.82	789.41	1,576.83	788.92	14
7	908.55	454.78					V	1,497.79	749.40	1,480.76	740.89	1,479.78	740.39	13
8	1,005.60	503.30					P	1,398.72	699.86	1,381.70	691.35	1,380.71	690.86	12
9	1,092.63	546.82			1,074.62	537.81	S	1,301.67	651.34	1,284.64	642.83	1,283.66	642.33	11
10	1,220.69	610.85	1,203.66	602.34	1,202.68	601.84	Q	1,214.64	607.82	1,197.61	599.31	1,196.63	598.82	10
11	1,348.75	674.88	1,331.72	666.37	1,330.74	665.87	Q	1,086.58	543.79	1,069.55	535.28	1,068.57	534.79	9
12	1,477.79	739.40	1,460.77	730.89	1,459.78	730.39	E	958.52	479.76	941.49	471.25	940.51	470.76	8
13	1,590.88	795.94	1,573.85	787.43	1,572.87	786.94	I	829.48	415.24	812.45	406.73	811.47	406.24	7

14	1,719.92	860.46	1,702.89	851.95	1,701.91	851.46	E	716.39	358.70	699.37	350.19	698.38	349.70	6
15	1,848.96	924.98	1,831.93	916.47	1,830.95	915.98	E	587.35	294.18	570.32	285.67	569.34	285.17	5
16	1,920.00	960.50	1,902.97	951.99	1,901.99	951.50	A	458.31	229.66	441.28	221.14			4
17	2,033.08	1,017.04	2,016.06	1,008.53	2,015.07	1,008.04	L	387.27	194.14	370.24	185.63			3
18	2,132.15	1,066.58	2,115.12	1,058.07	2,114.14	1,057.57	V	274.19	137.60	257.16	129.08			2
19							R	175.12	88.06	158.09	79.55			1

Query 45252 Hit 1

MS/MS Fragmentation of VLVDGEEHVGFLK
Found in sp|Q9NPA0|CO024_HUMAN, UPF0480 protein C15orf24 OS=Homo sapiens GN=C15orf24 PE=1 SV=1
Match to Query 45252: 1728.912from(577.3113,3+)
Title: 580: Scan 1286 (rt=38.7193, f=3, i=200) [D:\lab212\membrane\Grace\Joyce\iTRAQ40.raw]
Data File:Submitted from 20120508-1(merge) by Mascot Daemon on JOYCE-VAIO
Monoisotopic mass of neutral peptide Mr(calc): 1728.912
Variable modifications:
K13 :iTRAQ4plex (K)
Ions Score: 50.39 Expect: 0.008

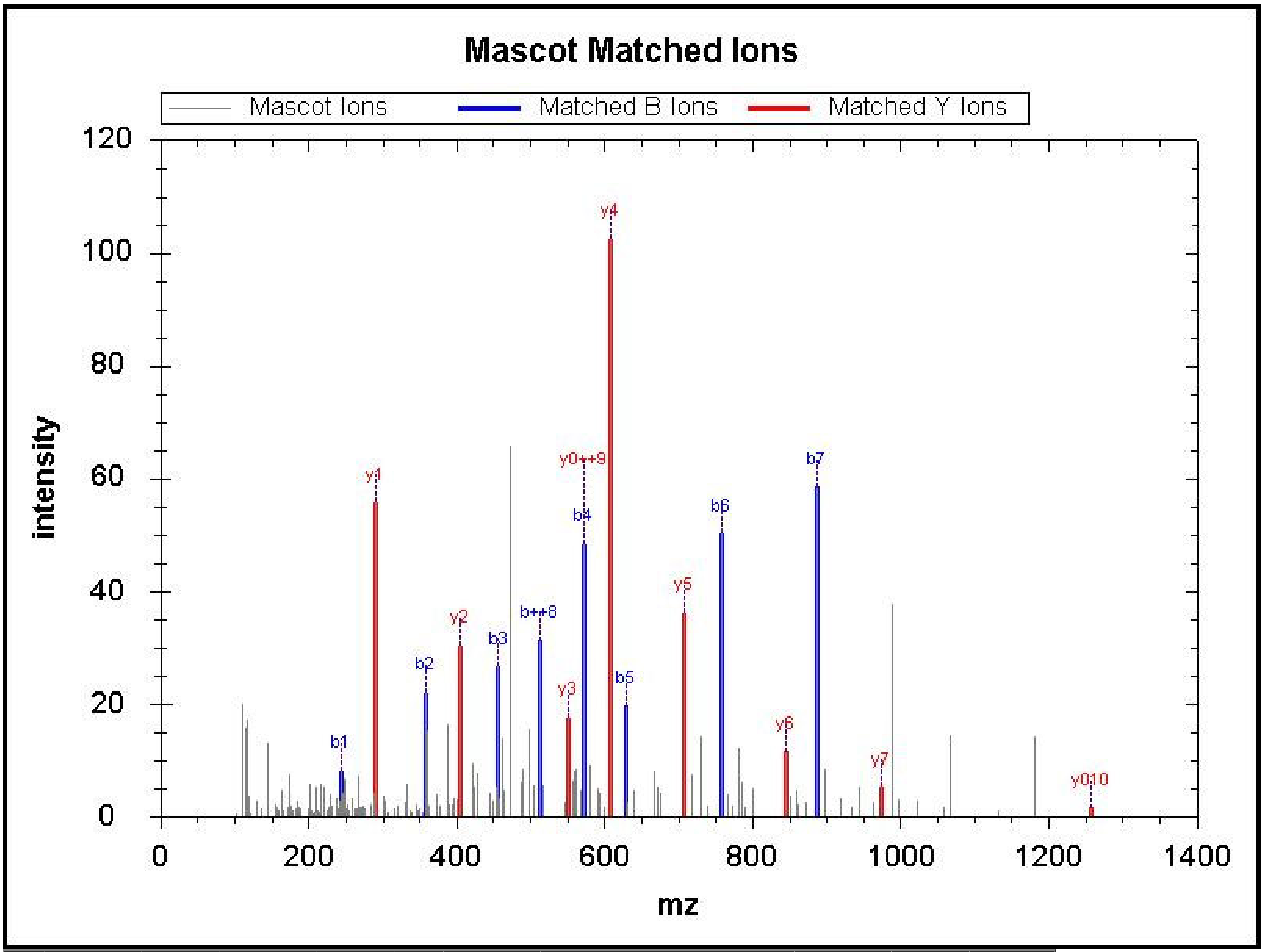

No	b	b++	b0	b0++	Seq	y	y++	y*	y*++	y0	y0++	RevNo
1	244.18	122.59			V							13
2	357.26	179.13			L	1,486.80	743.90	1,469.78	735.39	1,468.79	734.90	12
3	456.33	228.67			V	1,373.72	687.36	1,356.69	678.85	1,355.71	678.36	11
4	571.36	286.18	553.35	277.18	D	1,274.65	637.83	1,257.62	629.32	1,256.64	628.82	10
5	628.38	314.69	610.37	305.69	G	1,159.62	580.32	1,142.60	571.80	1,141.61	571.31	9
6	757.42	379.21	739.41	370.21	E	1,102.60	551.80	1,085.57	543.29	1,084.59	542.80	8
7	886.46	443.74	868.45	434.73	E	973.56	487.28	956.53	478.77	955.55	478.28	7
8	1,023.52	512.27	1,005.51	503.26	H	844.52	422.76	827.49	414.25			6

9	1,122.59	561.80	1,104.58	552.79	V	707.46	354.23	690.43	345.72			5
10	1,179.61	590.31	1,161.60	581.30	G	608.39	304.70	591.36	296.18			4
11	1,326.68	663.84	1,308.67	654.84	F	551.37	276.19	534.34	267.67			3
12	1,439.77	720.39	1,421.75	711.38	L	404.30	202.65	387.27	194.14			2
13					K	291.21	146.11	274.19	137.60			1

Query 73054 Hit 1

MS/MS Fragmentation of GQTVEDLLEVLSDIDEMSR
Found in sp|Q8N201|INT1_HUMAN, Integrator complex subunit 1 OS=Homo sapiens GN=INTS1 PE=1 SV=2
Match to Query 73054: 2308.123from(770.3816,3+)
Title: 898: Sum of 2 scans in range 2847 (rt=71.4021, f=4, i=487) to 2848 (rt=71.4275, f=4, i=488)
[D:\lab212\membrane\Grace\Joyce\iTRAQ19.raw]
Data File:Submitted from 20120508-1(merge) by Mascot Daemon on JOYCE-VAIO
Monoisotopic mass of neutral peptide Mr(calc): 2308.123
Variable modifications:
M17 :Oxidation (M), 63.99828
Ions Score: 50.33 Expect: 0.008

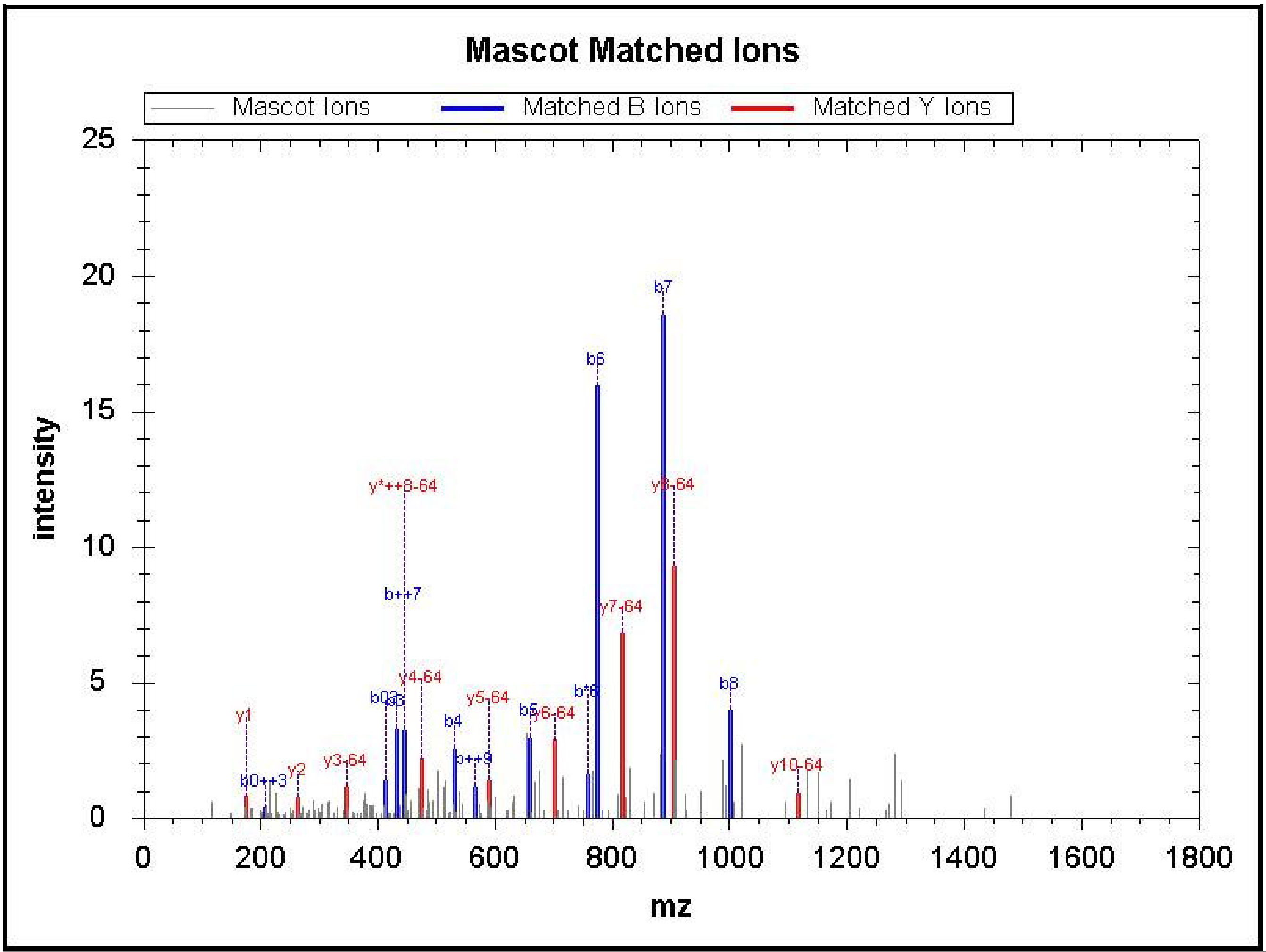

No	b	b++	b*	b*++	b0	b0++	Seq	y	y++	y*	y*++	y0	y0++	RevNo
1	202.13	101.57					G							19
2	330.19	165.60	313.16	157.09			Q	2,044.01	1,022.51	2,026.98	1,013.99	2,026.00	1,013.50	18
3	431.24	216.12	414.21	207.61	413.23	207.12	T	1,915.95	958.48	1,898.92	949.97	1,897.94	949.47	17
4	530.31	265.66	513.28	257.14	512.29	256.65	V	1,814.90	907.95	1,797.88	899.44	1,796.89	898.95	16
5	659.35	330.18	642.32	321.66	641.34	321.17	E	1,715.83	858.42	1,698.81	849.91	1,697.82	849.42	15
6	774.38	387.69	757.35	379.18	756.36	378.69	D	1,586.79	793.90	1,569.76	785.39	1,568.78	784.89	14
7	887.46	444.23	870.43	435.72	869.45	435.23	L	1,471.76	736.39	1,454.74	727.87	1,453.75	727.38	13

8	1,000.54	500.78	983.52	492.26	982.53	491.77	L	1,358.68	679.84	1,341.65	671.33	1,340.67	670.84	12
9	1,129.59	565.30	1,112.56	556.78	1,111.58	556.29	E	1,245.60	623.30	1,228.57	614.79	1,227.58	614.30	11
10	1,228.65	614.83	1,211.63	606.32	1,210.64	605.83	V	1,116.55	558.78	1,099.53	550.27	1,098.54	549.77	10
11	1,341.74	671.37	1,324.71	662.86	1,323.73	662.37	L	1,017.48	509.25	1,000.46	500.73	999.47	500.24	9
12	1,428.77	714.89	1,411.74	706.38	1,410.76	705.88	S	904.40	452.70	887.37	444.19	886.39	443.70	8
13	1,543.80	772.40	1,526.77	763.89	1,525.79	763.40	D	817.37	409.19	800.34	400.67	799.36	400.18	7
14	1,656.88	828.94	1,639.85	820.43	1,638.87	819.94	I	702.34	351.67	685.32	343.16	684.33	342.67	6
15	1,771.91	886.46	1,754.88	877.94	1,753.90	877.45	D	589.26	295.13	572.23	286.62	571.25	286.13	5
16	1,900.95	950.98	1,883.92	942.47	1,882.94	941.97	E	474.23	237.62	457.20	229.11	456.22	228.61	4
17	1,983.99	992.50	1,966.96	983.98	1,965.98	983.49	M	345.19	173.10	328.16	164.58	327.18	164.09	3
18	2,071.02	1,036.01	2,053.99	1,027.50	2,053.01	1,027.01	S	262.15	131.58	245.12	123.07	244.14	122.57	2
19							R	175.12	88.06	158.09	79.55			1

Query 53264 Hit 1

MS/MS Fragmentation of VNPDMNFEVFIHK
Found in sp|Q9HB90|RRAGC_HUMAN, Ras-related GTP-binding protein C OS=Homo sapiens GN=RRAGC PE=1 SV=1
Match to Query 53264: 1876.942from(626.6545,3+)
Title: 764: Sum of 2 scans in range 1665 (rt=47.2628, f=4, i=517) to 1666 (rt=47.2882, f=4, i=518)
[D:\lab212\membrane\Grace\Joyce\iTRAQ40.raw]
Data File:Submitted from 20120508-1(merge) by Mascot Daemon on JOYCE-VAIO
Monoisotopic mass of neutral peptide Mr(calc): 1876.942
Variable modifications:
K13 :iTRAQ4plex (K)
Ions Score: 50.32 Expect: 0.009

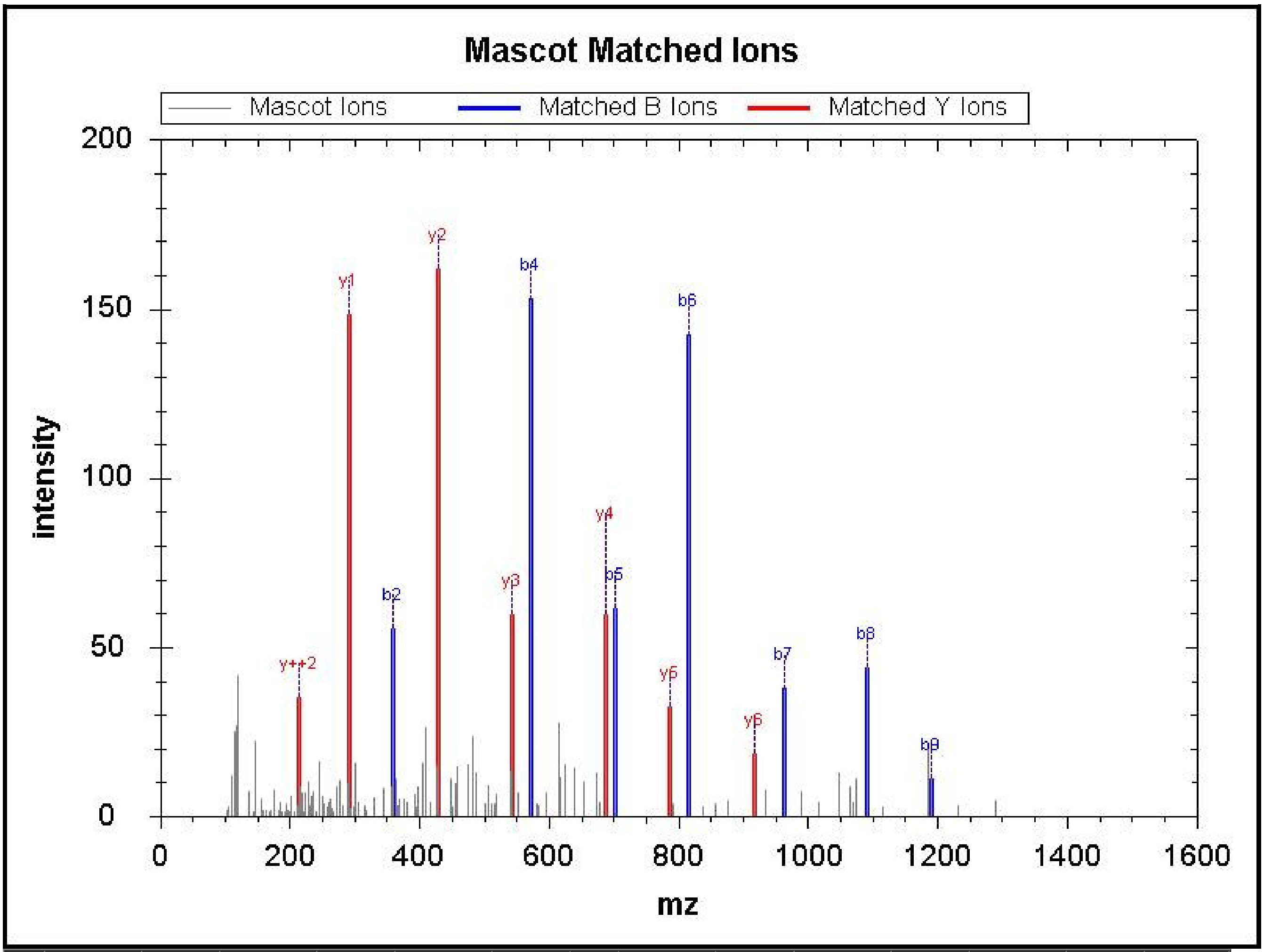

No	b	b++	b*	b*++	b0	b0++	Seq	y	y++	y*	y*++	y0	y0++	RevNo
1	244.18	122.59					V							13

2	358.22	179.61	341.19	171.10			N	1,634.81	817.91	1,617.79	809.40	1,616.80	808.90	12
3	455.27	228.14	438.25	219.63			P	1,520.77	760.89	1,503.74	752.37	1,502.76	751.88	11
4	570.30	285.65	553.27	277.14	552.29	276.65	D	1,423.72	712.36	1,406.69	703.85	1,405.71	703.36	10
5	701.34	351.17	684.31	342.66	683.33	342.17	M	1,308.69	654.85	1,291.66	646.34	1,290.68	645.84	9
6	815.38	408.20	798.36	399.68	797.37	399.19	N	1,177.65	589.33	1,160.62	580.81	1,159.64	580.32	8
7	962.45	481.73	945.43	473.22	944.44	472.72	F	1,063.61	532.31	1,046.58	523.79	1,045.60	523.30	7
8	1,091.49	546.25	1,074.47	537.74	1,073.48	537.25	E	916.54	458.77	899.51	450.26	898.53	449.77	6
9	1,190.56	595.79	1,173.54	587.27	1,172.55	586.78	V	787.49	394.25	770.47	385.74			5
10	1,337.63	669.32	1,320.61	660.81	1,319.62	660.31	F	688.43	344.72	671.40	336.20			4
11	1,450.72	725.86	1,433.69	717.35	1,432.71	716.86	I	541.36	271.18	524.33	262.67			3
12	1,587.77	794.39	1,570.75	785.88	1,569.76	785.39	H	428.27	214.64	411.25	206.13			2
13							K	291.21	146.11	274.19	137.60			1

Query 37666 Hit 1

MS/MS Fragmentation of HLEEIVHVEQGR
Found in sp|Q01433|AMPD2_HUMAN, AMP deaminase 2 OS=Homo sapiens GN=AMPD2 PE=1 SV=2
Match to Query 37666: 1588.849from(530.6238,3+)
Title: 335: Scan 764 (rt=26.8968, f=3, i=114) [D:\lab212\membrane\Grace\Joyce\iTRAQ_50_2.raw]
Data File:Submitted from 20120508-1(merge) by Mascot Daemon on JOYCE-VAIO
Monoisotopic mass of neutral peptide Mr(calc): 1588.849
Variable modifications:
Ions Score: 50.28 Expect: 0.008

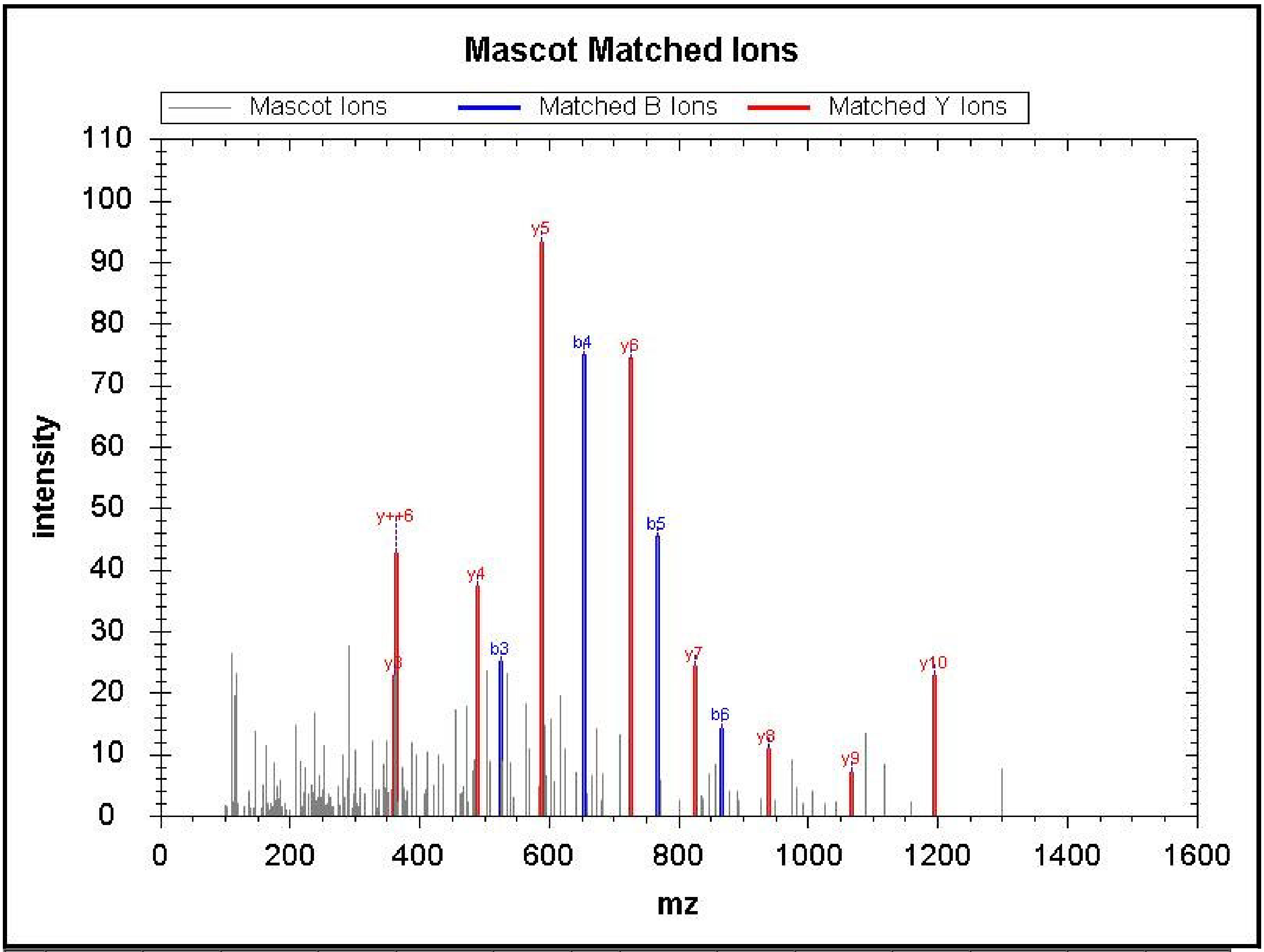

No	b	b++	b*	b*++	b0	b0++	Seq	y	y++	y*	y*++	y0	y0++	RevNo
1	282.17	141.59					H							12
2	395.25	198.13					L	1,308.69	654.85	1,291.66	646.34	1,290.68	645.84	11
3	524.29	262.65			506.28	253.65	E	1,195.61	598.31	1,178.58	589.79	1,177.60	589.30	10

4	653.34	327.17			635.33	318.17	E	1,066.56	533.79	1,049.54	525.27	1,048.55	524.78	9
5	766.42	383.71			748.41	374.71	I	937.52	469.26	920.49	460.75	919.51	460.26	8
6	865.49	433.25			847.48	424.24	V	824.44	412.72	807.41	404.21	806.43	403.72	7
7	1,002.55	501.78			984.54	492.77	H	725.37	363.19	708.34	354.67	707.36	354.18	6
8	1,101.62	551.31			1,083.61	542.31	V	588.31	294.66	571.28	286.15	570.30	285.65	5
9	1,230.66	615.83			1,212.65	606.83	E	489.24	245.12	472.22	236.61	471.23	236.12	4
10	1,358.72	679.86	1,341.69	671.35	1,340.71	670.86	Q	360.20	180.60	343.17	172.09			3
11	1,415.74	708.37	1,398.71	699.86	1,397.73	699.37	G	232.14	116.57	215.11	108.06			2
12							R	175.12	88.06	158.09	79.55			1

Query 52691 Hit 1

MS/MS Fragmentation of IQHAVQLATEPLEK
Found in sp|P49589|SYCC_HUMAN, Cysteine--tRNA ligase
Match to Query 52691: 1864.068from(622.3633,3+)
Title: 548: Scan 1226 (rt=37.2987, f=3, i=191) [D:\lab212\membrane\Grace\Joyce\iTRAQ_40_1.raw]
Data File:Submitted from 20120508-1(merge) by Mascot Daemon on JOYCE-VAIO
Monoisotopic mass of neutral peptide Mr(calc): 1864.068
Variable modifications:
K14 :iTRAQ4plex (K)
Ions Score: 50.27 Expect: 0.005

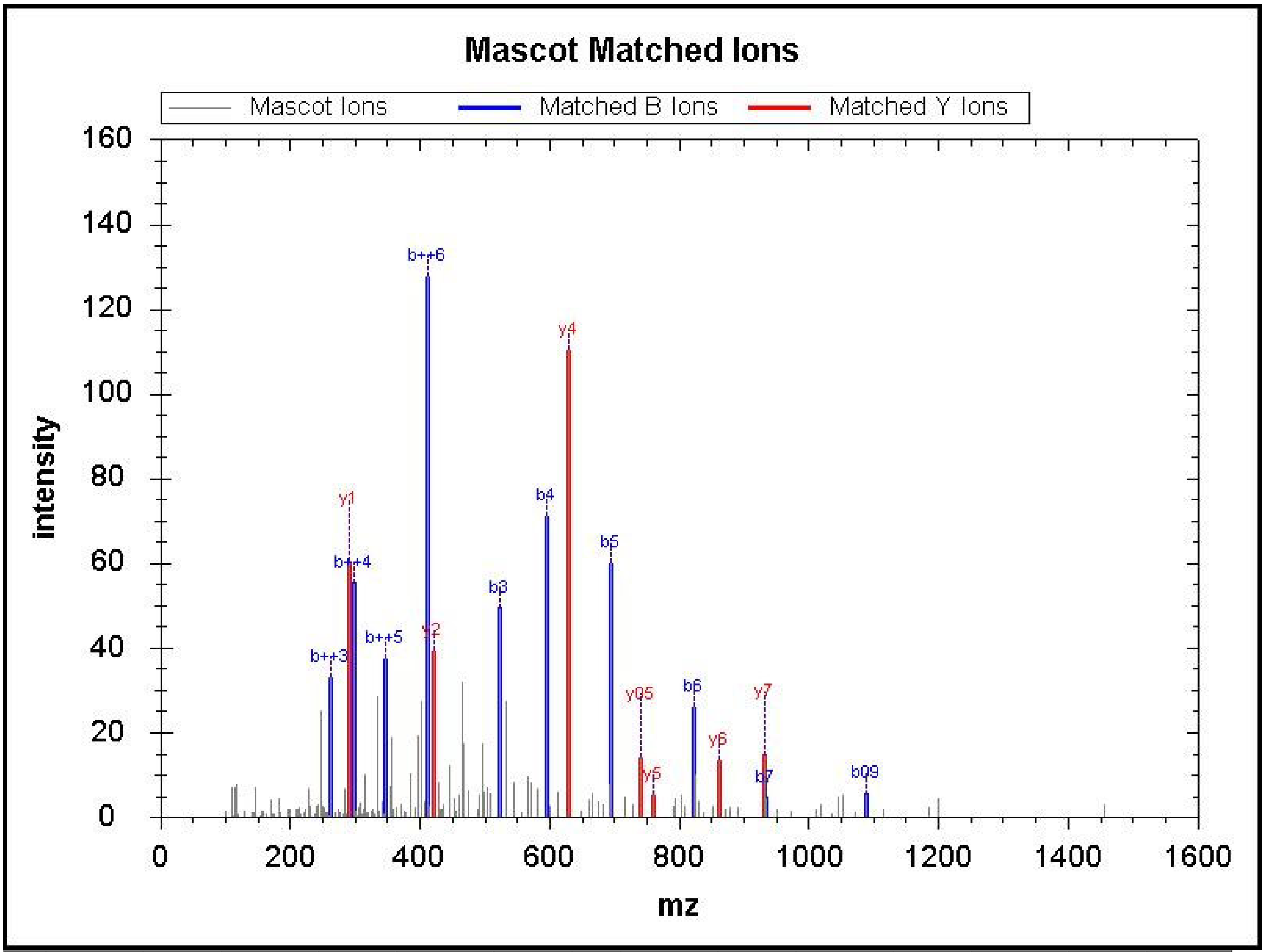

No	b	b++	b*	b*++	b0	b0++	Seq	y	y++	y*	y*++	y0	y0++	RevNo
1	258.19	129.60					I							14
2	386.25	193.63	369.23	185.12			Q	1,607.89	804.45	1,590.86	795.93	1,589.88	795.44	13
3	523.31	262.16	506.28	253.65			H	1,479.83	740.42	1,462.80	731.90	1,461.82	731.41	12
4	594.35	297.68	577.32	289.16			A	1,342.77	671.89	1,325.74	663.38	1,324.76	662.88	11
5	693.42	347.21	676.39	338.70			V	1,271.73	636.37	1,254.71	627.86	1,253.72	627.36	10

6	821.47	411.24	804.45	402.73			Q	1,172.66	586.84	1,155.64	578.32	1,154.65	577.83	9
7	934.56	467.78	917.53	459.27			L	1,044.61	522.81	1,027.58	514.29	1,026.60	513.80	8
8	1,005.60	503.30	988.57	494.79			A	931.52	466.26	914.50	457.75	913.51	457.26	7
9	1,106.64	553.83	1,089.62	545.31	1,088.63	544.82	T	860.48	430.75	843.46	422.23	842.47	421.74	6
10	1,235.69	618.35	1,218.66	609.83	1,217.68	609.34	E	759.44	380.22	742.41	371.71	741.43	371.22	5
11	1,332.74	666.87	1,315.71	658.36	1,314.73	657.87	P	630.39	315.70	613.37	307.19	612.38	306.70	4
12	1,445.82	723.42	1,428.80	714.90	1,427.81	714.41	L	533.34	267.17	516.31	258.66	515.33	258.17	3
13	1,574.87	787.94	1,557.84	779.42	1,556.86	778.93	E	420.26	210.63	403.23	202.12	402.25	201.63	2
14							K	291.21	146.11	274.19	137.60			1

Query 25904 Hit 1

MS/MS Fragmentation of LLLEQILNK
Found in sp|O43913|ORC5_HUMAN, Origin recognition complex subunit 5 OS=Homo sapiens GN=ORC5 PE=1 SV=1
Match to Query 25904: 1370.78from(686.3972,2+)
Title: 703: Scan 2170 (rt=55.1648, f=3, i=238) [D:\lab212\membrane\Grace\Joyce\iTRAQ_29_2.raw]
Data File:Submitted from 20120508-1(merge) by Mascot Daemon on JOYCE-VAIO
Monoisotopic mass of neutral peptide Mr(calc): 1370.78
Variable modifications:
K9 :iTRAQ4plex (K)
Ions Score: 50.24 Expect: 0.006

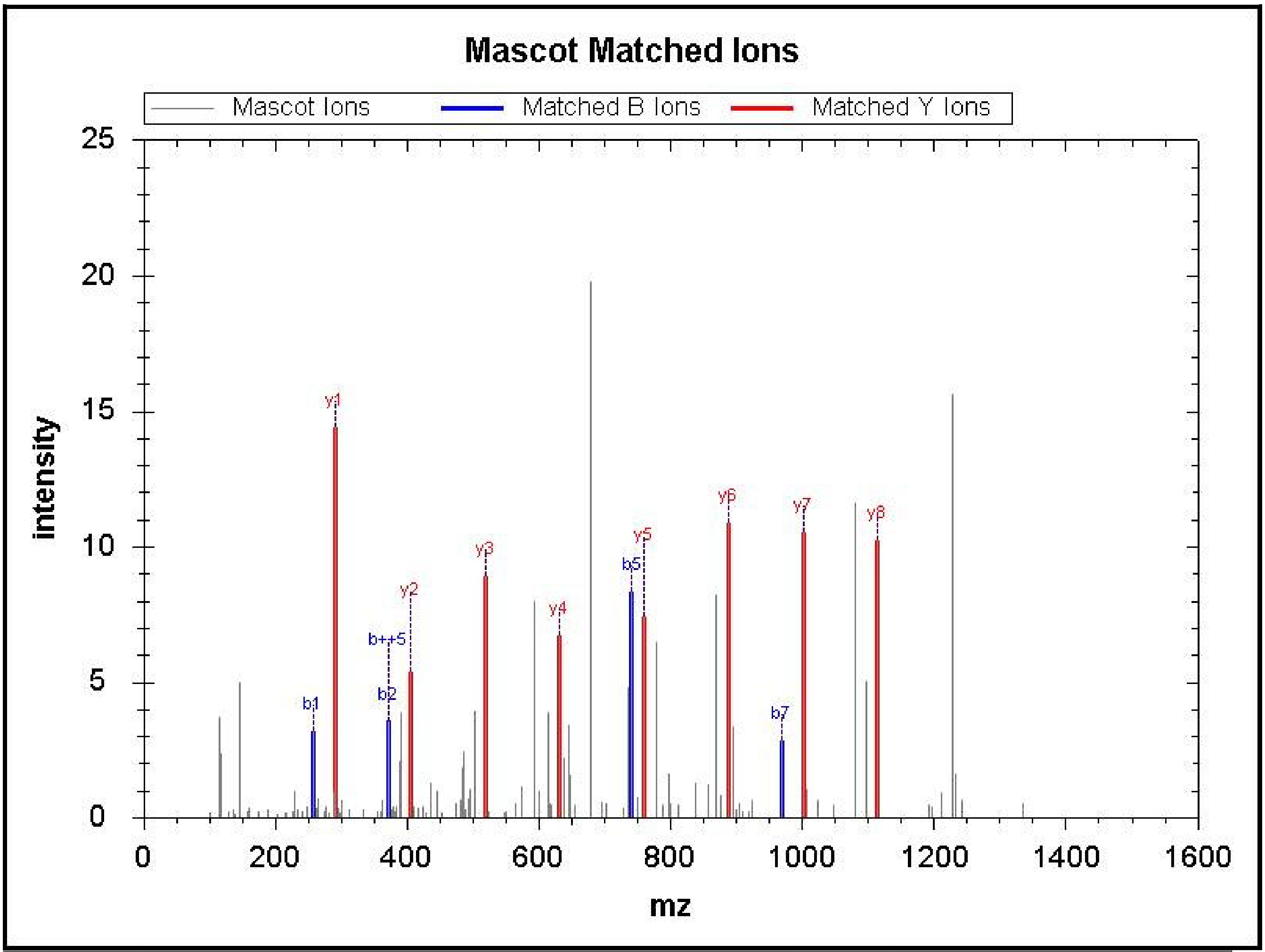

No	b	b++	b*	b*++	b0	b0++	Seq	y	y++	y*	y*++	y0	y0++	RevNo
1	258.19	129.60					L							9
2	371.28	186.14					L	1,114.70	557.85	1,097.67	549.34	1,096.68	548.85	8
3	484.36	242.68					L	1,001.61	501.31	984.58	492.80	983.60	492.30	7
4	613.40	307.21			595.39	298.20	E	888.53	444.77	871.50	436.25	870.52	435.76	6
5	741.46	371.23	724.44	362.72	723.45	362.23	Q	759.48	380.25	742.46	371.73			5

6	854.55	427.78	837.52	419.26	836.54	418.77	I	631.43	316.22	614.40	307.70			4
7	967.63	484.32	950.60	475.81	949.62	475.31	L	518.34	259.67	501.32	251.16			3
8	1,081.67	541.34	1,064.65	532.83	1,063.66	532.34	N	405.26	203.13	388.23	194.62			2
9							K	291.21	146.11	274.19	137.60			1

Query 40807 Hit 1

MS/MS Fragmentation of VLGQPHHELDSK
Found in sp|Q96DH6|MSI2H_HUMAN, RNA-binding protein Musashi homolog 2 OS=Homo sapiens GN=MSI2 PE=1 SV=1
Match to Query 40807: 1646.9from(549.9739,3+)
Title: 103: Scan 501 (rt=19.9529, f=2, i=67) [D:\lab212\membrane\Grace\Joyce\iTRAQ_51_2.raw]
Data File:Submitted from 20120508-1(merge) by Mascot Daemon on JOYCE-VAIO
Monoisotopic mass of neutral peptide Mr(calc): 1646.9
Variable modifications:
K12 :iTRAQ4plex (K)
Ions Score: 50.21 Expect: 0.008

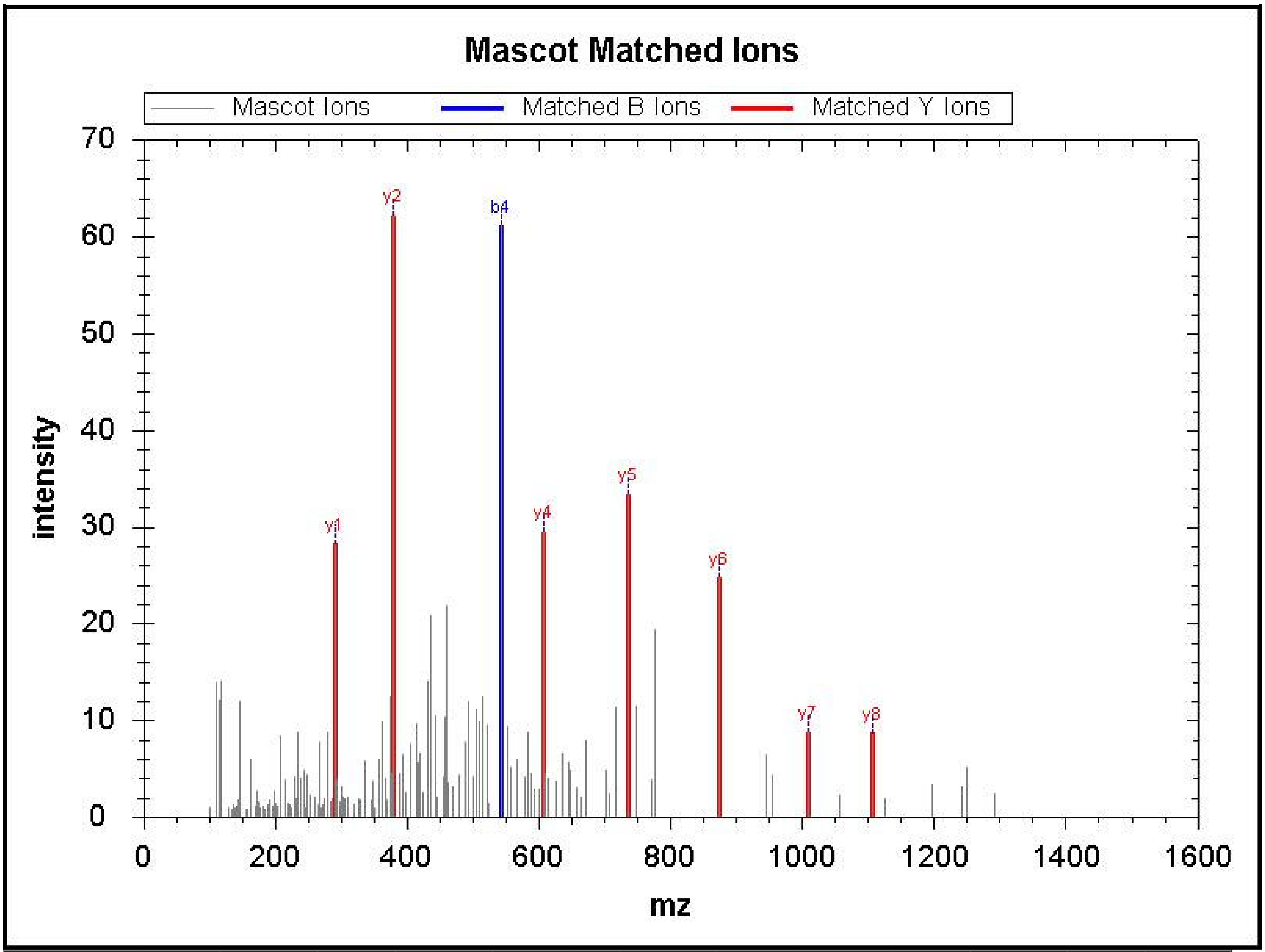

No	b	b++	b*	b*++	b0	b0++	Seq	y	y++	y*	y*++	y0	y0++	RevNo
1	244.18	122.59					V							12
2	357.26	179.13					L	1,404.74	702.87	1,387.71	694.36	1,386.72	693.87	11
3	414.28	207.65					G	1,291.65	646.33	1,274.62	637.82	1,273.64	637.32	10
4	542.34	271.67	525.32	263.16			Q	1,234.63	617.82	1,217.60	609.31	1,216.62	608.81	9
5	639.39	320.20	622.37	311.69			P	1,106.57	553.79	1,089.54	545.28	1,088.56	544.78	8
6	776.45	388.73	759.43	380.22			H	1,009.52	505.26	992.49	496.75	991.51	496.26	7
7	913.51	457.26	896.49	448.75			H	872.46	436.73	855.43	428.22	854.45	427.73	6
8	1,042.56	521.78	1,025.53	513.27	1,024.54	512.78	E	735.40	368.20	718.37	359.69	717.39	359.20	5
9	1,155.64	578.32	1,138.61	569.81	1,137.63	569.32	L	606.36	303.68	589.33	295.17	588.35	294.68	4
10	1,270.67	635.84	1,253.64	627.32	1,252.66	626.83	D	493.27	247.14	476.25	238.63	475.26	238.14	3

11	1,357.70	679.35	1,340.67	670.84	1,339.69	670.35	S	378.25	189.63	361.22	181.11	360.24	180.62	2
12							K	291.21	146.11	274.19	137.60			1

Query 20499 Hit 1

MS/MS Fragmentation of SEVAAENHLR
Found in sp|Q14554|PDIA5_HUMAN, Protein disulfide-isomerase A5 OS=Homo sapiens GN=PDIA5 PE=1 SV=1
Match to Query 20499: 1268.658from(423.8932,3+)
Title: 101: Sum of 2 scans in range 324 (rt=16.8319, f=4, i=63) to 325 (rt=16.8574, f=4, i=64)
[D:\lab212\membrane\Grace\Joyce\iTRAQ_41_2.raw]
Data File:Submitted from 20120508-1(merge) by Mascot Daemon on JOYCE-VAIO
Monoisotopic mass of neutral peptide Mr(calc): 1268.658
Variable modifications:
Ions Score: 50.15 Expect: 0.007

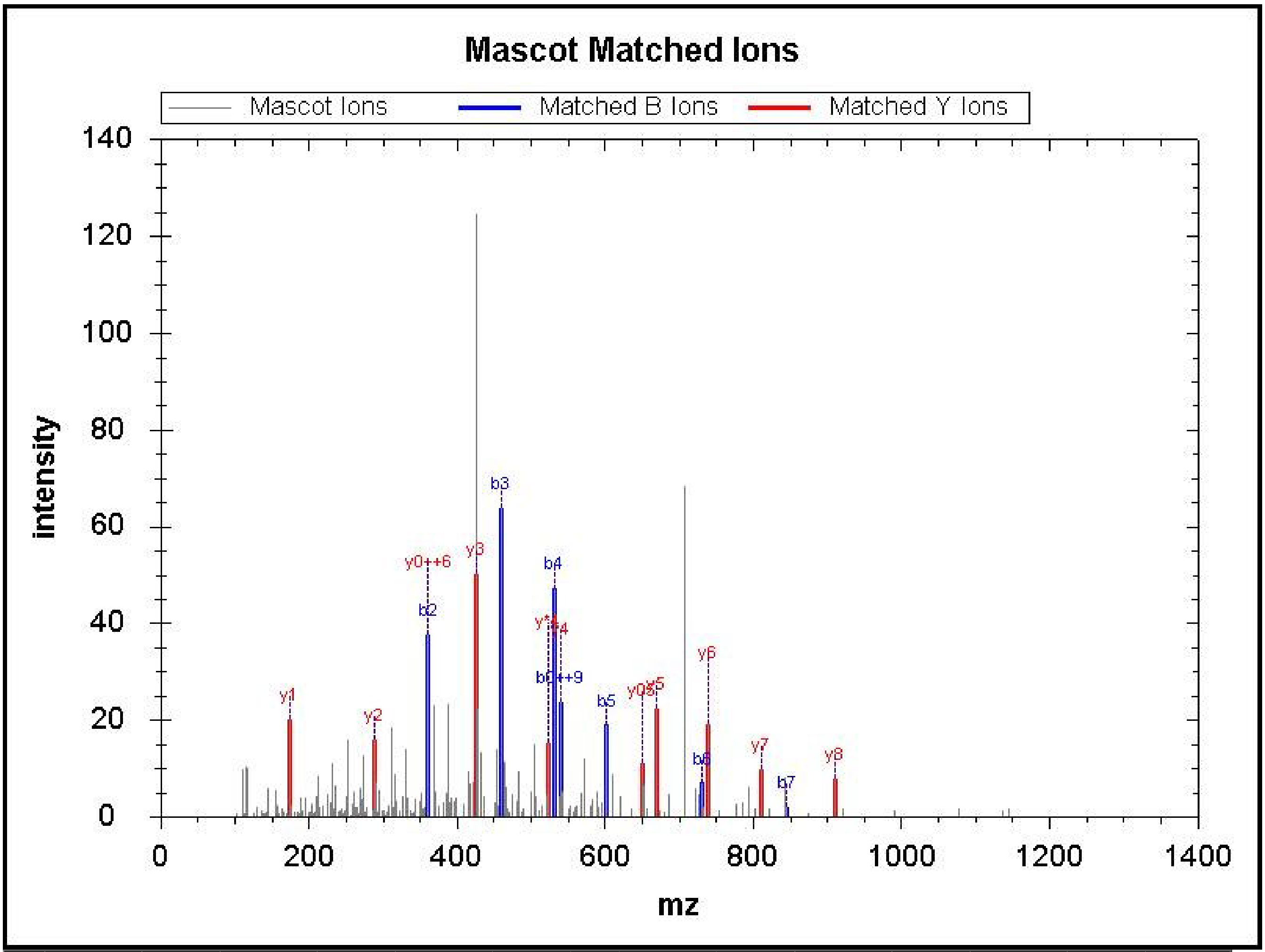

No	b	b++	b*	b*++	b0	b0++	Seq	y	y++	y*	y*++	y0	y0++	RevNo
1	232.14	116.57			214.13	107.57	S							10
2	361.18	181.10			343.17	172.09	E	1,038.53	519.77	1,021.51	511.26	1,020.52	510.76	9
3	460.25	230.63			442.24	221.62	V	909.49	455.25	892.46	446.74	891.48	446.24	8
4	531.29	266.15			513.28	257.14	A	810.42	405.71	793.40	397.20	792.41	396.71	7
5	602.33	301.67			584.32	292.66	A	739.38	370.20	722.36	361.68	721.37	361.19	6
6	731.37	366.19			713.36	357.18	E	668.35	334.68	651.32	326.16	650.34	325.67	5
7	845.41	423.21	828.39	414.70	827.40	414.20	N	539.30	270.16	522.28	261.64			4
8	982.47	491.74	965.44	483.23	964.46	482.73	H	425.26	213.13	408.24	204.62			3
9	1,095.56	548.28	1,078.53	539.77	1,077.54	539.28	L	288.20	144.61	271.18	136.09			2
10							R	175.12	88.06	158.09	79.55			1

Query 43405 Hit 1

MS/MS Fragmentation of SQLQDTLIHLIK
Found in sp|Q9NPI6|DCP1A_HUMAN, mRNA-decapping enzyme 1A OS=Homo sapiens GN=DCP1A PE=1 SV=2
Match to Query 43405: 1696.01 from(566.3438,3+)
Title: 850: Scan 1852 (rt=51.4046, f=3, i=292) [D:\lab212\membrane\Grace\Joyce\iTRAQ_40_1.raw]
Data File:Submitted from 20120508-1(merge) by Mascot Daemon on JOYCE-VAIO
Monoisotopic mass of neutral peptide Mr(calc): 1696.01
Variable modifications:
K12 :iTRAQ4plex (K)
Ions Score: 50.14 Expect: 0.004

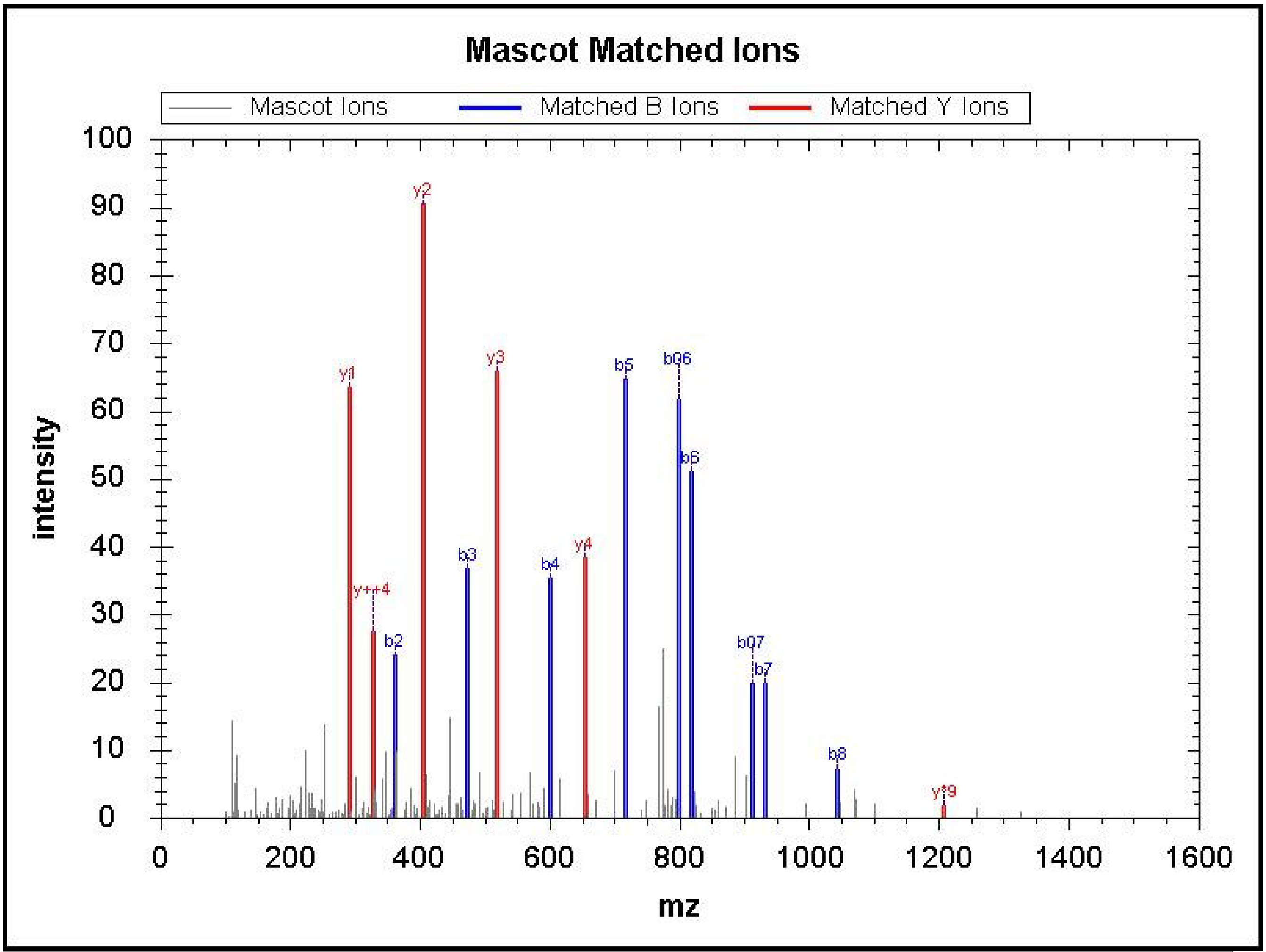

No	b	b++	b*	b*++	b0	b0++	Seq	y	y++	y*	y*++	y0	y0++	RevNo
1	232.14	116.57			214.13	107.57	S							12
2	360.20	180.60	343.17	172.09	342.19	171.60	Q	1,465.89	733.45	1,448.86	724.93	1,447.88	724.44	11
3	473.28	237.15	456.26	228.63	455.27	228.14	L	1,337.83	669.42	1,320.80	660.90	1,319.82	660.41	10
4	601.34	301.17	584.32	292.66	583.33	292.17	Q	1,224.74	612.88	1,207.72	604.36	1,206.73	603.87	9
5	716.37	358.69	699.34	350.18	698.36	349.68	D	1,096.68	548.85	1,079.66	540.33	1,078.67	539.84	8
6	817.42	409.21	800.39	400.70	799.41	400.21	T	981.66	491.33	964.63	482.82	963.65	482.33	7
7	930.50	465.75	913.47	457.24	912.49	456.75	L	880.61	440.81	863.58	432.30			6
8	1,043.59	522.30	1,026.56	513.78	1,025.57	513.29	I	767.53	384.27	750.50	375.75			5
9	1,180.64	590.83	1,163.62	582.31	1,162.63	581.82	H	654.44	327.72	637.42	319.21			4
10	1,293.73	647.37	1,276.70	638.85	1,275.72	638.36	L	517.38	259.20	500.36	250.68			3
11	1,406.81	703.91	1,389.79	695.40	1,388.80	694.90	I	404.30	202.65	387.27	194.14			2
12							K	291.21	146.11	274.19	137.60			1

Query 69579 Hit 1

MS/MS Fragmentation of WLIKEELEEMLVEK
Found in sp|P82933|RT09_HUMAN, 28S ribosomal protein S9
Match to Query 69579: 2220.247 from(741.0897,3+)

Title: 1079: Scan 2332 (rt=62.2758, f=3, i=369) [D:\lab212\membrane\Grace\Joyce\iTRAQ_43_1.raw]
Data File:Submitted from 20120508-1(merge) by Mascot Daemon on JOYCE-VAIO
Monoisotopic mass of neutral peptide Mr(calc): 2220.247
Variable modifications:
K4 :iTRAQ4plex (K)
K14 :iTRAQ4plex (K)
Ions Score: 50.12 Expect: 0.005

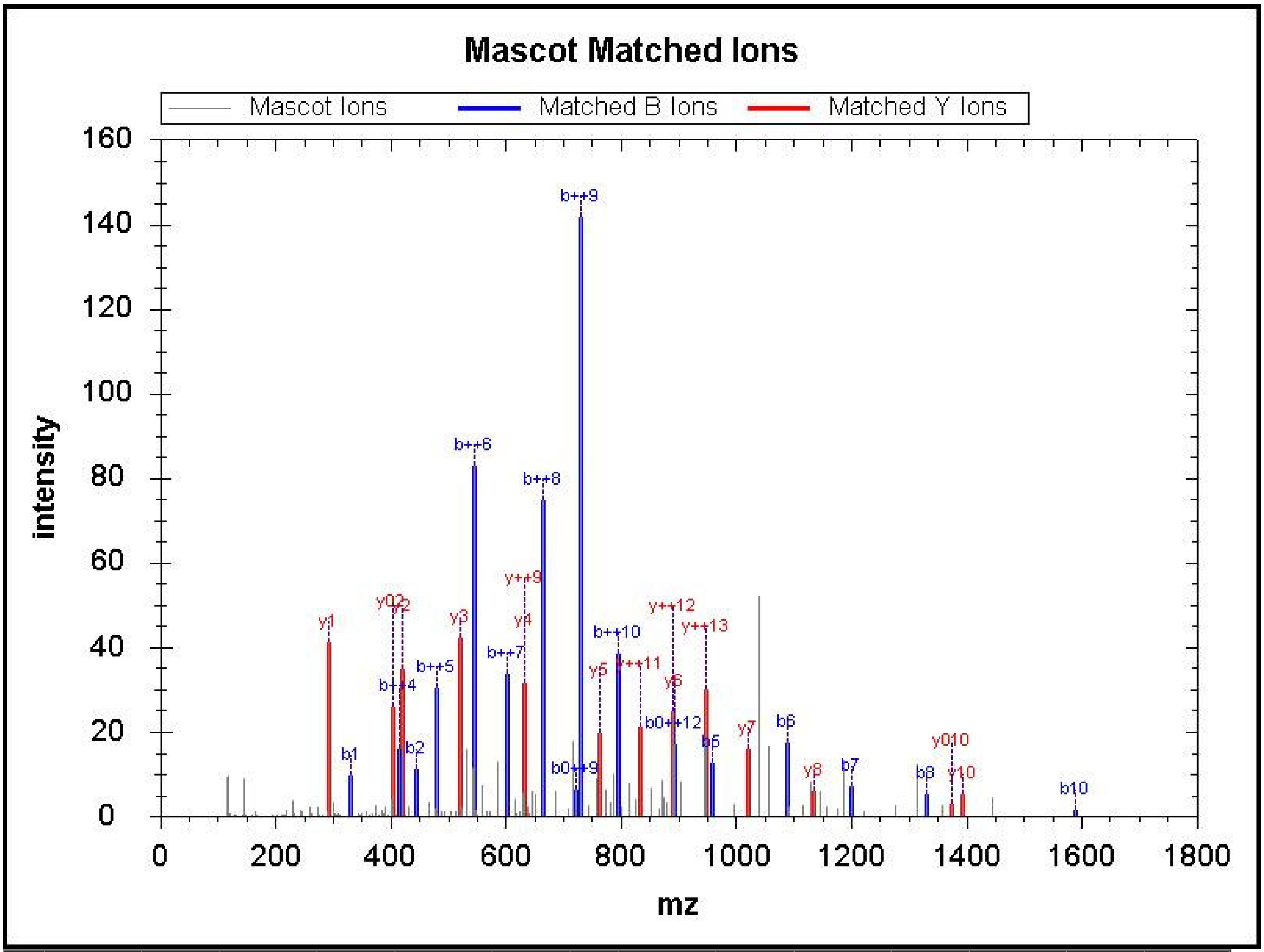

No	b	b++	b*	b*++	b0	b0++	Seq	y	y++	y*	y*++	y0	y0++	RevNo
1	331.19	166.10					W							14
2	444.27	222.64					L	1,891.07	946.04	1,874.04	937.53	1,873.06	937.03	13
3	557.36	279.18					I	1,777.99	889.50	1,760.96	880.98	1,759.98	880.49	12
4	829.55	415.28	812.53	406.77			K	1,664.90	832.95	1,647.88	824.44	1,646.89	823.95	11
5	958.60	479.80	941.57	471.29	940.59	470.80	E	1,392.70	696.86	1,375.68	688.34	1,374.69	687.85	10
6	1,087.64	544.32	1,070.61	535.81	1,069.63	535.32	E	1,263.66	632.33	1,246.64	623.82	1,245.65	623.33	9
7	1,200.72	600.87	1,183.70	592.35	1,182.71	591.86	L	1,134.62	567.81	1,117.59	559.30	1,116.61	558.81	8
8	1,329.77	665.39	1,312.74	656.87	1,311.76	656.38	E	1,021.54	511.27	1,004.51	502.76	1,003.53	502.27	7
9	1,458.81	729.91	1,441.78	721.39	1,440.80	720.90	E	892.49	446.75	875.47	438.24	874.48	437.74	6
10	1,589.85	795.43	1,572.82	786.91	1,571.84	786.42	M	763.45	382.23	746.42	373.72	745.44	373.22	5
11	1,702.93	851.97	1,685.91	843.46	1,684.92	842.96	L	632.41	316.71	615.38	308.20	614.40	307.70	4
12	1,802.00	901.50	1,784.97	892.99	1,783.99	892.50	V	519.33	260.17	502.30	251.65	501.32	251.16	3
13	1,931.04	966.03	1,914.02	957.51	1,913.03	957.02	E	420.26	210.63	403.23	202.12	402.25	201.63	2
14							K	291.21	146.11	274.19	137.60			1

Query 66941 Hit 1

MS/MS Fragmentation of IKPSQNTETLELLLK
Found in sp|Q9NVV4|PAPD1_HUMAN, Poly(A) RNA polymerase

Match to Query 66941: 2158.294from(720.4386,3+)
Title: 731: Scan 1644 (rt=46.6217, f=2, i=266) [D:\lab212\membrane\Grace\Joyce\iTRAQ_44_1.raw]
Data File:Submitted from 20120508-1(merge) by Mascot Daemon on JOYCE-VAIO
Monoisotopic mass of neutral peptide Mr(calc): 2158.294
Variable modifications:
K2 :iTRAQ4plex (K)
K15 :iTRAQ4plex (K)
Ions Score: 50.05 Expect: 0.002

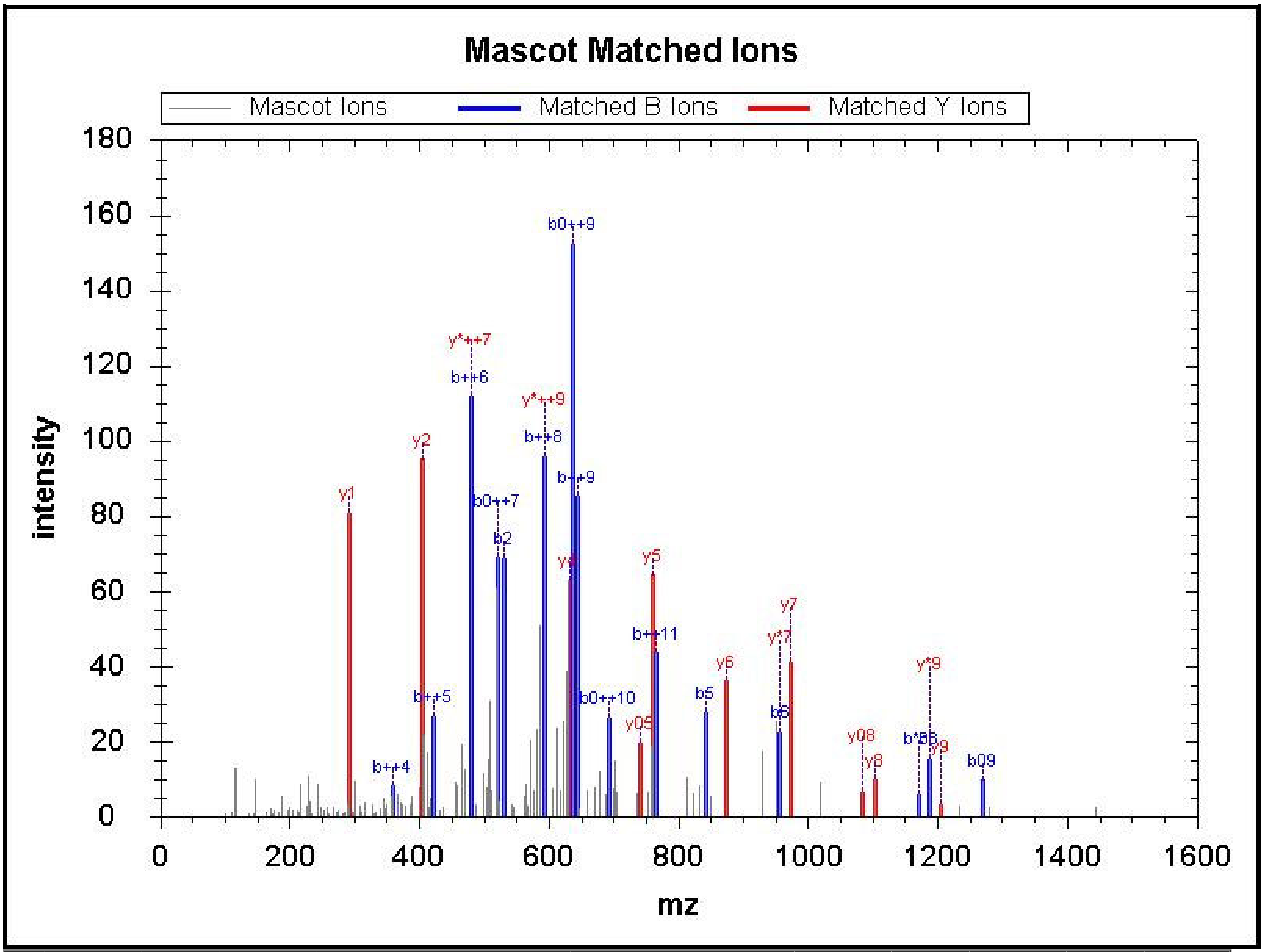

No	b	b++	b*	b*++	b0	b0++	Seq	y	y++	y*	y*++	y0	y0++	RevNo
1	258.19	129.60					I							15
2	530.39	265.70	513.36	257.19			K	1,902.11	951.56	1,885.09	943.05	1,884.10	942.56	14
3	627.44	314.23	610.42	305.71			P	1,629.92	815.46	1,612.89	806.95	1,611.91	806.46	13
4	714.48	357.74	697.45	349.23	696.46	348.74	S	1,532.87	766.94	1,515.84	758.42	1,514.85	757.93	12
5	842.53	421.77	825.51	413.26	824.52	412.77	Q	1,445.83	723.42	1,428.81	714.91	1,427.82	714.41	11
6	956.58	478.79	939.55	470.28	938.57	469.79	N	1,317.77	659.39	1,300.75	650.88	1,299.76	650.39	10
7	1,057.62	529.32	1,040.60	520.80	1,039.61	520.31	T	1,203.73	602.37	1,186.71	593.86	1,185.72	593.36	9
8	1,186.67	593.84	1,169.64	585.32	1,168.66	584.83	E	1,102.68	551.85	1,085.66	543.33	1,084.67	542.84	8
9	1,287.71	644.36	1,270.69	635.85	1,269.70	635.36	T	973.64	487.32	956.61	478.81	955.63	478.32	7
10	1,400.80	700.90	1,383.77	692.39	1,382.79	691.90	L	872.59	436.80	855.57	428.29	854.58	427.80	6
11	1,529.84	765.42	1,512.82	756.91	1,511.83	756.42	E	759.51	380.26	742.48	371.75	741.50	371.25	5
12	1,642.93	821.97	1,625.90	813.45	1,624.92	812.96	L	630.47	315.74	613.44	307.22			4
13	1,756.01	878.51	1,738.98	870.00	1,738.00	869.50	L	517.38	259.20	500.36	250.68			3
14	1,869.09	935.05	1,852.07	926.54	1,851.08	926.05	L	404.30	202.65	387.27	194.14			2
15							K	291.21	146.11	274.19	137.60			1

Query 29962 Hit 1

MS/MS Fragmentation of LNISEVDEQVR
Found in sp|Q01432|AMPD3_HUMAN, AMP deaminase 3 OS=Homo sapiens GN=AMPD3 PE=1 SV=1
Match to Query 29962: 1445.71 from(723.8621,2+)
Title: 257: Scan 670 (rt=24.4366, f=3, i=89) [D:\lab212\membrane\Grace\Joyce\iTRAQ_25_2.raw]
Data File:Submitted from 20120508-1(merge) by Mascot Daemon on JOYCE-VAIO
Monoisotopic mass of neutral peptide Mr(calc): 1445.71
Variable modifications:
Q9 :Deamidated (NQ)
Ions Score: 49.93 Expect: 0.008

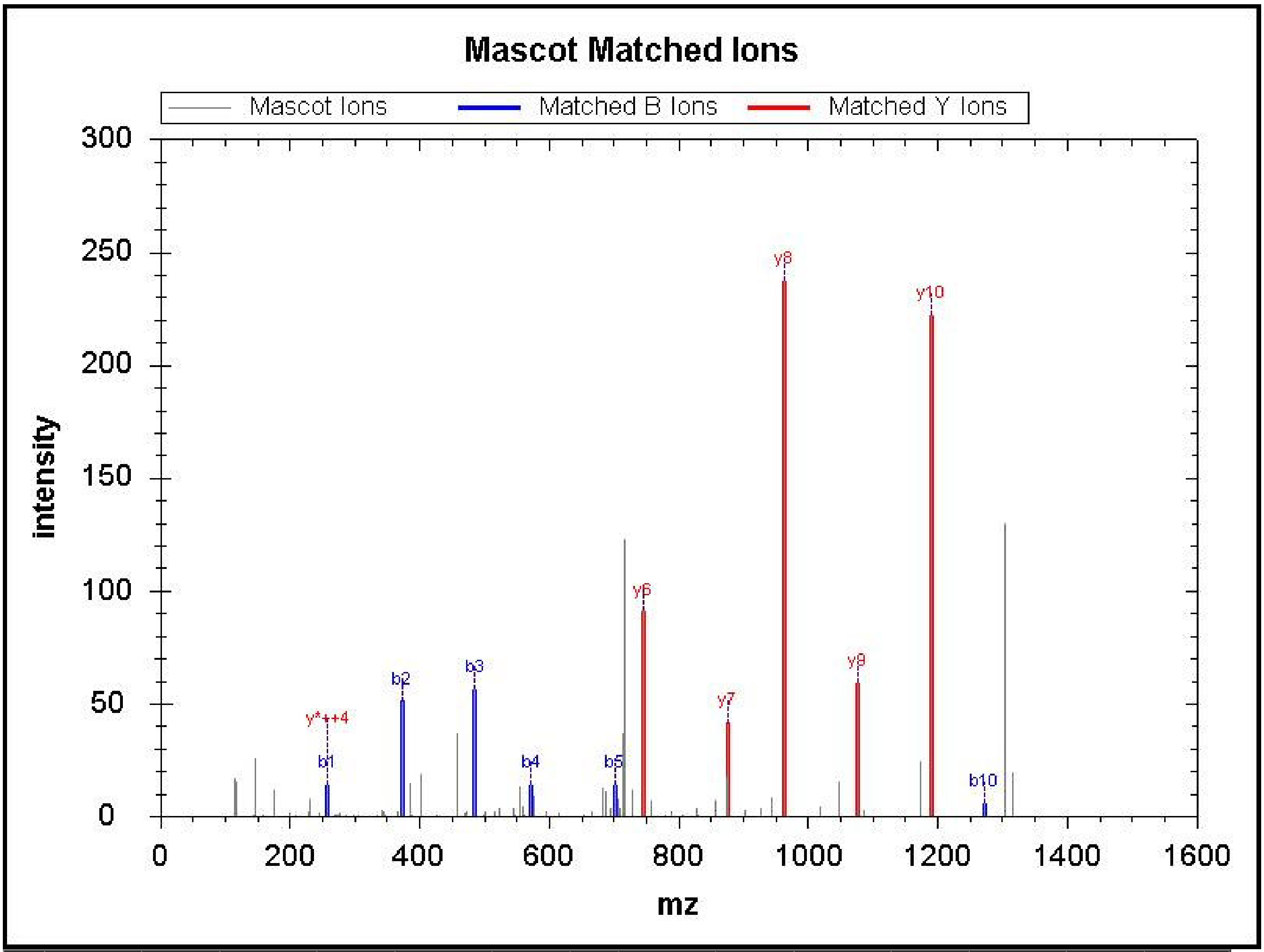

No	b	b++	b*	b*++	b0	b0++	Seq	y	y++	y*	y*++	y0	y0++	RevNo
1	258.19	129.60					L							11
2	372.24	186.62	355.21	178.11			N	1,189.57	595.29	1,172.54	586.78	1,171.56	586.28	10
3	485.32	243.16	468.29	234.65			I	1,075.53	538.27	1,058.50	529.75	1,057.52	529.26	9
4	572.35	286.68	555.33	278.17	554.34	277.67	S	962.44	481.72	945.42	473.21	944.43	472.72	8
5	701.40	351.20	684.37	342.69	683.38	342.20	E	875.41	438.21	858.38	429.70	857.40	429.20	7
6	800.46	400.74	783.44	392.22	782.45	391.73	V	746.37	373.69	729.34	365.17	728.36	364.68	6
7	915.49	458.25	898.46	449.74	897.48	449.24	D	647.30	324.15	630.27	315.64	629.29	315.15	5
8	1,044.53	522.77	1,027.51	514.26	1,026.52	513.76	E	532.27	266.64	515.25	258.13	514.26	257.63	4
9	1,173.58	587.29	1,156.55	578.78	1,155.56	578.29	Q	403.23	202.12	386.20	193.61			3
10	1,272.64	636.83	1,255.62	628.31	1,254.63	627.82	V	274.19	137.60	257.16	129.08			2
11							R	175.12	88.06	158.09	79.55			1

Query 48188 Hit 1

MS/MS Fragmentation of KLEEALMADILSR
Found in sp|Q9UBB5|MBD2_HUMAN, Methyl-CpG-binding domain protein 2 OS=Homo sapiens GN=MBD2 PE=1 SV=1
Match to Query 48188: 1776.007 from(593.0096,3+)
Title: 934: Sum of 2 scans in range 2029 (rt=55.4513, f=4, i=630) to 2030 (rt=55.4767, f=4, i=631)

[D:\lab212\membrane\Grace\Joyce\iTRAQ_43_1.raw]
Data File:Submitted from 20120508-1(merge) by Mascot Daemon on JOYCE-VAIO
Monoisotopic mass of neutral peptide Mr(calc): 1776.007
Variable modifications:
K1 :iTRAQ4plex (K)
Ions Score: 49.86 Expect: 0.007

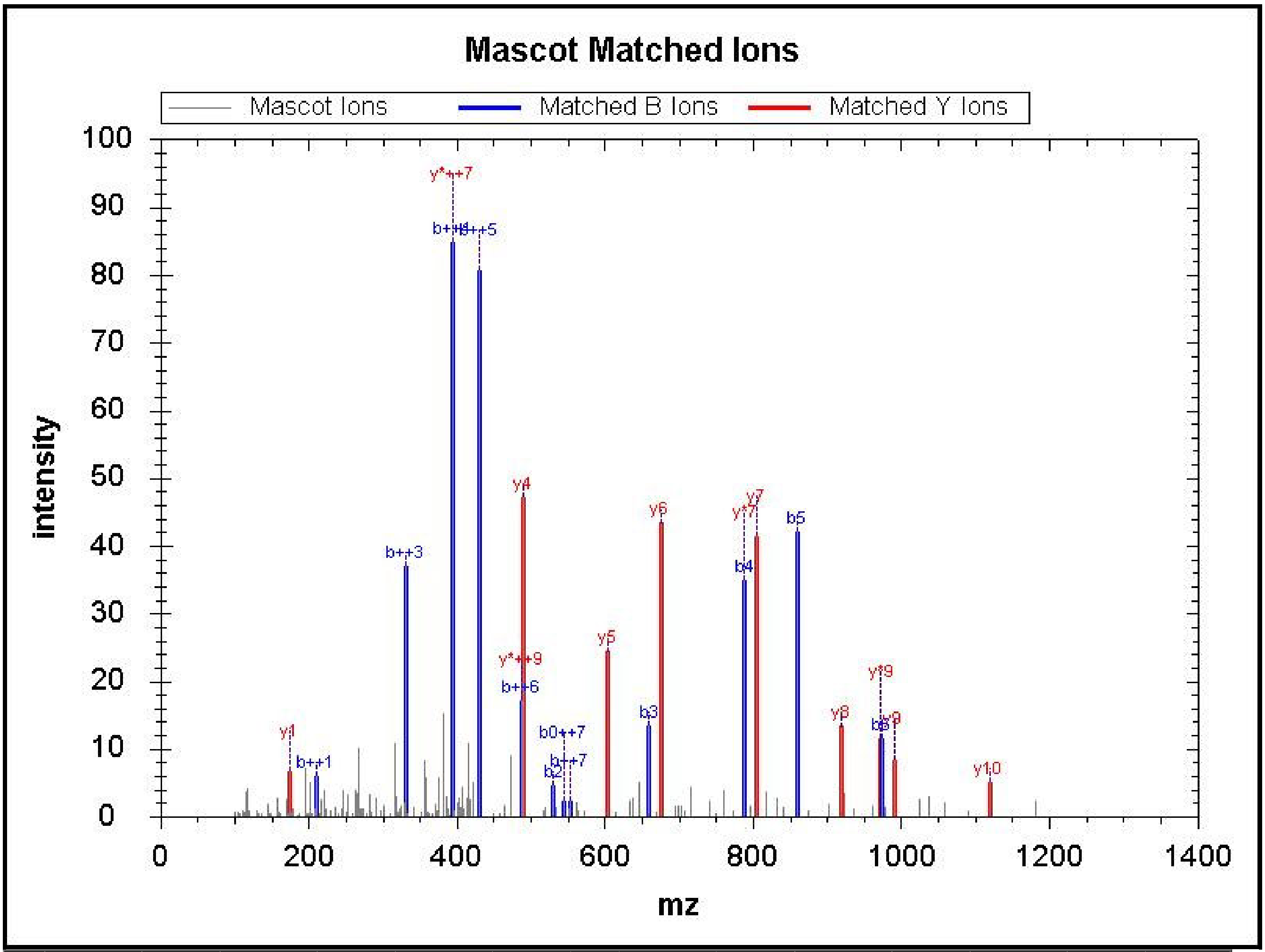

No	b	b++	b*	b*++	b0	b0++	Seq	y	y++	y*	y*++	y0	y0++	RevNo
1	417.31	209.16	400.28	200.64			K							13
2	530.39	265.70	513.36	257.19			L	1,360.71	680.86	1,343.69	672.35	1,342.70	671.86	12
3	659.43	330.22	642.41	321.71	641.42	321.21	E	1,247.63	624.32	1,230.60	615.81	1,229.62	615.31	11
4	788.48	394.74	771.45	386.23	770.47	385.74	E	1,118.59	559.80	1,101.56	551.28	1,100.58	550.79	10
5	859.51	430.26	842.49	421.75	841.50	421.25	A	989.54	495.28	972.52	486.76	971.53	486.27	9
6	972.60	486.80	955.57	478.29	954.59	477.80	L	918.51	459.76	901.48	451.24	900.50	450.75	8
7	1,103.64	552.32	1,086.61	543.81	1,085.63	543.32	M	805.42	403.22	788.40	394.70	787.41	394.21	7
8	1,174.67	587.84	1,157.65	579.33	1,156.66	578.84	A	674.38	337.70	657.36	329.18	656.37	328.69	6
9	1,289.70	645.35	1,272.67	636.84	1,271.69	636.35	D	603.35	302.18	586.32	293.66	585.34	293.17	5
10	1,402.79	701.90	1,385.76	693.38	1,384.77	692.89	I	488.32	244.66	471.29	236.15	470.31	235.66	4
11	1,515.87	758.44	1,498.84	749.93	1,497.86	749.43	L	375.24	188.12	358.21	179.61	357.22	179.12	3
12	1,602.90	801.95	1,585.88	793.44	1,584.89	792.95	S	262.15	131.58	245.12	123.07	244.14	122.57	2
13							R	175.12	88.06	158.09	79.55			1

Query 56216 Hit 1

MS/MS Fragmentation of FLVLATDGLWETMHR
Found in sp|Q9P0J1|PDP1_HUMAN, [Pyruvate dehydrogenase [acetyl-transferring]]-phosphatase 1
Match to Query 56216: 1932.005from(645.0089,3+)
Title: 1038: Sum of 2 scans in range 2298 (rt=61.1505, f=4, i=697) to 2299 (rt=61.1759, f=4, i=698)

[D:\lab212\membrane\Grace\Joyce\iTRAQ_33_1.raw]
Data File:Submitted from 20120508-1(merge) by Mascot Daemon on JOYCE-VAIO
Monoisotopic mass of neutral peptide Mr(calc): 1932.005
Variable modifications:
Ions Score: 49.7 Expect: 0.010

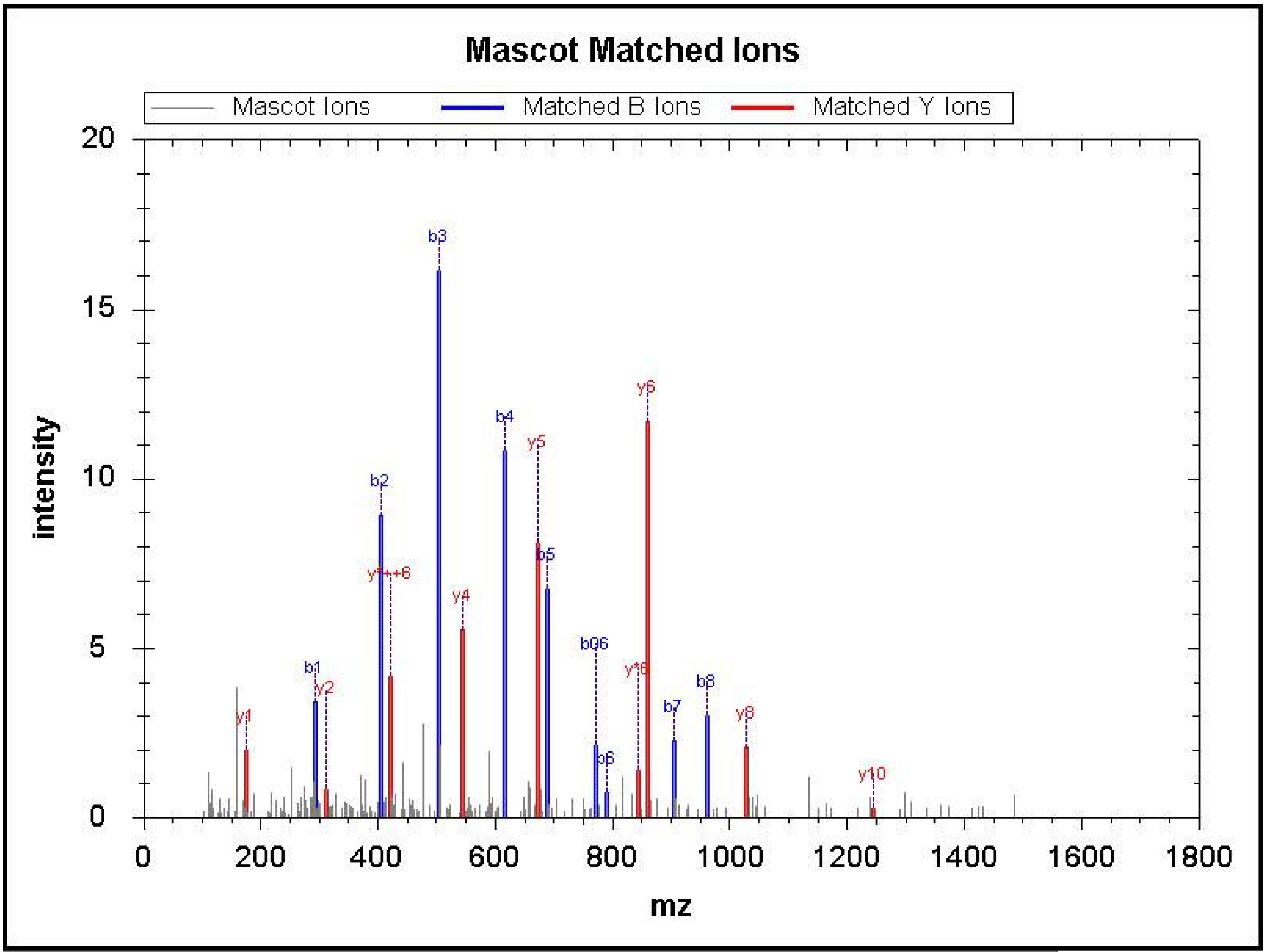

No	b	b++	b0	b0++	Seq	y	y++	y*	y*++	y0	y0++	RevNo
1	292.18	146.59			F							15
2	405.26	203.13			L	1,641.84	821.42	1,624.82	812.91	1,623.83	812.42	14
3	504.33	252.67			V	1,528.76	764.88	1,511.73	756.37	1,510.75	755.88	13
4	617.41	309.21			L	1,429.69	715.35	1,412.66	706.84	1,411.68	706.34	12
5	688.45	344.73			A	1,316.61	658.81	1,299.58	650.29	1,298.59	649.80	11
6	789.50	395.25	771.49	386.25	T	1,245.57	623.29	1,228.54	614.77	1,227.56	614.28	10
7	904.53	452.77	886.52	443.76	D	1,144.52	572.76	1,127.49	564.25	1,126.51	563.76	9
8	961.55	481.28	943.54	472.27	G	1,029.49	515.25	1,012.47	506.74	1,011.48	506.25	8
9	1,074.63	537.82	1,056.62	528.81	L	972.47	486.74	955.45	478.23	954.46	477.73	7
10	1,260.71	630.86	1,242.70	621.85	W	859.39	430.20	842.36	421.68	841.38	421.19	6
11	1,389.75	695.38	1,371.74	686.38	E	673.31	337.16	656.28	328.64	655.30	328.15	5
12	1,490.80	745.90	1,472.79	736.90	T	544.27	272.64	527.24	264.12	526.26	263.63	4
13	1,621.84	811.42	1,603.83	802.42	M	443.22	222.11	426.19	213.60			3
14	1,758.90	879.95	1,740.89	870.95	H	312.18	156.59	295.15	148.08			2
15					R	175.12	88.06	158.09	79.55			1

Query 65417 Hit 1

MS/MS Fragmentation of GVTDNVVDTVVHYVPLPR
Found in sp|O60240|PLIN1_HUMAN, Perilipin-1 OS=Homo sapiens GN=PLIN1 PE=2 SV=2

Match to Query 65417: 2123.15from(708.7241,3+)
Title: 902: Sum of 2 scans in range 2015 (rt=54.7534, f=4, i=605) to 2016 (rt=54.7788, f=4, i=606) [D:\lab212\membrane\Grace\Joyce\iTRAQ_31_2.raw]
Data File:Submitted from 20120508-1(merge) by Mascot Daemon on JOYCE-VAIO
Monoisotopic mass of neutral peptide Mr(calc): 2123.15
Variable modifications:
Ions Score: 49.7 Expect: 0.009

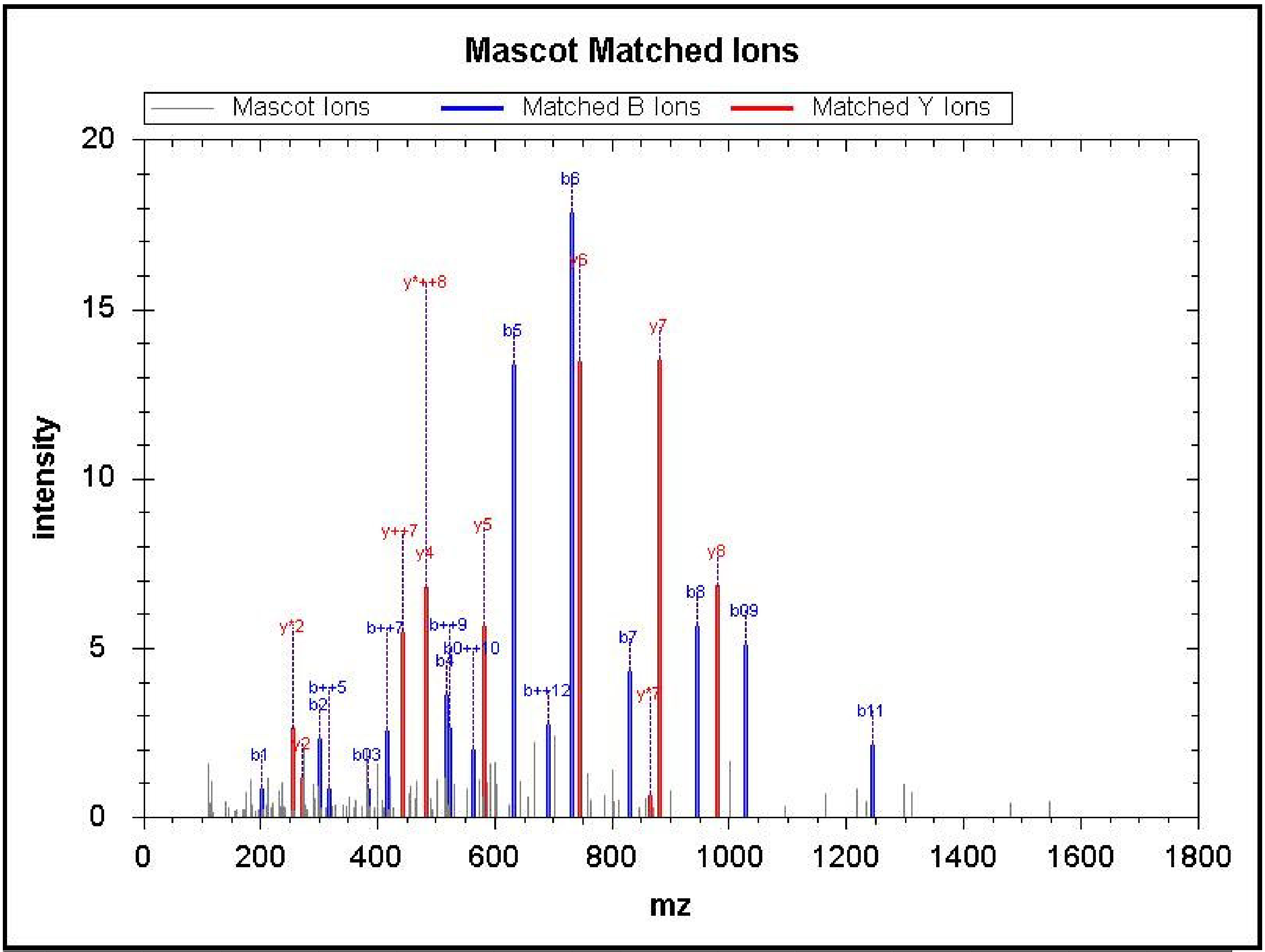

No	b	b++	b*	b*++	b0	b0++	Seq	y	y++	y*	y*++	y0	y0++	RevNo
1	202.13	101.57					G							18
2	301.20	151.10					V	1,923.03	962.02	1,906.01	953.51	1,905.02	953.02	17
3	402.25	201.63			384.24	192.62	T	1,823.96	912.49	1,806.94	903.97	1,805.95	903.48	16
4	517.27	259.14			499.26	250.14	D	1,722.92	861.96	1,705.89	853.45	1,704.91	852.96	15
5	631.32	316.16	614.29	307.65	613.31	307.16	N	1,607.89	804.45	1,590.86	795.94	1,589.88	795.44	14
6	730.39	365.70	713.36	357.18	712.37	356.69	V	1,493.85	747.43	1,476.82	738.91	1,475.84	738.42	13
7	829.45	415.23	812.43	406.72	811.44	406.23	V	1,394.78	697.89	1,377.75	689.38	1,376.77	688.89	12
8	944.48	472.74	927.45	464.23	926.47	463.74	D	1,295.71	648.36	1,278.68	639.85	1,277.70	639.35	11
9	1,045.53	523.27	1,028.50	514.75	1,027.52	514.26	T	1,180.68	590.85	1,163.66	582.33	1,162.67	581.84	10
10	1,144.60	572.80	1,127.57	564.29	1,126.59	563.80	V	1,079.64	540.32	1,062.61	531.81			9
11	1,243.66	622.34	1,226.64	613.82	1,225.65	613.33	V	980.57	490.79	963.54	482.27			8
12	1,380.72	690.87	1,363.70	682.35	1,362.71	681.86	H	881.50	441.25	864.47	432.74			7
13	1,543.79	772.40	1,526.76	763.88	1,525.78	763.39	Y	744.44	372.72	727.41	364.21			6
14	1,642.86	821.93	1,625.83	813.42	1,624.84	812.93	V	581.38	291.19	564.35	282.68			5
15	1,739.91	870.46	1,722.88	861.94	1,721.90	861.45	P	482.31	241.66	465.28	233.14			4
16	1,852.99	927.00	1,835.97	918.49	1,834.98	917.99	L	385.26	193.13	368.23	184.62			3
17	1,950.05	975.53	1,933.02	967.01	1,932.03	966.52	P	272.17	136.59	255.15	128.08			2

18							R	175.12	88.06	158.09	79.55			1

Query 21846 Hit 1

MS/MS Fragmentation of AFSQRSSLVR
Found in sp|Q86TJ5|ZN554_HUMAN, Zinc finger protein 554 OS=Homo sapiens GN=ZNF554 PE=2 SV=1
Match to Query 21846: 1293.677from(647.8459,2+)
Title: 254: Scan 663 (rt=24.2812, f=3, i=88) [D:\lab212\membrane\Grace\Joyce\iTRAQ_25_2.raw]
Data File:Submitted from 20120508-1(merge) by Mascot Daemon on JOYCE-VAIO
Monoisotopic mass of neutral peptide Mr(calc): 1293.677
Variable modifications:
Ions Score: 49.67 Expect: 0.009

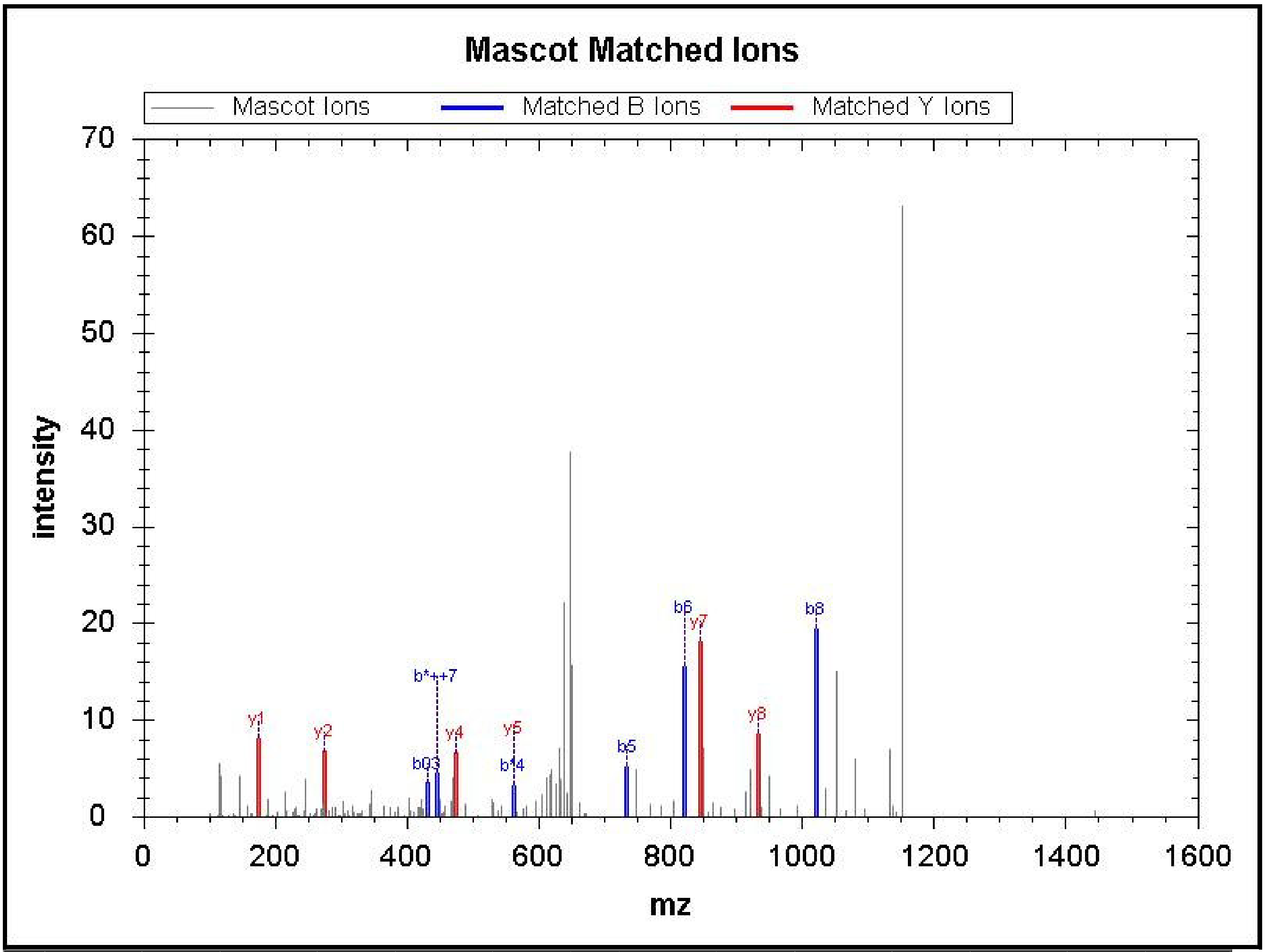

No	b	b++	b*	b*++	b0	b0++	Seq	y	y++	y*	y*++	y0	y0++	RevNo
1	216.15	108.58					A							10
2	363.21	182.11					F	1,079.60	540.30	1,062.57	531.79	1,061.59	531.30	9
3	450.25	225.63			432.24	216.62	S	932.53	466.77	915.50	458.25	914.52	457.76	8
4	578.31	289.66	561.28	281.14	560.29	280.65	Q	845.50	423.25	828.47	414.74	827.48	414.25	7
5	734.41	367.71	717.38	359.19	716.40	358.70	R	717.44	359.22	700.41	350.71	699.43	350.22	6
6	821.44	411.22	804.41	402.71	803.43	402.22	S	561.34	281.17	544.31	272.66	543.32	272.17	5
7	908.47	454.74	891.44	446.23	890.46	445.73	S	474.30	237.66	457.28	229.14	456.29	228.65	4
8	1,021.55	511.28	1,004.53	502.77	1,003.54	502.28	L	387.27	194.14	370.24	185.63			3
9	1,120.62	560.82	1,103.60	552.30	1,102.61	551.81	V	274.19	137.60	257.16	129.08			2
10							R	175.12	88.06	158.09	79.55			1

Query 56495 Hit 1

MS/MS Fragmentation of DGALHVIGSLAEILLK
Found in sp|O15397|IPO8_HUMAN, Importin-8 OS=Homo sapiens GN=IPO8 PE=1 SV=2

Match to Query 56495: 1936.16from(646.3941,3+)
Title: 1198: Sum of 2 scans in range 2752 (rt=70.8572, f=3, i=422) to 2753 (rt=70.8826, f=3, i=423) [D:\lab212\membrane\Grace\Joyce\iTRAQ_37_1.raw]
Data File:Submitted from 20120508-1(merge) by Mascot Daemon on JOYCE-VAIO
Monoisotopic mass of neutral peptide Mr(calc): 1936.16
Variable modifications:
K16 :iTRAQ4plex (K)
Ions Score: 49.66 Expect: 0.003

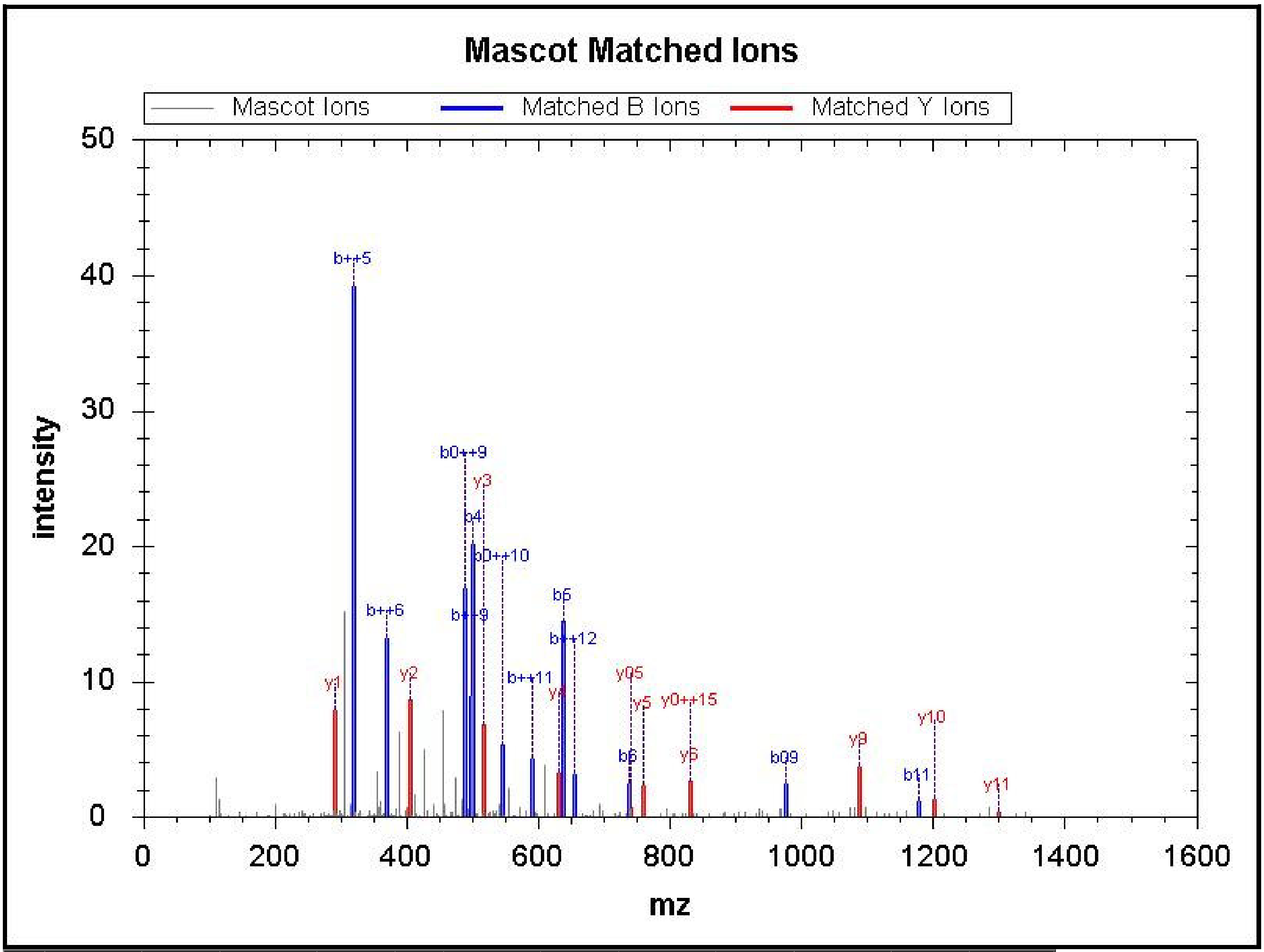

No	b	b++	b0	b0++	Seq	y	y++	y*	y*++	y0	y0++	RevNo
1	260.14	130.57	242.13	121.57	D							16
2	317.16	159.08	299.15	150.08	G	1,678.04	839.52	1,661.01	831.01	1,660.03	830.52	15
3	388.19	194.60	370.18	185.60	A	1,621.02	811.01	1,603.99	802.50	1,603.01	802.01	14
4	501.28	251.14	483.27	242.14	L	1,549.98	775.49	1,532.95	766.98	1,531.97	766.49	13
5	638.34	319.67	620.33	310.67	H	1,436.90	718.95	1,419.87	710.44	1,418.89	709.95	12
6	737.41	369.21	719.40	360.20	V	1,299.84	650.42	1,282.81	641.91	1,281.83	641.42	11
7	850.49	425.75	832.48	416.74	I	1,200.77	600.89	1,183.74	592.37	1,182.76	591.88	10
8	907.51	454.26	889.50	445.25	G	1,087.68	544.35	1,070.66	535.83	1,069.67	535.34	9
9	994.54	497.78	976.53	488.77	S	1,030.66	515.84	1,013.64	507.32	1,012.65	506.83	8
10	1,107.63	554.32	1,089.62	545.31	L	943.63	472.32	926.60	463.81	925.62	463.31	7
11	1,178.67	589.84	1,160.65	580.83	A	830.55	415.78	813.52	407.26	812.54	406.77	6
12	1,307.71	654.36	1,289.70	645.35	E	759.51	380.26	742.48	371.75	741.50	371.25	5
13	1,420.79	710.90	1,402.78	701.89	I	630.47	315.74	613.44	307.22			4
14	1,533.88	767.44	1,515.87	758.44	L	517.38	259.20	500.36	250.68			3
15	1,646.96	823.98	1,628.95	814.98	L	404.30	202.65	387.27	194.14			2
16					K	291.21	146.11	274.19	137.60			1

Query 37514 Hit 1

MS/MS Fragmentation of ILYEGTHLDPER
Found in sp|Q5RI15|FA36A_HUMAN, Protein FAM36A OS=Homo sapiens GN=FAM36A PE=1 SV=2
Match to Query 37514: 1585.82from(529.6139,3+)
Title: 432: Scan 1013 (rt=32.2635, f=2, i=152) [D:\lab212\membrane\Grace\Joyce\iTRAQ_37_2.raw]
Data File:Submitted from 20120508-1(merge) by Mascot Daemon on JOYCE-VAIO
Monoisotopic mass of neutral peptide Mr(calc): 1585.82
Variable modifications:
Ions Score: 49.57 Expect: 0.009

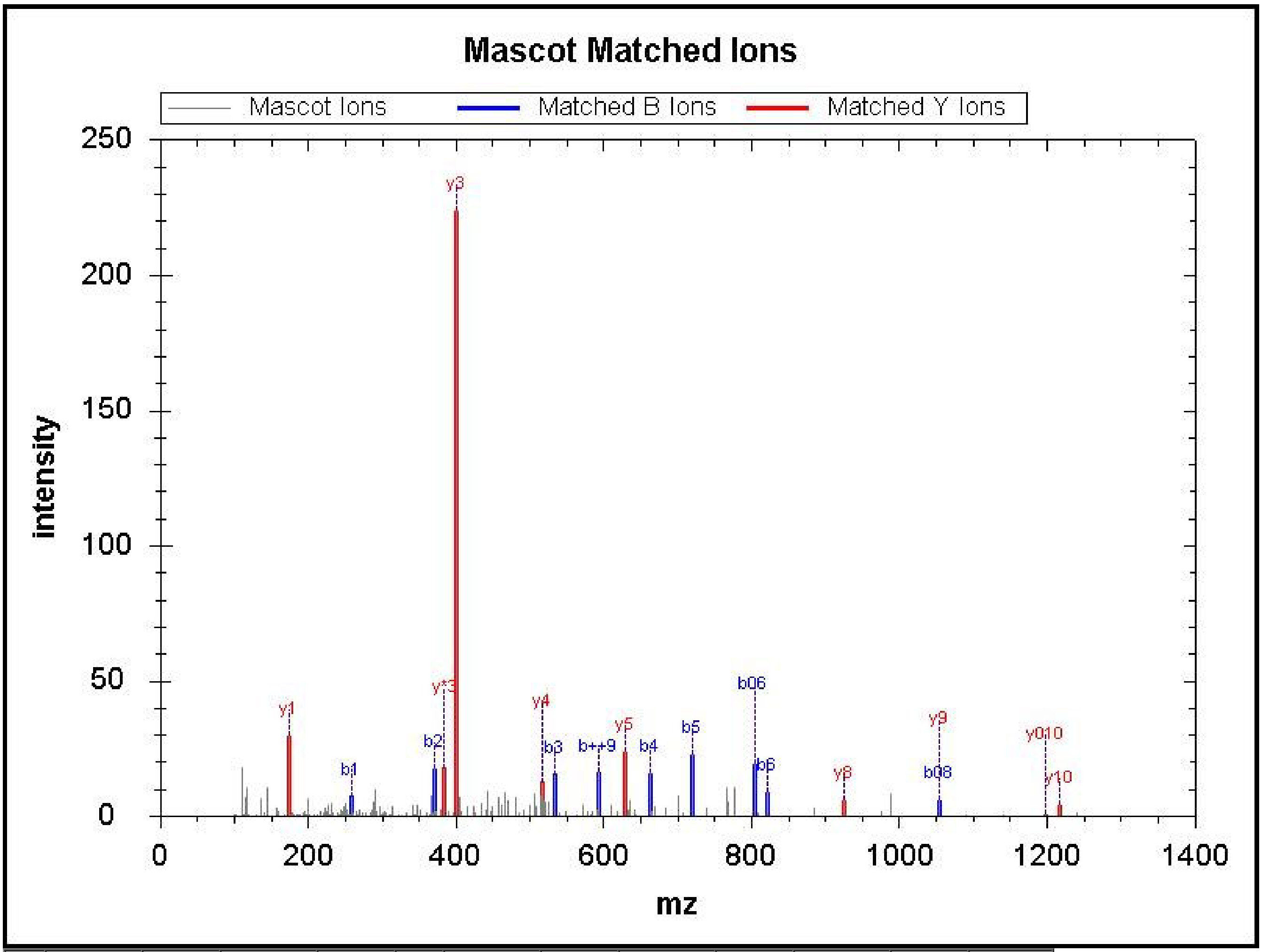

No	b	b++	b0	b0++	Seq	y	y++	y*	y*++	y0	y0++	RevNo
1	258.19	129.60			I							12
2	371.28	186.14			L	1,329.64	665.33	1,312.62	656.81	1,311.63	656.32	11
3	534.34	267.67			Y	1,216.56	608.78	1,199.53	600.27	1,198.55	599.78	10
4	663.38	332.20	645.37	323.19	E	1,053.50	527.25	1,036.47	518.74	1,035.49	518.25	9
5	720.40	360.71	702.39	351.70	G	924.45	462.73	907.43	454.22	906.44	453.73	8
6	821.45	411.23	803.44	402.22	T	867.43	434.22	850.41	425.71	849.42	425.21	7
7	958.51	479.76	940.50	470.75	H	766.38	383.70	749.36	375.18	748.37	374.69	6
8	1,071.60	536.30	1,053.58	527.30	L	629.33	315.17	612.30	306.65	611.31	306.16	5
9	1,186.62	593.81	1,168.61	584.81	D	516.24	258.62	499.21	250.11	498.23	249.62	4
10	1,283.68	642.34	1,265.66	633.34	P	401.21	201.11	384.19	192.60	383.20	192.11	3
11	1,412.72	706.86	1,394.71	697.86	E	304.16	152.58	287.13	144.07	286.15	143.58	2
12					R	175.12	88.06	158.09	79.55			1

Query 36622 Hit 1

MS/MS Fragmentation of EIVQDGDHMIIR
Found in sp|P09455|RET1_HUMAN, Retinol-binding protein 1 OS=Homo sapiens GN=RBP1 PE=1 SV=2

Match to Query 36622: 1568.812from(523.9446,3+)
Title: 391: Scan 927 (rt=30.3291, f=3, i=139) [D:\lab212\membrane\Grace\Joyce\iTRAQ_37_2.raw]
Data File:Submitted from 20120508-1(merge) by Mascot Daemon on JOYCE-VAIO
Monoisotopic mass of neutral peptide Mr(calc): 1568.812
Variable modifications:
Ions Score: 49.52 Expect: 0.009

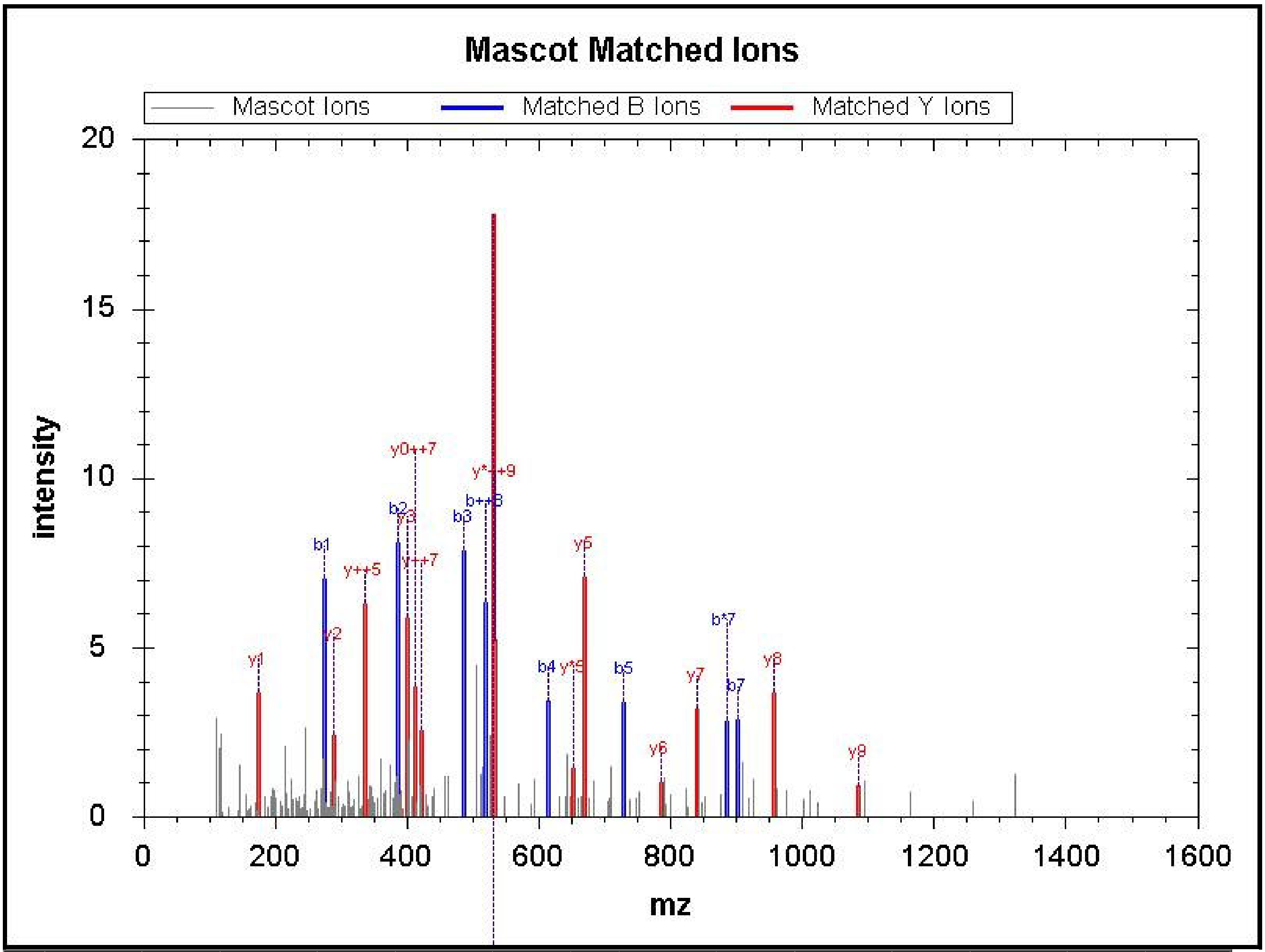

No	b	b++	b*	b*++	b0	b0++	Seq	y	y++	y*	y*++	y0	y0++	RevNo
1	274.15	137.58			256.14	128.57	E							12
2	387.24	194.12			369.23	185.12	I	1,296.67	648.84	1,279.65	640.33	1,278.66	639.83	11
3	486.30	243.66			468.29	234.65	V	1,183.59	592.30	1,166.56	583.78	1,165.58	583.29	10
4	614.36	307.69	597.34	299.17	596.35	298.68	Q	1,084.52	542.76	1,067.49	534.25	1,066.51	533.76	9
5	729.39	365.20	712.36	356.69	711.38	356.19	D	956.46	478.73	939.44	470.22	938.45	469.73	8
6	786.41	393.71	769.38	385.20	768.40	384.70	G	841.43	421.22	824.41	412.71	823.42	412.22	7
7	901.44	451.22	884.41	442.71	883.43	442.22	D	784.41	392.71	767.39	384.20	766.40	383.71	6
8	1,038.50	519.75	1,021.47	511.24	1,020.49	510.75	H	669.39	335.20	652.36	326.68			5
9	1,169.54	585.27	1,152.51	576.76	1,151.53	576.27	M	532.33	266.67	515.30	258.15			4
10	1,282.62	641.81	1,265.60	633.30	1,264.61	632.81	I	401.29	201.15	384.26	192.63			3
11	1,395.71	698.36	1,378.68	689.84	1,377.70	689.35	I	288.20	144.61	271.18	136.09			2
12							R	175.12	88.06	158.09	79.55			1

Query 79295 Hit 1

MS/MS Fragmentation of FKPGVISEELQDALGVTDK
Found in sp|Q6NZY4|ZCHC8_HUMAN, Zinc finger CCHC domain-containing protein 8 OS=Homo sapiens GN=ZCCHC8 PE=1 SV=2
Match to Query 79295: 2477.38from(826.8008,3+)
Title: 930: Sum of 2 scans in range 2014 (rt=54.9947, f=4, i=616) to 2015 (rt=55.0201, f=4, i=617) [D:\lab212\membrane\Grace\Joyce\iTRAQ_41_1.raw]
Data File:Submitted from 20120508-1(merge) by Mascot Daemon on JOYCE-VAIO

Monoisotopic mass of neutral peptide Mr(calc): 2477.38
Variable modifications:
K2 :iTRAQ4plex (K)
K19 :iTRAQ4plex (K)
Ions Score: 49.49 Expect: 0.006

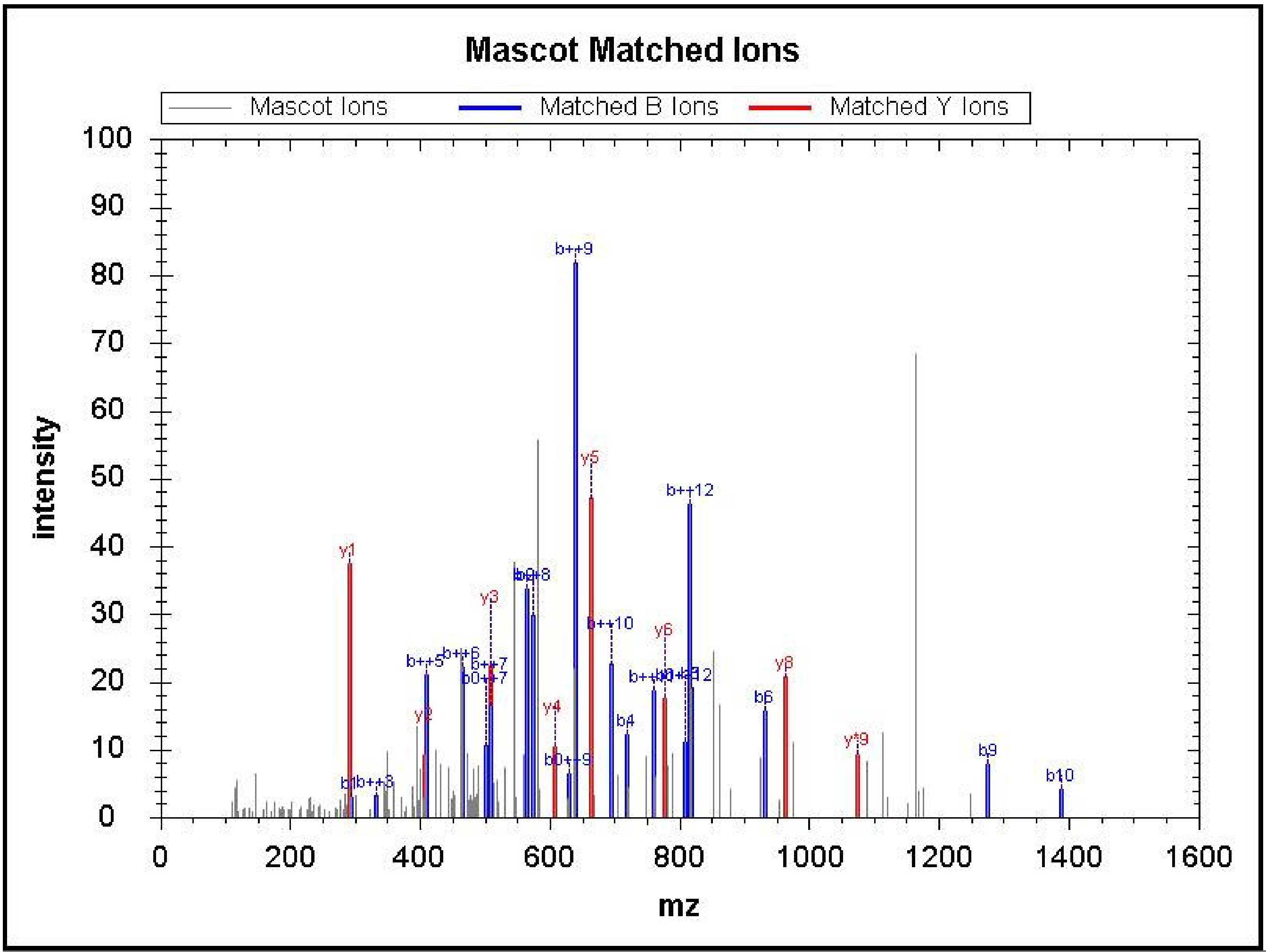

No	b	b++	b*	b*++	b0	b0++	Seq	y	y++	y*	y*++	y0	y0++	RevNo
1	292.18	146.59					F							19
2	564.37	282.69	547.35	274.18			K	2,187.21	1,094.11	2,170.18	1,085.60	2,169.20	1,085.10	18
3	661.43	331.22	644.40	322.70			P	1,915.01	958.01	1,897.99	949.50	1,897.00	949.01	17
4	718.45	359.73	701.42	351.21			G	1,817.96	909.48	1,800.93	900.97	1,799.95	900.48	16
5	817.52	409.26	800.49	400.75			V	1,760.94	880.97	1,743.91	872.46	1,742.93	871.97	15
6	930.60	465.80	913.57	457.29			I	1,661.87	831.44	1,644.84	822.93	1,643.86	822.43	14
7	1,017.63	509.32	1,000.61	500.81	999.62	500.32	S	1,548.79	774.90	1,531.76	766.38	1,530.78	765.89	13
8	1,146.68	573.84	1,129.65	565.33	1,128.67	564.84	E	1,461.76	731.38	1,444.73	722.87	1,443.74	722.38	12
9	1,275.72	638.36	1,258.69	629.85	1,257.71	629.36	E	1,332.71	666.86	1,315.69	658.35	1,314.70	657.85	11
10	1,388.80	694.91	1,371.78	686.39	1,370.79	685.90	L	1,203.67	602.34	1,186.64	593.83	1,185.66	593.33	10
11	1,516.86	758.93	1,499.83	750.42	1,498.85	749.93	Q	1,090.59	545.80	1,073.56	537.28	1,072.58	536.79	9
12	1,631.89	816.45	1,614.86	807.93	1,613.88	807.44	D	962.53	481.77	945.50	473.25	944.52	472.76	8
13	1,702.93	851.97	1,685.90	843.45	1,684.91	842.96	A	847.50	424.25	830.47	415.74	829.49	415.25	7
14	1,816.01	908.51	1,798.98	900.00	1,798.00	899.50	L	776.46	388.74	759.44	380.22	758.45	379.73	6
15	1,873.03	937.02	1,856.00	928.51	1,855.02	928.01	G	663.38	332.19	646.35	323.68	645.37	323.19	5
16	1,972.10	986.55	1,955.07	978.04	1,954.09	977.55	V	606.36	303.68	589.33	295.17	588.35	294.68	4
17	2,073.15	1,037.08	2,056.12	1,028.56	2,055.14	1,028.07	T	507.29	254.15	490.26	245.64	489.28	245.14	3
18	2,188.17	1,094.59	2,171.15	1,086.08	2,170.16	1,085.59	D	406.24	203.62	389.22	195.11	388.23	194.62	2
19							K	291.21	146.11	274.19	137.60			1

Query 31228 Hit 1

MS/MS Fragmentation of ELQHAALGGTATR
Found in sp|O14828|SCAM3_HUMAN, Secretory carrier-associated membrane protein 3 OS=Homo sapiens GN=SCAMP3 PE=1 SV=3
Match to Query 31228: 1467.788from(490.27,3+)
Title: 160: Scan 464 (rt=19.7768, f=2, i=67) [D:\lab212\membrane\Grace\Joyce\iTRAQ_38_1.raw]
Data File:Submitted from 20120508-1(merge) by Mascot Daemon on JOYCE-VAIO
Monoisotopic mass of neutral peptide Mr(calc): 1467.788
Variable modifications:
Ions Score: 49.48 Expect: 0.009

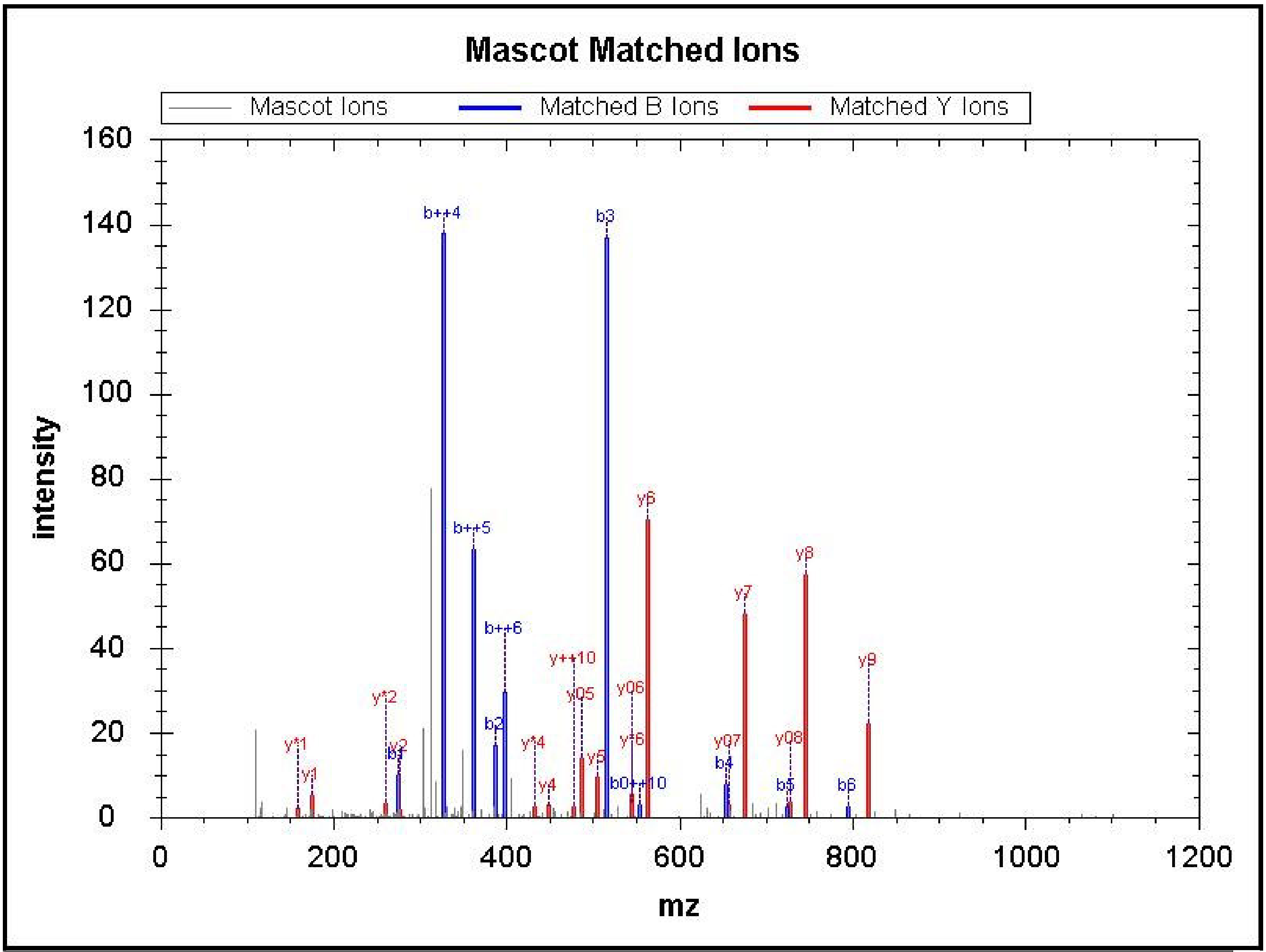

No	b	b++	b*	b*++	b0	b0++	Seq	y	y++	y*	y*++	y0	y0++	RevNo
1	274.15	137.58			256.14	128.57	E							13
2	387.24	194.12			369.23	185.12	L	1,195.65	598.33	1,178.63	589.82	1,177.64	589.33	12
3	515.29	258.15	498.27	249.64	497.28	249.15	Q	1,082.57	541.79	1,065.54	533.28	1,064.56	532.78	11
4	652.35	326.68	635.33	318.17	634.34	317.68	H	954.51	477.76	937.48	469.25	936.50	468.75	10
5	723.39	362.20	706.36	353.69	705.38	353.19	A	817.45	409.23	800.43	400.72	799.44	400.22	9
6	794.43	397.72	777.40	389.20	776.42	388.71	A	746.42	373.71	729.39	365.20	728.40	364.71	8
7	907.51	454.26	890.49	445.75	889.50	445.25	L	675.38	338.19	658.35	329.68	657.37	329.19	7
8	964.53	482.77	947.51	474.26	946.52	473.76	G	562.29	281.65	545.27	273.14	544.28	272.65	6
9	1,021.55	511.28	1,004.53	502.77	1,003.54	502.28	G	505.27	253.14	488.25	244.63	487.26	244.13	5
10	1,122.60	561.80	1,105.58	553.29	1,104.59	552.80	T	448.25	224.63	431.22	216.12	430.24	215.62	4
11	1,193.64	597.32	1,176.61	588.81	1,175.63	588.32	A	347.20	174.11	330.18	165.59	329.19	165.10	3
12	1,294.69	647.85	1,277.66	639.33	1,276.68	638.84	T	276.17	138.59	259.14	130.07	258.16	129.58	2
13							R	175.12	88.06	158.09	79.55			1

Query 31118 Hit 1

MS/MS Fragmentation of TTLLAGDIEHPR
Found in sp|Q07954|LRP1_HUMAN, Prolow-density lipoprotein receptor-related protein 1 OS=Homo sapiens GN=LRP1 PE=1 SV=2
Match to Query 31118: 1465.801 from(489.6076,3+)
Title: 385: Scan 916 (rt=30.0637, f=2, i=137) [D:\lab212\membrane\Grace\Joyce\iTRAQ_37_1.raw]
Data File:Submitted from 20120508-1(merge) by Mascot Daemon on JOYCE-VAIO
Monoisotopic mass of neutral peptide Mr(calc): 1465.801
Variable modifications:
Ions Score: 49.47 Expect: 0.009

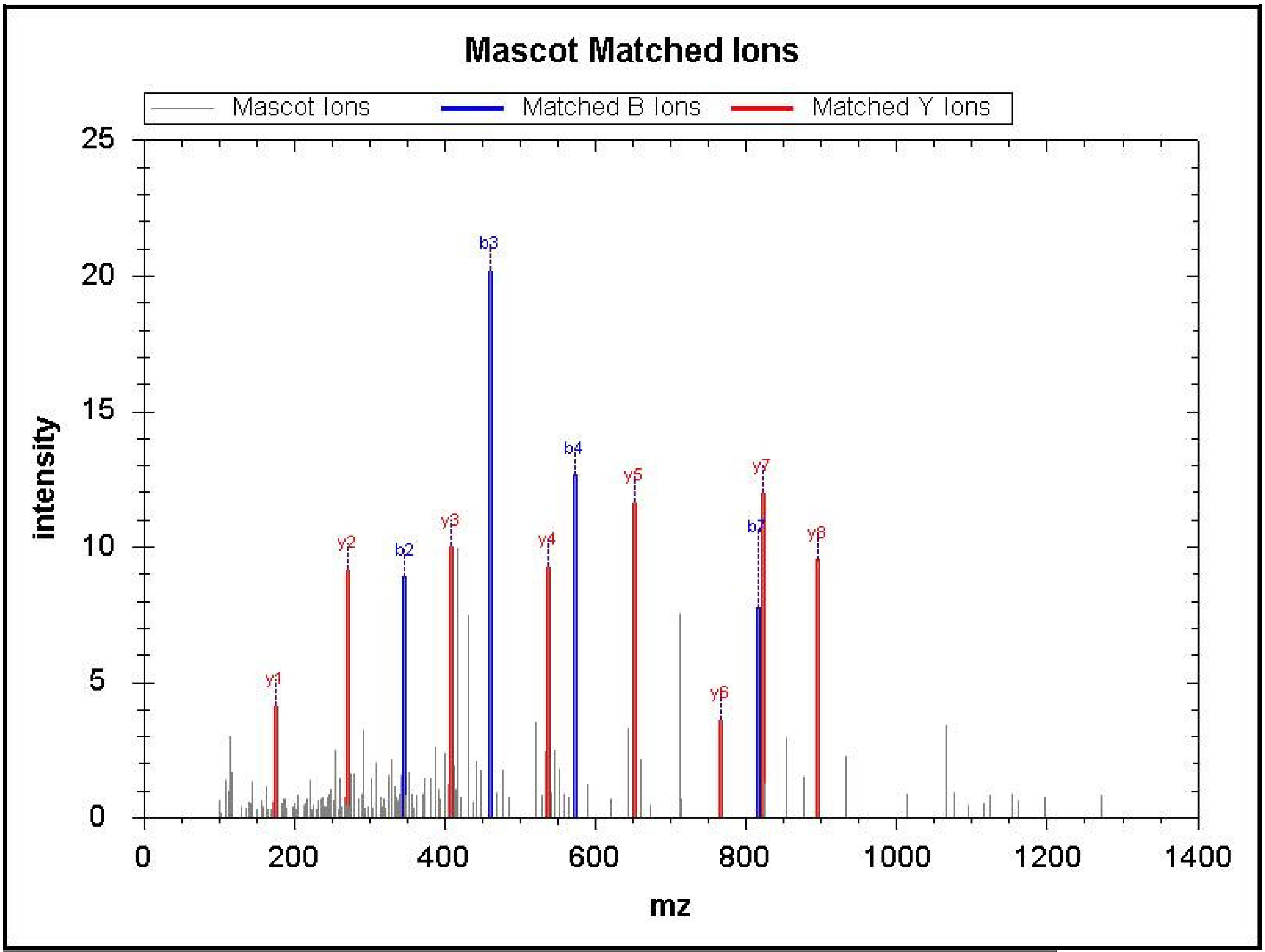

No	b	b++	b0	b0++	Seq	y	y++	y*	y*++	y0	y0++	RevNo
1	246.16	123.58	228.15	114.58	T							12
2	347.20	174.11	329.19	165.10	T	1,221.66	611.33	1,204.63	602.82	1,203.65	602.33	11
3	460.29	230.65	442.28	221.64	L	1,120.61	560.81	1,103.58	552.30	1,102.60	551.80	10
4	573.37	287.19	555.36	278.18	L	1,007.53	504.27	990.50	495.75	989.52	495.26	9
5	644.41	322.71	626.40	313.70	A	894.44	447.73	877.42	439.21	876.43	438.72	8
6	701.43	351.22	683.42	342.21	G	823.41	412.21	806.38	403.69	805.40	403.20	7
7	816.46	408.73	798.45	399.73	D	766.38	383.70	749.36	375.18	748.37	374.69	6
8	929.54	465.27	911.53	456.27	I	651.36	326.18	634.33	317.67	633.35	317.18	5
9	1,058.58	529.80	1,040.57	520.79	E	538.27	269.64	521.25	261.13	520.26	260.63	4
10	1,195.64	598.33	1,177.63	589.32	H	409.23	205.12	392.20	196.61			3
11	1,292.70	646.85	1,274.69	637.85	P	272.17	136.59	255.15	128.08			2
12					R	175.12	88.06	158.09	79.55			1

Query 56871 Hit 1

MS/MS Fragmentation of HMDFLSDFEMMLQR
Found in sp|Q96ST2|IWS1_HUMAN, Protein IWS1 homolog OS=Homo sapiens GN=IWS1 PE=1 SV=2
Match to Query 56871: 1942.889 from(648.6369,3+)

Title: 1116: Sum of 2 scans in range 2511 (rt=65.6578, f=2, i=413) to 2512 (rt=65.6832, f=2, i=414) [D:\lab212\membrane\Grace\Joyce\iTRAQ_38_2.raw]
Data File:Submitted from 20120508-1(merge) by Mascot Daemon on JOYCE-VAIO
Monoisotopic mass of neutral peptide Mr(calc): 1942.889
Variable modifications:
Ions Score: 49.39 Expect: 0.007

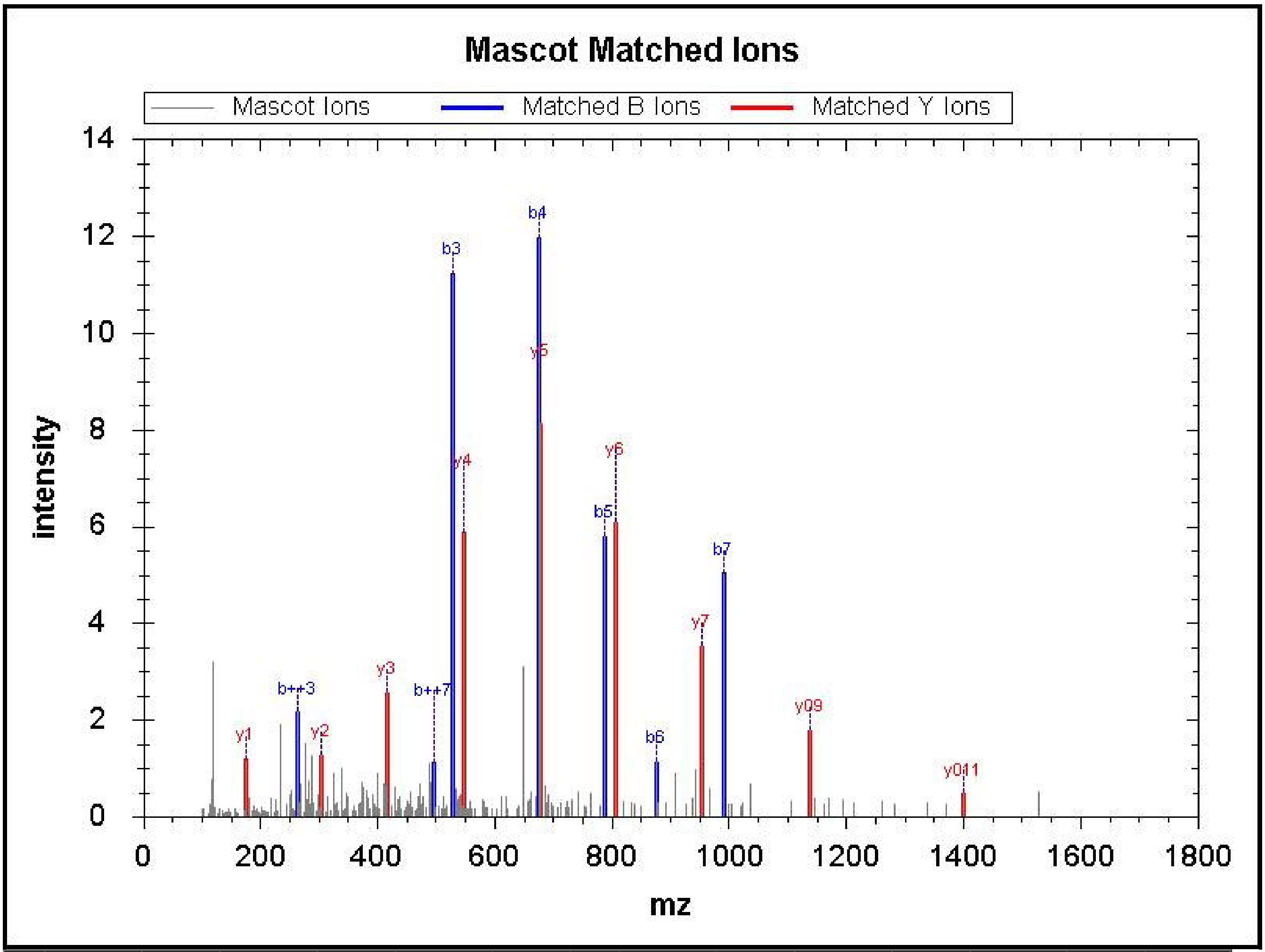

No	b	b++	b*	b*++	b0	b0++	Seq	y	y++	y*	y*++	y0	y0++	RevNo
1	282.17	141.59					H							14
2	413.21	207.11					M	1,662.73	831.87	1,645.71	823.36	1,644.72	822.86	13
3	528.24	264.62			510.23	255.62	D	1,531.69	766.35	1,514.67	757.84	1,513.68	757.34	12
4	675.30	338.16			657.29	329.15	F	1,416.67	708.84	1,399.64	700.32	1,398.65	699.83	11
5	788.39	394.70			770.38	385.69	L	1,269.60	635.30	1,252.57	626.79	1,251.59	626.30	10
6	875.42	438.21			857.41	429.21	S	1,156.51	578.76	1,139.49	570.25	1,138.50	569.75	9
7	990.45	495.73			972.44	486.72	D	1,069.48	535.24	1,052.45	526.73	1,051.47	526.24	8
8	1,137.52	569.26			1,119.51	560.26	F	954.45	477.73	937.43	469.22	936.44	468.73	7
9	1,266.56	633.78			1,248.55	624.78	E	807.39	404.20	790.36	395.68	789.37	395.19	6
10	1,397.60	699.30			1,379.59	690.30	M	678.34	339.67	661.32	331.16			5
11	1,528.64	764.82			1,510.63	755.82	M	547.30	274.15	530.28	265.64			4
12	1,641.72	821.37			1,623.71	812.36	L	416.26	208.63	399.24	200.12			3
13	1,769.78	885.39	1,752.76	876.88	1,751.77	876.39	Q	303.18	152.09	286.15	143.58			2
14							R	175.12	88.06	158.09	79.55			1

Query 13496 Hit 1

MS/MS Fragmentation of AAEEVEAK
Found in sp|Q9NX63|CHCH3_HUMAN, Coiled-coil-helix-coiled-coil-helix domain-containing protein 3
Match to Query 13496: 1133.619from(567.8168,2+)

Title: 64: Sum of 2 scans in range 254 (rt=15.0732, f=4, i=37) to 255 (rt=15.0986, f=4, i=38) [D:\lab212\membrane\Grace\Joyce\iTRAQ_31_2.raw]
Data File:Submitted from 20120508-1(merge) by Mascot Daemon on JOYCE-VAIO
Monoisotopic mass of neutral peptide Mr(calc): 1133.619
Variable modifications:
K8 :iTRAQ4plex (K)
Ions Score: 49.24 Expect: 0.011

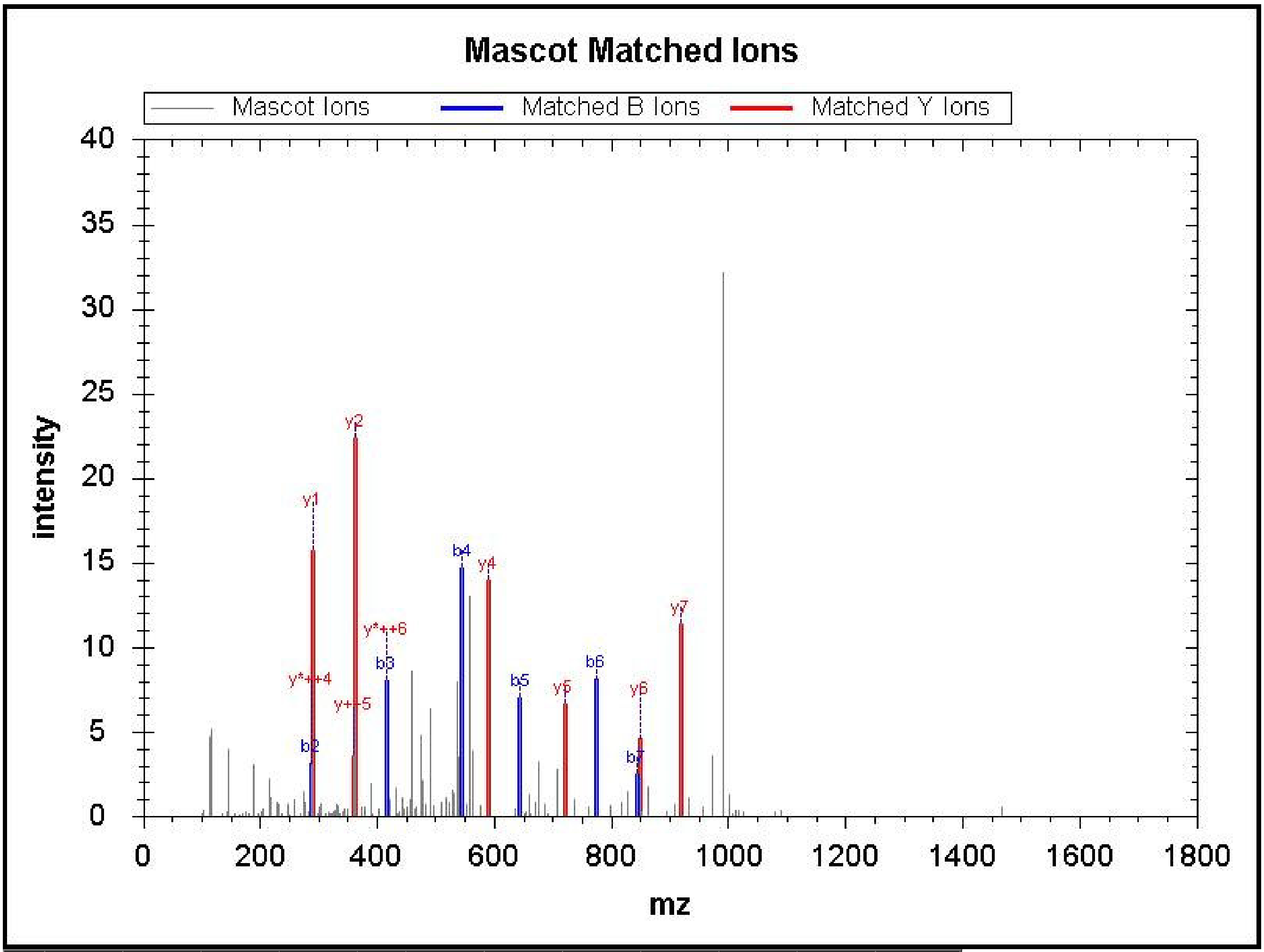

No	b	b++	b0	b0++	Seq	y	y++	y*	y*++	y0	y0++	RevNo
1	216.15	108.58			A							8
2	287.18	144.10			A	919.49	460.25	902.46	451.73	901.47	451.24	7
3	416.23	208.62	398.22	199.61	E	848.45	424.73	831.42	416.21	830.44	415.72	6
4	545.27	273.14	527.26	264.13	E	719.41	360.21	702.38	351.69	701.40	351.20	5
5	644.34	322.67	626.33	313.67	V	590.36	295.69	573.34	287.17	572.35	286.68	4
6	773.38	387.19	755.37	378.19	E	491.29	246.15	474.27	237.64	473.28	237.15	3
7	844.42	422.71	826.41	413.71	A	362.25	181.63	345.23	173.12			2
8					K	291.21	146.11	274.19	137.60			1

Query 61002 Hit 1

MS/MS Fragmentation of LHLLDQVFFQELLK
Found in sp|Q7Z6J4|FGD2_HUMAN, FYVE
Match to Query 61002: 2030.178from(677.7333,3+)
Title: 1159: Scan 2539 (rt=66.7029, f=2, i=415) [D:\lab212\membrane\Grace\Joyce\iTRAQ_39_1.raw]
Data File:Submitted from 20120508-1(merge) by Mascot Daemon on JOYCE-VAIO
Monoisotopic mass of neutral peptide Mr(calc): 2030.178
Variable modifications:
K14 :iTRAQ4plex (K)
Ions Score: 49.1 Expect: 0.005

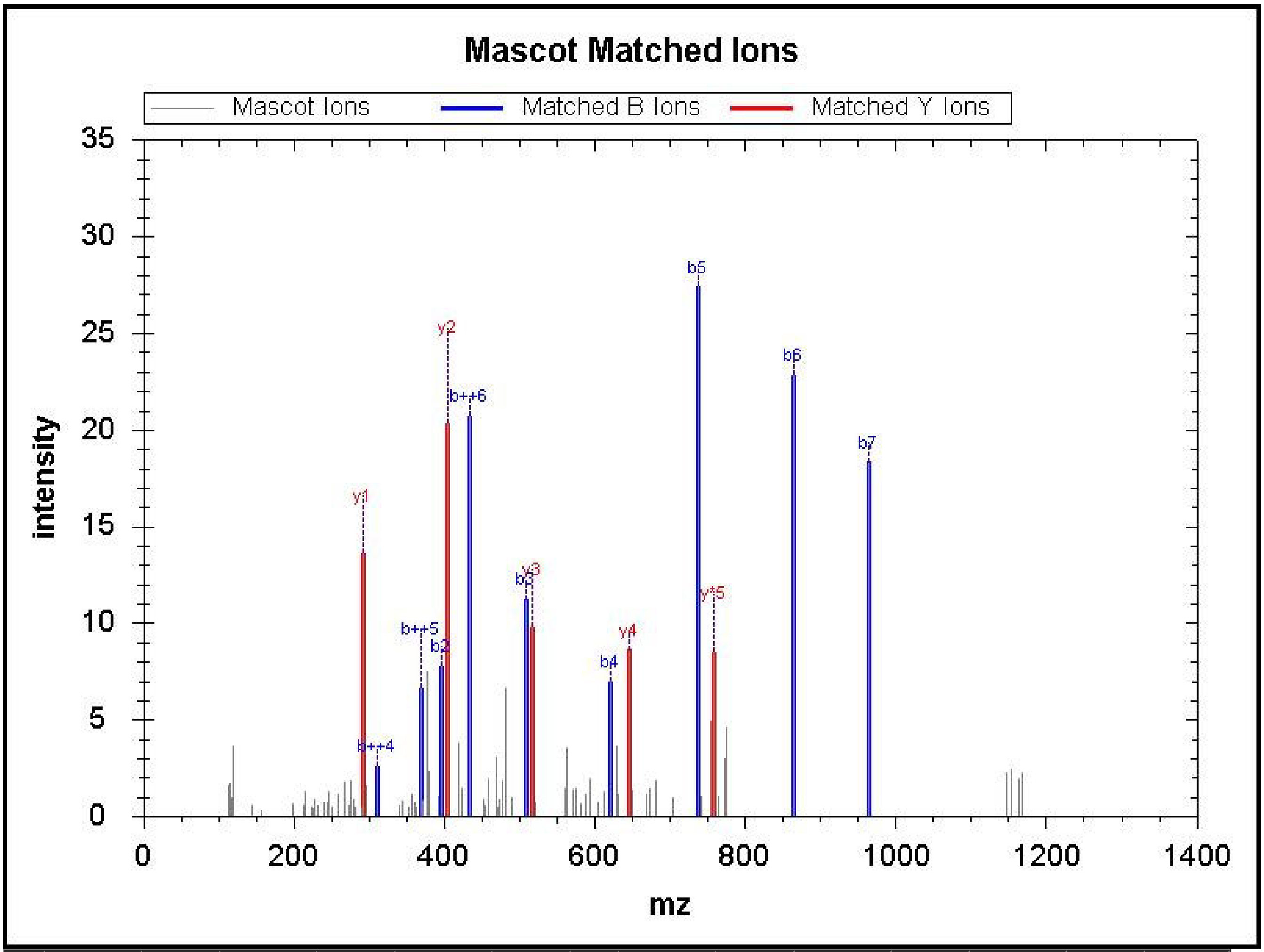

No	b	b++	b*	b*++	b0	b0++	Seq	y	y++	y*	y*++	y0	y0++	RevNo
1	258.19	129.60					L							14
2	395.25	198.13					H	1,774.00	887.50	1,756.98	878.99	1,755.99	878.50	13
3	508.34	254.67					L	1,636.94	818.98	1,619.92	810.46	1,618.93	809.97	12
4	621.42	311.21					L	1,523.86	762.43	1,506.83	753.92	1,505.85	753.43	11
5	736.45	368.73			718.44	359.72	D	1,410.77	705.89	1,393.75	697.38	1,392.76	696.89	10
6	864.51	432.76	847.48	424.24	846.50	423.75	Q	1,295.75	648.38	1,278.72	639.86	1,277.74	639.37	9
7	963.57	482.29	946.55	473.78	945.56	473.29	V	1,167.69	584.35	1,150.66	575.84	1,149.68	575.34	8
8	1,110.64	555.83	1,093.62	547.31	1,092.63	546.82	F	1,068.62	534.81	1,051.59	526.30	1,050.61	525.81	7
9	1,257.71	629.36	1,240.68	620.85	1,239.70	620.35	F	921.55	461.28	904.53	452.77	903.54	452.27	6
10	1,385.77	693.39	1,368.74	684.88	1,367.76	684.38	Q	774.48	387.75	757.46	379.23	756.47	378.74	5
11	1,514.81	757.91	1,497.79	749.40	1,496.80	748.90	E	646.43	323.72	629.40	315.20	628.41	314.71	4
12	1,627.90	814.45	1,610.87	805.94	1,609.89	805.45	L	517.38	259.20	500.36	250.68			3
13	1,740.98	870.99	1,723.95	862.48	1,722.97	861.99	L	404.30	202.65	387.27	194.14			2
14							K	291.21	146.11	274.19	137.60			1

Query 24609 Hit 1

MS/MS Fragmentation of SFEDIHHYR
Found in sp|P01116|RASK_HUMAN, GTPase KRas OS=Homo sapiens GN=KRAS PE=1 SV=1
Match to Query 24609: 1346.656from(449.8928,3+)
Title: 212: Scan 526 (rt=21.4652, f=3, i=73) [D:\lab212\membrane\Grace\Joyce\iTRAQ_53_2.raw]
Data File:Submitted from 20120508-1(merge) by Mascot Daemon on JOYCE-VAIO
Monoisotopic mass of neutral peptide Mr(calc): 1346.656
Variable modifications:
Ions Score: 48.96 Expect: 0.009

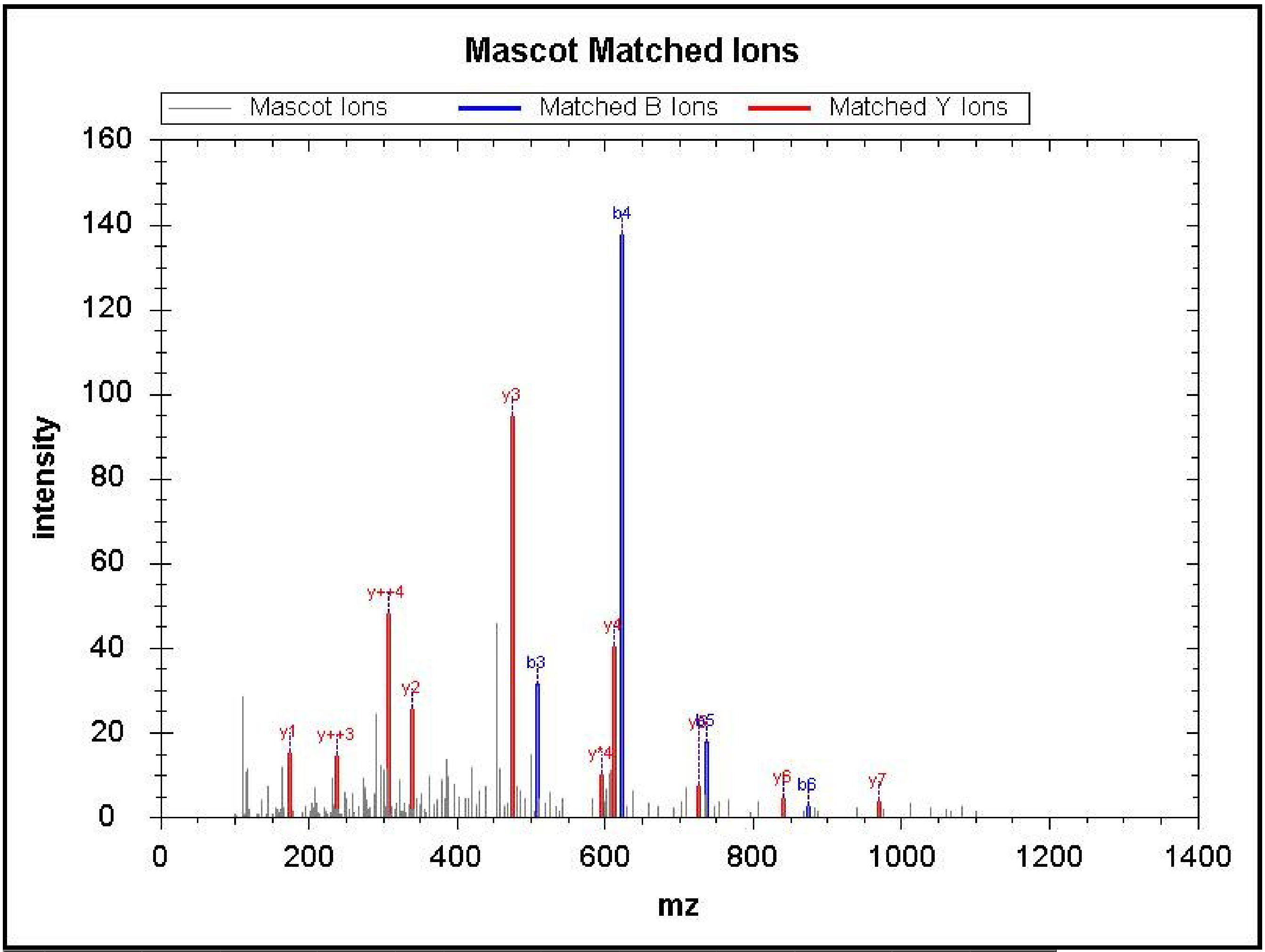

No	b	b++	b0	b0++	Seq	y	y++	y*	y*++	y0	y0++	RevNo
1	232.14	116.57	214.13	107.57	S							9
2	379.21	190.11	361.20	181.10	F	1,116.52	558.76	1,099.50	550.25	1,098.51	549.76	8
3	508.25	254.63	490.24	245.62	E	969.45	485.23	952.43	476.72	951.44	476.23	7
4	623.28	312.14	605.27	303.14	D	840.41	420.71	823.38	412.20	822.40	411.70	6
5	736.36	368.69	718.35	359.68	I	725.38	363.20	708.36	354.68			5
6	873.42	437.21	855.41	428.21	H	612.30	306.65	595.27	298.14			4
7	1,010.48	505.74	992.47	496.74	H	475.24	238.12	458.21	229.61			3
8	1,173.54	587.28	1,155.53	578.27	Y	338.18	169.59	321.16	161.08			2
9					R	175.12	88.06	158.09	79.55			1

Query 22205 Hit 1

MS/MS Fragmentation of TIQAHEGFVR
Found in sp|Q9NV06|DCA13_HUMAN, DDB1- and CUL4-associated factor 13 OS=Homo sapiens GN=DCAF13 PE=1 SV=2
Match to Query 22205: 1300.62from(434.5472,3+)
Title: 247: Sum of 2 scans in range 574 (rt=22.679, f=4, i=165) to 575 (rt=22.7044, f=4, i=166)
[D:\lab212\membrane\Grace\Joyce\iTRAQ40.raw]
Data File:Submitted from 20120508-1(merge) by Mascot Daemon on JOYCE-VAIO
Monoisotopic mass of neutral peptide Mr(calc): 1300.62
Variable modifications:
Ions Score: 48.85 Expect: 0.007

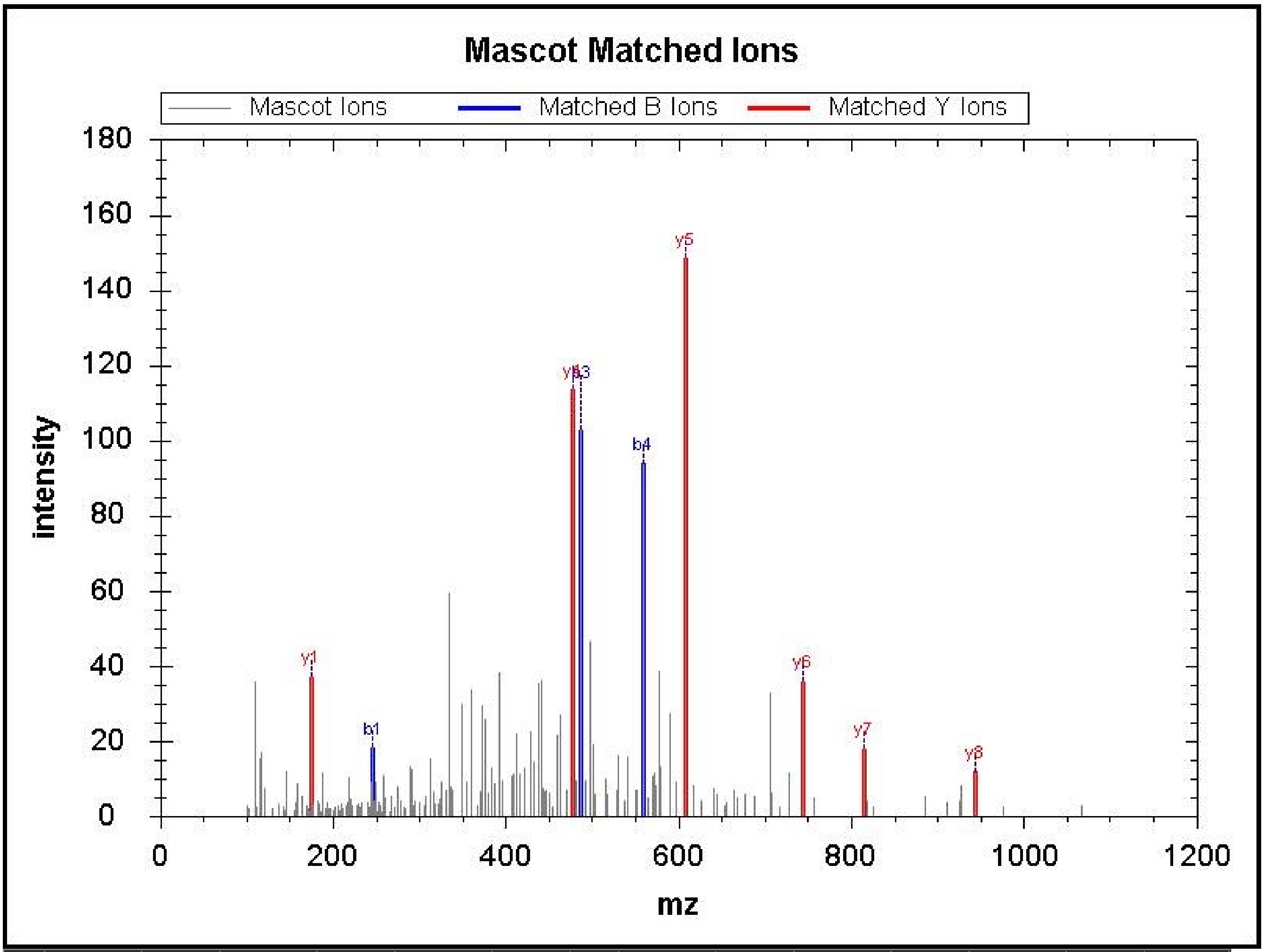

No	b	b++	b*	b*++	b0	b0++	Seq	y	y++	y*	y*++	y0	y0++	RevNo
1	246.16	123.58			228.15	114.58	T							10
2	359.24	180.12			341.23	171.12	I	1,056.56	528.78	1,039.53	520.27	1,038.55	519.78	9
3	487.30	244.15	470.27	235.64	469.29	235.15	Q	943.47	472.24	926.45	463.73	925.46	463.24	8
4	558.34	279.67	541.31	271.16	540.33	270.67	A	815.42	408.21	798.39	399.70	797.41	399.21	7
5	695.40	348.20	678.37	339.69	677.39	339.20	H	744.38	372.69	727.35	364.18	726.37	363.69	6
6	824.44	412.72	807.41	404.21	806.43	403.72	E	607.32	304.16	590.29	295.65	589.31	295.16	5
7	881.46	441.23	864.43	432.72	863.45	432.23	G	478.28	239.64	461.25	231.13			4
8	1,028.53	514.77	1,011.50	506.25	1,010.52	505.76	F	421.26	211.13	404.23	202.62			3
9	1,127.60	564.30	1,110.57	555.79	1,109.59	555.30	V	274.19	137.60	257.16	129.08			2
10							R	175.12	88.06	158.09	79.55			1

Query 64839 Hit 1

MS/MS Fragmentation of EKDGEFSVLQLVGMLR
Found in sp|P29317|EPHA2_HUMAN, Ephrin type-A receptor 2 OS=Homo sapiens GN=EPHA2 PE=1 SV=2
Match to Query 64839: 2108.155from(703.7255,3+)
Title: 1133: Sum of 2 scans in range 2436 (rt=64.5664, f=4, i=757) to 2437 (rt=64.5918, f=4, i=758)
[D:\lab212\membrane\Grace\Joyce\iTRAQ_40_1.raw]
Data File:Submitted from 20120508-1(merge) by Mascot Daemon on JOYCE-VAIO
Monoisotopic mass of neutral peptide Mr(calc): 2108.155
Variable modifications:
K2 :iTRAQ4plex (K)
Ions Score: 48.79 Expect: 0.010

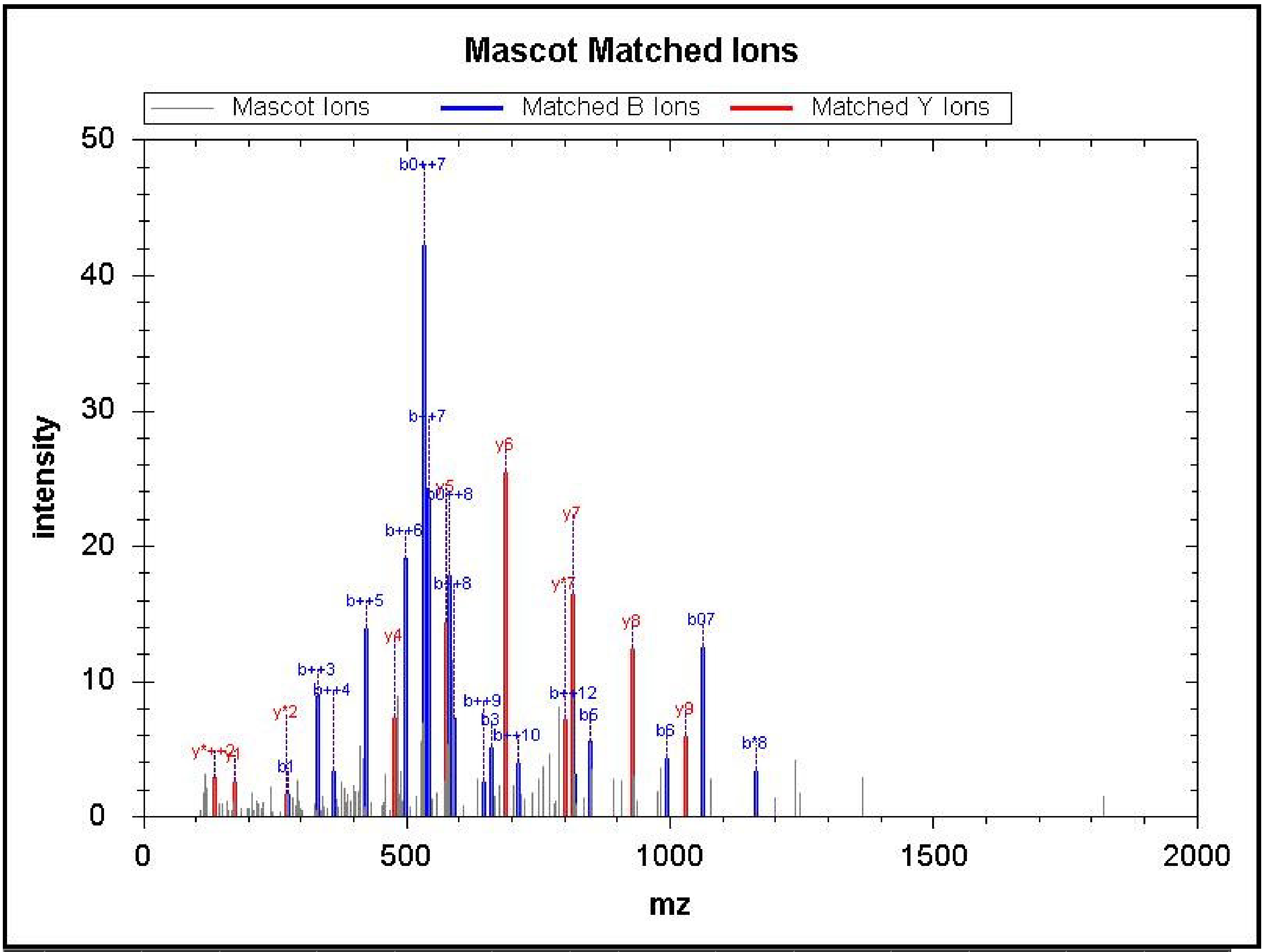

No	b	b++	b*	b*++	b0	b0++	Seq	y	y++	y*	y*++	y0	y0++	RevNo
1	274.15	137.58			256.14	128.57	E							16
2	546.35	273.68	529.32	265.16	528.34	264.67	K	1,836.02	918.51	1,818.99	910.00	1,818.01	909.51	15
3	661.38	331.19	644.35	322.68	643.37	322.19	D	1,563.82	782.41	1,546.79	773.90	1,545.81	773.41	14
4	718.40	359.70	701.37	351.19	700.39	350.70	G	1,448.79	724.90	1,431.77	716.39	1,430.78	715.89	13
5	847.44	424.22	830.41	415.71	829.43	415.22	E	1,391.77	696.39	1,374.74	687.88	1,373.76	687.38	12
6	994.51	497.76	977.48	489.24	976.50	488.75	F	1,262.73	631.87	1,245.70	623.35	1,244.72	622.86	11
7	1,081.54	541.27	1,064.51	532.76	1,063.53	532.27	S	1,115.66	558.33	1,098.63	549.82	1,097.65	549.33	10
8	1,180.61	590.81	1,163.58	582.29	1,162.60	581.80	V	1,028.63	514.82	1,011.60	506.30			9
9	1,293.69	647.35	1,276.67	638.84	1,275.68	638.34	L	929.56	465.28	912.53	456.77			8
10	1,421.75	711.38	1,404.72	702.87	1,403.74	702.37	Q	816.48	408.74	799.45	400.23			7
11	1,534.84	767.92	1,517.81	759.41	1,516.82	758.92	L	688.42	344.71	671.39	336.20			6
12	1,633.90	817.46	1,616.88	808.94	1,615.89	808.45	V	575.33	288.17	558.31	279.66			5
13	1,690.93	845.97	1,673.90	837.45	1,672.91	836.96	G	476.26	238.64	459.24	230.12			4
14	1,821.97	911.49	1,804.94	902.97	1,803.96	902.48	M	419.24	210.13	402.22	201.61			3
15	1,935.05	968.03	1,918.02	959.52	1,917.04	959.02	L	288.20	144.61	271.18	136.09			2
16							R	175.12	88.06	158.09	79.55			1

Query 24487 Hit 1

MS/MS Fragmentation of YGLLVGGAASHR
Found in sp|Q15274|NADC_HUMAN, Nicotinate-nucleotide pyrophosphorylase [carboxylating] OS=Homo sapiens GN=QPRT PE=1 SV=3
Match to Query 24487: 1343.744from(448.9219,3+)
Title: 383: Sum of 2 scans in range 909 (rt=29.9275, f=4, i=251) to 910 (rt=29.9529, f=4, i=252) [D:\lab212\membrane\Grace\Joyce\iTRAQ_37_2.raw]
Data File:Submitted from 20120508-1(merge) by Mascot Daemon on JOYCE-VAIO

Monoisotopic mass of neutral peptide Mr(calc): 1343.744
Variable modifications:
Ions Score: 48.74 Expect: 0.010

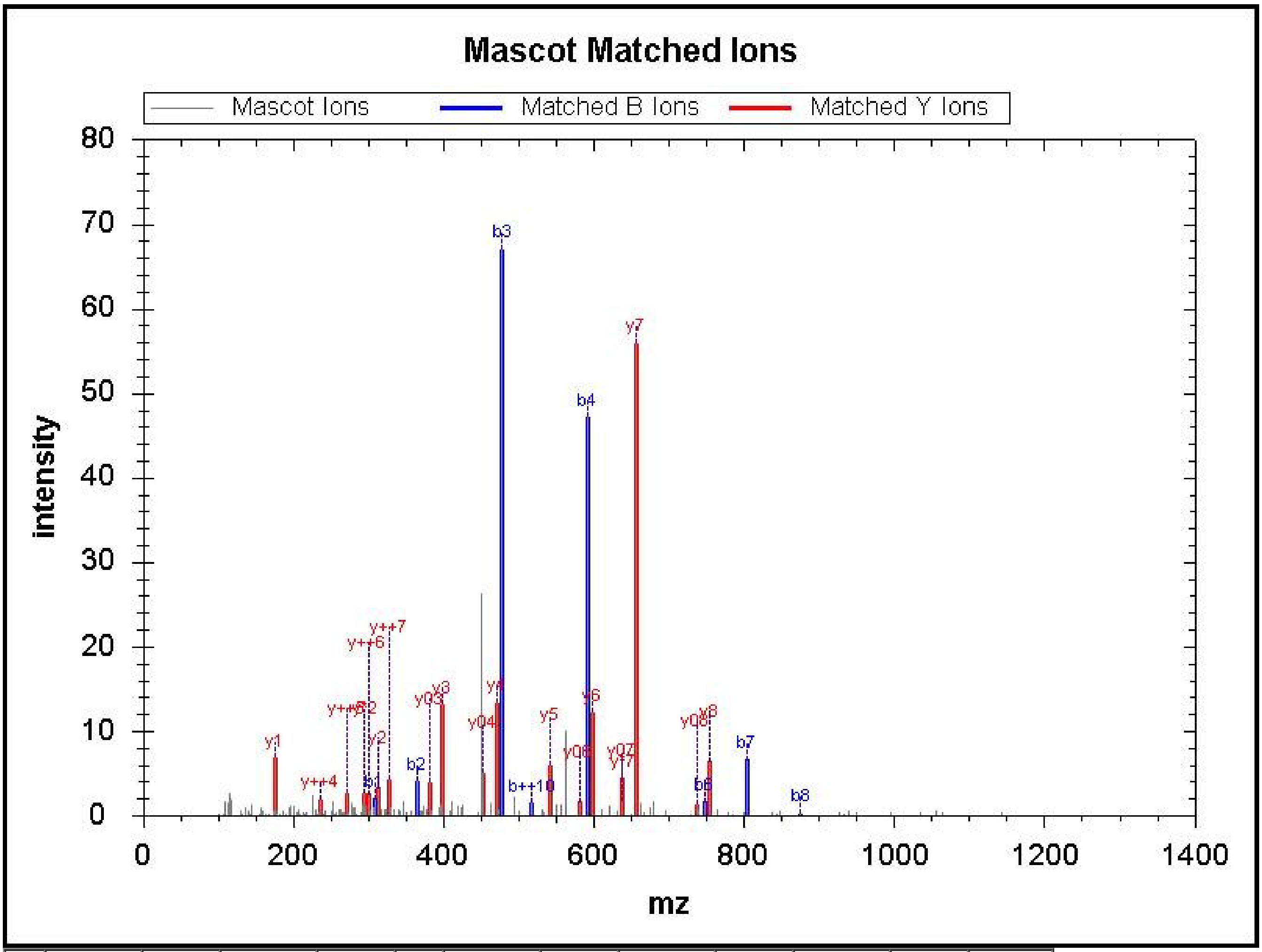

No	b	b++	b0	b0++	Seq	y	y++	y*	y*++	y0	y0++	RevNo
1	308.17	154.59			Y							12
2	365.19	183.10			G	1,037.59	519.30	1,020.56	510.78	1,019.57	510.29	11
3	478.28	239.64			L	980.56	490.79	963.54	482.27	962.55	481.78	10
4	591.36	296.18			L	867.48	434.24	850.45	425.73	849.47	425.24	9
5	690.43	345.72			V	754.40	377.70	737.37	369.19	736.38	368.70	8
6	747.45	374.23			G	655.33	328.17	638.30	319.65	637.32	319.16	7
7	804.47	402.74			G	598.31	299.66	581.28	291.14	580.30	290.65	6
8	875.51	438.26			A	541.28	271.15	524.26	262.63	523.27	262.14	5
9	946.55	473.78			A	470.25	235.63	453.22	227.11	452.24	226.62	4
10	1,033.58	517.29	1,015.57	508.29	S	399.21	200.11	382.18	191.60	381.20	191.10	3
11	1,170.64	585.82	1,152.63	576.82	H	312.18	156.59	295.15	148.08			2
12					R	175.12	88.06	158.09	79.55			1

Query 8386 Hit 1

MS/MS Fragmentation of LFVIGGK
Found in sp|Q8NAB2|KBTB3_HUMAN, Kelch repeat and BTB domain-containing protein 3 OS=Homo sapiens GN=KBTBD3 PE=2 SV=2
Match to Query 8386: 1020.653from(511.3337,2+)
Title: 548: Sum of 2 scans in range 1226 (rt=37.2906, f=4, i=369) to 1227 (rt=37.316, f=4, i=370) [D:\lab212\membrane\Grace\Joyce\iTRAQ_33_2.raw]
Data File:Submitted from 20120508-1(merge) by Mascot Daemon on JOYCE-VAIO
Monoisotopic mass of neutral peptide Mr(calc): 1020.653
Variable modifications:

K7 :iTRAQ4plex (K)
Ions Score: 48.68 Expect: 0.007

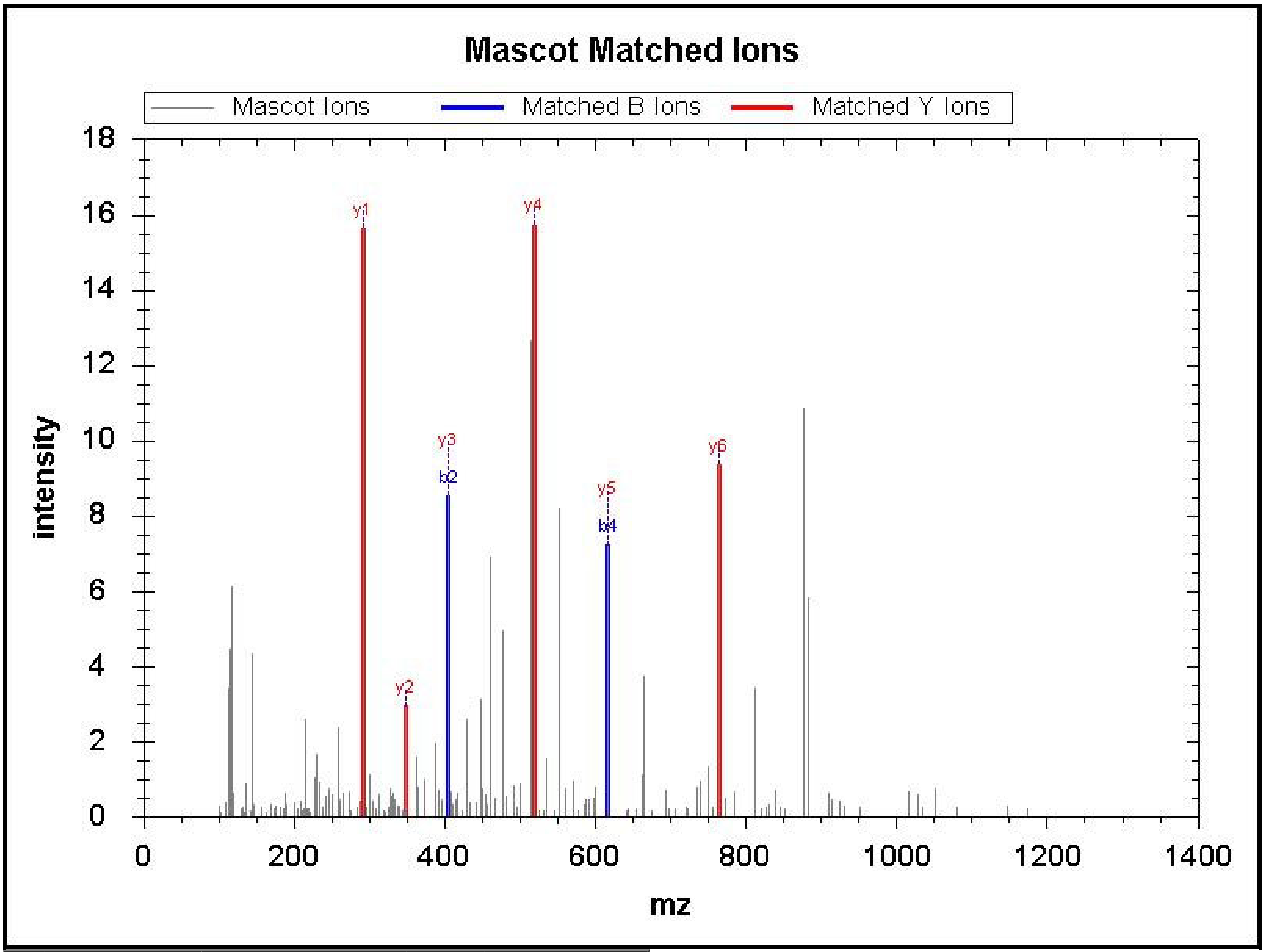

No	b	b++	Seq	y	y++	y*	y*++	RevNo
1	258.19	129.60	L					7
2	405.26	203.13	F	764.48	382.74	747.45	374.23	6
3	504.33	252.67	V	617.41	309.21	600.38	300.70	5
4	617.41	309.21	I	518.34	259.67	501.32	251.16	4
5	674.44	337.72	G	405.26	203.13	388.23	194.62	3
6	731.46	366.23	G	348.24	174.62	331.21	166.11	2
7			K	291.21	146.11	274.19	137.60	1

Query 55320 Hit 1

MS/MS Fragmentation of EEEESESSTSDDKR
Found in sp|Q7Z6M4|MTER2_HUMAN, mTERF domain-containing protein 2 OS=Homo sapiens GN=MTERFD2 PE=1 SV=3
Match to Query 55320: 1914.856from(639.2927,3+)
Title: 4: Sum of 2 scans in range 101 (rt=11.8732, f=2, i=14) to 102 (rt=11.8986, f=2, i=15)
[D:\lab212\membrane\Grace\Joyce\iTRAQ_44_2.raw]
Data File:Submitted from 20120508-1(merge) by Mascot Daemon on JOYCE-VAIO
Monoisotopic mass of neutral peptide Mr(calc): 1914.856
Variable modifications:
K13 :iTRAQ4plex (K)
Ions Score: 48.64 Expect: 0.007

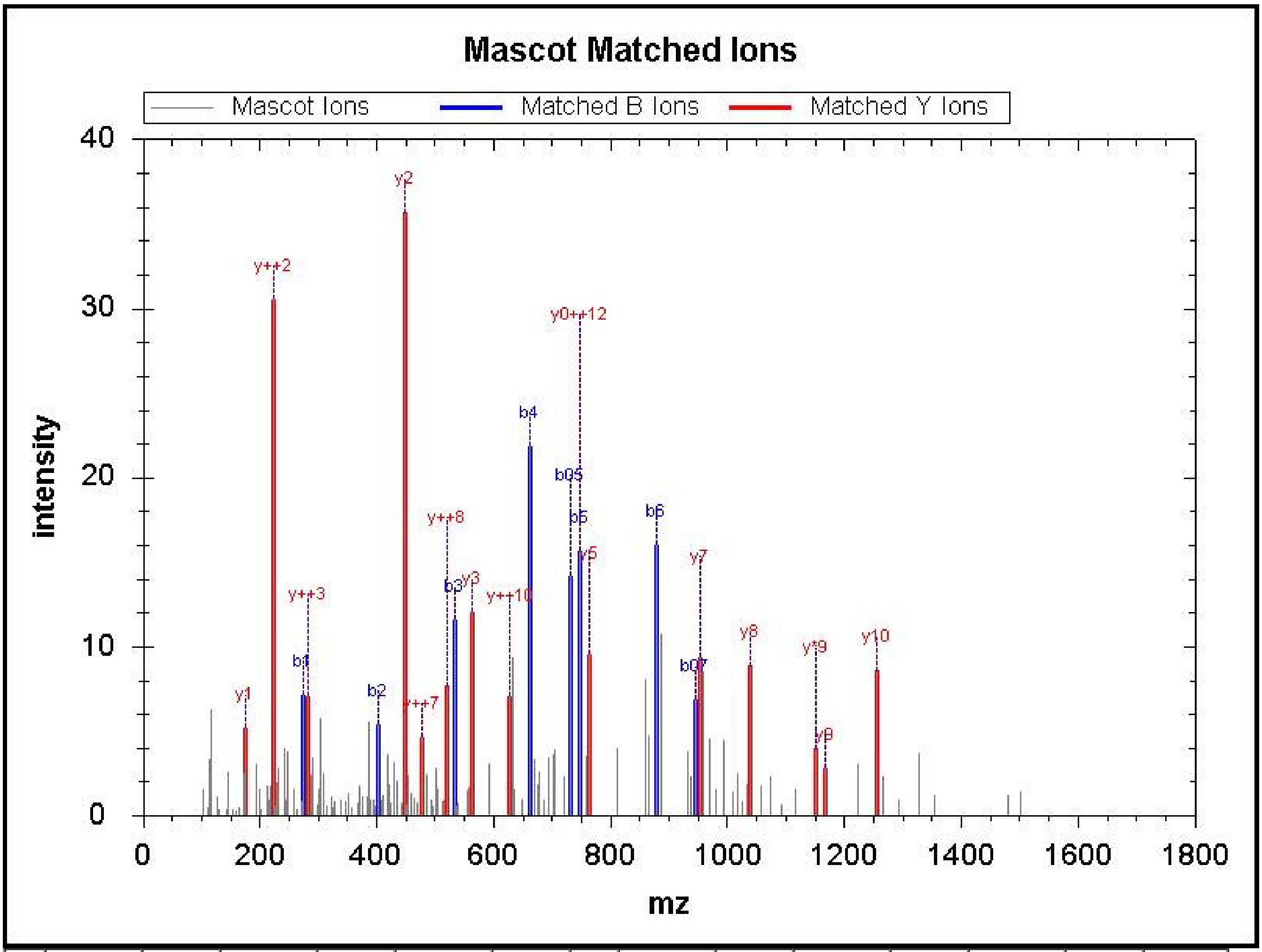

No	b	b++	b*	b*++	b0	b0++	Seq	y	y++	y*	y*++	y0	y0++	RevNo
1	274.15	137.58			256.14	128.57	E							14
2	403.19	202.10			385.18	193.10	E	1,642.72	821.86	1,625.69	813.35	1,624.71	812.86	13
3	532.24	266.62			514.23	257.62	E	1,513.67	757.34	1,496.65	748.83	1,495.66	748.34	12
4	661.28	331.14			643.27	322.14	E	1,384.63	692.82	1,367.60	684.31	1,366.62	683.81	11
5	748.31	374.66			730.30	365.65	S	1,255.59	628.30	1,238.56	619.78	1,237.58	619.29	10
6	877.35	439.18			859.34	430.18	E	1,168.56	584.78	1,151.53	576.27	1,150.55	575.78	9
7	964.39	482.70			946.38	473.69	S	1,039.51	520.26	1,022.49	511.75	1,021.50	511.26	8
8	1,051.42	526.21			1,033.41	517.21	S	952.48	476.74	935.46	468.23	934.47	467.74	7
9	1,152.47	576.74			1,134.46	567.73	T	865.45	433.23	848.42	424.72	847.44	424.22	6
10	1,239.50	620.25			1,221.49	611.25	S	764.40	382.70	747.38	374.19	746.39	373.70	5
11	1,354.53	677.77			1,336.51	668.76	D	677.37	339.19	660.34	330.68	659.36	330.18	4
12	1,469.55	735.28			1,451.54	726.27	D	562.34	281.68	545.32	273.16	544.33	272.67	3
13	1,741.75	871.38	1,724.72	862.86	1,723.74	862.37	K	447.32	224.16	430.29	215.65			2
14							R	175.12	88.06	158.09	79.55			1

Query 43782 Hit 1

MS/MS Fragmentation of NIVHNYSEAEIK
Found in sp|Q9Y6I3|EPN1_HUMAN, Epsin-1 OS=Homo sapiens GN=EPN1 PE=1 SV=2
Match to Query 43782: 1703.912from(568.9781,3+)
Title: 311: Sum of 2 scans in range 713 (rt=25.8318, f=4, i=212) to 714 (rt=25.8572, f=4, i=213)
[D:\lab212\membrane\Grace\Joyce\iTRAQ_42_2.raw]
Data File:Submitted from 20120508-1(merge) by Mascot Daemon on JOYCE-VAIO
Monoisotopic mass of neutral peptide Mr(calc): 1703.912
Variable modifications:
K12 :iTRAQ4plex (K)
Ions Score: 48.6 Expect: 0.012

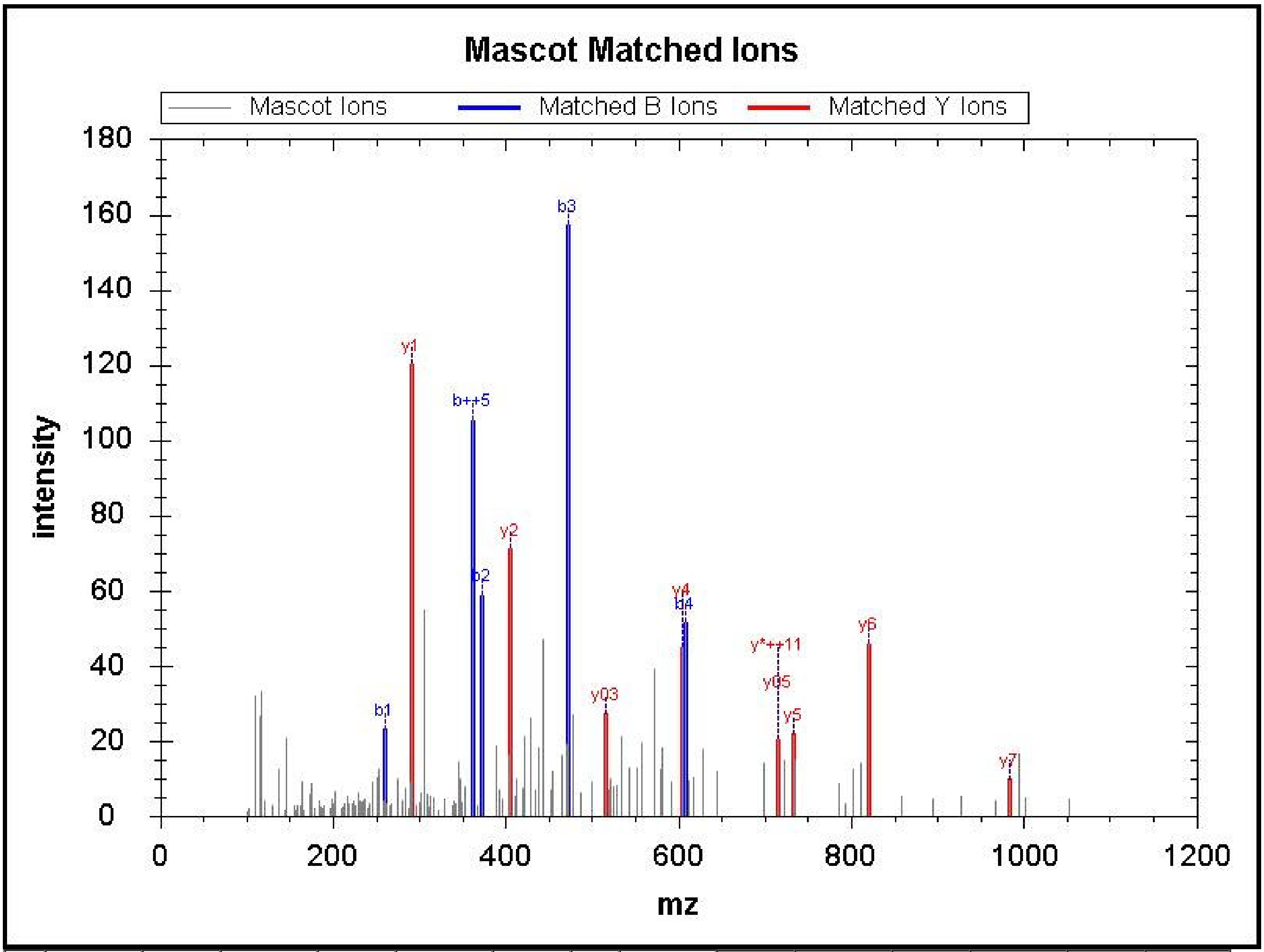

No	b	b++	b*	b*++	b0	b0++	Seq	y	y++	y*	y*++	y0	y0++	RevNo
1	259.15	130.08	242.13	121.57			N							12
2	372.24	186.62	355.21	178.11			I	1,446.77	723.89	1,429.74	715.38	1,428.76	714.88	11
3	471.30	236.16	454.28	227.64			V	1,333.69	667.35	1,316.66	658.83	1,315.68	658.34	10
4	608.36	304.69	591.34	296.17			H	1,234.62	617.81	1,217.59	609.30	1,216.61	608.81	9
5	722.41	361.71	705.38	353.19			N	1,097.56	549.28	1,080.53	540.77	1,079.55	540.28	8
6	885.47	443.24	868.44	434.73			Y	983.52	492.26	966.49	483.75	965.51	483.26	7
7	972.50	486.75	955.48	478.24	954.49	477.75	S	820.45	410.73	803.43	402.22	802.44	401.72	6
8	1,101.54	551.28	1,084.52	542.76	1,083.53	542.27	E	733.42	367.21	716.39	358.70	715.41	358.21	5
9	1,172.58	586.79	1,155.56	578.28	1,154.57	577.79	A	604.38	302.69	587.35	294.18	586.37	293.69	4
10	1,301.62	651.32	1,284.60	642.80	1,283.61	642.31	E	533.34	267.17	516.31	258.66	515.33	258.17	3
11	1,414.71	707.86	1,397.68	699.34	1,396.70	698.85	I	404.30	202.65	387.27	194.14			2
12							K	291.21	146.11	274.19	137.60			1

Query 90891 Hit 1

MS/MS Fragmentation of EIYLEVIHNLPDFELLSANTLEDR
Found in sp|Q8IXB1|DJC10_HUMAN, DnaJ homolog subfamily C member 10 OS=Homo sapiens GN=DNAJC10 PE=1 SV=2
Match to Query 90891: 2986.542from(996.5215,3+)
Title: 971: Sum of 2 scans in range 2777 (rt=68.6385, f=4, i=623) to 2778 (rt=68.6638, f=4, i=624)
[D:\lab212\membrane\Grace\Joyce\iTRAQ_29_2.raw]
Data File:Submitted from 20120508-1(merge) by Mascot Daemon on JOYCE-VAIO
Monoisotopic mass of neutral peptide Mr(calc): 2986.542
Variable modifications:
Ions Score: 48.56 Expect: 0.012

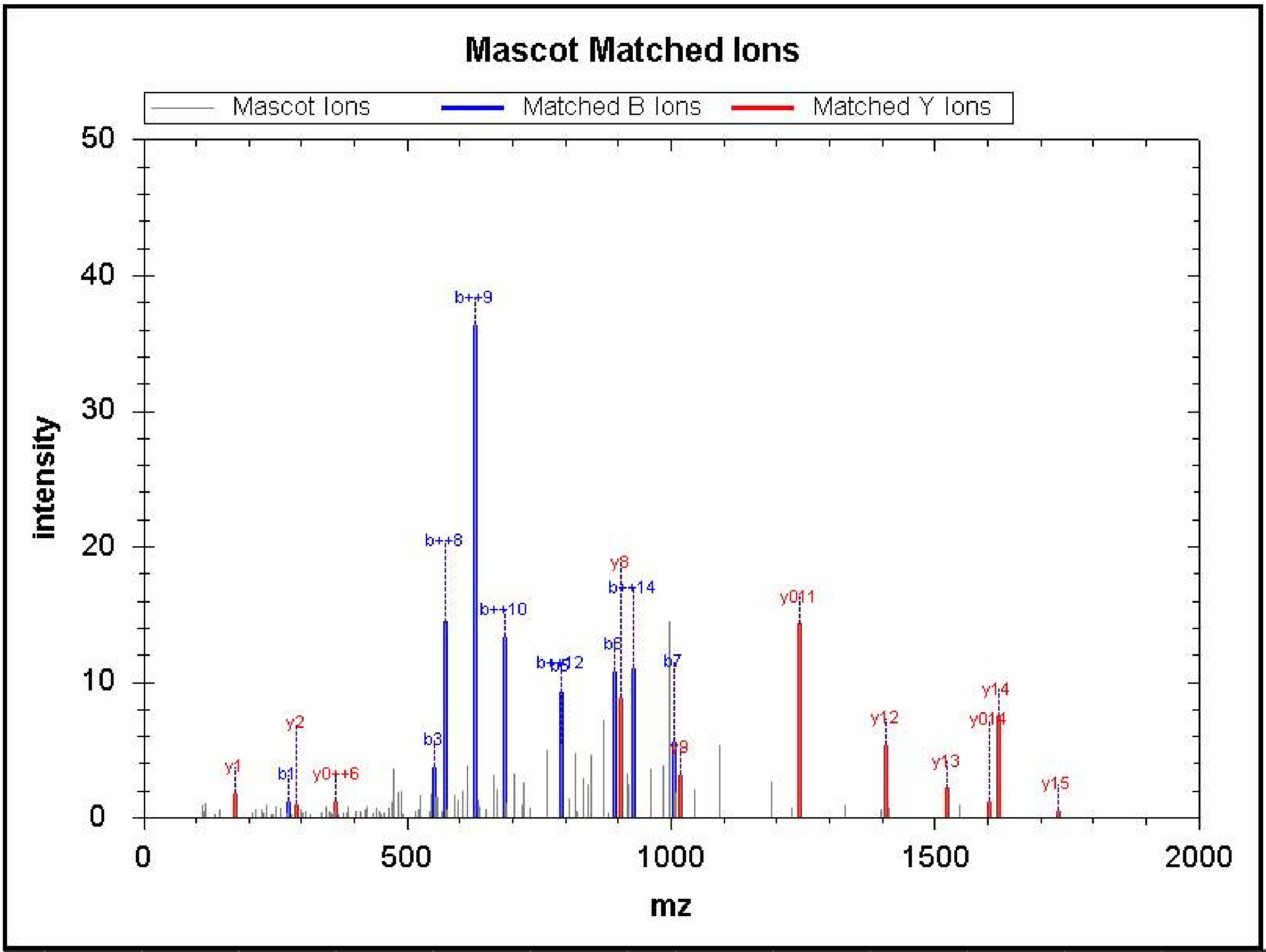

No	b	b++	b*	b*++	b0	b0++	Seq	y	y++	y*	y*++	y0	y0++	RevNo
1	274.15	137.58			256.14	128.57	E							24
2	387.24	194.12			369.23	185.12	I	2,714.40	1,357.71	2,697.38	1,349.19	2,696.39	1,348.70	23
3	550.30	275.65			532.29	266.65	Y	2,601.32	1,301.16	2,584.29	1,292.65	2,583.31	1,292.16	22
4	663.38	332.20			645.37	323.19	L	2,438.26	1,219.63	2,421.23	1,211.12	2,420.25	1,210.63	21
5	792.43	396.72			774.42	387.71	E	2,325.17	1,163.09	2,308.15	1,154.58	2,307.16	1,154.08	20
6	891.49	446.25			873.48	437.25	V	2,196.13	1,098.57	2,179.10	1,090.06	2,178.12	1,089.56	19
7	1,004.58	502.79			986.57	493.79	I	2,097.06	1,049.03	2,080.03	1,040.52	2,079.05	1,040.03	18
8	1,141.64	571.32			1,123.63	562.32	H	1,983.98	992.49	1,966.95	983.98	1,965.97	983.49	17
9	1,255.68	628.34	1,238.65	619.83	1,237.67	619.34	N	1,846.92	923.96	1,829.89	915.45	1,828.91	914.96	16
10	1,368.76	684.89	1,351.74	676.37	1,350.75	675.88	L	1,732.88	866.94	1,715.85	858.43	1,714.86	857.94	15
11	1,465.82	733.41	1,448.79	724.90	1,447.81	724.41	P	1,619.79	810.40	1,602.76	801.89	1,601.78	801.39	14
12	1,580.84	790.93	1,563.82	782.41	1,562.83	781.92	D	1,522.74	761.87	1,505.71	753.36	1,504.73	752.87	13
13	1,727.91	864.46	1,710.89	855.95	1,709.90	855.45	F	1,407.71	704.36	1,390.68	695.85	1,389.70	695.35	12
14	1,856.96	928.98	1,839.93	920.47	1,838.94	919.98	E	1,260.64	630.83	1,243.62	622.31	1,242.63	621.82	11
15	1,970.04	985.52	1,953.01	977.01	1,952.03	976.52	L	1,131.60	566.30	1,114.57	557.79	1,113.59	557.30	10
16	2,083.12	1,042.07	2,066.10	1,033.55	2,065.11	1,033.06	L	1,018.52	509.76	1,001.49	501.25	1,000.51	500.76	9
17	2,170.16	1,085.58	2,153.13	1,077.07	2,152.14	1,076.58	S	905.43	453.22	888.41	444.71	887.42	444.21	8
18	2,241.19	1,121.10	2,224.17	1,112.59	2,223.18	1,112.09	A	818.40	409.70	801.37	401.19	800.39	400.70	7
19	2,355.24	1,178.12	2,338.21	1,169.61	2,337.22	1,169.12	N	747.36	374.19	730.34	365.67	729.35	365.18	6
20	2,456.28	1,228.65	2,439.26	1,220.13	2,438.27	1,219.64	T	633.32	317.16	616.29	308.65	615.31	308.16	5
21	2,569.37	1,285.19	2,552.34	1,276.67	2,551.36	1,276.18	L	532.27	266.64	515.25	258.13	514.26	257.63	4
22	2,698.41	1,349.71	2,681.38	1,341.20	2,680.40	1,340.70	E	419.19	210.10	402.16	201.58	401.18	201.09	3
23	2,813.44	1,407.22	2,796.41	1,398.71	2,795.43	1,398.22	D	290.15	145.58	273.12	137.06	272.14	136.57	2

24							R	175.12	88.06	158.09	79.55			1

Query 89963 Hit 1

MS/MS Fragmentation of LGDYHFYGFGTDVDYETAFIHYR
Found in sp|Q9UBV2|SE1L1_HUMAN, Protein sel-1 homolog 1 OS=Homo sapiens GN=SEL1L PE=1 SV=3
Match to Query 89963: 2929.359from(733.3471,4+)
Title: 935: Scan 2023 (rt=55.2646, f=3, i=320) [D:\lab212\membrane\Grace\Joyce\iTRAQ_45_1.raw]
Data File:Submitted from 20120508-1(merge) by Mascot Daemon on JOYCE-VAIO
Monoisotopic mass of neutral peptide Mr(calc): 2929.359
Variable modifications:
Ions Score: 48.52 Expect: 0.010

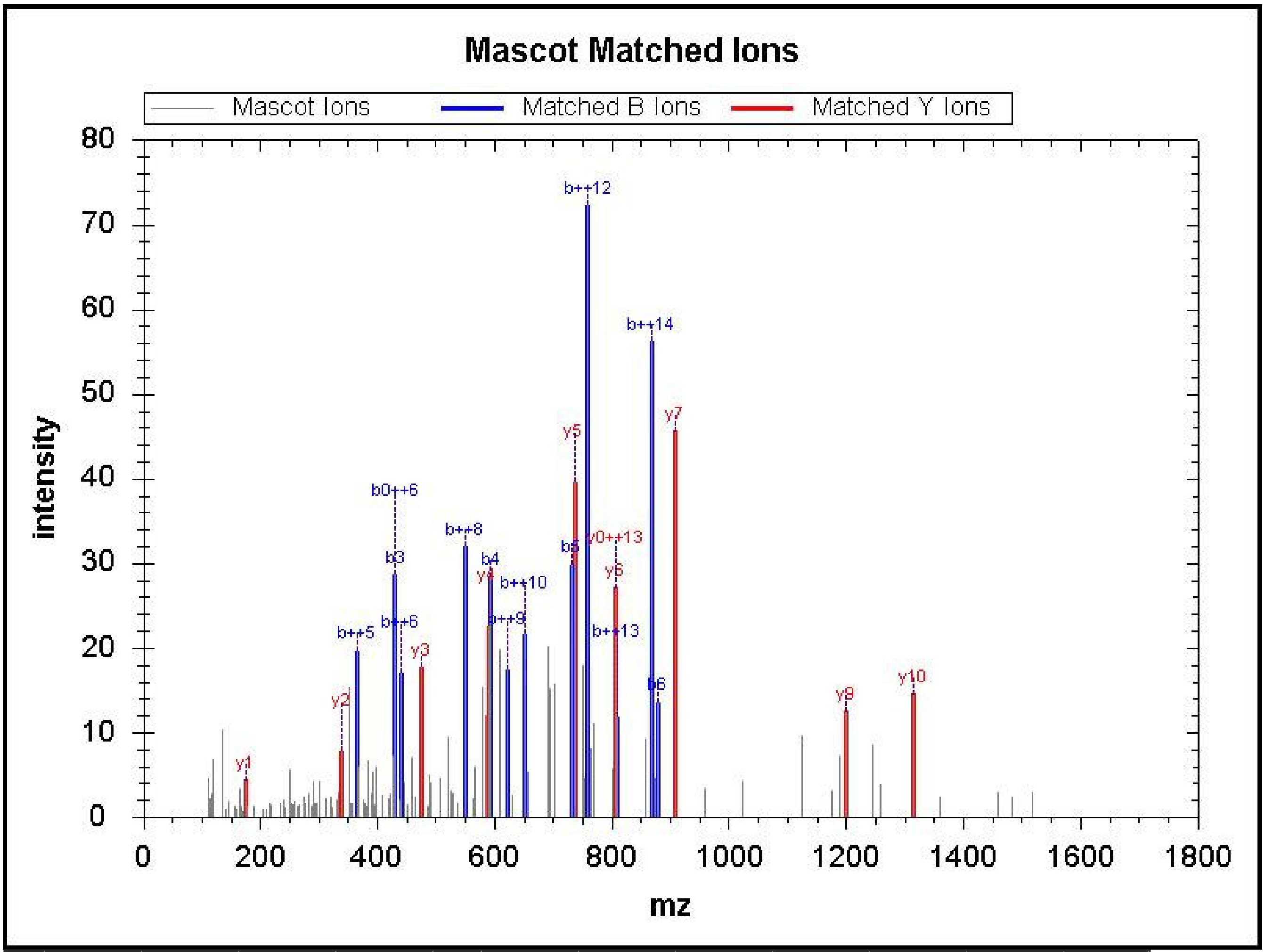

No	b	b++	b0	b0++	Seq	y	y++	y*	y*++	y0	y0++	RevNo
1	258.19	129.60			L							23
2	315.21	158.11			G	2,673.17	1,337.09	2,656.14	1,328.57	2,655.16	1,328.08	22
3	430.24	215.62	412.23	206.62	D	2,616.15	1,308.58	2,599.12	1,300.06	2,598.14	1,299.57	21
4	593.31	297.16	575.29	288.15	Y	2,501.12	1,251.06	2,484.09	1,242.55	2,483.11	1,242.06	20
5	730.36	365.69	712.35	356.68	H	2,338.06	1,169.53	2,321.03	1,161.02	2,320.05	1,160.53	19
6	877.43	439.22	859.42	430.21	F	2,201.00	1,101.00	2,183.97	1,092.49	2,182.99	1,092.00	18
7	1,040.50	520.75	1,022.49	511.75	Y	2,053.93	1,027.47	2,036.90	1,018.96	2,035.92	1,018.46	17
8	1,097.52	549.26	1,079.51	540.26	G	1,890.87	945.94	1,873.84	937.42	1,872.86	936.93	16
9	1,244.59	622.80	1,226.58	613.79	F	1,833.84	917.43	1,816.82	908.91	1,815.83	908.42	15
10	1,301.61	651.31	1,283.60	642.30	G	1,686.78	843.89	1,669.75	835.38	1,668.77	834.89	14
11	1,402.65	701.83	1,384.64	692.83	T	1,629.75	815.38	1,612.73	806.87	1,611.74	806.38	13
12	1,517.68	759.34	1,499.67	750.34	D	1,528.71	764.86	1,511.68	756.34	1,510.70	755.85	12
13	1,616.75	808.88	1,598.74	799.87	V	1,413.68	707.34	1,396.65	698.83	1,395.67	698.34	11

14	1,731.78	866.39	1,713.77	857.39	D	1,314.61	657.81	1,297.58	649.30	1,296.60	648.80	10
15	1,894.84	947.92	1,876.83	938.92	Y	1,199.58	600.30	1,182.56	591.78	1,181.57	591.29	9
16	2,023.88	1,012.45	2,005.87	1,003.44	E	1,036.52	518.76	1,019.49	510.25	1,018.51	509.76	8
17	2,124.93	1,062.97	2,106.92	1,053.96	T	907.48	454.24	890.45	445.73	889.47	445.24	7
18	2,195.97	1,098.49	2,177.96	1,089.48	A	806.43	403.72	789.40	395.21			6
19	2,343.04	1,172.02	2,325.03	1,163.02	F	735.39	368.20	718.37	359.69			5
20	2,456.12	1,228.56	2,438.11	1,219.56	I	588.33	294.67	571.30	286.15			4
21	2,593.18	1,297.09	2,575.17	1,288.09	H	475.24	238.12	458.21	229.61			3
22	2,756.24	1,378.62	2,738.23	1,369.62	Y	338.18	169.59	321.16	161.08			2
23					R	175.12	88.06	158.09	79.55			1

Query 81278 Hit 1

MS/MS Fragmentation of EVIQELEELGVGIGVVHAGYER
Found in sp|Q9Y2G8|DJC16_HUMAN, DnaJ homolog subfamily C member 16 OS=Homo sapiens GN=DNAJC16 PE=2 SV=3
Match to Query 81278: 2539.325from(847.4491,3+)
Title: 1088: Scan 2409 (rt=63.581, f=3, i=373) [D:\lab212\membrane\Grace\Joyce\iTRAQ_31_1.raw]
Data File:Submitted from 20120508-1(merge) by Mascot Daemon on JOYCE-VAIO
Monoisotopic mass of neutral peptide Mr(calc): 2539.325
Variable modifications:
Ions Score: 48.4 Expect: 0.013

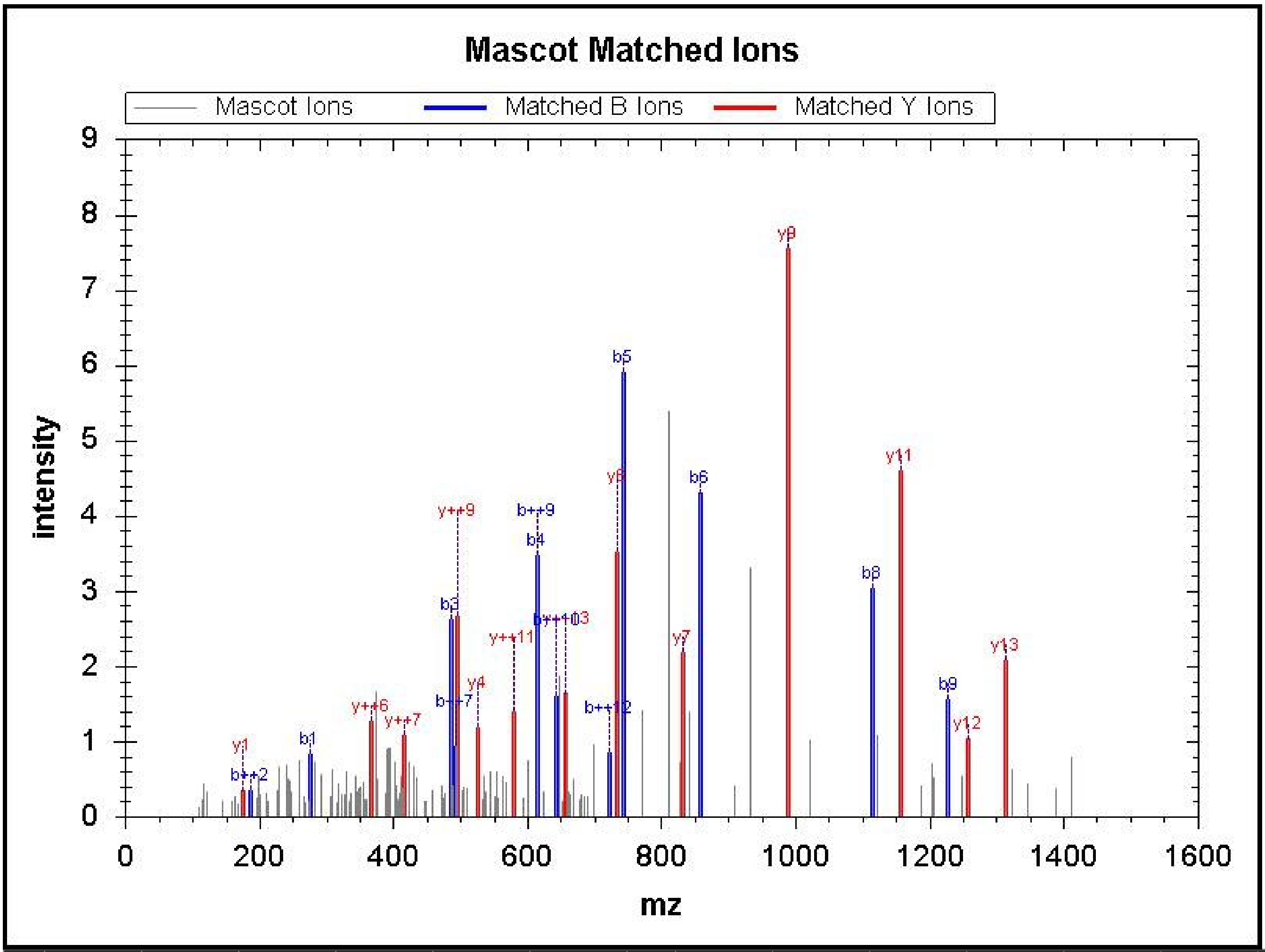

No	b	b++	b*	b*++	b0	b0++	Seq	y	y++	y*	y*++	y0	y0++	RevNo
1	274.15	137.58			256.14	128.57	E							22
2	373.22	187.11			355.21	178.11	V	2,267.20	1,134.11	2,250.18	1,125.59	2,249.19	1,125.10	21
3	486.30	243.66			468.29	234.65	I	2,168.13	1,084.57	2,151.11	1,076.06	2,150.12	1,075.57	20
4	614.36	307.69	597.34	299.17	596.35	298.68	Q	2,055.05	1,028.03	2,038.02	1,019.52	2,037.04	1,019.02	19
5	743.41	372.21	726.38	363.69	725.40	363.20	E	1,926.99	964.00	1,909.97	955.49	1,908.98	954.99	18

6	856.49	428.75	839.46	420.24	838.48	419.74	L	1,797.95	899.48	1,780.92	890.97	1,779.94	890.47	17
7	985.53	493.27	968.51	484.76	967.52	484.26	E	1,684.87	842.94	1,667.84	834.42	1,666.85	833.93	16
8	1,114.57	557.79	1,097.55	549.28	1,096.56	548.79	E	1,555.82	778.42	1,538.80	769.90	1,537.81	769.41	15
9	1,227.66	614.33	1,210.63	605.82	1,209.65	605.33	L	1,426.78	713.89	1,409.75	705.38	1,408.77	704.89	14
10	1,284.68	642.84	1,267.65	634.33	1,266.67	633.84	G	1,313.70	657.35	1,296.67	648.84	1,295.69	648.35	13
11	1,383.75	692.38	1,366.72	683.86	1,365.74	683.37	V	1,256.67	628.84	1,239.65	620.33	1,238.66	619.84	12
12	1,440.77	720.89	1,423.74	712.38	1,422.76	711.88	G	1,157.61	579.31	1,140.58	570.79	1,139.60	570.30	11
13	1,553.85	777.43	1,536.83	768.92	1,535.84	768.43	I	1,100.58	550.80	1,083.56	542.28	1,082.57	541.79	10
14	1,610.88	805.94	1,593.85	797.43	1,592.87	796.94	G	987.50	494.25	970.47	485.74	969.49	485.25	9
15	1,709.94	855.48	1,692.92	846.96	1,691.93	846.47	V	930.48	465.74	913.45	457.23	912.47	456.74	8
16	1,809.01	905.01	1,791.99	896.50	1,791.00	896.00	V	831.41	416.21	814.38	407.70	813.40	407.20	7
17	1,946.07	973.54	1,929.05	965.03	1,928.06	964.53	H	732.34	366.67	715.32	358.16	714.33	357.67	6
18	2,017.11	1,009.06	2,000.08	1,000.54	1,999.10	1,000.05	A	595.28	298.15	578.26	289.63	577.27	289.14	5
19	2,074.13	1,037.57	2,057.10	1,029.06	2,056.12	1,028.56	G	524.25	262.63	507.22	254.11	506.24	253.62	4
20	2,237.19	1,119.10	2,220.17	1,110.59	2,219.18	1,110.10	Y	467.22	234.12	450.20	225.60	449.21	225.11	3
21	2,366.24	1,183.62	2,349.21	1,175.11	2,348.23	1,174.62	E	304.16	152.58	287.13	144.07	286.15	143.58	2
22							R	175.12	88.06	158.09	79.55			1

Query 56433 Hit 1

MS/MS Fragmentation of VAHSDKPGSTSTASFR
Found in sp|Q9P0L0|VAPA_HUMAN, Vesicle-associated membrane protein-associated protein A OS=Homo sapiens GN=VAPA PE=1 SV=3
Match to Query 56433: 1935.007from(484.7591,4+)
Title: 56: Scan 403 (rt=17.7467, f=3, i=23) [D:\lab212\membrane\Grace\Joyce\iTRAQ_51_2.raw]
Data File:Submitted from 20120508-1(merge) by Mascot Daemon on JOYCE-VAIO
Monoisotopic mass of neutral peptide Mr(calc): 1935.007
Variable modifications:
K6 :iTRAQ4plex (K)
Ions Score: 48.3 Expect: 0.014

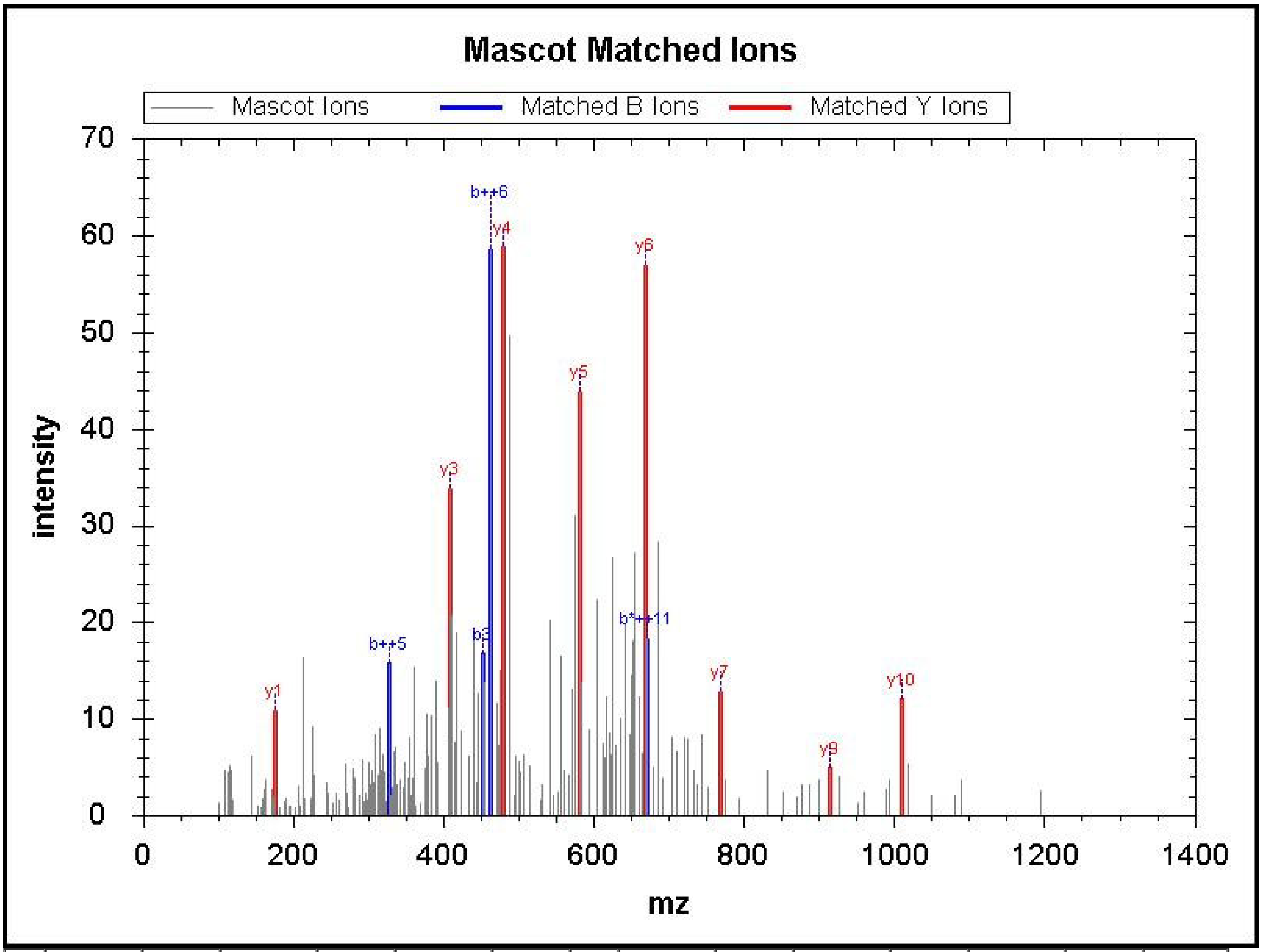

No	b	b++	b*	b*++	b0	b0++	Seq	y	y++	y*	y*++	y0	y0++	RevNo
1	244.18	122.59					V							16
2	315.21	158.11					A	1,692.84	846.92	1,675.82	838.41	1,674.83	837.92	15
3	452.27	226.64					H	1,621.81	811.41	1,604.78	802.89	1,603.79	802.40	14
4	539.31	270.16			521.30	261.15	S	1,484.75	742.88	1,467.72	734.36	1,466.74	733.87	13
5	654.33	327.67			636.32	318.66	D	1,397.71	699.36	1,380.69	690.85	1,379.70	690.36	12
6	926.53	463.77	909.50	455.26	908.52	454.76	K	1,282.69	641.85	1,265.66	633.33	1,264.68	632.84	11
7	1,023.58	512.29	1,006.56	503.78	1,005.57	503.29	P	1,010.49	505.75	993.46	497.24	992.48	496.74	10
8	1,080.60	540.81	1,063.58	532.29	1,062.59	531.80	G	913.44	457.22	896.41	448.71	895.43	448.22	9
9	1,167.64	584.32	1,150.61	575.81	1,149.63	575.32	S	856.42	428.71	839.39	420.20	838.41	419.71	8
10	1,268.68	634.85	1,251.66	626.33	1,250.67	625.84	T	769.38	385.20	752.36	376.68	751.37	376.19	7
11	1,355.72	678.36	1,338.69	669.85	1,337.71	669.36	S	668.34	334.67	651.31	326.16	650.33	325.67	6
12	1,456.76	728.89	1,439.74	720.37	1,438.75	719.88	T	581.30	291.16	564.28	282.64	563.29	282.15	5
13	1,527.80	764.40	1,510.77	755.89	1,509.79	755.40	A	480.26	240.63	463.23	232.12	462.25	231.63	4
14	1,614.83	807.92	1,597.81	799.41	1,596.82	798.91	S	409.22	205.11	392.19	196.60	391.21	196.11	3
15	1,761.90	881.45	1,744.87	872.94	1,743.89	872.45	F	322.19	161.60	305.16	153.08			2
16							R	175.12	88.06	158.09	79.55			1

Query 84824 Hit 1

MS/MS Fragmentation of YVSEVVIGAPYAVTAELLSHFK
Found in sp|Q99447|PCY2_HUMAN, Ethanolamine-phosphate cytidylyltransferase OS=Homo sapiens GN=PCYT2 PE=1 SV=1
Match to Query 84824: 2680.469from(894.4969,3+)
Title: 1216: Sum of 2 scans in range 2852 (rt=72.9157, f=2, i=482) to 2853 (rt=72.9411, f=2, i=483) [D:\lab212\membrane\Grace\Joyce\iTRAQ_32_2.raw]
Data File:Submitted from 20120508-1(merge) by Mascot Daemon on JOYCE-VAIO
Monoisotopic mass of neutral peptide Mr(calc): 2680.469

Variable modifications:
K22 :iTRAQ4plex (K)
Ions Score: 48.28 Expect: 0.008

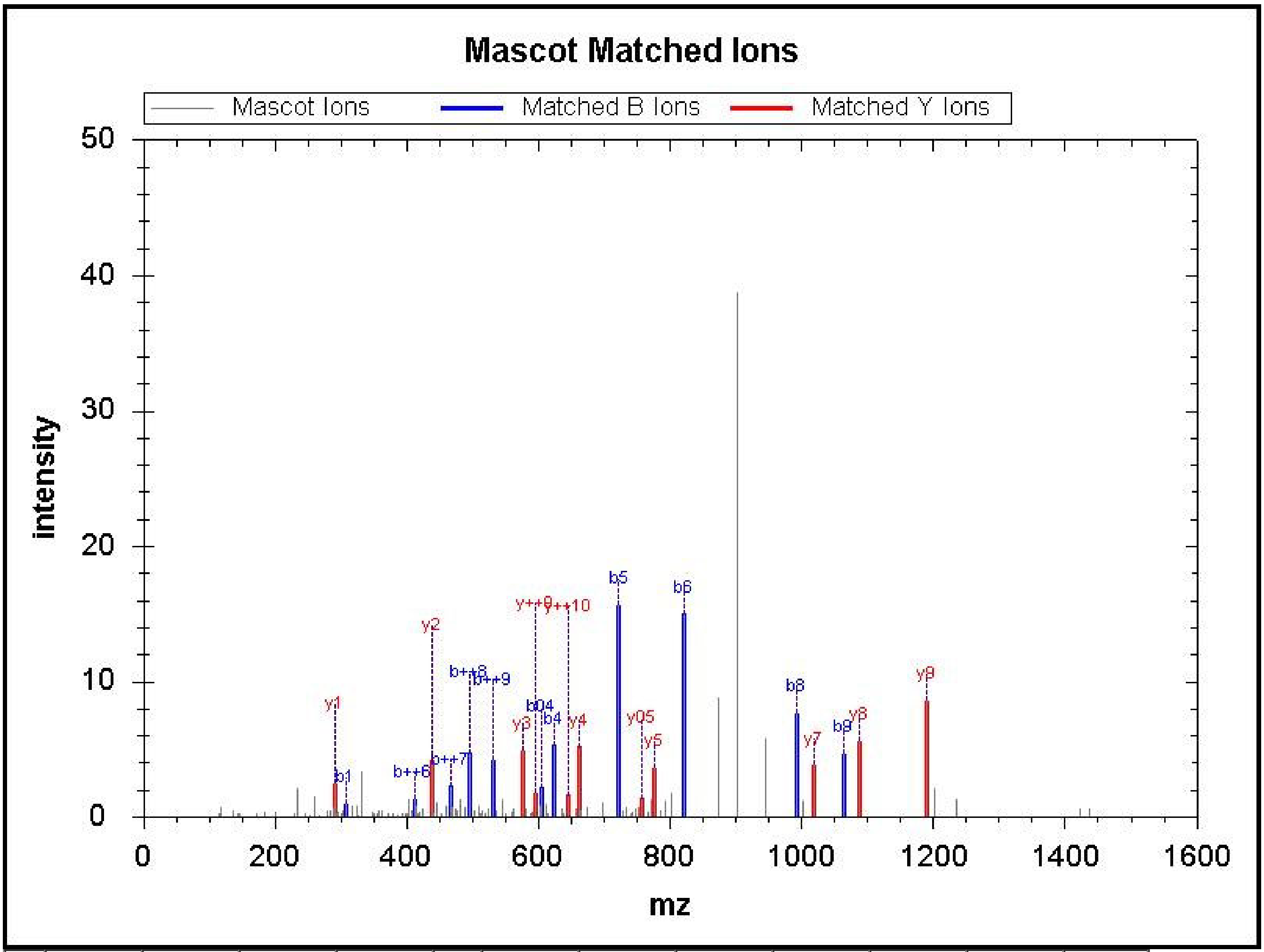

No	b	b++	b0	b0++	Seq	y	y++	y*	y*++	y0	y0++	RevNo
1	308.17	154.59			Y							22
2	407.24	204.12			V	2,374.31	1,187.66	2,357.29	1,179.15	2,356.30	1,178.66	21
3	494.27	247.64	476.26	238.63	S	2,275.25	1,138.13	2,258.22	1,129.61	2,257.23	1,129.12	20
4	623.32	312.16	605.31	303.16	E	2,188.21	1,094.61	2,171.19	1,086.10	2,170.20	1,085.61	19
5	722.38	361.70	704.37	352.69	V	2,059.17	1,030.09	2,042.14	1,021.58	2,041.16	1,021.08	18
6	821.45	411.23	803.44	402.22	V	1,960.10	980.55	1,943.08	972.04	1,942.09	971.55	17
7	934.54	467.77	916.53	458.77	I	1,861.03	931.02	1,844.01	922.51	1,843.02	922.02	16
8	991.56	496.28	973.55	487.28	G	1,747.95	874.48	1,730.92	865.97	1,729.94	865.47	15
9	1,062.60	531.80	1,044.58	522.80	A	1,690.93	845.97	1,673.90	837.45	1,672.92	836.96	14
10	1,159.65	580.33	1,141.64	571.32	P	1,619.89	810.45	1,602.86	801.94	1,601.88	801.44	13
11	1,322.71	661.86	1,304.70	652.85	Y	1,522.84	761.92	1,505.81	753.41	1,504.83	752.92	12
12	1,393.75	697.38	1,375.74	688.37	A	1,359.78	680.39	1,342.75	671.88	1,341.76	671.39	11
13	1,492.82	746.91	1,474.81	737.91	V	1,288.74	644.87	1,271.71	636.36	1,270.73	635.87	10
14	1,593.86	797.44	1,575.85	788.43	T	1,189.67	595.34	1,172.64	586.83	1,171.66	586.33	9
15	1,664.90	832.95	1,646.89	823.95	A	1,088.62	544.81	1,071.60	536.30	1,070.61	535.81	8
16	1,793.94	897.48	1,775.93	888.47	E	1,017.58	509.30	1,000.56	500.78	999.57	500.29	7
17	1,907.03	954.02	1,889.02	945.01	L	888.54	444.77	871.52	436.26	870.53	435.77	6
18	2,020.11	1,010.56	2,002.10	1,001.55	L	775.46	388.23	758.43	379.72	757.45	379.23	5
19	2,107.14	1,054.08	2,089.13	1,045.07	S	662.37	331.69	645.35	323.18	644.36	322.69	4
20	2,244.20	1,122.61	2,226.19	1,113.60	H	575.34	288.17	558.32	279.66			3
21	2,391.27	1,196.14	2,373.26	1,187.13	F	438.28	219.65	421.26	211.13			2

22					K	291.21	146.11	274.19	137.60			1

Query 50574 Hit 1

MS/MS Fragmentation of DLLALFNSFKPK
Found in sp|Q9H501|ESF1_HUMAN, ESF1 homolog OS=Homo sapiens GN=ESF1 PE=1 SV=1
Match to Query 50574: 1824.093from(609.0384,3+)
Title: 1057: Scan 2287 (rt=61.2607, f=2, i=371) [D:\lab212\membrane\Grace\Joyce\iTRAQ_43_1.raw]
Data File:Submitted from 20120508-1(merge) by Mascot Daemon on JOYCE-VAIO
Monoisotopic mass of neutral peptide Mr(calc): 1824.093
Variable modifications:
K10 :iTRAQ4plex (K)
K12 :iTRAQ4plex (K)
Ions Score: 48.26 Expect: 0.005

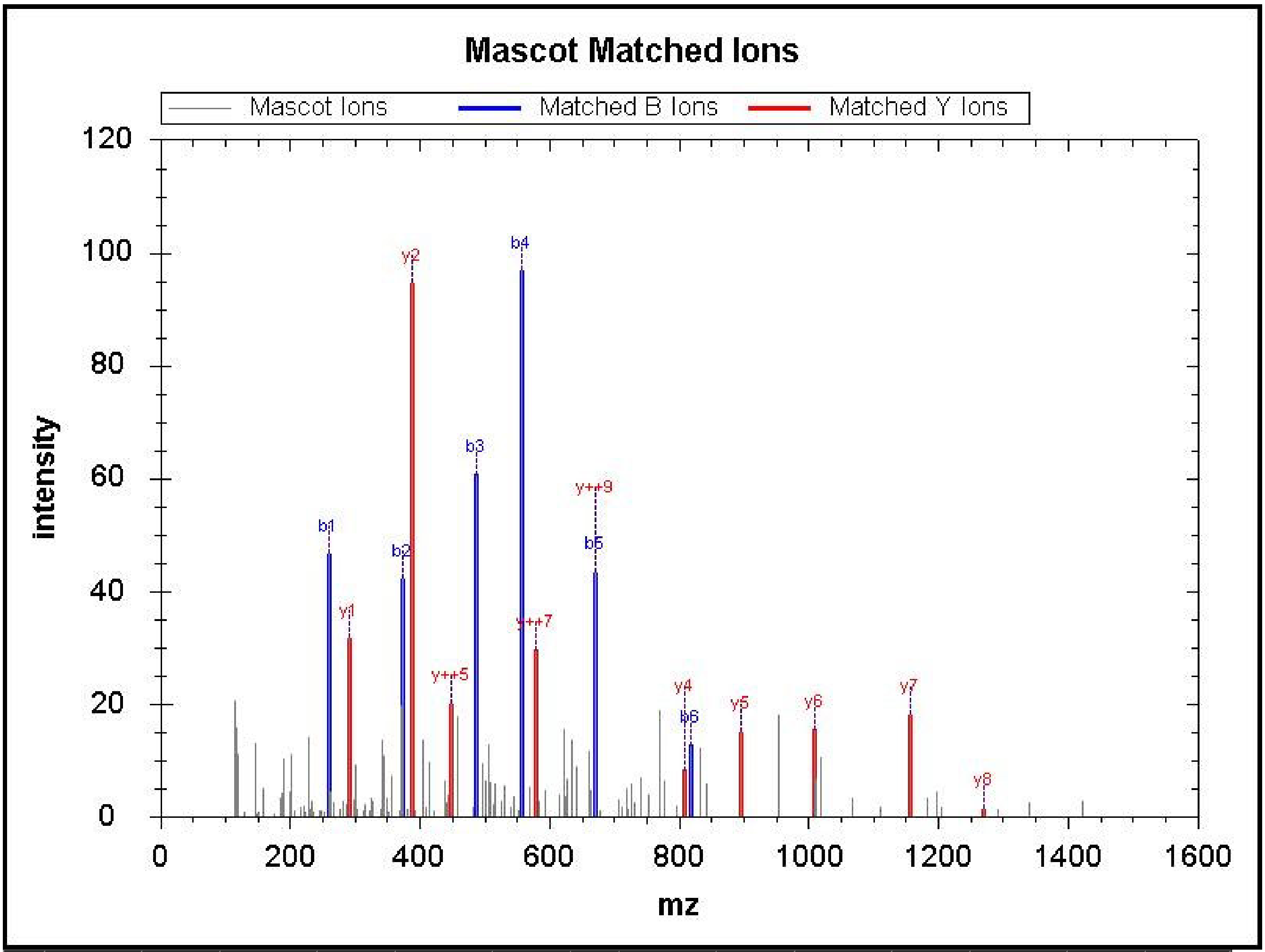

No	b	b++	b*	b*++	b0	b0++	Seq	y	y++	y*	y*++	y0	y0++	RevNo
1	260.14	130.57			242.13	121.57	D							12
2	373.22	187.11			355.21	178.11	L	1,565.97	783.49	1,548.94	774.97	1,547.96	774.48	11
3	486.30	243.66			468.29	234.65	L	1,452.88	726.94	1,435.86	718.43	1,434.87	717.94	10
4	557.34	279.17			539.33	270.17	A	1,339.80	670.40	1,322.77	661.89	1,321.79	661.40	9
5	670.43	335.72			652.41	326.71	L	1,268.76	634.88	1,251.73	626.37	1,250.75	625.88	8
6	817.49	409.25			799.48	400.25	F	1,155.68	578.34	1,138.65	569.83	1,137.67	569.34	7
7	931.54	466.27	914.51	457.76	913.53	457.27	N	1,008.61	504.81	991.58	496.29	990.60	495.80	6
8	1,018.57	509.79	1,001.54	501.27	1,000.56	500.78	S	894.57	447.79	877.54	439.27	876.55	438.78	5
9	1,165.64	583.32	1,148.61	574.81	1,147.63	574.32	F	807.53	404.27	790.51	395.76			4
10	1,437.83	719.42	1,420.81	710.91	1,419.82	710.42	K	660.46	330.74	643.44	322.22			3
11	1,534.89	767.95	1,517.86	759.43	1,516.88	758.94	P	388.27	194.64	371.24	186.12			2
12							K	291.21	146.11	274.19	137.60			1

Query 64308 Hit 1

MS/MS Fragmentation of LLVFATDDGFHFAGDGK
Found in sp|P05107|ITB2_HUMAN, Integrin beta-2 OS=Homo sapiens GN=ITGB2 PE=1 SV=2
Match to Query 64308: 2097.082from(700.0344,3+)
Title: 863: Sum of 3 scans in range 1906 (rt=52.3906, f=3, i=297) to 1908 (rt=52.4414, f=4, i=574)
[D:\lab212\membrane\Grace\Joyce\iTRAQ_37_2.raw]
Data File:Submitted from 20120508-1(merge) by Mascot Daemon on JOYCE-VAIO
Monoisotopic mass of neutral peptide Mr(calc): 2097.082
Variable modifications:
K17 :iTRAQ4plex (K)
Ions Score: 48.25 Expect: 0.014

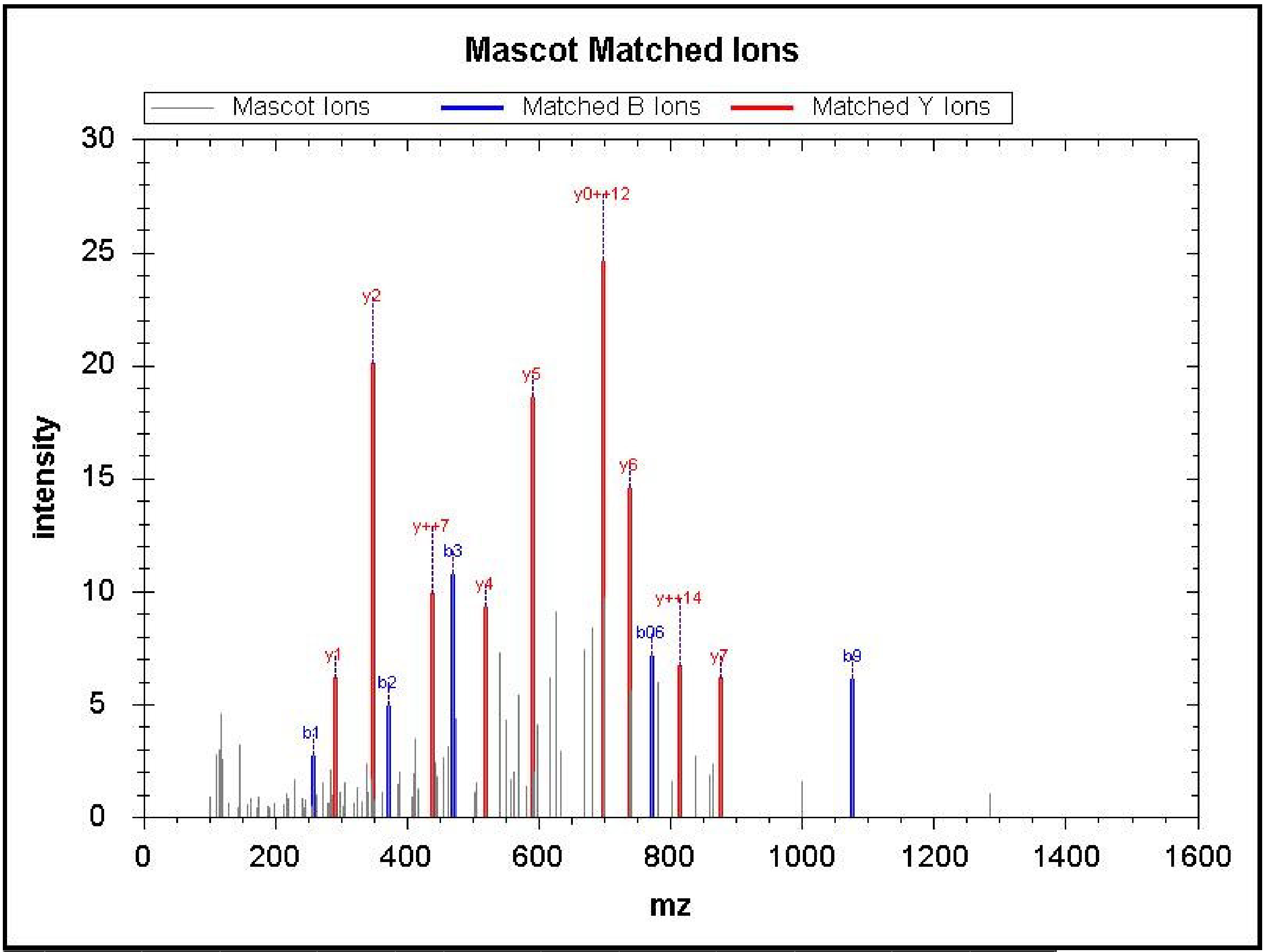

No	b	b++	b0	b0++	Seq	y	y++	y*	y*++	y0	y0++	RevNo
1	258.19	129.60			L							17
2	371.28	186.14			L	1,840.90	920.95	1,823.87	912.44	1,822.89	911.95	16
3	470.35	235.68			V	1,727.81	864.41	1,710.79	855.90	1,709.80	855.41	15
4	617.41	309.21			F	1,628.75	814.88	1,611.72	806.36	1,610.74	805.87	14
5	688.45	344.73			A	1,481.68	741.34	1,464.65	732.83	1,463.67	732.34	13
6	789.50	395.25	771.49	386.25	T	1,410.64	705.82	1,393.61	697.31	1,392.63	696.82	12
7	904.53	452.77	886.52	443.76	D	1,309.59	655.30	1,292.57	646.79	1,291.58	646.29	11
8	1,019.55	510.28	1,001.54	501.27	D	1,194.57	597.79	1,177.54	589.27	1,176.56	588.78	10
9	1,076.57	538.79	1,058.56	529.79	G	1,079.54	540.27	1,062.51	531.76	1,061.53	531.27	9
10	1,223.64	612.33	1,205.63	603.32	F	1,022.52	511.76	1,005.49	503.25	1,004.51	502.76	8
11	1,360.70	680.85	1,342.69	671.85	H	875.45	438.23	858.42	429.71	857.44	429.22	7
12	1,507.77	754.39	1,489.76	745.38	F	738.39	369.70	721.36	361.19	720.38	360.69	6
13	1,578.81	789.91	1,560.80	780.90	A	591.32	296.16	574.30	287.65	573.31	287.16	5
14	1,635.83	818.42	1,617.82	809.41	G	520.28	260.65	503.26	252.13	502.27	251.64	4

15	1,750.86	875.93	1,732.85	866.93	D	463.26	232.14	446.24	223.62	445.25	223.13	3
16	1,807.88	904.44	1,789.87	895.44	G	348.24	174.62	331.21	166.11			2
17					K	291.21	146.11	274.19	137.60			1

Query 7405 Hit 1

MS/MS Fragmentation of EGNPYK
Found in sp|Q9NZJ4|SACS_HUMAN, Sacsin OS=Homo sapiens GN=SACS PE=1 SV=2
Match to Query 7405: 995.5349from(498.7747,2+)
Title: 267: Sum of 2 scans in range 848 (rt=27.5637, f=4, i=171) to 849 (rt=27.5892, f=4, i=172)
[D:\lab212\membrane\Grace\Joyce\iTRAQ_34_1.raw]
Data File:Submitted from 20120508-1(merge) by Mascot Daemon on JOYCE-VAIO
Monoisotopic mass of neutral peptide Mr(calc): 995.5349
Variable modifications:
N3 :Deamidated (NQ)
K6 :iTRAQ4plex (K)
Ions Score: 48.19 Expect: 0.009

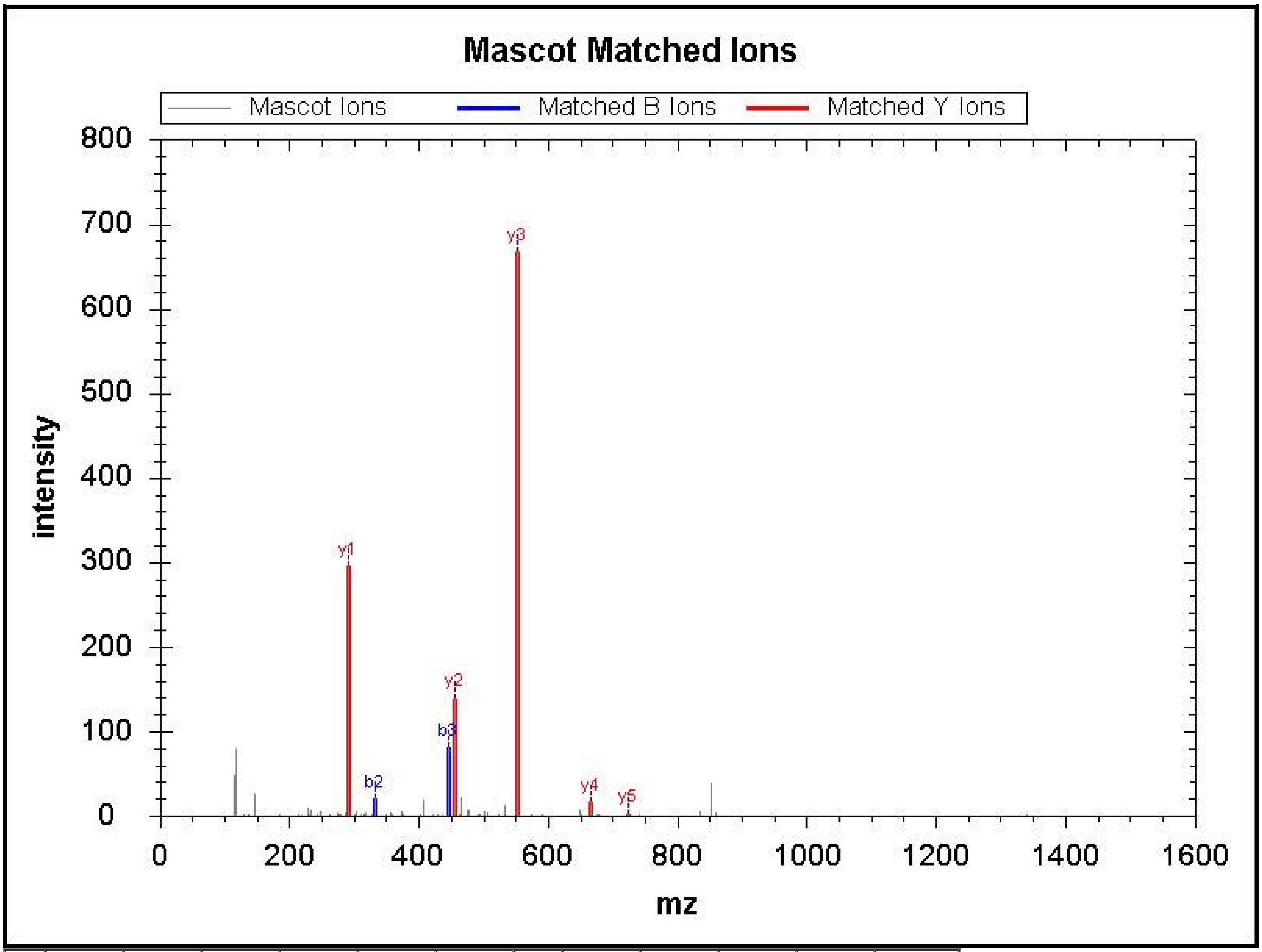

No	b	b++	b*	b*++	b0	b0++	Seq	y	y++	y*	y*++	RevNo
1	274.15	137.58			256.14	128.57	E					6
2	331.17	166.09			313.16	157.09	G	723.38	362.19	706.35	353.68	5
3	446.20	223.60	429.17	215.09	428.19	214.60	N	666.36	333.68	649.33	325.17	4
4	543.25	272.13	526.23	263.62	525.24	263.12	P	551.33	276.17	534.30	267.66	3
5	706.32	353.66	689.29	345.15	688.31	344.66	Y	454.28	227.64	437.25	219.13	2
6							K	291.21	146.11	274.19	137.60	1

Query 93590 Hit 1

MS/MS Fragmentation of KQDTTSTIISIASNVAGHPLVWDFVR
Found in sp|P97449|AMPN_MOUSE, Aminopeptidase N OS=Mus musculus GN=Anpep PE=1 SV=4

Match to Query 93590: 3142.697from(786.6815,4+)
Title: 866: Scan 2495 (rt=63.6754, f=2, i=436) [D:\lab212\membrane\Grace\Joyce\iTRAQ_46_1.raw]
Data File:Submitted from 20120508-1(merge) by Mascot Daemon on JOYCE-VAIO
Monoisotopic mass of neutral peptide Mr(calc): 3142.697
Variable modifications:
K1 :iTRAQ4plex (K)
Ions Score: 48.16 Expect: 0.008

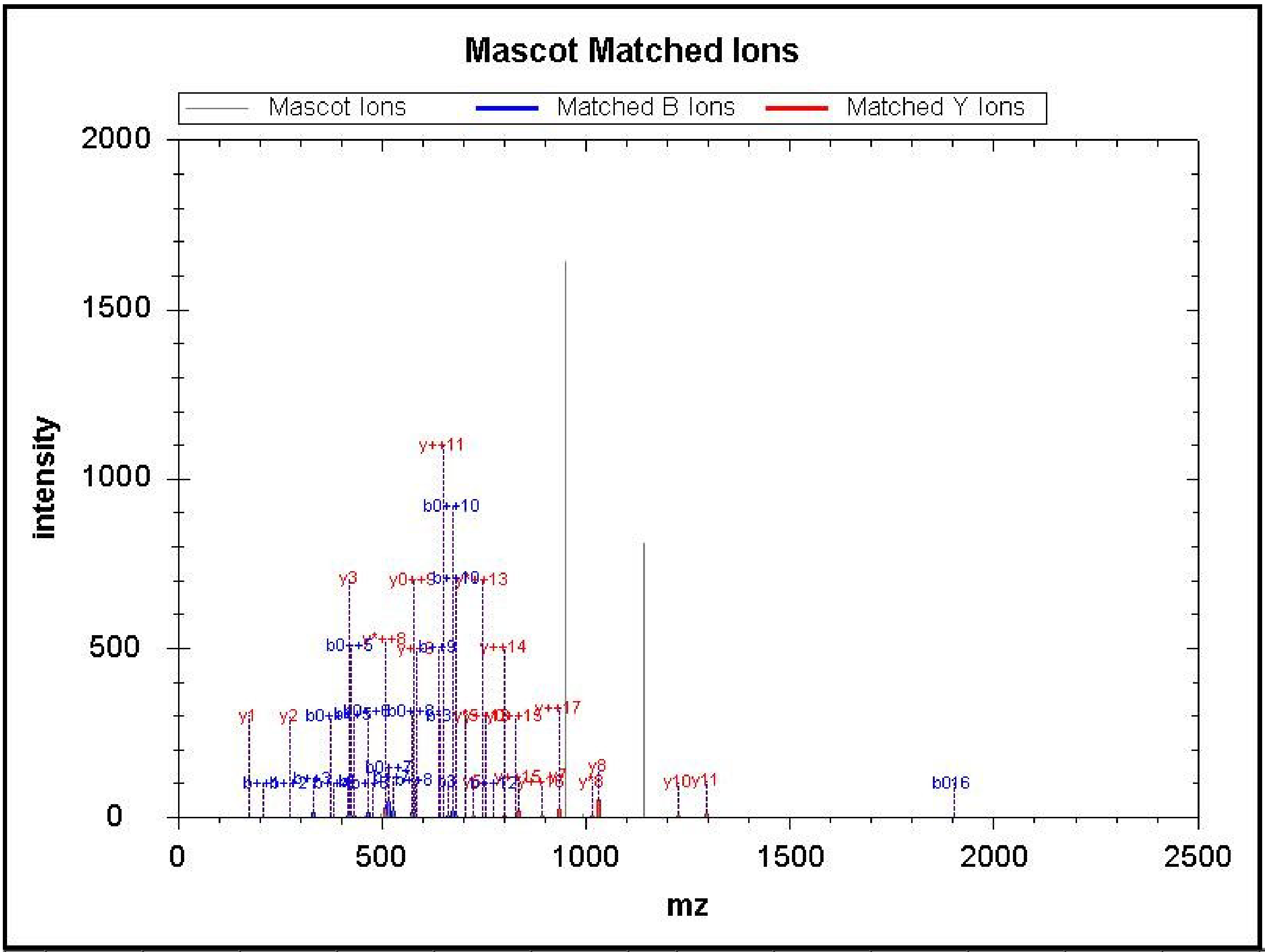

No	b	b++	b*	b*++	b0	b0++	Seq	y	y++	y*	y*++	y0	y0++	RevNo
1	417.31	209.16	400.28	200.64			K							26
2	545.36	273.19	528.34	264.67			Q	2,727.41	1,364.21	2,710.38	1,355.70	2,709.40	1,355.20	25
3	660.39	330.70	643.37	322.19	642.38	321.69	D	2,599.35	1,300.18	2,582.32	1,291.67	2,581.34	1,291.17	24
4	761.44	381.22	744.41	372.71	743.43	372.22	T	2,484.32	1,242.67	2,467.30	1,234.15	2,466.31	1,233.66	23
5	862.49	431.75	845.46	423.23	844.48	422.74	T	2,383.28	1,192.14	2,366.25	1,183.63	2,365.27	1,183.14	22
6	949.52	475.26	932.49	466.75	931.51	466.26	S	2,282.23	1,141.62	2,265.20	1,133.10	2,264.22	1,132.61	21
7	1,050.57	525.79	1,033.54	517.27	1,032.56	516.78	T	2,195.20	1,098.10	2,178.17	1,089.59	2,177.19	1,089.10	20
8	1,163.65	582.33	1,146.62	573.82	1,145.64	573.32	I	2,094.15	1,047.58	2,077.12	1,039.07	2,076.14	1,038.57	19
9	1,276.74	638.87	1,259.71	630.36	1,258.72	629.87	I	1,981.07	991.04	1,964.04	982.52	1,963.05	982.03	18
10	1,363.77	682.39	1,346.74	673.87	1,345.76	673.38	S	1,867.98	934.49	1,850.95	925.98	1,849.97	925.49	17
11	1,476.85	738.93	1,459.82	730.42	1,458.84	729.92	I	1,780.95	890.98	1,763.92	882.47	1,762.94	881.97	16
12	1,547.89	774.45	1,530.86	765.93	1,529.88	765.44	A	1,667.87	834.44	1,650.84	825.92	1,649.85	825.43	15
13	1,634.92	817.96	1,617.89	809.45	1,616.91	808.96	S	1,596.83	798.92	1,579.80	790.40	1,578.82	789.91	14
14	1,748.96	874.99	1,731.94	866.47	1,730.95	865.98	N	1,509.80	755.40	1,492.77	746.89	1,491.79	746.40	13
15	1,848.03	924.52	1,831.01	916.01	1,830.02	915.51	V	1,395.75	698.38	1,378.73	689.87	1,377.74	689.37	12
16	1,919.07	960.04	1,902.04	951.52	1,901.06	951.03	A	1,296.68	648.85	1,279.66	640.33	1,278.67	639.84	11
17	1,976.09	988.55	1,959.06	980.04	1,958.08	979.54	G	1,225.65	613.33	1,208.62	604.81	1,207.64	604.32	10
18	2,113.15	1,057.08	2,096.12	1,048.56	2,095.14	1,048.07	H	1,168.63	584.82	1,151.60	576.30	1,150.62	575.81	9

19	2,210.20	1,105.60	2,193.18	1,097.09	2,192.19	1,096.60	P	1,031.57	516.29	1,014.54	507.77	1,013.56	507.28	8
20	2,323.29	1,162.15	2,306.26	1,153.63	2,305.28	1,153.14	L	934.51	467.76	917.49	459.25	916.50	458.76	7
21	2,422.35	1,211.68	2,405.33	1,203.17	2,404.34	1,202.68	V	821.43	411.22	804.40	402.71	803.42	402.21	6
22	2,608.43	1,304.72	2,591.41	1,296.21	2,590.42	1,295.72	W	722.36	361.68	705.34	353.17	704.35	352.68	5
23	2,723.46	1,362.23	2,706.43	1,353.72	2,705.45	1,353.23	D	536.28	268.64	519.26	260.13	518.27	259.64	4
24	2,870.53	1,435.77	2,853.50	1,427.25	2,852.52	1,426.76	F	421.26	211.13	404.23	202.62			3
25	2,969.60	1,485.30	2,952.57	1,476.79	2,951.59	1,476.30	V	274.19	137.60	257.16	129.08			2
26							R	175.12	88.06	158.09	79.55			1

Query 6620 Hit 1

MS/MS Fragmentation of LELDALR
Found in sp|Q9ULX9|MAFF_HUMAN, Transcription factor MafF OS=Homo sapiens GN=MAFF PE=1 SV=2
Match to Query 6620: 972.6013 from(487.3079,2+)
Title: 290: Sum of 2 scans in range 1384 (rt=37.0885, f=4, i=171) to 1385 (rt=37.1138, f=4, i=172)
[D:\lab212\membrane\Grace\Joyce\iTRAQ_26_1.raw]
Data File:Submitted from 20120508-1(merge) by Mascot Daemon on JOYCE-VAIO
Monoisotopic mass of neutral peptide Mr(calc): 972.6013
Variable modifications:
Ions Score: 47.99 Expect: 0.009

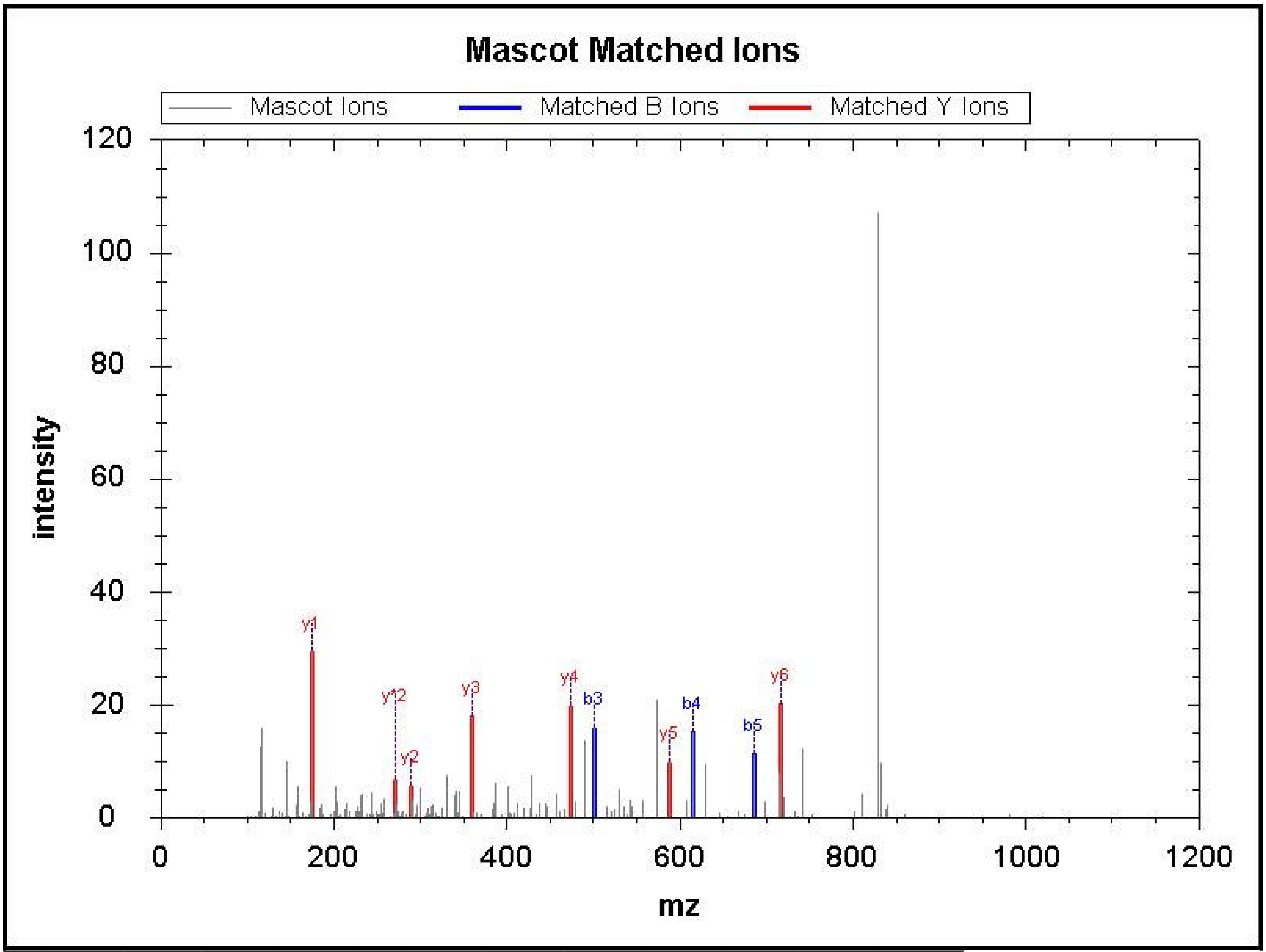

No	b	b++	b0	b0++	Seq	y	y++	y*	y*++	y0	y0++	RevNo
1	258.19	129.60			L							7
2	387.24	194.12	369.23	185.12	E	716.39	358.70	699.37	350.19	698.38	349.70	6
3	500.32	250.66	482.31	241.66	L	587.35	294.18	570.32	285.67	569.34	285.17	5
4	615.35	308.18	597.34	299.17	D	474.27	237.64	457.24	229.12	456.26	228.63	4
5	686.38	343.70	668.37	334.69	A	359.24	180.12	342.21	171.61			3
6	799.47	400.24	781.46	391.23	L	288.20	144.61	271.18	136.09			2

7					R	175.12	88.06	158.09	79.55			1

Query 55792 Hit 1

MS/MS Fragmentation of STLVHSLFLTDLYK
Found in sp|Q99719|SEPT5_HUMAN, Septin-5 OS=Homo sapiens GN=SEPT5 PE=1 SV=1
Match to Query 55792: 1924.092from(642.3713,3+)
Title: 850: Scan 1894 (rt=52.1693, f=2, i=311) [D:\lab212\membrane\Grace\Joyce\iTRAQ_39_1.raw]
Data File:Submitted from 20120508-1(merge) by Mascot Daemon on JOYCE-VAIO
Monoisotopic mass of neutral peptide Mr(calc): 1924.092
Variable modifications:
K14 :iTRAQ4plex (K)
Ions Score: 47.97 Expect: 0.009

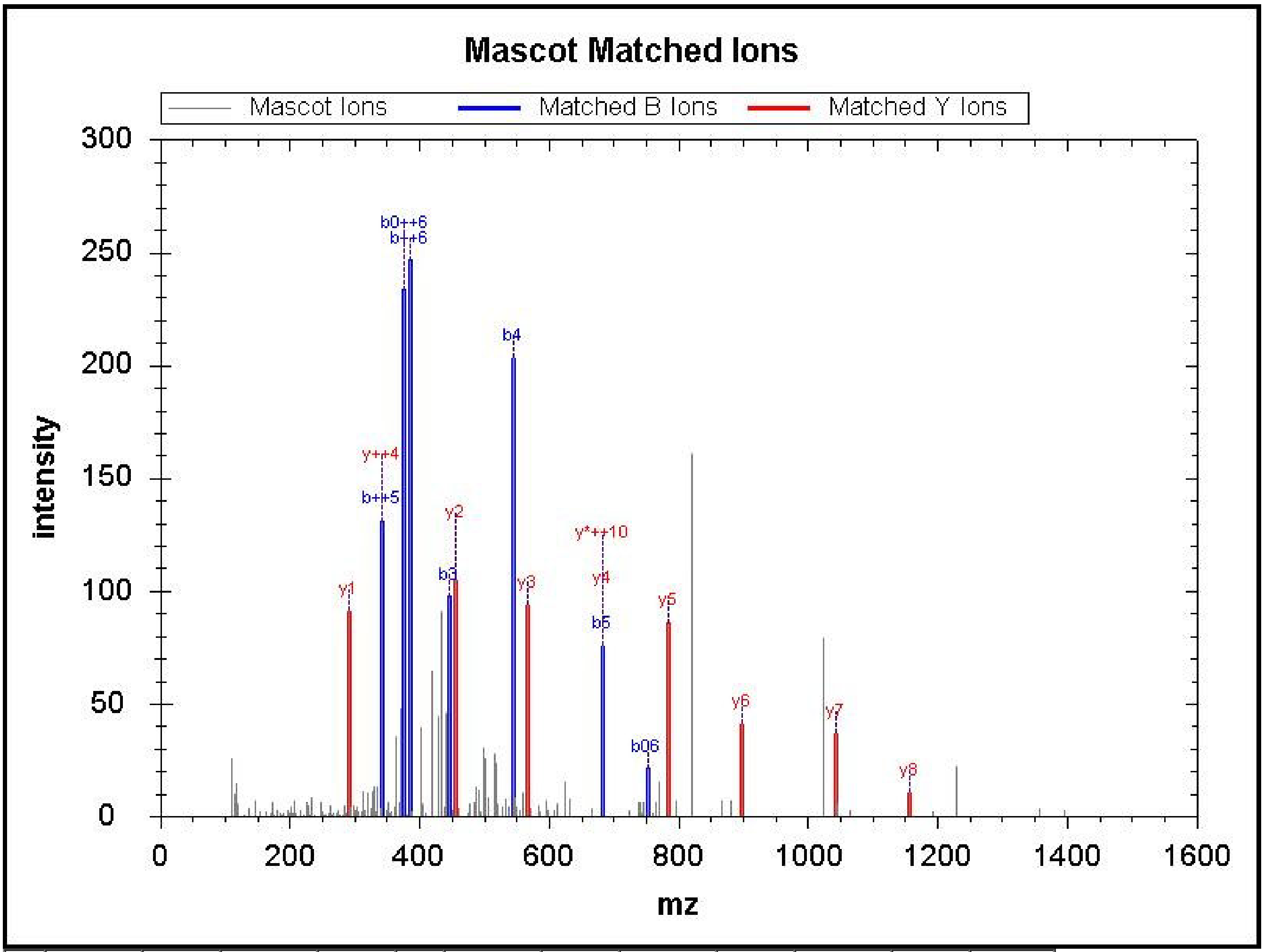

No	b	b++	b0	b0++	Seq	y	y++	y*	y*++	y0	y0++	RevNo
1	232.14	116.57	214.13	107.57	S							14
2	333.19	167.10	315.18	158.09	T	1,693.96	847.49	1,676.94	838.97	1,675.95	838.48	13
3	446.27	223.64	428.26	214.63	L	1,592.92	796.96	1,575.89	788.45	1,574.91	787.96	12
4	545.34	273.17	527.33	264.17	V	1,479.83	740.42	1,462.81	731.91	1,461.82	731.41	11
5	682.40	341.70	664.39	332.70	H	1,380.76	690.89	1,363.74	682.37	1,362.75	681.88	10
6	769.43	385.22	751.42	376.21	S	1,243.71	622.36	1,226.68	613.84	1,225.69	613.35	9
7	882.52	441.76	864.51	432.76	L	1,156.67	578.84	1,139.65	570.33	1,138.66	569.84	8
8	1,029.58	515.30	1,011.57	506.29	F	1,043.59	522.30	1,026.56	513.79	1,025.58	513.29	7
9	1,142.67	571.84	1,124.66	562.83	L	896.52	448.76	879.49	440.25	878.51	439.76	6
10	1,243.72	622.36	1,225.71	613.36	T	783.44	392.22	766.41	383.71	765.43	383.22	5
11	1,358.74	679.88	1,340.73	670.87	D	682.39	341.70	665.36	333.18	664.38	332.69	4
12	1,471.83	736.42	1,453.82	727.41	L	567.36	284.18	550.34	275.67			3

13	1,634.89	817.95	1,616.88	808.94	Y	454.28	227.64	437.25	219.13			2
14					K	291.21	146.11	274.19	137.60			1

Query 76132 Hit 1

MS/MS Fragmentation of FTDDTFDPELAATIGVDFK
Found in sp|Q9NP72|RAB18_HUMAN, Ras-related protein Rab-18 OS=Homo sapiens GN=RAB18 PE=1 SV=1
Match to Query 76132: 2389.193from(797.4051,3+)
Title: 959: Sum of 2 scans in range 2240 (rt=59.2722, f=4, i=625) to 2241 (rt=59.2976, f=4, i=626)
[D:\lab212\membrane\Grace\Joyce\iTRAQ_24_1.raw]
Data File:Submitted from 20120508-1(merge) by Mascot Daemon on JOYCE-VAIO
Monoisotopic mass of neutral peptide Mr(calc): 2389.193
Variable modifications:
K19 :iTRAQ4plex (K)
Ions Score: 47.95 Expect: 0.016

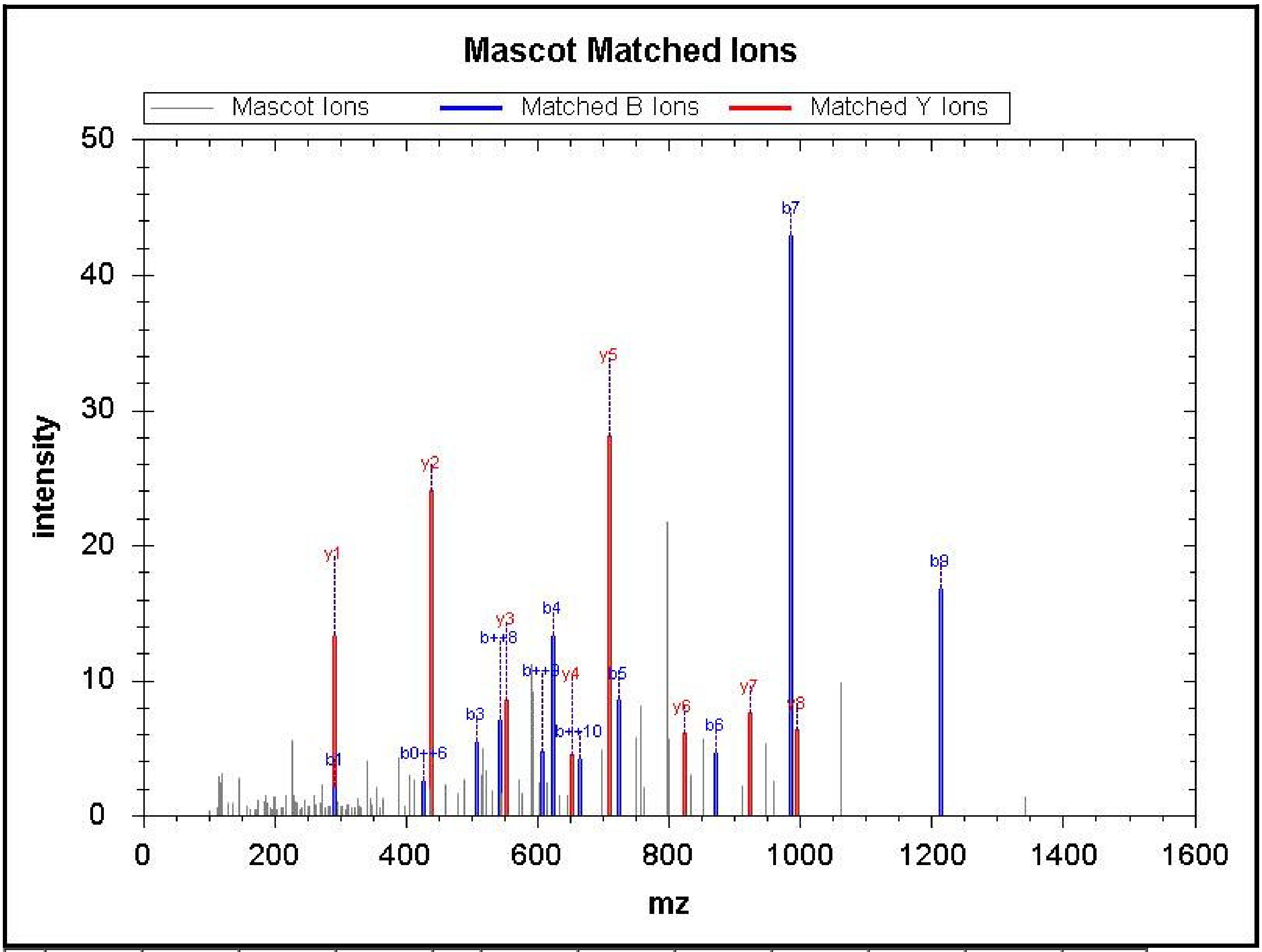

No	b	b++	b0	b0++	Seq	y	y++	y*	y*++	y0	y0++	RevNo
1	292.18	146.59			F							19
2	393.23	197.12	375.21	188.11	T	2,099.03	1,050.02	2,082.00	1,041.51	2,081.02	1,041.01	18
3	508.25	254.63	490.24	245.62	D	1,997.98	999.49	1,980.96	990.98	1,979.97	990.49	17
4	623.28	312.14	605.27	303.14	D	1,882.96	941.98	1,865.93	933.47	1,864.94	932.98	16
5	724.33	362.67	706.32	353.66	T	1,767.93	884.47	1,750.90	875.95	1,749.92	875.46	15
6	871.40	436.20	853.38	427.20	F	1,666.88	833.94	1,649.85	825.43	1,648.87	824.94	14
7	986.42	493.71	968.41	484.71	D	1,519.81	760.41	1,502.79	751.90	1,501.80	751.40	13
8	1,083.48	542.24	1,065.46	533.24	P	1,404.79	702.90	1,387.76	694.38	1,386.77	693.89	12
9	1,212.52	606.76	1,194.51	597.76	E	1,307.73	654.37	1,290.71	645.86	1,289.72	645.36	11
10	1,325.60	663.30	1,307.59	654.30	L	1,178.69	589.85	1,161.66	581.34	1,160.68	580.84	10
11	1,396.64	698.82	1,378.63	689.82	A	1,065.61	533.31	1,048.58	524.79	1,047.60	524.30	9

12	1,467.68	734.34	1,449.67	725.34	A	994.57	497.79	977.54	489.27	976.56	488.78	8
13	1,568.72	784.87	1,550.71	775.86	T	923.53	462.27	906.51	453.76	905.52	453.26	7
14	1,681.81	841.41	1,663.80	832.40	I	822.48	411.75	805.46	403.23	804.47	402.74	6
15	1,738.83	869.92	1,720.82	860.91	G	709.40	355.20	692.37	346.69	691.39	346.20	5
16	1,837.90	919.45	1,819.89	910.45	V	652.38	326.69	635.35	318.18	634.37	317.69	4
17	1,952.92	976.97	1,934.91	967.96	D	553.31	277.16	536.28	268.65	535.30	268.15	3
18	2,099.99	1,050.50	2,081.98	1,041.49	F	438.28	219.65	421.26	211.13			2
19					K	291.21	146.11	274.19	137.60			1

Query 98108 Hit 1

MS/MS Fragmentation of LNLQSVTEQSSLDDFLATAELAGTEFVAEK
Found in sp|Q9H089|LSG1_HUMAN, Large subunit GTPase 1 homolog OS=Homo sapiens GN=LSG1 PE=1 SV=2
Match to Query 98108: 3513.79from(879.4548,4+)
Title: 1283: Sum of 2 scans in range 2999 (rt=75.9602, f=4, i=849) to 3000 (rt=75.9856, f=4, i=850)
[D:\lab212\membrane\Grace\Joyce\iTRAQ_23_2.raw]
Data File:Submitted from 20120508-1(merge) by Mascot Daemon on JOYCE-VAIO
Monoisotopic mass of neutral peptide Mr(calc): 3513.79
Variable modifications:
K30 :iTRAQ4plex (K)
Ions Score: 47.94 Expect: 0.013

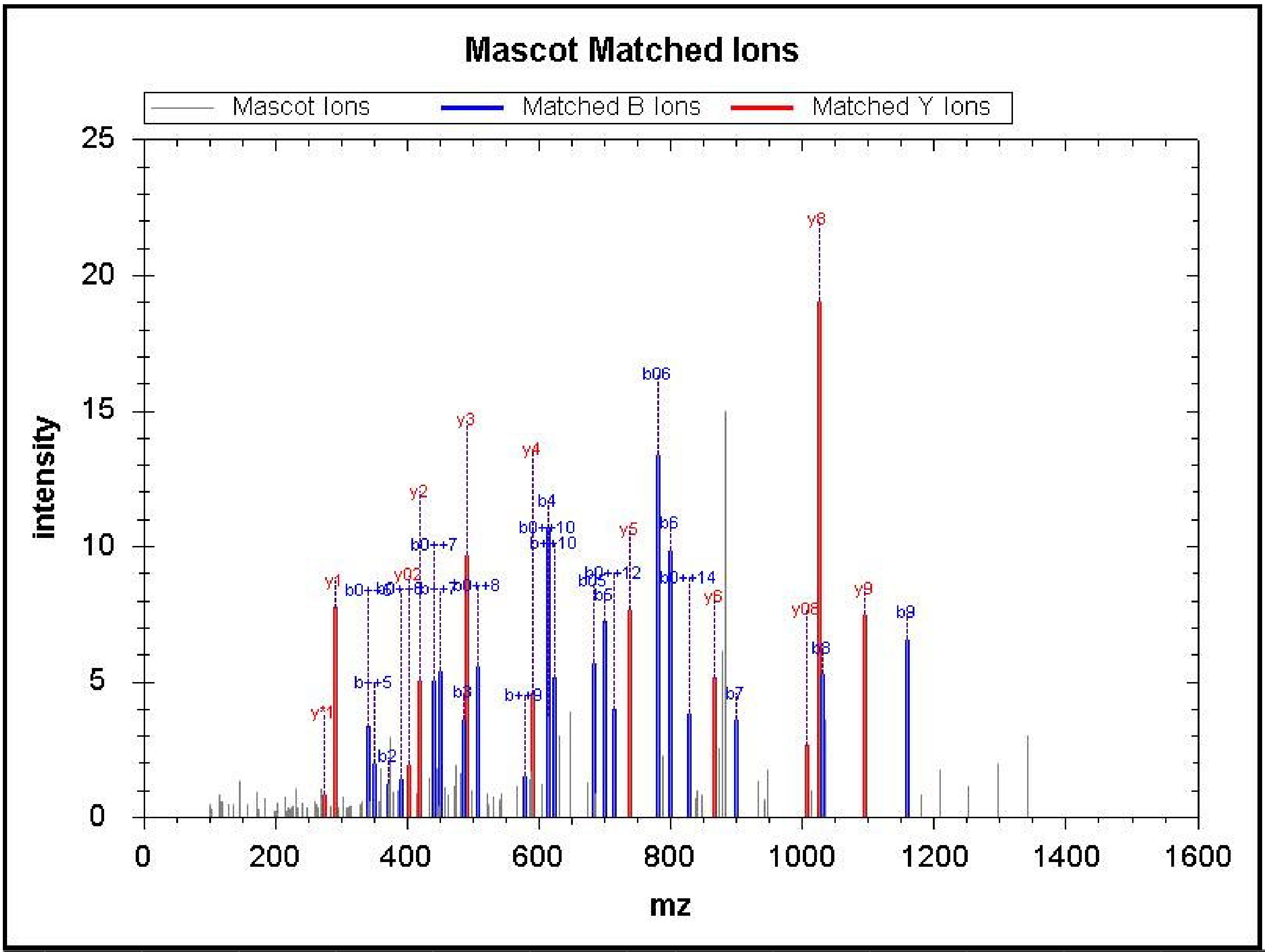

No	b	b++	b*	b*++	b0	b0++	Seq	y	y++	y*	y*++	y0	y0++	RevNo
1	258.19	129.60					L							30
2	372.24	186.62	355.21	178.11			N	3,257.62	1,629.31	3,240.59	1,620.80	3,239.61	1,620.31	29
3	485.32	243.16	468.29	234.65			L	3,143.57	1,572.29	3,126.55	1,563.78	3,125.56	1,563.29	28
4	613.38	307.19	596.35	298.68			Q	3,030.49	1,515.75	3,013.46	1,507.24	3,012.48	1,506.74	27
5	700.41	350.71	683.38	342.20	682.40	341.70	S	2,902.43	1,451.72	2,885.41	1,443.21	2,884.42	1,442.71	26

6	799.48	400.24	782.45	391.73	781.47	391.24	V	2,815.40	1,408.20	2,798.37	1,399.69	2,797.39	1,399.20	25
7	900.53	450.77	883.50	442.25	882.52	441.76	T	2,716.33	1,358.67	2,699.31	1,350.16	2,698.32	1,349.66	24
8	1,029.57	515.29	1,012.54	506.78	1,011.56	506.28	E	2,615.28	1,308.15	2,598.26	1,299.63	2,597.27	1,299.14	23
9	1,157.63	579.32	1,140.60	570.80	1,139.62	570.31	Q	2,486.24	1,243.62	2,469.22	1,235.11	2,468.23	1,234.62	22
10	1,244.66	622.83	1,227.63	614.32	1,226.65	613.83	S	2,358.18	1,179.60	2,341.16	1,171.08	2,340.17	1,170.59	21
11	1,331.69	666.35	1,314.67	657.84	1,313.68	657.34	S	2,271.15	1,136.08	2,254.12	1,127.57	2,253.14	1,127.07	20
12	1,444.78	722.89	1,427.75	714.38	1,426.77	713.89	L	2,184.12	1,092.56	2,167.09	1,084.05	2,166.11	1,083.56	19
13	1,559.80	780.41	1,542.78	771.89	1,541.79	771.40	D	2,071.04	1,036.02	2,054.01	1,027.51	2,053.02	1,027.02	18
14	1,674.83	837.92	1,657.80	829.41	1,656.82	828.91	D	1,956.01	978.51	1,938.98	969.99	1,938.00	969.50	17
15	1,821.90	911.45	1,804.87	902.94	1,803.89	902.45	F	1,840.98	920.99	1,823.95	912.48	1,822.97	911.99	16
16	1,934.98	968.00	1,917.96	959.48	1,916.97	958.99	L	1,693.91	847.46	1,676.89	838.95	1,675.90	838.45	15
17	2,006.02	1,003.51	1,988.99	995.00	1,988.01	994.51	A	1,580.83	790.92	1,563.80	782.40	1,562.82	781.91	14
18	2,107.07	1,054.04	2,090.04	1,045.52	2,089.06	1,045.03	T	1,509.79	755.40	1,492.77	746.89	1,491.78	746.39	13
19	2,178.10	1,089.56	2,161.08	1,081.04	2,160.09	1,080.55	A	1,408.74	704.88	1,391.72	696.36	1,390.73	695.87	12
20	2,307.15	1,154.08	2,290.12	1,145.56	2,289.14	1,145.07	E	1,337.71	669.36	1,320.68	660.84	1,319.70	660.35	11
21	2,420.23	1,210.62	2,403.20	1,202.11	2,402.22	1,201.61	L	1,208.66	604.84	1,191.64	596.32	1,190.65	595.83	10
22	2,491.27	1,246.14	2,474.24	1,237.62	2,473.26	1,237.13	A	1,095.58	548.29	1,078.55	539.78	1,077.57	539.29	9
23	2,548.29	1,274.65	2,531.26	1,266.14	2,530.28	1,265.64	G	1,024.54	512.78	1,007.52	504.26	1,006.53	503.77	8
24	2,649.34	1,325.17	2,632.31	1,316.66	2,631.33	1,316.17	T	967.52	484.26	950.50	475.75	949.51	475.26	7
25	2,778.38	1,389.69	2,761.35	1,381.18	2,760.37	1,380.69	E	866.47	433.74	849.45	425.23	848.46	424.74	6
26	2,925.45	1,463.23	2,908.42	1,454.71	2,907.44	1,454.22	F	737.43	369.22	720.40	360.71	719.42	360.21	5
27	3,024.52	1,512.76	3,007.49	1,504.25	3,006.51	1,503.76	V	590.36	295.69	573.34	287.17	572.35	286.68	4
28	3,095.55	1,548.28	3,078.53	1,539.77	3,077.54	1,539.28	A	491.29	246.15	474.27	237.64	473.28	237.15	3
29	3,224.60	1,612.80	3,207.57	1,604.29	3,206.59	1,603.80	E	420.26	210.63	403.23	202.12	402.25	201.63	2
30							K	291.21	146.11	274.19	137.60			1

Query 43237 Hit 1

MS/MS Fragmentation of EVTPVSSIPVETHR
Found in sp|Q9HD20|AT131_HUMAN, Probable cation-transporting ATPase 13A1 OS=Homo sapiens GN=ATP13A1 PE=1 SV=2
Match to Query 43237: 1693.913from(565.645,3+)
Title: 284: Sum of 2 scans in range 886 (rt=28.4176, f=4, i=183) to 887 (rt=28.4431, f=4, i=184)
[D:\lab212\membrane\Grace\Joyce\iTRAQ_34_1.raw]
Data File:Submitted from 20120508-1(merge) by Mascot Daemon on JOYCE-VAIO
Monoisotopic mass of neutral peptide Mr(calc): 1693.913
Variable modifications:
Ions Score: 47.91 Expect: 0.014

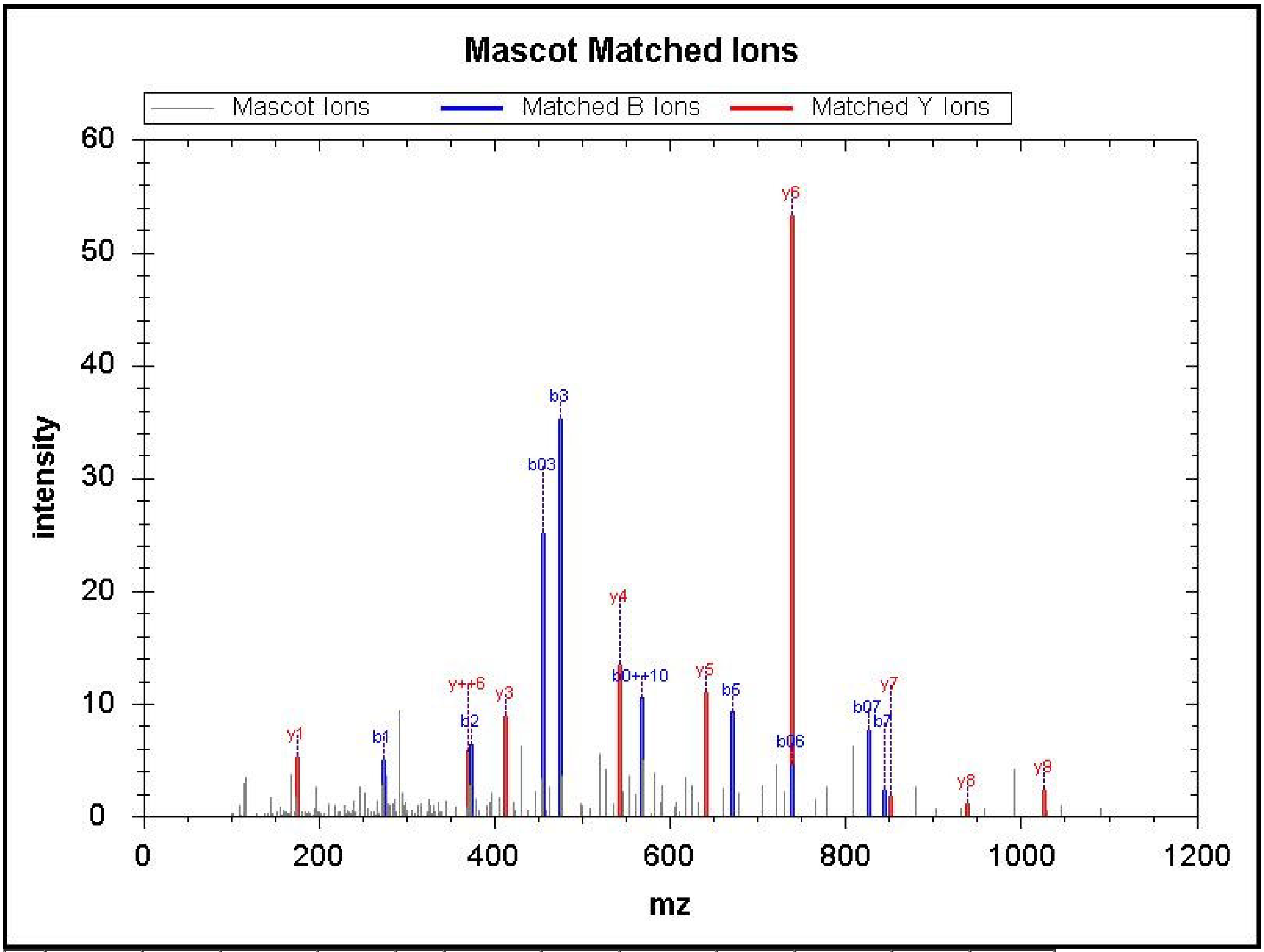

No	b	b++	b0	b0++	Seq	y	y++	y*	y*++	y0	y0++	RevNo
1	274.15	137.58	256.14	128.57	E							14
2	373.22	187.11	355.21	178.11	V	1,421.77	711.39	1,404.75	702.88	1,403.76	702.39	13
3	474.27	237.64	456.26	228.63	T	1,322.71	661.86	1,305.68	653.34	1,304.70	652.85	12
4	571.32	286.16	553.31	277.16	P	1,221.66	611.33	1,204.63	602.82	1,203.65	602.33	11
5	670.39	335.70	652.38	326.69	V	1,124.61	562.81	1,107.58	554.29	1,106.60	553.80	10
6	757.42	379.21	739.41	370.21	S	1,025.54	513.27	1,008.51	504.76	1,007.53	504.27	9
7	844.45	422.73	826.44	413.72	S	938.51	469.76	921.48	461.24	920.49	460.75	8
8	957.54	479.27	939.53	470.27	I	851.47	426.24	834.45	417.73	833.46	417.24	7
9	1,054.59	527.80	1,036.58	518.79	P	738.39	369.70	721.36	361.18	720.38	360.69	6
10	1,153.66	577.33	1,135.65	568.33	V	641.34	321.17	624.31	312.66	623.33	312.17	5
11	1,282.70	641.85	1,264.69	632.85	E	542.27	271.64	525.24	263.12	524.26	262.63	4
12	1,383.75	692.38	1,365.74	683.37	T	413.23	207.12	396.20	198.60	395.21	198.11	3
13	1,520.81	760.91	1,502.80	751.90	H	312.18	156.59	295.15	148.08			2
14					R	175.12	88.06	158.09	79.55			1

Query 64593 Hit 1

MS/MS Fragmentation of KFVFFNIPQIQYK
Found in sp|P82663|RT25_HUMAN, 28S ribosomal protein S25
Match to Query 64593: 2103.223from(702.0817,3+)
Title: 917: Scan 2023 (rt=55.1652, f=3, i=315) [D:\lab212\membrane\Grace\Joyce\iTRAQ_44_1.raw]
Data File:Submitted from 20120508-1(merge) by Mascot Daemon on JOYCE-VAIO
Monoisotopic mass of neutral peptide Mr(calc): 2103.223
Variable modifications:
K1 :iTRAQ4plex (K)
K13 :iTRAQ4plex (K)
Ions Score: 47.54 Expect: 0.007

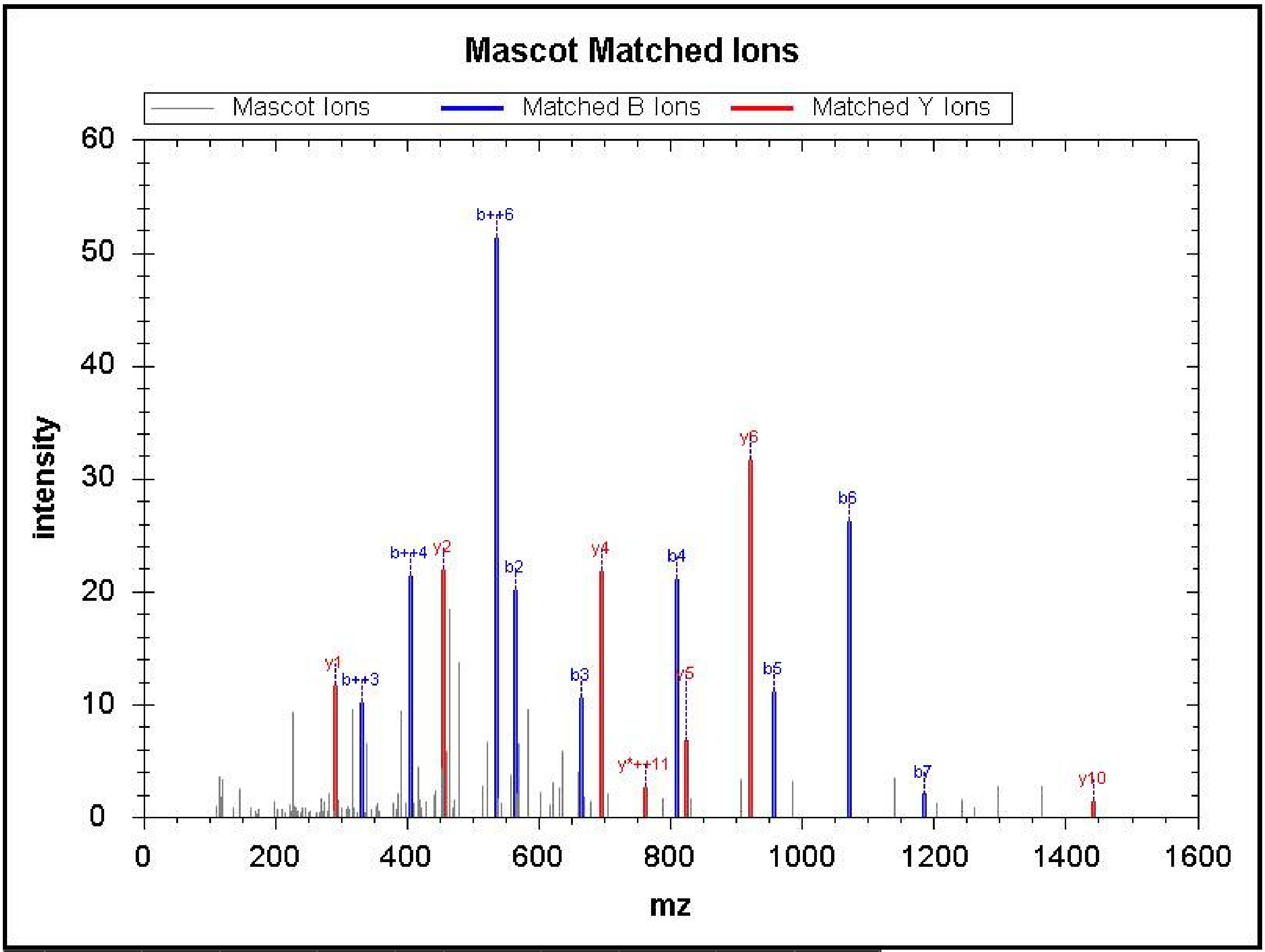

No	b	b++	b*	b*++	Seq	y	y++	y*	y*++	RevNo
1	417.31	209.16	400.28	200.64	K					13
2	564.37	282.69	547.35	274.18	F	1,687.93	844.47	1,670.91	835.96	12
3	663.44	332.23	646.42	323.71	V	1,540.86	770.94	1,523.84	762.42	11
4	810.51	405.76	793.49	397.25	F	1,441.80	721.40	1,424.77	712.89	10
5	957.58	479.29	940.55	470.78	F	1,294.73	647.87	1,277.70	639.35	9
6	1,071.62	536.32	1,054.60	527.80	N	1,147.66	574.33	1,130.63	565.82	8
7	1,184.71	592.86	1,167.68	584.34	I	1,033.62	517.31	1,016.59	508.80	7
8	1,281.76	641.38	1,264.73	632.87	P	920.53	460.77	903.51	452.26	6
9	1,409.82	705.41	1,392.79	696.90	Q	823.48	412.24	806.45	403.73	5
10	1,522.90	761.95	1,505.88	753.44	I	695.42	348.21	678.39	339.70	4
11	1,650.96	825.98	1,633.93	817.47	Q	582.34	291.67	565.31	283.16	3
12	1,814.02	907.52	1,797.00	899.00	Y	454.28	227.64	437.25	219.13	2
13					K	291.21	146.11	274.19	137.60	1

Query 45808 Hit 1

MS/MS Fragmentation of GMAFLHTLEPLIPR
Found in sp|Q13418|ILK_HUMAN, Integrin-linked protein kinase OS=Homo sapiens GN=ILK PE=1 SV=2
Match to Query 45808: 1737.972from(580.3312,3+)
Title: 845: Scan 2062 (rt=54.911, f=2, i=297) [D:\lab212\membrane\Grace\Joyce\iTRAQ_34_1.raw]
Data File:Submitted from 20120508-1(merge) by Mascot Daemon on JOYCE-VAIO
Monoisotopic mass of neutral peptide Mr(calc): 1737.972
Variable modifications:
Ions Score: 47.48 Expect: 0.013

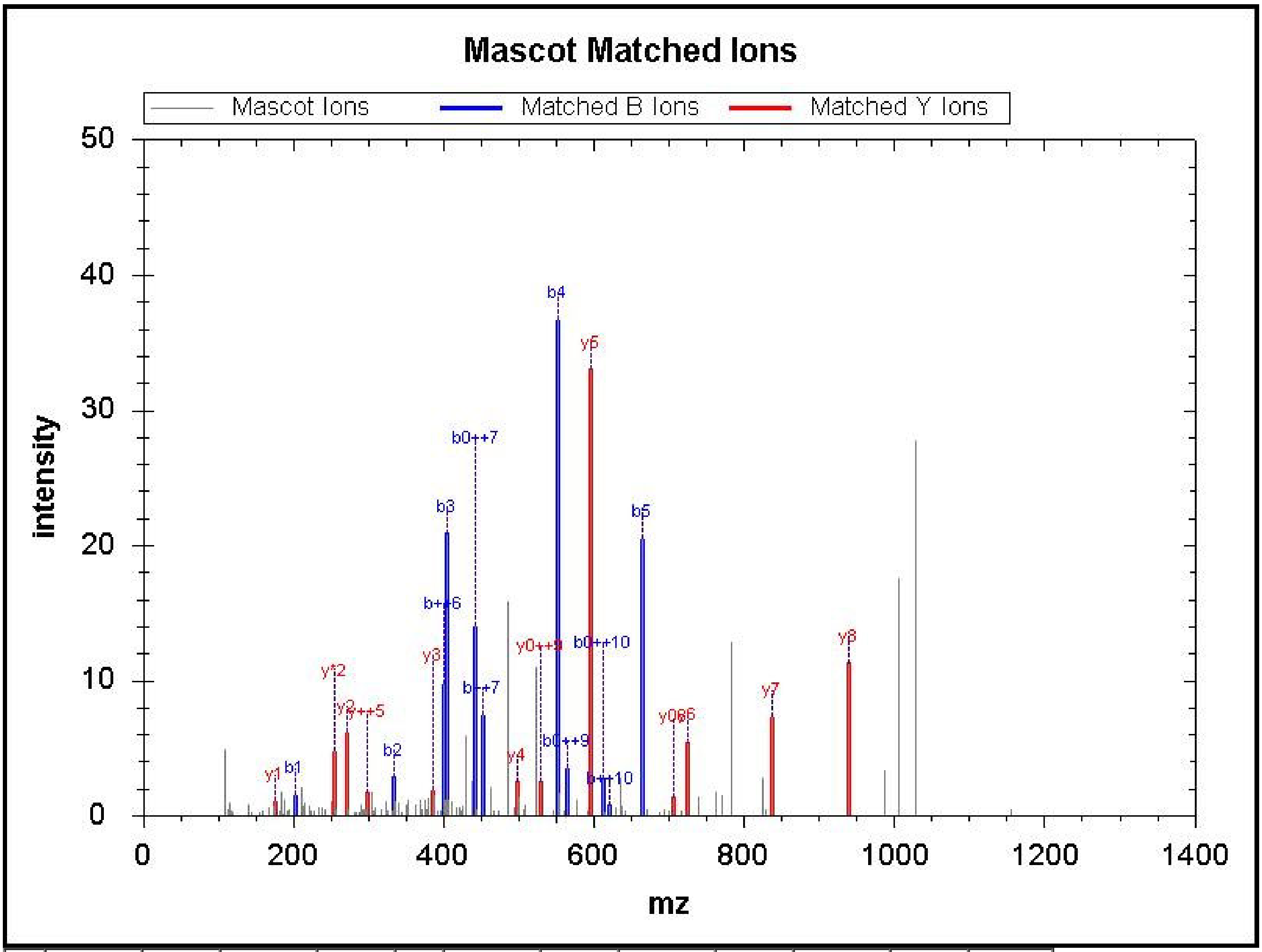

No	b	b++	b0	b0++	Seq	y	y++	y*	y*++	y0	y0++	RevNo
1	202.13	101.57			G							14
2	333.17	167.09			M	1,537.86	769.43	1,520.83	760.92	1,519.85	760.43	13
3	404.21	202.61			A	1,406.82	703.91	1,389.79	695.40	1,388.80	694.91	12
4	551.28	276.14			F	1,335.78	668.39	1,318.75	659.88	1,317.77	659.39	11
5	664.36	332.68			L	1,188.71	594.86	1,171.68	586.35	1,170.70	585.85	10
6	801.42	401.21			H	1,075.63	538.32	1,058.60	529.80	1,057.62	529.31	9
7	902.47	451.74	884.46	442.73	T	938.57	469.79	921.54	461.27	920.56	460.78	8
8	1,015.55	508.28	997.54	499.27	L	837.52	419.26	820.49	410.75	819.51	410.26	7
9	1,144.59	572.80	1,126.58	563.80	E	724.44	362.72	707.41	354.21	706.42	353.72	6
10	1,241.65	621.33	1,223.64	612.32	P	595.39	298.20	578.37	289.69			5
11	1,354.73	677.87	1,336.72	668.86	L	498.34	249.67	481.31	241.16			4
12	1,467.82	734.41	1,449.80	725.41	I	385.26	193.13	368.23	184.62			3
13	1,564.87	782.94	1,546.86	773.93	P	272.17	136.59	255.15	128.08			2
14					R	175.12	88.06	158.09	79.55			1

Query 49743 Hit 1

MS/MS Fragmentation of EFLDDLFSFMQK
Found in sp|Q99856|ARI3A_HUMAN, AT-rich interactive domain-containing protein 3A OS=Homo sapiens GN=ARID3A PE=1 SV=2
Match to Query 49743: 1806.914from(603.3121,3+)
Title: 1259: Scan 2793 (rt=72.2436, f=3, i=435) [D:\lab212\membrane\Grace\Joyce\iTRAQ_27_1.raw]
Data File:Submitted from 20120508-1(merge) by Mascot Daemon on JOYCE-VAIO
Monoisotopic mass of neutral peptide Mr(calc): 1806.914
Variable modifications:
K12 :iTRAQ4plex (K)
Ions Score: 47.46 Expect: 0.016

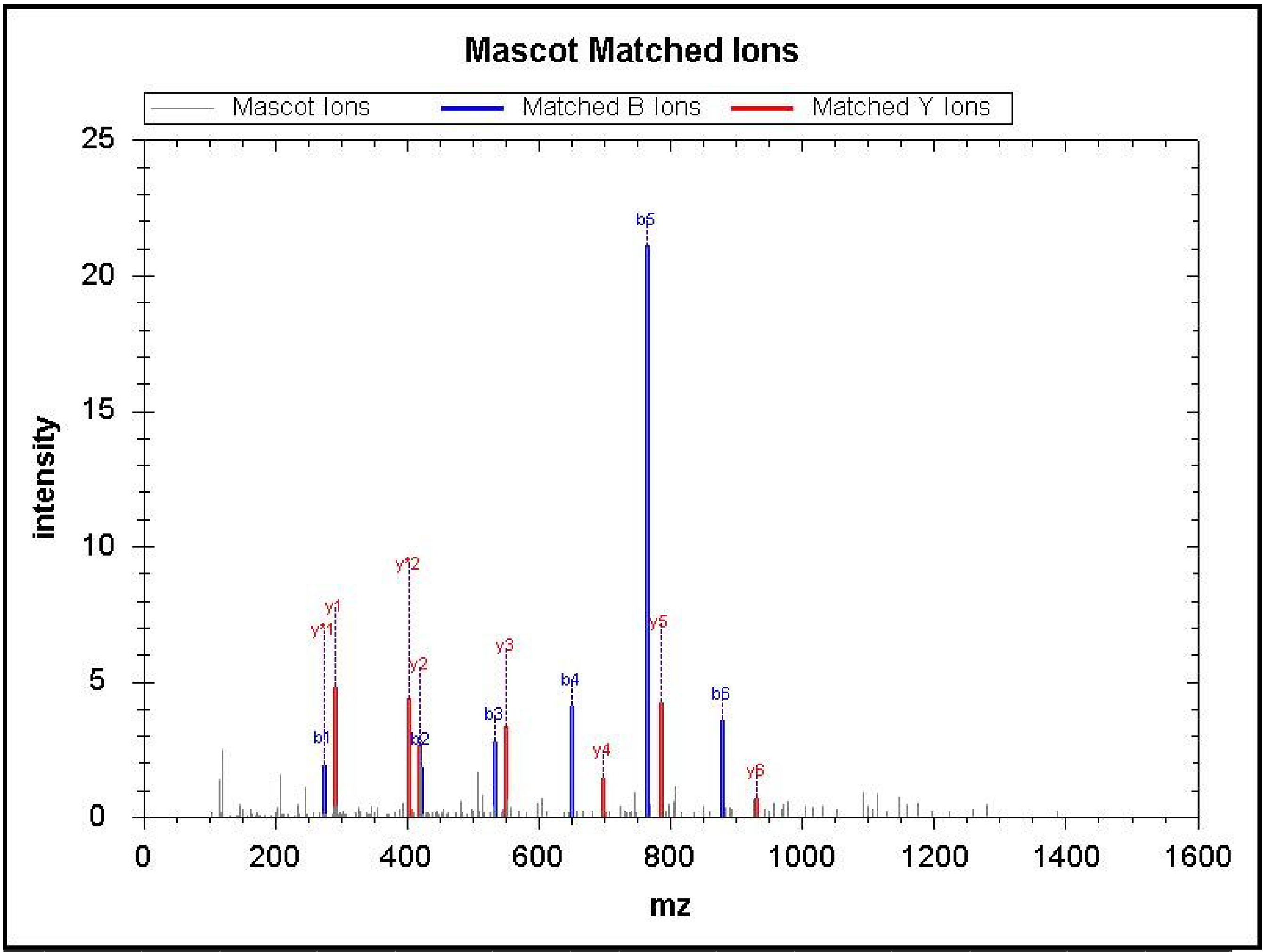

No	b	b++	b*	b*++	b0	b0++	Seq	y	y++	y*	y*++	y0	y0++	RevNo
1	274.15	137.58			256.14	128.57	E							12
2	421.22	211.11			403.21	202.11	F	1,534.77	767.89	1,517.75	759.38	1,516.76	758.89	11
3	534.30	267.66			516.29	258.65	L	1,387.70	694.36	1,370.68	685.84	1,369.69	685.35	10
4	649.33	325.17			631.32	316.16	D	1,274.62	637.81	1,257.59	629.30	1,256.61	628.81	9
5	764.36	382.68			746.35	373.68	D	1,159.59	580.30	1,142.57	571.79	1,141.58	571.30	8
6	877.44	439.22			859.43	430.22	L	1,044.57	522.79	1,027.54	514.27	1,026.56	513.78	7
7	1,024.51	512.76			1,006.50	503.75	F	931.48	466.25	914.46	457.73	913.47	457.24	6
8	1,111.54	556.28			1,093.53	547.27	S	784.41	392.71	767.39	384.20	766.40	383.71	5
9	1,258.61	629.81			1,240.60	620.80	F	697.38	349.19	680.36	340.68			4
10	1,389.65	695.33			1,371.64	686.32	M	550.31	275.66	533.29	267.15			3
11	1,517.71	759.36	1,500.68	750.85	1,499.70	750.35	Q	419.27	210.14	402.25	201.63			2
12							K	291.21	146.11	274.19	137.60			1

Query 89673 Hit 1

MS/MS Fragmentation of QEELGDLVDKEMAATSAAIETATAR
Found in sp|O00291|HIP1_HUMAN, Huntingtin-interacting protein 1 OS=Homo sapiens GN=HIP1 PE=1 SV=5
Match to Query 89673: 2907.476from(727.8762,4+)
Title: 1002: Sum of 2 scans in range 2394 (rt=62.3887, f=4, i=663) to 2395 (rt=62.4141, f=4, i=664)
[D:\lab212\membrane\Grace\Joyce\iTRAQ_34_1.raw]
Data File:Submitted from 20120508-1(merge) by Mascot Daemon on JOYCE-VAIO
Monoisotopic mass of neutral peptide Mr(calc): 2907.476
Variable modifications:
K10 :iTRAQ4plex (K)
Ions Score: 47.36 Expect: 0.017

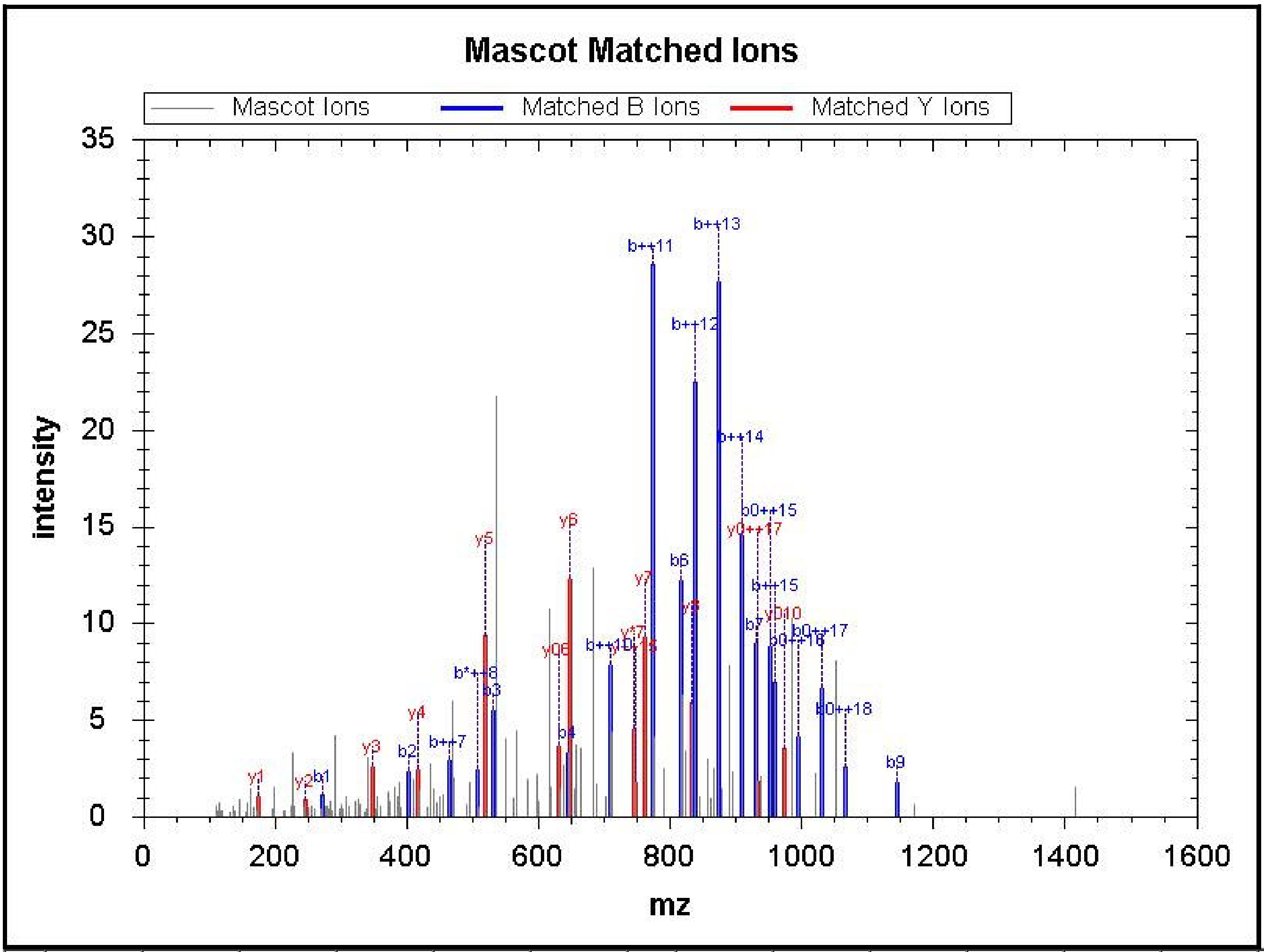

No	b	b++	b*	b*++	b0	b0++	Seq	y	y++	y*	y*++	y0	y0++	RevNo
1	273.17	137.09	256.14	128.57			Q							25
2	402.21	201.61	385.18	193.10	384.20	192.60	E	2,636.32	1,318.66	2,619.29	1,310.15	2,618.31	1,309.66	24
3	531.25	266.13	514.23	257.62	513.24	257.12	E	2,507.28	1,254.14	2,490.25	1,245.63	2,489.27	1,245.14	23
4	644.34	322.67	627.31	314.16	626.33	313.67	L	2,378.24	1,189.62	2,361.21	1,181.11	2,360.22	1,180.62	22
5	701.36	351.18	684.33	342.67	683.35	342.18	G	2,265.15	1,133.08	2,248.12	1,124.57	2,247.14	1,124.07	21
6	816.39	408.70	799.36	400.18	798.38	399.69	D	2,208.13	1,104.57	2,191.10	1,096.06	2,190.12	1,095.56	20
7	929.47	465.24	912.44	456.73	911.46	456.23	L	2,093.10	1,047.06	2,076.08	1,038.54	2,075.09	1,038.05	19
8	1,028.54	514.77	1,011.51	506.26	1,010.53	505.77	V	1,980.02	990.51	1,962.99	982.00	1,962.01	981.51	18
9	1,143.57	572.29	1,126.54	563.77	1,125.55	563.28	D	1,880.95	940.98	1,863.92	932.47	1,862.94	931.97	17
10	1,415.76	708.38	1,398.74	699.87	1,397.75	699.38	K	1,765.92	883.47	1,748.90	874.95	1,747.91	874.46	16
11	1,544.80	772.91	1,527.78	764.39	1,526.79	763.90	E	1,493.73	747.37	1,476.70	738.85	1,475.72	738.36	15
12	1,675.85	838.43	1,658.82	829.91	1,657.83	829.42	M	1,364.68	682.85	1,347.66	674.33	1,346.67	673.84	14
13	1,746.88	873.94	1,729.86	865.43	1,728.87	864.94	A	1,233.64	617.33	1,216.62	608.81	1,215.63	608.32	13
14	1,817.92	909.46	1,800.89	900.95	1,799.91	900.46	A	1,162.61	581.81	1,145.58	573.29	1,144.60	572.80	12
15	1,918.97	959.99	1,901.94	951.47	1,900.96	950.98	T	1,091.57	546.29	1,074.54	537.77	1,073.56	537.28	11
16	2,006.00	1,003.50	1,988.97	994.99	1,987.99	994.50	S	990.52	495.76	973.49	487.25	972.51	486.76	10
17	2,077.04	1,039.02	2,060.01	1,030.51	2,059.03	1,030.02	A	903.49	452.25	886.46	443.74	885.48	443.24	9
18	2,148.07	1,074.54	2,131.05	1,066.03	2,130.06	1,065.54	A	832.45	416.73	815.43	408.22	814.44	407.72	8
19	2,261.16	1,131.08	2,244.13	1,122.57	2,243.15	1,122.08	I	761.42	381.21	744.39	372.70	743.40	372.21	7
20	2,390.20	1,195.60	2,373.17	1,187.09	2,372.19	1,186.60	E	648.33	324.67	631.30	316.16	630.32	315.66	6
21	2,491.25	1,246.13	2,474.22	1,237.61	2,473.24	1,237.12	T	519.29	260.15	502.26	251.63	501.28	251.14	5
22	2,562.28	1,281.65	2,545.26	1,273.13	2,544.27	1,272.64	A	418.24	209.62	401.21	201.11	400.23	200.62	4
23	2,663.33	1,332.17	2,646.31	1,323.66	2,645.32	1,323.16	T	347.20	174.11	330.18	165.59	329.19	165.10	3

24	2,734.37	1,367.69	2,717.34	1,359.18	2,716.36	1,358.68	A	246.16	123.58	229.13	115.07			2
25							R	175.12	88.06	158.09	79.55			1

Query 75283 Hit 1

MS/MS Fragmentation of GRPITMFIPSDVDNYDDIR
Found in sp|Q9HC35|EMAL4_HUMAN, Echinoderm microtubule-associated protein-like 4 OS=Homo sapiens GN=EML4 PE=1 SV=3
Match to Query 75283: 2367.161from(790.061,3+)
Title: 738: Sum of 2 scans in range 1668 (rt=46.9589, f=4, i=495) to 1669 (rt=46.9843, f=4, i=496)
[D:\lab212\membrane\Grace\Joyce\iTRAQ_35_1.raw]
Data File:Submitted from 20120508-1(merge) by Mascot Daemon on JOYCE-VAIO
Monoisotopic mass of neutral peptide Mr(calc): 2367.161
Variable modifications:
Ions Score: 47.29 Expect: 0.017

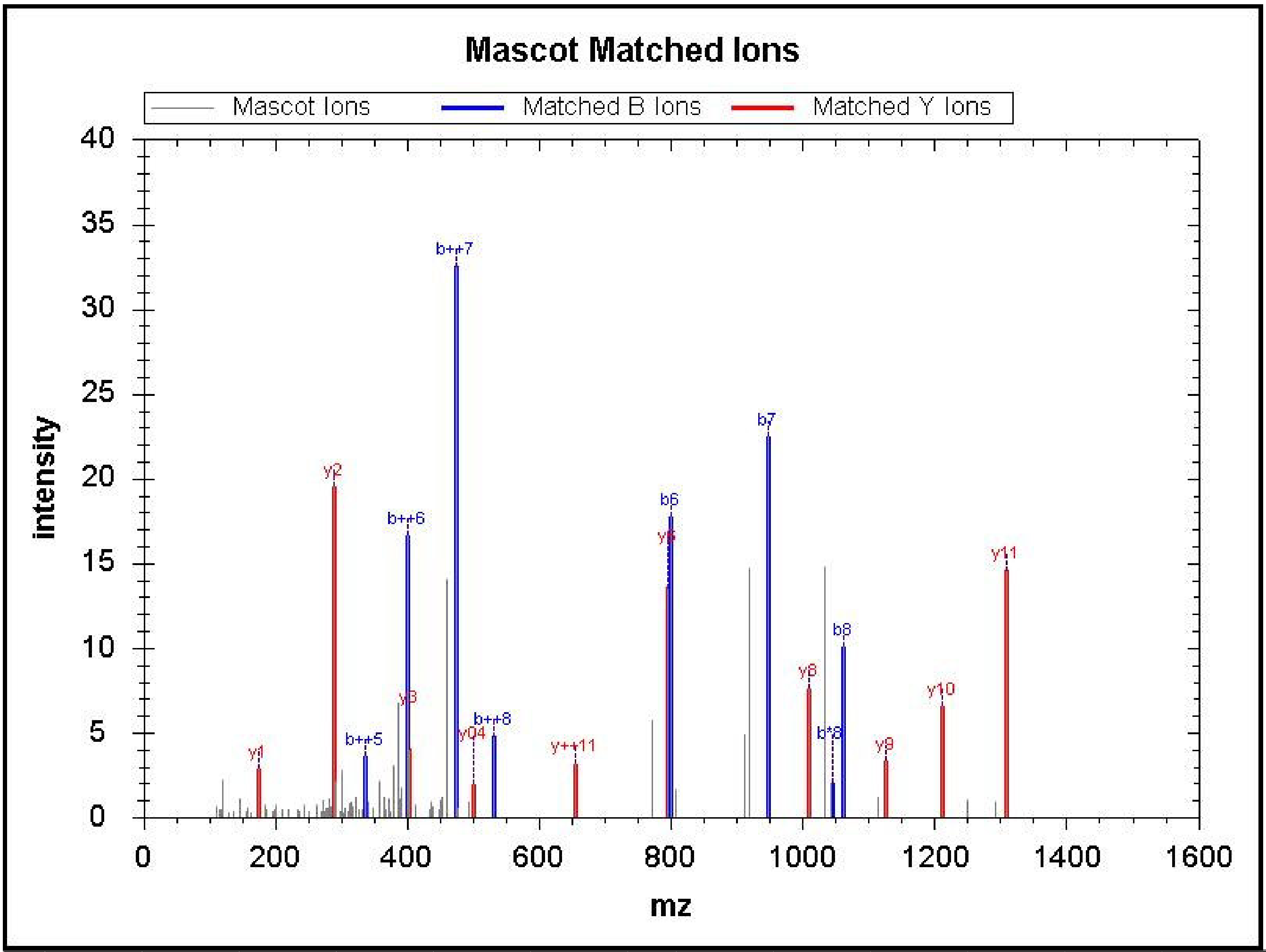

No	b	b++	b*	b*++	b0	b0++	Seq	y	y++	y*	y*++	y0	y0++	RevNo
1	202.13	101.57					G							19
2	358.23	179.62	341.21	171.11			R	2,167.05	1,084.03	2,150.02	1,075.51	2,149.04	1,075.02	18
3	455.28	228.15	438.26	219.63			P	2,010.95	1,005.98	1,993.92	997.46	1,992.94	996.97	17
4	568.37	284.69	551.34	276.17			I	1,913.90	957.45	1,896.87	948.94	1,895.88	948.45	16
5	669.42	335.21	652.39	326.70	651.41	326.21	T	1,800.81	900.91	1,783.78	892.40	1,782.80	891.90	15
6	800.46	400.73	783.43	392.22	782.45	391.73	M	1,699.76	850.39	1,682.74	841.87	1,681.75	841.38	14
7	947.53	474.27	930.50	465.75	929.51	465.26	F	1,568.72	784.86	1,551.70	776.35	1,550.71	775.86	13
8	1,060.61	530.81	1,043.58	522.30	1,042.60	521.80	I	1,421.65	711.33	1,404.63	702.82	1,403.64	702.33	12
9	1,157.66	579.33	1,140.64	570.82	1,139.65	570.33	P	1,308.57	654.79	1,291.54	646.28	1,290.56	645.78	11
10	1,244.69	622.85	1,227.67	614.34	1,226.68	613.85	S	1,211.52	606.26	1,194.49	597.75	1,193.51	597.26	10
11	1,359.72	680.36	1,342.69	671.85	1,341.71	671.36	D	1,124.49	562.75	1,107.46	554.23	1,106.47	553.74	9

12	1,458.79	729.90	1,441.76	721.39	1,440.78	720.89	V	1,009.46	505.23	992.43	496.72	991.45	496.23	8
13	1,573.82	787.41	1,556.79	778.90	1,555.81	778.41	D	910.39	455.70	893.36	447.19	892.38	446.69	7
14	1,687.86	844.43	1,670.83	835.92	1,669.85	835.43	N	795.36	398.19	778.34	389.67	777.35	389.18	6
15	1,850.92	925.97	1,833.90	917.45	1,832.91	916.96	Y	681.32	341.16	664.29	332.65	663.31	332.16	5
16	1,965.95	983.48	1,948.92	974.97	1,947.94	974.47	D	518.26	259.63	501.23	251.12	500.25	250.63	4
17	2,080.98	1,040.99	2,063.95	1,032.48	2,062.97	1,031.99	D	403.23	202.12	386.20	193.61	385.22	193.11	3
18	2,194.06	1,097.53	2,177.03	1,089.02	2,176.05	1,088.53	I	288.20	144.61	271.18	136.09			2
19							R	175.12	88.06	158.09	79.55			1

Query 62204 Hit 1

MS/MS Fragmentation of ELEELLESALDDFDK
Found in sp|P40855|PEX19_HUMAN, Peroxisomal biogenesis factor 19 OS=Homo sapiens GN=PEX19 PE=1 SV=1
Match to Query 62204: 2053.04from(685.354,3+)
Title: 1332: Sum of 2 scans in range 2953 (rt=75.8217, f=4, i=893) to 2954 (rt=75.8471, f=4, i=894)
[D:\lab212\membrane\Grace\Joyce\iTRAQ_27_1.raw]
Data File:Submitted from 20120508-1(merge) by Mascot Daemon on JOYCE-VAIO
Monoisotopic mass of neutral peptide Mr(calc): 2053.04
Variable modifications:
K15 :iTRAQ4plex (K)
Ions Score: 47.27 Expect: 0.018

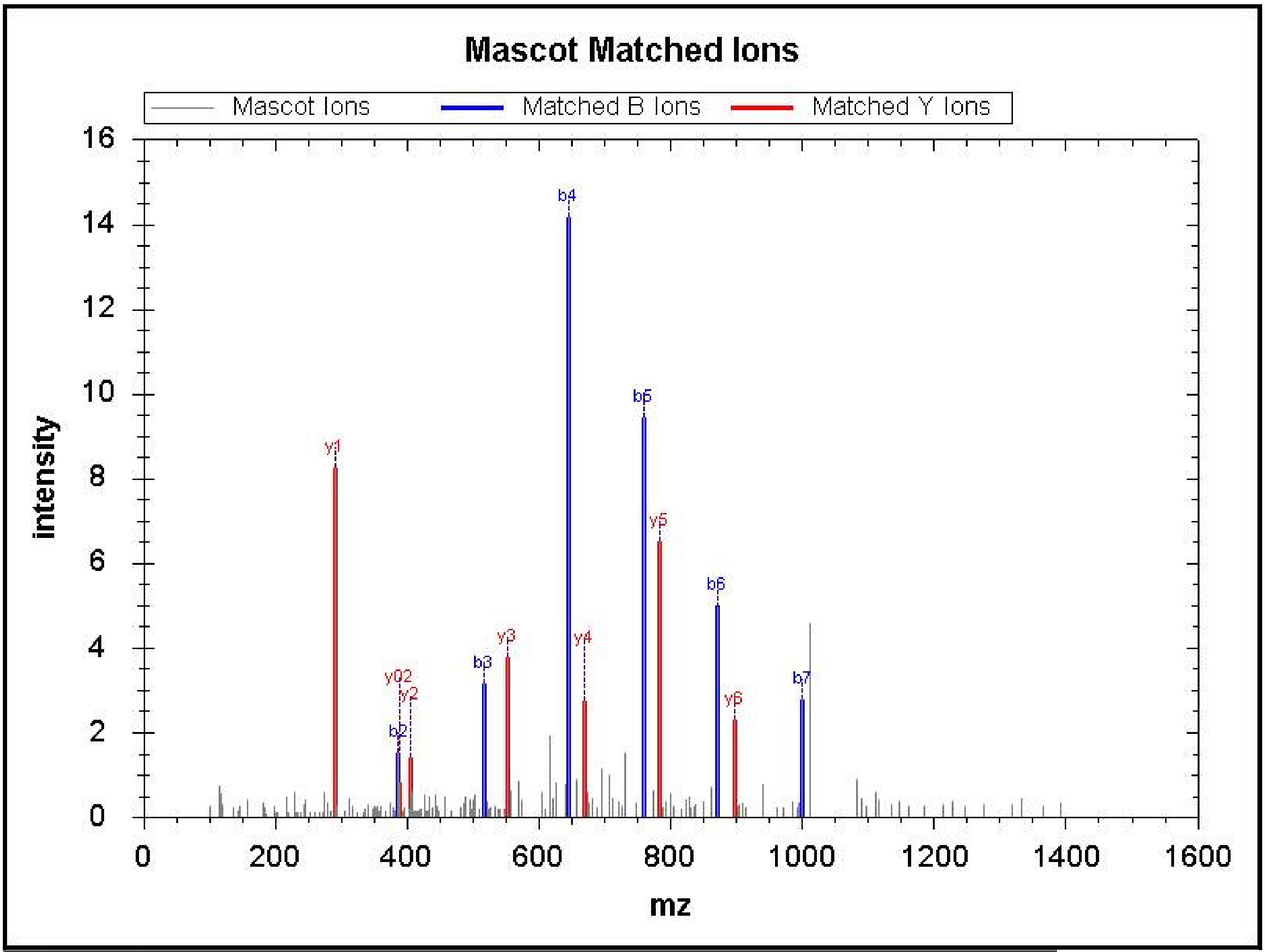

No	b	b++	b0	b0++	Seq	y	y++	y*	y*++	y0	y0++	RevNo
1	274.15	137.58	256.14	128.57	E							15
2	387.24	194.12	369.23	185.12	L	1,780.90	890.95	1,763.87	882.44	1,762.89	881.95	14
3	516.28	258.64	498.27	249.64	E	1,667.81	834.41	1,650.79	825.90	1,649.80	825.40	13
4	645.32	323.16	627.31	314.16	E	1,538.77	769.89	1,521.74	761.38	1,520.76	760.88	12

5	758.41	379.71	740.39	370.70	L	1,409.73	705.37	1,392.70	696.85	1,391.72	696.36	11
6	871.49	436.25	853.48	427.24	L	1,296.64	648.83	1,279.62	640.31	1,278.63	639.82	10
7	1,000.53	500.77	982.52	491.76	E	1,183.56	592.28	1,166.53	583.77	1,165.55	583.28	9
8	1,087.56	544.29	1,069.55	535.28	S	1,054.52	527.76	1,037.49	519.25	1,036.51	518.76	8
9	1,158.60	579.80	1,140.59	570.80	A	967.49	484.25	950.46	475.73	949.47	475.24	7
10	1,271.69	636.35	1,253.67	627.34	L	896.45	448.73	879.42	440.21	878.44	439.72	6
11	1,386.71	693.86	1,368.70	684.85	D	783.36	392.19	766.34	383.67	765.35	383.18	5
12	1,501.74	751.37	1,483.73	742.37	D	668.34	334.67	651.31	326.16	650.33	325.67	4
13	1,648.81	824.91	1,630.80	815.90	F	553.31	277.16	536.28	268.65	535.30	268.15	3
14	1,763.83	882.42	1,745.82	873.42	D	406.24	203.62	389.22	195.11	388.23	194.62	2
15					K	291.21	146.11	274.19	137.60			1

Query 65671 Hit 1

MS/MS Fragmentation of VITSAWWVYHHQVSK
Found in sp|O60524|NEMF_HUMAN, Nuclear export mediator factor NEMF OS=Homo sapiens GN=NEMF PE=1 SV=4
Match to Query 65671: 2128.071from(710.3641,3+)
Title: 603: Sum of 2 scans in range 1323 (rt=39.496, f=4, i=403) to 1324 (rt=39.5214, f=4, i=404) [D:\lab212\membrane\Grace\Joyce\iTRAQ_50_2.raw]
Data File:Submitted from 20120508-1(merge) by Mascot Daemon on JOYCE-VAIO
Monoisotopic mass of neutral peptide Mr(calc): 2128.071
Variable modifications:
K15 :iTRAQ4plex (K)
Ions Score: 47.24 Expect: 0.018

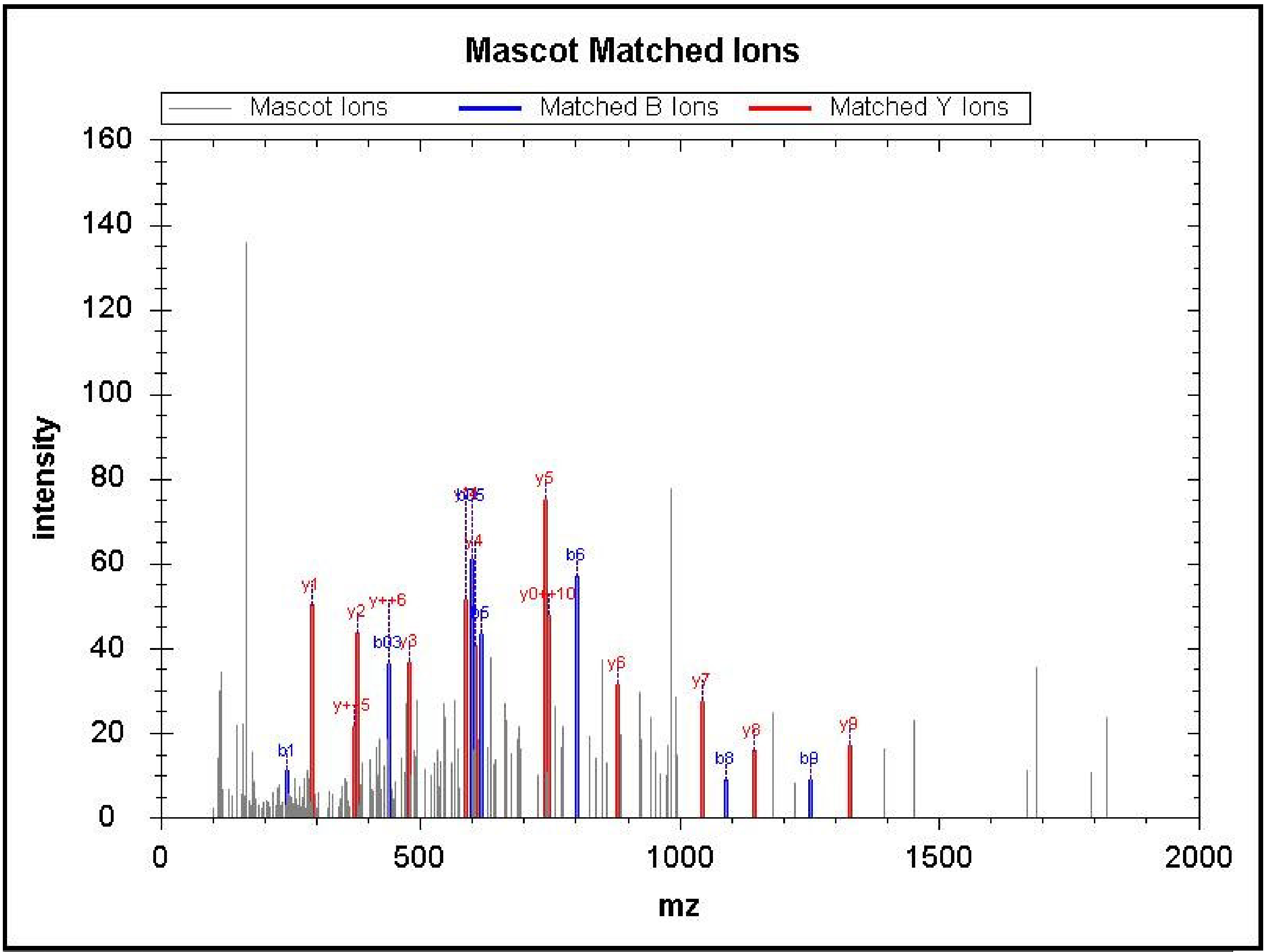

No	b	b++	b*	b*++	b0	b0++	Seq	y	y++	y*	y*++	y0	y0++	RevNo
1	244.18	122.59					V							15

2	357.26	179.13					I	1,885.98	943.50	1,868.96	934.98	1,867.97	934.49	14
3	458.31	229.66			440.30	220.65	T	1,772.90	886.95	1,755.87	878.44	1,754.89	877.95	13
4	545.34	273.17			527.33	264.17	S	1,671.85	836.43	1,654.82	827.92	1,653.84	827.42	12
5	616.38	308.69			598.37	299.69	A	1,584.82	792.91	1,567.79	784.40	1,566.81	783.91	11
6	802.46	401.73			784.45	392.73	W	1,513.78	757.39	1,496.76	748.88	1,495.77	748.39	10
7	988.54	494.77			970.53	485.77	W	1,327.70	664.36	1,310.68	655.84	1,309.69	655.35	9
8	1,087.61	544.31			1,069.60	535.30	V	1,141.62	571.32	1,124.60	562.80	1,123.61	562.31	8
9	1,250.67	625.84			1,232.66	616.83	Y	1,042.56	521.78	1,025.53	513.27	1,024.54	512.78	7
10	1,387.73	694.37			1,369.72	685.36	H	879.49	440.25	862.47	431.74	861.48	431.24	6
11	1,524.79	762.90			1,506.78	753.89	H	742.43	371.72	725.41	363.21	724.42	362.71	5
12	1,652.85	826.93	1,635.82	818.41	1,634.83	817.92	Q	605.37	303.19	588.35	294.68	587.36	294.19	4
13	1,751.91	876.46	1,734.89	867.95	1,733.90	867.46	V	477.32	239.16	460.29	230.65	459.30	230.16	3
14	1,838.95	919.98	1,821.92	911.46	1,820.94	910.97	S	378.25	189.63	361.22	181.11	360.24	180.62	2
15							K	291.21	146.11	274.19	137.60			1

Query 17394 Hit 1

MS/MS Fragmentation of LPLIFHLGR
Found in sp|P34059|GALNS_HUMAN, N-acetylgalactosamine-6-sulfatase OS=Homo sapiens GN=GALNS PE=1 SV=1
Match to Query 17394: 1208.752from(403.9246,3+)
Title: 846: Sum of 2 scans in range 1883 (rt=51.9289, f=4, i=565) to 1884 (rt=51.9543, f=4, i=566)
[D:\lab212\membrane\Grace\Joyce\iTRAQ_39_1.raw]
Data File:Submitted from 20120508-1(merge) by Mascot Daemon on JOYCE-VAIO
Monoisotopic mass of neutral peptide Mr(calc): 1208.752
Variable modifications:
Ions Score: 47.24 Expect: 0.008

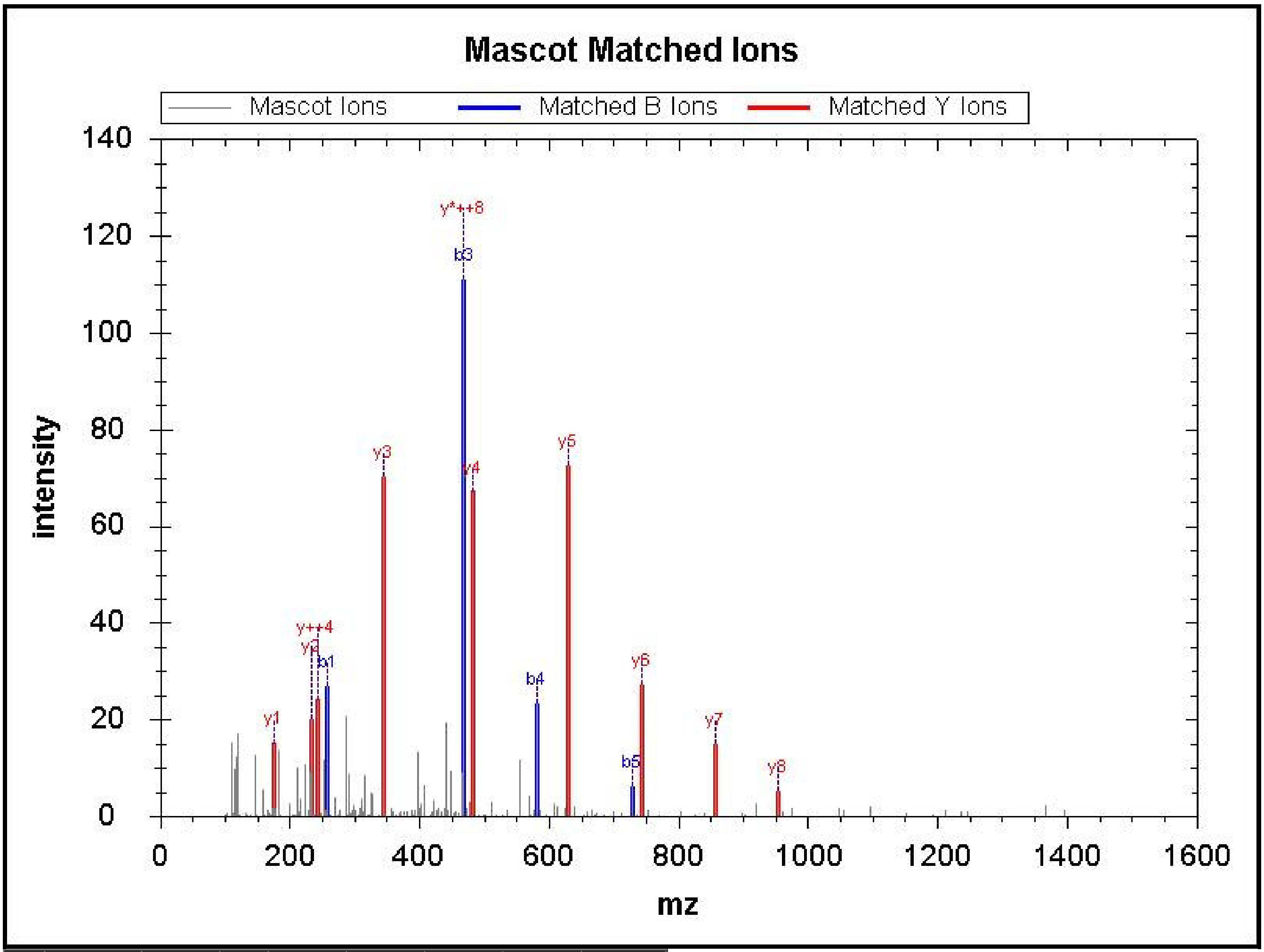

No	b	b++	Seq	y	y++	y*	y*++	RevNo

1	258.19	129.60	L					9
2	355.25	178.13	P	952.57	476.79	935.55	468.28	8
3	468.33	234.67	L	855.52	428.26	838.49	419.75	7
4	581.41	291.21	I	742.44	371.72	725.41	363.21	6
5	728.48	364.74	F	629.35	315.18	612.33	306.67	5
6	865.54	433.27	H	482.28	241.65	465.26	233.13	4
7	978.63	489.82	L	345.22	173.12	328.20	164.60	3
8	1,035.65	518.33	G	232.14	116.57	215.11	108.06	2
9			R	175.12	88.06	158.09	79.55	1

Query 31050 Hit 1

MS/MS Fragmentation of FDLLEELVAK
Found in sp|Q8WWC4|CB047_HUMAN, Uncharacterized protein C2orf47
Match to Query 31050: 1463.844from(732.9293,2+)
Title: 1033: Sum of 2 scans in range 2268 (rt=60.744, f=4, i=701) to 2269 (rt=60.7694, f=4, i=702) [D:\lab212\membrane\Grace\Joyce\iTRAQ_27_2_327.raw]
Data File:Submitted from 20120508-1(merge) by Mascot Daemon on JOYCE-VAIO
Monoisotopic mass of neutral peptide Mr(calc): 1463.844
Variable modifications:
K10 :iTRAQ4plex (K)
Ions Score: 47.2 Expect: 0.013

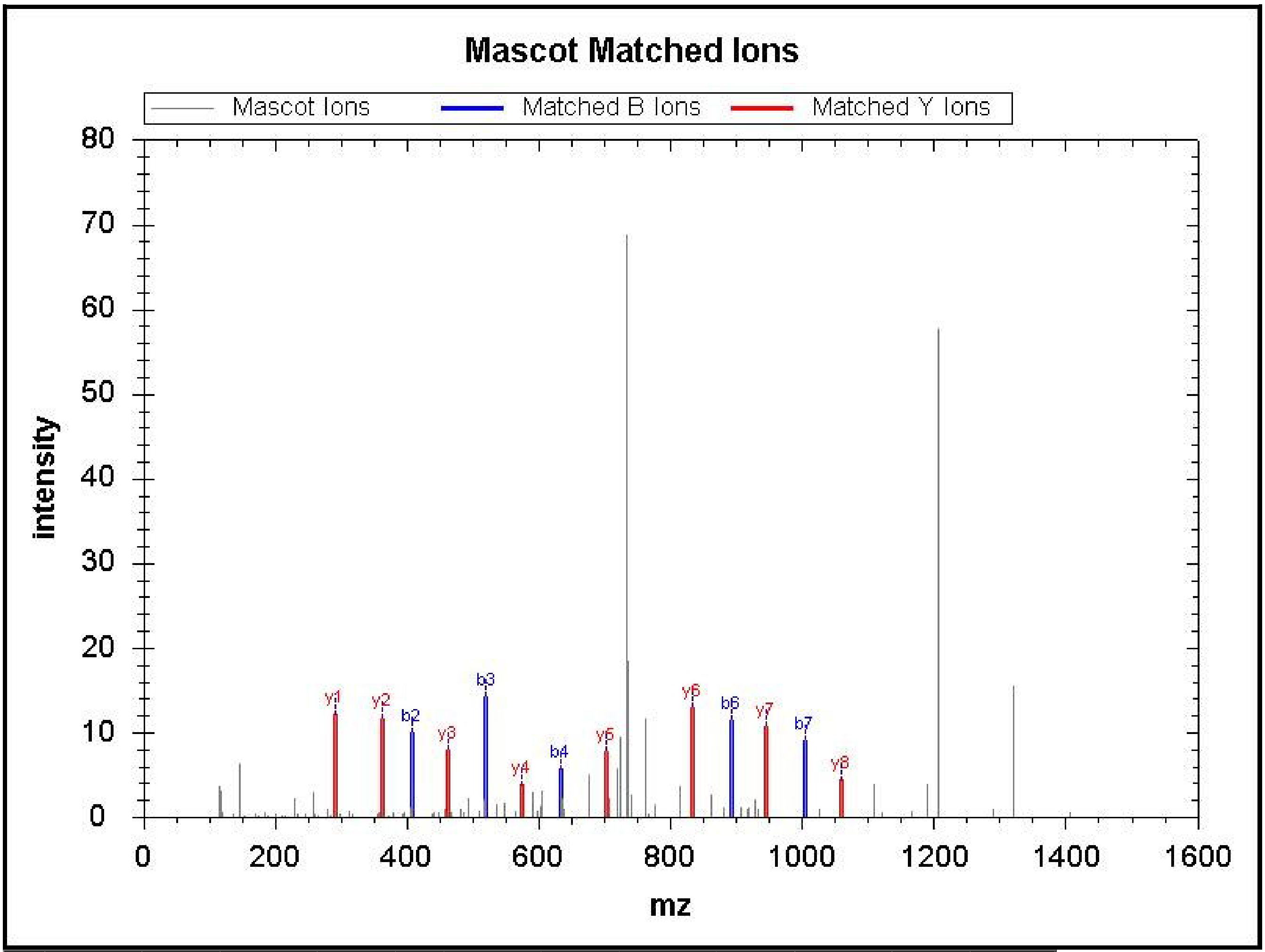

No	b	b++	b0	b0++	Seq	y	y++	y*	y*++	y0	y0++	RevNo
1	292.18	146.59			F							10
2	407.20	204.11	389.19	195.10	D	1,173.68	587.35	1,156.66	578.83	1,155.67	578.34	9
3	520.29	260.65	502.28	251.64	L	1,058.66	529.83	1,041.63	521.32	1,040.65	520.83	8
4	633.37	317.19	615.36	308.18	L	945.57	473.29	928.55	464.78	927.56	464.29	7

5	762.42	381.71	744.40	372.71	E	832.49	416.75	815.46	408.24	814.48	407.74	6
6	891.46	446.23	873.45	437.23	E	703.45	352.23	686.42	343.71	685.44	343.22	5
7	1,004.54	502.77	986.53	493.77	L	574.40	287.71	557.38	279.19			4
8	1,103.61	552.31	1,085.60	543.30	V	461.32	231.16	444.29	222.65			3
9	1,174.65	587.83	1,156.64	578.82	A	362.25	181.63	345.23	173.12			2
10					K	291.21	146.11	274.19	137.60			1

Query 35982 Hit 1

MS/MS Fragmentation of RPEEVALGLHHR
Found in sp|Q96ER9|CCD51_HUMAN, Coiled-coil domain-containing protein 51 OS=Homo sapiens GN=CCDC51 PE=1 SV=2
Match to Query 35982: 1556.863from(390.2231,4+)
Title: 195: Scan 462 (rt=20.2486, f=3, i=71) [D:\lab212\membrane\Grace\Joyce\iTRAQ_56_1_330.raw]
Data File:Submitted from 20120508-1(merge) by Mascot Daemon on JOYCE-VAIO
Monoisotopic mass of neutral peptide Mr(calc): 1556.863
Variable modifications:
Ions Score: 47.2 Expect: 0.014

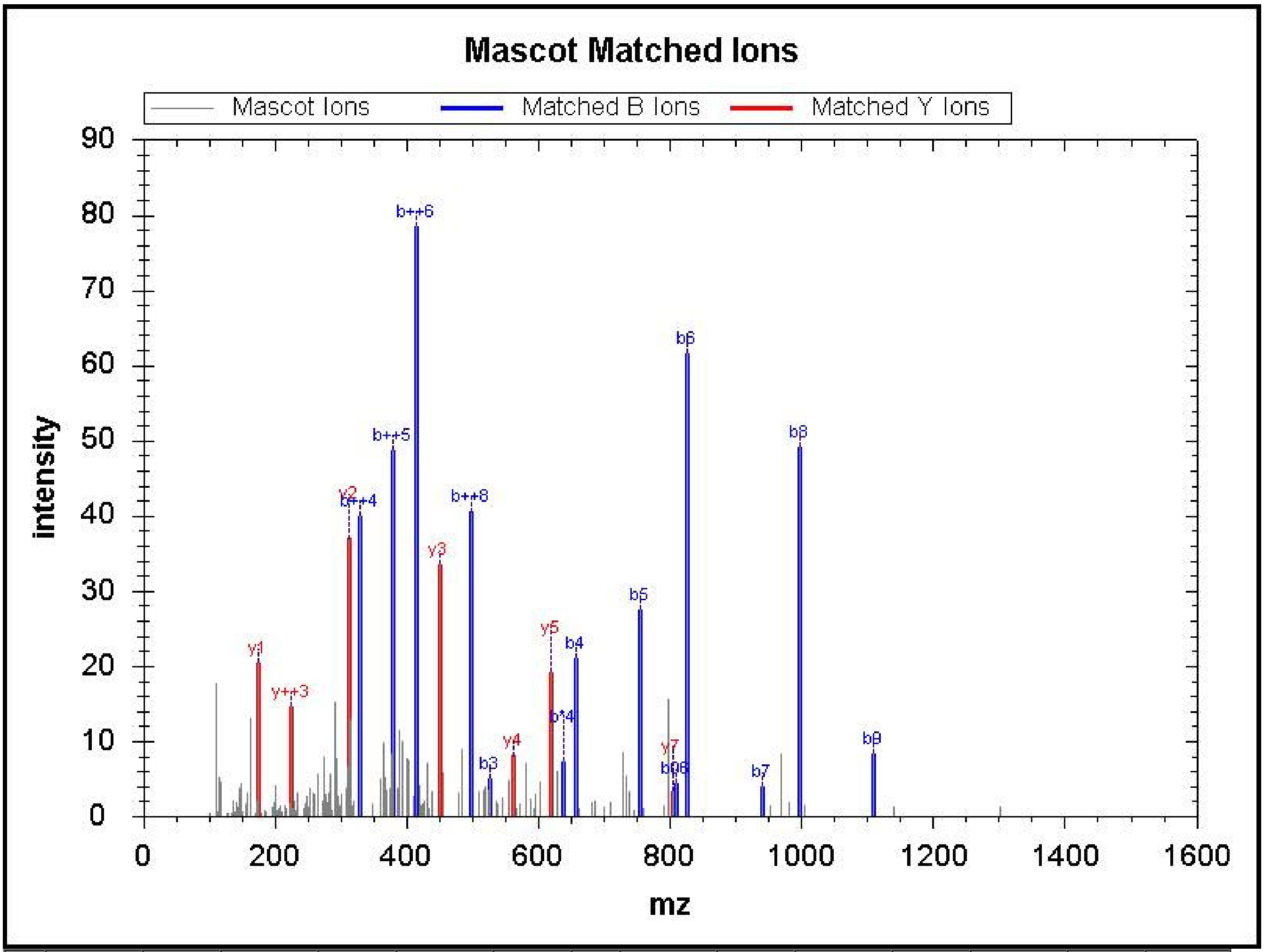

No	b	b++	b*	b*++	b0	b0++	Seq	y	y++	y*	y*++	y0	y0++	RevNo
1	301.21	151.11	284.18	142.60			R							12
2	398.26	199.64	381.24	191.12			P	1,257.67	629.34	1,240.64	620.83	1,239.66	620.33	11
3	527.31	264.16	510.28	255.64	509.30	255.15	E	1,160.62	580.81	1,143.59	572.30	1,142.61	571.81	10
4	656.35	328.68	639.32	320.16	638.34	319.67	E	1,031.57	516.29	1,014.55	507.78	1,013.56	507.29	9
5	755.42	378.21	738.39	369.70	737.41	369.21	V	902.53	451.77	885.51	443.26			8
6	826.45	413.73	809.43	405.22	808.44	404.73	A	803.46	402.24	786.44	393.72			7
7	939.54	470.27	922.51	461.76	921.53	461.27	L	732.43	366.72	715.40	358.20			6
8	996.56	498.78	979.53	490.27	978.55	489.78	G	619.34	310.17	602.32	301.66			5

9	1,109.64	555.33	1,092.62	546.81	1,091.63	546.32	L	562.32	281.66	545.29	273.15			4
10	1,246.70	623.85	1,229.68	615.34	1,228.69	614.85	H	449.24	225.12	432.21	216.61			3
11	1,383.76	692.38	1,366.73	683.87	1,365.75	683.38	H	312.18	156.59	295.15	148.08			2
12							R	175.12	88.06	158.09	79.55			1

Query 66724 Hit 1

MS/MS Fragmentation of NIFHLFHDVVPTYHK
Found in sp|O43264|ZW10_HUMAN, Centromere/kinetochore protein zw10 homolog OS=Homo sapiens GN=ZW10 PE=1 SV=3
Match to Query 66724: 2154.167from(539.549,4+)
Title: 809: Scan 1727 (rt=48.5562, f=3, i=268) [D:\lab212\membrane\Grace\Joyce\54-2.raw]
Data File:Submitted from 20120508-1(merge) by Mascot Daemon on JOYCE-VAIO
Monoisotopic mass of neutral peptide Mr(calc): 2154.167
Variable modifications:
K15 :iTRAQ4plex (K)
Ions Score: 47.2 Expect: 0.015

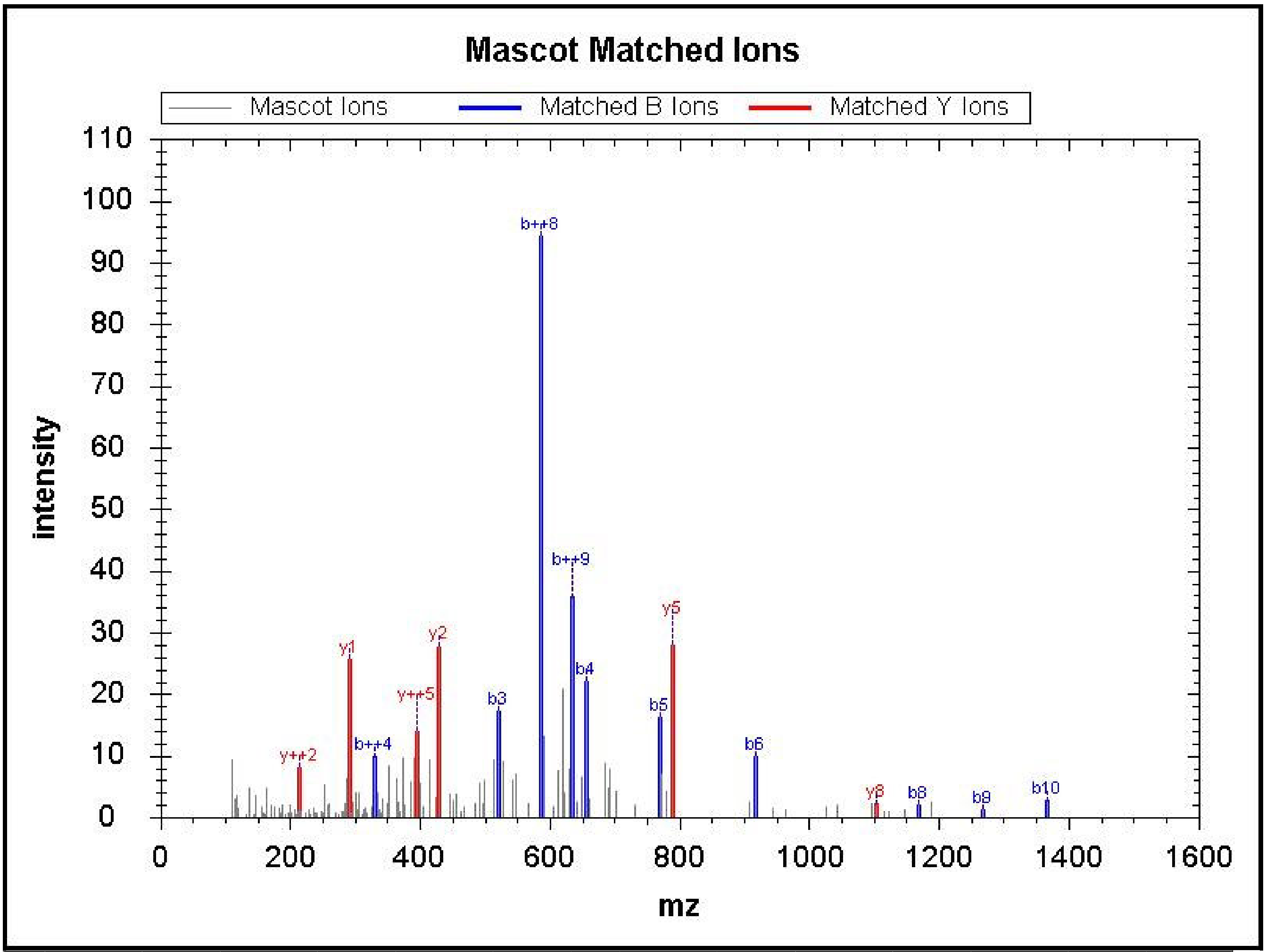

No	b	b++	b*	b*++	b0	b0++	Seq	y	y++	y*	y*++	y0	y0++	RevNo
1	259.15	130.08	242.13	121.57			N							15
2	372.24	186.62	355.21	178.11			I	1,897.02	949.02	1,880.00	940.50	1,879.01	940.01	14
3	519.30	260.16	502.28	251.64			F	1,783.94	892.47	1,766.91	883.96	1,765.93	883.47	13
4	656.36	328.69	639.34	320.17			H	1,636.87	818.94	1,619.85	810.43	1,618.86	809.93	12
5	769.45	385.23	752.42	376.71			L	1,499.81	750.41	1,482.79	741.90	1,481.80	741.40	11
6	916.52	458.76	899.49	450.25			F	1,386.73	693.87	1,369.70	685.35	1,368.72	684.86	10
7	1,053.58	527.29	1,036.55	518.78			H	1,239.66	620.33	1,222.63	611.82	1,221.65	611.33	9
8	1,168.60	584.80	1,151.58	576.29	1,150.59	575.80	D	1,102.60	551.80	1,085.57	543.29	1,084.59	542.80	8
9	1,267.67	634.34	1,250.64	625.83	1,249.66	625.33	V	987.57	494.29	970.55	485.78	969.56	485.29	7
10	1,366.74	683.87	1,349.71	675.36	1,348.73	674.87	V	888.51	444.76	871.48	436.24	870.50	435.75	6

11	1,463.79	732.40	1,446.77	723.89	1,445.78	723.39	P	789.44	395.22	772.41	386.71	771.43	386.22	5
12	1,564.84	782.92	1,547.81	774.41	1,546.83	773.92	T	692.38	346.70	675.36	338.18	674.37	337.69	4
13	1,727.90	864.45	1,710.88	855.94	1,709.89	855.45	Y	591.34	296.17	574.31	287.66			3
14	1,864.96	932.98	1,847.94	924.47	1,846.95	923.98	H	428.27	214.64	411.25	206.13			2
15							K	291.21	146.11	274.19	137.60			1

Query 41953 Hit 1

MS/MS Fragmentation of TVHAGEVGSAEVVK
Found in sp|P00813|ADA_HUMAN, Adenosine deaminase OS=Homo sapiens GN=ADA PE=1 SV=3
Match to Query 41953: 1669.926from(557.6494,3+)
Title: 234: Sum of 2 scans in range 603 (rt=23.1192, f=4, i=153) to 604 (rt=23.1446, f=4, i=154) [D:\lab212\membrane\Grace\Joyce\iTRAQ_41_2.raw]
Data File:Submitted from 20120508-1(merge) by Mascot Daemon on JOYCE-VAIO
Monoisotopic mass of neutral peptide Mr(calc): 1669.926
Variable modifications:
K14 :iTRAQ4plex (K)
Ions Score: 47.15 Expect: 0.014

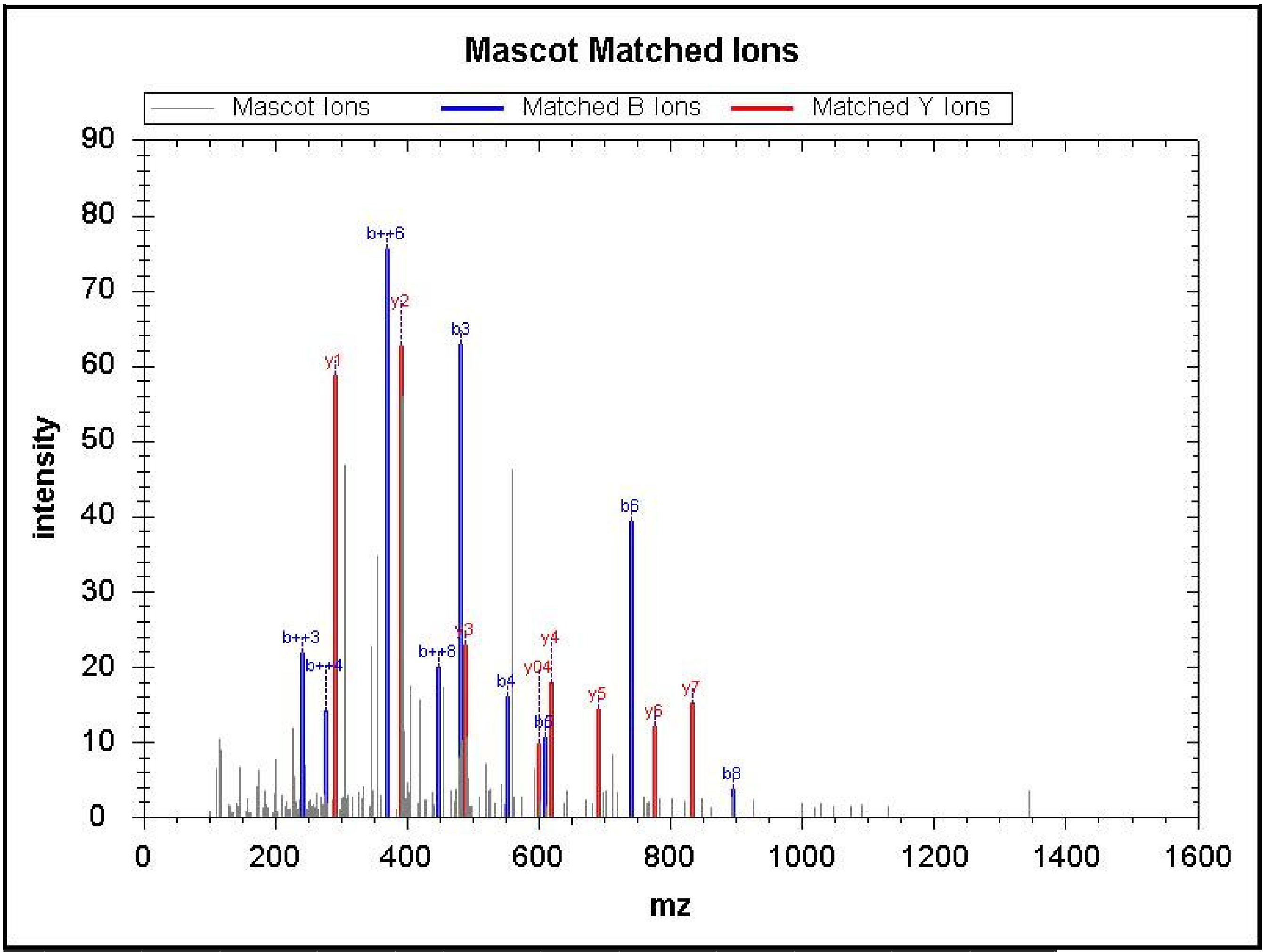

No	b	b++	b0	b0++	Seq	y	y++	y*	y*++	y0	y0++	RevNo
1	246.16	123.58	228.15	114.58	T							14
2	345.23	173.12	327.21	164.11	V	1,425.78	713.39	1,408.76	704.88	1,407.77	704.39	13
3	482.28	241.65	464.27	232.64	H	1,326.71	663.86	1,309.69	655.35	1,308.70	654.86	12
4	553.32	277.16	535.31	268.16	A	1,189.65	595.33	1,172.63	586.82	1,171.64	586.33	11
5	610.34	305.68	592.33	296.67	G	1,118.62	559.81	1,101.59	551.30	1,100.61	550.81	10
6	739.39	370.20	721.38	361.19	E	1,061.60	531.30	1,044.57	522.79	1,043.59	522.30	9
7	838.45	419.73	820.44	410.73	V	932.55	466.78	915.53	458.27	914.54	457.78	8
8	895.48	448.24	877.46	439.24	G	833.48	417.25	816.46	408.73	815.47	408.24	7

9	982.51	491.76	964.50	482.75	S	776.46	388.74	759.44	380.22	758.45	379.73	6
10	1,053.54	527.28	1,035.53	518.27	A	689.43	345.22	672.40	336.71	671.42	336.21	5
11	1,182.59	591.80	1,164.58	582.79	E	618.39	309.70	601.37	301.19	600.38	300.70	4
12	1,281.66	641.33	1,263.65	632.33	V	489.35	245.18	472.33	236.67			3
13	1,380.72	690.87	1,362.71	681.86	V	390.28	195.65	373.26	187.13			2
14					K	291.21	146.11	274.19	137.60			1

Query 27043 Hit 1

MS/MS Fragmentation of DVINVFHHLR
Found in sp|Q9UK58|CCNL1_HUMAN, Cyclin-L1 OS=Homo sapiens GN=CCNL1 PE=1 SV=1
Match to Query 27043: 1392.782from(465.2679,3+)
Title: 450: Sum of 2 scans in range 1229 (rt=36.361, f=4, i=295) to 1230 (rt=36.3864, f=4, i=296) [D:\lab212\membrane\Grace\Joyce\iTRAQ_51_2.raw]
Data File:Submitted from 20120508-1(merge) by Mascot Daemon on JOYCE-VAIO
Monoisotopic mass of neutral peptide Mr(calc): 1392.782
Variable modifications:
Ions Score: 47.02 Expect: 0.016

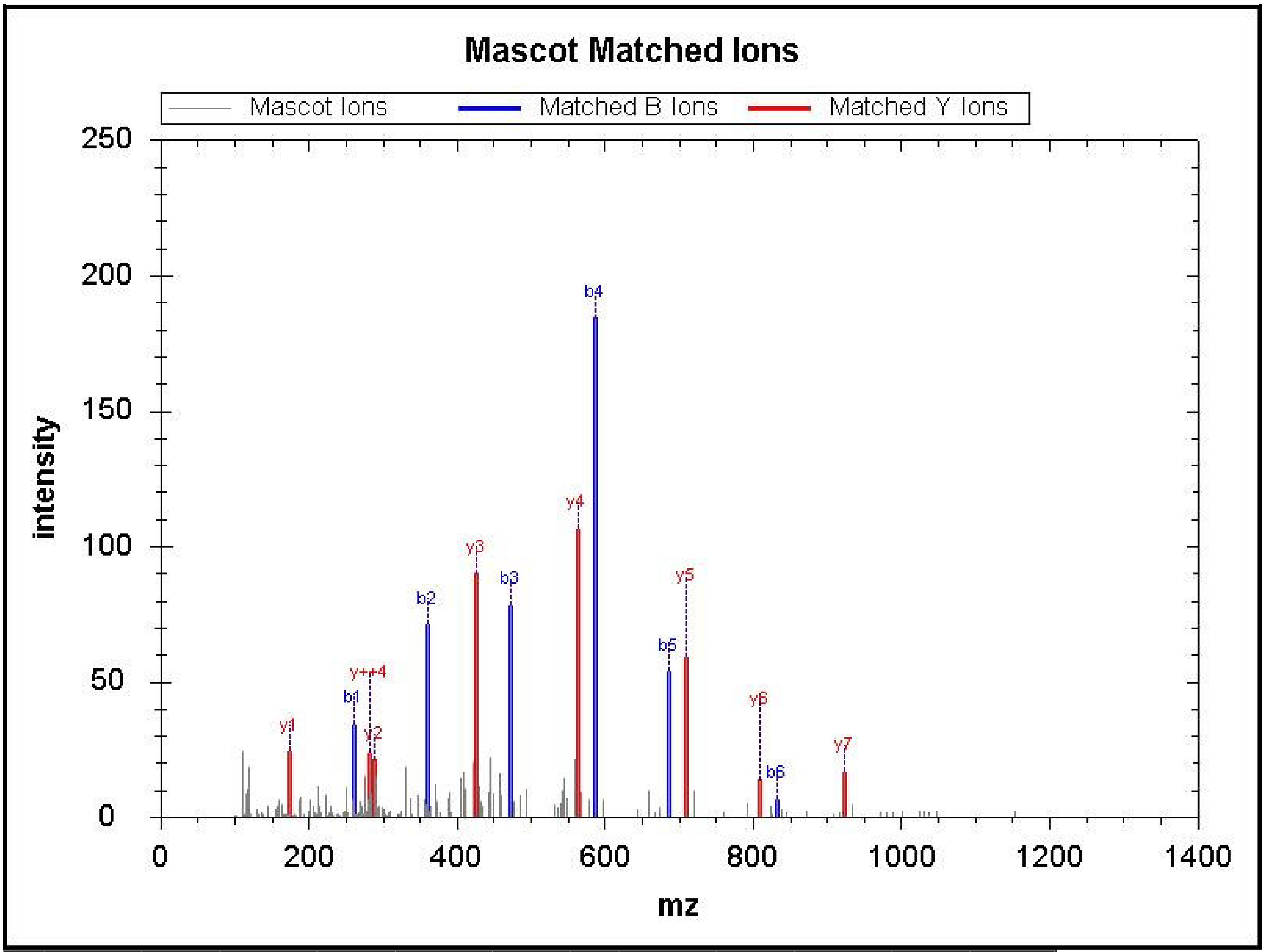

No	b	b++	b*	b*++	b0	b0++	Seq	y	y++	y*	y*++	RevNo
1	260.14	130.57			242.13	121.57	D					10
2	359.20	180.11			341.19	171.10	V	1,134.65	567.83	1,117.63	559.32	9
3	472.29	236.65			454.28	227.64	I	1,035.58	518.30	1,018.56	509.78	8
4	586.33	293.67	569.31	285.16	568.32	284.66	N	922.50	461.75	905.47	453.24	7
5	685.40	343.20	668.37	334.69	667.39	334.20	V	808.46	404.73	791.43	396.22	6
6	832.47	416.74	815.44	408.22	814.46	407.73	F	709.39	355.20	692.36	346.68	5
7	969.53	485.27	952.50	476.75	951.52	476.26	H	562.32	281.66	545.29	273.15	4
8	1,106.59	553.80	1,089.56	545.28	1,088.58	544.79	H	425.26	213.13	408.24	204.62	3

9	1,219.67	610.34	1,202.64	601.83	1,201.66	601.33	L	288.20	144.61	271.18	136.09	2
10							R	175.12	88.06	158.09	79.55	1

Query 38173 Hit 1

MS/MS Fragmentation of KEDFSEATLK
Found in sp|Q53FA7|QORX_HUMAN, Quinone oxidoreductase PIG3 OS=Homo sapiens GN=TP53I3 PE=1 SV=2
Match to Query 38173: 1598.893from(533.9717,3+)
Title: 264: Sum of 2 scans in range 732 (rt=25.6894, f=4, i=171) to 733 (rt=25.7148, f=4, i=172)
[D:\lab212\membrane\Grace\Joyce\iTRAQ_48_2.raw]
Data File:Submitted from 20120508-1(merge) by Mascot Daemon on JOYCE-VAIO
Monoisotopic mass of neutral peptide Mr(calc): 1598.893
Variable modifications:
K1 :iTRAQ4plex (K)
K10 :iTRAQ4plex (K)
Ions Score: 46.98 Expect: 0.014

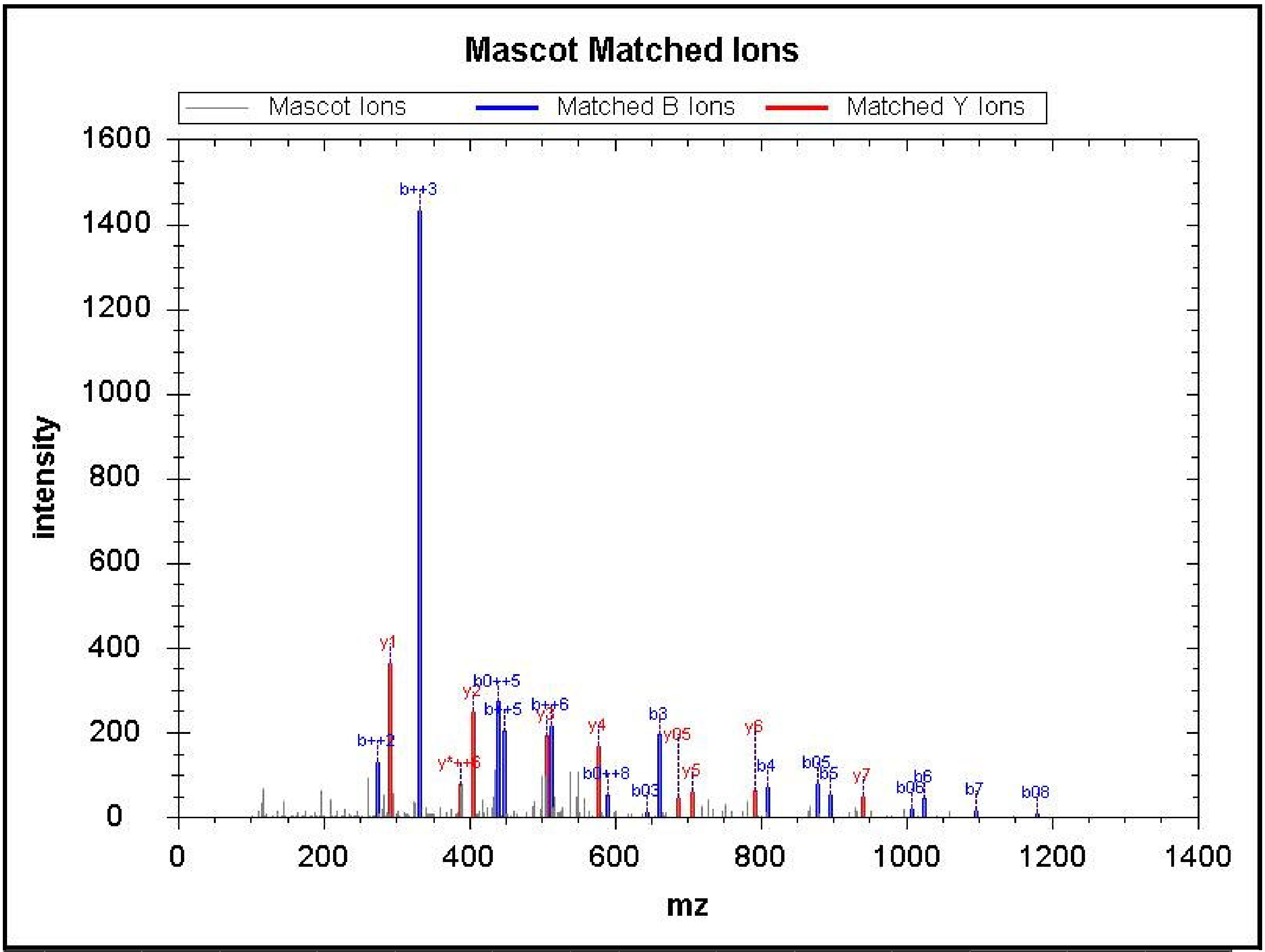

No	b	b++	b*	b*++	b0	b0++	Seq	y	y++	y*	y*++	y0	y0++	RevNo
1	417.31	209.16	400.28	200.64			K							10
2	546.35	273.68	529.32	265.16	528.34	264.67	E	1,183.60	592.30	1,166.57	583.79	1,165.59	583.30	9
3	661.38	331.19	644.35	322.68	643.37	322.19	D	1,054.55	527.78	1,037.53	519.27	1,036.54	518.78	8
4	808.44	404.73	791.42	396.21	790.43	395.72	F	939.53	470.27	922.50	461.75	921.52	461.26	7
5	895.48	448.24	878.45	439.73	877.47	439.24	S	792.46	396.73	775.43	388.22	774.45	387.73	6
6	1,024.52	512.76	1,007.49	504.25	1,006.51	503.76	E	705.43	353.22	688.40	344.70	687.42	344.21	5
7	1,095.56	548.28	1,078.53	539.77	1,077.55	539.28	A	576.38	288.70	559.36	280.18	558.37	279.69	4
8	1,196.60	598.81	1,179.58	590.29	1,178.59	589.80	T	505.35	253.18	488.32	244.66	487.34	244.17	3
9	1,309.69	655.35	1,292.66	646.83	1,291.68	646.34	L	404.30	202.65	387.27	194.14			2
10							K	291.21	146.11	274.19	137.60			1

Query 66530 Hit 1

MS/MS Fragmentation of TWITNSPMADLFVVWAR
Found in sp|Q92947|GCDH_HUMAN, Glutaryl-CoA dehydrogenase
Match to Query 66530: 2150.119from(717.7136,3+)
Title: 1266: Sum of 2 scans in range 2957 (rt=75.5663, f=4, i=826) to 2958 (rt=75.5917, f=4, i=827)
[D:\lab212\membrane\Grace\Joyce\20120320_iTRAQ_20-1.raw]
Data File:Submitted from 20120508-1(merge) by Mascot Daemon on JOYCE-VAIO
Monoisotopic mass of neutral peptide Mr(calc): 2150.119
Variable modifications:
Ions Score: 46.83 Expect: 0.019

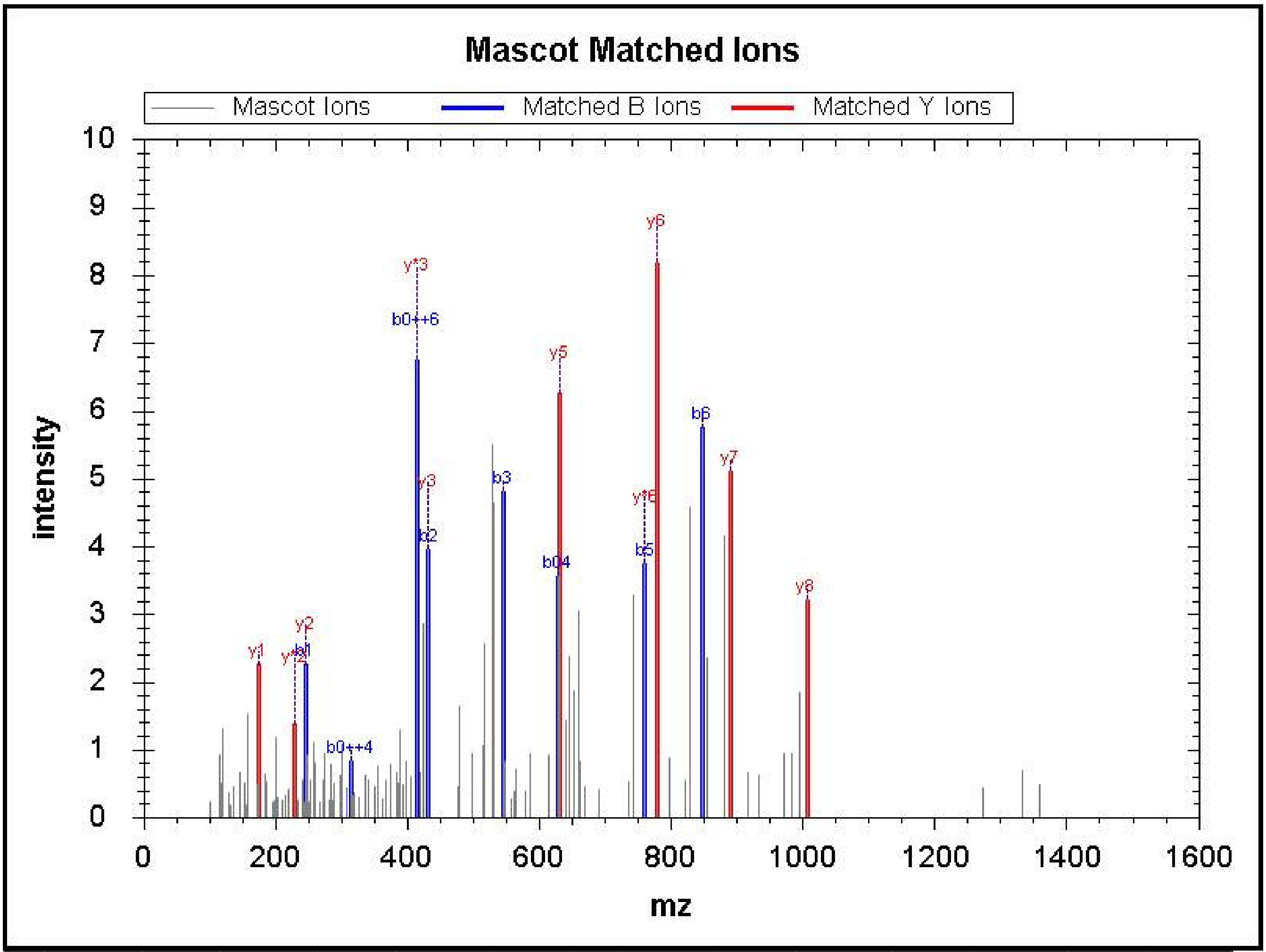

No	b	b++	b*	b*++	b0	b0++	Seq	y	y++	y*	y*++	y0	y0++	RevNo
1	246.16	123.58			228.15	114.58	T							17
2	432.24	216.62			414.23	207.62	W	1,905.97	953.49	1,888.94	944.97	1,887.96	944.48	16
3	545.32	273.16			527.31	264.16	I	1,719.89	860.45	1,702.86	851.93	1,701.88	851.44	15
4	646.37	323.69			628.36	314.68	T	1,606.80	803.91	1,589.78	795.39	1,588.79	794.90	14
5	760.41	380.71	743.38	372.20	742.40	371.70	N	1,505.76	753.38	1,488.73	744.87	1,487.75	744.38	13
6	847.44	424.23	830.42	415.71	829.43	415.22	S	1,391.71	696.36	1,374.69	687.85	1,373.70	687.36	12
7	944.50	472.75	927.47	464.24	926.49	463.75	P	1,304.68	652.84	1,287.66	644.33	1,286.67	643.84	11
8	1,075.54	538.27	1,058.51	529.76	1,057.53	529.27	M	1,207.63	604.32	1,190.60	595.81	1,189.62	595.31	10
9	1,146.57	573.79	1,129.55	565.28	1,128.56	564.79	A	1,076.59	538.80	1,059.56	530.28	1,058.58	529.79	9
10	1,261.60	631.30	1,244.57	622.79	1,243.59	622.30	D	1,005.55	503.28	988.53	494.77	987.54	494.27	8
11	1,374.68	687.85	1,357.66	679.33	1,356.67	678.84	L	890.52	445.77	873.50	437.25			7
12	1,521.75	761.38	1,504.73	752.87	1,503.74	752.37	F	777.44	389.22	760.41	380.71			6
13	1,620.82	810.91	1,603.79	802.40	1,602.81	801.91	V	630.37	315.69	613.35	307.18			5
14	1,719.89	860.45	1,702.86	851.94	1,701.88	851.44	V	531.30	266.16	514.28	257.64			4

15	1,905.97	953.49	1,888.94	944.97	1,887.96	944.48	W	432.24	216.62	415.21	208.11			3
16	1,977.01	989.01	1,959.98	980.49	1,959.00	980.00	A	246.16	123.58	229.13	115.07			2
17							R	175.12	88.06	158.09	79.55			1

Query 22644 Hit 1

MS/MS Fragmentation of DTDSSVASEVR
Found in sp|Q86X29|LSR_HUMAN, Lipolysis-stimulated lipoprotein receptor OS=Homo sapiens GN=LSR PE=1 SV=4
Match to Query 22644: 1308.619from(655.3168,2+)
Title: 107: Scan 450 (rt=19.2209, f=3, i=56) [D:\lab212\membrane\Grace\Joyce\iTRAQ_22_1.raw]
Data File:Submitted from 20120508-1(merge) by Mascot Daemon on JOYCE-VAIO
Monoisotopic mass of neutral peptide Mr(calc): 1308.619
Variable modifications:
Ions Score: 46.75 Expect: 0.013

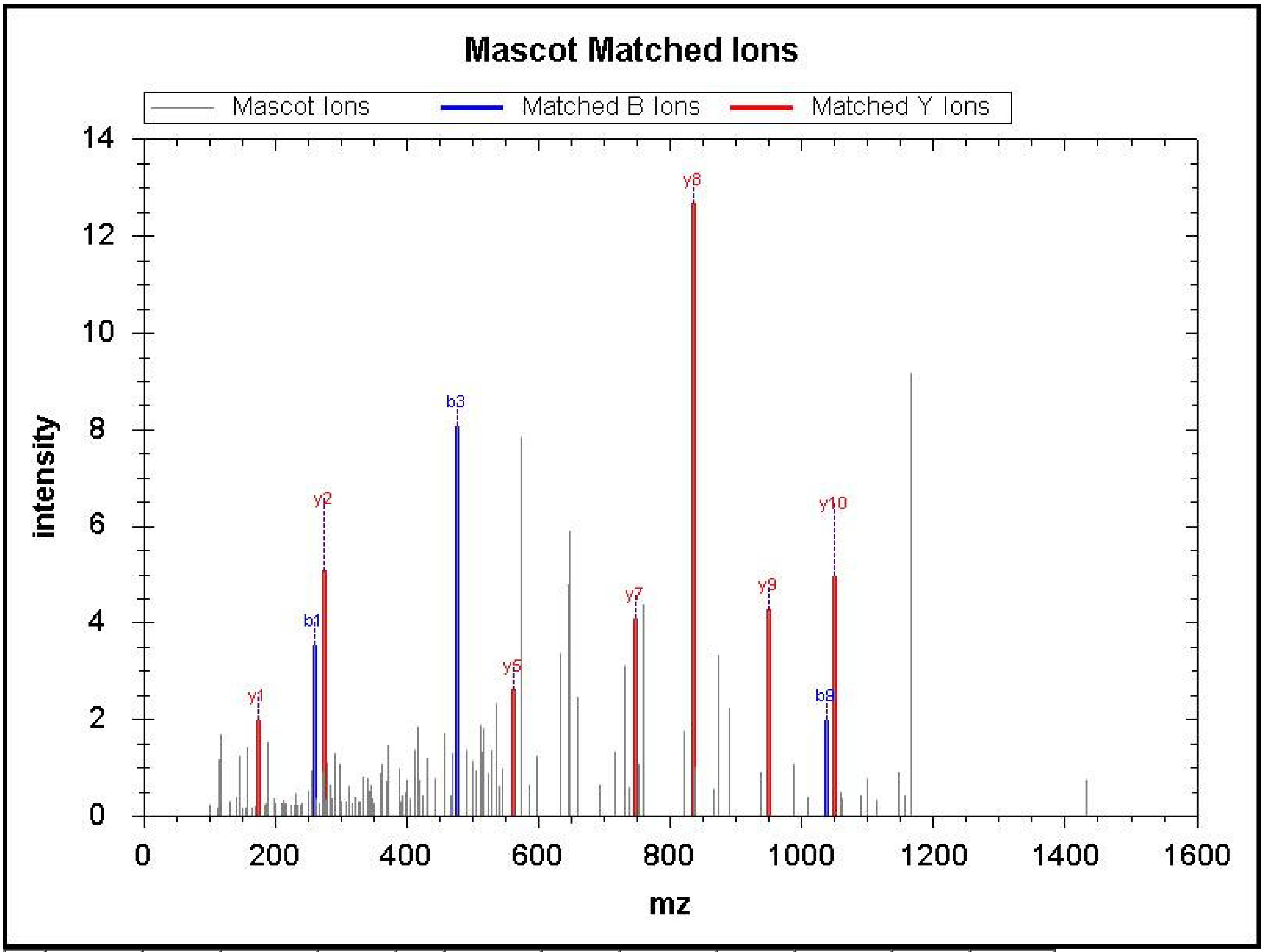

No	b	b++	b0	b0++	Seq	y	y++	y*	y*++	y0	y0++	RevNo
1	260.14	130.57	242.13	121.57	D							11
2	361.18	181.10	343.17	172.09	T	1,050.51	525.76	1,033.48	517.24	1,032.50	516.75	10
3	476.21	238.61	458.20	229.60	D	949.46	475.23	932.43	466.72	931.45	466.23	9
4	563.24	282.13	545.23	273.12	S	834.43	417.72	817.41	409.21	816.42	408.71	8
5	650.27	325.64	632.26	316.64	S	747.40	374.20	730.37	365.69	729.39	365.20	7
6	749.34	375.18	731.33	366.17	V	660.37	330.69	643.34	322.17	642.36	321.68	6
7	820.38	410.69	802.37	401.69	A	561.30	281.15	544.27	272.64	543.29	272.15	5
8	907.41	454.21	889.40	445.20	S	490.26	245.63	473.24	237.12	472.25	236.63	4
9	1,036.46	518.73	1,018.44	509.73	E	403.23	202.12	386.20	193.61	385.22	193.11	3
10	1,135.52	568.27	1,117.51	559.26	V	274.19	137.60	257.16	129.08			2
11					R	175.12	88.06	158.09	79.55			1

Query 52734 Hit 1

MS/MS Fragmentation of DLLVEGLAEILHQK
Found in sp|Q9Y3R5|DOP2_HUMAN, Protein dopey-2 OS=Homo sapiens GN=DOPEY2 PE=1 SV=5
Match to Query 52734: 1865.085from(622.7023,3+)
Title: 1103: Sum of 2 scans in range 2417 (rt=63.8596, f=3, i=378) to 2418 (rt=63.885, f=3, i=379)
[D:\lab212\membrane\Grace\Joyce\iTRAQ_37_1.raw]
Data File:Submitted from 20120508-1(merge) by Mascot Daemon on JOYCE-VAIO
Monoisotopic mass of neutral peptide Mr(calc): 1865.085
Variable modifications:
K14 :iTRAQ4plex (K)
Ions Score: 46.74 Expect: 0.010

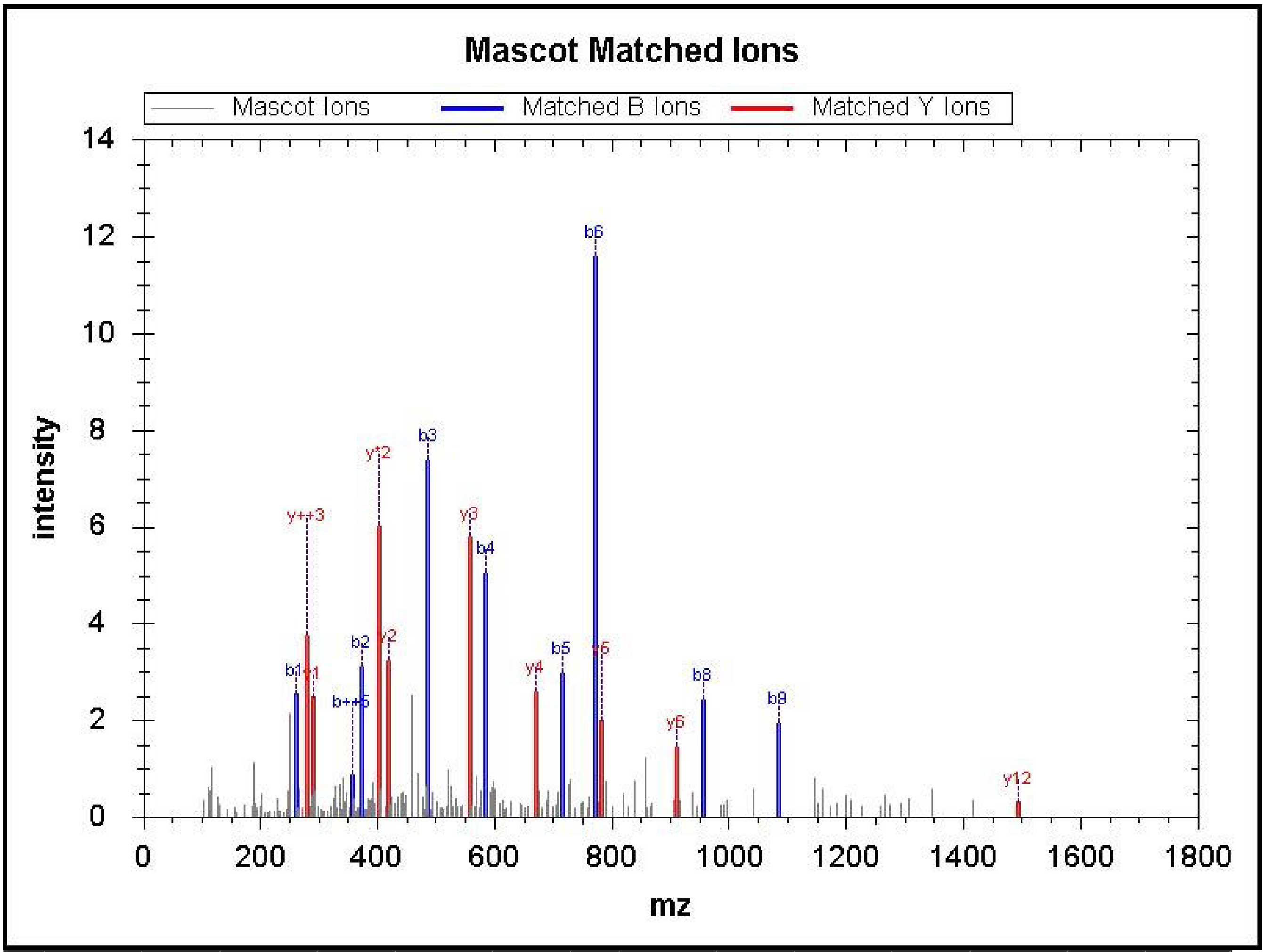

No	b	b++	b*	b*++	b0	b0++	Seq	y	y++	y*	y*++	y0	y0++	RevNo
1	260.14	130.57			242.13	121.57	D							14
2	373.22	187.11			355.21	178.11	L	1,606.96	803.99	1,589.94	795.47	1,588.95	794.98	13
3	486.30	243.66			468.29	234.65	L	1,493.88	747.44	1,476.85	738.93	1,475.87	738.44	12
4	585.37	293.19			567.36	284.18	V	1,380.80	690.90	1,363.77	682.39	1,362.79	681.90	11
5	714.42	357.71			696.40	348.71	E	1,281.73	641.37	1,264.70	632.85	1,263.72	632.36	10
6	771.44	386.22			753.43	377.22	G	1,152.69	576.85	1,135.66	568.33	1,134.68	567.84	9
7	884.52	442.76			866.51	433.76	L	1,095.66	548.34	1,078.64	539.82	1,077.65	539.33	8
8	955.56	478.28			937.55	469.28	A	982.58	491.79	965.55	483.28	964.57	482.79	7
9	1,084.60	542.80			1,066.59	533.80	E	911.54	456.28	894.52	447.76	893.53	447.27	6
10	1,197.68	599.35			1,179.67	590.34	I	782.50	391.75	765.47	383.24			5
11	1,310.77	655.89			1,292.76	646.88	L	669.42	335.21	652.39	326.70			4
12	1,447.83	724.42			1,429.82	715.41	H	556.33	278.67	539.31	270.16			3
13	1,575.89	788.45	1,558.86	779.93	1,557.88	779.44	Q	419.27	210.14	402.25	201.63			2

14							K	291.21	146.11	274.19	137.60			1

Query 43479 Hit 1

MS/MS Fragmentation of AALEAQNALHNMK
Found in sp|Q92879|CELF1_HUMAN, CUGBP Elav-like family member 1 OS=Homo sapiens GN=CELF1 PE=1 SV=2
Match to Query 43479: 1697.91from(566.9772,3+)
Title: 416: Scan 953 (rt=31.2394, f=3, i=148) [D:\lab212\membrane\Grace\Joyce\iTRAQ_42_2.raw]
Data File:Submitted from 20120508-1(merge) by Mascot Daemon on JOYCE-VAIO
Monoisotopic mass of neutral peptide Mr(calc): 1697.91
Variable modifications:
K13 :iTRAQ4plex (K)
Ions Score: 46.7 Expect: 0.017

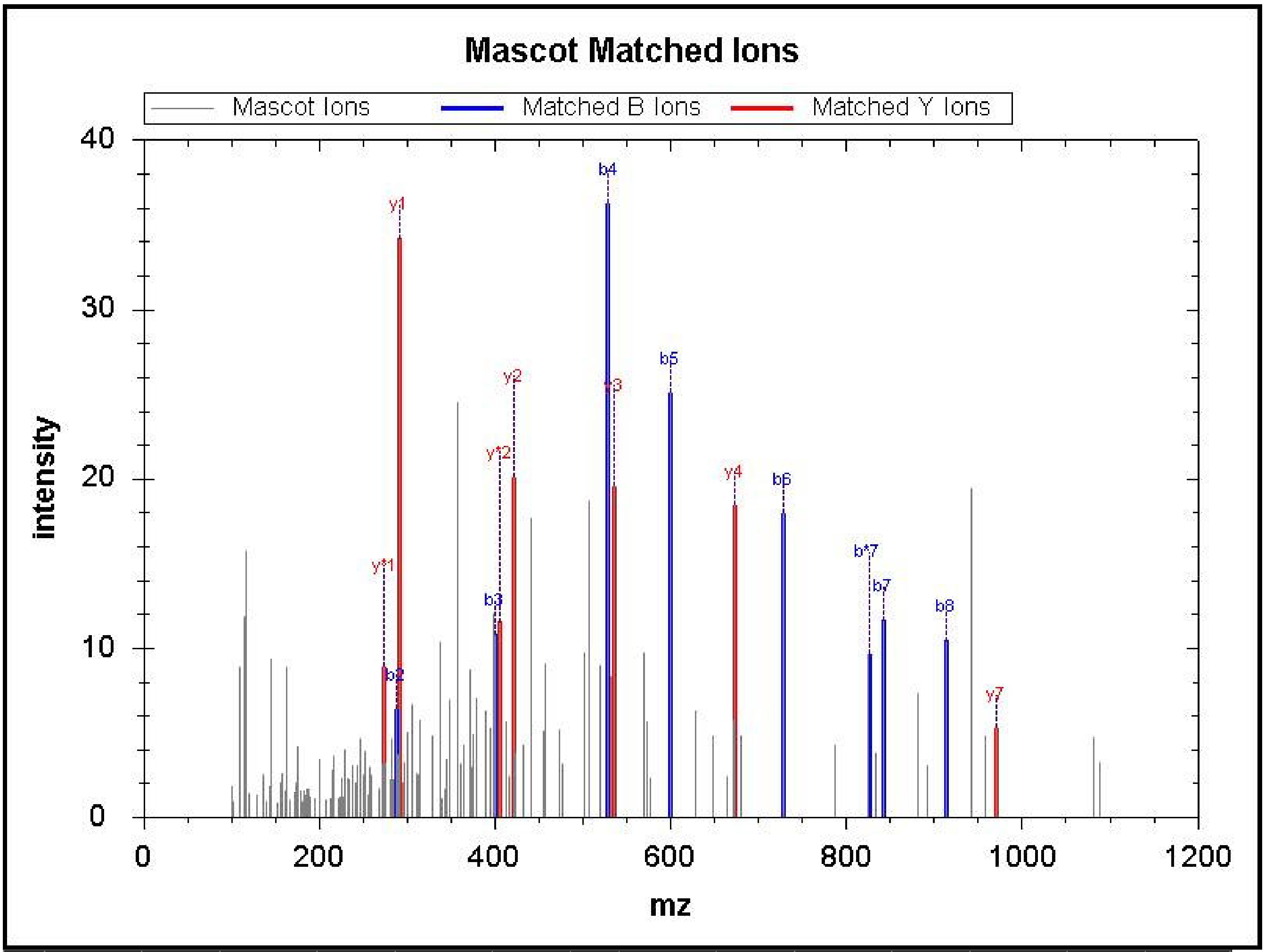

No	b	b++	b*	b*++	b0	b0++	Seq	y	y++	y*	y*++	y0	y0++	RevNo
1	216.15	108.58					A							13
2	287.18	144.10					A	1,483.78	742.39	1,466.75	733.88	1,465.77	733.39	12
3	400.27	200.64					L	1,412.74	706.88	1,395.72	698.36	1,394.73	697.87	11
4	529.31	265.16			511.30	256.15	E	1,299.66	650.33	1,282.63	641.82	1,281.65	641.33	10
5	600.35	300.68			582.34	291.67	A	1,170.62	585.81	1,153.59	577.30			9
6	728.41	364.71	711.38	356.19	710.40	355.70	Q	1,099.58	550.29	1,082.55	541.78			8
7	842.45	421.73	825.42	413.21	824.44	412.72	N	971.52	486.26	954.49	477.75			7
8	913.49	457.25	896.46	448.73	895.48	448.24	A	857.48	429.24	840.45	420.73			6
9	1,026.57	513.79	1,009.54	505.28	1,008.56	504.78	L	786.44	393.72	769.41	385.21			5
10	1,163.63	582.32	1,146.60	573.80	1,145.62	573.31	H	673.36	337.18	656.33	328.67			4
11	1,277.67	639.34	1,260.65	630.83	1,259.66	630.33	N	536.30	268.65	519.27	260.14			3
12	1,408.71	704.86	1,391.69	696.35	1,390.70	695.85	M	422.26	211.63	405.23	203.12			2
13							K	291.21	146.11	274.19	137.60			1

Query 33779 Hit 1

MS/MS Fragmentation of QVPDTSVQETDR
Found in sp|Q5J8M3|TMM85_HUMAN, Transmembrane protein 85 OS=Homo sapiens GN=TMEM85 PE=1 SV=2
Match to Query 33779: 1517.739from(759.8768,2+)
Title: 86: Sum of 2 scans in range 494 (rt=19.5161, f=4, i=41) to 495 (rt=19.5415, f=4, i=42)
[D:\lab212\membrane\Grace\Joyce\iTRAQ_23_2.raw]
Data File:Submitted from 20120508-1(merge) by Mascot Daemon on JOYCE-VAIO
Monoisotopic mass of neutral peptide Mr(calc): 1517.739
Variable modifications:
Ions Score: 46.57 Expect: 0.016

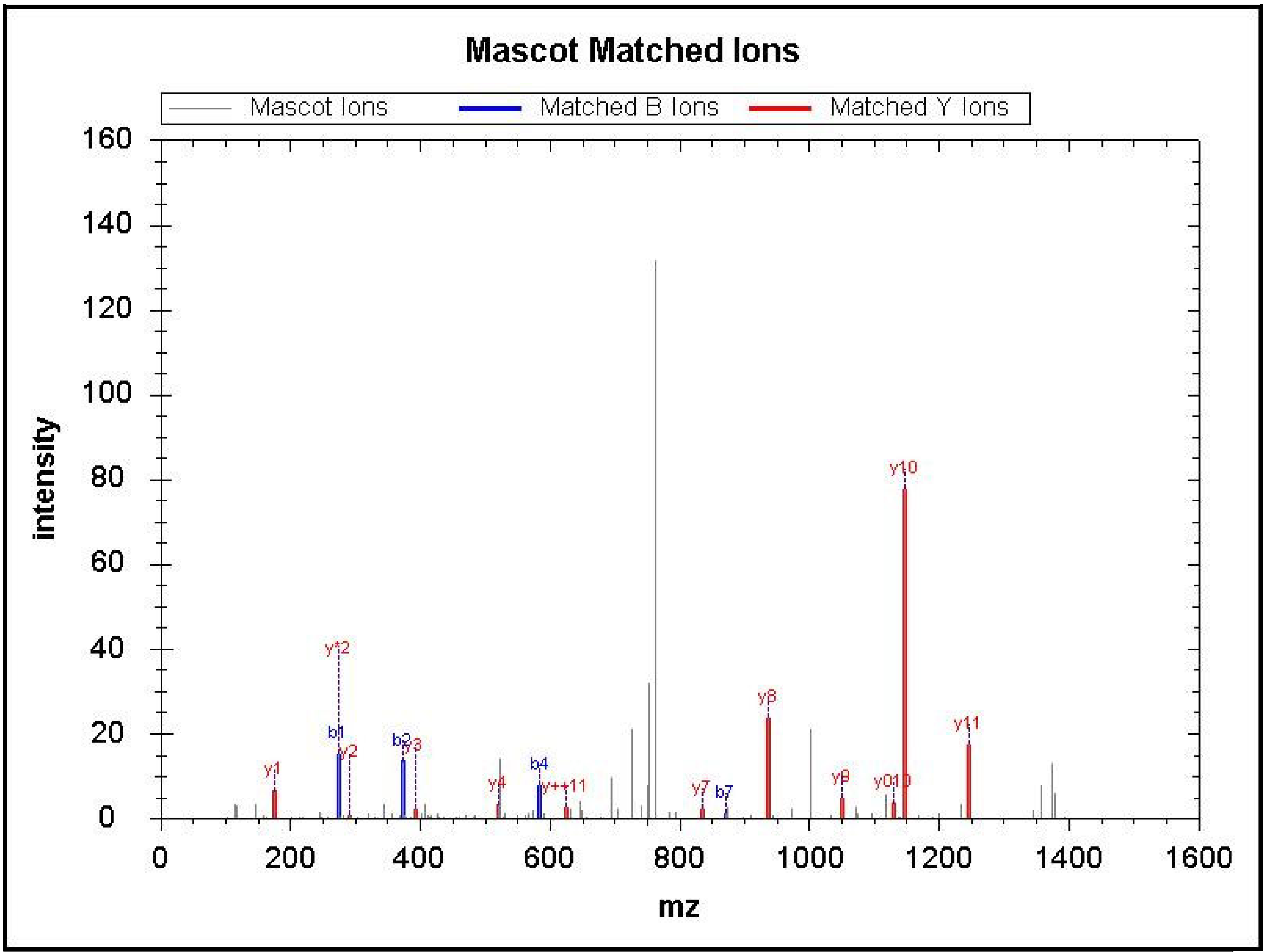

No	b	b++	b*	b*++	b0	b0++	Seq	y	y++	y*	y*++	y0	y0++	RevNo
1	273.17	137.09	256.14	128.57			Q							12
2	372.24	186.62	355.21	178.11			V	1,246.59	623.80	1,229.56	615.29	1,228.58	614.79	11
3	469.29	235.15	452.26	226.63			P	1,147.52	574.26	1,130.50	565.75	1,129.51	565.26	10
4	584.32	292.66	567.29	284.15	566.31	283.66	D	1,050.47	525.74	1,033.44	517.23	1,032.46	516.73	9
5	685.36	343.19	668.34	334.67	667.35	334.18	T	935.44	468.23	918.42	459.71	917.43	459.22	8
6	772.40	386.70	755.37	378.19	754.39	377.70	S	834.40	417.70	817.37	409.19	816.38	408.70	7
7	871.46	436.24	854.44	427.72	853.45	427.23	V	747.36	374.19	730.34	365.67	729.35	365.18	6
8	999.52	500.27	982.50	491.75	981.51	491.26	Q	648.29	324.65	631.27	316.14	630.28	315.65	5
9	1,128.57	564.79	1,111.54	556.27	1,110.55	555.78	E	520.24	260.62	503.21	252.11	502.23	251.62	4
10	1,229.61	615.31	1,212.59	606.80	1,211.60	606.30	T	391.19	196.10	374.17	187.59	373.18	187.10	3
11	1,344.64	672.82	1,327.61	664.31	1,326.63	663.82	D	290.15	145.58	273.12	137.06	272.14	136.57	2
12							R	175.12	88.06	158.09	79.55			1

Query 82374 Hit 1

MS/MS Fragmentation of DALHLLVFTTDDVPHIALDGK

Found in sp|P18084|ITB5_HUMAN, Integrin beta-5 OS=Homo sapiens GN=ITGB5 PE=1 SV=1
Match to Query 82374: 2577.407from(645.359,4+)
Title: 1001: Scan 2187 (rt=58.8698, f=3, i=340) [D:\lab212\membrane\Grace\Joyce\iTRAQ_44_2.raw]
Data File:Submitted from 20120508-1(merge) by Mascot Daemon on JOYCE-VAIO
Monoisotopic mass of neutral peptide Mr(calc): 2577.407
Variable modifications:
K21 :iTRAQ4plex (K)
Ions Score: 46.55 Expect: 0.014

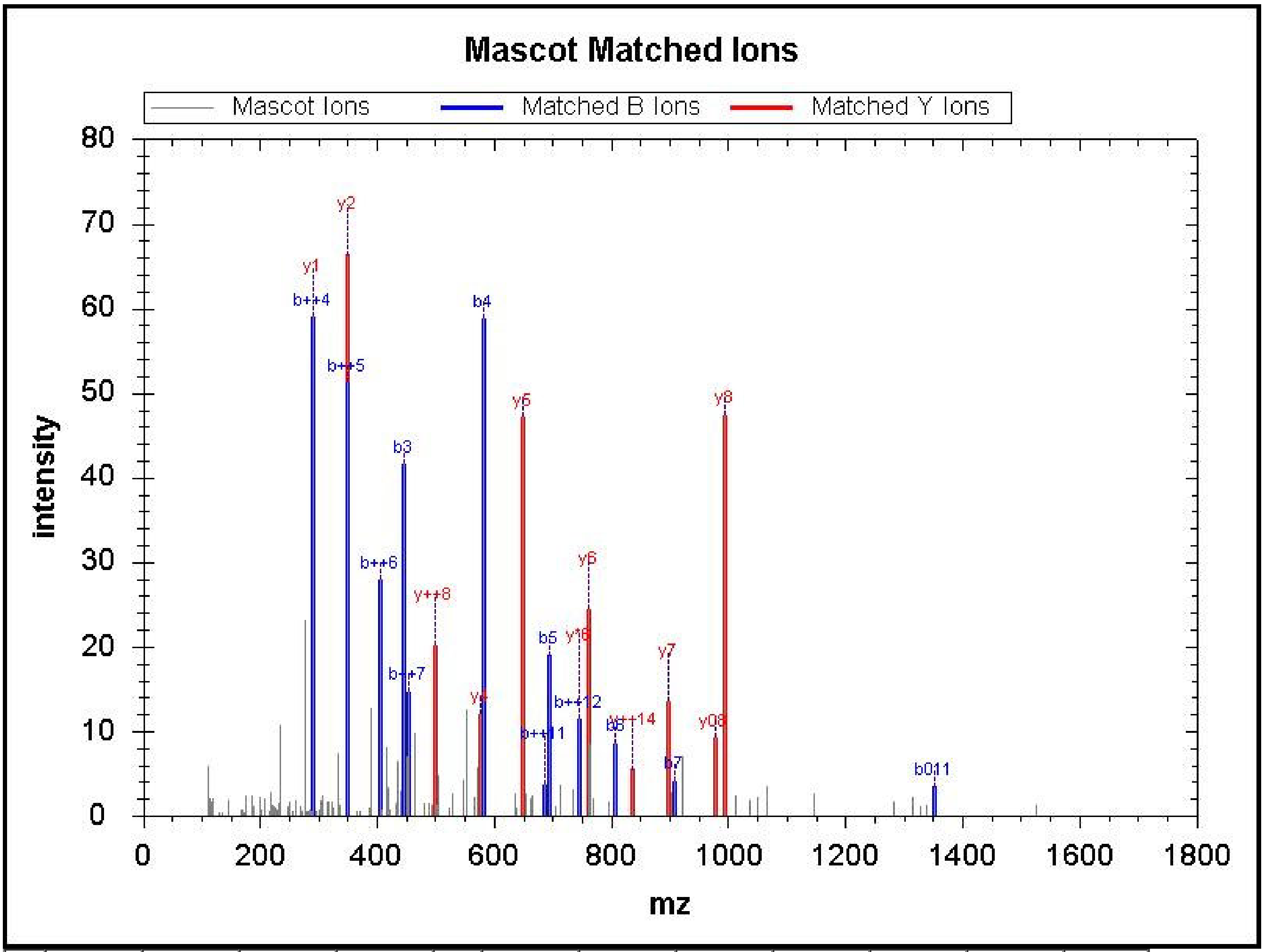

No	b	b++	b0	b0++	Seq	y	y++	y*	y*++	y0	y0++	RevNo
1	260.14	130.57	242.13	121.57	D							21
2	331.17	166.09	313.16	157.09	A	2,319.28	1,160.15	2,302.26	1,151.63	2,301.27	1,151.14	20
3	444.26	222.63	426.25	213.63	L	2,248.25	1,124.63	2,231.22	1,116.11	2,230.24	1,115.62	19
4	581.32	291.16	563.31	282.16	H	2,135.16	1,068.08	2,118.14	1,059.57	2,117.15	1,059.08	18
5	694.40	347.70	676.39	338.70	L	1,998.10	999.56	1,981.08	991.04	1,980.09	990.55	17
6	807.48	404.25	789.47	395.24	L	1,885.02	943.01	1,867.99	934.50	1,867.01	934.01	16
7	906.55	453.78	888.54	444.77	V	1,771.93	886.47	1,754.91	877.96	1,753.92	877.47	15
8	1,053.62	527.31	1,035.61	518.31	F	1,672.87	836.94	1,655.84	828.42	1,654.86	827.93	14
9	1,154.67	577.84	1,136.66	568.83	T	1,525.80	763.40	1,508.77	754.89	1,507.79	754.40	13
10	1,255.72	628.36	1,237.71	619.36	T	1,424.75	712.88	1,407.72	704.37	1,406.74	703.87	12
11	1,370.74	685.88	1,352.73	676.87	D	1,323.70	662.35	1,306.68	653.84	1,305.69	653.35	11
12	1,485.77	743.39	1,467.76	734.38	D	1,208.68	604.84	1,191.65	596.33	1,190.66	595.84	10
13	1,584.84	792.92	1,566.83	783.92	V	1,093.65	547.33	1,076.62	538.81	1,075.64	538.32	9
14	1,681.89	841.45	1,663.88	832.44	P	994.58	497.79	977.55	489.28	976.57	488.79	8
15	1,818.95	909.98	1,800.94	900.97	H	897.53	449.27	880.50	440.75	879.52	440.26	7
16	1,932.03	966.52	1,914.02	957.52	I	760.47	380.74	743.44	372.22	742.46	371.73	6
17	2,003.07	1,002.04	1,985.06	993.03	A	647.38	324.20	630.36	315.68	629.37	315.19	5

18	2,116.16	1,058.58	2,098.15	1,049.58	L	576.35	288.68	559.32	280.16	558.34	279.67	4
19	2,231.18	1,116.10	2,213.17	1,107.09	D	463.26	232.14	446.24	223.62	445.25	223.13	3
20	2,288.20	1,144.61	2,270.19	1,135.60	G	348.24	174.62	331.21	166.11			2
21					K	291.21	146.11	274.19	137.60			1

Query 70127 Hit 1

MS/MS Fragmentation of ETTGTGPNVYHENDTIAK
Found in sp|Q4G0F5|VP26B_HUMAN, Vacuolar protein sorting-associated protein 26B OS=Homo sapiens GN=VPS26B PE=1 SV=2
Match to Query 70127: 2234.11 from(745.7106,3+)
Title: 226: Scan 601 (rt=22.8682, f=3, i=80) [D:\lab212\membrane\Grace\Joyce\iTRAQ_38_1.raw]
Data File:Submitted from 20120508-1(merge) by Mascot Daemon on JOYCE-VAIO
Monoisotopic mass of neutral peptide Mr(calc): 2234.11
Variable modifications:
K18 :iTRAQ4plex (K)
Ions Score: 46.51 Expect: 0.021

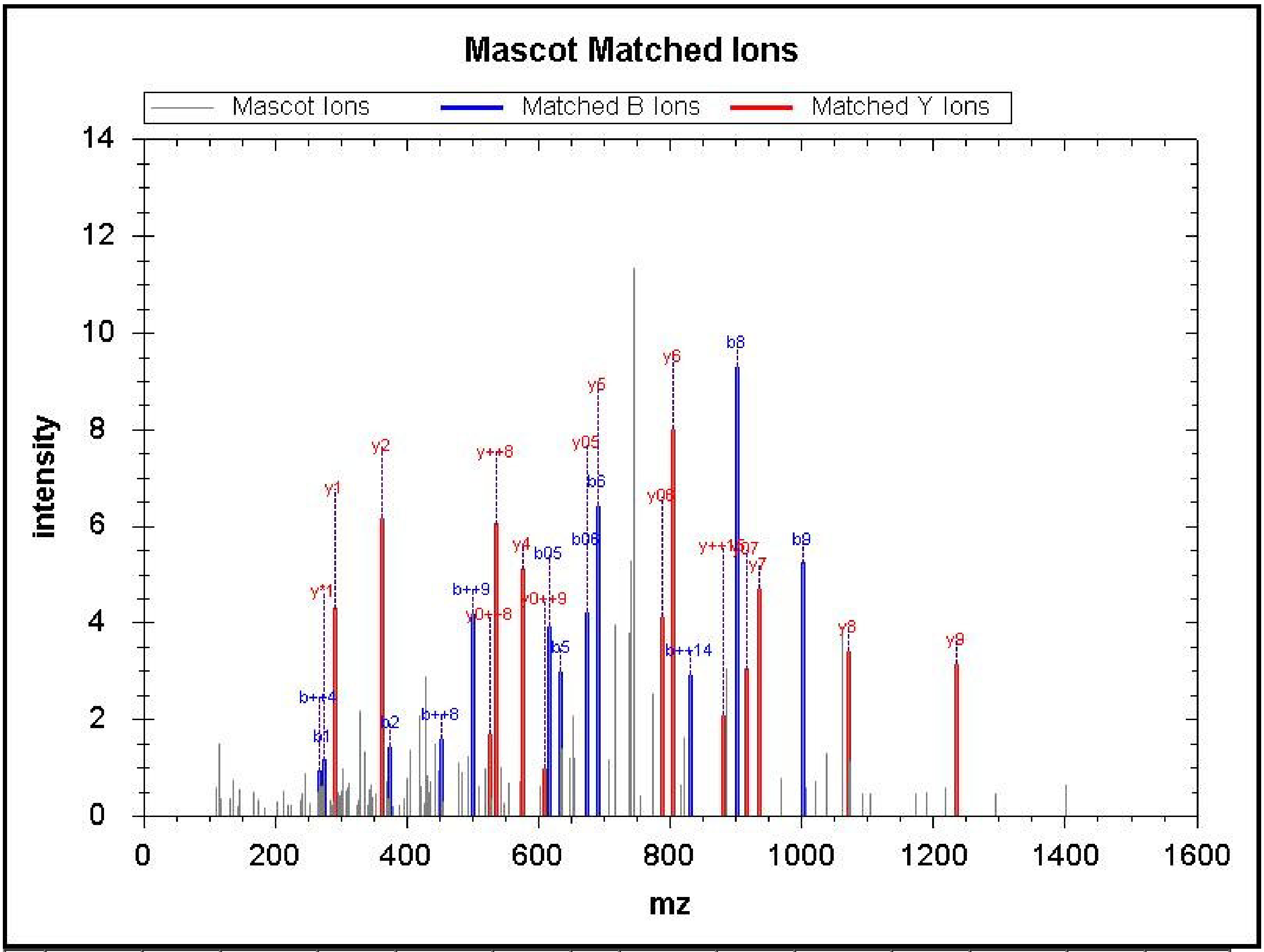

No	b	b++	b*	b*++	b0	b0++	Seq	y	y++	y*	y*++	y0	y0++	RevNo
1	274.15	137.58			256.14	128.57	E							18
2	375.20	188.10			357.19	179.10	T	1,961.97	981.49	1,944.94	972.97	1,943.96	972.48	17
3	476.25	238.63			458.24	229.62	T	1,860.92	930.96	1,843.89	922.45	1,842.91	921.96	16
4	533.27	267.14			515.26	258.13	G	1,759.87	880.44	1,742.85	871.93	1,741.86	871.43	15
5	634.32	317.66			616.31	308.66	T	1,702.85	851.93	1,685.83	843.42	1,684.84	842.92	14
6	691.34	346.17			673.33	337.17	G	1,601.80	801.41	1,584.78	792.89	1,583.79	792.40	13
7	788.39	394.70			770.38	385.69	P	1,544.78	772.89	1,527.76	764.38	1,526.77	763.89	12
8	902.43	451.72	885.41	443.21	884.42	442.72	N	1,447.73	724.37	1,430.70	715.86	1,429.72	715.36	11
9	1,001.50	501.25	984.48	492.74	983.49	492.25	V	1,333.69	667.35	1,316.66	658.83	1,315.68	658.34	10
10	1,164.57	582.79	1,147.54	574.27	1,146.55	573.78	Y	1,234.62	617.81	1,217.59	609.30	1,216.61	608.81	9

11	1,301.62	651.32	1,284.60	642.80	1,283.61	642.31	H	1,071.56	536.28	1,054.53	527.77	1,053.54	527.28	8
12	1,430.67	715.84	1,413.64	707.32	1,412.66	706.83	E	934.50	467.75	917.47	459.24	916.49	458.75	7
13	1,544.71	772.86	1,527.68	764.35	1,526.70	763.85	N	805.45	403.23	788.43	394.72	787.44	394.23	6
14	1,659.74	830.37	1,642.71	821.86	1,641.73	821.37	D	691.41	346.21	674.38	337.70	673.40	337.20	5
15	1,760.78	880.90	1,743.76	872.38	1,742.77	871.89	T	576.38	288.70	559.36	280.18	558.37	279.69	4
16	1,873.87	937.44	1,856.84	928.92	1,855.86	928.43	I	475.34	238.17	458.31	229.66			3
17	1,944.91	972.96	1,927.88	964.44	1,926.90	963.95	A	362.25	181.63	345.23	173.12			2
18							K	291.21	146.11	274.19	137.60			1

Query 70293 Hit 1

MS/MS Fragmentation of ITELRPFNSWEALFTK
Found in sp|Q9H4L7|SMRCD_HUMAN, SWI/SNF-related matrix-associated actin-dependent regulator of chromatin subfamily A containing DEAD/H box 1 OS=Homo sapiens GN=SMARCAD1 PE=1 SV=2
Match to Query 70293: 2239.225from(747.4157,3+)
Title: 1049: Scan 2291 (rt=61.2141, f=3, i=360) [D:\lab212\membrane\Grace\Joyce\iTRAQ_39_2.raw]
Data File:Submitted from 20120508-1(merge) by Mascot Daemon on JOYCE-VAIO
Monoisotopic mass of neutral peptide Mr(calc): 2239.225
Variable modifications:
K16 :iTRAQ4plex (K)
Ions Score: 46.47 Expect: 0.016

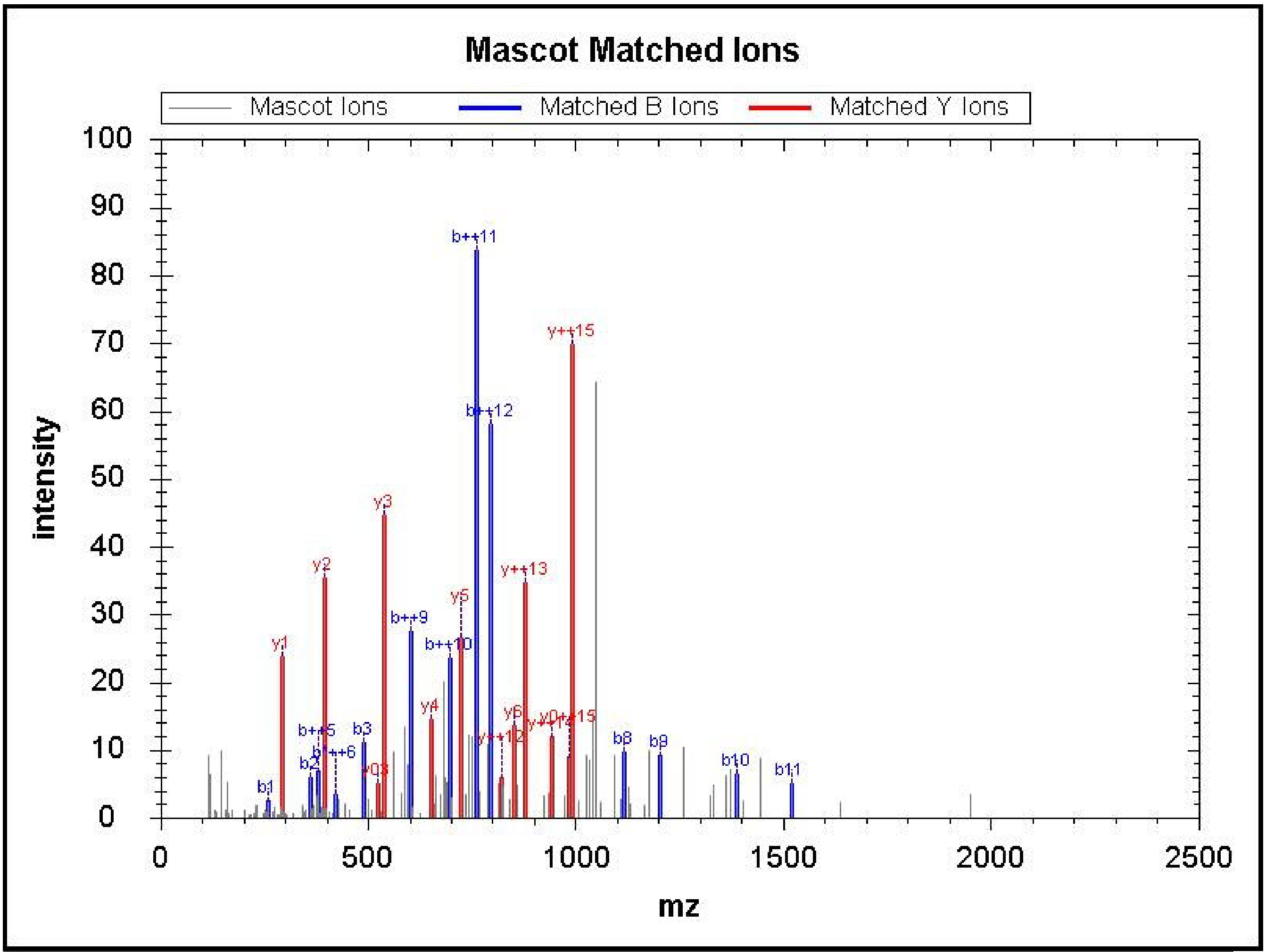

No	b	b++	b*	b*++	b0	b0++	Seq	y	y++	y*	y*++	y0	y0++	RevNo
1	258.19	129.60					I							16
2	359.24	180.12			341.23	171.12	T	1,983.05	992.03	1,966.02	983.51	1,965.03	983.02	15
3	488.28	244.65			470.27	235.64	E	1,882.00	941.50	1,864.97	932.99	1,863.99	932.50	14
4	601.37	301.19			583.36	292.18	L	1,752.96	876.98	1,735.93	868.47	1,734.94	867.98	13
5	757.47	379.24	740.44	370.72	739.46	370.23	R	1,639.87	820.44	1,622.84	811.93	1,621.86	811.43	12

6	854.52	427.76	837.49	419.25	836.51	418.76	P	1,483.77	742.39	1,466.74	733.88	1,465.76	733.38	11
7	1,001.59	501.30	984.56	492.79	983.58	492.29	F	1,386.72	693.86	1,369.69	685.35	1,368.71	684.86	10
8	1,115.63	558.32	1,098.61	549.81	1,097.62	549.31	N	1,239.65	620.33	1,222.62	611.81	1,221.64	611.32	9
9	1,202.66	601.84	1,185.64	593.32	1,184.65	592.83	S	1,125.61	563.31	1,108.58	554.79	1,107.60	554.30	8
10	1,388.74	694.88	1,371.72	686.36	1,370.73	685.87	W	1,038.57	519.79	1,021.55	511.28	1,020.56	510.79	7
11	1,517.79	759.40	1,500.76	750.88	1,499.78	750.39	E	852.49	426.75	835.47	418.24	834.48	417.75	6
12	1,588.82	794.92	1,571.80	786.40	1,570.81	785.91	A	723.45	362.23	706.43	353.72	705.44	353.22	5
13	1,701.91	851.46	1,684.88	842.94	1,683.90	842.45	L	652.41	326.71	635.39	318.20	634.40	317.71	4
14	1,848.98	924.99	1,831.95	916.48	1,830.97	915.99	F	539.33	270.17	522.30	261.66	521.32	261.16	3
15	1,950.02	975.52	1,933.00	967.00	1,932.01	966.51	T	392.26	196.63	375.24	188.12	374.25	187.63	2
16							K	291.21	146.11	274.19	137.60			1

Query 74619 Hit 1

MS/MS Fragmentation of DTPEDIVLEAPASGLAFHPAR
Found in sp|Q9H6Y2|WDR55_HUMAN, WD repeat-containing protein 55 OS=Homo sapiens GN=WDR55 PE=1 SV=2
Match to Query 74619: 2349.209 from(784.077,3+)
Title: 762: Sum of 2 scans in range 1769 (rt=49.0159, f=4, i=511) to 1770 (rt=49.0413, f=4, i=512)
[D:\lab212\membrane\Grace\Joyce\iTRAQ_30_1.raw]
Data File:Submitted from 20120508-1(merge) by Mascot Daemon on JOYCE-VAIO
Monoisotopic mass of neutral peptide Mr(calc): 2349.209
Variable modifications:
Ions Score: 46.44 Expect: 0.022

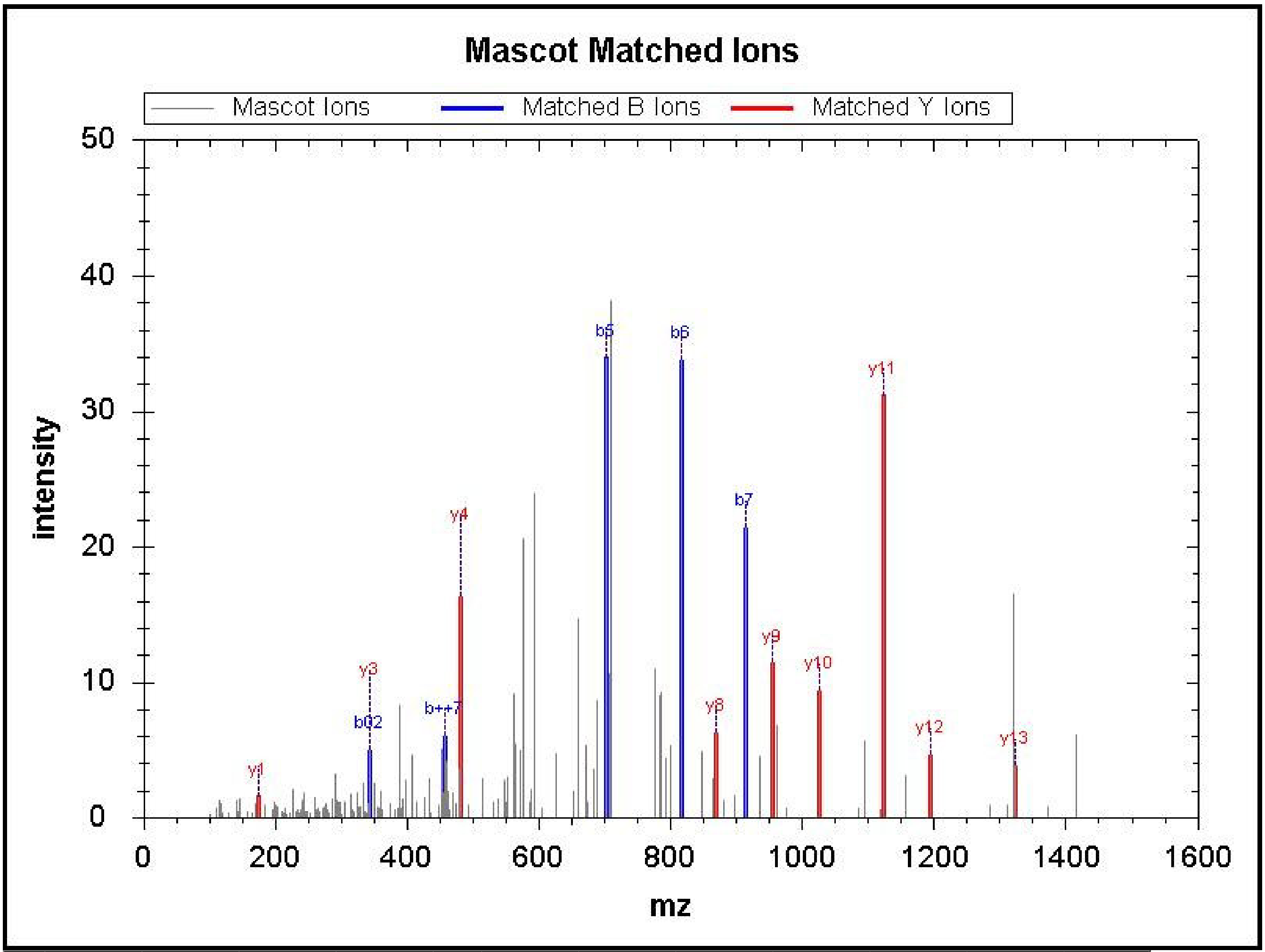

No	b	b++	b0	b0++	Seq	y	y++	y*	y*++	y0	y0++	RevNo
1	260.14	130.57	242.13	121.57	D							21
2	361.18	181.10	343.17	172.09	T	2,091.09	1,046.05	2,074.06	1,037.53	2,073.08	1,037.04	20
3	458.24	229.62	440.23	220.62	P	1,990.04	995.52	1,973.01	987.01	1,972.03	986.52	19

4	587.28	294.14	569.27	285.14	E	1,892.99	947.00	1,875.96	938.48	1,874.98	937.99	18
5	702.31	351.66	684.30	342.65	D	1,763.94	882.48	1,746.92	873.96	1,745.93	873.47	17
6	815.39	408.20	797.38	399.19	I	1,648.92	824.96	1,631.89	816.45	1,630.91	815.96	16
7	914.46	457.73	896.45	448.73	V	1,535.83	768.42	1,518.81	759.91	1,517.82	759.41	15
8	1,027.54	514.28	1,009.53	505.27	L	1,436.76	718.89	1,419.74	710.37	1,418.75	709.88	14
9	1,156.59	578.80	1,138.57	569.79	E	1,323.68	662.34	1,306.65	653.83	1,305.67	653.34	13
10	1,227.62	614.31	1,209.61	605.31	A	1,194.64	597.82	1,177.61	589.31	1,176.63	588.82	12
11	1,324.68	662.84	1,306.66	653.84	P	1,123.60	562.30	1,106.57	553.79	1,105.59	553.30	11
12	1,395.71	698.36	1,377.70	689.35	A	1,026.55	513.78	1,009.52	505.26	1,008.54	504.77	10
13	1,482.74	741.88	1,464.73	732.87	S	955.51	478.26	938.48	469.75	937.50	469.25	9
14	1,539.77	770.39	1,521.76	761.38	G	868.48	434.74	851.45	426.23			8
15	1,652.85	826.93	1,634.84	817.92	L	811.46	406.23	794.43	397.72			7
16	1,723.89	862.45	1,705.88	853.44	A	698.37	349.69	681.35	341.18			6
17	1,870.96	935.98	1,852.94	926.98	F	627.34	314.17	610.31	305.66			5
18	2,008.01	1,004.51	1,990.00	995.51	H	480.27	240.64	463.24	232.12			4
19	2,105.07	1,053.04	2,087.06	1,044.03	P	343.21	172.11	326.18	163.59			3
20	2,176.10	1,088.56	2,158.09	1,079.55	A	246.16	123.58	229.13	115.07			2
21					R	175.12	88.06	158.09	79.55			1

Query 46595 Hit 1

MS/MS Fragmentation of EYQDIEEMIFHR
Found in sp|O70423|AOC3_MOUSE, Membrane primary amine oxidase OS=Mus musculus GN=Aoc3 PE=2 SV=3
Match to Query 46595: 1752.831 from(585.2843,3+)
Title: 839: Sum of 2 scans in range 1866 (rt=51.4549, f=4, i=559) to 1867 (rt=51.4803, f=4, i=560) [D:\lab212\membrane\Grace\Joyce\iTRAQ_36_2.raw]
Data File:Submitted from 20120508-1(merge) by Mascot Daemon on JOYCE-VAIO
Monoisotopic mass of neutral peptide Mr(calc): 1752.831
Variable modifications:
Ions Score: 46.32 Expect: 0.017

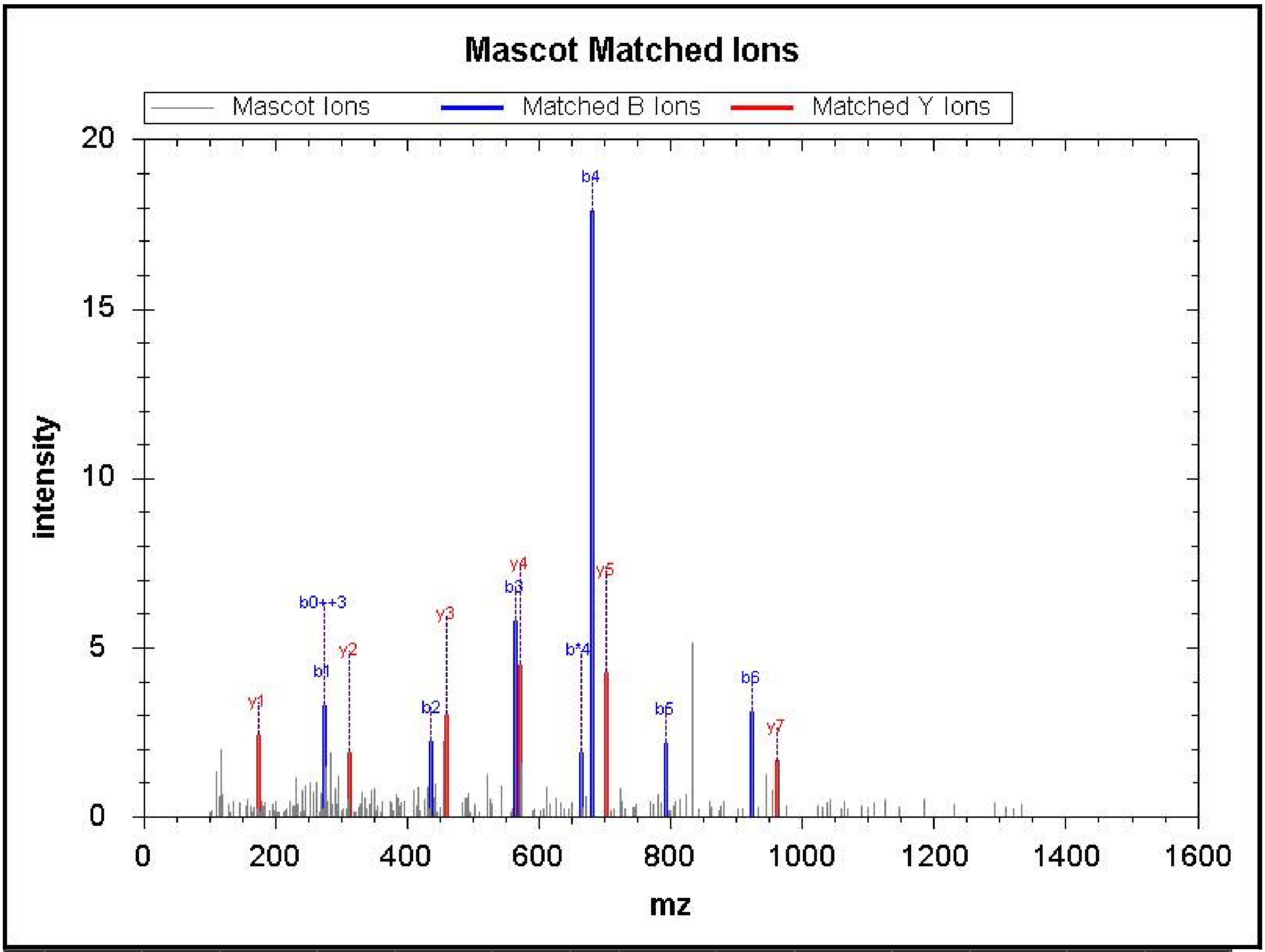

No	b	b++	b*	b*++	b0	b0++	Seq	y	y++	y*	y*++	y0	y0++	RevNo
1	274.15	137.58			256.14	128.57	E							12
2	437.22	219.11			419.20	210.11	Y	1,480.69	740.85	1,463.66	732.33	1,462.68	731.84	11
3	565.27	283.14	548.25	274.63	547.26	274.14	Q	1,317.63	659.32	1,300.60	650.80	1,299.61	650.31	10
4	680.30	340.65	663.27	332.14	662.29	331.65	D	1,189.57	595.29	1,172.54	586.77	1,171.56	586.28	9
5	793.38	397.20	776.36	388.68	775.37	388.19	I	1,074.54	537.77	1,057.51	529.26	1,056.53	528.77	8
6	922.43	461.72	905.40	453.20	904.42	452.71	E	961.46	481.23	944.43	472.72	943.45	472.23	7
7	1,051.47	526.24	1,034.44	517.73	1,033.46	517.23	E	832.41	416.71	815.39	408.20	814.40	407.71	6
8	1,182.51	591.76	1,165.48	583.25	1,164.50	582.75	M	703.37	352.19	686.34	343.68			5
9	1,295.59	648.30	1,278.57	639.79	1,277.58	639.30	I	572.33	286.67	555.30	278.16			4
10	1,442.66	721.84	1,425.64	713.32	1,424.65	712.83	F	459.25	230.13	442.22	221.61			3
11	1,579.72	790.36	1,562.70	781.85	1,561.71	781.36	H	312.18	156.59	295.15	148.08			2
12							R	175.12	88.06	158.09	79.55			1

Query 43336 Hit 1

MS/MS Fragmentation of QGNVLVISHQAVMR
Found in sp|O60825|F262_HUMAN, 6-phosphofructo-2-kinase/fructose-2
Match to Query 43336: 1694.925from(565.9824,3+)
Title: 386: Scan 937 (rt=30.5082, f=2, i=142) [D:\lab212\membrane\Grace\Joyce\iTRAQ_36_1.raw]
Data File:Submitted from 20120508-1(merge) by Mascot Daemon on JOYCE-VAIO
Monoisotopic mass of neutral peptide Mr(calc): 1694.925
Variable modifications:
Ions Score: 46.17 Expect: 0.019

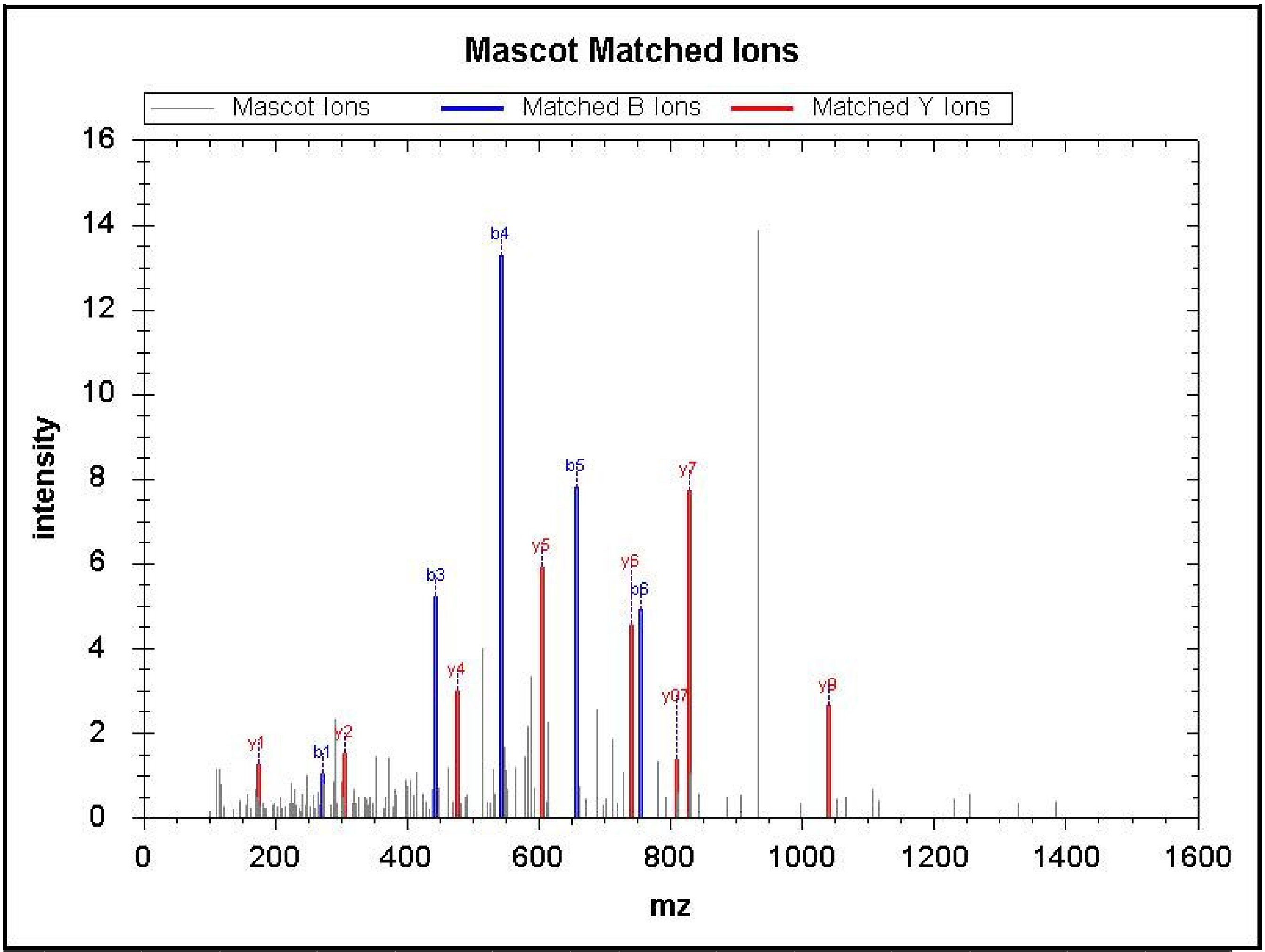

No	b	b++	b*	b*++	b0	b0++	Seq	y	y++	y*	y*++	y0	y0++	RevNo
1	273.17	137.09	256.14	128.57			Q							14
2	330.19	165.60	313.16	157.09			G	1,423.78	712.40	1,406.76	703.88	1,405.77	703.39	13
3	444.23	222.62	427.21	214.11			N	1,366.76	683.88	1,349.74	675.37	1,348.75	674.88	12
4	543.30	272.15	526.27	263.64			V	1,252.72	626.86	1,235.69	618.35	1,234.71	617.86	11
5	656.38	328.70	639.36	320.18			L	1,153.65	577.33	1,136.62	568.82	1,135.64	568.32	10
6	755.45	378.23	738.43	369.72			V	1,040.57	520.79	1,023.54	512.27	1,022.56	511.78	9
7	868.54	434.77	851.51	426.26			I	941.50	471.25	924.47	462.74	923.49	462.25	8
8	955.57	478.29	938.54	469.77	937.56	469.28	S	828.41	414.71	811.39	406.20	810.40	405.71	7
9	1,092.63	546.82	1,075.60	538.30	1,074.62	537.81	H	741.38	371.19	724.36	362.68			6
10	1,220.69	610.85	1,203.66	602.33	1,202.68	601.84	Q	604.32	302.67	587.30	294.15			5
11	1,291.72	646.37	1,274.70	637.85	1,273.71	637.36	A	476.26	238.64	459.24	230.12			4
12	1,390.79	695.90	1,373.77	687.39	1,372.78	686.89	V	405.23	203.12	388.20	194.60			3
13	1,521.83	761.42	1,504.81	752.91	1,503.82	752.41	M	306.16	153.58	289.13	145.07			2
14							R	175.12	88.06	158.09	79.55			1

Query 56454 Hit 1

MS/MS Fragmentation of WKDTDEADLVLAK
Found in sp|P45973|CBX5_HUMAN, Chromobox protein homolog 5 OS=Homo sapiens GN=CBX5 PE=1 SV=1
Match to Query 56454: 1935.069from(646.0303,3+)
Title: 567: Sum of 2 scans in range 1255 (rt=37.9625, f=4, i=381) to 1256 (rt=37.9879, f=4, i=382) [D:\lab212\membrane\Grace\Joyce\iTRAQ_45_1.raw]
Data File:Submitted from 20120508-1(merge) by Mascot Daemon on JOYCE-VAIO
Monoisotopic mass of neutral peptide Mr(calc): 1935.069
Variable modifications:
K2 :iTRAQ4plex (K)
K13 :iTRAQ4plex (K)

Ions Score: 46.15 Expect: 0.018

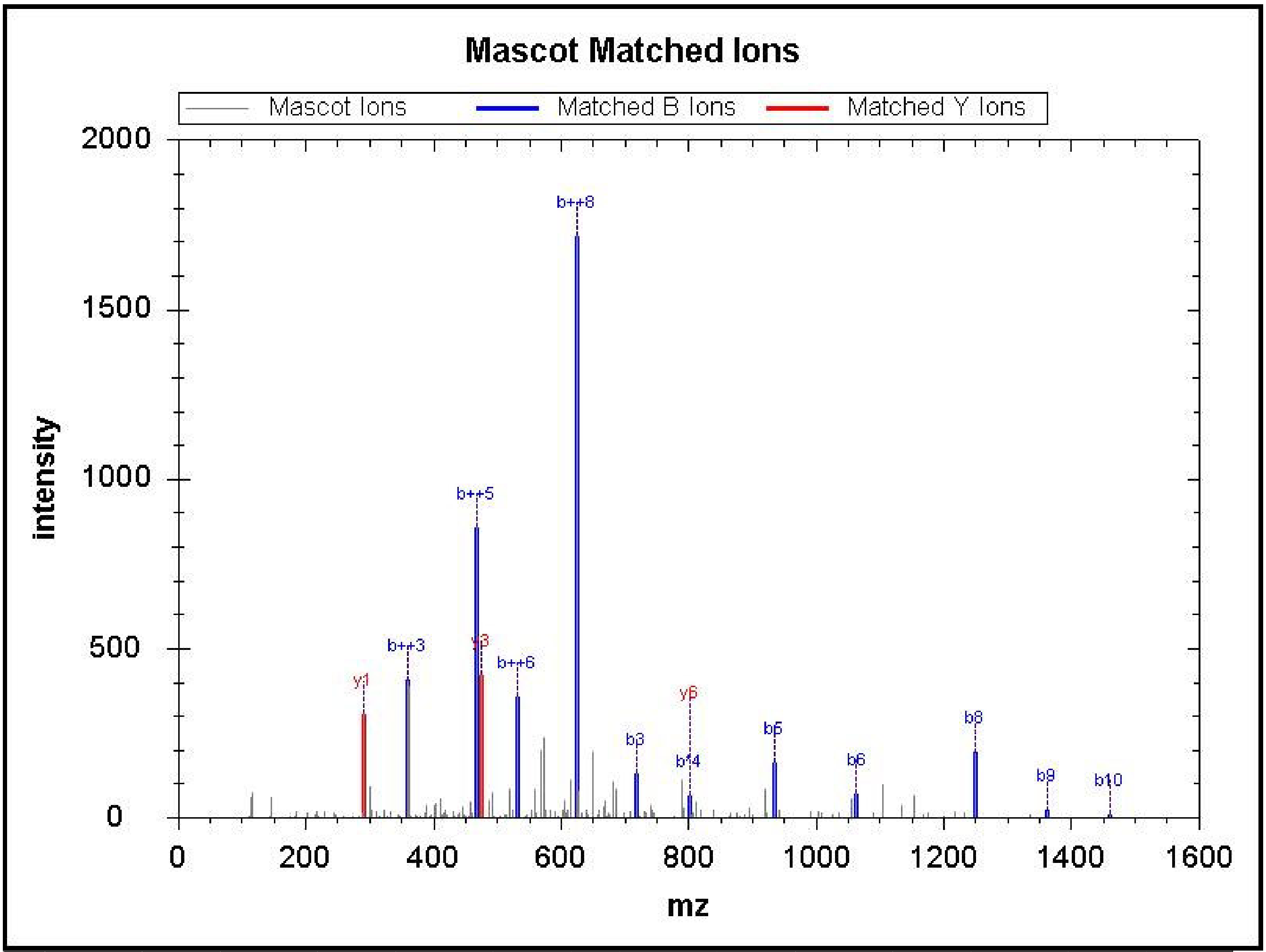

No	b	b++	b*	b*++	b0	b0++	Seq	y	y++	y*	y*++	y0	y0++	RevNo
1	331.19	166.10					W							13
2	603.39	302.20	586.36	293.68			K	1,605.89	803.45	1,588.87	794.94	1,587.88	794.45	12
3	718.41	359.71	701.39	351.20	700.40	350.70	D	1,333.70	667.35	1,316.67	658.84	1,315.69	658.35	11
4	819.46	410.23	802.43	401.72	801.45	401.23	T	1,218.67	609.84	1,201.64	601.33	1,200.66	600.83	10
5	934.49	467.75	917.46	459.23	916.48	458.74	D	1,117.62	559.31	1,100.60	550.80	1,099.61	550.31	9
6	1,063.53	532.27	1,046.50	523.76	1,045.52	523.26	E	1,002.60	501.80	985.57	493.29	984.58	492.80	8
7	1,134.57	567.79	1,117.54	559.27	1,116.56	558.78	A	873.55	437.28	856.53	428.77	855.54	428.27	7
8	1,249.59	625.30	1,232.57	616.79	1,231.58	616.30	D	802.52	401.76	785.49	393.25	784.50	392.76	6
9	1,362.68	681.84	1,345.65	673.33	1,344.67	672.84	L	687.49	344.25	670.46	335.73			5
10	1,461.75	731.38	1,444.72	722.86	1,443.74	722.37	V	574.40	287.71	557.38	279.19			4
11	1,574.83	787.92	1,557.80	779.41	1,556.82	778.91	L	475.34	238.17	458.31	229.66			3
12	1,645.87	823.44	1,628.84	814.92	1,627.86	814.43	A	362.25	181.63	345.23	173.12			2
13							K	291.21	146.11	274.19	137.60			1

Query 69344 Hit 1

MS/MS Fragmentation of SLEDLIFESLPENASHK
Found in sp|O95260|ATE1_HUMAN, Arginyl-tRNA--protein transferase 1 OS=Homo sapiens GN=ATE1 PE=1 SV=2
Match to Query 69344: 2216.161 from(739.7275,3+)
Title: 975: Sum of 2 scans in range 2140 (rt=57.6488, f=4, i=649) to 2141 (rt=57.6742, f=4, i=650) [D:\lab212\membrane\Grace\Joyce\iTRAQ_37_1.raw]
Data File:Submitted from 20120508-1(merge) by Mascot Daemon on JOYCE-VAIO
Monoisotopic mass of neutral peptide Mr(calc): 2216.161
Variable modifications:
K17 :iTRAQ4plex (K)

Ions Score: 46.09 Expect: 0.022

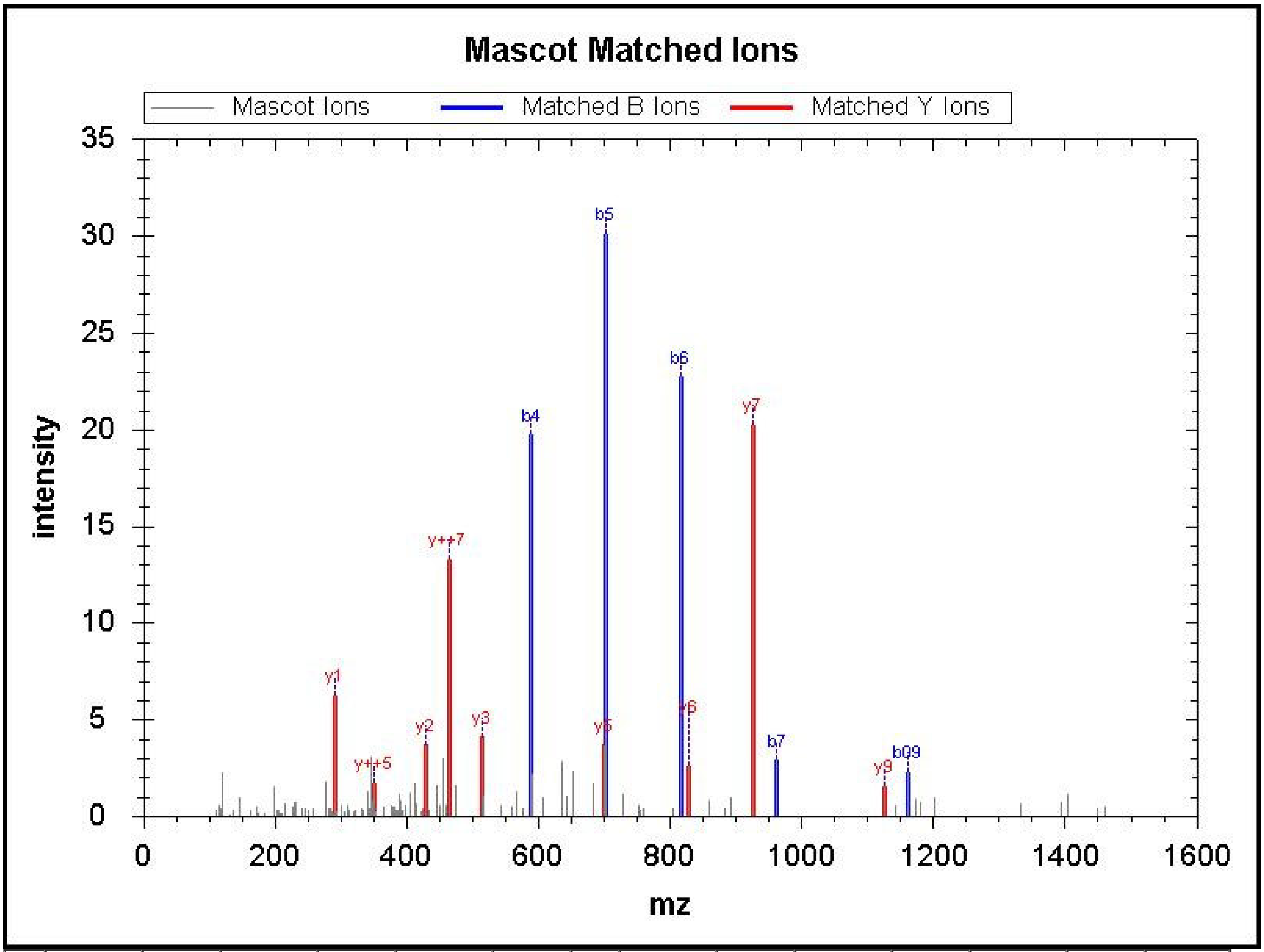

No	b	b++	b*	b*++	b0	b0++	Seq	y	y++	y*	y*++	y0	y0++	RevNo
1	232.14	116.57			214.13	107.57	S							17
2	345.23	173.12			327.21	164.11	L	1,986.03	993.52	1,969.00	985.01	1,968.02	984.51	16
3	474.27	237.64			456.26	228.63	E	1,872.95	936.98	1,855.92	928.46	1,854.94	927.97	15
4	589.29	295.15			571.28	286.15	D	1,743.90	872.46	1,726.88	863.94	1,725.89	863.45	14
5	702.38	351.69			684.37	342.69	L	1,628.88	814.94	1,611.85	806.43	1,610.87	805.94	13
6	815.46	408.24			797.45	399.23	I	1,515.79	758.40	1,498.77	749.89	1,497.78	749.39	12
7	962.53	481.77			944.52	472.76	F	1,402.71	701.86	1,385.68	693.34	1,384.70	692.85	11
8	1,091.57	546.29			1,073.56	537.29	E	1,255.64	628.32	1,238.61	619.81	1,237.63	619.32	10
9	1,178.61	589.81			1,160.60	580.80	S	1,126.60	563.80	1,109.57	555.29	1,108.59	554.80	9
10	1,291.69	646.35			1,273.68	637.34	L	1,039.57	520.29	1,022.54	511.77	1,021.55	511.28	8
11	1,388.74	694.88			1,370.73	685.87	P	926.48	463.74	909.45	455.23	908.47	454.74	7
12	1,517.79	759.40			1,499.77	750.39	E	829.43	415.22	812.40	406.70	811.42	406.21	6
13	1,631.83	816.42	1,614.80	807.90	1,613.82	807.41	N	700.39	350.70	683.36	342.18	682.38	341.69	5
14	1,702.87	851.94	1,685.84	843.42	1,684.85	842.93	A	586.34	293.68	569.32	285.16	568.33	284.67	4
15	1,789.90	895.45	1,772.87	886.94	1,771.89	886.45	S	515.31	258.16	498.28	249.64	497.30	249.15	3
16	1,926.96	963.98	1,909.93	955.47	1,908.95	954.98	H	428.27	214.64	411.25	206.13			2
17							K	291.21	146.11	274.19	137.60			1

Query 42907 Hit 1

MS/MS Fragmentation of LGVTNTIISHYDGR
Found in sp|P46087|NOP2_HUMAN, Putative ribosomal RNA methyltransferase NOP2 OS=Homo sapiens GN=NOP2 PE=1 SV=2
Match to Query 42907: 1688.894from(563.9719,3+)

Title: 513: Scan 1201 (rt=36.4637, f=2, i=185) [D:\lab212\membrane\Grace\Joyce\iTRAQ_36_2.raw]
Data File:Submitted from 20120508-1(merge) by Mascot Daemon on JOYCE-VAIO
Monoisotopic mass of neutral peptide Mr(calc): 1688.894
Variable modifications:
Ions Score: 45.99 Expect: 0.022

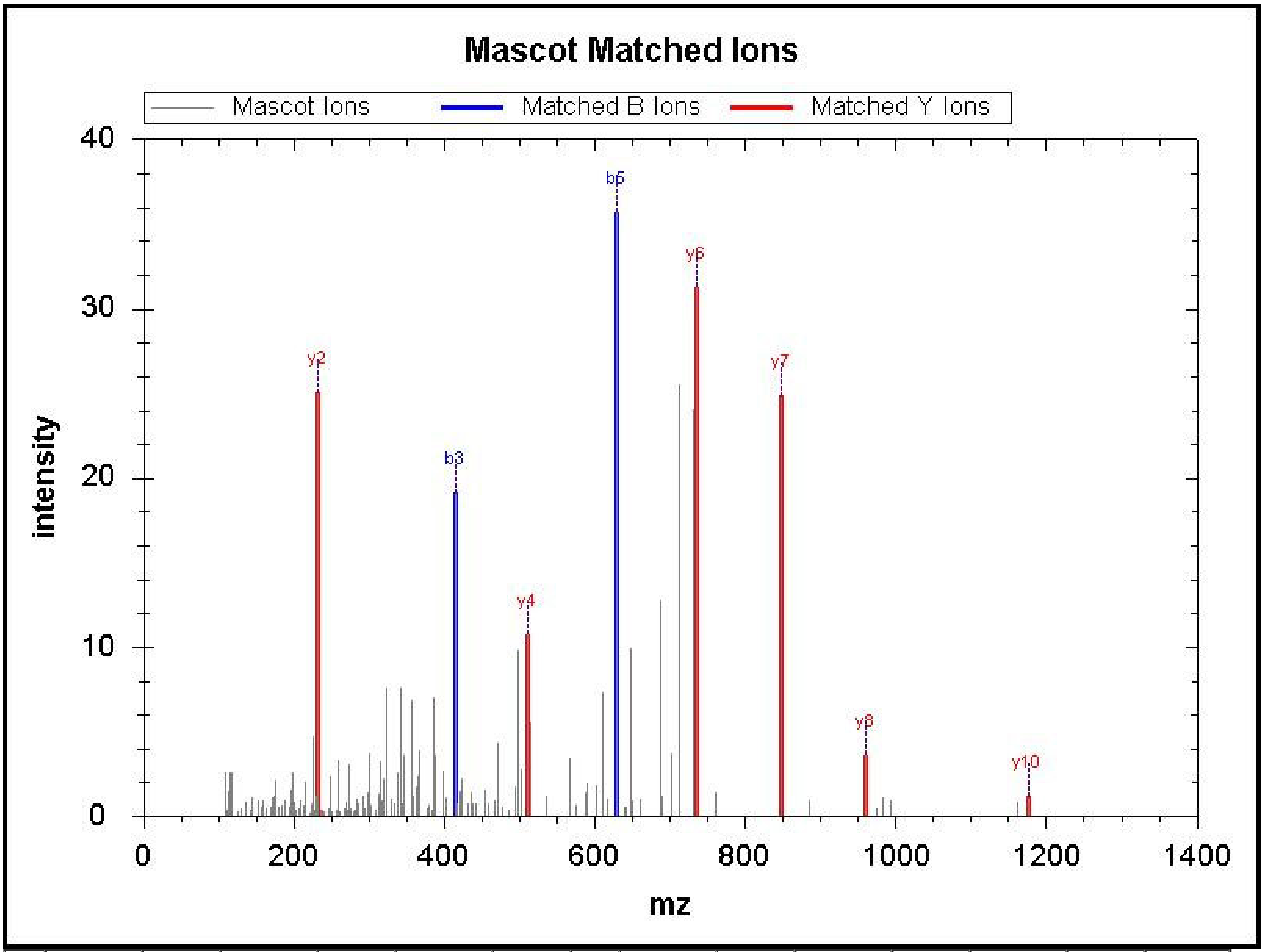

No	b	b++	b*	b*++	b0	b0++	Seq	y	y++	y*	y*++	y0	y0++	RevNo
1	258.19	129.60					L							14
2	315.21	158.11					G	1,432.72	716.86	1,415.69	708.35	1,414.71	707.86	13
3	414.28	207.65					V	1,375.70	688.35	1,358.67	679.84	1,357.69	679.35	12
4	515.33	258.17			497.32	249.16	T	1,276.63	638.82	1,259.60	630.30	1,258.62	629.81	11
5	629.37	315.19	612.35	306.68	611.36	306.19	N	1,175.58	588.29	1,158.55	579.78	1,157.57	579.29	10
6	730.42	365.71	713.39	357.20	712.41	356.71	T	1,061.54	531.27	1,044.51	522.76	1,043.53	522.27	9
7	843.51	422.26	826.48	413.74	825.49	413.25	I	960.49	480.75	943.46	472.24	942.48	471.74	8
8	956.59	478.80	939.56	470.29	938.58	469.79	I	847.41	424.21	830.38	415.69	829.40	415.20	7
9	1,043.62	522.31	1,026.60	513.80	1,025.61	513.31	S	734.32	367.66	717.30	359.15	716.31	358.66	6
10	1,180.68	590.84	1,163.65	582.33	1,162.67	581.84	H	647.29	324.15	630.26	315.64	629.28	315.14	5
11	1,343.74	672.38	1,326.72	663.86	1,325.73	663.37	Y	510.23	255.62	493.20	247.11	492.22	246.61	4
12	1,458.77	729.89	1,441.74	721.38	1,440.76	720.88	D	347.17	174.09	330.14	165.57	329.16	165.08	3
13	1,515.79	758.40	1,498.77	749.89	1,497.78	749.39	G	232.14	116.57	215.11	108.06			2
14							R	175.12	88.06	158.09	79.55			1

Query 51389 Hit 1

MS/MS Fragmentation of KGVEGLIDIENPNR
Found in sp|Q13442|HAP28_HUMAN, 28 kDa heat- and acid-stable phosphoprotein OS=Homo sapiens GN=PDAP1 PE=1 SV=1
Match to Query 51389: 1841.03from(614.6841,3+)
Title: 560: Scan 1276 (rt=38.3412, f=3, i=193) [D:\lab212\membrane\Grace\Joyce\iTRAQ_44_2.raw]

Data File:Submitted from 20120508-1(merge) by Mascot Daemon on JOYCE-VAIO
Monoisotopic mass of neutral peptide Mr(calc): 1841.03
Variable modifications:
K1 :iTRAQ4plex (K)
Ions Score: 45.92 Expect: 0.017

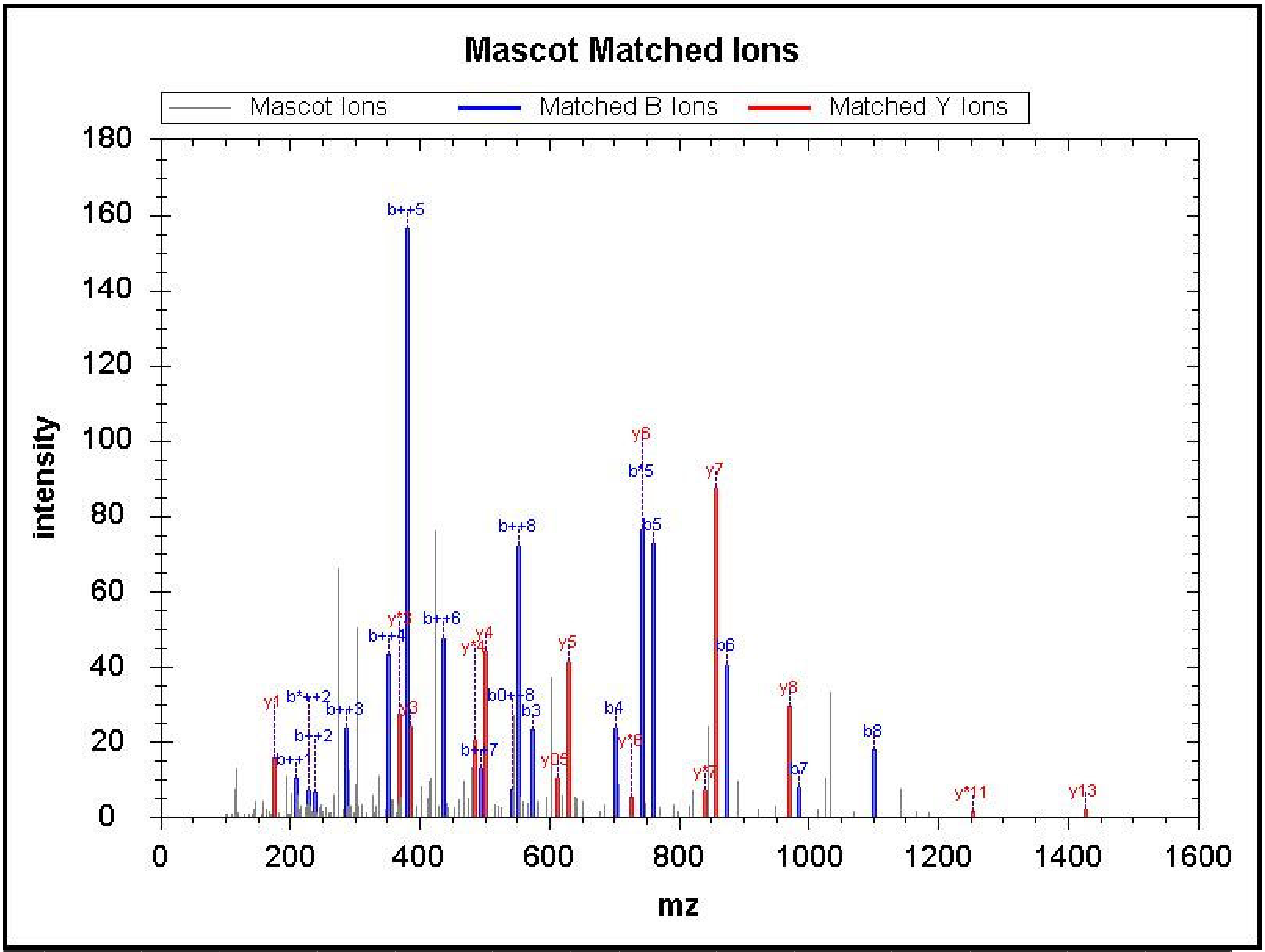

No	b	b++	b*	b*++	b0	b0++	Seq	y	y++	y*	y*++	y0	y0++	RevNo
1	417.31	209.16	400.28	200.64			K							14
2	474.33	237.67	457.30	229.15			G	1,425.73	713.37	1,408.71	704.86	1,407.72	704.36	13
3	573.40	287.20	556.37	278.69			V	1,368.71	684.86	1,351.69	676.35	1,350.70	675.85	12
4	702.44	351.72	685.41	343.21	684.43	342.72	E	1,269.64	635.33	1,252.62	626.81	1,251.63	626.32	11
5	759.46	380.23	742.43	371.72	741.45	371.23	G	1,140.60	570.80	1,123.57	562.29	1,122.59	561.80	10
6	872.54	436.78	855.52	428.26	854.53	427.77	L	1,083.58	542.29	1,066.55	533.78	1,065.57	533.29	9
7	985.63	493.32	968.60	484.80	967.62	484.31	I	970.50	485.75	953.47	477.24	952.48	476.75	8
8	1,100.66	550.83	1,083.63	542.32	1,082.64	541.83	D	857.41	429.21	840.38	420.70	839.40	420.20	7
9	1,213.74	607.37	1,196.71	598.86	1,195.73	598.37	I	742.38	371.70	725.36	363.18	724.37	362.69	6
10	1,342.78	671.89	1,325.76	663.38	1,324.77	662.89	E	629.30	315.15	612.27	306.64	611.29	306.15	5
11	1,456.83	728.92	1,439.80	720.40	1,438.81	719.91	N	500.26	250.63	483.23	242.12			4
12	1,553.88	777.44	1,536.85	768.93	1,535.87	768.44	P	386.21	193.61	369.19	185.10			3
13	1,667.92	834.46	1,650.89	825.95	1,649.91	825.46	N	289.16	145.08	272.14	136.57			2
14							R	175.12	88.06	158.09	79.55			1

Query 56949 Hit 1

MS/MS Fragmentation of DILLELVEHHLTPK
Found in sp|Q9D8Y7|TP8L2_MOUSE, Tumor necrosis factor alpha-induced protein 8-like protein 2 OS=Mus musculus GN=Tnfaip8l2 PE=1 SV=1
Match to Query 56949: 1944.131from(487.04,4+)

Title: 952: Sum of 2 scans in range 2158 (rt=58.0805, f=4, i=656) to 2159 (rt=58.1059, f=4, i=657)
[D:\lab212\membrane\Grace\Joyce\47_1.raw]
Data File:Submitted from 20120508-1(merge) by Mascot Daemon on JOYCE-VAIO
Monoisotopic mass of neutral peptide Mr(calc): 1944.131
Variable modifications:
K14 :iTRAQ4plex (K)
Ions Score: 45.89 Expect: 0.012

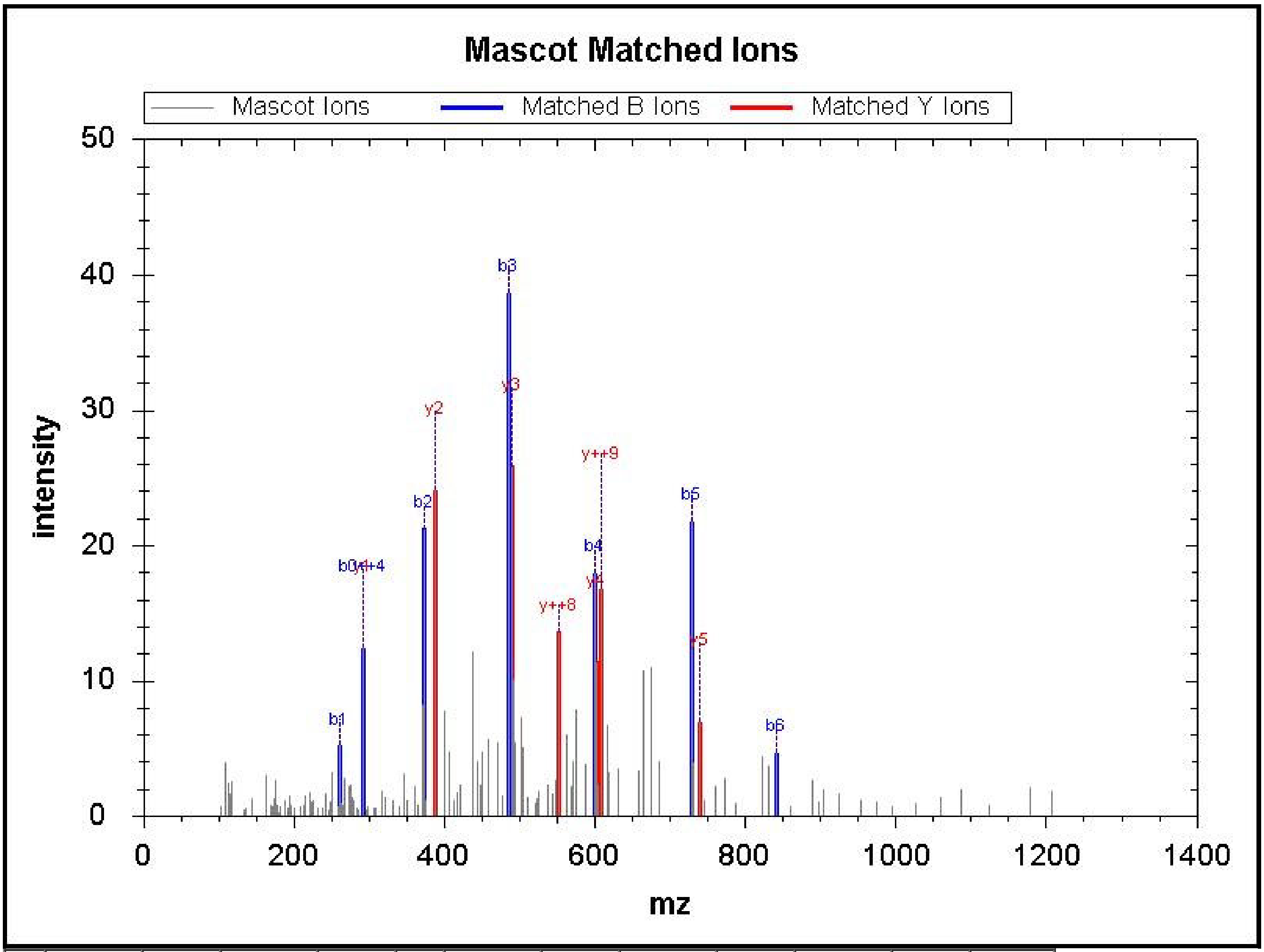

No	b	b++	b0	b0++	Seq	y	y++	y*	y*++	y0	y0++	RevNo
1	260.14	130.57	242.13	121.57	D							14
2	373.22	187.11	355.21	178.11	I	1,686.01	843.51	1,668.98	834.99	1,668.00	834.50	13
3	486.30	243.66	468.29	234.65	L	1,572.92	786.97	1,555.90	778.45	1,554.91	777.96	12
4	599.39	300.20	581.38	291.19	L	1,459.84	730.42	1,442.81	721.91	1,441.83	721.42	11
5	728.43	364.72	710.42	355.71	E	1,346.75	673.88	1,329.73	665.37	1,328.74	664.88	10
6	841.52	421.26	823.50	412.26	L	1,217.71	609.36	1,200.69	600.85	1,199.70	600.35	9
7	940.58	470.80	922.57	461.79	V	1,104.63	552.82	1,087.60	544.30	1,086.62	543.81	8
8	1,069.63	535.32	1,051.62	526.31	E	1,005.56	503.28	988.53	494.77	987.55	494.28	7
9	1,206.69	603.85	1,188.67	594.84	H	876.52	438.76	859.49	430.25	858.51	429.76	6
10	1,343.74	672.38	1,325.73	663.37	H	739.46	370.23	722.43	361.72	721.45	361.23	5
11	1,456.83	728.92	1,438.82	719.91	L	602.40	301.70	585.37	293.19	584.39	292.70	4
12	1,557.88	779.44	1,539.87	770.44	T	489.32	245.16	472.29	236.65	471.30	236.16	3
13	1,654.93	827.97	1,636.92	818.96	P	388.27	194.64	371.24	186.12			2
14					K	291.21	146.11	274.19	137.60			1

Query 61577 Hit 1

MS/MS Fragmentation of DIFGLGPVLLLDATALK
Found in sp|Q12894|IFRD2_HUMAN, Interferon-related developmental regulator 2 OS=Homo sapiens GN=IFRD2 PE=2 SV=3

Match to Query 61577: 2043.223from(682.0815,3+)
Title: 1302: Scan 2963 (rt=75.553, f=3, i=444) [D:\lab212\membrane\Grace\Joyce\iTRAQ_24_1.raw]
Data File:Submitted from 20120508-1(merge) by Mascot Daemon on JOYCE-VAIO
Monoisotopic mass of neutral peptide Mr(calc): 2043.223
Variable modifications:
K17 :iTRAQ4plex (K)
Ions Score: 45.78 Expect: 0.006

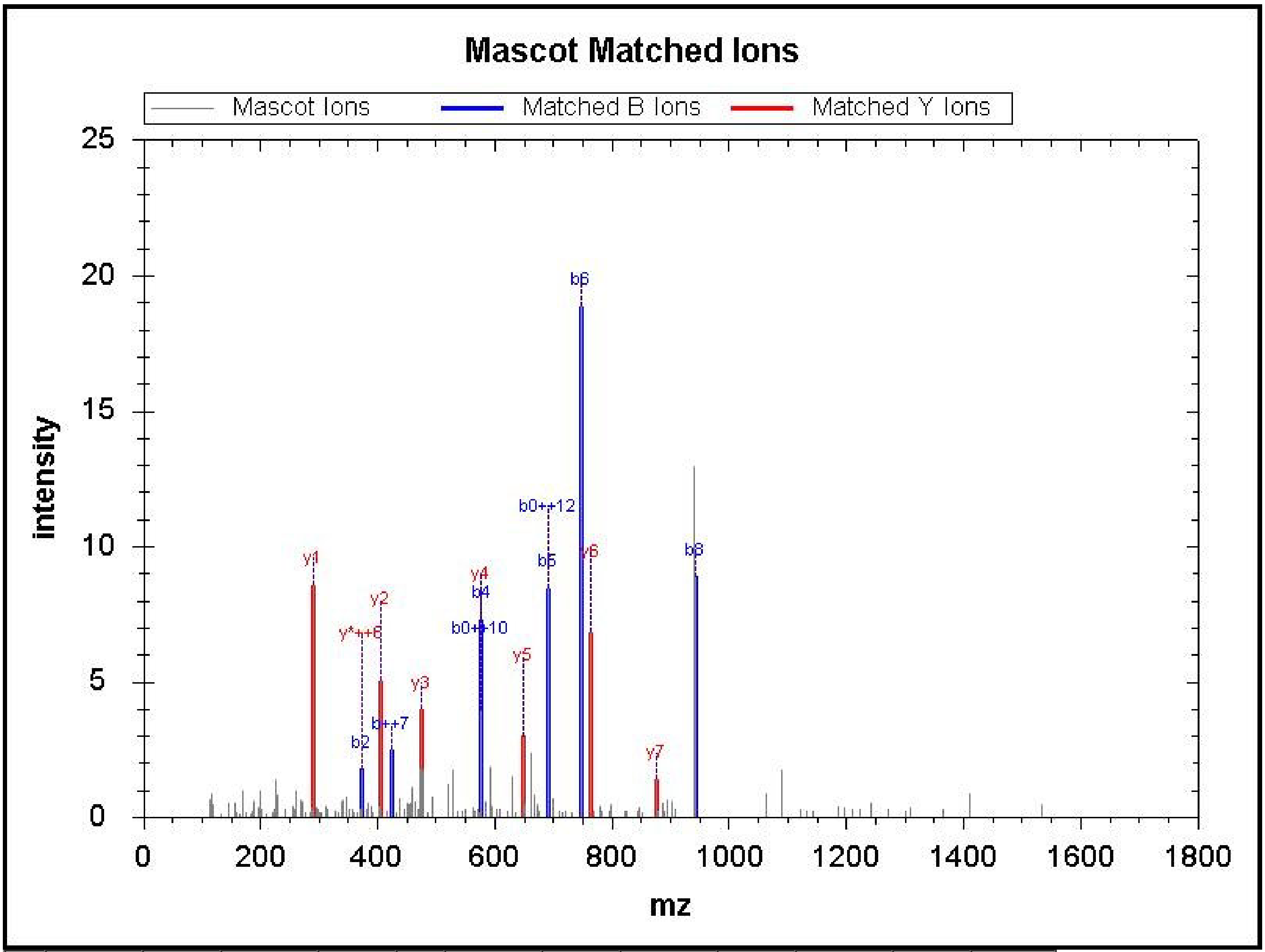

No	b	b++	b0	b0++	Seq	y	y++	y*	y*++	y0	y0++	RevNo
1	260.14	130.57	242.13	121.57	D							17
2	373.22	187.11	355.21	178.11	I	1,785.10	893.05	1,768.07	884.54	1,767.09	884.05	16
3	520.29	260.65	502.28	251.64	F	1,672.02	836.51	1,654.99	828.00	1,654.01	827.51	15
4	577.31	289.16	559.30	280.15	G	1,524.95	762.98	1,507.92	754.46	1,506.94	753.97	14
5	690.39	345.70	672.38	336.70	L	1,467.93	734.47	1,450.90	725.95	1,449.92	725.46	13
6	747.42	374.21	729.41	365.21	G	1,354.84	677.92	1,337.82	669.41	1,336.83	668.92	12
7	844.47	422.74	826.46	413.73	P	1,297.82	649.41	1,280.79	640.90	1,279.81	640.41	11
8	943.54	472.27	925.53	463.27	V	1,200.77	600.89	1,183.74	592.37	1,182.76	591.88	10
9	1,056.62	528.81	1,038.61	519.81	L	1,101.70	551.35	1,084.67	542.84	1,083.69	542.35	9
10	1,169.71	585.36	1,151.69	576.35	L	988.62	494.81	971.59	486.30	970.61	485.81	8
11	1,282.79	641.90	1,264.78	632.89	L	875.53	438.27	858.51	429.76	857.52	429.26	7
12	1,397.82	699.41	1,379.81	690.41	D	762.45	381.73	745.42	373.21	744.44	372.72	6
13	1,468.85	734.93	1,450.84	725.93	A	647.42	324.21	630.39	315.70	629.41	315.21	5
14	1,569.90	785.45	1,551.89	776.45	T	576.38	288.70	559.36	280.18	558.37	279.69	4
15	1,640.94	820.97	1,622.93	811.97	A	475.34	238.17	458.31	229.66			3
16	1,754.02	877.51	1,736.01	868.51	L	404.30	202.65	387.27	194.14			2
17					K	291.21	146.11	274.19	137.60			1

Query 34518 Hit 1

MS/MS Fragmentation of NVVALDTEVASNR
Found in sp|P01130|LDLR_HUMAN, Low-density lipoprotein receptor OS=Homo sapiens GN=LDLR PE=1 SV=1
Match to Query 34518: 1530.807from(766.4107,2+)
Title: 247: Sum of 2 scans in range 1104 (rt=32.1989, f=4, i=159) to 1105 (rt=32.2243, f=4, i=160)
[D:\lab212\membrane\Grace\Joyce\20120320_iTRAQ_20-2.raw]
Data File:Submitted from 20120508-1(merge) by Mascot Daemon on JOYCE-VAIO
Monoisotopic mass of neutral peptide Mr(calc): 1530.807
Variable modifications:
Ions Score: 45.77 Expect: 0.022

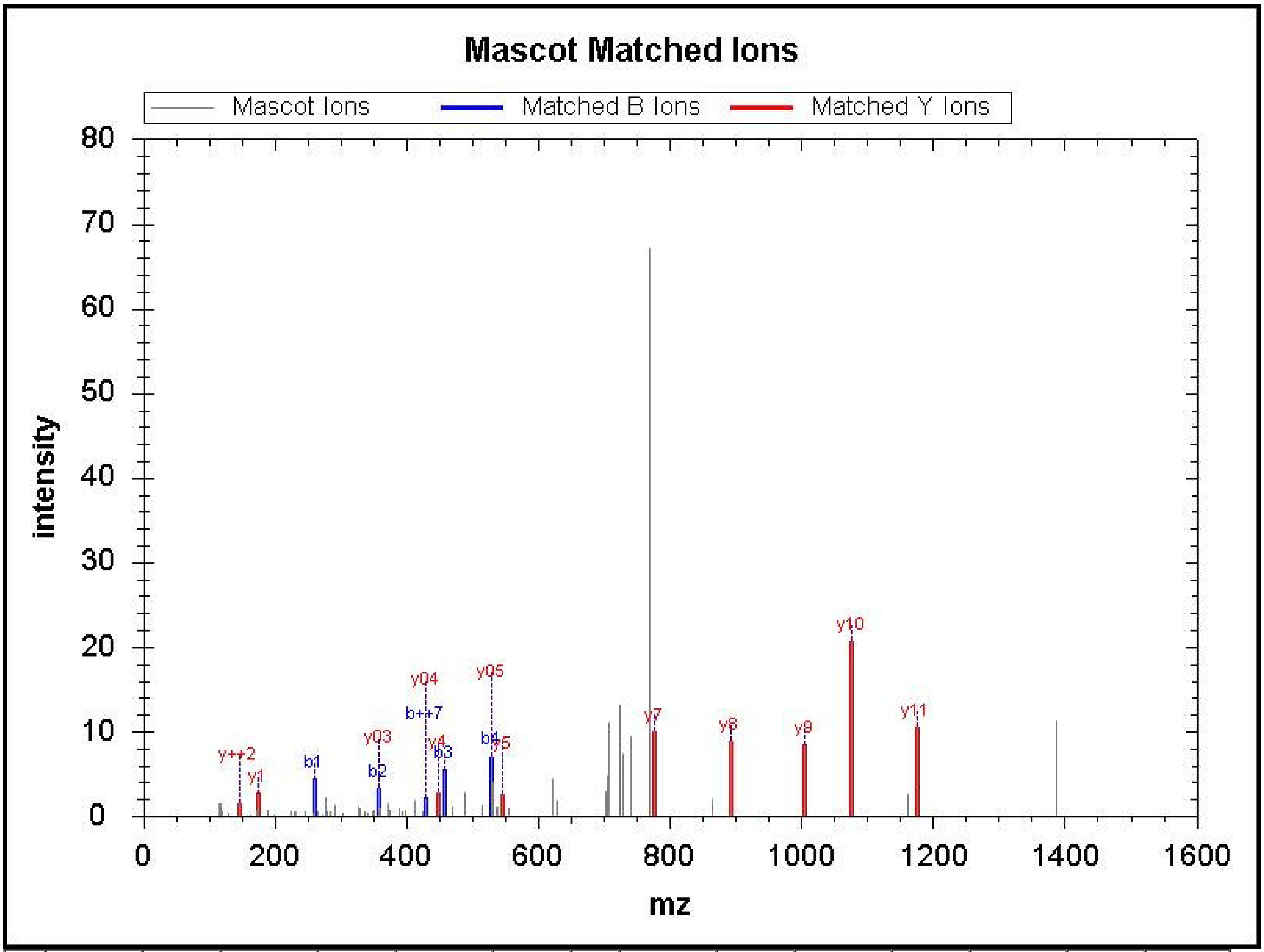

No	b	b++	b*	b*++	b0	b0++	Seq	y	y++	y*	y*++	y0	y0++	RevNo
1	259.15	130.08	242.13	121.57			N							13
2	358.22	179.61	341.19	171.10			V	1,273.67	637.34	1,256.65	628.83	1,255.66	628.34	12
3	457.29	229.15	440.26	220.63			V	1,174.61	587.81	1,157.58	579.29	1,156.60	578.80	11
4	528.33	264.67	511.30	256.15			A	1,075.54	538.27	1,058.51	529.76	1,057.53	529.27	10
5	641.41	321.21	624.38	312.70			L	1,004.50	502.75	987.47	494.24	986.49	493.75	9
6	756.44	378.72	739.41	370.21	738.43	369.72	D	891.42	446.21	874.39	437.70	873.41	437.21	8
7	857.48	429.25	840.46	420.73	839.47	420.24	T	776.39	388.70	759.36	380.19	758.38	379.69	7
8	986.53	493.77	969.50	485.25	968.52	484.76	E	675.34	338.17	658.32	329.66	657.33	329.17	6
9	1,085.60	543.30	1,068.57	534.79	1,067.59	534.30	V	546.30	273.65	529.27	265.14	528.29	264.65	5
10	1,156.63	578.82	1,139.61	570.31	1,138.62	569.81	A	447.23	224.12	430.20	215.61	429.22	215.11	4
11	1,243.67	622.34	1,226.64	613.82	1,225.65	613.33	S	376.19	188.60	359.17	180.09	358.18	179.60	3
12	1,357.71	679.36	1,340.68	670.84	1,339.70	670.35	N	289.16	145.08	272.14	136.57			2
13							R	175.12	88.06	158.09	79.55			1

Query 72827 Hit 1

MS/MS Fragmentation of SIFNSAMQEMEVYVENIR
Found in sp|Q9ULU4|PKCB1_HUMAN, Protein kinase C-binding protein 1 OS=Homo sapiens GN=ZMYND8 PE=1 SV=2
Match to Query 72827: 2303.105from(768.709,3+)
Title: 1233: Sum of 2 scans in range 2894 (rt=73.5931, f=4, i=815) to 2895 (rt=73.6185, f=4, i=816) [D:\lab212\membrane\Grace\Joyce\iTRAQ_23_2.raw]
Data File:Submitted from 20120508-1(merge) by Mascot Daemon on JOYCE-VAIO
Monoisotopic mass of neutral peptide Mr(calc): 2303.105
Variable modifications:
Ions Score: 45.66 Expect: 0.023

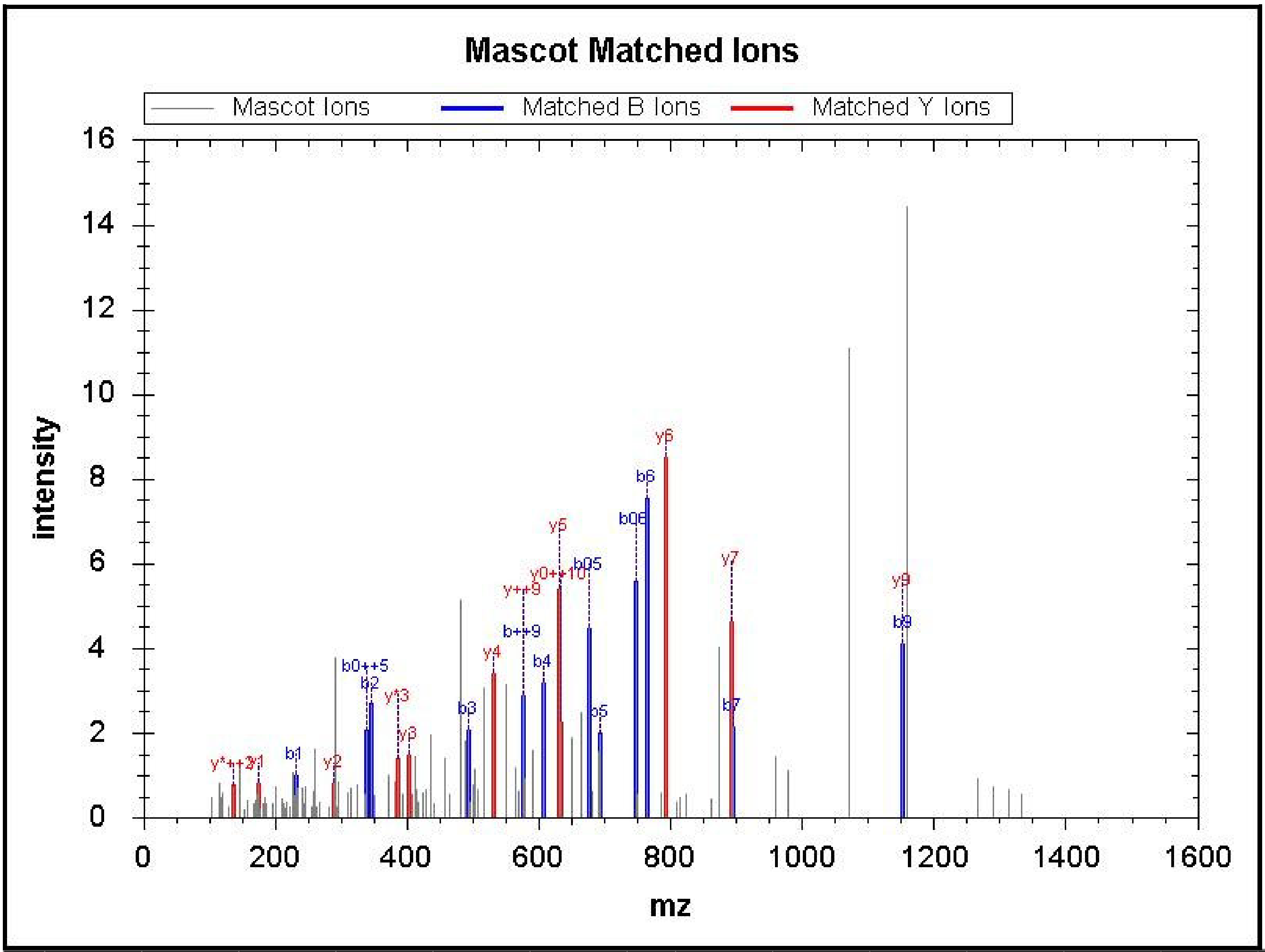

No	b	b++	b*	b*++	b0	b0++	Seq	y	y++	y*	y*++	y0	y0++	RevNo
1	232.14	116.57			214.13	107.57	S							18
2	345.23	173.12			327.21	164.11	I	2,072.98	1,036.99	2,055.95	1,028.48	2,054.97	1,027.99	17
3	492.29	246.65			474.28	237.65	F	1,959.89	980.45	1,942.87	971.94	1,941.88	971.45	16
4	606.34	303.67	589.31	295.16	588.33	294.67	N	1,812.83	906.92	1,795.80	898.40	1,794.81	897.91	15
5	693.37	347.19	676.34	338.67	675.36	338.18	S	1,698.78	849.89	1,681.76	841.38	1,680.77	840.89	14
6	764.41	382.71	747.38	374.19	746.40	373.70	A	1,611.75	806.38	1,594.72	797.87	1,593.74	797.37	13
7	895.45	448.23	878.42	439.71	877.44	439.22	M	1,540.71	770.86	1,523.69	762.35	1,522.70	761.86	12
8	1,023.50	512.26	1,006.48	503.74	1,005.49	503.25	Q	1,409.67	705.34	1,392.65	696.83	1,391.66	696.33	11
9	1,152.55	576.78	1,135.52	568.26	1,134.54	567.77	E	1,281.61	641.31	1,264.59	632.80	1,263.60	632.31	10
10	1,283.59	642.30	1,266.56	633.78	1,265.58	633.29	M	1,152.57	576.79	1,135.55	568.28	1,134.56	567.78	9
11	1,412.63	706.82	1,395.60	698.31	1,394.62	697.81	E	1,021.53	511.27	1,004.50	502.76	1,003.52	502.26	8
12	1,511.70	756.35	1,494.67	747.84	1,493.69	747.35	V	892.49	446.75	875.46	438.23	874.48	437.74	7
13	1,674.76	837.88	1,657.74	829.37	1,656.75	828.88	Y	793.42	397.21	776.39	388.70	775.41	388.21	6
14	1,773.83	887.42	1,756.80	878.91	1,755.82	878.41	V	630.36	315.68	613.33	307.17	612.35	306.68	5
15	1,902.87	951.94	1,885.85	943.43	1,884.86	942.94	E	531.29	266.15	514.26	257.63	513.28	257.14	4
16	2,016.92	1,008.96	1,999.89	1,000.45	1,998.91	999.96	N	402.25	201.63	385.22	193.11			3

17	2,130.00	1,065.50	2,112.97	1,056.99	2,111.99	1,056.50	I	288.20	144.61	271.18	136.09			2
18							R	175.12	88.06	158.09	79.55			1

Query 46275 Hit 1

MS/MS Fragmentation of RVPVLGSLLNLPGIR
Found in sp|Q9Y3E0|GOT1B_HUMAN, Vesicle transport protein GOT1B OS=Homo sapiens GN=GOLT1B PE=1 SV=1
Match to Query 46275: 1747.097from(583.3729,3+)
Title: 983: Sum of 2 scans in range 2169 (rt=58.3711, f=4, i=657) to 2170 (rt=58.3965, f=4, i=658)
[D:\lab212\membrane\Grace\Joyce\iTRAQ_39_1.raw]
Data File:Submitted from 20120508-1(merge) by Mascot Daemon on JOYCE-VAIO
Monoisotopic mass of neutral peptide Mr(calc): 1747.097
Variable modifications:
Ions Score: 45.65 Expect: 0.005

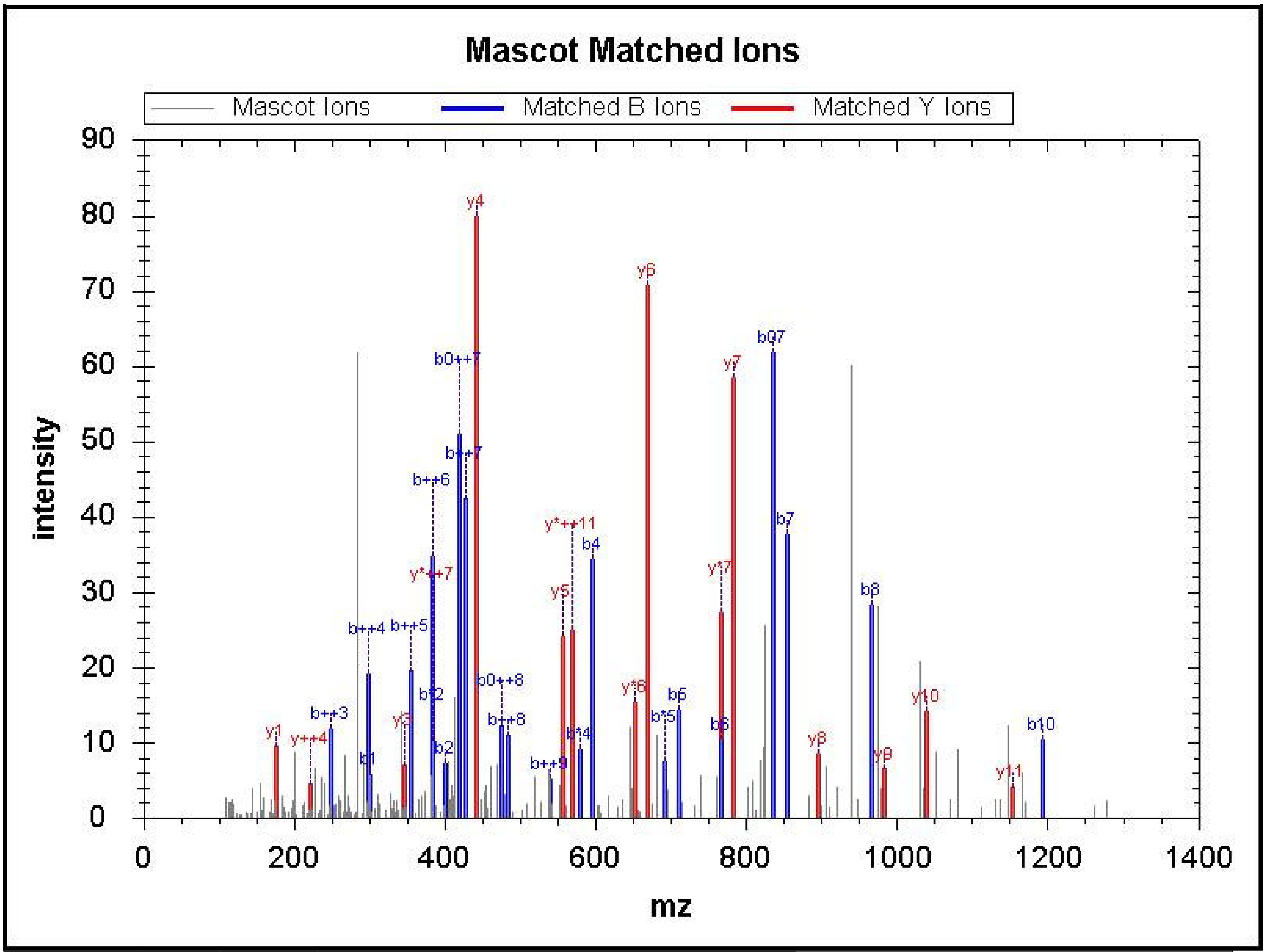

No	b	b++	b*	b*++	b0	b0++	Seq	y	y++	y*	y*++	y0	y0++	RevNo
1	301.21	151.11	284.18	142.60			R							15
2	400.28	200.64	383.25	192.13			V	1,447.90	724.45	1,430.87	715.94	1,429.89	715.45	14
3	497.33	249.17	480.31	240.66			P	1,348.83	674.92	1,331.80	666.41	1,330.82	665.91	13
4	596.40	298.70	579.37	290.19			V	1,251.78	626.39	1,234.75	617.88	1,233.77	617.39	12
5	709.48	355.25	692.46	346.73			L	1,152.71	576.86	1,135.68	568.35	1,134.70	567.85	11
6	766.51	383.76	749.48	375.24			G	1,039.63	520.32	1,022.60	511.80	1,021.62	511.31	10
7	853.54	427.27	836.51	418.76	835.53	418.27	S	982.60	491.81	965.58	483.29	964.59	482.80	9
8	966.62	483.81	949.60	475.30	948.61	474.81	L	895.57	448.29	878.55	439.78			8
9	1,079.71	540.36	1,062.68	531.84	1,061.70	531.35	L	782.49	391.75	765.46	383.23			7
10	1,193.75	597.38	1,176.72	588.86	1,175.74	588.37	N	669.40	335.21	652.38	326.69			6
11	1,306.83	653.92	1,289.81	645.41	1,288.82	644.91	L	555.36	278.18	538.33	269.67			5
12	1,403.89	702.45	1,386.86	693.93	1,385.88	693.44	P	442.28	221.64	425.25	213.13			4

13	1,460.91	730.96	1,443.88	722.44	1,442.90	721.95	G	345.22	173.12	328.20	164.60			3
14	1,573.99	787.50	1,556.96	778.99	1,555.98	778.49	I	288.20	144.61	271.18	136.09			2
15							R	175.12	88.06	158.09	79.55			1

Query 67781 Hit 1

MS/MS Fragmentation of VLGHVNNILISAVLPTAFR
Found in sp|Q8TB36|GDAP1_HUMAN, Ganglioside-induced differentiation-associated protein 1 OS=Homo sapiens GN=GDAP1 PE=1 SV=3
Match to Query 67781: 2177.28from(726.7671,3+)
Title: 1031: Scan 2230 (rt=59.9095, f=3, i=347) [D:\lab212\membrane\Grace\Joyce\iTRAQ_33_2.raw]
Data File:Submitted from 20120508-1(merge) by Mascot Daemon on JOYCE-VAIO
Monoisotopic mass of neutral peptide Mr(calc): 2177.28
Variable modifications:
Ions Score: 45.63 Expect: 0.008

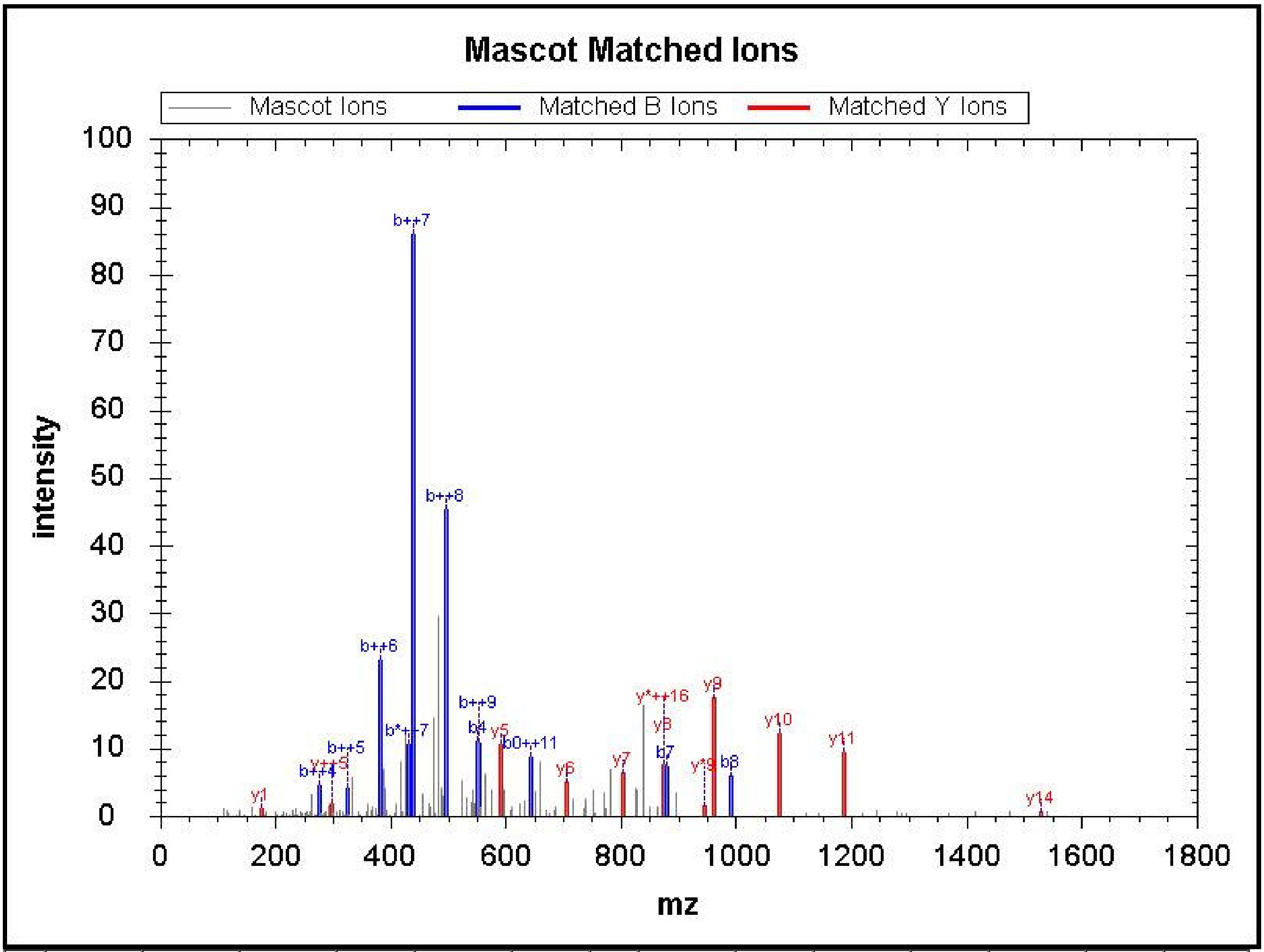

No	b	b++	b*	b*++	b0	b0++	Seq	y	y++	y*	y*++	y0	y0++	RevNo
1	244.18	122.59					V							19
2	357.26	179.13					L	1,935.12	968.06	1,918.09	959.55	1,917.11	959.06	18
3	414.28	207.65					G	1,822.03	911.52	1,805.01	903.01	1,804.02	902.52	17
4	551.34	276.17					H	1,765.01	883.01	1,747.99	874.50	1,747.00	874.00	16
5	650.41	325.71					V	1,627.95	814.48	1,610.93	805.97	1,609.94	805.47	15
6	764.45	382.73	747.43	374.22			N	1,528.88	764.95	1,511.86	756.43	1,510.87	755.94	14
7	878.50	439.75	861.47	431.24			N	1,414.84	707.92	1,397.82	699.41	1,396.83	698.92	13
8	991.58	496.29	974.55	487.78			I	1,300.80	650.90	1,283.77	642.39	1,282.79	641.90	12
9	1,104.66	552.84	1,087.64	544.32			L	1,187.71	594.36	1,170.69	585.85	1,169.70	585.36	11
10	1,217.75	609.38	1,200.72	600.86			I	1,074.63	537.82	1,057.60	529.31	1,056.62	528.81	10

11	1,304.78	652.89	1,287.75	644.38	1,286.77	643.89	S	961.55	481.28	944.52	472.76	943.54	472.27	9
12	1,375.82	688.41	1,358.79	679.90	1,357.81	679.41	A	874.51	437.76	857.49	429.25	856.50	428.76	8
13	1,474.89	737.95	1,457.86	729.43	1,456.88	728.94	V	803.48	402.24	786.45	393.73	785.47	393.24	7
14	1,587.97	794.49	1,570.94	785.98	1,569.96	785.48	L	704.41	352.71	687.38	344.19	686.40	343.70	6
15	1,685.02	843.02	1,668.00	834.50	1,667.01	834.01	P	591.32	296.17	574.30	287.65	573.31	287.16	5
16	1,786.07	893.54	1,769.04	885.03	1,768.06	884.53	T	494.27	247.64	477.25	239.13	476.26	238.63	4
17	1,857.11	929.06	1,840.08	920.54	1,839.10	920.05	A	393.22	197.12	376.20	188.60			3
18	2,004.18	1,002.59	1,987.15	994.08	1,986.17	993.59	F	322.19	161.60	305.16	153.08			2
19							R	175.12	88.06	158.09	79.55			1

Query 77791 Hit 1

MS/MS Fragmentation of LWNKDPLTGILYQINSK
Found in sp|Q61830|MRC1_MOUSE, Macrophage mannose receptor 1 OS=Mus musculus GN=Mrc1 PE=1 SV=2
Match to Query 77791: 2434.392from(812.4713,3+)
Title: 738: Sum of 2 scans in range 2282 (rt=58.4707, f=4, i=479) to 2283 (rt=58.4962, f=4, i=480) [D:\lab212\membrane\Grace\Joyce\iTRAQ_43_2.raw]
Data File:Submitted from 20120508-1(merge) by Mascot Daemon on JOYCE-VAIO
Monoisotopic mass of neutral peptide Mr(calc): 2434.392
Variable modifications:
K4 :iTRAQ4plex (K)
K17 :iTRAQ4plex (K)
Ions Score: 45.6 Expect: 0.010

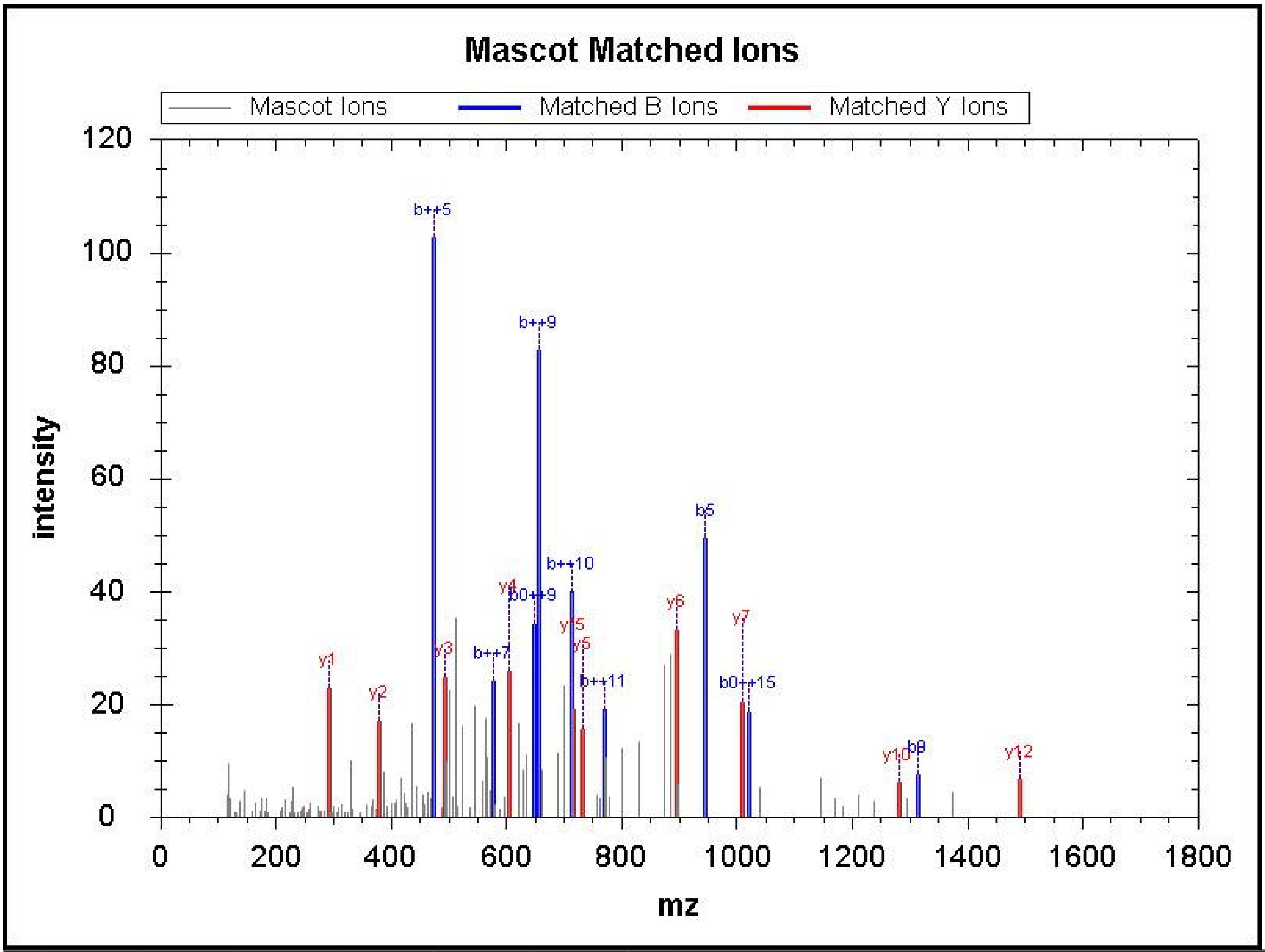

No	b	b++	b*	b*++	b0	b0++	Seq	y	y++	y*	y*++	y0	y0++	RevNo
1	258.19	129.60					L							17
2	444.27	222.64					W	2,178.22	1,089.61	2,161.19	1,081.10	2,160.21	1,080.61	16
3	558.32	279.66	541.29	271.15			N	1,992.14	996.57	1,975.11	988.06	1,974.13	987.57	15
4	830.51	415.76	813.49	407.25			K	1,878.09	939.55	1,861.07	931.04	1,860.08	930.55	14

5	945.54	473.27	928.51	464.76	927.53	464.27	D	1,605.90	803.45	1,588.87	794.94	1,587.89	794.45	13
6	1,042.59	521.80	1,025.57	513.29	1,024.58	512.79	P	1,490.87	745.94	1,473.84	737.43	1,472.86	736.93	12
7	1,155.68	578.34	1,138.65	569.83	1,137.67	569.34	L	1,393.82	697.41	1,376.79	688.90	1,375.81	688.41	11
8	1,256.72	628.87	1,239.70	620.35	1,238.71	619.86	T	1,280.73	640.87	1,263.71	632.36	1,262.72	631.86	10
9	1,313.75	657.38	1,296.72	648.86	1,295.74	648.37	G	1,179.69	590.35	1,162.66	581.83	1,161.67	581.34	9
10	1,426.83	713.92	1,409.80	705.41	1,408.82	704.91	I	1,122.66	561.84	1,105.64	553.32	1,104.65	552.83	8
11	1,539.91	770.46	1,522.89	761.95	1,521.90	761.46	L	1,009.58	505.29	992.55	496.78	991.57	496.29	7
12	1,702.98	851.99	1,685.95	843.48	1,684.97	842.99	Y	896.50	448.75	879.47	440.24	878.49	439.75	6
13	1,831.04	916.02	1,814.01	907.51	1,813.03	907.02	Q	733.43	367.22	716.41	358.71	715.42	358.21	5
14	1,944.12	972.56	1,927.09	964.05	1,926.11	963.56	I	605.37	303.19	588.35	294.68	587.36	294.19	4
15	2,058.16	1,029.59	2,041.14	1,021.07	2,040.15	1,020.58	N	492.29	246.65	475.26	238.14	474.28	237.64	3
16	2,145.19	1,073.10	2,128.17	1,064.59	2,127.18	1,064.10	S	378.25	189.63	361.22	181.11	360.24	180.62	2
17							K	291.21	146.11	274.19	137.60			1

Query 14134 Hit 1

MS/MS Fragmentation of SGLSDIILGK
Found in sp|Q9UBC2|EP15R_HUMAN, Epidermal growth factor receptor substrate 15-like 1 OS=Homo sapiens GN=EPS15L1 PE=1 SV=1
Match to Query 14134: 1145.654from(573.834,2+)
Title: 257: Scan 659 (rt=24.2052, f=3, i=90) [D:\lab212\membrane\Grace\Joyce\iTRAQ_31_1.raw]
Data File:Submitted from 20120508-1(merge) by Mascot Daemon on JOYCE-VAIO
Monoisotopic mass of neutral peptide Mr(calc): 1145.654
Variable modifications:
K10 :iTRAQ4plex (K)
Ions Score: 45.56 Expect: 0.023

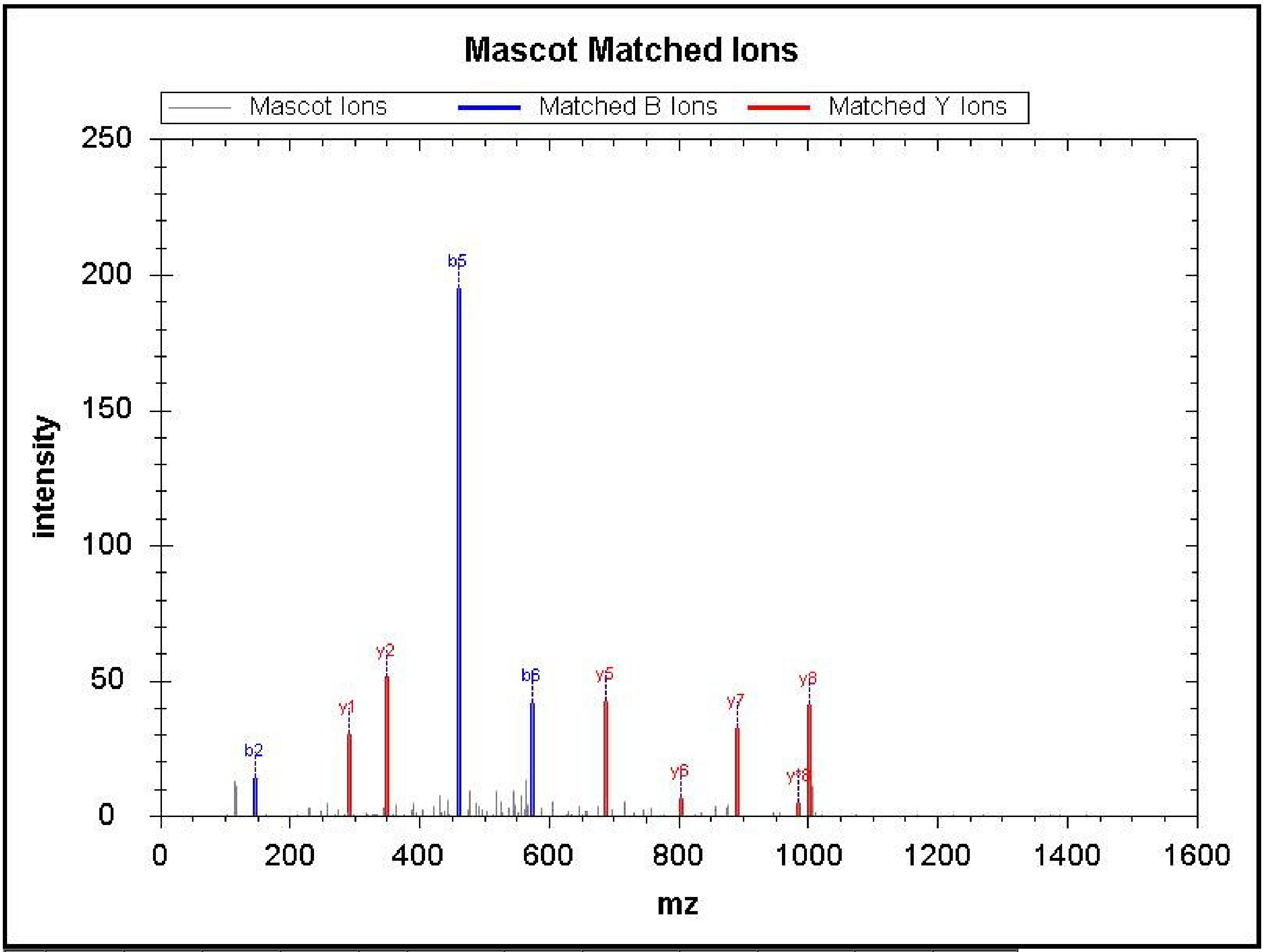

No	b	b++	b0	b0++	Seq	y	y++	y*	y*++	y0	y0++	RevNo

1	88.04	44.52	70.03	35.52	S							10
2	145.06	73.03	127.05	64.03	G	1,059.65	530.33	1,042.63	521.82	1,041.64	521.32	9
3	258.14	129.58	240.13	120.57	L	1,002.63	501.82	985.60	493.31	984.62	492.81	8
4	345.18	173.09	327.17	164.09	S	889.55	445.28	872.52	436.76	871.54	436.27	7
5	460.20	230.61	442.19	221.60	D	802.52	401.76	785.49	393.25	784.50	392.76	6
6	573.29	287.15	555.28	278.14	I	687.49	344.25	670.46	335.73			5
7	686.37	343.69	668.36	334.68	I	574.40	287.71	557.38	279.19			4
8	799.46	400.23	781.45	391.23	L	461.32	231.16	444.29	222.65			3
9	856.48	428.74	838.47	419.74	G	348.24	174.62	331.21	166.11			2
10					K	291.21	146.11	274.19	137.60			1

Query 57686 Hit 1

MS/MS Fragmentation of GMGLVNHVFTEDNLK
Found in sp|Q06203|PUR1_HUMAN, Amidophosphoribosyltransferase OS=Homo sapiens GN=PPAT PE=1 SV=1
Match to Query 57686: 1961.028from(654.6834,3+)
Title: 646: Scan 1431 (rt=41.9167, f=3, i=224) [D:\lab212\membrane\Grace\Joyce\iTRAQ_40_1.raw]
Data File:Submitted from 20120508-1(merge) by Mascot Daemon on JOYCE-VAIO
Monoisotopic mass of neutral peptide Mr(calc): 1961.028
Variable modifications:
K15 :iTRAQ4plex (K)
Ions Score: 45.52 Expect: 0.026

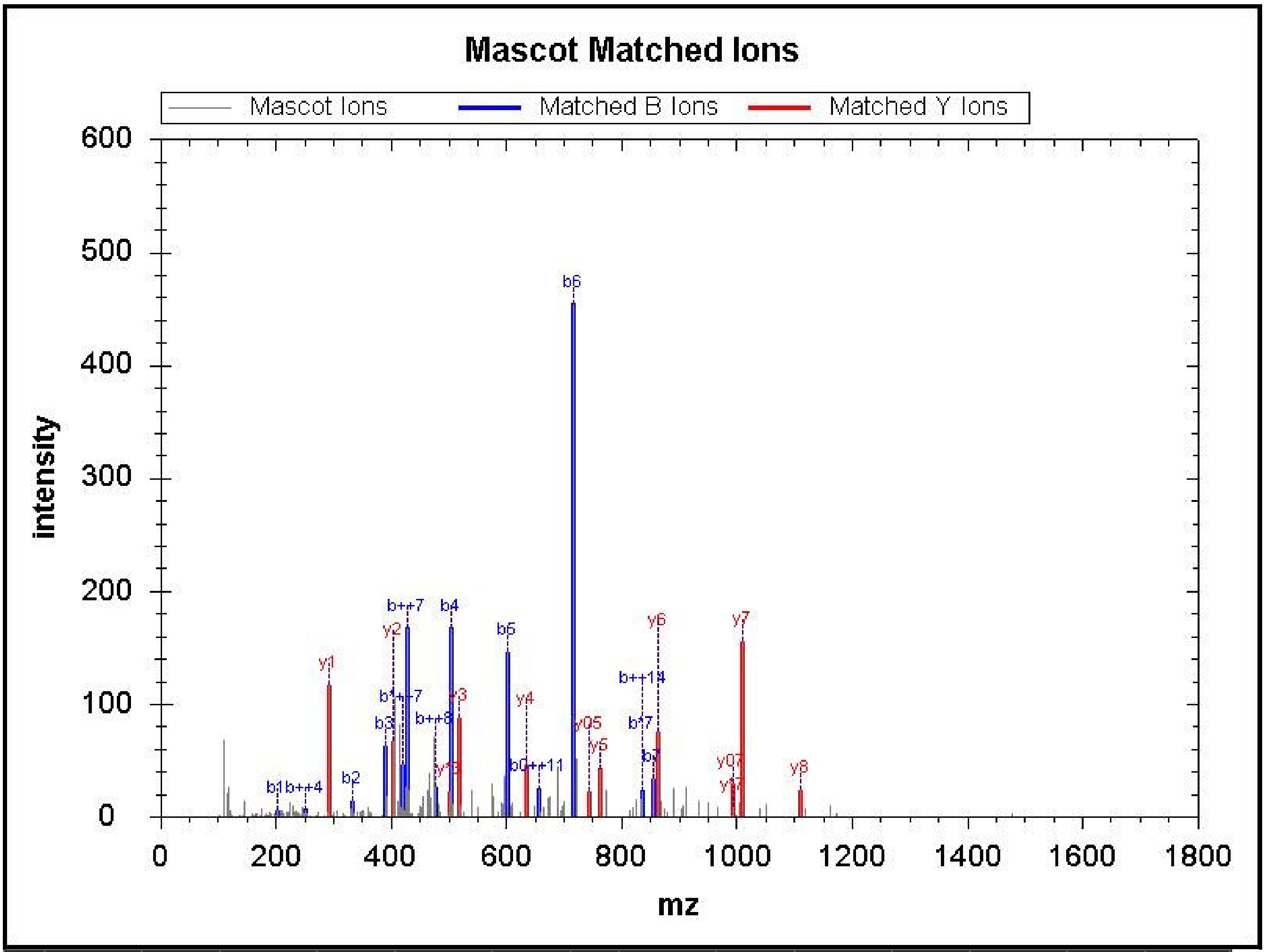

No	b	b++	b*	b*++	b0	b0++	Seq	y	y++	y*	y*++	y0	y0++	RevNo
1	202.13	101.57					G							15
2	333.17	167.09					M	1,760.91	880.96	1,743.89	872.45	1,742.90	871.95	14
3	390.19	195.60					G	1,629.87	815.44	1,612.85	806.93	1,611.86	806.43	13
4	503.28	252.14					L	1,572.85	786.93	1,555.82	778.42	1,554.84	777.92	12

5	602.35	301.68					V	1,459.77	730.39	1,442.74	721.87	1,441.76	721.38	11
6	716.39	358.70	699.36	350.18			N	1,360.70	680.85	1,343.67	672.34	1,342.69	671.85	10
7	853.45	427.23	836.42	418.71			H	1,246.65	623.83	1,229.63	615.32	1,228.64	614.83	9
8	952.52	476.76	935.49	468.25			V	1,109.60	555.30	1,092.57	546.79	1,091.59	546.30	8
9	1,099.58	550.30	1,082.56	541.78			F	1,010.53	505.77	993.50	497.25	992.52	496.76	7
10	1,200.63	600.82	1,183.61	592.31	1,182.62	591.81	T	863.46	432.23	846.43	423.72	845.45	423.23	6
11	1,329.67	665.34	1,312.65	656.83	1,311.66	656.34	E	762.41	381.71	745.38	373.20	744.40	372.70	5
12	1,444.70	722.85	1,427.67	714.34	1,426.69	713.85	D	633.37	317.19	616.34	308.67	615.36	308.18	4
13	1,558.74	779.88	1,541.72	771.36	1,540.73	770.87	N	518.34	259.67	501.32	251.16			3
14	1,671.83	836.42	1,654.80	827.90	1,653.82	827.41	L	404.30	202.65	387.27	194.14			2
15							K	291.21	146.11	274.19	137.60			1

Query 79330 Hit 1

MS/MS Fragmentation of QMNAFLEGFTELLPIDLIK
Found in sp|Q96PU5|NED4L_HUMAN, E3 ubiquitin-protein ligase NEDD4-like OS=Homo sapiens GN=NEDD4L PE=1 SV=2
Match to Query 79330: 2479.369from(827.4637,3+)
Title: 1216: Sum of 2 scans in range 3390 (rt=82.1473, f=2, i=467) to 3391 (rt=82.1727, f=2, i=468)
[D:\lab212\membrane\Grace\Joyce\iTRAQ_25_1.raw]
Data File:Submitted from 20120508-1(merge) by Mascot Daemon on JOYCE-VAIO
Monoisotopic mass of neutral peptide Mr(calc): 2479.369
Variable modifications:
K19 :iTRAQ4plex (K)
Ions Score: 45.51 Expect: 0.016

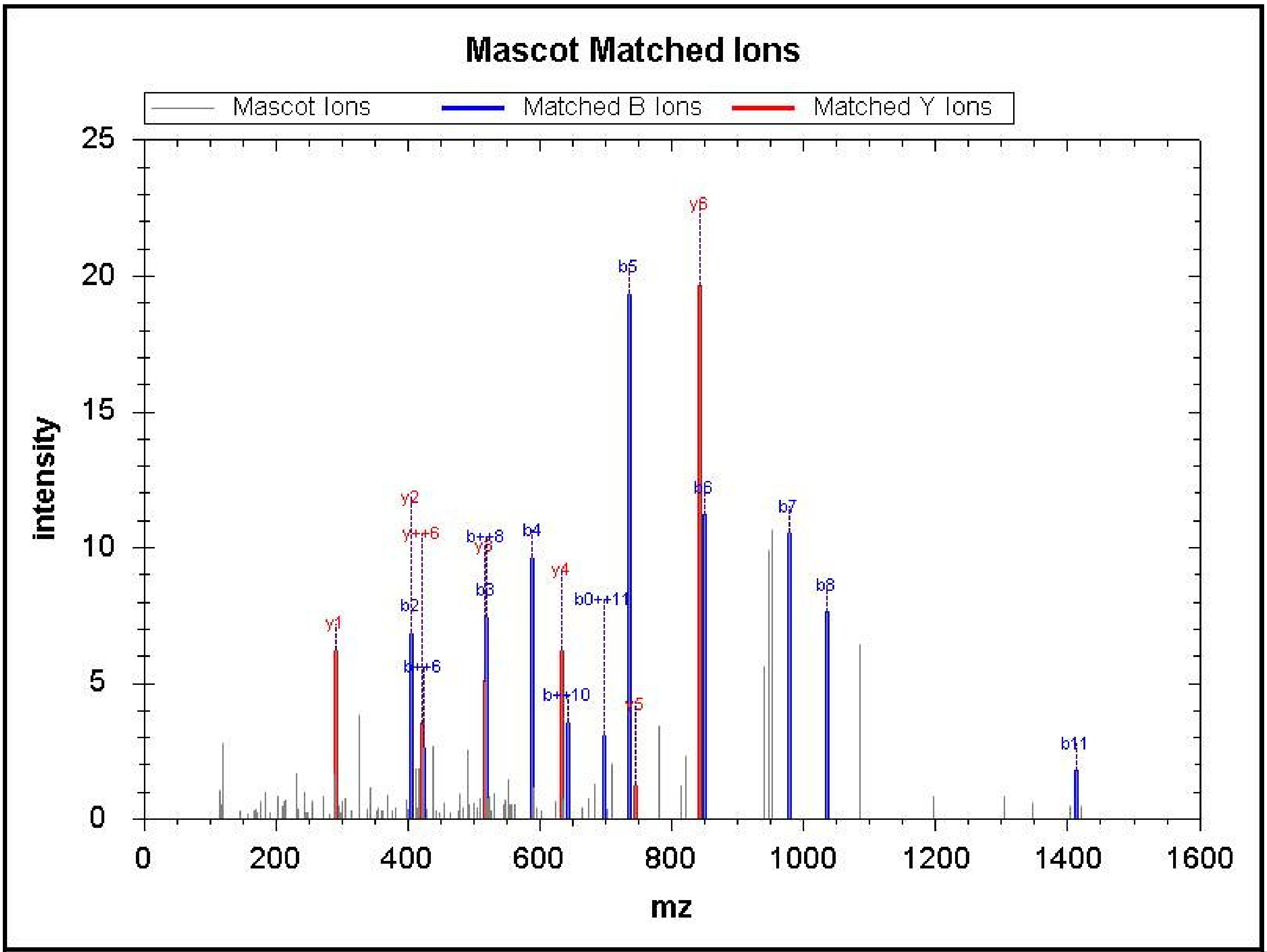

No	b	b++	b*	b*++	b0	b0++	Seq	y	y++	y*	y*++	y0	y0++	RevNo
1	273.17	137.09	256.14	128.57			Q							19
2	404.21	202.61	387.18	194.09			M	2,208.21	1,104.61	2,191.18	1,096.10	2,190.20	1,095.60	18

3	518.25	259.63	501.22	251.12			N	2,077.17	1,039.09	2,060.14	1,030.58	2,059.16	1,030.08	17
4	589.29	295.15	572.26	286.63			A	1,963.13	982.07	1,946.10	973.55	1,945.12	973.06	16
5	736.36	368.68	719.33	360.17			F	1,892.09	946.55	1,875.06	938.04	1,874.08	937.54	15
6	849.44	425.22	832.41	416.71			L	1,745.02	873.01	1,728.00	864.50	1,727.01	864.01	14
7	978.48	489.75	961.46	481.23	960.47	480.74	E	1,631.94	816.47	1,614.91	807.96	1,613.93	807.47	13
8	1,035.51	518.26	1,018.48	509.74	1,017.49	509.25	G	1,502.90	751.95	1,485.87	743.44	1,484.88	742.95	12
9	1,182.57	591.79	1,165.55	583.28	1,164.56	582.79	F	1,445.87	723.44	1,428.85	714.93	1,427.86	714.44	11
10	1,283.62	642.31	1,266.59	633.80	1,265.61	633.31	T	1,298.81	649.91	1,281.78	641.39	1,280.79	640.90	10
11	1,412.66	706.84	1,395.64	698.32	1,394.65	697.83	E	1,197.76	599.38	1,180.73	590.87	1,179.75	590.38	9
12	1,525.75	763.38	1,508.72	754.86	1,507.74	754.37	L	1,068.71	534.86	1,051.69	526.35	1,050.70	525.86	8
13	1,638.83	819.92	1,621.81	811.41	1,620.82	810.91	L	955.63	478.32	938.60	469.81	937.62	469.31	7
14	1,735.88	868.45	1,718.86	859.93	1,717.87	859.44	P	842.55	421.78	825.52	413.26	824.54	412.77	6
15	1,848.97	924.99	1,831.94	916.47	1,830.96	915.98	I	745.49	373.25	728.47	364.74	727.48	364.25	5
16	1,964.00	982.50	1,946.97	973.99	1,945.99	973.50	D	632.41	316.71	615.38	308.20	614.40	307.70	4
17	2,077.08	1,039.04	2,060.05	1,030.53	2,059.07	1,030.04	L	517.38	259.20	500.36	250.68			3
18	2,190.16	1,095.59	2,173.14	1,087.07	2,172.15	1,086.58	I	404.30	202.65	387.27	194.14			2
19							K	291.21	146.11	274.19	137.60			1

Query 27463 Hit 1

MS/MS Fragmentation of YIFHNFMER
Found in sp|Q14624|ITIH4_HUMAN, Inter-alpha-trypsin inhibitor heavy chain H4 OS=Homo sapiens GN=ITIH4 PE=1 SV=4
Match to Query 27463: 1399.688from(467.5699,3+)
Title: 635: Scan 1422 (rt=41.6552, f=2, i=231) [D:\lab212\membrane\Grace\Joyce\iTRAQ_41_1.raw]
Data File:Submitted from 20120508-1(merge) by Mascot Daemon on JOYCE-VAIO
Monoisotopic mass of neutral peptide Mr(calc): 1399.688
Variable modifications:
Ions Score: 45.51 Expect: 0.018

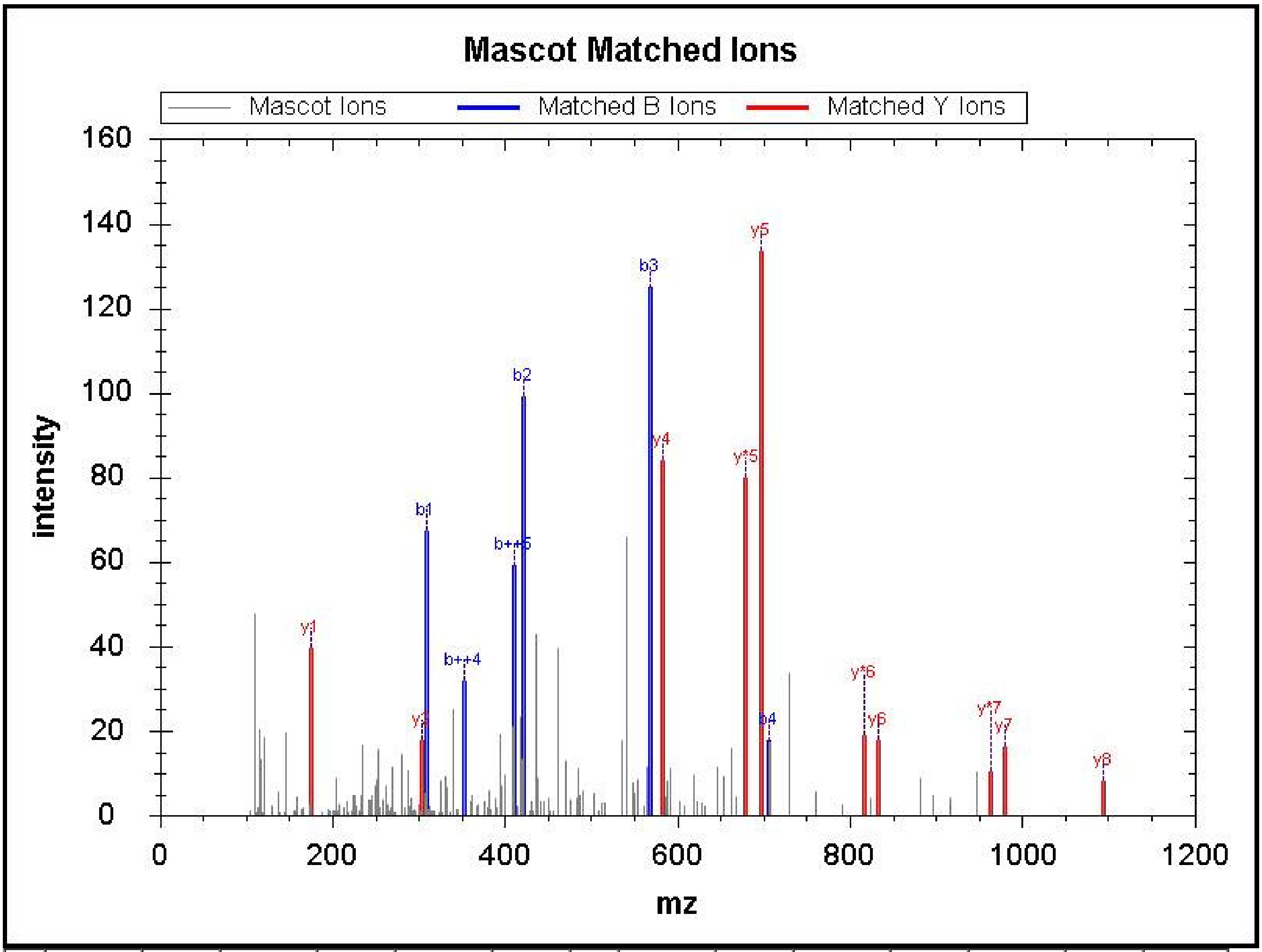

No	b	b++	b*	b*++	b0	b0++	Seq	y	y++	y*	y*++	y0	y0++	RevNo
1	308.17	154.59					Y							9
2	421.26	211.13					I	1,093.52	547.27	1,076.50	538.75	1,075.51	538.26	8
3	568.33	284.67					F	980.44	490.72	963.41	482.21	962.43	481.72	7
4	705.38	353.20					H	833.37	417.19	816.35	408.68	815.36	408.18	6
5	819.43	410.22	802.40	401.70			N	696.31	348.66	679.29	340.15	678.30	339.65	5
6	966.50	483.75	949.47	475.24			F	582.27	291.64	565.24	283.13	564.26	282.63	4
7	1,097.54	549.27	1,080.51	540.76			M	435.20	218.10	418.18	209.59	417.19	209.10	3
8	1,226.58	613.79	1,209.55	605.28	1,208.57	604.79	E	304.16	152.58	287.13	144.07	286.15	143.58	2
9							R	175.12	88.06	158.09	79.55			1

Query 58400 Hit 1

MS/MS Fragmentation of IKDLLQQMEEGLK
Found in sp|Q9NS86|LANC2_HUMAN, LanC-like protein 2 OS=Homo sapiens GN=LANCL2 PE=1 SV=1
Match to Query 58400: 1976.141 from(659.7211,3+)
Title: 711: Scan 2166 (rt=56.2634, f=2, i=383) [D:\lab212\membrane\Grace\Joyce\iTRAQ_46_1.raw]
Data File:Submitted from 20120508-1(merge) by Mascot Daemon on JOYCE-VAIO
Monoisotopic mass of neutral peptide Mr(calc): 1976.141
Variable modifications:
K2 :iTRAQ4plex (K)
K13 :iTRAQ4plex (K)
Ions Score: 45.47 Expect: 0.014

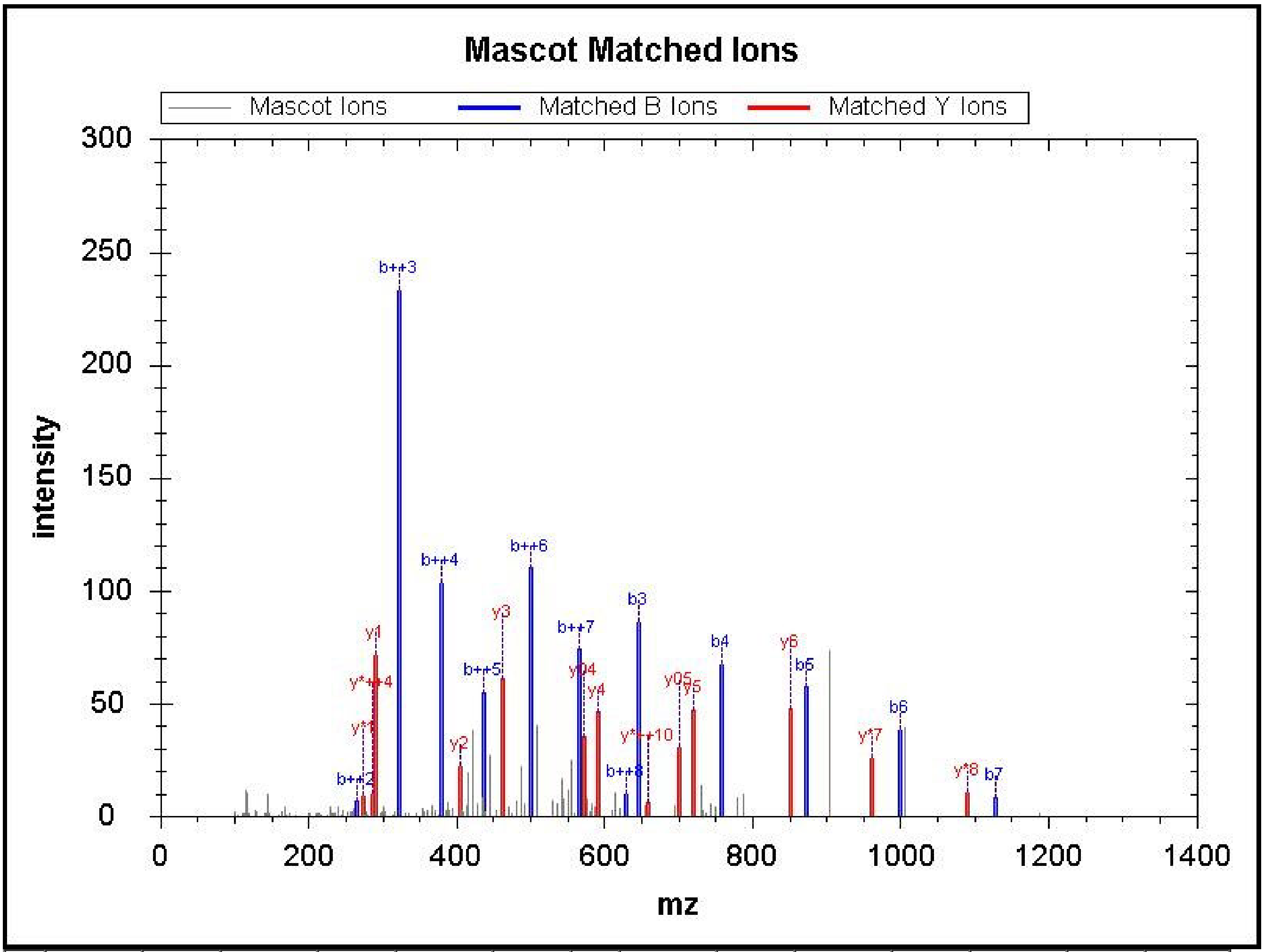

No	b	b++	b*	b*++	b0	b0++	Seq	y	y++	y*	y*++	y0	y0++	RevNo
1	258.19	129.60					I							13
2	530.39	265.70	513.36	257.19			K	1,719.96	860.48	1,702.93	851.97	1,701.94	851.48	12
3	645.42	323.21	628.39	314.70	627.41	314.21	D	1,447.76	724.38	1,430.73	715.87	1,429.75	715.38	11
4	758.50	379.75	741.47	371.24	740.49	370.75	L	1,332.73	666.87	1,315.70	658.36	1,314.72	657.86	10
5	871.59	436.30	854.56	427.78	853.57	427.29	L	1,219.65	610.33	1,202.62	601.81	1,201.64	601.32	9
6	999.64	500.33	982.62	491.81	981.63	491.32	Q	1,106.56	553.79	1,089.54	545.27	1,088.55	544.78	8
7	1,127.70	564.35	1,110.68	555.84	1,109.69	555.35	Q	978.50	489.76	961.48	481.24	960.49	480.75	7
8	1,258.74	629.88	1,241.72	621.36	1,240.73	620.87	M	850.45	425.73	833.42	417.21	832.44	416.72	6
9	1,387.79	694.40	1,370.76	685.88	1,369.78	685.39	E	719.41	360.21	702.38	351.69	701.39	351.20	5
10	1,516.83	758.92	1,499.80	750.40	1,498.82	749.91	E	590.36	295.69	573.34	287.17	572.35	286.68	4
11	1,573.85	787.43	1,556.82	778.92	1,555.84	778.42	G	461.32	231.16	444.29	222.65			3
12	1,686.93	843.97	1,669.91	835.46	1,668.92	834.97	L	404.30	202.65	387.27	194.14			2
13							K	291.21	146.11	274.19	137.60			1

Query 36916 Hit 1

MS/MS Fragmentation of AFVRPSGTEDVVR
Found in sp|O95394|AGM1_HUMAN, Phosphoacetylglucosamine mutase OS=Homo sapiens GN=PGM3 PE=1 SV=1
Match to Query 36916: 1575.844from(526.2888,3+)
Title: 283: Scan 719 (rt=25.5261, f=3, i=99) [D:\lab212\membrane\Grace\Joyce\iTRAQ_38_2.raw]
Data File:Submitted from 20120508-1(merge) by Mascot Daemon on JOYCE-VAIO
Monoisotopic mass of neutral peptide Mr(calc): 1575.844
Variable modifications:
Ions Score: 45.47 Expect: 0.025

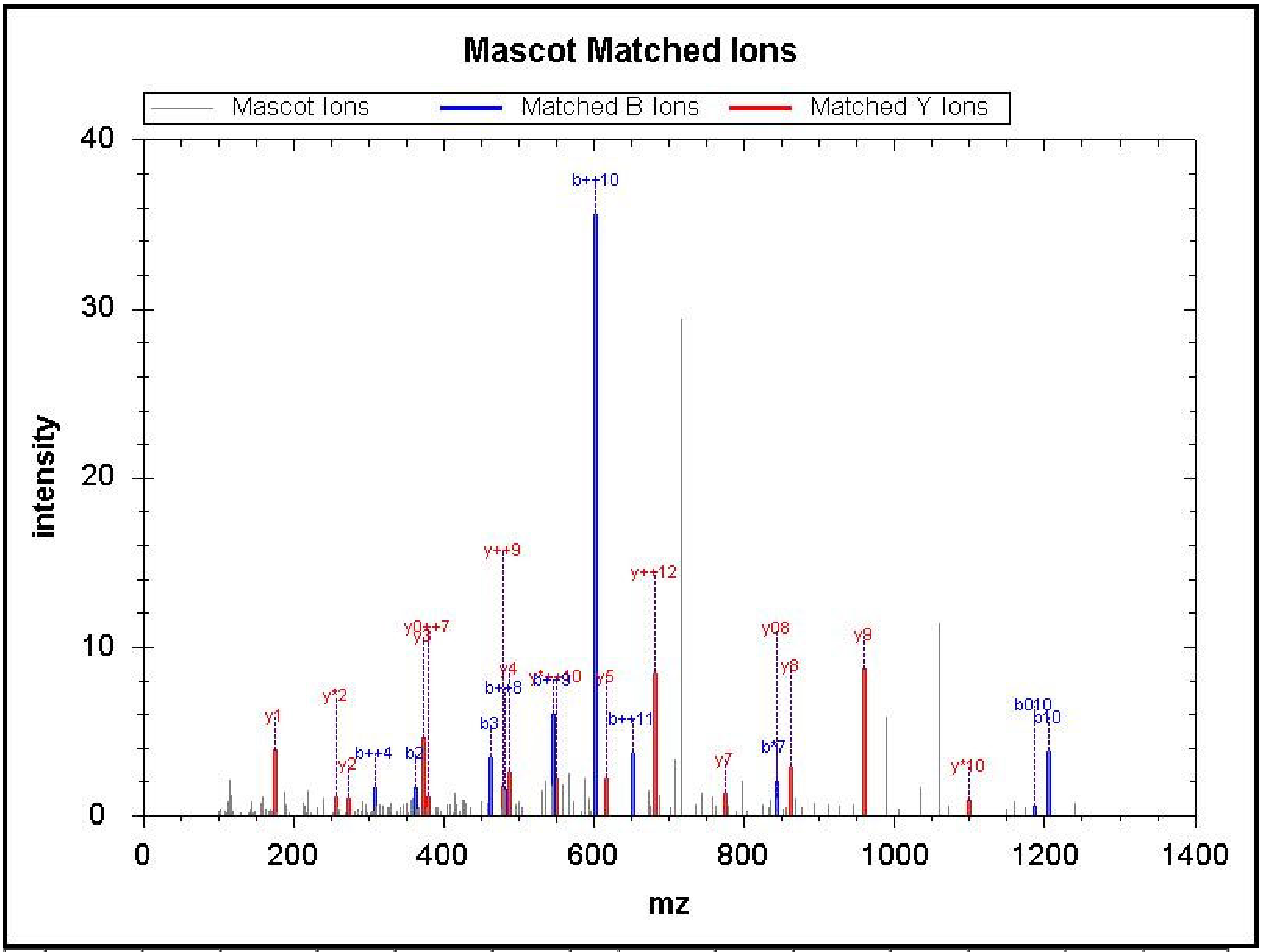

No	b	b++	b*	b*++	b0	b0++	Seq	y	y++	y*	y*++	y0	y0++	RevNo
1	216.15	108.58					A							13
2	363.21	182.11					F	1,361.72	681.36	1,344.69	672.85	1,343.71	672.36	12
3	462.28	231.65					V	1,214.65	607.83	1,197.62	599.31	1,196.64	598.82	11
4	618.38	309.70	601.36	301.18			R	1,115.58	558.29	1,098.55	549.78	1,097.57	549.29	10
5	715.44	358.22	698.41	349.71			P	959.48	480.24	942.45	471.73	941.47	471.24	9
6	802.47	401.74	785.44	393.22	784.46	392.73	S	862.43	431.72	845.40	423.20	844.42	422.71	8
7	859.49	430.25	842.46	421.74	841.48	421.24	G	775.39	388.20	758.37	379.69	757.38	379.20	7
8	960.54	480.77	943.51	472.26	942.53	471.77	T	718.37	359.69	701.35	351.18	700.36	350.68	6
9	1,089.58	545.29	1,072.55	536.78	1,071.57	536.29	E	617.33	309.17	600.30	300.65	599.31	300.16	5
10	1,204.61	602.81	1,187.58	594.29	1,186.60	593.80	D	488.28	244.65	471.26	236.13	470.27	235.64	4
11	1,303.68	652.34	1,286.65	643.83	1,285.67	643.34	V	373.26	187.13	356.23	178.62			3
12	1,402.74	701.88	1,385.72	693.36	1,384.73	692.87	V	274.19	137.60	257.16	129.08			2
13							R	175.12	88.06	158.09	79.55			1

Query 46054 Hit 1

MS/MS Fragmentation of EAFEEAGVLLLRPR
Found in sp|A8MXV4|NUD19_HUMAN, Nucleoside diphosphate-linked moiety X motif 19
Match to Query 46054: 1742.979from(582.0001,3+)
Title: 741: Sum of 2 scans in range 1674 (rt=47.0946, f=4, i=497) to 1675 (rt=47.12, f=4, i=498) [D:\lab212\membrane\Grace\Joyce\iTRAQ_35_1.raw]
Data File:Submitted from 20120508-1(merge) by Mascot Daemon on JOYCE-VAIO
Monoisotopic mass of neutral peptide Mr(calc): 1742.979
Variable modifications:
Ions Score: 45.41 Expect: 0.020

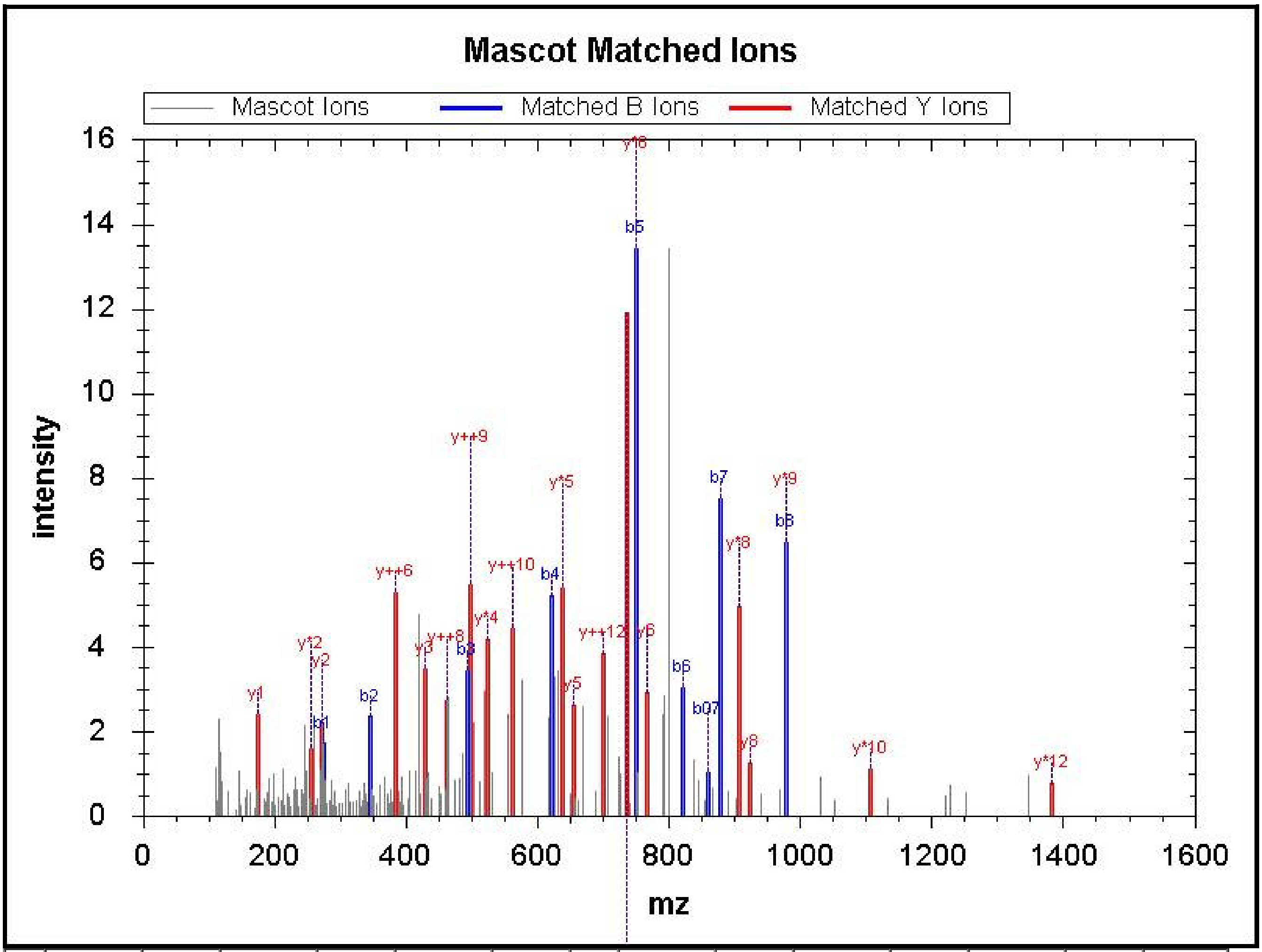

No	b	b++	b*	b*++	b0	b0++	Seq	y	y++	y*	y*++	y0	y0++	RevNo
1	274.15	137.58			256.14	128.57	E							14
2	345.19	173.10			327.18	164.09	A	1,470.84	735.93	1,453.82	727.41	1,452.83	726.92	13
3	492.26	246.63			474.25	237.63	F	1,399.81	700.41	1,382.78	691.89	1,381.80	691.40	12
4	621.30	311.15			603.29	302.15	E	1,252.74	626.87	1,235.71	618.36	1,234.73	617.87	11
5	750.34	375.67			732.33	366.67	E	1,123.69	562.35	1,106.67	553.84	1,105.68	553.35	10
6	821.38	411.19			803.37	402.19	A	994.65	497.83	977.63	489.32			9
7	878.40	439.70			860.39	430.70	G	923.61	462.31	906.59	453.80			8
8	977.47	489.24			959.46	480.23	V	866.59	433.80	849.57	425.29			7
9	1,090.55	545.78			1,072.54	536.78	L	767.52	384.27	750.50	375.75			6
10	1,203.64	602.32			1,185.63	593.32	L	654.44	327.72	637.41	319.21			5
11	1,316.72	658.86			1,298.71	649.86	L	541.36	271.18	524.33	262.67			4
12	1,472.82	736.92	1,455.80	728.40	1,454.81	727.91	R	428.27	214.64	411.25	206.13			3
13	1,569.88	785.44	1,552.85	776.93	1,551.87	776.44	P	272.17	136.59	255.15	128.08			2
14							R	175.12	88.06	158.09	79.55			1

Query 84451 Hit 1

MS/MS Fragmentation of EVAQQAVDADVHAVGVSTLAAGHK
Found in sp|P16332|MUTA_MOUSE, Methylmalonyl-CoA mutase
Match to Query 84451: 2660.409from(666.1095,4+)
Title: 524: Scan 1211 (rt=36.8681, f=3, i=184) [D:\lab212\membrane\Grace\Joyce\iTRAQ_44_1.raw]
Data File:Submitted from 20120508-1(merge) by Mascot Daemon on JOYCE-VAIO
Monoisotopic mass of neutral peptide Mr(calc): 2660.409
Variable modifications:
K24 :iTRAQ4plex (K)
Ions Score: 136.01 Expect: 0.000

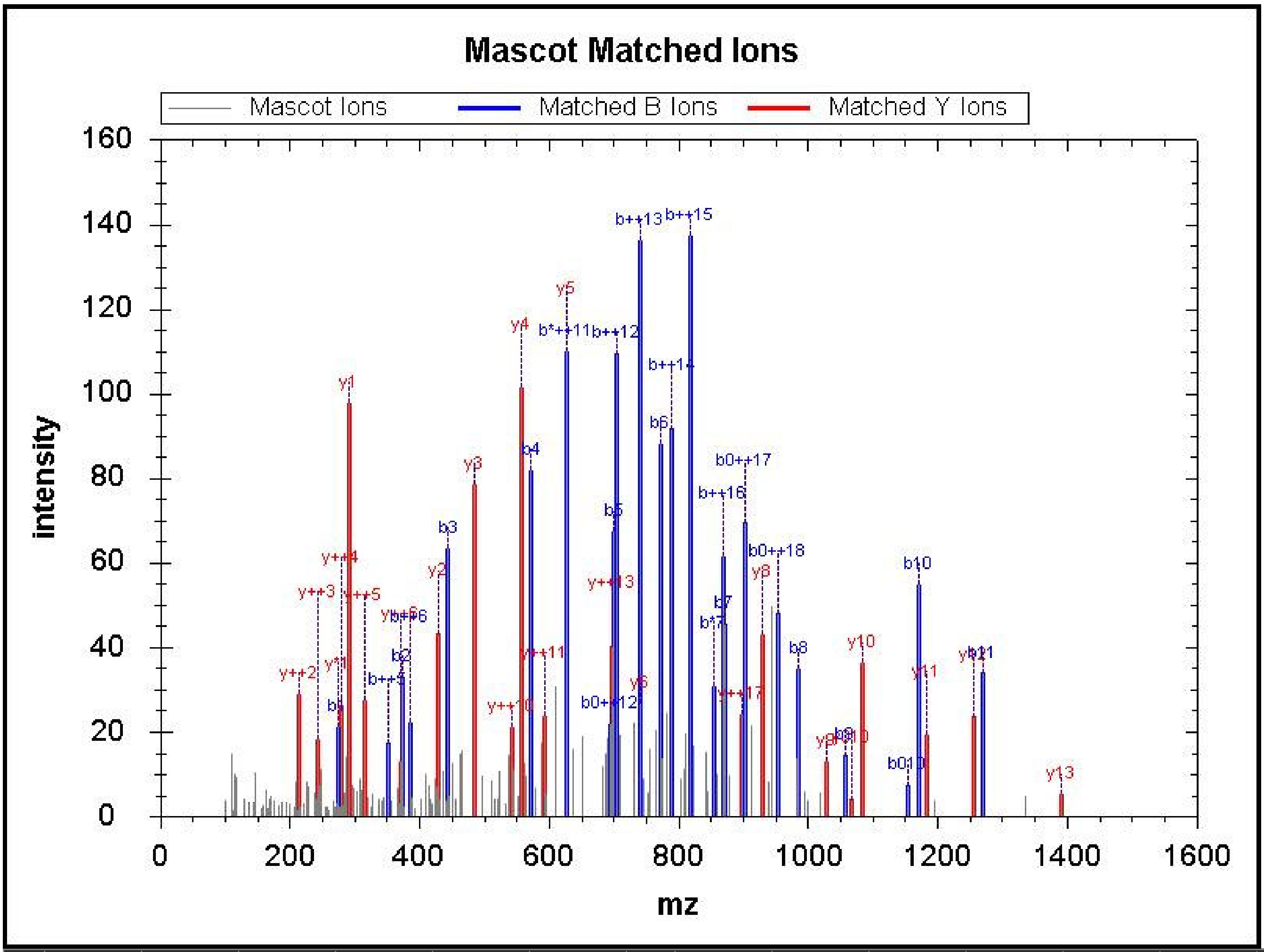

No	b	b++	b*	b*++	b0	b0++	Seq	y	y++	y*	y*++	y0	y0++	RevNo
1	274.15	137.58			256.14	128.57	E							24
2	373.22	187.11			355.21	178.11	V	2,388.27	1,194.64	2,371.25	1,186.13	2,370.26	1,185.64	23
3	444.26	222.63			426.25	213.63	A	2,289.21	1,145.11	2,272.18	1,136.59	2,271.20	1,136.10	22
4	572.32	286.66	555.29	278.15	554.31	277.66	Q	2,218.17	1,109.59	2,201.14	1,101.08	2,200.16	1,100.58	21
5	700.37	350.69	683.35	342.18	682.36	341.69	Q	2,090.11	1,045.56	2,073.08	1,037.05	2,072.10	1,036.55	20
6	771.41	386.21	754.39	377.70	753.40	377.20	A	1,962.05	981.53	1,945.03	973.02	1,944.04	972.52	19
7	870.48	435.74	853.45	427.23	852.47	426.74	V	1,891.02	946.01	1,873.99	937.50	1,873.00	937.01	18
8	985.51	493.26	968.48	484.74	967.50	484.25	D	1,791.95	896.48	1,774.92	887.96	1,773.94	887.47	17
9	1,056.54	528.78	1,039.52	520.26	1,038.53	519.77	A	1,676.92	838.96	1,659.89	830.45	1,658.91	829.96	16
10	1,171.57	586.29	1,154.54	577.78	1,153.56	577.28	D	1,605.88	803.45	1,588.86	794.93	1,587.87	794.44	15
11	1,270.64	635.82	1,253.61	627.31	1,252.63	626.82	V	1,490.86	745.93	1,473.83	737.42	1,472.85	736.93	14
12	1,407.70	704.35	1,390.67	695.84	1,389.69	695.35	H	1,391.79	696.40	1,374.76	687.88	1,373.78	687.39	13
13	1,478.74	739.87	1,461.71	731.36	1,460.73	730.87	A	1,254.73	627.87	1,237.70	619.35	1,236.72	618.86	12
14	1,577.80	789.41	1,560.78	780.89	1,559.79	780.40	V	1,183.69	592.35	1,166.66	583.84	1,165.68	583.34	11
15	1,634.83	817.92	1,617.80	809.40	1,616.81	808.91	G	1,084.62	542.82	1,067.60	534.30	1,066.61	533.81	10

16	1,733.89	867.45	1,716.87	858.94	1,715.88	858.45	V	1,027.60	514.30	1,010.58	505.79	1,009.59	505.30	9
17	1,820.93	910.97	1,803.90	902.45	1,802.92	901.96	S	928.53	464.77	911.51	456.26	910.52	455.76	8
18	1,921.97	961.49	1,904.95	952.98	1,903.96	952.49	T	841.50	421.25	824.47	412.74	823.49	412.25	7
19	2,035.06	1,018.03	2,018.03	1,009.52	2,017.05	1,009.03	L	740.45	370.73	723.43	362.22			6
20	2,106.09	1,053.55	2,089.07	1,045.04	2,088.08	1,044.55	A	627.37	314.19	610.34	305.68			5
21	2,177.13	1,089.07	2,160.11	1,080.56	2,159.12	1,080.06	A	556.33	278.67	539.31	270.16			4
22	2,234.15	1,117.58	2,217.13	1,109.07	2,216.14	1,108.58	G	485.30	243.15	468.27	234.64			3
23	2,371.21	1,186.11	2,354.19	1,177.60	2,353.20	1,177.10	H	428.27	214.64	411.25	206.13			2
24							K	291.21	146.11	274.19	137.60			1

Query 38682 Hit 1

MS/MS Fragmentation of DESTNVDMSLAQR
Found in sp|P50995|ANX11_HUMAN, Annexin A11 OS=Homo sapiens GN=ANXA11 PE=1 SV=1
Match to Query 38682: 1608.755from(805.3846,2+)
Title: 163: Scan 930 (rt=28.0072, f=3, i=57) [D:\lab212\membrane\Grace\Joyce\iTRAQ_22_2.raw]
Data File:Submitted from 20120508-1(merge) by Mascot Daemon on JOYCE-VAIO
Monoisotopic mass of neutral peptide Mr(calc): 1608.755
Variable modifications:
Ions Score: 106.56 Expect: 0.000

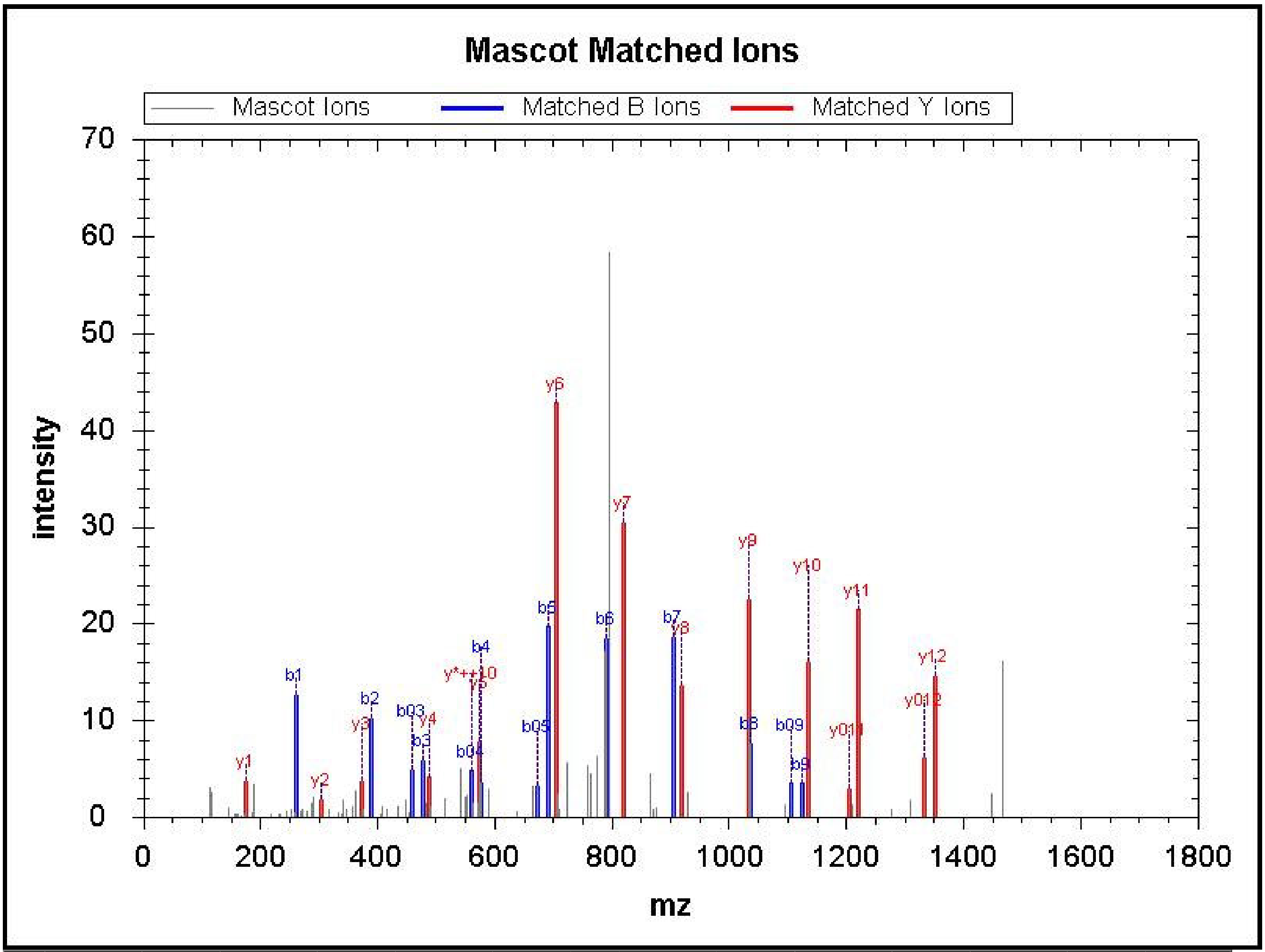

No	b	b++	b*	b*++	b0	b0++	Seq	y	y++	y*	y*++	y0	y0++	RevNo
1	260.14	130.57			242.13	121.57	D							13
2	389.18	195.09			371.17	186.09	E	1,350.63	675.82	1,333.61	667.31	1,332.62	666.81	12
3	476.21	238.61			458.20	229.60	S	1,221.59	611.30	1,204.56	602.78	1,203.58	602.29	11
4	577.26	289.13			559.25	280.13	T	1,134.56	567.78	1,117.53	559.27	1,116.55	558.78	10
5	691.30	346.15	674.27	337.64	673.29	337.15	N	1,033.51	517.26	1,016.48	508.75	1,015.50	508.25	9
6	790.37	395.69	773.34	387.18	772.36	386.68	V	919.47	460.24	902.44	451.72	901.46	451.23	8

7	905.40	453.20	888.37	444.69	887.39	444.20	D	820.40	410.70	803.37	402.19	802.39	401.70	7
8	1,036.44	518.72	1,019.41	510.21	1,018.43	509.72	M	705.37	353.19	688.34	344.68	687.36	344.18	6
9	1,123.47	562.24	1,106.44	553.73	1,105.46	553.23	S	574.33	287.67	557.30	279.16	556.32	278.66	5
10	1,236.55	618.78	1,219.53	610.27	1,218.54	609.78	L	487.30	244.15	470.27	235.64			4
11	1,307.59	654.30	1,290.56	645.79	1,289.58	645.29	A	374.21	187.61	357.19	179.10			3
12	1,435.65	718.33	1,418.62	709.82	1,417.64	709.32	Q	303.18	152.09	286.15	143.58			2
13							R	175.12	88.06	158.09	79.55			1

Query 85745 Hit 1

MS/MS Fragmentation of IHSEVVEDTEAVSAVQQLLDDER
Found in sp|Q96GA7|SDSL_HUMAN, Serine dehydratase-like OS=Homo sapiens GN=SDSL PE=1 SV=1
Match to Query 85745: 2725.35from(909.4572,3+)
Title: 908: Scan 2000 (rt=54.5787, f=2, i=316) [D:\lab212\membrane\Grace\Joyce\iTRAQ_34_2.raw]
Data File:Submitted from 20120508-1(merge) by Mascot Daemon on JOYCE-VAIO
Monoisotopic mass of neutral peptide Mr(calc): 2725.35
Variable modifications:
Ions Score: 105.8 Expect: 0.000

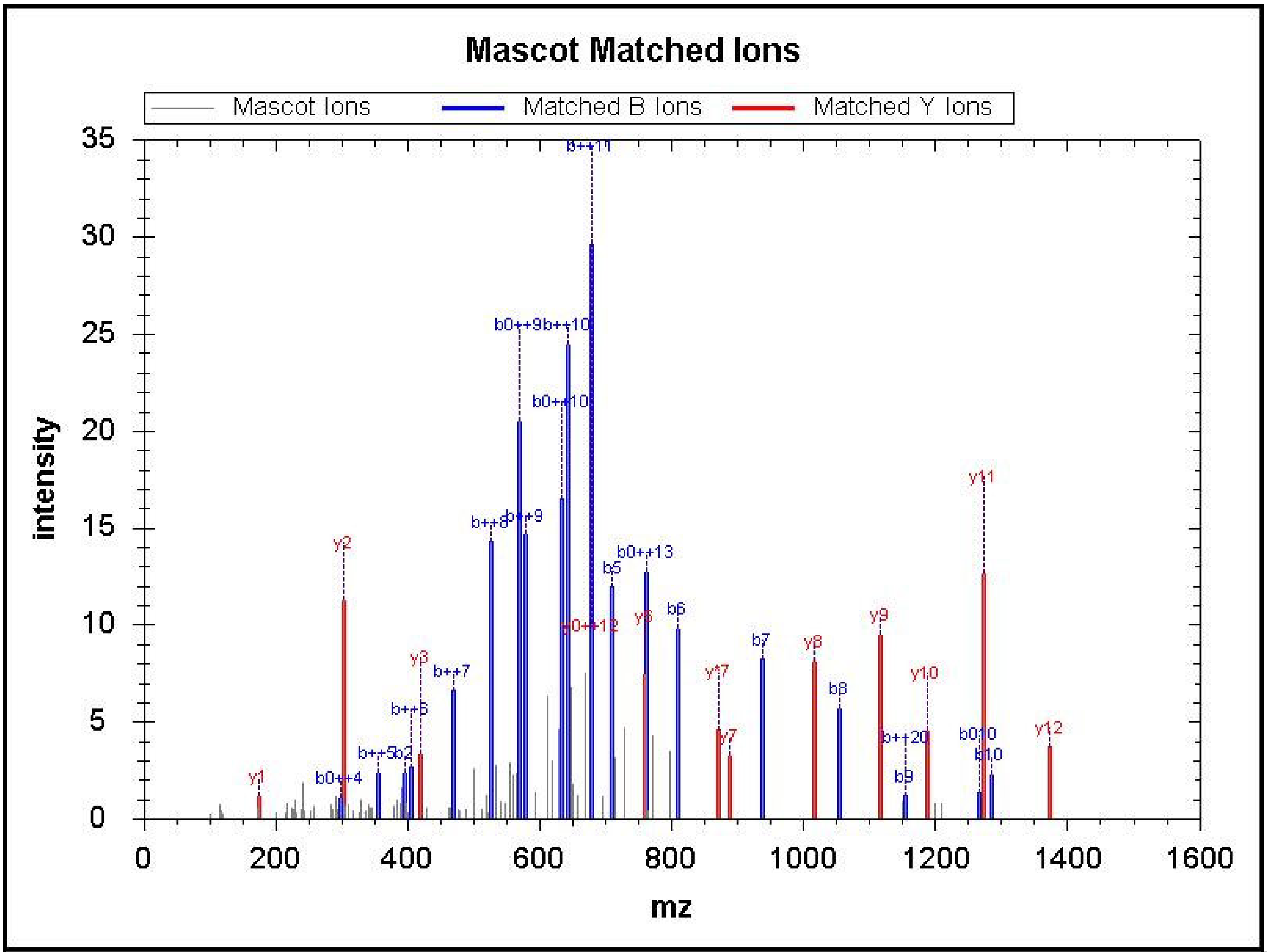

No	b	b++	b*	b*++	b0	b0++	Seq	y	y++	y*	y*++	y0	y0++	RevNo
1	258.19	129.60					I							23
2	395.25	198.13					H	2,469.17	1,235.09	2,452.15	1,226.58	2,451.16	1,226.09	22
3	482.28	241.65			464.27	232.64	S	2,332.11	1,166.56	2,315.09	1,158.05	2,314.10	1,157.56	21
4	611.33	306.17			593.32	297.16	E	2,245.08	1,123.05	2,228.06	1,114.53	2,227.07	1,114.04	20
5	710.40	355.70			692.38	346.70	V	2,116.04	1,058.52	2,099.01	1,050.01	2,098.03	1,049.52	19
6	809.46	405.24			791.45	396.23	V	2,016.97	1,008.99	1,999.95	1,000.48	1,998.96	999.98	18
7	938.51	469.76			920.50	460.75	E	1,917.90	959.46	1,900.88	950.94	1,899.89	950.45	17
8	1,053.53	527.27			1,035.52	518.27	D	1,788.86	894.93	1,771.83	886.42	1,770.85	885.93	16

9	1,154.58	577.79			1,136.57	568.79	T	1,673.83	837.42	1,656.81	828.91	1,655.82	828.42	15
10	1,283.62	642.32			1,265.61	633.31	E	1,572.79	786.90	1,555.76	778.38	1,554.78	777.89	14
11	1,354.66	677.83			1,336.65	668.83	A	1,443.74	722.38	1,426.72	713.86	1,425.73	713.37	13
12	1,453.73	727.37			1,435.72	718.36	V	1,372.71	686.86	1,355.68	678.34	1,354.70	677.85	12
13	1,540.76	770.88			1,522.75	761.88	S	1,273.64	637.32	1,256.61	628.81	1,255.63	628.32	11
14	1,611.80	806.40			1,593.79	797.40	A	1,186.61	593.81	1,169.58	585.29	1,168.60	584.80	10
15	1,710.87	855.94			1,692.86	846.93	V	1,115.57	558.29	1,098.54	549.77	1,097.56	549.28	9
16	1,838.93	919.97	1,821.90	911.45	1,820.91	910.96	Q	1,016.50	508.75	999.47	500.24	998.49	499.75	8
17	1,966.98	984.00	1,949.96	975.48	1,948.97	974.99	Q	888.44	444.72	871.42	436.21	870.43	435.72	7
18	2,080.07	1,040.54	2,063.04	1,032.02	2,062.06	1,031.53	L	760.38	380.70	743.36	372.18	742.37	371.69	6
19	2,193.15	1,097.08	2,176.13	1,088.57	2,175.14	1,088.07	L	647.30	324.15	630.27	315.64	629.29	315.15	5
20	2,308.18	1,154.59	2,291.15	1,146.08	2,290.17	1,145.59	D	534.22	267.61	517.19	259.10	516.20	258.61	4
21	2,423.21	1,212.11	2,406.18	1,203.59	2,405.20	1,203.10	D	419.19	210.10	402.16	201.58	401.18	201.09	3
22	2,552.25	1,276.63	2,535.22	1,268.11	2,534.24	1,267.62	E	304.16	152.58	287.13	144.07	286.15	143.58	2
23							R	175.12	88.06	158.09	79.55			1

Query 53024 Hit 1

MS/MS Fragmentation of SELEEQLTPVAEETR
Found in sp|P02649|APOE_HUMAN, Apolipoprotein E OS=Homo sapiens GN=APOE PE=1 SV=1
Match to Query 53024: 1873.943 from(937.9787,2+)
Title: 198: Sum of 2 scans in range 1367 (rt=37.0315, f=4, i=160) to 1368 (rt=37.0569, f=4, i=161)
[D:\lab212\membrane\Grace\Joyce\iTRAQ_19_2_330.raw]
Data File:Submitted from 20120508-1(merge) by Mascot Daemon on JOYCE-VAIO
Monoisotopic mass of neutral peptide Mr(calc): 1873.943
Variable modifications:
Ions Score: 103.35 Expect: 0.000

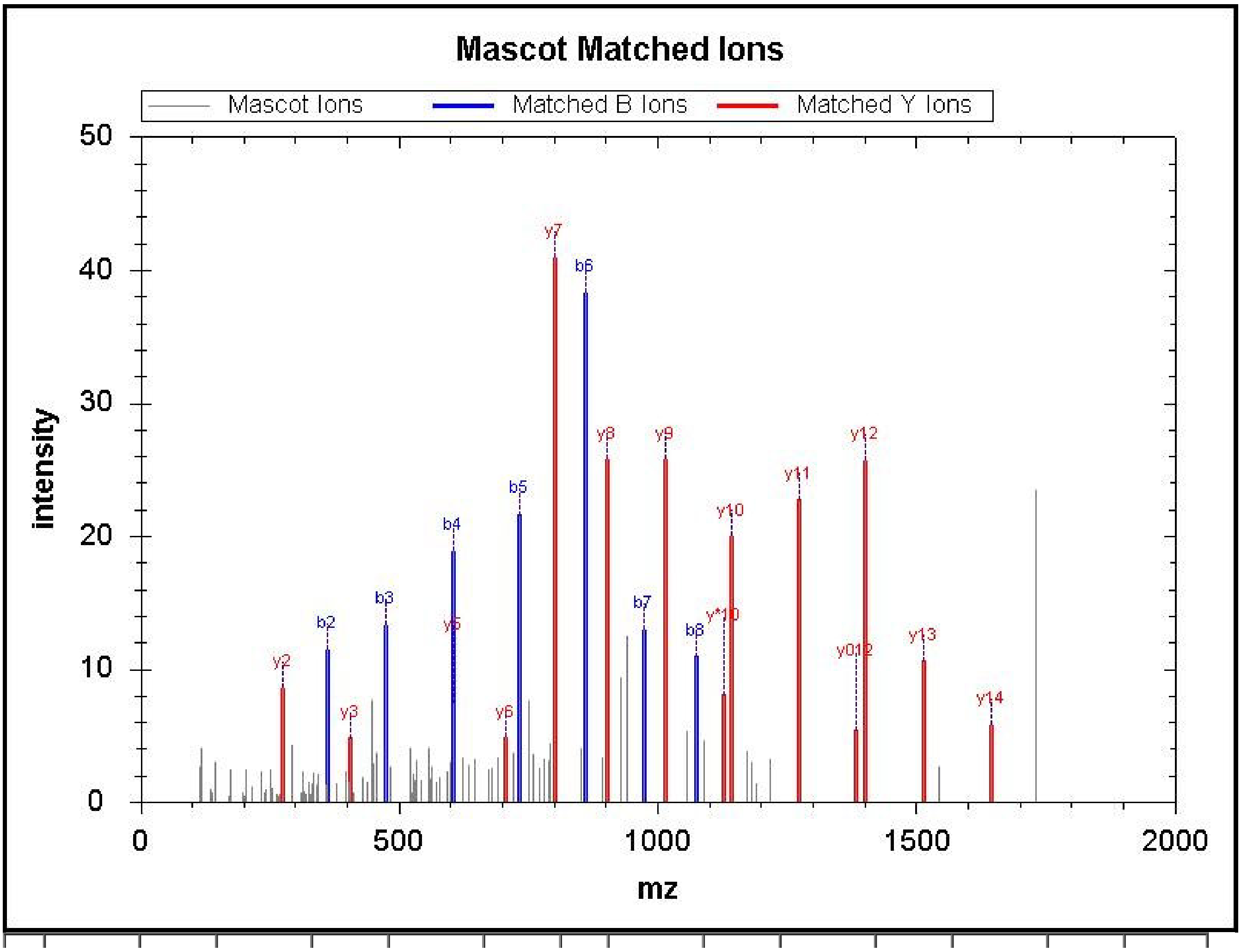

No	b	b++	b*	b*++	b0	b0++	Seq	y	y++	y*	y*++	y0	y0++	RevNo
1	232.14	116.57			214.13	107.57	S							15
2	361.18	181.10			343.17	172.09	E	1,643.81	822.41	1,626.79	813.90	1,625.80	813.40	14
3	474.27	237.64			456.26	228.63	L	1,514.77	757.89	1,497.74	749.38	1,496.76	748.88	13
4	603.31	302.16			585.30	293.15	E	1,401.69	701.35	1,384.66	692.83	1,383.67	692.34	12
5	732.35	366.68			714.34	357.67	E	1,272.64	636.83	1,255.62	628.31	1,254.63	627.82	11
6	860.41	430.71	843.39	422.20	842.40	421.70	Q	1,143.60	572.30	1,126.57	563.79	1,125.59	563.30	10
7	973.50	487.25	956.47	478.74	955.49	478.25	L	1,015.54	508.27	998.52	499.76	997.53	499.27	9
8	1,074.54	537.78	1,057.52	529.26	1,056.53	528.77	T	902.46	451.73	885.43	443.22	884.45	442.73	8
9	1,171.60	586.30	1,154.57	577.79	1,153.59	577.30	P	801.41	401.21	784.38	392.70	783.40	392.20	7
10	1,270.66	635.84	1,253.64	627.32	1,252.65	626.83	V	704.36	352.68	687.33	344.17	686.35	343.68	6
11	1,341.70	671.35	1,324.68	662.84	1,323.69	662.35	A	605.29	303.15	588.26	294.63	587.28	294.14	5
12	1,470.74	735.88	1,453.72	727.36	1,452.73	726.87	E	534.25	267.63	517.23	259.12	516.24	258.62	4
13	1,599.79	800.40	1,582.76	791.88	1,581.78	791.39	E	405.21	203.11	388.18	194.59	387.20	194.10	3
14	1,700.83	850.92	1,683.81	842.41	1,682.82	841.92	T	276.17	138.59	259.14	130.07	258.16	129.58	2
15							R	175.12	88.06	158.09	79.55			1

Query 32852 Hit 1

MS/MS Fragmentation of TLGDQLSLLLGAR
Found in sp|Q9UBG0|MRC2_HUMAN, C-type mannose receptor 2 OS=Homo sapiens GN=MRC2 PE=1 SV=2
Match to Query 32852: 1499.875from(750.9446,2+)
Title: 972: Sum of 2 scans in range 2227 (rt=59.2248, f=4, i=633) to 2228 (rt=59.2503, f=4, i=634)
[D:\lab212\membrane\Grace\Joyce\iTRAQ_23_1.raw]
Data File:Submitted from 20120508-1(merge) by Mascot Daemon on JOYCE-VAIO
Monoisotopic mass of neutral peptide Mr(calc): 1499.875
Variable modifications:
Ions Score: 98.7 Expect: 0.000

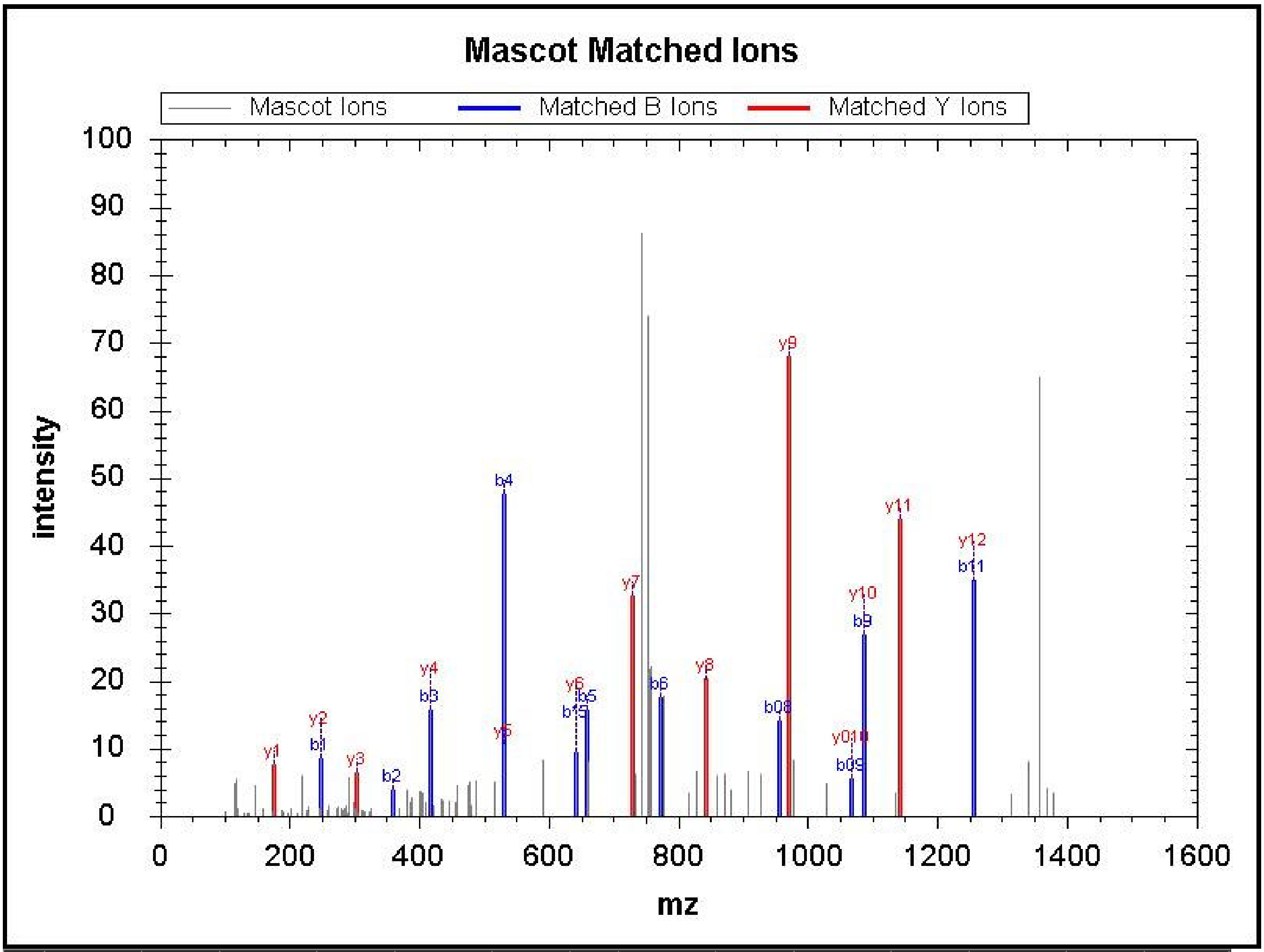

No	b	b++	b*	b*++	b0	b0++	Seq	y	y++	y*	y*++	y0	y0++	RevNo
1	246.16	123.58			228.15	114.58	T							13
2	359.24	180.12			341.23	171.12	L	1,255.74	628.37	1,238.71	619.86	1,237.73	619.37	12
3	416.26	208.63			398.25	199.63	G	1,142.65	571.83	1,125.63	563.32	1,124.64	562.82	11
4	531.29	266.15			513.28	257.14	D	1,085.63	543.32	1,068.60	534.81	1,067.62	534.31	10
5	659.35	330.18	642.32	321.66	641.34	321.17	Q	970.60	485.81	953.58	477.29	952.59	476.80	9
6	772.43	386.72	755.41	378.21	754.42	377.71	L	842.55	421.78	825.52	413.26	824.54	412.77	8
7	859.46	430.24	842.44	421.72	841.45	421.23	S	729.46	365.23	712.44	356.72	711.45	356.23	7
8	972.55	486.78	955.52	478.26	954.54	477.77	L	642.43	321.72	625.40	313.21			6
9	1,085.63	543.32	1,068.61	534.81	1,067.62	534.31	L	529.35	265.18	512.32	256.66			5
10	1,198.72	599.86	1,181.69	591.35	1,180.71	590.86	L	416.26	208.63	399.24	200.12			4
11	1,255.74	628.37	1,238.71	619.86	1,237.73	619.37	G	303.18	152.09	286.15	143.58			3
12	1,326.78	663.89	1,309.75	655.38	1,308.76	654.89	A	246.16	123.58	229.13	115.07			2
13							R	175.12	88.06	158.09	79.55			1

Query 82496 Hit 1

MS/MS Fragmentation of LVVTQEDVELAYQEAMMNMAR
Found in sp|P49903|SPS1_HUMAN, Selenide
Match to Query 82496: 2584.254from(862.4254,3+)
Title: 759: Scan 2544 (rt=63.5498, f=3, i=279) [D:\lab212\membrane\Grace\Joyce\iTRAQ_19_2_330.raw]
Data File:Submitted from 20120508-1(merge) by Mascot Daemon on JOYCE-VAIO
Monoisotopic mass of neutral peptide Mr(calc): 2584.254
Variable modifications:
Ions Score: 95.44 Expect: 0.000

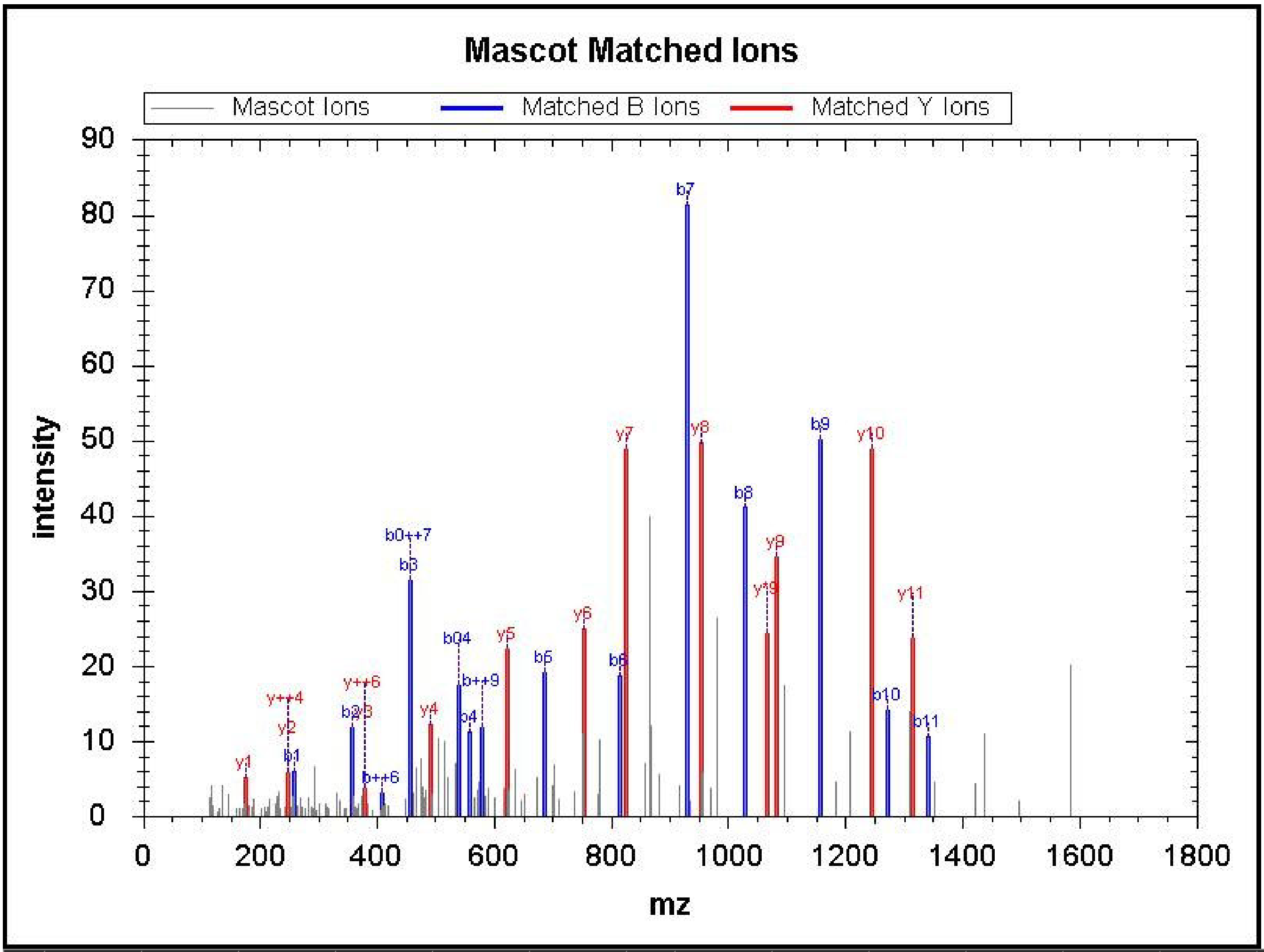

No	b	b++	b*	b*++	b0	b0++	Seq	y	y++	y*	y*++	y0	y0++	RevNo
1	258.19	129.60					L							21
2	357.26	179.13					V	2,328.07	1,164.54	2,311.04	1,156.02	2,310.06	1,155.53	20
3	456.33	228.67					V	2,229.00	1,115.00	2,211.97	1,106.49	2,210.99	1,106.00	19
4	557.38	279.19			539.37	270.19	T	2,129.93	1,065.47	2,112.90	1,056.96	2,111.92	1,056.46	18
5	685.44	343.22	668.41	334.71	667.43	334.22	Q	2,028.88	1,014.94	2,011.86	1,006.43	2,010.87	1,005.94	17
6	814.48	407.74	797.45	399.23	796.47	398.74	E	1,900.82	950.92	1,883.80	942.40	1,882.81	941.91	16
7	929.51	465.26	912.48	456.74	911.50	456.25	D	1,771.78	886.39	1,754.75	877.88	1,753.77	877.39	15
8	1,028.57	514.79	1,011.55	506.28	1,010.56	505.79	V	1,656.75	828.88	1,639.73	820.37	1,638.74	819.88	14
9	1,157.62	579.31	1,140.59	570.80	1,139.61	570.31	E	1,557.69	779.35	1,540.66	770.83	1,539.68	770.34	13
10	1,270.70	635.85	1,253.67	627.34	1,252.69	626.85	L	1,428.64	714.83	1,411.62	706.31	1,410.63	705.82	12
11	1,341.74	671.37	1,324.71	662.86	1,323.73	662.37	A	1,315.56	658.28	1,298.53	649.77	1,297.55	649.28	11
12	1,504.80	752.90	1,487.78	744.39	1,486.79	743.90	Y	1,244.52	622.76	1,227.50	614.25	1,226.51	613.76	10
13	1,632.86	816.93	1,615.83	808.42	1,614.85	807.93	Q	1,081.46	541.23	1,064.43	532.72	1,063.45	532.23	9
14	1,761.90	881.46	1,744.88	872.94	1,743.89	872.45	E	953.40	477.20	936.37	468.69	935.39	468.20	8
15	1,832.94	916.97	1,815.91	908.46	1,814.93	907.97	A	824.36	412.68	807.33	404.17			7
16	1,963.98	982.49	1,946.95	973.98	1,945.97	973.49	M	753.32	377.16	736.29	368.65			6
17	2,095.02	1,048.01	2,077.99	1,039.50	2,077.01	1,039.01	M	622.28	311.64	605.25	303.13			5
18	2,209.06	1,105.04	2,192.04	1,096.52	2,191.05	1,096.03	N	491.24	246.12	474.21	237.61			4
19	2,340.10	1,170.56	2,323.08	1,162.04	2,322.09	1,161.55	M	377.20	189.10	360.17	180.59			3
20	2,411.14	1,206.07	2,394.11	1,197.56	2,393.13	1,197.07	A	246.16	123.58	229.13	115.07			2
21							R	175.12	88.06	158.09	79.55			1

Query 37987 Hit 1

MS/MS Fragmentation of DIFSEVGPVVSFR

Found in sp|P33240|CSTF2_HUMAN, Cleavage stimulation factor subunit 2 OS=Homo sapiens GN=CSTF2 PE=1 SV=1
Match to Query 37987: 1594.846from(798.4301,2+)
Title: 843: Scan 2012 (rt=54.5108, f=3, i=304) [D:\lab212\membrane\Grace\Joyce\iTRAQ_21_2.raw]
Data File:Submitted from 20120508-1(merge) by Mascot Daemon on JOYCE-VAIO
Monoisotopic mass of neutral peptide Mr(calc): 1594.846
Variable modifications:
Ions Score: 92.95 Expect: 0.000

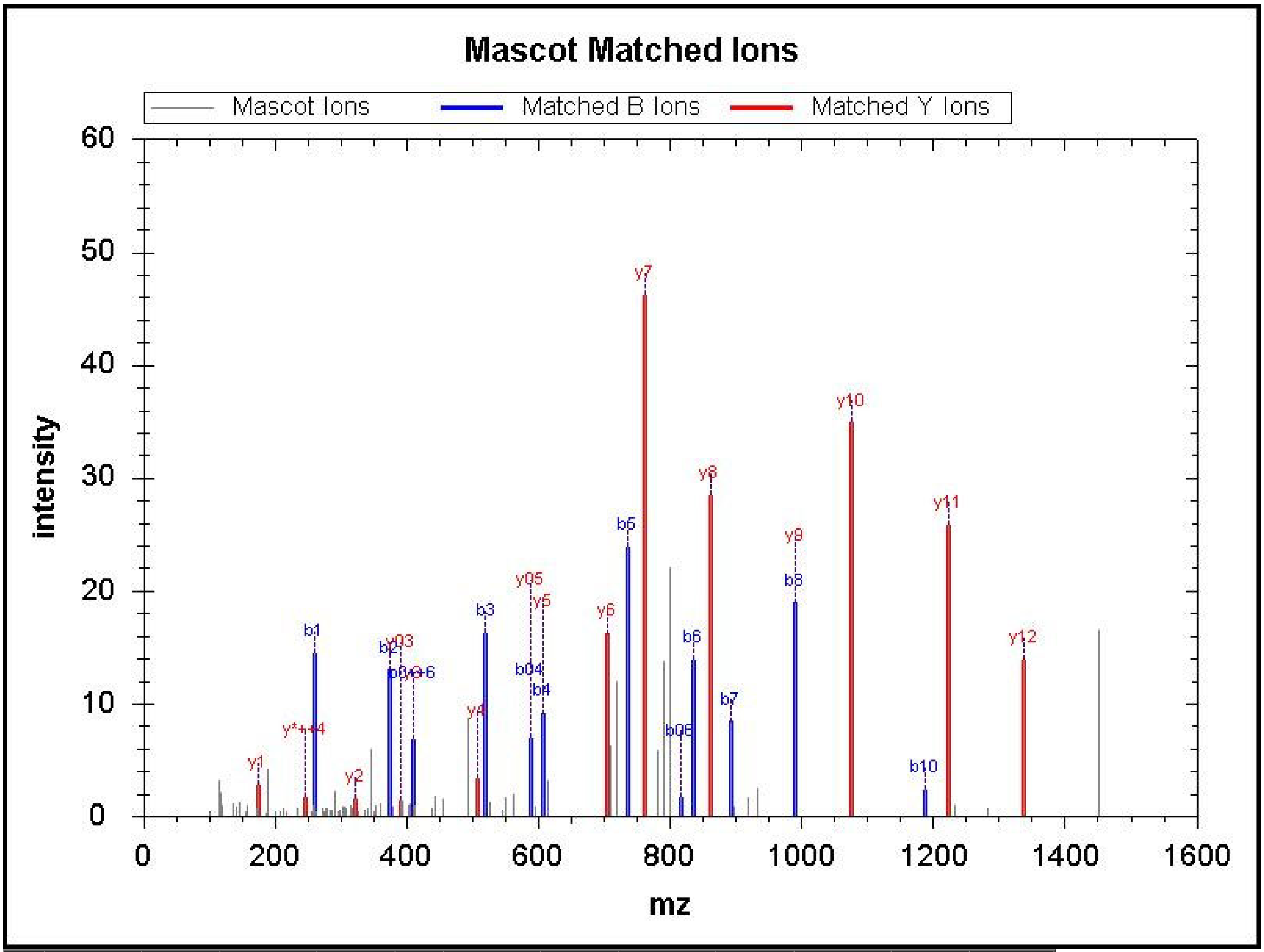

No	b	b++	b0	b0++	Seq	y	y++	y*	y*++	y0	y0++	RevNo
1	260.14	130.57	242.13	121.57	D							13
2	373.22	187.11	355.21	178.11	I	1,336.73	668.87	1,319.70	660.35	1,318.72	659.86	12
3	520.29	260.65	502.28	251.64	F	1,223.64	612.32	1,206.62	603.81	1,205.63	603.32	11
4	607.32	304.16	589.31	295.16	S	1,076.57	538.79	1,059.55	530.28	1,058.56	529.79	10
5	736.36	368.69	718.35	359.68	E	989.54	495.27	972.51	486.76	971.53	486.27	9
6	835.43	418.22	817.42	409.21	V	860.50	430.75	843.47	422.24	842.49	421.75	8
7	892.45	446.73	874.44	437.73	G	761.43	381.22	744.40	372.71	743.42	372.21	7
8	989.51	495.26	971.50	486.25	P	704.41	352.71	687.38	344.19	686.40	343.70	6
9	1,088.57	544.79	1,070.56	535.79	V	607.36	304.18	590.33	295.67	589.35	295.18	5
10	1,187.64	594.33	1,169.63	585.32	V	508.29	254.65	491.26	246.13	490.28	245.64	4
11	1,274.67	637.84	1,256.66	628.84	S	409.22	205.11	392.19	196.60	391.21	196.11	3
12	1,421.74	711.38	1,403.73	702.37	F	322.19	161.60	305.16	153.08			2
13					R	175.12	88.06	158.09	79.55			1

Query 49554 Hit 1

MS/MS Fragmentation of SAAMLGNSEDHTALSR
Found in sp|O60749|SNX2_HUMAN, Sorting nexin-2 OS=Homo sapiens GN=SNX2 PE=1 SV=2
Match to Query 49554: 1802.871from(601.9642,3+)

Title: 262: Sum of 2 scans in range 672 (rt=24.5437, f=4, i=173) to 673 (rt=24.5691, f=4, i=174) [D:\lab212\membrane\Grace\Joyce\iTRAQ_36_1.raw]
Data File:Submitted from 20120508-1(merge) by Mascot Daemon on JOYCE-VAIO
Monoisotopic mass of neutral peptide Mr(calc): 1802.871
Variable modifications:
Ions Score: 92.22 Expect: 0.000

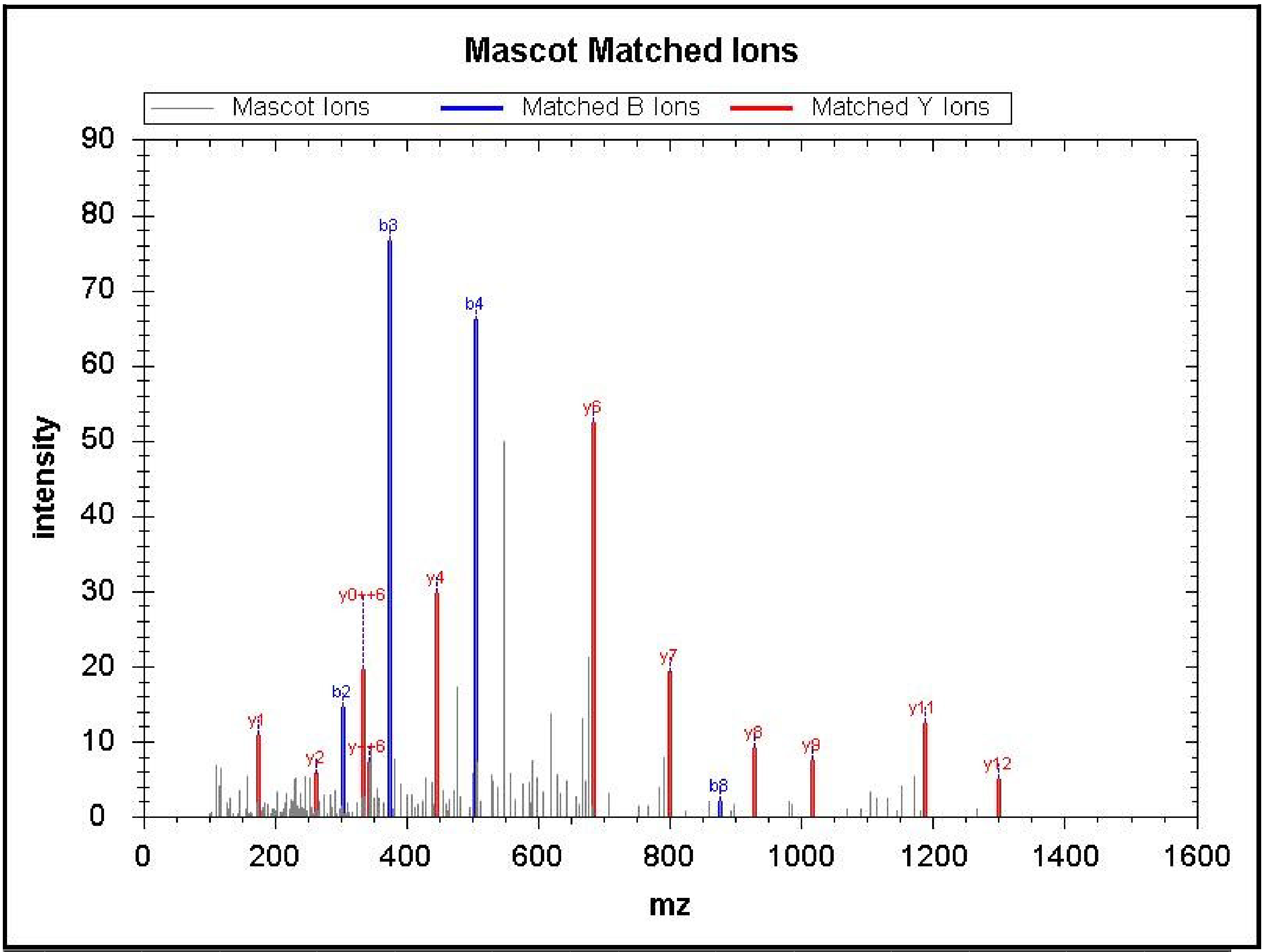

No	b	b++	b*	b*++	b0	b0++	Seq	y	y++	y*	y*++	y0	y0++	RevNo
1	232.14	116.57			214.13	107.57	S							16
2	303.18	152.09			285.17	143.09	A	1,572.74	786.88	1,555.72	778.36	1,554.73	777.87	15
3	374.22	187.61			356.21	178.61	A	1,501.71	751.36	1,484.68	742.84	1,483.70	742.35	14
4	505.26	253.13			487.25	244.13	M	1,430.67	715.84	1,413.64	707.33	1,412.66	706.83	13
5	618.34	309.67			600.33	300.67	L	1,299.63	650.32	1,282.60	641.80	1,281.62	641.31	12
6	675.36	338.18			657.35	329.18	G	1,186.54	593.78	1,169.52	585.26	1,168.53	584.77	11
7	789.40	395.21	772.38	386.69	771.39	386.20	N	1,129.52	565.27	1,112.50	556.75	1,111.51	556.26	10
8	876.44	438.72	859.41	430.21	858.43	429.72	S	1,015.48	508.24	998.45	499.73	997.47	499.24	9
9	1,005.48	503.24	988.45	494.73	987.47	494.24	E	928.45	464.73	911.42	456.21	910.44	455.72	8
10	1,120.51	560.76	1,103.48	552.24	1,102.50	551.75	D	799.41	400.21	782.38	391.69	781.40	391.20	7
11	1,257.57	629.29	1,240.54	620.77	1,239.55	620.28	H	684.38	342.69	667.35	334.18	666.37	333.69	6
12	1,358.61	679.81	1,341.59	671.30	1,340.60	670.80	T	547.32	274.16	530.29	265.65	529.31	265.16	5
13	1,429.65	715.33	1,412.62	706.82	1,411.64	706.32	A	446.27	223.64	429.25	215.13	428.26	214.63	4
14	1,542.73	771.87	1,525.71	763.36	1,524.72	762.87	L	375.24	188.12	358.21	179.61	357.22	179.12	3
15	1,629.77	815.39	1,612.74	806.87	1,611.76	806.38	S	262.15	131.58	245.12	123.07	244.14	122.57	2
16							R	175.12	88.06	158.09	79.55			1

Query 89469 Hit 1

MS/MS Fragmentation of RPDVVENQPDAASQLNVDASGNLAK
Found in sp|Q9NZL9|MAT2B_HUMAN, Methionine adenosyltransferase 2 subunit beta OS=Homo sapiens GN=MAT2B PE=1 SV=1
Match to Query 89469: 2895.495from(724.881,4+)
Title: 411: Scan 986 (rt=31.5413, f=3, i=142) [D:\lab212\membrane\Grace\Joyce\iTRAQ_38_2.raw]
Data File:Submitted from 20120508-1(merge) by Mascot Daemon on JOYCE-VAIO
Monoisotopic mass of neutral peptide Mr(calc): 2895.495
Variable modifications:
K25 :iTRAQ4plex (K)
Ions Score: 91.66 Expect: 0.000

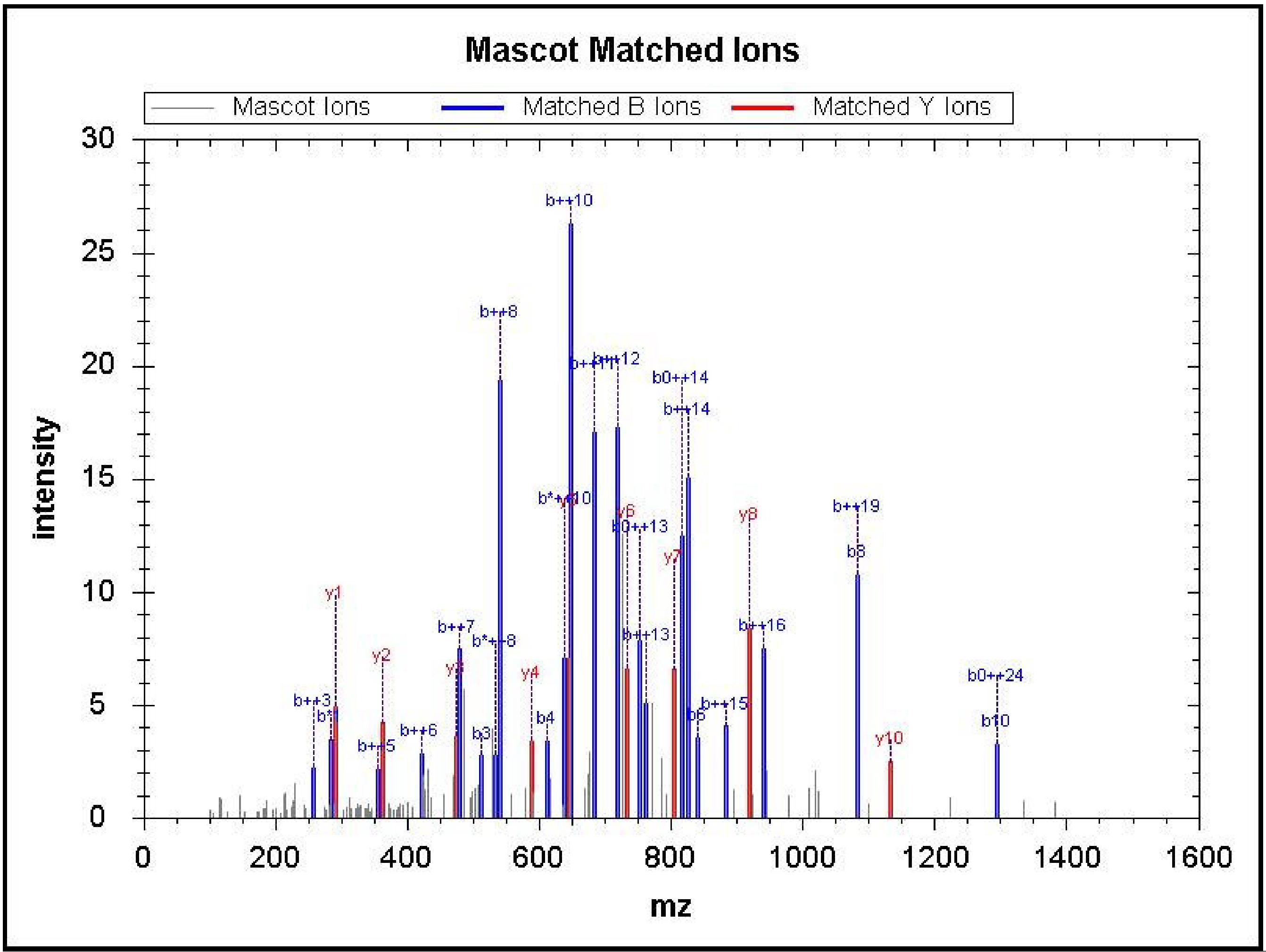

No	b	b++	b*	b*++	b0	b0++	Seq	y	y++	y*	y*++	y0	y0++	RevNo
1	301.21	151.11	284.18	142.60			R							25
2	398.26	199.64	381.24	191.12			P	2,596.30	1,298.65	2,579.27	1,290.14	2,578.29	1,289.65	24
3	513.29	257.15	496.26	248.64	495.28	248.14	D	2,499.24	1,250.13	2,482.22	1,241.61	2,481.23	1,241.12	23
4	612.36	306.68	595.33	298.17	594.35	297.68	V	2,384.22	1,192.61	2,367.19	1,184.10	2,366.21	1,183.61	22
5	711.43	356.22	694.40	347.70	693.42	347.21	V	2,285.15	1,143.08	2,268.12	1,134.56	2,267.14	1,134.07	21
6	840.47	420.74	823.44	412.23	822.46	411.73	E	2,186.08	1,093.54	2,169.05	1,085.03	2,168.07	1,084.54	20
7	954.51	477.76	937.49	469.25	936.50	468.75	N	2,057.04	1,029.02	2,040.01	1,020.51	2,039.03	1,020.02	19
8	1,082.57	541.79	1,065.54	533.28	1,064.56	532.78	Q	1,942.99	972.00	1,925.97	963.49	1,924.98	963.00	18
9	1,179.62	590.32	1,162.60	581.80	1,161.61	581.31	P	1,814.94	907.97	1,797.91	899.46	1,796.93	898.97	17
10	1,294.65	647.83	1,277.62	639.32	1,276.64	638.82	D	1,717.88	859.45	1,700.86	850.93	1,699.87	850.44	16
11	1,365.69	683.35	1,348.66	674.83	1,347.68	674.34	A	1,602.86	801.93	1,585.83	793.42	1,584.85	792.93	15
12	1,436.73	718.87	1,419.70	710.35	1,418.71	709.86	A	1,531.82	766.41	1,514.79	757.90	1,513.81	757.41	14
13	1,523.76	762.38	1,506.73	753.87	1,505.75	753.38	S	1,460.78	730.89	1,443.76	722.38	1,442.77	721.89	13
14	1,651.82	826.41	1,634.79	817.90	1,633.81	817.41	Q	1,373.75	687.38	1,356.72	678.87	1,355.74	678.37	12
15	1,764.90	882.95	1,747.87	874.44	1,746.89	873.95	L	1,245.69	623.35	1,228.67	614.84	1,227.68	614.34	11
16	1,878.94	939.98	1,861.92	931.46	1,860.93	930.97	N	1,132.61	566.81	1,115.58	558.29	1,114.60	557.80	10

17	1,978.01	989.51	1,960.98	981.00	1,960.00	980.50	V	1,018.56	509.79	1,001.54	501.27	1,000.55	500.78	9
18	2,093.04	1,047.02	2,076.01	1,038.51	2,075.03	1,038.02	D	919.50	460.25	902.47	451.74	901.49	451.25	8
19	2,164.08	1,082.54	2,147.05	1,074.03	2,146.06	1,073.54	A	804.47	402.74	787.44	394.23	786.46	393.73	7
20	2,251.11	1,126.06	2,234.08	1,117.54	2,233.10	1,117.05	S	733.43	367.22	716.41	358.71	715.42	358.21	6
21	2,308.13	1,154.57	2,291.10	1,146.05	2,290.12	1,145.56	G	646.40	323.70	629.37	315.19			5
22	2,422.17	1,211.59	2,405.15	1,203.08	2,404.16	1,202.58	N	589.38	295.19	572.35	286.68			4
23	2,535.26	1,268.13	2,518.23	1,259.62	2,517.25	1,259.13	L	475.34	238.17	458.31	229.66			3
24	2,606.29	1,303.65	2,589.27	1,295.14	2,588.28	1,294.64	A	362.25	181.63	345.23	173.12			2
25							K	291.21	146.11	274.19	137.60			1

Query 62397 Hit 1

MS/MS Fragmentation of ENLELILTQSVENVGVR
Found in sp|Q9BYD2|RM09_HUMAN, 39S ribosomal protein L9
Match to Query 62397: 2056.126from(686.3827,3+)
Title: 829: Sum of 2 scans in range 2320 (rt=59.597, f=4, i=551) to 2321 (rt=59.6224, f=4, i=552) [D:\lab212\membrane\Grace\Joyce\20120320_iTRAQ_20-2.raw]
Data File:Submitted from 20120508-1(merge) by Mascot Daemon on JOYCE-VAIO
Monoisotopic mass of neutral peptide Mr(calc): 2056.126
Variable modifications:
Ions Score: 90.38 Expect: 0.000

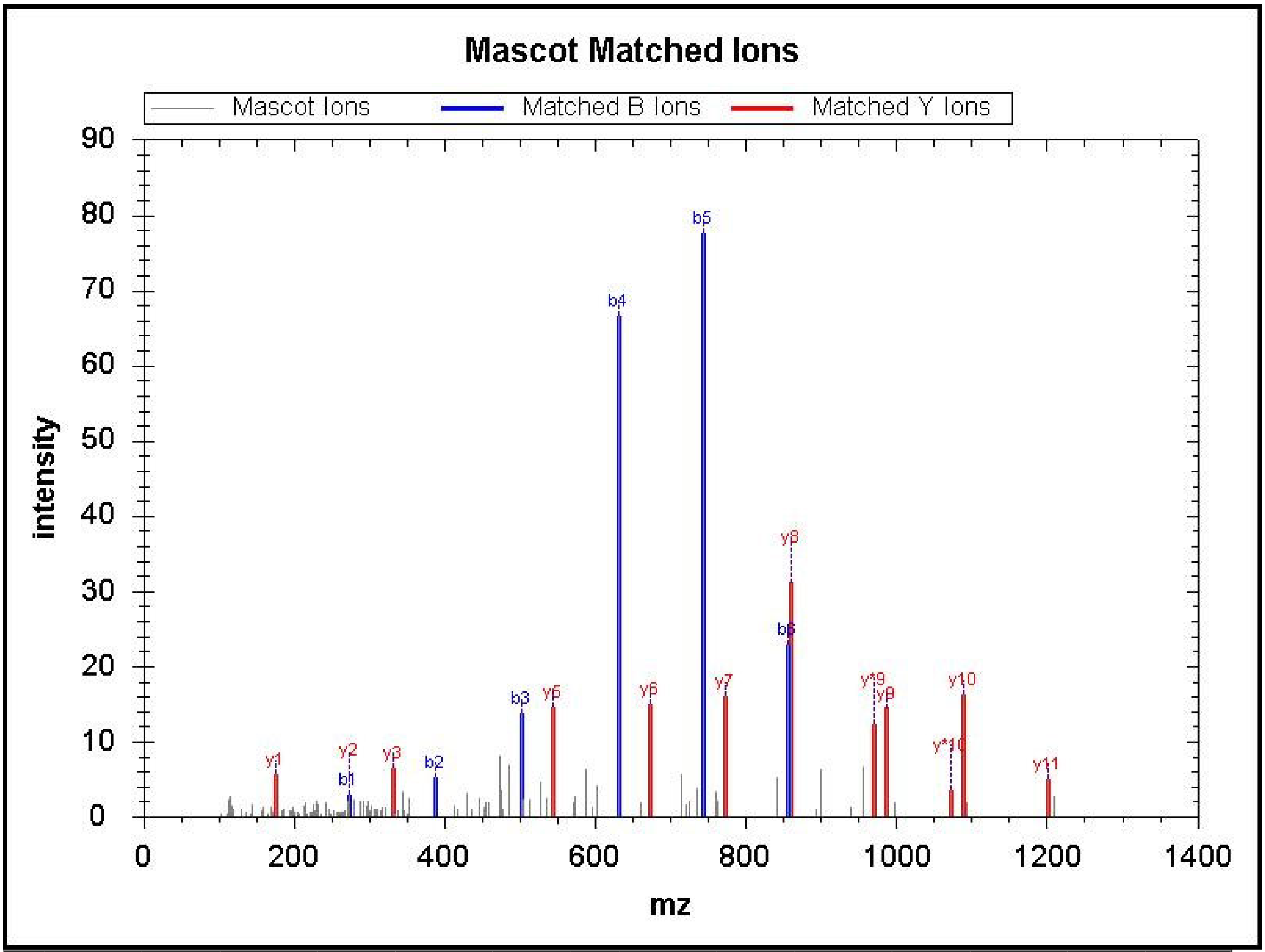

No	b	b++	b*	b*++	b0	b0++	Seq	y	y++	y*	y*++	y0	y0++	RevNo
1	274.15	137.58			256.14	128.57	E							17
2	388.19	194.60	371.17	186.09	370.18	185.60	N	1,783.99	892.50	1,766.96	883.99	1,765.98	883.49	16
3	501.28	251.14	484.25	242.63	483.27	242.14	L	1,669.95	835.48	1,652.92	826.96	1,651.94	826.47	15
4	630.32	315.66	613.29	307.15	612.31	306.66	E	1,556.86	778.94	1,539.84	770.42	1,538.85	769.93	14
5	743.41	372.21	726.38	363.69	725.40	363.20	L	1,427.82	714.41	1,410.80	705.90	1,409.81	705.41	13

6	856.49	428.75	839.46	420.24	838.48	419.74	I	1,314.74	657.87	1,297.71	649.36	1,296.73	648.87	12
7	969.57	485.29	952.55	476.78	951.56	476.29	L	1,201.65	601.33	1,184.63	592.82	1,183.64	592.33	11
8	1,070.62	535.81	1,053.59	527.30	1,052.61	526.81	T	1,088.57	544.79	1,071.54	536.28	1,070.56	535.78	10
9	1,198.68	599.84	1,181.65	591.33	1,180.67	590.84	Q	987.52	494.26	970.50	485.75	969.51	485.26	9
10	1,285.71	643.36	1,268.69	634.85	1,267.70	634.35	S	859.46	430.24	842.44	421.72	841.45	421.23	8
11	1,384.78	692.89	1,367.75	684.38	1,366.77	683.89	V	772.43	386.72	755.40	378.21	754.42	377.71	7
12	1,513.82	757.42	1,496.80	748.90	1,495.81	748.41	E	673.36	337.18	656.34	328.67	655.35	328.18	6
13	1,627.87	814.44	1,610.84	805.92	1,609.86	805.43	N	544.32	272.66	527.29	264.15			5
14	1,726.93	863.97	1,709.91	855.46	1,708.92	854.97	V	430.28	215.64	413.25	207.13			4
15	1,783.96	892.48	1,766.93	883.97	1,765.95	883.48	G	331.21	166.11	314.18	157.59			3
16	1,883.02	942.02	1,866.00	933.50	1,865.01	933.01	V	274.19	137.60	257.16	129.08			2
17							R	175.12	88.06	158.09	79.55			1

Query 97263 Hit 1

MS/MS Fragmentation of GNKPDFHLAMPTEQAEGFYNSFLEQLR
Found in sp|Q8TEA8|DTD1_HUMAN, D-tyrosyl-tRNA(Tyr) deacylase 1 OS=Homo sapiens GN=DTD1 PE=1 SV=2
Match to Query 97263: 3426.685from(857.6785,4+)
Title: 963: Sum of 2 scans in range 2316 (rt=60.901, f=4, i=643) to 2317 (rt=60.9264, f=4, i=644)
[D:\lab212\membrane\Grace\Joyce\iTRAQ_46_2.raw]
Data File:Submitted from 20120508-1(merge) by Mascot Daemon on JOYCE-VAIO
Monoisotopic mass of neutral peptide Mr(calc): 3426.685
Variable modifications:
K3 :iTRAQ4plex (K)
Ions Score: 88.98 Expect: 0.000

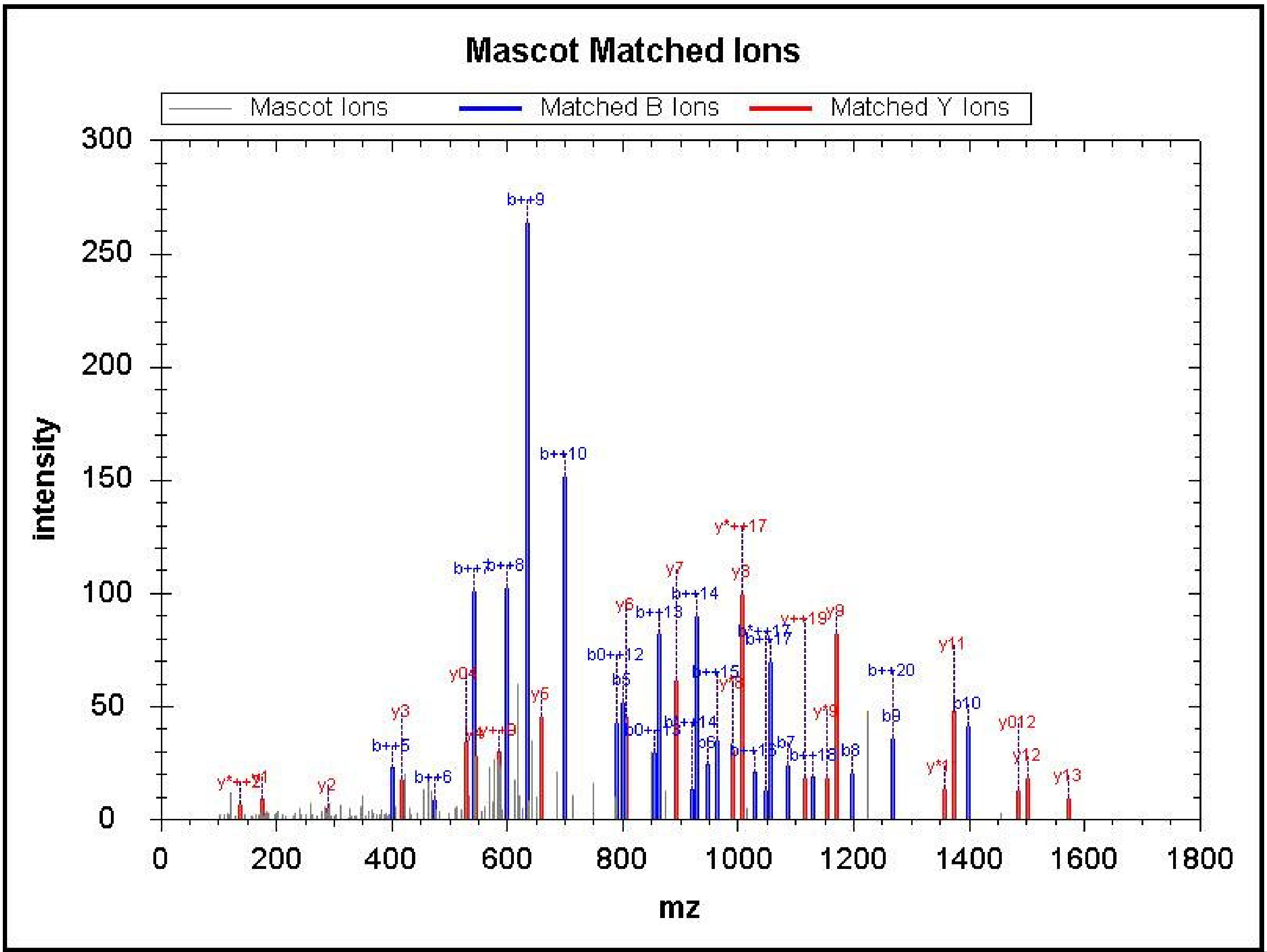

No	b	b++	b*	b*++	b0	b0++	Seq	y	y++	y*	y*++	y0	y0++	RevNo
1	202.13	101.57					G							27

2	316.17	158.59	299.15	150.08			N	3,226.57	1,613.79	3,209.55	1,605.28	3,208.56	1,604.79	26
3	588.37	294.69	571.34	286.18			K	3,112.53	1,556.77	3,095.50	1,548.26	3,094.52	1,547.76	25
4	685.42	343.22	668.40	334.70			P	2,840.33	1,420.67	2,823.31	1,412.16	2,822.32	1,411.67	24
5	800.45	400.73	783.42	392.22	782.44	391.72	D	2,743.28	1,372.14	2,726.26	1,363.63	2,725.27	1,363.14	23
6	947.52	474.26	930.49	465.75	929.51	465.26	F	2,628.25	1,314.63	2,611.23	1,306.12	2,610.24	1,305.63	22
7	1,084.58	542.79	1,067.55	534.28	1,066.57	533.79	H	2,481.19	1,241.10	2,464.16	1,232.58	2,463.18	1,232.09	21
8	1,197.66	599.33	1,180.64	590.82	1,179.65	590.33	L	2,344.13	1,172.57	2,327.10	1,164.05	2,326.12	1,163.56	20
9	1,268.70	634.85	1,251.67	626.34	1,250.69	625.85	A	2,231.04	1,116.03	2,214.02	1,107.51	2,213.03	1,107.02	19
10	1,399.74	700.37	1,382.71	691.86	1,381.73	691.37	M	2,160.01	1,080.51	2,142.98	1,071.99	2,142.00	1,071.50	18
11	1,496.79	748.90	1,479.77	740.39	1,478.78	739.89	P	2,028.97	1,014.99	2,011.94	1,006.47	2,010.96	1,005.98	17
12	1,597.84	799.42	1,580.81	790.91	1,579.83	790.42	T	1,931.91	966.46	1,914.89	957.95	1,913.90	957.46	16
13	1,726.88	863.95	1,709.86	855.43	1,708.87	854.94	E	1,830.87	915.94	1,813.84	907.42	1,812.86	906.93	15
14	1,854.94	927.97	1,837.91	919.46	1,836.93	918.97	Q	1,701.82	851.42	1,684.80	842.90	1,683.81	842.41	14
15	1,925.98	963.49	1,908.95	954.98	1,907.97	954.49	A	1,573.76	787.39	1,556.74	778.87	1,555.75	778.38	13
16	2,055.02	1,028.01	2,037.99	1,019.50	2,037.01	1,019.01	E	1,502.73	751.87	1,485.70	743.35	1,484.72	742.86	12
17	2,112.04	1,056.52	2,095.02	1,048.01	2,094.03	1,047.52	G	1,373.68	687.35	1,356.66	678.83	1,355.67	678.34	11
18	2,259.11	1,130.06	2,242.08	1,121.55	2,241.10	1,121.05	F	1,316.66	658.84	1,299.64	650.32	1,298.65	649.83	10
19	2,422.17	1,211.59	2,405.15	1,203.08	2,404.16	1,202.59	Y	1,169.59	585.30	1,152.57	576.79	1,151.58	576.30	9
20	2,536.22	1,268.61	2,519.19	1,260.10	2,518.21	1,259.61	N	1,006.53	503.77	989.51	495.26	988.52	494.76	8
21	2,623.25	1,312.13	2,606.22	1,303.61	2,605.24	1,303.12	S	892.49	446.75	875.46	438.23	874.48	437.74	7
22	2,770.32	1,385.66	2,753.29	1,377.15	2,752.31	1,376.66	F	805.46	403.23	788.43	394.72	787.45	394.23	6
23	2,883.40	1,442.20	2,866.37	1,433.69	2,865.39	1,433.20	L	658.39	329.70	641.36	321.18	640.38	320.69	5
24	3,012.44	1,506.73	2,995.42	1,498.21	2,994.43	1,497.72	E	545.30	273.16	528.28	264.64	527.29	264.15	4
25	3,140.50	1,570.75	3,123.48	1,562.24	3,122.49	1,561.75	Q	416.26	208.63	399.24	200.12			3
26	3,253.59	1,627.30	3,236.56	1,618.78	3,235.58	1,618.29	L	288.20	144.61	271.18	136.09			2
27							R	175.12	88.06	158.09	79.55			1

Query 77140 Hit 1

MS/MS Fragmentation of DLLEQMMAEMIGEFPDLHR
Found in sp|Q9H4I3|TRABD_HUMAN, TraB domain-containing protein OS=Homo sapiens GN=TRABD PE=2 SV=1
Match to Query 77140: 2418.153from(807.0583,3+)
Title: 1243: Sum of 2 scans in range 3182 (rt=78.9956, f=2, i=555) to 3183 (rt=79.021, f=2, i=556)
[D:\lab212\membrane\Grace\Joyce\iTRAQ_31_1.raw]
Data File:Submitted from 20120508-1(merge) by Mascot Daemon on JOYCE-VAIO
Monoisotopic mass of neutral peptide Mr(calc): 2418.153
Variable modifications:
Ions Score: 88.71 Expect: 0.000

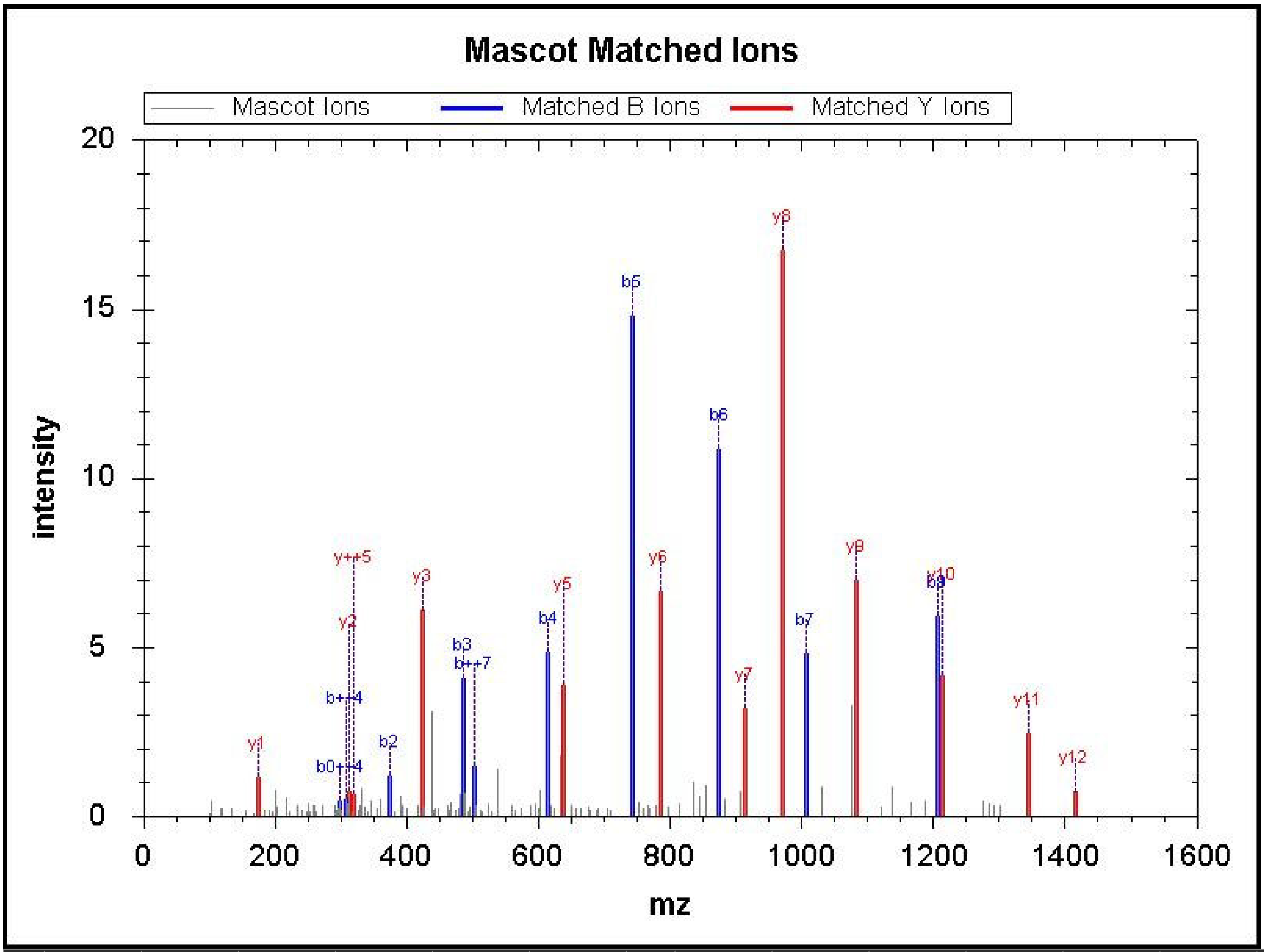

No	b	b++	b*	b*++	b0	b0++	Seq	y	y++	y*	y*++	y0	y0++	RevNo
1	260.14	130.57			242.13	121.57	D							19
2	373.22	187.11			355.21	178.11	L	2,160.03	1,080.52	2,143.00	1,072.00	2,142.02	1,071.51	18
3	486.30	243.66			468.29	234.65	L	2,046.94	1,023.98	2,029.92	1,015.46	2,028.93	1,014.97	17
4	615.35	308.18			597.34	299.17	E	1,933.86	967.43	1,916.83	958.92	1,915.85	958.43	16
5	743.41	372.21	726.38	363.69	725.40	363.20	Q	1,804.82	902.91	1,787.79	894.40	1,786.81	893.91	15
6	874.45	437.73	857.42	429.21	856.44	428.72	M	1,676.76	838.88	1,659.73	830.37	1,658.75	829.88	14
7	1,005.49	503.25	988.46	494.73	987.48	494.24	M	1,545.72	773.36	1,528.69	764.85	1,527.71	764.36	13
8	1,076.52	538.77	1,059.50	530.25	1,058.51	529.76	A	1,414.68	707.84	1,397.65	699.33	1,396.67	698.84	12
9	1,205.57	603.29	1,188.54	594.77	1,187.56	594.28	E	1,343.64	672.32	1,326.61	663.81	1,325.63	663.32	11
10	1,336.61	668.81	1,319.58	660.29	1,318.60	659.80	M	1,214.60	607.80	1,197.57	599.29	1,196.59	598.80	10
11	1,449.69	725.35	1,432.66	716.84	1,431.68	716.34	I	1,083.56	542.28	1,066.53	533.77	1,065.55	533.28	9
12	1,506.71	753.86	1,489.69	745.35	1,488.70	744.85	G	970.47	485.74	953.45	477.23	952.46	476.74	8
13	1,635.76	818.38	1,618.73	809.87	1,617.74	809.38	E	913.45	457.23	896.43	448.72	895.44	448.22	7
14	1,782.82	891.92	1,765.80	883.40	1,764.81	882.91	F	784.41	392.71	767.38	384.20	766.40	383.70	6
15	1,879.88	940.44	1,862.85	931.93	1,861.87	931.44	P	637.34	319.17	620.32	310.66	619.33	310.17	5
16	1,994.90	997.96	1,977.88	989.44	1,976.89	988.95	D	540.29	270.65	523.26	262.13	522.28	261.64	4
17	2,107.99	1,054.50	2,090.96	1,045.98	2,089.98	1,045.49	L	425.26	213.13	408.24	204.62			3
18	2,245.05	1,123.03	2,228.02	1,114.51	2,227.04	1,114.02	H	312.18	156.59	295.15	148.08			2
19							R	175.12	88.06	158.09	79.55			1

Query 60468 Hit 1

MS/MS Fragmentation of ENIASVLENYHTESK
Found in sp|Q6NVY1|HIBCH_HUMAN, 3-hydroxyisobutyryl-CoA hydrolase
Match to Query 60468: 2021.006from(674.6758,3+)

Title: 575: Sum of 2 scans in range 1275 (rt=38.4732, f=4, i=391) to 1276 (rt=38.4986, f=4, i=392)
[D:\lab212\membrane\Grace\Joyce\iTRAQ40.raw]
Data File:Submitted from 20120508-1(merge) by Mascot Daemon on JOYCE-VAIO
Monoisotopic mass of neutral peptide Mr(calc): 2021.006
Variable modifications:
K15 :iTRAQ4plex (K)
Ions Score: 87.95 Expect: 0.000

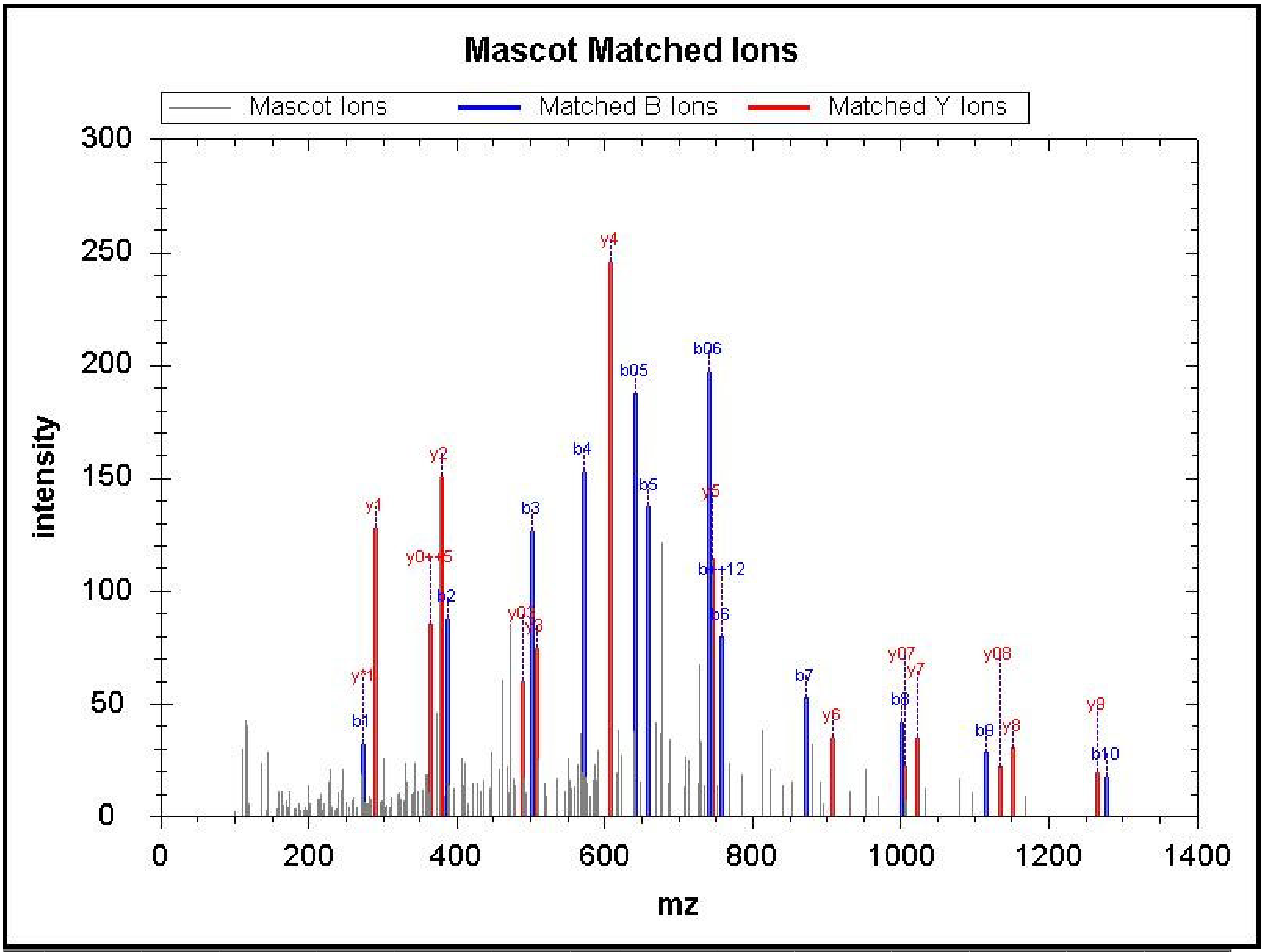

No	b	b++	b*	b*++	b0	b0++	Seq	y	y++	y*	y*++	y0	y0++	RevNo
1	274.15	137.58			256.14	128.57	E							15
2	388.19	194.60	371.17	186.09	370.18	185.60	N	1,748.89	874.95	1,731.87	866.44	1,730.88	865.95	14
3	501.28	251.14	484.25	242.63	483.27	242.14	I	1,634.85	817.93	1,617.82	809.42	1,616.84	808.92	13
4	572.32	286.66	555.29	278.15	554.31	277.66	A	1,521.77	761.39	1,504.74	752.87	1,503.76	752.38	12
5	659.35	330.18	642.32	321.66	641.34	321.17	S	1,450.73	725.87	1,433.70	717.36	1,432.72	716.86	11
6	758.42	379.71	741.39	371.20	740.41	370.71	V	1,363.70	682.35	1,346.67	673.84	1,345.69	673.35	10
7	871.50	436.25	854.47	427.74	853.49	427.25	L	1,264.63	632.82	1,247.60	624.30	1,246.62	623.81	9
8	1,000.54	500.78	983.52	492.26	982.53	491.77	E	1,151.54	576.28	1,134.52	567.76	1,133.53	567.27	8
9	1,114.59	557.80	1,097.56	549.28	1,096.58	548.79	N	1,022.50	511.75	1,005.48	503.24	1,004.49	502.75	7
10	1,277.65	639.33	1,260.62	630.82	1,259.64	630.32	Y	908.46	454.73	891.43	446.22	890.45	445.73	6
11	1,414.71	707.86	1,397.68	699.34	1,396.70	698.85	H	745.40	373.20	728.37	364.69	727.39	364.20	5
12	1,515.76	758.38	1,498.73	749.87	1,497.75	749.38	T	608.34	304.67	591.31	296.16	590.33	295.67	4
13	1,644.80	822.90	1,627.77	814.39	1,626.79	813.90	E	507.29	254.15	490.26	245.64	489.28	245.14	3
14	1,731.83	866.42	1,714.80	857.91	1,713.82	857.41	S	378.25	189.63	361.22	181.11	360.24	180.62	2
15							K	291.21	146.11	274.19	137.60			1

Query 72333 Hit 1

MS/MS Fragmentation of FYPGQAPSLAENFAEHVLR
Found in sp|Q9Y276|BCS1_HUMAN, Mitochondrial chaperone BCS1 OS=Homo sapiens GN=BCS1L PE=1 SV=1
Match to Query 72333: 2289.163 from(764.0615,3+)
Title: 974: Sum of 2 scans in range 2160 (rt=58.0317, f=4, i=647) to 2161 (rt=58.0571, f=4, i=648)
[D:\lab212\membrane\Grace\Joyce\iTRAQ_31_1.raw]
Data File:Submitted from 20120508-1(merge) by Mascot Daemon on JOYCE-VAIO
Monoisotopic mass of neutral peptide Mr(calc): 2289.163
Variable modifications:
Ions Score: 87.95 Expect: 0.000

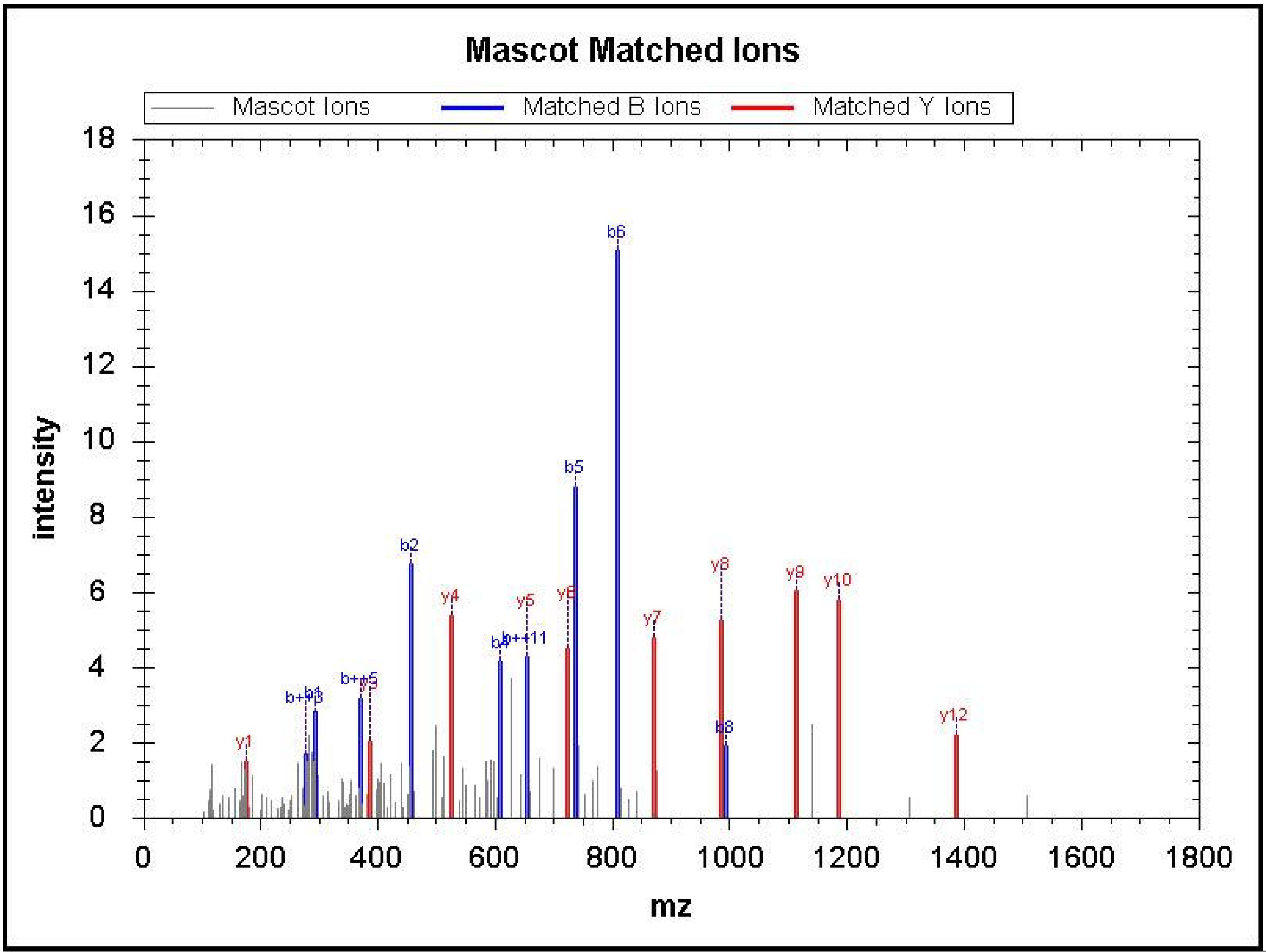

No	b	b++	b*	b*++	b0	b0++	Seq	y	y++	y*	y*++	y0	y0++	RevNo
1	292.18	146.59					F							19
2	455.24	228.12					Y	1,999.00	1,000.01	1,981.98	991.49	1,980.99	991.00	18
3	552.29	276.65					P	1,835.94	918.47	1,818.91	909.96	1,817.93	909.47	17
4	609.32	305.16					G	1,738.89	869.95	1,721.86	861.43	1,720.88	860.94	16
5	737.37	369.19	720.35	360.68			Q	1,681.87	841.44	1,664.84	832.92	1,663.85	832.43	15
6	808.41	404.71	791.38	396.20			A	1,553.81	777.41	1,536.78	768.89	1,535.80	768.40	14
7	905.46	453.24	888.44	444.72			P	1,482.77	741.89	1,465.74	733.38	1,464.76	732.88	13
8	992.50	496.75	975.47	488.24	974.49	487.75	S	1,385.72	693.36	1,368.69	684.85	1,367.71	684.36	12
9	1,105.58	553.29	1,088.55	544.78	1,087.57	544.29	L	1,298.69	649.85	1,281.66	641.33	1,280.67	640.84	11
10	1,176.62	588.81	1,159.59	580.30	1,158.61	579.81	A	1,185.60	593.30	1,168.57	584.79	1,167.59	584.30	10
11	1,305.66	653.33	1,288.63	644.82	1,287.65	644.33	E	1,114.56	557.79	1,097.54	549.27	1,096.55	548.78	9
12	1,419.70	710.35	1,402.68	701.84	1,401.69	701.35	N	985.52	493.26	968.49	484.75	967.51	484.26	8
13	1,566.77	783.89	1,549.74	775.38	1,548.76	774.88	F	871.48	436.24	854.45	427.73	853.47	427.24	7
14	1,637.81	819.41	1,620.78	810.89	1,619.80	810.40	A	724.41	362.71	707.38	354.20	706.40	353.70	6
15	1,766.85	883.93	1,749.82	875.42	1,748.84	874.92	E	653.37	327.19	636.35	318.68	635.36	318.18	5
16	1,903.91	952.46	1,886.88	943.95	1,885.90	943.45	H	524.33	262.67	507.30	254.16			4

17	2,002.98	1,001.99	1,985.95	993.48	1,984.97	992.99	V	387.27	194.14	370.24	185.63			3
18	2,116.06	1,058.53	2,099.04	1,050.02	2,098.05	1,049.53	L	288.20	144.61	271.18	136.09			2
19							R	175.12	88.06	158.09	79.55			1

Query 85886 Hit 1

MS/MS Fragmentation of LLFGHSTEGDILELVDGHFDTK
Found in sp|Q9UHY7|ENOPH_HUMAN, Enolase-phosphatase E1 OS=Homo sapiens GN=ENOPH1 PE=1 SV=1
Match to Query 85886: 2730.415from(911.1454,3+)
Title: 770: Scan 2291 (rt=59.0828, f=3, i=288) [D:\lab212\membrane\Grace\Joyce\iTRAQ_46_1.raw]
Data File:Submitted from 20120508-1(merge) by Mascot Daemon on JOYCE-VAIO
Monoisotopic mass of neutral peptide Mr(calc): 2730.415
Variable modifications:
K22 :iTRAQ4plex (K)
Ions Score: 85.64 Expect: 0.000

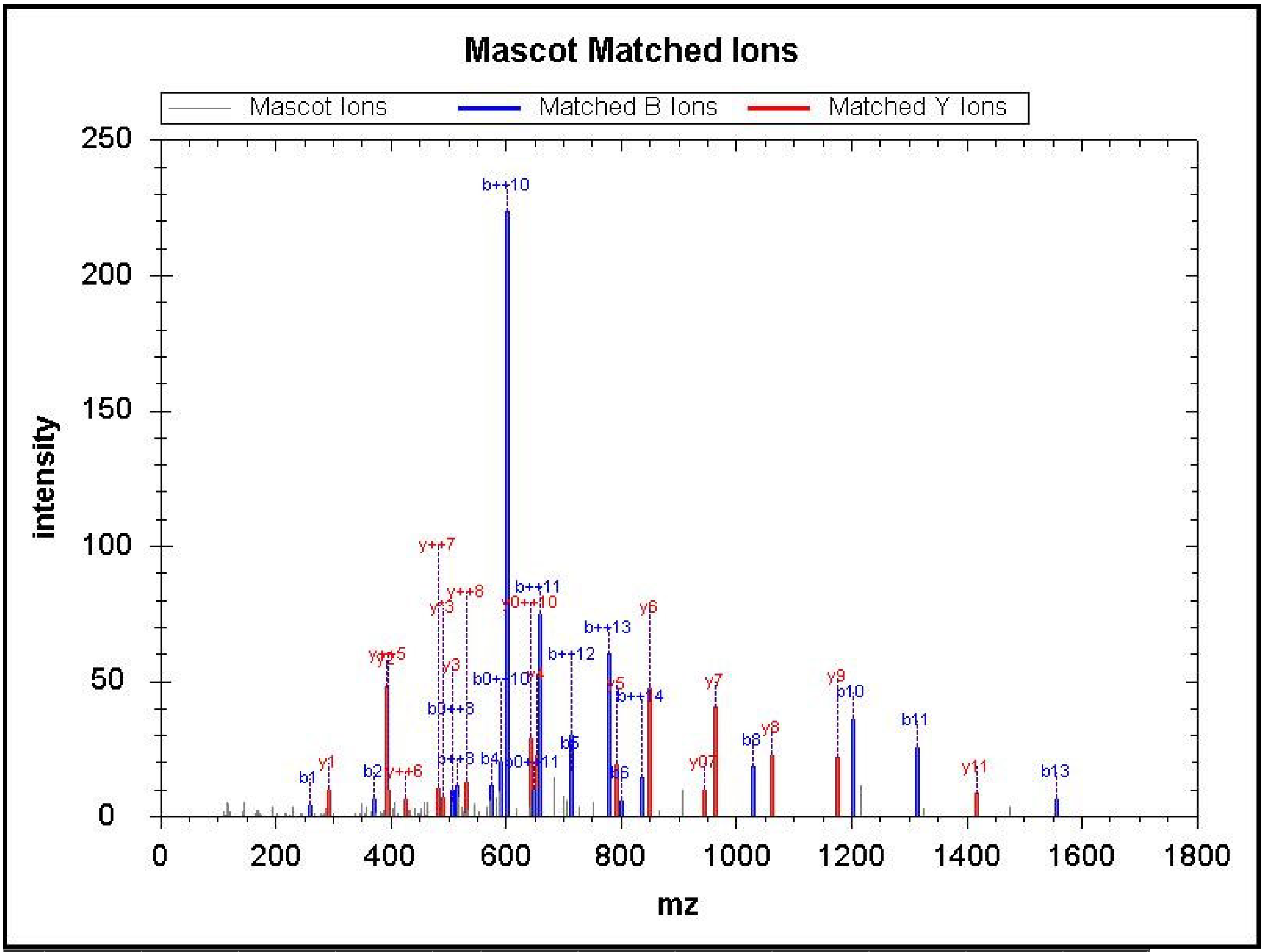

No	b	b++	b0	b0++	Seq	y	y++	y*	y*++	y0	y0++	RevNo
1	258.19	129.60			L							22
2	371.28	186.14			L	2,474.23	1,237.62	2,457.21	1,229.11	2,456.22	1,228.61	21
3	518.35	259.68			F	2,361.15	1,181.08	2,344.12	1,172.56	2,343.14	1,172.07	20
4	575.37	288.19			G	2,214.08	1,107.54	2,197.05	1,099.03	2,196.07	1,098.54	19
5	712.43	356.72			H	2,157.06	1,079.03	2,140.03	1,070.52	2,139.05	1,070.03	18
6	799.46	400.23	781.45	391.23	S	2,020.00	1,010.50	2,002.97	1,001.99	2,001.99	1,001.50	17
7	900.51	450.76	882.50	441.75	T	1,932.97	966.99	1,915.94	958.47	1,914.96	957.98	16
8	1,029.55	515.28	1,011.54	506.27	E	1,831.92	916.46	1,814.89	907.95	1,813.91	907.46	15
9	1,086.57	543.79	1,068.56	534.78	G	1,702.88	851.94	1,685.85	843.43	1,684.87	842.94	14
10	1,201.60	601.30	1,183.59	592.30	D	1,645.86	823.43	1,628.83	814.92	1,627.84	814.43	13
11	1,314.68	657.84	1,296.67	648.84	I	1,530.83	765.92	1,513.80	757.40	1,512.82	756.91	12

12	1,427.77	714.39	1,409.75	705.38	L	1,417.74	709.38	1,400.72	700.86	1,399.73	700.37	11
13	1,556.81	778.91	1,538.80	769.90	E	1,304.66	652.83	1,287.63	644.32	1,286.65	643.83	10
14	1,669.89	835.45	1,651.88	826.44	L	1,175.62	588.31	1,158.59	579.80	1,157.61	579.31	9
15	1,768.96	884.98	1,750.95	875.98	V	1,062.53	531.77	1,045.51	523.26	1,044.52	522.77	8
16	1,883.99	942.50	1,865.98	933.49	D	963.47	482.24	946.44	473.72	945.45	473.23	7
17	1,941.01	971.01	1,923.00	962.00	G	848.44	424.72	831.41	416.21	830.43	415.72	6
18	2,078.07	1,039.54	2,060.06	1,030.53	H	791.42	396.21	774.39	387.70	773.41	387.21	5
19	2,225.14	1,113.07	2,207.13	1,104.07	F	654.36	327.68	637.33	319.17	636.35	318.68	4
20	2,340.16	1,170.59	2,322.15	1,161.58	D	507.29	254.15	490.26	245.64	489.28	245.14	3
21	2,441.21	1,221.11	2,423.20	1,212.10	T	392.26	196.63	375.24	188.12	374.25	187.63	2
22					K	291.21	146.11	274.19	137.60			1

Query 91440 Hit 1

MS/MS Fragmentation of TKEEVAGTLEAVQTIQSITQALQK
Found in sp|Q0JRZ9|FCHO2_HUMAN, FCH domain only protein 2 OS=Homo sapiens GN=FCHO2 PE=1 SV=2
Match to Query 91440: 3017.702from(755.4329,4+)
Title: 1095: Scan 2403 (rt=63.6431, f=3, i=367) [D:\lab212\membrane\Grace\Joyce\iTRAQ_39_1.raw]
Data File:Submitted from 20120508-1(merge) by Mascot Daemon on JOYCE-VAIO
Monoisotopic mass of neutral peptide Mr(calc): 3017.702
Variable modifications:
K2 :iTRAQ4plex (K)
K24 :iTRAQ4plex (K)
Ions Score: 85.42 Expect: 0.000

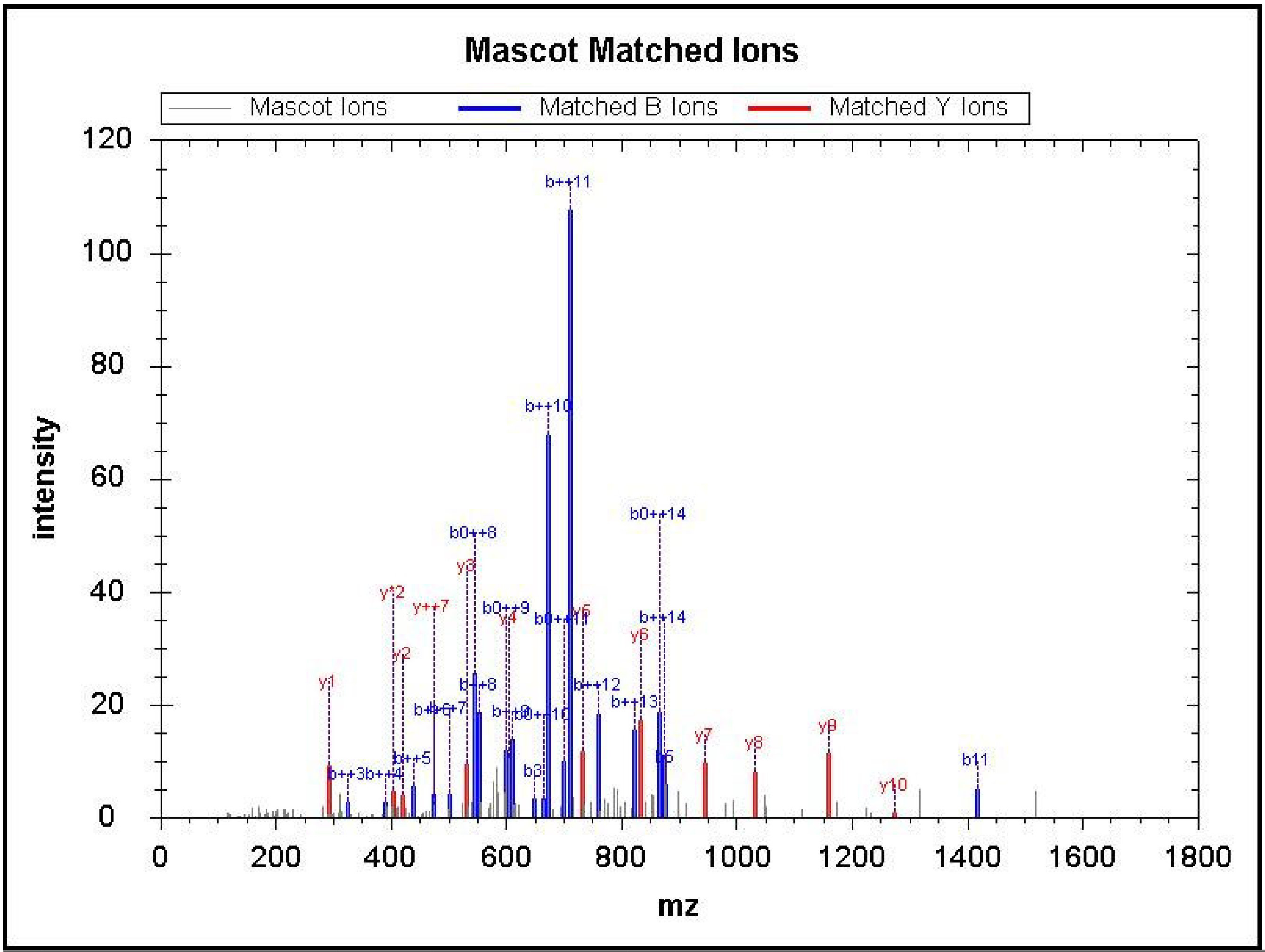

No	b	b++	b*	b*++	b0	b0++	Seq	y	y++	y*	y*++	y0	y0++	RevNo
1	246.16	123.58			228.15	114.58	T							24
2	518.35	259.68	501.33	251.17	500.34	250.68	K	2,773.55	1,387.28	2,756.53	1,378.77	2,755.54	1,378.28	23

3	647.40	324.20	630.37	315.69	629.39	315.20	E	2,501.36	1,251.18	2,484.33	1,242.67	2,483.35	1,242.18	22
4	776.44	388.72	759.41	380.21	758.43	379.72	E	2,372.32	1,186.66	2,355.29	1,178.15	2,354.30	1,177.66	21
5	875.51	438.26	858.48	429.74	857.50	429.25	V	2,243.27	1,122.14	2,226.25	1,113.63	2,225.26	1,113.13	20
6	946.54	473.78	929.52	465.26	928.53	464.77	A	2,144.20	1,072.61	2,127.18	1,064.09	2,126.19	1,063.60	19
7	1,003.57	502.29	986.54	493.77	985.56	493.28	G	2,073.17	1,037.09	2,056.14	1,028.57	2,055.16	1,028.08	18
8	1,104.61	552.81	1,087.59	544.30	1,086.60	543.81	T	2,016.15	1,008.58	1,999.12	1,000.06	1,998.14	999.57	17
9	1,217.70	609.35	1,200.67	600.84	1,199.69	600.35	L	1,915.10	958.05	1,898.07	949.54	1,897.09	949.05	16
10	1,346.74	673.87	1,329.71	665.36	1,328.73	664.87	E	1,802.01	901.51	1,784.99	893.00	1,784.00	892.51	15
11	1,417.78	709.39	1,400.75	700.88	1,399.77	700.39	A	1,672.97	836.99	1,655.94	828.48	1,654.96	827.98	14
12	1,516.85	758.93	1,499.82	750.41	1,498.84	749.92	V	1,601.93	801.47	1,584.91	792.96	1,583.92	792.47	13
13	1,644.90	822.96	1,627.88	814.44	1,626.89	813.95	Q	1,502.87	751.94	1,485.84	743.42	1,484.86	742.93	12
14	1,745.95	873.48	1,728.93	864.97	1,727.94	864.47	T	1,374.81	687.91	1,357.78	679.39	1,356.80	678.90	11
15	1,859.04	930.02	1,842.01	921.51	1,841.03	921.02	I	1,273.76	637.38	1,256.73	628.87	1,255.75	628.38	10
16	1,987.10	994.05	1,970.07	985.54	1,969.08	985.05	Q	1,160.68	580.84	1,143.65	572.33	1,142.66	571.84	9
17	2,074.13	1,037.57	2,057.10	1,029.05	2,056.12	1,028.56	S	1,032.62	516.81	1,015.59	508.30	1,014.61	507.81	8
18	2,187.21	1,094.11	2,170.18	1,085.60	2,169.20	1,085.10	I	945.58	473.30	928.56	464.78	927.57	464.29	7
19	2,288.26	1,144.63	2,271.23	1,136.12	2,270.25	1,135.63	T	832.50	416.75	815.47	408.24	814.49	407.75	6
20	2,416.32	1,208.66	2,399.29	1,200.15	2,398.31	1,199.66	Q	731.45	366.23	714.43	357.72			5
21	2,487.35	1,244.18	2,470.33	1,235.67	2,469.34	1,235.18	A	603.39	302.20	586.37	293.69			4
22	2,600.44	1,300.72	2,583.41	1,292.21	2,582.43	1,291.72	L	532.36	266.68	515.33	258.17			3
23	2,728.50	1,364.75	2,711.47	1,356.24	2,710.49	1,355.75	Q	419.27	210.14	402.25	201.63			2
24							K	291.21	146.11	274.19	137.60			1

Query 85432 Hit 1

MS/MS Fragmentation of DLNLAGTAEVGLAGYFMDHTVAFR
Found in sp|Q9NP81|SYSM_HUMAN, Serine--tRNA ligase
Match to Query 85432: 2711.352from(904.7912,3+)
Title: 1080: Scan 2450 (rt=64.1411, f=3, i=362) [D:\lab212\membrane\Grace\Joyce\iTRAQ_29_1.raw]
Data File:Submitted from 20120508-1(merge) by Mascot Daemon on JOYCE-VAIO
Monoisotopic mass of neutral peptide Mr(calc): 2711.352
Variable modifications:
Ions Score: 85.27 Expect: 0.000

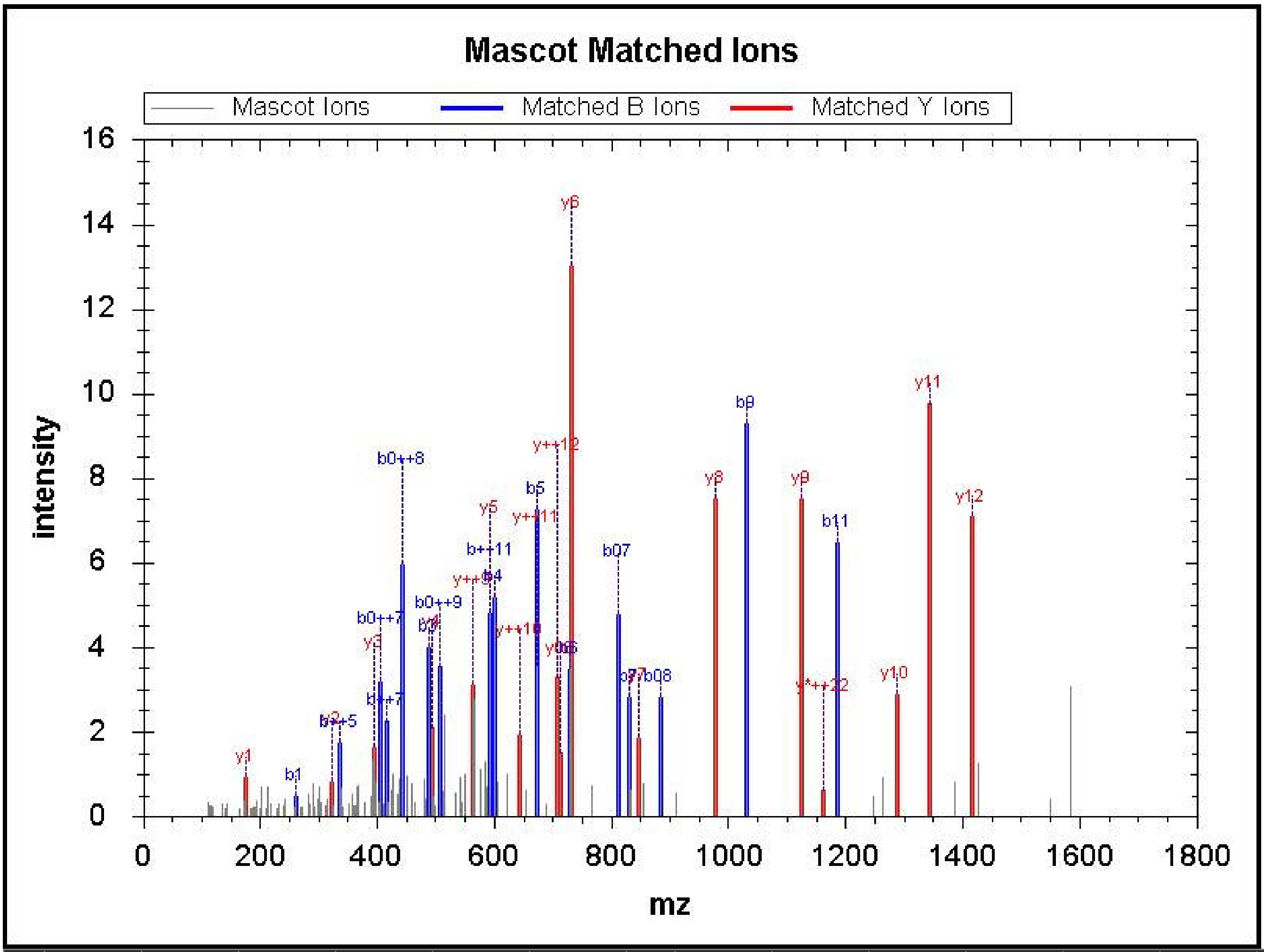

No	b	b++	b*	b*++	b0	b0++	Seq	y	y++	y*	y*++	y0	y0++	RevNo
1	260.14	130.57			242.13	121.57	D							24
2	373.22	187.11			355.21	178.11	L	2,453.23	1,227.12	2,436.20	1,218.60	2,435.22	1,218.11	23
3	487.26	244.14	470.24	235.62	469.25	235.13	N	2,340.14	1,170.58	2,323.12	1,162.06	2,322.13	1,161.57	22
4	600.35	300.68	583.32	292.16	582.34	291.67	L	2,226.10	1,113.55	2,209.07	1,105.04	2,208.09	1,104.55	21
5	671.38	336.20	654.36	327.68	653.37	327.19	A	2,113.02	1,057.01	2,095.99	1,048.50	2,095.01	1,048.01	20
6	728.41	364.71	711.38	356.19	710.40	355.70	G	2,041.98	1,021.49	2,024.95	1,012.98	2,023.97	1,012.49	19
7	829.45	415.23	812.43	406.72	811.44	406.23	T	1,984.96	992.98	1,967.93	984.47	1,966.95	983.98	18
8	900.49	450.75	883.46	442.24	882.48	441.74	A	1,883.91	942.46	1,866.88	933.95	1,865.90	933.45	17
9	1,029.53	515.27	1,012.51	506.76	1,011.52	506.27	E	1,812.87	906.94	1,795.85	898.43	1,794.86	897.94	16
10	1,128.60	564.80	1,111.58	556.29	1,110.59	555.80	V	1,683.83	842.42	1,666.80	833.91	1,665.82	833.41	15
11	1,185.62	593.32	1,168.60	584.80	1,167.61	584.31	G	1,584.76	792.89	1,567.74	784.37	1,566.75	783.88	14
12	1,298.71	649.86	1,281.68	641.34	1,280.70	640.85	L	1,527.74	764.37	1,510.71	755.86	1,509.73	755.37	13
13	1,369.74	685.38	1,352.72	676.86	1,351.73	676.37	A	1,414.66	707.83	1,397.63	699.32	1,396.65	698.83	12
14	1,426.77	713.89	1,409.74	705.37	1,408.76	704.88	G	1,343.62	672.31	1,326.59	663.80	1,325.61	663.31	11
15	1,589.83	795.42	1,572.80	786.91	1,571.82	786.41	Y	1,286.60	643.80	1,269.57	635.29	1,268.59	634.80	10
16	1,736.90	868.95	1,719.87	860.44	1,718.89	859.95	F	1,123.54	562.27	1,106.51	553.76	1,105.52	553.27	9
17	1,867.94	934.47	1,850.91	925.96	1,849.93	925.47	M	976.47	488.74	959.44	480.22	958.46	479.73	8
18	1,982.97	991.99	1,965.94	983.47	1,964.95	982.98	D	845.43	423.22	828.40	414.70	827.42	414.21	7
19	2,120.02	1,060.52	2,103.00	1,052.00	2,102.01	1,051.51	H	730.40	365.70	713.37	357.19	712.39	356.70	6
20	2,221.07	1,111.04	2,204.05	1,102.53	2,203.06	1,102.03	T	593.34	297.17	576.31	288.66	575.33	288.17	5
21	2,320.14	1,160.57	2,303.11	1,152.06	2,302.13	1,151.57	V	492.29	246.65	475.27	238.14			4
22	2,391.18	1,196.09	2,374.15	1,187.58	2,373.17	1,187.09	A	393.22	197.12	376.20	188.60			3
23	2,538.25	1,269.63	2,521.22	1,261.11	2,520.24	1,260.62	F	322.19	161.60	305.16	153.08			2

24							R	175.12	88.06	158.09	79.55			1

Query 90123 Hit 1

MS/MS Fragmentation of EVTSHFQVTLNDIQLQMEQHNER
Found in sp|P40222|TXLNA_HUMAN, Alpha-taxilin OS=Homo sapiens GN=TXLNA PE=1 SV=3
Match to Query 90123: 2939.437from(735.8665,4+)
Title: 743: Scan 1669 (rt=47.1845, f=2, i=270) [D:\lab212\membrane\Grace\Joyce\iTRAQ_44_1.raw]
Data File:Submitted from 20120508-1(merge) by Mascot Daemon on JOYCE-VAIO
Monoisotopic mass of neutral peptide Mr(calc): 2939.437
Variable modifications:
Ions Score: 85.16 Expect: 0.000

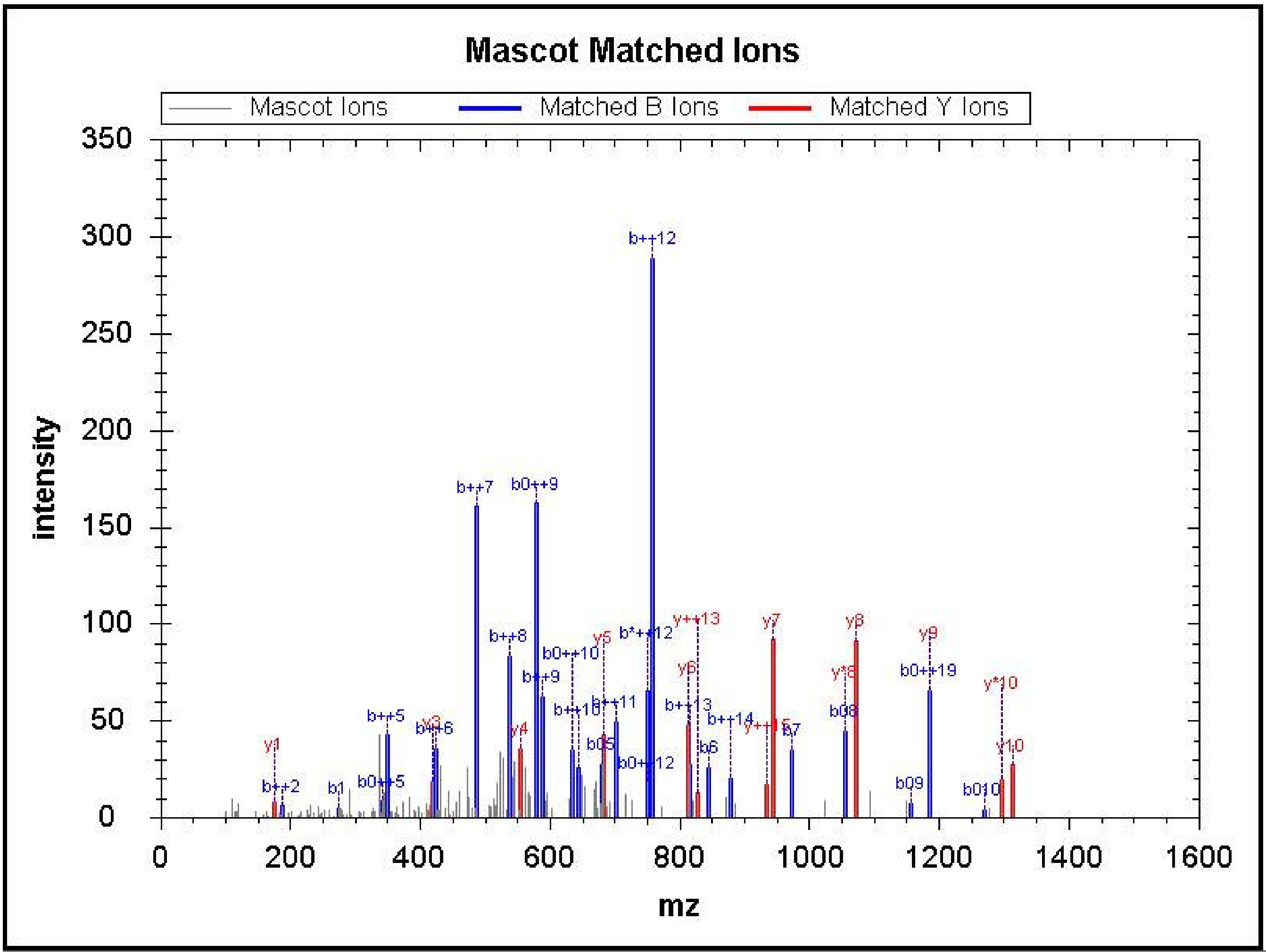

No	b	b++	b*	b*++	b0	b0++	Seq	y	y++	y*	y*++	y0	y0++	RevNo
1	274.15	137.58			256.14	128.57	E							23
2	373.22	187.11			355.21	178.11	V	2,667.29	1,334.15	2,650.27	1,325.64	2,649.28	1,325.15	22
3	474.27	237.64			456.26	228.63	T	2,568.23	1,284.62	2,551.20	1,276.10	2,550.22	1,275.61	21
4	561.30	281.15			543.29	272.15	S	2,467.18	1,234.09	2,450.15	1,225.58	2,449.17	1,225.09	20
5	698.36	349.68			680.35	340.68	H	2,380.15	1,190.58	2,363.12	1,182.06	2,362.14	1,181.57	19
6	845.43	423.22			827.42	414.21	F	2,243.09	1,122.05	2,226.06	1,113.53	2,225.08	1,113.04	18
7	973.49	487.25	956.46	478.73	955.48	478.24	Q	2,096.02	1,048.51	2,078.99	1,040.00	2,078.01	1,039.51	17
8	1,072.55	536.78	1,055.53	528.27	1,054.54	527.78	V	1,967.96	984.48	1,950.93	975.97	1,949.95	975.48	16
9	1,173.60	587.30	1,156.58	578.79	1,155.59	578.30	T	1,868.89	934.95	1,851.87	926.44	1,850.88	925.94	15
10	1,286.69	643.85	1,269.66	635.33	1,268.68	634.84	L	1,767.84	884.43	1,750.82	875.91	1,749.83	875.42	14
11	1,400.73	700.87	1,383.70	692.36	1,382.72	691.86	N	1,654.76	827.88	1,637.73	819.37	1,636.75	818.88	13
12	1,515.76	758.38	1,498.73	749.87	1,497.75	749.38	D	1,540.72	770.86	1,523.69	762.35	1,522.71	761.86	12
13	1,628.84	814.92	1,611.81	806.41	1,610.83	805.92	I	1,425.69	713.35	1,408.66	704.84	1,407.68	704.34	11

14	1,756.90	878.95	1,739.87	870.44	1,738.89	869.95	Q	1,312.61	656.81	1,295.58	648.29	1,294.60	647.80	10
15	1,869.98	935.50	1,852.96	926.98	1,851.97	926.49	L	1,184.55	592.78	1,167.52	584.26	1,166.54	583.77	9
16	1,998.04	999.52	1,981.02	991.01	1,980.03	990.52	Q	1,071.46	536.24	1,054.44	527.72	1,053.45	527.23	8
17	2,129.08	1,065.04	2,112.06	1,056.53	2,111.07	1,056.04	M	943.41	472.21	926.38	463.69	925.39	463.20	7
18	2,258.12	1,129.57	2,241.10	1,121.05	2,240.11	1,120.56	E	812.36	406.69	795.34	398.17	794.35	397.68	6
19	2,386.18	1,193.60	2,369.16	1,185.08	2,368.17	1,184.59	Q	683.32	342.16	666.30	333.65	665.31	333.16	5
20	2,523.24	1,262.12	2,506.22	1,253.61	2,505.23	1,253.12	H	555.26	278.14	538.24	269.62	537.25	269.13	4
21	2,637.28	1,319.15	2,620.26	1,310.63	2,619.27	1,310.14	N	418.20	209.61	401.18	201.09	400.19	200.60	3
22	2,766.33	1,383.67	2,749.30	1,375.15	2,748.32	1,374.66	E	304.16	152.58	287.13	144.07	286.15	143.58	2
23							R	175.12	88.06	158.09	79.55			1

Query 58674 Hit 1

MS/MS Fragmentation of QGTFHSQQALEYGTK
Found in sp|P53597|SUCA_HUMAN, Succinyl-CoA ligase [ADP/GDP-forming] subunit alpha
Match to Query 58674: 1982.015from(661.6788,3+)
Title: 54: Scan 848 (rt=26.1519, f=2, i=125) [D:\lab212\membrane\Grace\Joyce\iTRAQ_43_2.raw]
Data File:Submitted from 20120508-1(merge) by Mascot Daemon on JOYCE-VAIO
Monoisotopic mass of neutral peptide Mr(calc): 1982.015
Variable modifications:
K15 :iTRAQ4plex (K)
Ions Score: 83.82 Expect: 0.000

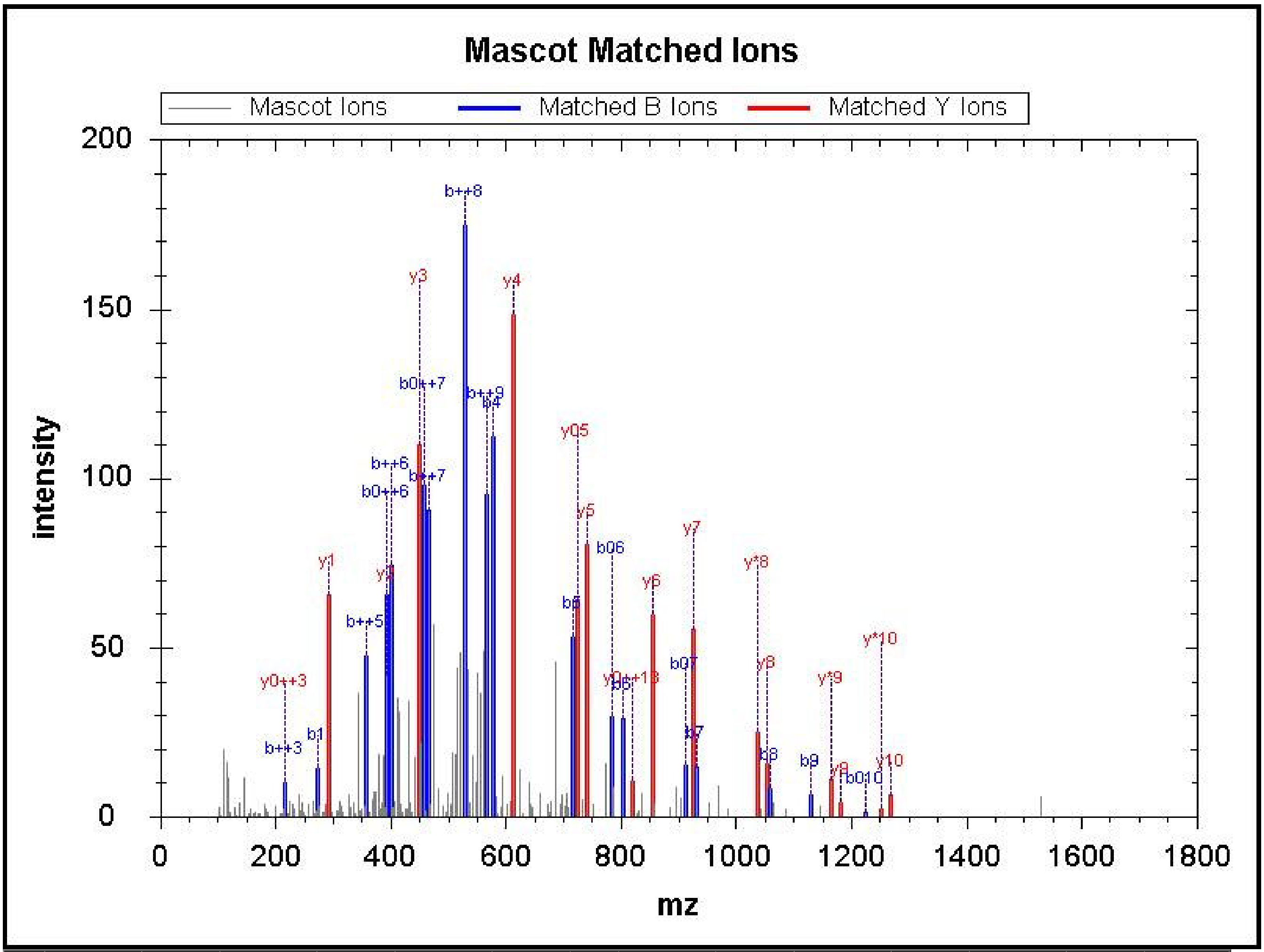

No	b	b++	b*	b*++	b0	b0++	Seq	y	y++	y*	y*++	y0	y0++	RevNo
1	273.17	137.09	256.14	128.57			Q							15
2	330.19	165.60	313.16	157.09			G	1,710.86	855.93	1,693.83	847.42	1,692.85	846.93	14
3	431.24	216.12	414.21	207.61	413.23	207.12	T	1,653.84	827.42	1,636.81	818.91	1,635.82	818.42	13
4	578.31	289.66	561.28	281.14	560.29	280.65	F	1,552.79	776.90	1,535.76	768.38	1,534.78	767.89	12

5	715.36	358.19	698.34	349.67	697.35	349.18	H	1,405.72	703.36	1,388.69	694.85	1,387.71	694.36	11
6	802.40	401.70	785.37	393.19	784.39	392.70	S	1,268.66	634.83	1,251.63	626.32	1,250.65	625.83	10
7	930.46	465.73	913.43	457.22	912.44	456.73	Q	1,181.63	591.32	1,164.60	582.80	1,163.62	582.31	9
8	1,058.51	529.76	1,041.49	521.25	1,040.50	520.76	Q	1,053.57	527.29	1,036.54	518.78	1,035.56	518.28	8
9	1,129.55	565.28	1,112.52	556.77	1,111.54	556.27	A	925.51	463.26	908.48	454.75	907.50	454.25	7
10	1,242.63	621.82	1,225.61	613.31	1,224.62	612.82	L	854.47	427.74	837.45	419.23	836.46	418.74	6
11	1,371.68	686.34	1,354.65	677.83	1,353.67	677.34	E	741.39	371.20	724.36	362.69	723.38	362.19	5
12	1,534.74	767.87	1,517.71	759.36	1,516.73	758.87	Y	612.35	306.68	595.32	298.16	594.34	297.67	4
13	1,591.76	796.38	1,574.74	787.87	1,573.75	787.38	G	449.28	225.15	432.26	216.63	431.27	216.14	3
14	1,692.81	846.91	1,675.78	838.40	1,674.80	837.90	T	392.26	196.63	375.24	188.12	374.25	187.63	2
15							K	291.21	146.11	274.19	137.60			1

Query 87723 Hit 1

MS/MS Fragmentation of IFHDLDMLTTVQEENEPVIYNR
Found in sp|P83111|LACTB_HUMAN, Serine beta-lactamase-like protein LACTB
Match to Query 87723: 2819.386from(940.8027,3+)
Title: 886: Sum of 2 scans in range 1927 (rt=53.0853, f=4, i=595) to 1928 (rt=53.1107, f=4, i=596)
[D:\lab212\membrane\Grace\Joyce\iTRAQ_33_2.raw]
Data File:Submitted from 20120508-1(merge) by Mascot Daemon on JOYCE-VAIO
Monoisotopic mass of neutral peptide Mr(calc): 2819.386
Variable modifications:
Ions Score: 83.6 Expect: 0.000

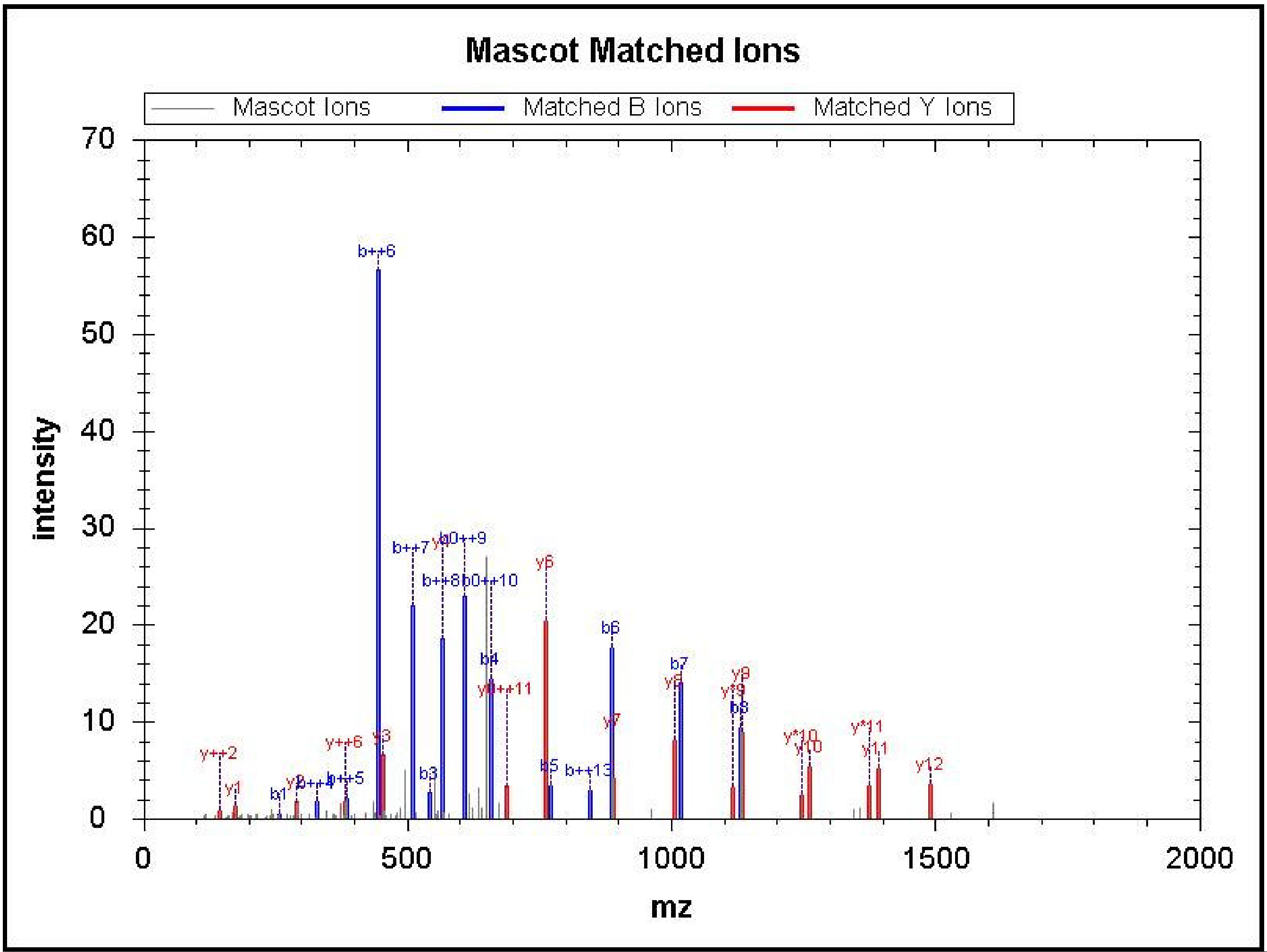

No	b	b++	b*	b*++	b0	b0++	Seq	y	y++	y*	y*++	y0	y0++	RevNo
1	258.19	129.60					I							22
2	405.26	203.13					F	2,563.21	1,282.11	2,546.19	1,273.60	2,545.20	1,273.10	21

3	542.32	271.66					H	2,416.14	1,208.58	2,399.12	1,200.06	2,398.13	1,199.57	20
4	657.35	329.18			639.34	320.17	D	2,279.09	1,140.05	2,262.06	1,131.53	2,261.08	1,131.04	19
5	770.43	385.72			752.42	376.71	L	2,164.06	1,082.53	2,147.03	1,074.02	2,146.05	1,073.53	18
6	885.46	443.23			867.45	434.23	D	2,050.98	1,025.99	2,033.95	1,017.48	2,032.96	1,016.99	17
7	1,016.50	508.75			998.49	499.75	M	1,935.95	968.48	1,918.92	959.96	1,917.94	959.47	16
8	1,129.58	565.30			1,111.57	556.29	L	1,804.91	902.96	1,787.88	894.44	1,786.90	893.95	15
9	1,230.63	615.82			1,212.62	606.81	T	1,691.82	846.42	1,674.80	837.90	1,673.81	837.41	14
10	1,331.68	666.34			1,313.67	657.34	T	1,590.78	795.89	1,573.75	787.38	1,572.77	786.89	13
11	1,430.75	715.88			1,412.74	706.87	V	1,489.73	745.37	1,472.70	736.85	1,471.72	736.36	12
12	1,558.81	779.91	1,541.78	771.39	1,540.80	770.90	Q	1,390.66	695.83	1,373.63	687.32	1,372.65	686.83	11
13	1,687.85	844.43	1,670.82	835.91	1,669.84	835.42	E	1,262.60	631.80	1,245.57	623.29	1,244.59	622.80	10
14	1,816.89	908.95	1,799.86	900.44	1,798.88	899.94	E	1,133.56	567.28	1,116.53	558.77	1,115.55	558.28	9
15	1,930.93	965.97	1,913.91	957.46	1,912.92	956.97	N	1,004.52	502.76	987.49	494.25	986.51	493.76	8
16	2,059.98	1,030.49	2,042.95	1,021.98	2,041.97	1,021.49	E	890.47	445.74	873.45	437.23	872.46	436.73	7
17	2,157.03	1,079.02	2,140.00	1,070.50	2,139.02	1,070.01	P	761.43	381.22	744.40	372.71			6
18	2,256.10	1,128.55	2,239.07	1,120.04	2,238.09	1,119.55	V	664.38	332.69	647.35	324.18			5
19	2,369.18	1,185.09	2,352.15	1,176.58	2,351.17	1,176.09	I	565.31	283.16	548.28	274.64			4
20	2,532.24	1,266.63	2,515.22	1,258.11	2,514.23	1,257.62	Y	452.23	226.62	435.20	218.10			3
21	2,646.29	1,323.65	2,629.26	1,315.13	2,628.28	1,314.64	N	289.16	145.08	272.14	136.57			2
22							R	175.12	88.06	158.09	79.55			1

Query 47910 Hit 1

MS/MS Fragmentation of HTLADNFNPVSEER
Found in sp|P20645|MPRD_HUMAN, Cation-dependent mannose-6-phosphate receptor OS=Homo sapiens GN=M6PR PE=1 SV=1
Match to Query 47910: 1771.863from(591.6283,3+)
Title: 423: Scan 1002 (rt=32.0729, f=3, i=141) [D:\lab212\membrane\Grace\Joyce\iTRAQ_39_1.raw]
Data File:Submitted from 20120508-1(merge) by Mascot Daemon on JOYCE-VAIO
Monoisotopic mass of neutral peptide Mr(calc): 1771.863
Variable modifications:
Ions Score: 83.43 Expect: 0.000

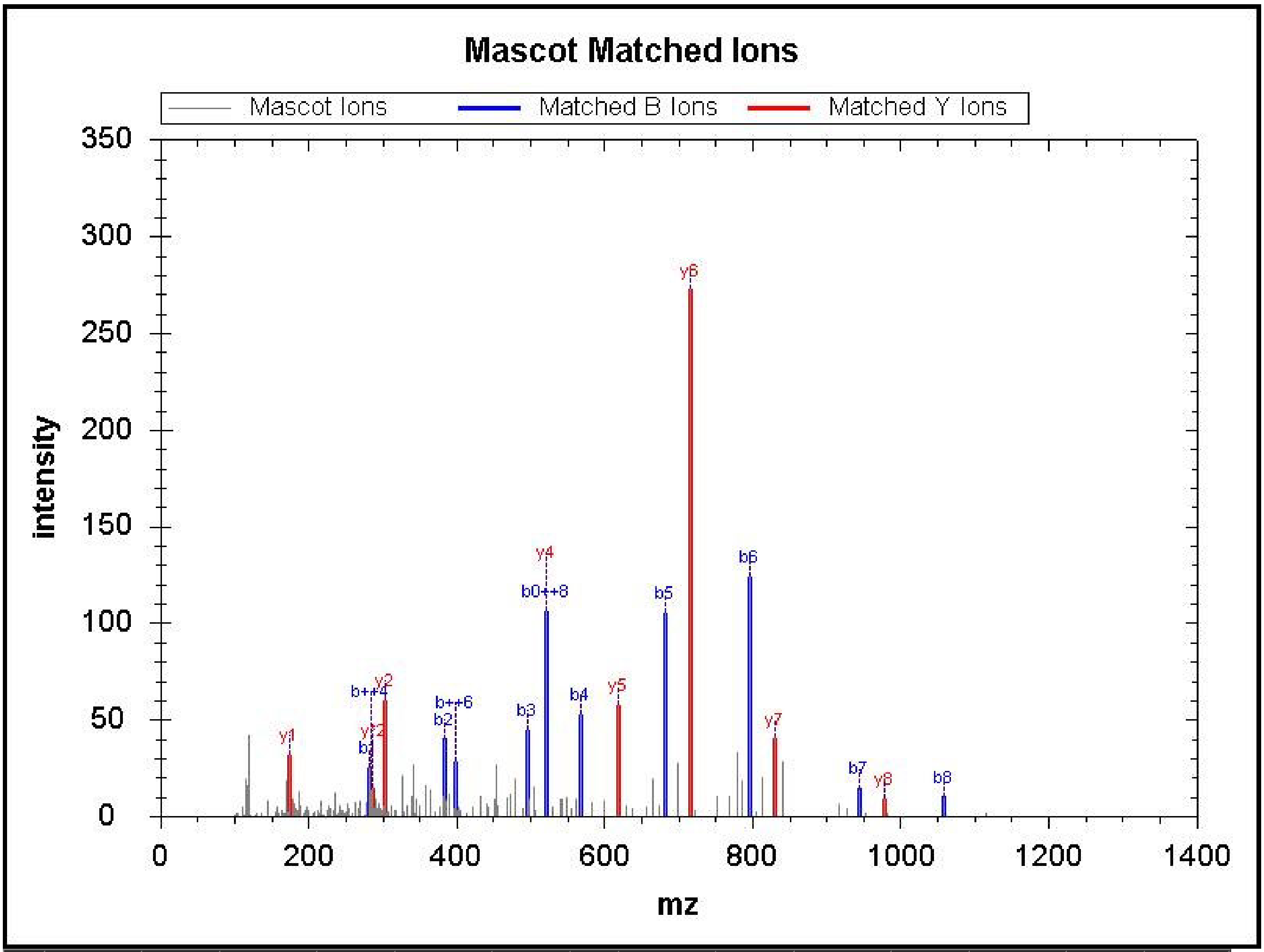

No	b	b++	b*	b*++	b0	b0++	Seq	y	y++	y*	y*++	y0	y0++	RevNo
1	282.17	141.59					H							14
2	383.22	192.11			365.21	183.11	T	1,491.71	746.36	1,474.68	737.84	1,473.70	737.35	13
3	496.30	248.65			478.29	239.65	L	1,390.66	695.83	1,373.63	687.32	1,372.65	686.83	12
4	567.34	284.17			549.33	275.17	A	1,277.58	639.29	1,260.55	630.78	1,259.57	630.29	11
5	682.36	341.69			664.35	332.68	D	1,206.54	603.77	1,189.51	595.26	1,188.53	594.77	10
6	796.41	398.71	779.38	390.19	778.40	389.70	N	1,091.51	546.26	1,074.49	537.75	1,073.50	537.25	9
7	943.48	472.24	926.45	463.73	925.46	463.24	F	977.47	489.24	960.44	480.72	959.46	480.23	8
8	1,057.52	529.26	1,040.49	520.75	1,039.51	520.26	N	830.40	415.70	813.37	407.19	812.39	406.70	7
9	1,154.57	577.79	1,137.54	569.28	1,136.56	568.78	P	716.36	358.68	699.33	350.17	698.35	349.68	6
10	1,253.64	627.32	1,236.61	618.81	1,235.63	618.32	V	619.30	310.16	602.28	301.64	601.29	301.15	5
11	1,340.67	670.84	1,323.64	662.33	1,322.66	661.83	S	520.24	260.62	503.21	252.11	502.23	251.62	4
12	1,469.71	735.36	1,452.69	726.85	1,451.70	726.36	E	433.20	217.11	416.18	208.59	415.19	208.10	3
13	1,598.76	799.88	1,581.73	791.37	1,580.75	790.88	E	304.16	152.58	287.13	144.07	286.15	143.58	2
14							R	175.12	88.06	158.09	79.55			1

Query 36661 Hit 1

MS/MS Fragmentation of VAELVHFLLLK
Found in sp|P43357|MAGA3_HUMAN, Melanoma-associated antigen 3 OS=Homo sapiens GN=MAGEA3 PE=1 SV=1
Match to Query 36661: 1568.99from(785.5024,2+)
Title: 1111: Sum of 2 scans in range 2384 (rt=63.4657, f=4, i=749) to 2385 (rt=63.4911, f=4, i=750) [D:\lab212\membrane\Grace\Joyce\iTRAQ40.raw]
Data File:Submitted from 20120508-1(merge) by Mascot Daemon on JOYCE-VAIO
Monoisotopic mass of neutral peptide Mr(calc): 1568.99
Variable modifications:
K11 :iTRAQ4plex (K)
Ions Score: 83.27 Expect: 0.000

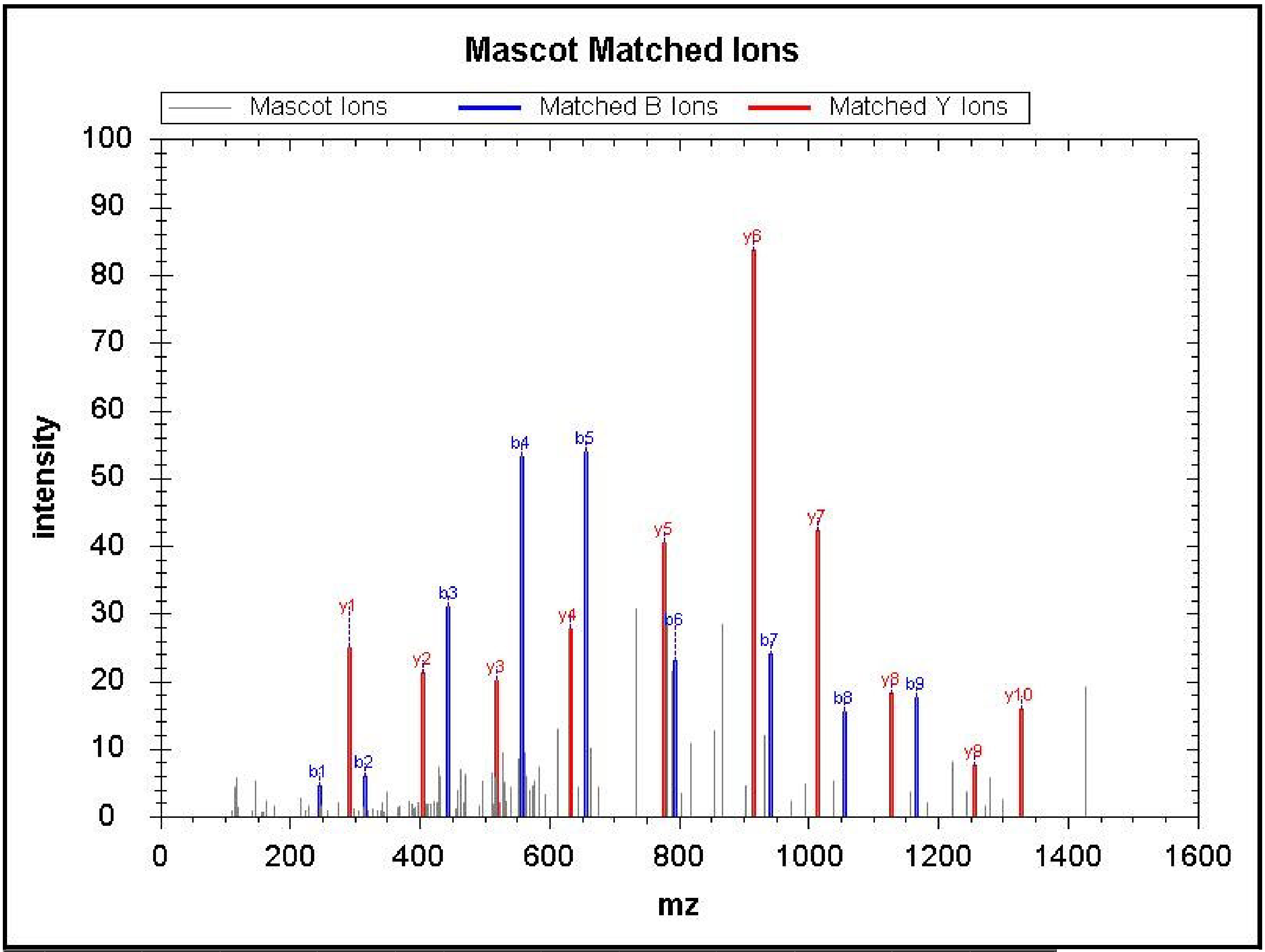

No	b	b++	b0	b0++	Seq	y	y++	y*	y*++	y0	y0++	RevNo
1	244.18	122.59			V							11
2	315.21	158.11			A	1,326.83	663.92	1,309.80	655.40	1,308.82	654.91	10
3	444.26	222.63	426.25	213.63	E	1,255.79	628.40	1,238.76	619.89	1,237.78	619.39	9
4	557.34	279.17	539.33	270.17	L	1,126.75	563.88	1,109.72	555.36			8
5	656.41	328.71	638.40	319.70	V	1,013.66	507.33	996.64	498.82			7
6	793.47	397.24	775.46	388.23	H	914.59	457.80	897.57	449.29			6
7	940.54	470.77	922.53	461.77	F	777.54	389.27	760.51	380.76			5
8	1,053.62	527.31	1,035.61	518.31	L	630.47	315.74	613.44	307.22			4
9	1,166.71	583.86	1,148.69	574.85	L	517.38	259.20	500.36	250.68			3
10	1,279.79	640.40	1,261.78	631.39	L	404.30	202.65	387.27	194.14			2
11					K	291.21	146.11	274.19	137.60			1

Query 61915 Hit 1

MS/MS Fragmentation of DKPVVLQFMDWILR
Found in sp|Q8VHL0|UT1_MOUSE, Urea transporter 1 OS=Mus musculus GN=Slc14a1 PE=1 SV=2
Match to Query 61915: 2047.158from(683.3932,3+)
Title: 1310: Sum of 2 scans in range 3070 (rt=77.8649, f=2, i=528) to 3071 (rt=77.8903, f=2, i=529)
[D:\lab212\membrane\Grace\Joyce\iTRAQ_39_1.raw]
Data File:Submitted from 20120508-1(merge) by Mascot Daemon on JOYCE-VAIO
Monoisotopic mass of neutral peptide Mr(calc): 2047.158
Variable modifications:
K2 :iTRAQ4plex (K)
Ions Score: 82.55 Expect: 0.000

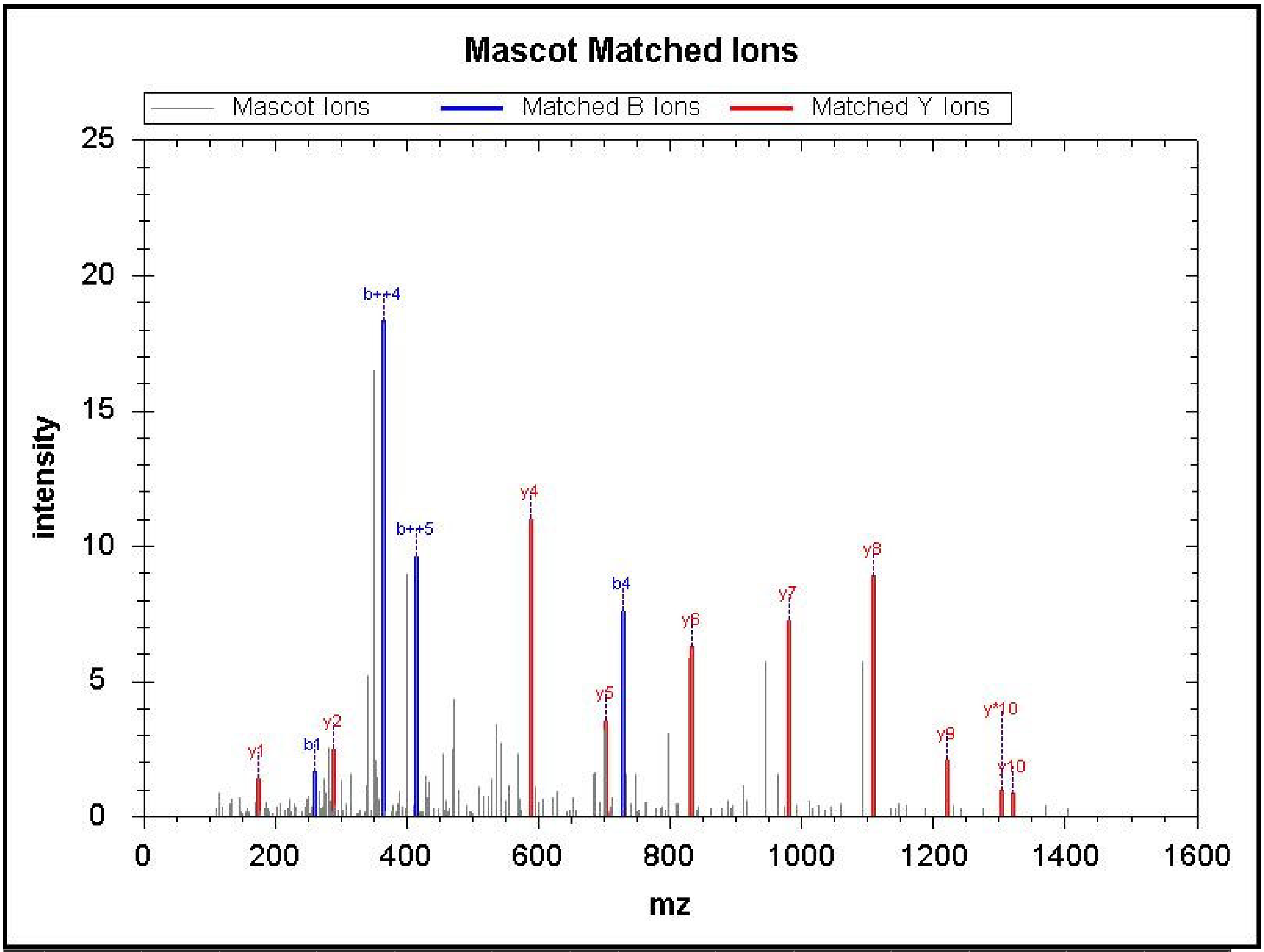

No	b	b++	b*	b*++	b0	b0++	Seq	y	y++	y*	y*++	y0	y0++	RevNo
1	260.14	130.57			242.13	121.57	D							14
2	532.33	266.67	515.31	258.16	514.32	257.67	K	1,789.03	895.02	1,772.00	886.51	1,771.02	886.01	13
3	629.39	315.20	612.36	306.68	611.38	306.19	P	1,516.83	758.92	1,499.81	750.41	1,498.82	749.92	12
4	728.45	364.73	711.43	356.22	710.44	355.73	V	1,419.78	710.39	1,402.76	701.88	1,401.77	701.39	11
5	827.52	414.27	810.50	405.75	809.51	405.26	V	1,320.71	660.86	1,303.69	652.35	1,302.70	651.85	10
6	940.61	470.81	923.58	462.29	922.60	461.80	L	1,221.64	611.33	1,204.62	602.81	1,203.63	602.32	9
7	1,068.67	534.84	1,051.64	526.32	1,050.65	525.83	Q	1,108.56	554.78	1,091.53	546.27	1,090.55	545.78	8
8	1,215.73	608.37	1,198.71	599.86	1,197.72	599.37	F	980.50	490.75	963.48	482.24	962.49	481.75	7
9	1,346.77	673.89	1,329.75	665.38	1,328.76	664.89	M	833.43	417.22	816.41	408.71	815.42	408.22	6
10	1,461.80	731.40	1,444.77	722.89	1,443.79	722.40	D	702.39	351.70	685.37	343.19	684.38	342.69	5
11	1,647.88	824.44	1,630.85	815.93	1,629.87	815.44	W	587.37	294.19	570.34	285.67			4
12	1,760.96	880.99	1,743.94	872.47	1,742.95	871.98	I	401.29	201.15	384.26	192.63			3
13	1,874.05	937.53	1,857.02	929.01	1,856.04	928.52	L	288.20	144.61	271.18	136.09			2
14							R	175.12	88.06	158.09	79.55			1

Query 80933 Hit 1

MS/MS Fragmentation of LDAITDEENDMLDLAYGLTDR
Found in sp|P10109|ADX_HUMAN, Adrenodoxin
Match to Query 80933: 2526.199from(843.0737,3+)
Title: 860: Sum of 2 scans in range 2756 (rt=68.3293, f=4, i=608) to 2757 (rt=68.3547, f=4, i=609) [D:\lab212\membrane\Grace\Joyce\iTRAQ_19_2_330.raw]
Data File:Submitted from 20120508-1(merge) by Mascot Daemon on JOYCE-VAIO
Monoisotopic mass of neutral peptide Mr(calc): 2526.199
Variable modifications:
Ions Score: 82.45 Expect: 0.000

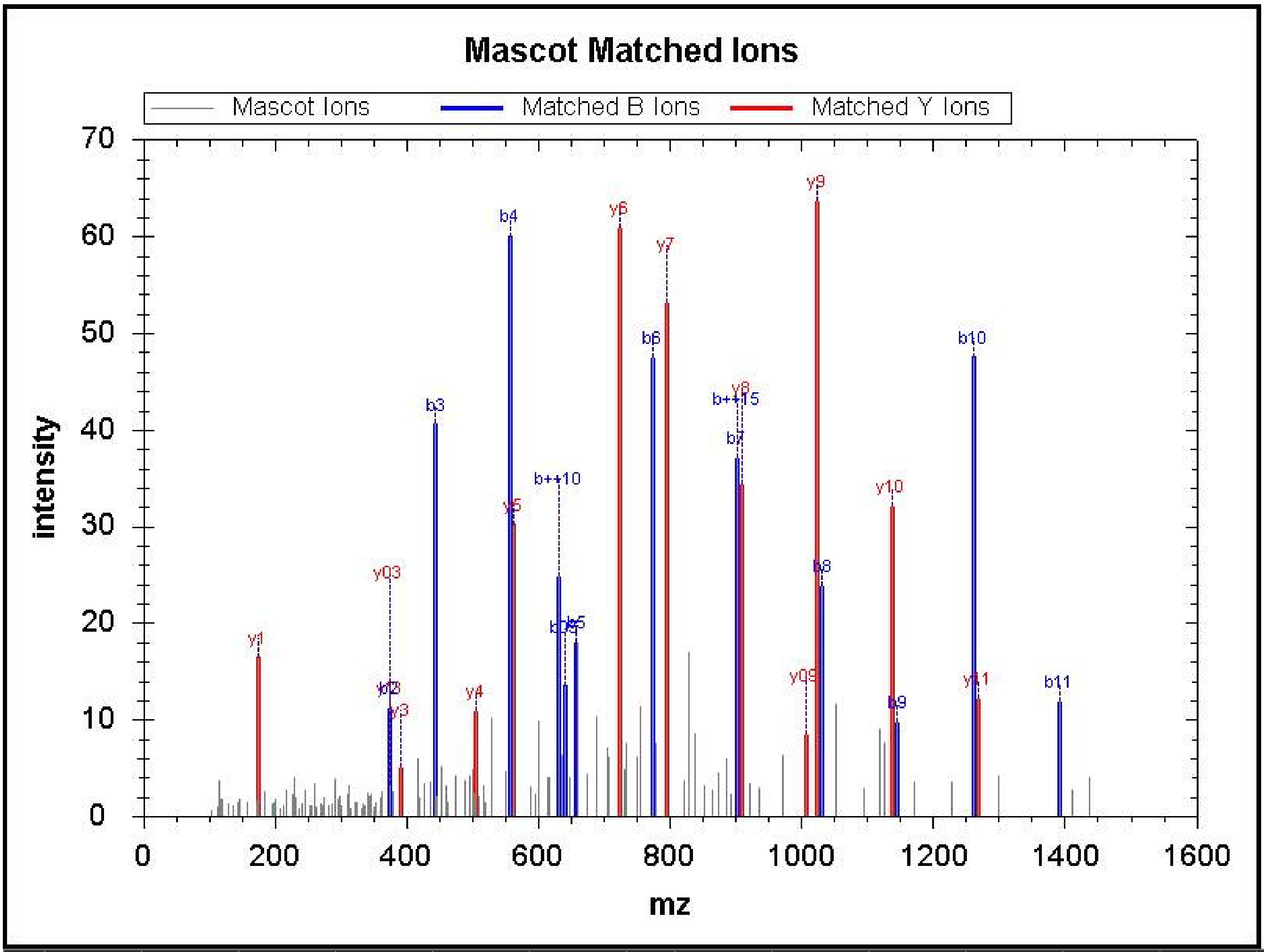

No	b	b++	b*	b*++	b0	b0++	Seq	y	y++	y*	y*++	y0	y0++	RevNo
1	258.19	129.60					L							21
2	373.22	187.11			355.21	178.11	D	2,270.01	1,135.51	2,252.99	1,127.00	2,252.00	1,126.50	20
3	444.26	222.63			426.25	213.63	A	2,154.99	1,078.00	2,137.96	1,069.48	2,136.98	1,068.99	19
4	557.34	279.17			539.33	270.17	I	2,083.95	1,042.48	2,066.92	1,033.96	2,065.94	1,033.47	18
5	658.39	329.70			640.38	320.69	T	1,970.86	985.94	1,953.84	977.42	1,952.85	976.93	17
6	773.42	387.21			755.41	378.21	D	1,869.82	935.41	1,852.79	926.90	1,851.81	926.41	16
7	902.46	451.73			884.45	442.73	E	1,754.79	877.90	1,737.76	869.39	1,736.78	868.89	15
8	1,031.50	516.25			1,013.49	507.25	E	1,625.75	813.38	1,608.72	804.86	1,607.74	804.37	14
9	1,145.54	573.28	1,128.52	564.76	1,127.53	564.27	N	1,496.71	748.86	1,479.68	740.34	1,478.69	739.85	13
10	1,260.57	630.79	1,243.54	622.28	1,242.56	621.78	D	1,382.66	691.83	1,365.64	683.32	1,364.65	682.83	12
11	1,391.61	696.31	1,374.59	687.80	1,373.60	687.30	M	1,267.64	634.32	1,250.61	625.81	1,249.62	625.32	11
12	1,504.70	752.85	1,487.67	744.34	1,486.69	743.85	L	1,136.59	568.80	1,119.57	560.29	1,118.58	559.80	10
13	1,619.72	810.37	1,602.70	801.85	1,601.71	801.36	D	1,023.51	512.26	1,006.48	503.75	1,005.50	503.25	9
14	1,732.81	866.91	1,715.78	858.39	1,714.80	857.90	L	908.48	454.75	891.46	446.23	890.47	445.74	8
15	1,803.84	902.43	1,786.82	893.91	1,785.83	893.42	A	795.40	398.20	778.37	389.69	777.39	389.20	7
16	1,966.91	983.96	1,949.88	975.44	1,948.90	974.95	Y	724.36	362.68	707.34	354.17	706.35	353.68	6
17	2,023.93	1,012.47	2,006.90	1,003.95	2,005.92	1,003.46	G	561.30	281.15	544.27	272.64	543.29	272.15	5
18	2,137.01	1,069.01	2,119.99	1,060.50	2,119.00	1,060.00	L	504.28	252.64	487.25	244.13	486.27	243.64	4
19	2,238.06	1,119.53	2,221.03	1,111.02	2,220.05	1,110.53	T	391.19	196.10	374.17	187.59	373.18	187.10	3
20	2,353.09	1,177.05	2,336.06	1,168.53	2,335.08	1,168.04	D	290.15	145.58	273.12	137.06	272.14	136.57	2
21							R	175.12	88.06	158.09	79.55			1

Query 81684 Hit 1

MS/MS Fragmentation of FVADEELVHLLLDEVVASAALR
Found in sp|Q9UID3|FFR_HUMAN, Protein fat-free homolog OS=Homo sapiens GN=FFR PE=1 SV=2
Match to Query 81684: 2552.398from(851.8067,3+)
Title: 1266: Sum of 2 scans in range 3230 (rt=80.0027, f=2, i=539) to 3231 (rt=80.0281, f=2, i=540)
[D:\lab212\membrane\Grace\Joyce\iTRAQ_29_1.raw]
Data File:Submitted from 20120508-1(merge) by Mascot Daemon on JOYCE-VAIO
Monoisotopic mass of neutral peptide Mr(calc): 2552.398
Variable modifications:
Ions Score: 82.01 Expect: 0.000

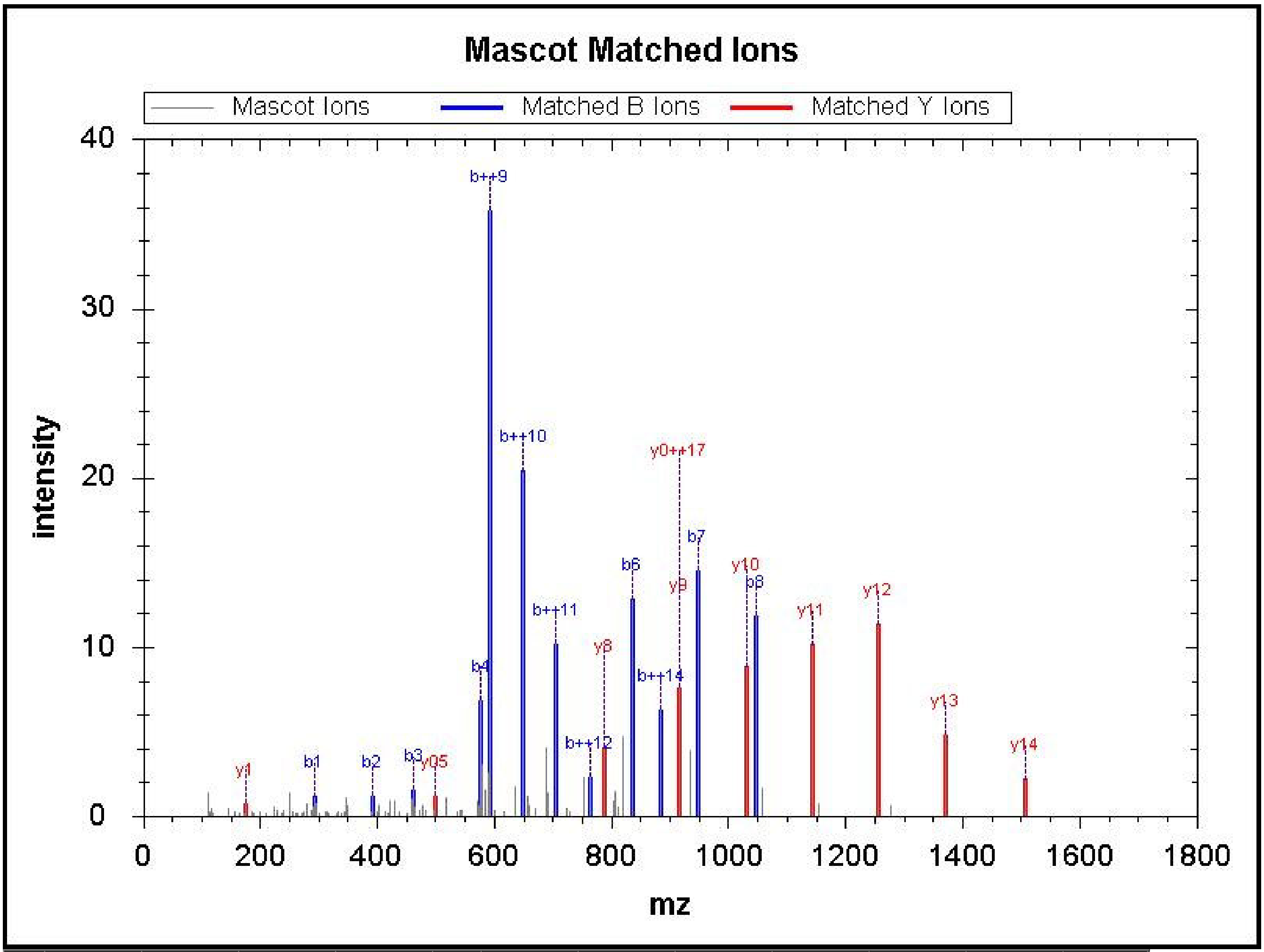

No	b	b++	b0	b0++	Seq	y	y++	y*	y*++	y0	y0++	RevNo
1	292.18	146.59			F							22
2	391.25	196.13			V	2,262.23	1,131.62	2,245.21	1,123.11	2,244.22	1,122.62	21
3	462.28	231.65			A	2,163.17	1,082.09	2,146.14	1,073.57	2,145.16	1,073.08	20
4	577.31	289.16	559.30	280.15	D	2,092.13	1,046.57	2,075.10	1,038.05	2,074.12	1,037.56	19
5	706.35	353.68	688.34	344.67	E	1,977.10	989.05	1,960.08	980.54	1,959.09	980.05	18
6	835.40	418.20	817.38	409.20	E	1,848.06	924.53	1,831.03	916.02	1,830.05	915.53	17
7	948.48	474.74	930.47	465.74	L	1,719.02	860.01	1,701.99	851.50	1,701.01	851.01	16
8	1,047.55	524.28	1,029.54	515.27	V	1,605.93	803.47	1,588.91	794.96	1,587.92	794.46	15
9	1,184.61	592.81	1,166.60	583.80	H	1,506.86	753.94	1,489.84	745.42	1,488.85	744.93	14
10	1,297.69	649.35	1,279.68	640.34	L	1,369.81	685.41	1,352.78	676.89	1,351.79	676.40	13
11	1,410.78	705.89	1,392.76	696.89	L	1,256.72	628.86	1,239.69	620.35	1,238.71	619.86	12
12	1,523.86	762.43	1,505.85	753.43	L	1,143.64	572.32	1,126.61	563.81	1,125.63	563.32	11
13	1,638.89	819.95	1,620.88	810.94	D	1,030.55	515.78	1,013.53	507.27	1,012.54	506.77	10
14	1,767.93	884.47	1,749.92	875.46	E	915.53	458.27	898.50	449.75	897.52	449.26	9
15	1,867.00	934.00	1,848.99	925.00	V	786.48	393.75	769.46	385.23	768.47	384.74	8
16	1,966.07	983.54	1,948.05	974.53	V	687.41	344.21	670.39	335.70	669.40	335.21	7

17	2,037.10	1,019.05	2,019.09	1,010.05	A	588.35	294.68	571.32	286.16	570.34	285.67	6
18	2,124.13	1,062.57	2,106.12	1,053.57	S	517.31	259.16	500.28	250.64	499.30	250.15	5
19	2,195.17	1,098.09	2,177.16	1,089.08	A	430.28	215.64	413.25	207.13			4
20	2,266.21	1,133.61	2,248.20	1,124.60	A	359.24	180.12	342.21	171.61			3
21	2,379.29	1,190.15	2,361.28	1,181.14	L	288.20	144.61	271.18	136.09			2
22					R	175.12	88.06	158.09	79.55			1

Query 63653 Hit 1

MS/MS Fragmentation of GAHLTALEMLTAFASHIR
Found in sp|Q96EL3|RM53_HUMAN, 39S ribosomal protein L53
Match to Query 63653: 2082.113 from(521.5355,4+)
Title: 1116: Sum of 2 scans in range 2499 (rt=65.764, f=4, i=766) to 2500 (rt=65.7894, f=4, i=767) [D:\lab212\membrane\Grace\Joyce\47_1.raw]
Data File:Submitted from 20120508-1(merge) by Mascot Daemon on JOYCE-VAIO
Monoisotopic mass of neutral peptide Mr(calc): 2082.113
Variable modifications:
Ions Score: 81.07 Expect: 0.000

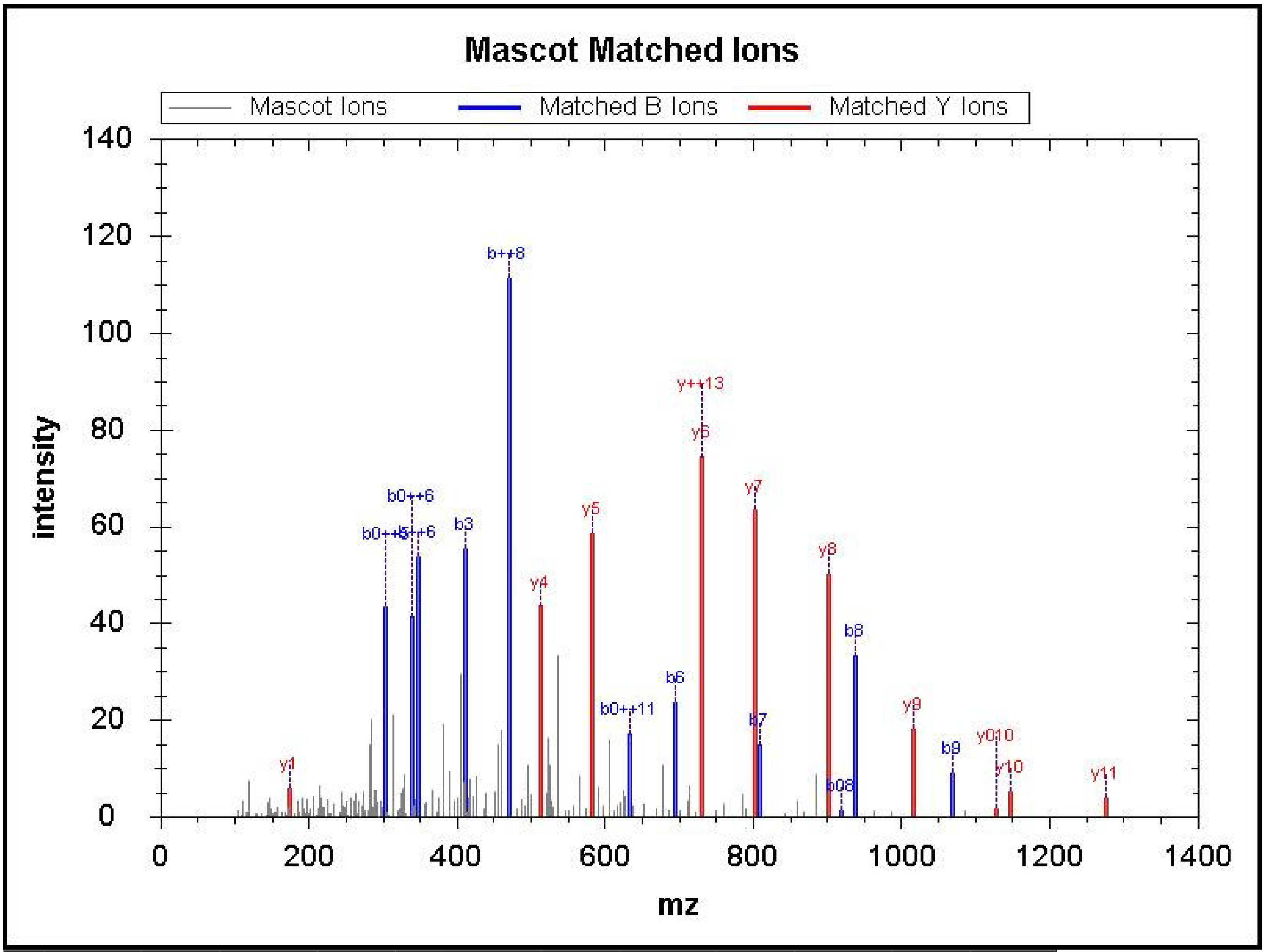

No	b	b++	b0	b0++	Seq	y	y++	y*	y*++	y0	y0++	RevNo
1	202.13	101.57			G							18
2	273.17	137.09			A	1,882.00	941.50	1,864.97	932.99	1,863.99	932.50	17
3	410.23	205.62			H	1,810.96	905.99	1,793.94	897.47	1,792.95	896.98	16
4	523.31	262.16			L	1,673.90	837.46	1,656.88	828.94	1,655.89	828.45	15
5	624.36	312.68	606.35	303.68	T	1,560.82	780.91	1,543.79	772.40	1,542.81	771.91	14
6	695.40	348.20	677.39	339.20	A	1,459.77	730.39	1,442.75	721.88	1,441.76	721.38	13
7	808.48	404.74	790.47	395.74	L	1,388.74	694.87	1,371.71	686.36	1,370.72	685.87	12
8	937.52	469.26	919.51	460.26	E	1,275.65	638.33	1,258.62	629.82	1,257.64	629.32	11

9	1,068.56	534.79	1,050.55	525.78	M	1,146.61	573.81	1,129.58	565.29	1,128.60	564.80	10
10	1,181.65	591.33	1,163.64	582.32	L	1,015.57	508.29	998.54	499.77	997.56	499.28	9
11	1,282.69	641.85	1,264.68	632.85	T	902.48	451.75	885.46	443.23	884.47	442.74	8
12	1,353.73	677.37	1,335.72	668.36	A	801.44	401.22	784.41	392.71	783.43	392.22	7
13	1,500.80	750.90	1,482.79	741.90	F	730.40	365.70	713.37	357.19	712.39	356.70	6
14	1,571.84	786.42	1,553.83	777.42	A	583.33	292.17	566.30	283.66	565.32	283.16	5
15	1,658.87	829.94	1,640.86	820.93	S	512.29	256.65	495.27	248.14	494.28	247.65	4
16	1,795.93	898.47	1,777.92	889.46	H	425.26	213.13	408.24	204.62			3
17	1,909.01	955.01	1,891.00	946.00	I	288.20	144.61	271.18	136.09			2
18					R	175.12	88.06	158.09	79.55			1

Query 72108 Hit 1

MS/MS Fragmentation of VLYFGDHLYSDLADLMLR
Found in sp|Q9H857|NT5D2_HUMAN, 5'-nucleotidase domain-containing protein 2 OS=Homo sapiens GN=NT5DC2 PE=1 SV=1
Match to Query 72108: 2284.168from(762.3968,3+)
Title: 1206: Sum of 2 scans in range 2722 (rt=70.3926, f=2, i=453) to 2723 (rt=70.418, f=2, i=454)
[D:\lab212\membrane\Grace\Joyce\iTRAQ_32_1.raw]
Data File:Submitted from 20120508-1(merge) by Mascot Daemon on JOYCE-VAIO
Monoisotopic mass of neutral peptide Mr(calc): 2284.168
Variable modifications:
Ions Score: 79.58 Expect: 0.000

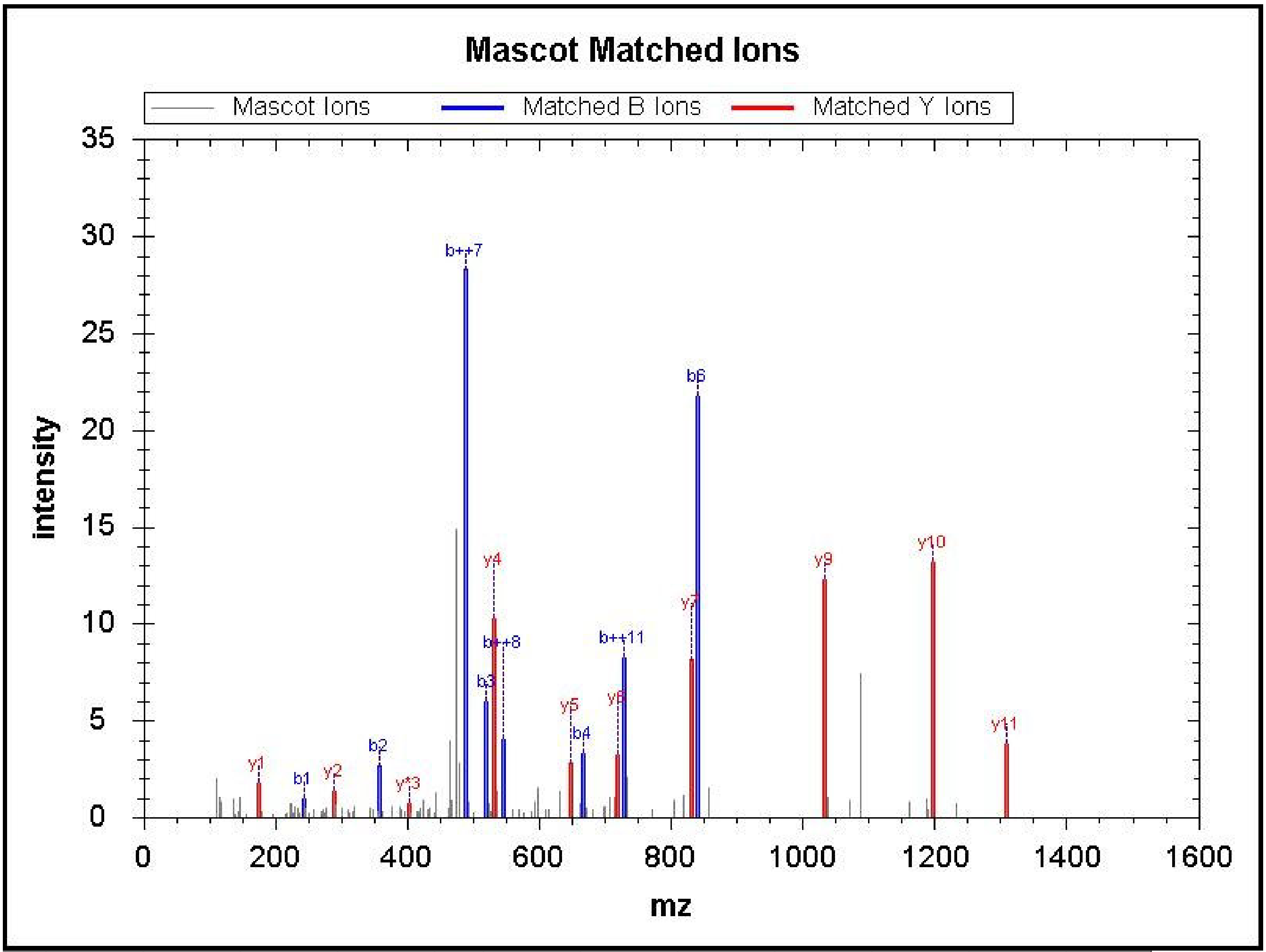

No	b	b++	b0	b0++	Seq	y	y++	y*	y*++	y0	y0++	RevNo
1	244.18	122.59			V							18
2	357.26	179.13			L	2,042.01	1,021.51	2,024.98	1,012.99	2,023.99	1,012.50	17
3	520.33	260.67			Y	1,928.92	964.96	1,911.89	956.45	1,910.91	955.96	16

4	667.39	334.20			F	1,765.86	883.43	1,748.83	874.92	1,747.85	874.43	15
5	724.42	362.71			G	1,618.79	809.90	1,601.76	801.39	1,600.78	800.89	14
6	839.44	420.22	821.43	411.22	D	1,561.77	781.39	1,544.74	772.87	1,543.76	772.38	13
7	976.50	488.75	958.49	479.75	H	1,446.74	723.87	1,429.71	715.36	1,428.73	714.87	12
8	1,089.58	545.30	1,071.57	536.29	L	1,309.68	655.34	1,292.66	646.83	1,291.67	646.34	11
9	1,252.65	626.83	1,234.64	617.82	Y	1,196.60	598.80	1,179.57	590.29	1,178.59	589.80	10
10	1,339.68	670.34	1,321.67	661.34	S	1,033.53	517.27	1,016.51	508.76	1,015.52	508.27	9
11	1,454.71	727.86	1,436.70	718.85	D	946.50	473.75	929.48	465.24	928.49	464.75	8
12	1,567.79	784.40	1,549.78	775.39	L	831.48	416.24	814.45	407.73	813.47	407.24	7
13	1,638.83	819.92	1,620.82	810.91	A	718.39	359.70	701.36	351.19	700.38	350.69	6
14	1,753.86	877.43	1,735.84	868.43	D	647.35	324.18	630.33	315.67	629.34	315.18	5
15	1,866.94	933.97	1,848.93	924.97	L	532.33	266.67	515.30	258.15			4
16	1,997.98	999.49	1,979.97	990.49	M	419.24	210.13	402.22	201.61			3
17	2,111.06	1,056.04	2,093.05	1,047.03	L	288.20	144.61	271.18	136.09			2
18					R	175.12	88.06	158.09	79.55			1

Query 63485 Hit 1

MS/MS Fragmentation of EVLEYLGNPANYPVSIR
Found in sp|Q9Y296|TPPC4_HUMAN, Trafficking protein particle complex subunit 4 OS=Homo sapiens GN=TRAPPC4 PE=1 SV=1
Match to Query 63485: 2077.102from(693.3746,3+)
Title: 593: Sum of 2 scans in range 2198 (rt=55.7559, f=4, i=428) to 2199 (rt=55.7813, f=4, i=429)
[D:\lab212\membrane\Grace\Joyce\iTRAQ_19_2_330.raw]
Data File:Submitted from 20120508-1(merge) by Mascot Daemon on JOYCE-VAIO
Monoisotopic mass of neutral peptide Mr(calc): 2077.102
Variable modifications:
Ions Score: 79.46 Expect: 0.000

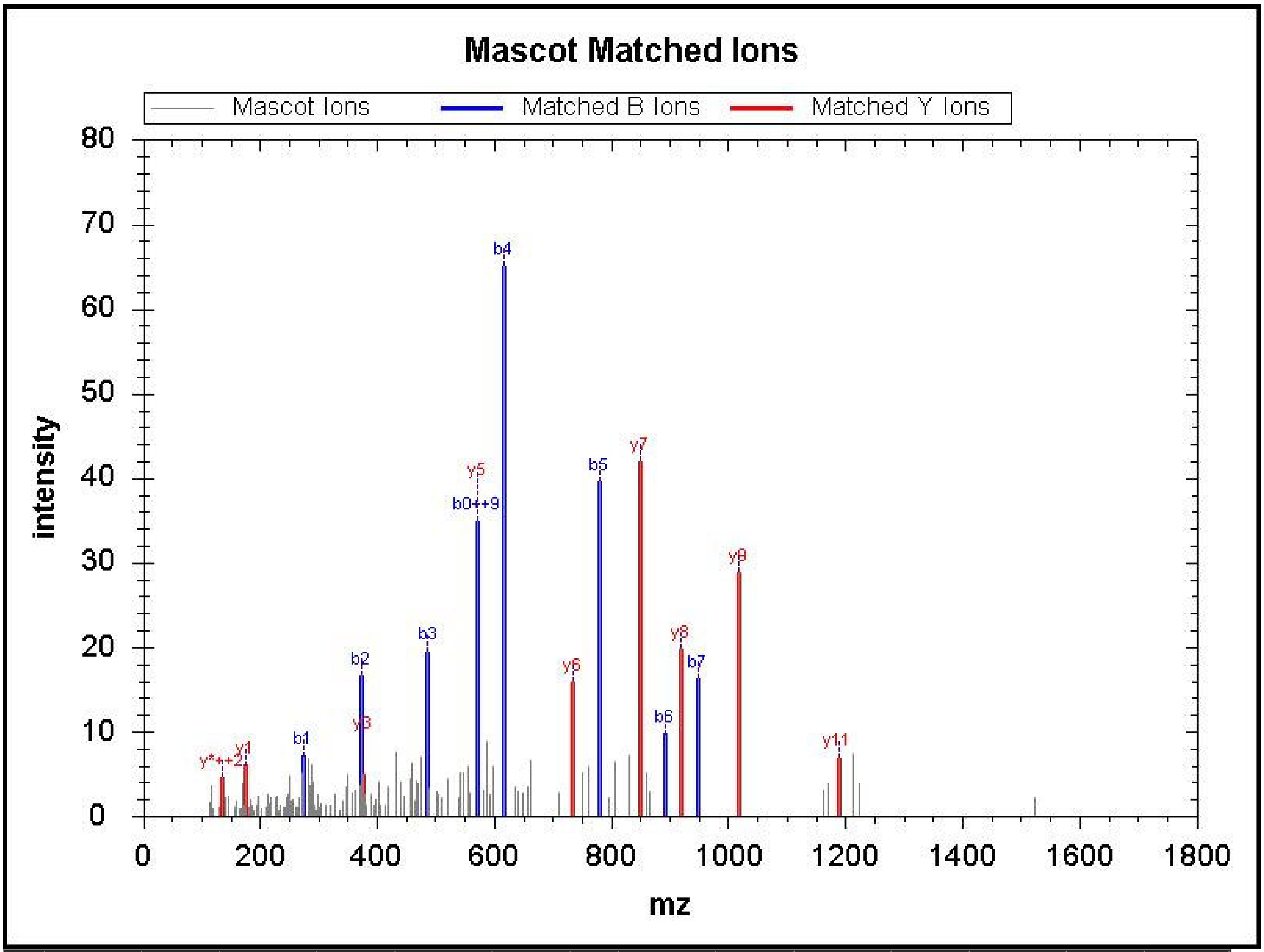

No	b	b++	b*	b*++	b0	b0++	Seq	y	y++	y*	y*++	y0	y0++	RevNo
1	274.15	137.58			256.14	128.57	E							17
2	373.22	187.11			355.21	178.11	V	1,804.96	902.98	1,787.93	894.47	1,786.95	893.98	16
3	486.30	243.66			468.29	234.65	L	1,705.89	853.45	1,688.86	844.94	1,687.88	844.44	15
4	615.35	308.18			597.34	299.17	E	1,592.81	796.91	1,575.78	788.39	1,574.80	787.90	14
5	778.41	389.71			760.40	380.70	Y	1,463.76	732.39	1,446.74	723.87	1,445.75	723.38	13
6	891.49	446.25			873.48	437.25	L	1,300.70	650.85	1,283.67	642.34	1,282.69	641.85	12
7	948.52	474.76			930.51	465.76	G	1,187.62	594.31	1,170.59	585.80	1,169.61	585.31	11
8	1,062.56	531.78	1,045.53	523.27	1,044.55	522.78	N	1,130.60	565.80	1,113.57	557.29	1,112.58	556.80	10
9	1,159.61	580.31	1,142.59	571.80	1,141.60	571.30	P	1,016.55	508.78	999.53	500.27	998.54	499.77	9
10	1,230.65	615.83	1,213.62	607.31	1,212.64	606.82	A	919.50	460.25	902.47	451.74	901.49	451.25	8
11	1,344.69	672.85	1,327.67	664.34	1,326.68	663.84	N	848.46	424.73	831.44	416.22	830.45	415.73	7
12	1,507.76	754.38	1,490.73	745.87	1,489.74	745.38	Y	734.42	367.71	717.39	359.20	716.41	358.71	6
13	1,604.81	802.91	1,587.78	794.39	1,586.80	793.90	P	571.36	286.18	554.33	277.67	553.35	277.18	5
14	1,703.88	852.44	1,686.85	843.93	1,685.87	843.44	V	474.30	237.66	457.28	229.14	456.29	228.65	4
15	1,790.91	895.96	1,773.88	887.44	1,772.90	886.95	S	375.24	188.12	358.21	179.61	357.22	179.12	3
16	1,903.99	952.50	1,886.97	943.99	1,885.98	943.49	I	288.20	144.61	271.18	136.09			2
17							R	175.12	88.06	158.09	79.55			1

Query 59379 Hit 1

MS/MS Fragmentation of AALEAIDELDLFGAHGGR
Found in sp|P46019|KPB2_HUMAN, Phosphorylase b kinase regulatory subunit alpha
Match to Query 59379: 1998.028from(667.0167,3+)
Title: 928: Sum of 2 scans in range 2039 (rt=55.4638, f=4, i=621) to 2040 (rt=55.4893, f=4, i=622) [D:\lab212\membrane\Grace\Joyce\iTRAQ_34_2.raw]
Data File:Submitted from 20120508-1(merge) by Mascot Daemon on JOYCE-VAIO

Monoisotopic mass of neutral peptide Mr(calc): 1998.028
Variable modifications:
Ions Score: 78.95 Expect: 0.000

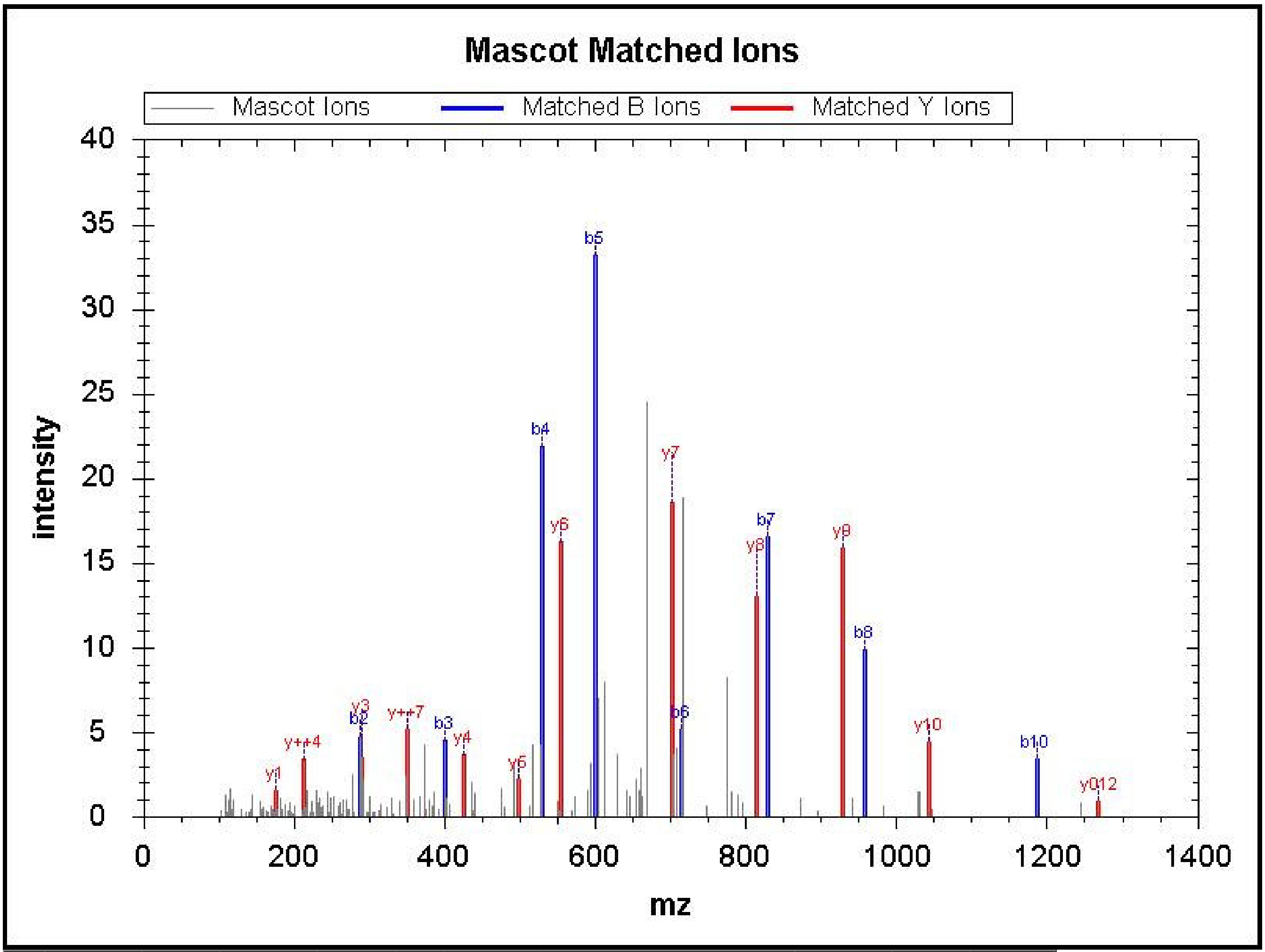

No	b	b++	b0	b0++	Seq	y	y++	y*	y*++	y0	y0++	RevNo
1	216.15	108.58			A							18
2	287.18	144.10			A	1,783.90	892.45	1,766.87	883.94	1,765.89	883.45	17
3	400.27	200.64			L	1,712.86	856.93	1,695.83	848.42	1,694.85	847.93	16
4	529.31	265.16	511.30	256.15	E	1,599.78	800.39	1,582.75	791.88	1,581.77	791.39	15
5	600.35	300.68	582.34	291.67	A	1,470.73	735.87	1,453.71	727.36	1,452.72	726.87	14
6	713.43	357.22	695.42	348.21	I	1,399.70	700.35	1,382.67	691.84	1,381.69	691.35	13
7	828.46	414.73	810.45	405.73	D	1,286.61	643.81	1,269.59	635.30	1,268.60	634.80	12
8	957.50	479.25	939.49	470.25	E	1,171.59	586.30	1,154.56	577.78	1,153.57	577.29	11
9	1,070.59	535.80	1,052.57	526.79	L	1,042.54	521.78	1,025.52	513.26	1,024.53	512.77	10
10	1,185.61	593.31	1,167.60	584.30	D	929.46	465.23	912.43	456.72	911.45	456.23	9
11	1,298.70	649.85	1,280.69	640.85	L	814.43	407.72	797.41	399.21			8
12	1,445.76	723.39	1,427.75	714.38	F	701.35	351.18	684.32	342.66			7
13	1,502.79	751.90	1,484.78	742.89	G	554.28	277.64	537.25	269.13			6
14	1,573.82	787.42	1,555.81	778.41	A	497.26	249.13	480.23	240.62			5
15	1,710.88	855.94	1,692.87	846.94	H	426.22	213.61	409.19	205.10			4
16	1,767.90	884.46	1,749.89	875.45	G	289.16	145.08	272.14	136.57			3
17	1,824.93	912.97	1,806.91	903.96	G	232.14	116.57	215.11	108.06			2
18					R	175.12	88.06	158.09	79.55			1

Query 69319 Hit 1

MS/MS Fragmentation of NTFWDVDGSMVPPEWHR
Found in sp|Q9UI09|NDUAC_HUMAN, NADH dehydrogenase [ubiquinone] 1 alpha subcomplex subunit 12 OS=Homo sapiens GN=NDUFA12 PE=1 SV=1
Match to Query 69319: 2216.029from(739.6837,3+)
Title: 792: Scan 1779 (rt=49.4788, f=2, i=279) [D:\lab212\membrane\Grace\Joyce\iTRAQ_35_2.raw]
Data File:Submitted from 20120508-1(merge) by Mascot Daemon on JOYCE-VAIO
Monoisotopic mass of neutral peptide Mr(calc): 2216.029
Variable modifications:
Ions Score: 78.01 Expect: 0.000

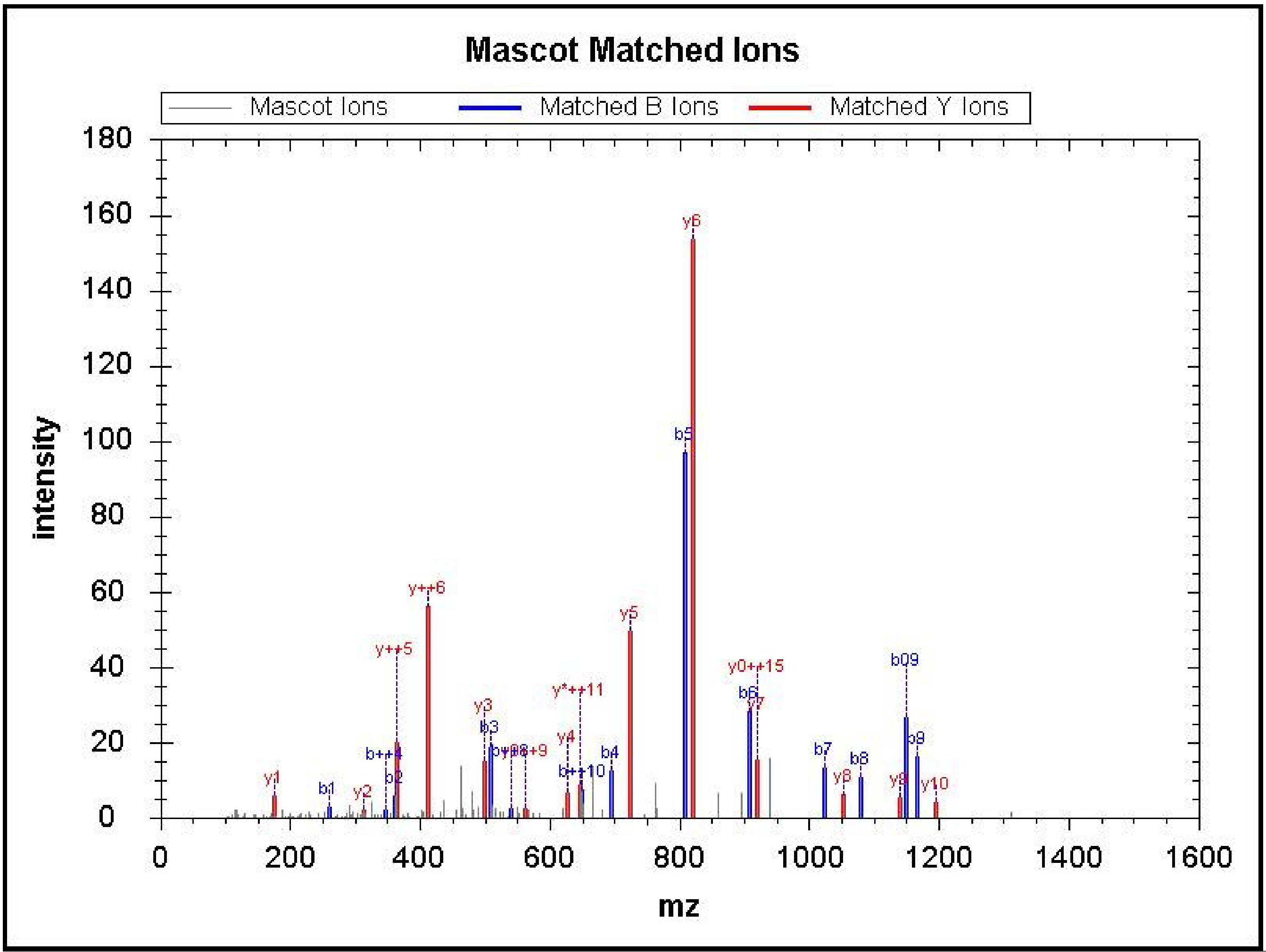

No	b	b++	b*	b*++	b0	b0++	Seq	y	y++	y*	y*++	y0	y0++	RevNo
1	259.15	130.08	242.13	121.57			N							17
2	360.20	180.60	343.17	172.09	342.19	171.60	T	1,958.89	979.95	1,941.86	971.43	1,940.87	970.94	16
3	507.27	254.14	490.24	245.62	489.26	245.13	F	1,857.84	929.42	1,840.81	920.91	1,839.83	920.42	15
4	693.35	347.18	676.32	338.66	675.34	338.17	W	1,710.77	855.89	1,693.74	847.38	1,692.76	846.88	14
5	808.37	404.69	791.35	396.18	790.36	395.69	D	1,524.69	762.85	1,507.66	754.34	1,506.68	753.84	13
6	907.44	454.23	890.42	445.71	889.43	445.22	V	1,409.66	705.34	1,392.64	696.82	1,391.65	696.33	12
7	1,022.47	511.74	1,005.44	503.23	1,004.46	502.73	D	1,310.59	655.80	1,293.57	647.29	1,292.58	646.80	11
8	1,079.49	540.25	1,062.46	531.74	1,061.48	531.24	G	1,195.57	598.29	1,178.54	589.77	1,177.56	589.28	10
9	1,166.52	583.77	1,149.50	575.25	1,148.51	574.76	S	1,138.55	569.78	1,121.52	561.26	1,120.54	560.77	9
10	1,297.56	649.29	1,280.54	640.77	1,279.55	640.28	M	1,051.51	526.26	1,034.49	517.75	1,033.50	517.26	8
11	1,396.63	698.82	1,379.61	690.31	1,378.62	689.81	V	920.47	460.74	903.45	452.23	902.46	451.74	7
12	1,493.69	747.35	1,476.66	738.83	1,475.67	738.34	P	821.41	411.21	804.38	402.69	803.39	402.20	6
13	1,590.74	795.87	1,573.71	787.36	1,572.73	786.87	P	724.35	362.68	707.33	354.17	706.34	353.67	5
14	1,719.78	860.39	1,702.75	851.88	1,701.77	851.39	E	627.30	314.15	610.27	305.64	609.29	305.15	4
15	1,905.86	953.43	1,888.83	944.92	1,887.85	944.43	W	498.26	249.63	481.23	241.12			3
16	2,042.92	1,021.96	2,025.89	1,013.45	2,024.91	1,012.96	H	312.18	156.59	295.15	148.08			2

17							R	175.12	88.06	158.09	79.55			1

Query 76984 Hit 1

MS/MS Fragmentation of HGLTTGATLISDQWLLTTAK
Found in sp|Q61646|HPT_MOUSE, Haptoglobin OS=Mus musculus GN=Hp PE=1 SV=1
Match to Query 76984: 2414.338from(805.7866,3+)
Title: 846: Sum of 2 scans in range 1870 (rt=51.7318, f=4, i=563) to 1871 (rt=51.7572, f=4, i=564)
[D:\lab212\membrane\Grace\Joyce\iTRAQ_39_2.raw]
Data File:Submitted from 20120508-1(merge) by Mascot Daemon on JOYCE-VAIO
Monoisotopic mass of neutral peptide Mr(calc): 2414.338
Variable modifications:
K20 :iTRAQ4plex (K)
Ions Score: 77.91 Expect: 0.000

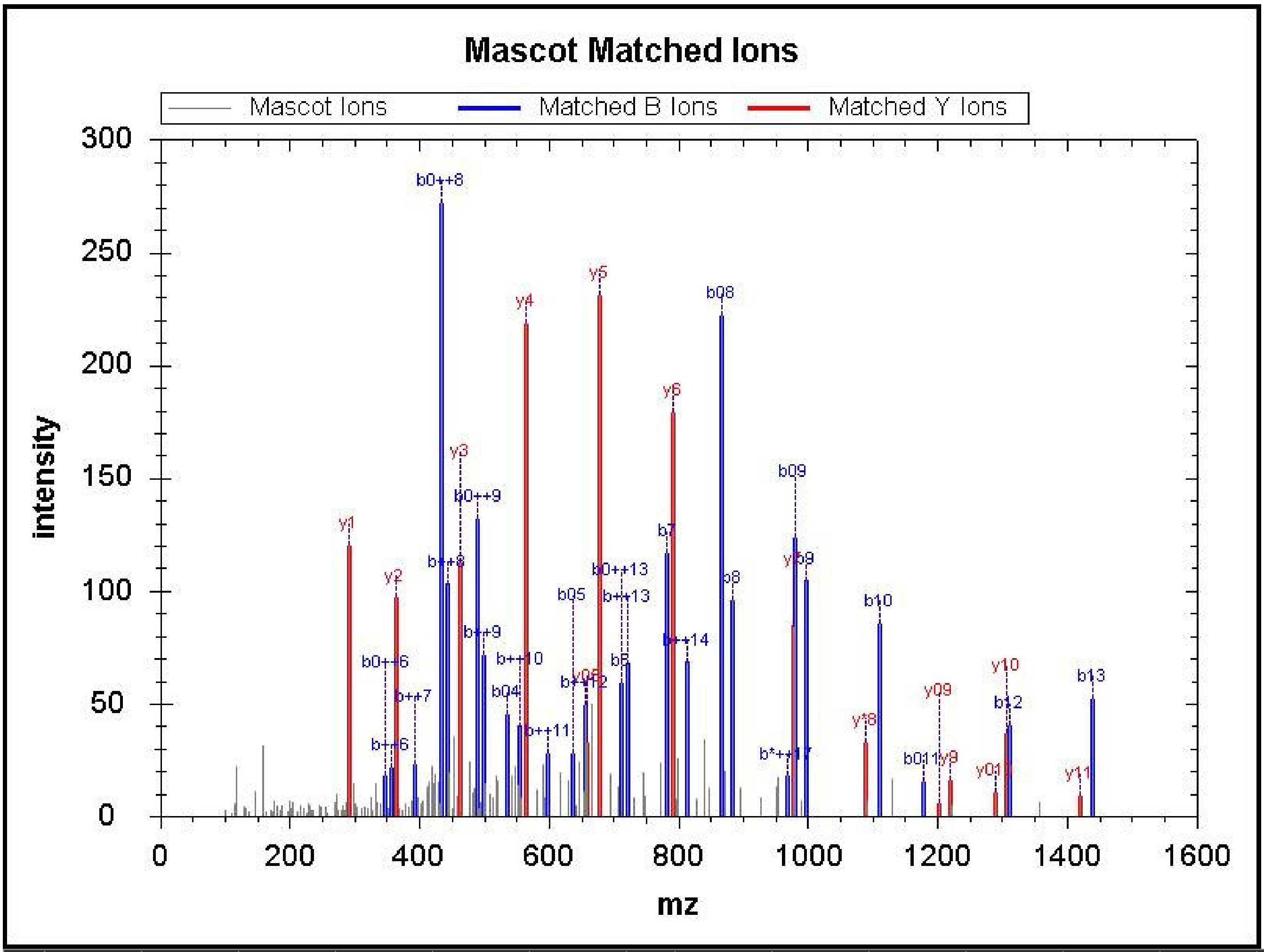

No	b	b++	b*	b*++	b0	b0++	Seq	y	y++	y*	y*++	y0	y0++	RevNo
1	282.17	141.59					H							20
2	339.19	170.10					G	2,134.19	1,067.60	2,117.16	1,059.08	2,116.18	1,058.59	19
3	452.27	226.64					L	2,077.17	1,039.09	2,060.14	1,030.57	2,059.16	1,030.08	18
4	553.32	277.16			535.31	268.16	T	1,964.08	982.54	1,947.06	974.03	1,946.07	973.54	17
5	654.37	327.69			636.36	318.68	T	1,863.03	932.02	1,846.01	923.51	1,845.02	923.02	16
6	711.39	356.20			693.38	347.19	G	1,761.99	881.50	1,744.96	872.98	1,743.98	872.49	15
7	782.43	391.72			764.42	382.71	A	1,704.97	852.99	1,687.94	844.47	1,686.95	843.98	14
8	883.48	442.24			865.46	433.24	T	1,633.93	817.47	1,616.90	808.95	1,615.92	808.46	13
9	996.56	498.78			978.55	489.78	L	1,532.88	766.94	1,515.85	758.43	1,514.87	757.94	12
10	1,109.64	555.33			1,091.63	546.32	I	1,419.80	710.40	1,402.77	701.89	1,401.79	701.40	11
11	1,196.68	598.84			1,178.66	589.84	S	1,306.71	653.86	1,289.69	645.35	1,288.70	644.85	10
12	1,311.70	656.35			1,293.69	647.35	D	1,219.68	610.34	1,202.65	601.83	1,201.67	601.34	9

13	1,439.76	720.38	1,422.73	711.87	1,421.75	711.38	Q	1,104.65	552.83	1,087.63	544.32	1,086.64	543.83	8
14	1,625.84	813.42	1,608.81	804.91	1,607.83	804.42	W	976.59	488.80	959.57	480.29	958.58	479.80	7
15	1,738.92	869.97	1,721.90	861.45	1,720.91	860.96	L	790.52	395.76	773.49	387.25	772.50	386.76	6
16	1,852.01	926.51	1,834.98	917.99	1,834.00	917.50	L	677.43	339.22	660.40	330.71	659.42	330.21	5
17	1,953.06	977.03	1,936.03	968.52	1,935.05	968.03	T	564.35	282.68	547.32	274.16	546.34	273.67	4
18	2,054.10	1,027.56	2,037.08	1,019.04	2,036.09	1,018.55	T	463.30	232.15	446.27	223.64	445.29	223.15	3
19	2,125.14	1,063.07	2,108.11	1,054.56	2,107.13	1,054.07	A	362.25	181.63	345.23	173.12			2
20							K	291.21	146.11	274.19	137.60			1

Query 89707 Hit 1

MS/MS Fragmentation of LHGGTPANFLDVGGGATVHQVTEAFK
Found in sp|Q9P2R7|SUCB1_HUMAN, Succinyl-CoA ligase [ADP-forming] subunit beta
Match to Query 89707: 2910.527from(728.639,4+)
Title: 529: Scan 1782 (rt=47.6093, f=2, i=321) [D:\lab212\membrane\Grace\Joyce\iTRAQ_46_1.raw]
Data File:Submitted from 20120508-1(merge) by Mascot Daemon on JOYCE-VAIO
Monoisotopic mass of neutral peptide Mr(calc): 2910.527
Variable modifications:
K26 :iTRAQ4plex (K)
Ions Score: 77.71 Expect: 0.000

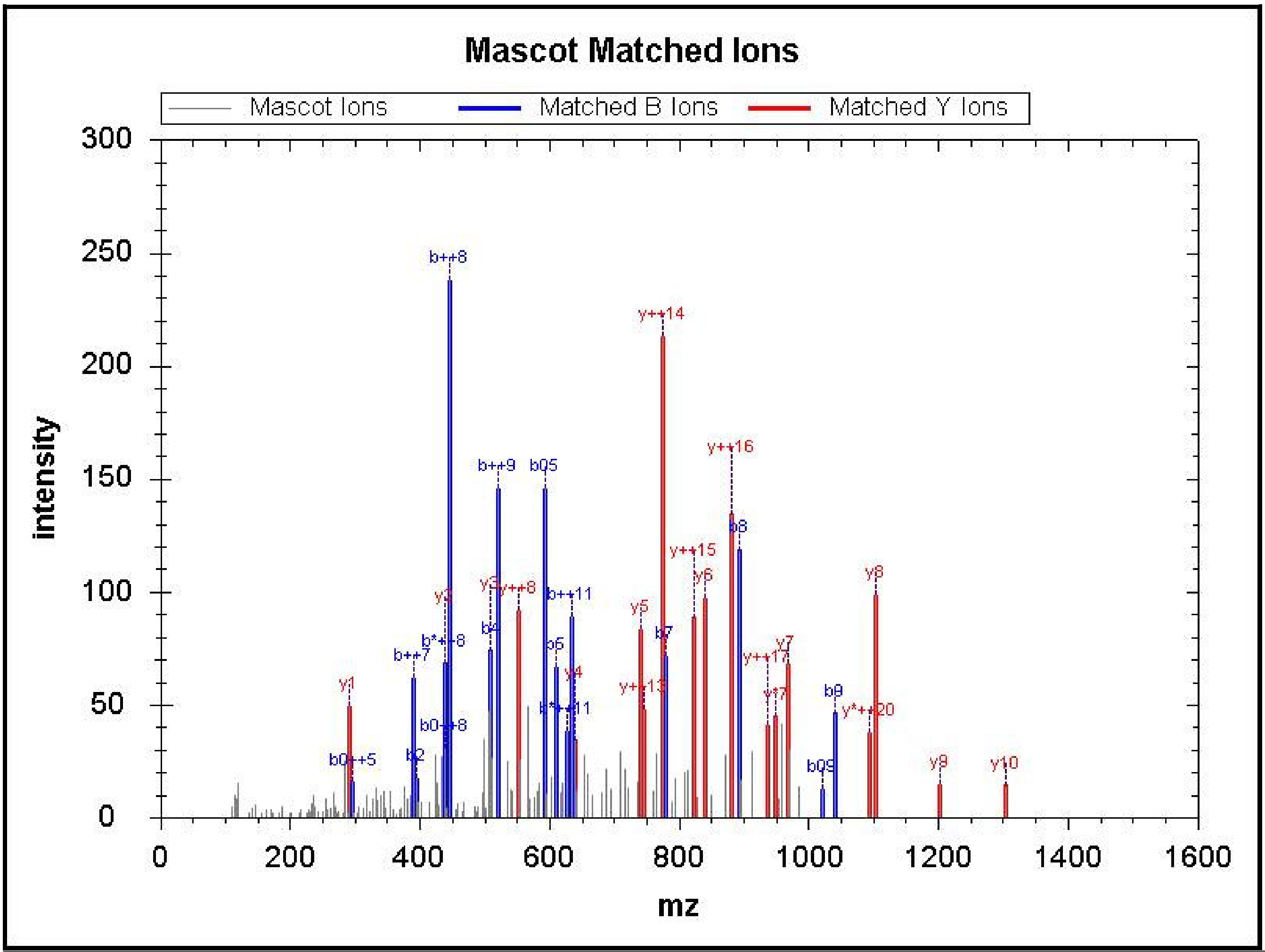

No	b	b++	b*	b*++	b0	b0++	Seq	y	y++	y*	y*++	y0	y0++	RevNo
1	258.19	129.60					L							26
2	395.25	198.13					H	2,654.34	1,327.68	2,637.32	1,319.16	2,636.33	1,318.67	25
3	452.27	226.64					G	2,517.29	1,259.15	2,500.26	1,250.63	2,499.27	1,250.14	24
4	509.30	255.15					G	2,460.26	1,230.64	2,443.24	1,222.12	2,442.25	1,221.63	23
5	610.34	305.68			592.33	296.67	T	2,403.24	1,202.12	2,386.22	1,193.61	2,385.23	1,193.12	22
6	707.40	354.20			689.39	345.20	P	2,302.19	1,151.60	2,285.17	1,143.09	2,284.18	1,142.60	21

7	778.43	389.72			760.42	380.71	A	2,205.14	1,103.07	2,188.12	1,094.56	2,187.13	1,094.07	20
8	892.48	446.74	875.45	438.23	874.47	437.74	N	2,134.10	1,067.56	2,117.08	1,059.04	2,116.09	1,058.55	19
9	1,039.54	520.28	1,022.52	511.76	1,021.53	511.27	F	2,020.06	1,010.53	2,003.04	1,002.02	2,002.05	1,001.53	18
10	1,152.63	576.82	1,135.60	568.30	1,134.62	567.81	L	1,872.99	937.00	1,855.97	928.49	1,854.98	928.00	17
11	1,267.66	634.33	1,250.63	625.82	1,249.64	625.33	D	1,759.91	880.46	1,742.88	871.95	1,741.90	871.45	16
12	1,366.72	683.87	1,349.70	675.35	1,348.71	674.86	V	1,644.88	822.94	1,627.86	814.43	1,626.87	813.94	15
13	1,423.75	712.38	1,406.72	703.86	1,405.73	703.37	G	1,545.81	773.41	1,528.79	764.90	1,527.80	764.41	14
14	1,480.77	740.89	1,463.74	732.37	1,462.76	731.88	G	1,488.79	744.90	1,471.77	736.39	1,470.78	735.89	13
15	1,537.79	769.40	1,520.76	760.88	1,519.78	760.39	G	1,431.77	716.39	1,414.74	707.88	1,413.76	707.38	12
16	1,608.83	804.92	1,591.80	796.40	1,590.81	795.91	A	1,374.75	687.88	1,357.72	679.37	1,356.74	678.87	11
17	1,709.87	855.44	1,692.85	846.93	1,691.86	846.43	T	1,303.71	652.36	1,286.69	643.85	1,285.70	643.35	10
18	1,808.94	904.97	1,791.91	896.46	1,790.93	895.97	V	1,202.66	601.84	1,185.64	593.32	1,184.65	592.83	9
19	1,946.00	973.50	1,928.97	964.99	1,927.99	964.50	H	1,103.60	552.30	1,086.57	543.79	1,085.59	543.30	8
20	2,074.06	1,037.53	2,057.03	1,029.02	2,056.05	1,028.53	Q	966.54	483.77	949.51	475.26	948.53	474.77	7
21	2,173.13	1,087.07	2,156.10	1,078.55	2,155.12	1,078.06	V	838.48	419.74	821.45	411.23	820.47	410.74	6
22	2,274.17	1,137.59	2,257.15	1,129.08	2,256.16	1,128.59	T	739.41	370.21	722.38	361.70	721.40	361.20	5
23	2,403.22	1,202.11	2,386.19	1,193.60	2,385.21	1,193.11	E	638.36	319.69	621.34	311.17	620.35	310.68	4
24	2,474.25	1,237.63	2,457.23	1,229.12	2,456.24	1,228.63	A	509.32	255.16	492.29	246.65			3
25	2,621.32	1,311.17	2,604.30	1,302.65	2,603.31	1,302.16	F	438.28	219.65	421.26	211.13			2
26							K	291.21	146.11	274.19	137.60			1

Query 85540 Hit 1

MS/MS Fragmentation of DGNLPDIVNSGSLHEFLVNLHER
Found in sp|Q6UW02|CP20A_HUMAN, Cytochrome P450 20A1 OS=Homo sapiens GN=CYP20A1 PE=1 SV=1
Match to Query 85540: 2718.386from(680.6038,4+)
Title: 935: Sum of 2 scans in range 2061 (rt=56.0249, f=4, i=628) to 2062 (rt=56.0504, f=4, i=629) [D:\lab212\membrane\Grace\Joyce\iTRAQ_44_1.raw]
Data File:Submitted from 20120508-1(merge) by Mascot Daemon on JOYCE-VAIO
Monoisotopic mass of neutral peptide Mr(calc): 2718.386
Variable modifications:
Ions Score: 77.63 Expect: 0.000

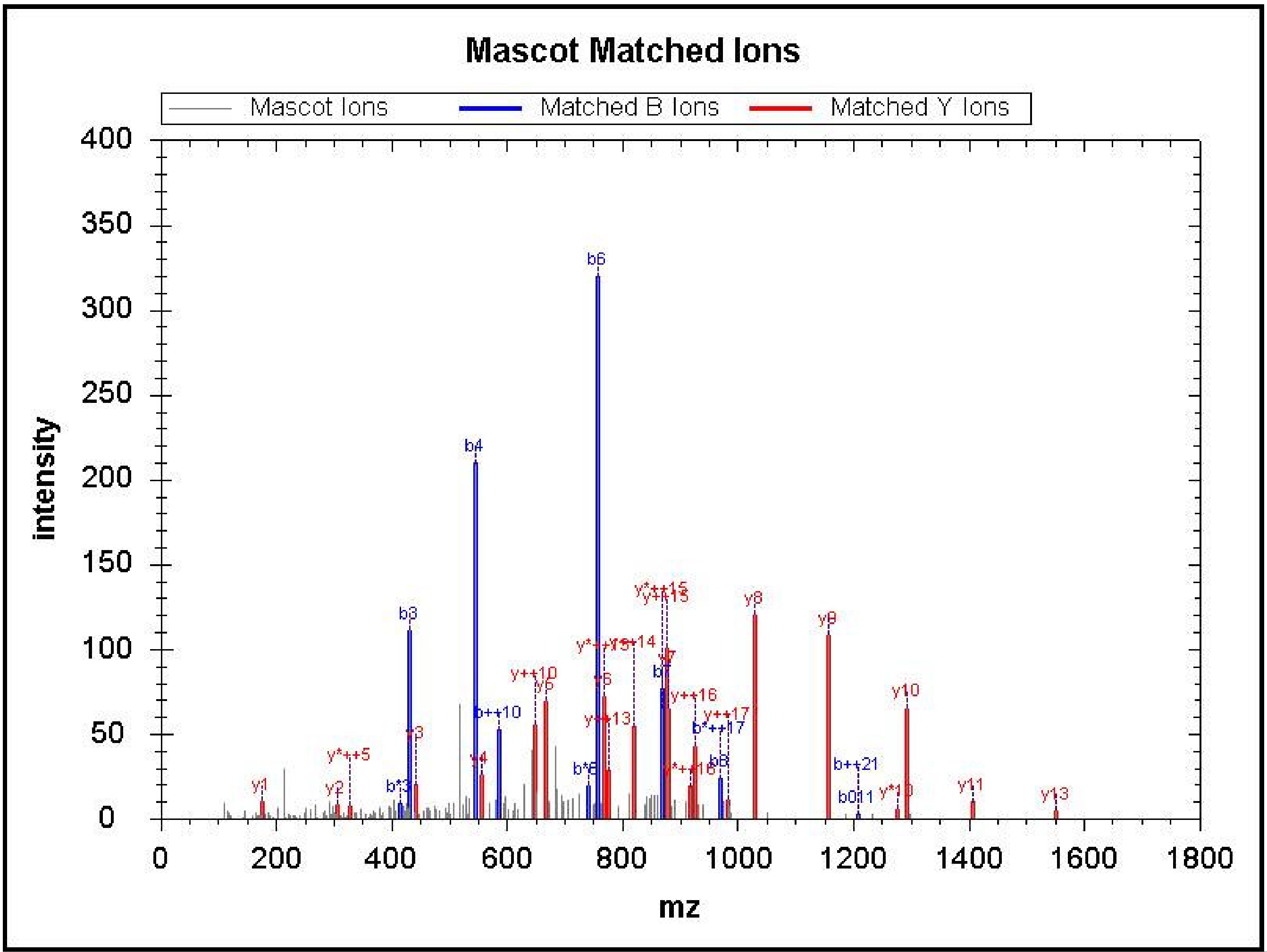

No	b	b++	b*	b*++	b0	b0++	Seq	y	y++	y*	y*++	y0	y0++	RevNo
1	260.14	130.57			242.13	121.57	D							23
2	317.16	159.08			299.15	150.08	G	2,460.26	1,230.64	2,443.24	1,222.12	2,442.25	1,221.63	22
3	431.20	216.10	414.17	207.59	413.19	207.10	N	2,403.24	1,202.12	2,386.21	1,193.61	2,385.23	1,193.12	21
4	544.28	272.65	527.26	264.13	526.27	263.64	L	2,289.20	1,145.10	2,272.17	1,136.59	2,271.19	1,136.10	20
5	641.34	321.17	624.31	312.66	623.33	312.17	P	2,176.11	1,088.56	2,159.09	1,080.05	2,158.10	1,079.56	19
6	756.36	378.69	739.34	370.17	738.35	369.68	D	2,079.06	1,040.03	2,062.04	1,031.52	2,061.05	1,031.03	18
7	869.45	435.23	852.42	426.71	851.44	426.22	I	1,964.03	982.52	1,947.01	974.01	1,946.02	973.52	17
8	968.52	484.76	951.49	476.25	950.51	475.76	V	1,850.95	925.98	1,833.92	917.47	1,832.94	916.97	16
9	1,082.56	541.78	1,065.53	533.27	1,064.55	532.78	N	1,751.88	876.44	1,734.86	867.93	1,733.87	867.44	15
10	1,169.59	585.30	1,152.57	576.79	1,151.58	576.29	S	1,637.84	819.42	1,620.81	810.91	1,619.83	810.42	14
11	1,226.61	613.81	1,209.59	605.30	1,208.60	604.80	G	1,550.81	775.91	1,533.78	767.39	1,532.80	766.90	13
12	1,313.65	657.33	1,296.62	648.81	1,295.63	648.32	S	1,493.79	747.40	1,476.76	738.88	1,475.78	738.39	12
13	1,426.73	713.87	1,409.70	705.36	1,408.72	704.86	L	1,406.75	703.88	1,389.73	695.37	1,388.74	694.88	11
14	1,563.79	782.40	1,546.76	773.88	1,545.78	773.39	H	1,293.67	647.34	1,276.64	638.83	1,275.66	638.33	10
15	1,692.83	846.92	1,675.80	838.41	1,674.82	837.91	E	1,156.61	578.81	1,139.58	570.30	1,138.60	569.80	9
16	1,839.90	920.45	1,822.87	911.94	1,821.89	911.45	F	1,027.57	514.29	1,010.54	505.77	1,009.56	505.28	8
17	1,952.98	977.00	1,935.96	968.48	1,934.97	967.99	L	880.50	440.75	863.47	432.24	862.49	431.75	7
18	2,052.05	1,026.53	2,035.03	1,018.02	2,034.04	1,017.52	V	767.42	384.21	750.39	375.70	749.41	375.21	6
19	2,166.09	1,083.55	2,149.07	1,075.04	2,148.08	1,074.55	N	668.35	334.68	651.32	326.16	650.34	325.67	5
20	2,279.18	1,140.09	2,262.15	1,131.58	2,261.17	1,131.09	L	554.30	277.66	537.28	269.14	536.29	268.65	4
21	2,416.24	1,208.62	2,399.21	1,200.11	2,398.23	1,199.62	H	441.22	221.11	424.19	212.60	423.21	212.11	3
22	2,545.28	1,273.14	2,528.25	1,264.63	2,527.27	1,264.14	E	304.16	152.58	287.13	144.07	286.15	143.58	2
23							R	175.12	88.06	158.09	79.55			1

Query 37126 Hit 1

MS/MS Fragmentation of TLAYIITELDER
Found in sp|Q9Y5K8|VATD_HUMAN, V-type proton ATPase subunit D OS=Homo sapiens GN=ATP6V1D PE=1 SV=1
Match to Query 37126: 1579.856from(790.9355,2+)
Title: 735: Sum of 2 scans in range 2462 (rt=62.8229, f=3, i=331) to 2463 (rt=62.8483, f=3, i=332) [D:\lab212\membrane\Grace\Joyce\iTRAQ19.raw]
Data File:Submitted from 20120508-1(merge) by Mascot Daemon on JOYCE-VAIO
Monoisotopic mass of neutral peptide Mr(calc): 1579.856
Variable modifications:
Ions Score: 77.57 Expect: 0.000

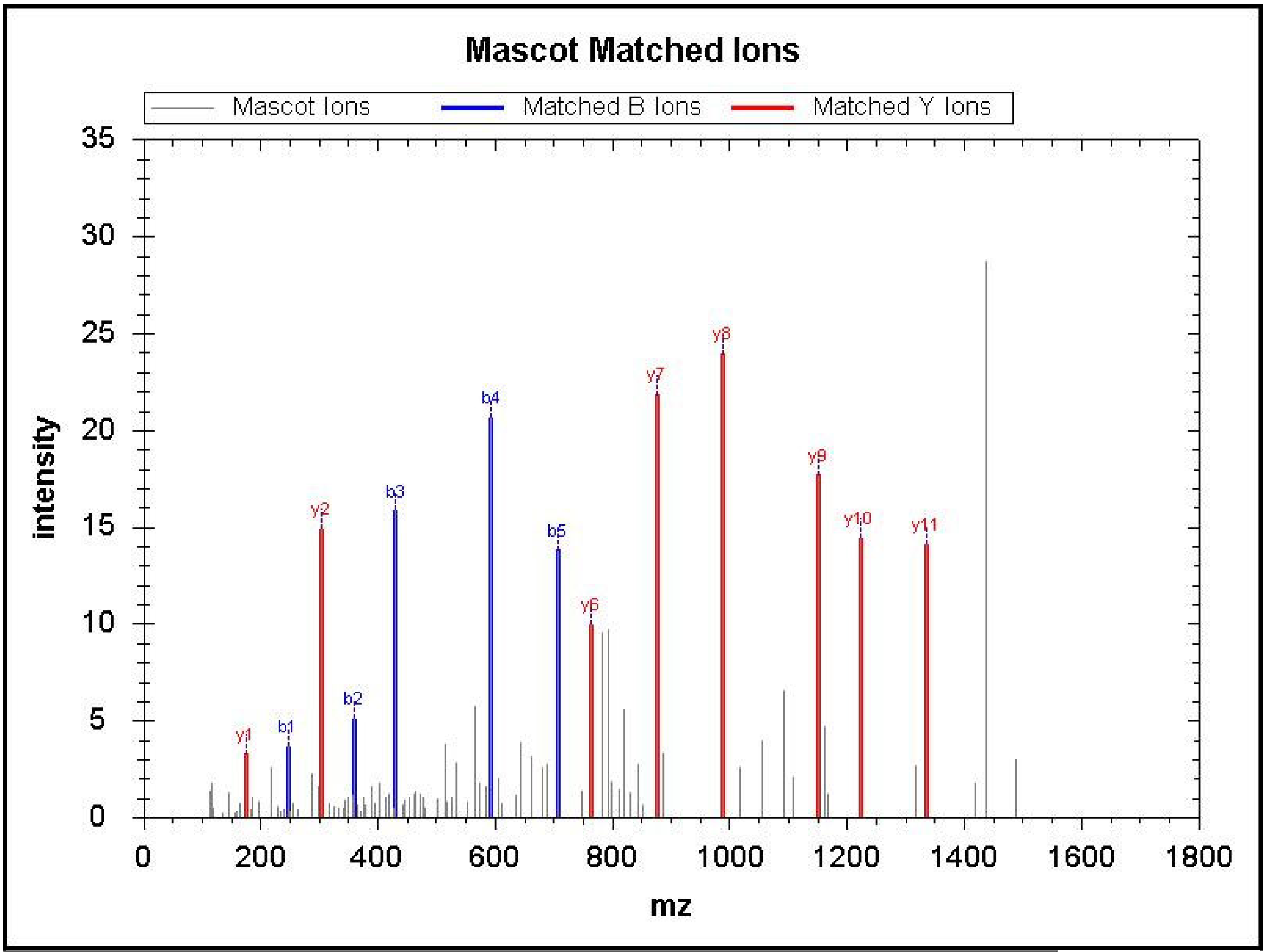

No	b	b++	b0	b0++	Seq	y	y++	y*	y*++	y0	y0++	RevNo
1	246.16	123.58	228.15	114.58	T							12
2	359.24	180.12	341.23	171.12	L	1,335.72	668.36	1,318.69	659.85	1,317.70	659.36	11
3	430.28	215.64	412.27	206.64	A	1,222.63	611.82	1,205.60	603.31	1,204.62	602.81	10
4	593.34	297.17	575.33	288.17	Y	1,151.59	576.30	1,134.57	567.79	1,133.58	567.30	9
5	706.43	353.72	688.41	344.71	I	988.53	494.77	971.50	486.26	970.52	485.76	8
6	819.51	410.26	801.50	401.25	I	875.45	438.23	858.42	429.71	857.44	429.22	7
7	920.56	460.78	902.55	451.78	T	762.36	381.69	745.34	373.17	744.35	372.68	6
8	1,049.60	525.30	1,031.59	516.30	E	661.32	331.16	644.29	322.65	643.30	322.16	5
9	1,162.68	581.85	1,144.67	572.84	L	532.27	266.64	515.25	258.13	514.26	257.63	4
10	1,277.71	639.36	1,259.70	630.35	D	419.19	210.10	402.16	201.58	401.18	201.09	3
11	1,406.75	703.88	1,388.74	694.88	E	304.16	152.58	287.13	144.07	286.15	143.58	2
12					R	175.12	88.06	158.09	79.55			1

Query 88288 Hit 1

MS/MS Fragmentation of EIENMILNDPDFQHEDLNFLTR

Found in sp|Q15067|ACOX1_HUMAN, Peroxisomal acyl-coenzyme A oxidase 1 OS=Homo sapiens GN=ACOX1 PE=1 SV=3
Match to Query 88288: 2846.373from(949.7982,3+)
Title: 927: Scan 2115 (rt=56.8103, f=3, i=314) [D:\lab212\membrane\Grace\Joyce\iTRAQ_30_1.raw]
Data File:Submitted from 20120508-1(merge) by Mascot Daemon on JOYCE-VAIO
Monoisotopic mass of neutral peptide Mr(calc): 2846.373
Variable modifications:
Ions Score: 76.94 Expect: 0.000

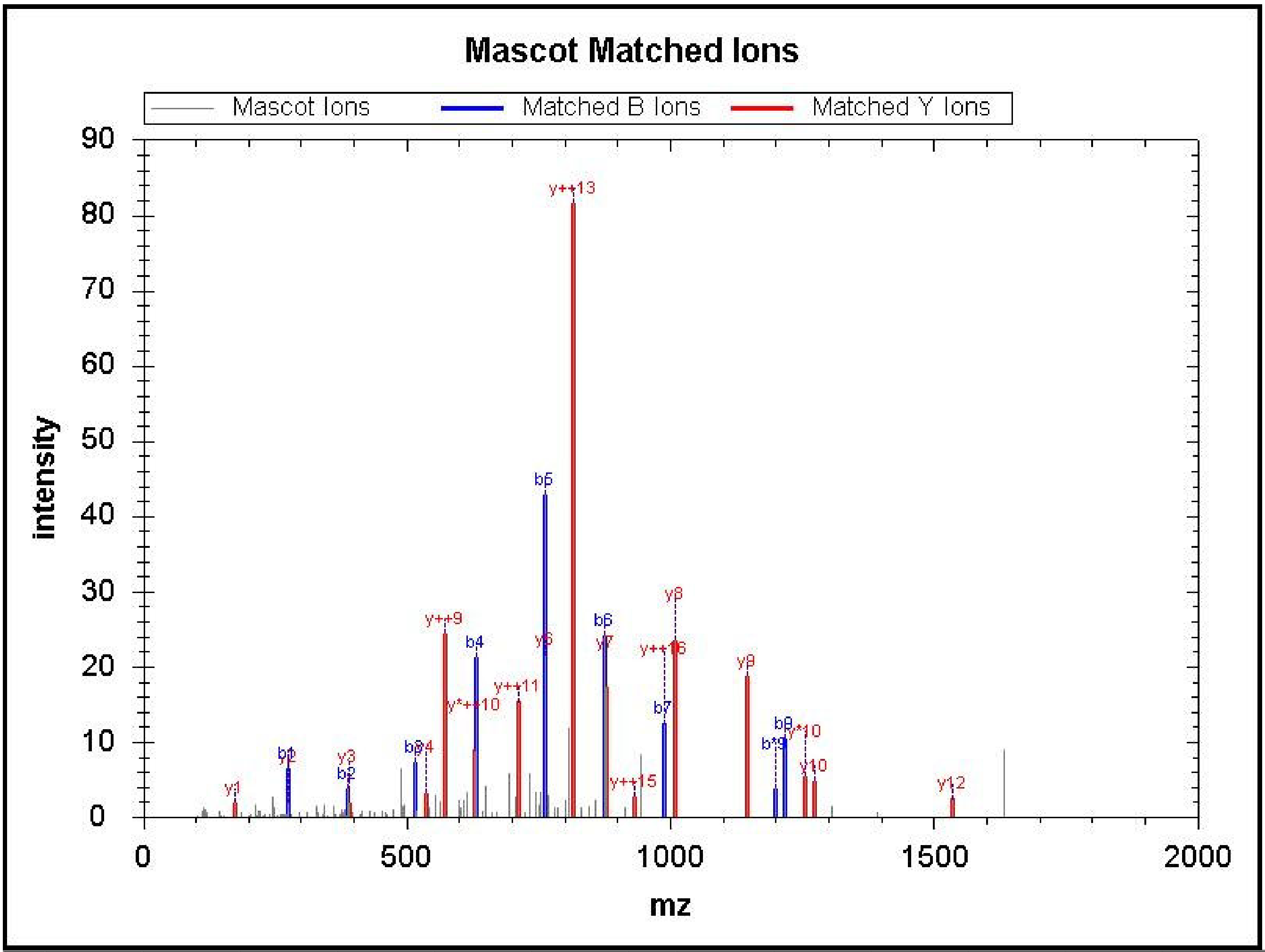

No	b	b++	b*	b*++	b0	b0++	Seq	y	y++	y*	y*++	y0	y0++	RevNo
1	274.15	137.58			256.14	128.57	E							22
2	387.24	194.12			369.23	185.12	I	2,574.23	1,287.62	2,557.20	1,279.10	2,556.22	1,278.61	21
3	516.28	258.64			498.27	249.64	E	2,461.15	1,231.08	2,444.12	1,222.56	2,443.13	1,222.07	20
4	630.32	315.66	613.29	307.15	612.31	306.66	N	2,332.10	1,166.56	2,315.08	1,158.04	2,314.09	1,157.55	19
5	761.36	381.18	744.34	372.67	743.35	372.18	M	2,218.06	1,109.53	2,201.03	1,101.02	2,200.05	1,100.53	18
6	874.45	437.73	857.42	429.21	856.44	428.72	I	2,087.02	1,044.01	2,069.99	1,035.50	2,069.01	1,035.01	17
7	987.53	494.27	970.50	485.76	969.52	485.26	L	1,973.94	987.47	1,956.91	978.96	1,955.92	978.47	16
8	1,101.57	551.29	1,084.55	542.78	1,083.56	542.28	N	1,860.85	930.93	1,843.82	922.42	1,842.84	921.92	15
9	1,216.60	608.80	1,199.57	600.29	1,198.59	599.80	D	1,746.81	873.91	1,729.78	865.39	1,728.80	864.90	14
10	1,313.65	657.33	1,296.63	648.82	1,295.64	648.32	P	1,631.78	816.39	1,614.75	807.88	1,613.77	807.39	13
11	1,428.68	714.84	1,411.65	706.33	1,410.67	705.84	D	1,534.73	767.87	1,517.70	759.35	1,516.72	758.86	12
12	1,575.75	788.38	1,558.72	779.86	1,557.74	779.37	F	1,419.70	710.35	1,402.67	701.84	1,401.69	701.35	11
13	1,703.81	852.41	1,686.78	843.89	1,685.80	843.40	Q	1,272.63	636.82	1,255.61	628.31	1,254.62	627.81	10
14	1,840.87	920.94	1,823.84	912.42	1,822.85	911.93	H	1,144.57	572.79	1,127.55	564.28	1,126.56	563.79	9
15	1,969.91	985.46	1,952.88	976.94	1,951.90	976.45	E	1,007.52	504.26	990.49	495.75	989.51	495.26	8
16	2,084.94	1,042.97	2,067.91	1,034.46	2,066.92	1,033.97	D	878.47	439.74	861.45	431.23	860.46	430.73	7
17	2,198.02	1,099.51	2,180.99	1,091.00	2,180.01	1,090.51	L	763.45	382.23	746.42	373.71	745.44	373.22	6
18	2,312.06	1,156.53	2,295.04	1,148.02	2,294.05	1,147.53	N	650.36	325.68	633.34	317.17	632.35	316.68	5

19	2,459.13	1,230.07	2,442.10	1,221.56	2,441.12	1,221.06	F	536.32	268.66	519.29	260.15	518.31	259.66	4
20	2,572.21	1,286.61	2,555.19	1,278.10	2,554.20	1,277.61	L	389.25	195.13	372.22	186.62	371.24	186.12	3
21	2,673.26	1,337.13	2,656.24	1,328.62	2,655.25	1,328.13	T	276.17	138.59	259.14	130.07	258.16	129.58	2
22							R	175.12	88.06	158.09	79.55			1

Query 41527 Hit 1

MS/MS Fragmentation of LEDILESINSIK
Found in sp|Q9Y3A6|TMED5_HUMAN, Transmembrane emp24 domain-containing protein 5 OS=Homo sapiens GN=TMED5 PE=1 SV=1
Match to Query 41527: 1660.946from(831.4803,2+)
Title: 1020: Sum of 2 scans in range 2232 (rt=59.7608, f=4, i=683) to 2233 (rt=59.7862, f=4, i=684) [D:\lab212\membrane\Grace\Joyce\iTRAQ_28_2_327.raw]
Data File:Submitted from 20120508-1(merge) by Mascot Daemon on JOYCE-VAIO
Monoisotopic mass of neutral peptide Mr(calc): 1660.946
Variable modifications:
K12 :iTRAQ4plex (K)
Ions Score: 76.82 Expect: 0.000

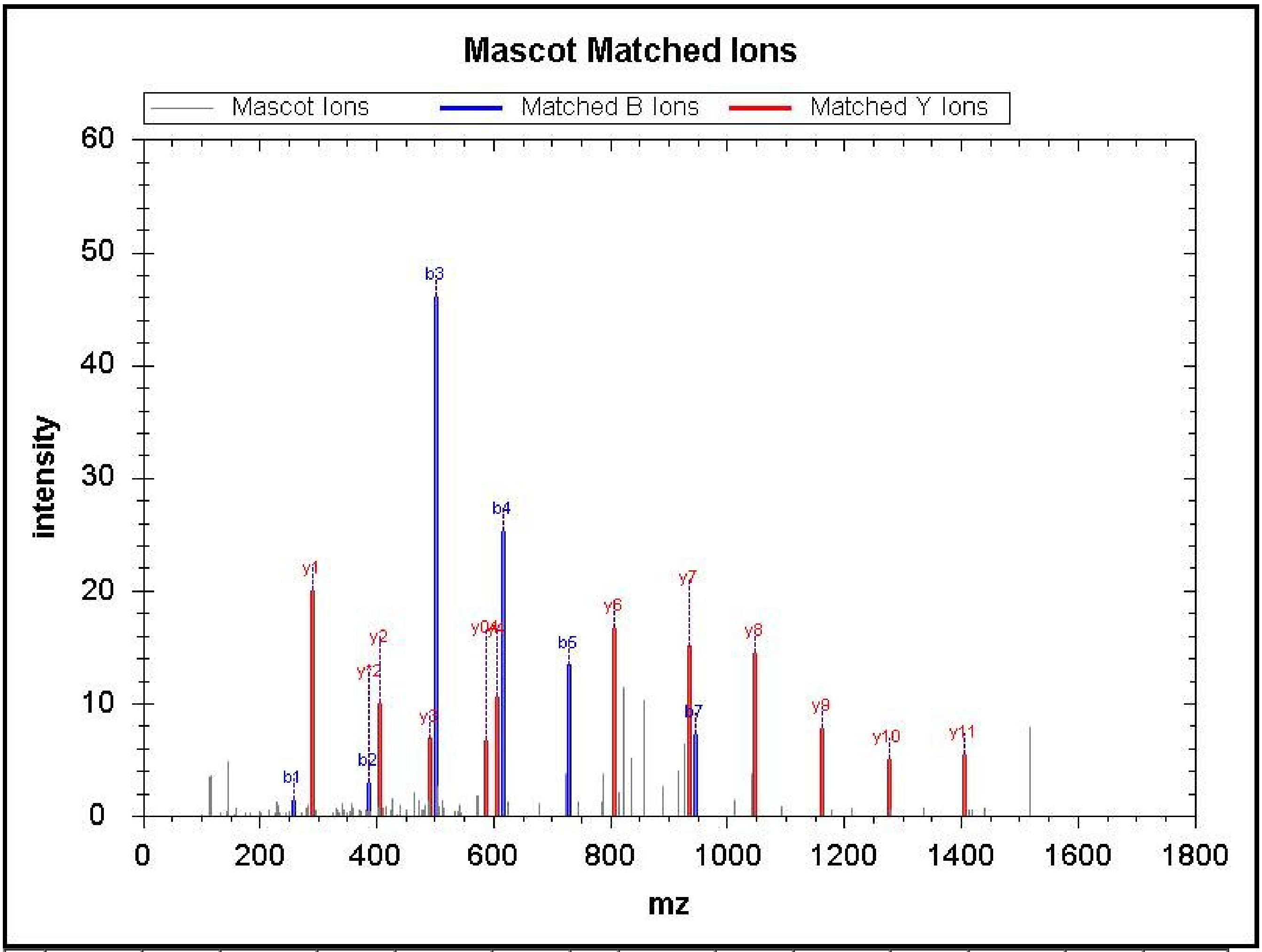

No	b	b++	b*	b*++	b0	b0++	Seq	y	y++	y*	y*++	y0	y0++	RevNo
1	258.19	129.60					L							12
2	387.24	194.12			369.23	185.12	E	1,404.77	702.89	1,387.74	694.38	1,386.76	693.88	11
3	502.26	251.64			484.25	242.63	D	1,275.73	638.37	1,258.70	629.85	1,257.72	629.36	10
4	615.35	308.18			597.34	299.17	I	1,160.70	580.85	1,143.67	572.34	1,142.69	571.85	9
5	728.43	364.72			710.42	355.71	L	1,047.62	524.31	1,030.59	515.80	1,029.61	515.31	8
6	857.47	429.24			839.46	420.24	E	934.53	467.77	917.51	459.26	916.52	458.76	7
7	944.51	472.76			926.50	463.75	S	805.49	403.25	788.46	394.74	787.48	394.24	6
8	1,057.59	529.30			1,039.58	520.29	I	718.46	359.73	701.43	351.22	700.45	350.73	5
9	1,171.63	586.32	1,154.61	577.81	1,153.62	577.31	N	605.37	303.19	588.35	294.68	587.36	294.19	4

10	1,258.66	629.84	1,241.64	621.32	1,240.65	620.83	S	491.33	246.17	474.30	237.66	473.32	237.16	3
11	1,371.75	686.38	1,354.72	677.86	1,353.74	677.37	I	404.30	202.65	387.27	194.14			2
12							K	291.21	146.11	274.19	137.60			1

Query 66321 Hit 1

MS/MS Fragmentation of ILDSFAAAPVPTTTLVLK
Found in sp|P18583|SON_HUMAN, Protein SON OS=Homo sapiens GN=SON PE=1 SV=4
Match to Query 66321: 2144.268from(715.7632,3+)
Title: 711: Scan 2213 (rt=56.0954, f=3, i=231) [D:\lab212\membrane\Grace\Joyce\iTRAQ_24_2.raw]
Data File:Submitted from 20120508-1(merge) by Mascot Daemon on JOYCE-VAIO
Monoisotopic mass of neutral peptide Mr(calc): 2144.268
Variable modifications:
K18 :iTRAQ4plex (K)
Ions Score: 76.43 Expect: 0.000

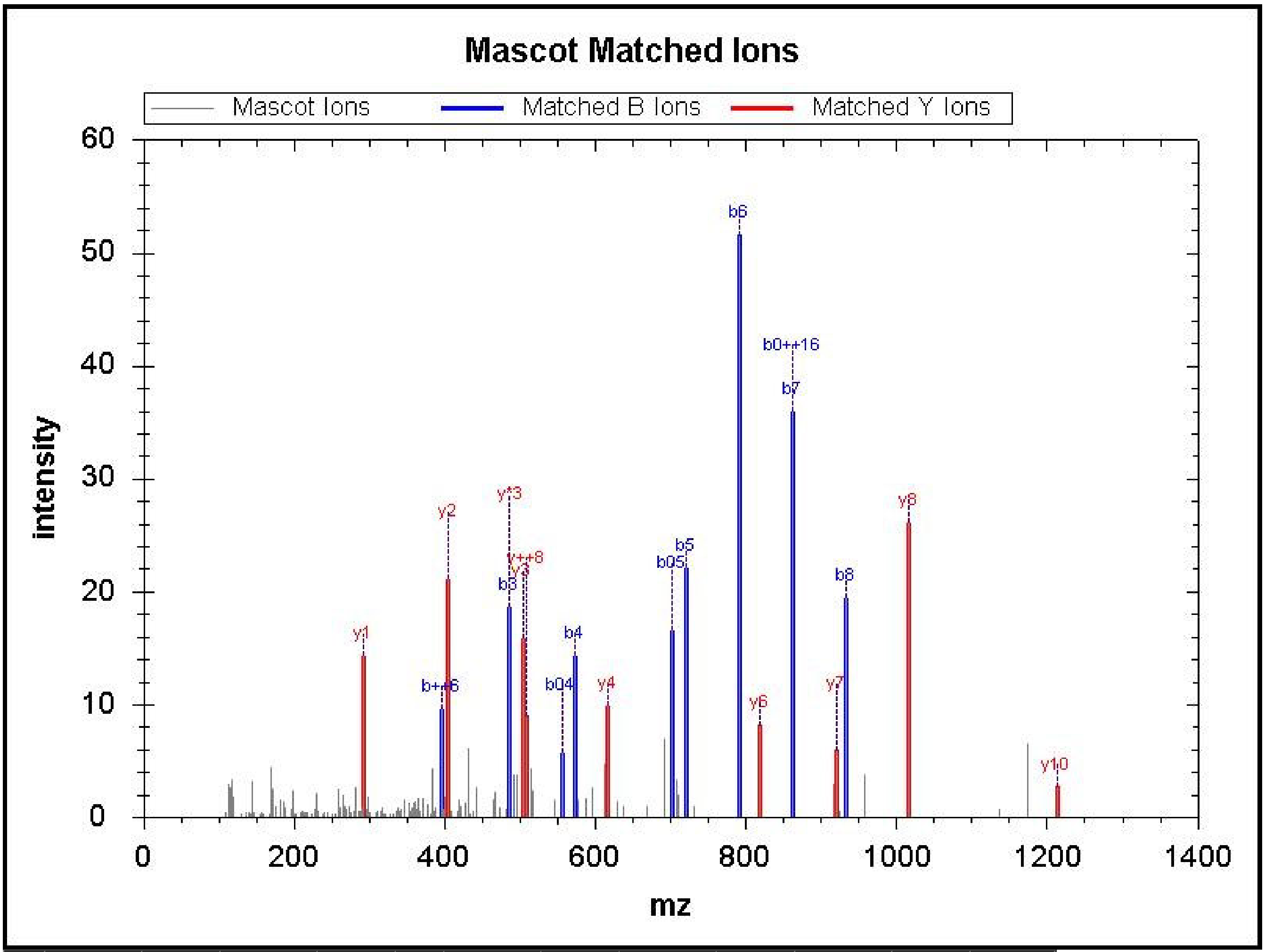

No	b	b++	b0	b0++	Seq	y	y++	y*	y*++	y0	y0++	RevNo
1	258.19	129.60			I							18
2	371.28	186.14			L	1,888.09	944.55	1,871.06	936.04	1,870.08	935.54	17
3	486.30	243.66	468.29	234.65	D	1,775.01	888.01	1,757.98	879.49	1,757.00	879.00	16
4	573.34	287.17	555.33	278.17	S	1,659.98	830.49	1,642.95	821.98	1,641.97	821.49	15
5	720.40	360.71	702.39	351.70	F	1,572.95	786.98	1,555.92	778.46	1,554.94	777.97	14
6	791.44	396.22	773.43	387.22	A	1,425.88	713.44	1,408.85	704.93	1,407.87	704.44	13
7	862.48	431.74	844.47	422.74	A	1,354.84	677.92	1,337.82	669.41	1,336.83	668.92	12
8	933.52	467.26	915.51	458.26	A	1,283.81	642.41	1,266.78	633.89	1,265.79	633.40	11
9	1,030.57	515.79	1,012.56	506.78	P	1,212.77	606.89	1,195.74	598.37	1,194.76	597.88	10
10	1,129.64	565.32	1,111.63	556.32	V	1,115.72	558.36	1,098.69	549.85	1,097.70	549.36	9
11	1,226.69	613.85	1,208.68	604.84	P	1,016.65	508.83	999.62	500.31	998.64	499.82	8

12	1,327.74	664.37	1,309.73	655.37	T	919.59	460.30	902.57	451.79	901.58	451.30	7
13	1,428.79	714.90	1,410.77	705.89	T	818.55	409.78	801.52	401.26	800.54	400.77	6
14	1,529.83	765.42	1,511.82	756.41	T	717.50	359.25	700.47	350.74	699.49	350.25	5
15	1,642.92	821.96	1,624.91	812.96	L	616.45	308.73	599.42	300.22			4
16	1,741.99	871.50	1,723.98	862.49	V	503.37	252.19	486.34	243.67			3
17	1,855.07	928.04	1,837.06	919.03	L	404.30	202.65	387.27	194.14			2
18					K	291.21	146.11	274.19	137.60			1

Query 73795 Hit 1

MS/MS Fragmentation of AQVLVEDISDILEEHAEK
Found in sp|Q5VV41|ARHGG_HUMAN, Rho guanine nucleotide exchange factor 16 OS=Homo sapiens GN=ARHGEF16 PE=1 SV=1
Match to Query 73795: 2325.229from(776.0835,3+)
Title: 1057: Sum of 2 scans in range 2325 (rt=61.764, f=4, i=707) to 2326 (rt=61.7894, f=4, i=708)
[D:\lab212\membrane\Grace\Joyce\iTRAQ_35_1.raw]
Data File:Submitted from 20120508-1(merge) by Mascot Daemon on JOYCE-VAIO
Monoisotopic mass of neutral peptide Mr(calc): 2325.229
Variable modifications:
K18 :iTRAQ4plex (K)
Ions Score: 76.08 Expect: 0.000

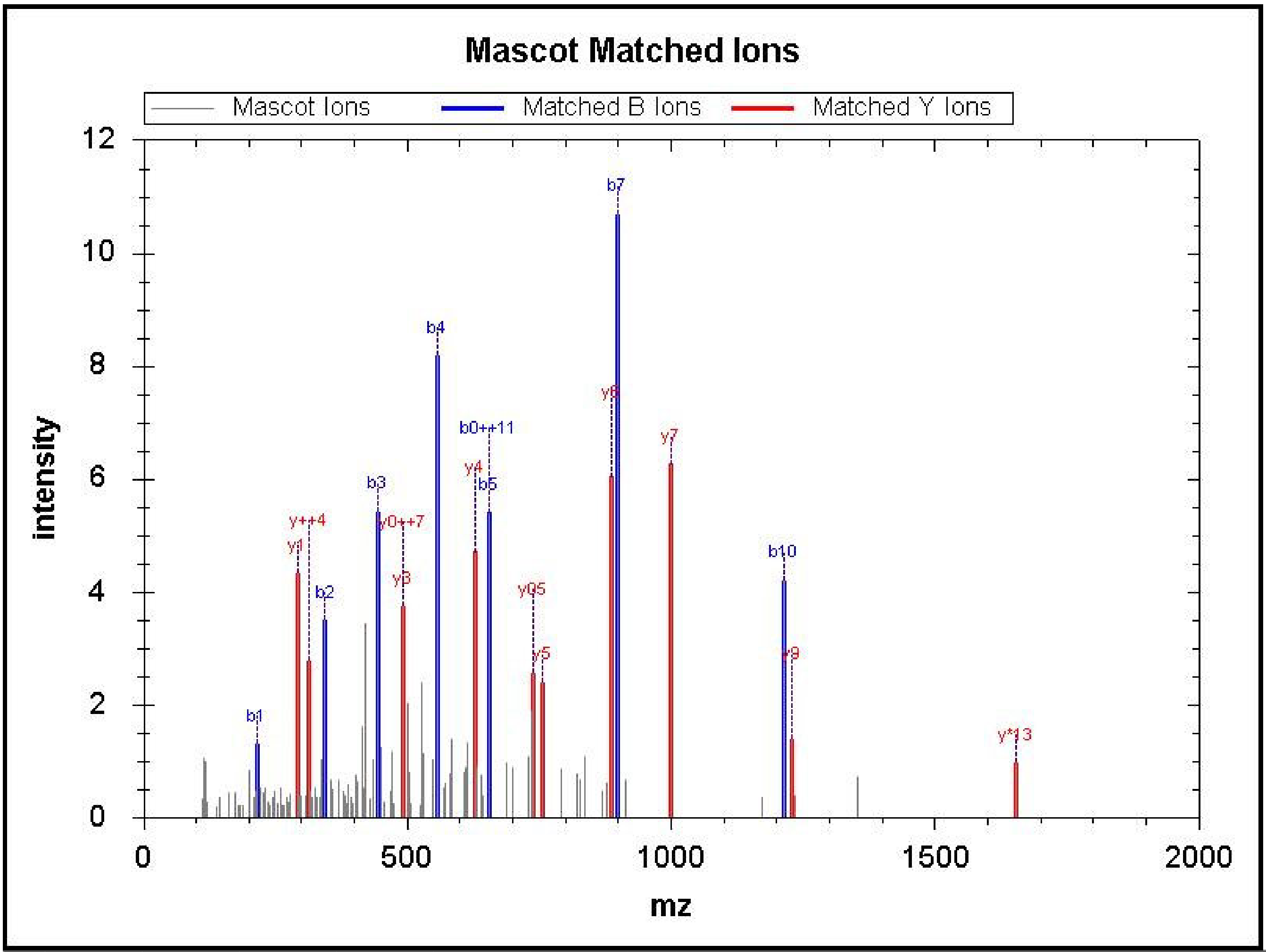

No	b	b++	b*	b*++	b0	b0++	Seq	y	y++	y*	y*++	y0	y0++	RevNo
1	216.15	108.58					A							18
2	344.21	172.61	327.18	164.09			Q	2,111.10	1,056.05	2,094.07	1,047.54	2,093.09	1,047.05	17
3	443.27	222.14	426.25	213.63			V	1,983.04	992.02	1,966.01	983.51	1,965.03	983.02	16
4	556.36	278.68	539.33	270.17			L	1,883.97	942.49	1,866.95	933.98	1,865.96	933.48	15
5	655.43	328.22	638.40	319.70			V	1,770.89	885.95	1,753.86	877.43	1,752.88	876.94	14
6	784.47	392.74	767.44	384.22	766.46	383.73	E	1,671.82	836.41	1,654.79	827.90	1,653.81	827.41	13

7	899.50	450.25	882.47	441.74	881.48	441.25	D	1,542.78	771.89	1,525.75	763.38	1,524.77	762.89	12
8	1,012.58	506.79	995.55	498.28	994.57	497.79	I	1,427.75	714.38	1,410.72	705.87	1,409.74	705.37	11
9	1,099.61	550.31	1,082.59	541.80	1,081.60	541.30	S	1,314.67	657.84	1,297.64	649.32	1,296.66	648.83	10
10	1,214.64	607.82	1,197.61	599.31	1,196.63	598.82	D	1,227.63	614.32	1,210.61	605.81	1,209.62	605.32	9
11	1,327.72	664.36	1,310.70	655.85	1,309.71	655.36	I	1,112.61	556.81	1,095.58	548.29	1,094.60	547.80	8
12	1,440.81	720.91	1,423.78	712.39	1,422.80	711.90	L	999.52	500.26	982.50	491.75	981.51	491.26	7
13	1,569.85	785.43	1,552.82	776.92	1,551.84	776.42	E	886.44	443.72	869.41	435.21	868.43	434.72	6
14	1,698.89	849.95	1,681.87	841.44	1,680.88	840.94	E	757.40	379.20	740.37	370.69	739.39	370.20	5
15	1,835.95	918.48	1,818.92	909.97	1,817.94	909.47	H	628.35	314.68	611.33	306.17	610.34	305.68	4
16	1,906.99	954.00	1,889.96	945.48	1,888.98	944.99	A	491.29	246.15	474.27	237.64	473.28	237.15	3
17	2,036.03	1,018.52	2,019.00	1,010.01	2,018.02	1,009.51	E	420.26	210.63	403.23	202.12	402.25	201.63	2
18							K	291.21	146.11	274.19	137.60			1

Query 58806 Hit 1

MS/MS Fragmentation of LGVPFQVLVATHAGLYR
Found in sp|Q96T60|PNKP_HUMAN, Bifunctional polynucleotide phosphatase/kinase OS=Homo sapiens GN=PNKP PE=1 SV=1
Match to Query 58806: 1984.135from(662.3854,3+)
Title: 910: Sum of 2 scans in range 2002 (rt=54.6296, f=4, i=609) to 2003 (rt=54.655, f=4, i=610) [D:\lab212\membrane\Grace\Joyce\iTRAQ_34_2.raw]
Data File:Submitted from 20120508-1(merge) by Mascot Daemon on JOYCE-VAIO
Monoisotopic mass of neutral peptide Mr(calc): 1984.135
Variable modifications:
Ions Score: 74.84 Expect: 0.000

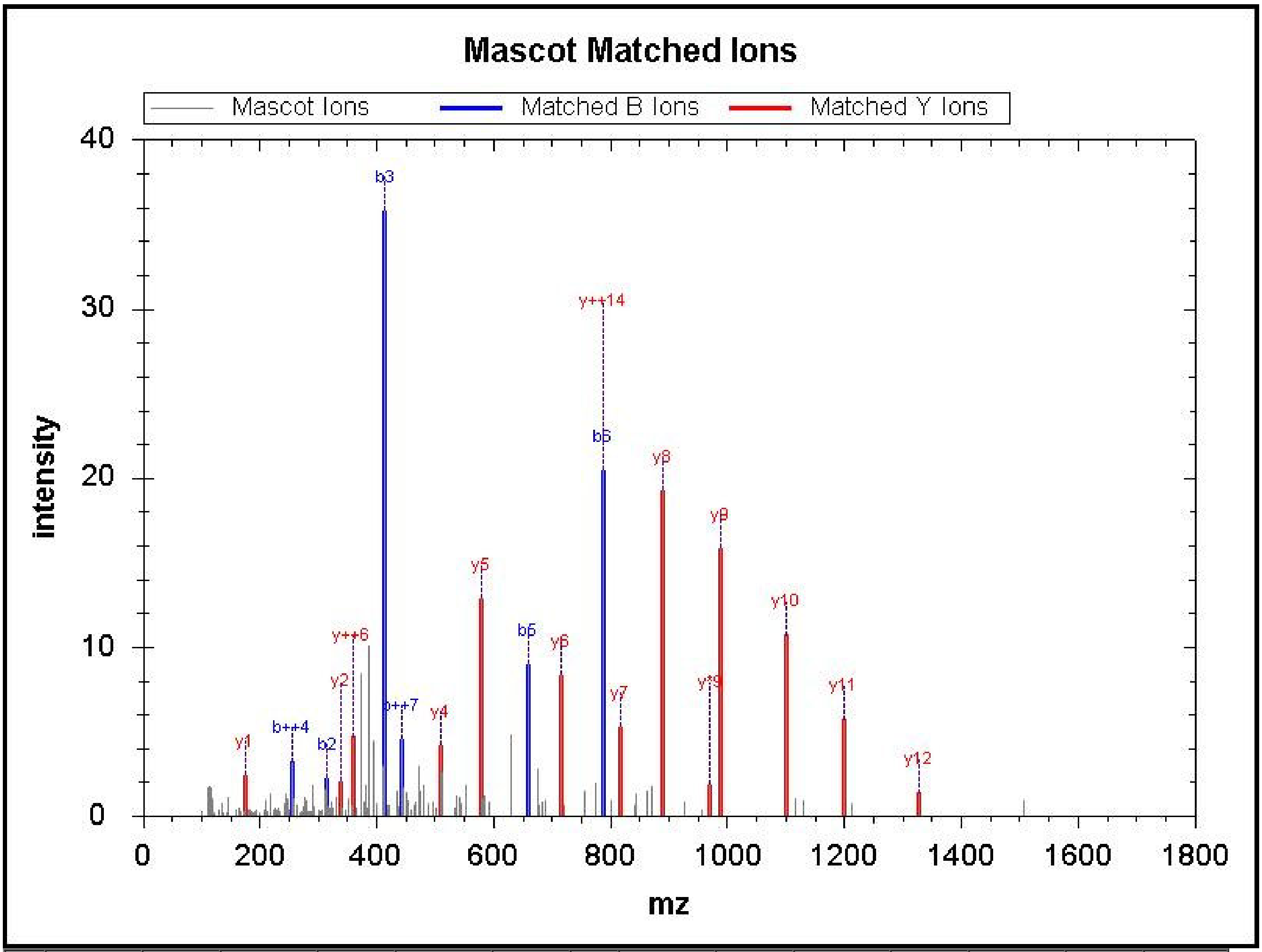

No	b	b++	b*	b*++	b0	b0++	Seq	y	y++	y*	y*++	y0	y0++	RevNo
1	258.19	129.60					L							17

2	315.21	158.11					G	1,727.96	864.48	1,710.93	855.97	1,709.95	855.48	16
3	414.28	207.65					V	1,670.94	835.97	1,653.91	827.46	1,652.93	826.97	15
4	511.34	256.17					P	1,571.87	786.44	1,554.84	777.92	1,553.86	777.43	14
5	658.40	329.71					F	1,474.82	737.91	1,457.79	729.40	1,456.81	728.91	13
6	786.46	393.74	769.44	385.22			Q	1,327.75	664.38	1,310.72	655.86	1,309.74	655.37	12
7	885.53	443.27	868.50	434.76			V	1,199.69	600.35	1,182.66	591.84	1,181.68	591.34	11
8	998.62	499.81	981.59	491.30			L	1,100.62	550.81	1,083.59	542.30	1,082.61	541.81	10
9	1,097.68	549.35	1,080.66	540.83			V	987.54	494.27	970.51	485.76	969.53	485.27	9
10	1,168.72	584.86	1,151.69	576.35			A	888.47	444.74	871.44	436.22	870.46	435.73	8
11	1,269.77	635.39	1,252.74	626.87	1,251.76	626.38	T	817.43	409.22	800.40	400.71	799.42	400.21	7
12	1,406.83	703.92	1,389.80	695.40	1,388.82	694.91	H	716.38	358.70	699.36	350.18			6
13	1,477.86	739.44	1,460.84	730.92	1,459.85	730.43	A	579.32	290.17	562.30	281.65			5
14	1,534.89	767.95	1,517.86	759.43	1,516.88	758.94	G	508.29	254.65	491.26	246.13			4
15	1,647.97	824.49	1,630.94	815.98	1,629.96	815.48	L	451.27	226.14	434.24	217.62			3
16	1,811.03	906.02	1,794.01	897.51	1,793.02	897.02	Y	338.18	169.59	321.16	161.08			2
17							R	175.12	88.06	158.09	79.55			1

Query 66682 Hit 1

MS/MS Fragmentation of VGLIHMLTHLAEALHQAR
Found in sp|Q9BWS9|CHID1_HUMAN, Chitinase domain-containing protein 1 OS=Homo sapiens GN=CHID1 PE=1 SV=1
Match to Query 66682: 2153.201 from(718.7408,3+)
Title: 819: Scan 2435 (rt=61.2623, f=2, i=360) [D:\lab212\membrane\Grace\Joyce\iTRAQ_52_2.raw]
Data File:Submitted from 20120508-1(merge) by Mascot Daemon on JOYCE-VAIO
Monoisotopic mass of neutral peptide Mr(calc): 2153.201
Variable modifications:
Ions Score: 74.17 Expect: 0.000

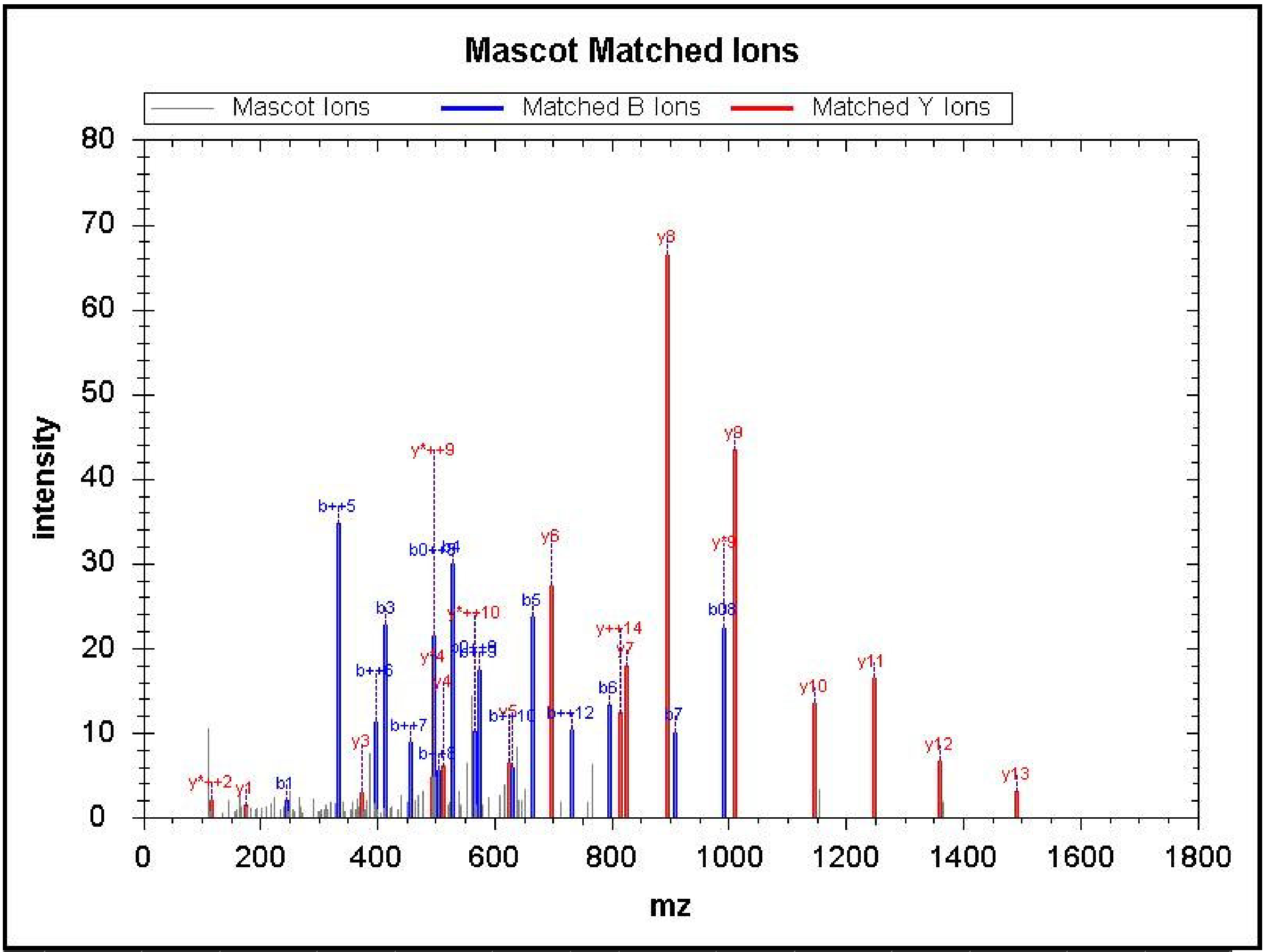

No	b	b++	b*	b*++	b0	b0++	Seq	y	y++	y*	y*++	y0	y0++	RevNo
1	244.18	122.59					V							18
2	301.20	151.10					G	1,911.04	956.02	1,894.01	947.51	1,893.03	947.02	17
3	414.28	207.65					L	1,854.02	927.51	1,836.99	919.00	1,836.01	918.51	16
4	527.37	264.19					I	1,740.93	870.97	1,723.91	862.46	1,722.92	861.96	15
5	664.43	332.72					H	1,627.85	814.43	1,610.82	805.91	1,609.84	805.42	14
6	795.47	398.24					M	1,490.79	745.90	1,473.76	737.39	1,472.78	736.89	13
7	908.55	454.78					L	1,359.75	680.38	1,342.72	671.86	1,341.74	671.37	12
8	1,009.60	505.30			991.59	496.30	T	1,246.67	623.84	1,229.64	615.32	1,228.65	614.83	11
9	1,146.66	573.83			1,128.65	564.83	H	1,145.62	573.31	1,128.59	564.80	1,127.61	564.31	10
10	1,259.74	630.37			1,241.73	621.37	L	1,008.56	504.78	991.53	496.27	990.55	495.78	9
11	1,330.78	665.89			1,312.77	656.89	A	895.47	448.24	878.45	439.73	877.46	439.24	8
12	1,459.82	730.41			1,441.81	721.41	E	824.44	412.72	807.41	404.21	806.43	403.72	7
13	1,530.86	765.93			1,512.85	756.93	A	695.39	348.20	678.37	339.69			6
14	1,643.94	822.47			1,625.93	813.47	L	624.36	312.68	607.33	304.17			5
15	1,781.00	891.00			1,762.99	882.00	H	511.27	256.14	494.25	247.63			4
16	1,909.06	955.03	1,892.03	946.52	1,891.05	946.03	Q	374.21	187.61	357.19	179.10			3
17	1,980.10	990.55	1,963.07	982.04	1,962.09	981.55	A	246.16	123.58	229.13	115.07			2
18							R	175.12	88.06	158.09	79.55			1

Query 40396 Hit 1

MS/MS Fragmentation of DPAAPEPEEQEER
Found in sp|Q969T4|UB2E3_HUMAN, Ubiquitin-conjugating enzyme E2 E3 OS=Homo sapiens GN=UBE2E3 PE=1 SV=1
Match to Query 40396: 1639.742from(820.8785,2+)
Title: 87: Sum of 2 scans in range 395 (rt=18.0766, f=4, i=43) to 396 (rt=18.102, f=4, i=44)

[D:\lab212\membrane\Grace\Joyce\iTRAQ_21_2.raw]
Data File:Submitted from 20120508-1(merge) by Mascot Daemon on JOYCE-VAIO
Monoisotopic mass of neutral peptide Mr(calc): 1639.742
Variable modifications:
Ions Score: 74.13 Expect: 0.000

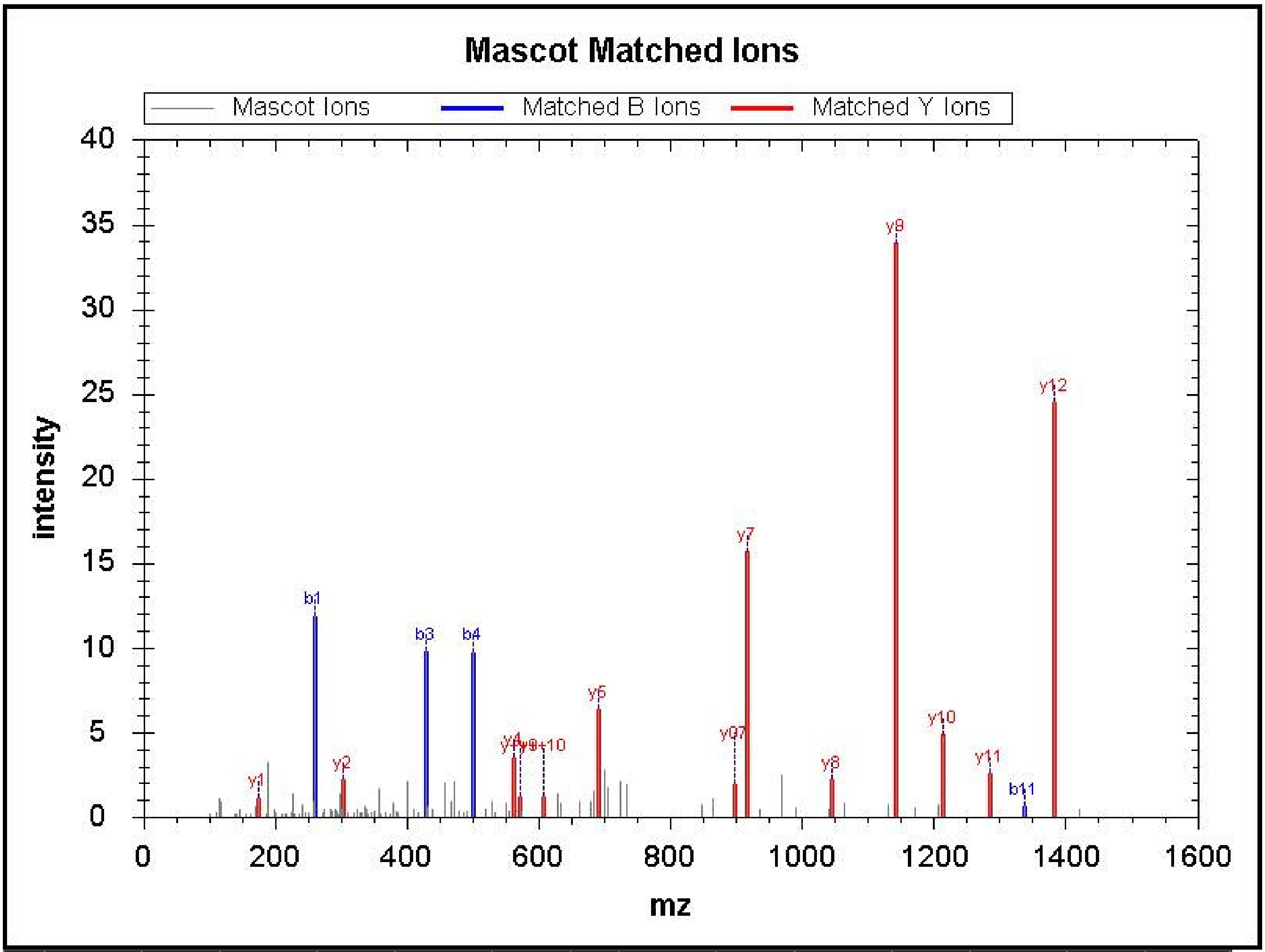

No	b	b++	b*	b*++	b0	b0++	Seq	y	y++	y*	y*++	y0	y0++	RevNo
1	260.14	130.57			242.13	121.57	D							13
2	357.19	179.10			339.18	170.09	P	1,381.62	691.32	1,364.60	682.80	1,363.61	682.31	12
3	428.23	214.62			410.22	205.61	A	1,284.57	642.79	1,267.54	634.28	1,266.56	633.78	11
4	499.26	250.14			481.25	241.13	A	1,213.53	607.27	1,196.51	598.76	1,195.52	598.26	10
5	596.32	298.66			578.31	289.66	P	1,142.50	571.75	1,125.47	563.24	1,124.49	562.75	9
6	725.36	363.18			707.35	354.18	E	1,045.44	523.23	1,028.42	514.71	1,027.43	514.22	8
7	822.41	411.71			804.40	402.70	P	916.40	458.70	899.37	450.19	898.39	449.70	7
8	951.45	476.23			933.44	467.23	E	819.35	410.18	802.32	401.66	801.34	401.17	6
9	1,080.50	540.75			1,062.49	531.75	E	690.31	345.66	673.28	337.14	672.29	336.65	5
10	1,208.56	604.78	1,191.53	596.27	1,190.54	595.78	Q	561.26	281.13	544.24	272.62	543.25	272.13	4
11	1,337.60	669.30	1,320.57	660.79	1,319.59	660.30	E	433.20	217.11	416.18	208.59	415.19	208.10	3
12	1,466.64	733.82	1,449.61	725.31	1,448.63	724.82	E	304.16	152.58	287.13	144.07	286.15	143.58	2
13							R	175.12	88.06	158.09	79.55			1

Query 56384 Hit 1

MS/MS Fragmentation of AIGIEPSLATYHHIIR
Found in sp|Q96EY7|PTCD3_HUMAN, Pentatricopeptide repeat-containing protein 3
Match to Query 56384: 1934.087from(484.529,4+)
Title: 575: Scan 1370 (rt=40.3204, f=3, i=210) [D:\lab212\membrane\Grace\Joyce\47_1.raw]
Data File:Submitted from 20120508-1(merge) by Mascot Daemon on JOYCE-VAIO

Monoisotopic mass of neutral peptide Mr(calc): 1934.087
Variable modifications:
Ions Score: 73.94 Expect: 0.000

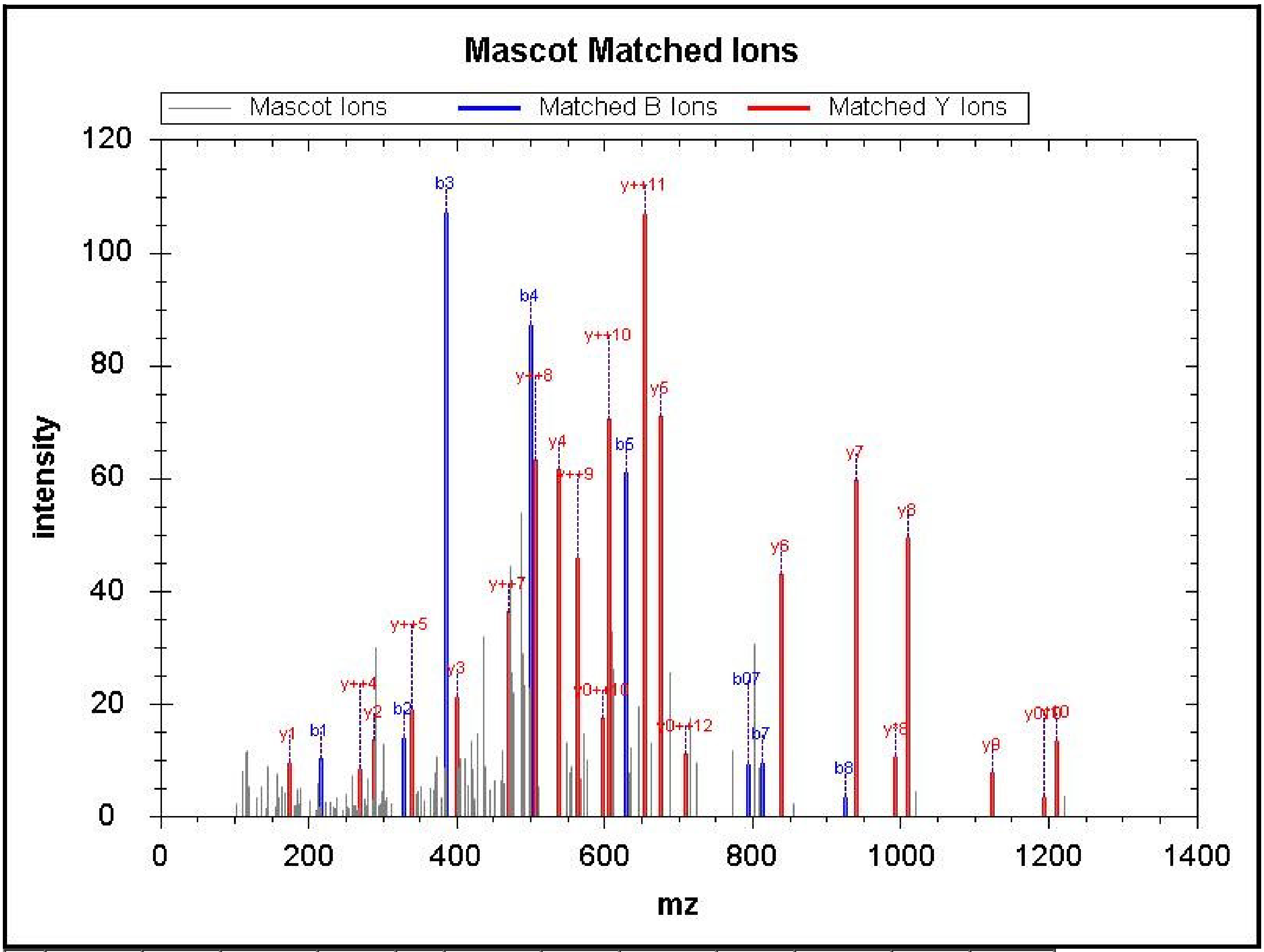

No	b	b++	b0	b0++	Seq	y	y++	y*	y*++	y0	y0++	RevNo
1	216.15	108.58			A							16
2	329.23	165.12			I	1,719.95	860.48	1,702.93	851.97	1,701.94	851.48	15
3	386.25	193.63			G	1,606.87	803.94	1,589.84	795.43	1,588.86	794.93	14
4	499.34	250.17			I	1,549.85	775.43	1,532.82	766.91	1,531.84	766.42	13
5	628.38	314.69	610.37	305.69	E	1,436.76	718.89	1,419.74	710.37	1,418.75	709.88	12
6	725.43	363.22	707.42	354.21	P	1,307.72	654.36	1,290.70	645.85	1,289.71	645.36	11
7	812.46	406.74	794.45	397.73	S	1,210.67	605.84	1,193.64	597.32	1,192.66	596.83	10
8	925.55	463.28	907.54	454.27	L	1,123.64	562.32	1,106.61	553.81	1,105.63	553.32	9
9	996.58	498.80	978.57	489.79	A	1,010.55	505.78	993.53	497.27	992.54	496.77	8
10	1,097.63	549.32	1,079.62	540.31	T	939.52	470.26	922.49	461.75	921.51	461.26	7
11	1,260.70	630.85	1,242.69	621.85	Y	838.47	419.74	821.44	411.22			6
12	1,397.75	699.38	1,379.74	690.38	H	675.40	338.21	658.38	329.69			5
13	1,534.81	767.91	1,516.80	758.91	H	538.35	269.68	521.32	261.16			4
14	1,647.90	824.45	1,629.89	815.45	I	401.29	201.15	384.26	192.63			3
15	1,760.98	880.99	1,742.97	871.99	I	288.20	144.61	271.18	136.09			2
16					R	175.12	88.06	158.09	79.55			1

Query 70132 Hit 1

MS/MS Fragmentation of FNSVVTNPQGHYNPSTGK
Found in sp|Q02105|C1QC_MOUSE, Complement C1q subcomponent subunit C OS=Mus musculus GN=C1qc PE=2 SV=1
Match to Query 70132: 2234.125from(745.7156,3+)

Title: 319: Scan 793 (rt=27.1951, f=3, i=111) [D:\lab212\membrane\Grace\Joyce\iTRAQ_38_1.raw]
Data File:Submitted from 20120508-1(merge) by Mascot Daemon on JOYCE-VAIO
Monoisotopic mass of neutral peptide Mr(calc): 2234.125
Variable modifications:
K18 :iTRAQ4plex (K)
Ions Score: 73.48 Expect: 0.000

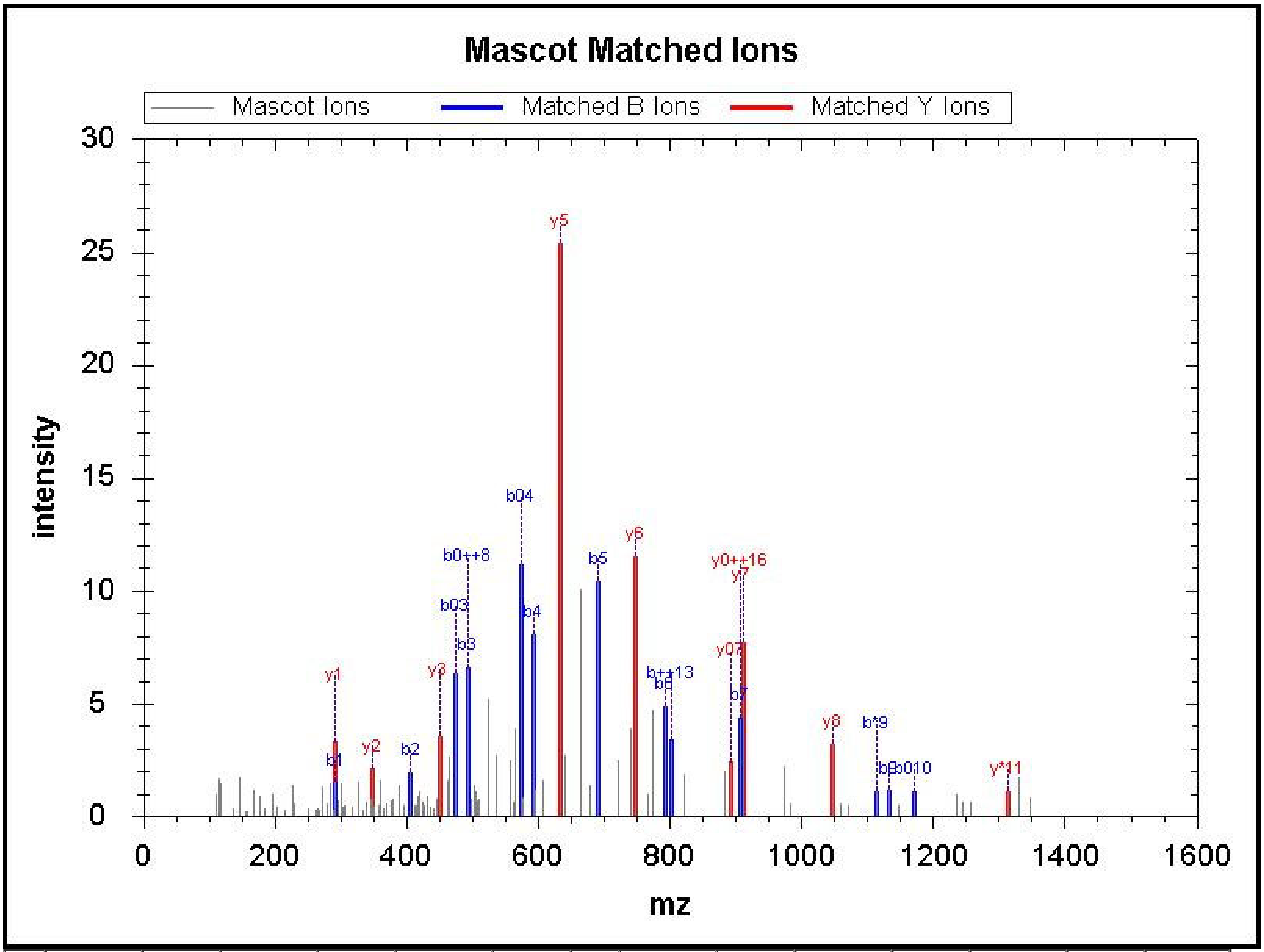

No	b	b++	b*	b*++	b0	b0++	Seq	y	y++	y*	y*++	y0	y0++	RevNo
1	292.18	146.59					F							18
2	406.22	203.61	389.19	195.10			N	1,943.97	972.49	1,926.94	963.97	1,925.96	963.48	17
3	493.25	247.13	476.23	238.62	475.24	238.12	S	1,829.93	915.47	1,812.90	906.95	1,811.92	906.46	16
4	592.32	296.66	575.29	288.15	574.31	287.66	V	1,742.89	871.95	1,725.87	863.44	1,724.88	862.95	15
5	691.39	346.20	674.36	337.69	673.38	337.19	V	1,643.83	822.42	1,626.80	813.90	1,625.82	813.41	14
6	792.44	396.72	775.41	388.21	774.43	387.72	T	1,544.76	772.88	1,527.73	764.37	1,526.75	763.88	13
7	906.48	453.74	889.45	445.23	888.47	444.74	N	1,443.71	722.36	1,426.68	713.85	1,425.70	713.35	12
8	1,003.53	502.27	986.51	493.76	985.52	493.26	P	1,329.67	665.34	1,312.64	656.82	1,311.66	656.33	11
9	1,131.59	566.30	1,114.57	557.79	1,113.58	557.29	Q	1,232.61	616.81	1,215.59	608.30	1,214.60	607.81	10
10	1,188.61	594.81	1,171.59	586.30	1,170.60	585.80	G	1,104.56	552.78	1,087.53	544.27	1,086.54	543.78	9
11	1,325.67	663.34	1,308.65	654.83	1,307.66	654.33	H	1,047.53	524.27	1,030.51	515.76	1,029.52	515.27	8
12	1,488.74	744.87	1,471.71	736.36	1,470.72	735.87	Y	910.47	455.74	893.45	447.23	892.46	446.74	7
13	1,602.78	801.89	1,585.75	793.38	1,584.77	792.89	N	747.41	374.21	730.39	365.70	729.40	365.20	6
14	1,699.83	850.42	1,682.80	841.91	1,681.82	841.41	P	633.37	317.19	616.34	308.67	615.36	308.18	5
15	1,786.86	893.94	1,769.84	885.42	1,768.85	884.93	S	536.32	268.66	519.29	260.15	518.31	259.66	4
16	1,887.91	944.46	1,870.88	935.95	1,869.90	935.45	T	449.28	225.15	432.26	216.63	431.27	216.14	3
17	1,944.93	972.97	1,927.91	964.46	1,926.92	963.96	G	348.24	174.62	331.21	166.11			2
18							K	291.21	146.11	274.19	137.60			1

Query 30729 Hit 1

MS/MS Fragmentation of EILVEESNVQR
Found in sp|P60510|PP4C_HUMAN, Serine/threonine-protein phosphatase 4 catalytic subunit OS=Homo sapiens GN=PPP4C PE=1 SV=1
Match to Query 30729: 1458.781from(730.3979,2+)
Title: 192: Scan 991 (rt=29.3788, f=2, i=105) [D:\lab212\membrane\Grace\Joyce\iTRAQ_22_2.raw]
Data File:Submitted from 20120508-1(merge) by Mascot Daemon on JOYCE-VAIO
Monoisotopic mass of neutral peptide Mr(calc): 1458.781
Variable modifications:
Ions Score: 73.28 Expect: 0.000

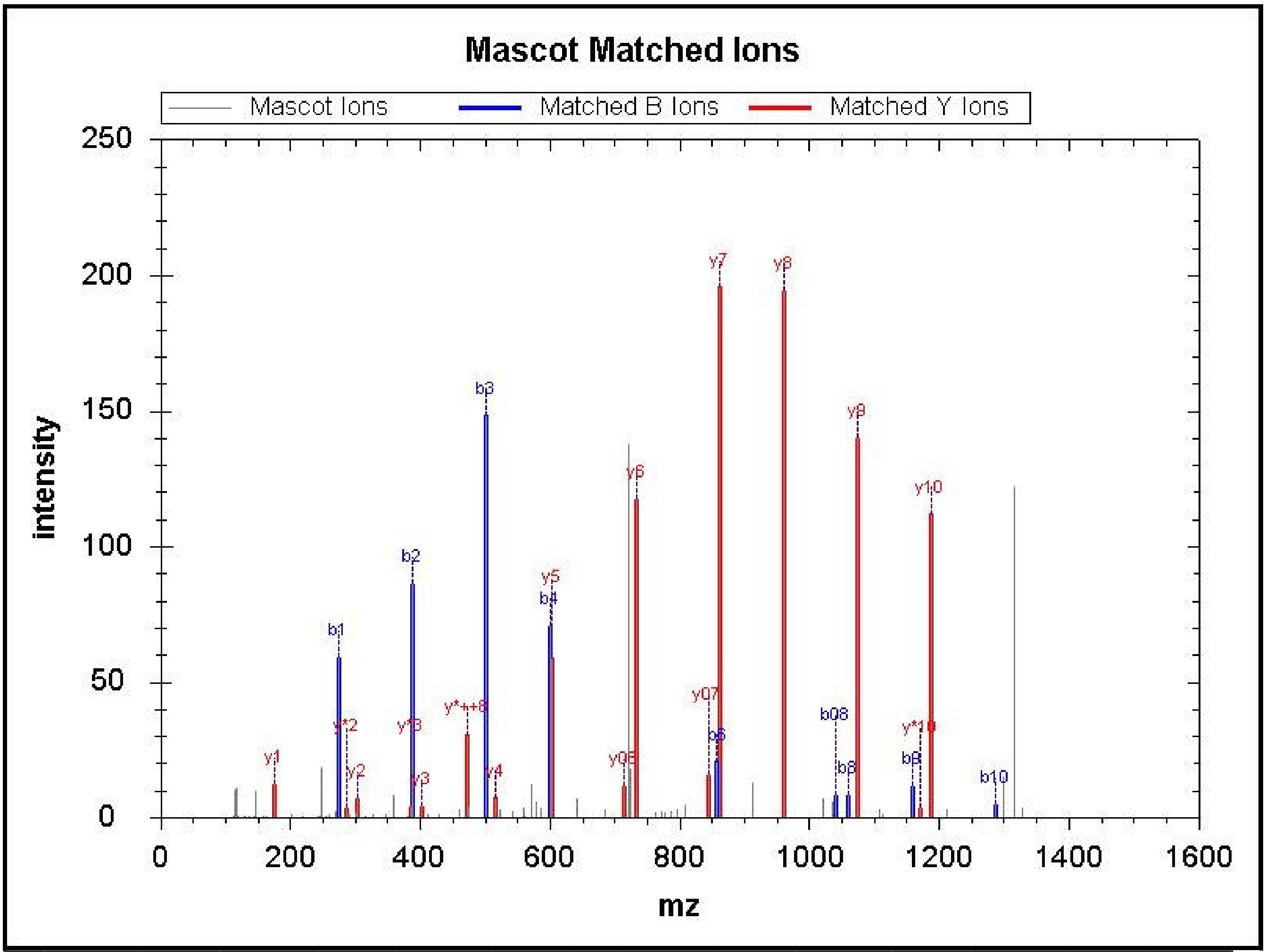

No	b	b++	b*	b*++	b0	b0++	Seq	y	y++	y*	y*++	y0	y0++	RevNo
1	274.15	137.58			256.14	128.57	E							11
2	387.24	194.12			369.23	185.12	I	1,186.64	593.83	1,169.62	585.31	1,168.63	584.82	10
3	500.32	250.66			482.31	241.66	L	1,073.56	537.28	1,056.53	528.77	1,055.55	528.28	9
4	599.39	300.20			581.38	291.19	V	960.47	480.74	943.45	472.23	942.46	471.74	8
5	728.43	364.72			710.42	355.71	E	861.41	431.21	844.38	422.69	843.40	422.20	7
6	857.47	429.24			839.46	420.24	E	732.36	366.69	715.34	358.17	714.35	357.68	6
7	944.51	472.76			926.50	463.75	S	603.32	302.16	586.29	293.65	585.31	293.16	5
8	1,058.55	529.78	1,041.52	521.26	1,040.54	520.77	N	516.29	258.65	499.26	250.13			4
9	1,157.62	579.31	1,140.59	570.80	1,139.61	570.31	V	402.25	201.63	385.22	193.11			3
10	1,285.68	643.34	1,268.65	634.83	1,267.67	634.34	Q	303.18	152.09	286.15	143.58			2
11							R	175.12	88.06	158.09	79.55			1

Query 74205 Hit 1

MS/MS Fragmentation of VGLTSEILNSFEHEFLSK
Found in sp|Q14181|DPOA2_HUMAN, DNA polymerase alpha subunit B OS=Homo sapiens GN=POLA2 PE=1 SV=2

Match to Query 74205: 2337.247from(780.0897,3+)
Title: 1159: Sum of 2 scans in range 2590 (rt=67.6027, f=2, i=430) to 2591 (rt=67.6281, f=2, i=431)
[D:\lab212\membrane\Grace\Joyce\iTRAQ_36_2.raw]
Data File:Submitted from 20120508-1(merge) by Mascot Daemon on JOYCE-VAIO
Monoisotopic mass of neutral peptide Mr(calc): 2337.247
Variable modifications:
K18 :iTRAQ4plex (K)
Ions Score: 73.28 Expect: 0.000

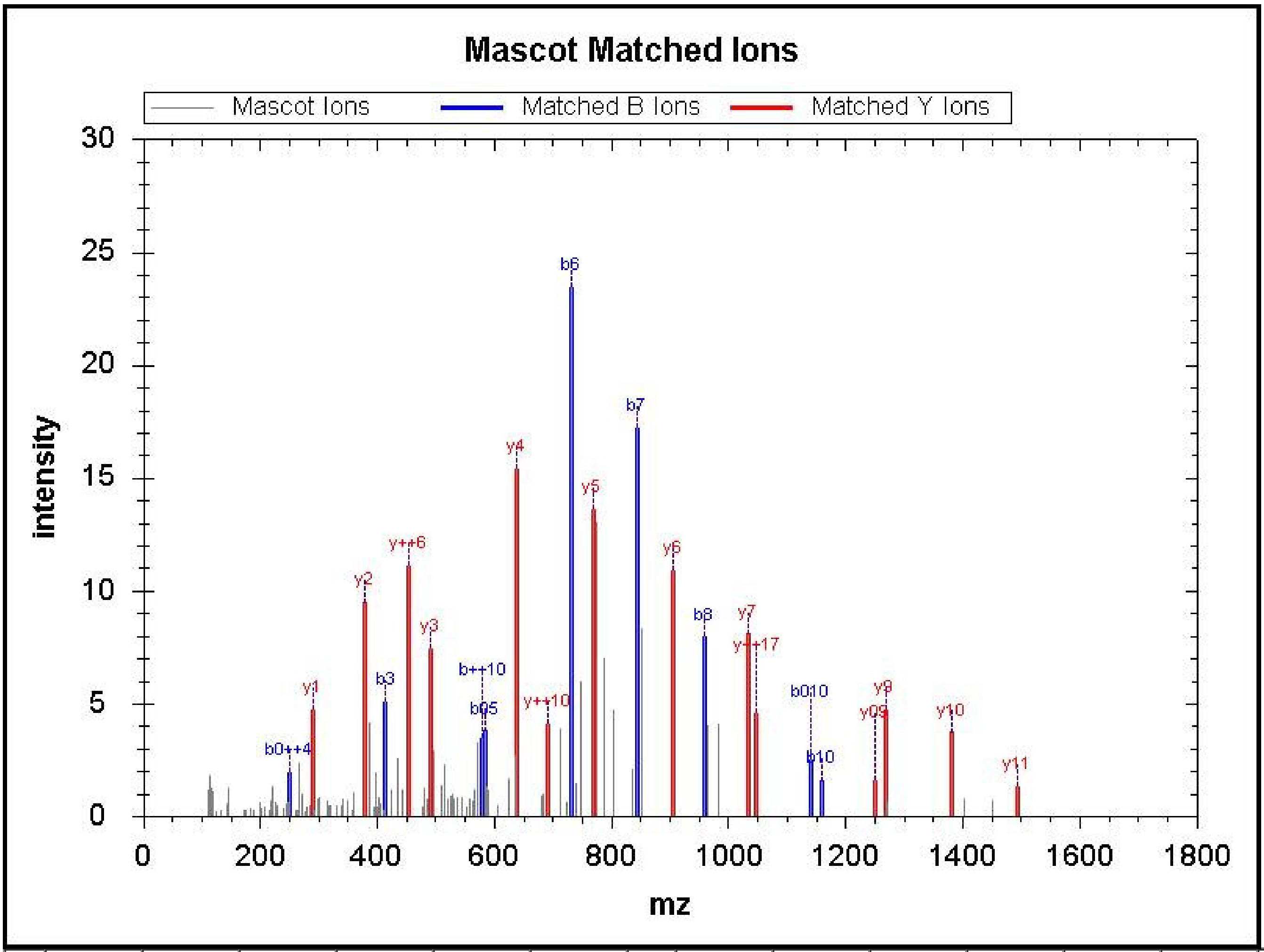

No	b	b++	b*	b*++	b0	b0++	Seq	y	y++	y*	y*++	y0	y0++	RevNo
1	244.18	122.59					V							18
2	301.20	151.10					G	2,095.08	1,048.05	2,078.06	1,039.53	2,077.07	1,039.04	17
3	414.28	207.65					L	2,038.06	1,019.53	2,021.03	1,011.02	2,020.05	1,010.53	16
4	515.33	258.17			497.32	249.16	T	1,924.98	962.99	1,907.95	954.48	1,906.97	953.99	15
5	602.36	301.69			584.35	292.68	S	1,823.93	912.47	1,806.90	903.96	1,805.92	903.46	14
6	731.41	366.21			713.40	357.20	E	1,736.90	868.95	1,719.87	860.44	1,718.89	859.95	13
7	844.49	422.75			826.48	413.74	I	1,607.85	804.43	1,590.83	795.92	1,589.84	795.43	12
8	957.57	479.29			939.56	470.29	L	1,494.77	747.89	1,477.74	739.38	1,476.76	738.88	11
9	1,071.62	536.31	1,054.59	527.80	1,053.61	527.31	N	1,381.69	691.35	1,364.66	682.83	1,363.68	682.34	10
10	1,158.65	579.83	1,141.62	571.31	1,140.64	570.82	S	1,267.64	634.33	1,250.62	625.81	1,249.63	625.32	9
11	1,305.72	653.36	1,288.69	644.85	1,287.71	644.36	F	1,180.61	590.81	1,163.59	582.30	1,162.60	581.80	8
12	1,434.76	717.88	1,417.73	709.37	1,416.75	708.88	E	1,033.54	517.28	1,016.52	508.76	1,015.53	508.27	7
13	1,571.82	786.41	1,554.79	777.90	1,553.81	777.41	H	904.50	452.75	887.47	444.24	886.49	443.75	6
14	1,700.86	850.93	1,683.83	842.42	1,682.85	841.93	E	767.44	384.22	750.42	375.71	749.43	375.22	5
15	1,847.93	924.47	1,830.90	915.96	1,829.92	915.46	F	638.40	319.70	621.37	311.19	620.39	310.70	4
16	1,961.01	981.01	1,943.99	972.50	1,943.00	972.01	L	491.33	246.17	474.30	237.66	473.32	237.16	3
17	2,048.05	1,024.53	2,031.02	1,016.01	2,030.04	1,015.52	S	378.25	189.63	361.22	181.11	360.24	180.62	2

No	b	b++	b*	b*++	b0	b0++	Seq	y	y++	y*	y*++	y0	y0++	RevNo
18							K	291.21	146.11	274.19	137.60			1

Query 76433 Hit 1

MS/MS Fragmentation of LLEEFVNHIQELQIMDER
Found in sp|O00471|EXOC5_HUMAN, Exocyst complex component 5 OS=Homo sapiens GN=EXOC5 PE=1 SV=1
Match to Query 76433: 2399.232from(800.7513,3+)
Title: 1163: Scan 2572 (rt=67.2852, f=2, i=409) [D:\lab212\membrane\Grace\Joyce\iTRAQ_33_1.raw]
Data File:Submitted from 20120508-1(merge) by Mascot Daemon on JOYCE-VAIO
Monoisotopic mass of neutral peptide Mr(calc): 2399.232
Variable modifications:
Ions Score: 72.75 Expect: 0.000

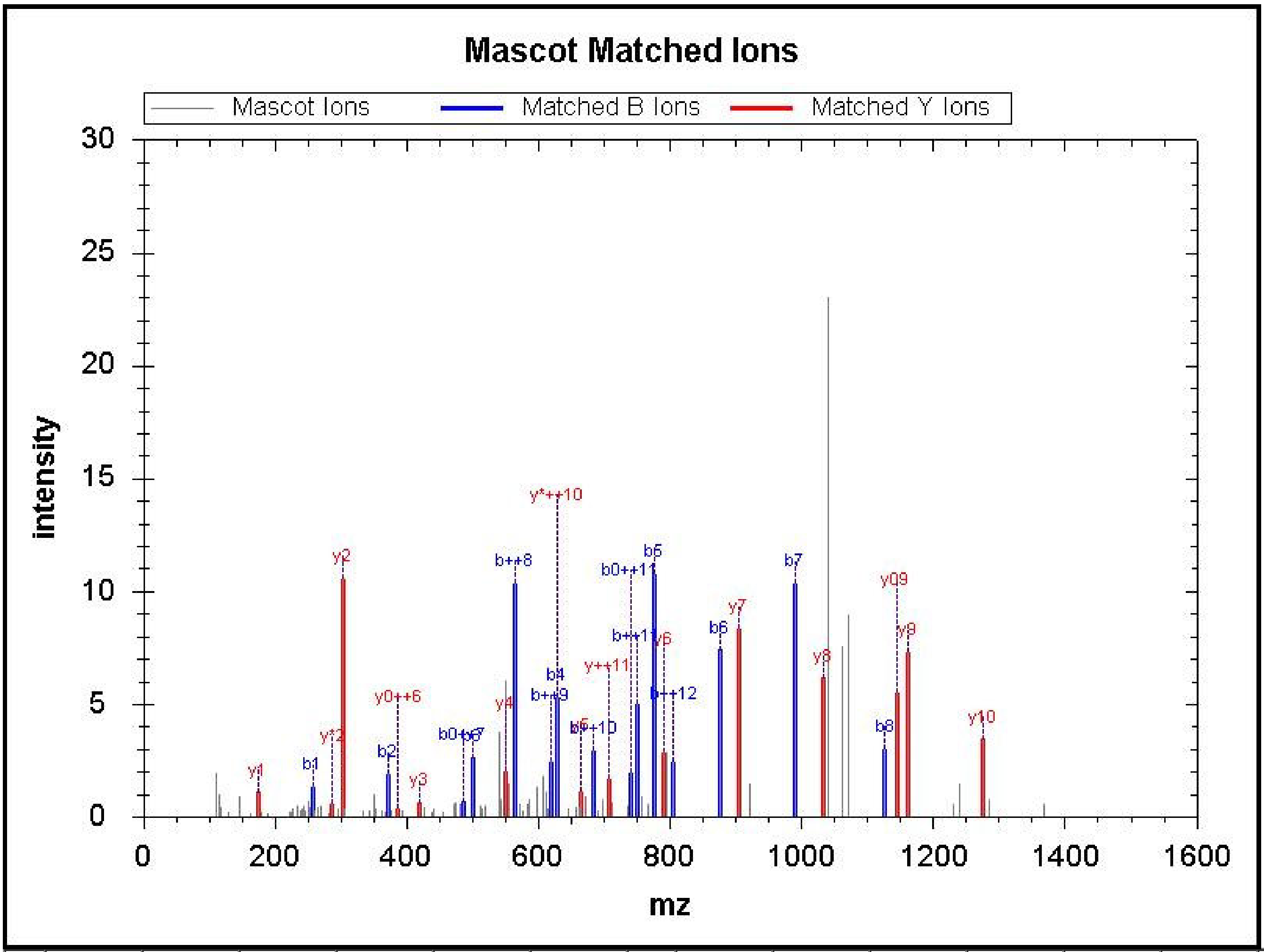

No	b	b++	b*	b*++	b0	b0++	Seq	y	y++	y*	y*++	y0	y0++	RevNo
1	258.19	129.60					L							18
2	371.28	186.14					L	2,143.05	1,072.03	2,126.02	1,063.51	2,125.04	1,063.02	17
3	500.32	250.66			482.31	241.66	E	2,029.96	1,015.49	2,012.94	1,006.97	2,011.95	1,006.48	16
4	629.36	315.18			611.35	306.18	E	1,900.92	950.96	1,883.90	942.45	1,882.91	941.96	15
5	776.43	388.72			758.42	379.71	F	1,771.88	886.44	1,754.85	877.93	1,753.87	877.44	14
6	875.50	438.25			857.49	429.25	V	1,624.81	812.91	1,607.78	804.40	1,606.80	803.90	13
7	989.54	495.27	972.52	486.76	971.53	486.27	N	1,525.74	763.38	1,508.72	754.86	1,507.73	754.37	12
8	1,126.60	563.80	1,109.57	555.29	1,108.59	554.80	H	1,411.70	706.35	1,394.67	697.84	1,393.69	697.35	11
9	1,239.69	620.35	1,222.66	611.83	1,221.67	611.34	I	1,274.64	637.82	1,257.61	629.31	1,256.63	628.82	10
10	1,367.74	684.38	1,350.72	675.86	1,349.73	675.37	Q	1,161.56	581.28	1,144.53	572.77	1,143.55	572.28	9
11	1,496.79	748.90	1,479.76	740.38	1,478.78	739.89	E	1,033.50	517.25	1,016.47	508.74	1,015.49	508.25	8
12	1,609.87	805.44	1,592.84	796.93	1,591.86	796.43	L	904.46	452.73	887.43	444.22	886.45	443.73	7
13	1,737.93	869.47	1,720.90	860.96	1,719.92	860.46	Q	791.37	396.19	774.35	387.68	773.36	387.18	6
14	1,851.01	926.01	1,833.99	917.50	1,833.00	917.01	I	663.31	332.16	646.29	323.65	645.30	323.15	5

15	1,982.05	991.53	1,965.03	983.02	1,964.04	982.53	M	550.23	275.62	533.20	267.10	532.22	266.61	4
16	2,097.08	1,049.04	2,080.05	1,040.53	2,079.07	1,040.04	D	419.19	210.10	402.16	201.58	401.18	201.09	3
17	2,226.12	1,113.57	2,209.10	1,105.05	2,208.11	1,104.56	E	304.16	152.58	287.13	144.07	286.15	143.58	2
18							R	175.12	88.06	158.09	79.55			1

Query 88597 Hit 1

MS/MS Fragmentation of DFVEAPSQMLENWVWEQEPLLR
Found in sp|P52888|THOP1_HUMAN, Thimet oligopeptidase OS=Homo sapiens GN=THOP1 PE=1 SV=2
Match to Query 88597: 2859.406from(954.1427,3+)
Title: 1204: Sum of 2 scans in range 2827 (rt=72.6365, f=4, i=784) to 2828 (rt=72.6619, f=4, i=785)
[D:\lab212\membrane\Grace\Joyce\20120320_iTRAQ_20-1.raw]
Data File:Submitted from 20120508-1(merge) by Mascot Daemon on JOYCE-VAIO
Monoisotopic mass of neutral peptide Mr(calc): 2859.406
Variable modifications:
Ions Score: 72.65 Expect: 0.000

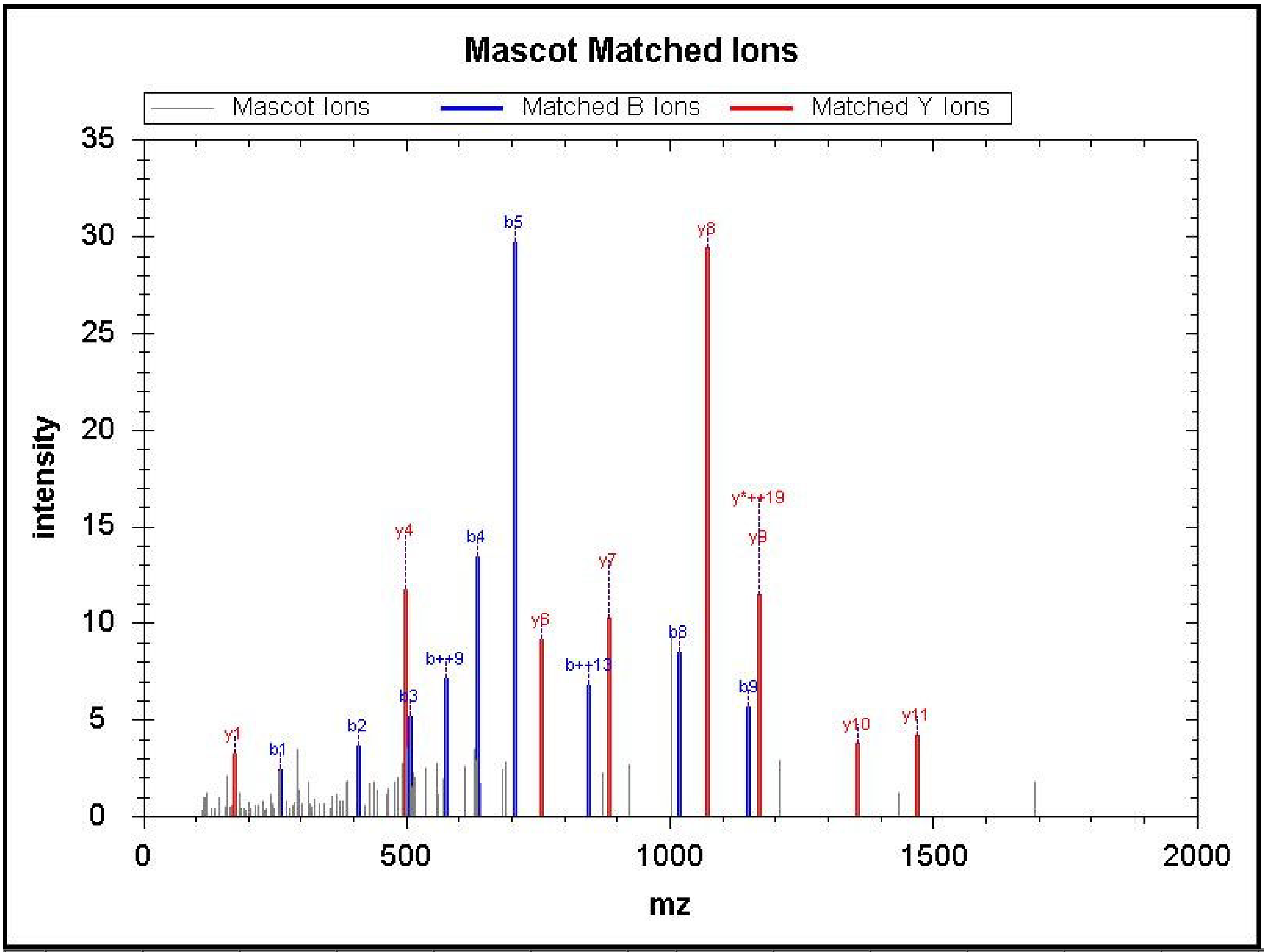

No	b	b++	b*	b*++	b0	b0++	Seq	y	y++	y*	y*++	y0	y0++	RevNo
1	260.14	130.57			242.13	121.57	D							22
2	407.20	204.11			389.19	195.10	F	2,601.28	1,301.14	2,584.25	1,292.63	2,583.27	1,292.14	21
3	506.27	253.64			488.26	244.63	V	2,454.21	1,227.61	2,437.19	1,219.10	2,436.20	1,218.60	20
4	635.32	318.16			617.31	309.16	E	2,355.14	1,178.08	2,338.12	1,169.56	2,337.13	1,169.07	19
5	706.35	353.68			688.34	344.67	A	2,226.10	1,113.55	2,209.07	1,105.04	2,208.09	1,104.55	18
6	803.41	402.21			785.40	393.20	P	2,155.06	1,078.04	2,138.04	1,069.52	2,137.05	1,069.03	17
7	890.44	445.72			872.43	436.72	S	2,058.01	1,029.51	2,040.98	1,021.00	2,040.00	1,020.50	16
8	1,018.50	509.75	1,001.47	501.24	1,000.49	500.75	Q	1,970.98	985.99	1,953.95	977.48	1,952.97	976.99	15
9	1,149.54	575.27	1,132.51	566.76	1,131.53	566.27	M	1,842.92	921.96	1,825.89	913.45	1,824.91	912.96	14
10	1,262.62	631.81	1,245.59	623.30	1,244.61	622.81	L	1,711.88	856.44	1,694.85	847.93	1,693.87	847.44	13

11	1,391.66	696.34	1,374.64	687.82	1,373.65	687.33	E	1,598.80	799.90	1,581.77	791.39	1,580.79	790.90	12
12	1,505.71	753.36	1,488.68	744.84	1,487.70	744.35	N	1,469.75	735.38	1,452.73	726.87	1,451.74	726.38	11
13	1,691.79	846.40	1,674.76	837.88	1,673.78	837.39	W	1,355.71	678.36	1,338.68	669.85	1,337.70	669.35	10
14	1,790.85	895.93	1,773.83	887.42	1,772.84	886.93	V	1,169.63	585.32	1,152.60	576.81	1,151.62	576.31	9
15	1,976.93	988.97	1,959.91	980.46	1,958.92	979.97	W	1,070.56	535.79	1,053.54	527.27	1,052.55	526.78	8
16	2,105.98	1,053.49	2,088.95	1,044.98	2,087.97	1,044.49	E	884.48	442.75	867.46	434.23	866.47	433.74	7
17	2,234.03	1,117.52	2,217.01	1,109.01	2,216.02	1,108.52	Q	755.44	378.22	738.41	369.71	737.43	369.22	6
18	2,363.08	1,182.04	2,346.05	1,173.53	2,345.07	1,173.04	E	627.38	314.19	610.36	305.68	609.37	305.19	5
19	2,460.13	1,230.57	2,443.10	1,222.06	2,442.12	1,221.56	P	498.34	249.67	481.31	241.16			4
20	2,573.21	1,287.11	2,556.19	1,278.60	2,555.20	1,278.11	L	401.29	201.15	384.26	192.63			3
21	2,686.30	1,343.65	2,669.27	1,335.14	2,668.29	1,334.65	L	288.20	144.61	271.18	136.09			2
22							R	175.12	88.06	158.09	79.55			1

Query 72806 Hit 1

MS/MS Fragmentation of DVQFAVQQVLQEEHFDAR
Found in sp|Q8R146|APEH_MOUSE, Acylamino-acid-releasing enzyme OS=Mus musculus GN=Apeh PE=2 SV=2
Match to Query 72806: 2302.143 from(768.3881,3+)
Title: 872: Sum of 2 scans in range 1927 (rt=52.8858, f=4, i=583) to 1928 (rt=52.9112, f=4, i=584) [D:\lab212\membrane\Grace\Joyce\iTRAQ_32_1.raw]
Data File:Submitted from 20120508-1(merge) by Mascot Daemon on JOYCE-VAIO
Monoisotopic mass of neutral peptide Mr(calc): 2302.143
Variable modifications:
Ions Score: 72.6 Expect: 0.000

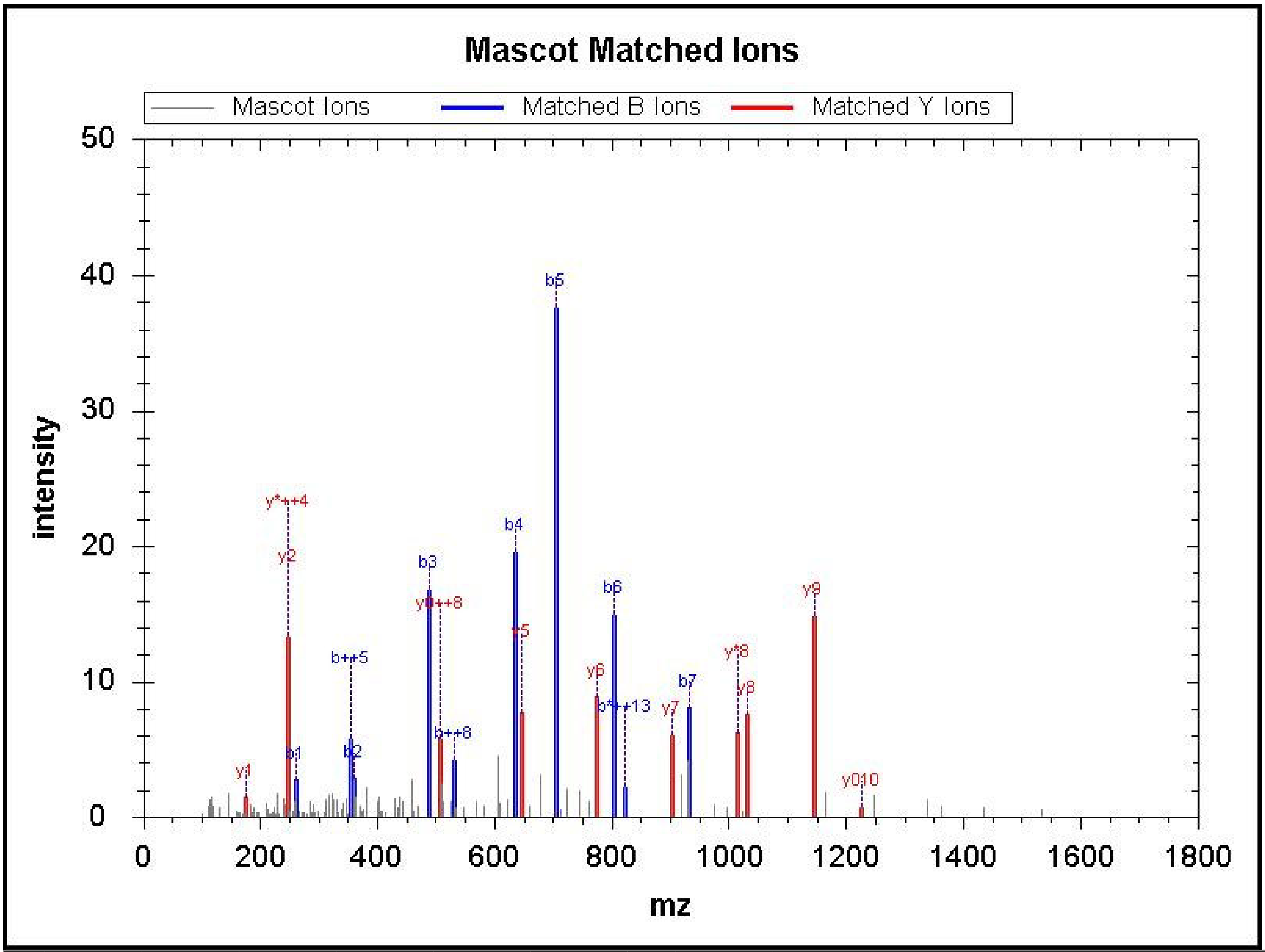

No	b	b++	b*	b*++	b0	b0++	Seq	y	y++	y*	y*++	y0	y0++	RevNo
1	260.14	130.57			242.13	121.57	D							18
2	359.20	180.11			341.19	171.10	V	2,044.02	1,022.52	2,027.00	1,014.00	2,026.01	1,013.51	17

3	487.26	244.14	470.24	235.62	469.25	235.13	Q	1,944.96	972.98	1,927.93	964.47	1,926.95	963.98	16
4	634.33	317.67	617.31	309.16	616.32	308.66	F	1,816.90	908.95	1,799.87	900.44	1,798.89	899.95	15
5	705.37	353.19	688.34	344.67	687.36	344.18	A	1,669.83	835.42	1,652.80	826.91	1,651.82	826.41	14
6	804.44	402.72	787.41	394.21	786.43	393.72	V	1,598.79	799.90	1,581.77	791.39	1,580.78	790.89	13
7	932.50	466.75	915.47	458.24	914.49	457.75	Q	1,499.72	750.37	1,482.70	741.85	1,481.71	741.36	12
8	1,060.55	530.78	1,043.53	522.27	1,042.54	521.78	Q	1,371.67	686.34	1,354.64	677.82	1,353.65	677.33	11
9	1,159.62	580.32	1,142.60	571.80	1,141.61	571.31	V	1,243.61	622.31	1,226.58	613.79	1,225.60	613.30	10
10	1,272.71	636.86	1,255.68	628.34	1,254.70	627.85	L	1,144.54	572.77	1,127.51	564.26	1,126.53	563.77	9
11	1,400.77	700.89	1,383.74	692.37	1,382.75	691.88	Q	1,031.45	516.23	1,014.43	507.72	1,013.44	507.23	8
12	1,529.81	765.41	1,512.78	756.89	1,511.80	756.40	E	903.40	452.20	886.37	443.69	885.38	443.20	7
13	1,658.85	829.93	1,641.82	821.42	1,640.84	820.92	E	774.35	387.68	757.33	379.17	756.34	378.67	6
14	1,795.91	898.46	1,778.88	889.95	1,777.90	889.45	H	645.31	323.16	628.28	314.65	627.30	314.15	5
15	1,942.98	971.99	1,925.95	963.48	1,924.97	962.99	F	508.25	254.63	491.22	246.12	490.24	245.62	4
16	2,058.00	1,029.51	2,040.98	1,020.99	2,039.99	1,020.50	D	361.18	181.10	344.16	172.58	343.17	172.09	3
17	2,129.04	1,065.02	2,112.02	1,056.51	2,111.03	1,056.02	A	246.16	123.58	229.13	115.07			2
18							R	175.12	88.06	158.09	79.55			1

Query 64045 Hit 1

MS/MS Fragmentation of SAFLLQNLLVGHPEHK
Found in sp|Q9NZL4|HPBP1_HUMAN, Hsp70-binding protein 1 OS=Homo sapiens GN=HSPBP1 PE=1 SV=1
Match to Query 64045: 2090.196from(697.7391,3+)
Title: 652: Scan 1803 (rt=48.8846, f=2, i=316) [D:\lab212\membrane\Grace\Joyce\iTRAQ_48_1.raw]
Data File:Submitted from 20120508-1(merge) by Mascot Daemon on JOYCE-VAIO
Monoisotopic mass of neutral peptide Mr(calc): 2090.196
Variable modifications:
K16 :iTRAQ4plex (K)
Ions Score: 72.32 Expect: 0.000

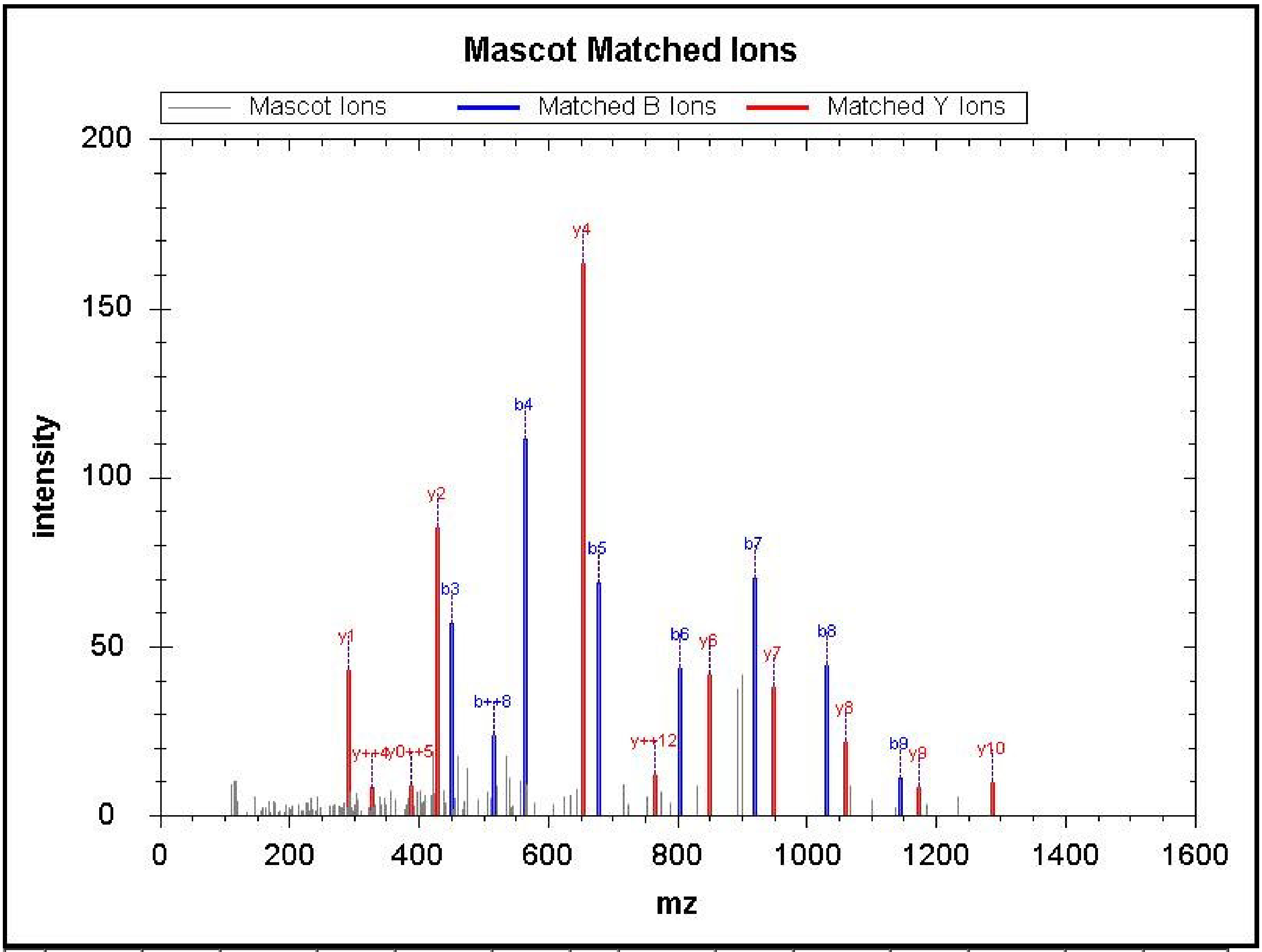

No	b	b++	b*	b*++	b0	b0++	Seq	y	y++	y*	y*++	y0	y0++	RevNo
1	232.14	116.57			214.13	107.57	S							16
2	303.18	152.09			285.17	143.09	A	1,860.06	930.53	1,843.04	922.02	1,842.05	921.53	15
3	450.25	225.63			432.24	216.62	F	1,789.02	895.02	1,772.00	886.50	1,771.01	886.01	14
4	563.33	282.17			545.32	273.16	L	1,641.96	821.48	1,624.93	812.97	1,623.95	812.48	13
5	676.41	338.71			658.40	329.71	L	1,528.87	764.94	1,511.85	756.43	1,510.86	755.93	12
6	804.47	402.74	787.45	394.23	786.46	393.74	Q	1,415.79	708.40	1,398.76	699.88	1,397.78	699.39	11
7	918.52	459.76	901.49	451.25	900.51	450.76	N	1,287.73	644.37	1,270.70	635.85	1,269.72	635.36	10
8	1,031.60	516.30	1,014.57	507.79	1,013.59	507.30	L	1,173.69	587.35	1,156.66	578.83	1,155.68	578.34	9
9	1,144.68	572.85	1,127.66	564.33	1,126.67	563.84	L	1,060.60	530.80	1,043.58	522.29	1,042.59	521.80	8
10	1,243.75	622.38	1,226.73	613.87	1,225.74	613.37	V	947.52	474.26	930.49	465.75	929.51	465.26	7
11	1,300.77	650.89	1,283.75	642.38	1,282.76	641.89	G	848.45	424.73	831.42	416.22	830.44	415.72	6
12	1,437.83	719.42	1,420.81	710.91	1,419.82	710.42	H	791.43	396.22	774.40	387.70	773.42	387.21	5
13	1,534.89	767.95	1,517.86	759.43	1,516.88	758.94	P	654.37	327.69	637.34	319.17	636.36	318.68	4
14	1,663.93	832.47	1,646.90	823.95	1,645.92	823.46	E	557.32	279.16	540.29	270.65	539.31	270.16	3
15	1,800.99	901.00	1,783.96	892.48	1,782.98	891.99	H	428.27	214.64	411.25	206.13			2
16							K	291.21	146.11	274.19	137.60			1

Query 72306 Hit 1

MS/MS Fragmentation of VLVHPPQDGEDEPTLVQK
Found in sp|Q16740|CLPP_HUMAN, Putative ATP-dependent Clp protease proteolytic subunit
Match to Query 72306: 2288.217from(763.7464,3+)
Title: 436: Sum of 2 scans in range 1038 (rt=32.7903, f=4, i=291) to 1039 (rt=32.8157, f=4, i=292) [D:\lab212\membrane\Grace\Joyce\iTRAQ_36_1.raw]
Data File:Submitted from 20120508-1(merge) by Mascot Daemon on JOYCE-VAIO
Monoisotopic mass of neutral peptide Mr(calc): 2288.217

Variable modifications:
K18 :iTRAQ4plex (K)
Ions Score: 72.24 Expect: 0.000

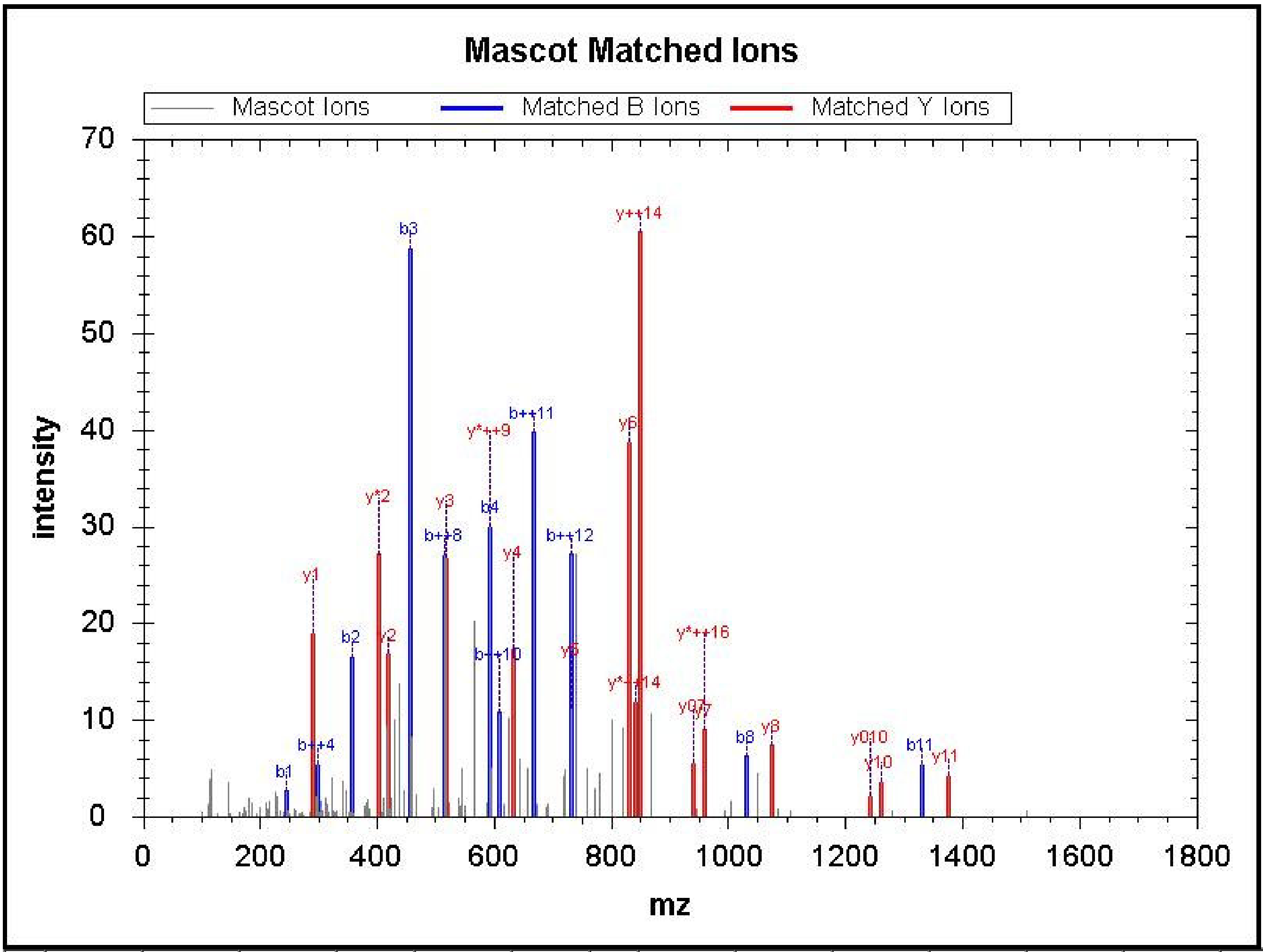

No	b	b++	b*	b*++	b0	b0++	Seq	y	y++	y*	y*++	y0	y0++	RevNo
1	244.18	122.59					V							18
2	357.26	179.13					L	2,046.06	1,023.53	2,029.04	1,015.02	2,028.05	1,014.53	17
3	456.33	228.67					V	1,932.98	966.99	1,915.95	958.48	1,914.97	957.99	16
4	593.39	297.20					H	1,833.91	917.46	1,816.88	908.95	1,815.90	908.45	15
5	690.44	345.72					P	1,696.85	848.93	1,679.82	840.42	1,678.84	839.92	14
6	787.49	394.25					P	1,599.80	800.40	1,582.77	791.89	1,581.79	791.40	13
7	915.55	458.28	898.53	449.77			Q	1,502.75	751.88	1,485.72	743.36	1,484.73	742.87	12
8	1,030.58	515.79	1,013.55	507.28	1,012.57	506.79	D	1,374.69	687.85	1,357.66	679.33	1,356.68	678.84	11
9	1,087.60	544.30	1,070.58	535.79	1,069.59	535.30	G	1,259.66	630.33	1,242.63	621.82	1,241.65	621.33	10
10	1,216.64	608.83	1,199.62	600.31	1,198.63	599.82	E	1,202.64	601.82	1,185.61	593.31	1,184.63	592.82	9
11	1,331.67	666.34	1,314.64	657.83	1,313.66	657.33	D	1,073.60	537.30	1,056.57	528.79	1,055.59	528.30	8
12	1,460.71	730.86	1,443.69	722.35	1,442.70	721.86	E	958.57	479.79	941.54	471.27	940.56	470.78	7
13	1,557.77	779.39	1,540.74	770.87	1,539.76	770.38	P	829.53	415.27	812.50	406.75	811.52	406.26	6
14	1,658.81	829.91	1,641.79	821.40	1,640.80	820.91	T	732.47	366.74	715.45	358.23	714.46	357.74	5
15	1,771.90	886.45	1,754.87	877.94	1,753.89	877.45	L	631.43	316.22	614.40	307.70			4
16	1,870.97	935.99	1,853.94	927.47	1,852.96	926.98	V	518.34	259.67	501.32	251.16			3
17	1,999.03	1,000.02	1,982.00	991.50	1,981.01	991.01	Q	419.27	210.14	402.25	201.63			2
18							K	291.21	146.11	274.19	137.60			1

Query 12562 Hit 1

MS/MS Fragmentation of SMLALLLGR

Found in sp|Q9BTE7|DCNL5_HUMAN, DCN1-like protein 5 OS=Homo sapiens GN=DCUN1D5 PE=1 SV=1
Match to Query 12562: 1116.685from(559.3495,2+)
Title: 856: Sum of 2 scans in range 2517 (rt=62.9506, f=4, i=555) to 2518 (rt=62.976, f=4, i=556)
[D:\lab212\membrane\Grace\Joyce\iTRAQ_24_2.raw]
Data File:Submitted from 20120508-1(merge) by Mascot Daemon on JOYCE-VAIO
Monoisotopic mass of neutral peptide Mr(calc): 1116.685
Variable modifications:
Ions Score: 71.9 Expect: 0.000

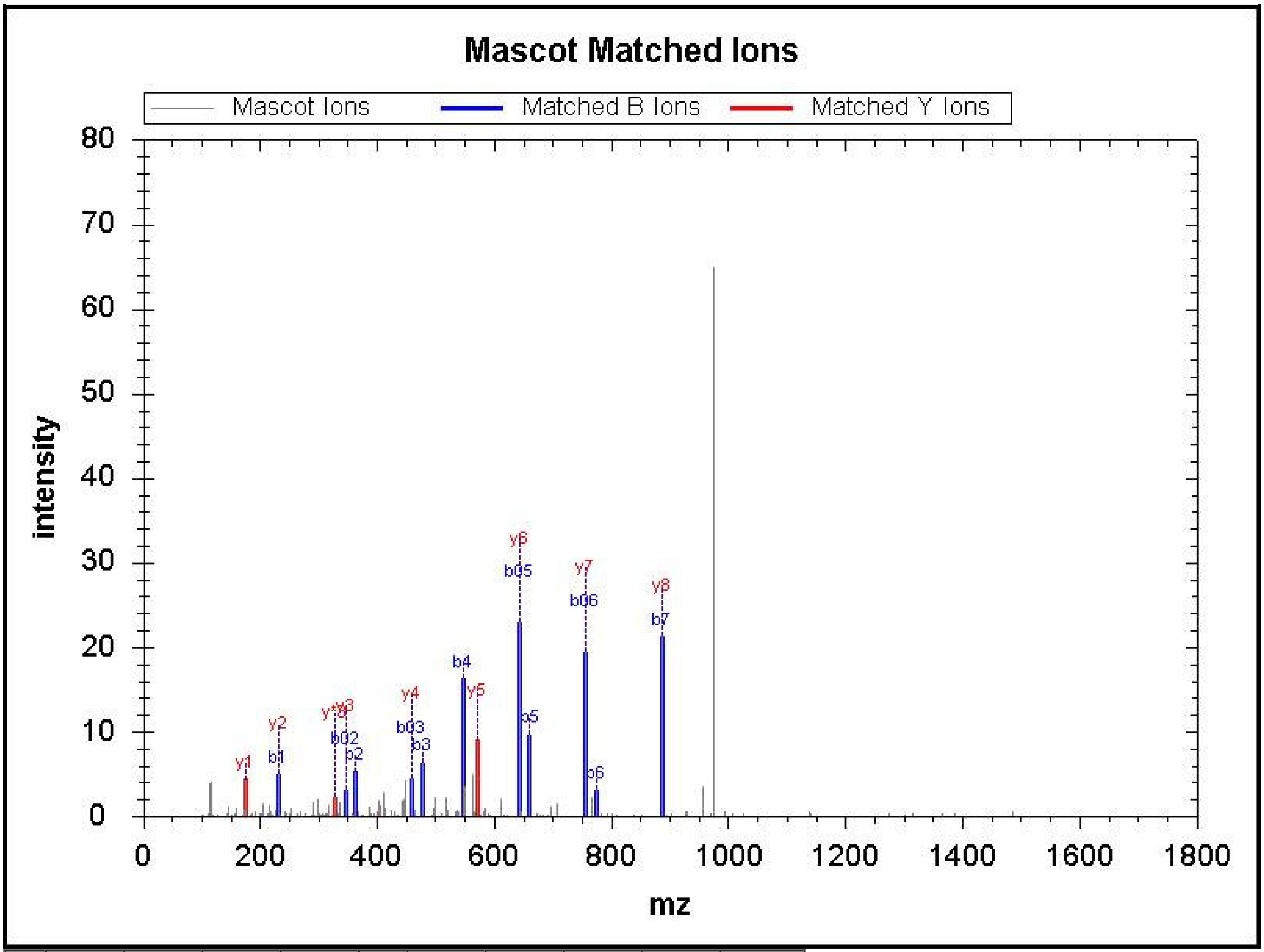

No	b	b++	b0	b0++	Seq	y	y++	y*	y*++	RevNo
1	232.14	116.57	214.13	107.57	S					9
2	363.18	182.09	345.17	173.09	M	886.55	443.78	869.53	435.27	8
3	476.27	238.64	458.26	229.63	L	755.51	378.26	738.49	369.75	7
4	547.30	274.16	529.29	265.15	A	642.43	321.72	625.40	313.21	6
5	660.39	330.70	642.38	321.69	L	571.39	286.20	554.37	277.69	5
6	773.47	387.24	755.46	378.23	L	458.31	229.66	441.28	221.14	4
7	886.56	443.78	868.54	434.78	L	345.22	173.12	328.20	164.60	3
8	943.58	472.29	925.57	463.29	G	232.14	116.57	215.11	108.06	2
9					R	175.12	88.06	158.09	79.55	1

Query 57782 Hit 1

MS/MS Fragmentation of NDSLAGVVIADNEYPSR
Found in sp|O15498|YKT6_HUMAN, Synaptobrevin homolog YKT6 OS=Homo sapiens GN=YKT6 PE=1 SV=1
Match to Query 57782: 1962.98from(655.3339,3+)
Title: 280: Sum of 2 scans in range 1540 (rt=40.9315, f=4, i=216) to 1541 (rt=40.957, f=4, i=217)
[D:\lab212\membrane\Grace\Joyce\iTRAQ_19_2_330.raw]
Data File:Submitted from 20120508-1(merge) by Mascot Daemon on JOYCE-VAIO
Monoisotopic mass of neutral peptide Mr(calc): 1962.98
Variable modifications:

Ions Score: 71.7 Expect: 0.000

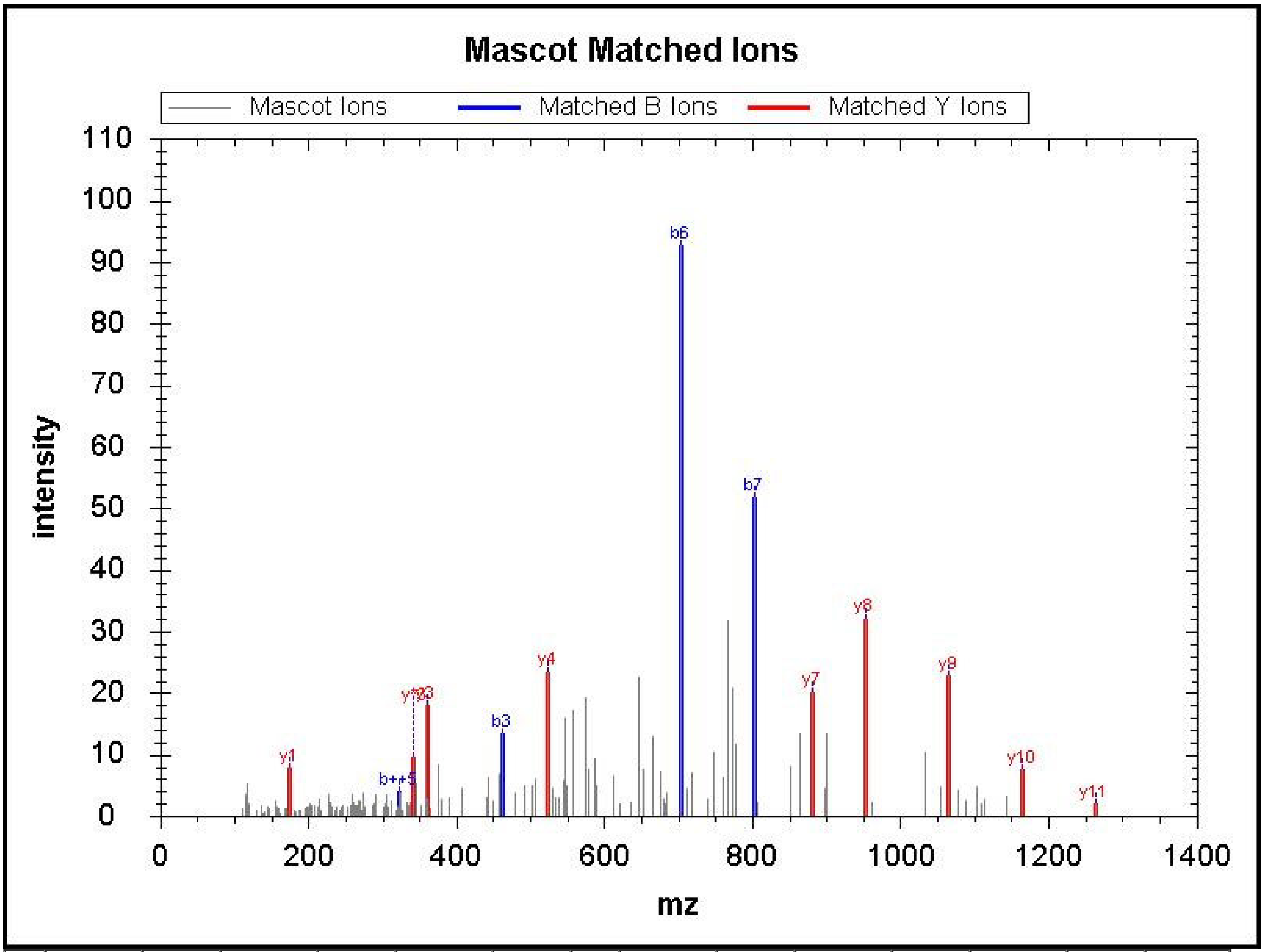

No	b	b++	b*	b*++	b0	b0++	Seq	y	y++	y*	y*++	y0	y0++	RevNo
1	259.15	130.08	242.13	121.57			N							17
2	374.18	187.59	357.15	179.08	356.17	178.59	D	1,705.84	853.42	1,688.81	844.91	1,687.83	844.42	16
3	461.21	231.11	444.18	222.60	443.20	222.10	S	1,590.81	795.91	1,573.79	787.40	1,572.80	786.90	15
4	574.30	287.65	557.27	279.14	556.28	278.65	L	1,503.78	752.39	1,486.75	743.88	1,485.77	743.39	14
5	645.33	323.17	628.31	314.66	627.32	314.16	A	1,390.70	695.85	1,373.67	687.34	1,372.69	686.85	13
6	702.35	351.68	685.33	343.17	684.34	342.68	G	1,319.66	660.33	1,302.63	651.82	1,301.65	651.33	12
7	801.42	401.21	784.40	392.70	783.41	392.21	V	1,262.64	631.82	1,245.61	623.31	1,244.63	622.82	11
8	900.49	450.75	883.46	442.24	882.48	441.74	V	1,163.57	582.29	1,146.54	573.77	1,145.56	573.28	10
9	1,013.57	507.29	996.55	498.78	995.56	498.29	I	1,064.50	532.75	1,047.47	524.24	1,046.49	523.75	9
10	1,084.61	542.81	1,067.59	534.30	1,066.60	533.80	A	951.42	476.21	934.39	467.70	933.41	467.21	8
11	1,199.64	600.32	1,182.61	591.81	1,181.63	591.32	D	880.38	440.69	863.35	432.18	862.37	431.69	7
12	1,313.68	657.34	1,296.66	648.83	1,295.67	648.34	N	765.35	383.18	748.33	374.67	747.34	374.17	6
13	1,442.72	721.87	1,425.70	713.35	1,424.71	712.86	E	651.31	326.16	634.28	317.65	633.30	317.15	5
14	1,605.79	803.40	1,588.76	794.88	1,587.78	794.39	Y	522.27	261.64	505.24	253.12	504.26	252.63	4
15	1,702.84	851.92	1,685.81	843.41	1,684.83	842.92	P	359.20	180.11	342.18	171.59	341.19	171.10	3
16	1,789.87	895.44	1,772.85	886.93	1,771.86	886.43	S	262.15	131.58	245.12	123.07	244.14	122.57	2
17							R	175.12	88.06	158.09	79.55			1

Query 74286 Hit 1

MS/MS Fragmentation of QLLGHMEDLEGDAQSVLDAR
Found in sp|Q9NX46|ARHL2_HUMAN, Poly(ADP-ribose) glycohydrolase ARH3 OS=Homo sapiens GN=ADPRHL2 PE=1 SV=1
Match to Query 74286: 2340.152from(781.058,3+)
Title: 846: Scan 1900 (rt=52.1799, f=2, i=296) [D:\lab212\membrane\Grace\Joyce\iTRAQ_33_1.raw]

Data File:Submitted from 20120508-1(merge) by Mascot Daemon on JOYCE-VAIO
Monoisotopic mass of neutral peptide Mr(calc): 2340.152
Variable modifications:
Ions Score: 71.62 Expect: 0.000

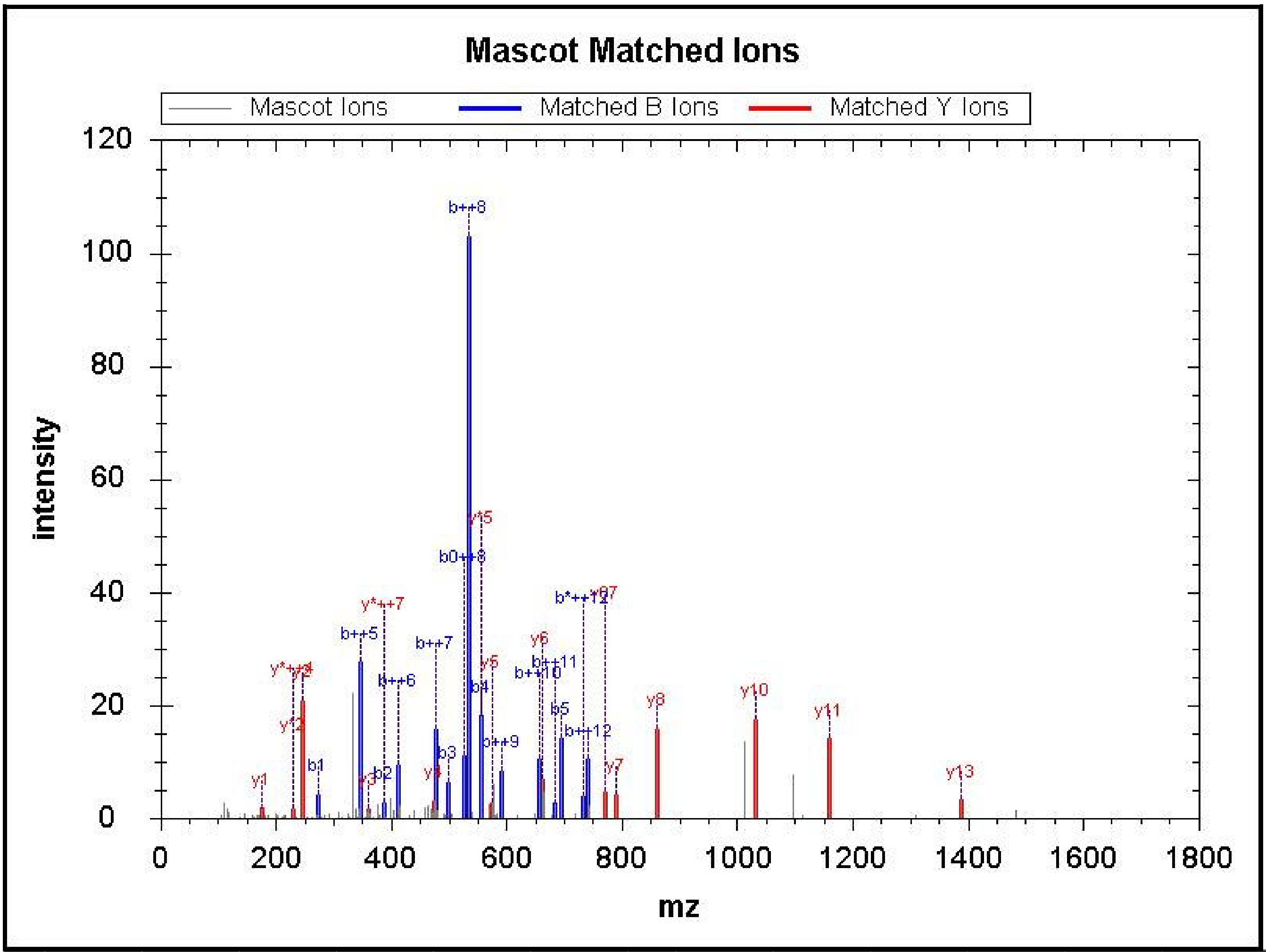

No	b	b++	b*	b*++	b0	b0++	Seq	y	y++	y*	y*++	y0	y0++	RevNo
1	273.17	137.09	256.14	128.57			Q							20
2	386.25	193.63	369.23	185.12			L	2,069.00	1,035.00	2,051.97	1,026.49	2,050.99	1,026.00	19
3	499.34	250.17	482.31	241.66			L	1,955.91	978.46	1,938.89	969.95	1,937.90	969.45	18
4	556.36	278.68	539.33	270.17			G	1,842.83	921.92	1,825.80	913.40	1,824.82	912.91	17
5	693.42	347.21	676.39	338.70			H	1,785.81	893.41	1,768.78	884.89	1,767.80	884.40	16
6	824.46	412.73	807.43	404.22			M	1,648.75	824.88	1,631.72	816.36	1,630.74	815.87	15
7	953.50	477.25	936.47	468.74	935.49	468.25	E	1,517.71	759.36	1,500.68	750.84	1,499.70	750.35	14
8	1,068.53	534.77	1,051.50	526.25	1,050.52	525.76	D	1,388.67	694.84	1,371.64	686.32	1,370.65	685.83	13
9	1,181.61	591.31	1,164.58	582.80	1,163.60	582.30	L	1,273.64	637.32	1,256.61	628.81	1,255.63	628.32	12
10	1,310.65	655.83	1,293.63	647.32	1,292.64	646.82	E	1,160.55	580.78	1,143.53	572.27	1,142.54	571.78	11
11	1,367.67	684.34	1,350.65	675.83	1,349.66	675.34	G	1,031.51	516.26	1,014.49	507.75	1,013.50	507.25	10
12	1,482.70	741.85	1,465.68	733.34	1,464.69	732.85	D	974.49	487.75	957.46	479.24	956.48	478.74	9
13	1,553.74	777.37	1,536.71	768.86	1,535.73	768.37	A	859.46	430.24	842.44	421.72	841.45	421.23	8
14	1,681.80	841.40	1,664.77	832.89	1,663.79	832.40	Q	788.43	394.72	771.40	386.20	770.42	385.71	7
15	1,768.83	884.92	1,751.80	876.41	1,750.82	875.91	S	660.37	330.69	643.34	322.17	642.36	321.68	6
16	1,867.90	934.45	1,850.87	925.94	1,849.89	925.45	V	573.34	287.17	556.31	278.66	555.32	278.17	5
17	1,980.98	990.99	1,963.96	982.48	1,962.97	981.99	L	474.27	237.64	457.24	229.12	456.26	228.63	4
18	2,096.01	1,048.51	2,078.98	1,039.99	2,078.00	1,039.50	D	361.18	181.10	344.16	172.58	343.17	172.09	3
19	2,167.05	1,084.03	2,150.02	1,075.51	2,149.04	1,075.02	A	246.16	123.58	229.13	115.07			2
20							R	175.12	88.06	158.09	79.55			1
