# Supplementary material for: Quantitative Proteomic Analysis of Human Lung Tumor Xenografts Treated with the Ectopic ATP Synthase Inhibitor Citreoviridin
Source: PLoS One. 2013 Aug 21;8(8):e70642. doi: 10.1371/journal.pone.0070642 (PMC3749231; doi:10.1371/journal.pone.0070642)
Supplement: Spectra S5 — The information of single-peptide-based protein identifications in the large-scale experiment (part 3). (PDF) [file pone.0070642.s022.pdf]

Spectra S5: The information of single-peptide-based protein identifications in the large-scale experiment (part 3).

Query 54670 Hit 1

MS/MS Fragmentation of EGHLPDLLSMIHIER
Found in sp|Q92536|YLAT2_HUMAN, Y+L amino acid transporter 2 OS=Homo sapiens GN=SLC7A6 PE=1 SV=3
Match to Query 54670: 1903.011 from(476.76,4+)
Title: 922: Sum of 2 scans in range 2096 (rt=56.6834, f=4, i=636) to 2097 (rt=56.7089, f=4, i=637) [D:\lab212\membrane\Grace\Joyce\47_1.raw]
Data File:Submitted from 20120508-1(merge) by Mascot Daemon on JOYCE-VAIO
Monoisotopic mass of neutral peptide Mr(calc): 1903.011
Variable modifications:
Ions Score: 70.93 Expect: 0.000

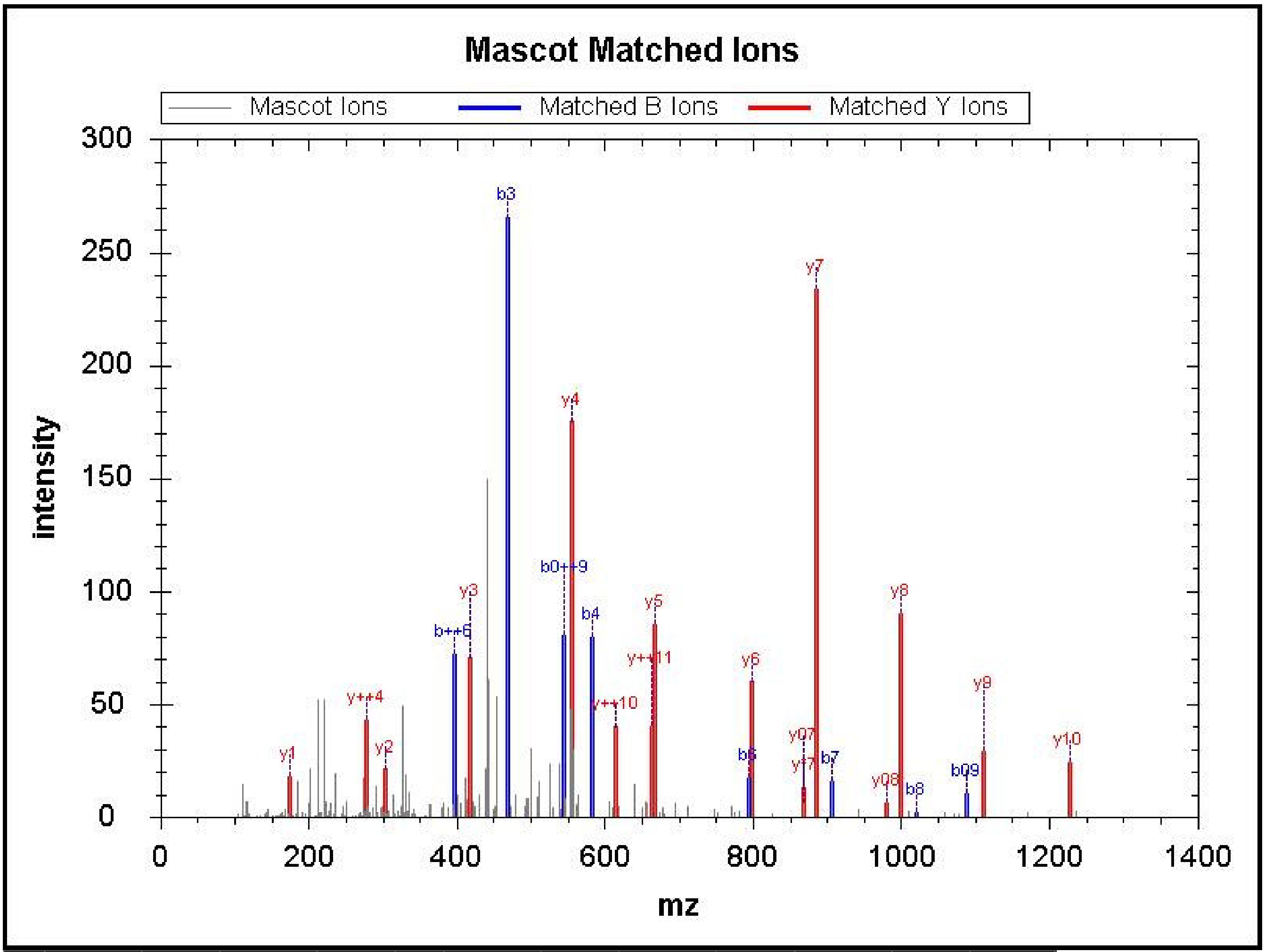

No	b	b++	b0	b0++	Seq	y	y++	y*	y*++	y0	y0++	RevNo
1	274.15	137.58	256.14	128.57	E							15
2	331.17	166.09	313.16	157.09	G	1,630.87	815.94	1,613.85	807.43	1,612.86	806.94	14
3	468.23	234.62	450.22	225.61	H	1,573.85	787.43	1,556.83	778.92	1,555.84	778.42	13
4	581.32	291.16	563.31	282.16	L	1,436.79	718.90	1,419.77	710.39	1,418.78	709.89	12
5	678.37	339.69	660.36	330.68	P	1,323.71	662.36	1,306.68	653.84	1,305.70	653.35	11
6	793.40	397.20	775.39	388.20	D	1,226.66	613.83	1,209.63	605.32	1,208.65	604.83	10
7	906.48	453.74	888.47	444.74	L	1,111.63	556.32	1,094.60	547.80	1,093.62	547.31	9
8	1,019.56	510.29	1,001.55	501.28	L	998.55	499.78	981.52	491.26	980.53	490.77	8
9	1,106.60	553.80	1,088.59	544.80	S	885.46	443.23	868.43	434.72	867.45	434.23	7
10	1,237.64	619.32	1,219.63	610.32	M	798.43	399.72	781.40	391.20	780.42	390.71	6
11	1,350.72	675.86	1,332.71	666.86	I	667.39	334.20	650.36	325.68	649.38	325.19	5
12	1,487.78	744.39	1,469.77	735.39	H	554.30	277.66	537.28	269.14	536.29	268.65	4
13	1,600.86	800.94	1,582.85	791.93	I	417.25	209.13	400.22	200.61	399.24	200.12	3
14	1,729.91	865.46	1,711.90	856.45	E	304.16	152.58	287.13	144.07	286.15	143.58	2

15					R	175.12	88.06	158.09	79.55			1

Query 85369 Hit 1

MS/MS Fragmentation of LDAVSEVLHSESSVFGQIENHLR
Found in sp|P20585|MSH3_HUMAN, DNA mismatch repair protein Msh3 OS=Homo sapiens GN=MSH3 PE=1 SV=4
Match to Query 85369: 2709.388from(904.1367,3+)
Title: 1122: Scan 2424 (rt=64.3459, f=2, i=393) [D:\lab212\membrane\Grace\Joyce\iTRAQ_43_1.raw]
Data File:Submitted from 20120508-1(merge) by Mascot Daemon on JOYCE-VAIO
Monoisotopic mass of neutral peptide Mr(calc): 2709.388
Variable modifications:
Ions Score: 70.85 Expect: 0.000

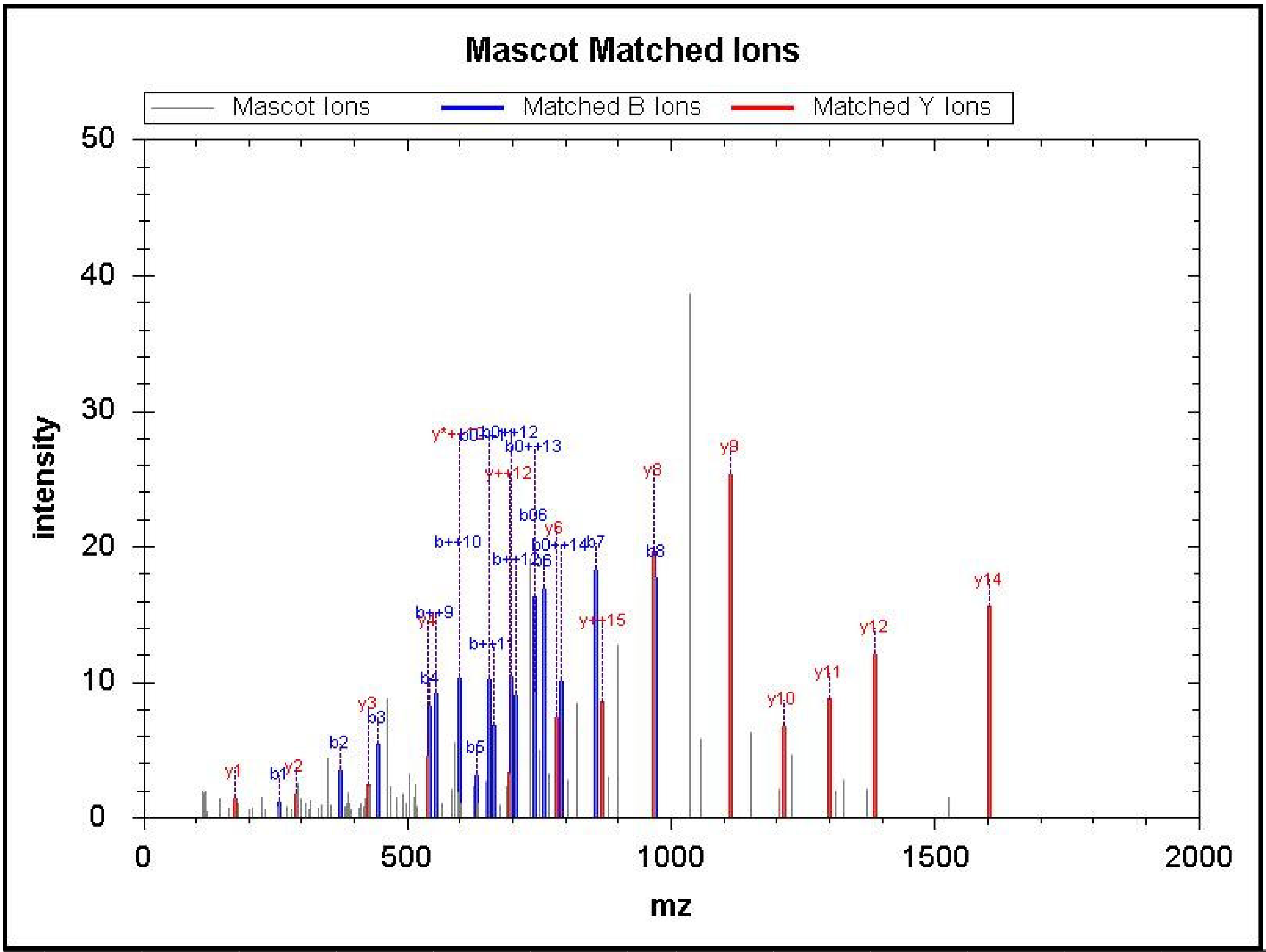

No	b	b++	b*	b*++	b0	b0++	Seq	y	y++	y*	y*++	y0	y0++	RevNo
1	258.19	129.60					L							23
2	373.22	187.11			355.21	178.11	D	2,453.21	1,227.11	2,436.18	1,218.59	2,435.19	1,218.10	22
3	444.26	222.63			426.25	213.63	A	2,338.18	1,169.59	2,321.15	1,161.08	2,320.17	1,160.59	21
4	543.33	272.17			525.32	263.16	V	2,267.14	1,134.07	2,250.11	1,125.56	2,249.13	1,125.07	20
5	630.36	315.68			612.35	306.68	S	2,168.07	1,084.54	2,151.05	1,076.03	2,150.06	1,075.53	19
6	759.40	380.20			741.39	371.20	E	2,081.04	1,041.02	2,064.01	1,032.51	2,063.03	1,032.02	18
7	858.47	429.74			840.46	420.73	V	1,952.00	976.50	1,934.97	967.99	1,933.99	967.50	17
8	971.55	486.28			953.54	477.27	L	1,852.93	926.97	1,835.90	918.46	1,834.92	917.96	16
9	1,108.61	554.81			1,090.60	545.80	H	1,739.85	870.43	1,722.82	861.91	1,721.84	861.42	15
10	1,195.64	598.33			1,177.63	589.32	S	1,602.79	801.90	1,585.76	793.38	1,584.78	792.89	14
11	1,324.69	662.85			1,306.68	653.84	E	1,515.75	758.38	1,498.73	749.87	1,497.74	749.38	13
12	1,411.72	706.36			1,393.71	697.36	S	1,386.71	693.86	1,369.69	685.35	1,368.70	684.85	12
13	1,498.75	749.88			1,480.74	740.87	S	1,299.68	650.34	1,282.65	641.83	1,281.67	641.34	11

14	1,597.82	799.41			1,579.81	790.41	V	1,212.65	606.83	1,195.62	598.31	1,194.64	597.82	10
15	1,744.89	872.95			1,726.88	863.94	F	1,113.58	557.29	1,096.55	548.78	1,095.57	548.29	9
16	1,801.91	901.46			1,783.90	892.45	G	966.51	483.76	949.48	475.25	948.50	474.75	8
17	1,929.97	965.49	1,912.94	956.97	1,911.96	956.48	Q	909.49	455.25	892.46	446.74	891.48	446.24	7
18	2,043.05	1,022.03	2,026.02	1,013.52	2,025.04	1,013.02	I	781.43	391.22	764.40	382.71	763.42	382.21	6
19	2,172.09	1,086.55	2,155.07	1,078.04	2,154.08	1,077.55	E	668.35	334.68	651.32	326.16	650.34	325.67	5
20	2,286.14	1,143.57	2,269.11	1,135.06	2,268.13	1,134.57	N	539.30	270.16	522.28	261.64			4
21	2,423.20	1,212.10	2,406.17	1,203.59	2,405.19	1,203.10	H	425.26	213.13	408.24	204.62			3
22	2,536.28	1,268.64	2,519.25	1,260.13	2,518.27	1,259.64	L	288.20	144.61	271.18	136.09			2
23							R	175.12	88.06	158.09	79.55			1

Query 70304 Hit 1

MS/MS Fragmentation of ERDEDDEDGDGDGDGATGK
Found in sp|P50579|AMPM2_HUMAN, Methionine aminopeptidase 2 OS=Homo sapiens GN=METAP2 PE=1 SV=1
Match to Query 70304: 2239.923from(747.6484,3+)
Title: 24: Sum of 2 scans in range 149 (rt=12.9478, f=4, i=12) to 150 (rt=12.9732, f=4, i=13)
[D:\lab212\membrane\Grace\Joyce\iTRAQ_44_2.raw]
Data File:Submitted from 20120508-1(merge) by Mascot Daemon on JOYCE-VAIO
Monoisotopic mass of neutral peptide Mr(calc): 2239.923
Variable modifications:
K19 :iTRAQ4plex (K)
Ions Score: 70.2 Expect: 0.000

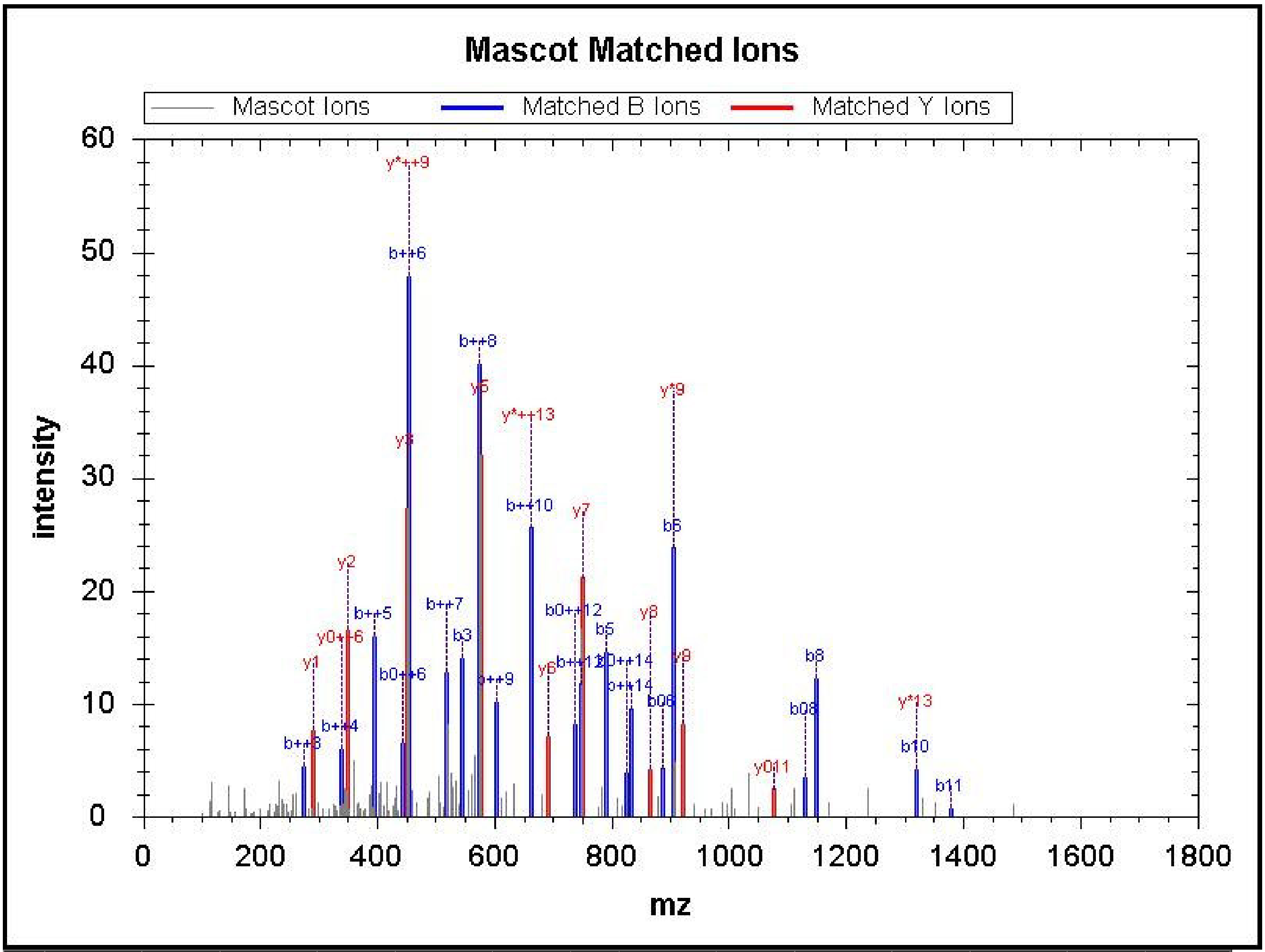

No	b	b++	b*	b*++	b0	b0++	Seq	y	y++	y*	y*++	y0	y0++	RevNo
1	274.15	137.58			256.14	128.57	E							19
2	430.25	215.63	413.23	207.12	412.24	206.62	R	1,967.78	984.39	1,950.76	975.88	1,949.77	975.39	18
3	545.28	273.14	528.25	264.63	527.27	264.14	D	1,811.68	906.34	1,794.65	897.83	1,793.67	897.34	17

4	674.32	337.66	657.30	329.15	656.31	328.66	E	1,696.65	848.83	1,679.63	840.32	1,678.64	839.83	16
5	789.35	395.18	772.32	386.67	771.34	386.17	D	1,567.61	784.31	1,550.58	775.80	1,549.60	775.30	15
6	904.38	452.69	887.35	444.18	886.37	443.69	D	1,452.58	726.80	1,435.56	718.28	1,434.57	717.79	14
7	1,033.42	517.21	1,016.39	508.70	1,015.41	508.21	E	1,337.56	669.28	1,320.53	660.77	1,319.55	660.28	13
8	1,148.45	574.73	1,131.42	566.21	1,130.44	565.72	D	1,208.51	604.76	1,191.49	596.25	1,190.50	595.76	12
9	1,205.47	603.24	1,188.44	594.72	1,187.46	594.23	G	1,093.49	547.25	1,076.46	538.73	1,075.48	538.24	11
10	1,320.49	660.75	1,303.47	652.24	1,302.48	651.75	D	1,036.47	518.74	1,019.44	510.22	1,018.46	509.73	10
11	1,377.52	689.26	1,360.49	680.75	1,359.51	680.26	G	921.44	461.22	904.41	452.71	903.43	452.22	9
12	1,492.54	746.78	1,475.52	738.26	1,474.53	737.77	D	864.42	432.71	847.39	424.20	846.41	423.71	8
13	1,549.56	775.29	1,532.54	766.77	1,531.55	766.28	G	749.39	375.20	732.36	366.69	731.38	366.19	7
14	1,664.59	832.80	1,647.56	824.29	1,646.58	823.79	D	692.37	346.69	675.34	338.18	674.36	337.68	6
15	1,721.61	861.31	1,704.59	852.80	1,703.60	852.30	G	577.34	289.17	560.32	280.66	559.33	280.17	5
16	1,792.65	896.83	1,775.62	888.32	1,774.64	887.82	A	520.32	260.66	503.29	252.15	502.31	251.66	4
17	1,893.70	947.35	1,876.67	938.84	1,875.69	938.35	T	449.28	225.15	432.26	216.63	431.27	216.14	3
18	1,950.72	975.86	1,933.69	967.35	1,932.71	966.86	G	348.24	174.62	331.21	166.11			2
19							K	291.21	146.11	274.19	137.60			1

Query 60599 Hit 1

MS/MS Fragmentation of IPEEFNVFNLIQEMR
Found in sp|Q05209|PTN12_HUMAN, Tyrosine-protein phosphatase non-receptor type 12 OS=Homo sapiens GN=PTPN12 PE=1 SV=3
Match to Query 60599: 2022.039from(1012.027,2+)
Title: 1261: Sum of 2 scans in range 2881 (rt=74.0959, f=4, i=845) to 2882 (rt=74.1214, f=4, i=846) [D:\lab212\membrane\Grace\Joyce\iTRAQ_21_2.raw]
Data File:Submitted from 20120508-1(merge) by Mascot Daemon on JOYCE-VAIO
Monoisotopic mass of neutral peptide Mr(calc): 2022.039
Variable modifications:
Ions Score: 70.06 Expect: 0.000

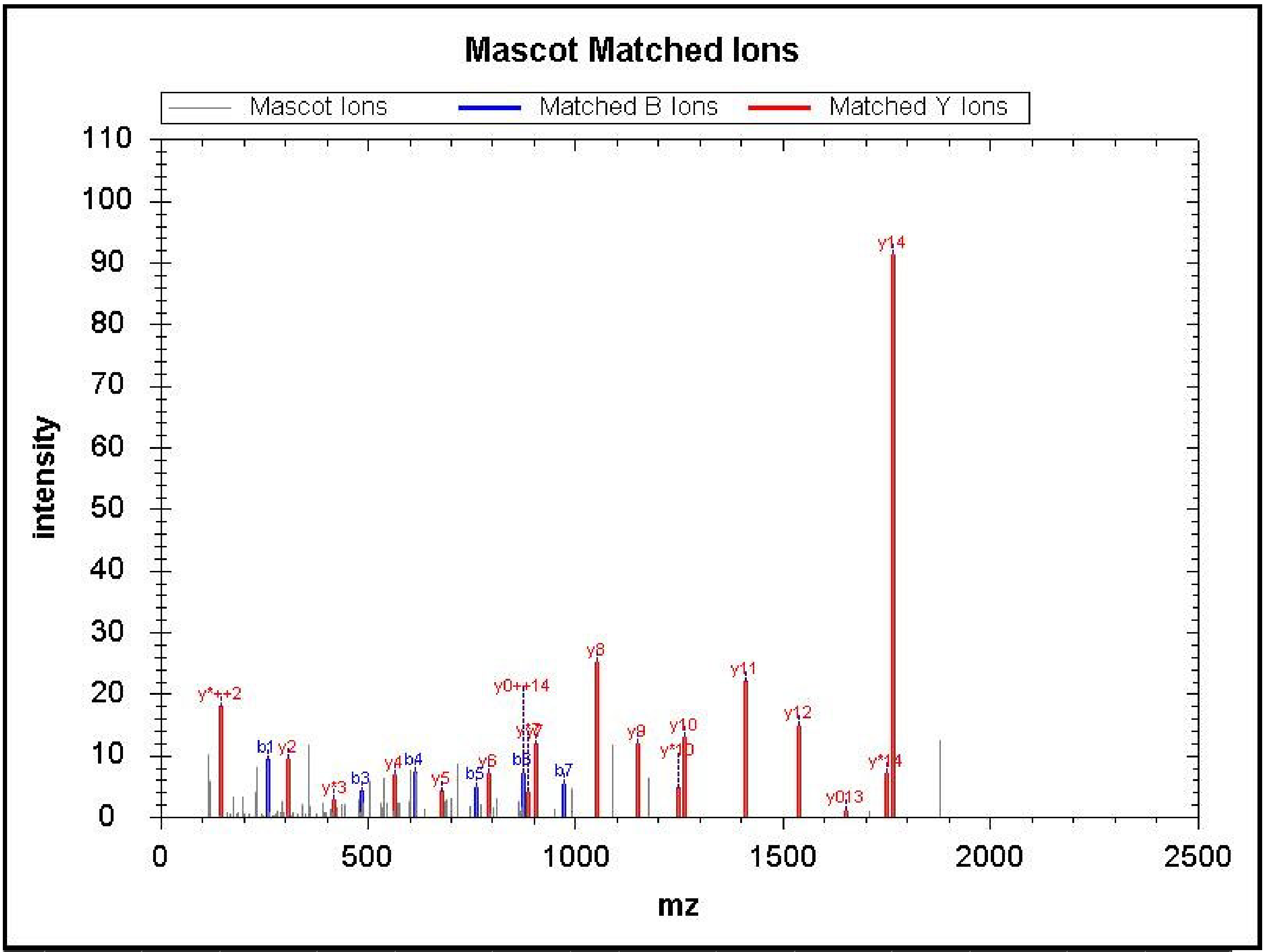

No	b	b++	b*	b*++	b0	b0++	Seq	y	y++	y*	y*++	y0	y0++	RevNo
1	258.19	129.60					I							15
2	355.25	178.13					P	1,765.86	883.43	1,748.83	874.92	1,747.85	874.43	14
3	484.29	242.65			466.28	233.64	E	1,668.80	834.91	1,651.78	826.39	1,650.79	825.90	13
4	613.33	307.17			595.32	298.16	E	1,539.76	770.38	1,522.74	761.87	1,521.75	761.38	12
5	760.40	380.70			742.39	371.70	F	1,410.72	705.86	1,393.69	697.35	1,392.71	696.86	11
6	874.44	437.72	857.42	429.21	856.43	428.72	N	1,263.65	632.33	1,246.62	623.82	1,245.64	623.32	10
7	973.51	487.26	956.48	478.75	955.50	478.25	V	1,149.61	575.31	1,132.58	566.79	1,131.60	566.30	9
8	1,120.58	560.79	1,103.55	552.28	1,102.57	551.79	F	1,050.54	525.77	1,033.51	517.26	1,032.53	516.77	8
9	1,234.62	617.81	1,217.60	609.30	1,216.61	608.81	N	903.47	452.24	886.45	443.73	885.46	443.23	7
10	1,347.71	674.36	1,330.68	665.84	1,329.70	665.35	L	789.43	395.22	772.40	386.70	771.42	386.21	6
11	1,460.79	730.90	1,443.76	722.39	1,442.78	721.89	I	676.34	338.68	659.32	330.16	658.33	329.67	5
12	1,588.85	794.93	1,571.82	786.42	1,570.84	785.92	Q	563.26	282.13	546.23	273.62	545.25	273.13	4
13	1,717.89	859.45	1,700.87	850.94	1,699.88	850.44	E	435.20	218.10	418.18	209.59	417.19	209.10	3
14	1,848.93	924.97	1,831.91	916.46	1,830.92	915.96	M	306.16	153.58	289.13	145.07			2
15							R	175.12	88.06	158.09	79.55			1

Query 73526 Hit 1

MS/MS Fragmentation of DVEGQDVVEAILAHLNTVPR
Found in sp|Q9UNS1|TIM_HUMAN, Protein timeless homolog OS=Homo sapiens GN=TIMELESS PE=1 SV=2
Match to Query 73526: 2318.232from(773.7515,3+)
Title: 1113: Sum of 2 scans in range 2514 (rt=65.7559, f=3, i=381) to 2515 (rt=65.7813, f=3, i=382) [D:\lab212\membrane\Grace\Joyce\iTRAQ_30_1.raw]
Data File:Submitted from 20120508-1(merge) by Mascot Daemon on JOYCE-VAIO
Monoisotopic mass of neutral peptide Mr(calc): 2318.232
Variable modifications:

Ions Score: 69.97 Expect: 0.000

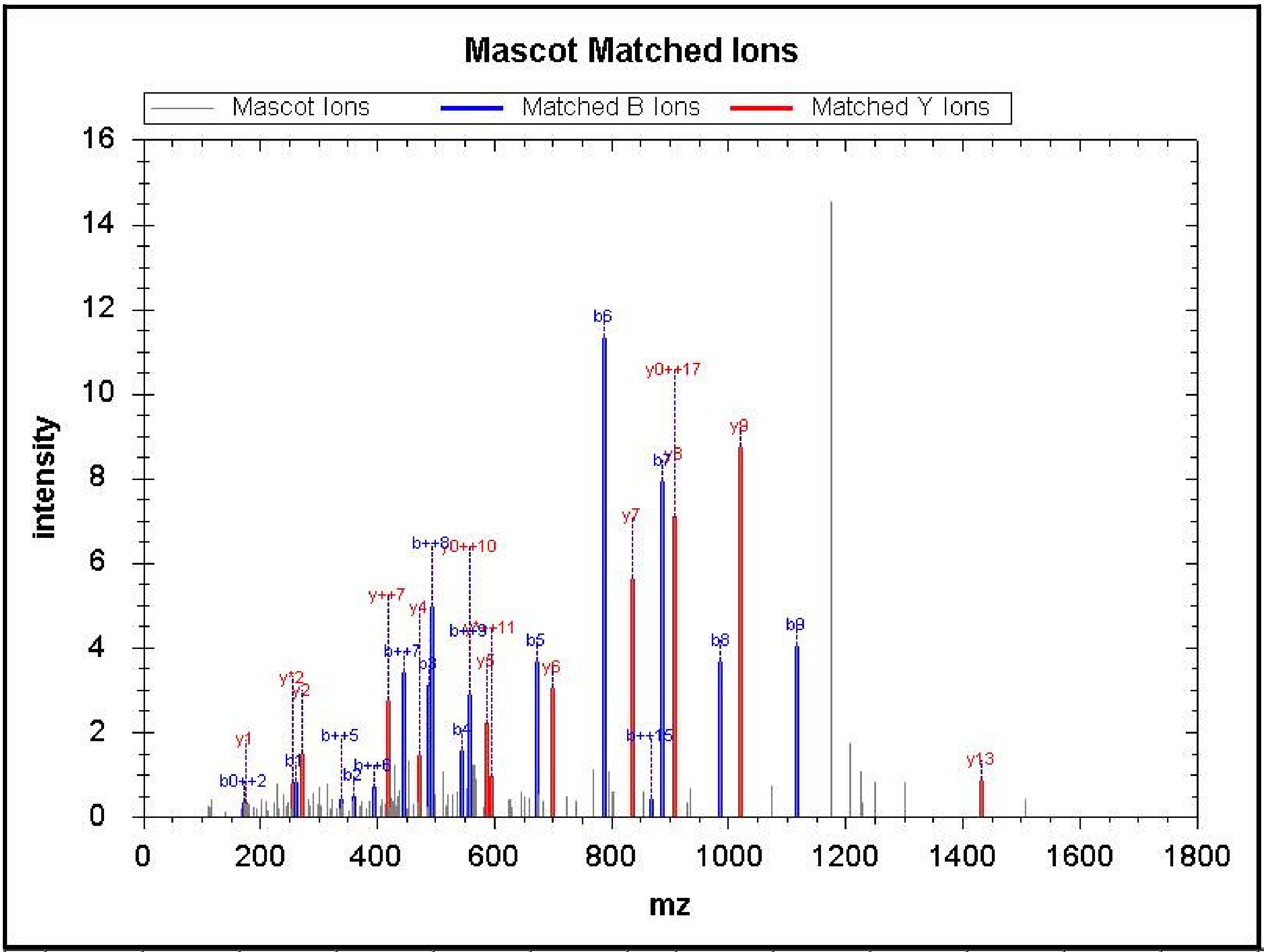

No	b	b++	b*	b*++	b0	b0++	Seq	y	y++	y*	y*++	y0	y0++	RevNo
1	260.14	130.57			242.13	121.57	D							20
2	359.20	180.11			341.19	171.10	V	2,060.11	1,030.56	2,043.09	1,022.05	2,042.10	1,021.55	19
3	488.25	244.63			470.24	235.62	E	1,961.04	981.03	1,944.02	972.51	1,943.03	972.02	18
4	545.27	273.14			527.26	264.13	G	1,832.00	916.50	1,814.98	907.99	1,813.99	907.50	17
5	673.33	337.17	656.30	328.65	655.32	328.16	Q	1,774.98	887.99	1,757.95	879.48	1,756.97	878.99	16
6	788.35	394.68	771.33	386.17	770.34	385.68	D	1,646.92	823.96	1,629.90	815.45	1,628.91	814.96	15
7	887.42	444.21	870.40	435.70	869.41	435.21	V	1,531.90	766.45	1,514.87	757.94	1,513.88	757.45	14
8	986.49	493.75	969.46	485.24	968.48	484.74	V	1,432.83	716.92	1,415.80	708.40	1,414.82	707.91	13
9	1,115.53	558.27	1,098.51	549.76	1,097.52	549.27	E	1,333.76	667.38	1,316.73	658.87	1,315.75	658.38	12
10	1,186.57	593.79	1,169.54	585.28	1,168.56	584.78	A	1,204.72	602.86	1,187.69	594.35	1,186.71	593.86	11
11	1,299.65	650.33	1,282.63	641.82	1,281.64	641.33	I	1,133.68	567.34	1,116.65	558.83	1,115.67	558.34	10
12	1,412.74	706.87	1,395.71	698.36	1,394.73	697.87	L	1,020.59	510.80	1,003.57	502.29	1,002.58	501.80	9
13	1,483.78	742.39	1,466.75	733.88	1,465.77	733.39	A	907.51	454.26	890.48	445.75	889.50	445.25	8
14	1,620.84	810.92	1,603.81	802.41	1,602.82	801.92	H	836.47	418.74	819.45	410.23	818.46	409.74	7
15	1,733.92	867.46	1,716.89	858.95	1,715.91	858.46	L	699.41	350.21	682.39	341.70	681.40	341.21	6
16	1,847.96	924.48	1,830.94	915.97	1,829.95	915.48	N	586.33	293.67	569.30	285.16	568.32	284.66	5
17	1,949.01	975.01	1,931.98	966.50	1,931.00	966.00	T	472.29	236.65	455.26	228.13	454.28	227.64	4
18	2,048.08	1,024.54	2,031.05	1,016.03	2,030.07	1,015.54	V	371.24	186.12	354.21	177.61			3
19	2,145.13	1,073.07	2,128.10	1,064.56	2,127.12	1,064.06	P	272.17	136.59	255.15	128.08			2
20							R	175.12	88.06	158.09	79.55			1

Query 96608 Hit 1

MS/MS Fragmentation of EKEKEDDEEEEDEDASGGDQDQEER
Found in sp|O60216|RAD21_HUMAN, Double-strand-break repair protein rad21 homolog OS=Homo sapiens GN=RAD21 PE=1 SV=2
Match to Query 96608: 3371.417from(843.8616,4+)
Title: 6: Sum of 2 scans in range 281 (rt=15.0451, f=2, i=25) to 282 (rt=15.0705, f=2, i=26) [D:\lab212\membrane\Grace\Joyce\iTRAQ_51_2.raw]
Data File:Submitted from 20120508-1(merge) by Mascot Daemon on JOYCE-VAIO
Monoisotopic mass of neutral peptide Mr(calc): 3371.417
Variable modifications:
K2 :iTRAQ4plex (K)
K4 :iTRAQ4plex (K)
Ions Score: 69.77 Expect: 0.000

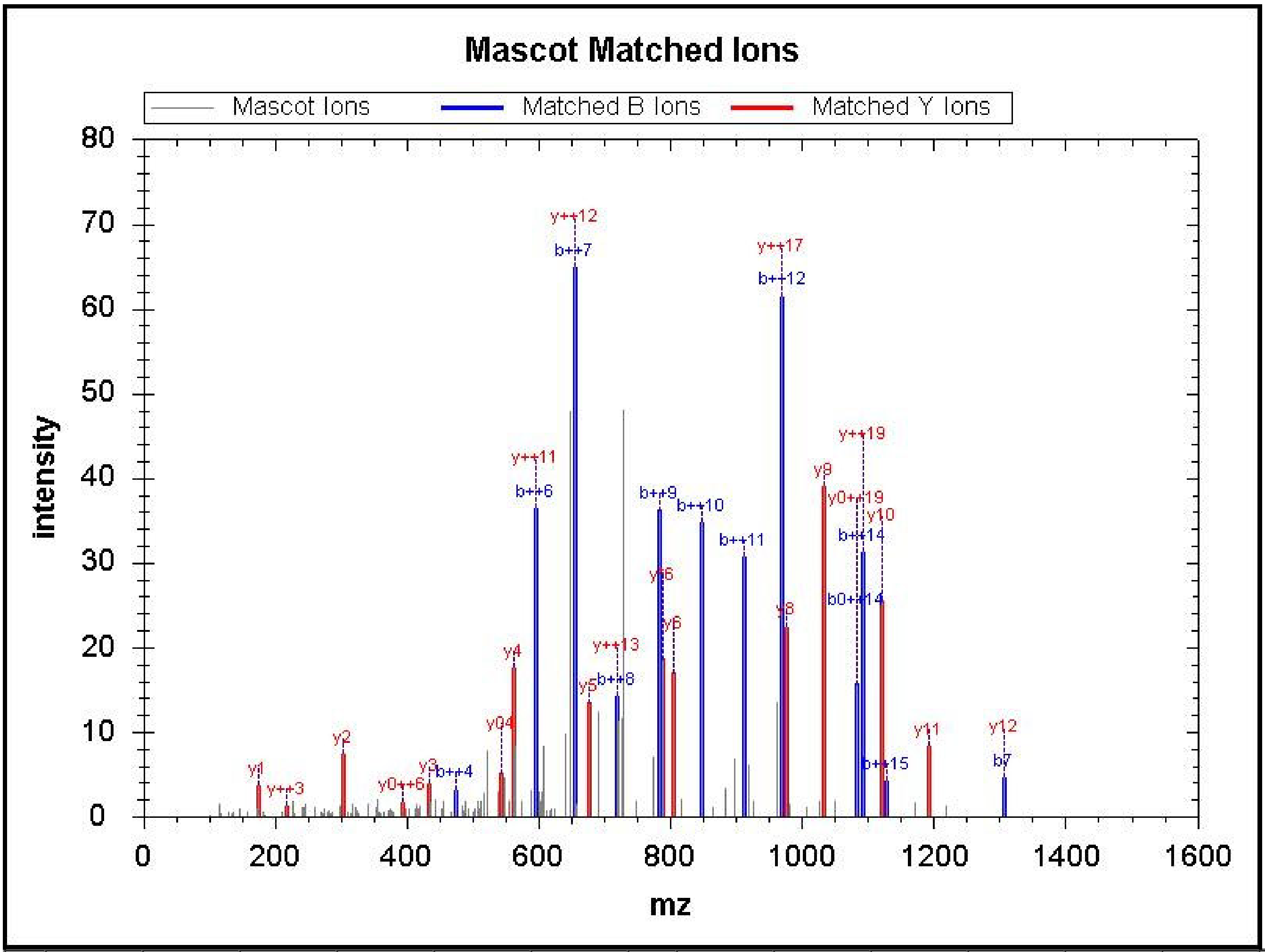

No	b	b++	b*	b*++	b0	b0++	Seq	y	y++	y*	y*++	y0	y0++	RevNo
1	274.15	137.58			256.14	128.57	E							25
2	546.35	273.68	529.32	265.16	528.34	264.67	K	3,099.29	1,550.15	3,082.26	1,541.63	3,081.28	1,541.14	24
3	675.39	338.20	658.36	329.69	657.38	329.19	E	2,827.09	1,414.05	2,810.06	1,405.54	2,809.08	1,405.04	23
4	947.59	474.30	930.56	465.78	929.58	465.29	K	2,698.05	1,349.53	2,681.02	1,341.01	2,680.04	1,340.52	22
5	1,076.63	538.82	1,059.60	530.31	1,058.62	529.81	E	2,425.85	1,213.43	2,408.82	1,204.92	2,407.84	1,204.42	21
6	1,191.66	596.33	1,174.63	587.82	1,173.65	587.33	D	2,296.81	1,148.91	2,279.78	1,140.39	2,278.80	1,139.90	20
7	1,306.69	653.85	1,289.66	645.33	1,288.67	644.84	D	2,181.78	1,091.39	2,164.75	1,082.88	2,163.77	1,082.39	19
8	1,435.73	718.37	1,418.70	709.85	1,417.72	709.36	E	2,066.75	1,033.88	2,049.73	1,025.37	2,048.74	1,024.88	18
9	1,564.77	782.89	1,547.74	774.38	1,546.76	773.88	E	1,937.71	969.36	1,920.69	960.85	1,919.70	960.35	17
10	1,693.81	847.41	1,676.79	838.90	1,675.80	838.40	E	1,808.67	904.84	1,791.64	896.32	1,790.66	895.83	16
11	1,822.86	911.93	1,805.83	903.42	1,804.84	902.93	E	1,679.63	840.32	1,662.60	831.80	1,661.62	831.31	15
12	1,937.88	969.44	1,920.86	960.93	1,919.87	960.44	D	1,550.58	775.80	1,533.56	767.28	1,532.57	766.79	14
13	2,066.93	1,033.97	2,049.90	1,025.45	2,048.91	1,024.96	E	1,435.56	718.28	1,418.53	709.77	1,417.55	709.28	13
14	2,181.95	1,091.48	2,164.93	1,082.97	2,163.94	1,082.47	D	1,306.51	653.76	1,289.49	645.25	1,288.50	644.76	12

15	2,252.99	1,127.00	2,235.96	1,118.48	2,234.98	1,117.99	A	1,191.49	596.25	1,174.46	587.73	1,173.48	587.24	11
16	2,340.02	1,170.51	2,322.99	1,162.00	2,322.01	1,161.51	S	1,120.45	560.73	1,103.42	552.22	1,102.44	551.72	10
17	2,397.04	1,199.02	2,380.02	1,190.51	2,379.03	1,190.02	G	1,033.42	517.21	1,016.39	508.70	1,015.41	508.21	9
18	2,454.06	1,227.54	2,437.04	1,219.02	2,436.05	1,218.53	G	976.40	488.70	959.37	480.19	958.39	479.70	8
19	2,569.09	1,285.05	2,552.06	1,276.54	2,551.08	1,276.04	D	919.38	460.19	902.35	451.68	901.36	451.19	7
20	2,697.15	1,349.08	2,680.12	1,340.57	2,679.14	1,340.07	Q	804.35	402.68	787.32	394.16	786.34	393.67	6
21	2,812.18	1,406.59	2,795.15	1,398.08	2,794.17	1,397.59	D	676.29	338.65	659.26	330.14	658.28	329.64	5
22	2,940.23	1,470.62	2,923.21	1,462.11	2,922.22	1,461.62	Q	561.26	281.13	544.24	272.62	543.25	272.13	4
23	3,069.28	1,535.14	3,052.25	1,526.63	3,051.27	1,526.14	E	433.20	217.11	416.18	208.59	415.19	208.10	3
24	3,198.32	1,599.66	3,181.29	1,591.15	3,180.31	1,590.66	E	304.16	152.58	287.13	144.07	286.15	143.58	2
25							R	175.12	88.06	158.09	79.55			1

Query 60047 Hit 1

MS/MS Fragmentation of GDAFDFNVALQDHFK
Found in sp|Q9NVZ3|NECP2_HUMAN, Adaptin ear-binding coat-associated protein 2 OS=Homo sapiens GN=NECAP2 PE=1 SV=1
Match to Query 60047: 2011.009from(671.3436,3+)
Title: 765: Scan 1715 (rt=48.1393, f=3, i=256) [D:\lab212\membrane\Grace\Joyce\iTRAQ_39_1.raw]
Data File:Submitted from 20120508-1(merge) by Mascot Daemon on JOYCE-VAIO
Monoisotopic mass of neutral peptide Mr(calc): 2011.009
Variable modifications:
K15 :iTRAQ4plex (K)
Ions Score: 69.76 Expect: 0.000

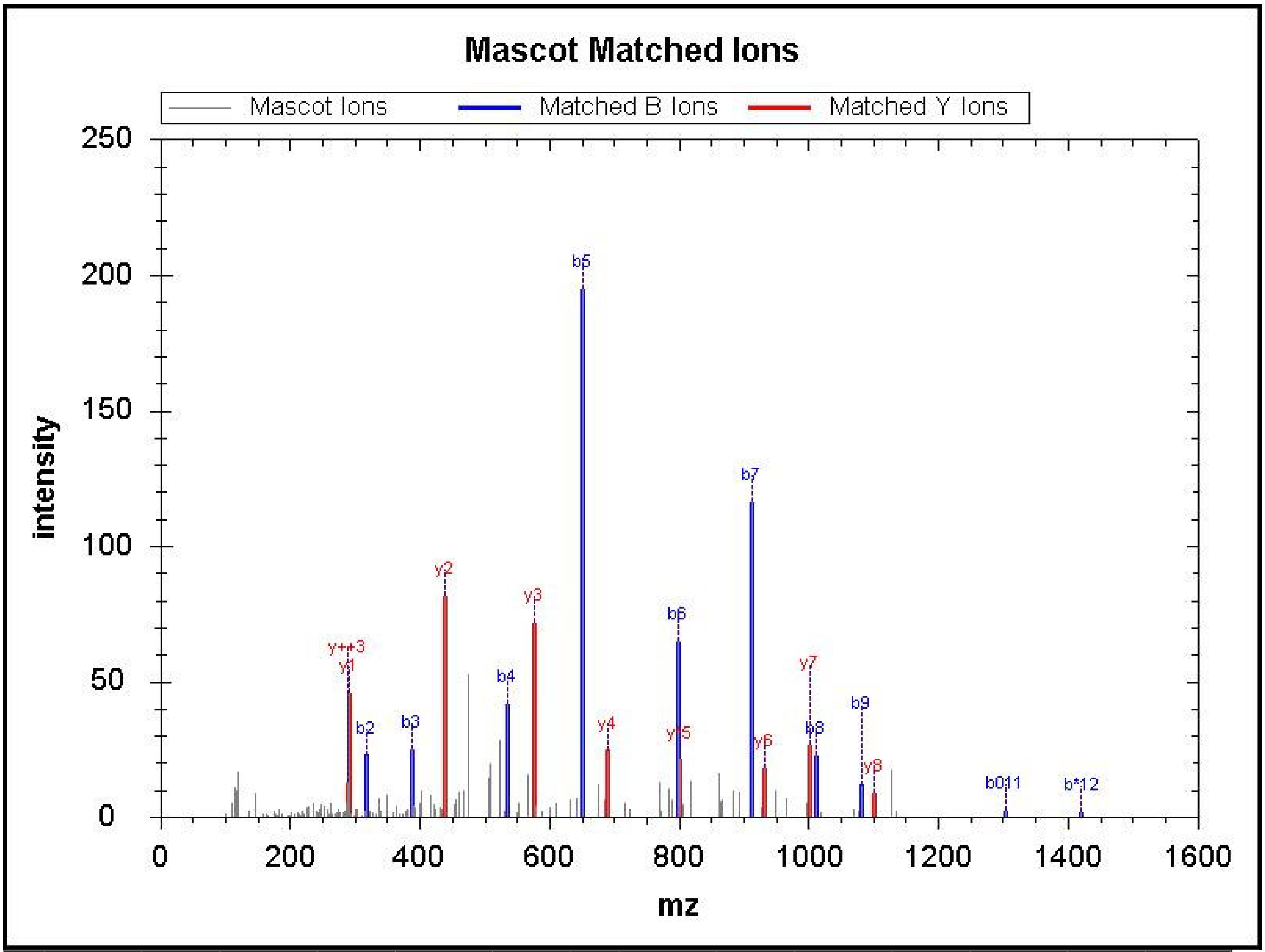

No	b	b++	b*	b*++	b0	b0++	Seq	y	y++	y*	y*++	y0	y0++	RevNo
1	202.13	101.57					G							15
2	317.16	159.08			299.15	150.08	D	1,810.89	905.95	1,793.86	897.43	1,792.88	896.94	14
3	388.19	194.60			370.18	185.60	A	1,695.86	848.43	1,678.83	839.92	1,677.85	839.43	13

4	535.26	268.14			517.25	259.13	F	1,624.82	812.92	1,607.80	804.40	1,606.81	803.91	12
5	650.29	325.65			632.28	316.64	D	1,477.76	739.38	1,460.73	730.87	1,459.74	730.38	11
6	797.36	399.18			779.35	390.18	F	1,362.73	681.87	1,345.70	673.35	1,344.72	672.86	10
7	911.40	456.20	894.37	447.69	893.39	447.20	N	1,215.66	608.33	1,198.63	599.82	1,197.65	599.33	9
8	1,010.47	505.74	993.44	497.23	992.46	496.73	V	1,101.62	551.31	1,084.59	542.80	1,083.61	542.31	8
9	1,081.51	541.26	1,064.48	532.74	1,063.50	532.25	A	1,002.55	501.78	985.52	493.26	984.54	492.77	7
10	1,194.59	597.80	1,177.56	589.29	1,176.58	588.79	L	931.51	466.26	914.49	457.75	913.50	457.25	6
11	1,322.65	661.83	1,305.62	653.32	1,304.64	652.82	Q	818.43	409.72	801.40	401.20	800.42	400.71	5
12	1,437.68	719.34	1,420.65	710.83	1,419.67	710.34	D	690.37	345.69	673.34	337.17	672.36	336.68	4
13	1,574.74	787.87	1,557.71	779.36	1,556.73	778.87	H	575.34	288.17	558.32	279.66			3
14	1,721.80	861.41	1,704.78	852.89	1,703.79	852.40	F	438.28	219.65	421.26	211.13			2
15							K	291.21	146.11	274.19	137.60			1

Query 85974 Hit 1

MS/MS Fragmentation of EKYPDLANFWHELHFVEK
Found in sp|Q8IVF7|FMNL3_HUMAN, Formin-like protein 3 OS=Homo sapiens GN=FMNL3 PE=1 SV=3
Match to Query 85974: 2733.436from(547.6944,5+)
Title: 1003: Sum of 2 scans in range 2075 (rt=56.4013, f=4, i=634) to 2076 (rt=56.4267, f=4, i=635)
[D:\lab212\membrane\Grace\Joyce\54-2.raw]
Data File:Submitted from 20120508-1(merge) by Mascot Daemon on JOYCE-VAIO
Monoisotopic mass of neutral peptide Mr(calc): 2733.436
Variable modifications:
K2 :iTRAQ4plex (K)
K18 :iTRAQ4plex (K)
Ions Score: 69.23 Expect: 0.000

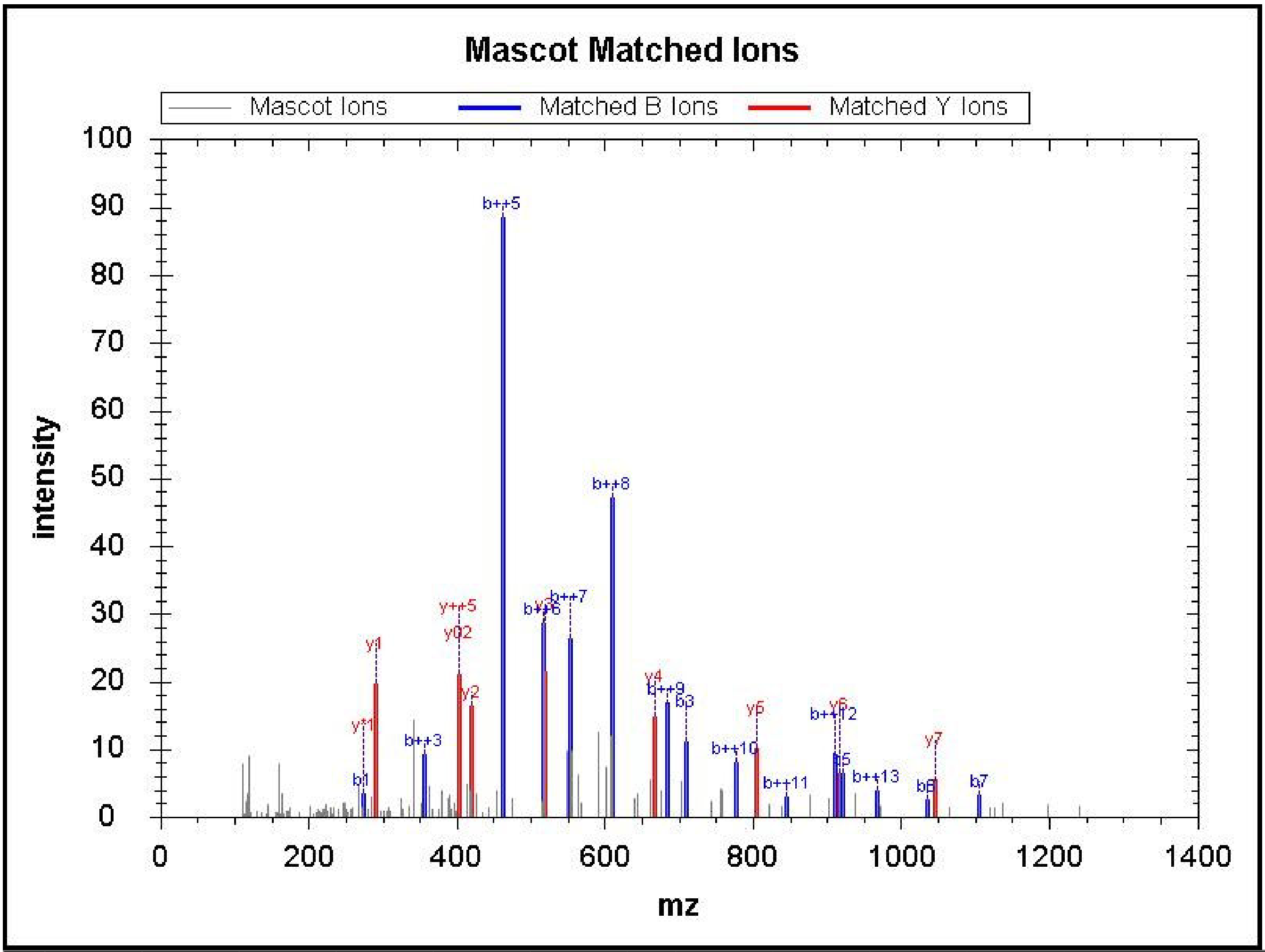

No	b	b++	b*	b*++	b0	b0++	Seq	y	y++	y*	y*++	y0	y0++	RevNo

1	274.15	137.58			256.14	128.57	E							18
2	546.35	273.68	529.32	265.16	528.34	264.67	K	2,461.29	1,231.15	2,444.26	1,222.64	2,443.28	1,222.14	17
3	709.41	355.21	692.39	346.70	691.40	346.20	Y	2,189.09	1,095.05	2,172.07	1,086.54	2,171.08	1,086.05	16
4	806.47	403.74	789.44	395.22	788.45	394.73	P	2,026.03	1,013.52	2,009.00	1,005.01	2,008.02	1,004.51	15
5	921.49	461.25	904.47	452.74	903.48	452.24	D	1,928.98	964.99	1,911.95	956.48	1,910.97	955.99	14
6	1,034.58	517.79	1,017.55	509.28	1,016.57	508.79	L	1,813.95	907.48	1,796.92	898.97	1,795.94	898.47	13
7	1,105.61	553.31	1,088.59	544.80	1,087.60	544.30	A	1,700.87	850.94	1,683.84	842.42	1,682.86	841.93	12
8	1,219.66	610.33	1,202.63	601.82	1,201.65	601.33	N	1,629.83	815.42	1,612.80	806.91	1,611.82	806.41	11
9	1,366.72	683.87	1,349.70	675.35	1,348.71	674.86	F	1,515.79	758.40	1,498.76	749.88	1,497.78	749.39	10
10	1,552.80	776.91	1,535.78	768.39	1,534.79	767.90	W	1,368.72	684.86	1,351.69	676.35	1,350.71	675.86	9
11	1,689.86	845.44	1,672.84	836.92	1,671.85	836.43	H	1,182.64	591.82	1,165.61	583.31	1,164.63	582.82	8
12	1,818.91	909.96	1,801.88	901.44	1,800.89	900.95	E	1,045.58	523.29	1,028.55	514.78	1,027.57	514.29	7
13	1,931.99	966.50	1,914.96	957.99	1,913.98	957.49	L	916.54	458.77	899.51	450.26	898.53	449.77	6
14	2,069.05	1,035.03	2,052.02	1,026.51	2,051.04	1,026.02	H	803.45	402.23	786.43	393.72	785.44	393.22	5
15	2,216.12	1,108.56	2,199.09	1,100.05	2,198.11	1,099.56	F	666.39	333.70	649.37	325.19	648.38	324.70	4
16	2,315.19	1,158.10	2,298.16	1,149.58	2,297.17	1,149.09	V	519.33	260.17	502.30	251.65	501.32	251.16	3
17	2,444.23	1,222.62	2,427.20	1,214.10	2,426.22	1,213.61	E	420.26	210.63	403.23	202.12	402.25	201.63	2
18							K	291.21	146.11	274.19	137.60			1

Query 51306 Hit 1

MS/MS Fragmentation of ALLADAQIMLDHLK
Found in sp|Q9JMH9|MY18A_MOUSE, Unconventional myosin-XVIIIa OS=Mus musculus GN=Myo18a PE=1 SV=2
Match to Query 51306: 1839.053from(614.025,3+)
Title: 1039: Sum of 2 scans in range 2318 (rt=61.5113, f=4, i=681) to 2319 (rt=61.5368, f=4, i=682)
[D:\lab212\membrane\Grace\Joyce\iTRAQ_38_1.raw]
Data File:Submitted from 20120508-1(merge) by Mascot Daemon on JOYCE-VAIO
Monoisotopic mass of neutral peptide Mr(calc): 1839.053
Variable modifications:
K14 :iTRAQ4plex (K)
Ions Score: 69.21 Expect: 0.000

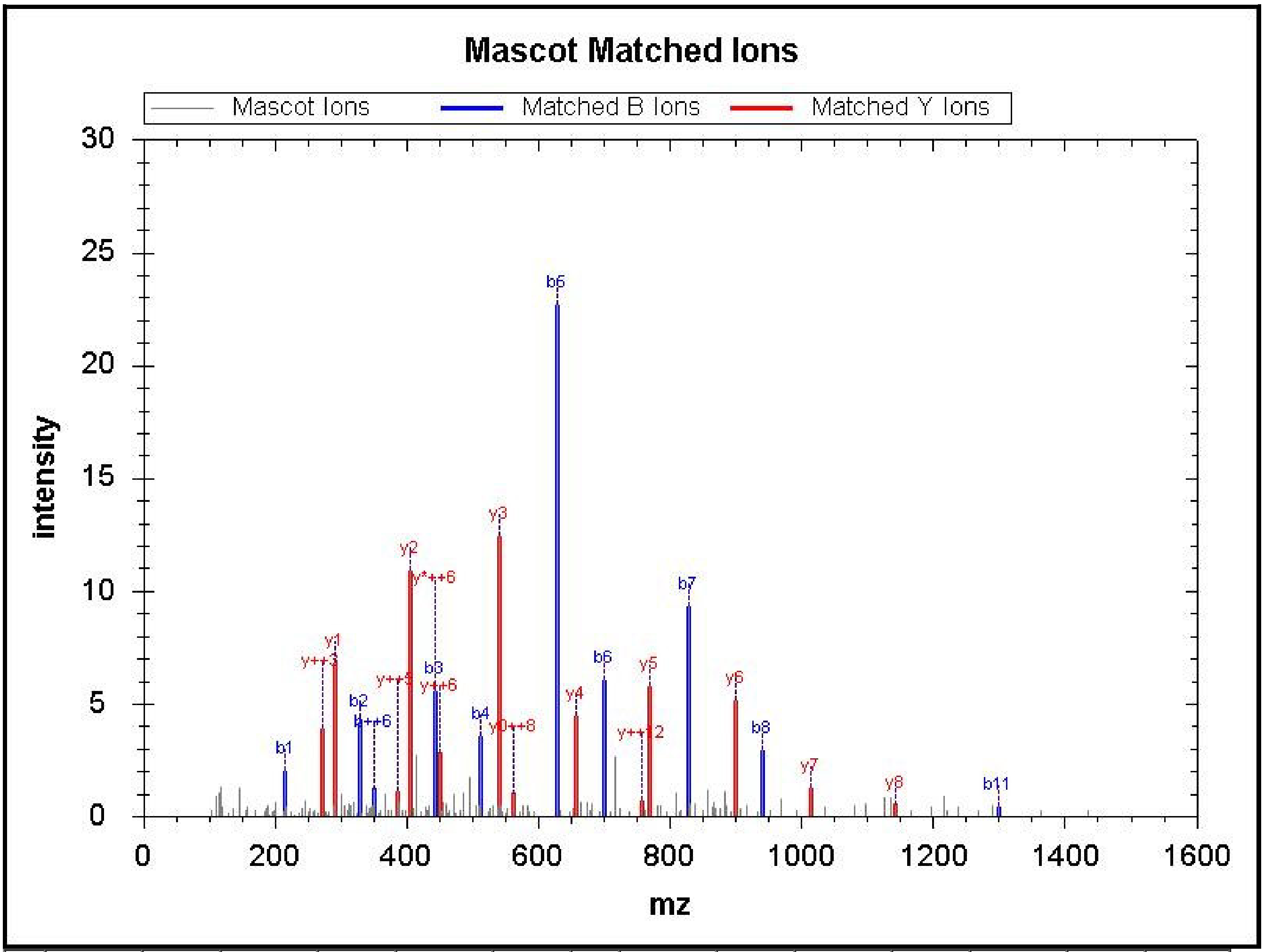

No	b	b++	b*	b*++	b0	b0++	Seq	y	y++	y*	y*++	y0	y0++	RevNo
1	216.15	108.58					A							14
2	329.23	165.12					L	1,624.92	812.96	1,607.89	804.45	1,606.91	803.96	13
3	442.31	221.66					L	1,511.84	756.42	1,494.81	747.91	1,493.83	747.42	12
4	513.35	257.18					A	1,398.75	699.88	1,381.73	691.37	1,380.74	690.87	11
5	628.38	314.69			610.37	305.69	D	1,327.72	664.36	1,310.69	655.85	1,309.71	655.36	10
6	699.42	350.21			681.41	341.21	A	1,212.69	606.85	1,195.66	598.33	1,194.68	597.84	9
7	827.47	414.24	810.45	405.73	809.46	405.24	Q	1,141.65	571.33	1,124.63	562.82	1,123.64	562.32	8
8	940.56	470.78	923.53	462.27	922.55	461.78	I	1,013.59	507.30	996.57	498.79	995.58	498.29	7
9	1,071.60	536.30	1,054.57	527.79	1,053.59	527.30	M	900.51	450.76	883.48	442.24	882.50	441.75	6
10	1,184.68	592.85	1,167.66	584.33	1,166.67	583.84	L	769.47	385.24	752.44	376.72	751.46	376.23	5
11	1,299.71	650.36	1,282.68	641.85	1,281.70	641.35	D	656.38	328.70	639.36	320.18	638.37	319.69	4
12	1,436.77	718.89	1,419.74	710.37	1,418.76	709.88	H	541.36	271.18	524.33	262.67			3
13	1,549.85	775.43	1,532.83	766.92	1,531.84	766.42	L	404.30	202.65	387.27	194.14			2
14							K	291.21	146.11	274.19	137.60			1

Query 22641 Hit 1

MS/MS Fragmentation of VVGALLHALK
Found in sp|P49222|EPB42_MOUSE, Erythrocyte membrane protein band 4.2 OS=Mus musculus GN=Epb42 PE=2 SV=3
Match to Query 22641: 1307.858from(654.9363,2+)
Title: 788: Sum of 2 scans in range 1770 (rt=49.4419, f=4, i=532) to 1771 (rt=49.4674, f=4, i=533) [D:\lab212\membrane\Grace\Joyce\iTRAQ_42_1.raw]
Data File:Submitted from 20120508-1(merge) by Mascot Daemon on JOYCE-VAIO
Monoisotopic mass of neutral peptide Mr(calc): 1307.858
Variable modifications:
K10 :iTRAQ4plex (K)
Ions Score: 69.14 Expect: 0.000

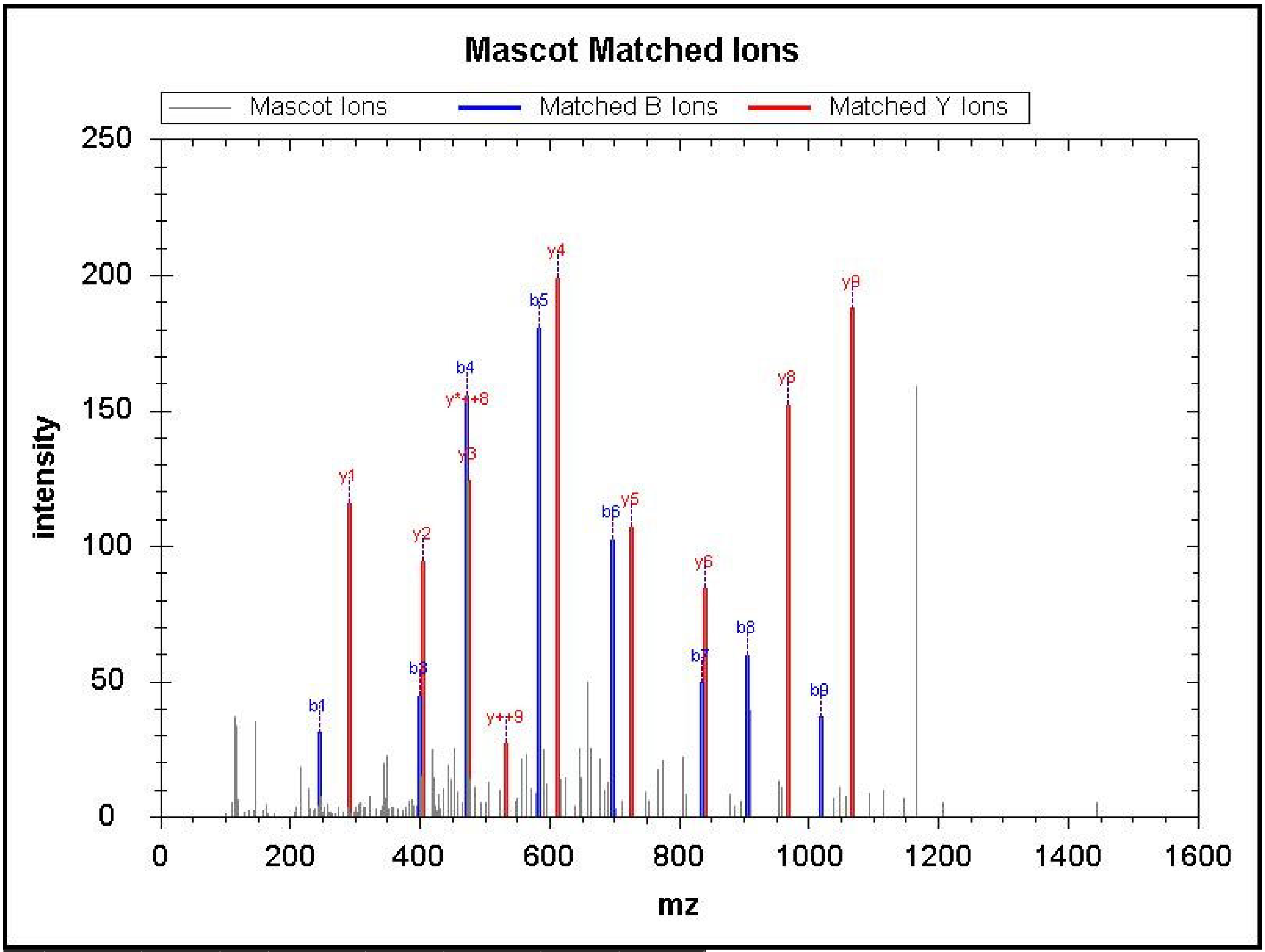

No	b	b++	Seq	y	y++	y*	y*++	RevNo
1	244.18	122.59	V					10
2	343.25	172.13	V	1,065.69	533.35	1,048.66	524.84	9
3	400.27	200.64	G	966.62	483.81	949.60	475.30	8
4	471.30	236.16	A	909.60	455.30	892.57	446.79	7
5	584.39	292.70	L	838.56	419.79	821.54	411.27	6
6	697.47	349.24	L	725.48	363.24	708.45	354.73	5
7	834.53	417.77	H	612.39	306.70	595.37	298.19	4
8	905.57	453.29	A	475.34	238.17	458.31	229.66	3
9	1,018.65	509.83	L	404.30	202.65	387.27	194.14	2
10			K	291.21	146.11	274.19	137.60	1

Query 96552 Hit 1

MS/MS Fragmentation of FSLQGELLAPDVDLPTHLGLHHHGEEAER
Found in sp|Q9BWH6|RPAP1_HUMAN, RNA polymerase II-associated protein 1 OS=Homo sapiens GN=RPAP1 PE=1 SV=3
Match to Query 96552: 3360.709from(673.1491,5+)
Title: 846: Scan 1777 (rt=49.6535, f=3, i=277) [D:\lab212\membrane\Grace\Joyce\54-1.raw]
Data File:Submitted from 20120508-1(merge) by Mascot Daemon on JOYCE-VAIO
Monoisotopic mass of neutral peptide Mr(calc): 3360.709
Variable modifications:
Ions Score: 68.98 Expect: 0.000

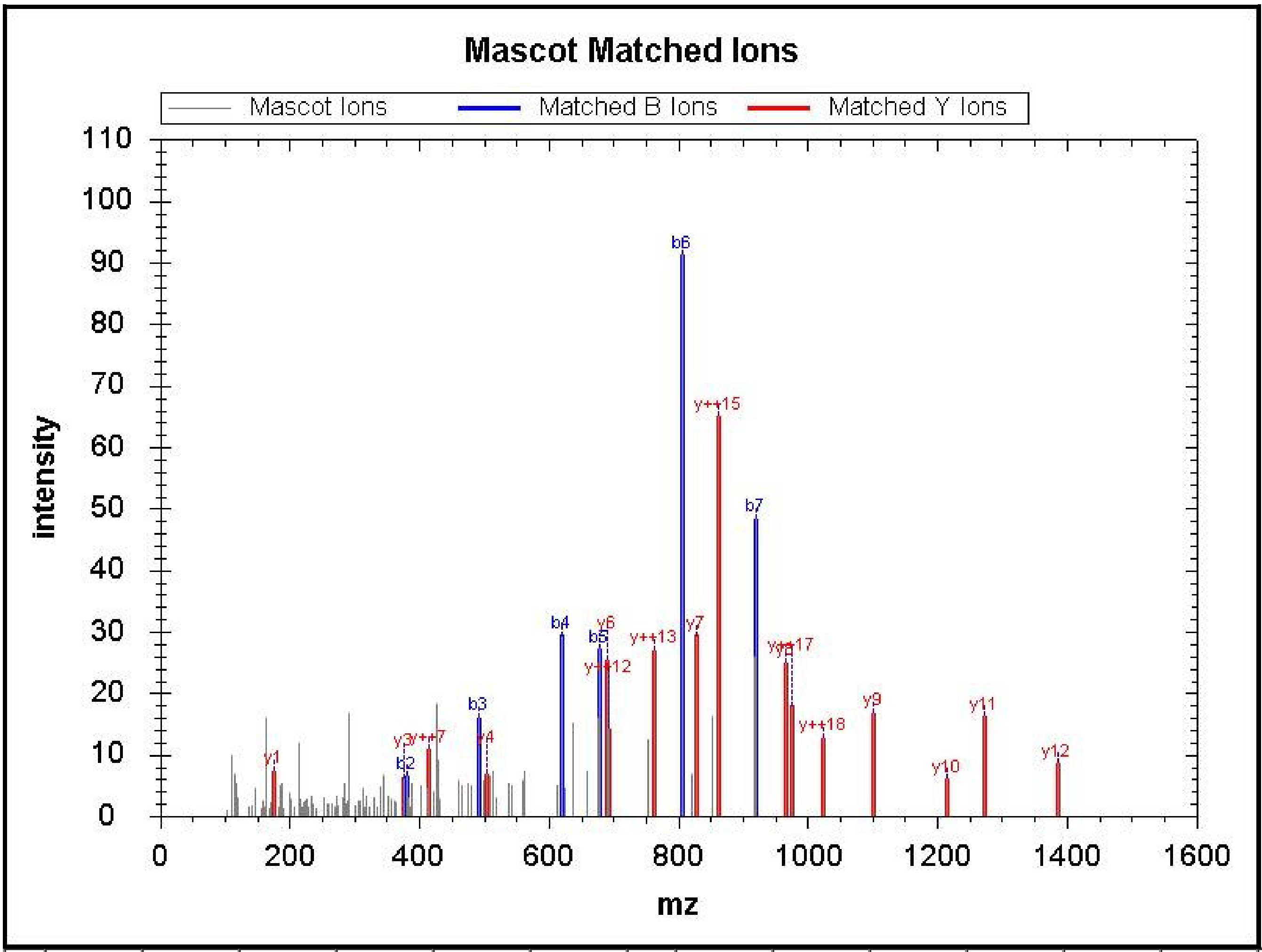

No	b	b++	b*	b*++	b0	b0++	Seq	y	y++	y*	y*++	y0	y0++	RevNo
1	292.18	146.59					F							29
2	379.21	190.11			361.20	181.10	S	3,070.53	1,535.77	3,053.51	1,527.26	3,052.52	1,526.77	28
3	492.29	246.65			474.28	237.65	L	2,983.50	1,492.25	2,966.48	1,483.74	2,965.49	1,483.25	27
4	620.35	310.68	603.33	302.17	602.34	301.67	Q	2,870.42	1,435.71	2,853.39	1,427.20	2,852.41	1,426.71	26
5	677.37	339.19	660.35	330.68	659.36	330.19	G	2,742.36	1,371.68	2,725.33	1,363.17	2,724.35	1,362.68	25
6	806.42	403.71	789.39	395.20	788.41	394.71	E	2,685.34	1,343.17	2,668.31	1,334.66	2,667.33	1,334.17	24
7	919.50	460.25	902.47	451.74	901.49	451.25	L	2,556.30	1,278.65	2,539.27	1,270.14	2,538.28	1,269.65	23
8	1,032.58	516.80	1,015.56	508.28	1,014.57	507.79	L	2,443.21	1,222.11	2,426.18	1,213.60	2,425.20	1,213.10	22
9	1,103.62	552.31	1,086.60	543.80	1,085.61	543.31	A	2,330.13	1,165.57	2,313.10	1,157.05	2,312.12	1,156.56	21
10	1,200.67	600.84	1,183.65	592.33	1,182.66	591.84	P	2,259.09	1,130.05	2,242.06	1,121.54	2,241.08	1,121.04	20
11	1,315.70	658.35	1,298.68	649.84	1,297.69	649.35	D	2,162.04	1,081.52	2,145.01	1,073.01	2,144.03	1,072.52	19
12	1,414.77	707.89	1,397.74	699.38	1,396.76	698.88	V	2,047.01	1,024.01	2,029.98	1,015.50	2,029.00	1,015.00	18
13	1,529.80	765.40	1,512.77	756.89	1,511.79	756.40	D	1,947.94	974.47	1,930.92	965.96	1,929.93	965.47	17
14	1,642.88	821.94	1,625.85	813.43	1,624.87	812.94	L	1,832.92	916.96	1,815.89	908.45	1,814.90	907.96	16
15	1,739.93	870.47	1,722.91	861.96	1,721.92	861.47	P	1,719.83	860.42	1,702.80	851.91	1,701.82	851.41	15
16	1,840.98	920.99	1,823.95	912.48	1,822.97	911.99	T	1,622.78	811.89	1,605.75	803.38	1,604.77	802.89	14
17	1,978.04	989.52	1,961.01	981.01	1,960.03	980.52	H	1,521.73	761.37	1,504.70	752.86	1,503.72	752.36	13
18	2,091.12	1,046.07	2,074.10	1,037.55	2,073.11	1,037.06	L	1,384.67	692.84	1,367.65	684.33	1,366.66	683.83	12
19	2,148.15	1,074.58	2,131.12	1,066.06	2,130.14	1,065.57	G	1,271.59	636.30	1,254.56	627.78	1,253.58	627.29	11
20	2,261.23	1,131.12	2,244.20	1,122.61	2,243.22	1,122.11	L	1,214.57	607.79	1,197.54	599.27	1,196.56	598.78	10
21	2,398.29	1,199.65	2,381.26	1,191.13	2,380.28	1,190.64	H	1,101.48	551.24	1,084.46	542.73	1,083.47	542.24	9
22	2,535.35	1,268.18	2,518.32	1,259.66	2,517.34	1,259.17	H	964.42	482.72	947.40	474.20	946.41	473.71	8
23	2,672.41	1,336.71	2,655.38	1,328.19	2,654.40	1,327.70	H	827.36	414.19	810.34	405.67	809.35	405.18	7

24	2,729.43	1,365.22	2,712.40	1,356.70	2,711.42	1,356.21	G	690.31	345.66	673.28	337.14	672.29	336.65	6
25	2,858.47	1,429.74	2,841.44	1,421.23	2,840.46	1,420.73	E	633.28	317.15	616.26	308.63	615.27	308.14	5
26	2,987.51	1,494.26	2,970.49	1,485.75	2,969.50	1,485.25	E	504.24	252.62	487.21	244.11	486.23	243.62	4
27	3,058.55	1,529.78	3,041.52	1,521.27	3,040.54	1,520.77	A	375.20	188.10	358.17	179.59	357.19	179.10	3
28	3,187.59	1,594.30	3,170.57	1,585.79	3,169.58	1,585.29	E	304.16	152.58	287.13	144.07	286.15	143.58	2
29							R	175.12	88.06	158.09	79.55			1

Query 86056 Hit 1

MS/MS Fragmentation of EPGLFDVVIINDSLDQAYAELK
Found in sp|Q16774|KGUA_HUMAN, Guanylate kinase OS=Homo sapiens GN=GUK1 PE=1 SV=2
Match to Query 86056: 2736.452from(913.158,3+)
Title: 1221: Sum of 2 scans in range 2792 (rt=71.7096, f=4, i=803) to 2793 (rt=71.735, f=4, i=804) [D:\lab212\membrane\Grace\Joyce\iTRAQ_24_1.raw]
Data File:Submitted from 20120508-1(merge) by Mascot Daemon on JOYCE-VAIO
Monoisotopic mass of neutral peptide Mr(calc): 2736.452
Variable modifications:
K22 :iTRAQ4plex (K)
Ions Score: 68.95 Expect: 0.000

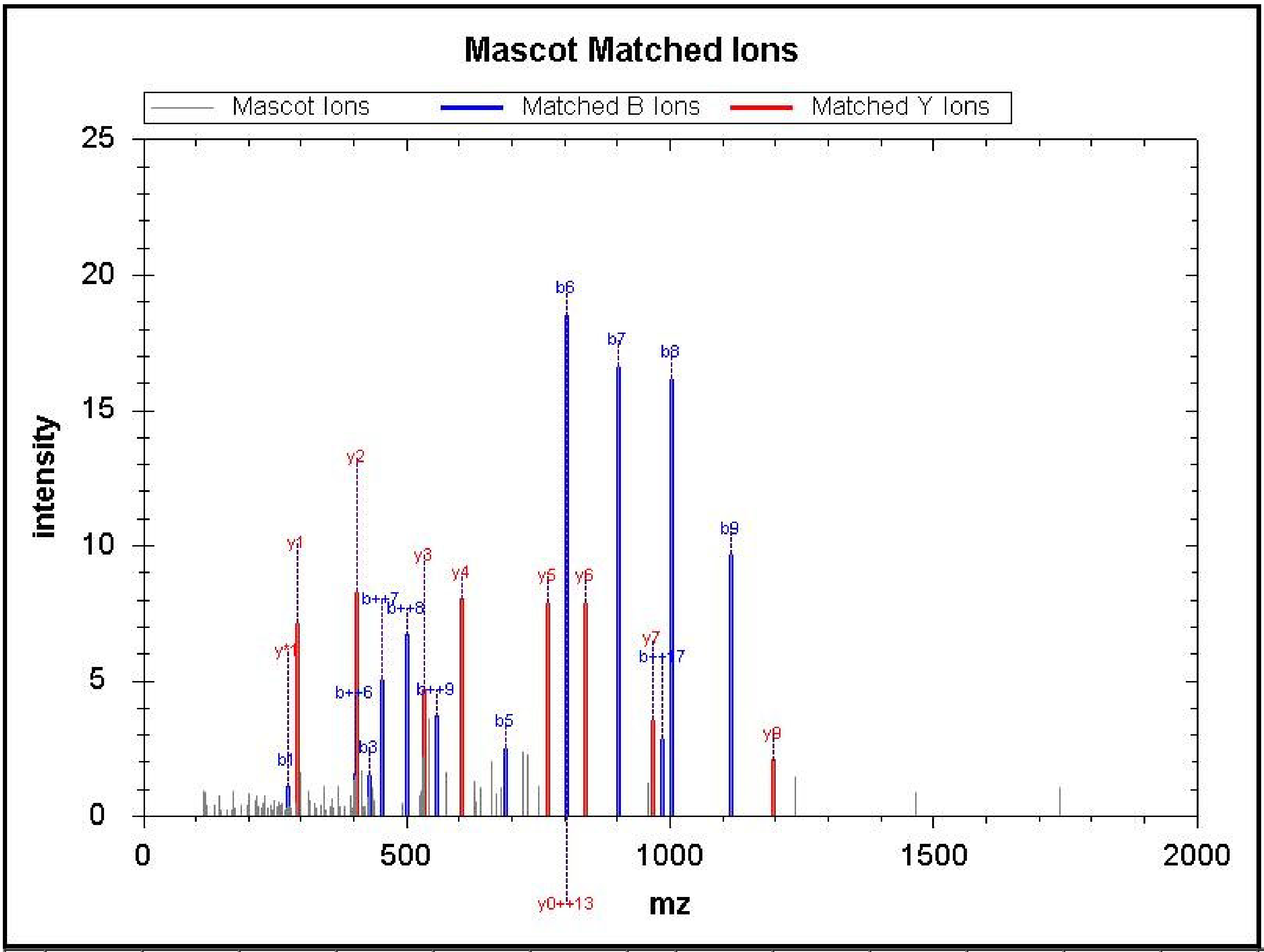

No	b	b++	b*	b*++	b0	b0++	Seq	y	y++	y*	y*++	y0	y0++	RevNo
1	274.15	137.58			256.14	128.57	E							22
2	371.20	186.11			353.19	177.10	P	2,464.31	1,232.66	2,447.28	1,224.14	2,446.30	1,223.65	21
3	428.23	214.62			410.22	205.61	G	2,367.26	1,184.13	2,350.23	1,175.62	2,349.25	1,175.13	20
4	541.31	271.16			523.30	262.15	L	2,310.23	1,155.62	2,293.21	1,147.11	2,292.22	1,146.62	19
5	688.38	344.69			670.37	335.69	F	2,197.15	1,099.08	2,180.12	1,090.57	2,179.14	1,090.07	18
6	803.41	402.21			785.39	393.20	D	2,050.08	1,025.54	2,033.06	1,017.03	2,032.07	1,016.54	17
7	902.47	451.74			884.46	442.74	V	1,935.06	968.03	1,918.03	959.52	1,917.04	959.03	16

8	1,001.54	501.27			983.53	492.27	V	1,835.99	918.50	1,818.96	909.98	1,817.98	909.49	15
9	1,114.63	557.82			1,096.62	548.81	I	1,736.92	868.96	1,719.89	860.45	1,718.91	859.96	14
10	1,227.71	614.36			1,209.70	605.35	I	1,623.83	812.42	1,606.81	803.91	1,605.82	803.42	13
11	1,341.75	671.38	1,324.73	662.87	1,323.74	662.38	N	1,510.75	755.88	1,493.72	747.37	1,492.74	746.87	12
12	1,456.78	728.89	1,439.75	720.38	1,438.77	719.89	D	1,396.71	698.86	1,379.68	690.34	1,378.70	689.85	11
13	1,543.81	772.41	1,526.79	763.90	1,525.80	763.40	S	1,281.68	641.34	1,264.65	632.83	1,263.67	632.34	10
14	1,656.90	828.95	1,639.87	820.44	1,638.89	819.95	L	1,194.65	597.83	1,177.62	589.31	1,176.64	588.82	9
15	1,771.92	886.47	1,754.90	877.95	1,753.91	877.46	D	1,081.56	541.29	1,064.54	532.77	1,063.55	532.28	8
16	1,899.98	950.49	1,882.96	941.98	1,881.97	941.49	Q	966.54	483.77	949.51	475.26	948.53	474.77	7
17	1,971.02	986.01	1,953.99	977.50	1,953.01	977.01	A	838.48	419.74	821.45	411.23	820.47	410.74	6
18	2,134.08	1,067.54	2,117.06	1,059.03	2,116.07	1,058.54	Y	767.44	384.22	750.42	375.71	749.43	375.22	5
19	2,205.12	1,103.06	2,188.09	1,094.55	2,187.11	1,094.06	A	604.38	302.69	587.35	294.18	586.37	293.69	4
20	2,334.16	1,167.58	2,317.14	1,159.07	2,316.15	1,158.58	E	533.34	267.17	516.31	258.66	515.33	258.17	3
21	2,447.25	1,224.13	2,430.22	1,215.61	2,429.24	1,215.12	L	404.30	202.65	387.27	194.14			2
22							K	291.21	146.11	274.19	137.60			1

Query 36868 Hit 1

MS/MS Fragmentation of AQAELVGTADEATR
Found in sp|P30049|ATPD_HUMAN, ATP synthase subunit delta
Match to Query 36868: 1574.799from(788.4069,2+)
Title: 160: Scan 923 (rt=27.846, f=2, i=94) [D:\lab212\membrane\Grace\Joyce\iTRAQ_22_2.raw]
Data File:Submitted from 20120508-1(merge) by Mascot Daemon on JOYCE-VAIO
Monoisotopic mass of neutral peptide Mr(calc): 1574.799
Variable modifications:
Ions Score: 68.9 Expect: 0.000

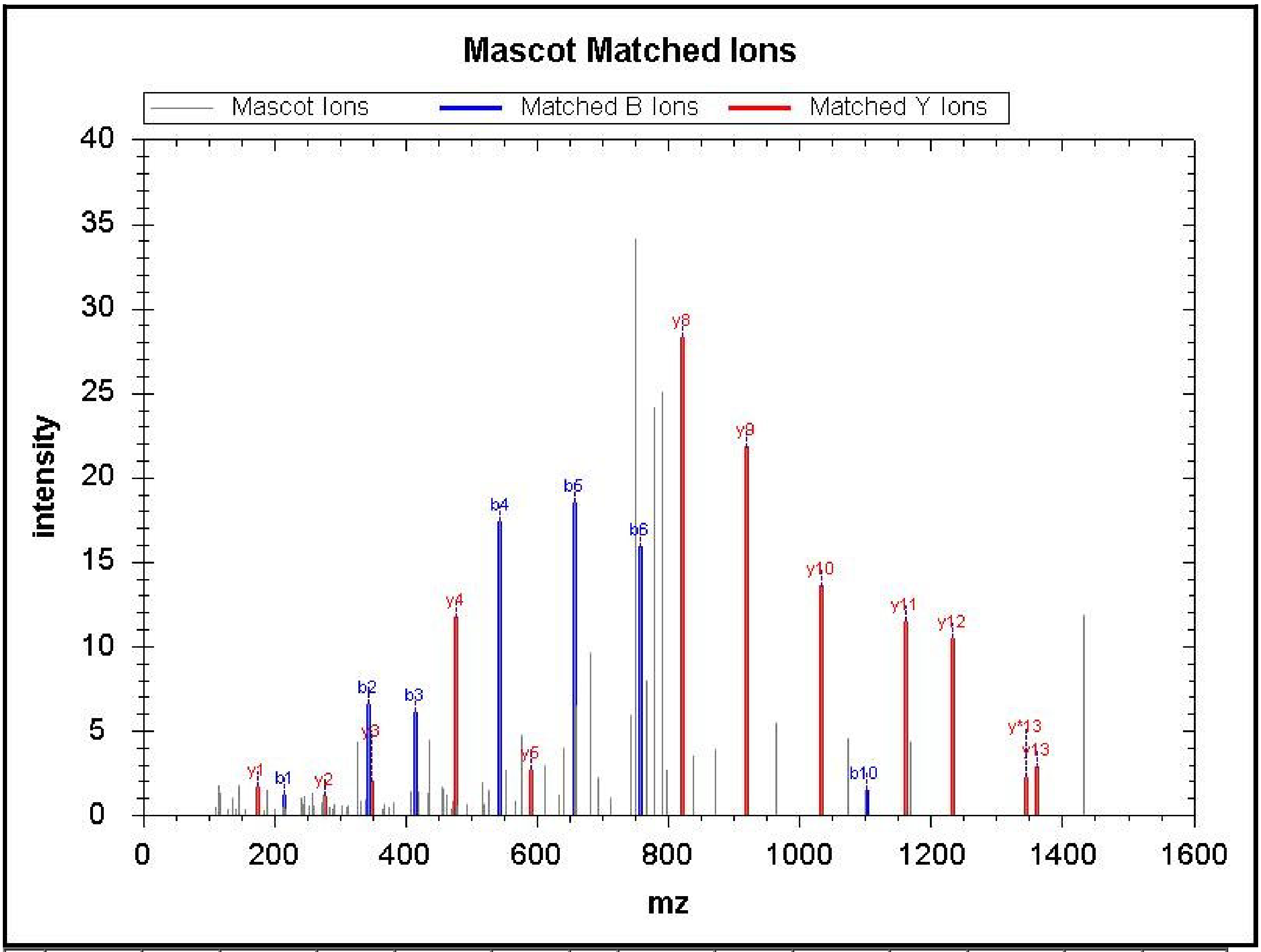

No	b	b++	b*	b*++	b0	b0++	Seq	y	y++	y*	y*++	y0	y0++	RevNo

1	216.15	108.58					A							14
2	344.21	172.61	327.18	164.09			Q	1,360.67	680.84	1,343.64	672.33	1,342.66	671.83	13
3	415.24	208.12	398.22	199.61			A	1,232.61	616.81	1,215.59	608.30	1,214.60	607.80	12
4	544.28	272.65	527.26	264.13	526.27	263.64	E	1,161.57	581.29	1,144.55	572.78	1,143.56	572.29	11
5	657.37	329.19	640.34	320.67	639.36	320.18	L	1,032.53	516.77	1,015.51	508.26	1,014.52	507.76	10
6	756.44	378.72	739.41	370.21	738.43	369.72	V	919.45	460.23	902.42	451.71	901.44	451.22	9
7	813.46	407.23	796.43	398.72	795.45	398.23	G	820.38	410.69	803.35	402.18	802.37	401.69	8
8	914.51	457.76	897.48	449.24	896.50	448.75	T	763.36	382.18	746.33	373.67	745.35	373.18	7
9	985.54	493.28	968.52	484.76	967.53	484.27	A	662.31	331.66	645.28	323.15	644.30	322.65	6
10	1,100.57	550.79	1,083.54	542.28	1,082.56	541.78	D	591.27	296.14	574.25	287.63	573.26	287.13	5
11	1,229.61	615.31	1,212.59	606.80	1,211.60	606.30	E	476.25	238.63	459.22	230.11	458.24	229.62	4
12	1,300.65	650.83	1,283.62	642.32	1,282.64	641.82	A	347.20	174.11	330.18	165.59	329.19	165.10	3
13	1,401.70	701.35	1,384.67	692.84	1,383.69	692.35	T	276.17	138.59	259.14	130.07	258.16	129.58	2
14							R	175.12	88.06	158.09	79.55			1

Query 78294 Hit 1

MS/MS Fragmentation of GLQVLLTPVLANILEADQEK
Found in sp|Q9UHD2|TBK1_HUMAN, Serine/threonine-protein kinase TBK1 OS=Homo sapiens GN=TBK1 PE=1 SV=1
Match to Query 78294: 2451.419from(818.147,3+)
Title: 1077: Sum of 2 scans in range 2982 (rt=73.4282, f=4, i=705) to 2983 (rt=73.4536, f=4, i=706)
[D:\lab212\membrane\Grace\Joyce\iTRAQ_24_2.raw]
Data File:Submitted from 20120508-1(merge) by Mascot Daemon on JOYCE-VAIO
Monoisotopic mass of neutral peptide Mr(calc): 2451.419
Variable modifications:
K20 :iTRAQ4plex (K)
Ions Score: 68.83 Expect: 0.000

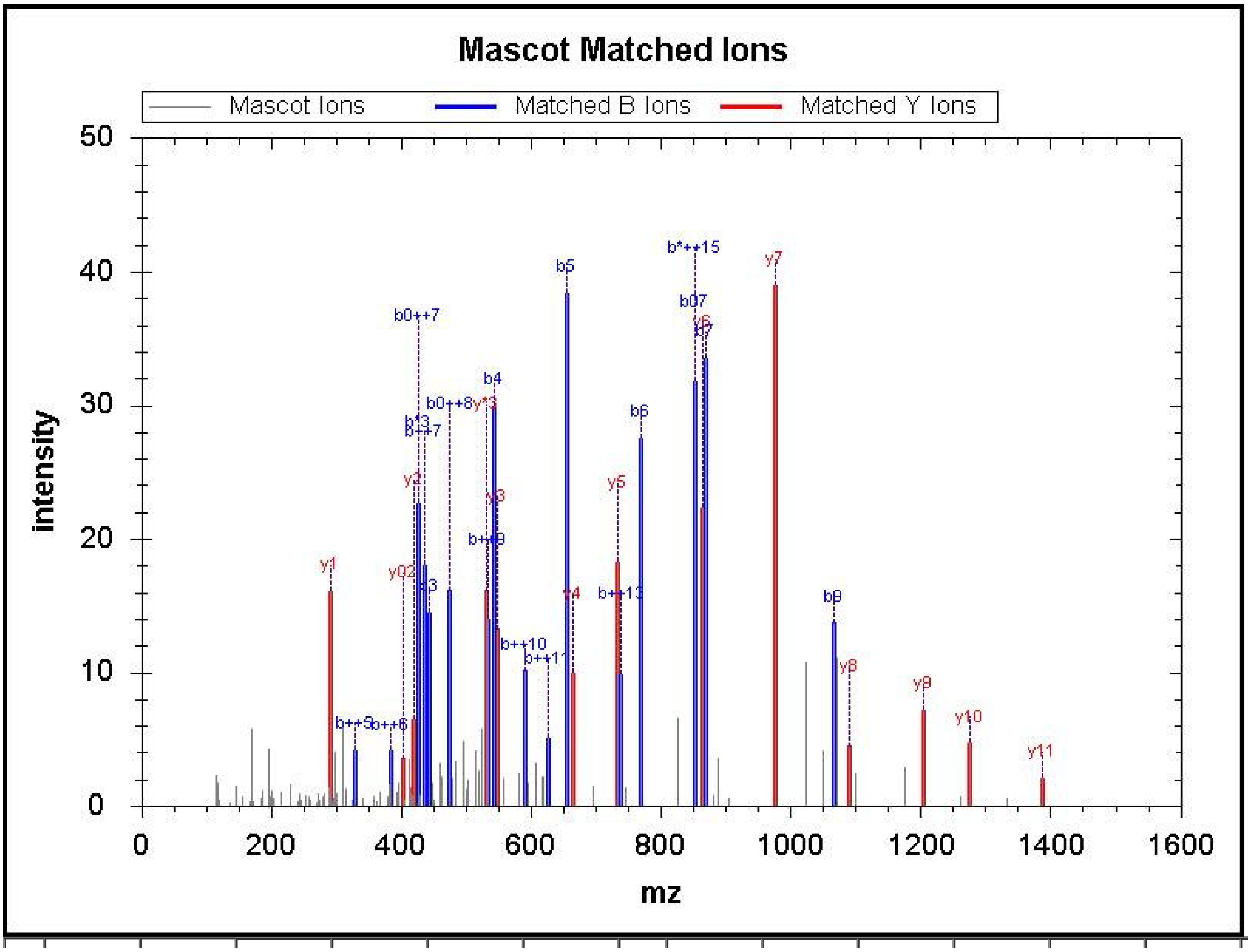

No	b	b++	b*	b*++	b0	b0++	Seq	y	y++	y*	y*++	y0	y0++	RevNo
1	202.13	101.57					G							20
2	315.21	158.11					L	2,251.30	1,126.16	2,234.28	1,117.64	2,233.29	1,117.15	19
3	443.27	222.14	426.25	213.63			Q	2,138.22	1,069.61	2,121.19	1,061.10	2,120.21	1,060.61	18
4	542.34	271.67	525.32	263.16			V	2,010.16	1,005.58	1,993.13	997.07	1,992.15	996.58	17
5	655.43	328.22	638.40	319.70			L	1,911.09	956.05	1,894.07	947.54	1,893.08	947.04	16
6	768.51	384.76	751.48	376.25			L	1,798.01	899.51	1,780.98	890.99	1,780.00	890.50	15
7	869.56	435.28	852.53	426.77	851.55	426.28	T	1,684.92	842.97	1,667.90	834.45	1,666.91	833.96	14
8	966.61	483.81	949.58	475.30	948.60	474.80	P	1,583.88	792.44	1,566.85	783.93	1,565.87	783.44	13
9	1,065.68	533.34	1,048.65	524.83	1,047.67	524.34	V	1,486.82	743.92	1,469.80	735.40	1,468.81	734.91	12
10	1,178.76	589.89	1,161.74	581.37	1,160.75	580.88	L	1,387.76	694.38	1,370.73	685.87	1,369.74	685.38	11
11	1,249.80	625.40	1,232.77	616.89	1,231.79	616.40	A	1,274.67	637.84	1,257.64	629.33	1,256.66	628.83	10
12	1,363.84	682.43	1,346.82	673.91	1,345.83	673.42	N	1,203.63	602.32	1,186.61	593.81	1,185.62	593.32	9
13	1,476.93	738.97	1,459.90	730.45	1,458.92	729.96	I	1,089.59	545.30	1,072.56	536.79	1,071.58	536.29	8
14	1,590.01	795.51	1,572.98	787.00	1,572.00	786.50	L	976.51	488.76	959.48	480.24	958.50	479.75	7
15	1,719.05	860.03	1,702.03	851.52	1,701.04	851.03	E	863.42	432.21	846.40	423.70	845.41	423.21	6
16	1,790.09	895.55	1,773.06	887.04	1,772.08	886.54	A	734.38	367.69	717.35	359.18	716.37	358.69	5
17	1,905.12	953.06	1,888.09	944.55	1,887.11	944.06	D	663.34	332.18	646.32	323.66	645.33	323.17	4
18	2,033.18	1,017.09	2,016.15	1,008.58	2,015.17	1,008.09	Q	548.32	274.66	531.29	266.15	530.31	265.66	3
19	2,162.22	1,081.61	2,145.19	1,073.10	2,144.21	1,072.61	E	420.26	210.63	403.23	202.12	402.25	201.63	2
20							K	291.21	146.11	274.19	137.60			1

Query 80791 Hit 1

MS/MS Fragmentation of YKGESVQGALILAELLSAVK
Found in sp|Q8NBN3|TM87A_HUMAN, Transmembrane protein 87A OS=Homo sapiens GN=TMEM87A PE=1 SV=3
Match to Query 80791: 2520.493from(841.1717,3+)
Title: 1201: Scan 2600 (rt=68.124, f=2, i=428) [D:\lab212\membrane\Grace\Joyce\iTRAQ_41_1.raw]
Data File:Submitted from 20120508-1(merge) by Mascot Daemon on JOYCE-VAIO
Monoisotopic mass of neutral peptide Mr(calc): 2520.493
Variable modifications:
K2 :iTRAQ4plex (K)
K20 :iTRAQ4plex (K)
Ions Score: 68.71 Expect: 0.000

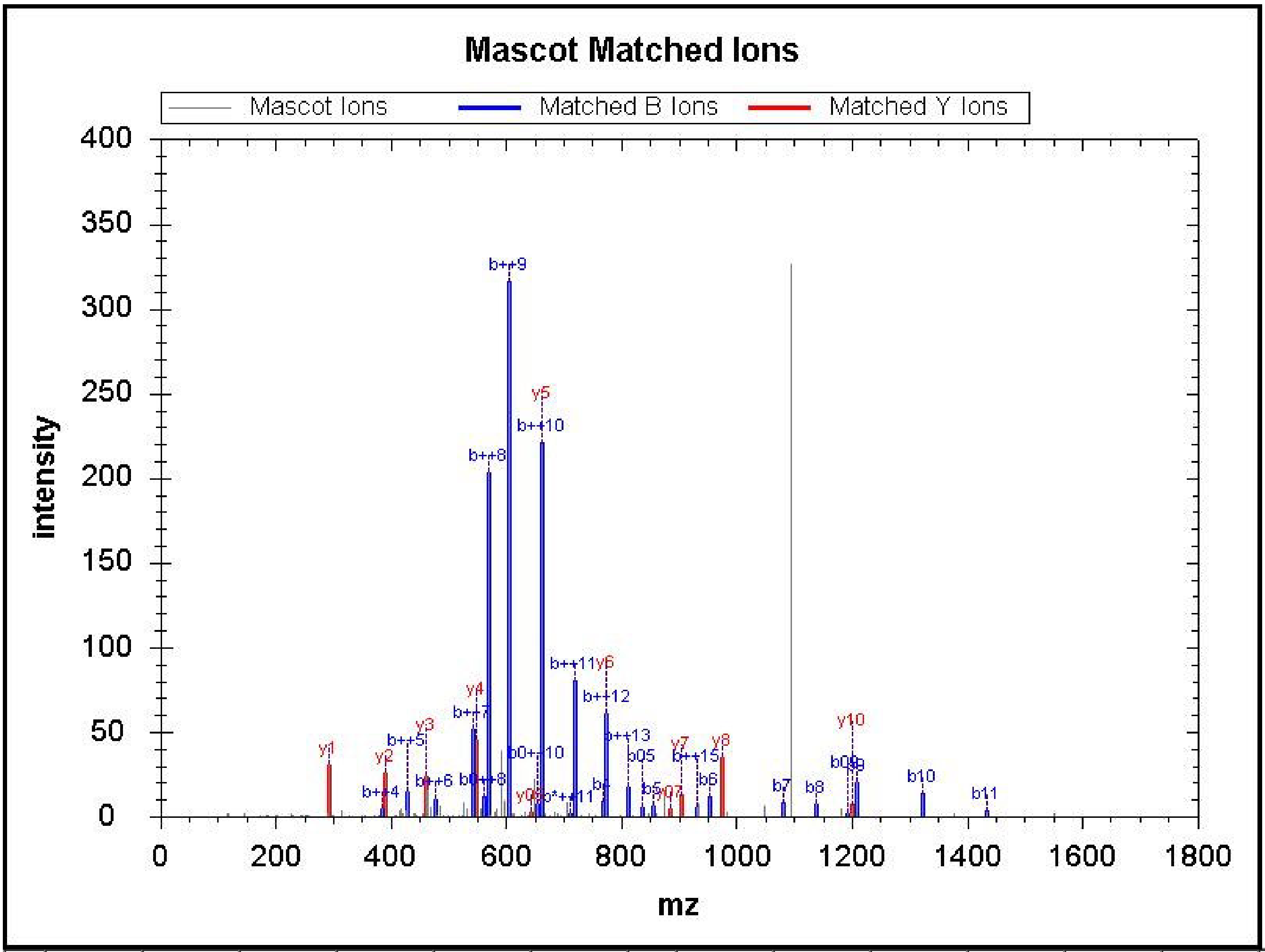

No	b	b++	b*	b*++	b0	b0++	Seq	y	y++	y*	y*++	y0	y0++	RevNo
1	308.17	154.59					Y							20
2	580.37	290.69	563.34	282.18			K	2,214.33	1,107.67	2,197.30	1,099.16	2,196.32	1,098.66	19
3	637.39	319.20	620.36	310.69			G	1,942.13	971.57	1,925.11	963.06	1,924.12	962.57	18
4	766.43	383.72	749.41	375.21	748.42	374.72	E	1,885.11	943.06	1,868.09	934.55	1,867.10	934.05	17
5	853.47	427.24	836.44	418.72	835.46	418.23	S	1,756.07	878.54	1,739.04	870.03	1,738.06	869.53	16
6	952.53	476.77	935.51	468.26	934.52	467.77	V	1,669.04	835.02	1,652.01	826.51	1,651.03	826.02	15
7	1,080.59	540.80	1,063.57	532.29	1,062.58	531.79	Q	1,569.97	785.49	1,552.94	776.98	1,551.96	776.48	14
8	1,137.61	569.31	1,120.59	560.80	1,119.60	560.31	G	1,441.91	721.46	1,424.88	712.95	1,423.90	712.45	13
9	1,208.65	604.83	1,191.63	596.32	1,190.64	595.82	A	1,384.89	692.95	1,367.86	684.44	1,366.88	683.94	12
10	1,321.74	661.37	1,304.71	652.86	1,303.72	652.37	L	1,313.85	657.43	1,296.83	648.92	1,295.84	648.42	11
11	1,434.82	717.91	1,417.79	709.40	1,416.81	708.91	I	1,200.77	600.89	1,183.74	592.37	1,182.76	591.88	10
12	1,547.90	774.46	1,530.88	765.94	1,529.89	765.45	L	1,087.68	544.35	1,070.66	535.83	1,069.67	535.34	9
13	1,618.94	809.97	1,601.91	801.46	1,600.93	800.97	A	974.60	487.80	957.57	479.29	956.59	478.80	8
14	1,747.98	874.50	1,730.96	865.98	1,729.97	865.49	E	903.56	452.29	886.54	443.77	885.55	443.28	7
15	1,861.07	931.04	1,844.04	922.52	1,843.06	922.03	L	774.52	387.76	757.49	379.25	756.51	378.76	6
16	1,974.15	987.58	1,957.13	979.07	1,956.14	978.57	L	661.44	331.22	644.41	322.71	643.43	322.22	5
17	2,061.18	1,031.10	2,044.16	1,022.58	2,043.17	1,022.09	S	548.35	274.68	531.33	266.17	530.34	265.67	4
18	2,132.22	1,066.61	2,115.19	1,058.10	2,114.21	1,057.61	A	461.32	231.16	444.29	222.65			3
19	2,231.29	1,116.15	2,214.26	1,107.64	2,213.28	1,107.14	V	390.28	195.65	373.26	187.13			2
20							K	291.21	146.11	274.19	137.60			1

Query 93886 Hit 1

MS/MS Fragmentation of SISHYHETLGEALQGVELEFSGLDIK
Found in sp|Q9HD45|TM9S3_HUMAN, Transmembrane 9 superfamily member 3 OS=Homo sapiens GN=TM9SF3 PE=1 SV=2

Match to Query 93886: 3159.674from(790.9257,4+)
Title: 1068: Sum of 3 scans in range 2404 (rt=63.6177, f=2, i=376) to 2414 (rt=63.8493, f=4, i=739) [D:\lab212\membrane\Grace\Joyce\47_1.raw]
Data File:Submitted from 20120508-1(merge) by Mascot Daemon on JOYCE-VAIO
Monoisotopic mass of neutral peptide Mr(calc): 3159.674
Variable modifications:
K26 :iTRAQ4plex (K)
Ions Score: 68.52 Expect: 0.000

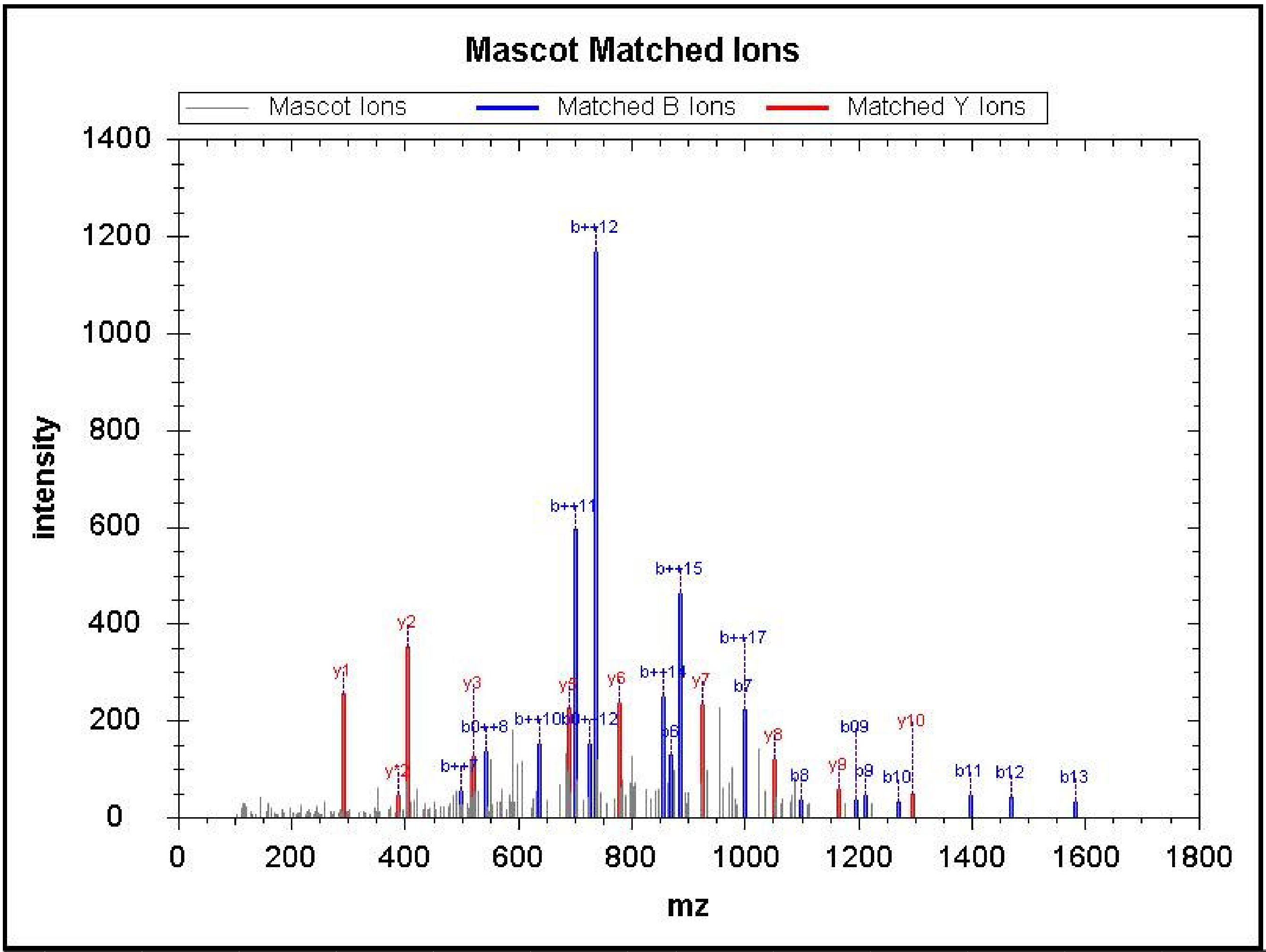

No	b	b++	b*	b*++	b0	b0++	Seq	y	y++	y*	y*++	y0	y0++	RevNo
1	232.14	116.57			214.13	107.57	S							26
2	345.23	173.12			327.21	164.11	I	2,929.51	1,465.26	2,912.48	1,456.74	2,911.50	1,456.25	25
3	432.26	216.63			414.25	207.63	S	2,816.42	1,408.71	2,799.40	1,400.20	2,798.41	1,399.71	24
4	569.32	285.16			551.31	276.16	H	2,729.39	1,365.20	2,712.36	1,356.69	2,711.38	1,356.19	23
5	732.38	366.69			714.37	357.69	Y	2,592.33	1,296.67	2,575.30	1,288.16	2,574.32	1,287.66	22
6	869.44	435.22			851.43	426.22	H	2,429.27	1,215.14	2,412.24	1,206.62	2,411.26	1,206.13	21
7	998.48	499.74			980.47	490.74	E	2,292.21	1,146.61	2,275.18	1,138.09	2,274.20	1,137.60	20
8	1,099.53	550.27			1,081.52	541.26	T	2,163.17	1,082.09	2,146.14	1,073.57	2,145.16	1,073.08	19
9	1,212.61	606.81			1,194.60	597.80	L	2,062.12	1,031.56	2,045.09	1,023.05	2,044.11	1,022.56	18
10	1,269.63	635.32			1,251.62	626.32	G	1,949.03	975.02	1,932.01	966.51	1,931.02	966.02	17
11	1,398.68	699.84			1,380.67	690.84	E	1,892.01	946.51	1,874.99	938.00	1,874.00	937.51	16
12	1,469.71	735.36			1,451.70	726.36	A	1,762.97	881.99	1,745.94	873.48	1,744.96	872.98	15
13	1,582.80	791.90			1,564.79	782.90	L	1,691.93	846.47	1,674.91	837.96	1,673.92	837.47	14
14	1,710.86	855.93	1,693.83	847.42	1,692.85	846.93	Q	1,578.85	789.93	1,561.82	781.42	1,560.84	780.92	13
15	1,767.88	884.44	1,750.85	875.93	1,749.87	875.44	G	1,450.79	725.90	1,433.76	717.39	1,432.78	716.89	12
16	1,866.95	933.98	1,849.92	925.46	1,848.94	924.97	V	1,393.77	697.39	1,376.74	688.88	1,375.76	688.38	11
17	1,995.99	998.50	1,978.96	989.99	1,977.98	989.49	E	1,294.70	647.85	1,277.67	639.34	1,276.69	638.85	10

18	2,109.07	1,055.04	2,092.05	1,046.53	2,091.06	1,046.04	L	1,165.66	583.33	1,148.63	574.82	1,147.65	574.33	9
19	2,238.12	1,119.56	2,221.09	1,111.05	2,220.11	1,110.56	E	1,052.57	526.79	1,035.55	518.28	1,034.56	517.79	8
20	2,385.18	1,193.10	2,368.16	1,184.58	2,367.17	1,184.09	F	923.53	462.27	906.51	453.76	905.52	453.26	7
21	2,472.22	1,236.61	2,455.19	1,228.10	2,454.21	1,227.61	S	776.46	388.74	759.44	380.22	758.45	379.73	6
22	2,529.24	1,265.12	2,512.21	1,256.61	2,511.23	1,256.12	G	689.43	345.22	672.40	336.71	671.42	336.21	5
23	2,642.32	1,321.66	2,625.30	1,313.15	2,624.31	1,312.66	L	632.41	316.71	615.38	308.20	614.40	307.70	4
24	2,757.35	1,379.18	2,740.32	1,370.66	2,739.34	1,370.17	D	519.33	260.17	502.30	251.65	501.32	251.16	3
25	2,870.43	1,435.72	2,853.41	1,427.21	2,852.42	1,426.71	I	404.30	202.65	387.27	194.14			2
26							K	291.21	146.11	274.19	137.60			1

Query 80672 Hit 1

MS/MS Fragmentation of GIGGIFFDDLDSPSKEEVFR
Found in sp|P36551|HEM6_HUMAN, Coproporphyrinogen-III oxidase
Match to Query 80672: 2515.287from(839.4363,3+)
Title: 907: Scan 2008 (rt=54.6505, f=3, i=307) [D:\lab212\membrane\Grace\Joyce\iTRAQ_36_2.raw]
Data File:Submitted from 20120508-1(merge) by Mascot Daemon on JOYCE-VAIO
Monoisotopic mass of neutral peptide Mr(calc): 2515.287
Variable modifications:
K15 :iTRAQ4plex (K)
Ions Score: 68.47 Expect: 0.000

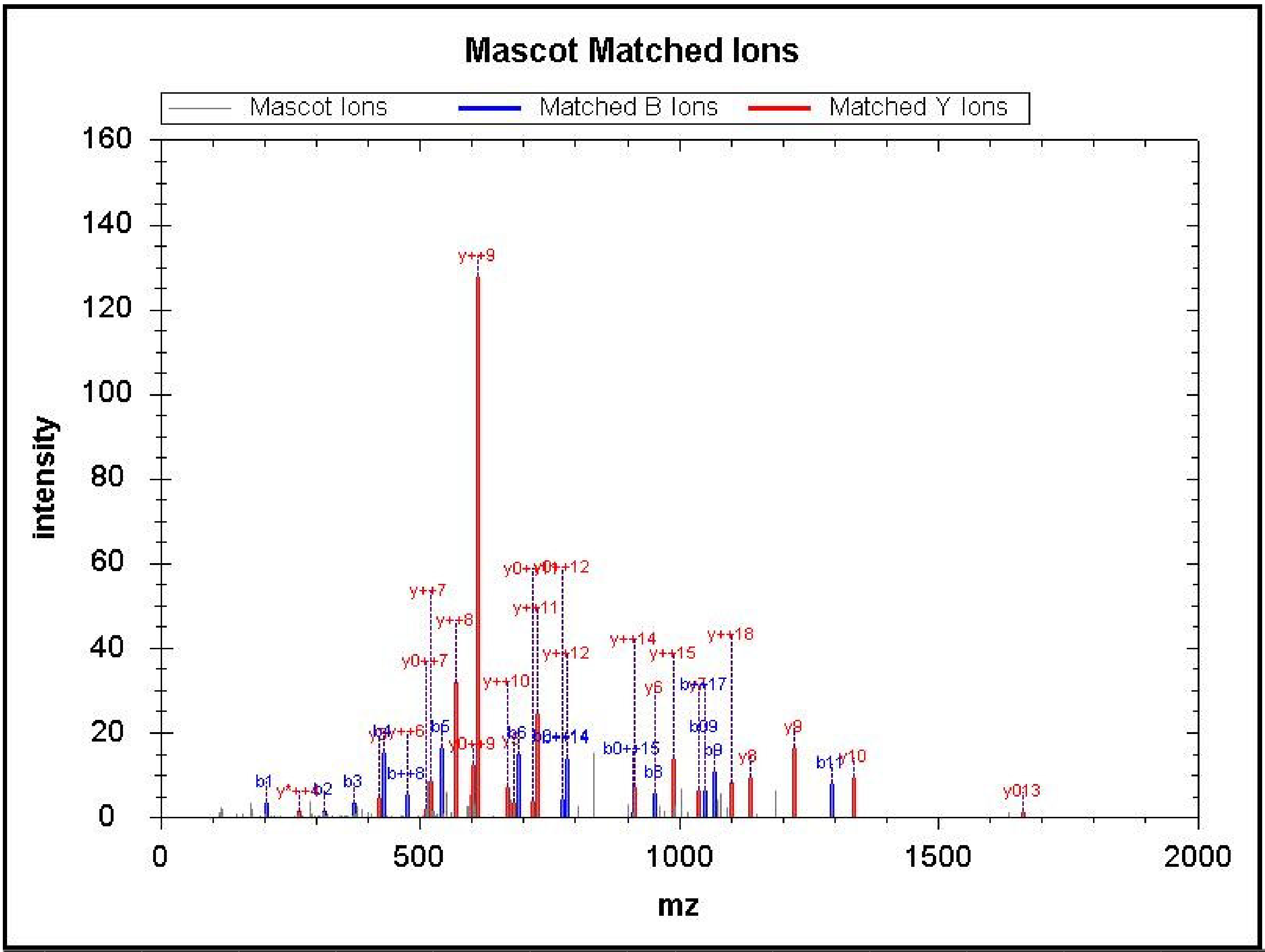

No	b	b++	b*	b*++	b0	b0++	Seq	y	y++	y*	y*++	y0	y0++	RevNo
1	202.13	101.57					G							20
2	315.21	158.11					I	2,315.17	1,158.09	2,298.14	1,149.57	2,297.16	1,149.08	19
3	372.24	186.62					G	2,202.08	1,101.55	2,185.06	1,093.03	2,184.07	1,092.54	18
4	429.26	215.13					G	2,145.06	1,073.03	2,128.04	1,064.52	2,127.05	1,064.03	17
5	542.34	271.67					I	2,088.04	1,044.52	2,071.01	1,036.01	2,070.03	1,035.52	16

6	689.41	345.21					F	1,974.96	987.98	1,957.93	979.47	1,956.95	978.98	15
7	836.48	418.74					F	1,827.89	914.45	1,810.86	905.93	1,809.88	905.44	14
8	951.51	476.26			933.49	467.25	D	1,680.82	840.91	1,663.79	832.40	1,662.81	831.91	13
9	1,066.53	533.77			1,048.52	524.76	D	1,565.79	783.40	1,548.77	774.89	1,547.78	774.39	12
10	1,179.62	590.31			1,161.61	581.31	L	1,450.77	725.89	1,433.74	717.37	1,432.76	716.88	11
11	1,294.64	647.83			1,276.63	638.82	D	1,337.68	669.34	1,320.66	660.83	1,319.67	660.34	10
12	1,381.68	691.34			1,363.67	682.34	S	1,222.65	611.83	1,205.63	603.32	1,204.64	602.83	9
13	1,478.73	739.87			1,460.72	730.86	P	1,135.62	568.32	1,118.60	559.80	1,117.61	559.31	8
14	1,565.76	783.38			1,547.75	774.38	S	1,038.57	519.79	1,021.54	511.28	1,020.56	510.78	7
15	1,837.96	919.48	1,820.93	910.97	1,819.95	910.48	K	951.54	476.27	934.51	467.76	933.53	467.27	6
16	1,967.00	984.00	1,949.97	975.49	1,948.99	975.00	E	679.34	340.17	662.31	331.66	661.33	331.17	5
17	2,096.04	1,048.52	2,079.02	1,040.01	2,078.03	1,039.52	E	550.30	275.65	533.27	267.14	532.29	266.65	4
18	2,195.11	1,098.06	2,178.08	1,089.55	2,177.10	1,089.05	V	421.26	211.13	404.23	202.62			3
19	2,342.18	1,171.59	2,325.15	1,163.08	2,324.17	1,162.59	F	322.19	161.60	305.16	153.08			2
20							R	175.12	88.06	158.09	79.55			1

Query 74215 Hit 1

MS/MS Fragmentation of IDFSDIMVLGDINTKPK
Found in sp|P55011|S12A2_HUMAN, Solute carrier family 12 member 2 OS=Homo sapiens GN=SLC12A2 PE=1 SV=1
Match to Query 74215: 2337.306from(780.1094,3+)
Title: 973: Sum of 2 scans in range 2108 (rt=57.1074, f=4, i=646) to 2109 (rt=57.1329, f=4, i=647) [D:\lab212\membrane\Grace\Joyce\iTRAQ_41_1.raw]
Data File:Submitted from 20120508-1(merge) by Mascot Daemon on JOYCE-VAIO
Monoisotopic mass of neutral peptide Mr(calc): 2337.306
Variable modifications:
K15 :iTRAQ4plex (K)
K17 :iTRAQ4plex (K)
Ions Score: 68.46 Expect: 0.000

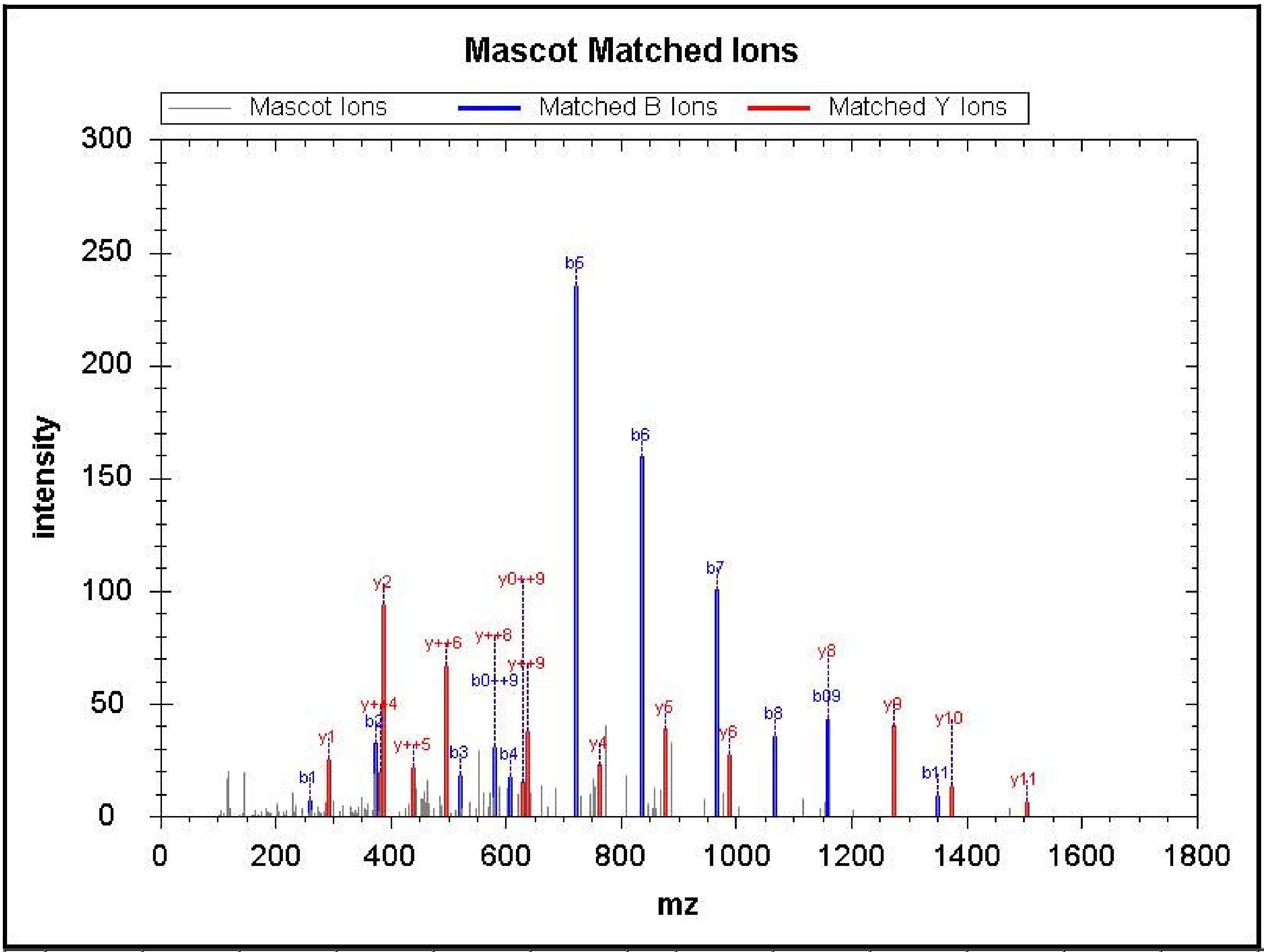

No	b	b++	b*	b*++	b0	b0++	Seq	y	y++	y*	y*++	y0	y0++	RevNo
1	258.19	129.60					I							17
2	373.22	187.11			355.21	178.11	D	2,081.12	1,041.06	2,064.09	1,032.55	2,063.11	1,032.06	16
3	520.29	260.65			502.28	251.64	F	1,966.09	983.55	1,949.07	975.04	1,948.08	974.54	15
4	607.32	304.16			589.31	295.16	S	1,819.02	910.02	1,802.00	901.50	1,801.01	901.01	14
5	722.35	361.68			704.34	352.67	D	1,731.99	866.50	1,714.97	857.99	1,713.98	857.49	13
6	835.43	418.22			817.42	409.21	I	1,616.96	808.99	1,599.94	800.47	1,598.95	799.98	12
7	966.47	483.74			948.46	474.73	M	1,503.88	752.44	1,486.85	743.93	1,485.87	743.44	11
8	1,065.54	533.27			1,047.53	524.27	V	1,372.84	686.92	1,355.81	678.41	1,354.83	677.92	10
9	1,178.62	589.82			1,160.61	580.81	L	1,273.77	637.39	1,256.75	628.88	1,255.76	628.38	9
10	1,235.65	618.33			1,217.64	609.32	G	1,160.69	580.85	1,143.66	572.33	1,142.68	571.84	8
11	1,350.67	675.84			1,332.66	666.83	D	1,103.67	552.34	1,086.64	543.82	1,085.66	543.33	7
12	1,463.76	732.38			1,445.75	723.38	I	988.64	494.82	971.61	486.31	970.63	485.82	6
13	1,577.80	789.40	1,560.77	780.89	1,559.79	780.40	N	875.56	438.28	858.53	429.77	857.54	429.28	5
14	1,678.85	839.93	1,661.82	831.41	1,660.84	830.92	T	761.51	381.26	744.49	372.75	743.50	372.25	4
15	1,951.05	976.03	1,934.02	967.51	1,933.03	967.02	K	660.46	330.74	643.44	322.22			3
16	2,048.10	1,024.55	2,031.07	1,016.04	2,030.09	1,015.55	P	388.27	194.64	371.24	186.12			2
17							K	291.21	146.11	274.19	137.60			1

Query 98837 Hit 1

MS/MS Fragmentation of KLMHQAALLGGLIQDAPNYGWEVAQPVPHDWR
Found in sp|Q9NNW7|TRXR2_HUMAN, Thioredoxin reductase 2
Match to Query 98837: 3625.833 from(907.4654,4+)
Title: 1023: Sum of 2 scans in range 2253 (rt=60.327, f=4, i=688) to 2254 (rt=60.3524, f=4, i=689) [D:\lab212\membrane\Grace\Joyce\iTRAQ_42_1.raw]
Data File:Submitted from 20120508-1(merge) by Mascot Daemon on JOYCE-VAIO

Monoisotopic mass of neutral peptide Mr(calc): 3625.833
Variable modifications:
M3 :Oxidation (M), 63.99828
Ions Score: 68.33 Expect: 0.000

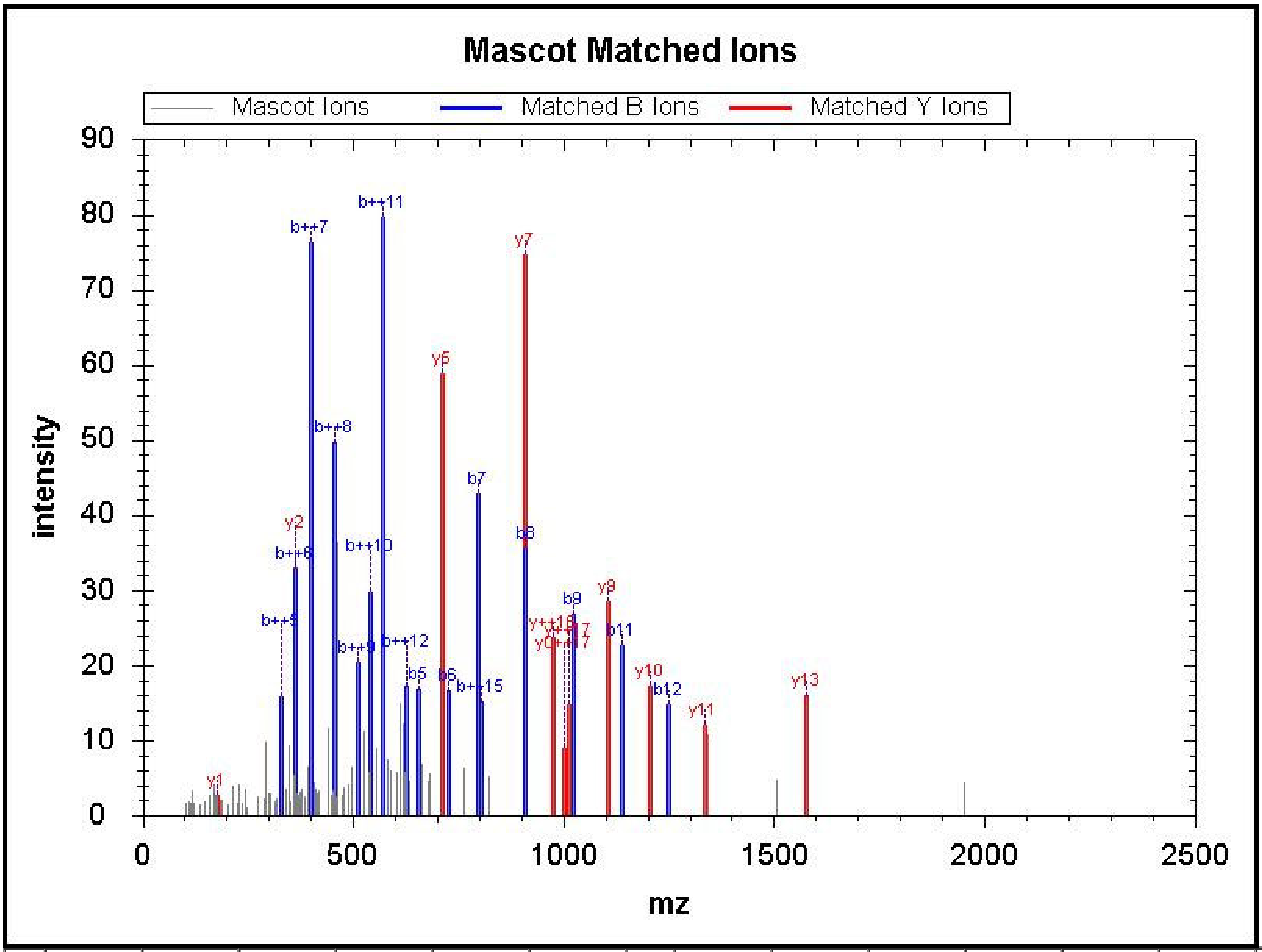

No	b	b++	b*	b*++	b0	b0++	Seq	y	y++	y*	y*++	y0	y0++	RevNo
1	129.10	65.05	112.08	56.54			K							32
2	242.19	121.60	225.16	113.08			L	3,498.74	1,749.87	3,481.71	1,741.36	3,480.73	1,740.87	31
3	389.22	195.11	372.20	186.60			M	3,385.65	1,693.33	3,368.63	1,684.82	3,367.64	1,684.33	30
4	526.28	263.64	509.25	255.13			H	3,238.62	1,619.81	3,221.59	1,611.30	3,220.61	1,610.81	29
5	654.34	327.67	637.31	319.16			Q	3,101.56	1,551.28	3,084.53	1,542.77	3,083.55	1,542.28	28
6	725.38	363.19	708.35	354.68			A	2,973.50	1,487.25	2,956.47	1,478.74	2,955.49	1,478.25	27
7	796.41	398.71	779.39	390.20			A	2,902.46	1,451.74	2,885.44	1,443.22	2,884.45	1,442.73	26
8	909.50	455.25	892.47	446.74			L	2,831.43	1,416.22	2,814.40	1,407.70	2,813.42	1,407.21	25
9	1,022.58	511.79	1,005.55	503.28			L	2,718.34	1,359.67	2,701.32	1,351.16	2,700.33	1,350.67	24
10	1,079.60	540.31	1,062.58	531.79			G	2,605.26	1,303.13	2,588.23	1,294.62	2,587.25	1,294.13	23
11	1,136.62	568.82	1,119.60	560.30			G	2,548.24	1,274.62	2,531.21	1,266.11	2,530.23	1,265.62	22
12	1,249.71	625.36	1,232.68	616.84			L	2,491.22	1,246.11	2,474.19	1,237.60	2,473.20	1,237.11	21
13	1,362.79	681.90	1,345.77	673.39			I	2,378.13	1,189.57	2,361.10	1,181.06	2,360.12	1,180.56	20
14	1,490.85	745.93	1,473.82	737.42			Q	2,265.05	1,133.03	2,248.02	1,124.51	2,247.04	1,124.02	19
15	1,605.88	803.44	1,588.85	794.93	1,587.87	794.44	D	2,136.99	1,069.00	2,119.96	1,060.48	2,118.98	1,059.99	18
16	1,676.92	838.96	1,659.89	830.45	1,658.90	829.96	A	2,021.96	1,011.48	2,004.94	1,002.97	2,003.95	1,002.48	17
17	1,773.97	887.49	1,756.94	878.97	1,755.96	878.48	P	1,950.92	975.97	1,933.90	967.45	1,932.91	966.96	16
18	1,888.01	944.51	1,870.98	936.00	1,870.00	935.50	N	1,853.87	927.44	1,836.85	918.93	1,835.86	918.43	15
19	2,051.07	1,026.04	2,034.05	1,017.53	2,033.06	1,017.04	Y	1,739.83	870.42	1,722.80	861.90	1,721.82	861.41	14
20	2,108.10	1,054.55	2,091.07	1,046.04	2,090.09	1,045.55	G	1,576.77	788.89	1,559.74	780.37	1,558.76	779.88	13

21	2,294.18	1,147.59	2,277.15	1,139.08	2,276.16	1,138.59	W	1,519.74	760.38	1,502.72	751.86	1,501.73	751.37	12
22	2,423.22	1,212.11	2,406.19	1,203.60	2,405.21	1,203.11	E	1,333.66	667.34	1,316.64	658.82	1,315.65	658.33	11
23	2,522.29	1,261.65	2,505.26	1,253.13	2,504.28	1,252.64	V	1,204.62	602.81	1,187.60	594.30	1,186.61	593.81	10
24	2,593.32	1,297.17	2,576.30	1,288.65	2,575.31	1,288.16	A	1,105.55	553.28	1,088.53	544.77	1,087.54	544.28	9
25	2,721.38	1,361.19	2,704.36	1,352.68	2,703.37	1,352.19	Q	1,034.52	517.76	1,017.49	509.25	1,016.51	508.76	8
26	2,818.43	1,409.72	2,801.41	1,401.21	2,800.42	1,400.72	P	906.46	453.73	889.43	445.22	888.45	444.73	7
27	2,917.50	1,459.26	2,900.48	1,450.74	2,899.49	1,450.25	V	809.41	405.21	792.38	396.69	791.39	396.20	6
28	3,014.56	1,507.78	2,997.53	1,499.27	2,996.55	1,498.78	P	710.34	355.67	693.31	347.16	692.33	346.67	5
29	3,151.61	1,576.31	3,134.59	1,567.80	3,133.60	1,567.31	H	613.28	307.15	596.26	298.63	595.27	298.14	4
30	3,266.64	1,633.82	3,249.61	1,625.31	3,248.63	1,624.82	D	476.23	238.62	459.20	230.10	458.21	229.61	3
31	3,452.72	1,726.86	3,435.69	1,718.35	3,434.71	1,717.86	W	361.20	181.10	344.17	172.59			2
32							R	175.12	88.06	158.09	79.55			1

Query 20253 Hit 1

MS/MS Fragmentation of GIAWWTDK
Found in sp|Q9NV96|CC50A_HUMAN, Cell cycle control protein 50A OS=Homo sapiens GN=TMEM30A PE=1 SV=1
Match to Query 20253: 1263.686from(632.8505,2+)
Title: 658: Sum of 2 scans in range 1505 (rt=43.2853, f=4, i=443) to 1506 (rt=43.3107, f=4, i=444)
[D:\lab212\membrane\Grace\Joyce\iTRAQ_33_1.raw]
Data File:Submitted from 20120508-1(merge) by Mascot Daemon on JOYCE-VAIO
Monoisotopic mass of neutral peptide Mr(calc): 1263.686
Variable modifications:
K8 :iTRAQ4plex (K)
Ions Score: 68.21 Expect: 0.000

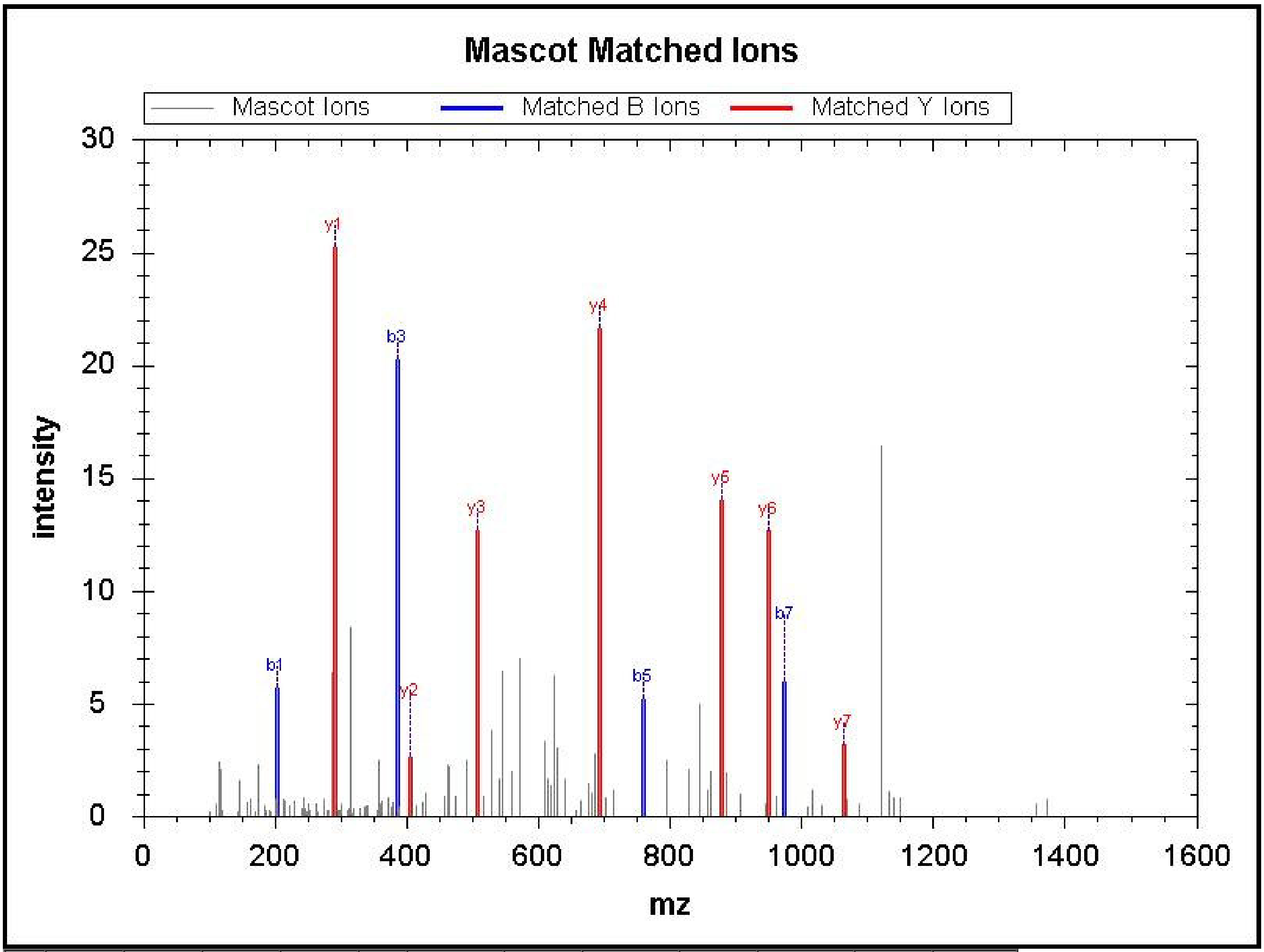

No	b	b++	b0	b0++	Seq	y	y++	y*	y*++	y0	y0++	RevNo
1	202.13	101.57			G							8

2	315.21	158.11			I	1,063.57	532.29	1,046.54	523.78	1,045.56	523.28	7
3	386.25	193.63			A	950.49	475.75	933.46	467.23	932.47	466.74	6
4	572.33	286.67			W	879.45	440.23	862.42	431.71	861.44	431.22	5
5	758.41	379.71			W	693.37	347.19	676.34	338.67	675.36	338.18	4
6	859.46	430.23	841.45	421.23	T	507.29	254.15	490.26	245.64	489.28	245.14	3
7	974.49	487.75	956.47	478.74	D	406.24	203.62	389.22	195.11	388.23	194.62	2
8					K	291.21	146.11	274.19	137.60			1

Query 82709 Hit 1

MS/MS Fragmentation of GQIQEAIALINSLHPELLDTNR
Found in sp|Q9NWU2|CT011_HUMAN, Protein C20orf11 OS=Homo sapiens GN=C20orf11 PE=1 SV=1
Match to Query 82709: 2588.411from(863.8108,3+)
Title: 1100: Scan 2508 (rt=65.5539, f=2, i=400) [D:\lab212\membrane\Grace\Joyce\iTRAQ_30_2.raw]
Data File:Submitted from 20120508-1(merge) by Mascot Daemon on JOYCE-VAIO
Monoisotopic mass of neutral peptide Mr(calc): 2588.411
Variable modifications:
Ions Score: 68 Expect: 0.000

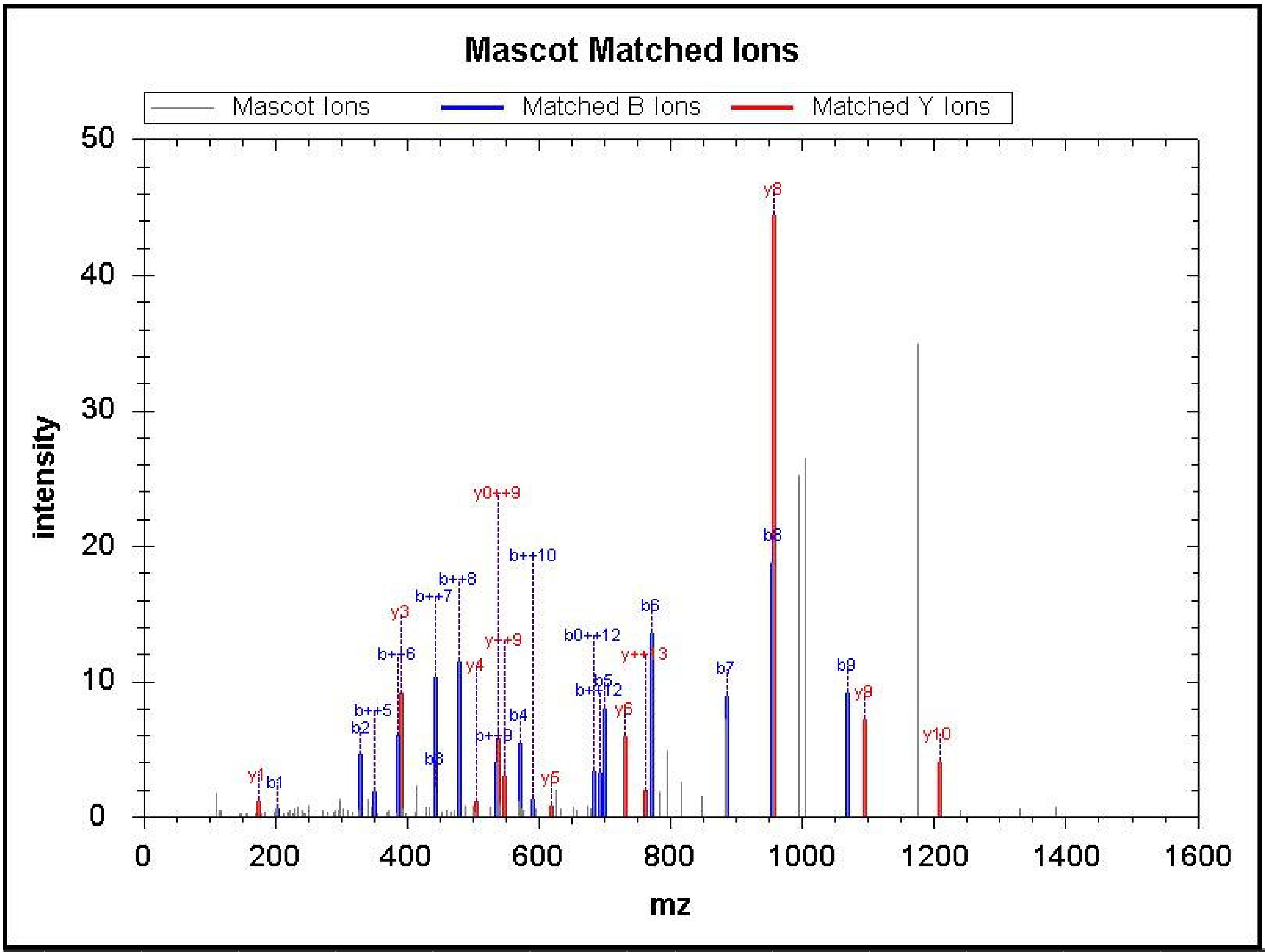

No	b	b++	b*	b*++	b0	b0++	Seq	y	y++	y*	y*++	y0	y0++	RevNo
1	202.13	101.57					G							22
2	330.19	165.60	313.16	157.09			Q	2,388.29	1,194.65	2,371.26	1,186.13	2,370.28	1,185.64	21
3	443.27	222.14	426.25	213.63			I	2,260.23	1,130.62	2,243.20	1,122.11	2,242.22	1,121.61	20
4	571.33	286.17	554.31	277.66			Q	2,147.15	1,074.08	2,130.12	1,065.56	2,129.14	1,065.07	19
5	700.37	350.69	683.35	342.18	682.36	341.69	E	2,019.09	1,010.05	2,002.06	1,001.53	2,001.08	1,001.04	18
6	771.41	386.21	754.39	377.70	753.40	377.20	A	1,890.04	945.53	1,873.02	937.01	1,872.03	936.52	17

7	884.50	442.75	867.47	434.24	866.49	433.75	I	1,819.01	910.01	1,801.98	901.49	1,801.00	901.00	16
8	955.53	478.27	938.51	469.76	937.52	469.26	A	1,705.92	853.47	1,688.90	844.95	1,687.91	844.46	15
9	1,068.62	534.81	1,051.59	526.30	1,050.61	525.81	L	1,634.89	817.95	1,617.86	809.43	1,616.88	808.94	14
10	1,181.70	591.35	1,164.67	582.84	1,163.69	582.35	I	1,521.80	761.40	1,504.78	752.89	1,503.79	752.40	13
11	1,295.74	648.38	1,278.72	639.86	1,277.73	639.37	N	1,408.72	704.86	1,391.69	696.35	1,390.71	695.86	12
12	1,382.78	691.89	1,365.75	683.38	1,364.77	682.89	S	1,294.68	647.84	1,277.65	639.33	1,276.66	638.84	11
13	1,495.86	748.43	1,478.83	739.92	1,477.85	739.43	L	1,207.64	604.33	1,190.62	595.81	1,189.63	595.32	10
14	1,632.92	816.96	1,615.89	808.45	1,614.91	807.96	H	1,094.56	547.78	1,077.53	539.27	1,076.55	538.78	9
15	1,729.97	865.49	1,712.95	856.98	1,711.96	856.48	P	957.50	479.25	940.47	470.74	939.49	470.25	8
16	1,859.01	930.01	1,841.99	921.50	1,841.00	921.01	E	860.45	430.73	843.42	422.21	842.44	421.72	7
17	1,972.10	986.55	1,955.07	978.04	1,954.09	977.55	L	731.40	366.21	714.38	357.69	713.39	357.20	6
18	2,085.18	1,043.09	2,068.16	1,034.58	2,067.17	1,034.09	L	618.32	309.66	601.29	301.15	600.31	300.66	5
19	2,200.21	1,100.61	2,183.18	1,092.10	2,182.20	1,091.60	D	505.24	253.12	488.21	244.61	487.23	244.12	4
20	2,301.26	1,151.13	2,284.23	1,142.62	2,283.25	1,142.13	T	390.21	195.61	373.18	187.10	372.20	186.60	3
21	2,415.30	1,208.15	2,398.27	1,199.64	2,397.29	1,199.15	N	289.16	145.08	272.14	136.57			2
22							R	175.12	88.06	158.09	79.55			1

Query 94533 Hit 1

MS/MS Fragmentation of SISQYLHAGPPVYFVLEEGHDYTSSK
Found in sp|O15118|NPC1_HUMAN, Niemann-Pick C1 protein OS=Homo sapiens GN=NPC1 PE=1 SV=2
Match to Query 94533: 3211.609from(803.9096,4+)
Title: 885: Sum of 2 scans in range 1962 (rt=53.7937, f=4, i=596) to 1963 (rt=53.819, f=4, i=597)
[D:\lab212\membrane\Grace\Joyce\iTRAQ_44_1.raw]
Data File:Submitted from 20120508-1(merge) by Mascot Daemon on JOYCE-VAIO
Monoisotopic mass of neutral peptide Mr(calc): 3211.609
Variable modifications:
K26 :iTRAQ4plex (K)
Ions Score: 67.64 Expect: 0.000

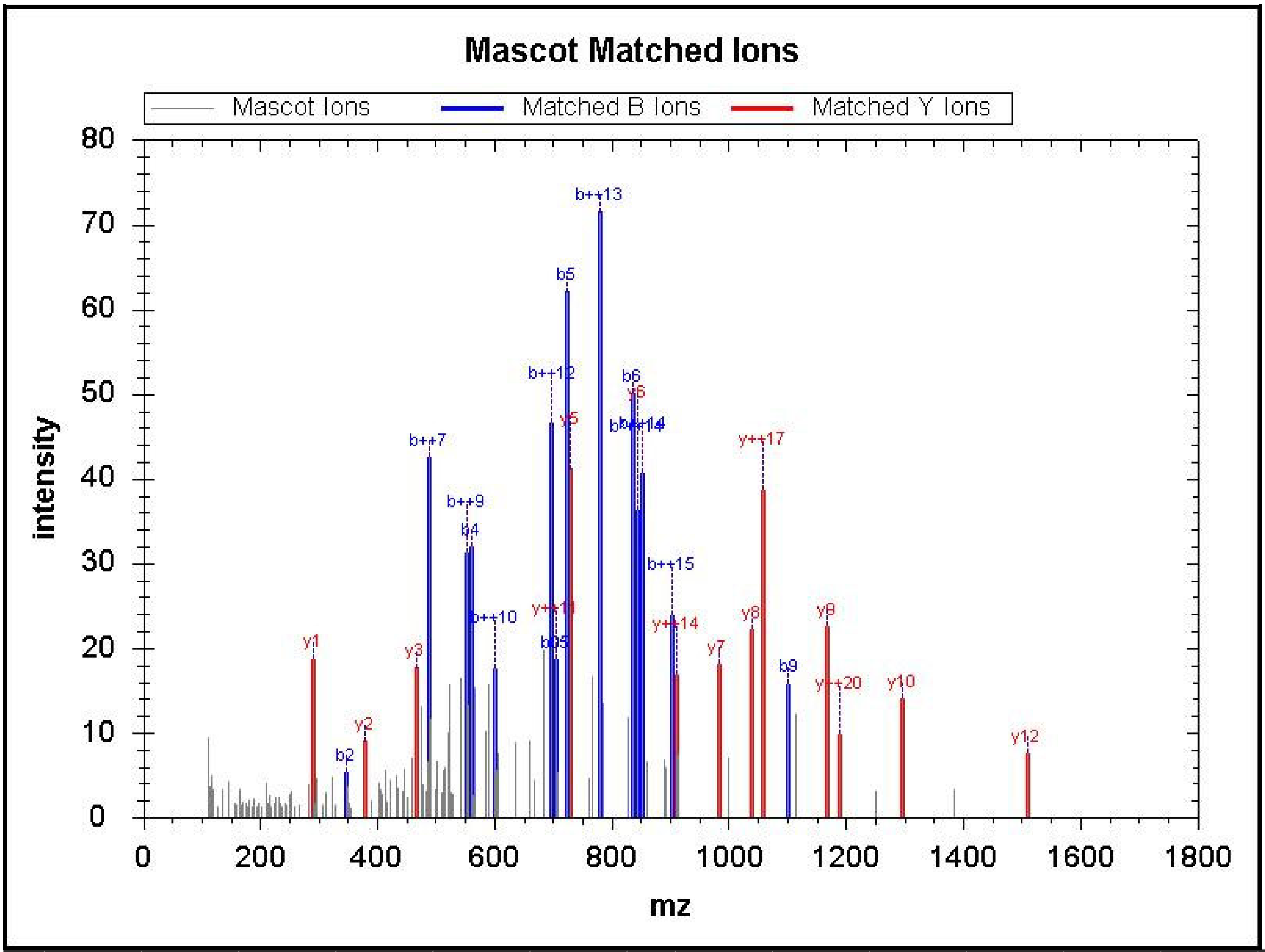

No	b	b++	b*	b*++	b0	b0++	Seq	y	y++	y*	y*++	y0	y0++	RevNo
1	232.14	116.57			214.13	107.57	S							26
2	345.23	173.12			327.21	164.11	I	2,981.48	1,491.24	2,964.45	1,482.73	2,963.47	1,482.24	25
3	432.26	216.63			414.25	207.63	S	2,868.40	1,434.70	2,851.37	1,426.19	2,850.39	1,425.70	24
4	560.32	280.66	543.29	272.15	542.31	271.66	Q	2,781.36	1,391.19	2,764.34	1,382.67	2,763.35	1,382.18	23
5	723.38	362.19	706.35	353.68	705.37	353.19	Y	2,653.31	1,327.16	2,636.28	1,318.64	2,635.29	1,318.15	22
6	836.46	418.74	819.44	410.22	818.45	409.73	L	2,490.24	1,245.62	2,473.22	1,237.11	2,472.23	1,236.62	21
7	973.52	487.26	956.50	478.75	955.51	478.26	H	2,377.16	1,189.08	2,360.13	1,180.57	2,359.15	1,180.08	20
8	1,044.56	522.78	1,027.53	514.27	1,026.55	513.78	A	2,240.10	1,120.55	2,223.07	1,112.04	2,222.09	1,111.55	19
9	1,101.58	551.29	1,084.55	542.78	1,083.57	542.29	G	2,169.06	1,085.03	2,152.04	1,076.52	2,151.05	1,076.03	18
10	1,198.63	599.82	1,181.61	591.31	1,180.62	590.82	P	2,112.04	1,056.52	2,095.01	1,048.01	2,094.03	1,047.52	17
11	1,295.69	648.35	1,278.66	639.83	1,277.68	639.34	P	2,014.99	1,008.00	1,997.96	999.48	1,996.98	998.99	16
12	1,394.75	697.88	1,377.73	689.37	1,376.74	688.88	V	1,917.93	959.47	1,900.91	950.96	1,899.92	950.47	15
13	1,557.82	779.41	1,540.79	770.90	1,539.81	770.41	Y	1,818.87	909.94	1,801.84	901.42	1,800.86	900.93	14
14	1,704.89	852.95	1,687.86	844.43	1,686.88	843.94	F	1,655.80	828.41	1,638.78	819.89	1,637.79	819.40	13
15	1,803.95	902.48	1,786.93	893.97	1,785.94	893.48	V	1,508.73	754.87	1,491.71	746.36	1,490.72	745.87	12
16	1,917.04	959.02	1,900.01	950.51	1,899.03	950.02	L	1,409.67	705.34	1,392.64	696.82	1,391.66	696.33	11
17	2,046.08	1,023.54	2,029.06	1,015.03	2,028.07	1,014.54	E	1,296.58	648.79	1,279.56	640.28	1,278.57	639.79	10
18	2,175.12	1,088.07	2,158.10	1,079.55	2,157.11	1,079.06	E	1,167.54	584.27	1,150.51	575.76	1,149.53	575.27	9
19	2,232.15	1,116.58	2,215.12	1,108.06	2,214.14	1,107.57	G	1,038.50	519.75	1,021.47	511.24	1,020.49	510.75	8
20	2,369.20	1,185.11	2,352.18	1,176.59	2,351.19	1,176.10	H	981.48	491.24	964.45	482.73	963.47	482.24	7
21	2,484.23	1,242.62	2,467.20	1,234.11	2,466.22	1,233.61	D	844.42	422.71	827.39	414.20	826.41	413.71	6
22	2,647.29	1,324.15	2,630.27	1,315.64	2,629.28	1,315.15	Y	729.39	365.20	712.36	356.69	711.38	356.19	5
23	2,748.34	1,374.67	2,731.32	1,366.16	2,730.33	1,365.67	T	566.33	283.67	549.30	275.15	548.32	274.66	4

24	2,835.37	1,418.19	2,818.35	1,409.68	2,817.36	1,409.19	S	465.28	233.14	448.25	224.63	447.27	224.14	3
25	2,922.41	1,461.71	2,905.38	1,453.19	2,904.40	1,452.70	S	378.25	189.63	361.22	181.11	360.24	180.62	2
26							K	291.21	146.11	274.19	137.60			1

Query 40192 Hit 1

MS/MS Fragmentation of TFGIWTLLSSVIR
Found in sp|Q9UKR5|ERG28_HUMAN, Probable ergosterol biosynthetic protein 28 OS=Homo sapiens GN=C14orf1 PE=1 SV=1
Match to Query 40192: 1635.945from(818.9797,2+)
Title: 1385: Scan 3194 (rt=80.892, f=3, i=498) [D:\lab212\membrane\Grace\Joyce\iTRAQ_22_1.raw]
Data File:Submitted from 20120508-1(merge) by Mascot Daemon on JOYCE-VAIO
Monoisotopic mass of neutral peptide Mr(calc): 1635.945
Variable modifications:
Ions Score: 67.35 Expect: 0.000

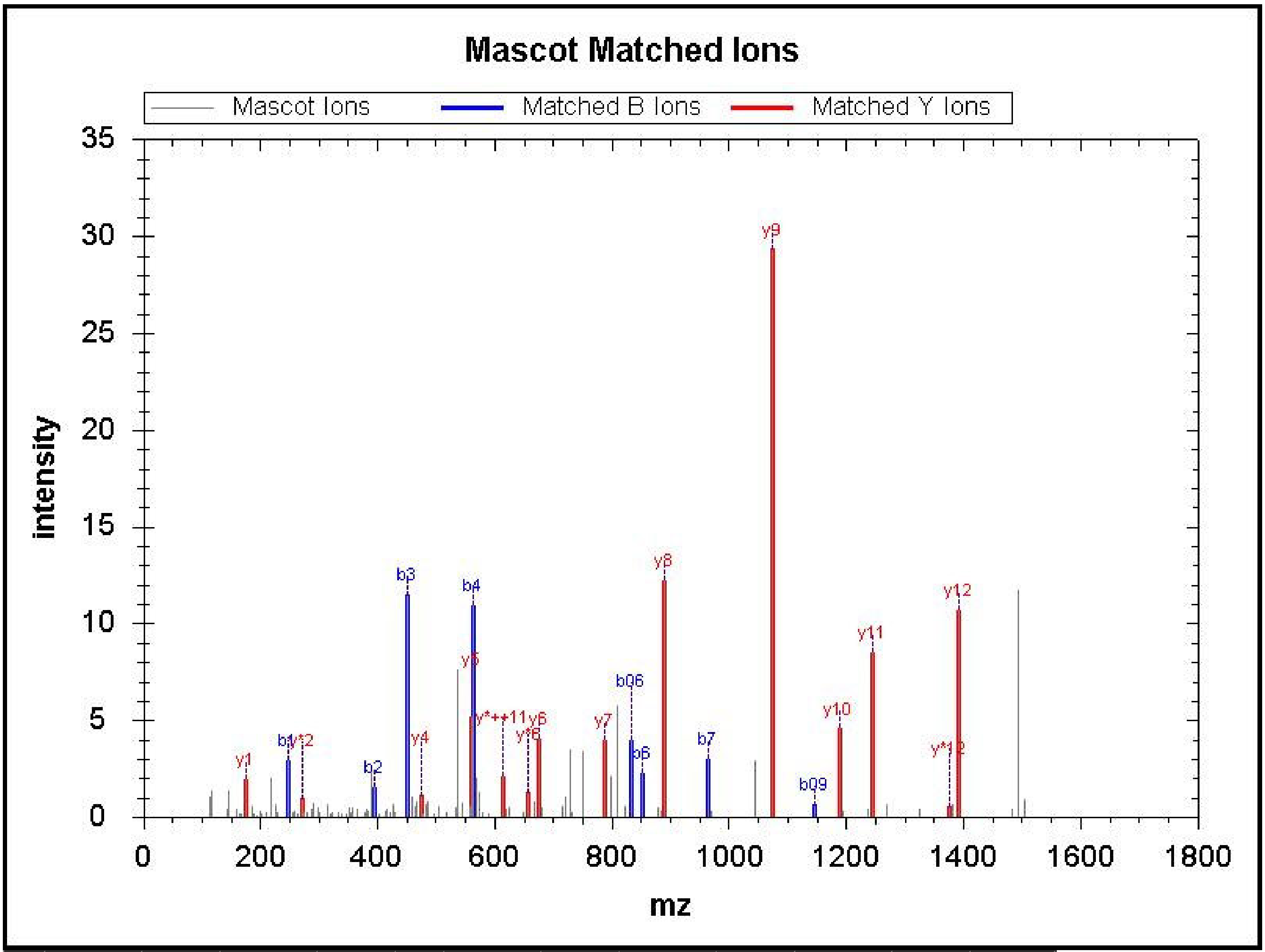

No	b	b++	b0	b0++	Seq	y	y++	y*	y*++	y0	y0++	RevNo
1	246.16	123.58	228.15	114.58	T							13
2	393.23	197.12	375.21	188.11	F	1,391.80	696.41	1,374.78	687.89	1,373.79	687.40	12
3	450.25	225.63	432.24	216.62	G	1,244.74	622.87	1,227.71	614.36	1,226.73	613.87	11
4	563.33	282.17	545.32	273.16	I	1,187.71	594.36	1,170.69	585.85	1,169.70	585.36	10
5	749.41	375.21	731.40	366.20	W	1,074.63	537.82	1,057.60	529.31	1,056.62	528.81	9
6	850.46	425.73	832.45	416.73	T	888.55	444.78	871.52	436.27	870.54	435.77	8
7	963.54	482.27	945.53	473.27	L	787.50	394.26	770.48	385.74	769.49	385.25	7
8	1,076.63	538.82	1,058.62	529.81	L	674.42	337.71	657.39	329.20	656.41	328.71	6
9	1,163.66	582.33	1,145.65	573.33	S	561.34	281.17	544.31	272.66	543.32	272.17	5
10	1,250.69	625.85	1,232.68	616.84	S	474.30	237.66	457.28	229.14	456.29	228.65	4
11	1,349.76	675.38	1,331.75	666.38	V	387.27	194.14	370.24	185.63			3

12	1,462.84	731.92	1,444.83	722.92	I	288.20	144.61	271.18	136.09			2
13					R	175.12	88.06	158.09	79.55			1

Query 42592 Hit 1

MS/MS Fragmentation of VILHGEHAVVHGK
Found in sp|Q03426|KIME_HUMAN, Mevalonate kinase OS=Homo sapiens GN=MVK PE=1 SV=1
Match to Query 42592: 1682.983from(562.0017,3+)
Title: 171: Scan 396 (rt=18.7496, f=2, i=67) [D:\lab212\membrane\Grace\Joyce\iTRAQ_55_2_330.raw]
Data File:Submitted from 20120508-1(merge) by Mascot Daemon on JOYCE-VAIO
Monoisotopic mass of neutral peptide Mr(calc): 1682.983
Variable modifications:
K13 :iTRAQ4plex (K)
Ions Score: 67.26 Expect: 0.000

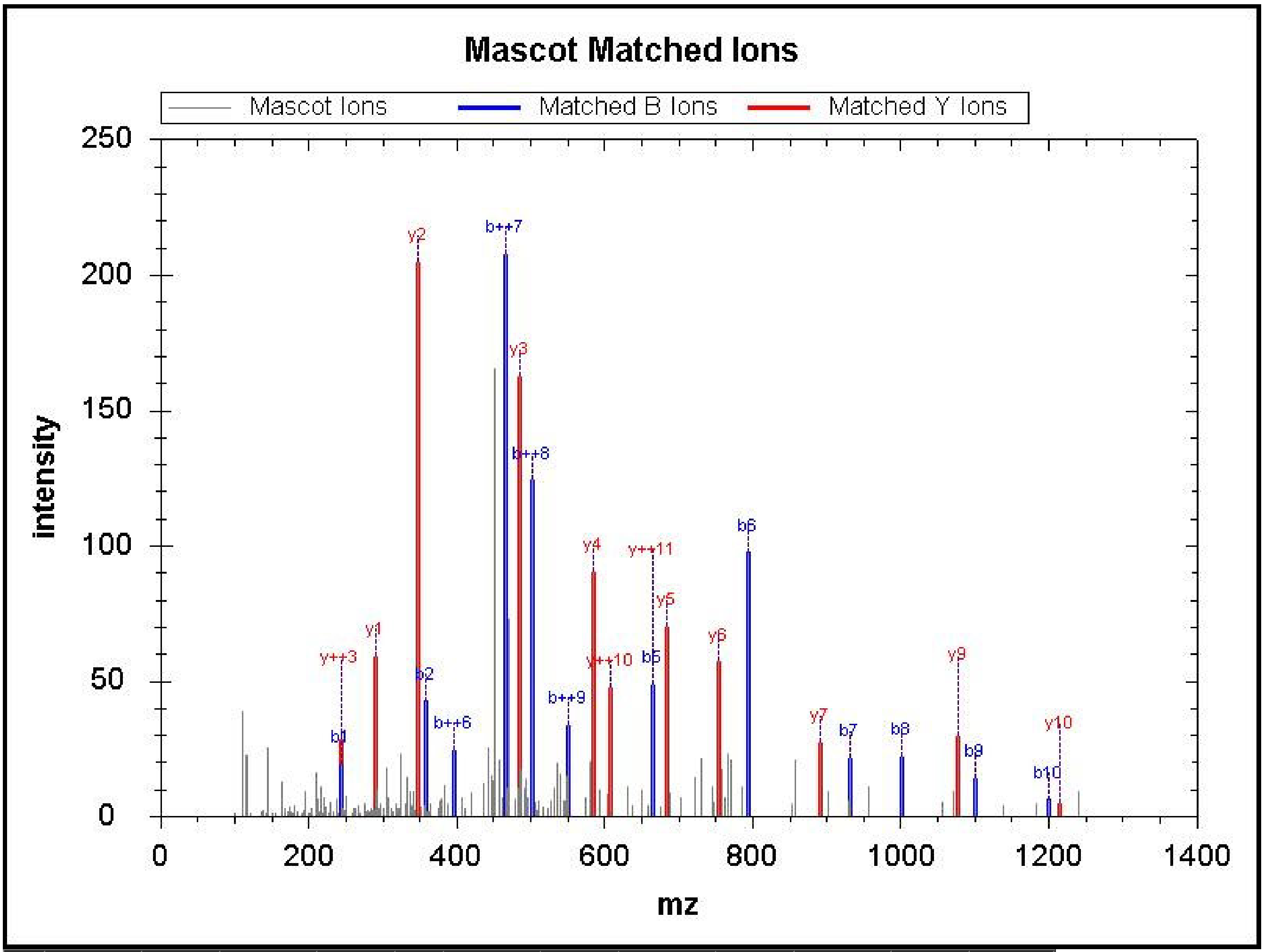

No	b	b++	b0	b0++	Seq	y	y++	y*	y*++	y0	y0++	RevNo
1	244.18	122.59			V							13
2	357.26	179.13			I	1,440.82	720.91	1,423.79	712.40	1,422.81	711.91	12
3	470.35	235.68			L	1,327.74	664.37	1,310.71	655.86	1,309.72	655.37	11
4	607.40	304.21			H	1,214.65	607.83	1,197.62	599.32	1,196.64	598.82	10
5	664.43	332.72			G	1,077.59	539.30	1,060.57	530.79	1,059.58	530.29	9
6	793.47	397.24	775.46	388.23	E	1,020.57	510.79	1,003.54	502.28	1,002.56	501.78	8
7	930.53	465.77	912.52	456.76	H	891.53	446.27	874.50	437.75			7
8	1,001.56	501.29	983.55	492.28	A	754.47	377.74	737.44	369.22			6
9	1,100.63	550.82	1,082.62	541.82	V	683.43	342.22	666.41	333.71			5
10	1,199.70	600.35	1,181.69	591.35	V	584.36	292.69	567.34	284.17			4
11	1,336.76	668.88	1,318.75	659.88	H	485.30	243.15	468.27	234.64			3

12	1,393.78	697.39	1,375.77	688.39	G	348.24	174.62	331.21	166.11			2
13					K	291.21	146.11	274.19	137.60			1

Query 94374 Hit 1

MS/MS Fragmentation of AADHLEALAAIEDFFLEHEALGISMAK
Found in sp|Q13144|EI2BE_HUMAN, Translation initiation factor eIF-2B subunit epsilon OS=Homo sapiens GN=EIF2B5 PE=1 SV=3
Match to Query 94374: 3199.65from(800.9197,4+)
Title: 1270: Scan 2787 (rt=72.332, f=2, i=458) [D:\lab212\membrane\Grace\Joyce\iTRAQ_45_1.raw]
Data File:Submitted from 20120508-1(merge) by Mascot Daemon on JOYCE-VAIO
Monoisotopic mass of neutral peptide Mr(calc): 3199.65
Variable modifications:
K27 :iTRAQ4plex (K)
Ions Score: 67.18 Expect: 0.000

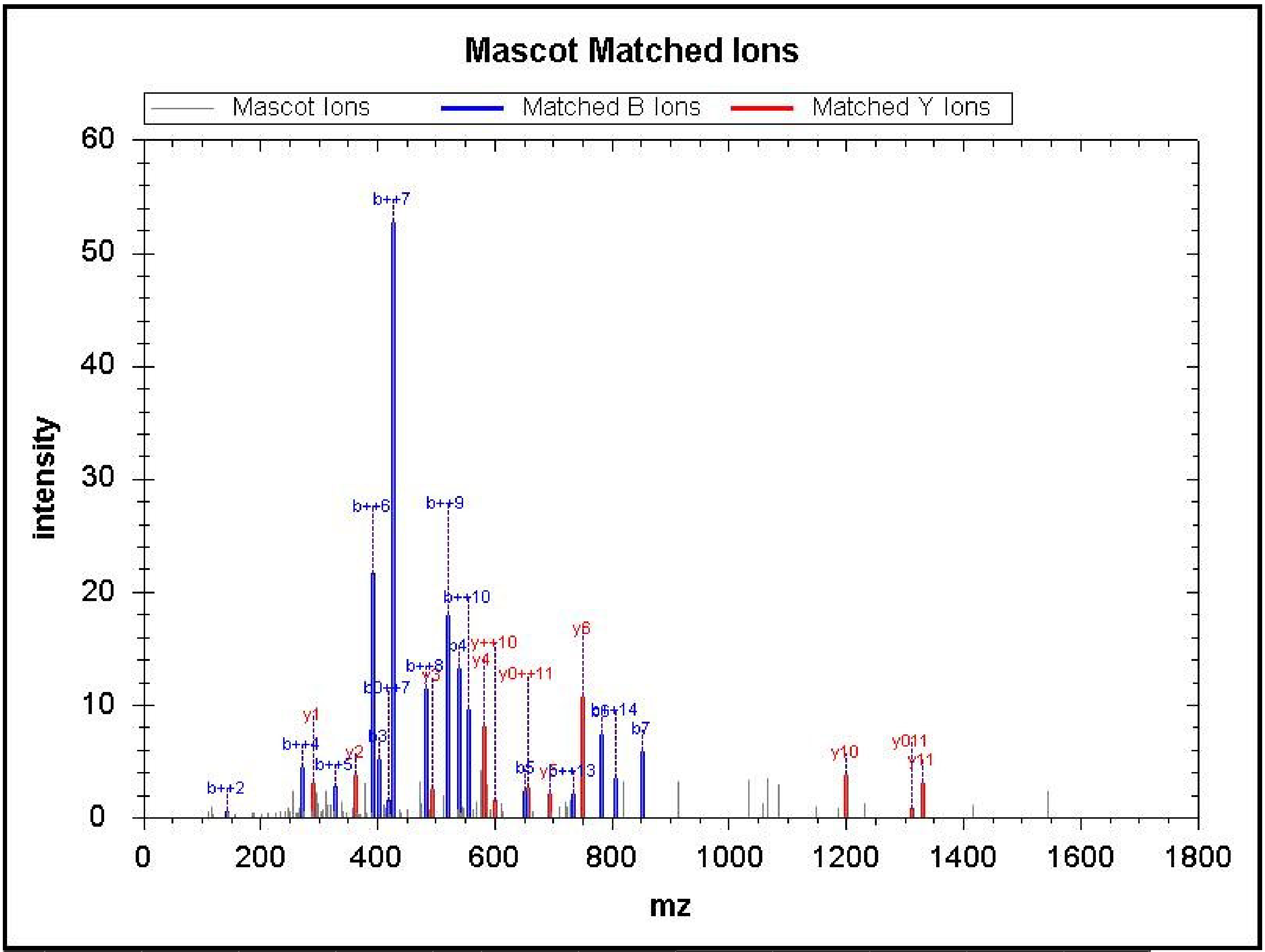

No	b	b++	b0	b0++	Seq	y	y++	y*	y*++	y0	y0++	RevNo
1	216.15	108.58			A							27
2	287.18	144.10			A	2,985.51	1,493.26	2,968.49	1,484.75	2,967.50	1,484.26	26
3	402.21	201.61	384.20	192.60	D	2,914.48	1,457.74	2,897.45	1,449.23	2,896.47	1,448.74	25
4	539.27	270.14	521.26	261.13	H	2,799.45	1,400.23	2,782.42	1,391.72	2,781.44	1,391.22	24
5	652.35	326.68	634.34	317.68	L	2,662.39	1,331.70	2,645.36	1,323.19	2,644.38	1,322.69	23
6	781.40	391.20	763.39	382.20	E	2,549.31	1,275.16	2,532.28	1,266.64	2,531.30	1,266.15	22
7	852.43	426.72	834.42	417.71	A	2,420.27	1,210.64	2,403.24	1,202.12	2,402.25	1,201.63	21
8	965.52	483.26	947.51	474.26	L	2,349.23	1,175.12	2,332.20	1,166.60	2,331.22	1,166.11	20
9	1,036.55	518.78	1,018.54	509.78	A	2,236.14	1,118.58	2,219.12	1,110.06	2,218.13	1,109.57	19
10	1,107.59	554.30	1,089.58	545.29	A	2,165.11	1,083.06	2,148.08	1,074.54	2,147.10	1,074.05	18
11	1,220.68	610.84	1,202.67	601.84	I	2,094.07	1,047.54	2,077.04	1,039.03	2,076.06	1,038.53	17

12	1,349.72	675.36	1,331.71	666.36	E	1,980.99	991.00	1,963.96	982.48	1,962.98	981.99	16
13	1,464.75	732.88	1,446.73	723.87	D	1,851.94	926.48	1,834.92	917.96	1,833.93	917.47	15
14	1,611.81	806.41	1,593.80	797.41	F	1,736.92	868.96	1,719.89	860.45	1,718.91	859.96	14
15	1,758.88	879.94	1,740.87	870.94	F	1,589.85	795.43	1,572.82	786.91	1,571.84	786.42	13
16	1,871.97	936.49	1,853.96	927.48	L	1,442.78	721.89	1,425.75	713.38	1,424.77	712.89	12
17	2,001.01	1,001.01	1,983.00	992.00	E	1,329.70	665.35	1,312.67	656.84	1,311.68	656.35	11
18	2,138.07	1,069.54	2,120.06	1,060.53	H	1,200.65	600.83	1,183.63	592.32	1,182.64	591.82	10
19	2,267.11	1,134.06	2,249.10	1,125.05	E	1,063.59	532.30	1,046.57	523.79	1,045.58	523.30	9
20	2,338.15	1,169.58	2,320.14	1,160.57	A	934.55	467.78	917.52	459.27	916.54	458.77	8
21	2,451.23	1,226.12	2,433.22	1,217.11	L	863.51	432.26	846.49	423.75	845.50	423.26	7
22	2,508.25	1,254.63	2,490.24	1,245.62	G	750.43	375.72	733.40	367.21	732.42	366.71	6
23	2,621.34	1,311.17	2,603.33	1,302.17	I	693.41	347.21	676.38	338.69	675.40	338.20	5
24	2,708.37	1,354.69	2,690.36	1,345.68	S	580.32	290.67	563.30	282.15	562.31	281.66	4
25	2,839.41	1,420.21	2,821.40	1,411.20	M	493.29	247.15	476.27	238.64			3
26	2,910.45	1,455.73	2,892.44	1,446.72	A	362.25	181.63	345.23	173.12			2
27					K	291.21	146.11	274.19	137.60			1

Query 36271 Hit 1

MS/MS Fragmentation of KIEQELTAAK
Found in sp|Q9H444|CHM4B_HUMAN, Charged multivesicular body protein 4b OS=Homo sapiens GN=CHMP4B PE=1 SV=1
Match to Query 36271: 1561.94from(521.654,3+)
Title: 338: Scan 874 (rt=29.144, f=3, i=130) [D:\lab212\membrane\Grace\Joyce\47_1.raw]
Data File:Submitted from 20120508-1(merge) by Mascot Daemon on JOYCE-VAIO
Monoisotopic mass of neutral peptide Mr(calc): 1561.94
Variable modifications:
K1 :iTRAQ4plex (K)
K10 :iTRAQ4plex (K)
Ions Score: 66.91 Expect: 0.000

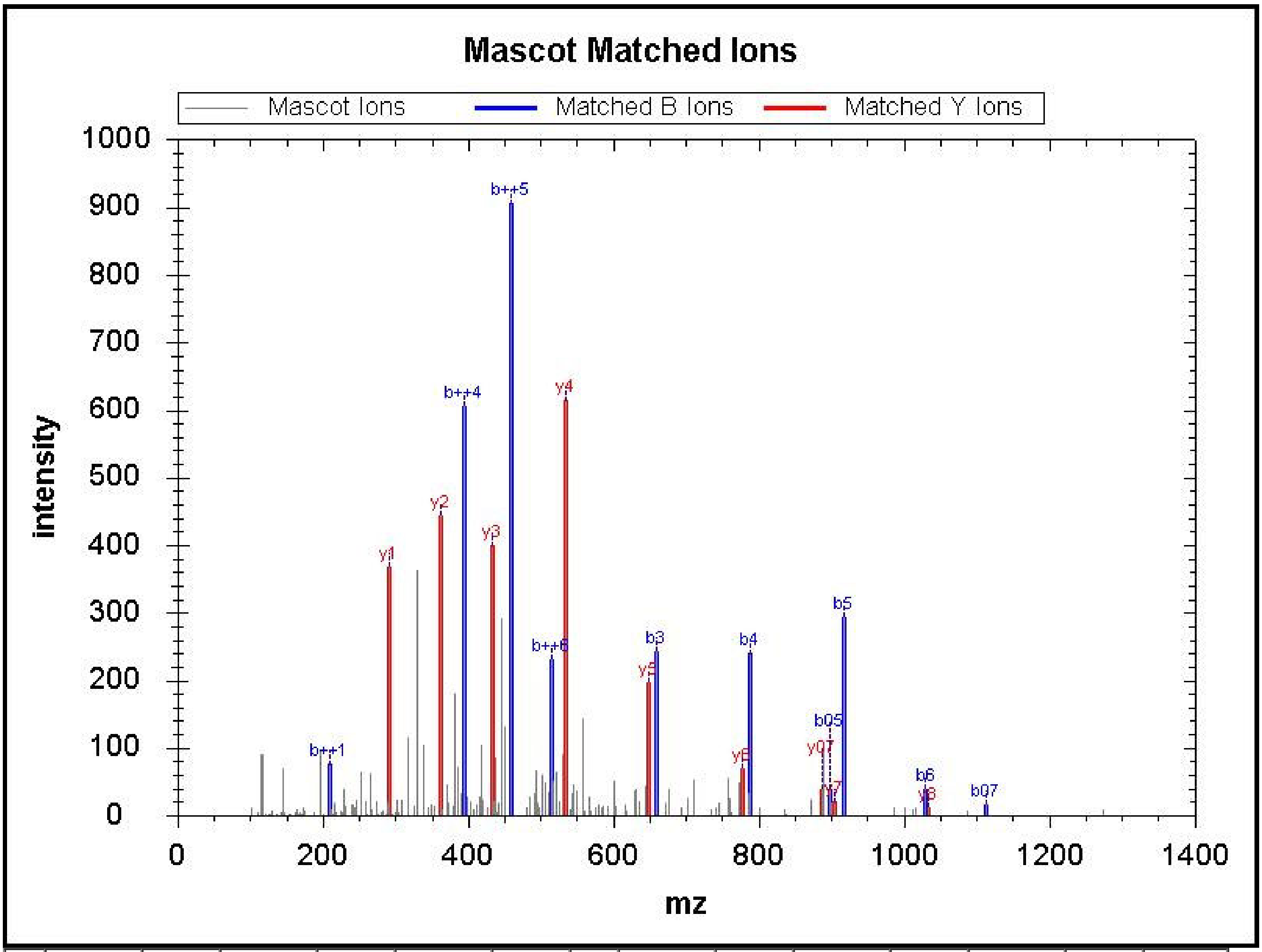

No	b	b++	b*	b*++	b0	b0++	Seq	y	y++	y*	y*++	y0	y0++	RevNo
1	417.31	209.16	400.28	200.64			K							10
2	530.39	265.70	513.36	257.19			I	1,146.65	573.83	1,129.62	565.31	1,128.64	564.82	9
3	659.43	330.22	642.41	321.71	641.42	321.21	E	1,033.56	517.29	1,016.54	508.77	1,015.55	508.28	8
4	787.49	394.25	770.47	385.74	769.48	385.24	Q	904.52	452.76	887.50	444.25	886.51	443.76	7
5	916.53	458.77	899.51	450.26	898.52	449.77	E	776.46	388.74	759.44	380.22	758.45	379.73	6
6	1,029.62	515.31	1,012.59	506.80	1,011.61	506.31	L	647.42	324.21	630.39	315.70	629.41	315.21	5
7	1,130.67	565.84	1,113.64	557.32	1,112.66	556.83	T	534.34	267.67	517.31	259.16	516.33	258.67	4
8	1,201.70	601.36	1,184.68	592.84	1,183.69	592.35	A	433.29	217.15	416.26	208.63			3
9	1,272.74	636.87	1,255.71	628.36	1,254.73	627.87	A	362.25	181.63	345.23	173.12			2
10							K	291.21	146.11	274.19	137.60			1

Query 91854 Hit 1

MS/MS Fragmentation of QGQAITLVTQYDIHLVHAIEEQIK
Found in sp|Q9Y6V7|DDX49_HUMAN, Probable ATP-dependent RNA helicase DDX49 OS=Homo sapiens GN=DDX49 PE=1 SV=1
Match to Query 91854: 3034.672from(759.6753,4+)
Title: 1130: Sum of 2 scans in range 2433 (rt=64.507, f=4, i=761) to 2434 (rt=64.5324, f=4, i=762)
[D:\lab212\membrane\Grace\Joyce\iTRAQ_45_1.raw]
Data File:Submitted from 20120508-1(merge) by Mascot Daemon on JOYCE-VAIO
Monoisotopic mass of neutral peptide Mr(calc): 3034.672
Variable modifications:
K24 :iTRAQ4plex (K)
Ions Score: 66.8 Expect: 0.000

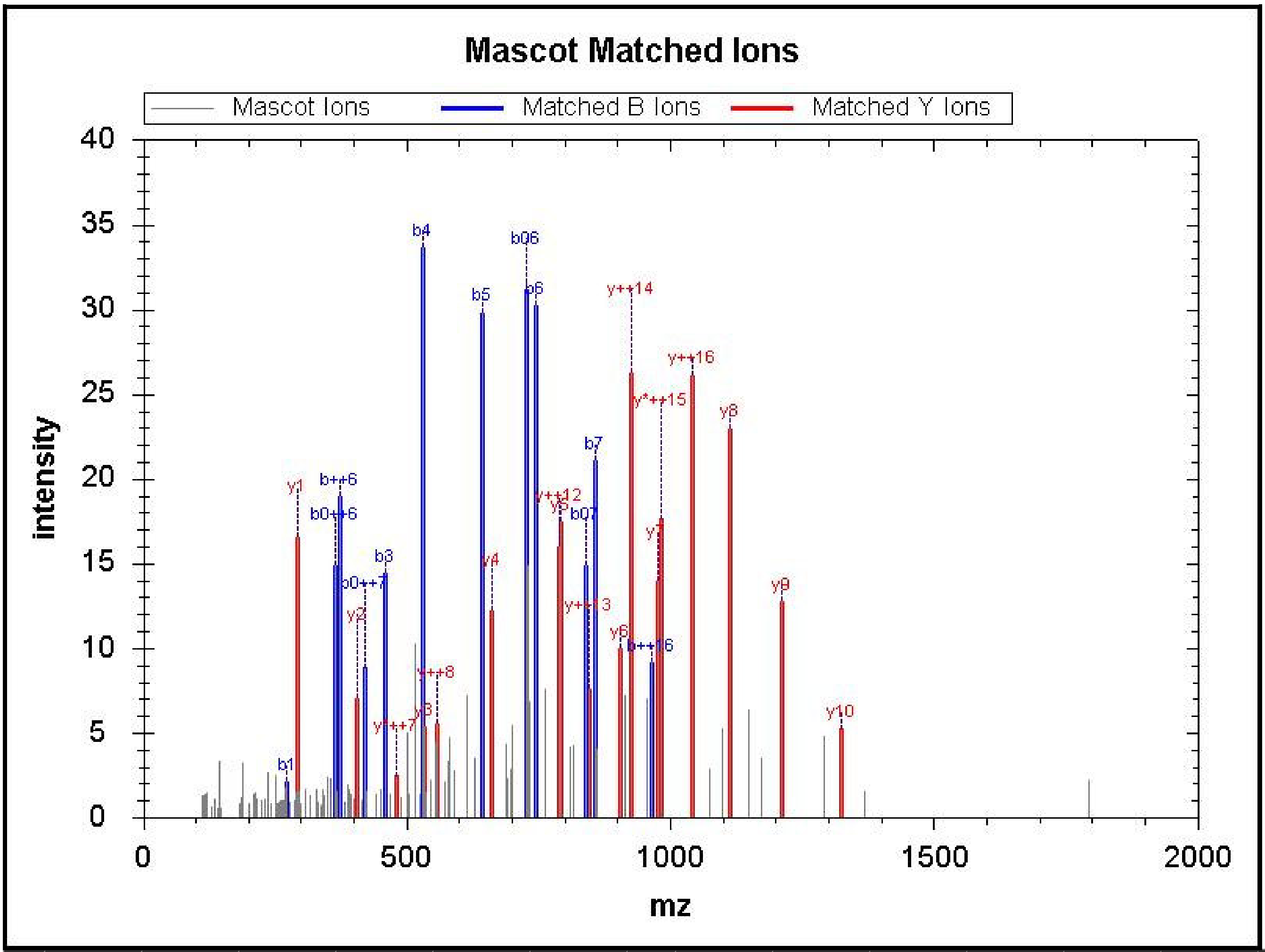

No	b	b++	b*	b*++	b0	b0++	Seq	y	y++	y*	y*++	y0	y0++	RevNo
1	273.17	137.09	256.14	128.57			Q							24
2	330.19	165.60	313.16	157.09			G	2,763.52	1,382.26	2,746.49	1,373.75	2,745.51	1,373.26	23
3	458.25	229.63	441.22	221.11			Q	2,706.49	1,353.75	2,689.47	1,345.24	2,688.48	1,344.75	22
4	529.29	265.15	512.26	256.63			A	2,578.44	1,289.72	2,561.41	1,281.21	2,560.43	1,280.72	21
5	642.37	321.69	625.34	313.17			I	2,507.40	1,254.20	2,490.37	1,245.69	2,489.39	1,245.20	20
6	743.42	372.21	726.39	363.70	725.41	363.21	T	2,394.31	1,197.66	2,377.29	1,189.15	2,376.30	1,188.66	19
7	856.50	428.75	839.47	420.24	838.49	419.75	L	2,293.27	1,147.14	2,276.24	1,138.62	2,275.26	1,138.13	18
8	955.57	478.29	938.54	469.77	937.56	469.28	V	2,180.18	1,090.60	2,163.16	1,082.08	2,162.17	1,081.59	17
9	1,056.62	528.81	1,039.59	520.30	1,038.61	519.81	T	2,081.11	1,041.06	2,064.09	1,032.55	2,063.10	1,032.06	16
10	1,184.68	592.84	1,167.65	584.33	1,166.66	583.84	Q	1,980.07	990.54	1,963.04	982.02	1,962.06	981.53	15
11	1,347.74	674.37	1,330.71	665.86	1,329.73	665.37	Y	1,852.01	926.51	1,834.98	917.99	1,834.00	917.50	14
12	1,462.77	731.89	1,445.74	723.37	1,444.76	722.88	D	1,688.95	844.98	1,671.92	836.46	1,670.93	835.97	13
13	1,575.85	788.43	1,558.82	779.92	1,557.84	779.42	I	1,573.92	787.46	1,556.89	778.95	1,555.91	778.46	12
14	1,712.91	856.96	1,695.88	848.44	1,694.90	847.95	H	1,460.83	730.92	1,443.81	722.41	1,442.82	721.92	11
15	1,825.99	913.50	1,808.97	904.99	1,807.98	904.49	L	1,323.78	662.39	1,306.75	653.88	1,305.76	653.39	10
16	1,925.06	963.03	1,908.03	954.52	1,907.05	954.03	V	1,210.69	605.85	1,193.66	597.34	1,192.68	596.84	9
17	2,062.12	1,031.56	2,045.09	1,023.05	2,044.11	1,022.56	H	1,111.62	556.32	1,094.60	547.80	1,093.61	547.31	8
18	2,133.16	1,067.08	2,116.13	1,058.57	2,115.15	1,058.08	A	974.56	487.79	957.54	479.27	956.55	478.78	7
19	2,246.24	1,123.62	2,229.21	1,115.11	2,228.23	1,114.62	I	903.53	452.27	886.50	443.75	885.52	443.26	6
20	2,375.28	1,188.15	2,358.26	1,179.63	2,357.27	1,179.14	E	790.44	395.72	773.42	387.21	772.43	386.72	5
21	2,504.33	1,252.67	2,487.30	1,244.15	2,486.32	1,243.66	E	661.40	331.20	644.37	322.69	643.39	322.20	4
22	2,632.39	1,316.70	2,615.36	1,308.18	2,614.37	1,307.69	Q	532.36	266.68	515.33	258.17			3
23	2,745.47	1,373.24	2,728.44	1,364.72	2,727.46	1,364.23	I	404.30	202.65	387.27	194.14			2
24							K	291.21	146.11	274.19	137.60			1

Query 65874 Hit 1

MS/MS Fragmentation of ASLAAILEHSLFSTEQK
Found in sp|Q14149|MORC3_HUMAN, MORC family CW-type zinc finger protein 3 OS=Homo sapiens GN=MORC3 PE=1 SV=3
Match to Query 65874: 2132.173from(711.7318,3+)
Title: 1057: Scan 2308 (rt=61.5961, f=2, i=376) [D:\lab212\membrane\Grace\Joyce\iTRAQ_39_2.raw]
Data File:Submitted from 20120508-1(merge) by Mascot Daemon on JOYCE-VAIO
Monoisotopic mass of neutral peptide Mr(calc): 2132.173
Variable modifications:
K17 :iTRAQ4plex (K)
Ions Score: 66.51 Expect: 0.000

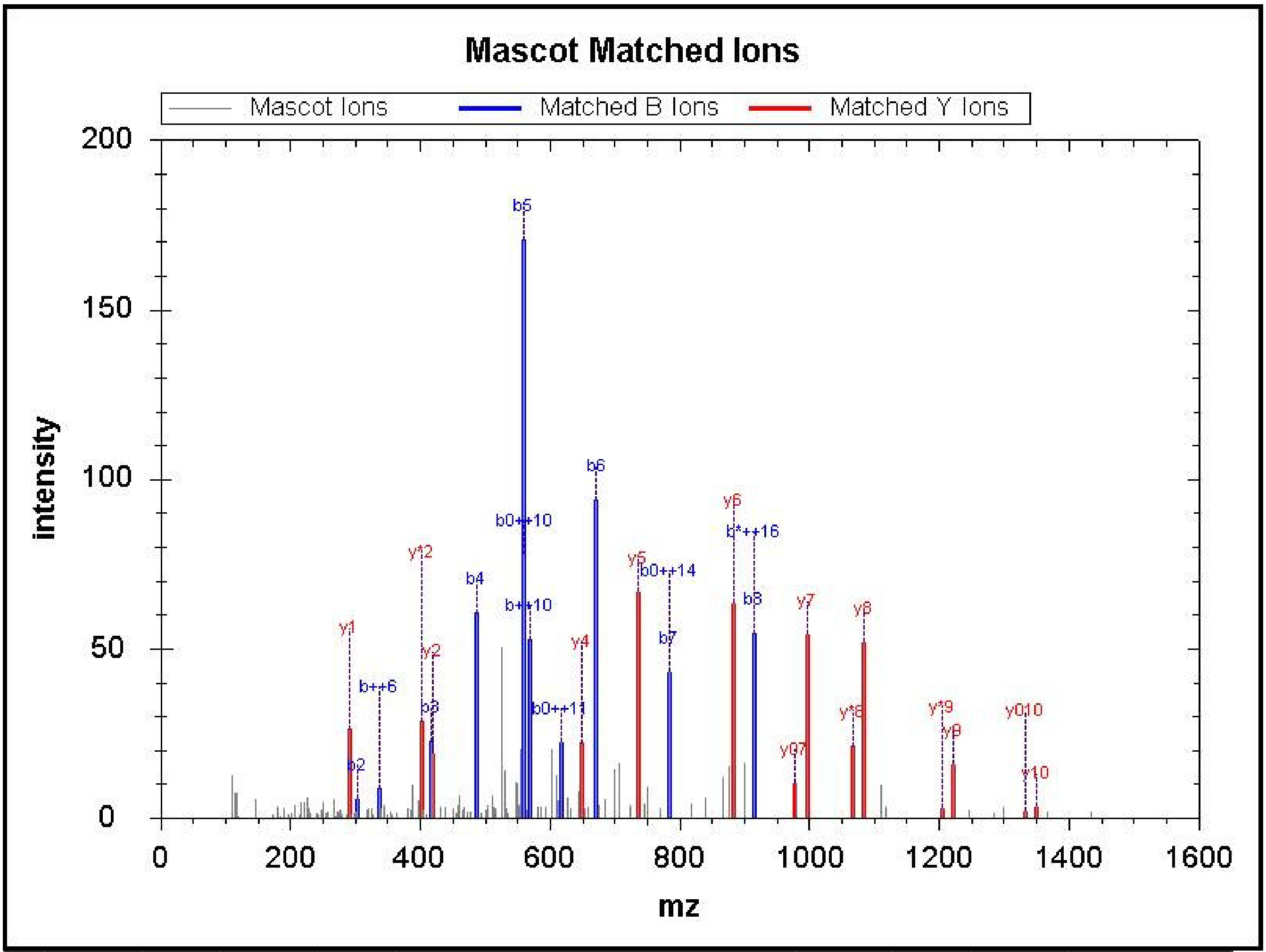

No	b	b++	b*	b*++	b0	b0++	Seq	y	y++	y*	y*++	y0	y0++	RevNo
1	216.15	108.58					A							17
2	303.18	152.09			285.17	143.09	S	1,918.04	959.52	1,901.01	951.01	1,900.03	950.52	16
3	416.26	208.63			398.25	199.63	L	1,831.01	916.01	1,813.98	907.49	1,813.00	907.00	15
4	487.30	244.15			469.29	235.15	A	1,717.92	859.47	1,700.90	850.95	1,699.91	850.46	14
5	558.34	279.67			540.33	270.67	A	1,646.89	823.95	1,629.86	815.43	1,628.88	814.94	13
6	671.42	336.21			653.41	327.21	I	1,575.85	788.43	1,558.82	779.92	1,557.84	779.42	12
7	784.50	392.76			766.49	383.75	L	1,462.77	731.89	1,445.74	723.37	1,444.76	722.88	11
8	913.55	457.28			895.54	448.27	E	1,349.68	675.34	1,332.66	666.83	1,331.67	666.34	10
9	1,050.61	525.81			1,032.60	516.80	H	1,220.64	610.82	1,203.61	602.31	1,202.63	601.82	9
10	1,137.64	569.32			1,119.63	560.32	S	1,083.58	542.29	1,066.55	533.78	1,065.57	533.29	8
11	1,250.72	625.86			1,232.71	616.86	L	996.55	498.78	979.52	490.26	978.54	489.77	7
12	1,397.79	699.40			1,379.78	690.39	F	883.46	442.24	866.44	433.72	865.45	433.23	6
13	1,484.82	742.92			1,466.81	733.91	S	736.40	368.70	719.37	360.19	718.39	359.70	5
14	1,585.87	793.44			1,567.86	784.43	T	649.36	325.19	632.34	316.67	631.35	316.18	4

15	1,714.91	857.96			1,696.90	848.96	E	548.32	274.66	531.29	266.15	530.31	265.66	3
16	1,842.97	921.99	1,825.95	913.48	1,824.96	912.98	Q	419.27	210.14	402.25	201.63			2
17							K	291.21	146.11	274.19	137.60			1

Query 36723 Hit 1

MS/MS Fragmentation of IIADIFEYTAK
Found in sp|P22033|MUTA_HUMAN, Methylmalonyl-CoA mutase
Match to Query 36723: 1570.884from(786.4494,2+)
Title: 938: Scan 2099 (rt=56.6345, f=3, i=318) [D:\lab212\membrane\Grace\Joyce\iTRAQ_28_1_327.raw]
Data File:Submitted from 20120508-1(merge) by Mascot Daemon on JOYCE-VAIO
Monoisotopic mass of neutral peptide Mr(calc): 1570.884
Variable modifications:
K11 :iTRAQ4plex (K)
Ions Score: 66.41 Expect: 0.000

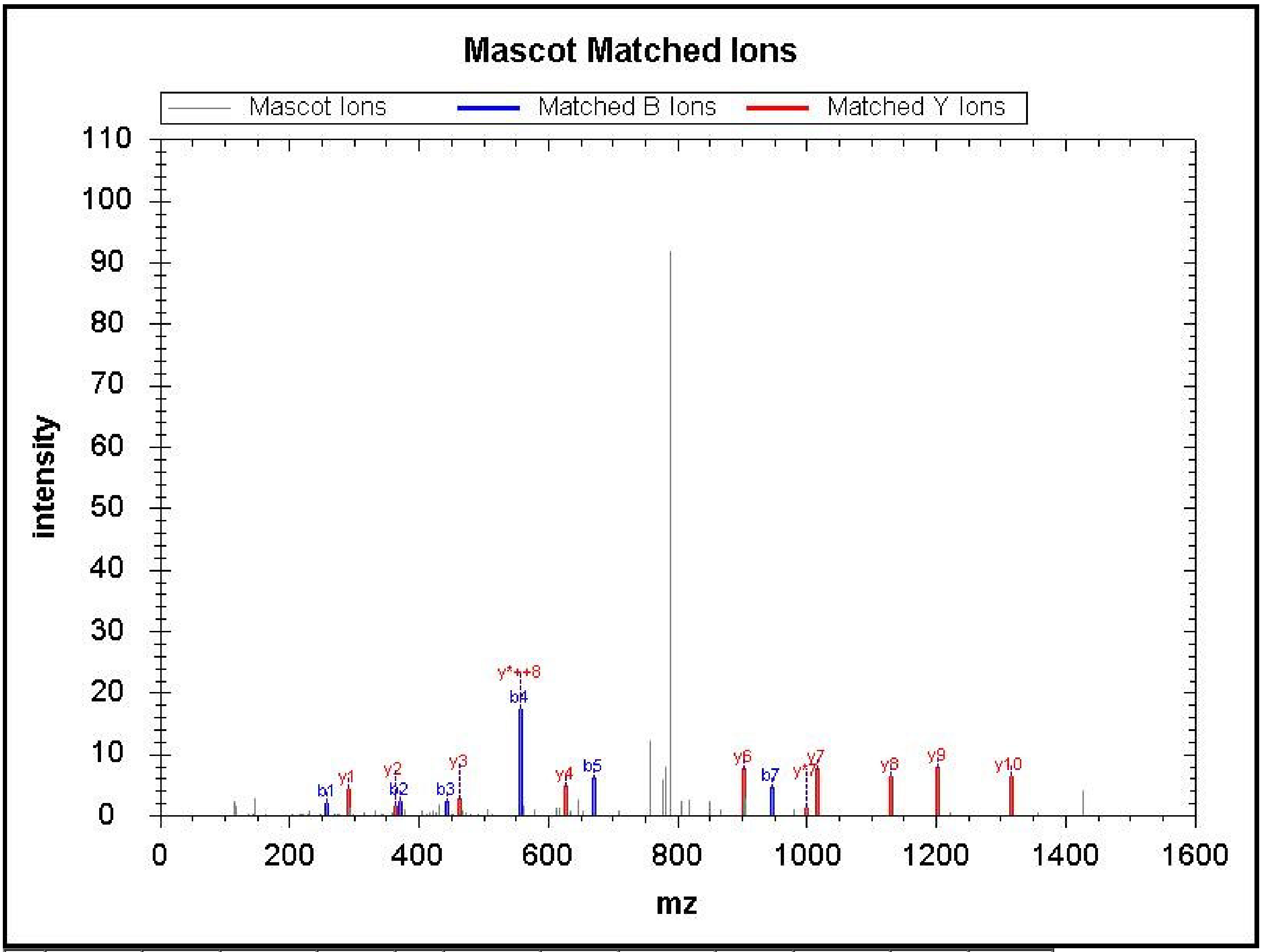

No	b	b++	b0	b0++	Seq	y	y++	y*	y*++	y0	y0++	RevNo
1	258.19	129.60			I							11
2	371.28	186.14			I	1,314.71	657.86	1,297.68	649.34	1,296.70	648.85	10
3	442.31	221.66			A	1,201.62	601.31	1,184.60	592.80	1,183.61	592.31	9
4	557.34	279.17	539.33	270.17	D	1,130.58	565.80	1,113.56	557.28	1,112.57	556.79	8
5	670.43	335.72	652.41	326.71	I	1,015.56	508.28	998.53	499.77	997.55	499.28	7
6	817.49	409.25	799.48	400.25	F	902.47	451.74	885.45	443.23	884.46	442.74	6
7	946.54	473.77	928.53	464.77	E	755.41	378.21	738.38	369.69	737.39	369.20	5
8	1,109.60	555.30	1,091.59	546.30	Y	626.36	313.69	609.34	305.17	608.35	304.68	4
9	1,210.65	605.83	1,192.64	596.82	T	463.30	232.15	446.27	223.64	445.29	223.15	3
10	1,281.68	641.35	1,263.67	632.34	A	362.25	181.63	345.23	173.12			2

11					K	291.21	146.11	274.19	137.60			1

Query 87153 Hit 1

MS/MS Fragmentation of HLAGYEQQDAHEFLIAALDVLHR
Found in sp|Q9UPT9|UBP22_HUMAN, Ubiquitin carboxyl-terminal hydrolase 22 OS=Homo sapiens GN=USP22 PE=1 SV=2
Match to Query 87153: 2789.443from(698.368,4+)
Title: 1039: Scan 2214 (rt=59.5665, f=3, i=349) [D:\lab212\membrane\Grace\Joyce\iTRAQ_50_2.raw]
Data File:Submitted from 20120508-1(merge) by Mascot Daemon on JOYCE-VAIO
Monoisotopic mass of neutral peptide Mr(calc): 2789.443
Variable modifications:
Ions Score: 66.23 Expect: 0.000

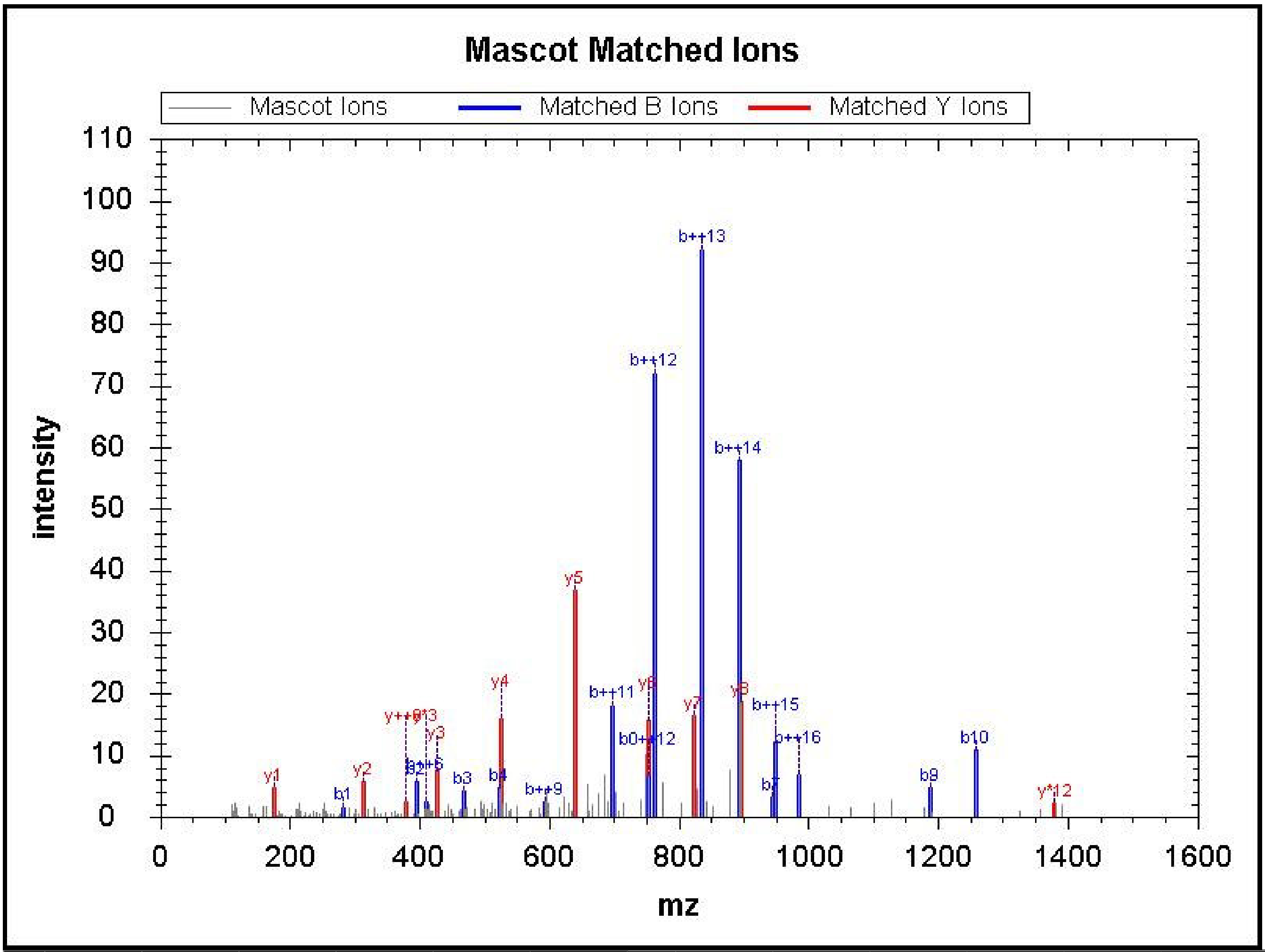

No	b	b++	b*	b*++	b0	b0++	Seq	y	y++	y*	y*++	y0	y0++	RevNo
1	282.17	141.59					H							23
2	395.25	198.13					L	2,509.28	1,255.15	2,492.26	1,246.63	2,491.27	1,246.14	22
3	466.29	233.65					A	2,396.20	1,198.60	2,379.17	1,190.09	2,378.19	1,189.60	21
4	523.31	262.16					G	2,325.16	1,163.08	2,308.14	1,154.57	2,307.15	1,154.08	20
5	686.37	343.69					Y	2,268.14	1,134.57	2,251.11	1,126.06	2,250.13	1,125.57	19
6	815.42	408.21			797.41	399.21	E	2,105.08	1,053.04	2,088.05	1,044.53	2,087.07	1,044.04	18
7	943.48	472.24	926.45	463.73	925.46	463.24	Q	1,976.03	988.52	1,959.01	980.01	1,958.02	979.52	17
8	1,071.53	536.27	1,054.51	527.76	1,053.52	527.27	Q	1,847.98	924.49	1,830.95	915.98	1,829.97	915.49	16
9	1,186.56	593.78	1,169.53	585.27	1,168.55	584.78	D	1,719.92	860.46	1,702.89	851.95	1,701.91	851.46	15
10	1,257.60	629.30	1,240.57	620.79	1,239.59	620.30	A	1,604.89	802.95	1,587.86	794.44	1,586.88	793.94	14
11	1,394.66	697.83	1,377.63	689.32	1,376.65	688.83	H	1,533.85	767.43	1,516.83	758.92	1,515.84	758.43	13
12	1,523.70	762.35	1,506.67	753.84	1,505.69	753.35	E	1,396.79	698.90	1,379.77	690.39	1,378.78	689.90	12
13	1,670.77	835.89	1,653.74	827.37	1,652.76	826.88	F	1,267.75	634.38	1,250.73	625.87	1,249.74	625.37	11
14	1,783.85	892.43	1,766.83	883.92	1,765.84	883.42	L	1,120.68	560.85	1,103.66	552.33	1,102.67	551.84	10

15	1,896.94	948.97	1,879.91	940.46	1,878.93	939.97	I	1,007.60	504.30	990.57	495.79	989.59	495.30	9
16	1,967.97	984.49	1,950.95	975.98	1,949.96	975.49	A	894.52	447.76	877.49	439.25	876.50	438.76	8
17	2,039.01	1,020.01	2,021.98	1,011.50	2,021.00	1,011.00	A	823.48	412.24	806.45	403.73	805.47	403.24	7
18	2,152.09	1,076.55	2,135.07	1,068.04	2,134.08	1,067.55	L	752.44	376.72	735.41	368.21	734.43	367.72	6
19	2,267.12	1,134.06	2,250.09	1,125.55	2,249.11	1,125.06	D	639.36	320.18	622.33	311.67	621.35	311.18	5
20	2,366.19	1,183.60	2,349.16	1,175.09	2,348.18	1,174.59	V	524.33	262.67	507.30	254.16			4
21	2,479.27	1,240.14	2,462.25	1,231.63	2,461.26	1,231.14	L	425.26	213.13	408.24	204.62			3
22	2,616.33	1,308.67	2,599.31	1,300.16	2,598.32	1,299.66	H	312.18	156.59	295.15	148.08			2
23							R	175.12	88.06	158.09	79.55			1

Query 76347 Hit 1

MS/MS Fragmentation of QLAENLVVIGGTSMLPGFLHR
Found in sp|Q9NZ32|ARP10_HUMAN, Actin-related protein 10 OS=Homo sapiens GN=ACTR10 PE=1 SV=1
Match to Query 76347: 2395.311 from(799.4443,3+)
Title: 998: Sum of 2 scans in range 2213 (rt=59.216, f=4, i=669) to 2214 (rt=59.2414, f=4, i=670) [D:\lab212\membrane\Grace\Joyce\iTRAQ_31_2.raw]
Data File:Submitted from 20120508-1(merge) by Mascot Daemon on JOYCE-VAIO
Monoisotopic mass of neutral peptide Mr(calc): 2395.311
Variable modifications:
Ions Score: 66.1 Expect: 0.000

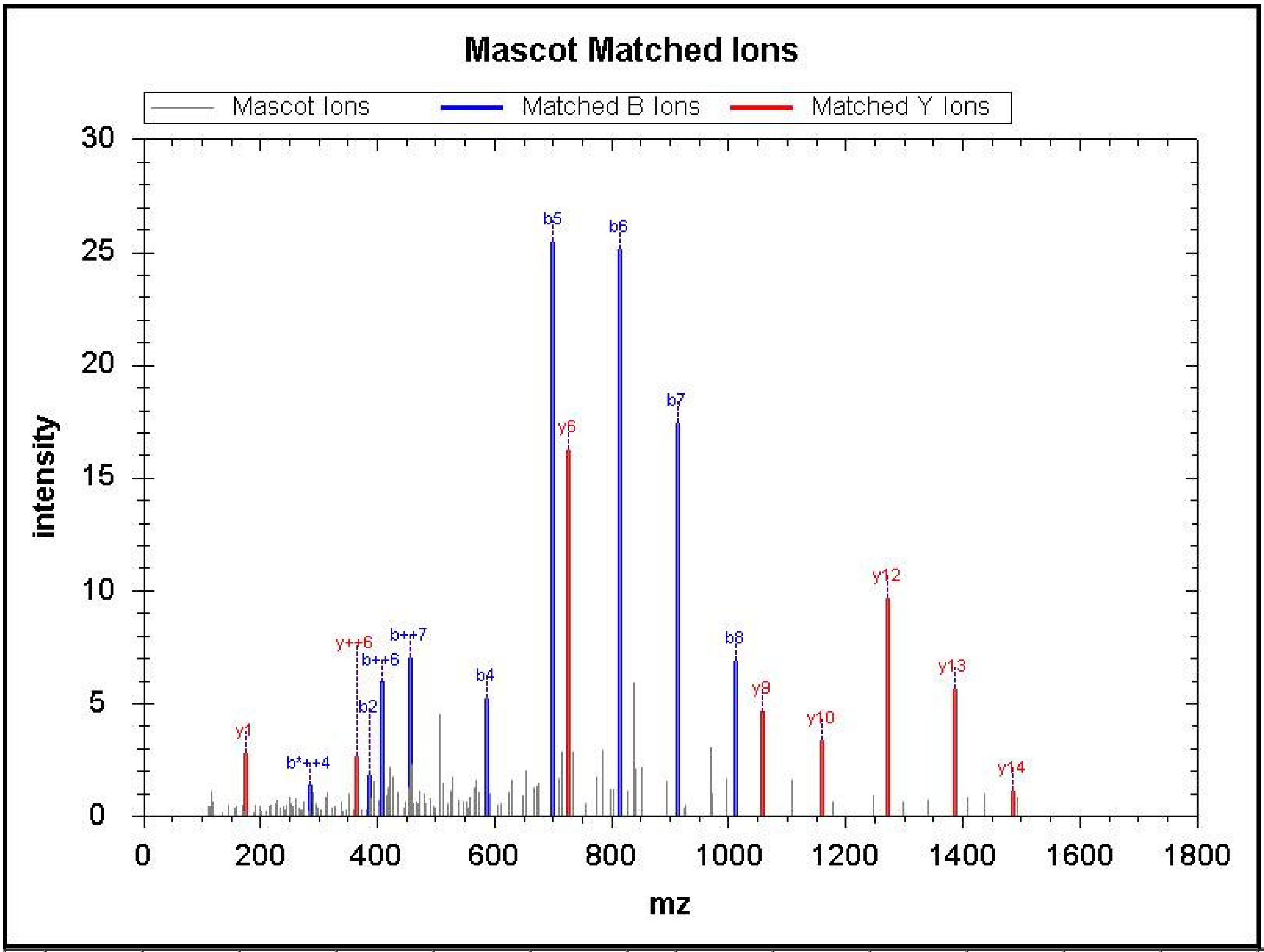

No	b	b++	b*	b*++	b0	b0++	Seq	y	y++	y*	y*++	y0	y0++	RevNo
1	273.17	137.09	256.14	128.57			Q							21
2	386.25	193.63	369.23	185.12			L	2,124.16	1,062.59	2,107.14	1,054.07	2,106.15	1,053.58	20
3	457.29	229.15	440.26	220.63			A	2,011.08	1,006.04	1,994.05	997.53	1,993.07	997.04	19
4	586.33	293.67	569.31	285.16	568.32	284.66	E	1,940.04	970.52	1,923.02	962.01	1,922.03	961.52	18
5	700.37	350.69	683.35	342.18	682.36	341.69	N	1,811.00	906.00	1,793.97	897.49	1,792.99	897.00	17

6	813.46	407.23	796.43	398.72	795.45	398.23	L	1,696.96	848.98	1,679.93	840.47	1,678.95	839.98	16
7	912.53	456.77	895.50	448.25	894.52	447.76	V	1,583.87	792.44	1,566.85	783.93	1,565.86	783.43	15
8	1,011.60	506.30	994.57	497.79	993.58	497.30	V	1,484.80	742.91	1,467.78	734.39	1,466.79	733.90	14
9	1,124.68	562.84	1,107.65	554.33	1,106.67	553.84	I	1,385.74	693.37	1,368.71	684.86	1,367.73	684.37	13
10	1,181.70	591.35	1,164.67	582.84	1,163.69	582.35	G	1,272.65	636.83	1,255.63	628.32	1,254.64	627.82	12
11	1,238.72	619.86	1,221.70	611.35	1,220.71	610.86	G	1,215.63	608.32	1,198.60	599.81	1,197.62	599.31	11
12	1,339.77	670.39	1,322.74	661.88	1,321.76	661.38	T	1,158.61	579.81	1,141.58	571.29	1,140.60	570.80	10
13	1,426.80	713.90	1,409.78	705.39	1,408.79	704.90	S	1,057.56	529.28	1,040.53	520.77	1,039.55	520.28	9
14	1,557.84	779.43	1,540.82	770.91	1,539.83	770.42	M	970.53	485.77	953.50	477.25			8
15	1,670.93	835.97	1,653.90	827.45	1,652.92	826.96	L	839.49	420.25	822.46	411.73			7
16	1,767.98	884.49	1,750.95	875.98	1,749.97	875.49	P	726.40	363.71	709.38	355.19			6
17	1,825.00	913.00	1,807.97	904.49	1,806.99	904.00	G	629.35	315.18	612.33	306.67			5
18	1,972.07	986.54	1,955.04	978.03	1,954.06	977.53	F	572.33	286.67	555.30	278.16			4
19	2,085.15	1,043.08	2,068.13	1,034.57	2,067.14	1,034.08	L	425.26	213.13	408.24	204.62			3
20	2,222.21	1,111.61	2,205.19	1,103.10	2,204.20	1,102.60	H	312.18	156.59	295.15	148.08			2
21							R	175.12	88.06	158.09	79.55			1

Query 19052 Hit 1

MS/MS Fragmentation of MDAEHPELR
Found in sp|Q12765|SCRN1_HUMAN, Secernin-1 OS=Homo sapiens GN=SCRN1 PE=1 SV=2
Match to Query 19052: 1240.515from(414.5121,3+)
Title: 211: Sum of 2 scans in range 500 (rt=21.0105, f=4, i=141) to 501 (rt=21.0359, f=4, i=142) [D:\lab212\membrane\Grace\Joyce\iTRAQ40.raw]
Data File:Submitted from 20120508-1(merge) by Mascot Daemon on JOYCE-VAIO
Monoisotopic mass of neutral peptide Mr(calc): 1240.515
Variable modifications:
Ions Score: 65.93 Expect: 0.000

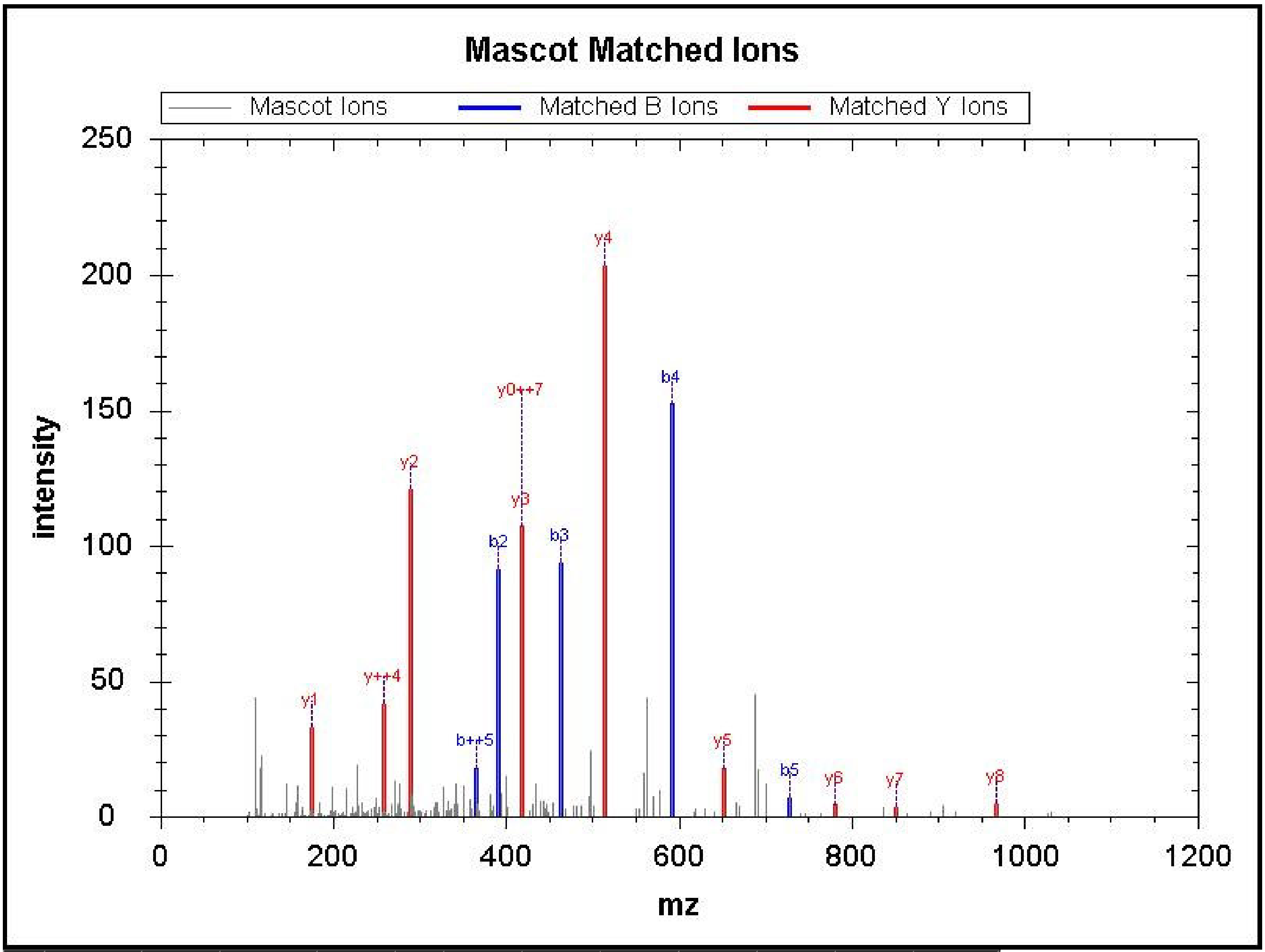

No	b	b++	b0	b0++	Seq	y	y++	y*	y*++	y0	y0++	RevNo
1	276.15	138.58			M							9
2	391.18	196.09	373.17	187.09	D	966.46	483.74	949.44	475.22	948.45	474.73	8
3	462.21	231.61	444.20	222.61	A	851.44	426.22	834.41	417.71	833.43	417.22	7
4	591.26	296.13	573.25	287.13	E	780.40	390.70	763.37	382.19	762.39	381.70	6
5	728.32	364.66	710.30	355.66	H	651.36	326.18	634.33	317.67	633.35	317.18	5
6	825.37	413.19	807.36	404.18	P	514.30	257.65	497.27	249.14	496.29	248.65	4
7	954.41	477.71	936.40	468.70	E	417.25	209.13	400.22	200.61	399.24	200.12	3
8	1,067.49	534.25	1,049.48	525.25	L	288.20	144.61	271.18	136.09			2
9					R	175.12	88.06	158.09	79.55			1

Query 56590 Hit 1

MS/MS Fragmentation of AHEGEIEDLALGPDGK
Found in sp|Q9HCU5|PREB_HUMAN, Prolactin regulatory element-binding protein OS=Homo sapiens GN=PREB PE=1 SV=2
Match to Query 56590: 1937.996from(647.0059,3+)
Title: 474: Sum of 2 scans in range 1072 (rt=33.9237, f=4, i=328) to 1073 (rt=33.9491, f=4, i=329) [D:\lab212\membrane\Grace\Joyce\iTRAQ_42_2.raw]
Data File:Submitted from 20120508-1(merge) by Mascot Daemon on JOYCE-VAIO
Monoisotopic mass of neutral peptide Mr(calc): 1937.996
Variable modifications:
K16 :iTRAQ4plex (K)
Ions Score: 65.86 Expect: 0.000

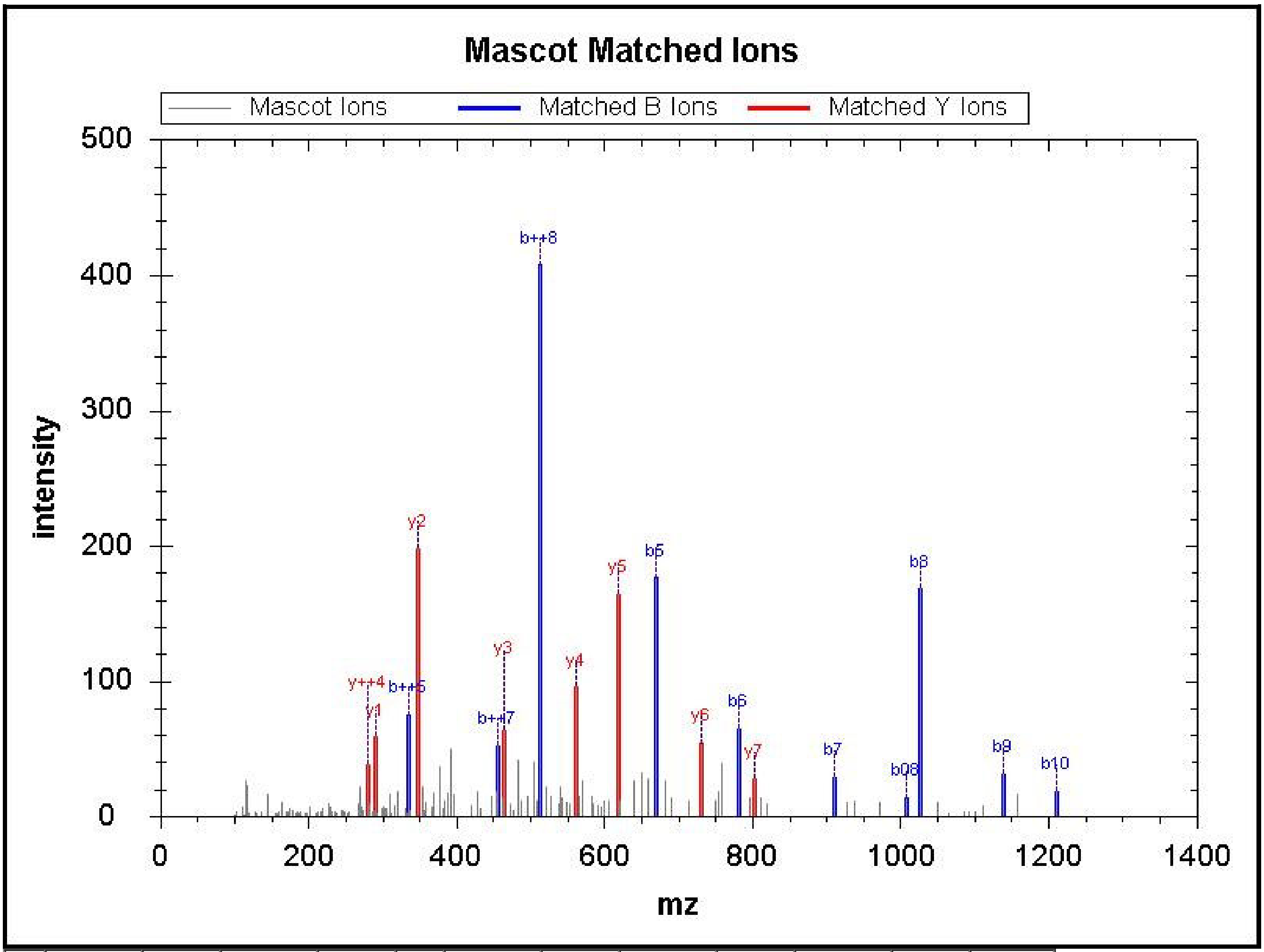

No	b	b++	b0	b0++	Seq	y	y++	y*	y*++	y0	y0++	RevNo
1	216.15	108.58			A							16
2	353.21	177.11			H	1,723.86	862.43	1,706.84	853.92	1,705.85	853.43	15
3	482.25	241.63	464.24	232.62	E	1,586.80	793.91	1,569.78	785.39	1,568.79	784.90	14
4	539.27	270.14	521.26	261.13	G	1,457.76	729.38	1,440.73	720.87	1,439.75	720.38	13
5	668.31	334.66	650.30	325.65	E	1,400.74	700.87	1,383.71	692.36	1,382.73	691.87	12
6	781.40	391.20	763.39	382.20	I	1,271.70	636.35	1,254.67	627.84	1,253.69	627.35	11
7	910.44	455.72	892.43	446.72	E	1,158.61	579.81	1,141.59	571.30	1,140.60	570.80	10
8	1,025.47	513.24	1,007.46	504.23	D	1,029.57	515.29	1,012.54	506.78	1,011.56	506.28	9
9	1,138.55	569.78	1,120.54	560.77	L	914.54	457.77	897.52	449.26	896.53	448.77	8
10	1,209.59	605.30	1,191.58	596.29	A	801.46	401.23	784.43	392.72	783.45	392.23	7
11	1,322.67	661.84	1,304.66	652.83	L	730.42	365.71	713.40	357.20	712.41	356.71	6
12	1,379.69	690.35	1,361.68	681.34	G	617.34	309.17	600.31	300.66	599.33	300.17	5
13	1,476.75	738.88	1,458.73	729.87	P	560.32	280.66	543.29	272.15	542.31	271.66	4
14	1,591.77	796.39	1,573.76	787.38	D	463.26	232.14	446.24	223.62	445.25	223.13	3
15	1,648.79	824.90	1,630.78	815.90	G	348.24	174.62	331.21	166.11			2
16					K	291.21	146.11	274.19	137.60			1

Query 85971 Hit 1

MS/MS Fragmentation of ITHEVDELTQIIADVSQDPTLPR
Found in sp|P36954|RPB9_HUMAN, DNA-directed RNA polymerase II subunit RPB9 OS=Homo sapiens GN=POLR2I PE=1 SV=1
Match to Query 85971: 2733.428from(912.15,3+)
Title: 1128: Scan 2499 (rt=65.683, f=2, i=401) [D:\lab212\membrane\Grace\Joyce\iTRAQ_32_2.raw]
Data File:Submitted from 20120508-1(merge) by Mascot Daemon on JOYCE-VAIO
Monoisotopic mass of neutral peptide Mr(calc): 2733.428
Variable modifications:

Ions Score: 65.56 Expect: 0.000

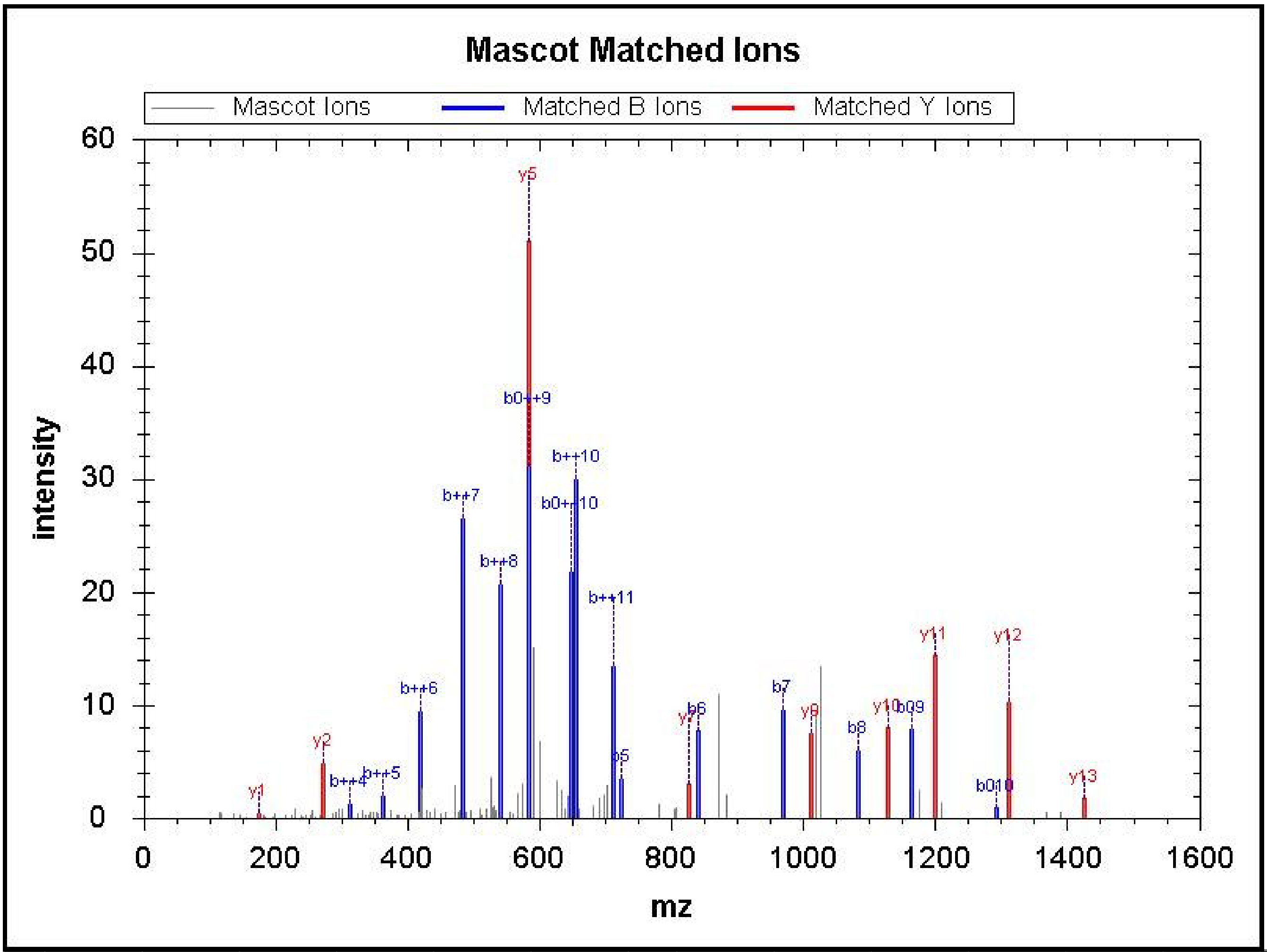

No	b	b++	b*	b*++	b0	b0++	Seq	y	y++	y*	y*++	y0	y0++	RevNo
1	258.19	129.60					I							23
2	359.24	180.12			341.23	171.12	T	2,477.25	1,239.13	2,460.23	1,230.62	2,459.24	1,230.12	22
3	496.30	248.65			478.29	239.65	H	2,376.20	1,188.61	2,359.18	1,180.09	2,358.19	1,179.60	21
4	625.34	313.17			607.33	304.17	E	2,239.15	1,120.08	2,222.12	1,111.56	2,221.13	1,111.07	20
5	724.41	362.71			706.40	353.70	V	2,110.10	1,055.56	2,093.08	1,047.04	2,092.09	1,046.55	19
6	839.44	420.22			821.43	411.22	D	2,011.03	1,006.02	1,994.01	997.51	1,993.02	997.02	18
7	968.48	484.74			950.47	475.74	E	1,896.01	948.51	1,878.98	939.99	1,878.00	939.50	17
8	1,081.56	541.29			1,063.55	532.28	L	1,766.96	883.99	1,749.94	875.47	1,748.95	874.98	16
9	1,182.61	591.81			1,164.60	582.80	T	1,653.88	827.44	1,636.85	818.93	1,635.87	818.44	15
10	1,310.67	655.84	1,293.64	647.33	1,292.66	646.83	Q	1,552.83	776.92	1,535.81	768.41	1,534.82	767.91	14
11	1,423.76	712.38	1,406.73	703.87	1,405.74	703.38	I	1,424.77	712.89	1,407.75	704.38	1,406.76	703.89	13
12	1,536.84	768.92	1,519.81	760.41	1,518.83	759.92	I	1,311.69	656.35	1,294.66	647.84	1,293.68	647.34	12
13	1,607.88	804.44	1,590.85	795.93	1,589.87	795.44	A	1,198.61	599.81	1,181.58	591.29	1,180.60	590.80	11
14	1,722.90	861.96	1,705.88	853.44	1,704.89	852.95	D	1,127.57	564.29	1,110.54	555.77	1,109.56	555.28	10
15	1,821.97	911.49	1,804.95	902.98	1,803.96	902.48	V	1,012.54	506.77	995.52	498.26	994.53	497.77	9
16	1,909.00	955.01	1,891.98	946.49	1,890.99	946.00	S	913.47	457.24	896.45	448.73	895.46	448.24	8
17	2,037.06	1,019.03	2,020.04	1,010.52	2,019.05	1,010.03	Q	826.44	413.72	809.42	405.21	808.43	404.72	7
18	2,152.09	1,076.55	2,135.06	1,068.03	2,134.08	1,067.54	D	698.38	349.70	681.36	341.18	680.37	340.69	6
19	2,249.14	1,125.07	2,232.12	1,116.56	2,231.13	1,116.07	P	583.36	292.18	566.33	283.67	565.35	283.18	5
20	2,350.19	1,175.60	2,333.16	1,167.09	2,332.18	1,166.59	T	486.30	243.66	469.28	235.14	468.29	234.65	4
21	2,463.27	1,232.14	2,446.25	1,223.63	2,445.26	1,223.14	L	385.26	193.13	368.23	184.62			3
22	2,560.33	1,280.67	2,543.30	1,272.15	2,542.32	1,271.66	P	272.17	136.59	255.15	128.08			2

23							R	175.12	88.06	158.09	79.55			1

Query 55984 Hit 1

MS/MS Fragmentation of ETSSDVALASHILTALR
Found in sp|O00273|DFFA_HUMAN, DNA fragmentation factor subunit alpha OS=Homo sapiens GN=DFFA PE=1 SV=1
Match to Query 55984: 1927.048from(643.3566,3+)
Title: 861: Scan 1928 (rt=52.852, f=3, i=296) [D:\lab212\membrane\Grace\Joyce\iTRAQ_32_2.raw]
Data File:Submitted from 20120508-1(merge) by Mascot Daemon on JOYCE-VAIO
Monoisotopic mass of neutral peptide Mr(calc): 1927.048
Variable modifications:
Ions Score: 64.71 Expect: 0.000

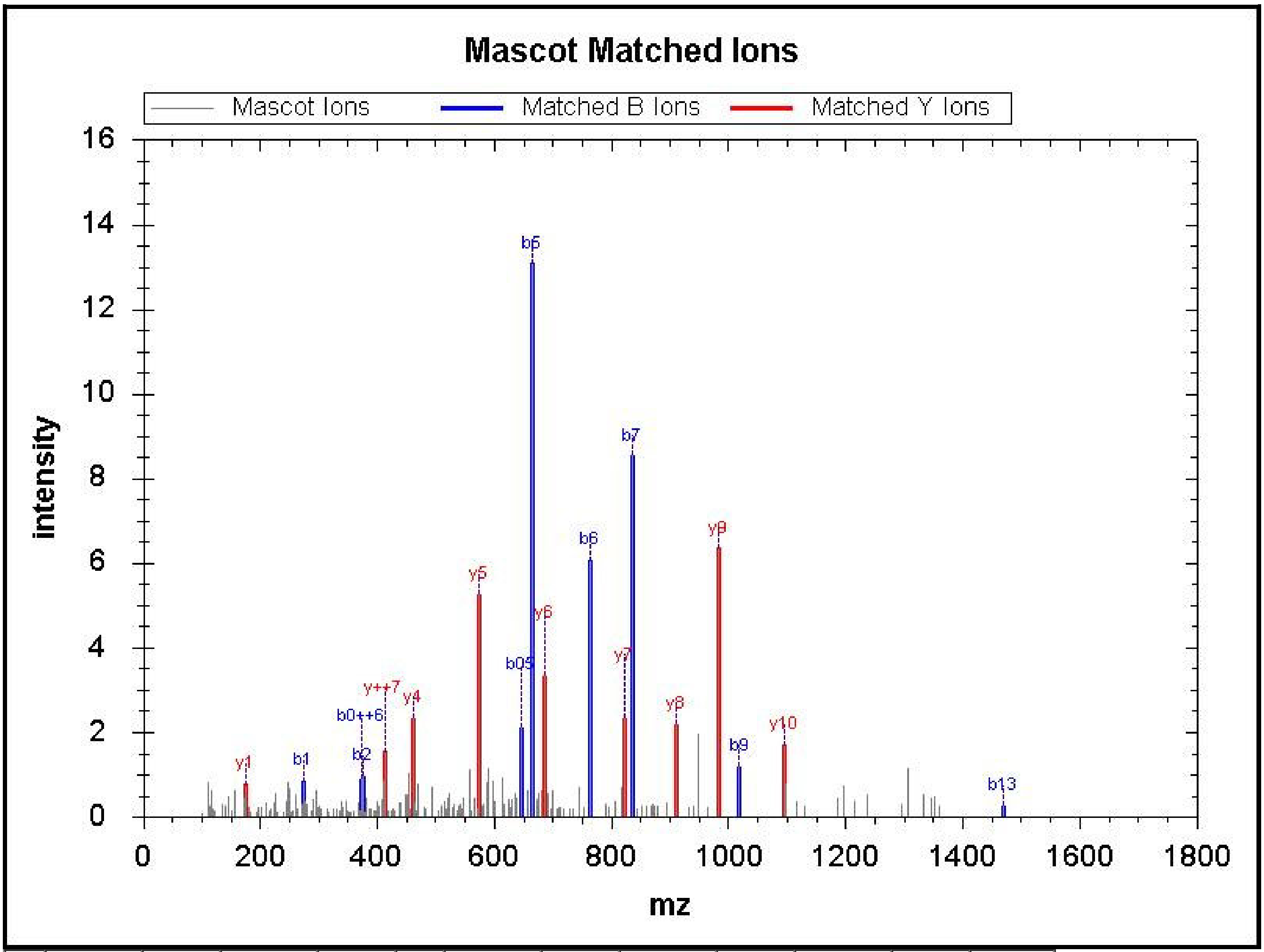

No	b	b++	b0	b0++	Seq	y	y++	y*	y*++	y0	y0++	RevNo
1	274.15	137.58	256.14	128.57	E							17
2	375.20	188.10	357.19	179.10	T	1,654.91	827.96	1,637.89	819.45	1,636.90	818.95	16
3	462.23	231.62	444.22	222.61	S	1,553.86	777.44	1,536.84	768.92	1,535.85	768.43	15
4	549.26	275.14	531.25	266.13	S	1,466.83	733.92	1,449.81	725.41	1,448.82	724.91	14
5	664.29	332.65	646.28	323.64	D	1,379.80	690.40	1,362.77	681.89	1,361.79	681.40	13
6	763.36	382.18	745.35	373.18	V	1,264.77	632.89	1,247.75	624.38	1,246.76	623.89	12
7	834.40	417.70	816.39	408.70	A	1,165.71	583.36	1,148.68	574.84	1,147.69	574.35	11
8	947.48	474.24	929.47	465.24	L	1,094.67	547.84	1,077.64	539.32	1,076.66	538.83	10
9	1,018.52	509.76	1,000.51	500.76	A	981.58	491.30	964.56	482.78	963.57	482.29	9
10	1,105.55	553.28	1,087.54	544.27	S	910.55	455.78	893.52	447.26	892.54	446.77	8
11	1,242.61	621.81	1,224.60	612.80	H	823.51	412.26	806.49	403.75	805.50	403.26	7
12	1,355.69	678.35	1,337.68	669.34	I	686.46	343.73	669.43	335.22	668.45	334.73	6
13	1,468.78	734.89	1,450.77	725.89	L	573.37	287.19	556.35	278.68	555.36	278.18	5
14	1,569.82	785.42	1,551.81	776.41	T	460.29	230.65	443.26	222.13	442.28	221.64	4

15	1,640.86	820.93	1,622.85	811.93	A	359.24	180.12	342.21	171.61			3
16	1,753.95	877.48	1,735.93	868.47	L	288.20	144.61	271.18	136.09			2
17					R	175.12	88.06	158.09	79.55			1

Query 56161 Hit 1

MS/MS Fragmentation of WSLALGLPLPLGEHAGR
Found in sp|Q96F86|EDC3_HUMAN, Enhancer of mRNA-decapping protein 3 OS=Homo sapiens GN=EDC3 PE=1 SV=1
Match to Query 56161: 1930.088from(644.3699,3+)
Title: 990: Scan 2143 (rt=57.9495, f=3, i=333) [D:\lab212\membrane\Grace\Joyce\iTRAQ_33_2.raw]
Data File:Submitted from 20120508-1(merge) by Mascot Daemon on JOYCE-VAIO
Monoisotopic mass of neutral peptide Mr(calc): 1930.088
Variable modifications:
Ions Score: 64.57 Expect: 0.000

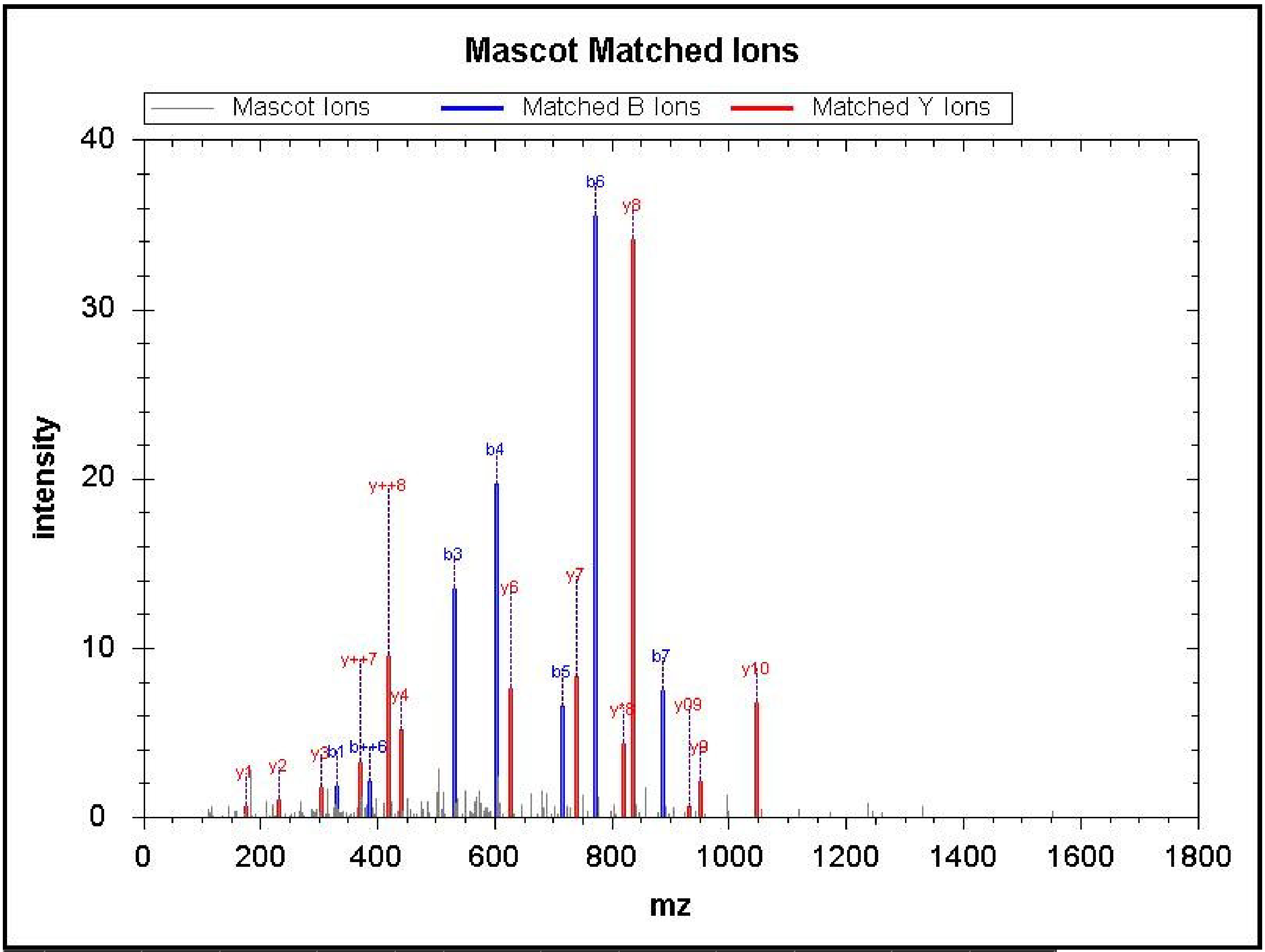

No	b	b++	b0	b0++	Seq	y	y++	y*	y*++	y0	y0++	RevNo
1	331.19	166.10			W							17
2	418.22	209.61	400.21	200.61	S	1,600.92	800.96	1,583.89	792.45	1,582.91	791.96	16
3	531.30	266.16	513.29	257.15	L	1,513.89	757.45	1,496.86	748.93	1,495.87	748.44	15
4	602.34	301.67	584.33	292.67	A	1,400.80	700.90	1,383.77	692.39	1,382.79	691.90	14
5	715.43	358.22	697.42	349.21	L	1,329.76	665.39	1,312.74	656.87	1,311.75	656.38	13
6	772.45	386.73	754.44	377.72	G	1,216.68	608.84	1,199.65	600.33	1,198.67	599.84	12
7	885.53	443.27	867.52	434.26	L	1,159.66	580.33	1,142.63	571.82	1,141.65	571.33	11
8	982.58	491.80	964.57	482.79	P	1,046.57	523.79	1,029.55	515.28	1,028.56	514.79	10
9	1,095.67	548.34	1,077.66	539.33	L	949.52	475.26	932.49	466.75	931.51	466.26	9
10	1,192.72	596.86	1,174.71	587.86	P	836.44	418.72	819.41	410.21	818.43	409.72	8
11	1,305.81	653.41	1,287.79	644.40	L	739.38	370.20	722.36	361.68	721.37	361.19	7

12	1,362.83	681.92	1,344.82	672.91	G	626.30	313.65	609.27	305.14	608.29	304.65	6
13	1,491.87	746.44	1,473.86	737.43	E	569.28	285.14	552.25	276.63	551.27	276.14	5
14	1,628.93	814.97	1,610.92	805.96	H	440.24	220.62	423.21	212.11			4
15	1,699.97	850.49	1,681.95	841.48	A	303.18	152.09	286.15	143.58			3
16	1,756.99	879.00	1,738.98	869.99	G	232.14	116.57	215.11	108.06			2
17					R	175.12	88.06	158.09	79.55			1

Query 75173 Hit 1

MS/MS Fragmentation of AYQIDTVINLNVPFEVIK
Found in sp|Q9UIJ7|KAD3_HUMAN, GTP:AMP phosphotransferase
Match to Query 75173: 2363.321 from(788.781,3+)
Title: 1055: Sum of 2 scans in range 2363 (rt=62.5901, f=4, i=703) to 2364 (rt=62.6155, f=4, i=704) [D:\lab212\membrane\Grace\Joyce\iTRAQ_25_2.raw]
Data File:Submitted from 20120508-1(merge) by Mascot Daemon on JOYCE-VAIO
Monoisotopic mass of neutral peptide Mr(calc): 2363.321
Variable modifications:
K18 :iTRAQ4plex (K)
Ions Score: 64.51 Expect: 0.000

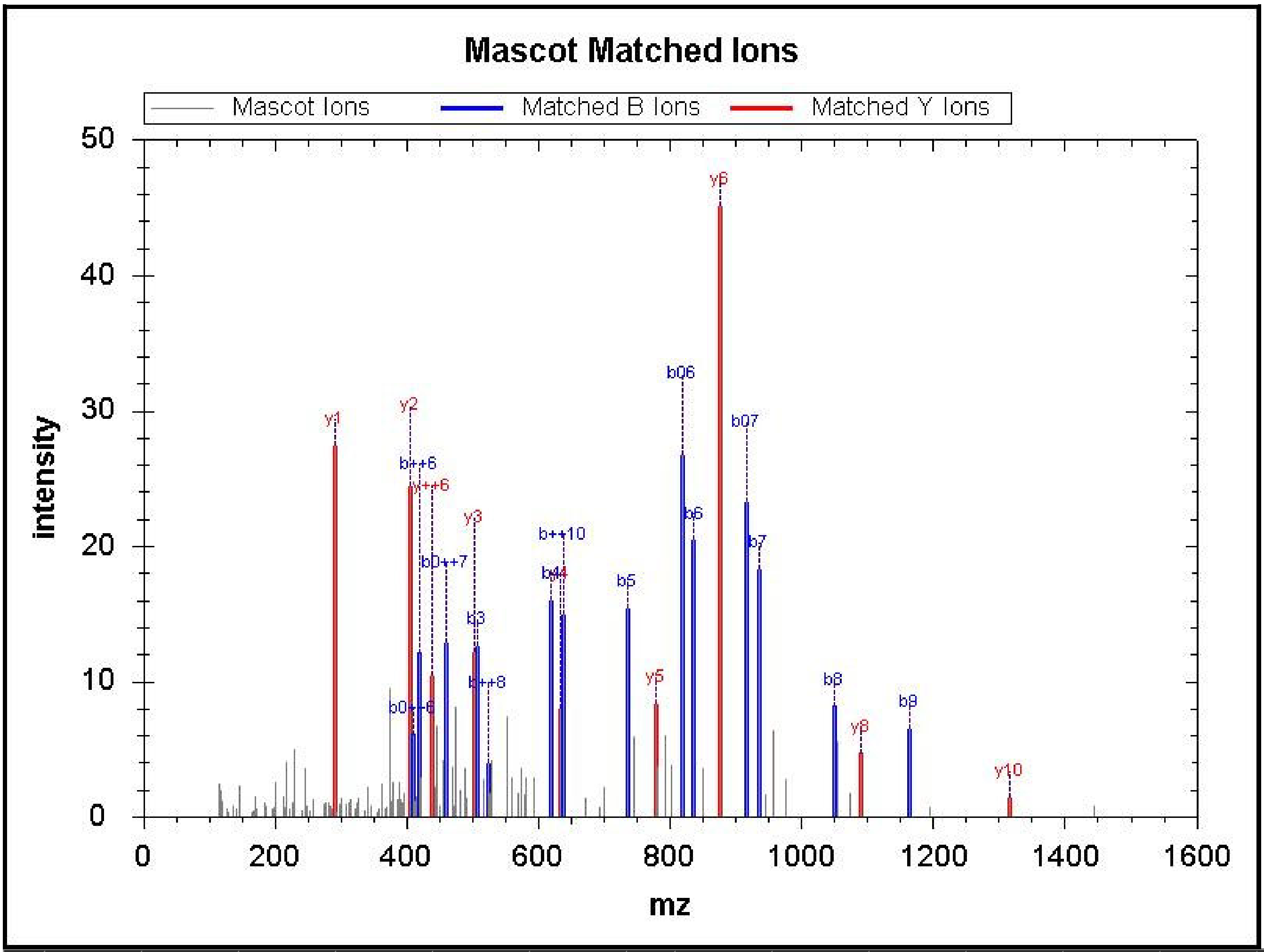

No	b	b++	b*	b*++	b0	b0++	Seq	y	y++	y*	y*++	y0	y0++	RevNo
1	216.15	108.58					A							18
2	379.21	190.11					Y	2,149.20	1,075.10	2,132.18	1,066.59	2,131.19	1,066.10	17
3	507.27	254.14	490.24	245.62			Q	1,986.14	993.57	1,969.11	985.06	1,968.13	984.57	16
4	620.35	310.68	603.33	302.17			I	1,858.08	929.54	1,841.05	921.03	1,840.07	920.54	15
5	735.38	368.19	718.35	359.68	717.37	359.19	D	1,745.00	873.00	1,727.97	864.49	1,726.99	864.00	14
6	836.43	418.72	819.40	410.20	818.42	409.71	T	1,629.97	815.49	1,612.94	806.98	1,611.96	806.48	13
7	935.50	468.25	918.47	459.74	917.48	459.25	V	1,528.92	764.96	1,511.90	756.45	1,510.91	755.96	12

8	1,048.58	524.79	1,031.55	516.28	1,030.57	515.79	I	1,429.85	715.43	1,412.83	706.92	1,411.84	706.43	11
9	1,162.62	581.81	1,145.60	573.30	1,144.61	572.81	N	1,316.77	658.89	1,299.74	650.38	1,298.76	649.88	10
10	1,275.71	638.36	1,258.68	629.84	1,257.70	629.35	L	1,202.73	601.87	1,185.70	593.35	1,184.72	592.86	9
11	1,389.75	695.38	1,372.72	686.87	1,371.74	686.37	N	1,089.64	545.32	1,072.62	536.81	1,071.63	536.32	8
12	1,488.82	744.91	1,471.79	736.40	1,470.81	735.91	V	975.60	488.30	958.57	479.79	957.59	479.30	7
13	1,585.87	793.44	1,568.84	784.93	1,567.86	784.43	P	876.53	438.77	859.50	430.26	858.52	429.76	6
14	1,732.94	866.97	1,715.91	858.46	1,714.93	857.97	F	779.48	390.24	762.45	381.73	761.47	381.24	5
15	1,861.98	931.49	1,844.96	922.98	1,843.97	922.49	E	632.41	316.71	615.38	308.20	614.40	307.70	4
16	1,961.05	981.03	1,944.02	972.52	1,943.04	972.02	V	503.37	252.19	486.34	243.67			3
17	2,074.13	1,037.57	2,057.11	1,029.06	2,056.12	1,028.57	I	404.30	202.65	387.27	194.14			2
18							K	291.21	146.11	274.19	137.60			1

Query 40209 Hit 1

MS/MS Fragmentation of TEESPASDEAGEK
Found in sp|P05114|HMGN1_HUMAN, Non-histone chromosomal protein HMG-14 OS=Homo sapiens GN=HMGN1 PE=1 SV=3
Match to Query 40209: 1636.772from(819.3934,2+)
Title: 11: Scan 218 (rt=13.8644, f=2, i=11) [D:\lab212\membrane\Grace\Joyce\iTRAQ_29_1.raw]
Data File:Submitted from 20120508-1(merge) by Mascot Daemon on JOYCE-VAIO
Monoisotopic mass of neutral peptide Mr(calc): 1636.772
Variable modifications:
K13 :iTRAQ4plex (K)
Ions Score: 64.45 Expect: 0.000

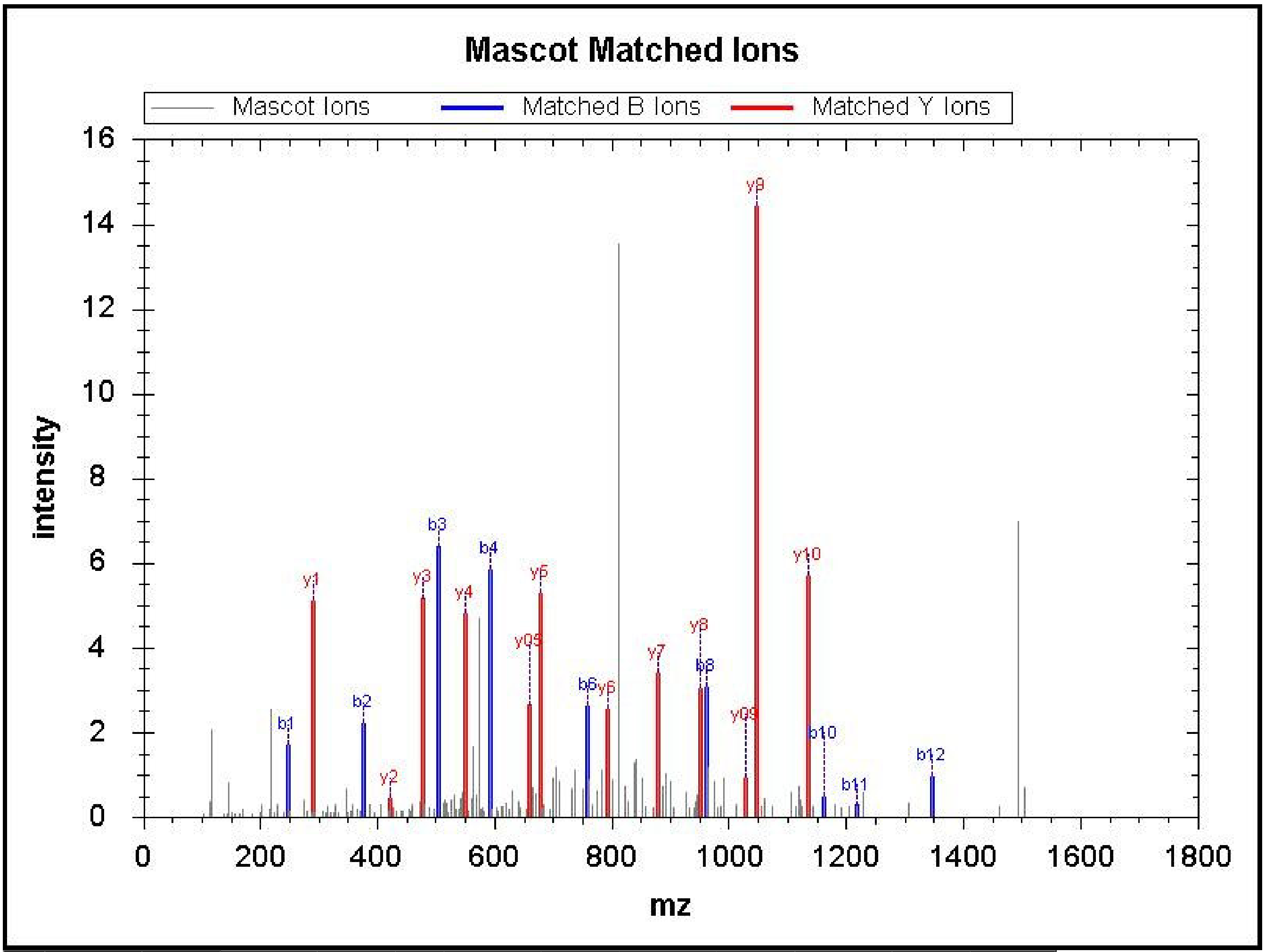

No	b	b++	b0	b0++	Seq	y	y++	y*	y*++	y0	y0++	RevNo
1	246.16	123.58	228.15	114.58	T							13
2	375.20	188.10	357.19	179.10	E	1,392.62	696.82	1,375.60	688.30	1,374.61	687.81	12

3	504.24	252.62	486.23	243.62	E	1,263.58	632.29	1,246.56	623.78	1,245.57	623.29	11
4	591.27	296.14	573.26	287.14	S	1,134.54	567.77	1,117.51	559.26	1,116.53	558.77	10
5	688.33	344.67	670.32	335.66	P	1,047.51	524.26	1,030.48	515.74	1,029.50	515.25	9
6	759.36	380.19	741.35	371.18	A	950.45	475.73	933.43	467.22	932.44	466.73	8
7	846.40	423.70	828.39	414.70	S	879.42	440.21	862.39	431.70	861.41	431.21	7
8	961.42	481.22	943.41	472.21	D	792.39	396.70	775.36	388.18	774.38	387.69	6
9	1,090.47	545.74	1,072.46	536.73	E	677.36	339.18	660.33	330.67	659.35	330.18	5
10	1,161.50	581.26	1,143.49	572.25	A	548.32	274.66	531.29	266.15	530.31	265.66	4
11	1,218.52	609.77	1,200.51	600.76	G	477.28	239.14	460.25	230.63	459.27	230.14	3
12	1,347.57	674.29	1,329.56	665.28	E	420.26	210.63	403.23	202.12	402.25	201.63	2
13					K	291.21	146.11	274.19	137.60			1

Query 59487 Hit 1

MS/MS Fragmentation of HPNILAYIDGLETEK
Found in sp|Q96KG9|NTKL_HUMAN, N-terminal kinase-like protein OS=Homo sapiens GN=SCYL1 PE=1 SV=1
Match to Query 59487: 2000.084from(667.702,3+)
Title: 872: Sum of 2 scans in range 1937 (rt=53.206, f=4, i=586) to 1938 (rt=53.2314, f=4, i=587)
[D:\lab212\membrane\Grace\Joyce\iTRAQ_42_1.raw]
Data File:Submitted from 20120508-1(merge) by Mascot Daemon on JOYCE-VAIO
Monoisotopic mass of neutral peptide Mr(calc): 2000.084
Variable modifications:
K15 :iTRAQ4plex (K)
Ions Score: 64.38 Expect: 0.000

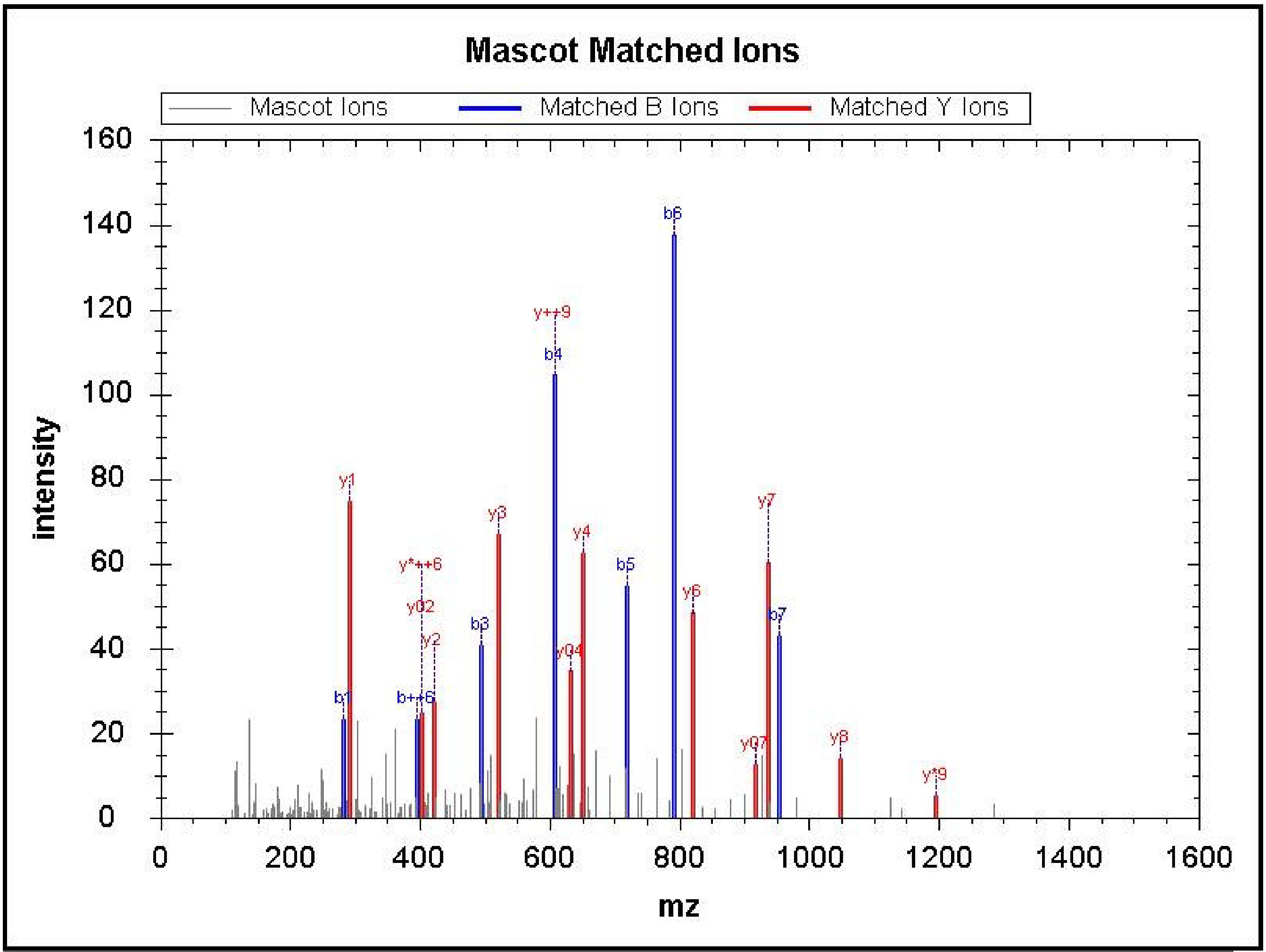

No	b	b++	b*	b*++	b0	b0++	Seq	y	y++	y*	y*++	y0	y0++	RevNo
1	282.17	141.59					H							15
2	379.22	190.11					P	1,719.93	860.47	1,702.90	851.95	1,701.92	851.46	14

3	493.26	247.14	476.24	238.62			N	1,622.88	811.94	1,605.85	803.43	1,604.87	802.94	13
4	606.35	303.68	589.32	295.16			I	1,508.83	754.92	1,491.81	746.41	1,490.82	745.91	12
5	719.43	360.22	702.41	351.71			L	1,395.75	698.38	1,378.72	689.86	1,377.74	689.37	11
6	790.47	395.74	773.44	387.22			A	1,282.66	641.84	1,265.64	633.32	1,264.65	632.83	10
7	953.53	477.27	936.51	468.76			Y	1,211.63	606.32	1,194.60	597.80	1,193.62	597.31	9
8	1,066.62	533.81	1,049.59	525.30			I	1,048.56	524.79	1,031.54	516.27	1,030.55	515.78	8
9	1,181.64	591.33	1,164.62	582.81	1,163.63	582.32	D	935.48	468.24	918.45	459.73	917.47	459.24	7
10	1,238.67	619.84	1,221.64	611.32	1,220.65	610.83	G	820.45	410.73	803.43	402.22	802.44	401.72	6
11	1,351.75	676.38	1,334.72	667.87	1,333.74	667.37	L	763.43	382.22	746.41	373.71	745.42	373.21	5
12	1,480.79	740.90	1,463.77	732.39	1,462.78	731.89	E	650.35	325.68	633.32	317.16	632.34	316.67	4
13	1,581.84	791.42	1,564.81	782.91	1,563.83	782.42	T	521.31	261.16	504.28	252.64	503.29	252.15	3
14	1,710.88	855.94	1,693.86	847.43	1,692.87	846.94	E	420.26	210.63	403.23	202.12	402.25	201.63	2
15							K	291.21	146.11	274.19	137.60			1

Query 72367 Hit 1

MS/MS Fragmentation of TQAVFNHTEDYVLLPDER
Found in sp|Q05048|CSTF1_HUMAN, Cleavage stimulation factor subunit 1 OS=Homo sapiens GN=CSTF1 PE=1 SV=1
Match to Query 72367: 2290.137from(764.3862,3+)
Title: 625: Sum of 2 scans in range 1437 (rt=41.7524, f=4, i=421) to 1438 (rt=41.7778, f=4, i=422) [D:\lab212\membrane\Grace\Joyce\iTRAQ_33_1.raw]
Data File:Submitted from 20120508-1(merge) by Mascot Daemon on JOYCE-VAIO
Monoisotopic mass of neutral peptide Mr(calc): 2290.137
Variable modifications:
Ions Score: 63.89 Expect: 0.000

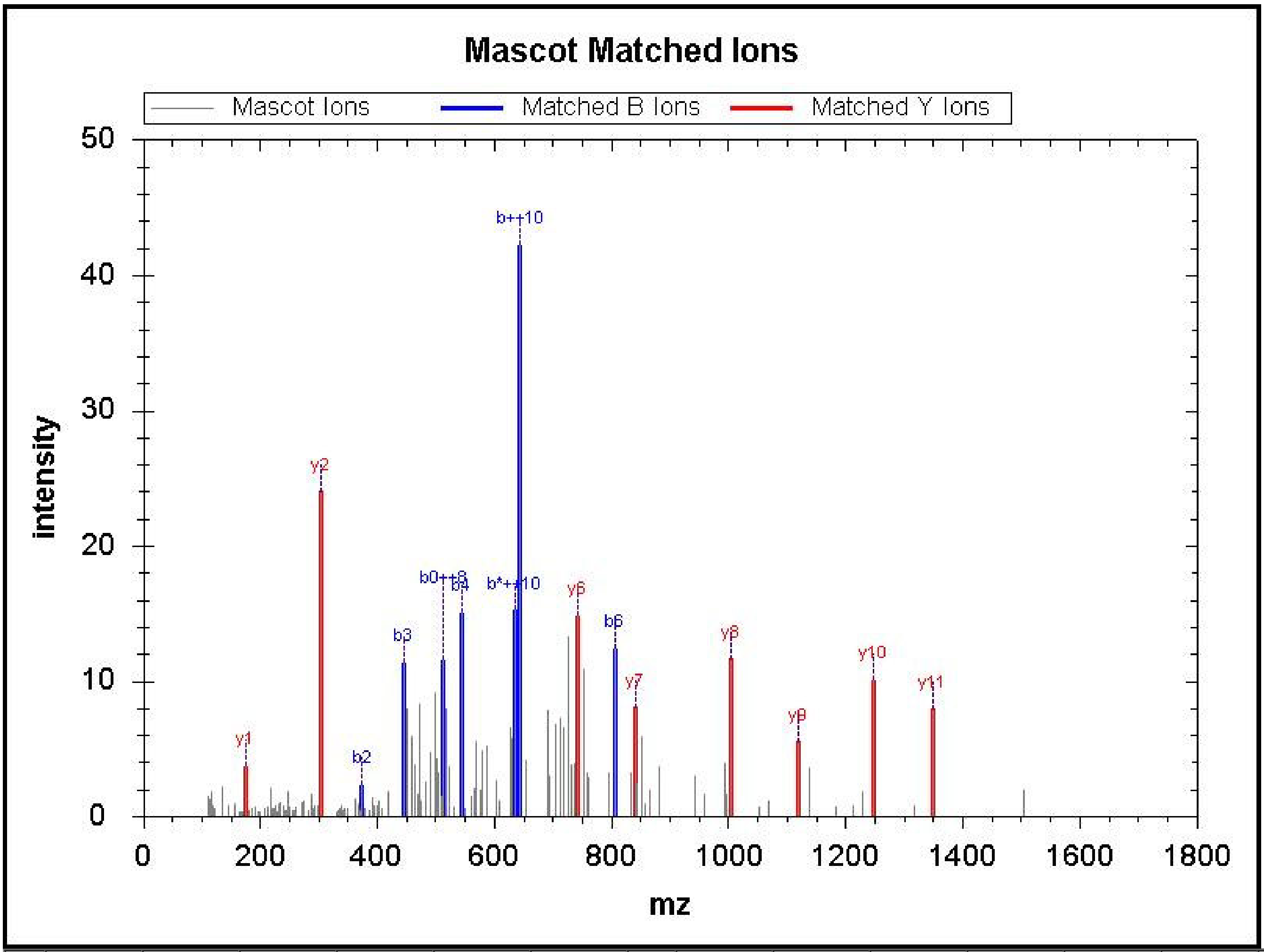

No	b	b++	b*	b*++	b0	b0++	Seq	y	y++	y*	y*++	y0	y0++	RevNo

1	246.16	123.58			228.15	114.58	T							18
2	374.22	187.61	357.19	179.10	356.21	178.61	Q	2,045.99	1,023.50	2,028.97	1,014.99	2,027.98	1,014.49	17
3	445.25	223.13	428.23	214.62	427.24	214.12	A	1,917.93	959.47	1,900.91	950.96	1,899.92	950.47	16
4	544.32	272.66	527.29	264.15	526.31	263.66	V	1,846.90	923.95	1,829.87	915.44	1,828.89	914.95	15
5	691.39	346.20	674.36	337.69	673.38	337.19	F	1,747.83	874.42	1,730.80	865.90	1,729.82	865.41	14
6	805.43	403.22	788.41	394.71	787.42	394.21	N	1,600.76	800.88	1,583.73	792.37	1,582.75	791.88	13
7	942.49	471.75	925.46	463.24	924.48	462.74	H	1,486.72	743.86	1,469.69	735.35	1,468.71	734.86	12
8	1,043.54	522.27	1,026.51	513.76	1,025.53	513.27	T	1,349.66	675.33	1,332.63	666.82	1,331.65	666.33	11
9	1,172.58	586.79	1,155.56	578.28	1,154.57	577.79	E	1,248.61	624.81	1,231.58	616.30	1,230.60	615.80	10
10	1,287.61	644.31	1,270.58	635.79	1,269.60	635.30	D	1,119.57	560.29	1,102.54	551.77	1,101.56	551.28	9
11	1,450.67	725.84	1,433.65	717.33	1,432.66	716.83	Y	1,004.54	502.77	987.51	494.26	986.53	493.77	8
12	1,549.74	775.37	1,532.71	766.86	1,531.73	766.37	V	841.48	421.24	824.45	412.73	823.47	412.24	7
13	1,662.82	831.92	1,645.80	823.40	1,644.81	822.91	L	742.41	371.71	725.38	363.20	724.40	362.70	6
14	1,775.91	888.46	1,758.88	879.94	1,757.90	879.45	L	629.33	315.17	612.30	306.65	611.31	306.16	5
15	1,872.96	936.98	1,855.93	928.47	1,854.95	927.98	P	516.24	258.62	499.21	250.11	498.23	249.62	4
16	1,987.99	994.50	1,970.96	985.98	1,969.98	985.49	D	419.19	210.10	402.16	201.58	401.18	201.09	3
17	2,117.03	1,059.02	2,100.00	1,050.51	2,099.02	1,050.01	E	304.16	152.58	287.13	144.07	286.15	143.58	2
18							R	175.12	88.06	158.09	79.55			1

Query 78098 Hit 1

MS/MS Fragmentation of IEEYNHVLIEETDQLWK
Found in sp|Q14241|ELOA1_HUMAN, Transcription elongation factor B polypeptide 3 OS=Homo sapiens GN=TCEB3 PE=1 SV=2
Match to Query 78098: 2446.264from(816.4286,3+)
Title: 881: Scan 1957 (rt=53.5917, f=3, i=295) [D:\lab212\membrane\Grace\Joyce\iTRAQ_39_1.raw]
Data File:Submitted from 20120508-1(merge) by Mascot Daemon on JOYCE-VAIO
Monoisotopic mass of neutral peptide Mr(calc): 2446.264
Variable modifications:
K17 :iTRAQ4plex (K)
Ions Score: 63.85 Expect: 0.000

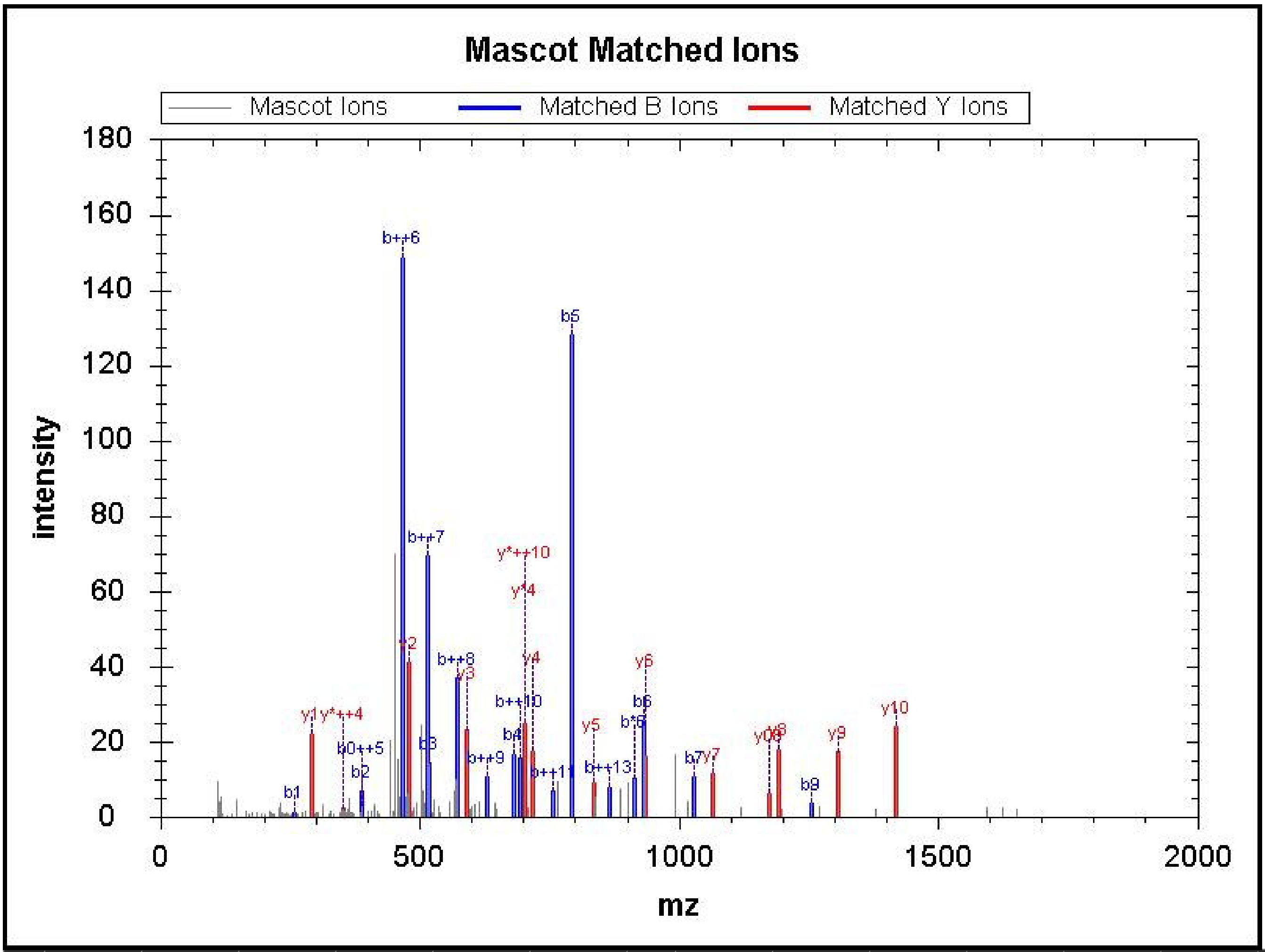

No	b	b++	b*	b*++	b0	b0++	Seq	y	y++	y*	y*++	y0	y0++	RevNo
1	258.19	129.60					I							17
2	387.24	194.12			369.23	185.12	E	2,190.08	1,095.55	2,173.06	1,087.03	2,172.07	1,086.54	16
3	516.28	258.64			498.27	249.64	E	2,061.04	1,031.02	2,044.01	1,022.51	2,043.03	1,022.02	15
4	679.34	340.17			661.33	331.17	Y	1,932.00	966.50	1,914.97	957.99	1,913.99	957.50	14
5	793.38	397.20	776.36	388.68	775.37	388.19	N	1,768.94	884.97	1,751.91	876.46	1,750.92	875.97	13
6	930.44	465.73	913.42	457.21	912.43	456.72	H	1,654.89	827.95	1,637.87	819.44	1,636.88	818.94	12
7	1,029.51	515.26	1,012.49	506.75	1,011.50	506.25	V	1,517.83	759.42	1,500.81	750.91	1,499.82	750.41	11
8	1,142.60	571.80	1,125.57	563.29	1,124.59	562.80	L	1,418.76	709.89	1,401.74	701.37	1,400.75	700.88	10
9	1,255.68	628.34	1,238.65	619.83	1,237.67	619.34	I	1,305.68	653.34	1,288.65	644.83	1,287.67	644.34	9
10	1,384.72	692.87	1,367.70	684.35	1,366.71	683.86	E	1,192.60	596.80	1,175.57	588.29	1,174.59	587.80	8
11	1,513.77	757.39	1,496.74	748.87	1,495.75	748.38	E	1,063.55	532.28	1,046.53	523.77	1,045.54	523.28	7
12	1,614.81	807.91	1,597.79	799.40	1,596.80	798.90	T	934.51	467.76	917.48	459.25	916.50	458.75	6
13	1,729.84	865.42	1,712.81	856.91	1,711.83	856.42	D	833.46	417.24	816.44	408.72	815.45	408.23	5
14	1,857.90	929.45	1,840.87	920.94	1,839.89	920.45	Q	718.44	359.72	701.41	351.21			4
15	1,970.98	986.00	1,953.96	977.48	1,952.97	976.99	L	590.38	295.69	573.35	287.18			3
16	2,157.06	1,079.03	2,140.04	1,070.52	2,139.05	1,070.03	W	477.29	239.15	460.27	230.64			2
17							K	291.21	146.11	274.19	137.60			1

Query 62484 Hit 1

MS/MS Fragmentation of GSLDPESIFEMMETGK
Found in sp|Q15024|EXOS7_HUMAN, Exosome complex component RRP42 OS=Homo sapiens GN=EXOSC7 PE=1 SV=3
Match to Query 62484: 2057.99from(687.004,3+)
Title: 1071: Sum of 2 scans in range 2394 (rt=63.2646, f=4, i=723) to 2395 (rt=63.29, f=4, i=724)
[D:\lab212\membrane\Grace\Joyce\iTRAQ_27_1.raw]
Data File:Submitted from 20120508-1(merge) by Mascot Daemon on JOYCE-VAIO

Monoisotopic mass of neutral peptide Mr(calc): 2057.99
Variable modifications:
K16 :iTRAQ4plex (K)
Ions Score: 63.64 Expect: 0.000

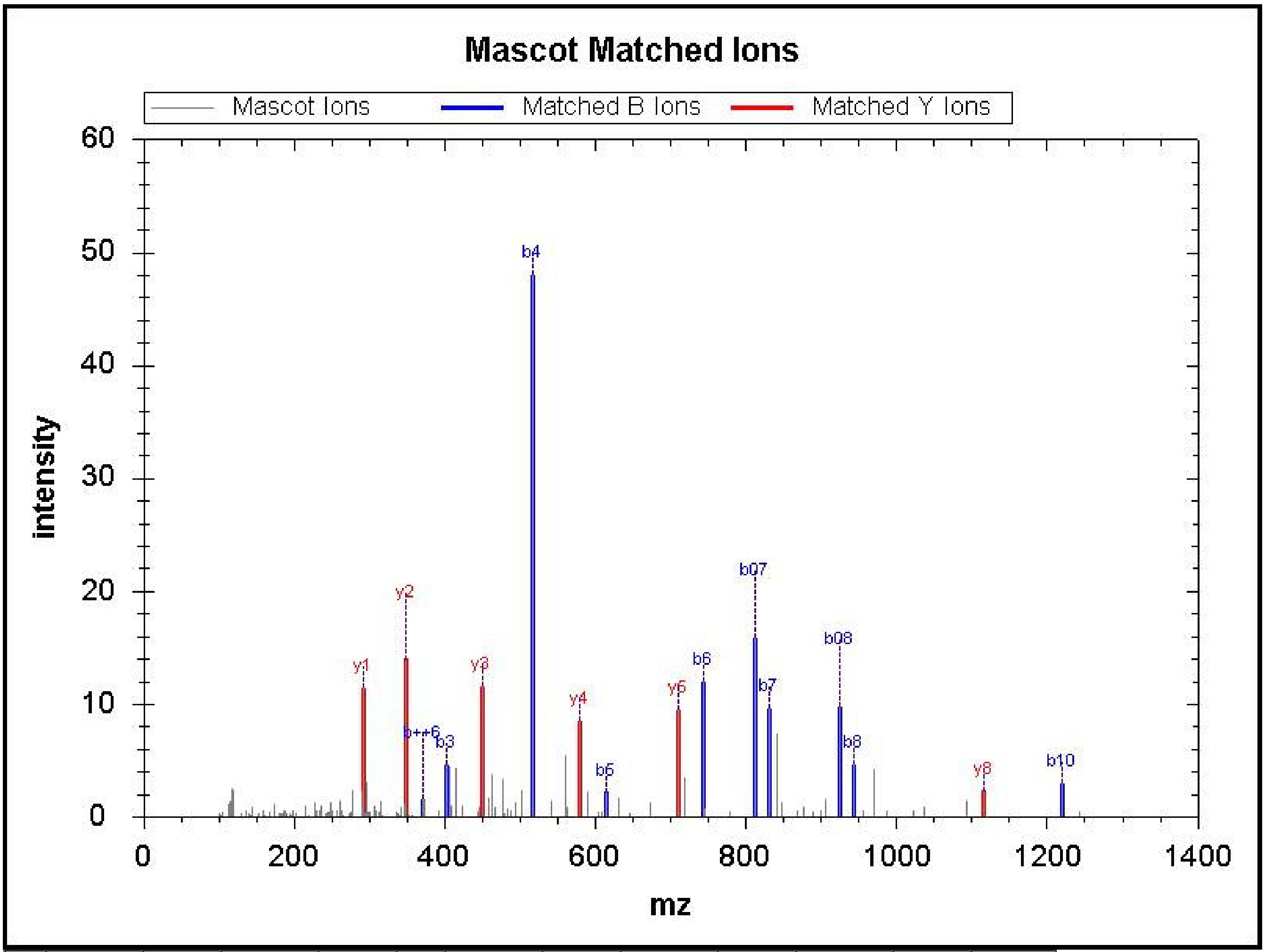

No	b	b++	b0	b0++	Seq	y	y++	y*	y*++	y0	y0++	RevNo
1	202.13	101.57			G							16
2	289.16	145.09	271.15	136.08	S	1,857.87	929.44	1,840.85	920.93	1,839.86	920.43	15
3	402.25	201.63	384.24	192.62	L	1,770.84	885.92	1,753.81	877.41	1,752.83	876.92	14
4	517.27	259.14	499.26	250.14	D	1,657.76	829.38	1,640.73	820.87	1,639.75	820.38	13
5	614.33	307.67	596.32	298.66	P	1,542.73	771.87	1,525.70	763.36	1,524.72	762.86	12
6	743.37	372.19	725.36	363.18	E	1,445.68	723.34	1,428.65	714.83	1,427.67	714.34	11
7	830.40	415.70	812.39	406.70	S	1,316.63	658.82	1,299.61	650.31	1,298.62	649.82	10
8	943.49	472.25	925.47	463.24	I	1,229.60	615.30	1,212.58	606.79	1,211.59	606.30	9
9	1,090.55	545.78	1,072.54	536.78	F	1,116.52	558.76	1,099.49	550.25	1,098.51	549.76	8
10	1,219.60	610.30	1,201.59	601.30	E	969.45	485.23	952.42	476.72	951.44	476.22	7
11	1,350.64	675.82	1,332.63	666.82	M	840.41	420.71	823.38	412.19	822.40	411.70	6
12	1,481.68	741.34	1,463.67	732.34	M	709.37	355.19	692.34	346.67	691.36	346.18	5
13	1,610.72	805.86	1,592.71	796.86	E	578.33	289.67	561.30	281.15	560.32	280.66	4
14	1,711.77	856.39	1,693.76	847.38	T	449.28	225.15	432.26	216.63	431.27	216.14	3
15	1,768.79	884.90	1,750.78	875.89	G	348.24	174.62	331.21	166.11			2
16					K	291.21	146.11	274.19	137.60			1

Query 86508 Hit 1

MS/MS Fragmentation of VLHNQLVLFHNAIAAYFAGNQK
Found in sp|P53367|ARFP1_HUMAN, Arfaptin-1 OS=Homo sapiens GN=ARFIP1 PE=1 SV=2

Match to Query 86508: 2755.524from(689.8882,4+)
Title: 878: Scan 2274 (rt=59.4983, f=2, i=392) [D:\lab212\membrane\Grace\Joyce\iTRAQ_48_1.raw]
Data File:Submitted from 20120508-1(merge) by Mascot Daemon on JOYCE-VAIO
Monoisotopic mass of neutral peptide Mr(calc): 2755.524
Variable modifications:
K22 :iTRAQ4plex (K)
Ions Score: 63.51 Expect: 0.000

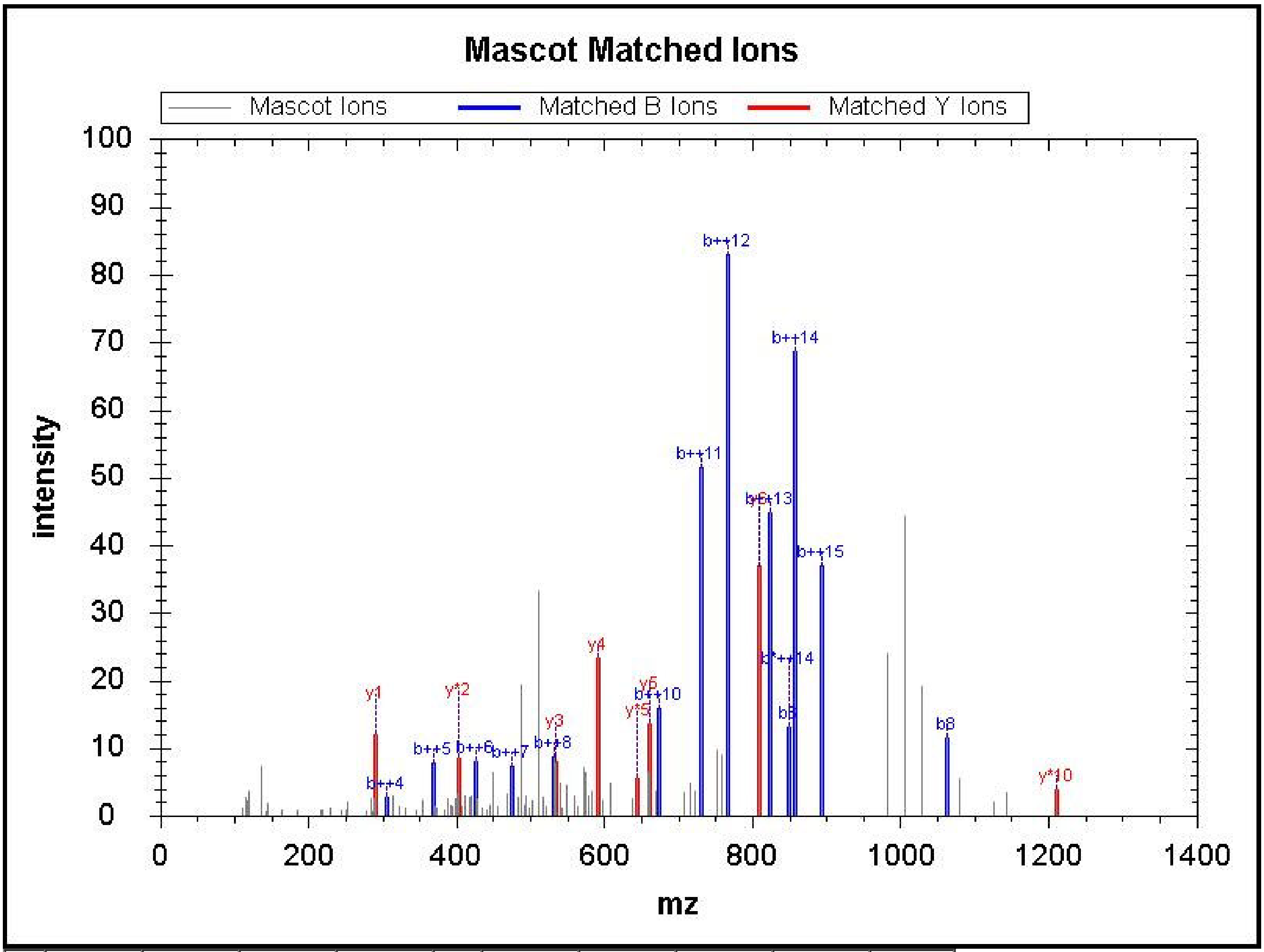

No	b	b++	b*	b*++	Seq	y	y++	y*	y*++	RevNo
1	244.18	122.59			V					22
2	357.26	179.13			L	2,513.35	1,257.18	2,496.33	1,248.67	21
3	494.32	247.66			H	2,400.27	1,200.64	2,383.24	1,192.13	20
4	608.36	304.69	591.34	296.17	N	2,263.21	1,132.11	2,246.18	1,123.60	19
5	736.42	368.71	719.40	360.20	Q	2,149.17	1,075.09	2,132.14	1,066.57	18
6	849.51	425.26	832.48	416.74	L	2,021.11	1,011.06	2,004.08	1,002.54	17
7	948.57	474.79	931.55	466.28	V	1,908.03	954.52	1,891.00	946.00	16
8	1,061.66	531.33	1,044.63	522.82	L	1,808.96	904.98	1,791.93	896.47	15
9	1,208.73	604.87	1,191.70	596.35	F	1,695.87	848.44	1,678.85	839.93	14
10	1,345.79	673.40	1,328.76	664.88	H	1,548.80	774.91	1,531.78	766.39	13
11	1,459.83	730.42	1,442.80	721.91	N	1,411.75	706.38	1,394.72	697.86	12
12	1,530.87	765.94	1,513.84	757.42	A	1,297.70	649.35	1,280.68	640.84	11
13	1,643.95	822.48	1,626.92	813.97	I	1,226.67	613.84	1,209.64	605.32	10
14	1,714.99	858.00	1,697.96	849.48	A	1,113.58	557.29	1,096.55	548.78	9
15	1,786.02	893.52	1,769.00	885.00	A	1,042.54	521.78	1,025.52	513.26	8
16	1,949.09	975.05	1,932.06	966.53	Y	971.51	486.26	954.48	477.74	7
17	2,096.16	1,048.58	2,079.13	1,040.07	F	808.44	404.73	791.42	396.21	6

18	2,167.19	1,084.10	2,150.17	1,075.59	A	661.37	331.19	644.35	322.68	5
19	2,224.22	1,112.61	2,207.19	1,104.10	G	590.34	295.67	573.31	287.16	4
20	2,338.26	1,169.63	2,321.23	1,161.12	N	533.32	267.16	516.29	258.65	3
21	2,466.32	1,233.66	2,449.29	1,225.15	Q	419.27	210.14	402.25	201.63	2
22					K	291.21	146.11	274.19	137.60	1

Query 33737 Hit 1

MS/MS Fragmentation of EVLLEAQDMAVR
Found in sp|Q9NWU5|RM22_HUMAN, 39S ribosomal protein L22
Match to Query 33737: 1516.808from(759.4111,2+)
Title: 444: Scan 1519 (rt=41.5458, f=3, i=155) [D:\lab212\membrane\Grace\Joyce\20120320_iTRAQ_20-2.raw]
Data File:Submitted from 20120508-1(merge) by Mascot Daemon on JOYCE-VAIO
Monoisotopic mass of neutral peptide Mr(calc): 1516.808
Variable modifications:
Ions Score: 63.43 Expect: 0.000

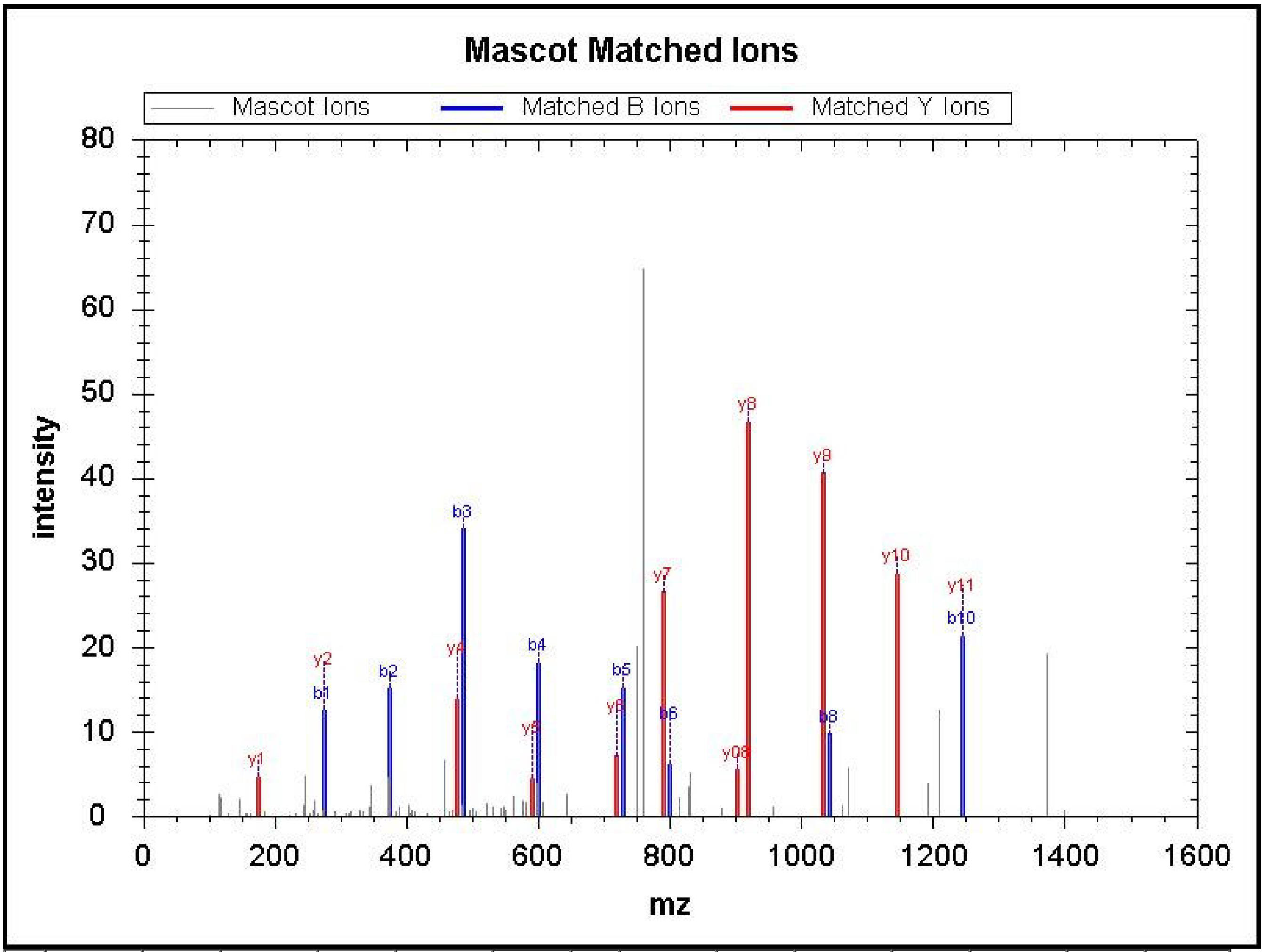

No	b	b++	b*	b*++	b0	b0++	Seq	y	y++	y*	y*++	y0	y0++	RevNo
1	274.15	137.58			256.14	128.57	E							12
2	373.22	187.11			355.21	178.11	V	1,244.67	622.84	1,227.64	614.32	1,226.66	613.83	11
3	486.30	243.66			468.29	234.65	L	1,145.60	573.30	1,128.57	564.79	1,127.59	564.30	10
4	599.39	300.20			581.38	291.19	L	1,032.51	516.76	1,015.49	508.25	1,014.50	507.76	9
5	728.43	364.72			710.42	355.71	E	919.43	460.22	902.40	451.71	901.42	451.21	8
6	799.47	400.24			781.46	391.23	A	790.39	395.70	773.36	387.18	772.38	386.69	7
7	927.53	464.27	910.50	455.75	909.52	455.26	Q	719.35	360.18	702.32	351.67	701.34	351.17	6
8	1,042.55	521.78	1,025.53	513.27	1,024.54	512.78	D	591.29	296.15	574.27	287.64	573.28	287.14	5
9	1,173.59	587.30	1,156.57	578.79	1,155.58	578.30	M	476.26	238.64	459.24	230.12			4
10	1,244.63	622.82	1,227.60	614.31	1,226.62	613.81	A	345.22	173.12	328.20	164.60			3

11	1,343.70	672.35	1,326.67	663.84	1,325.69	663.35	V	274.19	137.60	257.16	129.08			2
12							R	175.12	88.06	158.09	79.55			1

Query 34186 Hit 1

MS/MS Fragmentation of QGLQTTQAHLER
Found in sp|Q14C86|GAPD1_HUMAN, GTPase-activating protein and VPS9 domain-containing protein 1 OS=Homo sapiens GN=GAPVD1 PE=1 SV=2
Match to Query 34186: 1524.822from(509.2813,3+)
Title: 175: Scan 486 (rt=20.4445, f=2, i=84) [D:\lab212\membrane\Grace\Joyce\iTRAQ_39_1.raw]
Data File:Submitted from 20120508-1(merge) by Mascot Daemon on JOYCE-VAIO
Monoisotopic mass of neutral peptide Mr(calc): 1524.822
Variable modifications:
Ions Score: 63.17 Expect: 0.000

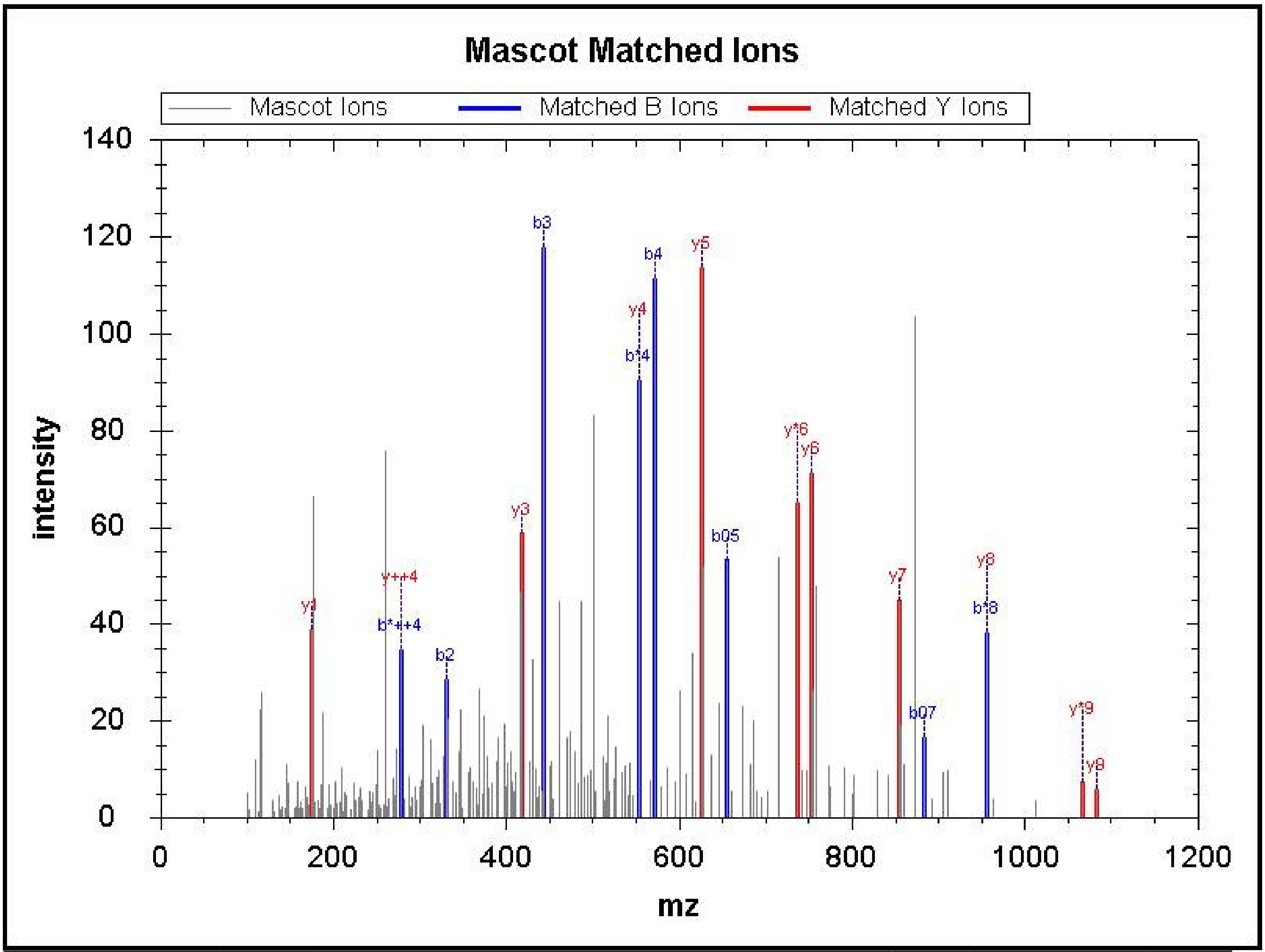

No	b	b++	b*	b*++	b0	b0++	Seq	y	y++	y*	y*++	y0	y0++	RevNo
1	273.17	137.09	256.14	128.57			Q							12
2	330.19	165.60	313.16	157.09			G	1,253.66	627.33	1,236.63	618.82	1,235.65	618.33	11
3	443.27	222.14	426.25	213.63			L	1,196.64	598.82	1,179.61	590.31	1,178.63	589.82	10
4	571.33	286.17	554.31	277.66			Q	1,083.55	542.28	1,066.53	533.77	1,065.54	533.28	9
5	672.38	336.69	655.35	328.18	654.37	327.69	T	955.50	478.25	938.47	469.74	937.48	469.25	8
6	773.43	387.22	756.40	378.70	755.42	378.21	T	854.45	427.73	837.42	419.21	836.44	418.72	7
7	901.49	451.25	884.46	442.73	883.48	442.24	Q	753.40	377.20	736.37	368.69	735.39	368.20	6
8	972.52	486.77	955.50	478.25	954.51	477.76	A	625.34	313.17	608.32	304.66	607.33	304.17	5
9	1,109.58	555.29	1,092.56	546.78	1,091.57	546.29	H	554.30	277.66	537.28	269.14	536.29	268.65	4
10	1,222.67	611.84	1,205.64	603.32	1,204.66	602.83	L	417.25	209.13	400.22	200.61	399.24	200.12	3
11	1,351.71	676.36	1,334.68	667.84	1,333.70	667.35	E	304.16	152.58	287.13	144.07	286.15	143.58	2
12							R	175.12	88.06	158.09	79.55			1

Query 40894 Hit 1

MS/MS Fragmentation of LPPVLSHPIFDNR
Found in sp|Q04206|TF65_HUMAN, Transcription factor p65 OS=Homo sapiens GN=RELA PE=1 SV=2
Match to Query 40894: 1647.923from(550.3148,3+)
Title: 703: Scan 1594 (rt=45.3132, f=3, i=239) [D:\lab212\membrane\Grace\Joyce\iTRAQ_35_2.raw]
Data File:Submitted from 20120508-1(merge) by Mascot Daemon on JOYCE-VAIO
Monoisotopic mass of neutral peptide Mr(calc): 1647.923
Variable modifications:
Ions Score: 62.94 Expect: 0.000

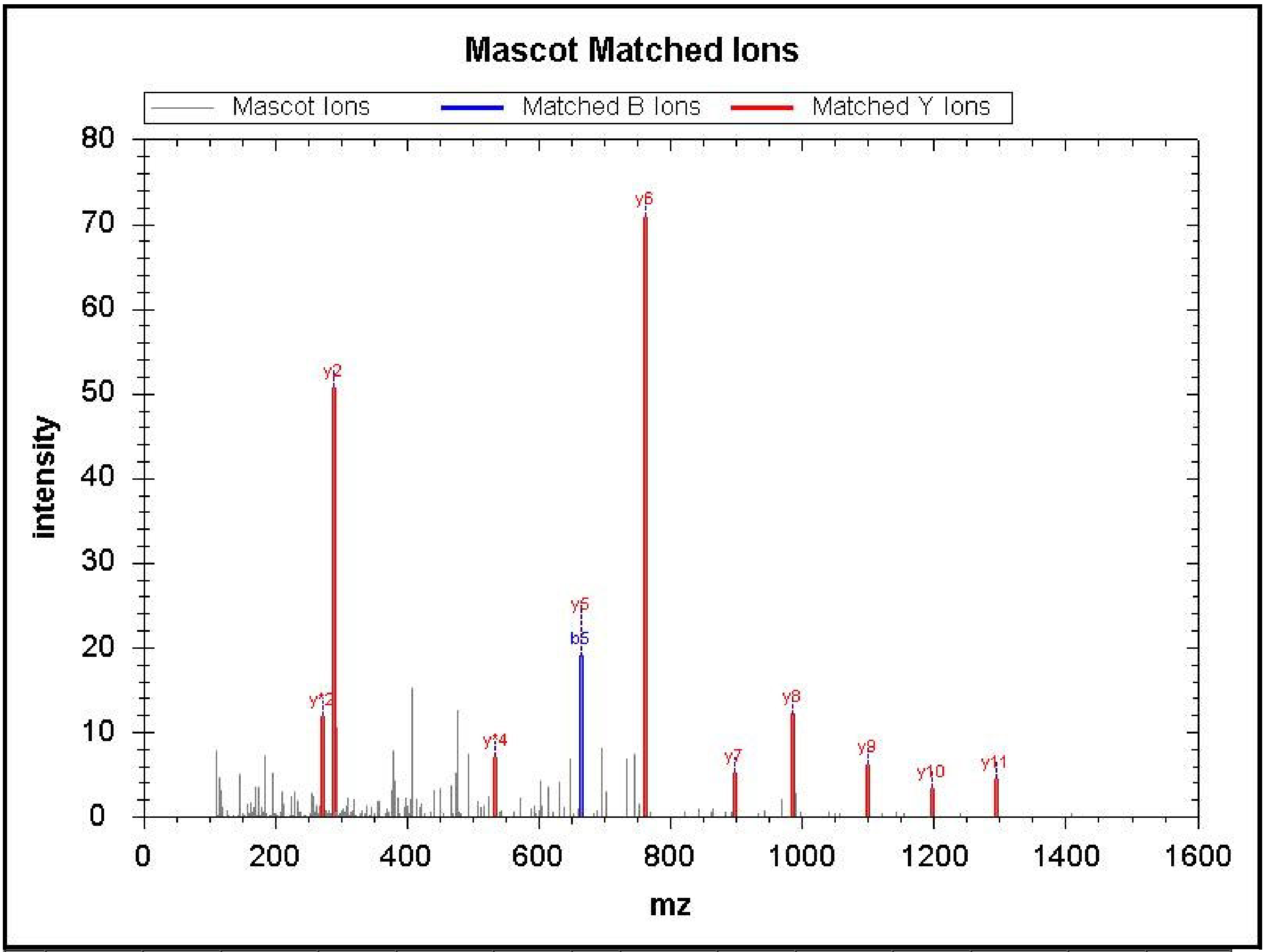

No	b	b++	b*	b*++	b0	b0++	Seq	y	y++	y*	y*++	y0	y0++	RevNo
1	258.19	129.60					L							13
2	355.25	178.13					P	1,391.74	696.38	1,374.72	687.86	1,373.73	687.37	12
3	452.30	226.65					P	1,294.69	647.85	1,277.66	639.34	1,276.68	638.84	11
4	551.37	276.19					V	1,197.64	599.32	1,180.61	590.81	1,179.63	590.32	10
5	664.45	332.73					L	1,098.57	549.79	1,081.54	541.27	1,080.56	540.78	9
6	751.48	376.25			733.47	367.24	S	985.48	493.25	968.46	484.73	967.47	484.24	8
7	888.54	444.77			870.53	435.77	H	898.45	449.73	881.43	441.22	880.44	440.72	7
8	985.60	493.30			967.58	484.30	P	761.39	381.20	744.37	372.69	743.38	372.20	6
9	1,098.68	549.84			1,080.67	540.84	I	664.34	332.67	647.31	324.16	646.33	323.67	5
10	1,245.75	623.38			1,227.74	614.37	F	551.26	276.13	534.23	267.62	533.25	267.13	4
11	1,360.77	680.89			1,342.76	671.89	D	404.19	202.60	387.16	194.08	386.18	193.59	3
12	1,474.82	737.91	1,457.79	729.40	1,456.81	728.91	N	289.16	145.08	272.14	136.57			2
13							R	175.12	88.06	158.09	79.55			1

Query 68822 Hit 1

MS/MS Fragmentation of LPPEVANFMQLVELDVSR

Found in sp|Q14160|SCRIB_HUMAN, Protein scribble homolog OS=Homo sapiens GN=SCRIB PE=1 SV=4
Match to Query 68822: 2200.166from(734.3958,3+)
Title: 1317: Sum of 2 scans in range 3033 (rt=77.3954, f=4, i=877) to 3034 (rt=77.4208, f=4, i=878)
[D:\lab212\membrane\Grace\Joyce\iTRAQ_21_1.raw]
Data File:Submitted from 20120508-1(merge) by Mascot Daemon on JOYCE-VAIO
Monoisotopic mass of neutral peptide Mr(calc): 2200.166
Variable modifications:
Ions Score: 62.77 Expect: 0.000

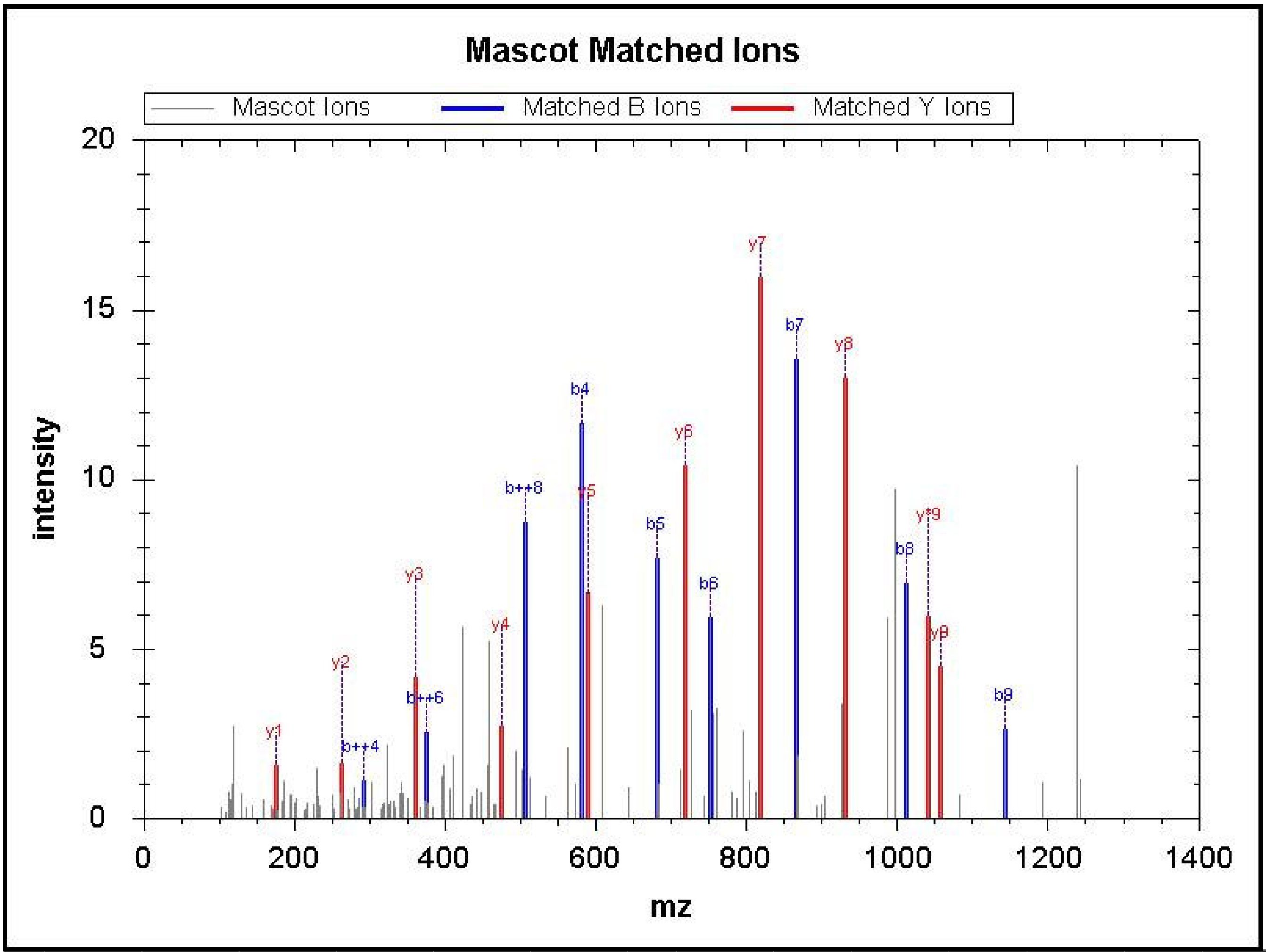

No	b	b++	b*	b*++	b0	b0++	Seq	y	y++	y*	y*++	y0	y0++	RevNo
1	258.19	129.60					L							18
2	355.25	178.13					P	1,943.99	972.50	1,926.96	963.99	1,925.98	963.49	17
3	452.30	226.65					P	1,846.94	923.97	1,829.91	915.46	1,828.93	914.97	16
4	581.34	291.17			563.33	282.17	E	1,749.88	875.45	1,732.86	866.93	1,731.87	866.44	15
5	680.41	340.71			662.40	331.70	V	1,620.84	810.92	1,603.81	802.41	1,602.83	801.92	14
6	751.45	376.23			733.44	367.22	A	1,521.77	761.39	1,504.75	752.88	1,503.76	752.38	13
7	865.49	433.25	848.46	424.74	847.48	424.24	N	1,450.74	725.87	1,433.71	717.36	1,432.73	716.87	12
8	1,012.56	506.78	995.53	498.27	994.55	497.78	F	1,336.69	668.85	1,319.67	660.34	1,318.68	659.84	11
9	1,143.60	572.30	1,126.57	563.79	1,125.59	563.30	M	1,189.62	595.32	1,172.60	586.80	1,171.61	586.31	10
10	1,271.66	636.33	1,254.63	627.82	1,253.65	627.33	Q	1,058.58	529.80	1,041.56	521.28	1,040.57	520.79	9
11	1,384.74	692.87	1,367.72	684.36	1,366.73	683.87	L	930.53	465.77	913.50	457.25	912.51	456.76	8
12	1,483.81	742.41	1,466.78	733.90	1,465.80	733.40	V	817.44	409.22	800.41	400.71	799.43	400.22	7
13	1,612.85	806.93	1,595.83	798.42	1,594.84	797.92	E	718.37	359.69	701.35	351.18	700.36	350.68	6
14	1,725.94	863.47	1,708.91	854.96	1,707.93	854.47	L	589.33	295.17	572.30	286.66	571.32	286.16	5
15	1,840.96	920.99	1,823.94	912.47	1,822.95	911.98	D	476.25	238.63	459.22	230.11	458.24	229.62	4
16	1,940.03	970.52	1,923.01	962.01	1,922.02	961.51	V	361.22	181.11	344.19	172.60	343.21	172.11	3

17	2,027.06	1,014.04	2,010.04	1,005.52	2,009.05	1,005.03	S	262.15	131.58	245.12	123.07	244.14	122.57	2
18							R	175.12	88.06	158.09	79.55			1

Query 85667 Hit 1

MS/MS Fragmentation of TPEDLNTIYSYLHGMEILSNLR
Found in sp|Q8TEU7|RPGF6_HUMAN, Rap guanine nucleotide exchange factor 6 OS=Homo sapiens GN=RAPGEF6 PE=1 SV=2
Match to Query 85667: 2722.372from(908.4647,3+)
Title: 1118: Sum of 2 scans in range 2538 (rt=66.2348, f=2, i=400) to 2539 (rt=66.2603, f=2, i=401)
[D:\lab212\membrane\Grace\Joyce\iTRAQ_30_1.raw]
Data File:Submitted from 20120508-1(merge) by Mascot Daemon on JOYCE-VAIO
Monoisotopic mass of neutral peptide Mr(calc): 2722.372
Variable modifications:
Ions Score: 62.57 Expect: 0.001

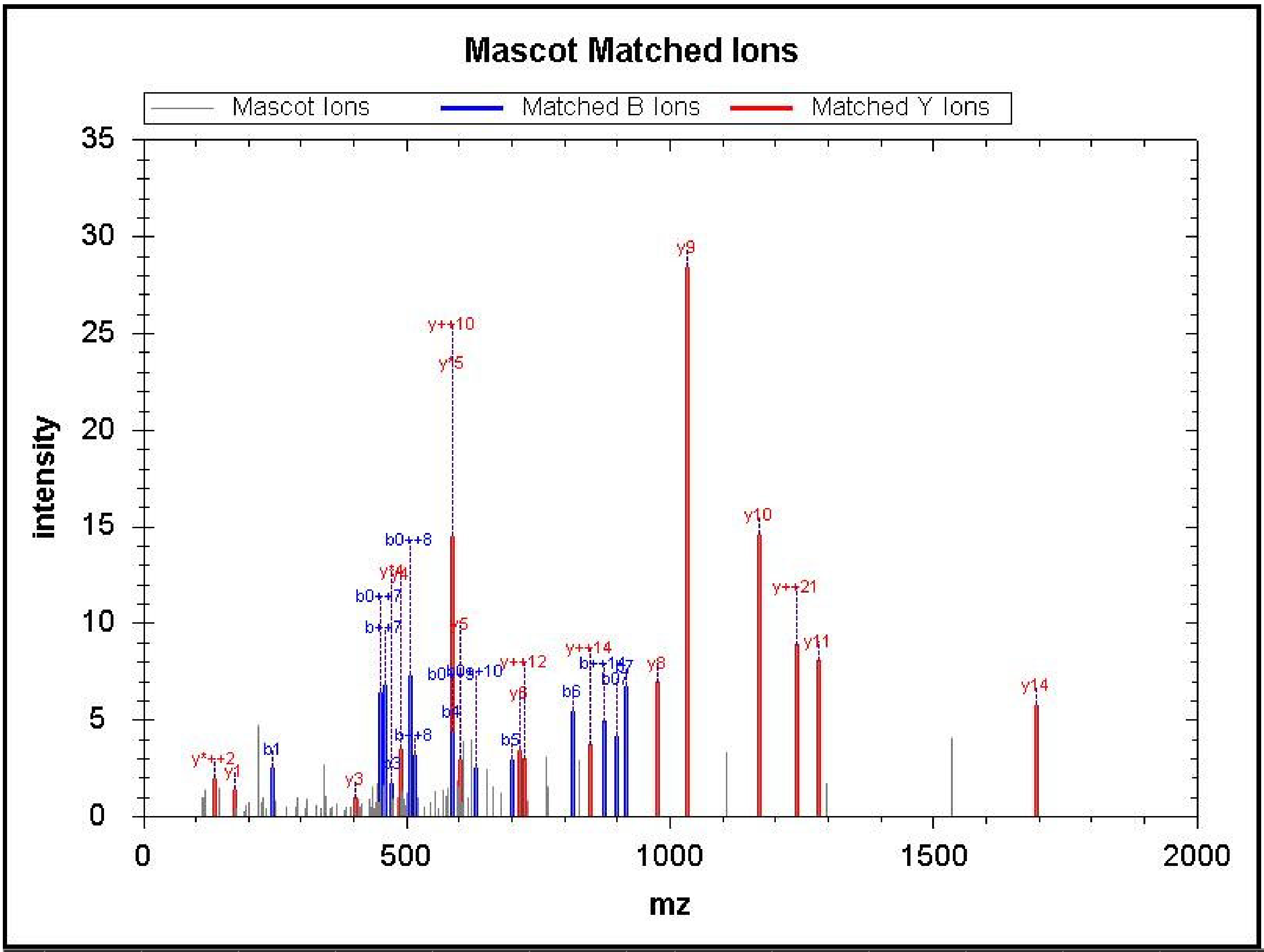

No	b	b++	b*	b*++	b0	b0++	Seq	y	y++	y*	y*++	y0	y0++	RevNo
1	246.16	123.58			228.15	114.58	T							22
2	343.21	172.11			325.20	163.10	P	2,478.23	1,239.62	2,461.21	1,231.11	2,460.22	1,230.61	21
3	472.25	236.63			454.24	227.62	E	2,381.18	1,191.09	2,364.15	1,182.58	2,363.17	1,182.09	20
4	587.28	294.14			569.27	285.14	D	2,252.14	1,126.57	2,235.11	1,118.06	2,234.13	1,117.57	19
5	700.36	350.69			682.35	341.68	L	2,137.11	1,069.06	2,120.08	1,060.55	2,119.10	1,060.05	18
6	814.41	407.71	797.38	399.19	796.40	398.70	N	2,024.03	1,012.52	2,007.00	1,004.00	2,006.02	1,003.51	17
7	915.45	458.23	898.43	449.72	897.44	449.23	T	1,909.98	955.50	1,892.96	946.98	1,891.97	946.49	16
8	1,028.54	514.77	1,011.51	506.26	1,010.53	505.77	I	1,808.94	904.97	1,791.91	896.46	1,790.93	895.97	15
9	1,191.60	596.30	1,174.57	587.79	1,173.59	587.30	Y	1,695.85	848.43	1,678.83	839.92	1,677.84	839.42	14
10	1,278.63	639.82	1,261.61	631.31	1,260.62	630.82	S	1,532.79	766.90	1,515.76	758.38	1,514.78	757.89	13
11	1,441.70	721.35	1,424.67	712.84	1,423.69	712.35	Y	1,445.76	723.38	1,428.73	714.87	1,427.75	714.38	12
12	1,554.78	777.89	1,537.75	769.38	1,536.77	768.89	L	1,282.69	641.85	1,265.67	633.34	1,264.68	632.85	11
13	1,691.84	846.42	1,674.81	837.91	1,673.83	837.42	H	1,169.61	585.31	1,152.58	576.80	1,151.60	576.30	10
14	1,748.86	874.93	1,731.83	866.42	1,730.85	865.93	G	1,032.55	516.78	1,015.52	508.27	1,014.54	507.77	9

15	1,879.90	940.45	1,862.88	931.94	1,861.89	931.45	M	975.53	488.27	958.50	479.75	957.52	479.26	8
16	2,008.94	1,004.98	1,991.92	996.46	1,990.93	995.97	E	844.49	422.75	827.46	414.23	826.48	413.74	7
17	2,122.03	1,061.52	2,105.00	1,053.00	2,104.02	1,052.51	I	715.45	358.23	698.42	349.71	697.44	349.22	6
18	2,235.11	1,118.06	2,218.09	1,109.55	2,217.10	1,109.05	L	602.36	301.68	585.34	293.17	584.35	292.68	5
19	2,322.14	1,161.58	2,305.12	1,153.06	2,304.13	1,152.57	S	489.28	245.14	472.25	236.63	471.27	236.14	4
20	2,436.19	1,218.60	2,419.16	1,210.08	2,418.18	1,209.59	N	402.25	201.63	385.22	193.11			3
21	2,549.27	1,275.14	2,532.24	1,266.63	2,531.26	1,266.13	L	288.20	144.61	271.18	136.09			2
22							R	175.12	88.06	158.09	79.55			1

Query 54815 Hit 1

MS/MS Fragmentation of NPEISHLLNNPDIMR
Found in sp|Q9UHD9|UBQL2_HUMAN, Ubiquilin-2 OS=Homo sapiens GN=UBQLN2 PE=1 SV=2
Match to Query 54815: 1905.989from(636.3369,3+)
Title: 617: Scan 1414 (rt=41.2579, f=3, i=210) [D:\lab212\membrane\Grace\Joyce\iTRAQ_35_2.raw]
Data File:Submitted from 20120508-1(merge) by Mascot Daemon on JOYCE-VAIO
Monoisotopic mass of neutral peptide Mr(calc): 1905.989
Variable modifications:
Ions Score: 62.12 Expect: 0.001

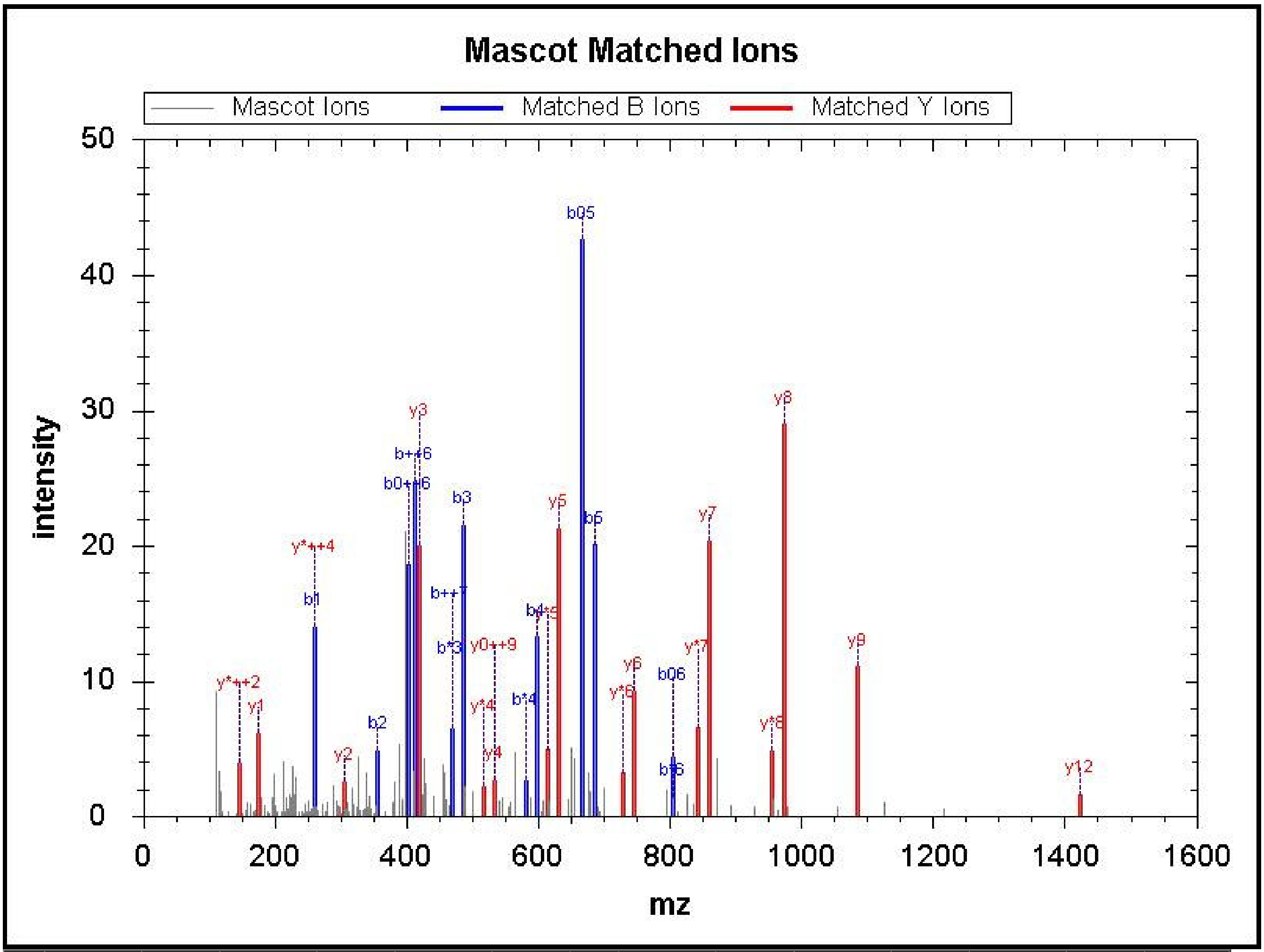

No	b	b++	b*	b*++	b0	b0++	Seq	y	y++	y*	y*++	y0	y0++	RevNo
1	259.15	130.08	242.13	121.57			N							15
2	356.21	178.61	339.18	170.09			P	1,648.85	824.93	1,631.82	816.41	1,630.84	815.92	14
3	485.25	243.13	468.22	234.61	467.24	234.12	E	1,551.79	776.40	1,534.77	767.89	1,533.78	767.40	13
4	598.33	299.67	581.31	291.16	580.32	290.66	I	1,422.75	711.88	1,405.73	703.37	1,404.74	702.87	12
5	685.36	343.19	668.34	334.67	667.35	334.18	S	1,309.67	655.34	1,292.64	646.82	1,291.66	646.33	11
6	822.42	411.71	805.40	403.20	804.41	402.71	H	1,222.64	611.82	1,205.61	603.31	1,204.63	602.82	10

7	935.51	468.26	918.48	459.74	917.50	459.25	L	1,085.58	543.29	1,068.55	534.78	1,067.57	534.29	9
8	1,048.59	524.80	1,031.56	516.29	1,030.58	515.79	L	972.49	486.75	955.47	478.24	954.48	477.74	8
9	1,162.63	581.82	1,145.61	573.31	1,144.62	572.82	N	859.41	430.21	842.38	421.69	841.40	421.20	7
10	1,276.68	638.84	1,259.65	630.33	1,258.67	629.84	N	745.37	373.19	728.34	364.67	727.36	364.18	6
11	1,373.73	687.37	1,356.70	678.86	1,355.72	678.36	P	631.32	316.17	614.30	307.65	613.31	307.16	5
12	1,488.76	744.88	1,471.73	736.37	1,470.75	735.88	D	534.27	267.64	517.24	259.13	516.26	258.63	4
13	1,601.84	801.42	1,584.81	792.91	1,583.83	792.42	I	419.24	210.13	402.22	201.61			3
14	1,732.88	866.94	1,715.85	858.43	1,714.87	857.94	M	306.16	153.58	289.13	145.07			2
15							R	175.12	88.06	158.09	79.55			1

Query 65641 Hit 1

MS/MS Fragmentation of EVFLPSTPGLGMHVEVK
Found in sp|Q7Z7H5|TMED4_HUMAN, Transmembrane emp24 domain-containing protein 4 OS=Homo sapiens GN=TMED4 PE=1 SV=1
Match to Query 65641: 2127.164from(710.0618,3+)
Title: 687: Sum of 2 scans in range 1562 (rt=44.5722, f=4, i=461) to 1563 (rt=44.5976, f=4, i=462) [D:\lab212\membrane\Grace\Joyce\iTRAQ_35_1.raw]
Data File:Submitted from 20120508-1(merge) by Mascot Daemon on JOYCE-VAIO
Monoisotopic mass of neutral peptide Mr(calc): 2127.164
Variable modifications:
K17 :iTRAQ4plex (K)
Ions Score: 61.78 Expect: 0.000

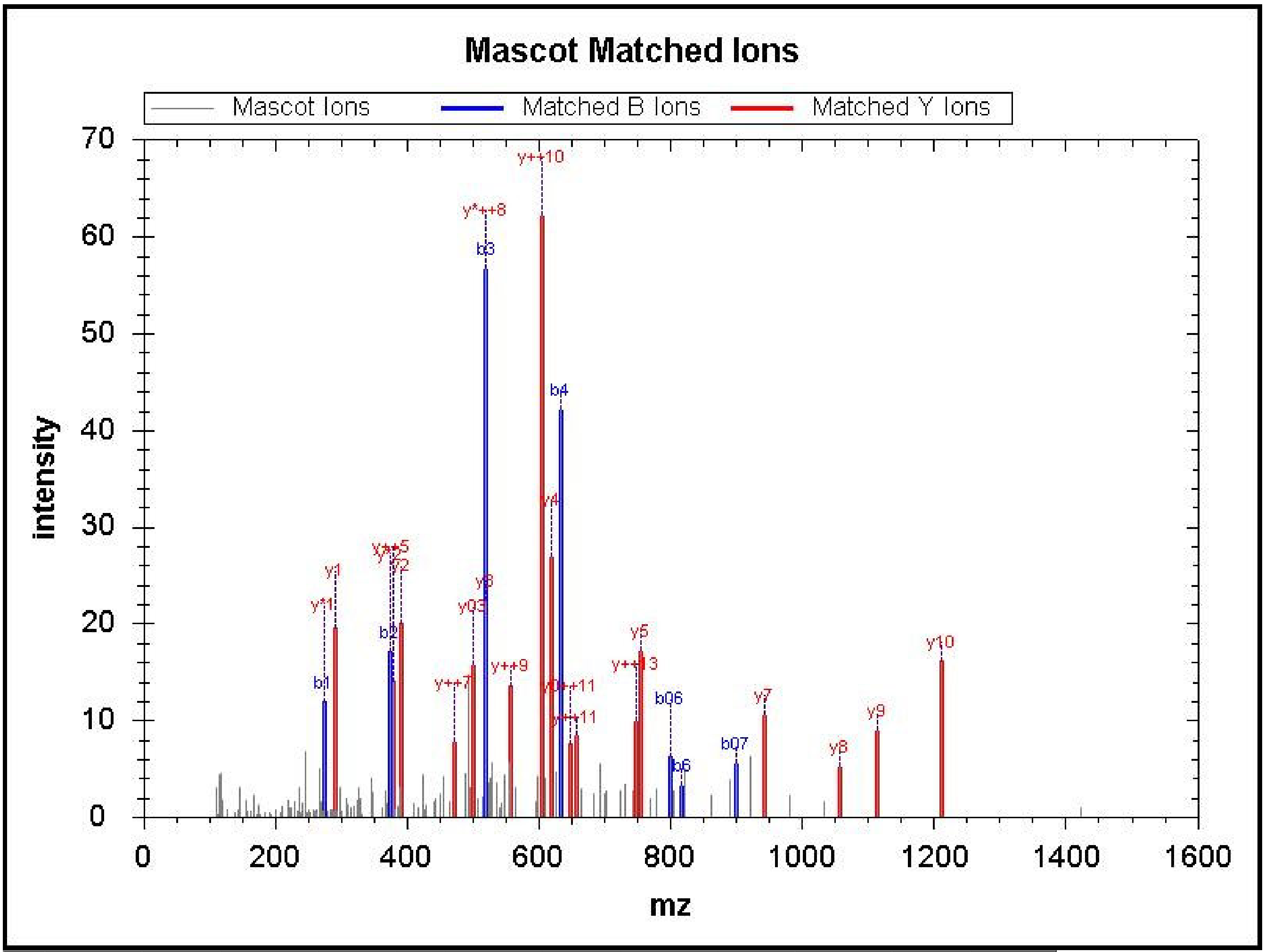

No	b	b++	b0	b0++	Seq	y	y++	y*	y*++	y0	y0++	RevNo
1	274.15	137.58	256.14	128.57	E							17
2	373.22	187.11	355.21	178.11	V	1,855.03	928.02	1,838.00	919.50	1,837.02	919.01	16

3	520.29	260.65	502.28	251.64	F	1,755.96	878.48	1,738.93	869.97	1,737.95	869.48	15
4	633.37	317.19	615.36	308.18	L	1,608.89	804.95	1,591.86	796.44	1,590.88	795.94	14
5	730.43	365.72	712.42	356.71	P	1,495.81	748.41	1,478.78	739.89	1,477.80	739.40	13
6	817.46	409.23	799.45	400.23	S	1,398.75	699.88	1,381.73	691.37	1,380.74	690.87	12
7	918.51	459.76	900.49	450.75	T	1,311.72	656.36	1,294.69	647.85	1,293.71	647.36	11
8	1,015.56	508.28	997.55	499.28	P	1,210.67	605.84	1,193.65	597.33	1,192.66	596.84	10
9	1,072.58	536.79	1,054.57	527.79	G	1,113.62	557.31	1,096.59	548.80	1,095.61	548.31	9
10	1,185.66	593.34	1,167.65	584.33	L	1,056.60	528.80	1,039.57	520.29	1,038.59	519.80	8
11	1,242.69	621.85	1,224.67	612.84	G	943.52	472.26	926.49	463.75	925.50	463.26	7
12	1,373.73	687.37	1,355.72	678.36	M	886.49	443.75	869.47	435.24	868.48	434.75	6
13	1,510.78	755.90	1,492.77	746.89	H	755.45	378.23	738.43	369.72	737.44	369.22	5
14	1,609.85	805.43	1,591.84	796.42	V	618.39	309.70	601.37	301.19	600.38	300.70	4
15	1,738.90	869.95	1,720.89	860.95	E	519.33	260.17	502.30	251.65	501.32	251.16	3
16	1,837.96	919.49	1,819.95	910.48	V	390.28	195.65	373.26	187.13			2
17					K	291.21	146.11	274.19	137.60			1

Query 69569 Hit 1

MS/MS Fragmentation of EVEVQLLPELEEMILHR
Found in sp|Q14667|K0100_HUMAN, UPF0378 protein KIAA0100 OS=Homo sapiens GN=KIAA0100 PE=1 SV=3
Match to Query 69569: 2220.195from(741.0722,3+)
Title: 1137: Sum of 2 scans in range 2624 (rt=68.0281, f=2, i=432) to 2625 (rt=68.0535, f=2, i=433)
[D:\lab212\membrane\Grace\Joyce\iTRAQ_30_2.raw]
Data File:Submitted from 20120508-1(merge) by Mascot Daemon on JOYCE-VAIO
Monoisotopic mass of neutral peptide Mr(calc): 2220.195
Variable modifications:
Ions Score: 61.77 Expect: 0.001

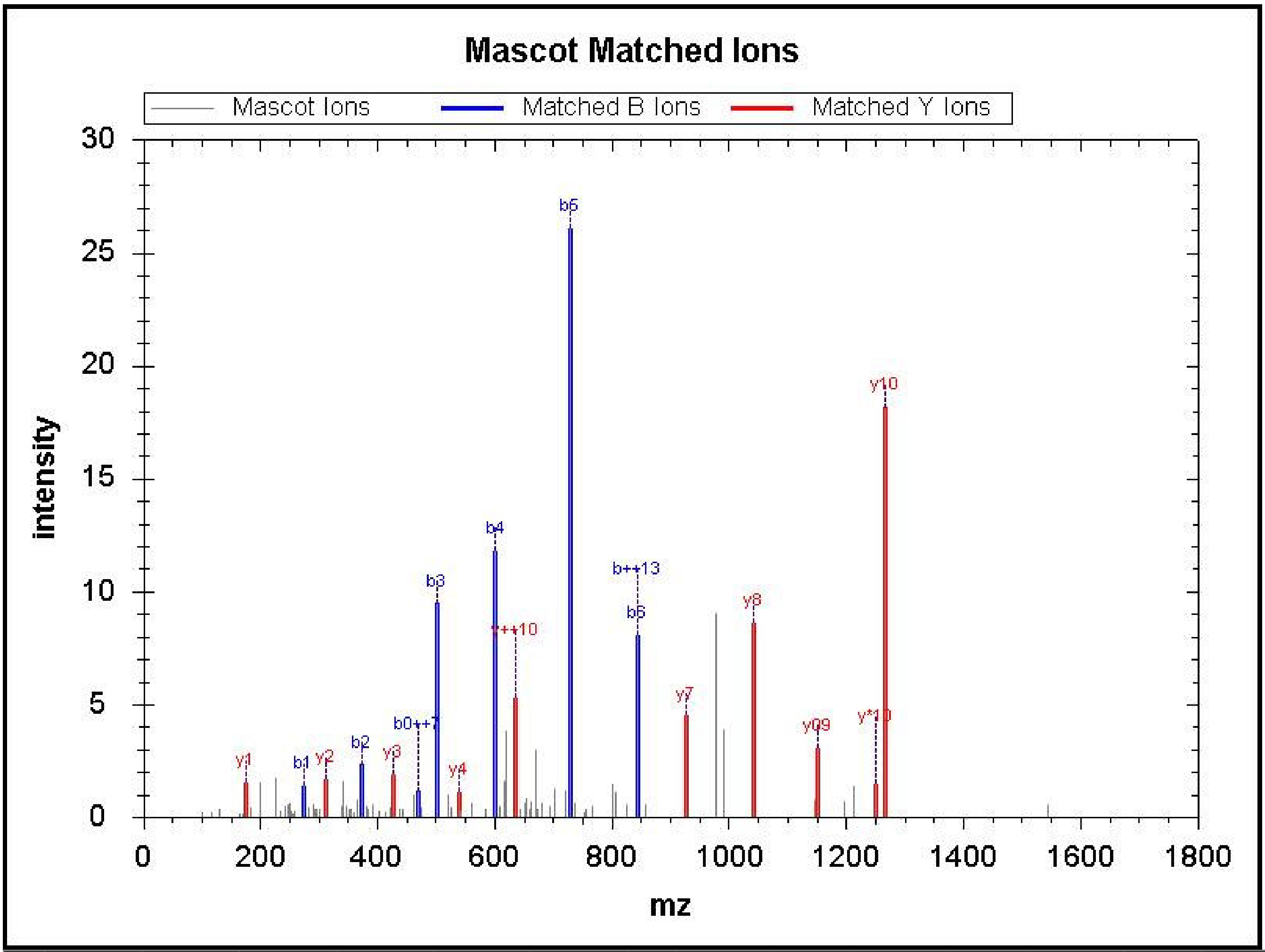

No	b	b++	b*	b*++	b0	b0++	Seq	y	y++	y*	y*++	y0	y0++	RevNo
1	274.15	137.58			256.14	128.57	E							17
2	373.22	187.11			355.21	178.11	V	1,948.06	974.53	1,931.03	966.02	1,930.05	965.53	16
3	502.26	251.64			484.25	242.63	E	1,848.99	925.00	1,831.96	916.48	1,830.98	915.99	15
4	601.33	301.17			583.32	292.16	V	1,719.95	860.48	1,702.92	851.96	1,701.94	851.47	14
5	729.39	365.20	712.36	356.69	711.38	356.19	Q	1,620.88	810.94	1,603.85	802.43	1,602.87	801.94	13
6	842.47	421.74	825.45	413.23	824.46	412.74	L	1,492.82	746.91	1,475.79	738.40	1,474.81	737.91	12
7	955.56	478.28	938.53	469.77	937.55	469.28	L	1,379.74	690.37	1,362.71	681.86	1,361.72	681.37	11
8	1,052.61	526.81	1,035.58	518.30	1,034.60	517.80	P	1,266.65	633.83	1,249.62	625.32	1,248.64	624.82	10
9	1,181.65	591.33	1,164.63	582.82	1,163.64	582.33	E	1,169.60	585.30	1,152.57	576.79	1,151.59	576.30	9
10	1,294.74	647.87	1,277.71	639.36	1,276.73	638.87	L	1,040.56	520.78	1,023.53	512.27	1,022.55	511.78	8
11	1,423.78	712.39	1,406.75	703.88	1,405.77	703.39	E	927.47	464.24	910.45	455.73	909.46	455.23	7
12	1,552.82	776.92	1,535.80	768.40	1,534.81	767.91	E	798.43	399.72	781.40	391.20	780.42	390.71	6
13	1,683.86	842.44	1,666.84	833.92	1,665.85	833.43	M	669.39	335.20	652.36	326.68			5
14	1,796.95	898.98	1,779.92	890.46	1,778.94	889.97	I	538.35	269.68	521.32	261.16			4
15	1,910.03	955.52	1,893.01	947.01	1,892.02	946.51	L	425.26	213.13	408.24	204.62			3
16	2,047.09	1,024.05	2,030.06	1,015.54	2,029.08	1,015.04	H	312.18	156.59	295.15	148.08			2
17							R	175.12	88.06	158.09	79.55			1

Query 62282 Hit 1

MS/MS Fragmentation of TFTDHMLMVEWNDK
Found in sp|O15382|BCAT2_HUMAN, Branched-chain-amino-acid aminotransferase
Match to Query 62282: 2053.991 from(685.6708,3+)
Title: 758: Scan 1665 (rt=47.2806, f=2, i=267) [D:\lab212\membrane\Grace\Joyce\iTRAQ_42_2.raw]
Data File:Submitted from 20120508-1(merge) by Mascot Daemon on JOYCE-VAIO
Monoisotopic mass of neutral peptide Mr(calc): 2053.991

Variable modifications:
K14 :iTRAQ4plex (K)
Ions Score: 61.62 Expect: 0.001

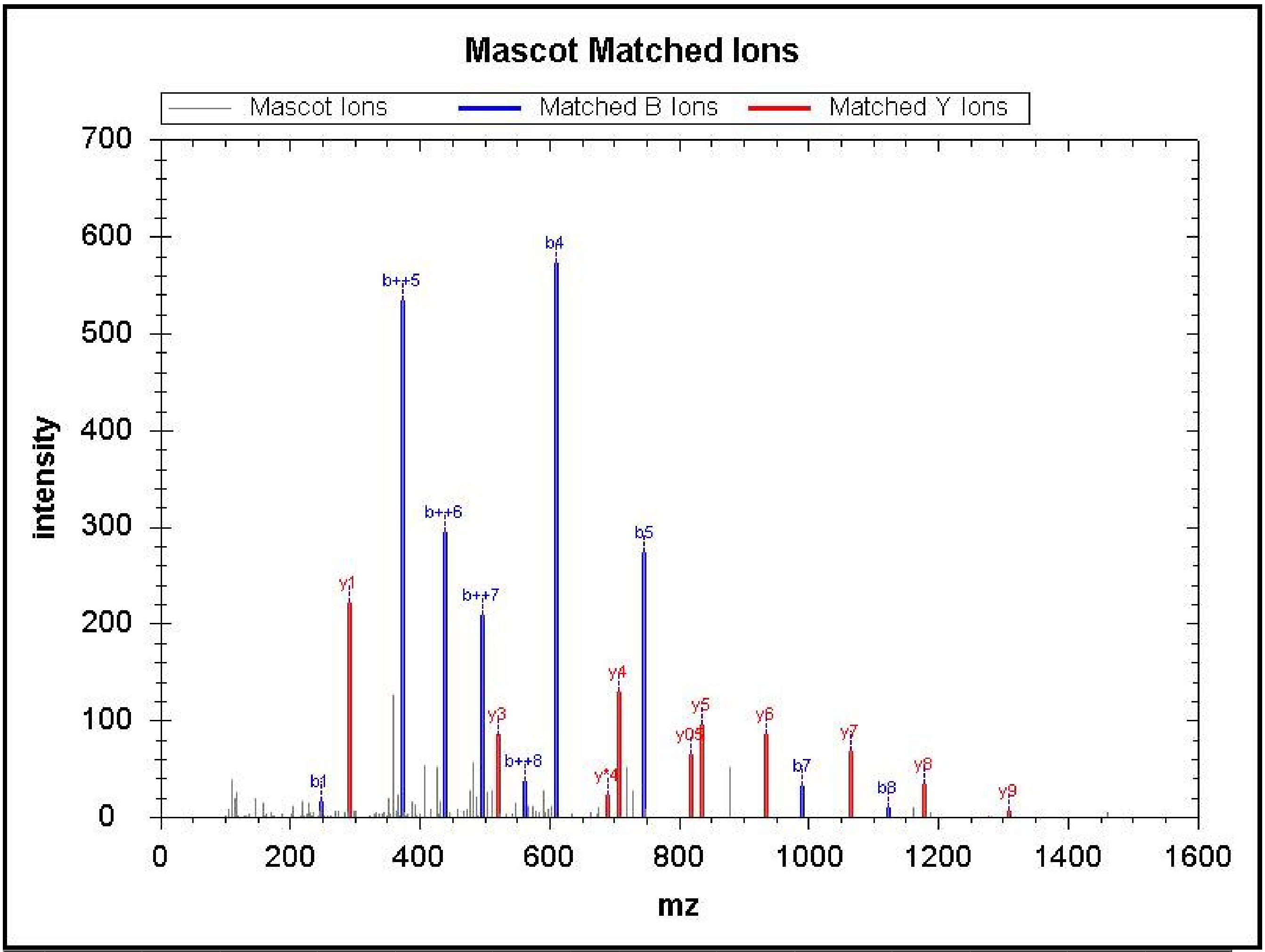

No	b	b++	b*	b*++	b0	b0++	Seq	y	y++	y*	y*++	y0	y0++	RevNo
1	246.16	123.58			228.15	114.58	T							14
2	393.23	197.12			375.21	188.11	F	1,809.84	905.42	1,792.82	896.91	1,791.83	896.42	13
3	494.27	247.64			476.26	238.63	T	1,662.77	831.89	1,645.75	823.38	1,644.76	822.89	12
4	609.30	305.15			591.29	296.15	D	1,561.73	781.37	1,544.70	772.85	1,543.72	772.36	11
5	746.36	373.68			728.35	364.68	H	1,446.70	723.85	1,429.67	715.34	1,428.69	714.85	10
6	877.40	439.20			859.39	430.20	M	1,309.64	655.32	1,292.61	646.81	1,291.63	646.32	9
7	990.48	495.75			972.47	486.74	L	1,178.60	589.80	1,161.57	581.29	1,160.59	580.80	8
8	1,121.52	561.27			1,103.51	552.26	M	1,065.52	533.26	1,048.49	524.75	1,047.50	524.26	7
9	1,220.59	610.80			1,202.58	601.79	V	934.48	467.74	917.45	459.23	916.46	458.74	6
10	1,349.64	675.32			1,331.62	666.32	E	835.41	418.21	818.38	409.69	817.40	409.20	5
11	1,535.71	768.36			1,517.70	759.36	W	706.36	353.69	689.34	345.17	688.35	344.68	4
12	1,649.76	825.38	1,632.73	816.87	1,631.75	816.38	N	520.28	260.65	503.26	252.13	502.27	251.64	3
13	1,764.78	882.90	1,747.76	874.38	1,746.77	873.89	D	406.24	203.62	389.22	195.11	388.23	194.62	2
14							K	291.21	146.11	274.19	137.60			1

Query 97565 Hit 1

MS/MS Fragmentation of WYQADSPPADLLLTEEEFLSFLHPEHSR
Found in sp|Q9BRK5|CAB45_HUMAN, 45 kDa calcium-binding protein OS=Homo sapiens GN=SDF4 PE=1 SV=1
Match to Query 97565: 3470.694from(868.6807,4+)
Title: 1303: Sum of 2 scans in range 2918 (rt=75.0288, f=4, i=847) to 2919 (rt=75.0542, f=4, i=848) [D:\lab212\membrane\Grace\Joyce\iTRAQ_39_2.raw]

Data File:Submitted from 20120508-1(merge) by Mascot Daemon on JOYCE-VAIO
Monoisotopic mass of neutral peptide Mr(calc): 3470.694
Variable modifications:
Ions Score: 61.61 Expect: 0.001

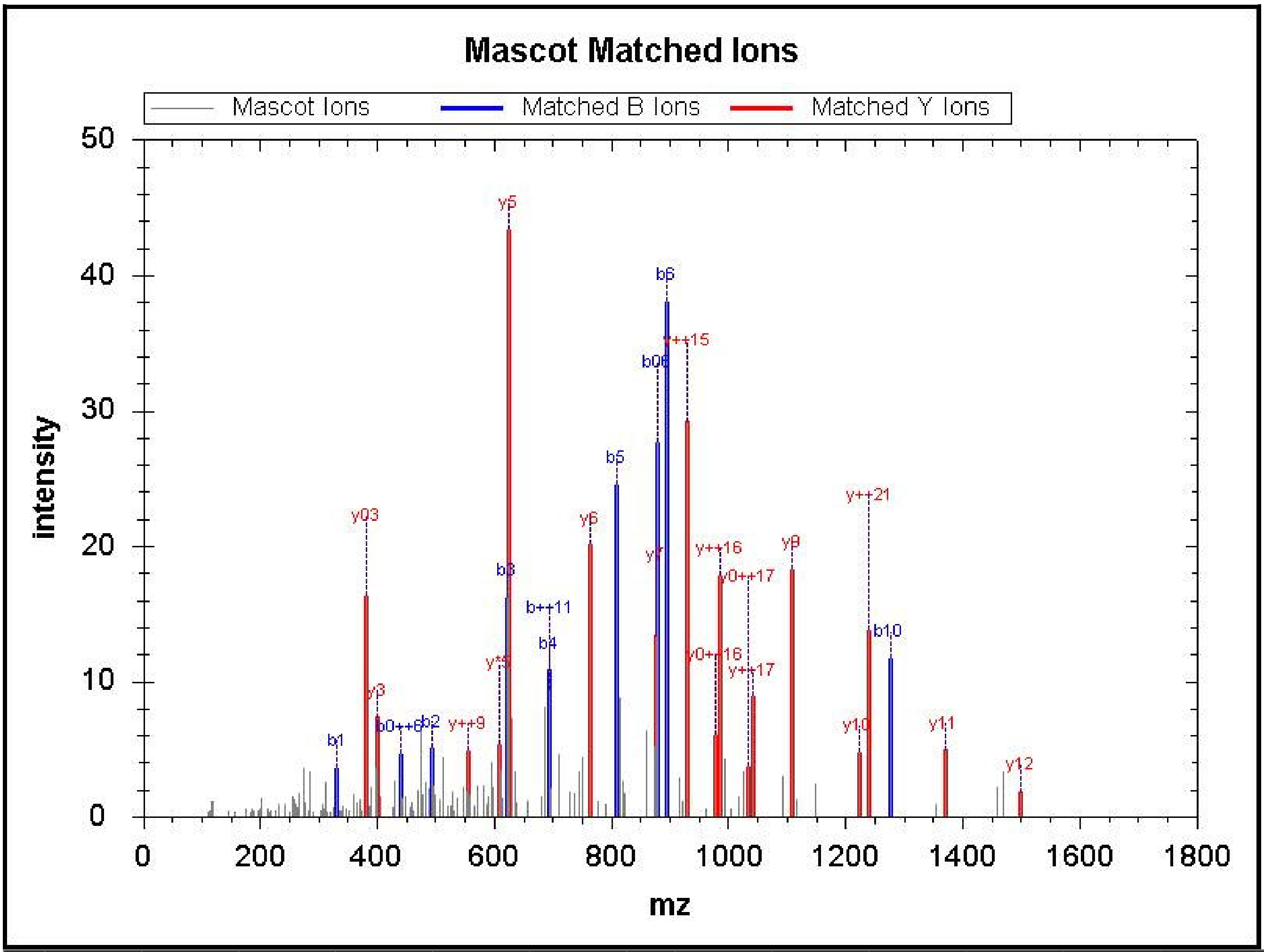

No	b	b++	b*	b*++	b0	b0++	Seq	y	y++	y*	y*++	y0	y0++	RevNo
1	331.19	166.10					W							28
2	494.25	247.63					Y	3,141.52	1,571.26	3,124.49	1,562.75	3,123.51	1,562.26	27
3	622.31	311.66	605.28	303.15			Q	2,978.45	1,489.73	2,961.43	1,481.22	2,960.44	1,480.72	26
4	693.35	347.18	676.32	338.66			A	2,850.39	1,425.70	2,833.37	1,417.19	2,832.38	1,416.70	25
5	808.37	404.69	791.35	396.18	790.36	395.69	D	2,779.36	1,390.18	2,762.33	1,381.67	2,761.35	1,381.18	24
6	895.41	448.21	878.38	439.69	877.40	439.20	S	2,664.33	1,332.67	2,647.30	1,324.16	2,646.32	1,323.66	23
7	992.46	496.73	975.43	488.22	974.45	487.73	P	2,577.30	1,289.15	2,560.27	1,280.64	2,559.29	1,280.15	22
8	1,089.51	545.26	1,072.49	536.75	1,071.50	536.25	P	2,480.25	1,240.63	2,463.22	1,232.11	2,462.24	1,231.62	21
9	1,160.55	580.78	1,143.52	572.27	1,142.54	571.77	A	2,383.19	1,192.10	2,366.17	1,183.59	2,365.18	1,183.09	20
10	1,275.58	638.29	1,258.55	629.78	1,257.57	629.29	D	2,312.16	1,156.58	2,295.13	1,148.07	2,294.15	1,147.58	19
11	1,388.66	694.83	1,371.63	686.32	1,370.65	685.83	L	2,197.13	1,099.07	2,180.10	1,090.55	2,179.12	1,090.06	18
12	1,501.74	751.38	1,484.72	742.86	1,483.73	742.37	L	2,084.04	1,042.53	2,067.02	1,034.01	2,066.03	1,033.52	17
13	1,614.83	807.92	1,597.80	799.40	1,596.82	798.91	L	1,970.96	985.98	1,953.93	977.47	1,952.95	976.98	16
14	1,715.88	858.44	1,698.85	849.93	1,697.87	849.44	T	1,857.88	929.44	1,840.85	920.93	1,839.87	920.44	15
15	1,844.92	922.96	1,827.89	914.45	1,826.91	913.96	E	1,756.83	878.92	1,739.80	870.40	1,738.82	869.91	14
16	1,973.96	987.48	1,956.94	978.97	1,955.95	978.48	E	1,627.79	814.40	1,610.76	805.88	1,609.78	805.39	13
17	2,103.00	1,052.01	2,085.98	1,043.49	2,084.99	1,043.00	E	1,498.74	749.88	1,481.72	741.36	1,480.73	740.87	12
18	2,250.07	1,125.54	2,233.05	1,117.03	2,232.06	1,116.53	F	1,369.70	685.35	1,352.67	676.84	1,351.69	676.35	11
19	2,363.16	1,182.08	2,346.13	1,173.57	2,345.15	1,173.08	L	1,222.63	611.82	1,205.61	603.31	1,204.62	602.81	10
20	2,450.19	1,225.60	2,433.16	1,217.08	2,432.18	1,216.59	S	1,109.55	555.28	1,092.52	546.76	1,091.54	546.27	9

21	2,597.26	1,299.13	2,580.23	1,290.62	2,579.25	1,290.13	F	1,022.52	511.76	1,005.49	503.25	1,004.51	502.76	8
22	2,710.34	1,355.67	2,693.31	1,347.16	2,692.33	1,346.67	L	875.45	438.23	858.42	429.71	857.44	429.22	7
23	2,847.40	1,424.20	2,830.37	1,415.69	2,829.39	1,415.20	H	762.36	381.69	745.34	373.17	744.35	372.68	6
24	2,944.45	1,472.73	2,927.43	1,464.22	2,926.44	1,463.72	P	625.31	313.16	608.28	304.64	607.29	304.15	5
25	3,073.49	1,537.25	3,056.47	1,528.74	3,055.48	1,528.25	E	528.25	264.63	511.23	256.12	510.24	255.62	4
26	3,210.55	1,605.78	3,193.53	1,597.27	3,192.54	1,596.78	H	399.21	200.11	382.18	191.60	381.20	191.10	3
27	3,297.59	1,649.30	3,280.56	1,640.78	3,279.58	1,640.29	S	262.15	131.58	245.12	123.07	244.14	122.57	2
28							R	175.12	88.06	158.09	79.55			1

Query 27994 Hit 1

MS/MS Fragmentation of SVIDYQTHFR
Found in sp|Q9Y3B3|TMED7_HUMAN, Transmembrane emp24 domain-containing protein 7 OS=Homo sapiens GN=TMED7 PE=1 SV=2
Match to Query 27994: 1408.724from(470.5819,3+)
Title: 403: Scan 921 (rt=30.424, f=2, i=148) [D:\lab212\membrane\Grace\Joyce\iTRAQ_40_1.raw]
Data File:Submitted from 20120508-1(merge) by Mascot Daemon on JOYCE-VAIO
Monoisotopic mass of neutral peptide Mr(calc): 1408.724
Variable modifications:
Ions Score: 61.6 Expect: 0.001

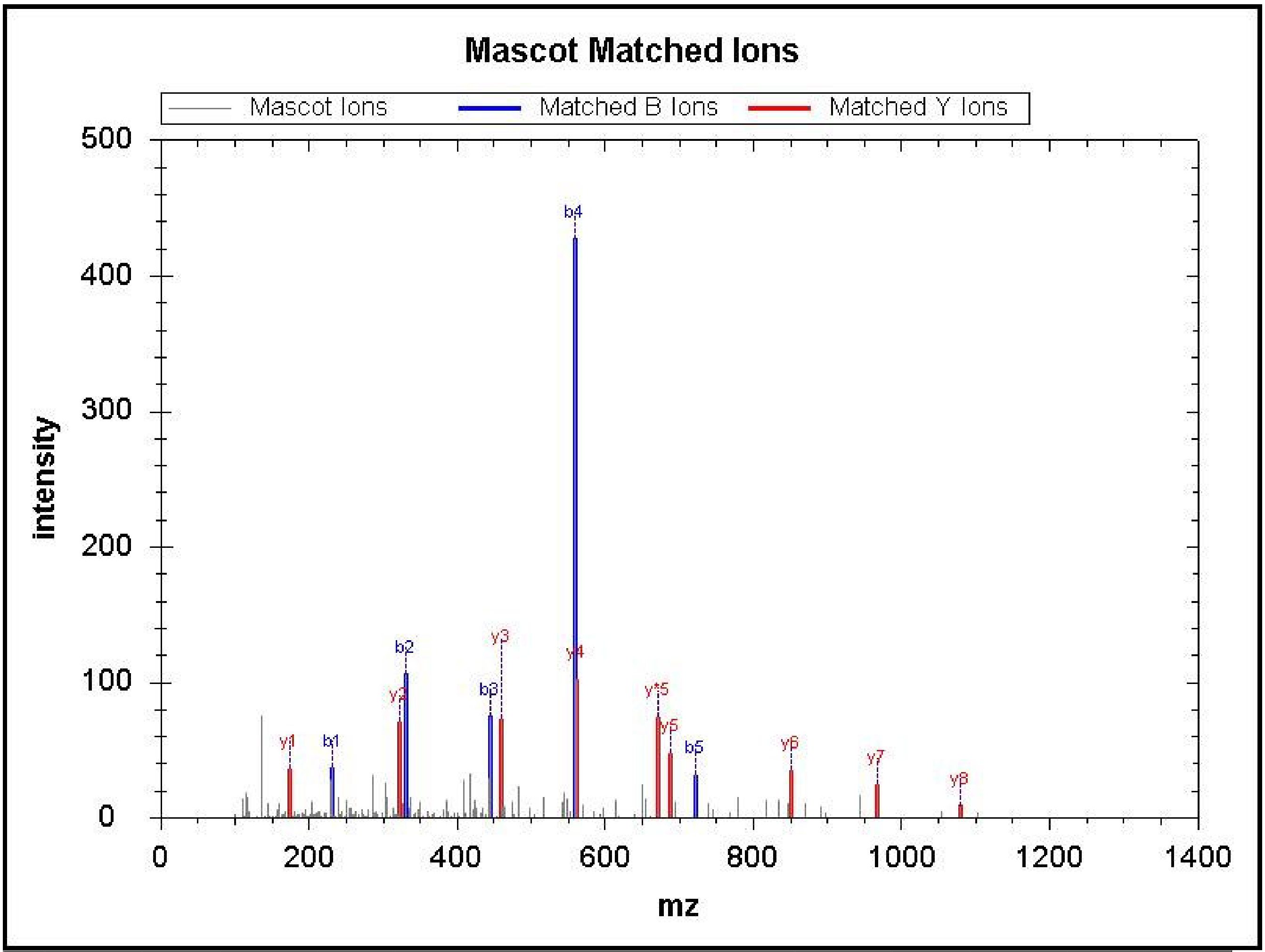

No	b	b++	b*	b*++	b0	b0++	Seq	y	y++	y*	y*++	y0	y0++	RevNo
1	232.14	116.57			214.13	107.57	S							10
2	331.21	166.11			313.20	157.10	V	1,178.60	589.80	1,161.57	581.29	1,160.58	580.80	9
3	444.29	222.65			426.28	213.65	I	1,079.53	540.27	1,062.50	531.75	1,061.52	531.26	8
4	559.32	280.16			541.31	271.16	D	966.44	483.73	949.42	475.21	948.43	474.72	7
5	722.38	361.70			704.37	352.69	Y	851.42	426.21	834.39	417.70	833.41	417.21	6
6	850.44	425.72	833.42	417.21	832.43	416.72	Q	688.35	344.68	671.33	336.17	670.34	335.67	5

7	951.49	476.25	934.46	467.74	933.48	467.24	T	560.29	280.65	543.27	272.14	542.28	271.65	4
8	1,088.55	544.78	1,071.52	536.27	1,070.54	535.77	H	459.25	230.13	442.22	221.61			3
9	1,235.62	618.31	1,218.59	609.80	1,217.61	609.31	F	322.19	161.60	305.16	153.08			2
10							R	175.12	88.06	158.09	79.55			1

Query 37436 Hit 1

MS/MS Fragmentation of NLLHQDAVDLFR
Found in sp|P57105|SYJ2B_HUMAN, Synaptojanin-2-binding protein OS=Homo sapiens GN=SYNJ2BP PE=1 SV=2
Match to Query 37436: 1583.848from(528.9565,3+)
Title: 623: Scan 1407 (rt=41.1296, f=3, i=213) [D:\lab212\membrane\Grace\Joyce\iTRAQ_37_1.raw]
Data File:Submitted from 20120508-1(merge) by Mascot Daemon on JOYCE-VAIO
Monoisotopic mass of neutral peptide Mr(calc): 1583.848
Variable modifications:
Ions Score: 61.37 Expect: 0.001

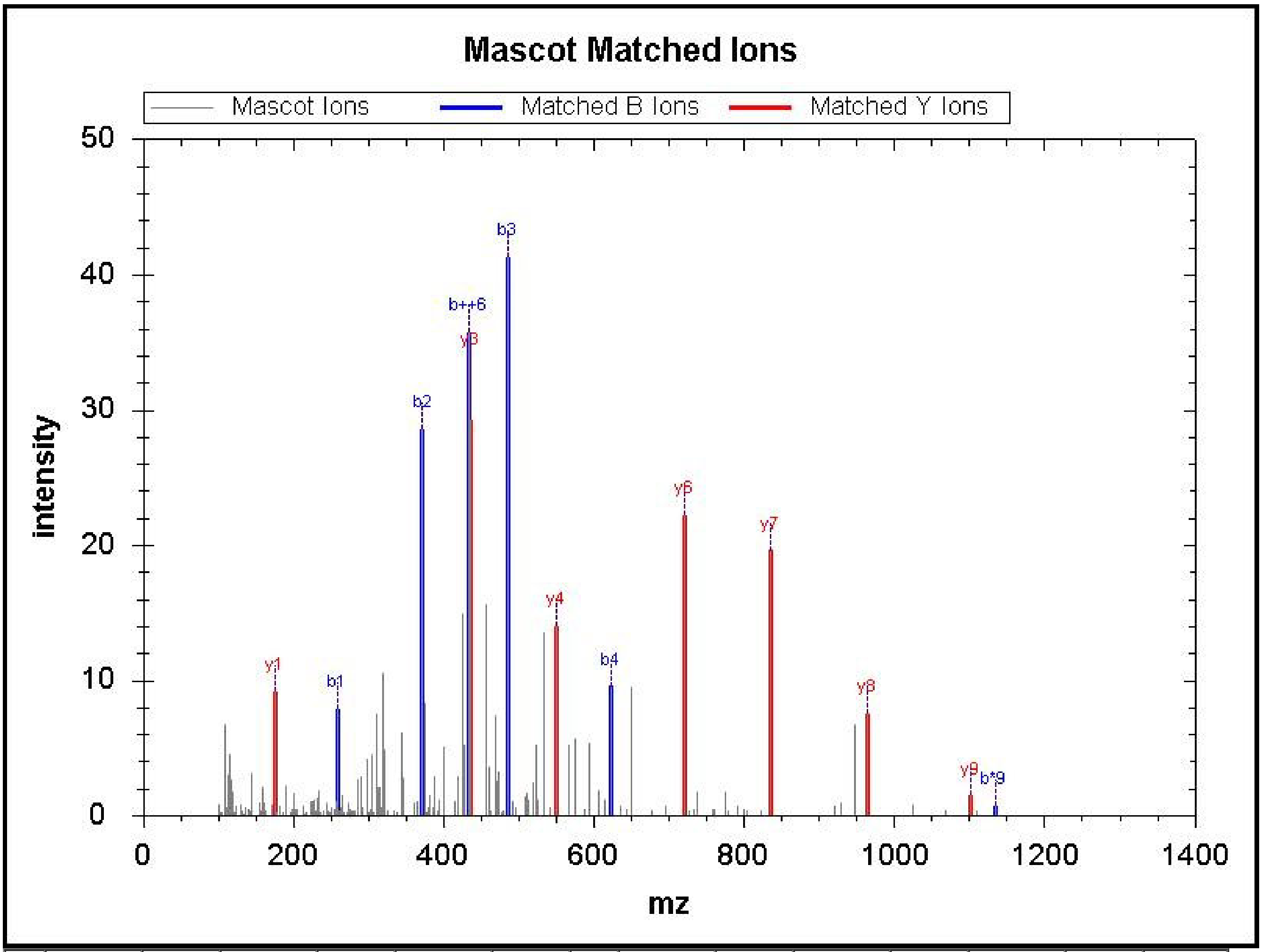

No	b	b++	b*	b*++	b0	b0++	Seq	y	y++	y*	y*++	y0	y0++	RevNo
1	259.15	130.08	242.13	121.57			N							12
2	372.24	186.62	355.21	178.11			L	1,326.72	663.86	1,309.69	655.35	1,308.71	654.86	11
3	485.32	243.16	468.29	234.65			L	1,213.63	607.32	1,196.61	598.81	1,195.62	598.31	10
4	622.38	311.69	605.35	303.18			H	1,100.55	550.78	1,083.52	542.26	1,082.54	541.77	9
5	750.44	375.72	733.41	367.21			Q	963.49	482.25	946.46	473.74	945.48	473.24	8
6	865.46	433.24	848.44	424.72	847.45	424.23	D	835.43	418.22	818.40	409.71	817.42	409.21	7
7	936.50	468.75	919.48	460.24	918.49	459.75	A	720.40	360.71	703.38	352.19	702.39	351.70	6
8	1,035.57	518.29	1,018.54	509.78	1,017.56	509.28	V	649.37	325.19	632.34	316.67	631.36	316.18	5
9	1,150.60	575.80	1,133.57	567.29	1,132.59	566.80	D	550.30	275.65	533.27	267.14	532.29	266.65	4
10	1,263.68	632.34	1,246.65	623.83	1,245.67	623.34	L	435.27	218.14	418.24	209.63			3

11	1,410.75	705.88	1,393.72	697.37	1,392.74	696.87	F	322.19	161.60	305.16	153.08			2
12							R	175.12	88.06	158.09	79.55			1

Query 81111 Hit 1

MS/MS Fragmentation of KGPNVVGPYGLLQPFADAMK
Found in sp|P03886|NU1M_HUMAN, NADH-ubiquinone oxidoreductase chain 1 OS=Homo sapiens GN=MT-ND1 PE=1 SV=1
Match to Query 81111: 2533.41 from(634.3597,4+)
Title: 997: Scan 2163 (rt=58.4665, f=2, i=351) [D:\lab212\membrane\Grace\Joyce\iTRAQ_43_1.raw]
Data File:Submitted from 20120508-1(merge) by Mascot Daemon on JOYCE-VAIO
Monoisotopic mass of neutral peptide Mr(calc): 2533.41
Variable modifications:
K1 :iTRAQ4plex (K)
K20 :iTRAQ4plex (K)
Ions Score: 61.22 Expect: 0.000

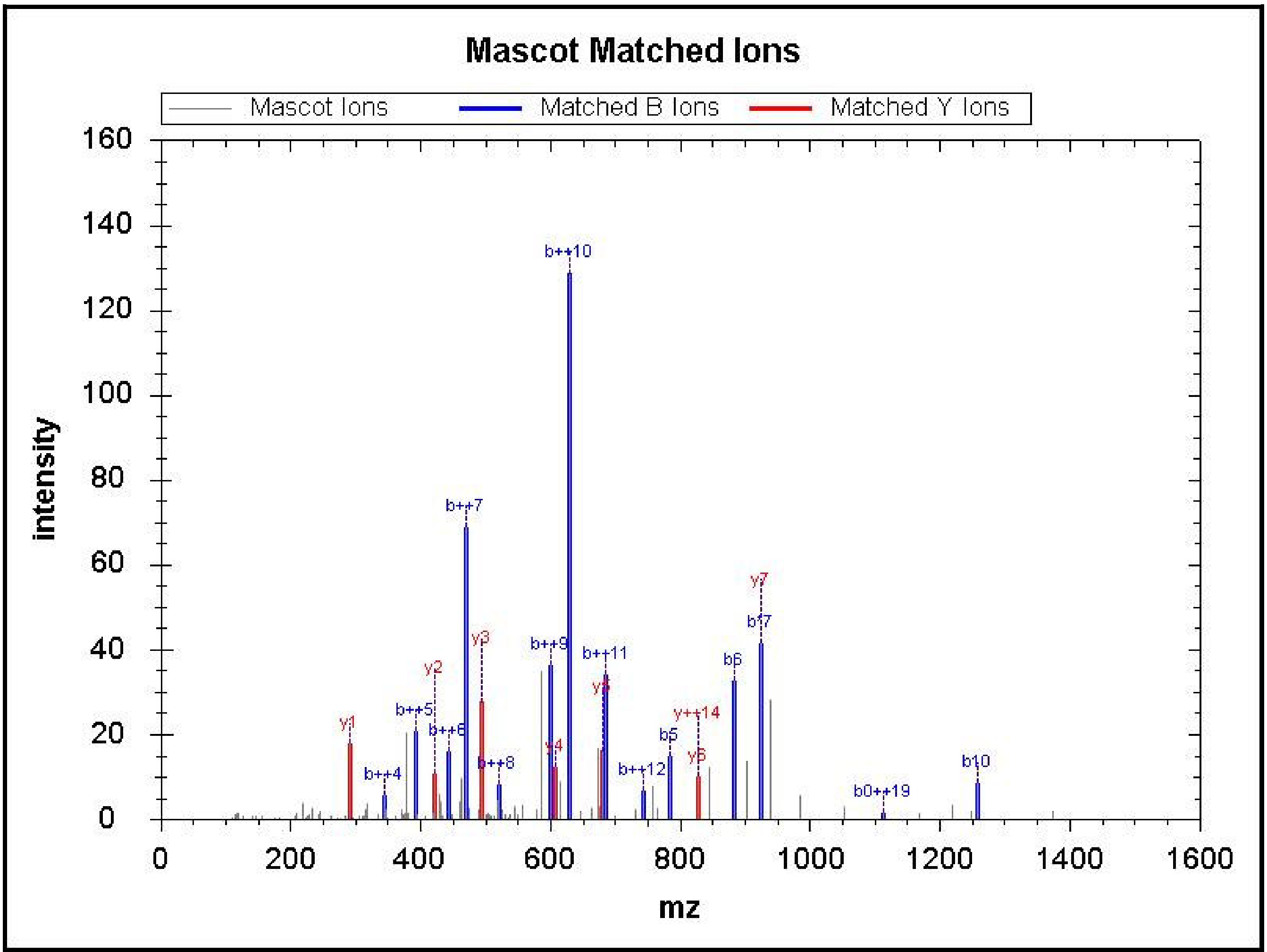

No	b	b++	b*	b*++	b0	b0++	Seq	y	y++	y*	y*++	y0	y0++	RevNo
1	417.31	209.16	400.28	200.64			K							20
2	474.33	237.67	457.30	229.15			G	2,118.12	1,059.56	2,101.09	1,051.05	2,100.11	1,050.56	19
3	571.38	286.19	554.35	277.68			P	2,061.10	1,031.05	2,044.07	1,022.54	2,043.09	1,022.05	18
4	685.42	343.22	668.40	334.70			N	1,964.04	982.53	1,947.02	974.01	1,946.03	973.52	17
5	784.49	392.75	767.47	384.24			V	1,850.00	925.50	1,832.97	916.99	1,831.99	916.50	16
6	883.56	442.28	866.53	433.77			V	1,750.93	875.97	1,733.91	867.46	1,732.92	866.96	15
7	940.58	470.79	923.56	462.28			G	1,651.86	826.44	1,634.84	817.92	1,633.85	817.43	14
8	1,037.63	519.32	1,020.61	510.81			P	1,594.84	797.92	1,577.82	789.41	1,576.83	788.92	13
9	1,200.70	600.85	1,183.67	592.34			Y	1,497.79	749.40	1,480.76	740.89	1,479.78	740.39	12
10	1,257.72	629.36	1,240.69	620.85			G	1,334.73	667.87	1,317.70	659.35	1,316.72	658.86	11
11	1,370.80	685.91	1,353.78	677.39			L	1,277.70	639.36	1,260.68	630.84	1,259.69	630.35	10

12	1,483.89	742.45	1,466.86	733.93			L	1,164.62	582.81	1,147.59	574.30	1,146.61	573.81	9
13	1,611.95	806.48	1,594.92	797.96			Q	1,051.54	526.27	1,034.51	517.76	1,033.53	517.27	8
14	1,709.00	855.00	1,691.97	846.49			P	923.48	462.24	906.45	453.73	905.47	453.24	7
15	1,856.07	928.54	1,839.04	920.02			F	826.42	413.72	809.40	405.20	808.41	404.71	6
16	1,927.10	964.06	1,910.08	955.54			A	679.36	340.18	662.33	331.67	661.35	331.18	5
17	2,042.13	1,021.57	2,025.10	1,013.06	2,024.12	1,012.56	D	608.32	304.66	591.29	296.15	590.31	295.66	4
18	2,113.17	1,057.09	2,096.14	1,048.57	2,095.16	1,048.08	A	493.29	247.15	476.27	238.64			3
19	2,244.21	1,122.61	2,227.18	1,114.09	2,226.20	1,113.60	M	422.26	211.63	405.23	203.12			2
20							K	291.21	146.11	274.19	137.60			1

Query 76676 Hit 1

MS/MS Fragmentation of ELHVGENQIEMLEAEHLK
Found in sp|Q9H9A6|LRC40_HUMAN, Leucine-rich repeat-containing protein 40 OS=Homo sapiens GN=LRRC40 PE=1 SV=1
Match to Query 76676: 2406.256from(602.5714,4+)
Title: 467: Scan 1413 (rt=40.1009, f=3, i=158) [D:\lab212\membrane\Grace\Joyce\iTRAQ_48_1.raw]
Data File:Submitted from 20120508-1(merge) by Mascot Daemon on JOYCE-VAIO
Monoisotopic mass of neutral peptide Mr(calc): 2406.256
Variable modifications:
K18 :iTRAQ4plex (K)
Ions Score: 61.04 Expect: 0.001

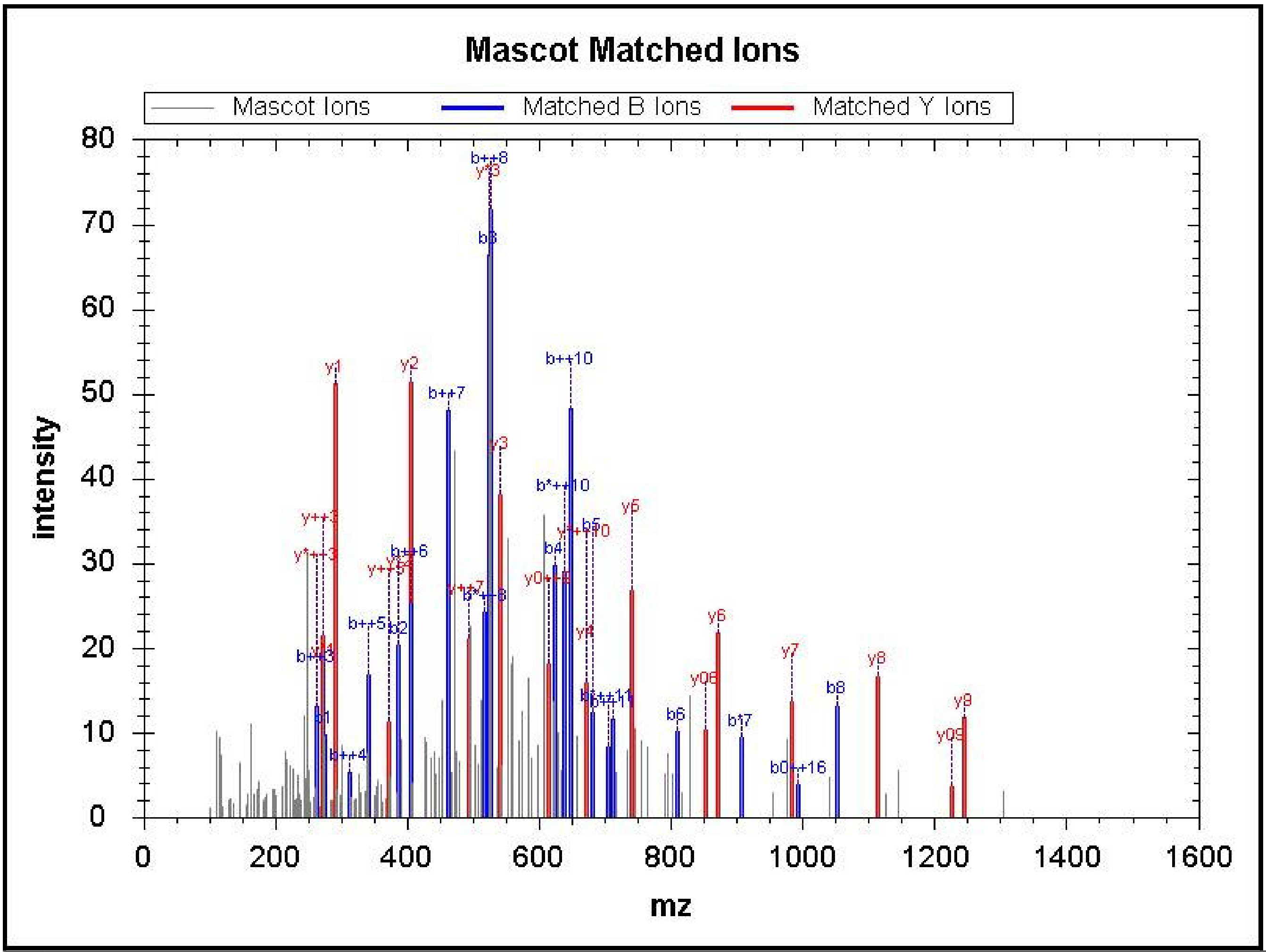

No	b	b++	b*	b*++	b0	b0++	Seq	y	y++	y*	y*++	y0	y0++	RevNo
1	274.15	137.58			256.14	128.57	E							18
2	387.24	194.12			369.23	185.12	L	2,134.11	1,067.56	2,117.08	1,059.04	2,116.10	1,058.55	17
3	524.29	262.65			506.28	253.65	H	2,021.02	1,011.02	2,004.00	1,002.50	2,003.01	1,002.01	16
4	623.36	312.19			605.35	303.18	V	1,883.97	942.49	1,866.94	933.97	1,865.95	933.48	15
5	680.38	340.70			662.37	331.69	G	1,784.90	892.95	1,767.87	884.44	1,766.89	883.95	14

6	809.43	405.22			791.42	396.21	E	1,727.88	864.44	1,710.85	855.93	1,709.86	855.44	13
7	923.47	462.24	906.44	453.73	905.46	453.23	N	1,598.83	799.92	1,581.81	791.41	1,580.82	790.91	12
8	1,051.53	526.27	1,034.50	517.75	1,033.52	517.26	Q	1,484.79	742.90	1,467.76	734.39	1,466.78	733.89	11
9	1,164.61	582.81	1,147.59	574.30	1,146.60	573.80	I	1,356.73	678.87	1,339.70	670.36	1,338.72	669.86	10
10	1,293.66	647.33	1,276.63	638.82	1,275.65	638.33	E	1,243.65	622.33	1,226.62	613.81	1,225.64	613.32	9
11	1,424.70	712.85	1,407.67	704.34	1,406.69	703.85	M	1,114.60	557.81	1,097.58	549.29	1,096.59	548.80	8
12	1,537.78	769.39	1,520.75	760.88	1,519.77	760.39	L	983.56	492.29	966.54	483.77	965.55	483.28	7
13	1,666.82	833.92	1,649.80	825.40	1,648.81	824.91	E	870.48	435.74	853.45	427.23	852.47	426.74	6
14	1,737.86	869.43	1,720.83	860.92	1,719.85	860.43	A	741.44	371.22	724.41	362.71	723.43	362.22	5
15	1,866.90	933.95	1,849.88	925.44	1,848.89	924.95	E	670.40	335.70	653.37	327.19	652.39	326.70	4
16	2,003.96	1,002.48	1,986.94	993.97	1,985.95	993.48	H	541.36	271.18	524.33	262.67			3
17	2,117.05	1,059.03	2,100.02	1,050.51	2,099.04	1,050.02	L	404.30	202.65	387.27	194.14			2
18							K	291.21	146.11	274.19	137.60			1

Query 65517 Hit 1

MS/MS Fragmentation of VTYVDFLVYDVLDQHR
Found in sp|P15626|GSTM2_MOUSE, Glutathione S-transferase Mu 2 OS=Mus musculus GN=Gstm2 PE=1 SV=2
Match to Query 65517: 2125.097from(709.3729,3+)
Title: 1126: Scan 2479 (rt=65.2564, f=3, i=387) [D:\lab212\membrane\Grace\Joyce\iTRAQ_32_1.raw]
Data File:Submitted from 20120508-1(merge) by Mascot Daemon on JOYCE-VAIO
Monoisotopic mass of neutral peptide Mr(calc): 2125.097
Variable modifications:
Ions Score: 60.9 Expect: 0.001

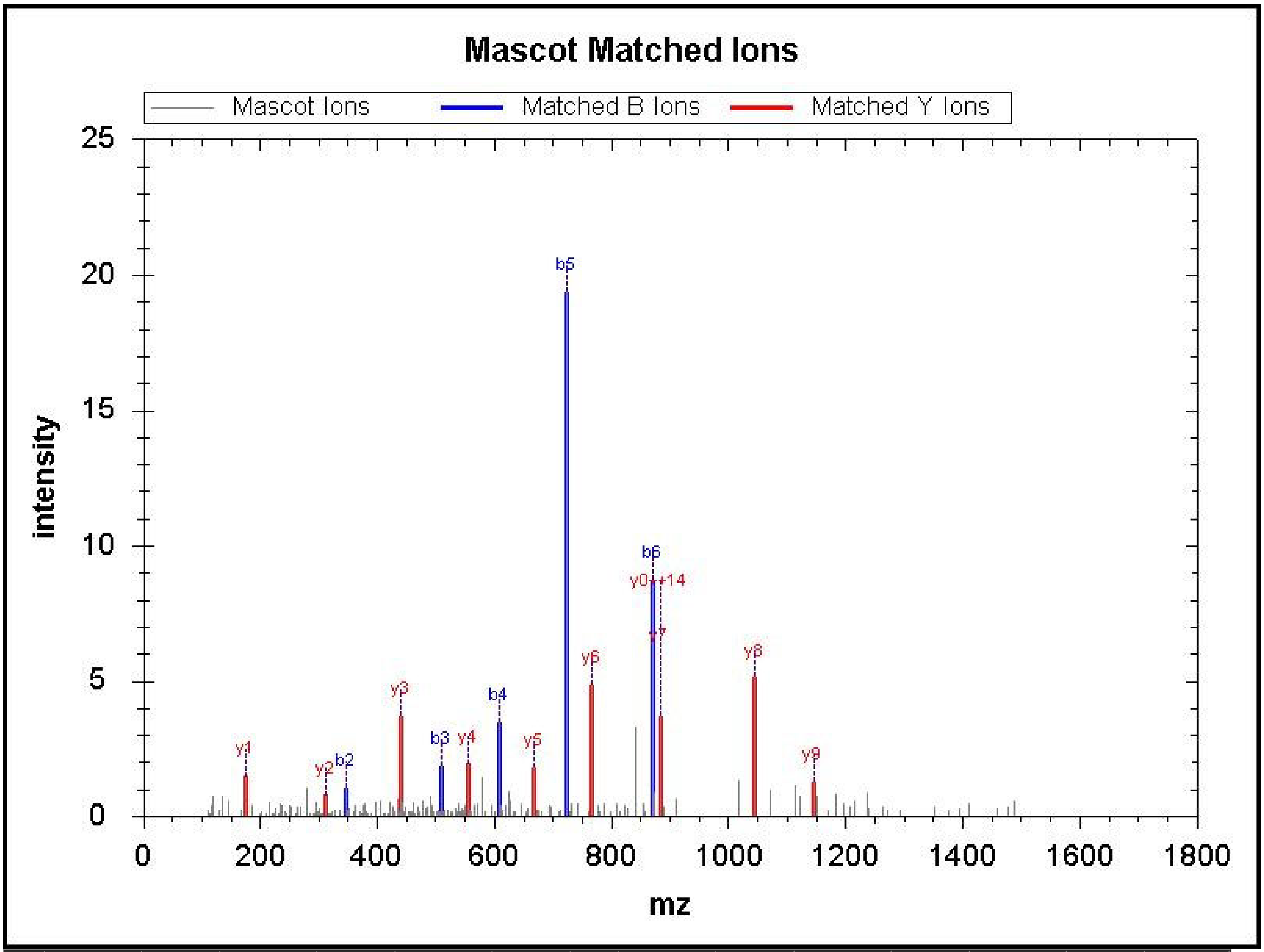

No	b	b++	b*	b*++	b0	b0++	Seq	y	y++	y*	y*++	y0	y0++	RevNo
1	244.18	122.59					V							16
2	345.23	173.12			327.21	164.11	T	1,882.93	941.97	1,865.91	933.46	1,864.92	932.97	15

3	508.29	254.65			490.28	245.64	Y	1,781.89	891.45	1,764.86	882.93	1,763.88	882.44	14
4	607.36	304.18			589.35	295.18	V	1,618.82	809.91	1,601.80	801.40	1,600.81	800.91	13
5	722.38	361.70			704.37	352.69	D	1,519.75	760.38	1,502.73	751.87	1,501.74	751.38	12
6	869.45	435.23			851.44	426.22	F	1,404.73	702.87	1,387.70	694.35	1,386.72	693.86	11
7	982.54	491.77			964.53	482.77	L	1,257.66	629.33	1,240.63	620.82	1,239.65	620.33	10
8	1,081.60	541.31			1,063.59	532.30	V	1,144.57	572.79	1,127.55	564.28	1,126.56	563.79	9
9	1,244.67	622.84			1,226.66	613.83	Y	1,045.51	523.26	1,028.48	514.74	1,027.50	514.25	8
10	1,359.70	680.35			1,341.68	671.35	D	882.44	441.73	865.42	433.21	864.43	432.72	7
11	1,458.76	729.89			1,440.75	720.88	V	767.42	384.21	750.39	375.70	749.41	375.21	6
12	1,571.85	786.43			1,553.84	777.42	L	668.35	334.68	651.32	326.16	650.34	325.67	5
13	1,686.87	843.94			1,668.86	834.94	D	555.26	278.14	538.24	269.62	537.25	269.13	4
14	1,814.93	907.97	1,797.91	899.46	1,796.92	898.97	Q	440.24	220.62	423.21	212.11			3
15	1,951.99	976.50	1,934.97	967.99	1,933.98	967.49	H	312.18	156.59	295.15	148.08			2
16							R	175.12	88.06	158.09	79.55			1

Query 89789 Hit 1

MS/MS Fragmentation of HLMELNALDKQEELTPLGVHLAR
Found in sp|Q9H2U1|DHX36_HUMAN, Probable ATP-dependent RNA helicase DHX36 OS=Homo sapiens GN=DHX36 PE=1 SV=2
Match to Query 89789: 2914.592from(729.6553,4+)
Title: 657: Scan 1661 (rt=46.0892, f=2, i=254) [D:\lab212\membrane\Grace\Joyce\iTRAQ_51_2.raw]
Data File:Submitted from 20120508-1(merge) by Mascot Daemon on JOYCE-VAIO
Monoisotopic mass of neutral peptide Mr(calc): 2914.592
Variable modifications:
K10 :iTRAQ4plex (K)
Ions Score: 60.88 Expect: 0.000

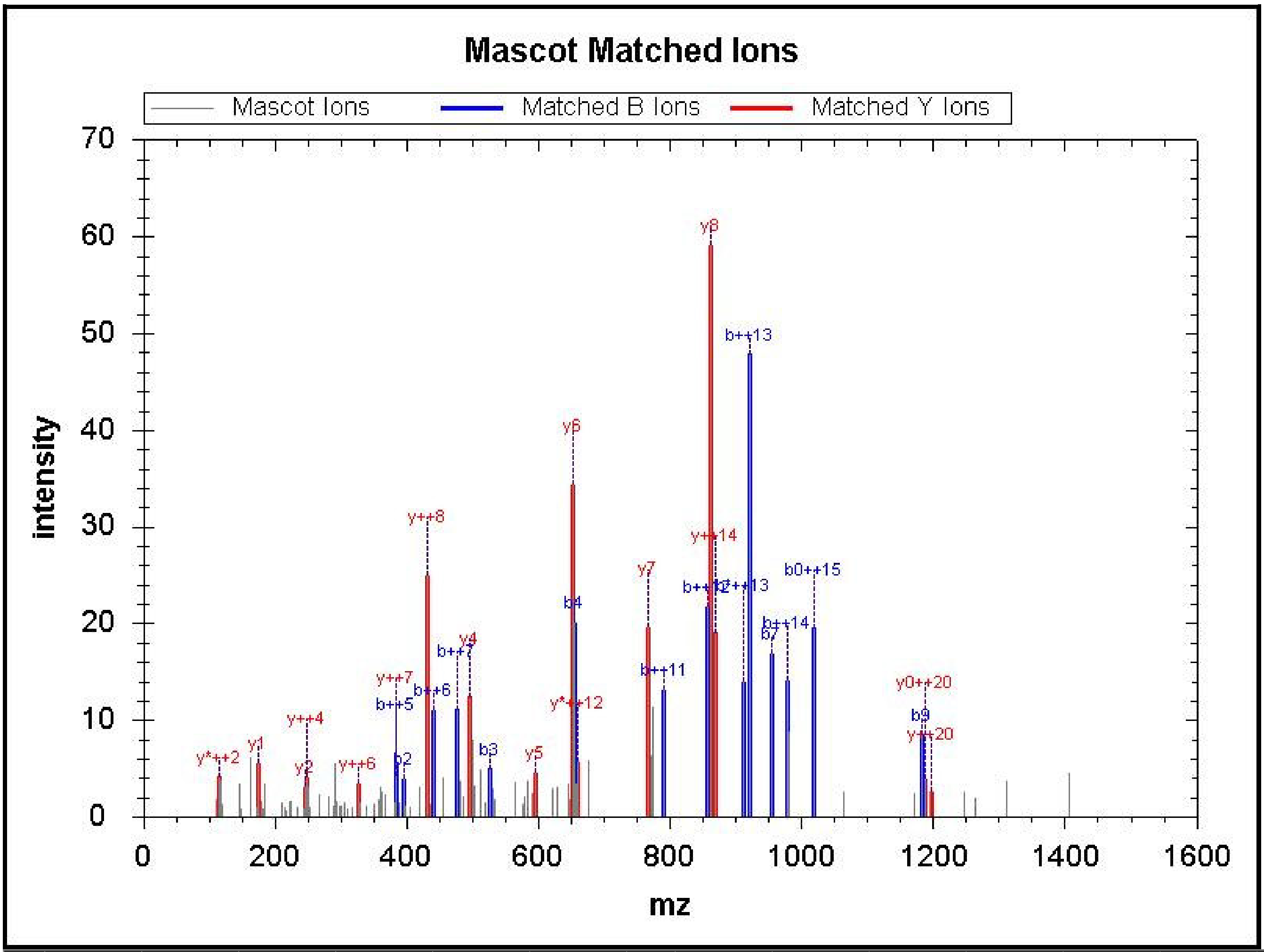

No	b	b++	b*	b*++	b0	b0++	Seq	y	y++	y*	y*++	y0	y0++	RevNo

1	282.17	141.59					H							23
2	395.25	198.13					L	2,634.44	1,317.72	2,617.41	1,309.21	2,616.43	1,308.72	22
3	526.29	263.65					M	2,521.36	1,261.18	2,504.33	1,252.67	2,503.35	1,252.18	21
4	655.34	328.17			637.32	319.17	E	2,390.32	1,195.66	2,373.29	1,187.15	2,372.31	1,186.66	20
5	768.42	384.71			750.41	375.71	L	2,261.27	1,131.14	2,244.25	1,122.63	2,243.26	1,122.14	19
6	882.46	441.73	865.44	433.22	864.45	432.73	N	2,148.19	1,074.60	2,131.16	1,066.09	2,130.18	1,065.59	18
7	953.50	477.25	936.47	468.74	935.49	468.25	A	2,034.15	1,017.58	2,017.12	1,009.06	2,016.14	1,008.57	17
8	1,066.58	533.80	1,049.56	525.28	1,048.57	524.79	L	1,963.11	982.06	1,946.08	973.55	1,945.10	973.05	16
9	1,181.61	591.31	1,164.58	582.80	1,163.60	582.30	D	1,850.03	925.52	1,833.00	917.00	1,832.01	916.51	15
10	1,453.81	727.41	1,436.78	718.89	1,435.80	718.40	K	1,735.00	868.00	1,717.97	859.49	1,716.99	859.00	14
11	1,581.87	791.44	1,564.84	782.92	1,563.86	782.43	Q	1,462.80	731.90	1,445.77	723.39	1,444.79	722.90	13
12	1,710.91	855.96	1,693.88	847.44	1,692.90	846.95	E	1,334.74	667.88	1,317.72	659.36	1,316.73	658.87	12
13	1,839.95	920.48	1,822.92	911.97	1,821.94	911.47	E	1,205.70	603.35	1,188.67	594.84	1,187.69	594.35	11
14	1,953.04	977.02	1,936.01	968.51	1,935.02	968.02	L	1,076.66	538.83	1,059.63	530.32	1,058.65	529.83	10
15	2,054.08	1,027.55	2,037.06	1,019.03	2,036.07	1,018.54	T	963.57	482.29	946.55	473.78	945.56	473.29	9
16	2,151.14	1,076.07	2,134.11	1,067.56	2,133.13	1,067.07	P	862.53	431.77	845.50	423.25			8
17	2,264.22	1,132.61	2,247.19	1,124.10	2,246.21	1,123.61	L	765.47	383.24	748.45	374.73			7
18	2,321.24	1,161.12	2,304.21	1,152.61	2,303.23	1,152.12	G	652.39	326.70	635.36	318.18			6
19	2,420.31	1,210.66	2,403.28	1,202.15	2,402.30	1,201.65	V	595.37	298.19	578.34	289.67			5
20	2,557.37	1,279.19	2,540.34	1,270.67	2,539.36	1,270.18	H	496.30	248.65	479.27	240.14			4
21	2,670.45	1,335.73	2,653.43	1,327.22	2,652.44	1,326.72	L	359.24	180.12	342.21	171.61			3
22	2,741.49	1,371.25	2,724.46	1,362.74	2,723.48	1,362.24	A	246.16	123.58	229.13	115.07			2
23							R	175.12	88.06	158.09	79.55			1

Query 100052 Hit 1

MS/MS Fragmentation of LNLLQSHPQEVMYFQPGEPFGSVEDDHIPFLR
Found in sp|Q9NXS2|QPCTL_HUMAN, Glutaminyl-peptide cyclotransferase-like protein OS=Homo sapiens GN=QPCTL PE=1 SV=2
Match to Query 100052: 3882.913from(971.7357,4+)
Title: 1018: Sum of 2 scans in range 2246 (rt=60.0071, f=3, i=346) to 2247 (rt=60.0325, f=3, i=347)
[D:\lab212\membrane\Grace\Joyce\iTRAQ_36_2.raw]
Data File:Submitted from 20120508-1(merge) by Mascot Daemon on JOYCE-VAIO
Monoisotopic mass of neutral peptide Mr(calc): 3882.913
Variable modifications:
Ions Score: 60.61 Expect: 0.001

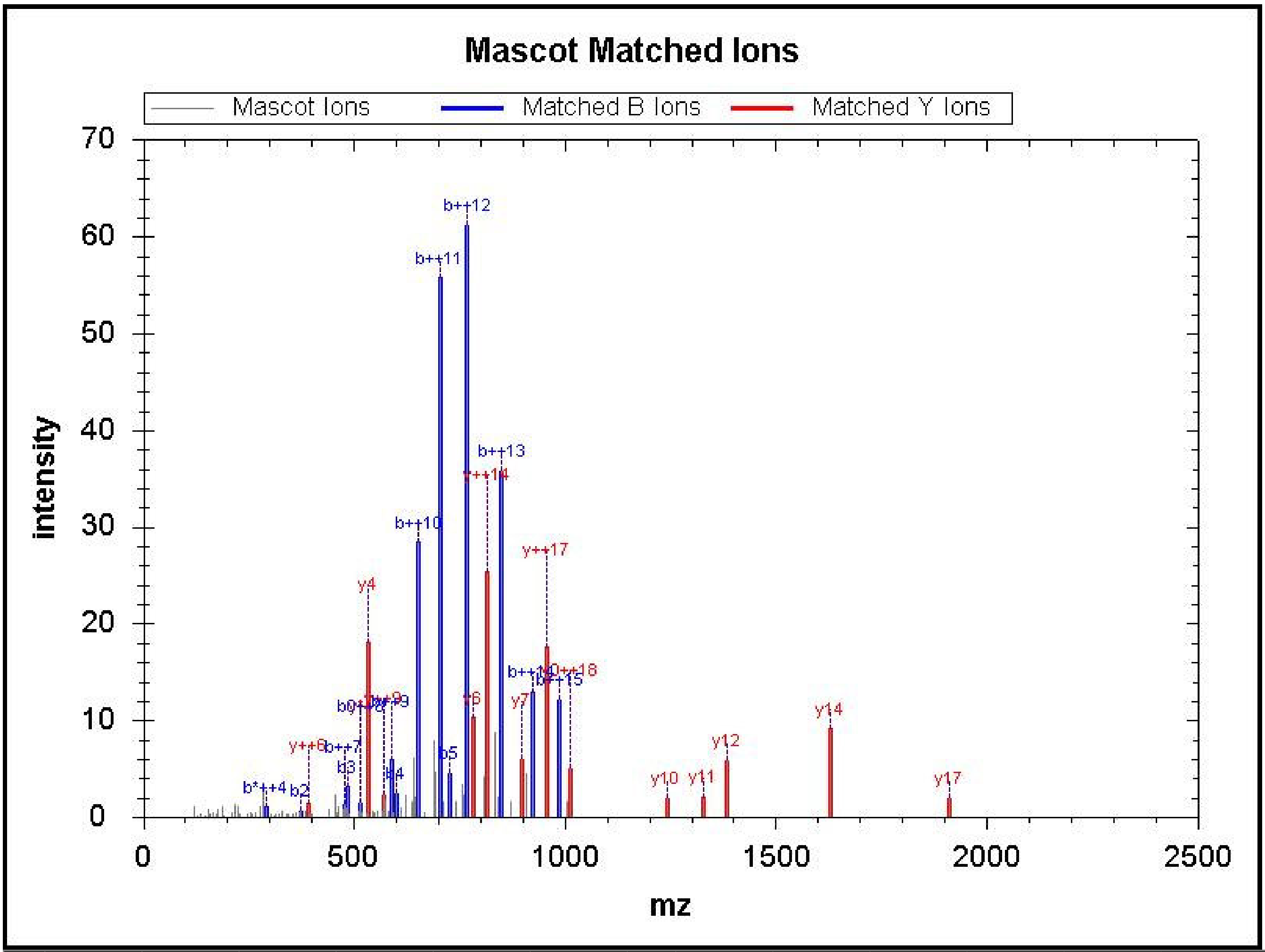

No	b	b++	b*	b*++	b0	b0++	Seq	y	y++	y*	y*++	y0	y0++	RevNo
1	258.19	129.60					L							32
2	372.24	186.62	355.21	178.11			N	3,626.74	1,813.87	3,609.71	1,805.36	3,608.73	1,804.87	31
3	485.32	243.16	468.29	234.65			L	3,512.69	1,756.85	3,495.67	1,748.34	3,494.68	1,747.85	30
4	598.40	299.71	581.38	291.19			L	3,399.61	1,700.31	3,382.58	1,691.80	3,381.60	1,691.30	29
5	726.46	363.74	709.44	355.22			Q	3,286.53	1,643.77	3,269.50	1,635.25	3,268.52	1,634.76	28
6	813.50	407.25	796.47	398.74	795.48	398.25	S	3,158.47	1,579.74	3,141.44	1,571.22	3,140.46	1,570.73	27
7	950.55	475.78	933.53	467.27	932.54	466.78	H	3,071.44	1,536.22	3,054.41	1,527.71	3,053.42	1,527.22	26
8	1,047.61	524.31	1,030.58	515.79	1,029.60	515.30	P	2,934.38	1,467.69	2,917.35	1,459.18	2,916.37	1,458.69	25
9	1,175.67	588.34	1,158.64	579.82	1,157.65	579.33	Q	2,837.32	1,419.17	2,820.30	1,410.65	2,819.31	1,410.16	24
10	1,304.71	652.86	1,287.68	644.34	1,286.70	643.85	E	2,709.27	1,355.14	2,692.24	1,346.62	2,691.25	1,346.13	23
11	1,403.78	702.39	1,386.75	693.88	1,385.77	693.39	V	2,580.22	1,290.61	2,563.20	1,282.10	2,562.21	1,281.61	22
12	1,534.82	767.91	1,517.79	759.40	1,516.81	758.91	M	2,481.15	1,241.08	2,464.13	1,232.57	2,463.14	1,232.08	21
13	1,697.88	849.44	1,680.85	840.93	1,679.87	840.44	Y	2,350.11	1,175.56	2,333.09	1,167.05	2,332.10	1,166.56	20
14	1,844.95	922.98	1,827.92	914.46	1,826.94	913.97	F	2,187.05	1,094.03	2,170.02	1,085.52	2,169.04	1,085.02	19
15	1,973.01	987.01	1,955.98	978.49	1,955.00	978.00	Q	2,039.98	1,020.49	2,022.96	1,011.98	2,021.97	1,011.49	18
16	2,070.06	1,035.53	2,053.03	1,027.02	2,052.05	1,026.53	P	1,911.92	956.47	1,894.90	947.95	1,893.91	947.46	17
17	2,127.08	1,064.04	2,110.05	1,055.53	2,109.07	1,055.04	G	1,814.87	907.94	1,797.84	899.43	1,796.86	898.93	16
18	2,256.12	1,128.57	2,239.10	1,120.05	2,238.11	1,119.56	E	1,757.85	879.43	1,740.82	870.92	1,739.84	870.42	15
19	2,353.18	1,177.09	2,336.15	1,168.58	2,335.17	1,168.09	P	1,628.81	814.91	1,611.78	806.39	1,610.80	805.90	14
20	2,500.25	1,250.63	2,483.22	1,242.11	2,482.23	1,241.62	F	1,531.75	766.38	1,514.73	757.87	1,513.74	757.38	13
21	2,557.27	1,279.14	2,540.24	1,270.62	2,539.26	1,270.13	G	1,384.69	692.85	1,367.66	684.33	1,366.67	683.84	12
22	2,644.30	1,322.65	2,627.27	1,314.14	2,626.29	1,313.65	S	1,327.66	664.34	1,310.64	655.82	1,309.65	655.33	11
23	2,743.37	1,372.19	2,726.34	1,363.67	2,725.36	1,363.18	V	1,240.63	620.82	1,223.61	612.31	1,222.62	611.81	10

24	2,872.41	1,436.71	2,855.38	1,428.20	2,854.40	1,427.70	E	1,141.56	571.29	1,124.54	562.77	1,123.55	562.28	9
25	2,987.44	1,494.22	2,970.41	1,485.71	2,969.43	1,485.22	D	1,012.52	506.76	995.49	498.25	994.51	497.76	8
26	3,102.46	1,551.74	3,085.44	1,543.22	3,084.45	1,542.73	D	897.49	449.25	880.47	440.74	879.48	440.25	7
27	3,239.52	1,620.26	3,222.50	1,611.75	3,221.51	1,611.26	H	782.47	391.74	765.44	383.22			6
28	3,352.61	1,676.81	3,335.58	1,668.29	3,334.60	1,667.80	I	645.41	323.21	628.38	314.69			5
29	3,449.66	1,725.33	3,432.63	1,716.82	3,431.65	1,716.33	P	532.32	266.67	515.30	258.15			4
30	3,596.73	1,798.87	3,579.70	1,790.35	3,578.72	1,789.86	F	435.27	218.14	418.24	209.63			3
31	3,709.81	1,855.41	3,692.78	1,846.90	3,691.80	1,846.40	L	288.20	144.61	271.18	136.09			2
32							R	175.12	88.06	158.09	79.55			1

Query 23131 Hit 1

MS/MS Fragmentation of EALGDAQQSVR
Found in sp|Q99615|DNJC7_HUMAN, DnaJ homolog subfamily C member 7 OS=Homo sapiens GN=DNAJC7 PE=1 SV=2
Match to Query 23131: 1316.683from(659.3486,2+)
Title: 135: Scan 522 (rt=20.556, f=3, i=48) [D:\lab212\membrane\Grace\Joyce\iTRAQ_24_1.raw]
Data File:Submitted from 20120508-1(merge) by Mascot Daemon on JOYCE-VAIO
Monoisotopic mass of neutral peptide Mr(calc): 1316.683
Variable modifications:
Ions Score: 60.43 Expect: 0.001

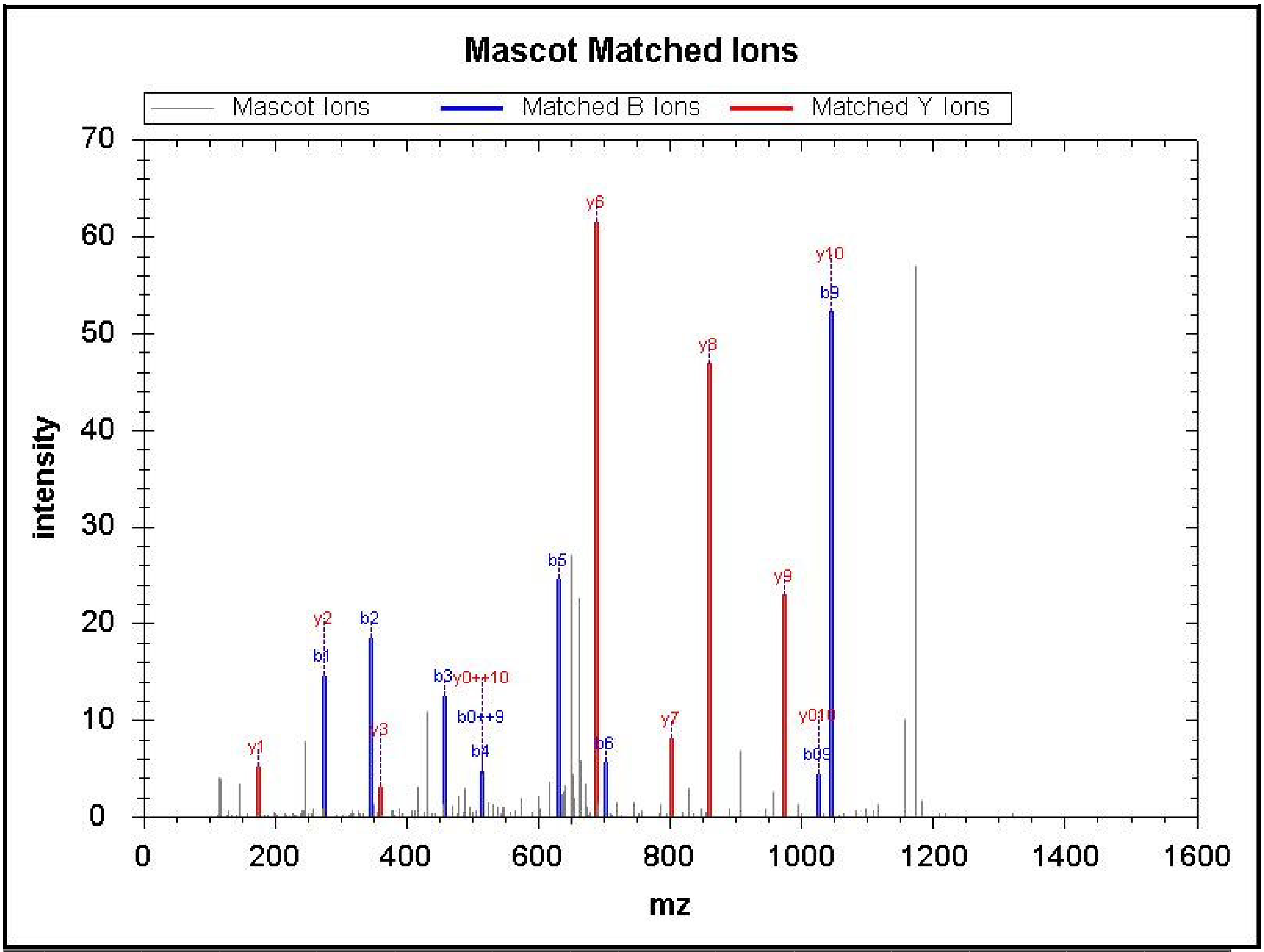

No	b	b++	b*	b*++	b0	b0++	Seq	y	y++	y*	y*++	y0	y0++	RevNo
1	274.15	137.58			256.14	128.57	E							11
2	345.19	173.10			327.18	164.09	A	1,044.54	522.78	1,027.52	514.26	1,026.53	513.77	10
3	458.27	229.64			440.26	220.63	L	973.51	487.26	956.48	478.74	955.50	478.25	9
4	515.29	258.15			497.28	249.15	G	860.42	430.71	843.40	422.20	842.41	421.71	8
5	630.32	315.66			612.31	306.66	D	803.40	402.20	786.37	393.69	785.39	393.20	7

No	b	b++	b*	b*++	b0	b0++	Seq	y	y++	y*	y*++	y0	y0++	RevNo
6	701.36	351.18			683.35	342.18	A	688.37	344.69	671.35	336.18	670.36	335.69	6
7	829.42	415.21	812.39	406.70	811.41	406.21	Q	617.34	309.17	600.31	300.66	599.33	300.17	5
8	957.48	479.24	940.45	470.73	939.47	470.24	Q	489.28	245.14	472.25	236.63	471.27	236.14	4
9	1,044.51	522.76	1,027.48	514.24	1,026.50	513.75	S	361.22	181.11	344.19	172.60	343.21	172.11	3
10	1,143.58	572.29	1,126.55	563.78	1,125.57	563.29	V	274.19	137.60	257.16	129.08			2
11							R	175.12	88.06	158.09	79.55			1

Query 85743 Hit 1

MS/MS Fragmentation of QAFLDELESSDLPVALLLAQHK
Found in sp|P51948|MAT1_HUMAN, CDK-activating kinase assembly factor MAT1 OS=Homo sapiens GN=MNAT1 PE=1 SV=1
Match to Query 85743: 2724.495from(909.1722,3+)
Title: 1078: Scan 2570 (rt=66.3008, f=2, i=388) [D:\lab212\membrane\Grace\Joyce\iTRAQ_34_1.raw]
Data File:Submitted from 20120508-1(merge) by Mascot Daemon on JOYCE-VAIO
Monoisotopic mass of neutral peptide Mr(calc): 2724.495
Variable modifications:
K22 :iTRAQ4plex (K)
Ions Score: 60.1 Expect: 0.000

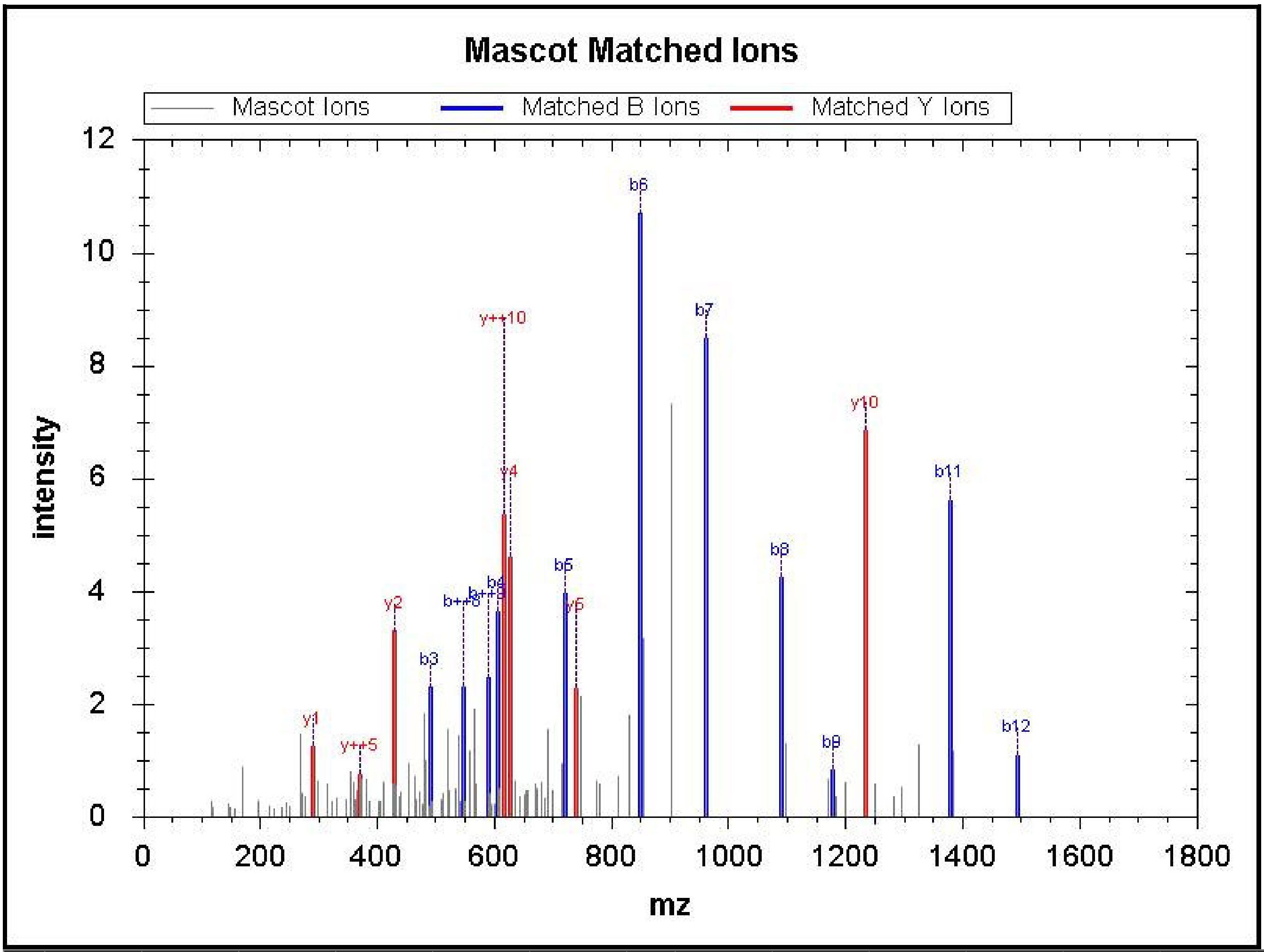

No	b	b++	b*	b*++	b0	b0++	Seq	y	y++	y*	y*++	y0	y0++	RevNo
1	273.17	137.09	256.14	128.57			Q							22
2	344.21	172.61	327.18	164.09			A	2,453.34	1,227.17	2,436.31	1,218.66	2,435.33	1,218.17	21
3	491.27	246.14	474.25	237.63			F	2,382.30	1,191.66	2,365.28	1,183.14	2,364.29	1,182.65	20
4	604.36	302.68	587.33	294.17			L	2,235.24	1,118.12	2,218.21	1,109.61	2,217.22	1,109.12	19
5	719.38	360.20	702.36	351.68	701.37	351.19	D	2,122.15	1,061.58	2,105.12	1,053.07	2,104.14	1,052.57	18
6	848.43	424.72	831.40	416.20	830.42	415.71	E	2,007.12	1,004.07	1,990.10	995.55	1,989.11	995.06	17
7	961.51	481.26	944.48	472.75	943.50	472.25	L	1,878.08	939.54	1,861.06	931.03	1,860.07	930.54	16
8	1,090.55	545.78	1,073.53	537.27	1,072.54	536.78	E	1,765.00	883.00	1,747.97	874.49	1,746.99	874.00	15

9	1,177.59	589.30	1,160.56	580.78	1,159.58	580.29	S	1,635.95	818.48	1,618.93	809.97	1,617.94	809.48	14
10	1,264.62	632.81	1,247.59	624.30	1,246.61	623.81	S	1,548.92	774.97	1,531.90	766.45	1,530.91	765.96	13
11	1,379.64	690.33	1,362.62	681.81	1,361.63	681.32	D	1,461.89	731.45	1,444.86	722.94	1,443.88	722.44	12
12	1,492.73	746.87	1,475.70	738.35	1,474.72	737.86	L	1,346.86	673.94	1,329.84	665.42			11
13	1,589.78	795.39	1,572.76	786.88	1,571.77	786.39	P	1,233.78	617.39	1,216.75	608.88			10
14	1,688.85	844.93	1,671.82	836.42	1,670.84	835.92	V	1,136.73	568.87	1,119.70	560.35			9
15	1,759.89	880.45	1,742.86	871.93	1,741.88	871.44	A	1,037.66	519.33	1,020.63	510.82			8
16	1,872.97	936.99	1,855.94	928.48	1,854.96	927.98	L	966.62	483.81	949.60	475.30			7
17	1,986.06	993.53	1,969.03	985.02	1,968.04	984.53	L	853.54	427.27	836.51	418.76			6
18	2,099.14	1,050.07	2,082.11	1,041.56	2,081.13	1,041.07	L	740.45	370.73	723.43	362.22			5
19	2,170.18	1,085.59	2,153.15	1,077.08	2,152.17	1,076.59	A	627.37	314.19	610.34	305.68			4
20	2,298.23	1,149.62	2,281.21	1,141.11	2,280.22	1,140.62	Q	556.33	278.67	539.31	270.16			3
21	2,435.29	1,218.15	2,418.27	1,209.64	2,417.28	1,209.15	H	428.27	214.64	411.25	206.13			2
22							K	291.21	146.11	274.19	137.60			1

Query 51411 Hit 1

MS/MS Fragmentation of GVEVTVGHEQEEGGK
Found in sp|P30042|ES1_HUMAN, ES1 protein homolog
Match to Query 51411: 1841.896from(614.9728,3+)
Title: 197: Scan 473 (rt=20.3969, f=2, i=76) [D:\lab212\membrane\Grace\Joyce\iTRAQ40.raw]
Data File:Submitted from 20120508-1(merge) by Mascot Daemon on JOYCE-VAIO
Monoisotopic mass of neutral peptide Mr(calc): 1841.896
Variable modifications:
K15 :iTRAQ4plex (K)
Ions Score: 59.97 Expect: 0.001

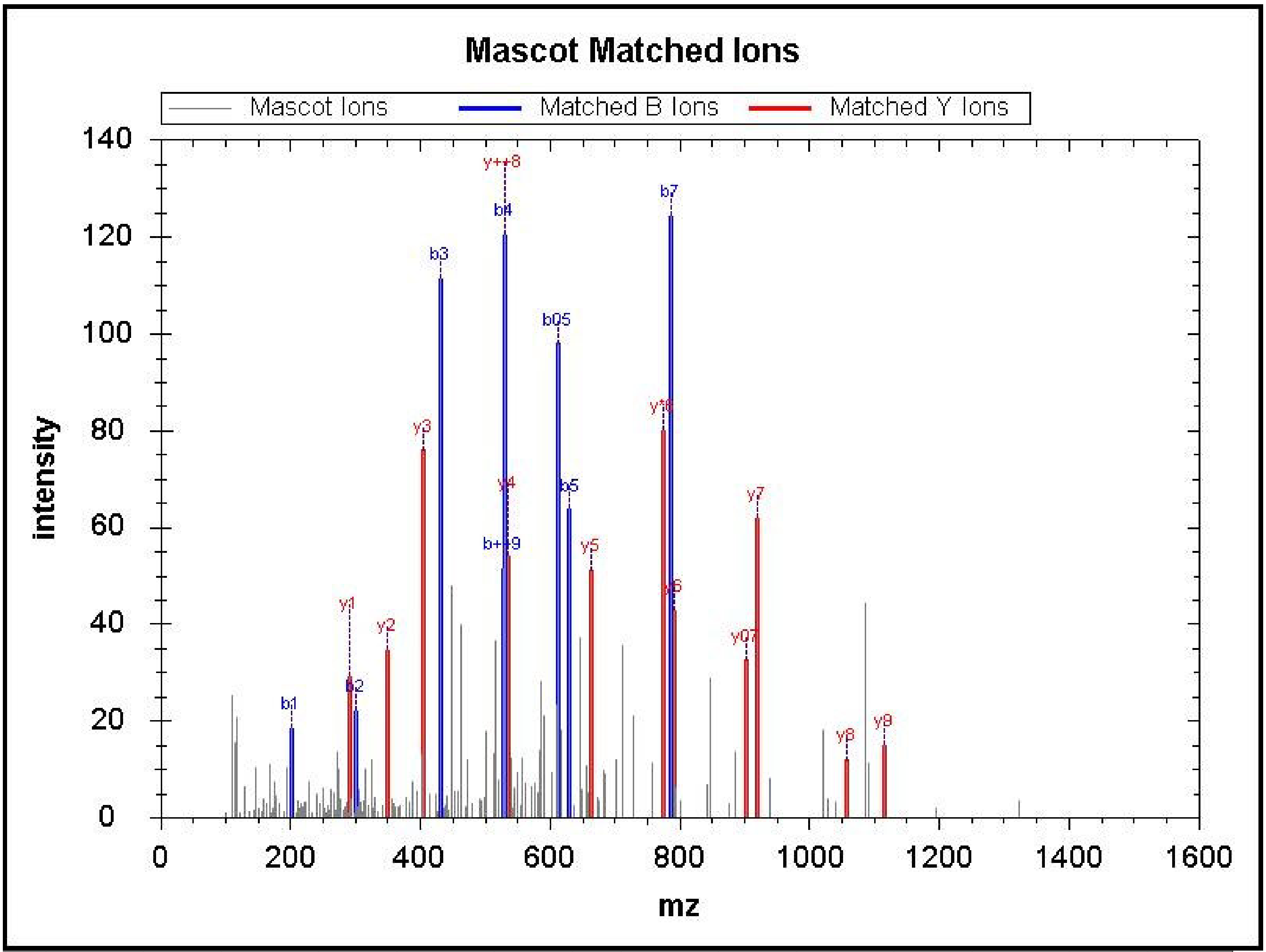

No	b	b++	b*	b*++	b0	b0++	Seq	y	y++	y*	y*++	y0	y0++	RevNo

1	202.13	101.57					G							15
2	301.20	151.10					V	1,641.82	821.41	1,624.79	812.90	1,623.81	812.41	14
3	430.24	215.62			412.23	206.62	E	1,542.75	771.88	1,525.73	763.37	1,524.74	762.87	13
4	529.31	265.16			511.30	256.15	V	1,413.71	707.36	1,396.68	698.84	1,395.70	698.35	12
5	630.36	315.68			612.35	306.68	T	1,314.64	657.82	1,297.61	649.31	1,296.63	648.82	11
6	729.43	365.22			711.42	356.21	V	1,213.59	607.30	1,196.57	598.79	1,195.58	598.29	10
7	786.45	393.73			768.44	384.72	G	1,114.52	557.77	1,097.50	549.25	1,096.51	548.76	9
8	923.51	462.26			905.50	453.25	H	1,057.50	529.26	1,040.48	520.74	1,039.49	520.25	8
9	1,052.55	526.78			1,034.54	517.77	E	920.44	460.73	903.42	452.21	902.43	451.72	7
10	1,180.61	590.81	1,163.58	582.29	1,162.60	581.80	Q	791.40	396.20	774.38	387.69	773.39	387.20	6
11	1,309.65	655.33	1,292.62	646.82	1,291.64	646.32	E	663.34	332.18	646.32	323.66	645.33	323.17	5
12	1,438.69	719.85	1,421.67	711.34	1,420.68	710.84	E	534.30	267.65	517.27	259.14	516.29	258.65	4
13	1,495.71	748.36	1,478.69	739.85	1,477.70	739.36	G	405.26	203.13	388.23	194.62			3
14	1,552.74	776.87	1,535.71	768.36	1,534.73	767.87	G	348.24	174.62	331.21	166.11			2
15							K	291.21	146.11	274.19	137.60			1

Query 77710 Hit 1

MS/MS Fragmentation of LSLQDSVATLALSHVTPHDER
Found in sp|Q8R2Y2|MUC18_MOUSE, Cell surface glycoprotein MUC18 OS=Mus musculus GN=Mcam PE=1 SV=1
Match to Query 77710: 2432.28from(609.0773,4+)
Title: 785: Scan 1746 (rt=48.9294, f=2, i=284) [D:\lab212\membrane\Grace\Joyce\iTRAQ_44_2.raw]
Data File:Submitted from 20120508-1(merge) by Mascot Daemon on JOYCE-VAIO
Monoisotopic mass of neutral peptide Mr(calc): 2432.28
Variable modifications:
Ions Score: 59.96 Expect: 0.001

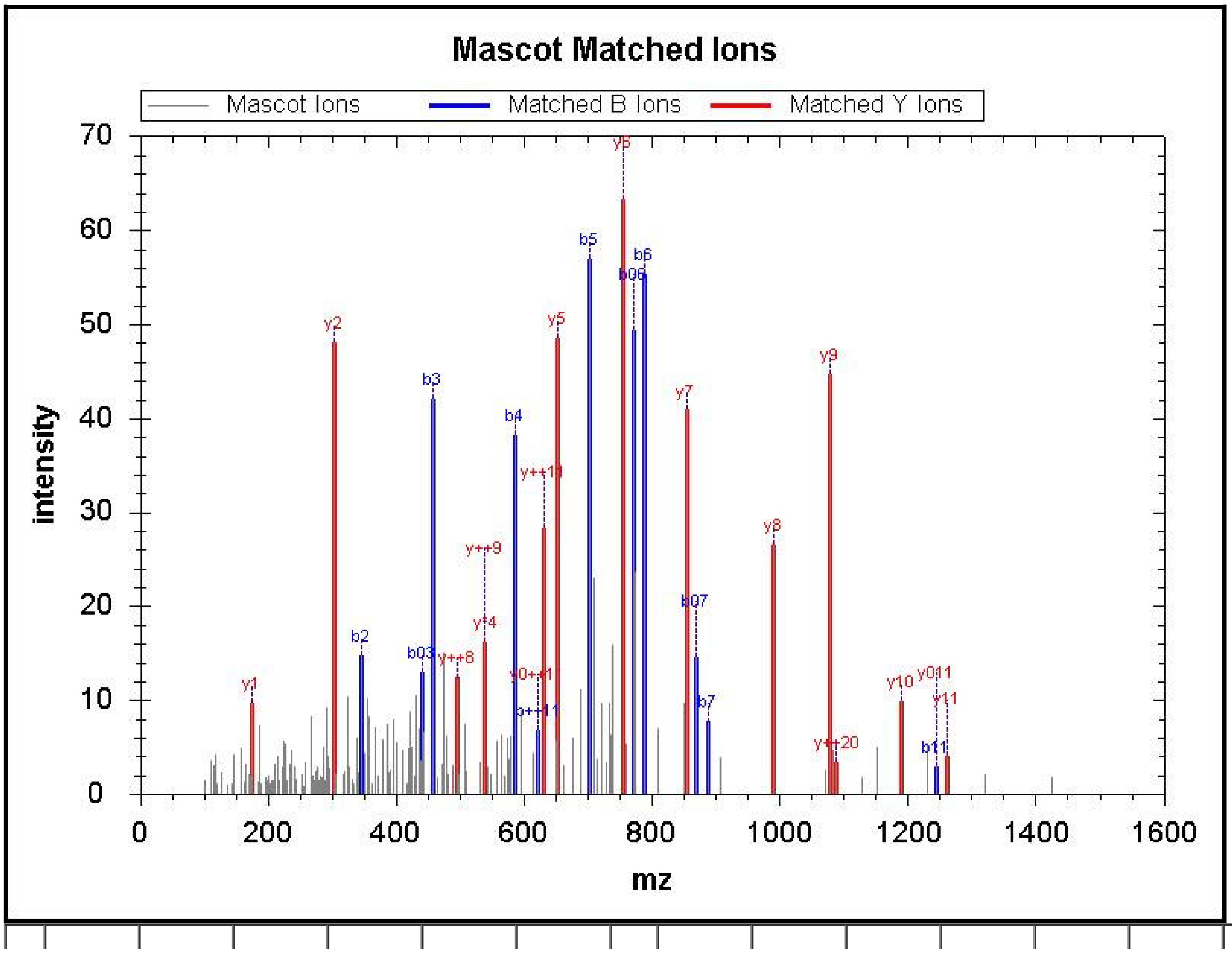

No	b	b++	b*	b*++	b0	b0++	Seq	y	y++	y*	y*++	y0	y0++	RevNo
1	258.19	129.60					L							21
2	345.23	173.12			327.21	164.11	S	2,176.10	1,088.55	2,159.07	1,080.04	2,158.09	1,079.55	20
3	458.31	229.66			440.30	220.65	L	2,089.07	1,045.04	2,072.04	1,036.52	2,071.06	1,036.03	19
4	586.37	293.69	569.34	285.17	568.36	284.68	Q	1,975.98	988.50	1,958.96	979.98	1,957.97	979.49	18
5	701.39	351.20	684.37	342.69	683.38	342.20	D	1,847.92	924.47	1,830.90	915.95	1,829.91	915.46	17
6	788.43	394.72	771.40	386.20	770.42	385.71	S	1,732.90	866.95	1,715.87	858.44	1,714.89	857.95	16
7	887.50	444.25	870.47	435.74	869.48	435.25	V	1,645.87	823.44	1,628.84	814.92	1,627.86	814.43	15
8	958.53	479.77	941.51	471.26	940.52	470.76	A	1,546.80	773.90	1,529.77	765.39	1,528.79	764.90	14
9	1,059.58	530.29	1,042.55	521.78	1,041.57	521.29	T	1,475.76	738.38	1,458.73	729.87	1,457.75	729.38	13
10	1,172.66	586.84	1,155.64	578.32	1,154.65	577.83	L	1,374.71	687.86	1,357.69	679.35	1,356.70	678.85	12
11	1,243.70	622.35	1,226.68	613.84	1,225.69	613.35	A	1,261.63	631.32	1,244.60	622.80	1,243.62	622.31	11
12	1,356.79	678.90	1,339.76	670.38	1,338.78	669.89	L	1,190.59	595.80	1,173.56	587.29	1,172.58	586.79	10
13	1,443.82	722.41	1,426.79	713.90	1,425.81	713.41	S	1,077.51	539.26	1,060.48	530.74	1,059.50	530.25	9
14	1,580.88	790.94	1,563.85	782.43	1,562.87	781.94	H	990.48	495.74	973.45	487.23	972.46	486.74	8
15	1,679.94	840.48	1,662.92	831.96	1,661.93	831.47	V	853.42	427.21	836.39	418.70	835.41	418.21	7
16	1,780.99	891.00	1,763.97	882.49	1,762.98	881.99	T	754.35	377.68	737.32	369.16	736.34	368.67	6
17	1,878.05	939.53	1,861.02	931.01	1,860.03	930.52	P	653.30	327.15	636.27	318.64	635.29	318.15	5
18	2,015.10	1,008.06	1,998.08	999.54	1,997.09	999.05	H	556.25	278.63	539.22	270.11	538.24	269.62	4
19	2,130.13	1,065.57	2,113.10	1,057.06	2,112.12	1,056.56	D	419.19	210.10	402.16	201.58	401.18	201.09	3
20	2,259.17	1,130.09	2,242.15	1,121.58	2,241.16	1,121.09	E	304.16	152.58	287.13	144.07	286.15	143.58	2
21							R	175.12	88.06	158.09	79.55			1

Query 92041 Hit 1

MS/MS Fragmentation of ESQELAQHAAEIGADGIAVIAPFFFK
Found in sp|Q9DCJ9|NPL_MOUSE, N-acetylneuraminate lyase OS=Mus musculus GN=Npl PE=2 SV=1
Match to Query 92041: 3046.603from(762.658,4+)
Title: 1224: Sum of 2 scans in range 2748 (rt=71.1388, f=3, i=425) to 2749 (rt=71.1642, f=3, i=426) [D:\lab212\membrane\Grace\Joyce\iTRAQ_33_2.raw]
Data File:Submitted from 20120508-1(merge) by Mascot Daemon on JOYCE-VAIO
Monoisotopic mass of neutral peptide Mr(calc): 3046.603
Variable modifications:
K26 :iTRAQ4plex (K)
Ions Score: 59.82 Expect: 0.001

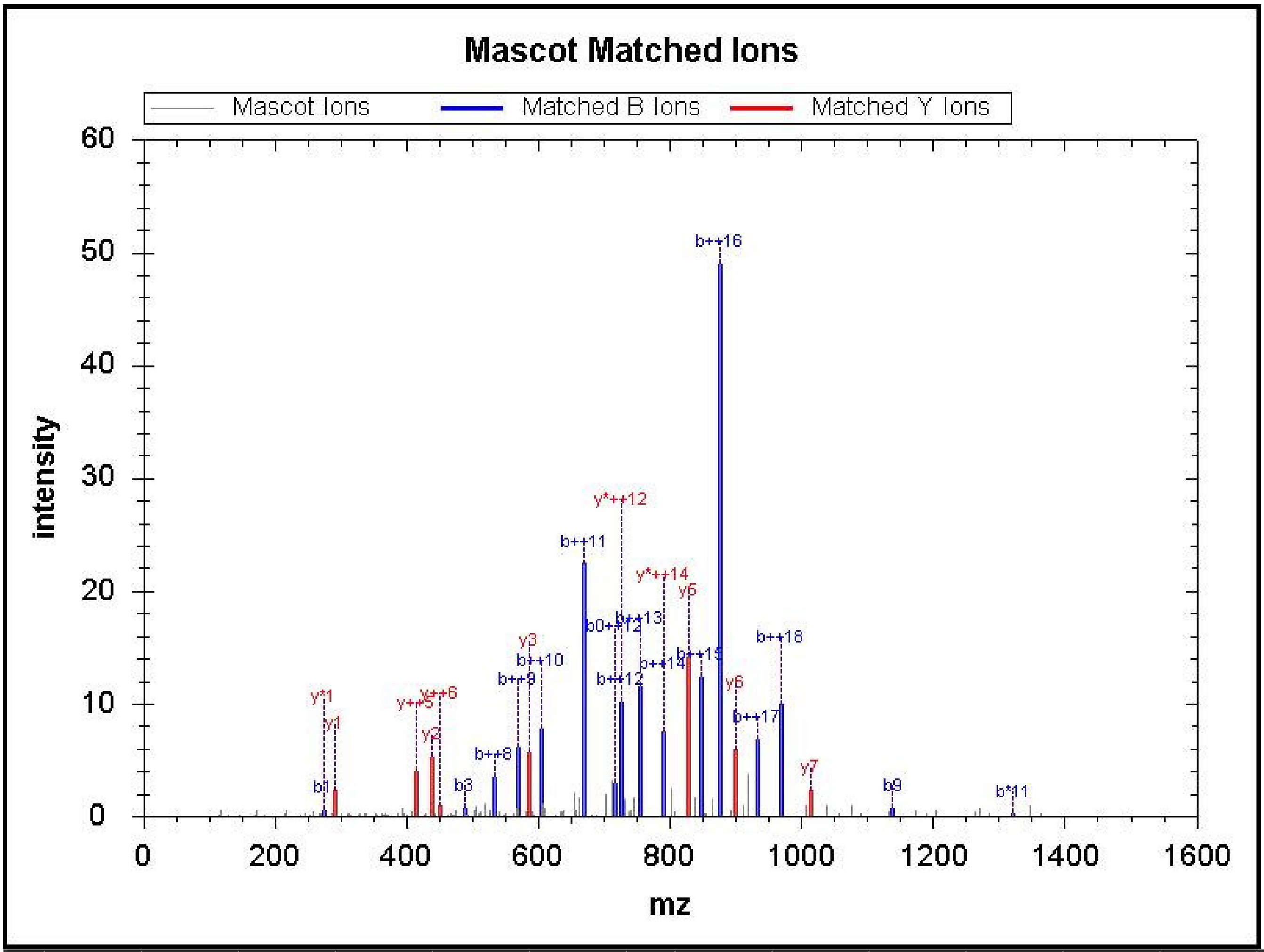

No	b	b++	b*	b*++	b0	b0++	Seq	y	y++	y*	y*++	y0	y0++	RevNo
1	274.15	137.58			256.14	128.57	E							26
2	361.18	181.10			343.17	172.09	S	2,774.46	1,387.74	2,757.44	1,379.22	2,756.45	1,378.73	25
3	489.24	245.12	472.22	236.61	471.23	236.12	Q	2,687.43	1,344.22	2,670.40	1,335.71	2,669.42	1,335.21	24
4	618.29	309.65	601.26	301.13	600.27	300.64	E	2,559.37	1,280.19	2,542.35	1,271.68	2,541.36	1,271.18	23
5	731.37	366.19	714.34	357.67	713.36	357.18	L	2,430.33	1,215.67	2,413.30	1,207.16	2,412.32	1,206.66	22
6	802.41	401.71	785.38	393.19	784.40	392.70	A	2,317.25	1,159.13	2,300.22	1,150.61	2,299.24	1,150.12	21
7	930.46	465.74	913.44	457.22	912.45	456.73	Q	2,246.21	1,123.61	2,229.18	1,115.09	2,228.20	1,114.60	20
8	1,067.52	534.27	1,050.50	525.75	1,049.51	525.26	H	2,118.15	1,059.58	2,101.12	1,051.07	2,100.14	1,050.57	19
9	1,138.56	569.78	1,121.53	561.27	1,120.55	560.78	A	1,981.09	991.05	1,964.07	982.54	1,963.08	982.04	18
10	1,209.60	605.30	1,192.57	596.79	1,191.59	596.30	A	1,910.05	955.53	1,893.03	947.02	1,892.04	946.53	17
11	1,338.64	669.82	1,321.61	661.31	1,320.63	660.82	E	1,839.02	920.01	1,821.99	911.50	1,821.01	911.01	16
12	1,451.72	726.37	1,434.70	717.85	1,433.71	717.36	I	1,709.97	855.49	1,692.95	846.98	1,691.96	846.49	15
13	1,508.75	754.88	1,491.72	746.36	1,490.74	745.87	G	1,596.89	798.95	1,579.86	790.44	1,578.88	789.94	14
14	1,579.78	790.40	1,562.76	781.88	1,561.77	781.39	A	1,539.87	770.44	1,522.84	761.93	1,521.86	761.43	13
15	1,694.81	847.91	1,677.78	839.40	1,676.80	838.90	D	1,468.83	734.92	1,451.81	726.41	1,450.82	725.91	12
16	1,751.83	876.42	1,734.81	867.91	1,733.82	867.41	G	1,353.81	677.41	1,336.78	668.89			11
17	1,864.92	932.96	1,847.89	924.45	1,846.91	923.96	I	1,296.78	648.90	1,279.76	640.38			10
18	1,935.95	968.48	1,918.93	959.97	1,917.94	959.47	A	1,183.70	592.35	1,166.67	583.84			9
19	2,035.02	1,018.01	2,017.99	1,009.50	2,017.01	1,009.01	V	1,112.66	556.83	1,095.64	548.32			8
20	2,148.11	1,074.56	2,131.08	1,066.04	2,130.09	1,065.55	I	1,013.59	507.30	996.57	498.79			7
21	2,219.14	1,110.07	2,202.12	1,101.56	2,201.13	1,101.07	A	900.51	450.76	883.48	442.25			6
22	2,316.20	1,158.60	2,299.17	1,150.09	2,298.18	1,149.60	P	829.47	415.24	812.45	406.73			5
23	2,463.26	1,232.14	2,446.24	1,223.62	2,445.25	1,223.13	F	732.42	366.71	715.39	358.20			4

24	2,610.33	1,305.67	2,593.31	1,297.16	2,592.32	1,296.66	F	585.35	293.18	568.33	284.67			3
25	2,757.40	1,379.20	2,740.37	1,370.69	2,739.39	1,370.20	F	438.28	219.65	421.26	211.13			2
26							K	291.21	146.11	274.19	137.60			1

Query 26012 Hit 1

MS/MS Fragmentation of SLDIYSFIK
Found in sp|Q9NR50|EI2BG_HUMAN, Translation initiation factor eIF-2B subunit gamma OS=Homo sapiens GN=EIF2B3 PE=1 SV=1
Match to Query 26012: 1372.784from(687.3993,2+)
Title: 632: Sum of 2 scans in range 2022 (rt=51.8335, f=4, i=411) to 2023 (rt=51.8589, f=4, i=412)
[D:\lab212\membrane\Grace\Joyce\iTRAQ_29_2.raw]
Data File:Submitted from 20120508-1(merge) by Mascot Daemon on JOYCE-VAIO
Monoisotopic mass of neutral peptide Mr(calc): 1372.784
Variable modifications:
K9 :iTRAQ4plex (K)
Ions Score: 59.75 Expect: 0.001

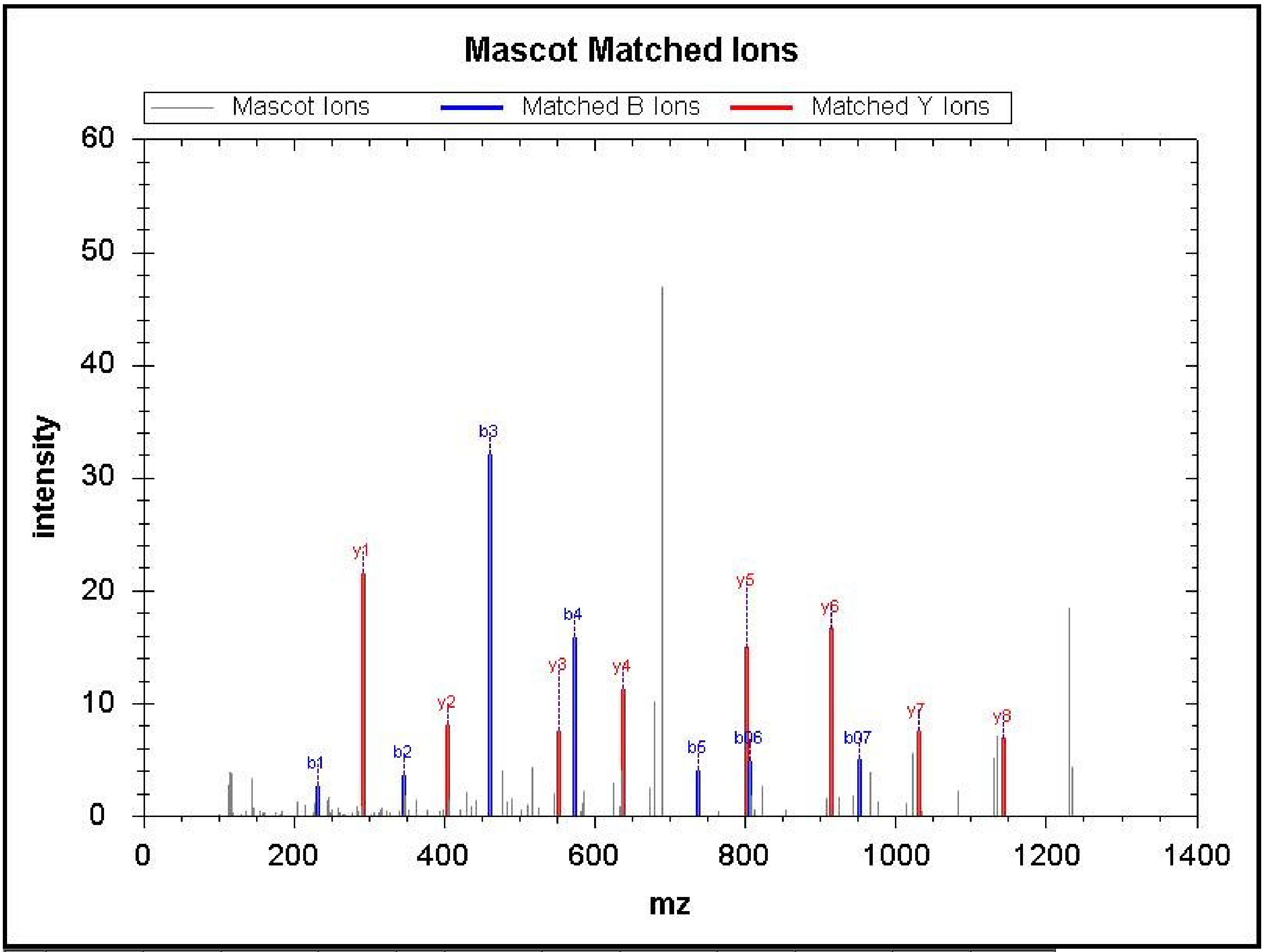

No	b	b++	b0	b0++	Seq	y	y++	y*	y*++	y0	y0++	RevNo
1	232.14	116.57	214.13	107.57	S							9
2	345.23	173.12	327.21	164.11	L	1,142.66	571.83	1,125.63	563.32	1,124.65	562.83	8
3	460.25	230.63	442.24	221.62	D	1,029.57	515.29	1,012.55	506.78	1,011.56	506.29	7
4	573.34	287.17	555.33	278.17	I	914.55	457.78	897.52	449.26	896.54	448.77	6
5	736.40	368.70	718.39	359.70	Y	801.46	401.23	784.44	392.72	783.45	392.23	5
6	823.43	412.22	805.42	403.21	S	638.40	319.70	621.37	311.19	620.39	310.70	4
7	970.50	485.75	952.49	476.75	F	551.37	276.19	534.34	267.67			3
8	1,083.58	542.30	1,065.57	533.29	I	404.30	202.65	387.27	194.14			2
9					K	291.21	146.11	274.19	137.60			1

Query 68299 Hit 1

MS/MS Fragmentation of AAPEINNLIEEATEFIK
Found in sp|Q8IVP5|FUND1_HUMAN, FUN14 domain-containing protein 1 OS=Homo sapiens GN=FUNDC1 PE=1 SV=1
Match to Query 68299: 2189.179from(730.7336,3+)
Title: 1231: Scan 2687 (rt=70.1812, f=2, i=432) [D:\lab212\membrane\Grace\Joyce\iTRAQ_27_2_327.raw]
Data File:Submitted from 20120508-1(merge) by Mascot Daemon on JOYCE-VAIO
Monoisotopic mass of neutral peptide Mr(calc): 2189.179
Variable modifications:
K17 :iTRAQ4plex (K)
Ions Score: 59.63 Expect: 0.001

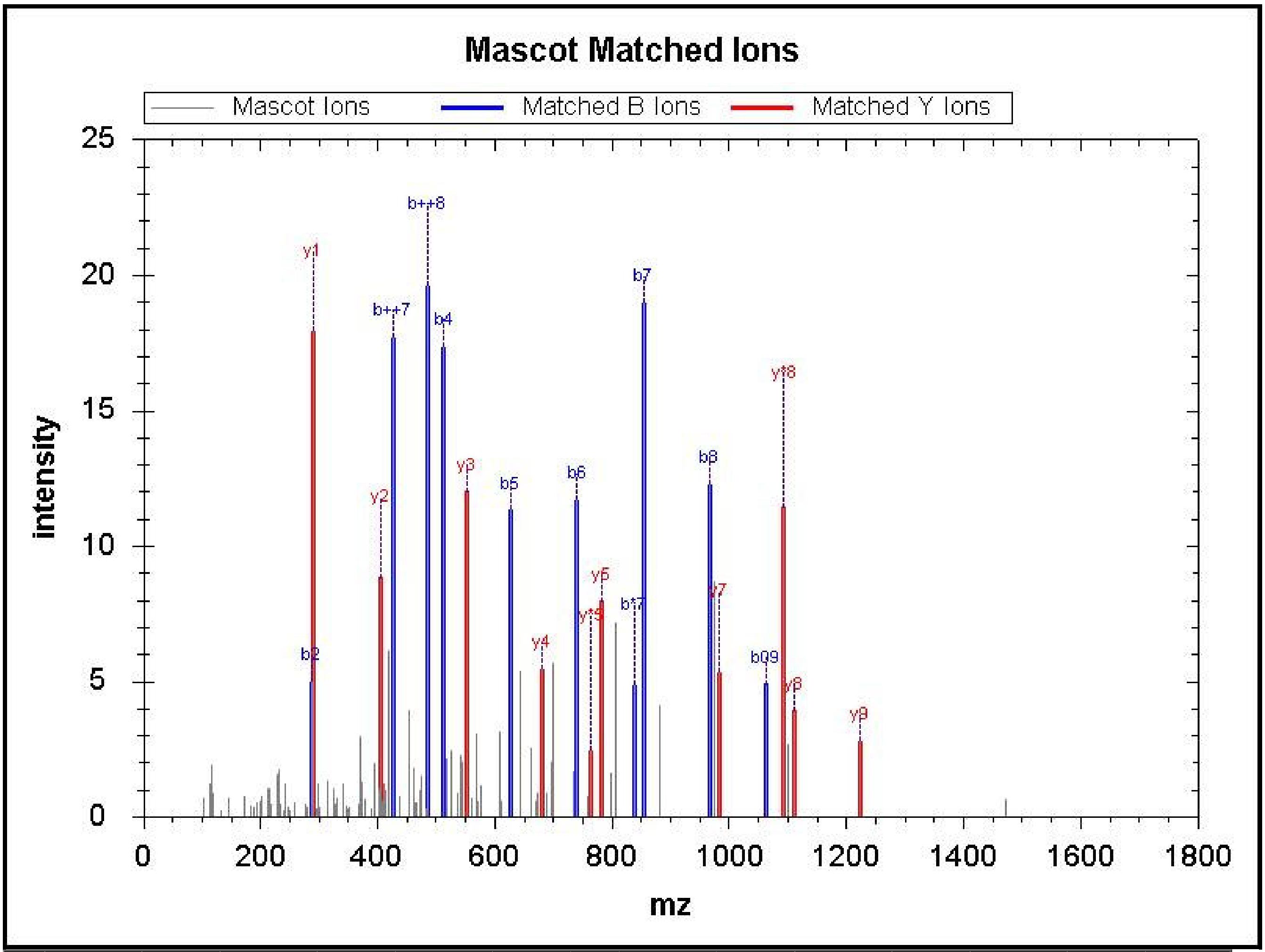

No	b	b++	b*	b*++	b0	b0++	Seq	y	y++	y*	y*++	y0	y0++	RevNo
1	216.15	108.58					A							17
2	287.18	144.10					A	1,975.05	988.03	1,958.02	979.52	1,957.04	979.02	16
3	384.24	192.62					P	1,904.01	952.51	1,886.99	944.00	1,886.00	943.51	15
4	513.28	257.14			495.27	248.14	E	1,806.96	903.98	1,789.93	895.47	1,788.95	894.98	14
5	626.36	313.69			608.35	304.68	I	1,677.92	839.46	1,660.89	830.95	1,659.91	830.46	13
6	740.41	370.71	723.38	362.19	722.40	361.70	N	1,564.83	782.92	1,547.81	774.41	1,546.82	773.92	12
7	854.45	427.73	837.42	419.21	836.44	418.72	N	1,450.79	725.90	1,433.76	717.39	1,432.78	716.89	11
8	967.53	484.27	950.51	475.76	949.52	475.26	L	1,336.75	668.88	1,319.72	660.36	1,318.74	659.87	10
9	1,080.62	540.81	1,063.59	532.30	1,062.61	531.81	I	1,223.66	612.34	1,206.64	603.82	1,205.65	603.33	9
10	1,209.66	605.33	1,192.63	596.82	1,191.65	596.33	E	1,110.58	555.79	1,093.55	547.28	1,092.57	546.79	8
11	1,338.70	669.85	1,321.68	661.34	1,320.69	660.85	E	981.54	491.27	964.51	482.76	963.53	482.27	7
12	1,409.74	705.37	1,392.71	696.86	1,391.73	696.37	A	852.49	426.75	835.47	418.24	834.48	417.75	6
13	1,510.79	755.90	1,493.76	747.38	1,492.78	746.89	T	781.46	391.23	764.43	382.72	763.45	382.23	5
14	1,639.83	820.42	1,622.80	811.91	1,621.82	811.41	E	680.41	340.71	663.38	332.20	662.40	331.70	4

15	1,786.90	893.95	1,769.87	885.44	1,768.89	884.95	F	551.37	276.19	534.34	267.67			3
16	1,899.98	950.49	1,882.96	941.98	1,881.97	941.49	I	404.30	202.65	387.27	194.14			2
17							K	291.21	146.11	274.19	137.60			1

Query 23385 Hit 1

MS/MS Fragmentation of AFLLESLLK
Found in sp|Q96JM3|CHAP1_HUMAN, Chromosome alignment-maintaining phosphoprotein 1 OS=Homo sapiens GN=CHAMP1 PE=1 SV=2
Match to Query 23385: 1320.825from(661.4197,2+)
Title: 995: Sum of 2 scans in range 2271 (rt=60.1113, f=4, i=661) to 2272 (rt=60.1367, f=4, i=662) [D:\lab212\membrane\Grace\Joyce\iTRAQ_29_1.raw]
Data File:Submitted from 20120508-1(merge) by Mascot Daemon on JOYCE-VAIO
Monoisotopic mass of neutral peptide Mr(calc): 1320.825
Variable modifications:
K9 :iTRAQ4plex (K)
Ions Score: 59.58 Expect: 0.000

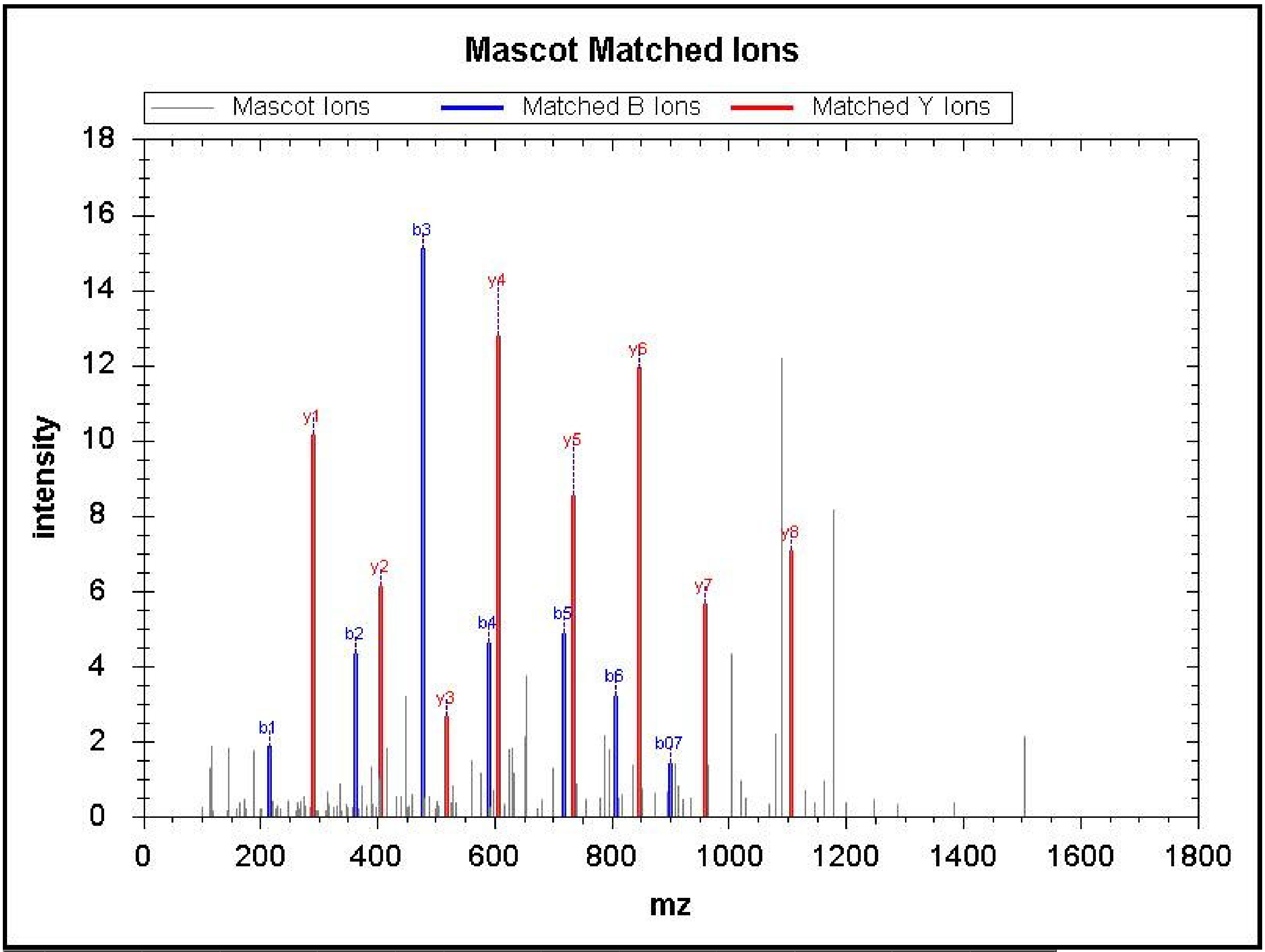

No	b	b++	b0	b0++	Seq	y	y++	y*	y*++	y0	y0++	RevNo
1	216.15	108.58			A							9
2	363.21	182.11			F	1,106.69	553.85	1,089.67	545.34	1,088.68	544.85	8
3	476.30	238.65			L	959.63	480.32	942.60	471.80	941.62	471.31	7
4	589.38	295.20			L	846.54	423.77	829.52	415.26	828.53	414.77	6
5	718.43	359.72	700.42	350.71	E	733.46	367.23	716.43	358.72	715.45	358.23	5
6	805.46	403.23	787.45	394.23	S	604.41	302.71	587.39	294.20	586.40	293.71	4
7	918.54	459.77	900.53	450.77	L	517.38	259.20	500.36	250.68			3
8	1,031.63	516.32	1,013.62	507.31	L	404.30	202.65	387.27	194.14			2
9					K	291.21	146.11	274.19	137.60			1

Query 74496 Hit 1

MS/MS Fragmentation of ALNQAESFIGEHHLDHLK
Found in sp|P16383|GCFC2_HUMAN, GC-rich sequence DNA-binding factor 2 OS=Homo sapiens GN=GCFC2 PE=1 SV=2
Match to Query 74496: 2346.241 from(587.5676,4+)
Title: 630: Scan 1378 (rt=40.792, f=2, i=220) [D:\lab212\membrane\Grace\Joyce\iTRAQ_54_1.raw]
Data File:Submitted from 20120508-1(merge) by Mascot Daemon on JOYCE-VAIO
Monoisotopic mass of neutral peptide Mr(calc): 2346.241
Variable modifications:
K18 :iTRAQ4plex (K)
Ions Score: 59.57 Expect: 0.001

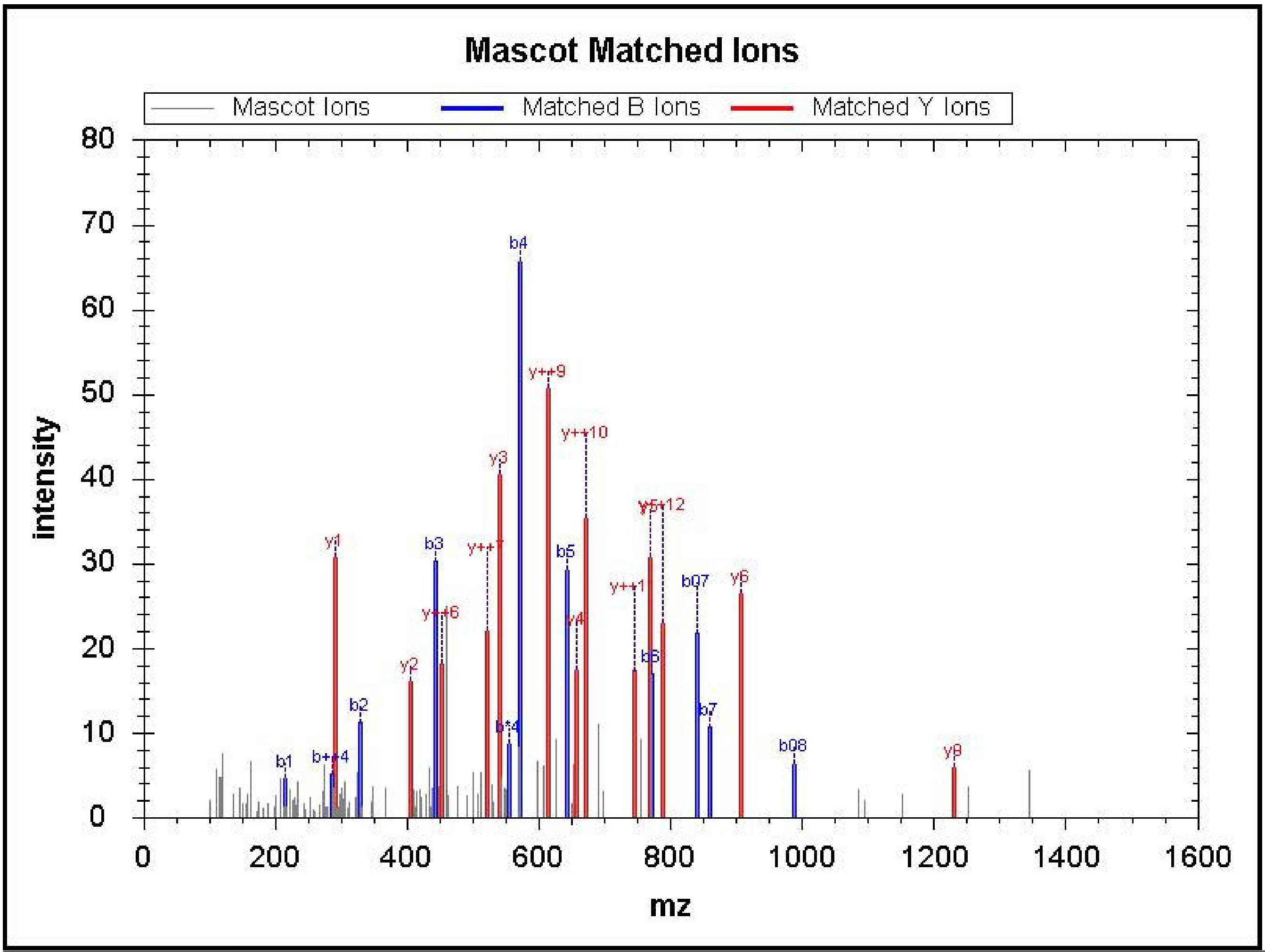

No	b	b++	b*	b*++	b0	b0++	Seq	y	y++	y*	y*++	y0	y0++	RevNo
1	216.15	108.58					A							18
2	329.23	165.12					L	2,132.10	1,066.55	2,115.07	1,058.04	2,114.09	1,057.55	17
3	443.27	222.14	426.25	213.63			N	2,019.02	1,010.01	2,001.99	1,001.50	2,001.01	1,001.01	16
4	571.33	286.17	554.31	277.66			Q	1,904.97	952.99	1,887.95	944.48	1,886.96	943.99	15
5	642.37	321.69	625.34	313.17			A	1,776.91	888.96	1,759.89	880.45	1,758.90	879.96	14
6	771.41	386.21	754.39	377.70	753.40	377.20	E	1,705.88	853.44	1,688.85	844.93	1,687.87	844.44	13
7	858.44	429.73	841.42	421.21	840.43	420.72	S	1,576.84	788.92	1,559.81	780.41	1,558.82	779.92	12
8	1,005.51	503.26	988.49	494.75	987.50	494.25	F	1,489.80	745.41	1,472.78	736.89	1,471.79	736.40	11
9	1,118.60	559.80	1,101.57	551.29	1,100.59	550.80	I	1,342.73	671.87	1,325.71	663.36	1,324.72	662.87	10
10	1,175.62	588.31	1,158.59	579.80	1,157.61	579.31	G	1,229.65	615.33	1,212.62	606.82	1,211.64	606.32	9
11	1,304.66	652.83	1,287.63	644.32	1,286.65	643.83	E	1,172.63	586.82	1,155.60	578.30	1,154.62	577.81	8
12	1,441.72	721.36	1,424.69	712.85	1,423.71	712.36	H	1,043.59	522.30	1,026.56	513.78	1,025.58	513.29	7
13	1,578.78	789.89	1,561.75	781.38	1,560.77	780.89	H	906.53	453.77	889.50	445.25	888.52	444.76	6
14	1,691.86	846.43	1,674.84	837.92	1,673.85	837.43	L	769.47	385.24	752.44	376.72	751.46	376.23	5
15	1,806.89	903.95	1,789.86	895.44	1,788.88	894.94	D	656.38	328.70	639.36	320.18	638.37	319.69	4

16	1,943.95	972.48	1,926.92	963.96	1,925.94	963.47	H	541.36	271.18	524.33	262.67			3
17	2,057.03	1,029.02	2,040.01	1,020.51	2,039.02	1,020.01	L	404.30	202.65	387.27	194.14			2
18							K	291.21	146.11	274.19	137.60			1

Query 34142 Hit 1

MS/MS Fragmentation of VLLDFLMEEK
Found in sp|Q9BSJ2|GCP2_HUMAN, Gamma-tubulin complex component 2 OS=Homo sapiens GN=TUBGCP2 PE=1 SV=2
Match to Query 34142: 1523.855from(762.9347,2+)
Title: 1043: Scan 2316 (rt=61.5244, f=3, i=353) [D:\lab212\membrane\Grace\Joyce\iTRAQ_28_1_327.raw]
Data File:Submitted from 20120508-1(merge) by Mascot Daemon on JOYCE-VAIO
Monoisotopic mass of neutral peptide Mr(calc): 1523.855
Variable modifications:
K10 :iTRAQ4plex (K)
Ions Score: 59.53 Expect: 0.001

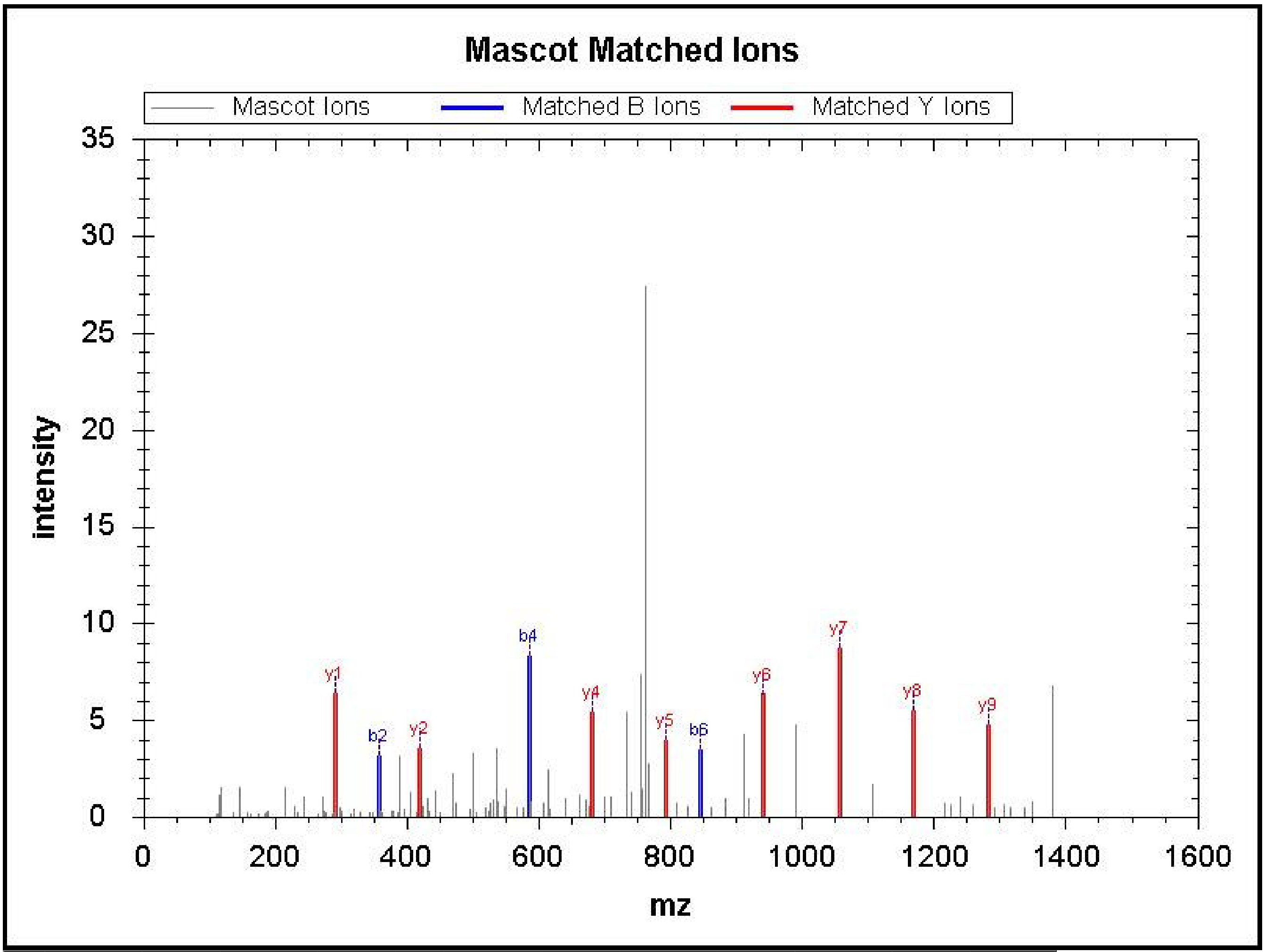

No	b	b++	b0	b0++	Seq	y	y++	y*	y*++	y0	y0++	RevNo
1	244.18	122.59			V							10
2	357.26	179.13			L	1,281.69	641.35	1,264.66	632.83	1,263.68	632.34	9
3	470.35	235.68			L	1,168.60	584.81	1,151.58	576.29	1,150.59	575.80	8
4	585.37	293.19	567.36	284.18	D	1,055.52	528.26	1,038.49	519.75	1,037.51	519.26	7
5	732.44	366.72	714.43	357.72	F	940.49	470.75	923.47	462.24	922.48	461.74	6
6	845.53	423.27	827.51	414.26	L	793.42	397.22	776.40	388.70	775.41	388.21	5
7	976.57	488.79	958.56	479.78	M	680.34	340.67	663.31	332.16	662.33	331.67	4
8	1,105.61	553.31	1,087.60	544.30	E	549.30	275.15	532.27	266.64	531.29	266.15	3
9	1,234.65	617.83	1,216.64	608.82	E	420.26	210.63	403.23	202.12	402.25	201.63	2
10					K	291.21	146.11	274.19	137.60			1

Query 77986 Hit 1

MS/MS Fragmentation of IMGLDLPDGGHLTHGFMTDK
Found in sp|P34896|GLYC_HUMAN, Serine hydroxymethyltransferase
Match to Query 77986: 2442.235from(611.566,4+)
Title: 782: Scan 1804 (rt=50.0998, f=3, i=280) [D:\lab212\membrane\Grace\Joyce\47_1.raw]
Data File:Submitted from 20120508-1(merge) by Mascot Daemon on JOYCE-VAIO
Monoisotopic mass of neutral peptide Mr(calc): 2442.235
Variable modifications:
K20 :iTRAQ4plex (K)
Ions Score: 59.48 Expect: 0.001

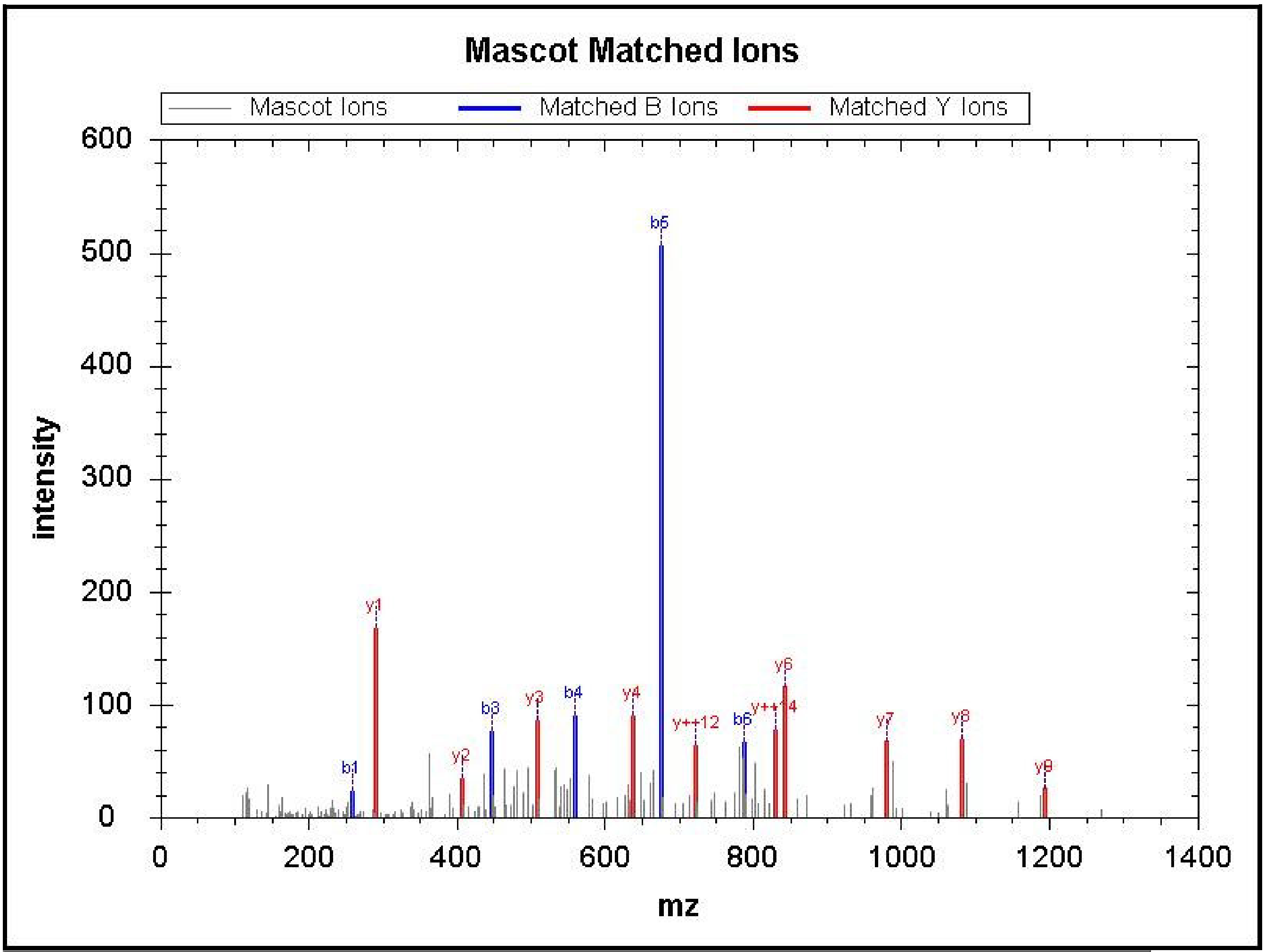

No	b	b++	b0	b0++	Seq	y	y++	y*	y*++	y0	y0++	RevNo
1	258.19	129.60			I							20
2	389.23	195.12			M	2,186.05	1,093.53	2,169.02	1,085.02	2,168.04	1,084.52	19
3	446.26	223.63			G	2,055.01	1,028.01	2,037.98	1,019.49	2,037.00	1,019.00	18
4	559.34	280.17			L	1,997.99	999.50	1,980.96	990.98	1,979.98	990.49	17
5	674.37	337.69	656.36	328.68	D	1,884.90	942.96	1,867.88	934.44	1,866.89	933.95	16
6	787.45	394.23	769.44	385.22	L	1,769.88	885.44	1,752.85	876.93	1,751.87	876.44	15
7	884.50	442.76	866.49	433.75	P	1,656.79	828.90	1,639.77	820.39	1,638.78	819.89	14
8	999.53	500.27	981.52	491.26	D	1,559.74	780.37	1,542.71	771.86	1,541.73	771.37	13
9	1,056.55	528.78	1,038.54	519.77	G	1,444.71	722.86	1,427.69	714.35	1,426.70	713.85	12
10	1,113.57	557.29	1,095.56	548.28	G	1,387.69	694.35	1,370.66	685.84	1,369.68	685.34	11
11	1,250.63	625.82	1,232.62	616.81	H	1,330.67	665.84	1,313.64	657.33	1,312.66	656.83	10
12	1,363.72	682.36	1,345.71	673.36	L	1,193.61	597.31	1,176.58	588.80	1,175.60	588.30	9
13	1,464.76	732.89	1,446.75	723.88	T	1,080.53	540.77	1,063.50	532.25	1,062.52	531.76	8
14	1,601.82	801.42	1,583.81	792.41	H	979.48	490.24	962.45	481.73	961.47	481.24	7

15	1,658.84	829.93	1,640.83	820.92	G	842.42	421.71	825.39	413.20	824.41	412.71	6
16	1,805.91	903.46	1,787.90	894.45	F	785.40	393.20	768.37	384.69	767.39	384.20	5
17	1,936.95	968.98	1,918.94	959.97	M	638.33	319.67	621.30	311.16	620.32	310.66	4
18	2,038.00	1,019.50	2,019.99	1,010.50	T	507.29	254.15	490.26	245.64	489.28	245.14	3
19	2,153.03	1,077.02	2,135.02	1,068.01	D	406.24	203.62	389.22	195.11	388.23	194.62	2
20					K	291.21	146.11	274.19	137.60			1

Query 54669 Hit 1

MS/MS Fragmentation of LLSAGEDTFVHIWK
Found in sp|Q9H977|WDR54_HUMAN, WD repeat-containing protein 54 OS=Homo sapiens GN=WDR54 PE=1 SV=1
Match to Query 54669: 1903.008from(635.3433,3+)
Title: 890: Sum of 2 scans in range 1926 (rt=53.1424, f=4, i=601) to 1927 (rt=53.1679, f=4, i=602)
[D:\lab212\membrane\Grace\Joyce\iTRAQ40.raw]
Data File:Submitted from 20120508-1(merge) by Mascot Daemon on JOYCE-VAIO
Monoisotopic mass of neutral peptide Mr(calc): 1903.008
Variable modifications:
K14 :iTRAQ4plex (K)
Ions Score: 59.33 Expect: 0.001

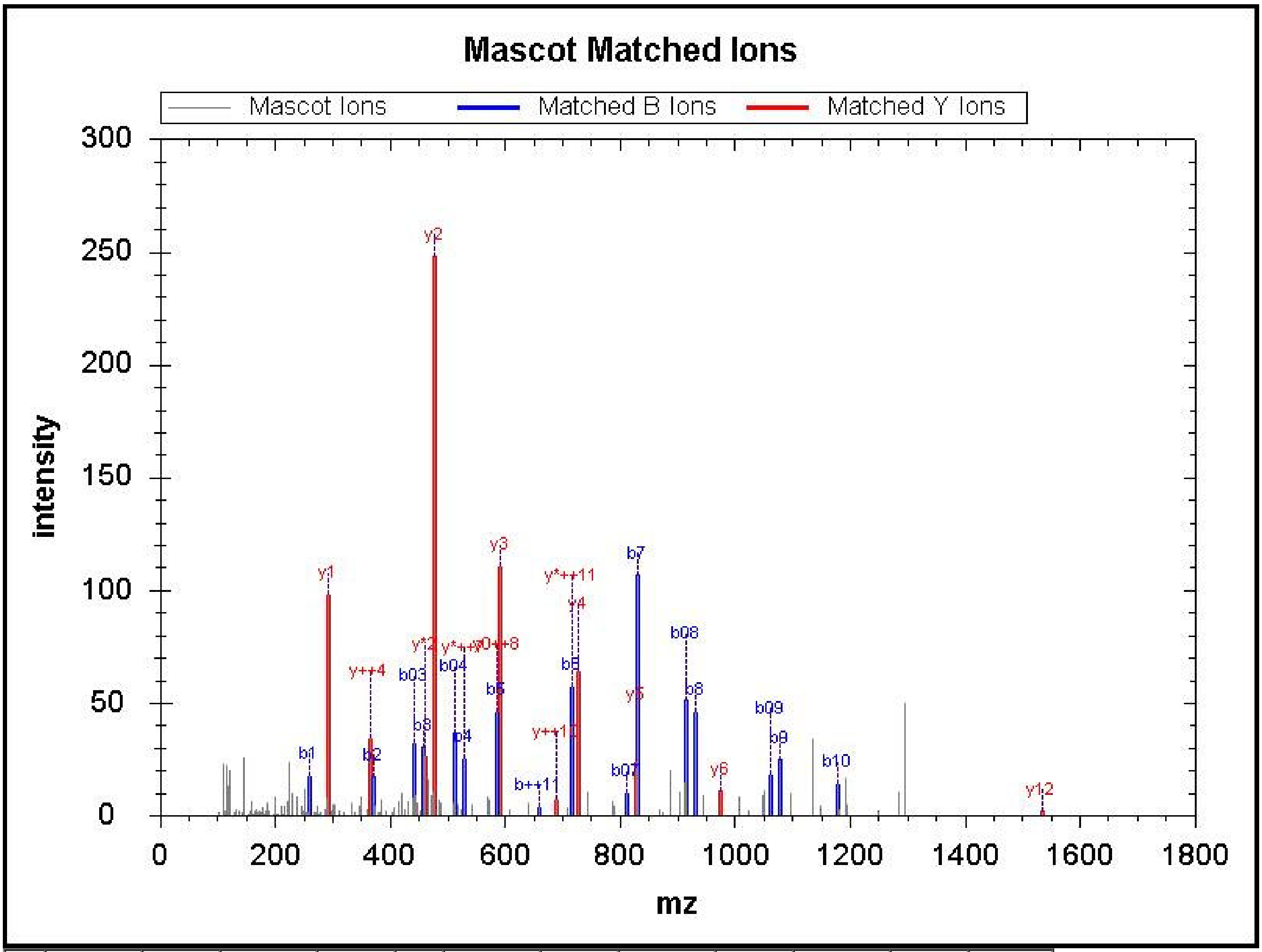

No	b	b++	b0	b0++	Seq	y	y++	y*	y*++	y0	y0++	RevNo
1	258.19	129.60			L							14
2	371.28	186.14			L	1,646.87	823.94	1,629.84	815.42	1,628.86	814.93	13
3	458.31	229.66	440.30	220.65	S	1,533.78	767.39	1,516.76	758.88	1,515.77	758.39	12
4	529.35	265.18	511.34	256.17	A	1,446.75	723.88	1,429.72	715.37	1,428.74	714.87	11
5	586.37	293.69	568.36	284.68	G	1,375.71	688.36	1,358.69	679.85	1,357.70	679.35	10
6	715.41	358.21	697.40	349.20	E	1,318.69	659.85	1,301.66	651.34	1,300.68	650.84	9
7	830.44	415.72	812.43	406.72	D	1,189.65	595.33	1,172.62	586.81	1,171.64	586.32	8

8	931.49	466.25	913.47	457.24	T	1,074.62	537.81	1,057.60	529.30	1,056.61	528.81	7
9	1,078.55	539.78	1,060.54	530.78	F	973.57	487.29	956.55	478.78			6
10	1,177.62	589.31	1,159.61	580.31	V	826.51	413.76	809.48	405.24			5
11	1,314.68	657.84	1,296.67	648.84	H	727.44	364.22	710.41	355.71			4
12	1,427.77	714.39	1,409.75	705.38	I	590.38	295.69	573.35	287.18			3
13	1,613.84	807.43	1,595.83	798.42	W	477.29	239.15	460.27	230.64			2
14					K	291.21	146.11	274.19	137.60			1

Query 69833 Hit 1

MS/MS Fragmentation of ALLEEAFDNLKDEMFR
Found in sp|P82094|TMF1_HUMAN, TATA element modulatory factor OS=Homo sapiens GN=TMF1 PE=1 SV=2
Match to Query 69833: 2228.143from(743.7216,3+)
Title: 1162: Sum of 2 scans in range 2670 (rt=69.0854, f=2, i=449) to 2671 (rt=69.1108, f=2, i=450) [D:\lab212\membrane\Grace\Joyce\iTRAQ_38_1.raw]
Data File:Submitted from 20120508-1(merge) by Mascot Daemon on JOYCE-VAIO
Monoisotopic mass of neutral peptide Mr(calc): 2228.143
Variable modifications:
K11 :iTRAQ4plex (K)
Ions Score: 59.27 Expect: 0.001

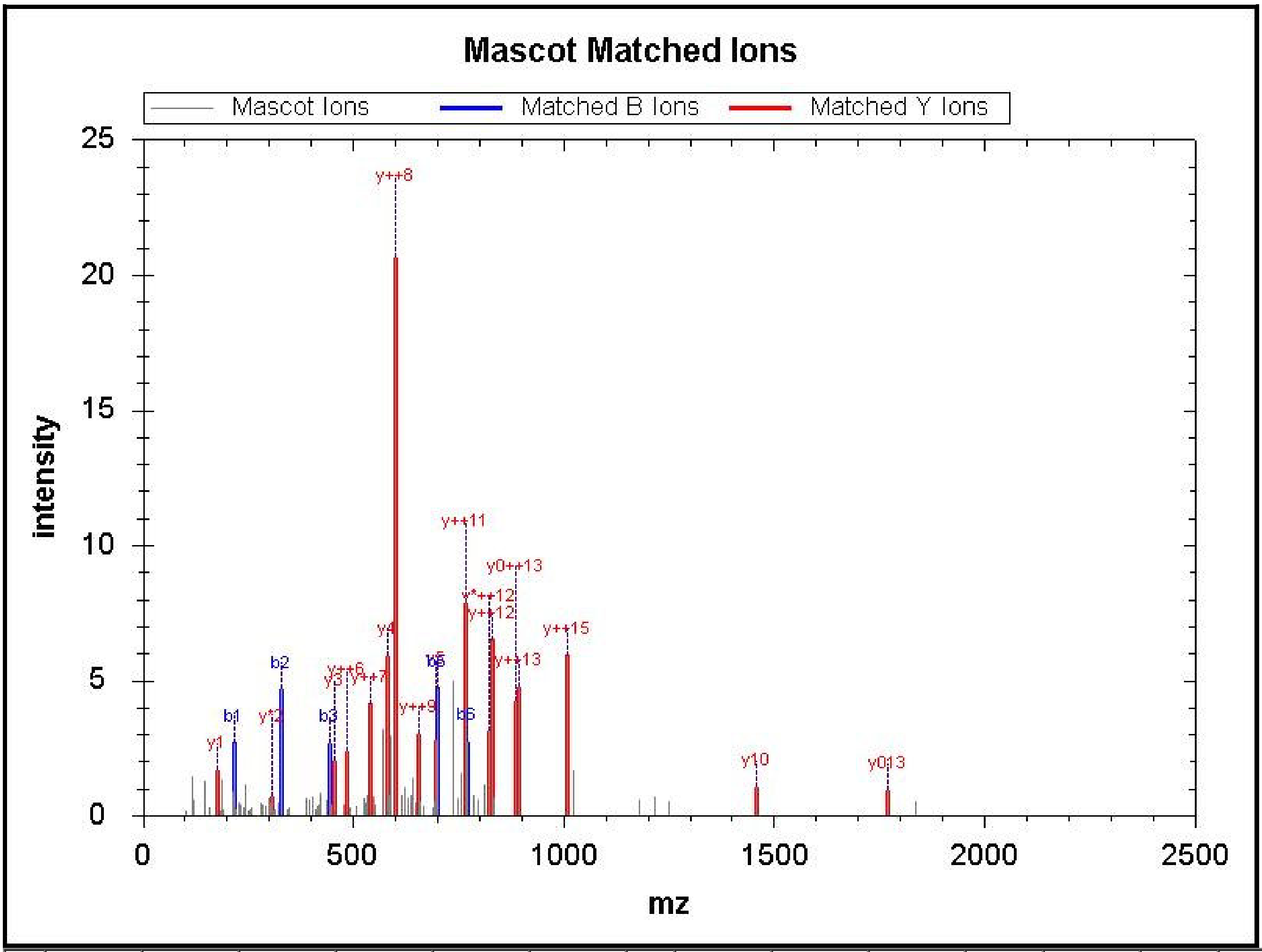

No	b	b++	b*	b*++	b0	b0++	Seq	y	y++	y*	y*++	y0	y0++	RevNo
1	216.15	108.58					A							16
2	329.23	165.12					L	2,014.01	1,007.51	1,996.98	998.99	1,996.00	998.50	15
3	442.31	221.66					L	1,900.92	950.97	1,883.90	942.45	1,882.91	941.96	14
4	571.36	286.18			553.35	277.18	E	1,787.84	894.42	1,770.81	885.91	1,769.83	885.42	13
5	700.40	350.70			682.39	341.70	E	1,658.80	829.90	1,641.77	821.39	1,640.79	820.90	12
6	771.44	386.22			753.43	377.22	A	1,529.75	765.38	1,512.73	756.87	1,511.74	756.38	11

7	918.51	459.76			900.49	450.75	F	1,458.72	729.86	1,441.69	721.35	1,440.71	720.86	10
8	1,033.53	517.27			1,015.52	508.26	D	1,311.65	656.33	1,294.62	647.81	1,293.64	647.32	9
9	1,147.58	574.29	1,130.55	565.78	1,129.56	565.29	N	1,196.62	598.81	1,179.59	590.30	1,178.61	589.81	8
10	1,260.66	630.83	1,243.63	622.32	1,242.65	621.83	L	1,082.58	541.79	1,065.55	533.28	1,064.57	532.79	7
11	1,532.86	766.93	1,515.83	758.42	1,514.85	757.93	K	969.49	485.25	952.47	476.74	951.48	476.25	6
12	1,647.88	824.45	1,630.86	815.93	1,629.87	815.44	D	697.30	349.15	680.27	340.64	679.29	340.15	5
13	1,776.93	888.97	1,759.90	880.45	1,758.92	879.96	E	582.27	291.64	565.24	283.13	564.26	282.63	4
14	1,907.97	954.49	1,890.94	945.97	1,889.96	945.48	M	453.23	227.12	436.20	218.60			3
15	2,055.03	1,028.02	2,038.01	1,019.51	2,037.02	1,019.02	F	322.19	161.60	305.16	153.08			2
16							R	175.12	88.06	158.09	79.55			1

Query 23952 Hit 1

MS/MS Fragmentation of MIETAQVDER
Found in sp|Q9UL25|RAB21_HUMAN, Ras-related protein Rab-21 OS=Homo sapiens GN=RAB21 PE=1 SV=3
Match to Query 23952: 1334.665from(668.3398,2+)
Title: 63: Scan 860 (rt=25.6063, f=2, i=72) [D:\lab212\membrane\Grace\Joyce\iTRAQ_24_2.raw]
Data File:Submitted from 20120508-1(merge) by Mascot Daemon on JOYCE-VAIO
Monoisotopic mass of neutral peptide Mr(calc): 1334.665
Variable modifications:
Ions Score: 59.13 Expect: 0.001

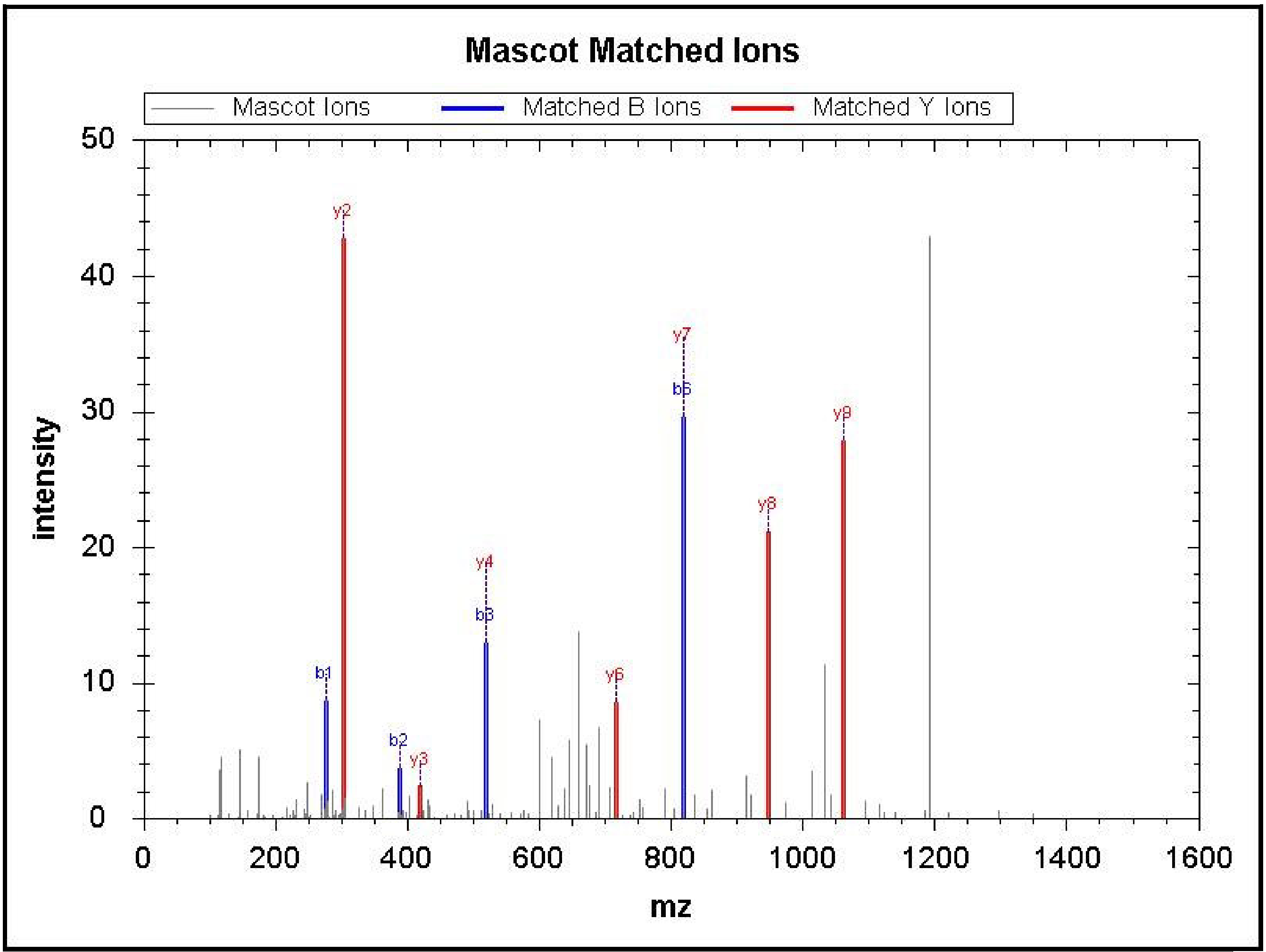

No	b	b++	b*	b*++	b0	b0++	Seq	y	y++	y*	y*++	y0	y0++	RevNo
1	276.15	138.58					M							10
2	389.23	195.12					I	1,060.53	530.77	1,043.50	522.25	1,042.52	521.76	9
3	518.28	259.64			500.27	250.64	E	947.44	474.23	930.42	465.71	929.43	465.22	8
4	619.32	310.17			601.31	301.16	T	818.40	409.70	801.37	401.19	800.39	400.70	7
5	690.36	345.68			672.35	336.68	A	717.35	359.18	700.33	350.67	699.34	350.17	6

6	818.42	409.71	801.39	401.20	800.41	400.71	Q	646.32	323.66	629.29	315.15	628.30	314.66	5
7	917.49	459.25	900.46	450.73	899.48	450.24	V	518.26	259.63	501.23	251.12	500.25	250.63	4
8	1,032.52	516.76	1,015.49	508.25	1,014.50	507.76	D	419.19	210.10	402.16	201.58	401.18	201.09	3
9	1,161.56	581.28	1,144.53	572.77	1,143.55	572.28	E	304.16	152.58	287.13	144.07	286.15	143.58	2
10							R	175.12	88.06	158.09	79.55			1

Query 59445 Hit 1

MS/MS Fragmentation of NPHPSSAFLNLIGFVSR
Found in sp|P53004|BIEA_HUMAN, Biliverdin reductase A OS=Homo sapiens GN=BLVRA PE=1 SV=2
Match to Query 59445: 1999.068from(667.3633,3+)
Title: 981: Sum of 2 scans in range 2168 (rt=58.2533, f=4, i=655) to 2169 (rt=58.2788, f=4, i=656) [D:\lab212\membrane\Grace\Joyce\iTRAQ_36_2.raw]
Data File:Submitted from 20120508-1(merge) by Mascot Daemon on JOYCE-VAIO
Monoisotopic mass of neutral peptide Mr(calc): 1999.068
Variable modifications:
Ions Score: 58.9 Expect: 0.001

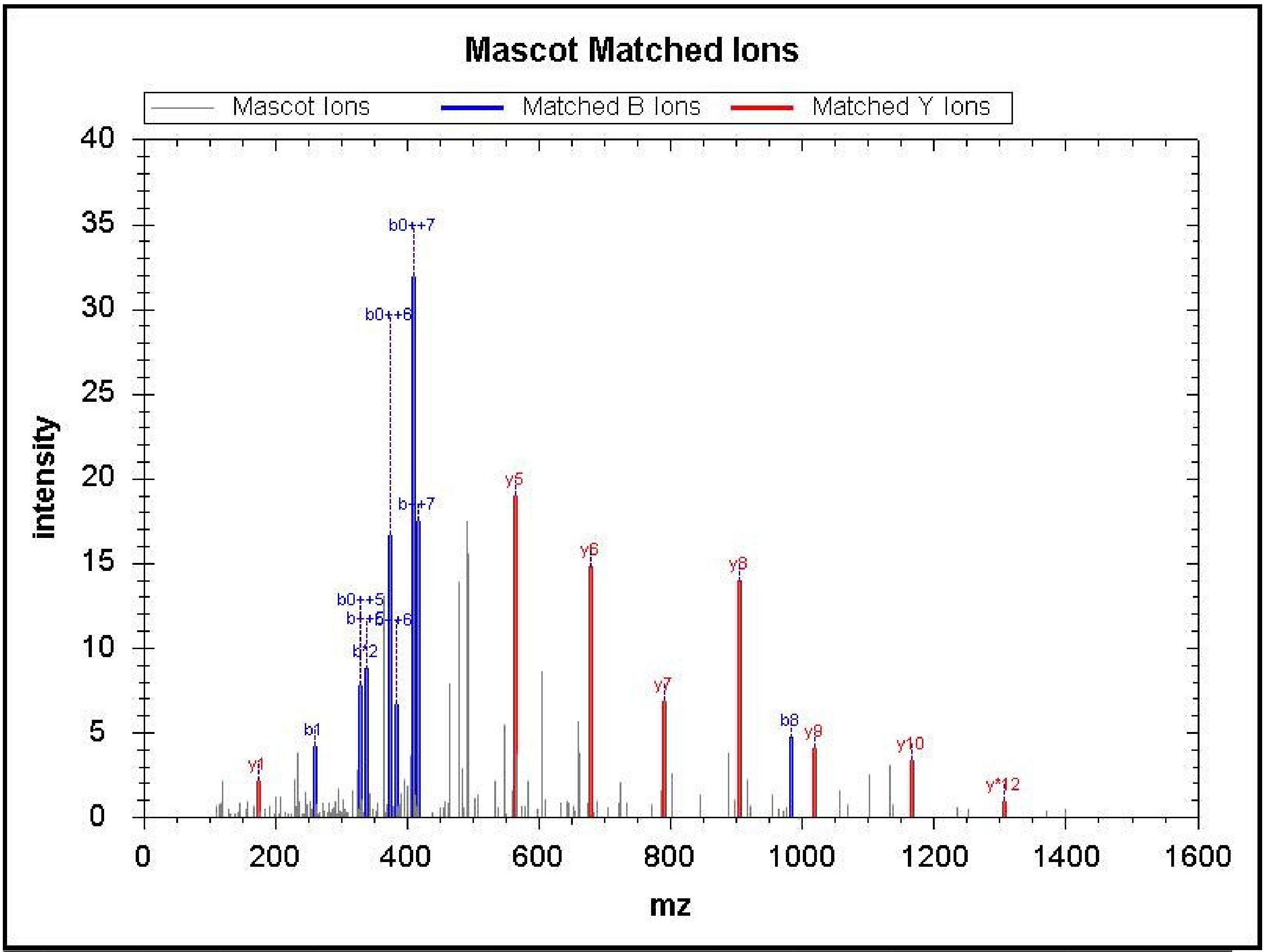

No	b	b++	b*	b*++	b0	b0++	Seq	y	y++	y*	y*++	y0	y0++	RevNo
1	259.15	130.08	242.13	121.57			N							17
2	356.21	178.61	339.18	170.09			P	1,741.94	871.47	1,724.91	862.96	1,723.93	862.47	16
3	493.26	247.14	476.24	238.62			H	1,644.89	822.95	1,627.86	814.43	1,626.87	813.94	15
4	590.32	295.66	573.29	287.15			P	1,507.83	754.42	1,490.80	745.90	1,489.82	745.41	14
5	677.35	339.18	660.32	330.66	659.34	330.17	S	1,410.77	705.89	1,393.75	697.38	1,392.76	696.89	13
6	764.38	382.69	747.35	374.18	746.37	373.69	S	1,323.74	662.37	1,306.72	653.86	1,305.73	653.37	12
7	835.42	418.21	818.39	409.70	817.41	409.21	A	1,236.71	618.86	1,219.68	610.35	1,218.70	609.85	11
8	982.49	491.75	965.46	483.23	964.48	482.74	F	1,165.67	583.34	1,148.65	574.83	1,147.66	574.33	10

9	1,095.57	548.29	1,078.54	539.78	1,077.56	539.28	L	1,018.60	509.81	1,001.58	501.29	1,000.59	500.80	9
10	1,209.61	605.31	1,192.59	596.80	1,191.60	596.31	N	905.52	453.26	888.49	444.75	887.51	444.26	8
11	1,322.70	661.85	1,305.67	653.34	1,304.69	652.85	L	791.48	396.24	774.45	387.73	773.47	387.24	7
12	1,435.78	718.39	1,418.76	709.88	1,417.77	709.39	I	678.39	339.70	661.37	331.19	660.38	330.70	6
13	1,492.80	746.91	1,475.78	738.39	1,474.79	737.90	G	565.31	283.16	548.28	274.64	547.30	274.15	5
14	1,639.87	820.44	1,622.85	811.93	1,621.86	811.43	F	508.29	254.65	491.26	246.13	490.28	245.64	4
15	1,738.94	869.97	1,721.91	861.46	1,720.93	860.97	V	361.22	181.11	344.19	172.60	343.21	172.11	3
16	1,825.97	913.49	1,808.95	904.98	1,807.96	904.48	S	262.15	131.58	245.12	123.07	244.14	122.57	2
17							R	175.12	88.06	158.09	79.55			1

Query 65678 Hit 1

MS/MS Fragmentation of TVLGSINWAFLGVDEAHR
Found in sp|O14647|CHD2_HUMAN, Chromodomain-helicase-DNA-binding protein 2 OS=Homo sapiens GN=CHD2 PE=1 SV=2
Match to Query 65678: 2128.118from(710.3798,3+)
Title: 1020: Sum of 2 scans in range 2206 (rt=59.372, f=4, i=685) to 2207 (rt=59.3974, f=4, i=686)
[D:\lab212\membrane\Grace\Joyce\iTRAQ_33_2.raw]
Data File:Submitted from 20120508-1(merge) by Mascot Daemon on JOYCE-VAIO
Monoisotopic mass of neutral peptide Mr(calc): 2128.118
Variable modifications:
Ions Score: 58.89 Expect: 0.001

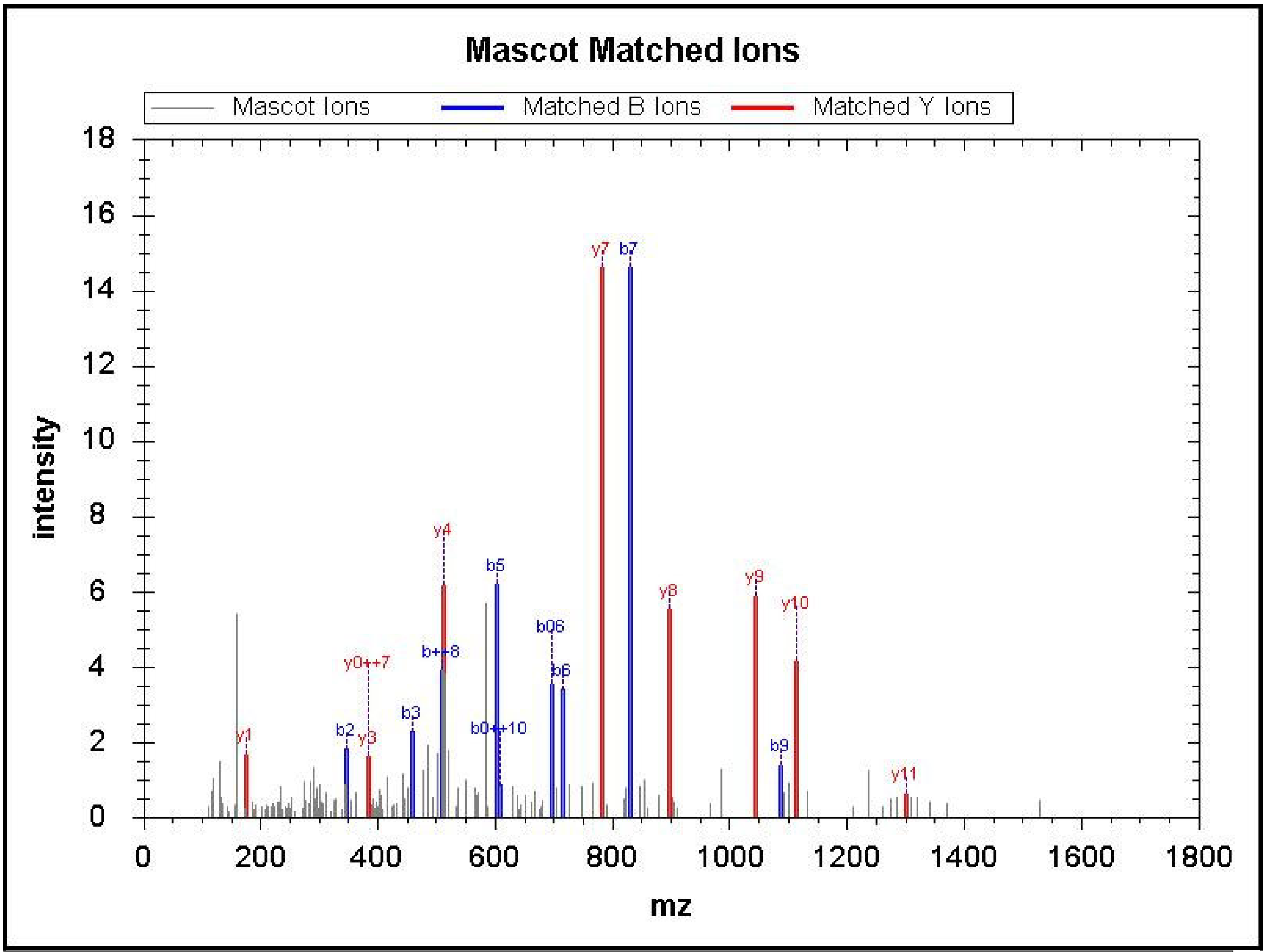

No	b	b++	b*	b*++	b0	b0++	Seq	y	y++	y*	y*++	y0	y0++	RevNo
1	246.16	123.58			228.15	114.58	T							18
2	345.23	173.12			327.21	164.11	V	1,883.98	942.49	1,866.95	933.98	1,865.97	933.49	17
3	458.31	229.66			440.30	220.65	L	1,784.91	892.96	1,767.88	884.44	1,766.90	883.95	16
4	515.33	258.17			497.32	249.16	G	1,671.82	836.42	1,654.80	827.90	1,653.81	827.41	15

5	602.36	301.69			584.35	292.68	S	1,614.80	807.90	1,597.78	799.39	1,596.79	798.90	14
6	715.45	358.23			697.44	349.22	I	1,527.77	764.39	1,510.74	755.88	1,509.76	755.38	13
7	829.49	415.25	812.46	406.74	811.48	406.24	N	1,414.69	707.85	1,397.66	699.33	1,396.68	698.84	12
8	1,015.57	508.29	998.54	499.77	997.56	499.28	W	1,300.64	650.83	1,283.62	642.31	1,282.63	641.82	11
9	1,086.61	543.81	1,069.58	535.29	1,068.60	534.80	A	1,114.56	557.79	1,097.54	549.27	1,096.55	548.78	10
10	1,233.67	617.34	1,216.65	608.83	1,215.66	608.34	F	1,043.53	522.27	1,026.50	513.75	1,025.52	513.26	9
11	1,346.76	673.88	1,329.73	665.37	1,328.75	664.88	L	896.46	448.73	879.43	440.22	878.45	439.73	8
12	1,403.78	702.39	1,386.75	693.88	1,385.77	693.39	G	783.37	392.19	766.35	383.68	765.36	383.19	7
13	1,502.85	751.93	1,485.82	743.41	1,484.84	742.92	V	726.35	363.68	709.33	355.17	708.34	354.67	6
14	1,617.88	809.44	1,600.85	800.93	1,599.87	800.44	D	627.28	314.15	610.26	305.63	609.27	305.14	5
15	1,746.92	873.96	1,729.89	865.45	1,728.91	864.96	E	512.26	256.63	495.23	248.12	494.25	247.63	4
16	1,817.96	909.48	1,800.93	900.97	1,799.94	900.48	A	383.21	192.11	366.19	183.60			3
17	1,955.01	978.01	1,937.99	969.50	1,937.00	969.01	H	312.18	156.59	295.15	148.08			2
18							R	175.12	88.06	158.09	79.55			1

Query 58360 Hit 1

MS/MS Fragmentation of EVPIPEHIDIYHLTR
Found in sp|Q9UG63|ABCF2_HUMAN, ATP-binding cassette sub-family F member 2 OS=Homo sapiens GN=ABCF2 PE=1 SV=2
Match to Query 58360: 1975.069from(494.7746,4+)
Title: 437: Scan 1590 (rt=43.2821, f=2, i=290) [D:\lab212\membrane\Grace\Joyce\iTRAQ_46_1.raw]
Data File:Submitted from 20120508-1(merge) by Mascot Daemon on JOYCE-VAIO
Monoisotopic mass of neutral peptide Mr(calc): 1975.069
Variable modifications:
Ions Score: 58.76 Expect: 0.001

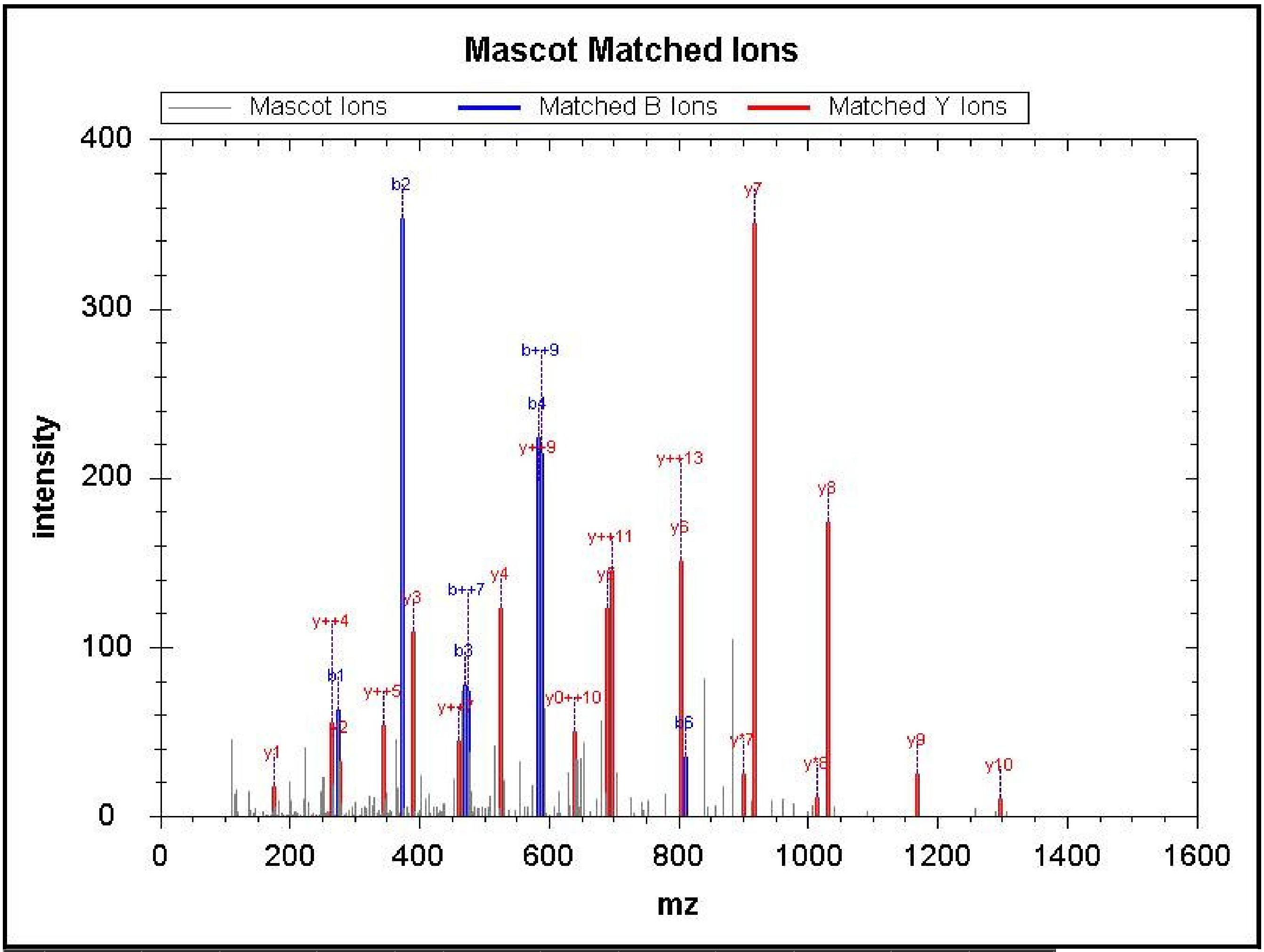

No	b	b++	b0	b0++	Seq	y	y++	y*	y*++	y0	y0++	RevNo

1	274.15	137.58	256.14	128.57	E							15
2	373.22	187.11	355.21	178.11	V	1,702.93	851.97	1,685.90	843.45	1,684.92	842.96	14
3	470.27	235.64	452.26	226.63	P	1,603.86	802.43	1,586.83	793.92	1,585.85	793.43	13
4	583.36	292.18	565.35	283.18	I	1,506.81	753.91	1,489.78	745.39	1,488.80	744.90	12
5	680.41	340.71	662.40	331.70	P	1,393.72	697.36	1,376.70	688.85	1,375.71	688.36	11
6	809.45	405.23	791.44	396.22	E	1,296.67	648.84	1,279.64	640.33	1,278.66	639.83	10
7	946.51	473.76	928.50	464.75	H	1,167.63	584.32	1,150.60	575.80	1,149.62	575.31	9
8	1,059.60	530.30	1,041.58	521.30	I	1,030.57	515.79	1,013.54	507.27	1,012.56	506.78	8
9	1,174.62	587.81	1,156.61	578.81	D	917.48	459.25	900.46	450.73	899.47	450.24	7
10	1,287.71	644.36	1,269.70	635.35	I	802.46	401.73	785.43	393.22	784.45	392.73	6
11	1,450.77	725.89	1,432.76	716.88	Y	689.37	345.19	672.35	336.68	671.36	336.18	5
12	1,587.83	794.42	1,569.82	785.41	H	526.31	263.66	509.28	255.15	508.30	254.65	4
13	1,700.91	850.96	1,682.90	841.95	L	389.25	195.13	372.22	186.62	371.24	186.12	3
14	1,801.96	901.48	1,783.95	892.48	T	276.17	138.59	259.14	130.07	258.16	129.58	2
15					R	175.12	88.06	158.09	79.55			1

Query 64660 Hit 1

MS/MS Fragmentation of AIIEEMLDLLEQSEGK
Found in sp|Q9NXJ5|PGPI_HUMAN, Pyroglutamyl-peptidase 1 OS=Homo sapiens GN=PGPEP1 PE=1 SV=1
Match to Query 64660: 2105.12from(702.7139,3+)
Title: 1370: Sum of 2 scans in range 3008 (rt=77.2983, f=2, i=491) to 3009 (rt=77.3237, f=2, i=492)
[D:\lab212\membrane\Grace\Joyce\iTRAQ_27_2_327.raw]
Data File:Submitted from 20120508-1(merge) by Mascot Daemon on JOYCE-VAIO
Monoisotopic mass of neutral peptide Mr(calc): 2105.12
Variable modifications:
K16 :iTRAQ4plex (K)
Ions Score: 58.63 Expect: 0.001

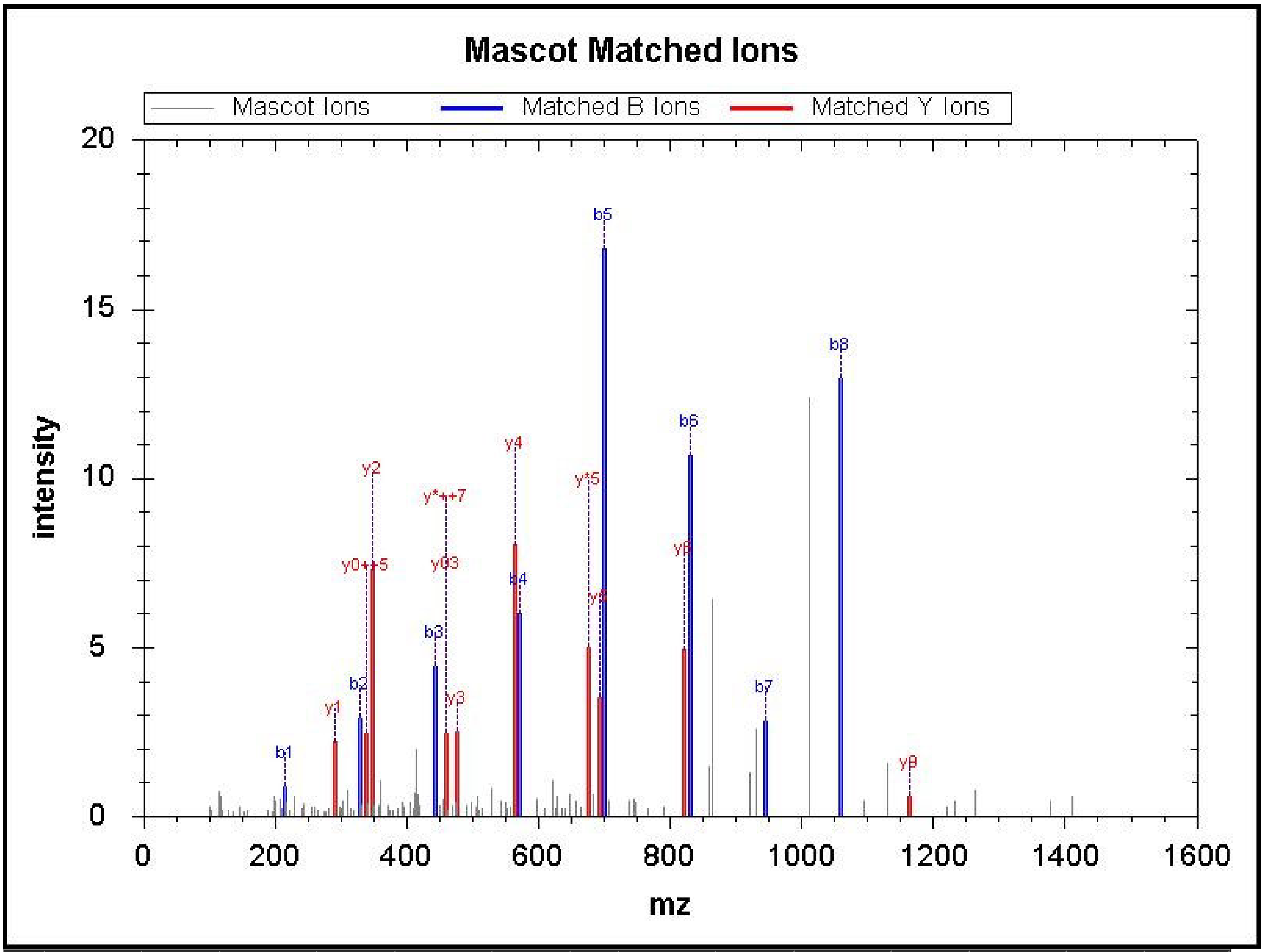

No	b	b++	b*	b*++	b0	b0++	Seq	y	y++	y*	y*++	y0	y0++	RevNo
1	216.15	108.58					A							16
2	329.23	165.12					I	1,890.99	946.00	1,873.96	937.48	1,872.97	936.99	15
3	442.31	221.66					I	1,777.90	889.45	1,760.87	880.94	1,759.89	880.45	14
4	571.36	286.18			553.35	277.18	E	1,664.82	832.91	1,647.79	824.40	1,646.81	823.91	13
5	700.40	350.70			682.39	341.70	E	1,535.77	768.39	1,518.75	759.88	1,517.76	759.39	12
6	831.44	416.22			813.43	407.22	M	1,406.73	703.87	1,389.71	695.36	1,388.72	694.86	11
7	944.52	472.77			926.51	463.76	L	1,275.69	638.35	1,258.66	629.84	1,257.68	629.34	10
8	1,059.55	530.28			1,041.54	521.27	D	1,162.61	581.81	1,145.58	573.29	1,144.60	572.80	9
9	1,172.64	586.82			1,154.62	577.82	L	1,047.58	524.29	1,030.55	515.78	1,029.57	515.29	8
10	1,285.72	643.36			1,267.71	634.36	L	934.50	467.75	917.47	459.24	916.49	458.75	7
11	1,414.76	707.88			1,396.75	698.88	E	821.41	411.21	804.39	402.70	803.40	402.20	6
12	1,542.82	771.91	1,525.79	763.40	1,524.81	762.91	Q	692.37	346.69	675.34	338.18	674.36	337.68	5
13	1,629.85	815.43	1,612.83	806.92	1,611.84	806.42	S	564.31	282.66	547.28	274.15	546.30	273.65	4
14	1,758.90	879.95	1,741.87	871.44	1,740.88	870.95	E	477.28	239.14	460.25	230.63	459.27	230.14	3
15	1,815.92	908.46	1,798.89	899.95	1,797.91	899.46	G	348.24	174.62	331.21	166.11			2
16							K	291.21	146.11	274.19	137.60			1

Query 32472 Hit 1

MS/MS Fragmentation of ASVDELFAEIVR
Found in sp|P61225|RAP2B_HUMAN, Ras-related protein Rap-2b OS=Homo sapiens GN=RAP2B PE=1 SV=1
Match to Query 32472: 1491.806from(746.9101,2+)
Title: 942: Scan 2285 (rt=59.8691, f=3, i=322) [D:\lab212\membrane\Grace\Joyce\iTRAQ_23_2.raw]
Data File:Submitted from 20120508-1(merge) by Mascot Daemon on JOYCE-VAIO
Monoisotopic mass of neutral peptide Mr(calc): 1491.806
Variable modifications:

Ions Score: 58.58 Expect: 0.001

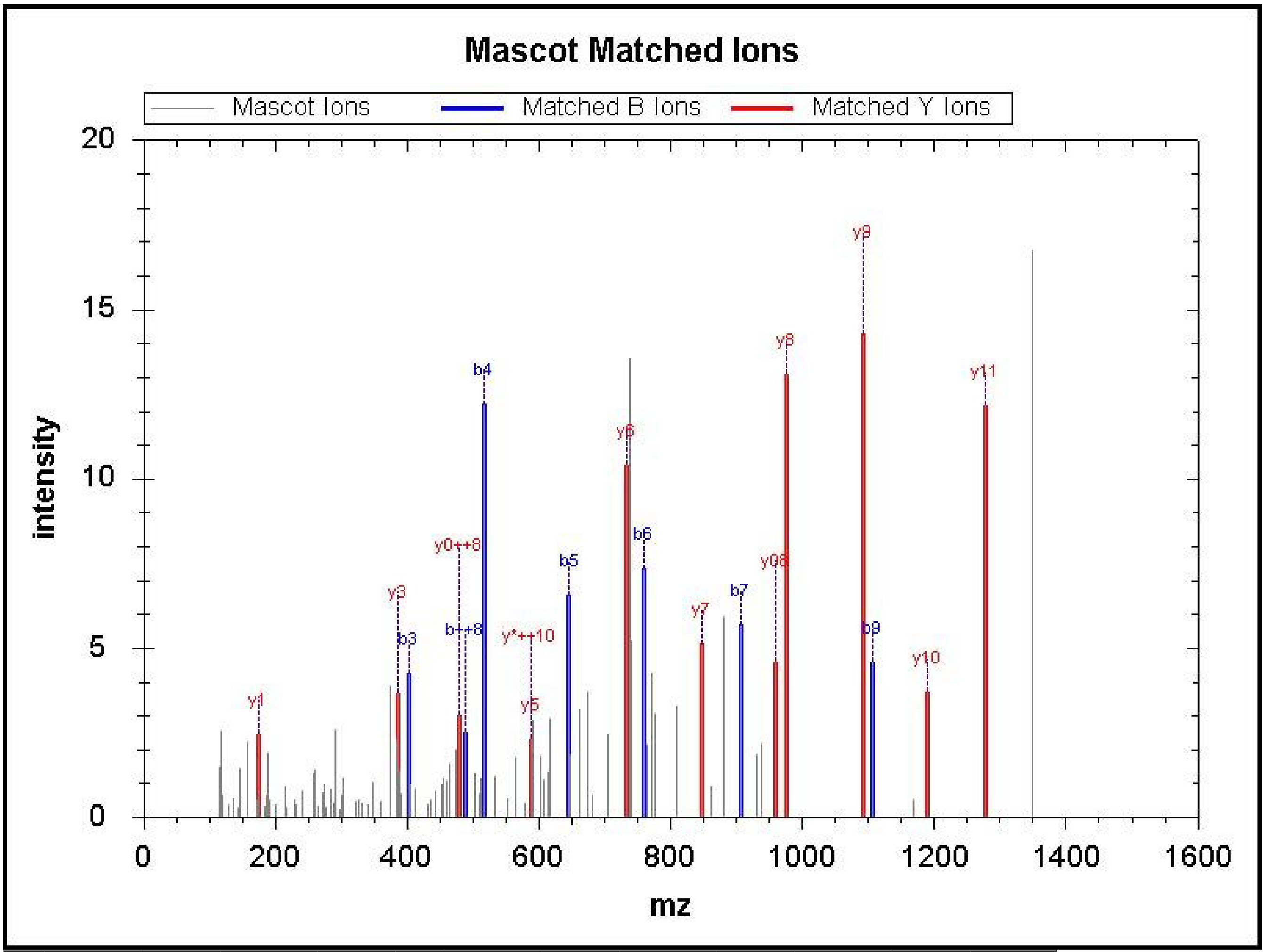

No	b	b++	b0	b0++	Seq	y	y++	y*	y*++	y0	y0++	RevNo
1	216.15	108.58			A							12
2	303.18	152.09	285.17	143.09	S	1,277.67	639.34	1,260.65	630.83	1,259.66	630.34	11
3	402.25	201.63	384.24	192.62	V	1,190.64	595.82	1,173.61	587.31	1,172.63	586.82	10
4	517.27	259.14	499.26	250.14	D	1,091.57	546.29	1,074.55	537.78	1,073.56	537.28	9
5	646.32	323.66	628.31	314.66	E	976.55	488.78	959.52	480.26	958.54	479.77	8
6	759.40	380.20	741.39	371.20	L	847.50	424.26	830.48	415.74	829.49	415.25	7
7	906.47	453.74	888.46	444.73	F	734.42	367.71	717.39	359.20	716.41	358.71	6
8	977.51	489.26	959.50	480.25	A	587.35	294.18	570.32	285.67	569.34	285.17	5
9	1,106.55	553.78	1,088.54	544.77	E	516.31	258.66	499.29	250.15	498.30	249.66	4
10	1,219.63	610.32	1,201.62	601.31	I	387.27	194.14	370.24	185.63			3
11	1,318.70	659.85	1,300.69	650.85	V	274.19	137.60	257.16	129.08			2
12					R	175.12	88.06	158.09	79.55			1

Query 63637 Hit 1

MS/MS Fragmentation of EFWEIVHSFTDEQK
Found in sp|Q05086|UBE3A_HUMAN, Ubiquitin-protein ligase E3A OS=Homo sapiens GN=UBE3A PE=1 SV=4
Match to Query 63637: 2082.012from(695.0114,3+)
Title: 984: Scan 2122 (rt=57.5546, f=2, i=342) [D:\lab212\membrane\Grace\Joyce\iTRAQ40.raw]
Data File:Submitted from 20120508-1(merge) by Mascot Daemon on JOYCE-VAIO
Monoisotopic mass of neutral peptide Mr(calc): 2082.012
Variable modifications:
K14 :iTRAQ4plex (K)
Ions Score: 58.56 Expect: 0.001

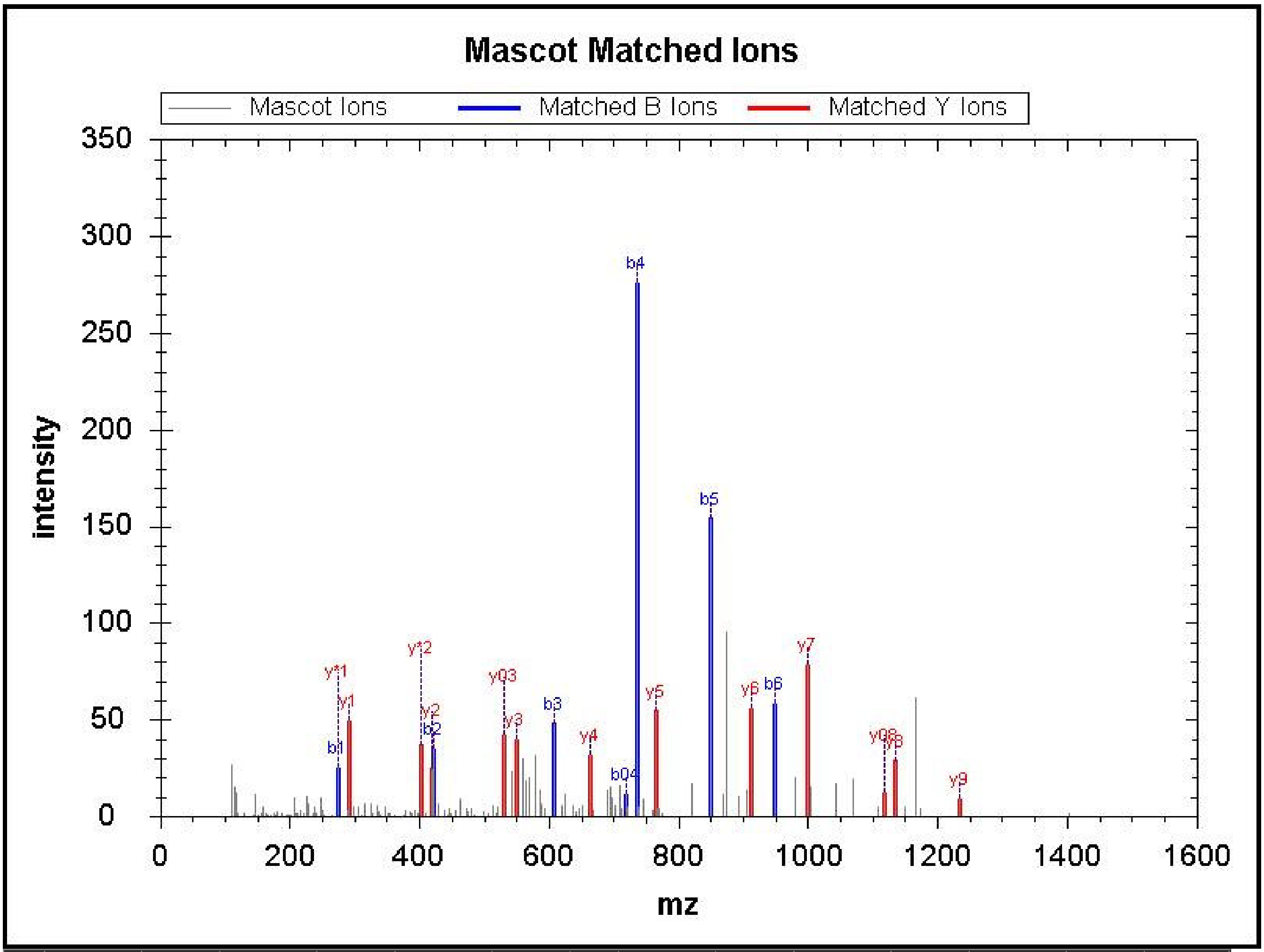

No	b	b++	b*	b*++	b0	b0++	Seq	y	y++	y*	y*++	y0	y0++	RevNo
1	274.15	137.58			256.14	128.57	E							14
2	421.22	211.11			403.21	202.11	F	1,809.89	905.45	1,792.87	896.94	1,791.88	896.44	13
3	607.30	304.15			589.29	295.15	W	1,662.82	831.92	1,645.80	823.40	1,644.81	822.91	12
4	736.34	368.67			718.33	359.67	E	1,476.75	738.88	1,459.72	730.36	1,458.73	729.87	11
5	849.43	425.22			831.42	416.21	I	1,347.70	674.35	1,330.68	665.84	1,329.69	665.35	10
6	948.49	474.75			930.48	465.75	V	1,234.62	617.81	1,217.59	609.30	1,216.61	608.81	9
7	1,085.55	543.28			1,067.54	534.28	H	1,135.55	568.28	1,118.52	559.77	1,117.54	559.27	8
8	1,172.59	586.80			1,154.58	577.79	S	998.49	499.75	981.46	491.24	980.48	490.74	7
9	1,319.65	660.33			1,301.64	651.33	F	911.46	456.23	894.43	447.72	893.45	447.23	6
10	1,420.70	710.85			1,402.69	701.85	T	764.39	382.70	747.36	374.19	746.38	373.69	5
11	1,535.73	768.37			1,517.72	759.36	D	663.34	332.18	646.32	323.66	645.33	323.17	4
12	1,664.77	832.89			1,646.76	823.88	E	548.32	274.66	531.29	266.15	530.31	265.66	3
13	1,792.83	896.92	1,775.80	888.41	1,774.82	887.91	Q	419.27	210.14	402.25	201.63			2
14							K	291.21	146.11	274.19	137.60			1

Query 69312 Hit 1

MS/MS Fragmentation of KPIDYTILDDIGHGVK
Found in sp|Q9NYB9|ABI2_HUMAN, Abl interactor 2 OS=Homo sapiens GN=ABI2 PE=1 SV=1
Match to Query 69312: 2215.264from(554.8232,4+)
Title: 702: Scan 1532 (rt=44.1989, f=2, i=245) [D:\lab212\membrane\Grace\Joyce\iTRAQ_50_2.raw]
Data File:Submitted from 20120508-1(merge) by Mascot Daemon on JOYCE-VAIO
Monoisotopic mass of neutral peptide Mr(calc): 2215.264
Variable modifications:
K1 :iTRAQ4plex (K)
K16 :iTRAQ4plex (K)
Ions Score: 58.56 Expect: 0.001

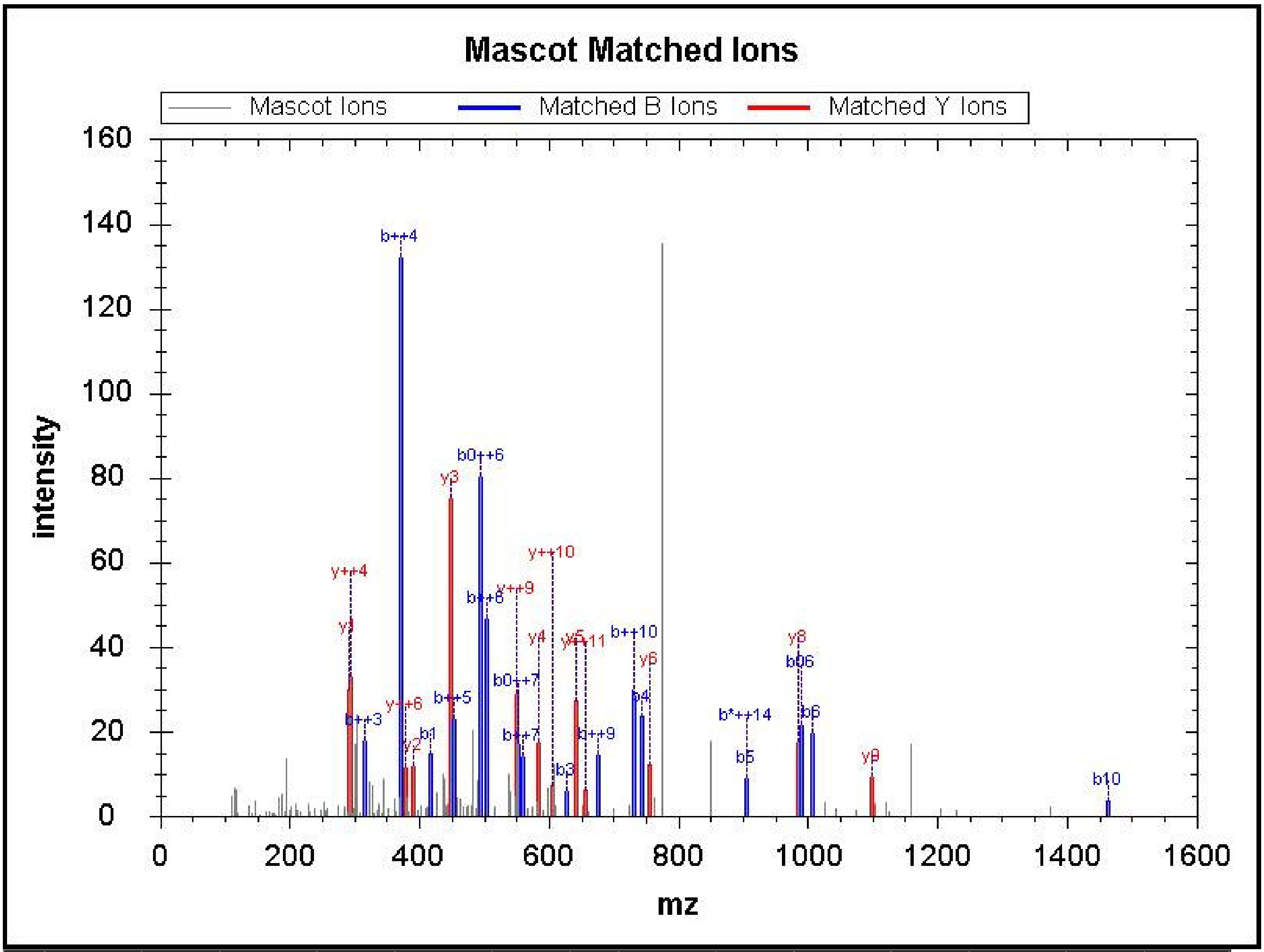

No	b	b++	b*	b*++	b0	b0++	Seq	y	y++	y*	y*++	y0	y0++	RevNo
1	417.31	209.16	400.28	200.64			K							16
2	514.36	257.68	497.33	249.17			P	1,799.97	900.49	1,782.94	891.97	1,781.96	891.48	15
3	627.44	314.23	610.42	305.71			I	1,702.91	851.96	1,685.89	843.45	1,684.90	842.96	14
4	742.47	371.74	725.44	363.23	724.46	362.73	D	1,589.83	795.42	1,572.80	786.91	1,571.82	786.41	13
5	905.53	453.27	888.51	444.76	887.52	444.27	Y	1,474.80	737.90	1,457.78	729.39	1,456.79	728.90	12
6	1,006.58	503.79	989.55	495.28	988.57	494.79	T	1,311.74	656.37	1,294.71	647.86	1,293.73	647.37	11
7	1,119.67	560.34	1,102.64	551.82	1,101.65	551.33	I	1,210.69	605.85	1,193.66	597.34	1,192.68	596.84	10
8	1,232.75	616.88	1,215.72	608.37	1,214.74	607.87	L	1,097.61	549.31	1,080.58	540.79	1,079.60	540.30	9
9	1,347.78	674.39	1,330.75	665.88	1,329.77	665.39	D	984.52	492.77	967.50	484.25	966.51	483.76	8
10	1,462.80	731.91	1,445.78	723.39	1,444.79	722.90	D	869.50	435.25	852.47	426.74	851.49	426.25	7
11	1,575.89	788.45	1,558.86	779.93	1,557.88	779.44	I	754.47	377.74	737.44	369.22			6
12	1,632.91	816.96	1,615.88	808.44	1,614.90	807.95	G	641.39	321.20	624.36	312.68			5
13	1,769.97	885.49	1,752.94	876.97	1,751.96	876.48	H	584.36	292.69	567.34	284.17			4
14	1,826.99	914.00	1,809.96	905.49	1,808.98	904.99	G	447.30	224.16	430.28	215.64			3
15	1,926.06	963.53	1,909.03	955.02	1,908.05	954.53	V	390.28	195.65	373.26	187.13			2
16							K	291.21	146.11	274.19	137.60			1

Query 70180 Hit 1

MS/MS Fragmentation of HYLLSQGWWDEEQEK
Found in sp|P12694|ODBA_HUMAN, 2-oxoisovalerate dehydrogenase subunit alpha
Match to Query 70180: 2235.086from(746.036,3+)
Title: 711: Scan 1601 (rt=45.6575, f=3, i=247) [D:\lab212\membrane\Grace\Joyce\iTRAQ_44_1.raw]
Data File:Submitted from 20120508-1(merge) by Mascot Daemon on JOYCE-VAIO
Monoisotopic mass of neutral peptide Mr(calc): 2235.086

Variable modifications:
K15 :iTRAQ4plex (K)
Ions Score: 58.47 Expect: 0.001

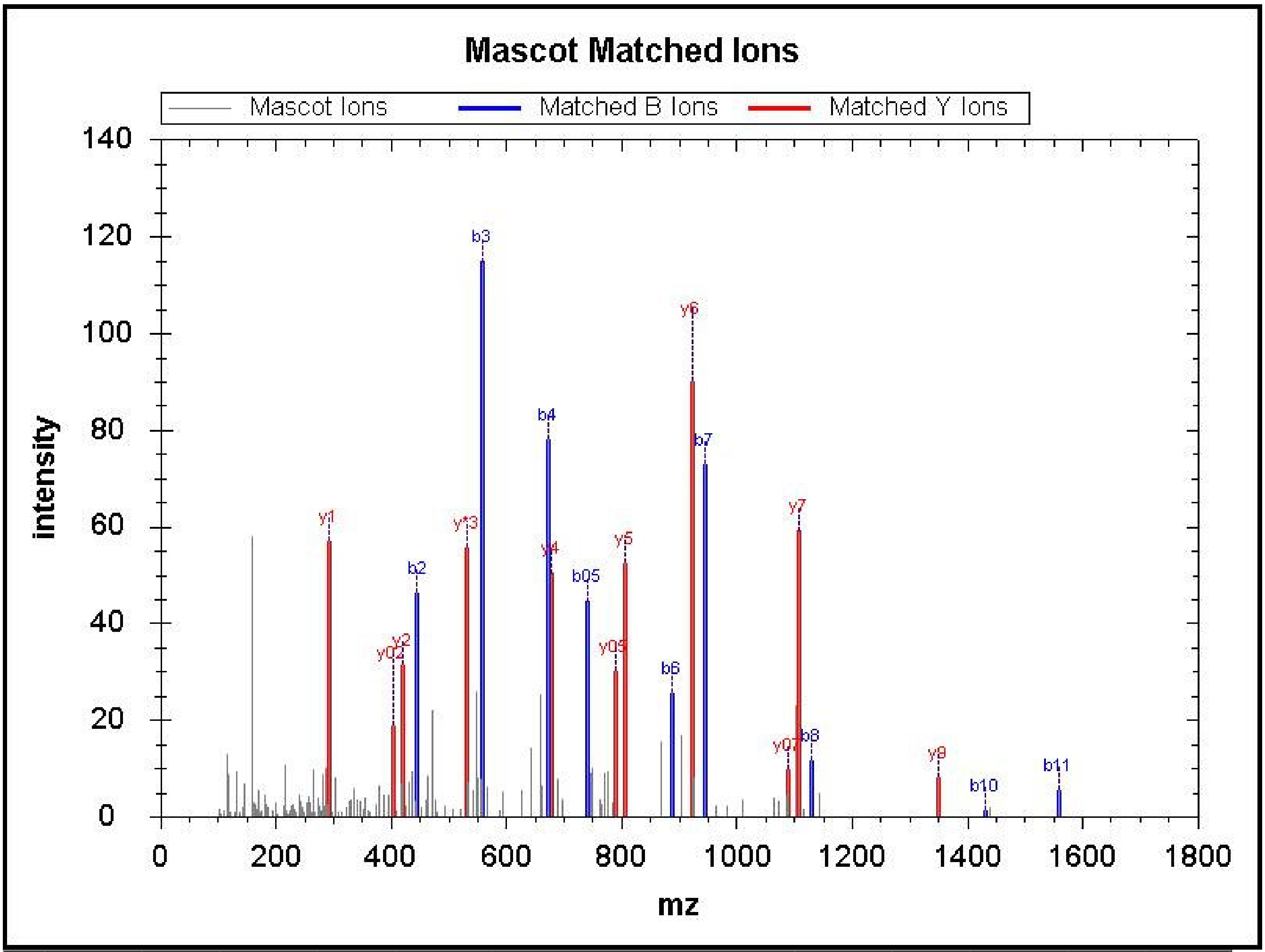

No	b	b++	b*	b*++	b0	b0++	Seq	y	y++	y*	y*++	y0	y0++	RevNo
1	282.17	141.59					H							15
2	445.23	223.12					Y	1,954.93	977.97	1,937.90	969.46	1,936.92	968.96	14
3	558.32	279.66					L	1,791.87	896.44	1,774.84	887.92	1,773.86	887.43	13
4	671.40	336.20					L	1,678.78	839.90	1,661.76	831.38	1,660.77	830.89	12
5	758.43	379.72			740.42	370.71	S	1,565.70	783.35	1,548.67	774.84	1,547.69	774.35	11
6	886.49	443.75	869.46	435.24	868.48	434.74	Q	1,478.67	739.84	1,461.64	731.32	1,460.66	730.83	10
7	943.51	472.26	926.49	463.75	925.50	463.25	G	1,350.61	675.81	1,333.58	667.29	1,332.60	666.80	9
8	1,129.59	565.30	1,112.56	556.79	1,111.58	556.29	W	1,293.59	647.30	1,276.56	638.78	1,275.58	638.29	8
9	1,315.67	658.34	1,298.64	649.83	1,297.66	649.33	W	1,107.51	554.26	1,090.48	545.74	1,089.50	545.25	7
10	1,430.70	715.85	1,413.67	707.34	1,412.69	706.85	D	921.43	461.22	904.40	452.70	903.42	452.21	6
11	1,559.74	780.37	1,542.71	771.86	1,541.73	771.37	E	806.40	403.70	789.37	395.19	788.39	394.70	5
12	1,688.78	844.90	1,671.76	836.38	1,670.77	835.89	E	677.36	339.18	660.33	330.67	659.35	330.18	4
13	1,816.84	908.92	1,799.81	900.41	1,798.83	899.92	Q	548.32	274.66	531.29	266.15	530.31	265.66	3
14	1,945.88	973.45	1,928.86	964.93	1,927.87	964.44	E	420.26	210.63	403.23	202.12	402.25	201.63	2
15							K	291.21	146.11	274.19	137.60			1

Query 77146 Hit 1

MS/MS Fragmentation of HQLGGPMWAEPIHDLDFVGR
Found in sp|Q9NXH9|TRM1_HUMAN, tRNA (guanine(26)-N(2))-dimethyltransferase OS=Homo sapiens GN=TRMT1 PE=1 SV=1
Match to Query 77146: 2418.207from(605.5591,4+)
Title: 599: Sum of 2 scans in range 1926 (rt=50.8615, f=4, i=367) to 1927 (rt=50.8869, f=4, i=368)

[D:\lab212\membrane\Grace\Joyce\iTRAQ_46_1.raw]
Data File:Submitted from 20120508-1(merge) by Mascot Daemon on JOYCE-VAIO
Monoisotopic mass of neutral peptide Mr(calc): 2418.207
Variable modifications:
Ions Score: 58.46 Expect: 0.001

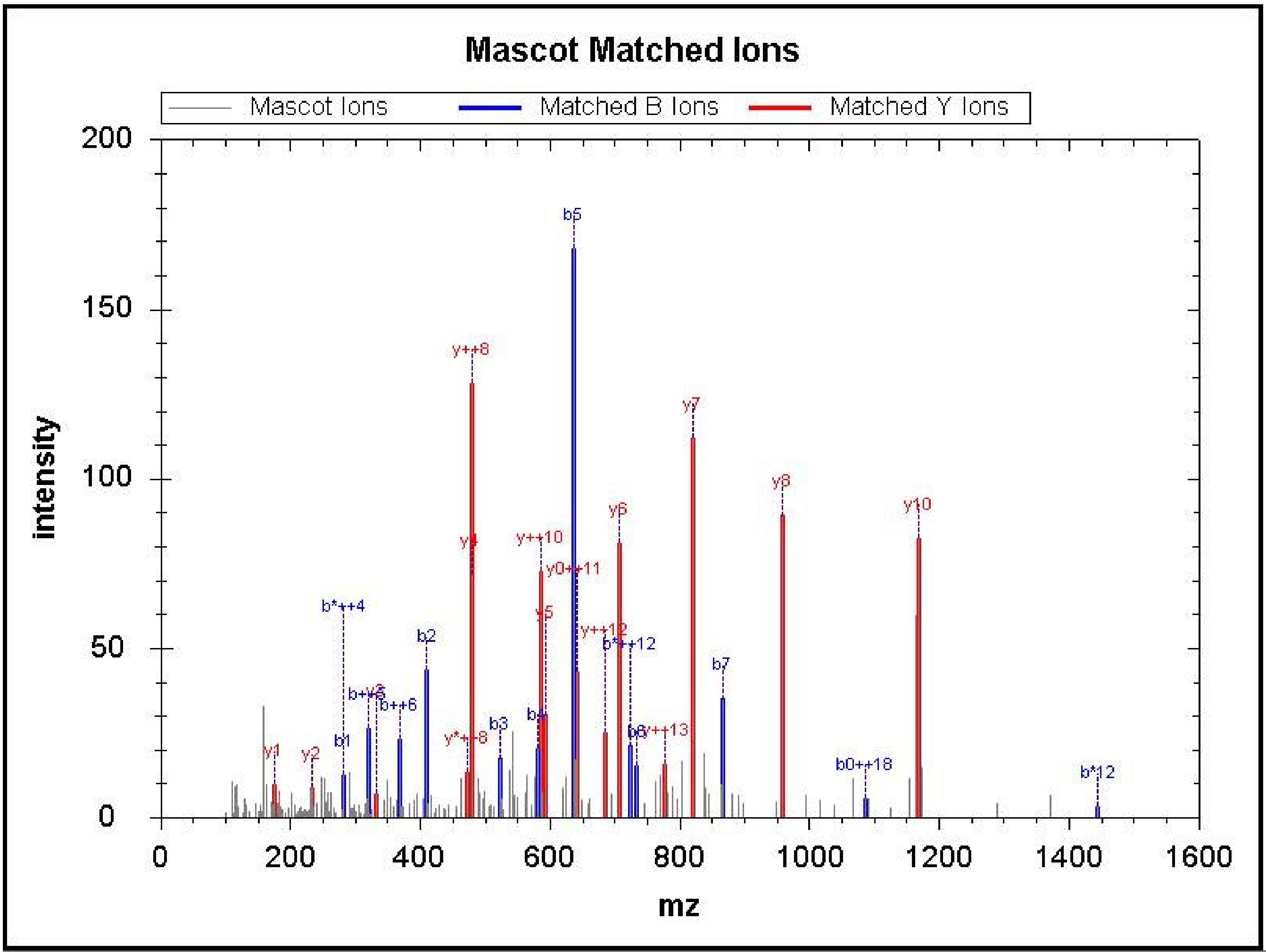

No	b	b++	b*	b*++	b0	b0++	Seq	y	y++	y*	y*++	y0	y0++	RevNo
1	282.17	141.59					H							20
2	410.23	205.62	393.20	197.10			Q	2,138.05	1,069.53	2,121.02	1,061.01	2,120.04	1,060.52	19
3	523.31	262.16	506.28	253.65			L	2,009.99	1,005.50	1,992.96	996.99	1,991.98	996.49	18
4	580.33	290.67	563.31	282.16			G	1,896.91	948.96	1,879.88	940.44	1,878.90	939.95	17
5	637.35	319.18	620.33	310.67			G	1,839.88	920.45	1,822.86	911.93	1,821.87	911.44	16
6	734.41	367.71	717.38	359.19			P	1,782.86	891.94	1,765.84	883.42	1,764.85	882.93	15
7	865.45	433.23	848.42	424.71			M	1,685.81	843.41	1,668.78	834.90	1,667.80	834.40	14
8	1,051.53	526.27	1,034.50	517.75			W	1,554.77	777.89	1,537.74	769.38	1,536.76	768.88	13
9	1,122.56	561.79	1,105.54	553.27			A	1,368.69	684.85	1,351.66	676.34	1,350.68	675.84	12
10	1,251.61	626.31	1,234.58	617.79	1,233.60	617.30	E	1,297.65	649.33	1,280.63	640.82	1,279.64	640.33	11
11	1,348.66	674.83	1,331.63	666.32	1,330.65	665.83	P	1,168.61	584.81	1,151.58	576.30	1,150.60	575.80	10
12	1,461.74	731.38	1,444.72	722.86	1,443.73	722.37	I	1,071.56	536.28	1,054.53	527.77	1,053.55	527.28	9
13	1,598.80	799.90	1,581.78	791.39	1,580.79	790.90	H	958.47	479.74	941.45	471.23	940.46	470.74	8
14	1,713.83	857.42	1,696.80	848.90	1,695.82	848.41	D	821.42	411.21	804.39	402.70	803.40	402.21	7
15	1,826.91	913.96	1,809.89	905.45	1,808.90	904.95	L	706.39	353.70	689.36	345.18	688.38	344.69	6
16	1,941.94	971.47	1,924.91	962.96	1,923.93	962.47	D	593.30	297.16	576.28	288.64	575.29	288.15	5
17	2,089.01	1,045.01	2,071.98	1,036.49	2,071.00	1,036.00	F	478.28	239.64	461.25	231.13			4
18	2,188.08	1,094.54	2,171.05	1,086.03	2,170.07	1,085.54	V	331.21	166.11	314.18	157.59			3
19	2,245.10	1,123.05	2,228.07	1,114.54	2,227.09	1,114.05	G	232.14	116.57	215.11	108.06			2

20							R	175.12	88.06	158.09	79.55			1

Query 21672 Hit 1

MS/MS Fragmentation of QVAQASHSYR
Found in sp|O43819|SCO2_HUMAN, Protein SCO2 homolog
Match to Query 21672: 1289.66from(430.8941,3+)
Title: 48: Scan 160 (rt=13.3691, f=3, i=20) [D:\lab212\membrane\Grace\Joyce\iTRAQ_42_2.raw]
Data File:Submitted from 20120508-1(merge) by Mascot Daemon on JOYCE-VAIO
Monoisotopic mass of neutral peptide Mr(calc): 1289.66
Variable modifications:
Ions Score: 58.24 Expect: 0.001

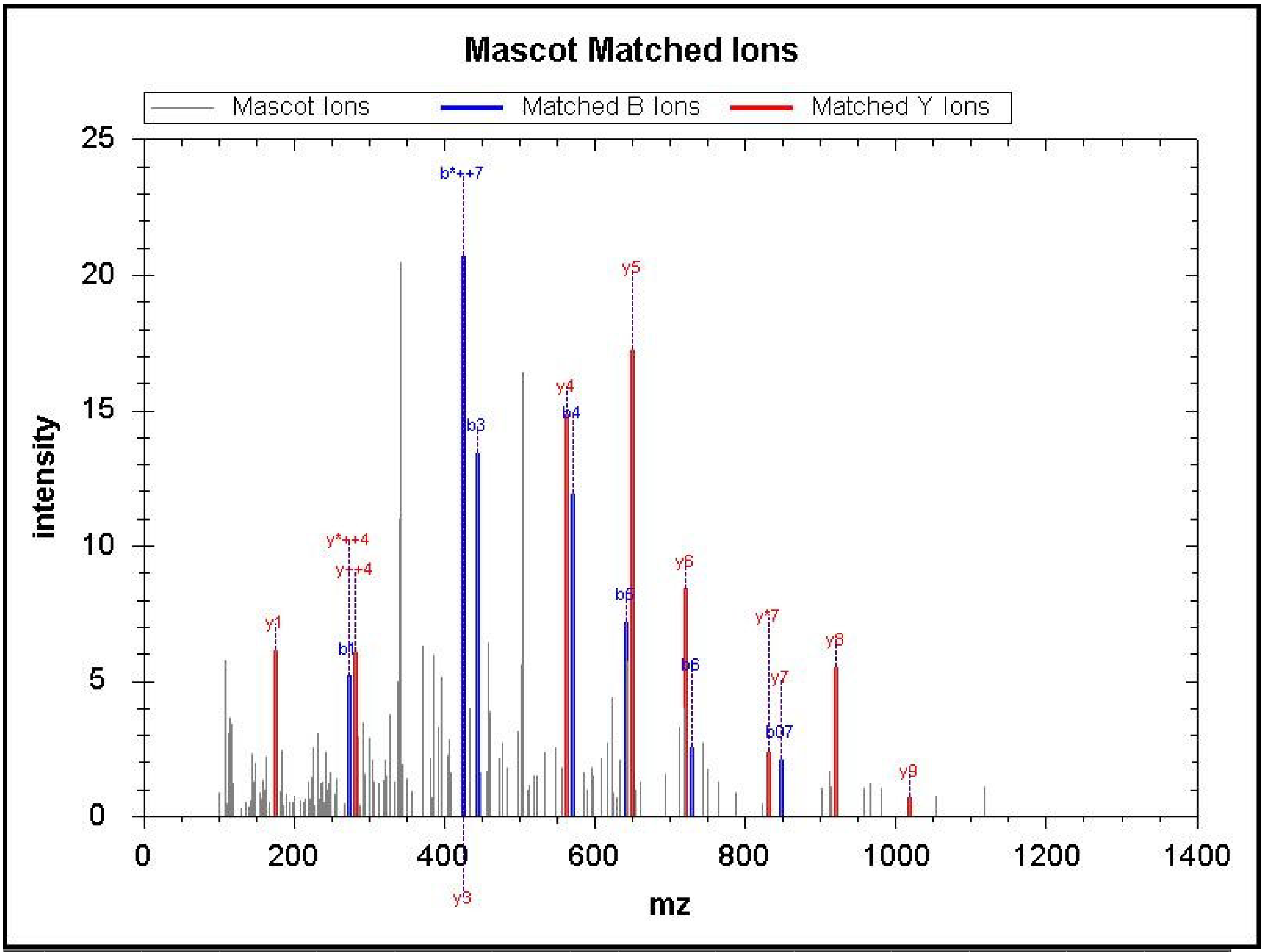

No	b	b++	b*	b*++	b0	b0++	Seq	y	y++	y*	y*++	y0	y0++	RevNo
1	273.17	137.09	256.14	128.57			Q							10
2	372.24	186.62	355.21	178.11			V	1,018.51	509.76	1,001.48	501.24	1,000.50	500.75	9
3	443.27	222.14	426.25	213.63			A	919.44	460.22	902.41	451.71	901.43	451.22	8
4	571.33	286.17	554.31	277.66			Q	848.40	424.70	831.37	416.19	830.39	415.70	7
5	642.37	321.69	625.34	313.17			A	720.34	360.67	703.32	352.16	702.33	351.67	6
6	729.40	365.20	712.37	356.69	711.39	356.20	S	649.31	325.16	632.28	316.64	631.29	316.15	5
7	866.46	433.73	849.43	425.22	848.45	424.73	H	562.27	281.64	545.25	273.13	544.26	272.63	4
8	953.49	477.25	936.47	468.74	935.48	468.24	S	425.21	213.11	408.19	204.60	407.20	204.11	3
9	1,116.56	558.78	1,099.53	550.27	1,098.54	549.78	Y	338.18	169.59	321.16	161.08			2
10							R	175.12	88.06	158.09	79.55			1

Query 71888 Hit 1

MS/MS Fragmentation of ILEAHQNVAQLSLTEAQLR
Found in sp|Q8K1B8|URP2_MOUSE, Fermitin family homolog 3 OS=Mus musculus GN=Fermt3 PE=1 SV=1

Match to Query 71888: 2277.256from(760.0926,3+)
Title: 617: Scan 1423 (rt=41.43, f=2, i=219) [D:\lab212\membrane\Grace\Joyce\iTRAQ_33_1.raw]
Data File:Submitted from 20120508-1(merge) by Mascot Daemon on JOYCE-VAIO
Monoisotopic mass of neutral peptide Mr(calc): 2277.256
Variable modifications:
Ions Score: 58.12 Expect: 0.001

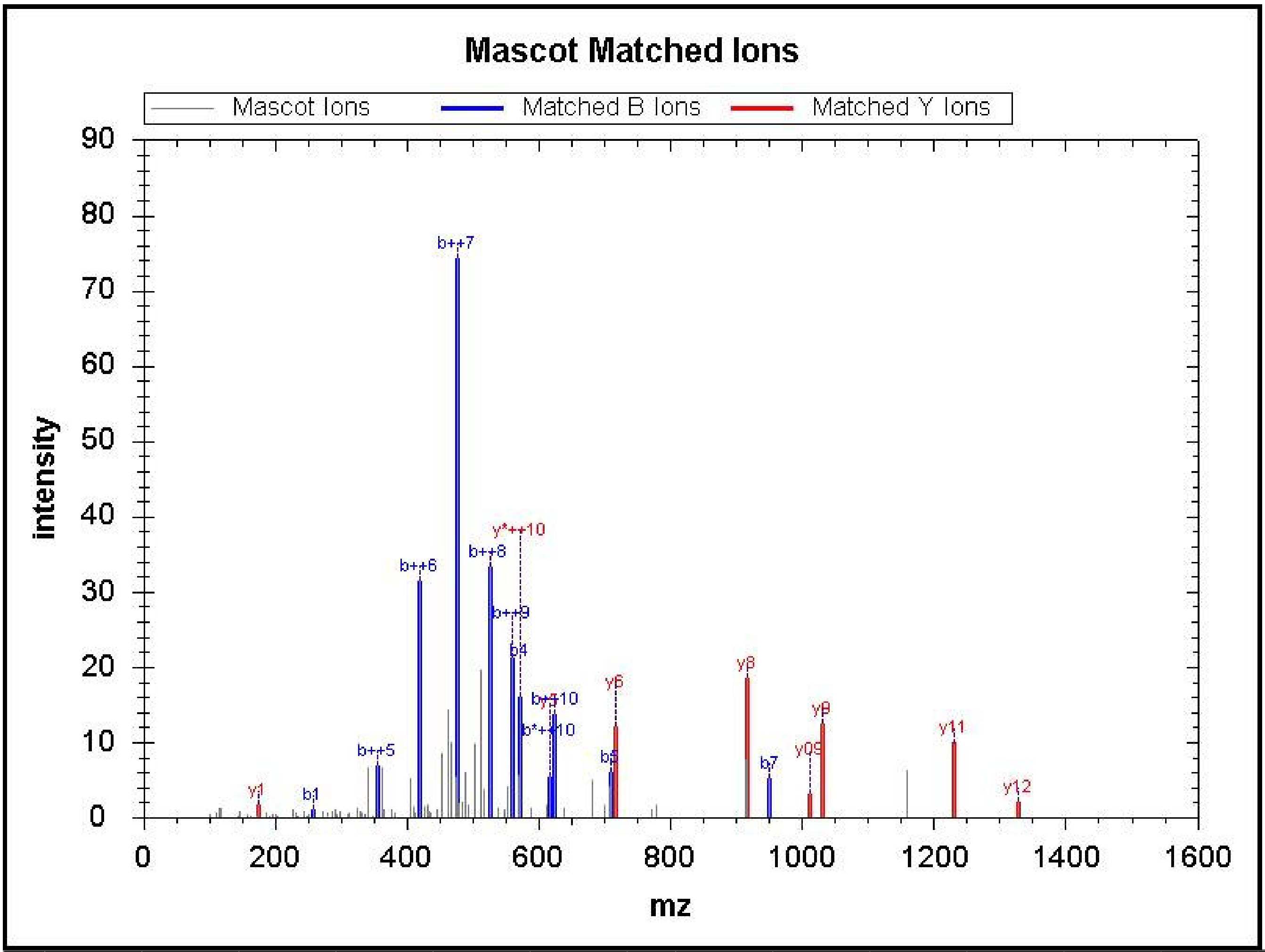

No	b	b++	b*	b*++	b0	b0++	Seq	y	y++	y*	y*++	y0	y0++	RevNo
1	258.19	129.60					I							19
2	371.28	186.14					L	2,021.08	1,011.04	2,004.05	1,002.53	2,003.07	1,002.04	18
3	500.32	250.66			482.31	241.66	E	1,907.99	954.50	1,890.97	945.99	1,889.98	945.50	17
4	571.36	286.18			553.35	277.18	A	1,778.95	889.98	1,761.92	881.47	1,760.94	880.97	16
5	708.42	354.71			690.41	345.71	H	1,707.91	854.46	1,690.89	845.95	1,689.90	845.46	15
6	836.47	418.74	819.45	410.23	818.46	409.74	Q	1,570.85	785.93	1,553.83	777.42	1,552.84	776.93	14
7	950.52	475.76	933.49	467.25	932.51	466.76	N	1,442.80	721.90	1,425.77	713.39	1,424.79	712.90	13
8	1,049.59	525.30	1,032.56	516.78	1,031.58	516.29	V	1,328.75	664.88	1,311.73	656.37	1,310.74	655.87	12
9	1,120.62	560.82	1,103.60	552.30	1,102.61	551.81	A	1,229.68	615.35	1,212.66	606.83	1,211.67	606.34	11
10	1,248.68	624.84	1,231.66	616.33	1,230.67	615.84	Q	1,158.65	579.83	1,141.62	571.31	1,140.64	570.82	10
11	1,361.77	681.39	1,344.74	672.87	1,343.76	672.38	L	1,030.59	515.80	1,013.56	507.28	1,012.58	506.79	9
12	1,448.80	724.90	1,431.77	716.39	1,430.79	715.90	S	917.51	459.26	900.48	450.74	899.49	450.25	8
13	1,561.88	781.44	1,544.86	772.93	1,543.87	772.44	L	830.47	415.74	813.45	407.23	812.46	406.73	7
14	1,662.93	831.97	1,645.90	823.46	1,644.92	822.96	T	717.39	359.20	700.36	350.68	699.38	350.19	6
15	1,791.97	896.49	1,774.95	887.98	1,773.96	887.48	E	616.34	308.67	599.31	300.16	598.33	299.67	5
16	1,863.01	932.01	1,845.98	923.50	1,845.00	923.00	A	487.30	244.15	470.27	235.64			4
17	1,991.07	996.04	1,974.04	987.52	1,973.06	987.03	Q	416.26	208.63	399.24	200.12			3
18	2,104.15	1,052.58	2,087.13	1,044.07	2,086.14	1,043.57	L	288.20	144.61	271.18	136.09			2

19							R	175.12	88.06	158.09	79.55			1

Query 76034 Hit 1

MS/MS Fragmentation of LSLVDVSQVSVVHTLQTDFR
Found in sp|Q8WW59|SPRY4_HUMAN, SPRY domain-containing protein 4 OS=Homo sapiens GN=SPRYD4 PE=1 SV=2
Match to Query 76034: 2386.287from(796.4363,3+)
Title: 958: Scan 2128 (rt=57.3078, f=3, i=327) [D:\lab212\membrane\Grace\Joyce\iTRAQ_31_1.raw]
Data File:Submitted from 20120508-1(merge) by Mascot Daemon on JOYCE-VAIO
Monoisotopic mass of neutral peptide Mr(calc): 2386.287
Variable modifications:
Ions Score: 58.03 Expect: 0.001

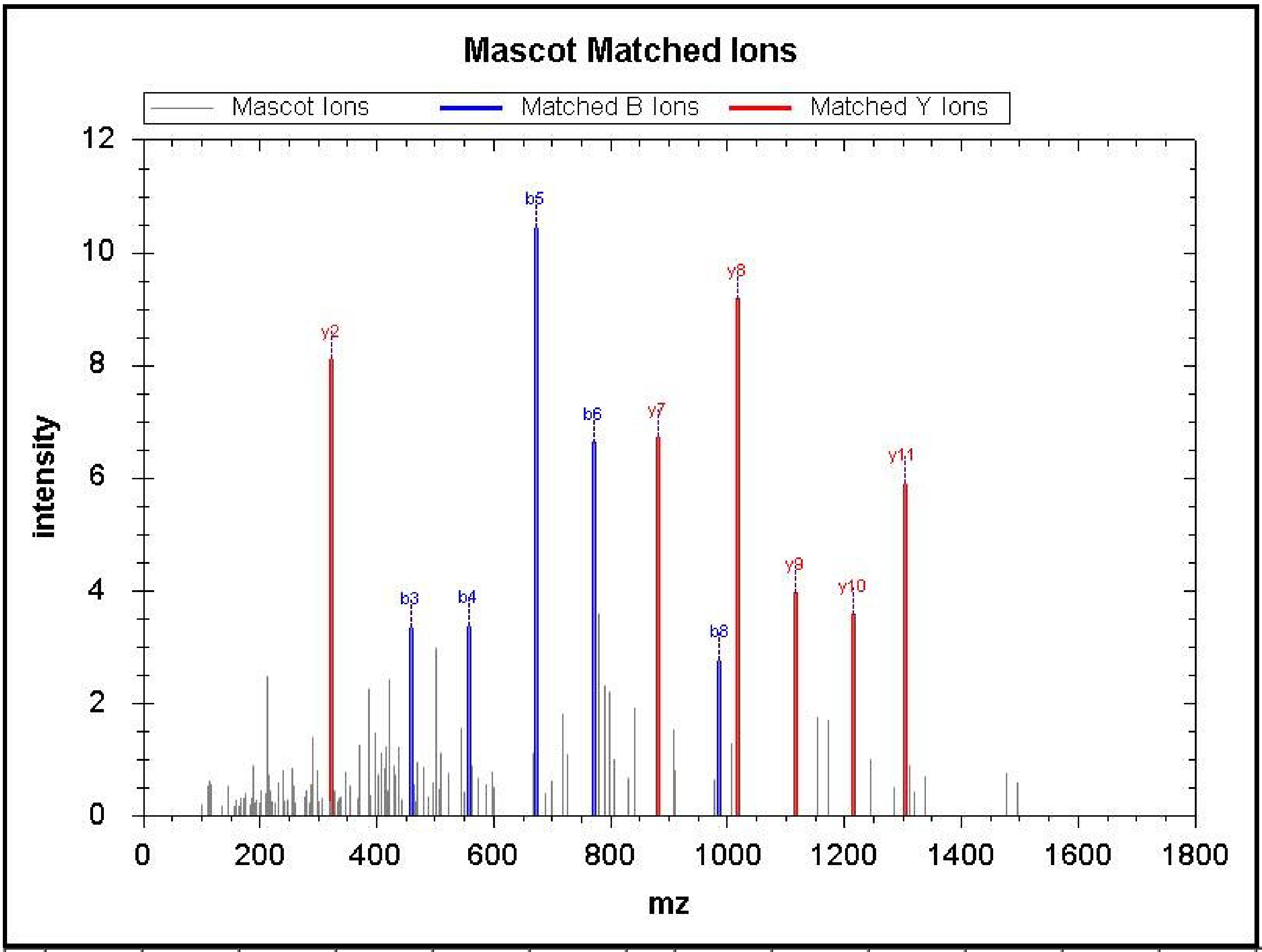

No	b	b++	b*	b*++	b0	b0++	Seq	y	y++	y*	y*++	y0	y0++	RevNo
1	258.19	129.60					L							20
2	345.23	173.12			327.21	164.11	S	2,130.12	1,065.56	2,113.09	1,057.05	2,112.11	1,056.56	19
3	458.31	229.66			440.30	220.65	L	2,043.09	1,022.05	2,026.06	1,013.53	2,025.08	1,013.04	18
4	557.38	279.19			539.37	270.19	V	1,930.00	965.50	1,912.98	956.99	1,911.99	956.50	17
5	672.40	336.71			654.39	327.70	D	1,830.93	915.97	1,813.91	907.46	1,812.92	906.97	16
6	771.47	386.24			753.46	377.23	V	1,715.91	858.46	1,698.88	849.94	1,697.90	849.45	15
7	858.51	429.76			840.49	420.75	S	1,616.84	808.92	1,599.81	800.41	1,598.83	799.92	14
8	986.56	493.79	969.54	485.27	968.55	484.78	Q	1,529.81	765.41	1,512.78	756.89	1,511.80	756.40	13
9	1,085.63	543.32	1,068.61	534.81	1,067.62	534.31	V	1,401.75	701.38	1,384.72	692.86	1,383.74	692.37	12
10	1,172.66	586.84	1,155.64	578.32	1,154.65	577.83	S	1,302.68	651.84	1,285.65	643.33	1,284.67	642.84	11
11	1,271.73	636.37	1,254.71	627.86	1,253.72	627.36	V	1,215.65	608.33	1,198.62	599.81	1,197.64	599.32	10
12	1,370.80	685.90	1,353.77	677.39	1,352.79	676.90	V	1,116.58	558.79	1,099.55	550.28	1,098.57	549.79	9
13	1,507.86	754.43	1,490.83	745.92	1,489.85	745.43	H	1,017.51	509.26	1,000.48	500.75	999.50	500.25	8
14	1,608.91	804.96	1,591.88	796.44	1,590.90	795.95	T	880.45	440.73	863.43	432.22	862.44	431.72	7

15	1,721.99	861.50	1,704.97	852.99	1,703.98	852.49	L	779.40	390.21	762.38	381.69	761.39	381.20	6
16	1,850.05	925.53	1,833.02	917.02	1,832.04	916.52	Q	666.32	333.66	649.29	325.15	648.31	324.66	5
17	1,951.10	976.05	1,934.07	967.54	1,933.09	967.05	T	538.26	269.63	521.24	261.12	520.25	260.63	4
18	2,066.13	1,033.57	2,049.10	1,025.05	2,048.11	1,024.56	D	437.21	219.11	420.19	210.60	419.20	210.11	3
19	2,213.19	1,107.10	2,196.17	1,098.59	2,195.18	1,098.10	F	322.19	161.60	305.16	153.08			2
20							R	175.12	88.06	158.09	79.55			1

Query 17889 Hit 1

MS/MS Fragmentation of AFLLSLAALR
Found in sp|Q8IV08|PLD3_HUMAN, Phospholipase D3 OS=Homo sapiens GN=PLD3 PE=1 SV=1
Match to Query 17889: 1217.763from(609.8889,2+)
Title: 983: Scan 2251 (rt=59.7623, f=3, i=339) [D:\lab212\membrane\Grace\Joyce\iTRAQ_23_1.raw]
Data File:Submitted from 20120508-1(merge) by Mascot Daemon on JOYCE-VAIO
Monoisotopic mass of neutral peptide Mr(calc): 1217.763
Variable modifications:
Ions Score: 57.94 Expect: 0.001

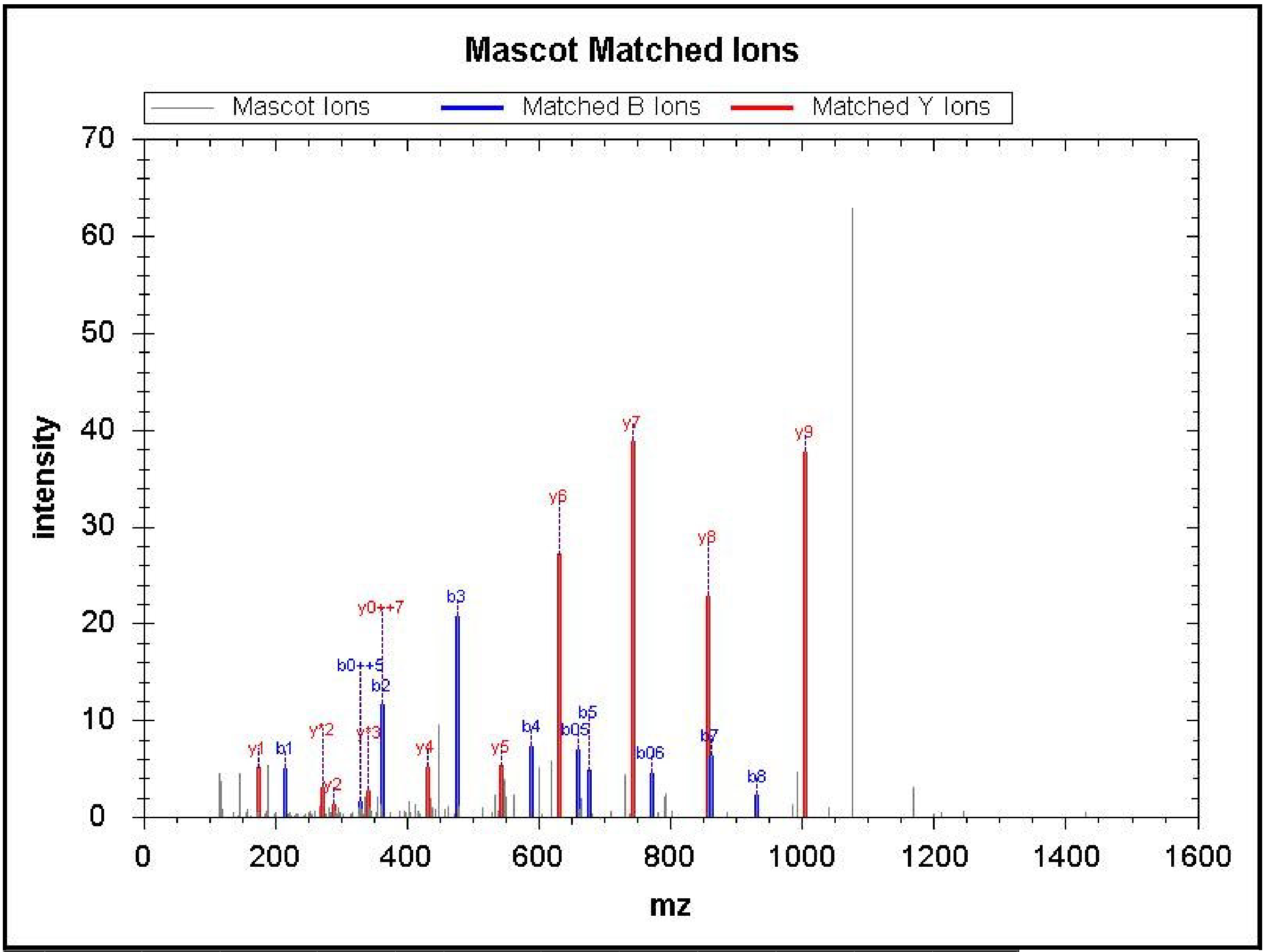

No	b	b++	b0	b0++	Seq	y	y++	y*	y*++	y0	y0++	RevNo
1	216.15	108.58			A							10
2	363.21	182.11			F	1,003.63	502.32	986.60	493.81	985.62	493.31	9
3	476.30	238.65			L	856.56	428.78	839.53	420.27	838.55	419.78	8
4	589.38	295.20			L	743.48	372.24	726.45	363.73	725.47	363.24	7
5	676.42	338.71	658.40	329.71	S	630.39	315.70	613.37	307.19	612.38	306.70	6
6	789.50	395.25	771.49	386.25	L	543.36	272.18	526.33	263.67			5
7	860.54	430.77	842.53	421.77	A	430.28	215.64	413.25	207.13			4
8	931.57	466.29	913.56	457.29	A	359.24	180.12	342.21	171.61			3
9	1,044.66	522.83	1,026.65	513.83	L	288.20	144.61	271.18	136.09			2

10					R	175.12	88.06	158.09	79.55			1

Query 83695 Hit 1

MS/MS Fragmentation of DKEWQEQFLIPNLALIDK
Found in sp|Q9UFN0|NPS3A_HUMAN, Protein NipSnap homolog 3A OS=Homo sapiens GN=NIPSNAP3A PE=1 SV=2
Match to Query 83695: 2631.465from(878.1622,3+)
Title: 997: Sum of 2 scans in range 2198 (rt=59.0853, f=4, i=670) to 2199 (rt=59.1107, f=4, i=671)
[D:\lab212\membrane\Grace\Joyce\iTRAQ_42_1.raw]
Data File:Submitted from 20120508-1(merge) by Mascot Daemon on JOYCE-VAIO
Monoisotopic mass of neutral peptide Mr(calc): 2631.465
Variable modifications:
K2 :iTRAQ4plex (K)
K18 :iTRAQ4plex (K)
Ions Score: 57.94 Expect: 0.001

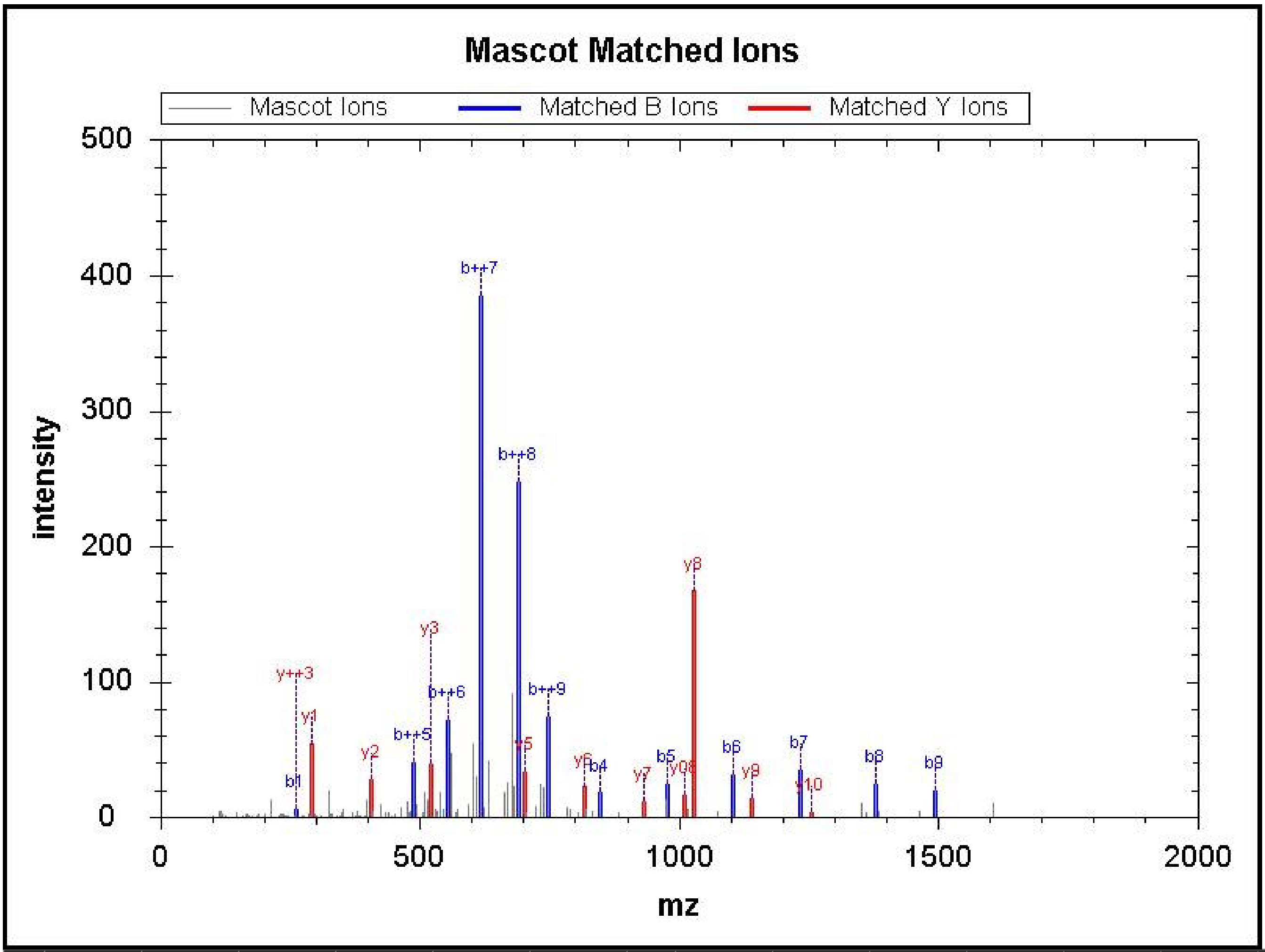

No	b	b++	b*	b*++	b0	b0++	Seq	y	y++	y*	y*++	y0	y0++	RevNo
1	260.14	130.57			242.13	121.57	D							18
2	532.33	266.67	515.31	258.16	514.32	257.67	K	2,373.34	1,187.17	2,356.32	1,178.66	2,355.33	1,178.17	17
3	661.38	331.19	644.35	322.68	643.37	322.19	E	2,101.15	1,051.08	2,084.12	1,042.56	2,083.13	1,042.07	16
4	847.46	424.23	830.43	415.72	829.44	415.23	W	1,972.10	986.55	1,955.08	978.04	1,954.09	977.55	15
5	975.51	488.26	958.49	479.75	957.50	479.26	Q	1,786.02	893.52	1,769.00	885.00	1,768.01	884.51	14
6	1,104.56	552.78	1,087.53	544.27	1,086.55	543.78	E	1,657.96	829.49	1,640.94	820.97	1,639.95	820.48	13
7	1,232.62	616.81	1,215.59	608.30	1,214.60	607.81	Q	1,528.92	764.96	1,511.90	756.45	1,510.91	755.96	12
8	1,379.68	690.35	1,362.66	681.83	1,361.67	681.34	F	1,400.86	700.94	1,383.84	692.42	1,382.85	691.93	11
9	1,492.77	746.89	1,475.74	738.37	1,474.76	737.88	L	1,253.79	627.40	1,236.77	618.89	1,235.78	618.40	10
10	1,605.85	803.43	1,588.83	794.92	1,587.84	794.42	I	1,140.71	570.86	1,123.68	562.35	1,122.70	561.85	9
11	1,702.90	851.96	1,685.88	843.44	1,684.89	842.95	P	1,027.63	514.32	1,010.60	505.80	1,009.62	505.31	8

12	1,816.95	908.98	1,799.92	900.46	1,798.94	899.97	N	930.57	465.79	913.55	457.28	912.56	456.79	7
13	1,930.03	965.52	1,913.01	957.01	1,912.02	956.51	L	816.53	408.77	799.50	400.26	798.52	399.76	6
14	2,001.07	1,001.04	1,984.04	992.52	1,983.06	992.03	A	703.45	352.23	686.42	343.71	685.44	343.22	5
15	2,114.15	1,057.58	2,097.13	1,049.07	2,096.14	1,048.57	L	632.41	316.71	615.38	308.20	614.40	307.70	4
16	2,227.24	1,114.12	2,210.21	1,105.61	2,209.23	1,105.12	I	519.33	260.17	502.30	251.65	501.32	251.16	3
17	2,342.26	1,171.64	2,325.24	1,163.12	2,324.25	1,162.63	D	406.24	203.62	389.22	195.11	388.23	194.62	2
18							K	291.21	146.11	274.19	137.60			1

Query 27637 Hit 1

MS/MS Fragmentation of LWNLLMPTK
Found in sp|Q8IZ81|ELMD2_HUMAN, ELMO domain-containing protein 2 OS=Homo sapiens GN=ELMOD2 PE=1 SV=1
Match to Query 27637: 1402.823from(702.4189,2+)
Title: 931: Scan 2140 (rt=57.156, f=3, i=312) [D:\lab212\membrane\Grace\Joyce\iTRAQ_29_1.raw]
Data File:Submitted from 20120508-1(merge) by Mascot Daemon on JOYCE-VAIO
Monoisotopic mass of neutral peptide Mr(calc): 1402.823
Variable modifications:
K9 :iTRAQ4plex (K)
Ions Score: 57.77 Expect: 0.001

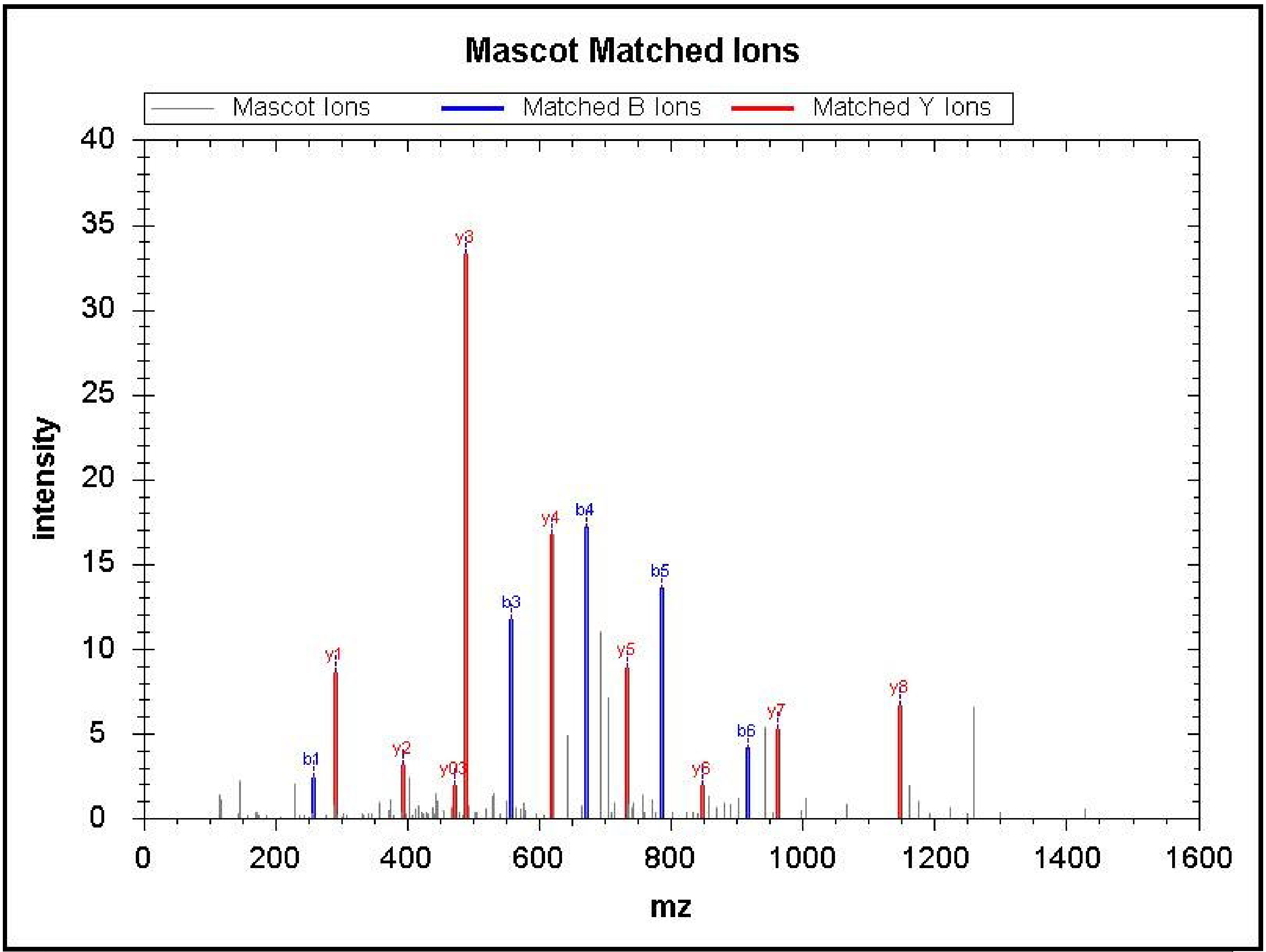

No	b	b++	b*	b*++	b0	b0++	Seq	y	y++	y*	y*++	y0	y0++	RevNo
1	258.19	129.60					L							9
2	444.27	222.64					W	1,146.65	573.83	1,129.62	565.31	1,128.64	564.82	8
3	558.32	279.66	541.29	271.15			N	960.57	480.79	943.54	472.27	942.56	471.78	7
4	671.40	336.20	654.37	327.69			L	846.52	423.77	829.50	415.25	828.51	414.76	6
5	784.48	392.75	767.46	384.23			L	733.44	367.22	716.41	358.71	715.43	358.22	5
6	915.52	458.27	898.50	449.75			M	620.36	310.68	603.33	302.17	602.35	301.68	4
7	1,012.58	506.79	995.55	498.28			P	489.32	245.16	472.29	236.65	471.30	236.16	3

8	1,113.62	557.32	1,096.60	548.80	1,095.61	548.31	T	392.26	196.63	375.24	188.12	374.25	187.63	2
9							K	291.21	146.11	274.19	137.60			1

Query 71343 Hit 1

MS/MS Fragmentation of GANIQLLDLPGIIEGAAQGK
Found in sp|P55039|DRG2_HUMAN, Developmentally-regulated GTP-binding protein 2 OS=Homo sapiens GN=DRG2 PE=1 SV=1
Match to Query 71343: 2265.292from(756.1047,3+)
Title: 1107: Sum of 2 scans in range 2431 (rt=64.1646, f=4, i=731) to 2432 (rt=64.19, f=4, i=732)
[D:\lab212\membrane\Grace\Joyce\iTRAQ_26_2.raw]
Data File:Submitted from 20120508-1(merge) by Mascot Daemon on JOYCE-VAIO
Monoisotopic mass of neutral peptide Mr(calc): 2265.292
Variable modifications:
K20 :iTRAQ4plex (K)
Ions Score: 57.6 Expect: 0.001

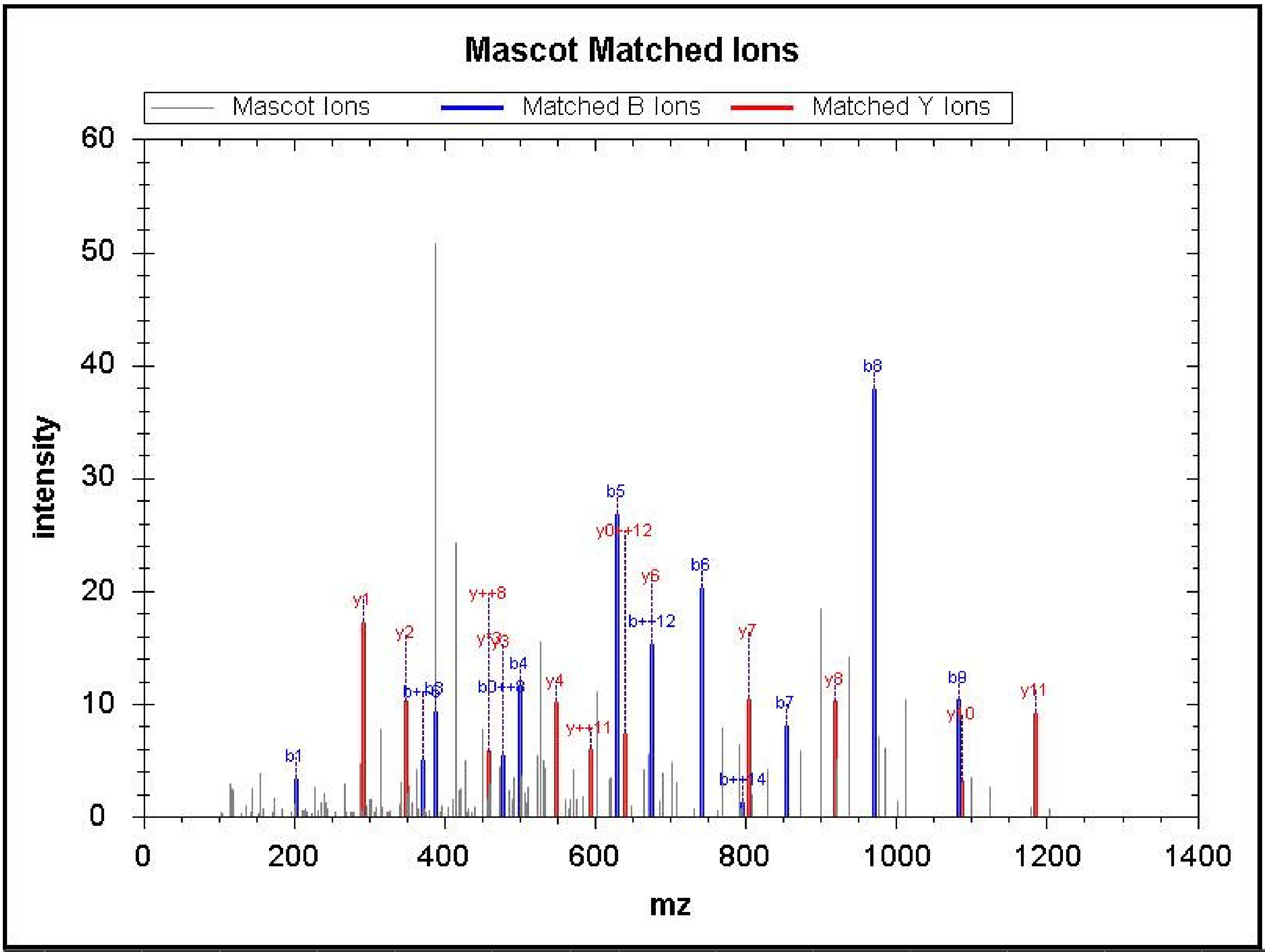

No	b	b++	b*	b*++	b0	b0++	Seq	y	y++	y*	y*++	y0	y0++	RevNo
1	202.13	101.57					G							20
2	273.17	137.09					A	2,065.18	1,033.09	2,048.15	1,024.58	2,047.17	1,024.09	19
3	387.21	194.11	370.18	185.60			N	1,994.14	997.57	1,977.11	989.06	1,976.13	988.57	18
4	500.29	250.65	483.27	242.14			I	1,880.10	940.55	1,863.07	932.04	1,862.09	931.55	17
5	628.35	314.68	611.33	306.17			Q	1,767.01	884.01	1,749.99	875.50	1,749.00	875.01	16
6	741.44	371.22	724.41	362.71			L	1,638.95	819.98	1,621.93	811.47	1,620.94	810.98	15
7	854.52	427.76	837.50	419.25			L	1,525.87	763.44	1,508.84	754.93	1,507.86	754.43	14
8	969.55	485.28	952.52	476.76	951.54	476.27	D	1,412.79	706.90	1,395.76	698.38	1,394.78	697.89	13
9	1,082.63	541.82	1,065.61	533.31	1,064.62	532.81	L	1,297.76	649.38	1,280.73	640.87	1,279.75	640.38	12
10	1,179.69	590.35	1,162.66	581.83	1,161.67	581.34	P	1,184.68	592.84	1,167.65	584.33	1,166.66	583.84	11
11	1,236.71	618.86	1,219.68	610.34	1,218.70	609.85	G	1,087.62	544.32	1,070.60	535.80	1,069.61	535.31	10

12	1,349.79	675.40	1,332.76	666.89	1,331.78	666.39	I	1,030.60	515.80	1,013.57	507.29	1,012.59	506.80	9
13	1,462.88	731.94	1,445.85	723.43	1,444.86	722.94	I	917.52	459.26	900.49	450.75	899.51	450.26	8
14	1,591.92	796.46	1,574.89	787.95	1,573.91	787.46	E	804.43	402.72	787.41	394.21	786.42	393.71	7
15	1,648.94	824.97	1,631.91	816.46	1,630.93	815.97	G	675.39	338.20	658.36	329.69			6
16	1,719.98	860.49	1,702.95	851.98	1,701.97	851.49	A	618.37	309.69	601.34	301.17			5
17	1,791.01	896.01	1,773.99	887.50	1,773.00	887.01	A	547.33	274.17	530.31	265.66			4
18	1,919.07	960.04	1,902.05	951.53	1,901.06	951.03	Q	476.29	238.65	459.27	230.14			3
19	1,976.09	988.55	1,959.07	980.04	1,958.08	979.55	G	348.24	174.62	331.21	166.11			2
20							K	291.21	146.11	274.19	137.60			1

Query 87223 Hit 1

MS/MS Fragmentation of VTNVGSLLLTPQENESLFTFLGK
Found in sp|O00401|WASL_HUMAN, Neural Wiskott-Aldrich syndrome protein OS=Homo sapiens GN=WASL PE=1 SV=2
Match to Query 87223: 2794.528from(932.5166,3+)
Title: 1149: Scan 2641 (rt=68.302, f=2, i=418) [D:\lab212\membrane\Grace\Joyce\iTRAQ_24_1.raw]
Data File:Submitted from 20120508-1(merge) by Mascot Daemon on JOYCE-VAIO
Monoisotopic mass of neutral peptide Mr(calc): 2794.528
Variable modifications:
K23 :iTRAQ4plex (K)
Ions Score: 57.44 Expect: 0.001

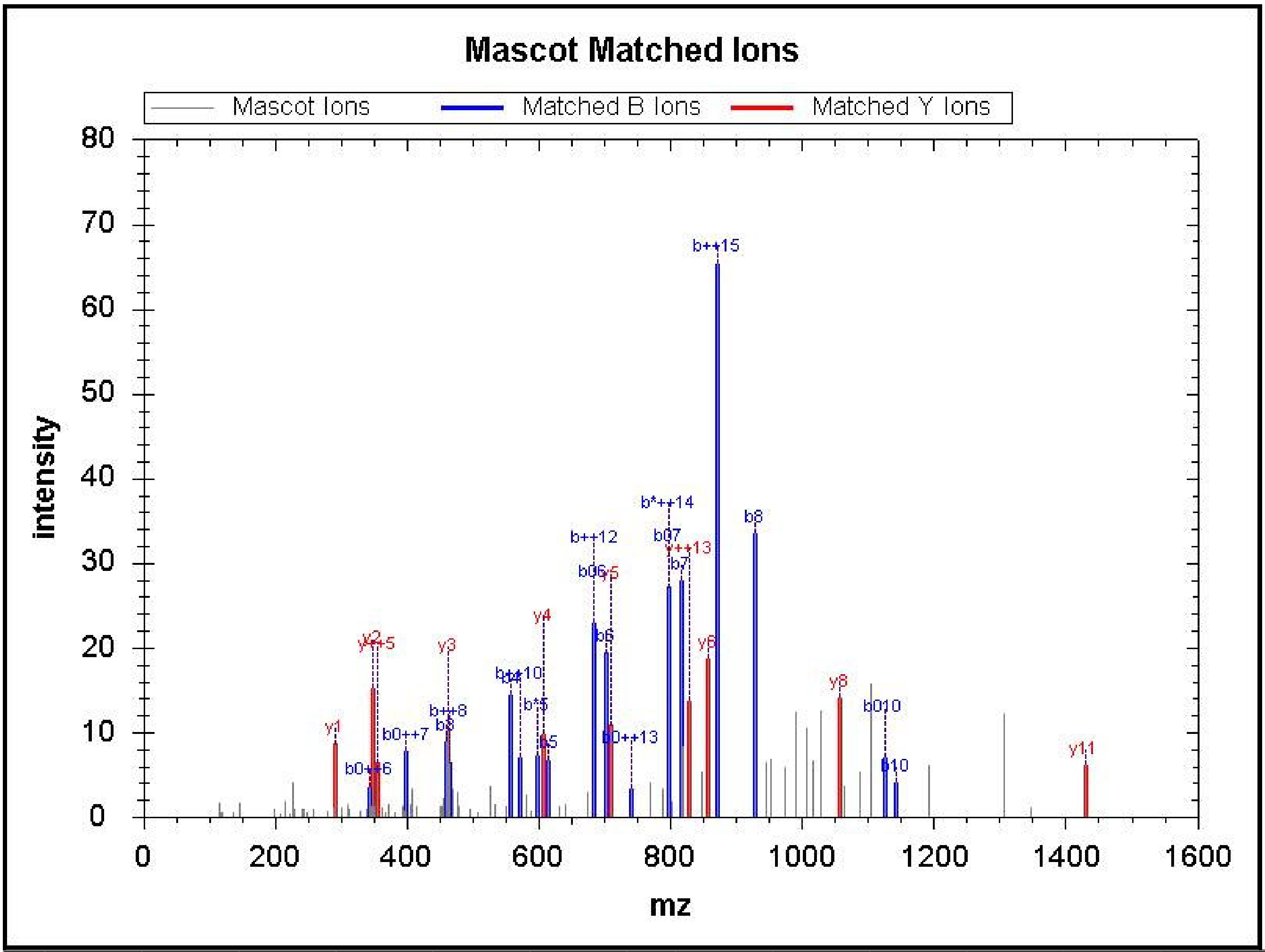

No	b	b++	b*	b*++	b0	b0++	Seq	y	y++	y*	y*++	y0	y0++	RevNo
1	244.18	122.59					V							23
2	345.23	173.12			327.21	164.11	T	2,552.37	1,276.69	2,535.35	1,268.18	2,534.36	1,267.68	22
3	459.27	230.14	442.24	221.62	441.26	221.13	N	2,451.33	1,226.17	2,434.30	1,217.65	2,433.31	1,217.16	21
4	558.34	279.67	541.31	271.16	540.33	270.67	V	2,337.28	1,169.14	2,320.26	1,160.63	2,319.27	1,160.14	20
5	615.36	308.18	598.33	299.67	597.35	299.18	G	2,238.21	1,119.61	2,221.19	1,111.10	2,220.20	1,110.61	19

6	702.39	351.70	685.36	343.19	684.38	342.69	S	2,181.19	1,091.10	2,164.17	1,082.59	2,163.18	1,082.09	18
7	815.47	408.24	798.45	399.73	797.46	399.24	L	2,094.16	1,047.58	2,077.13	1,039.07	2,076.15	1,038.58	17
8	928.56	464.78	911.53	456.27	910.55	455.78	L	1,981.08	991.04	1,964.05	982.53	1,963.07	982.04	16
9	1,041.64	521.32	1,024.62	512.81	1,023.63	512.32	L	1,867.99	934.50	1,850.97	925.99	1,849.98	925.49	15
10	1,142.69	571.85	1,125.66	563.34	1,124.68	562.84	T	1,754.91	877.96	1,737.88	869.44	1,736.90	868.95	14
11	1,239.74	620.38	1,222.72	611.86	1,221.73	611.37	P	1,653.86	827.43	1,636.83	818.92	1,635.85	818.43	13
12	1,367.80	684.40	1,350.78	675.89	1,349.79	675.40	Q	1,556.81	778.91	1,539.78	770.39	1,538.80	769.90	12
13	1,496.84	748.93	1,479.82	740.41	1,478.83	739.92	E	1,428.75	714.88	1,411.72	706.36	1,410.74	705.87	11
14	1,610.89	805.95	1,593.86	797.43	1,592.88	796.94	N	1,299.71	650.36	1,282.68	641.84	1,281.70	641.35	10
15	1,739.93	870.47	1,722.90	861.96	1,721.92	861.46	E	1,185.66	593.34	1,168.64	584.82	1,167.65	584.33	9
16	1,826.96	913.98	1,809.94	905.47	1,808.95	904.98	S	1,056.62	528.81	1,039.59	520.30	1,038.61	519.81	8
17	1,940.05	970.53	1,923.02	962.01	1,922.04	961.52	L	969.59	485.30	952.56	476.78	951.58	476.29	7
18	2,087.11	1,044.06	2,070.09	1,035.55	2,069.10	1,035.06	F	856.50	428.76	839.48	420.24	838.49	419.75	6
19	2,188.16	1,094.58	2,171.14	1,086.07	2,170.15	1,085.58	T	709.44	355.22	692.41	346.71	691.43	346.22	5
20	2,335.23	1,168.12	2,318.20	1,159.61	2,317.22	1,159.11	F	608.39	304.70	591.36	296.18			4
21	2,448.31	1,224.66	2,431.29	1,216.15	2,430.30	1,215.66	L	461.32	231.16	444.29	222.65			3
22	2,505.34	1,253.17	2,488.31	1,244.66	2,487.33	1,244.17	G	348.24	174.62	331.21	166.11			2
23							K	291.21	146.11	274.19	137.60			1

Query 73337 Hit 1

MS/MS Fragmentation of SIVQELQQWAHEFADVK
Found in sp|E9Q555|RN213_MOUSE, E3 ubiquitin-protein ligase RNF213 OS=Mus musculus GN=Rnf213 PE=2 SV=1
Match to Query 73337: 2315.219from(772.747,3+)
Title: 1246: Scan 2738 (rt=71.1743, f=3, i=425) [D:\lab212\membrane\Grace\Joyce\iTRAQ_39_1.raw]
Data File:Submitted from 20120508-1(merge) by Mascot Daemon on JOYCE-VAIO
Monoisotopic mass of neutral peptide Mr(calc): 2315.219
Variable modifications:
K17 :iTRAQ4plex (K)
Ions Score: 57.35 Expect: 0.002

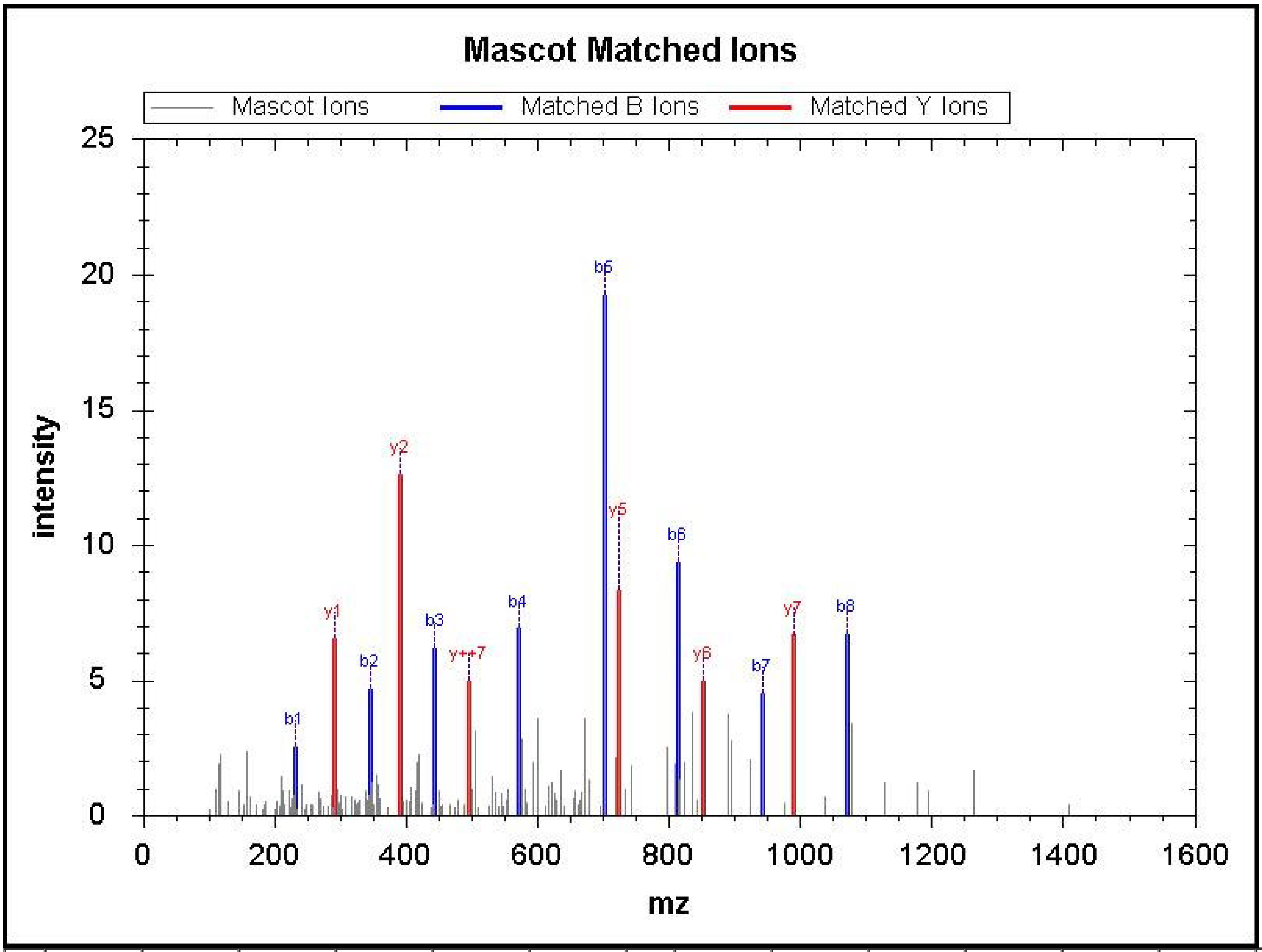

No	b	b++	b*	b*++	b0	b0++	Seq	y	y++	y*	y*++	y0	y0++	RevNo
1	232.14	116.57			214.13	107.57	S							17
2	345.23	173.12			327.21	164.11	I	2,085.09	1,043.05	2,068.06	1,034.53	2,067.08	1,034.04	16
3	444.29	222.65			426.28	213.65	V	1,972.00	986.51	1,954.98	977.99	1,953.99	977.50	15
4	572.35	286.68	555.33	278.17	554.34	277.67	Q	1,872.94	936.97	1,855.91	928.46	1,854.93	927.97	14
5	701.40	351.20	684.37	342.69	683.38	342.20	E	1,744.88	872.94	1,727.85	864.43	1,726.87	863.94	13
6	814.48	407.74	797.45	399.23	796.47	398.74	L	1,615.84	808.42	1,598.81	799.91	1,597.82	799.42	12
7	942.54	471.77	925.51	463.26	924.53	462.77	Q	1,502.75	751.88	1,485.72	743.37	1,484.74	742.87	11
8	1,070.60	535.80	1,053.57	527.29	1,052.59	526.80	Q	1,374.69	687.85	1,357.67	679.34	1,356.68	678.84	10
9	1,256.68	628.84	1,239.65	620.33	1,238.67	619.84	W	1,246.63	623.82	1,229.61	615.31	1,228.62	614.82	9
10	1,327.71	664.36	1,310.69	655.85	1,309.70	655.35	A	1,060.55	530.78	1,043.53	522.27	1,042.54	521.78	8
11	1,464.77	732.89	1,447.75	724.38	1,446.76	723.88	H	989.52	495.26	972.49	486.75	971.51	486.26	7
12	1,593.81	797.41	1,576.79	788.90	1,575.80	788.41	E	852.46	426.73	835.43	418.22	834.45	417.73	6
13	1,740.88	870.95	1,723.86	862.43	1,722.87	861.94	F	723.42	362.21	706.39	353.70	705.41	353.21	5
14	1,811.92	906.46	1,794.89	897.95	1,793.91	897.46	A	576.35	288.68	559.32	280.16	558.34	279.67	4
15	1,926.95	963.98	1,909.92	955.46	1,908.94	954.97	D	505.31	253.16	488.28	244.65	487.30	244.15	3
16	2,026.02	1,013.51	2,008.99	1,005.00	2,008.00	1,004.51	V	390.28	195.65	373.26	187.13			2
17							K	291.21	146.11	274.19	137.60			1

Query 83357 Hit 1

MS/MS Fragmentation of RLEGDTALQGVSHIIVDEVHER
Found in sp|Q6P158|DHX57_HUMAN, Putative ATP-dependent RNA helicase DHX57 OS=Homo sapiens GN=DHX57 PE=1 SV=2
Match to Query 83357: 2616.379from(524.2831,5+)
Title: 741: Scan 1620 (rt=46.1591, f=3, i=253) [D:\lab212\membrane\Grace\Joyce\iTRAQ_51_1.raw]
Data File:Submitted from 20120508-1(merge) by Mascot Daemon on JOYCE-VAIO
Monoisotopic mass of neutral peptide Mr(calc): 2616.379

Variable modifications:
Ions Score: 57.34 Expect: 0.002

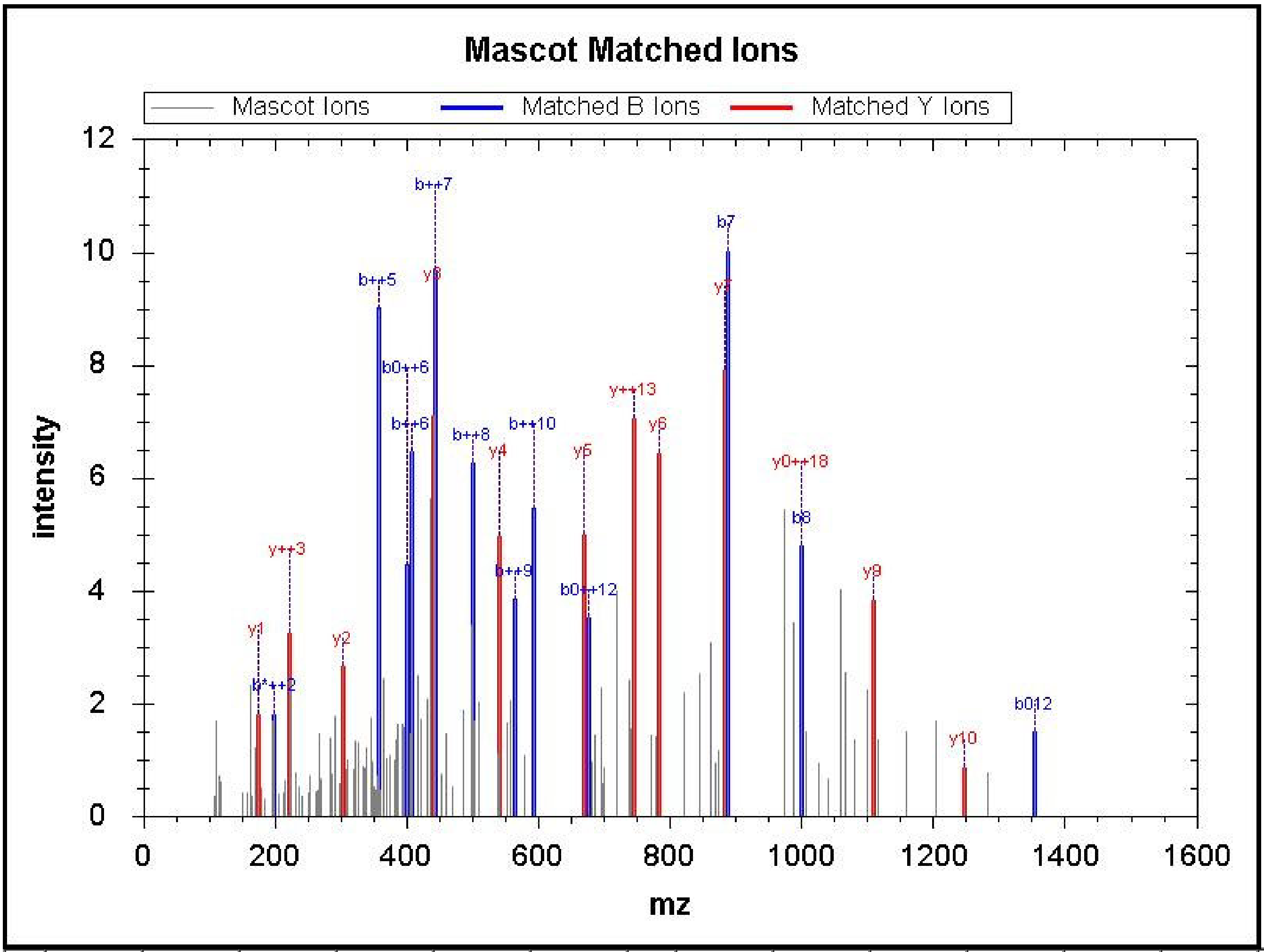

No	b	b++	b*	b*++	b0	b0++	Seq	y	y++	y*	y*++	y0	y0++	RevNo
1	301.21	151.11	284.18	142.60			R							22
2	414.29	207.65	397.27	199.14			L	2,317.18	1,159.09	2,300.15	1,150.58	2,299.17	1,150.09	21
3	543.34	272.17	526.31	263.66	525.33	263.17	E	2,204.09	1,102.55	2,187.07	1,094.04	2,186.08	1,093.55	20
4	600.36	300.68	583.33	292.17	582.35	291.68	G	2,075.05	1,038.03	2,058.02	1,029.52	2,057.04	1,029.02	19
5	715.39	358.20	698.36	349.68	697.37	349.19	D	2,018.03	1,009.52	2,001.00	1,001.01	2,000.02	1,000.51	18
6	816.43	408.72	799.41	400.21	798.42	399.71	T	1,903.00	952.01	1,885.98	943.49	1,884.99	943.00	17
7	887.47	444.24	870.44	435.73	869.46	435.23	A	1,801.96	901.48	1,784.93	892.97	1,783.94	892.48	16
8	1,000.55	500.78	983.53	492.27	982.54	491.78	L	1,730.92	865.96	1,713.89	857.45	1,712.91	856.96	15
9	1,128.61	564.81	1,111.59	556.30	1,110.60	555.80	Q	1,617.83	809.42	1,600.81	800.91	1,599.82	800.42	14
10	1,185.63	593.32	1,168.61	584.81	1,167.62	584.32	G	1,489.78	745.39	1,472.75	736.88	1,471.77	736.39	13
11	1,284.70	642.86	1,267.68	634.34	1,266.69	633.85	V	1,432.75	716.88	1,415.73	708.37	1,414.74	707.88	12
12	1,371.73	686.37	1,354.71	677.86	1,353.72	677.37	S	1,333.69	667.35	1,316.66	658.83	1,315.68	658.34	11
13	1,508.79	754.90	1,491.77	746.39	1,490.78	745.90	H	1,246.65	623.83	1,229.63	615.32	1,228.64	614.83	10
14	1,621.88	811.44	1,604.85	802.93	1,603.87	802.44	I	1,109.59	555.30	1,092.57	546.79	1,091.58	546.30	9
15	1,734.96	867.98	1,717.94	859.47	1,716.95	858.98	I	996.51	498.76	979.48	490.25	978.50	489.75	8
16	1,834.03	917.52	1,817.00	909.01	1,816.02	908.51	V	883.43	442.22	866.40	433.70	865.42	433.21	7
17	1,949.06	975.03	1,932.03	966.52	1,931.05	966.03	D	784.36	392.68	767.33	384.17	766.35	383.68	6
18	2,078.10	1,039.55	2,061.07	1,031.04	2,060.09	1,030.55	E	669.33	335.17	652.30	326.66	651.32	326.16	5
19	2,177.17	1,089.09	2,160.14	1,080.57	2,159.16	1,080.08	V	540.29	270.65	523.26	262.13	522.28	261.64	4
20	2,314.23	1,157.62	2,297.20	1,149.10	2,296.22	1,148.61	H	441.22	221.11	424.19	212.60	423.21	212.11	3
21	2,443.27	1,222.14	2,426.24	1,213.63	2,425.26	1,213.13	E	304.16	152.58	287.13	144.07	286.15	143.58	2

22							R	175.12	88.06	158.09	79.55			1

Query 40010 Hit 1

MS/MS Fragmentation of LISSDGHEFIVK
Found in sp|Q15369|ELOC_HUMAN, Transcription elongation factor B polypeptide 1 OS=Homo sapiens GN=TCEB1 PE=1 SV=1
Match to Query 40010: 1631.913from(544.9782,3+)
Title: 524: Sum of 2 scans in range 1186 (rt=36.3552, f=4, i=350) to 1187 (rt=36.3806, f=4, i=351)
[D:\lab212\membrane\Grace\Joyce\iTRAQ_41_1.raw]
Data File:Submitted from 20120508-1(merge) by Mascot Daemon on JOYCE-VAIO
Monoisotopic mass of neutral peptide Mr(calc): 1631.913
Variable modifications:
K12 :iTRAQ4plex (K)
Ions Score: 57.05 Expect: 0.002

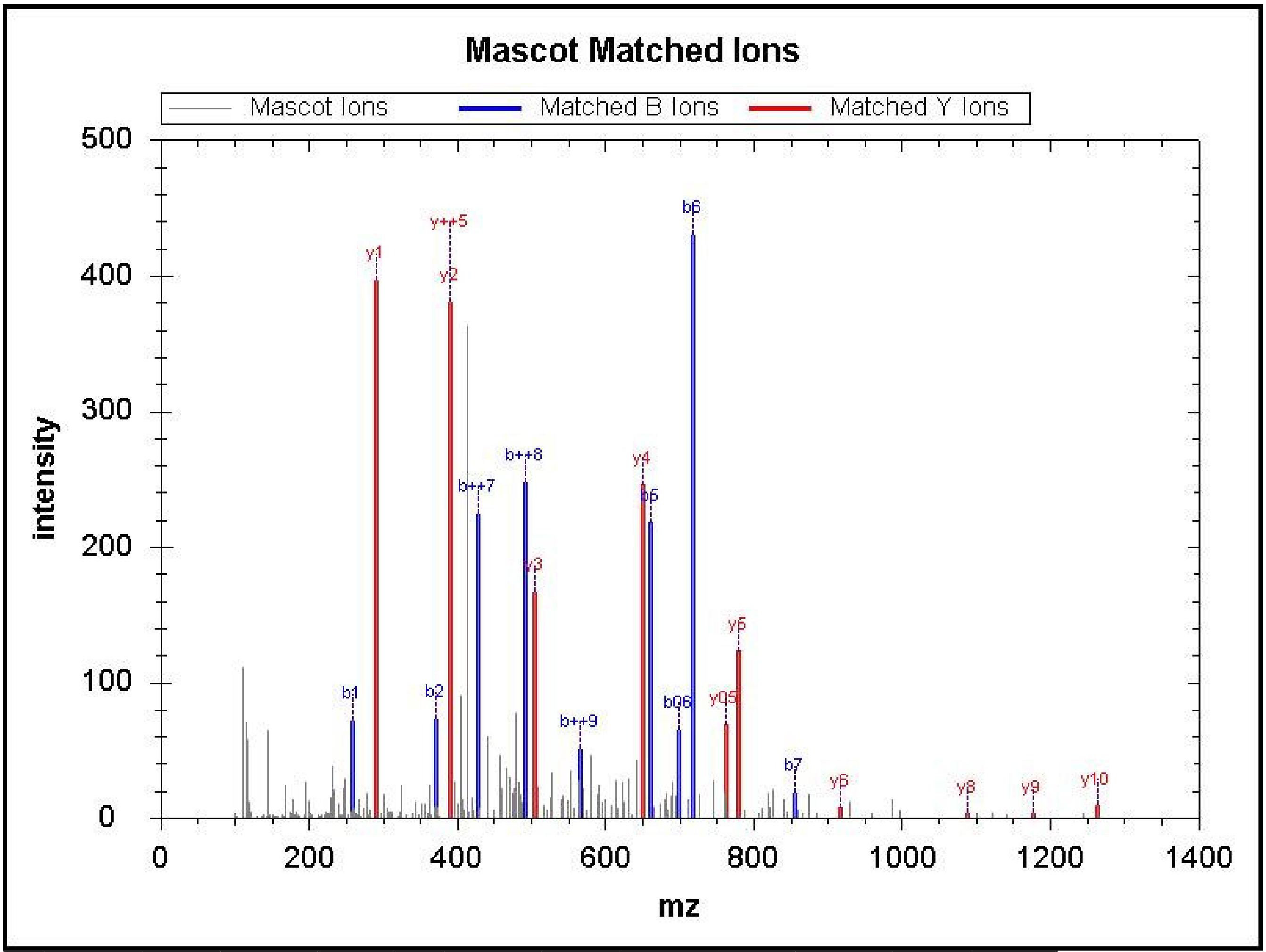

No	b	b++	b0	b0++	Seq	y	y++	y*	y*++	y0	y0++	RevNo
1	258.19	129.60			L							12
2	371.28	186.14			I	1,375.73	688.37	1,358.71	679.86	1,357.72	679.37	11
3	458.31	229.66	440.30	220.65	S	1,262.65	631.83	1,245.62	623.32	1,244.64	622.82	10
4	545.34	273.17	527.33	264.17	S	1,175.62	588.31	1,158.59	579.80	1,157.61	579.31	9
5	660.37	330.69	642.36	321.68	D	1,088.59	544.80	1,071.56	536.28	1,070.58	535.79	8
6	717.39	359.20	699.38	350.19	G	973.56	487.28	956.53	478.77	955.55	478.28	7
7	854.45	427.73	836.44	418.72	H	916.54	458.77	899.51	450.26	898.53	449.77	6
8	983.49	492.25	965.48	483.24	E	779.48	390.24	762.45	381.73	761.47	381.24	5
9	1,130.56	565.78	1,112.55	556.78	F	650.44	325.72	633.41	317.21			4
10	1,243.64	622.33	1,225.63	613.32	I	503.37	252.19	486.34	243.67			3
11	1,342.71	671.86	1,324.70	662.85	V	390.28	195.65	373.26	187.13			2
12					K	291.21	146.11	274.19	137.60			1

Query 96192 Hit 1

MS/MS Fragmentation of LAASEAATAISHQAIQILGGMGYVTEMPAER
Found in sp|P16219|ACADS_HUMAN, Short-chain specific acyl-CoA dehydrogenase
Match to Query 96192: 3329.687from(833.429,4+)
Title: 1178: Sum of 2 scans in range 2596 (rt=67.8367, f=4, i=791) to 2597 (rt=67.8621, f=4, i=792) [D:\lab212\membrane\Grace\Joyce\iTRAQ_28_1_327.raw]
Data File:Submitted from 20120508-1(merge) by Mascot Daemon on JOYCE-VAIO
Monoisotopic mass of neutral peptide Mr(calc): 3329.687
Variable modifications:
Ions Score: 56.88 Expect: 0.002

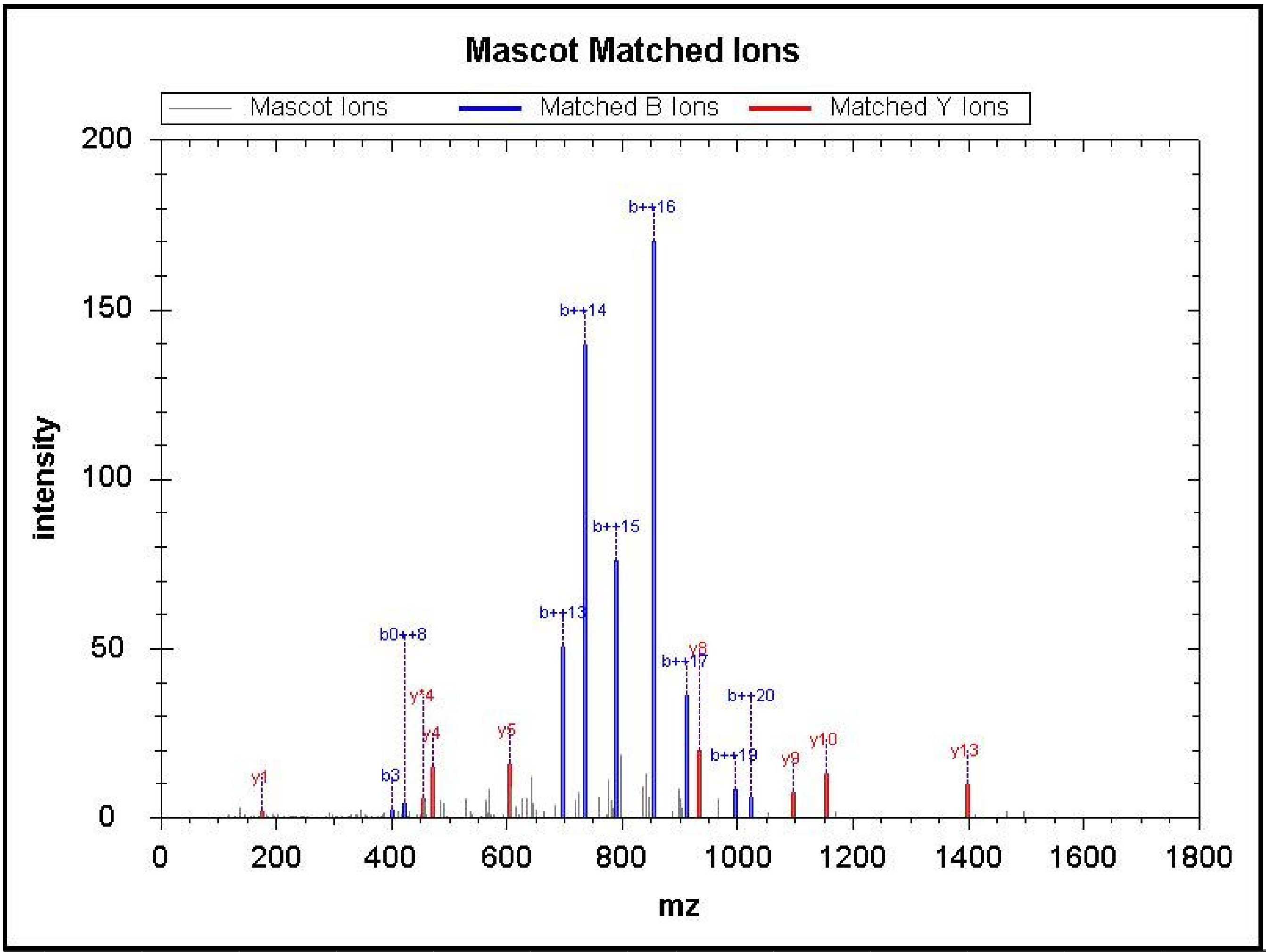

No	b	b++	b*	b*++	b0	b0++	Seq	y	y++	y*	y*++	y0	y0++	RevNo
1	258.19	129.60					L							31
2	329.23	165.12					A	3,073.51	1,537.26	3,056.48	1,528.74	3,055.50	1,528.25	30
3	400.27	200.64					A	3,002.47	1,501.74	2,985.44	1,493.23	2,984.46	1,492.73	29
4	487.30	244.15			469.29	235.15	S	2,931.43	1,466.22	2,914.41	1,457.71	2,913.42	1,457.22	28
5	616.34	308.67			598.33	299.67	E	2,844.40	1,422.70	2,827.38	1,414.19	2,826.39	1,413.70	27
6	687.38	344.19			669.37	335.19	A	2,715.36	1,358.18	2,698.33	1,349.67	2,697.35	1,349.18	26
7	758.42	379.71			740.41	370.71	A	2,644.32	1,322.66	2,627.30	1,314.15	2,626.31	1,313.66	25
8	859.46	430.24			841.45	421.23	T	2,573.29	1,287.15	2,556.26	1,278.63	2,555.27	1,278.14	24
9	930.50	465.75			912.49	456.75	A	2,472.24	1,236.62	2,455.21	1,228.11	2,454.23	1,227.62	23
10	1,043.59	522.30			1,025.57	513.29	I	2,401.20	1,201.10	2,384.17	1,192.59	2,383.19	1,192.10	22
11	1,130.62	565.81			1,112.61	556.81	S	2,288.12	1,144.56	2,271.09	1,136.05	2,270.11	1,135.56	21
12	1,267.68	634.34			1,249.67	625.34	H	2,201.08	1,101.05	2,184.06	1,092.53	2,183.07	1,092.04	20
13	1,395.73	698.37	1,378.71	689.86	1,377.72	689.37	Q	2,064.03	1,032.52	2,047.00	1,024.00	2,046.02	1,023.51	19
14	1,466.77	733.89	1,449.75	725.38	1,448.76	724.88	A	1,935.97	968.49	1,918.94	959.97	1,917.96	959.48	18
15	1,579.86	790.43	1,562.83	781.92	1,561.85	781.43	I	1,864.93	932.97	1,847.90	924.46	1,846.92	923.96	17

16	1,707.91	854.46	1,690.89	845.95	1,689.90	845.46	Q	1,751.85	876.43	1,734.82	867.91	1,733.84	867.42	16
17	1,821.00	911.00	1,803.97	902.49	1,802.99	902.00	I	1,623.79	812.40	1,606.76	803.88	1,605.78	803.39	15
18	1,934.08	967.55	1,917.06	959.03	1,916.07	958.54	L	1,510.70	755.86	1,493.68	747.34	1,492.69	746.85	14
19	1,991.10	996.06	1,974.08	987.54	1,973.09	987.05	G	1,397.62	699.31	1,380.59	690.80	1,379.61	690.31	13
20	2,048.13	1,024.57	2,031.10	1,016.05	2,030.12	1,015.56	G	1,340.60	670.80	1,323.57	662.29	1,322.59	661.80	12
21	2,179.17	1,090.09	2,162.14	1,081.57	2,161.16	1,081.08	M	1,283.58	642.29	1,266.55	633.78	1,265.57	633.29	11
22	2,236.19	1,118.60	2,219.16	1,110.08	2,218.18	1,109.59	G	1,152.54	576.77	1,135.51	568.26	1,134.52	567.77	10
23	2,399.25	1,200.13	2,382.22	1,191.62	2,381.24	1,191.12	Y	1,095.51	548.26	1,078.49	539.75	1,077.50	539.26	9
24	2,498.32	1,249.66	2,481.29	1,241.15	2,480.31	1,240.66	V	932.45	466.73	915.42	458.22	914.44	457.72	8
25	2,599.37	1,300.19	2,582.34	1,291.67	2,581.36	1,291.18	T	833.38	417.19	816.36	408.68	815.37	408.19	7
26	2,728.41	1,364.71	2,711.38	1,356.20	2,710.40	1,355.70	E	732.33	366.67	715.31	358.16	714.32	357.67	6
27	2,859.45	1,430.23	2,842.42	1,421.72	2,841.44	1,421.22	M	603.29	302.15	586.27	293.64	585.28	293.14	5
28	2,956.50	1,478.76	2,939.48	1,470.24	2,938.49	1,469.75	P	472.25	236.63	455.22	228.12	454.24	227.62	4
29	3,027.54	1,514.27	3,010.51	1,505.76	3,009.53	1,505.27	A	375.20	188.10	358.17	179.59	357.19	179.10	3
30	3,156.58	1,578.79	3,139.56	1,570.28	3,138.57	1,569.79	E	304.16	152.58	287.13	144.07	286.15	143.58	2
31							R	175.12	88.06	158.09	79.55			1

Query 70984 Hit 1

MS/MS Fragmentation of QLDNILAAVHDVLNESSK
Found in sp|Q96Q15|SMG1_HUMAN, Serine/threonine-protein kinase SMG1 OS=Homo sapiens GN=SMG1 PE=1 SV=3
Match to Query 70984: 2253.214from(752.0787,3+)
Title: 1109: Sum of 2 scans in range 2425 (rt=64.0622, f=4, i=731) to 2426 (rt=64.0877, f=4, i=732)
[D:\lab212\membrane\Grace\Joyce\iTRAQ_37_2.raw]
Data File:Submitted from 20120508-1(merge) by Mascot Daemon on JOYCE-VAIO
Monoisotopic mass of neutral peptide Mr(calc): 2253.214
Variable modifications:
K18 :iTRAQ4plex (K)
Ions Score: 56.88 Expect: 0.002

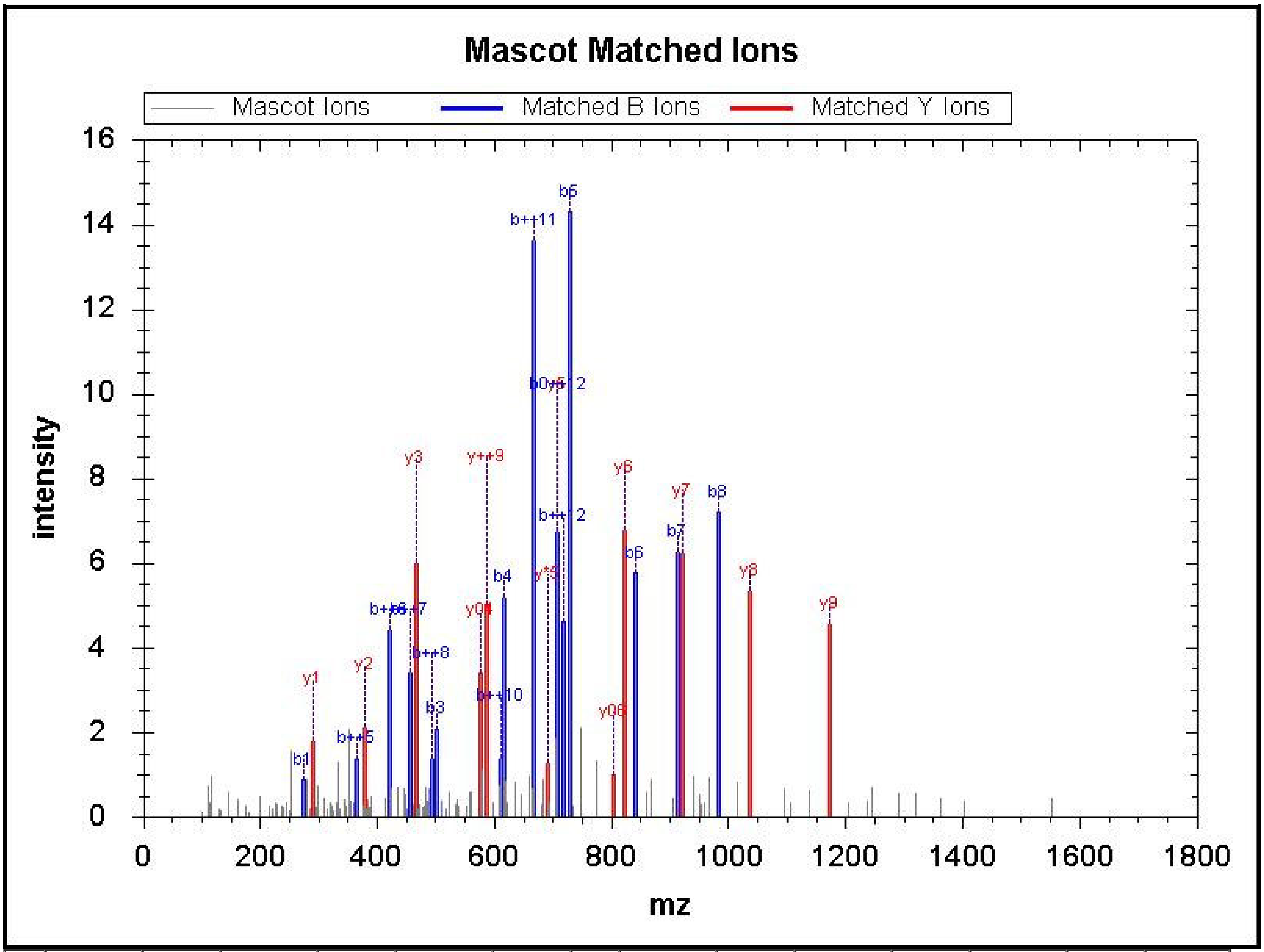

No	b	b++	b*	b*++	b0	b0++	Seq	y	y++	y*	y*++	y0	y0++	RevNo
1	273.17	137.09	256.14	128.57			Q							18
2	386.25	193.63	369.23	185.12			L	1,982.07	991.54	1,965.04	983.02	1,964.06	982.53	17
3	501.28	251.14	484.25	242.63	483.27	242.14	D	1,868.98	935.00	1,851.96	926.48	1,850.97	925.99	16
4	615.32	308.16	598.30	299.65	597.31	299.16	N	1,753.96	877.48	1,736.93	868.97	1,735.95	868.48	15
5	728.41	364.71	711.38	356.19	710.40	355.70	I	1,639.91	820.46	1,622.89	811.95	1,621.90	811.46	14
6	841.49	421.25	824.46	412.74	823.48	412.24	L	1,526.83	763.92	1,509.80	755.41	1,508.82	754.91	13
7	912.53	456.77	895.50	448.25	894.52	447.76	A	1,413.75	707.38	1,396.72	698.86	1,395.73	698.37	12
8	983.56	492.29	966.54	483.77	965.55	483.28	A	1,342.71	671.86	1,325.68	663.34	1,324.70	662.85	11
9	1,082.63	541.82	1,065.61	533.31	1,064.62	532.81	V	1,271.67	636.34	1,254.64	627.83	1,253.66	627.33	10
10	1,219.69	610.35	1,202.67	601.84	1,201.68	601.34	H	1,172.60	586.81	1,155.58	578.29	1,154.59	577.80	9
11	1,334.72	667.86	1,317.69	659.35	1,316.71	658.86	D	1,035.54	518.28	1,018.52	509.76	1,017.53	509.27	8
12	1,433.79	717.40	1,416.76	708.88	1,415.78	708.39	V	920.52	460.76	903.49	452.25	902.51	451.76	7
13	1,546.87	773.94	1,529.84	765.43	1,528.86	764.93	L	821.45	411.23	804.42	402.71	803.44	402.22	6
14	1,660.91	830.96	1,643.89	822.45	1,642.90	821.96	N	708.36	354.69	691.34	346.17	690.35	345.68	5
15	1,789.96	895.48	1,772.93	886.97	1,771.95	886.48	E	594.32	297.66	577.29	289.15	576.31	288.66	4
16	1,876.99	939.00	1,859.96	930.48	1,858.98	929.99	S	465.28	233.14	448.25	224.63	447.27	224.14	3
17	1,964.02	982.51	1,946.99	974.00	1,946.01	973.51	S	378.25	189.63	361.22	181.11	360.24	180.62	2
18							K	291.21	146.11	274.19	137.60			1

Query 44604 Hit 1

MS/MS Fragmentation of LGWGSGWDVLLPK
Found in sp|Q99575|POP1_HUMAN, Ribonucleases P/MRP protein subunit POP1 OS=Homo sapiens GN=POP1 PE=1 SV=2
Match to Query 44604: 1714.966from(858.4901,2+)
Title: 986: Sum of 2 scans in range 2253 (rt=59.7039, f=4, i=655) to 2254 (rt=59.7294, f=4, i=656)

[D:\lab212\membrane\Grace\Joyce\iTRAQ_29_1.raw]
Data File:Submitted from 20120508-1(merge) by Mascot Daemon on JOYCE-VAIO
Monoisotopic mass of neutral peptide Mr(calc): 1714.966
Variable modifications:
K13 :iTRAQ4plex (K)
Ions Score: 56.4 Expect: 0.002

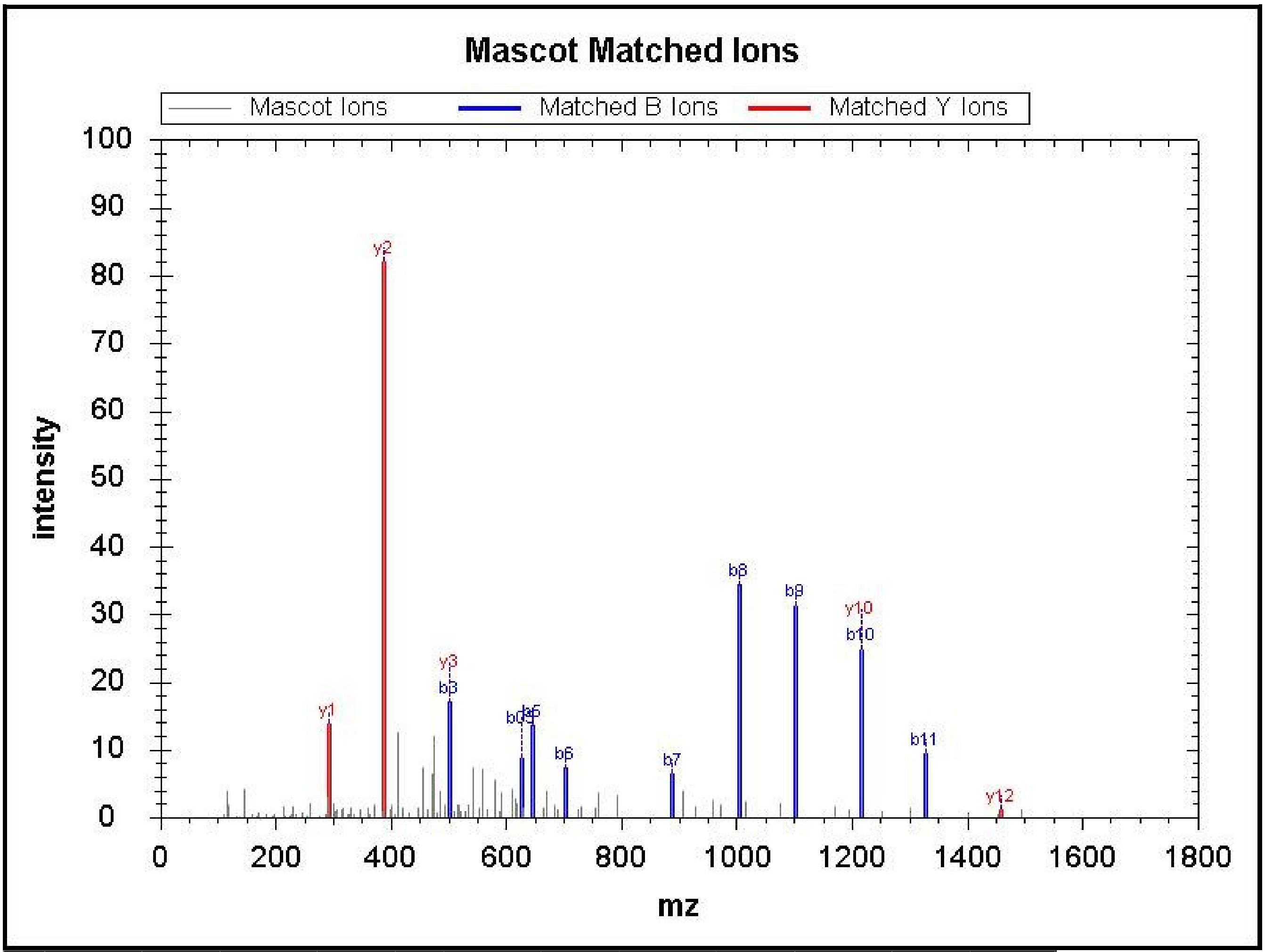

No	b	b++	b0	b0++	Seq	y	y++	y*	y*++	y0	y0++	RevNo
1	258.19	129.60			L							13
2	315.21	158.11			G	1,458.79	729.90	1,441.76	721.38	1,440.78	720.89	12
3	501.29	251.15			W	1,401.76	701.39	1,384.74	692.87	1,383.75	692.38	11
4	558.32	279.66			G	1,215.69	608.35	1,198.66	599.83	1,197.67	599.34	10
5	645.35	323.18	627.34	314.17	S	1,158.66	579.84	1,141.64	571.32	1,140.65	570.83	9
6	702.37	351.69	684.36	342.68	G	1,071.63	536.32	1,054.61	527.81	1,053.62	527.31	8
7	888.45	444.73	870.44	435.72	W	1,014.61	507.81	997.58	499.30	996.60	498.80	7
8	1,003.48	502.24	985.46	493.24	D	828.53	414.77	811.50	406.26	810.52	405.76	6
9	1,102.54	551.78	1,084.53	542.77	V	713.50	357.26	696.48	348.74			5
10	1,215.63	608.32	1,197.62	599.31	L	614.44	307.72	597.41	299.21			4
11	1,328.71	664.86	1,310.70	655.85	L	501.35	251.18	484.33	242.67			3
12	1,425.76	713.39	1,407.75	704.38	P	388.27	194.64	371.24	186.12			2
13					K	291.21	146.11	274.19	137.60			1

Query 56950 Hit 1

MS/MS Fragmentation of LVISHEEELIPLHK
Found in sp|O14646|CHD1_HUMAN, Chromodomain-helicase-DNA-binding protein 1 OS=Homo sapiens GN=CHD1 PE=1 SV=2
Match to Query 56950: 1944.131 from(649.051,3+)
Title: 612: Scan 1373 (rt=40.5718, f=2, i=219) [D:\lab212\membrane\Grace\Joyce\iTRAQ_54_2.raw]

Data File:Submitted from 20120508-1(merge) by Mascot Daemon on JOYCE-VAIO
Monoisotopic mass of neutral peptide Mr(calc): 1944.131
Variable modifications:
K14 :iTRAQ4plex (K)
Ions Score: 56.36 Expect: 0.001

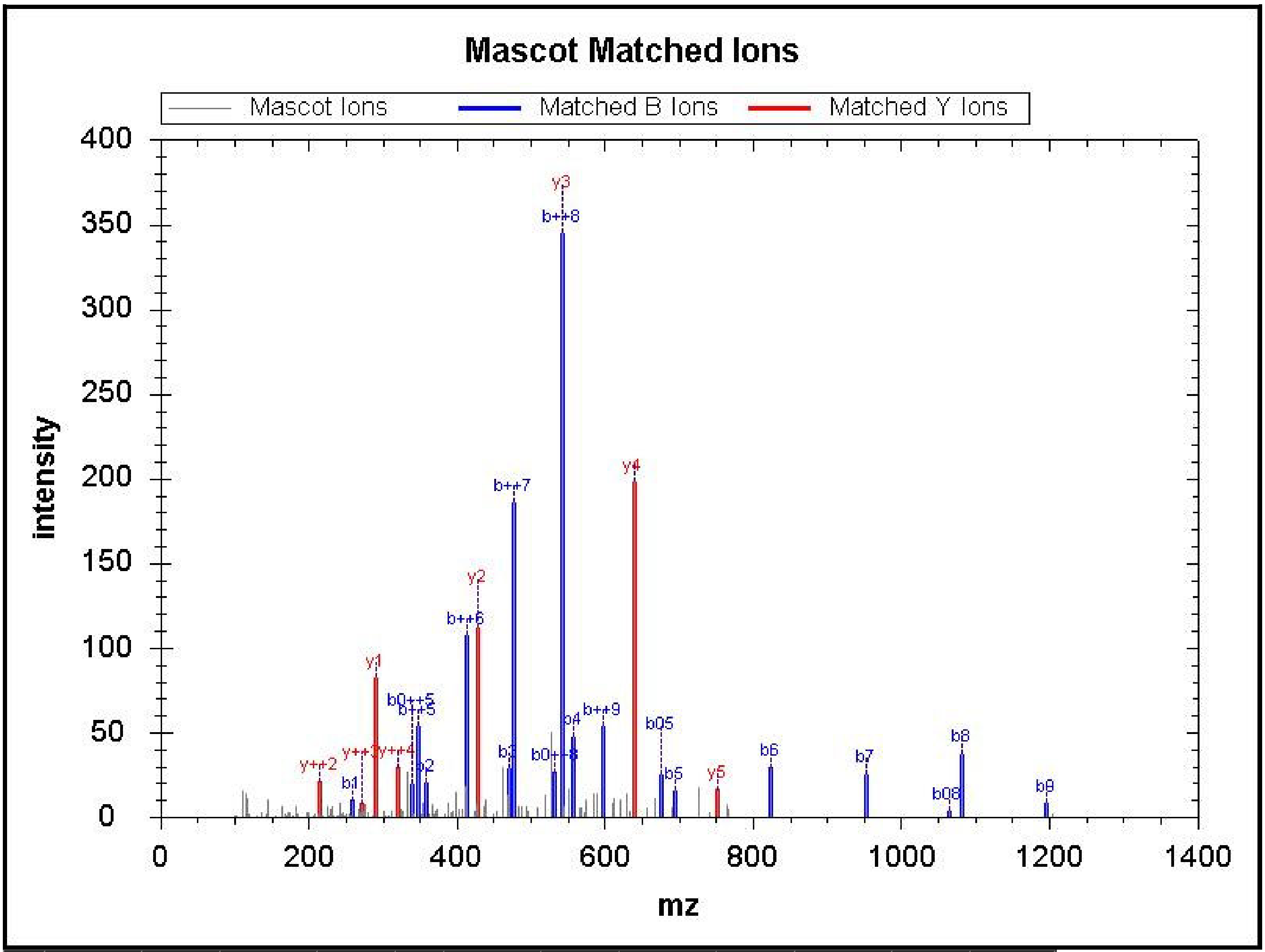

No	b	b++	b0	b0++	Seq	y	y++	y*	y*++	y0	y0++	RevNo
1	258.19	129.60			L							14
2	357.26	179.13			V	1,687.95	844.48	1,670.92	835.97	1,669.94	835.47	13
3	470.35	235.68			I	1,588.88	794.94	1,571.85	786.43	1,570.87	785.94	12
4	557.38	279.19	539.37	270.19	S	1,475.80	738.40	1,458.77	729.89	1,457.79	729.40	11
5	694.44	347.72	676.43	338.72	H	1,388.77	694.89	1,371.74	686.37	1,370.75	685.88	10
6	823.48	412.24	805.47	403.24	E	1,251.71	626.36	1,234.68	617.84	1,233.70	617.35	9
7	952.52	476.76	934.51	467.76	E	1,122.66	561.84	1,105.64	553.32	1,104.65	552.83	8
8	1,081.56	541.29	1,063.55	532.28	E	993.62	497.31	976.59	488.80	975.61	488.31	7
9	1,194.65	597.83	1,176.64	588.82	L	864.58	432.79	847.55	424.28			6
10	1,307.73	654.37	1,289.72	645.36	I	751.49	376.25	734.47	367.74			5
11	1,404.79	702.90	1,386.78	693.89	P	638.41	319.71	621.38	311.20			4
12	1,517.87	759.44	1,499.86	750.43	L	541.36	271.18	524.33	262.67			3
13	1,654.93	827.97	1,636.92	818.96	H	428.27	214.64	411.25	206.13			2
14					K	291.21	146.11	274.19	137.60			1

Query 38852 Hit 1

MS/MS Fragmentation of KDVLETFTVK
Found in sp|P21926|CD9_HUMAN, CD9 antigen OS=Homo sapiens GN=CD9 PE=1 SV=4
Match to Query 38852: 1610.965from(537.9957,3+)

Title: 563: Scan 1257 (rt=37.9892, f=3, i=193) [D:\lab212\membrane\Grace\Joyce\iTRAQ_47_1.raw]
Data File:Submitted from 20120508-1(merge) by Mascot Daemon on JOYCE-VAIO
Monoisotopic mass of neutral peptide Mr(calc): 1610.965
Variable modifications:
K1 :iTRAQ4plex (K)
K10 :iTRAQ4plex (K)
Ions Score: 56.19 Expect: 0.001

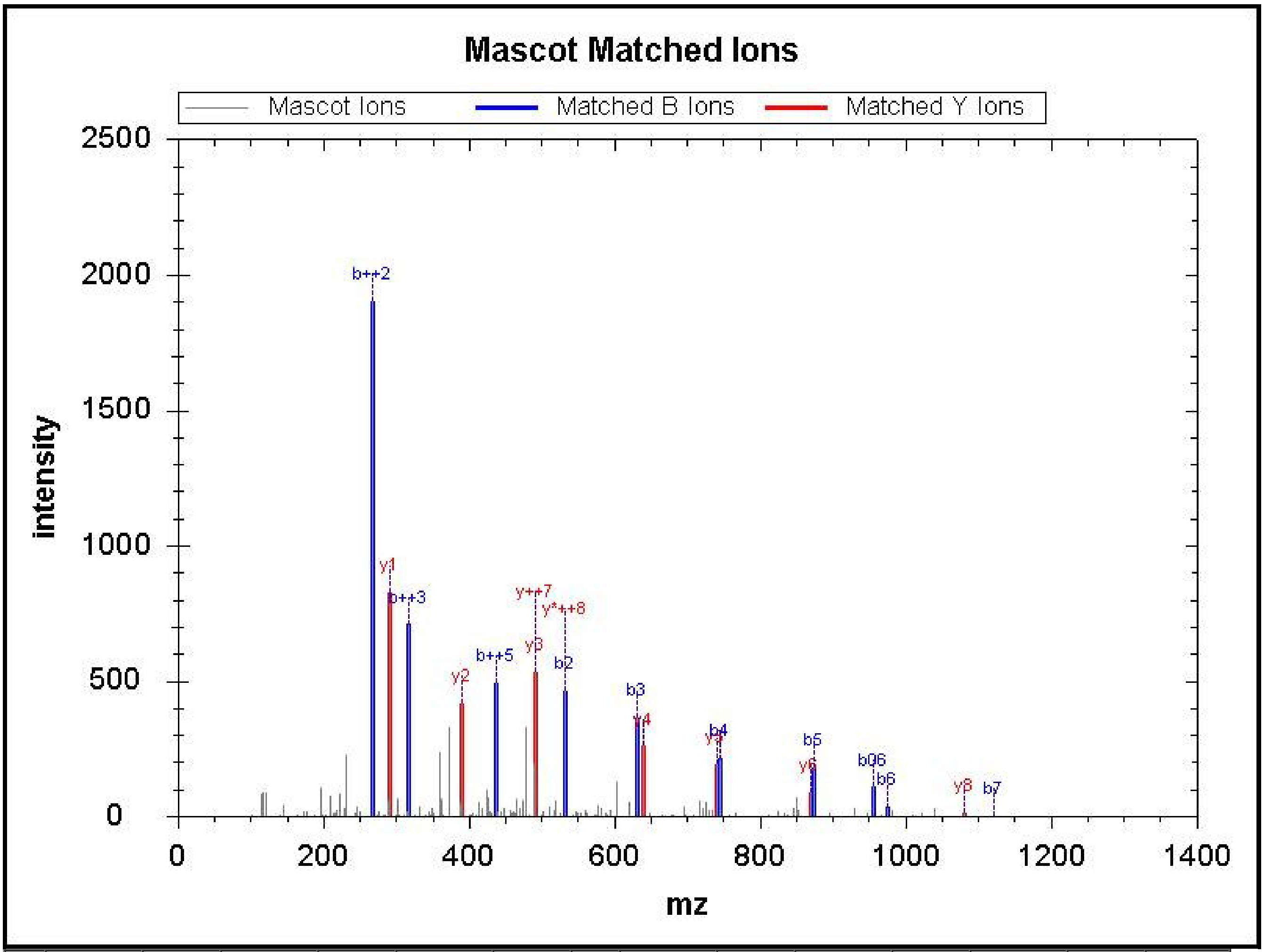

No	b	b++	b*	b*++	b0	b0++	Seq	y	y++	y*	y*++	y0	y0++	RevNo
1	417.31	209.16	400.28	200.64			K							10
2	532.33	266.67	515.31	258.16	514.32	257.66	D	1,195.67	598.34	1,178.64	589.82	1,177.66	589.33	9
3	631.40	316.20	614.38	307.69	613.39	307.20	V	1,080.64	540.82	1,063.62	532.31	1,062.63	531.82	8
4	744.49	372.75	727.46	364.23	726.48	363.74	L	981.57	491.29	964.55	482.78	963.56	482.29	7
5	873.53	437.27	856.50	428.75	855.52	428.26	E	868.49	434.75	851.46	426.24	850.48	425.74	6
6	974.58	487.79	957.55	479.28	956.57	478.79	T	739.45	370.23	722.42	361.71	721.44	361.22	5
7	1,121.64	561.33	1,104.62	552.81	1,103.63	552.32	F	638.40	319.70	621.37	311.19	620.39	310.70	4
8	1,222.69	611.85	1,205.67	603.34	1,204.68	602.84	T	491.33	246.17	474.30	237.66	473.32	237.16	3
9	1,321.76	661.38	1,304.73	652.87	1,303.75	652.38	V	390.28	195.65	373.26	187.13			2
10							K	291.21	146.11	274.19	137.60			1

Query 72045 Hit 1

MS/MS Fragmentation of LASGEITQDDFLVVVHQIR
Found in sp|O94913|PCF11_HUMAN, Pre-mRNA cleavage complex 2 protein Pcf11 OS=Homo sapiens GN=PCF11 PE=1 SV=3
Match to Query 72045: 2283.224from(762.0819,3+)
Title: 849: Sum of 3 scans in range 1902 (rt=52.2638, f=2, i=297) to 1905 (rt=52.34, f=4, i=572)
[D:\lab212\membrane\Grace\Joyce\iTRAQ_32_2.raw]
Data File:Submitted from 20120508-1(merge) by Mascot Daemon on JOYCE-VAIO
Monoisotopic mass of neutral peptide Mr(calc): 2283.224

Variable modifications:
Ions Score: 56.09 Expect: 0.002

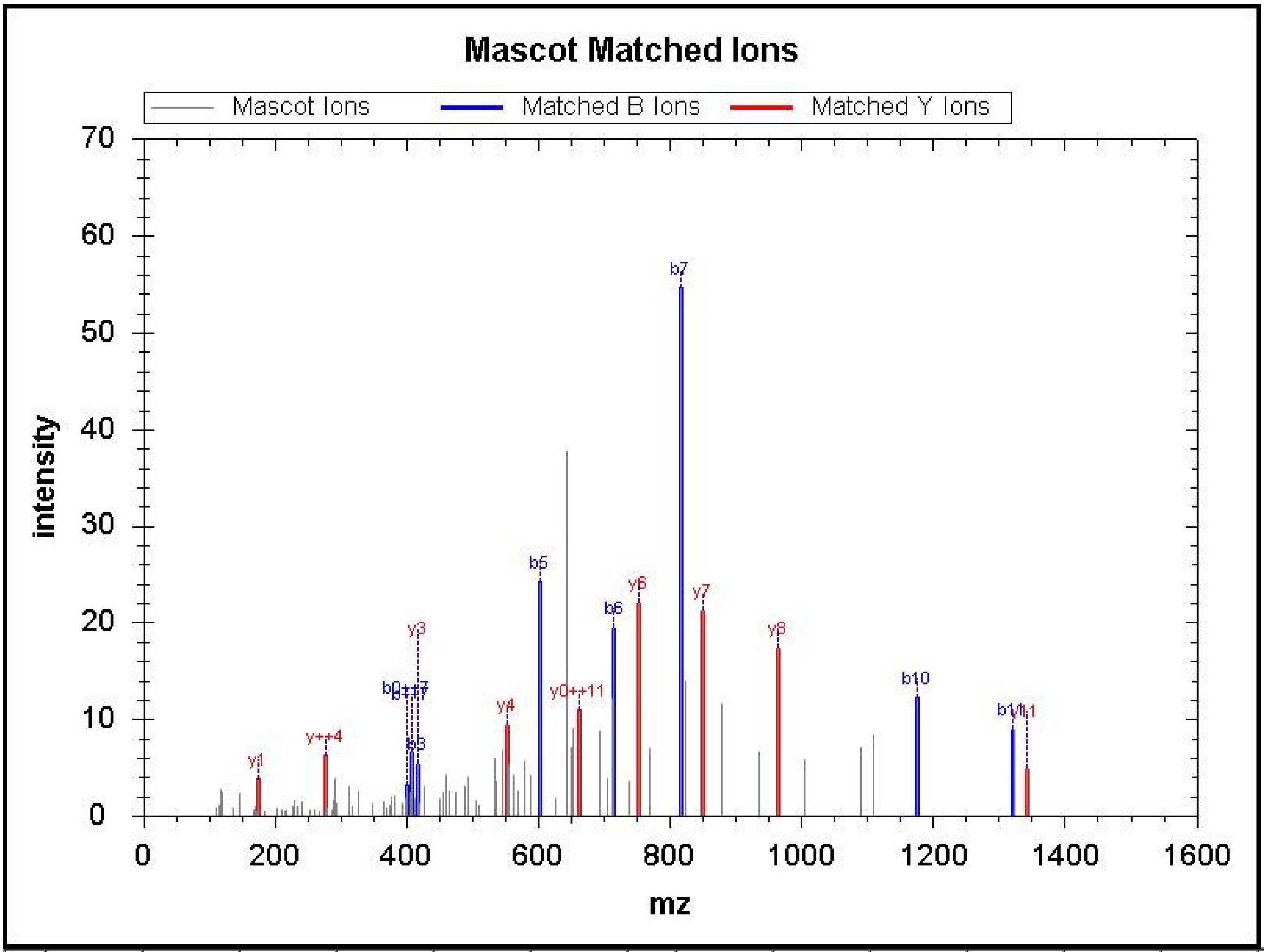

No	b	b++	b*	b*++	b0	b0++	Seq	y	y++	y*	y*++	y0	y0++	RevNo
1	258.19	129.60					L							19
2	329.23	165.12					A	2,027.06	1,014.03	2,010.03	1,005.52	2,009.05	1,005.03	18
3	416.26	208.63			398.25	199.63	S	1,956.02	978.51	1,938.99	970.00	1,938.01	969.51	17
4	473.28	237.15			455.27	228.14	G	1,868.99	935.00	1,851.96	926.48	1,850.98	925.99	16
5	602.33	301.67			584.32	292.66	E	1,811.97	906.49	1,794.94	897.97	1,793.95	897.48	15
6	715.41	358.21			697.40	349.20	I	1,682.92	841.96	1,665.90	833.45	1,664.91	832.96	14
7	816.46	408.73			798.45	399.73	T	1,569.84	785.42	1,552.81	776.91	1,551.83	776.42	13
8	944.52	472.76	927.49	464.25	926.51	463.76	Q	1,468.79	734.90	1,451.76	726.39	1,450.78	725.89	12
9	1,059.54	530.28	1,042.52	521.76	1,041.53	521.27	D	1,340.73	670.87	1,323.71	662.36	1,322.72	661.86	11
10	1,174.57	587.79	1,157.54	579.28	1,156.56	578.78	D	1,225.71	613.36	1,208.68	604.84	1,207.69	604.35	10
11	1,321.64	661.32	1,304.61	652.81	1,303.63	652.32	F	1,110.68	555.84	1,093.65	547.33			9
12	1,434.72	717.87	1,417.70	709.35	1,416.71	708.86	L	963.61	482.31	946.58	473.80			8
13	1,533.79	767.40	1,516.77	758.89	1,515.78	758.39	V	850.53	425.77	833.50	417.25			7
14	1,632.86	816.93	1,615.83	808.42	1,614.85	807.93	V	751.46	376.23	734.43	367.72			6
15	1,731.93	866.47	1,714.90	857.95	1,713.92	857.46	V	652.39	326.70	635.36	318.18			5
16	1,868.99	935.00	1,851.96	926.48	1,850.98	925.99	H	553.32	277.16	536.29	268.65			4
17	1,997.05	999.03	1,980.02	990.51	1,979.04	990.02	Q	416.26	208.63	399.24	200.12			3
18	2,110.13	1,055.57	2,093.10	1,047.06	2,092.12	1,046.56	I	288.20	144.61	271.18	136.09			2
19							R	175.12	88.06	158.09	79.55			1

Query 26944 Hit 1

MS/MS Fragmentation of HTAAPTDPADGPV
Found in sp|Q04941|PLP2_HUMAN, Proteolipid protein 2 OS=Homo sapiens GN=PLP2 PE=1 SV=1
Match to Query 26944: 1391.685from(696.8498,2+)
Title: 202: Scan 552 (rt=21.7533, f=3, i=73) [D:\lab212\membrane\Grace\Joyce\iTRAQ_27_1.raw]
Data File:Submitted from 20120508-1(merge) by Mascot Daemon on JOYCE-VAIO
Monoisotopic mass of neutral peptide Mr(calc): 1391.685
Variable modifications:
Ions Score: 56 Expect: 0.002

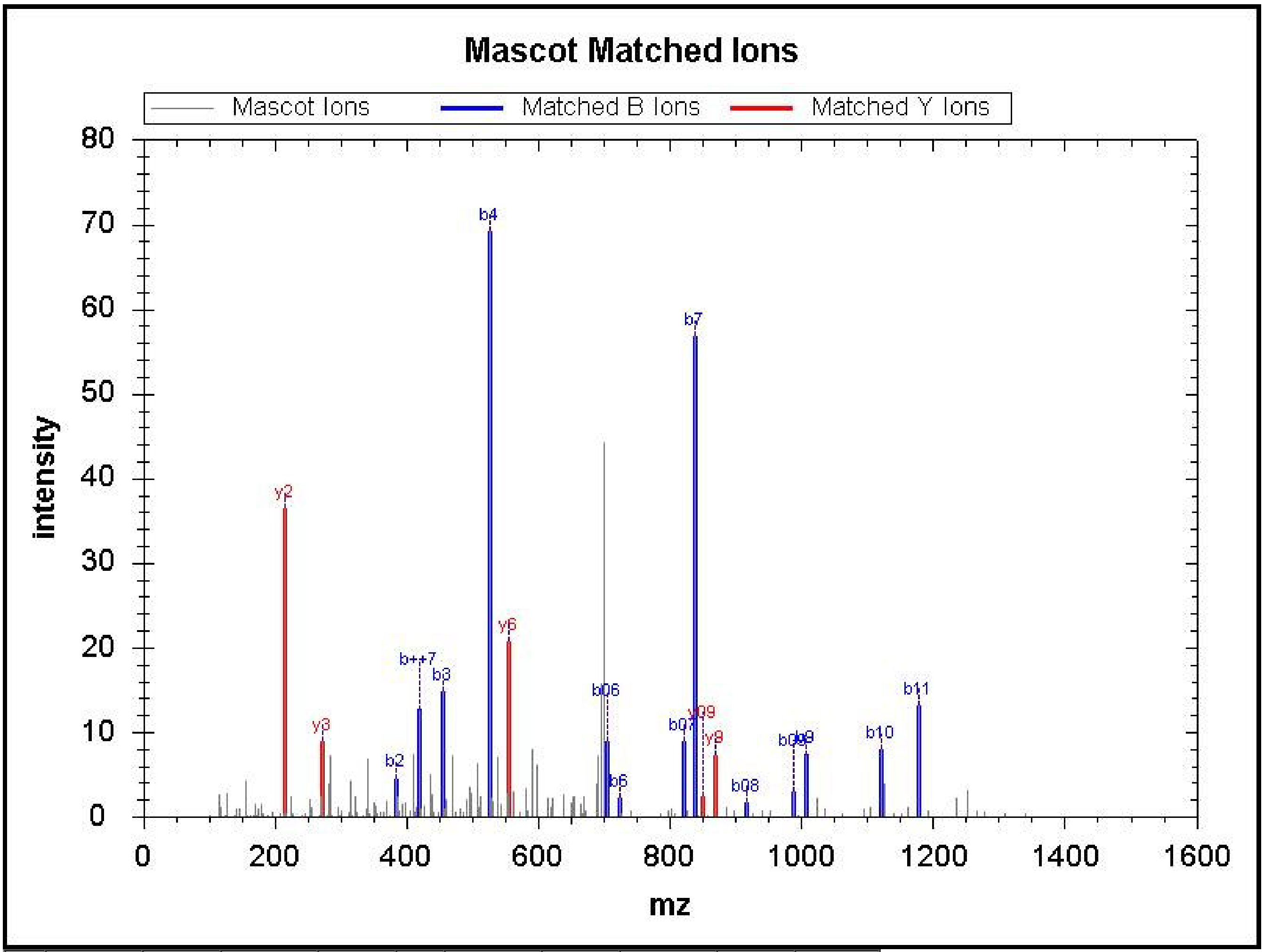

No	b	b++	b0	b0++	Seq	y	y++	y0	y0++	RevNo
1	282.17	141.59			H					13
2	383.22	192.11	365.21	183.11	T	1,111.53	556.27	1,093.52	547.26	12
3	454.25	227.63	436.24	218.62	A	1,010.48	505.74	992.47	496.74	11
4	525.29	263.15	507.28	254.14	A	939.44	470.22	921.43	461.22	10
5	622.34	311.68	604.33	302.67	P	868.40	434.71	850.39	425.70	9
6	723.39	362.20	705.38	353.19	T	771.35	386.18	753.34	377.17	8
7	838.42	419.71	820.41	410.71	D	670.30	335.66	652.29	326.65	7
8	935.47	468.24	917.46	459.23	P	555.28	278.14	537.27	269.14	6
9	1,006.51	503.76	988.50	494.75	A	458.22	229.62	440.21	220.61	5
10	1,121.53	561.27	1,103.52	552.27	D	387.19	194.10	369.18	185.09	4
11	1,178.56	589.78	1,160.55	580.78	G	272.16	136.58			3
12	1,275.61	638.31	1,257.60	629.30	P	215.14	108.07			2
13					V	118.09	59.55			1

Query 70699 Hit 1

MS/MS Fragmentation of HGYNMEQALGMLFWHK
Found in sp|Q9UKL0|RCOR1_HUMAN, REST corepressor 1 OS=Homo sapiens GN=RCOR1 PE=1 SV=1

Match to Query 70699: 2249.11from(750.7108,3+)
Title: 1018: Sum of 2 scans in range 2179 (rt=58.7672, f=4, i=677) to 2180 (rt=58.7925, f=4, i=678)
[D:\lab212\membrane\Grace\Joyce\iTRAQ_50_1.raw]
Data File:Submitted from 20120508-1(merge) by Mascot Daemon on JOYCE-VAIO
Monoisotopic mass of neutral peptide Mr(calc): 2249.11
Variable modifications:
K16 :iTRAQ4plex (K)
Ions Score: 55.93 Expect: 0.002

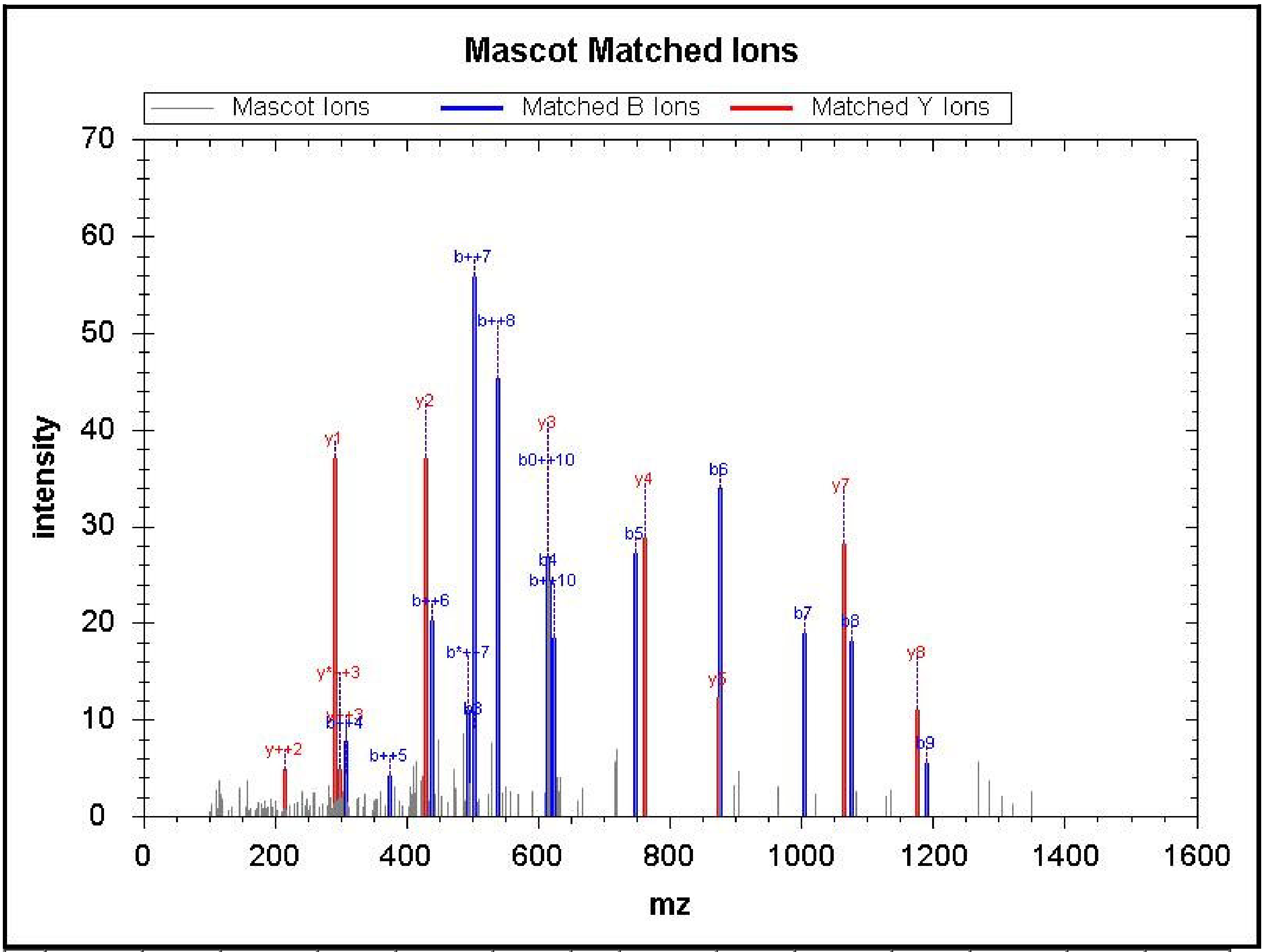

No	b	b++	b*	b*++	b0	b0++	Seq	y	y++	y*	y*++	y0	y0++	RevNo
1	282.17	141.59					H							16
2	339.19	170.10					G	1,968.96	984.98	1,951.93	976.47	1,950.95	975.98	15
3	502.25	251.63					Y	1,911.94	956.47	1,894.91	947.96	1,893.93	947.47	14
4	616.30	308.65	599.27	300.14			N	1,748.87	874.94	1,731.85	866.43	1,730.86	865.93	13
5	747.34	374.17	730.31	365.66			M	1,634.83	817.92	1,617.80	809.41	1,616.82	808.91	12
6	876.38	438.69	859.35	430.18	858.37	429.69	E	1,503.79	752.40	1,486.76	743.89	1,485.78	743.39	11
7	1,004.44	502.72	987.41	494.21	986.43	493.72	Q	1,374.75	687.88	1,357.72	679.36			10
8	1,075.47	538.24	1,058.45	529.73	1,057.46	529.24	A	1,246.69	623.85	1,229.66	615.33			9
9	1,188.56	594.78	1,171.53	586.27	1,170.55	585.78	L	1,175.65	588.33	1,158.63	579.82			8
10	1,245.58	623.29	1,228.55	614.78	1,227.57	614.29	G	1,062.57	531.79	1,045.54	523.27			7
11	1,376.62	688.81	1,359.59	680.30	1,358.61	679.81	M	1,005.55	503.28	988.52	494.76			6
12	1,489.71	745.36	1,472.68	736.84	1,471.69	736.35	L	874.51	437.76	857.48	429.24			5
13	1,636.77	818.89	1,619.75	810.38	1,618.76	809.89	F	761.42	381.21	744.39	372.70			4
14	1,822.85	911.93	1,805.83	903.42	1,804.84	902.92	W	614.35	307.68	597.33	299.17			3
15	1,959.91	980.46	1,942.89	971.95	1,941.90	971.45	H	428.27	214.64	411.25	206.13			2
16							K	291.21	146.11	274.19	137.60			1

Query 46037 Hit 1

MS/MS Fragmentation of KGEETEETSSSK
Found in sp|Q9UEE9|CFDP1_HUMAN, Craniofacial development protein 1 OS=Homo sapiens GN=CFDP1 PE=1 SV=1
Match to Query 46037: 1742.89from(581.9708,3+)
Title: 7: Sum of 2 scans in range 102 (rt=11.9238, f=2, i=16) to 103 (rt=11.9492, f=2, i=17)
[D:\lab212\membrane\Grace\Joyce\iTRAQ_49_2.raw]
Data File:Submitted from 20120508-1(merge) by Mascot Daemon on JOYCE-VAIO
Monoisotopic mass of neutral peptide Mr(calc): 1742.89
Variable modifications:
K1 :iTRAQ4plex (K)
K12 :iTRAQ4plex (K)
Ions Score: 55.82 Expect: 0.002

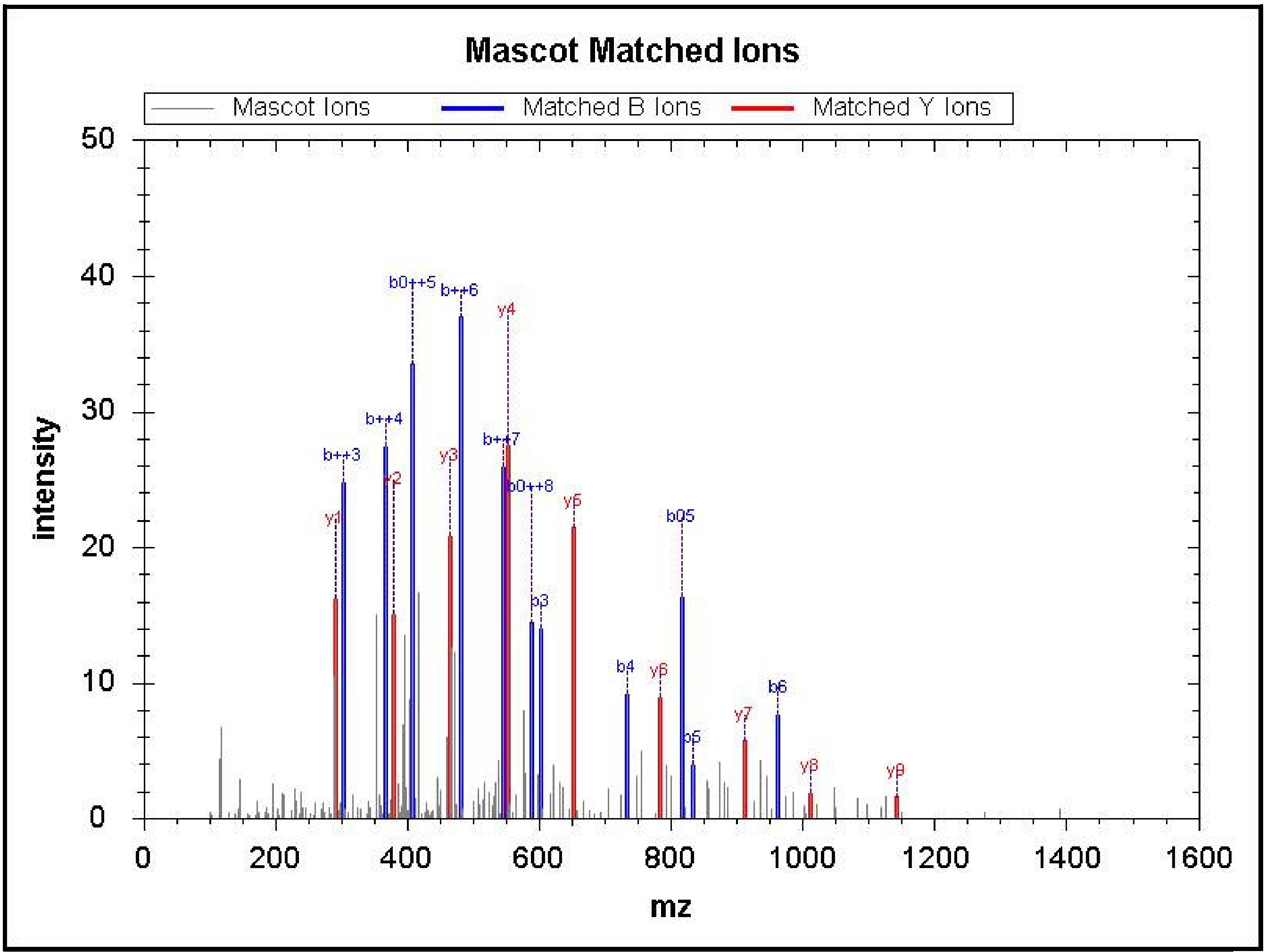

No	b	b++	b*	b*++	b0	b0++	Seq	y	y++	y*	y*++	y0	y0++	RevNo
1	417.31	209.16	400.28	200.64			K							12
2	474.33	237.67	457.30	229.15			G	1,327.60	664.30	1,310.57	655.79	1,309.59	655.30	11
3	603.37	302.19	586.34	293.68	585.36	293.18	E	1,270.58	635.79	1,253.55	627.28	1,252.57	626.79	10
4	732.41	366.71	715.39	358.20	714.40	357.70	E	1,141.53	571.27	1,124.51	562.76	1,123.52	562.27	9
5	833.46	417.23	816.43	408.72	815.45	408.23	T	1,012.49	506.75	995.46	498.24	994.48	497.74	8
6	962.50	481.76	945.48	473.24	944.49	472.75	E	911.44	456.23	894.42	447.71	893.43	447.22	7
7	1,091.55	546.28	1,074.52	537.76	1,073.54	537.27	E	782.40	391.70	765.37	383.19	764.39	382.70	6
8	1,192.59	596.80	1,175.57	588.29	1,174.58	587.80	T	653.36	327.18	636.33	318.67	635.35	318.18	5
9	1,279.63	640.32	1,262.60	631.80	1,261.61	631.31	S	552.31	276.66	535.28	268.15	534.30	267.65	4
10	1,366.66	683.83	1,349.63	675.32	1,348.65	674.83	S	465.28	233.14	448.25	224.63	447.27	224.14	3
11	1,453.69	727.35	1,436.66	718.84	1,435.68	718.34	S	378.25	189.63	361.22	181.11	360.24	180.62	2
12							K	291.21	146.11	274.19	137.60			1

Query 50363 Hit 1

MS/MS Fragmentation of LYHILTPVPPEELR
Found in sp|Q5SY16|NOL9_HUMAN, Polynucleotide 5'-hydroxyl-kinase NOL9 OS=Homo sapiens GN=NOL9 PE=1 SV=1
Match to Query 50363: 1820.034from(607.6852,3+)
Title: 654: Sum of 2 scans in range 1463 (rt=42.483, f=4, i=435) to 1464 (rt=42.5084, f=4, i=436)
[D:\lab212\membrane\Grace\Joyce\iTRAQ_34_2.raw]
Data File:Submitted from 20120508-1(merge) by Mascot Daemon on JOYCE-VAIO
Monoisotopic mass of neutral peptide Mr(calc): 1820.034
Variable modifications:
Ions Score: 55.33 Expect: 0.002

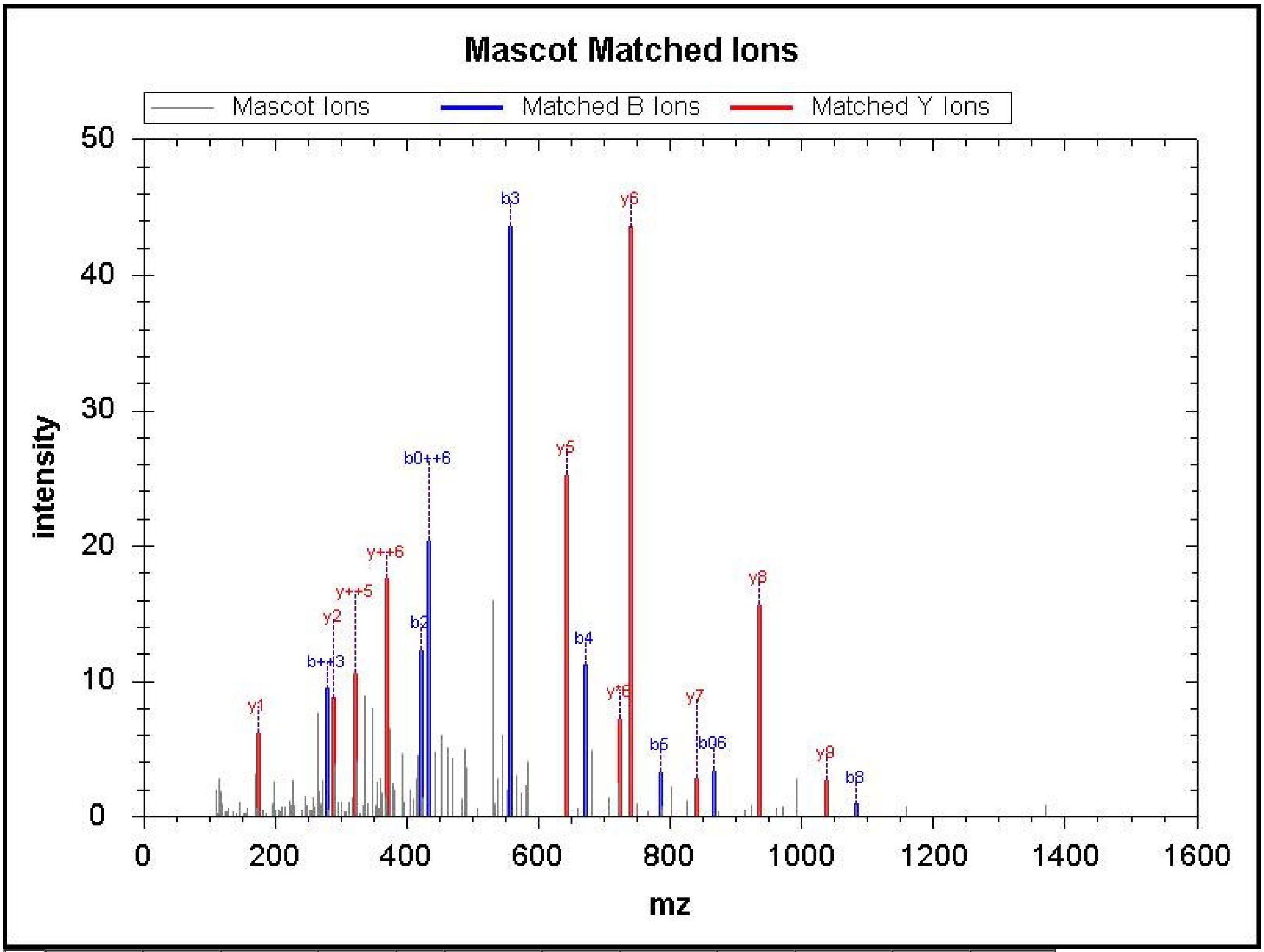

No	b	b++	b0	b0++	Seq	y	y++	y*	y*++	y0	y0++	RevNo
1	258.19	129.60			L							14
2	421.26	211.13			Y	1,563.85	782.43	1,546.83	773.92	1,545.84	773.42	13
3	558.32	279.66			H	1,400.79	700.90	1,383.76	692.39	1,382.78	691.89	12
4	671.40	336.20			I	1,263.73	632.37	1,246.70	623.86	1,245.72	623.36	11
5	784.48	392.75			L	1,150.65	575.83	1,133.62	567.31	1,132.64	566.82	10
6	885.53	443.27	867.52	434.26	T	1,037.56	519.28	1,020.54	510.77	1,019.55	510.28	9
7	982.58	491.80	964.57	482.79	P	936.51	468.76	919.49	460.25	918.50	459.76	8
8	1,081.65	541.33	1,063.64	532.32	V	839.46	420.23	822.44	411.72	821.45	411.23	7
9	1,178.71	589.86	1,160.69	580.85	P	740.39	370.70	723.37	362.19	722.38	361.70	6
10	1,275.76	638.38	1,257.75	629.38	P	643.34	322.17	626.31	313.66	625.33	313.17	5
11	1,404.80	702.90	1,386.79	693.90	E	546.29	273.65	529.26	265.13	528.28	264.64	4
12	1,533.84	767.43	1,515.83	758.42	E	417.25	209.13	400.22	200.61	399.24	200.12	3
13	1,646.93	823.97	1,628.92	814.96	L	288.20	144.61	271.18	136.09			2
14					R	175.12	88.06	158.09	79.55			1

Query 58152 Hit 1

MS/MS Fragmentation of LLPVLVHTFWIDTK
Found in sp|Q13505|MTX1_HUMAN, Metaxin-1 OS=Homo sapiens GN=MTX1 PE=1 SV=2
Match to Query 58152: 1969.158from(657.3934,3+)
Title: 1122: Sum of 2 scans in range 2461 (rt=64.8384, f=4, i=735) to 2462 (rt=64.8638, f=4, i=736)
[D:\lab212\membrane\Grace\Joyce\iTRAQ_37_1.raw]
Data File:Submitted from 20120508-1(merge) by Mascot Daemon on JOYCE-VAIO
Monoisotopic mass of neutral peptide Mr(calc): 1969.158
Variable modifications:
K14 :iTRAQ4plex (K)
Ions Score: 55.31 Expect: 0.001

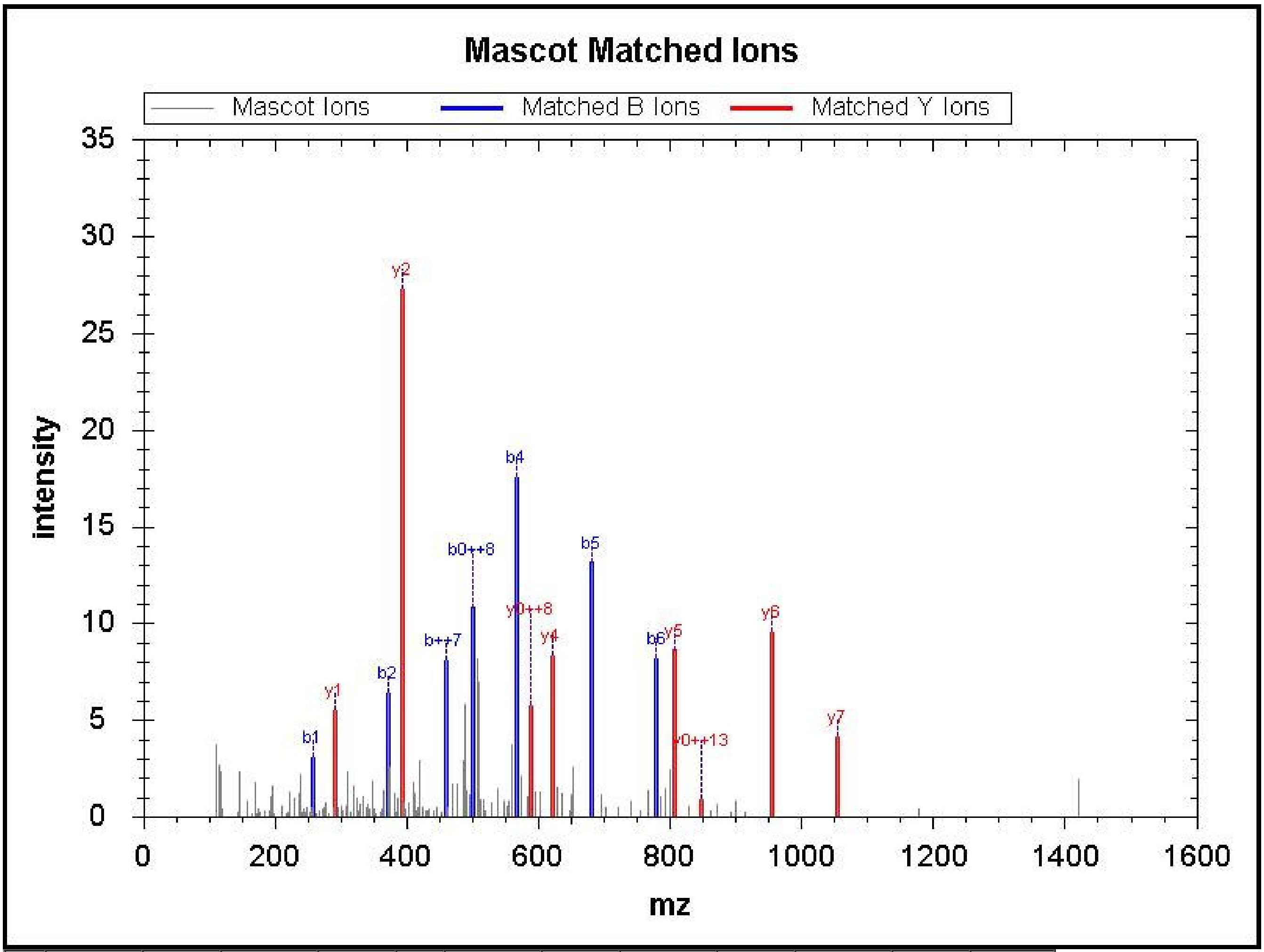

No	b	b++	b0	b0++	Seq	y	y++	y*	y*++	y0	y0++	RevNo
1	258.19	129.60			L							14
2	371.28	186.14			L	1,712.99	857.00	1,695.96	848.48	1,694.97	847.99	13
3	468.33	234.67			P	1,599.90	800.45	1,582.88	791.94	1,581.89	791.45	12
4	567.40	284.20			V	1,502.85	751.93	1,485.82	743.41	1,484.84	742.92	11
5	680.48	340.74			L	1,403.78	702.39	1,386.75	693.88	1,385.77	693.39	10
6	779.55	390.28			V	1,290.70	645.85	1,273.67	637.34	1,272.69	636.85	9
7	916.61	458.81			H	1,191.63	596.32	1,174.60	587.80	1,173.62	587.31	8
8	1,017.66	509.33	999.65	500.33	T	1,054.57	527.79	1,037.54	519.27	1,036.56	518.78	7
9	1,164.73	582.87	1,146.72	573.86	F	953.52	477.26	936.49	468.75	935.51	468.26	6
10	1,350.81	675.91	1,332.79	666.90	W	806.45	403.73	789.43	395.22	788.44	394.72	5
11	1,463.89	732.45	1,445.88	723.44	I	620.37	310.69	603.35	302.18	602.36	301.69	4
12	1,578.92	789.96	1,560.91	780.96	D	507.29	254.15	490.26	245.64	489.28	245.14	3
13	1,679.96	840.49	1,661.95	831.48	T	392.26	196.63	375.24	188.12	374.25	187.63	2
14					K	291.21	146.11	274.19	137.60			1

Query 75650 Hit 1

MS/MS Fragmentation of VNQWTTNVVEQTLSQLTK
Found in sp|P63172|DYLT1_HUMAN, Dynein light chain Tctex-type 1 OS=Homo sapiens GN=DYNLT1 PE=1 SV=1
Match to Query 75650: 2376.281from(793.101,3+)
Title: 1099: Scan 2455 (rt=64.6362, f=3, i=380) [D:\lab212\membrane\Grace\Joyce\iTRAQ_27_1.raw]
Data File:Submitted from 20120508-1(merge) by Mascot Daemon on JOYCE-VAIO
Monoisotopic mass of neutral peptide Mr(calc): 2376.281
Variable modifications:
K18 :iTRAQ4plex (K)
Ions Score: 55.21 Expect: 0.002

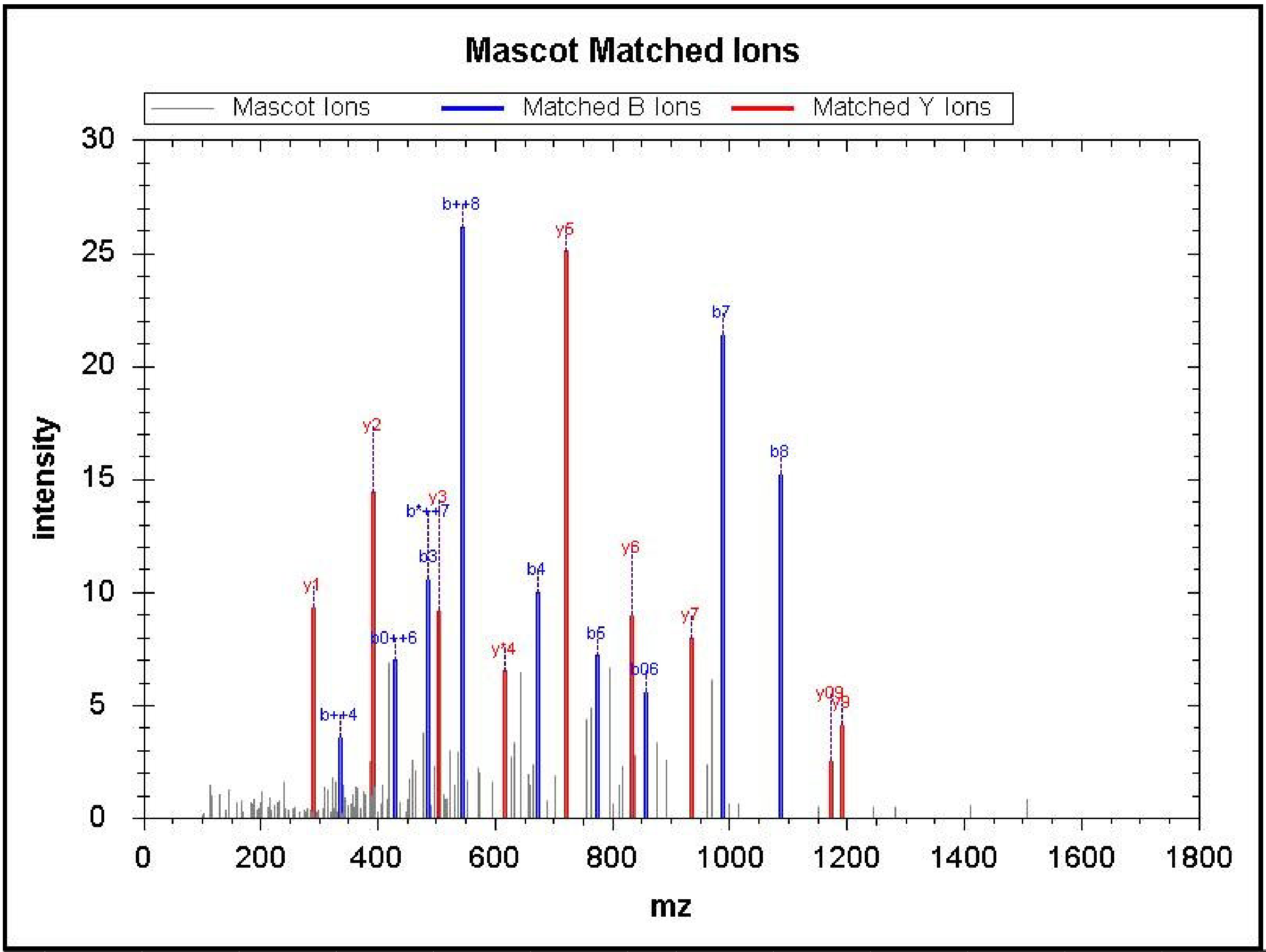

No	b	b++	b*	b*++	b0	b0++	Seq	y	y++	y*	y*++	y0	y0++	RevNo
1	244.18	122.59					V							18
2	358.22	179.61	341.19	171.10			N	2,134.13	1,067.57	2,117.10	1,059.05	2,116.12	1,058.56	17
3	486.28	243.64	469.25	235.13			Q	2,020.08	1,010.55	2,003.06	1,002.03	2,002.07	1,001.54	16
4	672.36	336.68	655.33	328.17			W	1,892.02	946.52	1,875.00	938.00	1,874.01	937.51	15
5	773.41	387.21	756.38	378.69	755.40	378.20	T	1,705.95	853.48	1,688.92	844.96	1,687.93	844.47	14
6	874.45	437.73	857.43	429.22	856.44	428.73	T	1,604.90	802.95	1,587.87	794.44	1,586.89	793.95	13
7	988.50	494.75	971.47	486.24	970.49	485.75	N	1,503.85	752.43	1,486.82	743.92	1,485.84	743.42	12
8	1,087.57	544.29	1,070.54	535.77	1,069.55	535.28	V	1,389.81	695.41	1,372.78	686.89	1,371.80	686.40	11
9	1,186.63	593.82	1,169.61	585.31	1,168.62	584.82	V	1,290.74	645.87	1,273.71	637.36	1,272.73	636.87	10
10	1,315.68	658.34	1,298.65	649.83	1,297.67	649.34	E	1,191.67	596.34	1,174.64	587.83	1,173.66	587.33	9
11	1,443.73	722.37	1,426.71	713.86	1,425.72	713.37	Q	1,062.63	531.82	1,045.60	523.30	1,044.62	522.81	8
12	1,544.78	772.89	1,527.76	764.38	1,526.77	763.89	T	934.57	467.79	917.54	459.27	916.56	458.78	7
13	1,657.87	829.44	1,640.84	820.92	1,639.86	820.43	L	833.52	417.26	816.49	408.75	815.51	408.26	6
14	1,744.90	872.95	1,727.87	864.44	1,726.89	863.95	S	720.44	360.72	703.41	352.21	702.43	351.72	5
15	1,872.96	936.98	1,855.93	928.47	1,854.95	927.98	Q	633.41	317.21	616.38	308.69	615.39	308.20	4

16	1,986.04	993.52	1,969.01	985.01	1,968.03	984.52	L	505.35	253.18	488.32	244.66	487.34	244.17	3
17	2,087.09	1,044.05	2,070.06	1,035.53	2,069.08	1,035.04	T	392.26	196.63	375.24	188.12	374.25	187.63	2
18							K	291.21	146.11	274.19	137.60			1

Query 78471 Hit 1

MS/MS Fragmentation of LLQLHITMPFSSPMEAELVR
Found in sp|O75638|CTAG2_HUMAN, Cancer/testis antigen 2 OS=Homo sapiens GN=CTAG2 PE=2 SV=2
Match to Query 78471: 2455.303from(819.4417,3+)
Title: 1027: Sum of 2 scans in range 2325 (rt=61.5303, f=4, i=685) to 2326 (rt=61.5557, f=4, i=686)
[D:\lab212\membrane\Grace\Joyce\iTRAQ_30_1.raw]
Data File:Submitted from 20120508-1(merge) by Mascot Daemon on JOYCE-VAIO
Monoisotopic mass of neutral peptide Mr(calc): 2455.303
Variable modifications:
Ions Score: 55.18 Expect: 0.002

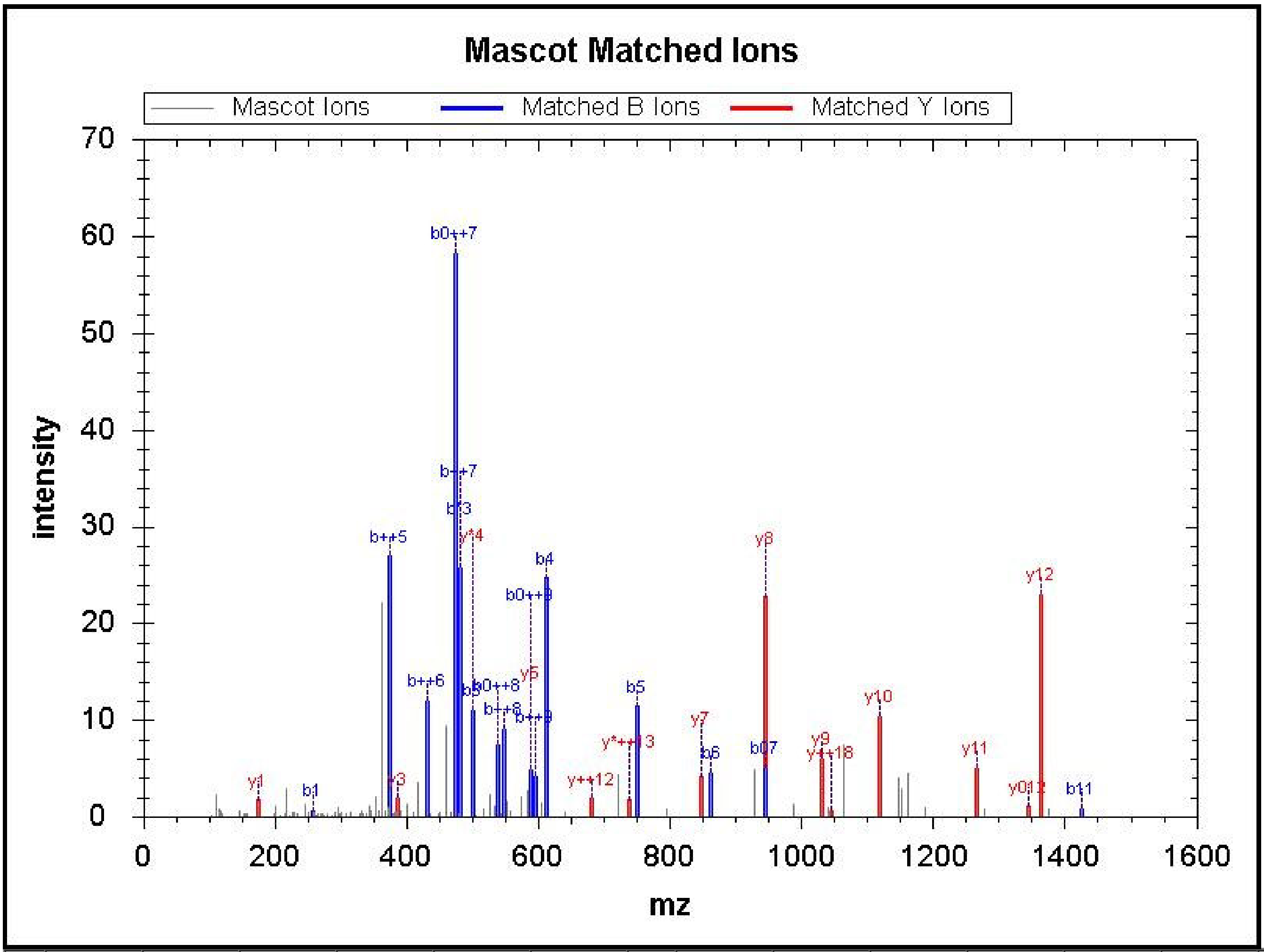

No	b	b++	b*	b*++	b0	b0++	Seq	y	y++	y*	y*++	y0	y0++	RevNo
1	258.19	129.60					L							20
2	371.28	186.14					L	2,199.13	1,100.07	2,182.10	1,091.56	2,181.12	1,091.06	19
3	499.34	250.17	482.31	241.66			Q	2,086.05	1,043.53	2,069.02	1,035.01	2,068.04	1,034.52	18
4	612.42	306.71	595.39	298.20			L	1,957.99	979.50	1,940.96	970.98	1,939.98	970.49	17
5	749.48	375.24	732.45	366.73			H	1,844.90	922.96	1,827.88	914.44	1,826.89	913.95	16
6	862.56	431.79	845.54	423.27			I	1,707.84	854.43	1,690.82	845.91	1,689.83	845.42	15
7	963.61	482.31	946.58	473.80	945.60	473.30	T	1,594.76	797.88	1,577.73	789.37	1,576.75	788.88	14
8	1,094.65	547.83	1,077.62	539.32	1,076.64	538.82	M	1,493.71	747.36	1,476.69	738.85	1,475.70	738.35	13
9	1,191.70	596.36	1,174.68	587.84	1,173.69	587.35	P	1,362.67	681.84	1,345.65	673.33	1,344.66	672.83	12
10	1,338.77	669.89	1,321.75	661.38	1,320.76	660.88	F	1,265.62	633.31	1,248.59	624.80	1,247.61	624.31	11
11	1,425.80	713.41	1,408.78	704.89	1,407.79	704.40	S	1,118.55	559.78	1,101.52	551.27	1,100.54	550.77	10

12	1,512.84	756.92	1,495.81	748.41	1,494.83	747.92	S	1,031.52	516.26	1,014.49	507.75	1,013.51	507.26	9
13	1,609.89	805.45	1,592.86	796.93	1,591.88	796.44	P	944.49	472.75	927.46	464.23	926.48	463.74	8
14	1,740.93	870.97	1,723.90	862.46	1,722.92	861.96	M	847.43	424.22	830.41	415.71	829.42	415.22	7
15	1,869.97	935.49	1,852.95	926.98	1,851.96	926.48	E	716.39	358.70	699.37	350.19	698.38	349.70	6
16	1,941.01	971.01	1,923.98	962.50	1,923.00	962.00	A	587.35	294.18	570.32	285.67	569.34	285.17	5
17	2,070.05	1,035.53	2,053.03	1,027.02	2,052.04	1,026.52	E	516.31	258.66	499.29	250.15	498.30	249.66	4
18	2,183.14	1,092.07	2,166.11	1,083.56	2,165.13	1,083.07	L	387.27	194.14	370.24	185.63			3
19	2,282.20	1,141.61	2,265.18	1,133.09	2,264.19	1,132.60	V	274.19	137.60	257.16	129.08			2
20							R	175.12	88.06	158.09	79.55			1

Query 32347 Hit 1

MS/MS Fragmentation of LLLSTLTLLSK
Found in sp|Q96EK6|GNA1_HUMAN, Glucosamine 6-phosphate N-acetyltransferase OS=Homo sapiens GN=GNPNAT1 PE=1 SV=1
Match to Query 32347: 1488.975from(745.4948,2+)
Title: 1060: Sum of 2 scans in range 2324 (rt=62.0053, f=4, i=719) to 2325 (rt=62.0307, f=4, i=720) [D:\lab212\membrane\Grace\Joyce\iTRAQ_27_2_327.raw]
Data File:Submitted from 20120508-1(merge) by Mascot Daemon on JOYCE-VAIO
Monoisotopic mass of neutral peptide Mr(calc): 1488.975
Variable modifications:
K11 :iTRAQ4plex (K)
Ions Score: 55.07 Expect: 0.000

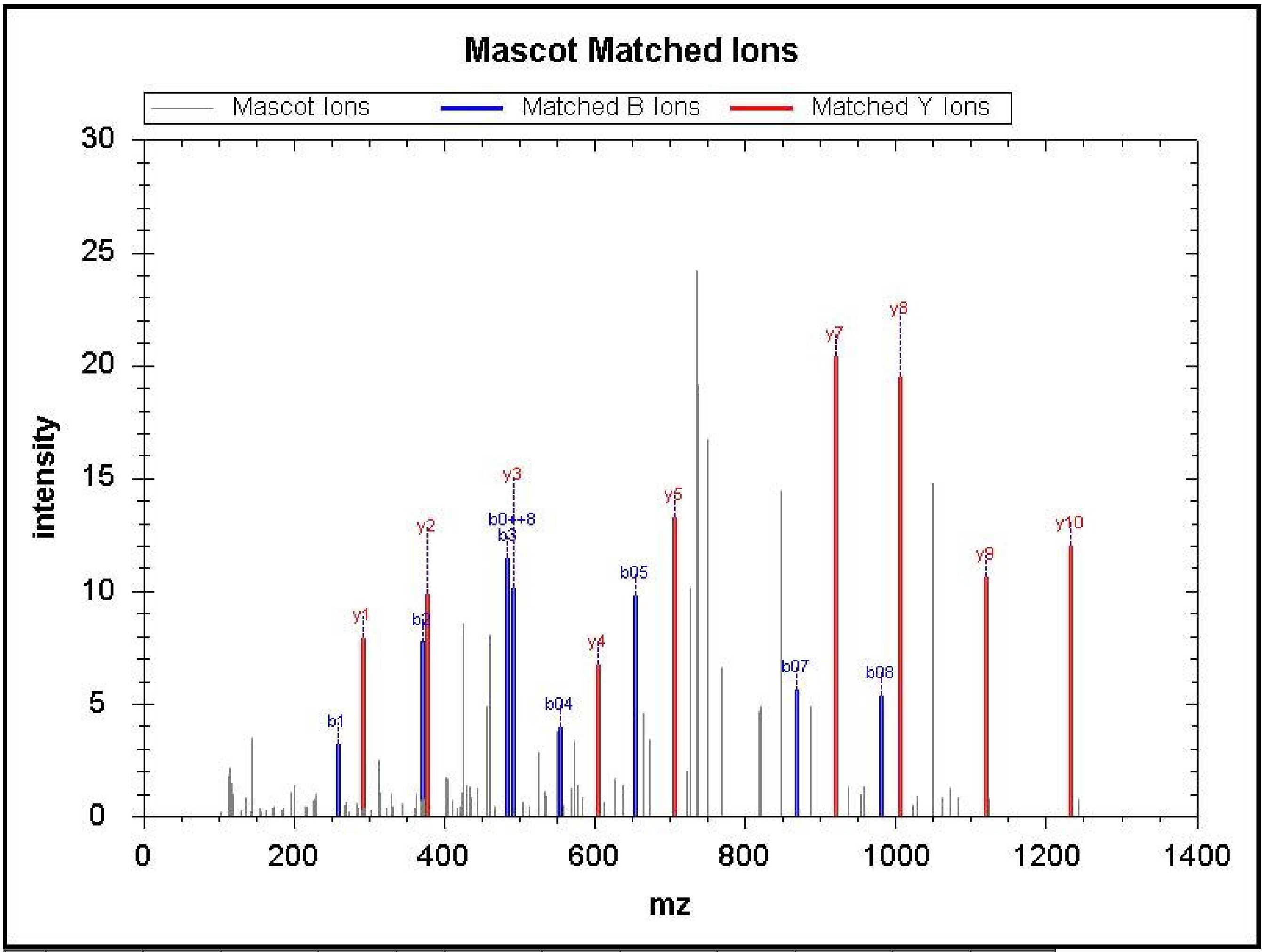

No	b	b++	b0	b0++	Seq	y	y++	y*	y*++	y0	y0++	RevNo
1	258.19	129.60			L							11
2	371.28	186.14			L	1,232.79	616.90	1,215.77	608.39	1,214.78	607.90	10
3	484.36	242.68			L	1,119.71	560.36	1,102.68	551.85	1,101.70	551.35	9
4	571.39	286.20	553.38	277.20	S	1,006.63	503.82	989.60	495.30	988.62	494.81	8

5	672.44	336.72	654.43	327.72	T	919.59	460.30	902.57	451.79	901.58	451.30	7
6	785.53	393.27	767.51	384.26	L	818.55	409.78	801.52	401.26	800.54	400.77	6
7	886.57	443.79	868.56	434.78	T	705.46	353.23	688.44	344.72	687.45	344.23	5
8	999.66	500.33	981.65	491.33	L	604.41	302.71	587.39	294.20	586.40	293.71	4
9	1,112.74	556.87	1,094.73	547.87	L	491.33	246.17	474.30	237.66	473.32	237.16	3
10	1,199.77	600.39	1,181.76	591.38	S	378.25	189.63	361.22	181.11	360.24	180.62	2
11					K	291.21	146.11	274.19	137.60			1

Query 46875 Hit 1

MS/MS Fragmentation of IHAFVQDTTLSEPR
Found in sp|Q8N1G2|MTR1_HUMAN, Cap-specific mRNA (nucleoside-2'-O-)-methyltransferase 1 OS=Homo sapiens GN=FTSJD2 PE=1 SV=1
Match to Query 46875: 1756.919from(586.647,3+)
Title: 454: Scan 1057 (rt=33.2589, f=3, i=160) [D:\lab212\membrane\Grace\Joyce\iTRAQ_37_2.raw]
Data File:Submitted from 20120508-1(merge) by Mascot Daemon on JOYCE-VAIO
Monoisotopic mass of neutral peptide Mr(calc): 1756.919
Variable modifications:
Ions Score: 55.02 Expect: 0.003

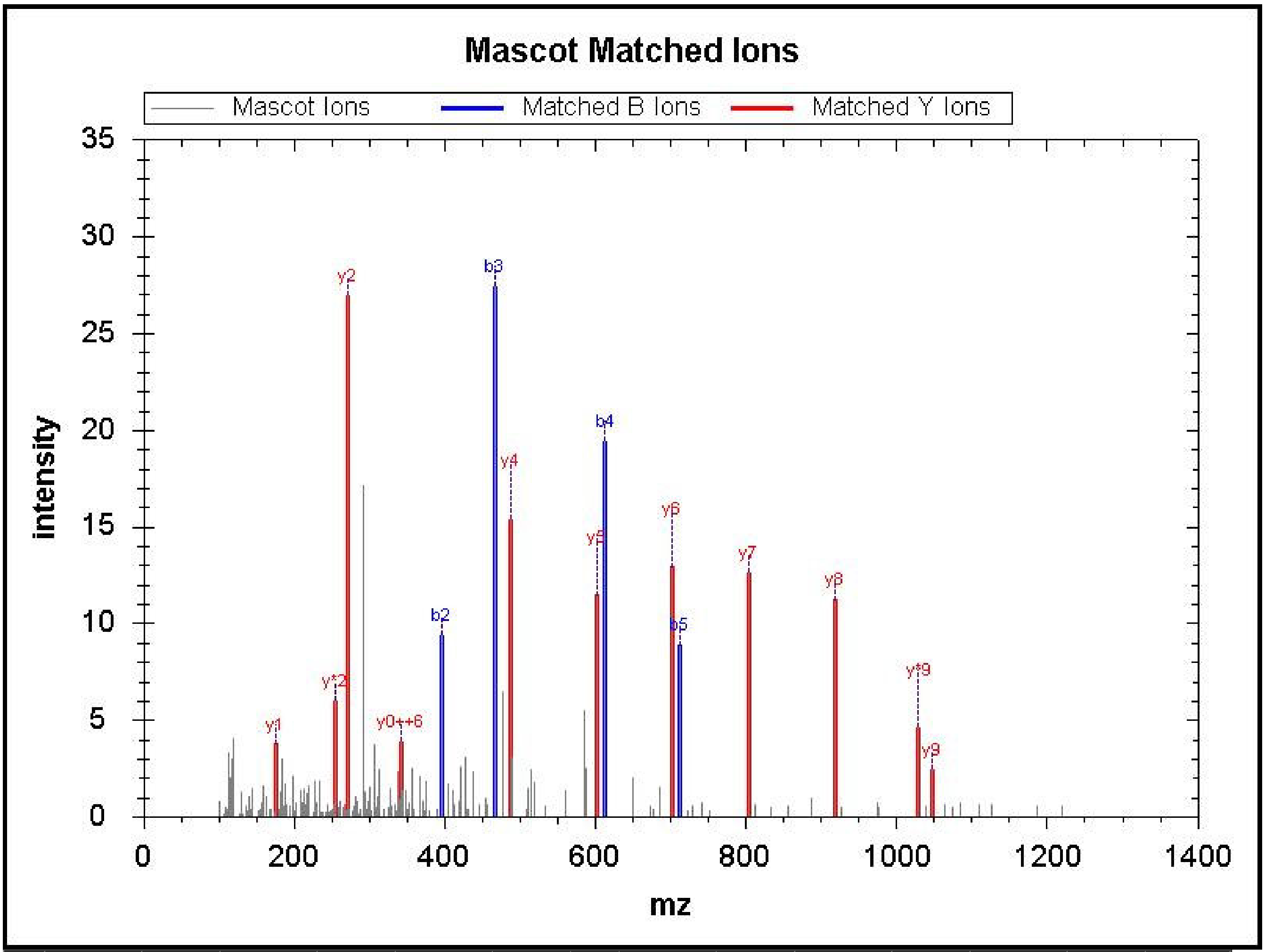

No	b	b++	b*	b*++	b0	b0++	Seq	y	y++	y*	y*++	y0	y0++	RevNo
1	258.19	129.60					I							14
2	395.25	198.13					H	1,500.74	750.88	1,483.72	742.36	1,482.73	741.87	13
3	466.29	233.65					A	1,363.69	682.35	1,346.66	673.83	1,345.67	673.34	12
4	613.36	307.18					F	1,292.65	646.83	1,275.62	638.31	1,274.64	637.82	11
5	712.43	356.72					V	1,145.58	573.29	1,128.55	564.78	1,127.57	564.29	10
6	840.48	420.75	823.46	412.23			Q	1,046.51	523.76	1,029.48	515.25	1,028.50	514.75	9
7	955.51	478.26	938.49	469.75	937.50	469.25	D	918.45	459.73	901.43	451.22	900.44	450.72	8

8	1,056.56	528.78	1,039.53	520.27	1,038.55	519.78	T	803.43	402.22	786.40	393.70	785.42	393.21	7
9	1,157.61	579.31	1,140.58	570.79	1,139.60	570.30	T	702.38	351.69	685.35	343.18	684.37	342.69	6
10	1,270.69	635.85	1,253.66	627.34	1,252.68	626.84	L	601.33	301.17	584.30	292.66	583.32	292.16	5
11	1,357.72	679.37	1,340.70	670.85	1,339.71	670.36	S	488.25	244.63	471.22	236.11	470.24	235.62	4
12	1,486.77	743.89	1,469.74	735.37	1,468.76	734.88	E	401.21	201.11	384.19	192.60	383.20	192.11	3
13	1,583.82	792.41	1,566.79	783.90	1,565.81	783.41	P	272.17	136.59	255.15	128.08			2
14							R	175.12	88.06	158.09	79.55			1

Query 84211 Hit 1

MS/MS Fragmentation of LHAATPPTFGVDLINELVENFGR
Found in sp|Q8TF05|PP4R1_HUMAN, Serine/threonine-protein phosphatase 4 regulatory subunit 1 OS=Homo sapiens GN=PPP4R1 PE=1 SV=1
Match to Query 84211: 2653.402 from(885.4745,3+)
Title: 1245: Sum of 2 scans in range 2976 (rt=75.3522, f=2, i=511) to 2977 (rt=75.3776, f=2, i=512) [D:\lab212\membrane\Grace\Joyce\iTRAQ_32_1.raw]
Data File:Submitted from 20120508-1(merge) by Mascot Daemon on JOYCE-VAIO
Monoisotopic mass of neutral peptide Mr(calc): 2653.402
Variable modifications:
Ions Score: 55.01 Expect: 0.003

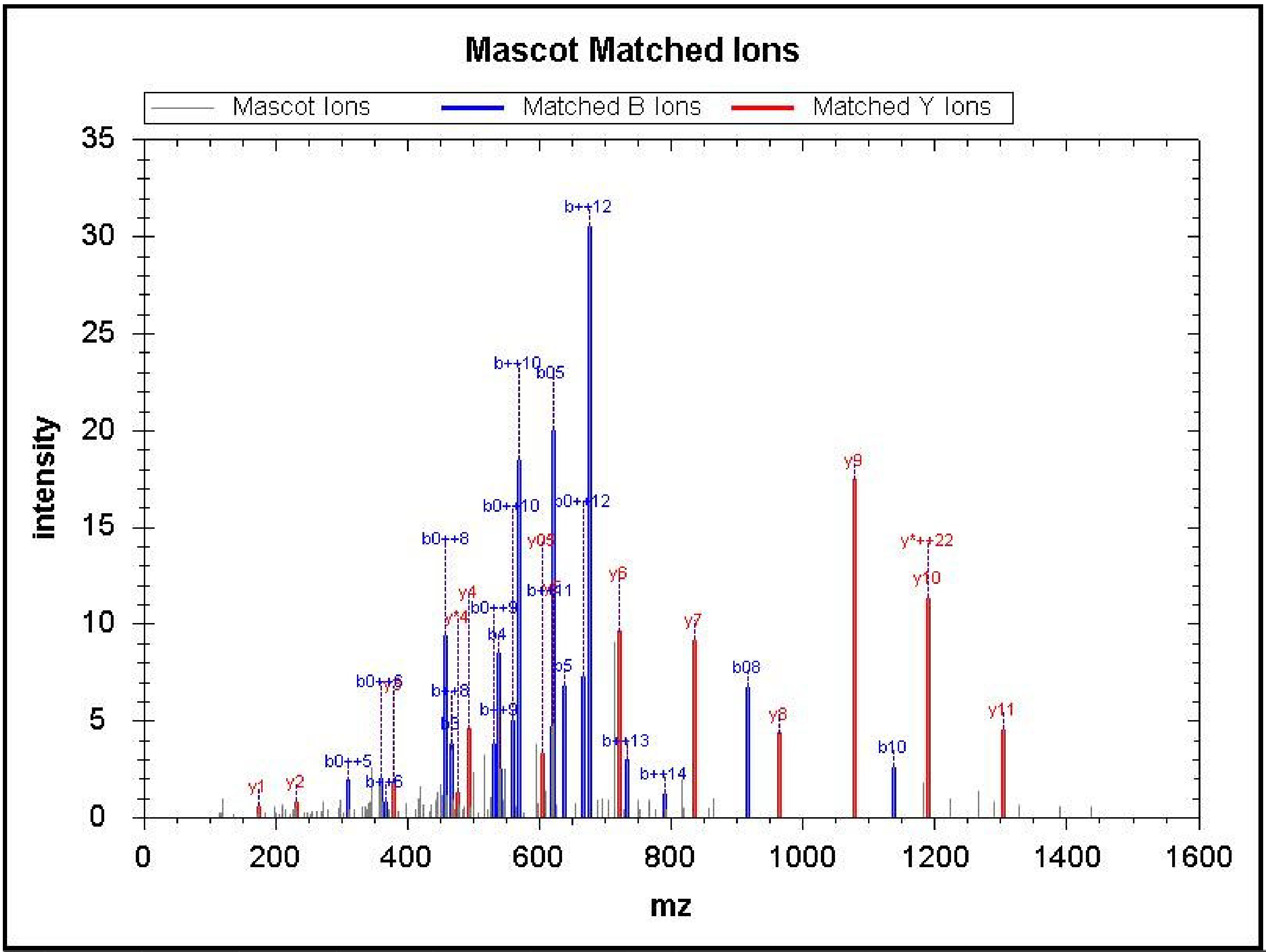

No	b	b++	b*	b*++	b0	b0++	Seq	y	y++	y*	y*++	y0	y0++	RevNo
1	258.19	129.60					L							23
2	395.25	198.13					H	2,397.22	1,199.11	2,380.19	1,190.60	2,379.21	1,190.11	22
3	466.29	233.65					A	2,260.16	1,130.58	2,243.13	1,122.07	2,242.15	1,121.58	21
4	537.33	269.17					A	2,189.12	1,095.07	2,172.10	1,086.55	2,171.11	1,086.06	20
5	638.37	319.69			620.36	310.69	T	2,118.09	1,059.55	2,101.06	1,051.03	2,100.08	1,050.54	19
6	735.43	368.22			717.42	359.21	P	2,017.04	1,009.02	2,000.01	1,000.51	1,999.03	1,000.02	18

7	832.48	416.74			814.47	407.74	P	1,919.99	960.50	1,902.96	951.98	1,901.98	951.49	17
8	933.53	467.27			915.52	458.26	T	1,822.93	911.97	1,805.91	903.46	1,804.92	902.97	16
9	1,080.60	540.80			1,062.59	531.80	F	1,721.89	861.45	1,704.86	852.93	1,703.88	852.44	15
10	1,137.62	569.31			1,119.61	560.31	G	1,574.82	787.91	1,557.79	779.40	1,556.81	778.91	14
11	1,236.69	618.85			1,218.68	609.84	V	1,517.80	759.40	1,500.77	750.89	1,499.79	750.40	13
12	1,351.71	676.36			1,333.70	667.35	D	1,418.73	709.87	1,401.70	701.35	1,400.72	700.86	12
13	1,464.80	732.90			1,446.79	723.90	L	1,303.70	652.35	1,286.67	643.84	1,285.69	643.35	11
14	1,577.88	789.44			1,559.87	780.44	I	1,190.62	595.81	1,173.59	587.30	1,172.61	586.81	10
15	1,691.92	846.47	1,674.90	837.95	1,673.91	837.46	N	1,077.53	539.27	1,060.51	530.76	1,059.52	530.26	9
16	1,820.97	910.99	1,803.94	902.47	1,802.96	901.98	E	963.49	482.25	946.46	473.74	945.48	473.24	8
17	1,934.05	967.53	1,917.02	959.02	1,916.04	958.52	L	834.45	417.73	817.42	409.21	816.44	408.72	7
18	2,033.12	1,017.06	2,016.09	1,008.55	2,015.11	1,008.06	V	721.36	361.18	704.34	352.67	703.35	352.18	6
19	2,162.16	1,081.58	2,145.13	1,073.07	2,144.15	1,072.58	E	622.29	311.65	605.27	303.14	604.28	302.65	5
20	2,276.20	1,138.61	2,259.18	1,130.09	2,258.19	1,129.60	N	493.25	247.13	476.23	238.62			4
21	2,423.27	1,212.14	2,406.25	1,203.63	2,405.26	1,203.13	F	379.21	190.11	362.18	181.59			3
22	2,480.29	1,240.65	2,463.27	1,232.14	2,462.28	1,231.65	G	232.14	116.57	215.11	108.06			2
23							R	175.12	88.06	158.09	79.55			1

Query 79296 Hit 1

MS/MS Fragmentation of DLTKPVVTISDEPDILYK
Found in sp|P82664|RT10_HUMAN, 28S ribosomal protein S10
Match to Query 79296: 2477.393 from(826.8051,3+)
Title: 665: Sum of 2 scans in range 1514 (rt=43.4424, f=4, i=435) to 1515 (rt=43.4678, f=4, i=436)
[D:\lab212\membrane\Grace\Joyce\iTRAQ_38_1.raw]
Data File:Submitted from 20120508-1(merge) by Mascot Daemon on JOYCE-VAIO
Monoisotopic mass of neutral peptide Mr(calc): 2477.393
Variable modifications:
K4 :iTRAQ4plex (K)
K18 :iTRAQ4plex (K)
Ions Score: 54.96 Expect: 0.001

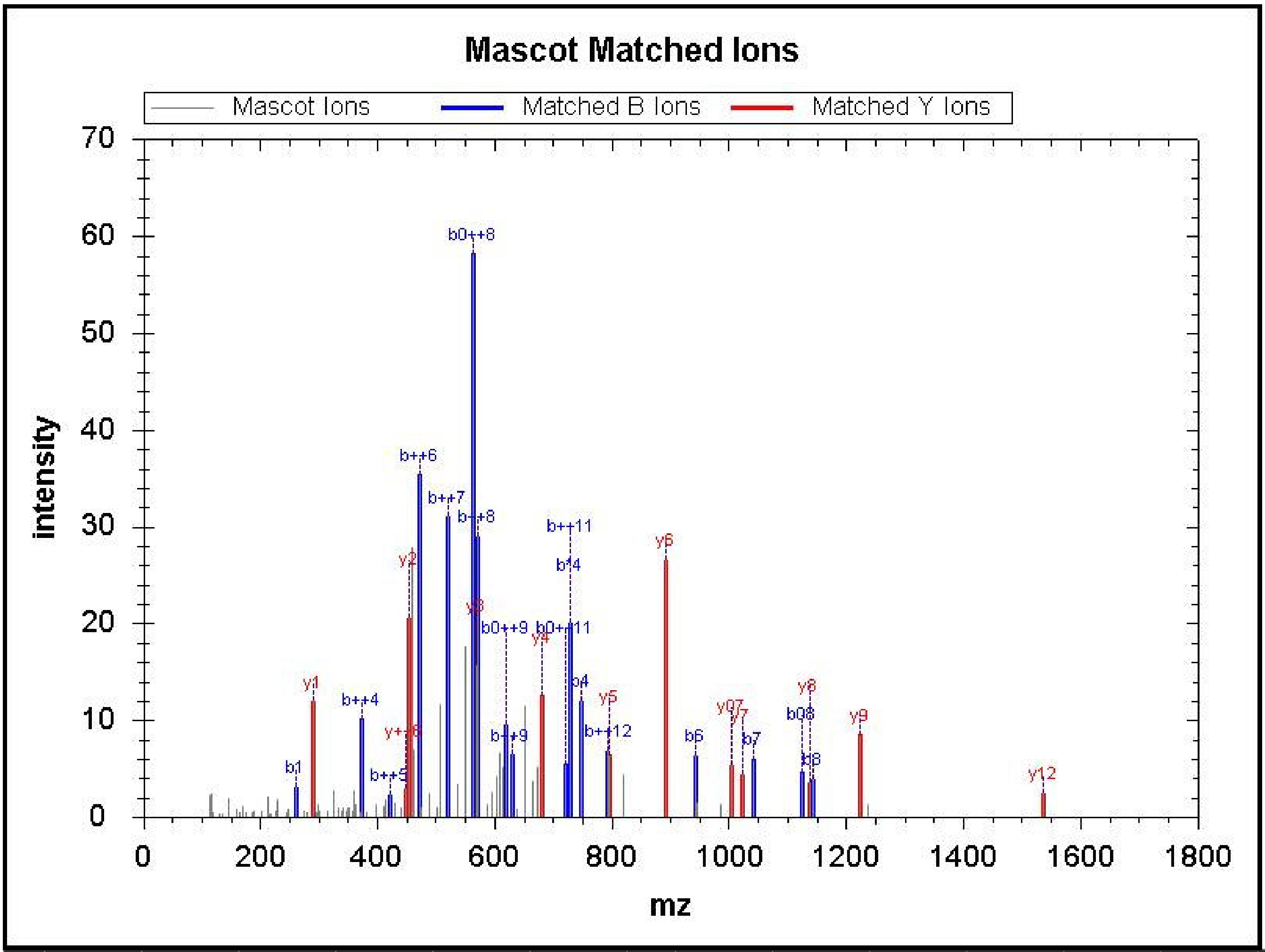

No	b	b++	b*	b*++	b0	b0++	Seq	y	y++	y*	y*++	y0	y0++	RevNo
1	260.14	130.57			242.13	121.57	D							18
2	373.22	187.11			355.21	178.11	L	2,219.28	1,110.14	2,202.25	1,101.63	2,201.27	1,101.14	17
3	474.27	237.64			456.26	228.63	T	2,106.19	1,053.60	2,089.17	1,045.09	2,088.18	1,044.60	16
4	746.47	373.74	729.44	365.22	728.45	364.73	K	2,005.15	1,003.08	1,988.12	994.56	1,987.14	994.07	15
5	843.52	422.26	826.49	413.75	825.51	413.26	P	1,732.95	866.98	1,715.92	858.46	1,714.94	857.97	14
6	942.59	471.80	925.56	463.28	924.58	462.79	V	1,635.90	818.45	1,618.87	809.94	1,617.89	809.45	13
7	1,041.65	521.33	1,024.63	512.82	1,023.64	512.33	V	1,536.83	768.92	1,519.80	760.40	1,518.82	759.91	12
8	1,142.70	571.85	1,125.68	563.34	1,124.69	562.85	T	1,437.76	719.38	1,420.73	710.87	1,419.75	710.38	11
9	1,255.79	628.40	1,238.76	619.88	1,237.78	619.39	I	1,336.71	668.86	1,319.69	660.35	1,318.70	659.85	10
10	1,342.82	671.91	1,325.79	663.40	1,324.81	662.91	S	1,223.63	612.32	1,206.60	603.80	1,205.62	603.31	9
11	1,457.85	729.43	1,440.82	720.91	1,439.83	720.42	D	1,136.60	568.80	1,119.57	560.29	1,118.58	559.80	8
12	1,586.89	793.95	1,569.86	785.43	1,568.88	784.94	E	1,021.57	511.29	1,004.54	502.77	1,003.56	502.28	7
13	1,683.94	842.47	1,666.91	833.96	1,665.93	833.47	P	892.53	446.77	875.50	438.25	874.52	437.76	6
14	1,798.97	899.99	1,781.94	891.47	1,780.96	890.98	D	795.47	398.24	778.45	389.73	777.46	389.23	5
15	1,912.05	956.53	1,895.03	948.02	1,894.04	947.52	I	680.45	340.73	663.42	332.21			4
16	2,025.14	1,013.07	2,008.11	1,004.56	2,007.13	1,004.07	L	567.36	284.18	550.34	275.67			3
17	2,188.20	1,094.60	2,171.17	1,086.09	2,170.19	1,085.60	Y	454.28	227.64	437.25	219.13			2
18							K	291.21	146.11	274.19	137.60			1

Query 63177 Hit 1

MS/MS Fragmentation of RQPDSYFSVLNAFIDR
Found in sp|Q9Y4P1|ATG4B_HUMAN, Cysteine protease ATG4B OS=Homo sapiens GN=ATG4B PE=1 SV=2
Match to Query 63177: 2071.067from(691.363,3+)
Title: 1059: Sum of 2 scans in range 2281 (rt=61.0739, f=4, i=707) to 2282 (rt=61.0993, f=4, i=708)

[D:\lab212\membrane\Grace\Joyce\iTRAQ_40_1.raw]
Data File:Submitted from 20120508-1(merge) by Mascot Daemon on JOYCE-VAIO
Monoisotopic mass of neutral peptide Mr(calc): 2071.067
Variable modifications:
Ions Score: 54.94 Expect: 0.003

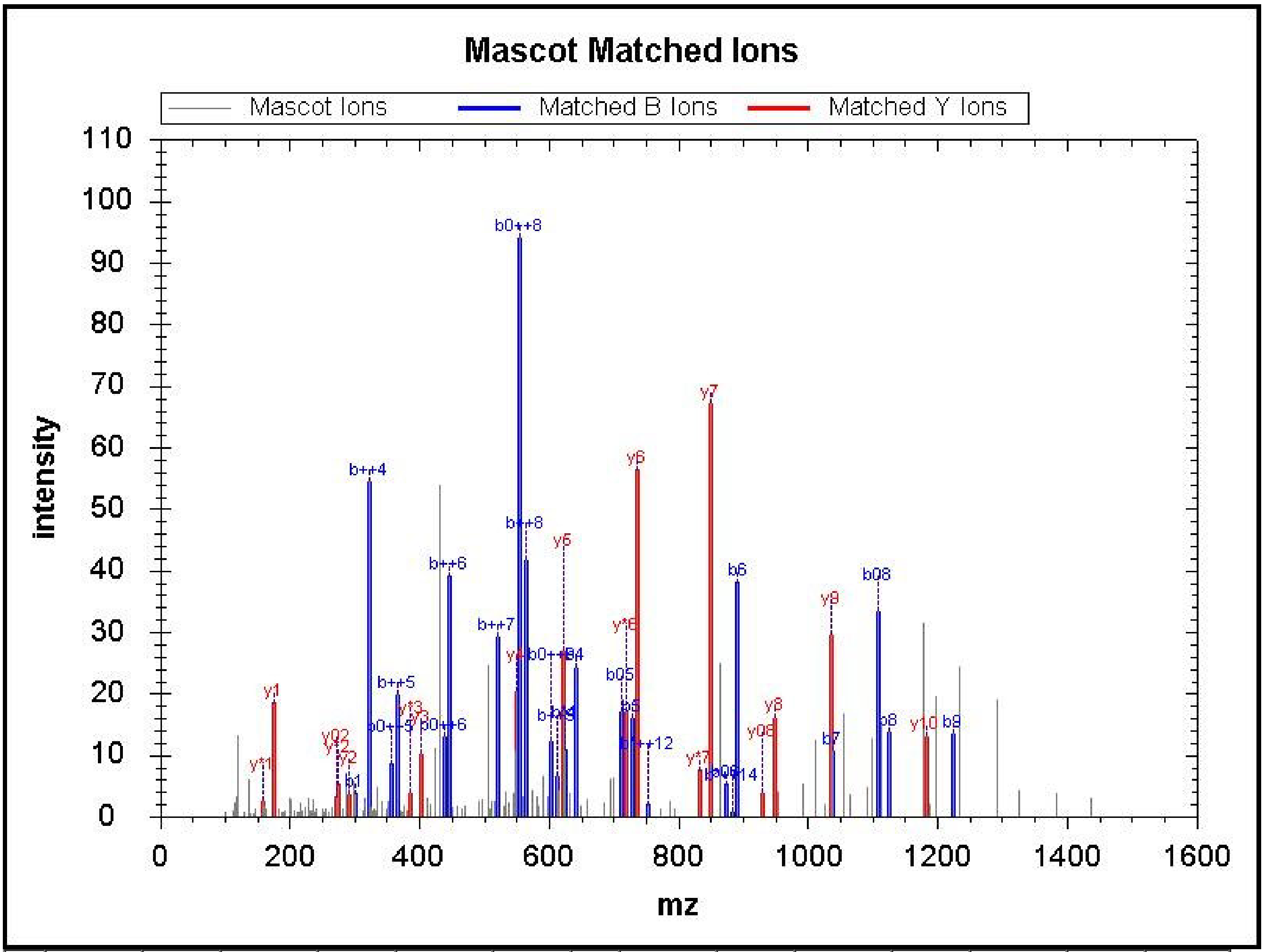

No	b	b++	b*	b*++	b0	b0++	Seq	y	y++	y*	y*++	y0	y0++	RevNo
1	301.21	151.11	284.18	142.60			R							16
2	429.27	215.14	412.24	206.62			Q	1,771.86	886.44	1,754.84	877.92	1,753.85	877.43	15
3	526.32	263.66	509.30	255.15			P	1,643.81	822.41	1,626.78	813.89	1,625.80	813.40	14
4	641.35	321.18	624.32	312.66	623.34	312.17	D	1,546.75	773.88	1,529.73	765.37	1,528.74	764.88	13
5	728.38	364.69	711.35	356.18	710.37	355.69	S	1,431.73	716.37	1,414.70	707.85	1,413.72	707.36	12
6	891.44	446.23	874.42	437.71	873.43	437.22	Y	1,344.69	672.85	1,327.67	664.34	1,326.68	663.85	11
7	1,038.51	519.76	1,021.49	511.25	1,020.50	510.75	F	1,181.63	591.32	1,164.60	582.81	1,163.62	582.31	10
8	1,125.54	563.28	1,108.52	554.76	1,107.53	554.27	S	1,034.56	517.79	1,017.54	509.27	1,016.55	508.78	9
9	1,224.61	612.81	1,207.59	604.30	1,206.60	603.80	V	947.53	474.27	930.50	465.76	929.52	465.26	8
10	1,337.70	669.35	1,320.67	660.84	1,319.69	660.35	L	848.46	424.73	831.44	416.22	830.45	415.73	7
11	1,451.74	726.37	1,434.71	717.86	1,433.73	717.37	N	735.38	368.19	718.35	359.68	717.37	359.19	6
12	1,522.78	761.89	1,505.75	753.38	1,504.77	752.89	A	621.34	311.17	604.31	302.66	603.32	302.17	5
13	1,669.85	835.43	1,652.82	826.91	1,651.84	826.42	F	550.30	275.65	533.27	267.14	532.29	266.65	4
14	1,782.93	891.97	1,765.90	883.46	1,764.92	882.96	I	403.23	202.12	386.20	193.61	385.22	193.11	3
15	1,897.96	949.48	1,880.93	940.97	1,879.95	940.48	D	290.15	145.58	273.12	137.06	272.14	136.57	2
16							R	175.12	88.06	158.09	79.55			1

Query 87532 Hit 1

MS/MS Fragmentation of QFHVLLSTIHELQQTLENDEK

Found in sp|Q6I9Y2|THOC7_HUMAN, THO complex subunit 7 homolog OS=Homo sapiens GN=THOC7 PE=1 SV=3
Match to Query 87532: 2809.488from(937.5034,3+)
Title: 971: Sum of 2 scans in range 2474 (rt=64.0117, f=4, i=637) to 2475 (rt=64.0371, f=4, i=638)
[D:\lab212\membrane\Grace\Joyce\iTRAQ_48_1.raw]
Data File:Submitted from 20120508-1(merge) by Mascot Daemon on JOYCE-VAIO
Monoisotopic mass of neutral peptide Mr(calc): 2809.488
Variable modifications:
K21 :iTRAQ4plex (K)
Ions Score: 54.9 Expect: 0.002

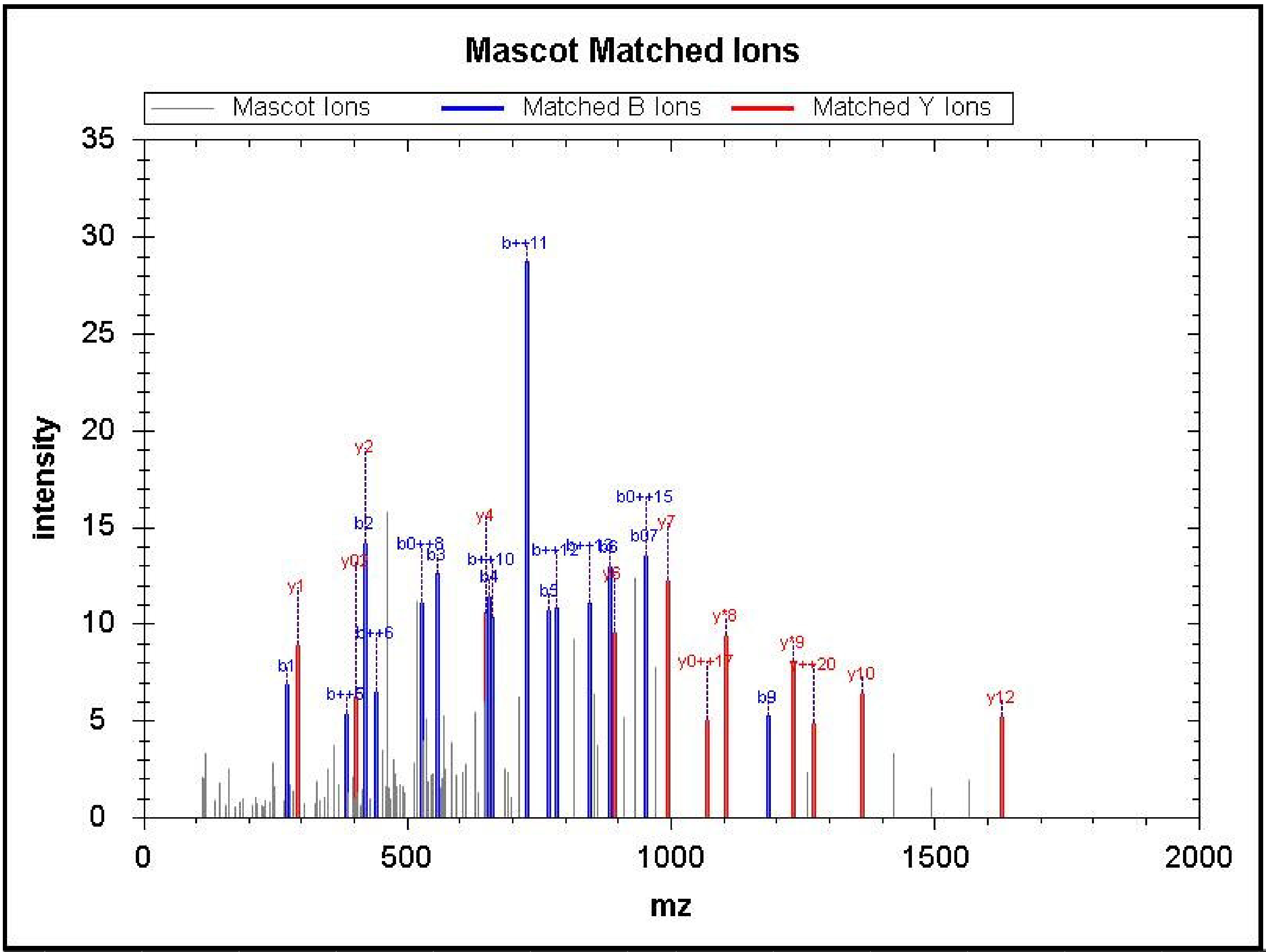

No	b	b++	b*	b*++	b0	b0++	Seq	y	y++	y*	y*++	y0	y0++	RevNo
1	273.17	137.09	256.14	128.57			Q							21
2	420.24	210.62	403.21	202.11			F	2,538.33	1,269.67	2,521.31	1,261.16	2,520.32	1,260.66	20
3	557.30	279.15	540.27	270.64			H	2,391.26	1,196.14	2,374.24	1,187.62	2,373.25	1,187.13	19
4	656.36	328.69	639.34	320.17			V	2,254.20	1,127.61	2,237.18	1,119.09	2,236.19	1,118.60	18
5	769.45	385.23	752.42	376.71			L	2,155.14	1,078.07	2,138.11	1,069.56	2,137.13	1,069.07	17
6	882.53	441.77	865.51	433.26			L	2,042.05	1,021.53	2,025.03	1,013.02	2,024.04	1,012.52	16
7	969.56	485.29	952.54	476.77	951.55	476.28	S	1,928.97	964.99	1,911.94	956.47	1,910.96	955.98	15
8	1,070.61	535.81	1,053.59	527.30	1,052.60	526.80	T	1,841.94	921.47	1,824.91	912.96	1,823.93	912.47	14
9	1,183.70	592.35	1,166.67	583.84	1,165.69	583.35	I	1,740.89	870.95	1,723.86	862.43	1,722.88	861.94	13
10	1,320.75	660.88	1,303.73	652.37	1,302.74	651.88	H	1,627.80	814.41	1,610.78	805.89	1,609.79	805.40	12
11	1,449.80	725.40	1,432.77	716.89	1,431.79	716.40	E	1,490.75	745.88	1,473.72	737.36	1,472.73	736.87	11
12	1,562.88	781.94	1,545.85	773.43	1,544.87	772.94	L	1,361.70	681.36	1,344.68	672.84	1,343.69	672.35	10
13	1,690.94	845.97	1,673.91	837.46	1,672.93	836.97	Q	1,248.62	624.81	1,231.59	616.30	1,230.61	615.81	9
14	1,819.00	910.00	1,801.97	901.49	1,800.99	901.00	Q	1,120.56	560.78	1,103.53	552.27	1,102.55	551.78	8
15	1,920.05	960.53	1,903.02	952.01	1,902.04	951.52	T	992.50	496.75	975.48	488.24	974.49	487.75	7
16	2,033.13	1,017.07	2,016.10	1,008.56	2,015.12	1,008.06	L	891.45	446.23	874.43	437.72	873.44	437.23	6

17	2,162.17	1,081.59	2,145.15	1,073.08	2,144.16	1,072.58	E	778.37	389.69	761.34	381.18	760.36	380.68	5
18	2,276.22	1,138.61	2,259.19	1,130.10	2,258.21	1,129.61	N	649.33	325.17	632.30	316.65	631.32	316.16	4
19	2,391.24	1,196.13	2,374.22	1,187.61	2,373.23	1,187.12	D	535.28	268.15	518.26	259.63	517.27	259.14	3
20	2,520.29	1,260.65	2,503.26	1,252.13	2,502.27	1,251.64	E	420.26	210.63	403.23	202.12	402.25	201.63	2
21							K	291.21	146.11	274.19	137.60			1

Query 25396 Hit 1

MS/MS Fragmentation of LPFLVMIIK
Found in sp|Q9Y6A4|CP080_HUMAN, UPF0468 protein C16orf80 OS=Homo sapiens GN=C16orf80 PE=1 SV=1
Match to Query 25396: 1360.876from(681.4454,2+)
Title: 1174: Scan 2610 (rt=68.1287, f=3, i=405) [D:\lab212\membrane\Grace\Joyce\iTRAQ_27_1.raw]
Data File:Submitted from 20120508-1(merge) by Mascot Daemon on JOYCE-VAIO
Monoisotopic mass of neutral peptide Mr(calc): 1360.876
Variable modifications:
K9 :iTRAQ4plex (K)
Ions Score: 54.89 Expect: 0.001

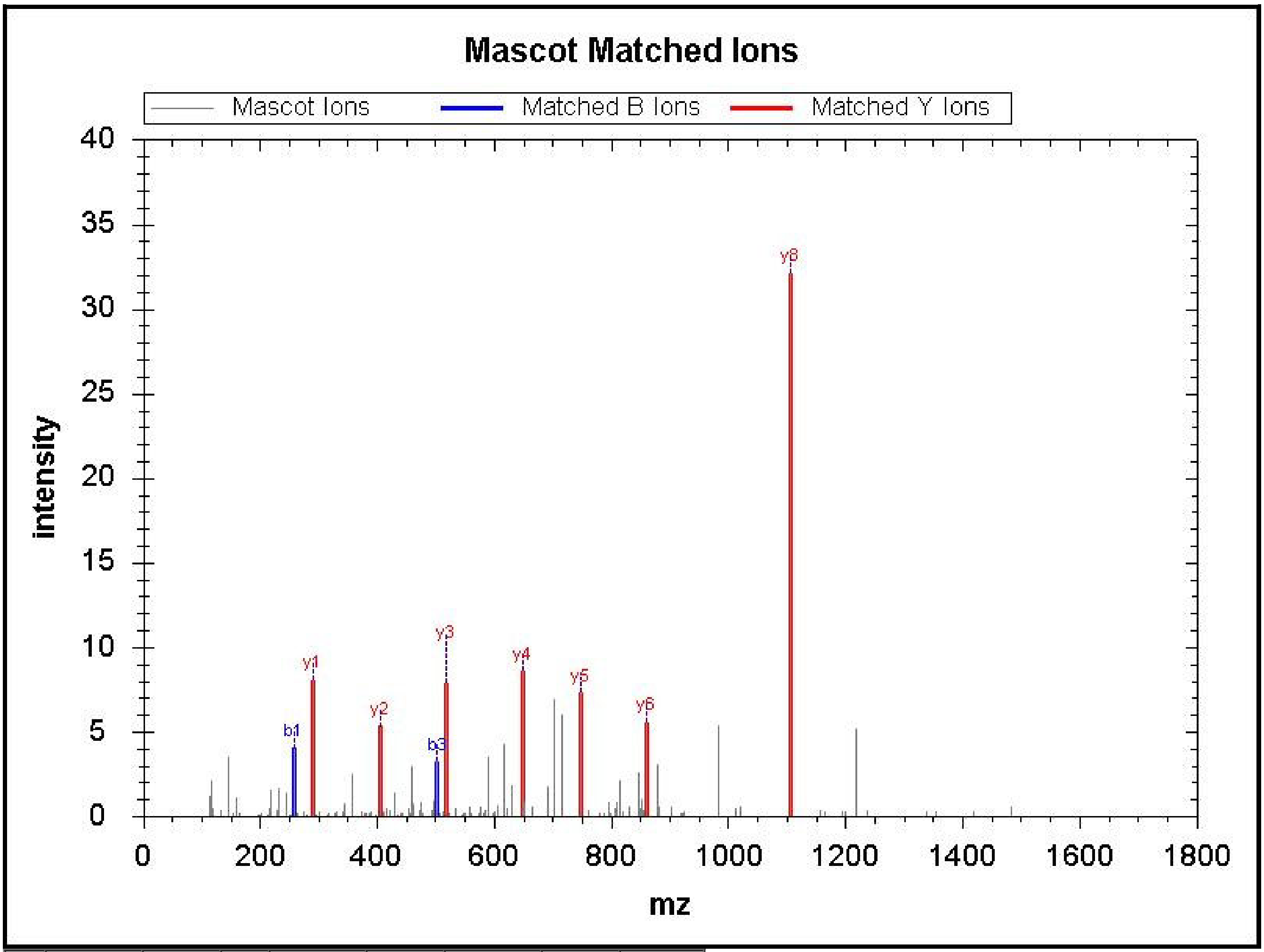

No	b	b++	Seq	y	y++	y*	y*++	RevNo
1	258.19	129.60	L					9
2	355.25	178.13	P	1,104.70	552.85	1,087.67	544.34	8
3	502.31	251.66	F	1,007.64	504.33	990.62	495.81	7
4	615.40	308.20	L	860.58	430.79	843.55	422.28	6
5	714.47	357.74	V	747.49	374.25	730.47	365.74	5
6	845.51	423.26	M	648.42	324.72	631.40	316.20	4
7	958.59	479.80	I	517.38	259.20	500.36	250.68	3
8	1,071.68	536.34	I	404.30	202.65	387.27	194.14	2
9			K	291.21	146.11	274.19	137.60	1

Query 93589 Hit 1

MS/MS Fragmentation of SGPPGEEAQVASQFIADVIENSQIIQK
Found in sp|Q96B26|EXOS8_HUMAN, Exosome complex component RRP43 OS=Homo sapiens GN=EXOSC8 PE=1 SV=1
Match to Query 93589: 3142.636from(786.6663,4+)
Title: 1260: Sum of 2 scans in range 2873 (rt=73.5336, f=4, i=829) to 2874 (rt=73.559, f=4, i=830)
[D:\lab212\membrane\Grace\Joyce\iTRAQ_24_1.raw]
Data File:Submitted from 20120508-1(merge) by Mascot Daemon on JOYCE-VAIO
Monoisotopic mass of neutral peptide Mr(calc): 3142.636
Variable modifications:
K27 :iTRAQ4plex (K)
Ions Score: 54.34 Expect: 0.003

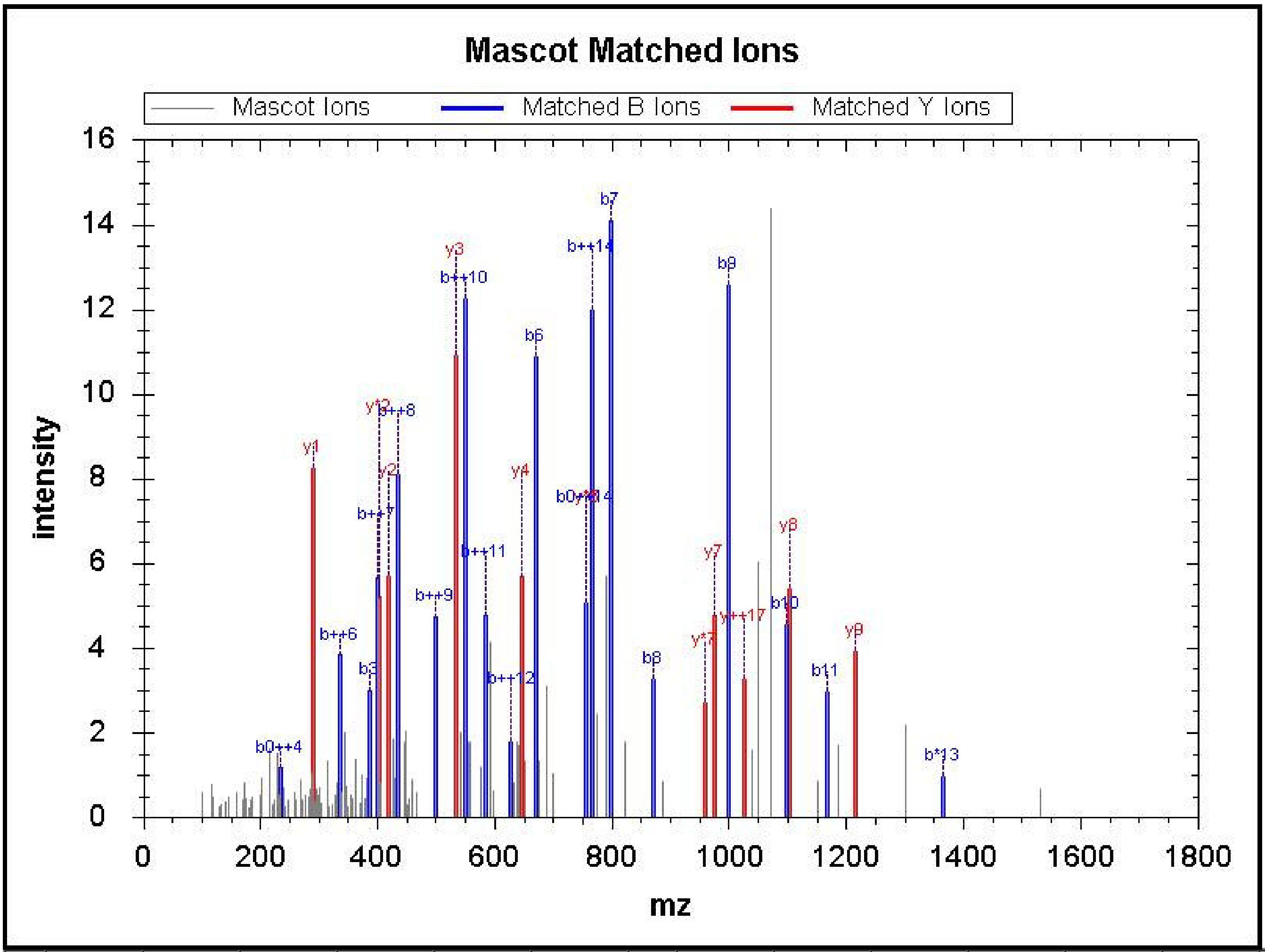

No	b	b++	b*	b*++	b0	b0++	Seq	y	y++	y*	y*++	y0	y0++	RevNo
1	232.14	116.57			214.13	107.57	S							27
2	289.16	145.09			271.15	136.08	G	2,912.51	1,456.76	2,895.49	1,448.25	2,894.50	1,447.75	26
3	386.22	193.61			368.21	184.61	P	2,855.49	1,428.25	2,838.46	1,419.74	2,837.48	1,419.24	25
4	483.27	242.14			465.26	233.13	P	2,758.44	1,379.72	2,741.41	1,371.21	2,740.43	1,370.72	24
5	540.29	270.65			522.28	261.64	G	2,661.39	1,331.20	2,644.36	1,322.68	2,643.37	1,322.19	23
6	669.33	335.17			651.32	326.16	E	2,604.36	1,302.69	2,587.34	1,294.17	2,586.35	1,293.68	22
7	798.38	399.69			780.36	390.69	E	2,475.32	1,238.16	2,458.29	1,229.65	2,457.31	1,229.16	21
8	869.41	435.21			851.40	426.20	A	2,346.28	1,173.64	2,329.25	1,165.13	2,328.27	1,164.64	20
9	997.47	499.24	980.44	490.73	979.46	490.23	Q	2,275.24	1,138.12	2,258.21	1,129.61	2,257.23	1,129.12	19
10	1,096.54	548.77	1,079.51	540.26	1,078.53	539.77	V	2,147.18	1,074.10	2,130.16	1,065.58	2,129.17	1,065.09	18
11	1,167.58	584.29	1,150.55	575.78	1,149.57	575.29	A	2,048.11	1,024.56	2,031.09	1,016.05	2,030.10	1,015.56	17
12	1,254.61	627.81	1,237.58	619.29	1,236.60	618.80	S	1,977.08	989.04	1,960.05	980.53	1,959.07	980.04	16
13	1,382.67	691.84	1,365.64	683.32	1,364.66	682.83	Q	1,890.05	945.53	1,873.02	937.01	1,872.03	936.52	15
14	1,529.74	765.37	1,512.71	756.86	1,511.72	756.37	F	1,761.99	881.50	1,744.96	872.98	1,743.98	872.49	14

15	1,642.82	821.91	1,625.79	813.40	1,624.81	812.91	I	1,614.92	807.96	1,597.89	799.45	1,596.91	798.96	13
16	1,713.86	857.43	1,696.83	848.92	1,695.85	848.43	A	1,501.83	751.42	1,484.81	742.91	1,483.82	742.42	12
17	1,828.88	914.95	1,811.86	906.43	1,810.87	905.94	D	1,430.80	715.90	1,413.77	707.39	1,412.79	706.90	11
18	1,927.95	964.48	1,910.93	955.97	1,909.94	955.47	V	1,315.77	658.39	1,298.74	649.88	1,297.76	649.38	10
19	2,041.04	1,021.02	2,024.01	1,012.51	2,023.03	1,012.02	I	1,216.70	608.85	1,199.68	600.34	1,198.69	599.85	9
20	2,170.08	1,085.54	2,153.05	1,077.03	2,152.07	1,076.54	E	1,103.62	552.31	1,086.59	543.80	1,085.61	543.31	8
21	2,284.12	1,142.56	2,267.09	1,134.05	2,266.11	1,133.56	N	974.58	487.79	957.55	479.28	956.56	478.79	7
22	2,371.15	1,186.08	2,354.13	1,177.57	2,353.14	1,177.08	S	860.53	430.77	843.51	422.26	842.52	421.76	6
23	2,499.21	1,250.11	2,482.19	1,241.60	2,481.20	1,241.10	Q	773.50	387.25	756.47	378.74			5
24	2,612.30	1,306.65	2,595.27	1,298.14	2,594.29	1,297.65	I	645.44	323.22	628.41	314.71			4
25	2,725.38	1,363.19	2,708.35	1,354.68	2,707.37	1,354.19	I	532.36	266.68	515.33	258.17			3
26	2,853.44	1,427.22	2,836.41	1,418.71	2,835.43	1,418.22	Q	419.27	210.14	402.25	201.63			2
27							K	291.21	146.11	274.19	137.60			1

Query 65226 Hit 1

MS/MS Fragmentation of LLQAGEENQVLELLIHR
Found in sp|Q9NPJ6|MED4_HUMAN, Mediator of RNA polymerase II transcription subunit 4 OS=Homo sapiens GN=MED4 PE=1 SV=1
Match to Query 65226: 2118.19from(707.0705,3+)
Title: 980: Scan 2176 (rt=58.4401, f=3, i=336) [D:\lab212\membrane\Grace\Joyce\iTRAQ_32_2.raw]
Data File:Submitted from 20120508-1(merge) by Mascot Daemon on JOYCE-VAIO
Monoisotopic mass of neutral peptide Mr(calc): 2118.19
Variable modifications:
Ions Score: 54.3 Expect: 0.002

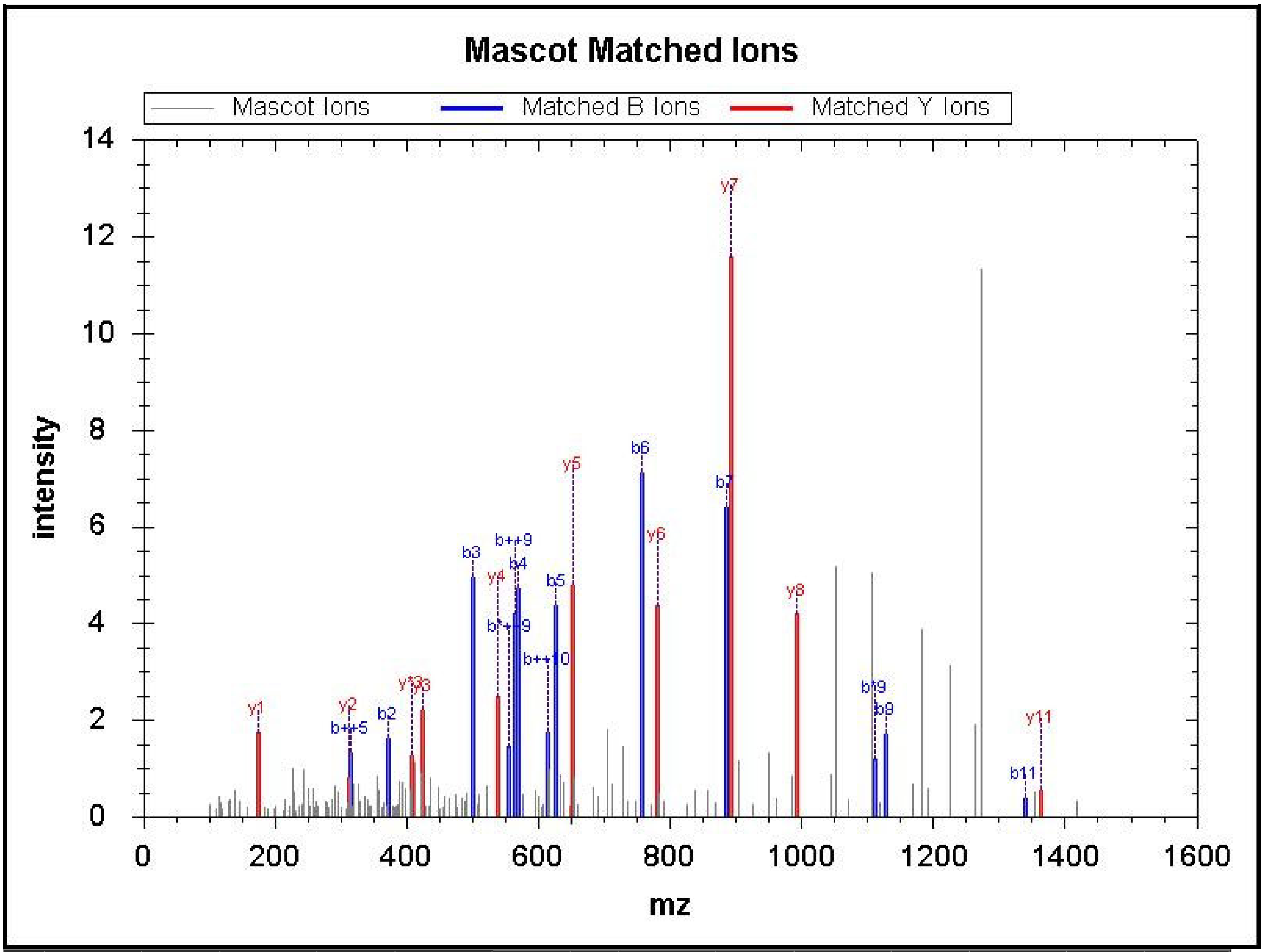

No	b	b++	b*	b*++	b0	b0++	Seq	y	y++	y*	y*++	y0	y0++	RevNo
1	258.19	129.60					L							17

2	371.28	186.14					L	1,862.01	931.51	1,844.99	923.00	1,844.00	922.50	16
3	499.34	250.17	482.31	241.66			Q	1,748.93	874.97	1,731.90	866.45	1,730.92	865.96	15
4	570.37	285.69	553.35	277.18			A	1,620.87	810.94	1,603.84	802.43	1,602.86	801.93	14
5	627.39	314.20	610.37	305.69			G	1,549.83	775.42	1,532.81	766.91	1,531.82	766.41	13
6	756.44	378.72	739.41	370.21	738.43	369.72	E	1,492.81	746.91	1,475.79	738.40	1,474.80	737.90	12
7	885.48	443.24	868.45	434.73	867.47	434.24	E	1,363.77	682.39	1,346.74	673.88	1,345.76	673.38	11
8	999.52	500.27	982.50	491.75	981.51	491.26	N	1,234.73	617.87	1,217.70	609.35	1,216.72	608.86	10
9	1,127.58	564.29	1,110.55	555.78	1,109.57	555.29	Q	1,120.68	560.85	1,103.66	552.33	1,102.67	551.84	9
10	1,226.65	613.83	1,209.62	605.32	1,208.64	604.82	V	992.63	496.82	975.60	488.30	974.61	487.81	8
11	1,339.73	670.37	1,322.71	661.86	1,321.72	661.37	L	893.56	447.28	876.53	438.77	875.55	438.28	7
12	1,468.78	734.89	1,451.75	726.38	1,450.77	725.89	E	780.47	390.74	763.45	382.23	762.46	381.73	6
13	1,581.86	791.43	1,564.83	782.92	1,563.85	782.43	L	651.43	326.22	634.40	317.71			5
14	1,694.94	847.98	1,677.92	839.46	1,676.93	838.97	L	538.35	269.68	521.32	261.16			4
15	1,808.03	904.52	1,791.00	896.00	1,790.02	895.51	I	425.26	213.13	408.24	204.62			3
16	1,945.09	973.05	1,928.06	964.53	1,927.08	964.04	H	312.18	156.59	295.15	148.08			2
17							R	175.12	88.06	158.09	79.55			1

Query 73704 Hit 1

MS/MS Fragmentation of DLIPIIAALEYNQWFTK
Found in sp|Q5VZK9|LR16A_HUMAN, Leucine-rich repeat-containing protein 16A OS=Homo sapiens GN=LRRC16A PE=1 SV=1
Match to Query 73704: 2322.286from(775.1025,3+)
Title: 1356: Scan 3013 (rt=77.2001, f=3, i=470) [D:\lab212\membrane\Grace\Joyce\iTRAQ_25_2.raw]
Data File:Submitted from 20120508-1(merge) by Mascot Daemon on JOYCE-VAIO
Monoisotopic mass of neutral peptide Mr(calc): 2322.286
Variable modifications:
K17 :iTRAQ4plex (K)
Ions Score: 53.99 Expect: 0.002

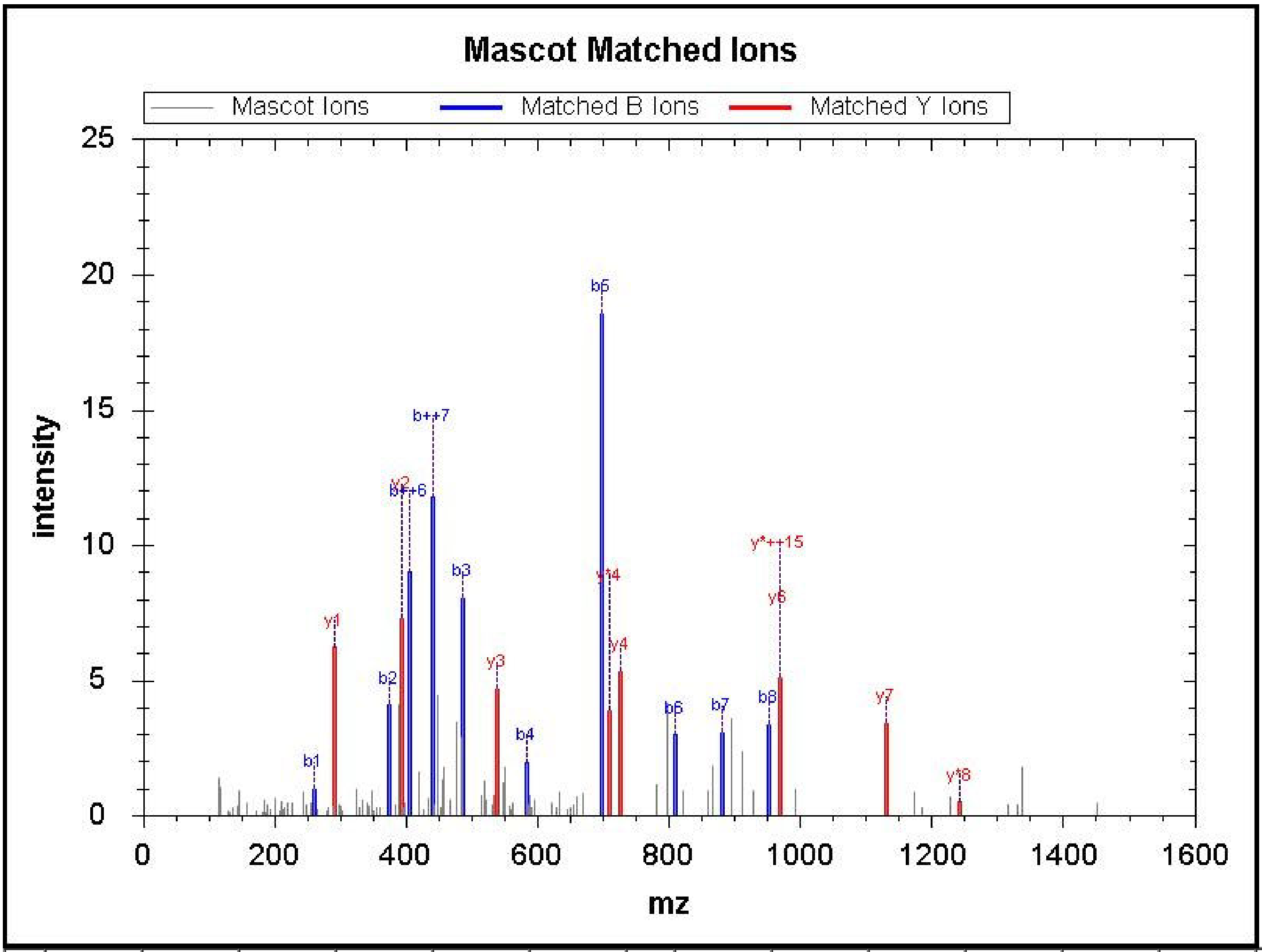

No	b	b++	b*	b*++	b0	b0++	Seq	y	y++	y*	y*++	y0	y0++	RevNo
1	260.14	130.57			242.13	121.57	D							17
2	373.22	187.11			355.21	178.11	L	2,064.17	1,032.59	2,047.14	1,024.07	2,046.15	1,023.58	16
3	486.30	243.66			468.29	234.65	I	1,951.08	976.04	1,934.05	967.53	1,933.07	967.04	15
4	583.36	292.18			565.35	283.18	P	1,838.00	919.50	1,820.97	910.99	1,819.99	910.50	14
5	696.44	348.72			678.43	339.72	I	1,740.94	870.98	1,723.92	862.46	1,722.93	861.97	13
6	809.53	405.27			791.51	396.26	I	1,627.86	814.43	1,610.83	805.92	1,609.85	805.43	12
7	880.56	440.78			862.55	431.78	A	1,514.78	757.89	1,497.75	749.38	1,496.77	748.89	11
8	951.60	476.30			933.59	467.30	A	1,443.74	722.37	1,426.71	713.86	1,425.73	713.37	10
9	1,064.68	532.85			1,046.67	523.84	L	1,372.70	686.85	1,355.68	678.34	1,354.69	677.85	9
10	1,193.73	597.37			1,175.72	588.36	E	1,259.62	630.31	1,242.59	621.80	1,241.61	621.31	8
11	1,356.79	678.90			1,338.78	669.89	Y	1,130.58	565.79	1,113.55	557.28	1,112.56	556.79	7
12	1,470.83	735.92	1,453.81	727.41	1,452.82	726.91	N	967.51	484.26	950.49	475.75	949.50	475.25	6
13	1,598.89	799.95	1,581.86	791.44	1,580.88	790.94	Q	853.47	427.24	836.44	418.72	835.46	418.23	5
14	1,784.97	892.99	1,767.94	884.48	1,766.96	883.98	W	725.41	363.21	708.38	354.70	707.40	354.20	4
15	1,932.04	966.52	1,915.01	958.01	1,914.03	957.52	F	539.33	270.17	522.30	261.66	521.32	261.16	3
16	2,033.09	1,017.05	2,016.06	1,008.53	2,015.08	1,008.04	T	392.26	196.63	375.24	188.12	374.25	187.63	2
17							K	291.21	146.11	274.19	137.60			1

Query 32394 Hit 1

MS/MS Fragmentation of AAEEEDEADPK
Found in sp|P20962|PTMS_HUMAN, Parathymosin OS=Homo sapiens GN=PTMS PE=1 SV=2
Match to Query 32394: 1490.701from(746.3575,2+)
Title: 29: Sum of 2 scans in range 224 (rt=14.1917, f=3, i=10) to 225 (rt=14.2171, f=3, i=11) [D:\lab212\membrane\Grace\Joyce\iTRAQ_30_2.raw]
Data File:Submitted from 20120508-1(merge) by Mascot Daemon on JOYCE-VAIO

Monoisotopic mass of neutral peptide Mr(calc): 1490.701
Variable modifications:
K11 :iTRAQ4plex (K)
Ions Score: 53.97 Expect: 0.002

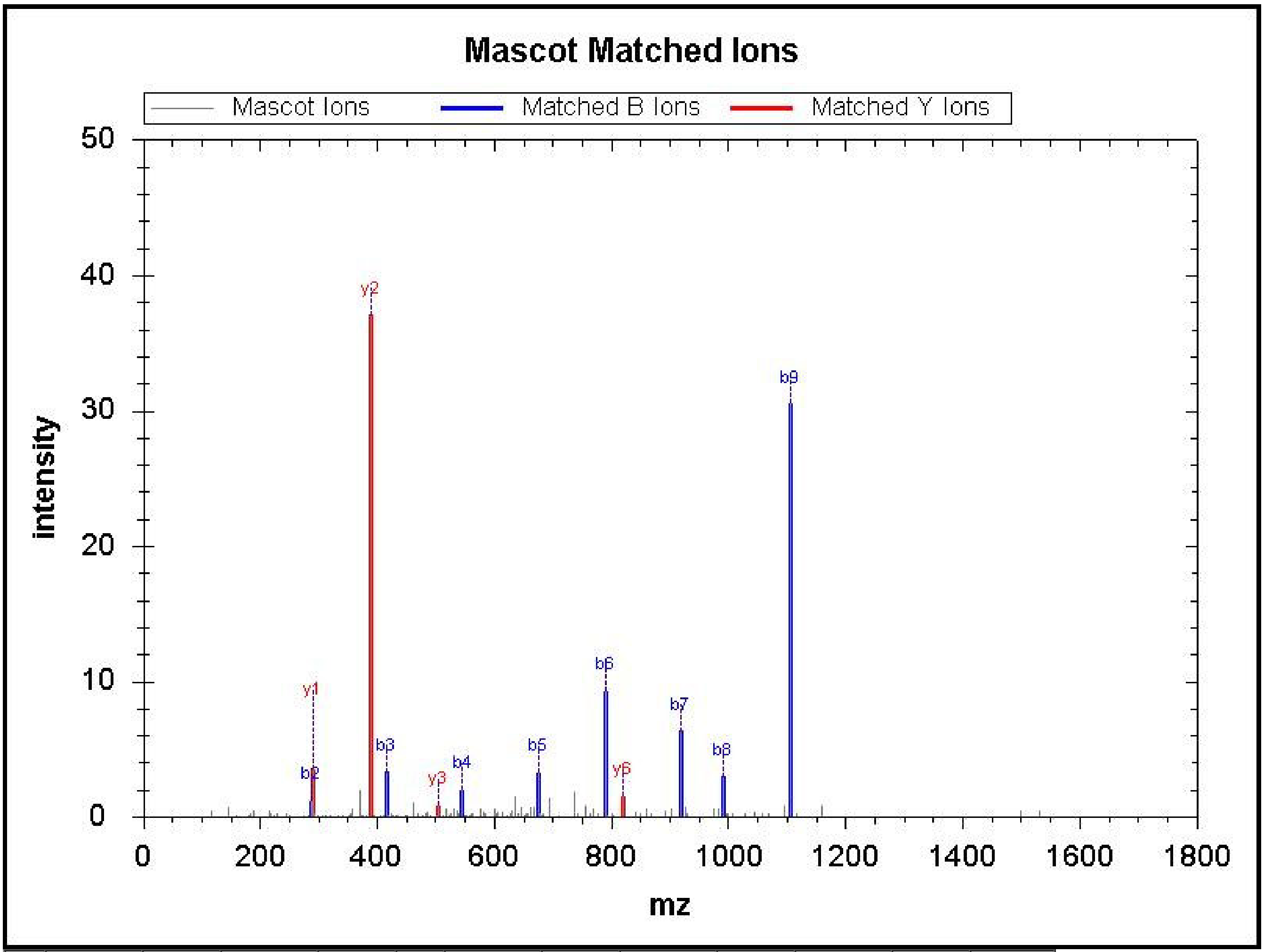

No	b	b++	b0	b0++	Seq	y	y++	y*	y*++	y0	y0++	RevNo
1	216.15	108.58			A							11
2	287.18	144.10			A	1,276.57	638.79	1,259.54	630.27	1,258.56	629.78	10
3	416.23	208.62	398.22	199.61	E	1,205.53	603.27	1,188.50	594.75	1,187.52	594.26	9
4	545.27	273.14	527.26	264.13	E	1,076.49	538.75	1,059.46	530.23	1,058.48	529.74	8
5	674.31	337.66	656.30	328.65	E	947.44	474.23	930.42	465.71	929.43	465.22	7
6	789.34	395.17	771.33	386.17	D	818.40	409.70	801.37	401.19	800.39	400.70	6
7	918.38	459.69	900.37	450.69	E	703.37	352.19	686.35	343.68	685.36	343.19	5
8	989.42	495.21	971.41	486.21	A	574.33	287.67	557.31	279.16	556.32	278.66	4
9	1,104.44	552.73	1,086.43	543.72	D	503.29	252.15	486.27	243.64	485.28	243.15	3
10	1,201.50	601.25	1,183.49	592.25	P	388.27	194.64	371.24	186.12			2
11					K	291.21	146.11	274.19	137.60			1

Query 52177 Hit 1

MS/MS Fragmentation of DIPVTIHNLEEYLR
Found in sp|Q14669|TRIPC_HUMAN, Probable E3 ubiquitin-protein ligase TRIP12 OS=Homo sapiens GN=TRIP12 PE=1 SV=1
Match to Query 52177: 1854.992from(619.3381,3+)
Title: 832: Sum of 2 scans in range 2033 (rt=54.2633, f=4, i=553) to 2034 (rt=54.2887, f=4, i=554)
[D:\lab212\membrane\Grace\Joyce\iTRAQ_34_1.raw]
Data File:Submitted from 20120508-1(merge) by Mascot Daemon on JOYCE-VAIO
Monoisotopic mass of neutral peptide Mr(calc): 1854.992
Variable modifications:
Ions Score: 53.92 Expect: 0.003

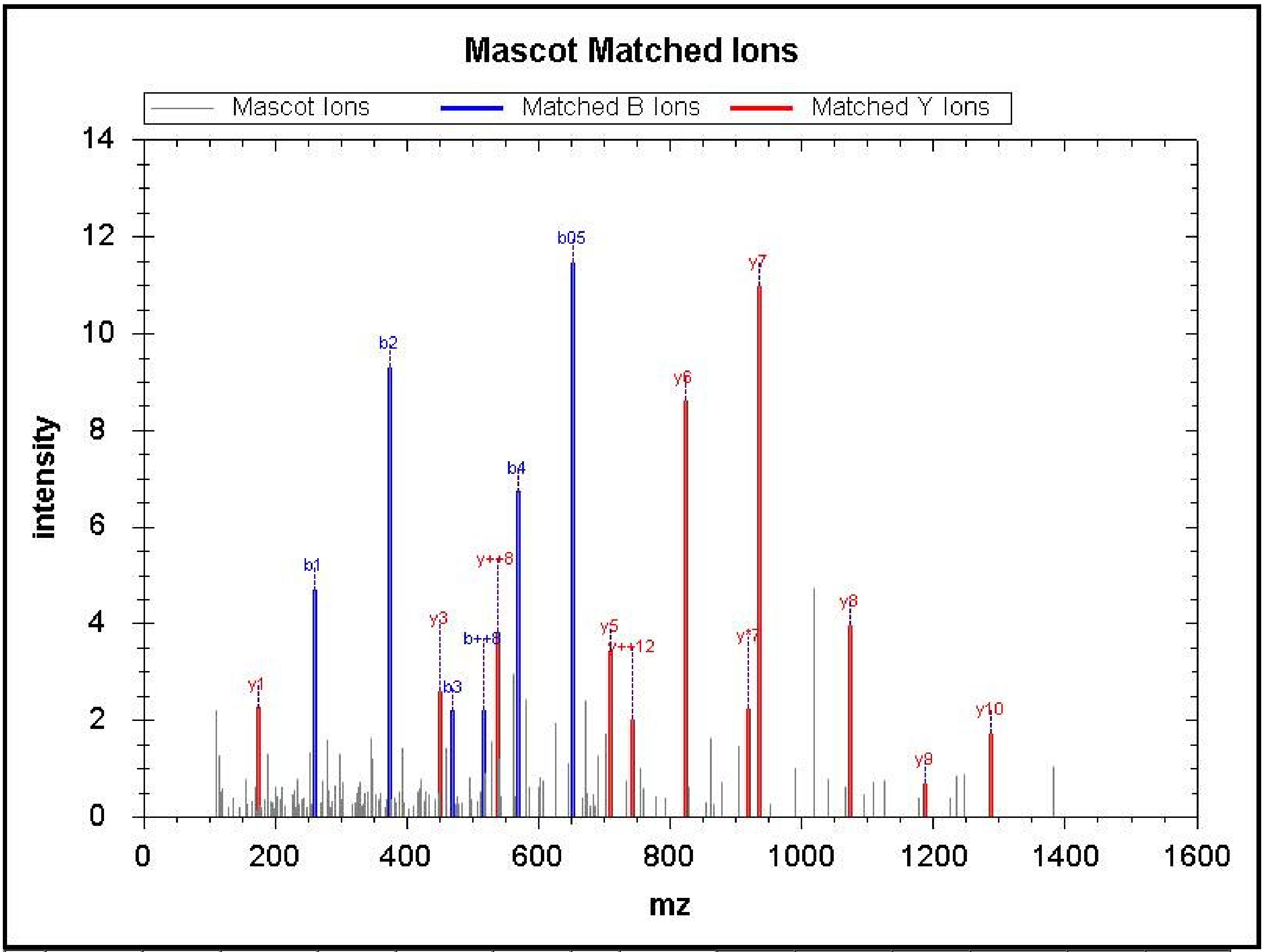

No	b	b++	b*	b*++	b0	b0++	Seq	y	y++	y*	y*++	y0	y0++	RevNo
1	260.14	130.57			242.13	121.57	D							14
2	373.22	187.11			355.21	178.11	I	1,596.87	798.94	1,579.85	790.43	1,578.86	789.94	13
3	470.27	235.64			452.26	226.63	P	1,483.79	742.40	1,466.76	733.89	1,465.78	733.39	12
4	569.34	285.17			551.33	276.17	V	1,386.74	693.87	1,369.71	685.36	1,368.73	684.87	11
5	670.39	335.70			652.38	326.69	T	1,287.67	644.34	1,270.64	635.83	1,269.66	635.33	10
6	783.47	392.24			765.46	383.23	I	1,186.62	593.81	1,169.59	585.30	1,168.61	584.81	9
7	920.53	460.77			902.52	451.76	H	1,073.54	537.27	1,056.51	528.76	1,055.53	528.27	8
8	1,034.58	517.79	1,017.55	509.28	1,016.56	508.79	N	936.48	468.74	919.45	460.23	918.47	459.74	7
9	1,147.66	574.33	1,130.63	565.82	1,129.65	565.33	L	822.44	411.72	805.41	403.21	804.42	402.72	6
10	1,276.70	638.85	1,259.68	630.34	1,258.69	629.85	E	709.35	355.18	692.32	346.67	691.34	346.17	5
11	1,405.74	703.38	1,388.72	694.86	1,387.73	694.37	E	580.31	290.66	563.28	282.14	562.30	281.65	4
12	1,568.81	784.91	1,551.78	776.39	1,550.80	775.90	Y	451.27	226.14	434.24	217.62			3
13	1,681.89	841.45	1,664.87	832.94	1,663.88	832.44	L	288.20	144.61	271.18	136.09			2
14							R	175.12	88.06	158.09	79.55			1

Query 34582 Hit 1

MS/MS Fragmentation of TIEYLQPNPASR
Found in sp|Q99961|SH3G1_HUMAN, Endophilin-A2 OS=Homo sapiens GN=SH3GL1 PE=1 SV=1
Match to Query 34582: 1531.809from(766.9116,2+)
Title: 379: Sum of 2 scans in range 1057 (rt=32.8761, f=4, i=241) to 1058 (rt=32.9015, f=4, i=242) [D:\lab212\membrane\Grace\Joyce\iTRAQ_21_1.raw]
Data File:Submitted from 20120508-1(merge) by Mascot Daemon on JOYCE-VAIO
Monoisotopic mass of neutral peptide Mr(calc): 1531.809
Variable modifications:
Ions Score: 53.9 Expect: 0.004

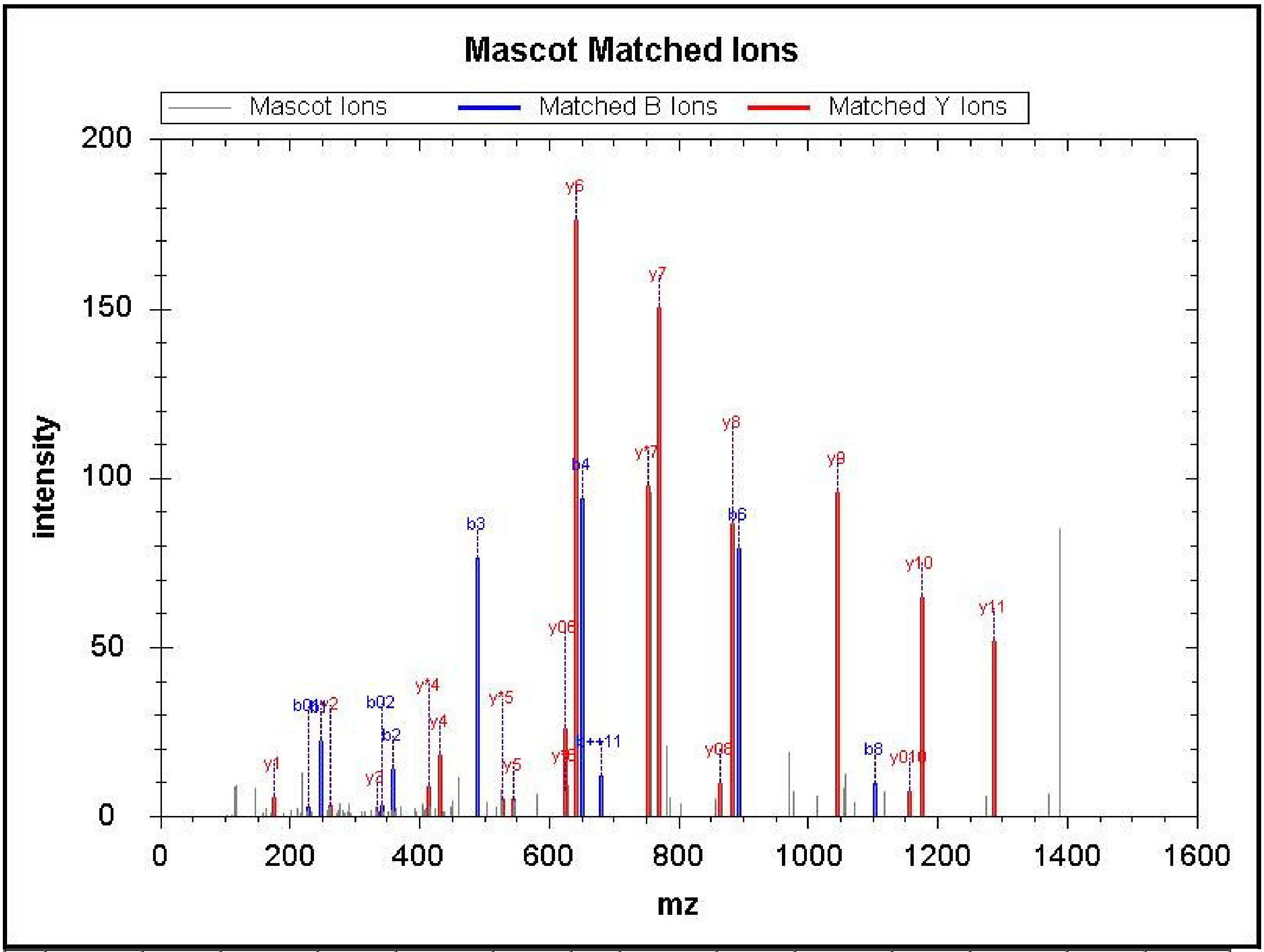

No	b	b++	b*	b*++	b0	b0++	Seq	y	y++	y*	y*++	y0	y0++	RevNo
1	246.16	123.58			228.15	114.58	T							12
2	359.24	180.12			341.23	171.12	I	1,287.67	644.34	1,270.64	635.83	1,269.66	635.33	11
3	488.28	244.65			470.27	235.64	E	1,174.59	587.80	1,157.56	579.28	1,156.57	578.79	10
4	651.35	326.18			633.34	317.17	Y	1,045.54	523.27	1,028.52	514.76	1,027.53	514.27	9
5	764.43	382.72			746.42	373.71	L	882.48	441.74	865.45	433.23	864.47	432.74	8
6	892.49	446.75	875.46	438.24	874.48	437.74	Q	769.40	385.20	752.37	376.69	751.38	376.20	7
7	989.54	495.27	972.52	486.76	971.53	486.27	P	641.34	321.17	624.31	312.66	623.33	312.17	6
8	1,103.59	552.30	1,086.56	543.78	1,085.57	543.29	N	544.28	272.65	527.26	264.13	526.27	263.64	5
9	1,200.64	600.82	1,183.61	592.31	1,182.63	591.82	P	430.24	215.62	413.21	207.11	412.23	206.62	4
10	1,271.68	636.34	1,254.65	627.83	1,253.66	627.34	A	333.19	167.10	316.16	158.58	315.18	158.09	3
11	1,358.71	679.86	1,341.68	671.34	1,340.70	670.85	S	262.15	131.58	245.12	123.07	244.14	122.57	2
12							R	175.12	88.06	158.09	79.55			1

Query 61938 Hit 1

MS/MS Fragmentation of LTQLGTFEDHFLSLQR
Found in sp|P00533|EGFR_HUMAN, Epidermal growth factor receptor OS=Homo sapiens GN=EGFR PE=1 SV=2
Match to Query 61938: 2048.085 from(683.7023,3+)
Title: 809: Sum of 2 scans in range 1983 (rt=53.1379, f=4, i=537) to 1984 (rt=53.1633, f=4, i=538) [D:\lab212\membrane\Grace\Joyce\iTRAQ_34_1.raw]
Data File:Submitted from 20120508-1(merge) by Mascot Daemon on JOYCE-VAIO
Monoisotopic mass of neutral peptide Mr(calc): 2048.085
Variable modifications:
Ions Score: 53.71 Expect: 0.004

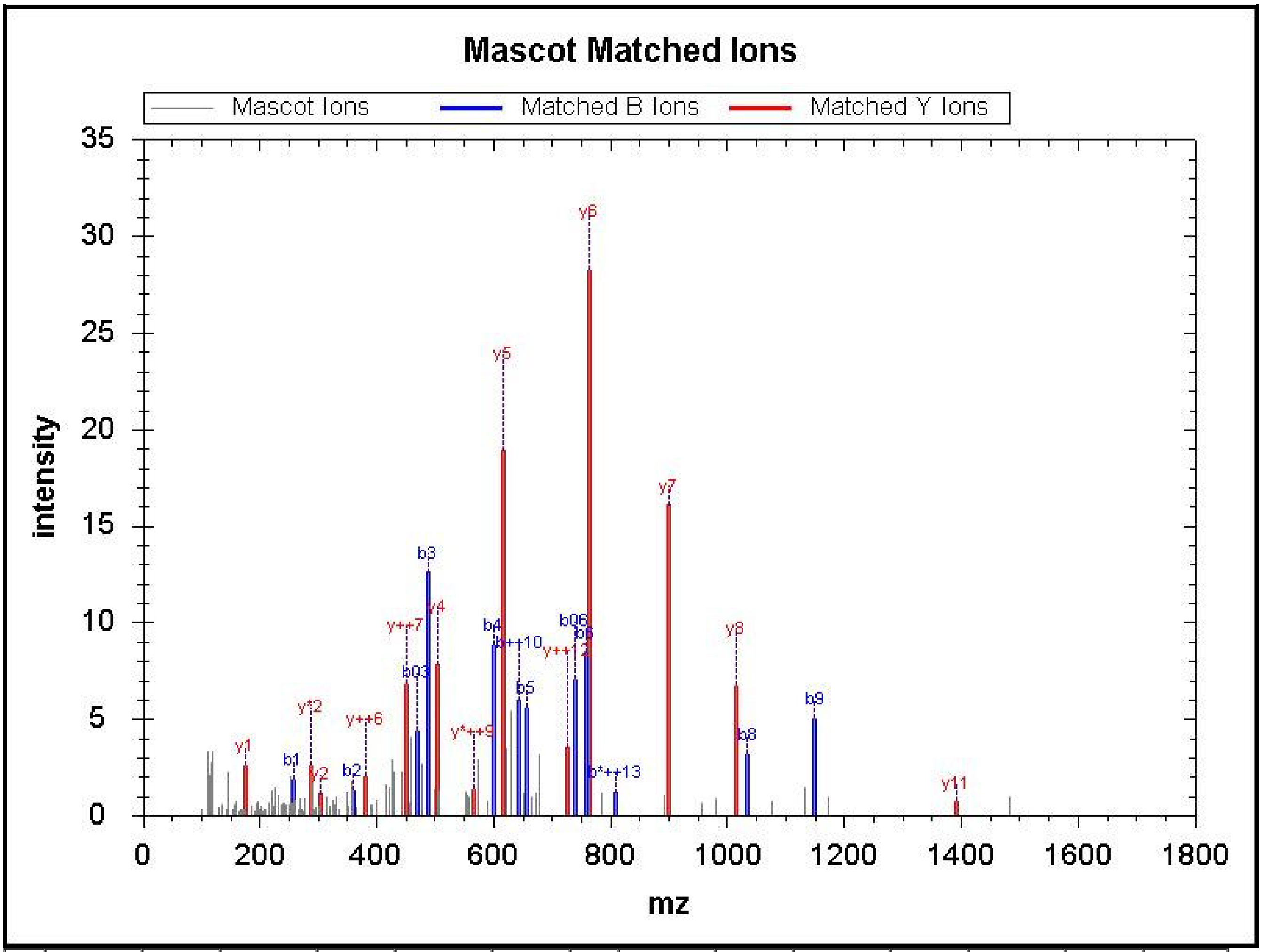

No	b	b++	b*	b*++	b0	b0++	Seq	y	y++	y*	y*++	y0	y0++	RevNo
1	258.19	129.60					L							16
2	359.24	180.12			341.23	171.12	T	1,791.90	896.45	1,774.88	887.94	1,773.89	887.45	15
3	487.30	244.15	470.27	235.64	469.29	235.15	Q	1,690.85	845.93	1,673.83	837.42	1,672.84	836.93	14
4	600.38	300.70	583.36	292.18	582.37	291.69	L	1,562.80	781.90	1,545.77	773.39	1,544.79	772.90	13
5	657.41	329.21	640.38	320.69	639.39	320.20	G	1,449.71	725.36	1,432.69	716.85	1,431.70	716.35	12
6	758.45	379.73	741.43	371.22	740.44	370.72	T	1,392.69	696.85	1,375.66	688.34	1,374.68	687.84	11
7	905.52	453.26	888.49	444.75	887.51	444.26	F	1,291.64	646.33	1,274.62	637.81	1,273.63	637.32	10
8	1,034.56	517.79	1,017.54	509.27	1,016.55	508.78	E	1,144.57	572.79	1,127.55	564.28	1,126.56	563.79	9
9	1,149.59	575.30	1,132.56	566.79	1,131.58	566.29	D	1,015.53	508.27	998.51	499.76	997.52	499.26	8
10	1,286.65	643.83	1,269.62	635.32	1,268.64	634.82	H	900.50	450.76	883.48	442.24	882.49	441.75	7
11	1,433.72	717.36	1,416.69	708.85	1,415.71	708.36	F	763.45	382.23	746.42	373.71	745.44	373.22	6
12	1,546.80	773.90	1,529.78	765.39	1,528.79	764.90	L	616.38	308.69	599.35	300.18	598.37	299.69	5
13	1,633.83	817.42	1,616.81	808.91	1,615.82	808.42	S	503.29	252.15	486.27	243.64	485.28	243.15	4
14	1,746.92	873.96	1,729.89	865.45	1,728.91	864.96	L	416.26	208.63	399.24	200.12			3
15	1,874.98	937.99	1,857.95	929.48	1,856.97	928.99	Q	303.18	152.09	286.15	143.58			2
16							R	175.12	88.06	158.09	79.55			1

Query 10412 Hit 1

MS/MS Fragmentation of LSGFSFK
Found in sp|P29966|MARCS_HUMAN, Myristoylated alanine-rich C-kinase substrate OS=Homo sapiens GN=MARCKS PE=1 SV=4
Match to Query 10412: 1072.618from(537.3165,2+)
Title: 592: Scan 1318 (rt=39.3607, f=3, i=200) [D:\lab212\membrane\Grace\Joyce\iTRAQ_33_2.raw]
Data File:Submitted from 20120508-1(merge) by Mascot Daemon on JOYCE-VAIO
Monoisotopic mass of neutral peptide Mr(calc): 1072.618
Variable modifications:

K7 :iTRAQ4plex (K)
Ions Score: 53.62 Expect: 0.003

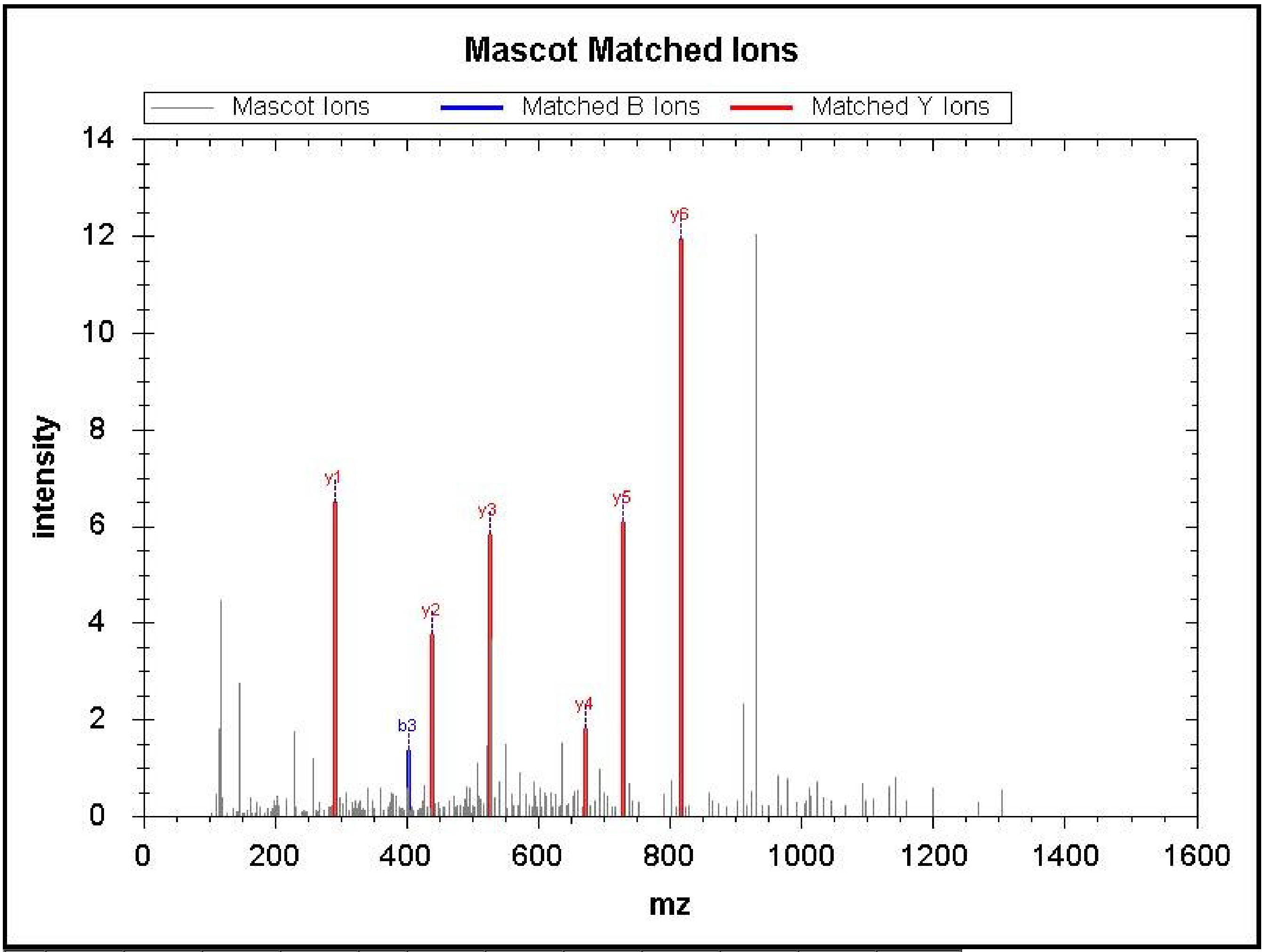

No	b	b++	b0	b0++	Seq	y	y++	y*	y*++	y0	y0++	RevNo
1	258.19	129.60			L							7
2	345.23	173.12	327.21	164.11	S	816.44	408.72	799.41	400.21	798.43	399.72	6
3	402.25	201.63	384.24	192.62	G	729.41	365.21	712.38	356.69	711.39	356.20	5
4	549.32	275.16	531.30	266.16	F	672.38	336.70	655.36	328.18	654.37	327.69	4
5	636.35	318.68	618.34	309.67	S	525.32	263.16	508.29	254.65	507.30	254.16	3
6	783.42	392.21	765.41	383.21	F	438.28	219.65	421.26	211.13			2
7					K	291.21	146.11	274.19	137.60			1

Query 41696 Hit 1

MS/MS Fragmentation of AQAAAPASVPAQAPK
Found in sp|P47914|RL29_HUMAN, 60S ribosomal protein L29 OS=Homo sapiens GN=RPL29 PE=1 SV=2
Match to Query 41696: 1664.949from(555.9902,3+)
Title: 195: Sum of 2 scans in range 534 (rt=21.3518, f=4, i=123) to 535 (rt=21.3772, f=4, i=124)
[D:\lab212\membrane\Grace\Joyce\iTRAQ_27_1.raw]
Data File:Submitted from 20120508-1(merge) by Mascot Daemon on JOYCE-VAIO
Monoisotopic mass of neutral peptide Mr(calc): 1664.949
Variable modifications:
K15 :iTRAQ4plex (K)
Ions Score: 53.34 Expect: 0.003

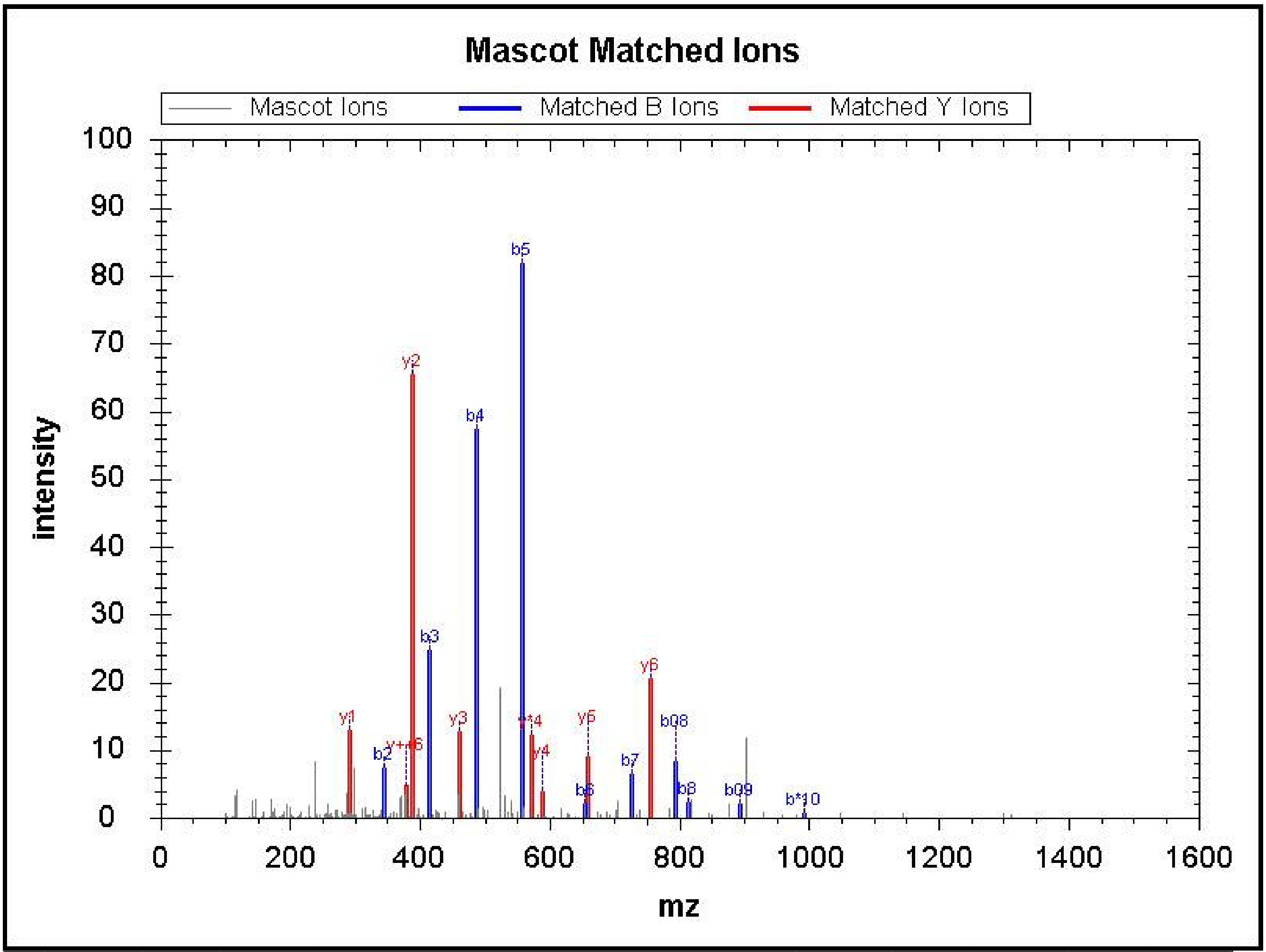

No	b	b++	b*	b*++	b0	b0++	Seq	y	y++	y*	y*++	y0	y0++	RevNo
1	216.15	108.58					A							15
2	344.21	172.61	327.18	164.09			Q	1,450.81	725.91	1,433.79	717.40	1,432.80	716.91	14
3	415.24	208.12	398.22	199.61			A	1,322.75	661.88	1,305.73	653.37	1,304.74	652.88	13
4	486.28	243.64	469.25	235.13			A	1,251.72	626.36	1,234.69	617.85	1,233.71	617.36	12
5	557.32	279.16	540.29	270.65			A	1,180.68	590.84	1,163.65	582.33	1,162.67	581.84	11
6	654.37	327.69	637.34	319.17			P	1,109.64	555.33	1,092.62	546.81	1,091.63	546.32	10
7	725.41	363.21	708.38	354.69			A	1,012.59	506.80	995.56	498.29	994.58	497.79	9
8	812.44	406.72	795.41	398.21	794.43	397.72	S	941.55	471.28	924.53	462.77	923.54	462.28	8
9	911.51	456.26	894.48	447.74	893.50	447.25	V	854.52	427.76	837.49	419.25			7
10	1,008.56	504.78	991.53	496.27	990.55	495.78	P	755.45	378.23	738.43	369.72			6
11	1,079.60	540.30	1,062.57	531.79	1,061.59	531.30	A	658.40	329.70	641.37	321.19			5
12	1,207.66	604.33	1,190.63	595.82	1,189.64	595.33	Q	587.36	294.19	570.34	285.67			4
13	1,278.69	639.85	1,261.67	631.34	1,260.68	630.84	A	459.30	230.16	442.28	221.64			3
14	1,375.74	688.38	1,358.72	679.86	1,357.73	679.37	P	388.27	194.64	371.24	186.12			2
15							K	291.21	146.11	274.19	137.60			1

Query 86725 Hit 1

MS/MS Fragmentation of SILTDNPTWIIDPIDGTTNFVHR
Found in sp|P29218|IMPA1_HUMAN, Inositol monophosphatase 1 OS=Homo sapiens GN=IMPA1 PE=1 SV=1
Match to Query 86725: 2768.418from(923.8132,3+)
Title: 995: Scan 2207 (rt=59.0809, f=2, i=348) [D:\lab212\membrane\Grace\Joyce\iTRAQ_31_1.raw]
Data File:Submitted from 20120508-1(merge) by Mascot Daemon on JOYCE-VAIO
Monoisotopic mass of neutral peptide Mr(calc): 2768.418
Variable modifications:
Ions Score: 53.34 Expect: 0.004

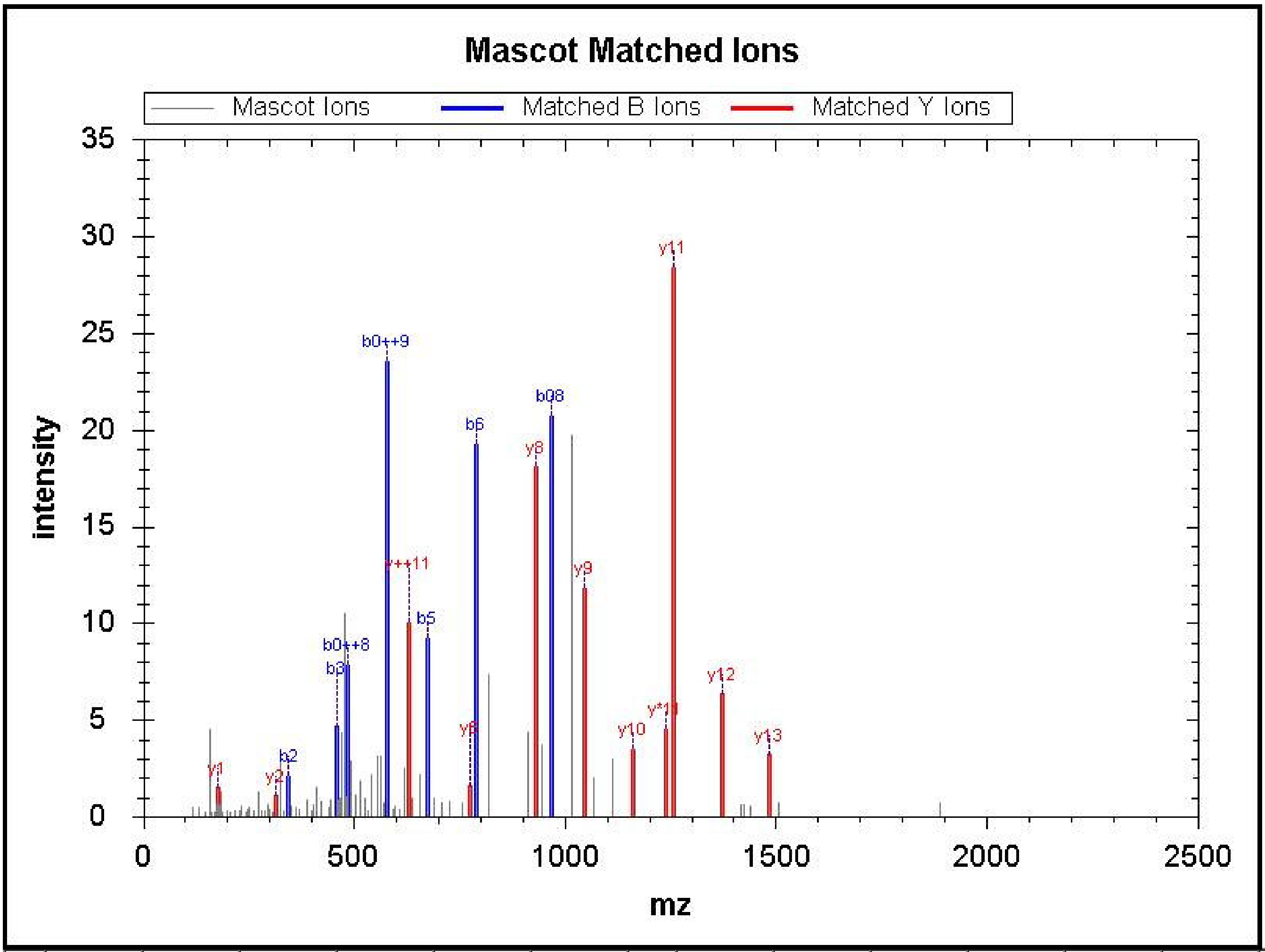

No	b	b++	b*	b*++	b0	b0++	Seq	y	y++	y*	y*++	y0	y0++	RevNo
1	232.14	116.57			214.13	107.57	S							23
2	345.23	173.12			327.21	164.11	I	2,538.30	1,269.65	2,521.27	1,261.14	2,520.29	1,260.65	22
3	458.31	229.66			440.30	220.65	L	2,425.21	1,213.11	2,408.19	1,204.60	2,407.20	1,204.11	21
4	559.36	280.18			541.35	271.18	T	2,312.13	1,156.57	2,295.10	1,148.06	2,294.12	1,147.56	20
5	674.38	337.70			656.37	328.69	D	2,211.08	1,106.05	2,194.06	1,097.53	2,193.07	1,097.04	19
6	788.43	394.72	771.40	386.20	770.42	385.71	N	2,096.06	1,048.53	2,079.03	1,040.02	2,078.05	1,039.53	18
7	885.48	443.24	868.45	434.73	867.47	434.24	P	1,982.01	991.51	1,964.99	983.00	1,964.00	982.50	17
8	986.53	493.77	969.50	485.25	968.52	484.76	T	1,884.96	942.98	1,867.93	934.47	1,866.95	933.98	16
9	1,172.61	586.81	1,155.58	578.29	1,154.60	577.80	W	1,783.91	892.46	1,766.89	883.95	1,765.90	883.45	15
10	1,285.69	643.35	1,268.66	634.84	1,267.68	634.34	I	1,597.83	799.42	1,580.81	790.91	1,579.82	790.42	14
11	1,398.77	699.89	1,381.75	691.38	1,380.76	690.89	I	1,484.75	742.88	1,467.72	734.37	1,466.74	733.87	13
12	1,513.80	757.40	1,496.78	748.89	1,495.79	748.40	D	1,371.67	686.34	1,354.64	677.82	1,353.65	677.33	12
13	1,610.85	805.93	1,593.83	797.42	1,592.84	796.93	P	1,256.64	628.82	1,239.61	620.31	1,238.63	619.82	11
14	1,723.94	862.47	1,706.91	853.96	1,705.93	853.47	I	1,159.59	580.30	1,142.56	571.78	1,141.57	571.29	10
15	1,838.97	919.99	1,821.94	911.47	1,820.96	910.98	D	1,046.50	523.75	1,029.47	515.24	1,028.49	514.75	9
16	1,895.99	948.50	1,878.96	939.98	1,877.98	939.49	G	931.47	466.24	914.45	457.73	913.46	457.24	8
17	1,997.03	999.02	1,980.01	990.51	1,979.02	990.02	T	874.45	437.73	857.43	429.22	856.44	428.72	7
18	2,098.08	1,049.54	2,081.06	1,041.03	2,080.07	1,040.54	T	773.41	387.21	756.38	378.69	755.39	378.20	6
19	2,212.13	1,106.57	2,195.10	1,098.05	2,194.11	1,097.56	N	672.36	336.68	655.33	328.17			5
20	2,359.19	1,180.10	2,342.17	1,171.59	2,341.18	1,171.10	F	558.31	279.66	541.29	271.15			4
21	2,458.26	1,229.63	2,441.24	1,221.12	2,440.25	1,220.63	V	411.25	206.13	394.22	197.61			3
22	2,595.32	1,298.16	2,578.29	1,289.65	2,577.31	1,289.16	H	312.18	156.59	295.15	148.08			2
23							R	175.12	88.06	158.09	79.55			1

Query 95577 Hit 1

MS/MS Fragmentation of GGGGGGSPGPTAGPEPLSLPGILHFIQHEWAR
Found in sp|Q9NRL3|STRN4_HUMAN, Striatin-4 OS=Homo sapiens GN=STRN4 PE=1 SV=2
Match to Query 95577: 3292.695from(824.1811,4+)
Title: 1285: Sum of 2 scans in range 2818 (rt=73.0891, f=3, i=454) to 2819 (rt=73.1145, f=3, i=455)
[D:\lab212\membrane\Grace\Joyce\iTRAQ_42_2.raw]
Data File:Submitted from 20120508-1(merge) by Mascot Daemon on JOYCE-VAIO
Monoisotopic mass of neutral peptide Mr(calc): 3292.695
Variable modifications:
Ions Score: 53.31 Expect: 0.004

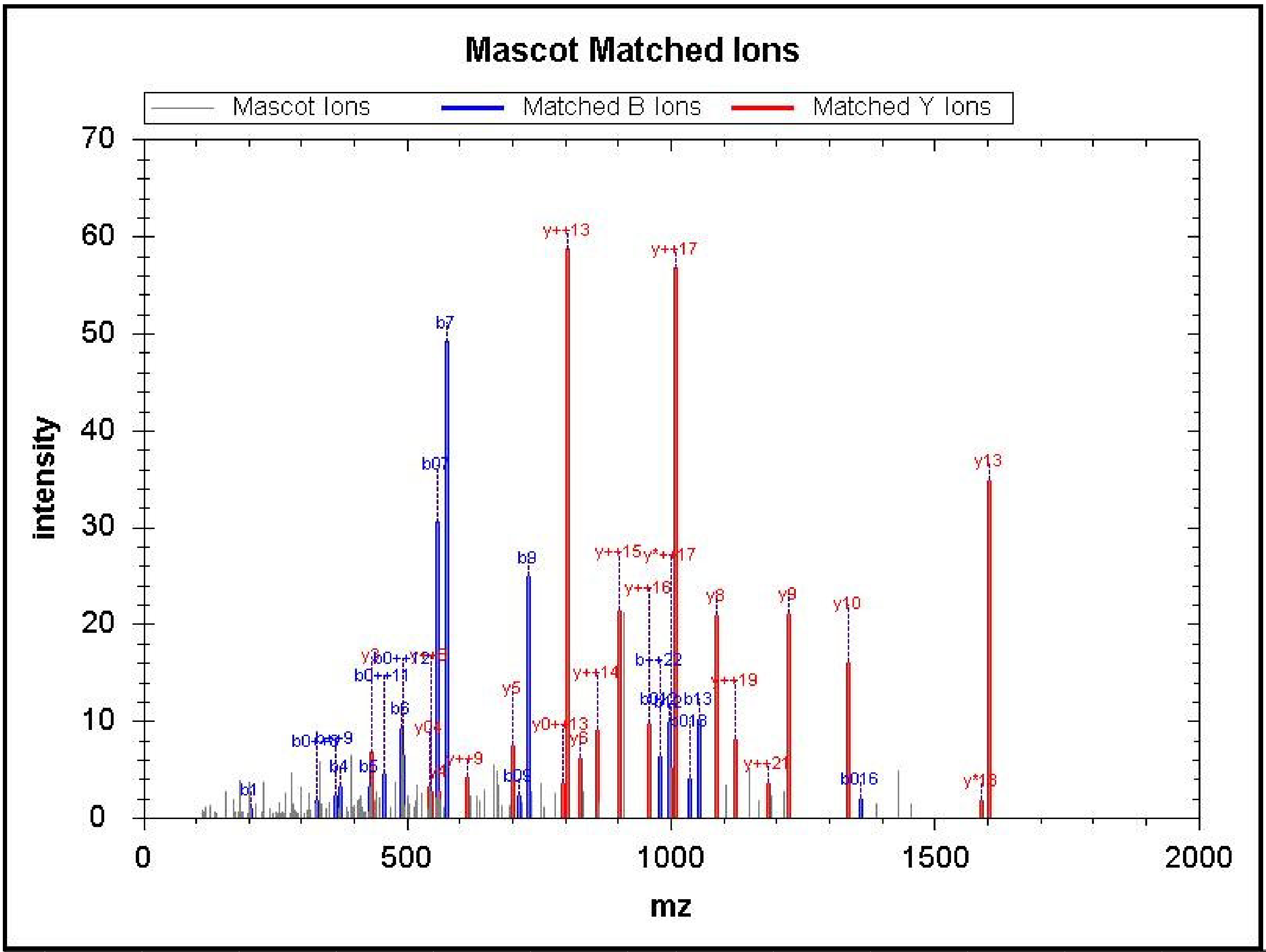

No	b	b++	b*	b*++	b0	b0++	Seq	y	y++	y*	y*++	y0	y0++	RevNo
1	202.13	101.57					G							32
2	259.15	130.08					G	3,092.57	1,546.79	3,075.54	1,538.28	3,074.56	1,537.78	31
3	316.17	158.59					G	3,035.55	1,518.28	3,018.52	1,509.76	3,017.54	1,509.27	30
4	373.20	187.10					G	2,978.53	1,489.77	2,961.50	1,481.25	2,960.52	1,480.76	29
5	430.22	215.61					G	2,921.51	1,461.26	2,904.48	1,452.74	2,903.50	1,452.25	28
6	487.24	244.12					G	2,864.48	1,432.75	2,847.46	1,424.23	2,846.47	1,423.74	27
7	574.27	287.64			556.26	278.63	S	2,807.46	1,404.23	2,790.44	1,395.72	2,789.45	1,395.23	26
8	671.32	336.17			653.31	327.16	P	2,720.43	1,360.72	2,703.40	1,352.21	2,702.42	1,351.71	25
9	728.34	364.68			710.33	355.67	G	2,623.38	1,312.19	2,606.35	1,303.68	2,605.37	1,303.19	24
10	825.40	413.20			807.39	404.20	P	2,566.36	1,283.68	2,549.33	1,275.17	2,548.35	1,274.68	23
11	926.44	463.73			908.43	454.72	T	2,469.30	1,235.16	2,452.28	1,226.64	2,451.29	1,226.15	22
12	997.48	499.24			979.47	490.24	A	2,368.26	1,184.63	2,351.23	1,176.12	2,350.25	1,175.63	21
13	1,054.50	527.76			1,036.49	518.75	G	2,297.22	1,149.11	2,280.19	1,140.60	2,279.21	1,140.11	20
14	1,151.56	576.28			1,133.55	567.28	P	2,240.20	1,120.60	2,223.17	1,112.09	2,222.19	1,111.60	19

15	1,280.60	640.80			1,262.59	631.80	E	2,143.14	1,072.08	2,126.12	1,063.56	2,125.13	1,063.07	18
16	1,377.65	689.33			1,359.64	680.32	P	2,014.10	1,007.55	1,997.08	999.04	1,996.09	998.55	17
17	1,490.74	745.87			1,472.72	736.87	L	1,917.05	959.03	1,900.02	950.52	1,899.04	950.02	16
18	1,577.77	789.39			1,559.76	780.38	S	1,803.97	902.49	1,786.94	893.97	1,785.95	893.48	15
19	1,690.85	845.93			1,672.84	836.92	L	1,716.93	858.97	1,699.91	850.46	1,698.92	849.97	14
20	1,787.90	894.46			1,769.89	885.45	P	1,603.85	802.43	1,586.82	793.92	1,585.84	793.42	13
21	1,844.93	922.97			1,826.92	913.96	G	1,506.80	753.90	1,489.77	745.39	1,488.79	744.90	12
22	1,958.01	979.51			1,940.00	970.50	I	1,449.78	725.39	1,432.75	716.88	1,431.76	716.39	11
23	2,071.09	1,036.05			2,053.08	1,027.05	L	1,336.69	668.85	1,319.66	660.34	1,318.68	659.84	10
24	2,208.15	1,104.58			2,190.14	1,095.57	H	1,223.61	612.31	1,206.58	603.79	1,205.60	603.30	9
25	2,355.22	1,178.11			2,337.21	1,169.11	F	1,086.55	543.78	1,069.52	535.26	1,068.54	534.77	8
26	2,468.31	1,234.66			2,450.29	1,225.65	I	939.48	470.24	922.45	461.73	921.47	461.24	7
27	2,596.36	1,298.69	2,579.34	1,290.17	2,578.35	1,289.68	Q	826.40	413.70	809.37	405.19	808.38	404.70	6
28	2,733.42	1,367.21	2,716.40	1,358.70	2,715.41	1,358.21	H	698.34	349.67	681.31	341.16	680.33	340.67	5
29	2,862.47	1,431.74	2,845.44	1,423.22	2,844.45	1,422.73	E	561.28	281.14	544.25	272.63	543.27	272.14	4
30	3,048.54	1,524.78	3,031.52	1,516.26	3,030.53	1,515.77	W	432.24	216.62	415.21	208.11			3
31	3,119.58	1,560.29	3,102.55	1,551.78	3,101.57	1,551.29	A	246.16	123.58	229.13	115.07			2
32							R	175.12	88.06	158.09	79.55			1

Query 16602 Hit 1

MS/MS Fragmentation of VVVPPLPGK
Found in sp|O60493|SNX3_HUMAN, Sorting nexin-3 OS=Homo sapiens GN=SNX3 PE=1 SV=3
Match to Query 16602: 1192.78from(597.397,2+)
Title: 460: Sum of 2 scans in range 1096 (rt=34.0359, f=4, i=307) to 1097 (rt=34.0613, f=4, i=308)
[D:\lab212\membrane\Grace\Joyce\iTRAQ_28_1_327.raw]
Data File:Submitted from 20120508-1(merge) by Mascot Daemon on JOYCE-VAIO
Monoisotopic mass of neutral peptide Mr(calc): 1192.78
Variable modifications:
K9 :iTRAQ4plex (K)
Ions Score: 53.18 Expect: 0.001

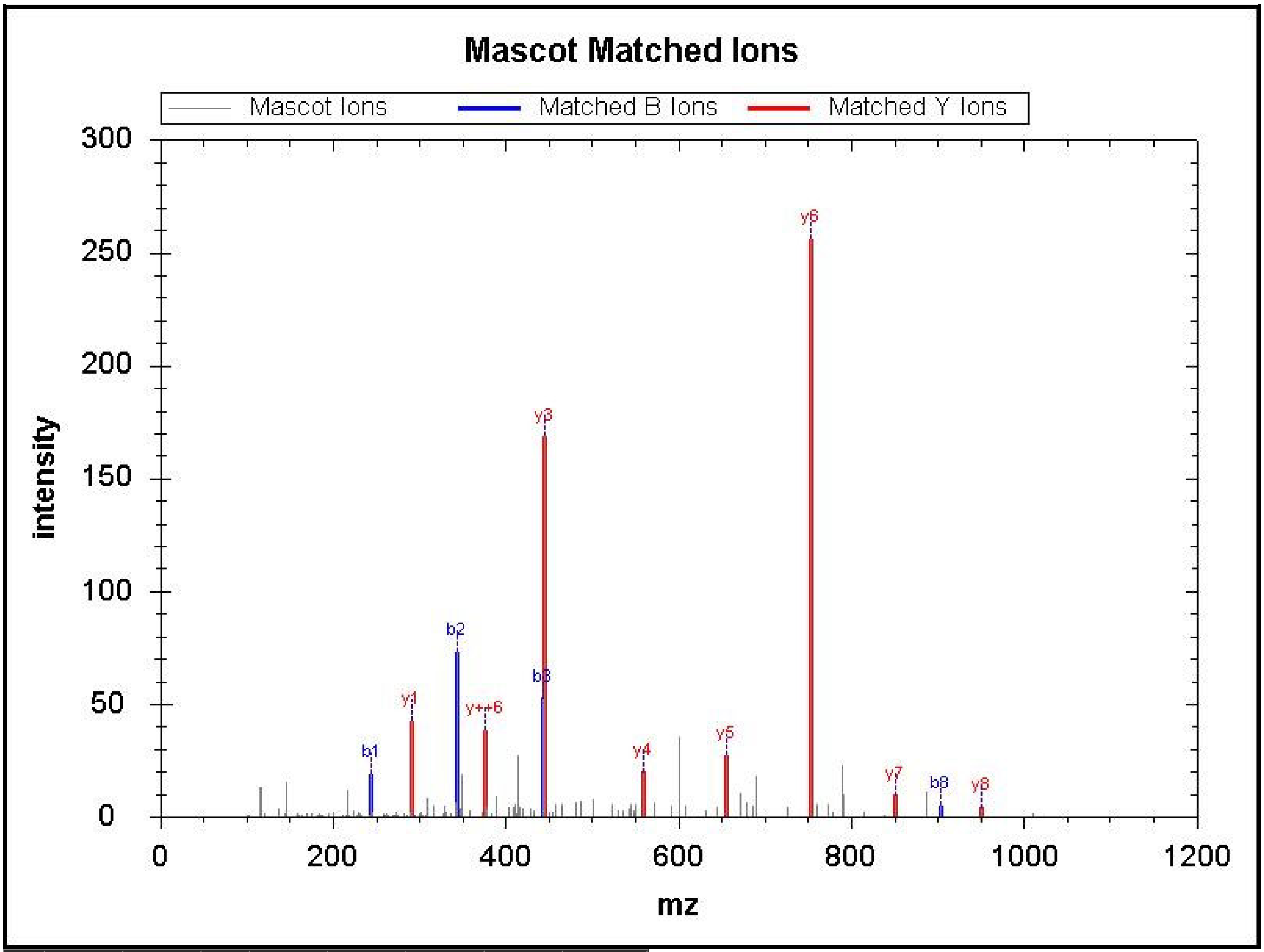

No	b	b++	Seq	y	y++	y*	y*++	RevNo
1	244.18	122.59	V					9
2	343.25	172.13	V	950.62	475.81	933.59	467.30	8
3	442.31	221.66	V	851.55	426.28	834.52	417.76	7
4	539.37	270.19	P	752.48	376.74	735.45	368.23	6
5	636.42	318.71	P	655.43	328.22	638.40	319.70	5
6	749.50	375.26	L	558.37	279.69	541.35	271.18	4
7	846.56	423.78	P	445.29	223.15	428.26	214.63	3
8	903.58	452.29	G	348.24	174.62	331.21	166.11	2
9			K	291.21	146.11	274.19	137.60	1

Query 31871 Hit 1

MS/MS Fragmentation of LQVEEVHQLSR
Found in sp|Q9NR28|DBLOH_HUMAN, Diablo homolog
Match to Query 31871: 1480.806from(494.6092,3+)
Title: 328: Scan 812 (rt=27.6222, f=3, i=114) [D:\lab212\membrane\Grace\Joyce\iTRAQ_38_1.raw]
Data File:Submitted from 20120508-1(merge) by Mascot Daemon on JOYCE-VAIO
Monoisotopic mass of neutral peptide Mr(calc): 1480.806
Variable modifications:
Ions Score: 53.13 Expect: 0.004

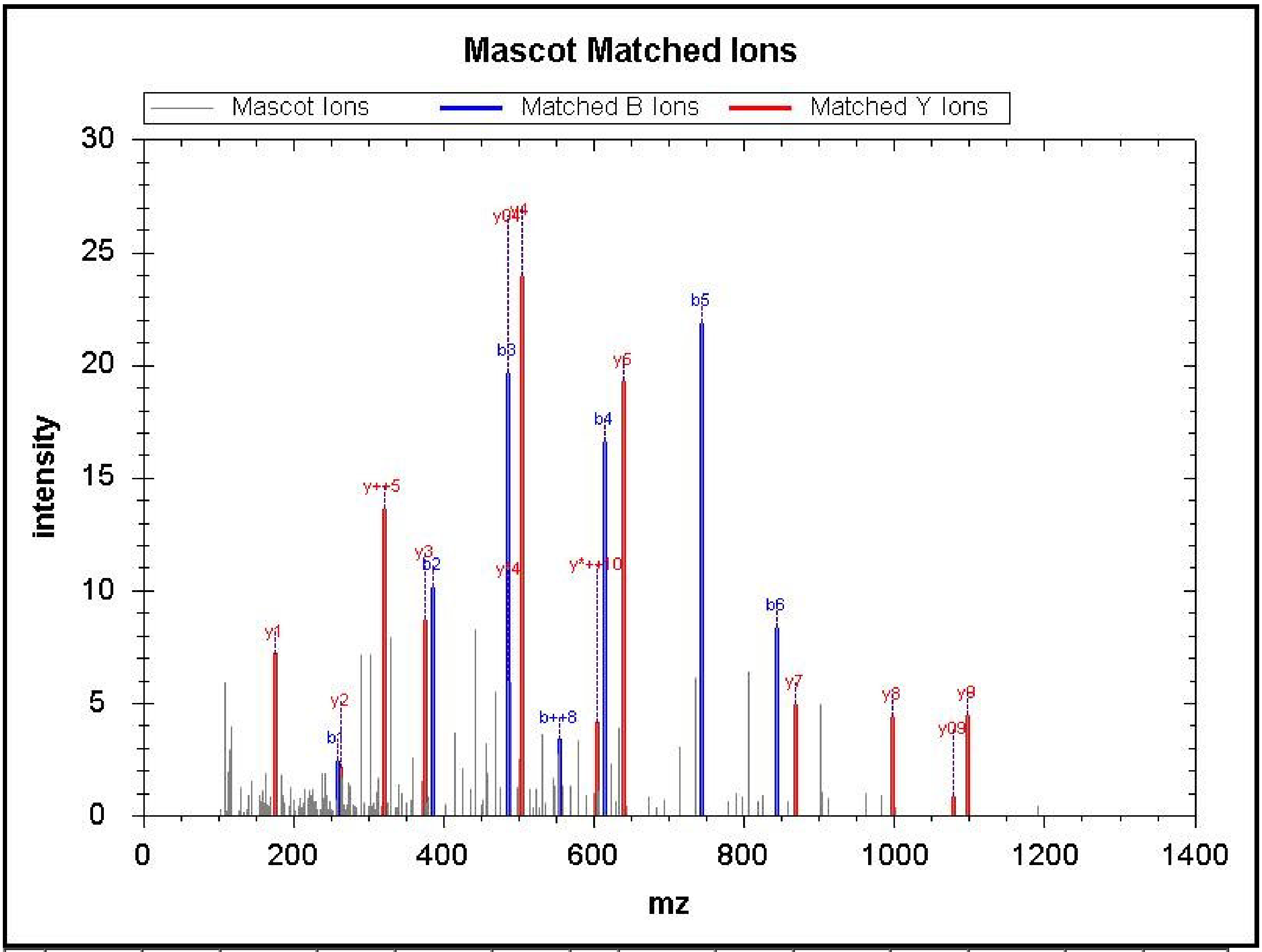

No	b	b++	b*	b*++	b0	b0++	Seq	y	y++	y*	y*++	y0	y0++	RevNo
1	258.19	129.60					L							11
2	386.25	193.63	369.23	185.12			Q	1,224.63	612.82	1,207.61	604.31	1,206.62	603.81	10
3	485.32	243.16	468.29	234.65			V	1,096.57	548.79	1,079.55	540.28	1,078.56	539.79	9
4	614.36	307.69	597.34	299.17	596.35	298.68	E	997.51	499.26	980.48	490.74	979.50	490.25	8
5	743.41	372.21	726.38	363.69	725.40	363.20	E	868.46	434.74	851.44	426.22	850.45	425.73	7
6	842.47	421.74	825.45	413.23	824.46	412.74	V	739.42	370.21	722.39	361.70	721.41	361.21	6
7	979.53	490.27	962.51	481.76	961.52	481.26	H	640.35	320.68	623.33	312.17	622.34	311.67	5
8	1,107.59	554.30	1,090.57	545.79	1,089.58	545.29	Q	503.29	252.15	486.27	243.64	485.28	243.15	4
9	1,220.68	610.84	1,203.65	602.33	1,202.67	601.84	L	375.24	188.12	358.21	179.61	357.22	179.12	3
10	1,307.71	654.36	1,290.68	645.84	1,289.70	645.35	S	262.15	131.58	245.12	123.07	244.14	122.57	2
11							R	175.12	88.06	158.09	79.55			1

Query 40679 Hit 1

MS/MS Fragmentation of EAILEYILHQK
Found in sp|Q9Y314|NOSIP_HUMAN, Nitric oxide synthase-interacting protein OS=Homo sapiens GN=NOSIP PE=1 SV=1
Match to Query 40679: 1643.895from(548.9721,3+)
Title: 875: Sum of 2 scans in range 1895 (rt=52.444, f=4, i=591) to 1896 (rt=52.4693, f=4, i=592) [D:\lab212\membrane\Grace\Joyce\iTRAQ40.raw]
Data File:Submitted from 20120508-1(merge) by Mascot Daemon on JOYCE-VAIO
Monoisotopic mass of neutral peptide Mr(calc): 1643.895
Variable modifications:
K11 :iTRAQ4plex (K)
Ions Score: 53.1 Expect: 0.004

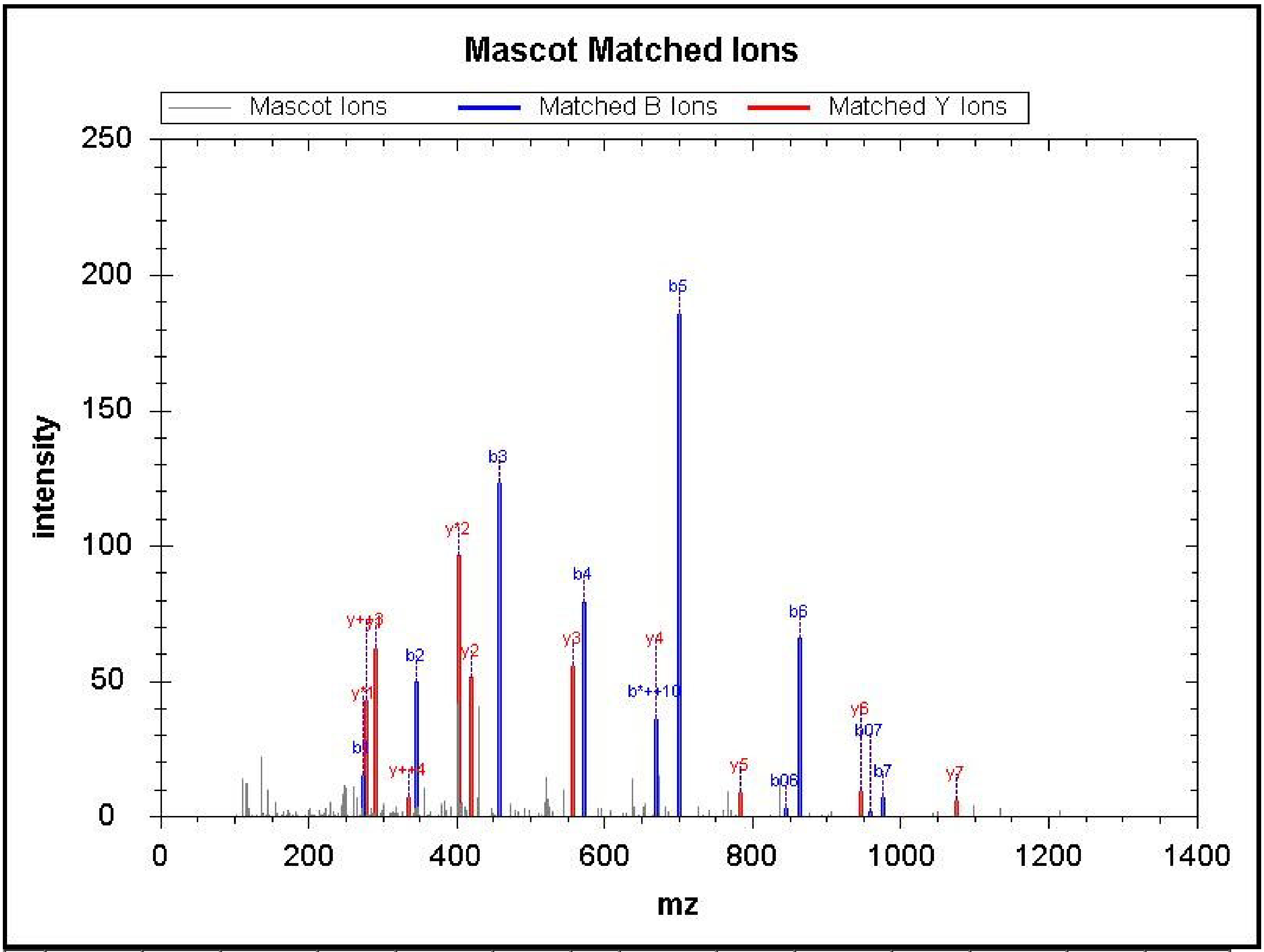

No	b	b++	b*	b*++	b0	b0++	Seq	y	y++	y*	y*++	y0	y0++	RevNo
1	274.15	137.58			256.14	128.57	E							11
2	345.19	173.10			327.18	164.09	A	1,371.81	686.41	1,354.79	677.90	1,353.80	677.40	10
3	458.27	229.64			440.26	220.63	I	1,300.77	650.89	1,283.75	642.38	1,282.76	641.89	9
4	571.36	286.18			553.35	277.18	L	1,187.69	594.35	1,170.66	585.84	1,169.68	585.34	8
5	700.40	350.70			682.39	341.70	E	1,074.61	537.81	1,057.58	529.29	1,056.60	528.80	7
6	863.46	432.24			845.45	423.23	Y	945.56	473.29	928.54	464.77			6
7	976.55	488.78			958.54	479.77	I	782.50	391.75	765.47	383.24			5
8	1,089.63	545.32			1,071.62	536.31	L	669.42	335.21	652.39	326.70			4
9	1,226.69	613.85			1,208.68	604.84	H	556.33	278.67	539.31	270.16			3
10	1,354.75	677.88	1,337.72	669.36	1,336.74	668.87	Q	419.27	210.14	402.25	201.63			2
11							K	291.21	146.11	274.19	137.60			1

Query 75851 Hit 1

MS/MS Fragmentation of GRPGSGTSGVDSLSNFIESVK
Found in sp|Q8IXT5|RB12B_HUMAN, RNA-binding protein 12B OS=Homo sapiens GN=RBM12B PE=1 SV=2
Match to Query 75851: 2381.311 from(794.7774,3+)
Title: 821: Sum of 2 scans in range 1783 (rt=49.921, f=4, i=555) to 1784 (rt=49.9464, f=4, i=556) [D:\lab212\membrane\Grace\Joyce\iTRAQ40.raw]
Data File:Submitted from 20120508-1(merge) by Mascot Daemon on JOYCE-VAIO
Monoisotopic mass of neutral peptide Mr(calc): 2381.311
Variable modifications:
K21 :iTRAQ4plex (K)
Ions Score: 53.08 Expect: 0.003

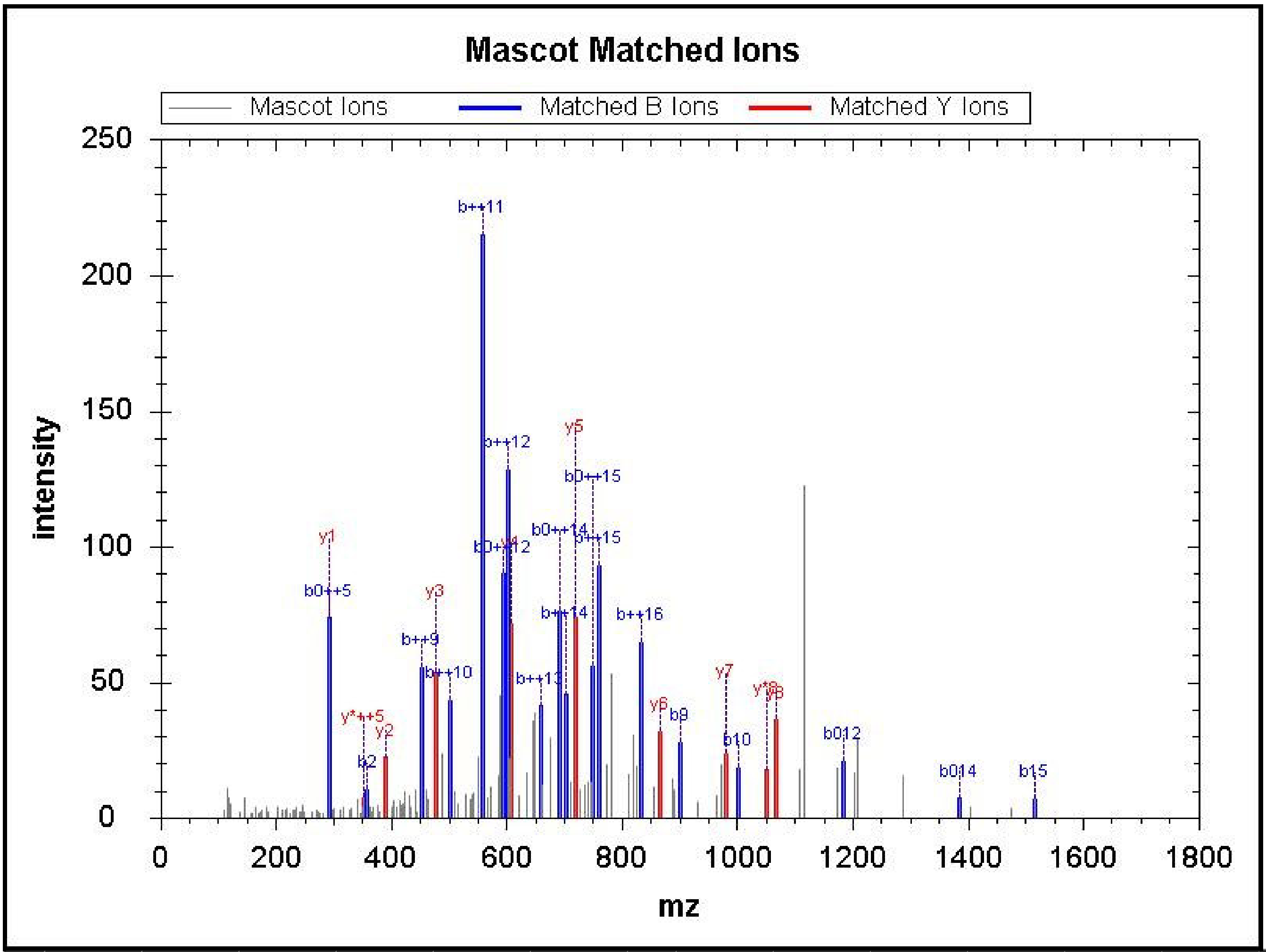

No	b	b++	b*	b*++	b0	b0++	Seq	y	y++	y*	y*++	y0	y0++	RevNo
1	202.13	101.57					G							21
2	358.23	179.62	341.21	171.11			R	2,181.13	1,091.07	2,164.10	1,082.55	2,163.12	1,082.06	20
3	455.28	228.15	438.26	219.63			P	2,025.03	1,013.02	2,008.00	1,004.50	2,007.02	1,004.01	19
4	512.31	256.66	495.28	248.14			G	1,927.97	964.49	1,910.95	955.98	1,909.96	955.48	18
5	599.34	300.17	582.31	291.66	581.33	291.17	S	1,870.95	935.98	1,853.92	927.47	1,852.94	926.97	17
6	656.36	328.68	639.33	320.17	638.35	319.68	G	1,783.92	892.46	1,766.89	883.95	1,765.91	883.46	16
7	757.41	379.21	740.38	370.69	739.40	370.20	T	1,726.90	863.95	1,709.87	855.44	1,708.89	854.95	15
8	844.44	422.72	827.41	414.21	826.43	413.72	S	1,625.85	813.43	1,608.82	804.92	1,607.84	804.42	14
9	901.46	451.23	884.43	442.72	883.45	442.23	G	1,538.82	769.91	1,521.79	761.40	1,520.81	760.91	13
10	1,000.53	500.77	983.50	492.25	982.52	491.76	V	1,481.80	741.40	1,464.77	732.89	1,463.79	732.40	12
11	1,115.56	558.28	1,098.53	549.77	1,097.55	549.28	D	1,382.73	691.87	1,365.70	683.35	1,364.72	682.86	11
12	1,202.59	601.80	1,185.56	593.28	1,184.58	592.79	S	1,267.70	634.35	1,250.67	625.84	1,249.69	625.35	10
13	1,315.67	658.34	1,298.65	649.83	1,297.66	649.33	L	1,180.67	590.84	1,163.64	582.33	1,162.66	581.83	9
14	1,402.70	701.86	1,385.68	693.34	1,384.69	692.85	S	1,067.59	534.30	1,050.56	525.78	1,049.57	525.29	8
15	1,516.75	758.88	1,499.72	750.36	1,498.74	749.87	N	980.55	490.78	963.53	482.27	962.54	481.77	7
16	1,663.82	832.41	1,646.79	823.90	1,645.81	823.41	F	866.51	433.76	849.48	425.25	848.50	424.75	6
17	1,776.90	888.95	1,759.87	880.44	1,758.89	879.95	I	719.44	360.22	702.42	351.71	701.43	351.22	5
18	1,905.94	953.47	1,888.92	944.96	1,887.93	944.47	E	606.36	303.68	589.33	295.17	588.35	294.68	4
19	1,992.97	996.99	1,975.95	988.48	1,974.96	987.99	S	477.32	239.16	460.29	230.65	459.30	230.16	3
20	2,092.04	1,046.53	2,075.02	1,038.01	2,074.03	1,037.52	V	390.28	195.65	373.26	187.13			2
21							K	291.21	146.11	274.19	137.60			1

Query 46878 Hit 1

MS/MS Fragmentation of GLHSQNFTQALLER

Found in sp|Q9Y4W2|LAS1L_HUMAN, Ribosomal biogenesis protein LAS1L OS=Homo sapiens GN=LAS1L PE=1 SV=2
Match to Query 46878: 1756.934from(586.652,3+)
Title: 534: Sum of 2 scans in range 1213 (rt=36.9269, f=4, i=351) to 1214 (rt=36.9523, f=4, i=352)
[D:\lab212\membrane\Grace\Joyce\iTRAQ_39_2.raw]
Data File:Submitted from 20120508-1(merge) by Mascot Daemon on JOYCE-VAIO
Monoisotopic mass of neutral peptide Mr(calc): 1756.934
Variable modifications:
Ions Score: 52.95 Expect: 0.004

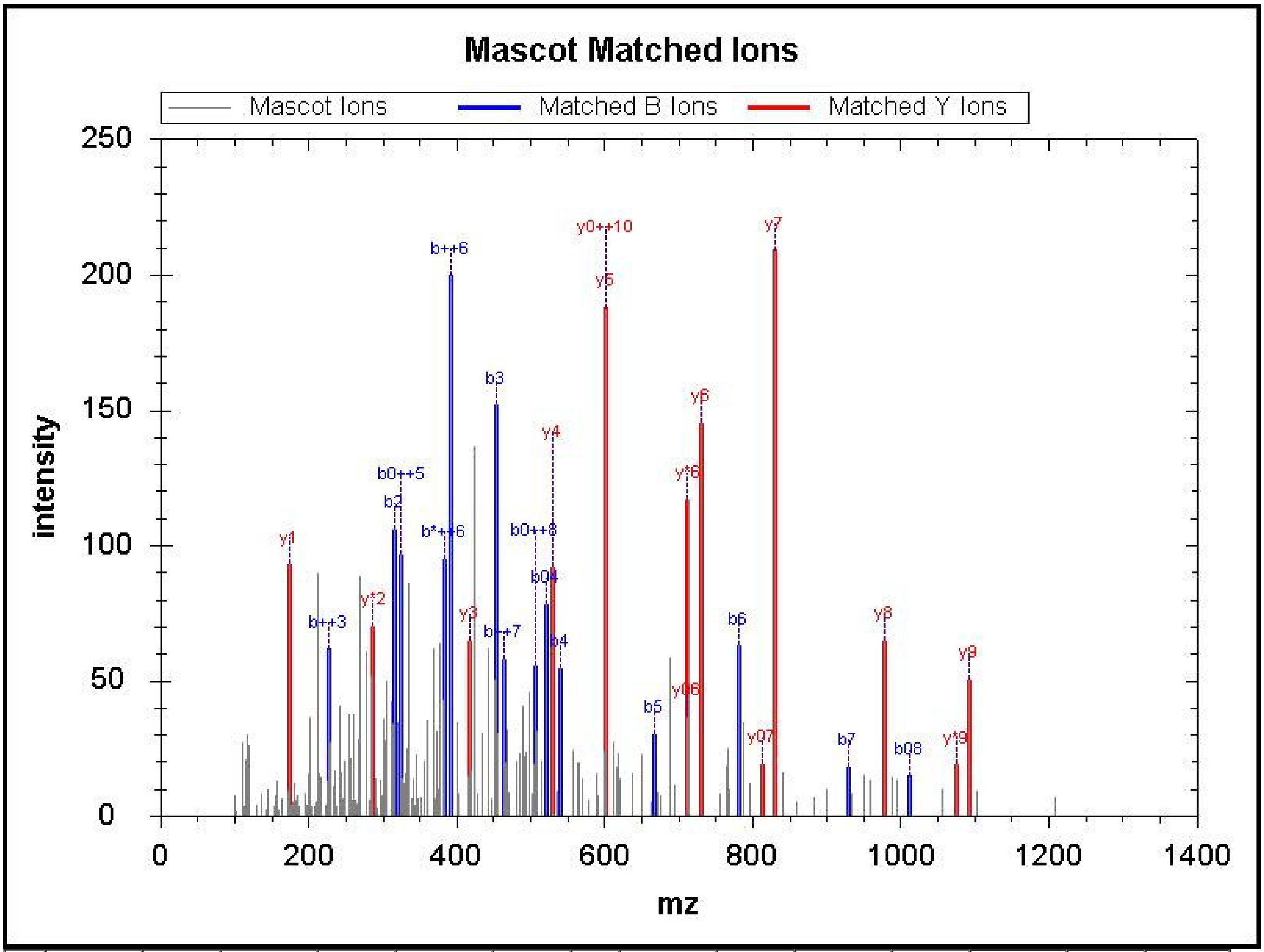

No	b	b++	b*	b*++	b0	b0++	Seq	y	y++	y*	y*++	y0	y0++	RevNo
1	202.13	101.57					G							14
2	315.21	158.11					L	1,556.82	778.91	1,539.79	770.40	1,538.81	769.91	13
3	452.27	226.64					H	1,443.73	722.37	1,426.71	713.86	1,425.72	713.37	12
4	539.31	270.16			521.30	261.15	S	1,306.67	653.84	1,289.65	645.33	1,288.66	644.84	11
5	667.36	334.19	650.34	325.67	649.35	325.18	Q	1,219.64	610.33	1,202.62	601.81	1,201.63	601.32	10
6	781.41	391.21	764.38	382.69	763.40	382.20	N	1,091.58	546.30	1,074.56	537.78	1,073.57	537.29	9
7	928.48	464.74	911.45	456.23	910.47	455.74	F	977.54	489.27	960.51	480.76	959.53	480.27	8
8	1,029.52	515.27	1,012.50	506.75	1,011.51	506.26	T	830.47	415.74	813.45	407.23	812.46	406.73	7
9	1,157.58	579.29	1,140.56	570.78	1,139.57	570.29	Q	729.43	365.22	712.40	356.70	711.41	356.21	6
10	1,228.62	614.81	1,211.59	606.30	1,210.61	605.81	A	601.37	301.19	584.34	292.67	583.36	292.18	5
11	1,341.70	671.36	1,324.68	662.84	1,323.69	662.35	L	530.33	265.67	513.30	257.16	512.32	256.66	4
12	1,454.79	727.90	1,437.76	719.38	1,436.78	718.89	L	417.25	209.13	400.22	200.61	399.24	200.12	3
13	1,583.83	792.42	1,566.80	783.91	1,565.82	783.41	E	304.16	152.58	287.13	144.07	286.15	143.58	2
14							R	175.12	88.06	158.09	79.55			1

Query 99878 Hit 1

MS/MS Fragmentation of IEIMPELADSLVPMEIKPGISLATVSAVLHTK

Found in sp|Q9NV88|INT9_HUMAN, Integrator complex subunit 9 OS=Homo sapiens GN=INTS9 PE=1 SV=2
Match to Query 99878: 3834.229from(959.5646,4+)
Title: 1254: Sum of 2 scans in range 2695 (rt=70.429, f=3, i=432) to 2696 (rt=70.4544, f=3, i=433)
[D:\lab212\membrane\Grace\Joyce\iTRAQ40.raw]
Data File:Submitted from 20120508-1(merge) by Mascot Daemon on JOYCE-VAIO
Monoisotopic mass of neutral peptide Mr(calc): 3834.229
Variable modifications:
K17 :iTRAQ4plex (K)
K32 :iTRAQ4plex (K)
Ions Score: 52.93 Expect: 0.000

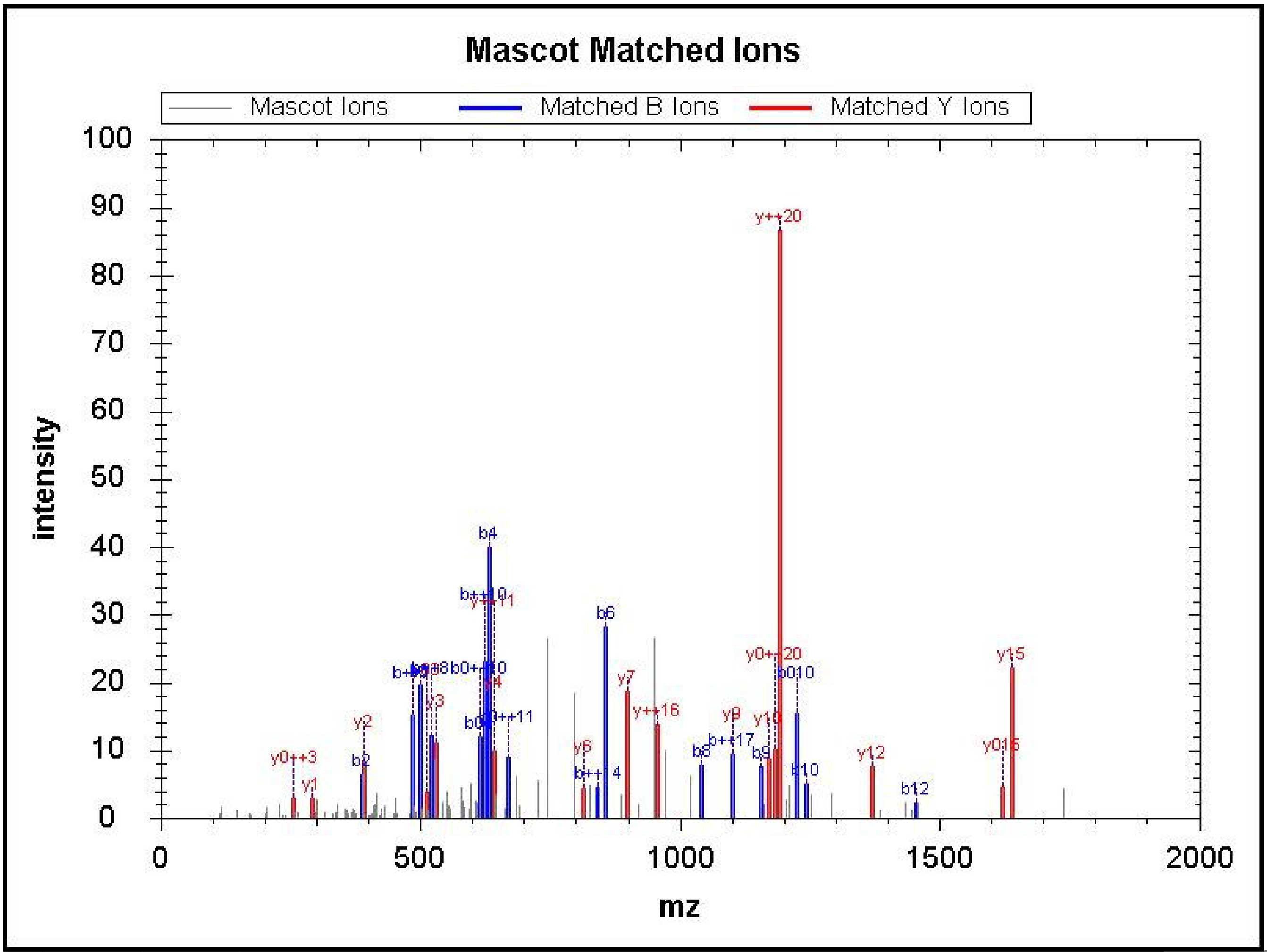

No	b	b++	b*	b*++	b0	b0++	Seq	y	y++	y*	y*++	y0	y0++	RevNo
1	258.19	129.60					I							32
2	387.24	194.12			369.23	185.12	E	3,577.98	1,789.49	3,560.96	1,780.98	3,559.97	1,780.49	31
3	500.32	250.66			482.31	241.66	I	3,448.94	1,724.97	3,431.91	1,716.46	3,430.93	1,715.97	30
4	631.36	316.18			613.35	307.18	M	3,335.86	1,668.43	3,318.83	1,659.92	3,317.85	1,659.43	29
5	728.41	364.71			710.40	355.70	P	3,204.82	1,602.91	3,187.79	1,594.40	3,186.80	1,593.91	28
6	857.46	429.23			839.45	420.23	E	3,107.76	1,554.38	3,090.74	1,545.87	3,089.75	1,545.38	27
7	970.54	485.77			952.53	476.77	L	2,978.72	1,489.86	2,961.69	1,481.35	2,960.71	1,480.86	26
8	1,041.58	521.29			1,023.57	512.29	A	2,865.64	1,433.32	2,848.61	1,424.81	2,847.63	1,424.32	25
9	1,156.60	578.81			1,138.59	569.80	D	2,794.60	1,397.80	2,777.57	1,389.29	2,776.59	1,388.80	24
10	1,243.64	622.32			1,225.63	613.32	S	2,679.57	1,340.29	2,662.55	1,331.78	2,661.56	1,331.28	23
11	1,356.72	678.86			1,338.71	669.86	L	2,592.54	1,296.77	2,575.51	1,288.26	2,574.53	1,287.77	22
12	1,455.79	728.40			1,437.78	719.39	V	2,479.46	1,240.23	2,462.43	1,231.72	2,461.45	1,231.23	21
13	1,552.84	776.92			1,534.83	767.92	P	2,380.39	1,190.70	2,363.36	1,182.18	2,362.38	1,181.69	20
14	1,683.88	842.44			1,665.87	833.44	M	2,283.33	1,142.17	2,266.31	1,133.66	2,265.32	1,133.17	19
15	1,812.92	906.97			1,794.91	897.96	E	2,152.29	1,076.65	2,135.27	1,068.14	2,134.28	1,067.65	18

16	1,926.01	963.51			1,908.00	954.50	I	2,023.25	1,012.13	2,006.23	1,003.62	2,005.24	1,003.12	17
17	2,198.21	1,099.61	2,181.18	1,091.09	2,180.20	1,090.60	K	1,910.17	955.59	1,893.14	947.07	1,892.16	946.58	16
18	2,295.26	1,148.13	2,278.23	1,139.62	2,277.25	1,139.13	P	1,637.97	819.49	1,620.94	810.98	1,619.96	810.48	15
19	2,352.28	1,176.64	2,335.25	1,168.13	2,334.27	1,167.64	G	1,540.92	770.96	1,523.89	762.45	1,522.91	761.96	14
20	2,465.36	1,233.19	2,448.34	1,224.67	2,447.35	1,224.18	I	1,483.90	742.45	1,466.87	733.94	1,465.89	733.45	13
21	2,552.40	1,276.70	2,535.37	1,268.19	2,534.39	1,267.70	S	1,370.81	685.91	1,353.79	677.40	1,352.80	676.90	12
22	2,665.48	1,333.24	2,648.45	1,324.73	2,647.47	1,324.24	L	1,283.78	642.39	1,266.75	633.88	1,265.77	633.39	11
23	2,736.52	1,368.76	2,719.49	1,360.25	2,718.51	1,359.76	A	1,170.70	585.85	1,153.67	577.34	1,152.69	576.85	10
24	2,837.56	1,419.29	2,820.54	1,410.77	2,819.55	1,410.28	T	1,099.66	550.33	1,082.63	541.82	1,081.65	541.33	9
25	2,936.63	1,468.82	2,919.61	1,460.31	2,918.62	1,459.81	V	998.61	499.81	981.58	491.30	980.60	490.80	8
26	3,023.66	1,512.34	3,006.64	1,503.82	3,005.65	1,503.33	S	899.54	450.28	882.52	441.76	881.53	441.27	7
27	3,094.70	1,547.85	3,077.68	1,539.34	3,076.69	1,538.85	A	812.51	406.76	795.48	398.25	794.50	397.75	6
28	3,193.77	1,597.39	3,176.74	1,588.88	3,175.76	1,588.38	V	741.47	371.24	724.45	362.73	723.46	362.24	5
29	3,306.85	1,653.93	3,289.83	1,645.42	3,288.84	1,644.93	L	642.41	321.71	625.38	313.19	624.39	312.70	4
30	3,443.91	1,722.46	3,426.89	1,713.95	3,425.90	1,713.45	H	529.32	265.16	512.29	256.65	511.31	256.16	3
31	3,544.96	1,772.98	3,527.93	1,764.47	3,526.95	1,763.98	T	392.26	196.63	375.24	188.12	374.25	187.63	2
32							K	291.21	146.11	274.19	137.60			1

Query 13488 Hit 1

MS/MS Fragmentation of NGDIVSSAAR
Found in sp|Q07968|F13B_MOUSE, Coagulation factor XIII B chain OS=Mus musculus GN=F13b PE=1 SV=1
Match to Query 13488: 1133.579from(567.7966,2+)
Title: 130: Sum of 2 scans in range 500 (rt=20.3521, f=4, i=65) to 501 (rt=20.3775, f=4, i=66)
[D:\lab212\membrane\Grace\Joyce\iTRAQ_22_1.raw]
Data File:Submitted from 20120508-1(merge) by Mascot Daemon on JOYCE-VAIO
Monoisotopic mass of neutral peptide Mr(calc): 1133.579
Variable modifications:
N1 :Deamidated (NQ)
Ions Score: 52.89 Expect: 0.004

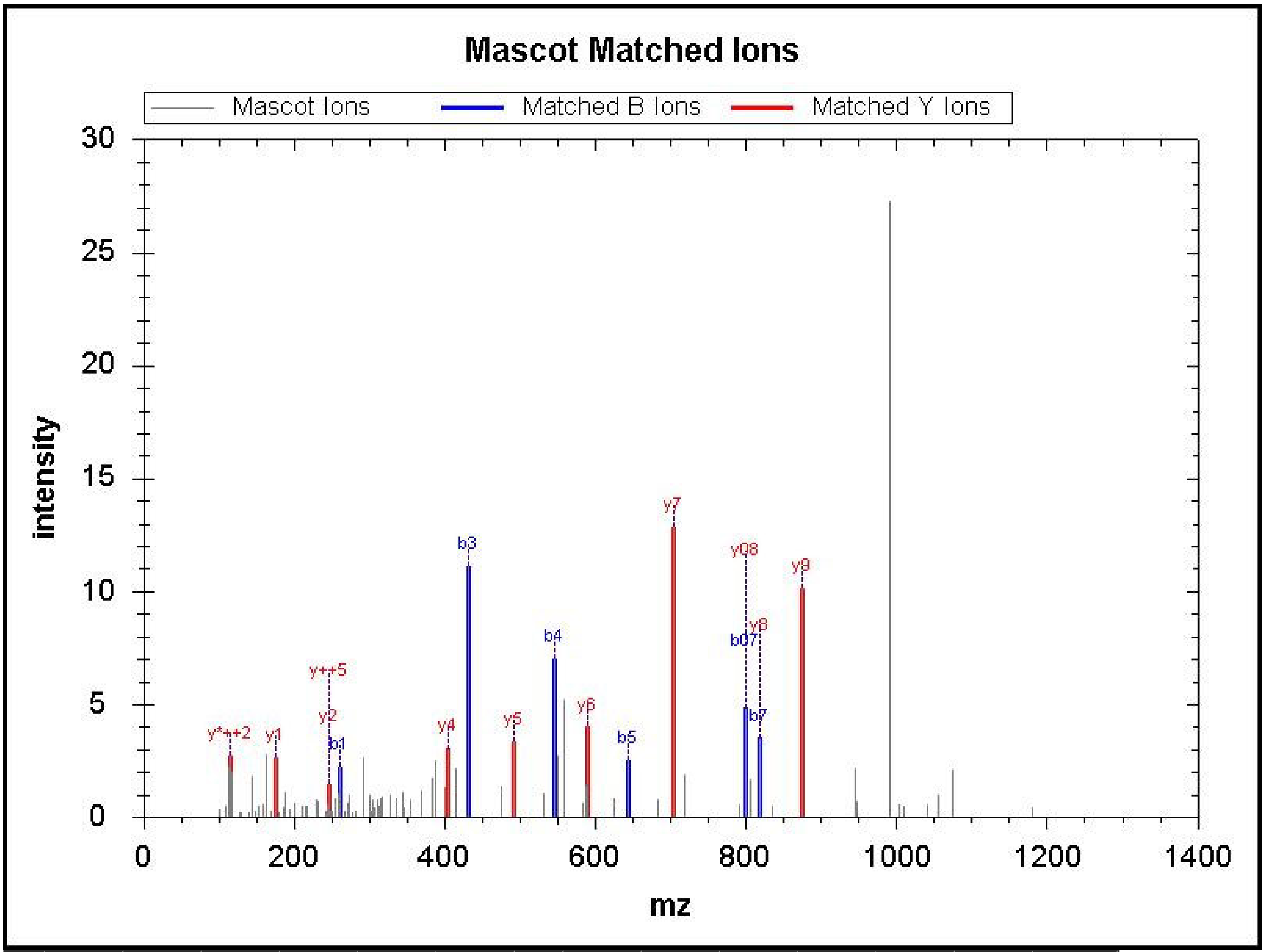

No	b	b++	b*	b*++	b0	b0++	Seq	y	y++	y*	y*++	y0	y0++	RevNo
1	260.14	130.57	243.11	122.06			N							10
2	317.16	159.08	300.13	150.57			G	875.46	438.23	858.43	429.72	857.45	429.23	9
3	432.18	216.60	415.16	208.08	414.17	207.59	D	818.44	409.72	801.41	401.21	800.43	400.72	8
4	545.27	273.14	528.24	264.62	527.26	264.13	I	703.41	352.21	686.38	343.70	685.40	343.20	7
5	644.34	322.67	627.31	314.16	626.33	313.67	V	590.33	295.67	573.30	287.15	572.32	286.66	6
6	731.37	366.19	714.34	357.67	713.36	357.18	S	491.26	246.13	474.23	237.62	473.25	237.13	5
7	818.40	409.70	801.37	401.19	800.39	400.70	S	404.23	202.62	387.20	194.10	386.21	193.61	4
8	889.44	445.22	872.41	436.71	871.43	436.22	A	317.19	159.10	300.17	150.59			3
9	960.48	480.74	943.45	472.23	942.46	471.74	A	246.16	123.58	229.13	115.07			2
10							R	175.12	88.06	158.09	79.55			1

Query 75619 Hit 1

MS/MS Fragmentation of INLLSHDYGDIVAQELLYR
Found in sp|Q5EB52|MEST_HUMAN, Mesoderm-specific transcript homolog protein OS=Homo sapiens GN=MEST PE=2 SV=2
Match to Query 75619: 2375.26from(792.7606,3+)
Title: 959: Sum of 2 scans in range 2131 (rt=57.3921, f=4, i=645) to 2132 (rt=57.4175, f=4, i=646) [D:\lab212\membrane\Grace\Joyce\iTRAQ_33_1.raw]
Data File:Submitted from 20120508-1(merge) by Mascot Daemon on JOYCE-VAIO
Monoisotopic mass of neutral peptide Mr(calc): 2375.26
Variable modifications:
Ions Score: 52.78 Expect: 0.004

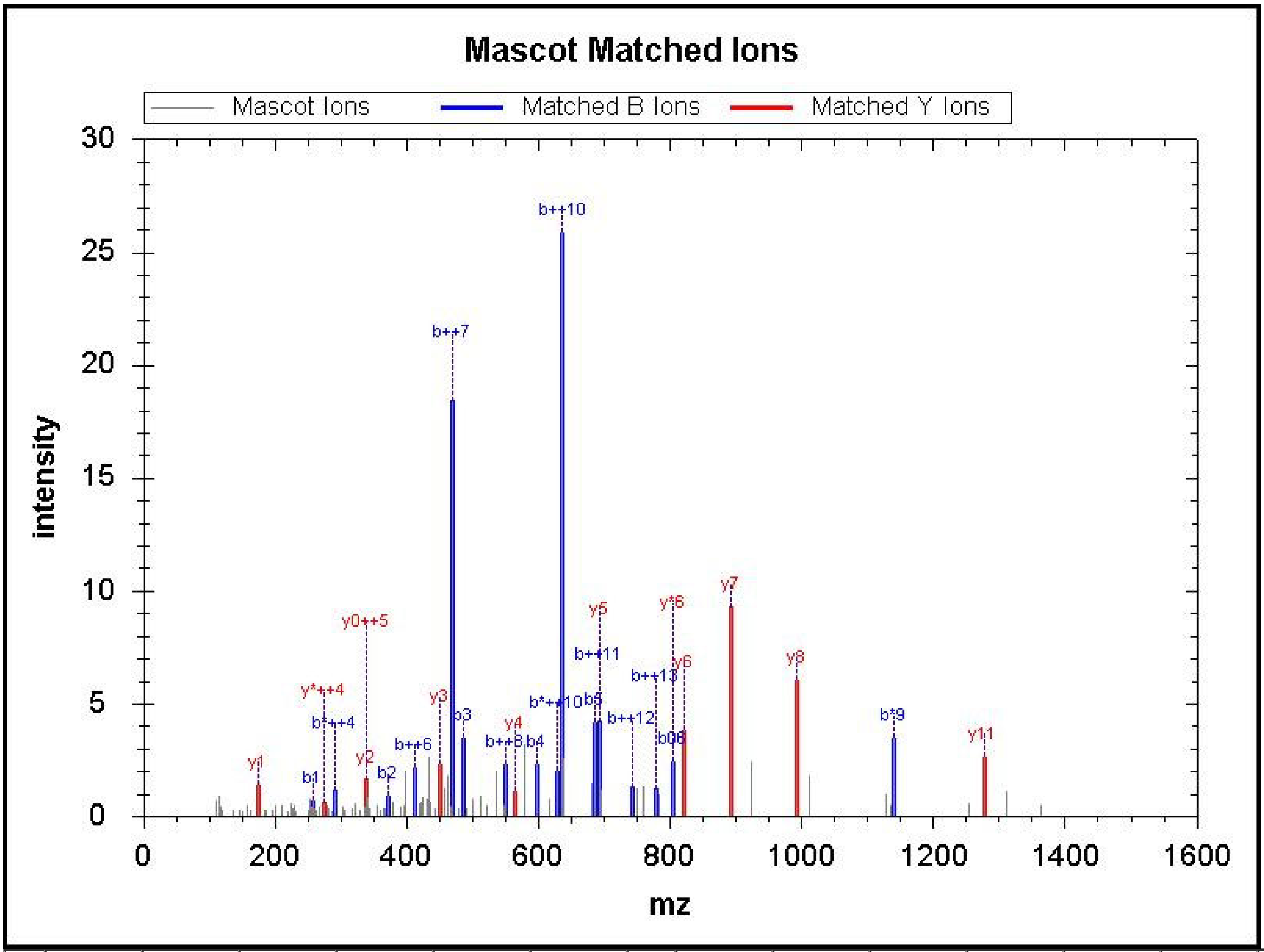

No	b	b++	b*	b*++	b0	b0++	Seq	y	y++	y*	y*++	y0	y0++	RevNo
1	258.19	129.60					I							19
2	372.24	186.62	355.21	178.11			N	2,119.08	1,060.04	2,102.06	1,051.53	2,101.07	1,051.04	18
3	485.32	243.16	468.29	234.65			L	2,005.04	1,003.02	1,988.01	994.51	1,987.03	994.02	17
4	598.40	299.71	581.38	291.19			L	1,891.95	946.48	1,874.93	937.97	1,873.94	937.48	16
5	685.44	343.22	668.41	334.71	667.43	334.22	S	1,778.87	889.94	1,761.84	881.43	1,760.86	880.93	15
6	822.50	411.75	805.47	403.24	804.48	402.75	H	1,691.84	846.42	1,674.81	837.91	1,673.83	837.42	14
7	937.52	469.26	920.50	460.75	919.51	460.26	D	1,554.78	777.89	1,537.75	769.38	1,536.77	768.89	13
8	1,100.59	550.80	1,083.56	542.28	1,082.58	541.79	Y	1,439.75	720.38	1,422.73	711.87	1,421.74	711.37	12
9	1,157.61	579.31	1,140.58	570.79	1,139.60	570.30	G	1,276.69	638.85	1,259.66	630.34	1,258.68	629.84	11
10	1,272.63	636.82	1,255.61	628.31	1,254.62	627.82	D	1,219.67	610.34	1,202.64	601.82	1,201.66	601.33	10
11	1,385.72	693.36	1,368.69	684.85	1,367.71	684.36	I	1,104.64	552.82	1,087.61	544.31	1,086.63	543.82	9
12	1,484.79	742.90	1,467.76	734.38	1,466.78	733.89	V	991.56	496.28	974.53	487.77	973.55	487.28	8
13	1,555.82	778.42	1,538.80	769.90	1,537.81	769.41	A	892.49	446.75	875.46	438.23	874.48	437.74	7
14	1,683.88	842.44	1,666.86	833.93	1,665.87	833.44	Q	821.45	411.23	804.42	402.72	803.44	402.22	6
15	1,812.92	906.97	1,795.90	898.45	1,794.91	897.96	E	693.39	347.20	676.37	338.69	675.38	338.19	5
16	1,926.01	963.51	1,908.98	954.99	1,908.00	954.50	L	564.35	282.68	547.32	274.17			4
17	2,039.09	1,020.05	2,022.07	1,011.54	2,021.08	1,011.04	L	451.27	226.14	434.24	217.62			3
18	2,202.16	1,101.58	2,185.13	1,093.07	2,184.15	1,092.58	Y	338.18	169.59	321.16	161.08			2
19							R	175.12	88.06	158.09	79.55			1

Query 71528 Hit 1

MS/MS Fragmentation of AMEAVLTGLVEAALGPEVLSR
Found in sp|Q9Y4R8|TELO2_HUMAN, Telomere length regulation protein TEL2 homolog OS=Homo sapiens GN=TELO2 PE=1 SV=2
Match to Query 71528: 2269.247from(757.4229,3+)
Title: 1406: Sum of 2 scans in range 3343 (rt=83.9733, f=3, i=504) to 3344 (rt=83.9987, f=3, i=505)

[D:\lab212\membrane\Grace\Joyce\20120320_iTRAQ_20-1.raw]
Data File:Submitted from 20120508-1(merge) by Mascot Daemon on JOYCE-VAIO
Monoisotopic mass of neutral peptide Mr(calc): 2269.247
Variable modifications:
Ions Score: 52.78 Expect: 0.003

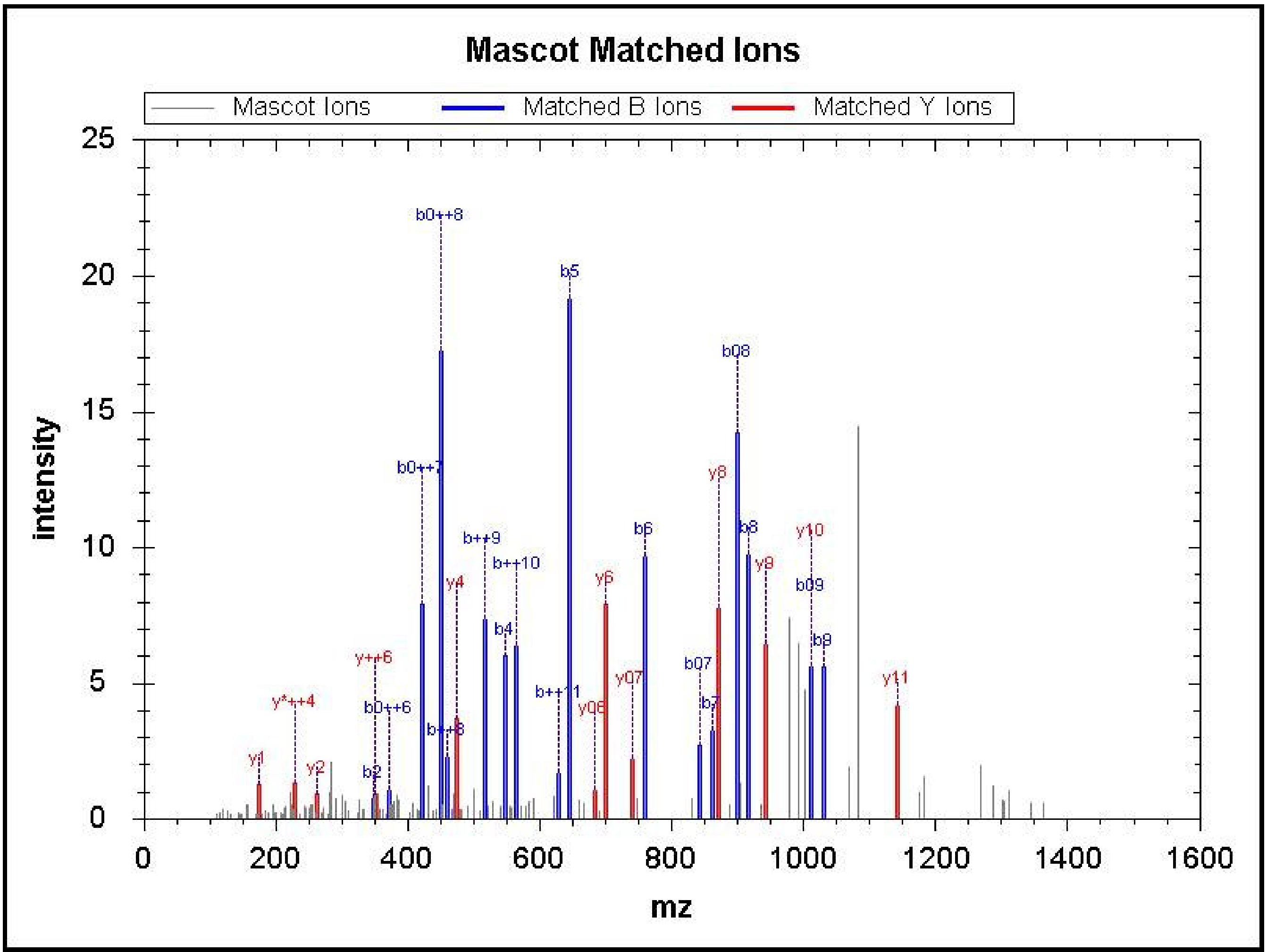

No	b	b++	b0	b0++	Seq	y	y++	y*	y*++	y0	y0++	RevNo
1	216.15	108.58			A							21
2	347.19	174.10			M	2,055.12	1,028.06	2,038.09	1,019.55	2,037.10	1,019.06	20
3	476.23	238.62	458.22	229.61	E	1,924.07	962.54	1,907.05	954.03	1,906.06	953.54	19
4	547.27	274.14	529.26	265.13	A	1,795.03	898.02	1,778.01	889.51	1,777.02	889.01	18
5	646.34	323.67	628.32	314.67	V	1,724.00	862.50	1,706.97	853.99	1,705.98	853.50	17
6	759.42	380.21	741.41	371.21	L	1,624.93	812.97	1,607.90	804.45	1,606.92	803.96	16
7	860.47	430.74	842.46	421.73	T	1,511.84	756.43	1,494.82	747.91	1,493.83	747.42	15
8	917.49	459.25	899.48	450.24	G	1,410.80	705.90	1,393.77	697.39	1,392.78	696.90	14
9	1,030.57	515.79	1,012.56	506.78	L	1,353.77	677.39	1,336.75	668.88	1,335.76	668.39	13
10	1,129.64	565.32	1,111.63	556.32	V	1,240.69	620.85	1,223.66	612.34	1,222.68	611.84	12
11	1,258.68	629.85	1,240.67	620.84	E	1,141.62	571.31	1,124.59	562.80	1,123.61	562.31	11
12	1,329.72	665.36	1,311.71	656.36	A	1,012.58	506.79	995.55	498.28	994.57	497.79	10
13	1,400.76	700.88	1,382.75	691.88	A	941.54	471.27	924.51	462.76	923.53	462.27	9
14	1,513.84	757.42	1,495.83	748.42	L	870.50	435.76	853.48	427.24	852.49	426.75	8
15	1,570.86	785.94	1,552.85	776.93	G	757.42	379.21	740.39	370.70	739.41	370.21	7
16	1,667.92	834.46	1,649.91	825.46	P	700.40	350.70	683.37	342.19	682.39	341.70	6
17	1,796.96	898.98	1,778.95	889.98	E	603.35	302.18	586.32	293.66	585.34	293.17	5
18	1,896.03	948.52	1,878.02	939.51	V	474.30	237.66	457.28	229.14	456.29	228.65	4
19	2,009.11	1,005.06	1,991.10	996.05	L	375.24	188.12	358.21	179.61	357.22	179.12	3

20	2,096.14	1,048.58	2,078.13	1,039.57	S	262.15	131.58	245.12	123.07	244.14	122.57	2
21					R	175.12	88.06	158.09	79.55			1

Query 58801 Hit 1

MS/MS Fragmentation of ELHAPLTVVADGLFSK
Found in sp|Q14534|ERG1_HUMAN, Squalene monooxygenase OS=Homo sapiens GN=SQLE PE=2 SV=3
Match to Query 58801: 1984.118from(662.3799,3+)
Title: 797: Sum of 2 scans in range 1799 (rt=49.7812, f=4, i=511) to 1800 (rt=49.8066, f=4, i=512) [D:\lab212\membrane\Grace\Joyce\iTRAQ_38_2.raw]
Data File:Submitted from 20120508-1(merge) by Mascot Daemon on JOYCE-VAIO
Monoisotopic mass of neutral peptide Mr(calc): 1984.118
Variable modifications:
K16 :iTRAQ4plex (K)
Ions Score: 52.72 Expect: 0.003

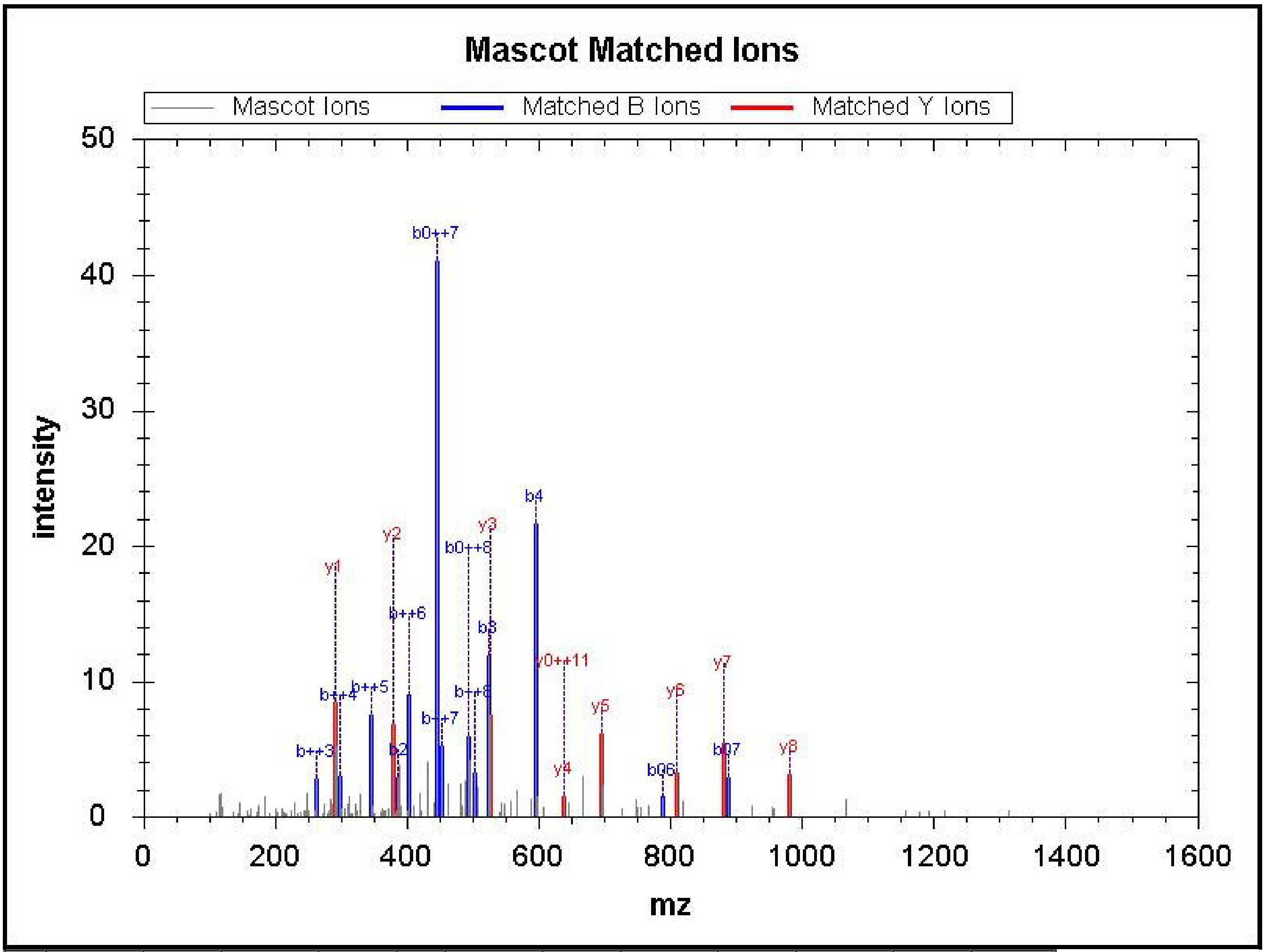

No	b	b++	b0	b0++	Seq	y	y++	y*	y*++	y0	y0++	RevNo
1	274.15	137.58	256.14	128.57	E							16
2	387.24	194.12	369.23	185.12	L	1,711.99	856.50	1,694.96	847.98	1,693.98	847.49	15
3	524.29	262.65	506.28	253.65	H	1,598.90	799.95	1,581.88	791.44	1,580.89	790.95	14
4	595.33	298.17	577.32	289.16	A	1,461.84	731.43	1,444.82	722.91	1,443.83	722.42	13
5	692.38	346.70	674.37	337.69	P	1,390.81	695.91	1,373.78	687.39	1,372.80	686.90	12
6	805.47	403.24	787.46	394.23	L	1,293.75	647.38	1,276.73	638.87	1,275.74	638.38	11
7	906.52	453.76	888.51	444.76	T	1,180.67	590.84	1,163.64	582.33	1,162.66	581.83	10
8	1,005.58	503.30	987.57	494.29	V	1,079.62	540.31	1,062.60	531.80	1,061.61	531.31	9
9	1,104.65	552.83	1,086.64	543.83	V	980.55	490.78	963.53	482.27	962.54	481.77	8
10	1,175.69	588.35	1,157.68	579.34	A	881.48	441.25	864.46	432.73	863.47	432.24	7
11	1,290.72	645.86	1,272.71	636.86	D	810.45	405.73	793.42	397.21	792.44	396.72	6

12	1,347.74	674.37	1,329.73	665.37	G	695.42	348.21	678.39	339.70	677.41	339.21	5
13	1,460.82	730.92	1,442.81	721.91	L	638.40	319.70	621.37	311.19	620.39	310.70	4
14	1,607.89	804.45	1,589.88	795.44	F	525.32	263.16	508.29	254.65	507.30	254.16	3
15	1,694.92	847.97	1,676.91	838.96	S	378.25	189.63	361.22	181.11	360.24	180.62	2
16					K	291.21	146.11	274.19	137.60			1

Query 86862 Hit 1

MS/MS Fragmentation of GIELAYLEQLHGQHEAWLIHK
Found in sp|Q16854|DGUOK_HUMAN, Deoxyguanosine kinase
Match to Query 86862: 2772.495from(694.1311,4+)
Title: 705: Scan 1761 (rt=48.3461, f=3, i=242) [D:\lab212\membrane\Grace\Joyce\iTRAQ_51_2.raw]
Data File:Submitted from 20120508-1(merge) by Mascot Daemon on JOYCE-VAIO
Monoisotopic mass of neutral peptide Mr(calc): 2772.495
Variable modifications:
K21 :iTRAQ4plex (K)
Ions Score: 52.52 Expect: 0.003

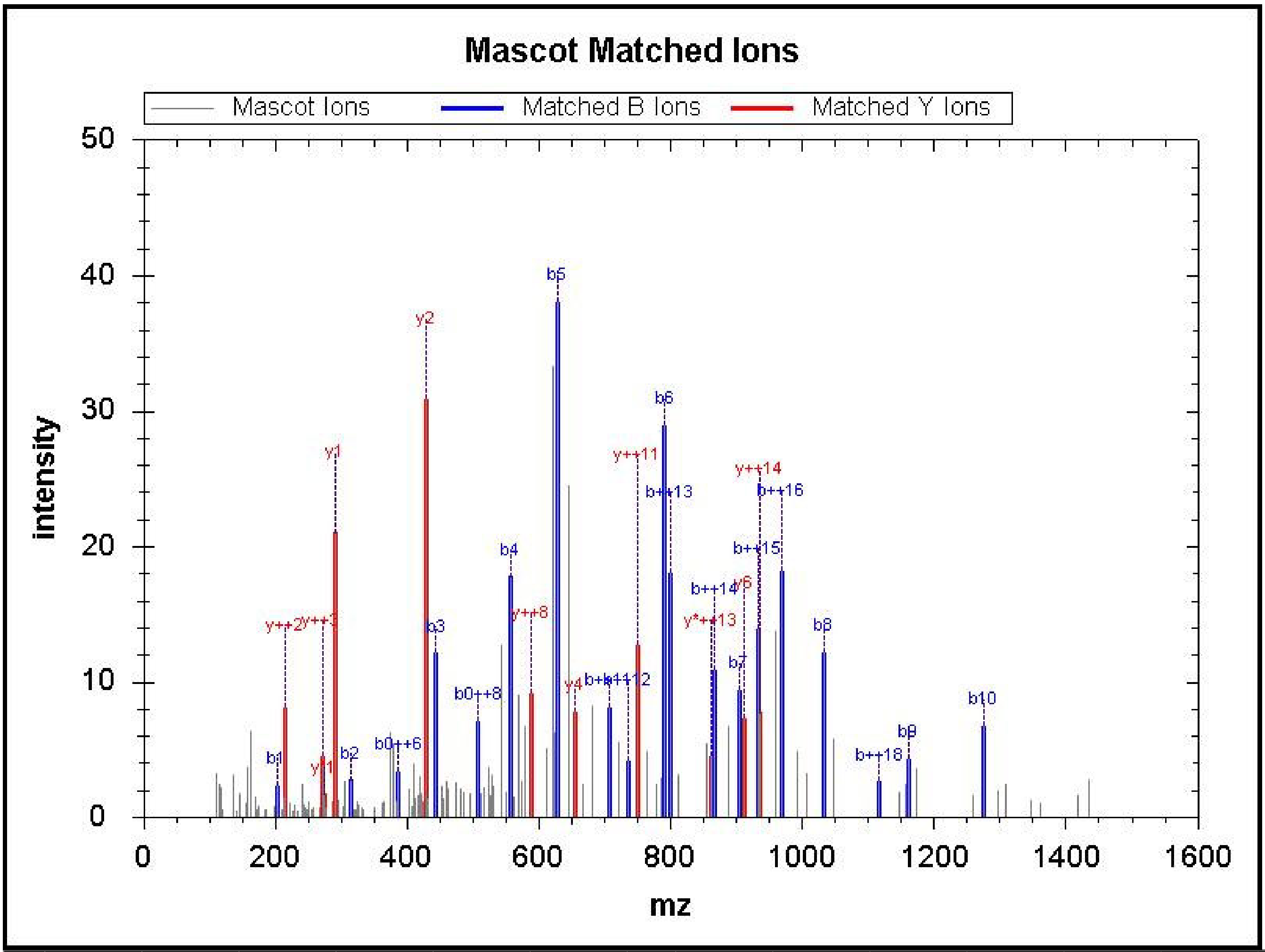

No	b	b++	b*	b*++	b0	b0++	Seq	y	y++	y*	y*++	y0	y0++	RevNo
1	202.13	101.57					G							21
2	315.21	158.11					I	2,572.38	1,286.69	2,555.35	1,278.18	2,554.37	1,277.69	20
3	444.26	222.63			426.25	213.63	E	2,459.30	1,230.15	2,442.27	1,221.64	2,441.28	1,221.15	19
4	557.34	279.17			539.33	270.17	L	2,330.25	1,165.63	2,313.23	1,157.12	2,312.24	1,156.62	18
5	628.38	314.69			610.37	305.69	A	2,217.17	1,109.09	2,200.14	1,100.57	2,199.16	1,100.08	17
6	791.44	396.22			773.43	387.22	Y	2,146.13	1,073.57	2,129.10	1,065.06	2,128.12	1,064.56	16
7	904.53	452.77			886.52	443.76	L	1,983.07	992.04	1,966.04	983.52	1,965.06	983.03	15
8	1,033.57	517.29			1,015.56	508.28	E	1,869.98	935.50	1,852.96	926.98	1,851.97	926.49	14

9	1,161.63	581.32	1,144.60	572.80	1,143.62	572.31	Q	1,740.94	870.97	1,723.92	862.46	1,722.93	861.97	13
10	1,274.71	637.86	1,257.68	629.35	1,256.70	628.85	L	1,612.88	806.95	1,595.86	798.43	1,594.87	797.94	12
11	1,411.77	706.39	1,394.74	697.88	1,393.76	697.38	H	1,499.80	750.40	1,482.77	741.89	1,481.79	741.40	11
12	1,468.79	734.90	1,451.77	726.39	1,450.78	725.89	G	1,362.74	681.87	1,345.71	673.36	1,344.73	672.87	10
13	1,596.85	798.93	1,579.82	790.42	1,578.84	789.92	Q	1,305.72	653.36	1,288.69	644.85	1,287.71	644.36	9
14	1,733.91	867.46	1,716.88	858.95	1,715.90	858.45	H	1,177.66	589.33	1,160.63	580.82	1,159.65	580.33	8
15	1,862.95	931.98	1,845.93	923.47	1,844.94	922.97	E	1,040.60	520.80	1,023.57	512.29	1,022.59	511.80	7
16	1,933.99	967.50	1,916.96	958.98	1,915.98	958.49	A	911.56	456.28	894.53	447.77			6
17	2,120.07	1,060.54	2,103.04	1,052.02	2,102.06	1,051.53	W	840.52	420.76	823.49	412.25			5
18	2,233.15	1,117.08	2,216.13	1,108.57	2,215.14	1,108.07	L	654.44	327.72	637.42	319.21			4
19	2,346.24	1,173.62	2,329.21	1,165.11	2,328.23	1,164.62	I	541.36	271.18	524.33	262.67			3
20	2,483.30	1,242.15	2,466.27	1,233.64	2,465.28	1,233.15	H	428.27	214.64	411.25	206.13			2
21							K	291.21	146.11	274.19	137.60			1

Query 87631 Hit 1

MS/MS Fragmentation of KVFSDVMEDLYNYINPHNGK
Found in sp|P23921|RIR1_HUMAN, Ribonucleoside-diphosphate reductase large subunit OS=Homo sapiens GN=RRM1 PE=1 SV=1
Match to Query 87631: 2815.423 from(704.863,4+)
Title: 1077: Scan 2334 (rt=62.2012, f=2, i=380) [D:\lab212\membrane\Grace\Joyce\iTRAQ_49_2.raw]
Data File:Submitted from 20120508-1(merge) by Mascot Daemon on JOYCE-VAIO
Monoisotopic mass of neutral peptide Mr(calc): 2815.423
Variable modifications:
K1 :iTRAQ4plex (K)
N18 :Deamidated (NQ)
K20 :iTRAQ4plex (K)
Ions Score: 52.34 Expect: 0.005

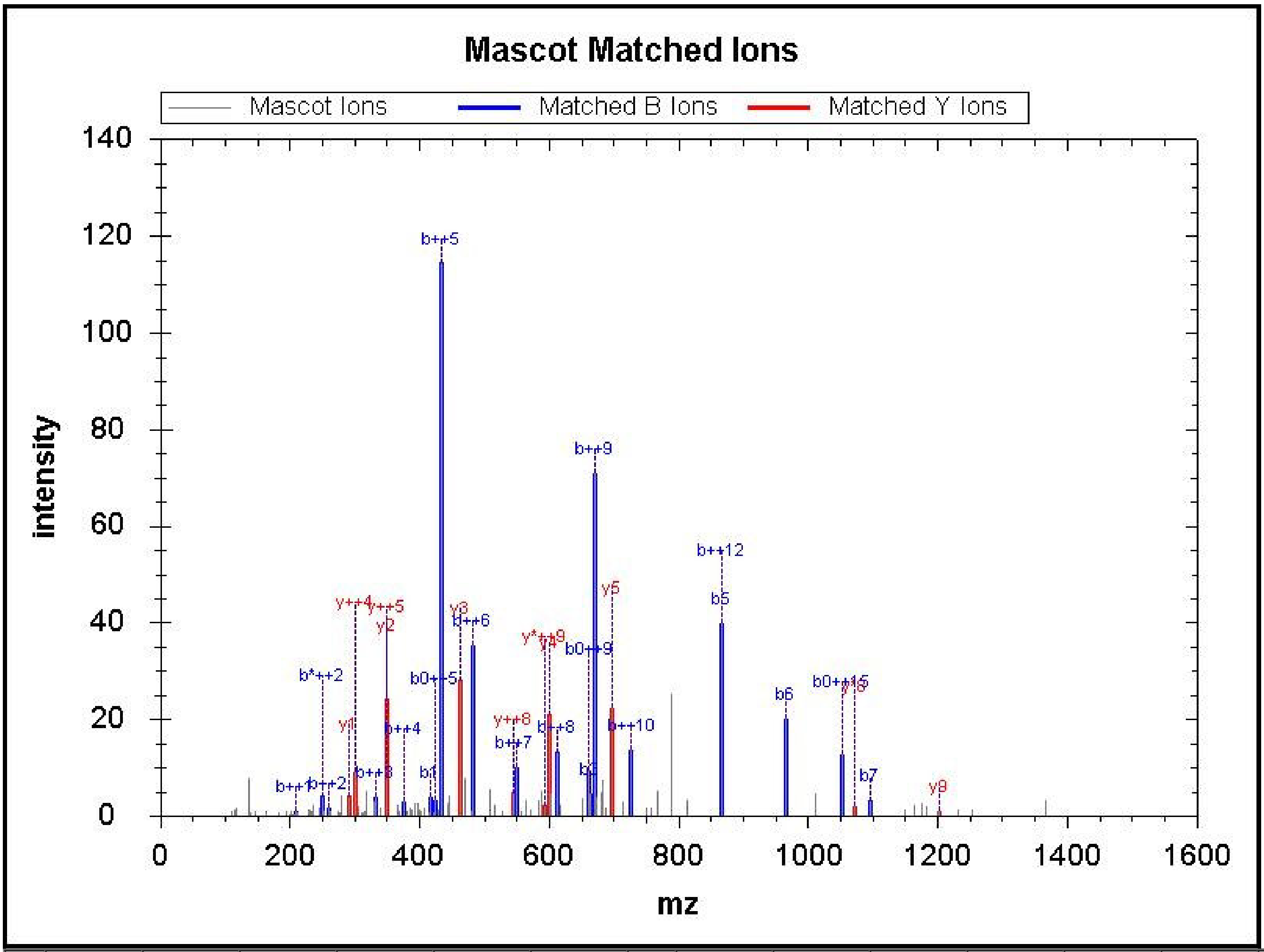

No	b	b++	b*	b*++	b0	b0++	Seq	y	y++	y*	y*++	y0	y0++	RevNo

1	417.31	209.16	400.28	200.64			K						20
2	516.37	258.69	499.35	250.18			V	2,400.13	1,200.57	2,383.10	1,192.06	2,382.12	1,191.56	19
3	663.44	332.23	646.42	323.71			F	2,301.06	1,151.03	2,284.03	1,142.52	2,283.05	1,142.03	18
4	750.48	375.74	733.45	367.23	732.46	366.74	S	2,153.99	1,077.50	2,136.97	1,068.99	2,135.98	1,068.49	17
5	865.50	433.25	848.48	424.74	847.49	424.25	D	2,066.96	1,033.98	2,049.93	1,025.47	2,048.95	1,024.98	16
6	964.57	482.79	947.54	474.28	946.56	473.78	V	1,951.93	976.47	1,934.91	967.96	1,933.92	967.47	15
7	1,095.61	548.31	1,078.58	539.80	1,077.60	539.30	M	1,852.87	926.94	1,835.84	918.42	1,834.86	917.93	14
8	1,224.65	612.83	1,207.63	604.32	1,206.64	603.83	E	1,721.83	861.42	1,704.80	852.90	1,703.81	852.41	13
9	1,339.68	670.34	1,322.65	661.83	1,321.67	661.34	D	1,592.78	796.89	1,575.76	788.38	1,574.77	787.89	12
10	1,452.76	726.89	1,435.74	718.37	1,434.75	717.88	L	1,477.76	739.38	1,460.73	730.87			11
11	1,615.83	808.42	1,598.80	799.90	1,597.82	799.41	Y	1,364.67	682.84	1,347.65	674.33			10
12	1,729.87	865.44	1,712.84	856.93	1,711.86	856.43	N	1,201.61	601.31	1,184.58	592.79			9
13	1,892.93	946.97	1,875.91	938.46	1,874.92	937.97	Y	1,087.57	544.29	1,070.54	535.77			8
14	2,006.02	1,003.51	1,988.99	995.00	1,988.01	994.51	I	924.50	462.75	907.48	454.24			7
15	2,120.06	1,060.53	2,103.03	1,052.02	2,102.05	1,051.53	N	811.42	406.21	794.39	397.70			6
16	2,217.11	1,109.06	2,200.09	1,100.55	2,199.10	1,100.06	P	697.37	349.19	680.35	340.68			5
17	2,354.17	1,177.59	2,337.15	1,169.08	2,336.16	1,168.58	H	600.32	300.66	583.30	292.15			4
18	2,469.20	1,235.10	2,452.17	1,226.59	2,451.19	1,226.10	N	463.26	232.14	446.24	223.62			3
19	2,526.22	1,263.61	2,509.19	1,255.10	2,508.21	1,254.61	G	348.24	174.62	331.21	166.11			2
20							K	291.21	146.11	274.19	137.60			1

Query 60201 Hit 1

MS/MS Fragmentation of SDPLLIGIPTSENPFK
Found in sp|Q9UBI6|GBG12_HUMAN, Guanine nucleotide-binding protein G(I)/G(S)/G(O) subunit gamma-12 OS=Homo sapiens GN=GNG12 PE=1 SV=3
Match to Query 60201: 2015.119from(672.7136,3+)
Title: 873: Sum of 2 scans in range 1941 (rt=53.1241, f=4, i=573) to 1942 (rt=53.1496, f=4, i=574) [D:\lab212\membrane\Grace\Joyce\iTRAQ_26_2.raw]
Data File:Submitted from 20120508-1(merge) by Mascot Daemon on JOYCE-VAIO
Monoisotopic mass of neutral peptide Mr(calc): 2015.119
Variable modifications:
K16 :iTRAQ4plex (K)
Ions Score: 52.28 Expect: 0.004

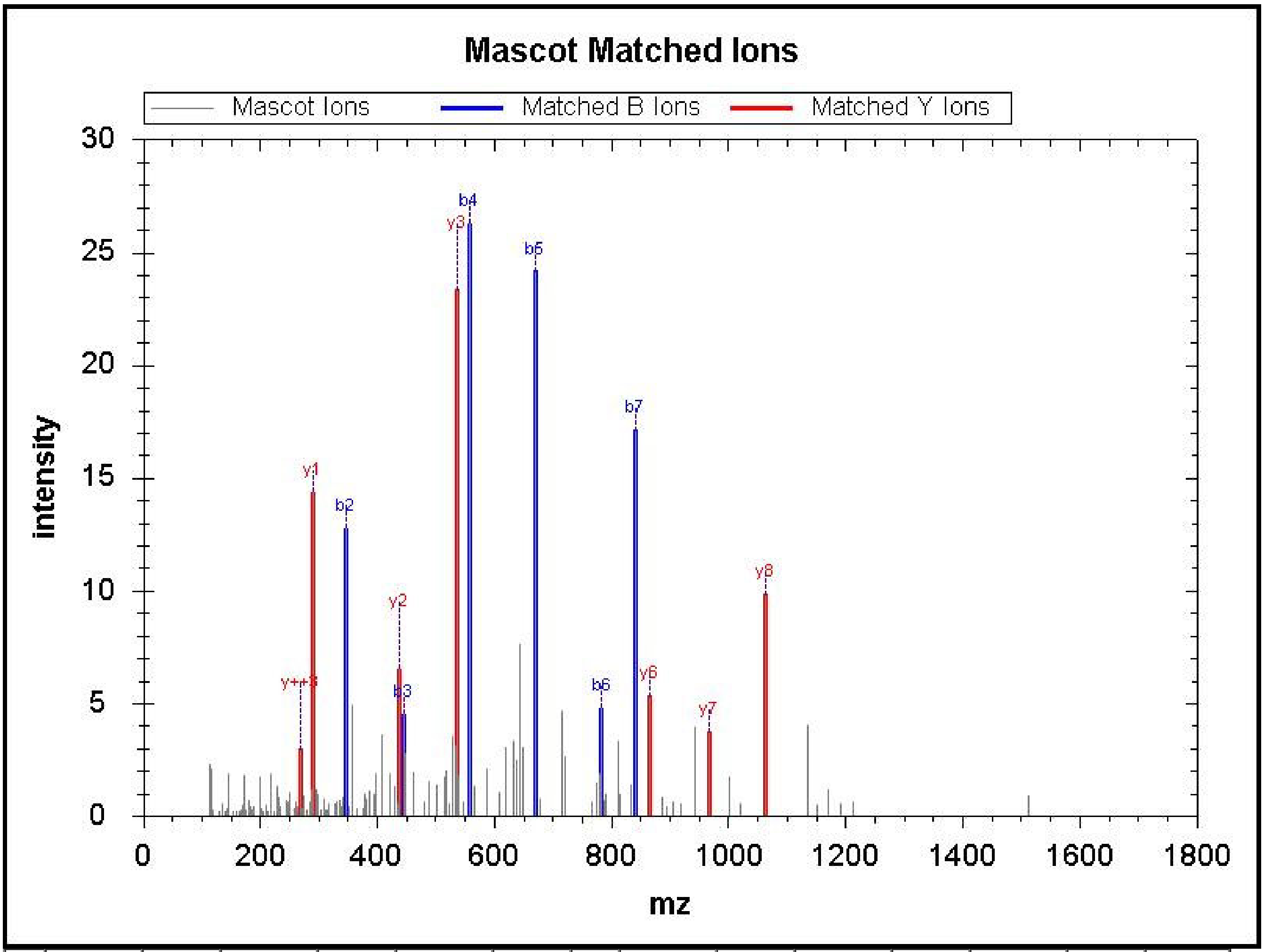

No	b	b++	b*	b*++	b0	b0++	Seq	y	y++	y*	y*++	y0	y0++	RevNo
1	232.14	116.57			214.13	107.57	S							16
2	347.17	174.09			329.16	165.08	D	1,784.99	893.00	1,767.97	884.49	1,766.98	883.99	15
3	444.22	222.61			426.21	213.61	P	1,669.96	835.49	1,652.94	826.97	1,651.95	826.48	14
4	557.31	279.16			539.29	270.15	L	1,572.91	786.96	1,555.89	778.45	1,554.90	777.95	13
5	670.39	335.70			652.38	326.69	L	1,459.83	730.42	1,442.80	721.90	1,441.82	721.41	12
6	783.47	392.24			765.46	383.23	I	1,346.74	673.88	1,329.72	665.36	1,328.73	664.87	11
7	840.49	420.75			822.48	411.75	G	1,233.66	617.33	1,216.63	608.82	1,215.65	608.33	10
8	953.58	477.29			935.57	468.29	I	1,176.64	588.82	1,159.61	580.31	1,158.63	579.82	9
9	1,050.63	525.82			1,032.62	516.81	P	1,063.55	532.28	1,046.53	523.77	1,045.54	523.28	8
10	1,151.68	576.34			1,133.67	567.34	T	966.50	483.75	949.47	475.24	948.49	474.75	7
11	1,238.71	619.86			1,220.70	610.85	S	865.45	433.23	848.43	424.72	847.44	424.23	6
12	1,367.75	684.38			1,349.74	675.38	E	778.42	389.71	761.40	381.20	760.41	380.71	5
13	1,481.80	741.40	1,464.77	732.89	1,463.79	732.40	N	649.38	325.19	632.35	316.68			4
14	1,578.85	789.93	1,561.82	781.42	1,560.84	780.92	P	535.34	268.17	518.31	259.66			3
15	1,725.92	863.46	1,708.89	854.95	1,707.91	854.46	F	438.28	219.65	421.26	211.13			2
16							K	291.21	146.11	274.19	137.60			1

Query 82968 Hit 1

MS/MS Fragmentation of EQLLEALQDLGLLQSGSISGLK
Found in sp|Q96DE0|NUD16_HUMAN, U8 snoRNA-decapping enzyme OS=Homo sapiens GN=NUDT16 PE=1 SV=2
Match to Query 82968: 2599.458from(867.4933,3+)
Title: 1023: Scan 2933 (rt=72.023, f=3, i=349) [D:\lab212\membrane\Grace\Joyce\iTRAQ_25_1.raw]
Data File:Submitted from 20120508-1(merge) by Mascot Daemon on JOYCE-VAIO
Monoisotopic mass of neutral peptide Mr(calc): 2599.458
Variable modifications:

K22 :iTRAQ4plex (K)
Ions Score: 52.24 Expect: 0.002

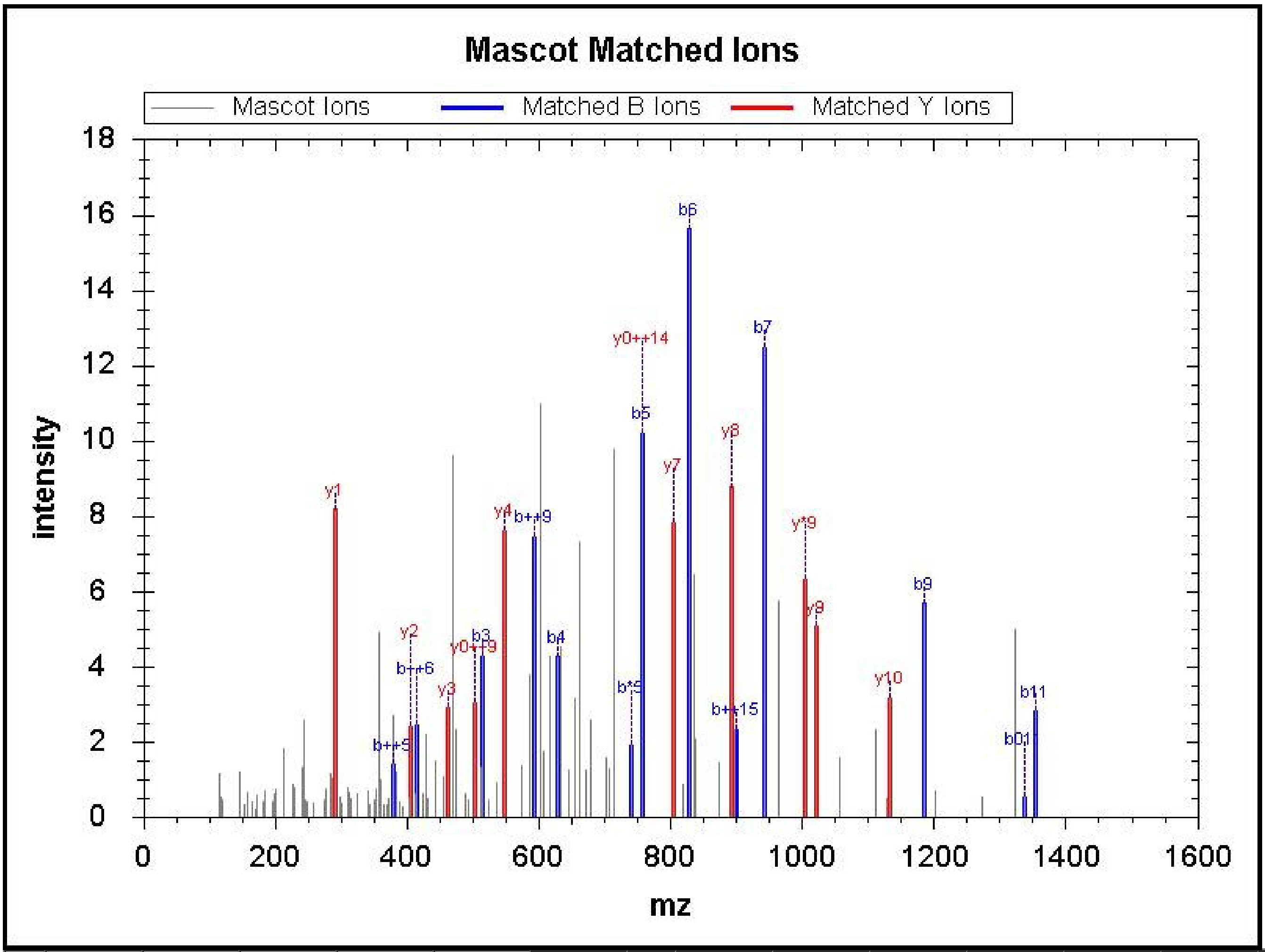

No	b	b++	b*	b*++	b0	b0++	Seq	y	y++	y*	y*++	y0	y0++	RevNo
1	274.15	137.58			256.14	128.57	E							22
2	402.21	201.61	385.18	193.10	384.20	192.60	Q	2,327.33	1,164.17	2,310.30	1,155.66	2,309.32	1,155.16	21
3	515.29	258.15	498.27	249.64	497.28	249.15	L	2,199.27	1,100.14	2,182.25	1,091.63	2,181.26	1,091.13	20
4	628.38	314.69	611.35	306.18	610.37	305.69	L	2,086.19	1,043.60	2,069.16	1,035.08	2,068.18	1,034.59	19
5	757.42	379.21	740.39	370.70	739.41	370.21	E	1,973.10	987.06	1,956.08	978.54	1,955.09	978.05	18
6	828.46	414.73	811.43	406.22	810.45	405.73	A	1,844.06	922.53	1,827.03	914.02	1,826.05	913.53	17
7	941.54	471.27	924.52	462.76	923.53	462.27	L	1,773.02	887.02	1,756.00	878.50	1,755.01	878.01	16
8	1,069.60	535.30	1,052.57	526.79	1,051.59	526.30	Q	1,659.94	830.47	1,642.91	821.96	1,641.93	821.47	15
9	1,184.63	592.82	1,167.60	584.30	1,166.62	583.81	D	1,531.88	766.44	1,514.85	757.93	1,513.87	757.44	14
10	1,297.71	649.36	1,280.69	640.85	1,279.70	640.35	L	1,416.85	708.93	1,399.83	700.42	1,398.84	699.93	13
11	1,354.73	677.87	1,337.71	669.36	1,336.72	668.87	G	1,303.77	652.39	1,286.74	643.88	1,285.76	643.38	12
12	1,467.82	734.41	1,450.79	725.90	1,449.81	725.41	L	1,246.75	623.88	1,229.72	615.36	1,228.74	614.87	11
13	1,580.90	790.95	1,563.88	782.44	1,562.89	781.95	L	1,133.66	567.34	1,116.64	558.82	1,115.65	558.33	10
14	1,708.96	854.98	1,691.93	846.47	1,690.95	845.98	Q	1,020.58	510.79	1,003.55	502.28	1,002.57	501.79	9
15	1,795.99	898.50	1,778.97	889.99	1,777.98	889.49	S	892.52	446.76	875.50	438.25	874.51	437.76	8
16	1,853.01	927.01	1,835.99	918.50	1,835.00	918.01	G	805.49	403.25	788.46	394.74	787.48	394.24	7
17	1,940.05	970.53	1,923.02	962.01	1,922.04	961.52	S	748.47	374.74	731.44	366.22	730.46	365.73	6
18	2,053.13	1,027.07	2,036.10	1,018.56	2,035.12	1,018.06	I	661.44	331.22	644.41	322.71	643.43	322.22	5
19	2,140.16	1,070.58	2,123.14	1,062.07	2,122.15	1,061.58	S	548.35	274.68	531.33	266.17	530.34	265.67	4
20	2,197.18	1,099.10	2,180.16	1,090.58	2,179.17	1,090.09	G	461.32	231.16	444.29	222.65			3
21	2,310.27	1,155.64	2,293.24	1,147.12	2,292.26	1,146.63	L	404.30	202.65	387.27	194.14			2

22							K	291.21	146.11	274.19	137.60			1

Query 66686 Hit 1

MS/MS Fragmentation of EHWNPAIVALVYNVLK
Found in sp|Q16537|2A5E_HUMAN, Serine/threonine-protein phosphatase 2A 56 kDa regulatory subunit epsilon isoform OS=Homo sapiens GN=PPP2R5E PE=1 SV=1
Match to Query 66686: 2153.21 from(718.7441,3+)
Title: 1176: Scan 2514 (rt=66.3898, f=3, i=398) [D:\lab212\membrane\Grace\Joyce\iTRAQ40.raw]
Data File:Submitted from 20120508-1(merge) by Mascot Daemon on JOYCE-VAIO
Monoisotopic mass of neutral peptide Mr(calc): 2153.21
Variable modifications:
K16 :iTRAQ4plex (K)
Ions Score: 52.14 Expect: 0.003

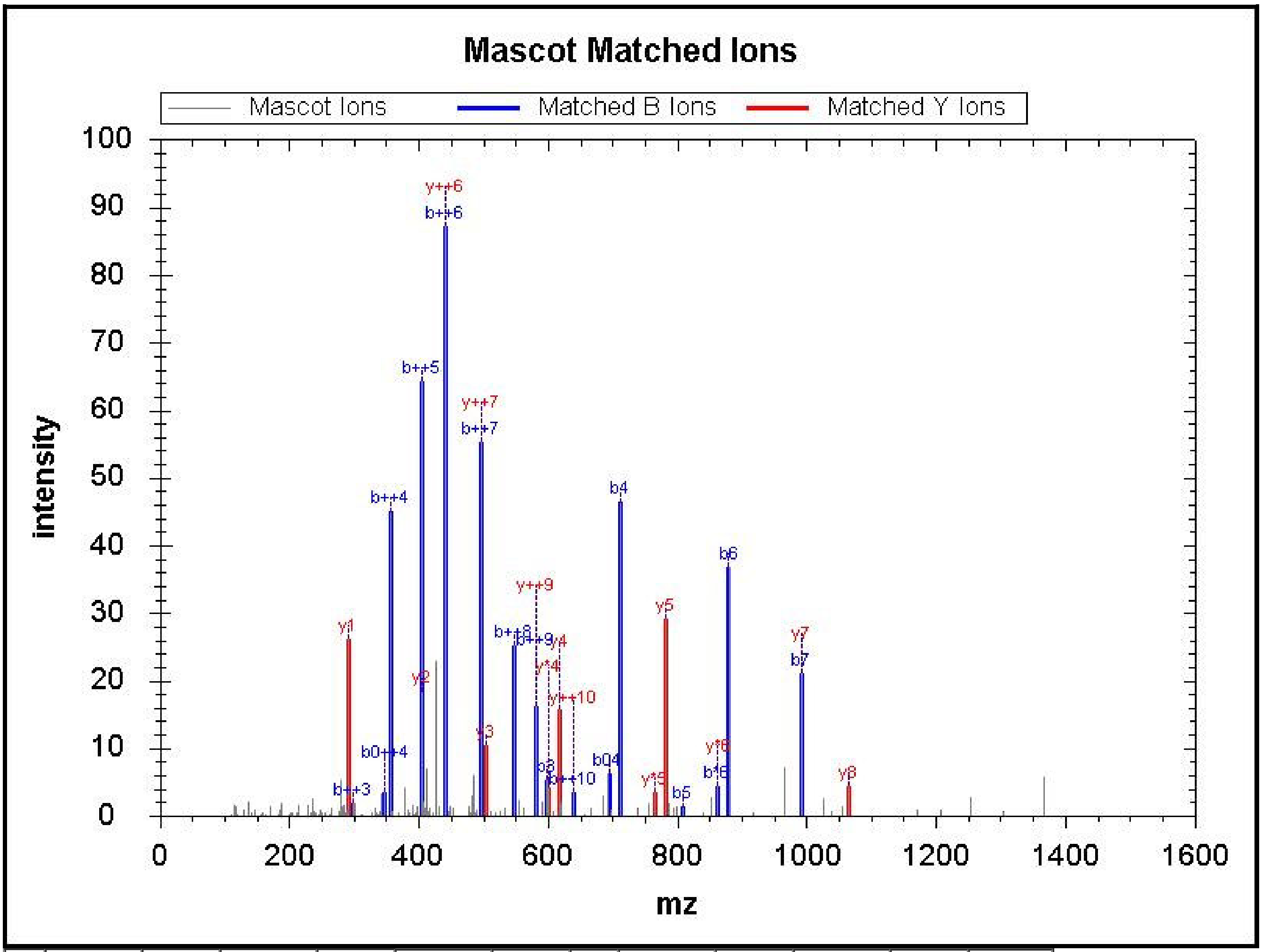

No	b	b++	b*	b*++	b0	b0++	Seq	y	y++	y*	y*++	RevNo
1	274.15	137.58			256.14	128.57	E					16
2	411.21	206.11			393.20	197.10	H	1,881.09	941.05	1,864.06	932.53	15
3	597.29	299.15			579.28	290.14	W	1,744.03	872.52	1,727.00	864.00	14
4	711.33	356.17	694.31	347.66	693.32	347.16	N	1,557.95	779.48	1,540.92	770.96	13
5	808.39	404.70	791.36	396.18	790.38	395.69	P	1,443.91	722.46	1,426.88	713.94	12
6	879.42	440.22	862.40	431.70	861.41	431.21	A	1,346.85	673.93	1,329.83	665.42	11
7	992.51	496.76	975.48	488.24	974.50	487.75	I	1,275.82	638.41	1,258.79	629.90	10
8	1,091.58	546.29	1,074.55	537.78	1,073.56	537.29	V	1,162.73	581.87	1,145.70	573.36	9
9	1,162.61	581.81	1,145.59	573.30	1,144.60	572.80	A	1,063.66	532.34	1,046.64	523.82	8
10	1,275.70	638.35	1,258.67	629.84	1,257.69	629.35	L	992.63	496.82	975.60	488.30	7
11	1,374.77	687.89	1,357.74	679.37	1,356.75	678.88	V	879.54	440.27	862.52	431.76	6
12	1,537.83	769.42	1,520.80	760.90	1,519.82	760.41	Y	780.47	390.74	763.45	382.23	5

13	1,651.87	826.44	1,634.84	817.93	1,633.86	817.43	N	617.41	309.21	600.38	300.70	4
14	1,750.94	875.97	1,733.91	867.46	1,732.93	866.97	V	503.37	252.19	486.34	243.67	3
15	1,864.02	932.52	1,847.00	924.00	1,846.01	923.51	L	404.30	202.65	387.27	194.14	2
16							K	291.21	146.11	274.19	137.60	1

Query 96626 Hit 1

MS/MS Fragmentation of GTHMQHAYDFYKPDMLSEYPIVDGK
Found in sp|Q01581|HMCS1_HUMAN, Hydroxymethylglutaryl-CoA synthase
Match to Query 96626: 3373.648from(844.4193,4+)
Title: 408: Scan 1686 (rt=44.1872, f=2, i=253) [D:\lab212\membrane\Grace\Joyce\iTRAQ_52_1.raw]
Data File:Submitted from 20120508-1(merge) by Mascot Daemon on JOYCE-VAIO
Monoisotopic mass of neutral peptide Mr(calc): 3373.648
Variable modifications:
K12 :iTRAQ4plex (K)
K25 :iTRAQ4plex (K)
Ions Score: 52.05 Expect: 0.006

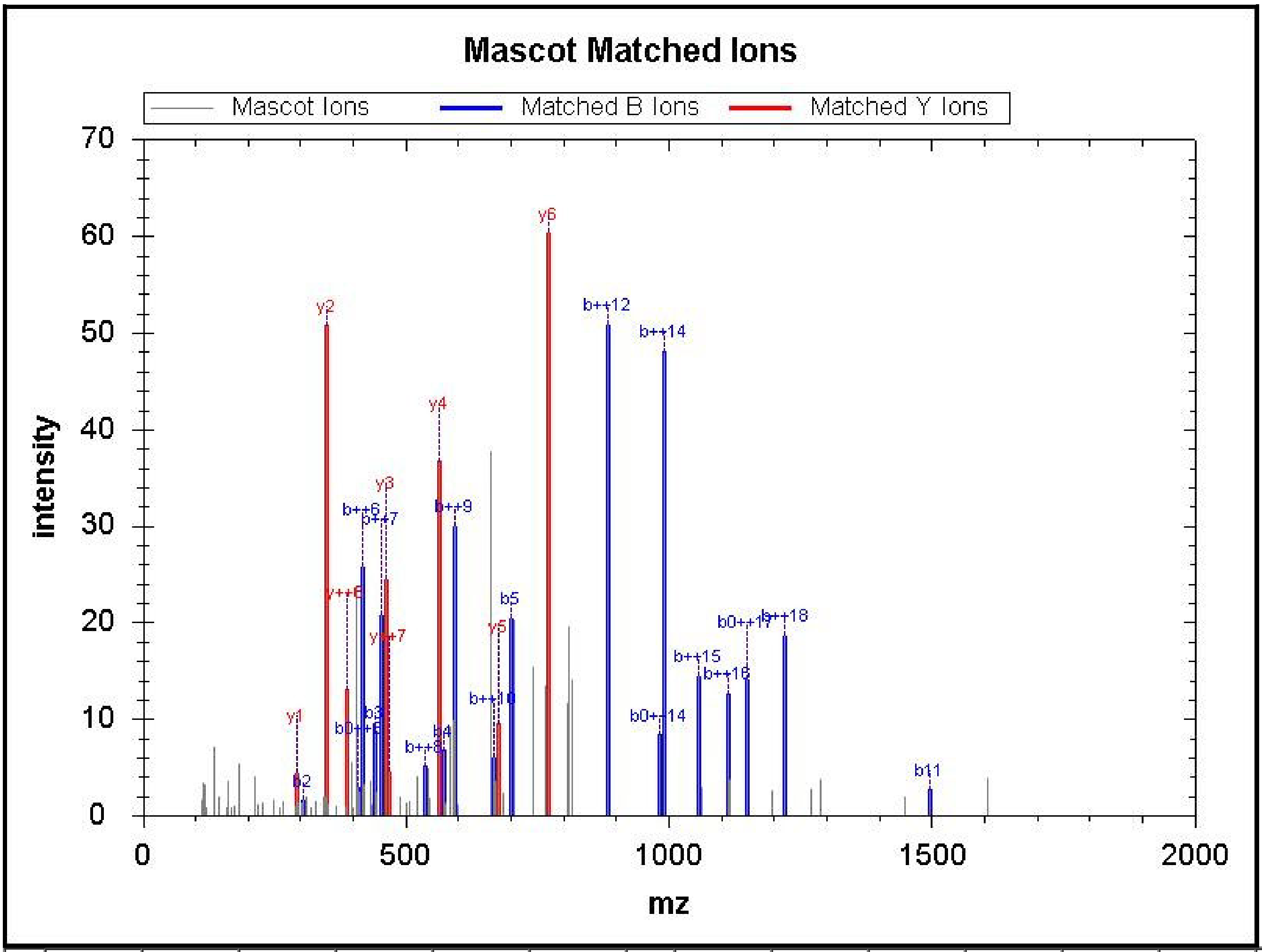

No	b	b++	b*	b*++	b0	b0++	Seq	y	y++	y*	y*++	y0	y0++	RevNo
1	202.13	101.57					G							25
2	303.18	152.09			285.17	143.09	T	3,173.53	1,587.27	3,156.50	1,578.76	3,155.52	1,578.26	24
3	440.24	220.62			422.23	211.62	H	3,072.48	1,536.75	3,055.46	1,528.23	3,054.47	1,527.74	23
4	571.28	286.14			553.27	277.14	M	2,935.42	1,468.22	2,918.40	1,459.70	2,917.41	1,459.21	22
5	699.34	350.17	682.31	341.66	681.33	341.17	Q	2,804.38	1,402.70	2,787.36	1,394.18	2,786.37	1,393.69	21
6	836.40	418.70	819.37	410.19	818.38	409.70	H	2,676.33	1,338.67	2,659.30	1,330.15	2,658.32	1,329.66	20
7	907.43	454.22	890.41	445.71	889.42	445.21	A	2,539.27	1,270.14	2,522.24	1,261.62	2,521.26	1,261.13	19
8	1,070.50	535.75	1,053.47	527.24	1,052.49	526.75	Y	2,468.23	1,234.62	2,451.20	1,226.11	2,450.22	1,225.61	18
9	1,185.52	593.27	1,168.50	584.75	1,167.51	584.26	D	2,305.17	1,153.09	2,288.14	1,144.57	2,287.16	1,144.08	17

10	1,332.59	666.80	1,315.56	658.29	1,314.58	657.79	F	2,190.14	1,095.57	2,173.11	1,087.06	2,172.13	1,086.57	16
11	1,495.65	748.33	1,478.63	739.82	1,477.64	739.33	Y	2,043.07	1,022.04	2,026.04	1,013.53	2,025.06	1,013.03	15
12	1,767.85	884.43	1,750.83	875.92	1,749.84	875.42	K	1,880.01	940.51	1,862.98	931.99	1,862.00	931.50	14
13	1,864.90	932.96	1,847.88	924.44	1,846.89	923.95	P	1,607.81	804.41	1,590.78	795.90	1,589.80	795.40	13
14	1,979.93	990.47	1,962.90	981.96	1,961.92	981.46	D	1,510.76	755.88	1,493.73	747.37	1,492.75	746.88	12
15	2,110.97	1,055.99	2,093.95	1,047.48	2,092.96	1,046.98	M	1,395.73	698.37	1,378.70	689.86	1,377.72	689.36	11
16	2,224.06	1,112.53	2,207.03	1,104.02	2,206.05	1,103.53	L	1,264.69	632.85	1,247.66	624.34	1,246.68	623.84	10
17	2,311.09	1,156.05	2,294.06	1,147.53	2,293.08	1,147.04	S	1,151.61	576.31	1,134.58	567.79	1,133.60	567.30	9
18	2,440.13	1,220.57	2,423.10	1,212.06	2,422.12	1,211.56	E	1,064.57	532.79	1,047.55	524.28	1,046.56	523.79	8
19	2,603.19	1,302.10	2,586.17	1,293.59	2,585.18	1,293.10	Y	935.53	468.27	918.51	459.76	917.52	459.26	7
20	2,700.25	1,350.63	2,683.22	1,342.11	2,682.24	1,341.62	P	772.47	386.74	755.44	378.22	754.46	377.73	6
21	2,813.33	1,407.17	2,796.30	1,398.66	2,795.32	1,398.16	I	675.42	338.21	658.39	329.70	657.41	329.21	5
22	2,912.40	1,456.70	2,895.37	1,448.19	2,894.39	1,447.70	V	562.33	281.67	545.31	273.16	544.32	272.66	4
23	3,027.43	1,514.22	3,010.40	1,505.70	3,009.42	1,505.21	D	463.26	232.14	446.24	223.62	445.25	223.13	3
24	3,084.45	1,542.73	3,067.42	1,534.21	3,066.44	1,533.72	G	348.24	174.62	331.21	166.11			2
25							K	291.21	146.11	274.19	137.60			1

Query 47779 Hit 1

MS/MS Fragmentation of YSHEVLSEENFK
Found in sp|Q7Z2W4|ZCCHV_HUMAN, Zinc finger CCCH-type antiviral protein 1 OS=Homo sapiens GN=ZC3HAV1 PE=1 SV=3
Match to Query 47779: 1768.892from(590.6379,3+)
Title: 405: Sum of 2 scans in range 932 (rt=30.7316, f=4, i=276) to 933 (rt=30.757, f=4, i=277)
[D:\lab212\membrane\Grace\Joyce\iTRAQ_43_1.raw]
Data File:Submitted from 20120508-1(merge) by Mascot Daemon on JOYCE-VAIO
Monoisotopic mass of neutral peptide Mr(calc): 1768.892
Variable modifications:
K12 :iTRAQ4plex (K)
Ions Score: 51.98 Expect: 0.005

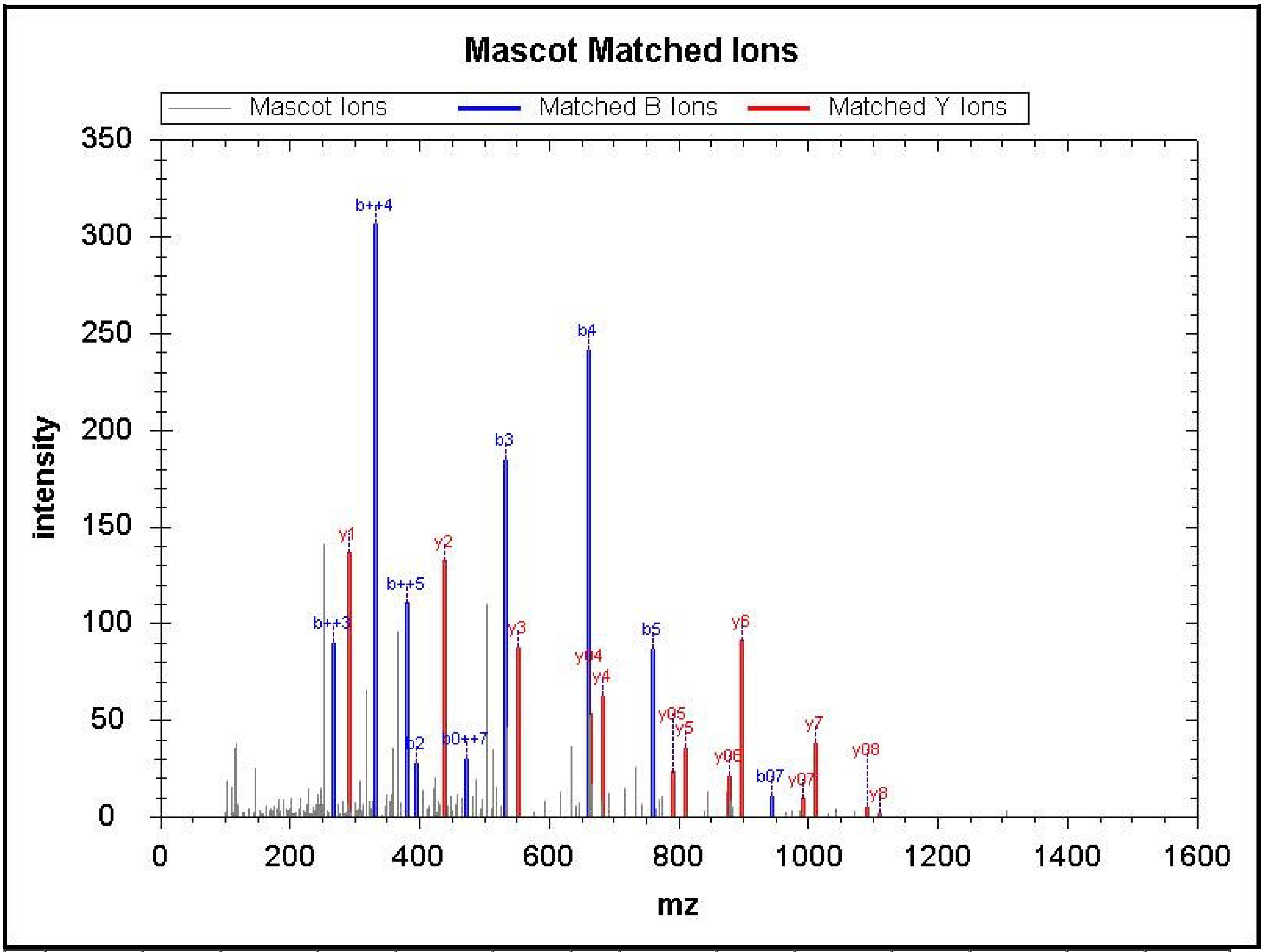

No	b	b++	b*	b*++	b0	b0++	Seq	y	y++	y*	y*++	y0	y0++	RevNo
1	308.17	154.59					Y							12
2	395.20	198.11			377.19	189.10	S	1,462.73	731.87	1,445.70	723.36	1,444.72	722.86	11
3	532.26	266.64			514.25	257.63	H	1,375.70	688.35	1,358.67	679.84	1,357.69	679.35	10
4	661.31	331.16			643.30	322.15	E	1,238.64	619.82	1,221.61	611.31	1,220.63	610.82	9
5	760.37	380.69			742.36	371.69	V	1,109.60	555.30	1,092.57	546.79	1,091.59	546.30	8
6	873.46	437.23			855.45	428.23	L	1,010.53	505.77	993.50	497.25	992.52	496.76	7
7	960.49	480.75			942.48	471.74	S	897.44	449.23	880.42	440.71	879.43	440.22	6
8	1,089.53	545.27			1,071.52	536.27	E	810.41	405.71	793.38	397.20	792.40	396.70	5
9	1,218.58	609.79			1,200.57	600.79	E	681.37	341.19	664.34	332.67	663.36	332.18	4
10	1,332.62	666.81	1,315.59	658.30	1,314.61	657.81	N	552.33	276.67	535.30	268.15			3
11	1,479.69	740.35	1,462.66	731.83	1,461.68	731.34	F	438.28	219.65	421.26	211.13			2
12							K	291.21	146.11	274.19	137.60			1

Query 66660 Hit 1

MS/MS Fragmentation of FNHPEIVQLVSELEAER
Found in sp|Q7Z4H3|HDDC2_HUMAN, HD domain-containing protein 2 OS=Homo sapiens GN=HDDC2 PE=1 SV=1
Match to Query 66660: 2153.127from(718.7164,3+)
Title: 946: Scan 2103 (rt=56.7804, f=3, i=321) [D:\lab212\membrane\Grace\Joyce\iTRAQ_35_2.raw]
Data File:Submitted from 20120508-1(merge) by Mascot Daemon on JOYCE-VAIO
Monoisotopic mass of neutral peptide Mr(calc): 2153.127
Variable modifications:
Ions Score: 51.88 Expect: 0.006

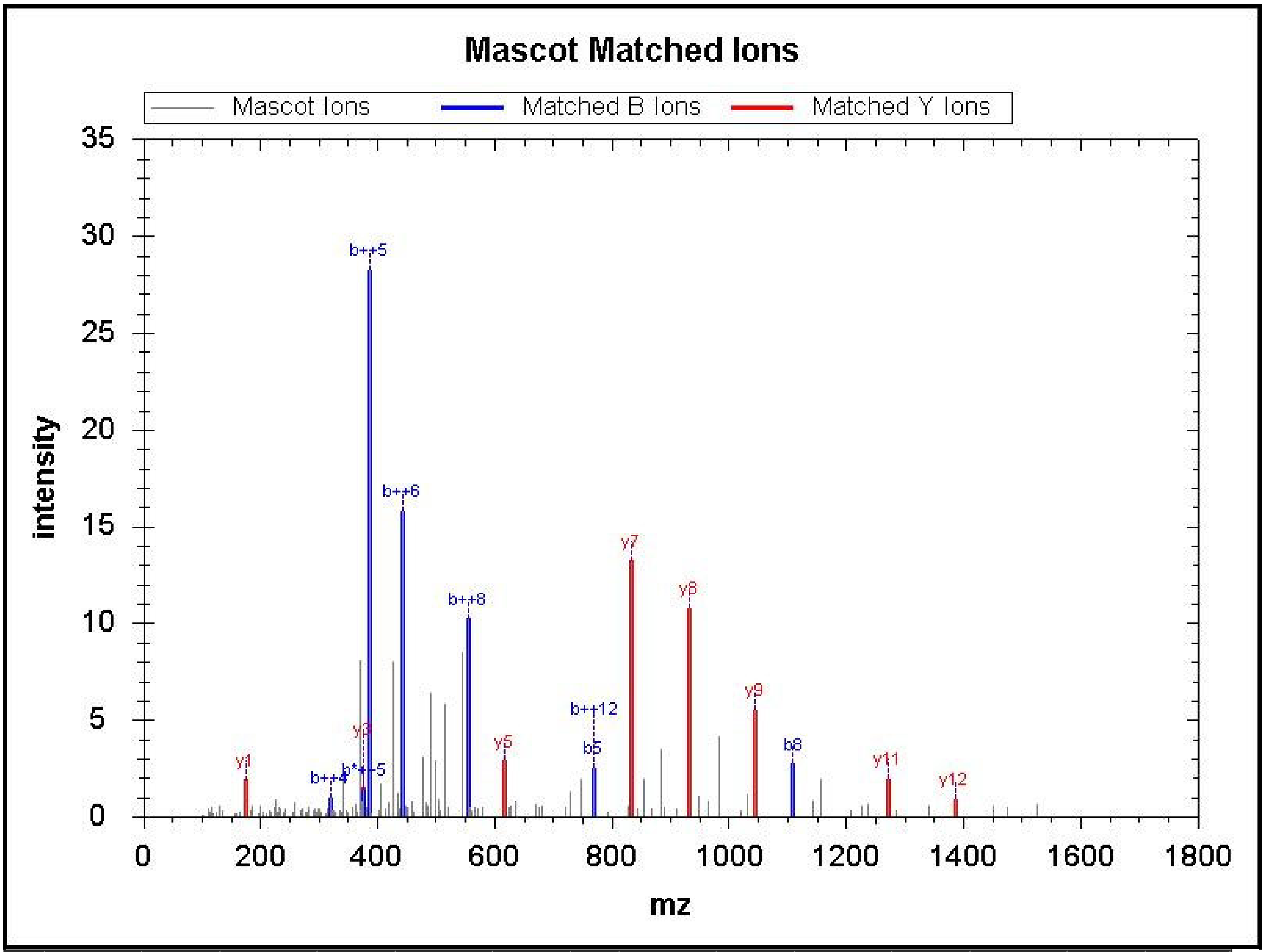

No	b	b++	b*	b*++	b0	b0++	Seq	y	y++	y*	y*++	y0	y0++	RevNo
1	292.18	146.59					F							17
2	406.22	203.61	389.19	195.10			N	1,862.96	931.98	1,845.93	923.47	1,844.95	922.98	16
3	543.28	272.14	526.25	263.63			H	1,748.92	874.96	1,731.89	866.45	1,730.91	865.96	15
4	640.33	320.67	623.31	312.16			P	1,611.86	806.43	1,594.83	797.92	1,593.85	797.43	14
5	769.37	385.19	752.35	376.68	751.36	376.19	E	1,514.81	757.91	1,497.78	749.39	1,496.80	748.90	13
6	882.46	441.73	865.43	433.22	864.45	432.73	I	1,385.76	693.39	1,368.74	684.87	1,367.75	684.38	12
7	981.53	491.27	964.50	482.75	963.52	482.26	V	1,272.68	636.84	1,255.65	628.33	1,254.67	627.84	11
8	1,109.59	555.30	1,092.56	546.78	1,091.58	546.29	Q	1,173.61	587.31	1,156.58	578.80	1,155.60	578.30	10
9	1,222.67	611.84	1,205.64	603.33	1,204.66	602.83	L	1,045.55	523.28	1,028.53	514.77	1,027.54	514.27	9
10	1,321.74	661.37	1,304.71	652.86	1,303.73	652.37	V	932.47	466.74	915.44	458.22	914.46	457.73	8
11	1,408.77	704.89	1,391.74	696.38	1,390.76	695.88	S	833.40	417.20	816.37	408.69	815.39	408.20	7
12	1,537.81	769.41	1,520.79	760.90	1,519.80	760.40	E	746.37	373.69	729.34	365.17	728.36	364.68	6
13	1,650.90	825.95	1,633.87	817.44	1,632.89	816.95	L	617.33	309.17	600.30	300.65	599.31	300.16	5
14	1,779.94	890.47	1,762.91	881.96	1,761.93	881.47	E	504.24	252.62	487.21	244.11	486.23	243.62	4
15	1,850.98	925.99	1,833.95	917.48	1,832.97	916.99	A	375.20	188.10	358.17	179.59	357.19	179.10	3
16	1,980.02	990.51	1,962.99	982.00	1,962.01	981.51	E	304.16	152.58	287.13	144.07	286.15	143.58	2
17							R	175.12	88.06	158.09	79.55			1

Query 29285 Hit 1

MS/MS Fragmentation of TSEDADELHK
Found in sp|Q9UKX7|NUP50_HUMAN, Nuclear pore complex protein Nup50 OS=Homo sapiens GN=NUP50 PE=1 SV=2
Match to Query 29285: 1431.711 from(478.2444,3+)
Title: 120: Scan 323 (rt=17.0072, f=3, i=45) [D:\lab212\membrane\Grace\Joyce\iTRAQ_43_1.raw]
Data File:Submitted from 20120508-1(merge) by Mascot Daemon on JOYCE-VAIO
Monoisotopic mass of neutral peptide Mr(calc): 1431.711

Variable modifications:
K10 :iTRAQ4plex (K)
Ions Score: 51.87 Expect: 0.005

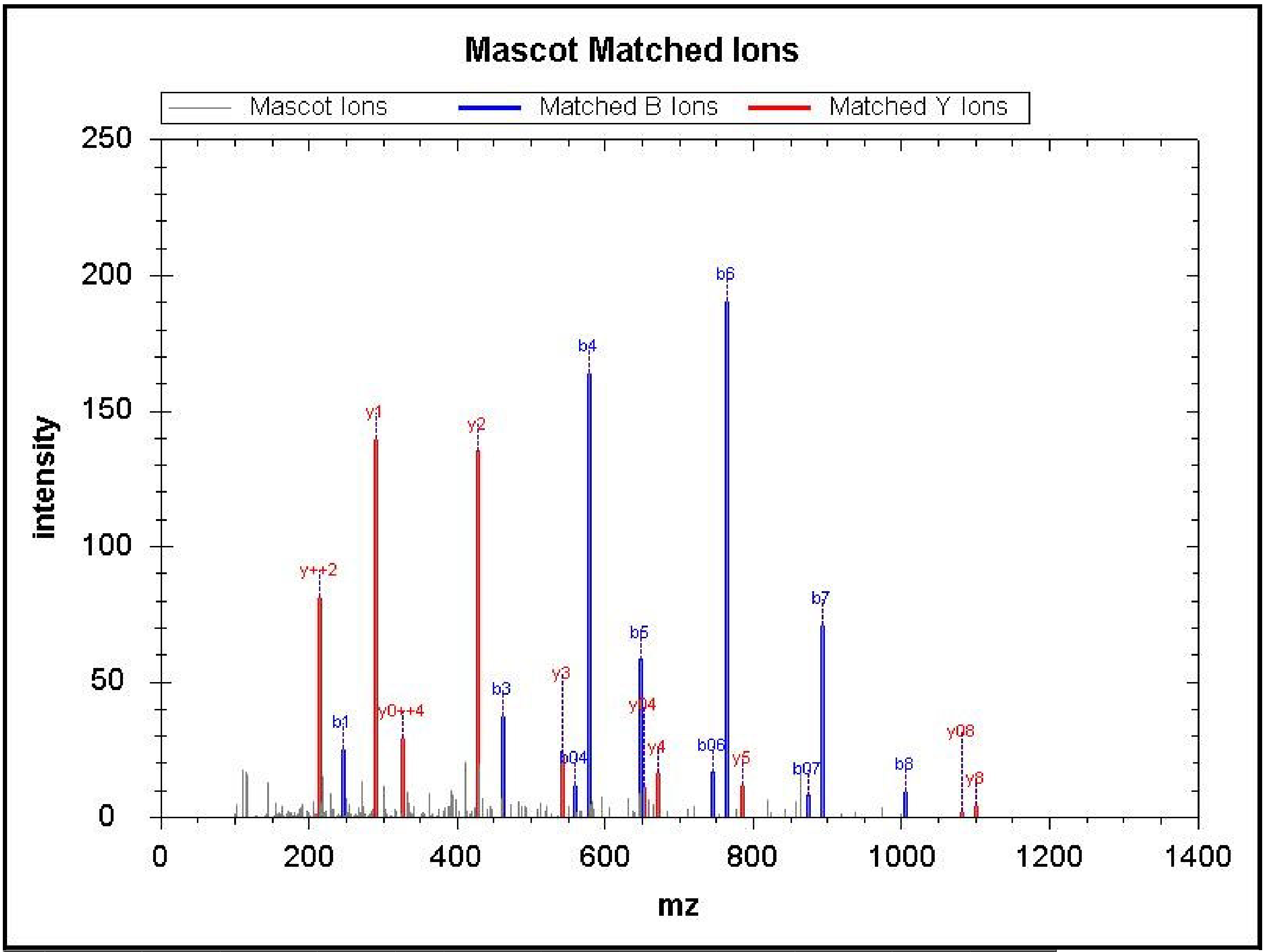

No	b	b++	b0	b0++	Seq	y	y++	y*	y*++	y0	y0++	RevNo
1	246.16	123.58	228.15	114.58	T							10
2	333.19	167.10	315.18	158.09	S	1,187.57	594.29	1,170.54	585.77	1,169.56	585.28	9
3	462.23	231.62	444.22	222.61	E	1,100.53	550.77	1,083.51	542.26	1,082.52	541.77	8
4	577.26	289.13	559.25	280.13	D	971.49	486.25	954.46	477.74	953.48	477.24	7
5	648.30	324.65	630.29	315.65	A	856.46	428.74	839.44	420.22	838.45	419.73	6
6	763.32	382.16	745.31	373.16	D	785.43	393.22	768.40	384.70	767.42	384.21	5
7	892.37	446.69	874.35	437.68	E	670.40	335.70	653.37	327.19	652.39	326.70	4
8	1,005.45	503.23	987.44	494.22	L	541.36	271.18	524.33	262.67			3
9	1,142.51	571.76	1,124.50	562.75	H	428.27	214.64	411.25	206.13			2
10					K	291.21	146.11	274.19	137.60			1

Query 66968 Hit 1

MS/MS Fragmentation of TELQGLIGQLDEVSLEK
Found in sp|Q9UL15|BAG5_HUMAN, BAG family molecular chaperone regulator 5 OS=Homo sapiens GN=BAG5 PE=1 SV=1
Match to Query 66968: 2159.187from(720.7364,3+)
Title: 1003: Scan 2213 (rt=59.2495, f=3, i=339) [D:\lab212\membrane\Grace\Joyce\iTRAQ_26_2.raw]
Data File:Submitted from 20120508-1(merge) by Mascot Daemon on JOYCE-VAIO
Monoisotopic mass of neutral peptide Mr(calc): 2159.187
Variable modifications:
K17 :iTRAQ4plex (K)
Ions Score: 51.65 Expect: 0.005

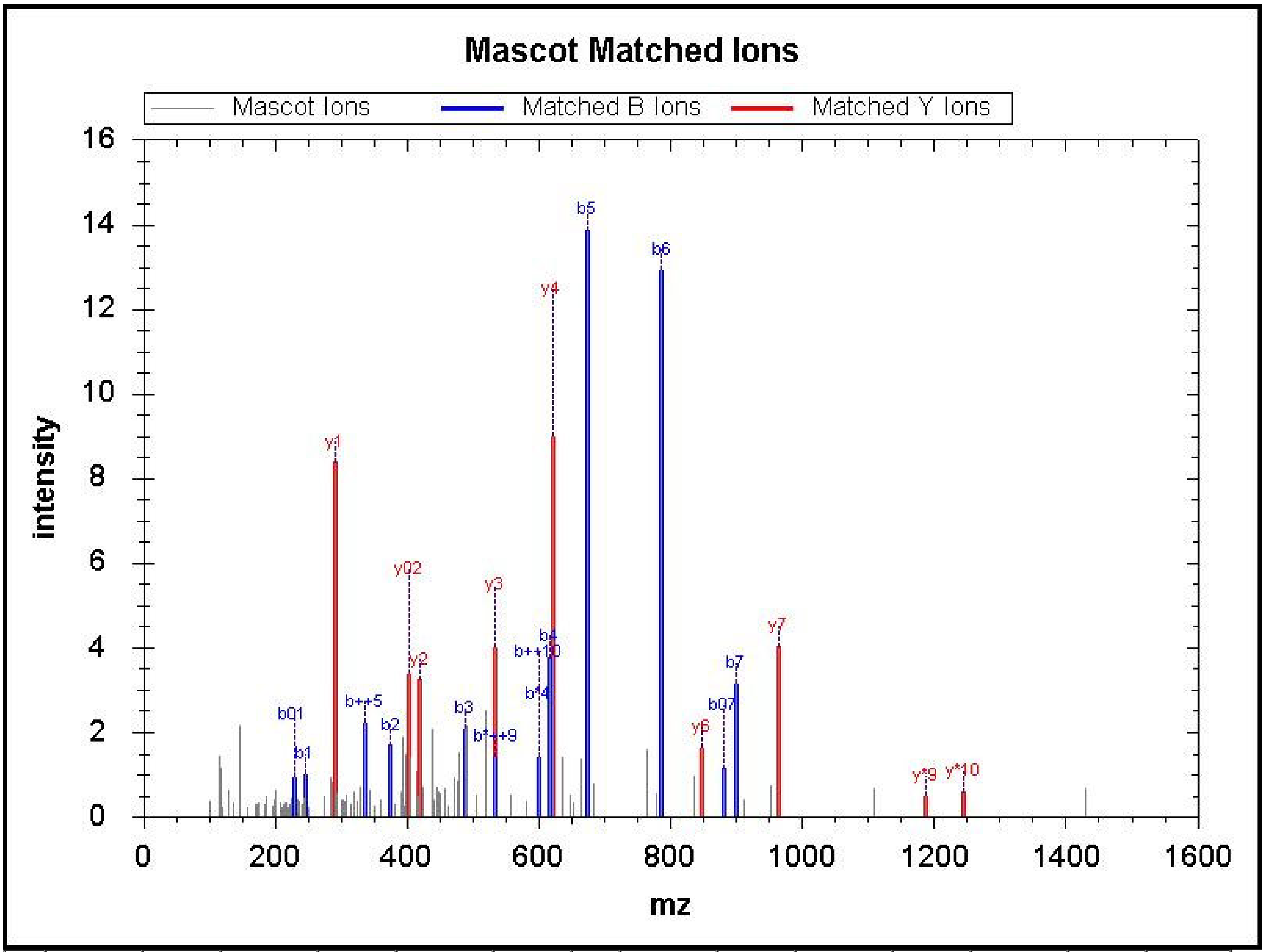

No	b	b++	b*	b*++	b0	b0++	Seq	y	y++	y*	y*++	y0	y0++	RevNo
1	246.16	123.58			228.15	114.58	T							17
2	375.20	188.10			357.19	179.10	E	1,915.05	958.03	1,898.02	949.52	1,897.04	949.02	16
3	488.28	244.65			470.27	235.64	L	1,786.01	893.51	1,768.98	884.99	1,768.00	884.50	15
4	616.34	308.67	599.32	300.16	598.33	299.67	Q	1,672.92	836.97	1,655.90	828.45	1,654.91	827.96	14
5	673.36	337.19	656.34	328.67	655.35	328.18	G	1,544.87	772.94	1,527.84	764.42	1,526.85	763.93	13
6	786.45	393.73	769.42	385.21	768.44	384.72	L	1,487.84	744.43	1,470.82	735.91	1,469.83	735.42	12
7	899.53	450.27	882.51	441.76	881.52	441.26	I	1,374.76	687.88	1,357.73	679.37	1,356.75	678.88	11
8	956.55	478.78	939.53	470.27	938.54	469.77	G	1,261.68	631.34	1,244.65	622.83	1,243.67	622.34	10
9	1,084.61	542.81	1,067.59	534.30	1,066.60	533.80	Q	1,204.65	602.83	1,187.63	594.32	1,186.64	593.83	9
10	1,197.70	599.35	1,180.67	590.84	1,179.69	590.35	L	1,076.60	538.80	1,059.57	530.29	1,058.58	529.80	8
11	1,312.72	656.87	1,295.70	648.35	1,294.71	647.86	D	963.51	482.26	946.48	473.75	945.50	473.25	7
12	1,441.77	721.39	1,424.74	712.87	1,423.76	712.38	E	848.48	424.75	831.46	416.23	830.47	415.74	6
13	1,540.83	770.92	1,523.81	762.41	1,522.82	761.92	V	719.44	360.22	702.42	351.71	701.43	351.22	5
14	1,627.87	814.44	1,610.84	805.92	1,609.86	805.43	S	620.37	310.69	603.35	302.18	602.36	301.69	4
15	1,740.95	870.98	1,723.92	862.47	1,722.94	861.97	L	533.34	267.17	516.31	258.66	515.33	258.17	3
16	1,869.99	935.50	1,852.97	926.99	1,851.98	926.49	E	420.26	210.63	403.23	202.12	402.25	201.63	2
17							K	291.21	146.11	274.19	137.60			1

Query 56750 Hit 1

MS/MS Fragmentation of DLHQGIEAASDEEDLR
Found in sp|Q9UKS6|PACN3_HUMAN, Protein kinase C and casein kinase substrate in neurons protein 3 OS=Homo sapiens GN=PACSIN3 PE=1 SV=2
Match to Query 56750: 1940.92from(647.9805,3+)
Title: 344: Scan 848 (rt=28.5138, f=3, i=120) [D:\lab212\membrane\Grace\Joyce\iTRAQ_36_2.raw]

Data File:Submitted from 20120508-1(merge) by Mascot Daemon on JOYCE-VAIO
Monoisotopic mass of neutral peptide Mr(calc): 1940.92
Variable modifications:
Ions Score: 51.62 Expect: 0.005

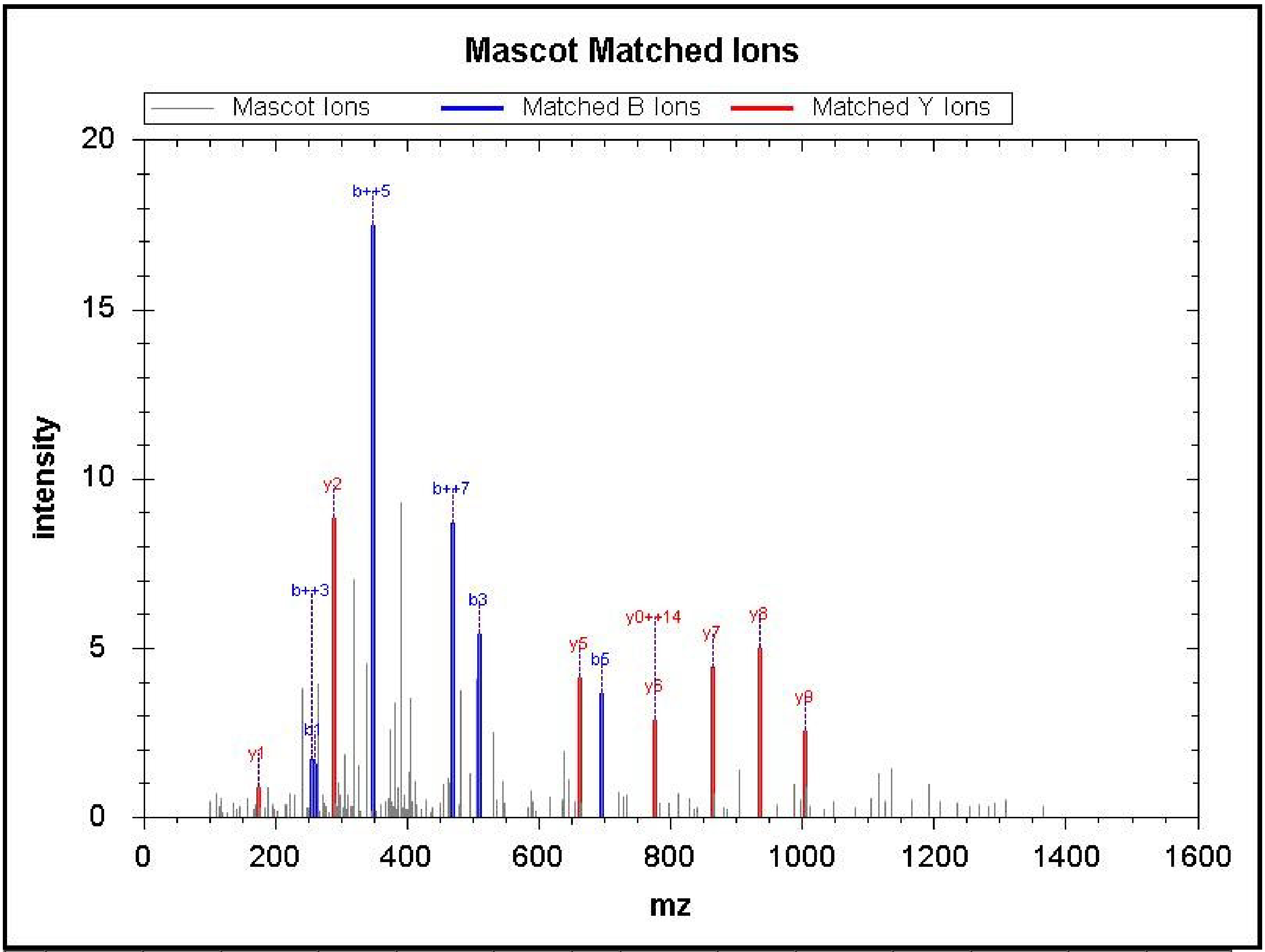

No	b	b++	b*	b*++	b0	b0++	Seq	y	y++	y*	y*++	y0	y0++	RevNo
1	260.14	130.57			242.13	121.57	D							16
2	373.22	187.11			355.21	178.11	L	1,682.80	841.90	1,665.77	833.39	1,664.79	832.90	15
3	510.28	255.64			492.27	246.64	H	1,569.71	785.36	1,552.69	776.85	1,551.70	776.36	14
4	638.34	319.67	621.31	311.16	620.33	310.67	Q	1,432.66	716.83	1,415.63	708.32	1,414.64	707.83	13
5	695.36	348.18	678.33	339.67	677.35	339.18	G	1,304.60	652.80	1,287.57	644.29	1,286.59	643.80	12
6	808.44	404.73	791.42	396.21	790.43	395.72	I	1,247.57	624.29	1,230.55	615.78	1,229.56	615.29	11
7	937.49	469.25	920.46	460.73	919.48	460.24	E	1,134.49	567.75	1,117.46	559.24	1,116.48	558.74	10
8	1,008.52	504.77	991.50	496.25	990.51	495.76	A	1,005.45	503.23	988.42	494.71	987.44	494.22	9
9	1,079.56	540.28	1,062.53	531.77	1,061.55	531.28	A	934.41	467.71	917.38	459.20	916.40	458.70	8
10	1,166.59	583.80	1,149.57	575.29	1,148.58	574.79	S	863.37	432.19	846.35	423.68	845.36	423.19	7
11	1,281.62	641.31	1,264.59	632.80	1,263.61	632.31	D	776.34	388.67	759.32	380.16	758.33	379.67	6
12	1,410.66	705.83	1,393.64	697.32	1,392.65	696.83	E	661.32	331.16	644.29	322.65	643.30	322.16	5
13	1,539.70	770.36	1,522.68	761.84	1,521.69	761.35	E	532.27	266.64	515.25	258.13	514.26	257.63	4
14	1,654.73	827.87	1,637.70	819.36	1,636.72	818.86	D	403.23	202.12	386.20	193.61	385.22	193.11	3
15	1,767.82	884.41	1,750.79	875.90	1,749.80	875.41	L	288.20	144.61	271.18	136.09			2
16							R	175.12	88.06	158.09	79.55			1

Query 78501 Hit 1

MS/MS Fragmentation of GYSFYLAHEFFDVLPVHK
Found in sp|Q7L592|MIDA_HUMAN, Protein midA homolog

Match to Query 78501: 2456.286from(615.0788,4+)
Title: 1048: Scan 2279 (rt=60.9907, f=3, i=357) [D:\lab212\membrane\Grace\Joyce\iTRAQ_54_2.raw]
Data File:Submitted from 20120508-1(merge) by Mascot Daemon on JOYCE-VAIO
Monoisotopic mass of neutral peptide Mr(calc): 2456.286
Variable modifications:
K18 :iTRAQ4plex (K)
Ions Score: 51.55 Expect: 0.006

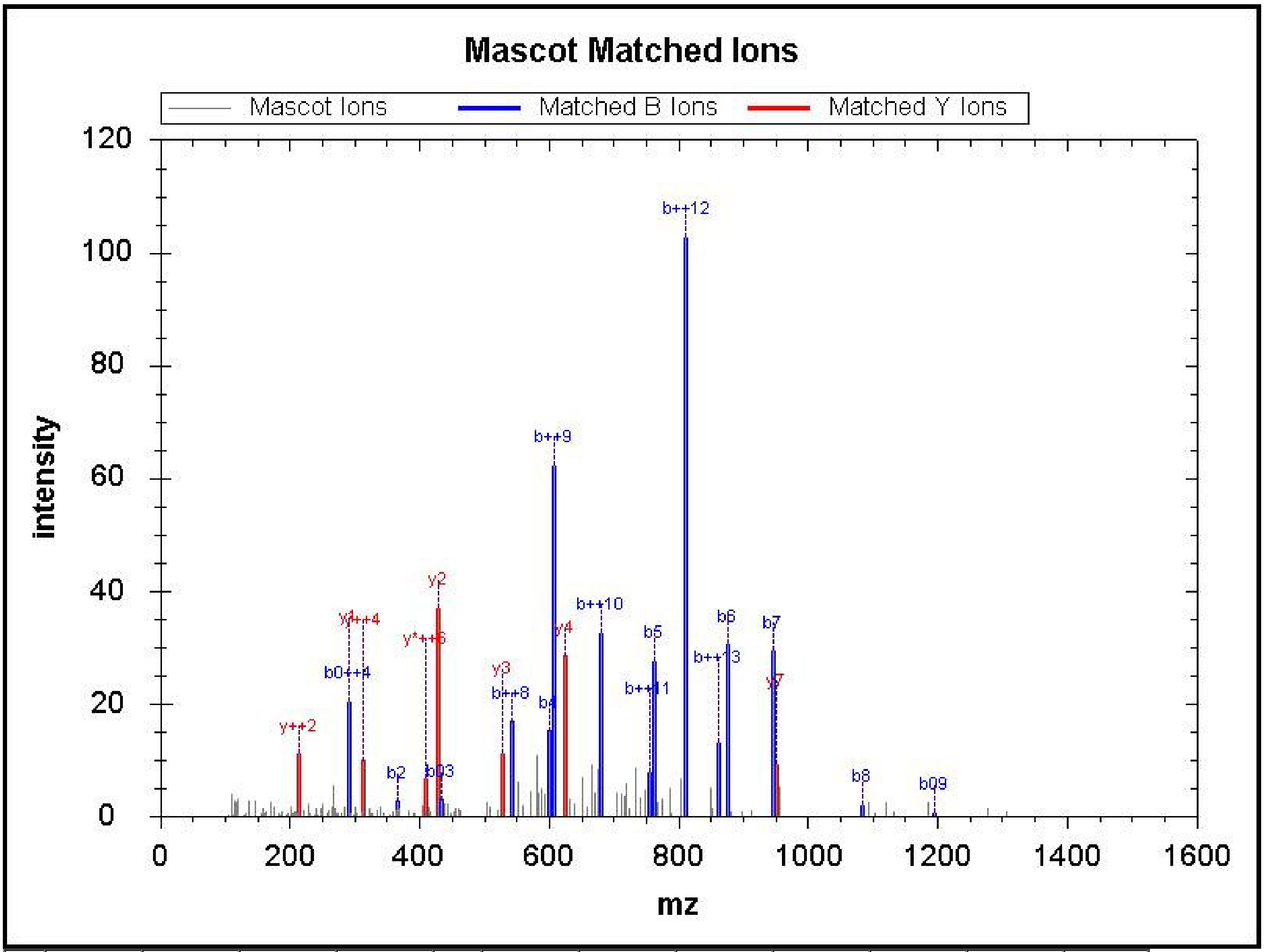

No	b	b++	b0	b0++	Seq	y	y++	y*	y*++	y0	y0++	RevNo
1	202.13	101.57			G							18
2	365.19	183.10			Y	2,256.16	1,128.58	2,239.13	1,120.07	2,238.15	1,119.58	17
3	452.23	226.62	434.22	217.61	S	2,093.10	1,047.05	2,076.07	1,038.54	2,075.09	1,038.05	16
4	599.29	300.15	581.28	291.15	F	2,006.07	1,003.54	1,989.04	995.02	1,988.06	994.53	15
5	762.36	381.68	744.35	372.68	Y	1,859.00	930.00	1,841.97	921.49	1,840.99	921.00	14
6	875.44	438.22	857.43	429.22	L	1,695.93	848.47	1,678.91	839.96	1,677.92	839.47	13
7	946.48	473.74	928.47	464.74	A	1,582.85	791.93	1,565.82	783.42	1,564.84	782.92	12
8	1,083.54	542.27	1,065.53	533.27	H	1,511.81	756.41	1,494.79	747.90	1,493.80	747.40	11
9	1,212.58	606.79	1,194.57	597.79	E	1,374.75	687.88	1,357.73	679.37	1,356.74	678.88	10
10	1,359.65	680.33	1,341.64	671.32	F	1,245.71	623.36	1,228.68	614.85	1,227.70	614.35	9
11	1,506.72	753.86	1,488.71	744.86	F	1,098.64	549.83	1,081.62	541.31	1,080.63	540.82	8
12	1,621.74	811.38	1,603.73	802.37	D	951.57	476.29	934.55	467.78	933.56	467.29	7
13	1,720.81	860.91	1,702.80	851.90	V	836.55	418.78	819.52	410.26			6
14	1,833.90	917.45	1,815.89	908.45	L	737.48	369.24	720.45	360.73			5
15	1,930.95	965.98	1,912.94	956.97	P	624.39	312.70	607.37	304.19			4
16	2,030.02	1,015.51	2,012.01	1,006.51	V	527.34	264.17	510.32	255.66			3
17	2,167.08	1,084.04	2,149.07	1,075.04	H	428.27	214.64	411.25	206.13			2

18					K	291.21	146.11	274.19	137.60			1
